# A Systematic Immuno-Informatic Approach to Design a Multiepitope-Based Vaccine Against Emerging Multiple Drug Resistant *Serratia marcescens*

**DOI:** 10.3389/fimmu.2022.768569

**Published:** 2022-03-14

**Authors:** Marcelo Silva Folhas Damas, Fernando Gabriel Mazur, Caio Cesar de Melo Freire, Anderson Ferreira da Cunha, Maria-Cristina da Silva Pranchevicius

**Affiliations:** ^1^ Departamento de Genética e Evolução, Universidade Federal de São Carlos, São Carlos, Brazil; ^2^ Centro de Ciências Biológicas e da Saúde, Biodiversidade Tropical – BIOTROP, Universidade Federal de São Carlos, São Carlos, Brazil

**Keywords:** *Serratia marcescens*, reverse vaccinology, multidrug resistance, computational approaches, subtractive proteomics

## Abstract

*Serratia marcescens* is now an important opportunistic pathogen that can cause serious infections in hospitalized or immunocompromised patients. Here, we used extensive bioinformatic analyses based on reverse vaccinology and subtractive proteomics-based approach to predict potential vaccine candidates against *S. marcescens*. We analyzed the complete proteome sequence of 49 isolate of *Serratia marcescens* and identified 5 that were conserved proteins, non-homologous from human and gut flora, extracellular or exported to the outer membrane, and antigenic. The identified proteins were used to select 5 CTL, 12 HTL, and 12 BCL epitopes antigenic, non-allergenic, conserved, hydrophilic, and non-toxic. In addition, HTL epitopes were able to induce interferon-gamma immune response. The selected peptides were used to design 4 multi-epitope vaccines constructs (SMV1, SMV2, SMV3 and SMV4) with immune-modulating adjuvants, PADRE sequence, and linkers. Peptide cleavage analysis showed that antigen vaccines are processed and presented *via* of MHC class molecule. Several physiochemical and immunological analyses revealed that all multiepitope vaccines were non-allergenic, stable, hydrophilic, and soluble and induced the immunity with high antigenicity. The secondary structure analysis revealed the designed vaccines contain mainly coil structure and alpha helix structures. 3D analyses showed high-quality structure. Molecular docking analyses revealed SMV4 as the best vaccine construct among the four constructed vaccines, demonstrating high affinity with the immune receptor. Molecular dynamics simulation confirmed the low deformability and stability of the vaccine candidate. Discontinuous epitope residues analyses of SMV4 revealed that they are flexible and can interact with antibodies. In silico immune simulation indicated that the designed SMV4 vaccine triggers an effective immune response. In silico codon optimization and cloning in expression vector indicate that SMV4 vaccine can be efficiently expressed in *E. coli* system. Overall, we showed that SMV4 multi-epitope vaccine successfully elicited antigen-specific humoral and cellular immune responses and may be a potential vaccine candidate against *S. marcescens*. Further experimental validations could confirm its exact efficacy, the safety and immunogenicity profile. Our findings bring a valuable addition to the development of new strategies to prevent and control the spread of multidrug-resistant Gram-negative bacteria with high clinical relevance.

## Introduction

The spread of antimicrobial resistance (AMR) is urgent, especially regarding bacteria (1). Once resistant strains emerge, the options for effective antibiotic therapy become limited and their alarming spread around the globe has not been followed by the development of novel antibiotics (2, 3). AMR produces significant impacts on human health around the world, causing troublesome levels of morbidity and mortality leading to dramatic economic consequences (4). It has been estimated that 10 million lives a year will be lost to AMR by 2050, and cumulative loss of world economies might be as high as $100 trillion (2, 5). AMR is a serious issue that demands an organized global action plan (4, 6, 7). Developing novel and integrated strategies are paramount to effectively fight AMR; these strategies include the development of monoclonal antibodies, new antibiotics, new diagnostics, new vaccines that target antibiotic-resistant bacteria, and increasing coverage of existing vaccines (3, 4, 8).

*Serratia* spp. is within the World Health Organization (9) global priority list of multidrug-resistant (MDR) bacteria that poses a major threat to human health around the world. Hence, there is an urgent need to development new and effective treatments and prevention strategies. *Serratia marcescens* is a Gram-negative *Enterobacteriaceae* species that has emerged as a neglected opportunistic human pathogen (10). This species can cause a variety of infections, including respiratory, bloodstream, skin, ocular, urinary, and catheter-related infections, as well as meningitis and sepsis in immunocompromised or critically ill patients, especially those in intensive care units (ICUs) and neonatal intensive care units (NICU). Studies have reported an increase in the number, and it of multidrug-resistant *S. marcescens* strains worldwide (11) and this increase has been related to severe outcomes (12) and a high mortality rate (13, 14).

Several studies and medical experiments have supported that *S. marcescens* may be promising for vaccine development. For instance, Field et al. (15) immunized adult mice with lipopolysaccharide (LPS) somatic antigen, or a heat-killed vaccine of *Serratia marcescen*s and observed a rapid presence of specific antibody-forming cells in the spleen, in the mesenteric nodes, and in the thymus. Kreger et al. (16) showed that the severity of experimentally induced corneal disease by *S. marcescens* is considerably reduced by immunization against either the lipopolysaccharide endotoxins or the proteases of the bacteria. Kumagai et al. (17) showed that the protection against an experimental *Serratia marcescens* infection in mice was enhanced by prior injection of formalin-killed or viable bacteria of the same strain. They suggested that the humoral immunity and T-cell-mediated immunity were associated with protection against systemic *Serratia* infection. Shi et al. (18) reported that *S. marcescens* vaccine was effective for malignant pleural effusion and presented tolerable toxic effects. In the late 19th century, William Coley developed a formulation containing *Streptococcus pyogenes* and *S. marcescens* called by various names, such as Coley’s fluid, Coley’s vaccine, mixed bacterial vaccine (MBV), Coley’s toxins, and Vaccineurin. This formulation was used to treat sarcoma in many countries until 1990 (19–21). In the 1970s, Coley’s mixture (MBV) was further investigated, and it has been used in clinical trials against different types of cancer presenting variable results (22–27). The recent interest in MBV is motivated by humoral and cellular immunity to cancer antigens, which has the ability to spontaneous induce antibody responses. The stimulation of the innate immune system produces a complex cascade of cytokines that contribute to the immune recognition of cancer, possibly inducing apoptosis (22).

Vaccination is one of the most effective means to efficiently, rapidly and affordably improve public health; it is also the most feasible way to eradicate a variety of infectious diseases (28). Current vaccine research has mostly focused on peptide and subunit vaccines instead of whole organism vaccines. This is because subunit vaccines contain specific immunogenic components of the pathogens responsible for the infection rather than the whole pathogen. Traditional approaches for vaccine production have also been considered less efficient than computational approaches for a variety of reasons, including inaccuracy, safety, stability, high cost, hypersensitivity, and specificity.

Reverse vaccinology (RV), subtractive proteomics (SP), and genomics studies have emerged as powerful computational tools that have revolutionized the identification of drug targets and potential vaccine candidates (29). These methodologies are able to identify *in silico* the complete repertoire of immunogenic antigens and druggable targets that an organism is capable of expressing without the need of culturing the microorganism (30). In addition, it reduces the dependence on conventional animal testing based screening for getting a potentially suitable candidate, minimizing the time consuming and cost of the vaccine and drug development processes (31). Since the first application of reverse vaccinology that was used to development of a vaccine against serogroup B *Neisseria meningitidis* (MenB) (32), this tool has been used in the identification of numerous promising vaccine candidates against many bacterial pathogens, including *Mycoplasma pneumoniae* (33), *Pseudomonas aeruginosa* (34)*, Mycobacterium tuberculosis* (30), *Acinetobacter baumannii* (35), and *Neisseria meningitidis* (36).

In this study, we have applied RV and SP based computational strategies and selected a new multi epitope-based vaccine candidate against *Serratia marcescens*, which can be used in further experiments to validate its efficacy, safety, and immunogenic profile.

## Material and Methods

**Figure 1:**
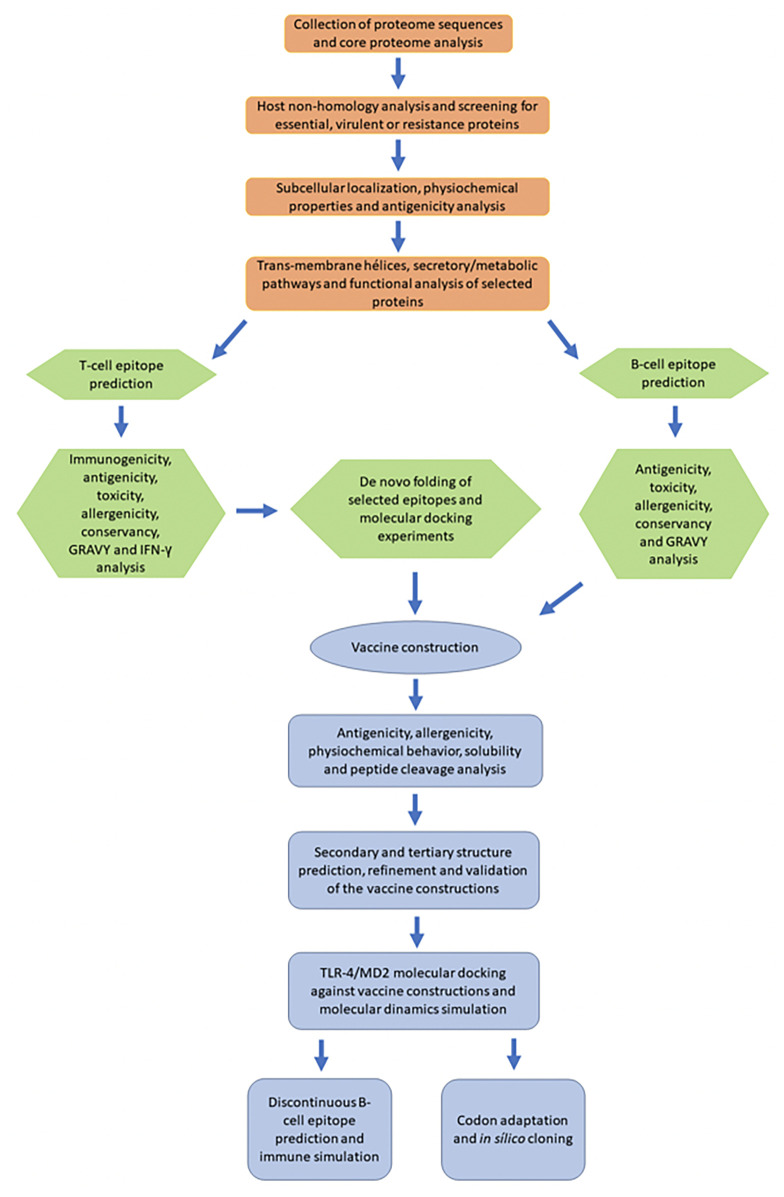
A schematic flowchart diagram showing the procedure used in the current study. Orange: subtractive proteome analysis. Green: identification, characterization, and selection of peptide epitopes. Blue: construction and analysis of the multiepitope vaccine.

Subtractive proteomics and reverse vaccinology approaches were used to identify potential vaccine candidates against the *S. marcescens* strain. A flowchart summarizing the methodology is shown in **Figure 1**.

### Data Collection of Proteome and Selection of Core Proteins

The proteome sequences of 49 *S. marcescens* were downloaded from the Genome Project database of the National Center for Biotechnology Information (NCBI) (https://www.ncbi.nlm.nih.gov/genbank/). Out of these proteomic sequences, one corresponded to the representative proteome of *Serratia marcescens* subsp. *marcescens* Db11 and 48 sequences were from *S. marcescens* associated with human infections. Bacterial Pan Genome Analysis (BPGA) tool (37) version 1.3 was used to identify core (conserved) protein families (**Supplementary Data Sheet 2** **)**. BPGA uses USEARCH as a default protein clustering tool with an identity cut off = 50%. Strain names, source of isolation, country, RefSeq assembly accession numbers, assembly levels, and references are shown in **Supplementary File Table S1**.

### Screening of Essential Proteins, Virulence Factors and Resistance Proteins

The identified core protein families related to 49 bacteria species were subjected to BLASTp searches against the Database of Essential Genes (DEG 10) providing the essential information of the proteins (35, 38–42). DEG is a database for essential genes that is frequently updated (43, 44). The parameters of the analysis were E-value ≤ 10^-4^ and bitscore ≥ 100 (**Supplementary Data Sheet 3**). The core proteins of *S. marcescens* were also subjected to BLASTp search against Virulence Factor database (VFdb) (http://www.mgc.ac.cn/VFs/) (45) and Microbial virulence DataBase (MvirDB) (http://mvirdb.llnl.gov/) **Supplementary Data Sheet 4** (46). In both databases, the E-value cut-off was set to ≤ 10^-4^ and bitscore ≥ 100. The resistance associated proteins were found through a BLASTp against two databases, ARG-ANNOT (Antibiotic Resistance Gene-ANNOTation), which provides protein sequences associated with antibiotic (47), and CARD (Comprehensive Antibiotic Resistance Database), a database of peer-reviewed antibiotic resistance determinants (**Supplementary Data Sheet 5**) (48). The E-value cut-off for both antibiotic resistance analyses was ≤ 10^-4^.

### Subtracting Gut-Human Homologous and Human Non-Homology Proteins

The identified essential, virulent or resistance associated proteins were filtered against the proteome of host *Homo sapiens* (taxid:9606), using BLASTp with E-value of ≤ 10^−4^ (**Supplementary Data Sheet 6**). The host non-homologue proteins were filtered against a custom protein database containing 79 human gut floral species [see supplementary Text 1 from (44, 49)]. For subtraction of homologous sequence between gut microbiota and *S. marcescens*, we carried out BLASTp analysis. The obtained hits with an E-value of ≤ 10^−4^ and similarity ≥ 50% were considered as gut-flora homologous proteins and excluded from further analyses (**Supplementary Data Sheet 7**).

### Prediction of Subcellular Localization

Prediction of selected proteins subcellular localization was done by using two different web servers: PSORTb v3.0.2 (50) algorithm (https://www.psort.org/psortb/) that determines different subcellular localization like cytoplasmic membrane, outer membrane, periplasm, extracellular, cytoplasmic, and unknown; and CELLO v2.5 (http://cello.life.nctu.edu.tw) (51), a web-based system which is also used for predicting protein subcellular localization.

### Physicochemical Property and Antigenicity Analysis of Proteins

Physicochemical properties such as number of amino acids and molecular weight were examined on the online servers Expasy ProtParam (52) (https://web.expasy.org/protparam/) and UniProt (https://www.uniprot.org/). Antigenicity of proteins was predicted using two online servers: VaxiJen v2.0 (53), (http://www.ddg-pharmfac.net/vaxijen/VaxiJen/VaxiJen.html) which predicts whether a protein could be a protective antigen based on physicochemical properties of amino acid sequence and has a threshold value ≥ 0.5; and AntigenPRO (http://scratch.proteomics.ics.uci.edu/), an alignment-free, sequence-based and pathogen-independent predictor of protein antigenicity with 79% accuracy and an area under curve (AUC) of 0.89 (54).

### Identification of Trans-Membrane Alpha-Helices and Secretory Pathway Analysis

To assess the proteins getting embedded in the plasma membrane and to subtract those being exported, we submitted the amino acid sequences from the outer membrane, periplasm and extracellular proteins of *S. marcescens* to the TMHMM v.2.0 (https://services.healthtech.dtu.dk/service.php?TMHMM-2.0) server, which predicted the topology of these proteins by the Markov method (55). Secretory pathway was analyzed using SignalP 5.0 (https://services.healthtech.dtu.dk/service.php?SignalP-5.0), a server based on deep neural network method that predicts signal peptide (SP) sequences and discriminates among three main types of SPs (56).

### Pathogen-Specific Pathways and Functionality Analysis of Selected Proteins

The comparison between metabolic pathways of *S. marcescens* and human pathways was done manually, using KEGG (Kyoto encyclopedia of gene and genome) pathway database. Proteins that play a role in unique and shared pathways in both pathogen and host were enlisted (**Table S2**) (35). Protein function prediction was made by three different servers: UniProt, KEGG Genes Database, and InterPro (https://www.ebi.ac.uk/interpro/), a server that provides family classification, biological process and molecular function of the protein (57).

### Prediction of T Cell and B Cell Epitope

The prediction of MHC-I epitopes was performed by three servers: IEDB Tepitool prediction (http://tools.iedb.org/tepitool/) server (58), NetMHCpan 4.1 BA (https://services.healthtech.dtu.dk/service.php?NetMHCpan-4.1), and NetCTLpan 1.1 (https://services.healthtech.dtu.dk/service.php?NetCTLpan-1.1). In the IEDB server, 27 different alleles that cover more than 97% of the global population were selected for MHC class I predictions (59). Identified T-cell epitopes having alleles with IC50 value ≤ 50 nM were considered of high binding affinity. The default prediction method was set as the IEDB recommended that uses the Consensus method consisting of ANN (Artificial neural network, also called as NetMHC, version 3.4), SMM (Stabilized matrix method), CombLib (Scoring Matrices derived from Combinatorial Peptide Libraries), and NetMHCpan (version 2.8). NetMHCpan 4.1 server predicts binding of peptides to any MHC molecule of a known sequence using artificial neural networks (ANNs). We used a threshold value IC50 ≤ 50 nM and a percentile rank ≤ 0.20 (34). NetCTLpan 1.1 server performs integrate prediction of peptide MHC class I binding, proteasomal C terminal cleavage, and TAP transport efficiency. In this analysis, the threshold value was set as 0.75 (35).

Predictions of MHC class II epitopes or HTL epitopes were made by Tepitool, using the IEDB recommended method. A set of the 26 most frequent human class II alleles from DP, DQ, and DR loci was used. Selection criteria was peptides with binding affinity ≤ 50nM for IC50. Prediction of linear B-cell epitopes or BCL epitopes for proteins was achieved by using IEDB server, ABCpred, and Bcepred. IEDB server predicted epitopes based on antigenicity (60), accessibility (61), linear epitope (Bepipred-1.0) (62) and sequential/conformational epitope (BepiPred-2.0) (63). ABCpred (https://webs.iiitd.edu.in/raghava/abcpred/) uses Artificial Neural Network (ANN) machine-learning to predict B-cell epitopes and has an accuracy of 65.93%. In this server, parameters were set to default. Bcepred (https://webs.iiitd.edu.in/raghava/bcepred/bcepred_instructions.html) predicted B-cell epitopes based on four amino acid properties (hydrophilicity, flexibility, polarity and exposed surface). We used a threshold of 2.38 that predicts epitopes with 58.7% accuracy

### MHC Class I Immunogenicity Determination

The MHC I immunogenicity prediction were assessed by the IEDB server (64) (http://tools.immuneepitope.org/immunogenicity/). A high score suggests a higher probability of stimulating an immune response. The epitopes with positive immunogenicity value were selected for further studies.

### Antigenicity, Toxicity, Allergenicity of Selected Epitopes

The epitopes of MHC Class I, MHC Class II and LB were screened for their antigenic properties by VaxiJen2.0. The threshold for MHC class I and MHC class II epitopes was set to ≥ 0.5, and to ≥ 0.70 for the B-cell epitopes (53). The antigenic B-Cell epitopes obtained, with 9 or more amino acids in length and those that overlapped with the amino acids sequences found in IEDB, ABCpred and Bcepred tools were selected for toxicity and allergenicity analyses. The toxicity prediction was carried out using ToxinPred (http://www.imtech.res.in/raghava/toxinpred/index.html), keeping all the parameters to default. This tool predicts the antigenic behavior of epitopes through their physicochemical properties and confirms that the specific immune responses in the host cell will only target the bacteria rather and not host tissue (65). Allergenicity analysis was conducted with AllerTOP v2.0 server (https://www.ddg-pharmfac.net/AllerTOP/feedback.py). This is a server based on the main physicochemical properties of proteins (66), presenting an accuracy of 88.7% (67).

### Conservancy, Hydrophobicity and IFN-Inducing Validation of Selected Epitopes

The conservancy of MHC Class I and MHC Class II selected epitopes within protein sequences were predicted using IEDB web server (68). For calculating the conservancy score, the sequence identity threshold was kept at 100%. Grand average of hydropathicity of MHC Class I. MHC Class II and LB epitopes were done using ProtParam (52) server. The GRAVY value is described by the sum of hydropathy values of all amino acids divided by the protein’s length (34). A negative value implies that protein contains hydrophilic properties whereas a positive GRAVY value indicates that the protein is hydrophobic (35). For further refinements, we investigated whether Helper T cell (HTL) epitope can induce IFN gamma immune response using the IFN epitope server (69) (http://crdd.osdd.net/raghava/ifnepitope/), an online tool with 82.10% accuracy. The server constructs overlapping sequences from which the IFN-γ epitopes are predicted. The default prediction method was set as “Motif and Support Vector Machine (SVM) hybrid” and “IFN-gamma vs. Non-IFN-gamma” model to predict IFN-γ-inducing peptides based on score. The higher the score, the higher the chance of inducing IFN-γ (70). Although the IFN epitope server has limitations regarding the number of residues that can be used for prediction (71), it is a common online prediction server used for vaccine design (70, 72–74). Therefore, the epitopes with positive results for the IFN-γ response were selected for further prediction.

### Predicting Three Dimensional (3D) Epitope Structure and Molecular Docking of the Selected Epitopes

The best-selected MHC class I and MHC class II epitopes were submitted to PEP-FOLD3 server (http://bioserv.rpbs.univ-paris-diderot.fr/services/PEP-FOLD3/), an online tool for generating *de novo* peptide 3D structure (75). The docking experiments were made using PatchDock (https://bioinfo3d.cs.tau.ac.il/PatchDock/php.php) tool. The obtained models were refined and re-scored by FireDock server (http://bioinfo3d.cs.tau.ac.il/FireDock/), that ranks the docked models by their global energy, and the lowest global energy represented the best prediction (76). The MHC class I epitopes were docked with HLA-A*0101 (PDB: 6AT9), HLA-A*0201 (PDB: 3UTQ), HLA-B*1501 (PDB: 1XR8), HLA-B*3501 (PDB: 1ZSD), HLA-B*3901 (PDB: 4O2E), HLA-B*5301 (PDB: 1A1M), HLA-B*5801 (PDB: 5IM7), HLA-B*4403 (PDB: 1SYS) alleles. The alleles used to MHC Class II epitopes were: HLA-DRB1*0101 (PDB: 2FSE), HLA-DRB1*0301 (PDB: 1A6A), HLA-DRB1*0401 (PDB: 2SEB), HLA-DRB1*1501 (PDB: 1BX2), HLA-DRB3*0101 (PDB: 2Q6W), HLA-DRB3*0202 (PDB: 3C5J) and HLA-DRB5*0101 (PDB: 1H15). The docked structures were visualized using PyMol tool (https://pymol.org/pymol.html?) (67). The epitopes that showed the best binding affinity were selected for vaccine construction.

### Vaccine Construction

Best binding peptides were selected for potential vaccine candidate. To construct the vaccine, CTL, HTL and BCL epitopes were linked together by GGGS, GPGPG and KK linkers. GGGS linkers were used to conjugate the Universal Pan HLA DR sequence (PADRE) sequence with CTL epitopes and the CTL epitopes among themselves. GPGPG linkers were used to conjugate the CTL epitopes with HTL epitopes and also the HTL epitopes with the other HTL. KK linkers were used to attach the HTL and BCL epitopes as well as the BCL epitopes among themselves (67). Adjuvants sequences were linked with the help of EAAAK linkers at both N- and C-terminus, and EAAAK linkers were also used to conjugate the PADRE sequence (AKFVAAWTLKAAA) (35). Five different adjuvant sequences were used to attach the PADRE sequence: 50s ribosomal L7/L12 protein (77), beta-defensin (78), HBHA protein (*M. tuberculosis*, accession number: AGV15514.1), and HBHA conserved sequence (79).

### Antigenicity and Allergenicity of Vaccine Constructs

VaxiJen 2.0 and ANTIGENpro server were used to determine the antigenicity of the vaccine constructs. AllerTOP and AlgPred (http://crdd.osdd.net/raghava/algpred/) servers were used to evaluate the allergen potential of the multi-epitope vaccine construct. Allergen prediction is based on similarity of known epitope of any of the known region of the protein. It uses MAST to search MEME/MAST allergen motifs and predict the allergen if it has a motif. AlgPred is an SVM module based program which uses amino acid or dipeptide composition for the prediction of allergen. The parameters (IgE epitope + MAST + SVM + ARPs BLAST) were combined to predict the allergenicity of vaccine constructs (35, 80).

### Solubility Prediction and Physiochemical Behavior Analysis of Vaccine Constructs

SOLpro of Scratch Protein predictor was used for vaccine solubility estimation. SOLpro performs a two-stage SVM architecture method based on multiple representations of the primary sequence (81). The overall accuracy of SOLpro is estimated in over 74% using multiple runs of ten-fold cross-validation (81). Vaccine constructs physiochemical properties were analyzed using Expasy ProtParam server, which determined the number of amino acids, molecular weight, theoretical isoelectric point (PI), instability and aliphatic index, and hydropathicity GRAVY values.

### Peptide Cleavage Analysis

Proteasomal cleavage is important for T Cell epitope presentation. This was analyzed by NetChop 3.1 (http://www.cbs.dtu.dk/services/NetChop/), a neural network-based method trained on MHC class I ligands produced by the human proteasomes (**Supplementary Data Sheet 8**) (82). Since cathepsins cleavage sites may play a vital role in the immune antigen presentation, cathepsin specific peptidase activity was analyzed with the SitePrediction (http://www.dmbr.ugent.be/prx/bioit2-public/SitePrediction/index.php) server for MHC class II epitopes (83).

### Secondary and Tertiary Structure Prediction of the Vaccine Constructs

The secondary structures of the multi-epitope vaccine constructs were generated using online tool PSIPRED 4.0 (http://bioinf.cs.ucl.ac.uk/psipred/), a web-based freely accessible online server that also predicts the transmembrane topology, transmembrane helix, fold and domain recognition (74). PSIPRED 4.0 has a Q3 secondary structure prediction precision of 84.2% (84). The 3D structures of multi-epitope vaccine constructs were predicted using the I-TASSER (Iterative Threading ASSEmbly Refinement) server (https://zhanglab.ccmb.med.umich.edu/I-TASSER/). I-TASSER is an integrated platform for automated protein structure and function prediction based on the sequence-to-structure-to-function paradigm. I-TASSER initial creates three-dimensional (3D) atomic models from several threading alignments and iterative structural assembly simulations starting from an amino acid sequence. In five community wide CASP (Critical Assessment of techniques for Structure Prediction) experiments, I-TASSER has been ranked best server for protein 3D structure prediction (70). Pymol program was used to visualize the modeled 3D structures.

### Refinement and Validation of Vaccines Constructs

The 3D structures of the constructed vaccines were refined using 3Drefine server (http://sysbio.rnet.missouri.edu/3Drefine/). 3Drefine server is based in optimization of the hydrogen bonding network and composite physics and knowledge-based force fields to give atomic-level energy minimization using the MESHI molecular modeling framework (85, 86). The validation process was performed using the PROCHECK’s Ramachandran plot analysis (https://servicesn.mbi.ucla.edu/PROCHECK/) (87) that analyzes the geometry of the refined vaccine construct and predict the best stereochemical quality of the construct (88); ProSA (https://prosa.services.came.sbg.ac.at/prosa.php) (89) that computes the overall quality score (Z score) for a specific 3D structure (90); and ERRAT server (http://services.mbi.ucla.edu/ERRAT/) (91) that analyzes the statistics of non-bonded interactions between different atom types (92).

### Protein-Protein Docking

Each vaccine construct was docked against TLR4-MD2 complex (PDB:3FXI). The docking experiments were made using ClusPro 2.0 (https://cluspro.bu.edu/login.php) and PatchDock (https://bioinfo3d.cs.tau.ac.il/PatchDock/php.php). ClusPro 2.0 ranks the cluster of docked complexes based on their center and lowest energy scores (93). PatchDock algorithm divides the Connolly dot surface representation of the molecules into concave, convex, and flat patches (94). ClusPro 2.0 and PatchDock were further analyzed by the PRODIGY tool of HADDOCK server (https://haddock.science.uu.nl/) and FireDock server (http://bioinfo3d.cs.tau.ac.il/FireDock/php.php), respectively. The PRODIGY server produces binding affinity score (95) and the FireDock server accesses the global energy of the docked complexes.

### Molecular Dynamic Simulation

After performing the protein-protein molecular docking, the best-scored vaccine construction (SMV4) complexed with TLR4-MD2 was subjected to molecular dynamic simulation by the online server iMODS (http://imods.chaconlab.org/) (96), using the parameters as default. This server predicts the dynamics simulation of the protein complex in terms of atomic B-factors, eigenvalue variance, deformability, elastic network, and covariance map. The deformability of a given protein mostly relies on the capability of each of its residues to deform. The eigenvalue is related with the energy that is required to deform the given structure; the lower the eigenvalue value, the easier the deformability of the complex (67, 97). Moreover, the eigenvalue of the given protein complex provides its motion stiffness (79).

### Discontinuous B Cell Epitopes

SMV4 vaccine construction selected was submitted to ElliPro server (http://tools.iedb.org/ellipro/) that predicts epitopes based upon solvent-accessibility and flexibility (98). The algorithms implemented in this analysis were approximation of the protein shape as an ellipsoid (99), protrusion index (PI) of residue (100), and neighboring residues clustering based on their PI values. The conformational B-cell epitopes with minimum score value set at 0.70 while the maximum distance was set as default.

### Immune Simulation of the Vaccine Construct

C-ImmSim server (http://150.146.2.1/C-IMMSIM/index.php?page=1) was used for the immune simulation study. It uses position-specific scoring matrix for immune epitope forecast and machine learning techniques to estimate immune interactions (101). The three mammalian anatomical regions to get simulated by the server were thymus (T cell), bone marrow (lymphoid and myeloid cell), and a lympathic organ to exhibit immune response (102). All parameters were kept as default at the time of vaccine introduction, and three injections were administered with the recommended intervals of 30 days. The time steps followed for three injections were 1, 90 and 180. The volume of simulation and the steps of the simulation were set at 10 and 600, respectively (103).

### Codon Adaptation and *In Silico* Cloning

Reverse translation and codon optimization were performed using Java Codon Adaptation Tool (JCat) server (http://www.prodoric.de/JCat) (104). The JCat output includes the codon adaptation index (CAI) and percentage GC content, which can be used to assess protein expression levels. CAI provides information on codon usage biases; CAI score >0.8 is considered a good score (105). The ideal GC content of a sequence should range between 30–70% (80). The *E. coli* strain K12 was chosen as host for cloning our vaccine construct. We avoided rho-independent transcription termination, prokaryote ribosome binding site, and restriction enzymes cleavage sites. Vaccine construct was cloned in pET28a (+) plasmid vector by adding XhoI and NdeI restriction sites at C and N terminus, respectively. The optimized sequence of the vaccine was inserted into the expression vector [pET-28a (+)] using Benchling webserver (https://www.benchling.com/).

## Results

### Pre-Screening of Primary Data

Primarily, we selected representative proteomes of 48 *S. marcescens* associated with human infections, and a *Serratia marcescens* subsp. marcescens Db11 as a reference strain for our vaccine prediction. The proteomes of all *S. marcescens* strains were retrieved from Genome Project database of the National Center for Biotechnology Information (NCBI). With the help of Bacterial Pan Genome Analysis (BPGA) tool the number of core proteins found from analyzing of the 49 proteomes was 2832 proteins.

### Screening of Essential, Virulence, Resistance and Non-Homology Against Human and Gut Flora Proteins

All the 2832 proteins were subsequently analyzed for essential, virulent and resistance functions. The analyses of the non-redundant proteins resulted in 1815 proteins. Of these proteins, we have found 879 essential proteins, 155 proteins contained virulence property, 98 were resistant proteins, 370 proteins were found to be virulence and essential, 70 were resistant and essential, 42 resistant and virulence, and 201 proteins were related with essential, virulence and resistance functions, 1106 were non-homologous with human proteins. Of these 1106 proteins, 20 were gut flora non-homologous proteins, and were used for subsequent analysis

### Subcellular Localization, Identification of Essential Proteins, Virulence Factors and Resistant Determinants

Next, the subcellular localization of 20 gut flora non-homologous proteins revealed that 2 proteins were outer membrane proteins, 6 periplasmic, and 2 extracellular (**Table 1**). Of these 10 proteins, 4 protein were essential (D-alanyl-D-alanine carboxypeptidase, 51.35 kDa; patatin-like phospholipase, 35.68 kDa; lipoprotein 11.82 kDa; helix-turn-helix domain-containing protein, 10.66 kDa), 4 virulence (phospholipase C, 79.68 kDa; spore coat U domain-containing protein, 33.28 kDa; protein of avirulence locus ImpE, 29.48 kDa; NADPH-dependent FMN reductase, 19.24 kDa; 1 was related to resistance (TonB-dependent receptor, 76.90 kDa), and 1 protein presented essential and virulent functions (MoaF domain-containing protein, 16.27 kDa) (**Table 1**).

**Table 1:** Predicted subcellular localization, physicochemical, antigenicity, trans-membrane alpha-helices and peptide signal analysis.

Ref. Sequence ^(1)^	Protein name ^(2)^	Length (amino acid) ^(3)^	Mol. Wt kDa ^(4)^	Signal peptide ^(5)^	Localization ^(6)^	Functional Discription ^(7,8,9)^	TMHMM ^(10)^	Antigenicity ^(11,12)^
	**Essential proteins**							
WP_041033700.1	* D-alanyl-D-alanine carboxypeptidase/D-alanyl-D-alanine-endopeptidase	489	51.35	Sec/SPIICleavage site (17 and 18, LAG-CS)	Outer membrane/Periplasm	Penicilin-binding protein/Serine endopeptidase activity	0	0.5856, 0.5066
WP_084827239.1	* Patatin-like phospholipase Family protein	323	35.68	Not identified	Outer membrane	Hydrolase activity/lipid catabolic process	0	0.6024, 0.8235
WP_004939944.1	Lipoprotein	108	11.82	Sec/SPIICleavage site (16 and 17, LSA-CA)	Periplasm	Lipoprotein with MoaF domain	0	0.5595, 0.4857
WP_047571040.1	Helix-turn-helix domain-containing protein	92	10.66	No identified	Periplasm	Uncharacterized conserved protein with HTH_43 domain	0	0.2089, 0.2900
	**Virulent proteins**							
WP_141960268.1	* Phospholipase C, phosphocholine-specific	715	79.68	Tat/SPICleavage site (31 and 32, (ALA-IP)	Extracellular	Membrane damaging toxin, phosphoric-diester hydrolase	0	0.4097, 0.6277
WP_048321499.1	* Spore coat U domain-containing protein	311	33.28	Sec/SPICleavage site (23 and 24, AFA-DC)	Extracellular	Involved in motility and biofilm formation	0	0.6887, 0.8719
WP_148123533.1	Protein of avirulance locus ImpE	273	29.48	Not identified	Periplasm	Signaling, type VI secretion system component	0	0.5895, 0.7594
WP_004940045.1	NADPH-dependent FMN reductase	183	19.24	Not identified	Periplasm	Electron transfer activity/FMN binding	0	0.4056, 0.7386
	**Resistance protein**							
WP_033636744.1	* TonB-dependent receptor	697	76.90	Sec/SPICleavage site (44 and 45, VNA-AE)	Outer membrane	Iron complex receptor protein, channel transporter of siderophores	0	0.6847, 0.7910
	**Essential and virulent protein**							
WP_099783007.1	MoaF domain-containing protein	150	16.27	Sec/SPICleavage site (26 and 27, ATA-AQ)	Periplasm	Exported protein with MoaF domain	1	0.5896, 0.9631

All data were analyzed using various server: 1, 2, 3 = NCBI/UniProt; 4 = Expasy; 5 = SignalP5.0; 6 = PSORTb/CELLO; 7, 8, 9 = Uniprot/KEGG/InterPro; 10 = TMHMM; 11 = Vaxijen, 12 = AntigenPRO. * proteins considered for further analysis.

### Peptide Signal, Trans-Membrane, and Antigenicity Prediction

Of these 10 proteins selected, analyses of presence of signal peptide/anchor resulted into 3 proteins with secretory signal peptides that are transported by the Sec translocon and cleaved by Signal Peptidase I (Sec/SPI), 2 proteins having lipoprotein signal peptides transported by the Sec translocon and cleaved by Signal Peptidase II (Sec/SPII), and 1 protein with Tat signal peptides transported by the Tat translocon and cleaved by Signal Peptidase I (Tat/SPI). Only 1 protein (MoaF domain-containing protein) contained 1 transmembrane helix (**Table 1**). VaxiJen v2.0 and AntigenPRO tools reveled 7 and 8 proteins with a good antigenic nature (>0.50) (**Table 1**), respectively. Of these, 2 essential proteins (D-alanyl-D-alanine carboxypeptidase, patatin-like phospholipase family protein), 2 virulent proteins (Phospholipase C, phosphocholine specific; spore coat U domain-containing protein), and 1 resistant protein (TonB-dependent receptor) presented antigenicity profile, had extracellular domain or were proteins located in the outer membrane. Therefore, these 5 protein were considered for further prediction of vaccine targets (**Table 1**).

### MHC Class-I Epitopes Prediction and Immunogenicity, Antigenicity, Toxicity, Hydropathicity and Conservancy Analysis of Selected Epitopes

The prediction of T-cell epitopes of MHC class-I of the 5 proteins (D-alanyl-D-alanine carboxypeptidase, patatin-like phospholipase family protein, Phospholipase C phosphocholine specific, spore coat U domain-containing protein, TonB-dependent receptor) had the sequence length 9 residues. Among the 284 predicted epitopes, the 123 common epitopes found in three servers were selected for immunogenicity analysis and resulted in 59 epitopes. From these, 31 epitopes were found to be antigenic and we found no epitopes with toxicity. Out of 31, 17 epitopes were non-allergenic. Epitope conservancy analysis found 14 peptides with a score of more than 50%. GRAVY analysis resulted in 7 peptides with negative value score, which suggests hydrophilic nature of peptides. For further analysis, we selected 7 MHC class-I epitopes (TPFGAGWSW, LEDRLVETL, SSNVNFPLY, FTIPLPGDR, QTYGAKIAR, SEYVWNYEL, YQFLKGWEL) that were found to be immunogenic, antigenic, non-allergenic, non-toxic, conserved, and with negative hydropathicity (**Table 2** **).** We excluded the patatin-like phospholipase family protein because its prediction analysis did not reach all the recommended parameters (**Table 2** **)**.

**Table 2:** Predicted MHC class-I epitopes and immunogenicity, antigenicity, toxicity, allergenicity, conservancy and hydropathicity analysis.

No.	Protein ID _(1);_ name _(2)_	Start _(3 4,5)_	End _(3,4,5)_	Epitope _(3,4,5)_	Tepitool Alleles _(3)_	NetMHCpan Alleles _(4)_	NetCTLpan Alleles _(5)_	Immunogenicity _(6)_	Antigenicity _(7)_	Toxicity _(8)_	Allergenicity _(9)_	Conservancy _(10)_	Hydropathicity _(11)_
1	WP_041033700.1; D-alanyl-D-alanine carboxypeptidase/endopeptidase	156	164	TPFGAGWSW	HLA-B*53:01	HLA-B*53:01	HLA-B*35:01, HLA-B*53:01	0.23238	0.5798	Non-toxin	Non-allergen	100%	-0.12
		471	479	LEDRLVETL	HLA-B*40:01	HLA-B*40:01	HLA-B*40:01	0.19452	0.7321	Non-toxin	Non-allergen	100%	-0.01
3	WP_141960268.1; Phospholipase C, phosphocholine-specific	74	82	FTIPLPGDR	HLA-A*68:01	HLA-A*68:01	HLA-A*68:01	0.05236	0.739	Non-toxic	Non-allergen	97.96%	-0.13
4	WP_048321499.1; spore coat U domain-containing protein	127	135	SSNVNFPLY	HLA-A*30:02	HLA-A*30:02	HLA-A*01:01, HLA-A*11:01, HLA-A*30:02	0.1275	0.983	Non-toxin	Non-allergen	63.27%	-0.08
5	WP_033636744.1; TonB-dependent receptor	469	477	QTYGAKIAR	HLA-A*68:01	HLA-A*31:01, HLA-A*68:01	HLA-A*68:01	0.00318	1.2908	Non-toxin	Non-allergen	100%	-0.69
		499	507	SEYVWNYEL	HLA-B*40:01	HLA-B*40:01	HLA-B*40:01, HLA-B*44:03	0.30533	1.4052	Non-toxin	Non-allergen	100%	-0.76
		608	616	YQFLKGWEL	HLAA*02:01, HLA-A*02:06	HLA-A*02:01, HLA-A*02:06	HLA-A*02:01, HLA-A*02:06	0.09418	0.561	Non-toxin	Non-allergen	100%	-0.34

All data were analyzed using various server: 1, 2 = NCBI/UniProt; 3 = IEDB Tepitool; 4 = NetMHCpan 4.1; 5 = NetCTLpan 1.1; 6 = IEDB server; 7 = VaxiJen 2.0; 8 = ToxinPred; 9 = AllerTOP v2.0; 10 = IEDB, 11 = GRAVY ProtParam.

### MHC-II Epitopes and Antigenicity, Toxicity, Conservancy, Hydropathicity, IFN-γ Analysis

The MHC-II binding prediction of the 5 proteins (D-alanyl-D-alanine carboxypeptidase, patatin-like phospholipase family protein, Phospholipase C phosphocholine specific, spore coat U domain-containing protein, TonB-dependent receptor) resulted in 415 MHC-II epitopes with higher affinity. From these, 196 were antigenic, and all were subjected to toxicity and allergenicity prediction. According with results, all selected epitopes were non-toxic and 114 had non-allergic nature. Conservancy analysis showed that 93 epitopes had score more than 50%, and GRAVY analysis revealed that 70 epitopes had a hydrophilic nature. Additionally, the 31 the best resultant epitopes of all analyses conducted were analyzed for their IFN-γ inducing. A total of 16 epitopes (4 from D-alanyl-D-alanine, 1 from patatin-like phospholipase family protein, 2 from Phospholipase C phosphocholine specific, 2 from spore coat U domain-containing protein and 7 from TonB-dependent receptor) had a IFN-γ inducing profile and were selected for molecular docking analysis (**Table 3**).

**Table 3:** Identification of MHC-II epitopes and antigenicity, toxicity, conservancy, hydropathicity and IFN-γ inducing profile prediction.

No.	Protein ID _(1)_; name _(2)_	Start _(3)_	End _(3)_	Epitope _(3)_	Alleles _(3)_	Antigenicity _(4)_	Toxicity _(5)_	Allergenicity _(6)_	Conservancy _(7)_	Hydropathicity _(8)_	IFN-γ inducing _(9)_
1	WP_041033700.1; D-alanyl-D-alanine carboxypeptidase/endopeptidase	7	21	WLLPAILALAGCSSS	HLA-DRB1*01:01	10.376	Non-toxin	Non-allergen	83.67%	-0.93	0.476
		170	184	AFAAPISALNYAFTP	HLA-DRB1*01:01	0.6842	Non-toxin	Non-allergen	75.51%	-0.89	0.228
		197	211	PGARAGAPGRVSFYP	HLA-DQA1*05:01/DQB1*03:01	10.191	Non-toxin	Non-allergen	61.22%	-0.1	0.329
		451	465	PLAFAIISNNYLVPG	HLA-DRB1*04:05/HLA-DRB1*04:01/HLA-DRB1*15:01HLA-DRB1*07:01/HLA-DRB1*01:01/HLA-DRB1*13:02	0.5584	Non-toxin	Non-allergen	100%	-0.92	0.089
2	WP_084827239.1; patatin-like phospholipase family protein	40	54	SGASAGAIAALLVGL	HLA-DQA1*05:01/DQB1*03:01	0.8684	Non-toxin	Non-allergen	100%	-0.71	0.416
3	WP_141960268.1; Phospholipase C, phosphocholine-specific	243	257	RQYRAASIQVGNPAR	HLA-DRB1*01:01	0.5796	Non-toxin	Non-allergen	97.96%	-0.93	0.131
		452	466	EKRFQVHEPNISAWR	HLA-DRB1*01:01	0.8644	Non-toxin	Non-allergen	100%	-1.34	0.673
4	WP_048321499.1; spore coat U domain-containing protein	117	131	SLNLLSLILISSNVN	HLA-DRB1*01:01	0.6343	Non-toxin	Non-allergen	97.96%	-0.82	0.041
		121	135	LSLILISSNVNFPLY	HLA-DRB1*13:02/HLA-DRB1*01:01	0.5934	Non-toxin	Non-allergen	63.27%	-1.05	0.41
5	WP_033636744.1; TonB-dependent receptor	125	139	NVGANAFLSGTRPRL	HLA-DRB5*01:01	0.7968	Non-toxin	Non-allergen	87.76%	-0.11	0.314
		129	143	NAFLSGTRPRLNLSL	HLA-DRB5*01:01, HLA-DRB1*01:01, HLA-DRB1*11:01	0.8159	Non-toxin	Non-allergen	87.76%	-0.03	0.136
		339	353	TDFNINRPTAYNIQY	HLA-DRB3*02:02, HLA-DRB1*13:02	0.6574	Non-toxin	Non-allergen	87.76%	-0.93	0.154
		372	386	ADSRLHGLAGLRYFH	HLA-DRB1*01:01	0.5227	Non-toxin	Non-allergen	100%	-0.27	0.494
		565	579	RWDFELFGNLGLLKT	HLA-DRB1*01:01	0.5005	Non-toxin	Non-allergen	87.76%	-0.03	0.108
		595	609	ARAPAYTANMGAKYQ	HLA-DRB3*02:02	0.9467	Non-toxin	Non-allergen	87.76%	-0.65	0.04
		606	620	AKYQFLKGWELSSNV	HLA-DRB1*01:01	0.7445	Non-toxin	Non-allergen	87.76	-0.41	0.63

All data were analyzed using various server: 1, 2 = NCBI/UniProt; 3 = IEDB Tepitool; 4 = VaxiJen 2.0; 5 = ToxinPred; 6 = AllerTop v2.0; 7 = IEDB; 8 = GRAVY ProtParam; 9 = IFN epitope.

### B-Cell Epitope Prediction and Antigenicity, Toxicity, Allergenicity and Hydropathicity Analysis

The prediction of linear B-cell epitopes for D-alanyl-D-alanine carboxypeptidase, patatin-like phospholipase family protein, Phospholipase C, TonB-dependent receptor and spore coat U domain-containing protein is showed in **Figure S1**. Antigenicity scale, and the most potent regions in epitopes found is showed in yellow (**Figure S1**). A total of 503 B cell epitopes were predicted by three servers, of which 236 epitopes were found to be antigenic. From these antigenic epitopes, we manually selected 23 epitopes that had regions overlapping with the amino acids sequences found in IEDB, ABCpred and Bcepred tools. These epitopes were subsequently tested to toxicity, allergenicity, conservancy and hydropathicity. This analysis resulted in 12 epitopes (TGEQRGDTL, SGDPTLHPDDL, GRKTQGKGD, QREVYSHRTTPRM, SSQRINTRTLGLRLDS, MAVANTDGSGD, TTVWDSTNKQSGAGT, QPEVRLRPTG, FAAQRHESVGN, AETKSNETYQD, DRQRRRSEADL, RLEREHRRRDG) non-allergen, non-toxic, conserved and having hydrophilic nature. All 12 epitopes were selected for further analysis and vaccine construction (**Table 4**).

**Table 4:** Identification of B-cell epitopes and antigenicity, toxicity, allergenicity and hydropathicity prediction of selected epitopes.

No.	Protein ID _(1)_; name _(2)_	Start	End	Lenght	Epitopes _(3,4,5)_	Antigenicity _(6)_	Toxicity _(7)_	Allergenicity _(8)_	Hydropathicity _(9)_	Conservancy _(10)_
1	WP_041033700.1; D-alanyl-D-alanine carboxypeptidase/endopeptidase	100	108	9	TGEQRGDTL	1.4316	Non-toxin	Non-allergen	1.49	51.02%
		117	127	11	SGDPTLHPDDL	0.7116	Non-toxin	Non-allergen	-1.02	100%
		331	339	9	GRKTQGKGD	2.7203	Non-toxin	Non-allergen	2.36	89,80%
2	WP_084827239.1; patatin-like phospholipase family protein	147	159	13	QREVYSHRTTPRM	0.7944	Non-toxin	Non-allergen	-2.05	100%
		214	229	16	SSQRINTRTLGLRLDS	17.872	Non-toxin	Non-allergen	-0.77	71.43%
3	WP_141960268.1; Phospholipase C, phosphocholine-specific	620	629	10	QPEVRLRPTG	1.3449	Non-toxin	Non-allergen	-1.23	93.88%
4	WP_048321499.1; spore coat U domain-containing protein	81	91	11	MAVANTDGSGD	1.8801	Non-toxin	Non-allergen	-0.28	63.27%
		263	277	15	TTVWDSTNKQSGAGT	1.023	Non-toxin	Non-allergen	-0.97	61.22%
5	WP_033636744.1; TonB-dependent receptor	5	15	11	FAAQRHESVGN	0.8087	Non-toxin	Non-allergen	-0.80	97.96%
		45	55	11	AETKSNETYQD	1.6267	Non-toxin	Non-allergen	-2.10	100%
		233	243	11	DRQRRRSEADL	1.2989	Non-toxin	Non-allergen	-2.47	87.86%
		429	439	11	RLEREHRRRDG	1.6679	Non-toxin	Non-allergen	-2.98	87.86%

All data were analyzed using various on line server: 1, 2 = NCBI/UniProt; 3, 4, 5 = ABCPred, Bcepred, IEDB; 6 = VaxiJen 2.0; 7 = ToxinPred; 8 = AllerTOP v2.0; 9 = GRAVY ProtParam; 10 = IEDB.

### Peptide Modeling and Molecular Docking Analysis

All the 7 MHC class I and 16 MHC class II T-cell epitopes were subjected to 3D structure generation by the PEP-FOLD3 server, and the predicted 3D structures found were docked with 8 MHC class I alleles and 7 MHC class II alleles, respectively. Among the epitopes, 5 MHC class I and 12 class II epitopes showed the best result with the lowest global energy of -34.89 and -70.54, respectively (**Table 5**) and were used in multi-peptide vaccine construction.

**Table 5:** Molecular docking of epitopes with HLA.

MHC Class I											
No.	Protein ID, name	Epitope	Global Energy								
			HLA-A*0101 (PDB: 6AT9)	HLA-A*0201 (PDB: 3UTQ)	HLA-B*1501 (PDB: 1XR8)	HLA-B*3501 (PDB: 1ZSD)	HLA-B*3901 (PDB: 4O2E)	HLA-B*4403 (PDB: 1SYS)	HLA-B*5301 (PDB: 1A1M)	HLA-B*5801 (PDB: 5IM7)	Average
1	WP_041033700.1 D-alanyl-D-alanine carboxypeptidase/endopeptidase	**TPFGAGWSW**	-54.89	-18.06	-34.65	-42.60	-13.15	-43.74	-38.83	-33.18	-34.89
		**LEDRLVETL**	-41.35	-22.48	-43.79	-24.36	-35.34	-16.97	-38.40	-23.62	-30.79
2	WP_141960268.1 Phospholipase C, phosphocholine-specific	**FTIPLPGDR**	-46.08	-24.55	-15.99	-24.14	-6.21	-15.41	-33.75	-23.74	-23.73
3	WP_048321499.1 spore coat U domain-containing protein	**SSNVNFPLY**	-56.28	-23.22	-32.65	-9.88	-19.00	-36.62	-37.51	-30.24	-30.68
4	WP_033636744.1 TonB-dependent receptor	**QTYGAKIAR**	-43.86	-4.69	-22.38	-19.36	-15.90	-25.60	-18.49	-26.75	-22.13
		**SEYVWNYEL**	-40.96	-17.05	-33.96	-25.68	-8.14	-15.25	-42.89	-39.22	-27.89
		**YQFLKGWEL**	-49.03	-18.24	-15.98	-8.29	-15.5	-25.71	-47.98	-35.74	-27.06
**MHC Class II**											
			**Global Energy**								
**No.**	**Protein ID, name**	**Epitope**	HLA-DRB1*0101 (PDB: 2FSE)	HLA-DRB1*0301 (PDB: 1A6A)	HLA-DRB1*0401 (PDB: 2SEB)	HLA-DRB1*1501 (PDB: 1BX2)	HLA-DRB3*0101 (PDB: 2Q6W)	HLA-DRB3*0202 (PDB: 3C5J)	HLA-DRB5*0101 (PDB: 1H15)	**Average**	
1	WP_041033700.1 D-alanyl-D-alanine carboxypeptidase/endopeptidase	**WLLPAILALAGCSSS**	-89.74	-79.65	-74.83	-68.81	-65.01	-43.20	-72.52	-70.54	
		**PGARAGAPGRVSFYP**	-74.58	-62.83	-62.02	-62.94	-41.19	-30.71	-64.83	-57.01	
		**LAVTFLKVSNNGYGE**	-66.12	-73.03	-51.81	-54.69	-36.28	-30.34	-61.55	-53.40	
		**PLAFAIISNNYLVPG**	-72.3	-57.61	-55.05	-62.76	-58.5	-47.77	-89.92	-63.42	
2	WP_084827239.1 patatin-like phospholipase family protein	**SGASAGAIAALLVGL**	-58.55	-72.12	-64.58	-75.20	-59.65	-41.55	-64.48	-62.30	
3	WP_141960268.1 Phospholipase C. phosphocholine-specific	**RQYRAASIQVGNPAR**	-61.17	-55.80	-47.44	-47.05	-13.96	-30.96	-69.12	-46.50	
		**EKRFQVHEPNISAWR**	-38.13	-38.77	-45.12	-8.34	-16.93	-24.41	-27.34	-28.43	
4	WP_048321499.1 spore coat U domain-containing protein	**SLNLLSLILISSNVN**	-88.21	-56.66	-52.15	-68.53	-29.65	-50.10	-89.29	-62.08	
		**LSLILISSNVNFPLY**	-93.62	-65.33	-50.97	-82.72	-16.16	-46.28	-45.35	-57.20	
5	WP_033636744.1 TonB-dependent receptor	**NVGANAFLSGTRPRL**	-65.95	-52.33	-55.04	-61.88	-20.96	-31.18	-58.32	-49.38	
		**NAFLSGTRPRLNLSL**	-74.06	-48.43	-59.42	-37.86	-35.10	-31.70	-35.66	-46.03	
		**TDFNINRPTAYNIQY**	-42.87	-44.67	-49.32	-32.67	-13.94	-34.32	-43.83	-37.37	
		**ADSRLHGLAGLRYFH**	-62.54	-47.66	-53.30	-61.08	-24.56	-25.95	-58.13	-47.60	
		**RWDFELFGNLGLLKT**	-45.56	-55.26	-56.56	-51.49	-16.14	-32.27	-63.07	-45.76	
		**ARAPAYTANMGAKYQ**	-45.59	-44.37	-51.96	-44.44	-22.36	-29.58	-34.58	-38.98	
		**AKYQFLKGWELSSNV**	-54.33	-61.70	-39.90	-59.14	-18.93	-25.68	-38.05	-42.53	

3D structures were generated by the PEP-FOLD3 server. The docking was performed using PatchDock online tool and the results were refined by FireDock online server.

### Construction of Multi-Epitope Peptide Vaccine, Physiochemical Properties and Antigenicity, Allergenicity, Solubility Analysis of Different Vaccine Constructs

We combined an adjuvant, PADRE sequence, CTL epitopes (MHC-I epitopes), HTL epitopes (MHC-II epitopes) and BCL epitopes (B-cell epitopes) in a sequential manner, and constructed four vaccines candidates, named SMV1, SMV2, SMV3 and SMV4. All designed vaccine proteins contained 5 CTL epitopes, 12 HTL, and 12 BCL epitopes. The vaccines differed each other only by adjuvant sequence, and the adjuvants used were 50s ribosomal L7/L12 protein, beta defensin, HBHA conserved sequence and HBHA protein (*M. tuberculosis*, accession number: AGV15514.1) (**Table 6** **)**. For vaccine construction, the adjuvant sequence was linked with PADRE sequence by EAAAK linker, GGGS linkers were used to join the PADRE sequence with the CTL epitopes and the CTL epitopes with the other CTL epitopes, GPGPG were used to linked the CTL epitopes with the HTL epitopes and also the HTL epitopes among themselves, and KK linkers were used to conjugate HTL with the BCL epitopes, the BCL with the other BCL epitopes, and BCL with the PADRE sequence. Each vaccine construct was finished by an additional GGGS linker.

**Table 6:** Characteristics of the constructed vaccines against *S. marcescens* strains.

Vaccine name/adjuvant	Sequence	Antigenicity _(1, 2)_	Allergenicity _(3,4)_	Amino acids length _(5)_	Mol. weight kDa _(5)_	pI _(5)_	Instability index _(5)_	Aliphatic index _(5)_	GRAVY _(5)_	SOLpro _(6)_
**SMV1/(HBHA)**	**EAAAK**MAENPNIDDLPAPLLAALGAADLALATVNDLIANLRERAEETRAETRTRVEERRARLTKFQEDLPEQFIELRDKFTTEELRKAAEGYLEAATNRYNELVERGEAALQRLRSQTAFEDASARAEGYVDQAVELTQEALGTVASQTRAVGERAAKLVGIEL**EAAAK** *AKFVAAWTLKAAA* **GGGS**TPFGAGWSW**GGGS**LEDRLVETL**GGGS**SSNVNFPLY**GGGS**SEYVWNYEL**GGGS**YQFLKGWEL**GPGPG**WLLPAILALAGCSSS**GPGPG**AFAAPISALNYAFTP**GPGPG**PGARAGAPGRVSFYP**GPGPG**PLAFAIISNNYLVPG**GPGPG**SGASAGAIAALLVGL**GPGPG**RQYRAASIQVGNPAR**GPGPG**SLNLLSLILISSNVN**GPGPG**LSLILISSNVNFPLY**GPGPG**NVGANAFLSGTRPRL**GPGPG**NAFLSGTRPRLNLSL**GPGPG**ADSRLHGLAGLRYFH**GPGPG**RWDFELFGNLGLLKT**KK**TGEQRGDTL**KK**SGDPTLHPDDL**KK**GRKTQGKGD**KK**QREVYSHRTTPRM**KK**SSQRINTRTLGLRLDS**KK**MAVANTDGSGD**KK**TTVWDSTNKQSGAGT**KK**QPEVRLRPTG**KK**FAAQRHESVGN**KK**AETKSNETYQD**KK**DRQRRRSEADL**KK**RLEREHRRRDG**KK** *AKFVAAWTLKAAA* **GGGS**	Vaxijen: 1.0377 ANTIGENpro: 0.835	Non-allergen	668	70.335	9.91	33.33	72.46	-0.525	0.967
**SMV2/(HBHA Conserved Sequence)**	**EAAAK**MAENSNIDDIKAPLLAALGAADLALATVNELITNLRERAEETRRSRVEESRARLTKLQEDLPEQLTELREKFTAEELRKAAEGYLEAATSELVERGEAALERLRSQQSFEEVSARAEGYVDQAVELTQEALGTVASQVEGRAAKLVGIEL**EAAAK** *AKFVAAWTLKAAA* **GGGS**TPFGAGWSW**GGGS**LEDRLVETL**GGGS**SSNVNFPLY**GGGS**SEYVWNYEL**GGGS**YQFLKGWEL**GPGPG**WLLPAILALAGCSSS**GPGPG**AFAAPISALNYAFTP**GPGPG**PGARAGAPGRVSFYP**GPGPG**PLAFAIISNNYLVPG**GPGPG**SGASAGAIAALLVGL**GPGPGR**QYRAASIQVGNPAR**GPGPG**SLNLLSLILISSNVN**GPGPG**LSLILISSNVNFPLY**GPGPG**NVGANAFLSGTRPRL**GPGPG**NAFLSGTRPRLNLSL**GPGPG**ADSRLHGLAGLRYFH**GPGPG**RWDFELFGNLGLLKT**KK**TGEQRGDTL**KK**SGDPTLHPDDL**KK**GRKTQGKGD**KK**QREVYSHRTTPRM**KK**SSQRINTRTLGLRLDS**KK**MAVANTDGSGD**KK**TTVWDSTNKQSGAGT**KK**QPEVRLRPTG**KK**FAAQRHESVGN**KK**AETKSNETYQD**KK**DRQRRRSEADL**KK**RLEREHRRRDG**KK** *AKFVAAWTLKAAA* **GGGS**	Vaxijen: 1.0449 ANTIGENpro: 0.851	Non-allergen	659	69.217	9.86	35.66	73.87	-0.510	0.974
**SMV3/(β-Defensin)**	**EAAAK**GIINTLQKYYCRVRGGRCAVLSCLPKEEQIGKCSTRGRKCCRRKK**EAAAK** *AKFVAAWTLKAAA* **GGGS**TPFGAGWSW**GGGS**LEDRLVETL**GGGS**SSNVNFPLY**GGGS**SEYVWNYEL**GGGS**YQFLKGWEL**GPGPG**WLLPAILALAGCSSS**GPGPG**AFAAPISALNYAFTP**GPGPG**PGARAGAPGRVSFYP**GPGPG**PLAFAIISNNYLVPG**GPGPG**SGASAGAIAALLVGL**GPGPG**RQYRAASIQVGNPAR**GPGPG**SLNLLSLILISSNVN**GPGPG**LSLILISSNVNFPLY**GPGPG**NVGANAFLSGTRPRL**GPGPG**NAFLSGTRPRLNLSL**GPGPG**ADSRLHGLAGLRYFH**GPGPG**RWDFELFGNLGLLKT**KK**TGEQRGDTL**KK**SGDPTLHPDDL**KK**GRKTQGKGD**KK**QREVYSHRTTPRM**KK**SSQRINTRTLGLRLDS**KK**MAVANTDGSGD**KK**TTVWDSTNKQSGAGT**KK**QPEVRLRPTG**KK**FAAQRHESVGN**KK**AETKSNETYQD**KK**DRQRRRSEADL**KK**RLEREHRRRDG**KK** *AKFVAAWTLKAAA* **GGGS**	Vaxijen: 1.1417 ANTIGENpro: 0.827	Non-allergen	554	57.867	10.36	31.21	66.68	-0.547	0.873
**SMV4/(50s ribosomal L7/L12 protein)**	**EAAAK**MAKLSTDELLDAFKEMTLLELSDFVKKFEETFEVTAAAPVAVAAAGAAPAGAAVEAAEEQSEFDVILEAAGDKKIGVIKVVREIVSGLGLKEAKDLVDGAPKPLLEKVAKEAADEAKAKLEAAGATVTVK**EAAAK** *AKFVAAWTLKAAA* **GGGS**TPFGAGWSW**GGGS**LEDRLVETL**GGGS**SSNVNFPLY**GGGS**SEYVWNYEL**GGGS**YQFLKGWEL**GPGPG**WLLPAILALAGCSSS**GPGPG**AFAAPISALNYAFTP**GPGPG**PGARAGAPGRVSFYP**GPGPG**PLAFAIISNNYLVPG**GPGPG**SGASAGAIAALLVGL**GPGPG**RQYRAASIQVGNPAR**GPGPG**SLNLLSLILISSNVN**GPGPG**LSLILISSNVNFPLY**GPGPG**NVGANAFLSGTRPRL**GPGPG**NAFLSGTRPRLNLSL**GPGPG**ADSRLHGLAGLRYFH**GPGPG**RWDFELFGNLGLLKT**KK**TGEQRGDTL**KK**SGDPTLHPDDL**KK**GRKTQGKGD**KK**QREVYSHRTTPRM**KK**SSQRINTRTLGLRLDS**KK**MAVANTDGSGD**KK**TTVWDSTNKQSGAGT**KK**QPEVRLRPTG**KK**FAAQRHESVGN**KK**AETKSNETYQD**KK**DRQRRRSEADL**KK**RLEREHRRRDG**KK** *AKFVAAWTLKAAA* **GGGS**	Vaxijen: 1.0210 ANTIGENpro: 0.818	Non-allergen	639	66.147	9.85	28.01	74.19	-0.389	0.957

The bolded sequence represent the linker sequences. The italic regions characterize PADRE sequences.1, 2: VaxiJen 2.0, ANTIGENpro; 3, 4: AlgPred,AllerTOP v2.0; 5, 6: ProtParam Expasy; 6: SOLPro. SMV, Serratia marcescens vaccine.

Each designed vaccine construct contained 668 (SMV1), 659 (SMV2), 554 (SMV3) and 639 (SMV4) residues long, while the molecular weight of each construction was found to be 70.335, 69.217, 57.867 and 66.147 kDa respectively. The theoretical pI of each construct ranged from 9.85 to 10.36, suggesting that the constructions have a negative charge if the pH is above the isoelectric point and vice versa. The computed instability index of constructions varied from 28.01 to 35.66 representing the stable nature of the vaccine proteins. The high aliphatic index range (66.68 to 74.19) of all vaccine constructs suggest the protein stability in several temperatures. The negative GRAVY value of the vaccine constructs revealed that all of them has a hydrophilic in nature. All four vaccine constructs showed good solubility (>0.873) during its heterologous expression in the *E. coli*. Therefore, all of the vaccine constructs showed be antigenic, non-allergenic, hydrophilic, stable and soluble. The sequence of vaccine constructs and their physiochemical properties are showed in **Table 6**.

### Peptide Cleavage Analysis

We investigated both proteasomal and cathepsin specific peptidase activity on the vaccine constructs. NetChop 3.1 server detected 17 proteasomal sites, which majority of them were close to the linkers. SitePrediction server provided 1 peptidase and 14 peptidase links with 99.9% and 99% specificity for cathepsin B, respectively; 1 peptidase and 2 peptidase links with 99.9% and 99% specificity for cathepsin D, respectively; 8 and 3 peptidase links with 99% specificity for cathepsins E and G, respectively; 2 peptidase links with 99.9% and 4 peptidase links with 99% specificity for cathepsin K, and 1 peptidase link with 99% specificity for cathepsin L. Our results indicates that these multi-epitope vaccine constructs might be processed and presented in context of MHC class molecule.

### Secondary Structure Prediction of the Constructed Vaccines

**Figure 2:**
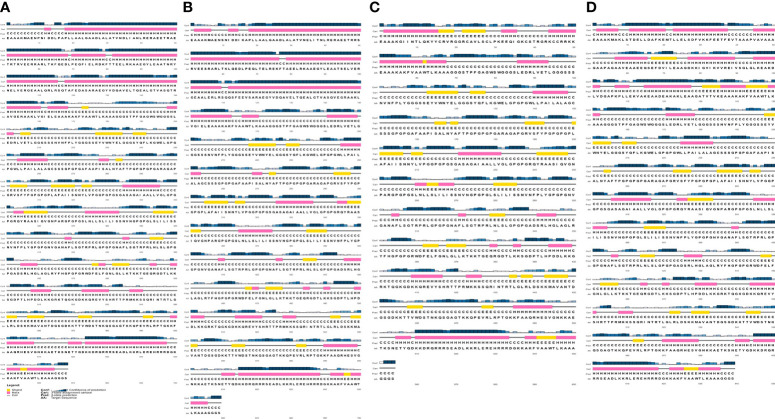
Secondary structure prediction of the constructed *S. marcescens* vaccines using PESIPRED 4.0 server. **(A)** SMV1, **(B)** SMV2, **(C)** SMV3, **(D)** SMV4.

The analyze of the secondary structure of vaccine constructs showed that SMV1 had 48.35% of amino acids in coil structure, 40.12% of amino acids in alpha helix, and the lowest percentage of the amino acids in beta sheet formation (11.23%). SMV2 had 49.75% of amino acids in coil structure, 38.56% in alpha helix region, and 11.69% of the amino acids in the beta sheet formation. SMV3 had the highest percentage of coil structure (55.05%), 27.62% of the amino acids in alpha helix region, and the highest percentage of the amino acids in the beta sheet formation (17.33%). SMV4 presented coil structure in 54.23%, 30.05% of alpha helix region, and 15.72% of the amino acids in the in beta sheet formation (**Figure 2**).

### 3D Structure Prediction of the Constructed *S. marcescens*

**Figure 3:**
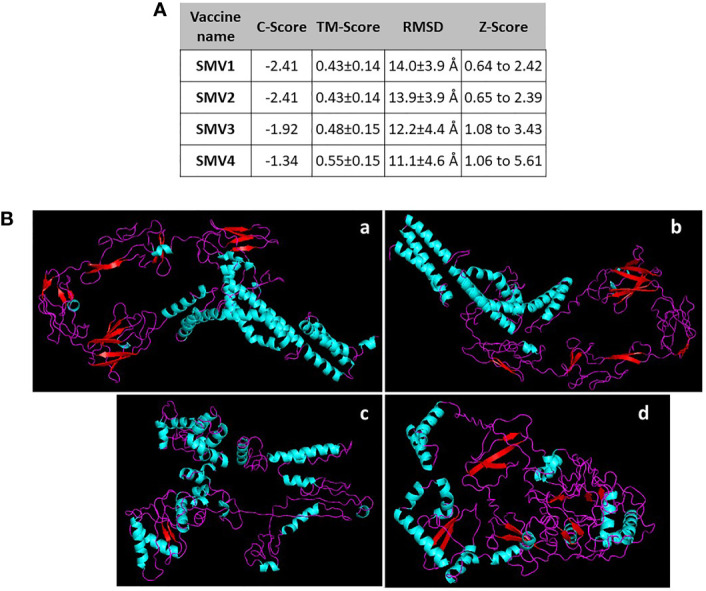
**(A)** Characteristics and **(B)** 3D structure prediction of the constructed S. marcescens vaccines using I-TASSER server. a SMV1, b SMV2, c SMV3, d SMV4.

The 3D structure was obtained by threading using I-TASSER web server. For each vaccine sequence was predicted five 3D models, and the first model of each construction was selected. All the model was ranked on their C-scores values, which measure similarity between the query and template based on the significance of threading template alignment and the query coverage parameters. C-score values ranges between -5 and 2, and a higher value represents a model with a higher confidence and correct topology. SMV1 presented a Z-Score ranging from 0.64 to 2.42 and a C-Score of -2.41. SMV2 showed a Z-Score ranging from 0.65 to 2.39 and a C-Score of -2.41. SMV3 had a C-Score of -1.92 and a Z-Score ranging from 1.08 to 3.43. SMV4 exhibited a Z-Score of 1.06 to 5.61 and the highest C-Score, -1.34 (**Figure 3A**). In addition to C and Z score, I-TASSER predicted the TM-score, a metric for measuring the similarity of two protein structures, and the root mean square deviation (RMSD) of atomic positions. TM-score obtained in vaccines constructs ranged from 0.43 ± 0.14 to 0.55 ± 0.15. SMV4 had a TM-score more than 0.5, indicating a higher accuracy in topology. For all vaccines tested the RMSD ranged from 11.1 ± 4.6Å to 14.0 ± 3.9 Å (**Figures 3A, B**).

### 3D Structure Refinement and Validation

**Figure 4:**
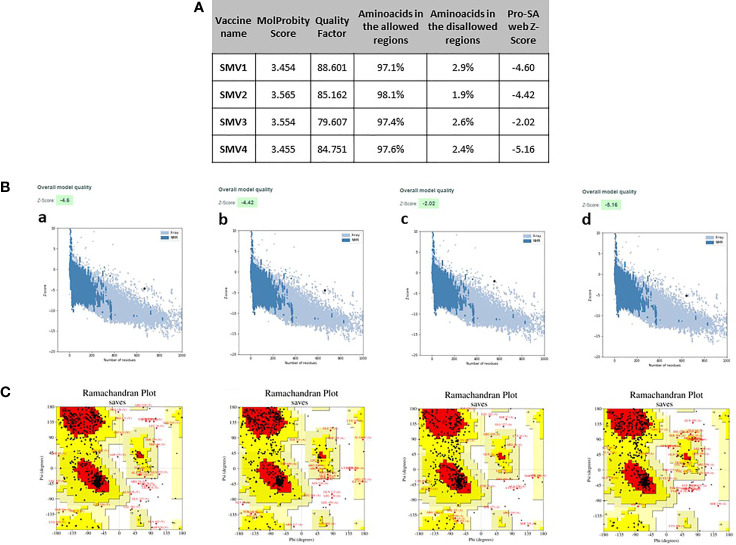
Refinement and validations characteristics of *S. marcescens* vaccine constructs **(A)** ProSA Z-score (highlighted as a black dot) **(B)** is displayed in a plot that contains the Z-scores of all experimentally determined protein chains currently available in the Protein Data Bank; Ramachandran plotanalysis **(C)**, indicating residues in the favored regions (red), allowed regions (yellow), generously allowed regions (light yellow) and disallowed regions (white). a: SMV1, b: SMV2, c: SMV3, d: SMV4.

The 4 vaccine constructs 3D model were refined using the 3Drefine server. 3Drefine server provided five refined models with different parameters, including the 3D refined score, GDT-TS, GDTHA, RMSD, MolProbity, and RWPlus. Higher GDT-TS, GDT-HA, and RMSD values, and lower 3D refine Score, RWplus, and MolProbity values indicate a higher quality for the models. The models number 1 in all 4 vaccine constructs presented lowest MolProbity score, which ranged from 3.454 to 3.565 (**Figure 4A**). Therefore, these were validated by PROCHECK’s Ramachandran plot, ERRAT and ProSA webserver. ERRAT score for 3D models of four vaccines were calculated as 88.601, 85.162, 79.607, and 84.751, respectively (**Figure 4A** **)**. The ProSA Z-Score for SMV1, SMV2, SMV3 and SMV4 were -4.60, -4.42, -2.02 and -5.16 respectively, indicating models were within the range of scores typically found for the native proteins of similar size (**Figures 4A, B**). Ramachandran plot analysis showed 97.1%, 97.4%, and 97.6% residues in allowed region for vaccine SMV1, SMV3 and SMV4, respectively. The SMV2 vaccine had 98.1% of residues in the allowed regions (**Figure 4C**). These analyses authenticated the reliability and stability of the predicted structures.

### Protein-Protein Docking

**Figure 5:**
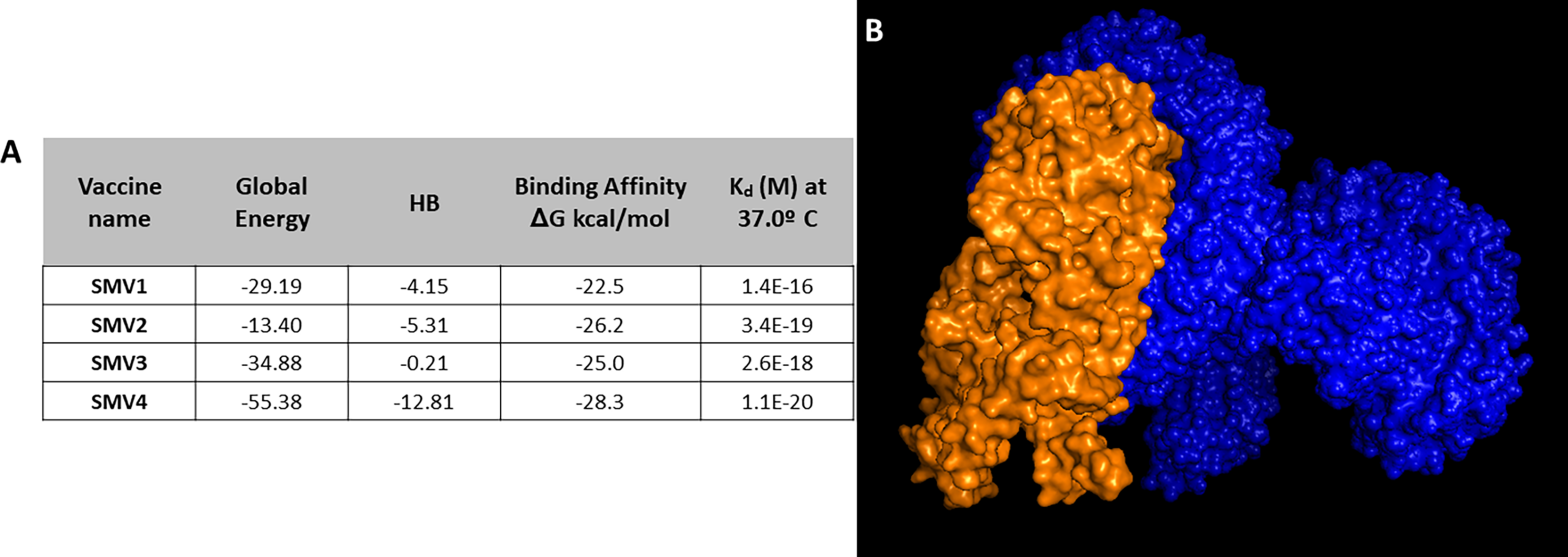
**(A)** Docking analysis of vaccine constructs. **(B)** 3D representation of SMV4 vaccine construct and TLR4-MD2 complex. The SMV4 vaccine construct is represented by orange color, and TLR4-MD2 complex is in blue. The docking was carried out by ClusPro 2.0 and PatchDock servers, and refined and re-scored by the PRODIGY tool of HADDOCK server, and FireDock server, respectively.

Docking analysis was performed between SMV1, SMV2, SMV3 and SMV4 vaccine constructs and TLR4-MD2 complex (PDB:3FXI), in order to find out the best constructed *S. marcescens* vaccine. SMV4 showed binding affinity -28.3 kcal/mol, a K_d_ of 1.1E-20 at 37°C, a global energy of -55.38, and an HB energy of -12.81 (**Figure 5A**). Since SMV4 showed superior results in the protein-protein docking study, it was considered as the best vaccine construct among the four constructed vaccines (**Figure 5B**).

### Molecular Dynamics Simulation

**Figure 6:**
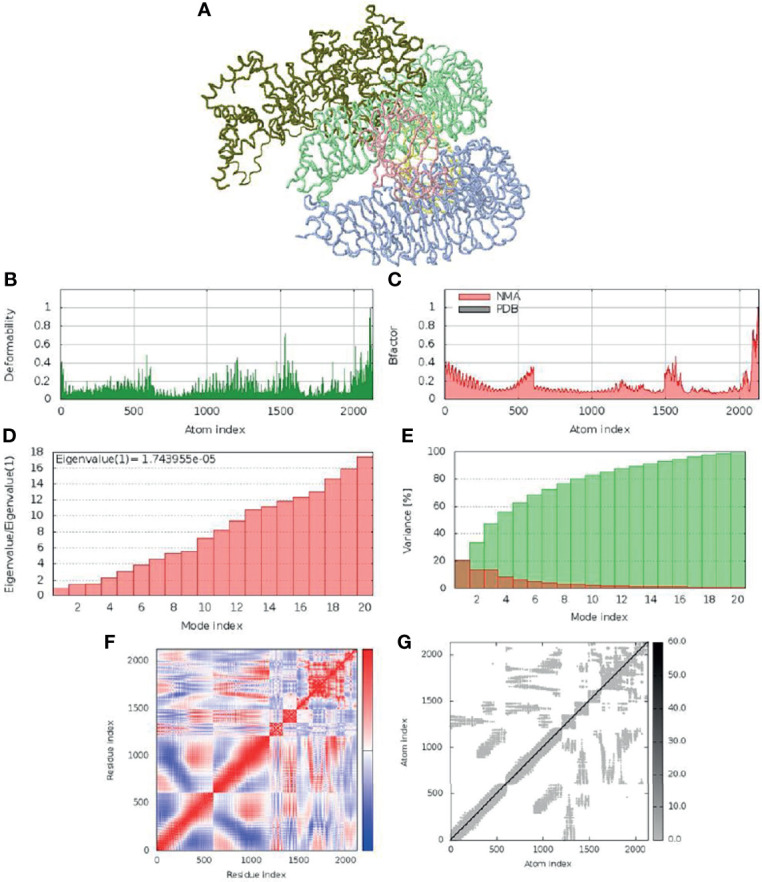
Molecular dynamic simulation of SMV4 and TLR4 docked complex. **(A)** NMA mobility. **(B)** deformability. **(C)** B-Factor. **(D)** eigenvalue. **(E)** variance (red: individual variance, green: cumulative variance). **(F)** co-variance map (correlated in red, uncorrelated in white, and anti-correlated in blue). **(G)** elastic network.

The molecular dynamics simulation and normal mode analysis (NMA) of SMV-4-TLR4 docked complex is showed in **Figure 6A**. Deformability graphs of the complex illustrates the peaks in the graphs, having regions of the proteins with high deformability (**Figure 6B**). The B-Factor graphs of the complexes provide easy understanding and visualization of the comparison between NMA and the PDB field of the docked complex (**Figure 6C**). The SMV4-TLR4 docked complex suggested that docked complex should be quite stable and should have relatively less chance of deformability (**Figures 6B, D**). In the variance graph (**Figure 6E**), red colored bars shows the individual variance and green colored bars represent the cumulative variance. Co-variance map of the complex showed a good amount of amino acid pairs in the correlated motion (**Figure 6F** **).** The elastic map (**Figure 6G**) of the complex describes the connection between atoms and darker gray regions shows stiffer regions.

### Discontinuous B Cell Epitopes

**Figure 7:**
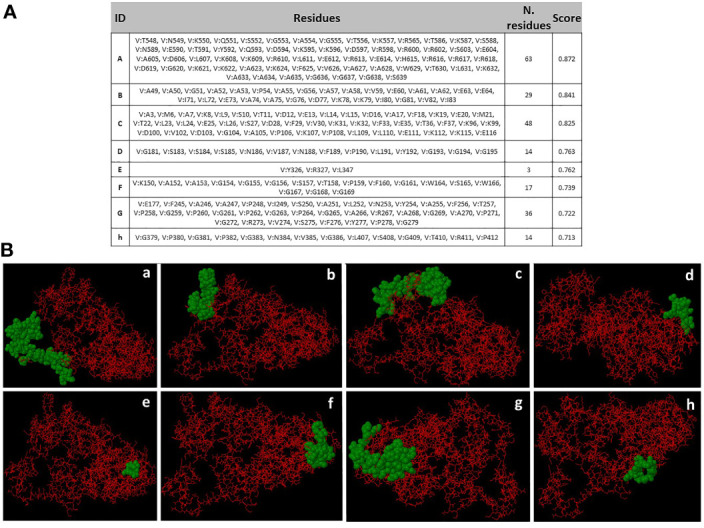
Conformational B-cell epitopes prediction. **(A)** Amino acid residues present in conformational epitopes, the number of residues and their scores. ID: Identification of Epitopes. **(B) a-h**: 3D representation of conformational B-cell epitopes of protein. The predicted epitope residues are represented by green color, and the bulk of the polyprotein is represented in red color.

Eight discontinuous B-cell epitopes with scores ranging from 0.713 to 0.872 were predicted by Ellipro online tool at IEDB. Shortest and longest discontinuous B cell epitope ranged from 3 to 63 residues long respectively (**Figure 7A** **)**. The amino acid residues present in conformational epitopes, the number of residues, their scores, and the 3D representation of conformational B-cell epitopes are shown in **Figure 7A, B**.

### Immune Simulation for Vaccine Efficacy

**Figure 8:**
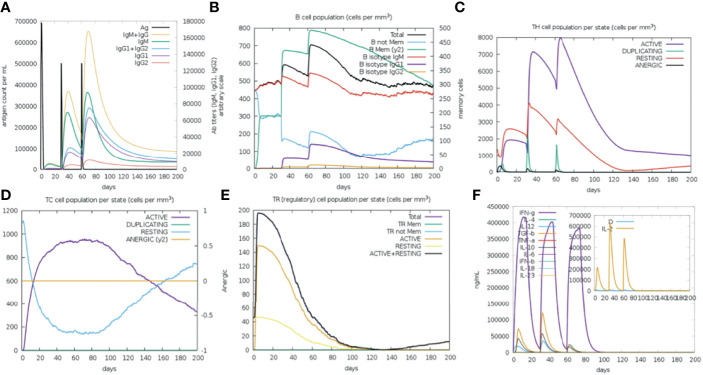
Immune Simulation with the SMV4 vaccine candidate using C-ImmSim server. **(A)** Immunoglobulin production in response to antigen injections; specific subclasses are showed as colored peaks. **(B)** B-cell populations after the three injections. **(C)** Generations of T-helper cells. **(D)** Generation of T-cytotoxic cell populations. The resting state characterizes cells not presented to the antigen, the anergic state indicates tolerance of the T-cells to the antigen. **(E)** Levels of T regulatory cells. **(F)** The main plot shows cytokine levels after the injections. The insert plot shows IL-2 level with the Simpson index, **(D)** shown by the dotted line. **(D)** is a measure of diversity. Increase in **(D)** over time indicates emergence of different epitope-specific dominant clones of T-cells. The smaller the **(D)** value, the lower the diversity.

The vaccine primary response was characterized by high levels of IgM, while the secondary and tertiary responses were higher than the primary reaction and distinguished by greater IgM + IgG, IgG1 + IgG2, IgG1 antibodies level, and a rapid clearance in antigen concentration (**Figure 8A**). B cell activation were found high, particularly B isotype IgM and IgG1, with prominent memory cell development (**Figure 8B**). The cell population of TH (helper) and TC (cytotoxic) cells were also found high along with memory development (**Figures 8C, D**). A significant levels of T regulatory (Treg cells) cells was found in the exposure to the SMV4, and a Treg cell reduction few days after antigen exposure (**Figure 8E**). The vaccine can induce both IFN-γ and IL-2 with a suitable Simpson Index (D) (**Figure 8F**), which is a measure of diversity.

### Codon Adaptation of the Final Vaccine Construct

**Figure 9:**
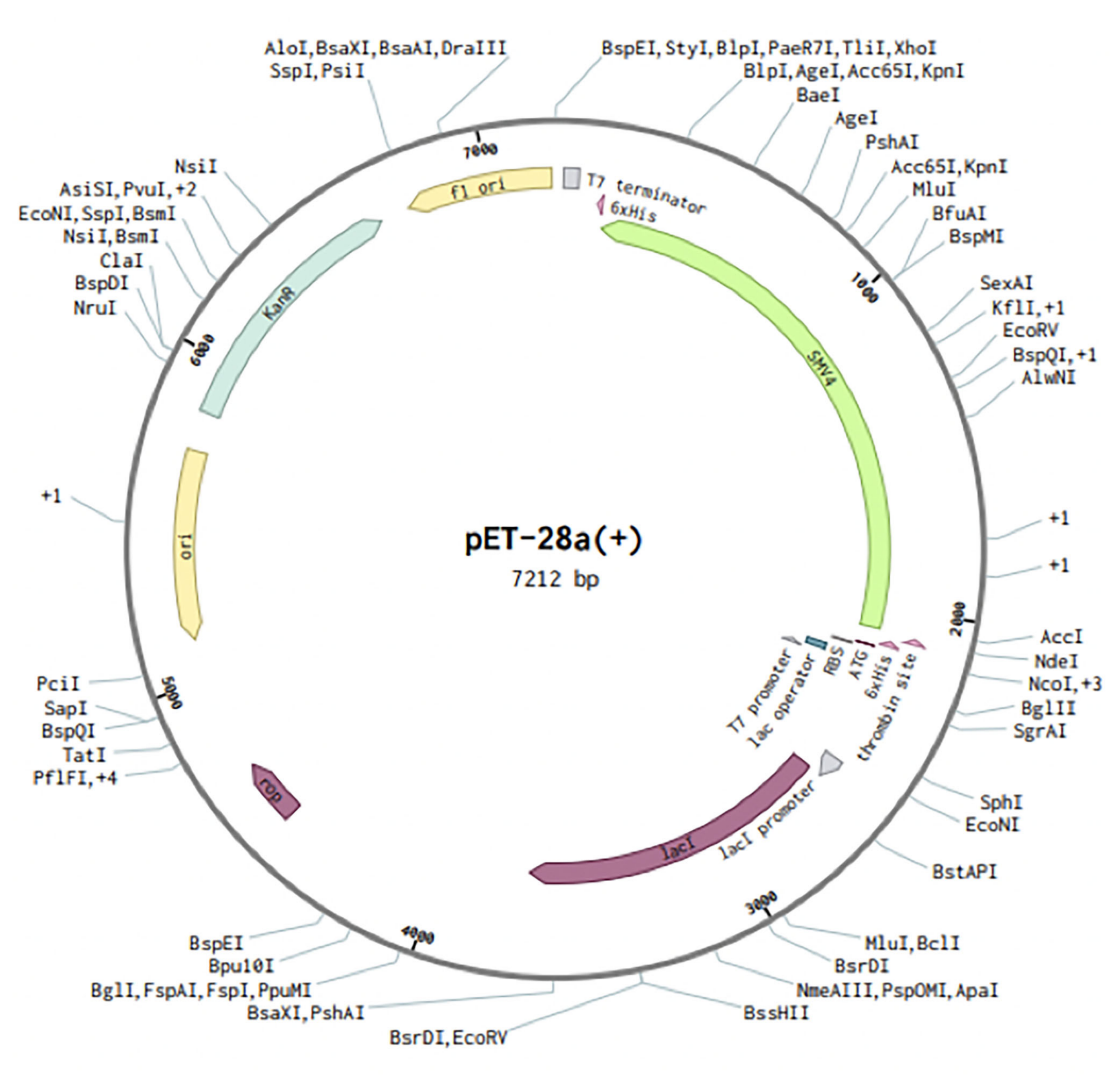
*In silico* restriction cloning of the multi-epitope vaccine sequence (SMV4) into the pET28a (+) expression vector. Green arrow represents the vaccine’s gene coding. The His-tag is located at the N-terminal end.

Codons of SMV4 construct were adapted as per codon utilization of *E. coli* expression system, and JCAT server was used to optimize the SMV4 codons according to *E. coli* K12. The optimized SMV4 construct had a length of 1917 pb; an ideal range of GC content 54.17% (30–70%), showing good probable expression of the vaccine candidate in the *E. coli* K12; and CAI value 0.958 (0.8–1.0), indicating a high gene expression potential. In the next step, the SMV4 sequence was cloned between XhoI and NdeI restriction sites at the multiple cloning-site of the pET28a(+) vector. The clone had a total length of 7212 bp (**Figure 9**).

## Discussion

Vaccine development is one of greatest advances to prevent global morbidity and mortality; not only does it halt the onset of different diseases, but it also labels a gateway for its elimination while reducing toxicity (74). Vaccines that prevent infections caused by MDR bacterial species have a number of potential benefits. They can be used prophylactically reducing antibiotic use, emergence and spread of AMR, incidence of sensitive and resistant infections, severity life-threatening diseases, sequelae remaining after infection resolution, and health care costs (3, 4, 8).

The main strategy in the present study was to design and construct a multiepitope-based vaccine against *S. marcescens*, a gram-negative rod frequently involved in diverse nosocomial infections and with systemic mortality rate in immunocompromised and intensive care patients (11, 13).

Using computational subtractive analysis, we enrolled non-redundant proteome of *S. marcescens* to find proteins which had essential, virulent, and resistance profile and, at the same time, were non-homologous from human and gut flora, antigenic, had extracellular domain and/or were secreted. The antigens used in vaccines do not need to be virulence factors, although virulence gene products are often immunogenic and responsible for acquired immunity that protects against the disease (106, 107). The exclusion of human and gut flora homologs is necessary to prevent autoimmunity in the host and to protect the symbiotic environment of the gut flora (44). Antigenicity of a protein means the potential to generate immune response against the organism to which the protein belongs, an essential factor to use the protein as a vaccine (82). Bacterial cell surface and secreted proteins are of interest for their potential as vaccine candidates because they are easily accessible and can significantly improve therapeutic target identification (39, 108).

After shortlisting, we identified five novel antigenic proteins of *S. marcescens* that were taken as suitable vaccine candidates. The first filtered antigenic protein was D-alanyl-D-alanine carboxypeptidase/endopeptidase, an essential membrane-associated protein and member of the penicillin binding proteins (PBPs), a family of proteins inhibited by ß-lactam antibiotics involved in peptidoglycan synthesis and remodeling (109). The second identified protein was patatin-like phospholipase family protein, an essential protein that has been associated with infection in host cells and phagosome escape of various pathogenic bacteria (110, 111). The third selected protein was phospholipase C, phosphocholine-specific (PLC-PC). PLCs are considered an important virulence factor that can be exported out of the cytoplasm to their functional locality through Tat or Sec pathway (112). In bacteria, PLCs have been related in a wide variety of cellular function during infection, including membrane lysis, intracellular signaling, lipid metabolism and/or pathogenicity-associated activity (113, 114). The fourth protein was also antigenic and identified like spore coat U domain-containing protein, a domain found in a bacterial family of the secreted pili proteins involved in motility and biofilm formation (115, 116). The fifth and last selected protein was TonB-dependent receptor, a family of beta barrel proteins located in the outer membrane that is associated to progressive antibiotic resistance, transport ferric–siderophore complexes, vitamins, nickel complexes, and carbohydrates (117–121).

Prado et al. (122) introduced seven proteins that can be considered as vaccine candidates against *S. marcescens* using reverse vaccinology and subtractive genomic approaches. Prediction of these proteins was based on non-host homologous proteins, subcellular localization (putative surface exposed, secreted; membrane), transmembrane helix, Signal IP, MHC-I and MHC-II adhesion probability, and essentiality. Some features are required to select a potential vaccine candidate, such as sub-cellular localization; presence of a signal peptide; transmembrane domain; and antigenic epitopes. In addition to recognizing antigenic and virulence factors, one of the main strategies behind identifying potential vaccine candidates is predicting epitopes that are likely to bind to major histocompatibility complex molecules on the antigen presenting cells within the host (123). Therefore, mapping of T-cell derived B-cell epitopes for antigenic proteins is a critical step for designing vaccines (39).

In addition to selecting five novel proteins as potential vaccine candidates against *S. marcescens*, we used the sequence these proteins to predict MHC-class-I, MHC class-II allele and B cell epitopes that would be capable of inducing effective cellular and humoral immunity. All selected antigenic epitopes were antigenic, so they could induce antigenic response; non-allergenic in nature, thus not be able to induce any allergenic reaction; conserved epitopes, which is an important feature for designing a broad spectrum vaccine; hydrophilic in nature, hence able to interact with water molecules; and non-toxic. We selected the IFN-γ inducing Helper T cell (HTL) epitopes since this cytokine plays a significant role in innate and adaptive immune responses, stimulates macrophages and natural killer cells, and provides an enhanced response to MHC antigens (124).

In addition to *S. marcescens* having extracellular proliferation, this bacterium is able to invade nonphagocytic cells, such as epithelial cells (125–127). After internalization, *S. marcescens* can control the autophagic traffic, generating an appropriate niche for survival and replication inside the host cell (126, 128). Efficient protection against intracellular pathogens is dependent on the induction of cellular immunity, including pathogen-specific cytotoxic T cell responses (129, 130). CTL epitopes are essential for coherent vaccine design (131, 132). Thus, we analyzed the immunogenicity of CD8+ T cell epitopes to ensure that the epitope vaccine could effectively activate CD8 T cell-mediated immune response. In humans, MHC molecules are known as human leukocyte antigens (HLAs), as they are highly polymorphic; the frequency of expression of diverse HLA alleles varies in ethnically different populations (28). Thus, the HLA specificity of T-cell epitopes must be an important criterion for epitopes selection (133). We used the molecular docking simulation to delineate the interactions between the targeted T cell epitopes and their respective HLA alleles. In the docking results, five MHC class-I and twelve MHC class-II epitopes produced global energies. This means they had the capacity to bind specifically with their targets.

A total of 4 multi-epitope vaccines (SMV1, SMV2, SMV3, SMV4) were constructed using five MHC class-I, twelve MHC class-II and twelve B cell epitopes; four different adjuvants HBHA protein (*M. tuberculosis*), HBHA conserved sequence, beta-defensin, L7/L12 ribosomal protein (13) along with PADRE; and four different linkers EAAAK, GGGS, GPGPG and KK, which were used to bind the adjuvant, CTL, HTL and B-cell epitopes, respectively. Adjuvant HBHA and L7/L12 ribosomal protein are agonists to the TLR4/MD2 complex while beta-defensin adjuvant can act as an agonist to TLR1, TLR2, and TLR4 (134). The PADRE peptide induces CD4+ T-cells that increase efficacy and potency of peptide vaccine (135). It also overcomes the problems caused by highly polymorphic HLA alleles (88). Linkers ensure effective separation of individual epitopes *in vivo* (136). After that, several predicted physiochemical and immunological properties showed that all the vaccine constructions were safe with no possible allergenicity, had the capability to induce immunity with high antigenicity, were hydrophilic and soluble during its heterologous expression in *E. coli*, which is important to many biochemical and functional studies (137), and had negative charge. Neutral or negatively charged molecules are preferred and a balance between its hydrophobicity and hydrophilicity is important in designing vaccine candidates (138). The molecular weight range (57.867 to 70.335) and the high pI value range (9.85 to 10.36) indicated the efficacy and stability of the vaccine constructs (138). In addition to evaluating the vaccine efficacy, the epitopes separated by linker were sensitive to both degradation proteasomal and cathepsin specific peptidase activity. Hence, our data showed that the chosen linkers and their distribution were suitable, and the epitope produced could be presented in the host immune system, processed, and induced in the host humoral and cellular immune pathway (139).

Secondary and tertiary structures are necessary for designing a vaccine candidate (140). Analyses of the secondary structure of all vaccine constructs showed that all the proteins mainly contained amino acids in coil, and in alpha helix structure. Natively unfolded protein regions and α-helical coiled-coils peptides have been identified as important “structural antigen” forms (70). After 3D modeling, the structure of the vaccine was refined, displaying suitable characteristics and high-quality structure.

Molecular docking is a widely used computer simulation approach to explore the binding affinity with a protein, a strategic tool in vaccine design (141). Our findings showed stable interaction and high affinity between the vaccine construct SMV4 and the TLR-4/MD2 complex. The interaction between the TLR4 and adjuvant enhance the immune response, while TLR3, TLR4 and TLR9 agonists have been used to improve vaccines against HBV, influenza, malaria and anthrax (142). Furthermore, the physical movement and stabilization of the docked complex were assessed by molecular dynamics simulation, which confirmed that SMV4-TLR-4/MD2 complex has low deformability and remains stable in a biological environment.

Various discontinuous epitope residues were predicted from SMV4 vaccine sequence and revealed that they can interact with antibodies. The most B-cell epitopes are discontinuous epitopes composed of amino acid residues located on separate regions of the protein, joined together by the folding of the chain (143). Thus, analysis of discontinuous epitope in the final vaccine construct is essential (88).

Immune simulation through repeated exposure to the antigen showed a consistent increase in the generated immune responses. There was a notable generation of T- cells as well as memory B cells, which is required for immunity, supporting a humoral response (124). The levels of IFN-γ and IL-2 increased after the first injection and got induced following repeated exposures to the antigen, which also contribute to the subsequent immune response after vaccination (144). Interleukin induction is needed for any kind of cellular immunity and the vaccine satisfies this criterion having good induction potentiality (82). Considering the designed vaccine is constituted of sufficient B- and T-cell epitopes, the Simpson index (D) value suggests that the vaccine can stimulate a large and diverse immune response.

When designing a multi-epitope vaccine candidate, the efficacious cloning and expression in a suitable vector is a critical stage (145). Codon optimization is essential because the genetic code’s degeneracy allows most of the amino acids to be encoded by multiple codons (70). In this context, codon optimization and in silico cloning were performed, and our data showed expression and translation efficiency of the SMV4 vaccine using pET-28a (+).

In conclusion, our study identified a potential SMV4 vaccine candidate against *S. marcescens* with the ability to stimulate both cellular and humoral immunity. The epitopes used in the vaccine construct are antigenic, non-toxic, and non-allergic. The SMV4 vaccine candidate were highly immunogenic, safe, non-toxic, stable, and had high affinity and stability of binding to TLR4 innate immune receptor, which is vital in recognition and processing by the host immune system. Altogether, our findings have the potential to provide a novel strategy for the protection against multidrug resistant Gram negative infection. Future experimental validation of the proposed vaccine candidate is required to establish its potency as well efficacy and safety.

## Data Availability Statement

The original contributions presented in the study are included in the article/**Supplementary Material**, further inquiries can be directed to the corresponding author.

## Author Contributions

M-CP, MD, and FM conceived this project. M-CP, MD, FM, CF, and AC aided with edition of the manuscript and analyzed data. M-CP and MD wrote the manuscript. All authors contributed to the article and approved the submitted version.

## Funding

This work was supported by Fundação de Amparo à Pesquisa do Estado de São Paulo-Brazil (FAPESP grants 2020/11964-4 and 2022/01316-0 to M-CP, and FAPESP grants 2018/20697-0 to AC). This study was partially financed by the Fundação de Amparo à Pesquisa do Estado de São Paulo-Brazil (FAPESP) as a fellowship to MD (FAPESP fellowship 2018/24213-7).

## Conflict of Interest

The authors declare that the research was conducted in the absence of any commercial or financial relationships that could be construed as a potential conflict of interest.

## Publisher’s Note

All claims expressed in this article are solely those of the authors and do not necessarily represent those of their affiliated organizations, or those of the publisher, the editors and the reviewers. Any product that may be evaluated in this article, or claim that may be made by its manufacturer, is not guaranteed or endorsed by the publisher.

## Supplementary Material

Supplementary Data Sheet S2 Core protein sequences.

Supplementary Data Sheet S3 Essential protein sequences.

Supplementary Data Sheet S4 Virulence protein sequences.

Supplementary Data Sheet S5 Resistance protein sequences.

Supplementary Data Sheet S6 Human non-homologue protein sequences.

Supplementary Data Sheet S7 Gut flora non-homologue protein sequences.

Supplementary Data Sheet S8 Cleavage sites predicted by NetChop 3.1.

## References

[1] Prestinaci F Pezzotti P Pantosti A. Antimicrobial Resistance: A Global Multifaceted Phenomenon. Pathog Global Health (2015) 109:309–18. doi: 10.1179/2047773215Y.0000000030 PMC4768623 26343252

[2] Jansen KU Anderson AS. The Role of Vaccines in Fighting Antimicrobial Resistance (AMR). Hum Vaccines Immunother (2018) 14:2142–9. doi: 10.1080/21645515.2018.1476814 PMC6183139 29787323

[3] Micoli F Bagnoli F Rappuoli R Serruto D. The Role of Vaccines in Combatting Antimicrobial Resistance. Nat Rev Microbiol (2021) 19:287–302. doi: 10.1038/s41579-020-00506-3 33542518 PMC7861009

[4] Bloom DE Black S Salisbury D Rappuoli R. Antimicrobial Resistance and the Role of Vaccines. Proc Natl Acad Sci USA (2018) 115:12868–71. doi: 10.1073/pnas.1717157115 PMC6305009 30559204

[5] AMR Review. Antimicrobial Resistance: Tackling a Crisis for the Health and Wealth of Nations (2014). Available at: https://amr-review.org/sites/default/files/AMR%20Review%20Paper%20-%20Tackling%20a%20crisis%20for%20the%20health%20and%20wealth%20of%20nations_1.pdf (Accessed February 6, 2022).

[6] Murray CJ Ikuta KS Sharara F Swetschinski L Robles Aguilar G Gray A. Global Burden of Bacterial Antimicrobial Resistance in 2019: A Systematic Analysis. Lancet (2022) 399:629–55. doi: 10.1016/s0140-6736(21)02724-0 PMC8841637 35065702

[7] World Health Organization (WHO). Antimicrobial Resistance (2021). Available at: https://www.who.int/news-room/fact-sheets/detail/antimicrobial-resistance (Accessed February 6, 2022).

[8] López-Siles M Corral-Lugo A McConnell MJ. Vaccines for Multidrug Resistant Gram Negative Bacteria: Lessons From the Past for Guiding Future Success. FEMS Microbiol Rev (2021) 45:fuaa054. doi: 10.1093/femsre/fuaa054 33289833

[9] World Health Organization (WHO). Global Priority List of Antibiotic-Resistant Bacteria to Guide Research, Discovery, and Development of New Antibiotics (2017). Available at: https://www.who.int/medicines/publications/WHO-PPL-Short_Summary_25Feb-ET_NM_WHO.pdf?ua=1 (Accessed February 6, 2022).

[10] Cristina ML Sartini M Spagnolo AM. Serratia Marcescens Infections in Neonatal Intensive Care Units (NICUs). Int J Environ Res Public Health (2019) 16:610. doi: 10.3390/ijerph16040610 PMC6406414 30791509

[11] Ferreira RL Rezende GS Damas MSF Oliveira-Silva M Pitondo-Silva A Brito MCA. Characterization of KPC-Producing Serratia Marcescens in an Intensive Care Unit of a Brazilian Tertiary Hospital. Front Microbiol (2020) 11:956. doi: 10.3389/fmicb.2020.00956 32670210 PMC7326048

[12] Kim B Jeon YD Kim JH Kim JK Ann HW Choi H. Risk Factors for Mortality in Patients With Serratia Marcescens Bacteremia. Yonsei Med J (2015) 56:348–54. doi: 10.3349/ymj.2015.56.2.348 PMC4329343 25683980

[13] Khanna A Khanna M Aggarwal A. Serratia Marcescens- A Rare Opportunistic Nosocomial Pathogen and Measures to Limit Its Spread in Hospitalized Patients. J Clin Diagn Res (2013) 7:243–6. doi: 10.7860/JCDR/2013/5010.2737 PMC3592283 23543704

[14] Engel HJ Collignon PJ Whiting PT Kennedy KJ. Serratia Sp. Bacteremia in Canberra, Australia: A Population-Based Study Over 10 Years. Eur J Clin Microbiol Infect Dis (2009) 28:821–4. doi: 10.1007/s10096-009-0707-7 19194731

[15] Field C Allen JL Friedman H. The Immune Response of Mice to Serratia Marcescens LPS or Intact Bacteria. J Immunol (Baltimore Md : 1950) (1970) 105:193–203. 4912960

[16] Kreger AS Lyerly DM Hazlett LD Berk RS. Immunization Against Experimental Pseudomonas Aeruginosa and Serratia Marcescens Keratitis. Vaccination With Lipopolysaccharide Endotoxins and Proteases. Invest Ophthalmol Visual Sci (1986) 27:932–9. 3519522

[17] Kumagai Y Okada K Sawae Y. The Effect of Humoral and Cell-Mediated Immunity in Resistance to Systemic Serratia Infection. J Med Microbiol (1992) 36:245–9. doi: 10.1099/00222615-36-4-245 1560446

[18] Shi H Zhu Y Xu L Liu Z You Y Meng Q. Serratia Marcescens Vaccine in the Treatment of Malignant Pleural Effusion. Zhonghua zhong liu za zhi [Chin J Oncol] (2002) 24:188–90. 12015046

[19] Woodward H. A Case of Infection in Man by the Bacterium Prodigiosum. Lancet (1913) 181:314–5. doi: 10.1016/S0140-6736(00)76133-2

[20] Kleef R Hager ED. Fever, Pyrogens and Cancer. In: Hyperthermia in Cancer Treatment: A Primer. Boston, MA: Springer US (2006). p. 276–337. doi: 10.1007/978-0-387-33441-7_21

[21] Mahlen SD. Serratia Infections: From Military Experiments to Current Practice. Clin Microbiol Rev (2011) 24:755–91. doi: 10.1128/CMR.00017-11 PMC3194826 21976608

[22] Karbach J Neumann A Brand K Wahle C Siegel E Maeurer M. Phase I Clinical Trial of Mixed Bacterial Vaccine (Coley’s Toxins) in Patients With NY-ESO-1 Expressing Cancers: Immunological Effects and Clinical Activity. Clin Cancer Res (2012) 18:5449–59. doi: 10.1158/1078-0432.CCR-12-1116 22847809

[23] Kempin S Cirrincione C Straus DS. Improved Remission Rate and Duration in Nodular Non-Hodgkin Lymphoma (NNHL) With the Use of Mixed Bacterial Vaccine (MBV). Proc Am Assoc Cancer Res (1981) 22:514–4.

[24] Kempin S Cirrincione C Myers J. Combined Modality Therapy of Advanced Nodular Lymphomas (NL): The Role of Nonspecific Immunotherapy (MBV) as an Important Determinant of Response and Survival. Proc Am Soc Clin Oncol (1983) 24:56.

[25] Kölmel KF Vehmeyer K Göhring E Kuhn B Wieding JU. Treatment of Advanced Malignant Melanoma by a Pyrogenic Bacterial Lysate. A Pilot Study. Onkologie (1991) 14:411–7. doi: 10.1159/000217017

[26] Tang ZY Zhou HY Zhao G Chai LM Zhou M Lu JZ. Preliminary Result of Mixed Bacterial Vaccine as Adjuvant Treatment of Hepatocellular Carcinoma. Med Oncol Tumor Pharmacother (1991) 8:23–8. doi: 10.1007/BF02988567 1645825

[27] Vas HFH Axelrod RS Burns MM Murasko D Goonewardene M. Clinical Results and Immunologic Effects of a Mixed Bacterial Vaccine in Cancer Patients. Med Oncol Tumor Pharmacother (1993) 10:145–58. doi: 10.1007/BF02989663 7513036

[28] Lata KS Kumar S Vaghasia V Sharma P Bhairappanvar SB Soni S. Exploring Leptospiral Proteomes to Identify Potential Candidates for Vaccine Design Against Leptospirosis Using an Immunoinformatics Approach. Sci Rep (2018) 8:1–15. doi: 10.1038/s41598-018-25281-3 29720698 PMC5932004

[29] Moxon R Reche PA Rappuoli R. Editorial: Reverse Vaccinology. Front Immunol (2019) 10:2776. doi: 10.3389/fimmu.2019.02776 31849959 PMC6901788

[30] Monterrubio-López GP González-Y-Merchand JA Ribas-Aparicio RM. Identification of Novel Potential Vaccine Candidates Against Tuberculosis Based on Reverse Vaccinology. BioMed Res Int (2015) 2015:11–4. doi: 10.1155/2015/483150 PMC4413515 25961021

[31] Naz A Obaid A Shahid F Dar HA Naz K Ullah N. Reverse Vaccinology and Drug Target Identification Through Pan-Genomics. London, GB: Elsevier Inc (2020) p. 317–33. doi: 10.1016/b978-0-12-817076-2.00016-0

[32] Pizza M Scarlato V Masignani V Giuliani MM Aricò B Comanducci M. Identification of Vaccine Candidates Against Serogroup B Meningococcus by Whole-Genome Sequencing. Science (2000) 287:1816–20. doi: 10.1126/science.287.5459.1816 10710308

[33] Rodrigues TCV Jaiswal AK De Sarom A Oliveira LDC Oliveira CJF Ghosh P. Reverse Vaccinology and Subtractive Genomics Reveal New Therapeutic Targets Against Mycoplasma Pneumoniae: A Causative Agent of Pneumonia. R Soc Open Sci (2019) 6:190907. doi: 10.1098/rsos.190907 31417766 PMC6689572

[34] Solanki V Tiwari M Tiwari V. Prioritization of Potential Vaccine Targets Using Comparative Proteomics and Designing of the Chimeric Multi-Epitope Vaccine Against Pseudomonas Aeruginosa. Sci Rep (2019) 9:1–19. doi: 10.1038/s41598-019-41496-4 30918289 PMC6437148

[35] Solanki V Tiwari V. Subtractive Proteomics to Identify Novel Drug Targets and Reverse Vaccinology for the Development of Chimeric Vaccine Against Acinetobacter Baumannii. Sci Rep (2018) 8:1–19. doi: 10.1038/s41598-018-26689-7 29899345 PMC5997985

[36] Serruto D Bottomley MJ Ram S Giuliani MM Rappuoli R. The New Multicomponent Vaccine Against Meningococcal Serogroup B, 4cmenb: Immunological, Functional and Structural Characterization of the Antigens. Vaccine (2012) 30:B87–97. doi: 10.1016/j.vaccine.2012.01.033 PMC3360877 22607904

[37] Chaudhari NM Gupta VK Dutta C. BPGA-An Ultra-Fast Pan-Genome Analysis Pipeline. Sci Rep (2016) 6:1–10. doi: 10.1038/srep24373 27071527 PMC4829868

[38] Luo H Lin Y Gao F Zhang CT Zhang R. DEG 10, an Update of the Database of Essential Genes That Includes Both Protein-Coding Genes and Noncoding Genomic Elements. Nucleic Acids Res (2014) 42:574–80. doi: 10.1093/nar/gkt1131 PMC3965060 24243843

[39] Hassan A Naz A Obaid A Paracha RZ Naz K Awan FM. Pangenome and Immuno-Proteomics Analysis of Acinetobacter Baumannii Strains Revealed the Core Peptide Vaccine Targets. BMC Genomics (2016) 17:732. doi: 10.1186/s12864-016-2951-4 27634541 PMC5025611

[40] Rashid MI Rehman S Ali A Andleeb S. Fishing for Vaccines Against Vibrio Cholerae Using *In Silico* Pan-Proteomic Reverse Vaccinology Approach. PeerJ (2019) 7:e6223. doi: 10.7717/peerj.6223 31249730 PMC6589079

[41] Asad Y Ahmad S Rungrotmongkol T Ranaghan KE Azam SS. Immuno-Informatics Driven Proteome-Wide Investigation Revealed Novel Peptide-Based Vaccine Targets Against Emerging Multiple Drug Resistant Providencia Stuartii. J Mol Graphics Modell (2018) 80:238–50. doi: 10.1016/j.jmgm.2018.01.010 29414043

[42] Vilela Rodrigues TC Jaiswal AK de Sarom A de Castro Oliveira L Freire Oliveira CJ Ghosh P. Reverse Vaccinology and Subtractive Genomics Reveal New Therapeutic Targets Against Mycoplasma Pneumoniae : A Causative Agent of Pneumonia. R Soc Open Sci (2019) 6:190907. doi: 10.1098/rsos.190907 31417766 PMC6689572

[43] Peng C Lin Y Luo H Gao F. A Comprehensive Overview of Online Resources to Identify and Predict Bacterial Essential Genes. Front Microbiol (2017) 8:2331. doi: 10.3389/fmicb.2017.02331 29230204 PMC5711816

[44] Naz K Naz A Ashraf ST Rizwan M Ahmad J Baumbach J. PanRV: Pangenome-Reverse Vaccinology Approach for Identifications of Potential Vaccine Candidates in Microbial Pangenome. BMC Bioinf (2019) 20:1–11. doi: 10.1186/s12859-019-2713-9 PMC6419457 30871454

[45] Chen L Xiong Z Sun L Yang J Jin Q. VFDB 2012 Update: Toward the Genetic Diversity and Molecular Evolution of Bacterial Virulence Factors. Nucleic Acids Res (2012) 40:641–5. doi: 10.1093/nar/gkr989 PMC3245122 22067448

[46] Zhou CE Smith J Lam M Zemla A Dyer MD Slezak T. MvirDB - A Microbial Database of Protein Toxins, Virulence Factors and Antibiotic Resistance Genes for Bio-Defence Applications. Nucleic Acids Res (2007) 35:391–4. doi: 10.1093/nar/gkl791 PMC1669772 17090593

[47] Gupta SK Padmanabhan BR Diene SM Lopez-Rojas R Kempf M Landraud L. ARG-Annot, a New Bioinformatic Tool to Discover Antibiotic Resistance Genes in Bacterial Genomes. Antimicrob Agents Chemother (2014) 58:212–20. doi: 10.1128/AAC.01310-13 PMC3910750 24145532

[48] Alcock BP Raphenya AR Lau TTY Tsang KK Bouchard M Edalatmand A. CARD 2020: Antibiotic Resistome Surveillance With the Comprehensive Antibiotic Resistance Database. Nucleic Acids Res (2020) 48:D517–25. doi: 10.1093/nar/gkz935 PMC7145624 31665441

[49] Jadhav A Shanmugham B Rajendiran A Pan A. Unraveling Novel Broad-Spectrum Antibacterial Targets in Food and Waterborne Pathogens Using Comparative Genomics and Protein Interaction Network Analysis. Infect Genet Evol (2014) 27:300–8. doi: 10.1016/j.meegid.2014.08.007 25128740

[50] Yu NY Wagner JR Laird MR Melli G Rey S Lo R. PSORTb 3.0: Improved Protein Subcellular Localization Prediction With Refined Localization Subcategories and Predictive Capabilities for All Prokaryotes. Bioinformatics (2010) 26:1608–15. doi: 10.1093/bioinformatics/btq249 PMC2887053 20472543

[51] Yu C-S Lin C-J Hwang J-K. Predicting Subcellular Localization of Proteins for Gram-Negative Bacteria by Support Vector Machines Based on N -Peptide Compositions. Protein Sci (2004) 13:1402–6. doi: 10.1110/ps.03479604 PMC2286765 15096640

[52] Wilkins MR Gasteiger E Bairoch A Sanchez JC Williams KL Appel RD. Protein Identification and Analysis Tools in the ExPASy Server. Methods Mol Biol (Clifton NJ) (1999) 112:531–52. doi: 10.1385/1-59259-584-7:531 10027275

[53] Doytchinova IA Flower DR. VaxiJen: A Server for Prediction of Protective Antigens, Tumour Antigens and Subunit Vaccines. BMC Bioinf (2007) 8:1–7. doi: 10.1186/1471-2105-8-4 PMC1780059 17207271

[54] Magnan CN Zeller M Kayala MA Vigil A Randall A Felgner PL. High-Throughput Prediction of Protein Antigenicity Using Protein Microarray Data. Bioinformatics (2010) 26:2936–43. doi: 10.1093/bioinformatics/btq551 PMC2982151 20934990

[55] Krogh A Larsson B Von Heijne G Sonnhammer ELL. Predicting Transmembrane Protein Topology With a Hidden Markov Model: Application to Complete Genomes. J Mol Biol (2001) 305:567–80. doi: 10.1006/jmbi.2000.4315 11152613

[56] Almagro Armenteros JJ Tsirigos KD Sønderby CK Petersen TN Winther O Brunak S. SignalP 5.0 Improves Signal Peptide Predictions Using Deep Neural Networks. Nat Biotechnol (2019) 37:420–3. doi: 10.1038/s41587-019-0036-z 30778233

[57] Mitchell AL Attwood TK Babbitt PC Blum M Bork P Bridge A. InterPro in 2019: Improving Coverage, Classification and Access to Protein Sequence Annotations. Nucleic Acids Res (2019) 47:D351–60. doi: 10.1093/nar/gky1100 PMC6323941 30398656

[58] Paul S Sidney J Sette A Peters B. TepiTool: A Pipeline for Computational Prediction of T Cell Epitope Candidates. Curr Protoc Immunol (2016) 114:18.19.1–18.19.24. doi: 10.1002/cpim.12 27479659 PMC4981331

[59] Fleri W Paul S Dhanda SK Mahajan S Xu X Peters B. The Immune Epitope Database and Analysis Resource in Epitope Discovery and Synthetic Vaccine Design. Front Immunol (2017) 8:278. doi: 10.3389/fimmu.2017.00278 28352270 PMC5348633

[60] Kolaskar AS Tongaonkar PC. A Semi-Empirical Method for Prediction of Antigenic Determinants on Protein Antigens. FEBS Lett (1990) 276:172–4. doi: 10.1016/0014-5793(90)80535-Q 1702393

[61] Emini EA Hughes JV Perlow DS Boger J. Induction of Hepatitis A Virus-Neutralizing Antibody by a Virus-Specific Synthetic Peptide. J Virol (1985) 55:836–9. doi: 10.1128/jvi.55.3.836-839.1985 PMC255070 2991600

[62] Larsen JEP Lund O Nielsen M. Improved Method for Predicting Linear B-Cell Epitopes. Immunome Res (2006) 2:2. doi: 10.1186/1745-7580-2-2 16635264 PMC1479323

[63] Jespersen MC Peters B Nielsen M Marcatili P. BepiPred-2.0: Improving Sequence-Based B-Cell Epitope Prediction Using Conformational Epitopes. Nucleic Acids Res (2017) 45:W24–9. doi: 10.1093/nar/gkx346 PMC5570230 28472356

[64] Calis JJA Maybeno M Greenbaum JA Weiskopf D De Silva AD Sette A. Properties of MHC Class I Presented Peptides That Enhance Immunogenicity. PloS Comput Biol (2013) 9:e1003266. doi: 10.1371/journal.pcbi.1003266 24204222 PMC3808449

[65] Gupta S Kapoor P Chaudhary K Gautam A Kumar R Raghava GPS. In Silico Approach for Predicting Toxicity of Peptides and Proteins. PloS One (2013) 8:e73957. doi: 10.1371/journal.pone.0073957 24058508 PMC3772798

[66] Dimitrov I Bangov I Flower DR Doytchinova I. AllerTOP V.2 - A Server for *In Silico* Prediction of Allergens. J Mol Model (2014) 20:2278. doi: 10.1007/s00894-014-2278-5 24878803

[67] Ullah MA Sarkar B Islam SS. Exploiting the Reverse Vaccinology Approach to Design Novel Subunit Vaccines Against Ebola Virus. Immunobiology (2020) 225:151949. doi: 10.1016/j.imbio.2020.151949 32444135

[68] Dash R Das R Junaid M Akash MFC Islam A Hosen SMZ. *In Silico*-Based Vaccine Design Against Ebola Virus Glycoprotein. Adv Appl Bioinf Chem (2017) 10:11–28. doi: 10.2147/AABC.S115859 PMC5367765 28356762

[69] Dhanda SK Vir P Raghava GPS. Designing of Interferon-Gamma Inducing MHC Class-II Binders. Biol Direct (2013) 8:1–15. doi: 10.1186/1745-6150-8-30 24304645 PMC4235049

[70] Bibi S Ullah I Zhu B Adnan M Liaqat R Kong WB. In Silico Analysis of Epitope-Based Vaccine Candidate Against Tuberculosis Using Reverse Vaccinology. Sci Rep (2021) 11:1–16. doi: 10.1038/s41598-020-80899-6 33441913 PMC7807040

[71] Shey RA Ghogomu SM Shintouo CM Nkemngo FN Nebangwa DN Esoh K. Computational Design and Preliminary Serological Analysis of a Novel Multi-Epitope Vaccine Candidate Against Onchocerciasis and Related Filarial Diseases. Pathogens (2021) 10:99. doi: 10.3390/pathogens10020099 33494344 PMC7912539

[72] Mahmud S Rafi M Paul GK Promi MM Shimu M Biswas S. Designing a Multi-Epitope Vaccine Candidate to Combat MERS-CoV by Employing an Immunoinformatics Approach. Sci Rep (2021) 11:15431. doi: 10.1038/s41598-021-92176-1 34326355 PMC8322212

[73] Behbahani M. In Silico Design of Novel Multi-Epitope Recombinant Vaccine Based on Coronavirus Surface Glycoprotein. bioRxiv (2020). doi: 10.1101/2020.03.10.985499

[74] McGuffin LJ Bryson K Jones DT. The PSIPRED Protein Structure Prediction Server. Bioinformatics (2000) 16:404–5. doi: 10.1093/bioinformatics/16.4.404 10869041

[75] Lamiable A Thévenet P Rey J Vavrusa M Derreumaux P Tufféry P. PEP-FOLD3: Faster De Novo Structure Prediction for Linear Peptides in Solution and in Complex. Nucleic Acids Res (2016) 44:W449–54. doi: 10.1093/nar/gkw329 PMC4987898 27131374

[76] Sarkar B Ullah MA Araf Y Islam NN Zohora US. Immunoinformatics-Guided Designing and in Silico Analysis of Epitope-Based Polyvalent Vaccines Against Multiple Strains of Human Coronavirus (HCoV). Expert Rev Vaccines (2021) 00:1–21. doi: 10.1080/14760584.2021.1874925 PMC7989953 33435759

[77] Lee SJ Shin SJ Lee MH Lee MG Kang TH Park WS. A Potential Protein Adjuvant Derived From Mycobacterium Tuberculosis Rv0652 Enhances Dendritic Cells-Based Tumor Immunotherapy. PloS One (2014) 9:1–11. doi: 10.1371/journal.pone.0104351 PMC4125215 25102137

[78] Mei H-F Jin X-B Zhu J-Y Zeng A-H Wu Q Lu X-M. Defensin 2 as an Adjuvant Promotes Anti-Melanoma Immune Responses and Inhibits the Growth of Implanted Murine Melanoma *In Vivo* . PloS One (2012) 7:e31328. doi: 10.1371/journal.pone.0031328 22348070 PMC3278441

[79] Sarkar B Ullah M. Designing Novel Subunit Vaccines Against Herpes Simplex Virus-1 Using Reverse Vaccinology Approach. bioRxiv (2020). doi: 10.1101/2020.01.10.901678

[80] Ali M Pandey RK Khatoon N Narula A Mishra A Prajapati VK. Exploring Dengue Genome to Construct a Multi-Epitope Based Subunit Vaccine by Utilizing Immunoinformatics Approach to Battle Against Dengue Infection. Sci Rep (2017) 7:1–13. doi: 10.1038/s41598-017-09199-w 28835708 PMC5569093

[81] Magnan CN Randall A Baldi P. SOLpro: Accurate Sequence-Based Prediction of Protein Solubility. Bioinformatics (2009) 25:2200–7. doi: 10.1093/bioinformatics/btp386 19549632

[82] Chaudhuri D Datta J Majumder S Giri K. In Silico Designing of Peptide Based Vaccine for Hepatitis Viruses Using Reverse Vaccinology Approach. Infect Genet Evol (2020) 84:104388. doi: 10.1016/j.meegid.2020.104388 32485330

[83] Verspurten J Gevaert K Declercq W Vandenabeele P. SitePredicting the Cleavage of Proteinase Substrates. Trends Biochem Sci (2009) 34:319–23. doi: 10.1016/j.tibs.2009.04.001 19546006

[84] Buchan DWA Jones DT. The PSIPRED Protein Analysis Workbench: 20 Years on. Nucleic Acids Res (2019) 47:W402–7. doi: 10.1093/nar/gkz297 PMC6602445 31251384

[85] Shuid AN Kempster R Mcguffin LJ. ReFOLD: A Server for the Refinement of 3D Protein Models Guided by Accurate Quality Estimates. Nucleic Acids Res (2017) 45:W422–8. doi: 10.1093/nar/gkx249 PMC5570150 28402475

[86] Kalisman N Levi A Maximova T Reshef D Zafriri-Lynn S Gleyzer Y. MESHI: A New Library of Java Classes for Molecular Modeling. Bioinformatics (2005) 21:3931–2. doi: 10.1093/bioinformatics/bti630 16105898

[87] Laskowski RA Hutchinson EG Michie AD Wallace AC Jones ML Thornton JM. PDBsum: A Web-Based Database of Summaries and Analyses of All PDB Structures. Trends Biochem Sci (1997) 22:488–90. doi: 10.1016/S0968-0004(97)01140-7 9433130

[88] Khan MT Islam R Jerin TJ Mahmud A Khatun S Kobir A. Immunoinformatics and Molecular Dynamics Approaches: Next Generation Vaccine Design Against West Nile Virus. PloS One (2021) 16:1–27. doi: 10.1371/journal.pone.0253393 PMC8211291 34138958

[89] Wiederstein M Sippl MJ. ProSA-Web: Interactive Web Service for the Recognition of Errors in Three-Dimensional Structures of Proteins. Nucleic Acids Res (2007) 35:407–10. doi: 10.1093/nar/gkm290 PMC1933241 17517781

[90] Sanami S Azadegan-Dehkordi F Rafieian-Kopaei M Salehi M Ghasemi-Dehnoo M Mahooti M. Design of a Multi-Epitope Vaccine Against Cervical Cancer Using Immunoinformatics Approaches. Sci Rep (2021) 11:1–15. doi: 10.1038/s41598-021-91997-4 34117331 PMC8196015

[91] Colovos C Yeates TO. Verification of Protein Structures: Patterns of Nonbonded Atomic Interactions. Protein Sci (1993) 2:1511–9. doi: 10.1002/pro.5560020916 PMC2142462 8401235

[92] Khatoon N Pandey RK Prajapati VK. Exploring Leishmania Secretory Proteins to Design B and T Cell Multi-Epitope Subunit Vaccine Using Immunoinformatics Approach. Sci Rep (2017) 7:1–12. doi: 10.1038/s41598-017-08842-w 28811600 PMC5557753

[93] Kozakov D Hall DR Xia B Porter KA Padhorny D Yueh C. The ClusPro Web Server for Protein-Protein Docking. Nat Protoc (2017) 12:255–78. doi: 10.1038/nprot.2016.169 PMC5540229 28079879

[94] Sarkar B Ullah MA Araf Y. A Systematic and Reverse Vaccinology Approach to Design Novel Subunit Vaccines Against Dengue Virus Type-1 (DENV-1) and Human Papillomavirus-16 (HPV-16). Inf Med Unlocked (2020) 19:100343. doi: 10.1016/j.imu.2020.100343

[95] Xue LC Rodrigues JP Kastritis PL Bonvin AM Vangone A. PRODIGY: A Web Server for Predicting the Binding Affinity of Protein-Protein Complexes. Bioinformatics (2016) 32:3676–8. doi: 10.1093/bioinformatics/btw514 27503228

[96] López-Blanco JR Aliaga JI Quintana-Ortí ES Chacón P. IMODS: Internal Coordinates Normal Mode Analysis Server. Nucleic Acids Res (2014) 42:271–6. doi: 10.1093/nar/gku339 PMC4086069 24771341

[97] Islam R Parvez MSA Anwar S Hosen MJ. Delineating Blueprint of an Epitope-Based Peptide Vaccine Against the Multiple Serovars of Dengue Virus: A Hierarchical Reverse Vaccinology Approach. Inf Med Unlocked (2020) 20:100430. doi: 10.1016/j.imu.2020.100430

[98] Ponomarenko J Bui HH Li W Fusseder N Bourne PE Sette A. ElliPro: A New Structure-Based Tool for the Prediction of Antibody Epitopes. BMC Bioinf (2008) 9:1–8. doi: 10.1186/1471-2105-9-514 PMC2607291 19055730

[99] Taylor WR Thornton JM Turnell WG. An Ellipsoidal Approximation of Protein Shape. J Mol Graphics (1983) 1:30–8. doi: 10.1016/0263-7855(83)80001-0

[100] Thornton JM Edwards MS Taylor WR Barlow DJ. Location of “Continuous” Antigenic Determinants in the Protruding Regions of Proteins. EMBO J (1986) 5:409–13. doi: 10.1002/j.1460-2075.1986.tb04226.x PMC1166746 2423325

[101] Sanches RCO Tiwari S Ferreira LCG Oliveira FM Lopes MD Passos MJF. Immunoinformatics Design of Multi-Epitope Peptide-Based Vaccine Against Schistosoma Mansoni Using Transmembrane Proteins as a Target. Front Immunol (2021) 12:621706. doi: 10.3389/fimmu.2021.621706 33737928 PMC7961083

[102] Rapin N Lund O Bernaschi M Castiglione F. Computational Immunology Meets Bioinformatics: The Use of Prediction Tools for Molecular Binding in the Simulation of the Immune System. PloS One (2010) 5:e9862. doi: 10.1371/journal.pone.0009862 20419125 PMC2855701

[103] Qamar MTU Shokat Z Muneer I Ashfaq UA Javed H Anwar F. Multiepitope-Based Subunit Vaccine Design and Evaluation Against Respiratory Syncytial Virus Using Reverse Vaccinology Approach. Vaccines (2020) 8:1–27. doi: 10.3390/vaccines8020288 PMC7350008 32521680

[104] Grote A Hiller K Scheer M Münch R Nörtemann B Hempel DC. JCat: A Novel Tool to Adapt Codon Usage of a Target Gene to Its Potential Expression Host. Nucleic Acids Res (2005) 33:526–31. doi: 10.1093/nar/gki376 PMC1160137 15980527

[105] Morla S Makhija A Kumar S. Synonymous Codon Usage Pattern in Glycoprotein Gene of Rabies Virus. Gene (2016) 584:1–6. doi: 10.1016/j.gene.2016.02.047 26945626

[106] Delany I Rappuoli R Seib KL. Vaccines, Reverse Vaccinology, and Bacterial Pathogenesis. Cold Spring Harbor Perspect Med (2013) 3:a012476. doi: 10.1101/cshperspect.a012476 PMC3633180 23637311

[107] Wassenaar TM Gaastra W. Bacterial Virulence: Can We Draw the Line? FEMS Microbiol Lett (2001) 201:1–7. doi: 10.1016/S0378-1097(01)00241-5 11445159

[108] Shanmugham B Pan A. Identification and Characterization of Potential Therapeutic Candidates in Emerging Human Pathogen Mycobacterium Abscessus: A Novel Hierarchical *In Silico* Approach. PloS One (2013) 8:e59126. doi: 10.1371/journal.pone.0059126 23527108 PMC3602546

[109] Rioseras B Yaguë P López-Garciá MT Gonzalez-Quinõnez N Binda E Marinelli F. Characterization of SCO4439, a D-Alanyl-D-Alanine Carboxypeptidase Involved in Spore Cell Wall Maturation, Resistance, and Germination in Streptomyces Coelicolor. Sci Rep (2016) 6:1–15. doi: 10.1038/srep21659 26867711 PMC4751497

[110] Cui Z Dang G Song N Cui Y Li Z Zang X. Rv3091, An Extracellular Patatin-Like Phospholipase in Mycobacterium Tuberculosis, Prolongs Intracellular Survival of Recombinant Mycolicibacterium Smegmatis by Mediating Phagosomal Escape. Front Microbiol (2020) 11:532371. doi: 10.3389/fmicb.2020.532371 PMC7517356 33042041

[111] Gaspar AH Machner MP. VipD is a Rab5-Activated Phospholipase A1 That Protects Legionella Pneumophila From Endosomal Fusion. Proc Natl Acad Sci USA (2014) 111:4560–5. doi: 10.1073/pnas.1316376111 PMC3970493 24616501

[112] Sinha AK Dutta A Chandravanshi M Kanaujia SP. An Insight Into Bacterial Phospholipase C Classification and Their Translocation Through Tat and Sec Pathways: A Data Mining Study. Meta Gene (2019) 20:100547. doi: 10.1016/j.mgene.2019.100547

[113] Dedieu L Serveau-Avesque C Kremer L Canaan S. Mycobacterial Lipolytic Enzymes: A Gold Mine for Tuberculosis Research. Biochimie (2013) 95:66–73. doi: 10.1016/j.biochi.2012.07.008 22819994

[114] Schmiel DH Miller VL. Bacterial Phospholipases and Pathogenesis. Microbes Infect (1999) 1:1103–12. doi: 10.1016/S1286-4579(99)00205-1 10572314

[115] Tomaras AP Dorsey CW Edelmann RE Actis LA. Attachment to and Biofilm Formation on Abiotic Surfaces by Acinetobacter Baumannii: Involvement of a Novel Chaperone-Usher Pili Assembly System. Microbiology (2003) 149:3473–84. doi: 10.1099/mic.0.26541-0 14663080

[116] Gollop R Inouye M Inouye S. Protein U, a Late-Developmental Spore Coat Protein of Myxococcus Xanthus, Is a Secretory Protein. J Bacteriol (1991) 173:3597–600. doi: 10.1128/jb.173.11.3597-3600.1991 PMC207979 1904442

[117] Li W Zhao Y Yu J Lin L Ramanathan S Wang G. TonB-Dependent Receptors Affect the Spontaneous Oxytetracycline Resistance Evolution in Aeromonas Hydrophila. J Proteome Res (2021) 20:154–63. doi: 10.1021/acs.jproteome.9b00708 32911932

[118] Zhang Z Jiang S Liu Y Sun Y Yu P Gong Q. Identification of Irea, 0007, 0008, and 2235 as TonB-Dependent Receptors in the Avian Pathogenic Escherichia Coli Strain DE205B. Vet Res (2020) 51:1–10. doi: 10.1186/s13567-020-0734-z 31973724 PMC6979363

[119] Gómez-Santos N Glatter T Koebnik R Świątek-Połatyńska MA Søgaard-Andersen L. A TonB-Dependent Transporter Is Required for Secretion of Protease PopC Across the Bacterial Outer Membrane. Nat Commun (2019) 10:1360. doi: 10.1038/s41467-019-09366-9 30911012 PMC6434023

[120] Schalk IJ Mislin GLA Brillet K. Structure, Function and Binding Selectivity and Stereoselectivity of Siderophore-Iron Outer Membrane Transporters. London, GB: Elsevier (2012). p. 37–66. doi: 10.1016/B978-0-12-394390-3.00002-1 23046646

[121] Koebnik R Locher KP Van Gelder P. Structure and Function of Bacterial Outer Membrane Proteins: Barrels in a Nutshell. Mol Microbiol (2000) 37:239–53. doi: 10.1046/j.1365-2958.2000.01983.x 10931321

[122] Prado LC da S Giacchetto Felice A Rodrigues TCV Tiwari S Andrade BS Kato RB. New Putative Therapeutic Targets Against Serratia Marcescens Using Reverse Vaccinology and Subtractive Genomics. J Biomolec Struct Dynam (2021) 30:1–16. doi: 10.1080/07391102.2021.1942211 34192477

[123] Leow CY Kazi A Ismail CMKH Chuah C Lim BH Leow CH. Reverse Vaccinology Approach for the Identification and Characterization of Outer Membrane Proteins of Shigella Flexneri as Potential Cellular-and Antibody-Dependent Vaccine Candidates. Clin Exp Vaccine Res (2020) 9:15–25. doi: 10.7774/cevr.2020.9.1.15 32095437 PMC7024733

[124] Shey RA Ghogomu SM Esoh KK Nebangwa ND Shintouo CM Nongley NF. *In-Silico* Design of a Multi-Epitope Vaccine Candidate Against Onchocerciasis and Related Filarial Diseases. Sci Rep (2019) 9:1–18. doi: 10.1038/s41598-019-40833-x 30867498 PMC6416346

[125] Barchiesi J Castelli ME di Venanzio G Colombo MI García Véscovi E. The PhoP/PhoQ System and Its Role in Serratia Marcescens Pathogenesis. J Bacteriol (2012) 194:2949–61. doi: 10.1128/JB.06820-11 PMC3370626 22467788

[126] Fedrigo G v Campoy EM di Venanzio G Colombo MI García Véscovi E. Serratia Marcescens Is Able to Survive and Proliferate in Autophagic-Like Vacuoles Inside Non-Phagocytic Cells. PloS One (2011) 6:e24054. doi: 10.1371/journal.pone.0024054 21901159 PMC3162031

[127] Hertle R Schwarz H. Serratia Marcescens Internalization and Replication in Human Bladder Epithelial Cells. BMC Infect Dis (2004) 4:16. doi: 10.1186/1471-2334-4-16 15189566 PMC441377

[128] Xiong Q Yang M Li P Wu C. Bacteria Exploit Autophagy For Their Own Benefit. Infect Drug Resist (2019) 12:3205–15. doi: 10.2147/IDR.S220376 PMC6792943 31632106

[129] Ozawa Y Suda T Nagata T Hashimoto D Nakamura Y Enomoto N. Mucosal Vaccine Using CTL Epitope-Pulsed Dendritic Cell Confers Protection for Intracellular Pathogen. Am J Respir Cell Mol Biol (2009) 41:440–8. doi: 10.1165/rcmb.2008-0446OC 19202004

[130] Kaufmann SHE. Recent Findings in Immunology Give Tuberculosis Vaccines a New Boost. Trends Immunol (2005) 26:660–7. doi: 10.1016/j.it.2005.09.012 16246622

[131] Nain Z Karim MM Sen MK Adhikari UK. Structural Basis and Designing of Peptide Vaccine Using PE-PGRS Family Protein of Mycobacterium Ulcerans—An Integrated Vaccinomics Approach. Mol Immunol (2020) 120:146–63. doi: 10.1016/j.molimm.2020.02.009 32126449

[132] Chaudhri G Quah BJ Wang Y Tan AHY Zhou J Karupiah G. T Cell Receptor Sharing by Cytotoxic T Lymphocytes Facilitates Efficient Virus Control. Proc Natl Acad Sci (2009) 106:14984–9. doi: 10.1073/pnas.0906554106 PMC2736460 19706459

[133] Shi J Zhang J Li S Sun J Teng Y Wu M. Epitope-Based Vaccine Target Screening Against Highly Pathogenic MERS-CoV: An *In Silico* Approach Applied to Emerging Infectious Diseases. PloS One (2015) 10:1–16. doi: 10.1371/journal.pone.0144475 PMC4671582 26641892

[134] Azim KF Hasan M Hossain MN Somana SR Hoque SF Bappy MNI. Immunoinformatics Approaches for Designing a Novel Multi Epitope Peptide Vaccine Against Human Norovirus (Norwalk Virus). Elsevier B.V (2019). p. 103936. doi: 10.1016/j.meegid.2019.103936 31233780

[135] Ghaffari-Nazari H Tavakkol-Afshari J Jaafari MR Tahaghoghi-Hajghorbani S Masoumi E Jalali SA. Improving Multi-Epitope Long Peptide Vaccine Potency by Using a Strategy That Enhances CD4+ T Help in BALB/c Mice. PloS One (2015) 10:1–12. doi: 10.1371/journal.pone.0142563 PMC4640540 26556756

[136] Hajighahramani N Nezafat N Eslami M Negahdaripour M Rahmatabadi SS Ghasemi Y. Immunoinformatics Analysis and *In Silico* Designing of a Novel Multi-Epitope Peptide Vaccine Against Staphylococcus Aureus. Infect Genet Evol (2017) 48:83–94. doi: 10.1016/j.meegid.2016.12.010 27989662

[137] Chen Z Zhu Y Sha T Li Z Li Y Zhang F. Design of a New Multi-Epitope Vaccine Against Brucella Based on T and B Cell Epitopes. Epidemiol Infect (2021) 149:E136. doi: 10.1017/S0950268821001229 34032200 PMC8220514

[138] Chukwudozie OS Gray CM Fagbayi TA Chukwuanukwu RC Oyebanji VO Bankole TT. Immuno-Informatics Design of a Multimeric Epitope Peptide Based Vaccine Targeting SARS-CoV-2 Spike Glycoprotein. PloS One (2021) 16:1–25. doi: 10.1371/journal.pone.0248061 PMC7968690 33730022

[139] Couto J Seixas G Stutzer C Olivier NA Maritz-Olivier C Antunes S. Probing the Rhipicephalus Bursa Sialomes in Potential Anti-Tick Vaccine Candidates: A Reverse Vaccinology Approach. Biomedicines (2021) 9:1–18. doi: 10.3390/biomedicines9040363 PMC8067113 33807386

[140] Majid M Andleeb S. Designing a Multi-Epitopic Vaccine Against the Enterotoxigenic Bacteroides Fragilis Based on Immunoinformatics Approach. Sci Rep (2019) 9:1–15. doi: 10.1038/s41598-019-55613-w 31874963 PMC6930219

[141] Alom MW Shehab MN Sujon KM Akter F. Exploring E, NS3, and NS5 Proteins to Design a Novel Multi-Epitope Vaccine Candidate Against West Nile Virus: An *In-Silico* Approach. Inf Med Unlocked (2021) 25:100644. doi: 10.1016/j.imu.2021.100644

[142] Toussi DN Massari P. Immune Adjuvant Effect of Molecularly-Defined Toll-Like Receptor Ligands. Vaccines (2014) 2:323–53. doi: 10.3390/vaccines2020323 PMC4494261 26344622

[143] Palatnik-de-Sousa CB Soares I da S Rosa DS. Editorial: Epitope Discovery and Synthetic Vaccine Design. Front Immunol (2018) 9:826. doi: 10.3389/fimmu.2018.00826 29720983 PMC5915546

[144] Rahman MS Hoque MN Islam MR Akter S Ul Alam ASMR Siddique MA. Epitope-Based Chimeric Peptide Vaccine Design Against S, M and E Proteins of SARS-CoV-2, the Etiologic Agent of COVID-19 Pandemic: An in Silico Approach. PeerJ (2020) 8:e9572. doi: 10.7717/peerj.9572 33194329 PMC7394063

[145] Obaidullah AJ Alanazi MM Alsaif NA Albassam H Almehizia AA Alqahtani AM. Immunoinformatics-Guided Design of a Multi-Epitope Vaccine Based on the Structural Proteins of Severe Acute Respiratory Syndrome Coronavirus 2. RSC Adv (2021) 11:18103–21. doi: 10.1039/d1ra02885e PMC9033181 35480208

