# Supplementary material for: A Systematic Immuno-Informatic Approach to Design a Multiepitope-Based Vaccine Against Emerging Multiple Drug Resistant Serratia marcescens
Source: Front Immunol. 2022 Mar 14;13:768569. doi: 10.3389/fimmu.2022.768569 (PMC8967166; doi:10.3389/fimmu.2022.768569)
Supplement: Supplementary file 1 [file DataSheet_1.docx]

**Table S1.** *S. marcescens* strains used in this study.

| **N^0^.** | **Strains** | **Source of Isolation** | **Country** | **RefSeq assembly acession** | **Assembly level** | **Reference** |
| --- | --- | --- | --- | --- | --- | --- |
| 1 | 95 | Sputum | USA | GCA_003031545.1 | complete | Unpublished |
| 2 | 332 | Wound | USA | GCA_003186475.1 | complete | Unpublished |
| 3 | 1602 | Blood | China | GCA_009858195.1 | complete | Unpublished |
| 4 | 2838 | Body fluid | China | GCA_009909405.1 | complete | Unpublished |
| 5 | 3024 | Blood | China | GCA_009909365.1 | complete | Unpublished |
| 6 | 4201 | Sputum | China | GCA_009909345.1 | complete | Unpublished |
| 7 | 1140- | Body fluid | China | GCA_009909385.1 | complete | Unpublished |
| 8 | AR_0027 | Clinical/Patient associated | Unknown | GCA_002947235.1 | complete | Unpublished |
| 9 | AR_0091 | Clinical/Patient associated | Unknown | GCA_002996885.1 | complete | Unpublished |
| 10 | AR_0099 | Clinical/Patient associated | Unknown | GCA_002997125.1 | complete | Unpublished |
| 11 | AR_0121 | Clinical/Patient associated | Unknown | GCA_003071625.1 | complete | Unpublished |
| 12 | AR_0122 | Clinical/Patient associated | Unknown | GCA_003204405.1 | complete | Unpublished |
| 13 | AR_0123 | Clinical/Patient associated | Unknown | GCA_003071605.1 | complete | Unpublished |
| 14 | AR_0124 | Clinical/Patient associated | Unknown | GCA_003071565.1 | complete | Unpublished |
| 15 | AR_0130 | Clinical/Patient associated | Unknown | GCA_003071585.1 | complete | Unpublished |
| 16 | AR_0131 | Clinical/Patient associated | Unknown | GCA_003204075.1 | complete | Unpublished |
| 17 | BWH-23 | Blood | USA | GCA_003032415.1 | complete | Unpublished |
| 18 | BWH-35 | Sputum | USA | GCA_003031645.1 | complete | Unpublished |
| 19 | C110 | Sputum | China | GCA_009909425.1 | complete | Unpublished |
| 20 | CAV1492 | Respiratory | USA | GCA_001022215.1 | complete | Unpublished |
| 21 | CAV1761 | Peri-rectal | USA | GCA_003146705.1 | complete | Unpublished |
| 22 | FDAARGOS_65 | Endotracheal aspirate | USA | GCA_000783915.2 | complete | Unpublished |
| 23 | SER00094 | Sputum | USA | GCA_011769885.1 | complete | Unpublished |
| 24 | SM39 | Septicemia | Japan | GCA_000828775.1 | complete | Iguchi et al., 2014 |
| 25 | SMB2099 | Clinical/Patient associated | Germany | GCA_900029885.1 | complete | Unpublished |
| 26 | SmUNAM836 | Bronchial sample | Mexico | GCA_001294565.1 | complete | Sandner-Miranda et al., 2016 |
| 27 | U36365 | Urine | India | GCA_001672055.1 | complete | Unpublished |
| 28 | UMH1 | Bacteremia | USA | GCA_002220615.1 | complete | Anderson et al., 2017 |
| 29 | UMH10 | Bacteremia | USA | GCA_002220695.1 | complete | Anderson et al., 2017 |
| 30 | UMH11 | Bacteremia | USA | GCA_002220575.1 | complete | Anderson et al., 2017 |
| 31 | UMH12 | Bacteremia | USA | GCA_002220595.1 | complete | Anderson et al., 2017 |
| 32 | UMH2 | Bacteremia | USA | GCA_002220515.1 | complete | Anderson et al., 2017 |
| 33 | UMH3 | Bacteremia | USA | GCA_002220655.1 | complete | Anderson et al., 2017 |
| 34 | UMH5 | Bacteremia | USA | GCA_002220635.1 | complete | Anderson et al., 2017 |
| 35 | UMH6 | Bacteremia | USA | GCA_002220675.1 | complete | Anderson et al., 2017 |
| 36 | UMH7 | Bacteremia | USA | GCA_002220715.1 | complete | Anderson et al., 2017 |
| 37 | UMH8 | Bacteremia | USA | GCA_002220535.1 | complete | Anderson et al., 2017 |
| 38 | UMH9 | Bacteremia | USA | GCA_002220555.1 | complete | Anderson et al., 2017 |
| 39 | WVU-001 | Blood | USA | GCA_006838705.1 | complete | Unpublished |
| 40 | WVU-002 | Blood | USA | GCA_006842785.1 | complete | Unpublished |
| 41 | WVU-003 | Blood | USA | GCA_006711105.1 | complete | Unpublished |
| 42 | WVU-004 | Blood | USA | GCA_006711125.1 | complete | Unpublished |
| 43 | WVU-005 | Blood | USA | GCA_006711145.1 | complete | Unpublished |
| 44 | WVU-006 | Blood | USA | GCA_006711245.1 | complete | Unpublished |
| 45 | WVU-007 | Blood | USA | GCA_006711405.1 | complete | Unpublished |
| 46 | WVU-008 | Blood | USA | GCA_006711525.1 | complete | Unpublished |
| 47 | WVU-009 | Blood | USA | GCA_006716725.1 | complete | Unpublished |
| 48 | WVU-010 | Blood | USA | GCA_006716825.1 | complete | Unpublished |
| 49 | Db-11 | Insect pathogen | Sweden | GCA_000513215.1 | complete | Iguchi et al., 2014 |

**Table S2.** Metabolic pathways exclusive to pathogen and shared with host.

| **Pathway code** | **Pathways shared with host** |
| --- | --- |
| 00010 | Glycolysis/Gluconeogenesis |
| 00020 | Citrate cycle (TCA cycle) |
| 00030 | Pentose phosphate pathway |
| 00040 | Pentose and glucoronate interconversions |
| 00051 | Fructose and mannose metabolism |
| 00052 | Galactose metabolism |
| 00053 | Ascorbate and aldarate metabolism |
| 00061 | Fatty acid biosynthesis |
| 00071 | Fatty acid degradation |
| 00072 | Synthesis and degradation of ketone bodies |
| 00130 | Ubiquinone and other terpenoid-quinone biosynthesis |
| 00190 | Oxidative phosphorylation |
| 00220 | Argigine biosynthesis |
| 00230 | Purine metabolism |
| 00240 | Pyrimidine metabolism |
| 00250 | Alanine, aspartate and glutamate metabolism |
| 00260 | Glycine, serine and threonine metabolism |
| 00270 | Cysteine and methionine metabolism |
| 00280 | Valine, leucine and isoleucine degradation |
| 00290 | Valine, leucine and isoleucine biosynthesis |
| 00310 | Lysine degradation |
| 00330 | Arginine and proline metabolism |
| 00340 | Histidine metabolism |
| 00350 | Tyrosine metabolism |
| 00360 | Phenylalanine metabolism |
| 00380 | Tryptophan metabolism |
| 00400 | Phenylalanine, tyrosine and tryptophan biosynthesis |
| 00410 | beta-Alanine metabolism |
| 00430 | Taurine and hypotaurine metabolism |
| 00440 | Phosphonate and phosphinate metabolism |
| 00450 | Selenocompound metabolism |
| 00471 | D-Glutamine and D-glutamate metabolism |
| 00480 | Glutathione metabolism |
| 00500 | Starch and sucrose metabolism |
| 00511 | Other glycan degradation |
| 00520 | Amino sugar and nucleotide sugar metabolism |
| 00561 | Glycerolipid metabolism |
| 00562 | Inositol phosphate metabolism |
| 00564 | Glycerophospholipid metabolism |
| 00565 | Ether lipid metabolism |
| 00590 | Arachidonic acid metabolism |
| 00591 | Linoleic acid metabolism |
| 00592 | alpha-Linolenic acid metabolism |
| 00620 | Pyruvate metabolism |
| 00630 | Glyoxylate and dicarboxylate metabolism |
| 00640 | Propanoate metabolism |
| 00650 | Butanoate metabolism |
| 00670 | One carbon pool by folate |
| 00730 | Thiamine metabolism |
| 00740 | Riboflavin metabolism |
| 00750 | Vitamin B6 metabolism |
| 00760 | Nicotinate and nicotinamide metabolism |
| 00770 | Pantothenate and CoA biosynthesis |
| 00780 | Biotin metabolism |
| 00785 | Lipoic acid metabolism |
| 00790 | Folate biosynthesis |
| 00860 | Porphyrin and chlorophyll metabolism |
| 00900 | Terpenoid backbone biosynthesis |
| 00910 | Nitrogen metabolism |
| 00920 | Sulfur metabolism |
| 00970 | Aminoacyl-tRNA biosynthesis |
| 01040 | Biosynthesis of unsaturated fatty acids |
| 01100 | Metabolic pathways |
| 01200 | Carbon metabolism |
| 01210 | 2-Oxocarboxylic acid metabolism |
| 01212 | Fatty acid metabolism |
| 01230 | Biosynthesis of amino acids |
| 02010 | ABC transporters |
| 03010 | Ribosome |
| 03018 | RNA degradation |
| 03020 | RNA polymerase |
| 03030 | DNA replication |
| 03060 | Protein export |
| 03410 | Base excision repair |
| 03420 | Nucleotide excision repair |
| 03430 | Mismatch repair |
| 03440 | Homologous recombination |
| 04122 | Sulfur relay system |
| **Pathway code** | **Pathways exclusive to patoghen** |
| 00261 | Monobactam biosynthesis |
| 00281 | Geraniol degradation |
| 00300 | Lysine biosynthesis |
| 00332 | Carbapenem biosynthesis |
| 00361 | Chlorocyclohexane and chlorobenzene degradation |
| 00362 | Benzoate degradation |
| 00364 | Fluorobenzoate degradation |
| 00401 | Novobiocin biosynthesis |
| 00460 | Cyanoamino acid metabolism |
| 00473 | D-Alanine metabolism |
| 00521 | Streptomycin biosynthesis |
| 00523 | Polyketide sugar unit biosynthesis |
| 00525 | Acarbose and validamycin biosynthesis |
| 00540 | Lipopolysaccharide biosynthesis |
| 00550 | Peptidoglycan biosynthesis |
| 00622 | Xylene degradation |
| 00623 | Toulene degradation |
| 00624 | Polycyclic aromatic hydrocarbon degradation (smac) |
| 00625 | Chloroalkane and chloroalkene degradation |
| 00626 | Naphthalene degradation |
| 00627 | Aminobenzoate degradation |
| 00633 | Nitrotoluene degradation |
| 00643 | Styrene degradation (smac) |
| 00660 | C5-Branched dibasic acid metabolism |
| 00680 | Methane metabolism |
| 00791 | Atrazine degradation |
| 00903 | Limonene and pinene degradation |
| 00930 | Caprolactam degradation |
| 01053 | Biosynthesis of siderophore group nonribosomal peptides |
| 01054 | Nonribosomal peptide structures |
| 01110 | Biosynthesis of secondary metabolites |
| 01120 | Microbial metabolism in diverse environments |
| 01220 | Degradation of aromatic compounds |
| 01501 | beta-Lactam resistance |
| 01502 | Vancomycin resistance |
| 01503 | Cationic antimicrobial peptide (CAMP) resistance |
| 02020 | Two-component system |
| 02024 | Quorum sensing |
| 02030 | Bacterial chemotaxis |
| 02040 | Flagellar assembly |
| 02060 | Phosphotransferase system (PTS) |
| 03070 | Bacterial secretion system |

**
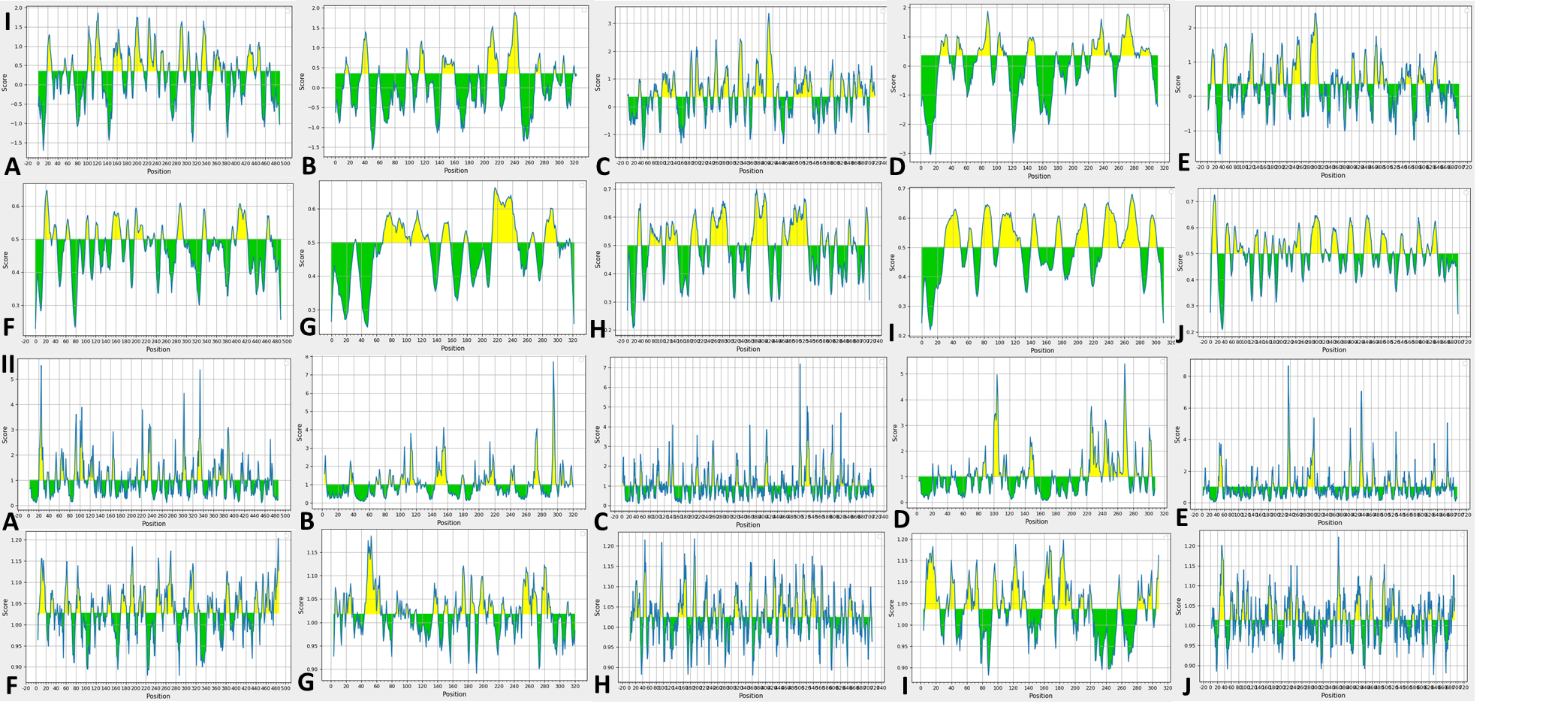
**

**Figure S1. Identification of B-cell epitopes of Proteins using IEDB server.** A and F) D-alanyl-D-alanine carboxypeptidase/endopeptidase, B and G) Patatin-like phospholipase family protein, C and H) Phospholipase C, phosphocholine-specific, D and I) Spore coat U domain-containing protein, E and J) TonB-dependent receptor**. I)** Bepipred 1.0 Linear Epitope prediction (A-E), and Bepipred 2.0 Linear Epitope prediction (F-J). **II)** Emini Surface Accessibility Prediction (A-E), and Kolaskar & Tongaonkar Antigenicity (F-J).
