# Supplementary material for: A Systematic Immuno-Informatic Approach to Design a Multiepitope-Based Vaccine Against Emerging Multiple Drug Resistant Serratia marcescens
Source: Front Immunol. 2022 Mar 14;13:768569. doi: 10.3389/fimmu.2022.768569 (PMC8967166; doi:10.3389/fimmu.2022.768569)
Supplement: Supplementary Data Sheet S2 — Core protein sequences. [file DataSheet_2.pdf]

>CORE_REP|Org17_Gene4397#
MDLLRFILRLPFTLVKGVCRLLGGGLSLLGRLLRPLVGNLSWRAPAWWAALPRGFLRLESGVDKHPKA
IGLGLLLLAGAAGGAFYGWHWWQNRPQPIEPAPMVVQEVSASLANPAPIDYTAARQAPQTVSLAFSGS
VAPIAAVGKTVSAGITLKPTAEGQWAWNNESTLVFTPKKPLPMGAKYDVTLEPATLLAPQVKLAKTRY
AFTVPAFGYRLGQAEYYRDPQNAQKRSAIFNLQFNAPVDVASFEKQISLGLKEGSATSEKKLNFSLVY
DEKKLNAWVHSEPLQALDQGGAVHLTVGKGVKSTAAGNAVEEAKSNWVKVPTLYSLALSDASAQVVDT
DGGKGQRALVVGFSDAVKDKELARAAKAWLLPQHDPSDAEAANDAEDFYPWTVDSVGKNVLAQATPLP
LTLNEAEEAYQPQFSFRFDAPAHRFMLLEIDNQLISAGGYKMPKKVYRVVEVPEFPKSLQFMSQGSLL
SVNGDKQISVAARNVAGLRLDIKRVIPSQLQHIVSFKSREYSSTEFNRLNDEYFTEHFKYQTALNNER
PGEVNYQGIDLSRYLSTNASSHRGVFLLTLSEWQPNRKPAAEEAGAEQEQEQEQDESAGEEQPDVGDS
RFVVVTDLGIVAKSSQDKTRDVFVQSIQSGAPVSGANVSVVAKNGVTLLSQTTGADGHVRFPALDVYT
NERQPVMFLVEKEGDVSFLPTGSYNDRGLDFSRFDVAGEQTPTDPRTLSSYLFSDRGVYRPGDTFNIG
LITRAADWRVGLAGVPVRAEIRDPRDKLMSTVPLTLGASGFNELSYTTDENSPTGEWNVYLYLIGKNN
DTSTLLGHTAVNVKEFEPDQLKVKLALTPDRQQGWVKPSELKASIDVQNLFGTPAQDRRVTTRLTLRP
MYPSFDRFPDYAFYENRQNSDGFETELEDRTTDEHGAADIPLDLKSYADATYQLQLLSEAFVAGGGRS
VAATARTLVSPYDYLIGVKADGDLGYINRDAERHLNVIAIDPALKQIAMPNLKQVLIEQKYISVLTKQ
DSGVYKYQSKMKEVQLSEQPLALGDQGAELRLATDKPGDFVLVIEDAQGKTLNRVAYSVAGNANLSRS
LDRNAELKLKLNQAEYQPGEEIEVAINAPYTGSGLITIEKDKVYAWQWFHTDTTSSVQTIRVPAGMEG
NGYINVQFVRDINSSEIFMSPLSYGVMPFKISTKARQNSLEVTAPEVIKPGENLTMTVKTDAPQQVAL
FAVDEGILQVARYRLKDPLEFFFSKRELNVSSSQILDLILPEFSKLMALTAAPGGDAGEGLDLNLNPF
KRKRDKPVAYWSGITEVSGEKQFVYPVPDYFNGKIRVMAISVTPEKIGKAQTAATVRDSFIMTPNVPA
MVAPGDEFDVSVGVSNNLEGLNGQSAAINVLLTPPPQLEVVGNATQSLTLAEKREGVIAFRLRAKAVL
GDAPLVFDARYGDQASRRTVSTSVRPAAPYRTQSVMGRMSGGSQNVDGLRQMFDAFAQRRAAVSNSPL
VLTSGLAQYLADYPYYCSEQIVSRSIPLMLQSRHPEMKGSLSQAEVSKQLRNLLGVLRARQNDSGAIG
AWRSSPDADPFVTPYVVQYLLEAKAAGYALPEGMLDEANGALRELAASGFDDLYLLRLRSWAVYLLTL
QGEVTTNSLAAVQDTLQKRYGEGWKTDLSALYLASSYRLLKMDDEAAALLQPSWQQLSKAYDSAWWTQ
NYFDPLVQDATRLYLITRHFPEKVASIPPQVLENMVKALKEERYTTYSSAMSILALESYSAQVAAQSA
NADALGIVQVGKSGGDPQRISELQGLFVQGQFNADATAVRFTNGGSAPAWYVVTQAGYDLNAPQKALS
RGIEIVREYTDEQGKPVTQVTLGQKINVHLKVRANSKEGQSNLAIVDLLPGGFEVVQQTPPEPADAGE
ESDDRGGWQSPLAATGSTWAPDYSDIREDRVIIYGSAGTEVQEFVYQIKATNTGSFIIPPAYGEAMYD
REVQAMSVGGGKLVVVPAGND
>CORE_REP|Org9_Gene4123#
MLYDKSQERDNCGFGLIAHIEGEPSHKVVRTAIHALARMQHRGAILADGKTGDGCGLLLQKPDRFFRM
VAEERGWRLAKNYAVGMMFLSQNEEEARLSRRIVEEELQNETLSIVGWREVPTNPDVLGEIALSSLPR
IEQIFVNAPAGWRPRDMERRLFVARRRIEKRVQDNSFYVCSFSNLVTIYKGLCMPADLPRFYLDLADL
RLESAICLFHQRFSTNTVPRWPLAQPFRYLAHNGEINTITGNRQWARARTYKFQTPLIPDLQAAAPFV
NETGSDSSSLDNMLELLLAGGMDLIRAMRLLVPPAWQNNPDMDGDLRAFFDFNSMHMEPWDGPAGIVM
SDGRYAACNLDRNGLRPARYVITKDKLITCASEVGIWDYQPDEVVEKGRVGPGELMVIDTRSGRILHS
AETDNDLKSRHPYKEWMEKNVKRLVPFEDLPDDQVGSRELDDAQLETYQKQFGYSSEELDQVIRVLGE
IGQEATGSMGDDTPFAVLSSRPRIVYDYFRQQFAQVTNPPIDPLREAHVMSLATSIGREMNVFCEAEG
QAHRLSFKSPILLYSDFKQLTTLEGEYYRAETLDLTFDPQQQDLEQTIRALCDEAERKVREGAVLLVL
SDRAIAPGRLPVPAPMAVGAVQTRLVEKSLRCDANLIVETASARDPHHFAVLLGFGATAIYPYLAYET
LAKLVDSQAIDKKYRDVMLNYRNGINKGLYKIMSKMGISTIASYRCSKLFEAVGLHRDLADLCFQGVV
SRIGGASFSDFQQDLQNLSKRAWLKRKPLEQGGLLKFVHGGEYHAYNPDVVNSLQKAVHSGEYSDYQA
YAKLVNERPVAMLRDLLAITPKGEPIPVDQVEPAESLFKRFDTAAMSIGALSPEAHESLAIAMNGLGG
FSNSGEGGEDPARYRTNKVSRIKQVASGRFGVTPAYLVNADVIQIKVAQGAKPGEGGQLPGDKVTPYI
ARLRYSVPGVTLISPPPHHDIYSIEDLAQLIFDLKQVNPKAVISVKLVSEPGVGTIATGVAKAYADLI
TIAGYDGGTGASPLSSVKYAGCPWELGLVETQQALVANGLRHKIRLQVDGGLKTGVDIVKAAILGAES
FGFGTGPMVALGCKYLRICHLNNCATGVATQDDKLRRDHYHGLPERVTNYFQFIARETREIMAQLGVS
QLVDLIGRTEFLTSLPGISAKQNKLDLSPLLKTATPHPGKAVYCTESSNPAFDKGLLNKELLAQAQPH
IEAKQGKTFYFDIRNTDRSVGAMLSGAIADVHGDQGMAADPIKAHFSGTAGQSFGVWNAGGVELTLTG
DANDYVGKGMAGGSIAVRPPIGSAFRSHEASIVGNTCLYGATGGKLFAAGRAGERFAVRNSGAITVVE
GIGDNGCEYMTGGIVCVLGKTGINFGAGMTGGFAYVLDEDGEFRKRVNPELVEVLDVDQLAIHEEHLR
GLITEHVQATGSSRAEEILANWPEWAPKFALVKPKSSDVKALLGHRSRSAAELRVQAQ

>CORE_REP|Org43_Gene4415#
MIERGKFRSLTLVNWNGFFARTFDLDELVTTLSGGNGAGKSTTMAAFVTALIPDLTLLHFRNTTEAGA
TSGSRDKGLHGKLRAGVCYSTLDVVNSRHQRVVVGVRLQQVAGRDRKVDIKPFTIQGLPTAVQPTELL
TQTVGERQARVLSLQELKERVEEMEGVQFKQFNSITDYHSLMFDLGVIPKRLRSSADRSKFYRLIEAS
LYGGISSAITRSLRDYLLPENSGVRKAFQDMEAALRENRMTLEAIRVTQSDRDLFKHLISEATSYVAA
DYMRHANERRIHLDGALALRSDLLGSRKQLAAEQYRHVEMARELSEQSGAESDLETDYQAASDHLNLV
QTAMRQQEKIERYEADLEELTYRLEEQNEVVAEASEQQAENEARAEAAELEVDELKSQLADYQQALDV
QQTRAIQYQQALQALERARALCQLPDLTADNAEQWLDTFQAREQEATEALLMLEQKLSVADAAHGQFE
QAYQLVGKIAGQVSRSEAWQCARELLRDWPSQQHLAECVQPLRLRLSELEQRLRSQQDAERLLQEFCK
RHGQEYQPDDLDMLQQELEERLEALSQNVSEAGERRMEMRQELEQIQQRIRELTARAPVWLAAQDALS
QLSDQSGEPLENSQQVTEYMQQLLERERETTVERDEVAARKREVEAQIERLSQPGGAEDQRLVTLAER
FGGVLLSEIYDDVTIDDAPYFSALYGPSRHAIVVPDLSLVREMLEGLEDCPEDLYLIEGDPQSFDDSV
FAVEEQDKAVVVKIADRQWRYSRYPEVPLFGRAARENRLEVLHAERETLAERYATLSFDVQKTQRSHQ
AFSRFIGTHLAVAFDADPEAEIRNLNARRGEIERALNNHEAQNQQQRQQYDQAKEGISALNRLMPLVS
VLNDETLQDRVDEIREELEEAQDAARHIQQHGVSLTKLEPLLSVLQSDPQQHEQLQQDYAQAQSVQRQ
AKQQAFALTEVVQRRAHFSYTDSAGMQNANNDLNDKLRQRLEQAEAERARAREQLRQYQTQFTQYSQV
LASLKSSYDAKRDMLKELSQELVDIGVQADANAEARARQRRDELHAALSNNRARRNQLEKQLTFCEAE
MDGLQKKLRKLERDYHQLREQVVTAKAGWCAVMRLVKDNGVERRLHRRELAYMDGDELRSMSDKALGA
LRLAVADNEHLRDVLRLSEDPKRPERKIQFYIAVYQHLRERIRQDIIRTDDPVEAIEQMEIELGRLTE
ELTAREQKLAISSKSVANIIRKTIQREQNRIRMLNQGLQAVAFGQVKSVRLNVNVREAHATLLDVLSE
QQEQHQDLFNSNRLTFSEALAKLYQRLNPQIDMGQRTPQTIGEELLDYRNYLEMEVEVYRGSDGWLRA
ESGALSTGEAIGTGMSILVMVVQSWEEESRRLRGKDISPCRLLFLDEAARLDAKSIATLFELCDRLEM
QLIIAAPENISPEKGTTYKLVRKVFQNHEHVHVVGLRGFASEPPALGTAPVETP
>CORE_REP|Org11_Gene2189#
MKDLLKFLKAQTKTEEFDAIKIALASPDMIRSWSFGEVKKPETINYRTFKPERDGLFCARIFGPVKDY
ECLCGKYKRLKHRGVICEKCGVEVTQTKVRRERMGHIELASPTAHIWFLKSLPSRIGLLLDMPLRDIE
RVLYFESYVVVEGGMTNLERRQILTEEQYLDALEEFGDEFDAKMGAEAIQALLKNMDLEAECEQLREE
LNETNSETKRKKLTKRIKLLEAFVQSGNKPEWMILTVLPVLPPDLRPLVPLDGGRFATSDLNDLYRRV
INRNNRLKRLLDLAAPDIIVRNEKRMLQEAVDALLDNGRRGRAITGSNKRPLKSLADMIKGKQGRFRQ
NLLGKRVDYSGRSVITVGPYLRLHQCGLPKKMALELFKPFIYGKLELRGLATTIKAAKKMVEREEAVV
WDILDEVIREHPVLLNRAPTLHRLGIQAFEPVLIEGKAIQLHPLVCAAYNADFDGDQMAVHVPLTLEA
QLEARALMMSTNNILSPANGEPIIVPSQDVVLGLYYMTRDCVNAKGEGMVLNGSKEAERVYRAGLASL
HARVKVRITEDVKNAEGEWTSQTSIIDTTIGRAILWMIVPKGLPYSIVNQPLGKKAISKMLNTCYRIL
GLKPTVIFADQIMYTGFAYAARSGASVGIDDMVIPAKKAEIIEEAETEVAEIQEQFQSGLVTAGERYN
KVIDIWAAANERVAKAMMENLSVEDVVNRDGEVEQQVSFNSIFMMADSGARGSAAQIRQLAGMRGLMA
KPDGSIIETPITANFREGLNVLQYFISTHGARKGLADTALKTANSGYLTRRLVDVAQDLVVTEDDCGT
HDGILMTPVIEGGDVKEPLRERVLGRVTAEDVLKPGTADILVPRNTLLNEKACDLLEENSVDSVKVRS
VVSCETDFGVCANCYGRDLARGHIINKGEAIGVIAAQSIGEPGTQLTMRTFHIGGAASRAAAESSIQV
KNKGSLKLSNVKFVMNAAGKLVITSRNTELKLIDEFGRTKESYKVPYGAVMGKGDGEEVNGGETVANW
DPHTMPVISEVSGFIRFADMVDGQTITRQTDELTGLSSLVVLDSAERTGSGKDLRPALKIVDAQGEDV
LIPGTDMPAQYFLPGKAIVQLEDGIQIGAGDTLARIPQESGGTKDITGGLPRVADLFEARRPKEPAIL
AEISGIISFGKETKGKRRLVISPLDGSDAYEEMIPKWRQLNVFEGEVVERGDVVSDGPESPHDILRLR
GVHAVTRYITNEVQEVYRLQGVKINDKHIEVIVRQMLRKGTIVSAGGSEFLEGEQAEVSRVKIANRQL
EAEGKIAATFSRDLLGITKASLATESFISAASFQETTRVLTEAAVAGKRDELRGLKENVIVGRLIPAG
TGYAYHQDRMRRRAQGEAPVVPQVSAEEATANLAELLNAGLGGSDDE
>CORE_REP|Org37_Gene101#
MVYSYTEKKRIRKDFGKRPQVLDIPYLLSIQLDSFQKFIEQDPEGQYGLEAAFRSVFPIQSYSGNSEL
QYVSYRLGEPVFDVKECQIRGVTFSAPLRVKLRLVIYEREAPEGTVKDIKEQEVYMGEIPLMTENGTF
VINGTERVIVSQLHRSPGVFFDSDKGKTHSSGKVLYNARIIPYRGSWLDFEFDPKDNLFVRIDRRRKL
PATIILRALNYTTEQILDLFFDKIVFEIRDNKLQMELVPERLRGETASFDIEANGKIYVEKGRRITAR
HIRQLEKDDIQSIEVPVEYIAGKVVAKDYIDTNTGELICAANMELSLDLLAKLSQSGHKRIETLFTND
LDHGAYISETLRVDPTNDRLSSLVEIYRMMRPGEPPTREAAESLFENLFFSEDRYDLSAVGRMKFNRS
LLRDEIEGSGILSKDDIIEVMKKLIDIRNGKGEVDDIDHLGNRRIRSVGEMAENQFRVGLVRVERAVK
ERLSLGDLDTLMPQDMINAKPISAAVKEFFGSSQLSQFMDQNNPLSEITHKRRISALGPGGLTRERAG

FEVRDVHPTHYGRVCPIETPEGPNIGLINSLSVYAQTNEYGFLETPYRRVRDGVVTDEINYLSAIEEG
NFVIAQANSNLDEEGRFVEDLVTCRSKGESSLFSRDQVDYMDVSTQQVVSVGASLIPFLEHDDANRAL
MGANMQRQAVPTLRADKPLVGTGMERAVAVDSGVTAVAKRGGVIQYVDASRIVIKVNEDEMYPGEAGI
DIYNLTKYTRSNQNTCINQMPCVNLGEPIERGDVLADGPSTDLGELALGQNMRVAFMPWNGYNFEDSI
LVSERVVQEDRFTTIHIQELACVSRDTKLGPEEITADIPNVGEAALSKLDESGIVYIGAEVTGGDILV
GKVTPKGETQLTPEEKLLRAIFGEKASDVKDSSLRVPNGVSGTVIDVQVFTRDGVEKDKRALEIEEMQ
LKQAKKDLTEELQILEAGLFARIHAVLVAGGIEADKLSKLPRDRWLELGLTDEEKQNQLEQLAEQYDE
LKSDFEKKLEAKRRKITQGDDLAPGVLKIVKVYLAVKRQIQPGDKMAGRHGNKGVISKINPIEDMPYD
ENGTPVDIVLNPLGVPSRMNIGQILETHLGMAAKGIGEKINQMLKQQQEVAKLREFIQKAYDLGDDVC
QKVDLNTFSDDEVLRLAENLKKGMPIATPVFDGAKETEIKKLLEMGGIPTSGQITLFDGRTGEQFERQ
VTVGYMYMLKLNHLVDDKMHARSTGSYSLVTQQPLGGKAQFGGQRFGEMEVWALEAYGAAYTLQEMLT
VKSDDVNGRTKMYKNIVDGDHRMEPGMPESFNVLLKEIRSLGINIELEDE
>CORE_REP|Org44_Gene3968#
MGTTTMGVKLDEATRDRIKSAAQRIDRTPHWLIKQAIFNYLERLESGTDIPEIPALAAAGQPEADDIM
PQAQEESHQPFLDFAEQILPQSVTRAAITAAYRRPETEAVPMLLEQARLPADLAQATHKMAYGIAEKL
RNQKSANGRAGMVQGLLQEFSLSSQEGVALMCLAEALLRIPDKPTRDALIRDKISNGNWHSHLGRSPS
LFVNAATWGLLFTGKLVSTHNEANLSRSLNRIIGKSGEPLIRKGVDMAMRLMGEQFVTGETIAEALAN
ARKLEDKGFRYSYDMLGEAALTEADAQAYLVSYQQAIHAIGKASNGRGIYEGPGISIKLSALHPRYSR
AQYERVMEELYPRLLSLTLQARQYDIGINIDAEEADRLEISLDLLEKLCFEPQLAGWNGIGFVIQAYQ
KRCPFAIDAVIDMAQRSRRRLMIRLVKGAYWDSEIKRAQMDGLEGYPVYTRKVYTDVSYLACARKLLS
VPNLIYPQFATHNAHTLSAIYHLAGNNYYPGQYEFQCLHGMGEPLYEQVVGKVADGKLNRPCRIYAPV
GTHETLLAYLVRRLLENGANTSFVNRIADATLPLDELVADPVSAVEALAASEGQIGLPHPRIPLPREL
YGEKRTNSSGLDLSNEQRLASLSSALLTSASHPWRAEPIIDAELDQGVEQPVINPAEPGDVVGYVREA
TENEVSRALDAAAAAGPIWFATPPTERAAILERAAELMESQLQSLLGILVREAGKTFNNAIAEVREAV
DFLHYYAGQVRDDFANDSHRPLGPVVCISPWNFPLAIFTGQIAAALAAGNSVLAKPAEQTPLVAAQAV
RILLEAGIPQGVLQLLPGQGETVGSTLVNDARVRGVMFTGSTDVAGILQRSIAGRLDPQGRPTPLIAE
TGGLNAMIVDSSALTEQVVTDVVASAFDSAGQRCSALRILCIQEDVAEHTLQMLRGAMAECRMGNPER
LSTDVGPVIDADAKTGIERHIQAMRAKGRKVYQAAKGSAQDEKEWARGTFIKPTLIELDSFDELQKEI
FGPVLHVVRFQRNNLDALVDQINAAGYGLTLGIHTRIDETIARVTERAKVGNLYVNRNMVGAVVGVQP
FGGEGLSGTGPKAGGPLYLYRLLANRPDDALQRTLHRQDEERPMEATARPQLLGALQSLEKWAVTSQQ
GELAALAQRYAELGQGGTVRPLPGPTGERNTYALLPRERVLCLADNEADALIQLAAVLAVGSSALWPE
AELQRNLFRRLPNDVQARIAFSKDWQQDKVEFDAAIYHGDADQLRTLCEQIAQRGGAIVSVQGFAHGE
TNILLERLLIERSLSVNTAAAGGNASLMTIG
>CORE_REP|Org31_Gene4431#
MEILRGSPALSAFRITKLLSRCQDAHLPVSDIYAEYVHFADVSAPLSAEEHAKLQRLLKYGPSLAEHA
PEGRLLLVTPRPGTISPWSSKATDIAHNCGLQQVVRLERGLAFYVKAPELTETQWRQLAALLHDRMME
TVFSELQQAEQLFAHHQPAPYQSVDVLGAGRAALEQANVRLGLALAQDEIDYLLNAFTGLGRNPTDIE
LYMFAQANSEHCRHKIFNADWIIDGEQQPKSLFKMIKNTYEQTPDYVLSAYKDNAAVMEGSQVGRFFA
APETGTYDYHQEDAHILMKVETHNHPTAISPWPGAATGSGGEIRDEGATGRGAKPKAGLVGFSVSNLR
IPGFEQPWEQDFGKPERIVTALDIMTEGPLGGAAFNNEFGRPALLGYFRTYEERVNSHNGVELRGYHK
PIMLAGGIGNIRADHVQKGEITVGAKLVVLGGPAMNIGLGGGAASSMASGQSDADLDFASVQRDNPEM
ERRCQEVIDRCWQLGDQNPILFIHDVGAGGLSNAMPELVSDGGRGGRFELRDILNDEPGMSPLEVWCN
ESQERYVMAIAPAQMAQFDEICRRERAPYAVIGEATEEQHLTLNDRHFDNQPIDMPLDVLLGKTPKMT
RDVTRLQASGEAVQRENITLADAVKRVLHLPAVAEKTFLITIGDRTVTGMVARDQMVGPWQIPVADCA
VTTASLDSYYGEAMSLGERAPVALLDFAASGRLAVGEALTNLAATEIGSLKRVKLSANWMAAAGHPGE
DAGLYEAVKAVGEELCPALGITIPVGKDSMSMKTRWQEGNEQREMTSPLSLVITAFARVEDVRHTVTP
QLRTDKGDSALLLIDLGNGHNALGATALAQVYRQLGDKPADVRNVAQLAGFFNAMQQLVADQALLAYH
DRADGGLLVTLAEMAFAGHCGVDVNLDGLGDDALAVLFNEELGAVIQVSAERLDDVKQAFAQHGLTDN
VHHIGSVQAGDRFVITQHGKALYSESRNTLRTWWAETTWQMQRLRDNPACADQEHQAKQDEQDPGLNV
KLTFAPQEDIAAPYIAKGARPKVAVLREQGVNSHVEMAAAFHRAGFDAVDVHMSDLLAGRRDLQDFHT
LVACGGFSYGDVLGAGEGWAKSILFNERVRDEFEAFFHRPQTLALGVCNGCQMMSNLRELIPGAEHWP
RFVRNLSDRFEARFSLVEVAASPSLLLQGMTGSRMPIAVSHGEGHVEVRDAAHLAALESHGLVALRFV
NNAGQVTETYPANPNGSPNGITAVTSANGRATVMMPHPERVFRTVSNSWHPEEWGEDSPWMRMFRNAR
KQLG

>CORE_REP|Org6_Gene4143#
MTKIAPQRLEPLSLPLFGERLIEASAGTGKTFTIGALYLRLLLGLGGDAAFPRPLTVEEILVVTFTEA
ATEELRGRIRDNIHGLRIACVRGKDAECKNPLFIALMEEIDDLSDAASQLLAAERQMDEAAIYTIHGF
CQRMLTHNAFESGMLFEQTLVQDELPLRRQACADFWRRHCYPLPLGVARAVSQEWSGPEALLADLSGY
LHGEAPALRRPPKDEETVLMRHEQIVARIDAIKAQWRAEAGDLEALIAQSGVDKRSYSSKHLPNWLNK
VGEWSGQETQDYQLPKELDKFRQSVLLEKTKKGEPPRHALFTAIDQLFDEPLTLRDLIMARALSEIRT
SIQQEKRQRAELGFDDLLSRLDGALQSGGGEQLALAIRQRYPVAMIDEFQDTDPQQYRIFQKLYVGRP
DCGLLLIGDPKQAIYAFRGADIFTYMRARSEVSAHYTLETNWRSSPSMVASVNHLFSQVEKPFLFGQI
PFIEVNAAEKNQGLAFELHGKPQPAMQFWLQQGEGAGVSDYQQLMARLCATQIRDWLSAGQQGLARLQ
NGKESRPVQASDITVLVRSRNEAALVRDALSALSIPSVYLSNRDSVFDTPEAKDLLWLLQAVLAPEQE
RTLRSAMATGLMGLDAPTLDGLSRDERAWDALVNEFDNYRTLWLRRGVLPMLSELMKARQLAENLLAS
AGGERRLTDVLHLGELLQEAAAQLDSEHALVRWLAQQIAQPNRQSDNQQLRLESDRHLVQVITIHKSK
GLEFDLVWLPFVGNFRQQQQALYHDRHSFQALLDLDANEESQAWAEEERLAEDLRLLYVALTRSVYHC
SIGIAPLFQGTRKKQGDTDLHRSALGYLVQGGQAGDAVYLQERLQQLAGGGIALSLVEPPDEEPWHPQ
AALAEALAAKSFTRRIQDFWRVTSYTGLQQHGASLMQDLLPRLDVDAAGERSEESEPALTPHTFPRGA
TPGTFLHSLFETLDFTQPLDEQWLLAQLQQQGFAEHWQPILLAWMQVLLNTPLTDSGVTLAALTPQHK
QAELQFYLPINRLLQAKELDALVKRYDPLSARCPALDFHQVQGMLKGFIDLVFCWQGKYYLLDYKSNW
LGEDSSAYTRPAMEQAMAEHRYDLQYQLYTLALHRYLRHRLPDYDYRRHFGGVIYLFLRGVDTAHPGN
GIFTCLPEFELVAGMDRLFSGEAAATEDGS
>CORE_REP|Org31_Gene4064#
MITTDGNNAVASVAYRTNEVIAIYPITPSSTMAEQADAWSGDGRQNIWGDIPRVVEMQSEGGAIATVH
GALQTGALSTSFTSSQGLLLMIPTLYKLAGELTPFVLHVAARTVATHALSIFGDHSDVMAVRQTGCAM
LCAGSVQEAQDFALISQTATLNARVPFIHFFDGFRTSHEINKIVPLSDDTLRQMLPQAAIDAHRSRAL
SPDHPVVRGTSANPDTYFQSREATNPWYDATGQHVIDAMAAFAALTGRHYRPFDYYGHPQAERVVVVM
GSAAGTCEEVIDTLLTRGEKVGVLKVRLFRPFSAQHMLGALPDSVRSVAVLDRTKEPGALAEPLYLDV
MTALAEAYSRGERATLPRVIGGRYGLSSKEFGPDCALAVFRELAQPQPRPRFTVGIFDDVTGLSLPLS
DEILPQRASLEALFYGLGSDGSVSATKNNIKIIGNATPLYAQGYFVYDSKKAGGLTVSHLRVSEQPIN
SAYLVSHADFIGCHQLQFIDKYQMVERLKPGGTFLLNTPYGADEVWSRLPQEVQALLHQRQARFYIIN
AAKLARECRLGARINTVMQMAFFHLTQILPSDVALQQLQDAIARSYSSKGQEIVERNWQALGATRAAL
TEIPLQPVDVSSPMRPPVVSDAAPDFVKTVTAAMLAGLGDALPVSAFPPDGTWPVGTTQWEKRNIAEA
IPIWQPDLCTQCNHCVAACPHSAIRAKVVQPAEMEHAPASLQSLDVKARDMRGQKYVLQVAPEDCTGC
NLCVEVCPAKDRQNPEIKAINMASRLDNLTAEKDNYDFFLQLPEIDPTQLERIDIRTSQLITPLFEYS
GACSGCGETPYIKLLTQLYGDRLLIANATGCSSIYGGNLPTTPYTTNAEGRGPAWANSLFEDNAEFGL
GFRLTVDQHRARVLRLLNALAPQLPERLVNALQMGDVAPEPRRKQIAELRTLLANLEGEDARQLAAGA
DYLVDKSIWLIGGDGWAYDIGFGGLDHVLSLTENVNVLVLDTQCYSNTGGQQSKATPLGAVTKFGEHG
KRKARKDLGVSMMMYGHVYVAQISLGAQLNQTVKAIQEAEAYPGPSLIIAYSPCEEHGYDLALSHDQM
KQLTATGFWPLYRFDPRRSAEGKAALALDSRPPNSSLSETLLKEQRFRRLNAQQPEVASALYQAAEKE
LQEKYDFLSLLAGKAEKSAAE
>CORE_REP|Org29_Gene3008#
MSTSDSRNRSSSPRYSLPDRAGDLRQLGQLTGAACAVECAEIVERHPGPVMLIAPDMQNALRLRDEIQ
QFTDQMVTTLSDWETLPYDSFSPHQEIISDRLSSLYHLPTMARGVIILPVNTLMQRVCPHEFLHGHAL
VMKKGQRLSRDKLRAQLEQAGYRSVDQVMEHGEFATRGALLDLYPMGSDEPYRIDFFDDEIDSLRIFD
VDSQRTLSEVEAINLLPAHEFPTDKNAIELFRSQWREQFEVRRDAEHIYQQVSKSAWPAGIEYWQPLF
FSQPLPSLFSYLPANTLIVNTGDLESAAERFWQDVNQRYESRRVDPMRPLLAPDTLWLRVDALFGELK
AWPRIALKTDELPAKAGNTNLDYHALPDLAVQAQHKSPLDNLRRFIEGFDGSVIFSVESEGRRETLQD
LLGRIKLAPALIQRLDQAETASRYMMVGAAEHGFLDGLRQRALICESDLLGERVSRRRQDNRRTINTD
TLIRNLAELHPGQPVVHLEHGVGRYVGLTTLEAGGIKAEYLILSYAGEDKLYVPVSSLHLISRYAGGA
DENAPLHKLGGDAWTRARQKAAERVRDVAAELLDIYAQRAAKAGFAFKHNREQYQLFCQSFPFETTPD
QEQAINAVLSDMCQPLAMDRLVCGDVGFGKTEVAMRAAFLAVENGKQVAVLVPTTLLAQQHFDNFRDR
FATWPIRIEMMSRFRSAKEQQQVLDDAAEGKVDIIIGTHKLLQSDLRWKDLGLLIVDEEHRFGVRHKE
RIKAMRADVDILTLTATPIPRTLNMAMSGMRDLSIIATPPARRLAVKTFVREYDNLVVREAILREVLR
GGQVYYLYNDVENIEKAAQRLAELVPEARIAIGHGQMRERDLERVMNDFHHQRFNVLVCTTIIETGID
IPSANTIIIERADRFGLAQLHQLRGRVGRSHHQAYAYLLTPNPKAMGTDAHKRLEAIASLEDLGAGFA
LATHDLEIRGAGELLGEDQSGQMTTVGFSLYMELLESAVDALKNGREPSLEDLTSSQTEVELRMPALL

PEDFIPDVNTRLSLYKRIASAKNDGELDELKVELIDRFGQLPDAARNLLQCAALRLHAQKLGIKRIES
NERGGFIEFGDNNRVDPGYLIGLLQGNPQVYRLDGPSKLKFTLDLADRQKRLTFTEDLLDAFREHTLA
A
>CORE_REP|Org49_Gene3454#
MFTVYHSNQLDLLKTLTSALIARDPLADPFQPEVVLVQSPGMAQWLQMQLAEQFGIAANIAFPLPATF
IWDMFTRVLPDIPKESAFSKDAMTWKLMWLLPEMLTQPAFAPLQHYLTDDGDKRKIHQLAGRVADLFD
QYLVYRPQWLESWQRGERIDGLAEAQQWQAPLWARLVEYTRELGQPEWHRANLYSRFIHALEQAKTCP
PGLPPRVFICGISALPPVYLEALQALGRHIDIHLMFTNPCRYYWGDIQDYAFLARLQSRKRRHYHQAR
EHGLFRQPDDAARLFDAEGQQQLSNPLLASWGKLGRDHLYLLSQMEGAQEVDAFVDIPADTMLHAVQR
DMLELEDHAVIGITAETLESSFSKRPLDENDRSLSLHACHSPQREVEVLHDQLLTMLAQDPSLTPRDI
IVMVADIDSYTPYIQAVFGNAPAERYLPFAISDRKARQAHPALQAFISLLDLPQSRFTSEQVLALLEV
PALAARFAIGEEGLRLLRHWVGESGVRWGLDDDNVRELDLPATGQHTWRFGITRMLLGYAMDSNAGDW
QGILPYDESSGLVAELAGQLADLLAQLSHWRQILSEARPLEAWLPLCRQLLDAFFAADSDTEVVLALI
EQQWQQAINFGLAARYPDEVPLTILRDDLAARLDQERISQRFLAGQINFCTLMPMRSIPFKVVCLLGM
NDGVYPRTLPPLGFDLMAQQVKRGDRSRRDDDRYLFLEAILSAQQRLYISFIGRSIQDNSPRYPSVLV
TELLEYLEQSYCLPGDEELSADDSARRVGEHLLKWHARMPFAAENFLPGSEEQSYAAEWLPAADGRGA
AHPAFNQPLPAEALQQISLEELLRFYRHPIRAFFQLRLGVSFILEETELPDEEPFTLDNLSRYQFNSQ
LLNTLIDGDDPERLFQRVRAAGGLPYGAFGEIYWQKQQEEMSELAEQVRAERAESHSLELDIDIAGVR
LSGWLHQVQDDGLLRWRPATLSAVDGILLWLEHLVYCCAGGTGESRMYGRKNSAWRFAALAPEEAQAQ
LAELLTGYQRGLSQPLLLLNKSGWAWLSQCYLPETQQIDWEEEAQIKARAKLLQAWQGDQRIPGEGED
PYVQRVFRQLDNEYLAQILAETERYLLPVARHNLG
>CORE_REP|Org9_Gene3622#
MPKRTDIKSILILGAGPIVIGQACEFDYSGAQACKALREEGYRVILVNSNPATIMTDPEMADATYIEP
IHWEVVRKIIEKERPDAVLPTMGGQTALNCALELERQGVLAEFGVTMIGATADAIDKAEDRRRFDVAM
KKIGLDTARSGIAHTMEEALAVAADVGFPCIIRPSFTMGGTGGGIAYNREEFEEICERGLDLSPTNEL
LIDESLIGWKEYEMEVVRDKNDNCIIVCSIENFDAMGIHTGDSITVAPAQTLTDKEYQIMRNASMAVL
REIGVETGGSNVQFSVNPKTGRLIVIEMNPRVSRSSALASKATGFPIAKIAAKLAVGYTLDELMNDIT
GGRTPASFEPSIDYVVTKIPRFNFEKFAGANDRLTTQMKSVGEVMAIGRTQQESLQKALRGLEVGATG
FDPKVSLDDPEALTKIRRELKDAGSDRIWYIADAFRAGLSVDGVFNLTNIDRWFLVQIEELVRLEEQV
ADAGINGLNKAFLRTLKRKGFADARLAKLAGVAESEIRKLRHSHGLHPVYKRVDTCAAEFATDTAYMY
STYEEECESNPTNDRPKVMVLGGGPNRIGQGIEFDYCCVHASLALREDGYETIMVNCNPETVSTDYDT
SDRLYFEPVTLEDVLEIVRIEKPKGVIVQYGGQTPLKLARELEAAGVPIIGTSPDAIDRAEDRERFQQ
AVNRLGLKQPANATVTAIEQAVEKAAGIGYPLVVRPSYVLGGRAMEIVYDETDLRRYFQTAVSVSNDA
PVLLDRFLDDAVEVDVDAICDGERVLIGGIMEHIEQAGVHSGDSACSLPAYTLSQEIQDVMRRQVEKL
AFELQVRGLMNVQFAVKDNEVYLIEVNPRAARTVPFVSKATGVPLAKVAARVMAGKTLVEQGVTEEII
PPYYSVKEVVLPFNKFPGVDPILGPEMRSTGEVMGVGRTFAEAFSKAMLGSNSGMKKQGRALLSVREG
DKARVVDLAASLLKQGFELDATHGTAVVLGEAGINPRLVNKVHEGRPHIQDRIKNGEYTYIVNTTAGR
QAIEDSKLIRRSALQYKVHYDTTLNGGFATAMALKADPTEQVTSVQEMHARIGK
>CORE_REP|Org10_Gene3917#
MTFPRFFIKRPIFAIVLSILTLIAGIVALFQLPLSEYPAVTPPTVQVTASYPGANPNVIAETVAAPLE
QAITGVEGMLYMSSQAATDGRMTLTVTFAQGTNADMAQIQVQNRVARALPRLPAEVQHQGVVTQKTSP
DILMVVHLLSPDQRYDPLYISNYAYLQVRDELSRIPGVSDVQVWGAGEYSMRLWLDPDLIAARGLTAG
DVIAAVREQNVQVAAGSVGQAPDTNAAFQVTVNTLGRLADEKQFGDIIIRTGSDGQVTRLRDVARIDM
GADAYALRSLLDGEPAVALQIIQSPGANALDVAQAVRATVKRLEGDFPAGLSSRIAYDPTVFVRASLE
SVVTTLLEAILLVVIVVVVFLRSWRASLIPLLAVPVSLVGTFAIMHLMGFSLNTLSLFGLVLSIGIVV
DDAIVVVENVERHIENGKTPQQAARLAMDEVTGPIVAITSVLAAVFIPTAFLSGLQGEFYRQFALTIA
ISTLLSALNSLTLSPALAGLLLRPHPAEHRAPGRIQRILQAAVRPFQRAPDAYANAVRKTVRVSGVAL
AIYGGLLVLTFFGFQAVPPGFVPMQDKYYLVGIAQLPNSASLDRTDAVVKQMSKIALAEPGVESVVAF
PGLSINGFVNVPNAAVMFVMLDPFKERATPDLAASAIAGRLQAKFADIPDGFLGVFPPPPVPGLGATG
GFKMQVEDRGGVGLESLVEHTRLLMVKATESGQVAGLMTSLDINAPQLDVVIDRTQAKSQGVSLADVF
ESLQIYLGSLYINDFNRFGRTYKVTAQADADHRMQAEAIGRLQVRNAAGDMLPLSSFVTVTPGSGPDR
IIRYNGYPSADISGGAAIGVSSGQAVALMEQLAKETLPEGMTVEWTDLTYQQKLAGNAALFIFPLCVL
LAYLILATQYNSWLLPLAVLLIVPMCLLSAMIGVWLLGGDNNVFVQIGLIVLVGLAAKNAILIVEFAR
GLEDEGANTLEAVIKACRLRLRPIVMTSIAFIAGVIPLIFASGAGAEMRHAMGVAVFAGMLGVTLFGL

FLTPVFYVVIRGLTARFEQYRTKGKANVRSESDEKSS
>CORE_REP|Org40_Gene1002#
MAKFFIDRPIFAWVIAIIVMLAGVLAIMKLPIAQYPTIAPPAVSISANYPGADAKTVQDTVTQIIEQN
MNGIDNLMYMSSTSDSSGSVTITLTFDSGTDPDIAQVQVQNKLSLATPLLPQEVQQQGLKVEKSSSSF
LMVAGFVSDDPNMTQDDIADYVASNIKDPISRSSGVGEVQLFGAQYAMRIWLDPNKLNNFQLTTTDVT
SAITEQNNQIAAGQLGGLPPVPGQQLNASIIAQTRLTSPEEFGKILLKVNTDGSQVRLRDVAHIERGA
ESYAVTARYNGKPAAGLGIKLATGANALNTAKGVKDELAKMAPFFPQGMKVVYPYDTTPFVKISINEV
VKTLIEAIILVFLVMYLFLQNFRATLIPTIAVPVVLLGTFAILAAFGFSINTLTMFGMVLAIGLLVDD
AIVVVENVERVMSEEGLPPKEATRKSMGQIQGALVGIAMVLSAVFVPMAFFGGSTGAIYRQFSITIVS
AMALSVLVALILTPALCATLLKPIPKGDHGVKTGFFGWFNRMFEKSTHHYTDSVGNILRSTGRYLIIY
LLIVVGMGLLFLRLPSSFLPDEDQGILLTMVQLPAGATESRTNKVLEEVSDYFLNKEKDNVVSVFTVA
GFGFNGNGQNNGLAFVSLKDWGERPGAGNKVEAIAGRAMGAFSQIKEGLVFPFNLPAIIELGTATGFD
FELIDQGGLGHEKLTEARNQLLGMVAQHPDVLVGVRPNGLEDTPQFKLIVDQEKAKALGVSITTINST
LSTALGGSYVNDFIDRGRVKKVYVQADAPFRMLPEDINKWYVRGTSGQMVPFSAFSSAKWEYGSPRLE
RYNGLPSMEILGQAAPGKSTGEAMNLMEQLASKLPSGIGYDWTGMSYQERLSGNQAPALYAISILVVF
LCLAALYESWSVPFSVMLVLPLGVIGALLAATMRGMNNDVYFQVGLLTTIGLSAKNAILIVEFAKDLM
EKEGKGLIEATLEAVRMRLRPILMTSLAFILGVLPLVISSGAGSGAQNAVGTGVMGGMITATVLAIFF
VPVFFVVVRRRFSKKNEDLEHSHPVEHH
>CORE_REP|Org28_Gene4189#
MQVMPPNAGGGPSRLFILRPVATTLLMVAILLAGIIGYRALPVSALPEVDYPTIQVVTLYPGASPDVV
TSAITAPLERQFGQMSGLKQMASQSSGGASVVTLQFQLALPLDVAEQEVQAAINAATNLLPSDLPYPP
IYSKVNPADPPILTLAVTSTAMPMTQVEDMVETRVAQKISQVTGVGLVTLAGGQRPAVRVKLNAAAVA
AYGLNSETIRAAISNANVNSAKGSLDGPTRSVTLSANDQMKSADDYRQLIVAYQNGAAIRLQDIATIE
QGAENTRLAAWANKQQAIVLNIQRQPGVNVITTADSIREMLPTLIKSLPKSVDVKVLTDRTTTIRASV
SDVQFELLLAIALVVMVIYVFLRNVPATIIPSVAVPLSLVGTFAAMYFLGFSINNLTLMALTIATGFV
VDDAIVVIENISRYIEKGEKPLDAALKGAGEIGFTIISLTFSLVAVLIPLLFMGDIVGRLFREFAVTL
AVAILISAVVSLTLTPMMCARMLSHESLRKQNRFSAASERFFDRVIAQYGQWLKTVLNHPWLTLGVAV
GTLALTVLLYLLIPKGFFPVQDNGIIQGTLEAPQSVSFSNMAERQQQVAAQILKDPAVESLTSFVGVD
GSNATLNSGRLQINLKPLSERSDRIPAIISRLQQQTAQFPGVKLYLQPVQDLTIDTQVSRTQYQFTLQ
AMSLDDLSLWVPQLMNELKQTPQLADVTSDWQDQGLVAYVNVDRDSASRLGVTMSDVDNALYNAFGQR
LISTIYTQANQYRVVLEHDVSATPGLAALNEIRLSGNDGAVVPLSAIAKIEERFGPLSVNHLDQFPSA
TVSFNVADGYSLGEAVDAVTQAEKNLNMPRDITTQFQGATLAFQAALGSTLWLILAAVVAMYIVLGVL
YESFIHPVTILSTLPTAGVGALLALMLAGSELDVIAIIGIILLIGIVKKNAIMMIDFALAAEREQGLS
ARDAIYQACLLRFRPILMTTLAALLGALPLMLSTGVGAELRHPLGVCMVGGLIMSQILTLFTTPVIYL
LFDKLARNTHRQPDTQELP
>CORE_REP|Org36_Gene4024#
MIPQISQAPGLVQRVLDFLEALKQNGFNGDTATSYADRLTMATDNSIYQLLPDAVVFPRSTADVALIA
RLAGEERFKTLTFSPRGGGTGTNGQSLNTGIVVDMSRHMNRILEINVEQGWVKVEAGVIKDQLNQYLR
PFGYFFSPELSTSNRATLGGMINTDASGQGSLVYGKTSDHVLGLRAVLLGGEMIDTRAMPTALAETIA
LEETAEGRIYRTVLSRCREQRALILEKFPKLNRFLTGYDLRHVLSDDLQTFDLTRILTGAEGTLAFIT
EARLDITPLPKVRRLVNVKYDSFDSALRNAPFMVEAKALSVETIDSKVLNLAREDIVWHSVNELIADV
PDKEMLGLNIVEFAGDDRALIDGQMETLCQRLDELIAQRQGGVIGYQICGDLAGIERIYNMRKKAVGL
LGNAKGRAKPIPFAEDTCVPPQHLADYIVEFRQLLDDHHLSYGMFGHVDAGVLHVRPALDMCDPQQEV
LMKQISDRVVALTAKYGGLLWGEHGKGFRAEYSPEFFGETLYEELRRIKAAFDPDNRLNPGKICSPLA
VDAPMMQVDAVKRGTFDRQIPVEVRTSFRGALECNGNGLCFNFDVRSPMCPSMKISGNRIHSPKGRAT
LVREWLRLLAEQGVDPLALEKQLPQQRVSLRGLIEKTRNSWHAGKGEYDFSHEVKEAMSGCLACKACS
TQCPIKIDVPGFRSRFLQLYHTRYLRPVSDYMVAGVESYTPLMARAPKVFNFFFRQPWLREMSRNAIG
MVDLPLLSSPTLRQQLSGHRATTLTLEQLEGLSAEQRADHVLIVQDPFTSYYDAKVVADFVRLVEKLG
YQPVLLPFSPNGKAQHVKGFLTRFARTARKTADFLNRVAQLGMPLVGVDPALVLCYRDEYREILGAER
GDFQVQLVHEWLQQRIADRAEQPATGEPWYLFGHCTETTALPASGQQWAAIFARFGAKLENVSVGCCG
MAGTYGHEAKNLQNSLGIYELSWHPTLQRLPRQRCLATGYSCRSQVKRIEGNGVRHPLQALLEMIE
>CORE_REP|Org10_Gene2662#
MRRQLARIAGLVLLAMCWAPLSWAAQGWQPLAEKINKSEHDPRQYEAIKLANGMTVLLVSDAQAPKSL
AALALPVGSLEDPNSQLGLAHYLEHMVLMGSKRYPEPENLSEFLKKHGGSHNASTASYRTAFYLEVEN

DALEPAVDRMADAIAEPLLDPGNADRERNAVNAELTMARSRDGMRMAQVGAETLNPAHPSARFSGGNL
DTLKDKPDSKLHDELTGFYKRYYSANLMMGVLYGNQPLPQLADIAAETFGRVPNHDASVPPITVPAVT
PEQQGIIIHYVPAQPRKQLKVEFRIDNNSAAFRSKTDTYISYLIGNRSKNTLSDWLQKQGLADAINAG
ADPMVDRNGGVFAISVSLTDKGLAKRDEVVAAIFNYLKMLRSEGIKQSYFDEISHVLNLDFRYPSITR
DMDYIEWLVDTMLRVPVEHALDAPYLADRYDPKAIAERLDAMTPQNARIWFVSPDEPHNKTAYFVNAP
YQVDKITPQHFTQWQQLGSGIALSLPALNPYIPDDFTLTKPSHEFKKPEMVVDKPGLRVLYMPSRYFA
DEPKADVTVAFRNAKTMDSARNQVLFSLTDYLAGLALDQLSYQASVGGLSFSTSPNNGLMFNANGFTQ
RLPQLLTALIEGYSSFTPTEDQLAQAKSWYLEQLDAAEKGKAFELAIQPVQMVSRVPYSERSERREVL
KTLTLKDVLAYRDSLLAEATPELLVVGNMSKQQVDTLASTLKHRLGCTGIEWWHGEDVVVDKNQLANL
QQVGSSTDSALAAVYVPTGYDEVTGMAYSSLLGQIIQPWFYSQLRTQEQLGYAVFAFPMSVGRQWGVG
FLLQSNSKQPAYLYQRYQDFYPKTEKRLRDMSEADFEQYKQALINELKQRPQTLSEEASRFANDFDRG
NFAFDTRQKLIAQVQQLTPAKLADYFHQAVIQPQGLAVLSQVSGSGQDKADYAAPKDWVTYPNASALQ
QILPRKVATP
>CORE_REP|Org10_Gene3237#
MEKTNSQLDTAYDPKQIEQKLYDHWENQGYFKPNGDTSQESFCIMIPPPNVTGSLHMGHAFQQTIMDT
MIRYQRMQGKNTLWQAGTDHAGIATQMVVERKIAAEEGKTRHDYGRDAFIDKIWQWKAESGGTITRQM
RRLGNSVDWERERFTMDEGLSNAVREVFVRLHKEDLIYRGKRLVNWDPKLRTAISDLEVENRESKGSM
WHLRYPLADGAKTAEGKDYLVVATTRPETVLGDTGVAVNPEDPRYKDLIGKEIILPLVGRRIRIVGDE
HADMEKGTGCVKITPAHDFNDYEVGKRHGLPMINILTFDGDIRQEAEVFNTLGEVCTDYCNEIPAEFR
GLERFAARKAVVAAFDQLGLLDEVKPHDLTVPYGDRGGVVIEPMLTDQWYVRTAPLAKVAVEAVEQGD
IQFVPKQYENMYFSWMRDIQDWCISRQLWWGHRIPAWYDVNGKVYVGRSEEEVRSENNLGADVVLTQD
EDVLDTWFSSGLWTFSTLGWPEQTEALKTFHPTSVMVSGFDIIFFWIARMIMLTMHFIKDENGKPQVP
FKTVYMTGLIRDDEGQKMSKSKGNVIDPLDMVDGISLEDLLEKRTGNMMQPQLAEKIRKRTEKQFPNG
IEPHGTDALRFTLAALASTGRDINWDMKRLEGYRNFCNKLWNASRFVLMNTEAHDCGFNGGEKVLSLA
DRWILAEFNRTVKAYREALDTYRFDLAANILYEFTWNQFCDWYLELTKPVVSNGSEAEQRGTRHTLIT
VLEALLRLAHPIIPFITETIWQRVKPLTGTTADTIMLQPFPAYDAALEDEQALNDLEWIKQTIIAVRN
IRAEMNIAPSKALDVLLRNCSADAQRRVQENQSFIARLARLESIALLPAGEKGPVSVTKLVDGAELLI
PMAGFIDKDAEIARLAKEMGKLDAEIASIEGKLANEGFVARAPEAVVAKERDRLAACKEGKVKLQEQQ
ATIAAL
>CORE_REP|Org14_Gene4616#
MKYLASFRTTLKISRYLFRVLAILLWSLGALLTTFYILNILHQKESDIRQEYNLNFDQAQGYIRHSAD
IIRDIKYMAENRLNGSVSGLDMFSGVIPGKGSPPQFFPLYPESNCALSTTYRSSLDSLSGLIQYWKEN
FVAAYDLNRVFFIGGDSLCMAEFGGGNASANRENMLKLLHERILKYRNAKNLDKDNNLYWISPSAQRP
DVGYLYVLTPLYIGNKLEALLGIEQTVRLEDFVTAGNLPIGVTLLDENNEPVLRLADGERYAAALNSY
PEEHAYFGYVDNYRDLILKKALPPSSLSIVYALPVKSVVERFKMLILNALLLNLLSAIVLFTLAWLFE
RKMFLPAEDNAFRLEEHEQFNRKIVASAPVGICILRISDGTNILSNELAHNYINLLTHEDRDRITRII
CEQQANFVDVMTSNNNNLQISFVHSRYRNEEVAICVLVDVSARVKMEESLQEMAAAAEQASQSKSMFL
ATVSHELRTPLYGIIGNLDLLQTKALPQGVDRLVNAMNNSSGLLLKIISDILDFSKIESEQLKIEPRE
FSCLEVITHIAGNYLPLVVKKRLGLYCFIEQNVPERIFGDPVRLQQVLSNLVNNAIKFTDTGCIVLQV
CTRGSYLEFSVRDTGVGIPEKEISRLFDPFFQVGTGVQRHFQGTGLGLAICEKLVNLMDGDVSVESEP
GLGSLFSIRIPLFNAQFPIPQASDTWQGRRLWLDIRNQRLESYLMAILGGYGADIQRYDGQETAAGEV
LLSDHPLMLDAPLLAQIQFSTEHIGPSQETRPGYWMHSTSTPRETLTLLNRLFGVGVGSGAAEALVQL
PVPAKASAADNGDIHLLVVDDHPINRRLLSDQLGSLGYQVVTANDGVDAPGVLKQHRVDIVLTDVNMP
NMDGYRLTQALRQMQFSAPVIGVTANALAEEKQRCLEAGMDNCLSKPVTLETLEQTLAYYSQQVRYSR
SEA
>CORE_REP|Org2_Gene3732#
MSPLSAALQAQAQQVVQRFQEVHGADSAFSEQEQWVLASSDFVSDALLAQPAWLATLREQPPAPGEWQ
HYAAWLQDELEEVRDEAQLMRTLRLFRRETLVRIAWAQAQGLCSTEETLLQLSGLAETLIVSARDWLY
QTCCREWGTPCNAAGEPQPLLILGMGKLGGGELNFSSDIDLIFAYPENGQTQGGRRELDNAQFFTRLG
QRLIKALDQQTIDGFVYRVDMRLRPFGDSGPLVMSFAALEDYYQEQGRDWERYAMVKARLMGGAEDAY
SQELRKTLRPFVFRRYIDFSVIQSLRNMKGMIAREVRRRGLKDNIKLGAGGIREIEFITQVFQLIRGG
REPALQGRSLLPTLQAVGELGLLEAEQVRALSAAYLFLRRLENLLQAIGDQQTQTLPQDALDQARLAY
GMGLADWPALMATLEVHMQAVRAVFDDLIGDDSPDVGEDPDYQHYHSLWQDALEENELAPLTPHLDEE
GRRQMLRTIADFRHDVDKRTIGPRGRDVLDQLMPRLLAEVCPRQDAPTALVRLAQLLLSIVTRTTYLE

LLVEYHAALSHLIRLCAASPMVANQLSRYPLLLDELLDPATLYQPVALDAYRSELRQYLLRVPEDDEE
QKLEALRQFKQAQQLRIAAADIAGALPVMKVSDHLTYLAEAIIDAVVQQAWSDMVARYGQPTHLQERE
GRGFAVIGYGKLGGWELGYSSDLDLVFLLDCPPEVMTDGDRCIDGRQFYLRLAQRVMHLFSTRTSSGI
LYEVDARLRPSGAAGMLVSTVEAFADYQQNEAWTWEHQALVRARIVHGDPALHQQFDAIRREILCKTR
DAETLKREVREMREKMRNHLGNKQRDLFDIKTDEGGITDIEFIAQYLVLRYAPGEPRLTRWSDNVRIF
ELMANYVIMPEEEARALTQAYVTMRDEIHHLALQEHSGKVGSELFTAEREQVRASWAKWLD
>CORE_REP|Org7_Gene4479#
MDNIEVRGARTHNLKNINLIIPRDKLIVVTGLSGSGKSSLAFDTLYAEGQRRYVESLSAYARQFLSLM
EKPDVDHIEGLSPAISIEQKSTSHNPRSTVGTITEIHDYLRLLFARVGEPRCPDHHVPLAAQTVSQMV
DNVLSQPEGKRLMLLAPVVKDRKGEHTKTLENLSAQGYIRARIDGEVCDLSDPPKLELQKKHTIEVVV
DRFKVRDDMAQRLAESFETALELSGGTAVVADMDDEKADELLFSANFACPICGYSMRELEPRLFSFNN
PAGACPTCDGLGVQQFFDPDRVVQNPELSLAGGAIRGWDRRNFYYFQMLRSLAEHYEFDVEAPFNTLS
ANVQKAVLSGSGKESIEFKYINDRGDTTVRRHPFEGVLHNMERRYKETESSAVREELAKFISNRPCAS
CHGTRLREEARNVFVEDTTLPEISDLSIGHAMTFFQNMKLSGQRAKIAEKVLKEIGDRLKFLVNVGLN
YLSLSRSAETLSGGEAQRIRLASQIGAGLVGVMYVLDEPSIGLHQRDNERLLETLIHLRNLGNTVIVV
EHDEDAIRAADHVIDIGPGAGVHGGQVVAEGTVDDIMAQPESLTGQFLSGKREIAIPAQRVQADPTKV
LKLSGARGNNLKDVTLTLPVGLFTCITGVSGSGKSTLINDTLFPIAQRQLNGATIAEPAPFREVTGLE
HFDKVIDIDQSPIGRTPRSNPATYTGIFTPVRELFAGVPESRSRGYTPGRFSFNVKGGRCEACQGDGV
IKVEMHFLPDIYVPCDQCKGKRYNRETLEVKYKGKSIHEVLEMTIEEARDFFDAVPALARKLQTLMDV
GLSYIRLGQSATTLSGGEAQRVKLARELSKRGTGQTLYILDEPTTGLHFADIQQLLAVLHQLRDQGNT
IVVIEHNLDVIKTADWIVDLGPEGGSGGGEILVAGTPETVAECEKSHTARFLKPLLEK
>CORE_REP|Org44_Gene2039#
MSDYKNTLNLPETGFPMRGDLAKREPGMLQRWYEQDLYGIIRTAKKGKKTFILHDGPPYANGSIHIGH
SVNKILKDIIIKSKGMAGFDSPYVPGWDCHGLPIELKVEQLYGKPGEKLTAAEFRQKCREYAAEQVEG
QKKDFIRLGVLGDWDRPYLTMDFKTEANIIRALGKIISNGHLLKGAKPVHWCTDCGSSLAEAEVEYYD
KTSPSIDVTFHAADAAAVAAKFGVSNFSGAISLVIWTTTPWTLPANRAISLHPDFTYQLVQVDGQCLI
LAAELVESVMKRAGITEWTVLGSCKGADLELLRFKHPFMGFDVPAIMGEHVTLDAGTGAVHTAGGHGP
DDFVISQKYGLEIANPVGPNGCYLTGTHPLLDGKFVFKANDLIVDLLREKGALLHVEKFLHSYPCCWR
HKTPIIFRATPQWFISMDQKGLRQQSLEEIKGVQWIPDWGQARIEMMVANRPDWCISRQRTWGVPMSL
FVHKETEQLHPRSVELMEEVAKRVEQDGIQAWWDLDAADILGADAADYVKVPDTLDVWFDSGSTHASV
VDVRPEFHGHSADMYLEGSDQHRGWFMSSLMISTAMKGKAPYKEVLTHGFTVDGQGRKMSKSIGNTVS
PQDVMNKLGGDILRLWVASTDYTGEIAVSDEILKRSADSYRRIRNTARFLLANLNGFEPSTDCVAPED
MVVLDRWAVGRALAAQQDIEQAYANYDFHEVVQRLMQFCSVEMGSFYLDIIKDRQYTAKSDSVARRSC
QTALYHIVEALVRWMAPIMSFTADEIWGFMPGKRAQYVFTEEWYDGLFGLAEGEPMNDAFWAELLKVR
GEVNKVLEQARADKRLGGSLEAAVTLYADSELAARLNSLQDELRFVLLTSAASVAPLAEAPADAQASE
LLKGLKIAFSTAPGEKCPRCWHYTTDIGLVAEHADICGRCVSNVAGDGEKRNFA
>CORE_REP|Org13_Gene2599#
MQNGAMKAWLDSSYLAGANQSYIEQLYEDFLTDPGSVEDSWRSIFQQLPTAGVKPDQLHSQTRDYFRR
LAKDSARYNTTITDPETDAKQVKVLQLINAFRFRGHQHANLDPLGLWQREQVPDLDPAYHNLTEADFQ
ETFNVGSFAIGKETMKLGDLYAALKQTYCGSIGAEYMHITNTEEKRWIQQRIESVVGRASFTADEKRR
FLNELTAAEGLERYLGAKFPGAKRFSLEGGDALVPMLKEMVRHAGKNGTREVVLGMAHRGRLNVLINV
LGKKPADLFDEFAGKHKEHLGTGDVKYHQGFSSDVETEGGMVHLALAFNPSHLEIVSPVVMGSVRARR
DRLDEARSNMVLPITIHGDAAITGQGVVQETLNMSQARGYEVGGTVRIVINNQVGFTTSNPLDARSTE
YCTDIAKMVQSPIFHVNADDPEAVAFVTRLALDFRNTFKRDVMIDLVCYRRHGHNEADEPSATQPVMY
QKIKKHPTPRKIYADVLTEQKVASLEDATEMVNLYRDALDRGDCVVEEWRPMNLHSFTWSPYLNHEWD
EEYPSKVEMKRLQELGRRISTVPEAIEMQSRVAKIYADRAEMAAGNKPFDWGAAETLAYATMADEGIP
IRISGEDAGRGTFFHRHAVVHNQKNGSVYVPLANIHSGQGEFKVWDSVLSEEAVLAFEYGYATAEPRT
LTIWEAQFGDFANGAQVVIDQFISSGEQKWGRMCGLVMLLPHGYEGQGPEHSSARLERYLQLCAEQNM
QVCVPSTPAQVYHMLRRQALRGMRRPLVVMSPKSLLRHPLAISSLDELANGTFLPAIGEIDDLDPKAV
KRVVLCSGKVYYDLLEQRRKNEQKDVAIVRIEQLYPFPHQAVQAVLEQYSHVHDFVWCQEEPLNQGAW
YCSQHNFREVVPFGASLRYAGRPASASPAVGYMSVHQKQQQTLVNDALNID
>CORE_REP|Org14_Gene3864#
MAQIAENPLILVDGSSYLYRAYHAFPPLTNSAGEPTGAMYGVLNMLRSLLLQYQPSHVAVVFDAKGKT
FRDDLFAEYKSHRPPMPDDLRAQIEPLHNMVKAMGLPLLVTPGVEADDVIGTLALEAEKAGHAVLIST

GDKDMAQLVTPNVTLINTMNNTILGPQEVCDKYGIPPELIIDFLALMGDSSDNIPGVPGVGEKTAQAL
LQGIGGLDALYGNLENIATLSFRGAKTMAAKLEQNKEVAYLSYKLATIKTDVELDLTCADLTVSAPDV
DTLQQLFKQYEFKRWLADVEAGVWLENKKGAGAKAAGGAKPAAAAAEAPKALAEAKLSQDGYVTILDE
ATFTDWLARLKKADVFAFDTETDGLDTLTANLIGLSFAIAPGEAAYLPVAHDYLDAPPQLDRAYVLEA
LKPLLEDDKALKVGQNLKFDMSLLARYGIEMRGIAYDTMLESYVLDSVGGRHDMDSLADRYLSHKTIT
FEEIAGKGKNQLTFNQIALEQAAPYAAEDADVTLQLHLAMWPQLKQSAELLTVFNEIEMPLLPVLSHI
ERTGVLIDPAILSAHSQELAKRLAELEAQAHELAEEPFNLASTKQLQAILYEKQKLPVLKKTPGGAPS
TNEEVLAELALDYPLPKVILEYRGLAKLKTTYTDKLPLMINPVSGRVHTSYHQAVTATGRLSSSDPNL
QNIPVRNEEGRRIRQAFIAPEGYRIVAADYSQIELRIMAHLSQDEGLLKAFAEGKDIHRATASEVFGV
PLDKVTGEQRRSAKAINFGLIYGMSAFGLARQLGIPRGEAQRYMDLYFERYPGVLDYMERTRQQASEQ
GYVSTLDGRRLYLPDVRSSNAMRRKAAERAAINAPMQGTAADIIKRAMIEVDAWLQGQEKPLVRAIMQ
VHDELVFEVHESVIEEASQRIRQLMEGSMTLAVPLKVDVGVGMNWDEAH
>CORE_REP|Org28_Gene3973#
MSNFNPSRRRFIKSAVIAGVSVYLAPLYSRAYAALFEQKILQSPNWDPQTKRVRFRIDGRAKVMGQKV
FARDIRAVDMPHWPQKQAHAFILRVTKADRLFEGVDLSLLGDDLQPDRLVTAEDLARDGLAFPAFYGD
DMLLPSGKTPAYLGQAVAILIYHDFARFRFAKDKLKFREETIKYGAVTGPLERDPWGSFRYVRVGGDQ
PFDDDRFSSLKDTPIFPVSMKKHLPVWPEGREGGKLDQEGMRYAGLIADEMANPPADWLVMSRRYTTQ
SIDTSALEPDNANGWFDAETQTLHLVVPTQSPQEVADEMPRMLAKRNPPVKQLILHPCYTVGYGSKDH
YNFPYYGAVAAMYGDGHPVRLANDRFEQFQTALKRHAFDMNYRIAVNRQTGVMQSFLGDMTADGGGRS
NFTPSVVMVGATAAQSIYYFPKSDLSSVGLASRAIDAGSARGYGTLQSMAATEMMVDELAAELKIDPI
EFRLRNVLKSGMKNTQGAIPAGAIRADEVLEKAAKHEMWLKRAERKAEFESRHPGKRYGVGFGCVQKD
FGTGAETSFARVELSEDGRITLHHSGAEMGTGMSTSQSVLCAQWLGKPADEAHFSVTDWSVLPMVTSG
DPYLMSQEEQDKLQTNPNWTPSYCSPSSASNSAYYFSHSTREAARLIFDHGLWPAAMALWQAGIGGGQ
AAPLVVRREDARWVEGGLTAAGMSVLSLELLAKTAYQMGGVTGAAVHVFNRWQWAEADFTLNGKSERL
PIDGMALRNAGGEFKPLARGQVYYPPTQRNNAAVTYYSAVGTLAEVAVDIATGQVELLNHHSIMECGN
LIVPELVSGQLQGGLAMGIGHALHEYLPLYEDGPGNGTWNFNRYHLPRASDVAVWKQSGDILPALSET
DPPKGMAEVVMIPIVAALVNAIADATGHRFRDLPVRAENIREVLQ
>CORE_REP|Org2_Gene3277#
MTKYSLRARMMILILAPTLLIGLLLSTFFVVHRYNELQEQLVDAGASIIEPLAVASEYGMTFRSRESV
RQLVSLLHRRHSDIVRSITVFDAQNNLFVTSNYHHNFAQLQLPKGVPLPTELMLTRRGDSLILRTPIL
SESQYPDETADGGSHPDNNLGYVAIELDLQSVRLQQYKEVFVSTLLLLLCMCIAILFAYRLMRDVTGP
IRNMVNTVDRIRRGQLDSRVEGYMLGELHMLKNGINSMAMSLTAYHEEMQQNIDQATSDLRETLEQME
IQNVELDLAKKRAQEAARIKSEFLANMSHELRTPLNGVIGFTRQMLKTDLSATQTDYLQTIERSANNL
LTIINDVLDFSKLEAGKLVLEHIPFALRETLDEVVVLLAPSAHDKGLELTLDVHNDVPEQVIGDSLRL
QQIITNLLGNAIKFTETGNIDIRVELRKQLDRRVEVEVQIHDTGIGISERQQSQLFQAFRQADASISR
RHGGTGLGLVITQKLVKEMGGDICFHSQLNRGSTFWFHITLDLNEGMLSLAPSLPDLSGKTLAYIESN
PTAAQATLNMLSITQLVITHSPTLGQLPPGHYDFLLAGVPIPFRDNMAQHEDKLLASLKLADRVILAL
PCQAQIDAELLKQQGALGCLIKPITSTRLFPLLRMEAPARLTAQPERKRLPLTVMAVDDNPANLKLIG
TLLGEQVEKTLLCESGEEALALARDNVLDLILMDIQMPKMDGIHASELIRQLPHHNSTPIVAVTAHAA
SGEREHLLQAGMDDYLAKPIDEKMLTRVLSRYHSGDVENAIADDAPLSLDWPLALRQAANKPDLARDL
LQMLLDFLPQVRERVQALLDGQHDDEILDLVHKLHGSCSYSGVPRLKQLCFYLERQLRQGVTNDELEP
EWLELLDEIELVIHAARAHLTQPA
>CORE_REP|Org38_Gene4841#
MLIPSKLSRPVRLQNTVIRDRLLAKLASAGNYRLTLVNCPAGYGKTTLIAQWAAGKADLGWYSLDESD
NQPERFASYLIAALQQASGGRCVKSEALSQKHQYASLSALFAQLFIELADWHQPLYLVIDDYHLITND
AIHEAMRFFLRHQPENLTLILLSRTLPPLGIANLRVRDQLLEMGTQQLAFTHQEAKQFFDCRLAAPME
QQDSSRLCDEVEGWATALQLIALSARQSASSAQLSAKRLAGLNASHLSDYLVDEVLDHVDADARAFLL
RCSVLRSMNDALIVRLTGEDNGQQRLEELERQGLFIHRMDDTGEWFNFHPLFASFLRQRCQWELALEL
PGLHRAAAEGWLALGYPAEAIHHALAASDVSMLRDILLQHAWSLFHHSELALLEECLNALPYERLIQN
PKLALLQAWLAQSQHRYSEVNTLLERAERTMREQKIEIDQTLHAEFDALRAQVAINAGKPEEAERLAT
EALKFLPLSSYYSRIVATSVTGEVHHCKGELARALPMMQQTEQMARRHQANHYALWALLQQSEILIAQ
GFLQAAYETQDKAFELIREQHLEQLPMHEFLLRIRAQILWSWSRLDEAEDAARTGLKILANYQPQQQL
QCIAMLAKCSLARGDLDNANTHLQRCETLLHGAHYHRDWLTNTDKSRVIHWQMTGDTTAAAQWLRHTE
KPGMADNHFTQGQWRNIARVQILLGQYDEAGVVLDELNENARRLRLVSDLNRNLLLSNQLYWLQERKG

EAQQALIEALSLANRTGFISHFVIEGEAMAQQLRQLIQLNTLPELEQHRAQRILRDINQHHRHKFAHF
DENFVDKLLTHPQVPELIRTSPLTQREWQVLGLIYSGYSNDQIAGELDVAATTIKTHIRNLYQKLGVA
HRQEAVQQAQQLLKMMGYGA
>CORE_REP|Org21_Gene3694#
MSHTTMLALQGLSCMNCAQRVKKALESRSDVEQADVNVHYAKVTGDAPDSALIDSVIAAGYQAEVAPH
ADTELQLSGLSCMHCVGTTRKALEAVPGVFATDVTIDGAKVYGDADPQTLIAAVEDAGYHAGVAGAVA
PKTEPLTDATPSLPDVQPAAQSPLPATDGADDSVQLLLSGMTCASCVNKVQLALQSVPGVEHARVNLA
ERSALVTGAADAQALVAAVEKAGYGAEMIQDETERRERQQQTARANMKRFSWQAALGLALGIPLMAWG
LFGGSMTLTPETQRPWLLVGVITLAVMVFAGGHFYRNAWRALMNGSATMDTLVALGTGAAWLYSIAVN
IWPDFFPMEARHLYYEASAMIIGLINLGHALEQRARQRSSQALERLLDLTPPTARLVTDDGERDIPLA
EVQLGMTLRLTTGDRVPVDGEIVQGEVWLDEAMLTGEAVPQQKGAGDTVHAGTVVDDGSVLFRAAAIG
SQTTLARIIKLVRQAQSSKPAIGQLADRVSAVFVPAVVGIALFSAAMWYFFGPQPQLVYTLVIATTVL
IIACPCALGLATPMSIISGVGRAAEFGVLVRDADALQQASQLDTLVFDKTGTLTEGKPQVVEILTFNQ
VSEQQAIGWAAALEQGSNHPLARAIMERAAGQTLPQVAQFRTLRGAGVSGEIDGVPVLLGNAALLEQH
QVATAELEAPMRALAERGVTPVLLAVNGKPAALFAIRDPLREDSVAALQRLHRQGYQLVMLTGDNPVT
ANAIAKEAGIDRVIAGVLPDGKAAAIKQLQAQGQRVAMVGDGINDAPALAQADVGIAMGGGSDIAIET
AAITLMRHSLHGVADAVALSKATLRNMKQNLFGAFIYNTLGIPIAAGVLYPLTGTLLSPVVAGAAMAL
SSITVVSNANRLLRFKPRK
>CORE_REP|Org38_Gene4244#
MKLKKLPRQLLGLFARGLPRRLVRRDSLLDSVGGAARDMPAGLAQQRLECAAAETMQLFERFHSHPEG
ITAHEAEQVRQRCGENVIDDQQKEAWWQHLWHCYRNPFNLLLTALGMISYATEDLTGALVIALMVLIS
TLLNFIQEARSNRAADALKAMVSNTATVIRSDALTGRSEHVELPIAQLVPGDIIKLAAGDMIPADLRV
LSAKDLFISQAALTGESLPVEKSAAPQALAADPLDCQNLCFMGTNVVSGTALAMVIGTGGGTYFGQLA
QRVTSQDEQPNAFQSGISKVSWLLIRFMLVMTPIVLLINGYTKGDWWEAALFALSVAVGLTPEMLPMI
VTSTLAKGAVKLSRQKVIVKRLDAIQNFGAMDILCTDKTGTLTQDKIVLERHTDVFGASSERVLRYAW
LNSFYQTGLKNLLDVAVLSCAEQNQQPQALQNYRKVDEIPFDFVRRRMSVVVAKDNEYHELVCKGALE
EMLAICSHVRHEDEVIPLSEALLVRIRRITDDLNQQGLRVVAVANKILPAQTHEYGVADESDLILEGY
VAFLDPPKESTAPALAALKQNGVTVKILTGDNELVAAKVCRDVGLEADHLLRGSEIEQMDDEQLAQAA
ARTTVFAKLTPLHKERIVKLLRRQGHVVGFMGDGINDAPALRAADIGISVDSAVDIAKEAADIILLEK
SLMVLEQGVIEGRRTFANMLKYIKMTASSNFGNVFSVLIASAFLPFLPMLPLHLLIQNLMYDISQIAI
PFDNVDDDQITQPQRWNSADLGRFMVFFGPISSIFDVLTFSLMWWVFKANTPEMQTLFQSGWFVEGLL
SQTLIVHMIRTRKIPFIQSRPSWPLCIMTLAVIATGIGLVFSPLAGFLQLQALPLGYFPWLVLILAGY
MVLTQCVKGWFVRRYGWQ
>CORE_REP|Org46_Gene1471#
MLVKLLTKVFGSRNDRTLRRMRKVVEQINRMEPDMEKLSDDELKAKTNEFRARLEKGESLESLIPEAF
AVVREASKRVFGMRHFDVQLLGGMVLNDRCIAEMRTGEGKTLTATLPAYLNALSGRGVHVVTVNDYLA
QRDAENNRPLFEFLGLSIGINLPGMPAPAKREAYAADITYGTNNEYGFDYLRDNMAFSPEERVQRKLH
YALVDEVDSILIDEARTPLIISGPAEDSSEMYIKVNKLIPKLIRQEKEDSDTFKGEGHFSVDEKARQV
HLTERGLILIEEMLVEAGIMDEGESLYSPTNIMLMHHVTAALRAHVLFTRDVDYIVKDGEVIIVDEHT
GRTMQGRRWSDGLHQAVEAKEGVEIQNENQTLASITFQNYFRLYEKLAGMTGTADTEAFEFSSIYKLD
TIVVPTNRPMIRKDMPDLVYMTEKEKIGAIIEDIRERTAKGQPVLVGTISIEKSEVVSRELTKAGIDH
KVLNAKFHAMEADIVAQAGQSGAVTIATNMAGRGTDIVLGGSWQAEVALLEAPTEEQIEAIKAAWKER
HDAVLAAGGLHIIGTERHESRRIDNQLRGRSGRQGDAGSSRFYLSMEDALMRIFASDRVSGMMRKLGM
KEGEAIEHPWVTKAIANAQRKVESRNFDIRKQLLEYDDVANDQRRAIYSQRNELLDVSDVSETIASIR
EDVFKSTIDNYITPQSLEEEWDIQGLEERLKNDFDLEMPIAQWLDKEPELHEETLRERILENAKEQYQ
RKEDVVGSEMMRNFEKGVMLQTLDSLWKEHLAAMDYLRQGIHLRGYAQKDPKQEYKRESFNMFATMLE
SLKYEVISVLSKVQVRMPEEVEALEQQRREEAERLAQHQQLSHHDENALVTEDPNAPATAERKVGRND
PCPCGSGKKYKQCHGRLQ
>CORE_REP|Org3_Gene3709#
MQNKQLTISSSNITRCFLLFIVLLTIGIGLYGYNYTNAWLAEKKYALNSIAGSLQKRIDTYRYMTYQV
YDKFGNAPAQNVDPGLQETRLRPDVYYIEKPHKKTDAVIFGSHDESTLAMIANISDYLDTRWGAKTEN
YAMYYLNGQDNSLSLITTQPLKELASRFRESYLTTSADERRAEMLQQANMLDERESFSDLRKQRFQNA
YSFSIRTTFNQPGHLATVIAFDLPINDIIPANLARANFLLQPDDVDLDDSTIPAETVLGTHATMSGGW
VEFSAALPNAPLKVVYRVSAINLAIDLLRNNIWLIAVNLLLLALSMLSIYFIRRQYIRPSENMAVELE

AERALNQEIVSSLPSGLLVYSFANNAVIASNKIAEHLLPHLSLQKIAHMAEQHHGVIQATVNNEVYEI
RIFRSQLSPDTYLFLMHDQDKEVMVNKRLQQARREYDKNVQARKLMLHNLGIELNQPVRQMHDLVDRL
HGRPDEEQQQALLGQLTAASASVLELIDNITLLTRLETQDWQPSREPFSPTAMIDELLLEALPALNQK
GLALFKHFQLDVEQNYIGDANALRKVISLLVHYAIITTACGKISLVVDHEPEHPDRLIFQINDTGSGI
SNEEISNLNYPFLSQTLVDRFNHGSGLTFFLCNQLCKKLNGQLDIRSKVDIGTRYTIRVAMEMEKKEP
QEQEKLFDGVTALLDVTSDEVRGIVTRLLQAYGADCLVADDRAVNRDYDVLLTDNPQRADDYTLLLAT
DEPGWQALDKRYIRVNYNLNGALIDAVLILIEQQMAALEQEESPLSLSSEDIQLYEKQLKSSDYYGLF
VDTVPDDVKKLYTEAGSSDFNALSQTAHRLKGVFAMLNLLPGKQLCESLEQRIAEGDAPEIENNISQI
DFFVSRLLKQGSQQHE
>CORE_REP|Org34_Gene4528#
MAVTNVAELNELVARVKKAQREYANFTQEQVDKIFRAAALAAADARIPLAKMAVEESGMGIVEDKVIK
NHFASEYIYNAYKDEKTCGILSEDDTFGTITIAEPIGLICGIVPTTNPTSTAIFKALISLKTRNGIIF
SPHPRAKNATNKAADIVLQAAIAAGAPKDIIGWIDQPTVELSNQLMHHPDINLILATGGPGMVKAAYS
SGKPAIGVGAGNTPVVVDETADIKRVVASILMSKTFDSGVICASEQSVIVVDAIYDAVRERFASHGGY
LLQGKELKAVQDIILKNGGLNAAIVGQSAPKIAEMAGIKVPANTKVLIGEVKLVDESEPFAHEKLSPT
LAMYRAKDFEDAVAKAEKLVAMGGIGHTSCLYTDQDNQTARIAYFGDKMKTARILINTPASQGGIGDL
YNFKLAPSLTLGCGSWGGNSISENVGPKHLINKKTVAKRAENMLWHKLPKSIYFRRGSLPIALEEVAT
DGAKRAFIVTDRFLFNNGYADQITKVLKSHGIETEVFFEVEADPTLSIVRKGAEQMNSFKPDVIIALG
GGSPMDAAKIMWVLYEHPETHFEDLALRFMDIRKRIYKFPKMGVKAKMIAITTTSGTGSEVTPFAVVT
DDTTGQKYPLADYALTPDMAIVDANLVMNMPKSLCAFGGLDAVTHALEAYVSVLANEYSDGQALQALK
LLKEYLPASYKEGAKNPVARERVHNAATIAGIAFANAFLGVCHSMAHKLGSEFHIPHGLANAMLISNV
IRYNANDNPTKQTAFSQYDRPQARRRYAEIADHLGLSAPGDRTAQKIEKLLAWLDELKTELGIPTSIR
EAGVQEADFLAKVDKLSEDAFDDQCTGANPRYPLIAELKQIMLDTFYGREFSEAVDEEAATPAAAKTA
VKKPRNNGSVNR
>CORE_REP|Org10_Gene4043#
MNQPAVKADLDPQETAEWLEAFEGVTDIDGRERAHFLLERMAEADQRKHGDFFSMVTTPYVNTIPAYK
QPTYPGDLAAEARINAFIRWNAMAMVLRAGKHSNVGGHIATYQSAAVLYDVGFTHFFRGRTDDFAGDM
VYIQGHSAPGIYGRAYLEGRIDEELLDNFRRESARRGLSSYPHPRLMPDFWQYPTVSMGLGPLTAAYQ
ARYMRYLEYRELKPHQGRKVWAFLGDGEMDQPESLAAIALGGREKLDNLIFVVNCNLQRLDGPVRGNG
KIIQELEGTFKAAGWQVIKVIWGSGWDKLLQKDRSGLLMQRMMECVDGDYQTFKSQSGAYVREHFFGK
YPELLELVADLSDDEIWALHRGGHDPQKVYAAYHQAVHTPGRPTVVLAKTVKGFGMGEAGEGQNINHQ
LKKMSQDAVKAFRDRLGLTISDAQLAEIPYLKPEPDSAAAKYITATRTALGGYIPARFGQSAPLAIPE
LSRFDGLLKGSGERNMSTTMAFVNILGTLLKDVNIGKLIVPIVPDESRTFGMEGLFRQIGIHSWLGQL
YTPQDAGQLSYYKEAKDGQILQEGINESGAISTWIAAGTAYSNHDVATIPFYIFYSMFGLQRVGDLAW
AAADARTKGFLLGATSGRTTLMGEGLQHDDGHSHVLSSVIPSCVSYDPTYAYELAVIVQSGMRRMFVE
QEDIYYYITLLNEGYPQPPMPAGVEDGIIQGAYLLKQSETTNQESPRAQLVASGAIMREALAAAELLA
ADFGVASDIWSATSLSELRRNGMAAERWNLLHPEEPPKVPYIQSLLAAHPGPVVVVTDYMKIVGDQIK
PFLPDRTFIALGTDGFGRSDTREALREFFEVNRHFIALAALKLLADEGRIARSEVNRAMALYGIAPDK
PDPAAVK
>CORE_REP|Org8_Gene2598#
MSSDLRETSLDKLVALNSEYYYYSLPLAAKQLGAIDRLPKSMKVLLENLLRHVDGDTVQVDDLKAIVA
WLQTGHADREIAYRPARVLMQDFTGVPAVVDLAAMREAVRRLGGNVEQVNPLSPVDLVIDHSVTVDEF
GDNNAFEDNVRIEMQRNHERYTFLRWGQKAFNRFRVVPPGTGICHQVNLEYLGQTVWHSDESGRRVAY
PDTLVGTDSHTTMINGLGILGWGVGGIEAEAAMLGQPVSMLIPDVVGFKLTGKLREGITATDLVLTVT
QMLRKHGVVGKFVEFYGDGLADLPLADRATIANMSPEFGATCGFFPVDDVTLGYMKLSGRSAEQIALV
EAYAKAQGMWRNPGDEPVFTSSLALDMSTVEASLAGPKRPQDRVALPNVPQAFKAATELDIGGHKAKT
DGKTFTLDGQQHELRDGAVVIAAITSCTNTSNPSVMMAAGLLAKNAVKKGLRSKPWVKTSLAPGSKVV
TDYFDSAKLTAYLEELGFNLVGYGCTTCIGNSGPLPDPIEQAIKEGDLTVGAVLSGNRNFEGRIHPLV
KTNWLASPPLVVAYALAGSMKIDLTKEPLGEGNDGQPVYLKDIWPSSQDIAQAVEEVRTEMFHKEYGE
VFDGDANWQAIQVTGSATYQWQEDSTYIRHPPFFSTMQVKPDPVQDIKDARILAILADSVTTDHISPA
GNIKRDSPAGRYLSEHGVAPQDFNSYGSRRGNHEVMMRGTFANIRIRNEMVPGVEGGYTRHIPSQQQL
SIYDAAMQYQQEKVPLAVIAGKEYGSGSSRDWAAKGPRLLGVRVVIAESFERIHRSNLIGMGILPLEF
PQGVTRKTLGLTGDEQISVGGLQQLQPGQTVPVHITYADGRKEVVDTRCRIDTGNELTYYENDGILHY
VIRKML

>CORE_REP|Org45_Gene2134#
MSDNLLQQAAPAVPMPPASPSTYGDEELTCPMLKQRLEQFQLWLAAAFDAGSSAESLVAARSDFIDRL
LRRLWTFYGFEDIPETALVAVGGYGRGELHPLSDIDVLVLSQRRLTEQQSQRVGEFITLLWDLKLEVG
HSVRTLEECLLEGLADLTVATNLIESRMICGDVALFLQMQKHIFSDGFWPSPQFFHAKINEQQERHQR
YHGTSYNLEPDIKSSPGGLRDIHTLLWVARRHFGATSLDEMVGFGFLTQAERNELNECQSFLWRIRFA
LHLVLPRYDNRLLFDRQLSVAQLLRYEGEGNEPVERMMKDFYRMTRRVSELNHMLLQLFDEAILALDA
TEKPRPLNDDFQLRGDLIDLRDETLFIREPQAIMRMFYLMVRNREIKGIYSTTVRQLRHARRHLKQPL
CTIPEARDLFMAILRHPGAVSRALVPMHRHSVLWAYMPQWGKIVGQMQFDLFHAYTVDEHTIRVLQKL
ESFADDQTRPRHPLCVELYPRLPHPELLLLAALFHDIAKGRGGDHSILGAEDVVEFAELHGLNSRETQ
LVAWLVRCHLLMSVTAQRRDIQDPTVIQQFSSEVQSETRLRYLVCLTVADICATNETLWNSWKQSLLR
ELYFATEKQLRRGMQNSPDLRERVRHHRLQALALLRMDNIDEEALHRIWSRCRADYFLRHSPNQLAWH
ARHLLAHDSTQPLVLVSRQATRGGTEIFIWSPDRPYLFAAVAGEMDRRNLSVHDAQIFTNRDGMAMDT
FIVLEPDGSPLAQDRHAAIRQALLQAITQREYQPPRVRRPSSKLRHFSVPTEVTFLPTHTDRRSYLEL
TALDQPGLLARVGEVFADLGLSLHGARISTIGERVEDLFILADGERRALDQETRRKLEQRLTEALTPN
DKM
>CORE_REP|Org43_Gene3871#
MNAFKLSALAALTATMGFLGGMGSAMADQQLVDQLSQLKLNVKMLDNRAGENGVDCAALGADWASCNR
VLFTLSNDGQAIDGKDWIIYFHSPRQTLRVDNDQFKIAHLTGDLYKLEPTAKFSGFPAGKAVEIPVVA
EYWQLFRNDFLPRWYATSGDAKPKMLANTDTENLDQFVAPFTGDQWKRTKDDKNILMTPASRFVSNAD
LQTLPAGALRGQIVPTPMQVKVHEQDADLRKGVSPDLSTLVKPAADVVNQRFALLGVPVQANGYPIKT
DIQPGKFKGAMAVPGAYELKIGKKEARVIGFDQAGVFYGLQSILSLVPTDGSGKIATLDASDAPRFQY
RGIFLDVARNFHKKDAVLRLLDQMAAYKLNKFHFHLSDDEGWRIEIPGLPELTEVGGQRCHDLSETTC
LLPQYGQGPDVYGGFFTRQDYIDIIKYAQARQIEVIPEIDMPAHARAAVVSMEARYKKLHAAGKEQEA
NEFRLVDPTDTSNTTSVQFFNRQSYLNPCLDSSQRFVDKVIGEIAQMHKEAGQPIKTWHFGGDEAKNI
RLGAGYTDKAKPEPGKGIIDQSNEDKPWAKSQVCQVMIKEGKVADMEHLPSYFGQEVSKLVKAHGIDR
MQAWQDGLKDAESSKAFATSRVGVNFWDTLYWGGFDSVNDWANKGYEVVVSNPDYVYMDFPYEVNPDE
RGYYWGTRFSDERKVFSFAPDNMPQNAETSVDRDGNHFNAKSDKPWPGAYGLSAQLWSETQRTDPQME
YMIFPRALSVAERAWHRAGWEQDYRAGREYKGGETHFVDTKTLEKDWLRFANILGQRELAKLDKGGVA
YRLPVPGARVAGGKLEANIALPGLGIEYSTDGGKQWQRYDAKAKPAVSGEVQVRSVSPDGKRYSRAEK
V
>CORE_REP|Org6_Gene4045#
MSEISRSVLFGKLDSLLFTSLESATAFCKLRGNPYVELAHWLHQLMQSPDGDLQQIVRHFALDEAQLA
RDIVEALDRLPRGASAISDLSEHIDSAVERAWVYGSLKFGAARIRGGHLQLGILKTYSLRHLLKAISP
QFERINADLLMEQFAAITAHSAENAEDAPAESRAENASGAARSGESVLAQYAQDLTARARDGEIDPVA
GRDEEIRQIIDILMRRRQNNPLLTGEAGVGKTAVVEGLALRIVAGDVPPQLRDVKLCLLDIGMLQAGA
GVKGEFEKRLQAVIDEVQSSPTPIILFIDEIHTLIGAGGAQGTGDAANLLKPALARGQLRTIGATTWS
EYKKYIEKDPALTRRFQVVQVHEPSEDKALLMLRSTVSPLEQHHRVLLLDEAVDAAVRLSHRYIPARQ
LPDKAVALLDTACARVAVSQHAEPAQVEDCRHRIDALQIELDIARREAKVGIGDPLRPQEIEAQLTAL
RLELEQLTERWQQELTLIQEIITLRAQLHRQEAEEPAGEETEAGPDADALRAQLGELQQQLSALQGEA
PLIFAAVDANIVAAVVADWTGIPLGRMVKNEIEAVLQLSDTLNQRVIGQRHALDLIARRVRTSRARLD
DPNKPVGVFLLAGPSGVGKTETALALAETLYGGEQNVITINMSEFQESHTVSTLKGAPPGYVGYGEGG
VLTEAVRRRPYSVVLLDEIEKAHPDVHEIFFQVFDKGWMEDGEGRHIDFRNTIIILTSNVGTDLIAGL
CSDPELLPEPEALSGALRQPLLSVFPAALLGRLLVVPYYPLTDATLGNIVRLQLGRIQRRLAENHDIV
CTFDDAVIEQIVSRCTEVESGGRMVDAILTNTLLPQISHTLLTGSANDQRYRQLHIALQNHEFICQFQ
A
>CORE_REP|Org12_Gene4140#
MSDLAREITPVNIEDELKNSYLDYAMSVIVGRALPDVRDGLKPVHRRVLYAMSVLGNDWNKPYKKSAR
VVGDVIGKYHPHGDSAVYDTIVRMAQPFSLRYMLVDGQGNFGSVDGDSAAAMRYTEVRMSKIAHELLA
DLEKETVDFVPNYDGTEQIPAVMPTKIPNLLVNGSSGIAVGMATNIPPHNLAEVVNGCLAYIDDENIS
IEGLMEHIPGPDFPTAAIINGRRGIEEAYRTGRGKIYLRARAEVEADAKTGRETIIVHEIPYQVNKAR
LIEKIAELVKEKRVEGISALRDESDKDGMRIVIEVKRDAVGEVVLNNLYALTQLQVTFGINMVALHQG
QPKLLNLKDILEAFVRHRREVVTRRTIFELRKARDRAHILEALAIALANIDPIIELIRRAPTPAEAKV
ALVAQPWDLGNVSAMLERAGDDAARPEWLEPEFGIRDGKYYLTEQQAQAILDLRLQKLTGLEHEKLLE
EYKELLNFIAELIFILESPERLMEVIREELVAVKELYNDGRRTEITANTSDINIEDLINQEDVVVTLS

HQGYVKYQPLSDYEAQRRGGKGKSAARIKEEDFIDRLLVANTHDTILCFSSRGRLYWMKVYQLPEASR
GARGRPIVNLLPLEADERITAILPVREYEEGRHVFMATASGTVKKTALTEFSRPRSAGIIAVNLNEGD
ELIGVDLTDGSNEVMLFSANGKVVRFPEAQVRSMGRTATGVRGINLGEGDSVISLIVPRGEGDILTVT
QNGFGKRTAVTEYPTKSRATQGVISIKVSERNGQVVGAVQVETSDQIMMITDAGTLVRTRVSEVSVVG
RNTQGVTLIRTAEDENVVGLQRVAEPVEDEELDSLEPGAEAVEEDTTPLDDGDDAAEPMDDENV
>CORE_REP|Org37_Gene3116#
MRVSPAGKLMRLQTLGLCISLALGVPSMAVFAAGDIQFNTDVLDVHDRENIDLSQFSRGGYIMPGTYG
MVVHVNKNDLQEQQVPFYAPEDDPNGSRACVTQVLTGQLGLKEDALKGVTWWHQGECLDEASIPGMEV
RGDLATSALYLSIPQAFLEYTAENWDPPSRWDEGIPGLLFDYNVNARTQKQHQGNGSSYSLSGNGTTG
LNLGAWRLRADWQGNVDHTTGSGQSTAQKLDWSRYYAYRAIPALRSKLTVGENYLDSGIFDSFRFTGA
SLMSDDNMLPPNLRGYAPEVVGIAKTNAKVVISQQGRVLYETQVAAGPFRIQDINDAVSGEMNVRVEE
QDGSVQEFTMNTATIPYLTRPGSVRFKLASGKPSDFQHHSRGPMFGTGEFSWGVSNGWSLYGGALVGG
DYNALSLGLGRDLMALGALSFDATQSRARLPQADGTLSGGSYRLSYSKNFDEYDSQVTFAGYRFSQED
FMSMSEYLDARYYGTRTGNGKEMYTVTFNKHFRDWGLSTYLNYSHETFWDRPANDRYNLTLSRYLDIG
SFRNVSLSLSAYRNKYNGVNDDGGYLSLSLPWGNSGSVGYSATVNRSDVTHQANYYDRLDEHNNYSMS
AGSSRSGASLSGYYNHEGDMARMSANASYQEGRHSAMGLSLQGGATLTMEGGALHRAGIPGGTRMLID
TNGVADVPVRGYGRTSNTNAWGKVVIGDVNSYYRNKASIDLNKLGDNAEATTSVVQATLTEGAIGYRQ
FDVIAGEKAMAVIKLADGSQPPFGATVMNARKQETGIVNDGGSVYLSGINAGDTMTVHWAGNAQCEVR
MPTPLPAEMLMNSLLLPCHPLSAQAPTHDGQTTAEDAPGAVTSTVPGRTVQPPSLSDKNRELF
>CORE_REP|Org20_Gene4791#
MNEQYSAMRSNVSMLGKLLGDTIKEALGEHILDRVETIRKLSKSSRAGNEAHRQELLSTLQNLSNDEL
LPVARAFSQFLNLTNVAEQYHSISPNGEAASNPEALAQLFSRLKDKKLSDKELQHAVSQLSIELVLTA
HPTEITRRTLIHKLVEVNTCLSQLDHNDLADYERNKIMRRLRQLVAQSWHTDEIRKHRPSPIDEAKWG
FAVVENSLWEGVPAFLREFNEQLENSIDYSLPAEAVPVRFTSWMGGDRDGNPNVTAEITRHVLLLSRW
KACDLFTRDIQVLVSELSMTECTPELRARAGGDEVQEPYREIMKQLRSQLMSSQAYLEGRLKGERVLK
PHDLLVNNEQLWEPLYACYQSLQACGMGIIANGQLLDTLRRVRCFGVPLVRIDVRQESTRHTEAIAEL
TRYLGLGDYESWSEADKQAFLIRELNSKRPLVPLKWQPSADTQEVLETCRVIAEAPQGSIAAYVISMA
RTPSDVLAVHLLLKEAGCPFALPVAPLFETLDDLNNADDVMTQLLNIDWYRGFIQGKQMVMIGYSDSA
KDAGVMAASWAQYRAQDALIKTCEKAGVALTLFHGRGGSIGRGGAPAHAALLSQPPGSLKGGLRVTEQ
GEMIRFKFGLPEATISSLALYAGAILEANLLPPPEPKKEWRALMDDLSDTSCRMYRGYVRENPDFVPY
FRAATPELELGKLPLGSRPAKRKPNGGVESLRAIPWIFAWTQNRLMLPAWLGAGAGLQEAVKAGKQAE
LEAMCRDWPFFSTRIAMLEMVFAKADLWLAEYYDQRLVDKSLWPLGQQLRDQLESDIKVVLTIANDAH
LMEDLPWIAESIALRNVYTDPLNVLQAELLHRSRQQEQPDARVEQALMVTIAGVAAGMRNTG
>CORE_REP|Org26_Gene4376#
MQQETPTTPTEARVKNKRRISPFWLLPFIALLIAGWLVYNNVQERGTTVTIDFQSAAGIVAGRTPVRY
QGVEVGTVQKISLSKDLRSIVVEASIKSDLEDSLREGTQFWLVTPKASLAGVSGLDALVGGNYIGMMP
GSGKEQTHFTALDTQPKYRLNTGELMIHLHADDLGSLNSGSLVYYRKIPVGKVYDYTISEGNKGVTID
VLIDRRFANLVKSNSRFWNVSGFKGDFSLSGATVQMESLAALVNGAIAFDSPADGQQAKGDQSYTLYP
DLAHSQRGVNILLDLPNGNSLSENRTPLMYQGLQVGTLTKLTLQQDSKVVGELTIDPSVVDLMRSGTR
IVMRSPRISLNDAKLSQLLTGTTLELVPGEGEPQQRFNVLDSSETLLQQPGVLTVTLNAPQSYGIDVG
QPLVVHGVKVGQILSRTLTAGGVVFTAAIDAQYRGLLHKDSKFVVNSRLDVKLGIDGMEVLGASAQEW
VDGGVRIIPGSKGEPGGQYPLYANSEKAEEGIVGNAPSTTLTLSATSLPDVQAGSVVLYRKFQVGEIV
NVRPKANEFEVDVYISPEYRKLLTRESIFWAEGGAKVQLNGSGLTVQASPLNRALKGAISFDNLQGVT
LNKGANRVLYASETAARAVGSQIMLRTYDASKLSAGMPLRYLGIDVGQVESLQLAPERNEVLAKAVLY
PEYVHTFARLGSRFSIVSPEISAAGVSNLDTLLQPYINVEPGRGRELRTFELQQASITDSRYLDGLSV
VLDAAETGSLQIGTPVLFRGVEVGTITGFYLGAMSDRVHVALRISKKYQHLVRNNSVFWLASGYNLQF
GLTGGVIKSGTFQQFIRGGIAFATPPTIPLAPKATPNKHFLLNPEEPKDWKTWGTAIPRD
>CORE_REP|Org38_Gene3259#
MTQQPQAKYRHDYRAPDYTTTDIDLDFSLDAETTRVTAVSKIKRQGAAGAPLVLDGEDLTLVSIQVDG
QPWSAYRQQDNQLIVEALPAQFTLTIVNDIHPAKNTALEGLYLSGDALCTQCEAEGFHHITYYQDRPD
VLARFTTRIVADKARYPFLLSNGNRIGQGELADGRHWVQWQDPFPKPCYLFALVAGDFDVLRDSFTTR
SGRKVALELFVDRGNLDRADWAMTSLKNSMKWDETRFGLEYDLDIYMIVAVDFFNMGAMENKGLNIFN
SKYVLAKAETATDKDYLNIEAVIGHEYFHNWTGNRVTCRDWFQLSLKEGLTVFRDQEFSSDLGSRSVN
RIDNVRVMRGAQFAEDASPMAHAIRPDKVIEMNNFYTLTVYEKGSEVIRMMHTLLGEENFQKGMQLYF

ERHDGSAATCDDFVQAMEDASNVDLSRFRRWYSQSGTPLLTVRDEYDAETQQYRLHVSQKTPPTADQP
EKLPLHIPLDIELYDSEGNVIALQKGGLPVNNVLNVTEAEQTFVFDGVAHKPVPSLLREFSAPVKLDY
PYSDQQLTFLMQHARNEFARWDAAQSLLATYIKLNVARHQQKQPLSLPLHVADAFRAVLLNETLDPAL
AAQILTLPSENEIAELFATIDPEAIAAVHEAIVRCLARELADEWLAVYHANKTDGYRVEHADIAKRAL
RNVCLGYLAFGEDAALADQLVSEQYRQADNMTDSLAALSAAVAAQLPCRDALLAAFDERWHQDGLVMD
KWFVLQGSSPSADVLSKVRALLQHRSFSLSNPNRTRSLIGGFASGNPAAFHAADGSGYQFLVEILSDL
NQRNPQIAARLIEPLIRLKRYDAGRQALMRKALEQLKGLENLSGDLYEKISKALDA
>CORE_REP|Org11_Gene135#
MGKALVIVESPAKAKTINKYLGSDYVVKSSVGHIRDLPTSGSASKKSADSTEDKAKKKVKKDEKAALV
NRMGVDPYHGWKAHYEILPGKEKVVAELKSLAENADHIYLATDLDREGEAIAWHLREVIGGDDKRFSR
VVFNEITKNAIQQAFKQPGELNIDRVNAQQARRFMDRVVGYMVSPLLWKKIARGLSAGRVQSVAVRLV
VERERDIKAFVPEEYWELHADLLAKGETALQMEVTHAHDKPFKPVNREQTHAAVKLLEKARYTVLDRE
DKPTSSKPGAPFITSTLQQAASTRLSFGVKKTMMMAQRLYEAGHITYMRTDSTNLSQDALNMVRGYIG
DNFGDKYLPKAPNQYSSKENSQEAHEAIRPSDVNVLAEQLKDMEADAQKLYQLIWRQFVACQMTPAQY
DSTTLTVKAGDYQLRAKGRTLRFDGWTKVMPALRKGDEDRTLPYVEIGSELDLQKLIPSQHFTKPPAR
YSEASLVKELEKRGIGRPSTYASIISTIQDRGYVRVESRRFYAEKMGEIVTDRLEENFRELMNYDFTA
RMEDGLDEVANNQAEWKAVLDEFFVDFSEQLETAEKDPEEGGMRPNQMVMTSIDCPTCGRKMGIRTAS
TGVFLGCSGYALPPKERCKTTINLVPEAEVLNILEGDDAETNALRARRRCQKCGTAMDSYLIDNQRKL
HVCGNNPACDGYEIEEGEFRLKGYDGPVVECDKCGSEMHLKMGRFGKYMGCTNENCKNTRKILRNGDV
APPKEDPVPLPELPCEKSDAYFVLRDGAAGVFLAANTFPKSRETRAPLVEELARFKDRLPEKLRYLAD
APVADAEGNKTLVRFSRKTKQQYVSSEKDGKATGWSAFYVDGKWVEGKK
>CORE_REP|Org7_Gene1835#
MLDRIIAHTPLGQEQLLFRSLDGIEALSTPFDFSIELLSTDARLDRKALLGQPLTLEIPTQGFLSAPR
YLNGKITAIAVSSEEIGGTRYAVYNLHVQPDLWPMTKDRNFRIFQEQTVPQIVKTLLAEHNVQLEDQL
TGDYRLWGYCVQYNESSFNFISRLMEQEGIYYYFKHEMGKHTLVLGDAPHHHQPYPGYEMIPYHLTPS
GGSTSEEGISQWTLSDRVTPGIYSLDDYDFRKPNAWLFQARQNPVSPTPGQIDVYDWPGRYTEHQQGE
FYARVRQEAWQAEHQQIRGTATAMGIAPGSTFTLYNAPHADDNREYLTLQASYHLKENRYASGDDQSS
EHRIDFIVLPADVPWHPPQQATWPKTHGPQTARVVGPAGESIWTDKYGRIKVKFHWDRFGPKDDGSSC
WVRVSSAWAGQGYGGVQIPRVNDEVVVDFINGDPDRPIVTGRVYNEASMPPWALPAAATQMGFMSRTK
DGTADNANALRFEDKAGAEQVWIQAERNMDTQVKNDESHTIANDHTHLVGGNQIKRVVLNQATGVKGE
SSALTGKTRSDAVVNAFTLGSGESLRLECGESVIELLADGQINITGTSFNITVKEDGAINTGGQLDLN
QPGGAARTAAPGGGHQAAIQSAVDQLFPNEEASGTPGKPVNAAPRAAAAAPASITQNAQSTTKPGRID
NRVVESVMASEGSAGEQGGRRELYGFRKGNGNAYDKILAARNQYGQGSAEEFEEVSKAMSASAKSAGA
LNFSDPGKQGAITSLAHMRGSSGAQAILNSMESGRIVKADTLTSEAIAKIESMSAESFQDNLLKARVE
YDRAIYGDTITTQGGKQYNWWARYGNGLQKRYAREAEEFLKLSNE
>CORE_REP|Org3_Gene2841#
MQEQYRPEDIESNVQLHWQEKQTFKVTEDDSKEKYYCLSMLPYPSGRLHMGHVRNYTIGDVISRYQRM
LGKNVLQPIGWDAFGLPAEGAAVKNNTAPAPWTYDNIEYMKNQLKLLGFGYDWDREIATCQPEYYRWE
QWFFTKLYEKGLVYKKTSAVNWCPHDLTVLANEQVIDGCCWRCDTKVERKEIPQWFIKITAYADQLLN
DLDTLESWPEQVKTMQRNWIGRSEGVEITFDVADSEEKLTVYTTRPDTFMGATYVAVAAGHPLAQQGA
RNNPALTDFIDECRNTKVAEAEMATMEKKGMPTGLFVVHPLSGEKLPVWVANFVLMEYGTGAVMAVPA
HDQRDWEFATKYDLPIKPVILNLDGSQPDVSAEAMTDKGALFKSGEFDGLDNEAGFNAIADKLVAKGV
GQRKVNYRLRDWGVSRQRYWGAPIPMVTLEDGTVMPTPEDQLPVILPEDVVMDGITSPIKADPEWAKT
TVDGQPALRETDTFDTFMESSWYYARYTCPQYDQGMLDPAAANYWLPVDQYIGGIEHAIMHLMYFRFF
HKLMRDAGLVDSDEPAKRLLCQGMVLADAFYYTGNSGERVWVSPVDATVERDDKGRIIKATDPQGREL
VYAGMSKMSKSKNNGIDPQEMVEKYGADTVRLFMMFASPAEMTLEWQESGVEGANRFLKRVWKLAYDH
VEKGAVQPLDVAALNEDQKALRRDLHKTIAKVTDDIGRRQTFNTAIAAVMELMNKLARAPQESEQDRA
LLQEALLAVVRMLYPFTPHVCFTLWQALGGEGDVDTAPWPVADEQAMVEDSKLVVVQVNGKVRAKITV
SADATEEQVRARAAEEHLVAKYLDGVTIRKVIYVPGKLLNLVVG
>CORE_REP|Org17_Gene960#
MRLDRLTNKFQLALADAQSLALGHDNQFIEPLHLMSALLNQEGGTVRPLLTSAGIDAGRVRTEIEQAL
SRLPQVEGTGGDVQPSHELVRVLNLCDKLAQKRADKFISSELFVLAVLEDRGSLTDLLKAAGATADKI
SKAIEQMRGGDSVEDQGAEDQRQALKKYTIDLTERAEQGKLDPVIGRDEEIRRTIQVLQRRTKNNPVL
IGEPGVGKTAIVEGLAQRIINGEVPEGLKHKRVLSLDMGALIAGAKYRGEFEERLKGVLNDLAKQEGS

VILFIDELHTMVGAGKADGAMDAGNMLKPALARGELHCVGATTLDEYRQYIEKDAALERRFQKVYVAE
PSVEDTIAILRGLKERYELHHHVQITDPAIVAAATLSHRYIADRQLPDKAIDLIDEAASSIRMQMDSK
PESLDRLERRIIQLKLEQQALNKESDDASKKRLDMLSDELGQKEREYSELEEEWKAEKASLSGTQNIK
AELEQAKITLEQARRVGDLGRMSELQYGKIPELEKQLAAATQAEGKSMKLLRNRVTDAEIAEVLARAT
GIPVARMLEGERDKLLRLEQELHSRVIGQDEAVSAVSNAIRRSRAGLSDPNRPIGSFLFLGPTGVGKT
ELCKALASFLFDSDDAMVRIDMSEFMEKHSVSRLVGAPPGYVGYEEGGYLTEAVRRRPYSVILLDEVE
KAHPDVFNILLQVLDDGRLTDGQGRTVDFRNTVVIMTSNLGSDLIQEHFGQMNYAQMKESVMEMVSHH
FRPEFINRIDEVVVFHPLGEKHIAAIAKIQLSRLYKRLEERGYEVTMTEPALALLSKTGYDPVYGARP
LKRAIQQEIENPLAQQILSGKLIPGKLVTLDVENDHIVARQ
>CORE_REP|Org44_Gene2350#
MKFVKYFLILAVCCIVLGAASIFGLYKYVEPQLPDVATLKDVRLQIPMQVYSADGELIAQYGEKRRIP
LKLDQIPPVMVHAFIATEDSRFYDHHGVDPVGIFRAASIALVSGHASQGASTITQQLARNFFLSPERT
LMRKIKEAFLAVRIEQMLTKDEILELYLNKIYLGYRAYGVGAAAQVYFGKDVSQLTLSEMATIAGLPK
APSTFNPLYSHDRAVARRNVVLSRMLDEHYITQAQYDQARSEDLVANYHAPEISFSAPYLSEMVRQEM
IKRYGENAYTDGYKVYTTVTKRLQLAAQESVRNNVLAYDMRHGYRGPSNVLWKVGEAAWDRKQIVDSL
KNLPNYGPLAPAVITAANPQEATAMLADGSSIALPMATMRWARPYRSDTQQGPTPKRVTDVVQAGQQV
WVRKVNDAWWLSQVPDVNSALVSINPNDGAVKALVGGFDFNQSKFNRVTQALRQVGSNIKPFLYTAAM
DKGLTLATILNDLPITRWDAGAGTDWRPKNSPPTYDGPIRLRQGLGQSKNVVMVRAMRAMGVDYAAEY
LQRFGFPAQNIVHTESLALGSASFTPMQLVRGYAVLANGGYLVDPYFITKIEDDNGNTVFEAKPKVVC
SSCNLPVIYGDTHRSAVLSDDNIENVATSQEGNNSTVPMPQLEQVTPAQVQQDGDQQYAPHVISTQLA
FLIHDALNSNIFGEPGWMGTAWRAGRDLKRHDIGGKTGTTNSSKDAWFSGYGPDTVTSVWIGFDDHRR
DLGRSTVSGAIPDQISGGEGGAKSAQPAWDDFMKTALEGIPEQKVTPPPGIISVTIDKSSGKLSGGGG
GSRSEYFIEGTQPTDYPSRDTGTTLTDPGGESHELF
>CORE_REP|Org29_Gene3641#
MNKPTQPAQDYLAALPLTAERSEALNPQTADDAQALEALHRQMGAADANVNSLSADDVALASVKPRIE
SAWPDAVSDDDFDTDAEGRAILKATPPIKRTTMFPEAWRTNPVARFWDSLLGRSPHNRHATKEEAEAE
NRWRVVGSMRRYVLLVLMLVQTGIATWYMKTILPYQGWALIDPIAMLDQDLMQSVLQLLPYVLQTGIL
ILFAVLFCWVSAGFWTALMGFLQLLIGKDKYSISSTIKGDEPINPAHRTALIMPICNEDVERVFAGLR
ATYESVAATGQLEHFDIYVLSDSYDPDICVAEQKAWMELCRDVDGHGRIFYRRRRRRVKRKSGNIDDF
CRRWGGEYSYMVILDADSVMSGECLTGLVRLMEANPNAGIIQSAPKASGMDTLYARVQQFATRVYGPL
FTAGLHFWQLGESHYWGHNAIIRVKPFIEHCALAPLPGEGSFAGSILSHDFVEAALMRRAGWGVWIAY
DLPGSYEELPPNLLDELKRDRRWCHGNLMNFRLFLVKGMHPVHRAVFLTGVMSYLSAPLWFMFLALST
ALQVVHTLMEPQYFLQPRQLFPVWPQWRPELAIALFSTTLVLLFLPKLLSIVLIWAKGAKEYGGAFRL
FISMLMEMLFSVLLAPVRMLFHTVFVVSAFLGWEVVWNSPQRDDDDTPWGEAFRRHGSQMLLGLVWAG
GMAWLDLRFLWWLSPIVFSLILSPFVSVLSSRATLGMKSKRAKLFLIPEEYNPPRELLATEEYLHLNR
NRALTNGFMHAVVNPSFNALATALATARHHLRATLDRNREERVNEALQLGPEKLVKGKRLELLSDPVT
LARLHQRVWLLPEGAAWREHYQQLPHNPLAHPTGRR
>CORE_REP|Org15_Gene823#
MYLYIETLKQRLDAINQLRVDRALAAMKPAFQRVYSLLPTLLHHHHPLMPGYLNGNVPHGICLYTPDE
TQQDYLNDLEDKWGSPFDKPASGELPITGVYSMGSTSSIGQSCSSDLDIWVCHQSWLDNEERTRLQQK
CSLLEKWAASMGVEVSFFLIDENRFRHNESGSLGGEDCGSTQHILLLDEFYRTAVRLAGKRILWNMVP
GEEEAHYDEYVLSLYAQGALTPNEWLDLGGLSSLSAEEYFGASLWQLYKSIDSPYKAVLKTLLLEAYS
WEYPNTQLLATDIKHRLHQGEIVSFGLDAYCMMLERVTRYLTDINDTTRLDLARRCFYLKVCEKLSLA
KACVGWRREILSQLVSEWGWSEERLAMLDNRANWKIERVREAHNELLDAMMQSYRNLIRFARRNNLSV
SASPQDIGVLTRKLYAAFEALPGKVTLVNPQISPDLSENDLTFIHVPVGRANRTGWYLYNQAPAMDSI
VSHQPLEYNRYLNKLVAWAYFNGLLTPQTRLHIKSGNLCDTAKLQELVADVSHHFPLRLPAPTPKALY
SPCEIRHLAIIVNLENDPTAAFRNQVVHFDFRKLDVFSFGQQQQCLVGSIDLLYRNSWNEVRTLHFSG
EQSVLEALKTILGKMHQDAAPPESVEVFCYSQHLRGLIRTRIQQLVSECIELRLSSTRLEPGRFKAVR
VAGQTWGLFFERLSVSVQKLENAVEFYGAISNNKLHGLSIKVETDQVHLPPVVDGFASEGIIQFFFED
TSDDKGFNIYILDESNRVEVYHHCEGSKEELVRDVSRFYSSSHDRFTYGSSFINFNLPQFYQIVQLDG
RTQVIPFRSNVLSSLCVTVADGAAQPLKQQFQLH
>CORE_REP|Org13_Gene4508#
MSRVKLAVVGNGMVGHRFIEDLLDKADKDQFEITVFCEEPRIAYDRVHLSSYFSHHTAEELSLVREGF
YEKHGVKVLVGERAITINRDEKVIHSNTGRTVYYDKLIMATGSYPWIPPIKGSDSQDCFVYRTIEDLN

AIEACARRSKRGAVVGGGLLGLEAAGALKSLGVETHVIEFAPVLMAEQLDPMGGDQLRRKIERMGVKV
HTGKNTQEIVNGGGTARKTMHFADGSLLEVDFIVFSTGIRAQDKLARQCGLEIGRRGGIAINDSCQTS
DPDVYAIGECAAWRDRTFGLVAPGYKMAQVAVDHLLGRENGFQGADMSAKLKLLGVDVGGIGDAHGRT
EGARSYVYLDESKEVYKRIVVSADNKTLLGAVLVGDTSDYGNLLQLALNGIELPENPDGLILPAHAGS
KPAIGVDSLPESAQICSCFDVSKGDIIQAVNKGCHTVAALKAETKAGTGCGGCIPLLTQVLNAELSKQ
GIEVNHHLCEHFAYSRQELFHLIRVEGIKSFETLLAKYGKGYGCEVCKPTVGSLLASCWNEYILKPQH
TPLQDTNDNFLGNIQKDGTYSVIPRSAGGEITPDGLLAIGQIAKEYNLYTKMTGSQRIGMFGAQKDDL
PAIWRKLLAAGFETGHAYAKALRMAKTCVGSTWCRYGVGDSVGFGVTLEHRYKGIRTPHKMKFGVSGC
TRECAEAQGKDVGIIATENGWNLYVCGNGGMKPRHADLLAADLDRETLVRYLDRFMMFYIRTADKLQR
TSVWLESLEGGIDYLRKVIIDDKLGINDQLEAEIARLRDAVICEWKETVEHPETQLRFAHFINSPLRD
PNVQVVAERDQHRPARPDERIPVTLIDTEESHA
>CORE_REP|Org46_Gene4129#
MSDHSMTSLLTIKKTPIIVILSVLWAKPVFSATEFNTDVLDIGERSKVDLSRFSDADYVMPGTYLLDI
KINQKTLPQRSIQYFPSPDNKSGSQVCLPPDLVEKMALKEDAARKVTLWHDNQCADIRGIKGATVSDR
ISGGVLAITIPQAWMKYSDPDWTPPEQWDDGIPGVLLDYNLSGQIGKQHHDNGTAESLSSYGTLGANL
GAWRLRADYQTDFNQQYGRRDSNFDWNQIYAYRALPMQAARLTLGETYLNSPVFDAYRFTGLNLASDE
RMLPPNLQGYAPEVRGIAKSNARITVSQEGRTLYQTTVPAGPFAIQDLSSSVRGKLDVKVEEQDGSVS
TFQVDTASIPYLTRPGYVRYNMALGKPSAYDHRTQGPVFSAGDFSWGLSNAWSLYGGALLGGDYNAWA
LGLGRDLNLFGALSVDATQSIARLPDEPSAKGMSFKVNYAKRFDELNGQITFAGYRFSQRKFMTMSQY
LQARYGDIDDRYSGRQKELYTVTASKTFMAEDSAQAITAYLTYSHQTYWDAGAQNRYGMSTSKLFDFG
GISNITASLAAYRTHYRGRTDDSAMLNFTVPIGEHNRLGYALQVNNRDVSQTATYTDNSDINNTWQVG
SGVTQSGKPTASGYYTHNASFGTLNANASYQQGSYSSIGGTFRGGLTATRHGVAAHQNAGNGGSRMML
DTNGVAGVPINNGRAYSNRFGLAVISDITSYYNTDTRIDVNKLADDVEATRAVVQGTLTEGAIGYRHF
EVVKGSKLLATIKLADGSEPPFGATVLSATGREIAVVNDGGSVYLTGVQPEERLDVAWEGRRQCRIAI
PGAAKPLDRLLLPCAKP
>CORE_REP|Org18_Gene3790#
MRVLKFGGTSVANAERFLRVADIMESNACQGQVATVLSAPAKITNHLVAMIDKTVAGQDILPNISDAE
RIFADLLSGLAQALPGFEYDRLKGVVDQEFAQLKQVLHGVSLLGQCPDSVNAAIICRGEKLSIAIMEG
VFRAKGYPVTVINPVEKLLAQGHYLESTVDIAESTLRIAAAAIPADHIVLMAGFTAGNDKGELVVLGR
NGSDYSAAVLAACLRADCCEIWTDVDGVYTCDPRTVPDARLLKSMSYQEAMELSYFGAKVLHPRTITP
IAQFQIPCLIKNTSNPQAPGTLIGKDSTDDAMPVKGITNLNNMAMINVSGPGMKGMVGMAARVFAVMS
RAGISVVLITQSSSEYSISFCVPQGELLRARRALEEEFYLELKDGVLDPLDVMERLAIISVVGDGMRT
LRGISARFFSALARANINIVAIAQGSSERSISVVVSNESATTGVRVSHQMLFNTDQVIEVFVIGVGGV
GGALIEQIYRQQPWLKQKHIDLRVCGIANSRVMLTNVHGIALDSWRDELAGAQEPFNLGRLIRLVKEY
HLLNPVIVDCTSSQAVADQYVDFLADGFHVVTPNKKANTSSMNYYQQLRAAAAGSHRKFLYDTNVGAG
LPVIENLQNLLNAGDELVRFSGILSGSLSFIFGKLDEGLSLSAATLQARANGYTEPDPRDDLSGMDVA
RKLLILAREAGYKLELSDIEVESVLPPSFDASGDVDQFLARLPELDKEFARNVANAAEQGKVLRYVGL
IDEGRCKVRIEAVDGNDPLYKVKNGENALAFYSRYYQPLPLVLRGYGAGNDVTAAGVFADLLRTLSWK
LGV
>CORE_REP|Org12_Gene2034#
MTSPFSYTSPTVSVEALKHSIAYKLMFIVGKDPAIANRHDWLNAVLFAVRDRMVERWLRSNRAQLSQD
VRQVYYLSMEFLIGRTLSNALLSMGIYQDIDNALNEMGLNLAELLEEENDPGLGNGGLGRLAACFLDS
LATLALPGRGYGIRYEYGMFKQNIVNGQQMESPDYWLEYGNPWEFPRHNTRYKVRFGGRVQQEGAKAR
WLETEEVVAIAYDQVIPGFDTDATNTLRLWGAQASNEINLGKFNQGDYFAAVEDKNHSENVSRVLYPD
DSTSSGRELRLRQEYFLVSATVQDILNRHWLMHKTFDNLADKIAIHLNDTHPVLSIPELMHRLIDEHK
FKWLDAWEVVEQVFSYTNHTLMSEALETWPLDMIGRILPRHLQLIFEINDHFLKMVQEVAPGDNDLLA
RVSIIDETNGRRVRMAWLAVVASHKVNGVSALHSELMVQSLFADFARLFPTRFCNKTNGVTPRRWLAL
ANPPLAAVLDDCIGQTWRTDLSQLSEIQANIDYPSFLQAVQRAKRQNKERLALYIAKTLNVVVNPDAL
FDVQIKRIHEYKRQLLNVLHVITLYNRLLDDPEIERVPRVVIFAGKAASAYYAAKQIIRLINDVAKVI
NNDPRVHTQLKVVFIPNYGVSLAQIIIPAADLSEQISLAGTEASGTSNMKFALNGALTIGTLDGANVE
MREHVGEENIFIFGNTAEQVEELRRNGYNPRQYYEQDPELHQALTQIATGVFSPEEPKRYGNLFDSLV
NLGDHYQLLADYRSYVDTQDKVDEVYLNQDEWTRRAVLNIANMGYFSSDRTIQEYADEIWHIQPVKL
>CORE_REP|Org26_Gene3156#
MNAIAVAGPVSGRQLHKFGGSSLADVKCYLRVAGIMAEYSQPGDMMVVSAAGSTTNQLINWLKLSQSD

RLSAHQVQQTLRRYHSDLISGLLPPESAEPLIAEFIQDLERLAVLLDGKVDEVCYAEVVGHGEIWSAR
LMAAVLNHLDMQAAWLDARDFLRAERAAQPQVDEGRSYPLLQQLLAQHPGKRLVVTGFISRNDAGETV
LLGRNGSDYSATQVGALAGAARVTIWSDVAGVYSADPRKVKDACLLPLLRLDEASELARLAAPVLHTR
TLQPVSGSDIDLQLRCSYQPEQGSTRIERVLASGTGAKIVTSHDDVCLIELHVAAQHDFKLAQKELDL
VLKRAQIKPLAVGIHPDRNRVQLCYTSEVVNSALAILQASALPGELHLREGLALVAMVGAGVCKNPLH
SHRFYQQLKDQPVEFIWQAEDGISLVAVLRQGPTALLIQGLHQSLFRAEKRIGLVLFGKGNIGSRWLE
LFAREQTNISARSGFEFILAGVVDSRRSLLNYEGLDASRALAFFEDEAQALDEESLFLWMRAHPFDDL
VVLDVTASEELAGQYLDFASYGFHVISANKLAGASCSDTYRQIRDAFAKTGRHWLYNATVGAGLPVNH
TVRDLRDSGDSILAISGIFSGTLSWLFLQYDGTVPFTELVDQAWQQGLTEPDPRVDLSGQDVMRKLVI
LAREAGYDIEPNQVRVESLVPAGCEQGSVDQFFENGEALNQQMQQRFEAASEMGLVLRHVARFDANGK
ARVGVEAVRPEHPLASLLPCDNVFAIESRWYRDNPLVIRGPGAGRDVTAGAIQSDLNRLAQLL
>CORE_REP|Org5_Gene4412#
MPVSAVLDELLAALQSAPQVLLHAPTGAGKSTWLPLQILAKAGLSGRIIMLEPRRLAAKNVAYRLAQQ
LGEEPGQTVGYRMRAESKSGPQTRLEVVTEGILTRMLQQDAELQGVSLVILDEFHERSLQADLALALL
LDVQQGLRDDLKLLIMSATLDNARLSQLLPAAPVVVSEGRSFPVERLYQPLASHQRLEDGVAAAVKRL
LAEQPGSLLLFLPGVAEINRVLERLTGEVASDTDLCPLYGALPLAQQQKAIQPAAVGRRKVVLATNIA
ETSLTIEGIRLVVDSGLERVARYDVRNGLTRLATQRISQASMVQRAGRAGRLEPGICWHLFAKEQAER
AAEHAEPDILQSDLTGFWLELLQWGCHDPAQLTWLDAPPAAALAAARALLHRLGATDDGGKLTAPGRQ
MAALGCEPRLATMLTAGAALSADGLATAALLAALLEEPPRGGQMDIGYWLSRPQPNWRQRAAQLAKRL
PQRAGQVDVDLAPRLLAPAFTDRIAQRRGQDGRYLLANGMGAAMNQDEALSRAPWLIVPSLLQGHNSP
DARILLALPVDIDALAAQLPAIATQRTAVEWDDEKGTLRAWKRQQIGRLTLRAQPLAKPADEELQQAL
LDWVRAQGLAVLNWEGAAEQLRVRLQCAQAWLPEAEWPAMDEEPLLAALEQWLLPSLNGVRDLRGLKQ
VNIAEALSRLLDWQQKQRLDNALPTHYTVPTGSRLPIRYEAGKPPALAVRLQEVFGEQRSPMLAEGRI
PVVLELLSPAHRPLQITGDLAAFWQGAYREVQKEMKGRYPKHVWPDDPATAVPTRRTKKYQ
>CORE_REP|Org44_Gene3424#
MSNSYDSSSIKVLKGLDAVRKRPGMYIGDTDDGTGLHHMVFEVVDNAIDEALAGHCSDIQVTIHADNS
VSVQDDGRGIPTGIHPEEGVSAAEVIMTVLHAGGKFDDNSYKVSGGLHGVGVSVVNALSEKLELVIRR
EGKVHEQTYSHGEPQAPLTVVGETEQTGTMVRFWPSHQTFTNVTDFEYDILAKRLRELSFLNSGVSIR
LKDKRTDREDHFHYEGGIKAFVEYLNKNKTPIHPNVFYFSTVKDDIGVEVALQWNDGFQENIYCFTNN
IPQRDGGTHLVGFRTAMTRTLNSYMEKEGYSKKAKVSATGDDAREGLIAVVSVKVPDPKFSSQTKDKL
VSSEVKTAVETLMNEKLVDYLMENPGDAKIVVGKIIDAARAREAARKAREMTRRKGALDLAGLPGKLA
DCQERDPALSELYLVEGDSAGGSAKQGRNRKNQAILPLKGKILNVEKARFDKMLSSQEVATLITALGC
GIGRDEYSPDKLRYHSIIIMTDADVDGSHIRTLLLTFFYRQMPEIIERGHVFIAQPPLYKVKKGKQEQ
YIKDDEAMDQYQIAIAMDGATLHTNASAPALGGEQLEKLVAEHYAVQKLIGRMERRYPRALLNNLIYQ
PTLNEGDLSDEAKVKTWIASLVQALNDKEQHGSSYDFVIFENRERQMFEPALRIRTHGVDTDYKLDFD
FIHGGEYRKICQLGEKLRGLIEDGAFIERGERRQPVDSFEQALEWLVKESRRGLSVQRYKGLGEMNPE
QLWETTMDPESRRMLRVTVKDAIAADQLFTTLMGDAVEPRRAFIEENALKAANIDI
>CORE_REP|Org25_Gene2031#
MSQPTLKKDAFLAALARQWQRFGLSSAQQMTQHQWWEAVSAALAEQLAAQPAPRKSAKPLRHVNYISM
EFLIGRLTANNLINLGWYDTVEQALAEQNVKLADLLEQETDPALGNGGLGRLAACFLDSMATVEQPAT
GYGLNYQYGLFRQSFQGGQQQEAPDNWQRESYPWFRHNAALAVDVGIGGKLEKQADGRELWRPAFTLR
GEAWDLPVLGYRNGVAQPLRLWQATHQHPFNLSDFNDGKFLQAEKQGVEADKLTKVLYPNDNHQAGKR
LRLMQQYFQCACSVADILRKHHQAGRKIEELPKYEVIQLNDTHPTIAIPEMLRILLDEHQLEWEAAWA
ITSNTFAYTNHTLMPEALECWDEKLVRSLLPRHFSIIKQINARFKKLVDKHWPGDEAVWAKLAVHHNK
QVRMANLCVVSGFAVNGVAQLHSDLVVKDLFPEYHQLWPNKFHNVTNGITPRRWLKQCNPALSGLIDE
TLKVEWASDLDALRGLEKFADDAAFRQRYQQIKRDNKVALAHYVHGVMGLTLDPDAIFDVQIKRLHEY
KRQHLNLLHILSLYRQLRDNPNLDIVPRVFLFGAKAAPGYYLAKNIIYAINQAAEKINHDPLVKDRLK
VAFIPDYRVSVAELMIPAADISEQISTAGKEASGTGNMKLALNGALTVGTLDGANVEIAEQVGEDNIF
IFGNTVEQVKAIVAKGYDPLSYRKKDKHLKAILDELASGAFSHGDKHAFDMMLHSLLEGGDPYLVLAD
FASYCQAQRRVDELYRDRDEWTRRTILNTARVGMFSSDRSIRDYQQRIWQAKR
>CORE_REP|Org37_Gene2911#
MHRPFCYTLLASSLWFSCGALAQPAGDLPLMPWPQQVEVTQPAGKLVLDHRLSLTLQGDDLGDALPRW
RQRIELQTGWTLAPAGEAKDGAAIKVVIKDRVAAQPLPGSDESYRLAVTPQGATLTANTRFGALRGME
TLLQLLQTDGQNTFLPLVDIRDVPRFPWRGVLLDSARHFLPLPDILRQLDGMAAAKFNVFHWHLTDDQ

GWRFASEHYPKLQQQASDGQFYTREQMRQVVAYATARGIRVVPEIDMPGHASSIAVAYPDLMSAPGPY
RMEREWGVHKPTLDPTRDEVYQFVDTIVGELAAIFPDPYLHIGGDEVDASQWRASPSIQAFMQKNGLA
DTHALQAYFNQKLEKILEKHQRQMVGWDEIYHPSLPRSIVIQSWQGQDSLGASAQDGYQGILSTGFYL
DQPQSTAYHYRNEILPQPLGIETAVQPGEQAQSWRFSMPRLKGSAVEGSFTLIEGKQGWRGFIDFNGK
SRRAVHDIVWRTPQQVTFRVDTWMGDTRPVFTLQQDKLSGYTLVGNVRYPTRGDKLAAVPAGKMPVVP
DEKGQANILGGEAALWAENVRAPLLDLKLWPRAFAVAERLWSAQDVTDESNMYRRLAAIDAWSVVSVG
LQQHAETAREFTRLSNSVQIAPLQILAEAVEPAQYYTRQHLKFQAGNYHQFEPLNRFADALPPESGAV
RDMHAQVAALLQDKRDKAAAQALRERLQRWQANGAALQTAIAGNRTLRDLAPVAQDVGALATLGLTLL
DRHQQGKPLSRAEAEQAQRRLDAAAQTRDEVVIAAVYPLEALLRGMQTAP
>CORE_REP|Org31_Gene1739#
MKFSELWLREWVNPAISSEALSDQITMAGLEVDGVDPVAGAFNGVVVGEVVECGQHPNADKLRVTKVN
VGGDRLLDIVCGAPNCRTGLKVAVATVGAVLPGDFKIKAAKLRGEPSEGMLCSFSELGISDDHDGIIE
LPLDAPIGTDIRDYLKLNDNTIEISVTPNRADCLGIIGVARDVGVLNQVALTEPDMSPVAATIDATLP
IRVDAPQACPRYLGRVVKGIDVKAPSPLWMREKLRRCGIRSIDAVVDVTNYVLLELGQPMHAFDLGRI
DGGIVVRMAEEGETLTLLDGNEAKLSADTLVIADHQKALAMGGIFGGEHSGVNGETQDVLLECAFFSP
LSITGRARRHGLHTDASHRYERGVDPALQYKSMERATRLLLDICGGQAGPVIDVTHENELPKCATITL
RREKLDRLIGHVVPSEQVSDILRRLGCQVTEQGDSWQAVAPSWRFDMEIEEDLVEEVARVYGYDNIPD
VPVRADLVMTQHREADLTLKRVKTMLVDHGYQEAITYSFVDPKVQALLHPNEEALILPSPISVEMSAM
RLSLWTGLLSAVVYNQNRQQTRLRLFESGLRFVPDSAADLGIRQDVMLAGVIAGHTHDEHWDLARKPV
DFYDLKGDLESVLELTGKLSEIQFRAEANPALHPGQSAAIYLHGERVGFIGVIHPELERKLDLNGRTV
VFELEWNKLASRAVPQAREISRFPANRRDIAVVVAENVPAEDILAECKKVGANQVVGVNLFDVYRGKG
VAEGDKSLAISLVLQDTARTLEEEEIAATVAKCVEALKQRFQASLRD
>CORE_REP|Org21_Gene1674#
MKKSFPTLLATMIWTALYSQHALADLAEQCMLGVPVYDKPLVSGDPNSQPVTINADDSRADYPKSALF
SGNVHIEQGNSTLTAKEVELNQTQKPGQTEPVRTVTATGDVHYSDNQIKLKGPKAWSNLNTKDTDVYE
GDYQMVGRQGRGDADKMKMRGANRYTILENGTFTSCLPGDDSWSVVGSEVIHDREEQVAEVWNARFRI
GGVPVFYSPYLQLPVGDKRRSGFLIPNAKYGSNNGFEFMLPYYWNIAPNYDATITPHYMSKRGLQWQT
EFRYLVQPGLGLMEFDWLPDDKEYGKDNDDSKRWLFYWNHNGVMDQVWRFNVDYTKVSDYKYFTDLDS
KYGSTTDGYATQKFSLGYANENWNATLSSKQFQIFDTTDRTRSDTYKVQPQLDLNYYKNDLGPFDFHI
YGQAAKFTSVNPYSPDATRLHMEPTLNLPLTNGWASLNTEAKLMATHYQQDIPDGFAANYESRKSTQN
NPVTAPNLDNSVNRVLPQFKVDGKLVFERPMIWAEGATQTLEPRVQYLYVPYRDQSNIYTYDTTLLQT
DYSGLFRDRTYSGLDRIASQNRVSTGLTTRIYDDALVERFNASVGQIYYFSRSRTGDQVTGYDNNDDT
GSVAWAGDTYWKIDDRWGLRGGLQYDTRLNSVSLGNGVVEYRQDAERVVQLNYRYATPEYIQTALNTK
TVPAFQDGISQVGITGSWPIADRWAVVGAYYYDTRAKQSADQLVGLKYNTCCWAVTLGYERKITDWNN
SNNTSVYDNRVSFNVELRGLSSDHSLGSAEMLRSGILPYQRAF
>CORE_REP|Org6_Gene3848#
MTALQQGFLLTRHWRDTSAGTEVEFWLATDAGPRQLRLPPQTSVAFIPAEHRQRAELLLREERQVELK
PLALTDFHHRPMLGLYCRQHRQLIKLEKLLREGGVAVYEADIRPPERYLMERFITAPVWFNGQGDGNG
PLLSGQMKPAPEYRPTLKLVSLDIETTAHGELYSIALEGCGQRQVYMLGPANGGGAPLDFDLEYCASR
PQLLERLNAWLERHDPDAIIGWNLVQFDLRVLQKHAERYQIPLRLGRGGNLLEWREHGFKQNHFFAAA
AGRLIIDGIEALKSATWNFPSFSLEYVSQELLGEGKAIDNPYQRMAEIDRRFAEDKPALARYNLKDCE
LVTRIFAKADLLNFLLERATVTGLAADRSGGSVAAFSHLYMPRMHRLGFVAPNLGEQPEEHSPGGFVM
DSQPGLYDSVLVLDYKSLYPSIIRTFLIDPVGLVEGMRHPSDADSVPGFRNARFSRSKHCLPAIVEQI
WQGREAAKRQHNKPLSQALKIIMNALYGVLGSSGCRFFDPRLASSITLRGHEIMRQTRELIEAEGYQV
IYGDTDSTFVWLKQPHDEQQAAQIGRALVQRVNAWWQQHLQQQFGLENALELEFETHYSRFLMPTIRG
AEQGSKKRYAGLIARADGEEEMVYKGLETVRTDWTPLAQQFQQQLYQRIFKQQPYQDYVRDYVGKTLN
GDFDDQLVYRKRLRRKLDDYQRNVPPHARAARIADDYNRQQGRPLQYQNGGWISYVMTVAGPEPLETR
HSPIDYQHYLERQLQPVADAILPFLHDDFTTLVTGQMGLF
>CORE_REP|Org6_Gene4193#
MNDPLSRIIATELQARPEQVDSAIRLLDEGNTVPFIARYRKEVTGGLDDTQLRQLETRLGYLRELEDR
RQTILKSIDEQGKLTEQLAGAINATLSKTELEDLYLPYKPKRRTRGQIAIEAGLEPLADTLWQDPQQQ
PEQLAERYVDADKGVADVKAALDGARYILMERFAEDAALLAKVRDYLWKNAHLVSKVVEGKEEEGAKF
RDYFDHHEPISQVPSHRALAMFRGRNEGVLQLALNADPQFEEAPRESQAELIIINHLNLRLNNAPADA
WRKAVVNWTWRIKVLLHLETELMGTVRERAEDEAINVFARNMHDLLMAAPAGMRATMGLDPGLRTGVK

VAVVDATGKLVATDTVYPHTGQAAKAAAIVAALCIKHNVELVAIGNGTASRETERFYLDLQKQFGDVR
AQKVIVSEAGASVYSASELAAQEFPDLDVSLRGAVSIARRLQDPLAELVKIDPKSIGVGQYQHDVSQS
QLAKKLDSVVEDCVNAVGVDLNTASVPLLTRVAGLTRMMAQNIVNWRDENGRFSNREQLLKVSRLGPK
AFEQCAGFLRINHGDNPLDASTVHPEAYPVVQRILAATEQALQDLMGNASTVRSLKAVDFTDDKFGVP
TVTDILKELEKPGRDPRPEFKTATFAEGVETLNDLQPGMILEGSVTNVTNFGAFVDIGVHQDGLVHIS
SLADKFVEDPHTVVKAGDIVKVKVMEVDLQRKRIALSMRLDEQPGEGSPRRGGNAPAQTRDNANRSAG
GNKAKPRNAAPAGNSAMGDALAAAFGKKR
>CORE_REP|Org37_Gene4361#
MKKLTIGLIGNPNSGKTTLFNQLTGARQRVGNWAGVTVERKEGHFTTPQSDVRLVDLPGTYSLTTISE
QTSLDEQIACHYILSGDADLLINVVDASNLERNLYLTLQLLELGIPCIVALNMLDIATSQHIDIDVAA
LSARLGCPVVPMVSTRADGIGVLKQMIDNHHINEQQALVNYPPLLLKAVATLSDAMPQTLPAVQRRWL
ALQMLEGDIYSHRLAGPAVALLPAAIQALQQQQQQQEDPALVIADARYQSIAALCDAVSNSQQAMPNR
LTEMLDKVILNRWLGVPIFLLVMYLMFLLAINIGGALQPIFDIGSAAIFIQGIQWLGYTLHFPDWLTV
FLAQGIGGGINTVLPLVPQIGMMYLFLSFLEDSGYMARAAFVMDRLMQALGLPGKSFVPLIVGFGCNV
PSIMGARTLDAQRERLITIMMAPFMSCGARLAIFAVFAAAFFGQDGAGVVFSLYMLGIAVAILTGLVL
KYTIMRGEASPFVMELPVYHVPHLKSLLLQTWQRLKGFVLRAGKVIVVASMFIGGLNSFSFSGKTVDN
INDSALASVSKVLTPLLQPMGVHSDNWQATVGLVTGAMAKEVVVGTLNTLYTAEHINKEAFDAANFNL
LDELGGALNETWDGLKNTFSLSVLSNPIEASKGDGEMGVGSMGVMSSKFGSGISAYSYLIFVLLYVPC
VSVMGAIARESSRGWMTFSILWGLNVAYSLATLFYQVATFNQHPQYSLTAILVVLAVNLLVLFGLRRA
RSRVTVRLGNATPAACCQGAKGSCH
>CORE_REP|Org30_Gene995#
MNQSLLVTKRDGRKERINLDKIHRVIDWAAEGLHNVSVSQVELRSHIQFYDGIKTADIHETIIKAAAD
LISRDAPDYQYLAARLAIFHLRKKAYGQFEPPKLHAHVTRMVEMGKYDKHLLEDYSAEEFEQMDSFID
HWRDMNFSYAAVKQLEGKYLVQNRVSGEIYESAQFLYMLVSACLFSNYPRETRLDYVKRFYDAISTFK
ISLPTPIMSGVRTPTRQFSSCVLIECGDSLDSINATSSAIVKYVSQRAGIGINAGRIRALGSPIRGGE
AFHTGCIPFYKHFQTAVKSCSQGGVRGGAATLFYPMWHLEVESLLVLKNNRGVEGNRVRHMDYGVQLN
RLMYQRLIKGEDITLFSPSDVPGLYDAFFADQDEFERLYTQYEKDDSIRQKRVKAVELFSLMMQERAS
TGRIYIQNVDHCNTHSPFDPQIAPVRQSNLCLEIALPTKPLEDVNDENGEIALCTLSAFNLGAIDSLD
DLEELATLAVRSLDALLDYQDYPIKAAHRGAMGRRTLGIGVINFAYYLAKNGVRYSDGSANNLTHKTF
EAIQYYLLKASNRLAQEQGACPWFKETTYSQGILPIDTYKKDLDAVCSEPLHYDWETLRKEIQETGLR
NSTLSALMPSETSSQISNATNGIEPPRGHISIKASKDGILRQVVPEYERLKDNYELLWEMPSNDGYLQ
LVGLMQKFIDQSISANTNYDPTRFPGGKVPMKQLLKDLLTTYKFGVKTLYYQNTRDGAEDAQEDLQPA
KAGDDDCESGACKI
>CORE_REP|Org23_Gene1253#
MTELNEKLANAWEGFSKGDWQNEVNVRDFIQKNYTPYEGDESFLAGATQATTTLWDKVMEGIKLENRT
HAPVDFDTNVAATITSHDAGYIAKELETIVGLQTDAPLKRALIPFGGIKMVEGSCKVYGRELDPQLKK
VFTEYRKTHNQGVFDVYTKDILNCRKSGVLTGLPDAYGRGRIIGDYRRVALYGIDFLMADKLNQFKSL
QEKLENGEDLEMTIQLREEIAEQHRALAQIKEMAAKYGYDISGPATNAQQAVQWTYFGYLAAVKSQNG
AAMSFGRVSTFLDVFIERDIKAGKLTEEQAQELIDHLVMKLRMVRFLRTPEYDELFSGDPIWATESLA
GMGVDGRTLVTKNSFRFLNTLYTMGPSPEPNMTILWSEKLPLNFKKFAAKVSIDTSSVQYENDDLMRP
DFNNDDYAIACCVSPMIVGKQMQFFGARANLAKTMLYAINGGVDEKLKMQVGPKEAPMMDEVLDYDKV
MARMDHFMDWLAKQYVTALNIIHYMHDKYSYEAALMALHDRDVYRTMACGIAGLSVAADSLSAIKYAK
VTTIRDEDGLAIDFKVEGEYPQFGNNDARVDDIACDLVERFMKKIQKLRTYRNAVPTQSVLTITSNVV
YGKKTGNTPDGRRAGAPFGPGANPMHGRDQKGAVASLTSVAKLPFAYAKDGISYTFSIVPNALGKDDD
VRKANLAGLMDGYFHHEASIEGGQHLNVNVMNREMLLDAMENPEKYPQLTIRVSGYAVRFNSLTKEQQ
QDVITRTFTQTM
>CORE_REP|Org9_Gene770#
MLNQELELSLNMAFARAREHRHEFMTVEHLLLALLSNPAAREALEACTVDLAALRQELEAFIEQTTPT
LPAGEEERDTQPTLSFQRVLQRAVFHVQSSGRSEVSGANVLVAIFSEQESQAAYLLRKHDVSRLDVVN
FISHGTRKDEPGQAPNAENPVNEEQSGGEDRMENFTTNLNQLARVGGIDPLIGRDRELERAIQVLCRR
RKNNPLLVGESGVGKTAIAEGLAWRIVQGDVPEVMADCTLYSLDIGSLLAGTKYRGDFEKRFKALLKQ
LEQDQNSILFIDEIHTIIGAGAASGGQVDAANLIKPLLSSGKIRVIGSTTYQEFSNIFEKDRALARRF
QKIDITEPTAEETVQIINGLKTKYEAHHDVRYTAKAIRAAVELSVKYINDRHLPDKAIDVIDEAGARS
RLMPASKRKKTVNVADIESVVARIARIPEKTVSASDRDVLRNLGDRLKMLVFGQDQAIEALTEAIKMS

RAGLGHERKPVGSFLFAGPTGVGKTEVTVQLAKAMDIELLRFDMSEYMERHTVSRLIGAPPGYVGYDQ
GGLLTDAVIKHPHAVVLLDEIEKAHPDVFNLLLQVMDNGTLTDNNGRKADFRNVILVMTTNAGVRETE
RKSIGLVQQDNSTDAMEEIKKVFTPEFRNRLDNIIWFNHLSTEVIQQVVDKFIVELQAQLDAKGVSLE
VSDEARDWLSVKGYDRAMGARPMARVMQENLKKPLANELLFGSLVDGGSVKVELDKDKKQLTYHFLSA
AKRKADEGAVH
>CORE_REP|Org33_Gene642#
MDEQLKQSALDFHQFPVPGKIQVSPTKPLATQRDLALAYSPGVAAPCLEIAEDPLAAYKYTARGNLVA
VISNGTAVLGLGNIGALAGKPVMEGKGVLFKKFSGIDVFDIEVDEHNPDKLIDIIAALEPTFGGINLE
DIKAPECFYIEQKLRERMKIPVFHDDQHGTAIITTAAVLNGLRVVKKNISDVRLVVSGAGAASIACLN
LLVALGLRQQNITVCDSKGVIYKGRDANMEQTKAAYAIEDNGQRTLGDAIPNADIFLGCSGPGVLTQD
MVKTMARDPLIMALANPEPEILPPLAKAVRPDAIICTGRSDYPNQVNNVLCFPFIFRGALDVGATTIN
EEMKLACVHAIADLALAEQSDVVASAYDDQDLSFGPEYIIPKPFDPRLIVKIAPAVAKAAMDSGVATR
PIEDFDAYVEKLAEFVYKTNLFMKPIFSQARKEVKRVVLAEGEEERVLHATQELVSQGLAFPILVGRP
SVIEMRLKKLGLQLTPGKDFEVVNNESDPRFNEYWGEYYQIMKRRGVSQEQARRAVIGNPTLIAAIML
HRGEADAMICGTIGSYHEHYDVVKNVFGFREGAHVAGAMNALLLPSGNTFIADTYVNDDPTPEQLAEI
TLMAAETVRRFGIEPKVALLSHSSFGSSDCPAARKMRKTLELVNELAPELEIDGEMHGDAALVESIRH
DLMPDSPLKGSANLLIMPNMEAARISYNLLRVSCSEGVTVGPVLMGVAKPVHILTPIASVRRIVNMVA
LAVVEAQTEPL
>CORE_REP|Org37_Gene3353#
MKISLDLAIFAVICGILPLLILPRLPEPWLQWPMLFVACLLLRTRWPICRYLACLGLGFIWAVFNAGS
LLGQMERLSCMPDVTAVAQVSSVALEPAASKQTLMRIERVDGHWLVPALAFTTTWAPERQRLCAGQRW
QLKLRLRPVHGKLNEGGFDSQRWAIAQRQPLTAQVRQARLLDGDCGLRQRIISHAETNIGELRYKAVL
LALAFGERTALEQALRTLMLKTGIAHLMAISGLHVAMVAILFWAVLRALQFFLPAHLIGYRFPLVAGW
VATLIYVWLVGAQPPAVRTVLAMTLWMLLRLRGVHCSSWQVWLWCVGLILLCDPLAVLSDSFWLSVLA
VGCLIFWFEWAPLGERFRSAWYWAPVRWLHIQLGMTLLLVPMQVALFLGLTLTSLPANLWAVPIVSLV
TVPLILLAVIGGVFPSLSYGLWWLADFTLSGVFVPLHYLQRGWVDLGAASLLASIAGWLIVICWRFHW
WWRYAPGLATIAICCVLWRGKEPGYRWRVDMLDVGHGLAMVIEQNGKGILYDTGDRWPAGSAAERHIL
PMLNWRGIELEQIIISHAHLDHIGGLSTVQSAFPQATVRSPIRGEGHLPCVAGERWRWQSLQFEVLWP
PKTLKRPVNDDSCVIRIDDGKYSLLLTGDAEKKAEAQLIRLRRDRLAATVLQVGHHGSRTSSTPPFLR
AVNPEVALASASRYNKWRLPARKVVARYRANGITWRDTTRSGQLSVLFFDNDWQIKGFREQLMPRWYH
QRFGVEGDNE
>CORE_REP|Org48_Gene2830#
MLTRLREIVEKVAAAASLTDALDLLVNETCLAMDTEVCSIYLADNDRRCYYLMATRGLKKPRGRTIAL
AFDEGVVGLVGRRAEPINLADAQSHPSFKYVPQVKEDRFRSFLGVPIIHRRQLLGVLVVQQRELRQFD
ESEESFMVTLATQMAGILSQSQLNAIFGQYRQTRVRALAASPGVAVAEGWQDSSQPSLDQVYRASTLD
TASERERLTLALEEAGAEFRRFSKRFAASSQKESAAIFDLYSHLLNDARLKRELFAEIDNGSVAEWAV
KQVIEAFAEQFAKLQDTYMRERGSDLRALGQRLLFHLDDTTQGATQWPARFVLVADELTATLLAEVPQ
DRLVGVVVRDGAANSHAAILVRAMGVPTVMGADIQPSLLSQRLLIVDGYRGELLVDPEPVLVQEYQRL
ISEEQELSKLAEDDVEQPAQLKSGERVQVMLNAGLSPEHEQLLGGRVDGVGLYRTEIPFMLQSGFPSE
EEQVAQYQGMLQLYPNKPVTLRTLDIGADKQLPYMPISEENPCLGWRGIRITLDQPEIFLIQVRAMLR
ANAGTGNLGILLPMVTSLEEVDEAKRLIDRAGREVEEVLGYAIPKPKIGVMLEVPSMIFLIPHLAGRV
DFISVGTNDLTQYLLAVDRNNTRVASLYDSLHPAMLQVLKLIAEQGKAAGLQLSLCGELAGDPMGALL
LVGMGYRNLSMNGRSVARIKYLLRHIDLADAEVLALRVLNTQMTTEVRHLVAAFMERRGMGGLIRGGR
>CORE_REP|Org13_Gene2736#
MKSFNRSGIYLAVMSAMLPGAALAADATDVGTISVKGQSLGGGMMVQDDSAKARSTVTKEAMDKMPSA
ANAIDKLKYTPGLNVNSNDASGLSGVDYTMRGMNSDQIGLSMDGIPINDSGNYAVYPNLLGDAENLEE
VFVTQGSSEADGPHIGSSGGNIGLVTRRPAKDFGGFVKQTLGSNSLSKTFARLDTGEYNGFSNWLSYS
HTEAKKWRGEGRLYSDKFEMNSLYEDGNGNSSNLVMKYNRQNNTNYNTLSKAQFQNDGRDTDYVTTPE
YNNKGQLNKYYKIERNPFENFTLSFTQKLQLRDNLSLTLQPYYYWGNGGSFNGQTASVLSNTSSKAGQ
YDLSNLKSNTYYRPSWTQTWRPGITTKLKWDINEQHSLDVGYWYERARQLQTQPFISIKGDGNPSQIW
GQPGGSDQVKDANGNTVQGRNQYTITPAQKVWLQDTWFATPDWTFVGGLAYQYVERKGDNRGSLYNVP
EKRKATYHEFLPNFSASYKVNQENQVFYNLTRNMRTPPNYVLYNVGDSLSTKPELSWNHELGWRFQQE
DMLLSATLFYMRYSDRQISTTNSAGDYEMMNIGNVENKGLELEWSGQLPHNFNYYTSYTYTESKQKSD
IVSNGGLPLPTSGKEVPNVPKNLLNMTLGYDDGLYYGSVSGKYVSSFYGDLTNDEKIGGRTVFDLAAG

VHLPVDKKIVKSAALRFGISNLFDKEYLTSVRTTTFNAAPYGGVKASTPYYNVGEERTFSVSLEATF
>CORE_REP|Org2_Gene3241#
MKRKHLWVLNPCLLAMLAPAAWAEDQKTGNEEQLVVSASRSHRSVAEMAQTTWVIESQEIEQQVQGGK
EIKDMLAQLIPGMDVSGQGRTNYGMNIRGRSMMVMIDGVRLNSSRSDSRQLDSIDPFNIDHIEVISGA
TSLYGGGSTGGLINIVTKKGQPEQQVELQIGGKTGFGGHNDHDENVAAAVSGGNDNASGRLSVSYQRY
GGWYDGKGNEVLIDNTQTSLQYSDRLDVMGTGTLNIDDHQQLQLTTQYYKSQSDGDHGLFLGENFAAV
TGNAKAYNSGSLDSDRIPGTERHLINLQYSNTDFLGQDLVAQVYYRDETLTFYPFPTLAGKAPNYYVS
SIGASQQKTDFYGGKLTLNSKPVDALTLTYGIDAEHESFNANQQFFNLAKAQQSGGMTLENAYSTGRY
PSYTTSNLASFLQASYDINPIFTLSGGVRYQYTENKIDDFVGYNQQQAIATGAAASADAIPGGKTDYN
NALFNAGLLAHLTERQQTWFNFSQGFEIPDPGKYYGNGTYALNGGHYQLLKSVNVGDSRLEGIKVNAY
ELGWRYTGDNLRTQIAAYYSLSDKSIAINKTDMTINVNADKRRIYGVEGAVDYFFEDSDWSAGTNFNV
IRSETKVNGEWKKLVVDTASPSKVTAYVGWAPGDWNLRLQSQQTFDVSDDGDYTKANSTQGRKIDGYN
TLDFLGSYALPVGKISFSVENLLDKEYTTVWGQRAPILYSPTYGSPELYSYKGRGRTFGLNYSVLF
>CORE_REP|Org16_Gene928#
MVAVRSAHLNTAGEFALDEWIAGLGLPNPQSCERLAATWRYCEQQTQNHPDASLLLWRGLEMVEILST
LSMDNDSMRAALLFPLVDAGIVQEETLTEAFGNGIVALVHGVRDMDAIRQLKATQNDSMASEQVDNVR
RMLLAMVEDFRCVVIKLAERIAHLREVKDAPEDERVLAAKECSNIYAPLANRLGIGQLKWELEDFCFR
YLHPEEYKRIAKLLHERRIDREQFIDDFVAGLRAEMAKEGIRVEIYGRPKHIYSIWRKMQKKHLAFDE
LFDVRAVRIVAERLQDCYAALGIVHTHFRHLPDEFDDYVANPKPNGYQSIHTVVLGPRGKTVEIQIRT
RQMHEDAELGVAAHWKYKEGAGVTVRSGYEERIAWLRKLIAWQEEMADSGEMLDEVRSQVFDDRVYVF
TPKGDVVDLPAGSTPLDFAYHIHSDVGHRCIGAKIGGRIVPFTYQLKMGDQIEIITQKQPNPSRDWLN
PNLGYVTTSRGRSKIHNWFRKQDRDKNILAGRQMLDNELEHLGISLKEAEKLLIPRYNMNSLDEVLAA
IGGGDIRLNQMVNYLQGKFNKPSAEEQDREALRQLVQQKAPPPTRNKDNGRVVVEGVGNLMHHIARCC
QPIPGDDIVGFITQGRGISIHRADCDQLVDLQSHAPERIVDAVWGESYSSGYSLVVRVMANDRSGLLR
DITTILANEKVNVLGVASRSDTKKQLATIDMDIEIYNQQVLSRVLAKLNQLPDVIDAKRLHGN
>CORE_REP|Org10_Gene3143#
MPTKRLSSSAAKQGRLPVSALAITVAAALGTLAMPAFSADAKPAAKEDTITVVGGSNSAQQESAWGPV
GTYVAKRSATGTKTDTPIEKNPQSVSVVTREEMDMRQPDTVKSALAYTPGVMIGNRGASTAYDAVNIR
GFSSVGTNMYLDGLKLQDDNYSIYQIDPYFLERAEVLRGPSSVLYGKSNPGGVVALVSKRPTTETLRE
VQFKMGTDNLFQTGFDFSDALDDAGVYSYRLTGVARDEDQQQVGEKSKRYAIAPSFSWRPDDRTSLTF
LSSFQDDPSVGFYGWLPKEGTVQNGVNGKLPTSFNDGEPGYNNISRKQQMVGYAFEHAFDDVWTVRQN
LRYSKMDVDYRSIYGLGIDPDNSAELKRGVMNSKEHMSSFAVDTQAQAKFATGQVDHIVLMGVDYMRM
RNDVVYQYGSASNLNVIAPQYGNRSYTITGGASQVNRQEQTGLYVQDQAEWNNWVLTMGGRYDWSDTN
STNRLNQNSVSKQQDKQFTGRAGLNYVFENGIAPYVSYSESFEPTSGTDFSGNTFAASKGKQYEAGVK
YAPKDRPITASLALYQLTKTNNKVADPNPEHAFASILGGEIRSRGVELEAKAALTANLNILGSYTYTN
TEYTKDTTLQGNTPAAIPKHMASLWADYTFHETAISGLTLGSGVRYVGSSYGDEANTFKVKDYTVFDA
AIKYDLARFNLPGSSIGINVNNLFDKEYVSSCFATYGCYWGAERQVVATATFRF
>CORE_REP|Org40_Gene4145#
MRLSTLVLAIGMALGSQAQAAETQPHADHSALPSGQAKEATVTGEANQHDKQKTQNPFFYQSRLPFQA
PPFNLIKESDYAPAIEAGIKQKREEVEKIANNPAKPNFKNTFVALEQAGSLLTRVMNVFGAMTSANTS
DALQKLDEETSPMLAALNDDIMLNGKLFARIKAIYQDRDALKLDPESRRLVEVTYKNFELAGANLSDA
DKAKLKALNQEAATLSTQFTNKLLAASKNGALAITDQAKLDGLSEGELAAAAQAAAERKLDKQWLLVL
QNTTQQPLLQSLKDRDTRQALFDASWTRAEKGDGNDTRQTLSRLAKVRAEQAKLLGYPNYAAWKLQNQ
MAKTPDAALSFMRNIVPAATARAEREAKDIQAVIDQQKGDFKVQAWDWQFYAEQVRKAKYDLDESQIK
PYFELNNVLNNGVFYAANLLYGISFKERKDIPVYQPDVKVYEVFDKDGKSLALFYTDYFKRDNKGGGA
WMSNFVDQSKLNGTKPVIYNVANFTKPAPGQPALLSYDDVITMFHEFGHALHGMFADQEYPSLSGTNT
ARDFVEFPSQFNEHWVSDPKVFSHFAKHYQTGETMPQELVDKIKKADKFNKGYSMTELLSAALLDMHW
HMLTADQPQQDVDKFEAESLQKDKVDLSYVPPRYRSSYFQHIWGNGYAAGYYAYLWTEMLADDAFQWF
TEHGGLTAENGQRFRDMILSRGNSQDLEKLYIDWRGKEPSIEPMLINRGLKDE
>CORE_REP|Org5_Gene3766#
MTTESKCPFSGGKQPAPQNGPTNQDWWPNQLSLKPLHQHSPLSDPMDKDFNYADAFNSLDLAAVKQDL
HALMTDSQEWWPADFGHYGGLFIRMAWHSAGTYRIGDGRGGAGEGQQRFAPLNSWPDNVSLDKARRLL
WPIKQKYGRNISWADLIILTGNVALESMGFKTFGYAGGRADTWEPDDVYWGSEKIWLELSGGPNSRYS
GDRDLENPLAAVQMGLIYVNPEGPDGNPDPVAAARDIRETFARMAMNDEETVALIAGGHTFGKTHGAG

PASNVGADPEAAGLESQGLGWHSTFGTGVGKDAITSGLEVTWTTTPTQWNHDFFRHLFEYEWELSQSP
AGAHQWVAKDIGETIPDAFDPNKKRRPTMLTTDLSLRFDPAYEKISRRFYEHPEELADAFARAWFKLT
HRDMGPRPRYLGPEVPQEELIWQDPIPAVDHPLIDEQDIAALKNAVLASGLPVSALVSTAWASASSFR
GSDKRGGANGARIRLAPQKDWAVNQPAQLAATLATLESIQRTFNDAQAGGKRVSLADLIVLAGAAGVE
QAAKNAGLALTVPFAPGRMDASQEQTDVDSFEAMEPLADGFRNFLKGKYRVPAETLLVDKAQLLTLTA
PEMTVLVGGLRVLGANVGGTPHGVFTQRPQALTNDFFVNLLDMGTTWHPVGEDGLFEGRDRRSGAVKW
TGTRVDLVFGSHAQLRALAEVYGSADAQEKFAHDFVAAWNKVMNLDRFDLA
>CORE_REP|Org2_Gene2457#
MPVVHVALPVPLARTFDYLLPPGMQPVAGARVGVPWGRQHAIGIVTGCSDTSELPLDKLKPIDSVIDA
ESLFSPSLWRILRWASDYYHYPIGEVLFHALPILLRQGKPAEAAPLWQWFATEEGRATPPESLKRAPK
QQQALAALLQRPVYRHQVSQLELTESALQALRAKGLIDLRAQVADTHDWRPNFAVLGERLRLNTEQAT
AVGAIRSEDEQFAAWLLAGVTGSGKTEVYLSVLENVLAKGRQALVLVPEIGLTPQTIARFRERFNAPV
DVLHSGLNDSERLAVWLRARSGEAAIVIGTRSALFTPFRQLGVIIIDEEHDSSYKQQEGWRYHARDLA
VFRAREEDIPMVMGSATPALETLHNVQLGKYRQLKLTQRAGNAKPATQHLIDLKGLPLKVGLSQPLLK
SMQHHLKAGNQVMLFLNRRGYAPALLCHECGWIAECQRCDHYYTFHQHQRQLRCHHCDSQRPVPHQCP
QCGSTHLVSVGVGTEQLEQELAPLFPDTPITRIDRDTTSRKGALEQHLADIHRGEARILIGTQMLAKG
HHFPDVTLVALLDVDGALFSADFRSAERFAQLYTQVSGRAGRAGKQGEVLLQTHHPEHPLLQVLLQQG
YDAFAKQTLAERNSVFLPPYTSHIIVRAEDHDNQQAPLFLQQLRNLLEASPLKDDSLWVMGPVPALQS
KRGGRFRWQLLLQHPTRRVLQQLMKSSLPLIGTLPQTRKVKWTLDVDPIDS
>CORE_REP|Org17_Gene2248#
MLYQGETLQLHWLDNGIAELVFNAPGSVNKLDTRTVASLGEALTVLENQPELKGLLLRSTKAAFIVGA
DITEFLSLFAAPAEKLQEWLVFANNVFNRLEDLPVPTISAINGYALGGGCECILATDFRVASPDARIG
LPETKLGIMPGFGGSVRLPRLLGNDSALEIIAAGKDVSAKDALKVGLVDAVVAPEKLAEAALNMLQQA
IDGKLNWRAARQPKLEPLKLSPIEAAMSFTTAKGMVLQTAGKHYPAPMTAVKTIEAAAKLGRDEALKL
ETASFVPLARSNEARALVGIFLNDQFVKGQAKKLAKNVDAPKQAAVLGAGIMGGGIAYQSALKGVPVI
MKDISDKSLTLGMNEAAKLLNKQLERGKLDGLKMAQVLSTIQPTLDYAGIERAQVIVEAVVENPKVKA
AVLSEVENLIGEDTVLASNTSTIPINHLAKSLKRPQNFCGMHFFNPVHRMPLVEIIRGEQTSDETIAK
VVAYASRMGKTPIVVNDCPGFFVNRVLFPYFAGFSLLLRDGADFRQIDKVMEKQFGWPMGPAYLLDVV
GIDTAHHAQAVMAAGFPERMSKDYRDAIDVMFDNQRFGQKNQLGFYRYSQDNKGKPRKDNDEQTDALL
AEVSQPRQTISDEEIVARMMIPMINEVVRCLEEKIVASPAEADMALVYGIGFPPFHGGAFRYLDTLGT
ANYVELAQRYAHLGALYQVPAGLRAKAERNESYYPVATPLSDVATRQPA
>CORE_REP|Org40_Gene2964#
MKNNALSVMVAEQDEKLEWERLIGPLWDNRWRIAVVTGVAGMLGVAYALLATPVYQATAVVQVEKQLS
GDSLLRETLDSSMMGQNSATQDEVTLAKSRYVLGKTVDTLGLTVRVSPDYFPVFGKGFARLSGEKPPV
LSIATLTTPADMEGEALTLTVRDGQHYELSYDGSKLFSGVVGQPVAQGGWNMTVSALDASPGASFTVV
KVARQEAVDDLRKYLDVVPGGKDSGIMTFTLPSEDPQSAEAMLKNITDNYLQQNVDRKTEEAQRMLAF
LQEQLPQTQTSLNNAETQLNQFRQQNDSVDLSLEAKSVLDTQVQLEAQLNELTFKEAEISKLYTRAHP
AYRALLEKRATLEAEKARLGKQVQTLPKMQQEILRLTRDVQVDQQVYMQLMNKQQELSISKAGTVGNV
RIIDEAETALRPIKPQKMLIVLLALLLGAGGGAIVVLLRAAFHRGINDIDTLEKRGINVYATVPLSPW
QVKRNREQRQLLPRSGGRRLPILAVAEPTDLSVEAIRSLRTSLHFAMMEAKNNILMVSGASPESGKSF
TSTNLAVVVAQAGQRVLLIDADMRKGFLHRWLADDGHQGLSDMLVGNVMAEQAVRKTAIANLDFVPRG
QVPPNPSELLMHRRFADFLRWAGQNYDLVLIDTPPILAVTDAAIVGNHAGTSLLVVRFEVNTVKQIET
SMRRFEQNGVAIKGVILNGVVKKAATDMSYYNFAYPSHREDHPQAGE
>CORE_REP|Org12_Gene2380#
MKREGILKHIPWMLLGILGAACLGVVALRRGEHISALWIVVASVAVYLVAYRYYSLYIATKVMKLDAG
RATPAVVNNDGLNYVPTNKNVLFGHHFAAIAGAGPLVGPVLAAQVGYLPGTLWLLGGVVLAGAVQDFM
VLFISSRRNGASLGEIIKKEMGPIPGTIALFGCFLIMIIILAVLALIVVKALAESPWGVFTVCSTVPI
ALFMGIYMRYLRPGRVGEVSIIGIVLLVAAIWFGGVVAHDPYWGPALTFKDTTITFTLIGYAFVSALL
PVWLILAPRDYLATFLKIGVIVGLAIGIVILNPELKMPAVTQFVDGTGPVWKGTLFPFLFITIACGAV
SGFHALIASGTTPKLLANETDARFIGYGAMLMESFVAIMALVAASIIEPGLYFAMNTPPAALGITMPD
LHRLGTEDAPMIMASLKDVTVHAAAAVSSWGFVISPEQILQTATDIGEPSVLNRAGGAPTLAVGIAHV
FHQIIPGANMGFWYHFGILFEALFILTALDAGTRSGRFMLQDLLGNFVPFLKKTDSLVAGIVGTAGCV
GLWGYLLYQGVVDPLGGVKSLWPLFGISNQMLAAVALVLGTVVLIKMKRTQYIWVTVLPAVWLLICTT
YALGLKLFSDNPQLEGFFFLAGEYKRKIAEGGAELSAQQIANMNHIVVNNYTNAGLSILFLLVVYSII

FYGVKTAMAAHKNPKRTDQETPYVPVPQAAPTDGVTEGEVKVSTQH
>CORE_REP|Org34_Gene2798#
MTQTTANQHPRDRMRGRIDRGVQAAVTASGLMVLMTLMLIFVYLLFAVLPLFKPASLGQAQPLPIAVS
APALALGMDVQQRVGYRIDAQGAGQFYRLTPAPSGQAQTPLVQQTLLAKPALLAQAAGERDLFALAQA
NGRLVVARADFATAENGRPQWQFPLGQQPLALDPQNKPLKLLSLADAHRGQYLLAGVTDDRRLVFGRF
SPDRPPQFSERPLEHDGEQLVLTPDGRQLYLLTGNRLARYQIDGAQLQLRETRTLGEHAPYQMTALPG
GSALLIKGADGNLREWFEVEKDRRWRLTPVQHFDHGADGQELTVAEPYRRVFATLRPDGGFSLFSAIQ
PQPLLNTRLGAEVRQMAFAPRGDGLLLESAQGWQRYALDNPYPDVTWRSLWGKVWYENYPQPAYVWQS
TSGEDSYQPKFSLMPVIFGTFKAAAYAMLFAIPLALAGAIYTAYFMTAGLRRVIKPAIEVMGALPTVV
IGLVAGIWLAPIIEQYLLAVLALPLLLAAAVLLCGALTHRFMPRCRPGVDLLLLLPLLALTVWLAFSL
GPWLEVALFGEPLHFWLGDNYDQRNALVVGVAMGFALVPIIFSLAEDALFSVPATLSQGSLALGATQW
QTVIKVVLPSASAGIFSALMIGFGRAVGETMIVLMATGNTPIIDGSLFQGLRALAANIAIEMPEAVSG
SSHYRVLFLTALVLFVFTFVFNTLAEAVRLRLRKRYTPNQEAP
>CORE_REP|Org16_Gene1935#
MSTTSLIQPDRELFSYKPYWAECYGTAPFLPMSRAEMDQLGWDSCDVIIISGDAYVDHPSFGMAIIGR
MLEAQGFRVGIIAQPDWSNKEDFMRLGKPNLFFGITAGNMDSMINRYTADRKLRHDDAYTAGNVGGKR
PDRATLVYSQRCKEAYKDVPIVLGGIEASLRRIAHYDYWSDTVRRSVLVDSKADMLIYGNGERPLVEV
AHRLAAGEKIGDIHDIRNTAVMRKEALPGWSGVDSTRLDKPGRIEPIPNPYGEDLPCADGAKPKEPEA
KPVTVRAAKPKPWEKTYVLLPSFEKVKGDKVLYAHTSRILHHETNPGCARALMQKHGDRYVWINPPAI
PLTTPEMDSVFALPYQRVPHPSYGQDRIPAYDMIRFSVNIMRGCYGGCSFCSITEHEGRIIQSRSEDS
IVREIEEIRDKVPGFTGVISDLGGPTANMYMLRCTNPRAEQTCRRASCVYPEICTYMDTNHEPTIKLY
RRARSLEGIKKILIASGVRYDLAVEDPRYIKELATHHVGGYLKIAPEHTEEGPLSKMMKPGMGSYDRF
KQLFDHYSKQAGKEQYLIPYFISSHPGTRDEDMVNLALWLKKNRFRLDQVQNFYPSPMANSTTMYYSG
KNPLSKVGYKSEDVVVPRGERQRRLHKALLRYHDPANWALIRTALEEMGLKHLIGSRRECLVPAPSID
EQREAKRLQRHTRPALTKHTDINRQRTPSNRPPRKPIRKAKP
>CORE_REP|Org6_Gene3182#
MKELKIATSASLVATIETLRQIVRVEQTDCTDVAAAVVSVADVNAGILARLQATGFDIPTFVAVEGDE
HLSPDYLPFVSGVFALLAGASKPFYMAQLEAAADAYEQALLPPFFKTLKTYVEMENSTFACPGHQGGE
FFRKHPAGRQFFDFYGETLFRSDMCNADVKLGDLLIHEGSAKDAQKHAARVFNADKTYFVLNGTSAAN
KVVTNALLTRGDLVLFDRNNHKSNHHGALIQAGATPIYLETARNPFGFIGGIDAHCFDERYLREQIRE
VAPEKAAAARPFRLAIIQLGTYDGTIYNARQVIDTIGHLCDYILFDSAWVGYEGFIPMLKECSPLLLE
LDEHDPGIFVTQSVHKQQAGFSQTSQIHKKDDHIKGQKRHCNHKHLNNAFMLHASTSPFYPLFAALDV
NAKMHAGPAGRRMWMDCVKLGIETRKQLLTRCSQLKPFIPQQVAGKDWQDYDTDLIANDARFFTFVPG
ETWHGFEGYAQDQYLVDPCKLLLTTPGIDAATGQYTEFGVPATILANFLRENGIVPEKCDLNSILFLL
TPAENPAKMEQLVEMLAQFERYVEEDAPLSVVLPTVYRKNEQRYRGYSIRRLCQEMHDLYVSFDVKQL
QKEMFRQDHFPPVVMNPQDANVEFIRDNVELVPIGQAEGRIAAEGALPYPPGVLCVVPGEVWGGAVQR
YFLALEEGINRLPGFSPELQGVYIEKKDHGWKRIFGYMIKQ
>CORE_REP|Org28_Gene2061#
MSFENALHEQRAKPSAFQLTIRPDNIGVITIDVPGDKVNTLKAEFVEQVNDVLIRAQQHTALEGLVIV
SGKPDSFIAGADITMIAACTSAKEAETLAKKGQSTLAQIAAFPVPVVAAIHGACLGGGLELALACHGR
VCSLDDKTALGLPEVQLGLLPGSGGTQRLPRLVGAAKALDMILTGKHIRARQALRMGLVDDAVPQSIL
LQTAIERVKQGWKHQRELPWQDRLLNGPLGKNLLFSIVRKKTLAKTHGNYPAAERIIQVVRSGLDHGS
ASGYEAEARAFGELAMTPQSAALRSLFFASTALKKERGGNAQPRALHRVGILGGGLMGGGIACVTATR
GGLPVRIKDINETGINHALKYSWDVLGKRVRSKRMRPAERQKQMMLISGSTDYTGFEQVDIVVEAVFE
DLALKQQMVAEVEANCATHTVFASNTSSLPIGRIAEKAQRPQQVIGLHYFSPVDKMPLVEVIPHAGTS
EETIATTVALAHKQGKTAIVVGDSAGFYVNRILAPYINEAARCLLEGEPIESLDKALVDFGFPVGPIT
LLDEVGIDVGTKIIPVLVEALGPRFAAPAAFDAVLKDGRKGRKNGRGFYLYPSEGQQRQRRKRADTSL
YTLLGVTPKAHMLPATVAQRCVMMMLNEAARCLDEGVIRSARDGDVGAVFGIGFPPFLGGPFRYMDEL
GAEKVVKTLDYLRQQHGEHFAPCERLQRMAQQGERFYPLGS
>CORE_REP|Org29_Gene4668#
MRNNFLFGDKHDMNSLFASTARGLEELLKSELEALGAHDCKVVQGGVHFQGDDRLLYQSLLWSRLASR
ILLPLNEFRVHSDLDLYLGVQAIDWPSIFGVDKTFAVHFSGVNEEIRNSQYGALKVKDAIVDSFTRKL
DQRPTVAKQQPDIRVNVFLQRDMASVALDLSGEGLHQRGYRDLTGQAPLKENLAAAIVLRSGWQPGTP
MLDPMCGSGTLLIEAAMIAADRAPGLHRQHWGFTAWNGHNAELWREVTTEAQVRARRGLQETASRFFG

SDIDRRVIEMARGNARRAGVAELITFNVGDVARLTNPLPEGPHGTVISNPPYGERLESEPALIALHNM
LGRVMKSAFGGWQLSLFSASPELLSCLQLRAERQFKAKNGPLECVQKNYQLAANPAGGTTGGVQVAED
FANRLRKNLKKLDKWAKQQGIECYRLYDADLPEYNVAVDRYGSKVVVQEYAPPKTVDAQKARQRLFDV
INATLAVLELPSNQLILKTRERQKGKNQYEKLAQKGEFLLVEEYNAKLWVNLTDYLDTGLFLDHRIAR
RMLGEMSNGKDFLNLFAYTGTASVHAGLGGARSTTTVDMSRTYLEWAEKNLRANGLTGRQHRLIQADC
LSWLSNANEQFDVIFIDPPTFSNSKRMENTFDVQRDHLALMKDLKRLLRRNGTIMFSNNKRGFQMDMA
GLSALGLEAKEITAKTLSQDFARNRQIHNCWLVTHAGEGK
>CORE_REP|Org42_Gene4620#
MDVSDLLDSLNEKQREAVAAPRSNLLVLAGAGSGKTRVLVHRIAWLLSVENCSPYSIMAVTFTNKAAA
EMRHRIEHLIGTSQGGMWIGTFHGLAHRLLRAHHLEANLPQDFQILDSDDQLRLLKRIIKALNVDEKQ
WPPRQAMWYINGKKDEGLRPQHVETYNNPVEATWLRIYQAYQEACDRAGLVDFAELLLRAHELWLNKP
HILNHYRERFTNVLVDEFQDTNSIQYAWIRLLAGGNSNVMIVGDDDQSIYGWRGAQVENIQRFLKDFP
GAETIRLEQNYRSTSNILKAANTLIANNDGRMGKNLWTEGGEGEPISIYCAFNELDEARFVVNRIKTW
QDNGGALNDCAILYRSNAQSRVLEEALLQTAMPYRIYGGQRFFERQEIKDALAYLRLISNRNDDAAFE
RVVNTPTRGIGDRTLDVVRQAARDRQLTLWQATRELMHDKVLAGRAASALQRFIELVESLAHETADMP
LHVQTDRVIRDSGLFIMYEQEKGEKGQARIENLEELVTATRQFSYQDEDQDLMPLQAFLSHAALEAGE
GQADAYQDAVQLMTLHSAKGLEFPLVFIVGMEEGMFPSQMSLDEGGRLEEERRLAYVGVTRAMQKLTL
TYAETRRLYGKEVYHRPSRFIGELPEECVEEVRLRASVSRPVNHRRMGTPISENDTGYKLGQRVRHPK
FGEGTIVNLEGSGEHSRLQIAFPGEGIKWLVAAYARLETV
>CORE_REP|Org40_Gene2515#
MSRTIMLIPTGTSVGLTSVSLGVIRSMEQKGVRLSVFKPIAQPRTGDNALDQTTTIIRSSNSTITAAE
PLRMDYVEGLLSSNQQDVLMEEIVARYHENTKDAEVVLIEGLVPTRKHQFANALNYEIAKTLNAEIVF
VLALGNDSPAQLKERIELARTSFGGSKNKNITGVIINKLNAPVDDQGRTRPDLSEIFDDSTKASIAHV
DPAQLFANSPLPVLGCVPWSFDLIATRAIDMARHLKARVVNEGDIMTRRVKSVTFCARSIPHMLEHFR
PGSLLVTSADRPDVLVSACLAAMNGVEIGAILLTGGYAIDEPIKKLCERAFQTGLPVFMVDTNTWQTS
LSLQSFNLEVPADDHQRIEKVQNYVASHINTEWIDSLTATSERSRRLSPPAFRYELTELARKAGKRIV
LPEGDEPRTVKAAAICAERGIAECVLLGNPDEIQRVAAAQGVELGKGIEIVDPVAVRENYVPRLVELR
KSKGMTEVVAREQLEDNVVLGTLMLEQGEVDGLVSGAVHTTANTIRPPLQLIKTAPGSSLVSSVFFML
LPDQVLVYGDCAINPDPTAEQLSEIAIQSADSAAAFGIEPRVAMISYSTGNSGAGSDVEKVREATRLA
QEKRPDLIIDGPLQYDAAIMADVAKSKAPNSPVAGQATVFIFPDLNTGNTTYKAVQRSADLVSIGPML
QGMRKPVNDLSRGALVDDIVYTVALTAIQSSQADAAAAKA
>CORE_REP|Org24_Gene1133#
MIFSLLRALFRLLFRVRVEGDVQHFERQKLLITPNHVSFLDGVLLALFLPIKPVFAVYASIGESWFMR
WLRPYIDFVSLDPTKPMAIKQLVRMVEQGRPIVVFPEGRITVTGALMKIYDGAAFIAAKSGATVVPVR
IDGPEFSPFGRMAGVFKIRWFPQISIRILPPTTLPMPEAPRARERRALAGDRLMQIMMRARMDTREPQ
TLFHALLAAQHRYGRRKPCIEDIAFKEDSYQTLIKKSLGVSRILQRFTAEGEHVGLLLPNATITAAAI
FGASLRNRIPAMLNYTAGANGLNSAMLAAGIKTIVTSRQFLEKGKLTHLPEQVTQANWVYLEDLKDTV
TLADKLWILRHLLQPQRAALPQRPEDAALILFTSGSEGHPKGVVHSHASLLANVEQIRTIADFTPRDR
FMSSLPLFHSFGLTVGLLTPLITGSRIFLYPSPLHYRVVPELVYDRNCTVLFGTATFLNNYARFAHPY
DFARLRYVVAGAEKLADSTKQIYQDKYGIRILEGYGVTECAPVVSINVPLATKVGTVGRIMPQMEARL
IAVPGIDNGGRLQLKGPNIMKGYLRVERPGELEPPAAEDANGVLQPGWYDTGDIVSLDEQGYCTIRGR
VKRFAKLAGEMVSLESVELLAQRLSPEKMHAATAKGDSSKGEALVLFTTDPAITREALLRVARELGSP
ELAVPRDIRLLKTLPVLGSGKPDFVTLRHMAEQPESAR
>CORE_REP|Org41_Gene4663#
MPDLSRRDILRAAAIGSAFSLLPASIRKALAIPANNRTGTLRDVEHVVILMQENRSFDHYFGTLPGVR
GFSDRFTIPLPGDRHVWQQQGAERLVLPYHLDSKRGNAQRVTGTPHSWVDEQAAWDHGRMSAWPTYKT
PASMGYYRQHELPFQFALANAFTLCDAYHCAIHAGTNTNRLFHWTGTNGPSAADVAVVVNEWDSPGPA
EIGYQWTTYPERLEASGVSWKVYQFLPDNFTDNPLAGFRQYRAASIQVGNPARPPKDFNAFVPYRDAL
NEAAPLYKGNGNTLPAADGNDLDAMLAGFRADIQQGKLPQVSWIIAPAAYSEHPDPSSPVQGGWFTQE
ILNALTDNPEVWSKTVLLVNYDENDGFFDHMPSPSAPSLREDGSFAGKSTVPFDTEIFQHVAPPGSQD
QPPPDGRIYGPGPRVPMLVLSPWSRGGWVNSQVFDHTSVLQFLEKRFQVHEPNISAWRRAVCGDLTSA
FNFVDPNSEALPSLPVTSRHAADGLRQRQEQLPQVPLPSPAHQRLPHQRRQARPSRALPYQLHVEATV
VAEQRRVTLNLFNTGEQGAVFHVYDRRDLAQIPRRFTVEAGKAVSDDWQTEDEYHLWLLGPNGFHREL
RGALNRPQPEVRLRPTGRSLQLQLNNPGTEAIAVTLERCPYTQQGPWHITLPAGGSHQQSFDAHASGG

WYDLTLQSPGGWLRRLAGRLEDGEHSVSDPLMGQE
>CORE_REP|Org2_Gene4497#
MATTELTRPETGALDYHSLNAMLNLYDAEGRIQFDKDRLAARHYFLQHVNQNTVFFHNLEEKLRYLVE
EGYYEPQVLAQYEFPFIKQLFQQAYAKKFRFETFLGAFKYYTSYTLKTFDGKRYLERYEDRVCMVALT
LAAGDTGLAQDLVEEMISGRFQPATPTFLNCGKRQRGELVSCFLLRIEDNMESIGRAVNSALQLSKRG
GGVAFLLSNIREVGAPIKRIENQSSGVIPIMKMLEDAFSYANQLGARQGAGAVYLNAHHPDILRFLDT
KRENADEKIRIKTLSLGVVIPDITFELAKNNEEMYLFSPYDVERVYGVPFSEIAVSEKYREMVDDKRI
RKSRINAREFFQVLAEIQFESGYPYVMFEDTVNRENPIAGRINMSNLCSEILQVNRASTYHEDLSYDR
IGKDISCNLGSLNIAKTMDAPDFGKAIETAIRALTAVADMSDIRSVPSIAEGNRSARAIGLGQMNLHG
YLARERIFYGSEEGIDFTNLYFYTVAYHAIRASNRLAIERGGAFDGFEHSRYASGEYFDKYTERDWLP
QTERVRELFAAAGIAIPNRDDWRALRQSVMLHGLYNQNLQAVPPTGSISYINNATSSIHPIVSRIEIR
KEGKIGRVYYPAPYMTNDNLAYYQDAYEIGPEKIIDTYAAATQHVDQGLSLTLFFRDTATTRDINRAQ
IYAWRKGIKTIYYIRLRQMALEGTEVQGCVSCAL
>CORE_REP|Org38_Gene2989#
MNIIAIMGPTGVYYKDEPIRELHAALGAMGFQLVYPKNSGDLLKLIEANARICGVIFDWDDYSLELCS
EINELNEYLPLYAFINTHSTFDVSLHEMRMVLYFFEYGLNAADDIAQRIQQYTAEYIDTITPPLTKAL
FNYVREGKYTFCTPGHMAGTAFQKSPVGCLFYDFFGANTLKADISISVTELGSLLDHTGPHLEAEEYI
ARTFNAEQSYLVTNGTSTANKIVGMYSAPAGSTVLIDRNCHKSLCHLLMMSDIVPIYLRPLRNAYGIL
GGIPQREFTRASIAARVQETPNATWPVHAVITNSTYDGLLYNTDYIKQTLEVPSIHFDSAWVPYTNFH
PIYDGKSGMSGDRVPGKVFYETQSTHKLLAAFSQASMIHIKGDYDESTFNEAYMMHTTTSPHYGIVAS
METAAAMLRGNPGRRLINRSVERALHFRREVQRLREESDSWFFDIWQPEEIDEAQCWPLDPDDNWHGF
GQTDRDHMYLDPIKVTILTPGMNELGALEEEGIPAALVAKYLDERGIVVEKTGPYNLLFLFSIGIDKT
KAMSLLRGLTDFKRAYDLNLRVKNMLPDLYAEDPDFYRHMRIQDLAAGIHRLICQHDLPRLMQRAFDV
LPEMKLTPHQMFQEQVRGNVETCELDQLVGKVAANMILPYPPGVPLVMPGEMITEESRAVLDFLLMLC
SIGERYPGFETDIHGAKLTEDGRYLVKVLKAPQP
>CORE_REP|Org1_Gene4267#
MKPVVIKRDGCQVPFDEARIAQAIERAALAVGVVDADYCATVARVVAQRMELQPRVDIHEIQQAVENQ
LMVGEYKQLARAYIEYRHDRDVARELRGRLNQEIRGLVEQSNRALLNENANKDSKVIPTQRDLLAGIV
AKHYAKQHILPRDVVLAHERGEIHYHDLDYSPFFPMFNCMLIDLKGMLTNGFKMGNAEIEPPKSISTA
TAVTAQIIAQVASHIYGGTTINRIDEILAPFVDESFRKHLQVAEEWQIPDAKGYAMARTEKECYDAFQ
SLEYEVNTLHTANGQTPFVTFGFGLGTSWESRLIQRSILKNRIAGLGKNRKTAVFPKLVFAIRDGLNH
QYGDPNYDIKQLALECASKRMYPDILNYDQVVKVTGSFKTPMGCRSFLGTYEQDGELVHDGRNNIGVI
SLNLPRIALEAMGDESRFWALLDQRLQLAKKALMTRIARLEGIKARVAPILYMEGACGVRLKADDNIA
DIFKNGRASISLGYIGLHETINALFGGKNHVYDDEALRAKAVAIVARLRAAVDAWKDETGYGFSLYST
PSENLCDRFCRLDTADFGVVPGVTDKGYYTNSFHLDVEKKVNPYDKLDFEAPYPPLASGGFICYGEYP
NLQHNLKALEDVWDYSYTRVPYYGTNTPIDECYECGFTGEFSCTSKGFTCPKCGNHDSAKVSVTRRVC
GYLGSPDARPFNAGKQEEVKRRVKHLGNGQLG
>CORE_REP|Org43_Gene425#
MKDNNLFERINEKLTFSLRKRVPSILQSESSECGLACLAMIASYYGFNVDMLSLRQRFGISTQGATLG
TISQIASQIQLKTRALSLDIDEINQLKTPCILHWNMNHFVVLVKVQRAGFVIHDPAFGRRVIGLQEMS
NHFTGIALELWPDRAFQKETLKTRLRLLDLMKNIEGLPGTLLKIFALSIVIESVNLLLPVGTQLVTDH
VIQAHDYSLLTVICLGLIFFTLFRAVVSIARAWISIVLGTLTDIQWKTTLFEHLMKLPLDFFEKRHLG
DIQSRFSSLDAIRTTFTNNIVSGIIDGIMTVGLFAMMMVYGGWLVWVVAGFTLIYILIRMMTYRTYRQ
FSEEQIVKAAKANSHFMETLYGISTVKALGIKETRSSYWLNLNVDAANTNIKITRFNMMFGGINTFIT
TLDQVAILWLGAMMVIDNSMTLGMFMAFNAYRGQFSQRASSLIDLAIGLRMLSLHNERISDIVFTDAE
TESAPRQVFPSGTGIAIEVKNLTYQYDALSRPIFKDLNMRIAAGESVAVVGASGAGKTTLLKVMCGLL
SPTSGQVLADAMDIHKVGVNNYRNAIACVLQDDRLFSGSIAENISGFEVNANKELIMACAIHSNIHDE
IMQMPMGYETLIGELGNGISGGQKQRLFIARALYRRPSVLFMDEATSHLDVENESAINRAISSLNITR
VIVAHRKSTIDSADRVVVLGAESGAPAGGGE
>CORE_REP|Org8_Gene4201#
MNPLLFTSRRRIAAALWLSLGVAGAALAQPQPQPPLAERAPKALTAHGETRTDDYYWLRDDSRKEQKV
LNYLKAENRYTEQMMAPYQNLRATLYQEMLGRMSPDDRSVPYQLNGYRYQESYAAGKEFARYQRQALT
ADAPWQTLLDANQRAAGHAYYRLGAMDISRDNRRLAVAEDLQGRRQYRISLRELGSERWSPETLENTS
GNMVWANDNQTLFYVRNHPQTLLPYQVYRHQYGTPPAEDKLVYQENDPAFYLSLSRSSSRDYLILTIS

GNTTSEVRLIDASQPQREPQLFAARQNGREYYLDHYRGEFYLRSNHQDPNFGLYRTAAAGKPWQTLIA
PQAQHEVESFSLFRDWLVVQERANGLVQLRQISWDGKTERAIPFDDASYMAWLGYNPEPDSDRLRYGY
SAMTTPTRTYEWDLNKGERTLLKQQEVKGVDPSLYHSERIWIAARDGVKVPVSLVYRTSLFKNGHNPL
LVYGYGAYGMSMDPAFSANRISLLDRGFVYALIHVRGGGELGQRWYKQGKLTHKPNSFNDFIDATQAL
INDGYGQPGRIYAMGGSAGGLLMGAVINQAPQLYNAVVAQVPFVDVVTTMLDDSIPLTTGEYEEWGNP
HQPAAYALMKSYSPYDNVRRQHYPNLLVTSGLYDSQVQYWEPAKWVAKLRRFKQGDSLLLLSTDMTAG
HGGKSGRLARLENGALEYAFILAADRQAQK
>CORE_REP|Org12_Gene3625#
MNNNKRGWWCALPLAACATLPTWAAEKVASKEESLTVIGRKDADGVQSYQPLTSVTGTRSETNLLNVP
QAIDVVPQQVITDQAVSSLDEALYNVSGITQANTLGGTQDAVMKRGFGDNRDGSILRDGVRSVQARNF
TPTTERVEVLKGPASMLYGMGEPGGMINMITKKPQLQQHTHVEGWGSSFNGGGGQLDVTGPLGTSGFA
YRMIVDHDETDYWRNFGRNRQTVIAPSLMWYGENTTVRLAYEHMEYLVPFDRGTIIDSRTGKPVNTPR
DRRFDEAYNATRGDQDSITLQIDQTLNERWKSSLTYAYSRNSYSDNQARATALNPVTGVLSRQADSTA
NAVSHANAVQLTLNGDVDWGSINHQMLFGFDFEDNRTYRGDMIRGKKNSDFNIYHPVYGLMPPSTAVS
AKDSDQRENLTSYGWFMQDSIQLTDKWLVMGGLRYDAFDVYAGKGRPFQTNTDSSDGKLVPRAGVVYK
LTPYVSLYSSYTESFKPNSSIATQIDSLPPEQGKSWEVGGKLALPNGVTGTLALFDITKRNVMVNELV
EGETVTRTAGRVRSQGVELDVAGNITDSLSLIGSYAYTDARVVDDPDNKGKEMTNVARHTASLFLTQN
LGSLGLYSGDEVRIGAGARYVGRRPGDAANSFYLDNYTVADAFAAYTMPINGYRVKWQLNVKNLFDKT
YYPSSGGNLRVAVGEPREVVLRGSIDF
>CORE_REP|Org36_Gene2938#
MRHSQIKTADDRVYSARFEGAGESQPPFCFSPISRRAGLRVRRSLTIKQMATVSGVALVTICIFIVIQ
LFHFVQQRRDDYAQQLENIAHSVRQPLAEAVLRMDVPEAKKVLNTLLPVGILSRADIVLPNEFQALHA
NFPPERPVPTLIARLFELPIQISVPLYSLERVPANQQPLAYLVLQADSFRMYQFILSILSTMLSTYLL
LALILSVAITWCMNRLMVHPLRAMAKELENISQDEAPYHQLMLPALHQDDELGLLVRNYNRNQQTLAK
AHADMSRLSTRHPVTELPNPALLNALLEQHIASSLRPERFNLLVIGIETLHEASGVMSPAMREALLLA
LAKKLRGCIDENGVLAQLSNTEFAILAKGTERPFHAMQLARRIMAEINAPLTLEGLALRPNASIGIAH
YLNQGESAEQLLRSATSAMMSAHREGKNQILFFEPSLTERTQKRLTQESEILHGIEQRHFTLFLQPQI
DMQSNEVIGAEALLRWQQYDGSYTLPADVIPLAEELGVIVPLGNWVLEESCRILADWQQRGIELPLAV
NVSGIQMQDEAFVPHLKNLLAQYRIDPRKLLLEITETVRIDDLDRALALLRELHDLGLSIALDDFGMG
YSSLEYLNRLKSLPIDLIKIDRSFIQGLPADDAMVRIVSSISEVLALPVMAEGVENAEQRDWLLKHGI
RSGQGFLFARPLPREAFEAEFCRAAP
>CORE_REP|Org7_Gene773#
MLTPIIRKFQYGQHTVTIETGMMARQATAAVMVSMDDTAVFVTVVGQKKAKPGQSFFPLTVNYQERTY
AAGRIPGSFFRREGRPSEGETLTSRLIDRPIRPLFPDSFLNEVQVIATVVSVNPQVNPDIVAMIGASA
ALSLSGIPFNGPIGAARVGYINDQYVLNPTTDELKESRLDLVVAGTAGAVLMVESEADVLSEDQMLGA
VVFGHDQQQVVIENINALVAEAGKPKWDWQAPAVNEALHARVAELAEARLGDAYHITEKQERYAQVDA
IKDSVVETLLAQDETLDAGEIQDILGTVEKNVVRSRVLRGEPRIDGREKDMIRGLDVRTGVLPRTHGS
ALFTRGETQALVTATLGTARDAQNLDELMGEKTDSFLFHYNFPPYSVGETGMVGSPKRREIGHGRLAK
RGVLAMMPKPEDFPYTVRVVSEITESNGSSSMASVCGASLALMDAGVPIKAAVAGIAMGLVKEADNYV
VLSDILGDEDHLGDMDFKVAGSRDGITALQMDIKIEGITREIMQVALNQAKGARLHILGVMEQAISTP
RGDISEFAPRIHTIRINPDKIKDVIGKGGSVIRALTEETGTTIEIEDDGTVKIAATDGEKAKFAIRRI
EEITAEIEVGRIYQGKVTRIVDFGAFVAIGGGKEGLVHISQIADKRVEKVTDYLQMGQEVPVKVLEVD
RQGRVRLSIKEAMAPEAGSPAPEAE
>CORE_REP|Org36_Gene4690#
MARTTPIARYRNIGISAHIDAGKTTTTERILFYTGVNHKIGEVHDGAATMDWMEQEQERGITITSAAT
TAFWSGMAKQFEPHRVNIIDTPGHVDFTIEVERSMRVLDGAVMVYCAVGGVQPQSETVWRQANKYKVP
RIAFVNKMDRMGANFLKVVGQIKSRLGANPVPLQLAIGAEDKFTGVIDLVKMKAINWNEEDAGVTFEY
EDVPADMMDLAEEWRQNLIESAAEASEELMEKYLGGEELTEAEIKSALRQRVLNNEIILVTCGSAFKN
KGVQAMLDAVIEYLPAPTDVPAINGILDDGKDTPAERHASDDEPFSALAFKIATDPFVGNLTFFRVYS
GVVNSGDTVLNSVKSARERFGRIVQMHANKREEIKEVRAGDIAAAIGLKDVTTGDTLCDPDSPIILER
MEFPEPVISIAVEPKTKADQEKMGLALGRLAKEDPSFRVWTDEESNQTIIAGMGELHLDIIVDRMKRE
FNVEANVGKPQVAYREAIRAKITDVEGKHAKQSGGRGQYGHVVIDMYPLEPGSNPKGYEFINDIKGGV
IPGEYIPAVDKGIQEQLKSGPLAGYPVVDMGIRLHFGSYHDVDSSELAFKLAASIAFKEGFKKAKPVL
LEPIMKVEVETPEENTGDVIGDLSRRRGMLRGQESEVTGVKIHAEVPLSEMFGYATQLRSLTKGRASY

TMEFLKYDDAPNNVAQAVIEARGK
>CORE_REP|Org16_Gene630#
MYLFESLNLLIQRYLPEEQIKRLKQAYLVARDAHEGQTRSSGEPYITHPVAVACILAEMRLDHETLMA
ALLHDVIEDTPATYQDMEQLFGKSVAELVEGVSKLDKLKFQDKKEAQAENFRKMIMAMVQDIRVVLIK
LADRTHNMRTLGSLRPDKRRRIARETLEIYSPLAHRLGIHHLKTELEELGFEALYPNRYRVIKEVVKA
ARGNRKEMIQKILSEIEGRLTEAGIACRVSGREKHLYSIYLKMHLKEQRFHSIMDIYAFRVIVKEVDT
CYRVLGQVHSLYKPRPGRVKDYIAIPKANGYQSLHTSLIGPHGVPVEVQIRTEDMDQMAEMGVAAHWA
YKEREQGETGTTAQIRAQRWMQSLLELQQSAGSSFEFIESVKSDLFPDEIYVFTPEGRIVELPAGATP
VDFAYAVHTDIGHACVGARVDRQPYPLSQSLTSGQTVEIITAPGARPNAAWLNFVVSSKARAKIRQML
KNLKRDDSVGLGRRLLNHALGGSRKLAEIPQENIQHELDRMKLATLDDLLAEIGLGNAMSVVVAKNLQ
GDQSSLGTTSGVRNLAIKGADGVLITFAKCCRPIPGDPIIAHVSPGKGLVIHHESCRNIRGYQKEPEK
FMAVEWDKETEQEFIAEIKVDMFNHQGALANLTAAINAAESNIQSLNTEEKDGRVYSAFIRLTTRDRI
HLANIMRKIRIMPDVIKVNRNRN
>CORE_REP|Org9_Gene1317#
MGKHFAAQRHESVGNGEKAGMKAIGAFSSVFLGVCSLAIGNVNAAETKSNETYQDAETLLVTGEKVKR
SIFDTSSSVQVFDSNRIASMPDAVQIPDLLRMTPNVVDLGIGNELPTVRGIDGSGPNVGANAFLSGTR
PRLNLSLDGRSLTYNEQAFGPQSLWDLDRVEVFLGPQSYIQGRNAIAGAIVMASKDPTFEWESAFKGG
AGNQHSSQLAAMASGPLVEDQLAFRVSVDRQRRRSEADLPAYAPVGDPREVEATTARAKLLFNPAGLR
DLTTKLTFNHFGSTAPQNESLNPQPHPTNPRHDPRRAVFKSNMNSTIWDLAWEASDALTLENRVIYTD
FNINRPTAYNIQYAEIDGQEVHVEPVVRFGGADSRLHGLAGLRYFHGTQDEFVNIFGGSTFKDKTDTH
SAFAELTYALTPQVDVTAASRLEREHRRRDGGSQAVRIDFDETYTVFLPKLDVAWKPTDTQTYGAKIA
RGYNAGGGGITIGTPVVSYTYGSEYVWNYELYTRHHLKDANVVLTGNIFYNDYKDMQLPYSLGENSSV
IRNADKVETYGAEIGATWQPRWDFELFGNLGLLKTDIKKFSGSGVEGHELARAPAYTANMGAKYQFLK
GWELSSNVAFSDSYYSAYDNDSRGRIGSYWTANAQLAYTFDYGRATLYAKNLFDSDRREMVRSNDIYT
ATLQRGRLVGAAVELNF
>CORE_REP|Org17_Gene4022#
MPLTFSTRLRFSALSLAIACALPTVALAQNTSTTPSSSPATPAKKAKAADETMTVVATGNQRSSFEAP
MMVTVIEGSSPESQTAGTAADMLRRVPGITVTGSGRSNGQDLMMRGYDRRGVLTLVDGIRQGTDTGHI
NGTFLDPALVKRIEIVRGPSALLYGSGALGGVVSYETVDAADLLLPGHDSGFRVYGTAGSGDHSLGMG
ASAYGKTDNLDGLLSFGTRDVGNLRQGNGFDAPNDETINNVLAKGTWKIDDNQSLGGNLRYYNNSAQE
PKNPQTPASSAGNLMTNRSTIQRDAALSYKLKPVGQDWLDAEAKVYYSDVKINAHASGSEDEARKQTT
KGAKLENRTRLFADTFASHLLTYGTEAYKQEQTPGGATESFPHAKINFASGWLQDEITLRDLPVTLLA
GTRYDNYKGSSDGYADVDANKWSSRGAVSITPTDWLMLFGSYSQAFRAPTMGEMYNDSKHFSIPMGPT
TITNYWVPNPNLKPETNETQEYGFGLRFDDLLLADDSLQFKASYFDTKAKDYITTDVTMELGRGPRGP
YCISCTTFSTNIDRAKIWGWDATLSYKTSWFGWDLAYNRTRGKNEATGDWLSSINPDTVTSSLDVPLG
ETGLSAGWVATFAERATRVQTGTPEQGGYGVNDFYLSYKGRDRLQGVTTTVVLGNAFDKEYYSPQGVP
QDGRNAKLLVSYQW
>CORE_REP|Org2_Gene3654#
MWRRLIYHPEINYALRQTLVLCLPVLFGLLIGQLQLGLMFSLVPACCNIAGLDTPHKRFFKRLVVGGS
LFAFSSVLLQQALLWHVPLPALMLGLALLLGVTGEISPLHARLLPAALVAAIFALSTAGTVPIWQAPL
LYAIGTVWYGLFTWFWFKLWKEQPMRETLSQLYLELADYFEAKYSLLTQHTDPQTALPPLLVRQQKVM
DLISLLYQQLNFLPHANNLEQKRLQRAFQVAMDLQEHITVSLHLPEEVQKLVEQSQAEAIIRRNAQVI
AGRLRVVAHDILYHQHSKRFSMAHELAALEKMAAQHPDNPVGQFCYYHFSRIARLLRTQHPLYRRDLM
PGQHRLPFWPALASYLSFKSTALRNAARLGVTLAVGSSLGAVFNLPKPYWILLTIMLVSQNGYNATRV
RIQHRALGTIAGLLLAAGLLQLQLPEGETLSIMLVITLLAYLVSRKNYGLSVIGFTVTAVYTLQLLAL
NGSHFLVPRLIDTLIGCVLAFGGTIWLWPQWQSGLLRKNAHQALEHDQTALRLMLEQPEPDATALAYT
RMQVNQAHNALFTSLNQAMQEPGFASNYLADMRLWVTHSQFIVEHLNAMTILAREHYMLTPKLAEAYL
QTCEIALQSCQQRLEYDGPSSGNSGIMQPPDLHPEMPVTEMERHLRRILSHLSVMHTISSLAWRQRPH
HGIWLKRKLRDQ
>CORE_REP|Org44_Gene2080#
MTQQTFLVEIGTEELPPKALRSLAESFAANFTAELDNAGLEHGDVSWFAAPRRLALKVANLSAAQADR
EIEKRGPAIAQAFDAEGKPSKAAEGWARGCGITVDQAERLVTDKGEWLMYRAHVKGQSAQALLAGMVS
TALAKLPIPKLMRWGDSDVQFVRPVHTVTMLLGADLIPGTVLGIDSARTVRGHRFMGEAEFTLDNADQ
YPQILLERGKVVADYEARKALIKRDAELAAQKIGGKADLSDSLLEEVASLVEWPVVLTAKFEEKFLAV

PAEALVYTMKGDQKYFPVYDAAGKLLPNFIFVANIESKDPQQIISGNEKVVRPRLADAEFFFNTDRKK
RLEDNLPRLETVLFQQQLGTLRDKTDRIQALAGWVAGQIGADVNHATRAGLLSKCDLMTNMVFEFTDT
QGVMGMHYARHDGEAEDVAVALNEQYQPRYAGDALPQSLVACSLAIADKMDTLAGIFGIGQHPKGDKD
PFALRRAALGVLRIIVEKNLPLDLQTLTEEAVRLYGSKLTNAKVVDEVVEFMLGRFRAWYQEEGHAVD
TIQAVLARRPTKPADFDARVKAVSHFRTLEAAAALAAANKRVSNILAKSTETLNDSVRASVLKDAAEI
QLATHLVVLRDKLQPYFAAGNYQEALVELAALREPVDAFFDNVMVMADDAEVRVNRLTLLSKLRELFL
QVADISVLQ
>CORE_REP|Org3_Gene1504#
MTRKQRALFEPALVRTALIDAVKKLDPRVQWRNPVMFVVYIGSILTTAIWLAILTGQTDGAAAFTGSV
ALWLWFTVLFANFAEALAEGRSKAQAESLKGTKKTSWAKKLAGPRRDGATEKVAAESLRKGDIVLVEA
GDTIPCDGEVLEGGASVDESAITGESAPVIRESGGDFSSVTGGTRVLSDWLVVQCSVNPGETFLDRMI
AMVEGAKRRKTPNEIALTILLVALTIVFVLATATLFPFSQYSVEAAGSGSVVTITVLVALLVCLIPTT
IGGLLSAIGVAGMSRMLGANVIATSGRAVEAAGDVDVLLLDKTGTITLGNRQASEFLPAPGVKEQELA
DAAQLASLADETPEGRSIVVLAKQRFNLRERDLQALNATFVPFSAQTRMSGVNVQERMIRKGAVDAIR
RHVETNQGHFPRAVDDLVESVARTGGTPLVVAEGARVLGVVALKDIVKGGIKERFNELRKMGIKTVMI
TGDNPLTAAAIAAEAGVDDFLSEATPEAKLALIRQYQGEGRLVAMTGDGTNDAPALAQADVAVAMNSG
TQAAKEAGNMVDLDSNPTKLIEVVHIGKQMLMTRGSLTTFSIANDVAKYFAIIPAAFAATYPQLNALN
VMHLHSPASAIMSAVIFNALVIVFLIPLALKGVSYKPMSAAALLRRNLWLYGVGGLLVPFVGIKLIDL
ILVALHVAG
>CORE_REP|Org45_Gene1678#
MNKFVRLTAIAGLLWAGVSYGAETANIRIGQLPQLQQEPQHATVSERVTSRFTRSHYRQFALDAEFSG
KIFDRYLNMLDYSHNVLLASDVAQFAGKRNQVGEELKTGKLDTFYALFNLAQKRRFERYTYALSLLDK
PMNFTGNGTIDLDRSKAPWPKDKAELDSLWDAKVTYDELNLKLTGKTDKEIRDTLTKRYQFAIKRLTQ
SNSEDVFQLAMNAFAHEIDPHTNYLSPRNTEQFNTEMSLSLEGIGAVLQMDDDYTLINSMVPGGPAAK
SKAITVGDRIVGVGQAGKPMVDVIGWRLDDVVSLIKGPKGSKVRLEILPAGKGTKTRVVTLTRERIRL
EDRAVKMTIKTVGKEKVAVFDIPGFYVGLTDDVKVQLQKMAKQNVKSVIIDLRTNGGGALTEAVSLSG
LFIPSGPVVQVRDNNGKVREDADTDGVTYYKGPLVVLVDRFSASASEIFAAAMQDYGRALIVGEPTFG
KGTVQQYRSLNRIYDQMLRPEWPALGSVQYTIQKFYRVNGGSTQRKGVTPDILMPTGVDPAETGEAFE
DNAMPWDSINAATYSKTGDMAPFEPELLKDHQQRIAQNPEFQYIAQDIAHYKALKDKRNIVSLNLAVR
EKENHDDDATRLKRINERLERAGKKPLKSLDDLPKDYQEPDPYLDETVHIALELAHLEKDRPAQQPTP
AK
>CORE_REP|Org34_Gene3734#
MLSSTFVRTKAGRSKPVRLTAVIAAALFLAGCPSRAPQTPPANIQDEASASSDYYLQQLQQSSDDNKA
DWQLLAIRALLREGKLPQAGDQLNQLPKNLSGAQQTERQLLTAELQIANKSYVSARSSLGHLDSGALS
PNQKVRYYQAQIAANQGKASLPLIRAYIAQEPLLTGKPHQDNLDQTWQALLQLTPQEMNSLVINADEN
VLQGWLDLLRVYQDNKQDPDLLKAGIKDWQNRYPKNPAAKTLPARLNQVLNFTQASTSKIALLLPLNG
QAKVFADAIQQGFEAAKNGGSMPAPQPQAAQPASAPAQTAPADQAAAGDINANGAVSPSAQESQPAVT
AAQPAAPSSAPITPLQAANAQVKVYDTTSQPLAALLSQAQQDGATLVVGPLLKENVDQLSASTTTLNV
LALNQPETPKDNLNICYFALSPEDEARDAARHIWEQQKRQPLLLVPRGAFGDRVAKAFNQEWQKQGGQ
TVLRQDIGSAGELRQMVNSGGIRMTGTPMSSAPAPQSVTIAGLTIPAPPSDVPAATGGSVDAVYIVAT
QSQLTLIKPMIDMATSSRGKPAMYASSRSYQAGAGPDFRLEMEGLQFSDIPLLAGSNPQLLQQASARF
RNDYSLVRLYAMGMDAWTLANHFAEMRQLPGFQVSGTTGTLSASPNCVINRKLPWLQYRQGTVVPVS
>CORE_REP|Org15_Gene2698#
MTTRETSSKPQQDRLNPVVFFTSAGLILAFSLMTIFFTDFSGQWITRTLNWVSTTFGWYYLLAATLYI
VFVVFIAASRFGAIKLGPEQSKPEFSLMSWAAMLFAAGIGIDLMFFSVAEPVTQYMMPPEGDAQTLEA
ARQAMVWTLFHYGLTGWSMYALMGIALGYFSYRYNLPLTIRSALYPIFGKRINGPIGHSVDIAAVLGT
IFGIATTLGIGVVQLNYGLKVLFEIPENLTVQGSLILLSVIMATISVTSGVNKGIRILSELNVLLALG
LILFVLFFGDTEFLLNALVLNVGDYVNRFMGMTLNSFAFDRPVEWMNNWTLFFWAWWVAWSPFVGLFL
ARISRGRTIRQFVVGTLIIPFVFTLLWLSIFGNSALYQIIHGNAEFAQEVMQFPERGFYSLLAQYPGF
TFSASVATITGLLFYVTSADSGSLVLGNFTSRLADINNDAPNWLRIFWSVAIGLLTIGMLMTDGVPAL
QKTTVIMGLPFSFVIFFVMAGLYKSLRVEDYRKASALSTLAPVPVSSHDVLNWKQRLSRVMNYPGTQY
TQKMLDKVCRPAMQDVARELELRGAKVEFSEVPPTEDERLNHLELLVHLGEEQNFIYQIWPMRYSVPG
FTYRARSGKSHYYRLETFLMEGTQGNDLMDYSKEQVIGDILDQYEKHLNFLHIHREAPGGTLTFPDM
>CORE_REP|Org22_Gene2298#

MNILSYLQKVGRALMVPVATLPAAAILMGVGYWIDPVGWGGDNALAALFIKSGSAIIDHMAVLFAIGV
AYGMSKDKDGSAALTGFVGFLVLTTLCSPAAVSMIQKIPLDQVPAAFGKIENQFVGILVGIISAEVYN
RFSGVELPKALSFFSGRRLVPILISFLMILVAYILMFVWPVVFGALVSFGEHIQKLGSVGAGIYAFFN
RLLIPVGLHHALNSVFWFDVAGINDIPNFLGGQQSIEAGKAVVGITGRYQAGFFPIMMFGLPGAALAI
YHCARPENKAKVLGIMMAGAFAAFFTGITEPLEFSFMFVAPVLYVLHAILTGISVFIAASMHWIAGFG
FSAGLVDMVLSSRNPLATHWYMLIPQGLVFFVIYYVVFRFTINKFNLMTPGRELAVAGDETDGYDVNV
NSNAGKDENETTTLARRYVGAIGGSDNLTGIDACITRLRLNVKDSALVNDALAKRLGASGVIRLNKQS
VQVIVGTRAELIASAMRNVIAAGPVAAAAAPAAAPAAEAKSQAVPNAPKTALETLVAPVTGEVVALDQ
VPDEAFASKAVGDGLAIRPTDNIVVAPADGTVVKIFNTNHAFCLETDKGAEIVVHMGIDTVALEGQGF
KRLVEEGAEVKAGQPILELDLDYLNANARSMISPVVVSNSDDYAGLAALASGSVVAGQTKLYEIQK
>CORE_REP|Org25_Gene4140#
MSMDISDFYQTFFDEADELLADMEQHLLELDPLAPDIEPLNAIFRAAHSIKGGAATFGFSVLQETTHL
LENLLDGARRQEMSLSTEIINLFLETKDIMQEQLDAYKTSQQPDAESFEYICQALRQLALEAQQQQEA
PAAQPAVQQPVAQPSAAPAAIEGGMRISLNGLKASEIPLMLEELGNLGEVKDPHQTEHSLDVTLLTSA
SEDDISAVLCFVLEPEQISFSTPTQSAPAVAEANLPETVAAPEPEPEPAPAPIASAAAPAAKPAAAAA
EAPKARAKASESTSIRVAVEKVDQLINLVGELVITQSMLAQRSGTLDPVNHGDLLNSMSQLERNARDL
QESVMSIRMMPMEYVFSRYPRLVRDLAGKLNKQVELTLQGSSTELDKSLIERIIDPLTHLVRNSLDHG
IEEPATRIAAGKSAVGNLVLSAEHQGGNICIEVIDDGAGLNREKILAKAASQGLAVSDSMSDEEVGML
IFAPGFSTAEQVTDVSGRGVGMDVVKRNIQEMGGHVEIRSQAGKGTTIRILLPLTLAILDGMSVKVND
EVFILPLNAVMESLQPQAEDLHPLAGGERVLQVRGEYLPLVELYRVFEVEGAKTDATQGIVVILQSAG
RRYALLVDQLIGQHQVVVKNLESNYRKVPGISAATILGDGSVALIVDVSALQTLNREKRLTDAAA
>CORE_REP|Org2_Gene2711#
MNLPFLEWSRTPWGKATGGQWRYALRNSLAMCLALWVAFVLELDEPYWALTSAAVVSFPTIGGVISKS
IGRIFGSLVGAAASVAIAGHCLNDPWLFTLFIAAWIGLCTYVSNHYQNNVSYAFALAGYTAAIIAFGT
VNVTDTQQIFDIAQARVCEVITGILCGGLMMMILPSTSDGEALLTSLRRMQLRLLEHAAMLWQPEITA
QMRTSHEGVIGQILTMNLLRIQAFWSHYRLRRQNNLLNYLLHQQLRMTSVISSLRRMLLNWPDRPANL
MPVLTQLLDELRDPATDKYRLARLLQQIAPQEATDYRHRAFWLRLRDFCWLYLRCNRWLRRLESATAV
SDLQPPRVTALARHTDSYEAAYNGLRTFLCIVIGCAYWINTQWDAGSAALTLTAISCVLYSSTPSPIN
SVSTLLKAVLLLSVACFVVKFGLMIQIDDFWVFCAFLFPILVTMQMLKLQNPPYAALWGQLIVFMGSF
LTVSNPPSYDYQSFINDNIAKIVGVLLAGLAFQILRPSSDKRKSRRIIRALRRDFIDQLSKRPQQSES
QFESLIYHRISQLNQSQDQDARTWLLRWGVVLLNCSHIVWQLRDWQTRSDPLSAVRNVCIHCLRGIMT
EKGVQHSSLDATLQELLRMSNALAHHPEQAARDLAGLIWRLYCSLQQLQQAMGQPTAGPAVSGG
>CORE_REP|Org13_Gene4551#
MPADQGPELLNAHFGTQSPHWRLAFDSNALELSAVKGKAHVAVAFSAMEAAKIRRLTGVTASLELTIT
LAGEPLHLHLVGRRVNNLEWAGTASAFSDTQSVARDLVHGLSFAEQVVSEANSVIVIVDQHGRIQRFN
RLSEEYTGLREHEVIGKNVFQLFMSPEEAAASRRNIAGFFRNGSSYEVERWVKTVKGERLFLFRNKFV
HSGSGKNEVYLICSGTDITEERRAQERLRVLANTDLITGLPNRNAIQDKINHAIATRGEESFGLVYLD
LDNFKKVNDAYGHMFGDRLLVEVALAILGCLSPDQVLARLGGDEFLVLAPQTDRERLQTLAPQTDRER
LQTLAQRIIDRLKTPFRIGLIEVYTGCSIGIALCPEHGNDLDSLIRSADTAMYVAKEHGKRTYTVFSP
EMNKRVAEYMWLDTNLRKGLEQNQLVLYYQPKIDARSGEVHSVEALVRWDSPERGLIPPLQFISYAEE
SGLIGPLGQWVLQTAAGQAAQWQEQGLNLRVAVNLSARQLADDSIVNDLLGVLRRHRMAPCLLDFELT
ESSLIEDENRARALITRLRELGAQVHLDDFGTGYSSLAQLARIPLDAIKLDKSFVRGVNFNPVSQSLV
RAIVAAAEALAFRVIAEGVETESENHFLDEVGVDEKQGFLFARPMLPEQLEHWLQSYRPHSPSA
>CORE_REP|Org34_Gene2953#
MAQVAKKLLVTCALPYANGSIHLGHMLEHIQADIWVRYQRMRGHEVHFICADDAHGTPIMLKAQQLGV
KPEEMIAEMSQEHQQDFAGFGISYDNYHSTHSDENRELSTLIYSRLKENGFIKNRTISQLYDPEKGMF
LPDRFVKGTCPKCKSPDQYGDNCEVCGATYSPTELIDPKSVVSGATPVMRDSEHFFFDLPAFSEMLQA
WTRSGALQEQVANKMQEWFESGLQQWDISRDAPYFGFEIPDAPGKYFYVWLDAPIGYMGSFKNLCDKR
GDLDFDEFWRKDATTELYHFIGKDIVYFHSLFWPAMLEGSNFRKPTNLFVHGYVTVNGAKMSKSRGTF
IKAGTYLQHLDADCLRYYYAAKLSSRIDDIDLNLEDFVQRVNADIVNKVVNLASRNAGFINKRFGGKL
ADSLADPALYQTFVDAAQSIAEAYASREFSRAIREIMALADLANRYVDEQAPWVVAKEEGRDADLQAI
CSMGINLFRVLMTYLKPVLPSLTERAEAFLNAELSWDAIPQPLLGHQVNAFKALFNRIDLDKVSEMVN
ASKEDMAAAKPVTGPLADDPIQETITFDDFAKVDMRIALIKSADFVEGSDKLLKLQLDLGGELRQIFS
GIRSAYPDPKALEGRLTIMVANLAPRKMRFGVSEGMVMAAGPGGKEIFLLSPDSGAQPGMQVK

>CORE_REP|Org22_Gene3407#
MRLNPSQQQAVEFVTGPCLVLAGAGSGKTRVITNKIAHLIHHCGYQARHIAAVTFTNKAAREMKERVS
QTLGRKEARGLMISTFHTLGLEIIKREYVALGMKSNFSLFDDQDQLALLKELTEKWLESDKTLVAQLI
STISNWKNDLIDPQRAAELARSERDKLFAHCYGLYHAHMRACNVLDFDDLILLPTLLLQRNEEVRERW
QQRIRYLLVDEYQDTNTSQYELVKLLVGNRARFTVVGDDDQSIYSWRGARPQNLVLLKEDFPALQVIK
LEQNYRSSERILKAANILIANNPHVFEKRLFSELGYGEELKVVTANNEDHEAERVVGELIAHHFVKKT
NYGDYAILYRGNHQSRVFEKMLMQNRIPYKISGGTSFFSRPEIKDLLAYLRVLTNPDDDSAFLRIVNT
PKREIGPATLQKLGEWANQRNKSLFHASFDLGLSQHLTGRGLESLQRFTHWLGGIAQQAEREPVAAVR
DLIRGVDYESWLFETSTSPKAAEMRMKNVNTLFGWMTEMLEGNDLDEPMTLTQVVTRFTLRDMMERGE
SEEELDQVQLMTLHASKGLEFPYVFLVGMEEGLLPHQSSIDEDNVDEERRLAYVGITRAQKELIFTLC
RERRQYGELVRPEPSRFLLELPQDDLAWETERKVVSPQERMQKGQSHLANIRAQLAKAKGGN
>CORE_REP|Org1_Gene3018#
MESIIQQINQLRATLRHHEYQYHVLDAPEVPDAEYDRLMRELRELESAHPELITADSPTQRVGAAPLA
AFDQVRHEVPMLSLDNVFDEESFLAFYKRVQDRLKSSDPLTFCCELKLDGLAVSLLYEDGELVRAATR
GDGTTGENITTNVRTIRAIPLRLTGDNIPRRLEVRGEVFMPQAGFEQMNEEARRKDGKVFANPRNAAA
GSLRQLDPRITAKRPLTFFCYGVGLLEGGELPRSHFERLMQFKAWGLPVSDRAQRRTGSEEVLAFYRQ
VEQDRAQLGFDIDGVVIKIDDIDLQETLGFVARAPRWATAFKFPAQEQITVVREVEFQVGRTGAITPV
ARLEPVLVAGVTVSNATLHNADEIERLGLRIGDTVIVRRAGDVIPQVVGVLEDRRPQDAREVVFPLHC
PVCGSDVERVEGEAVARCTGGLICGAQRKEALKHFVSRRALDVEGMGDKIIEQLVDKEYVKNPADLFR
LSAGILTGLDRMGPKSAQNLVNALEKSKQTTFARFLYALGIREVGEATAANLAAHFGSLEKLFAADIE
ALKEVPDVGEVVAKHTRNFLDEALNQQVINELVGAEIGIHWPAPVVVAAEEIDSPFAGKTVVLTGSLS
QLSRDEAKDRLTALGAKVSGSVSKKTDLVIAGEAAGSKLAKAQELGIAVIDEAEMIRLLGE
>CORE_REP|Org37_Gene4277#
MSNYPHLLAPLDLGFTTLKNRVLMGSMHTGLEELPDGPQRLAAFYAERAAAGVALIVTGGIAPNDKGV
VYRGGSTLNSEAQLPHHRPVTEAVHRAGGKIALQILHAGRYSYQPHPVGPSALQAPINPFAPSALSEA
EIEQTIADFARCAALAQQAGYDGVEVMGSEGYLINQFLAARTNQRDDRWGGSFTNRMRFAVEIVRAVR
QAVGAKFILIYRLSMLDLVEDGSSWQEIEQLALAVEQAGATIINTGIGWHEARIPTIATMVPRAGFSW
VTRKLMGKVGIPLITTNRINDPAVAEQVLADGCADMVSMARPFLADAAFVQKAAEGRADEINTCIGCN
QACLDQIFEGKLTSCLVNPRACRETEMPLTMAEKPKTLAVIGAGPAGLAFATTAASRGHQVTLFDAAD
QIGGQFNIAKQIPGKEEFHETLRYFRRQLALREVKVRLGVKVEAADLSEFDEVILACGIMPRTPDIPG
IGHAKVLSYLDVLRDKKPVGQRVAIVGAGGIGFDTAEYLSQHGVSSSQDQAEFNREWGIDGRLEQRGG
LAAQGPQAPRAARQIYLLQRKTSKVGEGLGKTTGWIHRASLAMRGVKMLNSVSYRLIDDEGLHITRAE
QDSCLPVDTVVICAGQEPRRELQQPLQAMGKTVHLIGGADVAAELDARRAIDQGTRLAMAL
>CORE_REP|Org42_Gene1946#
MSKLFKLHSEFKPAGDQPEAIRKLEEGLEDGLAHQTLLGVTGSGKTFTIANVIADLNRPTMVLAPNKT
LAAQLYGEMKEFFPENAVEYFVSYYDYYQPEAYVPSSDTFIEKDASVNEHIEQMRLSATKALLERRDV
VVVASVSAIYGLGDPDLYLKMMLHLTQGMIIDQRSILRRLAELQYSRNDQAFQRATFRVRGEVIDIYP
AESDELALRVELFDEEVERLSLFDPLTGQIEQVVPRFTIYPKSHYVTPRERIMQAMEEIKVDLADRRK
VLLANNKLLEEQRLTQRTQFDLEMMNELGYCSGIENYSRYLSGRAEGEPPPTLFDYLPADGLLVVDES
HVTIPQIGAMFKGDRARKETLVEYGFRLPSALDNRPLRFEEFEALAPQTIYVSATPGKYELEKSGGDL
IDQVVRPTGLLDPIVEVRPVATQVDDLLSEIRKRAAINERVLVTTLTKRMAEDLTEYLEEHGERVRYL
HSDIDTVERVEIIRDLRLGEFDVLVGINLLREGLDMPEVSLVAILDADKEGFLRSERSLIQTIGRAAR
NLNGKAILYGDRITDSMAKAIGETERRRAKQQAYNEANGIVPQGLNKKIGDILQIGQPVNRAKSKGKG
KAADGGASLQNLTPKALDQKIRDLEAQMYTHAQNLEFEQAAALRDQIHQLREQFIAIS
>CORE_REP|Org16_Gene4255#
MSSRKELANAIRALSMDAVQKANSGHPGAPMGMADIAEVLWRDYLNHNPTNPHWADRDRFVLSNGHGS
MLIYSLLHLTGYDLPMRELENFRQLHSKTPGHPEYGYTPGVETTTGPLGQGIANAVGFAIAERTLAAQ
FNRPGHDIVDHHTYAFMGDGCMMEGISHEVCSLAGTLKLGKLTAFYDDNGISIDGHVDGWFTDDTALR
FEAYGWHVVRNVDGHNPDAIKAAIEAARKVTDKPSLLMCKTVIGFGSPNKAGTHDVHGAALGAAEVAA
TREALGWKYAAFEIPQDIYAQWDAKEAGQAKEAAWNDKFAAYAKAFPELAAEFKRRMNGELPADWKAD
AKAFVEKLQANPANIASRKASQNALEAFGKVLPEFLGGSADLAPSNLTMWSGSKALNVDPAGNYIHYG
VREFGMTAITNGIALHGGFLPYSATFLMFVEYARNAVRMAALMKLRNVFVYTHDSIGLGEDGPTHQPV
EQLASLRVTPNMSTWRPCDQVESAVAWQYGIERNDGPTTLVFSRQNLTQQPRSAEQLANVYRGGYVLK
DCAGTPDVILIATGSEVGITVEAADKLTAAGRKVRVVSMPSTDAFDKQDAAYRESVLPAAVTARVAVE

AGIADYWYKYVGLNGAIVGMTTFGESAPAEQLFAEFGFTVDNVVAKAQALLK
>CORE_REP|Org27_Gene1425#
MLGKLTLDAVPYHEPIIMVTVAAIIVGGLAVLALLTYFGKWKWLWSEWLTSVDHKKIGIMYIIVAMVM
LLRGFADAIMMRSQQALASAGEAGFLPPHHYDQIFTAHGVIMIFFMAMPFVVGLMNVVVPLQIGARDV
AFPFLNSLSFWFFVVGVVLINISLGVGEFAQTGWLAYPPLSGKEYSPGVGVDYWIWSLQISGLGTLLT
GVNFFATILKMRAPGMPMMKMPVFTWAALCTNVLIIVSFPILTVTIALLTLDRYLGTHFFTNDMGGNM
MMYINLIWAWGHPEVYILVLPVFGVFSEVTATFSRKRLFGYTSLVWATIAITVLSFIVWLHHFFTMGS
GANVNAFFGIATMIISIPTGVKIFNWLFTMYQGRIKLNSAMLWTVGFIITFSVGGMTGVLLAVPGANF
VLHNSLFLIAHFHNVIIGGVVFGCFAGLTYWFPKSFGFTLNEKWGIRAFWFWIIGFFTAFMPLYALGF
MGMTRRISQNINPEFHPLLLVAAGGAALIACGILCQLIQIFVSIRDREQNRDLTGDPWGARTLEWSTS
SPPPFYNFAVVPQIHDRDEFWDMKEKGEAYKKPAKYEPIHMPKNTGAGVIIAFFSLVFGFAMIWEIWW
MALAGFIGMIVVWIGKSFDHDVDYYVQVDEIERIENQHYEQIRKAGVNHVN
>CORE_REP|Org13_Gene4198#
METPRYSKLAALVVASLSATAALAAPQNDTQDTMVVTASGFQQKIQDSAASISVIPRQQIEDKAYRDV
TDALKDVPGVVVTGGASSSDISIRGMSSKYTLILVDGKRVDTRSTRPNSDNAGIEQGWLPPLEAIERI
EVVRGPMSSLYGSDAMGGVINVITRKTSRTEWKGSLHGDATIQENRNSGDLFQTNAYASGPLVEGLLG
LRVNGLLSRRAEDKIVNGYNEQRMRSGTAVFTLTPDEKNEFDFEIGRSLQDRNSTPGKSVVAERCSKG
KCTPTEVSESLYTRTNYALTHNGYYDFGNSTSYVQREETGNPGRNMKAYNTIFNTQNQFELGSHMLNL
GGQYRYEKLGDGGNQLESAQGLSKLTRWSWALFAEDEWALTNDFSLTSGIRMDRDENFGSHWTPRMYG
VWHLTEQWTLKGGVSAGYKSPDLRQSSPNWGQVTGGGVRKGIIVGNPDLQPEKSLSEEIGLMWDSLKG
VNAGVTVFNTDFKDKITEVRRCEDTPDCKIGNDVYDFISDRVNVDKANMRGVEATFGWQINKDWKWNT
NYTYTSSEQKSGEFQGKALNQMPKHMLNTVLDWRATQDLSLWSRVNFRSKTSQYLSRTSMATSTPSYT
FVDAGLSYQAAKNLQLTGGVYNILDKTVDYDHFRTTLDGRRYTVGMTYNF
>CORE_REP|Org1_Gene3971#
MPLKITCMPRPAALAVALLCSMTLPAQAYDQLYVFGDSLSDTGNNGRFTYDGSQHLLYDEALAQRIGA
ALVASDNGGENYAAGGAVAVPGLNPVNNTQDQVQRYLNRVNGQADGDGLYIHWIGGNDLAAAALNAAT
APGVAYNSAAAAAAQVHSLLNAGAGTVIVPTVPNIGSTPQLMELIIQQALPPVQGAIQAAYATLNSV
ATPDNASRTQAIHAALAAAAKQGSAIPQVQQAIAAQLIAAYDSLSAQAAQLTDFYNQSEDRLLAQGGG
NIVRVDVNKLFAEAIANPAQFGFANTAGMACPAGVSSAVCRSDMPGFDAGQSYLFSDHFHPSPQAHLL
IADYIQAVLDGPAQVVALNQATAAMARDSRATLDSRFQQLRNGDNPQGSLGVFGGYAGQHYDYADNRA
AGDGNATTHNLTVGVDYQLTDSWLIGALIAGSNDDQHPSSRFDYKARGLLLSAFSSLALFEQGWVNAD
LHYATMDYDDIRRSMRLGPLTRTETGSTTGKQWGARVTAGYDFPIASYLTTGPVAQFAWDYSRVSGYS
EDGDDSTAMRFNDQTYHSQIGALGWRLDTQFGVFNPYAEVSYQHQFGDDVYRAGGGLKSTQTSFTRDS
AGQDKNWVDVTLGANMPLTDRVSAFATVSQTGGLSSGEQFMYNVGVSARF
>CORE_REP|Org16_Gene430#
MSDDLLIHRPPVAGESLSLRSMQEVAMNDRNASKMLRTYNVAYWGNNYYDVNELGHISVCPDPDVPQA
RVDLAELVKTRQQDGQRLPALFCFPQILQHRLRSINAAFKRARESFGYQGGYFLVYPIKVNQHRRVIE
SLVNSGEPLGLEAGSKAELMAVLAHAGMTRSVIVCNGYKDREYIRLALIGEKLGHKVYLVIEKMSEIN
LVLEEAERLNVIPRLGVRARLASQGSGKWQSSGGEKSKFGLAAVQVLKLVETLREAGRLDSLQLLHFH
LGSQLANIRDIATGVRESARFYVELHKLGVNIQCFDVGGGLGVDYEGTRSQSDCSVNYGLNEYANNVI
WGIGDACNEHGLPHPTVITESGRAVTAHHTVLVSNVIGVERNEFSEPQPPEADAPRALESMWETWLEM
NEPENRRSLREWLHDSQMDLHDVHTQYAHGMLDLTKRAWAEQLYLNICNKIQQQLDPSNRAHRPIIDE
LQERMADKFYVNFSLFQSMPDAWGIDQLFPVLPLEGLDKPPEGRAVLLDITCDSDGTIDHYVDGDGVA
TTMPMPPYDPENPPALGFFMVGAYQEILGNMHNLFGDTASVDVYVFPDGSVETELSDEGDTVADMLEY
VQLDPSALLSKFRDQVKETDLDSELQAQFLEEFEAGLYGYTYLEDE
>CORE_REP|Org2_Gene1789#
MMPELGSFLLCLALAIALLLSIYPQWGAARQDSRMMAVARPLTYGMFAAIALAFLCLVHAFVVNDFTV
AYVATNSNTQLPVYYRIAATWGAHEGSLLLWVLLLSCWSLAVALCSRAMPQDAVARVLSVMGMITAGF
LLFIIMTSNPFTRTLPNFPIDGSDLNPLLQDIGLIFHPPLLYMGYVGFSVAFAFAIASLMAGRLDTAW
ARWSRPWTTAAWVFLTLGIVLGSAWAYYELGWGGWWFWDPVENASFMPWLAGTALMHSLAVTEKRGTF
KAWTVLLAITAFSLCLLGTFLVRSGVLVSVHSFASDPARGMFILAYLVIVIGGSLLLYAVKGGQVRSR
VQHETFSRETFLLGNNVLLIAAMLVVLLGTLLPLVHKQLGLGSISIGEPFFNTMFTWLMAPLALLLGI
GPLVRWRRDEPSKLWRRLGVALLATLALSILLPWLLQDSIAGMTVVGLIMALWVIILTLMELHERATH
RHGFWRGLRQLSRSHWGMVLGHLGVAVTVIGIAFSQNYSVERDVRMKAGDSVDIHNYHFVFRDVHDIR

GPNYSGGVGIIDVTRNGKPEATLRAEKRYYSVARSMMTEAAIDGGFSRDLYAALGEELEDGSWAVRLY
YKPFVRWIWFGGVFMAIGGLLCILDPRYRMSKKLKREGKLETQP
>CORE_REP|Org15_Gene2244#
MMKHPLTLSALALLVCASAQAATVDLRVLETTDLHSNMMDFDYYKDTPTDKFGLVRTASLIQQARQQA
ANAVLVDNGDIIQGSPLGDYMAAKGLKPGDVHPVYKAMNTLDYVVGNIGNHEFNYGLDYLKNAIAGAK
FPYINANVIDAKTQKPLFTPYIIVDTPVKDRDGKAHTLRIGYIGFVPPQILVWDKANLQGKVTVDDIT
ATAKRYVPQMRKQGADLVVAIPHSGLSSEPYKAMAENSVYYLSQVPGIDAIMFGHAHAVFPSKDFANI
KGADIDKGLLNGVPAVMPGQWGDHLGVVDLQLNNDGGSWKVTAAKAEARPIYDKENKKSLAAEDAALV
KVLADDHKGTREFVSQPIGKSDGNMYSYLALVQDDPTVQIVNNAQKAYVEHYIQGDPDLADLPVLSAA
APFKVGGRKNDPASFVEVEKGQLTFRNAADLYLYPNTLVVVKASGKEVKEWLECSAGQFNQIDVNSAK
PQGLINWDGFRTYNFDVIDGVNYQIDVSQPARYDGECQLINAKAERIKQLTFNGKPIDPNATFLVATN
NYRAYGGKFAGTGDKHIAFASPDENRSVLAAYITAETQRHGAVRPQADNNWRLANFSSKQPLDIRFET
SPSDKATKFIKEHAQYPMSAAGSDSIGFAVYRIDLSGK
>CORE_REP|Org36_Gene3393#
MSNVNPPRARKAQREAAQQFIDTLQGMAFPNSRRIYLQGSHSDIQVPMREIQLSPTLVGGSKDNPQYE
QNEAIPVYDTAGPYGDPQAELDVHAGLAKLRAGWIDARGDTATLSGASSGFTQQRLADEGLDHLRFEH
LPLPRKALPGKCVTQLHYARAGIVTPEMEFIAIRENMGRERIRGEVLRHQHPGQSWGANLPDNITPEF
VRREVAAGRAIIPANINHPEAEPMIIGRNFLVKVNANIGNSAVTSSIEEEVEKLVWSTRWGADTVMDL
STGRYIHETREWILRNSPVPIGTVPIYQALEKVNGVAENLTWAMFRDTLLEQAEQGVDYFTIHAGVLL
RYVPMTAKRLTGIVSRGGSIMAKWCLSHHQENFLYQHFREICEICAAYDVSLSLGDGLRPGSIQDAND
EAQFAELHTLGELTKIAWEYDVQVMIEGPGHVPMQMIRRNMTEELEHCHEAPFYTLGPLTTDIAPGYD
HFTSGIGAAMIGWFGCAMLCYVTPKEHLGLPNKEDVKQGLITYKIAAHAADLAKGHPGAQIRDNAMSK
ARFEFRWEDQFNLALDPATARAYHDETLPQESGKVAHFCSMCGPKFCSMKISQEVRDYAAAQEAAKPI
EVQLTGMEKMSAEFRARGSELYHSAGASTLQEETSND
>CORE_REP|Org17_Gene1483#
MAALLQLNGIRRSYRSGEQTVEVLKGISLSIDAGEMVAIMGASGSGKSTLMNILGCLDKPSAGVYRVA
GQDVATLSDDALAQLRREHFGFIFQRYHLLPHLSAAHNVEVPAVYAGLGKAARRERAEALLRRLGLGE
RVNYRPSQLSGGQQQRVSIARALMNGGQVILADEPTGALDSHSGEEVMAILKQLCAQGHTVILVTHDP
AVARQAERIIEIRDGEIIADSRPAPSEDAQAKPLTLAAAAPSWRQMGGRFREALVMAWRAMAANKMRT
ALTMLGIIIGIASVVSILVIGDAAKQMVLADIKSIGTNTVDIYPGKDFGDDDPTYRQSLKYGDLDALR
EQPYISALSPSISSSMRLRLGNVDAAANVNGVSEQFFRVYGMSFTQGVGIDPMQVQSQAQTVVIDANT
QRRLFPHQKNVVGEVILVGNMPATVVGVAKEKQSMFGSSKTLNVWVPYSTMANRLMGNSYFDSITVRI
RDGYDSKEAEQQLSRLLTLRHGKKDFFTYNMDSLVQTAEKTTRTLQLFLTLVAVISLVVGGIGVMNIM
LVSVTERTREIGIRMAVGARSGDVLQQFLIEAVLVCLVGGALGITLSFAIGLAVQLVLPGWQISFPPA
ALLSAFLCSTGIGVVFGYLPARNAARLNPIDALARE
>CORE_REP|Org1_Gene4227#
MTTEFETSFADLGLSAPIISALNDLGYEKPSPIQAECIPHLLNGRDVLGMAQTGSGKTAAFSLPLLHN
LQADLKAPQILVLAPTRELAVQVAEAMTDFSKHMNGVNVVALYGGQRYDVQLRALRQGPQIVVGTPGR
LLDHLKRGTLNLSNLSGLVLDEADEMLRMGFIEDVETIMAEIPAEHQTALFSATMPEAIRRITRRFMK
EPQEVRIQSSVTTRPDISQSYWSVYGMRKNEALVRFLEAEDFDAAIIFVRTKNATLEVAEALERSGYS
SAALNGDMNQALREQTLERLKDGRLDILIATDVAARGLDVERISLVVNYDIPMDSESYVHRIGRTGRA
GRAGRALLFVENRERRLLRNIERTMKLTIPEVELPNAELLGERRLAKFAAKVQQQLESSDLDLYRALL
TKLQPEEELDMETLAAALLKMAQGERPLILPPDPVFKPRQRREFNDRDDRRGDRGDRRDSRDSRDGDR
PRRERRDVGEMQLYRIEVGRDDGVEVRHIVGAIANEGDISSRYIGNIKLFASHSTIELPKGMPGEILN
HFTRTRILNKPMNMQLLGDAQPFERRERRDGGNGGERRGNGNGRPFNGERREGGPRRSFGERREGGNG
GERRGGNYNRDGKPAPRRDDSAPAAPRRRFGDA
>CORE_REP|Org42_Gene213#
MNIFDHYRQRYEAAKDEEFTLQEFLTICRQDRSAYANAAERLLMAIGEPVMVDTALESRLSRLFSNRV
IARYPAFEEFYGMEEAIEQIVSYLKHAAQGLEEKKQILYLLGPVGGGKSSLAERLKALMQRVPIYTLS
ANGERSPVNDHPLCLFNPQEDASILEKEFNIPSRYLGTIMSPWAAKRLHEFGGDITKFKVVKVRPSIL
EQIAIAKTEPGDENNQDISALVGKVDIRKLENHAQNDPDAYGYSGALCRANQGIMEFVEMFKAPIKVL
HPLLTATQEGNYNGTEGISALPFNGIILAHSNESEWVTFRNNKNNEAFLDRVYIVKVPYCLRVSEEIK
IYDKLLDHSELTHAPCAPGTLETLARFSVLSRLKEPENSSIYSKMRVYDGESLKDTDPKAKSYQEYRD
YAGVDEGMNGLSTRFAFKILSRVFNFDHAEVAANPVHLFYVLEQQIEREQFPQDLAEKYLEHLKGYLI

PKYAEFIGKEIQTAYLESYSEYGQNIFDRYVTYADFWIQDQEYRDPDTGQLFDRESLNAELEKIEKPA
GISNPKDFRNEIVNFVLRARANNNGRNPNWTSYEKLRTVIEKKMFSNTEELLPVISFNAKTSTDEQKK
HDDFVDRMMEKGYTRKQVRLLCEWYLRVRKSS
>CORE_REP|Org45_Gene2188#
MFQDNPLLAQLKQQLHSQTPRVEGVVKGTEKGFGFLEVDGQKSYFIPPPYMKKVMHGDRIIATLHTEK
EREIAEPETLVEPFLSRFVGRVQKRDDRLSIVPDHPLLKEAIPCRPVRELTHNFQAGDWAVAEMRRHP
LKGDRGFNADLTQFITNGEDHFAPWWVTLARHNLEKEAPEMLALSEPAAASAREDLTALEFVTIDSAS
TEDMDDALYVTDNGDGSLQLTIAIADPTAYVEQGSKLDDIARVRAFTNYLPGFNIPMLPRDLSDNLCS
LRPNERRQVLACRVTIAADGALGEDIQFFAAEIESKAKLVYDEVSDWLEGIAGWQPPSDAIAQQVTLL
KRVCDARNAWRHQHALVFKDRPDYRFVLGEKGDVLEIVTEQRRSANRIVEECMIAANVCAAIVLRDRL
GFGVYNVHTGFDPALVEQAVTVLQANGVEAEADKLLTLDGFCELRRHLDAQPTQFLDSRIRRFQTFAE
ISTTPGPHFGLGLEAYATWTSPIRKYGDMVNHRLLKAIINQQPAEKPQDDVTVQLAERRRLNRMAERD
VGDWLYARFLQDKAGTDTRFNAEIIDVTRGGLRVRLLDNGAVAFIPAPFIHAVRDEMQCSQETGTVQI
KGEVVYRQSDTLQVTIAEVRMETRSVIARPAA
>CORE_REP|Org6_Gene4205#
MILRHPPIKFGCFSHDNYKNTLLAVVSSVTAFSGWAQDNTTATNGDNLVVTANRFPQPVSSVLAPTSV
VTRNDIDRWQAKSLTDVMRRLPGVDIAQNGGLGQQSSLFIRGTNSSHVLVLIDGIRLNQAGVSGSSDL
SQIPISLVQKVEYIRGPRSAVYGSDAIGGVVNIITTREKNGTTLAAGVGSNGYQSYDASTQQPLGDST
VATVAGNYTYAKGYNVIANLPDSFGNPAQPDRDGFMSKSLYGGIEHKFNEAFSGFVRGYGYDNRTAYD
GNYSYSDPAHLDALPDTRQLYSQSWDSGLRYQDGIYATQLIASYSHTKDYNYDPKYGPYSASATLDDS
TQYNVQWGNTFQVAQGHISTGVDWQNQKIEPGTAYITDSKSQRNTGLYLTAQQQVDAFTLEGAVRGDD
NSQFGWHGTWQTSVAWEFVEGYRAIASYGTAFKAPNLGQQYGSFGGNPDLKPEESKQWEGGFEGLTGP
VTWRVTGYRNDIDNLISYASSGSGSAYYNVNQARIKGVEATVSFDTGPLTHQIGYDYVDPRNAKTNEV
LLRRAKQQVKYELDWQLYDFDWAVTYQYLGERYDGDYSGYTTRTVKLGGVSLWDLAVSYPVTSHLTVR
GRIANLFDKDYETAYGYATPGREYYLTGSYTF
>CORE_REP|Org5_Gene4467#
MATQEKQLDVICLGRIAVDFYAQQIGARLEDAGTFAKYLGGSSGNVAYGTAIQGLKSGMLARVGDEHM
GRFLREELHRVGADTRCLITDKQRLTGLVILGIKDQETFPLIFYRENCADMALTPDDIDEAYIASARA
LAITGTHLSHPNTRAAVLKALEYARRHGLRTALDIDYRPVLWGLTSLGDGETRFVESDQVTRELQEVL
HHFDLIVGTEEEFHIAGGSTDTLTALKNVRRATAATLVCKRGAQGCSVFEGEIADGWEQVKLHAGVRV
EVLNVLGAGDAFMSGLLRGYLNDEGWDQACRYANACGALVVSRHGCAPAMPTKRELDDYLLRERQVTR
PDRDARLNHLHRVTTRKQQWPELCVFAFDHRKQLADMAREAGVGEERIPRLKTLLLTAAQQAAAQAGL
NGNSGILADTTYGQAALNEITGQGWWIGRPVELPSSRPLRLEHGNIGSQLIDWPQEHVVKCLVFYHPH
DAAELRREQDELIADVYRGCCKSGHELLLEVILPDNNPDKDERHYLEMIEHFYRLGIQPDWWKLPPLS
AENWRRVGALIDTYDPYCRGVLILGLDSPEAVLKAGFAAAADARWVKGFAVGRTIFGQPSRRWLQGEI
DDTTLIAQVKQKYLTLIGFWRQYRPQAGGAH
>CORE_REP|Org31_Gene252#
MAKNLILWLVIAVVLMSVFQSFGPSESNGRRVDYSTFMSELTQDQVREARINGREINVTKKDSNKYTT
YIPVNDPKLLDTLLTKNVKVVGEPPEEPSLLASIFISWFPMLLLIGVWIFFMRQMQGGGGKGAMSFGK
SKARMLTEDQIKTTFADVAGCDEAKEEVSELVEYLREPSRFQKLGGKIPKGVLMVGPPGTGKTLLAKA
IAGEAKVPFFTISGSDFVEMFVGVGASRVRDMFEQAKKAAPCIIFIDEIDAVGRQRGAGLGGGHDERE
QTLNQMLVEMDGFEGNEGIIVIAATNRPDVLDPALLRPGRFDRQVVVGLPDVRGREQILKVHMRRVPL
AADIDASVIARGTPGFSGADLANLVNEAALFAARGNKRVVSMVEFEKAKDKIMMGAERRSMVMTEAQK
ESTAYHEAGHAIIGRLVPEHDPVHKVTIIPRGRALGVTFFLPEGDAISASRQKLESQISTLYGGRLAE
EIIYGPEKVSTGASNDIKVATSIARNMVTQWGFSEKLGPLLYAEEEGEVFLGRSVAKAKHMSDETARI
IDQEVKSLIERNYTRARSLLMENMDILHSMKDALMKYETIDAPQIDDLMNRKDVRPPAGWDDANKGNS
SDNGGTPKAPTPVDEPRTPTPGNTMSEQLDK
>CORE_REP|Org26_Gene2931#
MAALQALAGKFTHLTVGKKLGLGFALLLLLAVIIAGTGAQYLHIIESRADRIDFSNRLNEEINQAKYN
RAMYGQTYRPEYIQNNRANIENAVKLIDHGQALDWDAQSRKDLQRLVTLIGEYQQQQKEFEQAVTAKD
AVRQSWNMSEVQASLSQVERQLTNGDLQLAFIQLNQKLTQVRYGARGLLLSLNKETEAPLMAAIDDAR
DAASALSRRVSDAQRPQLQPLLAALDDYKNRIAAYLPAYEHEQQISRRLGERAQAIGMLVNAFMQDEL
AQTHNDINLAQLQMGITTLIAIIAGVLIAWRITLQITRPLHSTLAMAERIAGGDLRQAQTSTRRDELG
QLLNAVAAMSQNLRTMIEKIQMGVSQVSTASAEIAAGNTDLSSRTEQQAAAVEETAASMEQLTATVKQ

NADNAHHANQLATDASQTAQQGGKLVENVVSTMRDISSSSQRIAEITTLINGIAFQTNILALNAAVEA
ARAGEQGRGFSVVASEVRSLASRSAQAAKEIEGLIAESVSRVKTGTELVESTGNTMEQIVRSVTHVRD
IMAEIAAASDEQTRGIAQIGQAIVEMDHTTQQNAALVEESAAAADSLEEQADMLLQSVSVFRLAEQTE
PAAVKAAAPKAPAVKPAAANAAAEENWTTF
>CORE_REP|Org5_Gene1386#
MVKVDKRRLLAMGLCLTAFSGAALADSLDAQRQRYQQIKQAWDGNQMGVVAQLMPTLRDYPLYPYLEY
RELTQDLSQAGFSEVNDFIKRNPTLPPAKSLAPRFVNELARREDWRTLLAFSPQPPKPVAARCNYYYA
KWATGDQQAAWSGADELWLNGKTLPGACDRLFSVWRGAGKQTPLDILARMKLALKEGNSSLVSNLYSQ
LPADYQTMGNALVRLQNDPTTVEAFARSVGPTDFTRAATGIAFERLARQDVENARAMIPTLARLQKMS
DDERLGLEEAVAWRLMGSDATYEQAQWRDRVILRSRSPSLLERRVRMALGNGDRQGVATWLARLPEAS
RNKDEWRYWRASQLMDEGKRAEGEEILRNLMTERGFYPMAAAQKLNATYPVMVAVAAKPRASLVDGPE
VARVRELMYWNMDNLARSEWGSYVASRSRPEQEALARYAFEQKWADLSVQATIVGKLWDHLEERFPVA
WPQEFRRATDDKGITTSYAMAIARQESAWNPKAQSPVGASGLMQVMPRTAQHTVQMFNIPGYVGPSQL
FDPQTNITIGTSYLESVYQQFGRNRILSSAAYNAGPSRVNTWLGNSAGRIDPVAFIESIPFSETRGYV
KNVLAYDAFYRYLTHRPAKVLTDAEWQRRY
>CORE_REP|Org31_Gene3983#
MALLKETIRDHSAEERLFIRRAGVALALVVVCFGALIVNLYRLQIRQHGFYQTRSNQNDIKMLPIAPS
RGLIFDRNGTPLVRNVTLYRIEITPSKISDMAALLQALTPIVDLTPEDISAFRDDMHHNSRYKPVTLK
AGLSDTEVARFAVNQYRFDGVTIDTYQQREYPYGAQLAHVLGYVSKINDSDLKRLDKAGLSENYAADR
NIGKQGIEAYYEAELHGTTGYQEVEVDNHGRVIRLLKEQPPKAGKNIYLTLDLPLQQYIESVLKGQRA
AVVVEDPRDGGILAMVSSPSYDPNPFVKGIGYQAYKALLTNPDLPLINRVTQGLYPPASTVKPYMAVS
ALFAGVITPTTTFFGAPTWTLPGTERRYRDWLKTGHGMLNVTKAIEESADTFFYQVAYEMGIDRIHHW
LSQFGYGQSTGIDLNEEYRGVLPSRDWKLKVHKKGWYQGDTVSVGIGQGYWVATPIQMVKALTTLINN
GQVKTPHLLYSLQQGNRVTRYPPPAKTAQIGDPNSPYWGIVRNGMYGMANLPNGTGYKLFHTAPYQIA
AKSGTSQVFSLKQNQTYNAKMIPVRLRDHIFYTLFAPYKNPRVAMALILENGGGNGVVAGPTARAILD
HIFDPANAPQPGDAGQSKPQLNDSADVQR
>CORE_REP|Org19_Gene3023#
MFSPDIKVKVQNFGRFLSNMVMPNIGAFIAWGIITALFIPTGWLPNETLAKLVGPMITYLLPLLIGYT
GGRLVGGERGGVVGAITTMGVIVGADMPMFLGSMIAGPLGGWAIKHFDRWVDGKIKSGFEMLVNNFSA
GIIGMLLAILAFLGIGPLVEVLSKLLAAGVHVMVKNNLLPLASIFVEPAKILFLNNAINHGIFSPLGI
QQATEVGKSVFFLIEANPGPGMGVLMAYMFFGRGSAKQSAGGAAIIHFLGGIHEIYFPYVLMNPRLLL
AVILGGMTGVFTLTMLNGGLVSPASPGSILAVLAMTPKGAYFANIAAVCAAFAVSFVVSAFLLKTSKV
KEDDDLEAATRRMQEMKSQSKGGAATPASVDGDLSTVRKIIVACDAGMGSSAMGAGVLRKKVADAGLK
NISVTNSAINSLPDDVDLVITHRDLTERAMRHAPQAQHISLTNFLDSKLYSDLVDRLLAANKTSDNQQ
KVLGALDDSFEAGESNLFKLSESNVFLNLQASDKEQAIRFAGEQLVKGGYVEAEYVPAMLEREKLTST
YLGESIAVPHGTIEAKDRVLRTGVVFCQYPQGVRFGEEEDEVARLVIGIAARNNEHIQVITSLTNALD
DESVIERLANTTSVQEVLDLLGGKKAG
>CORE_REP|Org35_Gene218#
MTDDFATDGALAQAIKGFKPREPQRQMAQAVTEAINFKQELVVEAGTGTGKTFAYLAPALRADRKVII
STGSKALQDQLYARDLPTVAKALKYKGKLALLKGRSNYLCLERLEQQSMAGGELAGQTLIDLVQLRKW
SSQTKEGDISTCSEVAEDSFVWPLVTSTNDNCLGSDCPLYQDCFVVKARRRAMDADVVVVNHHLFLAD
MVVKEGGFAELIPEAEVMIFDEAHQIPDIASQYFGQQLTSRQLLDLAKDITIAYRTEVRDAAQLQKSA
DRLSLSTQDFRLNLGEPGFRGNLRDVLGEPNVQRALLLLDDALELCYDVMKLSLGRSALLDAAFERAT
LYRARLKRLKAVTEPGYSYWYECNSRHFVLALTPLTVADRFREMLDEKPGSWIFTSATLSVNDQLGHF
TERLGLTKAKTLLLPSPFDYAKQALLCVPRFLPSPNQPGGARQLARMLRPLIEANNGRCFFLCTSHQM
MRELAEEFRATMTLPVLLQGETSKGQLLAQFVAAGNALLVATSSFWEGVDVRGDALSCVIIDKLPFTS
PDDPLLKARIEDCRLRGGDPFNDVQLPDAVITLKQGVGRLIRDTDDRGVLVICDNRLVMRPYGEVFLN
SLPPTPRTRDLKQAIAFLQAADTSAT
>CORE_REP|Org5_Gene3577#
MIVFSSLQIRRGIRVLLDNATATVNPGQKVGLVGKNGCGKSTLLSLLKGEIAADGGSFTFPGNWALAW
VNQETPALDVPAIEYVIDGDREFRQLEAELQAANDRNDGHAIATLHGKLDAIDAWTIRSRAASLLHGL
GFSNEQLQSPVRDFSGGWRMRLNLAQALVCRSDLLLLDEPTNHLDLDAVIWLERWLKSYPGTLVLISH
DRDFLDPIVDKILHIEQQTINEYTGNYSSFERQRATKLAQQQSLYQHQQEKVAHLQSYIDRFRAQATK
AKQAQSRIKMLERMELIAPAHVDNPFTFSFRPPESLPNPLLRMDKVSAGYGDKVILKSIKLNLVPGSR

IGLLGRNGAGKSTLIKLLAGTLEPLSGEIGLAKGIKLGYFAQHQLEFLRADESPLQHLSRIAPRVLEQ
QLRDYLGGFGFQGDKVSEVTERFSGGEKARLVLALIVWQRPNLLLLDEPTNHLDLDMRQALTEALIDF
EGALVVVSHDRHLLRSTTDDLYLVHDGQVEPFEGDLDDYQQWLVDLQRQESQQDAPEKESGGNSAQAR
KEQKRREAEFRTQTQPLRKQIAKLEQQMEKLGAELAAVEEQLADPALYDISRKAELTDCLQKQSQAKS
ALEETEMTWLDAQEQLEQLTQAFEA
>CORE_REP|Org40_Gene254#
MGKIIGIDLGTTNSCVAIMDGAKARVLENAEGDRTTPSIIAYTQDGETLVGQPAKRQAVTNPENTLFA
IKRLIGRRFQDEEAQRDKAIMPYKIVEADNGDAWLEVKGQKMAPPQISAEVLKKMKKTAEDYLGEPVT
EAVITVPAYFNDAQRQATKDAGRIAGLEVKRIINEPTAAALAYGLDKEVGNRTIAVYDLGGGTFDISI
IEIDDVDGEKTFEVLATNGDTHLGGEDFDSRLINYLVEEFKKDQGIDLRNDPLAMQRLKEAAEKAKIE
LSSAQQTDVNLPYITADATGPKHMNIKVTRAKLESLVEDLVARSIEPLKVALKDAGLSVSDIQDVILV
GGQTRMPMVQKKVADFFGKEPRKDVNPDEAVAVGAAVQGGVLAGDVKDVLLLDVTPLSLGIETMGSVM
TPLITKNTTIPTKHSQVFSTAEDNQSAVTIHVLQGERKRASDNKSLGQFNLDGIQAAPRGMAQIEVTF
DIDADGILHVSAKDKNTGREQKITIKASSGLNEDEIQKMVRDAELNAEADRKFEELVQTRNQADHLIH
GTRKQLEEAGDKLPAEDKTAIEAALKDLEAAVKGEDKAEIEAKTQALVQVSGKLLEMAQAQQAQQGAD
AGADNAAQKDDDVVDAEFEEVKDKK
>CORE_REP|Org2_Gene2907#
MSLISMSGAWLSFSDAPLLDNTEIHIEDNERVCLVGRNGAGKSTLLKILGKEIPLDDGRVIYEQDLIV
ARLQQDPPRNIGGSVFDFVAEGVAEQAEHLKAYHAISHLVESDPSEKNLARMAQIMEILDHQGLWQLD
SRISEVLLQLGLNGDAELSSLSGGWLRKAALGRALVSSPRVLLLDEPTNHLDIETIDWLEGFLKEFDG
SIVFISHDRSFIRNMATRIVDLDRGKLVSWPGNYDLYLQSKEEALRVEELQNAEFDRKLAQEEVWIRQ
GIKARRTRNEGRVRALKALRVERSERREVMGTAKMQVEEATRSGKIVFELEDVNYQVGEKVLVRGFSA
QVQRGDKIALVGPNGCGKTTLLKLMLGQLKADSGRVHCGTKLEVAYFDQHRADLDPERTVMDNLAEGK
QEVVVNGRPRHVLGYLQDFLFHPKRAMTPVKALSGGERNRLLLAKLFLKPSNLLILDEPTNDLDVETL
ELLEELIDGYQGTVLLVSHDRQFVDNSVTECWIFEGNGVINAFVGGYYDAHHQRATAKPIRQAAPSAS
KPAAEKKAEQPKKAAAKLSYNLLRELEQLPQRLEQLEAEIEALQAQMSDADFFTRPHSETQQVLTALA
NAEQALEQAFARWEELEAMKNG
>CORE_REP|Org41_Gene3684#
MKKIIFASVLGLSGNAMAAETVNITLLGTSDLHGTFVPWDYASDTENLAGSLSQIATQVKKVRAEQPN
LILVDAGDTIQGNFVETFKHEAVSPMMLGLNALNYDVWVMGNHEFDFGLPVLATPLKQFKGAALAGNI
VWDNGKPYLPAYTIVERQGVKIGIIGMDTPMTAEFAKGTDRIKGLNFTDPVQAVKQVIRQIDGQVDAI
VLVAHMGIDNENQRPGTGVADIANANPELAAIVAGHMHVKIDKAVVNGVIITEPDKYGRALSRIDLQF
ERRDGKFTLIDKNSYTYSIKGMTPDSAMQALYQPYHDILRANANRVVAKLSGSDLVPTDEFRGIPQVH
VQDTGISALFQQAARHYAPLAQVIALQIDNDRAKLDVGDIKAKDIAFNYQYAGGEITVYQLNGKALKR
YMEWSAGYFNQLQPGDVTYSFNPARRSSKYSTNDFFDGVTYTIDLRQPAGSRIVDLRLADGTPVTDDM
PIRLGMNSYRMGHLTQKGGALEGQSFPVLFDSKAQYGEEEGTIRHLTLRYLTEVKHGHYQGVPPQRWK
LIGMEGYEPQRAIVKQLLNEGVIQVPTTDDGRYTNVASINVKDALFSNADDYRATLTSLEQQRQAATD
PVQQRRLQDRIALIKALNDF
>CORE_REP|Org11_Gene1936#
MTQSSYNADAIEVLSGLEPVRRRPGMYTDTTRPNHLGQEVIDNSVDEALAGHAKRIDVILHADQSLEV
IDDGRGMPVDIHPEEGVPAVELILCRLHAGGKFSNKNYQFSGGLHGVGISVVNALSKRVEVNVRRDGN
VYGIAFENGDKVQDLTVTGTCGKRNTGTSVHFWPDEQFFDSPRFSVSRLTHLLKAKAVLCPGVEIYFI
DKVNNTEQRWCYQDGLTDYLMEAVNGLITLPEAPFVGNFAGDTEAVDWALLWLPEGGELLTESYVNLI
PTMQGGTHVNGLRQGLLDAMREFCEFRNILPRGVKLSAEDIWDRCAYVLSVKMQDPQFAGQTKERLSS
RQCAAFVSGVVKDAFSLWLNQNVQAAEQLAELAISSAQRRLRAAKKVVRKKLTSGPALPGKLADCTSQ
DLAMTELFLVEGDSAGGSAKQARDREYQAIMPLKGKILNTWEVSSDEVLASQEVHDISVAIGIDPDSE
DLSQLRYGKICILADADSDGLHIATLLCALFVRHFRSLVKGGHVYVAMPPLYRIDLGKEVFYALDEEE
KAGVLEQLKRKKGKPNVQRFKGLGEMNPLQLRETTLDPNTRRLVQLTVAEDDVDQTLAVMDMLLAKKR
SEDRRNWLQDKGDMAELAV
>CORE_REP|Org44_Gene1901#
MDSKLLDYYNRELAYLREMGAEFAEQYPKVAGRLGMRGIDVADPYIERLMEGFAFLTSRVQLKMDAEF
PRFSQRLLEIIYPNYLSPTPSMAIAELQPDSSKGDISNGFVVPRGTMMDSQTLKKSGITCSYATAHDV
TLQPVRIAAVELGGIPADIPLASLGLQHSGCVSALRIRLECYESVTLNNLQLDQLMFYLAGPDMQAQQ
LLELLMQHSVGLVCQTVEAQPQRRALALDGLRQEGFAAEQALLPNDLRNFEGYRLLQEYFAFPARFQF

FSVNGLRPLLQSVREGKKALRQFEIVVLLDRHDAALERVVDAAHLALHCTPVINLFPKVAERIAINEK
NHEYHLVVDNIRPLDYEVFSVQRLGGSASEKRYEQEFRPFYSTLSADDGNYGAYFSLRREQRTLSEHA
RRYGTRTGYAGSEVFVSLVDERQSPWHSDLKYLTADVLCTSRDLPLMLLQQDQGNFVMPDSIPIKQVS
LRKGPTPPRPALAEGMITWRLISQLQLNYLSMMDGDPEQGAASLRQLLGLYGNLSEPAIAKQIQGVRH
CNLRPVYRRVPEPGPIVFARGIAIDLTVDEQAFSGNSPYLLGSVLERLFSRLVAMNTFTEMTLSSQQR
GEIAHWQARMGKRTLI
>CORE_REP|Org43_Gene4171#
MPIQVLPPQLANQIAAGEVVERPASVVKELVENSLDAGATRIDIDIERGGAKLIRIRDNGCGIGKDDL
ALALARHATSKISTLDDLEAIVSLGFRGEALASISSVSRLTLTSRTADQSEAWQAYAEGREQAVTVKP
AAHPVGSTLEVLDLFYNTPARRKFMRTEKTEFGHIDEVVRRIALARFDVAINLSHNGKLIRQYRAAKE
ESQHERRLGSICGPAFLQHALNIDWQHGDLSIRGWVADPDGARQLGEMQYCYVNSRMMRDRLINHAIR
QAYQDQLKDDQQPAYVLYLEVDPHQVDVNVHPAKHEVRFHQARLVHDFIYQAVTTVLQQAGQTPSLPL
AETPDEAPAPVWQQENRVAAGGNHFSQPAPRRETPPPAGAARERAPQPGWQTAGGYQKREGELYGKLM
RPAAEPQAEAAPEAPSRPPLFPPVKAAVETPLASGQHSFGRVLMIHPPCYALIEQCQQPALLNLTVAE
RWLRQAQLNPPTEGLRPQPLLIPVKLTLDKNEAAAIARHQALLTMMGLDLQTDHGRVTLRAVPLPLRQ
QNLQKLIPELLGYLAEHQEMSPAVLATWLARRLGSEHEQWNTSQAIQLLTDVERLCPQLVKSPPSGLL
QPVDLQAALAALKHD
>CORE_REP|Org42_Gene1473#
MMDNLRAAANHVVLKIILALIILSFVLTGVGNYLIGGSGDYAAKVNGQTIERAQLEQAFQSERSRMQQ
QLGDQFSALAGNEGYMQQMRRQVLSQLIDNMLLDQYAKKLGLAVSDDQIKDAIRKAPYFQTNGQFDNA
KYLDLIGRMGYTADNFAQSMRQQLVNQQVIQAFGDSGFVLPSESQAMAALVLQERDVRLATIDLKALQ
AKQSAGDDELKAYYDQNKNSFIAPEQVKVSYIPLDAASMQDKVKVSEEDISAYYDQHKSSYGQPERKN
YSVIQLKTEAEANAALDELKKGADFATLAKEKSTDIISRRTGGELGWLEPETTADELKQANLTEKGQL
SGVVKSSVGYLIVRLNDIEPEKVKPLSEVHDAIAKQVQQEKAVDAYYALQQKVSEAATSDNESLASAE
EAAGVKAAQSDWFTRDNIPAALNFKPVVQAIFDGSLIGENGAPGSNSDVITVDGDRAFVVRVSGHKPE
GIEPFDQVKDRVAELVKRNKALQAAKLQGEKLLVELKQGKGDEAMKAAGLSFGAVQKMARAPEDSQLV
ESVFALPHPQDGKPVYGMSQDRQDNVVLIALDAVKPGTLPEDEMKTFVGKMEEGATGVSFDSLLASLR
KEAEIKMGAAEQQPQ
>CORE_REP|Org20_Gene2054#
MTDVLLRPAASGGPSLPPQRVLTVRDLSISFPQPDGAVAAVRNLSFDLDRGETLAIVGESGSGKSVTS
LGLMRLVEQGGGRIVGGVMTLRRRDGALLDLAQASQSTLRTVRGADMAMIFQEPMTSLNPVFPVGEQI
AESLRLHQGMDRRSARQEALRMLDLVRIPEAKEVLGRYPHQLSGGMRQRVMIAMALSCKPALLIADEP
TTALDVTIQAQILQLIRVLQREMQMGVIFITHDMGVVAEIADRVLVMRRGEQVEQNRVRELFAAPQQA
YTRALLAAVPKLGAMADRPLPAKFPLPGGEGDTAPQDTVPPGAAPILQVEHLVTRFDLRGGLFNRVTR
RVHAVENVSFDLYPGETLGLVGESGCGKSTTGRSLLKLVDSQSGTITFAGRRIDQLKGPALQHLRRDI
QFIFQDPYASLDPRLTVGFSIMEPLLVHNVMRGREAEQRVAWLLERVGLLPEHARRYPHEFSGGQRQR
ICIARALALNPKVVIADEAVSALDVSIQAQIVNLLLDLQREFGVAFLFISHDMAVVERISHRVAVMYL
GQIVEIGPRQAVFDNPQHPYTRKLMAAVPVADPAHAHKRQPLPADEIPSPVRALGDEPVTAPLVQVGA
GHFVARHPIAGAF
>CORE_REP|Org14_Gene3724#
MNHHASLIITNGKFHTVDRQNPTAQAVAIRDGKFLAVGSESEVMQHAGPETQVIDLHGHTAIPGLNDS
HLHLIRGGLNYNLELRWEGVPSLADALRMLKEQALRTPSPQWVRVVGGWTEFQFAERRMPTLDEINQA
APDTPVFILHLYDRALLNRAALKVVGYTKDTPNPPGGEIQRDANGNPTGMLIAKPNAMILYATLAKGP
KLPLEQQVNSTRQFMRELNRLGLTSAIDAGGGFQNYPEDYQVIAELHEKKQLTIRIAYNLFTQRPKQE
LEDFELWTDMLKPGQGTDFYRHNGAGEMLVFSAADFEDFLQPRPDLPPGTEDELERVVRHLVEHRWPF
RLHATYDESISRMLDVFEKVNRDIPFNGLHWLFDHAETITERNIERVKALGGGIAVQHRMAFQGEYFA
DRYGIEATKHTPPVAKMLAAELPVGLGTDATRVASYNPWTALYWLVSGRTVGGMAMYDDSARLDRETA
LMLWTQGSAWFSTEQGKKGQIKVGQLADVAVLSQDYFSVPEEQIKGIESVMTVVDGKVVYAAGSFSPL
APPPLPVLPEWSPVTQVPGHYRSAPPSAAAKVGVLSQAHQCCGPCGVHAHQHDIARRSSIPVSDENAF
WGAFGCSCFSF
>CORE_REP|Org26_Gene4567#
MSMKGQETRGFQSEVKQLLHLMIHSLYSNKEIFLRELISNASDAADKLRFRALSAPELYAGDGELRVR
LSFDKEQRTLTIADNGIGMRREEVIENLGTIAKSGTKAFLESIGSDQAKDSQLIGQFGVGFYSAFIVA
DKVTVRTRAAGAADDEGVFWESAGEGDYTIADITKETRGTEITLHLREGEDEYLDAWRLRSVIGKYSD

HIALPVEIESKNEEDDTVTWEKINKAQALWTRSKADVTDEEYKEFYKHIAHDFTDPLSWSHNRVEGKQ
EYTSLLYIPAQAPWDMWNRDHKHGLKLYVQRVFIMDDAEQFMPNYLRFVRGLIDSNDLPLNVSREILQ
DSRVTQNLRGALTKRVLQMLDKLAKDDAEGYQKFWQQFGLVLKEGPAEDHGNQEAIAKLLRFASTHGD
SSAQTVSLEEYVGRMAEGQEKIYYITADSYAAAKSSPHLELFRKKGIEVLLLSDRIDEWMMSYLTEFD
GKPFQSVSKADETLDKLADETEEQKAAEKQLEPFIERVKTLLGERVKDVRLTHRLTDTPAIVVTDADE
MSTQMAKLFAAAGQQAPEVKYIFELNPEHALVKRASDVGDNEHFAEWIDLLLDQALLAERGTLEDPNL
FIRRMNKLLSA
>CORE_REP|Org26_Gene2093#
MSLDIAKYPTLALAENPEELRSLPKESLPKLCDELRQYLLNSVSRSSGHFASGLGTVELTVALHFVYN
TPFDHLVWDVGHQAYPHKILTGRRDRIATIRQKNGLHPFPWRAESEYDVLSVGHSSTSISAGLGMAVA
AEREGKGRRTVCVIGDGAITAGMAFEAMNHAGDINPDMLVVLNDNEMSISENVGALNNHLAQLLSGKL
YSTLREGGKKVLAGVPPIKELVKRTEEHLKGMVVPGTLFEELGFNYIGPVDGHDVQGLVATLKNMRDL
KGPQLLHIMTKKGRGYAPAEKDPISFHAVPKFDPASGTLPKSAGGLPTYSKIFGDWLCETAAKDSSLM
AITPAMREGSGMVQFSRDYPQQYFDVAIAEQHAVTFAAGLAIGGYKPVVAIYSTFLQRAYDQLIHDVA
IQNLPVMFAIDRGGIVGADGQTHQGAFDLSFMRCIPTMVIMTPSDENECRQMLYTGYHYNDGPSAVRY
PRGTGTGAALEPLNLLPIGKGVARRQGEKIAILNFGTLLPEAAQAAEALNATLVDMRFVKPLDEQLVL
ELAASHETLVTLEENAIMGGAGSGVNELLMAKRRPVPVLNLGLPDSFVSQGSQEELRADLGLDAAGIQ
RQIETWLAQ
>CORE_REP|Org15_Gene3079#
MKYDTLASEILAGVGGRDNVKSLVHCATRLRFKLRDDRRANAAALKKNPGVIMVVESGGQFQVVVGNH
VAEVFDAVNRVGGLAEGASSGSDADGKKDNLLSRFIDVVSGIFTPLLGVMAASGVLKGLLALSLACGW
LLESSGAFKMLFAASDALFYFFPIMLGYTAGKKFGGNPFVTMAIGGALTHPLMMAAFEAAQQPGAVRE
YFFGIPLTFINYSSSVIPIIFAAWVSCRLEPLFNRVIHSALRNFITPLLCLAITVPLTFLLIGPAATW
LSHLLANGYQAIYAFNPIIAGAFMGAMWQVCVIFGLHWGLVPLMINNLSVLGRDTMVPLLLPAVMGQV
GATLGVMLRTRDAKLRALSGSAIGAGIFGITEPAVYGVTLPNKRPFIFGCIGGALGGAVIGYFHTSVY
SFGLVNVFTFAQIIPNGGIDATVWGAIGGTLLSFVFAALASYLFGVAPAEETAQPEAAAPLNRKQAIL
SPIAGDIVPLEQVNDATFASGLLGKGVAIAPLQGRVVAPVSGSVASLFKTKHAIGIESDDGAEILIHV
GIDTVKLDGAHFTAHVREGERIAPGDLLIEFDQAAIHAAGYDTTTPIIISNSDDYVDVLTSGLSPVQE
QAPLLTLLR
>CORE_REP|Org32_Gene3931#
MQQALDYFNQLNQDYLDVHRAKEELFWQNYMGTGDEGVSARFSAAESAYKRFIAEPRRLAEIRNLLAG
LETLPQEAQRDALIHGLQGWLRFFDCNAIEDPQAQVLLDQIIHAESDLYSRRKGYQVTHLNADGQRVA
ASLGELLTNQATNPNEDYRRSSQQALRDLEQWLLHNGLPELIGLRNRFARQMGYRNYFDYKVNKTERM
TPEQLFAILDRFERETREANARSLQQLAADKGSQALEPWNVRFASAGDVTRQLDPYFPFSRSLERWVD
SFKRLHIGFGGAEMNLDLLVRKGKYENGFMHGPVPPFVRQGEWVPARINFTSLAQPGQVGSGAYGLNT
LFHEGGHAAHFANIRQNAPCFSQEFPPTSMAYAETQSMFCDSLLDDADWLKRYAKNAAGEAVPDALIE
AGIAARQPMRAFNERHILLVPYFEWALYQWDDEQRTPEAITALAREVEQKILGISGSPRPTLAIPHLL
SLESACSYQGYLLAMMAVEQTRHFFLQRDGYLTDNPAIGPDLAQHYWLPGNSVSHDDTLRSLTGEGFN
PDYLAQACNQTVAQAWHEAQQTMAAAAKRPQPPADFDLQAHIRVVDGETVLADNAEGDERMCRDFAAA
IEARLP
>CORE_REP|Org48_Gene4550#
MDNHHMMIEGLIYLGSAALFVPIAVRLGLGSVLGYLIAGCIIGPWGLKLVSDAESILTFAEIGVVLML
FIIGLELDPKRLWTLRASVFGGGSIQMVGCGLALSAFCYFLGLNWKVALLIGLTLALSSTAIAMQAMS
ERNLTPSPIGRSAFAVLLFQDIAAIPLVAMIPLLASSGATTTLGAFVLSAAKVVGALTMVVLLGRYVT
RPLLHFVARSGMREVFSAVALFLVFGFGILLEMAGLSMAMGAFLAGVLLASSEYRHALESDIQPFKGL
LLGLFFIGVGMSIDFGTLFHHPLLIASLLLGFMLIKAALLWLIGPLLGVPKRQRGLFAILLGQGSEFA
FVIFSAAQLAGVLPVEWAKSLTLAVALSMAATPLLLVIAAQLEKNAPKEERPADVIDDENASVIIAGF
GRFGQIAGRLLLANGVHTVVLDHDPDHIETLRKFDTKVFYGDATRADLLEAAGAAHAKVLINAIDDVE
DSLALTELARQHFPHLKVVARARDVDHWYQLRQLGVEKPERETFESSLRIGRETLELLGLDAYEAREK
ADMFRRYNLKMLEDTLENYQDTEFRIASLQRAKEMLSAAIEQDQNRLSRVQQTGWRGSIDGKAPEDEV
VEAKG
>CORE_REP|Org1_Gene3287#
MIALLEQAVALGALRPLDVQFARVVANEDEPDILLAAACLSAEAGAGHVCLMLEQLQADTLFEGRQPA
LALAVWEAVGRPDSARWQQRLAASAAVGDGSGATPLVLRGPRLYLQRMWQNEGEVAAFIGGEGESLAV

PEEALRAILDRLFGTASDEPDWQKIAAAVAATRRIAVISGGPGTGKTTTVAKLLAALVQLDESARLRI
QLAAPTGKAAARLTESLGSASRQLALTPAQQALFPTEAATLHRLLGAQPNSQRMRYHRGNRLHLDVLV
VDEASMVDLPMMARLIAALPDRARVIFLGDRDQLASVEAGAVLGDICRFAEQGYSDARAAELSRLTGC
SIEGRQADAEAVVRDSLCLLRKSYRFDARSGIGQLALAVNAGAGDRALAALNGSFGDVAGYALATSEE
YQALLDACVAGYRDYLRLTAEGADAATVLAAFGRFQVLCALREGPFGVAGLNERIELGLQRAGLIDRK
PGVLGRWYRGRPVMIGRNDSALGLFNGDIGITLPDEHGDLRVHFQLPDGSIKSVQPSRLPAHETAYAM
TVHKSQGSEFDHTVLVLPNHFLPVLTRELVYTAITRARKQLSLYATETVLLRAIRTPTQRRSGLAERL
QATE
>CORE_REP|Org49_Gene2135#
MALLQISEPGLSAAPHQRRLAAGIDLGTTNSLVATVRSGQAETLADEQGRHLLPSVVHYQADAQRVGW
EARQQAAQDPANTVSSIKRMMGRSLADVQQRYPNLPYQFQASDNGLPLIVTAGGPVNPVGVSADILRA
LSARAQAALEGELDGVVITVPAYFDDAQRQGTKDAARLAGLHVLRLLNEPTAAAIAYGLDSGQEGVIA
VYDLGGGTFDISILRLSRGVFEVLATGGDSALGGDDFDHLLADWLREQAGVADRSDHGVQRQLLDAAI
AAKIALSDADSVRVEVAGWQGEVTRAQFEALIAPLVKRTLMACRRALKDAGVAADEVLEAVMVGGSTR
VPLVREQVGAFFGRTPLTSIDPDKVVAIGAAIQADILVGNKPDSDMLLLDVIPLSLGLETMGGLVEKV
IPRNTTIPVARAQEFTTFKDGQSAMMIHVLQGERELVQDCRSLARFTLRGLPPLPAGGAHIRVTFQVD
ADGLLSVTAMEKSTGVEASIQVKPSYGLSDSEIAGMIKDSMANAQSDVGARKLAEQRVEAARVLESLQ
GALASDAALLSEAESQAIAAATQALQQAVQGEDPAAIEDAIKTLDAQTQDFAARRMDASIRRALAGHS
VDEV
>CORE_REP|Org25_Gene3079#
MLNRYPLWKYLMLIVVILVGLLYALPNIYGEDPAVQITGARGVAASETTLDQVRTVLEKDNIASKSIA
LENGAILARFKDPDVQLRAREALVTELGDKFVVALNLAPATPTWLAMLGAEPMKLGLDLRGGVHFLME
VDMDTALSKLQEQTMDTLRSELREKGIPYASIRKLDNNGVEVRFRDDAARDQAISYIGPRQRDLVLSA
NGANTMKASLTDARLSEAREYAVQQNITILRNRVNQLGVAEPLVQRQGSDRIVVELPGIQDTARAKEI
LGATATLEFRLVNTNADATAAANGRVPGDSEVKYTRDGQPIVLYKRVILTGDHITDSTSSTDEYNQPQ
VNISLDSAGGTSMSNFTKDNIGKPMATLFVEYKDSGKKDANGRAVLVKQEEVINVANIQSRLGNSFRI
TGIGNPNEARQLSLLLRAGALIAPIQIVEERTIGPTLGQQNITQGLEACLWGLVASIVFMVVWYRKFG
VIATTALVANLVLIVGVMSLLPGATLTMPGIAGIVLTLAVAVDANVLINERIKEELKNGRSVQQAIHE
GYKGAFSSIVDANITTLITAVILYAVGTGSIKGFAITTAIGVATSMFTAIVGTRAIVNLLYGGKRINK
LSI
>CORE_REP|Org5_Gene4124#
MGIFSYKDLDEKASKALFSDALAISTYAYHNIDNGFDEGYHQTGFGLGLPLTLVTALIGSTQSQGGLP
GIPWNPDSEQAAQQAVNNAGWSVISAAQLGYAGKTDARGTYYGETAGYTTAQAEVLGKYDSEGNLTGI
GISFRGTSGPRESLIGDTIGDVINDLLAGFGPKGYADGYTLKAFGQLLGDVAKFAQAHGLSGEDVVVS
GHSLGGLAVNSMAAQSDANWGGFYAQSNYVAFASPTQYEAGGKVINIGYENDPVFRALDGTTLTGASL
GVHDAPHASATNNIVNFNDHYASDAWNLLPFSILNIPTWLSHLPFFYQDGLMRVLNSEFYSLTDKDST
IIVSNLSNVTRGNTWVEDLNRNAETHSGPTFIIGSDGNDLIKGGKGNDYLEGRDGDDIFRDAGGYNLI
AGGKGHNIFDTQQALKNTEVAYDGNTLYLRDAKGGITLADDISTLRSKETSWLIFNKQVDHQVTAAGL
KSDSGLKAYAAATAGGDGDDVLQARSHDAWLFGNAGNDTLNGHAGGNLTFVGGSGDDILKGVGNGNTF
LFSGDFGRDQLYGFNATDKLVFVGTEGASGNIRDYATQQNDDLVLAFGHSQVTLIGVSLDHFNTDQVV
LA
>CORE_REP|Org29_Gene3489#
MSDPVEACGFTLSEWQHHYQTRPAGERLACVSATIETLIAGLNPDDNAWLYLATPAQREQQYRQLEQL
LAAVDGDLSRLPLFGVPFAIKDNIDVGGWPTSAACPAFTYQAAADATVVANLRAAGAIALGKTNLDQF
ATGLVGTRSPYGAVVNSFDSRYVSGGSSSGSASVVARGLVPFALGTDTAGSGRVPAGFNNIVGLKPTK
GRLSNRGVVPACRLNDTVSVFALTVADAAQVAELASGFDEADPYSRPDPHTAPADIPAAPRFAIPAQL
EFFGDVQAERAFHRALAQLQAGGATLEPLDFAPFRTLAEQLYYGPWVAERTVAIEQVLEASPQAIDPV
VRGIVGNGLGYSACDAYKAEYLRAELARQIAQRLAPFDALMVPTAPTIRTLAEMAQEPVLFNSQFGTY
TNFTNLADLSALALPGPLREDGLPAGITLIAPAWHDRALAAFGLRWQRQSALPLGATGRALPPQPAPA
PAPSSGHVRLAVVGAHLSGMPLNVQLTQRDAVRVEQTVTAPCYRLYALADTEPPKPGLARVAQGAAIR
LELWDIPLARFGEFVAEIPAPLGIGTLLLADGRRVKGFICEAWALEGATDITEFGGWRDYLASLKGHE
HV
>CORE_REP|Org23_Gene2753#
MEQNPQSQLKLLVTRGKEQGYLTYAEVNDHLPEDIVDSDQIEDIIQMINDMGIQVMEEAPDADDLLLA

ENSNSTDEDAEEAAAQVLSSVESEIGRTTDPVRMYMREMGTVELLTREGEIDIAKRIEDGINQVQCSV
AEYPEAITYLLEQYDRVEAGEARLSDLITGFVDPNAEEDIAPTATHIGSELSSEEQDDDEEDEDAEDD
DTEDDNSIDPELARQKFAELRDQYEATRLVIKKNGRSHASAADEILKLSEVFKQFRLVPKQFDFLVNS
MRTMMDRVRTQERIIMKLCVEQCKMPKKNFVTLFAGNETSDSWFEAAVAMAKPWSEKLKDVAEDVQRS
LQKLRQIEEETGLTIEQVKDINRRMSIGEAKARRAKKEMVEANLRLVISIAKKYTNRGLQFLDLIQEG
NIGLMKAVDKFEYRRGYKFSTYATWWIRQAITRSIADQARTIRIPVHMIETINKLNRISRQMLQEMGR
EPTPEELAERMLMPEDKIRKVLKIAKEPISMETPIGDDEDSHLGDFIEDTTLELPLDSATSESLRSAT
HDVLAGLTAREAKVLRMRFGIDMNTDHTLEEVGKQFDVTRERIRQIEAKALRKLRHPSRSEVLRSFLD
D
>CORE_REP|Org12_Gene4434#
MSTAAVINRELLAEQVLRDTFGYQQFRPGQQTIINAAIGGQDCLVVMPTGGGKSLCYQIPALVMDGLT
LVVSPLISLMKDQVDQLLAYGVSAACYNSTQTREEQLDVMAGCRNGNIKMLYIAPERLMMESFLNLLD
HCPPAMLAVDEAHCISQWGHDFRPEYRALGQLKQRFPSMPVIALTATADESTRGDIVRLLSLQDPLVQ
VSSFDRPNIRYTLVEKFKPLDQLWRFVQDQRGKSGIIYCNSRAKVEDTAARLQSRGLSVGAYHAGLDN
DRRAQVQEAFQRDDLQVVVATVAFGMGINKPNVRFVVHFDIPRNIESYYQETGRAGRDGLPAEAILLY
DPADMAWLRRCLEEKPAGQQLDIERHKLNAMGAFAEAQTCRRLVLLNYFGEGKHENCGNCDICLDPPK
RYDGLEDARKALSCVYRVGQRFGLGYIVEVLRGSNNQRIREYGHDKLPVYGIGRDQTTEHWTSVLRQL
IHLGFITQNIAMHSALQLTEAARPVLRGEVALQLAVPRIQSLKSRSSSANQKSYGGNYDRKLFAKLRK
LRKSIADEENIPPYVVFNDATLLEMAEQMPIKAGDLLSVNGVGQRKLERFGAPFMAMIRDHLDNDED
>CORE_REP|Org46_Gene2862#
MNDRFDAKAFLSTVTSQPGVYRMYDATGTVIYVGKAKDLKKRLASYFRQQVSSRKTETLVKNIAQIDV
TVTHTETEALLLEHNYIKLYQPRYNVLLRDDKSYPLIFLSADTHPRLAVHRGAKHAKGEYFGPFPNSY
AVRETLALLQKLFPIRQCENSVYRNRSRPCLQYQIGRCLGPCVAGLVSEEEYRQQVDYVRLFLSGKDQ
QVLHQLIARMEEASKLLNFEEAARIRDQIQAVRRVTERQFVSGDSDDLDVIGVAFDAGMACLHVLFIR
QGKVLGSRSYFPKVPGGTDMGEVVQTFVGQFYLQGSQARTLPGEILLDFSLPEKDLLAESLSELAGRK
IQIQSKPRGDRARYLKLARTNAATALTTKLSQQSTIHQRLAELAKVLNLTEINRMECFDISHTMGEQT
VASCVVFDGNGPVRAEYRRYNISGITPGDDYAAMTQVLKRRYGKALEEKKIPDVIFIDGGKGQLGMAI
EVFKSLNVTWDKNKPLLIGIAKGADRKAGLETLFFVPEGEGISLPPDSPALHVIQHIRDDSHNHAITG
HRQRRAKVRNTSALELIEGVGPKRRQVLLKYMGGLQPLLNASVEEIAKVPGISQALAEKIYNALKH
>CORE_REP|Org29_Gene3548#
MRRLLTTLAILLVVVVAGMSALVLLVNPNDFRGYMVKKVEQKSGYQLTLEGDLRWHIWPQLSILAGRM
TLTAPGAKAPVVSAENMRLDVKLLPLFSHQLFVKQVMLKNAVIRLTPDSEEHSQVDAPIAPAGSGTDA
ADAAWKFDIDNLRVVDSLLIWQRADNEQINVRDINLTLQQTEKRQAQLELSSRVNRDQRDLTFSMAAD
VDLQQFPRQLSAKVTQFNYQLAGADILNGGIQGEGNAQVVYQQTPAQIAVSQLNVSANNSQLTGDISA
TLGAVPGYVVNLNSANLDLDALSGWQSSTNTAEQPAVTSAPVIASQVDDRQQNLEALRDFNAQLNLQA
AQVTYRGMNVTQLAVAADNQHGLLTLHKLAGQLAGGDFSLPGTLDARGDKPVISVQPVLNQVELGTVL
KAFDMPQMLTGKFSMKGDLTGDRLSSQAFEHRWRGTAQLAMQDAQLHGLNIQQLIQQAVARNDNSVRG
QDSYQRYTEVKSVSAQASLNQGTVKLSGLTADSPLLALTGAGSIDMPGKQCDMALNVRVTGGWQGRGE
LIEQLQKTPIPLRVYGPWQQLNYQLQVDQVLRKTLQDRAKDALNKWAEKNKDSREGQDLKKLLDKL
>CORE_REP|Org30_Gene154#
MIENLRNIAIIAHVDHGKTTLVDKLLQQSGTFGERAEATERVMDSNDLEKERGITILAKNTAINWNGY
RINIVDTPGHADFGGEVERVMSMVDSVLLVVDAMDGPMPQTRFVTKKAFANGLKPIVVINKVDRPGAR
PDWVVDQVFDLFVNLDATDEQLDFPIIYASALNGIAGVDHTDMAEDMTPLYQAIVDHVSAPQVELEAP
FQMQISQLDYNNYLGVIGIGRIKRGKVKPNQQVTIIDSEGKTRNGKVGKVLGHLGLERIDSTLAEAGD
IIAITGLGELNISDTICDTNAVEALPALSVDEPTVTMFFNVNTSPFCGKEGKYVTSRQILDRLNKELV
HNVALRVEETDDADAFRVSGRGELHLSVLIENMRREGFELAVSRPKVIFREIDGRKQEPFENVTLDIE
EQHQGSVMQAMGERKADLKNMDPDGKGRVRLDYVIPSRGLIGFRNEFMTMTSGTGLLYSTFSHYDDVR
PGEVGQRQNGVLISNGQGKAVAFALFGLQDRGKLFLGHGAEVYEGQIIGIHSRSNDLTVNCLTGKKLT
NMRASGTDEATTLVPAIKMTLEQALEFIDDDELVEVTPTSIRIRKRHLTENDRKRASRGPKDA
>CORE_REP|Org14_Gene2948#
MLNAWHQPVPPFVVKQGQRLDITLWLQGDELPERVFLRAEPDNEEWLLVMKAQRHEGMRRYQASLTLN
EGEPTRRYCFKLLWADRQQWFGPQGWSPTPPGQLAQFAVDEPDNGPEWVADQLFYQIFPDRFASSGGE
HGIQSGSYRHHAAGAEVIRRDWQHPLEDRHAASTFYGGDLDGIGAKLPYLQQLGVTALYLNPIFTAPS
VHKYDTEDYYQVDPHFGGNAALQRLRVSTHKVGMKLVLDGVFNHTGDSHPWFDRHRQGENGACHHPDS

PYRGWFNFYPDGRALDWKGNASLPKLNFAEPQVAEAIYRGEGSVVRHWLRPPYSIDGWRLDVVHMLGE
NGGATGNLHHLAGIYQAVKQENPQAYVLGEHFGDARRWLHAGVEDAAMNYMGFALPVRAFLAGLDVAY
HPVRLDAAGCAQWMDGYRAGLPHGRQLIQFNQLDSHDTARFLTLLQGNAARMQMAAVWLLSWIGVPCL
YYGDEIGLDGGNDPFCRKPFPWDVGDWDRPLLALFQRMAALRKQSVALRRGGCQVLYASGETLVFVRL
YQQEQVLVALQRDGSGQAQLPYNPLLARGPWRRVEGRGELSETAGGLRLQLAEETATVWRCEG
>CORE_REP|Org47_Gene4848#
MINPTLSRVTQRIIHRSQASRAAYLARIEAARSQTVHRAQLACGNLAHGFAACQPNDKTALKNMVRSD
IAIITAYNDMLSAHQPYEHYPQRLKQALKAVGAVGQVAGGVPAMCDGVTQGQDGMELSLMSRDVIAMS
AAVGLSHNMFDGALFLGICDKIVPGLVMAALSFGHLPALFVPAGPMSSGLPNKEKVRVRQLYAEGKAD
RLALLEAEAASYHGIGTCTFYGTANTNQMVMEVMGLHLPGASFVHPDTPLRDALNDAAARQVTRLTDT
AGNYLPIGRLVDEKVVVNGIVSLLATGGSTNLTMHLVAMARAAGIIITWDDFSELSEAVPLLCRIYPN
GPADINQFQAAGGVPLVVRELLQHGLLHEDVHTVAGFGLHRYTQEPWLDNGQLVWREGVAGSLDASVI
ASVAQPFEHHGGTKVMAGNLGRAVMKTSAVPADNQIIEAPAVVFDSQHDIVPAFEAGKLDRDCVVVVR
FQGPQANGMPELHKLMPPLGVLMDRGFKVALVTDGRLSGASGKVPSAIHVTPEAYTGGLLAKVRDGDP
IRVNGRSGELQVLVDADELAQRTPCQPDLSAEHIGCGRELFGALRSQLSGAEQGACCITF
>CORE_REP|Org4_Gene4333#
MSVRIFAALVLSALSFGLQAEALNESYAFALLGEPKYATDFSHFDYVNPAAPKGGDVRLAAIGTYDNF
NRFATRGVPGERTDQLYDTLFATSDDEPGSFYPLIAESARFPADMRWVEVDINARARFHDGSPITAAD
VAFTFSKFMAEGVPQFRSYYKGGTVKAISRLTVRIEFPQPNKEAMLSLFGSLPILPAAFWQNHKLNEP
LNTPPLASGPYKIGDYRLGQYITYQRVRDYWAANLPVNRGRYNFDSIRYDYYLDDKVALEAFKAGAYD
FRIEPSPKSWATQYQGGNFARNYIIKQDETNQAAQNTRWLAFNIQKPLFADPRVREAITLAFDFNWMN
KALYYNAYQRADSYFQNTAYAARGYPDAAELALLAPLKGQVPPEVFTSIYQPPASDGSGNDRQNLLKA
TQLLKEAGWVVKNQKLVNAKTGQPFAFELMLLSGSNFQYVLPFRHNLQRLGIDMAIREIDASQYTRRM
RERDFDMMPTVYMAMPFPTANLRIIWDSEYINSSYNTPGVKDPAVDSLVRQIAEHQGDEKALLPLGRA
LDRVLTWNRYMLPMWYSNHDRYAYWDKFSTPPIRPAYAIGFDNWWYDVNKAARLPAQRQ
>CORE_REP|Org38_Gene1242#
MITNLLQYHLIHRIQHQITHRADRTAFRQWSPNGEFQLTWEEAAVRIDRIAAGLLALGAEVQERIGIF
ANNGMAWSLADLAILHLRGVSVPLYSTNTPAQAAFVINDADIRILFVGEQAQLDAAIALRGVCPQLRH
IIVFDQDADLRGCEIARHLSAFEHAADPAAFIAQRRQRIEECDLQDLFTLIYTSGTTGEPKGVMLDYR
NLAAQLYLHDERLTVGEEDVSLSFLPLSHVFERAWSFFVMHSGAQNVFLPNTDWVREAMGQVRPTLMC
AVPRFYEKIFSAVHEKVARAPWLRRALFHWAIVCGERKFLQERAGKPLGKLFELSHRWADKLVLSKLR
GILGGRVRFLPAAGAKLDDNVILFFQAMGVNIKYGYGMTETCATVSCWEEGHFRFGSIGKPLPEVEVR
IGEENEIQVRGPIVMRGYFNKPLETAATFTADGWLKTGDAGAIDEEGNLFITERLKDLMKTSGGKYIA
PQMLEGTLAQDRFIEQVAIIADARKFVSALIVPCFESLEEYAKSVNLKYQDRLELLRNGEILEMFEKR
LREMQKELARFEQVKKFTLLPAAFSMELGELTPTLKLRRKVILQRYQREIDSMYQEQG
>CORE_REP|Org47_Gene4278#
MLLKKGNSLRRLALSGAIACSLVSSFSASATVPALPVASAGMSVAQSRSELLAALPRGMDLHYLSTLA
PLYAANHMQPMWQDREAVQQFQQQLAELAMSGVQPQFTQWVKMLTDPALSEAGRDAVLSDAMLGYLQF
VSAIGANGNNWLYSNIPYKLGLPPTAVINQWQLAVRQARTLSYVNSLAPQHPQYAKMHQALRDMLADN
RPWPQVGSGPSLRPGQMSNDIPALREILTRTGMLAASAPEADPEPAVVSAKINEPDDGGLTVDEEKSR
VTVSPSAAPVTELTAEQTPPQIGSVVSDNLYTDELVEGVKRFQKWQGLTADGVIGVRTREWLNVSPKT
RAALLALNIQRLRILPGHVGTGIMVNIPNYSLTYYQNGNEVLSSRVIVGRPSRKTPLMSSALNNVVVN
PPWNVPTTLVREDIVPKAMRDGNYFQKHGYTVLSGWSNDAEVINPAMIDWSMISARNFPYRVRQAPGA
TNSLGRFKFNMPSSDAIYLHDTPNHSLFQKDIRALSSGCVRVNKASDLANMLLQDAGWNNSRVSSTLK
EGNTTYVNIRQRIPVKLYYLTAWVSDDGQPQFRTDIYNYDNTVRSGAQILAQAKKLMQ
>CORE_REP|Org28_Gene1320#
MEGSTLLTAILLFLFAAVVTVPIARRLGIGAVLGYLIAGIAIGPWGLGFIRDVDEILHFSELGVVFLM
FIIGLELNPSKLWELRRSIFGAGAGQVLITAAVLGALLYLTHFAWQAAVIGGVGLAMSSTAMALQLMR
EKGMNRNEGGQLGFSVLLFQDMAVIPALALIPILAGAGGTSDDWAKIALKVAAFGGMLIGGRFLLRPL
FRYIAASGVREIFTAAALLLVLGSALFMEALGLSMALGTFIAGVLLAESEYRHELEISIEPFKGLLLG
LFFISVGMVLNIGVLYTHLAEVLIGVLVLVTVKSGVLYGVSRLFGLRSSVRLQFAGVLSQGGEFAFVL
FSAAGAQKVLQPDQLSLLLVVVTLSMMTTPLLMQAIDRILARRYNAKDEDEETPYVEDDDPQVIIVGF
GRFGQVIGRLLMANKMRITVLERDVSAVGVLRRYGYKVYYGDATELELLRAAGAEKAKSIVITCNEPE
DTMEIVRLCQQHFPNLSILARARGRVEAHELLQAGVKQFSRETFSSALELGRKALMELGMHPHQAFRA

QQHFRRLDMRMLRELMPPHQGDVAQISRVKEARRELEELFHREMQKESRQFDGWDEYE
>CORE_REP|Org11_Gene4089#
MTTQAPPTSLLPLTPEQLARLQATIGDYSPTQLAWLSGYFWGMVNQQPGAVAIAPAAPAAAAAITIIS
ASQTGNARRLAEQLRDDLLAANLSATLVSAGDYKFKQIAQERLLVIVASTQGEGEPAEEAVALHKFLF
SKKAPKLNDTAFAVFGLGDTSYENFCQSGKDFDGKLAELGAERLVERVDADVEYQELAAAWRKQVVSV
LKARAPAESAAPGVLASGAVDLIDSSPYSKEQPLTAQLAVKQKITGRASDKDVRHIEIDLGDSGLRYQ
PGDALGVWFDNDPALVDELVQLLWLKGDEPVEVEGKTLPLAQALRSHFELTQNTTPIVDKYAALSRDE
TLIGLLADKAALQQYAHNTPIVDMVRQAPADLSAEQLVGLLRPLTPRLYSIASSQAENESEVHITVGV
VRYDIDGRARSGGASGFLADRLEEDGDVRVFIEHNDNFRLPANPETPVIMIGPGTGIAPFRAFMQQRD
ADGAGGKNWLFFGNPHFTEDFLYQVEWQRYVKDGLLTRVDLAWSRDQQHKIYVQDKLREQGAEVWRWI
QEGAHIYVCGDANRMAKDVENTLLELVAEHGGMDTEQADEFLSELRLERRYQRDVY
>CORE_REP|Org9_Gene2216#
MKHIRNFSIIAHIDHGKSTLSDRIIQICGGLSDREMAAQVLDSMDLERERGITIKAQSVTLDYKALDG
QTYQLNFIDTPGHVDFSYEVSRSLAACEGALLVVDAGQGVEAQTLANCYTAIEMDLEVVPVLNKIDLP
AADPDRAAQEIEDIVGIDATDAVRCSAKTGVGVPDVLERLVRDIPPPQGDPDAPLQALIIDSWFDNYL
GVVSLVRVKNGTLRKGDKIKVMSTGQVYNADRLGIFTPKQVDRDVLNCGEVGWLVCAIKDILGAPVGD
TLTQARQPADKALPGFKKVKPQVYAGLFPISSDDYESFRDALGKLSLNDASLFYEPESSTALGFGFRC
GFLGLLHMEIIQERLEREYDLDLITTAPTVVYEVETTGKEVIYVDSPSKLPPLNNIQELREPIAECHM
LMPQEYLGNVITLCVEKRGVQTNMVYHGNQVALTYEIPMAEVVLDFFDRLKSTSRGYASLDYNFKRFQ
ASDMVRVDVLINNERVDALALITHRDNSQYRGRELVEKMKDLIPRQQFDIAIQAAIGTHIIARSTVKQ
LRKNVLAKCYGGDVSRKKKLLQKQKDGKKRMKQVGNVELPQEAFLAILHVGKDGK
>CORE_REP|Org4_Gene492#
MTDLTTHDALPAWQTRDHLDDPVIGELRNRFGPEAFTVQATRTGMPVVWVKPDQLLEVMTFLRKQPKP
YVMLFDLHGVDERLRTHRDGLPAADFSVFYHLISIERNRDIMLKVALSEKDLHVPTATKVFPNANWYE
RETWEMFGITFDGHPHLSRIMMPQTWEGHPLRKDYPARATEFDPFVLTKQKEDLEMEALTFKPEDWGM
KRGTENEDFMFLNLGPNHPSAHGAFRIILQLDGEEIVDCVPDIGYHHRGAEKMGERQSWHSYIPYTDR
IEYLGGCVNEMPYVLAVEKLAGIKVPERVDTIRVMLSELFRINSHLLYISTFIQDVGAMTPVFFAFTD
RQKIYDLVEAITGFRMHPAWFRIGGVAHDLPRGWDRLLREFLDWMPKRLDSYVKAALKNSILKGRSIG
VASYNAKEALEWGVTGAGLRATGVEFDVRKWRPYSGYENFDFEVPVGDGTSDCYTRVMLKVEELRQSL
RILEQCLNNMPEGPFKADHPLTTPPPKERTLQHIETLITHFLQVSWGPVMPANESFQMIEATKGINSY
YLTSDGSTMSYRTRVRTPSFAHLQQIPSVIRGSLVSDLIVYLGSIDFVMSDVDR
>CORE_REP|Org41_Gene4075#
MDIKQKVKNMTLEEKIGQKIMLDFRYWDRNGSSNQDMTVPDEAIGKLIADNHVGGVILFANNLKDKQQ
INTLTAWYAAMKTHAGIRLFIGTDNEGGNVFRLPRGDYASFPGNMALAAAIEGGADEQLAVEQGKLMA
QDMRALHINTNFAPVVDVNTNPFNPVINVRAFSDDKNTVSRLAEKMVAGMKHQGLITAYKHFPGHGST
STDSHTGLPRVDRTREEAFAIDIAPYKQAIDRCAAPDMVMTAHIQYPALDNRQIDTRSGEKITVPATM
SHEIQTQILRNELGYAGVTISDALDMGAIAEHFSQQAAAENVFAAGVDIALMPVSIASPAQASLLPAL
IRYLADRVKTGHLSEADIDASVERILRLKLRHSLMDHSDRPCSNDVASSAHKLEKCIADRSITVVINR
HSLLPLKDKALRYFILTPWGEQASGIARVMAQEGYQNVVAAKETELSDAQVRKHIAGCDVFLLGTLST
RFTPAEQDGVVTSATGASNDSSPYPGWLKYAAEQGKKRVHLSLRAPYDIVNYAAEVEAAVVTYSYYGY
DSGVWRGPSMVSLAQVLTGKIKPQGKLPVNTWHDYDVETNTGKVAFPRGTGLSW
>CORE_REP|Org29_Gene2405#
MRTEYCGQLNLSHVGQEVTLCGWVNRRRDLGGLIFIDMRDREGIVQVFFDPDQKVAFDKAYDLRNEFC
IQIVGTVRARPDSQINKDMATGEVEVFAHALEIINRSEPLPLDSNQVNSEEARLKYRYLDLRRPEMAE
RLKTRAKITSFVRRFMDSHGFLDIETPMLTKATPEGARDYLVPSRVHKGKFYALPQSPQLFKQLLMMS
GFDRYYQIVKCFRDEDLRADRQPEFTQIDVETSFMTADQVREVMEKLARELWLDVKGVDLGDFPVMTF
AEAMRRFGSDKPDLRNPLELVDVADLVKDVEFKVFSGPANDAKGRVAAIRVPGGAQLTRKQIDEYGAF
VNIYGAKGLAWLKVNDRAAGMEGVQSPIAKFLSADVLEAVLARTNAQTGDILFFGADSFKIVTDAMGA
LRLKLGRDLALTQLDSWAPLWVVDFPMFEEDEEGGLAAMHHPFTSPRDMSPEELAAAPVNAIANAYDM
VINGYEVGGGSVRIHRSEMQQTVFSILGINEHEQREKFGFLLDALKYGTPPHAGLAFGLDRLVMLLTG
TDNIRDVIAFPKTTAAACLMTDAPSFANPASLQELAISVVKKAGAEQESE
>CORE_REP|Org8_Gene3621#
MKNAVRRGEVMSVLAAYRRGFWGIALFTAVINLLMLAPALYMLQVYDRVLPSGNRMTLAMLTLMVVGL
YLFMGLLEWVRSQVVIRLGAQMDMRLNQRVYDAAFETNLKTGNPLAGQALNDLTNLRQFATGNALFAF

FDAPWFPVYLLVVFLLHPWLGALASAGVIVLVLLAWLNQRVSQAPLAEAGRVALSATQQANGNLRNAE
AIAAMGMLTDLRLRWLRQHQQFLLLQNRASEKIAAVTAWSKTVRLALQSLMLGCGALLAVSGDITPGM
MIAGSILIGRVLGPIDQLIGAWKQWSSARQSLQRLEVMLAANPPRIPSLPLPTPGGALTVSQLTASAP
GGTAPVLHGVSFRLEAGEVLGVIGASGSGKTLLMRQLVGALTPISGDVRLDGADIQQWDKQQLGPHIG
YLPQDIQLFAGTLTDNIARFGQVDAEKVVVAAALAGVHQLILHLPKGYETELGEGGSGLSGGQRQRVA
LARALYGSPALVVLDEPNANLDREGEEALQRAIEALKARGTTIVLVTHKPAILATTDKLLVLTAGQVQ
HFGPSDAILKKLPGFAPAAAAAPANTGRSNGGFNVNYANFAKTASGERKV
>CORE_REP|Org36_Gene3179#
MRFFQNLQNDGISPRAQIRLLDNTFMRLVFSFTAVPFVGIPFAIWIYLLGDELGPTITWIIVYLLCAV
AIRIWHRRYLHEAKENDEDAVLRRWLPRINKVAFIHGLGISSLYLITPQTHNFDFFLLLNISIAAIVA
ANATHLTPVISTFTRFFFASWGLLNLGIICRLEDVMFIVLMLNLLYGFAIYRHALTSHAFFIQQALLE
EQSSRLAEQFRQAKEEAEQALLDKNQFLTTASHDLRQPVHAMGFLIEAIIHKNRDDSLTPQLLDLQQS
VRSVHLMFNSLLDLSKIESGNVRTAATHVDIGALLDSVITLFREEANSRALALRTWRPKRRISVMGDP
LLVRQSLINLIQNALRYTQQGGVLIAIRPRGAECLVEVWDTGVGIADEEKSKIFSPYYRPELAWKIDS
AGHGLGLAVVARCAKLMKVKYGMHSVEGKGSRFWMRFTQYIGEDKAPETAAAYDNTATPIRYAPLRGA
CLVVDDDPLVTSAWESLMSTWGITVRCAASAEEAFAIVDDGFTPFAVLCDQRLRSGESGFDILKALFE
RLPDVSGAMVSGEFNSQILQEAEQEGYLVLRKPLEPARLHALLTQWGAAS
>CORE_REP|Org43_Gene1979#
MVTNRQRYREKVSQMISWGHWFALFNILLALGLGSRYLFVTDWPASLLGRVYALVSLLGHFSFIVFAG
YLLVIFPLTFVVMSQRLLRFISAALATAGLTLLLVDSEVFSHFHLHLNPVVWDLVVNPDQSELSRDWQ
LMFICVPVIFLVEMLFGTWSWQKLRSLNRRRFGKPLAALFISAFFASHLIYIWADANFYRPITMQRAN
LPLSYPMTARKFLEKHGLLDQQEYERRLVQQGNPEAVAVEYPLSDLSYGDKGSGYNLLMIVVDGIRAK
DVAQDMPTLTRFAQENVRFSDHYSSGNHADTGLFGLFYGISPTYLDSVLAGRKPSALINALGEQGYQL
GLFSSDGFNASLYRQALLTDFSLPTPAPQSDAQTTQQWQRWLTDQGDKEPWFSYINFSGAEPAEGAKT
PAPADFIQRYRTGAQDVDSQIAQVLDTLKQRGLLDKTVVVITAEHGVEFNDSGKGQWGAGTAFNQAQL
QVPLVIHWPGTPAQTINKLTGHNDVMRTLMQRLLHVKTAPKDYSQGEDLFTAQRRNNWIATGDGNQLV
ITTPTQTLMLDNSGNYRVYDQNGDEIKDEKPQLALLLQVLTDVKRFIAN
>CORE_REP|Org7_Gene188#
MATVMKKVDAVIVGFGWVGAIMAKELTEAGLNVVALERGPMRDTYPDGSYPQVIDELTYNIRRKLFQD
LSKSTVTIRHNSSQTAVPYRQLAAFLPGTGVGGAGLHWSGVHFRVDPIELRMRSHYEERYGKNFIPKD
MTIQDFGVTYDELEPFFDKAEKVFGTSGTAWTIKGQKVAQKGGNRFAPDRSDDFPLPAQKNTYSAQLF
EKAALEVGYHPYNLPSANTSDSYTNPYGAQMGPCNFCGFCSGYACYMYSKASPNVNILPALRMEKRFE
LRTNANVLKVNLTDDKSRATGVNYVDAQGREIEQPADLVILGAFQFHNVHLMLLSGIGKPYDPVTGEG
VVGRNFAYQNMTTIKAFFDKDVFTNPFIGAGGNGVGVDDFNADNFDHAKEGFVGGSPFWVNQAGTKPI
SGLPTPPGTPAWGSKWKAAVADAYTHHVSMDAHGAHQSYRNNYLDLDPNYKNVFGQPLLRMTFDWQEN
DIKMAQFMYDKMAPIAKAMNPKLIAGSPKNANSHFDTTSYQTTHMNGGAVMGEDPKTSAVNRYLQSWD
VHNVFSIGASAFPQGLGYNPTGTVAALAYWSARAIREQYLKNPGPLVQA
>CORE_REP|Org5_Gene3887#
MNKVQKLWPTLKRLLAYGSPYRKPLGLAVLMLWIAAAAEVAGPILVSYFIDNYVAKGQLPLTIVGGLA
AAYILLELLAAALHYFQALLFNQAAVGVVQRLRTDVMDAALRQPLSAFDTQPVGQLISRVTNDTEVIK
DLYVMVVSTVLKSAALIGAMLVAMFSLDWRMALVAVCIFPAVFVVMGIYQYYSTPIVRRVRSYLADIN
DGFNEVINGMGVIQQFRQQVRFGERMSAASQSHYLARMQTLRLDGFLLRPLLSLFSALVLCGLLMLFG
FSGEGVIGVGVLYAFINYLGRLNEPLIELTSQQSILQQAVVAGERIFELMDRSQQSYGADDRPLAGGR
IDITDLSFAYRADKKVLQHISLAVPSRGFVALVGHTGSGKSTLANLLMGYYPVSEGEVRLDGRPISSL
SHRTLRQGVAMVQQDPVVIADSVLANVTLGRNIEEDAVWRALETVQLASLVRGFPQGIHTRLGEQGNN
LSVGQKQLLAMARVLVQAPQILILDEATANIDSGTEQAIQRALRAIREHTTLVVIAHRLSTIVDADSI
LVLHRGQAVEQGNHQQLLAQQGRYYQMYQLQLAGEQLAEAVREESQPA
>CORE_REP|Org19_Gene2614#
MRLFAQIGWYFRREWRRYLGAVVLLIVIAILQLLPPKLVGIIVDGVTEKQMSTGVLMAWLGLMIGTAI
VVYLLRYVWRVLLFGASYQLAVELRENFYRQLSRQNPAFYLRHRTGDLMARATNDVDRVVFAAGEGVL
TLVDSLVMGLVVLVVMSTQISWQLTVLALIPMPLMAIAIKYYGDQLHQRFKSAQAAFSSLNDQAQESM
TSIRMIKAFGLEDHQSNRFADVAAQTGAKNMHVARVDARFDPTIYIAIGASNLLAIGGGSWMVVNGSL
TLGQLTSFVMYLGLMIWPMLALAWMFNIVERGSAAYSRIRSLLDEAPAVQDGPQALPAGRGVLDVDIR
AFHYPENPHPALHDVALTLKPGQMLGLCGPTGAGKSTLLSLIQRQFDVDQGQIRYHGLPLPQVKLDDW

RSRLSVVSQTPFLFSDTVANNIALGHPGATQAQIEQAARLASVHEDILRLPQGYDTEVGERGVMLSGG
QKQRISIARALLLDAEILILDDALSAVDGRTEHQILHNLRSWGQDRTVIISAHRLSALTEAGEILVMQ
HGGVAQRGDHAALAAQPGWYRDMYRYQQLEAALDEAPENGEEALADE
>CORE_REP|Org40_Gene1860#
MQAWRRKWQSLPRGLVVLITALVIYVPLSFIIIQSFLSAPFFSPSKVFSLEAFEFIFTDPDFYKALKS
GFILAFGLVIIAIPLGGILAFLMVRTDLPGRRIIEPLILVPIFVSPMVLGFGYVVAAGPVGFFSLWAE
ALLGFVPWNIYSMASIVVIAGLTHVPHAYLYISSALRSVGSDVEEAARTAGATPLQVMTAVSLPMVRP
SILYAGVLLFFLGLEVFGLMLVLGDPEGNLVLATYLYQLTNKLGTPSYHLMAAVAVVLICITIPLVML
QRRLMRTANRFVTVKGKASQARALPLGKWRWVAGAVVMFWLTVTIGVPLIGVVLRAFISNWGVGVSVW
DELSINTFRTIWAQPNLLRAIVNSMAIGVIGGALAVACYLFIGIAMHRKPDGATRFLDYSVLVPRAVP
GLLAGLAFLWVFLFLPMWLDKSLKEGWLSVLPMAEWLRENVIVWLRSLRSTIFSVWLAYTVVWMAYGL
RLISSTLLQVGPELEEAARSAGASRGQITRHVTIPLSRYGLIGSWLLMFLIFEREYSTGVYLLSPGTE
TIGSMLVSLWAAGAIDIVAALSFINILLVVLGLGIALRFGVKLHD
>CORE_REP|Org2_Gene2928#
MKKTRQQQLTRWLKTQSSLAQRWLRLSMLLGLFSGLLIVAQAWLLASLLHALIIEHTPREQLIPSFIW
LAAAFALRALLSWLRERVGFRCGQVIRQRMRQQVLDKLQQLGPAWIQGKPAGSWASIIVEQIEDMQDY
YSRYLPQMYLAVFIPLLILIAVFPINWAAGIILLATAPLIPLFMVLVGMGAADANRRNFVALARLSGN
FLDRLRGLDTLRLFDRAQAETAQIAKSSEDFRSRTMEVLRMAFLSSGVLEFFASISIAVVAVYFGFSY
LGELNFGSYGLGVTLFSGFLVLILAPEFFQPLRDLGTFYHAKAQAVGAAEALETFLSAEGEQMGNGTR
QLAADQPLTLQANALEILSPNGVLLAGPLSFTLQPQQRVALVGLSGAGKSSLLNLLLGFLPYRGSLTV
NGVELRDLSAENWRQQLSWVGQNPHLPAQTLRANILLGCPQADEAQLQQAVEHAYVSELLPYLPQGLD
TEVGDNAARLSVGQAQRVAVARALIGPRRLLLLDEPAASLDAHSEQRVMQALNAASHQQTTLLVTHQL
EDTEDYDQIWVMDNGRIVQQGDYATLSAQPGLFATLIAHRRGEL
>CORE_REP|Org38_Gene1111#
MFLQKWSKPLTMAALLVSGSLYAASNPAVEAKNGMVVTSQHLASQVGVDILKMGGNAIDAAVAVGYAQ
AVVNPCCGNIGGGGFMTVHLADGTDTFINFRETAPAAASANMYLDADGKVKKDASLYGYLAAGVPGTV
LGMETAREKYGKLSREQVLAPAIRLAREGFVLTRADTDILDTTVARFKQDPESAKIFLRPDGSPLQPG
DKLVQTDLANTLEAIAKGGTDAFYKGKIPQAVEAAAKQGGGILTAADFANYKVTETPPITCSYRGYKF
VSAPPPSSGGVTLCEILNVVEGYDLKSMGFNSAAAIHTMTEAMRHAYMDRNTYLGDPEFIKNPIDRLV
SKSYAEQIRKKIVADKATPSENVQPGMEPHEKPETTHYSIVDHDGNAVSTTYTVNGRFGAVVIAPGTG
FFLNDEMDDFTVKVGEKNLYGLVQGTANSIAPGKRPLSSMSPTLVTKDNKIFMVLGSPGGSRIITITL
QTALNVIDHGMAPQEAVDAPRIHHQWLPDEVYYEQRGVSADTLKLLSGMGYKMVEQTPWGAAELILVG
LPGAAGVSPANSGNDSAVSGKVREGYLYGANDVRRPAGSAVGY
>CORE_REP|Org20_Gene235#
MKAARPGKLRRQEDQASFVSWRFALLCGCILLAMVGLMLRVAYLQVINPDRLVKEGDMRSLRVQEVPT
ARGMISDRAGRPLAVSVPVNAVWADPKELNERGGITLDSRWKALSDALNIPLDQLSNRINANPKGRFV
YLARQVNPAIGDYIHKLKLPGIYLRQESRRYYPAGQVTSHIIGVTNIDGQGIEGVEKSFDRWLTGQPG
ERTVRKDRFGRVIEDISSVDSQAAHNLVLSVDERLQALVYRELNNAVAFNKAESGTAVLIDVNTGEVL
AMANSPSYNPNNMAGTPKETMRNRAITDIFEPGSTVKPMVVMTALQNGVVRENSVLNTIPYRIQGHEI
KDVARYSELSLTGILQKSSNVGVSKLALAMPSSALVDTYSRFGLGKATNLGLVGESSGIYPKKQRWSD
IERATFSFGYGLMVTPLQLARVYATIGSLGVYRPLSITKVDPPVAGERVFPEPLVRTVVHMMESVALP
GGGGVKAAIKGYRIAIKTGTAKKVGPDGKYVNRYIAYTAGVAPASNPRFALVVVINDPQGGKYYGGAI
SAPVFGAIMGGVLRTMNVEPDALPTGDKSELVINKKEGSGGRS
>CORE_REP|Org28_Gene3029#
MTQTFIPGKDAALEDSIARFQQKLSDLGFNIEEASWLNPVPHVWSVHIRDRDCPLCFTNGKGASKKAA
LASALGEYFERLSTNYFFADFYLGRQIAEGDFVHYPNEKWFPIPEDDALPAGILDERLHAFYDPQQEL
SASDLVDLQSGNADRGVCALPFTRQSDQQTVYIPMNIIGNLYVSNGMSAGNTANEARVQGLSEVFERY
VKNRIIAESISLPAIPDEVLNRYPGVVEAIAKLEEEGFPILSYDASLGGNYPVICVVLFNPTNGTCFA
SFGAHPDFGVALERTVTELLQGRSLKDLDVFTAPTFDDEEVAEHTNLETHFIDSSGLISWDLFKQDAD
YPFVDWNFSGSTQEEFATLMSIFDKEDAEVYIADYEHLGVYACRIIVPGMSDIYPAEDLLLANNSMGA
HLRDTLLALPGSEWKPEEYLALIEQLDDEGLDDFTRVRELLGIASGKDNAWHTLRVGELKSMLALAGG
DLDQALIWTEWTQDFNASVLSPARSNYYRCLQTLLLLAQEPEREAAQYYTAFVKMYGQEAVDAASAAI
SGEERFNGLFAVDADLKALPAHQALLAAYEKLQAAKRRHWAKA
>CORE_REP|Org35_Gene3748#

MASNTLTNNRLEWQSLLPDVTPYQAIFDTAAQLAPVPFSAIQPRLENALTLFCHPQSPPRFMLLKAQE
TREYLELIANAVKPLLPQNTACRGSHYVIQDGKVSVEPASHGDEPFAAGGACVFQEWIEPEQLFGCVR
IHNGDITLQPGLVHQANGGILILSARALLAQPLLWLRLKQMIGQRQFHWVSPDETRPLPVAIPPMPLD
LRLIVVGDRHGLADFHDIEPELSEQAVYGEYEDDLQLTEVDDMAQWCGYVNGVIAERQLPMLAADAWL
PLIVQAVRYSGDQGILPLSPVWLGQQLSEAALYAEEDRITAKAFEAALNAREWRESYLAERMQDEIEL
GQILIETEGEVVGQINGLSVLDYPGHPRSFGEPSRISCVVHLGDGEFTDVERKAELGGNLHAKGMMIM
QAFVIAELDLDQQLPFSASIVFEQSYGEVDGDSASLAELCALISALSQQPITQQIAVTGSVDQFGNVQ
PIGGVNEKVEGFFEVCLRRGLTGKQGVILPVTNVRHLCLRQDVVDAVREGQFHLWAVESAAEALPLLT
GCLYSDEQQPNLLAAIQERIAQVSLQERRRPWPLRWLNWFNHG
>CORE_REP|Org1_Gene2813#
MAFTLRPYQLEAVEATINHFRRHPEPALIVLPTGAGKSLVIAELAKRARGRVLVLAHVKELVAQNHAK
YCAYGLEADIFAAGLQQKESAGKVVFGSVQSVARNLPLFDGAFSLLIVDECHRISDDDDSQYQQIIQH
LQKTNPQLRLLGLTATPYRLGKGWIYQYHYHGFTRGDSASLFRDCIYELPLRYMIKNGFLVPPERLDM
PIVQYDFSRLEARSNGLFSEAELNRELKRQNRVTPHIISQIVEYAEDRKGVMIFAATVEHAREIHGLL
PNGEAALVSAETPPAERDALIDAFKQQRLRYLVNVAVLTTGFDAPHVDLIAILRPTESVSLYQQIVGR
GLRLAPNKKDCLILDYAGNPHDLFTPEVGVSKPHGDSQPVQVFCPACGFANLFWGKCTENGDIIEHYG
RRCQGWLEDDDGHREQCDYRFRFKSCPHCGAENDIAARRCHQCQEVLVDPDDMLKAALKLKDALVLRC
GGMELQSGRDDKGEWLKATYYDEDGTSTSERFRLQTPAQRKAFEMLFLRPHQRAPGVPFAWHTAADVL
AQQQALRHPDFVVARKRGQFWQVREKVFDYQGRFRRANQLA
>CORE_REP|Org11_Gene3260#
MAAKLELLIAQTILQGFDAQYGRFLEVTAGAQQRFEQADWPAVQQAMKKRIHLYDHHVGLVVEQLKCI
TGQKYFDADFPSRVKAVYIDLLPDYPRFEIAESFFNSVYCRLFKHRDLTPDKLFVFSSQPERRFRDIP
RPLARDFTPNGDLPAMLRSVLSDLPLRLPWEDLARDIRDITQALQRAFSAPQLAGATFQIANELFYRN
KAAWLVGKLRLADGVYPFLLPIHHSESGALFIDACLTGKAEASIVFGFARSYFMVYAPLPAAMVEWLR
EILPGKTTAELYMAIGCQKHGKTECYREYLTFMAHSQEQFIIAPGVKGMVMLVFTLPSFDRVFKVIKD
EFAPQKEVTQAQVMACYQLVKEHDRVGRMADTQEYENFVVDKARLSPELLAELRREVPGKLEDLGDRI
VIKHLYMERRMTPLNLYLEQANDQQMRDAIEEYGNAIKQLAAANIFPGDMLFKNFGVTRHGRVVFYDY
DEICYMTEVNFRDIPPPRYPEDELASEPWYSIAPNDVFPEEFRHFLCGDRRIRQVFEELHSDLFTAEY
WRGLQQRIREGHVEDVFAYRKKQRFSQRSGAALPAATAAM
>CORE_REP|Org13_Gene2952#
MMNDKDLSTWQTFRRLWPMITPFKTGLIVAAIALIMNAAGDTLMLSLLKPLLDDGFGKTDSSVLVWMP
LAVIALMLMRGVTSFVSSYCISWVSGMVVMQMRRRLFGHMMRMPVAFFDQQSTGTLLSRITYDSEQVA
SSSSSALVTVVREGASIIGLFIMMFYYSWQLSVILIVLAPIVSIAIRLVSKRFRNISKNMQNTMGQVT
TSAEQMLKGHKEVLIFGGQQVETERFNSVSNRMRQQGMKLVSASSISDPIIQLIASLALAFVLFAASF
PSVMSTLTAGTITVVFSSMIALMRPLKSLTNVNAQFQRGMAACQTLFSILDMEQEKDTGTREVMRAKG
DIEFRNVTFYYPSKETPALRDINLKIAEGKTVALVGRSGSGKSTIANLLTRFYDIQEGEILMDGHDLR
EYTLASLRNQVALVSQNVHLFNDTIANNIAYARESEYSREQIEKAAEMAYAMDFINKMENGLDTVIGE
NGVMLSGGQRQRIAIARALLRDCPILILDEATSALDTESERAIQAALDELQKDRTSLVIAHRLSTIEK
ADEILVVEDGRIVERGEHAELLERQGAYAQLHRMQFGQ
>CORE_REP|Org37_Gene887#
MAGRIPRVFINDLLARTDIVDLIDARVKLKKQGKNYHACCPFHHEKTPSFTVNGEKQFYHCFGCGAHG
NAVDFLMNYDRLEFVETIEELATMHGLEVPYEAGTGPTQIERHQRQSLYQLMEQLSAFYQQSLQQSSG
APARSYLQQRGLSDDVIRHFAIGFAPAGWDNALKRFGRDADSRRALNDAGMLVTNDQGRSYDRFRERV
MFPIRDKRGRVIAFGGRVLGDGMPKYLNSPETEVFHKGRQLYGLYEAQQNHPTLQRLLVVEGYMDVVA
LAQFGIDYAVASLGTSTTAEHIQLLFRATDNVVCCYDGDRAGREAAWRALETALPYLNDGRQLRFMFL
PDGEDPDTLVRKEGKEAFEQRMEQAQPLSTFLFESLLPQVDLSSPDGRAKLSTLALPLITQVPGETLR
LYLRQELGNKLGLLDDSQLDKLMPKQAENANPYQAPQLKRTTMRILIGLLVQNPQLATLIPSLEGLEQ
TKQAGLPLFVELVQTCLAQPGLTTGQLLELYRDNKFSQQLETLATWNHMIVEDMVEQTFLDTLASLYD
SVLEQRLETLIAQARTRGLSPEEREEVRSLNQVLAKKN
>CORE_REP|Org3_Gene3058#
MRVLLPFLALYRRHSLLISLGILLAIVTLLASIGLLALSGWFLAASSLAGLAGLLTFNYMLPAAGVRG
AAIFRTAGRYAERVVSHDATFRVLSHLRVFTFSKILPLTPGGIARFRQAELLNRLVADVDTLDHLYLR
VISPLISAAVVILVVTYGLSWLDPALALTLGGILLLLLLLVPPVFYCAGKPIGGQLTALRGQYRTDLT
AWLQGQAELVVFGAVNDFRQTLNATEQRWQRRQWQQASLSGMAQALMILASGLTVTLLLWLSAAGIGG

DTQPGALIALFVFAALASFEALMPVAGAFQHLGQVIASATRVKQIIDRQPEVTFPAAGPAAADRAQLS
LQQLSFTYPDQPQPVLRDVTLEVAAGEHIALLGRTGCGKSTLLQLLTRAWRTDGGKILLNGEPLEDYD
EATLRRMTTVVSQRVHIFSDTLRENLRLAAPDADDARLSEVLRQVGLDKLLDSDGGLNAWLGEGGRQL
SGGEQRRLGIARALLHPAPLLLLDEPTEGLDAETEQQILALLRRHCQGKTLILVTHRLYGLEHLDRIC
VMDDGRIVEQGDHATLMRRQGRYARFRNRISNLAP
>CORE_REP|Org37_Gene3654#
MSTLLSAQSVGYDNAFGVLLSEISFSLKKGDRIGLIGDNGCGKSTLLQLLSGALPIHSGTVTLSHQCL
MARIEQHLPPELHASTLLDAVLAQLPAGQHLSERWRCEALLAELGFEPTSWTLTAGTLSGGQHTRLLL
ARALIRQPDLLLLDEPSNHLDLPTLLWLEQFLRSWSGSFVLVSHDRYLLDQVTNCTWILRDKTLQFFR
LPCSAARAALAEQDAADEHRRQAEQKEIDRVEKSAKRLATWGKVYDNEDLARKAKQMEKRVDRLKEEQ
TTLTAGSPWRLRLQGEALDADRLLALPQWAVRPAPDAPVLFSLEHLRVKSGDRIAIVGRNGCGKSSLL
RLLWQAYQHPAERPAIFHPRVRIGYYDQSLQQLRDEDTLSEALAQFAPLTEEQRKMALIGAGFPYLRH
HQQIRSLSGGERSRLLFVGLTLANHSLLLLDEPTNHLDMAGKEELAETLRQFAGAVILVTHDRMLIEQ
SCNRFWLIDQQKLDEWHDLAPVYQRLAGEAPALPTADKANAGGPTPDERLEGEEALLTTLFALESKLE
DDLARKPKHQKPALQARWRREIADITARLNLG
>CORE_REP|Org1_Gene3313#
MNATPLQKHAVWQLIKPFWVSEERWRAWMMLIAIVILSLGLVYISVLINQWNQVFYDALQNKNYPVFK
AQLWRFTYLALIFIVLAVYKIYLTQGLQMRWRRWMTEKFMGKWLAHQAYYHTEQQQIVDNPDQRIAED
LNVLTQYTLSLSLGLLSSLVTLFSFIDILWHVSGPMTFALGQHAITLSGYMVWFALLYAVLGSLLIWW
VGKPLVMLGFNQERYEANFRFGLIRIRENNDAIALYHGEPREAQQLGDRFDTIRSNWWAIMRITRRLN
IATNFYSQFAIVFPLLVAAPRYFSGAIQMGGLMQIASAFGQVQGALSWFIDAFNDLATWKACVNRLAG
FNAAVDQVHHQPRGIQLREEAAHPLTLDNLSLNLPDGQPLLAGAKMTLQRGDRLLIVGPSGCGKSTLL
RAIAGIWPYGAGAIGLPANANTLFLPQRSYIPIGTLREALSYPSQATQYSDEQLMRVLENCRLKHLQR
WLDTAANWSHRLSPGEQQRLAFARALLIRPSILFLDEATSALDDETEQLMYCLLVDELPDVTLISVAH
RNSVAKYHQTCWRFSRSEDQPARLALSPLPV
>CORE_REP|Org45_Gene427#
MISGILVSPGIAFGKALLLKEDDIVINRKKISADQVEQEVSRFLAGRAKASEQLEAIKTKAGETFGEE
KEAIFEGHIMLLEDEELEQEIIALIKDDLASADAAAYTVIEGQAKALEELDDEYLKERAADVRDIGKR
LLQNILGMPIVDLGSIQDEVILVATDLTPSETAQLNLDKVLGFITDLGGRTSHTSIMARSLELPAIVG
TSDVTKQVKNDDYLILDAVNNQIYVNPTADVIDQLKAAQNQYITEKNDLAKLKDLPAITLDGHQVEVC
ANIGTVRDVAGAERNGAEGVGLYRTEFLFMDRDSLPTEDEQFQAYKAVAEAMGSQAVIVRTMDIGGDK
DLPYMNLPKEENPFLGWRAIRIAMDRREILHAQLRAILRASAFGKLRIMFPMIISVEEVRDLKGEIET
LKAQLREEGKAFDESIEVGVMVETPAAAVIAHHLAKEVDFFSIGTNDLTQYTLAVDRGNELISHLYNP
MSPSVLGLIKQVIDASHAEGKWTGMCGELAGDERATLLLLGMGLDEFSMSAISIPRIKKIIRNTNFED
VKALAAQALAQPTAQDLMNCVNKFIEEKTLC
>CORE_REP|Org18_Gene4888#
MESAPILFEETAVTTNNKVRNVDVRAPRGTQLNAKSWLTEAPLRMLMNNLDPEVAENPHELVVYGGIG
RAARDWDCYDKIVETLKTLEEDETLLVQSGKPVGVFKTHSNAPRVLIANSNLVPHWATWEHFNELDAK
GLAMYGQMTAGSWIYIGSQGIVQGTYETFVEAGRQHYDGSLQGRWVLTAGLGGMGGAQPLAATLAGAC
SLNIECQQSRIDFRLKTRYVDEQANDLDDALARIKKYTSEGKAISIALCGNAAEILPELVRRGVRPDM
VTDQTSAHDPLNGYLPKGWSWEEYRQRAQTEPAKVVAAAKQSMADHVKAMLAFQQMGVPTFDYGNNIR
QMAKETGVDNAFDFPGFVPAYIRPLFCRGIGPFRWAALSGDPQDIYKTDAMVKELIPDDEHLHRWLDM
ARERISFQGLPARICWVGLGQRAKLGLAFNEMVRRGELSAPIVIGRDHLDSGSVSSPNRETEAMKDGS
DAVSDWPLLNAVLNTASGATWVSLHHGGGVGMGFSQHSGMVIVCDGTDEAAERIARVLHNDPATGVMR
HADAGYDIAIDCAREQGLNLPMVAATQGEKA
>CORE_REP|Org46_Gene3320#
MDQRFLKLILLLCSLFFLPQAAQASLFAPKGGSQFVPVDQAFAFDFKQQGSQVTLNWQIRPGYYLYRQ
QIKLVPQQATLGAFTLPEGLSHKDEFFGEVAIFKQQLNLQVPLQQAAANASLSVTYQGCAEAGFCYPP
ETRIIPLDAVSASDVAPLPAAAPAPTEQPTTPASLPFSPLWALLIGIGIAFTPCVLPMYPLISGIILG
RDRPQSSGRILALAVVYVQGMALTYTLLGLVVAAAGLQFQAALQHPYVLIGLSVLFIALALSMFGLYS
LQLPSALQTRLANWSNTQRGGSLTGVFLMGALAGLICSPCTTAPLSAILLYIAQSGNLWAGGGTLYLY
ALGMGIPLVIVTLFGNRLLPRSGPWMQYVKEAFGFVILALPVFLLERVIGDLWGLRLWSLLGLAFFGW
AFALSLRSARGWARALQLLLLAAVIAARPLQDWAFGAGQTQQAAQQHLAFARINNVEQLDLALQQAH
GKPVMLDLYADWCVACKEFEKYTFSDAAVQASLANAVLLQADVTANGAEQAALLKHLQVLGLPTILFF

DPAGQELTAQRVTGFMKAEAFNAHLQKVMR
>CORE_REP|Org23_Gene2786#
MAPSTKKSGKTYSTVRFGWICAGMLVCFFLLAFRVGYLQLLEHQQLADQADQRSIRTQVVPTNRAMIT
DRNDEALAVSVSSKDIVLDPKHILDTQTDTGNERWQSMANVLKIPLADIQHLIQSNAHKRFVYLARKV
EDDNAAYISKLHLTGVSAEQDFSRFYPMGQDAAGLIGIVGQDNQGLEGIELGFNPLLQGKNGLRVYQK
DGSGAVIGVLKSVDPVPPPNVTLSIDKFIQYVLYAQIRDGVVANQADSGCAVLVKIDTGEILGMASYP
SFNPNNYGSTPAKDIRNVCSSDSFEPGSTVKPVVVMVGLEHKLIRPDTVLDTTPYRVNGHLIKDVGHW
SKLTITGVLQKSSDIAVSHIALALPATVLPAVYRSFGLGRPTELGIGNESSGYLPQHRERWADIERAT
FSFGYGLRVTPLQMAREYAAIGSFGIYRPLSITKVTPPVMGQRILPADTVRSVVHMMESDALPGGSGV
SAAVPGYRLAIKTGTAEKMGPSGKYDGGYINYTAGVAPASDPQVALVVMVNNPKAGKHFGGSVAGPVF
GKIMAQVLEHMNILPDAQPLNVVSSVKS
>CORE_REP|Org34_Gene2353#
MEMLSGAEMVVRSLIDQGVKHVFGYPGGAVLDIYDALHTVGGIDHILVRHEQGAVHMADGYARATGEV
GVVLVTSGPGATNAITGIATAYMDSIPMVVLSGQVPSSLIGYDAFQECDMVGISRPVVKHSFLVKRTE
DIPAVLKKAFYLASSGRPGPVVIDLPKDIVGPAVRMPYAYPQDVSMRSYNPTVQGHRGQIKRALQTIL
AAKKPVMYVGGGAINAGCEAELLSLAEQLNLPVTSSLMGLGAFPGTHRQSVGMLGMHGTYEANKTMHH
ADVIFAVGVRFDDRTTNNLAKYCPDATVLHIDIDPTSISKTVDADIPIVGDAKQVLVQMLELLAQDEK
AQDHDALRDWWQSIEQWRARDCLGYDKHSGTIKPQAVIETLHRLTKGDAYVTSDVGQHQMFAALYYPF
DKPRRWINSGGLGTMGFGLPAALGVKLALPEETVVCVTGDGSIQMNIQELSTALQYNLPVVVVNLNNR
YLGMVKQWQDMIYSGRHSQSYMDSLPDFVKLAEAYGHVGIAIRTPDELESKLAQALAEKERLVFVDVT
VDETEHVYPMQIRGGSMDEMWLSKTERT
>CORE_REP|Org13_Gene3486#
MSDKHPGPLVVEGKLADAERMKKESHFLRGTIAEDLNDGLTGGFNGDNFLLIRFHGMYQQDDRDIRAE
RAEQKLEPRHAMMLRCRLPGGIISPQQWLGIDKFAQESTLYGSIRITNRQTFQFHGILKGNVKPVHQL
LNRLGLDALATANDVNRNVLCTSNPVESELHQEAYEWAKKISEHLLPRTRAYAEVWLDQEKVATTDEE
PILGPTYLPRKFKTTVVIPPQNDVDLHANDMNFVAIAENGKLVGFNLLVGGGLSIEHGNKKTYARQAS
EFGYIPLEHTLPVAEAVVTTQRDWGNRTDRKNAKTKYTLERVGVDVFRAEVEKRAGITFAPVRPYEFT
GRGDRIGWVKGIDDQWHLTLFIENGRLLDYPGRPLKTGMAEIAKIHKGDFRLTANQNVIIAGVPESEK
AKIEALARDHGLIDDEISEQRKNSMACVSFPTCPLAMAEAERFLPQFVTKVEGIMHRHGVGDEHIVLR
ITGCPNGCGRALLAELGLVGKAVGRYNLHLGGNREGTRIPRMYRENINEDEILSEIDLLVGRWAKERN
AGEGFGDFTVRAGIVKPVLDPARDFWE
>CORE_REP|Org26_Gene4103#
MSASITAGESRDNGLQAIWNRLRANPKIPLLVAASAAIAIVVALLLWVKSPDYRVLYSNLNDRDGGAI
VTQLTQMNIPYRFAENGAALLIPAEKVHETRLRLAQQGLPKGGAVGFELLDQEKFGISQFSEQINYQR
ALEGELSRTIESLGPVQNARVHLALPKPSLFVREQKSPSASVTLTLQPGRALDDGQINAIVYMVSSSV
AGLPPGNVTVVDQAGRLLTQSDGTGRDLNASQLKYANEVENGFQRRIEAILAPVVGSANVRAQVTAQI
DFATREQTDEQYQPNQQPDKAAIRSQQTSLSEQIGGPQVGGVPGALSNQPSAPATAPIETAKPATAAG
NNANANATATAQNAATTRSAAANGVPQNTRRDATTNYELDRTIRHTQQKAGTVQRLSVAVVVNYLGTD
KDGKPQPMSKEQLAQIEALVREAMGYSSSRGDTLNVVNTPFTDTQVTGGELPFWQSQSFIDRLIDAGR
YLLVLLVAWLLWRKLVRPQLQQRQAAQQAAVAAANAPAAKAVDSSKPSNEELAQRRKSQQRVSAEVQS
QRIRDLADKDPRVVALVIRQWMSNEI
>CORE_REP|Org16_Gene3693#
MEFDYIIIGAGSAGNVLATRLTEDADVSVLLLEAGGPDYRMDFRTQMPAALAFPLQGRRYNWAYETDP
EPHMNNRRMECGRGKGLGGSSLINGMCYIRGNAMDFDNWAKAPGLEDWTYLDCLPYFRKAETRDIGPN
DYHGGDGPVSVTTPKAGNNELFHAMVEAGVQAGYPRTDDLNGYQQEGFGPMDRTVTPKGRRASTARGY
LDQARSRPNLKIVTHALTDHIVFDGKRAVGVKYLQGDSNQLTHATARREVLLCAGAIASPQILQRSGV
GPAALLKSLDINVVHDLPGVGENLQDHLEMYLQYACKKPVSLYPALQWFNQPKIGAEWLFNGTGVGAS
NQFEAGGFIRSRAEFAWPNIQYHFLPVAINYNGSNAVKEHGFQAHVGSMRSPSRGRVQVKSKDPRQHP
SILFNYMATEQDWQEFRDAIRITREIMAQPALDEYRGREISPGPDVQTDEQLDAFVREHAETAFHPSC
SCKMGEDEMAVVDGQGRVHGMEGLRVVDASIMPLIITGNLNATTIMIAEKIADRIRQRAPLPRSTADY
YVAGDAPLPRSTADYYVAGDAPVRKQ
>CORE_REP|Org13_Gene4429#
MLRRDFIKLTAALGAASALPLWSRAAWAADRPALPVPPLLTPDAQGKIALALQAGETRWLPGAATKTW
GFNGALLGPAVKLQRGQPVTVDIKNSLAEASTVHWHGLEIPGDVDGGPQALIHPGATRTVNFTVDQPA

ATCWFHPHTHGKTGSQVIMGLAGLVLLEDEESAKLPLPKTWGQDDIPVILQDKRLGKDAQIEYRLDVM
SAAVGWFGDRMFTNGAQYPQHLAPRGWLRLRFLNGCNARSLNLAASDNRPLYVIASDGGFLAEPVKLT
ELPMLMGERFEVLVDASDGKAFDIVTLPVKQMGMTLAPFDQALPVLRIQPSLAQGIKTMPDSLVKLPT
LPATTGIQERWLQLMMDPQLDMLGMQALMDRYGHQAMAGMSMNHGATGGADMKGMEKGGMQSMDHGNM
KGMEKGGMQGMDHGNMGNMGNMKGMDHGNMAGMDHGGAQGKAKSFDFSHGNMINGKAFDMTKPMFAAK
RGQYEKWTISGEGDMMLHPFHIHGTQFRILSENGKPPAAHRSGWKDTVRVEGWRSEVLVRFDHPASSE
HAYMAHCHLLEHEDTGMMMGFTVAD
>CORE_REP|Org28_Gene2522#
MQSGNQAFINQLKDIVGGPQLLTGDRNTERYRKGFRSGEGQALAVAFPTSLLQLWRILQACVAADKIV
IMQAANTGLTEGSTPSGNDYDRDIVIVSTLRLDHIQVLDNGKQVVGFPGSTLHHLEKLLKPYGREPHS
VIGSSCIGASVIGGVCNNSGGSLVKRGPAYTEMALYAQLGEDGQLRLVNHLGIRLGDTPEEILTRLEK
GDYRPTDVEYGELRASDNEYASRVRDVDADTPSRFNADKRRLYEASGCAGKLAVFAVRLDTFEKEGKE
QVFYIGTNDTAVLTELRRHMLSQFENLPVAGEYMHRDIFDIAEVYGKDTFMMIDKLGTDQMPRFFTLK
GRMDASLNKLPLLPHNLTDRLMQGLSHLAPSHLPKRMKEYRERFEHHLLLKMAGPGVDEAQRYLTEYF
AQAEGAFFACTPDEGKKAFLHRFAAAGAAVRYHAVHADKVEDILALDIALRRNDTDWFETLPPEIDSQ
LVHKLYYGHFMCHVFHQDYVVKKGVDSHALKEKMLEILNRRGAEYPAEHNVGHLYHAKADLQAFYRTA
DPTNSFNPGIGKTSKRKGWAEGPR
>CORE_REP|Org42_Gene1552#
MELEYESKRPLYIPYAGPILLEFPLLNKGSAFTEEERSHFNLHGLLPEAVETIEEQVERAYRQYQDFK
NDNDKHIYLRNIQDTNETLFYRLLDSHLSEMMPIIYTPTVGEACEHFSDIYRRARGLFISYPNRDRID
DMLQNATKQNVKVIVVTDGERILGLGDQGIGGMGIPIGKLSLYTACGGISPAYTLPVVLDVGTNNPQR
LNDPLYMGWRHPRISGEEYHAFVEEFIQAVKRRWPNVLLQFEDFAQNNATPLLNRYRDEICCFNDDIQ
GTAAVTLGSLIAASRAAGSQLRDQTVTFLGAGSAGCGIAEQIIAQMKSEGLSEDEARARVFMVDRFGL
LTDKLPNLLDFQSKLVQKSDNLAGWETASDAISLLDVVRNAKPTILIGVSGQPGLFTEELIREMHKHC
ERPIVMPLSNPTSRVEARPEDIINWTDGAALVATGSPFAPVSYKDQLYPIAQCNNSYIFPGIGLGVLA
SGATRVTDAMLMAASRALADCSPLATDGHGALLPNIDDIQGVSKCIAMEVGKAAQLQGVAIVTSEDAL
SKAIEHNFWRPQYRSYKRTSF
>CORE_REP|Org20_Gene1894#
MLIDPSSKYRPFPPVALPDRQWPARTLRQAPRWCSSDLRDGNQALAEPMDNARKREFYQLLLQCGFKE
IEVAFPSASQTDFDFVRTLIDEQLIPDDVTIQVLTQSRDDLIDRTFEALQGAPRAIVHLYNATAPMFR
DIVFRQDKAATVALAVNGARRIRRQCEAQPDTAWCFEYSPETFCFTELEFALEICEAVAAVWQPGPQR
PMIINLPATVEVSTPNVYADQIEWFCRHFSRRADVTISVHPHNDRGTGVACAELALLAGADRVEGCLF
GNGERTGNVDLVTLALNLYTQGVAPGLDFSRLKQVVEVVEVVELCNQLPVHPRHPYAGELVFTAFSGS
HQDAIKKGFAAQRQRQDGWWQVPYLPLDPADVGCSYEAVIRVNSQSGKSGAAWLLEQNHGLALPRGLQ
IDFSQVVQRATDGSGKEMSGAQLWRLFRDTYGLVEQPRLQLLSYQTESHGVEAYSFNARVACEGEPLR
LQGAGNGLLSSAVDALRQRFGLPLAIEDYHEHTLGHQSDSRAVAYIRCSLPQGEATYGVGIDVDSASA
SLQALLNVAGRYLASTSARPG
>CORE_REP|Org9_Gene2282#
MATSGTPQPGTSFTGAQLIVHLLERQGITTVAGIPGGAALPLYDALSQSTRIHHVLARHEQGAGFMAQ
GMARANGKAAVCIASSGPGATNLVTAIADAKLDSIPLVCITGQVPSSMIGTDAFQEVDTYGISIPITK
HNHLVRDIRELPQVIGDAFRIAESGRPGPVWIDVPKDVQTATITLEELPPIAVPDAAPSFDPALVAQA
AAMINQAKRPILYLGGGIICAEAHRQALELAERAGLPTTMTLMALGAMPVEHPLSLGMLGMHAARSTN
FILQEADLLIVLGARFDDRAIGKTEQFCPNAAIIHVDIDRAELGKVKQANVAIHADVGQVLQQLLPQI
DTQPRSAWLSTVNDLKREFPFNMPNADDPLSHYGLVLAAARCVDDSAIITTDVGQHQMWVAQAYPLSR
PRQWLTSGGLGTMGFGLPAAIGAALAEPERKVLCFSGDGSLMMNIQEMATAVEHDLDVKIILMNNQAL
GLVHQQQTLFYQQRIFAAAYPKRTDFLKIAAGFGLDTCDLNAAEDPQAALAEAIQRPGPCLIHALIDI
NEKVFPMVPPGAANIDMIGE
>CORE_REP|Org5_Gene2562#
MNKVKSLSQQNLSLLLAIYIGIFLNLSVFYRRFDSLAHGIQGIKVISAVTEVIAIVLFTFFIMRLVSL
GGRLFYRIVASLLVLISVAASYYMTFFNVVIGYGIVVSVMTTDIDLSKEVVGLHFVLWMVALSALPLL
LIWKNSLRYTLIEQLKTPGHRIKPLLVLLAVVALVWLPLRMLDDEQSVQEKLSNVDLPSYGGVVAHSY
LPSNWLSALGLFAYTRYDESQDQSTMFDPGKHFTYVPPADIDDTYVVFIIGETTRWDHMGMLGYERDT
TPRLSKEKNLVAFRGESCDTSTKLSLRCMFVREGGTEDNPQRTLKEQNVFAVLKDLGFSSELFAMQSE
VWFYNNTEVNNYSFREMIASEKRNDGKAVDDMLLVDEMKESLARYPKGKHLVILHTKGSHYLYSQRYP

RSYARYQPECMGVDDSCTKAQLINAFDNTVLYTDSFIANVIDQVRDKKAIVFYAADHGESIGENTHLH
GTPREMAPPEQFRVPMIVWASDKFLENPQHLSAFEQLQAQQRIGKTHRHVELFDTILGCLGYTSPDGG
IVDKNNWCHLPQDKTAPASL
>CORE_REP|Org41_Gene1788#
MRKFNKPLLALLIGSTLCSAAQAAAPGKPTIAWGNTKFAIVEVDQAATAYNNLVKVKNAADVSVSWNL
WNGDTGTTAKILLNGKEAWSGPSTGSSGTANFKVNKGGRYQMQVALCNADGCTASDATEIVVADTDGS
HLAPLKEPLLEKNKPYKQNSGKVVGSYFVEWGVYGRNFTVDKIPAQNLTHLLYGFIPICGGNGINDSL
KEIEGSFQALQRSCQGREDFKVSIHDPFAALQKAQKGVTAWDDPYKGNFGQLMALKQARPDLKILPSI
GGWTLSDPFFFMGDKVKRDRFVGSVKEFLQTWKFFDGVDIDWEFPGGKGANPNLGSPQDGETYVLLMK
ELRAMLDQLSAETGRKYELTSAISAGKDKIDKVAYNVAQNSMDHIFLMSYDFYGAFDLKNLGHQTALN
APAWKPDTAYTTVNGVNALLAQGVKPGKIVVGTAMYGRGWTGVNGYQNNIPFTGTATGPVKGTWENGI
VDYRQIASQFMSGEWQYTYDATAEAPYVFKPSTGDLITFDDARSVQAKGKYVLDKQLGGLFSWEIDAD
NGDILNSMNASLGNSAGVQ
>CORE_REP|Org20_Gene424#
MNINVASLLNGNYILLLFVVLALGLCLGKLRLGSVQLGNSIGVLVVSLLLGQQHFAINTEALNLGFML
FIFCVGVEAGPNFFSIFFRDGKNYLMLALVMVGSAMVIAIGLGKLFHWDIGLTAGMLAGSMTSTPVLV
GAGDTLRNTIVNGPALLAAQDHLSLGYALTYLIGLVSLIFGARYLPKLQHQDLSTSAQQIARERGLDT
DSQRKVYLPVIRAYRVGPELVAWADGKNLRELGIYRQTGCYIERIRRNGILANPDGDAVLQVGDEISL
VGYPDAHARLDPSFRNGKEVFDRDLLDMRIVTEEIVVKNSNAVNKRLSQLKLTDHGCFLNRVIRSQIE
MPIDDSIVLNKGDVLQVSGDARRVKSVAEKIGFISIHSQVTDLLAFCAFFIIGLLIGQITIQFSNFSF
GIGNAAGLLMSGIMLGFLRANHPTFGYIPQGALNMVKEFGLMVFMAGVGLSAGAGIGHSLGAVGGQML
IAGLIVSLVPVIICFLFGAYVLRMNRALLFGAIMGARTCAPAMEIISDTARSNIPALGYAGTYAIANV
LLTLAGSLIVVLWPGILG
>CORE_REP|Org6_Gene2773#
MDKVWLKRYPADVPAEIDADRYSSLIEMFEHAVQRYADQPAFINMGEVMTFRKLEERSRAFAAYLQNE
LGLKKGDRVALMMPNLLQYPIALFGILRAGMVVVNVNPLYTPRELEHQLNDSGASAIVIVSNFAHTLE
KVVFNTQVKHVILTRMGDQLSAAKGTLVNFVVKYVKRLVPKYNLPDAISFRSALQRGRRLQYVKPDII
NADLAFLQYTGGTTGVAKGAMLTHRNMQANLEQAKAAYSPLFREGQELVVTALPLYHIFALTVNCLLF
IDLGGRNLLITNPRDIPGLVKELGKYPFTAMSGVNTLFNALLNNEEFHKLDFSTLRFSVGGGMSVQKA
VAEKWEKTTGKHLLEGYGLTECAPLVAGNPYDLKHYSGSIGLPVPSTDIRLVDDNGQDVPPGEPGELW
VKGPQVMLGYWQRPGATDEVLKDGWLATGDVVTVDEQGFVRIVDRKKDMILVSGFNVYPNEIEDVVSQ
HPKVLECAAIGVPSEVSGETVKICVVKKDASLTKEELLTHCRRHLTGYKVPKIVEFRDELPKSNVGKI
LRRELRDELKTPKPADAA
>CORE_REP|Org28_Gene4234#
MAQEKTGNDWQCGADLVVKNLEAQGVKHVFGIPGAKIDRVFDSLEDAPSIETVVVRHEANAAFMAAAV
GRLTGKAGVALVTSGPGSSNLITGLATATSEGDAVVAFGGAVKRADSLKQTHQSMDTVSMFRPVTKYC
AEVHAGSAISEVIANAFRRAEFGRPGASFVSLPMDIVNEPVSAPVLAGCRLPRMGAAAADDIQAAVKL
IRQAKCPVLLLGLQASRPENSEAVRHLLYRTHMPVVGTYQAAGVIDVNHFARFAGRVGLFNNQPADQL
LQKADLVVSVGYDPIEYDPCMWNSHGRLKLVHIDVLPADIDTCYRPDVELVGNISATLNMMTETFTEA
VCVPPEVELILTDLGRQRTELAERAARRGGMPIHPLRIVKELQDIVSDDVTLCVDMGSFHIWIARYLY
SFRARQLLISNGQQTMGVALPWAIGAALVRPGDKVVSISGDGGFMQSSMELETAVRLKNNIVHVIWVD
NAYNMVEMQELNKYQRKSGVEFGPIDFKAYAESCGAVGFAVQSVDDLRPMLRKAMAIQGPVVVAIPVD
YADNYKLMAQMNFSQMI
>CORE_REP|Org44_Gene4221#
MKTLLMVDSSLGQARGHLAKRMLEAAAAKTGLTLVESLQDAELVAVAGQSAPADAGLNGKLVYVGNVE
QAVREPDAFLARAKAEAETYQAPQAAVPVKAGGQKRIVAITACPTGVAHTFMAAEAIESEAKKRGWWV
KVETRGSVGAGNAITPEEVAAADLVIVAADIEVDLDKFAGKPMYRTSTGLALKKTAQELDKALAEAEV
FQPQQRGSAAPAGKKKEGNGPYRHLLTGVSYMLPMVVAGGLCIALSFVFGIKAFEVKGTLAAALMQIG
GGSAFALMVPVLAGFIAFSIADRPGLTPGLIGGMLAVSTGAGFLGGIIAGFLAGYVAKAISSKLRLPQ
SMEALKPILIIPLVASLITGLIMIYVVGTPVAKIMEGLTHWLQSLGTANAVLLGAILGGMMCTDMGGP
VNKAAYAFGVALLSSSVYAPMAAIMAAGMVPPLAMGLATLLARRKFPKSEQEGGKAALVLGLCFITEG
AIPFAARDPMRVLPCCIAGGALTGALSMAFGAKLMAPHGGLFVLLIPGAISPVLLYLVAIAAGTLLAG
VAYALLKRAEVPAASVA
>CORE_REP|Org36_Gene2303#

MKITIGSFILQQLHALNVDRIYGVPGDYNLSLLELLESDDRLAFIGNCNELNASYAADGYARMKGAGA
LIVTYGVGDLAALSGIAGAYAESSPVICIAGTPPLHAMKNHQLLHHTLGDGNFDNVMNCFKQFTVAQA
LITPENAAQEIPRVISRAWIEKKPVYLQLPSDICDVEIEITAAAAAPQLPASDKYNVQLAAMALLTKI
KRAQRPIMLIDQMVDRYQLQQRVIEVAHRFAIPLTNMPTAKCIIPEDTAGWMGGYSGNLSRPELYERM
AHSDCVLSFGVRLVDSTTGYFSQQIPAAAQVDIQPFSLKLDNTSYPAVAAADLLQALLDLSEDAPVQP
LAPLPDPREKLATPSDTPIDQAYLWQRIQRFIRADDVVVVENGTSGAAIGGMRMPGGVKVVNQPIWGS
IGYTLPALLGTLMAAPERRHLLFIGDGSFQLTAQEVSTLLRCEQKPIIFLINNDGYTIERYILGENSS
YNDIGPWDYAKLPAVLNTQAQPFSVAVETTQQLEMALERASRQDRLAFIEVKVPMMDTPPVMKEFCNR
CNSFNFGLTNPRRSA
>CORE_REP|Org8_Gene1820#
MIKKITALTLLVSTALSAETLPDSHMMQDMSMGESRRALQDSTREVNQLIEQRRYQQLKQQRLLAEPE
PAAPALPQSAQCLPIAGVYLQGVTLLSPADLSALSALPEQCISSNDINRLTRELTRLYVQKGYITARV
QIVRPNSQGELGLSVTEGFIEKIEGGDRWVNSRLLFPGLEGKPLKLTELDQGLDQANRLQSNTTKLDI
LPGRQVGGSVIRLRNQHAKPWLITAGTDNYGQKSTGRWLARATATLDSPFGLSDFVSLNANSTLENPA
HRYNRAYTLLYSLPYGAFTFSGFASFSSYENHQQLPHNVVKLHGQTQQYGLRSDYVFYRDHDQIDSLS
GQLTYKRIDNYFESVRLEVSSPTLTLAELSASHLQILPNGVFSANLSVEQGMPWLGAGRHPSSVHLDS
QFTKGKLFANLSQRLRLGDATYQLNNLFYGQYSRDPLPGVEWLSLTDRSAVRGFSRSTQSGDNGWYLQ
NTLSRSFNLGATTLTPRLGADVGRILPRQDNSGWRSSAGISTGATLRYQRALVDLEVSRGWILSNHAT
PEDPVQVLARFSYTF
>CORE_REP|Org49_Gene4607#
MHRKVPMLAQLTISNFAIVRELEIDFQPGMTAITGETGAGKSIAIDALGLCLGNRADGNVVRLGAARA
DICARFSLADTPSAREWLEQNQLDDSNECLLRRVINADGRSRGFINGTAVPLSQLRELGQRLIQIHGQ
HAHQLLLKPEHQKALLDAYADEPALLAAMSQAYQRWHQSCRELAHHQQQSIEREARKQLLQYQLKELN
EFAPQAGEFEQTDAEYKRLANSGQLLTLSQQALQLLADSDENNMLSQLYSAKHLLLELAGLDDKLSGL
LDMLEEASIQISEASDELRHYADRMDLDPNRLHELEQRLSRQISLARKHQVAPEELPQLHQQLLDEQQ
LLSQQESDHEHLNEAVTLHHQQALALAEQLHQKRLHYAAELTTLITDSMQALSMPHGKFNIDVRFEPQ
HLSAEGASRTEFCVSTNPGQPLQPLAKVASGGELSRIALAIQVITARKMETPALIFDEVDVGISGPTA
AIVGRLLRQLGESTQVMCVTHLPQVAGCGHQHLFVSKQTDGTVTETQMAPLDKRARLQELARLLGGSE
VTRNTLANAKELLAA
>CORE_REP|Org19_Gene3632#
MSTSVFNRRWAAVLLEALARHGVRHVCIAPGSRSTPLTLAAAANRSFICHTHFDERGLGHLALGLAKA
AREPVAVIVTSGTAAANLYPALIEAGLTGERLVFLTADRPPELIDCGANQAIRQNGLYSSHPTLAIDL
PRPTPDIPAAWLASSVDSAMARLQHGALHINCPFAEPLYGGDERHYADWSAALGDWWQSDRPWLQESE
THAAPLPQPDWFFWRQKRGVVLAGRMSAQEGAQVAEWAATLGWPLIGDVLSQTGQPLPCADLWLAHPQ
AQRVLQDAQLVVQFGGSLTGKRLLQWQAQCRPEEYWIIDELPGRLDPAQHRGRRLRAGVAQWLAQHPA
QPRQPWAAVLAVLADKALTAASTHLHDSFGEAQLAHRLPELLPENGQLFLGNSLVVRLIDALTPLPVA
YPVFSNRGASGIDGLISTAAGVQRATARPTLAVVGDLSALYDLNALALLRQSSAPTVLIVVNNNGGQV
FSLLPTPEEDRQRFYCMPQNVEFSHAAAMFQLGYARPENWNQLQQAVEQGWRRGGATLIELQVPPSAG
AESLQYLVQQMAVQ
>CORE_REP|Org14_Gene52#
MTESFAQLFEESLKEIETRPGSIVRGVVVAIDKDIVLVDAGLKSESAIPAEQFKNAQGELEIQVGDEV
DVALDAVEDGFGETLLSREKAKRHEAWITLEKAYEEAETVTGVINGKVKGGFTVELNGIRAFLPGSLV
DVRPVRDTLHLEGKELEFKVIKLDQKRNNVVVSRRAVIESENSAERDQLLENLQEGMEVKGIVKNLTD
YGAFVDLGGVDGLLHITDMAWKRVKHPSEIVNVGDEITVKVLKFDRERTRVSLGLKQLGEDPWVAIAK
RYPEGTKLTGRVTNLTDYGCFVEIEEGVEGLVHVSEMDWTNKNIHPSKVVNVGDVVEVMVLDIDEERR
RISLGLKQCKSNPWQQFAETHNKGDRVEGKIKSITDFGIFIGLDGGIDGLVHLSDISWNVAGEEAVRE
YKKGDEIAAVVLQVDAERERISLGVKQLAEDPFNNYLSMNKKGAIVTGKVTAVDAKGATVELAGGVEG
YLRASEASRDRIEDATLVLNVGDEVEAKFTGVDRKNRVVSLSVRAKDEADEKDAIATVNNKQEEGNFS
NAMAEAFKAAKGE
>CORE_REP|Org12_Gene4030#
MLNRMKVVTSLLLVLVLFGALQLISGGLFFSSLKSDKENFTVLQTIRQQQLQLSESRVDLLQARNSLN
RAGIRYMMDTNKIGSGATIDELLAKAKEELGEAERHYAAYEKIPQDPRQDPQSAERVKQQYDILYGAL
SELIQLLGEGKINAFFDQPTQSYQDNFEQSYNVYLEQNGKLYQIAVDGSNSSYNSAIWTLIVILVVVL
AVIVLVWTGIHHILVRPLNRMIDHIKQIAAGDLTQQIVVNSRNEMGVLAASLKHMQGELIETVSGVRQ

GADAIYSGASEIAAGNNDLSSRTEQQAASLEETAASMEQLTATVKQNAENARQASQLALSASETAQKG
GKVVANVVQTMHDIAGSSQKIADITGVIDGIAFQTNILALNAAVEAARAGEQGRGFAVVAGEVRNLAQ
RSAQAAKEIKGLIEDSVSRVDMGSVLVESAGETMGDIVNAVTRVTDIMGEIASASDEQSRGIDQVGQA
VAEMDRVTQQNASLVEESASAAAALEEQASMLTQSVAVFRLRSEGQEEFKAPVTNKATVTPVINHKKM
NASDLQDNWETF
>CORE_REP|Org49_Gene2198#
MAQYVYTMHRVGKVVPPKRHILKNISLSFFPGAKIGVLGLNGAGKSTLLRIMAGIDTDIEGEARPQPG
IKIGYLPQEPQLNLEHTVRESVEEALAEVVGALKRLDEVYALYAEEGADFDKLAAEQGRLEEIIQAHD
GHNLNAQLERAADALRLPDWDAKIAHLSGGERRRVALCRLLLEKPDMLLLDEPTNHLDAESVAWLERF
LHDFEGTVVAITHDRYFLDNVAGWILELDRGEGIPWEGNYSSWLEQKDARLAQEASAEAARRKSIEKE
LEWVRQGTKGRQSKGKARLARFEELNNTEYQKRNETNELFIPPGARLGDKVVEVSNLRKSYGDRLLID
DLSFSVPKGAIVGIIGPNGAGKSTLFRMMSGQEQPDSGSIVLGDTVKLASVDQFRDSMDGSKTVWEEV
SGGQDIMRIGNTEMPSRAYVGRFNFKGVDQGKRVGELSGGERGRLHLAKLLQVGGNVLLLDEPTNDLD
IETLRALENALLEFPGCAMVISHDRWFLDRIATHILDYQDEGKVEFFEGNFTEYEEYKKRTLGADALE
PHRIKYKKIAK
>CORE_REP|Org25_Gene4314#
MIMTQIYTVADYLLDRLAQIGIRHVFGVPGDYNLQFLDHVIAHPQITWVGCANELNAAYAADGYARCK
PAAALLTTFGVGELSALNGVAGSYAEYLPVIHVVGTPAQRAQRAGDLLHHSLGDGDFTHFARIAKEVS
VAQANLTADNAAREIDRLIDAALLEHRPVYLMLPSDVAEAPLPAKPAPLMLRQARLSRSALQAFIAAA
REKLQSARQVSLLADFLADRFGVETALDNWMQEVAIPHATLLLGKGVLDENRDGFVGTYSGGASAAAT
KALIENADVVITVGVRLTDTITGGFTHHLPAEKCIDIQPFEAWVGRQRFSRIPMCAAVQALHRLTLSL
ASRWPLPTIGRPALLEGDGEGLDQHAFWRQIQDFLRPGDIVLAEQGTACFGAATLTLPRGSRMIVQSL
WGSIGYTLPATFGVQTAEPQRRVVLLIGDGAAQLTVQELGSMLRDGLKPVIFVLNNQGYTIERAIHGP
EQSYNDIARWNWTQLPTALAGGRPVTALRVSQPEALRQALSEAAQGDRLAFIEVMLPKMDIPELLDTI
SRVIQSRNAAA
>CORE_REP|Org13_Gene4747#
MSNPTPWWQNGVIYQIYPKSFQDSTGNGYGDLAGVTRRLDYLQELGVDAIWLTPVYVSPQVDNGYDVA
DYCAIDPAYGTMADFEQLVAAAHRRGIRIVMDMVFNHTSTEHPWFKAAQDRHSPYRQFYVWRDGEGDT
PPNNWRSKFGGNAWQWHADSGQYYLHLFAVEQADLNWEHPPVREELKKVCQFWADKGVDGLRLDVINL
VSKQQDFPSDSQGDGRRFYTDGPRIHEFLQEMSRDVFQPRGLMTVGEMSSTTLEYCQQYAAQSGEELS
MTFNFHHLKVDYANGEKWTRAAPDYVELKQIFRHWQQGMHNRAWNALFWCNHDQPRIVSRFGDEGALR
VPAAKMLAMVLHGMQGTPYIYQGEEIGMTNPGFRAIEQYRDVESLNMYAELSAQGRSDAELLAILADK
SRDNGRTPMQWSAAPHAGFTTGTPWIGCAENYPQINADAALADLDSVFYAYRQLIILRKQYPLLTHGD
YQDLAPDHPALWCYQRSWNGQRLLVVANLSREPLAWAAEGVEASAQWRPLMSNYSDSADQPQALTLRP
FEAVWWLLED
>CORE_REP|Org6_Gene3350#
MSEAEARPTNFIRQIVDEDLASGKHTSVHTRFPPEPNGYLHIGHAKSICLNFGIAKDYQGQCNLRFDD
TNPVKEDIEFVESIKHDVEWLGFEWSGNVHYSSDYFDQLHQYAVELITKGLAYVDELSPEQIREYRGT
LTSPGKDSPYRDRSVEENLALFEKMRNGEFAEGAACLRAKIDMASPFIVMRDPVLYRIKFAEHHQTGD
KWCIYPMYDFTHCISDALEGITHSLCTLEFQDNRRLYDWVLDNITIPCHPRQYEFSRLNLEYAIMSKR
KLHQLVAEKIVEGWDDPRMPTVSGLRRRGYTAASIREFCLRIGVTKQDNNVEMVALESCIRDDLNENA
PRAMAVLDPVKIVIENMGDAVEMVTMPNHPNKPEMGSRDVPFSREIYIDRADFREEANKQYKRLVLGK
EVRLRNAYVIKAERVEKDEAGEITTIFCSYDAETLSKDPADGRKVKGVIHWVSAAHALPAEIRLYDRL
FSVPNPGAAEDFLSTINPESLVIKHGFVEPSLAAAQPEKAYQFEREGYFCADNRYSSADHLVFNRTVG
LRDTWAKIEG
>CORE_REP|Org19_Gene1452#
MCSIFGVLDLKSDPVELRKKALELSRLMRHRGPDWSGVYASDKAILAHERLSIVDVNNGAQPLYNAAH
THVLAVNGEIYNHQALRQQLSDRYAFQTGSDCEVILALYQEKGPDFLDDLQGMFAFALYDTEKDAYLI
GRDHLGIIPLYMGHDEHGNLYVASEMKALVPVCRTIKEFPAGSYLWSQDGEIREYYRRDWFDYDSVKD
NVTDATALRTALEESVKSHLMSDVPYGVLLSGGLDSSVISAITKKYAARRVEDQERSEAWWPQLHSFA
VGLEGSPDLRAAQEVANHLGTVHHEIHFTVQEGLDAIRDVIYHIETYDVTTIRASTPMYLMSRKIKAM
GIKMVLSGEGADEVFGGYLYFHKAPDAREFHEETVRKLLALHMFDCARANKAMSAWGVEARVPFLDKK
FLDVAMRINPKDKMCGNGKMEKHIVRECFESYLPASVAWRQKEQFSDGVGYSWIDTLKEVAAQQISDQ
QLETARFRFPYNTPTSKEGYLYREIFEELFPLPSAAECVPGGPSVACSSAKAIEWDESFKKMDDPSGR

AVGVHQAAYK
>CORE_REP|Org21_Gene2645#
MKRLCSALSAAALLLLPDLAYADAIGGEVHRQPLNIQAIVMFILFVGATLYITYWASKRTRSRQDYYT
AGGRITGLQNGLAIAGDFMSAASFLGISALVYTSGYDGLIYSIGFLIGWPIILFLIAERLRNLGRYTF
ADVASYRLQQKPIRTLSACGSLVVVALYLIAQMVGAGKLIQLLFGLNYHVAVILVGILMVLYVLFGGM
LATTWVQIIKAVLLLAGASFMALMVMKSVNFDFNTLFAEAVKVHPKGIAIMSPGGLVSDPISALSLGL
ALMFGTAGLPHILMRFFTVSDAKEARKSVFYATGFIGYFYILTFIIGFGAILLVSANPAFKDATGALL
GGTNMAAVHLANAVGGNFFLGFISAVAFATILAVVAGLTLAGASAVSHDLYASVMKNGKATERDELKV
SKITVVVLGLVAIALGILFEKQNIAFMVGLAFSIAASCNFPIIILSMYWSRLTTRGAMIGGWLGLLTA
VILMILGPTIWVQILGHEKPIYPYEYPALFSMFVAFVGTWLFSITDSSLAGQQERERFRAQFVRSQTG
LGISQGSSH
>CORE_REP|Org5_Gene3280#
MSTSRLQQQFIRLWQRCHGETTDTTLQDLAEVLSCSRRHVRSLLSAMQREGWLTWQAESGRGKRSRLT
FHYTGLALQQQRAEELLEQDRIDQLVQLVGDKNVVRQMLLSQLGRSFRQGKHILRVLYYRQLYNLLPG
SALRRSETHLARQIFNGLTRINEENGELESDLSHHWQALTPLHWRFYLRPAIHFHHGRELEMADVITS
LSRLTSQPLFSHIESVTSPTPFVIDVQLRSPDHWLPWLLGSVQAMILPREWRELPDFARHPVGTGPYR
MVRNHPSQMKIHAFDDYFGYRALIDEVNIWVLPEFSEELVHSGVQLQGDETGNSELESRLEEGCYFLL
FDQRSPLATDPAIRSWLCELINPISLLSHAGPLYQRYWSPAYGLLPRWHHNRTLAQQPKPAGLTELTM
THFNEHSEFHAIRQAIEPLLAQHGIRLIVQSVDYATWHQGDARSDLWLGSANFYLPLEFSLFATLYEL
PLVQHCMNEDLAQDAALWRANRLPLAEFCQRLVSNHQLHPLFHHWLQLHGQRSMRGVRMNTLGWFDFK
SAWFAPPEA
>CORE_REP|Org8_Gene2744#
MFLERIEIVGFRGINRLSLMLDDNTLLLGENAWGKSSLLDALTLLLAPEQKLYRFEAHDFHFPPGEES
AKERHLQVVFTFCEKDIGHAHLPRYRHLTPLWVKGEDGLSRIHYRCEGELADDGTVCTWRGFLDADGN
AFQLHHIEQLAHAIIRIHPVLRLRDARFIRRLRPSSLGDERKPDTQALAQQLDQLTRELVRNPQKLTN
GELRQGLAAMQQLLEHYFAEQSSQVTRPRRRGGEPEQDAWRALDGINRMVAEPNSRSMRLILLGMFST
LLQAKGDVKLDPHARPLLLVEDPETRLHPIMLSVAWGLLNQLPLQRITTTNSSELVSLVPVEHVCRLV
RESGRVATYRLGPRGLSAEDGRRIAFHIRFNRPSSLFARCWLLVEGETEVWLLNELARQCGYHFEAEG
VRVIEFAQCGLKPLLRFARRMGIEWHALVDGDEAGKKYANTVRSLLDNHEDNERDRLTALPAPDMEHF
MFREGFAPVYHRMASVPINAQMPVRKVILKAVHHSSKPDLAIEVAMQAGEWGTDSVPPLLKKMFSRVI
WLARGRAD
>CORE_REP|Org30_Gene2974#
MSDIALTVSMLALAAVIGLWMGNWKLYGVGLGIGGVLFGGILVGHFAQSGQINLNGDMLHFIQEFGLI
LFVYTIGIQVGPGFFSSLRVSGLRLNAFAVLLVLTGGVVAAAVHKLFDVPLPIILGVFSGAVTNTPAL
GAGQQILTDLGSDPALVDGMGMGYAMAYPFGICGILLVMWLIRLFFRINIEREAQAFESSLGNQRELL
HAINVAVRNPNLQGMAIKQVPLLNGEAIVCSRLKRGELLMVPAPHERLELGDYLHLVGKREDLENARL
VIGEEVDASLSTRGTALQVVRAVVTNEQVLGKKIRDLNLKQKYDVVISRLNRAGVELVAGSNVTLQFG
DILNLVGRPEAIDAVTAIVGNAQQKLQQVQMLPVFIGIGLGVLLGSIPLFVPGFPAALRLGLAGGPLV
AALILGRIGSIGKLYWFMPPSANLALRELGIVLFLAVVGLKSGGNFIDTLLHGEGLTWVGYGALITAI
PLLSVGILARTVGKMNYLTLSGMLAGSMTDPPALAFANGLHPTSGAAALSYATVYPLAMFLRIMSPQL
LAVLFWTL
>CORE_REP|Org34_Gene4683#
MLNLIRRLHFYIGLLVAPFIFVAALTGTLYVLTPQLEEALYRDALFTEPHGQARSLADQIAAARRAAG
DEARIYAVRPAPGVMDTTRVQFAGADLGASESRALFVDPYTLAIKGDMTVYGTSGVLPLRTWLDKLHS
SLLLGDLGRNYSELAASWLWVAALGGVALWLATRPKRKLKRAKGGFAASRHWHITLGVALLAGLLFFS
ATGLTWSQWAGGNIDALRANLGWLTPQVNTSLQADAPQTPADPHAEHRGMMPGMKMGPAAQANALNGD
WDRALHAARAAGIDAAKVELRQPKGEGKAWTVTEVDRSWPTQVDAVAIAPQSFAVIDQVRFDTFPLIA
KLTRWGIDAHMGILFGLPNQLLLAAFGLGLCLMIALGYRMWWSRRPAAGAQHPVHTLVAAWLALSLPA
RVVTLLFAAVLGYCLPVMGVSLAALLLVDVWRWRGQRQGQRDETPEAAARRAEVRKFRAAVATVAVAV
GCVMGRAILGGTVEQFQLPIGDWSAQMVIMQSLMVLLYTLVFTLLLSIPLWYFFLGEKKRGGEVASGI
PAGMPDIW
>CORE_REP|Org30_Gene2089#
MRFSLKTTACALAVSLTLLSGAASAWEKDKTYAITILHTNDHHGHFWQNDHGEYGLGAQKTLVDGIRQ
EVAAQGGSLLLLSGGDINTGVPESDLQDAEPDFRGMNLVGYDAMAIGNHEFDNPLSVLRQQEKWATFP

LLSANIYQKSTGQRLFKPYALFDKQGIKIAVIGLTTDDTAKIGNPEYFTDMEFRVPAQEAKQVVEQLR
KDEKPDVIIAATHMGHYDNGEHGSNAPGDVEMARSLPAGYLDMIVGGHSQDPVCMAGDNRKQADYVPG
TPCSPDRQNGTWIVQAHEWGKYVGRADFEFRNGELKLVHYQLIPVNLKKKVEKADGTSERVYYTQQIA
EDPTMMKLLTPFQEKGKAQLGVKIGSVNGKLEGDRSKVRFVQTNLARVMLAAQRERADADFAVMSGGG
VRDSIESGDITYKNVLKVQPFGNTLVHVDMKGSEVEQYLAVVANMKPDSGAYAQFANVSLVADGKGVS
EVKINGQPLQADKTYRMATLNFNALGGDGYPKLDGLPSYVNTGFIDAEVLKQYIEKHSPLDAAAYEPK
GEIVYR
>CORE_REP|Org42_Gene1239#
MLTLLHLLSSVALLVWGTHIVRTGIMRVYGANLRRVLSDSVEKKPLAFVSGIGVTALVQSSNATALLV
TSFVAQGLVGLAPALVIMLGADVGTALMARVLTFDLSWLSPLLILIGVFLFLSRKQMRIGQIGRVLIG
LGLIVLALELIVAAATPITQAAGVKVLFSSLTGDVMLDALTGALFAIVSYSSLAAVLLTATLTASGVI
SLKVALCLVIGANLGSGLLAMINASGQNAAGRRVALGSLLFKLVGCVLVLPFVSYLADVMAQLPGGSE
ELVIYFHVFYNLIRCLVMIPLAGPMATLCERLIVDVAADDPRMRPRHLDASALDTPTLALANAARETL
RMGDVVEHMMILQREVLHGKQGVDKEVRRLDDDVDVLYTAIKLYLAQIQKEDLGEEDSRRWAEIIEMA
LNLEQAGDIIERMAGDVGAKSHAARRAFSAEGLAELDGLHERLIGNLRLGLSVFLSGDVTSAKRLRRS
KHRFRILDRRYAHAHVDRLHQQNVQSIETSSLHLGLLGDMKRLNSLFCAVAYNVLDQDAKDDDRDWED
TPSTL
>CORE_REP|Org12_Gene4436#
MSSYFDGGETDVIIIGGGATGAGIARDCARRSLRCILLERHDIATGATGRNHGLLHSGARYAVTDGES
ARECIEENRILKRIAHHCIERTDGLFITLPQDSLDYQQQFIAACRQADIDAEAIDPQLALRLEPAANP
ALIGAVRVPDGTVDPFRLTAANMLDAREHGAQILTYHQVVGLLRSGDRVTGVRVYDHQNQRRYELHAS
VVVNAAGIWGQQIAEYADLRIRMFPAKGALLILGHRINNMVINRCRKPADADILVPGDTISLIGTTST
HIDYDQIDNMLVTPQEVDILIREGALLAPTLAQTRILRAYAGVRPLVASDDDPSGRNVSRGIVLLDHA
ARDGLEGFITITGGKLMTYRLMAEWATDKVCEKLGVNRPCTTAQDALPGSRQSAEETVRSVVSLPASI
RGSAIYRHGDRASQVPAADRLDTSLVCECEAVTAGEVRYAVNSLTVNNLVDLRRRTRVGMGTCQGELC
ACRAAGLLTRFNVSTPQQSIAQLSQFLNERWKGVRPIAWGDALRESEFTSWVYQGLCGLEAQADQEQE
ADDAI
>CORE_REP|Org18_Gene1231#
MKNWVKSGSPWIWLTAGSVAVSLLALIGILLLLAGQGMRYFWPSPVYQFELNQNGAGPVTVIGELYQQ
QSISRRQLTEAGVTPPGEAQSVERYLIKVGNREREGQDFRTLLASDIRSQSTPRSLLVLERDSHGTAY
GYLAGLLEDGQPLTGRNLGQALQQRLPQIAALSRQAHDIQFRDMARINQQFDALRLREKRLQRDDKLD
ARAQDAIKAERLELQRQYQLLSERLAGLNRDRQRDALLLRDMHGQTLTIPLSQVRDAWYPNAMNTSEK
LAHWGEQVKKFLTDSPREANTEGGVFPAIFGTVLMVILMSIVVMPFGVIAAVYLHEYAGNNLLTRVIR
IAVVNLAGVPSIVYGVFGLGFFVYMIGGTLDQLFYPESLPNPTFGTPGVLWAALTLALLTLPVVIVAT
EEGLSRIPTSLRQGSMALGASRAETLWRIVLPMAAPAMMTGLILAVARAAGETAPLMLVGVVKSVPVL
PVDEIFPYLHLERKFMHLSFQIYDMAFQSPSVEAARPLVFATAFLLVTIVVSLNLAAMGIRHSLRERY
RAWSQ
>CORE_REP|Org3_Gene1228#
MEIFFTILILILVVSLSGVVTRMLPFQVPLPLMQIAIGALLAWPHFGLHVDFDPELFLVLFIPPLLFA
DGWKTPTREFLHHGREILGLALVLVLITVVGVGYLIYAMVPGIPLVAAFALAAVLSPTDAVALSGIVG
KGRIPKPIMGVLEGEALMNDASGLVSLKFAIAVAMGTMVFTVGGATLEFLKVAIGGLLAGVAVTWTYS
KSLRVMSRWSGDDPATQIVFLLLLPFASYLIAEHIGVSGILAAVAAGMTISQSGVIRNAPLAMRLRAN
SVWAMLEFVFNGMVFIMLGLQLPGILETSILQAELDPTIQTWYLFADVATIYAALLVLRFTWLWVMKK
ASNRFMKKRPLQFGDYSTRELWVASFAGVRGAITLAGVLSIPLLLSDGTAFPARYQLVFIAAGVILLS
LIVGVLALPLLLRGVQVADKSASKQEERMAIAMAAEVAIESVNKMEERLVADTEENLDPQVLKEVSSR
VTGMLRRRIASKDDIENALAMENLERRFRLTALRAERGELYHLRATQKISNETLQKLLHDLDLLEALL
IEREG
>CORE_REP|Org27_Gene2983#
MSNKPFHYQDPFPLKKDDTEYYLLSRDHVSVSEFEGQEILKVAPEALTLLAQHAFHDASFMLRPAHQQ
QVADILKDPDASENDKYVALQFLRNSEIAAKGILPTCQDTGTAIIVGKKGQRVWTGGGDEAALSRGVY
NTYIQENLRYSQNAALDMYKEVNTGSNLPAQIDLYSVDGEEYKFLCIAKGGGSANKTYLYQETKALLS
PGKLKNYLVDKMRTLGTAACPPYHVAFVIGGTSAEATLKTVKLASTKYYDGLPTEGNEHGQAFRDLEL
EAELLQEAQNLGLGAQFGGKYFAHDIRVVRLPRHGASCPVGMGVSCSADRNIKGKINRDGIWLEKLEH
NPGKFIPQELRQAGEGEAIKVDLNRPMAEILKQLSQYPVSTRLSLSGTIIVGRDIAHAKLKERLDRGE

GLPQYVKDHPIYYAGPAKTPEGYASGSLGPTTAGRMDSYVDLLQSHGGSMIMLAKGNRSQQVTDACHK
HGGFYLGSIGGPAAVLAQQSIKSLECVEYPELGMEAIWKIEVEDFPAFILVDDKGNDFFQKIQAGQCS
SCLK
>CORE_REP|Org30_Gene3742#
MAAKDVKFGNDARVKMLRGVNVLADAVKVTLGPKGRNVVLDKSFGAPTITKDGVSVAREIELEDKFEN
MGAQMVKEVASKANDAAGDGTTTATVLAQSIITEGLKAVAAGMNPMDLKRGIDKAVVAAVEELKKLSV
PCSDSKAIAQVGTISANSDETVGKLIAEAMEKVGKEGVITVEEGTGLQDELDVVEGMQFDRGYLSPYF
INKPETGSVELESPFILLADKKISNIREMLPVLEAVAKAGKPLLIIAEDVEGEALATLVVNTMRGIVK
VAAVKAPGFGDRRKAMLQDIATLTAGTVISEEIGLELEKATLEDLGQAKRVVINKDTTIIIDGVGDEA
TIQGRVAQIRQQIEEATSDYDREKLQERVAKLAGGVAVIKVGAATEVEMKEKKARVEDALHATRAAVE
EGVVAGGGVALIRVAGKIAALKGDNEDQNVGIKVALRAMEAPLRQIVVNAGEEASVIANQVKAGEGSY
GYNAYSEEYGDMIAMGILDPTKVTRSALQYAASVAGLMITTECMVTDLPKADAPDMGAAGGMGGMGGM
GGMM
>CORE_REP|Org47_Gene3464#
MNGAQWVVQALRAQGVDTVFGYPGGAIMPVYDALYDGGVEHLLCRHEQGAAMAAIGYARATGKVGVCI
ATSGPGATNLITGLADALLDSVPVVAITGQVGSALIGTDAFQEIDVLGLSLACTKHSFLVESLDALPG
IMAEAFAIAAGGRPGPVLIDIPKDIQLAQGDLHPHLIPVDEVPAFPAAALAEAAELLAQAHKPMLYVG
GGVGMAQAVPALREFIAVTRMPNVATLKGLGAPDAQDPLYLGMLGMHGAKAANLAVQECDLLIAVGAR
FDDRVTGKLNAFAPHAKVIHMDIDPAEMSKLRQAHVALQGDLKALLPALQRPLNIAPWQQQVTALKAD
HACRYDHPGQPIYAPLFLRQLSARKPANSVVTTDVGQHQMWSAQHMTFERPENFITSSGLGTMGFGVP
AAVGAQIARPQDTVICVSGDGSFMMNVQELGTIKRKQLPLKIVLLDNQRLGMVRQWQQLFFDGRYSET
NLSDNPDFLMLAAAFGIPGQRISRKDQVEGALEALFNTEGPYLLQVSIDELENVWPLVPPGAGNETML
EEIS
>CORE_REP|Org22_Gene1819#
MTENNHSVADVEKIKRWSPVWIIPIVTALIGAWILFYHFSHQGPVVTLVTTTAEGLEAGKTKIKSRSV
DVGVVETVTLSDDLSKVMVQARLNAGMEKLLRQDSAFWVVKPQIGREGVSGLGTLLSGAYIELQPGSK
GKDGKDNYQLLDAPPLASPDAKGLRIVLDSEKSGQLNAGDPVLFRGYRVGSVETSYFDPKERAMRYQL
FITAPYDQLVTTNVRFWKDSGVAFDMSAQGMRVEMGSLTTLFSGGVSFDVPDGWDRGEPAKEKAEYQL
FDNQRSTQDSLYTVHKDYLLFFSDSVRGLQPGAPVEFRGIRLGTVAQVPFYKDGMAQRLDNDYRIPVL
IRIEPDRLHKQLGDNVDIEAHLKDAESRGMRASMKSANLLTGSLYIDLDFYPQEKPWKGPRELFGYPL
MPTTSGGLAQIQQKLMQTLDKINAMPINPMLNEATKTLAESQKTMKSTQQTMKSLNDIIASKEMQALP
QDMQKTLLELNRSMKGFQPGSPAYNKMVGDMQRLDQVLRELQPVLRTLNEKSNALVFEAAGSTDPQPK
KATK
>CORE_REP|Org11_Gene375#
MKNINPSQTAAWQALQQHYAQMKDVRIADLFAEDSDRFSRFSATFNDQMLVDYSKNRITQETLEKLQA
LAKETDLQGAIKSMFAGEKINRTEDRAVLHIALRNRSNSPILVDGKDVMPEVNAVLAKIKQFCARVIG
GEWKGYTGKPITDVVNIGIGGSDLGPYMVTEALRPYKNHLNMHFVSNVDGTHIAETLQPLNPETTLFL
VASKTFTTQETMTNAHSARDWFLSSAADQQHVAKHFAALSTNGKAVAEFGIDTDNMFEFWDWVGGRYS
LWSAIGLSIALSIGYDNFEQLLSGAHAMDKHFAETPAEKNLPVLLALIGIWYNNFFGAETEAILPYDQ
YMHRFAAYFQQGNMESNGKYVDRNGNPVDYQTGPIIWGEPGTNGQHAFYQLIHQGTKLVPCDFIAPAI
SHNPLGDHHAKLLSNFFAQTEALAFGKSLEVVEAEFAAQGKTPEQVKHVAPFKVFEGNRPTNSILLRE
ITPFSLGSLIALYEHKIFTQGAILNIFTFDQWGVELGKQLANRILPELAGSEKISSHDSSTNALINRF
KEWR
>CORE_REP|Org30_Gene1695#
MANNPRAGQPARQSDLINVAQLTSQYYVLQPEAGNAAHAVKFGTSGHRGSAQRHSFNEAHILAIAQAI
AEVRHQQGTTGPCYVGKDTHALSEPAFISVLEVLTANGVDVIVQENNGFTPTPAVSHAILCHNRRGGA
QADGIVITPSHNPPEDGGIKYNPPNGGPADTNLTSVIEKRANELLAQQLKGVQRQSLDKAWNSGHLHA
KDLVQPYVEGLVEVVDMPAIQRAGLKLGVDPLGGSGIAYWQRVAEHYKLDLTLVNDSIDQTFRFMHLD
HDGIIRMDCSSESAMAGLLALRDKFDLAFANDPDYDRHGIVTPKGLMNPNHYLAVAINYLFQHRPQWG
ADVAVGKTLVSSAMIDRVVADLGRKLVEVPVGFKWFVDGLFDGSLGFGGEESAGASFLRFNGQPWSTD
KDGIIMCLLAAEITAVTGENPQHHYDDLAKRFGAPSYNRIQAPATHAQKAALSKLSPEMVKASTLGGD
PITARLTAAPGNGASIGGLKVMTDNGWFAARPSGTEEAYKIYCESFLGAEHREKIEHEAVEIVSEVLA
SAK
>CORE_REP|Org23_Gene4329#

MLKMVLATARDRARLKEITSVLIRYGLQDVLRLLGLGALLRGVRGEPALQDAQTLPERLREALEALGP
TFVKFGQILATRSDLLDPAWTDELDRLHSQAAVLPWETLAPQIVADLGGEPEHLFAEFDRTPLAAASM
AQIYRARLHSGEQVVVKVLRPGLAKTIHADLRLLASLAETVEQQSPTLARYRPRQMVRALATALNHEL
DLTHEGHNCDRVAKMFAREPGVVVPKIYWQWSSPRLLVQEYLPGTAPENPQQLAAAGFDGPLLAQRGA
RAFMSMVLEHRLYHADPHPGNVMALSGDRVGFIDFGMVGQLSERRRNQLLLLQAIADRQSEGIVNTL
IAWSDSEPLDLMDLELAAQNFLDKQAATTLTLGKALTDLLVMAREHQLALPPDLVLLFKALITADGVL
HRLDPAFDIVATLKPMLQQTVLQRYAPDAVRQRLLTLGGEALDAGEELPQTLRLLVRRLKRGQLNAEI
NVKNLGQLSKALERAAVTLAIAIVTAAFALGLAPYLMHSSLRLWGIPLFPLLGSAACLAGVILLALRL
RR
>CORE_REP|Org5_Gene2427#
MSDKITVGEAIARTLEQYAVSAMYGIISIHNLPIADAVGQRDKIRFVPARGEAGAVTMADAHGRFSGL
GVALTSTGAGAGNAVGAMIEALNANTPLLHITGQVEKAYLDADAGFIHETRDQLGFLRACSKRAYRVN
SPEQAVAVIQRAILDAQTVPCGPVAVEIPIDIQSSLVSRSVLTEPLAPAPLPQADDAAVERLHQRLKQ
AKRPLLWLGGGALACGDAVRKLADAGVAVISSTHGRGILPDSHPRSLRAFHNSPSIEAILTQCDLTLV
AGSRLRSNETRTWTLPLPRPLVQIDIDPAAANRNYLADEQINGDCAALLAALAARLSPGEKVNAEWDA
EIAGAVQQAERALRQQSGEYAKLNDAIDAALPQDGLLVRDITVSGSVWGSRLFRAISPLCNIHSLAGA
IGMGLPMAIGTAIANPQRKVVGLVGDGGLALGLGELATMAQEQVNITLIIMNDGGYGVMRGIQDKYFA
GRQYYNELHTPAFTQIAEAMGLKAWKVDAASQFNGVLAEAINYPGPSVVEVDMKQVGPLTFAGPPQKT
LY
>CORE_REP|Org6_Gene287#
MASYFIGVDVGTGSARAGVFDLNGRMVGQASRAIDLYRPKADFVEQSSDNIWQAVCNAVRDAVNQADI
NPIQVKGLGFDATCSLVVLDKEGKPLTVSPSGRTEQNIIVWMDHRAIAQAERINATKHRVLDFVGGII
SPEMQTPKLLWLKQHMPTTWANAGYLFDLPDFLTWRATQDATRSLCSTVCKWTYLGHEQRWDKSYFQQ
IGLEDVLEHDAAKIGSDVKMMGEPLGHGLTQRAASEMGLIAGTAVSVSIIDAHAGTLGTLGATGVSGE
VADFNRRVALIGGTSTGHMAMSRTARFIGGVWGPYYSAILPEYWLNEGGQSATGALIDHVIQSHPCYQ
ELLAQAKTQGQTIYEVLNAILRRMAGEPENIAFLTQDIHMLPYFHGNRSPRANPTLTGILTGLKLSRT
PEDMALHYLATIQAIALGTRHIIETMNHSGYSIDTIMASGGGTKNPIFVQEHANATGCAMLLPEESEA
MLLGGAMMGTVAAGVFDTLPEAMSAMSRIGKTVTPQTNQIKSYYDRKYRVFHELYNDHMKYRRLMQEE
A
>CORE_REP|Org33_Gene227#
MTTNYIFVTGGVVSSLGKGIAAASLAAILEARGLNVTIMKLDPYINVDPGTMSPIQHGEVFVTEDGAE
TDLDLGHYERFIRTKMSRRNNFTTGRIYSDVLRKERRGDYLGATVQVIPHITNAIKERIIEGGEGHDV
VLVEIGGTVGDIESLPFLEAIRQMAVEVGREHTLYMHLTLVPYMAAAGEVKTKPTQHSVKELLSIGIQ
PDVLICRSDRAVPANERAKIALFCNVPEKAVISLKDVDSIYKIPGLLKSQGLDDYICKRFSLNAPEAN
LAEWEQVIYEEANPGGEVTIGMVGKYVELPDAYKSVIEALKHGGLKNRLTVNIKLIDSQDVETRGVEV
LKGLDAILIPGGFGYRGVEGKVMTARYARENNIPYLGICLGMQVALMEFARNVAGMENANSTEFMPDC
KYPVVALITEWRDEDGNVEVRSEESDLGGTMRVGGQQCNLSDNSLVRQLYGEPTIVERHRHRYEVNNM
LLKQIEAAGLRVAGRSADNKLVEIIELPNHPWFVACQFHPEFTSTPRDGHPLFAGFVKAAGEHQKRQV
K
>CORE_REP|Org39_Gene4197#
MQQTLKRTSLTLLISGALGAGAVNSSLAAEVPAGVQLAQQQNIVINNGSEVASLDPHKVEGVPESNII
LNLLEGLVSTDANGHVVPAAATSWENQNYQQWTFHLRPGAVWSDGSPVIAQDFVYSWQRLADPKIASP
YASYLQYTKVENIDDILTGKKSPQTLGVKALDDQTLQVTLSEPVPYFISMLSHTSLKPVKQAVVEKFG
DKWTLPANYVGNGAYRLKEWVVNERIVLERSPSYWNNKQTVINQATFLPITSEVSDVNRFRSGEIDIT
NSAIPPYLYVKMKREMPEQLHVNPYLCTFYYELNNKRAPFTDPRVRTAVKMTLDRDIIANKIMGQGQI
PAYSFTPTFTEGASFTQPAWAGWSQEQRNAEARKLLAEAGYSDAKPLKFSLLYNTSDQNKQQAIAAAS
MWKKNLGAEVTLRNQEWKTSLESRHQGQYDVARATWCGDYNEPSAFLNLVLSNSSINTVFYKSPAFDA
IMAATLKAPDEAARTALYQQAEAQLDKDSALVPVYYRVSARLIKPTVGGFTGKDPLDYTDVKNLYIIK
Q
>CORE_REP|Org13_Gene1535#
MKFTLLLHTSYVMQPSSEHVSDVLIVGSGAAGLSLALRLAQHCKVTVLSKGPLSEGATFYAQGGIAAV
FDETDSIASHVDDTLIAGAGLCDKEAVEFIAGNARHCVQWLIDQGVLFDTEVNAQGEEHYHLTREGGH
SHRRILHAADATGKEVETTLVGKASAHPNICVMERRNAVDLITSNKIGLPGTRRVVGAYVWNRELERV
ETYRAKTVVLATGGAAKVYQYTTNPDISSGDGIAMAWRAGCRVANLEFNQFHPTCLFHPQARNFLLTE

ALRGEGAYLKRPDGSRFMPDFDPRGELAPRDIVARAIDHEMKRLGADCMYLDISHKPAEFITQHFPMI
HEKLLTLGFDLTRQPIPIVPAAHYTCGGVMVDQHGRTDLDGLYAIGEVSYTGLHGANRMASNSLLECL
VYGWSAAEDILQRLPFIQQAKQVPHWDESRVDDADERVVIQHNWHELRLFMWDYVGIVRTTKRLERAL
RRINTLQAEIDEYYAHFRISNNLLELRNLVQVAELIVRSAMARKESRGLHYTLDYPDLLPEALPTILQ
P
>CORE_REP|Org29_Gene2085#
MDSQRNLLLIALLFVSFMIWQAWQTDNAPQPAAQTTQQTSNAVAGDAASQAVPASGQGKLITVNTDVL
SLTINTRGGDIEQAKLLAYPDTLGSSTPFQLLETTPSFVYQAQSGLTGKNGPDNPANGERPLYQAAQD
SYTLPEGQDELRIPLTYTDKDGAVYTKTFVLKRDHYAVGVDYSIDNKGATPLELTLFGQLKQTTELPK
HRDTGSSNFALHTFRGAAYSSSDDKYQKYAFDKDENLSVTTKDGWVAMLQQYFATAWVPATKGDNTFY
TAKPGDNLSTIGFKSTPVVVQPGAQQQLNATLWVGPELQDQMAQLAPHLDLTVDYGWLWFISQPLFKL
LKFIHGFIGNWGFSIIIITFIVRGIMYPLTKAQYTSMAKMRMLQPKLQAMRERIGDDKQRMSQEMMAL
YKAEKVNPLGGCLPLIIQMPIFLALYYMLMGSVELRHAPFALWIHDLSAQDPYYILPILMGVTMFFIQ
KMSPTTVTDPMQQKIMTFMPVIFTVFFLWFPSGLVLYYIVSNLVTILQQQLIYRGLEKRGLHSRDKKK
S
>CORE_REP|Org2_Gene2715#
MWLRQCLRCNSLGFTLATALFFTLFQNALFLHRAWSYITFDSVHSVIFAASMPVVIFCALNIIFSVLT
VPYLRKPLIIFFLLGSAAANYFMFSYGVVIDGNMMQNAFETNPQEATALLTPRMGLWLALLGILPAVA
VCFTQIRQTRPWWYMVGLRAANVMLSLAVILIVAALFYKDYASLIRNNKSVVKMLTPSNFVAGTIKFT
EQRYFTRNLPLVKIGEDARKGPLIAGQAKKTLVILVVGETARAENFSLGGYQRETNPRLKQDNVVYFK
NASSCGTETAISVPCMFSNMPRKEYDATQATHQEGMLDVLAHAGVSVLWRDNDGGCKGACDRVPHIDM
TKLKLPQDCDGEVCMDNALLYKLNDYINGLKDDGVIVLHQMGSHGPAYYRRSTPEFQTFSPTCNSNQI
QDCSHEQLVNTYDNSILYTDAMLDATIKLLRQYDDQFNTALVYLSDHGESLGENGMYLHGTPYVFAPS
QQTHVPFLMWMSADYQRNFGVDRQCLNALAEKDDVSQDNLFHTLLGMLNVQTREYQSRLDILQRCRNA
A
>CORE_REP|Org24_Gene4315#
MTGRKIQRGFRLALCAAAIGACMSSAMAAQVPPGTALAAKQEIVRHIKDEPASLDPIKAVGLPEAQLA
RDLFEGLVNQDANGKVIPGVATRWQTSDNQTYIFHLRKDARWSNGDPVTAKDFVYSWQRLVDPKNLSP
FAWFAQLAGIQNAEQIISGKLPADRLGVSAPDDYTLKVQLDKPVPYFVSLTANFSLFPVNKAVVEKYG
NDWTKVGNLVGNGAFKLQERVVNEKLVLTPNDHYWDHARTVLTKVTFVPINQESNATKRYLAGDIDIT
ESFPKNMYQKLLKDIPGQVYTPDQLGTYYYAFNTQRAPTNDVRVRQALSYAIDRKIIAEKVLGTGEKP
AYHFTPDVTAGFKPEVSLLQQQSQAELDAQAKALLQAAGYGPNNPLKLTLLYNTSESHQKIAIAVASM
WKKKLGIDVKLQNQEWKTYIDSRNTGNFDVIRASWVGDYNEASTFLSLLTSTHSGNIAKFKNADYDKL
LAQAGRETNPAAVTADYNKMEQIIADQAPIAPIYQYTNGRLIKPWVKGYPITNPEDVAYSQTMYIIKH
>CORE_REP|Org45_Gene3357#
MTPGELRRLYFIVRVFLSYGLDELIPKMRLTLPLRFGRRLLFWMPNRHKDKPLGERLRLALQELGPVW
IKFGQMMSTRRDLFPPHIADQLTLLQDRVAPFDGALARKHIELAMGGPLETWFGDFDQQPLASASIAQ
VHTARLKTTGQEVVLKVIRPDIGPIIKADVRLMYRLAGWVPKLLPDGRRLRPREVVREYEKTLLDELN
LLREAANAIQLRRNFDGSPMLYVPEVYSDYCRESVLVMERIYGIPVSDIATLERQGTNMKLLAERGVQ
VFFTQVFRDSFFHADMHPGNIFVSYEHPEDPCYIGIDCGIVGSLNKDDKRYLAENFIAFFNRDYRKVA
ELHVDSGWVPRDTNVEDFEFAIRTVCEPIFEKPLAEISFGNVLLNLFNTARRFNMEVQPQLVLLQKTL
LYVEGLGRQLYPQLDLWTTAKPFLESWLRDQVGIPAVVRALKEKAPFWAEKLPELPELFYDSLQQHKL
LQQSVDKLTNQMQAQRVRQGQSRYLFGVGATLLVSGTLLLLGQIEVFPAWMMAAGIVCWVIGWKRTT
>CORE_REP|Org35_Gene3691#
MSIAFTPWPAEFASRYRERGYWIDKPLTEILDRQANNDAPAIIDAQGSLTYRELQQRSDRLAAALLRR
GVKSGDTALVQLGNVAEFYIVFFALLKIGVAPVNALFSHQRSELNAYAEQIKPALLIADRRHALFADD
QFLSAFRDAHPSLRAVALRSQPEGELALAAWLEEASDGFVAQPSAADRVAFFQLSGGSTGTPKLIPRT
HNDYYYSIRRSVEICRFDAQTRYLCALPVAHNYPMSSPGVLGVFYGAGLVVFAADPDAAQCFRLIEQH
QINVTALVPPAVTLWLQAIEEWGGNAQLASLKLLQVGGAKLGETLAARIQNEIGCQLQQVFGMAEGLV
NYTRLDDDERHILTTQGRPMSPDDEVWVADDDGNPLPAGEIGRLMTRGPYTFRGYYQSPAHNADAFDA
DGFYCSGDLISISEDGYITVQGRQKDQINRGGEKIAAEEIENLLLRHSDVINAALVSMPDALMGEKSC
AYIIANAPLKPVVLRRHLREQGVADFKLPDRFIQVDSLPLTPVGKVDKKRLREQLDAQLQTQAQGD
>CORE_REP|Org33_Gene3258#
MVWTTHTVFNQPKPLGNSNLFLSDTPLREALQREQGGWDAEVLASLGQQLGTQESLELGRLANANPPE

LLRYDATGQRLDDVRFHPAWHILMQGLIANRVHNLPWQEDARIGSFVARAARFMLHAQVEAGTLCPVT
MTFGATPLLLQALPVEFRSWLTPLLSDRYDAHLLPGGQKRGLLIGMGMTEKQGGSDVLSNTTTAAPLG
ARGPGEAYRLVGHKWFFSVPQSDAHLVLAQAEGGLSCFFLPRILPDGSRNAIRLERLKDKLGNRSNAS
SEVEFQDATAWLLGDEGDGVRHILKMGGLTRFDCSLGSHGLMRRGLSVALYHALQRQAFGKLLIEQPL
MRQVLARMALRLEGHTALLFRLARAWESRSNEGELIYSRLLTPAAKYSICRQGMPFIAEAMEVLGGIG
YCEESELPRLYREMPVNSIWEGSGNIMCLDVLRSLHKLPGALEMLQFELQPVRGQNRLFDHAWRQWQQ
RARQPREEMGRLLTQQLFDLCCAAQLLQHASPQIADAWCHLTLDHRGESLLSAEVCELLLNRAIGG
>CORE_REP|Org25_Gene478#
MKSSLFSRYTCFVLSILLTLVFLIMMHNHPWFWLPALACGCLMVLGIYDLTQQRHAICRNYPIIGRLR
FFFEFIRPELRQYFLEQDNEEIPFSRTQRTLVYRRAKNEMGDKPFGTLLDVYQTGYECIGHSMRPVEA
ADPTSFRITIGGADCRQPYSASIFNISAMSFGALSANAIRALNLGAAKGNFYHDTGEGSISRYHRENN
GDLVWELGSGYFGCRTADGHFDPRRFAEQAQSPQVKMIEIKLSQGAKPGHGGILPAKKVDAEIAATRG
VPEGVDCISPASHSAFTTPLEMMQFIQQLRELSGGKPVGFKLCIGHPWEFVAIVKAMLHTRILPDFIV
VDGKEGGTGAAPLELSNYMGMPLREGLLFVHNTLVGCGLRDQIKIGASGKIISAFDIASVLVLGADWV
NSARGFMFAVGCIQSQSCHTNHCPTGVATQDPLRQKALVVPNKAERVYHFHQNTVKALADMLAAAGVS
RPEQLTSHHMLRRITPTEIKVYADIYYYLEPGALLQPEIKSEFYARMWRMATPNSFDQAISLPAA
>CORE_REP|Org15_Gene1755#
MSRSLSIALAQLNLLVGDIEGNTERMLQIVQEQQKAGADLVMFTELALSGYPPEDLLYRNDFYQRCDA
QLLRLQQASAETAILVGHPWREGDKLYNALSLFAEGRLLTRYFKQQLPNYGVFDEKRYFHAGNDTCVV
ELKGYRLGLLICEDLWFPEPIDAAKAAGAEIILSINASPYNREKPYIRKTLMAGHCQRTQLPLVYLNQ
VGGQDELIFDGCSKVFDAAGTMTHRLAAFDEQVTRLEFNELDVVPMADPAAELPQLAQVYQALVLAVR
DYVTKNGFKGAVLGLSGGIDSALTLAIAVDALGKDKVQALMMPFRYTADISIADAKEEAEILGVEFDI
VSIEPMFDAFMGQLSPMFAGTERDTTEENLQARCRGVVLMALSNKRRSIVLTTGNKSEMAVGYATLYG
DMAGGFDVLKDVPKTLVFKLSEYRNTVSYVIPQRVIDRPPSAELAPDQVDQDSLPPYDILDAILEGYV
ERDKSVADLVAEGFDEAIVRKVIRLVDINEYKRRQAAVGPRITARNFGKDRRYPITSGFGRKNW
>CORE_REP|Org29_Gene3812#
MQENERYDYIVVGGGSAGCVVAGLLAENTTARILLLEAGREDKNLFIRMPAGFPEIVGKLIWPYQSDD
EPHMDHRTMGIPQGRVLGGGSSVNGQLYVRGHAQDYDDWEQLDGCTGWGYRDVLPYFIKAECNQSLHD
AYHGDRGHLKVSDSGYRHPLSYAIVKAGQELGYPYTLDFNGKQQQGIGFYQTTTFQGQRCSTAAAYLT
PMRHRSHLHVRTGVRVERLLFERDRAVGVAYVDSHGKRRQAMAEREIVLCAGAIGTPKLLMLSGIGPA
DHLREIGIKPTLDSPLVGQRLQDHLHFSVVAALKHPISLLGQDKGVKALANGLEWLLFKRGICASNLL
ECGGFFATGDDERPDVQLMGLAAADNVDDKGRQAQSQHAVSLKLAHLRPRARGEVRLRDSNPRSLPDI
RLNYLAEEADVAAQIRAVRLGLRLFQAPALADVVERVLLPTPEQRSDEQLAEFVRQHGKTEYHPVGTC
RMGAEPADSVVDLQLRLHGISGVRIADASVFPRIPAGNTNAPTIMVAERAVDMMLASLEEQDDA
>CORE_REP|Org29_Gene4497#
MRVKGITPQDLAAYGIHDVSEIVHNPSYELLFKEETDPSLEGFERGVVTKLGAVSVDTGIFTGRSPKD
KYIVRDDITRDTVWWADQGKGKNDNKPLSQEVWADLKHLVTEQLSGKRLFVVDTFCGANADSRLKVRF
ITEVAWQAHFVKNMFIRPSDEELADFEPDFVVMNGAKCTNPNWQQQGLNSENFVAFNLTERMQLIGGT
WYGGEMKKGMFSMMNYLLPLKGIASMHCSANVGEKGDVAVFFGLSGTGKTTLSTDPKRQLIGDDEHGW
DDDGVFNFEGGCYAKTIKLSEEAEPDIYHAIKRDALLENVTVLADGSIDFNDGSKTENTRVSYPIYHI
QNIVKPVSKAGHATKVIFLTADAFGVLPPVSRLTANQTQYHFLSGFTAKLAGTERGVTEPTPTFSACF
GAAFLSLHPTQYAEVLVKRMQAAGAQAYLVNTGWNGTGKRISIKDTRGIIDAILSGEIDKAETVTLPI
FDLAMPTALPGVNPEILDPRNTYASLEQWQEKAQDLAERFITNFDKYTDTPAGAALVSAGPKL
>CORE_REP|Org8_Gene3880#
MSAANKPKITLWEFFQSLGKTFMLPVALLSFCGIMLGIGSSLSSRDVITLMPFIGHPIFQLIFTWMSK
VGSFAFSFLPVMFAIAIPLGMARENKGVAGFSGFVGFAVLNLGTNFYLTAAGVLPTSDPLVLKANNIQ
NILGIQSIDTGILGAVIVGIIVYRLHERFHTIRLPDALAFFGGTRFVPIVTTVVLGLCGLVIPLIWPW
FAAGITGLGWVINSAGAFGPMLFGTGERLLLPFGLQHILVALIRFTEAGGTMEVCGHSVSGALTIFQA
QLSCPTTTGFAESATRFLSQGKMPAFLGGLPGAALAMYHCAKPENRHKIKGLLISGVVACVVGGTTEP
IEFLFLFVAPFLYLIHALLTGLGFTVMALLGVTIGNTDGNIIDFVVFGILHGTATKWYLVPVVAAVWF
VGYYAIFRFAIQRFNIKTPGRESDSAVSQSAPTGAVGKSGYNVPAILAALGGPDNIITLDNCITRLRL
SVNDMSRVDDAVLKANRAIGVVHLNDHNLQVVIGPQVQSVKDELDSLIATAQPAAALQGATHV
>CORE_REP|Org31_Gene2710#
MAHFAQSPYFVLHQLTCQFADGETLFGPLDLAFDRQRCGLVGRNGVGKTQLLRLIAGRDRPGNGHVES

HAALAYVAQQPEIAADTTLAQLLGYGEVFAALARIEQGRPLADDIDRLEGRWDLNDRLQSAFAAAGLP
AFDPLRSACDLSGGERMRAALCGAFLGEADYLLLDEPTNHLDSAGRAWLYQQLERWQGGLLIASHDRQ
LLGRMERIVELTPGALRSYGGNYDDYRRQRDTEQQAARADLEHAREERRRTRARQQKEHDMSQRRSAQ
TLRVVDTLNIASFERVAYKSAAKESLGTLRKQHQDQRDSLDAAVREAYQRVEEEQPVLLALPGSEVSA
NKQVLVLEQLQLPFVSAPPLDLRIDGPMRVALTGPNGCGKSTLLKTVLGQLAPLAGHCHCPLSTAYLD
QTLSQLDPSLSVMEHLGLQDSPLVEGALRTRLAQLQLGADRIALPLGSLSGGERLKAALACALWRRQP
AQLLLLDEPTNHLDLASSLAIETALADFPGAMLVVSHDEDFLQALRPTHRLHRQADGWRLQAW
>CORE_REP|Org40_Gene4041#
MPKQRVPLKLSTSITLMVSAIIASVLLVVFALFFVQMSREGQDQLQQKAIAVANTLALSNTVIDGLQR
RDQSGAIQRFAEQVRHQNELLFVVVVDMQGIRYSHPKPWLIGKHFIGDDLAPALQGNVNSAINRGTLA
PALRVFVPVYDDQKQQIGVVALGIALDTVQRVVAESRWIIYWTIAFAALVGSLGTFFLVSALKRIMLG
FEPYEISNLFEQRNAMLQSIKEGVIAVDNESRITIVNDEAKRLLRQSGPVENLLLEAASKHWPAQLHL
AEVLASGEPLRDRQISFNGSELLTNTVPVIVNGQVTGAIATFRDKTEVSRLLQRLSGMAHYADALRVQ
SHEFMNKLHVILGMLHMKAYQQLENYIINTASNYQEEIGALLRKIHSPEVAGFFIGKISRAHEAGVEL
TIEENSLLPETDDAETTHVLISVLGNLIENAIDAIDGVEGHEIGLSFHHHDDQLHCIVSDDGPGIDPA
IAARIFEHGFSTKGTGRGIGLALIRSHLEKLGGSIDFESEPGELTQFFVHLPYQAKSRAHD
>CORE_REP|Org25_Gene4270#
MATPLLAIQDLSIAFRQGDAVTPVVNELSLQIAPAETLALVGESGSGKSVTALSILRLLPAPPVVYPG
GDILFNGNSLLHAPEAELRKVRGNQIAMIFQEPMVSLNPLHTIEKQLAEVLMLHRGLRRETARAEIVD
CLERVGIRQAKTRLQDYPHQLSGGERQRVMIAMAVLTRPKLLIADEPTTALDVTIQAQILTLLQELKQ
EMGMGLLFITHNLNIVRRLADNVAVMRQGRCVEQNGRAQLFSRPQHPYTRQLLAAEEVGEPLPLPAAA
SARPGDERPLLKVEDLQVRFPIRRGLLRRTVDYHYALKSLSFELRAGESVGLVGESGSGKSTTGLALL
RLLASQGAIWFDGEPLHPLTMKQMLPYRSRMQIVFQDPYSALNPRLNVQQIIAEGLEVHQRLNAEQRE
QRVIEVLQEVGLDPQLRHRYPTEFSGGQRQRIAIARALILQPQLLILDEPTSSLDKSVQAQILTLLKS
LQQRHRLAYLFISHDLQVVRSLCHQVIVLRQGEVVEQGDCRAIFAAPAADYTRQLLQLAD
>CORE_REP|Org17_Gene3634#
MANRRQPLIPRWLWPGLLAAGMILLVAALAMGSLWRHSPDSGWRGLWQDSYLWHVVRFTFLQALLSAL
ISVLPAILLARALYRRRFPGRQLLLRLCAMTLVLPVLVAVFGLLSVYGRQGWLATLCGWLGVEYDFSP
YGLQGILLAHLFFNLPLATRLLLQALENIPVEQRQLAAQLGMNGWQQFRFVEWPALRRQILPSGALIF
MLCFASFATVLSLGGGPQATTIELAIYQALSYDYDLGRAALLALIQLGCCLGLVLISQRLSQALPVGH
THAQRWRNPEDSLWRRISDFLLIAAALLLLLPPLLAVIADGANQAIISVLRQPVLWQALFTSLRIALG
AGALCVALTMMLLWSSRELKLRQQLRGGQALELSGMVILAMPGIVLATGFFLLLNDTIGLPQSPYALV
ILTNALMAVPYALKVLENPMRDLAERYNPLCLSLDIRGWRRLRLIELRALRRPLAQALAFACVLSIGD
FGVVALFGNEHFRTLPFYLYQQIGAYRSQDGAVTALLLLLLCFLLFTLIERLPGRHADA
>CORE_REP|Org41_Gene1672#
MTRSLGKSGVLKFGIGLIALTVAASVQAKTLVYCSEGSPEGFNPQLFTSGTTYDASSVPIYNRLVEFK
IGTTELQPGLAEKWDVSEDGKTYTFHLRKGVKWQSSKDFKPTRDFNADDVVFSFERQLDANNAYHKVS
GGSYEYFEGMDMPKLIAKIEKVDDNTVRFVLNRPEAPFLADLGMDFASILSAEYADVMMKAGTPEKVD
LNPIGTGPFQLLQYQKDSKILYKAFDGFWGTKPKIDRLVFSITPDASVRYAKLQKNECQVMPYPNPAD
IARMKQDKSINLMEQPGLNVGYLSFNVEKKPLDNLKVRQALTMAVNKQAIIDAVYQGAGQAAKNLIPP
TMWGYNDAVQDYAYDPAKAKALLKEAGMADGFSIDLWAMPVQRPYNPNARRMAEMIQADWAKIGVKAK
IVTYEWGEYLKRAKAGEHQTVMMGWTGDNGDPDNFFATLFSCAAAKDGSNYSRWCYKPFEDLIQPARA
ESNHDKRIELYKQAQVVMHDQAPALIVAHSTVYEPVRKEVKGYVVDPLGKHHFENVSVD
>CORE_REP|Org3_Gene4776#
MDLNDLSTRIGGDVLVNILSGQPRAASVRWLGATVLFTLFSSPAWAFSIDDVAKQAQDLAAKGFEAPK
SNLPSQFREMKFADYQQIQFNHDKAYWSKLKTPFKLEFYHQGMYFDTPVKINEVTSTSVKQIKYSPDY
FNFGSVKHDPESVKNLGFAGFKVLYPVNSADKNDEIMSLLGASYFRVVGKGQVYGLSARGLAIDTALP
SGEEFPRFREFWVERPKQGDKHLVIYALLDSPRATGAYRFTVIPGRDTTVDVESKVFLRDKVGKLGLA
PLTSMFLFGPNQPSPTLNYRPALHDSNGLSIHAGNGEWIWRPLNNPKHLSVSTYTVENPKGFGLLQRG
RNFKEYEDLDDRYDLRPSAWIEPKGDWGKGKVELVEIPTADETNDNIVAFWTPDTLPEAKKPLTLSYR
LNFTRDEDKLHSQDIAYVARTMRSTGDVKQSNLIREPDGSVAFLVDFVGPVLKGLDANTPVASQISIG
DNGEMVENNVRYNPVTKGWRLTVRLKVKDDKKPVEMRAALVNGDKTLSETWSYQLPANE
>CORE_REP|Org25_Gene3782#
MSDHITPSRTPDKPVIWTVSITRLFDLFRDISLEFDHLATITPIRLGFEEAVQHIRARLATEPCDAII

AAGSNGAYLKSRLSVPVILVKPGGFDLLQALSQARRTADRIGVITYKTPLPALMEFQQTFDLPLEQRS
YVTEEDARGQIAELKAAGIQAVVGAGLISDLAEEAGLTAIFLYSAATLRAAFSDALDVTRLMLGGAKR
GGDYAARDTLQPRYGLSDLQGDSPQMEQTRRTIMLYARSPAAVLIEGETGTGKELAAQAIHREYFSRR
GAPSRGATPPFVAINCGAIAESLLEAELFGYEEGAFTGSRRGGRRGLLETANGGTLFLDEIGEMPLHL
QTRLLRALEEKTITRVGGQQPVKVDFRVISATHTRLEQAIQQGDFRADLFYRLSALRLQLPPLRARGD
DIAMLAEHFLKQSLAALDAPLTEPLRAALAGCYAALGHYAWPGNLRELRNMMERVALMLSTGVAPSGE
TLQWLLPELAVAPPPETPAAPVSAHEALARCGGDHAAAARLLGISRTTLWRRLKKPH
>CORE_REP|Org49_Gene4383#
MTQQIAGTELRFTQGFAAAERQVLTDEAVEFLAELVGKFTPRRNELLAARVRWQQNIDRGELPGFISE
TTSIHEGDWQIRGIPQDLRDRRVEITGPVERKMVINALNANVKVFMADFEDSLAPSWDKVIDGQINLH
DAVNGTISYTNEAGKIYQLKPNPAVLIARVRGLHLPEKHVLWRDEAIPGGLFDFALYFYHNYRQLLAK
GSGPYFYLPKTQSWEEAAWWSDVFSFTEDRFDLPRGTIKATVLIETLPAVFQMDEILYHLRDHIVGLN
CGRWDYIFSYIKTLKNHGDRVLPDRQSVTMEKPFLSAYSRLLIKTCHKRGAFAMGGMAAFIPSKDAEK
NAWVLNKVRADKELEANNGHDGTWVAHPGLADTVMEVFGKALGDRQNQLEVLREQDAPISAAQLLEPC
DGERTEAGMRANIRVAVQYIEAWISGNGCVPIYGLMEDAATAEISRTSIWQWIHHEKSLSDGRPVTKA
LFRQMLQEEMLVVREEVGEARFNAGRYEEAARLMERITTQDELIDFLTLPGYELLA
>CORE_REP|Org29_Gene4329#
MRLKLSFQIKLFLCLVAFSCLLLTCIGAYTYYQLDAQLHRDLGARAQVQAREIALIPSLVDAVENNDA
ARIAALMKKIRASSDASYIVIGDNHARHLYHSEYEGRLGTPMIGGDNKEVLEGKSIISIRKGGIGVSL
RSKAPIVDENNRVIGIVSVGYLKSHIDNLNARTLTQIIGSIILLLIALFVFSWLLSKNLKRQMFWLEP
KEIALLVRQQKALLEAIYEGVIAIDPQLRIITINHAARELLDLHQPAAGLLGRPIGDVIQAQPNFFAA
AQLGQDTHDEVCRFNHVRVIASRVRIMQEQELQGWVISFRDKNDINTLSSQLSQVKRYADNLRIMRHE
QLNWTATLAGLLHMQRYDEAIRYVEAQSEGAQEILDFISQRFSSAALCGLLLGKYSSAREKGIELRFD
PACQLRQIPAALNETELMSIVGNLLDNAVEATLHYPAPHEAIELYISDGSDELVIEVADHGTGIAEEI
RDTLFEQGVTTKADKSDHGIGLHLVASHVAQAHGSIEVSDNEPHGAIFSIFIPK
>CORE_REP|Org37_Gene1031#
MLSTNNITMQFGSKPLFENISVKFGGGNRYGLIGANGCGKSTFMKILGGDLAPTGGNVFLDPNERLGK
LRQDQFAFEQYSVLDTVIMGHTELWAVKEERDRIYAMAEMSEEDGYKVADLEVAYGEMDGYTAEARAG
ELLLGVGIPVEQHYGPMSEIAPGWKLRVLLAQALFSDPEILLLDEPTNNLDIDTIRWLEQVLNERNST
MIIISHDRHFLNMVCTHMADLDYGELRVYPGNYDEYMTAATQARERLLADNAKKKAQINELQSFVSRF
SANASKSKQATSRARQIDKIQLEEVKASSRQNPFIRFDQDKKLFRNALEVEALTKGFDNGPLFSKLNL
MVEVGEKVAVLGANGIGKTTLLKTLVGDAQPDSGTVKWSENARIGYYAQDHEYEFDDTLTVFDWMSQW
KQEKDDEQAVRSVLGRLLFSQDDIKKKVKVLSGGEKGRMLFGKLMMQRPNILVMDEPTNHLDMESIES
LNMALEMYEGTLIFVSHDREFVSSLATRILEITPNKVIDFTGNYEDYLRSQGIV
>CORE_REP|Org23_Gene3579#
MQQPRPIRRALLSVSDKAGIVEFAEALSQRGVELLSTGGTARLLADAGLPVTEVSDYTGFPEMMDGRV
KTLHPKVHGGILGRRGQDDAVMNQHDIQPIDMVVVNLYPFAQTVARPDCSLEDAVENIDIGGPTMVRS
AAKNHKDVAIVVKSSDYAAIITEMDNNDGSLRYATRFDLAIKAFEHTAAYDSMIANYFGALVPAYHGE
TEQPSGRFPRTLNLNYIKKQDMRYGENSHQQAAFYIEEEVKEASVATAEQLQGKALSYNNIADTDAAL
ECVKEFAEPACVIVKHANPCGVAIGDDILAAYERAYQTDPTSAFGGIIAFNRELDAATAQAIISRQFV
EVIIAPNVTQEARSLLAAKQNVRVLACGQWQQRVAGLDFKRVNGGLLVQDRDLGMVTAADLRVVSERQ
PTEQELRDALFCWKVAKFVKSNAIVYARDNMTIGIGAGQMSRVYSAKIAGIKASDEGLEVKGSAMASD
AFFPFRDGIDAAAAVGITCVIQPGGSIRDDEVIAAANEHGIAMIFTDMRHFRH
>CORE_REP|Org17_Gene4674#
MSPSEFAREVSKRRTFAIISHPDAGKTTITEKVLLFGQAIQTAGTVKGRGSSQHAKSDWMEMERQRGI
SITTSVMQFPYRDSLVNLLDTPGHEDFSEDTYRTLTAVDCCLMVIDAAKGVEDRTRKLMEVTRLRDTP
ILTFMNKLDRDIRDPMEVMDEVERELKIACSPITWPIGCGKLFKGVYHLYKDETYLYQTGKGHTIQEV
RIVKGLNNPELDVAVGEDLAAQLRDELELVQGASHEFDQAAFLSGELTPVFFGTALGNFGVDHMLDGL
VAWAPAPMPRKTDTREVTAAEEKFTGFVFKIQANMDPKHRDRVAFMRVVSGRYEKGMKLRQVRTGKDV
VISDALTFMAGDRSHVEEAYPGDIIGLHNHGTIQIGDTFTQGEDMKFTGIPNFAPELFRRIRLRDPLK
QKQLLKGLVQLSEEGAVQVFRPIANNDLIVGAVGVLQFDVVVARLKSEYNVEALYESVNVSTARWVEC
DDVKKFEEFKRKNEINLALDGGDNLSYIAPTMVNLNLTQERYPDVTFRKTREH
>CORE_REP|Org2_Gene2285#
MLMTHLAASRYRYYRWLLAGAVGAAILLVSLYTRYYQEVKSIELSQHTLATRTVGKLNQLLTPAQLQA

ERSMDMLNQSCENVSSTLRFRAAQNQALRAMLLVKNGIIYCSSLFGARHYQLAAVMPSFVNSDARLAL
RPSLAVSKGLPTLVLWTPSPRDKTSGVLHVFNIELLSNFLLEPQEPYVQRVVLNVADSSLEYGRREIL
SRDTLTNDLRYTAGSALYPFSISLFGPQIGMLALSALPRHIPLALLISLLAAYVVYLLTANRMSLSYH
IGHAITHREFRVYCQPIIHSDTGRCAGVEMLLRWKKKRQGWISPDVFIPLAEQHELIIPLTRYLMSTV
TENLQLFPPRPSFYISINVAAEHFKTLNIIDDIRQIWLPAHPMPSLMLELTERTALSAIQYDQIRTLK
DMGIMLAIDDFGTGHSSLSYLKNLSPDVLKIDRGFTAAIGTDAVNATVTDTIITLAQRLKLKLVAEGV
ETEEQADYLRSREVNALQGYYFAKPMPIHVFPLWLQQYESRVRKAEEDPPEA
>CORE_REP|Org35_Gene4411#
MKRLIPTLLYGALATAFGAQAATPPDTLVVASSLEGIISLDPAESFETVSSANLVNLYQRLVASDRGA
PQKLAPDAAARWQAGADGRSLIFTLKPQQRFASGNPLRPEDVIYSLVRAVKLNKAPSFILAEFGWTPD
NVEQQLKKIGDNQVQLSWSANIGSELALRLLTANVASIVDEKLLSEHAQQGDFGNAWLRNHSAGGGPY
RVNNYVPHEALLFGRNDYAPSPARLKTVLFKNVSDAGTRRLLLLKGDADVAYDLGADQFDSLRKAPGV
RIEQADAPKVYYLGFNTGSKDNPALGNPALWQAARWLVDYQSIAERLLKGQYRVHQAFLPQGLDGALD
ERPFKLDVDKAKQILRDAGIAEGTSISLIVINQPPYTDIAQALQASFAQAGLRLDIHPVVESDLWGKM
RGRDFQAIFTYWGADYLDPNTNASAFAYNVPGGPKTLAWRTQWSIPAISAETRAAAAEGDSAARSARY
AALQRELQASSPYVLALQGQTLVALRDNVEDARVDIANSMLYLDRVNKTQP
>CORE_REP|Org39_Gene3774#
MEWIADPTIWAGLATLVVLEIVLGIDNLVFIAILADKLPKQQRDKARVVGLLLALVMRLALLASISWL
ATLTKPMFIVAEHPFSGRDLIMLVGGIFLLFKATMELNERLEGKDEEQHGARKGARFWPVVAQIVVLD
AVFSLDSVITAVGMVDHLAVMMIAVCIAIGLMLLASKPLTRFVNAHPTIVILCLSFLLMIGFSLVAEG
FGYHIPKGYLYAAIGFSVMIEALNQLAQFNRRRFLSKVRPLRERTAEAVLRMLSGKHEEAEVDSHSAN
LLADSDSENGEIFNQQERRMIERVLGMAQRTVSSIMTSRHDVEYLELNDPQEKLTQLLERNQHTRIVV
VESSASDEPLGVIHTIDVLKQQLAQAPLDLRALIRQPLIFPEQLTLLSALEQFRQAQTHFAFVVDEFG
SVEGIVTLTDVMETIAGNLPEAGEEVDARHDIQQNDDGSWIANGYMPLEDLVLYLPMPLEDKREYHTL
AGLLMEHSQRVPQEGEQIKIGDYLFEPLEVNSHRILKVKITPPPPPEDYEV
>CORE_REP|Org12_Gene2579#
MPLLFSIAPIVLLIWMMTKRNGVPSYLALPLTAAAVYAVQLLWFDASLRLLHANIITALVSTLTPITI
IAGAILLNKLMQVSGAENVVRRWLETISPNPVAQLMIIGWAFAFMIEGASGFGTPAAIAAPILVGLGF
NPLRVALLTLVMNSVPVSFGAVGTPTWFGFANLGLSDASLLEIGRQTALIHFIAGFVIPLLALRFIVS
WQDIRRNLPFILLSVLSCTLPYLLLAQVNYEFPALVGGAIGLALSVLLARGGIGLARSDKLQNAGQAV
PFLQVVKAMTPTLLLIAILIVTRVHQLGLKALLNNTTLLWQENLGWLGELRISQALIVELQQVLGTSA
AAGYKTLYVPALIPFLLVVVLCIPLFRLNGGQVRQMFSETGGRIARPFIALFGALVMVNLMMQGGDNA
PVILIGKALAALTGESWLLFSSFLGALGSFFSGSNTVSNLTFGGIQQSIAQSSGLDVNLTLALQSVGG
AMGNMVCLNNIIAVCSILGIGNAEGKIIRKTVLPMLAYGGIAAGMAAILTL
>CORE_REP|Org27_Gene1968#
MQSPFLSSLRVGLWLLCAWPLLAQAGPADEFAAANRAQQAKLLQAWAAEPDAARLPLLQGLQQETVAI
DGAKRAFIRQGDRYLPLEGEAAPVGEPKKVWLNNRLRILIANALSAQRLVSTDAAVRLQAATALQREA
QGEQLPLLTRRLAQEKDPQVRDALSIALANLQLTNASPQVRFNAVRLLGESGEPETRARLQALTDAAH
EPDAAVRAEAQRSLQRVQHRLMIGDLLGQAFSGLSLGSILLLAALGLAITYGLLGVINMAHGEMLMLG
AYSAYLVQGLFQQFAPHWLALYPLAALPVAFAITAGIGMLLERTVIRHLYGRPLETLLATWGISLVLI
QLVRVLFGAQNVEVANPAWLSGGVQLLPNLVLPWNRIAVIAFVLLVLALTWLLLNKTRLGMNVRAVTQ
NRAMAACCGVPTGRVDMLAFGLGSGIAGLGGVALSQLGNVGPELGQGYIIDSFLVVVLGGVGQLAGTV
AAAFGLGIVNKILEPQIGAVLGKILILVLIVLFIQKRPQGLFALKGRVID
>CORE_REP|Org10_Gene2914#
MRKLLVLIFSLSLFSVTQQAAAEEKNKTVDVVLIGGGIMSATLGTYLHELEPTWTIDMYERMNGVAEE
SSNGWNNAGTGHSAFSEMNYTPEKADGTIDISKAVKVNESFEISRQFWSYQVKNAVLKDPKSFINSVP
HMSFVWGDDNVNFLRKRYAALQHSTLFRGMEYSEDPAQIKQWAPLVMNGRDPAQKIAATRMPLGTDVN
FGVITHQLVDALSASDKFKLNLSHEVRDIKRNADQTWSVTVADLNRDGKETTVNAKFVFIGAGGASLT
LLQKSGIPEADGYGGFPVGGQFLVTTNPEIANQHLAKVYGLASVGSPPMSVPHLDTRMLDGKRVLLFG
PFATFSSKFLKNGSLFDLMHSLSTSNLMPMTHVGLDNFDLVKYLVGQLMMNDDDRFAALKEYFPDAKQ
ADWKLWTAGQRVQIIKKDADKGGVLQFGTEVVSSEDGSIAALLGASPGASTAAPIMLHLMETVFKDKV
ATPEWQSKLKEIIPSYGHKLNGDIEMTNKIRGYTSSVLGLNYIEVKPETN
>CORE_REP|Org49_Gene2329#
MQFAHAGTPARKKSKWWQSDALKWLVVSLFSLVTCYLIVLMYAQGEYLFAILTLILVSAGLYVFANRR

AYAWRYVYPGVAGMGLFVLFPLICTIAIAFTNYSSTNQLTFERAQSVLMQRQFQSGKTFTFGLYPAEN
QQWRLQLTPPDGEAPLISEPFRLDAAAPQTLKLTAQSAAPQGERATLRMITQNRQALSQLVAQLPDGG
ELRMSSLRQFSGTRPLYALDQDGSTLTNNQTQVRYRPNGDTGFYQAINADGGWAQETLSPGYTVTTGW
KNFLRVLQDEGIKKPFVSIFIWTIVFSVMTVILTVAVGMVLACVVQWEALKGKAIYRVLLILPYAVPS
FISILIFKGLFNQSFGEINMMLSHLFGIKPAWFSDPLTAKSMILIVNTWLGYPYMMILCMGLLKAIPD
DLYEASAMDGANPWQNFFRITLPLLIKPLTPLMIASFAFNFNNFVLIQLLTNGGPDMIGTTTPAGYTD
LLVSYTYRIAFEGGGGQDFGLAAAIATLIFLLVGALAILNLKASKMNFD
>CORE_REP|Org17_Gene901#
MTQNIHKHRILILDFGSQYTQLVARRVREIGVYCELWAWDVSEEQIREFNPSGIILSGGPESTTEAGS
PRAPDYVFNAGVPVLGVCYGMQTMAMQLGGHVQGSNEREFGYAQVEIVNESALLRDIEDAISPAGKPL
LDVWMSHGDKVTAIPSDFVTVASTDTCPFAIMANEEKRFYGVQFHPEVTHTRQGQRMLERFVLDICQC
EALWTPATIIEDAVERIREQVGEDHVILGLSGGVDSSVTAMLLHRAIGKRLTCVFVDNGLLRLNEAKQ
VMEMFGDHFGLNIVHVEAENRFLTALAGVDEPEAKRKIIGRVFVEVFDEEACKQEQVKWLAQGTIYPD
VIESAASATGKAHVIKSHHNVGGLPKEMKLGLVEPLKELFKDEVRKIGLELGLPYDMLYRHPFPGPGL
GVRVLGEVKKEYCDLLRRADAIFIEELHKADLYNKVSQAFTVFLPVRSVGVMGDGRKYDWVVSLRAVE
TIDFMTAHWAHLPYDFLGRVSNRIINEVDGISRVVYDISGKPPATIEWE
>CORE_REP|Org24_Gene232#
MSQQVIIFDTTLRDGEQALQASLSVKEKIQIALALERMGVDVMEVGFPVSSPGDFESVQTIARQIKNS
RVCGLARCVDKDIDVAAEALRVAEAFRIHVFLATSTLHIESKLKRSFDEVLEMAVRSVKRARNYTDDV
EFSCEDAGRTPIDNLCRVVEAAINAGATTINIPDTVGYTTPNQFGGIITTLYDRVPNIDKAIISVHCH
DDLGMAVGNSIAAVQAGARQVEGTLNGIGERAGNCSLEEVIMAIKVRQDIMNVHTNINHQEIFRTSQI
VSQLCNMPIPANKAIVGSNAFAHSSGIHQDGVLKNRENYEIMTPQSIGLKDVQLNLTSRSGRAAVKHR
MEEMGYQEQDYNLDTLYAAFLKLADKKGQVFDYDLEALAFINKQQEEPEHFSLGYFSVQSGSSIMATA
SVKLICGGEEKAEAATGNGPVDAVYQAINRITDYPIELVKYQLTAKGHGRDALGQVDIVVSYNGRRFH
GVGLATDIVESSAKAMVHVLNNIWRSQQVEKEKQRLQQNKHQNNQETV
>CORE_REP|Org8_Gene2192#
MRLEVFCEDRLGLTRELLDLLVSRSIDLRGIEIDPIGRIYLNFSQLDFDTFRALMAEIRRIAGVTDVR
TVSFMPSEREHRALRALLESMPEPVFSIDMKGKVELANPAAQALFSLSEDKIRNQTAGALIGGYNFSR
WLESEHTAPHAERVVIRSQDFLMDITPIYLEDQQQQPAAVGAVVMLKSTARMGRQLQNLSVNDDTEFD
HIVAVSAKMRHVLEQARKLAMLDAPLLIVGDTGTGKDILARACHLRSPRGKQPFLALNCAALPDDVVE
SELFGHAPGAYPNALEGKKGFFEQANGGSVLLDEIGEMSPRMQTKLLRFLNDGTFRRVGEEHEVHVDV
RVICATQKNLTELVQRGEFREDLYYRLNVLTITIPPLRERPQDIMPLTELFVARFADEQGVARPKLAS
DLGGFLSKYGWPGNVRQLKNAIYRALTQTEGYELRPQDIVLPEFEVEMSLGDEVLDGSLDDISKRFER
SVLTRLYRTYPSTRKLAKRLGVSHTAIANKLREYGLSSRKGGAEGEE
>CORE_REP|Org1_Gene4471#
MVVYHRIIDQDWLTLGQYDDYMTPSSLLAERISRLSSALESGLYERQEAIRLCLLAALSGESVFLLGP
PGIAKSLIARRLKFAFRNARSFEYLMTRFSTPEEVFGPLSIQALKDEGRYQRLTAGYLPEAEIVFLDE
IWKAGPAILNTLLTAINERRFRNGNSEEPIPLRLLVTASNELPEADNSLEALYDRMLIRLWLDKVQDK
QNFRSLLVNRQSESQNPVPAALSISDEEFLQWQPQIDKVALPEACFELIFQLRQRLDALEQAPYVSDR
RWKKALRLLQACAFFSGRDAIAPLDLMLLKDCLWHDLTSLKLLQQQIDQLLTESAYQQQSMLIQLQQI
HTRWLQDQQQQSDRQAISLVKKGGMFSRKPQYAPATPFAGGTLTLLLQKPLQLHDIQVNHLSIESSVL
DNWLQKGGDVRAKLNGIGFAQPIDLDVDDQLHLTVLDVSRQPSLLALPGKQATATPQALLDEMDALAQ
RLAEQRRAFSQHQPCLFTPAAGLAKIEASLLQVAEQVKQQQQQMRGQ
>CORE_REP|Org12_Gene4488#
MKAKLLPLFLLAALPTAALAATPPNTLVVVQSLDDIVSLDPAEANELSSIQTVPSLYQRLVQADRDDP
AKVAPVLAESWQGDAAAKTLTVKLRPQAAFASGNPLTADDVIFSYSRAVKMNKSPAFILNVLGWQPDN
IDAQLKKIDDHTVQLRWTADVSPAVALNILSTPIASIVDSKAALANVKDGDFGNAWLKMHSAGSGPFK
MRVYQPHQAIVLDANPTSPGDKPQLKNIIIKNVPDPATRRLLIQQGDADIARELGADQTDTLKNQPGV
KVLEIPSAEQNYLVFNTGNGANPLLNNPAFWEAARYLVDYQGITKDLLKGQYFVHQSFLPVGLPGALE
TNPFSYDPAKAKAILAKAGITHAAFTLDVENKLPFITIAQALQGSFAAAGVKIELLPAAGSQVYSRVR
ARQHQAAIRLWIPDYFDAHSNASAFAYNDGKSSTVAGLNGWQIPQLNKQTLAALAEADPAKRRALYTA
MQQELQRSSPYVFIDQAKTEVVLRDNVKGYQQGLNADMVYYDRVSK
>CORE_REP|Org37_Gene721#
MFDIVELSRLQFALTAMYHFLFVPLTLGMAFLLAIMETVYVLSGKQIYKDMTKFWGKLFAINFALGVA

TGLTMEFQFGTNWSYFSHYVGDIFGAPLAIEGLMAFFLESTLVGLFFFGWDRLSKVQHMAVTWFVALG
SNLSALWILVANGWMQNPIASDFNFETMRMEMVSFSELVLNPVAQVKFVHTVASGYTCGAMFVLGISS
YYLLKGRDIAFAKRSFAIAASFGMAAILSVIVLGDESGYEMGDVQKTKLAAIEAEWDTQPAPASFTLF
GIPDQDKMENSFSIQIPYALGLIATRSTDTQVTGLKDLMAQHEVRIRNGMKAYQLLEELRGGNTDPAV
RAEFNKTKQDLGYGMLLKRYTPNVTDATEAQIQLATKDSIPRVAPLYFAFRIMVACGVIMLLIIGLSF
WNVIRGRIGQKKWLHRAALYGLPLPWIAIESGWFVAEYGRQPWAIGEVLPTAVANSSLTAGDILFSMG
LICGLYTLFLVAELYLMFKFARLGPSSLKTGRYHFEQPTAAVQEAR
>CORE_REP|Org10_Gene1198#
MTTQGKFKKQLTLTDLTFIGLGAIFGSGWLFAASHVSSIAGPAGIYSWLIGGLAVLLLGIVYCELGAA
LPRAGGIIRYPVFSHGELMGYLLGFITLIAFSSLIAIEVVAARQYAAAWFPFLSQPGSGDPTAIGWLV
QLALLCFFFALNYYSVKTFAKSNNLISVLKFLVPLLVIVVLFSFFKPENLHSQGFAPFGSAGVEAAIS
AGGIIFAYLGLTPIISVASEVQRPQRTIPIALILSVVLSTIIYVLLQIAFLGSIPSEMLSGGWAGISQ
QFSLPFRDIAITLGMGWLAFLVVSDAIVSPSGTGNIYMNATPRVIYGWARAGTFFKLFTRVDSESGIP
RPALWLTFALSVFWTLPFPSWEKLIGVVSAALVLSYAIAPVTAAGLRRNAPDMPRPFRVRAFCVLGPL
SFIISALIVFWSGWDTVSWLLGLQILMFFIYILFKNQVPTHAVSLRQQIWSSLWLIVFYALVIGLSYL
SSFGGIGAIAHPWDTFTMAVIALAIYYWGAYTCLPQANFIGDEEE
>CORE_REP|Org33_Gene1456#
MMNTKPQLTLLKAQASYRGDPTTIFHQLCGARPATLLLESAEINSKQNLQSLLVIDSALRITALGHTV
SVQALTANGAALLPLLDEALPPEVRNQARPNGRELTFPAIDAVQDEDARLRSLSVFDALRTLLTLVDS
PADEREAVMLGGLFAYDLVAGFEDLPALRQDQRCPDFCFYLAETLLVLDHQRGTARLQASVFSEQASE
AQRLQQRLEQLQAELQQTPQPIPHQTLENMQLSCNQSDEEYGAVVSGLQEAIRQGEIFQVVPSRRFSL
PCPAPLAAYQTLKDNNPSPYMFFMQDDDFTLFGASPESALKYDAGNRQIEIYPIAGTRPRGRRADGSL
DLDLDSRIELEMRTDHKELAEHLMLVDLARNDLARICQAGSRYVADLTKVDRYSFVMHLVSRVVGTLR
ADLDVLHAYQACMNMGTLSGAPKVRAMQLIAASEGTRRGSYGGAVGYFTATGDLDTCIVIRSAYVEDG
IATVQAGAGVVLDSIPQAEADETRNKARAVLRAIATAHHAKEVF
>CORE_REP|Org35_Gene3082#
MIPDVSQALSWLEAHPQALKGIRRGIERETLRVTPNGTLATTGHPEKLGAALTHHWITTDFAEALLEF
ITPVDDNIDHLLTFLRDIHRYVARNIGDERMWPLSMPCFIEAEQDIELAQFGSSNIGRMKTLYREGLK
NRYGALMQTISGVHYNFSLPLEFWQAWAGVQDAESGKEQISAGYFRLIRNYYRFGWVIPYLFGASPAI
CSSFLKGRETNLPFERTEQGMCYLPYATSLRLSDLGYTNKSQSNLGITFNDLHTYVAGLKRAIATPSE
EFAKLGVKEGDRYLQLNSNVLQIENELYAPIRPKRVTQSGETPSDALLRGGIEYIEVRSLDINPFSPI
GVDAVQARFLDLFLVWCALADAPEMSSDELLCTRKNWNRVILEGRKPGQTIGIGCGASHEPLEKVGKA
LFADLRRVAEVLDGEAGDRQYQQVCDELVAAFDDPELTFSARILKAMKAEGTGRVGLQLAEQYRQTLL
EEPLEILNEAELDKEREASWQRQRDIEANDTLSFEAFLKQNGGS
>CORE_REP|Org3_Gene1399#
MKPEDFRADSKRPFTGAEYLKSLQDSREIYIYGERVKDVTTHPAFRNAAASVGQLYDALHDPASQDRL
CWNTDTGNGGYTHKFFRYARSPEEMRQQRDAIADWSRQSYGWMGRTPDYKAAFGCALGAYPEFYGQFA
DNARHWYKRIQETGLYFNHAIVNPPIDRHKPVNEVKDVYIQVEKETDAGIVVSGAKVVATNSALTHYN
FIGFGSAQVMGDNPDFALMFVAPMDAEGVKLISRASYELVAGATGSPFDYPLSSRFDENDAILIMDHV
LIPWENVLIYRDFDRCRRWSTQGGFARLFPLQACVRLAVKMDFITALLQKSLSCTGVLEFRGVQADLG
EVVAWRNLFWSLSDAMCAEATKWENGAYLPDSAALQTYRVMAPMAYTKVKHIIEKNVTSGLIYLPSSV
RDMNNPEIDKYLARYVRGSDGMDHVERIKILKLMWDAIGSEFGGRHELYEINYAGSQDEIRLQCLRHA
QGSGNMDRMMQMVDKCLADYDQHGWKVPHLRNNDDINQLDNLLK
>CORE_REP|Org43_Gene4940#
MSLAVIYSRAIIGVQAPSVTVEVHISNGLPGLTLVGLPETTVKEARDRVRSALINNGFTFPARRITVN
LAPADLPKEGGRYDLPIALAILAASEQLPLAPLARYEFLGELALSGALRAVRGAIPAALAAADAGRQL
VLSTDNAAEVGLIAQSQSHTAQHLLEVCAFLLGQGELPVAITPPAADNPHENADLRDIIGQEQAKRAL
EIAAAGGHNLLLIGPPGTGKTMLASRLTGLLPPLTEPEALESLAVASLQHPVLTALPWRQRPFRAPHH
SASMAALVGGGSLPRPGEISMAHNGVLFLDELPEFERKVLDALPEFERKVLDALREPLESGEIVISRA
NAKVCFPAKVQLIAAMNPSPTGHYQGMHNRASPQQVLRYLARLSGPFLDRFDLSIEVPLLPPGTLSKR
QTQGESSEQVRERVRQARTRQLERAGKINALLNNREVERDCVLQAADAEFLEVTLNALGLSVRAWQRI
LKVARTLADLAGDAEIGRRHLSEALSYRSMDRLLLQLHRSLE
>CORE_REP|Org9_Gene1572#
MQSSVNKSESRTFFGHPYPLGSLFFTEMWERFSFYGIRPLLILFMAATVYDGGLGLARENASAIVGIF

AGSMYLAALPGGWLADNWLGQRKAVWYGSILIALGHLSIALSAVMGTNLFFIGLMFIVLGSGLFKTCI
SVMVGTLYKKGDARRDGGFSLFYMGINIGSFIAPLISGWLIKSHGWHWGFGIGGIGMLVALVIFRVFA
VPAMKRYDREVGLDSTWNSPVAKKKGVGAWLLALAVGLAAVIVLIAQGTIVINPVEVASVLVYVIAAS
VTLYFIYLFAFAGLSRKERARLLVCFILLISAAFFWSAFEQKPTSFNLFANDYTNRMVGGFEIPAVWF
QSINALFIILLAPVFSWAWPALARNNVRPSSITKFVIGILCAAGGFGLMMLAAQNVLSNGGAGVSPMW
LVGSILMLTLGELCLSPIGLATMTLLAPERMRGQMMGLWFCASALGNLAAGLIGGHVKADQLDMLPDL
FARCSVALLICAAVLIVLIVPIRRMLENTQTKSAQKPATSA
>CORE_REP|Org46_Gene1409#
MLKKITVKAGLIALLSLMTMLLIMVSVIGVNAINEGSRSIHTLNQILGEELGSLANSSNLTLRARTAA
SLAVRQREIGQTDVSDATVGRIYGYLEQSNKEMARFVGVGTVTERGRELSNRLQNSYRAYLDQGVKPM
AAAIKAGKIDEYYHIQETRISALSIAFEKDLSDFRSFAMKLGAQQVYDAESNASTKISLIVVAGLLSV
LLAVLAWFALRVIILRPLDESIAQLEHIAGGDLTHEIRGEGDTEMGRLVRAMQRMQQALASSVSKVRD
ASSQIDTGSRELAAGNLHLAQRTEESAASLEETAASMEQLTSTVKMNAENCEQANQLALSVSDIANQG
SEVVSQVMSKMQAITDSSRRIADIISVMDGIAFQTNILALNAAVEAARAGEQGRGFAVVAGEVRNLAQ
RSAQSAKEIKGLIEASQNRVQEGEQMVESAAQTMSGITGEVGRVTALMREISAATREQSSGIEQVNLA
VAQMDQVAQQNAALVEESAAATRSLEDQAQLLAQSMAAFKL
>CORE_REP|Org24_Gene3686#
MPLKNTATNKPQEIAAIDLGSNSFHMVIARVVNGALQVLGRLKQRVHLADGLDSNNVLSEEAIERGLA
CLALFAERLQGFPADNVTIVGTHTLRQAVNAEVFLKRAAKVIPYPIEIIAGQEEARLIFMGVEHTQPE
KGRKLVIDIGGGSTELVIGEDFEPLLAESRRMGCVSFAQLFFPGGEISKNNFRRARLAAAQKLETLAW
QYRIQGWQYALGASGTIKAAHEVLVAMGEKDGLITLDRLEMLAEQVLQFKSFSSLSLPGLSEDRQSVF
VPGLAILCGVFDALAIRDLRLSDGALREGVLYEMEGRFRHQDIRSRTAKSLADHYNIDREQAKRVLET
TELLYSQWMAQNTKLAHPQLEALLKWAAMLHEVGLSINHSGMHRHSAYILQNSNLPGFNQEQQLLLSA
LVRFHRKAIKLEELPRLNLFKKKHYLPLIQLLRLSTLLNNQRQSTTTPETLRLTTDDNHWTLRFPAGY
LAQNNLVQLDFEREQAYWNDVVGWKLLIEEEGSQNEQRSA
>CORE_REP|Org19_Gene2732#
MDTSLIYGIGGVAIGMLLGWLIASLRVQQANAQHETELRLLEQALQQAQQETAARQEALQRHEQQLRQ
SELELRNLHSQLAAGHEKLQQLNHWRNECELLNQELRAQREVNSAQEAELREVTIRLEETRMAAEEKQ
RLLINSEQRLTTQFENLANRIFEHSGRKVDEQNKQSLDRLLLPLREQLDGFRRQVQDSFGQEARERHT
LTHEIRNLQQLNAQMAREAINLTKALKGDNKTQGNWGEVVLSRVLEASGLREGHEYETQVNVRVDHQS
RMQPDVIVRLPQGKDVVIDAKMSLIAYERYFNGEDEVEREAALSEHIASLRGHIRMLGRKDYQQLPGL
RSLDYVLMFIPVEPAFLLAIDREPELISEALKHNIMLVSPTTLLVALRTITNLWRYEHQSQNAQRIAD
RAAKLYDKMRLFVDDMSALGQSLDKAQGSYRQAMNKLSEGRGNLIGQIEGFRALGVEVKRPINPLLAQ
QAGAQHDEAEEANDDDVAALPQTKDDDDTAGEPGFVSHG
>CORE_REP|Org33_Gene4650#
MFTRVANFCRKVLTRDDKLPRDDAAAGDKNVVREERAAAPSRPAPQPRAADNGSNAPARRSTPRKRPP
FPIAESNDSMTVIPREQHSISRKDISENALKVLYRLNKSGYEAYLVGGGVRDLLLGKKPKDFDITTNA
TPEQVRKLFRNCRLVGRRFRLAHVMFGPEIIEVATFRGHHEQNQESDKNSSQQAQNGMLLRDNIFGSI
EEDAQRRDFTINSLYYGVADFTLRDYVGGLNDLKQGVIRLIGDPETRYREDPVRMLRAVRFAAKLDMR
ISEETAEPIPRLASLLHEIPPARLFEESLKLLQAGYGFQTYLKLCEYQLFQPLFPLIARNFTPNHDTP
MERILAQVLKNTDHRLQNDMRVNPAFLFAAMLWYPLLEHAQKLAQESGLAYYDAFALAMNDVLDEQCR
SLAIPKRITTLVRDIWQLQLRLSRRQGKRAHKLMEHPKFRAAYDLLALRAEVEDNQEMLRLAEWWGEF
QDATPARQKAMLSTLGDDPAPRRARQRRPRRRTPRKEGA
>CORE_REP|Org47_Gene3450#
MSSNKKPMVLVILDGYGHREERQDNAILNAGTPVMDRLWREQPHTLIAASGLDVGLPDGQMGNSEVGH
VNLGAGRIVYQDLTRLDKAIADGDFFANPVLTAAVDKAVAAGKAVHIMGLLSPGGVHSHDEHILAMIK
LAAQRGAKAVYLHAFLDGRDTPPRSAEAPLQRCRDAFAALGVGRIASLIGRYYAMDRDNRWDRVQLAY
DLLTAAKGDAVAEDAIAGLQAAYQRGENDEFVRPTVIRAAGEADAAMQDGDALIFMNFRADRARQITR
AFVNADFDGFPRAKQVQFGDFVMLTEYAADIATACAYPPASLANTFGEWLMKHDKTQLRISETEKYAH
VTFFYNGGVEAPFKGEDRVLVNSPKVATYDLQPEMSAAELTDKLLSAIRSGKYDAIICNYPNGDMVGH
TGVYEAAVKAVETLDACIAQVVDAVRDVDGQLLITADHGNAEQMRDPATGQAHTAHTSLPVPLIYVGK
PARAVEGGKLSDIAPTLLTLMGMEIPQEMTGKPLFIVE
>CORE_REP|Org44_Gene705#
MAVSQPLPSAPCGAEYLRAVLRSPVYEVAQVTPLQAMSKISSRLGNTILVKREDRQPVHSFKLRGAYA

MIAGLDEEQKARGVVTASAGNHAQGVAFSGKRLGIKTLIVMPVSTADIKVDAVRGFGGEVLLHGANFD
EAKAKAIELSQQQGMTFVPPFDHPTVIAGQGTLAMELLQQDAHLDRVFVPVGGGGLAAGVAVLIKQLM
PQIKVIGVEAEDSACLRAALDAGHPVDLARVGLFAEGVAVKRIGDETFRLCREYLDDVITVDSDAICA
AVKDLFEDVRAIAEPSGALALAGLKKYVQQHNIQGERLAHVLSGANLNFHGLRYVSERCELGEQREAL
LAVTIPEQQGSFLKFCQLLGGRSVTEFNYRYADADNACIFVGVRLTRGHAERREIIDELNADGYQVVD
LSDDEMAKLHVRYMVGGRPSKPLRERLYSFEFPESPGALLKFLQTLGTHWNISLFHYRSHGTDFGRVL
AAFELAQSEPEFERHLQALGYDCHDETDNPAFRFFLQG
>CORE_REP|Org12_Gene3056#
MTMKTPPSLDEEPVRLNVPVFFGSAAVILLLGSLVVLFPAASKQWLNVAQSWVADVFGWYYMLLMVAC
MAFVFWLALSRFGHIRLSQDDEPPQFSYPSWVAMLFSSGIGIALVYYGAYEPLDHFLSPPEGSGGSVP
AARQAMALTFLHWGLHGWALYALIATALAYFAYCRGLPLALRSALYPIFGERIHGGVGHLVDSFGILV
TVISMVTNLGIGALLVNSGLFYLFDIPQSTGVLLALIVVMMVVATLAAVTGVEKGIAMLSNINVGFLC
LLLLFVFLAGPTLNLLNGMLQNLGDYLTSLVSRSFDMYLYGKARQWQGAWTLFYWAWWVAWAPFVGLF
IARISKGRTIRELIFGVLLIPLGFTLAWLSIFGNTAISLVLEGGQAILGQVAQSDPPMAVFKLFEYLP
YTQLTAGFVVVISFVLFLTPVDSGTLMIANLSCQGGSAHDDAPAWLRVFWAAVTTLVCAGLLYAGSFS
AMQTAVVLCGLPFSAVIVLYMVSLRKDLRGYAMTPAIP
>CORE_REP|Org42_Gene2826#
MEFLMDPSIWAGLLTLVVLEIVLGIDNLVFIAILADKLPPKQRDKARIIGLSLALLMRLGLLSVISWM
VTLTTPLFSVGDFSFSGRDLILLFGGVFLLFKATMELHERLEGQTHQDGANRGYAKFWAVVVQIVILD
AVFSLDAVITAVGMVNDLPVMMAAVVIAMAVMLLASKPLTNFVNAHPTIVVLCLSFLLMIGLSLIAEG
FGLHIPKGYLYAAIGFSILIELFNQIARRNFIKHQARRPMRERTAEAIMRLMGQQRAQQTDDAVTLPR
DETFAAEERYMISGVLTLASRSLRSVMTPRTDISWVDCERSREEVRAQLLDTPHSLFPVCRDSVDEIV
GVVRAKDLLVALEQGEDIAEFAARTPPIVVPETMDVIKLLAVLRRAKGRLVVVTNEFGVVQGLVTPLD
VLEAIAGEFPDEDETPDIVAEGDGWLVKGGTDLHSLEQALACDDLVSPTADYATLAGLLLAHYGQMPV
AGDSIELNHLRFDIVEVSEYRIELVRVTRVVLDETAHA
>CORE_REP|Org18_Gene4132#
MKIRSQVGMVLNLDKCIGCHTCSVTCKNVWTSREGMEYAWFNNVESKPGVGYPHAWEDQEKWKGGWIR
KINGKLEPRMGNRVGVLAKIFANPDVPALDDYYEPFDYDYQHLHTAKQGKHQPVARPRSLITGQRMNK
IESGPNWEEILGGEFEKRSQDKNFDNLQKAMYGQFENTFMMYLPRLCEHCLNPACVATCPSGAIYKRG
EDGIVLIDQDKCRGWRMCLTGCPYKKIYFNWKSGKSEKCIFCYPRIEAGQPTVCSETCVGRIRYLGVL
LYDADRIEQAAAVENDKDLYQSQLDIFLDPHDPKVIAQALADGVPQGVIEAAQQSPVYKMAMDWKLAL
PLHPEYRTLPMVWYVPPLSPIQSAADAGELAHSGVLPDVESLRIPVQYLANLLTAGDTEPVLLALKRM
LAMRHYKRAETVDGVVDTSALEQVGLSEAQAREMYRYLAIANYEDRFVVPSSHRELAREAFPESKGCG
FSFGDGCHGSDGKFNLFNSRRIDAIDVTAKTARPEDAS
>CORE_REP|Org43_Gene3942#
MQLNSTEISELIKQRIAQFNVVSEAHNEGTIVSVSDGIIRVHGLAEVMQGEMIALPGNRYAIALNLER
DSVGAVVMGPYADLAEGMKVKCTGRILEVPVGRGLLGRVVNTLGAPIDGKGPIENDGFSPVEAIAPGV
IERQSVDQPVQTGYKSVDAMIPIGRGQRELVIGDRQTGKTALAIDAIINQRDSGIKCVYVAIGQKAST
IANVVRKLEEHGALANTIVVVATASESAALQYLAPYAGCAMGEYFRDRGEDALIVYDDLSKQAVAYRQ
ISLLLRRPPGREAYPGDVFYLHSRLLERAARVNAEYVEAFTKGEVKGKTGSLTALPIIETQAGDVSAF
VPTNVISITDGQIFLESNLFNSGIRPAVNPGISVSRVGGAAQTKIMKKLSGGIRTALAQYRELAAFSQ
FASDLDDATRKQLSHGQKVTELLKQKQYAPMSVAQQSLVLFAAERGYLNDVEVAKVVSFEAALVAYAD
REHAELLNHINQTGNFNDEIEGKLKDILETFKKTQSW
>CORE_REP|Org27_Gene3371#
MIKIVVYLILAVTIAIIAARVLFRLPDISQRLPQAALPADPAAQLPARAAELMAAHPGLSGVVPLASG
HDAFASRLALARMAERSIDAQYYIWHNDTSGQILLKTLYDAAQRGVRVRLLLDDNGVAMDETLAALNA
QENVEIRLFNPSTVRTPKLAGYAFDFMRMNRRMHNKSYIVDGAVAIIGGRNIGDEYFQVGDENYFLDL
DVLSVGSVVAETAEVFDRYWNSASVFGVEQIIRGKGNLSAFLTQATATESSERARKLAVQLETSAVRF
RDGAVQPEFTQVELVADDPAKGLGKASRDRLMVTQLGKIIGGVGRQLDLVSAYFVPGREGASFFESLA
KQGKSIRVLTNAMNTTDVLVVHAGYAKYRRELLQAGVELFELKLRAGQPTGRKELKPLGLSGAALHAK
TFAIDDKRVFIGSFNFDPRSAHLNCEMGFLIDSPTLAADTRQLFDGPLEYAAYRPVLTPEGKMVWKEA
FEDGHTEVHQQEPGAGWVKRIILTVAGWLPIEWML
>CORE_REP|Org22_Gene3723#
MKHTFKRNALLAAVLLAAGTGPVWAAKDAVIAVASNFTTLDPYDANDTLSQAVAKSFYQGLFGFDKDM

KLVNVLADSYEVSKDGLTYTVKLRQGIKFHDGTAFNAEAVKINLDRASNPDSHLKRYNLFKMIDKTEA
VDADTVKIVLKAPFSAFVNNLAHPAAAIISPAALKQYGKEIGFHPVGTGPYQFVTWNQTDFVKVKKFD
GYWQPGLPKLDSITWRPVVDNNTRAAMLQTGEATFAFPIPYEQAKVLEGNAKLDVVAAPSILQRYISM
NVTQKPFDNPKIRQALNYAINKDALIKVAFAGYAVPAEGPVPPAIDFAARYKPWPYDPAKARELLKEA
GYPNGFTTTLWSSHNHSTAQKVLQFTQQQLAQVGVKVTVTAMDAGQRAAQVESVGVQDTGVRLFYTGW
SASTGEADWALSPLFSTQAAPPKQFNTAFYSNPQVDKDLTDALATTDRAEKQKLYQDAQDRIWADAPW
IFLATERLLSANSKQLSGFYVMPDTSFNFDNADLK
>CORE_REP|Org28_Gene2160#
MNLLKSLAAVSSMTMFSRVLGFARDAIVARVFGAGMATDAFFVAFKLPNLLRRIFAEGAFSQAFVPIL
AEYKSQQGEEATRTFIAYVSGLLTLVLAVVTVLGMLAAPWVIYITAPGFTDTPDKFALTSALLRITFP
YILLISLASLVGAILNTWNRFSIPAFAPTLLNVSMIGFALFAAPYFNPPVLALAWAVVVGGVLQLGYQ
LPHLRKIGMLVLPRLKLGDAGVWRVMRQMGPAILGVSVSQISLIINTIFASFLVSGSVSWMYYADRLM
EFPSGVLGVALGTILLPSLAKSFSSGNHDEYSRLMDWGLRLCFLLALPSAIALGILAKPLTVSLFQYG
KFSAFDAAMTQRALVAYSVGLMGLIVVKVLAPGFYSRQDIKTPVKIAIITLIMTQVMNLAFIGPLKHA
GLALSIGLAACLNASLLYWQLRKQKIFQPQPGWALFLTKLVIAVLVMSAVLIGVMWLMPAWDQGNMLE
RLLRLAAVVVAGVVAYFGVLAGLGFRPRDFARRVA
>CORE_REP|Org17_Gene1770#
MTTSTHDKVKDDKRLSDGPDWTFELLQVYLEQIDRVAKHYRLDTYPHQIEVITSEQMMDAYSSVGMPI
NYTHWSFGKKFIETEQRYKQGQQGLAYEIVINSNPCIAYLMEENTITMQALVMAHACYGHNSFFKNNY
LFRSWTDASSIVDYLLFARHYISQCEERYGVEEVERLLDSCHALMNYGVDRYKRPQKISLVEEKARQK
SREEYLQSQVNTLWKTLPRVEREESPEQARRYPSEPQENILYFIEKNAPLLEPWQREVLRIVRKVSQY
FYPQKQTQVMNEGWATFWHYTILNHLYDEGRVTERFMLEFLHSHTNVVYQPPYNSPYYNGINPYALGF
AMFQDIKRICQSPTEEDRYWFPDIAGKDWLDTLHFAMRDFKDESFISQFLSPKVMRDFRLFTVLDDDR
NNYLEIAAIHNEAGYRAIRQELSAQYNLSNHEPNIQIWNVDLRGDRSLTLRYIPQERAPLDKSRRDVL
KHLHRLWGFDVILEQQNEDGSVELLDRCPPRPTPL
>CORE_REP|Org22_Gene1710#
MKKSLVAVSVIVILGAAWTGASWYTGKQIEQHMGEVVDNANGQLKAYLPKAGVKLSYENYQRGLFSSK
VRYVLRSDGTDTSENAALKTGEEVAFLETIDHGPFPFAQLKKFNLLPSMASVHTELENTPAVKGLFDT
TKGKSLFTADSRISYSGDTSSAIDIIPLDYQKDKSSLKFSGATINADVSSDLKKFVLDANSDNIVFAS
PNEFGQNEQITFQGFNLKGNSNESKFGVKLGDQTMTLKQFKLTIDGKDTVALDGFNLVSKFGEQGSSN
IGGQIDYTMDALKVQGNDFGAGKLALKIDNVDGKALKDFADSYNRQTMALLQQGENLDPAVYEQQNAE
LLQKNLPLLLKGNPSLSIAPLSWKNSKGESTFTLDLALTDPSKAASPAQSPDQLIAQAVKKLDINLTI
PEAMATEVTAKTALLQGYNEEDAQKLAQQQVQGLAAMGQMFKLTTQKDGVIASTFHYADNQVDLNGNK
MSLQEFIGQFGLLGAPAEDGEPAQDAQPAPAPAQ
>CORE_REP|Org46_Gene4825#
MFRLTLATRLALSISLLTGWTATAGAADNAQLTQREDPYFLQAQAQLQQRLKQTPNTNRAKNVILVVG
DGMGFSTVTAARIFEGQQRGVDGESNVLAWEAFPYLAAAKTYSADAQITDSAPSAVAMTTGVKTINDL
MGLNHTATLNNCEDQQTNTVTTLWEMAATLGMATGAVTTATITHATPGATYAHIANRDWESDAKMPAA
ALAAGCRDIARQLVEMKYGNGLNVAMGGGRTNFLPDSVSDPEYPGKKGARKDGRDLTQAWLQRYGERG
QYVWNQAQFDKIEPGKVDHLLALFEPSHMNFEHDRRQDAAGEPSLAEMTGKAIDILARNPEGYLLLVE
GGRIDHGSHNGNAYRTLSDAVALNEAVKTIVDKVNLDETLVIVTGDHSHTLTIAGYAKRGNPILGISV
GVDGKPRLGSDGKPYTTVGFANGPGGTIPLTERPTLTMEQATAPDFIQPALVPLKSETHGGEDLGIYA
IGPWAHLFQGTVEENYTFHVMNYASRIGERLSER
>CORE_REP|Org38_Gene3633#
MAKASLLERQWVRALLALLSGAGGTLAFSPYDFWPAAIVSLFGLLAVTLNRTTKQSALLGFVWGFGLF
GSGINWVYVSIADFGGMPFAVNVFLVALLAAYLSLYTGLFAGLLTRLWPATRWWRLAIAAPALWQVTE
FLRGWVLTGFPWLQFGYSQINGPLKGIAPLLGVDAITFVLMAIAGLLVYAVNQRRLSAAVIAAALLLL
PWPLRQLQWFTPQTDKAVNVAMVQGNIAQSMKWDPKALVSTLQTYLDETRPYMGKAPIVIWPESAIPD
YEANQNGFLTMMDDLMRAKNSSLITGIVDVRATPQGQQIYNSAIVLGEPTPYRYPANDRYNKHHLVPF
GEFVPLETLLRPLAPFFDLPMSSFSRGDYVQPQLSVRGYNLTAAICYEIVLGQQVRDNFRPDTNFLLT
ISNDAWFGHSIGPWQHFQMARMRALELGRPLLRSTNNGVTAAVDANGDVIAEIPQFTRQVLEVKVTPT
TGVTPYARFGAAPLWVITLLLGGWALMLGLRRK
>CORE_REP|Org22_Gene1037#
MLLPWLILIPFIGGLLCWQLERFGTKVPRWIALIAMGLTLALSLQLWMQGGYTLTTPKGIPQWQSEFL

LPWIPRFGISIHLALDGLSLLMVVLTGLLGVLAILCSWREIQKYQGFFHLNLLWILGGVIGVFLAIDM
FLFFFFWEMMLVPMYFLIALWGHKASDGKTRITAATKFFIYTQASGLVMLIAILGLVFVHYNATGVWT
FDYEDLLQTPMSHNVQYLLMLGFFIAFAVKMPVVPLHGWLPDAHSQAPTAGSVDLAGILLKTAAYGLL
RFSLPLFPEASHEFAPIAMWLGVIGIFYGAWMAFAQTDIKRLIAYTSVSHMGFVLIAIYTGSQLAYQG
AVIQMIAHGLSAAGMFIICGQLYERLHTRDMRQMGGLWGRIKFIPALSLFFAVATLGMPGTGNFVGEF
MILFGSYQVVPVITVISTFGLVFASVYSLIMMQRAYYGAPKSDQPLQGMTARELFIILLLVVLLVLLG
VYPQPILDTSNAAMSNVQHWFGSSVSAISTTRP
>CORE_REP|Org46_Gene2457#
MSEPRIIAKAMKDGKPEQDLVILPALANRHGLITGATGTGKTVTLQKMAEQFSRIGVPVFLADVKGDL
SGIGAEGVPSEKLQARLAAIGVSDWQPQACTIIPWDIYGEKGHPIRATISDLGPLLLGRLLDLNEVQS
GVLQLVFKIADDNALLLLDMKDLRTMLQFVGDNAKQFQTQYGNISAASIGAIQRGLLTLEEQGADQFF
GEPMLDINDLMKTDANGHGVINLLAADKLINQPKLYSVFLLWLLAELFEHLPEVGDPEQPKLVFFFDE
AHLLFNDAPAALLTKIEQVVRLIRSKGVGIYFVTQNPLDIPDSVLGQLGNRVQHALRAFTPRDQKAVK
TAAQTLRANPAFDAETAITELGVGEALVSFLDEKGRPNVVERAMVIAPESKMGMLGAEGLNSAINKSP
LYGRYEDMIDRESAYEKLSSGGFATVGTPQQGQPQAQQQQQQAGGGLMDGLNELLFGSTGPRGGKRD
GIVQTAAKSMARDLGRQILRGVLGSITGGRKK
>CORE_REP|Org28_Gene971#
MRARQRRYSAKQQATAVAEVETQETENELDGLLKRSFRPRTNEASEAVRRAIGTLSEYANQGKVKVSQ
DVVLTIESLIAQIDEQLSQQMNNILHHKEFQKLESAWQGLSYLVDNTNVSETLKIRVLNISQDELTRN
LRRYRGSAWDQSPVFKQIYEQEYGQFGGEPFGCIIGDFEFDHSPMSVTLLTELAKISAASHCPFISAA
SPSLLQMSKWNELGNPRDIGKIFTTPEYASWRRLRESNDSRYLVLTMPRFLSRLPYGAKTNPIEEFAF
EEAVRPDMDDDFSWANSAYAMGVNINRAFHEYGWCSKIRGIESGGSVEELPAYAFPSDEGGYELTCPT
EVAISDRREQELSDAGFLPLVYRKHSDFAAFIGSCTMHAPAKYEDPDATANAKLSSRLPYIFATCRFA
HYLKCIVRDKIGSFRSRDDMQLWLNDWLMNYVDGDPSVSTEATKARRPLAAAEVRVEDVEDDPGYYRA
HFYLRPHYQLEGMTVSLRLVSKLPSAKKDGSR
>CORE_REP|Org25_Gene3146#
MDTWMYLTQGFEVALVPQNLIIALIGCFVGTIVGLLPGLGPINGVAILLPLAFALKLPAESALILLAT
VYIGCEYGGRISSILLNVPGDAAAIMTALDGYPMAQQGRAGVALSISAVSSFVGSMIAIGGIILFAPL
LARWSLAFGPAEYFALMVFAIACLGSMMSQNPLKSLLAALIGLGLATVGVDANTGVYRFTFDSVHLSD
GVQFIVVVIGLFSVSEILLMLESTSAGQKLVRKTGRLLFNRKEAAQCAGPTLRSSVIGFFVGILPGAG
ATIASALTYMTEKKISGNSDTFGKGDIRGVAAPEAANNASACGSFIPMLTLGVPGSGTTAVMMGALTL
YNITPGPAMFTEQPDIVWGLIAALLIANVILLIMNLPLVGLFTRMLTIPLWFLVPAIAAVSAVGVYAV
HSTTFDLLLMVGLGVFGYILRKMHFPMSPLILGFVLGEMLEQNLRRALSISNGEFGILWSSSIAQTLL
VLAVAVLALPPLLRLMRKRRQPAAEAKAKAE
>CORE_REP|Org15_Gene4380#
MPSPLAAIMISMKYRDLRDFLSLLEKRGELKRISQPIDPYLEMTEIADRTLRAGGPALLFENPKGYDM
PVLCNLFGTANRVAMGMGQEDISALREVGKLLAFLKEPEPPKGFRDLFDKIPKFKQVLNMPTKVLGSA
PCQEQVWQGDDVDLGRIPVMHCWPEDAAPLITWGLTVTRGPHKERQNLGIYRQQVLGKNKVIMRWLSH
RGGALDYQEWCQAHPGERFPVAVALGADPATILGAVTPVPDTLSEYAFAGLLRGNKTEVVKCLSNDLE
VPASAEIVLEGYIEPGEMAPEGPYGDHTGYYNEIDQFPVFTVTHITQRRNAIYHSTYTGRPPDEPAIL
GVALNEVFVPILQKQFPEIVDFYLPPEGCSYRLAVVTMKKQYAGHAKRVMMGVWSFLRQFMYTKFVIV
CDHDVNARDWNDVIWAITTRMDPARDTVLVENTPIDYLDFASPVSGLGSKMGLDATNKWPGETDREWG
RPIQMDEKVRARVDEIWDELAIFSDREPTL
>CORE_REP|Org26_Gene2774#
MSGIDFKQKISFQRPFSKPIEAEEEYDIVRQFESDRGRIVNSAAIRRLQQKTQVFPLERNAAVRSRLT
HSMEVQQVGRHIAKEILNRFKQAGRIDELGLTRLLDPFESIVEMACLMHDIGNPPFGHFGESAINDWF
AQRLDPASCGSEPGSHDRCQVATLRLHEGESDLNRLRSRIRQDLSHFEGNAQAIRMVYSLLKLNLTYA
QVGCILKYTRPAYWASDIPASHSYLMKKPGYYLAEEAFVDRLRRELHMGEFDRFPLTYIMEAADDISY
CVADLEDAVEKSIFTVEQLYQHLIQEWGEVAPGDLFDKTVASAFRKIDRGGARRSAEDQFFMYLRVFT
VARLVPHAAQRFIDNLDAVYQGSFNQALLEDSSPAYRLLKIFKNVAFKHVFNHPEVEQLELQGYRVIS
GLLDIYSPLLAMSLADFSQLVREDSHRAYPIETRLFHKLSTKHRLAYAEAVEGLQHLSPEQQAIREYY
FRARLLQDYISGMTDLYAYDEYRRLMAAE
>CORE_REP|Org48_Gene195#
MSEQQVQGADQALDLNNELQSRREKLAGLRENGIAFPNDFRRDSTSDKLHAAYGDKDNEELEALGVEV

TVAGRMMTRRIMGKASFVTLQDVGGRIQLYVARDDLAEGVYNEQFKKWDLGDILGARGKLFKTKTGEL
SIHCTELRLLTKALRPLPDKFHGLADQETRYRQRYLDLIANEESRNTFKVRSQVMSAIRNFMVERGFM
EVETPMMQVIPGGASARPFITHHNALDIDMYLRIAPELYLKRLVVGGFERVFEINRNFRNEGVSPRHN
PEFTMMELYMAYADYKDLIELTETLFRTLTEKVLGTSQVQYGDEVFDFGKPFEKLTMTEAIKKYRPET
DLADLADMGKAVAIAESIGIKVEKSWGLGRVVTEIFEEVAESHLIQPTFITEYPAEVSPLARRNDVNP
EITDRFEFFIGGREIGNGFSELNDAEDQAQRFADQVNAKDAGDDEAMFYDEDYVTALEHGLPPTAGLG
IGIDRMVMLFTNSHTIRDVILFPAMRPQK
>CORE_REP|Org36_Gene2733#
MAISGNTATQGAPLIALQQLSMTFGGQRALNAISLALMPGEVHCLAGTNGCGKSTLIKAIAGVYQPDD
GSRITIDGQTFGRLSPDQARAFGIQVIYQDLSLFPNLTVAENIAFEHNLHGLLGWYRPARLRRTAERL
LQELSFHLDLDKRVAELPIAQRQQVAICRALVAEARLVIMDEPTASLTRTEVNQLLRTVDYLKAKGIC
VVFVSHRLDEVLEISDRVTVIRDGNKIGTWPAAEITGDRLTELMTGLKLDYRLKSPSMNKDRVMLEAD
RLSRTGQYQDVSFRLHQGEVLGLCGLLGSGRTELALSLFGMTRPDSGKLYLDSKPVRFRGHEDAIKAG
IGYVSEDRLTLGLVQQQSVADNAVLTILDKLRGRFRLIDDYRKNRIVAEWIAKLGVRVADPEQAVSTL
SGGNQQKIVLAKWVLTQPRILILDSPTVGVDVGAKASIYQLIHLLAQEGIAILLISDEVPEVYYNCDR
VLHFSGGSVIGEYLPGQVSQQQLAEAVNA
>CORE_REP|Org25_Gene2809#
MKKLLPLLIGLSLGGFSAMSQAENLLQVYKQARESNPDLRKSAADRDAAFEKINEARSPLLPQLGLTA
GYDYTNGYRDSNGVNSNVTSGSLALTQTLFDMSKWRQLTLQEKSAGISDVTFQTAEQSLILNTATAYF
NVLKAIDTLSYTQAQKDAVYRTLDQTTQRFNVGLVAITDVQNARSNYDTVLAAEVSARNDLDNALETL
RQVTGAFYPELASLNTDRFSTQRPEAVNNLLKEAEARNLSLLSARLSQDLAREQIRAAQTGYMPTIDV
SASTGISNTKYNGSNTGGANAARYSDSDAGQNKVGISFNLPLYSGGATNSQVKQAQYGFVGASEQLES
AHRSVVQTVRSSFNNVNASISSINAYKQAVISAQSSLDAMEAGYQVGTRTIVDVLDATTTLYNAKRQL
SDARYTYLINQLNIKSALGTLNQNDLLLLNGALGKPVSTAPDAVAPQNRAQDAYADGYQDNAPMQQTA
APAPAATRASAPAVTTSQPARHSGNPFRN
>CORE_REP|Org29_Gene3810#
MSSQSKTFAQWRQQAAGLQIEGRAYIAGRYQDSTDGATFVDASPIDGRPLANIADCDEATVNHAVAAA
EQAFKAGDWARRTPAQRKATLLRLADLVAQHQEELALLESLDTGKTIRESLDMDMQDVQTAIRYYAEA
IDKVSGEVAPTGDAFHGMITLVPLGVVAVMTPWNNPLMIACWKIAPALAIGNSVVFKPSEKAPLTGIR
LAELTRMAGIPDGVFNVVTGGAQVGKTLALHPRVRAMAFTGSTQVAKQLLIYAGQSNMKRTFLEGGGK
NAHIIFADTPDLARAARFAALGFCANQGAVCASGTRLLVQSSIKDRFLTLLLDELKKWQPGHPLDPAT
AMGPLIDAQHQANVLRYIESARAEGASIISGGQADNNLLPGGHVLPPTLIDNATPEMTASQNEIFGPV
ASLMTFEDEEQAIRLANDSEYGLTVGFWTPDVAKVHRVARQLEAGTVWVNHFLTRDILSPFGGFKQSG
IGRDLSLHALPQYGEMKATWIALQDVDAH
>CORE_REP|Org23_Gene1416#
MSTANNQHPESVSLNAFKQPKAFYLIFSIELWERFGYYGLQGIMAVYLVKMLGMSEADSITLFSSFSA
LVYGFVAIGGWLGDKVLGAKRVIVLGALVLAAGYAMVAYSGHDIFWVYMGMATIAVGSGLFKANPSSL
LSTCYEKDDPRLDGAFTMYYMSVNIGSFFSMLATPWLAAKYGWSVAFSLSVVGMLITLVNFMMCHKWV
KENGSKPDFKPLHLPKLLMVLVGIVALIAVSSWLLHNQVIARWALALVSAGIVLVFAKETFALHGAAR
RKMIVAFLLMLEAVVFFVLYSQMPTSLNFFAIHNVEHSIFGIAFEPEQYQALNPFWIMLASPILAALY
NKMGDRLPMPHKFAFGMILCSCAFLVLPWGASFANEQGIVSVNWLILSYALQSIGELMISGLGLAMVA
QLVPQRLMGFIMGSWFLTTAAAALIAGKVAGLTAVPSDINDAHASLAIYSHVFMQIGIVTAVIAILMM
LTAPKLYRMTLDTTEDANQKAQEATAAH
>CORE_REP|Org43_Gene4105#
MTGHSEKAYADSLPHSVWPADWIRRSEPAAAASLGLSLYDLMLRAGQAAYVLARDTYPSCNHWLVLCG
HGNNGGDGYVVARLAKAAGVQVTVIACEGARPLPPEAAAAREAWLAAGGDILPANSRWPASVSLIIDG
LLGTGLGSAPRAPYDALIEAANRHSAPVVALDIPSGLLAESGAAPGAVVRAAHTVTFIGLKPGLLTGQ
ARDWVGQLHQNTLGLSDWLAKQPEQIQRLTAEALPRWLHPRRPCAHKGEHGRLLLVGGDRGFGGAIRM
AAEAALRSGAGLVRVLTHIEHVAPLLAARPELMAQALDETTLRQAMQWADVLVIGPGLGQAEWGKNAL
NVLQTSDKPALWDADALNLLALNPEKRQNRVMTPHPGEAARLLSCSTADIESDRLLAVRNLTARYGGV
AVLKGAGTLIADEQGQMAIADVGNAGMASGGMGDVLSGIIGGLLAQKLSLYDAACAGCVVHGAAGDRV
AERQGTRGMLATDLLPDILHFVNPEWAK
>CORE_REP|Org43_Gene333#
MEFSVKSGSPEKQRSACIVVGVFEPRRLSPIAEQLDKISDGYISALLRRGELEGKVGQTLLLHHVPNI

LSERILLIGCGKERELDERQYKQVIQKTINTLNDTGSMEAVCFLTELHVKGRNTYWKVRQAVETAKET
LYTFDQLKSNKVEPRRPLRKMVFNVPTRRELTSGERAIQHGLAVASGIKAAKDLGNMPPNICNAGYLA
SQARQLADAFSTNITTRVIGEQQMKELGMNAYLAVGAGSQNESLMSVMEYKGNPNPDAKPIVLVGKGL
TFDSGGISIKPADGMDEMKYDMCGAATVYGVMRVVAELNLPLNVIGVLAGCENMPGGRAYRPGDVLTT
MSGQTVEVLNTDAEGRLVLCDTLTYVERFEPELVIDIATLTGACVIALGHHITGLMSNHNPLAHELLG
ASEQAGDRAWRLPMADEYYEQLDSNFADMANIGGRPGGAITAACFLSRFTRKYTWAHLDIAGTAWRSG
KAKGATGRPVALLSQFLLNRAGLNGDD
>CORE_REP|Org37_Gene3954#
MSTATPSRLEMRNISIAFAGFNALQDVDFTLQGGSIHALVGANGAGKSTLMAILSGAHDHYRGEILID
GQAVAIHSPLQARRHGIHVVQQEVDVALIPTLSVAENIMLDWLNEPGHWLNWAELHRRAAQLLQQWAL
PLNPRRRLADCTLAEKQQVLLARALSHRCRFLVLDEPTAPLDRAESERLFNVVRRLQSEGIGIVFISH
RIHELSDICDRLTVLRDGRRVSEDPMRGLSGEQIVEKMLGHRLDDIFPPPRPPHAKRTLLQVQGLRDR
HKLRDVSLRLHEGEILGIAGLAGAGKTELCKALFGASAVQLERGELRGQPWAPRAPHLSVEQGLALVP
EERRKEGIFIDEAIPMNLSVSADDSFSRWSLFSRRQELRWAREIMQRLNIRASGPQQRLARLSGGNQQ
KVAIGKWLRGDAEVLIFDEPTKGVDIKAKQELFGLIDGLARAGKGVIYASGEFAELVGLCDRICVLWD
GRIVAELNAADIDEETLLLYSTGGTPA
>CORE_REP|Org39_Gene3471#
MTITHWINGEPAAGGERSQPVYDPATGQSAQEVQLADRATVERAIAAAEQAYPAWRDTPPLKRARIMM
KLKDLLEQHADAICQLITAEHGKVLSDALGELQRGIENIEYAGYVPELLKGEHSKEAGPGIDSWSEFQ
PLGVVAGITPFNFPAMVPLWMWPMAVACGNTFVLKPSERVPSAALYIARLAAEAGLPPGVLNVVNGDR
EAVETLLHDGRVKAISFVGSTPVAEHIYHTGCGQNKRVQALGGAKNHAVVLPDADIPGAVGALMGAAF
GSCGQRCMAIPLLVAVGDGTAEALIAGLRQQMAAMRVGPGSDNRNDMGPLVTQQHYQKVKGYIDQGVA
EGAELLVDGRELSVIGADGRPSQGYFLGPTLFDRVTPGMRIYQEEIFGPVLGVVRAASLPEAMALIDA
HEYGNGTCLFTRDGEAARYFSSRIQVGMVGINVALPVPVAYHSFGGWKRSLFGDLHAYGPDAVRFYTK
RKTVTQRWPASGDARRASFSFPSGQG
>CORE_REP|Org31_Gene1519#
MADSSREFLLEMTDICKSFPGVKALDNVNLRVRPHSIHALMGENGAGKSTLLKCLFGIYKKDSGSIVF
QGREIDFKSSKEALEHGVSMVHQELNLVLQRTVMDNMWLGRYPTKGLFVDQEKMLKDTQAIFDELDID
INPREKVGNLSVSQMQMIEIAKAFSYDAKIVIMDEPTSSLTEKEVNHLFTIIRKLKERGCGIVYISHK
MEEIFQLCDEITVLRDGQWIATQPLEGLDMDKIIAMMVGRSLSQRFPDRQNTPGEVILEVKNLTSLRQ
PSIRDVSFDLHQGEILGIAGLVGAKRTDIVETLFGIREKVAGTIKLHGKAIDNHSANEAINHGFALVT
EERRSTGIYAYLDVGFNSLISNIRNYKNKLGLLDNARMKSDTQWVIDAMRVKTPGHHTHIGSLSGGNQ
QKVIIGRWLLTQPEILMLDEPTRGIDVGAKFEIYQLMTELAKKGKGIIIVSSEMPELLGITDRILVMS
NGQVAGIVNTKQTSQNEILRLASLHL
>CORE_REP|Org16_Gene2622#
METKDLIVIGGGINGAGIAADAAGRGLSVLLLEAQDLACATSSASSKLIHGGLRYLEHYEFRLVSEAL
AEREVLLKLAPHIAFPMRFRLPHQPHLRPAWMIRIGLFLYDHLGKRTSLPGSKGLRFGPESVLKPELK
RGFEYSDCWVDDARLVVLNAQEVEKRGGEVRTRTKVTRAWRENGLWMVEAVDIDSGKTFTWRAKGLVN
ATGPWVKNFFDDGLKLKSPYGIRLIKGSHIVVPRVHDQPQSYILQNEDHRIVFVIPWNDEFSIIGTTD
VEYKGDPKDVKIDENEIAYLLKVYNDHFKKQLGRDDIVWTYSGVRPLCDDESDSPQAITRDYTLDVHD
EQGKAPLLSVFGGKLTTYRKLAEHAMEKLAHYYPGCGPAWTKNATLPGGDIDGDRDGYAAKLRRAHGW
LPEALARRYARTYGSQSELILAGANGLADLGEDFGHGLYEAELRYLTEKEWVVELADAIWRRTKLGMW
LDEAQQARVKAWLAEHAKTKALSLAS
>CORE_REP|Org44_Gene2139#
MTVEKKYIVALDQGTTSSRAVVLDHDANIVAVSQREFPQIYPKAGWVEHDPMEIWASQSSTLVEVLAK
ADISSDQIAGIGITNQRETTIVWEKETGKPIYNAIVWQCRRTADICEKLKRDGLEEYIRHNTGLVVDP
YFSGTKVKWILDNVEGARERAKRGELLFGTVDTWLVWKMTQGRVHVTDYTNASRTMMFNIHELDWDER
MLEVLDIPRAMLPKVRPSSEVYGQTNIGGKGGTRIPIAGIAGDQQAALYGQLCVQPGMAKNTYGTGCF
LLMNTGKEAVRSKNGLLTTIACGPRGEVNYALEGAVFIGGASIQWLRDELKLISDAADSEYFATKVKD
SNGVYVVPAFTGLGAPYWDPYARGAIFGLTRGANSNHIIRATLESIAFQTRDVLDAMQADANTRLQSL
RVDGGAVANNFLMQFQSDILGTRVERPEVRESTALGAAFLAGLAIGYWNDLDEVKSKAVIEREFRPSI
ETTERNYRYGGWKKAVARAQAWEDHE
>CORE_REP|Org8_Gene3095#
MKNLTLAQRLTLIFALLIVIGCAFSGWMQVRSSTQYSQAVIQRLSGNLAQHIADSNPLLGVNGPDPQA

VHTLFDQLMAVNPSVEVYLLDKQGAIIGNAAPAGHLKRQRVALAPLQALLDGAQMPVYGDDPRSADGR
KVFSVAPLKVDGRVEGYLYVVLLGEEYTALASNAQFNSAVRMALWTSGVMVLFSLLAGGFAFYWVTRP
IRRLTRQVNALDSGGIEAVQAYAALPAAPAGRDEVSQLQQAFHRMAQRLAEQWQTLAQQDRLRREFIA
NVSHDLRTPLTSLHGYLETLSVKAATLSDTERRRYLEIALAQSRKVGKLAQELFELARLEYGVVKPQK
EPFSLSELLQDVFQKFELAAEARNQRLHADIAPGIPPVFADLSMIERVLTNLLDNAIRHTPPGGDIGV
RLWRQEGRVMVQVSDSGPGIPQTLRADLFVRPSILSGARRPAGGLGLMIVRRILQLHDSDIQLIEQPQ
SGACFRFAIPPRESGTTVIARAGTAG
>CORE_REP|Org34_Gene3741#
MRSLSGDLLLQRLGEQPDDKLHKRLYNAIRTSILDGSLPPSSRLPASRDLAQELSLSRNTVLTVYEQL
LAEGYVSARAGSGTFVADTVPDSCLSTGGAPADGSGQPRRVELSERGATLLHHASASPKQWGAFIPGV
PDVNAFPHQLFSKIQARLSRRPAPQRLTYSNQGGSPELQHALVEYLRVARSVRCSPEQILITEGIHQA
IDLVTRMLCNPGDDAWIEEPGYWGIRNILRINALNICPLAVDEAGLVPPEQPTTPPRLIFVTPSHQYP
LGSVMSLARRQRLLALARNAGGWIVEDDYDSEFRFSGQPIPALQGLEADAPVIYIGTFSKTLYPALRL
GYVVLPPPLVQPLKTAHAELYRGGHLLIQSALAEFIQEGHYTAHIRRMRLLYARRRAFLTGLIEQHLG
KQALSEFNSNAGLHLILNLPDEADDVAIAAAAGAQGVLVRPLSRYYMLPNRRRGLLMGFACVPEEQMA
AAFTQLLACINVPAGGSLRSPTHYQA
>CORE_REP|Org26_Gene3602#
MNRQQRLMTFANPADLPGYQDVSKANLQARKPRDLKLCDSLQEAVRRSGLQDGMTISFHHAFRGGDLT
LNQVMETLAAMGFRNLTLASSSLTDCHAPLVEHIRHGVVSRIYTSGLRGPLADAVSRGLLAEPVQIHS
HGGRVNLIESGELRIDVAFLGVPACDEFGNANGYSGEACCGSLGYARVDAEAAGTVVLLTEQLVPYPH
HPASLAQDRVDLIVQLERVGDADKIGADATRMTSNPRELLIARRAAEVIAGSGYFTEGFSLQTGTGGA
SLAVTRFLEDKMRARGIRAAFALGGITSTMVDLHEKGLIGKLLDVQSFDRAAATSLARNPRHIEISAN
QYANFSSKGASVDRLDVVVLSALEIDTGFNVNVLTGSDGVLRGASGGHCDTAAAARLAIIVAPLVRGR
IPTLVEQVTTCVTPGSSIDILVTDHGIAVNPARPELAQRLREAGLEVVSIDWLRARALQLTGEPQPIA
FTDKVVAVVRYRDGSVIDVVHQVAE
>CORE_REP|Org7_Gene4589#
MQGTRIHLIVGGLLLAAASGSVQAEALQPDPAWQQGALANGFSWQILDTPQRPSDRVELRLIVNTGSL
VESSQQTGFAHLLPRLALTRSESFTAPQLRALWQQSVDNERPLPPAIISYDFTMYNLSLPNNRPDLLK
EALAWLADTTGKLAIDEHTVHAVMNSSINPVGTFPPNTKDAWWRYRLKGSTLLAHDPAQPAKRPVNLE
QLKKFYQQWYTPDAMTLFVVGKVDSRSLGEQINKAFSPLQGKRETPAPMPTLTPLPQQPVSLISEQVK
QDTLSIMWDSPWHPIRDSQSLLRYWRGDLAREALFWHLQQTLEKSDQKNLHLGFDCRVQYQRGQCAIH
LDTPNNNLNGSLGFIARELANVRDNGLSKEEFNALLAQKNDQLSKLFATYARTDTDVLMSQRLRSQQS
GVVDIAPELYQKLRQEFLSSLTLETLNQALKLQLSQDATLVLMQPKGEPEMSMKQLQDTYNGIMMPPP
AVVTEEAKPADTAVASPATDAAGAQ
>CORE_REP|Org8_Gene4219#
MRLSDHSALPAAVIRPGYARERVKPRMAHIGFGAFHRAHQAMYADRLASEHDSDWGYCEISMQHGRQK
IADLRQQDLLFSVAEMDDDGWRGRVIGVVRQALHLQTDGLAAVLAALTQPEIAIVSLTISEKGYCHHP
ASGRLMAEHPAIAHDIAHPAQPQSAPGLLLAALRLRRERGLPPFAVMSCDNMPANGRVTRQVLVELAQ
RQEPALADWVRQEVAFPSTMVDRIVPAVTPEARRQLRTWLGGVEDEVGVASEPFSQWVIEDRFPQGRP
AWEKVGAELVSDVLPYEEMKLRMLNGSHSFLAYLGYLAGYQHVGDCMQDADFVRAARHLMLAEQAPTL
QVPNVDLAQYADSLLQRYRNRALKHRTAQIAMDGTQKLPQRLLDAIRWHLARGSRFDCLALGVAGWMR
YVGGRDEQGEPIAISDPLRERLAALVAGSEEGRPRVLALLQLDSVFGRDLPDSPDFVHAVSHAYHQLL
SLGAKAAVAALPLTDREVSDDRHHA
>CORE_REP|Org45_Gene2871#
MKLRKKRHKPMHINDITIIDDSKLKKAITAAALGNAMEWFDFGVYGFVAYALGQVFFPGASPGVQMIA
ALATFSVPFLVRPLGGLFFGAMGDKFGRQKVLSITIIIMAVSTFCIGLIPSYASIGIWAPILLLLAKL
AQGFSVGGEYSGAAIFVAEYSPDRKRGFMGSWLDFGSIAGFVMGAGVVVLISSIVGEANFLDWGWRIP
FFIAAPLGLIGLYLRHALEETPAFQQHVDKMEKEDRNAIENPPKTSFKEIAAKHWKSLLVCVGIVIST
NVTYYMLLTYMPSYLSHNLHYSEDHGVLIIIAIMIGMLFVQPVIGLTSDRIGRKPFIIGGSIGLLALA
IPCFILINSNVIGLIFVGLLVLAVLLNSFTGVMASILPAMFPTHIRYSALAISFNISVLIAGATPTAA
AWLVEATGNLYMPAYYLMVVAVIGLITGLYMKETANKPLRGATPAASDRSEAKELLQETYDNIEQKVE
DINAQIAELEKKKQILIDQHPKLD
>CORE_REP|Org36_Gene1064#
MLHLFAGLDFHTGLMLILALLFVLFYEAINGFHDTANAVATVIYTRAMRSQLAVVMAGLFNFLGVMLG

GLSVAYAIVHLLPTDLLLNVSSAHGLAMVFSMLLAAIIWNLGTWYFGLPASSSHTLIGAIIGVGLTNA
LMTHTSVVDALNVPKMIGIFLSLLFSPLVGMMVAGVMVFALRRYWSGTKKRQRIHMTPAEREKVDGKR
KPPFWTRIALILSAIGVSFSHGANDGQKGIGLIMLVLIGVAPAGFVVNMNATGYDITRTRDAVTHLQQ
YYQQHGDALSHAVSLTPLVPSPDDEAAPNKPAEFHCDSSRAMPAIELAQGMLTNLQSYDQLTVEQRSH
LRRLLMCVTDTADKVAKLPETSSADQRFLKNLRQDLLQTVEYAPMWIIVAVALALSLGTMVGWKRVAT
TIGEKIGKKGMTYAQGVSAQMTAALSIGVASYTGMPVSTTHVLSSAVAGTMIVDGGGVQSKTVKNILL
AWVLTLPISILLSGALYWVALKLI
>CORE_REP|Org21_Gene4352#
MTIVPHFGLMIDTHLPTVIRPIMRHSLVTLFQQSPHTAGTLRDRLCGALRQAIHQTALSVGQRLPSSR
VLASDLGLSRVTVEAAYGQLEAEGYLQRRVGQGTFVAIIIAKSPPPATAAGIPRLSQRGQQIVQTGGC
RDPQQPRAFAAGSPDLRAFPLALWKQLTAQRLRLQGESLLRYGDPQGYLPLREAIAAHVNQTRGVICD
ARQVIVLTSSQQALQLIATLLLDSGDSVWMEEPGYTGARNAFISAGAALTPVAVDGDGLRADPSLPDP
RLIYLTPSHQYPTGAALSLTRRLALLALADRQQAWIVEDDYDSEFHYDGLPIPAMQGLDRRGRVLYLG
TFSKSLFPSLRLAYLIVPPTLVDAFVTARTVYDGHSAQLMQAVTAEFIRQGYFAAHIRYMRQLYRSRR
DTLLAEVHEKLGHFATPAPAAGGLQLSVWLPPGQEAALSRQAQRLGILTPGLTAQYQTAQTQRDGWLL
GFSALTPGEIRSAVERLAQIAVA
>CORE_REP|Org25_Gene2547#
MSDNTVAVLDSVSRFLDRQHGLYIDGQWRASAAEGRLAVYNPANGQQIATTADANEHDVAQAVESAHK
AFSEGVWAQRLPVERERILLRFADLVEQHAEELAQLETLEQGKSINIARAFEVGSTLNWMRYTAGLAT
KITGQTLDVSIPMPPGAKYQVYTRKEPIGVVAGIVPWNFPLMIGMWKVMPALAAGCSIVIKPSETTPL
TLLRIAELASEAGVPPGVFNVVTGRGTVCGKALTEHPLIAKVSFTGSTPVGKSIARAAADRLTRVTLE
LGGKNPAIVLQDADPQQVIEGLMLGSFLNQGQVCAASSRIYIEAPIYDRLVAGFEQAVKSLSVGPGMD
TGAQINPLVSLAHRNKVAAYLDDARAKNAELIGGAAGPDANGFYIPPTLVINPDDRLNLTREEVFGPV
VNLIRVASAEEALSKANDTDFGLTASLWTTSLQKAMAFTPRIQAGTVWVNTHTLIDPNMPFGGFKQSG
SGRDFGPDWLDAYTESKSVCIRY
>CORE_REP|Org33_Gene2051#
MTSAHSNTSAYDHLRALFSRLSRFGHLSAIAGWDMQTMMPPGGSKARSEALAELSVLQHQILTAQSTG
ELLDRAQQETLDELDRANLREMRRQYEDAVLLPASLVEAKSLAGARCEHAWRAQRPANDWDGFVENLR
EVVKLSREEAQIRAQAAGTSRYDALLNLYEPGMRSSDLDRIFGDLKTWLPDLLQRVVAKQANEPCQTP
QGPFNVDTQRQLSLSVMKLLGFNFDNGRVDVSAHPFCGGVPEDVRITTRYNEKEFLTALLGIVHETGH
ARYEQNLPRDLLGQPVALARSTAIHESQSLLFEMQLARGNEFLKILRPLVTAQFGEQPALEEANFIRL
NQRVKPGLIRVDADEVSYPAHVILRYEIEKALIEGDIEVEDIPALWNEKMHAYLGLDTIGNYRNGCMQ
DIHWTDGAFGYFPTYTLGAMYAAQLFHSVRQALPSLGEDIAAGNLQPLFHWLQHNIWRHGSRFPTETL
IANATGEALNPRYFRQHLENRYL
>CORE_REP|Org22_Gene4283#
MSTRKAVIGYYFIPTNQINNYTESDTSVVPFPVSNITPAKAKQLTHINFSFLDINSNLECAWDPATND
AKARDVVNRLTALKAHNPSLRIMFSIGGWYYSNDLGVSHANYVNAVKTPASRTKFAKSCVRIMKDYGF
DGVDIDWEYPQASEVDGFIAALQEIRTLLNQQTVTDGRQALPYQLTIAGAGGAFFLSRYYSKLAQIVA
PLDYINLMTYDLAGPWEKVTNHQAALFGDAAGPTFYNALREANLGWSWEELTRAFPSPFSLTVDAAVQ
QHLMMEGVPSAKIVMGVPFYGRAFKGVSGSNGGQYSSHSTPGEDPYPSTDYWLVGCEECVRDKDPRIA
SYRQLEQMLQGNYGYQRLWNDKTKTPYLYHAQNGLFVTYDDAESFKYKAKYIKQQQLGGVMFWHLGQD
NRNGDLLAALDRYFNAADYDDSQLDMGTGLRYTGVGPGNLPIMTAPAYVPGTIYAQGALVSYQGYVWQ
TKWGYITSAPGSDSAWLKVGRVA
>CORE_REP|Org37_Gene1051#
MTRSNVEMPNEVQAWVSEGRYKEGFFTQLATDELAKGINEEVVRAISAKRNEPEWMLEFRLEAYRAWL
QMEEPHWLKANYDRLNYQDYSYYSAPSCGSCDDACGSQPGAEQQPGAATEKDYLTSEVELAFNQLGVP
VREGSEVAVDAIFDSVSVATTYREKLAESGVIFCSFGEAIQEYPDLVRQYLGRVVPSNDNFFAALNAA
VASDGTFVYVPKGVRCPMELSTYFRINAAKTGQFERTILIADEGSYVSYIEGCSAPVRDSYQLHAAVV
EVILHKDAEVKYSTVQNWFSGGESKGGILNFVTKRALCEGAGSKMSWTQSETGSAITWKYPSVILQGD
NSIGEFFSVALTSGHQQADTGTKMIHIGKNTKSTIIAKGISAGHSENTYRGLVKILPGAENARNFTQC
DSMLIGPDSGAHTFPYVEARNNSAQLEHEATTSKIGDDQLFYCLQRGISEDDAISMIVNGFCKDVFSE
LPLEFAVEAQKLLAISLEHSVG
>CORE_REP|Org1_Gene2226#
MSVLRILALFAATAAALPALAKPAAHGMAVENGIRWQSCLNSGFQRWFDEAPPPGLRCGYLEVPLAYA

TPPAHAAQAGEQTVRLALTLQPATGPKKGSVVMISGGPGLPGINPYLGNDAHVAKLRKSYDIIGYDPR
GVGQSTPKISCQLAEGDETPTPDDNDVAGAENQTRTLIAACIKQTGADVLQHIGTDEAVNDLNAIRHA
LGEPGLTAVAYSYGTKVAALYAERFPKKTRALVLDGVVDLAEDDFTQRMNQERGFQQSFLRFAAYCGK
TDSCQLGGGANQALQRYHALLRKLHDQPFVTAAGYEISADDVLTVTRSLLLWPERWHELATVLRQLDA
GIAGQQVSDLIDESYSPDADDALNVITCADVANPTADPQQLRRQRQEINTAALYAHYLPLHEYPLEMC
DLWPYRGKDRPHTPVASAALPPLLFVAQRYDPTTPYRNAQVMAAAFKSPLITRERDGHTLALNGIDSC
VDESVVEYLLAPKKPRRDKVCR
>CORE_REP|Org46_Gene4641#
MSQSVLASETHRGHLQQIISGLSDGVILTDTDRTLLWANEAALAMHGVSHQKALGANAGEYAARFALR
YRNNHPLALEQYPLNRVADGETFTDVVVEVRQADDPDSFWVHRLRSLIITDAQGQPELLALILSDATE
WASAEQRFEKTFNANPAPAVICRLSDLRYVKVNQGFLDMTGYQREQVMGRSVYELDVLEQAEHKDLAI
QRLGEGATIPQMEAELKLPGGGSKLVVVAGQPLDINEDDCMLFTFTDLEPRRQAESALRESEERFAKA
FRLSPVPTLLCTAHERRVLDVNEAFTRTTEYDAEALIGKTVDEIQFIDDPEASRRLFAALEKSGNVEG
LDIRVRKKGSESIDCVASADAVSIHNAPCYLLVLMDITERKRSELELVSAIEEVMQDASWFSQTLIEK
LANVKSINRPDQAGLTASDLTPRERDVLELICEGLPDKKIAARLNLALNTIRNHVATVYSKLGVHSRS
EAIVWARERGLFTGGLAARNGK
>CORE_REP|Org46_Gene1367#
MLSSSTLYAAIDLGSNSFHMLVVREVAGSIQTLARIKRKVRLAAGLDQNNHLSHEAMQRGWQCLRLFS
ERLQDIPREQIRVVATATLRLASNADEFLQAAEQILGCPVQVISGEEEARLIYHGVAHTTGGPDRRLV
VDIGGGSTELVTGTGAQASQLYSLSMGCVTWLERYFSDRNLGQENFERAEQAAREMVRPIAPQLRQQG
WQICVGASGTVQALQEIMVAQGMDERITLPKLRQLKQRAIQCGKLEELEIEGLTLERALVFPSGLSIL
LAIFQELGIESMMLAGGALREGLVYGMLHLPVEQDIRIRTIRNLQRRYLIDTEQAERVSQLAANFSQQ
VANEWQLDARCRELLHSASLIHELGLSVDFKQAPQHAAYLIRHLDLPGFTPAQKKLLATLLQNQSNPL
DLSLLNQQNALPPRTAQRLCRILRLAIIFASRRRDDTLPAVRLRANQDDELTVILPPGWLEQHPLRAE
ALEQESHWQSYVHWTLRLEEQR
>CORE_REP|Org36_Gene4726#
MPRTAKVVDVPAIGELDRLAGQLSHQLAQALRQAIHRGDLKAGDLLPSTRRLSAALNLARGTVLEAFA
QLTAEGFLEPQPGSGTRVAHYQAPRRTPHANVQASSAVAVPLSSQAQHLSRFAAQARALPPVPFTVSV
PIGDTAPDDVWRRLGNRIRARGPGAPSGYGDPLGALPLREAICDYVRRSRSVNCTPQQILITSGTQQG
LYLAAQILLDAGDSAWVEDPAYAGITAIFDSLFRDRHMIRVPVADDGIDVAAGVRLAADARAAFVTPS
HQYPLGMPMSMAKRSALLTWAKERQAWIVEDDYDSEMRYAGHPFPSLQGLAPERTLYLGTFSKVLFPS
LRLGYAVVPPPLVDAFCGARILMDRHPPSADQHVLAAFIAEGYLDRHIRKMRGVYAEKRRVLIEAINT
HIPAELAVVQPCDQGMHMVLWLRKDLDDVRVAQCANEAGLALRAVSPMYAPGHGEPGLVLGLGGYADR
QVQQAVERLGQVILACAGSGA
>CORE_REP|Org35_Gene3248#
MNLSSRLHAHLARGKVGFPTTLASSVGVIMASPVILTVTSGFGIGGDTFALAMLIAFIMMQAQLTTFS
EAASLLPTSGSVYDYISCGMGRFFAITGALSAYLIVHIFAGTAETILSGIMALVNFEHLNTLMESHNA
SWMVGVGLVVVFGLLNAFGIEAFGKAEIVLTFAMWSTLVIFGIVGLLSPHAVPLEGWFGSTLSLNDPF
AVFSLIGMAMFMFVGCELVTPMAPEIKRCDRVIPRAMALGLCGVAVCMALYGAALSHQVENVVIDAAS
GTRLLETPMAIPAFAGQVMGQFGKYWLGVGLLLAGAATINTLMAAVPRILYGMALDGALPRMFAYLHP
RFKTPVVGILVAVLIPCVHAFAIQGNLDRIIPLVLAAVCAWGVAYLLVTCSVVILRVRRPDLPRAYKS
PWFPLPQIVSSVGIVLAIVYITPPGMNPSDIYIPFGWMIGLTAAYALFWTLCVQKVNPFKPVPVEQVL
ENAFAKGENEEAQFDRLTSLT
>CORE_REP|Org49_Gene1781#
MTKKTTLLQFFHWYYPDGGKLWQEAAERAPHLAELGITDLWLPPACKGASGGYSVGYDTYDLFDLGEF
DQKGSVATKYGDKAALEHAANTLREHGVGVLYDVVFNHKLGADEKEQVHVFKVDANNRNDIDDQGFDA
LAYTRFTFPGRQGVHSEFIWDYKCFSGVDYVEQPDDKGVFKIANDYGDDGWNDQVDDEKGNYDYLMGA
DVEFRNTAVTEELKYWARWLLESLPCDGFRLDAAKHIPAWFFKEWADHVRDSAQRDLLIVAEYWSHDL
SALQQYIELVDGKVMLFDVALHLKFHQASKQGDGFDMAQIFTDTLTAADPAHAVTLVANHDTQPLQSL
EAPVEPWFKPLAYALILLREQGVPCVFYPDLYGASYRDKGRDGGEYQIDMPAIPELEKLIAARQRFAN
GPQTDYFDDSHCVAFSRAGTAEAPGCVVVLTNGGESGKTVALGADLAHTAWRDFLGNRQEEITTDDQG
SAHFPVNAGSVSVWVPAASL
>CORE_REP|Org10_Gene547#
MIPVVALVGRPNVGKSTLFNRLTHTRDALVADFPGLTRDRKYGRAEIEGNEFIIVDTGGIDGTEDGVE

TRMAGQSLLAIEEADIVLFMVDARAGLMPADQGIAQHLRSRQKATFLVANKTDGLDPDTATADFYSLG
LGEVFAIAASHGRGVTQLIEHVLVPFVPEKPEDVELTEEEANAAYWAEQNGETLEGEEDEEPEEAFNP
QDLPIKLAIVGRPNVGKSTLTNRILGEERVVVYDMPGTTRDSIYIPMVRDEREYVLIDTAGVRKRGKV
TETVEKFSVIKTLQAIEDANVVLLVIDAREGISDQDLSLLGFILNSGRSLVIAVNKWDGMSEEDREHV
KEMLDLRLGFVDFARVHFISALHGSGVGNLFESVQEAYECATRRVNTSMLTKIMQMAVDDHQPPLVRG
RRVKLKYAHAGGYNPPIVVIHGNQVSDLADSYKRYLMNYFRRSLNVMGTPIRIQFKEGDNPFAGKRNL
LTPTQMRKRKRLMSHLKKSK
>CORE_REP|Org8_Gene3394#
MADRNLRDLLAPWVPTAPGRALREMTLDSRVAAAGDLFVAVVGHQTDGRRYIPQAIAQGVAAVIAEAD
GQAEDGAIVEMHGVPVIYLSQLNQRLSALAGRFYHQPGERLRLVGVTGTNGKTTTTQLLAQWSQLLGE
TSAVMGTVGNGLLGQVCPTENTTGSAVDVQHVLNELAEQGATFAAMEVSSHGLVQHRVAALPFAAAVF
TNLSRDHLDYHGDMANYEAAKWSLFAAHNVGQAIINADDEVGQRWLSKLPDAVAVTMQDNLQPGCHGR
WLKTTAVDYHDNGATVRFSSSWGDGEIESRLMGAFNVSNLLLALATLLSLGYPLEALVETGSRLQPVC
GRMEVFNAPGKPTVVVDYAHTPDALEKALEAARLHCQGQLWCVFGCGGDRDKGKRPLMGGIAEQFADR
VVITDDNPRTEEPRAIINDILTGLLDAGQALVIHGRAEAVTSAIMQAQEQDVVLVAGKGHEDYQLVGN
RRLDYSDRTTVARLLGVLA
>CORE_REP|Org46_Gene2335#
MQSTIKIHEQDNVAVALRDLAAGETVELDGERVQLAQPVARGHKFALGPIAKGEDIIKYGQPIGHALA
AIGPGEHIHSQNAKTNLSDLDSYRYQPQFPALPPQAADREVQLYRRAGGDVGIRNELWIVPTVGCVNG
IARQIQQRFLQQTQAEGIDGVHLFSHPFGCSQLGQDHANTRIMLQNMARHPNAGAVLVIGLGCENNQV
EAFRATLGMADERRLRFMVCQQQDDEVEAGLGLLHELYREMRHDRREPGRLSELKFGLECGGSDGLSG
ITANPLLGRFSDYVIANGGTTVLTEVPEMFGAERILMSRCRDGETFDKTVDMINDFKRYFIAHQQPIY
ENPSPGNKAGGITTLEEKSLGCTQKAGLSQVVDVLKYGERLRVPGLNLLSAPGNDAVATSALAGAGCH
MVLFSTGRGTPYGGFVPTVKLATNSELAAKKPHWIDFDAGGLVHGVAMETLLSRFVDLIVEIANGRPA
RNEANDFRELAIFKSGVTL
>CORE_REP|Org15_Gene4594#
MSSLHISQGSFRLSDTRTLTLEALAIQAGESWAFVGANGSGKSALARALADELVLLRGERRSDFQHAV
RISFEQLQKMVSDEWQRNNTDLLSADEDDTGRTAAEIIQEEVKDAARCERLAAQFGITALLTRRFKYL
STGETRKTLLCRALMPQPDLLILDEPFDGLDVHSRAQLAALLSELSAQGQTVVLVLNRFDEIPDFVRQ
VGVLADCTLTNRGPRSQVMADALVAQLAHSENLSGLALPETEDPAHKVALPADRPLIVLRDGVVSYND
RPILNHLDWQVNPGEHWQIVGPNGAGKSTLLSLITGDHPQGYSNDLTLFGRRRGSGETIWEIKRHIGY
VSSSLHLDYRVNTSVRNVVLSGFFDSIGIYQAVSDRQRQLTEQWLALLGLDGARGDAPFHSLSWGQQR
LALIARALVKHPALLILDEPLQGLDPLNRQLVRRFIDVLIGQGATQLLFVSHHAEDAPQCITHRLSFV
PDGEGGYGYQHQRLEAASV
>CORE_REP|Org7_Gene3966#
MTNNASPAPIAHRPLILIACMLAMFMSAIEATIVATAMPTIIGDLGGFSLLGWVFAVYLLSQAITIPI
YGRLADLYGRKRVFFFGATLFLLGSVLCGFAPDMYWLIGFRLLQGLGAGAIMPIASTIIGDIYSATER
PKVMGYLSSVWGVSAIIGPLLGAFIVQHLPWALVFWVNLPIGLLAMFFLWRYLPAHQPLRQHALDLAG
TAWLTLFVSALLLALLQMESLGWWVVPLFALAAAALALLVRQERRAVEPLFPLALWQSRVIVAGNIGG
LVIGAAMMGISAFLPTFIQGVMGGSPLEAGTTLALMSIGWPLASTLSGRLMLMTSYRATALLGALLLV
AGGLILLLLQPEGGLLWGRVAAFMVGAGMGLCNTTFLVSVQNAAHYSIRGIATACTVFTRMVGSAIGT
AILGATLNLNLQWRLPEIDDPVQRLMEPAVRQSMGSEALAQLTQQVAASLHWVFLVSALVSLLALAAA
MLIPARCRPQGEEEEAEQA
>CORE_REP|Org17_Gene1818#
MTMSTPMLVTFLVYIFGMVLIGLLAYRATNNFDDYILGGRSLGSVVTALSAGASDMSGWLLMGLPGAI
FLSGISESWIAIGLTIGAYLNWKLVAGRLRVHTEANNNALTLPDYFTSRFEDNSKLLRVISAIVILVF
FTIYCASGIVAGARLFESTFGMSYETALWAGAAATILYTFIGGFLAVSWTDTVQASLMIFALILTPVI
VIFAVGGIDTSMLVIQAQNPANLDMLKGLNFVAILSLLGWGLGYFGQPHILARFMAADSHRTIRSARR
ISMTWMILCLAGTIAVGFFGIAYFANNPDQAGNVSQNGERVFIELAMLLFNPWVAGVLLSAILAAVMS
TLSCQLLVCSSAITEDLYKAFLRKGASQRELVWVGRVMVLVVALVAIALAANPENRVLGLVSYAWAGF
GAAFGPVVLISVMWSRMTRNGALAGMLVGAVTVIVWKQYEWLGLYEIIPGFILGCLAIVVVSLMGRQP
SSTMTERFDQAEAEYKTV
>CORE_REP|Org31_Gene3285#
MSKHYLNIFTQRNSAILLLLGFASGLPLALTSGTLQAWMTVENIDLKTIGIFSLVGQAYVFKFLWSPF

MDRYTPPFLGRRRGWLLVSQLLLVAAIVAMGFMQPAQHLWWLAALAVLVAFCSASQDIVFDAYKTDLL
KAEERGAGAAISVLGYRLAMLVSGGLALWLADRYFGWQATYWLMAGLMLIGVAATLLAPEPDESIPAP
RTMEQAVVAPLRDFFGRNNAWLILLLIVMYKMGDAFAGSLSTTFLIRGVGFDAGEVGLVNKTLGLFAT
IVGALFGGVLMQRLSLFRALMLFGVLQAVSNLGYWILAVTDKSLLTMGSAIFLENLCGGMGTAAFVAL
LMTLCNRSFSATQFALLSALSAVGRVYVGPIAGWFVEAHGWPLFYLFSIAAALPGLLLGICRQTLEH
TQQHGDFMPRTEFSESYRWALRLLTLGCSLLGLWLLLIANALDWTQAPLLADRLLQVGAALSLLGVA
MGSTLDYLALRRTRLA
>CORE_REP|Org5_Gene1012#
MAQQDIKTSGQQAPGLRRELKARHLTMIAIGGSIGTGLFVASGATVSQAGPGGALLSYALIGLMVYFL
MTSLGELAAFMPVSGSFSTYGAKYVEEGFGFALGWNYWYNWAVTIAVDLVASQLVMSYWFPDTPGWIW
SALFLGLMFLLNYISVKGFGEAEYWFALIKVSTVIIFIAVGVLMIVGILKGGEHAGWQNWTIGDAPFA
GGFSAMIGVAMIVGFSFQGTELIGIAAGESENPGKNIPRAVRQVFWRILLFYIFAILIISLIIPYTDP
SLLRNDVKDISVSPFTLVFQHAGLLSAAAVMNAVILTAVLSAGNSGMYASTRMLYTLASEGKAPRIFA
KLSKGGVPRNALYATCVVAGLCFLTSMFGNQSVYLWLLNTSGMTGFIAWLGIAISHYRFRRGYMLQGR
DLNDLPYRSGFFPLGPIFAFVLCLIITLGQNYQAFLQDKIDWYGVTATYIGIPLFLLIWFGYKLSRGT
RVVKYSEMEFPKMDVK
>CORE_REP|Org42_Gene3971#
MSTSISATLKDPTLFREANYIDGQWLPAQAGRSIAIHNPANGELVGHVPAFGAEETARAIAAAKKALP
AWRALTAKERAGKLRRLFELMMENQDDLARIMTAEQGKPLAESRGEIAYAASFIEWFAEEGKRVYGDT
IPQPQAGRRIIVQKEPIGVFAAITPWNFPAAMITRKAGPGWAAGCTGVIRPASQTPFSALAIAVLAER
AGLPAGVCNVITGPSKGIGGELTANPDVRKLSFTGSTEVGAQLLAQCAPTIKKTSMELGGNAPFIVFD
DADLDAAVAGAVASKYRNAGQTCVCTNRFLVQDGVYDAFAAKLKAAVAKLKVGNGLDEGVTIGPLINP
DAVEKVREHIADAVEHGASVLLGGKPDALGGNFFTPTILTDVPRTAKIFREETFGPVAPLIRFNHEAD
AVELANDTPFGLAAYFYSRDIGRVMRVAEALEYGIVGINEGLISTEVAPFGGMKHSGLGREGSKYGIE
DYLEIKYLCLGDLGA
>CORE_REP|Org1_Gene3838#
MANYFNTLNLRQQLAQLGKCRFMARDEFADEAGYLKGKKVVIVGCGAQGLNQGLNMRDSGLDVAYALR
KEAIDEKRASWRKATENGFKVGTYEDLIPQADLVVNLTPDKQHSSVVRAVQPLMKDGAALGYSHGFNI
VEVGEQVRKDITVVMVAPKCPGTEVREEYKRGFGVPTLIAVHPENDPKGEGMAIAKAWAAATGGHRAG
VLESSFVAEVKSDLMGEQTILCGMLQAGSLLCFDKLVAEGTDPAYAEKLIQFGWETITEALKQGGITL
MMDRLSNPAKLRAYALSEQLKTIMAPLFQKHMDDIISGAFSSGMMADWAEDDVKLLTWREETGKTAFE
NAPQFEGKIGEQEYFDHGVLMVAMVKAGVELAFETMVDAGIIEESAYYESLHELPLIANTIARKRLYE
MNVVISDTAEYGNYLFANAAVPLLKDFMTTLQAGDLGKAVAGTAVDNAQLRDVNEAVRSHPIETVGRK
LRGYMTDMKRIAVAG
>CORE_REP|Org9_Gene4419#
MNTQQLAKLRTIVPEMRRVRHIHFVGIGGAGMGGIAEVLANEGYQISGSDLAPNPVTQQLSALGATIY
FNHRPENVLDASVVVVSTAISADNPEIVAAREARIPVIRRAEMLAELMRFRHGIAIAGTHGKTTTTAM
VSNIYAEARLDPTFVNGGLVKAAGTHARLGSSRYLIAEADESDASFLHLQPMVAVVTNIEADHMDTYQ
GDFENLKQTFINFLHNLPFYGRAVMCIDDPVVRELLPRVGRHITTYGFSEDADVRIEDYRQIGPQGHF
TLSRQDKPLLTVTLNAPGRHNALNAAAAVAVATEEGIDDEDILRALAGFQGTGRRFDFLGEFPLEPVN
GKAGSAMLVDDYGHHPTEVDATLKAARAGWPDKRLVMIFQPHRYTRTRDLYDDFANVLSQVDVLLMLD
VYAAGEAPIPGADSRSLCRTIRSRGKLDPILVSDADTVPETLAQLLQDEDLVLVQGAGNVGKIARKLA
ELKLQPPKKEEEHHG
>CORE_REP|Org14_Gene917#
MAVTSTAQACDLVIFGAKGDLARRKLLPSLYQLEKAGHIHPETRIIGVGRAEWDKKAYTEVVKEALGT
FMKEKLDDELWATLSARLDFCNLDVNDSKNFTKLGKMLDQKHRTTINYFAMPPSTFGAICKGLGEAKL
NHEPARVVMEKPLGTDLASSRVINDQVAEYFNESQVYRIDHYLGKETVLNLLALRFANSLFASNWDNR
TIDSVQITVAEEVGIEGRWGYFDQAGQMRDMIQNHLLQILTMIAMSPPADLTTDRIRDEKVKVLRSLR
RIDQTNVRETTVRGQYTAGFVQGKKVPGYLEEEGANKSSSTETFVSIRVDIDNWQWAGVPFYLRTGKR
LPTKCSEVVVYFKNPPLNLFSDSYQQLPQNKLTIRLQPDEGIEIQVLNKVPGLDHKHRLQTTKLDLSF
SETFNQEHVADAYERLLLETMRGIQALFVRRDEVEEAWKWVDSIMDAWKADNEAPKPYQAGTWGPVAS
VAMITRDGRSWNEFE
>CORE_REP|Org33_Gene2637#
MSRFGLQKLYIHGAYVDSTAGKTFNAINPANGEVLAEVQSAGAEDVDRAVASAASGQKVWAAMTAMAR

SRILRRAVDILRERNDELAALETLDTGKAMSETTAVDIVTGADVLEYYAGLIPAIEGEQIPLRETSFV
YTRREPLGVVAGIGAWNYPIQIALWKSAPALAAGNAMIFKPSEVTSLTALKLAEIYTEAGLPDGVFNV
VTGSGAEVGQYLTDHPGIAKVSFTGGVKTGKKVMANASGSTLKEVTMELGGKSPLIIFDDADLDRAAD
IAMMANFYSSGQVCTNGTRVFVPAALQAQFEAKILERVKRIRLGDPTDPQTNFGPLVSFAHMESVLRF
IESGKNSGARLLCGGERVTHGEFGKGAYVAPTVFSDCRDEMEIVREEIFGPVMSILSYQSEEEVVRRA
NDTTFGLAAGVVTNDLARAHRVIHQLEAGICWINTWGESAAEMPVGGYKQSGVGRENGLTTLEHYTQI
KSVQVELGEYASVF
>CORE_REP|Org38_Gene2223#
MSKLNPILASSAHSAEAYRQAIAQSSEAVVQWLQQPEMYQGKSVAELRERIQLDFTPQGLGNQAAIER
AIEYFLKDSLSVHHPQCVAHLHCPSLVISQAAEVLINATNQSMDSWDQSPSATLIEMKLIEWLRAQVG
YQPGDAGVFTSGGTQSNLMGLMLARDAFFARQGHSVQQDGLVGDLRKLKVFCSENAHFSVQKNMALMG
LGYQSVTLVKTDRFARMDVNDLAEKLAQAKANGEQVMAIVATAGTTDAGAIDPLRDIARLAAEQKIWV
HVDAAWGGALLLSEQYRDYLDGLELVDSITLDFHKQFFQTISCGAFLLKDERHYELMRYQAAYLNSEF
DEAQGVPNLVSKSLQTTRRFDALKLWMGLEALGQKQYAEIIDHGVSLAQQVARYIADQESLELVMQPQ
LASVLFRYRPAPLAAKGDAAVALFNQRIGDALLESGRANVGVTEFDGVTCLKMTLLNPIVTLEDIKLL
LALVEKTAQQLPA
>CORE_REP|Org6_Gene902#
MTAELLVNITPSETRVAYIDGGILQEIHIERESKRGIVGNIYKGRVSRVLPGMQAAFVDIGLDKAAFL
HASDIMPHTECVAGDERKNFHVRDIAELVRQGQDLMVQVVKDPLGTKGARLTTDITLPSRYLVFMPGA
AHVGVSQRIDSEAERERLKAIVAPHCDELGGFIIRTAAEGIGEEELAQDAAFLKRLWTKVMERKKRNQ
TKYKLYGELALAQRILRDFAGAALDRIRVDSRLTHDLLVEFTGEYIPDITNKLELYTGSQPIFDLYDV
ENEIQRALERKVELKSGGYLIIDQTEAMTTVDINTGAFVGHRNLDETIFNTNIEATQAIARQLRLRNL
GGIIIIDFIDMNNEEHRRRVLHSLEQALSKDRVKTTINGFSQLGLVEMTRKRTRESIEHVLCHDCPTC
NGRGTVKTVETVCYEILREIVRVHHAYDSDRFLVYASAAVGEALKSEESHALAEVEIFVGKQVKVQIE
PLYSQEQFDVVMM
>CORE_REP|Org15_Gene1885#
MERLNIFVAGRWREGRGEEMASVFPADGSVNARLRAANVEDVNEAVEAAEKAWRAPEWRGLVPHQRAS
ILYRVSNLILAQQEQLAELQTRDNGKPLAETRGLVASAAATARYFAAACEVLEGELPTPRSAEVMTLS
QYQPMGVIAAITPWNSPIASEMQKVAPALAAGNAVVLKPAEATPLMALKLAELFEQAGLPAGLLSVLP
GKGSVIGEALARHPLVKKIAFTGGTSTGRHLAHIAADKLIPTSLELGGKSPTIVLEDADLEQAARGIC
YGIFSSAGQACIAGSRLFVHRSLYQPLLARLTELTAGLRIGNPLVPGVHLGPLISAKHRQSVADYVAL
ARQEGGRVVIGGEAPADPQLASGSYYLPTIIEGLNNDARVCQEEIFGPVLVALPFDDERQLIEQANDS
VYGLAAGIWSRDFPRAMALAERLETGTVWVNTYKTFSISTPFGGFKESGLGREKGLNGIKAYMQQKSV
YLALSHQVNRWSD
>CORE_REP|Org22_Gene1680#
MTNRLTKTALAVLLLGTLNATALAPAQAESQDQLPDMGTSAGGTLSIGQELAMGDFYVRQLRASAPLI
NDPLLSQYINQLGNRLVASAYSVRTPFHFYLVRNDEINAFAFFGGNVVLHSALFRVSDNESQLASVLA
HEISHVTQRHLARAMEDQQRNAPLTWVGALGSILLAMANPTMGMAALSGTLAGTQQGMISFTQSNEQE
ADRIGIQVLQRAGFDPEAMPDFLQKLSDQSRYASKPPEMLLTHPLPDSRLSDARNRANQMPKHIVQSS
QDYLMAKVRALGMYSSEGYGLNEELLGSLSKGNVREQAAAKYGRAILFYEAKKYDDARNIIQPMLAQD
AKNVWLIDLMTDIDLGQKRAPQAIARLQAANAAQNNNPVLQLNLANAYVEGNQPAQASKILNRYTFAH
PDDPNGWDLLAQASAAQGLRDEELSARAESLALTGRLDQAIGLLSNASSLQKLGSLKQARYDARIDQL
RQLQQRFRQYQRS
>CORE_REP|Org3_Gene2772#
MLKTAGWLLPAILALAGCSSSGQRHEQTLSQALTALGQDKALIGASNGVMVRDAESGTVLYQAHAQQR
LAPASNMKMFTSLAAFGVLGADYRFETRLLTTGEQRGDTLRGDLYLQGSGDPTLHPDDLDTFAATLAQ
RGIRHIHGRLILDASAFDQTPFGAGWSWDDEPFAFAAPISALNYAFTPGGDINVVRVDVQPGARAGAP
GRVSFYPANDAVTLVNRTTTTGGDTALTFDRQPGSNRIVVSGTVAAQAEASSRLITVDQPARVVGALL
QNALRAHGITLRGNAEEGVTPAGARLLAEKTSPPLSRLAVTFLKVSNNGYGEVLTKAMGRKTQGKGDW
AAGLQAIGRFVQSQGIEAGAYRQVDGSGLSRMNQITPQQLTTLLLAARKQPWFADWYNALPIAGQPGL
LVGGTLRSRMVKSAAAGRAHAKSGSMTGVSSLSGYVDSATGRPLAFAIISNNYLVPGAEVKALEDRLV
ETLAACDATVVCR
>CORE_REP|Org21_Gene3032#
MTLLDETPDTRYLQLADTLAEAIRRGTLQPGDRLPSVRRCAQTHRVSINTVVSAYRTLEDRGLIEARP

QSGFYVRSTLPALKMASAPSSRIEPPADDVLALIDTVFAAQQNPAFTNIALACPQTSDFYPGGKLGRM
LSSQLRRQPGLIGQYPLPPGSLRLRQQIARRSMTLGMLLEPGDVVLTHGCMEALQLALRVTTKPGDCV
GLESPTYFYLLPLLASLGLKALEIPTDPQLGLSLDALELLLNEKRLNAVIAMPTVQNPLGCTMPLAAK
KRLARLMNDHQVPLIEDGLYAEIQFGGALSPAVKAFDRDGWVLFCSSFTKTLAPDFRVGWICGGRFHE
ALRKLKAVSSMSESQLLSETLATFLESGGYDHHLRNLRKRYAAQVDEARALIARHFPRGTLATQPAGG
FVFWVEFPPGVDSVALFHQLLEEQICLTPGTLYSPSGRYRNALRLSCCYPFNARYTQALARLGARACE
MSGLPPGIAQDG
>CORE_REP|Org20_Gene1502#
MHSQKKSADDKHAARRRWLDSHESGYHKSMGNRQIQMIAIGGSIGTGLFLGTGGRLELAGPALALVYL
VCGIFSFFILRALGELVLHRPSSGSFVSYAREFLGEKASYVAGWMYFLNWAMTGIVDITAVALYMHYW
GTFADVPQWLFALGALGIVATMNMIGVKWFAEMEFWFALIKVVAIALFLIVGVVFLGTGTPVAGHTTG
MHLITENGGMFPHGLLPALVLVQGVIFAFAGIELIGTAAGECKDPAKMMPKAINSVIWRIGLFYVGSV
VLLVLLLPWNAYQAGQSPFVTFFSKLGVPYIGTIMNIVVLTAALSSLNSGLYSTGRILRSLAMGGSAP
KLMAKMSSQQVPYAGILVTCGIYVIGVVLNYLVPSQVFEIVLNIASLGIIASWAFIIVCQMRLRKAVR
EGRAQPVSFKMPGAPFTSWLTLAFLLIVVVMMAFDYPNGTWTIATIPVLAVLLTLGWFGLRKRAQEVK
REQQAHEEQNPH
>CORE_REP|Org18_Gene2077#
MSYAPPRSGLSADQALDQLEALYDAAVDALRQAVSDFISHGTLPDAQARAAGLFVYPELRVSWDGQQS
GPNKTRAFGRFTHPGSYSTTVTRPQLFRHYLAEQLAMLEHDYAAHIEVAPSQQEIPFPYVIDGSSLAL
DRSMSAGIAQHFPTTELAQIGDETADGLYHATDSHFPLSHFDALRADFSLARLRHYTGTPVEHFQPFV
LFTNYTRYVDEFVRWACAQIADPASPYIALSSAGGTYITPETRAPEQAVSDLAWKNHQMPAYHLISRT
GQGITLINIGVGPSNAKTICDHLAVLRPSAWLMIGHCGGLRESQKIGDYVLAHAYLRDDHVLDAVLPP
DIPIPSIAEVQRALYDATKMVSGMPGEEVKQRLRTGTVVTTDDRNWELRYSASARRFNLSRAVAVDME
SATIAAQGYRFRVPYGTLLCVSDKPLHGEIKLPGQANRFYEGAISEHLQIGICAIDLLRAEGDRLHSR
KLRTFNEPPFR
>CORE_REP|Org18_Gene1162#
MSQTQEKIWKAIAPLAVLAILLLIPVPDGMPPQAWHYFAIFVAMIVGMILEPIPATAISFIAVTVSVL
SANWVLFGAQELAEPGFKAGKEALKWGLAGFSSTTVWLVFGAFIFALGYEATGLGRRIALFLVKFMGK
RTLTLGYAVVIIDILLAPFTPSNTARTGGTVFPVVKNLPPLFDSFPNDPSSRRIGGYLMWMMVVGTSI
SSSMFVTGAAPNVLGIEFVGKIAGVHISWMQWFLAFLPVGLLLLIVAPLISYYLYKPGVTHSSEVAAW
ADTALGEMGKLTRKEYTLIGLVLLSLCLWVFGGKVLDATAVCLLAVSLMLALHVVSWKEITKYSSAWN
TLVNLATLVVMANGLTRSGFIDWFAQTMSTHLDGFSPNMTVVALVLVFYFAHYLFASLSAHTATMLPV
ILAVGKGLPGVPMEQLSMLLVLSIGIMGVLTPYATGPGVIIYGCGYVKSKDYWRLGGILGVVYIAALL
LIGWPIMSLWY
>CORE_REP|Org9_Gene2396#
MLSLETLDLLLAIAEGEMIEEMIVGMLAAPQLSVFFKKFPALRRALDRDLPRWKLQLKERLQEAMVPP
ALAQEFYRYQQCQLENNTQFYHNLNDTMELLRQLVSPFYEQARALVDAADLPNHPLDDSFQTLFLQRW
RISLTLQATMLHHQLLEQEREQLMAELQERLALSGALEPLLSENDTAAGRLWDMSKGQLQRGDYQRLV
EYGNFLQQQPELKKLAQQLGRSYQAKAVQQQDAQPEPFRVMVQVPATLPEEVSGIHQSDDILRLLPPE
LATLGIEELEFEFYRRLLEKRLLSYRLQGDVWQEQILIRQVTHQQQDQQPRGPFIVCVDTSGSMGGFN
EQCAKAFCLALLRIALADNRRCYIMLFANQIVHYELTAASGIEQAVRFLGQQFRGGTDLAACLNATVT
KMAESGWFDADAVIISDFIAQRLPEEVIKKVKQQQQSHQQRFHAVAMSAYGKPGIMRIFDHIWRFDTG
LKSRLMRRWRR
>CORE_REP|Org39_Gene3379#
MQSTKKAIEITESNFAAAKTGYDAVADLLHYHERGNGIQINGKDSFSNEQAGLFITRENQTWNGYKVF
GQPVKLTFSFPDYKFSATNVAGDTGLSKFSAEQQQQAKLSLQSWADVANITFTEVAAGQKANITFGNY
SQDRPGHYDYGTQAYAFLPNTIWQGQDLGGQTWYNVNQSNVKHPATEDYGRQTFTHEIGHALGLSHPG
DYNAGEGNPTYRDASYAEDTRQFSLMSYWSETNTGGDNGGHYAAAPLLDDIAAIQHLYGANLSTRTGD
TVYGFNSNTGRDFLSTTSNSQKVIFAVWDAGGNDTFDFSGYTANQRINLNEKSFSDVGGLKGNVSIAA
GVTIENAIGGSGNDVIVGNAANNVLKGGAGNDVLFGGGGADELWGGSGQDTFVFSAASDSAPGASDWI
RDFQKGIDKIDLSFFNKEAQSSDFIHFVDHFSGAAGEALLSYNASNNVTDLSVNIGGHQAPDFLVKIV
GQVDVATDFIV
>CORE_REP|Org25_Gene4368#
MADVQVLSGLSCQQFINGQLIDGEGQQECIVNPANGETLIALTEASSAQVGSAVKAAQQAFSHWSRTT

PAQRATLLLRIADAIERQASQLAQLEALNCGKPLHQALNDDLPAAVDVFRFFAGAVRAQQGQLAGEYV
PGHTSMIRRDPIGVVASIAPWNYPLMMAAWKIAPALAAGNTVVFKPSEHTPLTILALVPALQEILPPG
VLNIVYGGGEGVGSQLVGHPQVRLVSVTGDIVTGQKILQAAAKSVKRTHLELGGKAPVIVCDDADLDE
VVNGIRTYGYYNAGQDCTAACRIYAQAGIYPKLVDALGEAVASLRFARKRDQDNEIGPLISSRQRDRV
ASFVERALSQPHIELITGAAAHSGPGFYYQPTLLAGCLQSDEIVQREVFGPVVSVTRFEHLAQAVEWA
NDSEYGLASSVWTQNIDRALHIAAHLQYGSTWINTHFTLASEMPHGGLKRSGYGKDLSSDSLQDYSVV
RHVMAKFKASF
>CORE_REP|Org2_Gene1032#
MLRIAKEALTFDDVLLVPAHSTVLPNTAELGTQLTKTIRLNIPMLSAAMDTVTESGLAIALAQEGGLG
FIHKNMSIERQAEEVSRVKKHESGVVTDPQTVTPSTTLQEVKELTARNGFAGYPVVTEDNELVGIITG
RDVRFVTDLTQPVTAVMTPKDRLVTVKEGEARDVVLQKMHEKRVEKALVVDDSFHLLGMITVKDFQKA
ERKPNACKDEHGRLRVGAAVGAGAGNEERVDALVAAGVDVLLIDSSHGHSEGVLQRIRETRAKYPDLQ
IVGGNVATAAGAKALAEAGVSAVKVGIGPGSICTTRIVTGVGVPQITAIADAVEALEGTGIPVIADGG
IRFSGDIAKAIAAGASCVMVGSMLAGTEESPGEIELYQGRSFKSYRGMGSLGAMSKGSSDRYFQTDNA
ADKLVPEGIEGRVAYKGMLKAIVHQQMGGLRSCMGLTGCATIDDLRTKAEFVRISGAGIQESHVHDVT
ITKESPNYRMG
>CORE_REP|Org17_Gene3814#
MITHDDSRWSDLFSGKNAASAIALSLGVALHAINILVATTILPSVVQDIGGLDLYAWNTTLFVVASIL
GSALSARLLSGYGARNAYLVASLFFIAGAGLCALAPSMPVMLVGRTVQGFGGGLIFALSYAMINLVFE
QRLWPRAMALISAMWGIATLVGPAVGGIFAELHAWRWAFGILLPIMALYAAFTFLILPKGQAQQAAAP
LPTAQLLLLTVAVLVVSAGSLAHSVWINLAGIALSLALMAWLMKREARSRTRLLPHGALRRGSSLAAL
YITVSLLVIGMTSEIFVPYFLQLLHGQSPLISGYIAATMAAGWTLSEILSSGWRGAGIRRAIVSGPLF
VLVGLLALAILMPTPSGGHWQALTPIVIALSLVGFGIGFGWPHLLTRILQVAPEADKDIAGASITTVQ
LFATAFGAALAGMIANLAGLNDPGGAAGAAGAASAARWLFLAFALAPLLAVFSAWRCAAIAPPAAETG
NFVPNPSSREC
>CORE_REP|Org4_Gene2195#
MTTFYTVISWLMVFGYWLLIAGVTMRILMKRRAVPSAMAWLLVIYILPLFGIVAYLSFGELHLGKRRA
ERAKAMWPSTARWLKELKESRRIFATEYSEVAEPLFQLCNRRQGIDGVKGNQLQLLTTTDDTLKALIR
DIELARHNIEMVFYIWQPGGLVDQVAESLMAAARRGVHCRLMLDSAGSLQFFRSPYPAMMRNAGIEVV
EALKVNLLRVFLRRMDLRQHRKVVLIDNYIAYTGSMNMVDPRYFKQDAGVGQWIDLMARMEGPVATTM
GIVYACDWEIETGKRILPPPPDVNIMPFEQESGHTIQVIASGPGFPEEMIHQALLTAVYSAREQLIMT
TPYFVPSDDLLHAICTAALRGVEVSIIVPRDNDSMMVRWASRAFFSELLEAGVKIYQFEGGLLHTKSV
LVDGQLSLVGTVNLDMRSLWLNFEITLVIDDDGFGSDLACVQEDYIARSQLLNAKEWLKRPFWHRLVE
RLFYFFSPLL
>CORE_REP|Org38_Gene3792#
MSELSQLSPQPLWDIFAKICSIPHPSYHEEALAQHILTWAKEKNLHAERDQVGNILLRKPATKGMENR
KPVALQAHLDMVPQKNNDTVHDFAKDPIQPYIAGEWVKARGTTLGADNGIGMASALAVLADDSVEHGP
LEVLLTMTEEAGMDGAFGLQPNWLQADILINTDSEEEGEIYMGCAGGIDFITTLPLQREAVPAGYQTL
KLTLKGLKGGHSGAEIHVGLGNANKLLARFLFAHAEALNLRVLDLNGGTLRNAIPREASAVVAVPAEK
ADALKALSQEFLAVLQNELSAKEKNITVLLEPTTSASQALSADSQQRFLALLNGTPNGVIRMSDAVKG
VVETSLNVGVVTTSENEAEIICLIRSLIDSGKDYVVEMLAALGQLAGANVAPKGGYPGWQPDADSPVM
HLVRELYQELFNKTPNIMVIHAGLECGLFKKPYPNMDMVSIGPTITGPHSPDEQVHIESVGLYWKLLT
SLLKAIPERA
>CORE_REP|Org12_Gene1018#
MGKAMNARINARHGLPLTVQLINLFMLAFLLSLVGILSRPIGSLSLFWPVNAILLGLLLRKPIYGTPL
GWLTTYLGMVAADLSTGEGWSLALWLNACNMSLIAVGYGIMLMLPQSQRRMGKPQAILYMFSASLAGA
AVASTLSVLRNDSLYNNTVVIAWLAWFSEQFSTTLLLLPVLMAAPRLKQLLRMQVRWRLKGCLPLLAL
LLSLAFSVYIGGPGAIAFPIPALLWCAVRYPLFPVTLLTLLTGMTEISSISANLVLYETPNNHNAFLD
TLMSARLGIAMLVMGPLILASSIAANRKLMRRLEHSANHDFLTGVLARSAMTRKAGELLEHKHRSKEA
VSLLLIDIDHFKQINDTHGHSAGDQVLASFAHIVRRELRHDQLFGRLGGEEFAIMLPRALAAQGVALG
EHLRRLVEQTELQAEGKQTLKITISVGVASLAMNEVKSLEQLMNMADIALYRAKSQGRNRVESFNVIN
GNSVEHILFR
>CORE_REP|Org18_Gene580#
MKRLKINYILIGVVTLLLALALWPNITWRSGQGGQLQEIISRGELRISTLNSPLTYFNTKQGPGGLDY

ELAKRFANYLGVKLVVIPHQNINDLFDDLDDDDADLLAAGLIYNQERLSRARTGPAYYSVSQQLVYRL
GTPRPKSFADIKGKLAVASGSAHVSTLKQLKQDKYPDLAWESSSDLTSKELLERVADGKLDYTLGDSV
TIALLQRIHPQLAVAFDVTDEEPVTWYLKRDGDDSLYAAMLDFYSQMVDDGTLARLEEKYLGHVGSFD
YVDTKTFLSAIDSVLPNFRPLFEKHANEIDWKLLAAIAYQESHWNPQATSPTGVRGLMMLTRATADGL
GVNDRLDPEESIQGGALYLQRLMAKVPDSVPEDERIWFSLAAYNMGWGHMLDARKLTKTQKGNPDSWV
DVKQRLPMLSQKRYYPQLTYGYARGREAYNYVENIRRYQVSLVGYLQEKERKAAQAAAEQEALGKGYP
TVMPELALNY
>CORE_REP|Org47_Gene4880#
MQTNSAAVENFAQHHEERRSSAFQNEVAHYLERHPATQYVDILLTDLNGSFRGKRIPVSGLKKLEKGS
YFPASVFAMDILGNVVEETGLGQELGEPDRICLPVPGSLTPSAADPQHIGQVLLTMLDEDGTPFDVEP
RNVLNRVWQALRQRGLFPVAAVELEFYLIDRQRDAEGDLQPPCAPGTQERNTQSQVYSVDNLNHFAEV
LNDIDALAKLQGLPADGAVAEASPGQFEVNLRHTDDILLACDHALALKRLVRLVAENHDMHATFMAKP
YEDHAGSGMHVHVSMQDGAGNNLFADDEGEDSPLLKQALAGMITLMPASMALLAPNVNAYRRFQPGMY
VPIQAAWGHNNRTVALRIPCGEPENHRVEYRVAGADANPYLVMAAILAGMLYGLDNALPLPEPVTGNG
LEQEGLPLPIRQSDALYEFEHQHALTHYLGERFTQVYHACKTDELLQFERRVTETEIDWMCVSSHRYC
KYLRVNKGVT
>CORE_REP|Org42_Gene1651#
MFNKNKKPFSLRARFLMATAGVILALSLSYGLVAVVGYIVSFDKTAFRLLRGESNLFFSLAQWKDNKL
TIAIPPDIDLNFPTLVFIYDDKGNLLWSQRKVPELEKLINKEWLEESGFYEIDTDTRVSSEVLGDNPK
AQDQLKNYDDTDQNALTHSVAVNTYAATPRLPALTIVVVDSIPQELQRSDVVWEWFSYVLLANLLLVV
PLLWLAAYWSLRPIKALVNQVGELENGERDQLDENPPSELRGLVRNLNILVRNERQRYTKYRTTLSDL
THSLKTPLAVLQSTLRSLRSGKQTTIEEAEPIMLDQIGRISQQIGYYLHRASINSGQTVLTREIHSVP
ALLDSLVVALNKVYQRKGVVITLDISPEVTFMGEKNDFMEVMGNVLENACKYCLEFVEITSLHSEKNL
TIVIDDDGPGIPESKRQLIFQRGQRVDTLRPGQGLGLSVAAEIIEQYDGEIVISDSPLGGARMQVTFA
RQHDTHHNE
>CORE_REP|Org29_Gene3840#
MTKNHCDRLNPGSAEGPNCAQTLQRGLSARHIQLISIGGAIGTGLFMGSGKTIALSGTSIVLTYAIVG
FFMFMVMRAMGELLLTRLDYRSFADFVSEYLGPRASFFLGWSYWLSWVVTCIADVVVCGGYVQYWLPN
VSPWLPALLTLGFLCLFNMLSVKMFGEAEFWFAMIKVVAIVALIATGAWMVFSGWTSPDGVTASLHNV
TDPAIFMPHGIFGFFAGFQIAIFSCTGIELLGTMSAETKNPEKVLPKAINVIPARIIVFYVCSMLTII
AVTSWSHISPDSSPFVMLFDRAGLPAAAAVINFVVLTSAMSSANSGVYSSTRMLYSLSMEKHAHGQFR
ILSRTTAIPIRSLLFSCFCMVAGTLLLVLVPNVMTLFTIVSTVAAILVVYSWGMILVAYLVYRQKRPD
LHADSNFKMPGGIAMAWLTLAFFAFTLVLMVFDRDTLIALCSMPLWFTTLGLIWRYRVRDSVTRESYV
FYQRGAEAE
>CORE_REP|Org29_Gene943#
MTITPQQLIALLPLLIVGLTVVVVMLGIAWRRDHFINATLTVIGLNLALLSLYFVGQAGPMDVTPLLR
VDGYSMFYTGLVLLASLATCTFAYPWLVGYPDNREEFYLLVLIAAMGGILLASANHLASLFIGIELIS
LPLFGLVGYAYRQKRPLEAAIKYMLLSAAASSFLLFGMALLYAESGDLSLAGLGKSLQENMMHQPLIL
AGMGMMIVGLGFKLSLVPFQLWTPDVYQGAPAPVSTFLATASKIAIFAVVMRLFLYAPAADNEALRMV
LSIIAFCSILFGNLMAISQTNIKRLLGYSSIAHLGYLLVALIAVQTHQLSLETAGVYLAGYLFSSLGA
FGVVSLMSSPYRGPDADSLFSYRGLFWHKPILSAVMTVMMLSLAGIPMTLGFIGKFFVIAMGVSAHLW
WLTGAVVVGSAIGLYYYLRVTVSLFLSAPESLQRDTPNNWALTAGGVVVLISAALVLLLGVYPQPLIS
LVQMAQPTF
>CORE_REP|Org13_Gene1039#
MKTINHWINGKNVASKEYFTTTNPANGEVLAEVASGGQLEIDQAVAAAKEAFPKWANTPMKERARLMR
RLGELIDQNVPQIAELETADTGLPIHQTKNVLIPRASHNFEFFAEVCQQMNGKTYPVDDKMLNYTLVQ
PVGVCALVSPWNVPFMTATWKTAPCLALGNTAVLKMSELSPLTADRLGELALEAGIPAGVLNVVQGYG
ATAGDALVRHKDVRAVSFTGGTATGRRIIESAGLKKFSMELGGKSPVLIFEDADIERALDAALFTIFS
INGERCTAGSRIFIQESIYPEFVKRFAERANRLRVGDPQDPNTQVGALISPQHWEKVSGYIRLGVEEG
ATLLAGGPDKPAGLSHGNFLRPTVLADVDNRMRVAQEEIFGPVACLLPFKSEEDGLRMANDVEYGLAS
YIWTQDVSKVLRLARGIEAGMVFVNTQNVRDLRQPFGGVKASGTGREGGEYSFEVFAEMKNVCISMGD
HPIPKWGV
>CORE_REP|Org46_Gene3566#
MYKFGVVGGGAAGIAFAYNFIKNKSVNHCKRPLSLTVFDKQGFKGGMAYSSDFDSHILNMSPENMSAD

IFDDAHFVDWIAMHFPQFCQDRYPPRWLYREYLDFIRDMTIYMAADSNVALNFMTAEVNTIAPLEAGY
RLTTTCGGAEQMNALVLCSGHNPPECLYPVDGVIAYQAHQDLPPINPYSSIGVIGCSLTAIDAIVELM
ERLGATDICALSRSGLFPSVQPALMRPPPEAFVEGVRRFVAGRDFIDAHQLVHAINAALARHYPGPER
LTVLSALGERRDCYRDLAESLDRARFAREHICSYLAAIHPAVCAAWVKMDANNRQVFMRFYNSSWMRN
RHAMPIKNANKIVAALESQRLRACGGLADISKGTDGFILRCHEARVHAQYLFNCTTPSYQLPPNRLSQ
QLLQDGLVQENLFGGVKCNPHTLKIADRRGREQHLYSLGAPAKGDLFYTSAMESITRDISKIVFNNPI
FNAKKAEA
>CORE_REP|Org38_Gene3198#
MLESANTRRPPFDREMVDIVDYVMKEAVDTPAAYRTAHYCLLDTLGCGLEALSYPACKKLMGPVVPGA
EVLNGSRVPGTRFQLDPVQAAFNIGTMIRWLDFNDTWLAAEWGHPSDNLGGILAVADWLSRQAVAAGK
APLTMRQVLIAMIKAHEIQGCLALENAFNRVGLDHVLLVKVASTAVVAQLLGLSREAILNAVSLAWVD
GQSLRTYRHAPNAGTRKSWAAGDATARAVRLALMAATGEMGYPSALTAPTWGFYDVSFNGKPFRFQRP
YGAYVMENVLFKISFPAEFHAQTAVEAAMTLHRQMQAAGKTAADIAAVSIRTHEACLRIIDKQGPLDN
PADRDHCIQYMVAIPLLFGRLTAADYEDRVAEDPRIDALRSKIHCHEDPAFTRDYHDPEKRSIANALT
VEFADGSRLDEVAVEYPIGHARRREEGIPLLIEKFKTNLARQFPALQQQRILEVSLDRQRLAQMPVNE
YLDLWVI
>CORE_REP|Org31_Gene1090#
MRSPFNWRFTPLFAVLLLAGCASTDNIAPQSTLMDPQSLQLAQPKVSSLAVSPQWWRALKDPQLDTLM
TQTLQSSPTLRQAAARVREAQSVVGEASAANGPNLDLNASTQRQRVPQNVNMGLGYPHKPIYSSSNSL
GLNLAYEFDWWGKYRNQVNAAKAQVNAARAEQEQAALTLTSSVASAYYQLQSNLALEKLLQQEVNNNE
RLTALRQQRYQAGLTGVDVPQQTQAQSDVAKQQILQLQSQIEQLRHQLAALAGQGPNAMQHLRQVPLP
ADNLMAPQGELTADLLGKRPDIAAQRQLVESYSQRVSAARKEFYPSLTISAFAGLMTTNTSGTSPNLF
EAASQAWNVMPAISLPIFHAGALRSKLGEESALYDEAVESYNQTILNAVQETADAITIQQSSAQQQLQ
AASAAQSMQQVYQVANARYQAGIIGRDDLLTSQTQLLQQQQAELNASSNLLQAKIGLIRALGGGYQAP
AAADSKA
>CORE_REP|Org12_Gene2435#
MKFIIKLFPEITIKSQSVRLRFIKILSTNIRNVLKQYDETLAVVRHWDHIEVRAKDENQRPIIADALT
RIPGIHHILEVEDRAYTDIHHIFEQTLEAYRAQLEGKTFCVRVKRRGKQAFNSQDVERYVGGGLNQHI
ESARVNLSRPQVTVNLEIEDDKLMLVKRRLEGIGGYPVGTQEDVLSLISGGFDSGVSSYMLMRRGCRV
HFCFFNLGGAAHEIGVKQVAHYLWNRFASSHKVRFVAIDFEPVVGEILEKVDDGQMGVVLKRMMVRAA
SQVAERYGVQALVTGEALGQVSSQTLTNLRLIDNASDTLILRPLISHDKEHIIKLAREIGTEDFAKTM
PEYCGVISKSPTVKAVKAKIEEEESHFDFSILDRVVSEAKNVDIRSIAEQTQEQVTEVETVAAFGADE
VILDIRSNDEQEEKPLQLEQVEVKALPFYKLSTQFGNLDQSKTYLLYCERGVMSRLQALYLLEQGFNN
VKVYRP
>CORE_REP|Org20_Gene2253#
MSTNNIINAADDAAIMPTIANKKILMGFWHNWAAGASDGYQQGQFANMNLTDIPAEYNVVAVAFMKGQ
GIPTFKPYNLSDAEFRRQVGVLNSQGRAVLISLGGADAHIELKTGDEDKLKDEIIRLVEVYGFDGLDI
DLEQAAIGAANNKTVLPAALKKVKDHYVAQGKNFIISMAPEFPYLQANGSYGDYINALDGYYDFIAPQ
YYNQGGDGIWVPEANNGAGAWIAQNNDAMKEDFLYYLTESLVTGTRGFIRIPASKFVIGLPSNNDAAA
TGYVIDKQAVYNAFSRLDAKNLSIKGLMTWSINWDNGKSKAGVAYSWEFKTRYAPLIQGGVTPPPGKP
NAPTALTVSELGATSLKLSWAAATGALPIASYTVYRNGTPIGQTAGLSLADSGLTPATQYSYFVTATD
SQGNTSLPSSALAVKTANDGTPPDPGAPEWQNNHSYQAGDVVSYKGKKYTCIQAHTSNAGWTPDAAFT
LWQLIA
>CORE_REP|Org19_Gene4298#
MSYQRKHNTGYILRICGIAALGGILFGYDTAVISGAIEALKTYFNLSPAETGWAVSNVVIGCVVGAFA
AGPLAARWGRKKALMLAALLFTVSAVGAALAPTFTWFVIYRIIGGLAVGIAATVSPMYMSEVSPKDMR
GRALSMQQFAIVFGQIVIFYVNFKIASLASEAWLVEMGWRWMFASGVIPCILFCILVFVIPESPRWNV
MMGRDDQALAMLTKVSNAAHAQNLLKEIKDSLQQDQQQRHRKLNYGDVRVRFILFVGCMIAMLQQVTG
VNVMMYYAPVVLKTVTENAQEALFQTIWIGVLQLVGSVIGAMLMDRMGRIPLMRYGTLGAIAGLLLTS
YALYTQATGYFALFGMLFFMVFYALSWGVGAWVLVSEIFPNRMRAQGMSIAVGCMWVANFAVSQSFPM
INDHPYLFSHFHGAFPMWIFAACCLFSYWFIGRYIPETKGVSLEKMEQVVLAKRHRHRHPLPDGKPLP
LENGKS
>CORE_REP|Org9_Gene2225#
MSLTFVSEQLLATNKLSHQDLYQVLGQLAERRIDYADLYFQSSYHEAWVIEDGIIKDGSYNIDQGVGV

RAVSGEKTGFAYADQITLNALQQSAQAARSIVREQGDGRAHTLGEIGYRALYPLLDPLQSLPREEKIA
LLHRVDKVARAADARVQEVNASITGVYEQVLVAATDGTLAADVRPLVRLSVSVLVEQDGKRERGSSGG
GGRFGYDYFLESVDGDVRADAYAKEAVRMALVNLGAVAAPAGNMPVVLGAGWPGVLLHEAVGHGLEGD
FNRRGTSVFSGHMGELVASELCTVVDDGTLQGRRGSLAIDDEGVPGQYNVLIENGVLKGYMQDKLNAR
LMGVAPTGNGRRESYAHLPMPRMTNTYMLAGQSTPEEIIASVEYGLYAPNFGGGQVDITSGKFVFSTT
EAYLIEKGRITKPVKGATLIGSGIEAMQQISMVGNDLALDKGVGVCGKEGQSVPVGVGQPTLKLDTLT
VGGTA
>CORE_REP|Org16_Gene217#
MSRRLRRTKIVTTLGPATDRDNNLEKIIAAGANVVRLNFSHGSPEDHQARADKVREIAAKLGRHVAIL
GDLQGPKIRVSTFKEGKIFLNVGDKFLLDANLSKGEGDKEKVGIDYKGLPADVVPGDVLLLDDGRVQL
KVLEVQGMKVFTEVTVGGPLSNNKGINKLGGGLSAEALTEKDKADIVTAAKIGVDYLAVSFPRTGEDL
NYARRLARDAGCNAKIVSKVERAEAVCTDEAMDDIILASDVVMVARGDLGVEIGDPELVGIQKKLIRR
ARTLNRAVITATQMMESMITNPMPTRAEVMDVANAVLDGTDAVMLSAETAAGQYPAETVAAMARVCLG
AEKIPSINVSKHRLDVQFDNIEEAIAMSSMYAANHLKGVTALIAMTESGRTALMMSRISSGLPIFAMS
RHEHTLNLTALYRGVTPVYFDSHKDGVIAANEAVNRLRDKGFLVSGDLVIVTQGDVMETVGTTNTSRI
LRVE
>CORE_REP|Org12_Gene3340#
MPQFDNAYYQQLPGFYTALNPTPLKDARLLYHSEPLARELGLDESWFTQDKTPIWAGESLLPGMQPLA
QVYSGHQFGVWAGQLGDGRGILLGEQVMADGSHRDWHLKGAGLTPYSRMGDGRAVLRSVIREFLASEA
LHHLGIPTTRALTIVTSQQPVYREQPERGAMLMRVAESHVRFGHFEHFYYRKQPEQVRQLADFVIARH
WPQLQDLAERYLLWFTDVVERTARLIAHWQTVGFAHGVMNTDNMSILGITIDYGPYGFLDDYQPGYIC
NHSDHQGRYAFDNQPAVALWNLHRLAQTLSGLMTTEQLQQALSAYEPALMRAYGEQMRAKLGFFTPTA
QDNDVLTGLLSLMAQEGRDYTRTFRLLSETEQQQAQSPLRDEFIDRAAFDAWYQQYRQRLQQEQVSDA
ERQRAMKAVNPRLILRNYLAQQAIEDAEKDDVGRLRRLHQALLRPFDDAPEYDDLAALPPDWGKHLEI
SCSS
>CORE_REP|Org38_Gene1257#
MKKTALVLSALAFSIGMAMGPVTASAAETASSSTQQLPSLAPMLEKVMPSVVSINVEGSTTVNTPRMP
QQFQQFFGEDSPFCQDGSPFQGSPMCQGAEQPGPDGQQPQGTQQKFQALGAGVVIDAAKGYVVTNNHV
VDNANKIQVQLSDGRRFDAKVIGKDPRSDIALIQLKDFKNLTAIKMADSDQLRVGDYTVAIGNPYGLG
ETATSGIVSALGRSGLNIENYENFIQTDAAINRGNSGGALVNLNGELIGINTAILAPDGGNIGIGFAI
PSNMVKNLTAQMVEYGQVKRGELGIMGTELNSELAKAMKVDAQRGAFVSQVMPKSSAAKAGIKAGDVI
VTMNGKAISSFASFRAEIGTLPVGSKMSLGIIRDGKPITIDVTLEQSAQTQVASGNIYTGIEGAELSN
TQVGNVKGVKVDSVKAGSAAARIGLKKGDVILGVNQQPIQNLGELRKILDSKPSVLALNIQRGDSQLY
LLAQ
>CORE_REP|Org29_Gene3593#
MTPQRVLVLGASGYIGQNLIPHLIEQGHQITAAARRIEWLQERNWPQVNCLYADLYRPETLSAALWEI
DTLYYLVHAMGDGDDFIEKERQAAENLRDALRNAGVKQVIFLGALQPNDDSSPHLAARRLTGEILRQS
GVPVTELRAGIVVGPGSAAFEVMRDMVYNLPVLTPPRWVRSKSSPVALENLLVYLADLLAHPAQEHRI
FDVAGPEYLSYQDMFKRFIALSGKRRWLIPIPLPTRLISVWFISLITSVPTPIARALIQGLKHDLPAD
GRPLQALIPQRLHTFDQAVVTTLQREQEVVDSADWGYDPAARARWRPGYGYYPKQAGCSLDTAATREA
LWQTVQLLGGEEGYFYANILWRIRARMDDMIGNGVVYGRPARATLALGDEIDGWKVITLKPLRQLALL
FGMKAPGLGRLSFSITDHGDRRTLDVRAWWHPAGFSGLLYWFAMMPAHLFIFRGMAKRIAELAESLDR
QRR
>CORE_REP|Org14_Gene1836#
MTRYATLAAILSQRIQQGLYPAGHRLPSVRALSQEHGVSISTVQQAYRLLEEQRLVEARPKSGYFVHT
RRAQAELPAMTAPVQRPVDISQWEQVLELVRSRPREGLIQLGRGMPDIAEPTMKPLIVALRNAARHGD
LRSLYYDSIQGVAALREQVARLLLDSGCQIGPDQLLITTGCQEAISAGLRAVCQPGDIVAVDSPCFHG
TMQTLKGLGIKALEIPTDPLTGVSLAALEMALEQWPIKAILLTPNCNNPLGYIMPDAHKQRLLTLAQR
HDAAIIEDDVYGDIAYHYPRPRTIKSFDEDGRVLLCSSFSKTLAPGLRVGWIAPGRYLERVLHMKFIG
SGATATQPQLAIAEFIRGGHYLQHLRRMRARYQQNRDRMTDLILKHFPAGTRVSRPRGGFMLWIELDE
AFDTLRLNRHLEQQGVQIAVGSIFSAAGKYRNCLRINYAPKLTAEIEQAVQRVGATIQALMPSGVLQP
QAD
>CORE_REP|Org43_Gene2089#
MISLKKWRLFPRSLRQLVLLAFLLVLLPLLVLAYQAYQSLDHLSAQAADINRTTLVDARRSEAMTSVA

LEMERSYRQYCVLVEPTLQKLYQNQRKQYSQMLDAHAPILPDERYYQTLRQLLTQLAAIKCHNSGPDQ
EASALLESFSRSNAEMVQATRAVVFSRGQQLQQAIAERGQFFGWQALLLFLVSVLLVVLFTRMIIGPV
KAVERMINRLGEGRALGSTASFKGPRELRSLAQRIIWLSERLAWLESQRHEFLRHISHELKTPLASMR
EGTELLADEVAGPLTSDQKEVVTILDNSSRHLQQLIEQLLDYNRKLADGPAEHENVELREMVDLVVAA
HSLPARAKMISTEIALEAEICWAEPTLLMRVLDNLYSNAVHYGKESGNIWIRSRQVGQRVQIDVANTG
TPIPEAERAMIFEPFFQGSHQRKGAVKGSGLGLSIAQDCIRRMRGELQLATVAGADVCFRIELPLTAE
NE
>CORE_REP|Org15_Gene2303#
MSKKGLTTAAGAPVVDNNNVITAGKRGPMLLQDVWFLEKLAHFDREVIPERRMHAKGSGAYGTFTVTH
DITRYTRAKIFSEIGKQTDMFIRFSTVAGERGAADAERDIRGFAMKFYTEEGNWDLVGNDTPVFYLRD
PLKFPDLNHVVKRDPHTNLRNPVYKWDFFSHLPESLHQLTIDFSDRGIPKSYRHMHGFGSHTFSFINA
ANERFWVKFHFRCEQGIENLMDEEAEAIIAKDRESSQRDLFDAIKRGDFPRWKLQIQIMPEHEASQTP
YNPFDLTKVWPHGDYPLIDVGFFELNRNPDNYFSEVEQVAMNPANVVPGISFSPDKMLQGRLFSYGDA
HRYRLGVNHHQIPVNGAKCPFHNYHRDGAMRVDGNSGNGATYEPNSFGLFQEQPDFSEPPLSIEGAAD
HWNHREDDDYYSQPRALFNLLSAEEHQRMFTRIAGELSQVPEHIQRRQVELFTKVHPDYGAGVAKALG
LK
>CORE_REP|Org3_Gene4830#
MTMKQLPKDFLWGGAVAAHQVEGGWDRGGKGPSIADVLSGGSHGVDRVMTDGVLDGYRYPNHEAVDFY
GRYKQDVALFAEMGFKCFRTSIAWTRIFPNGDEATPNEAGLQFYDDLFDELLKYGIQPVITLSHFEMP
YHLVKAYGGWKNRRVVEFFVRFSEVVMRRYREKVKYWMTFNEINNQSNYRYPLFGYCCSGVDYTREDN
PEQALYQVLHHQFVASAQVVKLGHQINPEFKIGCMLACVPFYPYSCKPDDVMYAVEAMHQRYLYTDVQ
MRGYYPSYLLRDWERKGLKIEMQPQDAQILREGCTDYIGFSYYMSNALQANAVDGSDGMFGFPGNVPN
PHVKASDWGWQIDPVGLRYSLNVLYERYQKPLFIVENGFGAFDKVEADGQINDDYRIDYLRAHIEEMK
KAVIEDGVDLIGYTPWGCIDCVSFTTGEYSKRYGFIYVDKHDDGTGTLERSRKKSFDWYRRVIASNGE
QL
>CORE_REP|Org47_Gene3329#
MSNNIRIEEDLLGTREVPADAYYGVHTLRAIENFYISNSKISDVPEFVRGMVMVKKAAAMANKELKTI
PRKIADVIIQACDEVLDKGKCMDQFPVDVFQGGAGTSLNMNTNEVLANIGLELMGHQKGEYQYLNPND
HLNKCQSTNDAYPTGFRIAVYASNQKLIDAINQLREGFDRKAKEFETILKMGRTQLQDAVPMTLGQEF
HAFSVLLNEETRSLHRTAELLLEVNLGATAIGTALNTPEGYQPLAVQKLAEVSGLPVVPAEDLIEATS
DCGAYVMVHSALKRLAVKLSKICNDLRLLSSGPRAGLNEINLPELQAGSSIMPAKVNPVVPEVVNQVC
FKVIGNDTCVTMAAEAGQLQLNVMEPVIGQAMFESIHILTNACYNLLEKCINGITANKEVCEHYVFNS
IGIVTYLNPFIGHHNGDIVGKICAETGKSVREVVLERGLLTEAELDDIFSVENLMHPAYKAKRYTDEN
EQ
>CORE_REP|Org41_Gene2421#
MKQGLQLRLSQQLAMTPQLQQAIRLLQLSTLELQQEIQLALESNPLLEQTDLHDEIDAKEIQETEGLD
TREALEQKDMPEELPLDATWDEIYTAGTPSGTGTDYSDDELPVYQGETTQTLQDYLMWQVDLTPFSDT
DAAIATSIVDAVDDTGYLTVPLEDILESLGDENVTLEEVEAVLKRVQRFDPIGVAARDLRDCLLVQLS
QYAKDTPYLAEARLIISDHLDLLANHDFRSLMRSTRLKEDTLKEAMLLIQSLDPRPGQSINTGESEYV
IPDVLVRKTQNTWTVELNGDSIPRLKINQQYAALGNSARSEADGQFIRSNLQEAKWLIKSLESRNETL
LKVTRCIVSQQQAFFEQGEEFMKPMVLADIAQAVEMHESTISRVTTQKFLHSPRGIFELKYFFSSHVN
TDSGGEASSTAIRALVKKLIAAENPAKPLSDSKLATLLSDQGIIVARRTVAKYRESLSIPPSNQRKQL
V
>CORE_REP|Org36_Gene3497#
MSYRLLALWLVCYAAAWTLLTVHLDPTLPYDAVEALNWGLNGEWGSPKNPWLVGAAMHPAIWLSWLPL
NVYWYGGHFIAIAIGMLGVWLLARRLSGSTLLAWMALLTLNLSGIINFDIIPYNDNYLLVMLWPWMAL
FFHMAISRSANWWPAFALVAGLAMMAKYSTFAFVYFVALSTLFVPQVRRCYRQPQFYLAVAIWLALVL
PNVFWLWNHDFAAFKWVDSQIKMQLNLDMLQSLLLVFYPSLVLWAILRRSGAVLAWPSALPMRVLLWI
YLLPLGIITFWFSFNVGGRLTEWLQPFFMLAPALLVGCVRQPPVRSLRAATLGLMCAALAVYLGYAAV
MLGNVRNAGQKMVGIKAFSAGVERQWQERYGVDLRYVGGEYLSQWMTVYAGSRPQTITRWSNHTRPNI
YNVNISYPQIAQHGVALFGRLGEDCAHSDFGGELAHWPKMRLDWQQTLTFRADPHADEQTLCVGFVRP
Q
>CORE_REP|Org4_Gene2540#
MYPDTQLYIDGQWRNALAGKTLPVTNPATDEIIGQVAHAATEDLDLALAATERGFTVWRDTAAHQRAN

LMRKAAALLRERANAIAAVMTQEQGKPVAQAKIEILNAADVIDWFAGEATRTYGQIIPSRARDVQQQT
LKLPVGPVAAFTPWNFPINQIVRKLSAALAAGCSIIVKGPEETPASPAELIKAFADAGIPAGVIALVY
GTPAEISEYLIPHPTIRKISFTGSTRVGKHLAALAGQHMKKATMELGGHAPVLIFDDADLDAAAKELA
QSKFRNAGQVCIAPTRFLIQQGVYEAFVEKFTAAVRELKLGNGLEDGVTMGPMVLGRSVDNIEALVQD
AVAHGAKACSGGKRVAGKGNFFEPTVLRDVPLSARAMSEEPFGPVALLRPFATYDEAIAEANRLPYGL
AAYAYSRNIATVNALGRDVESGMLSINHIGFGLPETPFGGVKDSGHGTEGGSEAIESYLETRFVTVAG
R
>CORE_REP|Org38_Gene899#
MRFSRIVSALACAFVLNANAAPVEEYTQYLPDGANLALVVQKIGAGSPTIDYHSQQMALPASTQKVLT
ALAALLQLGPDYRFTTTLESQGDIRDGVLRGNLIARFSGDPTFKRQSLRNMVAVLKKQGVRQISGDVL
VDTSVFASHDKAPGWPWNDLTQCFSAPPAAAIVDRNCFSVSLYSAPNPGDMAFIRVASYYPVNMFSQV
RTLARGSADAQYCELDVVPGELNRFTLTGCLTQRSEPLPLAFAIQDGASYAGAILKDELTQAGIQIDG
HLKRQTRPGITGTVIAQTQSAPLHDLLKIMLKKSDNMIADTVFRTIGHERFGVPGTWRAGSDAVRQVL
RQKAGIDLGNTIIADGSGLSRHNLLAPATMMQALQYIAQHDNELNFISMLPLSGYDGTLRYRGGLHEA
GVDGKVSAKTGALQGVYNLAGFITTASGQRMAFVQYLSGYAVPPEDQKQRRAPLVRFESRLYRDIYQN
N
>CORE_REP|Org18_Gene2559#
MSAATHNVKKVAEYRQRILTLLLNNKELVDGILGRPGDEHALSQSELLNQTAEITGLLDDMHAADLAD
LLEALPQDERMALWRLVGNSKRGQTLVEVAEPVWDSLIEEMSDKDLLKAIKTLDVDEQAYLAQYLPRN
LMGRLLTSLEPEQRAQVREMSQYAKDSVGWMMDFELVTVRPDVTLGAVHRFLRMRKTIPDATDKLFVT
DRKNTLLGELPLTAVLLNDPEIPVREVMDSDPATFQPEDKADEAAGAFERYDLISAPVVDAKGKLMGR
LTIEEIVDAVNEESDTNLRRMGGLSPEEDVFAPVSKAVKTRWAWLAINLCTAFIASRVIGLFEHTISQ
LVALAALMPIVAGIGGNTGNQTITMIVRALALHQIEVGNISRLMLRELGVAIINGVVWGGIMGVITWL
LYGDAAMGGVMTLAMLLNLLLAALMGVVIPMTMLKVGRDPAVGSSVLITALTDTGGFFIFLGLATLFL
L
>CORE_REP|Org3_Gene4038#
MATFLSEDFLLDSEFARRLYHDYAADQPIFDYHCHLPPQQIAENTRFSNLYDIWLRGDHYKWRAMRTN
GVAERLCTGDAGDREKFDAWAATVPHTIGNPLYHWTHLELRRPFGITGTLLSPATADEIWQRGNALLA
QDEFRARGIMRQMNVKMVGTTDDPIDDLRHHRAIADDHSFDIKVLPSWRPDKAFAIDAPGFNDYLRQL
EAAADTAIGRFSALCDALYKRMDHFAAHGCKVADHALDVVVFGEADEAALDAILLRRLNGELPAPEQN
AQFKSAVLLFLASEYRRRGWVQQYHIGALRNNNSRMLAAVGPDIGFDSINDRPLAEPLSRLLDAQARL
LDAQARQGGLPKTILYCLNPRDNEVIGTMIGNFQGEGTPGKMQFGSGWWFNDQKDGMQRQMTQLAQLG
LLSRFVGMLTDSRSFLSYTRHEYFRRILCQLIGRWVTDGEAPADLALLGEMVKNICFDNAKNYFAIEL
A
>CORE_REP|Org14_Gene1147#
MKVTLPDFRRAGVLVVGDVMLDRYWYGPTSRISPEAPVPVVKVDTIEERPGGAANVAMNIASLGANSR
LVGLTGIDDAARALSAKLNEVNVRCDFVSVPTHPTITKLRVLSRNQQLIRLDFEEGFSNVDPQPMLER
IQQALPQIGALVLSDYAKGALSQVQGMIQLARAAKVPVLIDPKGSDFERYRGATLLTPNLSEFEAVVG
HCKDEAELVARGMKLVADFELSALLVTRSEHGMTLLQPGVEPLHLPTQAQEVFDVTGAGDTVIGVLAA
SLAAGNSLEESCFLANAAAGVVVGKLGTSTVSPIELENAVRGRAETGFGVMTEAQLKTAVAQARQRGE
KVVMTNGIFDILHAGHVSYLANARKLGDRLIVAVNSDASTKRLKGEKRPVNALENRMIVLGALEAVDW
VVPFEEDTPQRLIADILPDLLVKGGDYKPEEIAGSAEVWANGGDVKVLNFEDGLSTTNIIKAIKDGRG
>CORE_REP|Org45_Gene4692#
MTTHLVWLRNDLRITDNKALHAACSDPEARVLAVFIATPQQWRQHEMAPRQAALIHASLQAVQQALAH
KGIALHCHSCADFAASIDWLADYCEREQVDALFYNRQYELNERRRDARLEQRLSGRVRCHGFDDSLLL
PPGSVLTGGGEMYKVYTPFRNAFLQRLTESDVSCLPAPKIRAGGALPAPEAPAAFDYPTAETGDGYPA
GEEAALQRLRAFCREQVQDYLRQRDLPALAGTSSLSPYLAIGTLSPRQCFNRLRAECPQLLEDRESGA
FAWLNELIWREFYRHLLMAYPDLCRHRPFIAWTDKVRWCDDAAKLHAWQRGETGYPIVDAAMRQLNAT
GWMHNRLRMISASFLVKDLLIDWRAGERYFMSQLLDGDLAANNGGWQWAASTGTDAAPYFRIFNPTTQ
GERFDPQGTFIRKWLPELADVPDNDIHHPHRWAEKQRCTLNYPLPIVDHKQARLETLAAFEAAKRGES
>CORE_REP|Org16_Gene2983#
MINIVVVSHSALLARGVEQLARQMMRGDGCKLALAAGVDDEQHPIGTDAVKVMEAIEAVADGDGVLVL
MDLGSALLSAETALDLLDPDLAAKVRLCAAPLVEGTLAAVVAANSGASLEQVVAEAQGALQAKQAQLG
EASPTAKSVALPLAQGKSVTWTVQNPHGLHARPAARLVETLAPFKAELVLEKQGQCVDPRSLNQLALL

QVRHGDTVRLIADGAQADEALAAFKALAEQHFGETVSERQQPSLHGIPVAESVTSGPVFQAHSFWPPT
VDRRIGADEVLGEQQRLREALQHTLSDLNRLAERTGTLIGKPQAAIFGAHSMLLDDPDLQQAAYTRIA
QQLCCAEQAWRQVLGAIAEEYRELDDDYMRARELDVRDMLRRTLCHLQGLPLPAMALAEPSILVMDEL
MPSEVVMLDRRLVLGICLSGGNALSHSAILAKAMGIPMVVGMQDCLSKTRSGQKAMLDAARGVLQLSH
>CORE_REP|Org21_Gene1518#
MDYLPIFADLKQRPVLVVGGGDVAARKVDLLQRAGAEIRIVAQSLSPELELQRQRGQLLWLGKTFDPP
QLDDVFLAIAATDDNALNAAVFAEADKRRVLANVVDDQPRCSFIFPSIIDRSPLVVAVSSSGQAPVLA
RLLREKLEALLPASLGQMAQVAGRWRGQVKQRLASIGERRRFWEKTFGGRFATLVANGQTAQAERQLE
QDLQSFAAGDEGAQGEIALVGAGPGDVGLLTLRGLQVMQQADVVLYDHLVSGEILDLVRRDAERICVG
KRAGAHSVIQEETNRLLVELAQQGKRVVRLKGGDPFIFGRGGEELQVAAAAGIPFQVVPGVTAAAGAT
AYAGIPLTHRDHAQSVTFITGHCRPDGDGLDWADLARARQTLAIYMGTMKAADISQRLIAHGRAADTP
VAVISRGTRADQQVQIGTLDQLEHLAQRAPLPALLVIGEVVELHHQIAWFGHQSQTEGAARPAVVNLA
>CORE_REP|Org25_Gene4261#
MKDNDRQNTFYIHDYETFGKSPSLDRPAQFAGVRTDMDFNIIEEPLVIYCAPADDYLPEPEAVMITGI
TPQVARAKGVNEAEFTRQIHQAFSVAGTCILGYNNIRFDDEVSRNIFYRNFYDPYAYSWQNGNSRWDL
LDVMRACYALRPDGIVWPENEDGFPSFRLEHLTRANGVEHTQAHDAMSDVYATIAMAKLVKQAQPRLF
DFLLQHRNKHKLNALIDVADMTPLVHVSGMFGAARGNTSWVSPLAWHPDNKNAVIMCDLAGDMTPLLT
LSAEQLRERLYTRRDDLAPDQAPVPIKLVHINKCPVLAPAKTLLTENAERLGIDRQACLQNLQLLKQH
PEVREKVVALFAEAEPFKGSEDVDARLYDGFFSDADKAAMRIIQQTKPQNLPALDLAFSDGRMKELLF
RFRARNYPNTLDDAEQRRWLQHRQEALSAERVQSYLLQLESLYNLHEGDKEKTALLKALFDYGKELVG
>CORE_REP|Org34_Gene4434#
MNNAIAQQIADQGGVESYLHAQQHKSLLRFLTCGSVDDGKSTLIGRLLHDTRQIYEDQLSTLHSDSKR
IGTQGEKKLDLALLVDGLQAEREQGITIDVAYRYFSTEKRKFIIADTPGHEQYTRNMATGASTCDLAI
LLIDARKGVLDQTRRHSFIATLLGIRHLVVAVNKMDLVDYQEAVFEQFKQDYLTFAQQLPGDLDIKFV
PLSALDGDNVASESAHMPWYSGPTLLEVLESVDVISERENQPLRFPVQYVNRPNLDFRGYAGTLSAGV
VRVGQRVKVLPSGVESSVARIVTFDGDLQEAVPGEAITLVLKDEVDISRGDLLVDAGESLQAAQSALV
DVVWMAEQPLVPGQSYDIKIAGKKTRARVESIRHQVEINTLTQHPADTLPLNGIGLVELTFDEPLVLD
SYQNNHDTGGLIFIDRMSNVTVGAGLVRETLQAASAARGEFSAFELELNALVRKHFPHWGARDLLGGR
>CORE_REP|Org31_Gene3880#
MQPAVSLIAGAVLSALLCSSAIAAETSANTDGLTERAARGTLTEPGGARRLAGDQTAALKASLSDKTV
KNVILLIGDGMGDSEITAARNYAEGAGGYFKGIDALPLTGQYTHYSLDKKTHKPDYVTDSAASATAWA
TGVKTYNGALGVDVNGKDQPTLLEIAKAAGKATGNVSTAELQDATPAALVSHVTSRKCYGPEETSEKC
AANALENGGRGSITEQLLKTRADVTLGGGAKSFNQLAKSGEWQGKSLKDQAAAQGYQWVSNADELQAV
TLANQQKPLLGLFADGNMPVRWLGPKASYHGNLDKPAVTCENNPARTAATPTLAAMTEKAIALLKDNT
NGFFLQVEGASIDKQDHAANPCGQIGETVDLDEAVQKALAFARADGNTLVIVTADHAHSSQIVAADAK
APGLTQTLTTKDGAPMTLSYGNSEEESQGHTGTQLRVAAYGPHAANVVGLTDQTDLFFTMRDAMGIK
>CORE_REP|Org5_Gene4763#
MRVKMSNLYPVIMAGGTGSRLWPLSRELFPKQFLALCNEFSMLQTTVMRLKGLEIINPLVICNEEHRF
IVAEQLRQITRLSHNIILEPVGRNTAPAIALAALQAVSSGDDPLMLVLAADHVIQDEAIFRDAVNQAI
PYAEAGKLATFGIVPTGPETGYGYIQKGASVDGSSICGVSRFVEKPNLETAQQYLASGDYLWNSGMFL
FKASRYLEELGRFRPDILDACKQSLAHLTPDMDFIRVDRDAFIACPDESVDYAVMEQTADAVVVPLDA
GWNDVGSWSALWEISEKDTKGNSTFGDVLEHNCSNNYIRAEHKLVAAVGVTNLVVVETKDAVLIADKD
NVQDVKEIVNQLKRQKRSESKQHREVYRPWGKHDAIAQGDRFQVRRITVKPGEKLSLQMHHHRSEHWV
VVSGTAKVHTNGKMQLISENESVYIPLGVEHSLENPGKIPLDLIEIQSGAYLGEDDIVRIGDSAQHN
>CORE_REP|Org49_Gene720#
MAGKKPTNKTNADETRERSRDRQMEGLKMPPHSLEAEQSVLGGLMLDNERWDNVAERVVANDFFSRPH
RLIFTEMQRLLEMSKPIDLITLSESLEQKGELDSVGGFAYLAELSKNTPSAANIGAYADIVRERAVVR
EMISVANEIADAGYDPQGRSSEDLLDLAESRVFQIAENRASKDEGPKGIERILEDTVSRIEQLYQQPH
DGVTGVDTGYQDLNKKTAGMQKSDLIIVAARPSMGKTTFAMNLCENAAMTQEKPVLIFSLEMPGEQIM
MRMLASLSRVDQTRIRTGQLDDEDWARISSTMGILLEKRNMYIDDSSGLTPTEVRSRARRIFREHDGL
SLIMIDYLQLMRVPALSDNRTLEIAEISRSLKALAKELQVPVVALSQLNRSLEQRADKRPVNSDLRES
GSIEQDADLIMFIYRDEVYHENSDLKGIAEIIIGKQRNGPIGTVRLTFNGQWSRFDNYAGPQYDDE
>CORE_REP|Org24_Gene3871#
MTKKLHIKTWGCQMNEYDSSKMADLLNSTHGFEWTENAEEADVLLLNTCSIREKAQEKVFAMLGRWRL

LKEKNPSVIIGVGGCVASQEGELIRSRAPCVDVVFGPQTLHRLPEMINHVQGTRSPVVDISFPEIEKF
DRLPEPRAEGPTAFVSIMEGCNKYCTFCVVPYTRGEEVSRPSDDVLFEIAQLAAQGVREVNLLGQNVN
AYRGATHDGDICSFAELLRLVAAIDGIDRIRFTTSHPIEFTDDIIAVYEDTPELVSFLHLPVQSGSDR
ILTMMKRAHTALEYKAIIRKLRKARPAIQLSSDFIVGFPGESQADFEQTMNLIADVNFDVSFSFIYSS
RPGTPAADMVDDVSEEEKKQRLYILQDRINQQALQFSRRMLGTVQRILVEGTSRKSVMELAGRTECNR
VVNFEGTPDMIGQFVDVEITEVLTNTLRGAVVRTEQQMDLRVHESPQSVIARTRKENALGVGIYQP
>CORE_REP|Org22_Gene4669#
MFPVPPTTKWVGVVNTELQSESSRLLASSNAGSPGWWLTVARRGTPWVEPAGNGRWRTTFFWRDPQGC
ELTSAYRRVWININCLTDHHQPNPPQSLQRLAGTDVWYWQTELSGAWRGSYCFIPCFDERPPAFSGDD
AHANMHNLRHWWHQVFASATPDLLNPYRSWQSASGHSVSGLHMPDAPPQPVWRSFDEYEIASGRCTPP
LPARLQRHTWQSERLGNSRDVWIYTTGDSKPAERPLAILLDGQFWAKQMPVWEPLMQLTREGALPEAV
YVLIDIIDLPHRSRELTCKDDFWLAVQEELMPQLADWAPHSGKPADTVVAGQSFGGLASLYAGLRWPQ
RFGAVIAQSGSYWWPRRDMLQLPSIPDDACWLMQQVERHGLGNHGALKVFMEAGSQEKLVHRVSGEMA
ARLSDAGHRVHYRVVEGGHDALCWRSGLTDGLQAVWASAFATAYPASATATATARGTHDGKPESVR
>CORE_REP|Org8_Gene2092#
MIARLLSNYIDTHMKAKAIFLASVLLVGCQSSRQDAPAPEQHAQSLSSAGQDGEAGEYTANGRASSAR
WLDNNSPAAQQDLWNFISDELKMEVPENSRIRDQKRKYLKSKSYLHDVTLRAEPYMYWIVGQIKKRNM
PMELVLLPIVESAFDPHATSSANAAGLWQIVPQTGRNYGLKNNQWYDGRRDVVASTTAALNMMQRLNR
MFNGDWLLTVAAYNSGEGRVMQAVKANKRQGKPTNFWALSLPRETSIYVPKMLALSDIIKNSKKYGVK
LPKTDETRALARIDVGQQIQLTQAAEMAGLSVTKMKAYNPGYKKGVTAPNGPHYIMVPKGHAEQLKDS
LADGQIAVTQPTTQLAKNSGLTGGSSYKVRSGDTLSGIAKRLNVKTSDLQSWNNLRAKSAIKVGQTLQ
VASNTGSNSSITYQVRKGDSLASIARRHGVDINDVMRWNSTLAKGNLQPGLKLTLFVGNKLTPDT
>CORE_REP|Org46_Gene2007#
MSQNVYQFIDLQRVDPPKKPLKIRKIEFVEIYEPFSETQAKAQADRCLSCGNPYCEWKCPVHNYIPNW
LKLANEGRIMEAADLAHQTNSLPEVCGRVCPQDRLCEGSCTLNDEFGAVTIGNIERYISDKAIEMGWK
PDMSHVQPTGKRVAIVGAGPAGLACADVLTRNGVKAVVYDRHPEIGGLLTFGIPAFKLEKEVMVKRRG
IFSEMGIEFQLNTEVGKDVSMETLLSEYDAVFLGVGTYQSMRGGLENEEAQGVYDALPFLIANTKQLM
GYEADQHEPYVSMEGKRVVVLGGGDTAMDCVRTSVRQGATQVICAYRRDEANMPGSKREVKNAREEGV
DFQFNLQPLSIELNSAGRVAGVKMVRTQLGAPDANGRQAAEQVPGSEHVIDADAVVMAFGFRPHRMDW
LAAHDVQLDKQGRILAPEGSDNAFQTSNPKIFAGGDAVRGSDLVVTAIAEGRKAADGIMNYLEV
>CORE_REP|Org13_Gene2318#
MTRYEQLAQQIREQIQNRVWRAGDKLPSLRESGKRAGLSLMTVVQSYQLLESQGWIVARPQSGYYVAA
RPQPLPQPSRGEKLLLSEQVDINAFIFDVLQACKDPDIVPFGSAFPDATLFAQPKLARALSSVARKFT
PHSSLANLPPGNDALRRHIAQRYALSGMQVAPDEIVITAGAMESLSLSLQAVTQPGDYVAIESPAFYG
ALQALERLRLKAVAIATHPQDGIDLDALQQAVEQYPIKACWLMTHFQNPQGATLPEANKQRLVALLRD
RQISLIEDDVYGELYFSAERPLPAKALDSGGQILHCSSFSKCLAPGFRVGWVAAGRYAQQIQRLQLMS
TVSTSVPTQMALADYLLHGGYDTHLRRLRRLLAQRQSAMRQAIAHHFPPTVKVSQPDGGYFLWLELDP
ALSSMELYRQALSRGISIAPGRMFTTGDHFNHCFRLNASFEWNDRFEEAIKTLAKLIRGLAAAG
>CORE_REP|Org34_Gene4429#
MRIHILGICGTFMGGLAMLARSLGHDVTGSDANVYPPMSTLLENQGIDLIQGYDPAQLDPAPDLVIIG
NAMTRGNPCVEAVLERGIPYVSGPQWLHDAVLRDRWVLAVAGTHGKTTTAGMATWILEACGYQPGFVI
GGVPGNFDVSARLGGSPFFVIEADEYDCAFFDKRSKFVHYSPRTLIMNNSPRTLIMNNLEFDHADIFD
DLKAIQKQFHHLVRLVPGKGKIILPDNDSHLKQVMAMGCWSEQELVGEEGTWRAQKLTPDASHYAVFL
DGEQVGEVNWALVGEHNMHNGLMAIAATRHVGVQPADACRALGDFINARRRLELRGEANGVTVYDDFA
HHPTAILATLAALRGKVGGTARILAVLEPRSNTMKMGISKNDLAPSLGRADEVFLFQPHHIPWQVAEV
ADACVQPAHWSADLDTLVDMVVKTAQPGDHILVMSNGGFGGIHDRLLDALAKKAGTEGDLLIVQ
>CORE_REP|Org11_Gene3175#
MTQSARSMAGLPWIAAMAFFMQALDATILNTALPAIAQSLGRSPLAMQSAVISYTLTVAMLIPVSGWL
ADRFGTRRVFIFAVTLFTLGSLLCALSPTLSALVASRVLQGIGGAMMMPVARLALLRAYPRSELLPVL
NFVTMPGLVGPILGPLLGGWLVTYATWHWIFLINIPIGLLGIFYARKYMPDFTTPKRRFDFLGFMLFG
LSLVLISTGLELFGERVLASYVSLGILLSGFVMLFGYITHARRHPQPLIGLDLFKTRTFSVGIAGNVA
SRLGTGCVPFLMPLMLQVGFGYTAIVAGCMMAPTAIGSLMAKSTVTQVLRWFGYRKTLVGITVIIGVL
IAQFALQSPGMPLWLMILPLFVLGMAMSTQFTAMNTISLADLNDANASAGNSVLAVTQQLSISFGVAI
SAAVLRFYESLSLGTMIDHFHYTFITMGIVTVASALVFMLLRRKDGRNLISGQESKKEAKAAS

>CORE_REP|Org21_Gene1305#
MKIKTRFAPSPTGYLHVGGARTALYSWLFSRHAGGEFVLRIEDTDLERSTQDAIDAIMDGMNWLNLDW
DEGPYFQTKRFDRYNAVIDEMLEQGTAYKCYCSKERLEALREKQMENGEKPRYDGHCRDSQCSHTDDE
PHVVRFRNPQEGSVIFDDKIRGPIEFSNQELDDLIIRRTDGSPTYNFCVVVDDWDMEITHVIRGEDHI
NNTPRQINILKALGAPVPEYAHVSMILGDDGKKLSKRHGAVGVMQYRDDGYLPQALLNYLVRLGWSHG
DQEIFSIDEMKEFFTLEAINKSASAFNTEKLQWLNHHYINHMPAEEVAVHLAWHVEQLGIETRNGPEL
KDIVKLLGERCKTLKEMAESCRYFYEDFSEFDADAAKKHLRPVARQPLEAVRAKLAAITVWTPENVHD
AIQGTADELGVGMGKVGMPLRVAVTGAGQSPGMDVTVHAIGQKRSLQRIDMALAYIAEREAQA
>CORE_REP|Org40_Gene1896#
MSLSRRQFLQASGLALCAGAVPLRAEASGTQTPLPIPPLLESRRGQPLFLTLQRAHWAFMDNRKAAVW
GINGMYLGPTVRVYSGDDVKLIYSNRLQEPVAMTISGLQVPGTLMGGAPRMMSPNVDWSPVLPIRQAA
ATCWYHANTPNRMAPHVYNGLAGLWLVEDAVSKALPLPNHYGVDDFPLIIQDKRFDNFGTPQYDAPSQ
GGFVGDTLLVNGVQNPYVDVSRGWVRLRLLNASNARRYTLQLSDGRPFNVIASDQGFLPAPVAVQQLS
LAPGERREVLIDMSKGEEVTITAGEAAGIMDRLRGLFEPSSILVSTQVLTLRPTGLLPLVTDNLPMRL
LADQLLDGSASRTRDFRLGDGGAGINGAIWDMNRIDVQAQQGTWERWNIHADTPQSFHIQGVQFLIKR
VNGAQPMAEDRGWKDTVWVDGDVELLVYFNQPTSEHFPFLYYSQTLEMADRGTAGQLMVQPTM
>CORE_REP|Org45_Gene4578#
MSKVKQQDIDRLIVLVGGRENIATVSHCITRLRFVLNDPSKASPKEIEELPMVKGCFTNAGQFQVVIG
TDVGDYYQALIASTGVNEADKEQAKVAARQNMTWTERTISHFAEIFFPLLPALISGGLILGFRNVIGD
IPMSGGQTLAQMHPAWKTIYDFLWLLGEAIFMFLPVAICWSTVKKMGGTPVLGIVLGVTLVSPQLMNS
YLLGQQTPEVWNFGWFVIQKVGYQAQVIPSILAGMALGWIETRLKKIVPDYLYLVVVPVVSLLLAVFL
AHALIGPFGRMIGDGVAWAVKAVMTGSFAPVGAALFGFLYAPLVITGVHQTTLAIDMQMIQSMGGTPV
WPLIALSNIAQASAVLGIIIISRKANEREISVPAAISAYLGVTEPAMYGINLKYRFPMLCAMIGSAIA
GLFCGLDGVMANGIGVGGLPGILSIKPQFWLIYSLAILVAIVIPLVLTIMVYKRKAARGELPV
>CORE_REP|Org39_Gene2538#
MASISSLGIGSGLDLNGLLDKLTKAEQQRLTPYTTQQTSYNAQLTAYGTLKGALEKFDNLSKDLAKPE
FFNNTTATKHDQFTVTTTDKSVPGNYSIEVLKLAQPQTLTTQTPIADQQAKLGTPGSSDRSISITAGN
PPKETKIPLGDDQTSLVEMRDAINKSKSGVTASIMRVGDNDYQLALSSTTPGEKNTIAVQVNNDDKLG
AILNYDPKKPPKDGSTAMKQTVPGQDAEIIVNGTKIKRSTNSIADALQGVTLDLKTTTKSGEPQNLVI
GIDKSGSADKIKEWVDNYNSLLDTFNSLTKYTPVKSGEAQNAKNGALLGDNTLRGIQSSIKSALSSAQ
DNPELKGLGNLGITTNVKTGKLEIDSTKLNKAIDEKPEQVANFFAGNGKDTGMATQIHNDIQSYIKAG
GIIENSTKSINTNLDRLNIQITTVTASIQNTIDRYKQQFVQLDTMMSKLSSTGNYLQQQFSAK
>CORE_REP|Org24_Gene1650#
MKKTKIVCTIGPKTESEEMLTNLLNAGMNVMRLNFSHGDYEEHGNRIKNMRAVMAKTGINAGILLDTK
GPEIRTMKLEGGKDASLVAGQTFTFTTDQSVIGNSERVAVTYAGFAADLKIGNTVLVDDGLIGMEVTN
VTENEVVCKVLNNGDLGENKGVNLPGVSIQLPALAEKDKRDLIFGCEQGVDFVAASFIRKRSDVLEIR
EHLKAHGGEQIQIISKIENQEGLNNFDEILEASDGIMVARGDLGVEIPVEEVIFAQKMMIEKCNRARK
VVITATQMLDSMIKNPRPTRAEAGDVANAILDGTDAVMLSGESAKGKYPLEAVNIMATICERTDRVMP
SRIDALNDRRKLRITEAVCRGAVETAEKLDAPLIVVATSGGKSAKSVRKYFPNAVILALTTNETTAHQ
LVLSKGVIPQMVKEIASTDDFYRIGKEAALASGLAQKGDVVVMVSGALVPSGTTNTASVHVL
>CORE_REP|Org34_Gene2045#
MQRGIVWIVDDDSSIRWVLERALTGAGLSCATFEGGNDVLEALATQTPDVLLSDIRMPGIDGLALLKQ
IKQRHPMLPVIIMTAHSDLDAAVSAYQQGAFDYLPKPFDIDEAVALVERAISHYQEQQQPVRSQPASD
PAADIIGEAPAMQDVFRIIGRLSRSSISVLINGESGTGKELVAHALHRHSPRAKSPFIALNMAAIPKD
LIESELFGHEKGAFTGANQIRQGRFEQADGGTLFLDEIGDMPLDVQTRLLRVLADGQFYRVGGYAPVK
VDVRIIAATHQNLELRVQEGKFREDLFHRLNVIRVHLPPLRERREDIPRLARHFLQIAAKELGVEAKN
LHPETETALTRLPWPGNVRQLENTCRWLTVMAAGQEVLIQDLPSELFETAAPESPSHSLPDSWATLLA
QWADRALRSGHQNLLSEAQPEMERTLLTTALRHTQGHKQEAARLLGWGRNTLTRKLKELGME
>CORE_REP|Org43_Gene4892#
MTKKLSYLAITTGLFLSTSAAAASGDGSIEARLNALEQRLAQAEQRAAQAETRATAAERRAQQLEQRT
ASTERQTAQVVQRAAALETQSSPTSALKLNGFNDLKLYGDVEFNLDGASRSGQLTSLKGSDHKDWKPG
NKERWDINGRILVGLDGYRRNPDGNFSGFSVQPLADMSGKMNLDDAAFFFGNEKNWQTKIGRFEAYDM
FPLNQDTFIQYSGNTANDLYADGFGYIYMMKEGRGRSSSGGNLMLSKYAGDVYFELNTLVEDGTSLFQ
DNSYHGNALENKKNVAYLRPVIAWKKDALSIAAAMESNVVNNAYGYQDSQGRFVDQSKRNGYGMTMSW

NNSAANPDNGVVANLSTAYLDASGEQDFTAGVNVLWRRFELGYIYAHNNIKEFNTAGIAADIHNPLSE
PGNYDIHTVHASYQIPNIMNMKNFNLYLGAYVSLLEANADNKIANGDNDQRYGMRARFKYFF
>CORE_REP|Org15_Gene1015#
MSAEHVLTMLNEHEVKFVDLRFTDTKGKEQHVTIPAHQVNADFFEEGKMFDGSSIGGWKGINESDMVL
MPDASTAVLDPFFEEPTLIIRCDILEPGTMQGYDRDPRSISKRAEDFLRSSGIADTVLFGPEPEFFLF
DDIRFGSSIRGSHVAIDDIEGAWNSGTKYDGGNKGHRPAVKGGYFPVPPVDSSQDLRSTMCLTMEEMG
LVVEAHHHEVATAGQNEVATRFNTMTKKADEIQIYKYVVHNVAHAFGKTATFMPKPMFGDNGSGMHCH
MSLSKNGTNLFAGDKYGGLSETALFYIGGIIKHAKAINALANPTTNSYKRLVPGYEAPVMLAYSARNR
SASIRIPVVASPKARRIEARFPDPAANPYLCFAALLMAGLDGIINKIHPGDAMDKNLYDLPPEEEAEI
PKVAGSLDEAMAALNEDREFLTRGGVFTDDAIDAYIELRKEEMDRVRMTPHPVEFELYYSV
>CORE_REP|Org27_Gene3126#
MQPSAPAAGQFKRSMKARHLVMLSLGGVIGTGLFFNTGYIISTTGALGTLLAYLIGALVVYLVMLCLG
ELSVAMPETGAFHVYASRYLGPATGYTVAWLYWLTWTVALGSSLTAAGFCMQYWFPQSPVWLWCLIFC
VAIFLLNVVTTRFFAESEFWFSLIKVVTILAFIILGGAAMFGLLPMKDGTPAPFLHNLTASGWLPHGT
LPILMTMVAVNFAFSGTELIGIAAGETENPEKVVPLAIRTTVIRLMLFFIGTVFVLAALIPMDQAGIV
KSPFVLVFERIGVPYAADIFNFVILTAILSAANSGLYASGRMLWSLAHQRTLPAYFARVNARGIPINA
LTFSMLGGVLALLTSVIAPDTVFVALSAISGFAVVAVWLSICAAHFAFRRAYLRSGQPISGLKYRAPG
YPLTPILGFALCLLACIGLAFDPEQRIALYCGLPFVALCYLTYFLTRRAGQKTALGEQHVG
>CORE_REP|Org18_Gene4521#
MEELSLIKQALRAVMNGQPRALRDPHRPAWHLAPSVGLLNDPNGFIQHNGVYHLFYQWNPLGCDHRNK
CWGHWQSTDLLRWEHQPIALAPGACYDSHGCYSGSAVVAEDKITLIYTGNVKFPDGSRTAYQCLAQES
DRSEYRKLGPVLPLPEGYSGHVRDPKVWRHQDAWYMVLGARDLQDRGKVLLLRSCDLRDWQALGEIAG
SGLNDLGEFGYMWECPDLFSLDGDDVLICCPQGLAPQPERYLNRYQAGYLLGKLDYRQAAFSHGEFRE
LDAGFEFYAPQTTQAEDGRRLLFGWMGVPEQDEEAHPTRRYGWIHTMTCPRELSLRHGRLYQRPAREL
QQLRGERAGWQGHADDAPAYALGAAELQLTPQGAFSAAFGDAMTLSWDGERLQLTRASLADGRPEHRY
WHGPVTHLQLLFDRSSVEIFINHGEAVMSARYFPAAEPQLRLNGSAPLALEYWPLTPCMLE
>CORE_REP|Org34_Gene1174#
MSKQQIGVVGMAVMGRNLALNIESRGYTVSIFNRSGDKTDEVIAENPGKNLAPYYTVEEFVESLEKPR
RILLMVKAGEATDKTIASLTPHLDKGDILIDGGNTYYQDTIRRNRELSDQGFNFIGTGVSGGEEGALK
GPSIMPGGQKEAYELVAPILEKIAAVAEGEPCVTYIGADGAGHYVKMVHNGIEYGDMQLIAEAYSLLK
QALNLSNEQLAETFAEWNKGELNSYLIDITKDIFTKKDEEGKYLVDVILDEAANKGTGKWTSQSSLDL
GEPLSLITESVFARYLSSLKDQRVAASKVLTGPKVAPFSGDKAEFIEKVRRALYLGKIVSYAQGFSQL
KAASKENNWDLHYGEIAKIFRAGCIIRAQFLQKITDAYAADADIANLLLAPYFKQIADEYQQALRDVV
AYAVQNGIPTPTFSAAIAYYDSYRSAVLPANLIQAQRDYFGAHTYKRIDKEGVFHTEWME
>CORE_REP|Org46_Gene1604#
MTAQQFVSPNEIRARFSHAMSDMYQKEVPLYGDLLELVAETNRQVLREDAALAHQLQITGEIERLAME
RHGAIRVGTADELATLRRLFRVMGMAPVGYYDLSVAGVPVHSTAFRAVHEDALQISPFRVFTSLLRLE
LIEDPALRALAERLLARRRIFTDRALELIALQEAQGGLNEVQAQEFVEQALETFRWHNQATVSAEEYR
QLHDQHRLIADVVAFKGPHINHLTPRTLDIDRVQQAMPGRGITPKAVIEGPPRRRRAILLRQTSFKAL
EERVDFVEHDGRAVAGHHTARFGEIEQRGVALTTKGRELYDRLLQATNDALQGPPSEKNAERYYQLLE
ENFRAFPDDYATLREQQLAWFRYFPTECGLAAKESLDKHSTLEQLIEKEHVRFQPLVYEDFLPVSAAG
IFQSNLGDSRHAQYNAASSRAAFEQALGSEVIDELALYQQTQQRSLEACAQALGLATLTS
>CORE_REP|Org47_Gene3139#
MQQQSPTTAPDNKLKRGLSTRHIRFMALGSAIGTGLFYGSADAIKMAGPSVLLAYLIGGIVAFIIMRA
LGEMSVNNPQASSFSRYAQDYLGPMAGYITGWTYCFEILIVAIADVTAFGIYMGVWFPEVPHWIWVLS
VVLIIGAINLISVKVFGELEFWFSFFKVATIIIMIAAGIGIIIWGIGNGGQPTGIHNLWSNGGFFSNG
FIGMILSLQLVMFAYGGIEIIGITAGEAKDPKKSIPKAINSVPWRILVFYVGTLFVIMSIYPWNQVGT
NGSPFVLTFQHMGITVAAGILNFVVITASLSAINSDVFGVGRMLHGMAEQGHAPKMFSKVSKRGIPWV
TVVVMMLALLLAVYLNYIMPESVFLVIASLATFATVWVWIMILFSQIAFRRSLSKEQVKQLAFPLRGG
VFTSVVAIVFLVFIIGLIGYFPTTRVSLYAGLVWVVLLLAGYWFKVNHQKKRAPLATQQD
>CORE_REP|Org41_Gene316#
MLGLDALELARIQFAFTVSFHIIFPAITIGLASYLAVLEGLWLKTHNEAYRELYHFWSKIFAVNFGMG
VVSGLVMAYQFGTNWSFFSEFAGSITGPLLTYEVLTAFFLEAGFLGVMLFGWNRVGPGLHFFATCMVA
LGTLISTFWILASNSWMQTPQGHEIINGQVVPVDWLKVIFNPSFPYRLLHMSTAAFLSSAFFVGASAA

WHLLRGRDTPAMRKMLSMAMWMALIVAPVQALIGDAHGLNTLKHQPAKIAAIEGHWENPPGEATPLIL
VGWPDMQREETRFKLEVPYLGSLILTHSLTEQVPALKSFPPEDRPNSTVVFWSFRIMVGLGMLMILAG
VWSLWLRWRGGLYQSRPFLYFILWMGPSGLLALLAGWFTTEIGRQPWVVYGLLRTKDAVSAHGDLHMS
ISLLAFIIVYCSVFGVGYSYMMRLIRKGPQPHEHQEDNTEGRPARPLSAVNDTLDDRS
>CORE_REP|Org15_Gene4429#
MSMSSSELDAARQTASEPRASELIYRLEDRPPLPQTLFAAGQHLLAMFVAVITPALLICQALGLPAQD
TQHIISMSLFASGLASILQIKTWGPVGSGLLSIQGTSFNFVSPLIMGGLALKNGGADVPTMMAALFGT
LMVASCTEIFLSRVLHLARRIITPLVSGIVVMIIGLSLIQVGLTSIGGGYAAMNDHSFGSPKNLLLAG
AVLAVIILLNRQRNPYLRVASLVIAMAVGYLLAWAMDMLPADAPAAPTAAITIPTPLYYGLGFDWNLL
LPLMLIFMVTSLETIGDITATSDVSEQPVSGPLYMKRLKGGVLANGLNSMLSAVFNTFPNSCFGQNNG
VIQLTGVASRYVGFVVALMLIALGLFPAVAGFVQHIPEPVLGGATIVMFGTIAASGVRIVSRERLNRR
AIMIMALSLAVGMGVSQQPLILQFAPDWLKTLLSSGIAAGGITAIVLNLVFPQEHEKK
>CORE_REP|Org34_Gene943#
MAKTLYQKLYDAHVVYEAPNETPLLYIDRHLVHEVTSPQAFDGLRAMGRKVRQPGKTFATMDHNVSTQ
TKDINASGEMARIQMQELIKNCAEFGVSLYDLNHPFQGIVHVIGPEQGMTLPGMTIVCGDSHTATHGA
FGSLAFGIGTSEVEHVLATQTLKQGRAKTMKIEVTGDAAEGITAKDIVLAVIGKTGSAGGTGHVVEFC
GKAIEALSMEGRMTLCNMAIEMGAKAGLVAPDDTTFAYLKGRQFAPTGENWEQAVAYWRTLKSDADAQ
FDTVVTLRAEEIAPQVTWGTNPGQVIAVNQAIPAPESFNDPVERASAEKALAYMDLKPGIKLTDVPID
KVFIGSCTNSRIEDLRAAAAIAKGRKVASGVQAIVVPGSGPVKAQAEAEGLDKIFIEAGFEWRLPGCS
MCLAMNNDRLNPGERCASTSNRNFEGRQGRGGRTHLVSPAMAAAAAVAGHFADIRDIH
>CORE_REP|Org5_Gene1561#
MSVVPVVDVLQGRAAVDSEVTVRGWVRTRRDSKAGISFLAVYDGSCFDPLQAVVNNSLPNYQDEVLHL
TTGCSVEVTGKVVASPGEGQSFELQATAINVVGWVDDPDTYPMAAKRHSIEYLREVAHLRPRTNLIGA
VARVRHTLAQAIHRFFHENGYFWVSTPLITASDTEGAGEMFRVSTLDLENLPRTDKGAVDFSQDFFGK
EAFLTVSGQLNGETYACALSKVYTFGPTFRAENSNTSRHLAEFWMIEPEVAFATLDDVAGLAESMLKY
VFQAVLDERADDLKFFAERVDKDAISRLERFVSSDFAQVDYTDAIEILLASGQTFENPVSWGIDLSSE
HERYLAEKHFQAPVVVKNYPKDIKAFYMRMNEDGKTVAAMDVLAPGIGEIIGGSQREERLDMLDQRLE
EMGLNKEDYWWYRDLRRYGTVPHSGFGLGFERLIAYVTGVQNVRDVIPFPRTPRNASF
>CORE_REP|Org29_Gene1696#
MTDDINRNEAWKVESTGIDRVPDSEQTGKPIELFWIWSAANIGILGVVYGAIIVGFGLSFLQSVLAAL
VGVASFVLVGLTSFAGKRGRTSTLTLSRVIFGLKGNVAPTLFSWINLMGWEAVNVITGTLTLAALFEA
FGLGSSHLLTALSLLLFGGLTVVVSLLGQNTVVWMQSWFSRIFGTMTLIVVLYILFNTEWSQVLALPS
GSWLTGFLPAVSVIAAGTGISWAIAGADYSRYQSPRSSSKSIFAAVMGGACLPLMLLMLAGILLSVQL
PDLASAANPIALIGSVLPAWMAIPYLLAATAGIVTIAVLSLYSASLNLLTIGVQVKQWLAVSIDAVVV
LGIALYVLFISGDFMGPFISFLVFCGVFLAAWEAIFLLDYLCLRRRHGYDGNALYGLHGQNRGVRKAP
LFCWFLGALCGLLVTKTGFIDGPLAKGLFADSSLGLFVSFAVSLIAYGLYLTQRREHQ
>CORE_REP|Org49_Gene4620#
MRHWKKKLGLTALTALVLSSMLGAGVFSLPQNMAQVASPAALLLGWGITGVGILFLAFAMLLLTRLRP
DLDGGIFTYAKEGFGELVGFCSAWGYWLCAVIANVSYLVIVFAALSIFTDRGGSVILGDGNTWQALIA
ESALLWIVHALVLRGVQTAASINLAATLAKLLPLGMFAVLAAIAFKMDVFTLDFKGIALGKPVWEQVK
DTMLITLWVFIGVEGAVVVSARARNKKDVGRATMLAVLSALAVYLMVTLLSLGVVPRSELAEMRNPSM
AVLMVELIGPWGDVLIAAGLIISVCGAYLSWTIMAAEVPLLAAQHGAFPRVFGKQNRHHAPSSSLWLT
NIAVQLALVLIWLTGSNYNSLLTIASEMILVPYFLVGAFLFKVAYRRRDKRLIFAATGACVYGLWLLY
ASGLMHLLMSVLLYAPGLLVFMYARRGHRDINLLNRLEKSSIFLLLAATLPAGWLMLH
>CORE_REP|Org23_Gene1424#
MAGVRIEKDSMGPIEVAADKLWGAQTQRSLAHFRISSEKMPTALIHALALTKRAAAQVNVDLGLLPAE
RGAAIINAADEVLADRHADQFPLSIWQTGSGTQTNMNMNEVLANRASELLGGVRGEERKVHPNDDVNK
SQSSNDVFPTAMHVAAVIALREHLIPELKVLHKTLSDKAEAYRDIVKIGRTHLQDATPLTLGQEISGW
AAMLAHNLKHIEDSIPHIAELALGGTAVGTGLNTHPEYAVRVAKALAELTHQPFVTAPNKFEALATCD
ALVQGHGALKGLAASLMKIANDVRWLASGPRCGIGEISIPENEPGSSIMPGKVNPTQCEAMTMLCAQV
LGNDVAVNIGGASGNFELNVFRPMVIHNYLQSIRLLADGMQGFNEHCAVGIEPNRDRITQLLNESLML
VTALNTHIGYDKAAEIAKKAHKEGLTLKAAALKLGYLTEAQFDEWVRPEAMVGSMQK
>CORE_REP|Org17_Gene161#
MQQHYQFDAIVIGSGPGGEGAAMGLVKQGARVAVIERYNNVGGGCTHWGTIPSKALRHAVSRIIEFNQ

NPLYNNSRTLSATFPDILRHADNVISQQTRMRQGFYERNQCKLFAGDARFIDANTVSVSYMDGTQDTI
RADHIVIACGSRPYHPASVDFNHPRIYDSDSILELSHEPRHVIIYGAGVIGCEYASIFRGLNVKVDLI
NTRDRLLAFLDQEMSDSLSYHFWNNGVVIRHNEEFEKIEGTEDGVIVHLKSGKKVKADCLLYANGRTG
NTDSLGLENVGLESDSRGLLKVNSMYQTALSHIYAVGDVIGYPSLASAAYDQGRIAAQAIASGEASGH
LIEDIPTGIYTIPEISSVGKTEQELTAMKVPYEVGRAQFKHLARAQIAGMNVGSLKILFHRDTLQILG
IHCFGERAAEIIHIGQAIMEQKGEGNTIEYFVNTTFNYPTMAEAYRVAALNGLNRLF
>CORE_REP|Org44_Gene4511#
MGMNFPLVINVLVFVALLLLLAQTRHKQWSLAKKVLVGLVVGVVFGLGLQLVYGSDNPVLKESISWFN
IVGNGYVQLLQMIVMPLVFASILSAVAKLHNASSLGKISVLTIGTLLFTTLISALVGVLVTNLFGLTA
EGLVQGAQESARLTAIQTNYVGKLADLTVPQMVLSFIPKNPFADLTGASPTSIISVVIFATFLGVASL
QLLNDDKPKGERVLVAIDTLQAWVMKLVRLVMKLTPYGVLALMTKVVAGSNIHDIVKLGSFVVASYIG
LGIMFVVHAALLAFTGVNPLKFFRKVWPVITFAFTSRSSAASIPLNVEAQTRRLGVPESIASFSASFG
ATIGQNGCAGLYPAMLAVMVAPTVGINPLDPVWIATLVGIVTISSAGVAGVGGGATFAALIVLPAMGL
PVTLVALLISVEPLIDMGRTALNVNGSMAAGTITSQLMKQTDKTVMDSEDEVELAHR
>CORE_REP|Org8_Gene458#
MSLSLWQQCLARLQDELPATEFSMWIRPLQAELSDNTLALYAPNRFVLDWVRDKYLNNINGLLNDFCG
TDAPLLRFEVGSKPITQVISQTVTASVSSAPAAPAARTAAPSRPSWDNAAAQPELSYRSNVNPKHTFD
NFVEGKSNQLARAAARQVADNPGGAYNPLFLYGGTGLGKTHLLHAVGNGIMARKANAKVVYMHSERFV
QDMVKALQNNAIEEFKRYYRSVDALLIDDIQFFANKERSQEEFFHTFNALLEGNQQIILTSDRYPKEI
NGVEDRLKSRFGWGLTVAIEPPELETRVAILMKKADENDIRLPGEVAFFIAKRLRSNVRELEGALNRV
IANANFTGRAITIDFVREALRDLLALQEKLVTIDNIQKTVAEYYKIKVADLLSKRRSRSVARPRQMAM
ALAKELTNHSLPEIGDAFGGRDHTTVLHACRKIEQLREESHDIKEDFSNLIRTLSS
>CORE_REP|Org25_Gene687#
MINSLTARIFAIFWFTLALVLMLVLMVPKLDSRQMTSLLDSEQRQGLMLEQHVEAELQNDPANDLMWW
RRLFRAIDKWAPPGQRLLLVTSEGRVIGAQRNEMQIVRNFIGQSDNSDHPKKKKYGRVELVGPFAVRD
GEDNYQLYLIRPANSPQSDFINLMFDRPLLLLIVTMLISAPLLLWLAWSLAKPARKLKNAADDVARGN
LKQHPELEAGPQEFLATGASFNQMVSALERMMNAQQRLISDISHELRTPLTRLQLATALMRRRHGEGH
ELARIETEAQRLDSMINDLLALSRGQQKGELAREQLKANELWADVLDNARFEAEQMGKQLEIAAPPGP
WTLFGNASALDSALENIVRNALRYSHTRIAVAFSADNQGVTIQVDDDGPGVSAEDREQIFRPFYRTDE
ARDRESGGTGLGLAIVEAAVNQHRGWVKAEDSPLGGLRLVLWLPLHHQRLSSKTEQ
>CORE_REP|Org18_Gene4752#
MAWFLPRFDVNDNSMTPQERRATWGLGTVFSLRMLGMFMVLPVLTTYGMALNGASEALIGIAIGIYGL
AQAVFQIPFGLVSDRIGRKPLIVGGLLIFALGSVIAAATDSIWGVILGRALQGSGAIAAAVMALLSDL
TREQNRTKAMAFIGVSFGITFAIAMVLGPIITHALGLHALFWMIAVLALAGIVITLAVVPSADTHLLN
RESSIVRGSFRKVLSNSRLLKLNFGIMCLHILLMSSFVALPLAMEKAGLAASEHWIVYLVTMLVSFAA
VVPFIIYAEKYRRMKQVFMGCVAVLFCAEVLLWLSGARLWGIIAGVQLFFIAFNVMEAILPSLISKES
PAGYKGTAMGVYSTSQFIGVAIGGSLGGWLYGLQGAGLVFIAGAVLAAVWFLVSSTMKEPPYVSSLRI
TLSELAVKDSALESRLKAQPGVAEAIVVPEERSAYVKVDTKQTNRGQLEALVNSL
>CORE_REP|Org48_Gene3914#
MENPQQPGRRAFLSQTGKLTTACAVIGLTGGMAQAASPGGEPCAPTPMTLTDRHYCLSEVRLEDGFEY
DGETVIGTRTALYTLEIKDGKIAAIHAANAALPAGVPRYKAQGRLLLPAFRDMHIHLDKTFYSGPWQA
PRPRQGKTIMDMIALEQTLIPTLLPTSQQRAENLIALLQSKGSTVARSHCNIDPVSGLKSLEHLQRAL
ENHRADFSCEIVAFPQHGLLHSKVDALMREAMQMGVQYVGGLDPTNVDGAMEKSLDAMFQIALDTGKG
VDIHLHETSPAGVAAINYMIATVEKNPALRGKVTISHAFALTTLTPGELAETATRLAAQQITIASTVP
IGGLMMPLPQLSEKGVFVMTGTDSVIDHWSPFGTGDILEKANLYAQLYRGSDEYHLSRAMAISTGGVL
PLDDKGQRAWPKAGDAAEFVLVNASCSAEAVARLPARSATFHQGRLVAGQVSKA
>CORE_REP|Org3_Gene2638#
MEYQFADGFWWGSATSAPQSEGAAARDGKSRNIFDYWYEIAPERFHDRVGPAEASTFYDHFRTDIGLL
KTLGHNTFRTSISWSRLIPDGDGEVNPQAVAFYNAMIDELLAQGITPFINLYHFDMPLCMQQRGGWES
RAVVEAYARYADICFGLFGDRVTHWFTFNEPIVPVEAGYLNDLHYPCVVDFKRAVTVAYHSVLAHAMA
VQRFRARELPGSIGIILNLSPTYPRSDAPADRQAAHDADLLLNRSFLDPVAKGRYPAALLQLLERHGL
MPHCEPQDAPLIEGGVVDILGVNYYQPRRVQAKEGRRAEGPIASPEDLFSYYAMPGRKINPHRGWEIY
EKGLYDILMDLKENYGNLPCYISENGMGVEGEEAFIGADGRVEDDYRIDFIREHLKWLHRALAEGSQC
KGYHLWTFIDCWSWLNAYKNRYGLVRLDRADQRRTIKKSGYWFAEAARRNGFD

>CORE_REP|Org8_Gene3333#
MYQATVGQRGYRFGDLRQLMAKASPARSGDYLAEVAAQSAEERMAARIALADLPLKAFLQQTLVPYEQ
DEVTRLIIDSHDAAAFEPISHLTVGDFRDWLLSEQADSAMLAQVAAGITPEMAAAVSKIMRNQDLILV
AKKCRVVTRFRNTIGLPGHLSVRLQPNHPTDSLQGIAASMLDGLLYGSGDAVVGINPASDSLPLLEKL
NYMLDDVIQRFAIPTQSCVLTHVTNTLRLMERGAPVDLVFQSIAGTEAANRGFGISLALLAEAQQAAL
SLRRGTLGDNVMYFETGQGSCLSANAHHGVDQQTCEARAYAVARHFSPLLINTVVGFIGPEYLYDGKQ
IIRAGLEDHFCGKLLGLPLGCDVCYTNHAEADQDDMDTLLTLLAAAGLTFLIGVPGADDIMLNYQSTS
FHDALYIRELLGLKHAPEFAAWLAAMNITDERGRLRDAAANHPLLLALQGERT
>CORE_REP|Org37_Gene1287#
MLKIFNTLSRQKEEFKPIHAGKVGMYVCGVTIYDLCHIGHGRTFVAFDVVARYLRYLGYSLNYVRNVT
DVDDKIIRRAAENHETCDQLTERMLAEMHADFDALLIDRPDQEPRATQHIAEIIEITQRLIDRDHAYV
ASNGDVMFSIDSDPQYGLLSRQDLDQLQAGARVEIDDVKRNPMDFVLWKMSKPGEPSWQSPWGPGRPG
WHIECSAMNCKQLGTHFDIHGGGSDLMFPHHENEIAQSSCAHDGPYVNYWMHSGMVMIDKEKMSKSLD
NFFTIRDVLGHYDAETVRYFLMSGHYRSQLNYSEENLKQARTALERLYTALRGTDADAAPAGGEAFEA
RFREAMDDDFNTPEAYSALFDLAREVNRLKGEDMAAANGMAAELRKLANVLGLLQQEPEQFLQGGAQV
DDGEVAEIEALIKQRNEARAAKDWALADAARDRLNEMNIVLEDGPQGTTWRRK
>CORE_REP|Org31_Gene850#
MMTDKVRIDTLVANSLNGNNETYLARQAEFESNVRSYPRKLPLAIAKAQGVWITDVENNQYLDCLAGA
GTLALGHNHPDVLQSIQNVITSGLPLHTLDLTTPLKDRFSDYLLSLLPGEGKEYCLQFCGPSGADAVE
AALKLAKKHTGRSGVISFSGGYHGMTHGALSVTGNLSPKAAINGMMPEVQFMPYPHEYRCPLGIGGEA
GVKALTYYFDNLINDVESGVRKPAAVILEAVQGEGGVNPAPAEWLQRIRKVTQEHGILLIIDEVQAGF
ARTGKLFAFEHAGIEPDIIVMSKAVGGGLPLAVLGIKKEFDAWEPGHHTGTFRGNQLAMATGLTTLQY
LKEHQVADKVAAQGEWLKGKLAELQKRYPVIGHVRGLGLMIGIEIVKPNEAQDHMGCYPADGELSALL
QKKCFESGLILERGGRNGCVLRLLPSLLITNDELGIFLDKFEQALLAAGVKPV
>CORE_REP|Org14_Gene3002#
MSYRSKVAIVYLLGFFVDLINMFIANVAYPAIGQAMRASVSQLAWVSNGYILGLTLVIPLSAWLAQRI
GGRRVFLLSLALFMLATFGAGNADSIGALIGWRTLQGMGGGLLIPIGQTLTYQLYRSHERAGLSAAIM
LVGLLAPALSPALGGWLVDRLDWRWVFFANLPLAALALALAALWLRAETSATAVRKPLDGKGLLSACA
ALTLLLLGLTRLSEAGHQASGAALLAAGLLVLAYYLRHSLRTPQPLLNLRLVGDPLLRNAMGVYLCIP
GLFIGVSLVAMLYLQNQLGMPAAQVGGLMLPWALASFLAITLTGKTFNRLGPRPLLIAGCLLQGAGML
TLAQIDQAGQHAWQIAAFALMGFGGSLCSSTAQSSAFLQIPDAQLADASALWNINRQLSFCLGVALLS
LLLNLLLTGLPPAAAYRTCFILAGASVFIPLLLCLRLANRAIVRQLNAQQDAL
>CORE_REP|Org10_Gene2637#
MSETTLAPSQTADAALAADERLATKEGRSQFWRATFSCWLGTAMEYVDFALYGLAAGMVFGDVFFPEA
TPLVALLASFATYSVGFVARPIGALVFGWIGDRKGRRVVLITTVALMGLSTTLIGLIPSYAQIGVWAP
ACLVILRFAQGFGAGAELSGGAVMLAEYAPAKRRGLVASIIAIGSNSGTLLASLVWLLVLQLDKEDLM
SWGWRIPFLASILIAGAALYLRRHVRETPVFERELQQNHQRMLDAAQAAPDTRSYLQRTKAFWVMLGL
RIGENGPSYLCQGFIVGYVAKVLMVDKSVPALAVLIASLCGFLVIPLAGWLSDRFGRRITYRWFCLLL
VLYAFPAFWLLDSREPAIVISVIVVGMCIASLGIFGVQAAYGVELFGVKNRYSKMAFAKELGSILSGG
TAPLIATALLSGFGHWWPVACYFVVMAAIGLITTFFAPETRGRDLNLPQDAA
>CORE_REP|Org12_Gene295#
MATGKIIQVIGAVVDVEFPQDAVPKVYDALEVENGTEKLVLEVQQQLGGGVVRCIAMGTSDGLRRGLK
VNNLDHPIEVPVGKATLGRIMNVLGQPIDMKGDIGEEERWAIHRAAPSYEELSSSQELLETGIKVMDL
ICPFAKGGKVGLFGGAGVGKTVNMMELIRNIAIEHSGYSVFAGVGERTREGNDFYHEMTDSNVLDKVS
LVYGQMNEPPGNRLRVALTGLTMAEKFRDEGRDVLLFVDNIYRYTLAGTEVSALLGRMPSAVGYQPTL
AEEMGVLQERITSTKTGSITSVQAVYVPADDLTDPSPATTFAHLDATVVLSRNIASLGIYPAVDPLDS
TSRQLDPLVVGQEHYDVARGVQSILQRYQELKDIIAILGMDELSEEDKLVVSRARKIQRFLSQPFFVA
EVFTGSPGKFVSLKDTIRGFKGIMDGDYDHLPEQAFYMVGTIEEAVEKAKKL
>CORE_REP|Org46_Gene2224#
MSFETLGLSAEIVRAVEEQGYREPTPIQRQAIPVVLEGRDLMASAQTGTGKTAGFTLPLLQLLSKHDH
PVKGRRPVRALILTPTRELAAQIGENVDAYSKHLRLRSLVVFGGVSINPQMMKLRGGVDILVATPGRL
LDLEHQNAVDLSKIEILVLDEADRMLDMGFIHDIRRVLAKLPAKRQNLLFSATFSDDIKALANKLLHN
PASVEVARRNTASEQIEQSVHFVDKKRKRELLSQMIGEGDWKQVLVFTRTKHGANHLAEQLNKDGITA
AAIHGNKSQGARTRALADFKDGRIRVLVATDIAARGLDIDQLPHVVNYELPNVPEDYVHRIGRTGRAE

RTGEAISLVCVDEHKLLRDIERLLKREIPRIALPGYEPDPTIKAEPIINGRQGGGRGAPRGNGGGQRS
GNGGGQRSGNANGGQRENRGNGNGNGNARPQGDGQRRSGAPSRRPRNRKPAE
>CORE_REP|Org12_Gene2801#
MSTVSFSSLPLPAEQLANLNELGYAEMTPVQAAALPAILQGRDVRAKAKTGSGKTAAFGIGLLNSIVV
GQVATQALVLCPTRELADQVSKELRRLARFTQNIKILTLCGGQPMGPQLDSLVHAPHIVVGTPGRIQE
HLRKKTLQLDELKVLVLDEADRMLDMGFADDIDDVISYTPPQRQTLLFSATYPAGIERISARVQRQPL
SVEVDDGEAQASIEQRFYETTRDQRPALLVSAIRYHQPASCVVFCNTKRDCQTVLEALEARGISALAL
HGDLEQRDRDQVLVRFANRSCRVLVATDVAARGLDIKELELVVNYELAFDPEVHVHRIGRTGRAGMSG
LAISLCTPQEMARAHAIEDYLQMSVDWSPVSELSGAANGSLEAEMVTLCIDGGRKAKIRPGDILGALT
GDAGLTAAEVGKIDMFPVHAYVAIRKASARKALQQLQQGKIKGKSCKVRLLK
>CORE_REP|Org33_Gene3049#
MKKTLGVFLPLYTTTLLLLLGSGLLTTYVSLRLASIHVSSALIGAIIAANYIGLVIGGKVGHFLIARV
GHIRAYVACAGIITAAVLGHGLTEFIPAWVALRLVIGLCMMCQYMVLESWLNDQAESNQRGMVFGFYM
AATYLGMSLGQIVLMLQSNLGITTLLVIALCFALCLVPIALTTRTNARHMSPAPMELRYFVGAIPKVL
ATTLVIGMVVGSFYGLAPVYASLQSLTTQQTGLFMALAIFAGLVAQFPLSWLSDRYSRPLLMRLNAIL
LIVAALPLALLPHIDFPLLLAVGFVVSMLQFTLYPLVVALANDLIEPERRVSLAACLLMAFGVGASIG
PLAVGALIEPLGGNILYAFFALCGVLLAALSRTAKAEEPQLAQDAPVPHIPLPDSLASSPLSPALNPT
FDEQIIHDTMPPPEAAPDVDEPQPEEQEAEPLPQGADPQEDTGLKKAHAML
>CORE_REP|Org13_Gene3724#
MSEALTCFKAYDIRGKLGSELNEDIAYRIGRAYGEYLRPKTMVLGGDVRLTSESLKLALARGLQDSGT
DVIDIGLSGTEEIYFATSHLKVDGGIEVTASHNPMDYNGMKLVREESKPISGDTGLRDIQRLAENNSF
PAVNDAARGGYQQLSILDAYVQKLLSFVALDNFTRPLKLVINSGNGAAGHVIDAIEARFKNAGLPVEF
IKVHHAPDGNFPNGIPNPLLPECRQDTTDAVLKHGADMGIAFDGDFDRCFLFDERGNFIEGYYIVGLL
AEAFLEKSPGSRIIHDPRLSWNTIDIVEKAYGIPVMSKTGHAFIKERMRKEDAVYGGEMSAHHYFRDF
YYCDSGMIPWLLVAELLCIKGRSLGELVNDRVAAYPASGEINSSLNNPKEAIGRVLGKYEMEADAVDH
TDGISVEYDNWRFNLRSSNTEPVVRLNVESRANVELMQEKTEEILQLLRSE
>CORE_REP|Org40_Gene1872#
MDGQQQGDQLKRGLKNRHIQLIALGGAIGTGLFLGIAQTIKMAGPSVLLGYAIGGFIAFLIMRQLGEM
VVEEPVAGSFSHFAYKYWGNFAGFASGWNYWVLYVLVAMAELTAVGIYVQYWWPEIPTWVSAAVFFLA
INAINLANVKVYGEMEFWFAIIKVVAIIGMIVFGAYLLFSGMGGPEATVTNLWAQGGFFPNGVMGLVM
AMAVIMFSFGGLELVGITAAEADNPQKSIPKATNQVIYRILIFYIGSLAILLSLYPWGKVVEGGSPFV
LIFHALNSNLVATVLNVVVLTAALSVYNSCVYCNSRMLYGLAQQGNGPKSLLKVDGRGVPVVAIGISA
LATALCVLINYLIPGRAFELLMALVVSALVINWAMISLAHLKFRAAKNREGVVPKFKAFWYPFSNYLC
LLFMAGILVIMYLTPGIQISVLLIPVWVAILAVGYAIKQRSQRVDGVTSR
>CORE_REP|Org32_Gene89#
MKIIILGAGQVGGTLAENLVGENNDITVVDTDSGRLRQLQDKFDLRVVQGHGSHPRVLREAGAEDADM
LVAVTNSDETNMIACQIAYSLFNTPNRIARIRAPEYIRESEKLFLPEAVPIDHLISPEQLVIDYIYKL
IEYPGALQVVNFAEGKVSIAAVKAYYGGPLVGNALSSMREHMPHIDTRVAAIFRQDRPIRPQGSTIIE
AGDEVFFVAASQHIRAVMSELQRLEKPYKRIMIVGGGNVGAGLAAKLEKDYNVKLIERNQQRAAELAE
QLHDTIVFYGDASDQELLAEEHVEQVDVFIAITNDDEANIMSAMLAKRMGAKKVMVLIQRRAYVDLVQ
GSVIDIAISPQQATISALLGHVRKADIVSVSSLRRGVAEAIEAIAHGDESTSKVVGRIVEDIKLPPGT
TIGAIVRGDDVIIANGNSKIEQGDHVIMFITDKKFVPDVERLFQPSPFFL
>CORE_REP|Org5_Gene2995#
MALWGGRFTQAADQRFKQLNDSLRFDYRLAEQDIVGSVAWSKALVTVNVLTATEQQQLEQALNALLTE
VQADPLAIVQSDAEDIHSWVEQKLIEKVGDLGKKLHTGRSRNDQVATDLKLWCKQQIGDLHQAIVQLQ
QALVETAEANQDAVMPGYTHLQRAQPVTFAHWCLAYVEMLARDESRLQDTLKRLDVSPLGSGALAGTA
YPIDREQLAGWLGFASATRNSLDSVSDRDHVLELLSNAAISMVHLSRFAEDLIFFNSGEAAFVELSDR
VTSGSSLMPQKKNPDALELIRGKCGRVQGALTGMMMTLKGLPLAYNKDMQEDKEGLFDALDTWMDCLQ
MAALVLDGIQVKRPRCQEAAEQGYANATELADYLVAKGVPFREAHHIVGEAVVEAIRQGKPLEALPLA
DLQQFSATIGDDVYPILALQSCLDKRAAKGGVAPQQVAAAIAAAKQRLA
>CORE_REP|Org1_Gene3914#
MSEQTIVWDLALIQKYNYSGPRYTSYPTALEFNQCYDEAAFQRAAARYPERPLSLYVHIPFCHKLCYF
CGCNKLVTRQTHKADEYLNVLAQEIASRAPLFAGRKVGQMHWGGGTPTYLDKAQISRLVALLREHFDF
LPDAEMSIEVDPREIELDVLDHLRAEGFNRLSMGVQDFNKQVQQLVNREQDEAFIFALIERAKALGFR

STNIDLIYGLPKQTPESFAFTLQRVAELNPDRLSVFNYAHMPNLFAAQRKIKDADLPGAQQKLDILQQ
SIAFLTDAGYQFIGMDHFAHPDDELAIAQREGKLHRNFQGYTTQGDSDLLGLGVSAISMLGDSYAQNQ
KELKRYYDSVQAQGNALWRGLALTDDDCLRRDLIKTLICNFRLAYQQLERQYGIDFTAYFAEDLQLLA
PFERDGLVERDEQGIRVTPRGRLLIRNICMCFDRYLRQQARSQQFSRVI
>CORE_REP|Org22_Gene2005#
MSNSAMSVVILAAGKGTRMYSDLPKVLHPLAGKPMVQHVIDAAMKLGAQHVHLVYGHGGDLLKSTLTD
GALNWVLQAEQLGTGHAMQQAAPHFADDEDVLMLYGDVPLISVDTLQRLLAAKPQGGIGLLTVKLDDP
SGYGRIVREQGNVVGIVEHKDASEAQRQINEINTGILVANGRDLKRWLGMLNNDNAQGEFYITDIIAL
AHADGKKIETVHPSRLSEVEGVNNRLQLATLERVFQSEQSEKLLLAGVMLLDPARFDLRGELVHGRDI
TIDANVIIEGHVKLGDRVKIGAGCVLKNCVIGDDCEISPYSVLEDSVLEADCTVGPFARLRPGAELAA
GAHVGNFVEMKKARLGKGSKAGHLSYLGDAEIGDDVNIGAGTITCNYDGANKHKTVIGDGVFVGSDTQ
LVAPVSVGKGSTIAAGTTVTRDIAENELVLSRVKQVHIQGWQRPVKKKS
>CORE_REP|Org11_Gene3462#
MPAYFAPRALLPEGWAHNVRLDVDAQGYLTQVTANAEPQGCIRLHGDAVPGMPNLHSHAFQRAMAGLA
EVAGNPQDSFWTWRDLMYRLVQRLTPEQVEVVARQLYIEMLKGGYTQVAEFHYLHHDADGKPYADRGE
MTGRLSEAAHQAGIGMTMLPVLYSYAGFGAQPAQPGQKRFIQDAESYLHQQQVIARQLADRPFQNQGL
CFHSLRAVELGQMQQILAASDRTLPVHIHIAEQQKEVNDCLAWSGQRPVAWLYDHLPVDQRWCLVHAT
HLDRDELEALARSRAVAGLCLTTEANLGDGIFPGDAYLQQQGRWGIGSDSHVSLNVVEELRWFEYGQR
LRDQRRNRLTTPEQSAVGDVLYLQALQGGAQACGAPIGRLQSGYRADWLVLDGDDPYLAAAPDASILN
RWLFAGGKEQIRDVFVGGRQVIDQGRHALQQQSSAEFLQVLKTFQQEAQ
>CORE_REP|Org49_Gene4060#
MSENTAETQAEIISAPRAGGKKVIFAASFGNALEFFDFGIYNFFVIYISVLFFPPSSDPHLALLLAFA
TFGVSFFMRPLGGILIGAYADRCGRKPAMILTISLMSLGTAMIGFAPTYASAGYWGTVTLVAARLIQG
VAAGGEVGASMSLLVESAPPNRRGFYSSWSLATQGIATVVGGVTALALSAALPALSGEPDAMAEWGWR
IPFFIGVALAPLGCWLRLGLESDRPSVPRAHEPPAQLRKHSRAVVLGVMLTIGATVATYISMYYLGTY
AVKYLGMAQAYGYAAMLLAGLVTFGGSLLVGHWCDRYGRLPLIRWSRIAILIVALPAFWWLSAVPHPA
VLLLIVALLVGLTTLGSVPTMLMISELFPQRIRALGFALVYSLGVAIFGGFAQYIASQSIALSGSLLA
PAVYLMLATLASLALLPFQVGVQAVGRAITLRVSMRTSAMVTCKVSLWR
>CORE_REP|Org7_Gene2401#
MRRLRFSPRSSFARTLLLIVTLLFVSLVTTYLVVLNFAILPSLQQFNKVLAYEVRMLMTDRLQLEDGT
LLEVPPAFRREIYRELGISLYTNSAAEESGLRWAQHYQFLSQQMAQQLGGPTDVRVEVNKNSPVVWLK
TWLQPDIWVRVPLTEIHQGDFSPLFRYTLAIMLLAIGGAWLFIRIQNRPLVELEHAALQVGKGIIPPP
LREYGASEVRSVTRAFNQMASGVKQLADDRTLLMAGVSHDLRTPLTRIRLATEMMSAEDGYLAESINK
DIEECNAIIEQFIDYLRTGQEMPTESSDLNAILGEVVAAESGYERVIETALSPGELMMNVHPLSIKRA
AVNMVVNAARYGNGWIKVSSGRELQRGWFQVEDDGPGIKPDELKHLLQPFVRGDSARSTSGTGLGLAI
VQRIIDAHDGELDIGTSERGGLLIRAYIPLPMEKKESTNGHQTARETA
>CORE_REP|Org20_Gene3244#
MHSNIIGFNPLLAELPASPTRAFHKELEHYLQRYPQTEQLDIYLHDLNGQLRGKRLPIAEAFGLEKGC
YFPLSIYALDLHGRVIEESGLGQRAGEPDRLCLPVPGTLRPCARDPERHAQLLLTMQNADGGACELEP
RVVLQRVLKRLHERNCFPVVAAELEFYLQDPQHPAAAETCPTQSFAVDAPERHHALLSDIERHARLQN
LPLTGVVAEAASGQYELNLHHSRRVLEACDQIMALKRLTRQIAEQHHQHACFMAKPCAHAAGSGLHFH
ISLQNEHGENLLTGAPGELSDNMQQAMAGMLALMPASMAILAPNINAFRRFRPGMHVPLRASWGHNNR
TVALRLPCADSANQRIEYRLAGADANPYLALAVMLGGLLHGLEQPLPLPPAANGCESDNAAPLPLGQQ
EALALFRHSDPLRELLGPAFCTLWHTCKNAELRRFEEQVTAAELGWML
>CORE_REP|Org34_Gene2699#
MSTSLLLLIAVLGVVLLLLMVIKAKVQPFVALLVVSLLVALASGIPTGEVMKVMTAGMGGVLGSVTII
IGLGAMLGRMIEHSGGAESLAQRFSQGLGPKHTVAALTLAAFILGIPVFFDVGFIILAPIIYGFAKVA
KVSPLKFGLPMAGVMLTVHVALPPHPGPVAAAGLLNADIGWLTIIGLAICIPVGVIGYFAANYLNRKT
YPLSIEVLEQLQLAAPEPRPEGQAPLSDRINPPGAGLVAALIVIPIAIIMLGTVSATLLPAGSALRDA
LSLLGSPAVALMIALLLAFYFLALRRGWSLQHASDVMGAALPTAAVVILVTGAGGVFGKVLVESGVGK
ALAEVLTAIGLPLVPAAFIISLALRASQGSATVAILTTGGLLSEAVSGLNQLQLVLVTLATCFGGLGL
SHVNDSGFWIVTRYLGLSVADGLKTWTVLTTLLGLSGFLFTWLLWLAV
>CORE_REP|Org1_Gene1887#
MTIKAIDGGPAGKPQLRKSLKLWQVVMMGLAYLTPMTVFDTFGIVSGLTDGHVPTSYLLALAGVLFTA

ISYGKLVRQFPTAGSAYTYAQKAINPHVGFLVGWSSLLDYLFLPMINTLLAKIYLTALFPEVPPWVWV
VGFVILITAINLKSVNLVANFNTLFVLAQVAIILVFIYLVVRGLHNGEGMGTVWSLRPFLSENAHLLP
IITGATILCFSFLGFDAVTTLCEETPDAAKVIPRAIFLTALYGGVIFISVSFFIQLFFPSIQRFHQPD
AALPEIALYVGGKLFQSIFLCVTFINTLASGLASHASVSRLLYVMGRDNVFPEKFFGYIHPKWRTPAL
NVLMVGLVALSALSFDLVTATALINFGALVAFTFVNLSVISHFFIREGRNKSWKDRFNFLFLPLVGAL
TVGVLWLNLEKSSLTMGLIWATLGFGYLAWLTRRFRQPPPQLERQPQQ
>CORE_REP|Org25_Gene3923#
MELSSLTAVSPVDGRYGDKVSALRPIFSEYGLLKFRVQVEVRWLQKLAACAEIKEVPAFDADANAFLD
KIVAEFNEEDAQRIKTIERTTNHDVKAVEYFLKEKVAAVPALHAVSEFIHFACTSEDINNLSHALMLQ
SARQDVVLPYWRKIIDALKGLALEYRDIPLLSRTHGQPATPSTVGKEFANVAYRMERQYRQLERVEIM
GKINGAVGNYNAHIVAYPEVDWHQFSEAFVTSLGITWNPYTTQIEPHDYIAELFDCVARFNTILIDFD
RDIWGYIALNHFKQKTIAGEIGSSTMPHKVNPIDFENSEGNLGLANAVLGHLAGKLPVSRWQRDLTDS
TVLRNLGVGLGYALIAYQATLKGISKLEVNQTHLLDELDHNWEVLAEPIQTVMRRYGIEKPYEKLKEL
TRGKRVDAAGMQAFIDGLALPEEEKTRLKAMTPANYIGRATTLVDELK
>CORE_REP|Org5_Gene4424#
MDITATANALLPLLGGKENIASAAHCATRLRLVLVDDSKVDKEAIGKLDGVKGCFSNAGQIQVIFGTG
LVNKVHAEFIKAAGVSESSKAEAADIAAKKLNPLQRIARLLSNIFVPIIPAIVASGLLMGLLGMVKTY
GWADANSALFIMLDMFSSAAFIILPILIGFTAAREFGGNPYLGATLGGILTHPALTNAWGVAEGFHTM
NFFGLEIAMIGYQGTVFPVLLTVWFMSLLEKRLRKVIPNALDLILTPFLTVVISGFVALLFIGPAGRA
LGDGISFVLSTLIAHAGWFAGLLFGGLYSAIVITGIHHSFHAVEAGLLGNPNIGVNFLLPIWSMANIA
QGGACLAVYFKTRDAKIKAIAVPSAFSAMLGITEAALFGINLRFVKPFLAALAGGALGGAWVVANHVG
MNAVGLTAIPGMAIVQASSLVSYIIGLAIAFGSAFALSLLLKYKTDAQ
>CORE_REP|Org6_Gene3716#
MVSVFDIFKIGIGPSSSHTVGPMKAGKQFVDDLIAHQQLQDTTRVVVDVYGSLSLTGKGHHTDIAIIM
GLAGNLPHDVDIDSIPGFIRDVEQRGRLPLANGHHEVDFPLHGGMNFHSDNLPLHENGMRIRAFADER
LLHSKTYYSIGGGFIVDEEHFGQSAEGATPVPYPFKSAHDLQQHCKETGLSLSGLVMQNELALRSKAD
IDAHFADVWQVMSAGIERGINTEGLLPGPMKVPRRAAALRRILVTGDKNNIDPMNVVDWINMFALAVN
EENAAGGRVVTAPTNGACGIIPAVLAYYDKFIRPVNANSYTRYFLASGVIGALYKMNASISGAEVGCQ
GEVGVACSMAAAGLTELLGGSPAQVCIAAEIAMEHHLGLTCDPLAGQVQVPCIERNAISAVKAVNAAR
MAMRRTSEPRVCLDKVIETMYETGKDMNAKYRETSQGGLAIKVVACN
>CORE_REP|Org48_Gene2921#
MTLAQFGGLFVVYLVSLTFILTLTYQEFRRVRFNFNVFFSLLYLLTFYFGFPLTCLLVFQFDVEVVPV
EFLLYALLSATAFYAIYYVTYKTRLRKRRSQPRAALFTMNRVETHMTWVLLALVAIGTVGIFFMQNGF
LLFKLNSYSQIFSSDVSGVALKRFFYFFIPAMLVVYFLRQDLRAWFLFLVATVAFGILTYVIVGGTRA
NIIIAFALFLFIGIVRGWITLWMLAAAGIFGVVGMFWLALKRYSLDVSGAEAFYTFLYLTRDTFSPWE
NLALLLQNYDKIDFQGLAPILRDFYVFIPTWLWPGRPDVVLNSANYFTWEVLNNHSGLAISPTLIGSL
VVMGGALFIPLGAILVGMIIKWFDWLYELGKTEPNRYKAAILQGFCFGAVFNIIVLAREGVDSFVSRV
VFFCIIFGACLVLAKLLYWLFDTAGLIKARVTRARALASPPRANGLL
>CORE_REP|Org18_Gene3234#
MKILSPLALSLAALLTAGCGNALKSDYRAPQVNYPTSWQHAADNAAPTPFDWRDFHDPELDRWLQQVM
DSNNDLAVAVLRVYRARLEAERVGISTAPDVNASLNSGINRPLSESSAWNKTSGATLSTSYEVDLWGK
LARQRDAAEWASQASEQDLQTARLTLLANAATNYWRIGFLNQQIGVSQASIAYAKQTLRLANARYRAG
SISALDVVNAEQNVLTQESRLLALQHDRQQALNEQAVLLGAPTGQATIAPARLPTTAMPQINTGIPAS
VLSRRPDLSAKELRLRAALANVDEKRLQYYPAFSLTGSLGASSSALLEFLRNPTGSLGASLTLPFLQW
RQMGVDIKIARNDYEQQVLEFRQALYKAMGDVNNALSLRAQLRAQETQLQASLALARKSERLNEVRYR
QGAVTITDWLNAQEQRRQAELAVDENRFAQYQNLAKIYLEFGGSSAP
>CORE_REP|Org18_Gene1706#
MAIPHSVIEMIGNTPMLELTRFDTGPCRLFVKLENQNPGGSIKDRVALSMIEQAERDGKLQPGGTIIE
ATAGNTGLGLALVAALKGYKLLLVVPDKMSREKIFHLRALGVEVLLTRSDVGKGHPAYYQDYAKRLAG
EIPGAFYIDQFNNPANPAAHTTTTAPELWRQMEHDVDAIVVGVGSGGTLGGLSRYFAEVSPQTEFVLA
DPAGSILADYLDNGRIGEAGSWLVEGIGEDFVPPLSDFDQVRNAYRIGDAEAFTTARDLLRKEGVLAG
SSTGTLLAAALRYCRAQTEPKRVVTFVCDSGNKYLSKMYNDHWMLEQGLLSKPQHGDLRDLIAYRHDE
GAAVSAAPDDTLAIVHARMRLYDISQLPVLEGDRVVGLIDEWDLLNAVQADAAHFSLPAGSAMTKQVQ
TLQKEAGYDELQATFNHGHVAVVLDGERFLGLITRTDVLNAWRQKLR

>CORE_REP|Org28_Gene2410#
MITHISPLGSMDLLSQLEVDMLKRTASSDLYRLFRNCSLAVLNSGSQTDNSKQLLSRYETFDINVLRR
ERGVKLELVNPPEEAFVDGRIIRSLQANLFAVLRDILFVHGQIASAGRFQHLNLENSAHITNLVFSIL
RNARTLHLDEDPNMVVCWGGHSINENEYLYARKVGSQLGLRELNICTGCGPGAMEAPMKGAAVGHAQQ
RYRNSRFIGMTEPSIIAAEPPNPLVNELVIMPDIEKRLEAFVRIAHGIIIFPGGVGTAEELLYLLGIL
MNPENSEQVLPLILTGPKESADYFRVLDEFIMNTLGDEARRHYTIIIDDPAEVARQMKQAMPLVKENR
RNTGDAYSFNWSIRIAPDLQLPFEPTHENMANLNLYPNQPPEQLAAALRRAFSGIVAGNVKENGIHAI
EQFGPYKLHGEPQMMKQMDSLLQGFVAQHRMKLPGSAYVPCYEIVA
>CORE_REP|Org36_Gene1404#
MSTTDTIVAQATPPGRGGVGILRISGSQAKDVAQALLGKLPKPRYADYLPFRDATGATLDQGIALWFP
GPNSFTGEDVLELQGHGGPVILDLLLKRVLALPDVRIARPGEFSERAFLNDKLDLAQAEAIADLIDAS
SEQAARSAMNSLQGAFSTRIHQLVEALTHLRIYVEAAIDFPDEEIDFLSDGKIEAQLNDVMADLDSVR
GEARQGSLLREGMKVVIAGRPNAGKSSLLNALAGREAAIVTDIAGTTRDVLREHIHIDGMPLHIIDTA
GLRDASDEVERIGIERAWNEIEQADRVLFMVDGTTTAATEPAEIWPEFMARLPGTLPITVVRNKADIT
GETLGLTEVNNHSLIRLSARTGEGVDVLRDHLKQSMGFTSNMEGGFLARRRHLQALELAAQHLVQGKE
QLVSAYAGELLAEELRQAQLALSEITGEFTSDDLLGRIFSSFCIGK
>CORE_REP|Org32_Gene2304#
MMQETVLHKIVRDKAQWIAARQQQQPLAGFQNDIVPSERSFYHALQGTRTAFILECKKASPSKGLIRE
SFDPVEIATVYKDFASAISVLTDEKYFQGSFDFLPLVSKTVSQPVLCKDFIIDPYQIYLARYYQADAI
LLMLSVLTDEQYRQLAAVAHSLNMGVLTEVISEEELQRAIALEARVVGINNRDLRDLSIDLDRTRQLA
PRVPHGVTVISESGINNYGQIRELSHYANGFLIGSALMSEPDLRAAVRRVILGDNKVCGLTRPQDAAA
AYQAGAIYGGLIFVGRSPRYVDITRAREVISGAPLKYVGVFCDAQVETVALTVERLGLHAVQLHGAED
QAYISALRARLPADCRIWKALSVKDRVPARDLQYVDRYLLDNGAGGTGQRFDWSVLQDEDLTNVMLAG
GLRADNCVEAAKLGCAGLDFNSGVESEPGIKDPARLASVFQTLRAY
>CORE_REP|Org41_Gene3508#
MIPVSLQTLAEVLSAELIGADCQIVEVTTDTRKVTAGCLFVALKGERFDAHDFAADAVAAGAGALLVS
KRLLVDAPQLVVQDTRLALGQLGAWVRQQVPARVVALTGSSGKTSVKEMTAAILRECGEVLYTAGNFN
NDIGVPLTLLRLEPQHDFAVIELGANHIGEIAYTTALTRPQTALVNNLAAAHLEGFGSLAGVAQAKGE
IFTGLPADGVAIINADNNDWPHWQSMLGGKTVWRFSPQAAAGVDFFADNVRVNGAGTQFTLHSPFGTA
EIALPLPGRHNVANALAATALATSVGATLEAVRQGLKQLQAVPGRLFPVALAEGKLLLDDSYNANVGS
MTAAAQVLAEMPGYRVMVVGDMAELGAEAEECHRQVGEAARLAGVDKVISVGGLSRVLSEASGNGEHY
QDKTAVIARVAELLSEHAVITVLIKGSRSAAMEQVVRALQEKAPC
>CORE_REP|Org14_Gene947#
MTARLGRWLSSLDTLEKRIARAPMVRRYGRLTRATGLVLEATGLQLPLGATCLIERHDAGEVQEVESE
VVGFNGQRLFLMPLEEVEGIVPGARVYARIAPEGQSAGKQLPLGPALLGRVLDGSAKPLDGLPSPETG
YRAPLITAPFNPLQRTPIEQVLDVGVRTINGLLTVGRGQRMGLFAGSGVGKSVLLGMMARYTQADVIV
VGLIGERGREVKDFIENILGAEGRARSVVIAAPADVSPLLRMQGAAYATRIAEDFRDRGQHVLLIMDS
LTRYAMAQREIALAIGEPPATKGYPPSVFAKLPALVERAGNGISGGGSITAFYTVLTEGDDQQDPIAD
SARAILDGHVVLSRRLAEAGHYPAIDIEASISRAMTSLIDEEHYRRVRTFKQMLASYQRNRDLISVGA
YAAGSDPLLDKAMTLYPQMEAYLQQGIFERSGYDEACQQLQQLIV
>CORE_REP|Org39_Gene373#
MSNSQANHAAKPTGGLDGYFKISARGSSVRQEVVAGLTTFLAMVYSVIVVPSMLGKAGFPPAAVFVST
CLVAGLGSLLMGLWANLPMAIGCAISLTAFTAFSLVLGQHISIPVALGAVFLMGVLFTIISVTGIRAW
ILRNLPMGVAHGTGIGIGLFLLLIAANGVGLVVKNPLDGLPVALGAFTSFPVVMTLVGLAVIFGLEKL
RVPGGILLVIIAISIIGLIFDPAVKYQGLFAMPSLADADGKSLIFSLDIMGALQPVVLPSVLALVMTA
VFDATGTIRAVAGQANLLDKDGQIINGGKALTSDSLSSIFSGLVGAAPAAVYIESAAGTAAGGKTGLT
ATVVGILFLLILFLSPLAYLVPVYATAPALMYVGLLMLSNVTKLDFNDFVDAMAGLLCAVFIVLTCNI
VTGIMLGFSSLVIGRIFSGEWRKLNIGTVLIAVALVAFYAGGWAI
>CORE_REP|Org12_Gene4291#
MKRLSLRLRLILIFSLLALLTWCTASVVAWVMSRNTINEVFDTQQMLFAKRLATANLGDLLADESARS
LPKTKKLVHHGKRGEQDDDALAFAIFDRDGKMLLNDGENGADFLFDGEREGFTDGERKGDDDSWRLVW
LTSPDGRYRIVVGQEWDYRRDMALGMVTGQLVPWLATLPVLMLLIALMVGRELRPLRAVAAGLRRRAP
DDATPLDARQVPTEVRPLVDALNALFARINALLVRERRFTSDAAHELRSPLAALRVQTEVVQLAGDDA
PMREHALDNLTVGIDRATRLVDQLLTLSRLDSLLDLAELAPIDWNDLVTMTLAEQDRQAHAAGVTLRY

EHRGTPPPRQGETLLLSLLLRNLLDNAVRYTPQGGVVTVTLSERSLTVEDDGPGVTAEHLARLGERFY
RPPGQEQTGSGLGLSIVQRIAGLHGLQISFANRSAGGFVARLAL
>CORE_REP|Org39_Gene1040#
MSTTDDSYLVAKDKAADIPLREKWWRILDNYKVGIIPLPFFILAGVLILLDCLEGKLPSDIVVMVATL
AFFGFACGEFGKRLPLIGKMGAAAICATFIPSALVHYGLLPDVVVESTTKFYKSTNILYLYICCIIVG
SIMSMNRQVLIQGFLRIFIPMLCGEIVGMLVGMGVGMALGLEPFQIFFFLILPIMAGGVGEGAIPLSM
GYAAILHMEQGVALGRILPIVMLGSLTAIVIAGLLNQLGKRYPHLTGEGSLMPSKQGDSDMNAVEKIS
GKVDVSTIACGALLAILLYMVGMLLHRLIGLPAPVGMLFAAVAVKLAHGVSPRIQEGSQVVYKFFRTA
VTYPILFAVGVAITPWQELVDAFTVQNLIVIVTTVSALVATGFFVGKKIGMHPIDVAIVSCCQSGQGG
TGDVAILTAGNRMSLMPFAQIATRIGGAINVSLSLLVLAKFFV
>CORE_REP|Org3_Gene230#
MVSVLWNLVAFLIALGILITVHEFGHFWVARRCGVRVERFSIGFGRALWRRTDRQGTEYVIALIPLGG
YVKMLDERVESVAPEMRHQAFNNKTVWQRAAIISAGPIANFLFAILAYWLVFIIGVPSFRPVIGEIAP
QSIAAKAEISSGMELKSVDGIETPDWESVRLALVAKIGDAQTEVGVAPFGSSQVVTKTLDLRQWNFEP
DKQDPVVALGIMPRGPQIESVLAEVQPNSAAQKAGLQAGDRIVKVDGQPLGRWQTLVKRIHDGPGQPL
ALEIERNGAPLSLTLIPDTKPVGKDKSVGFAGIIPKVLPLPDEYKTIRQYGPFPALYQAGDKTWQLMR
LTVNMLGKLITGDVKLNNLSGPISIAQGAGASAGVGFVYYLMFLALISVNLGIINLFPLPVLDGGHLL
FLAIEKLKGGPVSERVQDYSYRIGSIVLVLLMGLALFNDFSRL
>CORE_REP|Org49_Gene2784#
MPHDNNVEIRGFIDDQPFSRYQWLILVLCFLAVALDGFDTAIIGFIATSLVQEWGIEKTSLGPVMSAA
LVGLAVGALAAGPLADRIGRKKVLVISLLLFGGFSLLTAFAGSLSTLTLLRFLTGLGLGAAMPNAATL
MSEYAPQRCRALMVNLMFCGFPLGSSLGGFSSAWLIPHFGWQSVMVLGGVMPLLLALVLIAALPESAR
FMVARGYAAERIAKVLKRIAPLPKTPHFMLQEEGQIKAASPLGMIFSRRYLLGTLMLCLTYFMGLMIF
YLLTSWLPLLIRETGASVTQASLITALFPLGGGLGVLIIGWLMDRMNPHKVVAVGYLLTGLFVGIIGF
VYSYPPLMAITVFIAGTCMNGAQSSMPALAAGFYPTQSRATGVAWMLGLGRFGGILGAMSGGALMQMQ
LSFSTIFTLLAIPALIAALALIAKHLSGYPALPAPLNKNAVRE
>CORE_REP|Org4_Gene4199#
MFIGFDYGTANCSVAVMRDHGPELLTLENNEPYLPSMLCAPTREAVSECLHRHWQVPTGSEENQQLLR
RAISYNREEDIPVGGDSVLFGLQALAHYMEDPEEVYFVRSPKSFLGANGLKPQQIALFEDLVCAMMFH
IKRQAENVLQAGIDQAVIGRPINFQGIGGEEANRQAQGILQRAAERAGFKEIEFQFEPVAAGLDFETT
LSEEQTVLVVDIGGGTTDCSVLLMGPQWRDRADRQQSLLGHSGCRVGGNDLDIMLAFKQLMPLFGLGG
ETGKGIALPALPYWNAVATNDVPAQNDFYSAANGRVLRDLILDAAEPEKVKRLLKVYQQRLSYRLVRA
AEESKIALSGQTAISAPLSFVQADLAESISQDQLADAISQPLMRIQEQVSAALASSQTAPQVIYLTGG
SARSPLLRAALQQQLPGIPIVGGNDFGSVTAGLARWAQTLFR
>CORE_REP|Org9_Gene4194#
MTKHYDYLAIGGGSGGIASINRAAMYGQKCALIEAKELGGTCVNVGCVPKKVMWHAAQIAEAIHQYGP
DYGFDTIVNAFDWKKLVANRTAYIDRIHNSYDNVLGKNKVDVIKGFARFVDAHTVEVNGELITADHIL
IATGGRPSHPAIPGAEYGIDSDGFFELDAMPKRVAVVGAGYIAVEIAGVLNALGAETHLFVRKHAPLR
SFDPMIVETLVEVMNTEGPSLHTESVPKAIVKNADGSLTLQLENGKAFTVDCLIWAIGREPATDNLNL
GVTGVKTNEQGYIDVDKFQNTNVKGIYAVGDNTGAVELTPVAVAAGRRLSERLFNNKPDEHLDYSNIA
TVVFSHPPIGTVGLTEPEAIEKFGADNVKVYKSSFTAMYSAVTQHRQPCRMKLVCAGKEEKIVGLHGI
GFGMDEILQGFAVAVKMGATKKDFDNTVAIHPTAAEEFVTMR
>CORE_REP|Org22_Gene1689#
MFTPELLSPAGTLKNMRYAFAYGADAVYAGQPRYSLRVRNNEFNHENLAQGINEAHALGKKFYVVVNI
APHNAKLKTFLRDLKPVIDMGPDALIMSDPGLIMMVREAFPQMDIHLSVQANAVNWATVKFWKQMGLT
RVILSRELSLEEIAEIRAEVPDMELEIFVHGALCMAYSGRCLLSGYINKRDPNQGTCTNACRWQYKAE
EGKEDDTGNIVHLHEPIPVQTVEPTLGIGAPTDKVFMLSEAQKPGEYMSAFEDEHGTYIMNSKDLRAI
QHVERLTQLGVHSLKIEGRTKSFYYCARTAQVYRRAIDDAAAGKPFDPTLLTTLEGLAHRGYTEGFLR
RHVHEAQQNYDYGSSLSERQQFVGEFTGVRRDGWAEVDVKNKFSLGDSVEMMTPGGNVVFTLESMQNK
KGEPIEVAPGNGHIVYLPIPQDIDLNYALLIRNLAEENSVGA
>CORE_REP|Org41_Gene3516#
MAQFYSPKRRVTTRQSSQIFTVTVSDLDPFGQGVARHDGKAVFVPGVLPGEQAEIQLTEDKRQFAKGK
LKRLLSRSPQRVTPRCPHFGVCGGCQQQHADEALQQQSKAAALVRMIARETGVTPQQEPVIAGPEYGY
RRRARLSLAWQPKLQRLMMGFRQAASSELVAVEHCPVLRPELEQLLAPVRACLSGLQAVRRLGHVELV

LADNGPLLVLRHLDALKEADRQALLAFGERENVAVYLAPDSDRVEKLCGDTPYYQVDGLRLDFSPRDF
IQVNDAVNQQMVAQALEWLDIQPNDRVLDLFCGMGNFTLPLARRAAAVVGIEGVATLVANGQYNAHKN
KLNNASFFHENLEDDVARQPWAAQGFDKVMLDPARAGAAGVMSHIVKLAPARVVYVSCNPTTLARDSK
VLLSAGYRLARVRMLDMFPHTGHLESMALFINERAPEVAAK
>CORE_REP|Org24_Gene3288#
MIKIRKGLDLPIAGAPVQAIQDGPNIQHVALLGEEYVGMRPSMLVQEGDTVKKGQALFEDKKNPGVFF
TAPASGRIAAINRGERRVLQSVVIALENGGDDQLEFAHYPLGELAQLPREQVESELIASGLWTALRTR
PFSKTPSPGSEPRAIFVTAMDTRPLAADPQVIIAEQQAAFNAGLVVLARLTAGKVHVCHAAGASVGQQ
SGPQIAYSEFTGPHPAGLVGTHIHFLEPVSLKKTVWHIGYQDVIAIGTLFTTGQLDTRRVVALAGPQV
AQPALLRTRLGASLDELTAGRLKNGENRVISGSVLNGMHATGPNAWLGRFHSQVSVLEEGRDKELFGW
IVPSPNKFSITRTTLGHFLKNKLFAFSTTTNGGERAMVPIGNYERVMPLDILPTLLLRDLLAGDSDSA
QALGCLELDEEDLALCTFVCPGKYEYAPVLREVLTKIEQEG
>CORE_REP|Org19_Gene367#
MLDKIVIANRGEIALRILRACKELGIKTVAVHSAADRDLKHVLLADETVCIGPAPSVKSYLNIPAIIS
AAEITGAVAIHPGYGFLSENADFAEQVERSGFIFIGPKAETIRLMGDKVSAINAMKKAGVPCVPGSDG
PLTDDMDKNRAFAKRIGYPVIIKASGGGGGRGMRVVRSDKDLEQSINMTKAEAKAAFNNDMVYMEKYL
ENPRHIEIQVLADGQGNAIYLAERDCSMQRRHQKVVEEAPAPGITSEMRRYIGERCSKACVEIGYRGA
GTFEFLYENGEFYFIEMNTRIQVEHPVTEMITGVDLIKEQLRIAAGQPLSIKQEEVKIHGHAVECRIN
AEDPNTFLPSPGKITRFHAPGGFGVRWESHIYAGYTVPPYYDSMIGKLITFGENRDVAIARMKNALAE
LIIDGIKTNVELQQKIMNDENFQHGGTNIHYLEKKLGLQET
>CORE_REP|Org38_Gene883#
MEYAVSVESFLSSLQRHNPHQPEYLQAVREVFTSLWPFIERNPRYREQALLERLVEPERIIQFRVSWV
DDRGQVQVNRAFRVQFNSAIGPYKGGMRFHPSVNLSILKFLGFEQTFKNALTTLPMGGGKGGSDFDPK
GKSQGEIMRFCQALMTELYRHLGPDTDVPAGDIGVGGREVGFMAGMMKKLSNNTACVFTGKGLSFGGS
LIRPEATGYGLVYFTDAMLQRHGLGFEGMRVAVSGSGNVAQYTIEKALELDARVITVSDSGGTLVDED
GFTTEKLAHLAEIKNQRYGRVADYARERGLTYLAGQQPWNVPVDIALPCATQNELDLEAAQTLIRNGV
KAVAEGANMPTTIQATDAFLDAGVLFAPGKAANAGGVATSGLEMAQNAARIGWRAEKVDVRLQHIMAD
IHHACVEYGGEGKQTHYVHGANIAGFVKVAEAMLAQGVL
>CORE_REP|Org16_Gene3986#
MKVTVFGIGYVGLVQAAVLAEVGHDVMCIDVDERKVKNLKKGNIPIFEPGLTPLVQQNYEAGRLHFTT
DAKAGVAHGNIQFIAVGTPPDEDGSADLKYVTAVARTIAEHMTDRKVVIDKSTVPVGTADKVRQVMAE
TLAKRGSNVAFDVVSNPEFLKEGAAVADCMRPERIVIGTDNKEVIEPIRELYEPFNRNHDRMIMMDIR
SAELTKYAANCMLATKISFMNEMSNLAEMLGADIEKVRQGIGSDSRIGYHFIYPGCGYGGSCFPKDVQ
ALIRTAEQIGYQPKLLQAVEQVNYQQKDKLNSFIKDYFGSDLKGKTFALWGLAFKPNTDDMREASSRV
LMEQLWAAGATVQAYDPEAMNEVQRIYGQRDDLKLMGTKEAALHGADALVICTEWQNFRAPDFDVIKS
ALKQPVIFDGRNLYDPERLENRGFTYYAIGRGASIKPVI
>CORE_REP|Org21_Gene2387#
MKQINAELACDKNTASSESSGLINQLRQIDIGAVPIALFITICAIVAISAYANFLPKNMIGGLAVIMT
LGFALAQLGKSIPVLRDIGGPAILCLMVPSVLVYFNVFQPNVMGTVHLLMKDANLLYFVIASLVVGSI
LGMNRVILIQGMIRMFVPLVVGTVTAVITGLLVGKLFGFTFYHTFFFIIVPIIGGGIGEGILPLSLAY
SAILGATPDVYVAQLAPAAVLGNIFAIVTAGVLARIGMQRKALSGDGMLIRSAQENAMFAIKEQSGNV
DFQLMGGGLLVICAFFIVGGLFEHIVHIPGPVLMILFAVLCKYCRVIPASMETGAHSFYKFVSTALVW
PLMIGLGMLYVPLESVVAVFSVGYVVVCGSVVLSMGLVSFLIAPYLKMFPVEAAIVTCCHSGLGGTGD
VAILSASNRMGLMPFAQIATRIGGASTVIGATLLLGWLV
>CORE_REP|Org43_Gene4143#
MHNTKTRLALLVGCMALSANLWADAQPVQATLAGHALLPVKSAVSTPKDAPSDLQQSGKYTSGKRVTE
LGSVAGKSADRLTGFGLPIDGQPLQGHSGIKHMPDGTYWVLTDNGFGSKANSPDAMLYLNHYKIDFKD
GTVAPLKTVFLHDPDKKVPFHIINESTEKRYLTGSDFDPESFQFADDALWIGEEFGPYLIKADLNGKV
LAVFDTQVDGKVVKSPDNPTLTLPGAPDGKQNFQVARSKGFEGMAASPDGSKLYPLLEGALWDGEQFE
NVGGKRYLRVLEFDVKQQAWTGRSWQYVLEDNQNAIGDFNMIDATHGLVIERDNGEGTPDKACAAGAP
TDNCFSQVAKFKRVYKIAFSDANVGKPVEKLGYIDLMNIQDPNKLARKPLNDGVLTFPFFTIENVDVV
DANHIIVGNDNNFPFSSSRQPNMADDNEFILLDVKDFLK
>CORE_REP|Org6_Gene4262#
MPTGRFVGRIAEGFVWRYGGGLPAKKQYCYTYDFLTFRRKFTRYAGITRLMDDLNEGLRTPGAIMLGG

GNPAQIPEMEAYFKQLCQEMLDQGKLTEALCNYDGPQGKDALLKALANLLRDELGWEISPQNIALTNG
SQSAFFYLFNLFAGRYADGSRRRVLFPLAPEYIGYADAGLDEGLFVSAKPNIELLPDGQFKYHVDFEH
LTIGDDIGMICVSRPTNPTGNVITDEELMKLDLLAQQRDIPLVIDNAYGVPFPGIIFSDATPLWNPNT
ILCMSLSKLGLPGSRCGIVIADEKVITALTNMNGIISLSPGSMGPAMATEMIARGDLLRLSNEVIRPF
YKQRVEHTIEIIRRYLSPERCLIHKPEGAIFLWLWFKDLPITTEVLYQRLKKRGVLMVPGHYFFPGLE
HDWPHTHQCMRMNYVPDPEKIERGVAILAEEIERAHQEG
>CORE_REP|Org39_Gene3088#
MSQKNLLTKSALAAAVAIISSNVSAAGFQLNEFSAAGLGRSYSGEGAMADTAASASRNPALLMMYTRP
ELSIGAVFIDPDVDITGKSPSGASLDAKNIAPTAWVPNLHYVHPINDQFAVGGSVTSNYGLATEFNDG
YTAGAYGGKTDLETLNLNLSGAYRLDQHFSFGLGFDAVYARAKLERYAGELPKLLAGQGLQTGQLTPQ
QAGMIAQIPGDTQISHLKGDKWGFGWNAGLLYEVDENNRYGFTYRSEVKIKFDGDYKSSLPAQYNPIT
QALGVGLPWGTSGSTIPGSLDLNLPEMWELSGYNKVAPQWAIHYSLAYTSWSQFQELRATGSNGQTLF
QKHEGFRDAYRIALGTTYFYDDNWTFRGGVAFDDSPVPAQNRSISIPDQDRFWVSAGTSYAFNKDASV
DVGVSYMHGQKVTINEGPYTFNSVGKAWLYGANFNYRF
>CORE_REP|Org30_Gene3692#
MAAENHPLNSLDAEGLEAHWMPFTGNRNFKAQPRIITQAAGAYYTSHDGRKIFDGLSGLWCCGLGHGR
QEITDAAQRQLATLDYSPAFQFGHPLSFELANKIKALTPAGLDYVFFTGSGSEAADTSLKMARAYWRA
KGQAGKTCFIGREKGYHGVNFGGISVGGIAGNRKTFGAGAEADHLPHTLLAGNAFSRGMPQQGAELAE
ELNRIIALRDASTIAAVIVEPFSGSAGVIVPPVGYLQRLREICTQHDILLIFDEVITAFGRCGAMTGA
EAFGVTPDIMNIAKQVTNGAQPMGAVVVRPEIYQTFMNGGEPDYQLEFPHGYTYSAHPVSCAVALATL
DILQREQMVERVQALAPYFERAVHSLQGAKHVADIRNIGLAAGITLAARPGEPARRPFEAAMRCWESG
FYVRYGGDTLQLAPPFISSEAQVDALINAVGDALNATE
>CORE_REP|Org47_Gene2010#
MKVVTQVAEQRKALEQAVSQALELARAGSDAAEVAVTKSTGISVSTRFGEVENVEFNSDGALGITVYH
RQRKGSASSTDLSPDAIARTVQAALDIARYTSEDPCAGPAEKDLLAFEAPDLDLFHPIELDAERGIEL
AARAEQASLAADKRITNTEGGSFNSHYGIKVFGNSHGMLQSYCSSRHSLSSCVIAEQDGDMERDYAYT
IGRAMGDLQSPEWVGQECARRTLARLAPRKLSTMKAPVLFASEVATGLFGHLVGAISGSSVYRKSTFL
LDSLGKQILPAWLTVEEHPHLLKGLASTPFDSEGVRTQRRDIVKDGVLQTWLMTSYSARKLGLHSTGH
AGGIHNWRIAGQGADFAGMLKQLGTGLVVTELMGQGVSGVTGDYSRGAAGFWVENGEIQYPVSEITIA
GNLKDMLRNIVSVGSDIETRSNIQCGSVLLPEMKIAGQ
>CORE_REP|Org13_Gene871#
MDFFLQLAVILACLLYGARKGGIALGLLGGIGLMILVFGFHLQPGKPPVDVMLVIIAVVAASATLQAS
GGLDVMLQIAERMLRRNPRYVSIIAPFVTCILTILCGTGHVVYTILPIIYDVAIKNNIRPERPMAASS
IGAQMGIIASPVSVAVVSLVAMLSSYTFNGRHLEFLDLLSITIPSTLCGILAIGIFSWFRGKDLDKDP
EFQKFISVPENHRYVYGDAATLLDRVLPRSNWIAMWIFLATIALVAVLGAFSDLRPSFGGKPLSMVLV
IQMCMLMAGALIVIITRTNPASISKNEVFRSGMIAIVAVYGVAWMAETMFGAHLAQIEATLGVLVKEY
PWAYALILLLVSKFVNSQAAALAALVPVALAIGVNPAYIVASAPACYGYYILPTYPSDLAAIQFDRSG
TTRIGRFVINHSFILPGLIGVSVSCVFGWILAAAFGFL
>CORE_REP|Org39_Gene1516#
MSERKYFGTDGIRGKVGDSPITPDFVLKLGWAAGKVLARHGSRKIIIGKDTRISGYMLESALEAGLAA
AGLSASFTGPMPTPAVAYLTRTFRAEAGIVISASHNPFYDNGIKFFSIDGAKLPDNVEEAIEAEMEKP
LTCVESAELGKASRIIDAAGRYIEFCKGTFPSELSLKGLKIVVDCANGATYHIAPSVLRELGATVIAI
GVEPDGMNINEKCGATDVRQLQERVLKEKAHVGLAFDGDGDRVMMVDHLGNKVDGDQILYIIAREGLR
QGQLRGGAVGTLMSNMGLELALKQLGIPFARAKVGDRYVLEKLQELGWRIGAENSGHVILLDKTTTGD
GIVAGLQVLTAMVRNHMSLHDLCSGMKLLPQILVNVRFAGDHNPLESDAVRKVTEQVEAELAGRGRVL
LRKSGTEPLIRVMVEGEDEQQVTALAHRIADAVKSAG
>CORE_REP|Org22_Gene3871#
MKAFVPALVLSAVSFSVWAQDATVSSELIKRGEYLARAGDCVACHTDGKSGKTFAGGLAMETPIGTIY
STNITPDKKTGIGDYSFEDFDNAVRKGVAKNGSTLYPAMPYPSFALVKEEDMRAMYAYFMHGVQPVEQ
ANKDSDIPWPLSMRWPLSIWRGMFAPSPADFVADAKADPVIERGRYLVEGLGHCGACHTPRSITMQEK
ALSNGESDDYLSGSNAPIDGWVASSLRSDRKDGLGSWSEAELTEFLKTGRNDKAIVFGGMSDVVEHSL
QYLSDDDLTAIARYLKSLPPKDGKQQAAPVEDSVAKDLWRGDDSKPGAALYVDNCAACHRTDGVGYKR
AFPSLKGNPVVQTEDATSLIHIVLTGNTTPAVQGAVSNITMPPFGWRLNDQQVADVVNFIRTSWGNSA
KPVSASDVADVRKDRSMIRDEKAMGSAEVPDHPDAKK

>CORE_REP|Org31_Gene3088#
MREKAFFALSRVTRSANAGSRPLPERTAPRTFTAMCASVFCAGVLLLPLAGQAAEDNKSQLKDIQQSI
AEKEKAVKQQQQQRSSLQDQLRQQEKTIAQASRQLRDTQSTLTQLGKDIAGLNASIAKLQKQQSTQQN
LLAKQLDAAFRQGQHSAVQLILSGEESQRSERILAYFGYLNEARQKTIEELKQTRAELAKQKTTLVAK
QGQQKSLLGEQQTQQQKLEQARGARKKTLTALEASLEKDQQRLVELRQNEARMRDKIARAEREARARA
EREAREAAKVREQVRIKEQQAKKTGTTYKPSEADRSLMARTGGLGRPAGQLMWPVRGRTLHGFGEQQQ
GELRWKGMVIEAREGSEVKAVADGRVLLADWLQGYGLMVVVEHGKGDMSVYGYNQSALVNVGAQVRAG
QPIALVGTSGGQGTPSLYFEIRRQGQAVNPLPWLGR
>CORE_REP|Org41_Gene971#
MSEMTPREIVSELDSYIIGQNKAKRAVAIALRNRWRRMQLNEMLRHEVTPKNILMIGPTGVGKTEIAR
RLAKLANAPFIKVEATKFTEVGYVGKEVDSIIRDLTDAAIKMVRMQSIEKNRTRAEELAEERILDVLI
PPAKNNWGQPEEHQEPSAARQAFRKKLREGQLDDKEIEIDLAAAPMGVEIMAPPGMEEMTNQLQSMFQ
NLGGQKQKPRKVKIKEAFKLLVEEEAAKLVNPEELKEQAIEAVEQHGIVFIDEIDKICKRGGQSSGPD
VSREGVQRDLLPLVEGCTVSTKHGMVKTDHILFIASGAFQTANPSDLIPELQGRLPIRVELQALTTED
FERILTEPSASLTEQYKALMGTEGVNIEFTADGIRRIAEAAWQVNESTENIGARRLHTVLERLMEDIS
YDASEINGQSITIDADYVRSHLDELVADEDLSRFIL
>CORE_REP|Org1_Gene1537#
MSTHIGEPQDSYIEEIPQDERRFTRMGWLVVGIGLFGFLAWAAFAPLDKGVASPGSVTVSGNRKTVQA
PASGIIKNIAVKEGDKVKAGEVLVQLSQVQAQAQVDSLRDQYYTTLATEGRLLAERDGLSSVTFSPIF
TQIKDQPRVAEIIALQTQLFASRRQGLQSEIDGYKQSMDGIRFQLKGLQDSRVNKQIQLSSLREQMNS
MKQLAADGYLPRNRYLEVQRQFAEVNSSIDETVGRIGQLQKQLQESQQRIDQRFADYQREVRTQLAQT
QMDASEFRNKLQMADFDLGNTAITSPVDGTVVGLNIFTQGGVVGAGDHLMDVVPSQATLVVDSRLKVD
LIDKVYNGLPVDLMFTAFNQNKTPKIPGTVTLVSADRLVDKANGEPYYQMQVTVSPEGMKMLSGEDIK
PGMPVEVFVKTGSRSLLSYLFKPILDRAHTSLTEE
>CORE_REP|Org42_Gene2581#
METLASLYNDHLAELQKRAREVLERNKLDALLIHSGELQKVFLDDHSYPFKVNAHFKAWVPVTSVPNC
WLWIDGVNKPKLWFYSPVDYWHSVEPLPDSFWTKSLELMPLANADAIAQQLPPQRERVGYIGYAQQRA
RDLGIPSENINPKAVLNYLDFHRSIKTGYELACMREAQKTAVTGHRAAHEAFLSGMSEFDINLAYLTA
TGHRDTDVPYDNIVALNEHASVLHYTKLDHQPPAESLSFLIDAGAEYNGYAADLTRTYAAQSGSEFAH
LVKDLNGEQLALIDTIKTGVRYTDYHVQMHQRIAKLLKNHKLVNGLSEEAMVETGITTPFLPHGLGHP
LGLQVHDAAGFMQDEQGTHLAAPAKYPFLRCTRVLQPGMVLTIEPGLYFIESLLAPWRSGEFKQHFAW
DRIDALKPYGGIRIEDNIVIHEKRIENMTRDLNLA
>CORE_REP|Org36_Gene3361#
MWSFLKSRPDAPQVTDQRQIDASYKYWRIQLMCTMYIGYAAFYFTRKSFNFIMPAMLSDLGLTMSDVG
ILGTLFYITYGCSKFISGMISDRSNPRYFMGLGLIMTGVLNIFFGLSSSLLMLGTLWILNAFFQGWGW
PPCSKILTSWYSRSERGSWWAIWNTSHNVGGALIPLLVGFISLHFSWRYGMIIPGIIGVVLGLLMCWR
LRDKPSTLGLPSVGKWRNDAMELVQESEGQGLSNREIIKRYVLTNKYIWLLAVSYVLVYIVRTAINDW
GNLYLTQEKGYSLMTANSAISLFEVGGFIGSLVAGWGSDKLFRGNRGPMNLIFAIGIFLSVAALWLMP
GVTYLLQACCFFAIGFFIFGPQMLIGMAAAECSHKDAAGAATGFVGLFAYLGAALSGYPIARVMEIWH
WNGFFVVISIAACLSALFLLPFLRAQTPALKTANA
>CORE_REP|Org12_Gene282#
MLNSILLILFLIAVSAFFSLSEISLAASRKIKLKLMADEGNVNAARVLKLQETPGIFFTVVQIGLNAV
AILGGIVGDAAFSPTFKVLFDRFLSPELAEQVSFICSFVLVTSLFILFADLTPKRIGMIAPETVAVRI
INPMRFSIMIFRPLVWFFNGMANLIFRMFKLPMVRKDDITSDDIYAVVEAGALAGVLRKQEHELIENV
FELESRTVPSSMTSRESVVYFDLRESEESIKEKVSTHPHSKFLVCDGHIDQVVGYVDSKDLLNRVLGN
QSLVLSSGVQIRSALIVPDTLTLSEALESFKTAGEDFAVILNEYALVVGIITLNDVMTTLMGDLVGQG
QEEQIVARDESSWLIEGGTPIDDVMRVLDIDEFPQAGNYETIGGFMMYMLRKIPKRTDFVKYAGYKFE
VVDIDSYKIDQLLVTRLSDKPAAVLPKAPDDTPAA
>CORE_REP|Org12_Gene91#
MAKQPGLDFQSAKGGLGELKRRLLFVIGALIVFRIGSFIPIPGIDATVLAKLLEQQRGTIIEMFNMFS
GGALSRASIFALGIMPYISASIIIQLLTVVHPALAEIKKEGEAGRRKISQYTRYGTLVLAIFQSIGIA
TGLPNMPGMQGLVLNPGFAFYFTAVVSLVTGTMFLMWLGEQITERGIGNGISIIIFAGIVAGLPPAVA
HTIEQARQGDLHFLLLLLVAVLVFAVTFFVVFIERGQRRIVVNYAKRQQGRRVYAAQSTHLPLKVNMA
GVIPAIFASSIILFPATIASWFGGGTGWNWLTTISLYLQPGQPLYVLLYASAIIFFCFFYTALVFNPR

ETADNLKKSGAFVPGIRPGEQTAKYIDKVMTRLTLVGAMYITFICLIPEFMRDAMKVPFYFGGTSLLI
VVVVIMDFMAQVQTLMMSSQYESALKKANLKGYNR
>CORE_REP|Org11_Gene3941#
MRGRLFWKILLGFWLTFLIMTQALWVAFSLYGDRYVPPENAMARRVIGLQLTSAATQLRSGGMPALEA
LMRDWPEDDRRLLSVTPMTQPPPPAPEEPVFEGRRMPKAISAWVQTGEGQGYWLSYDVRGLREEYRPE
RRSHFFNIPAPMLWVGGLGGLLFSAVLAWNLTRPMRQLRGGLDRVAQGDLSVRLFPNMRRRHDELSDV
ARDFDTMAERLELLVSAREQLLHDVSHELRSPLARLQLAIGLARQNAGNVEASLKRIEHESGRLDKMI
GELLALSRTEHSSLPDEEYFDLYGLVDAVVSDARYEAQVPGVDIVLQAESDVEYTVKGNAELMRRAVD
NIVRNALRFSSHGQRVTVTVALSRVDNQFQIAVSDQGPGVEEAKLSSIFDPFVRVKSAQSGKGYGLGL
AITRKVVLAHGGQVEARNGDREGLVITLRIPRWSS
>CORE_REP|Org27_Gene3797#
MSDNATNQRQLRLGAILHGASGNMSAWRHPDATADASINLEFNIATAKKAEQGKFDFVFVADGLYINE
KSIPHFLNRFEPLTLLAALSAATDKIGLVGTLSTSYSDPFTVARQFASLDHLSNGRAGWNVVTSPLEG
SAKNFSRKEHPEHSLRYRIAGEFLDVAKGLWDSWEDDAFVRNKASGEFFRAGKLHTLNHQGEFFSVQG
PLNIGRTPQGRPILFQAGASEDGKRLAAQHADAIFTHHDTLEQAQDFYQDVKRQLVEQGREPDDLRIF
QGVSVIVGDDDADVERQYQETARLVSIENALNYLGRYFEHYDFARHPLDAPFPDIGDLGQNSFRSTTD
AIKRSARERHLTLRQVALEAASPRPVFSGTPEAVADGLQRWFDGEAADGFIISGGTPNAFGHFVDRVV
PVLQQRGLFRQAYHGDTLREHLGLKRPLNRFTQ
>CORE_REP|Org29_Gene813#
MKERSTELVQGFRHSVPYINAHRGKTFVIMLGGEAIEHENFSNIVNDIGLLHSLGIRLVVVYGARPQI
DANLAQHNYEPIYHKHTRVTDAHTLELVKQAAGLLQLDITARLSMSLNNTPLQGAHINVVSGNFIIAQ
PLGVDDGIDYCHSGRIRRIDEDAIHRQLDSNAIVLIGPVAVSVTGESFNLTSEEVATQLAIKLKAEKM
IGFCSSQGVTDAEGNILSELFPNDAQKRLEELEEGGDYHSGTVRFLRGAVKGCRSGVRRSHLISYQED
GALVQELFSRDGIGTQIVMESAEQVRRATINDIGGILELIRPLEQQGILVRRSREQLEMEIDKFTIIE
RDNLTIACAALYPFLEEKIGEMACVAVHPDYRSSSRGEMLLQRVENQARQMGLKKLFVLTTRSIHWFQ
ERGFTPAEVDVLPMQKQALYNYQRRSKILLADL
>CORE_REP|Org22_Gene2495#
MFLAQEIIRKKRDGQPLSEAEIRFFINGIRDNVVSEGQIAALAMTIYFHDMSMPERVALTMAMRDSGT
VLDWKSLALNGPIVDKHSTGGVGDVTSLMLGPMVAACGGYVPMISGRGLGHTGGTLDKLEAIPGFNIF
PDDNAFRKIIQDVGVAIIGQTSSLAPADKRFYATRDITATVDSIPLITASILAKKLAEGLDALVMDVK
VGSGAFMPTYALSQDLAQAIVGVANGAGCKTTALLTDMNQVLASSAGNAVEVREAVRFLTGEYRNPRL
LEVTLALCVEMLLSGGLAQDEADARAKLQAVLDNGKAAEVFGRMVAAQQGPIDFVERYDSYLPAATLS
KPVYAEKPGIISAMDTRALGMAVVSLGGGRRRASDAIDYSVGLTEVARLGDKVDAQQPLAMIHANDEE
SWQQAADAVRSAMTLSDKAPEATPVVYKRITQA
>CORE_REP|Org17_Gene3316#
MTKSLSPKDIIALGFMTFALFVGAGNIIFPPLVGLQSGEHLWPAALGFMVTAVALPVIAVIALARVGG
SISLLTGPIGRTAGLLLATVCYLALGPLFATPRTANVSFALGIAPFTGDGALPQFIYSLLFFTLAMVV
SLYPGRLLDNVGHILAPLKILALAALGIAALVWPAGAPVSAVGSYQAAAFSTGFVQGYQTMDTLSALM
FGSIIVTAARSRGVSDSGLLLRYTLWASLIAGVGLTLVYICMFKLGAGSGSLVGSGAQDGAAILHAYV
QHTFGDLGSVFMAVLMFIACLVTAVGMTCACADFFSRYLPLSYRALVVILALFAMLVSNMGLANLIRV
SLPVLTAIYPPCIALVLLSFSQNRWRSARRVFAPVIATSLVFGLADGLKASSFSGLLPAWFDKLPLAE
QGLVWLQPTLLVLLLAAAYDRLRSAESVKAAS
>CORE_REP|Org25_Gene39#
MSEFSQTVPELVAWARKNDFSISLPTERLAFLLAIATLNGERLDGEMSEGELVDAFRHVSKGFEQTHE
TVAMRANNAINDMVRQRLLNRFTSELADGNAIYRLTPLGIGITDYYIRQREFSTLRLSMQLSIVAQEL
KRAADAADEGGDDFHWHRNVFAPLKYSVAEIFDSIDMTQRVMDEQQQSVKNDIAALLSKDWRAAISSC
EMLLSETSGTLRELQDTLDAAGDKLQANLLRIQDATLGNVELGFVDKLVFDLQSKLDRIISWGQQAID
LWIGYDRHVHKFIRTAIDMDKNRVFAQRLRQSVQTYFDHPWALTHANADRLLDMRDEELALRSEEVTG
ELPPDLEFEEFSEIREQLAAMIEEALKVYQEQQMPLNLAAVMRDYLAQYPRARHFDVARLVVDQAVRL
GVAEADFSGLPAEWQAINDYGAKVQAHVIDKY
>CORE_REP|Org1_Gene4394#
MIMSKSNNKMGVVQLTILTAVNMMGSGIIMLPTKLAEVGTISIVSWLVTAVGSMALAYAFAKCGMFSR
KSGGMGGYAEYAFGKSGNFMANYTYGVSLLIANIAIAISAVGYGTELFDTTLSPLGICIATIGVLWLA
TVANFGGARITGKISGITVWGVIIPVVGISVIGWYWFSGSAYVAAWNPHQVPTFEAIGASISMTLWAF

LGLESACANTDVVENPERNVPIAVLGGTLSAAVIYIISTNVIAGIVPNMDLANSTAPFGLAFSHMFNP
TVGKIIMALMVMSCVGSLLGWQFTIAQVFKSSADSGFFPKIFSKLSKADAPVKGMLTIVVIQSGLSLM
TISPSLNKQFNVLVNLAVVTNIIPYILSMAALVIIQKVAKVPDNKARIANIIAGIGALYSFYALYSSG
EEAMMWGAIATFLGWTLYGIVSPRFELAGKKG
>CORE_REP|Org34_Gene3838#
MADYQGRKVVIIGLGLTGLSCVDFFMARGVTPRVMDTRIAPPGLDKLPESVERHLGDLNQDWLLAADL
IVASPGVALATPALSAAADAGVEIVGDVELFCREAQAPIVAITGSNGKSTVTTLVGEMAKAAGWAVGV
GGNIGLPALSLLRQECQLYVLELSSFQLETTYSLRAAAATILNVTEDHMDRYPFGLQQYRGAKLRVYE
NAAVCVVNADDALTMPVRGADERCVSFGADVGDYHLNRQQGETWLRVRGEKVLNTREMKLTGRHNYTN
ALAALALADAVNIPRASSLKALTTFTGLAHRFQLAWEHNGVRWINDSKATNVGSTEAALNGLQVDGTL
HLLLGGDGKSADFSPLARYLQGDNVRLYCFGHDGAQLAQLRPEVATLTETMEQAMRTIAGRVQPGDMV
LLSPACASLDQFRNFEVRGDEFARLAQELGG
>CORE_REP|Org5_Gene4512#
MLTYAIRKTTLCVALTLAVSTQALAATEIPFWHSMEGELGKEVDSLADRFNQSHSDVKIVPVYKGNYE
QSLAAGIAAYRSGKAPAILQVYEVGTATMMASKAIKPVYEVFKEAGINFDESVFVPTVAGYYTDNKSG
HLLSQPFNSSTPVLYYNKDAFKKAGLNPDQPPKTWQELAADTAKLREAGMKCGYASGWQGWIQLENFS
AWHGVPFASENNGFGGTNAKLEFNKPLQVKHIALLEAMNKKGDFTYFGRKDESTEKFYNGDCAITTAS
SGSLADIKHYAKFNYGVGMMPYDADAKNAPQNAIIGGASLWVMNGKDAATYKGVAEFLQYLAQPEIAA
EWHQKTGYLPITTAAYDLTKQQGFYDKNPGADVAARQMLNKPPLPFTKGLRLGNMPQIRSVVDEELEG
VWTGKKTPQQALDAAVQRGDVLLRRFEASTK
>CORE_REP|Org31_Gene1229#
MPLVIVAGGVALLLLLMIRFKLNGFISLVLVALAVGIAQGMPVDKVIGSIKAGVGGTLGSLALIMGFG
AMLGKLLADCGGAQRIATTLIDKFGRKHIQWAVVLTGFTVGFALFYEVGFVLLLPLVFTIAASARIPL
LYVGVPMAAALSVTHGFLPPHPGPTAIATIFHADMGKTLLYGTLLAIPTVILAGPVYARFLKGIDKPV
PEGLYNPKTFTEAEMPSFGVSVATSLVPVILMALRAVAEMVLPKGHSLLRFAEFFGDPVMATLIAVLI
AIFTFGLNRGRTMDEVMGTITDSIKIIAMMLLIIGGGGAFKQVLVDSGVEQYIAGLMEGSNVSPILMA
WSIAAALRLALGSATVAAITAGGIVAPLIATTGVSPELMVIAVGSGSVIFSHVNDPGFWLFKEYFNLS
IMETIKSWSVLETIISVCGLVGCLLLATVV
>CORE_REP|Org46_Gene2536#
MTQQEFNNRRQALLAKMAPASAAVIFSAPETTRSADSDYPYRQNSDFWYLTGFNEPEAVLVLIKSDET
HNHSVLFNRVRDLTAEIWFGRRLGQDAAPAKLGVDRALPFDEINDQLHLLLNGLDVVYHAQGEYAYAD
QILFGALDKLRKGFRQNLQAPATVTDWRPWLHDMRLFKSPEELAVMRRAGEISALAHTRAMEKCRPGM
FEYQLEAEIHHEFTRLGARYPSYNTIVGSGENGCILHYTENESQMRDGDLVLIDAGCEYQGYAGDITR
TFPVNGKFSKPQRAVYDIVLASLQRALELFKPGTSIREVNDEVVRIMVVGLVELGVLKGEVDQLIAEQ
AHRQFFMHGLSHWLGLDVHDVGHYGTPSRDRLLEPGMVLTVEPGLYIAPDADVPAEYRGIGIRIEDDI
VITADGNENLTATVVKDADAIEALMAAARS
>CORE_REP|Org4_Gene762#
MKSVKISLAWQILIALVLGIIVGAVLHNQTESREWLVSNILSPAGDIFIRLIKMIVVPIVISTLIVGI
AGVGDAKKLGRIGLKTIIYFEVITTVAIVVGLTLANVFQPGHGIDMSTLTTVDISQYEKTTEQVQSGS
HSLVATILSLIPSNVFSSMAKGDMLPIIFFSVLFGLGLSSLPKETKEPLLKVFKAVSESMFKVTHMIM
RYAPIGVFGLIAVTVANFGFASLLPLAKLVVLVYFAIAFFALVVLGAVARLCNLRIWTLIRILKDELI
LAYSTASSETVLPRIIEKMEAYGAPKSITSFVVPTGYSFNLDGSTLYQSIAAIFIAQLYGIELSLGQE
IVLVLTLMVTSKGIAGVPGVSFVVLLATLGSVGIPLEGLAFIAGVDRILDMARTALNVVGNALAVLVI
AKWEHQFDRKKALAYETEFLAQKVKPANQA
>CORE_REP|Org14_Gene489#
MLERLSWKRLALELALFCLPALLLGLIFGYLPWFLLASALAALVWNFYNQLKLSHWLWIDRSMTPPPG
RWSWEPLFYGLYQMQQRNRRRRRELALLIKRFRSGAESLPDAVVMTTVEGNIFWCNGLAQHLLGFRWP
EDNGQHILNLLRYPEFSHYLQQQEFSRPLTLQLNNEHYVEFRVMPYSEGQLLMVARDVTQMRQLEGAR
RNFFANVSHELRTPLTVLQGYLEMMGDEEQDGSLRSKALSTMQEQTRRMDGLVKQLLTLSRIEAAPNV
DMNERVDIPLMLRVLQREAQSLSGGNHEITFRVNEQLNVFGNEDQLRSAVSNLVYNAVNHTPKGTHIE
VSWQQTAHGAQFQVSDNGPGIAAEHLPRLTERFYRVDKARSRQTGGSGLGLAIVKHALSHHDARLEIL
SEPGIGTRFIFTLPNRLIVPAALSENAVKN
>CORE_REP|Org46_Gene3778#
MSKALPLVTRHGDRIAIVNGLRTPFAKQATAYHGIPAVDLGKTAVSELLARSGIDPALIEQLVFGQVV

QMPEAPNIAREIVLGTGMSVHTDAYSVSRACATSFQAIANVAESIMAGSISVGIAGGADSSSVLPIGV
SKALARTLVDVNKARTLSQRLKLFSRLKFRDLMPVPPAVAEYSTGLRMGDTAEQMAKSHGITREAQDA
LAHRSHELAAKAWREGWLRDEVMTAYVPPYRAQLSEDNNIRKDSSLASYAKLKPAFDRKHGSVTAANS
TPLTDGAAAVLMMSESRAKELGLQPLGYLRSFAFAAIDVWEDMLLGPSYATPLALDRAGIGLADLTLI
DMHEAFAAQTLANLKMFASEEFAREKLGRSQAIGEVDMDKFNVLGGSIAYGHPFAATGARMITQTLHE
LKRRGGGLGLTTACAAGGLGAAMIVEVEG
>CORE_REP|Org2_Gene1916#
MSALKIAVIGGGSSYTPELVDGLIQRIEELPVTELALVDVEPGRQKVETIAALTRRMLARHGLEQVKV
SVHFALDDAIRGAAFVLTQFRVGQLPARAADERLGLKYQLLGQETTGVGGFAKALRTIPVMLDIARRV
EKLAPDAWIINFTNPAGIVTEAVSRFTKAKIIGLCNVPISMHHMIANMLQAPYADVQLRFAGLNHMVW
VHQVTQQGRDVTAKVIDMLCDGAALTMNNIKEEPWQPDFLRALGAIPCPYHRYFYQTREMLAEEMAAA
GERGTRAEQVMQVEKELFELYADPQLNTKPEQLSFRGGSFYSEVALELIRAIHNNLGTQLVVNTANRG
AIHGLPDDAVIEINCIVDAQGAHPLTFGPLPEPMQALTQQVKAYERLTIEAAVHGDRRSGLLALIANP
LVGNANLAQPLLEEVLTINAPYLPQFR
>CORE_REP|Org35_Gene4197#
MSTFNTPIDWATCSAERQAELLMRPAIAASDSITRTVNEILDNVKANGDRALRDYSAHFDKAEVSALR
VSAEQIAAAAARLGDDIKQAMAVAVANVETFHNAQRLPPVDVETQPGVRCQQVTRPIDSVGLYIPGGS
APLFSTVLMLATPARIAGCRRVVLCSPPPIADEILYAAQLCGVQEVFQVGGAQAIAALAFGTESVPRV
AKIFGPGNAFVTEAKRQVSQRLDGAAIDMPAGPSEVLVIADAGATPAFVASDLLSQAEHGPDSQVILL
TPDAAMAQAVADAVESQLAELPRADTARQALASSRLIVARDLPECVAISNRYGPEHLIIQTRNARDLV
ESITSAGSVFLGDWSPESAGDYASGTNHVLPTYGYTATCSSLGLADFQKRMTVQELTPQGFSNLAATI
ETLAAAEQLIAHKNAVTLRVAALKEQA
>CORE_REP|Org6_Gene2323#
MTVEIELKFIAAPQAVAALPHWLAAWPHQHSAPQKLTNIYFETADNFLRSHDMGLRIRGFDDRYEMTI
KTAGKVVGGLHQRPEYNVAIAEPQLALAQFPADIWPQDCDITALQQALQPLFRTDFVREKWVVTYGQS
EIEIGLDQGEVRAGGLSEALSEIELELLKGNTADLLALAGELAAHDGLRQGSLSKAARGYHLAQGNPP
REVRPLRVLKPAAKATVEQGMIAAFELALSHWQYHEELWLRGDAQARASVIEAVGAIRQALVIFGGLV
PRKASADLRARLTELEPLLADKTAQPETLCYSAVYLQCKLALTSWLVSGAWRPFIDAKAQAKLDGSFK
RFSDIMLGRSGAELKEAFSRTLNEDEYQEQLPRLTRQISALVLLSGAYPDEQTGPYIEAWRELQAALS
ERRQGWYEASRKQALSHAPFWLNGALR
>CORE_REP|Org11_Gene2002#
MSTSRSQQIQQLEQEWKSARWEGITRPYSAEDVINLRGSVNPECTLAQNGAAKLWALLNGKARKGYVN
CLGALTGGQALQQAKAGVEAIYLSGWQVAADANSAAAMYPDQSLYPVDSVPKVVERINNTFRRADQIQ
WANQIEPGSKGYTDYFLPIVADAEAGFGGVLNAFELMKAMITAGAAGVHFEDQLAAVKKCGHMGGKVL
VPTQEAIQKLVAARLAADVLGVPTLVIARTDADAADLLTSDCDPYDSAFVTGERTAEGFFRTHAGVEQ
AISRGLAYAPYADMVWCETSTPDLDAAQRFADAIHAKYPGKLLAYNCSPSFNWKKNLDDQTIARFQQA
LSDMGYKYQFITLAGIHSMWFNMFDLAHAYAQGEGMKHYVEKVQQAEFAAVDRGYTFASHQQEVGTGY
FDKVTTVIQGGASSVTALTGSTEEQQF
>CORE_REP|Org8_Gene797#
MKFPGKRKSKHYFPVSARDPLLQQAQPESEISTSYIVGIDQTLVDIEAKVDDAFVQRYGLSLGHSLVI
EDDVAEALYQELNENNLVTHQFAGGTIGNTMHNYSVLADDRSVLLGVMCSNVKIGSYAYRYLCNTSSR
TDLNYLQGVDGAIGRCFTLIGESGERTFAINPGQMNQLRPESIPEEVIAGASALVLSSYLVRCKEGEP
IKDATLQAIEYAKKHDVPVVLTLGTKYVIADNPQWWRDFLKENVTILAMNEDEALELTGLSDPLTASD
MALEWVDLVLCTAGPNGLYMAGYTEEANKRQTQHPLLPGHIAEFNRYEFSRAMRREHCENPLRVYSHI
APYMGGPEKIMNTNGAGDGALSALLHDIAANGYHRHNVPNSSKHVRSYLTYSSLAQVCKYANRVSYQV
LNQHSPRLTRGLPEREDSLEESYWER
>CORE_REP|Org24_Gene58#
MRDAATTPSLTERTNIAIGAALAGRKRGAFTPLLFAGPAVIASIAYMDPGNFATNIQAGAKYGYSLLW
VVVMANLIAMLFQALSAKLGIVTNRNLAEMCRDQFSRPVVIGMWLLSEVAAMATDLAEFLGGAIALAL
LFHMPLLAGMGVTAVITYALLMVEKKGFRPVELMIGGLVGIIALCYLVEMFIVPVDWQAAGLGMVTPQ
LPDAQALTIAVGIIGATVMPHAIFLHSGLTQHRSPASDNGERRKLLRFSNIEVVIALALAGLVNIAMV
IMASSAFHAGNSDVAEIGTAYHTLTPLFGAAAAGIFLASLIASGISSSVVGTMAGQMIMQGFVGFRIP
VWVRRLVTMVPAFIVVAMGVNATDALVYSQVVLSLALPAPMIALVMFTRRRDIMGEFANGRWTSLAAV
VGTVVIVLLNVVLLLQTFGVDIPGLG

>CORE_REP|Org8_Gene817#
MQVSVETTQGLGRRLSITVPADTIKQAVKKELINAAKSVRIDGFRKGKVPMNIVEQRYGASVRQDVLG
EAMQRSFVDAIIKEKINPAGAPNYVPGEYKEGEDFTFAVEFEVYPEVELKGLENIEVEKPVVEVNDED
VDAMLDTLRKQQATWKETDRAAEAEDRVTVDFTGSIDGEEFEGGKASDFVLAMGQGRMIPGFEEGLVG
HKAGEEFSIDVNFPEDYHAENLKGKAAKFAIVLKKVEERELPELTEEFIKRFGVADGSVAGLRTEVRK
NMERELKGAVRNRIKSQAIDGLVSANEIDVPAALIDGEIDVLRRQAAQRFGGNEKQALELPRELFEEQ
AKRRVVVGLLLGEVISTNDLKADEDRVKTLIEEMASAYEDPSEVIEFYSKNKELMNNMRNVALEEQAV
EALLAKAKVTEKATTFSELMNQTQQA
>CORE_REP|Org38_Gene3496#
MLLRFSQLTTHKGAELSAIEHAVPMIVFSPDGTVLRANDLFLSTLGFQRDDVIGRHHRIFCDPNYVAS
PLYREHWETLNKGQPITDTIKRIAKNGEAVWLQGTYAPVLNKQGKVVEIVKIASEVTERVTQAQEHRS
LLAALNRSMAMISFTPQGTIVSANDNMLALMGYRLEEACGQSHAVLCPPAFAASDDYRRHWQRLARGE
FITGRFERVNRRGERVWLEASYNPILDNDGQVVKVVKIAQDITRLMQQQQHEEEMVRNAHHLSLDTDR
QAAQGAVIVQQAVKGMQQVEAAARETSDVVTELGKCSQQIGTIVEAIRKIASQTNLLAINASIEAAHA
GEHGRGFAVVANEVRTLAEQSRKAATEIERMTKSIQQGVAAAIAGMATCVEQAGGGVALTHDAGEVIN
QVNIGMHDVVKLMQAFTSVKQGDALH
>CORE_REP|Org10_Gene3824#
MNQQTLDARRSRQALLAGSVGNFIEWYEFGVYGFLATVIAANFFTLQGENEVTSLILTYAAFALAFFC
RPIGAVIFGRIGDRIGRRPTLIAVLLLMTLATALIGVMPTYASIGVAAPLLLTLLRMFQGLFAGGEFG
GAVSLMTEFAPKGKRGLFGAWQSLTVALGLLAGAGLVALLAALLSVQQLHDWGWRIPFLLALPMGAVA
LWLRLKLEETPTFTQAQQAAEHSAAPQEASLGGVAKTILIGIGRMMGWSAAGYTFLVVMPSYLQTSLH
ATFQQALVATVLANVGFALTILPAGIISDKLGRKTVMLTAVAAVILFTFPLLHLLQDAQSSLWAKGLV
VMIAGAVVGLLAGPGPAMLAEMFPTRVRYTGLGLAYSLSNAVFSGSAGLIITGLIKQTGNIDIPAYYV
VATSVVSLFALMTLRRDDHLRSLNER
>CORE_REP|Org46_Gene2700#
MKLSSIAIALSAVLALAGCDNSAVISPEQQMGPDPTLPAAQDFLMPPMQVPKGVGWQQNQMPKVAEGL
KIDKVADGLLHPRQLLTLPNGDVLVVEANGPGTEAVSTPKQLIAGLVKGQSGKGGKGGNRITLLRPTA
DGSWEKHVFLEGLDSPFGVQLIGNTLYVANTGNIMQYAYQPGETRISDAGKELADLPDTINHHWTKAL
LASPDGKKLYVGVGSNSNITENGLAVEYRRAAVLEVDTASGASRIFASGLRNPTGLQWEPHSGKLWAI
VNERDEIGADLVPDYLTSVQDGGFYGWPYSYFGQHVDRRVQPARPDLVAKAIKPDYALSSHVAPLGLL
FYTANALPAEYRGGAFVSEHGSWDRSPLNGYRVSYVAFEQGKPVGKLKAVVTGFVSDDEKELYGAPVG
LAIDKTGALLIADDVGNTVWRVKSK
>CORE_REP|Org43_Gene3676#
MKRLVHLHHYRALLRSLLIAVFIGVAAALAVWLFHRSMIGLEWLLLGNADGSLVAAAAALPGWRRALT
PALGGLAAGLLLYIHQRYRHQRPAAPTDYMEAIETGNGKLDTGASLVKCLASLLVVSSGSAIGREGAM
ILLAALVGSLFAQRFTHEKEWKLWVACAAAAGMASAYHAPLAGSLFIAEILFGTLMLASLGPVVIAAV
SALLMTNLLNGGQAPLYLVTPLAAPLPTQYLLMALVGVVAGAGGPLFLWLMTASGRAFRSLRLKPPLQ
LALGGLIVGLLSLLFPQVWGNGYSVVQALLIAPPGVLLLGAILVCKLLAILASSGSGAPGGVFTPTLF
VGAALGSIIGQLFGLWPGMDAAVPLLLALTGMATLLAATTHAPIMAALMVFEMTGEYALLPGILLACV
IATTVSRGLRPISVYHTAPLPKREV
>CORE_REP|Org36_Gene1083#
MNNEIESLTYYAATKKYDLRFPTLEEDLDVDVVIIGGGFSGINTALELAEKGITNIAILEGRYLGYGG
TGRNGGQVMAGIGHDLEKIKRHVGPAGLETIFKISNLGAGIIRERIKKYDIDADFCFGYGYLGSNARQ
EKTLRGWLKEFKAVAPQEEIELYTGAEVKQVVGSDAYTCALKHMGGGHVHSLNLLLGEAKALSGYGVK
IFENSSVLNVEYGPRITVRTAMGSVRANKMLWACNGFLNGMEPQIYRKTINTYAFQLATEPLSDDLIR
QISPIRGAYSDIRPVIDYYRVTNENRLLFGSATRLIEYFPSDLKAWNRNLMLKVFPYLKDVKIDLAWG
GPLCCSANLFPQIGTLPDRDNVFYVQGYSGFGVTPSHIVCKVLAEGMSEGSDRYDLMSSIPHVNIFGK
DKLRRVMTTAGKVWHQTSGYWKGRR
>CORE_REP|Org47_Gene2030#
MIKIEDYPLTRVPQDKRVSFLSVAIVHMGMLTALDQFMLGAVLGNSMTLIDAFTAIFVGSLIFGVVTY
GLGLAGMREGISGSLLARWCGFGRLGSVLIGVVVAVSLLGWFGIQNAIFAKSLDFALGNKLGFGLAAG
LSGTLLTILVAFGFKALRIAARIAVPMFIMLVAYISVTALSGHNLQEIIQLAPPGEPLTISAGITIVV
GGAIVASLMTPDLTRYSKNGKHVLGVTLFTIIAGEFVVNGLAILIAKTLGTADVVTIMSQAAGGAGLL
VVVFSTLRVNDLNLYSSSLGIVNAVEGITGKKLKYTYTTLVIGILGTTLSVLGILDRFVDFLTVLGVV

FPPIIGIMLVDYYLLRSHRKILDESRRTGQLPSETPTIGWAAIVASIVGGAVGLATEWGVPTINSLVA
ASLLYWVLKLAFSRAQKPLASQKSL
>CORE_REP|Org30_Gene1669#
MTTPVRSAAAMPSTLAANDDAAAAPKVKRSTLNNKLPYIERGTPQFMRVTLALFSAGLATFALLYCVQ
PILPVLSQDFGVSPAESSLSLSVSTGLLAIGLMFTGPLSDAIGRKSVMVVALLLAAVCTLICAFMTSW
HGILLMRALIGLSLSGVAAVGMTYLSEEIHPSFVAFSMGLYISGNSIGGMSGRLVTGVLTDFFSWRVS
LGVIGLFALAAACMFWRILPASRHFRASSLRPRTLLINFKLHWHDKGLPLLFAEGFLLMGSFVTLFNY
IGYRLLADPYHLSQAIVGLLSVVYLTGSYSSPKAGALTSRFGRGPVLLASIVIMLIGILITALPQVPA
IFIGMMLFTAGFFAAHSVASSWIGRRARRAKGQASSLYLFCYYVGSSVAGTLGGVFWHSFGWNGVAAF
ISLMLLLALLVVHYLKRLPEAARL
>CORE_REP|Org41_Gene448#
MDTTQTGTIASAASGSSSTWRKTDTMWMLGLYGTAIGAGVLFLPINAGIGGLIPLIIMAIIAFPMTFF
AHRGLCRFVLSGKNPGEDITAVVEEHFGITAGKLITLLYFFAIYPILLVYSVAITNTVDSFITHQLGM
TSPPRAILSLILIVGLMTIVRFGEQAIVKTMSILVFPFVAVLMLLAVYLIPNWTGAIFENVSLSGSGT
GMGHGLIMTLWLAIPVMVFSFNHSPIISAFAVAKREEYGADAEKKCSRILAYAHIMMVLTVMFFVFSC
VLSLTPENLAEAKAQNISILSYLANHFNNPMIEYIAPVIAFVAITKSFLGHYLGAREGFNGLVAKSMK
SRGKTVSTAKLNRMTAIFMLVTTWIVATLNPSILGMIETLGGPIIAMLLFLMPMYAIRKVPAMRKYSG
HISNVFVVVMGLIAISAIVFSLLG
>CORE_REP|Org12_Gene4019#
MSNKAGPNNGPHLHSQGAASPSRRRLLQGLGLGALALGGTRLAQAAGNAAEPLATPQDERWQKQPFYG
RHQAGVLTPQQAAMMLVAFDVLATDKAGLERLFRLLTDRIAFLTHGGKAPEVDAKLPPLDSGIMGPEI
YPDNLTITVSVGASLFDERFGLQAHKPLKLQKMTRFPNDALDAGLCHGDLLLQICANTNETALHALRD
IIKHSPDLLSVRWKREGFISAHAARSKGKETPINLLGFKDGTANPKTDDKSLMDKVVWVGDSVGEPAW
TAGGSYQAARIIRFRVEFWDRTPLQEQETIFGREKHSGAPLGMKHEHDEPNYANDPDGKVIPMDAHIR
LANPRTPESQSNLMLRRGYSYSLGVSNAGQLEMGLLFVCYQADLEKGFLTVQKRLNGEALEEYIKPIG
GGYFFVLPGVKENGDYLASGLLKA
>CORE_REP|Org12_Gene2228#
MGKNVVVLGTQWGDEGKGKVVDLLTERAQYVVRYQGGHNAGHTLVINGEKTVLHLIPSGILRENVTSI
IGNGVVLAPDALMKEMGELEARGIPVRERLLLSEACPLILPYHVALDNAREKARGAKAIGTTGRGIGP
AYEDKVARRGLRVSDLFNKETFAVKLKEIVDYHNFQLVNYYKVEAVDYQATLDYVLSIADILTAMVVD
VSELLDGARKRGDLIMFEGAQGTLLDIDHGTYPYVTSSNTTAGGVATGSGIGPRYVDYVLGIVKAYST
RVGAGPFPTELFDETGEYLCKQGNEFGATTGRRRRTGWLDAVAVRRAVQINSLSGFCLTKLDVLDGLK
EVKICVGYRMPDGREVDTTPLAAEGWEGIEPIYEIMPGWSETTFGVKEHSKLPQAALNYIKRIEEVTG
VPVDIISTGPDRSETMILRDPFDA
>CORE_REP|Org13_Gene2566#
MLGDPMALSALVTLVVIALWASARLPEYLVALLFFAAVMVLQLAPAAVTFSGFASSAFWLVLSGFVLG
TAIRSTGLADRLANRLLPHLAGSWPRLVGGVVAISYALAFVMPSNMGRITLLMPIVMALADRAGLGEG
SRGRIGLALAVGFGTFQLSASILPANVPNLVMSGAAESAYGSHFAYLSYLLLHAPVLGIAKGALLTLC
ICGLFRARPQPVAAEAAQTPLSAAEWRLIALLGVTVLLWMTDSWHGIAPAWIGLAAACVCLLPRIGFL
NGEQFAAGVNVRTCIYVAGILGLTALVSHSGLGDRLGGALLAMMPDLHGKPFTAFGSLVGITALLNFV
VTANGVPALFTPLAQALSAGSGLPLLTVLMTQVIGYATPLLPYQASPIVVAMGMGKVPAREGLKLCLL
LAALSFALLVPLDYLWFRLLGWIP
>CORE_REP|Org27_Gene4319#
MRKIKGLRWYMIGLVTIGTVLGYLTRNAIAVAAPTLEDTLHITTQQYSYIVAAYSACYTVMQPVAGYV
LDVLGTKVGYAMFAILWALFCMGTALANSWGGLALARGAVGMAEAAMIPAGLKASSEWFPAKERSIAV
GYFNVGSSIGGMIAPPLVVWAIVAHSWEMAFVITGVLSLIWAICWLLFYKHPKDQKKLSQEERRYILE
GQEAQHQTSNAKKMSAWQIVRNRQFWGIALPRFLAEPAWGTFNAWIPLFMFKAYGFNLKEIAMFAWMP
MLFADLGCIVGGYLPPLFQKYFKVNLIVSRKLVVTMGAVLMIGPGMIGLFTSPYAAIALLCVGGFAHQ
ALSGALITLSSDVFGRNEVATANGLTGMAAWTASTLFALVVGALADTLGFSPLFAALSVFDILGAIVI
WTVLQNRPAAEPQPATLQPSPARS
>CORE_REP|Org19_Gene2793#
MKAIITNALWLMLERVSLSLSGIFVSIYVARYLGPAQFGALNYLLATIAIVVPLVQLGADSIIFNRVA
RRQPSGIRLMLASIRLRRRLFLVVALPILIWSYFSQTPASQLMTLLLLVSAYFSIQDVYKIYYDARLQ
SKRNTLINNLALLLSIGLRLALVSAALPLVWFAVPYILSSAVPYLVRLWLFRGEAAASPRVTPRQARR

YGRYLLKVGLPLAISSLSIVIYTRIDQIMLGNLVGEQAVGWFSAATTLSQGWVFVPMALITSLMPGIA
SCRDPLEQEYRIRVLYLVVLGLSLPVLLGLWWFAHPAIALLYGAAFQPAASILAICTLTSLFSVMGTV
SYRSIVLFAGYRFIAIKMPLVAVANVVMNLLLIPRYGLIGAAVSTLLAEFISFFVLNSLFRGGKITRL
QLTCFYCLPRLVSKLRREHVKHPG
>CORE_REP|Org28_Gene1687#
MSKIVKVIGREIIDSRGNPTVEAEVHLEGGFVGLAAAPSGASTGSREALELRDGDKSRFLGKGVLKAV
AAVNGPIAQAVLGKDAKDQANIDKIMIDLDGTENKSNFGANAILAVSLAAAKAAAASKGMPLYEHIAE
LNGTPGKFSMPLPMMNIINGGEHADNNVDIQEFMIQPVGAKTLKEAVRIGSEVFHHLAKVLKAKGMNT
AVGDEGGYAPNLGSNAEALAVIAEAVKAAGYELGKDVTLAMDCAASEFYKDGKYVLAGEGNKAFTSEE
FTHFLEDLTKQYPIVSIEDGLDESDWAGFAYQTKVLGDKIQLVGDDLFVTNTKILKEGIEKGIANSIL
IKFNQIGSLTETLAAIKMAKDAGYTAVISHRSGETEDATIADLAVGTAAGQIKTGSMSRSDRVAKYNQ
LIRIEEALGDRAPFNGLKEVKGQ
>CORE_REP|Org41_Gene2975#
MNQRLDIIGIGLGPSNLSLAALGSEIEGFTGQFLERKPHFSWHPGMILADCSMQTNFLKDLVSAVAPT
NRYSFLNYLVKNRKFYRFLTTEQRTASREEFADYLTWAAGGMDSLAFNQDVQQIEFDDRQRQFVVTTS
NKVFHAKHVSIGIGKKIKLPDCVTAQSDRCFHASEMMLRNPDLTGKRVAIVGGGQSGADLFLNIFKGE
WGQPDQLDWISRRNNYNALDEAAFANEYFTPDYVESFYSLDSAAKRHMLAEQKMTSDGITSESLLAIY
RAMYHRFDVLREKLWVRLLPSRSLTAVKHTLDNAYQLETRHHLDHGEEAFKADVVIFATGYQTATPEF
LEPLAHRLLTTADGEYRIAPDFTFEWEGPAENCLFAMNASMHNHGIADPQLSLMAWRSARILNRALDH
KPFDLGTTPTAIQWRSESVPHAF
>CORE_REP|Org26_Gene1535#
MLDPNLLRNELDAVAVKLARRGFKLDLDLLRSQEERRKVLQVETETLQAERNSRSKSIGAAKARGEDI
EPLRREVNELGDKLDAAKAALDALQSEIRDYALTLPNLPDDAVPDGKDDSENLEVARWGEPRQYDFAV
RDHVDLGEMAGGLDFAAAVKLTGSRFVVMKGQIARMHRALSQFMLDLHTEQHGYLEAYVPYLVNHATL
YGTGQLPKFGEDLFHTRPLEEEADSSNYALIPTAEVPLTNLVRDEIVEEETLPLKMTAHTPCFRAEAG
SYGRDTRGLIRMHQFDKVEMVQIVRPEDSMDALEELTGHAEKVLQLLNLPYRKVLLCTGDMGFGACKT
YDLEVWLPAQDTYREISSCSNMWDFQARRMQARCRSKADKKPRLVHTLNGSGLAVGRTLVAVLENYQQ
ADGRIQVPEVLRPYMGGLEYIG
>CORE_REP|Org16_Gene4716#
MMTTLRKLPLALAVAAGVLSTQALAVDFHGYARSGIGWTGSGGEQQCFKATGAASKYRLGNECETYAE
LKLGQEVWKEGDKSFYFDTNLAYSVSQRSDWEDVTPGFREVNVQGKNLIEWLPGSTLWAGKRFYQRHD
VHMIDFYYWDISGPGAGLENIDLGFGKLSAAVTRNSESGGSYGYLDNEWKQRPTVNDTFDVRLAGLEL
NPGGTLELGVDYGRANAQDNYRLADGASKDGWMFTAEHTQSILNGYNKFVLQYATDSMTSQNNGRNEG
ATIDNNGKMIRVLDHGAIDFNDQWALMYVAMFQDIDRDNNNGTTWYTVGVRPMYKWTPIMSTLLEAGY
DNVKSQRTGDRNGQYKVTLAQQWQAGNSIWSRPAIRLFATYAKWDEKWGYATGDDTGYNAGTAYNDTS
MHTFSRGKDDEVTFGAQMEIWW
>CORE_REP|Org36_Gene672#
MTDKKATLTINDSEAPIELGVLTPTLGPDVLDVRALGSKGYFTFDPGFTSTASCESKITFIDGDKGVL
LHRGFPIEQLAKESSYLEVCYILLYGETPTPEEFETFKTTVTRHTMIHDQITHLFRGFRRDSHPMAVL
CGVTGALAAFYHDALDVNNERHREITAFRLLSKMPTVAAMCYKYSLGQPFVYPRNDLSYAGNFLHMMF
ATPCEEYVVNPVLERAMDRILILHADHEQNASTSTVRTAGSSGANPFACIAAGIASLWGPAHGGANEA
ALKMLEEIKTVEHIPEFIKRAKDKNDSFRLMGFGHRVYKNYDPRATVMRETCHEVLKELNKKDDNLLQ
VAMELEHIALNDPYFIEKKLYPNVDFYSGIILKAMGIPSSMFTVIFAIARTIGWIAHWNEMHDEGIKI
ARPRQLYTGYAERDFKSQLKNK
>CORE_REP|Org19_Gene444#
MKQAFRVALGFLILWASVLHAEVRIEITQGVDSARPIGVVPFKWAGPGTPPEDIGKIVGADLRNSGKF
NPIDVARMPQQPTSASEVTPAAWTALGIDAVVVGQVQPGADGSYLISYQLVDTSGSPGTVLAQNQYKV
TKQWLRYSAHTASDEVFEKLTGIKGAFRTRIAYVVQTNGGKFPYELRVADYDGYNQFTVHRSPEPLMS
PAWSPDGSKLAYVTFESGRSALVVQTLANGAIRQIASFPRHNGAPAFSPDGSKLAFALSKSGSLNLYV
MNLGSGQITQLTDGRNNNTEPTWFPDGQSLAFTSDQGGRPQIYKISASGGAAQRLTWEGSQNQDSEVS
SDGKFLVMVSTNSGAQHIAKQDLGSGAVQVLTGTFLDETPSIAPNGTMVIYSSTQGMGSVLQLVSTDG
RFKARLPATDGQVKFPAWSPYL
>CORE_REP|Org23_Gene3530#
MIMKRLLSMLGLSSVTLVPSLALAAPAVADKADNAFMMICTALVLFMTLPGIALFYGGLLRGKNVLSM

LTQVTVTFALVCVLWVLYGYSLAFSEGNAVFGGFETAMLKGIGIDSVTGSISQMIHVAFQASFACITV
ALVVGGFAERIRFSAVLIFAILWFTLSYLPIAHMVWGGGYLATDGALDFAGGTVVHINAASAGLIGAY
LLGKRAGFGKEAFKPHNLPMVFTGASILYIGWFGFNAGSASAANGIAALAFLNTVVATAGAILSWTFA
EWVLRGKPSLLGACSGMIAGLVAVTPAAGTVGVGGALIIGLVGGVAGLWGVVTLKKWLKVDDTCDVFG
VHGVCGIVGCLLTGVFTASSLGGTGYAEGVTMGHQVWIQLLSVVITLVWSSVAAFIAFKIAGAMVGLR
VPEEQEREGLDVNSHGESAYNQ
>CORE_REP|Org4_Gene2396#
MKLYNLKDHNEQVSFAQAIKQGLGKQQGLFFPLELPEFELTEIDQLLEQDFVTRSSRILSAFIGEEVP
EAALKKRVQAAFEFPAPVAKVTEDVSCLELFHGPTLAFKDFGGRFMAQMLAEVAGDQPVTILTATSGD
TGAAVAHAFYGLKNVRVVILYPQGKISPLQEKLFCTLGGNIHTVAIDGDFDACQALVKQAFDDRELKD
ALHLNSANSINISRLLAQICYYFEAVAQLPQEARNQLVISVPSGNFGDLTAGLLAKSLGLPVKRFIAA
TNANDTVPRFLTSGQWQPHATVATLSNAMDVSQPNNWPRVEELFRRKVWQLKALGHAAVSDETTKETM
RELAELGYISEPHAAIAYRALRDQLQEGEFGLFLGTAHPAKFKESVEAILGQELSLPKALALRADLPL
LSHTLPAGFAELRKFLMALPA
>CORE_REP|Org14_Gene4700#
MKNNYNLRSIAAKAIGQVLDQGQSLSTVLPTLQTAISDKDRGLLQELCFGTLRVLPQLEWCIQQLMAK
PLTGKQRTLHYLLMVGLYQLLYTRIPAHAVLAETVEGAVALKRPQLKGLINGVLRQFQRQQEELLQRA
ANNDSRYLHPSWLLKRIQQAYPANWEQIVDANNQKPPMWLRVNRLHHTREAYLQLLTDAGIAAEPHSD
YADAVRLLAPCAVTELPGFADGWVTVQDASAQGCVDLLDPQDGEQILDLCAAPGGKTTHILEAAPKAH
VMAVDIDEQRLARVKENLQRLRLHAEVKLGDGRTPQQWCGDKQFDRILLDAPCSATGVIRRHPDIKWL
RRDRDIAELAALQADILEAVWPHLKSGGVMVYATCSILPDENSSQIAAFLQRHADAKLVETGDAQRPG
RQNIPHPEDGDGFFYAKLIKM
>CORE_REP|Org19_Gene1398#
MSLHSKSESLYAQAQQLIPGGVNSPVRAFTGVGGVPLFIERADGAYLFDADGKAYIDYVGSWGPMVLG
HNHPAIRDAVIEAAQRGLSFGAPTEMEVKMAQLVTELVPTMDMVRMVNSGTEATMSAIRLARGYTNRD
KIIKFEGCYHGHADCLLVKAGSGALTLGQPNSPGVPADFAKHTLTCTYNDLDSVRAAFEQYPSEIACI
IVEPVAGNMNCVPPLPEFLPGLRALCDKYGALLIIDEVMTGFRVALAGAQSYYGVEPDLTCLGKIIGG
GMPVGAFGGRRDVMDALAPTGPVYQAGTLSGNPIAMAAGYACLTEVSQVGVHQTLTELTEMLAAGLLH
AAQEENIPLVVNNVGGMFGLFFTDAPAVTCYQDVMQCDVERFKRFFHLMLEEGVYLAPSAFEAGFMSV
AHSKEDIQRTIDAARRCFAKL
>CORE_REP|Org20_Gene1068#
MTRRAIGVSERPPLLQTIPLSFQHLFAMFGATVLVPILFKINPATVLLFNGIGTLLYLFICKGKIPAY
LGSSFAFISPVLLLLPLGYEVALGGFIMCGVLFCLVALIVKKAGTGWLDVMFPPAAMGAIVAVIGLEL
AGVAANMAGLLPAEGTSADSTTITISLVTLAVTVLGSVLFRGFLAIIPILIGVLVGYALSFFMGVVDL
TPIREAHWFALPTFYTPRFEWFAIFTILPAALVVIAEHVGHLVVTANIVKKDLIRDPGLHRSMFANGI
STVFSGFFGSTPNTTYGENIGVMAITKVYSTWVIGGAAVLAILLSCIGKLAAAIQAVPVPVMGGVSLL
LYGVIGASGIRVLIESKVDYNKAQNLILTSVILIIGVSGAKVHIGAAELKGMALATIVGIGLSLLFKV
ISLFRKEEEVLDAPDEPAKQK
>CORE_REP|Org44_Gene4636#
MSQSVIHGGLDVKRSLLDAVLAGLIALIVFGPIVGIVLNGYSFNFEPHRLVWIIVAVMAGRLLLSLFL
QTAPGRRVLARFDGGNDGVYVRPLGYKSNLRWLLPLMAAIALLFPFVATKYLLTVAILGLIYVLLGLG
LNIVVGLAGLLDLGYVAFYAIGAYGLALGYQYLGLGFWAMLPLGALMAALAGALLGFPVLRMHGDYLA
IVTLGFGEIIRLVLNNWMSLTGGPNGVSVPAPTVFGLEFGRRAKDGGVPIHEFLGIDYNPNLKFIFIY
AVLFLVVLLVLYIKHRLTRMPIGRAWEALREDEIACRSLGLNHVLVKLSAFMLGASTAGLAGVFFATY
QGFVNPTSFTFFESALILAIVVLGGMGSTLGVVLAAFVLTVAPELLRSFAEYRVLLFGMLMVAMMVWR
PRGLVRISRASFAERKGVAP
>CORE_REP|Org13_Gene3894#
MAGLPTNSNSNALQQLYRLFEGRGGERSPHALAHWQQALRLGWPTRKHENWKYTPLESLLEQQFLDPQ
PAPVSAEQFEALALGIDACRLVFIDGRYSAALSDGDLGDYQFELTAYGTPQALPEPIQPEIFLHLTES
LAQETSLIRLPAGKAPARPLYLLHISSGRGATGEVNTVHHRHHLEIGRGAEAEVIEHYVSLGEAAHFT
GARLTANVADNAGLLHCKLAFESQPSYHFAHNDLVIGRDARVKSDSFLLGAGLTRHNTSAQLNGEGAN
LVINSLVLPVGKEICDTRTYLEHNKGYCESRQLHKTVVSDRGKAVFNGMIKVAKHAIKTDGQMTNHNL
LLGKVAEVDTKPQLEIYADDVKCSHGATVGRIDEEQLFYLQSRGIDKHAAQQMIIFAFAAELTEGIAN
DTIRERVLARIAQRLPGEAA

>CORE_REP|Org25_Gene1949#
MSKTHLTEQKFSDFALHPLVLEALEKKGFHNCTPIQALALPLTLSGRDVAGQAQTGTGKTLAFLASTF
HYLLTHPAKQDRQTNQPRALIMAPTRELAVQIHSDAEALSQSTGLKLGLAYGGDGYDKQLKVLESGVD
ILIGTTGRLIDYTKQNYVDLGAMQVVVLDEADRMYDLGFIKDIRWLFRRMPAADQRLNMLFSATLSYR
VRELAFEQMNNAEYVEVEPEQKTGHRIKEELFYPSNEEKMRLLQTLIEEEWPDRAIIFANTKHRCEDI
WGHLAADGHRVGLLTGDVAQKKRLRILDDFTKGNLDILVATDVAARGLHIPAVTHVFNYDLPDDCEDY
VHRIGRTGRAGASGHSISLACEEYALNLPAIETYIDHSIPVSKYNSDALLTDLPAPKRLSRPRGGNGP
RRNSAPRRGGAPRSNRKRSS
>CORE_REP|Org39_Gene1339#
MKTTIFKSLYFQVLTAITLGILLGHFYPDLGAEMKPLGDGFVKLIKMIIAPVIFCTVVTGIAGMESMK
AVGRTGAIALLYFEIVSTIALIIGLVIVNLVQPGAGMNVDPGTLDAKAVAVYAEQAQQQGIIPFLLDI
IPGSVIGAFASGNILQVLLFAVLFGFALHRLGEKGQLIFNVIDSFSRVIFGIINMIMRLAPLGAFGAM
AFTIGKYGVGTLVQLGQLIVCFYITCILFVVVVLGSIARANGFSIFKFVNYIKEELLIVLGTSSSESA
LPRMLDKMEKLGCKKSVVGLVIPTGYSFNLDGTSIYLTMAAVFIAQATNTHMDIMHQVTLLVVLLLSS
KGAAGVTGSGFIVLAATISAVGHLPLAGLALILGIDRFMSEARALTNLVGNGVATVVVAKWCDQLDEK
QLKDTLNNKNAGADKTQPSA
>CORE_REP|Org49_Gene1844#
MQTQFFNVTVEQCACQQTAPDNLVRIIAAGQTFFFYRDDFSDSENLLATLATGDRVKIGAHRLQDGSY
WLHWLLHETKGRLEPDRTLKYKLKYFALLVLGAALAGGFPAAFFIMDGEDSWLTIVLFFIAVIAGLVG
IGMVLFVGSELLLIGHRGRRRLLKALDRVLQGQDVTPPGSLSLKIPGIRYRAVSADAASVPTTQQTEF
PLPDGVEASGIETVNALSYELRHYEIPRQVQYLDTYAWRQKETHFALTTRALNVPNTKLHPVFRRQHP
LFIAKGDPLQVLYCENDTVPELPTVRGIINHRDRQTYLIGGRRYFTESGLNLCARVVFILGAIVTCFV
LGLAVADNADTPAGFYLDAWTLQRLGSELPPILLLMMLMVSGLALLIEGFAQICRLYSFDQHRTFKTF
KYLRNLRRRLWQQDQVTELK
>CORE_REP|Org37_Gene3352#
MVDSLTLHPVALVNGTVNLPGSKSVSNRALLLAALAKGTTRLTNLLDSDDVRHMLNALQTLGVNYQLS
ADRTVCEVTGVAGPLVAGQPLELFLGNAGTAMRPLAAALCLGEGDVVLTGEPRMKERPIGHLVDALRQ
GGAQIDYLEQTDYPPIRLRGGFQGGDVTVDGSVSSQFLTALLMTAPLAPQDTQIHIKGELVSKPYIDI
TLHLMRTFGVSVSHDNYRVFHIQGRQTYLAPGDYLVEGDASSASYFLAAAAIKGGTVRVTGIGRKSVQ
GDTKFADVLEKMGARITWGDDFIECSRGELRGIDMDMNHIPDAAMTIATAALFAEGPTTIRNIYNWRV
KETDRLAAMATELRKVGAEVDEGEDYIHVVPPAKLQFAEIGTYNDHRMAMCFSLVALSDTPVTILDPK
CTAKTFPDYFEQLARISQPA
>CORE_REP|Org43_Gene4524#
MPTGATKLGGWLLMAAMLGVSGCAKPPEPEKPPQPAPPVTPMRGIWLATVMGLDWPPAASLKAETAPE
RIRLQQQALTDALDDMVKTGINTVYFQVKPDGTALWRSDILPWSEVLTGTVGQDPGYDPLAFMLKEAH
RRGIKVHAWLNPYRVSMNTRQQTIDALNQTLQSPPASVYVLHPDWIRTANDRFALDPGLPDVRNWITG
VVAEVVKNYDVDGIQFDDYFYYETPQSPLDDEKTYREYGKGFADKASWRRDNTLQLIKQVSATVRALK
PAVAFGVSPAGVWRNKADDPAGSATQAGAPSYDAAYADTRQWVKLGLLDYIAPQLYWPFDREIVRYDV
LANWWAEVVKDTPVRLYAGVALYKVGTPSASEPAWTVDGGVPELKRQLDLNESLPGMGGTILFRQRYL
TEPQTDKAVEYLRTRWKTGQ
>CORE_REP|Org24_Gene1642#
MSTISPDSGTLTAAQPAKWNKTDTVWMFGLYATAVGAGTLFLPINAGLNGPLVLLLMALFAFPLTYLP
HRALSRFVLSGSSRDGNIHDVVVEHFGVLAGKIIMMLYLMAFFPIVLVYSISITNALDSFLIHQFHVA
PLPRIWLSLAVVVVLNLVLLRGKDSIVAAMGMLVFPLLVFLMGISLYLMPSWNTANFVHGLAATRFDT
PGLWHSLWLAVPVMVFSFSHAPIISSFASTQKSLYGDKAERRCARIMRYSYVLICVTVLFFVFSCVLS
LSHEDMQQAKDQNITVLTTLANKFSNPLIAYLGPVMAMLAMAKSYLGTSLGVTEGATSLIDGVTRAVG
KPLSSRMTHRISAVSLFLLTWAATVWNPSALHIIETISGPLIAAILFILPMYAVRAVPAMRKYRAASN
VFVLVMGLIALSALIYGLV
>CORE_REP|Org26_Gene3371#
MARPSFFLDFSLLRSNAHFRAIFCARMLSVFSLGMLAVGVPIQIQAMTGSTLQVGVAVALDGVGMFIG
LMLGGVLADRYDRRKLILFARGTCGLGFVALSLNAFAPAPSLLALYLLAAWDGFFGALGMTALMAVIP
LLVGRENLAAAGALSMVTVRIGAILAPALGGIIIVFGGVGLAFAVAAAGTLGTLVPLVRLPTLLPQQQ
EPEHPLRALASGFQFVWRNKVVGSVVLLGMLMSIVGAVRVLFPALAQDAYHVGASSIGLMYSAVPLGA
MLGALTSGWVGRFSRPGVLILVAAIVAFTAIASLGLFSHLAPALLALVCYGYANAIASLLQFMLIQSN

TPDHLLGRVNSLGTAQDVTGDSIGALGLGVLGRVFTPLMSVLSFGAFAAVLGVLVAFSVRTLRQCRPA
DALVEHDEPAPATSAAADN
>CORE_REP|Org36_Gene128#
MVRFENKDPLMLARQLPIKSVALILAGGRGSRLKDLTSTRAKPAVHFGGKFRIIDFALSNCLNSGIRR
IGVITQYQSHTLVQHIQRGWSFLNEEMNEFVDLLPAQQRLSTEHWYKGTADAVYQNLDIIRRYEAEYV
VILAGDHIYKMDYSRMLIDHVEKGAQCTVACLPVPRSEAGEFGVMKVDESDRIIEFLEKPADPPAMPG
NPDMSLASMGIYIFNAAYLFQLLEEDMSTPGSSHDFGKDLIPKITAQQAAWAHPFTLSCVTSNPDLPP
YWRDVGTLDAYWRANLDLASVTPELDMYDRAWPIRTHMEPLPPAKFVQDRSGSHGMTMNSLVSGGCIV
SGSVVVHSVLFPRVRVNSFCTIDSTVLLPDVNVGRSCRLRRCIIDRACHIPEGMVIGENADEDSKRFY
RSEGGIVLVTREMLSKL
>CORE_REP|Org18_Gene3825#
MLLRLYQVLLYLIQPLIWLRLLLRSRKAPAYRKRWAERYGFCAGKVVPGGIMLHSVSVGETLAAIPLV
RALRHRYPALPITVTTMTPTGSERVQSAFGKDVHHVYLPYDLPSSMNRFLDQVNPKLVIIMETELWPN
LINALHQRQIPLVIANARLSARSAAGYKKIGGFMRDMLRRITLIAAQNQEDGDRFIELGLKRSQLAVT
GSLKFDISVTPELAARAVTLRRQWAPRRPVWIATSTHDGEETILLEAHRKLLEKHPDLLLILVPRHPE
RFPTAKELVQKAGFSYTLRSSGEIPSGSTQVVIGDTMGELMLLYGIADLAFVGGSLVERGGHNPLEAA
AHAIPVLMGPHTFNFKDICAKLSQAEGLITVTDVDSLVKEVETLLTDEDYRRYYGRHAVEVLYQNQGA
LQRLLQLLEPHLPPRSH
>CORE_REP|Org3_Gene1406#
MQLIMSLVGMAVLIAIAVLLSSNRRAIKLRTVAWAFIIQVGIGALVLYVPLGRSILGSMSNGVANVIA
YGNQGISFIFGGLVSDKMFEVFGGGGFVFALRVLPVIVFFSSLIAVLYYLGIMQLVIRVLGGGLQKLL
GTSRTESLSATANIFVGQTEAPLVVRPYIATMSQSELFAVMCGGLASVAGSVLAGYAQMGVPLEYLIA
ASFMAAPGGLLFAKLMVPETEQTHDKDDAMKLIAEEDRPANVIDAAASGAASGMQLALNVGAMLLAFI
ALIALLNGILGGIGGWFDYPQLSLELILGWVFSPIAFLIGVPWSEAMTAGSFIGQKIIVNEFVAYMNF
GAYLRPDDVVAAEGLQVLSAHTKAIISFALCGFANLSSVAILLGGLGSMAPNRRHDIARFGLKAVAAG
TLSNLMSATIAGFFLAL
>CORE_REP|Org27_Gene2506#
MTTITALRVEDIRFPTSLGLDGSDAMNPDPDYSAAYVILETDRADLSGHGLTFTIGRGNEICCAAIRA
LEHQIVGERLEDIAADMGAFWRRFTSDSQLRWIGPDKGAIHLATGAVVNAVWDLWAKSVGKPVWRLVA
EMTPEELVRCIDFRYITDCITPQEALALLKQRAEGKEERLARLLQEGYPCYTTSAGWLGYPDDKLRRL
CQEAVDAGFSYLKLKVGRDLEDDIRRVRIAREVIGPDRRLMIDANQVWEVDEAIPWVKHLAFAKPWFI
EEPTSPDDVEGHRRIREGVAPVKVATGEMCQNRIMFKQFIMRGAVDVVQIDACRLGGVNEVLAVMLMA
AKYQLPVCPHAGGVGLCEYVQHLSMIDYLCIAGTHEGRVIEYVDHLHEHFVDPCVINGAAYMPPSRPG
YSIEMHSSSIEQYRFRG
>CORE_REP|Org7_Gene1179#
MPQFDYLKTSIKQKGCTLQQVADASGMTKGYLSQLLNDKIKSPSAQKLEALHRFLGLEFPRKEVKVGV
VFGKFYPLHTGHIYLIQRACSQVDELHVILCHDEPRDRELFENSSMSQQPTVSDRLRWLLQTFKYQKN
IHIHSFDEQGIEPYPHGWNVWSDGMKAFMEQKGIVPSFIYSSEAQDAPRYREHLGIETILVDPERSFM
NISGNQIRQDPFRYWDYIPTEVKPFFVRTVAILGGESSGKSTLVNKLANIFNTTSAWEYGRDYVFSHL
GGDEMALQYSDYDKIALGQAQYVDFAVKYANKVAFIDTDFVTTQAFCKKYEGREHPFVQALIDEYRFD
LVILLENNTPWVADGLRSLGSTADRLAFQRLLEEMLRANNIEYVHVESSDYEERFLRCVELVQQLLAA
DAGRLANAPAQRHAAG
>CORE_REP|Org39_Gene3672#
MSVTASDLAFDQRHIWHPYTSMSRPLPCYPIESASGVELQLADGRRLVDGMSSWWAAIHGYNHPHLNQ
AASRQLEKMSHVMFGGITHPAAISLCRRLVAMTPEALQCVFLADSGSVAVEVSLKMALQYWQARGERR
QRILTLRHGYHGDTFGAMSVCDPDNSMHSLYQGYLAPHLFATAPQCRFDEEWREEDIAPFAALLEQHA
GEVAAVILEPVVQGAGGMRIYHPTYLKRVRELCDRHQVLLIADEIATGFGRTGKLFACEHAQVVPDIL
CLGKALTGGYMTLSATLTTRHVAETISNGAAGCFMHGPTFMGNPLACAVADASLALLAENRWQAQVSA
IEAQLKQELLPLAALPKVADVRVLGAIGVVEMREPVDVAGLQRGFVERGVWIRPFGKLIYLMPPYIIE
AEQLSRLTAAVAAAAR
>CORE_REP|Org29_Gene4300#
MAKNIQAIRGMNDYLPEETALWQRIEGTLKQVLGSYGYSEIRLPIVEQTPLFKRAIGEVTDVVEKEMY
TFEDRNGESLTLRPEGTAGCVRAGIEHGLLYNQEQRLWYIGPMFRYERPQKGRYRQFHQLGAEVFGLQ
GPDIDAELILLTARWWKALGIAEHVKLELNSIGSLEARANYRDALVAFLEQHVEVLDEDCKRRMYSNP

LRVLDSKNPEVQALLNDAPRLSEYLDEESRAHFAGLCELLAQAGIPYTVNERLVRGLDYYNRTVFEWV
TTSLGAQGTVCAGGRYDGLVEQLGGRATPAVGFAMGLERLVLLVQAVNPEFKAPSAIDVYVISSGAGT
QSAAIQLAEQVRDAAPQLKLMTNYGGGNFKKQITRADKWGARIALILGESEVAAQQVVVKDLRSGEQE
TLAQSEVAARLALMLG
>CORE_REP|Org2_Gene1799#
MASSNLIKQLQERGLVAQVTDEEALAERLAQGPIALYCGFDPTADSLHLGHLVPLLCLKRFQLAGHKP
VALVGGATGLIGDPSFKAAERKLNTTDTVNEWVEKIRKQVSPFLDFDCGSNSAIAANNYDWFGGMNVL
TFLRDIGKHFSVNQMINKEAVKQRLNRDDSGISFTEFSYNLLQGYDFSELYNRHQVELQIGGSDQWGN
ITSGIDLTRRQHQKQVFGLTVPLITKADGTKFGKTEGGAVWLAPEKTSPYKFYQFWINTADADVYRFL
KFFTFMSLEDINALEEEDKNSGKAPRAQYVLAEEVTGMVHGAEGLAAAKRITQSLFSGALHDMTEADF
AQLAQDGMPTIKLERDADLQQALVNAELVPSRGQARTMIGSNAVTINGEKQSDAEYRFSDADRLFGRY
TLLRRGKKHYCLVDWQ
>CORE_REP|Org5_Gene2120#
MEKLSYASDSSTTAWATYLQQIDRVAPYLGELSRWVDTLRHPKRALIVDIPLQMDDGTIRHFEGFRVQ
HNLSRGPGKGGIRFHPDVDLNEVMALSAWMTIKCAAVNLPYGGAKGGIRVDPFKLSEGELERLTRRYT
SEIGFIIGPQKDIPAPDVGTNAKVMAWMMDTYSMNHGTTITGVVTGKPIHLGGSLGREKATGRGVFVT
GSEVAKRLGVQIEGAKVAVQGFGNVGSEAARLFVGVGARVVTIQDHSATLFNADGIDLAALTEYQTKH
KQIAGFPGASEIESEAFWSVDMDILIPAALEGQITRERAEILSAKLVLEGANGPTFPEADDILRSRNI
TVVPDVICNAGGVTVSYFEWVQDMASYFWSESEINERMDKIMTDAMVHVWNKAAEKECSLRTAAYIVA
CERILTARKERGIYPG
>CORE_REP|Org19_Gene479#
MTDKRKDGSGKLLYCSFCGKSQHEVRKLIAGPSVYICDECVDLCNDIIREEIKEVAPHRERSALPTPH
EIRHHLDDYVIGQEQAKKVLAVAVYNHYKRLRNGDTSNGIELGKSNILLIGPTGSGKTLLAETLARFL
DVPFTMADATTLTEAGYVGEDVENIIQKLLQKCDYDVQKAQRGIVYIDEIDKISRKSDNPSITRDVSG
EGVQQALLKLIEGTIAAVPPQGGRKHPQQEFLQVDTSKILFICGGAFAGLDKVIGQRVNTGSGIGFGA
TVKGESEKATEGELLLQAEPEDLIKFGLIPEFIGRLPVVATLSELSEDALIQILKEPKNALTKQYQAL
FNLEGVELEFRDEALNAIAKKAMARKTGARGLRSIVEGALLDTMYDLPSMDSVDKVVIDESVIAGQSK
PLLIYGKPEAQASGE
>CORE_REP|Org13_Gene3315#
MAQVINTNSLSLMAQNNLNKSQSSLGTAIERLSSGLRINSAKDDAAGQAISNRFTANIKGLTQASRNA
NDGISLAQTTEGALNEVNDNLQNIRRLTVQSQNGSNSSSDLQSIQDEISQRLSEINRISEQTDFNGVK
VLSADQKLTIQVGANDGETIDIELKDINAKTLGLDKFSVADPIDTTKIGTTKLTAVDKMGAPTINADS
KAATPKNSDGKLYSTDDGAGNVAYFVKSTDGGIYDATVAADGKVSWDSSTATTKATAGMKETSQVKVG
TTNISGLTGSDELRTYTDPAGKSGYVVKGKDDNGNDAYFKATVDASGKVTKGAQQSTDPKTADPLATL
DKALSQVDSLRSSLGAVQNRFDSVINNLNSTVNNLSASQSRIQDADYATEVSNMSRANILQQAGTSVL
AQANQSTQNVLSLLR
>CORE_REP|Org21_Gene3509#
MRFDVVVIGGGLAGLSCAIAVAEQGKRCAVVSSGQSALYFSSGSLDLLARLPDGTPVEMPLAALPQLA
QQAPQHPYALIGPGRVAALSAAAQRLLARCGLQLQGEGANNHLRITPLGTRRATWLSPQAIPTLPLTG
QLPWRRIAVIGIEGFLDFQPQMAADSLSRELGVETEVAYLHMPALDRLRNNPSEFRAVNIARVLDLME
SLPPMAEELRRLAGEADALFLPACLGLEDDASLAVLQDAVGKPIRLLPTLPPSVPGMRLHQALRRRFQ
QLGGVFIPGDSVLRAECEAGRVTGLYTRNHGDIPLRAQQVVLASGSFFSNGLVADFDGVREPIFGLDV
HSRADRADWSRRELFAPQPYLQFGVRTDGRLRAMKQGMPFDNLYAIGAVAGGYDPLQQGCGAGVSLIG
ALHVAQQIAAEENA
>CORE_REP|Org33_Gene2400#
MNKATVAAKRWWYIMPIVFITYSLAYLDRANFSFASAAGINEDLGITKGMASLLGALFFLGYFFFQIP
GAIYAERRSVKKLIFWCLILWGGCASLTGVVSNIPMLAAIRFILGVVEAAVMPAMLIYISNWFTKSER
SRANTFLILGNPVTVLWMSVVSGYLIHAFGWREMFIIEGIPAVIWAFCWWVLAKDKPAQAGWLSAEEK
LALQQQLDEEQKGIKAVRNYGEAFRSRNVILLCVQYFAWSIGVYGFVLWLPSILRSGMQMGMVEAGWL
SAVPYLAATIAMIVVSWASDKMQNRKLFVWPLLLIGALAFFGSYAVGTNHFWISYGLLVVAGAAMYAP
YGPFFAIIPEMLPKNVAGGAMALINSMGALGSFFGSWFVGYLNGATGSPAASYMFMAIALVVAVVLTL
IVKPARNEIQPQLA
>CORE_REP|Org38_Gene942#
MQRRRFIKAFALSAAAVGLGLAWSAQAADTIKVGILSSLSGTMAISETPLKDVALMTIDEINAKGGVL

GKKLEPVVVDPASNWPLFAEKARQLLSQDKVAAVFGCWTSVSRKSVLPVFEELNGLLFYPVQYEGEEM
SPNVFYTGAAPNQQAIPAVEYLLSEDGGGAKRFFLLGTDYVYPRTTNKILRAFLHSKGVQDKDIEEVY
TPFGYSDYQTIVANIKKFAAGGNTAVISTINGDSNVPFYKELANQGVKATDVPVIAFSVGEEELRGID
TKPLVGNLAAWNYFESLDNPTNKQFVSQWKAYAKAHNLPNYATAVTNDPMEATYVGLHMWAQAVEKAG
TTDVDKVRAAMAGQTFAAPSGFTLTMDKTNHHLHKPVMIGEIEGNGQFNVVWQTEAPVRAQPWSPYIA
GNDKKPDYPVKGGK
>CORE_REP|Org18_Gene1079#
MAYFIDRRLNGKNKSMVNRQRFLRRYKSQIKQSIAEAINKRSVTDVDSGESVSIPTDDINEPMFHQGR
GGTRHRVHPGNDHFVQNDRVERPQGGAGGGGGQGNASQDGEGQDEFVFQISKDEYLDLLFEDLALPNL
KKNQYKQLTEFKTHRAGYTANGVPANISVVRSLQNSLARRTAMTAGKRRALHELEDALTQLENTEPVQ
LLEEERLRKEIAELRKKIESVPFIDTFDLRYKNYERRPEPSSQAVMFCLMDVSGSMDQATKDMAKRFY
ILLYLFLSRTYKNVEVVYIRHHTQAKEVDEQEFFYSQETGGTIVSSALKLMNDVVEERYDPAQWNIYA
AQASDGDNWADDSPLCHELLAKKILPMVRYYSYIEITRRAHQTLWREYEDLQARFDNFAMQHIREPDD
IYPVFRELFRKQTV
>CORE_REP|Org34_Gene4576#
MSENTASQIAAPQNAQASGRTILLFLALALMSALLNSSAPTPLYPLYQQQLTLSSVSLTVIYGAYAAG
VLISLFGVGNLAGKVKDLRSMIVPALLVVLSGALLFAQADTFAMMLMARLLAGVGTGALTGAANIALV
RFGPRDGGKNAALIATLSFTTGLALGPIFSGIALQTGFHPTTLPFVFIMVMAAVAALGVMFSWPRGVV
TAPSHVTSAETEKSSLLDGLRATGGKFFVCAGALFICWALAASILAIGPGVAETLLGLHARGVFGYAI
AVYLLIAGISQILSRRVNARHSLLFGCLAQVLAAVVFTMAIQWHSLGLAAVGLVVAGYAYGAIFVGSA
TLVNLISPPTSHARLLSLFYVIAYIANWVPILLGVVVDRVDLNQATHLLFLGSTVVCLLLAWKTSRVG
FLANYFVMNIYFYN
>CORE_REP|Org36_Gene4636#
MKNAELNQRRQDATPRGVGVMCGFYAERAENATLWDVEGKEVIDFASGIAVLNTGHRHPKVIAAIEKQ
LQAFTHTAYQIVPYESYVSLAERINQRAPIAGPCKTAFFTTGAEAVENAVKIARAYTGRPGLITFGGG
FHGRTYMTMALTGKVAPYKLGFGPFPGSVFHGQYPNALYGVTTEDAMNSLDRLFKADIDPKQVAAIVL
EPVQGEGGFNVAPAEFMQALRALCDQHGILLIADEVQTGFARTGKLFAMEHYSVKPDLITMAKSLAGG
MPLSAVAGRAEVMDAPAPGGLGGTYAGNPLAVAAAHAVLDVIEEEQLCQRAQRLGQHLVEVLQQARKT
SPAIADVRAQGSMVAVEFNDPATGKPSADITRQVQQKAMEEGLLLLSCGVNGNVIRFLYPLTIPDDQF
TKAMGILSRALAH
>CORE_REP|Org36_Gene1379#
MTTVSTLGALVALAVAIVLILRKVPPAYGMIAGALAGGLCGGADLVQTVTLMIGGAQGITNAVMRILA
AGVLAGVLIESGAAHTIAETIVRKVGETRALLALAVATLILTAVGVFIDVAVITVAPIALSIAQKAGI
SRAAILLAMIGGGKAGNVMSPNPNTIAAADNFHVPLTSVMMAGIVPGLCGLVVAYLLARRLSDKGSKV
MAEELTQHAEGARPGFAAAISAPLVAILLLSLRPIAGIAVDPLIALPAGGLAGALLMGRIRQCNHFMV
SGLSRMAPVAIMLLGTGTLAGIIANSALKDVLINGLTHTGLPAWLLAPLSGALMSMATASTTAGTAVA
SGVFSSTLLELGVSGLAGAAMIHAGATVLDHLPHGSFFHATGGSVNMAVHERLKLLPYETLVGFTIAA
ISALMFGVFNLAG
>CORE_REP|Org44_Gene49#
MQLGVIADDFTGATDIASFLVRNGMPTVQLNGVPTRDLPLTSEAVVISLKTRSCPAEMAVSQSLAALR
WLQAQGCQQFYFKYCSTFDSTAQGNIGPVLDALLAELGETRTVISPALPVNGRTVYQGYLFVGEQLLN
ESGMRHHPVTPMEDAHLGRLIERQGRGKAALIAWPIVARGPEAVAAALAAVNDPAVRYVVLDALSEQD
LLTQGVALREMKLVSGGSGLAIGLARDLAQRHGARGESAQAGMPLVGPAVVLSGSCSVMTNSQVAAYR
QQAPARAVDLSACFTDLESYVRTLTDWVDAQRDAPLAPMIYATTEPQTLQRIQAQYGDKASSERVEQL
FAALAAALKAKGFTRFIVAGGETSSIVAQTLGVEAFHIGPTISPGVPWVRDTRQPLSLALKSGNFGDI
QFFARAQQEFRHD
>CORE_REP|Org29_Gene2731#
MNNSPGQRSRLSEENRLLIILFFVFGCVFVDRLTISFLFPMIAADLKLSNVHLGTLSAVLALTWALSG
AGLGAIADRFNIRKPMLILSILVFSLFSALSGLVSGFAMLLIFRALMGIAEGPVLPIAQSLMVEKSQP
QRRGFNMGLIQGAAPGLLGGIIAPPLIIYLAQKWGWSMAFHLTAVPGIILAWLIYRYVNGKKDPAFSA
APAAGKAANKAAYGELFKIKNVALCILISCVFVTWFMIIITFTPNFLVTDRGFSEGTMGGIMSAIGAA
WVFWGVAVPAISDRLGRKPTLIFFSLLAVCCPLFLSYVDNPWLLGVLVFLSYTGLGCFTLFMATIPSE
TVSPARIATALGLVMGIGEVIGGCLAPFIAGLIADRYGLVSVMWLAAAGAVCAGVLSCFLDETAPAVV
SRRAVKPASVTDV

>CORE_REP|Org5_Gene3558#
MPRPLHDTSTALNAANLLALPQRFGCPVWAYDADIISQRIAQLRHFDTIRFAQKACSNIHILRLMREQ
GVKVDSVSLGEIERALQAGFQPGGEPSEIVFTADVLDHATLARVSELKIPVNAGSIDMLDQLGQVSAG
HPVWLRINPGFGHGHSQKTNTGGENSKHGIWYADLPQAVEKIRRYGLKLVGVHMHIGSGVDYQHLERV
CDAMVQQVIDLGHDISAISAGGGLSIPYQYGEEAIDTEHYFGLWNRARERIAAHLGHPVQLEIEPGRF
LMAEAGVLVAEVRAVKDMGSRHFVLVDAGFNDLMRPAMYGSYHHISLLPADGRDTSGQALRETVIAGP
LCESGDVFTQQAGGGVETRELPPVQIGDYLVFHDTGAYGASMSSNYNSRPLLPEVLFENGEPRLIRRR
QTIEELIALELI
>CORE_REP|Org20_Gene4243#
MSFDTISVIGLGYIGLPTAAAFASRKKKVVGVDVNQHAVDTINRGAIHIVEPDLDKVVKDAVDGGFLR
AVTKPLAADAFLIAVPTPFKGDHEPDLAYVEAAAKSLAPVLKKGDLVILESTSPVGATEQMADWLAQA
RSDLSFPQQAGEAADVNIAYCPERVLPGQVMVELIQNDRVIGGMTPKCSERASALYKIFLEGECVITN
SRTAEMCKLTENSFRDVNIAFANELSLICAEQGINVWELIRLANRHPRVNILQPGPGVGGHCIAVDPW
FIVAQNPQQARLIHTARLVNDGKPLWVVDRVKAAVADCLAATDKRASEVKIACFGLAFKPNIDDLRES
PAVEVVHLIAEWHVGETLAVEPNVEQLPKSLAGHVTLTPIAEALQQADVIVMLVDHQQFKAIRPEEIK
QSWVVDTKGVWR
>CORE_REP|Org22_Gene1226#
MTLLALGINHKTAPVSLRERVTFSPESIDEALTSLLQQPLVQGGVVLSTCNRTELYLSVEQQEHMHEQ
LVAWLCAYHNLRPEEVKKSLYWHQGNDAVSHLMRVASGLDSLVLGEPQILGQVKKAFAESQRGQSLSG
ELERLFQKSFSVAKRVRTETDIGASAVSVAFAACTLARQIFESLADLNVLLVGAGETIELVARHLREH
KVRHMIIANRTRERAQLLADEVGAEVITLPEIDERLADADIIISSTASPLPIIGKGMVERALKARRNQ
PMLLVDIAVPRDIEPEVGKLANAYLYSVDDLHAIIQSNLAQRKAAAVQAESIVQQESTNFMAWLRSQG
AVETIRDYRSQADQIRAEMEAKALAAIAQGANVEQVIHELAHKLTNRLIHAPTKSLQQAAGDGDVERL
QLLRDSLGLDQH
>CORE_REP|Org24_Gene371#
MDKFRVQGRTRLSGEVSISGAKNAALPILFAALLAEEPVELQNVPKLKDIDTTIKLLNQLGTKIERNG
SVFVDASGVNEFCAPYDLVKTMRASIWALGPLVARFGRGQVSLPGGCAIGARPVDLHITGLEQLGAEI
KLEEGYVKASVEGRLKGAHIVMDKVSVGATVTIMSAATLATGTTVIENAAREPEIVDTANFLNTLGAK
ISGAGSDKITIEGVERLGGGVYRVLPDRIETGTFLIAAAVSGGKVMCRNTRPDTLDAVLAKLREAGAD
IEVGEDWISLDMHGKRPKAVTVRTAPHPGFPTDMQAQFSLLNLVAEGTGVITETIFENRFMHVPELIR
MGAHAEIESNTVICHGVEQLSGAQVMATDLRASASLVIAGCIADGVTVVDRIYHIDRGYERIEDKLRA
LGANIERVKGE
>CORE_REP|Org2_Gene3295#
MAAVATPGHVYPMLAIARHLIAQGHQVRVMTGALFRERAEVAGASFVPFDAQVDFDYRHLEEHFPERA
ALPPGNAQMALALKDFFAAPIPLLDRQLRATIAAEKTDLLMVENCFYGVLPLLQSAARPPVFGIGVTP
LSYSSRDAIFYGPRIPPALLPQALTREQLVDEETRVLIDDVQQSFDAALLQAGGRALDRPFTDALIGG
CERFLQLATTALEYERDDLPSGVRFVGPLRSGGQPAAEETLWEADDRRPLVIVTQGTLANVDLHQLIV
PTLQALAHLPVRVLATTGGRATEGLMDALPGNARVREFISFERWLPETALLITNGGYGSISYALDSGV
PLIVAGTGEDKLEAAARVVAAGCGISLHTSTPSAEQILAAATRILQQPIYRQRAALVREDYARHDALT
AIANEVAAITA
>CORE_REP|Org25_Gene896#
MSMLEQMGKAAKQASWQLAVLSTAKKNQVLSVMADRLEANSEAILLANEQDMAQARATGMSEALLDRL
LLTPARLAAIANDVRQVCRLNDPVGHVLDGNLLDSGLKLERRRVPLGVIGVIYEARPNVTIDVASLCL
KTGNAVILRGGKETHNTNQATVKVIQQALEQCGLPAAAVQAIDSPDRALVNELLRLDRYVDMLIPRGG
AGLHKLCREQSTIPVITGGIGVCHTYVDADVDFDKALTVIENAKIQRPSACNSLETLLVNRSIAAEFL
PALSAKMAAVGVTLHAAENALPLLQGGPATVVPVNAEDYDDEWLSLDLNVLLVDDIDQAIDHIRTHGT
NHSDAILTRSLSSAEHFVRAVDSSAVYVNASTRFTDGGQFGLGAEVAVSTQKLHARGPMGLDALTTYK
WIGYGDDLVRS
>CORE_REP|Org21_Gene461#
MNLTELKNTPVSELITLGENMGLENLARMRKQDIIFSILKQHAKSGEDIFGDGVLEILQDGFGFLRSG
DSSYLAGPDDIYVSPSQIRRFNLRTGDTISGKIRPPKEGERYFALLKVNEVNYDKPENARSKILFENL
TPLHANSRLRMERGNGSTEDLTARVLDLAAPIGRGQRGLIVAPPKAGKTMLLQNIAQSIAYNHPDCVL
MVLLIDERPEEVTEMQRLVKGEVIASTFDEPASRHVQVAEMVIEKAKRLVEHKKDVIILLDSITRLAR
AYNTVVPASGKVLTGGVDANALHRPKRFFGAARNVEEGGSLTIIATALVDTGSKMDEVIYEEFKGTGN

MELHLARKIAEKRVFPAIDYNRSGTRKEELLTTSEELQKMWILRKIIHPMGEIDAMEFLINKLAMTKT
NDEFFDMMKRS
>CORE_REP|Org7_Gene4391#
MTTQDIDARAGRAGETVAENPQQRVRWSVPIALFACVLLAFFDKISIAALFSDSEFQQALGIGFDPAR
LGLLMSAFLFSYGISSMLLSGIGDRLNPVKVLIGMMVVWGVLMVLMGLVRSYHAMMTLRILLGIAEGP
LLPMAYAIIRQAFPPQLQARATMLWLLGTPLGAALGFPVTLYILNTFDWQATFFFMAFLTLPVMLLVL
FGMRHLNVSRPAAAAKPAVSERQQHRRELLRSPHFWMICLFNIAFLTYLWGMNGWLPSYLIKGKGIHL
EHAGYLSSLPFIAMLLGEVLGAWLSDKLDRRALACFLSLCGAGLGLAVVLHLQGTYSVIAAMAFSTFM
WGAGAPNIFALLAKATSSKVSATAGGIFNGLGNFAGALAPVLMGALIAATGNMDNGLLFLVVMAFVGC
LILLPLLRKY
>CORE_REP|Org8_Gene364#
MIKSTDRKLVVGLEIGTAKVSALVGEVLPDGMVNIIGVGSCPSRGMDKGGVNDLESVVKCVQRAIDQA
ELMADCQISSVYLALSGKHISCQNEIGMVPISEEEVTQDDVENVVHTAKSVRVRDEHRILHVIPQEYA
IDYQEGIKNPVGLSGVRMQAKVHLITCHNDMAKNIVKAVERCGLKVDQLIFAGLAASYAVLTEDEREL
GVCVVDIGGGTMDMAVYTGGALRHTKVIPYAGNVVTSDIAYAFGTPPTDAEAIKVRHGCALGSIVSKD
ENVEVPSVGGRPPRSLQRQTLAEVIEPRYTELLNLVNDEILQLQEQLRQQGVKHHLAAGIVLTGGAAQ
IDGLAACAQRVFHTQVRIGQPLNITGLTDYAQEPYYSTAVGLLHYGKESHLSGETEVEKRASVGNWFK
RINSWLRKEF
>CORE_REP|Org34_Gene1011#
MQQQITRWLEQFGLEFGGVMSLLMVLGLIVLISVVIHLVLHRVVLAALQRRGQQSQRVWQQAITQYKL
FQRVALLLQGVIISIQATLWLQSGSQTQAVIVTAAQVWILAFTLLSLFSLLDTLLALLRQSPIANQLP
LRGIFQGLKLVAAILIGIMIVSLLMGKSPLLLLSGLGAMTAVLMLVFKDPILGLVAGIQLSANDMLKI
GDWLEMPKYGADGAVTDIGLTTVKVRNWDNTVTTIPTYALISDSFKNWRSMSESGGRRIKRSLNIDTG
SVHFLSEEEQRRLQRNPLLHSYLNVKTQELSQHNQEIAVDLASPLNGRRLTNLGTLRAYLEAYLRAHP
RIHQNMTLMVRQLAPTPEGLPLEIYAFTNTTVWAEYESIQADIFDHILAVIDEFGLRVHQTPTGNDLR
GMLQQSANAS
>CORE_REP|Org22_Gene1047#
MLKREMNIADYDAELWRAMEQEVVRQEEHIELIASENYTSPRVMQAQGSQLTNKYAEGYPGKRYYGGC
EYVDIVEQLAIDRAKELFGADYANVQPHSGSQANFAVYTALLQPGDTILGMNLAHGGHLTHGSPVNLS
GKLYNVVPYGIDDKGQIDYDDLAKQAQTHKPKMIIGGFSAYSGVVDWAKMREIADSIGAYLFVDMAHV
AGLIAAGVYPNPVPHAHIVTTTTHKTLAGPRGGLILAKGGDEELYKKLNSAVFPGGQGGPLMHVIAGK
AVALKEAMEPEFKVYQQQVAVNAKAMVDVFLQRGYKVVSGGTHNHLFLLDLVDKNLTGKEADAALGRA
NITVNKNSVPNDPKSPFVTSGVRIGTPAVTRRGFKEADVRELAGWICDVLDNINDEATIERTKKKVLD
ICARLPVYA
>CORE_REP|Org27_Gene1608#
MESKVVVPAEGKKITVDAQGKLVVPHNPIIPFIEGDGIGVDVTPAMIHVVDAAVKKAYHGERKISWME
IYTGEKSTHVYGKDVWLPDETLDLIRDYRVAIKGPLTTPVGGGIRSLNVALRQQLDLYVCLRPVRYYQ
GTPSPVKQPELTDMVIFRENAEDIYAGIEWKAGSAEADKVIKFLRDEMGVKKIRFPEQCGIGVKPCSE
EGTKRLVRAAIEYAITNDRDSVTLVHKGNIMKFTEGAFKDWGYELAREEFGGELIDGGPWLKIKNPNT
GKEIVVKDVIADAFLQQILLRPAEYDVIACMNLNGDYISDALAAQVGGIGIAPGANIGSDCALFEATH
GTAPKYAGQDKVNPGSIILSAEMMLRHMGWFEAADLIVKGMEGAIAAKTVTYDFERLMEGAKLLKCSE
FGDAIVEHM
>CORE_REP|Org45_Gene2458#
MPNGHPVRRPLGRLYTALWGGCLLMLSQSAAARFAIPGYELVYTAPVETALQADDLRNTAEVWREMFD
AAKTRIDLGQFYVANQDGSLLDGVLQHLKAAGERGVKIRFLLEEKGLRMSTAETLEQLKAIPNLELRI
IPYQKLSGGILHAKYLLVDGEQAFVGSQNFDWRALEHIHETGLRISDAKVVGQIQAIFEQDWQAQALL
AQDKPVPALPDSPPTAQPQGNYLAASPRAYNPAGVIDSQAELPRLLAGAKRRVRVQVMDYAPLSFGPE
RSRPFYAVIDNALRSAAARGVQIELMVANWNTKKPDIAWLKSLALVPNVQIKVVTIPPASSGFIPFAR
VIHSKLMTIDDEIAWVGTSNWTGGYLDNSRNLELVMHSAAMSGRLDKLYQQLWNSVYAEPLRLDYDYP
PPKPGGES
>CORE_REP|Org13_Gene2630#
MAIKTRLKVMIFLQFFIWGAWLVTLGSYMINTLHFSGMQVGMVYSAKGIAALIMPGLAGIIADRWMKA
NSLYALCHLLGALALYCAAQVEQPMLMFWVMLFNAMVYMPTIALSNAISYFCLEKHGFDTVKDFPPVR
VYGTVGFILAMWLVGFSQIELSNLQLYLASAASLLLSAYSLTLPRCPTNRAAESKSWVSVLGLDALVL

FRQRRMALFFLFAMLLGAALQITNTFGNPFLHDLSLNPLYQDSLSVRYPAVLLSLSQISEVFFILTIP
FFLRRYGIKQVMLISMAAWTLRFLFLAYGTPVGFGFVLLLLSMIVYGCAFDFFNISGAIFVEKEADHR
IRASAQGLFMTMVNGLGAYLGALASGEVVDFFSHDGVKDWQSIWLVFALYTLVLGIVFALSFNYRHSP
QESAVAAQ
>CORE_REP|Org37_Gene1395#
MAWNQPGNNGQDRDPWGSSNNNGGNSGGNNKGGRDQGPPDLDDIFRKLSKKLSGFGGGKGSNSNSGGT
GTSGPGFSGRIIGIAAVAVVVIWAASGFYTIKEAERGVVTRFGKFSHLVQPGLNWKPTFIDDVRPVNV
ESVRELAASGVMLTSDENVVRVEMNVQYRVTNPEAYLFSVVNADDSLSQATDSALRGVIGKYSMDRIL
TEGRTVVRNDTQRMLEETIRPYNMGITLLDVNFQAARPPEEVKASFDDAIAARENEQQYIREAEAYAN
EVQPRANGQAQRLLEDSKAYKDRTILEAQGEVARFAKLLPEYKSAPQITRERLYIETMEKVLGHTRKV
LVSDKGNNLMVLPLDQMLRGQGAAPESGNKDTSLIRLNPNPAPAANSSTPRTSGGSIMDQRRANAQRD
DTTRVGRE
>CORE_REP|Org4_Gene1771#
MSLAKASIWTAGSTLIKIGVGLLVVKLLAVAFGPSGVGQAGNFRQLITVLGVLSGAGIFNGITKYVAE
YHQDPQRLRLAVGTASSIVLGFSTLLALVFLFAAEPISIGLFGHADYVDVVRAVAFIQMGIAYANLFM
AVLKGYRDAMGNALAVIGGSLIGVVAYYLCFTLGGYEGALAGLALVPALVVLPAGLMLWRRKTLPLRY
LAPRWDKLVAGNLGKFTLMALITSVTLPVAYVMMRNLLAAHYGWDEVGIWQGVSSISDAYLQFITASF
TVYLLPTLSRLTDKGAISREIVRSLKFVLPAVAAASFMVWVLRDFAIWLLFSDKFVAMRDLFAWQLVG
DVLKVGAYVFGYLVIAKASLRFYILTEVSQFLLLTVFSHWLIPLHGALGAAQAYMATYIVYFALCSCV
FLIYRRRV
>CORE_REP|Org8_Gene2144#
MKIHAITAPLSKARHQRCCECDLLFMLPPLSGNQAAYCPRCNAKVVHGRDWSMTRLTAMAITMLLLMP
FAFTEPLISIRLLGTRIDASLLEGIWQMSRQGDPLTASMVAFCTLGAPLTLALSLLYLRFGHALGMNL
RPVLLMLERLKEWVMLDIYLIGMAVAAIKVQDYADIQAGSALIAYLSLTLLSILTLIHANLEQLWERY
YPQEQPEGPPAALHICLSCHYTGYPDARGRCPRCHVPMCHRQPYSLQKTWAALIAAMILLIPANLLPI
SIIYANGVRLEDTIFSGVVSLATSGNVPIAAIVFIASVLVPFTKVIVLITLLLSIHFKTSHSLKTRIR
LLRLVTWIGRWSMLDLFVIALMMSLVNRDQLLSFTMGPAAFYFGSAVILTILAVEWLDSRLIWDAHAT
GNADYTD
>CORE_REP|Org40_Gene2806#
MKTTLPPAARLGRQALLFPLCLVLFEFATYIGNDMIQPGMLAVVADFNAGEEWVPTSMTAYLAGGIFL
QWLLGPLSDRRGRRPVMLAGVAFFIVSCLAILLVTTIEQFIAMRFLQGIGLCFIGAVGYATIQESFEE
SVCIKITALMANVALIAPLLGPLAGAALIHVAPWQSMFVLFAALAAIAFYGLWKAMPETATLQGEAFS
AANLWRDYRQVLGNRRFLCGALAIGFASLPLLAWIAQSPVILISGESLSTLDYGLLQIPVFGALILGN
LTLARLTGKNSVERLIKLGAGPMLLGLLIAALATQFSSHAYLWMTAGLSLYAFGIGLANAGLYRLTLF
SSNVSKGTVSATMGMLSMMVFTVGIELAKVAYVWGGSGLFNLFNLISGLCWLTLVALFLGKRRNGDPT
PQPTGAV
>CORE_REP|Org17_Gene1564#
MAGASLSFLTALRFSRGRKRGGMVSLISVISTIGIALGVAVLIVGLSAMNGFERELKNRILAVVPHGE
LEPVNQPFSGWPSILQRVEKVPGIVAAAPYINFTGLMENGAQLRAVEVKGVDPQQESRLSALPQYVQG
DAWANFKPGEQQVILGKGVADALGVKQGAYVTVMIPNSDPEMKLLQPKRIRLHVTGILQLSGQLDHSL
ALVPLADAQQYLDMGDSVTGIALKMNDVFAANKLVRDAGEVTNAYIYIKSWIGKYGYMYRDIQMIRAI
MYLAMVLVIGVACFNIVSTLVMAVKDKSADIAVLRTLGAKDGFIRAIFIWYGLLAGLLGSLSGVVIGV
IASLQLTNIIRGIEKLVGHSFLSGDIYFIDFLPSELHWLDVLIVLATAIVLSLLASWYPARRASRIDP
ARVLSGQ
>CORE_REP|Org24_Gene3286#
MAQANLSEILFKPKFKHPETSTLVLRARNKVSAAMHSALEGDTTGSWYRMLNPLLWAWRGVSPIEIHE
VLARIAASDAERSNPQRLDTVVGYRNGNWIYEWIHQGMQWQQRATEQQDPLLGGQYWLKAASLYSIAG
YPHLKGDELAEQAEMLANRAYEEAALLLPYQLKELEFRIEGGASVTGFLHMPEKGEAPFPTVLMCGSL
DTLQTDYHRLFRDYLAPHGIAMLTLDMPSIGSSCKWKLTQDSSYLHQQVLIQLASVPWVDHQRVSAFG
FRFGANVAVRLAYLEPQRLRGVACLGPVVHRLLCDSSTQLNVPDMYMDVLASRMGMANASDNVLKVEL
NSYSLKMQGLLGRRCPTPMISGYWERDTLSPKEESQLIVTSSVSGKLVAIPRTPVYDSFHRALLQMSG
WLKDKMR
>CORE_REP|Org37_Gene2232#
MSTDTIQKLARPSVLGGAMIIAGTAVGAGMFSIPIVTSGVWFSGSVALLVYTWACMLLSGLMILEATL

HYPSGASFNTMVKDLLGKGWNAVNGLSVAFVLYILTYAYISAGGSIIAHTLEGIVGVGQTTAGLVFAL
VVAFIVWLSTRAVDRLSTILIGGMVITFVMSVGDMFTHVQPAVLFNTGDDQASYLPYALAALPYLLTS
FGYHGNIPGLVKYYHKDSGSVVRSLVYGTLLALAIYILWQYVIQGNIARDAFKQVIAEGGNIGSLLKQ
MGNVSSSQTVSQLLNAFSYMALASSFLGVSLGLFDYLADFCKFKDDAVGRSKTALVTFVPPTLAALLF
PNGFLYAIGFAGLAATIWAVIVPALMARASRRRYPQAGYRAPGGNGVILFVILFGLINAAAHILSLFG
LLPVFH
>CORE_REP|Org27_Gene2383#
MASLVIDDIPQTIVNVKRQLRQALPNYRDVFLALEENIRGQVEQIRRELAAGHNPVPQIDAEDILQQR
VSEQQIALIKQRGACAIRGVFPRAKAEGWNREIGDYLERNNFVERLKNAAEDNYFGKLAASKPQIYGI
YWSKPQVEARQDARMHAVQVFLNSLWATESNGKQHFDPTRVATYADRTRRRPPNSSSLGLSPHVDSGT
IERWLDENFRYVYRHVFSGDWQQYDPFAADGRTEVREIASPAVCSMFRTFQGWTALTPQRTNAGTLNL
VPIANAMAYVLLRALQDDVADDDLCDAAPGRALSISEKWHPLLLSGISPIPDLEPGDTVFWHCDVIHA
VENEHNGEFDSNVMYIAAAPGCEKNDAYLQRQLPSFIDGRTPPDFAPDDFEVDFDGRATADLLTPLGK
AQLGMG
>CORE_REP|Org47_Gene3642#
MQQRQRWFGVIALLFLIVIAYADRVNIAVMLVNPDFLQHFQLGGDRAHQGTLMTVFLLGYGLSAMLLT
PFLETLMGYRRALTLSVVLWALLTAASPLAGSLMLLCVVRALLGVSEGPLFSLKTMYIGDHFAADERG
KPNAVSALGVSLGLVLGFPLVSFLMTHFGWAMSFHLLALLNLLLGLALVRLFVHPLPLSSSSRPADPQ
PVLNRVWRTFALAWRTPMLGWILLIEIATLSYLWGSSAWLPAYLTDEKGFSIKQMGWMAALPFIVSIA
SKYLGGVLLDRIRPYQAPLIFVCGGAATALCIYGVMHSHQLGWIAFFLLAANACWGAQGAAIPTLLQH
YARPEAVGSAYGLINGIGNLFSAFVPMAMGMVMASQGKVSSGFAVLIASQLLTLLAGGALFGRMLLAQ
QMKRA
>CORE_REP|Org31_Gene1596#
MSKRRVVVTGLGMLSPVGNTVESTWNALLAGQSGISLIDHFDTTAYATKFAGLVKNFNSEDFISRKDA
RKMDAFIQYGIAAGMQAMQDAGLDITEANASRIGAAIGSGIGGLGLIEENHSSLVNGGPRKISPFFVP
STIVNMIAGHLTIMYGMRGPSISIATACTSGVHNIGHAARIIAYNDADVMLAGGAEKASTPLGVGGFG
AARALSTRNDNPQAASRPWDKDRDGFVLGDGAGMMVLEEYEHAKKRGAKIYAEVVGFGMSSDAYHMTS
PPENGAGAALAMENALLDAGVTPSQIGYINAHGTSTPAGDQAEAQAVKSVFGADAERVLVSSTKSMTG
HLLGAAGAIESIFTVLALRDQAVPPTINLDNPDEGCDLDFVPHEARQVSDMEFSLCNSFGFGGTNGSL
IFRRV
>CORE_REP|Org4_Gene3927#
MSDNISVSPALAAGVRAPAPAKSLSFLEGGAMIVGTNIGAGVLSIAYASSKAGFLPLLFWLVLVGSLT
TITMLYVAESTLRTRAHLQLSGLAKRYVGGLGAWLMFASVCVNSVGALTAYMTGSGKLLQSLFGISPA
LGSLLFFVPAAGVLYLGLKAIGRGEKFISIGMVVMLTALVAATLLKDTTQMRNLLDGDWRYMVPVFNV
VVFCFSAQYIVPEMARGFADKPEQLPKAIIVGMVVTFILLAAVPMSVIALSGLGDISDVATISWGQAL
GQWAFFSANIFALCAMLTSYWGLGGSFLTNIFDKFRLGNDELPLRRFGVLLLVVVPPFVLAYSGLVSF
VNALYFAGVFSGVILSIMPMLILRGARKHGDQTPRWQCNWITHPLLQVSIVLLYLASAVYAIASLLGY
LPSGW
>CORE_REP|Org49_Gene2373#
MGLKNYFEKIEHHFTPGGKLEKWYPLYEATTTVFYTPGTVTRGASHVRDAIDLKRMMILVWLAVFPAM
FWGMYNVGQQAIPALHHLYSGDELQQVLAGDWHYRLAQWLGASLAADAGWVSKMVLGACYFLPIYAVV
FVVGGFWEVLFAIIRKHEVNEGFFVTSILFALIVPPTLPLWQAALGITFGVVVAKEIFGGTGRNFLNP
ALAGRAFLFFAYPAQISGDLVWTSADGFSGATPLAQWSAGGAHSLSNVATGQSISWMDAFLGNIPGSI
GEVSTLMILIGGAIILFGRVASWRIVAGVMLGMVASALLFNAIGSDTNPMFAMPWYWHLVLGGFAFGM
IFMATDPVSASFTNKGKWWYGILIGVMCVLIRVVNPAYPEGMMLAILFANLFAPLFDYLVVQANIKRR
KARGE
>CORE_REP|Org6_Gene3647#
MDHCHTSDLISLEQALEKMLGQIAPLQQTESVALTAAAGRITAAPVVSPLDVPPFANSAMDGYAVRLA
DLAANAPLPVAGKAFAGAPFDGQWPANSCVRIMTGAPIPAGAEAVVMQEQAEVSDQGVRFTAPVEAGQ
NIRLAGEDIRQGASVLPAGVKLGAAQLPLLASLGVAEVQVMRKLKVAVFSTGDELQPVGQPLQAGQIY
DTNRFAVRLMLEQMGCEVLDLGIIRDDRDALRAAFEQADSQADVVISSGGVSVGEADYTKQMLDELGQ
VSFWKLAIKPGKPFAFGKLGHAWFCGLPGNPVSAALTFYQLVQPLLAKLAGHSDWRLPPRLRARALTP
LKKSPGRLDFQRGVFSSNAAGELEVSTTGHQGSHVFSSYSQGNCFIVLERERGSVAAGETVEIEPFNA
LLRS

>CORE_REP|Org11_Gene2984#
MSVITEKKNHATPGKAMLASVTGYAMDGFDLLILGFMLPAISIELGLTSSAAGSLVTWTLIGAVLGGV
IFGHLSDRFGRIRVLTITILMFSLFTGLCAVAQGYWDLLAYRTLAGIGLGGEFGIGMALIAEAWPAEK
RNRASAYVGMGWQLGVLAAAFLTPLLLEHIGWRGMFLVGLLPALASFLIRRTLGEPEAFVRQKDAGQP
LSFLQRLRLLFKDRATSKASIGIFILCSVQNFGYYGLMIWMPTYLAKNFGFSLTKSGLWTAVTVVGMT
FGIWLFGMLADRFARWKIFVLYQVGAVVMVIGYAQLSDPMLMLFAGAVMGMFVNGMIGGYGALISDTY
PVQARATAQNILFNLGRGVGGLGPLVIGALVTQVSFTAAISLLAAIYLLDIYATLFLLPKKQGAGDTL
GAIG
>CORE_REP|Org30_Gene792#
MAKVSLEKDRIKFLLVEGVHQSTVDNLRAAGYTNIEYHKGALDTESLKASIRDAHFVGIRSRTHLTEE
VFAAAEKLVAVGCFCIGTNQVDLKAATKRGIPVFNAPFSNTRSVAEMVLGELLLMLRGIPAANAKAHR
GVWHKLAVGSYEARGKKLGIIGYGHIGTQLGILAEGLGMKVFFYDIENKLPLGNAQQVRHLSDLLNMS
DVVTLHVPETLATKNMMGAEELALMKPGAILINASRGTVVDIPALCDALASNHLAGAAIDVFPEEPAT
NSDPFNSPLCEFDNVLLTPHIGGSTQEAQENIGDEVAGKLAKYSDNGSTLSAVNFPEVSLPAHGPNAS
RLLHIHENRPGVLTQINQIFAEEGVNIAAQYLQTGPEIGYVVIDIEAETARADAALQRMKAIDGTIRA
RLLF
>CORE_REP|Org21_Gene3877#
MASVAEPVNWKRNFFVAWVGCFLTGAAFSLVMPFLPLYVETLGVTGHQALNMWSGLLFSITFLFSAIA
APFWGALADRRGRKLMLLRSALGMAIVMVLMGMAQTVWQFLALRAVLGLLGGFIPNANALIATQVPRN
RSGWALGTLSTGGVGGALIGPLIGGLLADLYGLRPVFYITAAVLFVCFVLTLLYVKEQFTPVQKRDML
HARQVFASLKNPKLVLSLFVTTMIIQIATGSIAPILTLYVRDLAGATHNLAFISGLIASVPGVAALMS
APRLGKLGDRIGPERILICMLIVSVLLLIPMAFVQTPWQLGVLRFLLGAADGALLPAVQTLLIYNCTN
QVAGRIFSYNQSFRDVGNVSGPLLGAAVSAGYGFRAVFGVTALVVLFNAGYSWWCLRRRPGYMREDTL
QEEQ
>CORE_REP|Org40_Gene1910#
MQKLLQRITQGSLVKQIMVGLVAGILVALVSPAAAAAVGLLGSLFVGALKAVAPVLVLILVMASIANH
KQGQKTNIRPILFLYLLGTFAAALVAVVVSFIFPSTLALTTGATDITPPGGIVEVMKGLLMSVVANPF
HALINANYIGILAWAVGLGLALRHASETTKGLINDMSHAVTLVVRAVIRCAPLGIFGLVASTLAETGF
GVLWGYAQLLMVLLGCMLLVALVLNPLIVYWKIRRNPYPLVFACLRESGVTAFFTRSSAANIPVNMEL
CKKLNLNEDTYSVSIPLGATINMAGAAITITVLTLAAVNTLGIAVDVPTALLLSVVAALCACGASGVA
GGSLLLIPLACNMFGIPNDVAMQVVAVGFIIGVLQDSAETALNSSTDVLFTAAACQAEDQRLANEDPL
KVR
>CORE_REP|Org36_Gene1032#
MLNSRVVETTSGRSARKIKLSLMGPAFIAAIGYIDPGNFATNIQAGASFGYSLLWVVVWANVMAMLIQ
LLSAKLGIATGKNLAEHIRDRFPRPAVWAYWVQAEIIAMATDLAEFIGAAIGFKLLLGVSLLQGAVLT
GIATFLILMLQKRGQKPLELVIGGLLLFVAAAYIVELAFSQPQLAPLLKGMALPDLPNGDAVFLAAGV
LGATIMPHVIYLHSSLTQIGGEHDKAERYAATKVDVAVAMTIAGFVNLAMMATAAAAFHFSGHSGIAD
LDVAYLTLQPLLGQAAATVFGLSLVAAGLSSTVVGTLAGQVVMQGFVHFHIPLWLRRAVTMLPSFIVI
MLGMDATRILVLSQVLLSFGIALALVPLLAFTGNRALMGEMVNGPVVQNLGKLIVLVVVGLNGYLLIG
SVL
>CORE_REP|Org2_Gene3767#
MKKRLAVLILLVAIVVIALLWWRENRRYDGPVQQVTAGAEQIARGRYLAQAADCAACHTASGGAPLAG
GYPLETPFGTIYGSNLTPSADHGIGRWTRDDFFLALTQGVAPGGRHLYPAMPYTSYKGMSRQDADDIY
AYLMTRPAVDVAIPANEMPFPFNQRMALIGWNLLFRSQDPLPASSQGSSPQWQRGRYLVDVLGHCGEC
HTPRGALGQMDLAKPMQGGDLGRFMAPDITPHGLAQRGWTPQDVSRFLSTGLAPQGSAFSEMHMVVDL
STRHLTPEDHQALALYLMGEQPPAAVPVKMGQGSDAGRMAYLDQCAGCHAREGEGKPHVAPAMRDNAT
LRQVDGKNLIVSVLDGLPAQQFPNGESMQSMPGFGERLSDADVAELVNYLRVTWGGLPADITAEQVKA
LRK
>CORE_REP|Org4_Gene1648#
MRLIHTSDWHLGQYFFTKSRAAEHQAFLNWLIEQIEQQQVDALIVAGDLFDTGSPPSYARELYNRFVV
ELQRTGCQLVVLGGNHDSVATLNESRELLSCLNTTVIASAQGSLEQQIVVLNRRDGQPGAVLCAIPFL
RPRDLLTSRAGESGAQKQQALQEAIAEHYQALYQAACERRERLGLPLPIVATGHLTTVGVTTSDSVRD
IYIGTLDAFPAQAFPPADYIALGHIHRAQNVAKSEHIRYSGSPIPLSFDELGKAKSVFLVDFADGTLQ
QVEALTIPQFQPMQLIKGDLAEIERQLQQFADYAGELPVWLDIEVATQDYLSDIQRRIQLMADELPVE

VVLLRRSKEQRRQAIAQQDKETLNELSVNEVFERRLAQEADMPEARQRRMHQLFRQVVDAVQHNPEET
HP
>CORE_REP|Org42_Gene3550#
MFGWTPLQRNAAIASFSSWTLDAFDFFVLVFLLSDIAQSFHVGLEQVTLAILLTLAVRPIGALIFGRA
AEKYGRKPILMLNIVFFSVFELLSAAAPSLTVFLLLRVLYGVAMGGIWGVASSLAMETIPDRSRGLMS
GIFQAGYPFGYLLAAVVYGLLFETVGWRGMFVIGAAPILLLPFIYYCVQESPVWLAARERKESSALLP
VLKSHWKLCCYLVLLMAAFNFFSHGTQDLYPVFLKVQHGFDPKTVSIIAISYNIASIIGGVFFGSLSE
KIGRKKAIIIAALLALPVIPLWAFSSGSLMLGIGAFLMQFMVQGAWGVVPTYLTELVPANTRAVLPGF
VYQLGNLIASVNATLQATIAEHHGHNYGLAMAIVAGTVAVAIALLVFFGKDTRGKAITDAVKNPGVRA
NV
>CORE_REP|Org1_Gene4681#
MQSISRIILAFYSACGPEEHSVKRSVSVVIGGILLPLAAQAAETTPHFPTMTPPAIDAASYVLMDYTT
GQVLAAGNADERRNPASLTKLMTGLVIDHALDQHKIGLDDVVTVGKDAWAQGNPVFKGSSLMFLKPGD
RVTVRDLSRGIIIDSGNDACVAMADYVAGSQANFVKLMNEKSAQLGLQNTHFETVHGLDAPGQFTTAG
DLVVIARAIIMSEPAEYHMYSEKSLTWNGITQQNRNGLLWDKTLHVDGLKTGHTASAGFNIIASATEG
DRRLIAVVMGGKSSKGREEQARKLLSWGLRDFTTVHLFSAGQSLGEEPVWYGENHRLPVGSVQEQSLS
LPKNEADKLKAQYVINTARLEAPIGKGQTVGEIRISDNGQVVKTLPLVALQAVPQGGVFSRLVDYVKL
RL
>CORE_REP|Org24_Gene4604#
MNGHSGDVMMKKNLSTSLKKLTFSVGILLLAAPAVHAAEPPAPPQVDAKAYILMDYNSGKVLTEGNAD
TRLDPASLTKIMSSYVIGQAIKAGKIKPEDLVTVGKDAWAPGNPALRGSSLMFIKPGDQVPVLELNKG
IVIQSGNDASIALADYVAGSQDSFVGLMNNYAKSLGLQNTHFLTVHGLDAEGQYSTARDMALLSQALI
RDVPDEYALHKEKEFTFNKIRQINRNRLLWSSNLNVDGIKTGYTSGAGHNLVASATDGPMRLISVVLG
APSDRVRFSESEKLLTWGFRFYETATPIKADKPFVTQKVWFGDVSEVPLGVAKDASVTIPKGQMKNLK
ASYKLTQPTLEAPLAKNQVVGTIDFQLDGKTIEQHPLVVMQEVKEGNFFSRIWDMVMMKLSQWFGGIF
G
>CORE_REP|Org43_Gene3254#
MRKKTLLLCLPLLFTGNALADAGGYQLEQVLVMSRHNLRAPLANNGSVLAQSTPKAWPAWETPGGQLT
TKGGVLEVYMGHYFNAWLKQTGLLPQEGCPTAGSVYVYANSLQRTVATAQFFSNGAFPGCDVSVHHQD
KMGEMDPTFNPIITDTGEAFNQQALAAMNAALGSLKLDASYQQLAKIIDYKDSAACKTDKHCDLTKEA
SVMSAVPGKEPGVSGPLRVGNSLVDAFMLQYYEGFPMKEVAWGKIATPHQWQQLAQLKDGYQDSLFTS
PVVAQNVAKPLLTYINNALLGERKPDAPKLTVLVGHDSNIASLLSAMQFQPYQLPQQYEKTPIGGKLV
FQRWRDAQNDRELLKIEYVYQSTEQLRKATPLTLQTPPQRVTLALKGCPIDKDGFCAWSDFEKTMKGI
L
>CORE_REP|Org37_Gene4035#
MNTKPANRSLIVLGTIICQMGLGTIYTWSLFNQPLVDKFHWGLADVATTFSITSFFLAFATLFAGKLQ
ERFGIRNLTLCSGILVGLGLIASAHVSSLDMIYLLAGVVVGFAVGIAYISTLSNLIKWFPANKGLISG
ISVGAFGSGSLLFKYVNAALIADVGVSGAFFYWGAIVMGLIVVGSLLLKEPVLATNAAQQGANGLGND
YSVRQMLATKEAYLLFTIFFAACMSGLYLIGIVKDMGVQLAGMDLATAANTVSAVAIFNTAGRIILGT
LSDKVGRMRVISFTMLVTVLAIVALSFMTLNHTLFFICVGAVAFCFGGNITVFPAIVGDFFGLKNHSK
NYGIIYQGFGLGALAGSFVAKYFGGFHATFMVIGVLSAASLLITLFIKAPKAVEAETAETTQTAELAK
A
>CORE_REP|Org49_Gene3307#
MSDKLLDPPCAALGRLPAPLVLLLAAASAFSVANVYYAQPLLDAIAHDFSISLAAVGMVITVTQLGCA
LALLLVVPLGDRLNRHWLLAGQQLGLIGALLLVGWAHSAPWLLAGMLLVGLLGTAMTQGLIAFAAALA
APQERGRVVGAAQGGVVLGLLLARTLSGALADVGGWRTVYFFSAGVTLVLLPILSRLLPAPRTAPSTL
SYPALLRSMLTLLLHDRTLQIRGMLALLMFGAFSLFWSSLVLPLSQAPFNFTHAAVGAFGLVGAVGAL
AAVRAGHLADRGLGQAASGVCLLLLTLAWLPLGLLGSGLVWLVAGIVLLDLAGQAIHVLNQSMIFSAH
PQSHSRLVGCYMLFYAVGSGLGAFAGTHMYAWAGWSGVCWLGAGVSLSALLFWRLTLRGMPPSAAAVE
Q
>CORE_REP|Org37_Gene3355#
MTTPIISLTAARALHLAAQGLLSPLKRQAKPDDVVSAIQRMGLLQIDTISVVARSPYLVLFSRLGAYQ
SEWLEQALAGRKLFEYWAHEACFLPIEDFGLLRHRMLAPHDMGWKYSADWVQQHQAAMDSLLRHIEQQ
GPVRSADFSAEKKGNSGWWDWKPEKRHLEILFTAGKLMVAERRNFHRVYDLTERLLPAWDDARHTLPA

ERARRQMLRRTCRYLGIFRAEWLADYYRLKRVAPKALLAELQEQGEITPVQVEGLEGPFYLHESLAEL
LPLAEQSKLKSTVTSLLSPFDPVVWDRRRALELFNFDYRLECYTPKEKRRYGYFTLPVLHRGELVGRI
DAKAHRRQGVFEIISFHAEPQVRFGKQRAQDIRQAIARTAKWHGAQRVALGDIPAALAAEWGAGWEVG
>CORE_REP|Org5_Gene3733#
MKRTFIMVLDSFGIGASEDAERFGDQGSDTLGHIAEVCARGEANVGRQGPLTLPNLSRLGLGKAAEES
TGNFPQGLDRNADIIGAYAYASELSSGKDTPSGHWEIAGVPVLFDWGYFKDEHNSFPQALLDKLVERA
KLPGYLGNCHSSGTVILDQLGEEHMKTGKPIFYTSADSVFQIACHEETFGLDRLYELCEIAREELTEG
GYNIGRVIARPFVGDKPGNFQRTGNRHDLAVEPPAPTVLKKLVDEKSGEVVSIGKIADIYANVGITKK
VKATGIDALFDATLIEMEKAGDNTIVFTNFVDFDSSYGHRRDVAGYAAALELFDRRLPELLKLVKDED
IIIFTADHGCDPTWPGTDHTREHIPVLVYGPKVKPGSLGHRETFADIGQTVANYFGLSPMDYGKSMF
>CORE_REP|Org18_Gene1127#
MAFSQAVSGLNAAATNLDVIGNNIANSATAGFKSGSVSFADMFAGSQVGLGVKVSGITQNFKGGTTTG
TSRALDIAINGNGFFRMQDKDGGIFYTRNGQFKLDENRNLTNMQGLQLTGYPAAGSPPTIQQGANPVP
LSIPEGMMNAKASTSGEMVTNLKSTHKVPENKTFDPTKQDSYNYVNTITAYDSLGNAHNINAYFVKTD
DNKWQVYTQDGSAAPVNAGTMEFSTSGNLVKTTSTNGAPGEFSMVIPMTAKDGAPAQNFTLSFAGSMQ
QNVGSDSVSKVAQDGYAAGEYTNFQINNDGTVVGIYSNQQTQVLGQIVMANFSNPEGLASQGDNVWQE
TGASGQPRVGLSGGGGFGKLTSGALESSNVDLSQELVNMIVAQRNYQSNAQTIKTQDSILQTLVSLR
>CORE_REP|Org26_Gene3175#
MEIILGVAMFTTIVMVLVLLILFAKSKLVNTGDIAVEINGDLDKSFHAPAGDKLLNVLSSQGIFVSSA
CGGGGSCGQCRVVIKEGGGDILPTELSHINKREAKEGCRLACQVNVKQNLKIELPEEIFGVKKWECEV
ISNDNKATFIKELKLKIPDGEDVPFRAGGFIQIEAPAHDISYADFDVPQEYRGDWDKFNLFRYRSTVN
DTTVRAYSMANYPEEKGIIMLNVRIATPPPNNPDVPPGIMSSYIWSLKAGDKVTISGPFGEFFAKDTD
AEMIFIGGGAGMAPMRSHIFDQLKRLNSKRKITFWYGARSLREMFYEDDFNQLQAENDNFTWHVALSD
PQPEDNWTGYTGFIHNVLLENYLRNHPAPEDCEFYMCGPPMMNAAVIKMLKDLGVEDENIMLDDFGG
>CORE_REP|Org23_Gene3525#
MMSTGGQAQIGNVDLVKQLNGAAVYRLIDQQGPISRIQIAELSQLAPASVTKITRQLLERGLIKEVDQ
QASTGGRRAISIVSETRHFHTVAVRLGRHDATITLYDMSGKSLSEEHYPLPERTQETLENALFAAIAQ
FIEANQRRLRELIAIAVILPGLVDPALGVVRYMPHISVSNWALVDNLQQRFNVTSFVGHDIRSLALAE
HYFGATRDCEDSILVRLHRGTGAGIIVNGQIFLGNNGNVGEIGHIQIDPLGERCHCGNFGCLETVAAN
AAIEQRVRQLLSQGYPSKLTLEDCGINAICKAANRGDLLASEVIEHVGRYLGKAVAIAINLFNPQKVV
IAGEITEADKVLLPAIQSCINTQVLKDFRKNLPVVTSELNHRSAIGAFALAKRAMLNGVLLQRLLES
>CORE_REP|Org41_Gene646#
MKRAVITGLGVVSSIGNNQQEVLASLQEGRSGITFSQELKDSGMRSHVWGQVKLDTTGLIDRKAVRFM
SDASIYAFLAMQEAIASSGLKEEEYQNNPRVGLIAGSGGGSPRFQVFGADAMRSPRGLKAVGPYVVTK
AMASGVSACLATPFKIHGVNYSISSACATSAHCIGNAVEQIQLGKQDIVFAGGGEELCWEMACEFDAM
GALSTKYNDTPEKASRTYDADRDGFVIAGGGGMVVVEELEHALARGAHIYAEIVGYGATSDGADMVAP
SGEGAVRCMKMAMQDLDAPIDYINVHGTSTPVGDVKELGAIREVFGDNTPAISSTKAMTGHSLGAAGV
QEAIYSLLMLEHGFIAPSINIETLDEQAAGMNIVTQPTQRELTTVMSNSFGFGGTNATLVMRKLAK
>CORE_REP|Org46_Gene4169#
MTDSSQSAMPPAKGSAVKGTAFSILGAISVSHLLNDMIQSLILAIYPILQADFHLSFVQIGMITLTYQ
LTASLLQPLIGYYTDKHPQPYSLPIGMGFTLSGLLLLSVASTFPLVLLAAALVGTGSSVFHPESSRVA
RMASGGRHGLAQSLFQVGGNFGSSLGPLLAALIIAPYGKGNVAWFTLAALLAIVVLLQVSKWYQHQHR
ATKGQPKSPSTLKALPKRTVVYSLGILLVLIFSKYFYLASISSYYTFYLIHKFGVSVQNAQIHLFAFL
FAVAAGTIIGGPLGDKIGRKYVIWGSILGAAPFTLVLPYASLYWTGILTVIIGVILASAFSAILVYAQ
ELIPGKVGMVSGLFFGFAFGMGGLGAAVLGYVADLTSIELVYQICAFLPLIGIITALLPNMEHKPQ
>CORE_REP|Org36_Gene712#
MQNQGIKKIVLAYSGGLDTSAIIPWLKENYGGCEVVAFVADIGQERSDLEGVEQKALQSGASECHVVD
LREEFIRDYVYPVLQTGALYEGSYLLGTSMARPIIAKAQVELALKVGADALCHGATGKGNDQVRFETT
YTALAPQLKVVAPWREWNLRSREALLDYLKERNIPTTASLEKIYSRDENAWHISTEGGVLESPWNAPN
KDCWVWTVDPQEAPDQPEQVTVTVEKGRVVAVNGKALSPYQCLETLNALGAKHGVGRIDIVENRLVGI
KSRGCYETPGGTIMVAALRAVEQLVLDRDSFKWREQLGLEMSYVVYDGRWFAPLRRSLQASAEALAEE
VNGEVVLQLYKGQVTATQKKSANSLYSEEFATFGEDEVYDHSHAGGFIRLFSLSSRIRALNEKKNK
>CORE_REP|Org14_Gene4570#
MTQENSIAPAANPLVDAANPLLNAISQIRQSATHANPAQLRQQLIDEMRRFEIRGQRANLPYEVIIGA

RYCLCTALDEAAALTPWGSNSVWSGSGLLVTFHNETWGGEKFFQLLAKLSQSPREHINLLELINYCLL
LGFEGRYRVMENGRSQLETMKQRLLQLIRSVRGGYAPPLSPHALDLPVQQKLWRPLVPLWACVALTGF
LASLLFIALNWRLGDNTSPVLAAIYQTNLPQVAIGNPAPAAPPTLSLKSFLRKEIAEGLVVVRDEAQQ
SVVILKGDGLFDSAATTVRANYIPVIDRIAAAMNGVSGKILVTGYSDNVPIRSARFASNWELSLARAE
AVSARLQKHLANPQRVKAEGRGESNPVAPNDNKVNRALNRRVEITLLVAPENTQAEINGLPQGTGK
>CORE_REP|Org26_Gene2805#
MTEKSAVSRSTFDQVILPVYAPAQFVPVRGQGSRVWDQQGKEYIDFSGGIAVTALGHCHPALVEALKR
QGETLWHTSNVFTNEPALRLASKLIDATFADRVFFANSGAEANEAAFKLARHYAITRHSPYKTKIIAF
YNAFHGRTLFTVSVGGQAKYSDGFGPKPADIVHVPFNDLAAVKAVMDDHTCAVVMEPIQGEGGITPVD
AGFLKGVRELCDQHQALLVFDEVQSGMGRSGKLFAYMHYGVTPDILTTAKALGGGFPVSAMLTTEDIA
SVMQVGTHGTTYGGNPLACAVAEAALDVINTPEVLSGIEQRHALYVQALQHIGDKYGIFTAIRGMGLL
IGAELTPHYHGRARDFLTAAAARGLMILNAGPNVIRFAPSLVVELQDIEAGMALFELAVQDVINA
>CORE_REP|Org45_Gene3367#
MKRRLAALILIALGLAAALLGRQQALRLASGWDQQVYIWQRVWTPQHADALARSRDLFGGLRVLALQV
HPREGWREIPADLSLLKQDGRPLWLVARLDGQLPQLDQEAIIQRLLALTQRWQAAGLPVTGVEIDHDA
ATARLPDYQRFLQQLRQRLPAALQLGITALPAWIGSPALPGVLQQADSSVLQVHAVLSPQQGLFDGSL
ALRWVRQYASVTPKPFRVALPAYGMALLGFDAQGAQVESESSLRVAGNGRELTVAPQQIADFLQTLAQ
QTPPQLRGIIWFRLPLADDRRAWSLATLRAVIERQPLNVDWQVKFRPQPQQNGLYDLIIHNNGPVDAP
LPQEVAIRADDCLAADAVGNYRLESAPQRQRFIRISGDQLRAGQSRPLGWLRCQQLTPGGTLVTP
>CORE_REP|Org20_Gene1490#
MSSVDILVPDLPESVADATVATWHKKPGDSVQRDEVLVEIETDKVVLEVPASEAGILDAIVEEEGATV
LSRQLLGRIRPGDSSGKPTAEKSQEKEATPAQRATASLEEESNDALSPAIRRLIAEHDLDAGAIKGTG
VGGRITREDVEAHLAKGGAAAKPAAAAEAAPQPALAGRSEKRVPMTRLRKRVAERLLEAKNSTAMLTT
FNEINMQPIMDLRKQYGEAFEKRHGVRLGFMSFYIKAVVEALKRFPEVNASIDGSDVVYHNYFDISIA
VSTPRGLVTPVLRDVDAMSMADIEKKIKELAVKGRDGKLTVEELTGGNFTITNGGVFGSLMSTPIINP
PQSAILGMHAIKDRPMAVNGQVVIQPMMYLALSYDHRLIDGKESVGYLVTVKEMLEDPARLLLDV
>CORE_REP|Org13_Gene4467#
MRNWQAPTRRTALFLALLAVTGPALHAEDAMNSTSSHFAYIGTYNPNGEGVYRVQVDPASGALSHTTL
VSKVPNPAQLTLGADGKTLYVASEVADFNGGKHGGITAYRVNPADGGLTQLNQVDSQGAGPVYLSSTP
DGRHLLVANYVSGSVAAFPIEADGSLGAASSVQQQQGPAGAAKPAAAVNGSFAISDHNGSHAHMIASD
PSGKFVFSTDLGLDRIYQWRFDAASGRLTPNDPPWIAASSAGAGPRHFVFHPDGETVLLVNEEASTLT
SYRFDRQKGTLKQLHAVSALPADYQGTSFASGLALSKNGDTLYVANRLHNSIAQFSVGAGGELKPVAE
TWTRGDYPRSLALSPDGRYLYALNQRSDNVTRFSVNPTDGKLSFVGGYLPIGSPSQMAFLPPPK
>CORE_REP|Org42_Gene2299#
MSWQQFKSQYLVRFWAPLPAVIAAGILSTYYFGMTGTFWAVTGEFTRWGGHVLQWFGLHPEQWGYFKV
IGLQGTPLERIDGRMIIGMFAGCIAAALWANNIKLRQPQHRIRIVQALLGGIIAGFGARLAMGCNLAA
FFTGIPQFSLHAWFFALATAAGSYFGAKFTLLPMFRIPVKLQKVKAAAPLTQKPEQARRRFRLGMAVF
GLAVAWSLWTLFDAPKLGIAMLFGIGFGLLIERAQICFTSAFRDLWITGRTHMAKAIIIGMAVSAIGI
FSYVQLGVAPKIMWAGPNAVLGGLLFGFGIVLAGGCETGWMYRAVEGQVHYWWVGLGNIIGATLLAYY
WDDLAPALATDYDKINLLDTFGPIGGLLVTYLLLALAFAAMLWWEKRFFRARPEAQVVNLRSLP
>CORE_REP|Org42_Gene666#
MKLPIYLDYSATTPVDPRVAEKMMQFLTLDGTFGNPASRSHRFGWQAEEAVDIARNQIAELVGADPRE
IVFTSGATESDNLAIKGAANFYQKKGKHIITSKTEHKAVLDTCRQLEREGFEVTYLAPQSNGIISLQD
LEAALRDDTILVSIMHVNNEIGVVQDIEAIGEMCRARGIIYHVDATQSVGKLPIDLSKLKVDLMSFSG
HKIYGPKGIGALYVRRKPRIRIEAQVHGGGHERGMRSGTLPVHQIVGMGEAYRIAKEEMTEEMARLRT
LRDRLWNGVKDMEEVYLNGDLEHGAPNILNVSFNYVEGESLIMALKDLAVSSGSACTSASLEPSYVLR
ALGMSDELAHSSIRFSLGRFTTEEEIDYTIQLVRKSIGRLRDLSPLWEMFKQGVDINSIEWAHH
>CORE_REP|Org43_Gene3862#
MKGFPSLINVLLASSLVLTIGRGVTLPFITIYLTEHFQLLPKSVGVILGVSFTLGIIASLYGGYLVDK
FSKNRLILLSIVLFALSFFAIPWIPRPGGVIVVLAILHTCYSVLSITIKACFADGLPVEQRIKAFSIN
YTLVNVGWAIGSALGVLVAGLSPLLPFYLSGGLALATVAALSLRLRGGEQRPAPSAAAPAALANFRQT
LAILRCDRRLIYFTLGSTLGAVVFGQFTGYLSQYLITVSSAEFAYKIIGLVMIVNAGIVIALQYLLSR
GMRQENMLRWLALGTLFFIVGLLGFMAAGQAVWLWLAAMAVFTLGEIIVIPVEYMFIDFIAPPHLKGS
YYGVQNLSALGGAINPVLCGVLLSYAAPPLMFVMLIAAALLSLLFFFLGHRLAEHAAAAAEDVR

>CORE_REP|Org24_Gene3677#
MAFWLIICVLLAGAVALLVVPAMRHGKQSAATRDALNKAFYQDRLSELEQDEQQGVVAERPELVKELQ
QNLLNDIPAEQAAQAKPINRWALVPGVLLLLAVTVGFYLKTGGLAQVLDWRLVQAQMPELRERVANER
AKPLSMEEIARLGLGLRTALQQDDRNINDWMMLGRVGMALNNATTATQAFAHAYQLDPNSLEVRLGYA
EVLTRSNDPEDNKQATQLLRRMIAENHGDPRVLSLLAFNAFEQGDFKQAIGAWQVMLQLLPANDPRAE
VIKRSIAQAKAQAGGENVKLNVTVSLSPQAAKALPAQGTLVISVTDGVSPVPVAVKQLPLSRFPLSFS
LDDGNAMMPERLLSAQHQVQVRVRLSQDGLATPQPGDWFGESSLQTFSGKEQVSVQINKQVTEK
>CORE_REP|Org5_Gene3028#
MSINDDGVYLIDGTRTAIGAYGGSLAHTRPDDMAATIIRSLLQRNPAAEAKLDEVVLGCANQAGEDNR
NVARMAALLSGLDPSVPAITLNRLCASGLDAVIYAAAKIRSGLSELILAGGVESMSRAPYVLSKGDTP
FDKGVKLYDTTLGWRFVNPRLAERYGSDPLGITAENVAQRQHISREEQDLFALASQQKARRAIDSGRL
AREITPVDVPADRKTMRTFAQDEFPRLSTLQQLQALKPAFSAGGSVTAGNASGINDGAAALLLASGRY
VKRQGWQPLAEIGDSAAAGVEPALMGLGPIASTERLLQRGGFTLNDFDIIEINEAFASQVLAALKHWR
IDAHDARVNPNGGAIALGHPLGMSGARLVLSAALELRDRQAGNALVTMCVGVGQGLSLNLKAV
>CORE_REP|Org4_Gene5062#
MENLGMPSLKLTPRRLTLIAVIVFIAVAIAIALTLYWQRPPQQDYVTAPARLGDIENAVLATGRLDAV
ERVNVGARVSGEVKSLKVKLGDRVTKGQPIADIDDLQQRNDLRNAEAALNVIKAELQAKQAQLKQAES
RFKRQRRMLNDEASSREDFETAEATLATTRAELLSLNARLVQAQIEVDKKKIDLGYTRVVAPMDGIVI
AVVTQQGQTVNSTQSAPTIVKLARLDMMTIKAQISEADITRISPGQKAYFTIFSDPDKRYDATLRTIE
LAPESVMKDDSLAGTSSASGSGTSNASVYYNALLDVPNPENRLRIAMTAQVSLLLGEAKNALLVPIQA
VHKTEGKVQQVQVLTQDQRLETREVTTGITNNVDIQILSGLKAGETVVLSQPAAKSAEDGIFL
>CORE_REP|Org5_Gene2696#
MTSKHIETTLIGAGRGKRYTQGSVNPVTQRASSLVFDSVAAKKHATAQRAHGELFYGRRGTLTHFALQ
DAMVELEGGAGCVLYPCGAAAVANAILSFVGAGDHLLVTGSAYEPTQDFCTHILGRMNVSTTYFDPLI
GADIATLIQPNTRVVFLESPGSITLEVQDIPAMVQAIRAVAPEVVIMIDNTWAAGVLFKALDFDIDIS
IQAGTKYLIGHSDYMLGTAVANARCWDRLREYSYLMGQMVDADTAYMASRGLRTLSVRLKQHERSSIE
VANWLAARPEVATVNHPALPSCKGHEFYRRDFSGCNGLFSFVLKERLDDAQLAAYLDNFSHFSMAYSW
GGFESLILANQPEELEAIRPAGGVDFSGTLVRLHIGLENVEDLIADLAAGFDRLNGVRQDGFR
>CORE_REP|Org36_Gene339#
MKNRTLGSVFIVAGTTIGAGMLAMPLAAAGVGFGVTLALLVGLWLLMCYTALLLVEVYQHEQADTGLG
TLAKRYLGGGGQWLTSFSMMFLMYALTAAYISGAGELLATSISQWTSQDFPVSLGVLLFTLVAGGVVC
IGTHSVDLFNRILFSAKVVFLVVMLGLMLPNIHQTNLMTLPLEQGLALSAIPVIFTSFGFHGSVPSIV
NYMGGNIRKLRWVFIIGSAIPLIAYIFWQLATLGSISSDTFVGILAQQAGLNGLLQAVRDAVASPHVE
LAVHLFADLALATSFLGVALGLFDFLADLFKRQDNVRGRLQTGAITFLPPLAFALFYPRGFVLALGFA
AIALSVLALLLPSLLVWKTRQKHQAQYRVWGGTPALALVFVCGVTVIAIQLGIASGMLPAVG
>CORE_REP|Org7_Gene3115#
MTQYASPILTSLLDTDAYKLHMQQAVFHRYPAISVAAEFRCRGDELLGEYADEIRAQVALMSQLTLTD
AEFAYLSSLPFFRQDYLSWLRTFRYDPQQVTIDNRDGKLQIRIAGPWREVIMWEVPLLAVISEVVHRR
RSPLATPEQAVAHLQTKLAQFKTLAGDLDLSRFKLMDFGTRRRFSQGVQQAIVSTLQAEFPYLSGTSN
YDLAHQLGLAPVGTQAHEWFQAHQQISPVLANSQRAALQAWLDEYPDQLGIALTDCITMDAFLRDFGP
QFAERYQGLRHDSGDPVEWGEKAIAHYQKLGIDPMSKTLVFSDNLDLEKALALYRHFYQRINLSFGIG
TRLTCDIPGVKPLNIVIKLVECKGKPVAKLSDSPGKTICQDQAFVRALRKAFDLPLVKKAS
>CORE_REP|Org15_Gene4578#
MSALHADGGAKAWLATFAVGLSTFTVVTAEMLPVGLLTPIVSTLNASIGRAGLLISLPALFAALFAPL
VVLGARRTDRRNLLAGFLLLLIAANLLAAAATSLALLFAARILLGFCIGGIWAIAGGLAERLVPPASV
GLALSIIFGGVAAASVFGVPLGVFLGEALGWRMAFLAVAVLAALTLLLLVCVLPPLPVTQAIGWRSFT
ALRANRRLLTGLLLTFLLVAGHFMAYTFVRPLLQTVAGIESRWVGPLLFAYGVAGIFGNFIAGQAAAK
RLRRTLALIALGLALAVLLLPLLGHAPLSGGAFLLLWGIAYGGVSVALMAWMLKAAPDAVEVASSLYI
ALFNLAISCGSLAGGLVVDAGGLTINGALSGLVLLLALAILMGTRRQRPKTAAKADSPPG
>CORE_REP|Org47_Gene2401#
MAIKLEVKNLYKIFGEHPERAFKLLDKGLTKDRLFEKTGLSLGVKDATLAIEEGEIFVIMGLSGSGKS
TLVRLLNRLIEPTRGQVLIDGEDIAKISDTALRTVRRNKISMVFQSFALMPHMNVLNNTAFGMELAGI
PLQERQEKALDALRQVGLENYALSYPDELSGGMRQRVGLARALANNPDILLMDEAFSALDPLIRTEMQ
DELVKLQAQHQRTIVFISHDLDEAMRIGDRIAIMQGGEVIQVGTPDEILNNPANDYVRTFFRGVDISH

VFSAKDIAQRRPVTLIRKTPGFGPRSALQLLRDEDRDYGYVVERGKKFIGVVSIESLKKALSANQTLD
DALLEAPAAVPADTPLSDLISLVAQAPCAVPVVCEEHNYLGIISKAMLLQALDKEGSANE
>CORE_REP|Org5_Gene2945#
MYQPVALFIGLRYMRGRASDRFGRFVSWLSTIGITLGVMALVTVLSVMNGFEKDLENNILGLMPQALI
TSPQGSVNPQQLPASEVQKLQGVTRVAPLTTGDVVLQSARSVAVGVMLGVNPDEADPLTPYLVNVKQQ
QLQPGQYNIIIGEQLAGQLGVKRGDSLRVMVPSASQFTPMGRIPSQRLFTLIGTFHANSEVDGYQLLV
NQQDASRLMRYPAGNITGWRLFLQQPLTVDTLSQQPLPAGTVWKDWRDRKGELFQAVRMEKNMMGLLL
SLIVAVAAFNIITSLGLLVMEKQGEVAILQTQGLTRRQIMSVFMVQGASAGIIGSLLGTLLGVLLATN
LNNLMPILGALIDGASLPVAVDPLQVTIIAVVAMAVSLLSTLYPSWRAAAVQPAEALRYE
>CORE_REP|Org32_Gene1843#
MQSFDVIIAGGGMVGLALACGLQGSGLRVAVLEQRQPEMAPPSEQPALRVSAINAASERLLQHIGVWD
DILQLRASAYNAMEVWDRDSFGKIAFRGDECGFSHLGHIIENSVIQQALWKRAESLSDITLITPAALK
QVAWGENDAFVTLEDGRMLTARLVIGADGAQSWLRQHADIPLTFWDYRHHALVATVRTEEPHQATARQ
IFHGDGILAFLPFSDPHLSSIVWSVTPEEAERLKRLEPEQFNRELAMTFDMRLGACSLESERLAFPLT
GRYARSFAAHRLALVGDAAHTVHPLAGQGVNLGFMDAAELISELRRLQRQGKDIGQHLYLRRYERRRK
HGAAVMLASMQGFRELFDGNHPAKKLLRDVGLRLADSLPGVKPKLVRQAMGLNDLPEWLA
>CORE_REP|Org9_Gene4259#
MLNHSVNSPSSRRWFNPLLLALVLIGLNMRPLLTSIGPLLPTLRQATGLSFGGAALLTTLPVLMMGLM
ALAGGAINRLFSERSAVALSLLAIGSGALWRELAPGSVQLLMSAVLGGLGIGVIQAVMPGIIKHHFLN
SMALVAGLWSAALMGGGGLGAAITPWLMSAGHDWHSALAWWALPALVALLAWWPVSRGLSRLGAAGEN
RGPSLLRNRRAWLLGAYFGLINGGYTSLIAWLPPYYMQLGWQPQASGSLLALMTFGQVVGALLLPALA
RNHDRRPLLLLALMMQLIGFIGLIYLPQTLPWLWVLVSGLGLGGAFPLCLVLALDHLHQPAAAAGRLV
AFMQGVGFLLAGVTPYLSGLLRDYSGGFVLDWQIHALLVVVLIAITWRFHPHSYRRAFAE
>CORE_REP|Org33_Gene2721#
MSLHQDHSFESCIKCTVCTTYCPVAKVNPLYPGPKQAGPDGERLRLKDPALYDEALKYCTNCKRCEVA
CPSDVKIGDIIQRARADFAQSKPTLRDAILSHTDIMGSLSTPFAPIVNAATGLKPVRKLLDKALKIDH
RRELPKYSFGTFRRWYRQQAQAQQRYAEQVAFFHGCFVNYNHPQLGKDLIRVFNALDIGVQLLKREKC
CGVPLIANGFIEQAKKQARVNAESLHETVLERGIPVVATSSSCTFTLRDEYPHLLDVDTTPVRDRVEL
ATRYLYRLINQGRSLPLKHTPLRVAYHTPCHMEKMGWTAYTLELLRQIPGLELVVLDSQCCGIAGTYG
FKSENYATSQGIGAPLFRQIEESGVDLVVTDCETCKWQIEMSTSKRCEHPITLLAQALA
>CORE_REP|Org30_Gene2796#
MKARRLFLWQAFDAQGALRRGELMSDEKRQVSRLLIEQGMQPWRIGHGKRVTPGQWRGEPLIHFTRQL
ATLLQAGLPLVNTLQLLAAEHPSAAWRCLLRQLAEQVREGQPLSETLAAQPGVFPLIYRQLIAIGELT
GNLDRSCLQLAQQQEAQLLLRRKVTKALRYPLFICAVALLVSVLMLVMVLPEFAKVYQSFDAPLPWFT
QGLLGLSALLIAVGPYLALLLGALLFGYCRWLHPRPPWRRREQAALLRLPLIARLVSGGALSQTFRIL
TMTQRAGLTLVEGLNAAALAADHLLYRQALEQVQRQLAEGEAFHHALALQPLFPPLCRQLVRVGEESG
SLDVLLDKLAQWYERQTHELADTLAQTLEPLLMLVVGGIVGALVIAMYLPIFQLGSVLG
>CORE_REP|Org10_Gene158#
MSASFYQQLEQQLAATRSEGLFKEERIITSAQQADIAVADGSHVINFCANNYLGLANHPALIAAAKSG
MDSHGFGMASVRFICGTQDSHKQLEQKLAAFLGMEDAILYSSCFDANGGLFETLLGPEDAIISDALNH
ASIIDGVRLCKAKRYRYANNDMTELAAQLKQAKADGARHILIATDGVFSMDGVIANLKGVCDLADEYQ
ALVMVDDSHAVGFVGANGRGTHEYCEVMGRVDIITGTLGKALGGASGGYTAAKKEVVEWLRQRSRPYL
FSNSLAPAIVAASIKVLELLEEGDALRDRLWANARLFREKMTAAGFTLAGADHAIIPVMLGEAKLAQE
FANALLKEGIYVTGFFYPVVPKGQARIRTQMSADHTPEQIERAVAAFTRIGKDLGVIA
>CORE_REP|Org40_Gene547#
MLEPITSEHTVSENNSLTTPSVNVEQPAAAKINLLDLNRQQMREFFAEMGEKPFRADQVMKWIYHYCC
DDFEQMTDINKVLRGKLQRVAEIRAPEVAEEQRSADGTIKWAIKVGDQQVETVYIPEADRATLCVSSQ
VGCALECKFCSTAQQGFNRNLRVSEIIGQVWRAAKIIGALKVTGQRPITNVVMMGMGEPLLNLNNVVP
AMEIMLDDFGFGLSKRRVTLSTSGVVPALDKLGDMIDVALAISLHAPNDTIRDEIVPINRKYNIETFL
SAVRRYLEKSNANQGRVTVEYVMLDHINDSTDDAHQLAEVLKDTPCKINLIPWNPFPGAPYGRSSNSR
VDRFSKVLMEYGFTTIVRKTRGDDIDAACGQLAGEVIDRTKRTLKKKMAGEPINVRAV
>CORE_REP|Org43_Gene3934#
MLRVEMLSTGDEVLHGQIIDTNAAWLADYLFQQGVPMSGRETVGDSLSALIEILQERSHIADVLIVNG
GLGPTSDDLSALAAAKACGVELIERADWLARMEAYFAERGRPMAPSNRKQAQIPANAEMLDNPVGTAC

GFALQLNQCLMFFTPGVPSEFKVMVEQQIVPRLRQRFTLPAPPLCLRLTTFGTSESDLAAELDSLPLP
PDVVLGYRSSSPIIELKLTGPENRRAEMELAWRRVREVAGDNCIFEGTVGLPATLTQRLNQQGLKLAL
SEQFSGGLINLQLQSEGAPLAGGELLPAHCIETLETTLARARSLAELTGAPLALAVSAMRDEQVSIAL
HTPRGGIGQTVRYRASRHGLRLRQESVAMLALDMLRRWLNGGQACGKNAWLEVVEIIE
>CORE_REP|Org32_Gene314#
MLRVLFLFLVVIASVVLGPMLAGHQGYVLIQTDNYNIETSVTGLVIMLVLLFVVLLAIEWILRRIFRT
GARTRGWFIGRKRTRARQQTKAALIKLAEGDYKQVEKLMTRNADHAEQPVVNYLLAAEAAQQRGDDFR
TNQYLERAAEAADTDQLPVDITRVRIQLAQGENHAARHGVDRLLNQAPRHPEVLRLAEQAYLRTGAYA
SLLEVLPSMRKIELHSEEQLQALQQQAYIGLMNQAMADEGSEGLKRWWKDQSRKTRHEVPLQIAMVEH
LIECNDHELAQEIVLDSLKRQYDERLVLLLPRLKSGNPEQLEKALRQQMKQHGATPLLNSTLGQLLMK
HGEWQQAADAFREALKQRPDAYDYAWLADTLEKLHRSDEAAQMRREGLMLTLKQNGGE
>CORE_REP|Org5_Gene782#
MQQNRTSHLGLIFILGLLSMLMPLAIDMYLPSMPVIAAQFGVESGSVQMTLSAYMLGFAFGQLFYGPM
SDSIGRKPVILWGTLIFAIAGCACAMAQSIDQLIGLRFLHGLAAAAASVVINALMRDMFTKDEFSRMM
SFVILVMTIAPLLAPMIGGALLLWFSWHAIFWTMGAAALIGSLLVALFIKETLPKERRQRFHLRTTLG
NFGSLFRHKRVLSYMLASAFSFAGMFSFLSAGPFVYIELNHVSPQHFGYYFALNIVFLFLTTLINSRN
VRRFGAVKMFKLGLLVQLAMGLWLLAVSAVGLGFWALVIGVAVYLGCIAMISSNAMAVILDDFPHMAG
TASSLAGTLRFSIGALVGAVLSMAPGKSAWPMVTSMALCSIVAVLFYVYASRPRDRAA
>CORE_REP|Org22_Gene1065#
MTRSITLARTLALSALATLVLSSSAFAKIEEGKLVIWINGDKGYNGLAEVGKKFEKDTGIKVTVEHPD
KLEEKYPQVAATGDGPDIIFWAHDRFGGYAQSGLLAEIHPSKAFQDKLFPFTWDAVRYDGKLIGYPIA
VEALSLIYNKDLVKQPPKTWEEIPALDKQLRANGKSAIMWNLQEPYFTWPIIAADGGYAFKYENGKYN
IKDVGVANAGSQAGLQFIVDLVKNKHINADTDYSIAEAAFNKGQTAMTINGPWAWNNIEQSKINYGVT
LLPTFKGKPSKPFVGVLTAGINAASPNKELATEFLENYLLTNEGLADVNKDKPLGAVALKSYQEALAK
DPKIAATMQNSQNGEIMPNIPQMSAFWYAERSAVINAVSGRQTVKAALDDVQTRITK
>CORE_REP|Org16_Gene1641#
MEHAPVSRSTAWLRVVILAVSAFIFNTTEFIPVGLLSDIAASFSMQTEQVGLIITIYAWIVAAASLAC
MLLTSKIERRKLLIGVFMLFIASHVLTAVAWDFTTLVISRAGVALAHSVFWSITASLAIRVAPPGKKA
QALSLLAGGTALAMVLGLPLGRVVGQLLGWRMTFIGIAVCATLALVLLWRLLPVLKSEHSGSLASVPL
LFKRPALVALYMLTIIVVTAHFTAYSYIEPFIQTVAGLSENFTTLMLLLFGAAGIVGSLLFSRYSERF
PSGFFIGAIVLLALSLLLLLPAAGSESHLTVLCIFWGMAIMAIGLSMQAKVLSLAPDATDVAMAIFSG
LYNFGIGSGALLGNQVSLHLGMGNIGFVAAPLALIALGWCLLSVYRSERLQQHHSR
>CORE_REP|Org7_Gene3423#
MTVRLFLAKGREKSLLRRHPWVFSGAVQRVEGKALSGETIDILDSQGKWLARGAYSPESQIRARVWTF
QQDEEINIDFFIRRLQQAQSWRDWVAQRDGLDGYRLIAGESDGLPGITIDRFQNFLVLQLLSAGAEYQ
RPALLSALQHCYPECSIYDRSDVAVRKKEGLPLAQGPVLGDLPPELLPITEHGMKLLVDIQQGHKTGF
YLDQRDSRLAARNYSAGRRVLNCFSYTGAFAVSALMGGCAQVISVDTSQAALDIARQNVELNKLELNK
AEFVRDDVFQLLRNYRAQGEKFDLIIMDPPKFVENKNQLASACRGYKDINMLALQLLNPGGILLSFSC
SGLMPTDLFQKILADAAVDAGRDVQFIEQFRQAADHPVIATYPEGLYLKGFACRVM
>CORE_REP|Org14_Gene889#
MTLLNPYFGEFGGQYVPQILMPALKQLEEAFVSAQRDPAFQAEFIDLLKNYAGRPTALTLCKNLTAGS
NTKLYLKREDLLHGGAHKTNQVLGQALLAKRMGKTEIIAETGAGQHGVASALACALLGLKCRIYMGAK
DVERQSPNVFRMRLMGAEVIPVHSGSATLKDACNEALRDWSGSYETAHYMLGTAAGPHPYPTIVREFQ
RMIGEETKAQVLEREGRLPDAVIACVGGGSNAIGMFADFIDDADVGLIGVEPAGLGIETGQHGAPLKH
GHVGIYFGMKAPMMQTAEGQIEESYSISAGLDFPSVGPQHAYLNSTGRAEYVSITDDEALEAFKALSR
HEGIIPALESSHALAHALKMIRETPQKEQILVVNLSGRGDKDIFTVHDILKARGEI
>CORE_REP|Org17_Gene4241#
MATLTTENQTFSGFEYAEQSTFLYRSEFRSLSAHGVFERIETPVFGGEQEGSALAQHIRQALARAKAA
GQAAPVVVGAIPFDTRRPSCLYIPEESRFVANDSFIRAARPMLQQPHRLVACTSIPDEPRFKHAVAEA
VSRFKQGKLDKAVLSRILDIELEQPVAGHRILNNLMVQNPTGYHFSLPLADGSVLIGASPELLIRKQG
GEIHTNPLAGSARRQDDPQQDRLGSERLMRSTKDKYEHKLVIDDIRRHLTPLCATLSVPSGPSLLSTG
TMWHLSTRIRGELLNPALNVMQLACLLHPTPALCGFPTESARQLIAALEPHDRGLFSGIVGWCDANGD
GEWAIVIRSGLLRGNRVRLFAGAGIVAASTPQSEWMETTAKLGTMLNAFGLNSGAL
>CORE_REP|Org34_Gene3799#

MLDSQTIATVKSTIPLLAATGPKLTAHFYDRMFAHNPELKDIFNMSNQRNGDQRQALFDAICAYAANI
ENLAALLPAVERIAQKHTSFNIQPEQYQIVGGHLLATLDEMFSPGQEVLDAWGKAYGVLANVFIQREE
QIYQQSETDNGGWRDLRAFRILKKQPQSDVICSFVLAPVDGGRVADFKPGQYLAVYIKHDSLEHQEIR
QYSLTTSPNGEFYRIAVKREDQGKVSNYLHQQAQEGDVIDIAPPHGDFFLDVATTTPVALISAGVGQT
PMLGMLNTLHDSQHQAQVHWLHAAENGSVHAFADEVADIAGRMPNLSRHVWYREPGADDVEGRDYHSR
GLMDLSALQGSLADPQMHYYFCGPVAFMQFVGKQLLEMGVEAERIHYECFGPHKVL
>CORE_REP|Org34_Gene3464#
MTKHLARQRLVYAVVLGLLAALGPLCTDLYLPALPEMAGELNTSTAAAQLSLTTGLLGLGVGQLIFGP
YSDKLGRMRPLLLSLILLLGASLWCALAPTIDQLLIARLLQGIAGAGGAVISRAIARDLYAGHELTRF
FALLMLVNGLAPIVAPVLGGVMLQVMNWRGIFGVLAAIAVLLFSLSALKLRESLPVERRSQGGILAML
MSLGGLLTQRYFMGLCLTQGFVMAGMFAYIGASPFVLQQIYGLSPQMFSLCFAINGVGLIIAAQLASR
LSARWGERRVLRGGLTLAAVASLLLLLAAALHAPLVVLLVPLFFSVAVIGIVGPTASSLAMQSQGDKA
GSASALIGVCMFALGACAVPLTGLGGTSGLSMALTIVGCYAIAILLFGLLARRNDA
>CORE_REP|Org9_Gene4009#
MTSNTSQPLGLHRIKFHGKGGEYFAIWLVNALLTIVTLGIYSAWATVRRRRYFYGNTELDGDRFDYHA
QPLQILKGRLLVIGGLIVFYILLAVTPLLGLLALLVLLALLPWIVIRSWRYNAIMSSYRGVRFNYVCR
TGRAYWALLFCPLLLIIGLYVALIILLSIGSGFESINAIGLMLVLTLLLAVPAFAAVNGIISALQHDL
YVNNLFFGNTPFIAELKKSAFIKFALIGLLIFLPFMLAALVCMGSFILSLYQMVLMGMLTDETADLLV
LENIGSLLLMMVVLLIGALIAGSYQVVAQRNYLFNQATLNGNVKLHSSMQTLPYMGLLITNTLITLFS
LGFAVPVAEVRHARYLAECTAVEGDLALLDIAAHQETANSAVAEEALQALDLGGSF
>CORE_REP|Org13_Gene2514#
MRPALSLRHLTLPAIMAGFVAVLVGYTSSAAIIFQAAAAAGATPAQIGGWLSALGIAMGVTSLGLSLY
YRTPILTAWSTPGAALLVTSLPGTPINEAIGVFIFASGLILLCGVTGLFARLMDYIPQAISAAMLAGI
LLRFGLDAFASLQLNFPLSAGMGLAYLLSRRYQPRYAIVLTLAAGLAIAALQGNIQLTQHAPAFAMPE
FIAPHFSWPTLLGIGVPFFVVTMASQNAPGIATLQAAGYRVPTSPLIAWTALTALLLAPFGGFSVCIA
AITAAICMGPDVHPDPQRRYMGAVAAGGFYLLAGLFGGAIGLLFSALPVALIHTIAGLALLGTIGGSL
QRALHDEKQRDAALIAFLITASGVTLLGIGSAFWGIVGGAIAHLLLSLPRRGTAA
>CORE_REP|Org38_Gene4435#
MPNQPNSSFNAGGRTRAFALGQRLSGVALLAALLAGCDNSVAHNAPPPPPVVSAASVVVKPISQWDAF
NGRVEAVQSVQLRPRVSGYIERVNYTEGDEVKKGQVLFIIDDRTYRAAREQAQAELVRARNQAALARS
ESSRTEKLIGTQAISQEVWEQRRSSAAQAQSNVLAAQAQLDMAQLNLDFTRVTAPIDGRASRAMITAG
NLVTAGDSASVLTTLVSLDKVYVYFDVDEATFLRYQQQGRHDVRLPVKVGLVGEDGTPHQGLVDFTDN
QLNAGTGTIRMRALLDNRDRRFTPGLFARVQMPGSAEFNAMLIDDKAVMTDQNRKFVYIVDKDGKAQR
RDIDVGRMAEGLRIVQKGLVNGDRVIVDGMQKVFMPGMPVDAKNVAMTTTASALN
>CORE_REP|Org35_Gene1276#
MNKNRGLTPLAAVLMLSGSLVLTGCNDKETQQQGAQQQAPEVGVVTLKAEPLNITTDLPGRTAAYRIA
EVRPQVSGIILKRNFVEGSDIKAGTSLYQIDPATYQASYDSAKGDLAKAQASASIARVTVNRYKPLLG
TSYISKQDYDNAVSTLQQADAAVVAAKAAVETARINLAYTKVTSPISGRIGKSAVTEGALVSNGQATA
LSTVQQLDPMYVDVTQSSTDFLRLKQELASGALKQENGKAKVKLMLENGTEYAQEGTLEFSDVTVDET
TGSITIRALFPNPNDTLLPGMFVRARLDEGVRSDALLVPQQGVTRNPRGDATALVVGADNKVELRTLK
ADQAIGDKWLVTDGLKAGDRVIVTGLMKVHPGAQVKVQEVDTQAQKQPQSEAQKS
>CORE_REP|Org18_Gene426#
MSHIAHFALAIVVVAILALLVCRDRKSIRIRYVIQLLVIEVLLAYFFLHSEAGLGFVKGFAALFDKLL
GFAGQGTDFVFGGMGDKGLAFFFLKVLCPIVFISALIGILQYIKVLPFIIRIIGTVLSKVNGMGKLES
FNAVSSLILGQSENFIAYKDILGKMSEKRMYTMAATAMSTVSMSIVGAYMTMLDAKFVVAALVLNMFS
TFIVLSLVNPYDTNKEEELHLGNLHEGQSFFEMLGEYILAGFKVAIIVAAMLIGFIALIAALNGVFSA
IFGLSFQEILGYFFYPFAWIMGIPKHEALQVGSIMATKLVSNEFVAMMELQKVSAELSPRSLGILSVF
LVSFANFSSIGIVAGAIKGLNEHQGNVVSRFGLKLVYGSTLVSILSASIAGLVLG
>CORE_REP|Org34_Gene2112#
MKTEGLLAQRIVNVKSSAIRELLKHSKMEHVISLAGGIPSDALFDFEGLSIATQQAITEQPKSAFQYG
LTEGSPLLRERICALCAERGVTAGAEEVMVTAGSQQALDLVMRAIVNPGDVFVVERPTYLAALQTLEL
AEANIMSVSSDSDGMVVEELAELLKTQRIKGVYVVPNFGNPSGITLSAARRELLVKLAAEHNFLIIED
DPYGELRFTEERHPTLHQVSQQVLGNTDHIIYTSTFSKILAPGLRLGWAILPPFLLHKVAIIKQAADL
HASALSQSIVECYLGLDRLPAQIDKIRAAYKQKGEILAGLVEQELGDYITFDKPKGGMFLWARFRQPF

NATEWLNTTLQQGVVFVPGEYFFSDNPDRSTFRLSFATATEQQMQEAVARLRRSL
>CORE_REP|Org39_Gene141#
MRKLENFHLLVMLILLVAVGQMAQTIYVPVIADIAHDLSVRTGAVQRVMAAYLLTYGFSQLIYGPISD
RIGRRPVILTGMMIFLVGALGALLSTNLTMLVAASAIQGMGTGVAGVMARTMPRDLYAGTALRYANSL
LNMGILVSPLLAPVIGGALAMVFGWRACYAFLLALCACVAFAMFRWLPETRPVQTEKRRMLASFRQLL
GDSTFSCYLVMLIGALAGIAVFEASCGVLMGGVLGLSGLTVSILFILPIPAAFFGAWYAGRDGKTFHT
LMWHSVISCLLAGAMMWIPGWFGVMNIWTLIVPAALFFFGAGMLFPLATTGAMEPFPYLAGAAGALVG
GMQNMGSGLATWLSAMLPQTGQFSLGLLMFAMALLILLCWWPLSNRMQHQGHTA
>CORE_REP|Org14_Gene3162#
MERLRRQHNAQWYHETQSSVRGDAPLEPQAATLRDRFLLGLGAFADEALNAALSARAGVFNASLAGYH
TLFPDQVSLSRYVTLSPYDRLSTALTVAQVTGVQSLCSHYAARLAPLHSPDASRESNIRLAQITQYAR
QLASQPTLICRRALQQLGDVGLSPADIVTFSQTIGFVSYQARVVAGVAALAGRPAVVVPGFPNVEDAD
GVDFSADELSWTARLPTIDTEAAAAEQLDVLDQSHPQARAESYYLLLAHDAAALRERNGVFNGINAEG
YGLSGRLKALATLAVSRINGSRYCAATVAHELQDDGLTQALFAGLPQGLAHTDDTVKRAVIHTAAELT
RAPEKFTPQSVQPLFNSGLNQAQALEVILTAALYAWENRLRQTLGDAEPAQPAQ
>CORE_REP|Org29_Gene2914#
MSLRSLYLLSLLAAGGSAQAMSAGEYVAKAGDCTACHTAPGGAELAGGMKFPTPLGAIYATNITPDKL
HGIGAYSFEEFDRAMRQGVAKDGHRLYPAMPYTSYAKMSAEDMRALYDYLMNEVPAQNVANRDSDISW
PLSMRWPLAVWNQLFHDDQPYQADPQQSAEWNRGAYLVQGAGHCGSCHTPRGWAMQEKGLDGKEPVFL
SGAELDGWYASNLRGLPPEEVTALLKTGRSRHAAVAGPMSEVVTHSTQYLSDGDLNAIAVYLRSLAPE
TAAKAAAPAVQANNPGGQATYAMYCSTCHGNKGEGTDFAIPALAGNATVTADNPLTALRVVLEGAHTP
ATQHAMAFDMPAYGWALNDRQAADLMSYLRGSWGNQAAPVTVQQVQDARQLQAK
>CORE_REP|Org28_Gene2530#
MLNNKDKPASSPWPAIFSLTVACFVMVTTEFLPIGLLTNIAPSLGVSTGTAGLMVTMPGIVAAVAAPA
LSLISGRLDRRLLMLGLSLLLIVSNLVAALAVNFPMMLLGRVLLGICVGGFWSFAANYGRHLVPEANQ
GRATALILSGISVGAVCGVPAGALIGDLFGWRAAFFGGAALAVGVLLAQLRLLTSVPPSRPVTPRDLV
LPLRLPMARIGLIAIVLLFIGHFAAYTYLRPLLQQVFVLSPSAISLQLLAYGAIGLLGTFLGERLGEY
SLRATFILIAAMLAAILIVSPLLSGLGGATLMVMVWGLAFGAVPVCATNWMFAAVPQAPEAGQALLVC
VVQIALASGALLGGEVVDWQGVSSAMLFGGALILSAALVFGLSLRSGAIGAKQC
>CORE_REP|Org39_Gene558#
MSKEKFERTKPHVNVGTIGHVDHGKTTLTAAITTVLAKTYGGSARAFDQIDNAPEEKARGITINTSHV
EYDTPTRHYAHVDCPGHADYVKNMITGAAQMDGAILVVAATDGPMPQTREHILLGRQVGVPFIIVFMN
KCDMVDDEELLELVEMEVRELLSAYDFPGDDLPVIRGSALKALEGEAEWEAKIIELAEALDSYIPEPE
RAIDKPFLLPIEDVFSISGRGTVVTGRVERGIIKVGEEVEIVGIKDTVKSTCTGVEMFRKLLDEGRAG
ENVGVLLRGIKREEIERGQVLAKPGSIKPHTQFESEVYILSKDEGGRHTPFFKGYRPQFYFRTTDVTG
TIELPEGVEMVMPGDNVNMVVTLIHPIAMDDGLRFAIREGGRTVGAGVVAKVIA
>CORE_REP|Org28_Gene3510#
MQQREVVIVAATRTPVGSFHGALAPLTAVELGTAAVQGLLAQSGVAPQQIDEVILGQVLTAGCGQNPA
RQTALNAGLPSTTPGLTINKVCGSGLKAVHLAVQAIRSGDAEAVIAGGQESMSRSPYLMTGARAGLRL
GHAQMVDSVIHDGLWDAFNDYHMGITAENLAEKYAISREEQDWFALRSQQKAQAAQQAGRFAQEITPV
TVPQPKGEALRVERDEQPRDTSLEALARLRPAFRKEGTVTAGNASSLNDGAAVVLLMSAEKAAALRLP
VLARIAGYASSGVDPAIMGIGPAPAARRCLEKAGWRLEEVDLIEANEAFAAQALAVGKELGWEAERVN
VNGGAIALGHPIGASGCRILVSLLYEMQRREVNKGLAMLCIGGGQGVALAVERA
>CORE_REP|Org46_Gene3522#
MNDSNRIRLTWISFFSYALTGALVIVTGMVMGNIAEYFNLPVSSMSNTFTFLNAGILISIFLNAWLME
IIPLKRQLIFGFVLMVLAVAGLMLGKSLTMFSLCMFILGVVSGITMSIGTFLITHMYAGRQRGSRLLF
TDSFFSMAGMIFPIVAAMLLARHIGWYWVYACIGLLYVGIFVLTLCSEFPVLGKKGADASQPAEKEKW
GIGVLFLSIAALCYILGQLGFIQWVPEYATKSFNMDIGQAGKLVSDFWTSYMVGMWVFSFILRFFDLQ
RIVTVLAALATGAMYLFVSTDNPEHLGYYIMALGFVSSAIYTTLITLGSLQTKVSSPKLVNFILTCGT
IGTMLTFVVTGPIVAKGGAHAALTTANGLYLAVFVMCLLLGFVTKHRSHGHVTH
>CORE_REP|Org39_Gene3651#
MKTSQNRYDAVVVGGGMVGAAAALGLAQAGWSVALLEYQAPQAFEAQSLPDLRISAIGCTSVGLLKQL
GAWQAVTAMRTAPYRRLETWEWASSRVAFDAVSLGLPELGFMVENRILQLALWQQFAQCANLTLLCPA
RLQSLQRADNAWQLTLDGGEALQARLVVGADGANSQVRKLAAIGTNGWQYRQACMLITVDTGAPQQDV

TWQRFFPSGPRAFLPLYDSWASLVWYDSPQRIRQLQAMPPAQLEREIAAAFPARLGPVKVHAAGSFPL
TRRHAQRYVLPGLALLGDAAHTINPLAGQGVNLGYRDVDALLNVLSDAREQGEDWSSEAVLLRYQRRR
RTDNLLMQSGMDLFYTAFSNNLAPLNVARNLALMAAQRAGKLKEHALKYALGL
>CORE_REP|Org2_Gene2691#
MFDFSTPIDRHGTWCTQWDYIADRFGSDDLLPFTISDMDFATAPCILDALQQRLQHGVLGYSRWQHED
FLGALRHWYQQRFNVGIDTATAVYGPSVIYMAAQLIRQWSVPGDYVVTHTPAYDAFYKVILANQRQLL
ACPLQKAGDDWRCDMAHLEALLARPQTKILLLCSPHNPTGKVWRRDELQQMAELCERHDVRVISDEIH
MDMVWGEHRHTPWSQVASGAWALLTSGSKSFNIPALTGAYGFISDAASREAYFQQLKARDGLSSPAVL
AVAAHVAAYRHGEPWLDALRDYLQDNLTYVAERLEQAFPALGWRPPQATYLAWIDLRPLAVDDRALQQ
VLIEREKVAIMPGFTYGEEGRGFLRLNVGCPRSKLEAGMDKLIAGLRLVLDEQ
>CORE_REP|Org46_Gene1185#
MTAENNLQLNRRILSVVMFTFVCYLTIGLPLAVLPGFVHDHLGYNSVLAGLIISAQYFATLFSRPHAG
RYADQLGPKKVVLFGLACCGASGLFYALAFGVDGYPWLSLLLLCVGRVFLGVGESFASTGSTLWGIGR
VGAMHTARVISWNGVATYGAMAAGAPLGVYLNQQWGLAGVAALIVLAVAVALLLASGKPDVSIAAGQR
IAFRAVFGRIWAYGLGLAMGTVGFGVIATFITLYYADKGWSGAAFSLTLFSCAFVGIRLIFSNVINRH
GGLKVTLASFLVEIVGLLLIWQAGEPWMVQTGALLAGAGFSLVFPALGVEAVKQVPPQNQGTALGTYS
AFLDLALGITGPLAGLLIGQAGVPSIYLAAALLVALGVLLTLRLLQRSRAIQE
>CORE_REP|Org8_Gene3446#
MQKMTNAVQNYAWGSHDALTRLYGIANPDNQPMAELWMGAHPKSPSRVPGADGELRSLRDLIDEDQPK
QLGANVASRFGELPFLFKVLCADQPLSIQVHPSKAAAEVGFAKENAAGIPLSAAERNYKDPNHKPELV
FALTPFLAMNGFRELADIVSLLQPIAGAHHDIAAFLQQPDTAHLATLFASLLTMSGEQKSLALGVLKA
ALNNQQGEPWDTVRFIAGFYPDDSGLFSPLLLNVVQLAPGEAMFLYAETPHAYLKGVALEVMANSDNV
LRAGLTPKFIDVPELLANLQFRPQPASGLLTQPEQRGNELFFPIPVEDFAFSLHDLTAAPQALAQRSA
AIVFCVAGEATLEKSGQRLTLKPGESCFIGAFESPVNVSGSGRIARVYNQLA
>CORE_REP|Org12_Gene3114#
MLTIGTALRPSATRVMLLGSGELGKEVAIECQRLGLEVIAVDRYPDAPAMHVAHRSHVINMLDGDALK
AVIEQERPDYIVPEIEAIATAMLVELERQGHRVVPCAEATRLTMNREGIRRLAAEELALPTSSYRFAD
SEIAFRQAVEHIGYPCIVKPVMSSSGKGQSLIRTPEQLQSAWDYAQQGGRAGGGRVIVEGLVRFDFEI
TLLTISAVDGVHFCEPIGHRQEDGDYRESWQPQRMSATALSRAQAIAEKVVKALGGFGLFGVELFVCG
DDVIFSEVSPRPHDTGMVTLISQDLSEFALHVRAFLGLPIGAIRQFGPSASAVILPQLTSTDVRFSGL
ENALRGHNQLRLFGKPEIAGQRRLGVALATADTTEQAVEQAKQAATAVVVNG
>CORE_REP|Org12_Gene4273#
MSVIIVGGGMAGATLALAISSLTQGRMAVDLVEATRPDDRSHPGFDARAIALAQGTCQQLARIGVWPA
LRDCATPITQVHVSDRGHAGFVNLQAQDYQVDALGQVIELHDAGQRLFALLAKAPGVTLHCPARVVDV
IRTAERAEVLLDNGQRLRGQLLVAADGSRSALAQACNMQWRQEDYPQFATIANVTTAEDPQGRAFERF
TRYGPLALLPMSQGRSSLVWCHAREDRAQVDAWDDERFIAELQQAFGWRLGRILKAGKRHSYPLGLLT
ADRHVSHRLALVGNAAQTLHPIAGQGFNLGLRDVMSLAETLAEVADSREDAGGYALLSRYQQRRQNDQ
RATIGVTDGLIHLFANRYGPLVIGRNLGLMAMARLPAIRDAFAKRTLGWVER
>CORE_REP|Org15_Gene3296#
MMMLPLYKRPLIILMLFLFSALGFYFLSPEQRYAPWQWNSGDAALPPVADYDFIKNYQAGDGALLVTN
KRVEVYNRLGYFNWSGDYVSQLLFAGPRGLRMLGRLQESVNDAVCSGQRCVLFLTEGRRLVDLQRGSV
GKLLPWQEADNDSRPTLVSGKLAALRSSDGLYFVSAEGIFYSPDFGAHWHQTLDLPALLEERQLLGQD
REESDASTLAYASARAAMPETFTAVDGDRVLFWMNPVAGSGALHIVVNGKTGEVTSQHWLAVRVKESA
QSPDGGIYLIAQTLDRGLYQILRHQPDGQLKVMLETGYKSLYRLWAGNDKLVAQTDNGDGHLSLIFDL
RSSEVRYRQPLDYVYKGLDDARQTVIEPESHYGDDGETPALPYFVRYRSTTP
>CORE_REP|Org6_Gene1492#
MLELLFLLLPVAAAYGWYMGRRSAQQDKQQEANRLSREYVAGVNFLLSNQQDKAVDLFLDMLKEDSNT
VEAHLTLGNLFRSRGEVDRAIRIHQALMESASLTFEQRLLAVQQLGRDYMAAGLYDRAEDMFSQLTDE
ADFRVSALQQLLVIHQATSDWQKAIDVAEKLVKLGKEKQRVEIAHFYCELALQAMGSDDLDRAMSLLK
RADAADKQCARVSIMFGRIYMAQNDYAKAAESLQRVLSQDKELVSETLPMLQECYTHLPEQQHNWADF
LKRCVEENTGATADLMLAEIIEQHEGRDVVQVYINRQLQRHPTMRVFYRLMDYHLADAEDGRAKESLL
LLRDMVGEQIRTKPRYRCHKCGFTAHSLYWHCPSCRAWSSVKPIRGLDGQ
>CORE_REP|Org47_Gene2881#
MTLADYNGHLVTLCLMATGTFAIGTDAFIVAGVLSDISDTFAVSPAQAGQLISVFALAYMLFAPLTAW

LLGNVNRKHILQLALVLFIAGNLACAWATSYLQISLGRVLAALGAACYTPQAAAAAVGLVAEKRRGLA
ISIVYGGMTLAIALGIPFGTFLAKLIGWREIFLFIALLGAIALLGLSLALRAIAPPGKHSLKERLAPL
RQKAVLTTLLITFFAVCSEHIVYSYVSVLLKNTQFGPQAILPLALLVFGIGAVIGNFASGALTDALGS
KFVLLFSVAIQTLSLFLLAFYVTSPWWVLAIFLVWGITGWMYLVPIQHHLLSLSKRFGALTVSLNSSV
LYAGIAAGGMLGGLTLYALPAHYLPLFSLPLGAIALLLTLLFFRGETGNE
>CORE_REP|Org41_Gene3790#
MNSHPLPRIATLDCVRGIAILGILLLNISAFGLPKAAYLNPAYLGMPSSRDAWTWALLDLFAQAKFLA
MFALLFGAGLQMLLRRGKSWIRARLSWLVLFGLAHAIFLWDGDILLAYGLIGLVCWRMIREAKETFQL
LKTGVVLYLIGVAVLLLLGFVSHGEPGSFWQPGVAELQYEKFWKLQGGFEAWRSRADLLSSSLLAIGA
QYGWELAGLMLFGAGLMRSGWLRGSYSSGYYLRQAAWLLPLSVLIQLPAVALQWQVHWDYRWSGFLLQ
VPRELGAPLQAMGYLALCYGFWPALSRMRIAHCLTLVGRMALSNYLLQTLICTTLFYRFGLYAQFDRL
QLLAFVPLVWLANLAFSALWLRYFAQGPMEWLWRKLTQLAAGAAEPKTQK
>CORE_REP|Org25_Gene4390#
MSASAETQNPQQPSGKKKQRKFWLLLLTVIFIVIGVAYLVYWFLVLRHHQETDDAYVSGNQVQIMAQV
SGSVNSVNFDNTDYVKQGDVLLTLDPTDAEQAFERAKTGLANSVRQTHQLIINSKQYQANIALRKTDL
SKAENDLKRRVVLGSVDAIGREELQHARDAVDSAKAALEVAVQQYNANQAMVLNTPLEQQPAIQQAAA
QMRDAWLALQRTKVISPITGYVSRRSVQVGAQIAAGSPLMAVVPADHIWVDANFKETQIANMRIGQPA
KVVSDVYGDDVVYQGKVVGIDMGTGSAFSLLPAQNATGNWIKVVQRLPVRIELDAKQVADHPLRIGLS
TLVTVDTANLDGRVLSDVVRDKPLYQSDALALNLAPVNQLIADVIHANAG
>CORE_REP|Org37_Gene3603#
MILHWLTGEATENHRRQIATRTIFFLAGLGMAAWAPLIPFVKARLGIDDGTLGLLLFCLAAGSMAIMP
FAGYLIAKLGCRTVLLGAGALLCIDLPLLALLDAPLLMGAALGVFGAVNGIMDVAMNSQAIVVERESG
QAKMSGFHGFYSLGGIAGAGGVSLLLLLGIVPALAIGLIALLIAILLPIVASDLLAQGGIGERCRGGV
RLALAHGKILFIALLCFFVFLTEGAMLDWSALFLHAERGVAKSQAGMGFTLYAVAVACGRLYGDRLIG
IIGRYRTLLFGSLCAATGLLLTVTVPLAWAAFGGLMMIGLGIANIVPILFNAVGNQKQVPPGQAFPAV
TLVGYIGLLTGPALIGFIAKYTTLTLAFGCTLLCLVLVSISARAVTRSSH
>CORE_REP|Org40_Gene2659#
MTESVKERINVFWFLPTHGDGRYLGTAQGGRPVDLPYLQQVALAADNLGYYGVLIPTGKSCEDSWLVA
AALAPITRRLRYLVAVRPGLQPPSLAARMAATLDRLSDGRLLINVVTGGDPVENKGDGIFLSHAERYE
VTQEFLTVYQRLLQGEKVDFSGKHIRVEGAELLFPPVQENGPPLYFGGSSPEAIDIAAGQIDTYLTWG
EPLAQVAEKLAAVRQRAERQGRKLSYGIRLHVIVRETEDEAWAAAERLIAHLDDDTIAAAQQIFARMD
STGQRRMSELHGGSRESLRIGPNLWAGVGLVRGGAGTALVGNPQQVAARIREYQALGIDNFILSGYPH
LEEAHRFAELVMPLLPLAQSAHQTARTINTGPFGETIGGDRRPGPAQREG
>CORE_REP|Org47_Gene663#
MNLHEYQAKQLFARYGMPAPTGYACTTPREAEEAASKIGSGPWVVKCQVHAGGRGKAGGVKVVNSKED
IRAFAEAWLGKRLVTYQTDALGQPVNQILVEAATDIDKELYLGAVVDRATRRIVFMASTEGGVEIEKV
AEETPELIHKMTIDPLAGPQPYQGRELAFKLGLTGKQVSQFAKIFMGLATLFLERDLAMVEINPLVIT
KQGDLVCLDGKLGADGNALFRQPELREMRDPSQEDERESRAAQWELNYVALDGNIGCMVNGAGLAMGT
MDIVKLHGGEPANFLDVGGGATKERVTEAFKIILSDDKVKAVLVNIFGGIVRCDLIADGIIGAVAEVG
VNVPVVVRLEGNNAELGAKKLADSGLNIIAATSLTDAAQQVVAAVEGK
>CORE_REP|Org27_Gene3222#
MKVNYPLLALAVGAFGIGTTEFSPMGLLPTIAKGVDVSIPMAGMLISAYAVGVMVGAPLMTLLLSHRA
RRSALIFLMAIFTLGNVLSAIAPDYTTLMLSRIITSLNHGAFFGLGSVVAASVVPKEKQASAVATMFM
GLTIANIGGVPAATWLGETIGWRMSFLATAGLGVIAMLGLWFSLPKGSAGARPDVKRELSVLVRPQVL
TALLTTVLGAGAMFTLYTYISPVLQHITEATPLFVTTMLVLIGVGFSIGNYLGGKFADRSESATLKGF
LLLLVAIMLLIPLLARSDIGAAVSMMIWGAATFAVVPPLQMRVMRVASEAPGLSSSVNIGAFNLGNAL
GAAAGGAVVSAGLGYSFVPVMGAIIAGLALLLVLFTSRTAAKVVYANG
>CORE_REP|Org27_Gene2744#
MTNIIRQFLRQEAAGGIILIAAAIVALIMANTPAQGIYQAFLNLPVMVKIASLEIAKPLLLWINDGLM
AIFFLVVGLEVKRELMQGSLSGRDKAVFPAIAALGGMLAPALIYLLFNGADEVTRQGWAIPAATDIAF
ALGVMALLGNRVPTSLKVFLLALAIIDDLGVIIIIALFYTHEVSMVALGVAAAATVLLAVMNWRGVGK
TSLYMMVGLVLWVAILKSGVHATLAGVIVGFMIPLNVKKGPSPSETLEHELHPWVAFMILPLFAFANA
GVSLQGVSLEGLTSLLPVGIAAGLFIGKPLGIFLFSLLAVKMGIARLPEGIGFKQVFAVSVLCGIGFT
MSIFIASLAFGDADAALSTYSRLGILLGSTAAAVVGYGLLRLALPRVR

>CORE_REP|Org30_Gene2720#
MKRIAITERPDWREKATEFGFRFHTMYGEPYWCEDAYYQFTLAQIEEIESATAELHQMCLQVVEKVVG
SDELMAKFCIPKHTWEFVRSSWRTNQPSLYSRLDLAYDGVNPPKLLENNADTPTSLYEAAFFQWLWLE
DQINAGKLDPESDQYNSLQEKLIERFAELKAHHGFGLLHLACCQDSEEDRGTVQYLQDCAQEAGLPTE
FLFMEEIGLGEKGQFTDLQDQVIGNLFKLYPWEFMLREMFSTKLEDAGVRWLEPAWKSIISNKALLPL
LWEMFPNHPNLLPAYFAQDDHPPMDHYVTKPLFSREGANIQIVENGQEVARVDGPYGEEGMIVQQFHP
LPKFEDSYTLIGSWLVNDQPCGIGLREDRELITQDLSRFYPHTILG
>CORE_REP|Org11_Gene4365#
MTRKPATIAVRSGLNDDEQYGCVVPPIHLSSTYNFTDFNQPRAHDYSRRGNPTRDVVQRALAELEGGA
GAVMTGSGMSAIHLVTTVLLKPGDLLVAPHDCYGGSYRLFDSLSKRGAYRVLFVDQGNEEALQQALAQ
KPKLVLIESPSNPLLRVVDIAAICAAAHAAGALTVVDNTFLSPALQQPIELGADLVVHSCTKYLNGHS
DVVAGAVIAKDPELAVELAWWANNIGVTGGAFDSYLLLRGMRTLSPRIKAAQQNAEAIVGYLQQQPLV
KKLYHPSLPENPGHEIARRQQRGFGAMLSFELDGDEAVLRRFLSALELFTLAESLGGVESLISHAATM
THAGMAAEARAAAGISESLLRISVGIEDSEDLIADLERAFQAAATR
>CORE_REP|Org22_Gene2034#
MSKKLTGFEKKRRWGWLWLLLLGIILGAALLAGTATVFHKTSDTAFCVSCHTMQQPLAEYQGSVHFQN
TKGIRAECADCHVPHQPIDYLWTKIRAVKDIYGEMVGTIDTPEKYEAHKLAMAQSVWKTLKENDSATC
RSCHSYDAMDITAQRPEARLQHPVAIKQGETCIDCHKGVAHILPDMSGETQAGAAELAKAAALTAPDA
TTLYTIATEPFFLGANDSHNAGNLMPSTEVQVVKQEGDKVLATVSGWQQDGVSEVFYAAQGKRILSVL
LGEDARQQLKTASTQTDAETGLVWHQVSLQVWLPRKQLIDDQQKIWRYAADMMSANCTGCHGLTALDR
FNANQWIGVIKGMAPRTSLTQEQLRVLTQYVQKHASDMPPAAPAKL
>CORE_REP|Org11_Gene3606#
MTIAVKHRVLLTLFMLLLLAAFGLPFLSYAPNRLLSGKSLSLFSLLHGPALWLLLPLAALAILSLLPP
TRGRALLAALAACGVLTLAFWISGQAAGHLAQEGSRLARTSWGSGCWLTMALSLLIAADAMARLTASH
LWRMLGNALVLVPPALLLFGHQLDQLSLLKEYHNRQEVFDAALLQHLTILLATMAPALAIGVPLGVLC
FRSERWQRPIFSALNIIQTVPSIALFGLLIAPLAGLATAVPWLAEHGVSGIGMAPAIVALVLYALLPL
VRSVVAGLQSVPAGVIESATGMGLTRGQIFLRVQLPLALPLFLTGVRILAVQTVGMAVVAALIGAGGF
GAIVFQGLLSSALDLVLLGVIPVVMMAVIVDSLFKFWVSILDVSRR
>CORE_REP|Org14_Gene3265#
MSTSALIPESKLPSLGTTIFTQMSALAQQHQAINLSQGFPDFDGPDYLKERLAWHVAQGANQYAPMTG
VAPLREAIADKTAELYGWQPDAGSEVTVTAGATEALFAAISALVRPCDEVVCFDPSYDSYAPAVTLAG
GILKRIALQPPAFAVDWPAFAAALSPRTRLVIVNTPHNPSATAWQAEDMQQLWHAIAEREIYVLSDEV
YEHICFAKGGHASVLAHPQLRQRAIAVSSFGKTFHMTGWKVGYCVAPAALSAEVRKVHQYLTFSVNTP
AQLALADSLRAEPEHWRQLPAFYRAKRDRFVQALASSRLEILPCAGTYFLLADYGAISDLDDVAFCHW
LTEHVGVAAIPLSVFCADPFPHKLIRLCFAKQDATLDAAAERLCRL
>CORE_REP|Org41_Gene2469#
MTDNPLLVLRERISALDLKLLALLAERRELAIEVGKTKLHSHRPIRDKERERDLLDALIAAAKPYDLD
GFYVTRLFQLIIEDSVLTQQALLQHQLNPVSQHSARIAFLGPKGSYSHLAARQYAARHFDQLIECGCQ
KFQDIFTQVETGQADYAILPIENTSSGSINEVYDLLQHTSLSIVGELTNPINHCVLIAGDSDLSQIET
VYSHPQPFQQCSQFLNRFPHWKIEYTESTAAAMEKVAKLNSPKVAALGSEAGGALYGLQVLEHNLANQ
QQNITRFIVLARKAIDVSEQVPAKTTLIMATGQQSGALVEALLVLRDNGIIMTKLESRPINGNPWEEM
FYIDVQANLRADAMQKALRDLAPITRSLKVLGCYPSDTVVPVNPS
>CORE_REP|Org40_Gene2189#
MAMERVRAGWRCFSHAFDKECRVAFRSPVVHWLSWIFPLILFALISSNFSEGTLLDLPVSVVDSDHSP
LSKSLTRRLDAGSHAHVEAYGGGLPESLSRLRSAQDYALLYIPPDFEANALSGKQPSVVMYYNALFYG
AGLYSTQDFGGLMNEINASTRSIIATEMGKTLPPLADVTLSYGSLFNASGSYIYYQQFAATIHLLQLF
AVTCMIYVLARSKSLLQAKPFSLALLGKLAPYTLCFTTLLMVEIAALVGIFDARVSGNPLFMLMIGLF
YVMAAQSIGLLLYTFTGSTITAYSLIGILVSIAMTFSGMAVPELSMPLPARIISNIEPLTHALYAMFD
VFLRQVHASAIFSVCALLAVYPLVAALLVRNRLPARLAKEGEAG
>CORE_REP|Org40_Gene2960#
MQSACSSRSKLPDVGTTIFTVIGQLSAEHQALNLSQGAPNFAGDPQLIEATAQAMRAGHNQYAPMSGV
AALRAALAEKAERLYGARYDADEEITVIASASEGLYSAISALVHPGDEVIYFEPAFDSYAPIVRLQGA
TPVAIKLSLQDFRVDWDEVAAAINGKTRMIIVNTPHNPTGAVFDAQDIDRLTALTRDTDIVILSDEVY
EHVVFDGDIHHSMARYPQLAERSVIVSSFGKTYHVTGWRVGYCLAPAALMDEIRKVHQFMVFSADTPM

QYAFAAALANPQSYLGLAAFYQQKRDLLASALQDSRFELLPSRGSFFMLARFSGFSHESDNDFAVRLI
REAKVATIPLSAFYSDGTDTGLIRLSFSKDNETLLEGARRLSQV
>CORE_REP|Org21_Gene393#
MFEPMELTNDAVIKVIGVGGGGGNAVEHMVRERIEGVEFFAVNTDAQALRKTAVGQTIQIGSGITKGL
GAGANPEVGRNSAEEDREALRAALDGADMVFIAAGMGGGTGTGAAPVVAEVAKDLGILTVAVVTKPFN
FEGKKRMAFAEQGIAELSKHVDSLITIPNDKLLKVLGRGISLLDAFGAANDVLKGAVQGIAELITRPG
LMNVDFADVRTVMSEMGYAMMGSGVACGEDRAEEAAEMAISSPLLEDIDLSGARGVLVNITAGFDLRL
DEFETVGNTIRAFASDNATVVIGTSLDPEMNDELRVTVVATGIGMDKRPEITLVTNKQASQPVMDHRY
QQHGMSPLPQEVKPAAKVVNDQAAQPNKEPDYLDIPAFLRKQAD
>CORE_REP|Org2_Gene2196#
MAKHLFTSESVSEGHPDKIADQISDAVLDAILEQDPKARVACETYVKTGMVLVGGEITTSAWVDIEEI
TRKTVREIGYVHSDMGFDANSCAVLSAIGKQSPDINQGVDRTDPLEQGAGDQGLMFGYATNETDVLMP
APVTYAHRLVQRQSEVRKNGTLPWLRPDAKSQVTFQYDDGKIVGIDAVVLSTQHSEDIALKDLQEAVM
EEIIKPVLPAEWLTAGTKYHINPTGRFVIGGPMGDCGLTGRKIIVDTYGGMARHGGGAFSGKDPSKVD
RSAAYAARYVAKNIVAAGLADRCEIQVSYAIGVAEPTSIMVETFGTEKVPTEQLTLLVREFFDLRPYG
LIQMMDLLQPIYRETAAYGHFGREHFPWEATDKAALLRDAAGLK
>CORE_REP|Org14_Gene2276#
MGKTTAVTTTVSALCLTVLCGLAQAADLPQTLGKGEGRLDIIAWPGYIERGQSDKNYDWVTQFEKQTG
CAVNVKTAATSDEMVSLMAKGGYDLVTASGDASLRLIFGKRVQPINTALIPNWKNIDPRLLNGAWYTV
DGKTYGTPYQWGPNLLMYNTKTFPTPPDSWAVIFQQQNLPDGKSNQGRVQAYDGPIYIADAALFLKAT
QPQLGISDPYQLNEEQYQAALKLLRSQHALIHRYWHDTSVQMSDFKNEGVVASSAWPYQANALKGEGQ
PIATVFPKEGVTGWADTTMLHADAKHPSCAYQWMNWSLEPKVQGDVAAWFGSVPASPAGCKASALLGE
KGCETNGFNQFDKIAFWKTPQAQGGKFVPYSRWTQDYIAIMGGR
>CORE_REP|Org6_Gene3980#
MKSGRYIGVMSGTSLDGIDVVLAAIDDRMVAQQASYSHPMPMALKKEILGMCQGQQTTLAAVGRLDAQ
LGTLFGEAVLGLLKQTGIDAEQVTAIGCHGQTVWHEPEGDARFSMQLGDNNRIAALTNITTVGDFRRR
DMAYGGQGAPLVPAFHQALLGHPVERRMVLNVGGIANLSLLLPGTAVRGFDTGPGNMLMDAWVWRHRA
QPYDQDGGWAMQGRVCLPLLQQMLADPYFAQPAPKSTGREYFNIAWLERQLAGLPAMAPVDVQATLTE
LTAVSISEQVQLAGGCERLLVCGGGARNTLLMARLSALLPGTEVGLTDDFGVSGDDMEALAFAWLAFR
TLSGQAGNLPSVTGASRETVLGGIYPVLPLGAVSSVRIAGEGFF
>CORE_REP|Org12_Gene1014#
MDSLSVDELAQKKDRWYRIVEEMLAEAGVAINGPRAWDIRVHNPALFKRILQEGSLGFGESYMDGWWE
CERLDMLFTRILQAGVDERLPKSLSDIARIAYARLFNRQSRKRAWQVGKEHYDIGNDLFRAMLDPYMQ
YSCGYWKEAQTLEQAQQAKLRMICEKLQLKPGMTLLDIGCGWGGLAQFAAQNYGVSVHGVTISAEQQK
LAQARCAGLDVEILLQDYRDLDRQFDRIVSVGMFEHVGPKNYETYFSVAARNLKPDGLFLLHTIGSNQ
TDLNVDAWIDKYIFPNGCLPSVRHIAEASEGRFVMEDWHNFGADYDRTLMAWLENFKRAWPDLMGGYS
ERFERMFTYYLNACAGAFRSRNIQLWQVLFSPAGVEGGVRVYR
>CORE_REP|Org7_Gene1444#
MSAYSRPVLLLLCGLLLLTVSIAVLNTLVPLWLTHAQLSTWQVGMVSSSYFSGNLLGTLVAGKLIQRV
GFTRSYHLSCLLFAAATAGMVLSIDFWSWLGWRFFAGVGCAWIWVIVESALLRSGNLSNRGQLLAAYM
IVYYLGTVTGQLLLSMTSTELLHVVPWVTAIVISAMLPMLFARVNRHEDEPQQAAVWTMLRRRSARLG
INGCIISGIVLGSLYGLMPLYLSHQGMSDANVGYWMALLVSSGIVGQWPVGRLADRYGRLLVLRIQVF
VVILASVAMLGNYAMAPSLFILGCAGFTLYPVAMSWACEKAMPHELVAMNQALLMSYTIGSLLGPSMT
ALLMQNYSDRVLFVMIAAVALVYLLMLLKKQKPDRHHTPFAAA
>CORE_REP|Org15_Gene1063#
MAMKLRVMTQAVALGLAIGSASFAAQAEITLLKQDPQAGDPLSRLNFTVGGSIRPQFNNMTGDGDKGS
YKRNGFDGGTRFRFAADYYLFDDISWISYYELGVNIPALFDWDHHYADGARNTSRRMLYTGLKSNTWG
QMTFGQQNSVYYDVVGAKTDIWDYDMLAQAPGNGINGDYDGSYRSRKMLKYKNRFGDADVYASYLFSD
SDYLPGNGLRYKRKGGGSLGVDYHITPDLTWGTAWNYTRAEMRNPSTSGSKSYDQNIFGTAISWKPDN
WTLTFGGGYYNDFLTTKKADVNNYFAGDAWGIEYLAGYTVPVGQYAVKSVMPYFMGDRLEYITGRNYQ
RIDNGLGVTVQFDYGFRVDVEHVLTSSTDNLGDMTVVRLRYDF
>CORE_REP|Org7_Gene2853#
MSWQQRIEQALAERRFNAAYRRRQTTEGGNGRQIRLGDRLYLNFSGNDYLGLSQDARVIAAWQQGAQR
YGVGSGGSGHVTGFSAAHQALEEQLAAWLGYPRALLFISGYAANQAVLAALMQKGDRILADRLSHASL

LEAAAQSPAELRRFQHNQPQALADLLAKPCDGQRLAVTEGVFSMDGDGAPLAELHRLTRAAGAWLMVD
DAHGIGVRGEQGRGSCWQQGVRPELLVATFGKAFGVSGAAVLCDEATAEYLLQFARHLIYSTAMPPAQ
ACALQAALVCIREGDDLRARLQDNIRRFRQGAAPLALTLTDSDTAIQPLLVGDNQRALDLATRLRECG
LWVSAIRPPTVPPGGARLRITLTAAHQPQDIDRLLEVLNDVSQ
>CORE_REP|Org2_Gene4595#
MKMKLPPFIELYRALIATKSISATDAGLDQSNEALINLLAGWFADLGFRVDVQPVPESRHKFNLLASI
GEGSGGLLLAGHTDTVPYDEGRWTRDPFTLTEHDNKLYGLGTADMKGFFAFILDAVRDIDASKLTKPL
YILATADEETTMAGARYFAASTAIRPDFAIIGEPTSLQPVRAHKGHMANAIRIVGQSGHSSDPARGVN
AIDLMHESIGRLMELRKTLQERYNNPAFAVPYPTMNFGHISGGDAANRICACCELHLDIRPLPGMTLD
NINELMHQALEPVSQRWPGRLTIEELHASVPGYECPTDHRMVAVIEELLGTRTQVVNYCTEAPFVQQV
CPTLVLGPGSIDQAHQPDEYIDTAFIEPTRKLLGQLVNHFCRQ
>CORE_REP|Org29_Gene693#
MAEDSDLEKSEAPTPHRLEKAREDGQIPRSRELTSVLMLLSGLAIILMSGSNMAQQLAAMLTQGLNFD
HGMVSNDKQMLRQLGMLLRQAVLALLPIMAGLVLVALAAPMLLGGILFSGKSIKFDLKRLNPLSGLKR
IFSTQVLAELLKGILKATLVGWVTGLYLWHNWAAMLHLMTQQPLDALGNALQMILFCGFLVVLGLTPM
VAFDVFYQLWSHFKKLKMTKQDIRDEFKDQEGDPHVKGRIRQQQRAIARRRMMADVPKADVIVTNPTH
YAVALQYNDKKMSAPKVLAKGAGEIALRIRELGAEHRIPMLEAPPLARALYRHSEIGQHIPATLYAAV
AEVLAWVYQLRRWRREGGLIPKKPERLPVPEALDFARESDSDG
>CORE_REP|Org14_Gene4877#
MTTVEELCLRFIAGAGPVRRAAGRAGHPPQLASQIVDQYAEHIFYNSGATGMALVVIDGNQVVNRSFG
DTKPGNNLRPRPDSLIRIASITKLMTSEVMVKMAAAGQVKLTDPLRKYAPKGAYVPAYNAGQPITLLN
LATHTSSLPREQPGKKPPKTPVFTWPTKAQRWQWLAHANVTVPPGVRAAYSNLAYDLLADALSRAAGK
PYNALLKEKITAPLGMVDTTLTPSPEQCSRLMVAAAGPSACRDTTAAAGSGGVYSTPRDMQRWMQQFL
SSSASGPRKATAASEQTMYFQRHDLVSLKGMDVPGQADALGLGWVYMAPKDGLPGIIQKTGGGGGFIT
YMAMIPAKNVGVFVVVTRSELSKFTNMSDPVNRLVSDLAANKS
>CORE_REP|Org14_Gene3402#
MAFNFDQWVDRSHSDSVKWDKYRGSDIIPLWVADSDFTSPPAVIEALQRRVAHGVFGYTHPSPDLIEV
FTRRMVERYGWHIKPEWIIFLPGLVCGLNLCVRACTEEHQSTLAPSPIYPPFRKAAKFAGREHLAVPL
KATGQRWVLDFSSLHDRLSGNEKLLLLCNPQNPGGTVYRRDELLQHHQFAREHALIVCSDEIHCELLL
EPGVRHIPFATLNDDAAQRSVTLMSPSKTFNLAGLGASLAIVPNEALRQKLKRARSGIVPEVNLLALV
AAQAAYQYGQPWLDEQLIYLRANRDRLIKRINAMPGLTLLPVEATYLAWIDCSALPVDNPHQFFERAG
VGLSAGLDFGDRRFVRLNFGCRWALLDEALDRMARACAALPG
>CORE_REP|Org39_Gene1042#
MSNRPLTIGLVAGETSGDILGAGLIRALKAQIPDARFVGVAGPLMQAEGCEAWYEMEELAVMGVVEVL
ERLPRLLKIRKDLTRRFGELRPDVFVGIDAPDFNITLEGRLKQRGIRTIHYVSPSVWAWRQKRVFKIG
KATDLVLAFLPFEKAFYDRFNVPCRFIGHTMADAMPLQPDRLAARAQLGIDPQARCLALLPGSRGAEV
EMLSADFLKTAQLLRTRYPELEVVVPLVNAKRREQFERIKAEVAPDLTVHLLNGQGREAMIASDAALL
ASGTAALECMLAKCPMVVGYRMKPFTFWLAQKLVKTPYVSLPNLLAGREIVTELLQHDCVPDKLAAAV
MPLLEESPQTEALKQTFLTLHQSIRCGADEQAAQAVLELAKA
>CORE_REP|Org9_Gene4300#
MIKSALLVLEDGTQFHGRAIGAEGTAVGEVVFNTSMTGYQEILTDPSYSRQIVTLTYPHIGNVGTNAS
DEESSAVHAQGLVIRDLPLIASNYRNEESLSDYLKRHNIVAIADIDTRKLTRLLREKGAQNGCIIAAD
SPDAALALAKAQGFPGLKGMDLAKEVTTQEAYSWQQGSWTLEGDLPEAKTAAELPFHVVAYDYGAKRN
ILRMLVDRGCRLTVVPAQTPADDVLKMNPDGIFLSNGPGDPEPCDYAIAAIKQFLETDIPVFGICLGH
QLLALASGAKTMKMKLGHHGGNHPVKDLDNNTVMITAQNHGFAVDENNLPANLRVTHKSLFDHTVQGI
HRTDKAAFSFQGHPEASPGPHDAAPLFDHFIELIETYRSNAK
>CORE_REP|Org37_Gene771#
MLQSRFIFCRITTLLMMQAQQFAQVVLDWYQRYGRKTLPWQLEKTAYQVWLSEVMLQQTQVATVIPYF
QRFMARFPNVRALAEAPLDEVLHLWTGLGYYARARNLHKAAQTIVAQHGGEFPTTFAEIADLPGVGRS
TAGAVLSLALGQHYPILDGNVKRVLARCYAVEGWPGKKEVENRLWKISEEVTPAQGVGQFNQAMMDLG
AMVCTRSKPKCELCPLNVGCLSYANHSWANYPGKKPKQTLPEKTAYFLLLQHGDRVWLEQRPAVGLWG
GLFCFPQFSARVDLELWLQQRGLKGKRLEQLTAFRHTFSHFHLDIVPMWLSLDAAGSGMDEGAGLWYN
LAQPPSVGLAAPVDRLLQQLAKQPPIQQNLYGDSAIDEELA
>CORE_REP|Org37_Gene2802#

MKALHFGAGNIGRGFIGKLLADAGVELTFADVNQTVLDLLNSRKSYAVHVVGEQERVENVNNVSAVNS
GSEAAVALIAEADLVTTAVGPQILGKIAGTIARGLVLRHQQGNVQPLNIIACENMVRGTSQLKQHVFA
ALPQDEQAWVEQHVGFVDSAVDRIVPPADSSDPLEVTVETFSEWIVDQTQFKGQPPAIAGMELTDNLM
AFVERKLFTLNTGHAITAYLGQQAGLQTIRDAILDPAIRRVVKGAMEESGAVLIKRYGFDADKHAAYI
NKILGRFENPYLHDDVERVGRQPLRKLSAGDRLIKPLLGTLEYGLPHANLIQGIAAAMSYRSEQDPQA
LELAELLNTLGPKAALAQISGLPAESEVVEEAVAVYNAMHK
>CORE_REP|Org15_Gene1328#
MAKFDTLTVHAGYTPDATGAVMPAIYATSTYAQPAPGEHTGYEYSRSANPTRTALESAIAELEGGSRG
YAFASGLAACSTVLELLDKDSHLIAVDDLYGGTYRLLEKVRSRTAGLRVTYVSPADLAGLEQAIEPDT
KMIWVETPTNPLLKLADLSAIAAIAKKHQLISVADNTFASPYLQRPLDLGFDVVVHSATKYLNGHSDV
VAGVAAVGNNPALAEQLGFLQNAVGGILDPFSSFLTLRGIRTLALRMQRHSDSALRIAQWLESQPQVE
NVYYPGLPSHPQHELAARQMTRFGGMISVRLKGDDAYARRVIQRSRLFTLAESLGGVESLISQPFSMT
HASIPLEQRLETGITPQLVRLSVGIEDVEDLIADLQQALAE
>CORE_REP|Org31_Gene2655#
MVLQSTRWLALSYFTYFFSYGIFLPFWGVWLKGEGIAPETIGMLLGAGLVARFLGSLLIAPRVKDPSH
LVSALRLLALLTLAFAVGFCFGNGWGWLMLVIAGFNLFFSPLVPLTDALAATWQKQIRMDYGRVRLWG
SLAFVIGSALTGQLVAVWGHNAILYSLIFSVLAMLLGMLLKPSVMPQGEARARSGTERSLWALLKEGP
VWRFLLCVTLLQGAHAGYYSFGSIYWQEAGYSASTIGYLWSLGVVAEVIIFASSNVLFRRWNARNLLL
LSACCGVLRWSLMAYSTELGWLLLIQILHCGTFTVCHLAAMRFIAARQGQEVIQLQAVYSALAMGGGI
AVMTVIAGFLFEHWQGGVFWVMAAVAVPALFIRPPAVSVSR
>CORE_REP|Org1_Gene2196#
MSTRRTFIKQLSAVAGVSLTASLGIPLRGHAKAALNPAWRMPDEGEPQQRAFLAFGAQRAIWGGFTAD
VQAAQGRIARAIADFQPLTVFCRGNERQLAEATCGSHNVSYVVTELDDIWMRDIGANFVVNDAGELGA
VDFNFNGWGDKQQHARDARLAGFVARRYGVAQPRRSALVGEGGGIEVDGHGTGIMTESSWVNANRNPG
WSRDRVEQELKAMLGLRKIIWLPGIKGRDITDAHVDFYARFVRPGVVVANLDTDPASYDHAVTQAHLA
ILRAATDADGRTLQVHTLSPPRAPRESRFSRRNPDFAAGYINYFVINGAVIAPEFGDLQADKAAFELL
SALYPQRKVVQLEIDAIAAGGGGGGIHCVTSQLPVHGKPDQ
>CORE_REP|Org14_Gene1134#
MLKLPPLSLYIHIPWCVQKCPYCDFNSHALKGDVPHQEYVDHLLADLDADLPLAGGREISTIFIGGGT
PSLLSAEAMQALLDGVRARIRVADDAEITMEANPGTVEADRFSGYQRAGVNRISIGVQSFSAEKLTRL
GRIHGPEEAKRAATLATGLGLRSFNLDLMHGLPDQSLEEALDDLRQAIALNPPHLSWYQLTIEPNTLF
SSRPPVLPDDDALWDIFERGHQLLSAAGYQQYETSAYAKPGYQCQHNLNYWRFGDYLGIGCGAHGKVT
FSDGRILRTAKTKHPRGFMRGDYMDKQHEVAAADRPFEFFMNRFRLLEAAPRADFVNYTGLAESVIRP
QLDEALAKGYLEETAEHWQITEKGKLFLNSLLELFLADDE
>CORE_REP|Org33_Gene3257#
MKLYWQTFVKVLLGMLERPVWLMLILSLCIMSMVYANRTVWDLPVGVVDQDHSTASRKLIRQLDATSK
IAIETYDSLEQAQRDLGWRKLFAVIIMPVDLEKKILSGQNIVVPVYGDATNRLANGQIQQDVVAAYQQ
LLTEYNNGLLLRSGFSERQAQILLTPILGQTLDVFNPGISFAAIIFPGLLVMLLQHSLLIACIRVNIA
MKSMPGGKAPLAAHLGGLTALLPIWLFLSIVLFVLWPWVLGYRQTANIAELLLLTFPFLLAVLGLGKL
VTECLRSVEMIYLTLAFITTPIFYLSGTIWPLQSMPAWVRAISYSIPSTWGTKAIAGVNQMGLSLNEV
WGDVVMMLVLGVVYTLLGFGVGFLRNSVALRGMFRKRRAS
>CORE_REP|Org44_Gene3596#
MSQLDLGTQQLELERYPQQEESTQLQAWEAADEYLLQQLENVDIGGRPVLIFNDNFGTLACALHAHRP
YSVSDSYMSQLATRHNLKLNGLDPEQVTLLDSLAELPAAPAVVLIRVPKALALLEQQLRALRHVVTED
TLIVAGAKARDVHTSTMQLFEKVLGPTRTSLAWKKARLIFCQAADIVPPAAAAETTDWPLDGTDWLIH
NHANVFSRGSLDIGARLFMEHLPRGLNGHIVDLGCGNGVIGLTALAQNPEAQVTFVDESYMAVASSEL
NVEHNLPQELDRCQFEVNNSLAGIERESVQAVLCNPPFHQQHAITDHTAWQMFCDAKRCLQVGGELRI
VGNRHLDYHQKLKRLFGNCTLVASNKKFVILRAVKSGARR
>CORE_REP|Org9_Gene697#
MFDYEVLRFIWWVLVGVLLIGFAVTDGFDMGVGILVRIIGKTDTERRVMINSIAPHWDGNQVWLITAG
GALFAAWPMVYAAAFSGFYVAMILVLAALFFRPVGFDYRSKLESSRWRNMWDWGIFIGSFVPAVVFGV
AFGNLLQGVPFHMDEYMRLFYTGNFFQLLNPFGLLAGVVSLTMLVTQGATYLQMRTTGEIHLRSRAAA
QIATLIMAVCFLLAGVWLVKGIDGFVVTSALDTLAESNPMRKEVAHQAGAWLINFNKYPLLWALPALG
VVLPLFTILFSRLEKGALAFVTSSLTIACVILTAGVTMFPFVMPSSTVPNVSLTMWDATSSLLTLKVM

TVVAAIFVPIVLAYTSWSYYKMFGRLDKNYIENNKHSLY
>CORE_REP|Org5_Gene2948#
MIKLENLTKQFMQKNGTPFNAVDNINLDVPEGEICVLLGPSGCGKTTTLKMINRLIEPTGGTILVNGE
DTSALDTVSLRRKIGYVIQQIGLFPNMTIEENITVVPRMLGWDKKRCHDRAEELMSMVALDPKRFLHR
YPKEMSGGQQQRIGVIRALAADPPVLLMDEPFGAVDPINRETIQNEFLDMQRQLKKTVMLVSHDIDEA
LKLGDRIAVFRQGKIVQNASADELLARPANDFVASFVGQDRTLKRLLLVQAGDVADQQETVTVRRETP
LVEAFGLMDDIDARSVTVVDADGKPLGYVKRREARGAPGVCADSLHRFRVTARAEENLRVVLSKLYEH
NTSWMPIVDEDGRYSGEISQDYIADYLSSGRTRRVLTPQ
>CORE_REP|Org12_Gene3061#
MYDLVIRGARLADDTLIDLAVKDGKIASVGRLAADISAVRQLDLAGNCRLSAGWIDAHVHCYPESPIY
HDEPDRVGVASGVTSVVDAGSTGADDIDAFYQLTRSAKTNVFAFLNISRIGLLRQNELAELTDIDKRE
AGQAIANHPGFIIGIKARMSSSVVGKNGTRPLVLAKEIQQENRQLPLMVHIGNNPPDLDEIADLLTRG
DIITHCYNGKPNRILTPAGTLRESIQRALRRGVLLDVGHGTASFSFEVARQAITLGILPHTISSDIYC
RNRMNGPVHSLATVMSKFFSVGLTLPQVIACVTENAASALQLSGKGRLEPGYDADFTLFELRREPQVF
ADAEGQTAEGEQLLVPLAAVVAGEILLTEQGEASHVFDL
>CORE_REP|Org29_Gene2782#
MKRNILAVVIPALLAAGAANAAEIYNKDGNKLDLYGKVDGLHYFSKDKGNDGDQTYVRFGFKGETQIT
DQLTGYGQWEYNVQSNHSESQGTEGTKTRLGFAGLKFADYGSFDYGRNYGVLYDVEGWTDMLPEFGGD
TYTNSDNFMTGRTNGVATYRNNNFFGLVDGLNFALQYQGKNQNDGRDIKKQNGDGWGISSTYDIGEGV
SFGAAYASSNRTDAQKNKSNERGDKADAWTVGAKYDANNVYLAAMYAETRNMTPYGGNNSLKDGTTSC
ADTQNNSCGGFASKTQNFEVTAQYQFDFGLRPEVSYLQSKGKNMNVPGAGSDQDLVKYVSVGTTYYFN
KNMSTYVDYKINLLDDNAFTKAAGIATDDIVAVGLVYQF
>CORE_REP|Org7_Gene4138#
MSTPLFRRKALHAALLAIPAFALSIDALAAEVPQVKVTVNDKQCEPMQLTVPAGKTQFVVHNTSQKNV
EWEILKGVMVVEERENIAPGFTQKMTATLEAGEYDMTCGLLSNPKGKLTVIAAANGATDGKPNALDLV
GPIAEYKVYVTKEVDGLVKQTKLFTDAVKAGNVEQARKLYAPTRQHYERIEPIAELFSDLDGSIDARE
DDYEKKSADPNFTGFHRLEKALFADNSTKDMGKYADRLYHDTVELQKRVSELTFPPSKVVGGAAGLIE
EVAASKISGEEDRYSRTDLWDFQANVDGAQKIVNLLRPLLVKANKPLLDKIDANFKTVDTILAKYQTK
DGYESYEKLTDADRNALKGPITTLAEDLSQLRGVLGLD
>CORE_REP|Org5_Gene259#
MIVNNVKLVLDDQVVQGSLEMQDGVIRSFADGPSRLPQALDGDGGWLLPGLIELHTDNLDKFFTPRPN
VDWPAHSAMSSHDALMVANGITTVLDAVAIGDVRDGGHRLENLQKMIDAVIHSQRAGVNRAEHRLHLR
CELPHESTLPLFEQLMDKPGVSLVSLMDHSPGQRQFASREKYREYYQGKYHLNDQQMSEYEEQQVALS
ARWAAPNREAIAAHCRARRISLASHDDATAAHVAESCALGSAIAEFPTTEAAARASHQQGLQVLMGAP
NIVRGGSHSGNVAAHHLAALGVLDILSSDYYPASLLDAAFRLAADERNAYDLPQAVRMITRNPARALD
LQDRGTIAEGLRADLVLARPHGEHVYVQNVWRQGRQVF
>CORE_REP|Org44_Gene4288#
MINPKVKALAIIVYGAMLTGCAIAPGQHLTTDSKNVVKQEDSDFQIDELVNIYPLTPSLIAKLRPVKV
VAQPNVLLEQATKNYEYRIGVGDVLNITVWDHPELTTPAGQYRSASDTGNWVQSDGTIFYPYIGKVKV
SGKTASQVRSEISSRLTQYIESPQVDVNIAAFRSQKAYITGEVEKSGQQPITNIPLTVLDAINAAGGL
SANADWRNVVLTHNGKEQILSLQKLMQNGDLTQNQLLYPGDIIYVPRNDDLKVFVMGEVKSPATLKMD
RSGMTLTEALGNSAGLDQNTADATGVFVIRPLRGTQGKKIADIYQLNMADATAMVMGTEFHLQPYDVV
YVTAAPVVRWNRVIVQLAPTISSFNNLTEASLRIRNWP
>CORE_REP|Org10_Gene2654#
MKGRWGKYLLGGLLVAVLAGCSSKPTDRGQQYKDGRLDQSLELVNQPNAKGSPVNAKDYSDQLMEIKY
ASPSLFNRNNSTYQAVQSWMASGADTRMLSQYGLSAYQMEGVDNYGNVQFTGYYTPVVQARYTQQGEF
RYPLYRMPPKGRGRLPDRAGIYSGALDDRYIIAYTNSLMDNFMMEVQGSGYVDYGNGQPLVFFGYGGK
NGHAYRSIGKVLIDRGEVAKADMSMQAIRQWADTHSAAEVRELLEQNPSFVFFRPEAFAPVKGASAVP
LIAKASVASDRSLIPAGTTLLAEVPLLDNKGKFTGKYEMRLMVALDVGGAIKGQHFDMYQGIGPDAGH
SAGYYNHYGRVWVLKNNGGGQLFSANQSNGSGSLLATR
>CORE_REP|Org49_Gene4083#
MSDLALPIVFMLFIVVAEAVALQWGRREPVNWHDLVFNLNSGHIMLWLFRGLEITCYGYVAAHFSLGL
LDAWPPLLMWLFALLAWDFGFYWLHRLHHRFRVLWAVHVVHHQGEHFNLSLGVRNSWYSSLTSIPFFL
LLALAGVPLSVFVTVSIFHYSIQLFNHNALTPKLGVLEKILVTPAHHRVHHVKDMAYSNKNFGGSFIF

WDKLFGTFCPALPTTPFSYGVGGDRPSANPFWASNLPFLRYFRLAWRPAPGRPRDRRSALSVFSGAML
LFSLVVGYVYQYGYGYGDISWPQMALLVLLALGSVALGGMTEGRPWASAVWLLIALGMPLLFIGYLGW
PQRYWHIAMAAVALHALCVALGWGRVAAPAAVEEPHG
>CORE_REP|Org21_Gene1915#
MLRQTNLLAEATARQIVQRAMGIISHSVNVMDSNGVIIASGNPQRLFQRHEGAVLALAENRVVEIDRA
TAEHLKGVRPGINLPFSFRNQRVGVIGISGEPAEVRAYAELVKMAAEMMVEQAALLDQHQWEKRYREE
LANQLLQPQPNNASLEAMAAYLGLDLRQARIVWIVELQEAQPHLLRELLAELEATQRDALIAITGFNE
MTLLRPACMAQGEWSLKLERQQAQRLQNQLKHRFRVRLIVGGFYDDPQSAYRSSLTARATQAMAQRLK
LRHATLFYHDYPLPSLLCDLGEDWRAQELGRPWRTLGEQDEKGVLRGTLRHYFSQNCDQTQTAAQLHI
HVNTLRYRLQRIEAITGMKINQLTDALRLYIGMLMHD
>CORE_REP|Org35_Gene2070#
MTDDFSSRWQQLDWDDITLRINGKTARDVERALNADKLTRDDFMALISPAAAPYLEPLAQRAQLLTRQ
RFGNVVSFYVPLYLSNLCANDCTYCGFSMSNRIKRKTLDAAEIARECEAIKALGFEHLLLVTGEHQTK
VGMDYFRQHIPAIRRHFSSLMMEVQPLAQEEYAELKTLGLDGVLVYQETYHPATYLQHHLRGQKQDFH
WRLATPDRLGRAGIDKIGLGALIGLSHSWRTDCYLLAEHLFYLQQTYWQSRYSISFPRLRPCAGGIEP
ASIMSEPQLVQLICAFRLFAPDVELSLSTRESPYFRDHMIPVAINSVSAGSKTQPGGYADDVPPELEQ
FEPHDGRTPQQVAEAISNAGLQPVWKDWDDYLGRSAQ
>CORE_REP|Org8_Gene2746#
MNDAIPRPQAKSQKVFTPLLEIRNLTKTFDGQNAVEDVSLTIYKGEIFALLGPSGCGKSTLLRMLAGF
EQPTEGQIVLDGQDMSHVPPYQRPINMMFQSYALFPHMTVEQNIAFGLKQDKMPRAEIAERVAEMLAL
VHMQEFAKRKPHQLSGGQRQRVALARSLAKRPKLLLLDEPMGALDKKLRDRMQLEVTDILERVGVTCV
MVTHDQEEAMTMAGRIAIMNRGKFVQIGEPEEIYEHPNSRFSAEFIGSVNVFDCVLQERHDDALILQS
PGLRHAIKVDPDASVVDGVPIQVALRPEKILLCEQVPEDGCNFAVGEVAHISYLGDLSIYHVKLHSGQ
IISAQLQNGHRFRKGMPTWGDEVRLCWETDSCVVLTV
>CORE_REP|Org13_Gene3783#
MTKGSKQREFFLDSIRAYLMLLGIPFHLSLIYSSHAWAVNSASPSFSLTVLNDFIHAFRMQVFFVISG
YFSYMLYERYERHEWLKVRLERVAIPLLSSIPLITVPQFFMLKYFTDKLNGWDEFSLYQKINVTVWEL
VSHLWFLLTLSLLTCACFYLFRKIKRQTREHIPALINRLRGLGNISLFFFIYAVCYSAAHRIILIVAP
QLLSNGLFNFVVMETMFYLPFFILGAYAFKYVWLKEIFLRRSPLALLGSLLLFIAYMLNQKLLAHSSY
MFELEVVIKTLMGILMTNVVFSFGHNLLNYHSPRITYLVNASLFIYLVHHPLTLIYGAFVTPYIGNNL
LGFLLGLLFVFSLAFALYELHKRIPVLRFLFSGKPQ
>CORE_REP|Org49_Gene1267#
MIPFNAPPVVGTELEYMQAAMGSGKLCGDGGFTRRCQQWMEQRFGSAKVLLTPSCTASLEMAAILLDI
QPGDEVIMPSFTFVSTANAFVLRGAKVVFVDLRPDTMNIDETKIEAAITDKTRAIVPVHYAGVACEMD
TIMALAKKYNLFVVEDAAQGVMSTYKGKALGTIGHIGCFSFHETKNYTAGGEGGATLVNDPALIDRAE
VIREKGTNRSQFFRGQVDKYTWRDIGSSYLMSDLQAAYLWGQLEVAERINQRRLALWQKYYDSFLPLA
RSGRIELPVIPADCVHNAHMFYIKLRDIEERTAFIDYLKEAEIMAVFHYIPLHDCPAGERFGRFAGED
RYTTQESARLVRLPLFYNMSDVNQRTVINTILSFFA
>CORE_REP|Org11_Gene2837#
MKQVRVGLIGTGYIGRCHAIAYAQAPTVFPLKGDLVLEMLAEVTPELAAQRAAEFGFARSTGDWRQLV
ADPAIDVVDICAPNFLHKEMALAAIRHGKHVYSEKPLALDAADAAEMVQAARAKGVKTLVGFNYMKNP
TSQLAREIIANGEIGEVVHFYGTHNEDYLADPRTPIDWHCRRASAGLGALGDLAAHIVNMAHYLVGDI
VAVSGDMQTVIKQRPDPQDPARLLPVENEDQASALLRFAGGAMGTIETSRIACGRKMGLTYVVTGTKG
TLSYTQERMAELKLYRHDEPVERQGFKTLLVGPKHPDYAAFCISAGHGIGFNDQKTVEIRDLVNGIAA
GDRMWPDFEEGWKVSCVLDAIAASAEQRCWLEINRD
>CORE_REP|Org16_Gene2773#
MKVLTVFGTRPEAIKMAPLVHALAQDEAFDARVCVTAQHREMLDQVLRLFEITPDYDLNIMKPGQGLT
EITCRILEGLKGVLEDFKPDVVLVHGDTTTTLATSLAAFYQRIPVGHVEAGLRTGNLYSPWPEEANRK
LTGHLAMYHFAPTENSRQNLLRELLPDNRIFVTGNTVIDALFWVRDRVMSDAALRAGLAQRYPFLDAD
KKLILVTGHRRESFGGGFERICSALAEIASNHPEVQVVYPVHLNPNVSEPVNRILKGIDNVMLIEPQD
YLPFVYLMTQAYMILTDSGGIQEEAPSLGKPVLVMRDTTERPEAVDAGTVRLVGTDVAKIVEAVTRLL
TDESEYHAMSRAHNPYGDGHACQRILEALKNHQVKL
>CORE_REP|Org5_Gene674#
MAYTTFSQTKNDQLQEPMFFGQSVNVARFDQQKHEIFEKLIEKQLSFFWRPEEVDVSRDRIDYQALPE

HEKHIFISNLKYQTLLDSIQGRSPNVALLPLISIPELETWVETWSFSETIHSRSYTHIIRNIVNDPAL
VFDDIVTNEEIKKRAKDISGYYDDLIEMTSYYHLLGEGTHQVNGKTVTVNLRALKKQLYLCLMSVNAL
EAIRFYVSFACSFAFAERELMEGNAKIIKLIARDEALHLTGTQHMLNLMRSGADDPEMAEIAEECQQQ
CYDLFVLAAQQEKEWAEYLFRDGSMIGLNKDILCQYVEYITNIRMQAVGLGLPFETRSNPIPWINAWL
VSDNVQVAPQEVEVSSYLVGQIDSEINADDLSDFEL
>CORE_REP|Org1_Gene1596#
MHCALYTAGTCRSCQWLEKPYPQQLADKQHHLQSLLAGRDVAQWLQPAAGELSAFRNKAKMVVSGSVE
RPLLGMLHRDGTPVDLSDCPLYPAGFAPMFAVLKSFIARAGLTPYNVARKRGELKYLLLTESTLDGGV
MLRFVLRSETKLAQLRAALPWLQQQLPQLKVISANIQPVHMAIMEGEREIALTEQQALEEQFNQVPLF
IRPQSFFQTNPQVAADLYATARDWVRALGIDSMWDLFCGVGGFGLHCAQPQTRLTGIEISAEAIACAR
QSAQRLGLLHVDFQALDSTRFATAEGQVPQLVLVNPPRRGIGQALCDYLSRMAPDYILYSSCNAESMA
KDIETLPGYRIERVQLFDMFPHTAHYEVLTLLVRRL
>CORE_REP|Org3_Gene4268#
MKAFRLAIVRQKYRPDGGAERFVSRALEALEQQDLDLNVITREWQGDANPNWHIHLCNPLKLGRISRE
RGFAVAARALWQKEHFDLVQSHERIPGCDIYRAGDGVHRRWLLQRARLLPEWRRKWLFSNRYHRYVMC
AERAMYAAPELKAVICNAEMIKQEIIADFGVPAEKITVIYNAIDNQKFPPADEALRQRLREQYQIPQQ
AHCLIFVGSGFERKGLAAAIRAVAATDSHLLVVGKDKAEKRYRALAQSLGCGDRVHFMGVQKQTLPFY
QAADALLLPTLYDPFPNVILEAMSCGLPVITSTTCGGAEFITPGQNGFVTDALDVPAIAEAIRALPRQ
ALGAEMGAAARATILPHDAQRLSQQLISLYRKLLTP
>CORE_REP|Org30_Gene3769#
MSVSHIIANRQTWFGHGSIRQLPPLLLADPQPTLLFSCRSFLNGPVYAGLRESLAPLFIGTEIVSHEA
SPQEIDAWVARWRGQARRVVAIGGGSVLDAAKAFSALVEHPLPTLRYMEKVGDSKISGATLPLIAIPT
TAGTGSEVTQNAVITDTQVSKVKASLRHNNFVPHTAILDPQLLAGAPDKVLAYCAIDAFTHLFEAYLS
KTAGAMTRDMSLSGIRHFLAAWPALNRSDAAREAIMQASYLGGLTLSATGLGVIHGIAGEIGALRDYH
HGQVCGRLLLPFLALLENSEQPQQRALMAELAARLYPHWQGSPESYLTDFITRHAIAPFWQDDLPISG
QELAVALDKSNSKNSWIDYAPAQRQRMIEEAFRVE
>CORE_REP|Org38_Gene2779#
MTCPVIELAQQLIKRPSLSPNDEGCQQLMIDRLQAIGFTVEAMDFEDTQNFWAWRGEGQTLAFAGHTD
VVPTGDEKRWDNPPFEPAIRDGMLYGRGAADMKGSLAAMVVAAERFVAANPNHRGRLAFLITSDEEAS
ATHGTVKVVEALMARNERLDYCLVGEPSSTERVGDVVKNGRRGSITANLHIHGVQGHVAYPHLADNPV
HRAMPALNELVAIEWDRGNEFFPPTSMQIANVQAGTGSNNVIPGDFYVQFNFRFSTELTDAMIKQRVE
ELLERHQLNYSIEWRLSGQPFLTSRGALVDAVVNAVEHYSELTPQLLTTGGTSDGRFIAQMGAQVVEL
GPVNATIHKVNECVNAADLQLLSRMYQRIMEQLIA
>CORE_REP|Org22_Gene1273#
MKYELQTTDGRARRGRLIFDRGVVETPAFMPVGTYGTVKGMTPEEVKETGAQILLGNTFHLWLRPGQE
IMKLHGDLHDFMQWHGPILTDSGGFQVFSLGAMRKIKEEGVYFRNPINGDKVFLSPEKSMEIQYDLGS
DIVMIFDECTPYPADWDYAKRSMEMSLRWAERSRKRFDELENKNALFGIIQGGVYEDLRDVSVKGLVD
IGFDGYAVGGLAVGEPKEDMHRILEHVCPQIPEDKPRYLMGVGKPEDLVEGVRRGIDMFDCVMPTRNA
RNGHLFVTDGVVKIRNAKHKDDTSPLDKDCDCYTCRHYSRAYLHHLDRCNEILGARLNTIHNLRHYQR
LMAGLRQAIEEGKLEQFVADFYGRIGKPIPPLNA
>CORE_REP|Org23_Gene4129#
MGTGQKADVIVIGAGIIGAACAWRLAREGYRVSVVDDRRAGATAAGMGHLVCMDDNPAELALSAYSLR
LWREVTGRMPQACAWRGCGTLWLADKTDEMGIAEQKRARLAEQGVTAELLTAEQIAAVEPMLRHGLAG
GLRVPADGIVYAPLVARWLLDDGSAAIDVVHGEATALEEGAVRLADGRRLAAPLVVLACGLRANSLLP
QALLHAKKGHLAITDRYPPRVRHQLVELGYGASAHASDGTSVAFNVQARPTGQWLIGSSRQFDAVDSA
LDMPVLAAMLKRAQHFLPALAQMNIIRCWAGLRAASADGLPLLGAHPRHSWLWLALGHEGLGVTTALG
SAALITAQIGRQTPEIDDSPYLAARAFAVEELPV
>CORE_REP|Org5_Gene1821#
MEMIKTRAAVAWGPNQPLKIEEVELMPPQKGEVLVRIVATGVCHTDAYTLSGKDPEGVFPAILGHEGG
GVVEAVGEGVTSVAVGDHVIPLYTPECGECKFCKSGKTNLCQAIRATQGKGLMPDGTTRFFKDGKPIF
HYMGTSTFSEYTVVPEISLAKINKEAPLEEVCLLGCGVTTGMGAVMNTAKVQPGDTVAIFGLGGIGLS
AIIGAQMAGAGRIIGIDINTSKFELARKLGATDLINPKDYDKPIQEVIVELTDGGVDFSFECIGNVNV
MRSALECCHKGWGESVIIGVAGAGEEISTRPFQLVTGRVWRGSAFGGVKGRSQLPGIVERYLDGEFAL
NDFITHTMGLEQINEAFDLMHEGKSIRSVIHFDQ

>CORE_REP|Org3_Gene1416#
MAKKDYYEILGVSKTADEREIKKAYKRLAMKYHPDRNQEQDAEIKFKEVKEAYEVLTDDQKRAAYDQY
GHAAFEQGGMGGGGFGGGADFSDIFGDVFGDIFGGGRRQRASRGSDLRYNMELTLEEAVRGVTKEIRI
PTLEECDVCHGSGAKPGSSPVTCPTCHGQGQVQMRQGFFTVQQACPHCHGRGQIIKDPCNKCHGHGRV
EKSKTLSVKIPAGVDTGDRIRLAGEGEAGEHGAPAGDLYVQVQVKAHPIFEREGNNLYCEVPINFAMA
ALGGEIEVPTLDGRVKLKVPSETQTGKLFRMRGKGVKSVRGGSQGDLLCRVVVETPVNLNDKQKQLLR
ELEESLGGPSGDKNSPRSKSFFDGVKKFFDDLTR
>CORE_REP|Org48_Gene2807#
MRPEEIALRMRGVTGLQTVVASRIVQQLSQMGCEPRRVLHELGLNERQQTQFNQLDPGYLSASLRWLE
LPAHRMLNYGAAGYPERLAQIDDAPLFLLIEGDPQALLHPQLAMVGSRQFSHYGERWANHFAEELARC
GFTITSGLAIGIDGICHRAALAAGGCTIAVLGSGLGNIYPRRHRRLAEQIVEQGGAVISDHLVTDLPL
ADHFPRRNRIISGLSLGVLVIEASLRSGTLVTARYALEQGREVFALPGPLGNPMSEGTHWLIQQGAHL
VTGPKDIAELLGSGLQWLSLNENTTICASQAEVELPFADVLANVGDEVTPVDVVAERAGQPVPVVVIK
LLELELAGWIAAVPGGYVRIRRASHVRRTHVLV
>CORE_REP|Org48_Gene4084#
MNYQLITTDAGLQQVCEQARKHAQIALDTEFVRTRTYYPQLGLIQLYDGEQLSLIDPLPIKQWQPFID
LLADTAVVKFLHAGSEDLEVFLNAFKTLPTPMIDTQILAAFTGRPLSCGFATLVAEYMKVELDKSESR
TDWLARPLTERQCVYAAADVFYLLPMAKQLVQETEEAGWTAAAHNECLLLCQRRSETLAPEVAYREIS
NAWQLRPRQLGCLQKLAEWRLRQARERDLAVNFVVREENLWQVARYMPSSLGELDSLGLSGPEIRYHG
KTLLALVAEAEALEESELPAPLANLIDQPGYKKVFKDIKAAIATVSEQSGLSSELLASRRQINQLLNW
HWKLKDGESRPELISGWRGDLLMAPLQDILKDY
>CORE_REP|Org1_Gene791#
MHNQAPINRRKSTRIYVGKVPIGDGAPIAVQSMTNTRTTDVEATVNQIKALERVGVDIVRVSVPTMDA
AEAFKLIKQQVNVPLVADIHFDYRIALQVAEYGVDCLRINPGNIGNESRIRSVVDCARDKNIPIRIGV
NGGSLEKDLQEKYGEPTPEALLESAMRHVDILDRLNFDQFKVSVKASDVFLAVQSYRLLASRIDQPLH
LGITEAGGARSGSVKSAIGLGMLLSEGIGDTLRISLAADPVEEVKVGFDILKSLRIRARGINFIACPT
CSRQEFDVIGTVNALEQRLEDIITPMDVSIIGCVVNGPGEALVSTMGVTGGHKKSGFYEDGVRQKERF
DNEQMIDQLEAKIRAKAAMMDESNRITVNLLEK
>CORE_REP|Org11_Gene2297#
MDYQLDLDWNDFLQRYWQKRPVILKRGFKNFIDPISPDELAGLAMENEVDSRLVSHQDGRWQVAHGPF
ESFDHLSENNWSLLVQAVDHWHEPSAALMRPFRQLPDWRMDDLMISFSVPGGGVGPHLDQYDVFIIQG
TGRRRWRVGEKTPLKQHCPHPDLLQVEPFDAIIDEEMEPGDILYIPPGFPHEGYALENALNYSVGFRA
PNGRELVSGFADYVLARELGSKRYGDPDVKLREHPAEILPQEVDALRQMMLDLVQQPEHFQHWFGEFI
SQSRHELDLAPPEPPYQAGEIYELLQQGEALQRLGGLRVLRVGDRCFVNGELIDTDQLQAADALCQNF
SVDAALLGDAVDDPSFLALLTALVNSGYWYFND
>CORE_REP|Org15_Gene2669#
MSSIYEKYGLKQVINTSGRMTILGVSTPRQDVIDAVDYGLNHYFEIKDLVNKTGAYIADLLNVEDAVI
VSCASAGIAQSVAAVIVKDNANLLVNLHSAPIAVPREIVLPRGHNVNFGAPVDTMVALGGGKVVEAGY
ANECSPEQIEACITPQTAAILYVKSHHCVQKSILSVEQAAVVARKHNLPLIVDAAAEEDLMCYYQMGA
DLVIYSGAKAIEGPTSGLVIGKKQYVEWVKLQSGGIGRAMKVGKEGILGLTQAIESYLTLEKTTGQQM
VERMTPFLDSLNTLTGVSARTVWDSAGRDIARAEIAFDEAVLGRSTYDIVQALKTGDIAIYFRGYKAN
EGKIEVDVRSVDERQLMTVFTCIKNLFTEKQA
>CORE_REP|Org34_Gene1297#
MMSKGISKGFFILILFIVTAAFLDVLGPYYSSVLWAVILAVIFHPVKKKLKQYVGERNGVVSLLTVAL
ICLIVFTPLAIIASSLAMEFNVIYTKLQTNNSQLPTMLADFMRHLPGWAQRFLAEHNLNSAAEIQKQL
SDVALKGGQYLAGSVFLIGKGTFNFVVGFGVMLYLLFFLLKDGPYLVNLALEALPLSQHVKHHLFVKF
AAVSRATVKGTVVVAVVQGALGGLAFYFTGIDGSLLWGALMAFLSIIPAVGSAIIWVPAVIYFFATGM
LWKAIFLVVFFVVVIGLVDNILRPLLVGKDTKMPDYLILISTLGGMEIYGINGFVIGPLIAALFIACW
NILSGRDSEENIEEIDEEFIEEGKNHPDAAAE
>CORE_REP|Org46_Gene2025#
MGYRIPITLGNIEPLASKPFRPGKMALVCEGGGQRGIFTAGVLDEFQRAGFNPFDLLIGTSAGAQNLS
AYICGQVGYARRVITRYTTSAQFFNPLRFVRGGHLIDLDWLVDTAAAQMPLAMDVAERHFTDGREFLL
CACRSDDFEPTYLPAQRERWLPAIKASSAIPGFYRQGVELDGISYQDGGISDAIPVEEAYRRGADTIV
VIRTVPSQMYYTPQWMKRMEHWLSDSSLQQMVRILQHHEQSYHRIQRFIEKPPGKLRIFEIFPPKPLA

SNALGSRLGSLNQDYHLGRRCGRYFLATVGQWMAQPEPDGMKVKKALSRRLIQPENVRMPSPIVTDIV
MPSDLGAQPEAAVVAPKIILPGDLPPGEGGAL
>CORE_REP|Org15_Gene3309#
MRNFLILLVLSLWFGVCSAQAKTVTDITGRQVVVPDNPQRIVVGESRMLYTLALLEPGNPAQRIVGWP
ADLARFDAQSWQLYTQQFPQIAQIPVISGNNFRQVNVESLLQLKPDLVILARYAREDGDNDRLVSALN
KAGVAVIYVDLRIDLLNNTVPSVRLLGDVLNRRQRAEQFIQFYQQHMAVIQQRLAGYQGPKPKVMLHL
HLGRRETCCTTAAHGNLGDLLTFAGGDNIANASVKGVYGELNPETILSANPDVYLATGMAGPQGKRLS
DLRLGPQVSRQEAADSFRRLIAKQPMLTNLQAVKNHRAWSVWHNFYLSPYHVVLVEVFAKAFYPDLFA
DINPQQTFQQLYQQFLPLPFSGTYWSQLDNE
>CORE_REP|Org36_Gene3978#
MTETSSLTPLVELHALSKAFDGKTIIADLELAINHGEFLTILGPSGCGKTTVLRLIAGLEDADRGRIV
LDGQDITAIPAEHRHVNTVFQSYALFPHMSVFDNVAFGLRMQKVPAAELTPRVEEALRMVQLDTFAKR
RPGQLSGGQQQRVAIARAVVNKPKVLLLDESLSALDYKLRKQMQNELKALQRKLGITFVFVTHDQEEA
LTMSDRIVVMREGRIEQDGTPREIYEEPKNLFVASFIGEINIFDAVVLQRLDAQRVRANVEGRECDIY
ADLPVEPGQKLKVLLRPEDLRVEEVNDSAQHDGLIGYVRERNYKGMTLESVVELESGKTVMVSEFFNE
DDPDVDHSLNQKMAVTWVESWEVVLADEEIA
>CORE_REP|Org25_Gene2928#
MEYDLIVVGSGSVGAAAGYYATRAGLKVLMIDSAIPPHRNGSHHGDTRIIRHAYGEGEKYVPLVLRAQ
ALWNALIQQSGEELFQSCGVLNLGPQHSEFIRNAQLSAQKFRLNAQTLSAEQINQRWPEFRAPEGYVG
VFEPDAGFLRAELAITSLIKLAKEAGCSQLFNCPVNAVEPIDGGVEVITGEGRFTARKAVITAGTWVK
ALLPQLPIAPLRKVFSWHQADGRYSVNNRFPAFTVEAQDGTHYYGFPADNDGLKVGKHDGGQPMDAPE
QRKPFGSYASDGTEVFSFLRQFLPGVGVCLHGEACSYDMSPDEDFIIDTLPDCDRLMVISGLSGHGFK
FASALGEIAALFAQDKAPPVDVSSFGLKRFS
>CORE_REP|Org25_Gene326#
MKLTTGKALLAGCIAMAMSQAAMAKDIKVAIVGAMSGPVAQYGDMEFTGARQAIADINAKGGIKGDKL
VGVEYDDACDPKQAVAVANKVINDGIRYVIGHLCSSSTQPASDIYEDEGVIMITPAATNADLTTRGYK
MILRTTGLDSDQGPTAAKYILSDIKPKRIAVVHDKQQYGEGLARSVRDSLKKQGTEVAMFEGITAGDK
DFSTLVARLKKENIDFVYFGGYYPEMGQILRQAKQAGLTTRFMGPEGVGNSSLSNIAGAASEGMLVTL
PKRYDQVPANQPIVDALKAKKLDPTGPFVWTTYAALQSLTTGMERSGSQEPADIVKDLKTGKPVDTVM
GPLTWDDKGDLKGFEFGVFEWHANGTSTPIK
>CORE_REP|Org36_Gene3265#
MKRTTGFLFDERCFWHSTGLHATTLPVGGWVQPPSGAGHAESPETKRRMKNLMDVSGLSRQLTLLSAE
PATEEDLLRIHPAHYLQRFKQVSDSGGGLLGEEAPLGPGSYEIAKLSAGLACAAVEAVLQGELDNAYA
LSRPPGHHCLPDQSMGFCFLANIPIAIERAKARHGLGKVAVLDWDVHHGNGTQHIYWQRGDVLTLSLH
QDGCFPAGYSGERDRGEGDGAGCNVNVPMLAGAGDDGYLHAMRRIIIPALEKFEPELIIVACGYDANA
LDPLARMQLHSDSFRAMTALVQNAADRLCGGKLVMVHEGGYAESYVPFCGLAVMEQLSGVRTEVQDPL
LAFIQQQQPRDAFNRFQREALDELARQFGL
>CORE_REP|Org35_Gene519#
MTESQQKGSIWTKIHIDPTFLLLILALLVYSAFVMWSASGQDIGMMERKIGQIVMGLIVMAVMAQIPP
RVYESWAPYLYIFCVILLILVDAFGQISKGAQRWLDLGVVRFQPSEIAKIAVPLMVARFMNRDVCPPS
LKNTAIALVLIFLPTLLVAAQPDLGTSILIAASGLFVLFLSGMSWKLIAVAAVALAAFIPVLWFFLMH
GYQRDRVMMLLDPESDPLGAGYHIIQSKIAIGSGGLSGKGWLHGTQSQLEFLPERHTDFIFAVLAEEL
GLIGVLVLLALYLLVIIRGLMIAAKAQTTFGRVMVGGLMLILFVYVFVNIGMVSGILPVVGVPLPLVS
YGGSALIVLMAGFGIIMSIHTHRKMLSKSL
>CORE_REP|Org42_Gene1891#
MNAKSPEQTNPEVLRALVTGVLAAFEHPTLKNNLTALKAIHHCALLDDVLHIELTMPFAWQSGFEALQ
ASVGPELLRVTGASAIDWKLKHDITTLKRANGQAGIKGVRNIVAVSSGKGGVGKSSTAVNLALALAAE
GAKVGILDADIYGPSIPNMLGTEHERPTSPDGQHMAPIMAHGLATNSIGYLVTDDNAMVWRGPMASKA
LMQLLQDTLWPDLDYLVLDMPPGTGDIQLTLSQNIPVTGALVVTTPQDIALLDAAKGIVMFEKVHVPV
LGIVENMSVHICSNCGHHEPIFGTGGAEKLVQKYHSRLLGQMPLHISLREDLDRGTPTVISRPDSEFA
EMYRQLAGRVAAQMYWQGEAIPTEIAFRAL
>CORE_REP|Org32_Gene1732#
MCQTCMTEAKMAAGIPEIMKAVVAYAPKDYRLEQVPVPKIGPKEILVKIEACGICAGDVKAFEGAPSF
WGDEKQPAYIKAPMIPGHEFIGHVVGYGEGVEGFNLGDRVISEQIVPCWQCRFCNRGQYWMCEKHDLY

GFQKNVNGGMAEYMKFTKEAINYHVPADLPIEKAILIEPYACSFHAVQRANIKLGDVVVLAGAGTLGL
GMIGAIKKSGPSKLVVLDLSDERLALAKRFGADVTLNPTRDDVPAAVKAMTDGYGCDIYIEATGAQKS
VEQGLTLIRKLGTFVEFSVFKDPVTVDWSIISDRKELDVLGSHLGPYCYPLVIEGIANGDLPTEGVVT
HTLPLEQFAEGFELMKRGIGSIKVVLNPNL
>CORE_REP|Org35_Gene788#
MASVTLRSVYKAFGEAVISKDVNLTIEDGEFVVFVGPSGCGKSTLLRMIAGLEDITSGDLLIGEKRMN
EVPPSERGIGMVFQSYALYPHLSVADNMSFGLKLAGAKKAEINQRVNQVSEVLQLAHLLDRRPKALSG
GQRQRVAIGRTLVAEPDVFLLDEPLSNLDAALRVQMRIEISRLHKRLQRTMIYVTHDQVEAMTLADKI
VVLDAGRVAQVGKPLELYHYPANRFVAGFIGSPKMNFLPVKVTAAEPRQVQVELPNRQLVWLPVEGAG
VQPGANLSLGIRPEHLLPGEASEVRLTGDVQVVEQLGNETQIHIQIPAIRQNLVYRQNDVVLVEEGAT
FAIGLPPHRCHLFREDGTACKRLHQEPGV
>CORE_REP|Org21_Gene2062#
MYHDEFYMARAFELARLGRFTTAPNPNVGCVIVRDGEIVGEGYHLRAGEPHAEVHALRMAGDKARGAT
AYVTLEPCSHHGRTPPCADALVAAGVTRVVAAMQDPNPQVAGRGLYKLQQAGVEVRHGLMLAEAEAVN
LGFLKRMRTGFPYVQLKLGASLDGRTAMASGESQWITSPEARQDVQRLRAQSAAILSTSATVLADDPS
LTVRWDELDAETQRLYPRDNLRQPLRILLDSQNRITPQHRVVQQPGATWLARLQADAQTWPQDVEQFI
CPAHGGGVDLVVMMMLLAKRQVNSIWVEAGASLAGALLQAGLVDELILYIAPKLLGDNGRGLCHLPGL
ERLADAPEFVFSDVRQVGPDLRLRLRAKH
>CORE_REP|Org23_Gene4698#
MKTLLLTGATGFLGGAVLEKLLIENQSINYLLLMRADDAQQGLARIRTNMEKFNIDANLLSKITIENI
LLGDLSEPAEFLTDARINNVTHVINCAAVASFGNNPLIWKVNVEGTLAFAERMAQVPGLKRFLHVGTA
MSCSPEPGSLVAESGEFEEDAEHLVEYTRSKSTIEQLMRQRCPQMPLTIARPSIVVGHTRLGCQPSSS
IFWVFGMALMLRKFMCSLQDNIDVIPVDYCADALVMLMNSETLENDVYHISAGEERSVSFAEIDRAMA
AALEKAPVGDEYAQVTYDALVKMRRQLKDIFGPCNERLMLKAMRLYGSFAMLNVRFSNEKILKLGMPK
PPRFTDYIAGCVQSTRGLSIQQQMVVDFK
>CORE_REP|Org4_Gene2616#
MVTQRKKWLSGVVAGLLMAASVTASAEEKTLHVYNWSDYIAPDTLAKFQKETGIKVVYDVFDSNEVLE
GKLMAGSTGYDLVVPSSNFLERQSQAGIFEPLDKSKIPNYKNLDPEMLKLVAHNDKDNKYGIPYMMVT
TGIGYNVDKVKAVLGKDAPVNSWDLIFKPENLEKLKSCGVSFLDAPSEVYATVLHYLGKDPNSTNAAD
YTGAANDLLLKLRPNIRYFHSSQYINDLANGDICVAIGWSGDVMQAANRAKEAKNGVNVAYAIPKEGA
LTYFDMFAMPADAKNKDVAYQFLNFLLKPDVMADISNHVYYANAVKDSTPLVNAEVRDNPNVYPPADV
RAKLFTLNVQSPKLDRVITRAWTKVKSGK
>CORE_REP|Org12_Gene4502#
MTQRAQDLGIIIGQGTPGPLNAITDVPGVRVGHASLHADLADGRSVRTGVTVIEPRPGSARHAPCFAG
VHVLNGNGDATGLEWVREAGLLTSPIAFTNTHSVGVVRDALIALEREALPAGDNAVYWNMPVVMETFD
GLLNDINGFHVKAEHVREAQQAAQGGLPQEGAVGGGSGMICHEFKGGSGTASRRLPADQGGWTVGAIV
QANHGKRESLLVGGYPVGRHLGHIPSPFTPQLPHPGMGSIVVALATDAPLLPHQCARLAQRASIGIAR
TGGGTEDSSGDIFIAFATGNDGLPPADYANKGAFTTPLRMVNNDYISALFAAAAEAVEEAIVNALLAA
NTVSGNGHRAEGLSAEQLVTALEKAGWRR
>CORE_REP|Org19_Gene4738#
MRVITLSRLYVHPVKSLRGLQLSYAQVGSSGLAFDRNFMITEPDGTFITARQYPQMVLFTPALLPDGL
FLSAPDGESAAIRFSDFTAAPQPTEVWGNHFTALIAPDEINRWLSGYFQRDVQLRWLGPELTRRVKKH
PEIPLTFADGYPYLLINQASFNDLQRRCPGSIKLEQFRPNLVVSGAAAWAEDGWQVIRVGDVMFDLVK
PCSRCVLTTVSTERGRKHPSGEPLSTLQKFRSADNGDIDFGQNMIARSSGIIRVGDTVEVLSTKPPRP
YGAGKVVESVQAPQDSEHSVTIEYEGKVFTGNNQQILLEQLEQQGIRVPYSCRAGICGSCRITLLSGE
VAPLKKSALGDNGTILCCSCIPKSDLTLA
>CORE_REP|Org12_Gene3248#
MSDTTQTQDPWATAPADAGAAPAHDAAANAGDAWSSAPPPAAHDAAGQGADWLSSAPAQPEHFSLLDP
FHKAWVPFDSWVTQGIDWLVLHFRPLFQGIRVPVDMILSGFQQLLLGMPAPIAILVFSLLAWQVSGLG
MGAATLLSLVAIGAIGAWSQAMVTLALVLTALFFCILIGLPLGIWLARSKHAAKVIRPLLDAMQTTPA
FVYLVPIVMLFGIGNVPGVVVTIIFALPPIVRLTILGIKQVPEDLIEAAESFGASPRQLLFKVQLPLA
MPTIMAGVNQTLMLALSMVVIASMIAVGGLGQMVLRGIGRLDMGLAAVGGVGIVILAIILDRLTQSLG
RDRRSKGIGRWYRRGPIGLLTRPFIKQA
>CORE_REP|Org22_Gene3622#

MIRCHDTYQGDAGCILPPPIFDARKSGLSFAKRTYLPRVAGLGLGFICVCAALYPLAPPTAVWLLLAF
HGFLWPHLAYRLACRAKDPFKAEIRNLLIDSAFGGFWAAMMAFNALPAIVILSMMSMNNIASAGKALF
VKGLAIQLAAAALTGALLGFPFHPHSTPLQIYLCLPMIYLYPTLLGLVTYRTAKRLAEKKQELQRIST
RDGLTGLYNRRHWEHLLHRQFDSCRRYQDNATLILMDIDRFKTINDTFGHALGDEALAALAEELLIGL
RNVDIVGRYGGDEFGAVLPNTSAEQAETVLRRIQQRLDVVIFKEAPQLRLQISAGIANYHPALGGYLD
WLKAADGALYRAKQNGRNRLETAAPTGD
>CORE_REP|Org30_Gene3551#
MYQDFESELNWAMCHMPRTRAAVAALPNLHGVRLACNMHLDLKMAPLVAGLLDKGAAIFLTTCNPTTV
QNDVVAWLERRGAQAYAWRDMNAGEWSESFDRALAWQPTHLCEMGADLTTRLHQSPNGPQIVAGLEAT
GSGISRLNGVAPRYPIFNWDDLPVKEGLHNRHMVGLTAWHTFFQTTHLTLHEKRVLVIGYGLVGQGTA
AAARAYGGQVMVAEIDPARALQARYDGWQVVDLASAVAQADVIATATGAKNVLSAQHLQQAQDGVFIL
NVGHVAEEIDVGFLQGLPHHEPMPYVNAYQLNEKTVYLLANGSMFNLTAGYGDSLNAFDVTLAVMAAG
IGHIVGAGARQTPGLYLLPQSAWQPAL
>CORE_REP|Org1_Gene3278#
MNKIALYCRPGFEKECAAEITDKAAQLEIYGFARVKEHSGYVLFECYQPDDADRLAREIPFRELIFAR
QMIVVGELLRDLPPEDRVSPIVGMLIGVVDRGGELRVEVPDTNESKELMKFCRKLTVPLRAAMREQKV
LMARENPTRPVVHVFFIAPGCCYVGYSYSNNNSPFYMGIPRLRFPADAPSRSTLKLEEAFHVFIPADE
WDERLASGMHAVDLGACPGGWTYQLVKRSMMVHSVDNGPMAPSLMETGQVTHHRADGFKFEPNSSKIY
WLVCDMVEKPAKVTSLMIQWLVKGWCREAIFNLKLPMKKRYEEVSQNLMSIREALGAAGISVEVHAKQ
LYHDREEITIHVRRMWSAVPGRRDERD
>CORE_REP|Org28_Gene2540#
MNESYVLAEVSNENQTLVAVVQQDHRAAYFYIYPAEAYSDRYQVRACWLRNLAAAPLQEDRAALEQGQ
PPMLAAEYCRNLEGEAPLNPEGLMVVWSESDDGAALWYYGQLLAVIPGWSLYIDHSVCYSASCIKESP
LAYPLGSASTNTQYAQAESTRQFWRSWQREEGNPWPKMQRDFQARYEPHFGPSVKYYAIDQGKWPPMA
ITQHERDGIYYFLTMGVSIRPMPWVEILFNDEASRYRRMEMAIAIDSQYMTEDNAVQMASALAGFAHA
PWARLTWIGEGHTLESDVAPLGYEGYILSSSFYPHSAHLALPQQYGDPVNLFWASPVFTAERQLAHAT
PNGGHDLVNRLREQGVDHIFRPRQPVC
>CORE_REP|Org49_Gene3991#
MSDRNYLLLTPGPLTTSKTVKEAMLFDSCTWDEDYNLGVVQRIRQQLVALATPSAGYTSVLLQGSGSF
AVEGVLGTAIGPQDKLLIVNNGAYGARMIEMARLMDIDHHAFDCGEVNEPDVAAMEAVLKSDARISHI
AMVHCETTTGMLNPLQKVAGLAARNGKTFIVDAMSSFGGIPLDVDGLGIDFLISSANKCIQGVPGFAF
VIARRSELEKCAGRSRSLSLDLYAQWRCMEDQAGKWRFTSPTHTVLAFAQALKELEQEGGIAARHRRY
QTNQRRLVAGMRELGFETLLDDALHSPIITAFYSPKADTYRFAEFYQRLKQQGFVIYPGKVSQSDCFR
IGNIGEIYPQDIERLLAAVGQAMYWNQ
>CORE_REP|Org23_Gene2684#
MTPENLPIERYDDQLAEKVARLKTLMSPFAAPEPEVFRSPVDHYRMRAEFRIWHDADDMYHIMFDQQT
KQRIRVDQFPAASELINRLMTALIAALKPEPILRHKLFQIDYLSTQSGKIIASLLYHRKLDDVWQRRA
EQLRDDLRAQGFDLQLIGRASKTKIMLDQDYVDEVLPVAGRDMIYRQVENSFTQPNAAMNVQMLEWAL
AVTAGSKGDLLELYCGNGNFSLALARNFERVLATEIAKPSVAAAQYNIAANHIDNVQIIRMAAEDFTQ
AMNGVREFNRLKGIDLSGYNCETIFVDPPRSGLDEETVKMVQAYPRILYISCNPETLCANLETLQATH
RVSRLALFDQFPYTHHMECGVLLEKHR
>CORE_REP|Org1_Gene1987#
MKTEKLLSPLKVGAVTLPNRVFMAPLTRLRSIEPGDIPTPLMAEYYAQRASAGLIVTEATQISFQAKG
YAGAPGLHTPEQIAAWKHITQAVHDKNGHIAVQLWHVGRISHASLQPGGQAPVAPSAINADTRTTVRD
ETGAWVRVPTSTPRALETSEIPGIVNDFRQATANARDAGFDFIELHAAHGYLLHQFMSPASNQRTDQY
GGSIENRTRLTLEVVDATIAEWGSEHIGIRISPLGPFNGLDNGEDQEEAALYLVEELNKRNIAYLHIS
EPDWAGGKPYSDAFRDSVRAHFKGVIVGAGAYTAEKAEALIEKGFIDAVAFGRSYIANPDLVERFRQH
APLNEPKPETFYGGGAEGYTDYPFLAK
>CORE_REP|Org14_Gene1997#
MKSQHDPGRSTKSRHTEYSLIFPIAALVVLNLWSSTANFPLIVGINILALVGILSSAFSVVRHADVLA
HRLGEPYGSLILSLSVVILEVSLISALMATGDAAPALMRDTLYSIIMIVSAGLVGFALLLGGRKFATQ
YVNLGGIKQYLMAIFPLAVIVLVFPSALPGGNFSTGQALLVALISAAMYGVFLVIQTKTHQSLFVYEH
EDDDGDPHHGKPSSHSSAWHAAWLIVHLIAVIAVTKFNANPLEGLLTKMNAPAQFTGFLVALLILSPE
GLGALRAVLNNQVQRAMNLFFGSVLATISLTVPAVTIIATLTGQTLIFGLQTPHIVVMLTVLLLCQLS

FSTGRTNVLNGTAHLALFAAYMMTIFA
>CORE_REP|Org10_Gene965#
MPILRLFHPMPFRLLSLAALLALAGCTSKTTTTAPAARPADVKARLMTLIPANAGDRQGWATDITAAF
AAQGIEPSDENLCSVLAVTEQESTFQANPQVPGLPKIAWKEIDRRADQMHIPNFLVHTALKISSSNGQ
SYSERLDKVRTEKDLSDIFDDMIDRVPMGQKLFGHLNPVHTGGPMQVSIAFAEVHAKGYPYPVDGSIR
REVFSRRGGMYFGIMHLLGYPADYSRPIYRFADFNAGWYASRNAAFQNAVSRASAIPLALDGDLIDFG
SDKPGSTELAVRTLGKRLNMSDSAIRRALEKGDTAGFADTELYKKVYALAGGKLPREMLPGIQLESPK
ITRKLTTAWFAKRVDERRQRCMARAGG
>CORE_REP|Org39_Gene576#
MNGSQTLVVKLGTSVLTGGSLRLNRAHIVELVRQCAQQHAAGHRIVIVTSGAIAAGREHLGYPELPAT
IASKQLLAAVGQSRLIQLWEQLFSIYGIHVGQMLLTRADLEDRERFLNARDTMTALLDNRIVPVINEN
DAVATAEIKVGDNDNLSALAAILAGADKLLLLTDQQGLYTADPRNNPQAELIREVHGIDDALRAIAGD
SVSGLGTGGMGTKLQAADVACRAGIDVVIAAGSKPGVVADVIEGKPVGTRFHALETPLENRKRWIFGA
PPAGEITVDDGAVEAMMARGSSLLPKGIREVKGDFSRGEVIRIRNLAGRDLAHGVSRYNSDAMRMIAG
HHSQEISEILGYEYGPVAVHRDDMIVS
>CORE_REP|Org45_Gene2965#
MPRLTLLAGLLLCSGLLHAAPTVSQLQDGLEHPWSLAFLPAEQGLLITERPGRLRLWQQGKGLSPPIA
GVPQVYAEGQGGLLEVLPAPDFAASRRVYLSFAEPGDGGKAGTAVGYGRLSDDGARLENFKVIFRQLP
KLSVGNHFGGKLAFDRQGYLFIALGENNQRPTAQETDKLQGKLVRLTAEGAVPPDNPWVGQAGKRPEV
WSYGHRNPQGLALNPWSGAIWEHEHGPRGGDELNLPLPGKNYGWPLATYGINYSGQPIPEAKGERVPG
TEQPLHYWRVSPGLSGMAFYDGQRFPAWRHSLFIGALAQKELIRLTLEGDKVVAEERLLGDRGERIRE
VRSGPDGYLYLLTDERNGKLLKVGAS
>CORE_REP|Org27_Gene1708#
MPLPQSRYDLPRIIFGVLFIAIMIVACFWVIQPFILGFAWAGMVVIATWPLLLKLQKLLWGRRSLAVL
VMTLLLILLFILPISLLISSVVDNSAPLIAWASSPGKLHIPDLAWLQSVPMIGDRLYTSYHTLVNAGG
AALLAKVQPYFGQTATWFVAQAAHIGRLLLHCALMLLFSALLYARGEQVALGIRHFAVRLGSARGDAA
VLLGGQAIRAVALGVVVTALVQSVLGGIGLAVSGIPAATLLTMLIFICCVAQLGPLLVLVPAIIWLYW
HGDTTWGTVLLVWSCVVATLDNVLRPVLIRMGADLPLLLILSGVIGGLLAFGMIGLFIGPVVLAVSYR
LLTAWMDEAPEPTTAPEQVIEDLEKR
>CORE_REP|Org32_Gene3103#
MLRIIQSPGKYIQGANALAVVGEYAKSLADHYLVIADDFVMQLAGDTLMGSLQQHGVKHHAARFNGEC
CRKEIDRLGRELQAHGCRGVIGVGGGKTLDTAKAVAHYQQLPVVLIPTIASTDAPTSALSVLYTEQGE
FAEYLIYPRNPDMVVMDSSIIANAPVRLLVAGMGDALSTYFEAQACFDAQATSMAGGKSTLAALSLAR
LCYDTLLAEGVKAKLAVEAGVVTEAVERIIEANTYLSGIGFESSGLAAAHAIHNGFTVLEECHHLYHG
EKVAFGTLAQLVLQNSPMAQIETVLAFCHDIGLPITLAQMGVTGDATAKMMAVAEASCAAGETIHNMP
FKVTPAGVQAAILTADRLGAAWLQRH
>CORE_REP|Org29_Gene2996#
MERITVTLGERSYPITIAAGLFNDPASFMPLKAGEQAMLVTNQTLAPLYLERVRQVLEQGGVVVDQVI
LPDGEQYKSLAVLEQVFSALLEKPHGRDTTLIALGGGVVGDLTGFAAACYQRGVRFIQVPTTLLSQVD
SSVGGKTAVNHPLGKNMIGAFYQPASVVVDLDCLKTLPTRELSSGLAEVIKYGIILDRAFFVWLENNI
DALMALDMQALAYCIRRCCELKAEVVAADERESGLRALLNLGHTYGHAIEAEMGYGVWLHGEAVAAGM
VMAAETAHRLGQFSVEDIERIKTLLLRAGLPVCGPQEMTPESYLPHMLRDKKVLAGELRLVLPTAIGA
AEVRGGVGHELVLASIAACLPEQQRN
>CORE_REP|Org29_Gene4139#
MRSPSPAVTPKRPALLNWLLYSPLHADDEPFQRQLRLTLTPSPLTPWLNAVGPAALLVGYAWLNQRLV
GIGLLLLLLLLTAGRAGLAQRRQPAWPDAMLVTVLLWMLLVGASAALAMLSGRFVLILFAGLTITALA
CWLMQRHAAAPRFALLEVIALTLPYLLAAPLSRVQNLFVLADLAPLWLVLAHGMIARYHRQRVQHVTL
AWEKQQASHRDRLTGFLNRAGGEVVMRSICRPAAAQTISHLFILEFGPLAALYQSHGVQIGDDVLRTV
GERLKTLIRPSDYVCRYTGGLFLILVHDLPYGAESEFLARIVPPLESPYDFGAFGEVNMQLNAGILAL
TQDYATVEDLMSSAQQALAEAKGGKK
>CORE_REP|Org46_Gene381#
MLKKWFIALCVGLVCLPAAAERIRDLVTVQGVRDNALIGYGLVVGLDGSGDQTMQTPFTTQSLSNMLS
QLGITVPPGTNMQLKNVAAVMVTAKLPPFSRAGQNIDVVVSSMGNAKSLRGGTLLMTPLKGVDNQVYA
LAQGNVLVGGAGAAAGGSSVQVNQLAGGRISNGATIERELPTTFGSGGVLNLQLNDEDFTLAQQISDA

INRQRGGGTATPLDARTIQVLVPQGNSSQVRFLAEIQNITVNVGAMDAKVIINSRTGSVVMNRDVILD
SCAVAQGNLSVVVDRQNTVSQPTTPFGGGQTVVTPNTQISVQQQGGSLQKVNASANLNNVIRALNALG
ATPIDLMSILQAMQSAGCLRAKLEII
>CORE_REP|Org33_Gene2599#
MSDNSQKKVIVGMSGGVDSSVTAYLLQQQGYQVAGLFMKNWEEDDDEEYCSAATDLADAQAVCDKLGI
ELHTVNFAAEYWDNVFELFLEEYKAGRTPNPDILCNKEIKFKAFLEFAAEDLGADFIATGHYVRRQDV
DGKSRLLRGVDGNKDQSYFLYTLSHEQVAQSLFPVGELEKPEVRRIAEQLELVTAKKKDSTGICFIGE
RKFRDFLGRYLPAQPGPIVSVDGQTVGEHQGLMYHTLGQRKGLGIGGMKDSSEDPWYVVDKDVANNVL
VVAQGHDHPRLMSVGLIAQQLHWVDRLPLSGPFRCTVKTRYRQQDIPCTVTPLDDERIEVRFDEPVSA
VTPGQSAVFYQGEICLGGGIIEQRLA
>CORE_REP|Org45_Gene4890#
MFEINPVKNRIQDLSERTAVLRGYLDYDAKKERLEEVNAELEQPDVWNEPERAQALGKERAALEAIVE
TIDQLEQGGEDVGGLLELAVEADDEETFNEAVAELDQLMSKLDQLEFRRMFSGEYDSADCYLDIQAGS
GGTEAQDWASMLLRMYLRWAEAKGFKTEVIEESDGDVAGLKSATIKIIGDYAFGWLRTETGVHRLVRK
SPFDSGGRRHTSFSSVFIYPEVDDDIDIEINPADLRIDVYRASGAGGQHVNKTESAVRITHLPTNIVV
QCQNDRSQHKNKDQAFKQLRAKLYEFEMQKKNADKQTMEDNKSDIGWGSQIRSYVLDDSRIKDLRTNV
ETRNTQAVLDGDLDKFIEASLKAGL
>CORE_REP|Org22_Gene4792#
MAGSSESGARHFALNLAVLRRLFCTMAALLLFASQPLRAEPALERMSNPLRVATPGGNFSYYVAHEHS
AQPRNALVVMHGHPRDAVKTLQAAIDAAQAAGAGGDTLLAAPLFQVPEKLAVHCHSAGLHQPQDGDAL
WRCGSWIEGGLDNAGKTGSFNAMDNLLADMKRRWPSLQTITVAGFSAGAQFVQHYVGFAHPPGGVRLR
YVVADPGSWLYFDHLRPQPINGGSCGSETACRFHWQTLNAGQCPQANRWKYGLESLPAHLHRTADAAR
RRYAAADISYLAAADDTGTAPGAYYRILDKSCAAQLQGPYRLQRALAYADYDRRYLAPDKPHRLTIVP
GCGHNVACVFPAPAARQALFPINAD
>CORE_REP|Org29_Gene4375#
MAKQTPLYDQHVACGARMVDFHGWMMPLHYGSQLDEHHAVRQDAGMFDVSHMTIVDLHGARTREFLRY
LLANDVAKLTQPGKALYTGMLNASGGVIDDLIVYFLTEDYFRLVVNSATRDKDLAWIEEHAAPYGVAL
TVRDDLALIAVQGPQAKERAGTLFTPEQKSAVEGMKPFFGVQAGELFIATTGYTGEAGYEIALPKEQA
VDFWQKLLAAGVKPAGLGARDTLRLEAGMNLYGQEMDEGVSPLAANMGWTIAWQPEDRRFIGREALEQ
QREQGTEQLVGLIMTEKGVLRNELPVRFTDAAGQTHEGVITSGSFSPTLGFSIALARVPAGIGEQAIV
QIRNREMPVKVTKPGFVRAGKPLTN
>CORE_REP|Org2_Gene4375#
MNLLTMSTEILFVFLFSLAFLFVARKAAKRIGLVDKPNYRKRHQGLIPLVGGISVYAGLCFAFWISEQ
PIAHAKLYLTCAGILVFVGALDDRFDISVKIRALVQALVGIAMMVFAGLYLRSFGHVLGDWEMLLGPF
GYLVTLFAVWAAINAFNMVDGIDGLLGGLSCVSFGALGLLLYLSGHHEMAFWCFAMIATIVPYILLNL
GILGRRYKVFMGDAGSTLIGFTAIWLLLQSSQGKAHSINPVTALWIIAIPLMDMIAIMYRRLRKGMSP
FSPDRQHIHHLIMRAGFTPRQAFVLITLAAALLAAVGVIGERLTFIPEWVMLALFLLAFFLYGYCIKR
AWRVARYIKRIKRRLRRSSDNKQVS
>CORE_REP|Org15_Gene4579#
MNARSYQELLNSKQRLALFLFLIMNAASSVFTLLFPFRDTPAFTLPLLCIPLFCLVAALFSLQTPRKY
LCKLNLFAGVLGLLWAAHIYVKSQYCLPNNQDFLLISLFSIFFISAISLTDNFTAFCLHAVPSAMVIL
ALDGMHNTLRILFTTLLPIIAFSIHHLMLKRSEIFTHALVANLYNERDKFNNLSMIDPLTGLYNRRGL
ENKITMLLEPQTGHHYVLLLDIDHFKVYNDSYGHAMGDRALVQVAVAIRDAVRSRDIVVRYGGEEFLV
LLTNVHEGYAAQLAERVRQRVAELNIPHGASPQHSGTLTLSAGISALEKLDIESAIGAADAALYLAKH
SGRNNIQLAQNVEPALLQPQELTR
>CORE_REP|Org23_Gene2628#
MIIIRYLVRETLKSQIAILFILLLIFFCQNLVRVLGDAVDGNIPTNLVLSLLALGVPKMAQLILPLSL
FLGLLMTLGRLYTESEITVMHACGLGKRTLIIAAMILALLTSAIAAVNVFWAGPWASRYQDVVVNEAK
ANPSIAGLAEGQFKPSQDGNAVLFIGNVKGSTFNDVFLAQLRPNGNQRPSVVVAEHGNIVQQKDGSQV
VTLDKGTRFEGTALLRDFRITDFTDYKAVIGHRTVAADNTESEQMSMQTLWESDDPDARAELHWRLTL
VVSVALMALLVVPLSVVNPRQGRVLSMLPAILLYLIFFLLQTSLRSNAGKGKLDPMLWLWLVNGVYFA
IALALNLWDTVPMRKLRARLRGAA
>CORE_REP|Org5_Gene1614#
MGFKCGIVGLPNVGKSTLFNALTKAGIEAANFPFCTIEPNTGVVPMPDPRLDKLAEIVKPQRILPTTM

EFVDIAGLVKGASKGEGLGNQFLTNIRETEAIGHVVRCFENDNIIHVNNKVDPAEDIDVINTELALSD
LDTCERAIHRVQKKAKGGDKDAKAELAALEKCLPHLENAGMLRALDLSDEDKAAIKYLSFLTLKPTMY
IANVNEDGFENNPYLDTVRKIADAEGSVVVAVCAAVESDIAELEDEDRDEFMAELGLEEPGLNRVIRA
GYELLNLQTYFTAGVKEVRAWTIPVGATAPQAAGKIHTDFEKGFIRAQTISYDDFITYKGEQGAKEAG
KMRSEGKDYIVKDGDVMNFLFNV
>CORE_REP|Org29_Gene4570#
MKKTAIALAVALAGFATVAQAAPKDNTWYTGAKLGWSQYHDTGFYGNGYQNGIGNGPTHKDQLGAGAF
LGYQANQYLGFELGYDWLGRMPYKGSENNGAFKAQGVQLAAKLSYPITDDLDIYTRLGGMVWRADSKA
NYTTGVSAGQRLSAHDTGVSPLAAVGVEYALTKNWATRLDYQFVSNIGDAGTVGARPDNTMLSLGVSY
RFGQDDVVAPVAPAPAPAPVVETKRFTLKSDVLFNFNKATLKPQGQQALDQLYTQLSSMDPKDGSVVV
LGYTDAVGSAQYNQKLSEKRAQSVVDYLVSKGIPSDKISARGMGKADPVTGNTCGYKAGRATKAQIDC
LAPDRRVEIEVKGIKDVVTQPQG
>CORE_REP|Org30_Gene3480#
MMKKISATVAAITLLGSVSAYAAFPAGYPADYQKWVDGAKKEGKVVIYSTTDTKAAAPLIQGFEALYP
GIKVEYNDMNSTELYNRFISEQAAGGTSGDVVWSSSMDTILKLAGDYAQEYASPEQAQLPKWAVWQDK
VYGTTYEPVVFIYNKRLIPQGDVPDSHAALAKLIAGQTDKFKKKVTTYDIEKSGVGFMLSVQDFKADP
NYFKTLADVAKGGLAVQSSTGTMMERVSSGENLIGFNILGSYAEARAKSDPSLGIVYPKDYTLVLSRV
SFISKEAGNPNAAKLWFDYVLSEKGQSILANQADIPSIRNDIEGKNDIDGMTKLLGNALKPIPVDDSL
LEYLQPAKRLDYIKQWRAAAAK
>CORE_REP|Org32_Gene3251#
MSIEKLARANVRELTPYQSARRLGGKGDVWLNANEYPIAPEFQLTAQTFNRYPECQPAQVIERYAAYA
GVKKEQVLVSRGADEGIELLIRAFCEPGKDAILFCPPTYGMYAVSAETFGVERRTVAAKEDWQLDLPA
IADSLDNVKLIYVCSPNNPTGNLIDPDSLRSLLELAKGKAIVAVDEAYIEFCPQATVAGWLSDYPHLA
ILRTLSKAFALAGLRCGFTLANEDLIALLLKVIAPYPLSTPVADIAAQALSEEGIRTMRQRVTDIAAT
RSWLQQQLEKCACVEQVFASDSNYLLARFTAASNVFKSLWDQGIILRDQNKQPGLSGCLRITIGTRDE
CQRVVDALSALPGANPTRQEPM
>CORE_REP|Org6_Gene4336#
MLLRHIRYFLAVAEQGNFTRAAEALHVSQPTLSQQIKQLEDALGAPLFDRSGRRVQLTDAGEAWMRYA
RLALQDLDAGARAIHDVATLARGHLRLAMTPTFTAYLVGPAIDAFYRRYPGITLSIEEMAQERIEVLL
AQDRLDLGIAFEMAQSAEVEATPLFSETLELMVGADHPLAARRRPLTLAEWRHLPLALLSGDFATRQF
IDRYCTQLGFRPLVAVEANALGAIVEIVRRGQLATLLPAAIARENRQLKKVALVNAMPARQAVLLQRQ
GAYRSAAAQAFIAVLQQQGVTPTPPALHHPQQMHQSETEANQRADDDEALAAAGIAQGERAGDAAQQV
DKGDDKQRGPQKRQRRDQARAG
>CORE_REP|Org37_Gene1468#
MYVAVKGGEKAIEAAHQLQEQLRRGDDALPAIGAEQIEQQLGLAVDRVMTEGGIYDRELAALAIKQAS
GDLVEAIFLLRAYRTTLPRLAVSEPLASENMRLERRISAVYKDLPGGQVLGPTYDYTHRLLDFALLAE
GEAPRAPQADEPLPDNCSHVFDLLCRQQLAREEQDDGSTPDDITRNPPVYPCSRSARLQQLVRGDEGF
LLALGYATQRGYGRNHPFAGEIRTGHVSVEIVPEELGFAIDIGEILLTECEMVNGFVDPKDHPPHFTR
GYGLVFGRAERKAMAMALVDRALQSPDYGEGVAGPAQDEEFVLAHADNVEAAGFVSHLKLPHYVDFQA
ELELLKRLRQEYSERQEARDE
>CORE_REP|Org25_Gene1948#
MTTLIHVLGSDIPHHNQTVLRFFNDVLAPRLPAEQTRHFMVAAKDVAALGDFPALNIEPYADKKSLAA
AVIARAQADRDARFFLHGQFNPGLWLALLSGKIKAHQVSWHIWGADLYEDATSWKFRLFYLLRRIAQG
RVGNVFATRGDVIHYQQRHARVPASLLYFPTRMDPALTDVHVEKNLAGPMTILVGNSGDRSNRHIEAL
QAIHQQFGADVRVILPMGYPANNDAYIEQVRAAGLPLFGEKNLQLLTQQVAFEDYLNILRACDLGYFI
FNRQQGIGTLCLLIQFGVPFVLSRQNPFWQDLAEQHLPVLFYGDSLDEAVVREAQRQLAAVDKQTIAF
FNPNYVDGWQQALALAAGEHS
>CORE_REP|Org12_Gene4009#
MKPLRPLAYRCDDSGLHRALMQAAQHYLAANGDHRFADGGMLAKVTLLLALCGLCYGLSLQQQNGWAF
FACYFGFIFIGMFLTVNVVHDASHNAFFRRPWANRWLNALVSVPLGLDPDCWRVRHVLFHHAHNNIEH
YDPDIDANGVLRQTPFQRWRPFMRAQRYYWPLVAALTFPYYIWLFDWLDRAGRTRVAARMAQQGVRGW
CGFLAGKAAHLLLALAIPCWLLPPAIGIGQILLVYLLSQMLSSLLFVMLIIGTHWAKANFYQAPAQGT
MPHGWYHHVFATTFDWLTRPRWLGYWLGGANLHLTHHLFPHWSHRHYPALSRIIGEVAPRFGIDYRLL
ELEELLRLQQRFLSAMGRKPY

>CORE_REP|Org21_Gene1210#
MAGNSIGQIFRVTTFGESHGVALGCIVDGVPPGIPLTEADLQHDLDRRRPGTSRYTTQRREPDQVRIL
SGVFEGVTTGTSIGLIIENTDQRSQDYSAIKDVFRPGHADYTYEQKYGLRDYRGGGRSSARETAMRVA
AGAIAKKYLQQKFGVQVRGYLAQIGDVTCELKDWDQVEQNPFFCPDPDKLEALDELMRALKKEGDSIG
AKVSVIAENVPVGLGEPVFDRLDADLAHALMSINAVKGVEIGDGFAVVTKRGSENRDEITPEGFQSNH
AGGILGGISSGQPVVAHLALKPTSSIMVPGRTINRQGEAVEMVTRGRHDPCVGIRAVPIAEAMMAIVL
MDHLLRQRAQNGDVVSDVPRW
>CORE_REP|Org40_Gene4117#
MKFRPVSPTAAKGLHIASSPFTHNQQSTSRIMLWVMLACIPGIAAQIWFFGYGVLIQVALAAIVALAA
EGAILKLRKLPVRSRLADNSALLTALLLGISLPPLAPWWMIVIGTFFAIVIAKQLYGGLGQNPFNPAM
VGYVVLLISFPVQMTSWLPPDELRATALPFHDTLLAIFSGHTSQGATLHALQMGVDGISQATPLDGFK
TGLRSGHSVEQVLQQPLFGGALAGIGWQWVNLGFLAGGLFMLARRLIHWQIPFSMLAAIAFCSGLAWW
LDPAHQASPLIHLFSGASMLGAFFIATDPVSASTTPKGRLIYGALIGVLVWLIRVYGGYPDGVAFAVL
LANITVPLIDHYTQPRVYGHR
>CORE_REP|Org45_Gene2934#
MTQVYNFSSGPAMLPVEVLRRAEQELCNWHGLGTSVMEISHRSKEFIAVAEQAEQDLRDLLKVPSNYK
VLFCHGGARAQFAALPLNLLGDKATADYIDGGYWAHSAIKEAEKYCAPNVIDVKTRIDGLSGIKPMKE
WQLSDDAAYVHYCPNETIDGVAIDETPDFGDKVVIGDYSSTILSRPLDVSRFGVIYAGAQKNIGPAGL
TLVIVRDDLLGKARKEVPSILDYTVLAENDSMFNTPPTFAWYLSGLVFKWLKEQGGLVEMQKRNQAKA
ELLYATIDKSDFYRSQVAIANRSWMNVPFQLVDAALDKVFLSEAEAIGLQALKGHRVVGGMRASIYNA
MPLAGVQALTDFMADFERRHG
>CORE_REP|Org23_Gene2182#
MALTRLLIKDFRNIEAADLALAPGFNFLVGANGSGKTSVLEAVYTLGHGRAFRSLQAGRVIRHDQPEF
VLHGRIEGAERELSVGLSKSRQGDSKVRIDGSDGHKVAELAQLLPMQLITPEGFTLLNGGPKFRRAFL
DWGCFHNEPGFFTAWSNLKRLLKQRNAALRQVSRYAQIRAWDQELIPLAERISEWRAEYSDAIAADIT
ATCAQFLPEFGLSFSFQRGWDKESDYGELLERQFERDRALTYTAVGPHKADFRIRADGTPVEDLLSRG
QLKLLMCALRLAQGEFLTRQSGRRCLYLIDDFASELDTGRRRLLADRLKATQAQVFVSAVSAEQVTDM
AGEKGKMFRVEQGKIEVQPQD
>CORE_REP|Org2_Gene234#
MLVWLAEHLVKYYSGFNVFSYLTFRAIVSLLTALFLSLWMGPRVIKRLQEMSFGQVVRNDGPESHFSK
RGTPTMGGIMILTSITISVLMWAYPSNPYVWCVLFVLVGYGIVGFVDDYRKVVRKDTKGLIARWKYFW
QSVIALIVAFAMYAVGKDTPATELVVPFFKDVMPQLGLLYILLAYFVIVGTSNAVNLTDGLDGLAIMP
TVFVAAGFALVAWATGNMNFANYLHIPYLRHAGELVIVCTAIVGAGLGFLWFNTYPAQVFMGDVGSLA
LGGALGTIAVLLRQEFLLVIMGGVFVVETLSVILQVGSFKLRGQRIFRMAPIHHHYELKGWPEPRVIV
RFWIISLMLVLIGLATLKVR
>CORE_REP|Org35_Gene3278#
MNGKLKIQEIARQTGLSISTVSRVLAGKANTSAEARRKVLDCAQQNGILQGISSGRLMLNNVMVFAPQ
RAFDVRTDIFYYKVIQGIAAALMEHEVRIRYCGLEEQHSDGALFLEKMSDPHTEAALIIGIDDEHIHT
LAADLHKPCVLINCSDRQMRLDSVSPDHQLIGDYSASYLFQQGHSHILNLQCLRRHTMELRLAGIRQA
YARHHLPFDDGRHLVTTSGFGGEEAEQALTTYLDGLLGHSPLPTAILAGGDYMAVGAVKALNKRGLSV
PGDVSVMSTDGFNLAEIHDVPLTSVQVPRDELGYEAIQLLQRRMLRADAPPCNLLLHGRLAVRASVRR
ISPHKTAPAVSTHDHRLYDE
>CORE_REP|Org45_Gene3841#
MSVSTDPMTDAKGQLNAGVMGRYQHILRHRLLMMGVLALAILGSLLLDFTMGPSGLSLSSLWQTLLDP
AAADAGTRVIVWDIRLPYALMAVVVGFALGLAGAEMQTILNNPLASPFTLGVSSAAAFGAALAIVLGI
GIPGIPDQWFISANAFIFALFAALMLDGITRWTRVATSGVVLFGIALVFTFNALVSMMQFIASEDTLQ
GLVFWTMGSLARASWDKLGILFGVFAVLLPLSMMSSWKLTALRLGEDRAVSFGIDVRRLRLTTLLRIS
ILSALAVAFVGPIGFIGLVAPHIARMIFGEDHRFYLPASALIGALVLSMASVASKNLVPGVIIPVGIV
TSLVGVPFFLSIILRHRGNV
>CORE_REP|Org29_Gene2094#
MKQNHPVVLVRKRKSHHAAHHGGSWKIAYADFMTAMMAFFLVMWLLAIASPQELTQIAEYFRTPLKVA
LTSGDKSSSESSPIPGGGDDPTQQHGLVRKQVDSPDKRAEELRLNKLREKLDELIESDPRLKALRPHL
LINMMDEGLRIQIIDSQNRPMFKTGSAQVESYMRDILRAIAPILNDLPNKISLSGHTDDIPYATGERG
YSNWELSADRANASRRELIAGGLAEGKVLRVVGMAATMSLKQHGADDAINRRITVLVLNKQTQEGIEH

ENAESNAMDIAQPDSLKQLAPSATAPASQTPESQAVTPTDQATLPPATDPVAQSQASPAIAPAGQAPE
QPVAAPAPTNRDSQPEVTP
>CORE_REP|Org36_Gene2461#
MKAATAVIDRRALRHNLQQVRRQAPQSRLIAVVKANAYGHGLLETAHTLQDADCYGVARIGEALMLRS
GGIVKPILLLEGFFSAEDLPVLVANNIETAVHSIEQLEALEQAELARPVPVWMKLDTGMHRLGVRPEH
AEAFYQRLCACRNVAQPVNIMSHFSRADEPESDTTLKQIACFEQFARGKPGQRSVAASGGTLLWPDAH
NEWVRPGIILYGVSPLDNGSGAEHGLQPAMTLKSSLIAVREHKAGEAVGYGGTWVSPRDTRLGVVAMG
YGDGYPRSAPTGTPILINGREVPIVGRVSMDMISVDLGPAAADKVGDEAVLWGPALPVERIAVCTGIS
AYELITKLTQRVAMEYIGD
>CORE_REP|Org19_Gene2479#
MRTQVKALLTGIILATSMVSAAQAADKVVIAHRGASGYLPEHTLPAKAMAYAQGADYLEQDLVMTKDN
ELVVLHDHYLDRVTDVAERFPDRARKDGRYYAIDFTLAEIKSLKFTEGFEIENGKKVQGYPGRFPMGK
SDFRVHTFQEEIEFVQGLNHSTGKNIGIYPEIKAPWFHKQEGKDISSKVLAVLKQYGYTGKNDNVYLQ
CFDANELKRIKNELEPKLGMDLKLVQLIAYNDWQETYEQKADGKWVEYDYDWMFKPGAMQKIAQYADG
IGPDYHMLVVADKSKPGHIVLTDMVKEAHASKLAVHPFTIRADALPKYVTDVNQLYDVIYNQAGVDGV
FTDFPDKGVQFLQKQGQHK
>CORE_REP|Org19_Gene798#
MSKIFDFVKPGVITGDDVQKVFAVAKENNFALPAVNCVGTDSINAVLEAAAKVRAPVIVQFSNGGAAF
IAGKGVKTDVPQGAAILGAISGAHHVHQMAEHYGVPVILHTDHCAKKLLPWLDGLLDAGEKHFAATGK
PLFSSHMIDLSEESLEENIEICSAYLKRMAKIGMTLEIELGCTGGEEDGVDNSHMDASALYTQPEDVA
YAYEKLNAISPRFTIAASFGNVHGVYKPGNVKLTPTILRDSQDYVSKKFNLPHNSLNFVFHGGSGSTD
AEIKESVGYGVIKMNIDTDTQWATWDGILQYYKANEAYLQGQLGNPKGADQPNKKYYDPRVWLRAAQT
SMVTRLEQAFKDLNAVDVL
>CORE_REP|Org23_Gene4578#
MKKILALLVIAPLLISCSGKKSEEINEAWIKDTNGFDILMGQFAHNIENIWGLNEVLIAGPKDYVKYT
DQYQTRSHINFDTGAITIETIATTDPAAHLRQAIISTLLMGDDPGSIDLYSDANDIQISKEPFLYGQV
LDNKGAPIRWEWRAAHFADYLLQTKLQKRTSGLHVIYSVTIQLVPNHLDKRAHKYLPMVRKASEKYGV
DESLILAIMQTESSFNPYAVSGSDALGLMQVVQHTAGKDVFQVRGKWGTPSRSYLFDPENNIDTGTAY
LAILQNNYLGGIQNPTSRRYAVITAYNGGAGSVLRVFSSDRTRAVGIINGMQPGDVYQTLTTKHPAAE
SRRYLVKVNTAQKSYRRK
>CORE_REP|Org8_Gene3145#
MSQPLTVTGVQKAPRLALSIGALALLALLVMPFMTLLPADHPLAVSTYTLTLAGKILCYAVVAVALDL
VWGYAGLLSLGHGLFFALGGYAMGMYLMRQAAGDGLPAFMAFLSWNELPWFWSGTQHFAWALCLIVLV
PGLLAFLFGYFAFRSKIKGVYFSIMTQALTYAGMLLFFRNETGFGGNNGFTGFTTLLGFPITAAGTRV
ALFLATVLLLAASLAIGFALARSKFGRVLTAVRDAENRLTFCGYDPKGFKLFVWTLSAVLCGLAGALY
VPQVGIINPGEMSPTNSIEAAIWVALGGRGTLVGPLLGAGIVNGAKSWFTMAIPEYWQFFLGLMFIVV
TLFLPKGVIGLLRRRKSS
>CORE_REP|Org46_Gene737#
MQKDALNNVHISAEQVLITPEELKNQFPLSADDENEIATARNTIANILQGRDHRLLVVCGPCSIHDPD
AALDYARRLKTLAADLSDQLYIVMRVYFEKPRTTVGWKGLINDPYMDGSFDVEAGLHIARRLLLDLVG
MGLPLATEALDPNSPQYLGDLFSWSAIGARTTESQTHREMASGLSMPVGFKNGTDGSLGTAINAMRAA
AMPHRFVGINQAGQVCLLQTQGNPDGHVILRGGKTPNYSAEHVAACEKQMLEAGLHPSLMIDCSHGNS
NKDYRRQPAVAESVVEQIKAGNRSITGIMLESHLHEGNQSSEQPRADMRYGVSVTDACINWESTETLL
RHMHQELGAALTARTGEK
>CORE_REP|Org47_Gene4637#
MTAPSAFARELGLRYPIVQGPMNGASPPALAVAVSNAGALGSCAAALFSPAVILERVQQIRAQTAAPF
NINLFLLDEQHPDLAELKRAQHLLRPFREALGLSEPPIPTQFAENNRDQIAALLEAAPPVASFTFGVL
PRATVTQFKKAGSRVIGTATTVAEARAWEAAGADFVCVSGAEAGGHRPTFLGDIEQSCVGLMALLPQV
AAAVKIPVIAAGGIMNGRGIAAARLLGAQAARLLGAQAAQLGTAFLCSPESGIAEAWRAALSNAGDDS
TRLTRAFSGRPARGIVNDFMRQMRAEEAQILPYPVQNALTGDIRQAAAKAGRGDFMSLWAGQGVGLAR
PMPAAELVATLAAELEAV
>CORE_REP|Org5_Gene1945#
MPRPITATMHLGAIENNLQVVRRFAPGAKVWAVVKANAYGHGIKHVWRSMAQTDGFAMLDLAEAVLLR
ESGWQGPILLLEGFFQPQDLALLDRYRLTTAVHSDWQLAAIADATLSAPLNVYLKVNSGMNRLGFAPE

RLHEVWRRAQAIANIGELTLMSHFATADGPEGVTQQMATIEAAAADIPLPRCLANSAATLWHSSTHGS
WVRPGIILYGASPSGCWNDVAATGLQPAMTLSSEIIGIQQLKSGDRVGYGGRYSAAGAQRIGVVACGY
ADGYPRHAPTGTPVWVDGVLTRTLGTVSMDMLAVDLTPCPQVELGAEVELWGKRLPVDEVATAAGTLG
YELLSALAARVPVAIEA
>CORE_REP|Org14_Gene2709#
MATDTTHTYPERRFPLFGLPRLVPGLALTGALTALAVWAGDIPWVAELGLGALTLAILFGILVGNTLY
PRWQTVCHGGVQLAKQRLLRLGIILYGFRLTFQQIADVGASGIIIDALTLTTTFLLACWLGKKVFGID
SQTAMLIGAGSSICGAAAVMATEPVLKADSSKVAVAVSTVVVFGTLAIFAYPWLYQLNEHFQWLPFSQ
ETFGIYAGSTIHEVAQVVAAGHAIGPDAENAAVIAKMIRVMMLAPFLLLLSGYISRGGAGKAEKSAIT
IPWFAVLFIAVAGLNSFNLLPATLVQHLITADTWMLAMAMAALGLTTHISAVRQAGMKPILLATLLFV
WLLVGGGAINQLVQHWL
>CORE_REP|Org29_Gene701#
MFGVLDRYIGKTIFNTIIMTLFMLVSLSGIIKFVDQLRKVGQGEYTALSAGMYTLLSVPKDIEIFFPM
AALLGALLGLGQLATRSELVVMQASGFTRLQIAGSVMKTAIPLVLLTMAIGEWVAPQGEQMARNYRAQ
QMYGGSLLSTKSGLWAKDGNDFIYIERVSGDKELSGVNIYHFNDQRRLETVRYAATASFENGLWQLSQ
VDTSDLTNPKQVTGTQTLTGEWKTNLTPDKLGVVALDPDSLSISGLHNYVKYLKQSGQESNRYQLNMW
SKIFSPLSVAVMMLMALSFIFGPLRSVPMGIRVVTGISFGFLFYVLDQIFGPLSMVYSMPPVLGALLP
SMLFLLISVYMLLKRK
>CORE_REP|Org33_Gene2430#
MKKLINQVESVLEEQLQGLAEAHPELLVHADPVFVTRADAPVAGKVAILSGGGSGHEPMHCGFVGEGM
LDGACPGEIFTSPTPDKMYECGQAIDGGAGVLLLIKNYTGDVLNFETATELLHDSGVAVATVLVDDDV
AVKDSLFTAGRRGVANTVLMEKLLGAAAARGDDLDSVVTLGHRINNHGHSIGIALGACTVPAAGKPSF
TLAENEMEFGVGIHGEPGIARRPFTTLNAAVDDMFHTLIDHGHYQRTIRVWDRQQGEWRDEMQTKQPL
TRGDRAIALVNNLGATPLSELYGVYHRLAERCAEAGIIIERNLVGSYCTSLDMQGVSITLLNVDDELL
SLWDAPVKTPALRWGC
>CORE_REP|Org19_Gene495#
MLGDAVFNRVKRSDHKAISEISAFLRSNDLNIDTTVEIFITVTQHDKLVACGGIADNIIKCVAISPLM
RGEGLALALATELVNLAYERHHTQLFIYTKVQNEPLFRQCGFYPIATVPGIVVLMENSPCRLKRYAAQ
LASQRRPGDTIGSIVMNANPFTRGHQYLVRQAAKRCDWLHLFLVKENTSRFSYEDRRRLVLAGTADIP
NLTVHEGSQYVISRATFPCYFIKDQGVADDCYTEIDLKIFRQYLAPALGITHRFVGNEPFCAVTAKYN
RDMRYWLETPALPSPPIALVEIERLQYQGTAISASWVRKLLAAGDFHAAAPLVPPDTLYYLQDLQTQR
RAKAAPHPFESAQSGE
>CORE_REP|Org45_Gene3361#
MAGLKLQAVTKSYDGKTPVIKQIDLDVADGEFIVMVGPSGCGKSTLLRMVAGLERTTSGDIYIDTRRV
TDLEPKDRGIAMVFQNYALYPHMSVYDNMAYGLKIRGFGKDHIRQRVEEAARILELEPLLKRKPRELS
GGQRQRVAMGRAIVREPAVFLFDEPLSNLDAKLRVQMRLELQQLHRRLKTTSLYVTHDQVEAMTLAQR
VIVMNKGVAEQIGTPSEVYQRPASLFVAGFIGSPAMNLLPGTLSADGGQLLLADGMALPLPAAKPQWA
GRPLTLGIRPEHIQLVAQGQGVPLQLQTLELLGADNLAHGQWGGHGVIARLSHETLPAAGSTLYLQLP
AQALHFFDTDSGLRMD
>CORE_REP|Org17_Gene3358#
MFSRFYLKTSLLALALGGVFSVCAAQPVKDAPMGRFAAEQTRHIATYFPGRMAGSPAELLTADYLKQQ
FGKMGYQSDIRSVNTRYLYTSKDGKKNWNNVTASSVIAARNGSSPKQVVIVAHFDTYTPQSDEDLDNN
LGGLTLQGVDDNASGIGVMLELAERLKDIPTAYGLRFVATSAEEIGSLGAQNYLQRMSAEEKSNTVLV
INLDSLITGDRLYFNAGRNTPPQMAKRSRDRALDIAHRYGIAAATNPGNAQHPQGTGCCSDQEVFDAA
GIPVLSVEATNWSLGDKDGYQQRAVSPHFPQGITWHRPQYDNLQYLDRYLPGRIDKRSRDSVQILLPL
IKELAQAHPPKAQKKK
>CORE_REP|Org3_Gene3945#
MIELSVENLHLTYGDNPVLKGVSMDLKRGEVVSLLGPSGSGKTTLLRAVAGLEKPSQGRIVIGNNAVY
NGSARSEIPAEERNLGLVFQSYALWPHKTVFENVAYPLKLRKIASAEITLRVQAVLDQLGLGHLAKRH
PHQLSGGQQQRVAIGRALVYNPPVILLDEPLSNLDAKLREEARVFLRELIIKLGLSALMVTHDQNEAM
AISDRILLLNNGKIEQQGTPQEMYGSPTTLFTAEFMGSNNRLPGKIVALEGDRARIEGKDWALWGKAG
EGVQVGQEGSAVIRVERVRLGEDPQGNQLELPLLTSMYLGDRWEYLFRTVAEDFVVRAYGHEARDRAL
CRLSLPAEHLWIFPKA
>CORE_REP|Org47_Gene2739#

MNDTAQPLQVALIGYGFVGKAFHAPLINAVPGLELSVVASRDAAKVHADLPHAQVIADPLEAIRRPEV
DLVVIASPNDTHAPLALAALEAGKHVVVDKPFTLDLAQARTLIAAAEKHDRLLSVFQNRRWDSDYLGV
KQVIEQGLIGKVTHFESHIDRYRPEVRVRWREQNLPGSGLWFDLGPHMVDQALQLFGLPHSVQANIAT
LRPNAQVNDWAHVVLNYPTHRVILHGSMLVAGGVSRFTVHGEKGSVVKTRADGQESQLLAGVTPGSAE
WGKDDDAMSLFIGTEPVRTLPTPNGDQRQYYFQVRDALRGAGSNPVTAAQALAVMAVLEAATLAAETG
VTQPLPLTEAEIAAW
>CORE_REP|Org26_Gene4161#
MRLDRKISSLELLTYRHYRIVHGVRIGLAFILTFLLIRLLAVPEGTWPLITLVVVMGPISFWGNVLQR
ALQRIAGTVFGAASGLIALYLELYSLPLMLAWCGVVMFVCGYLTLGKRPYMALLVGITLAVVCGAGAG
DMHTALWRSGDVIFGSLLALLFTSIYPQRAYILWRMQMADCLQTAARLYGAYFSPNVIERPRLEPQLK
DLLNQVVKLRSLIVPASKETRIPKAVFEAVQTLSRNLVCTLELMADAYWASRETHFIMLNARTLRSTQ
LLTLSSLESLAVLMREGPQAQRQAAAGQLAEIAGELKTLMQEVSLGQHGEAPIYGYVWLSMELAQQLE
ELGDLLLVCTAAGRE
>CORE_REP|Org14_Gene463#
MKPVCVLGNGQLGRMLRQAGEPLGIAVYPVGIDAEPEAVPYQNSVITAEIERWPETALTRELATHSAF
VNRDIFPRLADRLTQKQLLDQLGLATAPWQLLASAAEWPQVFAALGELAIVKRRVGGYDGRGQWRLRP
GQEAELPADAYGECIVEQGINFSGEVSLVGARGHDGRSVFYPLTHNLHEDGILRTSVALPQPNPALQQ
QAEQMLAAILNELNYVGVMAMECFIVGDRLLINELAPRVHNSGHWTQNGASISQFELHLRAILGLPLP
QPVVSTPSVMVNLIGTAVNEQWLSLPLVHLHWYEKEVRPGRKVGHLNLNDPSAADLRQALQALAPLLP
GEYQSGLAWAQQKLA
>CORE_REP|Org11_Gene1224#
MPVLHNRISNEELKARMLAETEPRTTVSFYKYFSIDDPKAFRDSLYVQFEKLKVFGRIYVAKEGINAQ
ISVPQHHFDAFKAALFASHPALDQVRLNIALEDDGKSFWVLRLKVRERIVADGIDDESFDPSNVGEYL
QADRVNQMIDDPNTVFVDMRNHYEYEVGHFENAIEVPSDTFRDQLPMAVEMLQDSKDKNIVMYCTGGI
RCEKASAYMLHKGFKNIYHVEGGIIEYTRKAKEQGLPLKFIGKNFVFDERMGERISDDVIANCHQCGA
PCDTHTNCKNDGCHLLFIQCPSCAAKFEGCCSEICREELKLPREEQRARRAGRENGVKIFNKSKGLLQ
TTMHIPAPEEEGPAR
>CORE_REP|Org13_Gene3304#
MTSKPEQIRQRVKRGELIAGEDLHGLSFAGMDLAGGMFNELNLSGVNFSDCDLRDSVFSDCRLEHAQF
ARANLKQTAFNQCAMPGGRFCESHIELTMFNDCRLEQSDFSRLPLNQSHWMSCQLAGANFSATQHDRT
TFYESPLDGAALNHARLSLVTFFRLNLCKTEFEGVDFDRVTFFECDHRGKSYAGQRLIACQFTDNQLD
DVDFSQATLRQSNFKGASLRRANLTGVQAQQSLWLEANLTQAQCRSGQFDQAIFSEATLDAANFSQAR
LYQCVFQRSRAARCDFSDSDLTYADFCYADIGAADFRRARFMRTRMHRAHQQQTRWGDRSGILERDEE
LYAAETWSAQRQSRI
>CORE_REP|Org39_Gene3369#
MKRILVTGGAGFIGSAVVRHIIEATDDSVVVVDKLTYAGNLESLAVVAESERYAFEQVDICDRAELDR
VFAQYQPDVVMHLAAESHVDRSIDGPAAFIETNVVGTYTLLEAARHYWQPLAAEKKQAFRFHHISTDE
VYGDLHGTDDLFTETTPYAPSSPYSASKASSDHLVRAWLRTYGLPTLVTNCSNNYGPYHFPEKLIPLV
ILNAVAGKPLPVYGNGAQVRDWLYVEDHARALYQVVTEGVVGETYNIGGHNERKNIDVVQTICELLEE
LAPNKPQGVANYRDLITYVKDRPGHDMRYAIDAGKIDRELDWRPQETFESGLRKTVVWYLNNETWWRR
VQDGSYAGERLGLSE
>CORE_REP|Org39_Gene1106#
MSQKILFIDRDGTLIAEPPEDFQVDRLDKLALEPDVIPSLLALQQAGYQLVMITNQDGLGTASFPQET
FDPPHNLMMQILSSQGIQFADVLICPHLPADNCDCRKPKTALVKGYLEPGVLNAAHSYVIGDRPTDVQ
LAENMGIQGLRYQRGVLGWKEIVRQLTLRDRHARVNRVTKETQIDVNVWLDREGGSKIKTGVGFFDHM
LDQIATHGGFRMEIDVKGDLYIDDHHTVEDTGLALGEALNKALGDKRGIARFGFVLPMDECLARCALD
ISGRPHLEYKAEFNYQRVGDLSTEMVEHFFRSLSYTMGCTLHLKTKGKNDHHRVESLFKVFGRTLRQA
IRVEGNTLPSSKGVL
>CORE_REP|Org20_Gene2219#
MLELDFSQQLGDLNLNVRADLPAQGITAIFGLSGAGKTSLINAIGGLTRLQQGRIALNGRTLVDTAAG
LCLPPEKRRIGYVFQDARLFPHYRVRGNLQYGMAAGMRAQFNTIVELLGIGPLLNRLPLTLSGGEKQR
VAIGRALLTAPELLLMDEPLASLDLPRKRELLPYLERLAQDVNIPILYVSHSLDEILRLAEQVMVLDR
GEVRAFGGLEAVWASSALRPWLQREDQSSVLRVSVIEHHQRYAMTALALGDQRLWVSGIDAELGTQLR
IRINAADVSLVLQPPVNSSIRNVLPAKVSESLDVDGQVEVKLAVGEHVLWARITPWARDELAIRPGQW

LYAQVKSVSISRESR
>CORE_REP|Org34_Gene239#
MSGKPKRLMVMAGGTGGHVFPGLAVAHHLMAQGWQVRWLGTADRMEADLVPKHGIEIDFIRISGLRGK
GLKAQLTAPLRIWQAVRQAKAIMRSYQPDVVLGMGGYVSGPGGLAAWQCGIPVVLHEQNGIAGLTNRW
LARIAAKVMQAFPGAFPNAEVVGNPVRTDVLALPLPAERLQGREGPIRVLVIGGSQGARVLNQTVPEV
AARLGDRITLWHQVGKGALETVLRDYERVGQTQHKVTEFIDDMAAAYAWADVVVCRSGALTVSEIAAA
GLPAIFVPFMHKDRQQYWNARPLEEAGAAKIIEQPQFNADVVAELLASWDRKTLLAMAEKARAVAIPD
ATERVAAELVRLAK
>CORE_REP|Org31_Gene3705#
MNALITKGKRAAFPLMLLSTLLFSNTLLAQTAPEVLRKPVGKGAYEMAYSPSENALYLATSQSRKLDK
GGIVYRLDPTTLDVTQIIHNDIKPFGAAVNAKTGTLFFGNTVNNSVTAIDAKTGDVKGRLVLDARKRS
ETVKPLAPRELVADADSDTLYITGLGESSVVWVVDGKDLTLRATVTDTGKYGTGLALDAAAKRLYVTN
ADGELVTIDTQSNKVLSRKKLDESKEHFFLNISLDTATHRAFITDSKQPQVLVVDTRNGNILSKIDVP
ESLAVLFNPARNEVYVTHRQAGEVSVIDAKSYKVLNTIKTPTHPNSLALSPDGQTLYVSIKQASSREK
EATAPDDVIRVALK
>CORE_REP|Org21_Gene3528#
MTELKNDRYLRALLRQPVDVTPVWMMRQAGRYLPEYKATRAQAGDFMSLCKNAELACEVTLQPLRRYA
LDAAILFSDILTIPDAMGLGLYFEAGEGPRFSSPVTCRADVDKLPVFDPEVELGYVMNAVRTIRRELK
GEVPLIGFSGSPWTLATYMVEGGSSKAFTKLKKMMYAEPATLHLLLDKLADSVILYLNAQIKAGAQSV
MVFDTWGGVLTGRDYREFSLHYMHKIVDGLLRENEGRRVPVTLFTKGGGQWLEAMAATGCDALGLDWT
TDIADARRRVGDKVALQGNMDPSMLYASPARIAEEVETILAGFGHGNGHVFNLGHGIHQDVPPEHAGA
FVEAVHAHSAKYHR
>CORE_REP|Org7_Gene1147#
MISMRRRLLLMLALILLVTQLISAFWLWHESQEQISFLVDETLSAKVRSERVDTEIAEAIASLLAPSL
IMMIVTLLASFWAISWITRPLNQLQQRLEKRSADNLTPLPITSDSQEMVAVTNALNQLFSRLDNTIQQ
ERLFTADAAHELRTPLAGIRLHLELMEKQGVKGSQALIARIDQLMHTVEQLLMLSRAGQDFASGHYQH
FDWVADVIQPLREELDEMTAQRGQTLAWQLPAAAAVNGDPVLLRLLLRNLVENAHRYGPEGGAIQVRL
TPQDRGYLLQVIDDGPGIKEEMVGELTQAFRRMDQRYGGSGLGLNIVIRIVQLHQGRLTLENRRDARG
LNAQCWLPEKALK
>CORE_REP|Org25_Gene1048#
MKRLCASVLTSAIVLSSQMSWAADTDLAALEQAAKKEGEVNSVGMPDSWANWKGTWQDLLSKYGLKHV
DTDMSSAQEIAKFDAEKNNATADIGDVGAAFGPVAVQKGVTQPYKPSTWDQVPEWAKDKDGHWALAYT
GTIAFIINKQQVKDIPHSWADLLKGSYQVTIGDVGTASQAASGVLAATYAMGGNEKNLKPGLEFFGKL
AKAGRLSLSNPVIASLEKGEVQVGVVWDFNGLNYRDQIDKTRFEVLIPSDGSITSGYTTIINKYAKHP
NAAKLAREYIFSDAGQINLARGYARPIRAEHLTLPDDVKAKLLPAEQYKNAHPIADPAAWEQSAKALP
RLWQENVIMFMQQ
>CORE_REP|Org18_Gene3303#
MPQSLNAPEHAPRAWPLWKPILFLLVVAVGLYYVKWQPYYGKAFVAADSHSIGKSILADGAASPWLAA
WQYALVYFTAVWKAALLGVLLGSLVQVLIPRDWLARTLGHRRFSGTVLGALIALPGMMCTCCAAPVAA
GMRRQSVSSGAALAFWLANPVLNPATLVFMGFVLGWPFAAIRLVAGVVMVLGIAWLVQRVTANDPQPA
APQPLVMTAQNEEQPFLVRWGKALWALFWSTIPIYVLAVLALGAARVWLFPHADGAVDNSLLWIIGLA
IVGCLFVIPTAAEIPIVQTMMLAGMGAGPALALLMTLPAVSLPSLLMLNKAFSARALCLTAALVALCG
VLTGVVGMGLGLV
>CORE_REP|Org5_Gene1767#
MGRTRVTDSSLRLAVLLAMLVIILAGVKAAADIVVPFLLAVFLAMVLNPLVTMLERRRVPRILGVTLL
VTAVIVVVMLFIGMLGASLNEFARSLPQYRGMMIEKLRELQHYADRFNISLSSEAMLQYVDPSAAMNL
VTRMLGHLSGAMTNVFLLLMTVVFMLFEVQLLPYKLQQALDKPNEGLAAMRRALDGVTRYLVIKTIIS
LATGVIVWIFLAAVGVRFAFIWGLLAFLLNYIPNIGSVLAAIPPLIQALLFNGLGDALVVAGGFIAVN
MVIGNILEPRVMGRGLGLSTLVVFLSLIFWGWLLGPVGMLLSVPLTIVARIALETTEGGYRLAVILGD
GRPPRQPPAAPE
>CORE_REP|Org24_Gene1188#
MFAKLLRSVVIGLIVAGLLLAALPVLRSSNGLFAEKTENTSDETPVSYNKAVRRAAPAVVNIYNRNLN
GAANVLSLGSGVIMNERGYIITNRHVIKDAQQITVVLQDGRRYEALLVGSDGLTDLAVLKIDPGNLPV
IPTNKNRVAHVGDVVLAIGNPYNLGQTVTQGILSATGRISMSTTGRQTFLQTDASINRGNSGGALVNS

LGELIGINTLTYDKITDGETPEGLGFAIPIELATKIMDKLIRDGRVIRGYFGIQGKEIIPLRSSNSGI
DRLQGIIVTEITPNGPASSAGFQINDIIINVDNKPAVSVLETMDQVAEIRPGTEIPVIVLRDGKRITL
KMTVGEFPEDNN
>CORE_REP|Org15_Gene3416#
MSHAHYLHPEQRDRIRRLYQRWHWRLELPTWGIMAAVYGGWFGVAHYWQALGPWLGAPLLILLTTGYM
SLQHELIHGHPTRRPRVNQLFGLLPLAVWYPYGLYRDSHLRHHRNEHLTDPHEDPESYYFSAAQWRRY
PRLLPLLAAVRNTLIGRVLLGPALDIAATLAAAVKALAGGAWRVWAMWLVHLSLLAALLHWLQAQGIG
TAFYLLAISYPALALTKVRSFFEHRAVDAPQARSIINEAGWPWRLLFLNLNYHLVHHDLPGLPWYGLR
EVYLAEREAYQRRSQGFVAQGYGEWLRDYALTPIDVGVHPLSAGERREERGARPKKKFIARWKRVSQD
FPTELAHEPENI
>CORE_REP|Org6_Gene1135#
MNVATQEILLEPADNQRLLSLCGPFDDNIKQLERRLGIEINRRDNRFKLVGKNLCVVAAADILRHLYV
DTAPIRGVIPDIDPEQIHLAIKESRVLEQVADSVPDYGKAVTIKTKRGMVKPRTPNQAQYIANILDHD
ITFGIGPAGTGKTYLAVAAAVDALERQEIRRILLTRPAVEAGEKLGFLPGDLSQKVDPYLRPLYDALF
EMLGFERVEKLIERNVIEVAPLAYMRGRTLNDAFIILDESQNTTIEQMKMFLTRIGFNSKAVITGDVT
QIDLPRNQKSGLRHAVEVLSDVEELSFNFFHSEDVVRHPVVARVVIAYEAWEAAEQKRKDAIAEQRKR
EALAASEQETP
>CORE_REP|Org43_Gene3638#
MNVPTPTERTLSSAPHAAYASGFKRIAGFGIGLALLLLCIIASLMLGSKAIPFHTVWLSLQGAASGSD
STIILNARVPRTLAGILAGMALGAAGALIQALTRNPLADPGVLGINAGASFAVVIGIMFFGAATTESY
MAYAFVGAAVTTLLVYVIGTLAGGRINPVRLTLAGVAIGAVLLGITTGLSLIDPQTFDQLRFWQAGTL
DIRTLATLPVTAPAILLGCLLTLLIARPLNTIGMGEDLAIALGARVVLTQAIAVLAITLLCGAATATV
GPISFIGLMVPHIARWWVGPDQRWILPYSMLLAPILLLCADVVGRLLAAGELRVSIVAAFIGAPVLIW
LVRRKKTLGGL
>CORE_REP|Org47_Gene4611#
MAAELSASAGAARRYWRWGGRLLGGALSLALTLLGLLLFTFMLSHLAPIDPALQVAGDHASEATYAQV
RHELGLDQPLPVQFWRYLVHLAHGDLGISRITAQPVLSDLLRTFPATVELATCAIILGALCGITLAFL
AVLKPGSWLDNAARLLSLIGYSVPIFWLSLLGLLLFYATLHWSAGPGRLDDIYLYSMEPRSGFVLIDS
WLSGDRDMFYNAIGHLWLPVVALALLSMAGITRLLRAAMLEECNKEYVTLARSKGAGRLRILLRHVFP
NVLGTLITVLSLSYASLLEGAVLTETVFAWPGVGRYLTSALFAADTPAILGATLLIGTCFVLLNALAD
ALTYLVDPRTR
>CORE_REP|Org15_Gene2366#
MNPETTSDKHTPAVDNELDIRGLCCTLWRGKPWIIGIAVLFAAVALIVSYLVKQEWSATAITDRPTVN
ALGGYYSQQQFLRNLDVRTLPAAAAGDQPSIADEAYNEFIMQLAAYDTRRDFWLQSDYYKQRQEGDAR
ADAALLDELINNILFTPRDDKKVPNDGVKLTAETAADANRLLRQYVAFASHRAALHLNEEIQGAWAAR
TTSMKAQVKRQEAVAESVYKRELNTTQQALKIAESQGISRTQTDTPAEQLPDSDLFLLGRPMLQARLE
GLQASGPTYDLDYDQNRAMLATLNVGPTLDEKFQTYRYLRTPEEPVKRDSPRRVFWLILWGAMGALVG
AGVALARRPRS
>CORE_REP|Org19_Gene4814#
MQHLDVTVVSQAISWLQQQPVWLCTVLSTYGSSPRSPGALMAATRDGRYSGSLSGGCVEEDFLRRVAA
GEYQAASQVIRYGEGGMTPNVALPCGGVLDVLIEYLPASEASIAYLRRIAGALEGHHALIKRLTLPNA
CHSLEQSHFTSATQVERRLEQITLHIAAAPRLLIAGLSSVALYCADFAVALGFEVLVCENRPEALDNF
AAELKPGVTLLRQFPAKFIEEGGCHANTAVVALTHDPRMDDLTLMEAIHTPAFYIGAMGSLRNSARRR
QRLQQIAEFTPQELERIHAPIGLPLGSKTPAEIALAVMAAIVQQKNRLPAAGRRRAQCSGTTGTRCCS
SRCSSSFPLSE
>CORE_REP|Org5_Gene2193#
MAYSLQKSTVAKVVGISLVMMLAACSSDQRYKRQVSGDEAYLDAAPLKALNAPSGMILPVQSGNYDVP
ATTLQGNVGKQLDIRPPVQPLALLSGSRAQYAGDSGTLLLENSPQNQNLWSRVVSLLQAKNIAIASRQ
DAGQTLTTDWVKWNRLDEDNQYEGRYQISVQQQGYQQALVVKSVGLQQQGKTEQVTDQSEIQRYNGMM
INTLIEGLDKQDNLASSQTASRFGALDVQSGADDTGLPMLVVRGPYTVVWDRLPPALEKLGMKVGDRS
RPQGTVAVTYKSLSSSDWDALGVKDPELAEGDYKLQVGDLNNRTSLQFIDPKGKPLTQSKNDALVAAF
QSAFSKTSVN
>CORE_REP|Org1_Gene1275#
MTEFNPIDHPHRRFNPLSGQWVLVSPHRAKRPWQGQQETVPTETLPAHDPDCFLCPGNARVTGDRNPD

YRGTYVFTNDFAALMSDTPPAPDSHDPLMRSQSARGVSRVICFSPDHSKTLPELTLPALEQVVTTWQA
QTEELGKHYPWVQLFENKGAAMGCSNPHPHGQVWANSFLPNEAEREDRLQHDYFREHASPLLLDYAQR
ELAAGERIVVNTEHWLAVVPYWAAWPFETLLLPKTAVQRITDLSAAQSRDLALALKKLTSRYDNLFQC
SFPYSMGWHGAPFNGADNRHWQLHAHFYPPLLRSASVRKFMVGYEMLAETQRDLTAEQAAERLRAVSD
IHYREAGAQA
>CORE_REP|Org26_Gene3305#
MSKNKLSKGQQRRVQANHQRRLKRADNKPEPDDSQLGEPQEGVVISRFGMHADVEAPDGTQHRCNIRR
TLRSLVTGDRVVWRPGLGAHEGVKGIVEAVHERTSVLTRPDFYDGVKPIAANINQIVIVSAILPELSL
NIIDRYLVACETLEVEPLIVLNKIDLLDAEARKLVDGMMDIYRKIGYRVLEVSSQTREGMPEFEQALA
GRISIFAGQSGVGKSSLLNALLPPSEEQILVNQVSDVSGLGQHTTTAARLYHFQHGGDVIDSPGVREF
GLWHLEPEQITRGFVEFRDYLGGCKFRDCRHDTDPGCAIRAAMEKGDIAEERFDNYHRILESMAQVKV
RKNFTDAAD
>CORE_REP|Org30_Gene945#
MSKIRVLCVDDSALMRQLMTEIVNGHADMEMVATAPDPLVARDLIKKFNPQVLTLDVEMPRMDGLDFL
EKLMRLRPMPVVMVSSLTGKGSEITLRALELGAVDFVTKPQLGIREGMLAYSELIAEKIRTAARARLP
QRSNSPAPAILSHAPLLSSEKLIAIGASTGGTEAIRQVLQPLPATSPALLITQHMPPGFTRSFAERLN
KLCQITVKEAEDGERVLPGHAYIAPGDRHLELARSGANYQVKLHDGPAVNRHRPSVDVLFRSVAQYAG
RNAVGVILTGMGNDGAAGMLEMHRAGAYTLAQNEASCVVFGMPREAIAGGGVSEVVELDRMSQRMLAQ
IAGGQALRI
>CORE_REP|Org12_Gene3136#
MTDIVQLLGKEAEDLLQHRCTTIPAENLYLPGDDFVDRVMIDNNRPNSVLRSMQTLFNHGRLAGTGYL
SILPVDQGIEHSAAASFAANPLYFDPKNIVELAIEAGCNCVASTYGVLASVSRRYAHKIPFLVKLNHN
ETLSYPTQYDQTLYASVEQAFNMGAVAVGATIYFGSEQSRRQIEEISIAFERAHELGMVTVLWAYLRN
PAFNKDGVDYHSSADLTGQANHIAATIGADIVKQKMAENNGGYRAVKFGYTDDRVYNKLTTDHPIDLV
RYQLANCYMGRAGLINSGGAAGENDLQESVRTAVINKRAGGMGLILGRKAFKKSMKDGVALINAVQDA
YLDKKVTIA
>CORE_REP|Org33_Gene2369#
MLYQLSVIFVLAVAWVLIARRAALVVGLVDKPNARKQHLGHIPLVGGIAVYLTLTLMTLWQPAWLPDS
AVYLLCVTALVVLGVLDDRFDLPVAPRVMVQGGIALAMMLAAGMQLSSLGYVWGHQEVMLGYGALLLT
PLAVWGAINAYNMVDGIDGQLGALSCVTFVALAILFGLGGREDLALWCLGLIVALAAYLLFNLSLFGA
RNKIFMGDAGSMVIGFSVLWLVLLATQGPQSVMRPVTALWLIAIPLMDMVTVMVRRLLRRQSPFKAGR
DHLHHILMRRGLNARQALAMSTMLAVTLACVGICSEVLKLQESLMLVAFLLCFCGYFAMLREPRQATV
LRDNPSVGR
>CORE_REP|Org12_Gene3606#
MTLVAKLRALAMKNWLTGVALSATLLLTGCGPEQVDLMGKTMGTSYSIRYVTGEDTPSAREMQAEIDK
RLEQVNDQMSTYRPDSELSRFNASRDIDRPFPVSPATAEVVREALRINRVTDGALDVTVGPLVNLWGF
GPEGRPDKVPSEAELAHRRAWTGADKLSVQGNALVKNIPELYVDLSSIAKGYGVDVVAEYLQSQHVQN
YMVDIGGEVRTRGRNGEQKPWRIAIERPTAGTQQQAQLVIQPGEMSIATSGDYRNYFEQDGVRYSHTI
DPITGRPINHRLVSITVLSPTCMTADGLSTGLNVMGPERGLALANLLGIPVFMIVKTADGFEERYSDA
FKPYLKKRS
>CORE_REP|Org20_Gene4467#
MSKELALRPALPWRQQLFDFLYKWGMLLTVAALIALFGLASDNFLDANNIINILRSIAIVTVIAIGVS
ISLSVGGFDLSVGSTASLANALVISLFVWHGFGTTGAIVVTLLLCTLVGLFNALLIVVFRIPDMLATL
ASLFVIQGVAMTYSYGGSITQNMVLPNGDMAEGLIPEVFSALGQVPVIVLIMLAVTVAVQLFLSLTKH
GRRMYAIGGNPEAARLSGIRTVRYRVAAYVISSWLAALGGILLASRIGSSQVNAGGGYLMDAVAAAYI
GFSLAGAGKPNALGTLIGAVILGVLQNGLVMLSVPYYAMDIIKGLVLALALAITYIQKRCSSPAHARC
AAFPFNSSG
>CORE_REP|Org38_Gene2549#
MKILVIGPSWVGDMMMSQSLYRTLKAEYPTAEIDVMAPAWCRPLLARMPEVNQALAMPLGHGALGLGE
RRRLGRALRANRYDRAYVLPNSFKSALVPFFADIPQRTGWRGEMRYGLLNDVRVLDKAAFPLMVQRYV
ALAYDKGRVQRADDLPQPLLWPQLRVSDEEIAETTSAFNLTDSRPIVGFCPGAEFGPAKRWPHYHYAA
LAQRLIESGYQIALFGSAKDHEAGEQIRAALQDDARDFCLNLAGKTQLEQAVILIAACRAVVSNDSGL
MHVAAALNKPLIALYGPSSPDFTPPLSDKARVIRLISGYHKVRKGDAEQGYHQSLIDIQPQQVLDALT
PLLVASEE

>CORE_REP|Org46_Gene2850#
MTAQPQVLKIRRPDDWHIHLRDDEMLKTVVPYTSQVFGRAIVMPNLVPPVTTVAAARAYRDRILAAVP
QGHNFTPLMTCYLTNSLAASELVNGFEQGVFTAAKLYPANATTNSSHGVSDVTGIYPLFEQMQKIGMP
LLIHGEVTDPAVDIFDREARFIEQVMEPIRQQFPELKIVFEHITTKEAAQYVQAGNRFLGATITPQHL
MFNRNHMLVGGIRPHLFCLPILKRNVHQEALRQAVASGSDRFFLGTDSAPHLKHRKESSCGCAGCFNA
PNAIPAYAAVFEQLGALAHFEAFCSLNGPRFYGLPLNEDFIELQRVPTTQPEEIALGNESVIPFLAGE
TLNWSLKD
>CORE_REP|Org40_Gene4771#
MSQKNFVELRNVSKRFGSNTVIDNITLTIPRGQMVTLLGPSGCGKTTILRLVAGLEKPSDGQIFIDGE
DVTHRSIQQRDICMVFQSYALFPHMSLGENVGYGLKMLGIPRAEVKARVQEALAMVDLAGFDDRYVDQ
ISGGQQQRVALARALILKPKVLLFDEPLSNLDANLRRSMREKIRELQKQFDITSLYVTHDQSEAFAVS
DTVLVMNKGHIMQMGSPQDLYRQPASRFMASFMGDANLFPAGFSADHVDISGYRLPRPAHFAAEGAGT
VGVRPEAITLSEHGDESQRCVIQHVAYMGPQYEVTVAWHGQQILLQVNATRLQPNVGEQYYLEIHPYG
MFMLADAA
>CORE_REP|Org5_Gene898#
MEFISVYGLFLAKVATVVLAIAALAILAVSLGQRKSRQKGELQLTDLGEQYREMQRDMRLARMDAAEQ
KVWLKQFKKQTKADDKLKKQRAKSGAVEVAKPCLYVLDFKGSMDAHEVTSLREEISAVLAVASAQDEV
LLRLESPGGVVHGYGLAASQLERLRKGGIRLTVAVDKVAASGGYMMACVADRIVAAPFAIIGSIGVVA
QIPNFHRLLKKNDIDVELHTAGQFKRTLTLFGENTEQGREKFREDLNETHELFKRFVHEQRPSLDIDS
VATGEHWFGSQAKEKGLIDAIGTSDDLLIAELDNHEVIAVRYSRRKRLMDRFTGSAAESVDRLLLRWW
QRGEKPLL
>CORE_REP|Org22_Gene3644#
MILEGAYQLPGRAAREAARLLVNESGEGVTLQRQDEQRYIPLADITVSAALGNIPLTLTFSDGARFVP
DDDAAFRRWFYQRRSPGWVHRLERHKRGVALALLMTLFTVVAYVYVVLPWASSEIAMRIPTSVEQTLG
EHTQTFLSQSGFEPSALPAERQQALQQLFRQVMPAEMREERTPLSLKLMAVPGAPNAFMLPDGTLVLS
DSLVALAKNDDALAAVMLHEMGHHAHRHPMRMLVRSSLVALTLMWMTGDVSGIGDTLLQSASFVNEMQ
FSRGMEREADAFAIAQMQRQGRSLAEMAAIYRALAQAPERETADGWTLPAWLSTHPAMQERLEAVNEA
MAQPPRQQ
>CORE_REP|Org17_Gene2268#
MTSFTLSSDDWLTRETQFAAFATGPLLDFWRQREEGEFSGVDGVPIRFVRFRSARHQRVVVVSPGRIE
SYVKYPEVAYDLFHCGYDVVIVDHRGQGRSGRLLADTHRGHVVNFADYVDDFEQLWLREVESRGYRQR
FALAHSMGGAILAQFLQRRPQAFDAAAFCAPMFGIQLPMPGWLADRILDWVETRPAIRDYYAVGTGQW
RPLPYVVNVLTHSRERYRRSLRYYADYPELQVGGPTYHWVRESIRAGRQIIAQAGKITTPLLLLQAGE
ERVVDNRSHQAFCQALSDAGRPCEGGLPWVINGARHEILFERDAMRAEALNAILRFFAQHLGGALPPT
TPSEVRT
>CORE_REP|Org3_Gene3461#
MTSPAIRLTQYSHGAGCGCKISPKVLETILHSEREKFVDPRLLVGNETRDDAAVYDIGNGVGIISTTD
FFMPIVDDPFDFGRIAAANAISDVYAMGGKPIMAIAILGWPIAKLPPEVAQQVIDGGRFACQQAGIAL
AGGHSIDAPEPIFGLAVTGVVNTERVKKNSAAQAGAKLYLTKPLGIGVLTTAEKQSKLRTEHQGLATE
VMCRLNKPGADFAEVAGVTAMTDITGFGLLGHLSEVCQGSGLQATVWFDRVPKLPDVEAYIAEGCVPG
GTGRNFESYGHLVGEMSDLQRQLLCDPQTSGGLLLAVLPEAEAQVLAIAGQHGITLNAIGELHVASAD
KPLIEVV
>CORE_REP|Org29_Gene14#
MFKKFRGMFSNDLSIDLGTANTLIYVKGQGIVLNEPSVVAIRQDRAGSPKSVAAVGHDAKQMLGRTPG
NIAAIRPMKDGVIADFFVTEKMLQHFIKQVHSNSFMRPSPRVLVCVPVGATQVERRAIRESAQGAGAR
EVFLIEEPMAAAIGAGLPVSEATGSMVVDIGGGTTEVAVISLNGVVYSSSVRIGGDRFDEAIINYVRR
NYGSLIGEATAERIKHEIGSAYPGDEVREIEVRGRNLAEGVPRGFTLNSNEILEALQEPLTGIVSAVM
VALEQCPPELASDISERGMVLTGGGALLRNLDRLLMEETGIPVVVAEDPLTCVARGGGKALEMIDMHG
GDLFSEE
>CORE_REP|Org7_Gene2737#
MTANTTSSEQSLQDYLDQGLRGMWYPVLASWEVGNNPVGITRLEQQIVVWRDGEGQIHALEDRCPHRG
ARLSMGWNLGDRIACWYHGVEVGGDGTVKDVPAVDRCPLVGQKCLRAYPAKEAYGAVFLYFGVTADEA
PAELTFPQELADEASYSHFLCTASWNCNYQYALENVMDPMHGTYLHSSSHSMAEGDRKADMGLEPTDS
GFIFKKNGQIGVNFDWVEFGNSGAYWMRLSIPYKKRFGPGGHFWIIGMVVPEDKDHCRVFFWRIRKVK

DWQRDMWRFMYRNRLESLHWDVLEQDRIVLENMAPNARGREYLYQHDVGLSRLRRLMQKEAQKQLATL
REREAAQ
>CORE_REP|Org15_Gene3628#
MKKWSHLLAAGMMALSFCSANASDGKTLYFYNWTEYVPPGLLEQFTKETGIKVIYSTYESNESMYAKL
KTYKDGAYDLVVPSTYFIAKMSKEGMLQKIDKSKLSHFKDLDPTLLNKPFDPNNDYSIPYIWGATAIG
VNSDAQDPSTVTSWADLWQPQYKGRLLLTDDAREVFQMALLKLGYSGNTTDPKEIEAAYNELQKLMPN
VLAFNSDNPGNPFMEGEVNVGMVWNGSAFVARQAGTPLEIVWPKEGGIFWMDSLAIPANARNVEGALK
LIDFLLRPEIAVQVAETIGYPTPNLAAKKLLSPEIANDPSLYPDKTVIEHGEWQNDVGDASTLYESYF
QKLKAGR
>CORE_REP|Org8_Gene1779#
MASVKKNKRITISDIATLAGVSKSTASLVLNGRSKEYRVSDDTRDRVLALAHEHHYQPSIHARSLRSN
RSHTLGLVVPEMTNYGFAVISRELETLCREAGLQLLIACTDENPAQEMMAVNSLVQRQVDGLIVASSQ
LNDAEYQKINAGLPVVQMDRLIAGSELPLVITDSVNSTADLVEKVARQHPDEIYFLGGQPRISPTRDR
LAGFQLGLERAGITCKPEWIINGNYHPSSGYEMFAQLCAQLGRPPKALFTAACGLLEGVLRYLTQHQL
MESDIHLCSFDDHYLFDCMTLKIDTVAQDCLALAQHSFDQVTALIDERPLEQSALYLPGRIHWRHAGS
RALLAGE
>CORE_REP|Org11_Gene1048#
MLTMSTAAAPDGEPYQLTQLQNAAGMTVTLMDWGATWLSAVLPLKSGEKRELLLGCRSPADYPRQGAY
LGATVGRYANRIANASLPIDGTPHALAANQGVHQLHGGPDGFHARRWRRVQQDAQQVCYALHSAEGDQ
GFPGNLDVQVCYRLTPDNRLEISYLAQVDRPCPVNLTNHAYFNLDGAGTDARAQRLQLFADRYLPVDA
EGIPCADLTPVDDSGMDFRQPKTLLQDFLRDRDQQRVKGYDHAFLLHRTCGALESPAAHLWSADGQVQ
MSVFTDAPALQLYSGNFLAGTPARDGGSYANHAGVALESEFLPDSPHHPEWPQPDCWLQPGSRYRSAT
HYQFYPI
>CORE_REP|Org34_Gene2352#
MRIEEDLKLGFKDVLIRPKRSTLKSRSEVELERQFTFKHSGCSWSGVPIIAANMDTVGTFRMAEALAS
FDVLTAVHKHYSVEQWADFVQRMPESVLRHVMVSTGTSEADFTKMQQILALSPALKFICIDVANGYSE
HFVAFLQKAREACPNHVICAGNVVTGEMVEELILSGADIVKVGIGPGSVCTTRVKTGVGYPQLSAVIE
CADAAHGLGGQIVSDGGCSVPGDVAKAFGGGADFVMLGGMLAGHDECEGTVVEEKGEKFMLFYGMSSE
SAMKRHVGGVAEYRAAEGKTVKLPLRGEVEFTVRDILGGLRSACTYVGAERLKELTKRTTFIRVAEQE
NRVFGSK
>CORE_REP|Org9_Gene3885#
MTRSARRTVLAAWLALGGVLMPGAPQAAQEVTLAAQEVTLAASGRDPAQFPAYVVQLKRRAREQGISQ
GTLDRAFAQIHFVDRVIKADRNQPEQKVTLDDYLRRVMSPAKVRQGRELYRQRQAQWARASERYRVPG
RYIIALWGMESAYGKIQGREDVVSALATLAFEGRREAFFSQELMAALRIVEQGHVGDTPLKGSWAGAM
GQCQFMPSSFLRYAADGDGDGRIDIWNNIDDVFASTASYLSKEGWQPGIGWGREVKLPAGFKRSELGL
KDAQARSVNAWQQRGVRRADGSALPHAVQRGWIIAPDDLQGRTFLVYDNFRTLMHWNRSYYFAIAVGM
MADAIGH
>CORE_REP|Org39_Gene2294#
MQYHRIPHSSLEVSVLGLGTMTFGEQNTEADAHAQLDYALAAGVNLIDTAELYPVPPRPETQGLTESY
IGSWIKARGNREKIVLASKVSGPVRGTDSSIRPQQALDRKNIRAALDASLKRLNTDYLDLYQLHWPQR
ATNCFGKLNYQYTDDKATVTLLETLEALTEQVRAGKIRYIGVSNETPWGVMRYLQLAEKHELPRIVSI
QNPYSLLNRSFEIGLAEISQHEGVELLAYSSLAFGTLSGKYLNGAKPAGARNTLFTRFNRYSGQQTQL
AIAEYVALAKKHGLDPSQMALAFVRQQPFVASTLLGATTVEQLKINIDSLDVVLDEDVLQALEEIHTR
FTIPAP
>CORE_REP|Org47_Gene3432#
MKLMRTTVASIVAATFSLTTVSAFAAASLTGAGATFPAPVYAKWADSYQKETGNKVNYQGIGSSGGVK
QIVANTVDFGASDAPLSDDKLAADGLFQFPTVIGGVVLAVNIPGIKSGELTLDGKTLGDIYLGNVKKW
NDPAITKLNPGVKLPDQNIAVVRRADGSGTSFVFTSYLSKANAQWKEKIGAGSTVNWPTGLGGKGNDG
IAAFVQRLPGSIGYVEYAYAKQNNLAYTKLVSADGKPVSPTEESFSNAAKGVDWSKTFAQDLTDQKGD
NVWPITSTTFILVHKEQKNPAQGAEVLKFFDWAYETGAKQANELDYATLPAEVVEQVRAAWKTNVKDS
SGKALY
>CORE_REP|Org16_Gene4632#
MSALTPASEVMLRHSDEFIERRVLFAGDLQDSLPAQFEAADVRVHTQQYHHWQLLNRTMGDNVQFGLT
VDPAFVADCDTLVYYWPKSKQEAQFQLCNLLALLPVGADVFVVGENRSGVRSAEPTLEGHATLVKIDS

ARRCGLYHGRLDAQTEFDLNDWWDSYQLHDLEVKTLPGVFSRDGLDVGSSLLLSTLEKHMKGKVLDIG
CGAGVMASVMSKLSPKVKLTLSDVNAAAVESSRATLAANGIEGEVIVSNVYSDITGRFDMIISNPPFH
DGLQTSLTAAETLIRGAVKHLPIGGRLRIVANAFLPYPDILDATFGSHEVLAQNGRFKVYQATVGRPP
RAPKKK
>CORE_REP|Org40_Gene2778#
MSNVKIEKPLSADSTGKGGLFSGLSGKMPKDTGIFIVMIGIALIFEILGWYMRDQSFLLNPNRLLLIV
LQVAIIGIIAVGVTQVIITTGIDLSSGSLIALTAVVAASLAQTSDSISPMYPGLLDLPAAIPIGAGIG
VGIVCGFINGFLITRTGIPPFIATLGMMVSARGLAQYYTKGNPVSFLSDGFTSIGQGAMPVIIFLVIA
VIFHIALKHTRYGKYIYAIGGNMTSARVSGINVNKYLVTVYTIAGGLAGLAGVVLAARVSSGQSSMGM
SYELDAIAAAVIGGSSLMGGVGRITGTLIGAVILGLIKSGFTFIGVDSYIQDIIKGVIIVAAVSIDMR
RNRKKH
>CORE_REP|Org1_Gene4118#
MSTESASLKNHNTFALPVNAAHLIMADRIELMLKVWQQTRKRQEPLLILGEGSNVLFLEDFSGTVMVN
QLKGIDVREDNDAWYLHVSSGENWHDLVQYTLQAGICGLENLALIPGLAGSAPIQNIGAYGVELKDVC
EYVDLLDFSTGAIDRIPAAECGFGYRESIFKHRFQTGHVIVGLGLRLNKQWQPKLSYGDLAKLEPTTV
TPLQVFESVCAMRRSKLPDPRETGNAGSFFKNPLVNAEKAAELVAKYPGMPHYPQQDGQVKLAAGWLI
DQCELKGYRIGGAAVHRQQALVLVNIDNAHSQDVVALARHVRKTVADKFGVWLEPEVRFIGATGELNA
VAVLS
>CORE_REP|Org4_Gene1178#
MTDKTSLSYKDAGVDIDAGNALVDRIKGVVKQTRRPEVMGGLGGFGALCALPQKYREPVLVSGTDGVG
TKLRLAMDLKRHDTIGIDLVAMCVNDLVVQGAEPLFFLDYYATGKLDVDTAASVITGIAEGCKQSGCA
LVGGETAEMPGMYHGEDYDVAGFCVGVVEKSEIIDGSKVQSGDALIALGASGPHSNGYSLVRKILEVS
NTDPTATQLEGKPLADHLLAPTKIYVKSVLELIEKADVHAIAHLTGGGFWENIPRVLPEGMQAVIDEA
SWQWPAVFTWLQQAGNVSRHEMYRTFNCGVGMVIALPEAEVETAIALLTAAGEKAWKIGKLTASSDEQ
QVVIN
>CORE_REP|Org18_Gene4103#
MANIRDVARLAGVSISSVSNLLNNRSHQMSAQTRERIEQAMATLGYRPARTAALPAPQAKIIGLLLPS
IVNPSFSALAHAVDGAARAHRYRVLLGNAYRQEQEEAAFIDDMFLHGVRGIIVAASDIRQTHFVRAAE
RGMKIVSYDSPFAEPMATDTRLFDSVSMDNIAAGRLAAQHLLERGCRHIVFATEATLTVGRSHKIDGF
LSALGHSLSERQRVIEGKANSAYGDTEMFELGLTLAPRVLALTPRPDGIVAINDALGIGLMVGLRAAG
VQVPADISVIGIDNIALADLAEPGLTSVRPPLAEMAQLMVERLIGRINDDAQPPGEFLFPPTVISRRS
VKAAG
>CORE_REP|Org33_Gene3792#
MPPVHPITIRDVAKRAGVSVATVSRVLNHSALTSKETREQVLQAVAELGYRPNANAQALATQSSDTLG
VVVMDVSDPFFGALVKAVDTVAQKHHKYLLIGNSYHQAGKERHAIEVLIRQRCNALIVHAKALSDAEL
IGFLEQVPGMVLINRIIPGYEPRCVGLDNVCGAEMAMRLLLSQGHRRIGYLGSNHPIEDGPLRQQGYA
QAMAAAGLATPDNWRAYGSPDLQGGEAAMVELLGRNLQLSAVFAYNDAMAAGAMAVLKENGITVPQHF
SLIGFDDIPIARYTSPKLTTVRYPIVSMATLATELALQGAAGLAEPQAAHLFMPTLVRRHSVAPWQSE
ATVTL
>CORE_REP|Org22_Gene1341#
MADRIHWTVGQAQALFDKPLLELLFEAQTVHRQHFDPRQVQVSTLLSIKTGACPEDCKYCPQSSRYKT
GLESERLMQVEQVLESARKAKANGSTRFCMGAAWKNPHERDMPYLQQMVQGVKAMGMETCMTLGTLDG
TQAERLAEAGLDYYNHNLDTSPEFYGSIITTRSYQERLDTLDKVRDAGIKVCSGGIVGLGETVRDRAG
LLVQLANLPKPPESVPINMLVKVKGTPLADNDDVDPFDFIRTIAVARIMMPSSYVRLSAGREQMNEQT
QAMCFMAGANSIFYGCKLLTTPNPEEDKDLQLFRKLGLNPQQTATEHGDNQQQQALAKQLLNADTAEF
YNAAP
>CORE_REP|Org10_Gene4169#
MSLPHTLHIGRPGGVINWRMPLRLLLVNLSLLALCLAMAVAALCYGTLQLSLEQVFAALSGEAPKNLV
TVVTQWRLPRIAMALLLGGALGMSGAIFQSIIRNPLGSPDVIGFNMGAYTGALIAITLFNGGYYYIAG
GALAGGILAALAIYLLAWRQGIAGFRLIIVGIAISAVLVSTNTWLIITASLERAMDAAMWQAGSLNGM
TWQKAQPATAFIVLAAAAALLMGKRLQLLEMGDDTARALGVNAEGSRLWLMLFGVTLTAAVTATAGPI
SFIALAAPQIARRLAGQSSVTLTSSALMGAALLLSADVVSQHLFAPIQLPVGVVTVCIGGLYLIWLLI
REARR
>CORE_REP|Org43_Gene3170#

MIRIYPEQLAAQLREGLRACYLLSGNEPLLLQESQDLLRQAAQQQQFSEHYSISLDAHTDWDAIFGIC
QAMSLFASRQTLLLIFPENGPTAPIGEQLTKLATLLHEDILLILRGPRLTKAQENSAWFKALSPHGAL
VSCQTPEQAQLPRWVATRAKAMKLELDDAANQLLCYCYEGNLLALSQALERLSLLHPDGKLTLPRVEQ
AVNDAAHFTPFHWLDALLAGKSKRAWHILQQLQQEDVEPVILLRTLQRELLLLLTLQRRMASAPLRTL
FDQHKVWQNRRPLVTQALQRLSSAQLQQAVQLLTQIELTLKQDYGQSVWPELETLSMLLCGKPLATSF
TDAH
>CORE_REP|Org26_Gene2881#
MGNYLKFRPDGTAVGLLALLVAVVLAFSLLMPGRFFSGATFTSVAFQLPELGLLTLAMFIPILSGGLN
LCIIASANLTSLLMAWLFISYLPPDAGLGLQALWLVLALAAAMLLAVTIGAATGALVAYVGAHPILVT
LATMTTVNGIGIYLTRGAALSGMPEIVRFIGAERVLGVPVPLLIFLAVAVLLALFLQKTRLGKCIYMS
GSNINATHFSGVNTHRVLIAIYTLSSLLCVIAGLVMMARFNSARMGYGDSYLLLTVLAIILGGTDPFG
GFGRVSGVVLALIVLQVIATGLNLMNVSPHFSLAMWGAVLIAVLALKFFRHRYRQRRAMRRSAAQARA
AAGH
>CORE_REP|Org22_Gene2198#
MMIIRPIERRDLADLLTLAGKSGIGLTSLPQNEDTLSARIERALKTWQGELPQSDQCYLFVLEDSERR
QAVGVCAIEVAVGLAEPWYSFRVGTQVHASKQLNVYKSVPTLFLSNDHTGHSELCTLFLDPDYRHGEN
GKLLSKVRFLFIAAFRERFSRRLIAEMRGFSDENGRSPFWESVGHHFFSIEFAKADYLSGTGQKAFIA
ELMPKHPLYVDFLAEDAQKVIGEVHPQTLPARRLLEAEGLSYQGYVDIFDGGPTLEAEIDHIRAVKQS
RLVKVVLDDTPMRADAPVHLVANDNYQNYRALLVNADLYDDRLHINAATAAALGVEQGSPVRVLPLIA
QEKA
>CORE_REP|Org13_Gene3324#
MKRINRYYDAAKAHHTPEGFRNPEPSQRQEGDLQRWQDERKRQGLPRPPQQGYAQFTERWWQPADLSG
SDDSIWWLGHASMLLRLGGRYILIDPVLSERASPLSFYGPKRRTPAPLTVEQLPAVDAVLISHNHYDH
LDRRTVRQLARRFPQAEFIVPLGLKRWFRRYRLKVHELDWWQSLSLGELTVYATPARHWSMRTLWDRN
RSLWCGWVIHHPALRFYFSGDSGYSARLAEIGQRLGPFDVAALPIGAYAPRWFMQEQHMDPQQSVALY
RELNQPRAIPIHWGVFELADESLDEPPQQLNLALSEAGLEQHQFHPLKIGERIALQDSQQALSIRPVV
ERKE
>CORE_REP|Org23_Gene1311#
MTRLTLALDAMGGDFGPCVTVPASLQALASNLQLHLLLVGNPDVISPLLAHADPVLLERLQVVPAESV
IAGDAKPSQAIRASRGTSMRIALEQLSSGNAQGCVSAGNTGALMGLAKLLVKPLDGIERPALMTAIPN
QQRSKTVVLDLGANVECDSTMLVQFAVMGAVMAEEVIGIAQPRVALLNIGEEETKGLDNIREAAAVLK
NTPAINYIGYLEGNELLTGKTDVLVCDGFVGNVTLKTMEGVVRVFLSLLKSSGDGNKQAWWLKLLGRW
LQKRVVKRFGHLNPDQYNGACLLGLRSTVVKSHGAANPHAFAVAIEQAVQAVQRQVPERIAARLEAVL
PKSD
>CORE_REP|Org24_Gene2291#
MSPSSQQNRRFLLASRPHGEPTAANFRLDTVPAPQPGAGQLVLRTVYLSLDPYMRGRMSDAPSYAPPV
EIGQVMVGGTVSRVAASQHPDFNVGDWVLGYDGWQDYALSDGSGLRNLGPHLPQPSRLLGVLGMPGFT
AYMGLLDIGQPQAGETLVVAAASGAVGSVVGQIGKLKGCRVVGVAGGAEKCRYVVEELGFDACIDHRA
PDFAEQLAAACPKGIDIYYENVGGAVFDAVLPLLNTKARIPVCGIIAHYNATGLPAGPDRLPLLEGLI
LRKRIRMQGFIIFDDYGSRFDEFLQQMSSWVEEGKIKFREDIVDGLEQAPQAFIGLLQGKNFGKLVIR
VADE
>CORE_REP|Org12_Gene1068#
MKKTWVTTLIASGIALATLSGAAHAKGRLVVYCSATNEMCEAETKAFGEKYDVKTAFIRNGSGSTLAK
VDAEKKNPQADVWYGGTLDPQSQAGEMGLLQPYKSPNLEQVMTQFRDPAKLKGNYSSAVYVGILGFGV
NTQRLKEKNLPVPKCWKDLTKPEYKGEIQIADPQSSGTAYTALATFAQLWGDDQAFAYLKQLNANVSQ
YTKSGIAPARNAARGETAIGIGFLHDYSLEKEQGAPLELISPCEGTGYEIGGVSILKGARNLDNAKLF
VDWVLSKEAQELAWKKGKSYQILTNTTADTSPNSLKLDDLKLINYDMDKYGSTEVRKALINKWVSEVK
MGK
>CORE_REP|Org48_Gene1493#
MSIKKITITDVAQQAGVSVTTVSLVLSGKGRISPTTVEKVNQAIEQLGYVRNRQAATLRGAESGVIGL
ILRDICEPFYAEMTAGLSEALEAHDKLLFLTQSGRDGQGLQRAFDALLAQGVDGIVLAGGIRAAAGLK
EKAAEQGVPLVCVARSSGLEGVDVVRPDNMQAAKLATEFLIKRGHSQIAYLGGQSDSLTRAERLGGFC
ATLVQYGLPFRSEWIVECDCRQREAAEAAEQLLRHYPNITAIVCHKASVALGAYFGLTRSGRSIGSDG
VDAYYGRQVALIGFGDVPEAELTEPPLTFVSSSAREVGRSAAARLLQRIGDADLPAQNVILPPTLIRR

GSA
>CORE_REP|Org47_Gene1406#
MAHIITQSTAHREDWLHQLADVITDPDELLQLLSLNTHPELPQGRDARRLFALRVPRAFAARMRPGDA
NDPLLRQVLTAREEFINAPGFTTDPLDEQRSVVPGLLHKYRNRALLLVKGGCAVNCRYCFRRHFPYQD
NQGNKNNWRQALDYIRQHPELDEIIFSGGDPLMAKDSELEWLVGELEAIPHLKRLRIHTRLPVVIPAR
ITPALCRLLSASRLQVLMVTHINHANEIDRDLQSAMAQLRLAGVTLLNQSVLLRDVNDDADTLAALSN
ALFDAGILPYYIHVLDKVQGAAHFMVSDDEARAIMQALLSKVSGYLVPRLTREVGGEPSKTPIDLRLM
QE
>CORE_REP|Org45_Gene3184#
MQALNTTSLTLQDNRLWILDQQALPQEKRWRACDSVEELVGHIHSLRVRGAPLIGLSASLLLALLAER
GLPRAELERALHTLRAARPTAVNLMNNLDRMKLALAEPDWAPAMVNEALRLVEEDRRLCDRIADHGAG
LVKPGSRLLTHCNTGGLATAGVGTAIGVLLHAHRQGKVQQVWVDETRPLLQGGRLTAWELGELGIPYR
LICDSMAASLMAQGQVDAVWVGADRIAANGDVANKIGTYSLAVLAHYHGIPFYVAAPHTTHDPHCPDG
AAIPIEQRAAAEVTGVSGSFGACQWAPNDAPVYNPAFDVTPAKLISGWIFDSGVITPQQVEAGIFQRA
LG
>CORE_REP|Org48_Gene4050#
MVQRLAEKMHKLDFDDEVTGLIYGHVFRSGEVPARLNSQQVCQAYQALPPGDGFVWLHLNLNHAAAEK
WLKNHFAISDFFFHEVRHGSHTTRIERQGDDLFAVLNDVIFHPEDSNPETATLWLYCCRGLVVTVRHK
PVRLIERLLGRLTALQLASSTELLAHLLEEQEDVLEQVVRQANQYVDTIEDRLLTSRVKRNRAELGRM
RRMLLRFQRLLAPEPAALFRLLNRPPTWLSREVVQDLRQFTEEFTVVLNDLASLTERIRLLQEELSAK
LMEQNNRTLYTLTVITVLALPINIVAGFFGMNVGGIPLASNHHGFILLVLLVGSFTLIAGYLAFRRRD
ET
>CORE_REP|Org33_Gene1231#
MTIAVQFIDVSRTFGDVRAVDRVSIDIQDGEFFSMLGPSGSGKTTCLRLIAGFEQLTSGSIRIHGQEA
ANLPPYQRDVNTVFQDYALFPHMSVLENVAYGLMVKGVAKRERLARAQEALESVALGFVAERKPAHLS
GGQRQRVALARALVNRPRVLLLDEPLGALDLKLREQMQGELKKLQRQLGITFIFVTHDQSEALSMSDR
VAVFNNGRIEQVDTPRELYMRPKTPFVAEFVGTSNVVRSELAQRLLGESRTFSIRPEHIRLLEHGGAA
QDEIQVQGTLQEIHYQGAATRYEIALNGGEKLLVSQANPQWIAEGQQRQIGQPIVACWPRAAMVPLLE
ER
>CORE_REP|Org42_Gene845#
MKALSKLKAEEGIWMTDVPQPELGHNDIMIKIRKTAICGTDVHIYNWDEWSQKTIPVPMVVGHEYVGE
VVAIGQEVKGFSIGDRVSGEGHITCGHCRNCRGGRTHLCRNTVGVGVNRPGSFAEYLVIPAFNAFKIP
DNISDELASIFDPFGNAVHTALSFDLVGEDVLVSGAGPIGIMAAAVCKHVGARHVVITDVNEYRLELA
RKMGVTRAVNVSKENLNDVMAELGMTEGFDVGLEMSGAPPAFRTLLNAMNHGGRIAMLGIPPSDMSID
WNQVIFKGLFIKGIYGREMFETWYKMAALIQSGLDLTPIITHRFSIDEFQQGFDAMRSGKSGKVVLSW
D
>CORE_REP|Org28_Gene1284#
MATIKDVAKRAGVSTTTVSHVINKTRFVAEETKAAVWAAIKELHYSPSAVARSLKVNHTKSIGLLATS
SEAPYFAEVIEAVENSCYSKGYTLILCNSHNNLDKQRAYLAMLAQKRVDGLLVMCSEYPDQLLGMLED
YRNIPMVVMDWGAARGDFTDTIIDNAFEGGYLAGRYLIERGHRDIGAIPGQLSRNTGGGRHQGFMKAL
QEAHIDIREEWIVQGDFEPESGYKAMHQILSQKQRPTAVFCGGDIMAMGAICAADELGLRVPQDISVI
GYDNVRNARYFTPALTTIHQPKERLGEMAFTMLLDRIISKREESQVIEVHPKLIERRSVADGPFIDYR
R
>CORE_REP|Org39_Gene2443#
MPAQRMRSVIPPYMLRRIIEHGNAPQRDCALHTLNHVQSLLGNKPLRSPAEKNARAGEALRDIYDAQN
GTQLPGKQVRKEGQPSNHDVAVDEAYDYLGVTYDFFWQAYRRNSLDNQGLPLVGSVHYGKEYQNAFWN
GQQMVFGDGDGEIFNRFTIAIDVVGHELAHGVTESEAGLIYYQQSGALNESLSDVFGSLVKQFHLQQT
ADKADWLIGAGLLAKGIKGKGLRSMSAPGTAYDDPLLGKDPQPASMKDYIQTKEDNGGVHLNSGIPNR
AFYLAATALGGFAWEKAGYVWYDTVCDKALPQNADFATFARATVKHALARFDQSVADKVQQAWHQVGV
E
>CORE_REP|Org44_Gene3565#
MKPIFSRGPSLQLRLFLAVIAAIGLIVADSRLGTFVKIRNYMDTAVSPFYFLANGPRKVLDSVSETLA
TRQQLELENRALRQELLLKNSDILLLGQFKQENARLRELLGSPLRQDEHKMVTQVISTGSDPYSDQVV
IDKGSDNGVYEGQPVISDKGVVGQVVAVAKVTSRVLLICDASHALPIQVLRNDIRVIAAGSGCADDLQ

LEHLPNNTDIRVGDVLVTSGLGGRFPEGYPVAVVSSVKVDNQRAYTVIQARPTAGLQRLRYLLLLWGA
DRNGDMPLPPDEVHRVANERLMQMMPQVLPPAGSVGPQLPAPATGVAPQTTAPASVQPQAQPAAAGVV
P
>CORE_REP|Org3_Gene3147#
MKKRKLKVLSIIVVLVLALLFWGYQKIERFADTPLAIQQETIFKLPAGTGRVALEGLLVRDKLVRNGR
WFQWLLKLEPELAEFKAGTYRFTPGMTVRQMLKLLASGKEAQFTARFIEGSRLRDWQQVLQQSKYLKH
TLAGKSEAEIAAALGIPAGETPEGHLYPDTYQYTAGMSDIALLKRAHVRMNKALQAAWAGRDTSLPYK
TPEELLTMASIVEKETAVPEERSKVASVFVNRLRIGMRLQTDPTVIYGMGESYNGNITRKDLETPTPY
NTYVIAGLPPTPIAMPGEASLQAAANPAKTPYLYFVADGKGGHTFTTNLASHNQAVRMYRQALKEKNE
K
>CORE_REP|Org14_Gene1844#
MIVLSNVCKTFDSTQGRVVAVDNVSLAVEAGQIYGIIGYSGAGKSTLIRLLNGLETPTSGRIDVGGFD
IARAKGSHLRQARLKISMVFQHFNLLWSRTVSQNIAFSMQIAGVPKAQIAPRVAELIALVGLQGREDA
YPSQLSGGQKQRVGIARALANNPSVLLCDEATSALDPQTTDAILDLLLDINRQLKLTIVLITHEMHVV
RKICHRVAVMENGRIVEEGPVLDVFTRPQQPITRQFVKQVSQYADTEESFNPLLTAHLPGAIFKLTFV
GVQTHQAVISEVIRRYALTINILHGKISHTLNGSFGELYIHAEGNEQQVADMLSLLHERDIAVEVIQH
D
>CORE_REP|Org10_Gene3362#
MKRALSLMGMVFATVLAGTQAASATEYPLPPPDSRLIGENTTYTVPNDGRPLEAIAADYKIGLLGMLE
ANPGTDPFLPKPGTVLTIPTQMLLPDTKREGIIVNLAELRLYYYPKGENKVIVYPIGIGQTGMHTPLE
VTSISQKIPNPTWTPTANIRKRYQSQGVTLPAVVPAGPENPMGLFALRLAMGRGEYLIHGTNANFGIG
MRVSSGCIRLRPTDIEALFNQVPRGTRVQVINDPVKISVEPDGKRYVEVHQPLSRVESDDPQTMPIAL
SKAEKAFAADGQTDRAVFDSAVVRRSGMPVLVNVGESPSEVSLTPAAAPAAAPAANKSPFKAAPISSV
N
>CORE_REP|Org33_Gene2736#
MLSIRLADLAQQLDAQLHGDGDLVITGIASMHSAQPGQITFLSNSRYQEQLSSCQASAVVLTEADLPH
CRTAALVVKNPYLTYARMAQLMDTTPAPAQDIAPSAVISPEAQLGHNVAIGANAVIESGAVLGDNVVI
GPGCFIGKHARIGAGTRLWANVTIYHAVEIGQRCLIQSGTVIGADGFGYANERGEWIKIPQLGTVIIG
DRVEIGACTTIDRGALDNTQIGNGVIIDNQCQIAHNVVIGDNTAVAGGVIMAGSLKIGRYCQIGGASV
INGHMEIADKVVVTGMGMVMRPITEPGVYSSGIPLQPNKVWRKTAALVMNIDEISKRLKAVERKVGKD
>CORE_REP|Org49_Gene4201#
MKQNRVKNLVLKGWLAAALLASGAASAAELLNSSYDVSRELFVALNPGFEQQWNQQHPNDKLTIKQSH
AGSSKQALAILQGLRADVVTYNQVTDVQILHDRGQLIPADWQARLPNNSSPFYSTMAFLVRKDNPKGI
HTWNDLVRDDVKLVFPNPKTSGNGRYTYLAAWGAASQADGNDAAKTRAFMTRFLKNVLVFDTGGRGAT
TTFVERGLGDVLISFESEVNNIRKQYGEDKYEVIVPPVDILAEFPVAWIDKNVERNGTEQAAKAYLNY
LYSPAAQQVITSFYYRVYDQKAMVAAKGQFPDTQLFRVEDQFGGWPQVMKTHFATGGELDQLLAAGRK
>CORE_REP|Org25_Gene2731#
MTLNIGVIGTGAIGRDHIRRCSKVLQGARVVAVNDINRDNAAKAVNDLQLDARVYDSGHDLIKAADVQ
AVLVTSWGPSHEEFVLAAIAAGKPVFCEKPLAVTAQGCKNIVDAEAKHGKRLVQVGFMRPYDQGYRAL
KQVLTSGQIGEPLMLHCAHRNPTVGEAYTTDMAITDTLIHEIDVLRWLLDDDYVSVQVVFPRKSPKAF
PHLKDPQIVLFETAKGTRIDVEIFVNCQYGYDIQCEVVGETGIAKLPEPSSVQLRSGARLSTEILTDW
KDRFIDAYDVELQGFINDVAAGQLTGPSAWDGYAAAVTADACVAAQLSGEIVPVTLPARPAFYNKPSA
>CORE_REP|Org16_Gene1626#
MKKIGFLSFGHWSPSAQSGTRSAADALLQSIDLAVAAEDLGADGAYFRVHHFARQLGSPFPLLAAIGA
KTQRIEIGTGVIDMRYENPLYMVEDAGAADLIAGGRLQLGISRGSPEQVIDGWRYFGYAPADGETDAD
MARRHTEVFLEALRGEGFAEPNPQPMFPNPPGLLRPEPFSAGLRDRIWWGAGSNATAVWAAKLGMHLQ
SSTLKNDETGEPFHIQQAKQIRAYRAAWQEAGHTHEPRVSVSRSIFALVDQRDRNYFGGSGKEGDQLG
YIDAQTRAIFGRSYAAEPDALIEQLAQDEAIAEADTLLLTVPNQLGVDYNAHVIESILTHVAPALGWR
>CORE_REP|Org4_Gene4172#
MSLNFSPPTTSRRHWVFGLWPLLPFTLALAAAALLAWHYWPQLLMQSVVWQKALHQQMAGLLQQVKAA
PQQAGLALMLFSLGYGILHALGPGHGKVVIATYLATHPARLKSSLKLTFAASLVQGGVAIALVTLMLV
VLQLSSRQLHQSSFWLEKGSFILVMLLGVLLSWRALKRLFAAIKAMRPAPALRINSLTPLAADHVHSA
HCGCGHRHLPSDSELQAGSDWRTQAAIVLAMGMRPCSGAILVLLFSKVIGVFGWGVISALAMAFGTSL
TISMLALLVHYSRRLAVRLSRSRARAAWSAVAWGALALAGGLILLAAGLLLYVSAQPEFGGGIRPFSR

>CORE_REP|Org21_Gene1241#
MSYAFPGTFPGRRMRRVRRHDFSRRLVAENQLTVNDLIYPVFVMEGSNRQEEVASMPGVSRMTIDLLV
KEAETIAKLGVPVISLFPVIEPGLKSLHAEEAYNPEGLVQRTVRALKDAVPELGILTDVALDPYTTHG
QDGVIDEQGYVINDVTKDILVRQALSHAEAGAEIVAPSDMMDGRIGAIRDRLELQGLVNTQIMAYSAK
YASCYYGPFRDALGSSGNLKGGNKKTYQMDPANSDEALQEIAQDLQEGADMVMVKPGMPYLDVVRRVK
DTFGVPTFAYQVSGEYAMHMAAIQNGWLQEQPAVMESLMCFKRAGADGVLTYFAKRVAQWLHDDAMRR
>CORE_REP|Org31_Gene3765#
MSTGNAFYQRHFLRLMDFTPAELQALLRLSADLKQAKKQGQEQRRLQGKNIALIFEKDSTRTRCSFEV
AAFDQGAQVTYLGPSGSQIGHKESMKDTARVLGRLYDGIQYRGYGQALVETLAEYAGVPVWNGLTDEF
HPTQLLADLLTVQEHLPGKALSEVKLAYIGDARNNMGNTLLEAAALAGMDLRLVAPKACWPQPELVAE
CQALAQQTGAKLTLTEDIAEGVQDADFLYTDVWVSMGEPKETWQERIALLRPYQVNMAMLKLTGNPNV
KFLHCLPAFHDDQTTLGKQMAQQYDLHGGMEVTDEVFESAHSVVFDQAENRLHTIKAVLVATLSETL
>CORE_REP|Org10_Gene2404#
MLQFILRRLGLVIPTFIGITLLTFAFVHMIPGDPVTIMAGERGISAERHAQLMAEMGLDKPLYQQYFS
YVSNVLHGDLGTSLKSRISVWSEFVPRFQATLELGFCAMLFAVLVGIPVGVLAAVKRGSVFDHTAVGI
SLTGYSMPIFWWGMMLIMLVSVQLNLTPVSGRISDTVFLDDSQPLTGFMLIDTLIWGEPGDFIDAVMH
MILPAIVLGTIPLAVIVRMTRSSMLEVLGEDYIRTARAKGVSRMRVIVVHALRNALLPVVTVIGLQVG
TMLAGAILTETIFSWPGLGRWLIDALQRRDYPVVQGGVLLVACMIILVNLLVDVLYGVVNPRIRHKK
>CORE_REP|Org24_Gene1275#
MNTVNASMTVIGAGSYGTALAITLARNGHTVVLWGHNPAQVQTLQQDRCNQAFLPDVPFPDTLLLEAD
LARALAASRDVLVVVPSHVFGDVLRQLKPHLRPDARIVWATKGLEAETGRLLQDVAREALGEAIPLAV
LSGPTFAKELAAGLPTAIALAATDAQFADDLQQLLHCGKSFRVYSNPDFIGVQLGGAVKNVIAIGAGM
SDGIGFGANARTALITRGLAEMSRLGSALGADPSTFMGMAGLGDLVLTCTDNQSRNRRFGIMLGQGKG
VQEAQDSIGQVVEGYRNTKEVLALAQRHGVEMPITEQIYQVLYCHKDAREAALSLLGRARKDEKPSA
>CORE_REP|Org18_Gene4598#
MLDVAGYELDAEEREILKHPLVGGLILFTRNFHDAEQLRELVRQIRAASHDRLVVAVDQEGGRVQRFR
EGFTRLPAAQSFAALHDAQEGGRLAQEAGWLMAAEMIAQDIDISFAPVLDIGHGSAAIGERSFHSDPQ
QALAMAERFILGMHSAGMKTTGKHFPGHGAVSADSHKETPRDPRPLAQIREHDMAIFRELINRQLLDA
VMPAHVIYTEADPRPASGSPYWLQQILRRELGFDGVIFSDDLSMEGAAIMGSYAERGQAALDAGCDMI
LVCNHREGAVSVLDNLSPVKAEKVKRLYHRGQFTRQELRDSERWQQAHKALSALSERWEEHKQRSQG
>CORE_REP|Org21_Gene2358#
MNFQQQYLYWLAGAVLLIVAVMSFRDRANPRRITTGLFWGLYGLVFLVGDWTYRLLGDVLGEGSNEKR
MLHIIVGGVVVVMALIAGFGGVRLGSYHQRTPQEREASAKRLGNKLFIPALAIPVVTVIGVLLFNNVP
ALQTAVFGSGNHATLITLFSMTVGCLIGLAIAVKMTHEKALQPVQEARRLLDSIGWAFILPQILATLG
LLFTVAGVGTAISHLTQEYLAVDNRFIAVAVYAIGMAVLTMVMGNAFAAFPIVTAGIGIPILVLQHGG
NPAVMAAIGMFSGYCGTLMTPMAANFNIVPAALLELPDKNAVIKAQVPTGVLLLLVNVFLLYFLMFL
>CORE_REP|Org25_Gene3878#
MPPLFPRLSFNRFGDPAQVLELQQTSRPLLRPGQRLLQMRYAPINPSDLIPIHGQYAHRIALPQVPGY
EGVGVVVNPQNGHSTGRRALAVAGNGSWQTFVTLPEDRVVWVPDDIDDACAAQIYINPLTCWVLLTQW
LPLSAGDVLLLNGGGSAVSQLLAQLTALRGIRLAVVVRNAAHRQALLAAGAWRVIEAPQLAEMTNFGA
RAAIDCIGGEDGLQLARAVRTGGDFVALGLLSGRQVDWRRVVDELKLRASLFHLRKWNAQAAPAQWQT
AFYQLFQLLRRGQLALRPPAAIYPLRQYAAALHHAAQPGVNGKIFLTPTTSETIAVDELLFNGVPNK
>CORE_REP|Org32_Gene2866#
MAVLVTGGAGYIGSHTVLALLEHGEDVVVLDNLSNSSDESLRRVEKLAGRSAQFYQGDILDAECLHRI
FEAHAISAVIHFAGLKAVGESTRKPLEYYQNNVTGTLVLLEEMRRAGVHKFIFSSSATVYGTPEQVPL
TETSRVGGTTNPYGTSKLMVEQILQDFAKAEPQFSITALRYFNPVGAHESGMIGEDPNGIPNNLMPYI
AQVAIGKLEKLSIFGDDYPTQDGTGVRDYIHVMDLAEGHLKAIEHIDEHQGFTVYNLGTGVGYSVLEM
LHAFEKASGRNVAYQIVPRREGDIAECWSAPELAFKELGWKATRDLDAMMRDAWNWQKNNPRGYRPG
>CORE_REP|Org6_Gene3849#
MPLPPYFSGVQRGQKYLPCLLLLCAAPAFAKPTLTVYTYDSFAADWGPGPAVKKAFEAECDCELKFVA
LEDGVSLLNRLRMEGKNSAADVVLGLDNNLLQAAQQTGLFAPSDVDTAKLTVPGGWQDATFVPYDYGY
FAFVYNKEKLKNPPKSLQELVSGDRNWKVIYEDPRTSTPGLGLLLWMQKVYGDKAPAAWQQLAKKTVT
VTKGWSEAYGLFLKGEGDLVLSYTTSPAYHLIEEKKDLYAAATFSEGHYLQVEVAGKLKAAKQPELAE
RFMRFMVTPAFQNTIPTGNWMYPVINTPLPVGFEQMNVPQTALQYSAEEVATQRGGWIRAWQTAVSR

>CORE_REP|Org17_Gene2886#
MLIDLRSDTVTRPSAAMRQAMAQAEVGDDVYGDDPTVNALEAEAVRLSGKEAALFLPSGTQANLVALL
SHCQRGDEYLVGQQAHNYKYEAGGAAVLGSIQPQPIEADTDGTLPLDKLAAAIKPDDIHFARTRLLSL
ENTISGRVLPQAYLQQAWQFTRERQLALHIDGARIFNAAVALNLPLKEIVQYCDTFTICLSKGLGAPV
GSLLCGSEAFIQRALRWRKMTGGGLRQAGILAAAGLYALEHNVARLREDHDNAVWLEQQLRQIGVEIA
EPGAQTNVLYLRQTPALAAKLGPWMRERGVLISSGPLTRILTHLDVSRQDLQRVVELWREFLQQHA
>CORE_REP|Org37_Gene944#
MSTIEHPQLQTGAGVKTSPLLDVKDLRVTFSTPDGDVTAVNDLNFDLRAGETLGIVGESGSGKSQTAF
ALMGLLASNGRIGGSAKFNGREILNLPENQLNKLRAEEISMIFQDPMTSLNPYMRVGEQLMEVLMLHK
KMSKSEAFEESVRMLDAVKMPEARKRMRMYPHEFSGGMRQRVMIAMALLCRPKLLIADEPTTALDVTV
QAQIMTLLNELKREFNTAIIMITHDLGVVAGICNKVLVMYAGRTMEYGSAREVFYQPSHPYSIGLLNA
VPRLDAEGEALLTIPGNPPNLLRLPKGCPFQPRCPYAMEQCASAPPLEQFGEGRLRACFKPVEALV
>CORE_REP|Org43_Gene4625#
MRVLVTGATSGLGRNAAQWLLEAGHQVRATGRDERAGATLRQLGAEFCALDLAQATPQQCRELVTDCE
WVWHCAAKSSPWGGKAEFHRINAAATDKLAEAAGRCGVRRFVHISTPAIYFDFQPHYDLDEGYRARRF
ANHYAASKYAAEQRLLAQAKIYPQTTYIMLRPRGLFGPHDRVIVPRLLQQLERDRGVLRLPGGGQALL
DLTFAPNVVHAMDLASRQRGLPSGAVYNITNHQPQRLAEMLDALLRQELGLSYRLQAVPYPLLHTLAG
GMELWATLSGKEPLLTRYSVAAVHFDMTLSQTRAIEELGYRPRYSMEEGIRLTGEWLRRQGGGQHG
>CORE_REP|Org49_Gene3579#
MSTGSPMITLRRVAVCTMVSLWLAGCTNNASTSAPISSVGGGGAVPSGNNNGGAQQASPEGRIVYNRS
YNAIPKGSYSGGDTYTVKRGDTLFYIAWITGNDFRDLAQRNNIPEPYSLNVGQTIQLGNGSANGGGGM
LATTDATQGGVPKPPSTSQIQTATVDSQSTNAYSENSGKQNVGKMLPAAGAAAVGTAAAPVTAPEAAP
PVSSTVSNSAPVSTWRWPTDGKVIDNFSSSEGGNKGVDIAGSRGQPIFATADGRVVYAGNALRGYGNL
IIIKHNDDYLSAYAHNDTMLVREQQEVKAGQKIATMGSTGTSSVRLHFEIRYKGKSVNPLRYLPQR
>CORE_REP|Org11_Gene2791#
MQKKSIYVAYTGGTIGMQRSDHGYIPVSGHLQRQLALMPEFHRPEMPDFTIHEYAPLIDSSDMTPEDW
QHIADDIKQNYDRYDGFVILHGTDTMAFTASALSFMLENLAKPVIVTGSQIPLAELRSDGQTNLLNAL
YLAANHPVNEVSLFFNNKLFRGNRTTKAHADGFDAFASPNLPPLLEAGIHIRRQHGIDSPACNGALRV
HDITPQPIGVVTIYPGISGAVVRNFLLQPVKALILRSYGVGNAPQKAELIDELRAASERGIVVVNLTQ
CISGRVNMEGYATGNALAHAGVISGFDMTVEAALTKLHYLLSQPLTPEQIRALMQQDLRGELSING
>CORE_REP|Org36_Gene177#
MTIRIAINGFGRIGRSVLRALYESGRRAEISVVAINELANAEGMAHLLKYDSSHGRFAWDVRQECDTL
SVGDDAIRLLHQPAVEQLPWGELGVDVVLDCSGVYGSRADGEAHLAAGAKKVLFAHPGGNDLDATIVF
GVNHQTLLAEHRIVSNASCTTNCIIPVIKLLDDAYSIESGTVTTIHSAMNDQPVIDAYHADLRRTRAA
SQSIIPVDTKLAAGITRIFPQFCDRFEAISVRVPTINVTAIDLSVSVSAAVKVAEVNQLLQKAARESF
RGIVDYTELPLVSIDFNHDPHSAIVDGTQTRVSGQHLIKTLVWCDNEWGFANRMLDTTRAMAASGF
>CORE_REP|Org45_Gene984#
MKAAVVTKNHTVDIQDKVLRPLKHGEAALKMECCGVCHTDLHVKNGDFGEVPGITLGHEGIGVVSAVG
EGVTSLKVGDRASVAWFYQGCGHCEYCVSGNETLCRSVKNAGYSVDGGMAEECIVVADYAVKVPDGLD
SFAASSITCAGVTTYKAVKISDIKPGQWIAIYGLGGLGNLALQYAKNVFNAKVIAIDVNDGQLEFAKQ
IGADLAINSKTQNAEEIIQQQTGGAHAAVVTAVAKAAFNSAVNAVRAGGKVVAVGLPPESMDLSIPRL
VLDGIQVVGSLVGTREDLKEAFQFAAEGKVTPKVTKRPLGDINAIFDEMKAGTIRGRMVIDLGMAK
>CORE_REP|Org10_Gene4402#
MDEFKPEDDLRPDSSDRRPTRSRKPAAAPRFAVSRQHLMIGIGILVLLLLIIGIGSALKTPTKHEAAQ
ESGAQNGAARDINLSGSSSLTTANNGVPGGTTDTHDNNGVSATQPQQPQNVSVPPISGTPTEAQTQPQ
QGGAQQRVDLPGNNMADALSQQQGQVDAATQGMTGAASTLPTAPATVMSGAAAREATRPVQGTAPQQH
KTPAKTAATKPTATQHKSPTTVYTPPAKPSSTAKAGAVASSGSSLQSAPGSHYTLQLSSASRSDTLNA
YAKQQKLQNYLVYATKRDGKPWYVLVSGNYASSAEAKRAIASLPADVQAKKPWVRPVHQVQQDLKK
>CORE_REP|Org3_Gene4266#
MKNILIIRRDNIGDLVCTTPLIEGVKIAYPDAKVYLLINKVSQDVVKNNPHLEKVFVYKKAKHKAKNE
TTLGVYFERLMIFLKLRKIKFDAVILANPVPCKYSLRLAKMAGATHIIGADLGTKDIHRPFRKDDFNG
QHQVEHTYSYLSAITDQSIPIPPVRVFLTPEERQLAAQRLQERLPPVERVCAVHISSRSPKRRWPVER
YAEIINRLVADPHTGVLIFWSPQGTLAPDDIGDQQRAEQLLALCQNERVALYPTASVRELLGGFDLCD
RVLCSDGGQMHLAAALNKDMVVFFGDTNKEQWHPWMNDYQILQSESGDCIDISVDDVWGCIKYNNA

>CORE_REP|Org45_Gene4005#
MNSLRLLISDSYDPWFNLAVEECIFREMTTQKILFLWRNAETVVIGQSQNPWKECNTRRMEQDGIRLA
RRSSGGGAVFHDLGNTCFTFMAGKPGYDKSVSTDIILQALRQLGVAAGASGRNDLVMETADGPRKISG
SAYRETQDRGFHHGTLLLNADLERLANYLNPDPKKLQAKGITSVRSRVANLAEFLPGISHEQVCDAIV
QAFFAHYGETAEPEIISPDVFPDLPDFAAQFAKQSSWEWNFGKAPAFSHLLNERFVWGGVDLFFDVEK
GAIVRAQVFTDSLNPAPLQRLADMLVGCPYRSEPVAACCDRLMADYPQQAAELAELRQWLSETIR
>CORE_REP|Org4_Gene2477#
MRVLGIETSCDETGIAVYDDQTGLLANQLYSQVKLHADYGGVVPELASRDHVRKTVPLIQAALKEANL
TPADIDGVAYTAGPGLVGALLVGATVGRALAFAWNVPAVPVHHMEGHLLAPMLEDNPPAFPFVALLVS
GGHTQLISVTGIGEYELLGESIDDAAGEAFDKTAKLLGLDYPGGPMLSKMAQQGTEGRFTFPRPMTDR
PGLDFSFSGLKTFAANTIRSNGNDDQTRADIARAFEDAVVDTLAIKCKRALEQTGFKRLVMAGGVSAN
RTLRAKLAEMMHKRGGEVFYARPEFCTDNGAMIAYAGMVRLKSGANPELSVSVRPRWPLAELPAV
>CORE_REP|Org13_Gene3248#
MSLDIDQIALHQLVKRDEQTLDVVLRDSLLPANAAVEEMMAELHRVYSAKSKALGLFNEGSELAEALR
TCRKDDKDFLAFSRAATGRLRDELAKYPFAEGGVVLFGQYRYLAVEYLLIAVLNSRNSMRVNEELDIN
TTHYLDINNADIVARIDLTEWETNPESTRYLTFLRGRVGRKVADFFMDFLAASEGLDTKAQNRGLLQA
VDDYCADAQLDKNERQSVRQQVYSYCNEQLQAGEEIELQALSQELSPVGEKDFMQFSSEQGYELEESF
PADRGTLRQLTKFAGSGGGISLNFDAMLLGERIFWDAATDTLTIKGTPPNLRDQLQRRTGSGSGK
>CORE_REP|Org13_Gene4482#
MTKDNHAPTYAANRFSIAPMLDWTDRHCRYFHRLLTKETLLYTEMVTTGAIIHGKGDYLAYSEEEHPV
ALQLGGSDPAALAHCAKLAEQRGYDEINLNVGCPSDRVQNGMFGACLMGQATLVADCIKAMRDVVSIP
VTVKTRIGIDDQDSYAFLCDFIQTVAGRGECDMFTIHARKAWLSGLSPKENREVPPLDYPRVYQLKRD
FPALTIAINGGVKTLEEAQQHLQHLDGVMMGREAYQNPGILAQVDSELFGAQTAVPDSVAIVEALYPY
IERELSSGTYLGHITRHILGLFQGVPGARQWRRHLSENAHKPGADARVVEQALALVRQPRVEMA
>CORE_REP|Org5_Gene1792#
MYYPLIRKALFQLDPERAHEVTFRQLSRITGTPLAFLVRQSVPTKPVSCMGLSFKNPLGLAAGLDKNG
ECIDAFGAMGFGHVEVGTVTPRPQPGNDKPRLFRVIEAEGLINRMGFNNHGVDNLVENVKKSHFGGIL
GINIGKNKDTPVEQGKDDYLICMDKVYPYAGYIAINISSPNTPGLRSLQYGEALDDLLAAIKNKQQEL
HARHHKYVPVAVKIAPDLSEDELIQIADSLVRHNIDGVIATNTTLDRKLIQGLNYCEQAGGLSGRPLQ
SRSTEVIRRLSTELQGRLPIIGVGGIDSLTAAREKMEAGASLLQIYSGFIYHGPRLIKDIVTHI
>CORE_REP|Org47_Gene3808#
MSDGWNIALLGATGAVGEALLELLQERQFPVGELYPLASERSAGANVRFNGKSLLVQNAEEFDWSQAQ
LAFFVAGSEASARYAEEAGNMGCLVIDTSGLFAMEPDVPLVVPGVNPQVLADYRNRNIVAVADSMVSQ
LLTAIKPLTEQAGLSRLHVTTLMSVSSRGKAAVDDLAGQSARLLNGIPAEEGVFGKQLAFNLLPLIAD
EQGSVREERLIVDQVRKVLQDEGLPISVSCVQSPVFYGHAQVVHLEALRPLSAEEARSELEEVEDIQL
SEEDDYPTQVTDASGSDALSIGCLRNDYGIPELLQFWTVADNVRFGGALMAVETAERLVQEQMY
>CORE_REP|Org17_Gene60#
MKVLVTGATSGLGRNAVEYLRRQGIKVRATGRNQAMGGLLEKMGAEFIHADLTNLISSQAKAMLADVD
VLWHCSSFTSPWGTEEAFELANVRATRRLGEWAAAYGVAQFIHISSPAIYFDYHHHRNVTEDFRPQRY
ANEFARSKAAGEQVIQQLALSNPQTHFTILRPQGLFGPHDKVMLPRLLQMIKRYGNLLLPRGGAAMVD
MTYLENAVHAMWLATLKEDTPSGRAYNITNQQPRPLRTVVQQLIDDLGMKCRIRSVPYPMLDMMARGM
ERLGSKSEKEPVLTHYGVAKLNFDLTLDTTRAQQELGYQPIVSLEEGIARTARWLKDHGKLHGL
>CORE_REP|Org37_Gene2662#
MVKVGINGFGRIGRNVLRAALGRSDFEVVAINDLTDSKTLAHLLKYDTLSGTLAARVEAGDNQLLLDG
RPIQVFSQRDPAEIPWSSVGVDVVIEATGFFTDKAKAEVHITHGGAKRVIISAPAKNDDITIVMGVND
QLYDPALHKVVSNGSCTTNGLAPAAQVLHQAFGIEYGLMNTTHAYTNSQALHDQPEKDLRGARAAAES
IVPYSSGAAKALGKVIPDLDGRLTGYSLRVPVPVVSIVDLTVTLKRPATVEEINAAFRAAAASGPLKG
ILGYSDEPLVSSDYRGDARSSIIDGLSTLVIGGNLVKVLAWYDNEWGFSNRLVDLALLMEKRGL
>CORE_REP|Org24_Gene3190#
MKRELAIEFSRVTEAAALAGYKWLGRGDKNAADGAAVHAMRIMLNNVDIDGRIVIGEGEIDEAPMLYI
GEQVGTGQGDAVDIAVDPIEGTRMTAMGQSNALAVLAVGDRGAFLHAPDMYMEKLVVGPAARGAIDLN
LPLAENLQNVAARLGKPLSQLTVIVLAKPRHDGVIAQMQQLGVRVFAIPDGDVAASILTCMPESEVDV
MYGIGGAPEGVISAAVIRALDGDMQARLLPRHEVKGDSAENRRIGEQELARCREMGIEAGQALRLDQM
ARNDNVIFSATGITKGDLLEGISRQGNMATTETLLIRGKSRTIRRIRSTHYLDRKDPALHEFLL

>CORE_REP|Org40_Gene2850#
MKFLVTGAAGFIGYHVAERLLTAGHQVVGIDNLNDYYDVGLKMARLDRLADKPGFRFIKLDLADREGM
AALFAEHQFQRVIHLGAQAGVRYSLVNPLAYADANLIGHLNVLEGCRHNKVEHLLYASSSSVYGLNRK
LPFATEDSVDHPVSLYAATKKANELMSHSYSHLYSLPTTGLRFFTVYGPWGRPDMALFKFTKAILAGE
SIDVYNHGEMHRDFTYIDDITEAIVRLQAVIPQADPSWSVEQGSPATSSAPYHVYNIGNNTPVKLMEY
ITALEQALGVTARKNMLPMQPGDVMDTSADTAELYRDIGFKPETSVEEGVKRFVDWYKAFYQVQ
>CORE_REP|Org2_Gene3995#
MTLSLRQLFIAFIATSSLLLSGCGPDDQSDGQKPSAPAADSSWPRTIDSAKGKFTLEKPPQRIVSTSV
TITGTLLAIDAPVVASAATSPNPLVADKQGFFTQWSEVAKQRHVERLYQVEPNAEAVAAAAPDLIVVA
ATGGDSALKLYDQLSAIAPTLVLDYGDKSWQQLASELGEITGHEAGAKQAEDRFEQRVEQVKQAIALP
PQPTTPLVYADNGREAMLWTPGSAQGKLLTQLGFQLATPPESAKGNTSMGKRHDIIQISGEKMAEGLN
GKTLILFATNERKVQEVLTNPFLKHLEPVEQRHVYAVGNDTFRLDYYSATNMLNQIERLFKKP
>CORE_REP|Org10_Gene1229#
MNWYPWLNGPYRQLIGQYADGRGHHALLLHAAAGNGDDALAYGLSRWLICQQRNGEKSCGECHSCRLM
LAGNHPDYHVLAPEKGKSNLGIEPIRQVIETLYAHAQQGGAKVIWLPQAEQLTEAAANALLKTLEEPP
EKTYFLLGCREPSRLMATLRSRCLYWHLASPDEQLSLQWLGRQAAGSQTDRLTALRLHDGAPLAAEQL
LQPQQWQQRSALCTALSAALPQRDMLSLLPVLNHEDVAERLHWLCALLVDAMKWQQGAHHYVLNQDQQ
PLVHQLASVLSSASLQQIVQQWLTCRHQLLSVVGVNRELLLTEQLLRWEQMLGAAGYSHPHSL
>CORE_REP|Org29_Gene883#
MSKPIVFSGAQPSGELTIGNYMGALRQWVQMQDDYDCIYCIVDLHAITVRQDAEKLRKATLDTLALYL
ACGIDPEKSTIFVQSHVPEHTQLSWVLNCYTYFGELSRMTQFKDKSARYAENINAGLFSYPVLMAADI
LLYQTNQVPVGEDQKQHLELSRDVGQRFNALYGDVFKVPEPFIPKSGARVMSLQEPTKKMSKSDDNRN
NVIGLLEDPKAVTKKIKRAMTDSEEPPVVRYDVVNKAGVSNLLDILAGVTGKSIAQLEAEFEGQMYGH
LKGAVAEAVSGMLGELQERYHRFRNDEAYLQQVMRDGAAKARARAQETLAKVYQAVGFVPPQA
>CORE_REP|Org21_Gene2754#
MSYTILTGDRATGPLHLGHFVGSLRQRVELQYQHNQTVMVADLQGLTDNGNNPQKISANVLNVVADYL
AVGIDPHKTTICLQSALPALAELTMYYLNLVSVARLERNPTVKNEIAEKDFARRLPAGFLIYPVSQAA
DITAFGATHVPVGEDQLPMLEQTNEIVRRFNHIVGQPILTECQPLLSNVGRLPGLDGQGKMSKSRGNA
IQLGAGADEVHKAVMSMFTDPGHLNVSDPGRVEGNMVFTYLDAFCEDAALVADLKAHYRRGGLGDVKI
KRLLEDCLQSLLEPIRTRRAEFIADKGELTRILQHGTRRAHQVSQQTLQQVKTALGLDFFTLA
>CORE_REP|Org47_Gene3979#
MTTQCRSPELLPLKIIATGAALPPNRVASSTLDARLGKPAGYVEKRSGIVYRYHADDDASQAELAAAA
LQDALARSTIPAASIDLLISASAIAVQALPCSAAHILKIAGLAPGTPGFDINSSCVSFISALQVAAGL
LNAGTYRRIAIVSADLASRGIDWQHEESSLIFGDGAACAIVERGDGTGGILASLVETYPAGSELCEIR
AGGTRRNPRAGMCEQDFLFHMQGKPLFRQASALIEDYLDRLLSASGLTLGQIATVVPHQASHLSLEHM
RKRLHVSSEALVDIYRHHGNQVAASIPTALHAAVTTGRFNPGQPVMLIGTAAGLALAGMVLLP
>CORE_REP|Org9_Gene2618#
MPTSRTFTLLLQHQRYRDKRQIGLLALCVAVALLFSLCAGDQWIWPSEWFSDRAQLFVWQLRLPRALA
VMLVGAALAVAGAVMQALFENPLAEPGLLGVANGAGVALVLTVLLGQGLLPVALMSAAAIAGALAMTF
LLLGFARRRRLTNARLLLVGVALGIVCSALMTWAVYFSTSLDLRQLMYWMMGGFGGVDWRQKWLVLAL
LPVLLWLCGQGKALNLMALGEVQARQLGLSLHLWRNLLVLAIGWLVGVSVALAGVIGFVGLVIPHILR
LIGLTDQRYLLPACALAGAGVLLVADVVARIALLAAELPIGVVTATLGAPLFIWLLTRAKGVR
>CORE_REP|Org48_Gene1728#
MGDSILSQAEIDALLNGDSAGDEPEAIVGKESEVKPYDPNTQRRVVRERLHALEIINERFARQFRMGL
FNLLRRSPDITVGPIKIQPYHEFARNLPVPTNLNLVHLNPLRGTALFVFAPSLVFIAVDNLFGGDGRF
PTKVEGREFTPTEQRVIKRMLRLALDAYGDAWSAIYKIDVEYVRAEMQVKFTNITTSPNDIVVTTPFQ
VEIGALTGEFNICIPFAMIEPLRELLTNPPLENSRQEDSHWRETLVKQVQHSELELIANFVDIPMRLS
KVLKLQPGDVLPIDKPERLIAHVDGVPVLTSQYGTLNGQYALRVEHLINPILNALSEEQPNE
>CORE_REP|Org49_Gene1607#
MRCRTSPQLAIIGLLVLLTLLALVAANLGALTLSFRTLWREPFSDAAWHIWLNIRLPRVLLAVVIGCA
LAVSGAVMQGLFRNPLADPSLLGISSGGALFVALFIVMPLALPVTIALYGHMLAAFLGSLLVSLLIYG
ISRSGHGNLSRLLLAGIAINALCMAAIGVLSYVSSDQQLRQFSLWMMGSLSQSQWPTLAVSASLILPA
ALLTLLQARRLNLLQLGDEEAHYLGVNVQRAKLQLLLLSALLIGAAVAMSGVIGFVGLVVPHLVRMRL
GGDHRWLLPCSALGGACLLLVSDTLARTLVAPAEMPVGLMTSLIGGPYFLWLVMRQRERAGG

>CORE_REP|Org13_Gene1386#
MIEADRLISAEPINEEEILDRAIRPKLLTEYVGQPHVREQMEIFIQAAKQRGDALDHLLIFGPPGLGK
TTLANIVANEMGVNLRTTSGPVLEKAGDLAAMLTNLEPHDVLFIDEIHRLSPVVEEVLYPAMEDYQLD
IMIGEGPAARSIKLDLPPFTLVGATTRAGSLTSPLRDRFGIVQRLEFYQVADLQHIVSRSAGCLGLEL
SDEGAHEVARRARGTPRIANRLLRRVRDFAEVRANGVISGSVAAQALDMLNVDAEGFDYMDRKLLLAI
IDKFTGGPVGLDNLAAAIGEERETIEDVIEPFLIQQGFIQRTPRGRLATQHAYRHFGLEREA
>CORE_REP|Org14_Gene263#
MKTLGEFIVEKQHDFSHATGELTALLSAIKLGAKIIHRDINKAGLVDILGTSGVSNVQGEVQMKLDLY
ANEKLKAALKARGEVAGIASEEEDEIVIFDGERAENAKYVVLMDPLDGSSNIDVNVSVGTIFSIYRRI
TPVGTPVTEEDFLQPGSAQVAAGYVVYGSSTMLVYTTGYGVHAFTYDPSLGVFCLSHEKVRFPASGNM
YSINEGNYIKFPLGVKKYIKYCQEQDEATQRPYTSRYIGSLVADFHRNLLKGGIYIYPSTASHPQGKL
RLLYECNPMAFLAEQAGGKASDGKNRILDITPVKLHQRAPFFVGTKSMVEDAERFIAENPDE
>CORE_REP|Org22_Gene3907#
MSWLQRLRIDKFLLVLILVVIVASLFPCEGIWKTFFEHLTTAAIALLFFMHGAKLSREAIVAGMSHWK
LHLLVFLSTFALFPLLGLAMNLLVPGIMTPTVYLGFLYLCALPATVQSAIAFTSAAGGNVAAAICSAS
ASSILGVFLSPLLVGALMHTQGGNTDVLHAIGSIILQLMVPFVVGHLARPLIGKWVDRHRKLINLTDR
SSILLVVYTAFSAAVVEGIWHRIDGWSLLTILVMSLVLLTVVLVINTYAARWLGFNTADEITIVFCGS
KKSLANGIPMANVLFPAAAVGAMVLPLMIFHQVQLMVCAVLAQRYARKTAKQRAEAEALLAK
>CORE_REP|Org4_Gene397#
MKLDEIARLAGVSRTTASYVINGKAKQYRVSDKTVEKVMAVVREHNYHPNAVAAGLRAGRTRSIGLVI
PDLENTSYTRIANYLERQARQRGYQLLIACSEDQPDNEMRCIEHLLQRQVDAIIVSTALPPEHPFYQR
WANDPLPIIALDRALDREHFISVVGADQEDAFALAQELRTFPAESVLYLGALPELSVSFLREQGFRQA
WQEDPRHVDYLYANSYEREAAGALFAEWLKTHPMPQALFTTSFSLLQGVMDVTLKQRGRLPTDLAIAT
FGDHELLDFLECPVLAVAQRHRDVAERVLELVLASLDEPRKPKPGLTRIRRNLFRRGSLSRK
>CORE_REP|Org47_Gene4045#
MRIGHHQLTNCLIAAPMAGITDRPFRTLCHAMGAGMAVSEMLSSNPEVWRTDKSRLRMVHSDEPGIRS
VQIAGCDPDDMAAAARINVTSGAQIIDINMGCPAKKVNRKLAGSALLQYPDLVKRILHAVVDAVDVPV
TLKIRTGWAPEHRNCVEIAQLAEDCGIQALTIHGRTRACLFNGDAEYDSIRAVKQSVSIPIIANGDIT
DPHKARAVLDYTGADALMIGRAAQGRPWIFREIQHYLDTGELLPPLPLGEVKRLLIGHIRELHGFYGQ
GKGFRIARKHVSWYLQEHAPNDQFRRTFNAIEDASEQLEALEAYFENLSVKKELTELCSNNA
>CORE_REP|Org36_Gene1608#
MSKANPNATIVDIARRARVTNITVSRAFNKPELVKPETRERIHAIAKELNYVPNAFAQGLKSSSSQII
GIVTSSMYNPFYSGLIKTVSRIARQQGYQIMLFDTDGSEEAEMRAIQALFGYKARGILLSAVRDDKRY
RPAYLELAEVYGVPLILIDRDLYDQQLSGVFLDNREIGVLAGRYLAEQPEQKLLIIGGPADSEITLTR
TAGIVAALQGSGREIHIINGDYDFTSQESEVRAYLAQPENRPDYIIGLNGIITLGAIAICHEMGLYEQ
VKFFSIDEPPRAGAYGLHIPGVYHDTQKLGEIAAELLFSAINSPRGELPVRREFFTGSLLNR
>CORE_REP|Org14_Gene271#
MRKSFVVIVLAVLVVLYASLFVVQEGQRGIVLRFGKVLRDGENKPLVYAPGLHLKIPFIETVKNLDAR
IQTMDNQADRFVTSEKKDLIVDSYLKWRISDFSRYYLATGGGDVSQAEVLLKRKFSDRLRSEIGRLDV
KDIVTDSRGKLMSDVRDALNTGTVGDGEEVATTEADDAIASAAARVERETTGKQPQVNPNSMAALGIE
VIDVRIKQINLPAEVSDAIYQRMRAEREAVARRLRSQGQEEAEKLRASADYEVTRTLAEAERQARITR
GEGDAEAAKLFANAFSQDPDFYAFIRSLRAYEASFKDNQDVLVLSPDSDFFRYMKSPDTLRK
>CORE_REP|Org23_Gene2135#
MLNTLIVGASGYAGAELTAYLNRHPHMNITALAVSAQSADAGKLLSDLHPQLKGIVDLPLQPLTDVAK
AAQGIDVVFLATAHEVSHDIAPAFLAAGCVVFDLSGAFRVQDAGFYSQYYGFEHQHGALLEQAVYGLA
EWQSDKIKQAQLIAVPGCYPTAAQLALKPLIEKQLLNLDQWPVINATSGVSGAGRKASMTTSFCEVSL
QPYGIFTHRHQPEIAAHLGVPVIFTPHLGNFPRGILETITCRLKAGVTAQDVAAAYHAAYDDKPLVRL
YDQGVPALKAVVGLPFCDIGFAVQGEHLIAVAVEDNLLKGAAAQAVQCLNIRFGFPETQSLL
>CORE_REP|Org34_Gene2861#
MLRFTRSALLCAALAAFNAGAAETPTFGPELQGFHYPYPLQQFSFTSQGQPLKMGYMDVPPEGAANGR
TALLLHGKNFCAATWQDTIKALSKAGYRVIAPDQIGFCSSTKPAHYQYSFQQLAQNTHGLLQNLNISK
AIVIGHSTGGMLATRYSLMYPQAVEQLVMVNPIGLEDWKAKGVPWRSVDQWFARELNTTAEGIRNYEQ
HTYYGGRWKPEYDRWVDMLAGLNNGPGHRLVAWNSALIYDMIFTQPVYYEFKDLQTPTTLMIGTADTT
AIGSDIAPPAVKAKIGHYAVLGKQAAKLIPHATLIEFAGLGHAPQMEEPARFHQALLKALQP

>CORE_REP|Org41_Gene3489#
MSIKAIAVDPQNPAGFIEISPGMPVPGQYDLLVEVKAVSVNPVDTKVHAGLQKSGLQQPRILGWDASG
IVVGVGSGVSGFKPGDEVWYAGDITRPGSNSSHQLIDSRIAAHKPRSLNWAESAALPLTALTAWEALF
EHLNIQDAPEHKTLLIIGGAGGVGSLAIPLAALRSKVKVIATASRPESAAWCRERGADLVVDYRDLKG
NLAQHGIEQVDYILCLNDTDGHWPAMAELVAPLGHICTIVENAQPLDQNALKLKSAALHWEFMFTRSM
FTTPDIAQQGKILQQMAQLLDEGKLSTTLSETLHGLSVDTLTAAHRQLLGGHMQGKLVIAY
>CORE_REP|Org4_Gene4595#
MSLMLSVIVPMHNVGELLEPFLASLLAQREQRLEVIIVNDGSTDGSGETAHRYAAAHPHIRVIDQANA
GVSNARNAGLALARGKYVAFPDADDLLAPDMYSTLLEQAERHQLDVMQCNGERYFASEDELQPIFPEA
RLQTTDVITGVQWFERALKSRKFIHVVWLAIYRLDFIRRHRLYFEPGLHHQDIPWTTEVMFNAQRVKY
LSTPLYRQRVHDKSISNRRRTGKANVDYQRHYMKIVEMLERLNLRYAGSIAIRPAFHWQITREALGIC
HSIRREPDPVAQNQITEEFYQRGIDRAMFANARGVKQGWHVMLWRHRLKQWRSDNGCSQVA
>CORE_REP|Org43_Gene2703#
MASLKDVAKLAGVSLMTVSRAINDPGKLRPETYRRVKQAIDRLDYVPDLSARRIRGDGNRVQTLGVLA
LDTATTPFSVEMILSIEKTARERGWNSFVVNLFADDNAEQTVDLLLAHRPDGVIFTTMGLREVTLPAK
LLDKKLVLANCVSPAHSIASYIPDDEQGQYDATRTLIAKGYRAPLCIHLPADTLAAGLRRRGLERAWR
EAGRDVEQLRQYHLDLSAGDQSYRDCVALLERHFSAGRRDCDVVVCGNDRIAFLAYQVLLAQGWRIPQ
QVAVLGYDNMVGTGELFLPALTTVQLPHYELGRLAALHVIERREQRDTVKVPCPLLERGSL
>CORE_REP|Org14_Gene641#
MATMKDVARLAGVSTSTVSHVVNNNRFVSDSVRDKVMAAVEQLNYAPSALARSLKLNQTRTIGMLVTA
SNNPFYAEVVRGVERSCYERGYSLILCNTEEDAARMNRSMETLLQKRVDGLLLMCTENHRPSQDALSR
YPSLPIVMMDWAPFEGANDIIQDNSLLGGEMATDHLIACGYRKIACIAGPQDKTTARHRLEGYRNAMR
RAGLPVPPGYEVHCDFEFEGGVNAMRQLLALDEPPHAVFAGNDAVAVGVYQALYQAGLSVPQDMAVMG
YDDIELARYLAPPLSTIHQPKDSLGELAIDALINRLQNPERAPQVLVLTPELVERASVGRR
>CORE_REP|Org45_Gene2110#
MDNHSARRVTRADVARVAGTSVAVVSYVINNGPRPVAEATRLRVLAAIEQTGYRPNDIARALASGSTQ
TYGLVVPDISNPFFATLARALQQEAFSRGRVLLLGDAGDDRQREYELINNLLRRQVDGLLYTSVDRHP
WFDLIRASGTPCVMIDTIDSQAGVCAIRVDERDAACQATRHLLQHGYRDIGIFIGPLTMLNAQDRLNG
WRDALLEAGIAPRDAWIFEAPYTRQGGYQATQRLVQGPRPRAVFTSNEQQALGCLSALAEHGLRAPDD
LALICFNGTQQSEFSVPPLSAVEQPIDAMAKRAIAMLAAGAAPAELHEFAFQLRIRRSCGC
>CORE_REP|Org7_Gene812#
MTAVTDKKVLLEVADLKVHFDIHDDKQWFWQPPKTLKAVDGVTLRLFEGETLGVVGESGCGKSTFARA
IIGLVKATSGRVAWLGKDLLGMSDADWRKTRSDIQMIFQDPLASLNPRMTIGEIIAEPLRTYYPKMPR
QEVKDKVKAMMLKVGLLPNLINRYPHEFSGGQCQRIGIARALILEPKLVICDEPVSALDVSIQAQVVN
LLQQLQREMGLSLIFIAHDLAVVKHISDRVLVMYLGHAVELGTYDEVYHNPQHPYTKALMSAVPIPDP
DKEKEKQIQLLEGELPSPINPPSGCVFRTRCPIAGPECAKTRPLLEGSFRHAVSCLKVDPL
>CORE_REP|Org7_Gene2047#
MRTTGIWALALTTLIGSQSAIAADLPGKGIAVQPVQSTISEETFQTLLVSKALEKLGYDVKEPREVDY
NVAYTSIASGDATFIAVNWDPLHADQYKAAGGDAKFYREGVYVNGAAQGYLIDKKTAEQYHITNVEQL
KDPKIAKLFDTNGDGKADLTGCTPGWGCEAVINHHIKAYGLSNTVEHNQGNYAAMIADTITRYKEGKP
VLYYTWTPYWVSDVMVPGRDVVWLQVPFSSLPGEQKNVDTKLPNGANYGFPVNTMRIAANKQWAEANP
AAAKLFAIMKLPIADINAQNLRMHEGQASEADIQNHVNGWIKAHQATFDGWVKTAAEAAKP
>CORE_REP|Org21_Gene3842#
MTQPTPLCPNRMQVHSIRRETADVWTLNLICDVFYPYQAGQFALVSIRNSEETLRAYTLSSSPGQSRF
LSISVRCLPDGVGSRWLTQEVKPGNTLWLSDAQGEFSCERHPADRYLMLAAGCGVTPIVSMCRWLTAN
RPACDIAAIVNVRTPADTIFADQWRALCAAHPQLRLTLMAERDLQPGYLSGRIDEQTLRQAAPDIAER
TVMTCGPAPYMEQVEQLCRQLGVPAERFHKEQFHTPTTQADATEGLTLRAARPLREFRVPVGSTLLAA
MEANALPVNAACRAGVCGSCKTRILEGDYTTTSTMTLSAEEVAQGYVLACSCRLQGDVTLA
>CORE_REP|Org47_Gene1273#
MNKHPKLTEADVTPESVFHQRRKVLQALGITAASLALPTGAQADLLSWFKGNDRPKAPPGKALEFSKP
AAWQAQLAMTPEDKVTGYNNFYEFGLDKADPAANAGGLKTEGWKVRIDGEVAKPITLDIDDLMKRFPL
EQRIYRMRCVEAWSMVVPWIGFELGKLIKLAEPNGNARYVAFQTLYDPEQMPGQKDRFIGGGLKYPYV
EGLRLDEAMHPLALLTVGVYGKTLPPQNGAPLRLITPWKYGFKGIKSIVHIRLVRDRPPTTWNQSAPN
EYGFYANVNPHVDHPRWSQATERFIGSGGILDVKRQPTLLFNGYADQVASLYRGMDLRENF

>CORE_REP|Org34_Gene1466#
MQPILEKLYRAESMSQQESQQLFSAIVRGELEPSQLAAALISMKVRGERPEEIAGAAKALLDDAQPFP
RPDYPFADIVGTGGDGTNSINISTASAFVAAACGAKIAKHGNRSVSSRSGSSDLLAAFGIRLDLPAEE
ARKALDDLGVCFLFAPQYHTGFRHAMPVRQQLKTRTLFNVLGPLINPARPPLALIGVYSPELVLPIAE
TLRVLGYQRAAVVHGGGMDEVAIHAPTHVAELNNGEISSYQLTPQSFGLETYPLEALLGGTPEENRDI
LARLLQGKGEPAHAAAVAANVALLLKLFGHEDLRQNARQALDMINSGQAYERVIALAARG
>CORE_REP|Org47_Gene4772#
MIDPRLPLTDIHRHLDGNIRAQTILDLGRQFNLALPANDLESLRPHVQITHAEPDLVSFLQKLDWGVA
VLGDLDACRRVAYENVEDAANAGLHYAELRFSPYYMAMKHQLPVAGVVEAVIDGIRSGCRDRDIDIRL
IGIMSRTFGEAACLQELEGLLALRDGITALDLAGDELGFPGSLFLNHFNRARDAGLRITVHAGEAAGP
ESIWQAIRELGAERIGHGVKAVEDPALMDFLAEHGIGIESCLTSNIQTSTVPSLAQHPLATFLRHGVL
ASINTDDPAVQGIEIEHEYRVAAPQAGLTPEEIRTAQENGLKLAFLSEQEKQALRAKVQG
>CORE_REP|Org44_Gene2053#
MAGKLFSLRNAALLALALSAASAYAVDVTVAYQTSAEPAKVAQAENSFAKRSGATVDWRKFDSGSSVL
RALASGDVQIGNIGSSPLAVAASQKLPIEVFLIASQLGSSEALVVKNDIKSPQDLIGKRIAVPFISTT
HYSLLASLKHWGIKPEQVKILNLQPPAIAAAWQRGDIDGAYVWAPVVNELAKQGKVLTDSAQVGEWGS
PTLDVWVVRKDFAEKHPDVVTAFAASALQAQKAYLAAPEQWLKDPSHLSTLARLSGVPEAQVPELVKG
NRYLPVADQVAQLGQPVDKAIRDTAEFLKQQGKIPQVDSDYSAYVTDRFVKQVQAAPQS
>CORE_REP|Org17_Gene4548#
MKKKRPVLQDVADKVGVTKMTVSRYLRNPDQVSAALQQKIAVALDELGYIPNRAPDILSNATSRAIGV
LLPSLTNQVFAEVLRGIESVTDAHNYQTMLAHYGYLPEREEERLTSLLSYNIDGLILSERHHTPRTLK
MIEVAGIPVVELMDCVSPCIDLAVGFNNFEAARQMTQQIIAHGHRHVVYFGARQDERTLIKQQGYEQA
MRESGLEPHSIMTARSSSYSAGGELLRVAQRDYPQIDSIFCTNDDLAIGAAFECQRQGLSIPQDMAIA
GFHGHDIGQVMVPKLASVLTPRERMGQIGAERLLARLRGETVCPRMVDVGFTVIPGGSI
>CORE_REP|Org6_Gene3318#
MKQIVYVASPESQQIHVWQLDDAGALALLQTVDVPGQVQPMTLHPDKTHLYVGVRPAFGIVSYRIEAD
GTLQQAGMAPLPGSPTHISTDLQGRYLFSASYSGNCASISPIGHDGVVTAPIQQIDGLSAPHSANIDP
TNQLVLVPCLKEDRIRLFNLSLAGQLTPHAQDAVDIAAGAGPRHMAFHHNDKFAYCVNELNGTVDVLA
ISENGGKYSVTQTLDIMPSDFDGTRWAADIHITPNGRFLYTSDRTASILTLFSVSEDGGTLSVAGYYP
TETQPRGFNIDHSGRFVISSGQKSDHIAVHEIDQASGKLTTLARYPVGKGPMWVSVLAK
>CORE_REP|Org31_Gene3749#
MELLCPAGNLPALKAAVDNGADAVYIGLKDDTNARHFAGLNFTEKKLQEAVDYVHRHGRKLHIAINTF
AHPDGYARWQRAVDMAAQLGADALILADLAMLEYAAQRYPQLERHVSVQASATNEEAIRFYQRNFDVG
RVVLPRVLSMHQVKQLARTSPVPLEVFAFGSLCIMAEGRCYLSSYLTGESPNTVGACSPARFVRWQQT
PQGMESRLNDVLIDRYQDHENAGYPTLCKGRYLVDDVRYHALEEPTSLNTLELLPELLAANIASVKIE
GRQRSPAYVSQVARVWRQAIDRCQADPAAYRADAAWMETLGAMSEGTQTTLGAYHRKWQ
>CORE_REP|Org39_Gene204#
MTIKVGINGFGRIGRIVFRAAQERSDIEIVAINDLLDAEYMAYMLKYDSTHGRFNGTVEVKDGHLVVN
GKTIRVTAEKDPANLKWNEVGVDVVAEATGIFLTDETARKHITAGAKKVVLTGPSKDATPMFVRGANF
DKYAGQDIVSNASCTTNCLAPLAKVINDNFGIVEGLMTTVHATTATQKTVDGPSHKDWRGGRGASQNI
IPSSTGAAKAVGVVLPELKGKLTGMAFRVPTPNVSVVDLTVRLEKAATYEEIKKAIKDAAEGSMKGVL
GYVEDDVVSTDFNGEVLTSVFDAKAGIALNDNFVKLVSWYDNETGYSNKVLDLIAHISK
>CORE_REP|Org46_Gene2042#
MHSNKRVVITPGEPAGVGPDLVAALAQQDWPVELVVCADPALLLERAKRLGLPLTLRDYQPQQPAEAQ
RAGTLTVLPVPLAHPVTAGELNVGNSAYVVETLARACDGCLNGEFAALITGPVNKGVINDAGVPFIGH
TEFFADRSRCDRVVMMLATEELRVALATTHLPLLAVPGAITQQSLFEVIRILDHDLKTKFGIARPHIY
VCGLNPHAGEGGHMGHEEIDTIIPALDALRAEGIHLVGPLPADTLFQPKYLQDADAVLAMYHDQGLPV
LKYQGFGRAVNITLGLPFIRTSVDHGTALELAGTGTADVGSFQTALNLAIKMIINCNE
>CORE_REP|Org2_Gene127#
MPLLDIRNLTIEFMTAEGPVKAVDRVSMTLTEGEVRGLVGESGSGKSLIAKAICGVTKDNWRVTADRF
RFDDIDLLQLSPRERRRLVGHNVSMIFQEPQSCLDPSESIGRQLAQAIPGWTYKGHWWQRFNWRKRRA
IELLHRVGIKDHDDIMGSFPYELTEGECQKVMIAIALANQPRLLIADEPTNAMEPTTQAQIFRLLARL
NQNNNTTILLISHDLQMMSKWADRVNVLYCGQTVESAQCEELLAAPHHPYTQALIRAMPDFGRSLPHK
SRLNTLPGAIPSLEHLPIGCRLGPRCPYAQKKCIETPRLRPVKNHFFACHFPLNMEEQ

>CORE_REP|Org3_Gene3928#
MAHNIPFPPRQPSASTHLPLTLISLEDWALVTLNGPDTVKYLQGQVTADIDALAADQHVLCAHCDAKG
KMWSNLRLFHRGEGFAYLERRSVLDSQLAEIKKYAVFSKVTIAADNDAVLLGVAGFQARAALADLFAT
LPSAEHPVVQDGETTILHFNAPAERFLLVTRPTVAEQLIGKLHDQAELNDSGQWLALEIEAGYPVIDA
ANSAQLIPQATNLQALEGISFSKGCYTGQEMVARAKFRGANKRALYWLEGKAARAPQAAEDLELQLGE
NWRRTGTVLASSQLADGRLWVQVVMNNDLDADSKLRVREDAASQLAIKPLPYPLAEEK
>CORE_REP|Org30_Gene2750#
MQQRKLGSHGPLVSALGLGCMGMSDFYSTGADRQEAIATLHRALELGVTLLDTADMYGPHTNEELVGE
AIKGKRQQVFLATKFGILRDPADPSARGVSSRPEYIRRSVEGSLRRLGVEEIDLYYQHRVDPQVPIED
VVGTMADLIREGKIRHIGLSEASVATLERAHKVHPITALQTEYSLWTRDAEQGVLAACERLGIGFVPY
SPLGRGFLTGAIRRPEDLAEDDFRRGNPRFQGENFARNLALVEKVGELAAQKGVKPSQLALAWVLAQG
EHIVPIPGTKRRRYLEENVAAAEITLSAAELAAIDAVFPLSAAAGDRYGAESMTYING
>CORE_REP|Org31_Gene600#
MKLAIYSTKQYDRKYLELVNQQFGYELEFFDFLLSKKTAKTAAGCKAVCIFVNDDGSREVLEELAALG
VEILALRCAGFNNVDLDAAKELGIKVVRVPAYSPEAVAEHAVGMMMCLNRRIHRAYQRTRDANFSLEG
LIGFNMHNRTAGVIGTGKIGVATMRILKGFGMKLLAYDPFPSEQALELGAEYVDLKTLYAQSDVITLH
CPLTPENHHLLNADAFAMMKNGVMVINTSRGALIDSTAAIDALKQQKIGALGMDVYENERDLFFEDKS
NDVIQDDVFRRLSACHNVLFTGHQAFLTEEALTSISQTTLQNISQLDRGEACPNQLNA
>CORE_REP|Org47_Gene1511#
MACGEFDLIARYFDRFKRVRRDVQLGIGDDCALLAVPEKQLVAVSTDTLVAGVHFLPDIDPADLGYKA
LAVNLSDLAAMGADPAWLSLALTLPEVNESWLKAFSDSLFDQLNYYGMQLIGGDTTRGPLSMTLTIQG
LIPAGRALTRSGARIGDWIYVTGTLGDSAAGLAILQDRLAVTETTARDYLVARHLRPQPRVLQGQALR
DLASSAIDISDGLISDLKHILKASDCGARIVLDELPMSQALSSHADAEQALRWALAGGEDYELCFTVP
EINRGALEVALSHLGADYTCIGQIGPLSEGIRYYRDDEAVELDWAGFDHFNAEPGTHG
>CORE_REP|Org42_Gene2589#
MAYLNVTRLNKHYGQTQVFQDIDFTAEEGEFVTLLGPSGCGKSTLLRCLAGLTPVDSGQILLQGQDLV
PLAPQKRGIGMVFQSYALFPNMTVEGNVAFGLKMQKLAAGQIGQRVQEVLALVELSDLAKRYPHQLSG
GQCQRVALARSLVTRPRLLLLDEPLSALDARIRKHLREQIRRIQRELNLTAIFVTHDQEEALTLSDRI
VLMNKGQIVQSGDAETLYTQPADAFAAGFIGNYNLLTAEQAAQLTGRSYVGKVAIRPESIGLLPAGQG
IGGVILGHSLLGNVVRYRIQVRGVELLVDVLNRSVADLRPDGEQIGLHLEPVVLREVA
>CORE_REP|Org46_Gene889#
MNLQQQLAQFPRLDLVGTATPLEKLSRLSDYLGREIYLKRDDVTPMAMGGNKLRKLEFLAADALRQGA
DTLVTAGAIQSNHVRQTAAVAAKLGLHCVALLENPIDTRAENYLTNGNRLLLGLFNAEVVMCEALHDP
QQQLAELATRLEAQGFRPYVVPVGGSNALGALGYVQCALEIAEQSQRSNVAFSSVVVASGSAGTHAGL
AVGLQQLLPETELIGVTVSRTVIDQLPKVEQIQKALACSLNIDELAPIALWDDYFAPQYGMPNEEGMA
AVQLLAQQEGVLLDPVYTGKAMAGLIDGIAQRRFRDEGPILFVHTGGAPALFAYHPQV
>CORE_REP|Org5_Gene1524#
MNKKVFTLAAMAAAMMFGAAAHADTRIGVTIYKYDDNFMSVVRKAIEKDAKASPDVTLLMNDSQNDQS
KQNDQIDVLLAKGVKALAINLVDPAAAPVVIDKARANDIPVVFYNKEPSRKALDSYEKAYYVGTDSKE
SGVIQGELIAKHWQANPAWDLNKDGQIQFVLLKGEPGHPDAEARTTYVVKTLNEKGIKTQQLQMDTAM
WDTAQAKDKMDAWLSGPNANKIEVVIANNDAMAMGAVEALKAHNKTTIPVFGVDALPEALALVKSGAM
AGTVLNDADNQAKATFELAKNLAAGKPATEGTQYKIENKVVRIPYVGVDKDNLSQFVK
>CORE_REP|Org22_Gene2932#
MKKQFIQKQQQISQVKSFFSRQLEQQLGLIEVQAPILSRLGDGTQDNLSGSEKAVQVKVKTLPDATFE
VVHSLAKWKRKTLGSYDFGAGEGLYTHMKALRPDEDRLTPIHSVYVDQWDWERVMGDGERSLDYLKST
VRSIYAAIKATEAEVSREHGLTPFLPEQIHFVHSETLLQRYPDLDAKGRERAIAKELGAVFLIGIGGK
LSHGKSHDVRAPDYDDWTTPAADGLAGLNGDIVVWNPVLQDAFELSSMGIRVDAAALKHQLALTGDEE
RMKLEWHQSLLRGDMPQTIGGGIGQSRLVMLLLQLSHIGQVQCGVWSPEVRGAVEGLL
>CORE_REP|Org20_Gene2905#
MSYPASPSRYQDMEYRRCGRSGLKLPAVSLGLWHNFGDATLYDNARGLIRCAFDRGITHFDLANNYGP
PPGAAEENFGRILNADLRAWRDELIVSSKAGYTMWPGPYGDWGSKKYLVASLDQSLRRMGLEYVDIFY
HHRPDPQTPLEETMAALDLLVRQGKALYVGLSNYPAERARQAFDILQRLGTPCVIHQPKYSMLERGPE
TALLDTLAEHGVGSIAFSPLAGGLLTDRYLHGVPQDSRAASGSRFLQPEQLTAERLDKVRRLDALARQ
RGQKLSQMALAWVLRGDRVTSVLIGASKNAQIEDAVGMLANRHFSEEELAQIEKILL

>CORE_REP|Org47_Gene1344#
MRKWGVGLTLVLLASGAMAKDIQLLNVSYDPTREFYQAYNTAFSKHYQAETGDKVTVRQSHGGSGKQA
TSVINGIEADVVTLALAYDVDAIAERGRIEKDWIKRLPDNSAPYTSTIVFLVRKGNPKQIHDWPDLIK
PGVSVITPNPKTSGGARWNYLAAWGYALHHNNNDKAKAQDFVKNLYKNVEVLDSGARGATNTFVERGI
GDVLIAWENEALLAEKELGKDKFEIITPSESILAEPTVSVVDKVVDKRGTRDVATAYLKYLYSPEGQT
IAAQHYYRPRDAAVAAKFAGQFPQLKLFTVDDTFGGWTEAQKVHFATGGVFDEISKR
>CORE_REP|Org8_Gene900#
MSQNQPLLQAIDLKKHYPVKKGLFAPERLVKALDGVSFTLERGKTLAVVGESGCGKSTLGRLLTMIEV
PTGGELYYQGQDLLKPDVSAEKLRRQKIQIVFQNPYGSLNPRKKVGQILEEPLLINTSLSAAERREKA
LEMMAKVGLKTEHYDRYPHMFSGGQRQRIAIARGLMLNPDVVIADEPVSALDVSVRAQVLNLMMDLQQ
ELGLSYVFISHDLSVVEHIADEVMVMYLGRCVEKGSKEAIFNNPRHPYTQALLSATPRLNPDMRRERI
KLTGELPSPMNPPPGCAFNARCRRAFGTCVQLQPQLKQYGEQMVACFAVDQDEHPGA
>CORE_REP|Org44_Gene57#
MQGSVTEFLKPRLVDIEQVSSTHAKVTLEPLERGFGHTLGNALRRILLSSMPGCAVTEVEIDGVLHEY
STKEGVQEDILEILLNLKGLAVRVQGKDEVILTLNKSGIGPVTAADITHDGDVEIVKPQHVICHLTDE
NAAISMRIKVQRGRGYVPASARIHSEEDERPIGRLLVDACYSPVERIAYNVEAARVEQRTDLDKLVIE
METNGTIDPEEAIRRAATILAEQLEAFVDLRDVRQPEVKEEKPEFDPILLRPVDDLELTVRSANCLKA
EAIHYIGDLVQRTEVELLKTPNLGKKSLTEIKDVLASRGLSLGMRLENWPPASIADE
>CORE_REP|Org21_Gene2443#
MIGIEQPAVARRAAGPSLGKRWEKLLHHPAVLPFIGFAVLFVLMSLLNDSFLSVNNLTNVARQVSINA
IIAVGMTCAILTGGIDLSVGPVMALAGSVAAGLMLAAVPIPLAMVAALAVGALFGLANGACIAYLRMP
PIIVTLASMGIARGLALLYTGGYPISGLPDVFSFFGRGTVLGIQVPILIMLGVYVLAWLMLNQLPFGR
YVYAMGGNEEAARLSGIRVPRYKMLVYVISGLTAALAGLVLTSRLMSGQPNAGEGFELDAIAAVVLGG
AAISGGRGAIVGTLVGAMMLGVLNNGLNLMNVSPYIQNVVKGGIILAAIYLSSVRRK
>CORE_REP|Org26_Gene583#
MDKSLLARLAGRHEFYLGLLVLLLAIGLSAGTDEFLTLGNLTDVATSYAILGILACGLFVVLIAGGID
ISFPAVTAIAQYVMASWVIQHGGSFPLAFALAIGVGLLLGLVNGFLVYWLKVPAIIITIATLNLFYGL
LVYATNGTWLYGFPDWFMNGINWFSFQGADGYDYGLTLPLLCLLATIVVTGVLMNRTRLGRQIYAMGG
NRDAASRLGLNLLRLHFCVYGYMGILAGVAAVVQAQITQSVAPNSLLGFELTVLAAVVLGGTSMSGGR
GSLTGTLLGVVLLAFLQNGLTLLSVSAYWHQVFSGAIILISISTTAWNEKRKLLKEI
>CORE_REP|Org2_Gene2735#
MPRVEPIKKVSVVIPVYNEQESLPALLERTTAACKQLSQPYEIILVDDGSSDNSADMLTAAAEKPDSH
VIAVLLNRNYGQHSAIMAGFNQVTGDLVITLDADLQNPPEEIPRLVSVAEEGYDVVGTVRANRQDSWF
RKSASRVINMMIQRATGKSMGDYGCMLRAYRRHIVEAMLHCHERSTFIPILANTFARRTTEIDVRHAE
REFGDSKYSLMKLINLINLMYDLITCLTTTPLRLLSVVGSIVALSGFVLALVLIALRLLLGPEWAAGG
VFTLFAVLFTFIGAQFVGMGLLGEYIGRIYTDVRARPRYFVQKVVGAQQGHNTQEEE
>CORE_REP|Org6_Gene934#
MGQLVDGVWQDIWYDTKSTGGRFKRSTAQFRNWVTADGQPGEHGAGGFQAEAGRYHLYVSLACPWAHR
TLLMRKLKGLEQIIPVSVVHPLMLENGWTFGQDFPETTGDPLYHADFLYQIYLRADPHYSGRVTVPVL
WDKQRQTIVSNESADIIRMFNSAFDGVGARAGDYYPTDLRGKIDELNGWIYDQVNNGVYKAGFATSQE
AYDEAVNGVFNALERLEQILGQHRYLTGDRLTEADLRLWTTLVRFDPVYVTHFKCDKHRISDYLNLYG
FLRDIYQMPGIAETVSLPHIRNHYYRSHATINPHGIISIGPAQDLDEPHGRDQRFG
>CORE_REP|Org20_Gene1136#
MRRFSLKPRGNEGYLAWVLLLTVIVFSLLSEQFLTVQNLLDLCESYAVSGIFALGLFVVLVTGGIDIS
FAAVASVVQYLIATLATHYGLASPAGSILLALAIGAALGMVNALLIYCLRIVSIIVTISMQALLFGML
MWLTNGRSLYALPDWWTLPRSVLPFQLGEQSYQLGLPTLVMLAVALLTWLLLNKTHLGRQLFAVGGDA
ESARRIGIRVGLLHLFAYGYLGVMAAIGGLVQVYRMGEVVPNALVGGELDVLAAAVLGGASLNGGKGS
VIGTLMGVFLIGVLKNGLNLIGVSSYFMNVVIGLVIVAAITVTHYKKRKETDVGFA
>CORE_REP|Org41_Gene1531#
MSNIELQPGFDFQQAGKEVLQIEREGLAQLDSYINADFTRACETIAACGGKVVVMGMGKSGHIGCKIA
ATFASTGTPSFFVHPAEASHGDLGMVTPQDIVLAISNSGESSEILALIPVLKRQQITLICMTNNPESS
MGKAADIHLCIKVPQEACPLGLAPTTSTTATLVMGDALAVALLKARGFTPEDFALSHPGGALGRKLLL
RVSDIMHSGDEMPHVSADASLRDALLEITRKNLGLTVICDDLMKIAGIFTDGDLRRVFDMGINLHEAK
IADVMTPGGVRVRPNILAVDALNLMQQRHITALLVADGDQLLGVVHMHDMLRAGVV

>CORE_REP|Org32_Gene2967#
MNNSAFNFDTLSPDLIMDALEGIGLRVDSGLTALNSYENRVYQFMDEDRRRYVVKFYRPERWSAAQIG
EEHQFALDLAGAEIPAVAPLALQGTTLHMHGGFFFTLFPSVGGRQYEIDNLDQLEWVGRFLGRIHQVG
GERLFAERPTMGIEEYLIAPRQALADCELLPAAQREAFLQATDNLIAAIKPHWHLNWQPRRLHGDCHP
GNILWRDGPLFVDLDDARNGPAVQDLWMLLHGERRDQLMQLDVLLEAYGEFAEFDQRELALIEPLRAM
RMVYYLAWVARRWQDPAFPKSFPWMAESDFWLSQTAAFTEQVKLLQEPPLQLTPMY
>CORE_REP|Org10_Gene2062#
MKFNLALLNACVVSACMLSTTPVFAADKYEIAVVAKVTGIPWFNRMEVGVNEAAKKLDVNAYQTGPST
PDPAAQVKVIEDLIAKNVNAIIVVPNDAKVLEPVLKKARDKGIVVLTHESPDQQIGQWDIETIDSEKY
AQANVDELAKDMGGKGGYAIYVGSLTVPLHNAWADFAIKYQKEKYPDMFEVTSRLPVAESIDKSYATT
LDLMKTYPQMKGIIGFGSLGPIGAGQAVAKKRAKDKIAVVGIAMPAQAAPYLMRGDIKKALLWDPKDA
GYALVTVADQLLQGKEVNKDLSIEGLGKADVDMEHKVIRFNKILEVTKDNAKSLGF
>CORE_REP|Org22_Gene3003#
MTPSLRAATVQFQHRANDKNYNLSTMERFITEAAEQQIQLLAFPEMCITGYWHVRHLSDAEVRALAEP
IASSPSLARIRPLAERYNMAIGVGLIELGDDGRCYNAYAVCLPDGELHVHRKLHAFEHPAIASGDRYT
VFDTPWGVRVGVLICWDNNLVENVRATALLGAEVLIAPHQTGGTHSRSPHGMKPIPQALWQRRAEDPQ
AIEAAFRGEHGRGWLMRWLPSRAHDNGLFLLFSNGVGRDDDEIRTGNAMILDPYGRIVAETWAAEDRM
VSAELDLTLIPLSTGRRWIYGRRPELYGLLTEPQGYERDARSARFSTQPTGRGGR
>CORE_REP|Org2_Gene3678#
MRLIIDCDPGNGVPGANVDDGLALALALAAKPQLQLELISIVAGNTPREVGFTVATDLLAQSGYQVPV
ALGAARALSEPPEPWRAHLDRPIADPKLAALWRDLPAPSLANAPVPDAAIAIGELICRHPGEITLAAI
GPLTNVAHAMQLYPQMAQAVKEIVIMGGVFNVEGYIKDTNFGLDPEAARLVLNSGAAITLAPLDVTTQ
TMLTQADLAALTQPDTPLCRYLRATTQPWIDYSRHTRHLPGCWIHDALVIAWLLAPQLVTTEMFHVDV
ALEGALTRGSSRRWRPDSLRLTVGMPAPQGKPVRVMQQVDNARLLALIGATLARG
>CORE_REP|Org2_Gene1452#
MDSKSMVVNCVAYKAGQRLGEVTIDDISEVVKQPDTFIWLGLRQPEPAFMRKVQEEFGLHDLAIEDAL
CAHQRPKLETYGDSLFIVVKTAQWGENDEIEYGETHFFVGKNFLVTVRHGASPSYAPIRAKAEENRKQ
MCRGPGFALYSVLDFVVDNYRSVVTRFESTIENIEANMFQSEFDQAAIENVYTLRRHLLALRNAALPM
DEICNQLIRLHEEVIPKELRAYVRDVQDHAHQVVSNIDDMREMLTNAMHVNLALVTVKQNEVVKRLAG
WGAILAIPTVIFSLYGMNFADMPELKFPWAYHATLGVTAVGCIFLYQKLKKSGWL
>CORE_REP|Org11_Gene3964#
MAKRIQFSATGGPEVLQYVDFTPLDPAAGEVQIENKAIGINYIDTYVRSGLYAPASLPSGLGTEAAGV
VTKVGAGVSAIKPGDRVVYAQSALGAYSEIHNVSAERVALLPGNLSFEQGAASFLKGLTVYYLLRQTY
DVQPGEVFLFHAASGGVGLIACQWAKALGARLIGSVGSDEKAALAKQAGAWATINYHKEDIAQRVAEL
TQGEKVGVVYDSVGKSTWLASLDSLKRRGLMVSFGNASGPVTGVDLALLNQKGSLYVTRPSLNGYITN
RAELQYASNELFSLIGSGAIRVEVKDEQKFALADAQRAHQVLESRSTSGSSLLIP
>CORE_REP|Org28_Gene4581#
MIDLLPLTLEGNEPVEWQGETPQLRWRWQGEGVLELTPRQPYRQAMVMSAGVHGNETAPIELLNQLVG
DLLTGRLPLRVRLLVVLGNPAAMRAGKRYLHSDMNRMFGGRYRNFAASGETVRAQQLERALAAFFDGE
QAARFHYDLHTAIRESRLPRFGILPFQKRPYSEPMLKLLDAADLDALVVHSAPGGTFSHYSSEHLNAA
SCTLELGKARPFGSNDLQQFAAIDLALQAAVSEGPLPARAADEIRVFRVMHSLIKHSEDFKLHLDDDT
ANFTELKPGMLLCEQPQEEYRVGQEGAWILFPNPTVALGLRAGMLLSEVSRSTLY
>CORE_REP|Org18_Gene3741#
MNGVNRSAALYRYSLPMEAGVVLRNQRLKTRDGWVVQLRQGEREGWGEIAPLPEFSRETLAQAEQAAL
DWLQAWLAGNEPDDSALPSVAFGISCAQAELEQRLPTQADFRKAPLCTGDPDELFETLNALPGEKVAK
VKVGLYEAVRDGMIVNVLLEALPDLRLRLDANRSWTRAKADGFAKYVNPAWRDRIAFLEEPCKTRDES
RDFARATGIAIAWDESVREADFVVQAEPGVAAIVIKPTLVGSLARCQALVAQAHAAGLTAVISSSIES
SLGLTQLARIARWLTPDTVPGLDTLGLMQAQVLRPWPGSALPLLDASALECLWRR
>CORE_REP|Org7_Gene185#
MPHLAELVANAKAAVEDAQDVAALDLVRVEYFGKKGHFTLQMQSLRDVPAEDRPAAGAVINQAKQEVQ
DALNARKNALESAALNARLAAETIDVSLPGRRMENGGLHPVTRTIDRIETFFGELGFSVATGPEIEDD
YHNFDALNIPGHHPARADHDTFWFDATRLLRTQTSGVQIRTMKNQQPPIRIIAPGRVYRNDYDQTHTP
MFHQMEGLIVDKDISFTNLKGTLHDFLNNFFEEDLQVRFRPSYFPFTEPSAEVDVMGKNGKWLEVLGC
GMVHPNVLRNVGIDPEVYSGFAFGMGMERLTMLRYGVTDLRAFFENDLRFLKQFK

>CORE_REP|Org38_Gene2987#
MSAYIRIEKGASGWGGPLELPIEPGKKVVYITAGTRPSIVDRLSELTGWPAVDGFKEGEPPAEEIGVA
VIDCGGTLRCGLYPKRRIPTVNIHATGQSGPLAQFILEDIYVSGVRDHNISRIDATENPPAAAAVAAP
PKARDYDTSKKITEQSDGLLAKVGMGMGSVVAVFFQAGRDTIDTVLKTILPFMAFVSALIGIIMASGL
GDFIAHGLTPLANSPIGLVTLALICSFPLLSPFLGPGAVIAQVIGVLVGVQIGLGHIPPQLALPALFA
INAQAACDFIPVGLSLAEAKQDTVRVGVPSVLVGRFLTGAPTVLIAWAASAFIYQ
>CORE_REP|Org25_Gene4545#
MVTLEDVAALAGVSRATVSRVVNGDSNVKAPTREKVERAVAQLGYTPNPAARALASSHSNTLGLVTTS
YRGGFFGALMDFVQTEAESHGKQLLVTQGRNSAENEWQAIQRLFSLRCDGVILHVRFLSDDRLRQLAA
EQRDFVLLDRLVPGLEARCVTFDHPLASRMATQQLLDAGHRRIACISGPRERPSSRLRLQGFEEAMQA
ANIEPVACLEGVYDLESGYRCADRLLRQAAPPSAIYCCNEEMAIGALLAINEHRLRVPQDISLICYDS
GERAPFVRPALSSVHFPISEMAQYAARRLIDPATPTHRFEPTIINRDSIVTVRK
>CORE_REP|Org24_Gene288#
MALLNVDKLSVHFGDEGTPFRAVDRISYSVEQGQVVGIVGESGSGKSVSSLAIMGLIDFPGKVMADKL
EFNGQDLRKISEKERRQLVGSEVAMIFQDPMTSLNPCYTVGYQIMEALKVHQGGNRRTRRQRAIDLLT
QVGIPDPASRLDVYPHQLSGGMSQRVMIAMAIACRPKLLIADEPTTALDVTIQAQIIELLLDLQQREN
MALLLITHDLALVAEAAHHIIVMYAGQVVESGKAAEIFRAPRHPYTQALLRALPEFAADKARLASLPG
VVPGKYDRPTGCLLNPRCPYANERCRNEEPELRSIPGRQVKCHTPLDDAGRPTV
>CORE_REP|Org22_Gene2986#
MIERIWSGGSLLYLALLPFSWLYGLLSWLIRLSYRCGLRKSWRAPVPVVVVGNLTAGGNGKTPMVIWL
VEHLQQRGYRVGVVSRGYGGKSAVYPLVLNQNTSTREAGDEPVLIYQRTGAPVAIAPKRAEAVQALLQ
QQPLDAIITDDGLQHYALQRDFELVVIDGVRRFGNGWWLPAGPMRERAARLGSVDACVANGGVAQAGE
IAMRLQARDAVNLLSGERRPAAELPRVVAMAGIGHPPRFFATLEKLNVEVVQEVAFADHQEYQQPQLT
GLVTAEQTLLMTEKDAVKCRAFAQPNWWYLPVDAVLPSAQAEQLLQDIESLLTK
>CORE_REP|Org30_Gene3714#
MPNTFRPLTGASNPHLQTLLPRLVRRRVLLKPHWQRLELPDGDFVDLAWSEDPAQARNKPRAVLFHGL
EGSFYSPYAHGLLNAWRERGWLGVVMHFRGCSGVPNRKQRIYHSGETEDARFFLRWLRDRYGEVPTAA
VGVSLGGNMLACYLAQQGADSLLQAAVVVSAPLMLEPCAYRMEQGFSRVYQRYLLGQLKQNATRKLLH
YPDTLPLKLSQLNGLRRIREFDDAITSRIHGFSDATDYYRRCSALPLLPAVQTPLLIIHAKDDPFMTP
EVIPDVAGLPRNIEYQLTEFGGHVGFVGGTLKKPYMWLEHRIPAWLSPYLDIPT
>CORE_REP|Org8_Gene2823#
MMKKVLALCLSGYSALAIAGFDVVALGVDGGVSDGNLTSYLIRSDNQPQYVALDAGSLLPGIAKGLEK
GSFPQVTAELAAPYTPQGHVFRQLIGSYFISHGHLDHVAGLIVGSPEDNKKTVYAQADTVLTLRNHYF
NWKAWPNFTDAGNGARLGTYRLQTVRPQQRFTLGLTGLTGVMYPLSHDRYPSSMLLISNGNEFFAYFG
DTGPDKVEQSRDLDTVWRALGPLVEQKKLKGMIIETSYPNGVEDKHLYGHLTPAWLLKELKNLTQYSG
GEGSLKGLPVVISHIKPSLKQGEDARAAIAQQLTQGNDLGVKFILMAQGDRQTF
>CORE_REP|Org43_Gene3526#
MKTAGKNLNQGSFGQGRAQWGKAFGRSLMASMVLVVGLAGSAQAAPASNPAVAESVAPTTAPAPAAAA
APESITPVNPAPTIQPPETRGMDLSVWGMYQHADAVVKAVMIGLVLASIVTWTILFSKGSELLRAKRR
LRREQLALAEARSLDEASELAQNFAPESVSAVLLNDAQNELELSAESNDNNGIKERTGFRLERRVAAY
SRNMGRGNGFLATIGAISPFVGLFGTVWGIMNSFIGIAHSQTTNLAVVAPGIAEALLATALGLVAAIP
AVVIYNIFARVISGHRAQVGDVAAQVMLLQGRDLDLAATAEAKRSQHAHQLRAG
>CORE_REP|Org3_Gene1124#
MANMFALILALATLVTGIIWCFERFKWAPARRAKIAAVNAQTAGAVDDKTLAKVAKQPGWVETGASVF
PVLLLVFVVRSFIYEPFQIPSGSMMPTLLIGDFILVEKYAYGIKDPITQTTLIETGHPKRGDIAVFKY
PLDPKLDYIKRVIGLPGDRITYDPVNKRVTVQPSCNSGQSCDTALAVTYADAQPSDFVQLFSRSGMGE
ASNGFYQIPLSDNVPPGGIRMRERQESLGNVTHRILTVPDAQDRVGAYYQQPGKPLAEWVVPAGHYFM
MGDNRDNSADSRYWGFVPEKNLVGKATAIWMSFEKQEGEWPTGVRFSRIGGIH
>CORE_REP|Org14_Gene3424#
MRALLLEQQDDQTLAHIKDIDSDRLPEGDVTVDINWSSLNYKDALAITGKGKIVRNFPMVPGIDFAGT
VRHSRDDRFKEGQSVVLTGWGVGEHHWGGLAEQARVSGEWLVPLPNGLDERKAMIIGTAGFTAMLCVM
ALEEGGVHPDDGDILVTGASGGVGSTAIALLAALGYRVTAISGRDSNTDYLKALGAKEVMPRDAYIDM
PRPLEKQLWAGAVDTVGDKLLARVLAQMNYNGTVAACGLAGGYQLPTTVMPFILRNVRLQGVDSVHTP
LHRRQTAWERLAGILPDSFYQQATHEIPLEQAPATAAALLNNQITGRTLVKIR

>CORE_REP|Org37_Gene194#
MAQQVQLTATVAESQLGQRLDQALAELFPDYSRSRIKEWILGDRVKVNGKTSNKPKEKVLGGETVAID
AQIEEEARWEPQDIALDIVYEDSDILVINKPRDLVVHPGAGNPDGTVLNALLHHYPEIADVPRAGIVH
RLDKDTTGLMVVAKTVPAQTRLVEALQAREITREYEAVAIGTMTAGGTVEEPIARHSTKRTHMAVHPM
GKPAVTHYRIMEHFRAHTRLRLRLETGRTHQIRVHMAYISHPLVGDPLYGGRPRPPKGASEAFINTLR
GFDRQALHATMLRLYHPISGIQMEWHAPLPQDMVDLINALKADTEEFKDQMDW
>CORE_REP|Org46_Gene3314#
MSETASWQPSAPIANLLKRAAILAEIRRFFADRGVLEVETPTMSQATVTDVHLFPFETRFVGPGAAEG
LTLYMMTSPEYHMKRLLAAGSGPIYQMGRSFRNEEAGRHHNPEFTMLEWYRPHYDMYRLMNEVDDLLQ
QVLDCENAETLSYQQAFLRHLDIDPLSAEKAQLREAAAKLDLSNIADTEEDRDTLLQLLFTVGVEPHI
GREKPAFVYHFPASQAALAEISTEDHRVAERFEVYFKGIELANGFRELTDGREQQQRFEQDNRKRAER
GLPQQPIDYNLLAALQHGMPECSGVALGVDRLVMLALGAESLSDVLAFPVGIA
>CORE_REP|Org23_Gene3300#
MNRDFTFTIKSSSFNEDYNPSESTRITTNFANLARGENRRENLRNTLVMINNRFNTLAHWDNPKADRY
SVELEIISVEMRVEDQGASFPVIEILKTNIIDKKTQKRIEGIVGNNFSSYVRDYDFSVLLPAHNKNTA
EFTIPDDFGDLHGNIFKHFVNSNEYHENFSKPPVICLSVSSKDTYHRTGNQHPVLGDEYRQDGASLTD
RYFKKMGLQVRYFMPKNSVAPLAFYFPGDLLSDYTDLELIGTISTMETFQKIYRPEIYNANSAAGQCY
QPSLNNQDHSLTKIVYDREERSQLAIEQGKFTEEQFIKPYKPLLEQWSAHYAL
>CORE_REP|Org41_Gene391#
MFLAIALLIVGLFLLVYGADRLVYGAAVISRSLGVPPLIIGMTIVGIGTSLPELIVSTTAALNGQIDM
AVGNVLGSNITNILLILGVAALIHPLAARSEILRRELPLMLAVTVLCGFVLMDGTLSRLDGVLLLAAA
AGFILLMLKIARLAQREGSDSLTMEQIAELPQDSSNTVAVLWLVLAFIILPLSSRMVVDNATVIAHYF
GLSELVVGLTIIAIGTSLPELATSIAGALKGEDDMAIGNIIGSNIFNTVIVLGVPALLSPGSVDTAAF
QRDYWVMLAASVLLSVLCIGRKHRIGHLAGALLLCGFIAYLAVLFFNPFSTFG
>CORE_REP|Org35_Gene2737#
MSNSLTYCDLPAEISQWPGLPLSLSGDEVMPLDYRAGNTGWLLYGRALDKARITQFQRKLGAAMVIVT
AWGVDDYQVVRLAGTLTPRAKLLAAESGLDVAPLGKIPHLRTPGLLVMDMDSTAIEIECIDEIAKLAG
VGEQVAEVTERAMRGELDFTASLRQRVGTLKGADANILKQVRDELPLMPGLTSLVGKLQAMGWHVAIA
SGGFTYYAEYLRNRLRLVAAAANELEIRDGKLTGEVLGPVVDAQFKADTLLRLAEKLEIPLAQTVAIG
DGANDLKMMQAAGLGIAYHAKPKVYEKAQVAIRHADLMGVLCILTGSLKHEVR
>CORE_REP|Org40_Gene2704#
MPKKTVDLSQPHAQHDIRAFLDALNAAGGKPMEQMKPKEARRVLEDAQRSVEVPLREVEISEKTIHVE
GQDILLQLVRPARVNEAPPVFMFFHGGGWVLGDFPTHERLVRDLVYSSGAAAVFVNYPRSPEVRYPEA
INLAYAATEWVAEYGEKINVDGSRLAVVGNSAGGNMAAVVSLMAKEKGIPALRCQILLWPVTRAQFDT
DSYHQFAEGHFLTRNMMKWFWDSYAPDKAQRKDIYASPLNATPEQLRGLPPALVQTAELDVLRDEGEA
YARLLDAAGVEVTATRYNGLIHDYGLLNPLAHVPAVHSAIHQAGRALKHYLS
>CORE_REP|Org19_Gene877#
MKLQQLRYIVEVVNHNLNVSSTAEGLYTSQPGISKQVRMLEDELGIQIFARSGKHLTQVTPAGQEIIR
IAREVLSKVDAIKAVAGEHTYPDKGSLYVATTHTQARYALPNVIKGFIERYPRVSLHMHQGSPTQIAE
AVSKGTADFAIATEALHLYDDLIMLPCYHWNRAVVVKPDHPLAGKSSISIEELAAYPIVTYTFGFTGR
SELDTAFNRAGLTPRIVFTATDADVIKTYVRLGLGVGVIASMAVDPVQDPDLVTVDASDIFTYSTTKI
GFRRSTFLRSYMYDFIQRFAPHLTRDVVDSAVALRSNEEIEAMFKDIKLPIK
>CORE_REP|Org49_Gene4224#
MNTEASQDKTVSMTTGQRLRQAREQLGLSQQTVAERLCLKMSTVRDIEEDSVSADLASTFVRGYIRSY
AKLVHLPEDELLPMLAKQAPLKMAKVAPMQSFSLGKRRKKRDGWLMSFTWLIVFVVIGLTGAWWWQNH
KAQQEEIATMADQSSAQLSQNNEGQSVPLTDGNADAGTNVPLTDNSATPADAGAAAQAPAATAQAPSA
AQEQPAVVSPSQTTLPETTPAAQAPLPTADAGVAAPAADPNALVMDFSADCWLQVSDASGKTLFSGTQ
KKGGKLNLAGTAPYKLTIGAPAAVQIQYQGKPVDLSRFVKSNRVARLTVAAQ
>CORE_REP|Org22_Gene2067#
MFNVVTQLENDAVEMRKVYAGAVRRQIEAGAPIIALEADLMSSMAMDGVHKDHPQHVINCGIMEANVI
GVAAGLSLTGRVPFVHTFTAFASRRCFDQLFMSLDYQRNNVKVIASDAGVSACHNGGTHMSFEDMGIV
RGLAHSVVLEVTDATMFADILRQLMDLRGFYWVRTIRKQATRIYQEGSRFTIGKGNLLRDGDDITLIA
NGIMVAEALKAAQMLAQQGVSAAVIDMFTLKPIDRELIKTYAAKTGRIVTCENHSIHNGLGSAVAEVL
AEECPAPMRRVGVKERYGQVGTQAFLQQEYGLTAEHILEAAGQLLQKQFSSQ

>CORE_REP|Org31_Gene2363#
MTKTNLITGFLGSGKTTTIRHLLAHKPEHERWAVLVNEFGEIGIDGALLADSGAVLKEIPGGCMCCVN
GLPMQVGLNMLLQQAKPDRLLIEPTGLGHPKQILSLLTQESYAGWIDLQATLCLLDARQLSQPRYRDN
ENFRDQLAAADVILASKSDTYQPEDRRALEAWRAQDPLRRPCYAIAQGEADVALLSLPRTNRTELPDA
QHHHGQAKTQGLAALRLPEHARWRRALNEGQGFTSCGWIFDGDTQFDTVGFMEWVRLAPVERAKGAVR
IPEGTLLINRQGQDLNIETRPVAPLDSRIELIHSENADWNALQSALFKIRLS
>CORE_REP|Org3_Gene1465#
MKYLKLGNTDLNVSRICLGCMTYGEPNRGNHAWTLPEESSRPLLKQALEAGINFFDTANSYSDGSSEE
ILGRALRDYARREDVVVATKVYFPLSNLERGLSRANIMQSIDDSLRRLGTDYVDLLQIHRWDYETPLE
ETLEALHDVVKAGKARYIGASSMYAWQFAKALYTADLHGWTRFVSMQDQYNLIQREEEREMHPLCTAE
GIAVLPWSPLARGRLTRPWGETTARLVSDQFGKSLYEETEGIDAIIAERVASLADERGVSRAQIALAW
LLNKPAVSAPIVGASRSEQLDDAIAAVDLSLSPQEVAELETAYVPHRVTGFE
>CORE_REP|Org19_Gene872#
MRDMRFYPEAWPLHTAFVIARGSRTEARVVVVEIEQQGVRGTGECTPYPRYGESEESVMAQLAEMAPA
IAQGITREQLLAQMPAGAARNALDSALWDLECRLHGQSLWQRSGVAAPAHIRMAQTVSIGTPEAMAFA
ARELERQGATLLKIKLDDHYISERLVAIRAAVPQATLIVDANESWQAEGLAARCQLLADLGVAMLEQP
LPAGDDAALEHFIHPLPICADESCHTRADLPRLAQRYQMVNIKLDKTGGLTEALALAQAAREQGFDIM
LGCMLCTSRAISAALPLAPAARFVDLDGPTWLARDVEPGLAFECGAIVDIAP
>CORE_REP|Org21_Gene1185#
MTLLEKLKAVTTVVADSGDMNAIREYHPEDATTNPSLILKAAAQPIYQPLIEQAVALARQQGGSQETQ
LINASDHLAVNIGVALLSQVPGKISTEVDARLSYDRGLCVSKARKLIRLYEHQGVDRSRVLIKLASTW
QGIKAAEELEREGIHCNLTLLFSFAQARACAEAGVWLISPFVGRIYDWYRDRNLLASDAPQDDPGVVS
VRRIFDYYKQHRYPTVIMGASFRKVEQVLALAGCDRLTISPALLDELATRDGELPTMLKPPVIANEAP
SPLSESEFNWLHNQDPMAVDKLAEGIRLFAQDQEKLEGLLKAAFAREAVGQV
>CORE_REP|Org27_Gene2677#
MMNSVGTPWLWGSFAAVIVVMLAIDLLLQGRKGAHTMSMKQAASWSLVWVSLSLLFNFGFWYYLNETA
GRAVADTQALAFLTGYLIEKALAVDNVFVWLMLFSYFAVPANLQRRVLIYGVLGAIVLRTIMIFAGSW
LVSQFQWLLYLFGAFLLFTGIKMALAKEDDSAIGDKPLVKWLRSHLRMTDNLEGERFFVRRNGILFAT
PLVLVLILVELSDVIFAVDSIPAIFAVTTDPFIVLTSNLFAIMGLRAMYFLLANVAERFSMLKYGLSV
ILVFIGIKMLIIDFFHIPIGVSLGVVAGILTLTLLINAWVNRRNDRLANKQP
>CORE_REP|Org31_Gene1684#
MSSMPIATQPTLYRIHPASFRDGNGDGVGDAHGMLAALPYLKALSIDGLLLPQTLAPEAEATVTAEGL
TLWYGDEANRVRNAVAPQRFVHGALALDVMPFSAEKLVAVLHARRATLADSLWSTGDADQPRVVSRWG
QGDLRSAAAFLTLLAMLPAPICLYQGEELGLPHAAGLQDPRGAQTPMPWHEAPEQVTAGEISWYQQVA
IEHRALAISRQQHDSHSTLRYCQALLALRRSPLIQRGELNAISQRDGVVRLLITHQDQCLEALINLQP
YTQAAAPSEATLPLAWQHGAQQEGHQWVLAGFASAIFTRHVNCESRGVTHG
>CORE_REP|Org1_Gene3115#
MSNANALLAFARETLEIELTEAQRLLARLDDNFVCACELLLNCRGKAVISGIGKSGHIGKKIAASLAS
TGTPSFFVHPAEALHGDLGMIGADDVVVFISYSGRAKELDLILPLLAENGIPVIAVTGGKESPLTQAA
ACVLDIGVEREACPMGLAPTSSAVNTLMMGDALAMALMRQRGFNAEDFARSHPGGSLGARLLNRVHHL
MRTGDRLPRVSESANVMEAMLELSRTGLGLVAVCDAQQRVVGVFTDGDLRRWLVKGNSLQDPLSPAIT
RPGYRLPEQWRAGEALEALHEQHISAAPVVDMDGVLVGALNLHDLHQAGIG
>CORE_REP|Org10_Gene2633#
MDTKQQVGVGISLALTTAVCWGALPIAMKEVLVVMEPFTVVWYRFTMAAIGLGVILAVRGKLPRLTMF
RQPRWLLLLAIATAGLLGNFVFFSSSLQYLSPTASQVIGQLSPVGMMFASVLILKERMRITQVIGALM
LICGLMLFFNVSLIEIFTRLTDYTLGVMLGVCAAMVWVSYGVAQKVLLRRLASPQILVMLYTLCAIAL
FPLAKPEVIFQLSGWQLACLLFCGANTLIGYGALAEAMARWQAAQVSALVTLTPLFTLLFSDLLALAW
PQAFAAPTLNIVGYVGAFVVVAGAMFSAIGHRWWPRRAEPPLVAPLKQPGE
>CORE_REP|Org22_Gene2100#
MTTVYESNRFAFQVSWGSILAGSAVALVTYLIFSVLGTAVGAQAVDMMQKGNPLSGFGTGTGIWLLVS
TLASLAAGAFVAGRTAPNRGGLHGLLSWAITTLLTTWLVVSLASGVVGLAGSAVGKGLSLAGSGLAAA
APNVGEGLKQQLDKKGISLDWGSLENQLNATLKQTGKPELDPARLEQKADRATADGKQSAMDAAADPA
RAASELKQWFDRVKQSGEPTLSAADKDALVNIVAARTGKSRDEASQIVDNYAQAYQQAVQKVEKLKAE
AEQKAREAADEAAKQLSRAAWGSLLVLLLGAALSAGVGRIAESTRRTLAVS

>CORE_REP|Org13_Gene3194#
MNYQFENLVFEGGGVKGIAYGGALELLEAKGIMPQIKQTSGASAGAIAALLVGLGCSSADVTKILSAM
DFKKFLDYNGGFFGTLQDAYRLFNQYGIAPGDYFYQWSRDIIKQYTGKPDITFEQFEAMKAAKGFKSI
YFIGANLNSGQREVYSHRTTPRMKVADGLRISMSFPFAFVAKNNTLGDLCIDGGMIDNYPVRLFDYDF
SATPPYIDSSSQRINTRTLGLRLDSAGEIAQAAGQAGPRTPVNNLFDFTLAVANVMLDIQTKVHLDSD
DWKRTVYVDTLDVGTLEFGISEEKKRALIESGRRGVERYFAWYDTAMKQAA
>CORE_REP|Org21_Gene1286#
MKKKTLFTLLLMLAAIALAILFRAHNQDLLLQGEVDAPEVIVASKAKGRVVERLIERGDDVKSGQLII
QLDSPELMAQLRSAQATRDEAKAQLELSLHGTREESIRNLRANLAQAEAQYRNAQNDYNRNLSVAGKG
YISKSELDASRRSRDTAFQQVQAAKANLDEGINGDRVEQRQQYAAALRAAEENLLQIQAQSDDLQVKA
PVDGEVGPIPAEVGELLNAGSPLVTLIRVPDAYFVFNLREDILAHVRKGDKVKLRVPALKDKMIDTEV
RYIAPLGDYATKRATRATGDFDLKTFEVRLYPSQPVDGLRPGMSTLWQWKE
>CORE_REP|Org20_Gene1105#
MTTVTSAPLVRAINWNIIEDDKDLEVWNRLTSNFWLPEKVPLSNDIPSWATLTPKEQQLTIRVFTGLT
LLDTIQNTVGAPALIADALTPHEEAVYSNISFMEAVHARSYSSIFSTLCQTPDVDDAYRWSEENRALQ
KKAGIILAHYRSDDPLLKKVASVFLESFLFYSGFYLPMYWSSRAKLTNTADLIRLIIRDEAVHGYYIG
YKFQKGLEKVDAARRRQVKNFAFDLLQDLYDNEVRYTEELYDGVGWTEDVKTFLHYNANKALMNLGYE
ALFPPAMAEVNPAILSALSPNADENHDFFSGSGSSYVIGKAVNTEDEDWDF
>CORE_REP|Org1_Gene4215#
MNLEQITELTAQDMAAVNATILEQLNSDVTLINQLGYYIISGGGKRIRPMIAVLAARALGYEGNKHVT
VAALIEFIHTATLLHDDVVDESDMRRGKATANAAFGNAASVLVGDFIYTRAFQMMTSLESLRVLALMS
EAVNVIAEGEVLQLMNVHDPDISEESYMRVIYSKTARLFEAAAQSSAILSGASAEQEKALQDYGRYLG
TAFQLIDDLLDYSADGSTLGKNTGDDLNEGKPTLPLLHAMHNGDDAQRDMIRGAIEQGNGRHLLEPVL
QAMQQCGSLEYTRQRAEEEADKAIAALQVLPASEHRTALEGLAHLAVQRDF
>CORE_REP|Org32_Gene1497#
MIEFGDFYQHIAKSPLSHWLDTLPAQLSAWQRESLHGKFKQWFNSVEHLPTLTPTRLDLLHGVRAEME
PGLSPGQLEGIEKMLRTLMPWRKGPFSLYGIDIDTEWHSDWKWDRVLPHISPLAGRTILDVGCGSGYH
LWRMIGAGAHFAVGIDPMQLFLCQFEAVRKLLGGDQRAHLLPLGIEQLPDLAAFDTVFSMGVLYHRRS
PLDHLYQLKNQLVAEGELVLETLVVEGDRHQVLVPGDRYAQMRNVYFIPSAEALKCWLEKCGFVDVKI
ADMCVTSLEEQRRTDWMTSESLAEFLDPNDRSKTVEGYPAPLRAVLTAKKP
>CORE_REP|Org47_Gene1607#
MGTAKHSKLLILGSGPAGYTAAVYAARANLSPVLITGMEQGGQLTTTTEVENWPGDAEGLTGPALMER
MREHAEKFQTEIVFDHINSVDLQQRPFRLFGDSGEYSCDALIIATGASARYLGLPSEEAFKGKGVSAC
ATCDGFFYRNQKVAVVGGGNTAVEEALYLSNIAAEVHLIHRRDSFRSEKILIDRLMEKVKSGNIVLHT
DHTLDEVLGDEMGVTGVRIRSTKAENETRELELAGVFIAIGHSPNTGIFGGQLELENGYIKVQSGIHG
NATQTTIPGVFAAGDVMDHIYRQAITSAGTGCMAALDAERYLDGIAGAEVC
>CORE_REP|Org12_Gene517#
MAQDYTVEQLNYGRKVYDFMRWDYLAFGISLLLLVASIVTMSVRGFNWGLDFTGGTVIEINLEKPANL
DLMRDTLEKAGFQDPIIQNFGSSRDVMVRMPPATGTAGQELGNKVIGVINDSVDKNATVKRIEFVGPS
VGSELAQTGGMALLVALICILIYVGFRFEWRLALGAVIALAHDVIITLGVLSLFHIEIDLTIVASLMS
VIGYSLNDSIVVSDRIRENFRKIRRGTPYEIMNVSLTQTLSRTLMTSGTTLMVVLMLYIFGGAMLQGF
SLAMLIGVSIGTVSSIYVASALALKLGMKREHMLQQKVEKEGADQPSILP
>CORE_REP|Org7_Gene2835#
MQNEKKSNVEFIPQFQKAFLYPRYWGVWLGTGLMAGVSLVPARLRDPVLGAIGTLAGKLAKGARRRAR
INLLYCLPELPESEREHIIDQMFACAPQSMVLMAELACTKPEKVLKRVRWHGEEVLDKIRAEGRNVIF
LVPHGWAVDVPAMLMAARGQPMAAMFHNQRNQLIDYLWNAVRRKFGGRMHARNDGIKPFISSVRQGYW
GYYLPDQDHGAEHSEFVDFFATYKATLPAVGRLMKVCRAAIVPLFPVYDGKTSMLDIYIREPMDDLAE
ADDPRIARRMNEEVENLVGPNPEQYTWILKLLKTRKEGEIEPYSRDDLYR
>CORE_REP|Org22_Gene2899#
MSKIYEDNSLTIGHTPLVRLNRIGNGRILAKVESRNPSFSVKCRIGANMIWDAEQRGVLTAGKELVEP
TSGNTGIALAFVAAARGYKLTLTMPETMSIERRKLLKALGANLVLTEGAKGMKGAIAKAEEIVATDPN
RYLILQQFSNPANPAIHEKTTGPEIWEDTDGEVDVFISGVGTGGTLTGVSRYIKNTKGKAITTVAVEP
TDSPVISQALAGEELKPGPHKIQGIGAGFIPGNLDLDLVDRVEKVSNDEAISMARRLMDEEGILAGIS
SGAAVVAAVKLAEEPAFADKTIVVILPSSGERYLSTALFADLFTEQELQQ

>CORE_REP|Org19_Gene2912#
MNPLFDAIVSAHQQLRPQVRVTPLERSVLLSQQLGCELYLKCDHLQHTGSFKFRGASNKLRLLDSEQR
RRGVIAASTGNHGQAVALAGQLMGVGVTVYAPETAASIKLDTIRALGGTVERVPGDALNAELAGEQAA
REQGKTYISPYNDEQVIAGQGTCGMELVEQLTGLDAVFVAVGGGGYIAGIGTVLRQLSPNTQLIACWP
ENATSMYSALEAGHIFPVEEQDTLSDGTAGGVEPGAVTFPLCQQLIDRKVLVSETEIKHAMRRIAASD
RWIIEGAAGVALAAAIKLAPEYQGKKVAVVLCGKNIVLEKYLKAIADAHP
>CORE_REP|Org28_Gene3714#
MSSQTIAAKRWFSKEWLLEQKSLIALLVLIAVVSSMSPNFFTLNNLFNILQQTSVNAIMAVGMTLVIL
TSGIDLSVGSLLALTGAVAASIVGFEVNALVAVAAALALGAAVGACTGMIVAKGKVQAFIATLVMMLL
LRGVTMVYTNGSPVNTGFTDVADTFGWFGIGRPLGVPTPIWIMAIVFIAAWYMLHHTRLGRYIYALGG
NEAATRLSGISVDKVKIIVYSLCGLLAALAGVIEVARLSSAQPTAGTGYELDAIAAVVLGGTSLAGGK
GRIVGTLIGALILGFLNNGLNLLGVSSYYQMIVKAVVILLAVLVDNKSNK
>CORE_REP|Org28_Gene4152#
MSMPLSFTSAVSSVAAIPTPRAAAETATATAAQPAAAPANPAPVASPSQNTLNAQALLNTLVGDISAA
ASTAAAAPGVTRGQQSQEGDYALALLAKDVYSLNGQGAAGFNRLSDSALLGFGIDPASLHDAGSGFQA
GIYSNDKQYVLAFAGTNDWRDWLSNVRQATGYDDVQYNQAVAAAKSAKAAFGDALVIAGHSLGGGLAA
TAALATGTVAVTFNAAGVSDYTLNRLGIDPAAAKKDAEAGGIRRYSEQYDMLTSTQESTSLIPDAIGH
NITLANNDTLTGIDDWRPSKHLDRSLTAHGIDKVISSMAEQKPWEAKANA
>CORE_REP|Org40_Gene4615#
MQAAIVTALGQAPVFGTFEEPQAQANEVPIDVLAAGIKQLDRATVAGTHYSSPKQLPIVPGTDGVGRT
ADGKRVYFASFRRPYGAMAERSVASWTVPVPEAVDDATAAALINPAFAAWLPLRWRADLQPGETVLII
GATGTSGKLAVAAARQAGAGRIVAAGRRLSVLEALGVDATVDLSLHGEALTQAFAAAAGPGGYQVIVD
YIWGPATEALLATLNNHDLSSYAGGRGIRLVNVGSMAAPDIRLPAAVLRSNQLQILGSGTGNFPPIPE
MQRYATEILTLAATGALTIETQDHALAEIAEVWDLNKKSDVRSVMRIAR
>CORE_REP|Org37_Gene820#
MSKPIQMERGVKYRDADKMALIPVKTVVTERQELLRKPEWMKIKLPADSTRIQGIKAAMRKNGLHSVC
EEASCPNLSECFNHGTATFMILGAICTRRCPFCDVAHGRPIAPDANEPEKLAQTIADMALRYVVITSV
DRDDLRDGGAQHFADCISAIRAKSPNIKIETLVPDFRGRMDRALEILTATPPDVFNHNLENVPRVYRQ
VRPGANYEWSLKLLERFKEAHPDIPTKSGLMVGLGETNAEIVEVMRDLRRHGVTMLTLGQYLQPSRHH
LPVQRYVSPAEFDEMKEEAMAMGFTHAACGPFVRSSYHADLQAKGMEVK
>CORE_REP|Org41_Gene1675#
MQVLIVKTSSMGDVLHTLPALTDALQAIPDIRFDWVVEEGFSQIPTWHPAVDRVIPVAIRRWRKNWFG
NDTRQQRCDFKRALQERRYDVVIDAQGLIKSAALITRIAKGNKHGPDCKSAREPFASWFYNVRHEIDK
QQHAVERTRELFAKSLGYDKPGSYGDYAIAARFLSRPPADAGQYLVFLHATTRDDKHWPEQNWRELIA
LTADSGLKIKLPWGAEHEHQRALRLAEGFSHVEVLPKLSLQQVAEVLAGAKGVVSVDTGLSHLTAALD
KPNITLFGPTDPGLIGGYGQNQHSLISPEKSMATIDADTAWQALQKVIA
>CORE_REP|Org1_Gene136#
MIIFTLRRILLLLITLFFLTLVSFSLSYFTPRAPLNGAALLDAYQFYFVSLLHWDFGVSSINGQAISE
QLREVFPATMELCLLAFALALFIGIPLGIIAGVLRGKWQDTAISTFALLGFSMPVFWLALLLMLFFSL
HLGWLPVSGRFDLLYQVKPITGFALIDAWLSDSPYRAEMIGSALRHMILPIAALAVAPTTEVVRLMRI
STDDVLSQNYIKAAATRGLSRFTIIRRHVLHNALPPIVPKLGLQFSTMLTLAMITEVVFSWPGLGRWL
INAIRQQDYAAISAGVMVVGTLVITINVLADILGAATNPLKHKEWYALR
>CORE_REP|Org11_Gene964#
MTRITRGLLLCLALLAGRGALAQPDGMLAGNLSSVGSDTLANLMALWAQDFSQHYPNVNLQIQAAGSS
TAPTALAAGAAQLGPMSRPMKAAEVSAFEHRYGYAPLAVPVAVDALVVLVHQDNPLRGLNLQQLDRIF
SATRRCGESKPLTRWGELGLSGDWATRSLQRFGRNSASGTYGYFKLRALCGGDFMPRVNELPGSASVV
QAVAGSLNGIGYASIGFRASGVRLLPLAESGEDYVAPTAANVRNDRYPLSRYLYIYINKAPNQPLEPL
TAAFLDRVLSTAGQSLVNHDGYLPLPPGALQKTRQALGLPPLAPAMMK
>CORE_REP|Org29_Gene475#
MKQEKHGVLLVNLGTPDAPTSSAVKRYLKEFLSDDRVVDTAPLIWWPILNGAILPIRSPRVAKLYQSV
WMEEGSPLLVYSRRQQRALAARMPNTPVELGMSYGSPSLAEAIDKLLAQGVTNLVVLPLYPQYSCSTS
AAVWDGVARVLKGYRRLPSVAFIRDYAEHPAYIAALQQSVERSFAEHGQPDRLVLSFHGIPKRYARLG
DDYPQRCEDTLRALSATLPLAPERVMMTYQSRFGREPWLTPYTDETLKGLPAQGVKHIQLICPGFSAD
CLETLEEIKEQNREIFLKAGGEKFEYISALNDEPAHIDMMQQLVAQRL

>CORE_REP|Org42_Gene1867#
MTLLSERTPEPPAKTPGTLKALFHRLLMAHGRKLVIALPYLWLTLLFMLPFLIVFKISLAELALAVPP
YTELMSWVDGKLNIALNFANYLQLTDDPLYIDAYLQSLRVAAVSTLCCLIIGYPLAWAVAHSKASTRN
ILLLLVILPSWTSFLIRVYAWMGILKNNGILNNFLMWLGVIDQPLVILHTNLAVYIGIVYSYLPFMVL
PIYTALIRLDYSLVEASLDLGARPLKTFFSVIVPLTRGGIIAGSMLVFIPAVGEFVIPELLGGPDSIM
IGRVLWQEFFNNRDWPVASAVATIMLLLLIVPILWFHKHQNKEMGGQA
>CORE_REP|Org46_Gene1306#
MEFKDYYATMGVEPNADLKTIKTAYRRLARKYHPDVSTEEDAESKFKELAEAYEVLKDEERRAEYDQI
RLHRNDPNFGRQARGDRGGYQQSASWHGGGADAQDFSDFFESMFGGRAAGGHRSASHSHGGHGFRGQD
LEMEVPLFLEETLHGQSREISYKLPVYDELGRQVSEASKTLNVKIPAGVGDGERIRLKGQGVAGVGGG
QNGDLYLVIRLAPHPLFEIDGHNLSIVAPLAPWEAALGASIEVPTLTGKIALTVPAGSQSGKRLRVKG
KGLAGKKEPGDLYVILKVVMPPKPNEKASALWRELAEQAAFNPRAEWE
>CORE_REP|Org31_Gene930#
MDKRKVAIIGGSGFIGTRLAKRLSARDDLTLVIVDKQPSAHFPALYQYGDVTQPQTLQEPLAGCDAII
NLAAEHQDNVDPISLYYQVNVEGARHVCMTASALNIRQIIFTSSVAVYGFVEQETDEGGRFDPFNHYG
KSKLEAEYVYEAWRKADSANKLTIIRPTVVFGEGNRGNVYNLFRQIASGRFVMIGSGNNMKSMAYVEN
IAARLEHALDQQSSYQVSNYVDKPDFTMNQLVDVISSSLGRENKTIRIPYVVGICGATALDVVSKISR
KKFPISRVRVQKFCARTQFKSNVQNNFVAPVELDSAIERTIKHEFSDR
>CORE_REP|Org49_Gene957#
MNANNNPPQAVFTPVTRHAIFIVATLSPVPAHLAAVRAWCGDIAAVVRSVGKRAPAGNLTCVCGFGSE
AWDTLFGAPRPRQLHPFSPIGSGERVAVATPGDILLHIRADEMDLCFELASQLVGKLGDAVTVVEEVH
GFRYFDQRAMIGFVDGTENPEGHEAFDYTVIGDEDAAFSGGSYVLVQKYLHDMQGWNSLSVETQEKII
GRHKQSNIELDEAVKPSSSHSSLTTITDEQGNEVKILRDNMPFGRPGLREFGTYFIGYARSPQPIEQM
LENMFIGRPAGNYDRLLDFSHAVTGTLFFVPSAPLLEALADRSGAELH
>CORE_REP|Org40_Gene445#
MAKYQNLLVAIDPNQDDQPALRRAVYLVKRNGGRIKAFLPIYDFSYEMTTLLSPDERTAMRQGVISQR
AAWINEQCRFYLNDGVPIEIKVVWHNRPFEAIIQEVLSGQHDLLLKMAHQHDRLESVIFTPTDWHLLR
KCPCPVWMVKDQPWPEGGKALVAVNLASEEPYHDPLNIKLVQETVELAQNVNQTEVHLVGAYPVTPIN
IAIELPDFDPSVYNDAIRGQHLIAMKALRQKFCIKEEFTHVEKGLPEEVIPDLAEHLQAGVVVLGTLG
RTGISAAFIGNTAEHVIDHLKCDLLVIKPENFNCPIEADEDDEHDDED
>CORE_REP|Org49_Gene1286#
MKDTKVPLKLIALLADGEFHSGEHLGESLGMSRAAINKHIQTIREWGLDVFTVPGKGYSLPGAIQLLE
AERILSLLEDKRVSVLPVVDSTNQYLLDRIAELHSGDACVAEYQQAGRGRRGRQWISPFGANLYLSMF
WRLEQGPAAAMGLSLVIGMVMAEVLQRLGAADVRVKWPNDLYLNDRKLAGILVELTGKTGDAAQLVIG
AGINLAMRDTNASAITQGWINLQEAGIQIDRNELAATLLNELRQSLKQFEIDGLAPFIGRWRTLDNFI
DRPVKLLIGERQIVGIARGIDSQGALLLEQDGEIKPFIGGEISLRSAE
>CORE_REP|Org49_Gene4442#
MTKPAFLITIDTEGDNLWQNHDRIATENTRFLPRFQALCEKYAFKPVYLTNYEMAMDPAYVEFARDVI
ARGAGEVGMHLHAWNSPPLTPLTDDDWRYKPYLIEYPADQIRAKVDHMTKLLEDAFQTKMLSHRAGRW
AFNEYYASLLLEYGYQVDCSVTPRVNWQFSPGNPQGNGGTDYSRFPSQAYFIDPQNIAKPGASALLEV
PMSIQYKHSALMNAFKQGYDRLRGKRRSPSVNWLRPSGGNVAKMIGVAEASLAQGHDYVEFMLHSSEF
MPGGSPTFKTEQDIEVLYRDLEQLFDWLQQRTVGMTLAEYYQKKTTAE
>CORE_REP|Org34_Gene4500#
MTIRDPFSFQIGSLPLLISIPHAGTQLTPAVEAGLTDDARPLPDTDWHIPQLYDFARAMGASVLVGNY
SRFVIDLNRPADDKPLYTTATTGLYPDVLFDGRPSFLPGKAPTDEERAGYLQQIWQPYHQQLQDELVR
LKAQHGYALLGEPLPQRSTAIPDGLYATLAEPHWLEVKDGNLAYLGSDEPLYQGEDGYAVSLSAHMPI
KLKWTGEAVEGEVGHVARRFLPVTANGDCLKHVQGLWYHPHYRTAFEIRGNRVHIGVGPAAQTGTLSP
LGQGRMLVECQDGPWLKRFCLYFRGYNVDLIANRSRMLTFRRSTVGRD
>CORE_REP|Org18_Gene1250#
MKQIWFSVCLLTGSLLYSSIAPAQPTASGALLQQMSSASRSLNYELAYISISKQGIESLRYRHAVIGN
VPLGQLLHMDGPRREVLQRGGGISYFEPGLEPFTLTGDHIVDALPAIVYADFTRLAKYYDFISVGSTR
IADRPCEVLRVVARDGSRYSYIVWMDEDTKLPLRVDLLDRDGETLEQYRVISFAVGADVQGAMQGLLK
ANLPPLLSLPAVENVKLSWSTGWLPAGVDEVARNRRKLPNVAVPVESRLYSDGLFSFSVNVSPAGSGA
GQQYYRQGRRTIQTEVRAGNEITIVGELPPATAKRIADSISFKVSPQ

>CORE_REP|Org44_Gene265#
MSLNFLDFEQPIAELEAKIDSLTAVSRQDEKLDINLDEEVQRLREKSVELTRKIFADLGAWQIAQLAR
HPRRPYTLDYIKHIFTDFEELAGDRAYADDKAIVGGIARLDGRPVMIIGHQKGRETKEKIRRNFGMPA
PEGYRKALRLMEMAARFKMPIITFIDTPGAYPGVGAEERGQSEAIARNLREMSRLNVPVICTVIGEGG
SGGALAIGVGDKVNMLQYSTYSVISPEGCASILWKSADKAPLAAEAMGITAPRLKELKLIDSVIPEPL
GSAHRDVPAMAAALKAQLLADLKDLDGLNDEELLNRRYQRLMNYGYC
>CORE_REP|Org3_Gene2961#
MDYTKIIKEIGRGKNHARDLDRQTAFELYQAMLAGAVPELELGGILIALRIKGEAEEEMLGFYQAMQQ
QVLPLQAPQGRPLPVVLPSYNGARKQANLTPLLALLLAKVGLPVVVHGVLDDSTRVTSAEIFRALGLP
WSEQAVHAQQRLDNGEAVFMPVSTLSAPLARQLGLRWRMGVRNSAHTLAKLATPFGEQDALRLASVSH
PEYVGRVASFFRQIGARGLLMHGTEGEAYANPLRCPQIHYIAGGEQRVLLERTLQDETPTLPAAKDAE
TTARWTERCLAGEVAIPQAIQRQVACCLVAAGESETLEQGLQRLRQA
>CORE_REP|Org16_Gene3401#
MSNAITPEQRQALKMAAHWYALLCDEHVTERQRQQWQAWHQQHDDHRWAWQRVEALQSQLQGVPGKFS
YRALDRADRQSAIDRRTLLKSLLLLLGVGGGGFFYQSPLGRELRADYRTATGEIKPIVLSDGTQLVLN
TASAVDVHYDDRQRLIRLHAGEISLVTGRDPRPLWVQSPQGAMRALGTRFLVRESDGETRLAVLEHAV
EAQLAQDAQQKRRVNAGEQISFSTTAFSDKQPATTEDGWLRGVLSVSQWRLDRVIAELARYRRGHLSC
DPAVAGLRVSGSFPLNDTDRALALLSQTLPVRLQSFTRYWLQIVPA
>CORE_REP|Org17_Gene317#
MAEYKPTIKAPGKNGDIIFSALVRLAALITLLLLGGIIVSLIFASWPSMQKFGFAFLWTKEWDAPAEQ
FGALVPIYGTVVTSLIALIIAVPVSFGIALFLTELAPNWLKRPLGIAIELLAAIPSIVYGMWGLFVFA
PLFAEYFQTPVGEVLSGIPIVGELFSGPAFGIGILAAGVILAIMIIPYIAAVMRDVFEQTPVMMKESA
YGIGCTTWEVIWRIVLPFTKNGVIGGVMLGLGRALGETMAVTFIIGNTYQLDSASLYMPGNSITSALA
NEFAEAESGVHTAALMELGLILFVITFIVLALSKLMIMRLAKNEGR
>CORE_REP|Org34_Gene1075#
MSDKIPIGISACLLGDAVRFDGGHKRLAFAVEQLAPYVRFEPVCPEMAIGLPTPRPALRLVKQAQPWP
AMRYSNDAGVDLTEEMRSFSAQRVAALQHLCGYIVCAKSPSCGLERVRVYSENGKDSRKNGVGLFTAE
LLRQMPWLPVEEDGRLQDAALRENFIERVYALYELNMLWRQGLTRGGLIAFHSRYKLSLLAHSQPAYR
ELGRFVADIHRWDSLEAFAVEYRSRLMALLAHKATRRNHTNVLMHVQGYFRRQLSAAQRQELAHLIDR
YRQGMQPLLAPIALLKHYMAEYPDRYLAEQRYFEPYPEALRLRYGH
>CORE_REP|Org5_Gene3696#
MPGFMMSLWHQIVLSLPLFVLIALGYGLIRWGKWPAGITDGLTRFVFSLALPAMLFRMMCDFSQRPAV
DARLLIAFFGSCLIVFVLGRLVARRLFRLDGVAGSVFALGGIFSNNVMLGLPIASVMLGEAAIPSVAL
VLVFNGLILWTLVTVSIEWARNGSPTLSGFVKTARSVLTNPLIVGILSGTLFSLTGLPLPAFVDRPIS
MLGQVAAPLSLVVLGMGLAEYRIGEGWRLSCAICVLKLLVQPLVIWLLAWMMDLPPMETRVVVLLGSM
AVGVNVYLMSRQFNTLTGPAATSLVMSTVLAAITTPLILTVMGVRA
>CORE_REP|Org18_Gene1916#
MEQLRGLYPPLAAYDSGWLDTGDGHRIYWELSGNPNGKPAVFIHGGPGGGISPHHRQLFDPERYKVLL
FDQRGCGRSRPHASLDNNTTWHLVADIERLREMAGVDQWLVFGGSWGSTLALAYAQTHPERVSEMVLR
GIFTLRKQELHWYYQDGASRFFPDKWERVLSILSDEERKDVIAAYRQRLTSADPQVQLEAAKLWSVWE
GETVTLLPSRESASFGEDDFALAFARIENHYFTHLGFLESDDQLLRNVPLIRHIPAVIVHGRYDMACQ
VQNAWDLAKAWPEAELHIVEGAGHSYDEPGILHQLMIATDRFAGK
>CORE_REP|Org28_Gene1656#
MQILLANPRGFCAGVDRAISIVERALELYGAPIYVRHEVVHNRYVVDSLRERGAVFIEEIAEVPDGSI
LIFSAHGVSQAVRAEAKARDLTMLFDATCPLVTKVHMEVARASRRGTEAILIGHAGHPEVEGTMGQYS
NPQGGMYLVESPEDVWKLQVKDESNLCFMTQTTLSVDDTSDVIDALRQRFPSIIGPRKDDICYATTNR
QEAVRNLAGDADVVLVVGSKNSSNSNRLAELAQRVGKPAYLIDSAADIQESWLSGARHIGVTAGASAP
DVLVQEVISRLKALGGMDVHEISGREENIVFEVPKELRVDVRQID
>CORE_REP|Org38_Gene1107#
MANNAKTAKSKALRGGWREQWRYAWMNAIKDMLRQPLATLLTVMVIAISLTLPSVCYIVWKNVSTAAS
QWYPTPQLTVYLDKSLDDDAALKVLDAIKAEAGVEKVNYLSREEARGEFRNWSGFGGALDMLEENPLP
AVAIVTPKMSFQSSDTLNTLRDRVAAVQGVEEVRMDDSWFARLAALTGLAGQIAAIIGVLMIVAVFLV
IGNSVRLSIFSRRDTINVMKLIGATDGFILRPFLNGGAMLGFAGALLSLVLSGALVWQLESVVAGVAK
VFGTTFTLHGLGWDEALLLLIISAMIGWIAAWLATVQHLRRFTPQ

>CORE_REP|Org33_Gene3861#
MSQLSYPLASARPLNGWQLMTALINGEKAPSNAWKKTSFRLKFLGRSLLNWRTTSGLLSTLASNPLLE
EILSAQPNLPCKLHRPYLAANMSKIECLFALRDHYDLIAQRMPLKMRLGHLGPQPFVLASAMGKNEAP
IALELAAIDKLNKEGEATLLLRNANGVMLAEITFALMHYQQQPTLFIGGLQGANHEVPHAEIQHTTKE
CHGLFPKRLVLEGICTLARHLGIRQIVAVGNATHIYQNWRYQSKKKDKLHADYDQFWLSMGAKPLDSG
YFLLPERIARKPIEEIASKKRAEYRRRYQLLDELEQGLAAHFCAR
>CORE_REP|Org14_Gene3071#
MRLRRSPLMIALLAAMALAGCHSKTATPVAATPATVNVQHLNGSTEVKKHPQRIVVLDYASLETLQLL
GVEPLALPGNRKNLPDSLKRYQDDKYLNAGTLFEPDMAVLRAAKPDLILIAGRASKAYDELNALAPTL
NMSVDPQDQLGSLKQRTLQLGELFDKQQQAQAAVDKLDAQIAAVKPQAAQAGRGLVVLFSGGKISAYA
PKSRFSFVYDALGFSSALQSDEKDVRGNKLTPEQVAKLNPDWLFVIDRDAATGRPNAVAPQKILTGTA
LKKTTAVKKGQVVYLPAAEVYLSGGIVTAQHVVERVSEALNHAAR
>CORE_REP|Org41_Gene1020#
MNFQQLKIIRESARCNYNLTEVANTLFTSQSGVSRHIRELEEELGIEIFIRRGKRLLGMTEPGKELLV
VAERILNDANNIRRLADVFSSNDSGQLHIATTHTQARYSLPGVIKEFRALYPRVRVVLNQGSPEEIVS
MLAAGEADIGIASERLMSDESLAAFPYYRWHHTILVPEGHELTRQPQVTLEMLSTLPLITYRQGITGR
AKLDAAFKAAGLTPDIALSAQDSDVIKTYVELGLGVGVLADMSYEKERDRGLVSLNAEHLFEPNTVWL
GLKRSQLQRNYAWRFIQLCNPTLSLTEIKDKVFSSQLDAVIDYQI
>CORE_REP|Org40_Gene2591#
MYTKILGTGSYLPVQVRTNADLEKMVDTSDEWIVTRTGIRERRIAAADETVATMSFQAAEKALEMAGV
AKEDIGLIVVATTTTTHAFPSAACLVQQMLGIKDCAAFDLAAACAGFTYALSVADQYVKNGAVKHALV
IGADVLSRTLDPEDRGTIILFGDGAGAVVLGASEAPGILSTHLHADGSYGSLLTLPYKDRQNQDKPAY
VTMAGNEVFKVAVTELARIVDETLQANNMDRSELDWLVPHQANLRIISATAKKLGMGMDKVVVTLDRH
GNTSAASVPSALDEAVRDGRIQRGQLVLLEAFGGGFTWGSALVRF
>CORE_REP|Org20_Gene4789#
MTLRDRLLALLVVVIWGVNFVVIKFGLQGMPPFLLAGMRFLLVALPAIFFIPRPTIPWKWLLLYGMTM
SFAQFAFLFVAIKVGMPAGLASLVLQAQVFFTLLLGVVLMGEKLRVNHFVGIAIASGGMLVLAQASLH
KPGSGGMLVLAQASLHKPGSGAVPLAGLLLTLAAAFSWALGNLTNKKILAGFPQRNILSLVVWSAPIP
VVPFLACSWLFDGPQAVLSSLGHVQVGTWLAVAYLAFAATLFGYSVWGSLLGRYETWRVAPLTLLVPL
VGLFAAWLLLDEALSPAQFGGALLVLGGMAVNTFGLPRRRAAIAR
>CORE_REP|Org43_Gene4535#
MAMIELRRLRAFVTVVEEGNITRAAERLFIQQPPLTRLLQGLEDELGVKLLQRLPRGVRVTEAGDVLF
EEARALLARAERLREAVQRAARGEQGHIAIGFTSSAALHPFVPNLLRRYRDILPGITTQLEEAGSGEL
MEALLEQRLDAAFVRSPANGIPGLSVEPVLSEPMIVALPLGHRLAQETQQPLPLAELAHEAFILYRRP
AGQGLYDAILAACHRAGFSPRIVQEAPRLPATLSLVGAGLGVSIVPGSMRRLGGDGIVYRTLAAEAQL
SAPLYLALRRSPASPIVERFRQLVLETVGAPDADVTTTARTANKK
>CORE_REP|Org28_Gene3967#
MIKLGIVMDPIDSINIKKDTSFAMLLEAQRRGYELHYMEMNDLYLHAGDGRARTRLLSVKEDKENWFS
FGSEQDLALHDLDVILMRKDPPFDTEYIYATYILERAEVKGTLVVNKPQSLRDCNEKLFTAWFPELTP
DTLVSRSAAHIRKFHQQHGDVILKPLDGMGGASIFRVKQDDPNLSVIIETLTEHGSRFCMAQNFLPAI
KDGDKRILVVDGEPVPYCLARIPAQGETRGNLAAGGRGEARPLSESDWKIARAVAPTLKEKGLIFVGL
DVIGDRLTEINVTSPTCAREIEAAFPISITGMLMDAIEKRLAAK
>CORE_REP|Org39_Gene1077#
MIKRDQSLATPYLQFDRTQWAALRDSVPLTLSEEEIVKLKGINEDLSLEEVAQIYLPLSRLLNFYISS
NLRRQAVLEQFLGTDGQKIPYVIGIAGSVAVGKSTTARLLQALLSRWPEHRSVELITTDGFLHPNKVL
NERGLMKKKGFPQSYDMHSLVKFVSEVKSGAKRVTAPVYSHLIYDVVPEGNKVIEQPDILILEGLNVL
QSGMDYPHDPHRVFVSDFVDFSIYVDAPETLLQSWYINRFLKFRQGAFSNPDSYFHHYSKLPEPEAIN
IATQLWNEINGLNLQQNILPTRERASLIMTKSANHAVESVRLRK
>CORE_REP|Org8_Gene701#
MKLKTTIIASALLSLTALSAHAAQELTPEKAAALKPFDRITITGRFNAINEAVDAVSRRADKLGADSF
YIQDSNNSNNGGNWRVTADLYHKDAPEVSKTPKYRVFNGVNELPKEEAYLLEPYDTVSVSGFYRSQPD
INDAISKEAKKKGAASFFIVRQVDANSGGNQFVTAYIYKADAPKRRVQSPDAIPADSDAGRAALAAGG
AAAAKVEIPGVASSGSPSREVGRFFETQSSTGQRYTVTLPNGTKIQEVNNVTAAQMVPFDSVTFTGHF
NSMTDVSTEVAKRAAEKGAKYYHVTRQWQNKSGGNLTVSADLFK

>CORE_REP|Org21_Gene463#
MLSAFKLDNRRLSRLELDDSDDLTSSLWVDLVEPEEGERERVQNELGQSLATRPELDDIEASARFFED
EDGLHIHSFFYFEDAEDHAGNSTVAFTIRDGRLYTLRERELPAFRLYRMRARNQTMLEGNAYELLLDL
FETKIEQLADEIENIYSDLEQLSRVIMEGHQGDEYDAALSTLAELEDIGWKVRLCLMDTQRALNFLVR
KARLPTGQLEQAREVLRDIESLLPHNESLFQKVNFLMQAAMGFINIEQNRIIKIFSVVSVVFLPPTLV
ASSYGMNFEFMPELKWSFGYPGAITLMILAGLAPYLYFKRKNWL
>CORE_REP|Org42_Gene789#
MRLKKYNKSIGMLSLIAATVLLSGCDMVLMNPKGAIGVEQRTLIITAIALMLIVVVPVIFMAFAFAWK
YRASNKDAKYSPNWAHSNKIEAVVWTIPIIIIAILGTITWKTTHELDPFKPIVTDKKPMTIEVVSLDW
KWLFIYPEQGIATVNELAFPKDVPVEFKITSNSVMNSFFIPQLGGQIYAMAGMQTKLHLIGNEAGAYK
GISSSYSGAGFSGMKFTAIVTPTEGDFDQWVAKVKASSKNLNTTDDFNKLAEPSENNPVEYFAAVKPN
LFKETIAKFMGDMDMHKGAGAHEGMDMSQGMDMGEHAAHAGAEE
>CORE_REP|Org15_Gene2458#
MQNRLTIKDIARLSGVGKSTVSRVLNNEGSVSPQTRERVEAVIRQQGFTPSKSARAMRGQSDKVVGII
VSRLDSPSENQAVRTMLPLLYQQGFDPIVMESQFETRLVQEHLHVLHQRNVDGVILFGFTGLTAAMLK
PWQEKMVVMVREYDGFSSVCYDDAGAVNLLMDRLYQQGHRHIGYLGVQLSDATTGQRRYQAYLDACER
LTLTPRATLGELSYQSGFQHAAEVIDAHTSALICASDSIALGAIKYLQQQPTRAIQVCAIGNTPLLSF
LFPDTLSVEFGYGSAGLLAAQQLLAQLSGEQGIRRLVVPSKLS
>CORE_REP|Org3_Gene542#
MPDMKLFAGNATPELAQRIANRLYTSLGDAAVGRFSDGEVSVQINENVRGGDIFIIQSTCAPTNDNLM
ELVVMVDALRRASAGRITAVIPYFGYARQDRRVRSARVPITAKVVADFLSSVGVDRVLTVDLHAEQIQ
GFFDVPVDNVFGSPILLEDMLQQNLENPIVVSPDIGGVVRARAIAKLLNDTDMAIIDKRRPRANVSQV
MHIIGDVAGRDCVLVDDMIDTGGTLCKAAEALKERGAKRVFAYATHPIFSGNAVDNIKNSVIDEVIVC
DTIPLSPEIKALKNVRTLTLSGMLAEAIRRISNEESISAMFEH
>CORE_REP|Org6_Gene518#
MAEYDSEIAMVKEPADIHLRSVDLNLLTVFDAVMQMQNITRAANSLGMSQPAVSNAVARLKVMFNDEL
FVRCGRGIQPTMRARQLFGPVRQALQLVQNELPGSEFEPLTSTRAFSLSLCSPLDLRLGAGIINHVKQ
IAPQLNLQIKSYINNNIERQLRYQDVEFVIGYSRFESAEFRSLAMFDDELVLAVAQAHPRIGEEVTPE
HMLAEQHAAVSLESFGSFSKPFYLDEPMLRAVTQQCTDLYSVLNMVSQTEMVAIAPAWLVRQQTEALK
IKAVPLCGNDNKATCYLSWHESSERDKGHQWMKSVLIEAGNPK
>CORE_REP|Org40_Gene167#
MYHLRVPVTEQELKEYYQFRWEMLRKPLHQPVGSEKDAYDAMAHHQMVVDEAGKIVAIGRLYINADNE
AAIRFLAVDPTLQDKGLGTLVAMTLESVARQEGVKRVVCSAREDAVDFFAKLGFVNQGEITAPQTTPI
RHFLMIKPVATLDDILHRPDWCGQLQQAWYEHIPLSEKMGVRISQYTGQRFVTTMPEIGNQNPHHTLF
AGSLFSLATLTAWGLIWLLLRERHLGGTIILADAHIRYSKPITGRPRAVADLGSLSGDLARLARGRRA
RVHAEVHLFGDEDKGAVFEGTYMVLPAEPDVPLDQGGSEALEE
>CORE_REP|Org2_Gene2309#
MAKKNKWLQRTLLASALLMAGPLSSASAAVVTSIRPLGFIASAIADGVTPTEVLLPDGASPHDFALRP
SDIQRLRSADLVLWVGPDMEAFLNKALVPISATRKLAISELPAVKPLLMKGEEDDDHDHAGEAHNHAD
DDHGHHHGEYNMHVWLSPEIAKVTAIAIHDRLLELMPQNKDKLDANLRQFENLLTQTDKNVGNMLTPV
QGKGYFVFHDAYGYFEKHYGLSPLGHFTVNPEIQPGAQRLHQIRTQLVEQKAVCVFAEPQFRPAVINA
VAKGTKVRSGTLDPLGIGIALGKDSYGKFLTQLSNQYVSCLK
>CORE_REP|Org33_Gene2258#
MDQVQAMRIFTRIVELGSFSRAAERLQLPRATVSNALKRLEQRLGVRLLIRTTRQVQVTSEGSLYYQR
CVQLLGALEEADTLFSHHKLQPSGKVRIDMPHSLARQIVIPALGDFYRRYPDITLALGANDTHVDLLR
EGVDCVLRAWETEDDSLVARRIAQLPQITCASPAYLQASGTPLDIDSLAPHRAVGYFSLASNRDYPLE
FCRGGKVELRELPARLSVSGADAYIAGARAGMGLIQAARYSLAPWLERGELVEVLADTPPPPMPIYIM
YPPGRFLAPRVRVLIDWLIWLFDQQKSGDMAVFPANARKAGK
>CORE_REP|Org8_Gene3849#
MPLISCAFHRSRPARAAALLRPFTLACLLLGAALVSQNALAEKKLRVVTTFTIIQDIAQNVAGDAAVV
ESITKPGAEIHDYQPTPRDIVKAQHADLILWNGMNLERWFQRFFENIKQVPAAVVTEGITPLPIREGP
YNGNPNPHAWMSPSNALVYIENIRKALVEHDPAHAETYNRNAKAYAEKIGALDAPLRERLARIPAAQR
WLVTSEGAFSYLAQDYQLKEVYLWPINADEQGSPQQVRRVIDAVRAHHIPVVFSESTISDKPAKQVAK
ETGAKYGGVLYVDSLSTRDGPVPTYIDLLNTTVQTIAKGFDQ

>CORE_REP|Org35_Gene2368#
MSDSLRIIFAGTPDFAARHLDALLSSEHQIVGVFTQPDRPAGRGNKLTPSPVKMLAEQHQLPVFQPKS
LRPEENQRLVADLNADVMVVVAYGLILPQAVLDMPRLGCINVHGSLLPRWRGAAPIQRSLWAGDSETG
VTIMQMDVGLDTGDMMHKIACPIESSDTSASLYDKLAQLGPQGMLTTLRQMADGSATREVQDESQVTY
AEKLSKEEARLDWTLSAAQLERCIRAFNPWPISYFTIDEQPVKVWQASVMAESANAEPGTVVHADKHG
IQVATADGILNLIQLQPAGKKPMSAQDLLNSRREWFTPGNRL
>CORE_REP|Org41_Gene3376#
MIELKHLRTLQALRNSGSLAGAAAQLHQTQSALSHQFSDLEQRLGFKLFVRKSQPLRFTAQGEILLQL
AEQVLPQIQQALQACHEPHQTTLRIAIECHSCIQWLTPALDNFRRRFPQVVMDFTSGVTFDPQPALQQ
GELDLVMTSDILPRSGLHYSPMFDFEVRLVLAPDHPLAGRPHIEPEDLSDETLLIYPVQRQRLDIWRH
FLQPAGVSPALKNVDNTLLLIQMVSARMGIAALPHWVVESFEQQGLVVTKTLGDGLWSRLYAAVRDGE
QRQAVTEAFIRSARQHACDHLPFVRDAARPGATCAKALAAGV
>CORE_REP|Org30_Gene813#
MSRPRRRGRDIHGVLLLDKPQGLSSNDALQKVKRLYNANRAGHTGALDPLATGMLPICLGEATKFSQY
LLDSDKRYRVIAKLGQRTDTSDADGQIVQERPVNFTQAQLDAALDTFRGDIQQVPSMYSALKYQGKKL
YEYARQGIEVPREARSITVYELQFIRWEGDELELEIHCSKGTYIRTITDDLGELLGCGAHVIYLRRLQ
VATYPIARMVTLEQLNALLAQAQEQEIAPGELLDPLLMPMDSPVENYPEVNLLPVVAGYVKQGQPVQV
AGAPASGLVRITEGEERKFIGVGDIAEDGRVAPRRLVVEHFD
>CORE_REP|Org44_Gene3052#
MKTIGFVVFPGFNLLDFAGPLAAFDNVSQFTDPPAYRCVAISPQGGMVASSAGVEIATQPCGDERFDT
LVVAGGSGNVMAAQSPALVAFLTTHSRRARRIASVCTGAFILAACGLLDGKRATTHWYHAARLQQSYP
RIRVDSNRIFIRDGDIWTSAGISAGIDLALALIEDDLGATLAAVVARQLVVYHRRPGGQSQYSLLLAL
NPSSDRMRAALSFAREHLHLPLSVADLADAACLSERQFGRLFRAETGQTPAKVIEQLRVEAARVRIEE
SAEPLEAIARSVGFSDPERMRRAFIRVFGLSPQAIRRLGRAG
>CORE_REP|Org5_Gene3088#
MFWKRCLLGAALAVMSLQAGAAAPQAKTPTPGVYRIMLGSFEVTALSDGIIRLPADKLLLNTTPQQIA
AGLAERHQSLPVVTSVNAYLINTGDKLVMIDSGAGQLLGDGLGKLVDNLRAAGYQPEQVDEIYLTHMH
PDHLGGLTHDGKAVFPNAVVRAASQDADFWLSAERLKQAKAQNKGNFEKAMAAIKPYQAAGHFKPFNG
DGELSPGIAAFAAHGHTPGHSVYQVTSQGKKLLLLGDLIHVAAVQMPHPKVAISFDSDAKAAVAQRLR
VFSDSARQSELVGGAHLSFPGLGYLNRQGEGYSWVPLNYGAL
>CORE_REP|Org9_Gene4148#
MMKKIILDCDPGHDDAIALLLAWGNPQIDLLAVTTVVGNQTLDKVTRNALAVARIANITGVPFAAGCP
RPLVRNIEVAPDIHGDSGLDGPVLPEPHLQLDSRHAVDLIIDTVMAHPPGSVTLVPTGGLTNIAMAVR
KEPRIAERVKEVVLMGGGYHVGNWSAVAEFNIKIDPEAAHIVFNEKWPLTMVGLDLTHQALATPAVCA
RIAALGTRPAAFVGELLAFFGRMYQQAQGFSAPPVHDPCAVAYVIDPSVMTVRKAPVDIELTGTLTLG
MTVADFRAPPPPDCHTQVAVKLDQDKFWDLVVDALERISEVE
>CORE_REP|Org43_Gene2252#
MSVIGIEKLEFGVEDLPTCEKFMHDFGLQPATQHWGEPQREFTTLSGARVVLHPLQSAALPAAFEGGS
TLRRMTWGVASPADLARLQPRLALMPGFRQVGEELECRDPNGMTLRFVVSRQQAVEVPVTPINQWGDV
RRIDQPSPVYSQAQPINIGHVVFFVDDLAATERFYRELLDFQVSDRYIDRAVFLRTQARGGHHNLFLL
KLPNRPRGLNHVAFTVRDIHEVIGGGIAMNKEQWSTFIGPGRHPISSAYFWYVNSPTGGAFEYYTNDD
YLTENWQPRELEHSLVSFTEWAVEGGIDHDTRRQHKKAEAL
>CORE_REP|Org43_Gene4137#
MHILNRQQILAAFDAETITPLLKQGFIAYSQQRVQQPPVQHFLFERADGDCCIKSAWLEGDELFVVKI
STGFYRNAEHGLPSNQGLMMAFSAQTGEPQALLQDEGWLTALRTALAGRIVAELCAPAHIQAIGIVGT
GLQARLQLQCLKPVTDCREVWVWGRDEQALAAYRRDAEAEGFRVRVTQNAAELAAHCQLIVTTTPSRE
PILQAADIRPGTHITAVGADTPGKQELATELVARADALLVDALTQCADYGEIATAYRQNRLISTPIVE
IGAVLAQGGRVRREPQHITIADLTGLAIQDLQIAKGVLAKV
>CORE_REP|Org31_Gene2245#
MSQSRPTLSSPTRECPVVDGVRQIQRIAVRNAEVGGIPINRALPTRERRTVGAWCFLDHAGPTVFNGT
SPGMDVGPHPHIGLQTFTWMIEGEVLHRDSLGSEQVIRPGQVNLMTAGRGIAHTEQSVGEQRRLHAAQ
LWIALPAEHADMAPRFDHYPDLPQWQNNGVNHRLLVGEFGAYRSPVFTLSPLIAIDLEWQEAARIELP
LRDDDEIGFLPLIGAFELNGETFSPDEFAYLGMKNNSIGLNAQKGSRGLLIGGAPLNEEILIWWNFVG
HSKAEITRAQHDWEQGAPRFPAVSGYSGERMTAPRLPWSDV

>CORE_REP|Org4_Gene2440#
MTETEMTPRPPAIFIMGPTASGKTALAIALRERLPVELISVDSALIYRGMDIGTAKPSAEELAQAPHR
LIDIRDPAEAYSAAEFRADALKEMADITAAGRIPLLVGGTMLYFKALLEGLSPLPSADPAVRERIERQ
AAEQGWEALHRQLQAIDPVAALRIHPNDPQRLSRALEVFFISGKTLTELTKISGESLPYQVHQFAIAP
TSRELIHQRIELRYHQMLAAGFETEARALFARGDLHTDLPSIRCVGYRQMWSYLSGEISYDEMVYRGI
CATRQLAKRQMTWLRGWESVHWLDSEKPGEALDSVIQVVSA
>CORE_REP|Org21_Gene771#
MSRRVATITLNPAYDLVGFCPQIERGEVNRVKTAGLHAAGKGINVAKVLKDLGIDVTVGGFLGKDNQD
GFQLLFSDLGIANRFQVVPGRTRINVKLTEKDGEVTDFNFSGFEVTPQDWDRFVSDSLSWLGQFDMVA
VSGSLPAGVDPDAFTDWMTQLRAKCPCIIFDSSREALVAGLKASPWLVKPNRRELEIWAGRPLPTLAD
VVEAAHALREQGIAHVVISLGAEGALWVNASGAWIAKPPSCEVVSTVGAGDSMVGGLIYGLLMRESSE
HTLRLATAVAALAVSQSNVGVTDRPQLAAMMARVDLKPFNQ
>CORE_REP|Org31_Gene1780#
MNIIYYHPLFNAQEWLAGIKQRLPQAEIREWQRGDERPADYALVWRPPHEMLANRRDLKAVFALGAGV
DAILDQERKHPGTLPAGVPLLRLEDTGMAQQMQEYALSYVLRYFRRFDEYQALQQRQEWQPLDPHSLD
DFTIGILGAGVLGQSVARKLTEFGFSVRCWSRSAKQIDGVQSFAGEAQRAAFLDGVKLLINLLPNTPE
TVGILNRELFAQLSSGAYLINIARGAHLVEADLLAALEQGQLAAATLDVFAREPLPQDHPFWRHPRVT
ITPHIAAITLPQQAMDQIAANIRALEAGHAPAGVVDRQRGY
>CORE_REP|Org32_Gene915#
MLENYKHTTVLLDEAVNGLNIRSNGIYIDGTFGRGGHSRLILSQLGPEGRLLAIDRDPQAIAAAKSIE
DPRFTIVHGPFSELSHYVRERELVGKIDGVLLDLGVSSPQLDDAERGFSFMRDGPLDMRMDPSTGLSA
ADWLMKAEADDIAWVLKTFGEERFAKRIARAIVERNRVEPMTRTKELADLIADASPFREKHKHPATRS
FQAIRIYINSELEEIERALDGALEVLAPQGRLSIISFHSLEDRIVKRFMRHHSRGAQVPAGIPLTEEQ
LRSMGGRTLKALGKMMPSEAEVADNPRARSSVLRIAERMPA
>CORE_REP|Org13_Gene4638#
MLDKIIRIATRQSPLALWQAHYVQQRLMACHPGLQVELVPMVTRGDVILDTPLAKVGGKGLFVKELEL
ALLEGRADIAVHSMKDVPVDFPAGLGLTTICEREDPRDAFVSNRFASLDQLPQGSVVGTSSLRRQCQL
RERRPDLIVRDLRGNVGTRLAKLDNGDFDAIILAVAGLKRLGLEQRIRSPLSAEECLPAVGQGAVGIE
CRLDDSVTRALLVPLNHAATETRVRAERAMNTRLEGGCQVPIGSYAELDGDSLWLRALVGAPDGSQMV
RGERRGPAAEAERMGVELAEELLARGAREILREVYQGNPPA
>CORE_REP|Org13_Gene1498#
MTRLSLDAIKIISTIKSTGSFSMAAEALHKTPSAISYRVSNIESKLCVKLFHRNGPMITLTDEGEFLL
QEGSWILNAVQDLESRVRNIPKLDNNIRLAVDTFFPLETLTQDIRDYIQHCPNANISVQREALNGTWD
ALKNNRADLIIAIGQIPDSVQAKTLMLGKLNFVLCVSPSHPFAAQRKPVCKKQRLNDIVVVIADSSHE
LPKRNHGTLPLQRQLVVCDVESNLALLKRGIGHAFLPPALIEKELASGELVTVPVEMQKGDEMIWLAW
HPASKGAGFSWWHERLTRKSDVYSLMGREVVRDGGYPWCHN
>CORE_REP|Org14_Gene2344#
MELIRGIHNIRARHHGCVLTIGNFDGVHRGHQALLEQLKQQGQRLGLPVMVMIFEPQPLEMFAADKAP
ARLTRLRDKANYLAQAGVDYLLCVKFDPRFAANTAQAFVAELLVEKLGVKFLMVGDDFRFGAGRQGDF
PLLQQAGKEYGFEVVSTPTFREGDRRISSTAIRTALSEDDLPLAETLLGHPYSISGRVVHGDELGRTI
GFPTANLPLKRLVAPVKGVYAVEVYGLGPQPLPGVANIGTRPTVAGVRQQLEVHLLDVTMDLYGRHIE
VVLRAKLRNEQRFASLDALKQQIANDVVTARKFFGLQTPV
>CORE_REP|Org40_Gene4471#
MSVNNPVARLLAEHPTLLLDGALATELEARGCDLTDPLWSAKVLIENPELIYQIHLDYFNAGAQCAIT
ASYQATPQGFLRRGLDQDQSLALIAKSVQLAQRARHDYLAAHPQAAPLLIAGSVGPYGAYLADGSEYR
GDYRLAQDDMIAFHRPRLAALAAAGVDLLACETLPSFAELQALLTLLQEFPTLGAWFAFTLRDSQHLS
DGTPLTEVLSALRGNPQVLAIGINCIALDKVAPALRQLGALADKPLLVYPNSGEHYDAVSKTWHACGG
EHGSLADQATEWSTLGAQLIGGCCRTTPQDIRAIAARCKK
>CORE_REP|Org32_Gene4672#
MQLPQLVNMFGADLQRRYGEKIHKLTLHGGFSCPNRDGTLGRSGCTFCNVASFADEAMQRQSIDEQLA
QQAARVDRARRYLAYFQAYTSTYAQIERLASMYRQALAQSAMVGLCVGTRPDCVPPAALDLLAGYREQ
GYEVWLELGLQTAHDKTLKRINRGHDFRCYQQTARLARQRGLKVCCHLIVGLPGETQRDHLLTLQRVV
ETGVDGIKLHPLHVVTGSTLARAWRAGRLSELALEDYAVSAGEMIRHTPGEVVYHRLSASARRPTLLA
PLWCENRWSGMQAVGAYLQQRGGQGSALDEKWRYRPGMPL

>CORE_REP|Org15_Gene3901#
MTENQQDNKRLQYNLNKLQKRLRRNVGEAIADFNMIEEGDRIMVCLSGGKDSYTMLEILRNLQQSAPI
NFSLVAVNLDQKQPGFPEHILPAYLEGLGVEYKIVEENTYSIVKDKIPEGKTTCSLCSRLRRGILYRT
ATELGATKIALGHHRDDILQTLFLNMFYGGKMKGMPPKLMSDDGKHVVIRPLAYCREKDIERFSIAKA
FPIIPCNLCGSQPNLQRQVIGDMLRDWDKRYPGRLETMFSAMQNVVPSHLSDINLFDFKGIHHGSAVV
DGGDLAFDREDIPMQPVGWQPEDSDDAAPAPARLDVLEIK
>CORE_REP|Org7_Gene2443#
MTIRNLAVIGECMIELSQQGAQLTRGFGGDTLNTAVYLARQMPEQTLRVHYVTALGTDSFSGDMLQAW
RQEKIETGLIQQFDNKLPGLYLIETDAAGERTFYYWRNDAAARYWLAGPQADALCERLAQFDYLYLSG
ISLAILAPADRMKLLALLRRCRANGGKVIFDNNYRPRLWQSREETQQAYREVLACTDIAFLTLDDEEL
LWGAQPIEQAVARTQALGVGEIVIKRGAHACLVFSMAGEKLEVPAIALPPERVVDTTAAGDSFSAGYL
AARLNGGSAQRAAQRGHLLAATVIQHRGAIIPAAMMPEA
>CORE_REP|Org30_Gene3715#
MAQKDYVSRGRAAGAKRKTPSRKKRSSPKVSKTVLALAAALLVVFVGGLYFITHNKPEDAPLLPAHTT
RPGNGLPPKPEERWRYIKELENRQIGVQTPTEPTAGGELNSKTQLTAEQRQLLEQMQADMQQRPTQLN
EVPYNDPGQANARSTRQQQQMQQQQMQQQPVQQQQQVSQPPRNPFNNGATTAPVQQHPQPKPTTQQPV
QVKQPEPKPQPKPEPKPEVKQETAKQETKPESKQKWMVQCGSFRATDQAESVRARLAFEGIESRITAG
GGWNRVVLGPYSSRAAADKTLSRLKGVGMSSCIPLSVGG
>CORE_REP|Org30_Gene1499#
MIHFEQVSKIFQGKPAVDDLTLHIAEGEFTVLIGTSGSGKSTTLKMINRLIEHDRGKILFAGEEIQSF
KPQDLRRRMGYAIQSIGLFPHWTVEENIATVPQLLKWPRARIRDRVTELLELLHLEPDLFRRRYPHQL
SGGQQQRVGVARALAADPEVLLMDEPFGALDPVTRAALQAEIARIHQLSGRTIVLVTHDIDEALGLAD
RLVLLDQGRVVQQGTPLALLTAPANDFVRDFFGRSDRGIKLLSLGTVAERVRPGAAEGEPIAAAMSLR
EALSVFVARGSDCLPVVDERGEALGVLHFNDLIAGQALS
>CORE_REP|Org26_Gene3903#
MFRGLKAFLLLTLSLLFCQRAFADCATTNGTVTLPGSSSFVVYNGQINAQGTAGLNCTGLGLSLLSQN
TVTVKVASTTNGMAVANTDGSGDKIAYLIYPDANYQYPYSIGQTIDYSSLNLLSLILISSNVNFPLYI
KTTAGANVRSGTYTDTINLIWNYHICGLGVLGLCIWWDGVNKVSTVSVSVAITKDCLIGTAPNVNFGS
MALVGQFNPVNQSITLTCTKTEGYNTYFTNGNNPVSGWRRMKSGTSNFMQYQIYLPNTTTVWDSTNKQ
SGAGTGLAQSIPYKAAVNAAQTEVAVGSYQDNLSFVVEY
>CORE_REP|Org28_Gene4384#
MSTNLSYALLPEMAVFVQVVESGSFSAAARKLGTSPSAVSRSVAKLEQALALQLLHRTTRKLRLSESG
EEAFAHCRTLLAAADAVMAIGGRGAVEPEGLVSVSVPKAVGRFVLHPHMPEFLRRYPKVDVRLRLEDR
YMDLIDDRVDLALRITDRPSPGLIGRQLMRIDHLLCATPHYLAQHGTPQHPHALAAHSCIYLGETPSD
AQWKFRRSGKTVTVNVRGRYAANHTGVRLDAVKQHIGIGSLPYFTARQALDDGEIVQVLPEWDFLSSY
HGGLWLLYAPNQYLPPKLRVFIDYLVACLAQEPQLKRLA
>CORE_REP|Org36_Gene3069#
MIKQTVCGLLLGAAITGQAGAAEKLTLVLDWYINPDHAPIMVAEQIGAFKAEGLDVKIVPPSDPALPP
RLVAAKQADLAITYQPQLHFFADQGLPLMRVGTLINTPLNTVIALDKNITSPADLKGKTVGYSVSGIE
QATLATMVEHEHLRPQDIKLINVNFQLTSALLAGQVDAVIGGYRNIEALELKLQGKTPVVFNVEDYGV
PTYDELIIVAHRDAVNEPKIRKFLAALKQGSDYLHAHPQDTWLAFAKAHPELNTELNKQAWQASLPLF
ARDPVKLDRARYQAYEQFLFDNKLIKKITPVEQYAVELD
>CORE_REP|Org26_Gene1351#
MANPLYHKHIISINDLSREDLELVLRTAAGLKANPQPELLKHKVIASCFFEASTRTRLSFETSMHRLG
ASVVGFADGSNTSLGKKGETLADTISVISTYVDAIVMRHPQEGAARMAAEFSGNVPVLNAGDGANQHP
TQTLLDLFTIQETQGRLSNLSIAMVGDLKYGRTVHSLTQALAKFEGNRFYFIAPDALAMPAYILKMLE
EKGIEYSLHSSIEEVVPELDILYMTRVQKERLDPSEYANVKAQFVLRAADLAGARANLKVLHPLPRID
EIATDVDKTPHAYYFQQAGNGIFARQALLALVLNADLAL
>CORE_REP|Org22_Gene3741#
MLINLSDIDLKLLRVFVAVAEAQGVSAAQETLLMNQSTISTHLASLETRLGFRLCQRGRSGFRLTPKG
ERMLIACRSLFNAARDFTRVSQSLNGLLTGDLQIGLVDNLVSLPGNPFSQAIKHFQRRHQDVQLQCRI
CSPNEIEQGLLHRQLDLGIGYFGQQLEALRYQPWLEETQAIYCSADHPLFAVEEPDREQIENARWVKR
GYLLAQQLCPIAPPHLAAVAHHMESVAHLVLSGSCLGYLPTHYAARWVEQGLLRQLGGAALSYRATLS
LVSRPVQPDEALNALLEDLTPITSSLRGQRPIHGKQRAG

>CORE_REP|Org15_Gene1826#
MKNFSIKITRIAITLILVLLGIAAVFKAWVFYTESPWTRDAKFTADVVAIAPDVSGLLTDVPVVDNQL
VKKGQVLFVVDRPRYEQALAEAGADVAYYQTLAAEKRREAGRRVKLGVQAMSQEEIDQSNNSLQTVQH
QLAKAIAARELAQLDLERTTVRAPADGWITNLNVHAGEYITRGSVAVALVKKDSFYILAYLEETKLNG
LNKGDRAEITPLGSNRIMHGTVDSVAAAVNNSSSTVNNKGLASIDSNLEWVRLAQRVPVKILLDAKDQ
QHPYPAGTTATVVIVGKNDRNADSGSPFVRLMHRLREFG
>CORE_REP|Org15_Gene3972#
MKSDLSALPAFVAVAEGGSFAAAAEKLHLTRSAVSKIVSRLEARLGVMLFMRTTRSLSLTDEGALYYE
HCRQALANVQAAENQLDSGKMQVSGRLRVSVPVLFGHLCIAPLLTALANEHPLLTLEISFSDRRIDLV
DEGFDLAVRIGELADSGSLVARRLGEHGMLLCASPDYVRRCGEPSTVEALSRHQAVGYLHAGAVLPWQ
LRGENGELQSFSPPAKMMMDDMQGIVDAISAGAGAGIAWLPEWLVRERLMAGTLVEIMRGESNLSFPV
NVVWPYMPYQPLKVRLAVDKLVAELPAKLALVPPPLSQR
>CORE_REP|Org32_Gene2221#
MRVLLAPMEGVLDSLVRELLTEVNDYDLCITEFLRVVDQLLPAKSFYRLCPELRHASRTPSGTLVRVQ
LLGQYPQWLAENAARAVELGSYGVDLNCGCPSKLVNGSGGGATLLKDPELIYQGAKAMRAAVPAHLPV
TVKVRLGWDSSSRSFEIADAVQQAGASELTVHGRTKEDGYKADRINWAAIGEIRQRLSIPVIANGEIW
DHQSAQDCLQATGCDAVMLGRGALNVPNLSRVVKYNEPRMPWPQVVELLQKYVHLEKQGDTGLYHVAR
IKQWLGYLRKEYDEATELFSEIRALTTSGDIARVICRS
>CORE_REP|Org24_Gene2431#
MDKIFVDEAVNELHTIQDMLRWTVSRFNAANIYYGHGTDNPWDEAVQLVLPSLFLPLDIPEDMHTARL
TSSERHRIVERVIRRVNERIPVAYLTNKAWFCGMEFYVDERVLVPRSPIGELINDRFSALIPHPPRHI
LDMCTGSGCIAIACGYAFPEAEVDAVDISSEVLAVTERNIQAHGVEHQVIPIRSDLFRDVPAIQYDLI
VTNPPYVDAEDMSDLPQEFRFEPELGLAAGSDGLKLVRRILACAPDYLTDDGVLICEVGNSMVHLMDQ
YPDIPFTWLEFENGGDGVFMLTKQQLVDCKEHFSLYRS
>CORE_REP|Org44_Gene875#
MKKVLSLTTLMAGAMMVFNAAAADAPKELNLGILGGQNATQQIGDNQCVKQFLDKELNVDTKLRNSSD
YSGVIQGLLGGKIDLVLSMSPSSFASVYIKDPKAVDIVGIAVDDVDQSRGYHSVVVVKAGSPYQKLED
LKGKAIGFADPDSTSGFLIPNQAFKKLFGGTVDNKYNNTFSSVTFSGGHEQDILGVLNGQFEGAVTWA
SMIGDYNTGYTSGAFTRMIRMDHPDLMKQIRIIWQSPLIPNGPILVSNSLPADFKAKVVTAIKKLDKD
DHQCFIKAMGGKQHIGDTTLAEYQTIIDMKRELTKGDR
>CORE_REP|Org1_Gene3510#
MSEWEAVTPVLVIGGAVGDLVMTLPRLPTSGEDIEAQPQARQIGGCAFNVARALTRLEVPVINGMPVG
NGEWGAAIEAAMGELGLEVLLRHGQMDNGWCLALVEPNGERTFITVSGCESQWNKAQLATLPLTEHTL
IYASGYELAGENGEALREWLTRMPFDQPRLIDPGPRIAQLSEDFFAMLSDSHTLLTLNRDEVAALCGA
GDAVEAAQRYAAARNLTLICRLDRDGAWICDGRTPPLHIPAYPVDVVDTIGAGDAHCAGLLAGLSAGW
PLPQAVDLANRVAACVVASLGAANPPDRHQLQQRFPQA
>CORE_REP|Org8_Gene3935#
MTDPDFNLLIALDALLTAGSVAGAARRLGLSPSAMSRTLSRLRAATGDPLLVRAGRHMVLTPYAETLR
ERARHAAFEARAVLRPAQGALDPAALDRTFTLRANDGFVEAFGPALIAAAAEQAPRVRLRFAPKPEKS
DRPLREGLVDLEVGVLGDMGPEIRLQALFRDRFVGVMRTAHPLAQQPEIDVADYAACGHVVASRSGRI
LGPVDAALAELGLARHIAAVVPSFPAALAVAQASNLLALLPASFLQAQPADGPLRVFELPVKTPPITV
SQMWHPRLDAEADHRWLRQLVLSVCRRQAQPPASDNVT
>CORE_REP|Org1_Gene2939#
MNMLFPLLAVLIWSINAVVSKLSATAIDPAAISLYRWLLALIALTPFVLPGVLRNWAQVRANWWKLMI
LGLLGMVLYQSLAYYAAHSVSALFMGIIVSLIPLLTILISIVLLRIAPTVGVLLGSILSFCGLIWLVS
GGQPGVLLQHGIGKGELMMLLATASYALYGVLTKRWAIALPNWQSLYVQILFGVLLLLPNFLMAQDVG
LNGHNLPLVLFAGIPASIIAPFLWIQGVMRLGANTASIFMNLAPVFTAAIAVLFLHEQLHGYHLIGGG
ITLLGVILSQRLRTPLTRKAKAKAKAPAANAESCKEQA
>CORE_REP|Org17_Gene4455#
MLLSHRHFICTLLALAIGFWLPTAVQARPDLERRIGVTVADSDSADYRFSDLRFTSADGQRRYRVRIA
QPRRAPAPDGYPTIYFLDGNAVLMELNASLLARLATAKRPPVLVMIGYDNDLRIDAAGRAYDYTLPLP
TGMTGMTGMKKSPQAGGGAEAFLQLIETRIKPAIAAKLAVDQQRQTLWGHSYGGLFVLHTLFAHPAAF
QHYIAVEPSLWWGNGMILQEAQQWAERHPTPAARLQLWVGLAERDRAAPPGVKSPALPANAAQMLAER
LAKLDGLTVGYREWPGLGHGAMLGAAIEPALNSVAYED

>CORE_REP|Org28_Gene1290#
MSIRIVPKEQLGAQREKSTTAENIPPLLFANLKSLYSRRADRLRQLAVDNPLGDYLNFAAELAQAQQH
ALHDNPLQLDLSEALAQGAASGKPPLDLSVFPRSEHWRKLLTSLIAELRPQAPEHILAVLDNLEKASA
HELELMADALLNREFGKVGSEKAPFLWAALSLYWAQMASLIPGKARAEYGEQRQFCPVCGSIPVSSMV
HIGTVNGLRYLHCNLCESEWHVVRVKCSNCEQTRDLNYWSLDSEQAAVKAESCGDCGTYLKILYQEKD
PQVEAVADDLASLVLDARMEEEGFGRSSINPFLFPAE
>CORE_REP|Org46_Gene3012#
MAITLSRGRSFAVALALLATGGLAQAADSVRVGSKIDTEGSLLGNLIVQVLEANGIQTTNKLQLGTTK
VLRGAITSGEIDIYPEYTGNGAFFFSDEKDPAWKNAQAGFEKVKQLDYDKNKIVWLDAAPANNTWTIA
IRQDVAEANHLKTLSDLGKWINGGGKFKLAASAEFIERPDALPAFQNAYGFKLNQDQLLSLAGGDTAV
TIKAAAEQTSGVNAAMAYGTDGPVAALGLQTLADPKGVQPIYAPAPIVREAALKAHANLPELLKPVFA
SLDGPTLQKLNAQIAVEGQDAKQVAAAYLKEKGFVKG
>CORE_REP|Org5_Gene2049#
MFPSKKHSQRATPLTSYQFSRLHTFECVARHLSFALAAQELSITPSAVSHRINLLEKELGFLLFQRFH
RRITLTPEGERMQWALDSSFNTLNQEILDIKNRELTGTLTLYSHPSLVQCLLLPRIGDFIAQHPTIHL
NILTGQEIINLANRGVDLAMYFGKLPSGRHLDEAFMQESMVPICTPQYAAAHSLYDAPENLAHCTLLH
DRYNSGEDEWQTWSQHFALGLDTDSKSMEFDRSDLAVLAATRHLGVAMGRLNLVQDWIKSGELIIPFT
DMTVPCEHCYFTSTISERQWPKILAFKQWIMKIAPLV
>CORE_REP|Org5_Gene1608#
MKLKKLIAASVLMCMLPASVLAKDIKIGVSMAYFDDNFLTILRQSMQNKMKADGNVSGQFEDAKGDIA
QQIQQIENFVSQGVDAIILNPVDTQGVKPMIKLAEKAKIPLVFVNRKPEVALPAGMAYVGSDSKLAGK
LQMEELAKLMNGKGNVMILMGELSSEATRDRTRGVEEVAANYPGIKIIDKQTAKFFRKEAVDVTTDWI
LSGQQIDAIASNNDEMAIGAILALKQAKKSGVLVAGVDGTPDALEFIKKGDLALSVFQDAKGQGEGAV
QTAVQLVKGEKVESNVLIPYQLITQANYQQFADKNKK
>CORE_REP|Org48_Gene2513#
MQVKKRALLGQLSDMDLRLLRVFKAVVDCGGMSAAELELNISLSTISKHIKDLEQRLGLTLCQRGREG
FAVTDEGLLIYQETVNLLAATEAFRRGVDEVHQRMGGQLHVAIFDHTVSNPQAQIGRAIALFSERAPE
VSLQMYVEPINTIERGVIDGQFQVGVIPMHRSAESLSYHSLFSERMFLYCGAQHELFSGPHETLNWDL
LHNYAFAGLGYHSPNMELSLQQHLHRKATGFAQESIATLILSGKYVGFLPDHYAAFFVAQNMMRAIKP
ALFRYHCEYSSVLRRSPVPQRVVKLFHECLLAAHGTA
>CORE_REP|Org13_Gene567#
MTQFAFVFPGQGSQTVGMLAELAAQFPIVEETFGEASSALGYDLWQLVQQGPAEELNKTWQTQPALLA
ASVAIFRVWQQQGGKAPALMAGHSLGEYSALVCAGVLDFKAAIRLVELRGKLMQEAVPEGTGAMYAII
GLDNDAIAKACEESAQGQVVSPVNFNSPGQVVIAGNKEAVERAGAACKAAGAKRALPLPVSVPSHCAL
MKPAADKLAVALQDITFNAPQVPVVNNVDVRTENDPEAIRSALVRQLYSPVRWTESVEFIAAQGVTSL
LEVGPGKVLTGLTKRIVDTLTAAAVNDTASLSAALEQ
>CORE_REP|Org1_Gene911#
MVKVYAPASIGNVSVGFDVLGAAVSPIDGTLLGDCVSVEAAETFTLQNAGRFVSKLPAEPKENIVYQC
WERFCQEIGREVPVAMRLEKNMPIGSGLGSSACSVVAGLMAMNEFCDRPLDKMTLLGLMGELEGRISG
SVHYDNVAPCYLGGLQLMLEEEGIISQEVPCFDDWLWVMAYPGIKVSTAEARAILPAQYRRQDCISHG
RYLAGFIHACHTRQPQLAAKLMQDVIAEPYRTRLLPGFAEARKAAQDIGALACGISGSGPTLFAVCND
GATAQRMAAWLQQHYLQNDEGFVHICRLDTAGARLLG
>CORE_REP|Org24_Gene644#
MIIVTGGAGMIGSNIIKALNDKGYRDILVVDNLKDGTKFVNLVDLDIADYIDKEDFIASIVAGDDLGD
IEAVFHEGACSATTEWDGKYMMDNNYQYSKDLLHYCLDREIPFLYASSAATYGGREEFIEEREYEAPL
NVYGYSKFLFDQYVREILPEADSQICGFRYFNVYGPREGHKGSMASVAFHLNTQINRGENPKLFAGSE
NFKRDFIYVGDVAAVNLWFWETGKSGIFNCGTGRAETFQAVADAVVDFHQKGAVEYIEFPEKLKGRYQ
AYTQADLTKLRAAGYDAPFKTVAEGVKEYMAWLNRTA
>CORE_REP|Org36_Gene4684#
MPIRVPDELPAVNFLRGENVFVMTSSRAKTQEIRPLKVLILNLMPKKIETENQFLRLLSNSPLQIDIQ
LLRIDSRESKNTPAEHLNNFYCDFEDIQNENFDGLIVTGAPLGLVDFCDVAYWPQIERVIDWAKHHVT
STLFVCWAVQAALNILYGIPKMTREVKLSGVYPHQTLQQHALLTRGFDESFLAPHSRYADFPTEVIRQ
YTDLDILAESEQAGAYLFASRDKRLAFVTGHPEYDTLTLAGEYCRDNEAGLDPAVPLNYFPDDNPALT
PKATWRSHGHLLFSNWLNYYVYQITPYDLRHMNPTLD

>CORE_REP|Org43_Gene3758#
MNEVALPPRRSLPDAQAWRGEMSDVGLDWVGMQGIALPLELAGKPLMAKVDAGINLRAEAAGERGIHM
SRLYLALDELTQGELTPQRIGRTLQAFLASQPEHSDRASLSIGGELLLSRPALLSPQRGWKAYPLRIE
ATLTDTLTLALTVGVPYSSTCPSSAALSRQLAQQQFQFDFEQAAERVSQRQVSEWLLEQGMPATPHSQ
RSWAWITVTPENERAFEPVKLIDRIEHALGTPVQTLVKRSDEQAFALANGQNLMFCEDAARRLYRMLR
SQCDYRAFSLRVEHQESLHAHNAVAELSWRGEHHAA
>CORE_REP|Org22_Gene1753#
MSEQLLYFLQQMFNGLTLGSTYALIAIGYTMVYGIIGMINFAHGEVYMIGSYVSFIVIAALMMLGIDV
GWLLIGAAFLVSIVIASAYGWSIERVAYKPVRNSKRLIALISAIGMSIFLQNYVSLTQGSRDLALPSL
VTGQWVLGESNGFAATISTMQLIIWLVTFLAMLALTLFIRYSRMGRACRACAEDLKMASLLGINTDRV
ISLTFVIGAVMAAVAGVLLGQFYGVINPYIGFMAGMKAFTAAVLGGIGSIPGAMIGGLVLGVAEALTS
AYLSTEYKDVVSFALLIVVLLIMPTGILGRPEVEKV
>CORE_REP|Org21_Gene3163#
METGKLVVLGSINADHILNIEQFPHPGETVIGKQYKVAFGGKGANQAVAAGRSGAEIAFIACVGADDI
GERVRRQLASDRIDTQPIEAIADSTTGVALIFVNAEGENVIGIDAGANAAVTPDYLARYQQKVIDADA
LLMQLESPLETVIAAARLAKQYHTQVILNPAPARELPDELLGMIDMITPNETEAQRLTGIAVDNDADA
ARAAQALHDKGIATVIITLGSRGVWLSENGNGKLVPGFKVQAVDTIAAGDTFNGALVTALLEGKIMAD
AVRFAHAAAAIAVTRPGAQPSVPWREEIDAFLQQQG
>CORE_REP|Org22_Gene2599#
MINRVWVLGDAVIDLVPENANGYLKCPGGAPANVAVGIARLGGDSAFIGRVGQDGFGAFLQQVLSDEG
VDIGHMRPDPEHHTSTVVVDLDLQGERSFTFMVQPSADLFLQPDDLPDFQRGEWLHLCSIALSQEPSR
SAAFAAMERIRAAGGRVSFDPNIREEVWRQPEALRPCLQKALLLADVVKLSREELAFISHLDDLENAI
RWMMQTYPLRLLLVTLGGDGVYVHDGQRLRHFRAAPVVPVDTTGAGDAFVAGLLAALARLHELPQEAQ
WPAVIAQAQACGALATTAKGAMTALPHADELAAFLR
>CORE_REP|Org4_Gene4035#
MKGKVCVFGSFNLDIVAGMARFPQPGESLIARNSMMGAGGKGANQATAALRAGARVHYIGKVGRDDFG
TFARRHLATAGFDAVTLFSTGDCPTGNALIYVAGEEAENMIAVDPGANLTVSEDEVRQCRPAIAAADI
LLTQLENNLPAIEQVIAIAREAQTFIILNPAPFQPVPDSLLAQVDMLTPNATECTLLTGVPVRDVASA
RQAAQVLHAKGIRLLIVTLGTQGALFSDGENSELIPAFPAQPKDTTGAGDAFNGALAAQLANQVPLAD
AVRFAAAYAAVCVERAGAAGSMPSYEEALERQRAFA
>CORE_REP|Org13_Gene3133#
MTKKADYIWFNGEMVPWAEAKVHVMSHALHYGTSVFEGVRCYGSHLGPVVFRHREHMQRLHDSAKIYR
MPVSQSVDELMEACRATLRKNNLVSAYIRPLVFVGDVGMGVNPPAGYKTDVIIAAFPWGAYLGEEALD
QGIDAMVSSWHRVAPNTIPTAAKAGGNYLSSLLVGSEARRHGYQEGIALDVHGYISEGAGENLFEVKD
GVIYTPPFTSSALPGITRDAIIKLAKDMGFEVREQVLSRESLYLADEVFMSGTAAEITPVRSVDGIQV
GIGKCGPVTKQIQQAFFGLFSGKTEDKYGWLDPVNP
>CORE_REP|Org18_Gene4444#
MLHKSVPLGIYEKALPPGEDWLTRLQLAGELGFDFVEMSIDESDGRLARLDWSTEQRLALVSAVAASG
VRVPSLCLSAHRRFPLGSADDTTRDRGLEIVRKAIRLAQDTGIRVIQLAGYDVYYQQSTAQTRARFRA
GLAQAVEMASRAQIMLAMEIMDYPLMNSISKALGYAHYLNNPWFQLYPDIGNLSAWDNDVPMELMAGR
GHIVAVHVKDTRPGEFKNVPFGEGVVDFVRCFETLLQSGYRGPYLLEMWSEKADDPLRAVRAVRAVRE
ARAWVVERMVEAGLEIRGEHAGATEKSGVPSQYGVA
>CORE_REP|Org17_Gene4464#
MNPPHALPDPGRINFRLLHYFRVVAEEMNFTQAARRLNMSQPPLSKHIKELESQLGVVLFKRTTRSMT
LTPAGRTLLRNVERLLDQADSALHQVQQMGRGEGGHMVVGMVGTSAWGGLIAALRRFSEQSVGATWSL
NELTPSQQITALQKRHIDIGVWREAQQQTLPGLTCQRLARESIAVVLPLDHPLAQQENIPLAALQNDS
FIVLPPHEASLGLYLHNLCLQQGFLPDVAYQVNEPQTLLALVAEGCGITLLPDSYGRIPWPGVRFCSL
QQAPPADLYAVYRTDSVTPVVQAFLATLTPSSSAAR
>CORE_REP|Org18_Gene2648#
MMTLRQIRHFIAVAETGSISAGAQAVFVSQSSLTLAIQQLETEIGVRLFDRHAKGMTLTHQGHQFLRQ
SYLILATVDNAKRSLQIGTESLTGKLTVGVTSLVAGYFLVELLTRFKSAYPNVTVQVVEDERPYIEHL
LVSGEIDIGVLILSNIEDRDALQTEVLMHSPYRLWLPPLHPLLEHESISLADVAKQPLIQLNADEMDV
HARRIWSRAGLKPEIAMKTASTEAVRSLVAAGMGVSIQPDMAYRAWSLEGNMIEARKLDDLLEPLDIG
LAWRRGSARPELVTPFLTIARENGSKHAAGLKHSI

>CORE_REP|Org13_Gene2245#
MHSISLRQIEIFRAVMTTGNLTEAAALLQTSQPTVSRELARFEKLIRLQLFDRVRGRLSPTVQGLRLF
EEVQRSYYGLDRIVNAAAGIRQFQQAQLSIVCLPVFSQSLLPAVCRPFIERYPEVSFSVIPQESPLLE
EWLSAQRHDLGLTETTLTPAGTERVTLMTLNEVCVLPTGHPLLVKDRLTPQDFAGQNFISLSSTDSYR
HLLDALFGEQGVERRMVMETHSAASVCAMVRAGVGVSIVNPLTALDYAGNGVHVRPFSIDVPFTVSLI
RPLHRPSSALVTAFIDHLHQQAAAFAARLAAAVRR
>CORE_REP|Org2_Gene3513#
MSQAVYFLLLPNVLSLDVSGPAETLRLAGQFSLHYLSPAPQIVCSIGMTLSGLQPLPERLEDGAILVL
PGVGDSQRYFAGEEAEQARRWLATLRPDLQRQRITLVCICSGALLAAQAGLLDGYQCTTHHDVIERIR
RQAPAAQVKENRIFVEDRGVYTSAGITTGIDLALHLVHRHCGSGRARDVARDMVVYFRRAGDDPQLSP
WLRYRNHLHPAVHRAQDVMAAEPEADWSVPQVAEKAHVSSRHLARLFRNHVGISVREYHEQLRLAVAQ
QRLQQGYGLEKAALAAGFSSGRQLRRAQQRQRSPL
>CORE_REP|Org47_Gene2528#
MNFRRLKYFVKIVDIGSLTQAAEVLHIAQPALSQQLATLEGELKQQLLIRTKRGVQPTEAGNILYAHA
QTILRQCEQAQSAVNSAGQAMSGQVSLGLASGSTAAQLALPLLQSLRDQQPGILLSLHENGGAALAGQ
VANQTLDMAMVYGAKMPAGLHAIALMREDLYLVATRAVPHPGNSVELLDVARLNLFLPREGDAVRNQL
EEAMALRKLAVNVVGEIESSGALSAAIASGLGATVLPESVARAMIGPAKAWMARINAPTMSVPLSLCM
SGQQALSAPALLVKDLLLSIAGGRSQEKRALALVR
>CORE_REP|Org21_Gene2741#
MKRYLDCSASDLADIGKADLLYAIRASEGRILVSETIAVTQPLLNNVTNAELAASQGADLLLLNLFDV
DHPHIAGLPADVPPQEALRTLQRLTGRVIGVNLEAVDPAFATEHNDFWQMTAGRAATAENARKLYQLG
ARMLVLTGNPNNGVSNQAIAVALKAIRDEVGDEMVLVTGKMHGAGIVRESGSQLIGEQDIALFVENGA
DIVLLPAPGTIPGMSQEKVAALINFAQRQGALAMTAIGTSQEGADVQTVRQIALMSKMAGADLHHIGD
TGYLGLALPENIFAYSVAIRGVRHTYSRMARSVNR
>CORE_REP|Org26_Gene1133#
MNYALELAQLTKTYAGGVKALRGIDLSVEAGDFYALLGPNGAGKSTTIGIISSLVNKTAGSVRVFGYD
IDKDIVNAKRQLGLVPQEFNFNPFETVLQIVVNQAGYYGVTRREAMARAEKYLNQLDLWGKRNERARM
LSGGMKRRLMIARALMHQPKLLILDEPTAGVDIELRRSMWGFLKELNAQGTTIILTTHYLEEAEMLCR
NIGIIQNGELVENTSMKGLLAKLKSETFILDLAAKSPLPKLDGYHSRLTDTSTLEVEVMREQGLNGLF
TQLSAQGVQVLSMRNKANRLEELFVTLVNGNGEKA
>CORE_REP|Org41_Gene3888#
MSAAKKSFISAMVPQIRLPEAVLIFITMIWGGTFLAVHHAMQVSGPFFFVGLRFATATLALTLFSLRV
LRGLTLYELKAGGLIGLAIMFGYSMQTVGLQTITSSQSAFITAMYVPIVPLLQWLVLGRFPGIMSWIG
ILLAFTGLMLLAAPSSTDMTLSLGEILTLAGTLGMAAEIILIGAFAGKVNIRRVTIVQLATASLTSFL
MMAPTGESPPPYSDYLLYSAIGLGLASALIQLTMNWAQRSVSPTRATVIYAGEPVWAGIVGRLAGERL
PGVALLGGALIVIGVVVSELRVRRKDKAAVATEAD
>CORE_REP|Org22_Gene4460#
MVTTLDNALLEEILQQVRPLIGQGKVADYIPALAEVPADRLAIAVCTVDGELFQAGDAAERFSIQSIS
KVLSLTLALTRYQEPEIWRRVGKEPSGLPFNSLLQLEMEQGKPRNPFINPGALVVCDMLQTRLSAPKQ
RMLEVVRQLAGEDDLAYDLRVARSEFEHSDRNAAIAYLMKSFGNFENDVITVLQTYFHYCALRMSCVE
LARSFVYLANHGRDLNGEAVISPLQARQINALMMTCGMYDGAGEFAYRVGLPGKSGVGGGIVAIVPGE
LSIVVWSPELDASGNSLAGTAALELLSQRIGRSIF
>CORE_REP|Org23_Gene1179#
MLSQQSRSVLPLIGALFTLYIVWGSTYFVIRLGVAQWPPLMMAGIRFLIAGIVLFSFLAWRGHALPTP
KQWLAAGAIGILLLAVGNGLVTVAEHQHVPSGIAAVMVATVPLFTLCFSLLWGMRNTKLEWAGIALGL
VGIVLLNTGNNLVGNPTGALLILLASASWAFGSVLGSRISLPAGPMAGAAEMLVAGVVLLVASRLSGE
HLAQMPSTGGFLALGYLIVFGSMLAISAYMFLLKNVRPAVATSYAYVNPVVAVLLGIGFAGESLAPRE
WLALVIIVAAVVLVTLGKYLFARPANVQAIDCERR
>CORE_REP|Org7_Gene3090#
MAGSSLLTLIDDIASLLDDVSLMSKMAAKKTAGVLGDDLALNAQQVTGVKADRELPVVWSVAKGSLIN
KAILVPLALLISAFAPWAITPLLMVGGAYLCYEGFEKVFHSLSPGKAAQEETADGPGANEDAAAYEKR
KVKGAIRTDFVLSAEIIAITLGTVAGATFSQQVIVLCGIAVVMTLGVYGIVAGIVRLDDLGLYLSRKR
SAVARSLGGGIVRAAPYLMKTLSIVGTVAMFMVGGGILTHGLPPVHHLFEDWASYTTVVPTFGHLLQG
VIPALLNVAFGLVAGGVVLAVVSALGAVRARFKA

>CORE_REP|Org40_Gene3137#
MPISLPSLDVLKTFVVVAQRLNFTHAARQLHLTQGAVSRQILGLEQRLGYPLFSRQARGLALTPQGAQ
LLAPVQQALGQLDEALTRAAAPPGALRIKCPTCAMRWVLPRIIRLQNERPDMHIELTASVSHGLDFST
EQFDAAVVFGRPPGKKLTAHLLFDEILTPVCTPTFLPPTPRLTDLTDKTLLHPTRDRRDWLRWLKAAG
ADALPSGKAQHFDTLDLAMSAALQGFGIAIGDLCLLEEDIQAQRIVTPFPLCVSSGAAYYLVYPERTV
APPTLTALVDFLAAEAADSRARLQNYLPMTCNAL
>CORE_REP|Org17_Gene3697#
MSEIVPSAAFADQLQIFRRRADRALLDFIVPLPFNDGNMVAAMRHGALLGGKRLRPFLVYTTGQMFGV
SLTNLDAPAAAVECIHAYSLIHDDLPAMDDDDLRRGQPTCHIKFGEANAILAGDALQTLAFSILADAE
MPDVALRDRLAMVSELATASGVAGMCGGQSLDLEAEGKRVDLQALEQIHRHKTGALIRAAVRLGALSA
GEAGRAALPQLDRYAAAVGLAFQVQDDILDVVGETEKIGKRQGADQQHGKSTYPALLGLDSAKAKAWD
LYQEALAALDTLAAQSYNTAPLRALASFIIERDN
>CORE_REP|Org33_Gene2755#
MLATHEYANDLILFALIVDCGSFSKAAESAGITSSVVSKRIGRLEKSLGARLLYRTTRSLTLTESGQA
LYQQAKEIGAKVQEALYAVSEKSEELTGTIRMSVPTISGELLLSESVAEFCALHPSLKVEMRLENRFV
DLVEEGIDLAIRTGTMPDSSLIARPIFDSRWVIVCSPGYLESHPEPRSAEDLLGHNCLTYTYQESGTA
NWLMKRPGRNEIYELQVNGNLSANNARAIRKAVIGGHGIAMVPRCMVYEDLQDGKLTEILAGHCGKVL
GIYAVYPYTRNLPLKTRLLIEHIIGSYQNISHYF
>CORE_REP|Org44_Gene480#
MSTLGHQSDNSLVSNAFGFLRFPLNFMPYDSDAEWVITGIPFDMATSGRAGGRHGPAAIRQVSTNLAW
EGNRWPWSFDLRDRLNVVDCGDIVFNFGDAQDMSDKLQAHAEKLLKAGKRMLSFGGDHFVTLPLLRAH
AKHFGKLALVHFDAHTDTYANGSKFDHGTMFYHAPNEGLIDPHHSVQIGIRTEFDHDNGFTVLDAAQV
NDRSVDDLLTQIKGIVGDMPVYLTFDIDCLDPAFAPGTGTPVIGGLTSDRALKLVRGMQSLNIVGMDV
VEVAPAYDQSEITALAAATLGLEMLYLQAAKKHA
>CORE_REP|Org40_Gene785#
MKSAKAFQLALLHPRYWLTWFGLALLFLLVQLPYPLLNRLGVWMGRTSMRFLKRRVTITRRNLELCFP
EMDEAQRERKVVGNFESLGMGLLETGMAWFWSDKRVKRWFNVSGINHLKMAQRDDRGVLVIGVHFMSL
ELGGRAMGLCQPMMAMYRPHNNKAMEWAQTKGRMRSNKAMIDRKDLRGMVHALKRGEAVWFAPDQDYG
PRGSVFAPLFAVDQAATTSGTFMLARMANPALVPVVLIRREGGRGYDLLIQPALEDYPLSDEQAAAAY
MNKVIEKEIMRAPEQYMWLHRRFKTRPAGAPSLY
>CORE_REP|Org17_Gene4172#
MSKLRVGVVGLGSIAQKAYLPILSQAADWTLVGCFSPNQQKAQPLCDSYRMACFPRLDSLAEQCDAVF
VHSSTASHFSVIGELLNRGVHVYVDKPLAETLEQGEQLLELAERRGKTLMVGFNRRFAPLYRQLKQQM
NQPASIRMDKHRADSVGPHDLRFTLLDDYLHVVDTTLWLAGGGEQLLSGSVRANAAGEMLYAEHHFAC
GDTLVTTSMHRRGGSQRESVQAVTDGARYQLTDMRHWLREDAQGELEQPVPGWQSTLVQRGFDGAVRH
FLCAVANQSAPETGGEQALVAQRVVERLLRDNSM
>CORE_REP|Org14_Gene622#
MDLTQLRMFCCVAETGSVARAAEQLHRVPSNLTTRLRQLEQELGADLFIREKQRLRLSPMGHNFLCYA
NRILALSDEAMSITHAGEPAGNFALGSMESTAATRLPSLLAAYHQRFSQVSLSLTTGTSGEIADRVRA
GTLAAALVDGPVPYDELNGCIAYPEHMVVISCLDHAPIHSAKDANGETLFAFRASCSYRLRLEAWFKR
EGARPGQIMEIQSYHAMLACVASGAGLAMIPHSVLSLLPGHERVRVHTLPPDVADTATWLLWRRDAFG
PNVRALKELIIEQTETAAVDESTPNDLSDVVDIA
>CORE_REP|Org17_Gene4328#
MSLPNIAEVKSFLLALQDHICTQLAQADGGAVFTEDQWTREEGGGGRSRVLTNGAVFEQAGVNFSHVS
GATLPASATAHRPELAGRSFQAMGVSLVIHPLSPYVPTSHANVRFFIAEKPGEAPVWWFGGGFDLTPF
YGFAEDAVHWHRTAAELCAPFGDEVYPKYKQWCDDYFFIKHRNEARGIGGLFYDDLNTPDFDHCFAFT
RAVGQGFLDAYLPIVEKRKALTWGERERQFQLYRRGRYVEFNLVWDRGTLFGLQTGGRTESILMSMPP
LVRWEYNYQPEGDSPEAALARDFLPVRDWLRETK
>CORE_REP|Org14_Gene600#
MKTLNKTRLRRSMLFVPGANAAMVSNAFIYQADALMFDLEDSVILREKDAARRLVYHALQHPLYQEVE
TIVRVNALDSAYGLADLQAVVRGGADIVRLPKTDSAQDVVDMEREIAAIEAACGRPVGSTGLLAAIES
AQGITNAVAIAHASPRLIGIALGAEDYVRNLRTERSPEGIELLFARCSLLQAARAAGIQAFDTVYSDA
NNEAGFLQEAALIKQLGFDGKSLINPRQIELLHNLYAPTAKEVAHAQRVVDAAEAAEREGRGVVSLNG
KMVDSPVIERARLVLERAALSGLREEPAQHGEEA

>CORE_REP|Org32_Gene2582#
MNYLKGLWLAVALCASTSAWAQTIGVSMAYFDQNFLTIIRQAIDKEAKARGITVQFEDARGDVGRQTD
QVQSFISAGVDAIIVDPVNSASTPVMTKMVQAAGVPLVYVNRTPGDAKLPQGVVFVGSDERESGTLQM
EELARLANYQGNVAVMIGNLTDAGALQRTKDVEQVVAKYPKMKVVQKQSANYSRSEGMDLMMNWLTNG
EAIDIVAANNDEMAIGAIMALQQAGKADKKVLIGGIDATPDGLKALASGKMQVTVFQDAVGQGKASVD
VAQRMINGEKLEPYYWIPFELVTPANQGKYAARP
>CORE_REP|Org47_Gene1024#
MLKFILRRLLEAIPTLFILITISFFMMRLAPGSPFTGERALPPEVMANIEAKYHLNDPIWKQYGHYLA
QLAQGDFGPSFKYKDYSVNDLVAGSFPVSAKLGLAAFLLAVVLGVSAGVVAALNQNTKWDYTVMGFAM
TGVVIPSFVVAPLLVLIFAITLKWLPGGGWNGGAPKFIILPMVALSLAYIASIARITRGSMIEVLHSN
FIRTARAKGLPMRRIIFRHALKPALLPVLSYMGPAFVGIITGSMVIETIYGLPGIGQLFVNGALNRDY
SLVLSLTILVGALTILFNAIVDVLYAVIDPKIRY
>CORE_REP|Org7_Gene3795#
MSHKTLSWSGVFPAVTTQFRNDFSLDLDATHTVIKNLVRDGVSGLVVCGTVGENTSMTVQEKLAVIEV
ARDAADGQVPVIAGIAEFTTAFAQNMAREAQKAGVDGIMVMPALVYSAKPHETAAHFRSVAGATDLPI
MVYNNPPIYKNDVTPDILTSLVDCENIVCFKDSSGDTRRFIDLRNEVGDRFVLFAGLDDVVLESIAVG
AQGWISGMSNAFPREGETLFRLAKEKRYEEALALYSWFMPLLHLDARPDLVQCIKLCEQRVGRGSAVT
RPPRLALQGETLSEINAVIDKALATRPALPDVGL
>CORE_REP|Org48_Gene2865#
MQKFDTKTFQGLILTLQDYWARQGCTIVQPLDMEVGAGTSHPMTCLRALGPEPMATAYVQPSRRPTDG
RYGENPNRLQHYYQFQVVIKPSPDNIQELYLGSLKELGLDPTIHDIRFVEDNWENPTLGAWGLGWEVW
LNGMEVTQFTYFQQVGGLECKPVTGEITYGLERLAMYIQGVDSVYDLVWSNGPLGVTTYGDVFHQNEV
EQSTYNFEYADVDFLFSCFEQYEKEAQSLLALEKPLPLPAYERILKAGHTFNLLDARKAISVTERQRY
ILRIRTLTKAVAEAYYASREALGFPMCNKKNEN
>CORE_REP|Org5_Gene1939#
MPAVNRKVRKAVIPVAGLGTRMLPATKAIPKEMLPLVDKPLIQYVVNECIAAGINEIVLVTHSSKNSI
ENHFDTSFELEAMLEKRVKRQLLDEVQSICPKGVTVMQVRQGNAKGLGHAIMCAYPMVGDEPVAVVLP
DVILDEYSADPKKDNLHEMLQRFETTGVSQIMVEPVPHKDVGNYGVADCKGVDLQPGESAPMVSVVEK
PSPDKAPSNLAIVGRYVLSADIWPLLAKTPPGAGDEIQLTDSIEMLMQQETVEAYHLKGVSHDCGNKL
GYMQAFVEYSMRHASLGKEFSQWLQQVVAADKK
>CORE_REP|Org8_Gene1191#
MIIRPHQNWFFRLFAWHGSVLSKITFRLSLNVLMSIVAVISYQWYEQLGVHLTIAPFSLLGIAIAIFL
GFRNNAGYSRFVEARNLWGSLLITERSLLRQIKSLLPDEPAVQQKVAKLLIAFSWSLKHQLRATDPTA
DLYHNLSSKELAEVIASPMPTNRILLMLGQEIGKLRRQGLLSDITFELLDNKLSELSHALGGCERLAS
TPVPFFAYTLILQRTVYLFCSLLPFALVTDLHYMTPFVSVFISYTFLSWDSLAEELEDPFGVSANHLPL
NAICNTIERNLLEMNDQSPLPPPMKPDEHFNLI
>CORE_REP|Org12_Gene3907#
MNALLYLAVVLIWGTTWIAITLQQEGPVAIPVSIAYRFAIAAAVMFAVLLLTRRLRRLAPRDHLFCVL
QGCCVFGFNFFCFYHAAAYISSGLESVIFSMAVLFNALNSLLFFRQRPSPNLLPAALLGLTGIVALFW
QDLAATRMAPELLKGIGLSALGTYGFSLGNMISTRHQRRGLDIFSTNTYAMTYGAMLMALIALAQGAS
FQMEYSSRYIGSLSYLAIFGSVIAFAAYFSLLGRIGAGAAAYSTLLFPLVALTISTIYEGYQWHFNAV
LGLCLILLGNLVMFAKPGLRWRRAGMPKQNSTL
>CORE_REP|Org11_Gene2832#
MDHHLLAIRVFNRVVETGGFTRAAESLGMPKATVTKLIQNLEDHLQTKLFQRTTRSVSVTREGECYYQ
NTVKWLADLEQMEGCLTESQSSPQGVLRIDTGGGTARRLLLPALPDFLARYPQIQIDLSVGDRVIDLI
SDSTDCVIRSGPLADSSLIARRLFDLDWVSCATPAYLALHGTPRHPCDLEQGFPMVHYRHPLNDRIHP
QRYAEHGKEIAIQQRSPVSINEGNALLAASLAGLGIIQIYRFMAQPHLDSGELVSLLHDWQPPPEQMY
VVYPSNRHLSGKLRAFIDWAVETFDSGKMSRTL
>CORE_REP|Org13_Gene586#
MFSLFKKTLPFIVAGGMLAASHGALAKQITIGMSFQEMNNDYFVTMKQALDQAAADIGAKVYVADARH
DVAKQIGDVEDMLQKKVDILLINPTDSVGVQSAVISAHKAGAVVVAIDAQAEGPLDSFVGSENYDAGF
QAGEYLAKALGGKGKVAILDGIPVVPILERVRGFEAAMKKYPDIKIVTKQNGKQERDTALTVTENMLQ
SAPDLAGIFSVNDVGALGALAAIESNGAKVKLVSVDGQPEAIKEILKPNSPFIATSAQFPRDQLRIAL
GIALARYWGATVPKTVPVKVKLIDRSNAAGFSW

>CORE_REP|Org3_Gene2084#
MIEIDAQRSRMARQAMAIATGNGYTPSPVPRVKILYVDRHCPRQPVMYEPGIVIIFQGHKVGYCGSKV
FQYDPRNYLLMTVPLPFECETFASPELPLVGLAVNIDTQMLQDLLIDIGDDDYLMQPRAESNGVNLAE
LTEALLCATERLLDVMAKPLDARVLGPQIVREILYYVLRGTCGASLQELVNRHTHFSQIAKALRRIEH
QYADNLNVEQLAGEVNMSVSAFHHNFKAVTNTSPLQYVKSYRLHKARLLMVHDGLKASTAAIRVGYES
ASQFSREFKRLFGMTPSDEVARLREANPLLLEG
>CORE_REP|Org20_Gene743#
MIKQRTLKRIVQATGVGLHTGKKVTLTMRPAPANTGVIYRRTDLNPPVDFPADAKSVRDTMLCTCLVN
EHDVRISTVEHLNAALAGLGIDNIVIEVDAAEIPIMDGSASPFVFLLLDAGIEELNSAKKFLRLKETV
RVEDGDKWAELSPHNGFRLDFTIDFNHPAIDASSQRYRLDFSAESFVRQISRARTFGFMRDIEYLQSR
GLALGGSFDCAIVVDDYRVLNEDGLRFEDEFVRHKMLDAIGDLFMCGHNIIGAFTAYKSGHALNNKLL
QAVLAKQEAWEYVTFQDEAEMPLAFKAPSTVLA
>CORE_REP|Org8_Gene1071#
MNIRDLEYLVALAEHRHFRRAADSCHVSQPTLSGQIRKLEDELGVMLLERTSRKVLFTQAGLLLVEQA
RTVLREVKVLKEMASQQGEAMSGPLHIGLIPTVGPYLLPQIIPTLHKTFPKLEMYLHEAQTHQLLAQL
DSGKLDCAILALVKETEAFIEVPLFDEPMKLAVYSDHPWAQRERVAMPDLAGEKLLMLEDGHCLRDQA
MGFCFQAGADEDTHFRATSLETLRNMVAAGSGITLLPSLAVPPQRERDGVCYLDCYKPEPKRTIALVY
RPGSPLRSRYEQLAEAIREHMQGYIDSALKQAV
>CORE_REP|Org44_Gene1547#
MRNRTLADLDRVVALGGGHGLGRVMSALSSLGSRLTGIVTTTDNGGSTGRIRRSEGGIAWGDTRNCLN
QLITEPSVASAMFEYRFSGNGELAGHNLGNLMLKALDHLSVRPLEAINLVRSLLKVDAALIPMSEQPV
DLMAHDHEGNHVYGEVNVDQLAHMPQELMLSPPVSATREALDAIAQADVILIGPGSFLTSLMPLLLLD
DLTQALRRSSASMIYIGNLGRELSVAAAALSLQDKLTLMEEKIGRRMIDALIVGPAVDASEVQDRVVI
QQPLEASDIPYRHDRQLLRQALDRALVALAARR
>CORE_REP|Org22_Gene1796#
MLALLTRLFPLWAILLSVAAYYTPTTFTGISPYVSPLLMLIMFAMGVTLRLDDFKRVLARPGPVAAGI
FLHYLIMPLAAWILAMLFRMPPDLSAGMVLVGSVASGTASNVMIYLAKGDVALSVTISAVSTLVGVFA
TPLLTRLYVDAKISVDIMGMLLSILQIVVIPIGLGLIVHHTLTKTVKRIEPLLPALSMVCILAIISAV
VAGSQSHIASVGLVVIVAVILHNGIGLLSGYWGGKLFGFDESTCRTLAIEVGMQNSGLAATLGKIYFS
PLAALPGALFSVWHNLSGSLLAGYWSGRPIKKK
>CORE_REP|Org3_Gene2035#
MKMSIKQLRAFLAVAHTLNFAQASERLNISQPALSLAIRGLEDALGGPLLLRTTRRVTLTPEGETFFP
MARQLLADWDNAEEAMRQRFTLQMGKVAIAAMPSFAGNPLPPILKAFRDRYAGINVAVHDVINEQVFE
MIREGRVEMGIAFEPEPSDTLHFTPLCRDRFLAVVPKDSALARKAQVSWKELLTLDFITLQRPSAVRL
LLEQELARSGRTLEVAFESHQLVTVGRMVANGLGASAVPALCEQQMDELGAVCVPLIGPIIERRVGLI
RLAQHQLSSAAQALATVIEREMAGSGAQPALRP
>CORE_REP|Org6_Gene2219#
MTDSQSAKTLVVTPAHPAAGHSAQGWMNGFLGMMIFSGSLPATRVAVLELDPLFLTAVRAAIAALLAL
CALLITRQARPTRADLPPLLFVALGVVIGFPLLTALALQHTTSAHSLVYIGLLPLATAGFGVLRGGER
PKPAFWAFSGVGAVLVGAFALSQGSGGAWQGDALMVGAVLLCGLGYAEGAVLSRRIGGWQAISWALVV
ALPLTLAATWLVAPAAWPVVSGAAWLSLVYVSVFSMWIGFIFWYRGLAQGGIAAVGQLQLLQPFFGLA
LAGWLLHETVQPMMIAVMAGVVVCVFGAKRFSR
>CORE_REP|Org23_Gene3194#
MSLPFDVHRLLPAFLAAAQAQNFSAAARQLGVTPAAVSKNIRALEEKLALRLFQRNTHNVLLTDEGKA
LLAQVAPLWQALAATLESAGGERQAPAGVVRVTMIPGFGRQMLMPLIPQFLARYPQIDLDLSLDARVV
NLVGEGFDVGIGSRVDPDSRLVARPLYPMHMVLAASPDYLARRGEPQTPHDLLRHDCLLHRNPANGRH
VKWQLRHQGETLALDLNGRLVVSRPEMLLDAALAGLGIVNLAHWYVEKHFVQGTLRPVLAECWPRPVQ
LWLYYASADLPPRVRVWVDFLLEHFRDRPTGD
>CORE_REP|Org39_Gene3100#
MKITLEELLAFTAVVDSGSVTAAADRLGQTTSGVSRALSRLETKLDATLLRRTTRRLSLTEEGLSFLA
PAREILRSVDQAEELMALRRRLPAGRLRVNAAAPFMAHVLVPMVAEFRRRYPQIELELDTDDRNIDLL
EKRADIAIRIGALRDSTLHARLLGNSRLRILASPDYLQRHGEPRGVEDLHRHCLLGFTYPESLNQWPL
RHRQARHFAIEPTISASSGETLRELALRGAGIVQLADFMTRRDREAGRLVPLLVRETLDVRQPIHAVY
YHDAQLAARLTCFLDYVSARLEGEPPEAAEGL

>CORE_REP|Org39_Gene3298#
MTHPGNYAVCIIGLGAMGMGAARSCLRAGLTTYGADLNPQALATLQQAGAKQTSTSACDFAAELDAVL
LLVVNAAQVKQILFGEQGLAPKLKPGTAVMVSSTISADDAKQIEQRLLDYGLPMLDAPVSGGAAKAEE
GQMTVMAAGADATFERLQPVLDAIAGKVYRIGETIGLGATVKIIHQLLAGVHIAAGAEAMALAARAGI
PLDVMYDVVTHAAGNSWMFENRMRHVVDGDYAPKSAVDIFVKDLGLVADTAKALHFPLPLASTAFTMF
TAASNAGYGKEDDSAVIKIFAGIDLPQKKEAL
>CORE_REP|Org1_Gene1117#
MRIGIDLGGTKIEVIALADDGRELFRHRIATPRHDYGQTLAAIAGLVKLAEDHTGELGSVGIGIPGTL
SPFTGRVKNANSVWLNGQPLDKDLSALLAREVRIANDANCLAVSEATDGAGAGAKTVFAVIIGTGCGA
GVALSGQAHSGGNGIAGEWGHNPLPWQDDDELRYARDVPCYCGKPGCIETFISGTGFATDYARLSGNA
LQGHEIMALSERGDALAEQAIVRYEMRLAKSLAHVINMLDPDVVVLGGGMSNVDRLYRTVPQRVKSWV
FGGECETPIRKAVHGDSSGVRGAAWLWPQQQR
>CORE_REP|Org19_Gene4376#
MQESHYVGRFAPSPSGDLHFGSLIAALGSYLQARAQRGQWLVRIEDIDPPREVPGAAARILSALEHYG
LHWDGQVIYQSQRHDAYRAALDLLQRQGLSYYCTCTRSRIQQIGGLYDGHCRDLQLGPQGAAIRLRQT
APVYGFHDRLQGELHADPALAGEDFIIRRRDGLFAYNLAVVIDDHFHGVTEIVRGADLIEPTVRQIAL
YRQLQAPVPAYVHLPLALGANGIKLSKQNHAPALPAGDPRPVLIAVLKFLRQPLPESWQDLDLPLLLS
WAVAHWRLENVPRQEAIPLDENTPAFSKEPW
>CORE_REP|Org6_Gene1333#
MEFKQLQDMALFALVAECGSFTAAAQRVGLPKSSVSQRISQLEQTLGLRLLNRTTRQLNLTFAGERYL
EHCQVMMSAAERADLALQRLRDNPSGRLRISTPAGLGATLVARLAADFQRQYPDVSLEVSVSDAMVDL
VQEGFDAALRTGKPQDSSLIGRRLGYAPRYLLAAPSYLAQHPPIEHPQQLQQHRCIAHRAWTAWNLRC
GDDYYRWQLPLAHTTDNLLYARECAIAGAGITLLPAFLSREVVAQKLLVEVLPAWRAEGNELYLVYPS
RKLNSAALACFIDVVLQHPAFDDYARELARE
>CORE_REP|Org6_Gene1631#
MERLKRMSVFAKVVEFGSFTAAARQLDMSVSSISQTVSKLENELQVKLLNRSTRSIGLTEAGKIYYQG
CRRMLQEVSEVHEQLYAFNNTPAGTLRIGSSSTMAQNVLANMTAEMMKEYPGLTVNLVTGIPAPDLIT
DGLDLVIRTGALQDSSLFSRRLGQMPMVVCAAKSYLIQHGTPQKPSDMVNFSWLEYSVRPDSEFELMS
PEGITTRISPQGRFVTNDSSTMIRWLKNGAGIAYAPLMWVIEEIKRGEIEILFKSYHSDPRPIYALYT
EKDKLPLKVQVCINYLTDYFERVAAVYQGYR
>CORE_REP|Org4_Gene3354#
MKITILGCGALGQLWLSRLYQQGHDVQGWLRVPQPFCAVNVIEPEGVSFNRNLPTNDPEHLAQSELLL
VTLKAWQVSSAVSALLPKLPPQCAILLLHNGMGTQEELPPNGQPILQGTTTHAARHEGSTIIHVATGI
THIGPSSPAAQHLSHLAEVLHQALPDVAWHNNIASANWRKLAVNCVINPLTALYGCRNGDLQRYPEQI
ETLCREVASVMAMEGYHTSCEGLMQYVMDVIHSTADNVSSMLQDIRNQRHTEIDYITGYLLRRARSHG
LALPENARLFELIKRKENEYERIGAGLPGTW
>CORE_REP|Org6_Gene1086#
MRLNLEALLILDALDRHGSFAAAAAALFKTPSALSYMVQKLENDLDITLLDRSGHRAKFTDTGKLMLE
KGRVLLRAAQDLEQQARYVENGWESEITLGIDASFPFARLLPLIDEFYRQHHHTRLRFSHEVLAGSWE
SLVYGCADIIIGAICEPPSRVGYAFSRLGQLDYVFAVAPQHPLAALPEPLPKDEIRQHRAVVVRDTSR
VNAPQSLNLLEEQDTLTVFGFDAKLQAQLAGLGCGYLPRSLAEPYLNSGELVAKRVESERCSDIAYFG
WRESASGLAAKWWRERLQRYADDGEAYPAAQ
>CORE_REP|Org43_Gene617#
MLWFKNLMVYRLSREVALNADEMEKQLSAFAFTPCGSQDMAKTGWVSPMGSHSDALTHAVNGQIVICA
RKEEKILPSPVIKQELQAKIERLEAEQHRKLKKTEKDALKDEVLHSLLPRAFSRFNQTFMWIDTVNDL
IMVDAASAKRAEDTLALLRKSLGSLPVVPLTMESPIELTLTEWVRSGEMPAGFAIQDEAELKAILEEG
GVIRCKKQNLISDEIAVHIEAGKLVTKLAVDWQERIQLMLSDDGSLKRLKFADTLREQNDDIDRDDFA
QRFDADFILMTSELAALIKNTIEALGGEAQR
>CORE_REP|Org24_Gene4092#
MQIKVLGSAAGGGFPQWNCNCDNCRGVRDHSISATRRTQSSIAVSDDGKNWVLCNVSPDICHQLLASP
ELNNPEVLRGTGIGAIVLTDSQIDHSAGLLNLREGCPHHVWCTPEVHDDLSTGFPVFPMLSHWNGGLI
HHPVTPGEPFRTMVCPNVRFTAIPLLSNAPPYSPFRDRPLPGHNVALFIEDTARGVGLLYAPGLGEPN
EALLPWLRRADCLLIDGTLWRDNELANAGVGRNTGKDMGHLALAEEHGLAALLATLPARRKILIHINN
TNPILNEDSVERQSLTAAGIEVSWDGMNIEL

>CORE_REP|Org4_Gene3643#
MTPIAPSLQQPVDAFLRYLKVERRLSPLTQLSYSRQLAALMRLAQEIGVTDWTALDAARVRMLAARSK
RAGLQSASLALRLSSLRSFLDWLVSQGVLHANPAKGIRTPRSGRHLPKNIDVDEMNQLLEIDLNDPLA
VRDRAMLEVMYGAGLRLSELVGLDCRHVDMAAGEVWVMGKGSKERKLPIGRTAVTWLEHWLAMRDLFG
PEDDAMFLSNQGRRISTRNVQKRFAEWGVKQGVNSHIHPHKLRHSFATHMLESSGDLRAVQELLGHAN
LTTTQIYTHLDFQHLANVYDAAHPRAKRGKS
>CORE_REP|Org4_Gene249#
MSEVKQHCGFIAIVGRPNVGKSTLLNQLLGQKVSITSRKPQTTRHRIMGIDTDGAYQAIYVDTPGLHI
EEKRAINRLMNRAASSSIGDVELVIFVVEGTNWTADDEMVVNKLRSLRCPVLLAINKVDNVTDKSKLL
PHIAFLSQQMNFLDVVPISAEKGMNVDTIAGIVRKLLPEAEHHFPEDYITDRSQRFMASEIIREKLMR
FLGEELPYSVTVEIEQFVANDRGGYDVHGLILVEREGQKKMVIGNKGAKIKTIGIEARQDMEQMFDAK
VHLELWVKVKSGWADDERALRSLGYVDDLK
>CORE_REP|Org46_Gene1493#
MLTDLNDLFFFASVVDHQGFAPAGRALGIPKSKLSRRVALLEERLGVRLIQRSTRRFSVTEVGQNYYA
HCKAMLVEAEAAQQAIEQTRAEPCGTVRMSCPVAILHTRVGSMVAAFMADYPKVTVHLEATNRRVDVV
GEGLDLAIRVRPPPLEDSDLVLKILAQRTWCVAASPALVRTLGPAHAPEDLRKYPTLDLGPARAQHQW
RLTGPQGERVEWEHTPRLVTDDMLMLRTAAIAGAGIVQLPAMMMRDDMLRGELVQLLPGWQPQGGVVH
AVYPSRRGLLPAVRLLLDYLGEQFTSIEEE
>CORE_REP|Org26_Gene2883#
MMRHFLGVDVGGTNTRLLLMDDDGEFSGYRKIATADWARQADPLAALGRLIAGHCQDRQVAQVMLGLP
GILSRDRSRVLSLPFIPALDAQPVAALLADLLALPVRMDKDVNHLLWWDLQQLPALPQVAVGLYLGTG
MGNSLWLNGNFYHGAHGAAGELGHIPWPGHQGECPCGKRGCVESLTSGHWLTGWARANAAQTPFERLF
ERHGEHPDLRRFVERLAQTIAIEMNVLDPERLILGGGVIAMSGFPLAQLEQEIRRHLREPQPAQGLAI
SISRLSDETGSKGACLAARRHFQLSREYPQ
>CORE_REP|Org10_Gene4419#
MDRFNQYRVFVQVAEMGSFIRAAHALEVPRASVSAAVQQLETQLGVRLLHRTTRQVRLTADGEQLLER
LRPLLAEVEDIDQSFQASQRQASGRLSVDVPSRIARRLIAPALPSLLRRHPHLQLVLGSADRAIDLVQ
EGVDCAVRVGDLHDSSLVMRPLGHIALINCASPAYLSEFGHPRQPADLAEGHWSIGYASPKTGRESPW
EYLTDDGHTQRLELPSRVVVNNAESYIACCSAGLGLMQIPRYDVQHLLDAGELVEVLPGYRAASMPIA
LIYPHRRQRSRRLAVFHEWFESLLQPHLER
>CORE_REP|Org41_Gene4645#
MSSAFTSSHDPLRRRLLMALALSPLLGSLPGRAADAPPDITRVAALEWLPIELLLALGVTPLAVADVH
NYNLWVAEPKLPATVVDVGQRTEPNLELLQQLQPSLVLLSQGYGPTPRKIQPIAPTMSFGFNDGSGKP
LTVARQSLLALGQRLGIESRAVNHLAQFDRFMQDARQRLQSYTRQPLLLFSLIDTRHALIIGQKSLFQ
EAMDQLGIRNAWQEQTDFWGTAVVGIERLATVRNARVIYLDHGNQAMMDKVSATPLWQSLPFVRQNQL
RQVPAVWFYGATLSTMRFCRLLAQAQERAA
>CORE_REP|Org18_Gene686#
MDRITAAEVFVTIVDRGSMIAAAETLEMSRAMVTRYLAQMEQWAGARLLHRTTRKLSLTDAGERTLER
CRQMLALAGEIDLVEEGQSDELRGLLRITCSQSLGQTALVGAVAQYLKRHPQVAVDLQMNNRAVNLVE
ERIDLALRITNELDPNLIARPLSTCASVVCAAPAYLAAHGTPRQPQDLALHNCLTYSYFGKSLWHFDA
QGVKSAVAVSGNLSANESVVLMAGTVQGAGISMQPYYSAAPLLASGELVELLPDYRPQSMGIYGIYTS
RRQMPATLRTMLDFLVEWFATDPQWQATLR
>CORE_REP|Org31_Gene884#
MMLTKKNSEALEHFSEKLEVEGRSLWQDARRRFMHNRAAVSSLFILVLITLFVVLAPMLSQFAYDDTD
WAMMSAAPSVESGHYFGTDSSGRDLLVRVAIGGRISLMVGVAAALVAVIVGTLYGAMSGYLGGKVDSV
MMRLLEILNSFPFMFFVILLVTFFGQNILLIFVAIGMVSWLDMARIVRGQTLGLKRKEFIEAALVCGV
STRNIVLRHIVPNVLGVVVVYASLLVPSMILFESFLSFLGLGTQEPLSSWGALLSDGANSMEVSPWLL
LFPAGFLVVTLFCFNFIGDGLRDALDPKDR
>CORE_REP|Org2_Gene2686#
MSSLLQLLPYFEAVARLGNFTRAASQLGVTPPAVSQNIQALENQLGVRLFHRTSRSVRLSDEGRLFYQ
KVSPAMSQIDVAADDVRALGAQPAGLLRITLPQLAASLLVMPHLAEFQRRYPDVQLELFTEDRFSDLV
LGSFDAGIRMHAMLQKDMIAVPIDNGQRRVLVASPDYLARCGVPATPDDLPVHHCLRYRFPGSGKLEP
WYFSLGDDERALDVSGSLIFNEDRLIKDAALAGLGIAQRFQGTVLQELAQGQLVEVLPDYASEASGFF
IYFPAGRHLPLKLRAFIDFMREQRERQHRW

>CORE_REP|Org41_Gene2769#
MKHWRRNAALKAMPLIDPNAVRTPWGEFWRRFRRQRAALVAGLFVLLLIAAALLAPYLAPFDAENYFD
YDRLNEGPSLMHWLGVDSLGRDIFSRILMGTRISLAAGVFSVLAGGAIGTLLGLLAGYYEGWWDRLTM
RVCDVLFAFPGILLAIGVVAIMGSGMANVIVAVAIFSIPAFARLVRGNTLVLKHLTYIESARSIGASD
WTIILRHILPGTLSSIVVYFTLRIGTSIITAASLSFLGLGAQPPTPEWGAMLNEARADMVIAPHVAIF
PSLAIFITVLAFNLLGDGLRDALDPKLKG
>CORE_REP|Org38_Gene1834#
MSEKLQKVLARAGHGSRREIETMIEAGRVSVDGKVAKLGDRVEVTPAMKIRLDGHVLSIKESEEVVCR
VLAYYKPEGELCTRSDPEGRPTVFDRLPKLRGSRWVAVGRLDVNTSGLLLFTTDGELANRLMHPSREV
EREYAVRVFGQVDDAKVKQLSKGVQLEDGPAAFRTISFQGGEGLNQWYNVTLTEGRNREVRRLWEAVG
VQVSRLIRVRYGDIDLPKGLPRGGWAELDLPAINYLRELVELKPETVSKMPVERERRRVKANQIRRAV
KRHSQVAGSGRRSAPGSKPSKSGKPSKRS
>CORE_REP|Org13_Gene2381#
MPQHSHAPALLILNGKGAGNEELRQAVKRLRAERITLHVRVTWEHGDAARYVAEAAQLGVGTVVAGGG
DGTINEVAAALVQLPAHNRPVLGILPLGTANDFAMACNIPPSPEQALQLAIKGRSVPIDLAKVNGERY
FINMATGGFGTRITTETPEKLKAALGGVSYFVHGLLRMDTLQADRCEIRGPDFRWAGEALVIGIGNGK
QAGGGQELCPSALINDGLLQLRLLIADELLPALVAALFNDEESNSILSAALPWLEIDAPHEMTFNLDG
EPLKGRHFRIEVLPQAIECRLPPNCALLG
>CORE_REP|Org34_Gene2652#
MKKVGLRVDVDTFSGTREGVPQLLDLFDKYDIQASFFFSVGPDNMGRHLWRLLRPKFLWKMLRSNAAS
LYGLDILLAGTAWPGRNISRALGPLMKRTAEAGHEVGLHAWDHQGWQAKVGKWSEAQLTEQVQRGVDA
LSASTGQPVKCSAVAGWRADTRVLEVKQRFGFHYNSDCRGTHPFRPVLSDGRHGTVQIPVTLPTFDEV
IGSEVSMADFNDYILRAIENDRGVPVYTIHTEVEGMSQAAMFEQLLQRARQQGIEFCPLSALLPQDLA
SLPLGRIKRAPFPGREGWLGCQTDVKDVT
>CORE_REP|Org41_Gene1437#
MDFSIVIPIYNSARYLPKTLKRVFNACQGFDYQVILVDDCSDEDDIKYIRQIAATNSNVMLHEKKKKS
NAAVSRNLGIKLAQSEIVFFLDSDDYFTTNYIIRRMNKHEDRHIDIIFGNYAEVSGDSVRDFNFHYKS
GSSGEDFLFLEMGDIRSSTISIRKKSDRRFLFPEFLNKHQDWGFLVNATNLGAHVAHDAGGGVFLDVG
RYGRMTAKLNLEASDRFINTFLSPSDRHISGFACKHLLASLYSENAQAFNYYSSRIVRRSLSGKFKMI
KWYGDCLARLGLFSVGGRLLRNVRQGFRR
>CORE_REP|Org27_Gene2648#
MVAVIIIGVFIVSFLYAHSRGKEKQKLTRQLFDHSTFMGPINMFMTGFSRLPARQPYFDEKVFPELQT
LTDNWQVIREEAVRLQHHIKKAQSHNDAGFNTFFKRGWKRFYLKWYSDSHPSAQTLCPKTVELLNRIP
SVKAAMFAELPPGSHLGKHRDPYAGSVRYHLGLMTPNDDRCFIEVDGEQHSWRDGEAVIFDETYYVHWA
QNATDQTRIILFCDVERPMKWRWAQAVNHWVGTTLMSAASSPNDDQDRTGGINRIFKYVNAVRDAGQR
LKARNRRGYYLLKWLVIAAIFAAIIIPSL
>CORE_REP|Org4_Gene2615#
MKIAILSRDGTLYSCKRLREAAEDRGHSIDIIDPLSCYMNINPAAPTIHYRGRQLERYDAVIPRIGSA
ITFYGTAVLRQFELLGSYPLNESVAITRARDKLRSLQLLARQGIDLPITGFAHSPDDTGDLIELVGGA
PLVVKLVEGTQGIGVVLAETRQAAESVIDAFRGLNAHILVQEYVREAQGSDVRCLVVGGRVVAAIERQ
AKPGEFRSNLHRGGTARKVTITARERAIAVKAASTLGLDVAGVDILRAERGPLVMEVNASPGLEGVET
TTGLDIAGMMIEYIEQRGRPGFRLKSGG
>CORE_REP|Org30_Gene1294#
MSQITESAVKGAPKPMTPFQEFWHYFKRNKGAVVGLVYIVLMLVIALGAGVLAPHAPADQFRDALLKP
PVWQEGGSWQYILGTDDVGRDVLSRLMYGARLSLLVGCLVVVLSLIMGVIFGLLAGYFGGVVDAIIMR
VVDIMLALPSLLLALVLVAVFGPSIVNASLALTFVALPHYVRLTRAAVLVEVNRDYVTASRVAGAGAL
RQMFVNILPNCLAPLIVQASLGFSNAILDMAALGFLGMGAQPPTPEWGTMLSDVLQFAQSAWWVVTFP
GLAILLTVLAFNLMGDGLRDALDPKLKQ
>CORE_REP|Org38_Gene325#
MAMQSQDIIRKSATNGFTPAPRARDHQEEVAKLIDVTTCIGCKACQVACSEWNDIRDEVGHNVGVYDN
PADLTAKSWTVMRFSEVEENGKLEWLIRKDGCMHCADPGCLKACPSEGAIIQYANGIVDFQSEHCIGC
GYCIAGCPFDVPRMNKDDNRVYKCTLCVDRVDVGQEPACVKTCPTGAIHFGTKEAMKQVAADRVSELN
TRGYQNAGLYDPAGVGGTHVMYVLHHADKPQLYHGLPDNPSISPAVTFWKGVWKPLAAIGFAATFAAS
VFHYVGVGPNRVEDEDEHDESHDEETRK

>CORE_REP|Org6_Gene3537#
MNDARYVEHLPIFLDVARLGSFSAAARRLGMVPSSLVRHIDALESALGATLFVRSTRGLLLTDAGELL
LTRAAALMTDITGIHAELSALNETPQGTLRISCLPTFGKTYVLPLLPTLAERYPQLSIDLDLTERQTD
PTQERLDAALRIGEQKDSALYASRIATQRWVMCASPAYVARYGLPSDLEALPQHRLIARYHKQQPACW
AQILDAALMSRCTMALRCDDFTAQRQAALLGLGIAFLPNWVVGPDVQNGQLVQMLEDPRHEQQGIYLL
RPMAKVSARLAAFTALLQQTLGQPPSWG
>CORE_REP|Org44_Gene4817#
MTALATLRDVGFEEWLGKINTACGRFCAKTLGPGFSGAMQEFRAHALRLSVVDVSQARLYRTPREIAR
SDGAHFFTVFQLRGSALMEQGESQTVLSPGDITLIDASRPSSFTFQRDSRQISLLLPRGCLPVPPPCA
QRLGAELSAVRLSRRLVLSSMQDPQLAAAESEAVLNALAALLRPALALEQARPEGQQPVFDKALALID
RHIQSAQLRPEWVAAELGVSLRSLYRVFARQGLVVAQYIRNRRLDLCAQALRSAAGQEKLAGVGLDWG
FADHSHFSTAFKQRFGMSPSEYRRQYQ
>CORE_REP|Org25_Gene625#
MLDKTRLRIAMQKSGRLSDESQELLARCGIKINLQQQRLIAFAENMPIDILRVRDDDIPGLVMDGVVD
LGIIGENVLEEELLSRRAQGEDPRYFTLRRLDFGGCRLSLATPLDAEYAGPQSLQDARIATSYPHLLK
QYLDKQGVRFKSCLLNGSVEVAPRAGLADAICDLVSTGATLEANGLREVEVIYRSKACLIQRDGEMPE
AKQQLIDRLMTRIQGVIQARESKYIMLHAPSEKLDEIVALLPGAERPTILPLAGAQNRVAMHMVSSET
LFWETMEKLKALGASSILVLPIEKMME
>CORE_REP|Org15_Gene1724#
MTQVQSGILLEHCRFAIFMEASVQGEFADLRQGCKQFCQTLSELQQQFPDARLGAVIAFGYDVWHDLS
SGQGAKELKPFTPLGKGLAPATQRDMLIHIQSLRHDVNFTLAQAALAAFGNVIKIEEETHGFRWVEER
DLSGFIDGTENPQGDQRPAVATIAEGEEDAGGSYVLVQRYEHNLRQWQRFTTEQQEQIIGRTKHDSEE
LPADRRPDTSHVSRVDLKENGKGLKILRQSLPYGTASGKHGLFFIAYCARLHNIEQQLLSMFGDLDGK
RDAMLRFSRAVTGSYYFAPSLTRLLSL
>CORE_REP|Org22_Gene2033#
MFFNLQRYSTHDGPGIRSVVFLKGCPLSCRWCQNPESRSRRADLLFDERLCLSGCTLCTERCPQGLRR
NEEALTLQRDVISADDYAALAAACPTGALSLCGSAVNPDDIMAEVMRDKPFYLRSGGGLTLSGGEPFM
QPEAAAELLRRGREAGIHTAVESCLHVPWRYIAPSLPWLDLLLADLKHTDEARFKAWTGGSARRVMNN
FRRLAAHGVPMTVRVPLIPDFNADRHSVRAIVDFAADEIGVSEIHFLPYHTLGINKYHLLGEPYRAAR
TPLDAPDLLAFAEAYAGAKGLTAILRG
>CORE_REP|Org33_Gene842#
MRMSHINFNHLYYFWQVCKAGSVVGAAEALFLTPQTITGQIKALEERLDGKLFKRQGRGLVPSELGQL
VFRYADKMFMLSQEMLDIVNYRKESNLLFDVGVADALSKRLVSQVLEAAVVVDNEQIHLRCFESTHEM
LLEQLSQHKLDMILSDCPVDSSQQEGLFSLKLGECGISFFCRQPAPELPFPACLEQRKLLIPGRRSML
GRKLLNWFNTQGIQVEILGEFDDAALMKAFGMYHNAIFVAPTLYAQDTYNDDNVVEIGRIDSVQEEYY
IIFAERMIQHPAVQRVCNKDFSALFSC
>CORE_REP|Org2_Gene3088#
MHSPSRARLPKLSAILAFETAARTGSLARAADTLALTAAAVSQQIRQLEQHLGITLFIRAKSGVTLTE
QGADYLAYVQEAFETLRVAQQHVERQRGKQALTVFALPALASKWLNPALGDWLAQCPDGDLRLHATHA
AVDFAHSAADFALCFGDQDYPLLEKVRLFQDRVQPVCSPALRDRGDWTQLPLIHVDWGKESQFLPGWH
EWFTAADRMPPARRGLTYNLTSLAIDAAVQGRGVLLGQRRLIGRELAAGQLVTLAEPALPLSKPYYVV
YPPRTLEKPGAAAFLAWLQTLASTDQA
>CORE_REP|Org40_Gene887#
MQQQDNALIEQFLDALWLERNLAENTLASYRLDLQALGAWLGQQNTTLLQAQALDLQAFLAERVDGGY
KATSSARLLSAMRRLFQYLYREKLRADDPTAQLASPKLPQRLPKDLSEAQVDALLQAPCVDQPLELRD
KAMLEVLYATGLRVSELVGLSISDVSLRQGVVRVIGKGNKERLVPLGEEAVYWIENYLEHGRPWLVNG
QTLDVLFPSTRCQQMTRQTFWHRIKHYAILAGIDSERLSPHVLRHAFATHLLNHGADLRVVQMLLGHS
DLSTTQIYTHVATERLKQLHQQHHPRA
>CORE_REP|Org18_Gene1667#
MIAGTPASAETLRQLKQQHPAIFAQQGRYLRTVGLIALAIVLYYVFFFLVFGITWPQFINGCQQLGRY
FLRMFVWHDFVNWPFMYYFQQIGITIAIVFAGTITASLIALPLSFFAARNVMSTPLLRPISVLVRRLL
DVLRGIDMAIWGLIFVRAVGMGPLAGVLAIVMQDVGLLGKLYAEGHEAVDKSPSRGLTAVGANGLQKH
RYGIFTQSFPTFLALSLYQIESNTRSAAVLGFVGAGGIGLVYAENMRLWNWDVVMFITLILVVVVMIM
DKVSSMLRNKYIIGEDIPLYQQKSQID

>CORE_REP|Org31_Gene3639#
MQHIIGISSRRLRISALALLLPAVSWAADTASLAVGPQYDTTHVYVERGKMDAFVDSILKTFGGTSTE
RVLVNVTPTPSETYSQLILTPAGSFSVFDFKTPIPHPFGTERNGFLVRDMDAAIRQARAAGADVQVAP
FDDPIGRDAVIQWPGGVNMQLYWHTKAPNYKPLLSVPENRLYLSAYRVDDFLKSYQAFSHATVVSDEQ
VSDTAIGRSDNGKIRQIELDSRFGKTRIFVTDGHLPYPFGHERTGYGVDDLPATLAKATASGAQVLWR
STAAERRASALVRFPGGYIAEIHQTAK
>CORE_REP|Org35_Gene2334#
MPRLLTPVIFIISVILTILVTVLCSIPITLAGIVKLVVPIPAVWRYISAFADFMMWCWCQGLALLLRI
NGQLRWDIEGLEGLDRKNWYLLISNHESWSDIVVLCVLFRNHIPMNKYFLKQQLAWVPFVGLACWALD
MPFMKRYSRAYLLKHPEKRGKDIETTRRSCEKFRQRPTTIVNFVEGSRFTEAKKIKSNSPYRNLLAPK
AAGIAFTLSALGNQFDKVLNVTLLYPENNQRPFLDMLCGRLTRIVVRIETLPIDETLHGDYFNDKQFK
RRFQLWLNTLWQEKDRLLDKLKRQYG
>CORE_REP|Org49_Gene1228#
MSFFHANQREALNQSLAELNGQINVSFEFFPPRTSEMEETLWQSIDRLSILKPKFVSVTYGANSGERD
RTHSIIKGIKERTGLEAAPHLTCIDASPAQLRDIAADYWNSGIRHIVALRGDLPPGGGKPDMYATDLV
ALLKDVGDFDISVAAYPEVHPEAKSAQADLINLKRKIDAGASRAITQFFFDVESYLRFRDRCVAAGID
VEIVPGILPVSNFKQLQRFATMTNVRVPSWMTSMFEGLDDDAETRKMVGANIAMDMVKILSREGVKDF
HFYTLNRAEMSYAICHTLGVRPVAAA
>CORE_REP|Org9_Gene3832#
MLRSDDVTPRAYKQVDVFTRTPLQGNPVAVVLEAEGLSSAQMLALARWTNLSETTFVLKPTHPAADYK
VRIFTTEKELPFAGHPTLGTAHALLEAGLTPKRPGTVMQECGVGLVAVDIQPEGTLAFAAPEVEFRPM
AAEETERLMAALRPAVMAASPLPVIAEMGIRWLMVRLPDAQSCLAVTPDQATIKRLQTSCNVDGVVIY
GACSATEPADYEMRAFMVECGALIEDPVTGSANACLARLLKANHFPDGSQTAQGYQVRQGTQLNRDGR
VSVRFIDGEPWIGGQCRTLIDGTLGI
>CORE_REP|Org23_Gene4581#
MEPNQKFKLLGFTRHGTIAANVMVLATGKTITMALNELADSEISEDLSRHELQALYRKLYGDNQQKTA
YELSDRHERSWYAYLIITVALSVIYIFSTLCGVKPIQIPVLNLITPPAIFIYPLTFILVDILNEFYGL
RLARRTIIISFIANLTFVLGVWVTTLVPSIPQWEYSETYNGIVHSIMAVLVASSAAYLISENVNSYLL
CKIKELTNSRYLFVRVITSTVVASAIDSVVFCTLAFYNVLSWDIIKTMILSQFLIKVVYALLGVGPIY
ATRSLFNRYINTEQAKGNEYAYPKAR
>CORE_REP|Org23_Gene336#
MRKSTGFIANIDICKEYDARYAADEVHYETFAGLAAFFGRDMQVHWHDCFFQVHFLETGKIELQLDDQ
HYSVQAPLFILTPPSVPHAFFTEPDSDGHVLTVRQELIWPLLERLYPGSNLALDMPGICLSLADAPQE
LTALSHYWALIRREFAQNLAGREQTLALLAQAVFTLLLRNTALEDSANSGVRGELQLFQRFNKMVDER
FREHLPVPEYAQALGVTESRLNDLCRRFANRPPKRLIFDRLLREAKRMLLFSACTVHETAYSLGFKDP
AYFARFFNRLEGCSPSTYRAAQHALS
>CORE_REP|Org42_Gene4579#
MNKDNVKLAIAPIGWTNDDMPDLGKENTFQQCVSEMALAGFTGSEVGSKYPRDPAVLKPMLDIRGIQI
CNAWFSTFFADGQKDKTIDEFINHMNFLHAMGARVIGCSEQSKSIQGTTKGVFEEKPYFSDEEWQRVA
DGYNELAKIAAGKGMQVCLHHHMGTGIQTTAEIDRYMSMVNDDVYLLFDTGHAYYSEGSQQAMMAILE
KYLPRINHVHLKDVRDEVVAEVKANKLSFLDGVKKGTFTVPGDGVIDFRPVFKLLDERGYKGWMVVEA
EQDPALANPFEYAVKARRYIREAAGI
>CORE_REP|Org20_Gene2931#
MVERIRLAPQGPEFSRMICGYWRLMEWGMSPQQLLTFIEQHVELGVTTADHADIYGGYACEQAFGDAL
RLKPALRQSLELVSKCGIATTAKPGNPIGHYITDRDHIVQSAEQSLRHLHTDYLDLLLIHRPDPLMDA
DDVAEAFVALHKSGKVRHFGVSNFTPAQFQLLQSRLPFSLATNQVEISPIHQPAILDGTLDQCQQLRI
KPMAWSCLGGGRLFNDVEFQPLRDELQRVAQEIGAETIEQVVYAWVMRLPSSPLPIIGSGKIERVRSA
LKAQKLVLNRQQWFRIRKAALGYDVP
>CORE_REP|Org19_Gene3737#
MFATLPVNALRTFESAARLRSFKLAAAELAVTPTAISHQIKALEQQLGFALFERVPRGVRLTPKGETL
FAGVHGALLDVAATLEGLRPQPSTGSLCVSVTHSFAALWLVPRLGRFYQAYPHYLVRLEACAEVIDLQ
QDASVDVAVRYSRAQYPALHQTARLEESFGVYAAPGLAAAEPENPVLITVKWGDSALYDSGWRDWCRA
AGVDWWQRHAAMRSYHEEHYALQAAVAGQGIVLASSVMVSDMVDNGLLVAYRPEVRVPGAAYSVLCAP
GRERHPPVRAFLAWLQQELPQGNGTK

>CORE_REP|Org47_Gene462#
MARDRALTLEALRVMDAIDRRGSFAAAADELGRVPSALSYTMQKLEEELDVVLFDRSGHRTKFTNVGR
MLLERGRVLLEAADKLTTDAEALARGWETHLTIVSEALSPPSLLFPLVDKLALKANTQVSIFTEVLAG
AWERLEQGRADIVIAPDMHFRASSEINTRKLYKVMSVYVAAPDHPIHQEPEPLSEVTRVKYRGIAVAD
TARERPVLTVQLLDKQQRLTVSTIEDKRLALLAGLGVATMPYPMVEKDIEAGRLRVVGPEYSREADII
MAWRRDSMGEAKAWFLREIPRLFAQR
>CORE_REP|Org9_Gene2634#
MEQLRAELSIVLGESISRLERVSEQPYAHMYSLYDRQGNAIPLMAKSFICQGIAQQEAYKLSMLARDG
DIRLPTVYGVVCTHQAPYKEILLIERLRGVSAEAPTRSPDRWNMLMEQIVDGILAWHRIDSHGSVGSV
DSTQENDWFCWYQQRVEVLWATVVNLTTPQLTMADRRLLYRTREALTHFFVGFDDPCVLVHGNLSLRS
MLKDPKSDQLLAMLNPGVVLWAPREYDLFRLCEAGMPSQLLFSYLRRAPVADAFLARRWLYVVWEAVG
RLIHTGKLERRPFDYASQQLLPWLAG
>CORE_REP|Org47_Gene129#
MYSGLLIILLPLIVGYLIPLRHRPLLVLINRLLSWMVYVILFFMGISLAFMEDLSSNLVLIFQYSAVF
FFCILCANLLLLALLERRMPWRSSHKQEKLPSRVHMALESLKLCGVVLGGFLLGLTQWQWLQFAHKGS
EYALIFLLLLVGIQLRNSGMTLRQIVLNRRGTLVALVVTVASLAGGALAAQLLGLPVKAGLAMASGFG
WYSLSGILITDAYGPVMGSAAFFNDLARELVAIMLIPTLVRRSRSTALGLCGATSMDFTLPVLQRSGG
LDMVPPAIVHGFLLSLLAPVLIALFS
>CORE_REP|Org12_Gene4213#
MNLGALVSETRNPATMGLDEMSTLDMVRCFNQEDRKVPEAIEKVLPAIAQAVDLAAAALKAGGRLIYL
GAGTSGRLGVLDASECPPTFGVPHGVVVGLIAGGPGALLKAVEGAEDDEALGEADLRALNLTAVDMVV
GLAASGRTPYVIGALRYARGLGCPTAAISCNPDSPIAHEAQVAISPVVGPEALTGSTRLKSGTAQKLV
LNMLSTGAMVKLGKVYQNLMVDVKATNVKLVDRACRIVVEATGAERAQAEAALAQTGFEVKPAILMIL
AGVSAEEAQQRLQRYDGYLRAALVR
>CORE_REP|Org46_Gene1345#
MSFFRRKKLPSAIKPGVTFGRLLYQRIDHDGLTMLAGHLAYVSLLSLVPLVTVVFALFAAFPMFSDIS
EQLKSFIFSNFVPAAGNVIQNYLEQFVANSNKMTAVGTCGLIVTALLLISSVDSVLNTIWRSKNKRPI
VFSFAVYWMVLTLGPLLVGASMAISSYLLSLNWLAQTGVNGLVDQVLRIFPLILSCASFWLLYCIVPT
VRVPPKDALIGALVAGLLFELGKKGFALYVTMFPSYQLIYGVLAVIPILFLWVYWSWCIVLLGAEITV
TIGEYRDYRRQKAEQQEQKPEGQQE
>CORE_REP|Org44_Gene1228#
MKRPDYRTLQALDAVIRERGFERAAQKLCITQSAVSQRIKQLENLFGQPLLVRTVPPRPTEQGQKLLA
LLHQVELLEEEWLGNDTGVDTPLLLSLAVNADSLATWLLPALKPVLADSPIRLNLQVEDETRTQERLR
RGEVVGAVSIQPQPLPSCLVDRLGALDYLFVASSAFAERYFPSGVTRSALLKAPAVAFDHLDDMHQAF
LQQNFDLSPGSVPCHIVNSSEAFVQLARQGTTCCMIPHLQIEKELASGELIDLTPGLYQRRMLYWHRF
APESRMMRKVTDALLEHGHQVLRQD
>CORE_REP|Org45_Gene2437#
MTNEDIFFIEELIEWVEIHLEKRPNLDEVARISGYSKWHLQRKFKRITGIQLATYIRSRILTRAAVAL
RITRRSIIDISDELGFDSQQTFTRMFKQRFGTTPNRYRSMTHWDVKNLMPRFNFDASYGAGYYPEVKR
LTLPEMQLVGFTRRLDFASEQELEYSSCMAMKDEIFNDFFKGLHVDCRRIYSIYSPHAGEGDELSSTL
VMAVDPEHKKDILSNYQIDTFHLPSREFISINHKGSAKECLQFFGYLMSHVMPGLKDEVRGSMEMEII
QTKEWNPESKLRQIDVDYTYLISID
>CORE_REP|Org24_Gene3483#
MAFNDSDDLLAQKAEQRMAQALATSQVEQDDYLDNQPAEALTRGDINRMAWRSLLLQASFNYERMQAG
GWLYQLIPGLRKIHRNPQDLANSMKMHMEFINVHPFDVTFLSGLVLAMEQNKEKISTIRAVKVALMGP
LGGIGDALFWLTLLPICAGIGASLALEGSLFGPIVFLLLFNLFHFGLRFGLAHYGYQAGTSALALLKT
HTRRISHAASIVGMTVIGALVASYVHLSTPLVMHAGKARVALQTDVLDKLMPNLLPLCFTLLIFFLMK
RGFSPVKLIGVTVAIGVAGKFIGIL
>CORE_REP|Org37_Gene4414#
MPELPEVETSRRGIEPYLVGHSIQYAVVRNARLRWPVSEQILTLSDRPVLSVQRRAKYLLIELENGWI
IVHLGMSGSLRMLREENEDEAGKHDHVDLVISNGMILRYTDPRRFGAWLWCEDLATSSVLAHLGPEPL
SEAFNGDYLYEKSRNKRTLIKPWLMDNKLVVGVGNIYASESLFSAGILPDRPAGSLSKAEAELLVKTI
KAVLQRSIEQGGTTLRDFLQSDGKPGYFAQELQVYGRAGEPCRACGTPIESAKHGQRSTFFAAAASAE
HHSAASFAISAWVTKSGRKGAISPP

>CORE_REP|Org31_Gene711#
MAMVQPKSQRLRLWITHILMLCFIALIMFPLLMVVAISLRSGNFATGSLIPETLSWDHWRLALGFSVT
HADGSVTPPPFPVLLWLWNSVKIAAITAIGIVTLSTTCAYAFARMRFRGKSTLLKSMLIFQMFPAVLS
LVALYALFDRLGQYIPFIGLNTHGGVIFAYMGGIALHVWTIKGYFETIDNSLEEAAALDGATPWQAFR
LVLLPLSVPILAVVFILSFIAAITEVPVASLLLRDVNSYTLAVGMQQYLNPQNYLWGDFAAAAVLSAI
PITAVFLLAQRWLVGGLTAGGVKG
>CORE_REP|Org4_Gene959#
MATMDMQNAVVLAESRRKMQAWRRQKNRLALFLSMATMAFGLFWLIWILIATVTKGFDGMSLALFTEM
TPPPNTAGGGLANAIAGSGLLILWATIFGTPLGIMAGIYLAEYGRKSWLAEVIRFINDILLSAPSIVV
GLFVYTIVVAKMEHFSGWAGIVALALLQVPIVIRTTENMLKLVPDTLREAAYALGTPKWRMISAITLK
ASVSGIITGVLLAIARIAGETAPLLFTSLSNQFWSTDLMQPIANLPVTIFKFAMSPFAEWQQLAWAGV
LLITLCVLLLNILARVIFAKKKHS
>CORE_REP|Org38_Gene118#
MDLRDLKLFLHLAESHHFGRTAKAMHVSPSTLSRQIQRLEEILGQPLFLRDNRTVQLTDAGEQLKEFA
QQTLLQYQQLKHSLGQHGPSLSGELRLFCSVTAAYSHLPPILDRFRAQHPLVEIKLTTGDAADAVDKV
QSNEADLGIAGRPETLPASVAFTKIGEIPLVLIAPALPCAVRSQAFADKPDWAEIPFILPEHGPSRKR
IELWFRRHRISNPLIYATVGGHEAIVSMVALGCGIALIPSVVVDNSPEPVRNRISQLDNISMVEPFEL
GVCVQKKRLSDPLIDAFWRLLHPR
>CORE_REP|Org38_Gene139#
MPFDNVYREKKMPSPLRYTWRIFYGDALAMIGFYGVIALLLLSLFGSLLAPYALDQQFLGYQLLPPSW
SRYGNVSFFLGTDDLGRDILSRLLTGTAATFGSALAVTLAAAFCGVILGVFAGVTHGLRSAVLNHILD
TLLSIPSLLLAIVVVAFIGPKLEHAMLAVWLALLPRMVRTIYSAVHDELEKEYVVAARLDGASTLQIL
WYAVMPNIAAVLVTEFTRALSMAILDIAALGFLDLGAQLPSPEWGAMLGDSLELVYVAPWTVMLPGAA
ILVSVLLVNLLGDGMRRAINAGVE
>CORE_REP|Org45_Gene2599#
MLVILGYLVVLGAVFGGYLIVGGHLGALYQPAEFLIIGGAGVGAFIVGNNGKAIKATLRALPRLMRRS
KYNKDLYMDLMALLFRLLAKSRQQGMLSLEFDIDNPQESEIFSNYPRILADNTLVEFITDYLRLMVSG
NMNAFEIEALMDEEIETYEQESEVPAGSLAMVGDSLPAFGIVAAVMGVVHALASADRPAAELGALIAN
AMVGTFLGILLAYGFISPLATLLRQKSAENVKMMQCIKVTLLSSLNGYAPQIAVEFGRKTLYTTERPS
FVELEEHVRRVKAPAQQVTEEEEA
>CORE_REP|Org40_Gene3438#
MTLRSPGLAFRQALNKEKPLQIAGAINANHALLAQRAGFQAIYLSGGGVAAGSLGLPDLGISTLDDVL
TDIRRITDVCPLPLLVDVDSGFGASAFNVARTVRSVSKAGAAALHIEDQVGAKRCGHRPNKAIVSTEE
MVDRIKAAVDARSDPDFVIMARTDALAVEGLEAAIERAQAYVAAGADMLFPEAITELGMYRRFAEATQ
VPILANITEFGATPLFTTDELRSARVDMALYPLSAFRAMNRAAEQVYRALREEGTQKNVIDTMQTRNE
LYESINYYLFEEKLDALFARQRDE
>CORE_REP|Org23_Gene3099#
MNIELRHLRYFIAVAEELHFGRAAERLRISQPPLSQQIQALEEMVGARLLARNNRNVSLTQAGEMFLK
EAYQVLDQVGRAAEKAARLDRGELGEMTIGFTSSAPFIGVVARSLRTFRQQSPQVHIKMREINTKQQI
EPLLNGELDLGVMRNTRLPEALHYQLLLREPLVAVVPEGHPLAETPGGGLRFQHLAQEPFVFFSREVG
TALYDEILLLLSKAGITPYITQEVGEAMTIIGLVSAGLGVSILPASFARVRVDGVRYLPLAEPDATTE
VWLVHHRRRPLTAAAQALMALMLK
>CORE_REP|Org5_Gene3514#
MRKPRLPPLGALRAFHAVAGCRSFKLAAEALGVSATAVSHQIKLLESVLACRVCERSAQGVSLTETGE
ILYAGTQRAFAALEQSVAQITRAQQPPALTVTTTSNFLTHWLVPRLADFKAEFPAIDLRLHTSVERVD
LSQRTVDVAIRYRETPESDLHCTLLHEDRFIVVASPALALERSEDLQRVTLFHVEHRQVPADAPTWEN
WRRRYGPEGLNVEAGLTFSDETHALQAAVAGQGVVIASRLLARDLLQRGVLAAPFETALPGANYYLVA
TEETAQRPDIIALREWLLRQMAAG
>CORE_REP|Org43_Gene3342#
MHYSPEALQAFVEAAALGSFSAAARKLRKSQSTVSTAIANLEADLALTLFDRSARQPVLTAAGRKVLG
HVQEILAASERLDALSIQLAGNVEPRLSMVFSDTYQPKHHDSLMQRFEQRYPEIELEWMIAEEGDVID
LLQSGRAHLGMVEVQKTYPPDIAFSRLPEQTEMGLFVAHQHPLAQVAAPTPEQLSSTRQLILNTYTGA
EAARQGGLVWSAPSYLLLLEMAEQGFGWAVLPHWLVQQYGHGRLTQLRPRGWPKLISVDAVWSKLTPP
GPAGYWMLERLLEDDAAQAAALRD

>CORE_REP|Org29_Gene3282#
MKRHIRKFISNQLSVYLPTFKTKLLYKRRFGKSLDLSTPVTLNEKIQYLKFKSYNNNALITQCADKYA
VREYVKSKGCEEILNELYFVCDSVDDIPWDSLPNKFVIKGNHGAGYNLICQDKKSLDKNLAGKTIDGW
MREDLWKRDVEFNYKNIKKKIIGEKYIDTPDGRGPDDYKVYCFHGVPHCMMLCIGRDKGVPKFVYFDR
DFKVLPYSQDSLELTTEEINSFVKPEGYDEIFEYAEKLAVPFDFVRADFYLSDGKVIFGELTFTPSAG
LDSDRLVSTDILFGNLLKLDNVKA
>CORE_REP|Org17_Gene124#
MKANSDELITFVTVVESGSFSRAAERLEQANSVVSRTVKKLESKLGVTLLNRTTRQISLTQEGENYFR
QVQKVLNDMAAAENALMESRQRPQGLLRVDAATPVVLHMLTPLVAEFRERYPEMSLSLVSSENFINLI
ERKVDIAIRVGELTDSTLKARKLMTSYRHVLASPAYLAQHGTPLTVEDLAHHCCIGFNDLPSLNRWPL
ACSDGSQLEITPGLTTNSGETQRHLCLHGNGIACLSDFMSDEDIKRGDLVPILVEATLPVAMPINAVY
YSDSAVSNRLRSFIDFVSEYLKR
>CORE_REP|Org14_Gene1282#
MDAKQTRQGIFFALAAYFMWGIAPAYFKLIQQVSADEILTHRIIWSFFFMLALITLGRNWPKVRAACQ
NRKRLLLLAVTALLIGGNWLLFIWAVNNHHMLEASLGYFINPLVNVLLGMLFLGERFRRMQWVAVALA
FTGVLVQLWQFGSLPIIGLGLAFSFAFYGLLRKKIAIDAQTGMLIETLWLLPVAAAYLFLFADSPTSH
LSANPWSLNLLLVAAGIVTTVPLLCFTAAATRLRLSTLGFFQYLGPTLMFLLAITFYGETVGQDKLVT
FGFIWAALILFTLDALYTQRKLR
>CORE_REP|Org29_Gene3419#
MPKQDVLKVVPARYPLRLIGALFSLFILAAIVQSVAGNARWEWGVFAEWFFAPAVLAGLGQTLLLTLL
GTLFSILFGTLLALARLSRSYLLASLAWGYIWLFRSLPLILVLIILYNFSYLYDAISLGIPFTSVVFA
SYPTIDILGQFAVAVLGLTLVQSAYTAEIIRGGILGVDYGQHEAAAALGLPGYRRTFRIILPQALRSI
IPTGFNEIISLAKGTSIVYVLALPELFYTIQVIYNRTQQVIPLLMVATVWYLFITTALSVIQYYIERY
FARGAVRELPPTPWQKLAGWLKR
>CORE_REP|Org1_Gene3926#
MKMKKLATLASAIALSATLSANAMAKDTIALVVSTLNNPFFVSMKDGAQQEANKLGYNLVVLDSQNNP
AKELANVQDLMVRAPKLLLINPTDSDAVGNAIKMANQAKIPVITLDRVASKGDVVSHIASDNRVGGKM
AGDFIAKKAGADAKVIQLEGIAGTSAARERGEGFKQSLDQNKFKLLASQPADFDRTKGLNVMQNLLTA
HPDVQAVFAQNDEMALGALRALQTAGKTDVIVVGFDGTADGVKAVEGGKLAATVAQRPDQIGVIGVET
ADKVLKGEKVPATIPVDLKLVTQ
>CORE_REP|Org28_Gene1805#
MDVRTLRYFVEVVRQQSFTRAAEKLFVTQPTISKMLRHLEEELECTLLIREGRKLRLTDSGQALYQRG
LTILDEFRQLEAELEDISSLKKGVLRLGIPPMVGRQIADLIRRFRQTYPGIELKISELGGLSVEQAVM
SGELDLAMTVLPFDSEQPLTFLPLLGHPMCVVAPRTPQWLNRTSINIAELADSPILIYNEDFALYKML
MKAFRQAGFEPQIAVRSGQWDFLASMVQAGVGIAMLPEPVCRWLDKENLVWLPLEPRMEWKIGLIWRQ
GSYLSHGAQAWIACCRDYWPPLK
>CORE_REP|Org32_Gene1116#
MSLSTSGRAPSALLPICLLIIAMVSIQSGASLAKSLFPIVGAEGITTLRLFIGTLILFIIFRPWRMRI
AAGSRLPLLIYGLSLGAMNYLFYLSLRTVPLGIAVALEFTGPLAVAMFSSRRPIDFLWVALAIAGLWF
LLPLGHGVGGIDPFGAACALGAGACWAVYIIFGQKAGGDHGPGTVALGSLIAALVFCPIGAWQTGSVL
FNVEILPIALAVAILSTALPYSLEIIALPKIPARTFGTLMSLEPAMAALSGMLFLGEHLSGVQWLALA
AIIAASMGSALTIRPKPRLESLS
>CORE_REP|Org24_Gene4547#
MSSSRPGFGCSWLPYALVLPQLLITAVFFLWPAGEALWYSVQSLDPFGLSSQFVGLDNFKQLFQDPYY
LDSFYTTLIFSFLVAGIGLAVSLFFAALVDYVLRGSRLYRTLMILPYAVATAVAAVLWIFLFNPGLGL
ITHFLNGLGYNWNHAQNSGQAMFLVVLASVWQQISYNFLFFLAALQSIPRSLVEAAAIDGAGPVRRFF
HLVLPLIAPVSFFLLVVNLVYAFFDTFPVIDAATGGGPVQSTTTLIYKIYREGFAGLDLSSSAAQSVI
LMLLVIGLTVIQFRFVERKVRYQ
>CORE_REP|Org21_Gene3382#
MKIAFDVDVIRDLGVTKMVQQVADWGYKYIEQSPHPQINPFYKHPKASREIMAEYKNALKAAGVEISS
FIVVYRWSGPGEDRRQAAVKNWRRMIEIAVEMGVQVINTELAGNPNEPEICEELWYRSMEELLPIVER
EGIRMEIQSHPWDFCELGNETADLVKSLRSDHVKYLYSVPHTFYYDKGQGDVAGMLQYAGDDLSHVLI
ADTMNHTKHCRYIVNPPGVDAAVHQHVGIGEGEVNFDALFQTLRDMEFANRSFKVGGESIICTSLFGY
PEKMPSQAVATRERIERELLGK

>CORE_REP|Org48_Gene3990#
MASFSGIWVAMVTPFNQDAVDLPAVKRLARHLLDAGIDGLVVCGSTGEAAALSKEEQLAVLDAVLEVA
PAHQVVMGLSGNNMAATLQMQQAIQLRDIAGVLIPAPYYIRPSQCGLIDYFTQLADASTVPVILYNIP
QRTGIAMELATLRRLARHPRITAIKDCGGNPDATMALIADGEIDVMTGEDNLILTTLCLGGTGAISAA
AHVHPERFVQLVQQVATGDLAAARSNFYELLPMIHQMFSFPNPAPVKTVLAQQGLIANELRSPMQVAP
QALQQQIAATQAQLQTAEALIG
>CORE_REP|Org49_Gene2811#
MFKKSLLTLAFTGVATLSTYATAADTLTMEVYNPGEKSVFPVSSEIISGKHEVALIDAQFQRNDAEEL
VKKIKATGKKLTTVYISHSDPDFYFGLDVIKAAFPEAKIIASPGTIKDINATKDGKVAYWGPILKDNA
PKTVIVPQPLQGDSFTIDGQKVEVKGLNGPTPDRTFVWIPALKAVVGGVAVAGDNIHPWIADNQSVES
RQHWQQTLKNIEALKPQVVVPGHFLPGAAQTLASVHFTQKYLTTLEAELPKAKDSAALIEAMKKHYPT
LKDESSLELSAKVLKGEMKWPQ
>CORE_REP|Org26_Gene1345#
MKPFLRWCYVATALMLAGCSNHDWRKDEVLAIPLQPTLQQEVILARMEQILASRALTDDERAQLLYER
GVLYDSLGLRALARNDFSQALAIRPDMPEVFNYLGIYLTQAGNFDAAYEAFDSVLELDPTYNYARLNR
GIALYYGGRFPLAQDDLQAFYQDDPNDPFRSLWLYLVEREIDPKKAEVALQQRYDKADRGQWGWNIVE
FYLGKISEKTLMERLKADATDNTSLAEHLSETDFYLGKHYLSLGDKDTASALFKLTVANNVHNFVEHR
YALLELALLGQEQDDLSESDQQ
>CORE_REP|Org47_Gene2986#
MHPRFHTAFSALPATLQSALQPYLDAPDFPAMFTAEQAAAIRTGCGLDDDALAFALLPLAAACSLTPI
SHFHVGAIARGQSGNLYFGANMEFSGAPLQQTVHAEQCAVTHAWLRGEPALASVTVNYTPCGHCRQFM
NELNSGGELQIRLPGRAPATLADYLPDAFGPKDLDVASLLMDEVDHGHQLALNDALAQAALAAANRSH
APYSNAHSGVALETADGIIYAGRYAENAAFNPSLPPLQAALILLNISGGDCLNIRRAVLAEPQEAIIS
QWDATRVTLAALGCQNVSRAAF
>CORE_REP|Org27_Gene3599#
MRSLPINLPAHRAEPACDFARAAFRALLVEVNLTPKPGLVDRHNTGAHRDMDLGHFYRSARAIGVWLP
RFIQRGREDAALPAEQQLARLRPLGLACENQMFRATGGINTHKGSVFSLGLLCTAFGRLQQQGRAIGA
EALCAEVAAMCRGLVDRELRRNNAGQTAGQRLFAAHGLSGARGEAEAGFRLVLDGALPLYRQRLAAGG
DEQRALLDSLLWLMAHNDDTNVASRGGLHGLRWLRRRAALLLAQGGLAGEAGLARLRRFDADCIARNL
SPGGSADLLIVTWLLAQLGAQE
>CORE_REP|Org32_Gene2606#
MKNVALFVGNDIFSWLVCQDLIAALRDECTFTVYFPLAKSAGRTQEPAVRRLGLYEREVLNDFVFPFV
GRNAAACEGAYQPPALFLAAAGVKAHRVLDINDAAFISSLGHMDGVISLRCYQKFSADYVRAFSRRGK
LLWNLHPGDLPRYRGVMTLFRAMMNGDRDCAVTLHEMDEHWDAGPVIARLPAELRHDLSFLENMMLLG
VQSGTFLARQLMRAEHSEAITAEKQGDSRYWGFPDAQTLAQAEQAGIELVDHDAVREQYLGLFVGDRF
HPLAGQFCAGFDDFVRAHGHD
>CORE_REP|Org46_Gene2357#
MKKQGKPFLNFKPLAVSPTRRQLLLSGLAVALLGVKSQRARAEGLLRNQHSKPAAKPAGAKKLVMIDP
GHGGIDSGAVGHEGSQEKHIVLEIANHVRRFLHERDHVEARLTREEDEFIPLFQRVEIAHQHQADLFI
SIHADGFTSPSASGASVFALSNRGASSAMARYLSNRENAADDVAGGKYKDQDNYLQQVLFDLVQTDTI
NNSLTLGRHVLGQIRPVHHLHSDSTEQAAFAVLKSPSIPSVLVETSFITNPNEERLLGTTAFREKIAR
AIADGIVNFFDYFDAHQRKPR
>CORE_REP|Org46_Gene2619#
MTDSNPPSTAFTLYDLHSHTTASDGYLTPTQLVQRAVEMRVGVLAITDHDTTAGLAEAAAAIAEQALP
LRLVNGVEISTLWENHEIHIVGLGMDVAHPALVALLAEQTERRNRRAQEIGVRLGKARIPDAYAGAQR
LAGAGAVTRGHFARYLVEIGVAGNMAQVFKKFLAKGKTGYVPPQWCTIEQAIDAIHQSGGQAVMAHPG
RYDLTAKWLKRLLAHFAEHGGDAMEVAQCQQAPHERSQLAKYAQDYRLLASQGSDFHQPCSWIELGRK
LWLPGGVEPVWRDWPQPGEAV
>CORE_REP|Org48_Gene1904#
MTTLEQCIGNTPLVKLQRLAAQAGSEVWVKLEGNNPAGSVKDRAALAMIQQAELRGEIKPGDVLIEAT
SGNTGIALAMIAALKGYTLKLLMPENMSLERQAAMRAYGAELILVSREQGMEGARDLALEMQRQGQGK
VLDQFNNLDNPYAHFTTTGPEIWRQTEGRITHFVSSMGTTGTITGVGGYLKSQNPQVQIVGLQPAEGS
SIPGIRRWSPAYLPGIFRPELVDQVLDIEQGDAEQTMRQLAQREGIFCGVSSGGAVAGALRIAAAHPG
SVVVAIVCDRGDRYLSTGVFD

>CORE_REP|Org22_Gene1174#
MPWIQLKLNTTGSQAEDLSDALVESGAVSVTFQDTHDNPVFEPLPGETLLWGDTDVIGLYDAETDMAE
VVAMLEQHPLLGAGFRHKIEQLEDKDWEREWMDNFHPMRFGERLWICPSWRDVPDPDAVNVMLDPGLA
FGTGTHPTTALCLQWLDGLDLAGKTVIDFGCGSGILAIAALKLGAARAIGIDIDPQAIQASRDNAQRN
GVSERLELYLPKDQPADLLADVVVANILAGPLRELAPLIGCLPKAGGHLGLSGVLASQASSVAQAYEE
KFTLDPVAEREEWCRITGRRK
>CORE_REP|Org29_Gene3925#
MLHQHTPAASGDELDLDGHGLTQLDESPLAGYALRKISLYDNQLTAFPASIFRHRNLRVLNISCNQLD
HLPPEIGQLQQLEMFDFGHNRASELPEELGQLHRLKYLYLSDNGFSELPRSLAQLQQLVYLNATDNHL
TALPQAIARLAALQELRLYNNRIGSLPAEIGQLRALRELHIMKNALTSLPAEMAQLGELEILDAANNA
LAELPQAFCRLPRLSELNLRFNQLTRLPDNIGELTALRSLDLRANRLSDLPESLGELSRLRKLDLRWN
DFTRTPKVVDILRARGCMVHI
>CORE_REP|Org42_Gene1816#
MNLATLFVLCRLVHFAAVMLMFGISLFTALLSPQRLSPILSRDVRPLLLAGTWIAGLSAVALLAIQAG
QMGDGWEDAWRLEVWWAVLGTTFGEVWRWHLGLSLLAILSLLLPERPRAQALALCSVLLLVSLAFIGH
AAMHEGALGALHRANHAVHLLAAGYWFGCLAPLLVCLRYLQPPQWRSDAITTLIRFSRWGHLAVAAVI
VTGIVNSLIILGGWPLNLSSPYLRLLLIKTALVALMVMVALANRYAIVPAMSRVPALAQRGVVLACWL
EVGLGMAVLLLVSLFATYAPV
>CORE_REP|Org24_Gene1069#
MFTGSIVALVTPMDDKGAVDRASLKKLIDYHVASGTAAIVSVGTTGESATLAHDEHVDVVLQTLELAD
GRIPVIAGTGANATAEAIALTTRFANTGVVGCLTVTPYYNKPTQEGLYQHFKAIAESTELPQILYNVP
SRTGCDMLPPTIARLAKIKNIVAVKEATGNLSRVSQIQVLVDDEDFILLSGDDASGLDFMQLGGKGVI
SVTANVAAREMAELCALAAQGKFAQARRLNQRLMPLHQDLFVEANPIPVKWACKALGLMATDTLRLPM
TPLSEAARPVVERALKSVGLL
>CORE_REP|Org48_Gene4426#
MKGIILAGGSGTRLHPITRGVSKQLLPIYDKPMIYYPMSVLMLAGIRDILIISTPEDLPSFERLLGNG
EQFGVNLSYAAQPKPEGLAQAFLIGEEFINGDSCCLVLGDNIFFGQSFSPKLKTVAARTEGATVFGYQ
VMDPERFGVVEFDGNFRALSIEEKPKKPKSDWAVTGLYFYDSQVVEFAKQVKPSERGELEITSINQMY
LERRELTVELLGRGFAWLDTGTHDSLLEASSFVQTVEKRQGFKIACLEEIAWRNGWLDDEGVKRAAQT
LAKTGYGKYLLDLLHARPRQY
>CORE_REP|Org35_Gene1585#
MRNRLPLNALRAFESSARHLNFTRAGLELRVTQAAVSQQVRMLEEQLGIQLFRRLPRGLDLTEEGQAL
LPVLSDAFDRIEAVLQQFEGGHFHEVLTVAVVGTFAVGWLMPRLAAFRAAHPFIDLRVLTHNNLVNLS
ADGMDFAIRFGEGLWPATRNIKLFDAPLTVLCSPAVAARLHTPKDLQHELLMRTYRQDEWERWFTAAQ
VTPWRINGPVFDSSRLMVEGALQCDGVALAPVSMFRRELAAGALQRPFAAEAALGAYWLTHLKSRDLT
PAMKAFIGWICREAEEEQRRD
>CORE_REP|Org19_Gene503#
MKKIALAAGVLLAASYSASSMADSKDSQYVSDWWHQSVNVVGSYHTRFGPQLNNDVYLEYEAFAKKDW
FDFYGYVDVPKFFGVGNTPDRGIWDKGSPLFMEIEPRFSIDKLTGTDLSFGPFKEWYFANNYIYDLGH
NADGRQNTWYMGLGTDIDTGLPMSLSMNIYAKYQWENYQAANENSWDGYRFKVKYFVPLTQVWGGNLS
YIGFTNFDFGSDLGKDSHWVDGTGKQVRTSNSIASSHILALNYDHWHYSFVARYFHNGGQWQDGADIG
TPQGPIKSTGWGYYLVVGYNF
>CORE_REP|Org7_Gene69#
MNKKFACIGIVGHPRHPSALATHEMLFHWLVARGYSVMVERQIAHDLGLKDAVTGSLADIGQRADLAV
VVGGDGNMLGAARVLARYDIKVIGVNRGNLGFLTDLDPDNALQQLADVLEGEYIDEQRFLLETIVHKE
HQQCRISTAINEVVLHPGKVAHMIEFEVYIDDRFAFSQRSDGLIIATPTGSTAYSLSAGGPILTPSLE
AIALVPMFPHTLSARPLVINGDSTIRLKFSQIGSDLEISCDSQIALPIQEGEEVLIRRSNFHLNLIHP
KDYSYFNTLSTKLGWSKKLF
>CORE_REP|Org20_Gene2724#
MIKGITLSVVASILFGAMYYFTSTLTPLNGEQVYGWRTLLTLPFLTLFMALSGDWRKVGDTLGWIGQR
PQRLLGLLLTSALLGVQLWLFLWAPLHGKALDVSLGYFLLPLTMVLAGRLIYRDRLSLLQKLAVACAM
VGVGNELYQAGGVSWPTLVVALGYPLYFILRRRFGTDNLGGLWCELALMLPAAAWFAFGDGGAAALPT
NAELYWRIPLLGVISAVALVCYILASRLLPFSLFGLLSYVEPVLLVIVALLLGESIGQSEWPTYLAIW
LAVLLLAAEGAQHLLRRQRV

>CORE_REP|Org41_Gene3095#
MKRLPISLAVAALLASPWAMAKTVDAVASFSILGDIVKQVGGDHVKVSTLVGPDGDPHSFEPSPQDGK
KLAQADVVFVSGLGLEGWIDRLVSASGYKGQVITASQGISTRQMEEDGKPITDPHAWNSMKNGVQYAT
NVMNALIAADPEDANYFRQRGADYIQQLQKLDLWAKTQFAAVPPQKRKVLTSHDAFGYFGQEYGVTFL
APVGFSTEAEASASDVAGLIKQIKQEKVNAYFIENQTDPRLVKQIAAATGAKAGGELYPEALSRAGGP
AATYEQAFKHNVDALLSSMK
>CORE_REP|Org38_Gene534#
MSDDHSQSNDSPSPKKGFFTLILNQLFHGEPKNRGDLVELIRDSEQNDLIDPDTRDMLEGVMDIAEQR
VRDIMIPRSQMVTLKRNQTLEECLDVIIDSAHSRFPVISEDKDHIEGILMAKDLLPFMRADSEPFSID
KVLRTAVVVPESKRVDRMLKEFRSQRYHMAIVIDEFGGVSGLVTIEDILELIVGEIEDEYDDEDDLDI
RQLSRHMYTVRALAPIEDFNEAFGTHFSDDEVDTIGGLVMQAFGHLPARGETIEIEGYLFKVAMADSR
RIIQVHVKIPDDSPPPKLED
>CORE_REP|Org35_Gene1137#
MKYALGPVLYYWPKNDIATFYQQAADSSADIIYLGESVCAKRREMKVGDWLALAREIARSGKQVVIST
LALLQAPSELNELKRYVENGEFLFEANDLGAVNMAAERGLPFVAGHALNCYNAYTLRLLRRQGMIRWC
MPVELSRDWLANLLTQCDELGFRHDFEVEVLSYGHLPLAYSARCFTARSENRGKDECETCCIKYPQGR
MMRSQEQQQVFVLNGIQTMSGYCYNLGNELPNMQGLVDIVRLSPQGAETLAQIDAFRANERGEQPLAL
TDHADCNGYWRRVAGLALVG
>CORE_REP|Org33_Gene634#
MSNHDQLHRYLFENYAVRGELVTVSETYQQILNNHDYPAPVQKLLGELLVATSLLTATLKFDGDITVQ
LQGDGPLKLAVINGNNRQEMRGVARTQAPIADDSTLHQMIGNGVMVITISPTEGERYQGVVGLEGETL
AECLEAYFRQSEQLPTRLFIRTGEAEGNAAAAGMLLQVLPAQDGNLDDFDHLVQLTNTVKSEELFGLP
ANEVLYRLYHQEEVTLYEPQDVQFRCTCSRQRCADALLTLPTDEVADMLEQDGNIDMHCDYCGSHYVF
DPVDVAALYAGNTGESDQLH
>CORE_REP|Org42_Gene2955#
MSIDLTFTDLSARAVQYNARASVEDFDACMAEYAALAQRARAQTPGIYDLRYGMSAAERLDLFPASAQ
PAPLLIFIHGGYWHSQRKEEACSMAAAFARRGVAVATLEYTLAPEATLAEIVHEVRSAVAWLYHHGAP
FGIDPARIFVSGSSAGGHLCGMLIADGWQQRYRLPPDAIKGALALSGLYDLRPLCDIYINDWLHLTPE
QAQALSPLFLLPAKSHAPQILLDVGQRETQGFKNQTQAYYDACLARGLDVQLLADRHCNHFTLVNELA
DAESAMFRQVIAMIDATAQS
>CORE_REP|Org13_Gene2565#
MKIGFAGLGGMGSAMAANLLQAGFALTVWNRSPQAAQPLVSAGAQQAERPEQLADADVLITMLADDAA
TQQVVVDSGLLQRMKPGALHINMATISVELAQRLTALHAEHGIGYLAAPVLGRVDVAAAGKLNILAAG
DSELLKQAQPLFDALGQKTWHFGADPAQANVVKIATNFTLASAIEAMAEGSALVRNYGVSGADYLQML
SSTVFAAPAYQGYGALIAAEKYSPAGFRLALGLKDVGLALAAGAESHTPMPFAGVLKDNFLDAMAQGD
ADLDWAALAKVAARRAGLK
>CORE_REP|Org18_Gene1164#
MSDAILRVEHLMMRFGGIKALNDVNLEVERGSITALIGPNGAGKTTVFNCLTGFYRASGGAILLNTHK
RPTDVIQVLGQKFRAGDWIRPKRLGSRLYYKMFGGTHLVNRAGLARTFQNIRLFREMSVVENLLVAQH
MQSNRNLIAGVLNTPGYRRAESAALDHAFYWLEVVDLVDCANRLAGEMSYGQQRRLEIARAMCTAPEM
ICLDEPAAGLNPVETATLSRIIRFLRQHHGITVLLIEHDMGMVMEISDRVIVLDHGDVIARGTPQEIQ
HNEAVIAAYLGADEEELAG
>CORE_REP|Org22_Gene2240#
MNEKIFTLPVSEQISPYISQRQLDELGVVVVSHPKVRAAVALQGAHLLAWQPSGEQPVIWLSNNTPFA
KGKAIRGGVPICWPWFGPVAQPSHGFARNQPWSLTAHDEDDNGVILTFTLKDNEQTRKLWPHAFTLIA
RFKLGEECEIELESHGDYQATAALHSYFQVGDIDRVSVAGLGEPYIDKVAGGAEARQTGEVTFVGQTD
RVYTRPEAFSLIRDPAFTRTIEVHHHHMSDVIAWNPGVELSCSMGDMPNDGYKTMVCVETGRVSKPLV
AAGEQPARLGVTFRSRKQA
>CORE_REP|Org32_Gene4334#
MMNERIPLHVLPTFAIAARLENLRAAAQQVHLTHGAVSQQIQLLEQAVGYPLFERRGRGVRLNAAGRE
LLAAVEPALQALLQGVARARRAATSQTLRISVLPSFAHYWLLPRLPAFHEACADIALDIDASLALQDL
SQRGFDAAIRIGSGQWTGLQAQRIATGDVLPVASPDMAREWRAAFESGGDIPLLEHDVSPWRDWFNAQ
GRPLCGRQQALFNDAGLLIRAAEQGFGIALAKKLLVQDALDAGRLVALAAPRRLSDDDVYLVWPQTAG
LTPAVTRLLQWLQRQLAAI

>CORE_REP|Org27_Gene3308#
MTDSVSNDRNPTPDVAEDNAFFPSPYSLSQYTSSKTDFAGASYPNAYRGGKWKVLMIASQERYLLMQN
GKFFSTGNHPVEMLLPMHHLDLAGFEIDIATPSGDPVKLEMWAFPQEDDAVKATYEKYRSQLKQPKKL
TEVVKDLDGGNYLGVFIPGGHGVLNDIPFSEEVKKTLHWAHDNDRYVISLCHGPAGLLAAGIGESKED
FLYRGYEMCVFPDSLDTGANIDIGYIPGPMPWLVGERLRERGVKIVNADITGKVHKDRRLLTGDSPLA
SNNLGKLAAETLLADVAAR
>CORE_REP|Org23_Gene1786#
MADVSAFNGAERPRVNWGKWTLIAIGTLFSVLLLVVPMMSIFAEAFSKGFGAMWSNLLDPDMLHAIWL
TVLIALITVPFNLVFGTLLAWLVTRFTFPGRQLLLTLIDIPFAVSPVVAGLIYLLFYGSNGLLGGWLD
AHNIQIMFSWPGMVLVTIFVTCPFVVRELVPMMLSQGSQEDEAAILLGASGWQMFRRVTLPNIRWALL
YGVVLTNARAIGEFGAVSVVSGSIRGETYSLPLQVELLQQDYNTVGSFTAAALLTLMAIVTLFLKSAL
QWRLERQNARLEREENHEH
>CORE_REP|Org28_Gene2548#
MGRITFDLEDLRSFVTGVELGSFAKAAERLGRSTSAVSAHLKKLEQQVGAPILRKAGRGMVMTEAGET
LLGYARRLLELNDEAAAAVRGLDLQGTVRLGLQEDFGETFLPQVLGSFARANPKVRIEARIARNAELI
DWVLKGQLDLSLAWDGGLSTPFHQALGQRQLHWIASPGFALAPWREGDEPLSLVMFDAPCLMRSAATQ
ALDRAGIPWRIAFTSRSLNGVWAAVNAGLGVTVRTAAGLPPGLAPLAPELLPALGQLGVVLHRAEDQP
SAAVQRLAQIVVERIGTNL
>CORE_REP|Org17_Gene4372#
MYTWSKRLRLSQTERSPRATHRAPLGKRPITWLGAVFFMPLLLAGCVDRAVSGGLSAQQQEAIPDTKV
IDYRTAACDTLWQLDDKDALDNALYWLRAMDCADRIGSTQARALAKTVPGDSWSGVFKQSILLGSAQP
TSGERRQMLDRINSYRMEFPGSLRPLTQLWRQQQMLQITLFDEKARYQHLQESSDSQIDSLRQSQARL
QSQLQDTSRKLENLTDIERQLSSRKQLQGEIPENGAGQQKGDAAGKSGAAAKGAETQDEPEKGTALPV
EPEDTYTPHPANKESHAQ
>CORE_REP|Org4_Gene806#
MSNSYLAFPKFDPVIFSIGPVSLHWYGLMYLVGFVFAMWLAVRRANKPGSGWTKDEVENLLYAGFLGV
FVGGRVGYVLFYNLPLFLDNPLYLFKVWDGGMSFHGGLMGVILVMFWFARRTKRTFFQVSDFIAPLIP
FGLGAGRLGNFINGELWGRVTTDTPWAMLFPSSRSEDVALAAADPSLLPLLNQYGVLPRHPSQLYELL
LEGVVLFIILNLFIRKPRPMGAVSGLFLIGYGAFRIIVEAFRQPDAQLGLFDGVISMGQILSIPMVVA
GVIMMIWAYRRRPQQQLS
>CORE_REP|Org21_Gene2881#
MDIKQLRALVALAEQGNYRQAASLLCISQPALSKQIQALETQLGVRLFERGRQGAVLTAGGQRLYPEA
QALVEQYQQFQRRARRVALGEAGRLALGFGLSSFHLAPQLVAAFRRRFPEVAIGLEDMPSERQYQLLL
QGELQVGFVRLPVTTPLCGAALLSDRLVLAAPGALALRADDLMARFNQLPLLQLTPKRGRGLSDQSLR
FIAAHRLTPNVVQQAGDIQTLLALVAAGVGVALLPHSITHIAPAGIDILPLSGEETEWQVGIAWDPQR
ADALRDNFIQTALAVQRA
>CORE_REP|Org44_Gene1287#
MEGSVTQSKWRAYSHLMRIDKPIGTLLLLWPTLWALWLAGKGVPPLSILLVFVLGVFLMRAAGCVVND
YADRAVDGYVKRTAGRPMPSGRVSAKEAKVLFVVLVLISFCLVLTLNAMTIWLSLAALALAWAYPFMK
RVTNLPQFVLGAAFGWGIPMGYAAVSESLPLSCWLLLLANICWTVAYDTLYAMVDRDDDLKIGIKSTA
ILFGRYDKLIVGLLQFATLLLLVWVGYLAQLGGAFYWSLLLAGALFIHQQKQIAGREREACFKAFLQN
NYVGLVVFIGIALSYLPT
>CORE_REP|Org30_Gene906#
MNTLDKIQSHLELLSKSERKVAEVILASPQTAIHSSIATLARMADVSEPTVNRFCRRLDTKGFPDFKL
HLAQSLANGTPYVNRNVEEDDSVDAYTSKIFESVMASLDTVKANLDIAAINRAVDLLTQAKKISFFGL
GASAAVAHDAMNKFFRFNIPVVYFDDIVMQRMSCMNSGEGDVVVLISHTGRTKNLVEMAHLARENDAT
VLAITSRDTPLAQAATLALLLDVPEDTDVYMPMVSRIAQLTLIDVLATGFTLRRGAKFRDNLKRVKEA
LKESRFDKGVVIPNSFDS
>CORE_REP|Org17_Gene530#
MSILIDKNTKVICQGFTGSQGTFHSEQAIAYGTKMVGGVTPGKGGTQHLGLPVFNTVREAVEATGATA
SVIYVPAPFCKDSILEAIDAGIKLIITITEGIPTLDMLTVKVKLDEAGVRMIGPNCPGVITPGECKIG
IMPGHIHLPGKVGIVSRSGTLTYEAVKQTTDAGLGQSTCVGIGGDPIPGSNFIDILKMFQQDPQTEAI
VMIGEIGGSAEEEAAAYIKEHVTKPVVGYIAGVTAPKGKRMGHAGAIIAGGKGTADEKFAALEAAGVK
TVRSLADIGDAVKAVLKR

>CORE_REP|Org6_Gene3586#
MLTNSSIRLNKYISESGICSRRDADRYIEQGNVFINGKRATVGAQVFAGDVVKVNGQLIEPRNEEDLV
LIALNKPVGIVTTTEDGERDNIADFVNHSKRIFPIGRLDKDSQGLIFLTNHGDLVNKILRAGNNHEKE
YLVTVNKPVTDEFIRGMGAGVPMLGTVTKKCKVKKEAPFVFRITLVQGLNRQIRRMCEHFGYEVTKLE
RTRIMNVSLKGLPLGEWRDLTDDELIELFKLIEGSSSEAKPAKKAPAKSAAARKPSAGGPKSADKAAA
PAGRKRFTQPGRKKKGR
>CORE_REP|Org16_Gene2285#
MDQAGIIRDLLSWLESHLDQPLSLDNVAAKAGYSKWHLQRMFKDITGNAIGAYIRARRLSKAAVALRL
TSRPILDIALQYRFDSQQTFTRAFKKQFAQTPALYRRAEDWNAFGICPPIRLGAFTLPQPEFVSLPDK
HLVGLTQSYSCTLEQITTVRTELRSQFWRQFLGDVETLPPVLYGLHHSRPSQEKDDEQEVLYTTALEP
DQVPDKVQEGQPLVLPGGEFAMFSYEGPTENLQDFILTVYGTCLPALQLTRRKGHDIERFYPKGERRP
HQAPIEIKCDYLIPIRR
>CORE_REP|Org23_Gene2399#
MWQAVSRLLSEHLGSAEIRERIELPGGDIHPAWRVSYGDNEVFVKCDAREQLPIFTAEADQLALLARS
KSVRVPEVYGVGSDRDYSFLLLEYQQLKPLDAHGAYCLGQQLAHLHQWSEQPQFGLDFDSDLTTTPQP
NAWQRRWSEFFAEQRIGWQLQLAAEKGMTFGDIDDIVDRVYLRLQHHQPQPSLLHGNLWPGNCAMTAN
GPILFDPASYWGDRECDLAMLPLYPELPPQIYDGYQSVWPLGAGFIERQPLYQLYYLLNRSNLFGGQH
LVAAQRAVEALLQPEAS
>CORE_REP|Org17_Gene1996#
MRWQGRRESDNVEDRRGQSSGLGGGGGGFRVPVRGKGGIVILVVVLVAGYYGIDLSPLLNGGNVAPQG
QQQSASISPKDDELAKFTSVVLASTEDNWKEIFQRMGKTYQPPKLVMYRGVTRTSCGTGQAAMGPFYC
PGDRTVYIDLSFYQDMKTKLGAGGDFAQAYVVAHEVGHHVQNLLGIEPKVRQMQQGASQAEVNRLSVK
MELQADCFAGVWGKYAEKQQMLEEGDLQAALNAAQAIGDDRLQQQSQGRVVPDSFTHGTSQQRYTWFK
QGFDSGDPNTCNTFASR
>CORE_REP|Org5_Gene4557#
MPRIDAHQHFWRYQPRHYPWIDERMRVLRQDFDPPRLRPLLQEQGFDGALAVQARPSEEETLALLALA
LAEQGEGVCGVVGWLDIAAPQLAQRLEALRPYSALRGVRHQVQDEADPAAWLARPEVKRGMQTLQRAG
YVYEILVTHRHLAAAAAFAARHDEHWLVLDHLGKPDIARGARHWAQQIRPLAALPHVACKLSGLITEA
PSGRWRAEELLPFFDAALEAFGPQRLMFGSDWPVCLLAGDYRQVVQLCERALSTLGAAEQAAIWGDTA
WRIYGLTESGYGSVSAR
>CORE_REP|Org28_Gene626#
MSAKIIDGKTIAQQVRNEVAEQVKQRLAAGKRAPGLAVVLVGENPASQIYVASKRRACDEVGFLSRSY
DLPAATSEAELLALIDQLNADEEIDGILVQLPLPAGIDNVKVLERIHPDKDVDGFHPYNVGRLCQRAP
KLRPCTPRGIVTLLERYNIDTYGLNAVVVGASNIVGRPMSMELLLAGCTTTVTHRFTKNLRHHVENAD
LLVVAVGKPGFIPGDWIKPGAIVVDVGINRLESGKVVGDVDFDAASERAAYITPVPGGVGPMTVATLI
QNTLQACEEYHDVQPK
>CORE_REP|Org34_Gene1848#
MKDLNVVDGINGAGNWLVKNQDLLIQYAVNIVAAIVILIIGSIVARVVGNALNRVMKLRGIDATVADF
LSAIVRYGVLAFTFIAVLGRVGVQTTSVIAVLGAAGLAVGLALQGSLSNFAAGVLLVIFRPLRVGEYV
DLGGVAGTVDQVQIFSTTLRTADNKTIVVPNGKIIAGNIINYSREPNRRVDIVVGVAYNADIDVVKKV
LGDVIAADKRIMHAKGVTVRLNEMAPSSLNFVTRSWTTNAEYWNVYFDLMENFKRALDAHNIGIPFPQ
MDVHLYRTEDASAKAE
>CORE_REP|Org36_Gene2971#
MGTQESHIKELLVWIEDNLTNPLSLDIVSAKSGYTKWYLQRMFKKQTGLSLASYIRARRLYLAAFALR
FTQKSILDISVEYQFDNQQTFSRCFKKHFAESPSVYRHARKQDFSNLVRSLAASQPGDIQVERVSIAR
GQYAFHGKQYAYHLDIEKLDKSHLPQRSALRGQFYTLLGERPTQTYSFTQLVPDGERVRVDYTLGVTT
EYPLREGVVLEPLPEIHGEFCRFRYSGKPVALNDHIIQIYTQVLPEMGLARGDGPDITVFSYSLSGKE
ELHLELQHLVPVVPLH
>CORE_REP|Org23_Gene3070#
MIRQWPSPAKLNLFLYITGRREDGYHLLQTLFQFLDYGDTLTIDPRQDDRIHLLTPVDGVPDEQNLIV
RAARLLQRYCDERGLQTAPRGADISIDKRLPMGGGLGGGSSNAATVLVALNELWRCGLGDDQLAALGL
SLGADVPVFVRGHAAFAEGIGERLQPAEPQEKWYLVAHPGVGIPTPVIFGDPELKRNTPVRSLSELLQ
APYANDCEPIARKRFREVEQLLSWLLEYAPSRLTGTGACVFAEFDTEIAARQVLNQAPEWLCGFVARG
VNVSPLHRIRSGRFES

>CORE_REP|Org12_Gene730#
MSQALQNLLDLLDLEKIEEGLFRGQSEDLGLRQVFGGQVVGQALYAAKQTVPDERSVHSFHSYFLRPG
DSSKPIVYDVETLRDGNSFSARRVSAIQHGKPIFYMTASFQSPETGFEHQNLMPDVPPPEGLLSESEI
AQKLAHMLPEKVREKFIGQKPIEMRPVKFHNPLKGSVEEPHRYVWFRANGSMPDDQRIHQYLLGYASD
FNFLPTALQPHGVGFLEPGMQVATIDHSMWFHRPFRMDDWLLYAVESSSASGARGFVRGQIYTRDGVL
VATTVQEGVIRQRDA
>CORE_REP|Org44_Gene232#
MQHKTLKYFSTVIVCAIAVCAGWWLWNYYMQSPWTRDGKVRAELVNITPEVSGRLEKISANDNQFVPA
GSLIFTLDPVPYQIALDNAEAAVAKAQSDLAKADHEAARRRGLPRNVISAEDLDESNLAAQAMKAAYK
AALANLEQAKWNLSKTKIYAPTDGYITNLQARVGNYANAGTPLVALVDVHSFYVLGYFEETKLKHIKE
GNKADIVLYNGNTPLQGEVESIGRAIYDQSVDSSNDLLMDVKPNVPWVRLAQRVPVRIKLLNVPADLT
LVAGTTCTISIHQRN
>CORE_REP|Org29_Gene904#
MATLLQEENTTSLEAIPSNSTATPRPTVLVFDSGVGGLSVYQEIRQLLPDLHYIYAFDNVAFPYGEKS
EEFIVERVLEIVGAVQQRHPLAIVVIACNTASTVSLPALRERFSFPVVGVVPAIKPAARLTVNGIVGL
LATRGTVQRSYTHELISRFATDCKIELLGSSELVELAEAKLHGEAVPLPVLKKILHPWLSMREPPDTV
VLGCTHFPLLAEELMQVLPEGTRLVDSGAAIARRTAWLISTQENLVSSQEENLAYCMALNEDTDALLP
VLQGYGFKSLKKLPL
>CORE_REP|Org33_Gene2793#
MTQKEIWYETLHTGFGQYFSVEKVLYREKTDHQDLVIFENPVLGRVMALDGVVQTTERDEFIYHEMLT
HVPLLAHGAAKKVLIIGGGDGGMLREVSRHPGVEQITMVEIDAGVVEFCRQYLPNHSAGAYDDPRFKL
VIDDGVNFVNQTDETFDVIISDCTDPIGPGESLFTSAFYEGCARCLNEGGIFVAQNGVCFLQQDEAVN
SHAKLSGYFSDVSFYQAAIPTYYGGIMTFAWASQNPALRQLDLPTLQQRFNQSGLHCRYYNPAIHVGS
FALPQYLLNALNVTR
>CORE_REP|Org9_Gene3396#
MNNQPLNCHIQPNTPFGAILTPQHPGQKIGELPVAALRALAQEHHLLVLRGFDSGFSEAEVLTRYAEQ
WGEIMMWPFGAVLDVKEHPDAKDHIFDSSYVPLHWDGMYKPTIPEFQLFHCVAAPSPDEGGCTTFVDT
TRLLANADEALLDQWLSISITYRIKQVVHYGGEVCSPLVVQHPNGRGLIMRYNEPPTEGKKFLNQHAL
EYHGVPEHQQEQFHHTLQQHLYDPRHYYAHQWQQGDVVVADNFSLLHGREGFTARSARHLQRVHIQSN
PVCANLALKPANAEA
>CORE_REP|Org21_Gene1636#
MEQQLLCYKTLPEWNSDTLPEAFRQRHNTQSGTWAKLTVLSGSLTFAMMTEDGATTETWQFSPESQPP
FIAPQQWHRIVSFSDDMICRLAFYCTPEDYYHKKYELTRTHSEVIEAAARIAPGKALDLGCGGGRNSL
YLNLKGFDVTAWDKHAPSIDRLNQIIDAEQLTRLSARVQDLNTHRFSGEYDFILSTVVMMFLERQQIP
PIVQNMQDSTVRGGHNLIVAAMDTEDYPCPLPFPFTFSPGELKHYYRDWGILKYNEDVGQLHKTDAAG
NRISLRFATLLARKL
>CORE_REP|Org10_Gene845#
MNTDFAHYYQRIRSKQKREALLWSLGLVVLYLGAGNLAEFNLHTVWVSIPHFFDYLAETVPTLHWHLL
FADGRTEGSLAYWGYRLNIQLPLIWETLQLALAATIFSVLVATVLAFLAAGNTYTPASVRLAIRTLVA
FLRTMPELAWAVMFVMAFGIGAIPGFLALALHTIGSLTKLFYESIETASNKPVRGLAACGATPLQRMR
FGLWPQVKPVFLSYSFMRLEINFRQSTILGLVGAGGIGQELMTNIKLDRYDQVSMTLLLIIVVVSVLD
YVSGELRKRVVEGAK
>CORE_REP|Org49_Gene272#
MAGAKEIRSKIASVQNTQKITKAMEMVAASKMRKSQDRMAASRPYAETMRKVIGHLALGNLEYKHPYL
DERDVKRVGYLVVSTDRGLCGGLNINLFKKLLAEMKAWSEKGVETDLALIGSKAASFFGSVGGNVVAQ
VTGMGDKPSLSDLIGPVKVMLQAYDEGRLDKLYIVSNKFVNTMSQEPQVLQLLPLPPADDEELKKTPW
DYLYEPDPKVLLDTLLRRYVESQVYQGVVENLASEQAARMVAMKAATDNGGSLIKELQLVYNKARQAS
ITQELTEIVSGASAV
>CORE_REP|Org28_Gene4729#
MLKHWPPLSALRGFEAAARLSSFHQAAEELHLTQSAISQQIRSLEAFLEQPLFFRTGRSVTLTDAGHD
LFSTAQVMLQQLAVGIRRLDQYRKPNQLIVNTTPAFARHWLMPRLGDFNRQHPQADLWLFTSFEPPNM
ATDSIDLAIRDDLSAQADCTFNVLCSDRLYPACHPSLLALAAEQRMTLHGEREMDWSHWTVAGGAHVG
QRDSGLNFSDPGLLLDAACQGLGIALVSQLLAQQARDAGLLQPLTEQRVRGANWAWLLHRDSEHNPLT
RHFCQWLQSALPAGA

>CORE_REP|Org7_Gene2122#
MRNANVALLQLCSGDQVRDNLAQIEQQIKQLNAGVKLVMTPENALLFANSAAYRQQAEKQGDGPLQNA
VRELARRYGVWLLVGSMPLVSRENPELITTSSLLFDEQGEIRARYDKLHMFDVDINDAHGHYRESDTY
QHGQHLTVVDTPVGRLGMTICYDLRFPALFQALRAQGAELISVPAAFTRVTGEAHWEILLRARAIENQ
CMILAPAQVGRHGPTRRTWGHTLAVDGWGKVLAENPDAVAALKVRVDAASLKNIRAQMPVLQHNRFQT
SLTAPSDKLSSNQE
>CORE_REP|Org44_Gene1699#
MKNILSIQSHVVFGHAGNSAAEFPMRRMGVNVWPLNTVQFSNHTQYGQWTGCVMPASHLTEIAQGIAN
IEQLQRCDAVLSGYIGSPEQGDHILEIVRQVKQANPNAWYFCDPVMGHPEKGCIVAPGVAEFHCRQAL
PCSDMMAPNLLELEMLSQMAVANVADAVQAARVLIAKGPRLVLVKHLARAGYHADCFEMLLVTADEAW
HISRPLVDFGARQPVGVGDLTSGLLLVDLLKGEALDKALEHVTAAVYEVMLTTQEMGEYELQVVAAQD
RIVQPLSEFKAVKL
>CORE_REP|Org12_Gene3831#
MKKSRKVFQNVVIVGVVAWLLLFVFMPNLMIIGTSFLTRDDANLVQMVFTLDNYRRLFDPLYAQVLLH
SLNMALIATLCCLVIGYPFAFILARLPQKVRPLLLFLLIVPFWTNSLIRIYGLKLFLSTRGYLNDALL
WIGVIDKPLRIMYTSEAVILGLVYILLPFMVMPLYSSIEKLDKSCLEASRDLGASKLQTFIRIIVPLT
MPGIIAGCLLVVLPAMGLFFVADLMGGAKNLLIGNVIKSQFLNIRDWPFGAATSICLTLVMGLLLLVY
YRAARLLNKKEDLA
>CORE_REP|Org45_Gene805#
MKTDSPFDLILPAATAKIAEDAGVYKATKHPLKTFYLAITAGVFISIAFVFYITATTGTAGVPFGLAK
LVGGICFSLGLMLVVVSGADLFTSTVLIVIAKASGRISWGQLGANWLNVYLGNLVGALFFVALIWFSG
EYMVANGQWGLNVLQTADHKLHHTFIEAVCLGILANLMVCLAVWMSYSGRTLTDKMLAMVLPVGMFVA
SGFEHSIANMFMIPMGIVVKHFATPEFWQAVGAVPEQFAHLTVSNFIIDNLIPVTIGNIIGGGLLVGL
TYWVIYLRGGREQH
>CORE_REP|Org21_Gene2638#
MAIPKLNDYALPTADELPQNKVTWQVEPQRAALLIHDMQQYFLNFWGEDSALIKQVVENIANLRRYCK
QQDIPVFYTAQPNQQSDEDRALLNDMWGPGLNKHPEQQAVTAALAPDEDDTVLVKWRYSAFHRSPLQE
ILQESGRDQLIICGVYAHIGCLTTAIDAFMRNIQPFMVADGLADFSRDEHLMALRYTAGRCGRVVTTA
SLLPAAGIASIDALRQQILPLLDEDSEDMGNDENLIDYGLDSVRIMELATRWRKIRGDIDFIALARNP
TIDSWWALLSEEKA
>CORE_REP|Org42_Gene254#
MSEVLTGYNLGYLDEQTKRMIRRAILKAVAIPGYQVPFGGREMPMPYGWGTGGIQLTASVIGRADVLK
VIDQGADDTTNAVSIRRFFQRVAGVETTERTPEATLIQTRHRIPETALREDQILIYQVPIPEPLRFIE
PRETETRKMHALEEYGVMQVKLYEDIARYGHIATTYAYPVKVNDRYVMDPSPIPKFDNPKMHMMPALQ
LFGAGREKRIYALPPFTKVESLDFDDHPFSVQQWDEPCALCGSRHSYLDEVVLDDRGNRMFVCSDTDY
CRQQLAQSSQEAQH
>CORE_REP|Org3_Gene3521#
MERTINLCPGIGASAHIIQHTELLFPSVYFEQPHLYLIQQGHKRVRWQQREVVAHAGELLIIDGGQTV
DIINGPSEEGVFSCQLLTCDPLLLTVQPPAEDSPAPMPFDAVLALRSLPCALKHSFETTSLALALRQR
FPTIIVRHKMLEILLWLAQFGIRFIHNEAKDLTQRVRRCLATDPHSIWTAAKVAESLSMSEVMLRRKL
SMENTALRNLMIDVRMSSALALLQSTDWPISAIAQHVGYESASRFAERFRKRFGFAPTAIRGHQRIME
PTSQGVETMATGET
>CORE_REP|Org48_Gene3876#
MTAGTPFSGKEPRVERSVFYISDGTAITAEVLGHAVLSQFPVTATTFTLPFVETEARARAVRQQIDDI
YNQTGVRPLVFYSIISPEVRDVIVQSQGFCQDIVQALVGPLQGELEVEPTPVPNRTHGLTASNLGKYD
ARIAAIDYTLAHDDGISLRNLDQAQVILLGVSRCGKTPTSLYLAMQFGIRAANYPFIADDMDNLHLPA
SLKPFQHKLFGLTIDPERLAAIREERRENSRYASLRQCRMEIAEVEALFRKNQIRYLNTTNYSVEEIS
TKILDILGMSRRMF
>CORE_REP|Org25_Gene2382#
MTQTYAKRFAQVFDYIDRHLDEALTVDKLSEVAHFSRFHFQRQFSAYCGISVWRYIQWMRLKRASYRL
AYNPLEPVIDIALDAGFQNPESFSRAFKQAFSQTPSQFRKQPAWIDWQQRFPEPKHRRKHPMKVDIVD
CPATPVAMLEHRGPSALVNETAARFIEWRKTSGLSPVRSSRTYGIAPHDPATTEAQDFRFYLCGEVTA
PIPEDNAFGVVNSMLPAGRCAVLRHLGSLDGLSESARYLYSEWLPASGEELRDFPLYFHYHNFVHEVA
EYELVTDLYLPLK

>CORE_REP|Org13_Gene1299#
MTKEMQTLALVPQGSLEAYIRAANAYPMLTAEEERELAERLHYQGDLDAAKQLILSHLRFVAHIARNY
SGYGLPQADLIQEGNIGLMKAVRRFNPEVGVRLVSFAVHWIKAEIHEYVLRNWRIVKVATTKAQRKLF
FNLRKTKQRLGWFNQDEVELVARELGVTSKDVREMESRMAAQDMTFDPTPDDEARDGQAMAPVLYLQD
KSSDFAEGIEEDNWESNAADKLAYALEGLDERSQHIIRARWLDDDNKSTLQELADQYGVSAERVRQLE
KNAMKKLKMAIEA
>CORE_REP|Org19_Gene1426#
MMLWHGLIDPFLSFGFMRRALMACLALSLSAAPLGVFLLLRRMSLVGDALSHAVLPGAAIGYLISGMS
LVAMGVGGFIAGLAVAMLSGLVSRRTPLKEDASFAGFYLGSLALGVTLVSLRGSSVDLLHVLFGSILA
VDAQAMLMVGAIASVSLLALAALYRALVIESFDVTFLRVNAPRRLALIHGLFLALVVVNLVAGFQILG
TLMSVGLMMLPAASARFWARNLPQTLATAMGIGALSSLIGLVWSYYASLPAGPAIVLSASVIFFVSIL
FGTRGGIYAFARR
>CORE_REP|Org9_Gene746#
MLAQAVVDINETNLHQTLEQSMSIPVLFYFWSERSQHCLQLTPVLDKLAAEYAGQFILAKVDCDAEQM
IASQFGLRSIPTVYLFKDGQPVDGFQGPQPEEAIRDLLQRFLPKEEDLKLAQAQELLAAGNAAEALPL
LKDAWQSSQQRSDIGLTLAETQIALSRSEDAEAVLATIPLQDRDTRYQGLVAQIELLKQAADTPEIQQ
LQQQVQQQPDNVELAVQLALQLHQVGRNEEALELLMGHLKKDLAAANGNARKTLMDILAALGTGDALA
AKYRRQLYSLLY
>CORE_REP|Org45_Gene695#
MVLMIVSGRSGSGKSVALRALEDMGFYCVDNLPVVLLPQLANTLAERNISAAVSIDVRNMPESPEVFE
YAMTQLPDSFSPQLLFLDADRNTLIRRYSDTRRLHPLSSKNLSLESAIDEESDLLEPLRSRADLIIDT
SEMSVHELAEMLRTRLLGKRERELTMVFESFGFKHGIPIDADYVFDVRFLPNPHWDPKLRPMTGLDKP
VASFLDRHTEVHNFIYQTRSYLEQWLPMLETNNRSYLTVAIGCTGGKHRSVYVAEQLADYFRSRGKNV
QSRHRTLEKRKQ
>CORE_REP|Org20_Gene1909#
MRSTSDYQPPVSLSPPSLVGLPEQEDVFAVEHLSRLCDGLAQQRPNNLRDLLNTLALIAPLLNAIPNV
VFFIKDAQARYLLANLTLARRCGFKTVTPLLGKTSADVFPAQLGSDYTEQDLRVLRHGVLIQDQLEMH
LYNGRETGWCLTQKLALYDAQGKIIGMAGISHDLQEARANHPAYQRLAAIDVHIRRHYARPIALEELT
ALTGLSVAQIERYCKRIFHLTPRQMIHKVRLEKATELLAGDLPITDIALQCGYTDHSAFSRQFKAMTG
STPRDFRLTLSA
>CORE_REP|Org9_Gene2151#
MKLSQLKFFCTVVEHKTIAAAARELHCVPSNVTLRLRELEESLGGELFFRDKNRLYVNPKGRLFYQQA
RDIVAQAERSKQLFAGEHQHGLLNLGALDFSLVSHLPARIARLRRLQPHLHINVLSRDSLVLERMLID
SDLDLAITDGPIEHPLLASQKAFDERLVLLMPADAGEPDAATLAPLEFYTFSRECSFRLKVDHWLASR
GLKPRMTLEMESYAAMAACVQAGCGVACVPGSLLPLILPAPGLKVVEMGEEGVSDLYFVWRRHQLSDE
LQTILAILARPG
>CORE_REP|Org15_Gene3670#
MDTLYQLLSEPFAYPFMQRAIVAAIVTGVVCAVLSCYLVLKGWSLMGDAISHAVLPGIVVAFVIGIPL
AIGAFLSGIFCAVATGYLKENSRVKEDTVMGIVFSGMFAFGLVLFSRIDTDQHLSHILFGNMLGITDG
ELKQTLLIAGLTLAVVLLKRKDFMLYCFDPHHARVIGLPVKLLHYGLLCLLAMTIVASLQAVGVILVI
AMLIAPGIIAFMLCRRFDRMLMVATVVSVFSCVLGTLISFHIDGATGPCIVIVQAVLFVIALLYGKLR
PLQRNQTALSDS
>CORE_REP|Org34_Gene1366#
MNPLFTPYLQRWQLEQDGKAFETHSSLLMPVRYRGEAAMLKIAREQEERFGGQLMCWWRGEGAAQVLA
WHEDGILLERAQGESSLAQLVRDGDDEQATAILCRAIAALHAPRAAPLPELIPLQEWFSSLWPAAQAH
GGMLRLSATTAAELLSSPRDESVLHGDIHHDNVLDFGERGWLAIDPKRLYGERGFDYANIFCNPNYGI
ATDPAIFQRRVEQVCRLAGLERRRLLQWILAWAGLSAAWFMEDGQAADIDFRVAELAARALDLPLPAG
DSGFILPVIERG
>CORE_REP|Org36_Gene3523#
MTLFTLLLVLAWERLFKLGEHWQLDHRLEVVFQRLHRVSLAQTLVMTAAWMVIVWGVLWLSHGLFFGV
VTLLLWIVIDLLCVGAGIKRKHYRAYLKAARQGDTHASDQMAEELALIHGLPVDCSEELRLRELQNAL
LWINFRYYLAPLFWFVVFGPYGPIALAGYAFLRAYQTWLARHNTPLERSQSGIDHLLNWLDWIPVRLA
GVAYALFGHGERALPAWFASLGDRHSSSYQVLTRLAQFSLARDPHMDPVQTPRAAVTLARKVTMIIVV
VVALLTIYGTLL

>CORE_REP|Org34_Gene1435#
MYLIANREMLLKAQRQGYAVPAFNVHNLETVQVVAETAAELRSPVIMAGTPGTFSYAGTDYLIGICQS
AAHRYDLPLALHLDHHEELDDIEHKVKSGIRSVMIDGSHLPFEQNIAKVAAAVALCHRYGASVEAELG
RLGGQEDDLIVDTADSFYTDPMAAREFVAATGIDSLAVAIGSAHGLYHGEPKLDFERLALIREQVDVP
LVLHGASGIPEAMVKRAISLGVCKVNVATELKIAFADAVKSYFSQHPDANDPRKYIVPGKLAMKEVVA
EKIRICGSSGML
>CORE_REP|Org12_Gene1894#
MIIIETLPMLRQQIRRWRQEGKRIALVPTMGNLHDGHMTLVDEARARADVVVVSIFVNPMQFDRPDDL
ARYPRTLQEDSEKLTRRGVDLVFAPAPAAVYPQGLEQQTYVDVPGISSILEGASRPGHFRGVSTIVSK
LFNLVQPDLACFGEKDYQQLALIRKMVADMGYDIDIVGVPTVRAKDGLALSSRNGYLTAEERKVAPQL
SKIMNALAQQLANGERQVEALLEQTAEQLRAAGFTPDELFIRDADSLQPLTVDSQRAVVLMAAWLGKA
RLIDNQQVDLTL
>CORE_REP|Org6_Gene1996#
MNRYPMFNPQLLLSFVAVCDSNSFTRAAERVFLSQSTVSQQVRRLEEMLGKPLFERSSHQVLLTEEGV
KLLSYARRIIALNEEAHDALTGIWRDGVLRIGMPEDFAVPTTELLAEFSREHPHLRLDVASGLSADLH
SAYAREELDLILVKQRRQQPPRAARPEPLLWLDSLAFPAIEQSPVPLAVFPLSGLYRDELCQALDNLG
KRWRIGYSSASLAALTAASAAGLGVTLLPAGCRLPTHRVLGAAEGLPPIDSFELALYYRDGAPAATLA
LAQRLTVFCGLI
>CORE_REP|Org22_Gene2790#
MHNDGRFDYHNALRYSSDELAKILSHCFENYIVRFVLDGPTFAARFGAEDISLNDSLIVTHRHEPVAV
ALIARRGQHSRVAAFSVRPEMRGQGLGKALMQRLVADARQRGDRRLSLEVIEGNEAALALYHRAGLRI
VRTLTGHQAPAEAPPGTTAGLQAVDPLTVSHRLTAEGATDLPWLIAPESLFKLPGKPQAYTLNRQAYA
VVMPGAEHCWLRLIYVPPQHRGQGHARALLAALQTRFAPLPLTANVFVPEVAAPFFTHLGWRQDPLRQ
FEMDMLLDSPQE
>CORE_REP|Org23_Gene3139#
MNKNKAVLLMLAIAMSGCAERSTAVSPATPPTDVTVSPPGVQPEMDASTRSKLREILALRAGWPAAQP
HGRTVDLISREFLGTPYLANRLIGSQNTPEQLVIDFRGLDCFTYIDYVEALSTARSEAEFVQRLVDIR
YVDGNIAFPQRKHFFTDWAQRPKKVAEDITAQLSPHAVSLVKNLNQKADGSSYLPGLPNVQRSVTYIP
SDNVDDKVLAQLRTGDYIGIYTNLAGLDVTHTGIFVMTDHGPVLRNASSRKANMQVVDSPFMDYVMAT
PGIVVLRSLSR
>CORE_REP|Org40_Gene2379#
MSTYLIGDVHGCFDELKSLLAQAAFDPERDQLWLTGDLVARGPASLDVLRYVRSLGPAVRMVLGNHDL
HLLAVYAGISRNKPKDRITPLLEAPDADELINWLRRQPVLQVDDEQKLVMAHAGITPQWDIDTAKMCA
REVEAVLSSDSYPLFLDAMYGDMPNNWAPELSGLARLRFSTNALTRMRYCFPNGQLDMICKDAPGSAP
APLKPWFELPRLVDPEYTIIFGHWASLEGKGTPEGVIGLDTGCCWGGDLTMLRWEDRRYFTQPANRGE
APDHAGRLAAS
>CORE_REP|Org8_Gene365#
MSQAALNARDREVDNGPRRSNGTQLAGMIFLLMVLGTVVWSGWAVLGWMKDASRLPLSRLVVTGERHY
TTNDDIRQAILALGAPGTFMTQDVDVIQQQIERLPWIKQASVRKQWPDELKIHLVEYVPVARWNDLHM
VDAEGKAFSVPAERAGKQKLPLLYGPEGSEQDVLEGYRAMSATLAASKYTLKMAAMTARHSWQLALDN
DVRLELGRDDRNGRLQRFIELYPVLQQQGQAESKRVSYVDLRYESGASVGWAPVLVDPQALGGQQNSN
QQQNQAQAKQQ
>CORE_REP|Org21_Gene3759#
MRLPSLWANAGLATLFVLLWSSGAIFSRWGLEHASAFAFLFFRCAIALAVLLAIGLWQRQCLPAPGTR
RRVALIGVLMIGSYQICYLLALEQGITPGVLATLLGVQPILTQFITERRCTGLRALGLALAMVGLTLV
VYQSLMVAHFSLSGMLFAFIALGCMTAGTLLQKGMAQPPLAVLPLQYAVGLLMCTLALPFEPLRVEWG
PAFVLPLLWMAVVISVLATFLLYRLIQRGNLVQVTSLFYLIPAATTLLDYLLLGHALAALSLLGMAGI
VLGVMLVFRRV
>CORE_REP|Org4_Gene1213#
MADITAALVKELRERTGAGMMDCKKALVESNGDIELAIENMRKSGAIKAAKKAGNVAADGVIKTKIEG
NYGIILEVNCQTDFVAKDAGFQAFADKVLDAAMAGKITDVDVLKAQFEEERVALVAKIGENINIRRVA
SLEGEVLGSYLHGARIGVLVAAKGADEELVKQIAMHIAASKPEFVKPEDVSAEVVEKEYQVQLDIAMQ
SGKPKEIAEKMVEGRMKKFTGEVSLTGQPFVMDPSKSVAQVLKEHNADVTNFIRFEVGEGIEKVETDF
AAEVAAMSKQS

>CORE_REP|Org19_Gene2883#
MMPNRLEPELDIYQYPEHLRACLAPLPKGPGVYLFHGESESLPLYIGKSVNVRSRVMAHFRAQDEAKM
LRQTRRISFIETAGELGALLLEAQLIKQQQPLFNKRLRRSKQLCALRLQADTVTIAHAKEIDFAVTPH
LYGLFANRHAALEKLRAIADEHRLCYGKLGIDKLPAGRACFRYSLRKCAGACCGVESAQEHAQRLGAA
LEQLRIACWPFAGRVALEEQGETLRQYHVIHNWFYLGSVSSLAQAQRLQSAASHFDSDGYKILCKPLM
AGDYRIIELP
>CORE_REP|Org28_Gene3430#
MSDNRISLSANDVKLIREQDFFNCKDFHLFIYNKVESATGLHQHDYYEFTIVLSGKCYQEINGKRVLL
ERGDFVFIPIGSHHQSFYEFGATKIFNVAVSKAFFEEHYLHQLPRCFVASQAYSLRSEFLAYIESVVS
SPQFREDDFAEFLETLTFYVISRIRHYKEENDGGDDIPQWLKNTLAGMHDKAMFGERALANMVALSGK
TQEYLTRAMRRYYHKTPMQVINEIRINFAKTQLEVTNSSVSDIAFDSGYGDVSLFIKNFKRLTDVTPG
NYRKKCYGPL
>CORE_REP|Org1_Gene218#
MPHQNVQRKVLRTICPDAKGLIAKITNICYKHELNIVQNNEFVDHRTGRFFMRTELEGIFNDNTLLAD
LDSALPEGSLRELHSTGRRRIVVLVTKEAHCLGDLLMKSAYGGLDVEIAAVIGNHDTLQTLVERFDIP
FHLVSHEGLTREQHDREMTAKIDQYQPDYVVLAKYMRVLTPAFVQHYPNQVINIHHSFLPAFIGARPY
HQAYERGVKIIGATAHYVNDNLDEGPIIMQDVIHVDHTYSAEDMMRAGRDVEKNVLSRALYQVLAQRV
FVYGNRTVIL
>CORE_REP|Org47_Gene2007#
MSTLLRIRQMYPTLAQNDRKLADFLLNNAEQARHLSSQKLAELAGISQSSVVKFAQKLGYKGFPALKL
ALSETLAQPQAEPVVTVHNHILSSDTLKIVGEKLLAEKQAALRATLDINSEERLHQALDMLRQARRVM
LIGIGASGLVAKDFSFKLLKIGVMAVAEPDMHVQLAAVQALDKRDLLLAISFSGERREINLAAEEARQ
AGARVLALTSFSPNGLQQRADHCLYTIAEEPHTRSAAISSSTAQYALTDLLFMALIQHDLDHARDRIK
HSEQLMKKLV
>CORE_REP|Org17_Gene2376#
MNERLNITPLGPYIGALVENVELARPLGDGQFEQLYHALLKHQVLFFRNQPITPLQQRDLAGRFGDLH
IHPVYPHATDVEEIIVLDTHDDNPPDNDNWHTDVTFIENPPLGAILAAKTLPATGGDTLWASGIAAYE
ALSAPFRTLLAGLRAEHDFTKSFPEHKHRGSEEEHQRWQLAVQKNPPLLHPVVRTHPVSGRQALFVNE
GFTTRIVDLAPKESDALLNFLFAHITKPEFQVRWRWQENDVAIWDNRVTQHYANADYLPQRRIMHRAT
ILGDKPFYKA
>CORE_REP|Org20_Gene2558#
MLNILVKNISKRLPDAWHHQVKYALYFKKLPHLSKPTGFSEKIMRRKIYPRSIYTTLSDKFKVREFIA
GLWGEEYLVELYAHGTELSYDMFRQLPNAFVLKANHGSGYNRLVFDKRQVSYAELYDLSNAWMRSNFY
EQSREKHYLDIEPCIMVERMLLDGEQVPNDIKFHCFNDNHEIRMFIQVDYQRFGTHRRDIFDVDWNRT
EIRISLPNADEPMPRPTRLDEMIRLARQTAQQFSYVRVDFYQVGEKVYFGELTFTPGAGLSKLMPKNI
EQEWGSYFTE
>CORE_REP|Org8_Gene1488#
MKIPKRLQPLVDDGLIDDVIRRLKSGKEADVFIVRCGDEIRCAKVYKEAEKRNFKQAVHYQEGRKVRN
SRDARAMSKGSKFGRQQQEEAWQNTEVDALYLLAKAGVRVPQPDICLDGVLLMELITDEEGLVAPRLS
DVILTPEQARADHALMMNYAVRMLCAGLVHGDLSEFNVLMDKDGPVIIDLPQVVDAAANNHAKSMFER
DINNLAEYYGQFAPELRDSQYAKEIWALYQEGNLTPDSALSGRFSEIQKRADVGSVLEEIQAASEEHQ
RRLMARNAEE
>CORE_REP|Org40_Gene2389#
MKLLRYGEPGQERPGMLDEQGRLRDLSQHIADVGGAALSPASLAKLRTLDSAALPLVEGQPRLGACVG
GIGKFICIGLNYADHAAETGAAIPEEPVVFNKWTSAVVGPYDRVEIPRGSQKTDWEVELGVVIGLGGR
YISEADAMRHVAGYCVINDVSEREYQIERGGTWDKGKGCDTFGPIGPWLVTADEIADPHSLNLWLEVD
GKRYQDGNTSTMIFRIPQIVSYLSRFMSLQPGDVISTGTPPGVGMGQKPQPIYLRAGQTMRLGIEGLG
EQRQQTVQA
>CORE_REP|Org45_Gene216#
MSQTTSPTLKGQCIAEFLGTALLIFFGVGCVAALKLAGASFGQWEISIIWGLGVAMAIYLTAAISGAH
LNPAVTLALWLFACFDGRKVVPYIIAQIAGAFCAAALVYGLYYNLFFDFEAAHHMVRGSNESLELAGI
FSTYPNAHISVGQAFLVEMVIAAILMCLILALTDDGNGIPRGPLAPLLIGILIAVIGASMGPLTGFAL
NPARDFGPKLFAYLAGWGKVAFTGARDIPYFLVPIFAPIVGCGLGAFGYRALIGRHLPCDVCVTEEEP
DAKTQQRKA

>CORE_REP|Org44_Gene1892#
MFVPFLIMFREGLEAALIVSLIASYLKRTQRSQWLGAVWVGVIVAAALCLALGIFINETTGEFPQKQQ
ELFEGIVAVVAVVILTYMVFWMRKVSKSVKVHLEGAIDQALSAGKGQGWALVAMVFFAVAREGLESVF
FLLAAFQQDVGAAAPIGAVLGLAAAIVLGMMIYWGGVKLHLAKFFKWTSLFILFVAAGLAAGAIRAFH
EAGLWNHFQDIAFDFSSTLSTHSLFGTLLEGILGYQEAPTVSEVAVYFLYLIPALIFFFLPQRAEPAA
APAQRKINH
>CORE_REP|Org39_Gene3109#
MNSPFLVTPQWLAQHINDENLVVIDVRMSPVGLTPKKDMLAEFERGHIPGAVYFDIDEVADKNTALPH
MLPTAAEFGAAVGKLGISERDTLVIYDEGNQFSAPRGWWTFRNFGARHVYVLDEGLNGWTALGQALET
GPAQPEPQTFNARFNADAVVDMRQVEQALGTPVQILDARAAPRFYAEAPEPRPGLHRGHIPGSINIPY
GELLENGRFKSLEALRQTFSDKGVDINGPIITSCGSGVTAAVLAFGLLSLGAPQVKLYDGAWSEWGQL
SGKQPIAKD
>CORE_REP|Org34_Gene1189#
MNNLPVVRSPWRIAILTVGFTFLYAPMLMLVIYSFNSSKLVTVWAGWSTRWYTELFHDSAMISAVGLS
LTIAAASATAAVVLGAIAAVVMVRFGRFRGSTGFAFMLTAPLVMPDVITGLSLLLLFVAMGHAFGWPS
ERGMFTIWLAHVTFCTAYVAVVISSRLREVDRSIEEAAMDLGAPPLKVFFVITLPMIAPALISGWMLA
FTLSLDDLVIASFVSGPGATTLPMLVFSSVRMGVNPEINALASLILLVVGILGLIAWWFMARSEKQRS
RELQRAARS
>CORE_REP|Org6_Gene3245#
MPPRTLERTRHELGDFLRQKREQLSPEAVGLPSGSGSRRRTPGLRREEVAALAGVGLTWYTWLEQGRE
INASVTFLENLARVLQLDAAGRYHLFLLAHQRPPVPVGHQWCQVSPLVRRLLDDLTLRPAYVMNLSWD
IIAWNPAAQRLFAIGERPAQERNMLWMLFADPQLNARLLEWRAQAPQILASFRRDYARAPEDEVMRQR
VQALEQVSPQFRQLWRQHDIHGRCQGRRSFTLPEIGPVTFDHASFIVDEENHLRLVMYSALPDEPASA
AFEALLRED
>CORE_REP|Org30_Gene693#
MIENRRGLDIFSHVMLIIGVLVVLFPLYVAFVAATLDDKQVFQVPMTLVPGGHLWENIRNIWQGGVGN
LKVPFSLLLLNSVIMALAITFGKIAVSVLSAYAIVYFRFPLRSLFFWLIFLTLMLPVEVRIFPTVEVI
SNLNLLDSYTGLTLPLMASATATFLLRQFFMTLPDELLEAARIDGAGPMRFFWDIVLPLSKTNLAALF
VITFIYGWNQYLWPILITSDASMGTAVAGIKSMISTSGAPTQWNQVMAAMILTLLPPLAVVLLMQRWF
VRGLVDSEK
>CORE_REP|Org31_Gene3888#
MGLTAVVRRLGVKEKISVACCQLALRVGEAEHNRALSAQAIRRAAQRGANVIVLPELVNSGYVLRDKA
EARALAEAEDGPSLSLWGALARELDVAIVAGFCERLPDGEVANSAALIDAQGVRAIYRKAHLWHEEST
IFTAGDRPPPVIETRFGRLAMIICYDLEFPEWVRLPALAGAQLLCAPVNWPLAPRPQGERPAEMVKAQ
ANAAVNRLFIAVCDRCETERGVAWIGGSVIVDADGYPLTQSLAGEGMVLASMDIGSADNKHIGRHNHV
HRDRLPMLY
>CORE_REP|Org24_Gene3142#
MSSYQDHQALSGLTLGKPTAYRDRYDASLLQAVPRSMNREPLGLYPDNLPFHGADIWTLYELSWLNAN
GLPQVAVGEISLNADSLNLIESKSFKLYLNSFNQTPFADWETVRSTLQRDLSACAQGEVSVTLFSVEQ
LEGTPIARLAGDCIDQQDIRIDNYEFNADYLLNAAGEEVVEEQLVSHLLKSNCLITHQPDWGSVQISY
RGGKIDREALLRYLVSFRHHNEFHEQCVERIFNDLMRYCRPQSLTVYARYTRRGGLDINPWRSNVEFA
PERGRLARQ
>CORE_REP|Org34_Gene1430#
MQQSSPLRSPSIDVISIQSQVVYGSVGNGIAYRALLKKGLEALQVPSVLFGCPPYYGKPHGGVISGEW
FGGFLDDLIARGVMKRTRAVIVGYLGDVMQCHILANWLQRVRSLNPQIKIYIDPVMGDYGEGIYVDER
IVNCYRSPFLRLANGLTPNGFELEQLCGRQLSSREQTQHAAQALLNDTTEWVLVTSAPGVAQHEDEVG
LMLVTRQETQCFTHPKVKSAVKGTGDLFAALLVSHLLHGAALDAAVVAAGGEVCDVLAEAAHFGWEEI
GSLRALKP
>CORE_REP|Org27_Gene1753#
MQLSSKVRMNRLFNNGKCLDVAIDHGIANEPDFLIGLEDIERVMGNLIAARPDAIQVNYGQADLLQRA
PQREKPALVMRTDVGNAYNAARHREMWAVLHNPEAPILAALQMDAAAVVVNLYMIPDEPGIFRQCVEN
IGRLRHACDRYAMPLMIEPLVMAPAGQGAAYGSLGDVEKIVPLVRLARELGADIIKADPTARVEDFHR
VVEAARCPTLVRGGGKGELGPVLEKSAALMAQGASGMVYGRNVYQHSNPSQVVRALMAIIHQGASGQQ
ALEIYHQQ

>CORE_REP|Org36_Gene3146#
MNKHLILDEPGDADSACLSRHAVWQRNDLAQAQQDWLAEEVPVALVYNGISHVVMMATPKDLAAFAIG
FSLSEGIIASPDDIYDIRQQPACNGIEVHVELSSRRFMQLKEKRRSLAGRTGCGVCGVEQLQEVAQPI
APLPFTQRFDLALLDRALAQLQDVQTVGKLTGCTHAAAWIQPDGALSGGCEDVGRHVALDKLLGYRSQ
QAWLQGAALVSSRASYEMVQKSAMCGVEILFAVSAATRLAVEVAERSNLTLVGFSKPGRATVYTHPQR
LWQSTPAA
>CORE_REP|Org49_Gene3127#
MTLSLELLEEHRMFGGWQQRYRHAAQSLNCNMTFSIYLPPPRDDNPPPVLYWLSGLTCNDENFTLKAS
AQRIAAELGLVLVMPDTSPRGDEVPNDEGYDLGQGAGFYLNATQAPWDRHFRMYDYISAELPALIEQH
FSVSGRQSIFGHSMGGHGALMMAFRNPQRFRSVSAFAPIVNPCQVPWGRKAFTAYLGSDESQWLQYDS
CHLLASGAEKMPVLIDQGDDDQFLADQLQPARLAELARQRDWPLTLRIQPGYDHSYFTIATFVEDHLR
FHAEHLFR
>CORE_REP|Org46_Gene1042#
MVDTTAQKKLTPADIRGVFLRSNLFQGSWNFERMQALGFCFSMVPVIRRLYPENNDDRKQAIKRHLEF
FNTHPYVAAPVLGVTMAMEEQRANGAPIDDGAINGIKVGLMGPLAGVGDPIFWGTVRPVFAALGAGIA
MSGSLLGPILFFVLFNLVRLLTRYYGVAYGYRKGVDIVNDMGGGFLQKLTEGASILGLFVMGALVNKW
THVNIPLVVSKITDQTGHTNVTTVQTILDQLMPGLVPLLLTFACMWLLRKKVNALWIIIGFFVIGIFG
YWIGLLGL
>CORE_REP|Org42_Gene1006#
MNKRELESAIVTDFSKRMSYGDYLCLDQLLDCQHPLSNPPHHDEMLFVIQHQTSELWMKLMLHELQAA
LRLVQQDKLSHCFKILARVKQIQRLLFEQWAVLETLTPSEYVEFRDVLGSSSGFQSHQYRSIEFLLGN
KNAAMLAVFSSDAEKHAALKAILEAPSLYDEYLLYLARHGLPIPQECLERDWTQPYQRNPDLLPAFKV
IYDNPQTYWEAYEMAEKLVDIEESFHLWRFRHMKTVERIIGFKSGTGGSSGVSFLKKALDLTFFPELL
DVRTEIGA
>CORE_REP|Org21_Gene3288#
MGTDNTLLTVERLAIGVPEPQPVALVKNISFSMGRERLALVGESGSGKSLTARALMGLLPPPLQLQAH
RLTLGDEDLTRLSERQWSRLRGDRVAMVMQDPKHALNPNQPIGRQVEEPLVLHTKLSRAERREKVLEM
LAAVGLPDPAALCRRYPHQLSGGMGQRVMLAIALINDPQLLIADEPTSALDHQMRDQVLQLIDNLVAQ
RNMGLILISHDLQQVAHHCERVLVMYKGELLDQLPAAELAQATHPYTRTLWACRPSRETRGKPLPVLD
RALLETLK
>CORE_REP|Org44_Gene792#
MLALSSSKRVLPGFGLSLGSSLFYTCLILLLPLTALVMQLAQMSLAQYWEVISNPQVVAAYKVTLLAA
GVASLFNAVFGMLMAWILTRYRFPGRSLLDGLIDLPFALPTAVAGLTLAGLFSTTGWYGQWLAHFDIK
VTFTWLGIAVAMAFTSLPFVVRTVQPVLEELGPEYEEAAETLGATRWQSFRRVVLPEVAPALLAGTAI
SFTRSLGEFGAVIFIAGNIAWKTEVTSLMIFVRLQEFDYPAASAIASVILAASLLLLFSINVLQSRFG
RRLGGGH
>CORE_REP|Org21_Gene921#
MKHRALALTLLASLTGLAAGAAHADKLDDIKQAGVVRIAVFDSNPPFGYIDPQSKKLVGYDVDVADAI
GKALGVKVELRATNPANRIPLLVSKKVDLIAANFTITDERAKEVNFSVPYFATGQKFIARKGVLKTPE
DIKKLRIGADKGTVQEITLRERFPTAKVISYDDTPLAFVALRNGNVQAITQDDAKLVGLLGNLPAAQK
AEFEISPFSLTKEYQGVGIPKGEDRLTAAVNETLIKLEKDGEAVKIYDRWFGPETQSAQPRGDFKIAP
LDQQPKA
>CORE_REP|Org47_Gene769#
MKSLFKVTLLATTMAFALNATQVMAADAAKPAEAAKPADAAAAPSTGKFKNDDEQAAYALGASLGRYM
DNSLKEQEKLGIKLDKDQLIAGVQDAFANKSKLNDADIEKTLQGFEARVKASAQAKMEQDAKDNETKG
AKYRDSFAKEKGVKKTESGLLYQVEKPGAGEAPKDSDTVVVNYKGTLTDGTEFDNSYTRGEPLSFRLD
GVIPGWTEGLKHIKKGGKIKLVIPPALAYGKTGVPGIPANSTLVFDVELLDVKAAPKADAKAEKPADA
KADAKAK
>CORE_REP|Org24_Gene3170#
MRYLVLLAALALGACSQAPVKAPANDVASLGSITDLHSGATLTPEQLLTRLAQQPRVILGEKHDNPYH
HQIEQWLVQQLPQRRPQGSVLMEMINPNQQAQVDKVKQWLQSDPTVRDGRVAELIAWQPGWKWALYGG
VTMAAMRAPYPLWSANLDRSEITAFYQQPQFPAGQLSAQPAVRKVLEETIRTSHGGKIEADQLRAMLA
IQQQRDRRMAERLLAAPTPALLIAGGYHAAKSVGVPLHVRDLQPAAALTVLMLAEPGVQVDANTADYL
WITPSVK

>CORE_REP|Org41_Gene649#
MKFVGAHVSASGGVDQAVIRAHELEATAFALFTKNQRQWKAAPLAADVIDKFKSACAQYGFGPGQILP
HDSYLINLGHPVAEALEKSREAFIDELQRCEQLGLTLLNFHPGSHLLQIDEDKCLARIAESINIALDK
TAGVTAVIENTAGQGSNLGFKFEHLAAIIDGVEDKSRVGVCIDTCHAFAAGYDLRTEEECERTFKQLG
DIVGFNYLRGMHLNDAKSEFNSRVDRHHSLGEGNIGKTVFSYIMRDPRFDNIPLILETVNPDIWAEEI
AWLKAQQ
>CORE_REP|Org28_Gene4260#
MKNWMLGLVALTASASASASAMALTPWQKIDHPVAGAPQAVGGFANGCIIGAQPLPLNSPNYQVMRTD
QRRYFGHPDLLAFIQRLSSQANQKALGTVLIGDMAMPAGGRFSSGHASHQSGLDVDIWLQLPRQRWSA
QQLLKPQPIDLVSGDGKQVVASQWQPQIESLIKLAAQDAEVTRIFVNPAIKQRLCLDAGADRAWLHKV
RPWFGHRAHMHVRLRCPAGSLECQEQDTPPPGDGCGAELASWFVPHQPNAKPGKSVPPPLPPTCQALL
DHHFSAE
>CORE_REP|Org28_Gene2350#
MVRVIAISNPRLALAFVDYMATQGIRLELRNSGEAAEIWLADDGHLEQVQHELQQFLVDPLNRRYQAA
SWQTGHTDAGLHYQSESYLHTLRSKAGPLTLGVMVLCIAVYILMQALGDDTVMYWLSWPQDSSQYTQL
WRWVSHAFLHFSLLHILFNLMWWWYLGGQMEKRLGAGKLFVLAVVSAFFSGWAQSLFSGALFGGLSGV
VYALMGYVWLTGERAPERGLMLPRGLMVFSVLWLVAGYFDILGMSIANAAHVAGLVLGLLMAFWDTRH
RAHNEQ
>CORE_REP|Org41_Gene130#
MNNPTQLSLLQDEIRHRYETLSKRLKQVARYILDNSNSIAFDTVASIAAQASVPPSTLIRFANAFGFS
GFNEMKQVFRQHLMEETVNYTERARLFRQTSTDDNVAPEKPAEILNVFTMVNAQALQQLAMQIAPEQL
DRAVELLNNAENIYVIGLRRSFSVASYLTYALRHLERRAFLIDGLGGMFTEQLSMVKPKDVVIAISYS
PYAREAVELVELGAKRGAQQIAITDSQVSPLAAFSDVCFVVREAQVDGFRSQVASMCLAQTLAVSLAL
NNARDE
>CORE_REP|Org24_Gene4551#
MQLTVRDMTLNLSHPQVMGILNVTPDSFSDGGRHNTLNQALLHAHALILAGATMIDIGGESTRPGAAE
VSEEEELERVVPVVEALAQRFEVFISVDTSKAGVIRESAHAGAHLINDIRSLQEPGALAAAAESGLPV
CLMHMQGQPRTMQQAPHYDDLIADVQAFFEHHIRRCNEAGITNQKLLLDPGFGFGKNLAHNYQLLARL
SEFHRFGLPLLVGMSRKSMIGQLLNVPPDQRVIGSVACAVIAAMQGAQIVRVHDVKETVEAMRVVEAT
LSAKGQ
>CORE_REP|Org4_Gene1450#
MTLALDRFCINRKIAPNLDLDSFFRLVKRCGLSKIELRNDMPSGKVTDDLSDAQLNALAAQYGIEIVT
INALGMFNRVDDPAALLQRAEALLAQAQAIHSRALVLCPHCSADDKRSEQQKRDDTLAALRLLAPLFA
RYGVQGYVEPLGFSISSLRSSLLTQALIRDSGAPYRIVLDTFHHYLSDVTQADFDAQIQVTQIGLVHL
SGVEDGRDKGALSDEERIMLSAGDRLESRRQVQNLERLGYTGVYAFEPFSSQLDSWSEADIEREIRHS
IALLQG
>CORE_REP|Org9_Gene3777#
MHTRHRLCQFRKSKHVLHQRFRSRIFHRDTMAAAELKKPFVVVLTGAGISAESGIRTFRAADGLWEEH
RVEDVATPEGFQRDPVLVQAFYNERRRQLQTEEIAPNAAHRALADLEEWLGENFLLVTQNIDNLHERA
GSKRVLHMHGELLKVRCTQSGQVFEWPGDLSVDDRCHCCQFPAPLRPHVVWFGEMPLGMDDIYQALSK
ADFFVAIGTSGHVYPAAGFVHEARLGGAYAMELNLEPSQVESQFDEKHYGLASEVVPRFVHKFLVGKV
ARADRP
>CORE_REP|Org13_Gene3749#
MKIWPGIIAAALLAGCQSPQQDTLVDRGAYQLETLHQAQGADQRIRFLVMHYTAEDFHSSLKTLTDEH
VSAHYLLPAHPQREHGKPTVYRLVPEAMRAWHAGTSAWRGRSNLNDTSIGIEIVNKGFTRSMLFTHWQ
PYTAEQIAVLIPLSRDIIQRYGIQPQDVVGHSDIAPQRKQDPGPLFPWRQLAQAGIGAWPDERDVQRL
LAGRDRHAPVPMAPLLEKLARYGYAIDPSWDARQQRNVVAAFQMHFRPDDVRGEPDAESEAIVDALLV
KYGAAR
>CORE_REP|Org41_Gene4511#
MIDKGQMLSRHDFSKVNWGILAAAALLFSVGAALLVLPLLSSIDESEVITLLQAGAGTVLLGCTLLAL
RHYLRPTLTYRLYEHGVRVFDGHHHKERFIPFEKIGDIYRFRGGKAFGGLFDVTAFRAGADQPWCTVF
SNVAHSWRLADVIVDQQLQQRGPLALNALYQGEVVPFHTVEGDARWLWQLLLGKRQGTPTETLRLSAT
LLATERGNVPLEQIRAVENHPQLGIRLLDGQGNVLFAINYDSLLSADLFIALLEHMIHNRIPAYHNPA
MTRPSV

>CORE_REP|Org11_Gene813#
MAQALLKLAQHDFPGQHAAASRKVLSVKGLGKAYKAQQRVLDDINFDLHAGEFVAVIGRSGAGKSTLL
HTLNGTIPSSCGEMLHFEDDGVAQDIAQLAGRQMRQWRARCGMIFQDFCLVPRLDVMTNVLLGRLSHT
STLKSFFKLFDDADRARAIELLQWLNMLPHALQRAEHLSGGQMQRVAICRALMQNPQILLADEPVASL
DPKNTRRIMDALQKISEDGIAVMVNLHSVELVKEYCSRVIGIAHGKIVFDGHPSQLNERILHQLYGEE
ANQIH
>CORE_REP|Org6_Gene4274#
MFRKKCRRAEERPDGKERATHPVSAGPWALPLALVIIWQIAVETGWLSNRILPAPSAVLAAFWRLSQS
GELWQHLSISSQRALIGFGIGGSIGLILGFITGLSRWGERLLDSSVQMIRNVPHLALIPLVILWFGID
ESAKIFLVALGTLFPIYLNTYHGIKNIDRGLLEMARSYGLSGFRLFTQVVLPGALPSIMVGVRFALGF
MWLTLIVAETISANSGIGYLAMNAREFLQTDVVVVAIVLYALLGKLADVGAQLLERVWLRWHPAYQLK
QGEAL
>CORE_REP|Org45_Gene1177#
MSAHHHDAAPSENELERLLKLAVTEPAHRPAFFRELLDATVLILGDSEQVQQDGDIALNADTPVNIQH
WEKQDGGSIIPFFTSLEALQKAVEDEQPFIAMPARVLFEITQGADLFLNPKAEYGKEFYPEEVAMLLA
TGGVVKPVEHYVDKETQILLGQPEEYPSAMVDALTTLFSQRKPVRRAFLALMHDQAADEKPNLLIGLE
VDAEPAEIEALINEAGSVASETAPNDEPVDFCLVSENERGISHYLISHTQPFYQRRWGSWLRNLIPST
DKTQ
>CORE_REP|Org49_Gene4138#
MSILTAPDGARIFYKDWGRGQPILFSHGWPLSADAWDNQLLFFGQSGFRVIAHDRRGHGRSDQTWHGN
DINQYADDLALLIETLDLRDLILVGHAIGGGEVARYIGRHGSDRVAKVVLVGAVPPLMLQTPVNPEGA
PMAAFDGIRSGVAGNRSQFFKDLAVPFYGLHCDEVESNAGLIDHFWLMAMQGGMKGQYDGVSAFSEVD
FTADLLAIDRPTLLVHGDADRIVPISASALKAAKLMQQAELNVYADGAHGLAQVDPDRFNADLLAFIQ
NSPF
>CORE_REP|Org20_Gene2744#
MGYVTTSDGVEIFYKDWGPKNGKVIYFHHGWPLSSDDWDAQMLFFVNKGFRVVAHDRRGHGRSSQVSE
GHDMDHYADDVAAVVKHLGVQGAMHVGHSTGGGEVVRYIVRHGEDKVSKAVLISAVPPLMVKTDANPQ
GTPKSVFDDFQAQLAANRAQFYYDVPAGPFYGYNRPGAKPSEAIIWNWWRQGMMGGAKAHYDGIVAFS
QTDFTEDLKKIAIPVLVIHGDDDQVVPYQDSGVLSAKLVQNGTLNTYKGAPHGIPTTHADQVNADLLA
FVNS
>CORE_REP|Org23_Gene2132#
MDTELLKTFLEVSRTRHFGRAAESLYLTQSAVSFRIRQLENQLGANLFTRHRNNIRLTPAGERLLPYA
ESLMNTWQLAKKEVVRSLQHTELSIGATASLWEAYLTPWLQALYQQREALQLEARVALRHSLVKQLHE
RQLDLLITTEPPKMDELASQQLSNFSLRLFSSAYRDKQAPMPYIKLEWGADFHQQESRMLEGDNVPVL
TTTSAHLTRQLLETTGGCAFLPSQWEKEYPQLVATTEIPPIIRPLYAVWLQNSDQQPLIRQLLKIPLN
TAA
>CORE_REP|Org44_Gene4808#
MQGVPQQFPFEKDCAQFRHLSHLPGVELYQAHIERYAFEPHTHDAFAIGTVDTGAERFRYRGAQHLAA
PGALVLMNPDELHTGEAETPGGWCYRMLYLAPEALEQLSGARSQWFTDAVRHDPRAAQRLSAILATLW
QTDDPLTLDGLLLEAVELLYPHIRTGQREKAEAAHRFEVVKSYLHDNFAEAVTLNQLAELVSLSPYHF
LRKFKAEYHVSPQQMLMAIRLSQAKRMLERGMPAAQVAAAAGLTDQAHLTRAFANRYGVTPVRFQKQV
KLG
>CORE_REP|Org48_Gene1124#
MSEAIDYYALPEQPVRNLTPVPARGLIEISNVSKFFGKHKALDDVSLTLQPGTVTVILGPSGSGKSTL
LRAINHLERVDEGFIRIDGDYVGYRRKGNRLYELKEKAILRQRINVGYVFQNFNLFPHLTVLENIIEA
PVVHKIHSRERAKAVAYELLDTVGLRHKADAYPRHLSGGQQQRIAIARALALNPKVILFDEPTSALDP
ELVGEVLDVIKGLADLGVTLVVVTHEIGFAREAADRVVFMVDGQIVEQGDARQVLSQPQHPRTVNFLN
KVL
>CORE_REP|Org23_Gene4045#
MSEKTPQFWRDPQLPFVEARAIADGRQACYSLHSHEFFSIGAITGGVSTYVNGERRMQVSAGDLVVIN
PQQAHACNPIADRRWSYIMFYLDLAWIGALQQELLGGEGERFVPFSRPLSRDPALFHGLNRLYALLTD
PLCSALEKQIAMVEYFSALQLGLGSGRQPETPPHARLEAAAAFIDAHCTRPLTLDDICRAAALSPSYL
IRAFRQRYGMTPHAYLVNRRVQHGHRLLKSGLPIAAAASESGFADQAHFQRTFKQLLAATPGQYQKPS
ANR

>CORE_REP|Org35_Gene2257#
MKTEHVMTLEQAQGGFAPAAAPVAASAIITDEQGIHAGQTTIPSQGDALPAYVAKPADHNGPFPIVLV
VQEIFGVHEHIQDLCRRLAKQGYLAIAPELYFRQGDANDYTDIGELFQQLVTKVPDRQVLSDLDHAAH
WAIRHGGDAGKLAITGFCWGGRITWLYAAHNPQLKAAVAWYGKLVGEKTLNSPKHPVDIATHLSTPVL
GLYGGQDSGIPLDTVENMRQAIRAANADAEIVVYPGVGHAFNADYRPSYNAEAAEDGWQRMLAWFAQH
GVK
>CORE_REP|Org36_Gene4626#
MESLFKLPVASGAAVRILQITDTHLFAGEHETLLGINTYRSYHAVLDAIQAQRRDVDLIVATGDLAQD
HSQEAYRHFAAGIAQLPAPCVWLPGNHDFQPAMVDALAAAGIAPSKQVLLGDRWQVLMLDSQVFGVPH
GELSEYQLEWMERCLQAHPERYTLLLLHHHPLPSGCTWLDQHSLRNPHMLGAILLRYPKVNTVVCGHI
HQDLDLEWQGRRLLATPSTCVQFKPHCTNFTIDDVSPGWRYLDLLPDGRVETQVFRLENDDFRPDMDS
DGY
>CORE_REP|Org14_Gene303#
MQFSKMHGLGNDFMVVDAVTQNVYFSPELIRRLADRHLGVGFDQMLVVEPPYDPELDFHYRIFNADGS
EVAQCGNGARCFARFVRLKGLTNKRDIRVSTQTGRMVLSVTDDDLVCVNMGEPNFDPQAVPFRAAKAE
KTYIMRAAEHTVLCGVVSMGNPHCVLQVDDVKTAKVELLGPVLEGHERFPERANIGFMQVVSRDHIKL
RVYERGAGETQACGSGACAAVAVGIQQELLSEEVHVELPGGSLHIRWKGPGNPLFMTGPATHVYDGFI
HL
>CORE_REP|Org14_Gene2931#
MKKVAIIGLGWLGMPLALSLMGRGYDVVGSKTTPDGVEAARMSGIECYQLELTPELECDPDDLESLLR
VDALVVTLPARRTVEGSENYFNAVRMLVDSAMAFGVPRVIFTSSTSVYGETAGTLREESPLRPVSPSG
RVLAELERWLHELPNTSVDILRLAGLVGADRHPGRFLAGKLDVKGGSQGVNLVHQDDVIAAIQLLLKL
PKGGHVYNLCAPRHPAKREFYPALAEQLHLEPPQFADEAEQDERLVDGNRICNELGFEYQYPDPARMP
VS
>CORE_REP|Org13_Gene2377#
MTTQQPIIKLHDGNLMPQLGLGVWQASIEETTRAVSKALEIGYRSIDTAAIYKNEEGVGAALQSSSLP
RSELFITTKLWNDDQGDPLAALETSLEKLRLDYVDLYLIHWPRPQQDQYVSAWRELIKLRDQGLVKSI
GVCNFHTPHLQRLLDETNVAPVVNQIELHPLLQQRQLRAWNATHHIATESWSPLAQGGEGVFDQPLIK
ALAEKYEKTPAQIVVRWHLDSGLIVIPKSVTPSRIRENFEVFDFKLDKDELGEIAKLDVGNRLGPDPD
TL
>CORE_REP|Org8_Gene2840#
MYRLAAFDMDGTLLMPDHRVGPETLAVLNQLVEREMVVTFATGRHYLDAQPIMAQLGLQGYLITGNGT
RVYDNRGQQLHATDLPAEIAEEVLHHHWRTRASMHVFRDEGWLTEFPVPEEMLRAHHLSGFRFQLADV
RRLPAFGNSKVCFVAPHEELLALQVQLRAQLGDEADLCFSAYDCLEVLPLGCNKGTALERLSRHLGLT
MADCMAFGDAMNDKEMLGAVGHGVVMGNALPQLKSLLPQLPVIGHCQQQAVAHYLQHWLRSPCLTYSP
EE
>CORE_REP|Org15_Gene101#
MAIVKCKPTSPGRRHVVKVVNPELHKGKPYAPLLEKLSKSGGRNNNGRITTRHIGGGHKQHYRLVDFK
RNKDGIPAVVERLEYDPNRSANIALVLYKDGERRYILAPKGLKAGDQIQSGVDAAIKAGNTLPMRNIP
VGSTVHNVEMKPGKGGQMARSAGAYVQIVARDGSYVTLRLRSGEMRKVPADCRATLGEVGNAEHMLRV
LGKAGAARWRGVRPTVRGTAMNPVDHPHGGGEGRNFGKHPVTPWGVQTKGKKTRSNKRTDKFIVRRRS
KK
>CORE_REP|Org38_Gene206#
MQQLQNVIESAFERRADITPANVDTVTREAVNQVIGLLDSGALRVAEKIDGQWVTHQWLKKAVLLSFR
INDNKVMDGAETRYYDKVPMKFADYDEARFQKEGFRVVPPATVRQGAFIARNTVLMPSYVNIGAYVDE
GTMVDTWATVGSCAQIGKNVHLSGGVGIGGVLEPLQANPTIIEDNCFIGARSEVVEGVIVEEGSVISM
GVYLGQSTRIYDRETGEIHYGRVPAGSVVVSGNLPSKDGSYSLYCAVIVKKVDAKTRGKVGINELLRT
ID
>CORE_REP|Org31_Gene2716#
MTDMHSLFIAFVLGVVEGLTEFLPVSSTGHMIIVGEWLGFTGDKAKTFEVIIQLGSILAVVVMFWRRL
FGLIGIHFGGKPVEHEGKTHGRLKLGHILLGMIPAVVLGLIFHDVIKSLFAPKNVMYALVVGGLLLLT
AEWLKPKKPRAEGLDDITYRQAFLIGCFQCLALWPGFSRSGATIAGGMLVGVNRYAASEFSFILAVPM
MIGASGLDLYKSLHFLTWGDLPMFAVGFVTAFVVALIAIKTFLSLIKRISFVPFAIYRFIVAAVVYMV
FL

>CORE_REP|Org34_Gene3451#
MSYRVIALDLDGTLLDNQKRILPQSLEALALAQARAAGVQVVVVTGRHHVAIHPFYQALQIDTPAICC
NGTYLYDFQQKKVLAADPLAKDQAKQVLQMLKQTDIHGLMYVDDAMLYQEPSGHVTRSLAWAETLPAA
QRPTLLQVGSLAQAADDAQAIWKFATSHADIPALREFADTVEKELGLACEWSWHDQVDIAKGGNSKGK
RLRQWVESQGLNMDQVVAFGDNYNDISMLEAVGLGVAMGNADDAIKERADLVIADNLQPGIAEVIRTR
VL
>CORE_REP|Org34_Gene2028#
MSSEELEQVWNSIKSEARALADCEPMLASFFHATLLKHENLGSALSYMLANKLATPIMPAIAVREVVE
EAYKSDNQMIVSAARDILAVRLRDPAVDKYSTPLLYLKGFHALQAYRIGHWLWQQGRQALAIYLQNQI
SVAFGVDIHPAATIGCGIMLDHATGIVIGETAVVENNVSILQSVTLGGTGKTSGDRHPKIREGVMIGA
GAKILGNIEVGKGAKIGAGSVVLQAVPPHTTAAGVPARIVGRPESDTPSMDMDQYFNGTNHGFEYGDG
I
>CORE_REP|Org12_Gene2905#
MKVQIPRRNLDIDQVPRPLFAVQSSTIEQDWEVEPHRHQKAQLIYTVRGMIRCEVENGLWLVPPQCAL
WMPGNVLHNAQGAGSTEAYCLFVDQHVVAGLPQSCCTLSVSPLLRELLLQATTFEPLYDEQGAEGRLT
AVLLDQLVAAPIENLHLPVSDDARIRQLTEGMLSCPADKSTLGQWAQRIGMSERSLSRTLQQQMGMSF
GHWRRQLHVMLALQRLTQGESVQTVALDLGYESASGFVTMFRKAVGKPPARYLAERNASGQPLGGAIS
M
>CORE_REP|Org31_Gene1399#
MQYWGKLLGVIVAIWSGAGFWGVVLGLIIGHMIDTARSNKRSRGFFTDQQTRQTLFFRTTFQVMGHLT
KSKGRVTEADIQIASLFMDRLQLHGEARTAAQQAFREGKQSQFPLRETLQQFRSICFGRFDLIRMFLE
IQIQAAFADGSLHPNERQVLYVIAEELGISHAQFDQFLSMMEGGRQFGGGQQGGYSQGGYQQAQRGPT
LDDACKVLGVRSSDDAATIKRAYRKLMSEHHPDKLVAKGLPPQMMEMAKQKAQEIQAAYDLIKREKGF
K
>CORE_REP|Org47_Gene1324#
MSSLLAKCQTPNAEGRIQHVTPENAGWRYVGFDVYRLAAGQSLQLECGDKELCLVLVAGIASVATLRA
EYPHIGKRMSPFERTPPYAVYVPHHDRIDVRAETDLELAVCSAPGSGHLPSRLITPADIGVERRGKGR
NQRLVHNILPDSEPADSLLVVEVYTDEGNTSSYPSHKHDREDSPDETYLEETYYHRIQPEQGFCMQRV
YTDDRTLDECMPVYNRDVVKVPRGYHPVATLAGYDNYYLNVMAGPVRQWKFTWEKDHAWINGDGYPAA
Q
>CORE_REP|Org10_Gene2985#
MFNQEDAFTCADVVRTRLPGFQPKAALILGSGLGALAEVMTETVTLDYAELPGFPVSGVAGHAGQLVA
GKLEGVPVLCMKGRGHFYEGRGMQVMTTAVRTFKLLGCEFLLATNAAGSLRRSVAPGSLVAITDHINF
MPESPLVGANDERFGPRFFSLANAYDRDLRAQLAAVAESAGIPLAEGVFAAYTGPNFETPAEIRMMQT
LGCDVVGMSIVPEVLAARHCGLKVLVVSAMTNYAEGLSDTPLSHEQTLSCAALAADDFMRLIRELFKT
L
>CORE_REP|Org22_Gene1610#
MSDSQIRIAIAGAGGRMGRQLIQAVQQAEGVVLGAALSRPGSSLVGVDAGELAGIGALGVKVSDSLEK
VANEFDILIDFTRPESTRGYLDFCVAHHKAMVIGTTGFDDAGKQAIRDAAQHIGIVFAANFSVGVNLV
LKLLEKAAQVMGNYTDIEIVEAHHRHKVDAPSGTALAMGEAIAGALGRDLKSCAVYAREGHTGERDPK
SIGFATIRAGDIVGEHTAMFADIGERVEITHKASSRMTFASGAVRAAAWLHNRDKGLFDMRDVLNLDQ
L
>CORE_REP|Org22_Gene3649#
MWGVLAASLFFLPFNRLIAWVILAASAGMGLYHGVLTPLSLSYLLAIVALAGLRHHFREQRNLAIAFE
GLVVAGCIALFLHLVPGIHNQLMIDGDKAGPLSAPFTMYYNFDKAMVPFLLFACLPTLFRTDKAEKSV
ASGSWIALIISVPALLLLAVALGGLKIELHAPAWILPFVMANLFFVCMAEEALFRGYLQQRLSQWLGA
WPALIVAALVFGAAHLAGGMLMVIFATLAGVIYGLAWMWSGRLWVPILFHFGLNLIHLLFFTYPLYQH
P
>CORE_REP|Org23_Gene4353#
MKSLASTLQGQSIAAAITAVENDIKAKPADADLRAALVQLLCLSGNWTRANAQLKSWQALKPIAQPTT
LLLMQSVNAELQRQAVFAGAAAPALLRQDQPWLQLLVQALHQDAQGAAEQAQTLRDEALEAAPAGAGQ
LTLAEGNQERQLSFDWLTDGDGRLGPVCELALNGVYYWLPFADIAAIQFQAPQSAIDLVWSHALVRLT
DGREQVCQLPARYPLAEGSDDALLLGKRTEWQPLGDGTHYAGLGLKTWLSESDEFPLHSLRQLSFDAS
A

>CORE_REP|Org26_Gene2222#
MSYQCPLCHQPLHFSSQRWRCDGNHQFDQAKEGYVNLLPVQHKRSKQPGDSAEMMQARRAFLDGGFYQ
PLQQQVAEWLDLALAADAGALLDIGCGEGYYTAAVAARLAQARNMAVYGLDVAKVAIRYAAKRYPAVS
FCVASSHRLPFADAALDAVLRIYAPCKAAELARVVKPGGVVVTVSPGPRHLYQLKEQVYQQAQLHAEQ
DEQFDGFECERKEALAYTMALPGAQAANLLQMTPFAWRATPEVQLRLAHGGEFECETDFVLALYRRRA
>CORE_REP|Org9_Gene1383#
MNNRVHQGHFARKRFGQNFLTDQFVIDSIVSAIHPQPGEAVVEIGPGLGALTEPVGARMDRMTVIELD
RDLATRLENHPRLKDKLTIHQQDAMTVNFAELAEEAGQPLRVFGNLPYNISTPLMFHLFSYTQAIRDM
HFMLQKEVVNRLVAGPNSKAYGRLTVMAQYYCNVIPVLEVPPTAFAPPPKVDSAVVRLVPHSVLPNPV
GDVRMLSRITTQAFNQRRKTIRNSLGDLFTPEQLTELGVDPSLRAENISVAQYCKLANWLSANPAPQQ
>CORE_REP|Org48_Gene2755#
MKKHLLMLAFASVATLASYGAAAATKLVVGASNVPHAEILEQAKPILAKEGIDLQIKRFQDYILPNTA
LASHDIDANYFQHVPYLNSVLKDHADDKSYDFVSAGAIHIEPIGIYSKKYKSLKDLPENGKIIMRDAV
AEEGRILSIFEQQGVIKLKPGVSKVDARITDVVENPKHLKFQANVEGALLPQMYNNNEGDAVVINANY
AIDAGLNPTKDPIAVESGENNPYANIITVHKADVNKPEIVALVKVLHSKPIQDFIREKYQGAVIPVNQ
>CORE_REP|Org8_Gene4403#
MEKFAVFGNPIGHSKSPRIHALFAAQTDIEHPYGTVLAPLDGFEISLQEFIRAGGQGANVTVPFKERA
YSAASELSERAAMAGAVNTLKVLPNGGLLGDNTDGIGLLTDLQRQQLIRPQDRILLVGAGGAARGVIL
PLLSFGCRLTITNRTFSRAQELADAFRHLGEISAVPMDQLGQQAFDLVINATASGISGEIPALPTGVV
NAQTRCYDMFYQQGVTPFLAWAQQQGVTEYADGLGMLVGQAAHAFLLWHGVMPEIEPVLRQLRCELAA
>CORE_REP|Org10_Gene1819#
MSLTFKSIATIGALIGTLALAGCGQDEKNPNHIKVGVIVGAEQQVAEVAQKVAKEKYGLDVELVTFND
YVLPNEALSKGDIDLNAFQHKPYLDQQIKDRGYKLVPVGSTFVYPIAGYSKKIKSLDELKEGSQIALP
NDPTNLGRSLLLLQKVGLIKLKDGVGLLPTVLDVTENPKNLKLVELEAPQLPRSLDDQQIALAVINTT
YASQIGLTPAKDGLFVEDKDSPYVNLLVAREDNKDAENVKKFVQAYQSDEVDAAANKIFNGGAVKGW
>CORE_REP|Org20_Gene834#
MLQKADNLVEVRDMSFSRGDRRIFEDINLTVPRGKVTAIMGPSGIGKTTLLRLIGGQLAPDSGEIWFD
GDNIPALSRRQLYDARKKMSMLFQSGALFTDLTVFENVAYPLREHSNLPEPLLRSTVLMKLEAVGLRG
AAQLMPNELSGGMARRAALARAIALDPEMIMFDEPFVGQDPITMGVLVKLIDELNHALGITCIVVSHD
VPEVLSIADYAYIVADHRVIAEGTTQQLQNNPDARVRQFLDGIADGPVPFRYPAGDYQTELLGLGSK
>CORE_REP|Org40_Gene2083#
MAIELIAIDMDGTLLDPQHQITPAVKQAIAAARRKGVHVVLATGRPYVGVQDYLRQLDIQGPGDFCIT
YNGALVLRAVDGACILQETLGFEDYLHFEQMAREFGVHYQAFDFDTLYTPNKDIGKYTIHEAEMTGIP
LKYRSVEEMDRQMRFPKVMMIDEPELLDRAIARIPAETRERYTILKSAPYFLEILHKNVDKGAGVRML
AEHLGVARENIMTLGDQANDTAMIEYAGVGVAMGNAIPELKAVAQFVTSANTEDGVARAIEKFVLNA
>CORE_REP|Org34_Gene2792#
MGVGAPPFQPTKKEHPLNFRWEIIQEYAPLFMEGAWMTIKCTIICVLLGTTWGLILGLGRLAQAPHGI
WKPILHYGVQWPVRIYISAFRGTPLFVQIMVVHFALVPLFINPRDGLLVTSGLMSVDFARALRADYGA
FLSCVVAITLNAGAYVSEIFRAGIQSIDRGQMEASRSLGMSYGKTMRQVILPQAFRRMLPPLGNNAIA
IVKDSSLASAIGLADLAYAARTVSGAYATYWEPYLTISLVYWVITFLLSLLVQHMEKRFGKSDSRT
>CORE_REP|Org9_Gene117#
MKKNRAFLKWAGGKYPLVDEIRRHLPAGDCLIEPFVGAGSVFLNTDYDAYILADINSDLINLYNIVKL
RTDDFVRDARTLFADEFNNSDQFYLLREEFNTSTEPYRRALLFLYLNRHCYNGLCRYNLRGEFNVPFG
RYKKPYFPEEELYWFAEKSRNATFVCEHYRDTMAKAVAGAVVYCDPPYAPLSATANFTAYHTNSFSIA
DQQSLAHLAHQLSVESQVPVLISNHDTELTRDWYQHAALYVVKARRTISRNILGRSKVNELLALYR
>CORE_REP|Org27_Gene597#
MARIIVVTSGKGGVGKTTSSAAIATGLAQKGKKTVVIDFDIGLRNLDLIMGCERRVVYDFVNVIQGDA
TLNQALIKDKRTENLYILPASQTRDKDALTREGVEKILNDLGEMDFDFVVCDSPAGIETGALMALYFA
DEAIITTNPEVSSVRDSDRILGILSSKSRRAEKGESPIKEHLLLTRYNPGRVSRGDMLSMEDVLEILR
IPLVGVIPEDQSVLRASNQGEPVILDAESDAGKAYDDTVCRLLGEERPFRFIEEEKKGFLKRLFGG
>CORE_REP|Org40_Gene798#
MGLMTPGSLPRLDVQHLDDEQTALAVNGLNLFYGDKQVLHDISLRIPKHRVTALIGPSGCGKSTLLRC
FNRMNDLVDNCRIEGDLQLNGAAISGAQIDVAALRRRVGMVFQRPNPFPKSIYENVVYGLRLQGVRDR
RLLDEAVERSLRAAALWHEVKDRLRENAFRLSSGQQQRLVIARAIAIEPEVLLLDEPTSALDPISTLT

IEELISALKQRYSVVLVTHNMQQAARVSDYTAFIHQGRLVEYNDTDAIFTSPRQRRTEDYITGRYG
>CORE_REP|Org43_Gene1620#
MSSIADFEFNTSPLSEGVILVSQSVRRDFPAADVERKLQQLVDEARAAVPADITQEQQLDALIELFYK
TWGFGGASGVYRLSDAIWLDKVLEGRQGTPVSLGTIFLHIANALDLPLLPVIFPTQLILRADWLDEEL
WLINPLNGETLSEHQLEVWIKGNLGLGAELEDDDLDESENIMVVRKMLDTLKAALMEEKQLEMALRTS
ETVLCFDPEDPYEIRDRGLIYAQLECNHIAISDLNYFVEQCPEDPVSEVIKVQIHSIEPKQVTLH
>CORE_REP|Org22_Gene1799#
MKTILVLLDGLNYRVAHDAMGYLQAECAAGRGRLYLLESELPSLSRPLYECILTGVTPVESGVVHNHV
SRLSHQQSVFHYARAAGLTTAAAAYHWFSELYNRTPFDAARDRHTDAPELPIQHGHFYYDDGYPDSHL
FDDAESLRLRHQPDFLLVHPMNIDDAGHRFGLSSPQYRNAARRADGSLSRYLPEWLAAGYQVLVTADH
GMNDDRSHGGVLPEERQVPLFVFGTGFSLDDADPQQTELCGTICDLLQAPHDKPRCRALLAQDAR
>CORE_REP|Org6_Gene3800#
MYHVVASDLDGTLLSPDHTLSPYAKETLKLLTQRGVHFVFATGRHHIDVAQIRDSLEISAFMITSNGA
RVHNTAGELIFSHNLDGDIARDLYGMLHDDPDITTNVYRNDDWFTNRESPEQEEFFQESVFKYQLFEP
GLLETDGVCKVYFTCEDHERLLQVEDAINARWGDRVNVSFSFPTCLEVMAGGVSKGHALEEVAKIIGY
TLQECIAFGDGMNDLEMLSMAGKGCIMRDAHQRLKDMLPELEVIGSNVDNAVPHYLRKMFLWNNH
>CORE_REP|Org15_Gene3666#
MKQIKAVILDWAGTTVDFGSFAPTQIFVEAFKQTFDIDISLAEARIPMGLGKWQHIEALGKLPAVDAR
WRQKLGRSMSHQDIDALYQAFMPLQIAKVIDFADPIEGVPQAIAALREQGIKIGSCSGYPRAVMEVLV
PAAAQRGYAPDYWVATDDLAAGGRPGPWMALQNVIALGIDAVAHCVKVDDAVPGIAEGLNAGMWSIGL
ALSGNEFGATWQEYRQMAAGEIEQRRTAAADKLYAAGAHYVIDTLAQLLGVIADINRRLANGERP
>CORE_REP|Org29_Gene4358#
MSAEQSVLNQNAEPDWKNYDDFARGIDTNRLPGTQDWRGKTLQIAFDNDGEITLRFSADRQRVLWAWG
GESGEDAYEEVQTSAARYFFNIPLQSPGNECLTLVLNRDSGRALLVRSTLLPEQTVEKGSRLKQSFHV
GRIVGVTPSGAAPHLTRELIGYRTLNVYSPNHYYEHFYVNTERYAWQNLRGEQFGHGDMDYATYYKFE
DEMFLFTFREKIIPVCSVFFFDFISGRCTGTFLGLDAAGRVLVSPAGAFISKMSYNAYPEGVQPL
>CORE_REP|Org23_Gene1532#
MERYQQLFTRLESSKEGAFVPFVTLGDPNPTLSLQIIDTLIEAGADALELGIPFSDPLADGPTIQSAT
LRAFAAGVTPTQCFEMLAAIRQKHPTIPIGLLMYANLVFHKGIDAFYQRCAEVGVDSVLVADVPFEES
APFRAAAIRHGIAPIFICPPNADDDLLREIASHGRGYTYLLSRAGVTGTESRAQLPLHHLVNKLREYH
AAPPLQGFGISEPEQVKAALQAGAAGAISGSAIVKIIEQHHANPAEMLTKLAAFVSNMKRATRA
>CORE_REP|Org24_Gene3723#
MPEQTVSFIKGRHGDIAVHDWGNDQPRYLALLVHGYGEHLGRYQYVARTLQAQGARVFGPDHLGHGLS
QGERVLIEDYDAVVDDVQRVVSHFRQRYPALPLVVIGHSMGGMIATRYVQRYGEEVRALVLSGPLLGD
RTAISDLAELPTIPDAPLDTATLARDPAVGAAYQEDPLVWHGPFKRPTLRAMQRILAAINAGPGFGAL
PTLWIHGDDDRLVLMSETQTALDRLRGDDFERMINAGGRHESFNETNKDRILKRVTDFIARALG
>CORE_REP|Org27_Gene1013#
MDWFVIGPEMLGVLFAVALLAGFIDSIAGGGGLLTVPALLAVGVPPAQALATNKLQSVGGSFSASLYF
IRRRAVNLNDQKLTIFLTLIGSIAGAILVQHMRADLLRQMLPLLVIGIGLYFLLMPRLGEEDRQRRLG
ALPFGLVAGGCVGFYDGFFGPGAGSFYALAYVTLCGFNLAKSTAHAKVLNFTSNVGGLALFIIGGKVV
WSIGLVMLVGQVLGARLGAHMVLTRGQKLIRPMIVIVSLVMSLKLLYDNHGAEIQQWLSALVHG
>CORE_REP|Org25_Gene785#
MHSERAPLGLKLAAWGGLVFLHFPLAIIAIYAFNTEDAAFSFPPKGFTLHWFNVAAGRQDIIDAVLLS
AQIACLATAIALVLGTLAAAALYRRDFFGKDSISLLLLLPIALPGIVTGLALLAAFKALNIEPGILTI
VVGHATFCVVIVFNNVIARFRRTSYSLIEASMDLGADGWQTFRYVILPNLGSALLAGGMLAFALSFDE
IIVTTFTAGHERTLPLWLLNQLGRPRDVPITNVVALSVMLLTMLPILGAYYLTKGGESVAGSGK
>CORE_REP|Org32_Gene4272#
MKFISFNINGLRARPHQLAAIIEQHQPDVIGLQETKVHDDMFPLEDVSQHGYHVFYHGQKGHYGVALL
TKAEPLAVRRGFPTDEEDAQRRIIMADLATPQGTLTVINGYFPQGESRDHPIKFPAKTRFYQDLQNYL
EQQLSAESPVLIMGDMNISPSDYDIGIGEDNRKRWLRTGKCSFLPEEREWMDRLLNWGLVDTYRHANP
GRSDEFSWFDYRSKGFDDNRGLRIDLLLASTPLASRCIATGIDYQTRGMEKPSDHAPVWAEFTL
>CORE_REP|Org21_Gene1917#
MGQLLRRLAAFLGLISPKRYAYPALDITLPGDRRLHLVGSIHMGTVDMSPLPSRLAARLQQADALIVE
ADITDSASPFDHAELQPALEQRLSAEEYQRLLALCHELGADPEAFVTLPGWQVALMMQARQAQRLGLR

AEYGVDYQLLQAARAQDKPVIELEGAQQQLAMLEQLPEGGIALLRDTLEHWHTNARLLQTMVSWWLDA
KPRGTLDTLPATFSAGLYDVLMHQRNRDWRRQLEALPAGDYVVAVGALHLYGEDNLPAMLQPQG
>CORE_REP|Org45_Gene4078#
MKAMQVTDELFTHPLPADKYRQQTDPVLQLDSVNVSFDGFRALTDLSLRIGVGELRCVIGPNGAGKTT
LMDVITGKTRPDSGRVFYDQTVDLTRLAPMQIAHAGIGRKFQKPTVFEALTVFENLEIAQKTRKSVWA
CLRARLSSEQRDRIDEMLKTLRLGHERHRPAGLLSHGQKQFLEIGMLLVQEPHLLLLDEPAAGMTDAE
TDYTAELFRELAGKHSLMVVEHDMGFVETIADRVTVLHQGQVLAEGSLAQVQADERVIEVYLGR
>CORE_REP|Org17_Gene3025#
MRLRFTSALWLLAFWLALPAGAAQRVISLAPNATELAYAAGMGEVLVAASAYSDYPPQAARLEQVASW
QGINLERVLALKPDLILAWRGGNPQRVLDQLAAFGIPIFYADADNLEGIAGLLDKLAQYSPHPDQAHQ
AAAGLRRQFAALRQQYADNPPRRVLLQFGTQPLFTSSGATLQSQVLALCGAQNVFADSRTPWPQISRE
QVLARQPQAIVITGGAKEAANVKAFWAPQLQVPVIALNEDWFNRGGPRLLLAAQQLCRQLAEIR
>CORE_REP|Org6_Gene848#
MSHRLHASHLKLGYDNKIIADDLSVAIPDGAFTVIVGPNACGKSTLLRALCRLLKPSAGEVMLDGKNI
SSFATKALARELGLLPQTSIAPDSITVADLVSRGRYPHQSLLKQWTQADKQAVEAAMAATNVSQLADR
SVDELSGGQRQRVWVAMALAQQTPLLLLDEPTTYLDIAHQIELLDLFRQLNRERGQTLIAVLHDLNHA
CRYADHIIAMRDGKIVAEGKPAEIITAELVERVFGMPCMIIDDPLSHTPLVIPRGRYHCDAPQA
>CORE_REP|Org48_Gene4298#
MLHCAADAYQNRKDDWGQDLSTGSKLARWRRIAVCLTLGLLLAGCSGKNTYNRDYDKLPKGSYTGKSY
TVKRGDTLYYIAWITDSEVSDLARINKIRPPYSLEVGQKLRLSGSAPTKTAATRRKTSSSAIAKQTPP
PGAARCWRWPTSGRIVQAYSNADGGNKGIDIGGKRGQPIYASAKGKVVYVGNQLRGYGNLIMIKHGED
FITAYAHNDTTLVRNGQDVKAGQKIGTMGSTGTDSVFLHFQIRYRATALDPQRYLPPQGSSPSC
>CORE_REP|Org22_Gene1393#
MSIPAFGLGTFRLQDQVVIDSVSTALELGYRAIDTAQIYENEAAVGQAIAASGVPRDELFITTKIWIA
NLAKGVLIPSLRDSLVKLQTSYVDLTLIHWPSPNDEVPVAEFMAELLEAKRLGLTRQIGVSNFTVDLM
QQAIDAVGADQIATNQIELSPFLQNEKVVAFARQHGIAITSYMTLAYGKALQEETIKRIAARHNATPA
QVVLAWALKLGYAVIPSSTKRENLESNLLAQQLLQLSDEDMAQIAALESNGRLVSPEGLAPDWD
>CORE_REP|Org8_Gene1598#
MQQTTTTATDRPEPHRQQREITRLCIQCALLLLQHGAESTVVEQLSTRLGLALGMDSVESSISANAVV
LTTLSHGACLTTTRKNVDRGINMQVVTEVQHIVILAEHRLADAHDVARRFERIRPLRYPRWLVVLMVG
LSCGCFSMLNGGGGDAFLVTFIASGAAMLVRQILTARQMNPLINFCLTAFVATSISGLLLRLPAFKDT
SSVAMAASVLLLVPGFPLINAVADMFKGHVNTGLARWAMASLLTLATCIGVVMAMSLWDLRGWS
>CORE_REP|Org15_Gene1754#
MHPMLTIAVRAARKAGNLIAKNYETPDAVEASQKGTNDFVTNVDRDAEHLIIDVIRKSYPQHSIVSEE
RGELIGEDRDVQWVIDPLDGTANFIKRFPHFSVSIAVRIKGRTEVAVVYDPMRNELFTATRGQGAQLN
GYRLRGTNAKDLDGTILATGFPFKVKQHATPYINIVGKLFTQCADFRRTGSAALDLAYVAAGRVDGFF
EIGLKPWDFAAGELLVRESGGLVTDFVGGHNHFSSGNVVAGNPRVVKAMLATMREELSEALKR
>CORE_REP|Org1_Gene1923#
MLIWSRTGRTLTCTLTITLFALFFCLPLAVILMSSLSEQWNGVLPSGFTLNHFRQAFSGASWDALVAS
LAIGFSASLFALLCGTWAALALRHVPERGRQLLGTLFFIPSAVPSVSIGLGMLVAFSQGPLQMNGTFL
IVPAAHFVLISAFTFGNVMAGLTRLPGDYENVAASLGASPLFRLRHVTLPMIAPYMISAFALSLSLSM
GELGATMMIYPPSWATLPVTIFSLTDRGSIANGATLTMILVAATLLLMLVLERIAQRLTPGAK
>CORE_REP|Org16_Gene971#
MSSEALSGPKALGAFLRAHRERITPEMIGLPSSSRRRTSGLRREELAQISGISATWYTWIEQGREVSI
SPYTLARIAKALRLGPAERHYLFTLARIADPEQEAHRETANDAVLQSVHQMTVPCYLLDVTWNVVAWN
PQAAALFRGWLDVANSPNLLHFMFFHPLAKTLVSDWEERARRVVAEFRAETSHHQNTEEMRAFVRNMT
HNSADFNHWWKQHDVMAREGGERAFEHPQQGALRYRQLTFHPAEHAGLKLVMLIPLPQLVTNS
>CORE_REP|Org25_Gene835#
MCELLGMSANVPTDICFSFTGLVQRGGRTGPHKDGWGITFYEGNGCRTFKDPQPSFNSPIARLVQDYP
IKSCAVVSHIRQANRGEVALENTHPFTRELWGRNWTYAHNGQLKGYRQLDTGTFRPVGQTDSEYAFCW
LLHQLALKYPRTPSQWPAVFRYIGLLASQLRKKGVFNMLLSDGRFVMAYCSTNLYWITRRAPFGKATL
LDQDVEIDFQQQTTPNDVVTVIATQPLTANETWHKIEPGEFALFHFGERLVLSEGIGVGRRAG
>CORE_REP|Org37_Gene3756#
MIELLLPGWLAGVLLAGAAGPLGSFVVWRRMSYFGDTLAHASLLGVAFGLLLDINPFYAVIAITLLLA

LALVWLERRPQLSVDTLLGILAHSALSLGLVVVALMSNVRVDLMAYLFGDLLSVTLSDILMIAGGVAV
VLLVLWWQWRDLLSMTISPELAHVDGVNLVRARTVLMLVTALTIGLAMKFVGALIITSLLIIPAATAR
RFARTPEQMAGVAVLLGMVAVTGGLTFSAFYDTPAGPSVVLCAAVLFTLSLFKKTTGLIKAGI
>CORE_REP|Org37_Gene448#
MRLIPLKDTAQVGKWAARHIVQRINAFKPTAERPFVLGLPTGGTPLEAYKHLIAMHKAGEVSFKHVVT
FNMDEYVGLPQEHPESYHTFMYRNFFDHVDIPRENINLLNGNAADVDAECRQYEEKIKSYGKINLFMG
GVGIDGHIAFNEPASSLASRTRIKTLTEDTRIANSRFFGGDVSLVPKYALTVGVGTLLDAEEVMILVT
GHAKAQALEAAVEGNINHMWTISCLQLHAKAVVVCDEPATMELKVKTVKYFRELEAESVKSL
>CORE_REP|Org14_Gene555#
MRFNNKMLALAALLFAAQASADTLESIDNCAVGCPTGGSSNVSIVRHAYTLNNNSTTKFANWVAYHIT
KDTPASGKTRNWKTDPALNPADTLAPADYTGANAALKVDRGHQAPLASLAGVSDWESLNYLSNITPQK
SDLNQGAWARLEDQERKLIDRADISSVYTVTGPLYERDMGKLPGTQKAHTIPSAYWKVIFINNSPAVN
HYAAFLFDQNTPKGADFCQFRVTVDEIEKRTGLIIWAGLPDDVQASLKSKPGVLPELMGCKN
>CORE_REP|Org22_Gene3010#
MGKSLHYLDVGSGFPLLLGHSYLFDLNMWAPQFEALAKRYRLIVPDLWGHGDSPALPAGRNSLSDIAA
DHLALMDHLDIEEFGIVGLSVGGMWGAELAALAPERVKVLALMDSYLGDETPEARQRYMGMLAAVEQA
GTITSPLLEYIAAQFYSDEVPDALSQPLLAHLQSLPADRLRESIVPLGRMIFGRPDKLPLLENITAAS
IVITGAQDKPRPQAEGQRMADLLGCQHVLIPNAGHISNKENPAAVNETLLAFLAENTTPLFW
>CORE_REP|Org48_Gene1788#
MIKWPWKATQPSQPQADTQAQWQDALAIPLLSPLNEQEQQRLVAVAGQILQQKRIVPLQGLQLTSQMQ
ARIALLFALPVLELGAECLDGFNEILLYPTPFVVEDEWQDEIGLVHSGPVVQSGQSWEQGPIVLNWQD
VQDSFDLSGFNLVIHEAVHKLDMRNGGVATGVPPIPLREVAAWEHDLHAAMESLQDEIDMVGEEAASM
DAYAATDAAECFAVLSEYFFSAPELLAERFPALYQHFCRFYRQDPLARLLRGQVENDAQWAD
>CORE_REP|Org41_Gene1783#
MMFAKVRRQWLLGVVGVALAAGLATQTYAADNLLQQVKQRGTLIVGLEGTYPPFSFQGEDGKLTGFEV
DFANALAEHLGVKAKLNPTKWDGMLASLDSKRIDVVINQVTLSDERKKKYDFSTPYTVSGIQALVKKG
NEGTITKPEDLKGKKVGVGLGTNYEQWLRANVQGVDVRTYDDDPTKYQDLRVGRINAILVDRLAALDL
VKKTGDTLAVAGPAFSRQESGVALRKNNPELLAAIDQAIAEMQKDGTMAKISEKWFGADVTK
>CORE_REP|Org22_Gene4310#
MKRINALTIAGTDPSGGAGIQADLKAFSALGAYGTSVITALVAQNTRGVQSVYYIDPAFVAAQLDSVF
SDVRIDSVKIGMLANADIVQAVAERLRHYRPEFVVLDTVMLAKSGDPLLAPEAVASIRRELLPLVSII
TPNLPEAAALLACAPAEDEAQMREQGRALLAMGCRAVLMKGGHLSESESPDWLFSAEGEQRFTAPRVA
TRHTHGTGCTLSAALAALRPRHADWAATVAAAKDYLQQALQQAGTLEVGHGIGPVHHFHAWW
>CORE_REP|Org28_Gene1191#
MEITTLQIVLIFIVACIAGMGSVLDEFQFHRPLVACTLIGFILGDMKTGIIIGGTLEMIALGWMNIGA
AVAPDAALASIISTILVIAGGQSVGAGIALAIPLAAAGQVLTIIVRTLTVAFQHAADSAAERGSLRAI
TWIHIGALLLQAMRIAIPAVIVAISVGTAGVHALLNSIPEVVTSGLNIAGGMIVVVGYAMVINMMRAG
YLMPFFYLGFVTAAFTNFNLVALGVIGVVMAVLYIQLSPKYNKSQVVQAGPANANDLDNELD
>CORE_REP|Org48_Gene2882#
MQDKLLNPGAAFALDNASFAVPGRVLLQPLSLSFPQGKVCGLIGHNGSGKSTLLKLLGRHQAPSGGQV
LLNRQPLAQWDSKSFARQVAYLPQQLPAAEGMTVRELVAVGRYPWHGALGRFGANDRQLVEEAISLVG
LKPFANRLVDSLSGGERQRAWLAMMVAQDSRCLLLDEPTSALDIAHQVEVLALIQRLSRERDLTVIAV
LHDINMAARYCDHLVALRGGEMIAQGGPLELMQGPVLEQIYGIPMGTLPHPSGGAPVSFVY
>CORE_REP|Org9_Gene4087#
MSSEAVFIQVGALAEGFAPHSNTLERQHGLAGSTLTLRFSDGATQRCRFTDEQTLEWGERCGVAYRAT
SIRPGVLFIDFLDPARANASITLVCDRNQGNFTAVYGQLPDEAQARLDAFSRVEQGLPLTAVEAEFRF
GTLDDADVAPPGFTDELIGMRNMYTYSPTERYEHIYLNDNFYAWQCLDGVEKGLADVDRCHYVKVAEQ
LYLFVWREKIIPTLGVVMIDLQGMRTDGKILGYQGSDFSALSNFAVGAHAQVLNTTRHPRG
>CORE_REP|Org36_Gene2125#
MYWINGQRHDALAPSDRGLQFGDGCFTTARVIDGNIELLPWHLERLQQAAQRLMLPATDWLAFEREMA
LAAESIPLGVVKAILTRGSGGRGYSPTGCENPTRIVARSSYPAHYLQWREQGITLALSPVALARNPLL
AGLKHLNRLEQVLIRAHLDQTAADEALVLDTAGMLVECCAANLFWRKGKAVFTPDLSQAGVAGLMRRR
VIALLAGSEYRLQCVSEPLETLADADEVLVSNALMPLLPVNAAQSWRYASRQLYDFLRPHC
>CORE_REP|Org2_Gene4668#

MMKPTTVTHLRQCKQEQRKFATLTAYDASFAKLFEEQGIKVLLVGDSLGMTLQGHDSTLPVTVADVAY
HTRAVRRGAPACLLLADLPFMSYATPEQTFANAAELMRAGANMVKLEGGSWLCDTVKMLAERAVPVCG
HLGLTPQSVNVFGGYKVQGRDELAAKQLLQDAQNLELAGIQLLVLECVPTELARQITEALSIPVIGIG
AGNGTDGQILVMHDAFGITGGHTPKFAKNFLAQSGDIRTAVQHYIQEVEQGLYPAAEHSFN
>CORE_REP|Org23_Gene3450#
MGPSSSASRGKAKKISFFIVIQIIVSGCGDHYDARALMENFDFLAIQTIYDFTSLFPLIFCYRRGLMH
TNKLANPGPLGLMGFGMTTVLLNLHNAGFFPLNSAIISMGIFFGGLAQILAGLLEYKKGNTFGMTAFT
AYGSFWLSLVGLLLLPRLGLAEATEAHVLGIYLALWGVFTLFMFFGTLAANRVLQFVFASLTLLFALL
AVGNITGNHALLTFAGYEGIICGASAIYLAMAEVLNEQYDRTVLPIGEPAPARVAASPVTV
>CORE_REP|Org34_Gene1812#
MKQYLDLMNKVLAEGTPKADRTGTGTLSIFGHQMRFNLQDGFPLVTTKKCHLRSIIHELLWFLNGDTN
TAYLRDNKVTIWDEWADENGDLGPVYGKQWRAWGAADGRQIDQLSNVLQQLKQDPDSRRIIVSAWNVG
ELDQMALAPCHAFFQFYVADGKLSCQLYQRSCDVFLGLPFNIASYALLVHMMAQQCDLEVGDFVWTGG
DTHLYSNHMEQTRLQLTREPRPLPKLVIKRKPASLFDYRFEDFEIEGYDPHPAIKAPVAI
>CORE_REP|Org36_Gene2710#
MKKIMMIGYGAMAKEVIARLPEGVEVGWILARAAHHAAIAEAFGGRVQALTHPEQCLQRPDLVLECAS
QQAVAEFGEAVLQRGWPLALISTGALADAALQQRLQQVCRRHHGQLIVLSGAVAGMDGLASAREGGLE
SVTYQASKSPASWRGSPAEQLIDLDAVSEAQVFFEGSAREAARLFPANANVAATIALNGLGMDATRVR
LQVDPATRRNTHRLQVCGDFGEFHIELSGTPLASNPKTSTLAALSAVQACRRLVDGGFIA
>CORE_REP|Org13_Gene3029#
MANEAKNDGIGKTLLVVLLLCLVCSVVVAGSAVGLKSKQQEQKLLDKQRNILDVAGLLQPKMESEQVK
RLYSERIEPRLVDLNSGEFVAGKAAAFDLGAALRDDAKSVALAASDDPAGIKRRSNQAEIYLVRDESG
QVNKIVLPVYGTGLWSMMYAFVALDNDGNTVKGITYYDQGETPGLGGEVENPSWRQQWVGKQLFDDNG
QPAIRVVKGGARQGDVHGVDGLSGATLTSNGVQHTFDFWLGEHGFGPFLKKVREGALKNG
>CORE_REP|Org3_Gene227#
MAISIKTPDDIQKMRVAGRLAAEVLEIIEPHVKPGVTTGELDRICHEYITNEQQAISACLGYHGFPKS
VCISVNEVVCHGIPSDDKTLKDGDIVNIDVTVIKDGFHGDTSKMFIVGKPTILGERLCRVTQESLYLA
LKMVKPGIRLRTLGKAIQQFVEAEKFSVVREYCGHGIGEVFHEEPQVLHYDADDGGVVLQAGMAFTIE
PMVNAGDYRIRTMKDGWTVKTKDRSLSAQYEHTIVVTDNGCEIMTLRKDDTIPNIITHEM
>CORE_REP|Org13_Gene1778#
MHKLKLHGFNNLTKSLSFCIYDICYAKTADDRDGYIAYIDEQYNANRLTEILSETCSIIGANILNIAR
QDYEPQGASVTILVSEEPIDPKDVDTSEHPGPLPNTVVAHLDKSHICVHTYPESHPEGGLCTFRADIE
VSTCGVISPLKALNYLIHQLESDIVTMDYRVRGFTRDVNGVKHYIDHEINSIQNFMSEDIKALYHMMD
VNVYQENIFHTKMLLKDFDLKHYLFNAAPEALSAVERKQITDLLWKEMQEIYYGRNIPPL
>CORE_REP|Org19_Gene410#
MADEQACKYLIPGLDRGLQLLLAFGEQHKEMTFAELHRLVDMPKATAYRVVQTLEHLGFLERNPRTNT
FALGIKVLRLGFEYIASLDVAQAGQPVIEQLRDRSQCSSHLAIRDGRDVIYIARVSAAGSQINQVSVG
TRLPVHQTSLGRMLLTSATRSEFEQLYPDAQLPGNAPGTPADRETLWQMVQQDKARGYVIGESFFRHG
ISSIVYPIFNREQRVEAVVSIMVPSDEIPKADRERLRMEVRDAAEKISGFLGAPPQANVG
>CORE_REP|Org12_Gene1885#
MNFQLEDRVAVVTGGSSGIGFETLKLLLAEGARVAFCGRDPDKLAGAEASLRADFPQAEILALRCDVL
DAQQVAQFAAQVTARFGGVDLLINNAGQGFVAHFDQTPREAWLHEAELKLFGVINPVQAFLPALERSA
IASITCVNSLLALQPEEHMIATSAARAALLNMTLTLSKELVDKGIRVNSILLGMVESGQWRRRFEERS
DKDQSWEQWTAAIAERRGIPMKRLGKPQEPAQALLFLASPLASFTTGAALDVSGGFNRHV
>CORE_REP|Org23_Gene4113#
MQDRNFDDIAEKFARNIYGTTKGKIRQAVVWQDLTGLLAQLPQLPQRPLRILDAGGGEGHMACQLAEL
GHQVLLCDLSGEMIQRAAQLAEQKGVSQNMQFVQSSAQDIAQHLEQPVDLILFHAVLEWIAEPEAALQ
ALCDCLTPGGALSLMFFNANGLLMRNVVLGNFQLVDPEVRRRRKRSLSPQYPHDPLLVYGWLEQLGMR
ISGKTGVRVFHDYLQSRQLQTQKFEELLALEQHYCRQEPYVSLGRYIHVMAHKPNLKDEL
>CORE_REP|Org13_Gene4492#
MSRAERLYHRTVTGLLLLILLILLLPLAATLIYALATQWGATILPDGFTLKWLTALWSDPRFLQALWH
SLLICFGTLLLSVVVILPAMFVIAYYFPKLDAVMNVLILLPFAVPPVVSAVGLMQLFAADPLPLLGTP
WILVGCYFTIALPFIYRAISNNMQAINLRDLMDAAHLLGASTWQAALLVVLPNLRKGGTIAVLLSFSF
LIGEFVFANLLVGSQYETLQVYLFNMRNGSGHFTSALVISYFAVVLLVTWLANLLNKNKG

>CORE_REP|Org16_Gene4625#
MSATYAANAFAGQVVLVTGGAQGIGLAIVSAFARLGAEVTIADVQLPQAQAAAQTLRDEGLSVQALAC
DLAEPGQIAELVAAVGERHQRLDVVIHNAAYFPLTPFAAIDAALLQRTLSVNLMAPFFLAQAALPWMR
HRGGGCILVTSSVTGPRVAYPGLAHYAASKAGVNGFIRAAALELAAENIRVNGVEPGMIRTPAMANLG
DAQVNQAIAASVPLGRLGEPADIAAAMVFLASPAAAYITGQTLVVDGGALLPETNSLLT
>CORE_REP|Org1_Gene240#
MPVFSIVALMAYLLSLGLIIPSLLRKNSAYRRLALVSAVVALICHAIALQQRIFDVSAGQNLSLLNIG
SIVSLIICSVMTFVASRDRGWFLLPIVYSFAMINLAFASFMPGEFITHLEASPELMVHIGLALFSYAT
LIIAALYALQLAWLDYLLKNKKLTFSADMPPLMSIERKMFHITQIGVVLLTLTLCTGLLYMDNLFSKE
NVHKAVLSIMAWFVYIVLLWGHYHEGWRGRRVVWFSFAGAFLLTLAYFGSRLIQQVMVR
>CORE_REP|Org35_Gene4529#
MIARPDALPGARAAACLTQFQQRPPLIHCLTNEVVQSLTANVLLALGASPAMVVEPQEAAQFSALADG
LLINVGTLNALRAESMRAAVAAANRAGKPWTLDPVAVGALSYRTAFAKQLLDEKPAAIRGNASEIMAL
SGLLASGRGVDSGDDSLVALPAARELARRSGAVVAVTGAVDYVTDGQRDWAIEGGSPLMTRVVGTGCA
LSAVVAAFCALPGDRLDNVATACRVMSHCGGLAARQAAGPGSFTPAFLDALYQLRGEDL
>CORE_REP|Org38_Gene1443#
MQAEILLTLKLQQKLFADPRRIALLKQVQHTGSISQGAKLAGISYKSAWDAINEMNQLAEQTVVERAT
GGKGGGGAHLTHYGQRLIQLYDLLGQVQQKAFDVLQQDDLPLDSLLAAISRFSLQTSARNQFFGTVIE
RDHQQVQQHLAILLNDGTTRLMAAVTQQSADRLQLTPGKEVLALIKAPWVRLSVETEEHTGADNALAG
VVAGIQPGAEHSEVLVTLAGGETLCATLTTAELQRLQLSVGAAVHALFNADHVIVATLC
>CORE_REP|Org6_Gene3678#
MSKPVHVNSWDSLRAFTDARIALGRTGASLPTDELLRFGLAHAQARDAVHQPFDSERLAADLHQAGWP
SLAVHSQAADRAAYLRRPDLGRRLASDSRSLLLGSPSRRVDLLLAVADGLSSKAVHRQALPLLQALRP
YLDTLGLSVSPVVLAHQARVALGDEIGECLQARAVAVLIGERPGLSSPDSLGIYLTWEPNARRTDAER
NCISNVRPEGLDYPQAAFRLAWLLEQAFQRRLSGIELKDESDNPALHNRVTPLYPQLGG
>CORE_REP|Org22_Gene2309#
MPEGPEIRRAADKLAAAVIDQPLTAVDFAFPQLKHYRQRLLGERIVAIEPRGKALLTHFSNGLTMYSH
NQLYGVWKVAAAGETPETKRDLRVRLETARSAILLYSASDITVGPREEIEQHPFLQRIGPDVLDMSLT
VDDVEERLLTPRFRRRQLGGMLLDQAFLAGLGNYLRAEILWQAELAPQHKPQDLTPEALRRLAEALLA
VPRLSYQTRGQADDNRHHGALFRFKVFHRSGEPCERCGGMIVRTELSSRPFYWCPGCQK
>CORE_REP|Org35_Gene1535#
MANADSDKQPDAVSSVMKVFGILQALGDEREIGITELSQRVMMSKSTVYRFLQTMKALGYVSQEGETE
KYALTLKLFELGAKSLQNVDLIRSADVQMRELSNHTRETIHLGALDEDGIVYIHKIDAMYNLRMYSRI
GRRNPLHSTAIGKVLLAWRERDEVAQILSQIEFTRSTEHTLTSAAELLPVLDRVRAQGYGEDAEEQEA
GIRCIAVPVFDRFGVAIAGLSISFPTLRFSEAAREEYVALLHVAARRISEQQGYHDYPF
>CORE_REP|Org34_Gene4658#
MKGYTDVLIILFKSIIIGGLVGVGVGAGAARMFHAPTTQGMGAFRTLGELNSCEGDPASHFSFGLGFF
FNAWASSVAAGSFTQDVDHRIIPHWGAAALMVKNRNLAQTLHDPKKMAIACGIIGMLVVAFLNTTASA
VPAALQVTAIKVLVPAANLLVNTVMPVIFWLAAIDAGRRSGFWGTIFGGLAQLIMGNAVPGLVLGILI
GKGVEESGWNKITKIMMAVIVLLFVLSGFFRGFDMKVLESFSLGVPGWLDAIHNTLSGK
>CORE_REP|Org36_Gene2477#
MITNLISGLLAPCFALYACAKSRRYWRQCRRLYTFQPIYRTSGALLAVELLTAVYHPNEPDKRQSPEQ
YFASLGVAQRLRVIQEQLALLQRWQALFIRHAVMVSVNIDGIALQALQRHSELQRQIAEMPYLRFELV
EHAETASNHPLQQIVGGERLWLDDFGSGLANFSAVGAWRYQYIKVARELFTLLKQSEEGVQLLGTLIT
MMNQHSDGVIVEGVETEQEWRLVQRSGALAAQGYYLSRPACFETLHSVPTLFAAPGAPA
>CORE_REP|Org42_Gene2137#
MSQGLRIEHFSAGYPKRQVIDDLSVPMLPRGQITVLLGPNGSGKSTLLRSLAGLNPAQGKLWLDDGDL
MQMPFARRAEKVVYLPQSLPAGVHLHVLESIIVAQRASGGRSNAGSEAEVMALLEQLGIAHLALSYLD
QLSGGQKQLVGLAQSLIRQPSLLLLDEPLSALDLNYQFHVMDLVRRETRKRNIVTVVVVHDINIALRH
GDHVLMLQDGDLIADGAPDQVITPQSLARVYGVRGRIERCSQGTPQVLIDGLVNQPTI
>CORE_REP|Org18_Gene263#
MIDQTAFIHPSAIVEEGAVIGANVHIGPFCYVGSQVEIGAGTVLKSHVVVNGITKIGCDNQIYQFASI
GEVNQDLKYAGEPTRVEVGDRNRIRESVTIHRGTAQGTGLTKVGNDNLLMVNVHVAHDCVVGNACVLA
NNATLAGHVEIDDHAIIGGMTAIHQFCIIGAHVMVGGCSGVAQDVPPFVIAQGNHATPFGVNAVGLKR

RGFDKDEMQAIRNAYKILYRSEKTLDEAKAEIEALAKEQPVVQQYLDFFTRSTRGIIR
>CORE_REP|Org3_Gene901#
MSLVDSLLRTLDFSPRGQVIAREADAWGEIIVSDHKDYRTLRFDGICEQSKMSLSNPAQPIHNYIKAM
LMAVAWQPPSSALILGLGGGSLLRALHALDPAMRLDVVELRAAVIAVARRYFTLPATDTISLRTADAM
DFVRPPVETRYDLIFSDLYSAFAMDPQQGTQTFLENCAARLNDGGWLVLNYHDLPDENSLLYHSLQRI
FGTVLFCVAPSGNVIIYATPAQVTLPLSALRSLAAGSGELFNCELGYLSRKIERLHFR
>CORE_REP|Org36_Gene1514#
MTSTPLSTTPLLSVNRLTHLYAPGKGFSDVSFDIYPGEVLGIVGESGSGKTTLLKSISARLAPQRGQI
LYRPQAGQEQDLYAMAESDRRRLLRTDWGVVHQHPLDGLRPQVSAGGNIGERLMAIGQRHYGDIRRQA
GQWLEDVEIPLSRLDDLPTTFSGGMQQRLQIARNLVTHPKLVFMDEPTGGLDVSVQARLLDLLRNLVV
EMQLAAVIVTHDLGVARLLAHRLLVMKQGEVVESGLTDRVLDDPHHPYTQLLVSSVLS
>CORE_REP|Org18_Gene4461#
MAENYYHVVAFTGEGLRGNPAGVCLLTQPQPLPAARLQAIANEIGLPETSFVWDDGDMTAIRWFTPQR
EVDLCGHGTLAAAHVMFSVVNPALQDIRFRSAGGELFVKRDAQDGERLVLDFPARPPQKVAEPAGLAA
LLGVQPQEVWQAKALMVVLENEAQVRALRPDIPALIALAGCAVIVTAPGDTVDFVSRYFTLDGGEDPV
TGSAHCTLMPYWTARLGRHRLQAQQVSARGGELFCELQGDRTLLGGYAHVFLHGTIAL
>CORE_REP|Org10_Gene1322#
MDNAACLTARELRYSLGTRRLINDVSLSLASGEMVAIIGPNGAGKSTLLRLLTGYLTPDCGECRLLDR
PLEHWAPQQLAKVRAVMRQYSDLAFPFSVEEVVSMGRSPHGKRDEHQAIQQVMEQTDCLALAQRDYRR
LSGGEQQRVQLARVLAQLWQPQPSPAWLFLDEPTSALDLYHQQHTLRLLRSLTRQQPLGVCCVLHDLN
LAALYADRILLLHQGRLVASGTPQEVLQTEILTRWYQADLGVVHHPEVSLPQVYLRQ
>CORE_REP|Org15_Gene3441#
MIPVERHQQILALVSERGVVSIAELTERLGVSHMTIRRDVQKLEEQGAVQSVSGGVQAPERVASEPSH
QTKEGMFGRQKIAIGRLAARQIPANSCIYLDAGTTTLALAKQIGERDDLTVVTNDFVIAGFLIEHSQC
RIIHTGGTVCRENRSCVGEAAAQALRGLFIDLAFISASSWSMRGLSTPNEDKVMVKKAIVEASRRRIL
LSDTSKYGKVATYLALPIAAFDAIITDEGLPAAAREAIEQAGIALLTAEKEEEEEEE
>CORE_REP|Org37_Gene4403#
MRHPLVMGNWKLNGSTHMVNELIAGLRNELSSVDGCGVAIAPPVMYLDQAKHALAGSRIALGAQNVDV
NLSGAFTGEVSANMLKDVGAQYIIIGHSERRTYHKESDEVIAEKFAVLKEAGLIPVLCIGETEAENAA
GKTEEVCARQIDAVLKTLGAPAMKGTVIAYEPVWAIGTGKSATPAQAQAVHKFIRDHIAKHDAAVAAE
IIIQYGGSVNDKNAAELFSQPDIDGALVGGASLKADAFAVIVKAAAAAKSLIALGLP
>CORE_REP|Org29_Gene3298#
MSYAFTDTHCHFDFPPFTGHEAESLARAASAGVQRIIVPTVTADRFARVLRLAQEHAPLFAALGLHPL
YIAQHHEPQLDQLATLLAERPRKLVAVGEIGLDLYMENPQFERQQSVLLAQLKLAKQHDLPVILHSRR
THDQLAAALRRMQLPRCGVVHGFAGSLSQAQAFIRLGYYIGVGGTITYERAQKTRGVMAQLPLEALLL
ETDAPDMPLAGYQGQPNRPERAAEVFQTLCELRPEPADEIAAHLQRNTQALFAMPDL
>CORE_REP|Org11_Gene3125#
MTAQILLHPSLAPLDGGINFRDFGGNGVADGRRIKRGLLFRSGSLERLTENDCTFLAGVPVRSVLDYR
DADEVQAKPDILWNGADYHHVPANPLSSEVNANLEKLTNETLAAFDARAFMLELYRRLPFGNAAYQRL
AQLLSNPGGGAIVQHCAVGKDRTGVGSALVLFALGADEATVLEDYLLTETTLATFREQMLDQLSIKLN
AAALEQFAYVLSAREEFLMTALGCIRQQYGSTDRWLEAEYGLGAAQREALQAFYLE
>CORE_REP|Org19_Gene3014#
MRNVRIDDIDHVTRAVIAIGTDYPPGHLLPMHSHRRAQLLYGATGVMHVFTQQGNWVVPPQHAVWLPP
QMPHAVRMVGVTTRSLYLEPGALPAERPQVCQVVSVTPLMRQLLMAAVDMPLEYAQEGRDGALATLLL
HELARLQPLPLHIPLPADPRLGELCRAFLQHPDAHDSAQRWAPRLYMSIRTFSRFFRAQTGLPFSQWR
QRACVVLALALLAEGRSVTQVAMEMGYDSSAAFSTMFRRVLGQAPSSYLTEDGRDG
>CORE_REP|Org7_Gene348#
MIPSLWIAKTGLDAQQTNMDVIANNLANVSTNGFKRQRAVFEDLLYQTMRQPGAQSSEQTTLPSGLQI
GTGVRPVATERLHSQGNLSQTNNSKDVAIKGQGFFQVMLPDGTQAYTRDGSFQIDQNGQLVTSSGFQV
QPAITIPANALSITVGRDGIVSVTQQGQTAAQQVGQLTLTTFVNDSGLESVGENLYQETESSGAPNES
TPGLNGAGLLYQGYVETSNVNVAEELVNMIQTQRAYEINSKAVSTSDQMLQKLTQL
>CORE_REP|Org37_Gene363#
MLLRALASLGRSGINTSASFGRAGLMLFNALIGRPEPGKQWPLLLKQLYSVGVQSLLIIMVSGLFIGM
VLGLQGYIVLTTYSAEASLGMMVALSLLRELGPVVTALLFAGRAGSALTAEIGLMKATEQISSLEMMA

VDPLRRIVAPRFWAGLISMPLLTIIFVAIGIWGGSVVGVDWKGIDSGFFWSAMQGAVEWKKDLLNCLI
KSVVFAITVTWIAIFNGYDAVPTSEGISRATTRTVVHSSLAVLGLDFVLTALMFGN
>CORE_REP|Org42_Gene3783#
MNRVVVITGGGTGVGAACARLLAAQGDRVFIIGRRPAPLAALAQEIGAQALVGDAASGESWNHALLPA
ILRDAGRIDCLIGSAGGMGFKRITEMTDAQWQGAMDSNLNSAFASARACLPELIKSGGNLLFVASIAS
LAAGPEVCGYVTAKHALIGLMRSIARDYGPLGVRANAVCPGWVTTPMADEEMRLLMDAHQISLEQAYQ
MVCRDVPLRRPASAEEIARVCRFLCSSEASIITGAALVADGGSTIVDVPTLAFTSL
>CORE_REP|Org43_Gene1624#
MIGRLLRGGFMTLVYAYLYIPIVILIVNSFNASRFGINWQGFTTKWYSTLLNNDSLLQAAGHSLTMAV
LSATFATLIGSLTAVALYRYRFRGKPFVGGMLFVVMMSPDIVMAISLLVLFMLLGISLGFWSLLFSHI
TFCLPFVVVTVYARLKGFDVKMLEAARDLGASEFTILRKIILPLAMPAVAAGWLLSFTLSMDDVVVSS
FVTGPSYEILPLKIYSMVKVGVSPEVNALATILLLLSLTLVIASQWVMRDRSPKAE
>CORE_REP|Org47_Gene3302#
MQLTFLGTGGAQQVPVFGCDCLICQRARREPAFRRRACSAMLNYQGETTLLDAGLPALERRFSAGQIQ
RFLLTHYHMDHVQGLFPLRWGCGNSIPVYGPPDAQGCDDLFKHPGILAFQPPLAPFATVELGGMRITP
LPLQHSKLTHGYLIQAAGAALAYLTDTVGLPPATADYLQNVALDLLVLDCSLPPQPQAPRNHNDLTRA
QETQRLLQPKRTLLTHISHHLDLWLLDNELPAGLELAFDHLSVSLGSSAADPTPAP
>CORE_REP|Org43_Gene3845#
MFDIGVNLTSSQFAKDRETVVERARAAGVTGMLITGTDLPESREAAKLAQQHAGYCWSTAGVHPHNAG
SWDERSTEQIYALAAGPEVVAIGECGLDFNRNFSTPEQQEAAFSAQLALAAELALPVFLHCRDAHARF
AELLTPWLDKLPAAVVHCFTGTAEELASCLSLGLSIGITGWVCDERRGLELRALLPQIPAERLLLETD
APYLLPRDLQPKPASRRNEPCFLPHLVHQVAVWRQEEPQWLGQKTDENARRLFRLV
>CORE_REP|Org44_Gene4837#
MNTSRMPALFLGHGSPMNVLEDNRYTRAWRALGESLPRPKAIVAVSAHWYTRGTAVTAMENPKTIHDF
GGFPQALFDTRYPAPGSPALAAQLQQMLAPVPVTADLGEWGLDHGTWGVLIKMYPNADIPVVQLSVDG
TQPAAYHYELGRKLAALRDEGVMIVASGNVVHNLRMVKWQGDTSAYPWAESFNSFVRDNLRYRGDNHP
LVDFMRHEGAALSNPTPEHYLPLLYVLGGWDGKEAISVPIDGIEMGALSMLSVQVG
>CORE_REP|Org13_Gene968#
MLTFDSAQLTVWLSHYFWPLLRILALISTAPIFSEKQISKKVKIGLGGLIVILIAPTLPASNIPIFSA
AGLWLAIQQILIGVALGLTMQFAFAAVRLAGEVIGMQMGLSFATFFDPSGGPNMPVLARLLNLLAMLL
FLSFDGHLWLISLLADSFHTLPIQTQPLNGNGFLVLTQVGSLIFINGMMLALPLICLLLTLNMALGLL
NRMTPQLSVFVIGFPVTMTFGIMTLGMMMPMLAPFCEHLFGEIFDRLAAVIGGMTF
>CORE_REP|Org29_Gene2205#
MACFGVSTQRWLLLLGVLSLTGCARSDALWGVVDKLCMANYQQKRDPAPCEQIYMPQGKAQGFSVLQN
PRYPYHFILVPTAPLSGIESPQLLARERTDYFGYAWLMRYRLAAEYGGPVPDDRLGMAINSAYGRSQN
QLHIHLTCLREDVRRQLQAERPYIQEQWRPLPDKLLRHTYYARRVMQPTAMGIYPIKDLADYFQLSPQ
QLAEYGVAVVPTTFADQRGFILLASKRGWDVGNRASVESLLDKTCAILPRESQDGR
>CORE_REP|Org48_Gene3395#
MMQVSLPMYGVGREQAESFWQVLRGKLLRLGLPAAPERLSWPDDLAQHWRREDLLLSQTCGYPLVSSL
PQVQLIGTYHYRVEGCDGPNYRSWLVVRAGDPGERLADFRGRTVAYNSTDSQSGYNGLRALIAPLAQD
GKFFGAAIASGAHYQSLKLIRSGQADIAAIDSISMELLRRAQPQALEGLKIIGRTAAVPGLPLITAAG
TPPEQVDILRAGARAMLDEAVSDRLLIGDFSLVPRSAYQIITVLEQQAAAQGVTAL
>CORE_REP|Org47_Gene3771#
MNEKHGCACARHLAQGFARQSINAGEGEIYQISLMSALIDGVYEGETTIAELLKHGDFGLGTFNHLDG
ELIAFDQEIHQLRADGSARPAGLQQQTPFAVVTFFQPSVSQQFDRPITKAQLHQCIDEQVASPNLFCA
VRVDGEFSHVETRTVPRQERPYRPMLEAIEEQPTFSFHQRRGTLVGFRSPDYMQGIGVAGYHEHFVTD
DRSGGGHVLDYQLDHGRLQFGVITRLNLQLPYDADFLRANLCPEDLDRAIRSAEG
>CORE_REP|Org36_Gene4482#
MLRQFYSQVVSVSKRSKADFTPRRGGFTFKQFFVAHDRCAMKVGTDGVLLGAWAPLGQARRVLDIGSG
SGLIALMLAQRSGDEVTIDAVELDEAAAGQARENAAESPWPQRIRVHAQDIHHYAQQHAAEYDLIVSN
PPYFEPAVACRDQARHNARYTETLTHDALLACAAQLLVEQGTFCVVLPHDIGAEFERLAQQNGWQTAA
KVNVSDRADTPLHRVLLALTRRETPLREQALAIKQADGCYTDDFLRLIADFYLFY
>CORE_REP|Org7_Gene4574#
MTTPTHSIHHAAAEGYQANADRYVKGRPDYPPEIAAWLRDVIGLHAGMTVIDLGAGTGKFTPRLLETG

AQVIAVEPVPQMLEKLSAALPQVKTLAGTADAIPLPDESVDAVVCAQSFHWFATPQALAEIQRILKPG
GKLGLVWNMRDARVGWVRKLNQIVDSHEGDAPRFYTGEWRKFFPFKGFEPLQEQVFMLGHRGAVEDVI
YNRVRSTSFIAALPQPQQEQVIDRLRQLVAEEEELRGKDTVTVPYQTKAYFTTKV
>CORE_REP|Org32_Gene2148#
MLKIADTTFTSRLFTGTGKFATPALMLEALAASGSQLVTMAMKRVDLRGGNDAILAPLQQLGVRLLPN
TSGAKTAAEAVFAARLAREALGTHWVKLEIHPDVKYLLPDPIETLKAAETLVKDGFVVLPYCGADPVL
CKRLEEVGCAAVMPLGAPIGSNRGLRTRDFLEIIIEQAKVPVVVDAGIGAPSHALAAMELGADAVLVN
TAIAVARDPVQMARAFRLALEAGELARSAGLGSSQRGAVASSPLTAFLSQPEEAQ
>CORE_REP|Org41_Gene3322#
MKKTPNYIDAISALLTLKGVGDRKHASTMANILNLQYNSAKQKLDGKRGITLDEVKKVFQYFNEPFAG
QRTHNGVFIMNSIHKRCNIEVDESPVTQADSDETYAYKKDDLFIISTNREHAADAPLYKVNKIDFLPA
PRIAILDNDGDILELLKKIASRYGIETETFQTADTMLAALEQQSFEAFILDWLLDFGETSERVVKKIK
DVLDPHARIIIILTGQLNHYEKNIGDMILHYDVHLVEKPTKPLIISSLLLSNLFFN
>CORE_REP|Org21_Gene1342#
MSEVAVVIGGGQTLGAFLSHGLAQAGYRVAVADLNADNANQVAQQINEAFGAGSACGFQADATDEQSV
IALAAAVDRAFGQANLLVYSAGIAKAAPITDFPLGDFDRSLQVNLVGYFLCAREFSRLMIRDGIAGRI
IQINSKSGKVGSKHNSGYSAAKFGGVGLTQSLALDLAEYGITVHSLMLGNLLKSPMFQSLLPQYAQKL
GIAPDQVEQYYIDKVPLKRGCDYQDVLNTLLFYASDKASYCTGQSINITGGQVMF
>CORE_REP|Org49_Gene4482#
MLTDALLIALLAGLAGVDLFDGLTHFHRPVVMGPLVGLILGDVYTGLLVGGTLELVWMGMVPLAGAQP
PNVVIGGVIGTAFAILTKADPKVAIGVAVPFSIAVQGCITLLFTLFSPMMHRCDRMVKELNWRGVERV
NYLGIGILFIFYFVVAFLPIYFGADAASAMVQKAPGWLLEGLAVAGGMMPAIGFSLLMKVMMKKTYVA
YFILGFISVTFLKLPILAVALGALAIALIDFFNTQRGAAEATARPAPQEDAEDGI
>CORE_REP|Org41_Gene875#
MSAGEVLTPQEYIGHHLTQLQVGTGFWSINLDSMFFSVVLGALFLVIFRKVAKNATSGVPGKLQTAVE
LVVGFVDSSVRDMYHGKSKVIAPLALTVFVWVFLMNLMDLIPVDFLPYLGTHVLGLPALRVVPTADVN
VTLSMALGVFILILFYSIKMKGVGGFVKELTMQPFNHPVFIPINLILEGVSLLSKPVSLGLRLFGNMY
AGELIFILIAGLLPWWSQWVLSLPWAIFHILIITLQAFIFMVLTIVYLSMASEEH
>CORE_REP|Org15_Gene1076#
MKALKTLFAAGCLLAAGSSLAAENSLRFGLEALYPPFESKSASGKLEGFDIELGDAVCAAAQLQCSWV
ETSFDSLIPALQARKFDAINSAMNVTEQRRQAIAFTDAIYQVPNRLIAKADSGLLPDAKALAGKHVGV
LQGSIQEIYAKTHWAPAGVDVVSYQDQNQVYLDLAAGRLDATLVMAPSGQSGFLSQPDGKGFAFVGEA
VRDDKILGEGIAFGLRKGDEALKKKLDAAIAKVKQQGTVTALSKKYFGDIDVTVK
>CORE_REP|Org37_Gene4465#
MIFDAVQPEKFFGAMLMPLPLLMLLMGLALLLLWFTRWQKAVRRFYAELAVLLLFSLQPVADRLLRPI
EAQYQTYRGNDPVSYIVVLGGGYTYNPDWAPSSNLLGNSLPRVTEGVRLYLAHPGARMVFTGASAGSM
QSNAATAALVAESLGVPRSDMVILREPRDTEEEAAQVAKLVGEQPFILVTSANHLPRAMRFFEAKGLH
PIPAPANQLAIDSPLNIWDRATPSSMFLGHTERAWYETLGSLWQWLKGADRAGAE
>CORE_REP|Org41_Gene1056#
MEFTETRRLYQQLAAELKQRIESGRYPVGDKLPAERYIAEEMNVSRTVVREAIIMLEVEGYVDVRKGS
GIHVVSNQQKHLVVPGDSIEFATAGPFELLQARQLIESNIAEFAATQVTKQDIVQLMEIQEHARKEDR
FRDSQWDLKFHVQVALATQNTAMATIVEKMWVQRVNNPYWKKLHEHIDERSIASWCDDHDQILKALMR
KDPYAAKLAMWQHLENTKQMLFNATSDDFEYNADRYLFAENPVVHLDGVSHEPK
>CORE_REP|Org22_Gene3704#
MPKFAANLSMMFNEVPFLERFAAAAAQGFSAVEFLFPYDYPAELLAEKLREHGLQQVLFNTAPGDAAA
GEWGLAALPGREQDARADIDRALAYAIALSCPSVHLMAGVVPAGADRRHYLDTFIANARYAADAFAPH
GVKVLIEALSPPVKPSYLFASQHQVAEAVAAIERPNVFIQFDFFHAQLVDGNISGLLETLAGRYAHIQ
IASVPDRHEPDEGELNYPWLFDRLDALGYPGWIGCEYRPRGDTAAGLGWLKPYR
>CORE_REP|Org14_Gene1496#
MLAKRIIPCLDVKDGQVVKGVQFRNHEIIGDIVPLAQRYAQEGADELVFYDITASSDGRVVDKSWVSR
VAEVIDIPFCVAGGIKSAEDASQILSFGADKISINSPALADPELISRLAERFGVQCIVVGIDTWFDSE
TGKYHVNQYTGDESRTRITQWETLDWVQEVQRRGAGEIVLNMMNQDGVRNGYDLQQLRRVREACKVPL
IASGGAGTMEHFLEAFRDADVDGALAASVFHKQIINIGELKRFLVEQGVEIRVC
>CORE_REP|Org13_Gene2734#

MLVIISPAKTLDYDSPLATERFTQPELLDKSQRLIKICRELTPAQIASLMSISDKLAGLNAARFSEWQ
PKFTPDNARQALLAFKGDVYTGLQAQDFNEADFDFAQQHLRMLSGLYGVLRPLDLMMPYRLEMGIKLE
NPKGKDLYSFWGDQITQKLNEALEQQGDDVVVNLASDEYFKAVKPAKLHGALIKPVFLDEKNGKFKVI
SFYAKKARGLMSRFIIKNRLTRSEQLVDFNLEGYAFDEAASQGNELVFKRPEQA
>CORE_REP|Org32_Gene2590#
MLLVDSHCHLDSLDYQTLHQNVDDALAKAKARDVGYVLAVATTLPGYRSMTELIGERNDVAFSCGVHP
LNLEEGYDYAELRRLAAAEQVVALGETGLDYFYQKDNLELQQDSFREHIRIGRDLNKPVIVHTREARA
DTLAILREENAQDCGGVLHCFTEDLTTAEALLDLGFYISFSGIVTFRNAEQLREVARYVPLDRILVET
DSPYLAPVPHRGKENQPAYVRDVAEYMAVLKGVSLEQLAEATTANFSRLFHLDL
>CORE_REP|Org12_Gene3522#
MKKDKQIMTIGEGQAIVKEAQRLLSAPSRRRFLRNGLTLGGIAMLTGCDLSDNANVEQALSRMSRLND
RVQGWLFNGDRLAPVYPELMITRPFPFNAFYAEEDAPDINGDDYRLEVAGLVQDKRAWSLPQLHRMAQ
VSQVTRHICVEGWSAIGKWGGVPFATFLKAIGADLSARYVSFKCADDYYTSIDMATALHPQTIIALTY
DGQILPRKYGYPMKLRMPTKLGYKNPKHIQVIEVTNRFPGGYWEDQGYNWFGGS
>CORE_REP|Org48_Gene1952#
MKPKQRQAAILEYLQRHGKTAVDALAEHFSTTGTTIRKDLTLLEDEGEVIRTYGGVVLSRDDGDQPID
RKTHINTEKKRHIASAAVALIADGDSLIFDAGSTVLQMVPHLAQFNNITVMTNSLTIVNALVELDNDQ
TILMPGGTYRKKSASFHGSLAESAFQQFSFDKLFIGADGVDLNAGVTTFNEVHNVSKAMCEAAGRIIL
LVDSSKFGRKSPNVVCELSAVDTLITDRDINPDYLAALQAKGINILLVGDPDE
>CORE_REP|Org27_Gene4360#
MSSRKSLSVVMIAKNEAGLLPDCLRSVEWADEIIVLDSGSEDDSVAIAESLGAKVFTHTDWQGFGKQR
QLAQSYASHDYVLMIDADERVTPELRQSIERVLNAPDDGAVYSCARRNLFLGRFMRHSGWYPDRVNRL
YANRRYRYNDDLVHESLNIGGAKVIPLNGDMLHLTCRDFFAFQRKQLRYAEEWATQRHRAGKRCGYLS
ILTHTLGAFVKTWLLRAGFLDGKQGLLLAVVNAQYTFNKYAALWALGRNYSEK
>CORE_REP|Org8_Gene1470#
MSGRIGRLLRDPLPWTLALLLALVFGMDHLRGLFAAWFPDLERPIYQQDSFIALVGAHLSLVAISSLI
AVAIGVAAGVAVTRRSGREFRSLVETVVAVGQTFPPVAVLAVAVPVMGFSEQPAIIALVLYGLLPILQ
GTLAGIESVPPATREIARGVGMSAWQILWRVELPLAAPVIVAGIRTSVIINIGTAAIASTVGTKTLGS
PIIIGLSGFNTAYVIQGAAVVALLAIITDMLFERWVRYLTAWRQQTLAATSAG
>CORE_REP|Org31_Gene1858#
MTLPARLAQGTPVTLESIGKGYGNRTVLDNIQLRISAGQFVAVVGRSGCGKSTLLRLLAGLEQPSSGA
LLSGNAPLAAAKEDTRLMFQDARLLPWKTVIDNVGLGLRGQWRDAALQALDAVGLADRARDWPAALSG
GQKQRVALARALIHRPRLLLLDEPLGALDALTRIEMQGLIETLWQQHGFTILLVTHDVSEAIALADRV
ILIEEGRIGLDLTLDLPRPRRKGSARLAELEAEVLERVLSPPATAASGRRAAN
>CORE_REP|Org38_Gene3465#
MKQQWTKWVGGIVLASGLVAQASAAEKITVFAAASLTNALQDIATQYQKGKDVQVVSSFASSSTLARQ
IEQGAPADLFISADQQWMDYAIDKQQMVKDTRYTLLGNELVLIAAKSDKQDKIAIDKQTDWAKLLNGG
RLAVGDPDHVPAGIYAKEALEHLGAWSALEPKLARANNVRSAMALVERGEAPLGIVYGSDAVASDKVK
VVGVFPEDSHKPVEYPMAIVKDRQNPTVSAFYTYLKSPEAAAIFEHYGFTPRK
>CORE_REP|Org27_Gene4526#
MNFAMKLHYQLLAAESDALPVLLIHGLFGNLDNLGVLARDLHKQHTVIKVDLRNHGLSPRADDMNYPA
MAQDLLALLDELQLEKAIVIGHSMGGKAAMALTAIAPERVAKLIVIDVAPVDYQTRRHDEIFAALKAV
SAAGITQRQAAAELMRDYLQEEGVIQFLLKSFHNGEWRFNLPVLIARYEDITGWQEVPPWPHPTLFIR
GGLSPYVQDSYRADIARQFPQARAHVVAGTGHWVHAEKPEAVLRAIHRFLGEA
>CORE_REP|Org9_Gene583#
MNITIASTSNQGGRASNQDQTGEVLGNRAACFVVCDGIAGFPGGDIAAKLARDTILQNFDGEKHLNAQ
SIRQHITRANAAIHQQQRQSDEYSKMGTTLVSLFIDRDYQLAYWAHAGDSRLYLFRRGYLHAVTTDHS
LIQQMQDAGYQTNGINSNLLYFALGLNEERDATYSDVLQLEDGDVFLLCTDGFWHSFSQAELEQSLHM
VNSPSEWIALMQQAWKKNNNSDNYSAIAVWIGSPQETTLLHSLADAERFLSRD
>CORE_REP|Org4_Gene3776#
MANHSIKGKTVLIAGGAKNLGGLIARDLAEQGAKAVVIHYNSAASQAEAGKTVAAIQAAGAQGVALQA
DLTTAGAVEKLFADTVATVGRPDIAINTVGKVLKKPMVDISEAEYDEMTAVNAKTAFFFLKEAGKHLN
DNGKICTLVTSLLGAFTPFYAAYAGTKAPVEHFTRAAAKEFGERGISVTAVGPGPMDTPFFYPAEGAD
AVAYHKTAAALSPFSKTGLTDIEDVVPFIRHLVSDGWWVTGQTILINGGYTTK

>CORE_REP|Org16_Gene4047#
MDLTYAPLLAWLLLVHLLADFPLQPLSWVEDKIQHRARSRFLVLHALLHGVLAAWVVAGFGLLHGGLS
SLQVLVSLLVIAVSHYLIDLLKVTVMTRLSPARSFLLDQGLHLAVIALLWLGLTPNAGELLAALGQQL
GRWQTGLVLVAYSLIYLPMSLLIGQLLAHWTPQMPPSAKADNDSLLRAGKQIGYLERTLILTFVLLGQ
IPAIGFLLAAKSIFRFGDLRQSDDKMRTEYVLLGTLFSFTLTIMLGLLVNKLL
>CORE_REP|Org24_Gene336#
MSENKLAVTELHKRYGDHEVLKGVSLAANAGDVISIIGSSGSGKSTFLRCINFLEKPSEGSISLNNED
IRMVRDKDGQLKVFDKKQLQLLRTRLTMVFQHFNLWSHMTVLENVMEAPVQVLGLSKADAHERAVRYL
DKVGIDERARGKYPVHLSGGQQQRVSIARALAMEPEVLLFDEPTSALDPELVGEVLRIMQKLAEEGKT
MVVVTHEMEFARHVSNHVIFLHKGLIEEQGPPAELFGNPKSPRLQQFLSGALK
>CORE_REP|Org46_Gene1332#
MNPLIIKLGGVLLDSEEALERLFTALDSYRQQHQRPLVIVHGGGCVVDELMKQLSLPVVKKNGLRVTP
ADQIDIITGALAGTANKTLLAWAIKHQINAVGLSLADGGSAVVTPLDPALGHVGNAQPGSPALLNTLL
SAGYLPVVSSIGITADGQLMNVNADQAATALAATLGADLILLSDVSGILDGKGQRIAEMTAQKAEQLI
AQGIITDGMVVKVNAALDAARTLGRPVDIASWRHADQLPALFNGVSIGTRILA
>CORE_REP|Org43_Gene2476#
MNQHNITNESLGLSMVLVVVAILISHREKLALEKDIIWSICRAVVQLIIVGYVLKYIFDLDNAVLTVL
MVLFICFNAAYNAKKRSKYVEHAFVTSFIAITTGAVLTLAVLVLTGSIEFTPMQVIPISGMIAGNAMV
AVGLCYTNLGQRFKSEQQKIQEMLSLGATPKFASAALIRDSIRASLIPTVDSAKTVGLVSLPGMMSGL
IFAGIDPVKAIKYQIMVTFMLLSTASLSTIIACYLAYRKFYNERHQLVVGNLK
>CORE_REP|Org16_Gene2512#
MMLATRILQQGEAGRPWLVWLHGLLGNNNEWRVIAARCPEWPSLAIDLPGHGDSVAVGCRGFDDISAQ
IAATLQLRNIERYWLVGYSLGGRIAMYHACHGRHDGLQGVIVEGGNPGLEDEQQRRDRCEQDARWAAR
FRSELIAEVLADWYQQPVFKELSHVHRQALIAARSVNSGPAIADMLEATSLGRQPYLAPQLRQLTVPL
RVLCGENDPKFQRLARDAGLPLRIVPQAGHNAHLANPQDFVAELQTFLVNPG
>CORE_REP|Org28_Gene3434#
MLKRLVLIGACLAATFSTAALAAESTYVVGSGGTYRPFEFENSQKQLEGFDIDIIKAVAKAEGFQIKL
INTPWEGIFATLNSGDRDIIISGITITDKRKQMVDFSAPYFPAEQAIVVPQDSTVDSIAALKALKVGV
VNSSTGDIVVSDVLGKNSTAIKRFDNTPLMLQELYEDGIGAAVGDVGVAKFYIKTHPEKAFKLVPDAK
FERQYFGIAVAKGNDELRNKINAGLKKIVADGTYAKIYQTWFDSNVPTLPAE
>CORE_REP|Org22_Gene536#
MTRLYWVALQSIWAKEVNRFARIWIQTLVPPVITMTLYFIIFGNLIGSRIGDMHGFSYMQFIVPGLIM
MAVITNSYANVASSFFSAKFQRNIEELLVAPVPTHVVIAGYVGGGVARGICVGVLVTIISLFFVPLQV
HAWWVIALTLLLTAILFSLAGLINAVFATTFDDISLIPTFVLTPLTYLGGVFYSLSLLPPFWQAVSKL
NPIVYMISGFRYGFLGINDVPLAFTMAVLVAFIAVFYLLSWYLIERGRGLRS
>CORE_REP|Org23_Gene2649#
MIEILYQDEHLVAVNKPSGWLVHRSWLDRHETRFMMQTVRDQLGQHVFTVHRLDRPTSGVLLMALSSE
VARLLSQQFEQHQVQKTYHAVVRGYVQEGATLDYPLTAELDKIADKHADADKGPQPAVTHYRPLAQVE
MPVAVGRYDSARYSLVELQPETGRKHQLRRHMAHLRHPIIGDSKHGDLRQNRGMAQHFGCPRLMLHAS
HLQLTHPVTGEPLQVTARWDEDWQGVMTQFGWAGVFPELAGVEFSAANGQDS
>CORE_REP|Org31_Gene2550#
MSVQGKTALVTGSTSGIGLGIASVLAAAGARVILNGFGDVAQAQAQVAQLGAAPGYHGADLGDAAQIA
DMMQYAESEFGGVDILVNNAGIQHVAPLDQFPVEKWNAILAINLSAVFHTCRLALPGMRERNWGRIIN
VASVHGLVASKDKSAYVAAKHGVVGLTKTLALETARTPVTCNAICPGWVLTPLVQQQIDKRIAAGTDP
QRARDELLAEKQPSQEFVTPEQLGELALFLCSDAAAQVRGAAWNMDGGWLAQ
>CORE_REP|Org44_Gene1862#
MSISNSHKNAVDRQFGEQANAYLTSAVHAQGKDLQRLAQLLEPHAGARLLDLGCGAGHASFTAAARVA
QVVAYDLSAQMLAVVEQAAAEKGLNNIQLQQGVAESLPFEDAGFDLVISRYSAHHWHDVGQALREVRR
VLKPGGRAIFMDVVSPGHPLLDIYLQTVEVLRDTSHVRNYAPGEWLTLLTEAGLVVREVTSDRLMLEF
GSWVARMRTPAHFVTAIRALQQSVSQEVAAHFAIQPDGTFTSDIMMFEVTKG
>CORE_REP|Org8_Gene1841#
MTALYWQTIGEGERDLVLLHGWGLNAEVWRCIQARLTPHFRLHLVDLPGYGRSQGFGALSLEQMTEIV
LAAAPPQAWWLGWSLGGLVASQAALMQPQRVSGLITVASSPCFAARDAWPGIRPDVLSGFQHQLSLDF
QRTVERFLALQTLGTESARQDARQLKAVVLNQPTPSVEVLNGGLEILRTADLRAPLAELNLPLLRIYG

YLDGLVPRKVAELLDAAWPNSTSQIVAKAAHAPFISHPDEFVTMIEAFIAAH
>CORE_REP|Org5_Gene3235#
MSKIALLFAMLVSMPMITACSASEQVPDAPVVKQQLLGSPVYIQIFKEERKLELYAKMGNEFRLVNTF
PICNFSGGLGPKRREGDFKSPEGFYSVDARHLKPDSKYYRAINIGFPNDYDKSQGYSGAYLMIHGECK
SIGCYAMTNTYMDEIYRYVEAAFAYGQSRVNISIYPFRMTEQNLKRHASSSYIAFWRQLKPGYDYFAK
NHQPPTMGVVNGQYVLGQPLMSSGGMMTQYASASPTPNTNPFAQNKPLTEVK
>CORE_REP|Org20_Gene2782#
MYKIDYNSYRSVASFGHRVRFLVLHYTAQNFADSVKSLTGKSVSAHYLVPDPTEATYQAAGFSGVRIF
NLVDENERAWHAGASQWGTRSNINDTSIGIEIVNLASGDGGNITFPPFNPQQIEAVTQLAQNILQRYP
DISPVNVVAHSDIAPGRKSDPGPQFPWQQLYQAGVGAWYDEATKQQRQQEYCRQGLPAQAELLKLFAQ
YGYDTSAANTAEGYRQLVRAFQLHFRQQKYDGVMDVETAAALRALVDKYAA
>CORE_REP|Org39_Gene1904#
MRSTLLRLPLLAGLLLAGQPQAFAAASILIWPIDPAIEDNQQATALWLENRDSKPVYMQIRVLGWQQT
GGKDDYRNQSEVVASPPVATILPGKRQLIRLIKQTPVAAGQERAYRILVDEVPIKDKDGAAPDKGAQM
GLKFQMRYSVPLFVSGKGVWTKQDFEHPRDYATANQPKLSYRLLQQNSQRWLDVRNDGIVHARLSQVS
IQGKPLNNGLLGYVLPGSQMRFALPPSGSFAAGKLQAMVNDNKQPVTIPSY
>CORE_REP|Org47_Gene3539#
MLNVNHLSAEYQGRPALRDVSFQIAAGQLVVVLGPSGCGKTTLLNLIAGFIEPSAGSITLDGTPVHGP
SAERGVVFQHEGLLPWRNVVDNVEFGLQLAGVGKAQRRQVAEQMLQRVGLAGYEQHFIWQLSGGMRQR
VGIARALAADPRLLLLDEPFGALDAFTREQMQELLLTIWRDTGKQVLLITHDIEEAVFLASELLLLSP
GPGQVVERLSLNFGQRYADGEACRVIKSDPEFIAQREYVLGKVFQQREAML
>CORE_REP|Org27_Gene2316#
MNIAKERGLTRLRRGLTVFAGLLLLWWLAAQSGIPAFLLPTPSAVAQALWDGRGYLAWHTLITASEIV
SGLALGVLLGAALALCMIFSPRLQRWLMPLVLTSQAIPVFALAPLLVLWFGFGMSAKVAMAVLVIFFP
VVSAFFDGLRRVNNDYLDLARTMRASRWAQLRHVRLMAALPAFGSGLRMAAAVAPIGAIIGEWVGSAE
GLGYVMLNANARMQTDVCFAALFILVLMTVLLWVAVDALLRRLIAWAPEND
>CORE_REP|Org41_Gene2476#
MSLRRGVFVCVALLAALAIALYYGLKPDHPDALWRIVSQQCLPNQQAHDNPAPCAQVDVPAGFVVFKD
RNGPLQYLLMPSAKITGIESPAVLDAATPNFFAQAWRARHVMAERYGKPIDDGDISLAINSEYGRTQN
QLHIHISCLLPAVKQRLAQIGPDFIEQWQPLPGGLLGHDYLGRRVTPAELEQQGAFRLLASGLPRADG
RMGSFGLAMTALPDGDFLLLATERSLLPFTLASAEEIQDHDCRLLTPPPRA
>CORE_REP|Org45_Gene3199#
MFIGIVSLFPEMFRAITDYGVTGRAVKNGLLSVQCWSPRDFTYDRHRTVDDRPYGGGPGMLMMVQPLR
EAIHAAKAAAGEGAKVIYLSPQGRKLDQTGVCELAANQKMILVCGRYEGIDERVIQTEIDEEWSIGDY
VLSGGELPAMTLIDSVARFIPGVLGHQASAEEDSFADGLLDCPHYTRPEVLEGMEVPPVLLSGNHAEI
RRWRLKQSLGRTWLRRPELLESLALTDEQAVLLADFQREHQARQQDYEGNV
>CORE_REP|Org29_Gene1215#
MKKLLPLALLLAAGSAGAQSHLDKVLQQKTLEVCTTGDYKPYTFLKEDGSYEGIDIDMAESLAKSLGA
KVKWVKTSWKTLTPDFASGKCDIAMGGISVTLERQKQVFFADKLDTDGKIPLVRCTDVKKYRTLEQIN
KPSVRLLEPAGGTNEAFVHAYLPKAKLTLTHDNMSIFQQLVDRKADVMITDASEALYQQKRYPKLCAV
NPTKPMQYGEKAYMLPRDDLSWKLYVDQWLHLAKATGEYQKIIDKWLAVKK
>CORE_REP|Org25_Gene3739#
MTSANDTVNKQAVASAFSRAAGSYDAAAELQRDVGERLLGMGSSHPGEQLLDAGCGTGYFSRMWRERG
KRVTALDLAPGMLEVARQRQAAHHYLLGDIEQVPLPDAAMDICFSSLVVQWCSDLPAALAELYRVTRP
GGVILFSTLAAGSLQELGDAWQQVDGERHVNAFLPLTQIRTACAAYRHELVTELRTLNYPDVMTLMRS
LKGIGATHLHQGREGGLMSRGRFAALQAAYPCRQGQFPLSYHLAYGVIYRE
>CORE_REP|Org43_Gene2307#
MELALTGNENGWWIVSHESKLWLPNGELPSGTAAVLGLQGHMARIIGEWEGAPVWLVRQAMPREMGSV
RQLLDQDRGLFQLAGRGVQLADFYRSHRFCGYCGHEMHLSRTESACLCGHCKERYYPQIAPCVIVAIR
RDDQILLAQHVRHRGGIHTVLAGFVEVGETLEQAVAREVMEESNIEIKNLRYVTSQPWPFPHSLMMAF
MADYHQGDLRHDPKELLNAGWYRYDQLPLLPPPGTVARRLIEDTVALCREE
>CORE_REP|Org36_Gene3981#
MSKETNQRLLDKLQQDLASAGAMPLYLRFNASVRQAIEQGLLNSGDFLPSERLFTEWLGISRITVRKA
LACLEQDGIIGRSRGYGTFIQPQRPEPKLFYSLADVKGFSREVIQQGRRPDTQWISRERMPAGAELAE

KLQLAAGTPIYKLKRIHLIDRRPMSVAVSYVVVTAIANVDEIGISLYDYFRRNKVEMGSLRSQVSAAM
ADDDIRQALRLSEPMPLLIVRQTLFDHHKKPIEYSESFCRSDMYEFTSES
>CORE_REP|Org20_Gene874#
MIPEKRVIRRIQSGGCAIHCQDCSISQLCIPFTLNAHELDQLDNIIERKKPIQKGQTLFKAGDELKSL
YAIRSGTIKSYTITEQGDEQITGFHLAGDLVGFDAIGGLKHPSFAQALETSMVCEIPFETLDDLSGKM
PNLRQQIMRLMSGEIKGDQDMILLLSKKNAEERLAAFVYNLSRRFAERGFSPREFRLTMTRGDIGNYL
GLTVETISRLLGRFQKSEILSVKGKYITIENADALSVLAGTPRINVSVNA
>CORE_REP|Org43_Gene1989#
MNNLFSLDNKTVLITGASRGIGFLLARGLAQQGAHILVNATTEEHARHAADRLREEGLRADAAAFDVT
DSQAVHAAIGRIETDIGAIDVLINNAGIQRRHPFTEFPEQDWDDIIAVNQKAVFIVSQTVARYMVPRR
RGKIVNIGSMQSELGRDTITPYAASKGAVKMLTRGMCVELARYNIQVNGIAPGYFKTEMTQALADDPA
FTAWLTQRTPAARWGDPQELIGAAVFLAADASSFVNGQLLFVDGGMSAAV
>CORE_REP|Org33_Gene417#
MAYSKIRQPKLSDVIEQQLEYLILEGTLRPGEKLPPERELAKQFDVSRPSLREAIQRLEAKGLLLRRQ
GGGTFVQTNLWQSFSDPLAELLADHPESQFDLLETRHALEGIAAYYAALRGTDEDLARIRDCHIVIQQ
AQDSGDLDAEADAVMQYQIAVTEAAHNVVLLHLLRCMGPMLEQNVRQNFELLYSRREMLAKVSSHRAG
IFEAIVAREPEKAREASHRHLAFIEEILLDLSREHTRRERSLRRLQQRKD
>CORE_REP|Org21_Gene4158#
MYEIAKRTAGGALLLLLMPLTVWVSGWHWQPGGNEPLLKALYWVTETVTSPWGILTSAILCGWFLWCL
RFRLKAAIGLALLLFAALVIGQGVKSLIKDRVQEPRPFVVWLEQTHGVDGKYFYSLHRKERSALVREQ
LQDQTLVPNWLRKHWQFETGFAFPSGHTMFAATWALLGVGLLWPRRHYKTVALLMVWATGVMGSRLLL
GMHWPRDLAMATLISWLLVVVTCWLAQRWFGPLTPPPQEQQEIAARKPEI
>CORE_REP|Org39_Gene1977#
MKHARIRHHGQVFNVQVDEQLRVTLPNGEVLQEREVEWLPPAQGTVFALGLNYADHASELEFKAPEEP
LVFLKAPNTLTGHRQVSVRPAGVEYMHYEAELVAVIGKTARNVSREHAMEYVAGYTLCNDYAIRDYLE
NYYRPNLRVKSRDTLTPIGPYIVDRDDVADPHRLALSTYVNGELRQRGSTADMIFDIPFLVAYLSEFM
TLQPGDMIATGTPKGLADVQPGDEVVVEIEGIGRLVNHIISEKDYEESLR
>CORE_REP|Org14_Gene1958#
MEKEPVKISKLKTLAIIASAMVCAQSAQAGTWSLGAGALVSPDAYRGYQDRVYPVPVIDYEGDDFYFR
TLTAGYYLWKDQENQLSIMGYYTPWGYRPGDSDDDRMKRLDKRRGTLMAGLAYSHHAEWGTLRTTFTG
DTLNYSNGLVGDVAYLYKFDLDALTLVPGVGVMWNSKNQNKYYYGVSANESRRSGLDSYTPGDSWAPY
LELSANYQINKSWNAFFLGRYVKLSDEVKDSPMVDKSYTGLLMTGVSYTF
>CORE_REP|Org39_Gene565#
MTYQSLLAPILSFLHCETPDAWIDAARRPENLQLLLTDHLVCELKAAQTGMWLIRRYVADKESGDALL
ALLRPYEAFLHEAQGAPDTLFRQGQFTRKILPKNGSAYGQDLADRMVLLIKEELHHFSQVLEIMQARG
IPYRKITASRYAKGMIREIRTHDPATLIDKLICGAYIEARSCERFAKLAPHLDDELNRFYVSLLRSEA
RHYQDYLTLAEQIAGGDISERVAHFGRLEAELILSPDSELRFHSGVPAAA
>CORE_REP|Org13_Gene1701#
MMNLTMTRKTVFLVGPLAAALTIGAVSLPAHAAIALDRTRVIFDGDLKTVSLNISNQNKQLPYLAQGW
IEDDRGNKIQSPFTVLPPVQRVEPGKPSQVKIQSLPAARQLPQDRETLYYFNLREIPPRSNKPNTLQI
ALQTRIKMFYRPAALAPKKNAAPWQEQLTLTRQGDKYVVNNPTPYYVTIVEASAGKGGKGAAGFEPLM
VAPKASAPLNVSAASLGNGPSLAYINDYGGRPQLNFRCAGNACQVVPVAK
>CORE_REP|Org12_Gene3675#
MDTQRYLQHIGFAGAARPDLPTLQQLHHRHMLSVPFENLSIIYHQGIQLAPEALFSKVVERNRGGFCY
ELNTLFALLLREIGFKVSFISGEIRARDGHFGPPYDHLALRVDLAEQAWLVDVGFGDSFLTPLKIVAA
EPQPQASGTFHLEQEGEYYLLERRNGDQRSHAKTLYRFTVQPRELHEFDEMCRFHSTSPQSHFTQRLV
CSRPTEHGRVTLSDMKLIVTEDHQRHETTLHSEEERRAALWQHFAIDLDR
>CORE_REP|Org48_Gene2804#
MGDLAGKKVFITGAEQGIGRATAERLIKAGCDIYFHYHSDESGPKALVALAHSLGQKAAYGYADLIDT
DETLRCVAAGAEFLGGIDILVNNVGGIVGRKWLGEIDRAFWQTVIDVNMTTMLNVTQSALPFLKAAAN
GASIVNLASQAGRAGGHSGSLVYSATKGAVLTWTRSLAAELGEHGIRVNAVAPGLILGTRFHNRHTTQ
ESAEETVRAIPLGRAGTPDDVARAIAFLAAEYDGFISGATLDINGGIYRM
>CORE_REP|Org33_Gene3522#
MMRILLSNDDGVSAPGIQVLAAALREFAEVQVVAPDRNRSGSSNALTLESPLRTQTLANGDIAVLQGT

PTDCVYLGVNALMHPAPDIVVSGINAGPNLGDDVIYSGTVAAAMEGRHLGLPALAVSLNGHQHYATAA
AITCRILRALQREPLRTGKILNINVPDLPLAEIRGLRVTRCGSRHPADKVFCQQDPRGQNLYWIGPPG
DKFDVGPDTDFAAVEQGYVAITPLQVDLTAYAAQEVVKTWLTKAEVSGEW
>CORE_REP|Org41_Gene3786#
MLPELTDAEALRYNRQIVLRGFDFDGQEKLKAARVLIAGLGGLGGLGCAAAPYLAAAGVGHLVLVDFD
TVSLSNLQRQILHRDDRIGQSKVASARQELSAINPHIRIDAIDGRLEDDAIAAEIAACNLVLDCTDNV
AARDALNRLCHAQQRPLVSGAAIRMEGQLSVFTYQPGEPCYRCLSRLFGDSALTCVEAGVMAPLVGTI
GTLQAMEAIKLLADYGQPLRGKLLMFDAMSMQFREMKLPKDPHCEVCGGE
>CORE_REP|Org38_Gene340#
MSQSDVFHLGLTKNDLQGAQLAIVPGDPQRVEKIAKLMENPVHLASHREFTTWRAELDGKAVIVCSTG
IGGPSTSIAVEELAQLGIRTFLRIGTTGAIQANINVGDVLVTTAAVRLDGASLHFAPMEFPAVADFAC
TTALVEAAKASGATTHIGVTASSDTFYPGQERYDTYSGRVVSRFKGSMEEWQAMGVMNYEMESATLLT
MCASQGLRAGMVAGVIVNRTQQEIPNAETMKNTESKAVQIVVDAARRLL
>CORE_REP|Org30_Gene769#
MTSTLHTLVRRPAVWLPAALLFISPAALAQLPGLISQPLANGGQSWSLPVQTLVLLTSLTFLPAMLLM
MTSFTRIIIVLGLLRNALGTPSAPPNQVMLGLALFLTFFIMSPVFDKVYQDAYLPFSQDKIGLEVALD
KGAQPLREFMLRQTRETDLALYARLANQPPLAGPEAVPMRILLPAYVTSELKTAFQIGFTVFIPFLII
DLVVASVLMALGMMMVPPATISLPFKLMLFVLVDGWQLLLGSLAQSFYS
>CORE_REP|Org48_Gene4564#
MEKLLIVNADDFGLSKGQNYGVVEAYQHGVVSSTTAMVNGGGVQHAAVLSRQYPGLPIGLHFVLTHGR
PLGAMPSLVNEHGELGKWLWHRAEAGELQLDEIQEELQRQFTRFMTLFGRAPTHIDSHHHVHMQPQIY
PLVEAFAQAQGLPLRLDREEAKRRGIALQTPYSTDAFDAGFYGEMISEALFLQRLARADEQGAESLEM
MCHPAFLDATILQSKYCHPRLVELDVLTAPTLKAAIAERGFMLGSFQDL
>CORE_REP|Org46_Gene2574#
MDNIFNRPVIGVVMCRYRLNGHLTQTLQEKYLNAVIAAGGLPIALPHALAEPEMLAELLPRLDGILLP
GSPSNVQPHLYGENGDEPDADPGRDALSLALIRQAFDRRIPLFAICRGMQEMVVATQGTLHRRLYELP
ELLEHREDHDLPLEQQYAPAHEVIVQEGGLLSQLIPDCNRFWVNSLHGQGAKTLGASVRIEAHAADGL
VEAISVRDQPFALGVQWHPEWNSDEYALSRLLFDGFITACRNYQKEKRP
>CORE_REP|Org49_Gene879#
MHYQPKQDLLEQRIILVTGAGDGIGREAALTYARFGARLVLLGRTESKLLAVQQEIAAQGGAPALVVT
LDLLHATPEQCRQIADDLARQIPRLDGVLHNAGLLGDIAPMADLSMTMWQEVMQVNVNATFMLTQALL
PLLLKSHAGSLVFTSSSVGRTGRADWGAYAVSKFATEGMMQVLADEYKNRNLRVNCINPGGTRTKMRA
SAFPHEDKNKLKTPADIMPLYLYLMGEDSRRKTGMSFDAQPNRKPGAAE
>CORE_REP|Org22_Gene4048#
MEHSDNGLFIKRERFEVLAILREICKQRTPLRVENQQHRFQSLLLSVGPDNIVFSGEEAASAIDGACT
IVIESNDAKIEFSVGQAELTEHQGVQACSTRLPQELVYIQRRRQFRITTPHWRQFFCTGEYPDGTPYE
LRIHDLSAGGVGLRFDGPLPECFQPGLQFKKALLDLGSYGSFKVNMELVVVNDDQETDDNDQVVHFSR
LSCRFLKLGLAMERKIQSAVFAFELDFNKKKNADSYQGQCAAPLIRLRP
>CORE_REP|Org1_Gene4498#
MKLKLWGMWLAAGLLAGLLAGCSGSAPEKEAQVSEAGQTRLQLGLEYLQQGDMDAARQNLEKALDAAP
QDYRTQLGMALYAQRNGENAEAEQRYRQALKLAPGNGTVLNNYGAFLCGLGQYVPAQQQFSAAALVPD
YGQVADSLENAGYCFLKAGQNDEARTLLSRALKVDPDKGTPLLAEAEKQFGEGKRAQSQLLLDVYQHV
LPASASSLWLQIRFAASAGRQDSVQRYGKQLARSFPQSKQYQQFLANEY
>CORE_REP|Org37_Gene3755#
MSTLITLKNISVAFGNRKVLSNISLSLQPGRILTLLGPNGAGKSTLVRVVLGLVKPTAGTLEREPDLR
IGYVPQKLHLDATLPLTVSRFMRLKPGVKKADILPALKRVHAAHLLDQPMQKLSGGENQRVLLARALL
NKPQLLVLDEPTQGVDVNGQLALYDLIDQLRKELGCAVLMVSHDLHLVMAKTDEVLCLNQHICCSGAP
EVVSMHPEFIAMFGNRGAEQLAVYRHHHNHRHDLQGRIVLKKTGSREA
>CORE_REP|Org37_Gene1653#
MSYTQPPARSTSGIHYFAEGWRLISRPGIKRYVVLPLLVNVLLMGSAFWWLFSRLGDWIPAMMSHVPD
WLQWLSYLLWPLAVISVLLVFSYLFSTITNLIAAPFCGLLAEQLEGSLTGKPLPDTGLLGIAKDLPRI
MAREWRKLMYYLPRALLLLALYFVPGIGQTVAPVLWFLFSAWMLAIQYCDYPFDNHKVSFADMRRALR
QHKTDNLQFGALVSLFTMIPILNLVILPVAVCGATAMWVDRYRSQFVR
>CORE_REP|Org30_Gene1016#

MNFRLTGVAFATVLLVGCASAPDNDPQGRSDPLEGFNRTMFDFNYNVLDPYILRPVAVAWRDYVPMPA
RNGISNFTSNLEEPASMVNAFLKGDPYRGMIHFNRFFLNTLLGMGGLIDVAGMANPKLAREEPNRFGS
TLGHYDVGYGPYVMLPGYGSFTLREDGGDFADTLYPMLSYLTFWMSAGKWVVEGIETRAQLLDSDGLL
RNSSDPYIMVREAYFQRHDFIANGGSLKPEENPNAKAIQGELDEIDSQ
>CORE_REP|Org44_Gene3259#
MPITRLENPRIYRQIADQLKRLIENNEFPPGSRLPSERDLAQQLQVSRASVREALIALEVIGLVDVKV
GNGVIVRSQTPLAPSQEPVMAQAGRNQWAEIDDELNIELDFNAELPPFSLLQTRLLIEPETAALAARH
ATDEELAGIRAAYEQNCRDNRAGSATHPGDRLFHIRIAQASGNPAYAFIIGHLLGHRYGSMFRVLQRH
YTPDDMPHRSELEHRAILAAIEARDVRGARKAMKVHLDEVIAIFARAQ
>CORE_REP|Org22_Gene4202#
MSIQMDFTGKRVWVTGAARGIGEQIARHFLTQGAEVVGFDREFANPDQPYPCVMLDISRPEQVEAVCR
QQLAENPRLDVLVNAAGILRMGNTEDLSVDDWHQCINVNASGAFYLFRAVLPHFKAQRSGAIVSIGSN
AAHVPRAQMAAYCASKAALTSLNHCVGLEMAPFGVRCNLVSPGSTDTPMQRGMWQTDDAQQRTIAGFP
EMFKLGIPLGKIARPDEIANAVLFLASDLASHITMQDIVIDGGATLAA
>CORE_REP|Org34_Gene2871#
MSSANQQEANPSSQGPRHVAIIMDGNGRWAKRQGKLRVFGHKAGVKSVRRAVSFAASHHLDALTLYAF
SSENWNRPVQEVSALMELFVRALDSEVKSLHKHNVRLRVIGDISRFSARLQERIRRSEALTENNDGLT
LNIAANYGGRWDIIQGVRELAEQVRVGELHPDQISEELLNERVCMSDLAPVDLVIRTGGEHRISNFLL
WQIAYAELYFTDVLWPDFDELVFEGALNAFAQRERRFGGTTPNGADAS
>CORE_REP|Org48_Gene151#
MKQTQRHDAIIELVRLQGYVSTEELVEHFDVSPQTIRRDLNDLADQNKIQRHHGGAALPSSSVNAAYN
DRKVMWSEEKARIAQRVASQIPDGATLFIDIGTTPEAVAHALMNHKNLRVVTNNLNVATLLTAKEDFR
LILAGGEVRTRDGGIMGEATLDFISQFRLDYGILGISGIDMDGSLLEFDYHEVRTKRAIIENSRCVML
VTDHSKFGRNAMVNLGNMNLIDYLFTDQLPPPSVMKIIEQYDVQLELC
>CORE_REP|Org6_Gene1508#
MIKLEVCCFSVDCALTAERAGADRIELCASQSEGGLTPSYGTLRLARERVAIPVHPIVRPRGGDFCYG
AVDFEVIKQDIRQIREMGFPGVVVGMLDDEGHIDLPRMREVMRLCEGMAVTFHRAFDMCQNPMVALEQ
LTELGVARILTSGQQQSAELGLPLLRDLRQASQGPVIMAGAGVRLSNLHKFVDIGLHELHSSSGHLVP
STMRYRKAGVTMCSDNEFDEFSHYCVDGEMVEAMKNALALVDPLAQSA
>CORE_REP|Org44_Gene3272#
MDIQVERLSAVIDAVASPRFYPSLLNWLEGFFAFDNAIVYAFERGRPPRCLIKTERDNSDAVNQIYQQ
GAYLQDPFYRALNDGGAGEVLTLRQLAPCGFYHSDYYRNFYRKTGWHDEAGVLLQLTPERGLGVFFGS
ARRTVAVRYPQRADLRSALTLVKSVARLHGEVVAAPAEADTGNDDGAQARYLLTPREREIVDLILAGC
GSQQIADRLFISLGTVKNHRKNIYGKLNIGSQAELFSLLLTAPQRRSA
>CORE_REP|Org45_Gene1769#
MAEQQTVLQLAAAMSDAPAPIYQRVKQAIVNQIRAGHWQPHQRVPSESELVAELGVSRMTINRALREL
TSEGFLIRMQGVGTFVAEAKAHTALLEVHNIADEIAARGHRHSSKILELKARAASEEEATALGIQPGQ
RLFYSQIVHYENDVPVQVEDRCVNPLVAPDYLKQDFDRVTPYTYLTQAAPLTAGEHIVEAVIPTRRER
ELLQLEDHEPGLLIHRRTWSGKTVVTSARLLYPGSRYQLFGRFTSEV
>CORE_REP|Org30_Gene1131#
MNGLLNGKRIVVTGAARGLGYSFAAAIAAAGAQVVMCDILADELAASGAALREQGAQVETQTIDLASP
DSIRSAFEKIAAGGGIDGLVNNAALATGVGGKTMMEYDIDLWDRVMQVNVRGTWLVSQAAVPLLARSP
HAKIVNVASDTALWGAPRLMAYVASKGALIAMTRSMARELGPQGICVNAIAPGLTRVEATEYVPAERH
QLYEQGRALAGAQHPDDVNGTVLYLLSPLADFVTGQLLPVNGGFVFN
>CORE_REP|Org1_Gene4517#
MNLISIPAFQDNYIWLLDDRQGRCIIVDPGEAQPVLEALQRLQLAPAAILLTHHHHDHVGGVAQIVAK
YPGLAVYGPQETADKGANHIVRDGDTFDIDGRQYRTIAVPGHTLGHVAFYSAPYLFCGDTIFSAGCGR
LFEGTAKQMYDSFQQLAQLPDNTLICCAHEYTLSNLKFARAILPEDREIETYQQHVEALRAKGQASVP
TTLQLERKINLFLRCHDADLQKKLGFNSPPESLHSVFSELRLRKDNF
>CORE_REP|Org33_Gene2987#
MSILEKVEEMKSENNTNNNDLKSSPIVVALDYADKNAALAFADRIDPQDCRLKVGKEMFTLFGPQLVR
DLHGRGFDVFLDLKFHDIPNTTAHAVAAAAELGVWMVNVHASGGARMMTAAKEALASFGVDAPLLIAV
TVLTSMEAEDLRGIGIEASPAEHAERLARLTRDCGLDGVVCSAHEAQRLKAACGQAFQLVTPGIRPEG
SAAGDQRRIMTPVQAQAAGVDYMVIGRPITQSADPAATLRAIRASLA

>CORE_REP|Org48_Gene3986#
MLQLVEVGVAGRLAPFTAQIDGGLQVHLIGPNGAGKSTLLARAAGMLPGQGEVCLDGRALSCYSGDEL
AHRRGYLSQQQPPVSLMPVFQYLALHRPAGAVQTEVEQAILYLCQRLKLVDKLSRMLTQLSGGEWQRV
RLAAVLLQVWPSVNPHSRLLLLDEPTNSLDVAQKVALDRLLREFCQSGRSALVCAHDLNHTLQQADRV
WLLHAGQLVAQGITREVMAPGLLSQIYEVDFHLQWVGDQRWIMTRTA
>CORE_REP|Org12_Gene1280#
MRFDNKVVVITGAGNGMGEAAARRFSAEGAIVVLADWAKEAVDKVAASLPKGRAMAVHIDVSDHVAVE
KMMNEVAEKLGRIDVLLNNAGVHVAGSVLETSVDDWRRIAGVDIDGVVFCSKFALPHLLKTKGCIVNT
ASVSGLGGDWGAAYYCAAKGAVVNLTRAMALDHGGDGVRINSVCPSLVKTNMTNGWPQEIRDKFNERI
ALGRAAEPEEVAAVMAFLASDDASFINGANIPVDGGATASDGQPKIV
>CORE_REP|Org1_Gene2546#
MDHAIYTAMGAARQTLEQQSITANNLANASTPGFRAQLAALRAVPVDGPSLATRTLVTASTPGADMSQ
GALNYTARPLDVALQQDGFLAVSLPGGGEAYTRNGNIQISSTGQLTVQGLPVMGDGGPIEVPPSAEIT
IAADGTISALNAGDPPNTIAQIGRLKLVKADAREVMRGDDGLFRLTPETQQQRGNQLQNDPQVRVMPG
VLEGSNVKPMETMVDMIANARRFEMQMKVIHSVDENEQRANSLLSMS
>CORE_REP|Org17_Gene4076#
MTQPLPLTPQAFEAALRAKGAYYHIHHPYHIAMHNGEATREQIQGWVANRFYYQTSIPIKDAAIMANC
LQPETRRKWVQRILDHDGYGGSEGGIEAWLRLGEAVGLQRDTLLSEQLVLPGVRFAVDAYVNFARRAC
WQEAACSSLTELFAPQIHQSRLDSWPQHYPWIDAAGYDYFRSRLGQANRDVEHGLALALDYCDTVEKQ
QRMLEILQFKLDILWSMLDAMSMAYTLNRPPYHSVTAARVWHNQRLV
>CORE_REP|Org49_Gene808#
MADQPQETTDFGFRTVARDEKQAMVADVFHSVAAKYDVMNDLMSFGIHRIWKRFTIDCSGVRRGQRVL
DLAGGTGDLAAKFSRMVGEQGQVVLADINDSMLKMGREKLRDRGIVGNINYVQANAEALPFPDNYFDC
ITISFGLRNVTDKDKALRSMFRVLKPGGRLLVLEFSKPLLAPLSKAYDAYSFHVLPKIGELVVKDPDS
YRYLAESIRMHPDQETLKGMMGNAGFENVTYFNLTGGIVALHRGFKF
>CORE_REP|Org6_Gene3279#
MKRLFRLLIILLVILLLAAVLWWFFGRGNPNALWQIVSQQCVPNQQQNNDPAPCLKVDLTEGYVLFKD
SKGPYHDLVMPTEKVSGIESPILQAERTPPYFAQAWNNREHISSELGKPLKDAWVSLAVNSKYGRSQN
QLHIHVACLRQDVYNALGQQAEQLDQQWRPLAVKLVGHQYLARKLAGTDLTQEDPFRLLQNYVVEQGD
SIGNYGLALAVSPQGEMLLLANRLKLTDLNLGSAGEIQDYRCEVAGN
>CORE_REP|Org49_Gene3616#
MYLLYRSRHLFSWLPALIVAGCLLFASQQAALKIQPRYDETAIELALVEPEPAPEPQPETPPEPQPEP
PPPEPEPIPEPVVPAPEPIVEAKPVPKPQPKPKPKPVKEKAKPVEKPKPTPAPAAPRALVSKPAAQPA
PATPAAPAAPKVNAQAIENGYLLALRRELEQRKRYPSGRQASLERPQGNVEVWLEVDRSGRVLSSGIA
NKAASMLLNRAAMSSLQSISSVKPFPSEAFAGQTTKRFTATFNYQAP
>CORE_REP|Org25_Gene748#
MAVTKLVLVRHGESQWNQENRFTGWYDVDLSDKGRTEAKAAGKLLKEEGFTFDFAYTSVLKRAIHTLW
NILDELDQAWLPTEKSWKLNERHYGALQGLNKAETAEKYGDEQVKQWRRGFAVTPPELTKEDERYPGH
DPRYASLSEQELPLTESLALTIDRVIPYWDEEILPRIKSGERVIVAAHGNSLRALVKYLDNLSEDEIL
ELNIPTGVPLVYEFDENFKPTKRYYLGNADEIAAKAAAVANQGKAK
>CORE_REP|Org3_Gene2569#
MSDVSPTVLFEHPLNEKMRTWLRIEFLLQQLHGQRALSETAIALTFFRTVSDLLDVLERGEVRTELLK
ELERQQQKLLAWADVPGVDMALIDQLRGQLKSRASALMAAPRLGQALREDRLIGLVRQRLNIPGGCCS
FDLPTLHMWMHAPQQDRDADVATWLQSLDPLNQALTMVLDLIRQSGPFRNQISLNGFFQDNAEGADLL
RLRLHLAPQLYPQISGHKTRYAIRFLPLDSENGVVPERLTFELACC
>CORE_REP|Org2_Gene1095#
MKIRTSLIALGIATLATGCQNLNTETLMQSGAQAFQAATLSNDDVKALSDKSCAEMDSKAQIAPADST
YAKRLNKIAAALGDNINGTPANYKVYVTKDVNAWAMANGCIRVYSGLMDMMNDNEVEGVLGHEMGHVA
LGHTRKAMQVAYGTVALRTAASSAGGVIGSLSQSQLADIGEKLVSAQFSQKQESEADDYSFDLLKKRG
IDPNGLATSFEKLAQMEAGRQSSMFDDHPSSQARAQHIRDRIAAEK
>CORE_REP|Org30_Gene1282#
MKTEIIVVTGAYGTDTVKQYGGQRALLPIIAGAGADGVEIRRELFAAGELDSLPALAQEIERQQLFAV
YSAPEALFTPQHTLNPNLAALLAEAQALNARQLKLSLGHFRPGFDFTELKVALEQHPVKLVVENDQTP
DCGILSMLNAFFHAAEDSHLPVSMTFDMANWLWVGQDAFAAAERLARHVGYVHVKAATQGPRGWRAVA

LDDTDGSWRDLLARLPHDAPRGIEFPLQGDDLEAVTRHYVNILRAE
>CORE_REP|Org27_Gene444#
MKIKNVICDIDGVLLHDNTPVPGADLFLARIQEQGMPLVVLTNYPSQTAQDLANRFAAAGLEVPESAF
YTSAMATADFLRRQEGKKAYVVGEGALIHELYKAGFTITDINPDFVIVGETRSYNWDMMHKAAYFVSN
GARFIATNPDSHGHGFSPACGALCAPIEKITGRKPFYVGKPSPWIIRAALNKMQAHSEETVIVGDNLR
TDILAGFQAGLETVLVLSGVSTLSDIETMPFRPSYVYPSVADITIF
>CORE_REP|Org10_Gene664#
MPITANALYRDSFNFLRNQLASILMLALLTAFISVLLNQAFSPDAEQLATLAATTSDFASSTGMGIQE
VIQQMTPEQQMVLLKVSAAATFSALVGNVLLVGGMLTLIRLVSQGQRTSALRALGASAPDLPRLLLLL
FICTLLIQLGLTLFVVPGVLMAIAFALAPVIASVDKKGVFASLKLSSKLAFANARVLVPAMMLWLAAK
LLVLFLVSHLSVLTPNVASVVLTALSNLVSALLLIYLFRLYMLLRG
>CORE_REP|Org1_Gene4545#
MRTALVTGASAGFGKAICHRLIAEGYRVIGAARRMEKLLLLRQELGEQFIPLPLDVTDPLSLDKAFEQ
VSEAPFSIDILVNNAGLALGIERAQQSNVQNWERMIATNITGLALVTHRVLPGMVEANSGLIINIGSI
AGTYPYPGGNVYGASKAFVKQFSLNLRADLAGTHVRVTNIEPGLCSGTEFSVVRLNGNMEAVEALYKD
VKAIAPEDIANTVFWIIQQPEHLNINTIEIMPVAQSSAALNVVRNL
>CORE_REP|Org14_Gene4232#
MMQIRSQRLQHEKKHLTPWHRHEGGQIYLLTRGMLAMELPGRQWAITGGTLGWLPPGCAHQALACGDV
AGWSLYLPVESVPEMPPLPQLPQLFTASALLQALVERIAQFPAGPLSAPQRRLLQVLLDEMHAASSAP
LQLPLPQDARLLNIARALLNDPASPRSQCDWAIWAGLSPRTLSRRFLQETGISFALWRQQARVLRSLE
GLSRGKAVGEVADACGYDNVSAYIAAFRHRFGVTPGAYFAPARQTE
>CORE_REP|Org26_Gene3049#
MTTAQPLHGKVAFVQGGSRGIGAAIVKRLASEGAAVAFTYAASADRAEAVASAVTAAGGKALAIKADS
ADAAALQQAVRQAVSQFGNLDILVNNAGVFTLGSTEELALDDLDRMLAVNVRSVFVASQEAARHMNDG
GRIIHIGSTNAERVPFGGAAVYAMSKSALVGLTKGMARDLGPRGITVNNVQPGPVDTEMNPDAGEFAE
QLKQLMAIGRYGKDEEIAGFVAYLAGPQAGYITGASLSIDGGFSA
>CORE_REP|Org39_Gene3392#
MLNDQEFLRYSRQLLLEDVGPEGQERLKRATVLIVGLGGLGSPASLYLAAAGVGTLLLADDDQLHITN
LQRQILYRSADTATGKAALAQRHLQALNPLVESIPLAQRLQGQALRDAVARADLVLDCCDNMATRHEV
NATCIAAAKPLISGSAVGFSGQLLVLEPPYAHGCYACLYPDQEEPQRNCRTAGVLGPVVGVIGTLQAL
EAIKMLAGMPSSLSGKLRLFDGKQQSWSTLQLSQARACPVCGGAA
>CORE_REP|Org15_Gene1348#
MSFIAIIPARYASTRLPGKPLADIHGKPMVVHVMERARESGASRVIVATDHPEVAKAVEAAGGEVCMT
SPDHHSGTERLAEVIAHYGFADDQIIVNVQGDEPLIPPVIVRQVAENLAGSQAGMATLAVPIDSAEEA
FNPNAVKVVMDAQGYALYFSRATIPWDRERFAASKESIGDSLLRHIGIYAYRAGFVRRYVSWAPSQLE
QIELLEQLRVLWYGEKIHVAVAKAVPSVGVDTPEDLQRVRDSIQP
>CORE_REP|Org16_Gene2779#
MVLRTDVGRILITGAGGRVATAFRQAMGDRYPLRLAERDVSLLRDRQPGDEVISFNIADLDACRAACA
NIDTVLHLAADPSPDADFCGSLMDNNILGTFNIFRAAKDAGCRRVVFASSAQAVEGYPLDYQIRPDDA
PKPKNLYGASKAFGEGIAAYFAHQEGLSALSVRIANFTTLSRGEQLSARDMSAFLSHRDAADLLERCI
RVEGVQYAVVHGVSNNRYKRLSLEETRRLLGYAPQDDAFSLLGFE
>CORE_REP|Org26_Gene3950#
MHKAARQRHLLDLLSERGQAAVAELAGAIGVSVDTVRRDLADLERQGLAQKHHGGAIALEPSDMPRQA
RAALLPQVKQRLGRAVAAQIPPGSTLMLDAGSTLLAVAQALRGPATVITASLDIAQCLSDRPEINLIL
LGGQWDARQRLFAGGATLALLARYRADIALLGACAVHAQLGLSAGEEADAEVKRAMLANSGERWLVAD
HMKLDRCEPHHVADLAQIQRLFTDRPWDNLDEQSLIELCVVADDR
>CORE_REP|Org6_Gene1423#
MSIQFWRMSIQLNGINCYYGAHQALFDITLECPAGETLVLLGPSGAGKSSLLRVLNLLEMPRSGQLQI
AGNQFDFRQAPGEKAIRELRQNVGMVFQQYNLWPHLTVVQNLIEAPCRVLGLTKAQAMERADKLLKRL
RLTDFADRFPLHLSGGQQQRVAIARALMMEPQVLLFDEPTAALDPEITAQIVSIIRELAGTGITQVIV
THEVEVARKTASRVVYMENGHVVEQGDSSHFTQPRTTEFANYLSH
>CORE_REP|Org43_Gene4812#
MMKVALVTGASRGVGRATALLLARQGYAVGVNYLRDESAARQVVAEIEAQGGKALALQADVADEAQVM
AMFSALDAGLGTLSALVNNAGILFRQANIEQLTAERINKVLGTNVTGYFLCCREAVKRMARRHGGQGG

AIVNVSSAASRLGAAGEYVDYAASKGAVDTLTIGLSREVAAQGIRVNGVRPGFIYTEMHASGGEPGRV
DRVKSSLPMQRGGQPQEVAEAIAWLLSDAASYVTGTFIEAAGGR
>CORE_REP|Org12_Gene812#
METKEIRRNNLRELMARYARQGVNQNEFATLVESSAPTLSQIIGEKSSRNLGDNLARRIEARLNLPKG
WFDVFHEKQLVRPFDNVAAESDFQPARLKPVVWEDTEQDKEEFVEIPLLDIDFSAGDGCYEIVDREEF
SLIFRRYYLHKMGVAVNAARIIRISGSSMEPRLQDGDVVGINTDDTRIREGKTYAIRHGNLLRVKVLI
EQPDGGVIIRSLNREEYQDEHLSYQQRKEQLVVLGRVFWSSSSW
>CORE_REP|Org9_Gene2696#
MSLTLLEIVALTGIFTLAGTVKGAIGLGLPTVSMGLLSLMMPPGQAAALLLLPSLITNLWQLLCGPRL
GALCRRLWPMMLCVTLGTLLSAGTLTAIDGRLAPLALGICLTGYALLGFTRFDWRVSAAAEPWLGPLC
GLLTGVLTGATGVFVIPAVPYLNALGLARDDLVQALGLSFTVSTLALAAGLAWHQALPGALLGVSLLA
LLPALAGMWLGGIARRHSSPLIFRRLFFIGLFILGVEIIWRSIN
>CORE_REP|Org45_Gene722#
MLSIKNLKVSVEGNEILKGLDLEIKPGEVHAIMGPNGSGKSTLSATLAGREEYEVTEGEVTFKGKDLL
ELDPEDRAGEGVFLAFQYPVEIPGVSNHFFLQTSVNAVRKYREQEPLDRFDFADFIEEKIALLDMPAD
LLTRSVNVGFSGGEKKRNDILQMAALEPDLCILDETDSGLDIDALKIVANGVNSLRDGKRAFIIVTHY
QRILDYIQPDYVHVLSQGRIVKSGDFSLVKQLEEQGYGWLTDQQ
>CORE_REP|Org21_Gene1592#
MQSQAADSLNPPAVSATFANGVVDSLPIVIGYVPVAFAFGLSAVKLGFSPLESIFFSCIIYAGASQFV
ITALLSAGMSLWVSALTVMAMDVRHLLYGPALRHRIVSRMSPGKTAMWAFGLTDEVFAAATARLMRNN
RSWSENWMLGIALCSWLSWVAGTALGALFGNGPLEQFPVIEASLAFMLPALFLSFLLAAFRRPQSLTI
AAALAGALLGVVLFSIPVAILAGIGAGCVAALFQPAPAEANHEH
>CORE_REP|Org14_Gene1934#
MNLRQQTILQLVNDRRRISVNELARASGVSEVTIRQDLNLLEKRSYLKRVHGSAVALESDDVDARMMS
NFTLKQRLAQYAAAQVNDGETIFIESGSANALLARYIAERKRITLITVSHYIANLLKETDCDVIVLGG
MYQKKSETVVGPLTRLCIQQVHFNKAFIGIDGFQAETGFTGRDMMRADVVSAVLAKGVENIVLTDSSK
FGQIQPNPLAQTGQISRVITDSRLALEYQHQLKRQGVQVELVNE
>CORE_REP|Org7_Gene2423#
MNKHPITLLTAAGLALSAVLPTADAAISLDRTRAVYVSDAKSISLNIVNENKELPFLAQSWLENEQHQ
KITSPLVVLPPLQRVEPSERSVVRITKTPEADRLPQDRESVFYFNLREIPPKSTKTNVMQLALQTQIK
LFYRPKAIVAPKGQVWQEKLVFRKSGGAITVDNPTPFYITLTGMTRQTQKQGGGAIGGFQPLMLKPKS
SESLKLQETGMNSFVITYINDYGGHPELRFVCNGGVCTAVPEKK
>CORE_REP|Org39_Gene3562#
MQSEEQRLIDGLFGRLKEAETKTGPRDLQAEQQINQHIREQPSAPYYMAQAMIIQEAALKQMDQRVKE
LEAQVAQLQQTANGQQSSGGFLAGLFGGGSRSTPSPREQYQAQQQNTAAWNNAQQGGYSQPQQPAYAQ
PQQAAPSRAGGFLGGALQTAAGVAGGVVLADMLTGMFRHSQPQEIVNIIEENPAQLDDSAMRNFDASN
NLDTFNNGDGGSFLNQDNGFQNANYQDDTDYADDDYSDDDDSFL
>CORE_REP|Org33_Gene1922#
MPNRDTLFSAPIAKLGDWTFDERVAEVFPDMIQRSVPGYSNIISMIGMLAERFVQPDSRVYDLGCSLG
AATLSMRRNIKVPGCNIVAVDNSPAMVERCRRHIDAFRADTPVDVIEADIRDIDIENASMVVLNFTLQ
FLEPADRLRLLEKIYRGLRPGGALVLSEKFSFEDAEVGELLFNMHHDFKRANGYSELEISQKRSMLEN
VMLTDSVEAHKARLHQAGFEHAEVWFQCFNFGSLIALKAGDAQ
>CORE_REP|Org9_Gene3663#
MRNLDLEQLINTELNAAAFQDYAPNGLQVEGRPHVQRIVTGVTACQALLDAAVAHQADAIIVHHGYFW
KNEVPAVRGMKRNRLKTLLTHDINLYGYHLPLDAHPVLGNNAQLAQTLGIRVIGDVEPLVPHGEFEQP
LTGEALQQRIENRLGRAVLHCGDNAPEHIRRVAWCTGGGQGFIDSAARFGVDAFISGEVSEQTIHSAR
EMGVHFFAAGHHATERGGVKALGDWLAQHHGFDVTFIDIPNPA
>CORE_REP|Org24_Gene951#
MEKISVIMPAYNAANSIKESILGVLNQRFTDYHLYVIDDASTDDTAEVVRPFIHDRLTYIRNEHNQGV
AETRNIGIEAANGDYIAFCDSDDVWLPNKLSRQASILQTRRYDVVCSHYYTFEDDLKLIKNTRGAEEL
IGYQDMLKSNWIGNLTGIYNQKRIGKVYQQKVGHEDYLMWLAVLQKARNGLAYCIPEPLACYRLSTHS
LSGNKIRAADWQWRIYRQHLGLSYQKSCYLFATYLFNAVVKRK
>CORE_REP|Org2_Gene2300#
MWKRLIISLFIIIAVLMGSAIALDRWISWKTAPYVYDELQALPHRQVGVVLGTAKYYRTGVINQYYRY

RIQGAINAYNSGKVKYLLLSGDNAQQSYNEPMTMRRDLIAAGVAPSDIVLDYAGFRTLDSIVRTRKVF
DTNDFIIITQRFHCERALFIALHMGIQAQCYAVPSPKDMMTVRAREIFARLGALTDLYILKREPRFLG
PLIPISAMHTVPEDAQGYPAVSPEQLVELEHKLKEEKQKAKQP
>CORE_REP|Org13_Gene3440#
MMLERICQLSREAGAAIMAVYDGEQPLDVAQKKDDSPVTAADLAAHHIIKRGLAALTPEVPLLSEEDP
PAWEERRNWTRYWLVDPLDGTKEFLHRNGEFTVNIALIEDGQAVMGVVYAPAIDVLYLAERGKAWKEE
KGVRQAIGVSNAHPPLVVVSRSHIDDELKDYLQQLGEHQTVSVGSSLKFCLVAEGKAQLYPRFGPTNI
WDTAAGHAVAVAAGAQIHDWQGKPLLYTPRESFLNPGFRVSLF
>CORE_REP|Org20_Gene435#
MKSIFKVSLAALSLAFAVSSHAADKLVVATDTAFVPFEFKQGDKYVGFDIDLWAAVAKELKLDYTLKP
MDFGGIIPALQTKNVDLALAGITITDERKKAIDFSDGYYKSGLLVMVNADNNSVKSIDDLNGKVVAVK
SGTGSVDYAKQHIKTKDLRQFPNIDNAYMELGTKRADAVLHDTPNILYFIKTAGAGKFKTVGDSLEAQ
QYGIAFPKGSDELREKVNGALKTLRENGTYNEIYKKWFGTEPK
>CORE_REP|Org28_Gene1333#
MAGHSKWANTKHRKAAQDAKRGKIFTKIIRELVTAAKLGGGDPDSNPRLRAAMDKALSNNMTRDTMNR
AIARGVGGDDDTNMETIIYEGYGPGGTAVMIECLSDNRNRTVAEVRHAFTKCGGNLGTDGSVAYLFTK
KGVITYAPGLDEDTVMEAALEAGAEDIVTYDDGAIDVFTAWESLGAVKDALTAAGFEAEAAEVSMIPS
TKADMDAETAPKLLRLIDMLEDCDDVQEVYHNGEISDEVAATL
>CORE_REP|Org28_Gene1440#
MISTTTRQIVLDTETTGMNKLGVHYEGHRIIEIGAVEVINRRLTGRNFHVYIKPDRLVDPEAYGVHGI
SDDFLADKPTFDQVADEFLDFIRGGELVIHNAAFDIGFMDHEFRMLQQGIPKTETFCTITDSLLMARR
LFPGKRNNLDALCSRYEIDNSKRTLHGALLDAEILAEVYLAMTGGQTSIAFQMEGDTQQNDAAQEIQR
IVRPATAMKVVYASDEEVKAHEARLDLVAKKGGSCLWRGAPAE
>CORE_REP|Org24_Gene2355#
MQTIPAAVLDDLAPQGVLRAAINYGNPVLAQAGAGGKPQGASVELAAALAQELGVALELVTYDAAGKV
FADLDSGAWNLAFMAIEPVRAAQIAFSEPYVIIEGTYLVANDAPYFEVAQLDRPEVRIAVGQGAAYDL
FLSRTLQQAQLVRAATSAEAIALFFDRGLEAAAGVRQPLLAAAAVHPGYRVLDGHFTAIRQAMAVPRQ
KTQGAAYVNDFIERCKANGLVKAALQRSGQGEVTVAPPAASAT
>CORE_REP|Org32_Gene1615#
MTSPLLPPGIQVRDLSLRFGQQIVFDRLSFDIAGGSFVALLGASGAGKTSLLKIIAGLAQASSGTVTG
SDGLPIAGRIAYMGQKDLLYPWLTVEENVALGSRLRGEVADRAWVAHLLERVGLAAHGRSLPAALSGG
MRQRAAIARTLYERQPIVLMDEPFSALDAITRAEIQSLAAELLAQNTVLLITHDPMEACRLSHRLLVL
SPWPLGLDDTHRISGQPPRAPDDADLLKSQAELLQQLVRAAQ
>CORE_REP|Org41_Gene1202#
MKLQPNYYRDRVCLNVLAGSKANAQDIYAAAEGHVLVGVLSKNYPDVDSAVTDMLRYARLIENALSVG
LGAGDPKQSAMVSLIAQQVQPQHVNQVFTGVGASRALLGQNDSVVNGLVSPTGRVGWVKISTGPLSAA
APDGIVPVETAIALLKDMGGSSIKYFPMGGLKCKDEYQYVAKACAEHDFMLEPTGGIDLENYEPIVEI
ALAAGVKRVIPHIYSSIIDAASGDTRPQDVKTLLAMTKKLVG
>CORE_REP|Org34_Gene2364#
MDLTGKRVLITAAGQGIGFTTARLFAAAGAEVIASDINLERLQGSAGIRALTLNVTDPAAIAAAAEAI
GPIDVLFNCAGVVHSGSILDCSEDQWAFALDLNVTAMFRMIRAFLPGMLARGKGSIINMSSVASSVKG
VPNRFAYSASKAAVIGLTRSVAADYVTQGIRCNAICPGTVESPSLRQRIAEQAREQGRSEQEVYQAFV
ARQPIGRIGTTEEIAQLALYLASDASSYTTGTVQIIDGGWSN
>CORE_REP|Org39_Gene2334#
MIVDLNADLGEGCANDQALLQLVSSANIACGFHAGDAQTMRQSVRWALQYGVAIGAHPSFPDRENFGR
TRMQLPPETVYAQVVYQLGALAAIARAEGGVMVHVKPHGMLYNQAAVEPALAEAIARAVQAVDPALRL
VGLAGSELIRAGEQLGLTTRQEVFADRGYQADGTLVPRGLPGALIDDDEQALAQTLEMVRHHRVRSVD
GVWTAVQAETVCLHGDGEHALAYARKLRDSFVQQGIRVSAEQ
>CORE_REP|Org31_Gene1303#
MTILVTRPSPSGEQLVSRLRALGRVAYHAPLIDFAPGGDLPQLPQALQQLNAGDLVFVLSQHSVNYAD
SVIGRAGLSWPAHLTYYAIGRTTGLALHRISSLPVEYPREREISETLLLLPALQKLAGKRALILRGNG
GRELLGTTLSERGADVSYYECYQRSPVHYDGSEQSAHWQRAGVDTLVVTSGEMLQQLYTLVPDYYRSS
WLLRCRLVVVSERLATLARDLGWRTIRVADNADNDALIRALQ
>CORE_REP|Org37_Gene4141#

MQHATQRVAIVTGASRGIGAAIAERLAADGFTVIINYSGNPAPADELVRKIEQAGGRALGAKADVSDA
AAVSRLFASAEQAFGGVDVLVNNAGVMALAPVADMRDEDADRLIDINLKGSFNTMREAAKRLRDNGRI
INFSSSVVGLLQPGYGMYAASKAAIEALTSVLAKELRGRNITVNAVAPGPTATGLFLDGKTPELIERL
AKMAPLERLGTPEDIAAAVAFLAGADGGWINGQTLRANGGII
>CORE_REP|Org37_Gene2847#
MEAKISVPQYELRGFSLWGFRDMAHCMDFLFDGGRVKQGTLVAMNAEKILKAEEDPALHALLDEAEYK
YADGISMVRSIRRKYPAADVSRVAGADLWEALMQRAGREGTPVFLVGGKPEVLAETEQKLRSQWNVNL
VGSQDGYFKPDQREALFERIRASGAVIVTVAMGSPKQEILMRDCRKVHPQALYMGVGGTFDVFTGHVK
RAPKVWQNLGLEWLYRLLSQPSRIGRQLKLLKFVGYYYSGKM
>CORE_REP|Org41_Gene591#
MSVTGRIHSFESCGTVDGPGIRFIVFFQGCLMRCLYCHNRDTWDTHGGKEVTVEELMKDAVAYRHFMN
ASGGGVTASGGEAILQAEFVRDWFRACHAEGINTCLDTNGFVRRYDPVIDELLDTTDLVMLDLKQMND
EIHQNLVGVSNHRTLEFARYLAKRNQRTWIRYVVVPGWSDDDKSAHLLGEFTKDMTNIEKIELLPYHE
LGKHKWVAMGEEYKLDGVHPPKAETMDRVKGILESYGHKVIY
>CORE_REP|Org34_Gene800#
MSIDWNWGIFLQQAPFGNTTYLGWIWSGFQVTVALSVCAWIIAFFVGSLFGILRTVPNRFLSALGTCY
VELFRNVPLIVQFFTWYLVIPELLPANIGTWFKSELDPNVQFFVSSMLCLGLFTAARVCEQVRAAIQS
LPRGQKAAGLAMGLTLPQTYRYVLLPNAYRVIVPPMTSEMLNLVKNSAIASTIGLVDMAAQAGKLLDY
SAHAYESFTAITLAYIGINAVIMLFMRLVEKKVQLPGNLGSK
>CORE_REP|Org34_Gene1265#
MYPVDLHMHTVASTHAYSTLHDYIAEAQQKGIKLFAITDHGPDMADAPHYWHFMNMHVWPRRVNGVGI
LRGIEANIKNLQGDIDCTGPMLTATDVIIAGFHEPVFAPQDKASNTEAMIAAMAQGDVHIISHPGNPR
YPIDIPAVAAAAAKYEVALELNNSSFTHSRKGSEANCRAIAAAVRDAGGWLALGSDSHVAFSLGNFEH
CERIIDEVGFPQERILNVSPRRLLDFLERRGKPAIAELADL
>CORE_REP|Org30_Gene3050#
MTRHWPYPHIVAHRGGGSLAPENTLAAIDVGARHGHKMIEFDAKLAQDGQIFLLHDDTLDRTSNGWGV
AGELPWDKLVQLDAGNWYSSAFKGERLPLLSEVAERCQEHGLMANIEIKPTTGSDDETGRVVALAARL
LWQGQTDPLLSSFSVDALAAAQRTVPDLPRGLLLEDWDDNWRELTERLDCVSLHIDHKALTAERVKAL
KDAGLRILVYTVNQPDRARLLLDWGVDCICTDRIDLIGPDF
>CORE_REP|Org40_Gene4448#
MWKWLHQLARPERLYHVCGRFIPWLGLAAAACLLLGWAWGFGFAPKDYQQGDSFRIIYIHVPAAMWSM
GIYASMAVAAFIGLVWQMKMSDTVVAAMAPIGAVFTFIALVTGSAWGKPMWGSWWVWDARLTSELVLL
FLYMGVIALYNAFEDRRLAGRAAGILVLVGVVNIPIIHFSVEWWNTLHQGSTNMQQSIAPSMRTPLRW
AILGYLLLFVTLTLMRLRNLILFQERQRPWVAGLVNKERQS
>CORE_REP|Org15_Gene2513#
MIIPALDLIDGNVVRLHQGDYGQQRDYGNDPLLRLQDYQQQGAQVLHLVDLTGAKDPAARQIPLLRKL
LAGVNVPVQVGGGIRNEQDVSALLEAGATRVVIGSTAVKQPQLVQSWFERYGADALVLALDVRIDAQG
VKRVAISGWQEDSDATLEQVVEQFLPYGLKHVLCTDISRDGTLAGSNVALYQAISRRYPQVAFQASGG
IGNLDDIAQLRGSGVAGVIVGRALLEGKFSVEEAIACWQNG
>CORE_REP|Org40_Gene4665#
MKPLLVMQTGDAPQAIRQELANFEGMFLQQGNIDAERAHIVHLPAGERPLPPAAYCGVVITGSPAMVT
ERLPWSEEAAEWLRQAMAIKLPLFGVCYGHQLLAYALGGEVDYHPQGMEVGTLEIELLPAAAEDRRLT
LLPPRFKANLIHSQSVLTPPAGAQTQTLARSQQDAHQILRYGDHALTTQFHPEFNGAVMSQYLQWLGE
LHPEQQARYQQQQQQVSDTPFSRLLLQGFVVSLGAQKAMAG
>CORE_REP|Org29_Gene4084#
MSRIFITGSVDGLGRAAAQTLLDEGHQVILHARAPGRLDAVRDLLERGAQAVIGDLSDVQQIRQLAEQ
VNRLGRPDAVIHNAGMFTGPQVMPVNVIAPYLLTALIERPKRIVYLSSSMHFDGVPELNGVDWLGGAA
GSYSDSKLFVTALAAAVARLWPDVISSAVDPGWVPTKMGGADAPDDLALGHVTQAWLVTSDEPQALAS
GGYWHHQQRFEPHAAVHDEAFQTALLAQLARAGGVSLPQA
>CORE_REP|Org10_Gene86#
MAEMQTLKIDVMRYNPESDAEPHFVTYAVPYDEQTSLLDALGYIKDNLAPDLSYRWSCRMAICGSCGM
MVNRVPKLACKTFLRDYVGGMKVEALGNFPIERDLVVDMTHFIESLEAIKPYIIGNDRKPEDGPNVQT
PAQMAKYHQFSGCINCGLCYAACPQFGLNPEFIGPAAITLAHRYNLDNRDHGKKQRMPQLNGQNGVWS
CTFVGYCSEVCPKHVDPAAAIQQGKVESAKDFMIAMLKPQ

>CORE_REP|Org46_Gene1948#
MFTHKAIAELNALELMVYNYVSKHKNQVMYMTIRELAEAAGVSTTTVLRFCKKMGCDGYSEFRIRFKL
YLEQSDAPPVDSGIGEILSFFKSVSNDEFNQLIDQAVQHIAAAERIIFVGISTSGALGKYGARFFSNV
GKFSTHIDDPYYPVNSDMYKNAVAIVLSVTGETEEILRLASQFSLHHCKIISITNNETSSLARLADFN
LSYHVPQHLIGGHHNITTQIPVLYIIETIGKRLGHINSAK
>CORE_REP|Org29_Gene862#
MLHNIRIVLVETSHTGNMGSTARAMKTMGLTNLYLVNPLIKPDSQAIALAAGASDVIGNATIVDTLDD
AIAGCSLVVGTSARSRTLPWPMLEPRECGVRAVHEGEHAPVALVFGRERVGLTNDELQKCHYHVAIPA
NPDYSSLNLAMAVQILAYEVRVAYLDRQQAGAPQLEETPYPLVDDLERFYQHLEQTLQRTGFIRPSHP
GQVMSRLRRLFTRARPEGQELNILRGMLTSIEKQDKHQGN
>CORE_REP|Org23_Gene3523#
MVSSMSNSLLSSEASELDLLNERPFTQTDHEILKSYEAVVDGLAMLIGGHCEIVLHALEDLNSSAVRI
ANGEHTGRKIGSPITDLALRMLHDMAGDDSSVSKAYFTRAKSGVLMKSVTIAIRNREQRVIGLLCINM
NLDVPFSQIMQTFMPPATQDVPSSVNFASSVDDLVAQTLEFTIEEVNADRSVSNNAKNRQVVLNLYEK
GIFDIKDAINQVADRLNISKHTVYLYIRQFKSGDLLGSDR
>CORE_REP|Org33_Gene684#
MAEFDLAALNALPKSGQALALAVVNGQLETLSAEQRVAWALEHLPGEFVLSSSFGIQAAVCLHLVTRI
RPDIPVILTDTGYLFPETYRFIDQLTDQLKLNLQVFRAEQSPAWQEARYGKLWEQGVEGIEKYNQINK
VEPMNRALETLGAQSWFAGLRREQSGSRANLPVLAVQRGVFKILPIIDWDNRKIYQYLTEHGLSYHPL
WEQGYLSVGDTHTTQKWEPGMSEEETRFFGLKRECGLHEG
>CORE_REP|Org11_Gene1725#
MKAIIVEDEFLAQEELSYLIKKHSNIDIVATFEDGLDVLKYLQTHQVDAIFLDINIPSLDGVLLAQNI
SKFAHRPSIVFITAYKEHAVEAFEIEAFDYILKPYHEARIVTMLQKLEALHHRPAGATEPASAPSRGS
HSINLIKDERIIVTDINDIYYAAADEKVTRVYTRREEFVMPMNITEFYGRLPEEHFFRCHRSYCVNLA
KIREIVPWFNNTYILRLSDLEFEVPVSRSKVKEFRKLMRL
>CORE_REP|Org23_Gene669#
MKKLLLAATMLAGMTFNATAAETIRFAASATYPPFESLDANNQIVGFDIDLANALCKQMQAQCTFTNQ
AFDSLIAALKFKKYDAVISGMDITPERSKQVAFTQPYYANSAIVIAQKGKFSSLADLKGKKLGMENGT
THQKYMQDKHPEINTVSYDSYQNAILELKNGRIDGVFGDTAVVNEWLKTNPQLAPVGEHITDAQYFGT
GLGIAVRPDNQALLAKLNAALDAIKADGTYKAINDKWFPQ
>CORE_REP|Org2_Gene3743#
MTDFMAWLRGHLAQGAVAPRYIQLAMAIETAIRQQVLAAEAFLPPERQMAEGLALSRVTVSKAMKLLE
EKGLILRQQGVGTRVAMHIGYSLDKDSGFTAQALRHGSSVSNRWLLRARVGAPAKAAAALGLTEGDEV
VKLRRLRLLNGSPVSLETTYIPPRFLPEPGQLEHSLYALWQSRGIVPEDKHFLLKAVSCSDEVAELLN
VPCGAPLLHITQTSRNAQGEALEFSDILCRSDVYEFEVNG
>CORE_REP|Org48_Gene782#
MSFEGKIVLVTGASRGIGRAIAETFVARGAKVIGTATSESGAEAISSYLGANGKGFMLNVVDAQSIDS
VLASIRAEFGEIDILVNNAGITRDNLLMRMKDDEWEDILDTNLTSVFRLSKAVMRAMMKKRFGRIITI
GSVVGTMGNAGQANYAAAKAGLIGFSKSLAREVASRGITVNVVAPGFIETDMTRALTDDQRAGILSSV
PANRLGDAKEIASAVAFLASDEAGYITGETLHVNGGMYMI
>CORE_REP|Org23_Gene2366#
MDNLNLNKHISGQFNAELEHIRTQVLTMGGLVEQQLTDAITAMHNQDGELAKRVIEGDAKVNMMEVAI
DEACVRIIAKRQPTASDLRLVMAIIKTISELERIGDVADKICRTALEKFSHQHQPLLVSLESLGRHTV
QMLHDVLDAFARMDLDEAIRIYREDKKVDQEYEGIVRQLMTYMMEDSRTIPSVLTALFCARSIERIGD
RCQNICEFIFYFVKGQDFRHLGGDALEKLLSPGGKDDKAD
>CORE_REP|Org48_Gene1283#
MNPESSLNQSSTTPQIKTTALYSLSASFYRWQVFGLLISGLVFLWLSRNEQLDWAISNYWYDAASGHF
PWQNNYWLDLINHRLLKQIVIVGAVLTLFWGLYRRSARLIVTMLLIGIGPLVVGILKATSAHSCPWDL
IEYGGKAMSFPLFGTVPALPGPGRCFPGGHASSGFAVMALFFLFYPQRPRLAWWCWCGGIALGMLMGF
GQIMRGAHFLTHNLWAGWWVWLSQLAIYWMISGYWRRKMR
>CORE_REP|Org23_Gene3458#
MALPDTTSSGMCVLSIDVASKTTIIKRNRVMSATALGMIIFAYLCGSISSAILVCRIARLPDPREHGS
GNPGATNVLRIGGRLAAAAVLVFDILKGMLPVWLAYKLDVPPLYLGLTAIAACLGHIYPVFFHFRGGK
GVATAFGAIAPIGWDLTGLMTGTWLLTVLLSGYSSLGAIVSALIAPFYVWWFKPQFTFPVAMLSCLIL

MRHHDNIQRLWRGQEGKIWGKFRKKKNQADEDNGDQPKDE
>CORE_REP|Org42_Gene3472#
MADLLLGVNIDHIATLRNARGTQYPDPVQAAFIAEQAGADGITVHLREDRRHITDRDVRLLRQTIQTR
MNLEMAVTDEMLDIAIELKPHFCCLVPEKREEVTTEGGLDVAGQQDKMSVAVERLAQAGILVSLFIDP
DHRQIDAAVAVGAPYIEIHTGAYAEAQGELAVQAELRRIAVAAAYAAEKGLKVNAGHGLTYHNVQPIA
ALPEMHELNIGHAIIGQAVMGGLPAAVADMKVLMREARR
>CORE_REP|Org19_Gene2545#
MRVHASIEPLVWESDFFQLESAKLHFDSSAAPVAEADLDAYALVQAKIPAYRLGWADALSTLGFRLVE
GEVDLVVNVAPESAMADAASAVAVRQAVPEDIPSLRAAAGEVFAASRFRAPWYDRADSGRFYAAWIEK
AVQGTFDHQCLLVLDSQGQPEGFVSLRDIGGQEMRIGLLAAFPGASGRGVGARLMTAAIAECRQQGMQ
RLRVATQVGNIAALRLYQRQGAVIESTAYWLYRGRHDSI
>CORE_REP|Org45_Gene2094#
MRIPRIYHPQPLTDRAEIALSEDAANHVGRVLRMSAGQALQLFDGSNQVFDAEIVRVDKKSVLVRLSD
GRVDDIESPLNLHLGQVISRGEKMEFTIQKSIELGVNVITPLFSERCGVKLDGERLAKKIQQWQKIAI
AACEQCGRNRIPEIREAMSLEAWCAEQDGSLKLNLHPRASHSINTLPQPVDRVRLLIGPEGGLSADEI
AMTTGHGFTDILLGPRVLRTETTALTAITALQVRFGDLG
>CORE_REP|Org18_Gene792#
MFTSQEGKKVPQVTFHTRQGDQWIDVTTDDLFKNKTVIVFSLPGAFTPTCSSSHLPRYNELSSVFKQH
GVDGILCVSVNDTFVMNAWKADQHAENITFVPDGNGEFTKGMNMLVEKADLGFGPRSWRYSMLVRDGV
VEKMFVEPNKPGDPFEVSDADTMLKYLAPEFKVQESVSLFTKPGCPFCAKAKQMLQERGIQYEEIVLG
KDATTVSLRAVSGRATVPQVFIGGRHIGGSDDLETFLSA
>CORE_REP|Org36_Gene866#
MKLKALAMAAMVGLGTLPMALQAAEVPEGPHVVTSGTASVDATPDIATLAIEVSVSSKDAAQAKKQVD
ERVAQYFDFLQKNNIEKKDISAANLRTQPEYDYLKTGESVLKGYRAVRQVQVTLRQLDKLNELLDGAL
KSGLNEIRAVELGVAKPDVYREQARQKAIENATQQAESLAKGFHAKLGPVYSIRYRVANYQPMPVARM
YKAAGAAAESDAAQTYEQQSIHFDDQVDVVFELQRNAAQ
>CORE_REP|Org31_Gene266#
MSEIIYGIHAVKALLERDPQRFLEVFILKGREDRRLQPLIAELEATGIVIQVANRQWLDDKVEGAVHQ
GIIARVREGRQYQENDLPGLLESVETPFLLVLDGVTDPHNLGACLRSADAAGVHAVIVPRDRSAQLNA
TAKKVACGAAENVPLIRVTNLARTLRLLQEMNVWVVGTAGEADHTLYQSKMTGPMALVMGAEGEGMRR
LTREHCDELISIPMAGTVSSLNVSVATGICLFEAVRQRG
>CORE_REP|Org4_Gene3913#
MRKSLGGWRRLRPGYWLKRGVIAILGLWVLGIAAFAFLPVPFSAVMVERQVSAWLSGDFGYVAHSDWV
SMDDISPQMALAVMAAEDQKFPDHWGFDVAAIEKALSHNEKRPTRIRGASTLSQQTAKNLFLWDGRSW
LRKGLEAGLTSGIELVWTKRRILTVYLNIVEFGDGVFGVEEASQRFFHKPAKRLTAAEAALLAAVLPN
PHRFRADAPSGYVIQRQQWIMRQMRQLGGEAFLSENKLD
>CORE_REP|Org19_Gene248#
MATPHINAEMGDFADVVLMPGDPLRAKYIAETFLEGAVEVNNVRGMLGFTGTYKGRRISVMGHGMGIP
SCSIYARELIAEFGVKKIIRVGSCGAVRDDIKLRDVVIGMGACTDSKVNRLRFKDNDYAAIADFDMVR
NAVDAAAAQGIPARVGNIFSADLFYTPDPDMFQVMKKYGILGVEMEAAGIYGVAAELYEEFGCKALTI
CTVSDHILRHEATTAAERQTTFNEMIVIALESVLLGDKA
>CORE_REP|Org27_Gene3644#
MNSLILPDSPLPPGVKACSTTRGGGVSLPPYDSLNLGAHVGDEEQAVKRNRERLVTVAGLPQMPVWLE
QIHGTRVVTLAGQAPADLRADAVYSNVRGQVCAVMTADCLPVLFCSQHGDEVAAAHAGWRGLCHGVLE
QTVAAFSAAPSQISAWLGPAIGPQQFEVGPEVRAAFIAEDAEAAAAFTPHGDKFLANIYLLARQRLLR
AGVQAIYGGDRCTVNEKSHFFSYRRDGITGRMASLIWLI
>CORE_REP|Org19_Gene218#
MTRMKYLVAAATLSLALAGCSTSKDAVPDNPPSEIYATAQQKLQDGNFKGAITQLEALDNRYPFGPYS
QQVQLDLIYAYYKAADLPMAQASIDRFMRLNPTHPNIDYVMYMRGLTDMALDDSALQGFFGVDRSDRD
PQHARAAFRDFSQLIQQYPNSQYATDANKRLVYLKDRLAKYELSVAEYYTKRGAYVAVVNRAEQMLRE
FPDTKATHDVLPLMENAYKQLQLNGQADKVAKVIAANPQ
>CORE_REP|Org44_Gene146#
MDGWERAFVLHGRPYSETSLMLDLFTEGHGRVRLLAKGARSRRSNLKGCLQPFTPLLVRWGGRGEVKT
LRNAEAVSLGLPLSGMMLYSGLYVNELLARVLEQETNYSVLFFDYLQCLQALAAEDSSPEQALRQFEL

ALLHHLGYGLDFLHCAGSGLPVDDAMTYRYREEKGFIASLVVDHYSFTGRELRALAERQFPDAETLRA
AKRFTRMALKPYLGGKPLKSRELFRQFVRKQPNTPVDDA
>CORE_REP|Org36_Gene1111#
MSSTNIEQVMPVKLAKALSNSLFPALDSQLRAGRHIGIDELDNHAFLMDFQDELEEFYTRYSVELIRA
PEGFFYLRPRSTTLIPRSVLSELDMMVGKILCYLYLSPERLAHEGIFSHQELYDELLSLADENKLLKF
VNQRSTGSDLDRQKLHEKVRTSLNRLRRLGMVYFMGNDSSKFRITEAVFRFGADVRSGDDPREAQLRM
IRDGEAMPVETSLSLNDENEAEDQQVDNAPDGAEDEQE
>CORE_REP|Org6_Gene3169#
MMELSRHPTTYPTRYQQIAAQLEQELRTQYRCGDYLPSEQQLAERYQVNRHTLRRAVDQLVERGWLQR
RHGVGILVLMRPYDYPLHANTRFSQNLFEQGSHPTSERLLAVLRPCNGHVASALSREEGEMVIHLRTL
RRVNGVPMSVIDHYLPDLEWWPVLQQFHSGSLHQFIEQHLRQPLARRQTRISARRAQAKESRLLEIAT
HAPLLCVRTLNVRSGGEDVAEYSVSLARADMIELTMEH
>CORE_REP|Org45_Gene3084#
MSFFSTSNILVHIPLGAGGYDLSWIEAIGTLFGLLCIWYASKEKIINYLFGLINVTLFAVIFFQIQLY
ASLLLQLFFFGANIYGWYAWSRQTQDNQAELQIRWLPLPKALAWAAVCIIGIGLMTFNIDRVFAWLTQ
IAVAVMQGLGLNVQMPELQPDAFPFWDSAMMVLSIVAMILMTRKYVENWLLWVVIDVISVAIFAYQGV
YAMALEYVILTLIALNGSWLWIKSAGRNGSNPLSSAP
>CORE_REP|Org21_Gene531#
MATLIAENLAKAYKGRKVVEDVSLKVKSGEIVGLLGPNGAGKTTTFYMVVGIVPRDAGRIVIDEEDIS
LLPLHARARRGIGYLPQEASIFRRLSVYDNLMAVLEIRPDLTSEQREDRAKELMEEFHISHLRDSLGQ
ALSGGERRRVEIARALAANPKFILLDEPFAGVDPISVIDIKKIIEHLRDSGLGVLITDHNVRETLDVC
ERAYIVSQGKLIAHGTPDAILADEQVKRVYLGEEFRL
>CORE_REP|Org29_Gene191#
MATNAKPVYQRILLKLSGEALQGAEGFGIDASVLDRMAQEVKELVELGIQVGVVIGGGNLFRGAGLAQ
AGMNRVVGDHMGMLATVMNGLAMRDALHRAYVNARLMSAIPLNGVCDNYSWAEAISLLRNNRVVIFSA
GTGNPFFTTDSAACLRGIEIEADVVLKATKVDGVYSADPVKNPDATLYEQLTYQDVLERELKVMDLAA
FTLARDHGLPIRVFNMNKPGALRRVVMGENEGTLISK
>CORE_REP|Org1_Gene1937#
MSNHKTNNSRRDFLLKSMTLIPAAVIGGSGVGALTAPAPAVAAPNTSTQNDYQPTFFTPEEWAFIRAA
VARLIPADERGPGALEAGVPEFIDRQMNTPYATGSIWYMQGPFNPDVPKEMGYQLPLVPKQIYNLGIS
DADAYSKKTAGKPFAELDGAQQDAMLQKFESGEAEFQQLPSKLFFSYLLQNTREGFFSDPIHGGNKDM
VGWKLINFPGARADFMDWVERGERYPFPPVSIRGERG
>CORE_REP|Org39_Gene699#
MATVSMRDMLKAGVHFGHQTRYWNPKMKPFIFGARNKVHIINLEQTVPMFNAALAELSKISSRKGKIL
FVGTKRAASEAVKDAANSCDQFFVNHRWLGGMLTNWKTVRQSIKRLKDLEIQSQDGTLDKLTKKEALM
RTRELAKLENSLGGIKDMGGLPDALFVIDADHEHIAIKEANNLGIPVFSIVDTNSNPDGVDFIIPGND
DAIRAVNLYLTAVAAAVREGRSQDLAVQAEEGFVEAE
>CORE_REP|Org37_Gene1550#
MLNALIVDDEPSARDNLRHLLEAEAEIAIIGECANAIEAISQIHRLQPDVVFLDIQMPRISGLEMVGM
LDPNRMPHIVFLTAYDEYAVQAFEEHAFDYLLKPAEPKRLSKTLQRLRQRSAPQDVAALEESAGYLKY
IPCTGHSRIYLLRFDEVLAIRSKLSGVFVVRSDGMECFTELTLRTLESRTPLVRCHRQYLVNLEQVRE
IRFEEGGAAEMIMSAGDPVPVSRRYLKALKEQLGLRG
>CORE_REP|Org27_Gene864#
MHKIVFVEDDPEVGKLIAAYLGKHDIEVLIEPRGDSAQARIAHEQPDLVLLDIMLPGKDGMTLCRDLR
PTFPGPIVLLTSLDSDMNHILSLEMGANDYILKTTPPAVLLARLRLHLRQHGQQPKEESVQPLTQHNA
LHFGLLCIDPVNRQVTLGEETVTLSTSDFDLLWELATHAGQIMDREALLQNLRGVSYDGMDRSIDVAI
SRLRRKLYDNALEPFRIKTVRNKGYLFAPNAWASVQQ
>CORE_REP|Org2_Gene2323#
MACHFARWTPASAVLDTQRLADEVIAATRTFSVKRRTRFLQGRILLAEMMFYLYGLPTLPPIATTPTG
RPCFADHQLPDFSLAYASNTVGVLLSDEGKVGLDIEVMRARGTRQSALQHAHQTPAESAWIGAQDDRL
EAETQLWSIRQSVLKISGLGNSGQSTLRLHPFSGHLRSSATPDVQVMSDADEYLSWACAGSPGLDRLL
CWRYEERGGLQKDGEISPRSPAASSRFVKLTGLKTPG
>CORE_REP|Org2_Gene1254#
MELFLLSNGKLSGEAELLGYAKSQLLAMIARRGIKSAVFIPYALIRYDYDQRAQELAQTLGIEVTSIH

HAASPAAAIAQAECILVSGGNTWLLNQMLHEQGLIVPIQRAVREREVPYVGWSAGCNVATPSIRTTND
MPVRCSVVLPALGLFPVQINPHYIDAHISGHMGETRDERLAEFCAINPSESVVALREGSLLHVEGNEL
RYFSANGQGFKVFRHGEETREYQDTRALAALVPFNCG
>CORE_REP|Org31_Gene334#
MNAESSSRVQNVDHQEIAKFEAVASRWWDLEGEFKPLHRINPLRLNYIMQRAGGIFDKQVLDVGCGGG
ILAESMAREGAKVTGLDMGAEPLQVARLHALESGMDVTYVQETVESHAQANPQRYDVVTCMEMLEHVP
DPASVVRACAQLVKPGGHVFFSTINRNTKAWLMAVIGAEYVLKMVPQGTHDHKKFIRPSELIGWVDGT
PLREKHMIGLHYNPITDHFKLGRNVDVNYMVHTQHEG
>CORE_REP|Org36_Gene774#
MISLKNVSKWYGHFQVLTDCTTEVKKGEVVVVCGPSGSGKSTLIKTVNGLEPIQQGDILVNGTPVNDK
KTNLAQLRAKVGMVFQHFELFPHLSIIDNLTLAQVKVLKRDKTASREKGLKLLERVGLSAHANKFPGQ
LSGGQQQRVAIARALCMDPIAMLFDEPTSALDPEMINEVLDVMVELANEGMTMMVVTHEMGFARKVAN
RVIFMDEGKIVEDRNKDDFFNNPESERAKDFLAKILH
>CORE_REP|Org14_Gene1135#
MLKKTGLALLLLAATAAQAHAEYQCSVKPQDDVIISPQSVQVKGASGDLQISPDGDVIRNGQALSLND
SQRQKAFSYQSALRKQLPWIDDGAQQHLEKARSALDKVIVKELGSNSNVRNRLTTLNGQLKQQMNRII
EHRSDGLTFHHKAIDQVEQDGRNIVQQSMGGVLQDSLNEMGVKQAANSGGNPLQAIMGNLGGLQKAIQ
NEWNNQEQDFQNFGRDVCNRVTALETQRKDLLKALK
>CORE_REP|Org24_Gene2256#
MISLKVIARLLDYPEQVLFDHQQALIEALEPASELDLHHSAQLILFIRRLCARPLLDAQADYCELFDR
GRATSLLLFEHVHGESRDRGQAMVDLMAQYRAAGLEIDSRELPDFLPLYLEYLASRSAAQAREGLQDI
APILALLGARLQQRESPYAVLFDLLLLLSGSEVQAQTLETQVAQEARDDTPQALDAVWEEEQVKFLGE
QGCASAQQTAHQRRFAGAVAPQYLDLTDALTGTKGR
>CORE_REP|Org26_Gene2019#
MLSLRSVNQFYGQNHTLWDINLELPRGQCTVLLGRNGVGKTTLVNCIMGHVPVVSGSMTWQPADQPPQ
NLLLQPMERRAALGISHVPQGRQLFSQLSVEENLQVAQMAGRGAPRRIPPLIYSLFPHLRQMRARRAG
DLCVGAQRQLAIGRALAQEPALLILDEPTAGVPPSIAADIGNVIRRLNRELGMTILLVEHQLPFVRRV
ADRFCLLDSGRTVAHGALAQLDEALIGAGLAGQEEG
>CORE_REP|Org39_Gene2055#
MEHPNSAPVLITGGARRIGLALAKAFLERGVPVIIAYRSEYPAMAELKTLGACCIQGDFSTHEGIYLF
ADRVRQVAPKLRAVIHNASAWQAESPEVPPEQVMAAMLQIHVYTPYLLNQLLESCLTGQGQAGADIIH
LTDYVVEKGSDKHIAYAASKAALDNMTRSFARKLAPEVKVNAIAPALIIFNAGDDEAYRQQALAKSLM
KVAPGESEVVNLVNYLLESRYVTGRTHGVDGGRPLR
>CORE_REP|Org49_Gene391#
MINDVISPEFDENGRAMRRIRSFVRRQGRLTKGQQHALENYWPVMGVEYQADAVDLAALFGREAPTVL
EIGFGMGASLVTMAGNNPQQNFLGIEVHSPGVGACLADAHEAKLSNLRVMCHDAVEVLENMIPDGSLD
MVQLFFPDPWHKARHNKRRIVQTPFVELVLRKLKTGGVFHMATDWQPYAEHMLEVMNGVAGYRNLSSD
NDYVPRPDSRPLTKFELRGQRLGHGVWDLMFERKE
>CORE_REP|Org6_Gene19#
MQENHKILVVDDDMRLRALLERYLTEQGFQVRSVANAEQMDRLLTRESFHLMVLDLMLPGEDGLSICR
RLRSQSNPMPIIMVTAKGEEVDRIVGLEIGADDYIPKPFNPRELLARIRAVLRRQANELPGAPSQEEA
VIAFGKFKLNLGTREMFREDEPMPLTSGEFAVLKALVSHPREPLSRDKLMNLARGREYSAMERSIDVQ
ISRLRRMVEEDPAHPRYIQTVWGLGYVFVPDGSKA
>CORE_REP|Org43_Gene2781#
MSTDSSAPTSLGWYGKLPSAGDFLQRRLPDPVVNNWAHWFHNGLVNLQRDAQGPNGHPFSNAPVWNFV
IPATLGSPYVQMGCLLPARDRVGRRYPICALRLFSLQEWRPQQLNMAASWYQQLGHTLLNGVRNGFSA
EQIDRTLQAIPALPSPPAEADSEILSIIGFQHPDVPGLGWQQAADCFDPAQYTSFWWTNQADGHPLYT
HVHSGNLTVQLFSLLFEPNGWARPGRGGQYPQMFD
>CORE_REP|Org45_Gene2275#
MKEHQGEMPHYLQIKDQLQARITRGALQAGDKLPSERELCAIFSTTRVTIRESLAQLEATGAIYRADR
RGWFVTPERLWLDPTQNTNFHRLCQEQGRAPRTALLSGEKTTVPLDVMQPLALEPFDQVYLLRRVRYA
DGRAICYCENHCLPQRVPELLSHDLNGSLTEVYQQHYALIYSNMHLSFYPTALPYRAANALGAMVGLP
ALLLRRLNYDQHGRILDFDIEYWRHDSLRIEVDTL
>CORE_REP|Org2_Gene3523#

MAVGIQSRGFARWLAPVLALLVVMQLTACGDKEPEQRKAFIDYLQNTVMRSGANIPTLSEDQKQKFGN
YAGDYAILVGFSQQLSKSVGASLTPALDQINQIRTAQDYLNKRDALQQSVGALNLLGQQIQSAKSQAD
TARVALKQPDDLKAVYNQAYDKIVTAPANALMPAIPTTAGFVQDLVQVGDFLQSQGNQVSFNNNGVQF
RTAQQVAQYNTMMSNLVAKQQNLLNAQKAVQSVTQ
>CORE_REP|Org14_Gene1392#
MKPAHTLQKLTGPQSWAELPWGEYYREALERQLQPWWPKLFGFHLLKMGMLSAELATDKCAISHQVNV
GLEGEGLQVIADAYQLPFAAKSVDACLLAHTLSYADDPHRMLREVDRVLIDDGWLVISSFNPFSLLGL
GKLVPGLRRRQPYVSRMFTQMRLLDWLSLLNYEVLYQARFHVLPWHRKGGKFLCTHLPALGCMSVIVA
RKRTLPLTPTPMKLGARKPSLSRAVGATKSYRKLP
>CORE_REP|Org7_Gene1738#
MVIKAQSPAGFAEEYIIESIWNNRFPPGSILPAERELSELIGVTRTTLREVLQRLARDGWLTIQHGKP
TKVNNFWETSGLNILETVARLDHKSVPQLIDNLLSVRTNIASIFIRAAVRNHPEQAQEVLAKAAEVED
QADAFNVLDYEIFRGLAFASGNPIYGLIFNGLKGLYTRVGRYYFSNPEARKLALTFYSKLSTLCHEKL
YDQVMDTVRNYGKDSGAIWQSMQGTMPSDLTEARR
>CORE_REP|Org18_Gene1779#
MINVLIVDDDPMVAELNKYYLSQVGGFHCQATVATLSQARALLADAGVSIDLVLLDIYMQQENGLDLL
PGLRELGEKTDVIIISSASDVNTVQKALHYGVVDYLIKPFQFSRFKEALSHYRQQSQLLAQREFSQAD
VDSLLRRQPGGQESKKLPKGLTSITLSTVCEWIEQQHDNEFSTDNLANAIGISRVSCRKYLIYLAESG
ILGTRILYGATGRPVYLYQLKPDAIAMLKEHCRPA
>CORE_REP|Org21_Gene2589#
MEDEYNLSNIIGKRLEAALGELGDLLWAYVVLSKKDIACIFGVTNYPSEWVKKYQEQGLQYIDPVVLT
ARNRLTPFAWDEQIMADAGLHFPELFEQARGFGVTHGYTFVLHDYNDNLVTLSFAFNVEQRAEAIQAL
TERKGDISVLLSSLHESYLALSPLSAKNAAALERNVRFTDRENEILYWASVGKTYQETAMILGIKTGT
IKFHMSNIVKKLGVTNARHAVRLGMELRLIKPVEY
>CORE_REP|Org4_Gene4784#
MQITNGENKARPEGLAERIYLQLKADIFEFRLLPGDRFSESDVAQRMAASRTPVRQALFRLEREGYVE
VLFRSGWQVRPFDFAYFEELYDLRIVLEQEAVKRLCEWPAGETPAALAALNRFWTEAPRLADGQAVSQ
HDEQFHQALVAAAGNGEMARIHRELTEKIRIIRRLDFTQQARVDATYREHAAILQAILQRQSGAAQAL
LGEHIAVSKAEVRKITLHMLHQARAQQPARSAQRF
>CORE_REP|Org39_Gene2549#
MSDRSALLDAVPHIQHGFGSKLALLPGHLLPYSATLPEKKQVHGIRIVDVLQPAQACGEADGFYTRQP
GILLSVLTADCLPVLFSRRDGGAIAAVHAGWRGLLDGILEQMAARIRQDGDTADWVASIGPAAGPCCY
EVDEALVENFKQRLPLPATLISPHYRHLDLAAIAEYKLAALGFAAVDRAGSCTICTPDVDPQRPQRFK
YTSYRRNSHRRAQDPTHPGIKGRNQYAGIIIAAG
>CORE_REP|Org25_Gene2235#
MKLEFSIYRYNPDVDDAPHMQDYTLEAEEGRDMMLLDALIQLKEKDPTLSFRRSCREGVCGSDGLNMN
GKNGLACITPVSSLRKGNGKIVIRPLPGLPVVRDLVVDMGQFYTQYEKIKPYLQNDGKNPPAREHLQS
PEQRAKLDGLYECILCACCSTSCPSFWWNPDKFIGPAGLLAAYRFLIDSRDTETQERLDDLDDAFSVF
RCHSIMNCVSVCPKGLNPTRAIGHIKSMLLQRGA
>CORE_REP|Org35_Gene254#
MQTPHILIVEDELVTRNTLKSIFEAEGYIVHEANDGAEMHNILSENDINLVIMDINLPGKNGLLLARE
LREQASVALMFLTGRDNEVDKILGLEIGADDYITKPFNPRELTIRARNLLSRTMNLGSLGEERRLVES
YKFNGWELDINSRSLISPAGEQYKLPRSEFRAMLHFCENPGKIQSRGELLKKMTGRELKPHDRTVDVT
IRRIRKHFESTPDTPEIIATIHGEGYRFCGDLEE
>CORE_REP|Org25_Gene2255#
MKTQWIERVRVGVARLWPHPLAVGKREMLISSVGAGLGLMLAGWISHFILGEVNLWFIAPMGASAVLL
FGVPNSPLAQPWSIVGGNALAATVGVSAGLLILDPGLACGVAAAVAIGLMFKLRCLHPPGGAVALTAI
LGGPGIHQMGYHFVLYPVLLNSVLLAALAILFNNLAGRRYPHALAPAEAKPANLPIDAVAITRADLHE
ALMEGDLFDIDEDDLQEILLRAEQLAHQRQSKAA
>CORE_REP|Org11_Gene2147#
MHFYRPLRPLAALTFDLDDTLYDNRPVIRQTEQQSVAFLQSYHPGLSSFQSADFHRLRQELREQEPEI
YHDVTQWRWRAIHLALSRQGLRDAEAAIGADAAMQNFALWRSRIEVPEATHATLKALAARYPLVAITN
GNVDPSLCGLDGYFQFVLRSGPDGRAKPYQDMYHLAVERLGVAPEQILHVGDDLTTDVAGALRAGLQA
CWINDRQRCLMQAADSRLLPHIEISQLASLTALL

>CORE_REP|Org13_Gene4007#
MKKAVAIRHVNFENLGILGSLLSLRGYQIDYYDAGRDDIRAINNEETDLLVVLGGPISAAQHFSGALR
YDFLDHELALVKQRLAQRRPTLGVCLGAQVIAQALGADVISLGVKEIGFAPLTTLAAEDDSPLAPLAD
TPVLHWHGDMFTIPEGARCLAGTAVCPHQAFDYQGFALGLQFHLEADYRDIERWLIGHACELELAGVA
PQSLREQAARHGTQLEQRARQVFARWLDNNEPRM
>CORE_REP|Org25_Gene885#
MNELQPLASAAGMTVGLAVCALILGLILAMLFAVWESSRWKAVSWLGTAWVTVLRGLPEILVVLFIYF
GSSQLLLMLSDGFTLNLGLFQLPIQLAIDNFEVSPFLCGVIALALLYSAYASQTLRGALKAVPQGQWE
SGQALGLGKAAIFFRLIMPQMWRHALPGLGNQWLVLLKDTALVSLISVNDLMLQTKSIATRTQEPFTW
YVIAAAIYLLVTLFSQYVIKRIELRATRFERGPV
>CORE_REP|Org21_Gene1433#
MKKGLMLLSLLVASVTGAAHADDAAIKKALASLGIQQADVQPSPVNGLKTVLTDSGVLYASEDGKHIL
QGPLFDVSGKEPVNVTNQLLSSKMDALKDQMIVYKAPKEKHVITVFTDITCGYCHKLHQQMKEYNDLG
ITVRYLAFPRQGLASQAEKDMKSIWCTADKAKAFDAAMKGDAISPATCKTDISKHYELGVQFGIQGTP
AIILENGMMIPGYQGPKEMAAMLDAHQAATKAGG
>CORE_REP|Org37_Gene809#
MRPAGRAPQQVRPLTLTRHYTKHAEGSVLVEFGDTKVLCTATVEEGVPRFLKGQGQGWITAEYGMLPR
STHSRNAREAAKGKQGGRTLEIQRLIARSLRAAVDLKKLGEFTITLDCDVLQADGGTRTASISGACVA
LADALNTLVANGKLKANPMKGMVAAVSVGIVNGEALCDLEYVEDSAAETDMNVVMMEDGRMIEVQGTA
EGEPFSHDELLALLALARGGIETIFQAQKAALAD
>CORE_REP|Org15_Gene1664#
MSETMLEFREVDVFYGPIQALRQVSLQVNAGETVALIGANGAGKSTLLMSIFGQPRIAGGQILFRGED
ISRRSTHFVASSGIAQAPEGRRIFPDMSVEENLLMGTITVGNRYLEEDLPRMFELFPRLKERRNQRAM
TMSGGEQQMLAIARALMSRPKLLLLDEPSLGLAPIVVRQIFGVLRELTRSGMTLFLVEQNANHALKLS
DRGYVMVNGQIRLTGSGEELLNDPQVRKAYLGGG
>CORE_REP|Org7_Gene2013#
MDIQNQPVQIMIVEDEPKLGQLLVDYLQAAGYATRWLTNGNEVVPTVHQHPPALILLDLMLPGADGLT
VCRELRRFSDVPIVMVTAKIEEIDRLLGLEIGADDYICKPYSPREVVARVKTILRRSYRPQENAREDD
LLHIDEPRFQASYQGQLLDLTPAEFRLLKTLASQPGNVFSREQLLNNLYDDYRVVTDRTIDSHIKNLR
RKLELIDGQKSFIRSVYGVGYRWEAEPCRLVNGV
>CORE_REP|Org39_Gene2390#
MKSVEEQSVAGLALDDDTVMQYLLQNPDFFIRNARQVEQMRVPHPVRGTVSLVEWHLARQRNHIERLE
EEITLLMEQASANEALFARLLHLQADLATADSLQDMLNRLQRWARGFGLAGATVRLFAERWKIGAPSD
FTHLALTRSAFEPFRIQRLGSEQHYLGGLNGPELLLLLPQAKQIGQIGSVALSMLGDDGELGMVIFSS
RDTQHYQQGMGTVMLNQLARMLPELLERWVERA
>CORE_REP|Org7_Gene2713#
MRLDKFLSQQLGISRALVARELRAKRVTVDGEVVKSGAVKLTPEQEVAFDGNPLQQQNGPRYFMLNKP
QGYVCSTDDPDHPTVLYFLDEPVAYKLHAAGRLDIDTTGLVLMTDDGQWSHRVTSPRHHCEKTYLVTL
EHPLAEDTAQRFAAGVQLHNEKDLTRPATLEQVDEHVVRLTISEGRYHQVKRMFAAVGNRVIELHRER
IGAIVLDDDLAPGEYRPLTEEEIASVGAPHLQD
>CORE_REP|Org30_Gene2612#
MQKLAELYRGKAKTVYTTENPDLLVLEFRNDTSALDGQRIEQFDRKGMVNNKFNHFIMSKLEEAGIPT
QMERMLSDNEVLVKKLDMVPVECVIRNRAAGSLVKRLGIEEGLVLNPPLFDLFLKNDAMHDPMVNESY
CETFGWVSKAHLARMRELSYRANDVLTQLFDDAGLILVDFKLEFGLFNGEVVLGDEFSPDGSRLWDKN
TLDKMDKDRFRQSLGGLIEAYEEVARRIGVKLD
>CORE_REP|Org46_Gene1803#
MDKPKRILIVEDDGDIAELLQLHLRDEGYAISHAADGNQGMAMLEQGGWDALILDLMLPGVDGLEICR
RARTMTRYTPIIIISARSSEVHRVLGLELGADDYLAKPFSMLELVARVKALFRRQEAMSRNLRMDAGV
LSFNDLTIDPIAREVHLHQQPVELTPREFDLLYFFARHPGQVFSRLSLLNQVWGYQHEGYEHTVNTHI
NRLRIKIERNPAEPERILTVWGMGYKFAAAPQE
>CORE_REP|Org38_Gene3476#
MISLRQLAIGYGATPLFPPLSGQFSAGSLTAVVGVNGAGKSTLLKTLAGLLPPVAGRLDFSGEKPPRK
AYLPQQAELDRQFPIAVSDLVAMGCWPQSGMFGGMNQRAASQVNEALASVGMSALAHSPVGELSGGQL
QRVLFARLLVQQAPLILLDEPFTGIDSATTQILLQVIAQLHQQGRTVIAVLHDMSMVAEHFPQVLLLT

PQACHWGAAERVLEQVPRYLAAERQPGLRVVAP
>CORE_REP|Org43_Gene2400#
MNLDWLLAPQYLSWLWHGFLLTLWLSACAGLAATLLGFVLAAMRDSSLRPLRWLAMGYSSLFRNTPLL
VQLFFWYFAAGQILPSAAMQWLNSAHQVGPLEWPSFEFLAGFFGLTLYSTAFIAEEIRSGIRGVAGGQ
KYAAQALGLTGWQAMRYVVLPQALKIALPPLLGQYMNVIKNSSLTMAIGVAELSYASRQVETETLRTF
QAFGVATVLYIAIIALLEGWGMWRQQRKPLGGH
>CORE_REP|Org44_Gene2260#
MTRRNLPLILLFNLLGVALFLSWFLPANHGGWFTLDSAIFFFFNRHLATDPAFLHLVAITNNRAFDVI
SLLAMGLLYLYFYLKQDAAGRRRLVITGVVMLLTAVVLNQLGHLLPVKHPSPTLTFDNIYRVSELTGI
PTKDASSDSFPGDHGMMLIIFSCFMLRYFGRRAFAVALLITLLFSLPRVMIGAHWFTDIAVGSLSVVL
VGASWVLMTPCSDWIVDRLNRLLPGKHRPGQP
>CORE_REP|Org49_Gene4380#
MSEAPTTVATICQTLNARIAAGEFAVGGKLPSERALSEQFATTRITLQEALGQLEAQGVIYRQVRRGW
FISPPRLIYNPLQRSHFHAMAQQQGRAAHTEAIDSSVVTLDAPLAGRLALPPGAEAYRIRRLRYIDGR
AVLYCEHYLNPAYFPGILDEDLTQSLTALYAARYGIHYGRVRFDMLPTLLPQQAAAMLKVTYGSPALF
ITRVNRDQHDRVIDCDLEYWRYDALHIDVEAQ
>CORE_REP|Org25_Gene3724#
MSRVLVLKSSILGEYSQSGKLVDFFVEQWREAHPEDTFTVRDLANPTLPELDGEVMAGFTAGDKPLTP
HQQSTLALSDELIAELKSHDTLIISAPMYNFNIPTQLKIYFDLIARAGQTFRYTSAGAEGLVTGKKAI
VISSRGGIHADTPTDLITPYVKLFLGFIGITDVEFVLAEGFAYGPEAAEKAAQDSRIAVAQKIPAGVA
VPASAPAPAPANVAQEAVSGGFLSNLLKKLFR
>CORE_REP|Org42_Gene2432#
MNHSAGTFPSVIAVLPAAGIGSRMQAECPKQYLTIGRHSIVEHAIHALLRHPRIERVIVAIGPEDRQF
EQLPIAQDPRVIVTEGGKQRADSVMAGLNLAGDADWVLVHDAARPCLHADDLERLLAITAHSKVGGIL
AAPVRDTMKRAEPGRETIAHTVDRQDLWHALTPQLFPLPLLKQCLQRALDEGANVTDEASALEHCGYH
PLLIAGRADNIKVTRPEDLALAAFYLTQLDN
>CORE_REP|Org5_Gene414#
MIFSPPLRSATLIKRYKRFLADVITPEGETFTLHCANTGAMTGCATPGDTVWYSTSDNPKRKYAHSWE
LTHTQQGHWICVNTLRANALVREAIEHNLINELSGYSKISGEVKYGGENSRIDLLLQAENRVNCYIEV
KSVTLLQQQRGYFPDAVTLRGQKHLRELLSVVESGQRAVLFFAVLHSGIEQVAPAHHIDERYAALLAQ
VRQLGVEVVCYGAKLSPDGIYLCDKLPFFID
>CORE_REP|Org37_Gene3701#
MTEHRPRSERGQLAVAGENYGSSLLGAPLLYFPAAVSGPETGLIIAGTHGDESAAIVTLSCALRSIAP
ERLRHHVVLAVNPDGCQLGLRANANGVDLNRNFPAANWRSGDTVYRWNSAAPVRDVKLSTGGRPGSEP
ETQALCHLIHRLKPHWVVSFHEPLACIEDPASSRLGVWLAHKFELPLVTSVGYETPGSFGSWCADLSL
PCITAEFPPISADDASERYIDAMTELLTYPN
>CORE_REP|Org11_Gene2639#
MSTIQQNLQDVRNRIAAAAQNCARAPEEVALLAVSKTKPVAAIEEAIAAGQRAFGENYVQEGVDKIRH
FAESPQGGELVWHFIGPLQSNKSRLVAEHFAWCHTVDRLRIAQRLSDQRPADMPPLNVLIQINISDEQ
SKSGIALSELPALAEALAALPNLTLRGLMAIPAPEADYQRQLAVFNQMNDAFLALRQRYPQADTLSMG
MTDDMAAAIAAGSTLVRIGTAIFGARDYSQA
>CORE_REP|Org24_Gene1986#
MLDSTQFPIVKRWPAQHPERLQLYSLPTPNGVKVSIMLEEIGLPYEAHLIEIGNNETWTPEFLALNPN
GKIPSIIDPDGPGGRPLPLFESGAILLYLAEKSGRFLPQDPAQRYETIQWVFFQMAAVGPMFGQLGFF
HKFAGREYEDKRPLERYKNESKRLLGVLETRLEGRDWIMGADYTIADISLLGWVRNLIGFYEARELVE
FDSFPRVAQWLERGLARPAVQRGLTIPARAA
>CORE_REP|Org37_Gene3786#
MTTETAATILLIDDHPMLRNGVKQLIGMDARLQVIAEASNGEQGVTLAEQHDPDLILLDLNMPGINGL
ETLDRLRQTDLSGRVVVFSVSNHEDDVVSALKRGADGYLLKDMEPEDLLKALHQAAAGQMVLSETLTP
ILAASLRENRPSADRDIQQLTPRERDILKLIAQGLPNKLIARRLTITESTVKVHVKHLLKKMKLKSRV
EAAVWVLQGKTVNRRRTAARFAPEWAAASGF
>CORE_REP|Org16_Gene1693#
MIEILQQYWQSLLWSDGYRFTGVAVTLWLLIASVVMGGLLAIPMAVARVSSLRWVRFPVWLYTYVFRG
TPLYVQLLVFYSGMYSLEIVRGTEFLNAFFRSGLNCTILALTLNTCAYTTEIFAGAIRAVPHGEIEAA

NAYGFSRFKMYRCIILPSALRTALPAYSNEVILMLHSTALAFTATVPDLLKIARDINAATYQPFYAFG
IAAVLYLIISYVLISLFRKAEKRWMAHVSH
>CORE_REP|Org8_Gene2858#
MLLGYGAGVAGLLALLLALLLAGILLAWWGWRYAAARCGRPRPPLSWGGYLLASALAIYPVAFALMLI
DIALQDLKSQRAEERWNRRSYLTLEATKPFGEITLPKGSWVNRQEPRQPGTEDSPITMNGVTAVRFPQ
PQSVAGVPIIAFEVLPPVMELASAHTFIRPSGQRVHCEAGWLVSFSLPPDKPAPQWEEWQAGLPAAAF
RPSDWIFRECFSGSPIAVLTLHNGEVRVLP
>CORE_REP|Org17_Gene3023#
MLTLEKLTYLYEHLPMRFDLRIQPGERVAVLGPSGAGKSTLLSLIAGFLPAASGRLLLNGEDHTATPP
AKRPVSMLFQENNLFAHLTVAQNIGLGLDPGLRLTAQQRQQREHIARQVGLEEHLDRLPAQLSGGQRQ
RAALARCLIRRRPILLLDEPFSALDPALRNEMLQLLQTVCEQRDLTLLMVSHNLDDAARIAPRTLLVV
DGRIYYDGPTQALLDGSAPEARVLGISGKA
>CORE_REP|Org3_Gene3193#
MAMNRYGQPIGEPMPDWQPARRPGGATLGGRFCSLAPLEPQRDYAALFEAFQLAPDGRDWTYLSIERP
DTPAAMLQHLETLQANPALVNLTVFDAASDAPVGTVALMRIDEANGVLEIGHVSWSPLMKQRSSATEA
IALLLRYAFDTLGYRRCEWKCDSHNAPSRQAALRFGFRYEGNFRFAVIVKGRSRDTDWFAITADRWPT
VRQALARWLSADNFDAQGRQIARLQVLRGE
>CORE_REP|Org1_Gene4257#
MTMQIRVEHISKTFVLHQQYGTRLPVLHDANLTVHGGECVVLHGHSGSGKSTLLRSLYANYLPDSGHI
WINHQGDWLDMVSADARQILAVRRHTLGWVSQFLRVIPRISALEVVMQPLLEQGVERAECRDRAEALL
AALNVPQRLWPLAPSTFSGGEQQRVNIARGFIVDYPILLLDEPTASLDSRNSAAVVQLIERAKARGAA
IVGIFHDEGVRQQVADRLYDMQAPQALEAL
>CORE_REP|Org32_Gene3897#
MSEPLVIVFPIYQGVTQLDFTGPLQFLRRMPGAEIIVASVDGADVESEGLHFTQLRPLPEIARCDVLC
VPGGSGCTQALQDEAFMQQIRRLGTDARYLTSVCTGSLILAAAGLLLGKRAACHWSMRDSLKLFGAIP
SAARVERDGNVISGGGVTAGIDFALALIAELHGEDTAQTIQLYLEYAPAPPFEGGTPELAPPHIYAKV
QAQMAESLARRRALVAHSDQAYTCPYASAT
>CORE_REP|Org19_Gene3530#
MTQPFSGFSQQGLNFLQQVRIENDKAWFDGNRDIYDRELLAPFRALVEQLAPGMLAIDPQFETRPAIG
KTLSRIHRDTRFSHDKSRYRSRMWLTFKRPSKDWKDAPVYFFELGPDMLRYGLGYYSANKPTMDLFRH
TLRQRPQPFLEVAACCRPPFELVGESYKRPLVKEQAAEIATWYNRKSFAVMVTDSEVEKLFSADLAPL
LAGAFLQLEPLYHWLMQVETMKQVDPADL
>CORE_REP|Org16_Gene2762#
MITCRTAQQCGQADFGWLQARYTFSFGHYFDPKLMGYASLRVLNQEVLAPGASFQPRTYPSVDILNLI
LQGEAEYRDSDGNTARAKAGEALLLATQPSLSYSEQNVSSDTPLTRLQLWLDACPERNNERVQRLTLP
TAGNLLLASPDGAQGSLQLRQQVWIHHLDLQPGEEHTLTINGPRAYLQSIHGTIAATGERHEEQRLTC
GDGAFVREENRLTIRAETPLRALLIDLPV
>CORE_REP|Org8_Gene519#
MAILPRQGQRWLAGMVMLTLSGCAYIPHKPLVDGATTAQPAPASAPMPNGSIFQTVQPMNYGYQPLFE
DRRPRNVGDTLTIVLQENVSASKSSSANASRNGASKFGVATSPRYLDGLLGNARADMDISGDSTFGGK
GGANANNTFNGTITVTVNQVLANGNLHVVGEKQIAINQGTEFIRFSGVVNPRTISGNNSVTSTQVADA
RIEYVGNGYINEAQTMGWLQRFFLNVSPF
>CORE_REP|Org8_Gene501#
MTILSCKVTSVEAITDTVYRVRLVPEQPFSFKAGQYLMVVMDERDKRPFSLASTPTQQDYIELHIGAS
ELNLYAMAVMDRILKEQAITVDVPHGDAWLREEGSRPLVLIAGGTGFSYARSILLTALEQQPDRDISI
YWGGRELKHLYDLSELEALSLQHPNLKVIPVVEQPEAEWRGRSGTVLSAVLQDFGTLAEHDIYIAGRF
EMAKIARERFCAERGALEAHMFGDAFSFI
>CORE_REP|Org47_Gene838#
MNTPAQLSLPLYLPDDETFASFYPGENPSLLAAIQSAVRQEHGSYIYFWSREGGGRSHLLHAACAELS
QKGEAVGYVPLDKRAYFVPEVLDGMEQLALVCIDNIECIAGDEEWEMAIFNLYNRILETGRTRLFITG
DRPPRQLNLRLPDLASRLDWGQIYKLQPLSDEEKLLALQLRGKLRGFELPEDVGRFLLKRLDREMRTL
FMTLDQLDRASITAQRKLTIPFVKEILGL
>CORE_REP|Org8_Gene359#
MKVGIIGAMEQEVTLLRDQIENRQTIQRAGCEIYTGQIGGVDVALLKSGIGKVSAAMGTTLLLEHCSP

DVVINTGSAGGLASTLRVGDIVVSEEVRYHDADVTAFGYEPGQMAGCPAAFVADDALIALAESCIKQL
DLHAVRGLICSGDAFINGAEPLARIRATFPRVAAVEMEAAAIAHVCHLFGTPFVVVRAISDVADSESH
MSFDEFLVVAAKQSTLMVNAMLQTLAKRG
>CORE_REP|Org29_Gene2561#
MSQSPIELKGSSFTLSVVHLHNSQPEVIRQALQEKVEQAPAFLKNAPVVINVATLDGDANWKELQQAV
AAAGLRVVGISGCRDERQKRAIARAGLPLLSEGKGQKMAAPEPAPAPVPAVADNAPAKTRIISTPVRS
GQQIYARNCDLIVTNSVSAGAELIADGNIHVYGMMRGRALAGASGDTQCQIFCTHLAAELVSIAGQYW
LSDQIPSDYVGQAVRLSLLDNALTIQPLN
>CORE_REP|Org3_Gene643#
MADFGAIRALAFDLDGTLVDSAPGLAAAIDMALEETGLPQAGEERVGTWIGNGADVLVQRALRWAEAE
GTPEQCSRLRERFDHFYAQTVDSGSRLFPQVQETLARLAAHGYPMALVTNKPTPFVAPLLAALGIGDY
FSLVIGGDDVTEKKPHPAPLYLVLGKLGLRAHELLFVGDSRNDIQAAQGAGCPSVGFTYGYNYGESIA
LSHPDRVLERFADLLPALGLSSLENQEI
>CORE_REP|Org24_Gene96#
MGQKVHPNGIRLGIVKPWNSTWYANTKEFADNLDSDFKVRQFLTKELSKASVSRIVIERPAKSIRVTI
HTARPGIVIGKKGEDVEKLRKVVADIAGVPAQINIAEVRKPELDAKLVADSITSQLERRVMFRRAMKR
AVQNAMRLGAKGIKVEVSGRLGGAEIARTEWYREGRVPLHTLRADIDYNTSEAHTTYGVIGVKVWIFK
GEILGGMAAVEQPEPAAQPKKQQRKGRK
>CORE_REP|Org18_Gene3273#
MSTITDTFIAPPCHDQIEILYQDDHLALINKPAGLLSLSGKNPQNLDSVHHRLVQNFPGCALVHRLDF
GTSGLMVVARNKAANAALCRQFSERTVSKVYSALLCGHLADNEGVIDAAIAKDPALFPLMSICALHGK
PARSRYRVVERFYRETEGGISLPLTRVQFTPETGRTHQLRIHSRQLGHPILGCDLYGGRLLPGTEQTP
RLMLHASELDFVHPVSGARIAARHAAPF
>CORE_REP|Org44_Gene1704#
MSEAKELIVQGLWKNNSALVQLLGMCPLLAVTSTVTNALGLGLATTLVLVLTNASISAVRRWVPSEVR
IPIYVMIIAAVVSIVQMLINAYAFGLYQSLGIFIPLIVTNCIVVGRAEAVAARKPVGLSALDGLAIGL
GATGVMVTLGSMRELLGNGTLFDGADQLLGGWAKSLRIEVVHFDSPFLLAMLPPGAFIGLGLLLAVKY
LIDEKMKARKARAVAVEPLLEQGRAEKA
>CORE_REP|Org7_Gene165#
MNKILLVDDDRELTSLLKELLEMEGFNIVVAHDGEQALSLLDSSVDLLLLDIMMPKKNGIDTLKELRQ
HHQTPVIMLTARGSELDRVLGLELGADDYLPKPFNDRELVARIRAILRRSNWSEQQQQVDSGAPTLDV
DGLQLNPGRQEASFDGQVLDLTGTEFTLLYLLAQHLGQVVSRELLSQEVLGKRLTPFDRAIDMHISNL
RRKLPDRKDGHPWFKTLRGRGYLMVSAT
>CORE_REP|Org2_Gene1833#
MAGLDKRVGSYQEALAGLTDNMTVLAGGFGLCGIPENLIEEIRRRGVRGLTVVSNNCGVDGFGLGRLL
ETHQIRKVVGSYVGENALFEQQALSGELEVDLTPQGTLAEKIRAGGAGIPAFFTATGYGTPIAEGKEV
REFNGRPYIMETAITGDFAIVKGWKADYFGNVIYRHTAQNFNPLMATAGRITVVEVEEIVAPGELDPA
AIHTPGIYVDRLIQGTFEKRIEQRTLRA
>CORE_REP|Org24_Gene473#
MKRAVVVFSGGQDSTTCLIQALQQYDEVHCVTFDYGQRHRAEIEVAQELSVALGAKAHKVLDVTLLNE
LAVSSLTRDNIPVPAYDPNQKNALPSTFVPGRNILFLTLAAIYAYQVEAEAVITGVCETDFSGYPDCR
DEFVKALNKAVTLGIARDIRFETPLMWLNKAETWALADYYHQLDRVRQDTLTCYNGIKGDGCGECAAC
NLRANGLTQYRANQAEVMAALKQKAGLA
>CORE_REP|Org41_Gene786#
MIHEIWWSLPLTLAVYFAARWLARKLNMPLLNPLLVSMAVIIPLLLLTGIPYERYFQGSKILNDLLQP
AVVALAFPLYEQLHQIRARWKSIIAVCFIGSLTAMISGGAIALWLGATPEIAASILPKSVTTPIAMAV
ADSLGGIPAISAVCVIFVGILGAVFGHTLFNLLKITTHSARGLAMGTASHALGTARCAEMDYQEGAFG
SLALVICGIITSLLAPFLFPVLLHLFG
>CORE_REP|Org5_Gene2413#
MLRRILFIAVFAAGAVQAHGINNFSQAKAAAAKINQDAPGSFYCGCRIDWQGKKGIPDLAGCGYQVRK
NAQRAQRIEWEHVVPAWQFGHQLQCWQDGGRKNCNKDATYRQIETDLHNLQPAIGEVNGDRNNFMYSQ
WNGGAGQYGQCPMKVDFKNKQAEPPARARGAIARTYFYMRDRYQLRLSRQQTQLFEVWNRQYPVSQWE
CQREARIAKVQGNHNPYIQQACQQRKS
>CORE_REP|Org30_Gene2257#

MLTRDFLQKADCKTAFGAIEESLLLTPEQRAASLACTLSRRPDHSPVWVFGYGSLMWNPVFESEEVRP
AILQGWHRAFCMRLTAGRGTLHQPGRMLALKEGGQTTGLAFRLPESTLREELELLWKREMITDCYVPT
WCELQLEGGEAVTALVFVMNPQHPLFEADTSHQVIAPLIASASGPLGTNAQYLFALDNELKHHGMQDD
CIGDLVRYVQQWLQQNRPGAFGAEATA
>CORE_REP|Org11_Gene4378#
MSPERYARICEMLATRQPDLTVCLEQVHKPHNVSAIIRTADAVGVHQVHAVWPTTRMRTLVSSAAGSN
SWVSVKTHPTIGDAVGHLKAQGMQILATNLSARAVDFREVDYTRPTCVLLGQEKTGITEEALALADQD
IVIPMIGMVQSLNVSVASALILYEAQRQRQNAGLYRRDNSMLDEEEQQRLLFEGGYPVLANVAKRKGL
PRPKIDEQGQVVASAEWWAAMQATVRK
>CORE_REP|Org14_Gene3426#
MKAQVRIALVGDYNPQAVAHQAIPVALQLTAAHLDIDVQPQWLPTETLTTPDILQNFDAIWVVPGSPY
RYDDGAFMAIRHARENDVPFLGSCGGFQYAIVEYARNVMGWHDAGHAETDSGGRLVIAPLSCSLVEKT
GDIVFQPDTRVAQAYGSLNTHEGYHCNFGVNPEFVADLERFPLIISGHDTEGDVRAIELPGRRFYVAT
LFQSERAALRGELSPLVVELVKTAANG
>CORE_REP|Org26_Gene2866#
MLTQLKQQVLEANLDLPRHKLVTFTWGNVSAVDRERGLVVIKPSGVEYEHMTAEDMVVVDLASGRTVE
GAKKPSSDTATHLALYREFADIGGIVHTHSRHATIWAQAGLDIPAWGTTHADYFYGAIPCTRLMTQDE
IEHDYELETGKVIIETFRRRDINPNAIPAVLVNAHGPFAWGKDAHNAVHNAVVLEEIAYMGIFSRQLT
PGIHSMQRELLDKHYLRKHGQNAYYGQ
>CORE_REP|Org11_Gene1728#
MKVLGLLGGMSWESTIPYYRTINEQVKARLGGLHSAKIVLYSVDFQEIERLQHQGDWDGAGRLLAEAA
VALRAAGAEAIVICTNTMHKVAEAVEQASGLPLIHIADATALQVREKNIRRVGLLGTRFTMEQTFYRG
RLQELHGIDVVTPVSADRDIVHRIIYDELCLGNIRDASRDEYRRIISDLEQQGAQGIIFGCTEIGLLV
SERDASVPVFDTTAIHARAAAAFALD
>CORE_REP|Org21_Gene749#
MILSEYEWQAVELSLKVSGLAVVCSLPFGILMAWVLVRCRFPGKALLDGVIHLPLVLPPVVVGYLLLI
AMGRRGVIGEWLYDWFGFSFSFSWRGAALASAVVAFPLMVRAIRLALEAVDTRLEQAARTLGANPWRV
FFTITLPLSLPGVIVGVVLAFARSLGEFGATITFVSNIPGETRTIPLAMYTLIETPGAEAAAARLCVI
AIVLSLASLMVSEWLARWGRKRMGV
>CORE_REP|Org31_Gene2612#
MKHEHFVVQSPATPAAQLILLFHGVGDNPVAMGEIGSYFAKEFPQAQVVSIGGPEAFGNGAGRQWFSV
QDVTEENRAARIADVMPQFVAVVRHWQQLSGVGYAGTALVGFSQGAIMALEALKAETRLAGRVVAFSG
RFAQLPEQAFGDSVVHLIHGAEDAVIAVQHAHAAAEALRAGGADFTLDVEENVGHAINQGMMNAALER
LHYYVPQRYWDEALSGKRGELIAFR
>CORE_REP|Org22_Gene620#
MARRILVVEDEAPIREMVCFVLEQNGYQPLEAEDYDSAVTRLSEPFPDLVLLDWMLPGGSGIQFIKHM
KREALTRDIPVMMLTARGEEEDRVRGLEVGADDYITKPFSPKELVARIKAVMRRISPMAVEEVIEMQG
LSLDPSSHRVMANDQALDMGPTEFKLLHFFMTHPERVYSREQLLNHVWGTNVYVEDRTVDVHIRRLRK
ALETSGHDKMVQTVRGTGYRFSTRY
>CORE_REP|Org19_Gene3893#
MSRAQRLLDLMQLLRSRRYPVAGHALAQTLGISMRTLYRDIATLQQQGAEIAGEAGVGYVLRPGFMLP
PLMFSQAETEALVLGMRWVSRRGDSQLASAAGQALAKIADVLPPALREELEANTLLIAPVQAPPVADE
LRVLIRDTIRRERKLHIDYLDLAGQRSERLLWPFALGFFEQLQVLVAWCELRQAFRHFRLDRIQAAMP
LEQRYPRGRRRLLKEWRLSEGIAEQ
>CORE_REP|Org49_Gene1435#
MSITPTNILIVEDEKEIRRFVRTALESEGLRVFESETLQRGLIEAGTRKPDLIILDLGLPDGDGLSYI
RDLRQWSAIPVIVLSARNAEEDKIAALDAGADDYLSKPFGIGELLARVRVALRRHSASQQESPLVSFS
AITVDLVNRRVLRNDEDLHLTPIEFRLLAELLANAGKVITQRQLLSHVWGPNYVEHSHYLRIYMGHLR
QKLEADPARPKHLLTETGVGYRFMP
>CORE_REP|Org21_Gene518#
MNDFILSLACFLATLALYFANKKLYRRRRTLLLMPLVLTPMILVLLLVVTHISYQDYIGETHWLLWLL
GPATIAFAVPVYENLHIIRRHWLSLTAGVTTAVLVAVYSSVWLARLLTLPEEVQRSLAVRSITTPFAL
EAAKQMGGQPDLVALFVVITGVFGMAVGDILFLRLAVRSRLAKGAGFGASSHGAGTARAYELGPQEGV
VSSLVMMLAGIITVVAAPLIGRLMW

>CORE_REP|Org19_Gene4193#
MQLNAQQQAAQRNLSYLLAEKLGQQILAGDYQAGSILPGEMELGEQFGVSRTAVREAVKMLAAKGMLL
PRPRIGTRVMPQSQWNFLDQDLLTWWMTRENFDQVMQHFLILRTSLEPQACALAANHASPQQTKLLAE
LMAEMRALHTQFDRERWIQVDTQFHQLIYEASGNPFLTSFANLFSSVYQSYFRAITGNEVIKLRHHQA
IVDAILAGDSAGALVACQILLKEKD
>CORE_REP|Org19_Gene1159#
MGSSHKNNESYFKGLITMMEHLSEPWGIKDQLSRHLYMNRAAYLYTNTPLNFDVAGKYDHEFPADWAD
SAADFIEHDKMTEAARDRVTVIETHYWYGKDSLTPFISEKLPVYNDDKQVIGVIWNAKPMNSLSPLKY
INQQKPSVLTTEVNNELFTRAELDVIFLMLQRFTVKEIAKIYNVSNKTIENRIYNIYQKANVHTQQQF
EEYCKYANLDNYIPDRLITKGIQFI
>CORE_REP|Org22_Gene200#
MTATAPVITVDGPSGAGKGTLCKALAESLGWRLLDSGAIYRVLALAALHHQVDITSEEALVPLAAHLD
VRFVAQDGKLQVILEGEDVSNEIRTETVGNTASQAAAFPRVREALLRRQRAFREAPGLIADGRDMGTV
VFPDAPVKIFLDASSEERAHRRMLQLQEKGFNVNFERLLAEIKERDDRDRNRPIAPLVPASDALVLDS
TSMSIEEVIRQALTYAQKVLALPQQ
>CORE_REP|Org23_Gene3913#
MSRLVLISNKQCDGKDLACAGQTLAADAEPGLWLGWNGDVQNFAQRPISCRQRAGYDQVTFPLSIEEF
RRHYQGYYHDGLWPVFHNQPEKAHFTPENYRAYRQLNRRFADIACEHLCPGDIVCIDDYQLLPCAQAL
KEQGLLNACAFFFHLPFPSAALLRRIPEHRQLIASLLFYDLIGFTTTDDRNAFLSCLAGEFPLEMLPD
DQIQANGHLFATGIFPAGINGRQIY
>CORE_REP|Org2_Gene1619#
MTGILQLFQAIGLGLVLLLPLANPLTTVALLLGLSGNMTREERNQQSLMASVYYVFCIMTVAFYAGQVV
MNTFGISIPGLRIAGGLIVAFIGFRMLFPQQSADEAPEVESKSHELRHKTSANIAFVPLAMPSTAGPG
TIAMIISSASSVKDNTLGFEPWVLTVAPVAIFLTVAVILWGCLLSSGAIMRLVGKSGIEAISRLMGFL
LVCMGVQFIINGVLEIISTYTPAAA
>CORE_REP|Org43_Gene3054#
MQLHIVLVAPARPENVGAAARAMKTMGFASLRIVDSEAHLQPAARWVAHGAGEILDGVQTFATLEQAL
ADVDFTVATTARSRARFHYYCTPPELLEQLSERKQWVGQAALVFGREDSGLTNEELALADLLTGVPMQ
ADYPSLNLGQAVMVYCYQLASLMGVNAAPQEAAAPEGQLRALRHRADALLDALEVGDDQKLRDWLHQR
LGALPQRDTAMLHTLLHDIEKKLAK
>CORE_REP|Org38_Gene2185#
MSHQTLDVLIVEDEPQLATLHAEFIEKNFNLRVVAYAATLAEARAKANEHQPRLILLDNFLPDGQGIE
LMEEPAVKNPACSVIFITAASDMHTCSQAIRNGAFDYIIKPVSYKRLRNSLERFMQFVQTQRTFKIID
QDNVDALYNLQSKQFSSEPSAKGIETNTLELVQALFIAQPAVAHAVEDVVEQVGISKTTARRYLEYCV
ATQFVRVEMLYGNIGHPRRLYRKA
>CORE_REP|Org19_Gene2584#
MTDMAALRAEQLRRAAEVIRYDDLPAEPPAFIAGADVGFEQEGAVTRAAIAILRYPSLELVEYQVARI
ATVMPYIPGFLSFREYPALLAAWGQLQQKPGLIFVDGHGISHPRRLGVASHFGLLVDVPTIGVAKKRL
CGKFAPLDAAVGALAPLEDKGEQLGWVWRSKARCNPLFISTGHRVSADSALAWVQRCMAGYRLPEPTR
WADAIASRRPAFQRWLQQHPEVSP
>CORE_REP|Org11_Gene1654#
MSAIYDWSVLALRNVYDRRIQGKPVLDSAALFPDAERFTAQWRQIREEALTVAHDLHNIPRFHEIMIQ
QESISANDARDWRMFIMKAYGQPIDRNLARCPTLAALIASSPDVLSASLSFLAPGKQVPPHRGPFRGI
LRGYLVLDMPKRADGVPAAVLKVDGREYRLHEGEFMLWDDTFEHEVWNDSPQVRTVLLLDIRRRDMPG
GLRLLSSAIIALVRLNVRWMQRQF
>CORE_REP|Org11_Gene1703#
MLQGYSQLIFEGALVTLELALSSVLLAVVIGLIGAGGKLSHNPFISGLFGAYTTLIRGVPDLVLMLLI
FYGLQIALNNITTLLGFSQIDIDPLGAGIITLGFIYGAYFTETFRGAYLAVPKGQIEAATAYGFSGAQ
IFRRILFPAMMRFALPGIGNNWQVILKATALVSILGLNDVVKATQLAGKGTYQPFFFAIVAGVVYLIF
TTVSNGVLLWLERRYSLGVKRAEL
>CORE_REP|Org21_Gene1456#
MKEKKDILRLDDIHYQIDNQVILDSVSFTLGEGEFKLITGPSGCGKSTLLKIISSLMDPTRGSLYFDG
QAIAEMSPEAYRKQVSYCFQTPALFGNTVYDNLALPYQIRQQSPDERKMKADLTRFGLPEAMLTKSIN
ELSGGEKQRVSLIRNLQFMPRVLLLDEITSALDEENKRNVNEIVHQLVAEHRLAVLWVTHDTEEIAHA

DEVITLRAHGAEQQEQQEQQHESA
>CORE_REP|Org25_Gene499#
MTDMNILDLFLKASLLVKLIMLILICFSVASWAIIIQRTRILNAATRDAEAFEDKFWSGIELSRLYQE
SQARRDSLTGSEQIFHSGFKEFARLHRANNHAPESVIEGASRAMRISMNRELETLETHIPFLGTVGSI
SPYIGLFGTVWGIMHAFIALGAVKQATLQMVAPGIAEALIATAIGLFAAIPAVMAYNRLNQRVNKLEQ
NYDNFMEEFTAILHRQAFSSDSK
>CORE_REP|Org20_Gene1090#
MATSLTWHDVIGKEKEQPYFVETLAFVAAERQAGKTIYPPQKDVFNAFRYTELADVKVVILGQDPYHG
PNQAHGLSFSVRPGVPAPPSLVNMYKELATDIPGFERPDHGYLQSWAEQGVLLLNTVLTVEGGRAHSH
ANLGWETFTDKVIAALNENREGVVFLLWGSHAQKKGNFIDRNRHHVLKAPHPSPLSAHRGFLGCRHFS
QANQLLEQQGLTPIDWMPRLPQA
>CORE_REP|Org38_Gene1209#
MARAKLKFRLHRTAIILICLALLVLLMQGASYFSLSHQMARSEQVEELAQTLTKQVAYSLAPLLDDDG
NNPRIDAILKQLTDHSRILDVSVYQLDGTLVSHAGEQISIRDRLSLDGKRAGSYFNHQLVESIQGKDG
PIGFIRITLDTHVLATESKQVDNTTNLLRLMILLALAIGIILARTLLQHRRSRWQQSPYLLTANTPLE
EGNTVEDDEDDAPEKKQEAPKQG
>CORE_REP|Org5_Gene817#
MSNPIVACWPGALAPREKLLMQGAAALSDAELLAIFLRTGLPGVHVMQLAEQLLRRFGSLYHLMSADH
QAFCSQKGLGDASYTQLQAIAELALRFFSSHLSQENAMLNPRVTQHYLQSLLAHREREVFLVLFLDNQ
HRVIRHQEMFAGTISSVVVYPREIVREALKANAAALILAHNHPSGKAEPSHADRLITEQVVKACQLLE
IRVLDHLVIGRGECVSFAERGWL
>CORE_REP|Org8_Gene2157#
MLSIRSRCWLCRQPLSLMRHGICSCCLRHLPAHPPCCPRCGLPAGKARTPCGRCLQRPPPWQRLVFVG
DYAAPLSQLVKRFKFHRAPELAPTLARLMLLRWQQARREQYLNRPDLILAVPLHASRCWRRGYNQSDL
LARPLARWLGCAYQPAALRRVRKTALQQRLSASARRRNLQRAFACTVPVAGRHIALVDDVVTTGSTVA
AIAALLRRQGAASVQIWCVCRTL
>CORE_REP|Org42_Gene4092#
MHNIDLEDRLAALSAAIADRTRARMLCLLMDGRAYTATELSAAVEVAPSTASAHLAKLLEQRLIACVK
QGRYRYFRLAGQPVAEALEGLMALAGVPRPSVKSSTPTTLQYARTCYDHMAGEVAVKLHDRLHALNWL
NGEEDYRLSDAGQAALARLGVDCSPAPTRRRFACGCLDWSERRSHLGGALGAALLAAFIHRGWIVRRL
DSRELQLTPAGKKALAAHFDLTV
>CORE_REP|Org46_Gene714#
MRAAFDEDKKLMAEKSDLNALSGRFRGFYPVVIDVETAGFNANTDALLEIAAVTLKMDEDGWLQRDET
LHFHVEPFEGANLQPEALAFNGIDPHNPLRGAVSEYDALHAIFKAVRKGLKDRGCNRAIIVAHNANFD
HSFLMAAAERAGLKRNPFHPFATFDTAALSGLVLGQTVLAKACIAAGMPFDSSQAHSALYDTEQTALL
FCELVNRWKRLGGWPLPAGDLAE
>CORE_REP|Org8_Gene2531#
MSITTLFESGLYGRLRRHAGSHWQDYVDHPFLQQLAAGTLPERAFRRYLTQDYLFLLHFARAYALLVS
KLRTLPEMRAATASLNGIVAELPLHVAYCAEWGLNEAQIAAQPEAAETMNYTRYVLDIGHAGDALDLL
AGLLPCVAGYAEIGLRLLHDPATQMEGNPYASWIRNYGDEGYLAGVRAAIELLETVGQQRGAQGRFTE
LAQIFTTATQLESAFWQMGLNAS
>CORE_REP|Org32_Gene73#
MNPIVINRLQRKLGYTFQQQELLLQALTHRSASSKHNERLEFLGDSILSFVIANALYHRFPRVDEGDM
SRMRATLVRGNTLAEMAREFDLGECLRLGPGELKSGGFRRESILADTVEALIGGVFLDSDIQTVERLI
LDWYRSRLDEISPGDKQKDPKTRLQEFLQGRHLPLPSYLVVQVRGEAHDQEFTIHCQVSGLSEPVVGT
GSSRRKAEQAAAEQALKKLELE
>CORE_REP|Org9_Gene1038#
MKILLVDDDLELGTMLSEYLTGEGFDATLVLTGKAGVEGALSGDYTAMILDIMLPDMSGIDVLRDVRK
KSRLPIIMLTAKGDNIDRVIGLEMGADDYMPKPCYPRELVARLRAVLRRFEERPQEADDEAAISFGEL
TLNPSTRSSEWRGKAFDLTASEFNLLELLLRAPDRVVSKDELSEKGLGRPREAYDRSVDVHISNIRQK
LSALAGSKLIIETVRSIGYRIR
>CORE_REP|Org28_Gene1107#
MVPEFDWPQIDTVLLDMDGTLLDLEFDSHFWLSLVPQALSERRAIPFDEARHIIEREYQAVQHTMNWY
CFDYWSERLDLDIYRMTSEVGSRARLREDTEPFLRALRDAGLQTILLTNAHPHSLAVKIEHTGLDRHL

DLLFSTHTFGYPKEDQRLWQAVQQHTGFNPQRTLFVDDGEPILDAARTFGIRYCLGVQNPDSSTAEKS
FQRHPSMRDYRLLIPALAKGEA
>CORE_REP|Org9_Gene1560#
MPLEVDGIIRGDRGSEPSRWQHASTKPLITLTWHHTIPWNCLRNVWNGLVAGQHWDALDEFMNLIGVP
NRAEVLKQIKNKNLQDRDGLHTLVTWQGWNIVEGPGNEYRAQGDDPGENFDGWSGKGMSTNQQATLQQ
VNVLYQVMAPLGSRALDAARQAPPITAEEAGVLQRTIKQTRPTLRGKEPIRWQEDMWHQVQPGKEAKH
FARWDTKPVWRKRLHSDLAQAG
>CORE_REP|Org40_Gene1162#
MTTENIDYASYVDHTLLAMDATEMQIAKLCEEAQQHNFYAVCVNSGYVPLAAQLLQESTVKVCSVIGF
PLGAGLTVAKAFEAKAAIAAGAQEIDMVINVGWLKSGLLDEVKADIAAVREVCAAIPLKVILETCLLS
DAQIVQVCEMCRELDVAFVKTSTGFSTGGAREEHVKLMRETVGSEMGVKASGAVRDRATAEKMIKAGA
TRIGTSSGVAIVSGAQPAAGSY
>CORE_REP|Org9_Gene1153#
MDIIKELIHALWQQDFETLANPSLVWTLYILLFMILFLENGLLPAAFLPGDSLLILVGVLIAKGTMGF
PLTIVILTVAASLGCWVSYIQGRWLGNTRTVQGWLSHLPAHYHQRAHNLFHRHGLSALLVGRFLAFVR
TLLPTIAGLSGLSNARFQFFNWMSGLLWVLILTTLGFALGKTPVFRKYEDQLMFCLMMLPLVLLVVGL
IGSLIVLWRKKRASGQNPGKGA
>CORE_REP|Org4_Gene59#
MQRILIVEDEQKTGRYLQQGLVEEGYQADLFNNGRDGLGAASKGQYDLIILDVMLPFLDGWQIISALR
ESGHEEPVLFLTAKDNVRDKVKGLELGADDYLIKPFDFTELVARVRTLLRRARSQAATVCTIADMTVD
MVRRTVIRSGKKIHLTGKEYVLLELLLQRTGEVLPRSLISSLVWNMNFDSDTNVIDVAVRRLRSKIDD
DFEPKLIHTVRGAGYVLEIREE
>CORE_REP|Org47_Gene894#
MQFLNQFFFDIYPYLAGAVFLIGSWLRYDYGQYSWRAGSSQMLDKKGMRLASNLFHIGIIGIFAGHFL
GMLTPHWMYEAFLPIDVKQKLAMIAGGACGLMTLIGGALLLKRRLTNPRVRATSSFADIMILTLLVVQ
VCLGLLTIPFSAQHMDGSEMMKLVAWAQAVVTFHAGASAHLEGVAIIFKLHMVLGMTLFVLFPFCRLV
HIWSAPVEYLTRRYQLVRNRR
>CORE_REP|Org13_Gene804#
MKKFLIAPSILSADFARLGEDTANVLAAGGDVVHFDVMDNHYVPNLTIGPMVCEALRNYGITAPIDVH
LMVKPVDRIVPDFAKAGASYISFHPEASEHVDRTIQLIKEHGCKAGLVFNPATPLSYLDYVMDKIDVI
LLMSVNPGFGGQSFIHGTLDKLRQVRKLIDDSGRDIRLEVDGGVKVDNIAEIAAAGADMFVAGSAIFG
QPDYRKVIDEMRSELAKVSHG
>CORE_REP|Org11_Gene655#
MAIAATTNESLDNTVIGNNSKNTNSQDLHNSFLTLLVAQLKNQDPTNPMQNNELTSQLAQINTVQGIE
KLNTTLGSISGQINSNQSLQATALIGHGVMVPGNNILVGSKDGKVSTTPFGVELERAADQVTATITNA
SGQVVRTIEIGGLTAGVHAFTWDGSLDDGSTAPDGAYKVAINAKGNGEQLVARSLHFGLVNGVIRDGN
GAKLDLGLAGNATLEDVRQIL
>CORE_REP|Org4_Gene1430#
MGKTDVVKAGNNKIVAAAYPWAEEIANSISHGIGLVFGIVGLVLLLVQAVNTGADATAITSYSLYGGS
MILLFLASTLYHAIPHQKAKHWLKKFDHCAIYLLIAGTYTPFLLVGLDSPLAKGLMAVIWGLALLGVL
FKLAFAHRFEALSLVTYLTMGWLSLIVIYQLVTRLEAGGVTLLAIGGVVYTLGVIFYASKRIRFGHAI
WHAFVLGGSACHFMAIYLYV
>CORE_REP|Org7_Gene3145#
MGRIAPRRRKTTRIYQPLLRTWLNISQRLKPGAAPTQEDAESPSEPPSANDKMQRVKALLAKVWHLPD
GFHWMEPLPYFHRRWVLIFGIILLLALLWPYSPERQPFPVSQQETSVPLQAELQNGGGATTPTNEPTP
AGNWQRYQIQPGQTLAQLFRDNNLPVNEVFAMAQVEGGDKPLSNMKAGQEVRIERDANGVINALSVTT
VDNSQALFRRQADGSYRRER
>CORE_REP|Org13_Gene460#
MYEFDWASIVPSFPYLLQGMVITLKITVTAIVVGILWGTVLAVMRLSPFKPISWFATLYVNLFRSVPL
VMVLLWFYLVVPSLLQQVLGLSPKTDIRLISAMVAFSLFEAAYYSEIIRAGIISISRGQSSAALALGM
THWQSMRLVILPQAFRAMVPLLLTQGIVLFQDTSLVYVLSLADFFRTASTIGERDGTQVEMILFAGFV
YFVISLAASALVSYLKKRTV
>CORE_REP|Org17_Gene1995#
MINAEMLTGRSTEHLAPLSGNHRLQPEAVNAFLAMQQAARAAGFDLQPASTFRDFDRQLAIWNGKFCG

QRPVLDKDSQPIDVAPLSAAERCEAILRWSALPGASRHHWGSDLDVYDPSLLPEGQKLQLEPWEYEEG
GYFAPLNQWLTAHMAEFGFYRPFTEDCGGVAVEPWHLSYRPLAQEAEHLLTPALLLAAWQDKEVAGAE
WLERHLPSIFSRFIRSKGKE
>CORE_REP|Org18_Gene1464#
MHHPDVYIGSIQPYEGGRPSAIAKRQVDGAIRLTPLGLEGDEQAEKSYHGGPDRALCHYPREHYAHWR
AQFPAQAEQFCAPAFGENISTEGLTEHNVFMGDIFRWGEALIQVTQPRSPCFKLNYHFAIEDISVLMQ
QSGRCGWLYRVVSPGAVSGDRPLALAARNSDVSVAEAIAIAWHMPFDAEQYRRLLAVAGLSASWSKTM
LTRLAEGRLEDFNRRLLGR
>CORE_REP|Org9_Gene4357#
MAPLLFARRALMAVGIAVGLSACSFHSDIPSFTASGFVADQGVIRLWRKDDEQHRPQVLVSVYSPYRG
PGTITTLYTYQGDVLRQIKRNDADGDRDSIQLRFADDGTVSFMQRQLATRREPLTSDEIALYQYQARR
ILEVSNALRAGKVKLLQGRWMQGEVQTCDGQRMKPGLDAASIVWIEKRARSSSRPVSVAWLEAPEGSE
LLLVANDDFCSWEPKEDQL
>CORE_REP|Org1_Gene324#
MIRFEQVSKAYLGGRQALQGVDFHLRPAEMAFLTGHSGAGKSTLLKLICGIERPSAGHIWFGGHDISR
LKNREVPFLRRQIGMIFQDHHLLLDRTVYDNVAMPLIIAGASTEDIRRRVSAALDKVGLLDKAKNFPI
QLSGGEQQRVGIARAVVNKPAVLLADEPTGNLDDALSEGILRLFEEFNRVGVTVLMATHDTGLIARRN
YRILTLSQGRMQGGAHHGQ
>CORE_REP|Org27_Gene2785#
MQYPINEMFQTLQGEGFFTGVPAIFIRLQGCPVGCSWCDTKHTWEKEANREVDLQRILVKTEESDAWG
NASAEQLLAVMRQQGYTARHVVITGGEPCIYDLTPLTELFEQHGYGCQIETSGTHEVRCSAKTWVTVS
PKVNMRGGMKVLDQALRRADEVKHPVARERDIEALDTLLATLQDDKARIIALQPISQKEEATRLCIET
CIARNWRLSMQTHKYLNIA
>CORE_REP|Org21_Gene1575#
MKKQRLSVAMAVLAAGLLLAGCSSKVTQTSQYSGFLSDYSKLQETTSPSGHKTLRWIDPNYKESNYRG
LYFQPVVYFPAEKPTTRVSQDTLNKIKAYATQRIKAALQNRFTILPSPAGSRVLVAKLAITAVSAENE
DMKFYEVVPVAAVVASTMAATGHRTQNTALYIEGELIDQDTGKTVMEVVRKAYGKTVNNDSTPVTADD
VKAAIDDIVTDITNFPKQG
>CORE_REP|Org17_Gene594#
MRVLVVEDNGLLRHHLSVQMREMGHQVDAAEDAKEADYFLQEHAPDIAIVDLGLPGEDGLSLIRRWRA
HQTKLPILVLTARESWQDKVAVLEAGADDYVTKPFHLEEVIARMQALMRRNSGLASQVIVLPPFQIDL
SRRELSVNDQQIKLTAFEYTIIETLIRNAGKVVSKDSLMLQLYPDAELRESHTIDVLMGRLRKKVQAE
YPHEVITTVRGQGYRFDAK
>CORE_REP|Org22_Gene2188#
MSVIALENLSVSHRQGYELRTVVHEVNLRIEPGECFGLVGPSGCGKSSLLWVLAGLNGSWQGGFELLG
RRLQPGQAFTGELRREVQMVFQDPYASLHPKHRLLRTLSEPLKLLKESDIERKVSAGFRQVGLDPRLL
DRYPHQLSGGQRQRVAIVRALLLRPKLLLLDEPTSALDMSVQAEILNLLNELKQAGDLTMVLVSHDAD
VIDHMCDRSVAMAHGRIIV
>CORE_REP|Org3_Gene2798#
MSTTFRVIDTETTSFEGGVVEIASVDIVDGVICNPMSDLVCPPEPISFEAMAIHHITEEMVAGAPPID
DVIARYLGADIYVAHNAAFDREKLPQITAPWICTLKLARKLWPEERHGNQYLRYRFGLKPDVPEGLYA
HRALYDCYVTATNLLYMNSIARWSISQMRDITARPSLLHTMQFGKHKGKTFVDIASDDPGYLRWALAN
MDLNEDQEFTIKHAMGALV
>CORE_REP|Org23_Gene2432#
MLTLTNVFTKMMNFKNVFRWHLPFLLLLGLFSLRAAAADTLLILGDSLSAGYRLPIERAWPTLLAEQW
QKKSGAPQLVNASISGDTAAQGLARLPALLKQHQPRWVLIELGANDGLRGFPAQDVQRDLGQIITLVQ
QAGAQPLLMQIRIPPNYGRRYTEAFSAVYPALAKQFNIPLLPFYMEQVVVKPEWMQDDGLHPNGDAQP
FIATWMAERLEPLVKHESH
>CORE_REP|Org23_Gene2642#
MIIVHHLNHSRSQRILWFLEELGVPYQVQRYERDPQTLLAPAALKKIHPLGKSPVIVDGDLTLAESGA
IIEYLQEAYDAQGMFMPTDFHARQQYRYWLHYAEGSLMPLLVMKLVFSRLGQPPIPWLLRPVAGAIGK
GVQREYLDKQIAPHCEYLEQHLNKGSWFVGNDFSAADIQMSFPLEAMAARGALDNCPKLRGFLQRIHA
RPAYQRALEQGGPYNLLS
>CORE_REP|Org3_Gene1527#

MIESLFPHLRLDQLWDATWETLYMTGIAGLATLVLGIVLGVLLFLTSKGQLWQNRAVYSLISVLVNVF
RSIPFIILIVLLIPFTKSLIGTILGADAALPALIVGAAPFYARLVEIALREVDKGVIEAARSMGAKNR
TLIFRVLLPESSPALVSGITVTLIALVSYTAMAGVIGAGGLGNLAYLEGFQRNHSDVTLVATLTILLI
VFVIQFIGDTLTRTLDKR
>CORE_REP|Org45_Gene1603#
MIEYLPEILKGLHTSLTLTVAALIVALVLSLLLTVILTLKTPILTPLVKIYVTLFTGTPLLVQIFLIY
YGPGQFPAIRDYPWLWNLLSQPWLCAMIALALNSAAYTTQLFYGAVRAIPAGQWQSCEALGMSRRQTL
RILLPFAFKRALSSYSNEVVLVFKSTSLAYTITLMEVMGYSQLMYGRTYDVMVFGAAGLVYLCVNGLL
TLLMRLVERRALAFERRN
>CORE_REP|Org9_Gene1124#
MHIRSSLKHYWQVRPRLLFSVGAGLLCFLLLPAQLSLLQRLMIGWNTLAWLYLLFLWRLMLVSTPQHI
RQIARRQDESASTVLALVSFGCLVSILAILFELSSAKQASDSLKTLHLALTGGTLAVSWLLLPTAFTM
HYAHQFYRQGAQQEPLPLLFPGNLTEPTYLDFAYFSFTIAVASQTADVAVGAAEVRKIALVQSVISFV
FNMLILGLSINVGAGLLG
>CORE_REP|Org28_Gene1317#
MKRTKNINQETFRKSWRSYRVAPVALAISAVFMLAGCEKSDETVSLYQNADDCSRANPSMSEQCTTAY
NNALKEAAKTAPKYATREDCVAEFGEAQCTQAPAPAQAGMAAESQSSGSFWMPLMAGYMMGRLMGGSG
FAQQPLFTSKNAASPANGKFVDASGKSYGPATAGGRTMTVPKTAMAPKPAVTNTVTRGGFGETVAKQT
SMQRSSATSNSGSRSMGG
>CORE_REP|Org1_Gene1214#
MTLLQPDKIILRQLGLQPYAPVSQAMHNFTDSRTETTPDELWLVQHHPVFTQGQAGKAEHVLMPGDIP
VVQSDRGGQVTYHGPGQQVMYVMVDLKRSKVGVRQLVTAIEDTVINTLAHFRLASRARPDAPGVYVGE
QKICSLGLRIRKGSSFHGLALNVAMDLSPFQRINPCGYAGMQMTQVSALAPGVGIEDIHPILVQEFVH
LLGYQTVELRNWNLHDYE
>CORE_REP|Org45_Gene1680#
MAYSQRIETAIFDMDGLLIDSEPLWLQAELDIFGALGLDLSDRHKLPDTLGLRIDLVVKMWYQAMPWQ
GVSLDEVSARIIERAIELVHETRPLLPGVRQALELCREQGLNIGLASASPLHMQRQVLKMFDLEGYFD
QLVSAEYLPYSKPHPEVYLIAAERLGSDPLRCITLEDSFNGMIATKAARMRSIVIPAAEYRHDPRWAL
ADHQLETLEQLTPAHFA
>CORE_REP|Org40_Gene1310#
MKTKIGCLTAILLLSGCAKDPQTASNISGSGTTRGGWLKPPPQAPVNRTGTPVAYNDYIRQAASNYGV
DETLIKAIIQVESGFNPNVVSTSNAVGLMQLKASTAGRDAYRMKGRNGQPSSRELKDPAVNIDLGTAY
INILQSQQLAGINNPQTLRYATIVSYVNGAGAMLRTFSSDKRVAVNRINQMSPDEFYQHIQKKHPAPQ
APRYLWKVTTAYQAMSQ
>CORE_REP|Org42_Gene3883#
MKFGLVVAALLSISMPAAATTLKLSPDIDLLVVDGKKMTGSLLKGADSLELDGGQHQLLFKVTKTVRS
GQHTQAYASLPLVATFNTQKISQVAIELPRIENDRDAQRFDRTLNYLVVDKDGNALPFRHDVLHPDSV
TFNTDLEKVMTDYNRQNRPASVPSFVQANAGNASALTLAGAPINAPTVTLKGENVSEQMLQYWFQQAD
KETQKRFLRWANKQPIR
>CORE_REP|Org14_Gene1933#
MELYLDTADVTAVKRLARILPLHGVTTNPSIVAKEGKPIWEVLPALRDALGGTGKLFAQVMAADAERM
VAEAALLSQRVPGLVVKIPATAEGLAAIKKLKTMSIPTLGTAVYGAGQGLLAALAGAEYVAPYVNRVD
AQGGDGIEMVHELQQLLSLHAPSAQVLAASFKTPRQALECLLAGCQAITLPVDVAEQFLSAPAVQAAV
EKFEQDWQGAFGSNLLG
>CORE_REP|Org25_Gene616#
MPTNPEHPTVLHALFGGTFDPIHYGHLRPVEALAAEVGLNRVTLLPNHVPPHRPQPEANAQQRLKMVE
LAIAGNPLFAVDDRELHRTTPSYTIETLETIRKERGAAQPLAFIIGQDSLLTLHKWHRWQALLDVCHL
LVLARPGYNDRMDTPELQQWLERHRTADPALLSRRPHGHIYLADTPELEISATEIRQRRHQGLNCDDL
LPRPVQRYIELQGLYR
>CORE_REP|Org37_Gene713#
MDIIKFIIDFILHIDVHLAELVAQYGMWVYAILFLILFCETGLVVTPFLPGDSLLFVAGALAALPTND
LNVHTMVALMVVAAILGDAVNYTIGRLFGEKLFSNPNSKIFRRSYLDKTHQFYEKHGGKTIILARFVP
IVRTFAPFVAGMGHMSYRHFAAYNVIGALVWVLLFTYAGYLFGDLPVVQENLKLLIVGIIIVSILPGV
IEIWRHKRAAARQQKQ

>CORE_REP|Org15_Gene2222#
MRILLIEDDKLIGDGIKAGLTKLGFNLDWFTDGAVGKNALGSAPYDAVILDLSLPGLDGLDLLRQWRQ
AGQDVPVLILTARDALEQRVSGLQSGADDYLCKPFALAEVAARLQALIRRRHGQLMPQLTHGNVVFDS
ATRSVSCNGEPVTLTPRELAVLELFLHNKGRVLARPLIQEKLYNWDDEVSSNAVEVHIHHLRRKLGNG
FIRTIHGVGYTLGDAP
>CORE_REP|Org37_Gene701#
MKLLVVEDDELLQQGLALALTGEGYVCDCAATAAEANSLLITSQYSMVILDLGLPDMDGAALLRQWRR
QQIDLPVLILTARDALEDRVDGLDAGADDYLVKPFALVELQARVRALLRRYQGHSDNLMQVDDLQLNL
SSQQVYLQQQPVEVTPKEFAILARLIMRAGQTVNRELLQQDLYTWQDDLGSNTLEVHIHNLRRKLGKD
RIRTVRGIGYRLEPSS
>CORE_REP|Org19_Gene942#
MHESIQLALDSAPFLLKGAILTLQLSLGGMAFGLLLGFLLALMRLSPLWPLAWLSRIYVSLFRGTPLI
AQLFMIYYGLPQFGIEFDPFPAALIGLSLNTAAYTSETLRAAISSIDKGQWEAAASIGMTRWQTLRRV
ILPQAARTALPPLGNSFIGLVKDTSLAATIQVPELFRQAQLITSRTLEVFTMYLAASLIYWVMATLLS
ALQNRLEAHVNRQDQE
>CORE_REP|Org1_Gene782#
MKKRIAIIAGAVSVALTLSACTTNPYTGESEVGKSGIGAGLGAALGAGVGVLSSSKKDRGKGALIGAA
AGAALGGGAGYYMDVQEAKLRDKMKGTGVSVTRQGDNIVLNMPNNVTFDSSSATLKPAGANTLTGVAM
VLKEYPKTAVNVVGYTDSTGSRSLNMNLSQQRADGVASALITQGVAANRIRTTGAGPDNPIASNSTAE
GKAQNRRVEITLSPLQ
>CORE_REP|Org47_Gene3623#
MTREKARVHRSYSYVAYELCYYAVLGGLALMVPLIIMTTLWLMPAAQAANDLDTYGADGVLHVRGALT
ESACRLEMDSARQDIRLDDIGTAELRRAGDRGEPVAFSLRLRDCLRSVGAGTRDERTGSLSWAPYQPA
VTIGFSAPADADNPQLVKLVGVSGIGLRVTDAAGRDARLGERGAPLLLEQGNNVLTYKVAPERTPAPL
RAGAYAATIDFRLNYD
>CORE_REP|Org14_Gene4764#
MDANLLHDIIFYVMYAALAIALVIIIERTLYFAYTQRQARRLEQALTPDVRRAADLPDELTQRNSLPM
SVIAPVLAQKHHAGDRAGDREAIGDLIDAQYLLSKPPMARGLWLLETVVTAAPLLGLLGTVMGIIETF
KALAASGVSEPSLVSAGMGTALYATGLGIAIALLCLVANNFLQSRMERINELLKVLLIRAGQPASRPE
SGSSEQWVDSGAPRYA
>CORE_REP|Org20_Gene4050#
MYSFTAQQRFTALVWLSLFHIAIITSSNYLVQLPITVFGFHTTWGAFTFPFIFLATDLTVRIFGAPLA
RRIILAVMVPALFISYVISTVTYQGEWQGFAALGSFNLFVARIAVASFMAYVLGQILDVHVFNRLRQR
SAWWVAPAAAMFFGNISDTLAFFFIAFYKSSDPFMANNWVEIALVDYSFKVMICLLFFLPMYGVLLNM
LLKRIAARSGNLQPG
>CORE_REP|Org36_Gene2160#
MFLTLVRRELKIACRKGSEIVNPLWFFLIVITLFPLGVGPEPQLLARIAPGIVWVAALLASLLSLERL
FRDDFLDGSLEQLLLLPTPLPMTVLGKVCAHWVVTGLPLLILSPLVALLLSLDMQTWLAVAGTLLLGT
PTLSLIGAIGVGLTVGLRKGGVLLSLLVLPLYIPVLIFATGAIDAASMGMPIDGYLAILGAMLAGSVT
LAPFATAAALRVSVH
>CORE_REP|Org30_Gene425#
MQFDWSAIWPAIPILLEGAKMTLWISVLGLIGGLIIGLVAGFARTYGGWIANHIALVFIEVIRGTPIV
VQVMFIYFALPMAFTDLRIDPFTAAVVTIMINSGAYIAEITRGAVLSIHNGFREAGLALGLSRRETIR
YVIMPLALRRMLPPLGNQWIISIKDTSLFIVIGVAELTRQGQEIIAGNFRALEIWSAVAVIYLIITLV
LSFVLRRLERRMKIL
>CORE_REP|Org43_Gene4420#
MKRMSTRRIAQAKNCFAALGAITTRSQFGGYGLLAEGVMFAVIAEGELYLRATASMEPAFRARGMVNM
VYSKRGVPITLRYYWVDESLWRERNELVGLAWQAVREARREQRLKAGDHGRLKALPNIDVNMERLLWR
AGIRNAYDLRLHGAKRSYLRLLKQQTNLGLRVLLSLGGAIAGYHQAALPAELRSELVRWFDHTMAMRR
HGHEPVIQGPSSGPE
>CORE_REP|Org45_Gene114#
MDRIVVSSSSRDSLLSTHKVLRNTYFLLSLTLAFSALTATASTMLGLPAPGLLLMLVGFYGLMFLTHK
LANSPAGILAAFALTGFMGYALGPILSSFLNAGAGDLIMLALGGTAAVFFCCSAYVLTTRKDMSFLSG
MMMAGFVVLLVAVIANLFLQIPALHLAISALFILFSAGAILWETSNIIHGGETNYIRATVSLYVSLYN

MFISLLSILGFARSN
>CORE_REP|Org32_Gene1151#
MDLALFDLDETLIDDDSASLWIRWLVGQGFAPAELELQEQQLMQLYYQGKLSMEDYMQATLAPLTGLS
VQTVAGWVQRYIRRDILPRVYPAARERLQWHRERGDCILVISATGEHLVAPIAEQLGADDALAIGVEI
SDGRFTGHTYGTMTYQQGKVIRLQHWLAQHPHLKFEHSHGYSDSLNDKAMLQFVDSATVINPDSELSA
LAAEHGWEVCRWER
>CORE_REP|Org24_Gene444#
MQQARYYLLGERAVVLELSPPVTLPSQQRIWALAEKLNHHPDVREVVPGMNNLTLLLHTPQADAEAML
ALLQQGWESKESLTPESRQVDIPVVYGGEQGPDLDEVARHTGMTPRQVVECHAAAAYVVYFLGFQPGF
SYLGGMPEQLATPRRAEPRLAVAAGSVGIGGGQTGIYPLVTPGGWQLIGRTPLALFNPHEMPPTLLRP
GDNVRFVPQKEGVC
>CORE_REP|Org46_Gene107#
MISVLLVDDHELVRAGIRRILEDIKGIKVVGEAQCGEDAVKWCRGNAVDIVLMDMNMPGIGGLEATRK
IVRYAPDVKVIMLTIHTENPLPAKVMQAGAAGYLSKGAAPQEVINALRSVHAGQRYIASDIAQQMALS
QLEPQAETPFSCLSERELQIMLMITKGKKVNEISEQLSLSPKTVNSYRYRMFSKLNISGDVELTHLAI
RHGLFNAETLLSSE
>CORE_REP|Org16_Gene1882#
MTQDELKKAVGWAALEYVTPGTIVGVGTGSTAAHFIDALGSIKHQIEGAVSSSDASTAKLKSLGIHVF
DSNEVDSLDIYVDGADEINGHMQMIKGGGAALTREKIIAAIAKKFICIVDASKQVDVLGKFPLPVEVI
PMARSYVARELVKLGGLPEYRQNVVTDNGNVILDVHNLSITDAIALENKINGIAGVVTVGLFANRGAD
VALVGTPEGVKVVK
>CORE_REP|Org36_Gene207#
MYQHRDWQGSLLDFPVNKVVCVGSNYADHIKEMGSAVSVEPVVFIKPETALCDIRQPVAIPKEFGAVH
HEVELAVLIGTPLKQANEDRVARAIAGYGVALDLTLRDLQAGFKKAGQPWEKAKAFDGSCPMSGFIPV
AEFGDPQNAELSLTVNDQLRQQGNTRDMITPILPLISYMSRFFTLRAGDIILTGTPQGVGPMASGDML
KISLNGKTLSTRVI
>CORE_REP|Org22_Gene2859#
MDWYSLTFFGDSMLLLPSGIIVFAILMFVCRDWKQTAQWALLFGGVGAIVCASKLAFMGWGIGIRELD
FTGFSGHSALSASIWPVMMWLLTGRFSTAVRKTAVLSGYALALLIGYSRLAIHVHSVSEVISGLALGG
AASSAFLWLQRNIAPPALSYRKIAAALLLPLLLINNGTIAPTQGLLEFIAVSIAPIDKPFTREDMHAN
VPVAYNTLNSRSAQ
>CORE_REP|Org23_Gene4202#
MFTGIVQGTAPLVAIDEKPNFRTHVIEMPTELLPGLELGASVAHNGCCLTVTAVEGNRVSFDLIKETL
RLTNLGDLALGDIVNIERAAKFNDEIGGHLMSGHIICTAEVAKIYTSENNRQVWLRMPDAELMKYVLH
KGFIGIDGISLTIGEVVNNRFCVHLIPETLDRTTLGKKRLGDKVNIEIDPQTQAVVDTVERVLANREA
TLAAAAAVAPAHKD
>CORE_REP|Org37_Gene1721#
MQKEKLSALMDGESFDSELLSSLSQDRTLQQSWQSYHLIRDTLRGDVGQVMHLDIADRVAAALEKEPA
RLVPSAVQESQPQPHTWQKMPFWDKVRPWASQITQIGMAACVSLAVIVGVQHYNQPAASSNASESPAF
TTLPIMGQASPVSLGVPADSFSTGSGQQQQVQEQRKRINAMLQDYELQRRLHSDQLQLEQSNPQQAAI
QVPGTQSLGMQQQ
>CORE_REP|Org10_Gene2915#
MKEVIEGFLKFQREAFVERTALFQQLATRQNPRTLFISCSDSRLVPELVTQREPGDLFVIRNAGNIVP
SFGPEPGGVTASVEYAVAALGVEDIVICGHSDCGAMTAIATCQCLDHLPTVAGWLRYADSAKAVNQAY
PHASDAARVASMVRENVIAQLNNIKTHPSVALALDQGRLALHGWVYDIASGAIEALDGETRRFVPLAT
HPEVTATPAIARF
>CORE_REP|Org9_Gene898#
MTELVREKLDLPAGKSKLLLHSCCAPCSGEVMEAIQASGIEYAIFFYNPNIHPQKEYLLRKEENIRFA
EKHGVPIIDADYDTDNWFARAKGMENEPERGIRCTMCFDMRFERTALYAHEHGYDTISSSLGISRWKN
MQQITDCGIRAAEKYPDLVYWDYNWRKKGGSSRMIEISKRERFYQQEYCGCVYSLRDTNLHRKAQGRP
LIKLGVLYYGDEE
>CORE_REP|Org24_Gene3291#
MSRINLENSGSTAQSVSDRLLTLLKTRGPQQASDAGKVLGTTGEAARQQFVKLAKEGLVEAVAETRGV
GRPVQLWHLTSAGNARFPDTHADLTVQLLRTVRDKLGEQALDVLIETREQENRINYKQAMIGAADLQE

RVARLTEIRCREGYMAEWRQEEDGSYLLVENHCPICAAATVCQGFCRAELSIFTEVLQAQVERAEHIL
AGSRRCAYRISLL
>CORE_REP|Org22_Gene1173#
MNQTLLSDFGTPTERVERAIDALRNGRGVMVLDDENRENEGDMIFAAETMTVEQMALTIRHGSGIVCL
CITEERRQQLELPMMVTNNSSQFQTAFTVTIEAAQGVTTGVSASDRLTTIRAAVADSAKPSDLNRPGH
VFPLRAQPGGVLSRRGHTEATIDLVSMAGFKPAGVLCELTNDDGSMAHAPEVIAFAKQHDMVVLTIED
LVAYRQAHEKKAS
>CORE_REP|Org18_Gene267#
MSEAMMWLMARGVWETVMMTFVSGFFGFVLGLPVGVLLYVTRPGQIIANNSLYKILSGLVNIFRSIPF
IILLVWMIPFTRMIVGTSIGLQAAIVPLTVGAAPFIARMVENALLEIPSGLVEAARAMGATPMQIIKK
VLLPEALPGLVNAATITLITLVGYSAMGGAVGAGGLGQIGYQYGYIGYNATVMNTVLVLLVVLVYLIQ
FCGDRIVKAVTHK
>CORE_REP|Org40_Gene2611#
MKKVGVVLSGSGVYDGTEIHEAVLTLLALDRAGAQAVCFAPDKPQCHVINHLSGEEMAESRNVLIESA
RIARGAVQPLALAEAAQLDALIVPGGFGAAKNLSNFAEAGAECWVDEDLARLTREMHKANKPIGLMCI
APALLPKLLDQQARLTIGNDPDLGEVIDAMGGEPVICPVDDIVVDSEHKIVTTPAYMLAPSIAQAALG
IDKLVARVLELSE
>CORE_REP|Org49_Gene2071#
MIEKSEFDVADLRREYTRGGLRRNDLTANPLELFERWLKQACDARLADPTAMCVATVDEHGQPYQRIV
LLKHFDEQGLVFYTNLGSRKAQQLAHNPHISLLFPWHMLDRQVIFLGQAERLSTLEVLKYFNSRPKDS
QIGAWVSQQSSRISARGVLESKFLELKQKFQQGEVPLPSFWGGFRVKFDSVEFWQGGAHRLHDRFLYQ
RDGNDWKIDRLAP
>CORE_REP|Org7_Gene3327#
MKSNWMQQIQTLIGQKAGAMGGAEGIGKLLAPTALGGLVGVLLANKSSRKLVGKFGKNALIIGGSAAV
GAVLWNKYKQRVKETHQDEPQFGLQTTPVDLRAKRLVQALVFAAKSDGHIDADEQRAIEHSLAQLQVG
EEAQGWVQEALDQPLNPALIARSVQNEDEALEVYYLSCLVIDVDHFMERGYLDALAQALKIPADVKQG
IESDVNEKKRELA
>CORE_REP|Org22_Gene3251#
MKGKMLLLIGLLCSLNARADDLATQIDSFIKGKFTGEPVQVKVRVRTPPAQWPACELPQLSLPPNARI
GGNVSISARCGQERRFIQTQVQVFGRYLVSARGISAGSRLTAADLTLKEGRLDTLPPRALTEASKALD
AVSLRNISPGQPLTLAMLRRAWIIKAGQPVQVTAQGEGFNISGAGKAMNNAAAEDSVRVRMASGQIVS
GVVGDDGAIRITL
>CORE_REP|Org15_Gene3097#
MTDITTPFPATPHKLGLYPVVDSVAWIARLLEAGVTTIQLRIKDLPDEQVEEDIAAAITLGRRYQARL
FINDYWRLAIRHGAYGVHLGQEDLDTTDLAAIHRAGLRLGVSTHDDAELARALAVKPSYIALGHIFPT
QTKDMPSAPQGLAELKRHIAGLADYPTVAIGGISIDRVPAVLACGVGSVAVVSAITQAPDWRAATAEL
LRLIEGKEPNDA
>CORE_REP|Org13_Gene4047#
MARHFGKLAVALAALFIGGQALAHGHHSHGKPLTEVEKKAEAGIFDDKDVKDRDLADWEGVWQSVYPY
LLNGDLDPVFKKKAEQDKSKTFEEVKAYYRKGYATDVDTIGIENGVMEFHRGGQSSACQYKYAGHKIL
TYVSGKKGVRYLFECQDAGSLAPKFVQFSDHIIGPRKSAHFHIFMGNTSQEALLKEMDNWPTYYPFQL
QTKEVVDEMLHH
>CORE_REP|Org6_Gene1037#
MNTLLYAWQNWAYIAGLTLEHLLLVGIAVGLAILIGVPLGVLIVRHKWLATPVLSLATLVLTVPSIAL
FGLMIPLFSLIGHGIGYVPAITAVFLYSLLPIVRNTHTALDNLPGGLREAGRGIGMTFWQRLRWVEIP
VALPVIFGGIRTAVVMNIGVMAIAAVIGAGGLGLLLLNGISSSDIRQLITGAVMISLLAIVLDWLLHR
LQIALTPKGIRS
>CORE_REP|Org8_Gene2280#
MLNTLAKLTAVAVLAIGLAACDNKDDTKPAVPPPDTKPTVTQPAPPPPPAPVETPPAPPGLNVSLQKG
KITFELPPGFSDQTLNSGIINDSTSTIQRFLDGKSRQSAVSSEVIPPDGMKLNTSDKMLKELTQSAIT
VLAERYQNIQTTKEENFSVGKQKFRRVDTEQTVNGQKVVSTLVLTVFNKRVVTLQMLSPAKTPEVHQA
LVQRIIDTLAVK
>CORE_REP|Org44_Gene1469#
MEPYNPPQDPLHILYQDEHIMVVNKPSGLLSVPGRAPENKDSLMTRIQADHPAAESVHRLDMATSGVI

VVALNKAAERELKRQFREREPKKSYIARVWGHMAHDEGLVDLPLICDWPNRPLQKVCFDTGKAAQTEY
QVLSRDADGSTRVKLTPITGRSHQLRVHMLALGHPILGDGFYAPPEAKAMAPRLQLHAQELRITHPAF
QTPMHFRAEPDF
>CORE_REP|Org14_Gene1970#
MKYELNAREARVIGCLLEKQVTTPDQYPLSLNAITLACNQKTNREPVMDLSESEVQQLLDLLLRKHFL
RTLSGFGNRVVKYEQRFCNSEFGQLKLSPAELAVIATLLLRGAQTPGELRTRTNRMHEFSDVSEVEQV
LQQLATREDGPFVVRLAREPGKRESRFMHLFSGQIDEAPPAAFDAEDDGELSARVSALESEVAELKRQ
LQQLLARDAND
>CORE_REP|Org38_Gene2396#
MTETTLHYIFDPLCGWCYGAAPLVKAAQSLPGLKVVPHAGGMMTGNNRRQITDEWRNYVIPHDKRIAE
MTGQPFGEAYFNGLLRDTTAVMDSEPPITAILAAEKLAGRGLDMLHRIQQAHYQEGRRIADTPVLEAL
AKELGLPSAAFIAEMRFNSGAPTAQHIAESRALLAKVQGQGFPTFALQDSEGQLHLLPAGNYLGNVEA
WKNLLGAAALA
>CORE_REP|Org6_Gene539#
MLQVYLVRHGETEWNAARRIQGQSDSPLTAMGEHQARLVARRVSKEGITHIITSDLGRTRRTAQIIAD
ACGCEVISDPRLRELHMGVLEERLIDSLTPQEEQWRKQMVDGTPDGRIPQGESMEELGERMRAALESC
LMLPEGSKPLLVSHGIALGCLISTVLGLPAYAERRLRLRNCSLSRVDHQQSPWLASGWIVETAGDVTH
LDMPALDELQR
>CORE_REP|Org38_Gene3396#
MHSTASDAFINSCLATITHLIPVSAGVFYLVDRDLRPDHYILHGMPDKTHQQYLNHFQQIDPLQPANF
HRQDITMVGMSPAAIADNRRYYHDFMLPNDMRDMTEIFIRQRKRIVAGVSLIRDTPFTDVERGRLRAV
LPLIELATRDLLPDSEAQLLTAKEQEIVNLVREGASNKRIALKLGISLSTVKTHMRNIFAKTDVVNRT
ELVAGGFLAHG
>CORE_REP|Org37_Gene2739#
MNKVLVTGIEPFDGDAVNPSWQVAQALAGEQIAGAEIAVLELPCVLGQANLQLIAAIEALQPLAVICL
GLAGGRAEISLERVAINLIDARIPDNAGKQPIDVPVVAGGPVGYFSTLPVKAAVQQLRRQGIPAGVSY
TAGTYNCNHIFYGLRHYLETQRAKVRGGFVHIPYSHTLAAAHPGKPSMALATMVEAVRTIVQVTLTVE
EDARFGDGAVH
>CORE_REP|Org16_Gene4075#
MTIDLFEDALPPPWREEIAPGAVVMHGFVRDHGPELLAAVQGVVAQVPWQHLTTPGGHVMSVAMSWCG
NGWTSDSRGYRYSERDARSGKRWPAIPPILMALADEAARQAGFGPFVPDSCLMNRYDPGSKLSLHQDK
DEHDFGAPIVSVSLGLPAVFQFGGLQRSDRARRIPLAHGDVVVWGGPSRLCFHGILPVKEGYHSLVGP
HRINITLRKAL
>CORE_REP|Org38_Gene1576#
MKYHLIPVTAFSQNCSLIWCENTQQAALVDPGGEAEKIKAEVAKQGVTITQILLTHGHLDHVGAAAEL
AEHYQVPIYGPDKEDAFWLDGLPAQSRMFGLEECAPLTPTRWLSEGDEMQVGEMKLKVLHCPGHTPGH
IVFINEQARLALVGDVLFNGGVGRSDFPRGDHQALIASIRTKLLPLGDDMRFIPGHGPMSTFGHERQT
NPFLREEPAVW
>CORE_REP|Org46_Gene767#
MTSKNYNYHVTHFVTSAPDIRHLPGDAGIEVAFAGRSNAGKSSALNTLTNQKSLARTSKTPGRTQLIN
LFEVEDGIRLVDLPGYGYAEVPEEMKRKWQRALGEYLQMRNSLKGLVVLMDIRHPLKDLDQQMIQWAV
DVGTPVLVLLTKADKLASGARKAQLNMVREAVLPFMGDIQVEAFSSLKKIGVDKLRQKLDTWFNEIPP
EVLPEDEAGE
>CORE_REP|Org31_Gene553#
MGQSLLDLSGFIKFFVGLFALVNPVGILPVFISMTSYQAEAGRNKTNLTANLSVAIILWTSLFLGEGI
LRMFGISIDSFRIAGGILVVTIAMSMISGKLGEDKQNKQEKSESAIRESIGVVPLALPLMAGPGAISS
TIVWSSRYHNWQSLLGFTVAIALFAFCCWLLFRAAPLLVRLLGQTGINVITRIMGLLLMALGIEFIVT
GIKAIFPGLL
>CORE_REP|Org10_Gene328#
MRDIPMPASDAATAGEIISRIGQLTRMLRDSMRELGLDQAIAQAAEAIPDARDRLDYVVTMTAQAAER
ALNCVEAAQPRQAELESGANALKGRWDEWFANPIELDDARSLVNDTRQYLDQVPGHTAFTNAQLLEIM
MAQDFQDLTGQVIKRMMDVVQEIEKQLLMVLMENMPEQPVKEKRPNDSLLNGPQLDQNGVGVIANQAQ
VDDLLDSLGF
>CORE_REP|Org47_Gene544#

MRIILLGAPGAGKGTQAQFIMEKYGIPQISTGDMLRAAVKAGSELGKQAKEIMDAGKLVTDELVIALV
KERIAQEDCRKGFLLDGFPRTIPQADAMKEAGINVDFVLEFDVPDELIVDRIVGRRVHAPSGRVYHVK
FNPPQVEGKDDVTGEELTTRKDDQEETVRKRLVEYHQMTAPLISYYSKEAAAGNTQYRKIDGTRKVAE
VSAELATILG
>CORE_REP|Org4_Gene2280#
MSLMLKGEKIDRNRFTGEKIENGSFMLCDFSGADLTGTEFIGCQFYDRESRQGGNFSRAILKDASFRS
CDLSMADFRHVDALGVEIRECRAQGADFRGASFMNMITSRTWFCSAYITKSNLSYANFAKVVLEKCEL
WENRWHGAQVLGASFSGSDLSGGEFSGFDWRAADVTQCDLSNAELGELDLRTTDLQGVKMDSHQAAQL
LERLGIAIIG
>CORE_REP|Org45_Gene513#
MSKFQLLDKDNSALIFIDHQPQMAFGVANIDRQQLKNNVVGLAKAGKIFNVPTLFTSVETESFSGYIW
PELLAVHPEITPIERTSMNSWEDAAFVKAVEATGRKKLVISALWTEVCLTFPALMALEAGYEVYVVTD
TSGGTSVDAHERSIDRMVQAGAVPVTWQQVLLEYQRDWARKDTYDAVMALVREHSGAYGMGVDYAYTM
VHHAPARTVK
>CORE_REP|Org18_Gene818#
MLESFGVLNLWTYLAGVVFIIILPGPNTLYVLKTGVSRGVRAGYTAALGVFIGDAILIFCAYIGVASL
IRTTPFLFTLVRFLGAIYLLFLGAKILYATFVQKAQAQAQQQQIEGGHSILRKSLTLSLTNPKAILFY
VSFFVQFIDFNYAHTGLSFTILALILEAVSFIYMSTLIFSGAMLAHFFNHKKGLAKLGNGLIGLLFLG
FATRLATLSS
>CORE_REP|Org29_Gene153#
MKAYQRQFIEFALNKQVLKFGEFTLKSGRTSPYFFNAGLFNTGRDLALLGRFYAEALMDSGIDFDLLF
GPAYKGIPIATTTAVALAEHHERDVPYCFNRKEAKTHGEGGSLVGSPLQGRVMLVDDVITAGTAIRES
MEIIGASGASLAGVLISLDRQERGRADISAIQEVERDYHCKVISIVTLKDLIAYLEEKPEMADHLAAV
RAYREQYGV
>CORE_REP|Org10_Gene1505#
MKNWKTSAEQILTAGPVVPVIVINKLEQAVPLAKALVAGGVRVLEVTLRTACGLEAIRAIAKEVPEAI
IGAGTVINPQQLREVTEAGAQFAISPGLTDALLQAATAGSIPLIPGISTVSELMLGMDYGLREFKFFP
AEANGGVKALQAIGGPFPQVRFCPTGGITPNNYRDYLALKSVLCIGGSWLVPADALESGDYARITELA
RSAVSGAAL
>CORE_REP|Org16_Gene1333#
MKVAIIGATGFVGRRVVDEALARGIQVTAIARQKKDLPEHANLTIALGDVADTAWLAGQLHGQDAVIS
AYNPGWGEDNLYEKTTRGAQQILTAVGQAGVKRLLVVGGAGSLEVAPGVELVDTPQFPENIRPGAQAV
RDLRNKLRNESALDWTYLSPAALLEPGKRTGQFRLGTTQLLMNGDAPASISVEDLAVAIVDEIEKPQF
IRAQFTAAY
>CORE_REP|Org33_Gene398#
MAVAANKRSVMTLFSGPTDIFSHQVRIVLAEKGVSVEIEQVEMDNLPQDLIDLNPYQTVPTLVDRELT
LYESRIIMEYLDERFPHPPLMPVYPVARGESRLMMLRIEKNWYSLMDKIEKSSGQEAESARRQLREEL
LAIAPIFGQAPYFMSEEFSLVDCYLAPLLWRLPQLGIELSGAGSKELKGYMTRVFERDAFLASLTEAE
REMRLQTRG
>CORE_REP|Org46_Gene2477#
MLANVKRSFAAAVVLMLALPAVQAADYRAGEQYTRLDKPVAAAPAVVEFFSFYCGPCYQFAETYRVGS
TVAQALPAGEKVTKYHVSLMGKLGNELTEAWAVATVLGVEDKIEGAMFDAVQKQRAVNSAEDIQRVFT
AAGIDAATYENARHSLLVKGLIAKQNEAVKAFEVRGTPSFYVAGKYKIDNAGMASTSVEGYAKEYAAV
VRHLLDTQP
>CORE_REP|Org30_Gene417#
MTDNPHQCVIIGIAGASASGKSLIASTLYRELREQVGDEHIGVIPEDSYYKDQTHLTMEERVKTNYDH
PSAMDHNLLFQHLQMLKAGKAIELPLYSYTEHTRKKETVHLEPKKVIILEGILLLTDIRLRQEMNFSI
FVDTPLDICLMRRMKRDVNERGRSMDSVMAQYQKTVRPMFLQFIEPSKQYADIIVPRGGKNRIAIDIL
KAKISQFFE
>CORE_REP|Org21_Gene925#
MNKQKRLEILTRLRDNNPHPTTELVYTTPFELLIAVLLSAQATDVSVNKATAKLYPVANTPAAMLALG
VDGVKEYIKTIGLFNSKAENVIKTCRMLLELHGGEVPEDRAALEALPGVGRKTANVVLNTAFGWATIA
VDTHIFRVCNRTHFAPGKNVDQVEEKLLKVVPAEFKVDCHHWLILHGRYTCIARKPRCGSCIIEDLCE
YKEKVYPES

>CORE_REP|Org1_Gene4207#
MVPFFDKSQTVDKASALPGRTTPMPVATLNVVTEHSMTQVPAGMEVAIFAMGCFWGVERLFWQQPGVY
STAAGYSGGYTPNPTYREVCSGQTGHAEVVRVVFDPQIVSYKQLLQVFWENHDPAQGMRQGGDVGTQY
RSAIYTLSPEQQAEAESSLQRFQQAMDAAGDKRVITTEIAPALPFYYAEDDHQQYLFKNPEGYCGLGG
IGVCLPPQG
>CORE_REP|Org6_Gene3942#
MKSKFVVIEGLEGAGKTTARDTVVNVLREHGVSDIVFTREPGGTPLAEKLRDLFKRGIDGELPTIKAE
VLMLYAARVQLVETVIKPALARGAWVVGDRHDLSSQAYQGGGRGVDPQLMASLRDTVLGDFRPDLTVY
LDLPPLVGLQRAQARGQLDRIEQEALPFFERTRARYLELAAQDETIVTVNAAQPLEQVTAAIRDCVGH
WLRQQEGAL
>CORE_REP|Org5_Gene2965#
MKKIVVLVSGQGSNLQALIDACQQDRIAAEIVAVFSNKAQAYGLQRAEAADIATQALDAKAYADRTAF
DAALADAIDQYQPDLVVLAGYMRILSPQFVQRYAGRMLNIHPSLLPKYPGLHTHRQAIDNGDSEHGTS
VHFVTEQLDGGPVILQAKVPIFADDEEDDVVERVQTQEHTIYPLVVSWFVDGRLAMRDGAAWLDGERL
PEQGHAAD
>CORE_REP|Org23_Gene3889#
MARALLVGATGLVGRELLQLLQSDPQITAIVAPTRTPLPPHGKLTNPVGDALFELLSSMQQPVDLVFC
CLGTTRQAAGSADAFRYVDYQLVVESALTGRRLGAQHCLVVSALGANAHSTFLYNRTKGEMEQALREQ
HWPRLTLVRPSMLVGDRPAPRLMERLTLPLFRLLPGKWRAVAAKDVAQTLLQQAFTPGEGVMVLESDR
LHCYRGSR
>CORE_REP|Org23_Gene3476#
MMNTRAQLAALLQPEFGIDGPPHAFRKATMEEAQAFENQVLEKLNAGKTVRSFLIAAVELLAEALNVL
VVQVFRKDDYAVKYAVEPLLSGSGPLGELSVRLKLMYGLGVISRHEYEDAELLMAMREELNHDGSEYR
FVDDEILGPFGELHCVAALPPVPTFLQPGEADEALIAMQRQRYQQMVRSTMVLSITDLIAGIGAKQPS
RLSPLGRG
>CORE_REP|Org44_Gene1074#
MSVANTTRAVNIAPPQQAAGQVVNLLPDGSLVVECEGRGWHCRRAASCLLTPALGDSVLVAGCGHQLW
AIAVLERAEPQSAARLSVAGDLHIETPSGSLSLHGAQALKLSGDAMTLQANSGDCQVDKMKYSGEELS
AFVSISRLVGKRCESLWHSVSQISHSLFRKVRQTEHVRAGQLDYQAEDYARIHARNTLITSKDITKLD
SEQIHVG
>CORE_REP|Org5_Gene3763#
MNTTHQSKRLVVLVGSPRRDGNSATLAQAILAGAAEAGTSASLHFLDDYLSGFLSDERHTPPPADRYS
ELFLEHFLPADGAVFCTPIYWYGMSAQTKAFFDRSFSFYSNAYPQAEQVHQRMSGKRIGLAVASEETY
PGAALGIVHQIQEFSRYTHSAFVGVVHGAGNQRGEIARDPRNPLQAARELGREFFTRPYSDYQMDSPR
STQVWEE
>CORE_REP|Org19_Gene1474#
MIVLIGSQKGGVGKSTKAVNIAGYLILKQGKTAIIVDADDQKSIMTWYNDRQNVEGLPHIPVVAASGK
IKETLLELDRHYDYVIVDTAGRDSAELRSGLLAADLFLSPLRPSQMDLDTIGYLSEMFSTAQEYNEKV
KGYIVLNMCPTNIFINEANEAAQVLSEYPEFTLVSHRLCDRKIYRDAWGEAITVHEANNEKAQSEIEC
LVKEVIL
>CORE_REP|Org22_Gene428#
MRKEKPIQRYSAPERINHWIVAFCFVFAAISGLGFFFPSFNWLMNIFGTPQLARILHPFVGVIMFAAF
LLMFLRYWKHNLINREDIVWAKNIHKIAMNEEVGDTGRYNFGQKCVFWAAIISLVLLLASGVVIWRPY
FAPSFSIPLIRIALLVHSLAAVGLIIVIMVHIYAALWVKGTITAMVEGWVPAAWAKKHHPRWYREVRE
KRQEDKP
>CORE_REP|Org40_Gene2220#
MMKLRRLLAAFGAVFSAGAIAHPHSFIDMNTTFVAKDQRLVGLKMVWVMDEITSADLLYDAKNAKSDS
EVWKKLAAEVMANVLGQHYFTDLYRDGKPVKYLNLPSEYHLSRQGNQAVLEFVLPLAEPQPLAGKPFE
LSTYDPTYFVDMTYKDQNALHLPPEMAQQCSYKLMTPQPNASLQAYALSLDKNDSPGEDLALGQQFAQ
RVTLQCR
>CORE_REP|Org16_Gene408#
MSQPITELYTDSEFFSPYAMSAFVVLTEKGIPFTVKPVDLAKEENKEAAYAALSLTRRVPTLAIGEFQ
LSESSAIAEYLEDIHPAHAVYPRDVKLRAKAREIQAWLRSDLLPIRAERSTEVVFNNGKFPPLSEAAQ
EAARKLIAAVEKLLSHGQDNLFREWCIADTDLALMLNRLVMHGDPVPERVRHYAHKQWQRPSVQAWLA

LSEKRRA
>CORE_REP|Org30_Gene1505#
MTTTIEQQAREEMVRLGASFFQRGYATGSAGNLSLLLPDGALLATPTGSCLGELQADRLSKVSLNGDW
LSGDKPSKEISFHRALYLNNPECKAVVHLHCTYLTALSCLQGLDVDNAIKPFTPYVVMRVGKVPVVPY
YRPGDERLAQDLARLAPTHRAFLLANHGPVVTGKDLRAAADNTEEMEDAARLIFTLGDRPIRYLTDDE
IAELRS
>CORE_REP|Org24_Gene15#
MVLGKPQTDPTLEWFLSHCHIHKYPSKSTLIHQGEKAETLYYIVKGSVAVLIKDEEGKEMILSYLNQG
DFIGELGLFEEGQERSAWVRAKTACEVAEISYKKFRQLIQVNPDILMRLSAQMASRLQVTSEKVGNLA
FLDVTGRIAQTLLNLAKQPDAMTHPDGMQIKITRQEIGQIVGCSRETVGRILKMLEDQNLISAHGKTI
VVYGTR
>CORE_REP|Org8_Gene887#
MVMKQYRVMIVDDHPLMRRGIKQLLGLDARFGVVAEAGNGSEAVALALQHAPDVILLDLNMKGMSGLD
TLRALRDEGVDARIIVLTVSDARSDLYALIDAGADGYLLKDSEPEQLLEHISAAAEGQNVISDAMADY
LLARSEQRDPFTALTERELDVLQEVARGLSNKQVAAQLHISEETVKVHIRNILRKLDVRSRVAATVMY
LEYKSH
>CORE_REP|Org48_Gene4541#
MKGTVFSVALNHRSQLDAWDQAFHQPPYQTPPKTPVWFIKPRNTHLANGGAIPFPAGETVQSGGTLAV
IIGDTVRKVPAAQVSRYLAGYALANDVSLPESSFYRPAIKAKCRDGFCPLGEIGLLENADRLEIVTEI
NGVEQDRWSTADLVRSVPELIAAISDFITLQPGDAVLIGTPHQRVDIKPGDEVTVRAAGLPTLTNRVT
QAGAAS
>CORE_REP|Org10_Gene148#
MGVRAQQKERTRRSLIEAAFSQLSAERSFASLSLREVSREAGIAPTSFYRHFRDVDELGLTMVDESGL
MLRQLMRQARQRIAKGGSVIRTSVSTFMEFIGNNPNAFRLLLRERSGTSAAFRAAVAREIQHFIAELA
DYLELENHMPRSFTEAQAEAMVTIVFSAGAEALDIDVEQRQQLEERLVLQLRMISKGAYYWYRREQEK
ASVSHV
>CORE_REP|Org23_Gene768#
MKKTTLMVLATMMAPMLASAHQAGDFLFRAGTATVRPNAGSDNVLGLGSLDAKNNTQLGLTFGYMVTD
NIGVELLAATPFRHKVTLGGTDLATVHQLPPTLMAQYYFGDKQDKLRPYLGVGVNYTTFFDEKFNDYG
KSAGLSDLDLKDSWGVAAQAGLDYNLDEHWMLNMSVWWMNIETKTRFNDANGGHHSFDTRLDPWVFMF
GAGYRF
>CORE_REP|Org4_Gene1541#
MNEKIDYHIEKYHFAPLDEAPRLAHQWSEVLNECRETQAGAEERLRIALLNVDYVTSFELPFRLLLVR
APQLIAGIREELPLSQKNVVFNGKRFGCVYSLKSDLSGVPEAFQYSLSTRIHRRAASGVDALPYREIA
KALKAPRERLRLALEQGLPVTALDGLFWFGIQRIAAEVRRLRKTGMAIVTAETEIFDTLTGTTRKVPV
YRLAES
>CORE_REP|Org37_Gene379#
MNRSQPSPVTFDKKDVEIIARETLYRGFFSLNLYRFRHRLFNGEMSPEITREIFERGHAAVLLPYDPV
RDEVVLIEQLRIAAVDTSNSPWLLEMVAGMIETGESVEDVCRREAQEEAGVVVGRCKPVLSYLASPGG
TSERLSIMVGEVDAGTAEGIHGLAEEHEDIRVHVVSREQAYRWVEEGAIDNAASVIALQWLALHHESL
RKEWAN
>CORE_REP|Org23_Gene2880#
MKTALLLIDLQNDFCPGGALAVTAGDAVIPVANQAIAACLARGEPVVASQDWHPANHRSFAVNSDAQV
GTLGELEGLPQVWWPVHCVQGSHGADFHPQLQRQHINAVFRKGQDTNIDSYSAFFDNGHRAQTELHGW
LQSQGVRRLAVMGLATDYCVKFSVLDALAAGYPTQVIVDGCRGVNLQPDDSERALQDMARAGAQLVTL
SQFLAN
>CORE_REP|Org12_Gene2927#
MGQDYALVVDDHPLVASGIANFLSTHCRFKQAHVVTNEENCYRHIRENGPPRLLVIDFWLSSGTALKL
LKEVKQRYPQVRILVVSGDENNDIWQKVHNAGGHGFVLKNEPPELFARAVFALNNNQEWFPEGNEAAI
KNNHDHLNKFNLTPRQLDVLTMMLRGLPNKRIATQLSISEPTVKEHISNILKKIGVNSRVEAITLLHG
KRDPSS
>CORE_REP|Org41_Gene2158#
MLEAKSLSCVRDERILFSELSFSVQPGDIIQVEGPNGAGKTSLLRILAGLARPDGGEVCWRGRSTLRD
RAGYQQDLLFIGHQPGIKAVLTPFENLQFYQAVRGTTDHPAIWRALEQVGLVGYEDLPVAQLSAGQQR

RVALARLWLSAAPLWILDEPLTAIDKQGVAELISLFEQHAQRGGMVLLTTHQDLAGVSQTVGKVRLAE
HDAGSL
>CORE_REP|Org11_Gene3912#
MSKKHWSNTELLHQTVTNPNIIVKGTHSYYSDCWDNGFERSVVRYLHGDAVSRQWQPLGDIDRLLIGD
YVCIAAEAVILMGGNHTHRIDWLSLYPFMETIKRAYRPKGDTRLGDGCWIGMRAMLMPGVSIGEGAIV
AAGSVVVGDVEPYAIVGGNPARFIRWRFAPEVIARLLALRLYDLSEADFAVVQPLLVDNDIAAVERAI
CNIKRY
>CORE_REP|Org2_Gene3023#
MFQPAAFREDDLDAQLALVRAHPLGLLVSHGEQGLTADPLPFLADVEQGQIRLRAHLSRANEHWRRLQ
HAAECLVIFQGLEGYVSPGWYPSKRQTGKVVPTWNYSVVQLYGVPTVMDDPAWLRRQLIDLTAQQEGG
RPAPWRLDDAPANYIAAQLKGIVGIEIAVTRREGKWKMSQNRSADDADGVIAGLREGDETQRRLAAEV
ERRRG
>CORE_REP|Org29_Gene2289#
MLNSMRKHGTTLAVFAAVTTGLTAVVYTLTKSTIAHQAALQQKALLDQVVPPENYDNVMQNECFLVSD
PALGNGAPHRLYLARKNGQPTAAALETTAPDGYSGAIKLLVGADFNGTVLGTRVIEHHETPGLGDKIE
LRISDWISFFSGKKIEGPDDKRWAVKKDGGMFDQFTGATITPRAVVNAVRRTALYMETLPPKLESLPA
CGASE
>CORE_REP|Org33_Gene329#
MPTIIMDSCSYTRLGLTDYLTSHGVKKRHINAIEDIDSLHEKCSKLNPSLVFINEDCFIHEANATERI
KRVISLHPDTLFFIFMAITNVHFDDYLYVRKNVIISSKSIKPETMNQLLSHYLERTSLRTEKSSLDQT
PVTLSQTESNMLRMWMSGQGTIQISDQMQIKAKTVSSHKGNIKRKIKTHNKQIIYHVVRLTDTLTSGI
FVNSR
>CORE_REP|Org40_Gene3440#
MSRESRYYYEPAAGHGLPHDPLNAIVGPRPIGWISSRSAAGLRNLAPYSFFNCFNYRPPIIGFASTGW
KDSVANIVETGEFVWNLATRPLAEAMNNSSITLPRGEDEFAFAGVTPLPGRRVKAERVAESPVNFECR
LTQCIQLQTAAGEQVETWLVLGEVVAVHLDPALLDEHGVYQTATAEPILRAGGPTAYYGISEQHRFDL
TRPSR
>CORE_REP|Org28_Gene2514#
MIRVILVDDHVVVRSGFAQLLNLEDDLDVVGQYSSAAAAWPALLRGDVNVAVMDIAMPDENGLSLLKR
LRAQKPQFRAIILSIYDSPTFVQSALDAGASGYLTKRCGPEELVQAVRSVDMGGHYLCADALRALRGG
ERPATALEVLTPREREIFDLLVKGDSVKEIAFKLDLSHKTVHVHRANVLGKLQCNSTIELVHFALDHQ
LLAGH
>CORE_REP|Org12_Gene3713#
MIDAATLLLFSGACVALALTPGPDMLLIASRSVSQGRRAGFASLAGIQLGTYCHALAAALGLSQLFVA
VPLAYDAVRMLGAAYLLYLAWKTLRSDSSLQASAGLQAVPTARIFRQGLFTNILNPKMALFVLALFPQ
FIDPRAGSVVVQMLALATVLNLVGLLINGGVILAVSGMKSRFAGRRFSARWPNYLLSGVFAGLACKLI
FDSRR
>CORE_REP|Org38_Gene2292#
MFKRLLMVALLVVAPLASAADQTNPYSMMQDAAQKTFSRLKNEQPKIKQDPNYLRSIVHQELMPFVQV
KYAGALVLGRYYKDATPAQREAYFTAFQAYLEQAYGQALAMYHGQTYQIAPEQPLGNADIVAIRVTII
DNGGRPPVRLDFQWRKNSKTGYWQAYDMIAEGVSMITTKQNEWASTLRTQGIDGLTKQLQAAAAQPIS
LDKKN
>CORE_REP|Org35_Gene679#
MIGLVGKKVGMTRIFTEDGVSIPVTVIEIEANRVTQVKSLDTDGYRAVQVTTGSKKANRVTKPEAGHF
AKAGVEAGRGLWEFRLEEGQEFAAGQEISVEIFADVKKVDVTGTSKGKGFAGTVKRWNFRTQDATHGN
SLSHRVPGSIGQNQTPGKVFKGKKMAGHLGDERVTVQSLDVVRVDAERNLLLVKGAVPGATGGNLIVK
PAVKA
>CORE_REP|Org26_Gene111#
MADSKEIKRVLLGPLFDNNPIALQVLGVCSALAVTTKLETAVVMTIAVTLVTAFSSFFISLIRHHIPN
SVRIIVQMAIIASLVIVVDQLLRAYAFEISKQLSVFVGLIITNCIVMGRAEAYAMKSPPIESFMDGIG
NGLGYGVILVLVGFLRELIGSGKLFGVPVLETVQNGGWYQPNGLFLLAPSAFFIIGLLIWVLRTLKPA
QIEKE
>CORE_REP|Org34_Gene4021#
MTFKYSRLDKAQAAVLLVDHQTGLLSLVRDQDPDKFKNNVLALADLAKYFNLPTILTTSFENGPNGPL

VPELKQTFPDAPYIARPGNINAWDNEDFVKAVKATGKKQLIIAGVVTEVCVAFPALSALEEGYEVFVI
TDASGTFNAITRDAAWDRMSQAGAQLMSWFGAACELHRDWRNDIEGLGNLFSQHIPDYRNLMTSFSLL
TSGKQ
>CORE_REP|Org29_Gene3704#
MKKFSVRNHNVKRFSKPNAAKPASPKGPRRIVLFNKPFDVLPQFTDEAGRATLKEYIPFPDVYAAGRL
DRDSEGLLVLTNDGQLQAQLTQPGKRTGKIYYVQVEGAPQDSDLAPLRSGVTLKDGPTLPAGVELVAE
PQWLWPRNPPIRERKSIPTSWLKITLYEGRNRQVRRMTAHIGFPTLRLIRYSMGNLSLGSLQPGEWKS
IDSL
>CORE_REP|Org33_Gene3157#
MAKNYYDITLAMAGISQAARLVQQLAHEGQCNREAFQTSLKSLLQMDPPSTLAVFGGEERNLLVGLET
LMGVLNANNKGPGAELTRYTISLMVLERKLNANKPAMNTLGERLGQLERQLAHFDLESDTIISALAGI
YVDVVSPLGPRIQVTGSPAILQNPQVQAKVRATLLAGIRAAVLWQQVGGSRLQLMFSRNRLFKQAQNI
VAHC
>CORE_REP|Org12_Gene237#
MVNKRMQTLLTQLRQQGIRDEKLLRAIEAVPRERFVDEALDHKAYENTALPIGSGQTISQPYMVARMT
ELLNLKPTSRVLEIGTGSGYQTAILAHLVQHVCSVERIKGLQWQAKRRLKQLDLHNVSTRHGDGWQGW
ASRGPFDAIIVTAAPPEIPQALVEQLDDGGILVLPVGEQAQTLKRIQRHGNDFVVDAVEAVRFVPLVK
GELA
>CORE_REP|Org27_Gene504#
MKIVEVKHPLVKHKLGLMRENDISTKRFRELASEVGSLLTYEATADLETEKVTIEGWCGPVEVDQIKG
KKITVVPILRAGLGMMEGVLEHVPSARISVVGVYRDEETLEPVPYFQKLVSNIEERMALVVDPMLATG
GSMIATIDLLKKAGCHSIKVLVLVAAPEGIAALEKAHPDVELYTASIDQCLNDKGYIVPGLGDAGDKI
FGTK
>CORE_REP|Org26_Gene1779#
MKLLVVDECCFTRVGIASYFADSGITTIKCCHSIEYATPLLASFQPSHILVNLSNQCRYNEADAQLLA
FMEASQSALLFIYLDTPYPYSETPMRIADNAFLFNKSILPLTLRTLRENPLALADDGEERSLFSPQEL
TVMKYWMAEMPNYRIAKKLQISSHTVYVHKRHITEKINARNRLEFYSLYNVLRYFYPPNTPTNSTPLA
LLAV
>CORE_REP|Org38_Gene4476#
MPIRKVSLLRLIPLASLVLAACTTTKPTGPATSPTSPQWRAHEQAVQQLSTYQTRGSFAYLSDQKKVY
ARFFWQQFSPERYRLLLTNPLGSTEMDLNVQKNVVQLTDNQGKRYVSDNPEEMIRKLTGMAIPLNNLR
QWMLGLPGEASDFALDDQYRLSKLTYQQGGQTWKVDYQGYSNDVQPTLPNRLELQQGDQRIKLKMDNW
TFK
>CORE_REP|Org5_Gene483#
MSYSGERDQFAPNMALVPMVVEQTSRGERSYDIYSRLLKERIIFLTGQVEDHMANLIVAQMLFLEAES
PEKDIYLYINSPGGVITAGMSIYDTMKFIKPDVSTICMGQACSMGSFLLTAGAKGKRFCLPNSRVMIH
QPLGGYQGQATDIEIHAREILKVKARMNELMAEHTGQPLEQIERDTERDRFMTAEEAVEYGLVDGILT
HRS
>CORE_REP|Org14_Gene3754#
MKQELHILLGKAVAFLLYCAFLAALVGLVFIDVHWLHNFVHETSLTEAAQELLLLAISGGFFAAAQWQ
SERRSAWMLVGGFFLCMLIREMDFAFDALWHGAWVWFALAVALACLVHAARHIAATVRGLAYFVTHPA
YGMMCAGLLCILVFSRLFGMSALWQTLMLDGYNRVVKNMVEEGCELLGYGLCLLATLSYMKTVFAPAD
AKA
>CORE_REP|Org32_Gene2065#
MKKIWLALVGMVMAFSASAAQFSDGTQYVTLDKPVTGEPQVLEFFSFYCPHCYQFEQVYHVSENVKKA
LPAGTKMTKYHVEFLGPLGKQLTQAWAVAMALGVEDKVSPLMFEAVQKTQTVQTPDDIRNVFVKAGVT
AADYDAAWNSFVVKSLVVQQEKAAEDLQLRGVPAVFVNGKYMVKNDGLDTSSMDAYVKQFADVVKFLS
QQK
>CORE_REP|Org14_Gene3696#
MPDIRFTLFDSDNFYQQGLRLLLQDYLHSLNECQRLYPQFSPLALQALDNIEIVFRTPEERWGCACCY
RSPYGIPRQRQLTLLILDDTAQQQVKNLSPLLSIHRRDSVYAIRLKLQMALEKFIQQPWMALHAADIW
KCQACRLAALSHCEKKVLGLMSSGMSACSIAGLLQRSQKTISAHKRSAMRKLNVRKNSELNRVLLNQR
ELN
>CORE_REP|Org11_Gene2836#

MLRVLGKASSINVRKVLWTCQELGLTLEREDWGSGFRSTQEPAFLALNPNGLVPVLIDGERVLWESNT
ICRYLVGREGRRDLLPQEPGARAAVEMWMDWQATDLNGAWRYVFMSRVRGHADYQDAASLAAAEREWN
RLMGLLDRHLAQHGPYAAGGTFTLADVVLGLSLNRWLMTPFERPNYAALAAYQQRLLQRPGYVQHGAN
GLP
>CORE_REP|Org25_Gene1098#
MAQGTLYIVSAPSGAGKSSLIQALLKTQPLYDTQVSVSHTTRDSRPGENHGEHYFFVSKDEFRRMIEQ
DAFLEHAEVFGNYYGTSRAAIEQVLSTGVDVFLDIDWQGAQQIRAKMPQARSIFILPPSKEELGRRLR
GRGQDSEEVIAKRMAQAVAEMTHYAEYDYLIVNDDFDLALSDLKTIIRAERLRLGRQLLRHDALISKL
LAD
>CORE_REP|Org48_Gene2344#
MQKKLDSLLAAAGIELPDQQKQQLLGYVGMLDKWNKAYNLTSVRDPQQMLVRHILDSIVVNPHLQGSR
FIDVGTGPGLPGIPLAIVRPDAHFTLLDSLGKRVRFLRQVQHELGLNNIEPVQSRVEAFPAEPPFDGV
ISRAFASLQDMLSWCHHLPAKGQGRFYALKGVRPDEELAHLPEGVSLESIVRLQVPELEGERHLVVLK
AN
>CORE_REP|Org5_Gene3276#
MLTEQQNNQLDWKKTDGLMPAIVQHAVSGEVLMLGYMTPEALAATEQSGNVTFFSRTKQRLWTKGESS
GHFLKVVSITPDCDNDTLLVLANPIGPTCHLGNSSCFHPAASDWTFLYQLEQLLAERKHASPDSSYTA
SLYASGTKRIAQKVGEEGVETALAATVNDREELTNEASDLIYHLLVLLQDQDLNLSAVIGRLRERHQK
KA
>CORE_REP|Org12_Gene3987#
MSQLFYIHPDNPQPRLISQAVDVLRKGGVIVYPTDSGYALGCMLEEKAAMERICRIRQLDGNHNFTLM
CRDLSELSTYAHVDNTAFRLIKNNTPGHYTFILKATKEVPRRLMNEKRKTIGLRVPSNPIALALLEAL
NEPMMSTTLMLPGNDFAESDPEEISDHLGKVVDLVIHGGFLGQQPTTVIDLTESTPEVVREGAGDAAP
FR
>CORE_REP|Org22_Gene1084#
MTLDWWLTYLLTTLILSLSPGSGAINTMSTGISHGYRGAAASIAGLQVGLSAHIVLVGIGLGALISQS
LLAFELLKWLGAAYLVWLGIQQWRAAGALDLHALAGSMPRRRLFRRAVLVNLTNPKSIVFLAALFPQF
ILPNQPQAEQYLVLGVTTVVVDILVMIGYATLATRIAGWLRTPRQMQLLNRVFGSLFILVAGLLATAR
KA
>CORE_REP|Org35_Gene1069#
MEVYTTENEQVDALRRFFAENGKALAVGVVLGIGALVGWRYWQSHENSNMMAASQSYQEASDRLAAGK
PDDVAAAEKFVQANGNSYGVLAALQLAKHFVEQNDFAKAEQQLALAQGQTKDDNLLAMIDLRLARVQL
QEKKLDEALKTLDGVKGEGWAAMMQDVRGDVLLAKGDAKGAREAYSKGIESNASQALQVLLRMKLNNL
SS
>CORE_REP|Org24_Gene262#
MTTPSFDSVEAQASYGIGLQVGQQLQESGLEGLQPEALLAGLRDALEGNAPAVPVDVVHRALREIHER
ADAVRRERQQAMAVEGQKFLDDNAKRDDVTLTESGLQFSVLEQGNGPIPSRQDRVRVHYTGRLINGDV
FDSSVERGQPAEFPVSGVIPGWIEALTLMPVGSKWQLYIPHNLAYGERGAGASIPPFSALVFDVELLE
IL
>CORE_REP|Org23_Gene771#
MPNSSSQLELLRSVADGIAALFFPNAEVVIHDLATNKIAYLANNLSKRKPGDDAGLEDFELDGGAGVT
GPYEKLNWDGKKMRSVSIAARDEQGKPSYLLCINLSTAMFEDARNALDMFLSVTRLQPQPQELFKDDW
QEKINTFLHDWLRRENAALGALSREQKRRLVSDLYHQGAFKAKSAADYIANVLSMGRATVYKHLRELK
QE
>CORE_REP|Org14_Gene638#
MLMLFLTVALVHLIALMSPGPDFFFVSQTAASRSRREAMMGVVGISLGIVVWAGVALMGLHLILQKMA
WLHQIIMVGGGIYLCWMGWQLLRSARAQQAQPAAEAQVALPKAGRSFIRGFLTNLSNPKAVIYFGSVF
SLFVGDSVGAGARWGLFLLIVAETFVWFSLVAVVFALPAMRRGYQRLAKWIDGVAGVLFTGFGLHLIF
TR
>CORE_REP|Org43_Gene141#
MARYLGPKLKLSRREGTDLFLKSGVRAIDSKCKIEQAPGQHGARKPRLSDYGVQLREKQKVRRMYGIL
ERQFRNYYKEATRLKGNTGENLLQLLEGRLDNVVYRMGFGATRAESRQLVSHKAVMVNGRVVNIASYQ
VSPNDVVSIREKAKKQSRVKASLELAEQREKPTWLEVDAAKMEGVFKRMPERTDLSADINEHLIVELY
SK

>CORE_REP|Org31_Gene2423#
MLGTMMTKSTHCNVDTREHLLATGETLSLRLGFTGMGLSELLATAGVPKGSFYHYFRSKEAFGEAMLQ
RYFAHYDAQMQALFADRRGDARHQLLGYYAQAISYHCRSECHNACLAVKLSAEVSDLSEPMRHALETG
TARVIGHLQEAIERGIAEGSLSVAMSPAATAETLYSLWLGASLRAKIRHSLAPLTSALESIELLLRPP
QA
>CORE_REP|Org33_Gene738#
MQKLRLICLAALSFSITWAAHAEDKRYISDELSTYVHSGPGNQYRIVGTLNAGEEVTLLSVNDSTNYG
QIRDPKGRTTWIPLDQLSQTPSLRTRVPELEQQVKTLTDKLANIDNTWNQRTSEMQEKVAGSDSTISS
LQKENQDLKNQLVVAQKKVNAVNLQLDDKQRTIILQWFMYGGGVAGVGLLLGLLLPHLIPRRKNNNRW
MN
>CORE_REP|Org41_Gene1077#
MTRLSPRAAAKKPPQAAAGQIRIIGGQWRGRKLPVPNSPGLRPTTDRVRETLFNWLAPVIQGARCLDC
FAGSGALGLEALSRYAGSATLLEFERPVAQQLEKNLALLQGKGVVINTNALSWLAGEGQPFDVVFLDP
PFRKGLLAETALLLEQRGWLADEAWIYVEAEAESAAADVPASWQLHREKVAGQVAYRLYIRSQEKTDH
AD
>CORE_REP|Org3_Gene854#
MLLSILYIIGITAEAMTGALAAGRRQMDMFGVIIIASVTAIGGGSVRDMLLGHFPLGWVKHPEYIVIV
AIAAIVTTWVAPLMRHLRRLFLVLDAVGLIVFSIIGAQVALDMGHSAIIAAIAAVITGVFGGVLRDMF
CNRIPLVFQKEIYAGISFSAAWLYIGLQHLSLPHNLVVILTLVAGLSARLIALRFRLGLPVFNYQHPD
H
>CORE_REP|Org18_Gene1226#
MTLALFAAFWAVSILLVITPGMDWAYVISAGIRGRVVVPAVAGLLFGHLLMIAIVVAGVGALLVGNPL
ALTALTLLGAAYLLWIGCNMLLHPPVPGAGEDQRSDSWLRWAGKGVCVSGLNPKVFLLFLALLPQFTD
ARAEWSVPMQMLALGVVHLISCGLVYLLVGFGSQSVLRTRPQAAKVVGRISGAAMIVIALGLLAEQAL
K
>CORE_REP|Org25_Gene188#
MLAVFLQGFALSAAMILPLGPQNVFVMNQGIRRQYHLMIASLCALSDIVLICAGIFGGSALLTRSPLL
LALVTWGGVAFLLWYGWGAFRSAFSPQPAQAAAQELAQSRWRIVVTMLAVTWLNPHVYLDTFVVLGSL
GGQLTADVRSWFALGAVSASAVWFFGLALLASWLAPWLNTQRAQRIINALVGLVMWGIALQLAWQGAN
L
>CORE_REP|Org48_Gene3180#
MLLTIARWLFQLPGKLFGLIFKNRLRIFLFFMLVAIAVLGAKRYLRAHQYDDEFSLAAPADYNRAIKR
EMPLREAREQCGGPLHDNAGQPWPASAGYLHQPTRLPATSLHRLTLDNQGNDFAVLVKLETPAAPQQL
AEVFIPPAGSFEVKINADAEHVMKIKNIKTGCMFRSRSFNLDKQRDWRMPLTLQKDGSLQLQSIGDRQ
F
>CORE_REP|Org31_Gene2631#
MSGDIINDLATLEQLYGKPAAPSVFKEVSYIHPAYRPFIEAAPFAALSTVGPDGMDVSPRGDPAGFIH
IEDEKTLLLPDRRGNNRIDSLRNILHDNRVALLLLIPGIGETLRINGRAEIVVAPAVLQRFAQRNQLP
RSVLRIRVEAVFFQCSRAILRSGLWEEGSRLECSALPSPGHILAAVSQTRFDGEEYDQALPQRLKDTL
Y
>CORE_REP|Org18_Gene2405#
MSTETLTNHNAAHAEHGHHDAGETKVFGFWIYLMSDCILFASLFATYAVLVNGTAGGPTGAHLFKLDF
VLVETFLLLFSSITYGMAMIAMNKGKVAGVNLWLFLTFLFGLGFIGMELFEFHEFISEGFGPDRSAFL
SSFFALVGTHGLHVSAGLVWIIVMMIQVSKFGLTATNKTRLMCLSLFWHFLDVVWICVFTVVYLLGAM
S
>CORE_REP|Org16_Gene3928#
MNTLDAIRQRRATKQFDTQYVMTLEEKKALLNIALQGAPSAFNLQHWRPLLIEDRAQREKIREAAWGQ
AQVTDASMLVVLCGDLSSWESQVKNVWAEAAEPVQQFMIPAVDQYYRGKPQVQRDEVMRSSGIFAQTL
MLAAKAQGYDSCPMDGFDFDAVGEIINKPAHYQIALMVAIGKAAGQPYPRIGKLPFDDVVKTDRFCSE
R
>CORE_REP|Org5_Gene1165#
MKTTNAQRKINIIKNIEYLMRTRGETKASFSNRSGLTRTTLYKILDGRVNNVQQSTVNRISDFFGVSC
EEIEDYDLEKLELLNETLSTEGNKNPSAIPVIPQSRYLAVSQRKIGQLVTEFPLTYFFGDESNMLAMK
IETEIKGSFIPGEIIIIKRPPVLICDSPLLYHSGKSGFFVVDDKDSESMDKRPQDTVQFLGYIVGERL

>CORE_REP|Org42_Gene2582#
MRPYPIPAEATCFTEEIKKSRFITLLAPTSGVEAAKTFIQQVRDEHPAARHHCWAFVAGSPDDSQQLG
FSDDGEPSGTAGKPILAQLMGSGIGEVTAVVVRYYGGVKLGTGGLVKAYGNGVQQALKQLALAQKVPE
AEYILQCDYAQLALVENLLQQTAGRILQGEYGAAVVLHLALPATEVELFGNKLRDLSRGNLQLTPISQ
>CORE_REP|Org29_Gene3336#
MTENPQLTALLAVCQWIGDKGWCPATGGNMSLRLDERQCLVTESGKDKGSLSAADFLLVDIADNHVPS
GRTPSAETGLHTLLYRLSAHTGAVLHTHSVNATVLSRVESGDALVLQGYEMQKSLAGQRSHLDSVAIP
IFDNDQDIPRLAARVAAYAEATPLQYGFLVRGHGLYCWGSQVAEARRHLEGLEFLFQCELQRRLLEAK
>CORE_REP|Org41_Gene1324#
MLVFWLDILGTAVFAVSGVLLAGKLRMDPFGVLVLGVVTAVGGGTIRDMALANGPVFWVKDPTDLVVA
MITCVLTLLLVRQPRRLPKWVLPVLDAVGLAVFVGIGVNKAFAAGTGPLVAICMGVITGVGGGIIRDV
LAREIPMILRTEIYATACIIGGIVHATAFYTFHMPLQQAMMLGMIITLAIRLAAIRWHLKLPAFILER
>CORE_REP|Org10_Gene3571#
MAYIVALTGGIGSGKTTVANAFARHGATLVDADVIARQVVEPGTPALAAIAEQFGNEMLLPDGALNRA
ALRQRIFSHPDDKTWLNQLLHPLIHRETQRQLAQAASPYALWVVPLLVENRLQDRADRVLVIDVDAET
QLARTIARDGVSREQAQSILAAQATRQQRLAVADDIIDNSGAAQGIEPQVAALHRRYLELAATAPQQD
>CORE_REP|Org10_Gene1508#
MIGRLRGNILEKQPPLVLLEANGVGYEVHMPMTCFYELPELGQEAIVFTHFVVREDAQLLYGFNDKQE
RALFRELIKVNGVGPKLALAILSGMSAQQFVSAVEREEITALVKLPGVGKKTAERLVVEMKDRFKGLN
GDLFNSSSEISLPSAADNAPEADAEAEAVSALVALGYKPQEASRMVSKIAKPGADCETLIRDALRAAL
>CORE_REP|Org35_Gene2480#
MSLTLTPEQRQTVHPLWLRLTHWLNALAMLIMVTSGWRIYNASPLFNFSFFNELTLGGWLGGALQWHF
AGMWLFGVNGLCYLLLNLASGRFKRKYWPLSPKQFLADVGAALRGRLQHADLRHYNMVQRVAYLSVML
LGVLSALSGLVLWKSVQFPLLRTLLGGYEAARYIHFFAMSALVAFVAIHLVMVALVPRTLLAMLRGR
>CORE_REP|Org22_Gene1526#
MKTPELILLQLKALGPHSAKMLAERLDITPMGIRQHLQSLEKRELVCYEEARSKVGRPTRYWSLTERG
HAFAAADQERLRASALQLFGGAGVEPLLSAREEQLYLRYAEELSAERSHQDRLARLVRLRQHDGYMAE
LFDHPHGALLVENHCPIGVAGSSCSSLCNSELQLFHRLFGNDYRVERTAHAISGSRHCAYLIRPREL
>CORE_REP|Org13_Gene1615#
MAEFTDRLRPLLLGALLCGGSALAATPLGVIEGTTGTVSFHGGLLSSPCSLTPDSRDQSVDLGDVSAR
DFRNAGDRSAPVRFRLSFRNCLLGARALADNPAGPRNGDAGRLYLQGEQAATLVFLGESDVDNPSLLK
LNGGVQGIGLRLQDQQGQALELNQRSRPYILQPGSNTLWFSASVESTQRAVMAAGFAAVAHIQVIYL
>CORE_REP|Org3_Gene839#
MSSAVPALDFGSMTQVIQFLMEIDKLKGVQRRTKVLGTQRQENSAEHSWHFAIAAMSLAPYAGDDVDI
QRVIQMALLHDIVEIDAGDVLVYDLAARAAIHDQEVAAARRLFGMLPEAQREYFTALWQEYEDGESAD
ARFALLLDRTMPMLMNLHNEGQSWVENGISLEQVLARNASIADVHPELWHHMEQHLRDAQRKGWLK
>CORE_REP|Org33_Gene3379#
MLFTPLLTGALETSLNNLLFRDRSMKAARQRLAGKVLRIELEELASPLVLVFSELRVDVLGQSEDSAD
CTVRSRIPDLLKLRDRQQLPVLMRSGELTVEGDIQVVQQLVGLLDLAEWDPAEWLAPYIGDIAAQGIT
QALGKGASLLKTGFMRRQQDMAEALTEEWRLAPRPLEVVWFNEEVDALARSAEALSARMDKLEGKR
>CORE_REP|Org37_Gene798#
MKALTTRQQEVYDLIRDHISQTGMPPTRAEIAMRLGFRSPNAAEEHLKALARKGVIEIVSGASRGIRL
LMEEEEGLPLIGRVAAGEPLLAQQHIEGHYQVDPSLFKPSADFLLRVNGMSMRDIGILDGDLLAVHKT
QDVRNGQVVVARIEDEVTVKRLKKHGNVVELLPENNEFQPIVVDLRQQNFTIEGLAVGVIRNGDWI
>CORE_REP|Org32_Gene2805#
MLDSAFVSYVTVMSITPGPNNLLLASSGVNFGLRRTLPMLFGICVGCAVQLAIIIPLLALALSWAGVI
RLPLAVIGCAYLLWLSWKLFQAASPDAKEQAQPMSLLNGALFQAVNPKAWLMVINVAILFTPREGVTF
VHTLSVIAGFALLNLPCVLLWAVLGDRLRNALRVTWKLRAFNGVMSGLMAATALWLLFDEWRAAFA
>CORE_REP|Org19_Gene1658#
MPFQPQFAQQRQAIRQLIRQRRRELTPGQQRFAADKIAERLVAHTHIKAAHSIAVFLSFDGELDTGPL
IEQLWTLGKRVYLPVLHPFSPGHLLFLRYAPETPLVRNRFNILEPRLDVRQVLPLGELDVVLTPLVAF
DHTGQRLGMGGGFYDRTLQNWRSGGPYPIGLAHDCQQVERLPIEHWDIPLPEILTPLCSWAWHEPK
>CORE_REP|Org5_Gene1666#
MKLFYKAGACSLSPHIVLREAGLDFTAEKVDLAQKKTESGADYLAINPKGQVPALLLDDGSLLTEGVA

IVQYLADRVPDRNLIPAAGTLSRYHAIEWLNYIATELHKGFSPLFNPKTPEEYQTIARAKLEQQFSYL
DSVLAKQHFLLGSRFSVADAYLFTVLRWAFALQFDVRKHAHLAAWFDRVAARPAVDAALNAEGLK
>CORE_REP|Org28_Gene634#
MQTSPLLESLMEALRCLPGVGPKSAQRMAFQLLQRDRSGGMRLAQALTRAMSEIGHCADCRTFTEQDV
CTICANPRRQQNGQICVVESPADIHAIEQTGQFAGRYFVLMGHLSPLDGIGPGDIGLDRLEQRLEKES
ITEVILATNPTVEGEATANYIAEMCGQYGVLASRIAHGVPVGGELEMVDGTTLSHSLAGRHAIKF
>CORE_REP|Org21_Gene2928#
MEQLNHFLFAWINATPASPEWMIDFATFLARDLIIIVPLLIVGLWLWGPQSQLASQRQVVAKTTIALL
FAMLAAATIGALLPHERPFVAGVGYTFLAHAPDSSFPSDHGTAIFTFALAFLFWHRVWSGVLLMIVAV
GIAWSRVYLGVHWPLDMVGGFLLGLVGCLFAQLVWNLFGDAIADKLSRLYRFLFAFAIRRGWVKE
>CORE_REP|Org3_Gene138#
MELVLKDAQSALTVSETTFGRDFNEALVHQVVVAYAAGARQGTRAQKTRAEVTGSGKKPWRQKGTGRA
RSGSVKSPIWRSGGVTFAAKPQDHSQKVNKKMYRGALKSILSELVRQDRLIVVEKFSVEAPKTKLLAQ
KLKDMALEDVLIVTGELDENLFLAARNLYKVDVRDVAGIDPVSLIAFDKVVMTADAVKQVEEMLA
>CORE_REP|Org3_Gene1410#
MANKTAIAMRCAKSPVPGFLGARLNIGRLLLIVLLVLLTLSGPARAADDPYQKRTHAVTTVVVGIISY
ARWSREPNPIRLCVTAPTQYAEGLFDPILLSAPRPIKAERVPFDSPTLSTGCDVIYLGNITAAQRQNF
MQRISGNSILSISENDSECSAGSAFCLQIDGDQASFKVNLDALARSGVRVHPNVLQLARKQAPPL
>CORE_REP|Org22_Gene2013#
MSVSALVCLAPGSEETEAVTTIDLLVRAGVKVTTASVAGDGELTIVCSRGVKLLADAPLVAIVDEPFD
AIVLPGGLKGAECFRDSPLLVEKVRQMHLQGNIVAAICAAPALVLQHHDLFPVGNMTGFPGLKDQIPA
DKWMERRVVYDARVNLLTSQGPGTAMEFALKLIDLLLGKTKAAEIAAQLVLIPGMYDYRDSEEN
>CORE_REP|Org7_Gene2388#
MEPSLFLSMLGFLWVAAITPGPNNLLLTTSGAHFGFMRSLWLMIGIMLGMQSILLLVAFGVGSLILVY
PSLHLILKILGSLYLLWLAWKVATAAYEKLETDVAPPQPVRLYQGWLLQFLNPKAWLMALGAVASFSL
AGDKYNGSIAAIALGILAVNIVAGVIWLAFGTVIGRLLRSKKAWIIFNVSMGLLTAACVLLIWH
>CORE_REP|Org42_Gene764#
MAKFTQHTGLVVPLDAANVDTDAIIPKQFLQKVTRTGFGQHLFNDWRFLDDAGQQPNPEFVLNKPRYK
GASILLARENFGCGSSREHAPWALTDYGFKVVIAPSFADIFYGNSFNNQLLPVTLSEQQVDELFKLVD
ANEGTEFVVDLENQTVNAGGKSYPFEIDSFRRHCMINGLDSIGLTLQHEADISRYEAQQPAFLN
>CORE_REP|Org49_Gene3493#
MNDFAQPKIGDNVTLNRTRLGQYVHLADDAILEEVEMGDYSYTAGHNQIFYATIGKFVSIASYARINP
GNHPTYQRIAQHHFTYRASEYGLGEDDAAFFDWRREHHVAVGHDVWIGHNAILMPGVSVGNGAVIGSA
AVVTKDVEPYSIVAGVAAKKIGMRFDDALIERIERSQWWHWDHATLQARLADFRDINRFAQKYL
>CORE_REP|Org44_Gene507#
MPKVGMQPIRRQQLIDATLAAVNEVGMHDATIAQIARRAGVSNGIISHYFKDKNGLLEATMRYLISHL
GEAVKLRLQALTDNSPAARLQAIVAGNFDDSQINSAAMKTWLAFWASSLHQPQLNRLQQVNGRRLYSN
LCAEFRRVMPQPQARLAAKGLAALIDGLWLRSALRGSAFNQAQALALTTEYITFQLRGQTPPGG
>CORE_REP|Org40_Gene522#
MVLVTRQAPDFTAAAVLGSGEIVENFNLKKHLNGKPAVLFFWPMDFTFVCPSELIAFDHRYEEFQKRG
VEVVGVSFDSEFVHNAWRKTPVDKGGIGEVKYAMVADVKREIQKAYGIEHPEAGVALRGSFLIDKDGI
VRAQVVNDLPIGRNIDEMIRTVDALQFHEEHGEVCPAQWEKGKAGMGASPDGVAKYLSENAAKL
>CORE_REP|Org37_Gene2799#
MADERCGWVTADPLYLEYHDKEWGAPTTDARELFEMLCLEGQQAGLSWITVLKKRENYRRAFHGFDPQ
RVAAMTTEDVENLLQDSGIIRHRGKIEAIITNAKAYLAMEAAGEDFVAFIWDFVGGRPQLNRWQALNQ
VPAKTEQSDAMSKALKKRGFKFIGSTICYAFMQASGLVNDHLTGCICYPKPRCSAPVARRISNG
>CORE_REP|Org34_Gene4490#
MEKKHEKYVLVALAAFTLSGCLSRPPEPDQPLPPVTVEPEQPTPPVEQPQPPTTEPVPQPPKIQQLDW
LGSVQPLVNQMLKADGVTAGSVLLLDSVKNNTNGSLQMGKATAALYKALASNSTFSVVPEAKLTSARQ
TLGLSADDSFGSRSKAIGLARIVGAQYVLYSDVSGDVKSPALDMQLMLVQTGEIVWSGNGAVKR
>CORE_REP|Org23_Gene1627#
MKKILVLKSSIMGNDSQTNNLIDHYLAARRAKGYEDQIVEHDLTALDLPVLDGELFDALRGAENISPR
AKATVALSDRLIAELKGSDLLLIGAPMYNLNVPTQLKNWFDLVARARVTFNYTATYPVGLVEGVNALV
FSSRGGIHAGQPTDAVTPYLRSVMGLMGIGDVQFIYAEGLDMKPHGLAQGLANARERIAELAG

>CORE_REP|Org42_Gene1324#
MTNTLVERARRYATKAHAAIDQRRKYTDDPYIVHPQAVMELVRSVPHTEEMLAAAWLHDTVEDTPTTL
GDIESHFGPKVAELVRMLTNVSRAEDGNRFERKNRDRRHSAGASPQAKTIKLADLIDNTRSLLDYDSH
FAQTYLIEKQRLLEVLTEGDPTLWRQASHIVEQGLLRLQQPPHNVPASWFEHARRRYRESDAA
>CORE_REP|Org20_Gene1585#
MTKVLVLYYSMYGHIESLAQAVAEGANRVNGVDVTIKRVPETMTPEAFAKAGGKQHQQAPVATPQELA
DYDGIIFGTPTRFGNMAGQMRTFLDQTGGLWASGALYGKVGSVFSSTGTGGGQEHTISSTWTTLAHHG
FIIVPIGYATPELFDVSQVRGGTPYGATTIAGADGSRQPSNEELTIARYQGEHVAKITAKLKS
>CORE_REP|Org41_Gene2106#
MRAVPYECQWASPELAADLIAGRMTLAQDENWARSGARDRAEYVEWANHVCGMACLKMVLSHRDGEAP
PLLELARRSLPYGAYVREGERIKGLIYAPFVEYVREQFALQAEVKVGLEAAELSALLSQYRYFIASVH
PSIRRPESVPPQRGGHLVLITAADTQSVTFHNPSGDSPATRERVQLPLNRFTPFFAGRGIAIA
>CORE_REP|Org12_Gene2101#
MAEKENTKRNRREEILQALAQMLESSDGSQRITTAKLAANVGVSEAALYRHFPSKTRMFDSLIEFIED
SLITRINLILQDEKETFNRLRLILLLILGFAERNPGLTRIMTGHALMFEQDRLQGRINQLFERIEAQL
RQVLKERKLREGKGFIVDETLLASQLLAFCEGMLSRYVRSEFRYRPTQEFDARWPLLAAQLQ
>CORE_REP|Org49_Gene112#
MEHYISLFVRAVFVENMALAFFLGMCTFLAVSKKVSTAFGLGIAVTIVLGISVPVNNLVYNLILRDGA
LVEGVDLSFLNFITFIGVIAALVQILEMILDRFFPSLYNALGIFLPLITVNCAIFGGVSFMVQRDYNF
AESVVYGFGSGTGWMLAIVAMAGIREKLKYANVPAGLRGLGITFITTGLMALGFMSFSGVQL
>CORE_REP|Org37_Gene3179#
MVSHVWGLLAHPSAEFEQIKSENESVSHLYTHHVLLLAAIPVVCAFIGTTQLGWLSGEGHAIRLDMFT
ALYTAVAFYLLMLAGVAAMGKVIHWMARRYESRPSLHRCMVFAGYVATPMFLSGIVALYPVVWLCLFV
GIIGLCYSGYLLNLGIPHFLNIDSKEGFIFSSSTFAIGILLLELLLGVTVLLWGVRFLVVLS
>CORE_REP|Org11_Gene1861#
MYRRDIPEQRKEQLINAAFETISVVGLAGVTLSQVAKEAGLSTGIVSHYFGDKEGLLSATMRKILRDL
RDAVAECRAQAASDSQSQLCAIIQGNFHPSQTNAISMRAWLDFWAASMHQPVLRRLQRANDRRLYSNI
CSQFRRELPLQQARDAARGLAAMIDGLWLRGSLAGDDTDLQQDYRIACDYVIQRLRAAPSAE
>CORE_REP|Org14_Gene2680#
MLSPNDSKQAKVQARRDQIVEAAKTSFRRHGFHAASMAEIAQGSQLSVGQIYRYFANKDAIIEEIVNR
IIASKMQRLENLGDHINLIAGTLAARTLFQQPGESETDHMLMLEVTAEATRNPVVAKMLSDAEARLFR
HVCHNLQRLYPDFSAEEIAARVEFIAVMSEGTGYRILTTQKADASLLRDLYQQAISHLFRKR
>CORE_REP|Org31_Gene956#
MPTPISVISGDLVDSRRSDTVNYLNELHSLLDRLRRDGRLHQVEIFRGDAFQAQAAPEDGLLLAVYIR
LALRATGAAHWDARVAVGLGSQRPDAAGYGSAFINSGSALDAMAKNCRLALKTDNDRTNAIVSDLLPM
LDHVIGRLSQTEARIVQARMFADSGAAVADQLQKAASTVSAALKRAAYEEIMRFIHAINRIV
>CORE_REP|Org19_Gene4712#
MMKWIPVALALLLSACSSSPQKTYYQLPALGAPAAVSSSSGASTRQLWLEHVSVADYLAQSGVVYQTN
DVQYVIAQNNLWASPLDQQLQQTLVTNLSSALPGWVVSSQPMSSDQDVLNVTISGFHGRFDGKAIVRG
EWVLNRQGRLIKRPFSLELKQGEDGYDALVRTLAEGWQKEAKSIALQLKKICFIFGRDIKAL
>CORE_REP|Org3_Gene1190#
MQKVVLATGNPGKVRELADLLADFGLDVVAQTDLGVESAEETGLTFIENAILKARHAAQVTGLPAIAD
DSGLAVDALGGAPGIYSARYAGEDADDRQNLDKLLAALKDVAPGRRGAQFHCVLVYLRHAQDPTPLVF
HGSWAGEITEQAAGEGGFGYDPVFYVPELGRTAAELSRDEKRAISHRGKALKLMLEAMRNA
>CORE_REP|Org14_Gene667#
MQLKRVAEAKLPTPWGDFLMVGFEELATGHDHLALVFGDISGEAPVLARVHSECLTGDALFSLRCDCG
FQLEAALEQIAEEGRGILLYHRQEGRNIGLLNKIRAYALQDKGADTVEANHQLGFAADERDFTLCADM
FKLLGVDAVRLLTNNPKKVEILTEAGINISERVPLIVGRNPKNEHYLATKAAKMGHLLDQK
>CORE_REP|Org38_Gene913#
MTEMISATVLLFLIMDPLGNLPIFMSVLKHLEPRRRRVVLIRELLIALLLMLIFLFAGEKILAFLNLR
TETVSISGGIILFLIAIKMIFPSQEGNSSGLSAGEEPFLVPLAIPLVAGPSILAALMLLSHQYPHQLP
HLVAALLIAWGLSAAILLMSNLFLRLLGSKGVSALERLMGLILVMLSTQMFLDGVRAYMKL
>CORE_REP|Org24_Gene1064#
MNKTSRTLFSLGLLSAAMFGVSQQANAHGYVESPASRAYQCKLQLNTQCGSVQYEPQSVEGLKGFPQA

GPADGHIASADKSTFFELDQQTPTRWNKLNLKTGPNSFTWKLTARHSTTSWRYFITKPNWDASQPLTR
ASFDLTPFCQFNDGGAIPAAQVTHQCNIPADRSGSHVILAVWDIADTANAFYQAIDVNLSK
>CORE_REP|Org24_Gene1121#
MIEPFIYPAATLIAGVDEVGRGPLVGAVVTAAVILDPAQPIVGLADSKKLSEKRRLALYDEIVAKALS
WSLGRAEPAEIDQLNILHATMLAMQRAVAGLHIAPDMVLIDGNRCPNLPMRSQAVVKGDSRVAEISAA
SILAKVTRDREMAALDSEFPDYGFAQHKGYPTAFHLERLAALGATEHHRRSFAPVKRALAL
>CORE_REP|Org25_Gene1287#
MNNIYLSMAAFALAASITPGPVNIVALSAGAQFGFGATQRHVFGATLGFTLLLVLLGLGMHQLLILWP
ALTRLIQWAGVAFLLFMAYRLAFDDGRLGNGEAQAPSCWYGALMQWLNPKAWLASVASLGAFVGDGDI
RLLWQFAGLYFVICYASVACWAYVGAMLRGLLRQARNVQRFNRAMAALLAASACYLLAEGF
>CORE_REP|Org26_Gene381#
MLDRIKVCFTESIQTQIAAAEALPDAISRAAMTLVQSLLNGNKILCCGNGTSAANAQHFAASMINRFE
TERPSLPAIALNADNVVLTAISNDRLHDEVYAKQVRALGHTGDVLLAISTRGNSRDIVKAVEAAVTRD
MTIVALTGYDGGELAGLLGQQDVEIRIPSHRSSRIQEMHMLTVNCLCDLIDNTLFPHQDD
>CORE_REP|Org4_Gene1977#
MTLRVAFIDDHDIVRSGFVQLLSLEADIQVVGEFSSAAQARAGLPGLEAEICICDISMPDGSGLDLLA
DIPSGIRVVMLSMHDNPALVEMALDRGASGFLSKRCKPEDLITAVRTVAGGGVYLMPEIAQQLARVRV
DPLTRREREIALLLAQGQEVREIAAALGLSPKTVHVHRANLFAKLGINNNVELARRMLNL
>CORE_REP|Org14_Gene1789#
MNVVILDTGCANLASVTYAVRRLGYQPEVSRDPEIVLRADKLFLPGVGTAQAAMDQLRERDLVELIKA
CTQPVLGICLGMQLLAASSEENGGVTTLGLIDTPVKQMTDFGLPLPHMGWNQVSAQAGHHLFRGIDDG
AYFYFVHSYAMPICPSTIAQANYGEPFTAAVQKDNFFGVQFHPERSGAAGAQLLKNFLEM
>CORE_REP|Org6_Gene1208#
MFLKRTLLACSLALIFPALPSYAEVGTESGNAAQAEKPGLWQRFTDNVAETWNNSPNHDLYIPAITWH
NRWTYDDEHIDKYNERPWGAGYGISRYDSDGDWHGIYIMAFKDSFNKWEPIGGYAYEKIWRPLDDKDF
RLGLGFTASVTARDNWNYIPIPAPLPLASIGYQRLTFQATYIPGTYNNGNVFFAWLRWQF
>CORE_REP|Org30_Gene1369#
MIGLLPSLNRAWRRAPSGYRPGAALDRLARNLEPYACERLAPGLLRLTLPQGPQIEIGEQVQGLFMAH
IVSHRFRLQGACAAQPPLTLDVVTGGWLRRRGVRYLLRQRHAAAQRVLDGLQRYPQIGETLAQLDFRR
VRLTVKDGRWQVDIEHFAASEVVSRLPAGRRYLRLDAEQRRLLLSSLLMIGQLMEKLNHE
>CORE_REP|Org4_Gene2206#
MAEDRHQQRQQRLKEQVDARIAAAQDTRGLLLVFTGNGKGKTTAAFGTVTRAVGHGMRAGVIQFIKGE
WPNGEKNLLQQHGVEFQVMATGFTWETQNKAGDTAACQAVWQHGKRMLADSSLDLVLLDEVTYMLTYD
YLELEELKAALLGRPAHQTVILTGRGCHRDLLELADTVTEMRPVKHAFDAGVKAQQGIDW
>CORE_REP|Org26_Gene1265#
MLYIFDLGNVIVDIDFKRVLGVWSNLSGTPLAVLSDRFTMGEVFQQHERGEISDEDFAGKLCDEMGIA
LSFEQFATGWQAVFVALRPEVIDIMQRLRNEGHRVVVLSNTNRLHCNYWPQHYPEVAAAADHLYLSQD
LGMRKPEAEIYQHVLAAENTAAADAVFFDDHPANVLAAQALGIKTVHVTDRDVVPAYFAQ
>CORE_REP|Org26_Gene420#
MSKNNVLVISECKYSYVGLSVLLKKHYGAYDIRLFSDFMHGGAKAEKITKHDIALIFTSQNLEQSVNV
IESLVSLHQQYNSKTRLLVFYDDERVVKLLSILGISVELVSTRIPLYSLQEKIQRLLESKEPGGIRVQ
KTRELSPAESDVIFNLLRGDSLLHIAEKRGTHPKTIFSQKYSAMKKLRLRSMSTIFVAGK
>CORE_REP|Org45_Gene3434#
MHKEISGVILAGGRATRMGGEDKGLVSIAGIALYQHVLARLRPQVASVAISANRNQARYQASGLPVIG
DLTPDFAGPLAGMLAGLKQAPNEWVAFVPCDVPDFPADLVAQLWRQKGAAPAAFASDGERDHPTLALL
HTQIAPQLADYLARGERKLMLFLHQINAQRVVFSGQQTAFHNLNTPEDCLHWQQARELKT
>CORE_REP|Org14_Gene739#
MLRNPIHLRLERLEAWQHLTFMASLCERMYPNYQMFCKQTEFGDPAVYRRILDLVWETLVVKDAKVNF
DSQLEKLEEAIPSAEDYDLYGVYPAIDACIALGELIHSRLGGETLEHAIAISETSIRTVAMLEMTQAG
KEMTDEELESLPAVEEEWDIQWEIFRLLDACEERDIDLIKGLRSDLREAGVSNIGINLAQ
>CORE_REP|Org35_Gene562#
MSSIKLIVGLANPGAEYAQTRHNAGAWYVDLLAQRHNQQLKEESKFFGYTARLNLAGNDVRLLVPTTF
MNLSGKAVLAMANFYRIEPNEILVAHDELDLPPGVAKIKLGGGNGGHNGLKDIQNKFGNNPNFYRLRI
GIGHPGDKNKVVGFVLGKPPASEQKMIDEAIDESLRCTEMLMKDGLEKTVQRLHSFKAQA

>CORE_REP|Org47_Gene318#
MDILRIGLVSVSDRASGGVYQDKGIPALEEWLSGALATPFKLETRLIPDEQTQIEQTLCELVDEMGCH
LVLTTGGTGPARRDVTPDATLAIADRVMPGFGEQMRQISLHYVPTAILSRQVGAIRKQALIINLPGQP
KSIKETLEGVKDEQGNAVVHGIFASVPYCIQLLDGPYVETHAAVVAAFRPKSARREINL
>CORE_REP|Org20_Gene995#
MSQPRTSDYPIDAQFIERWSPRAMANDAIDDQTLLSFFEAARWSPSAYNIQPWRFAYSKHGSDSWENY
LEFLIDFNRGWAQHASALVVIVSKTTSLNGERLVDNPTHAFDTGAAWASLAHQAHLKGWLTHCMSGIH
HDKIKAALQLPDDYAVHAMVAIGKPGDKARLPDFLQEKEAPSGRLPLGKTVSEGLNFTL
>CORE_REP|Org2_Gene1007#
MNIRPLLLGLPLLLTGCSSMSNFSWSSLSPFNWFGSSLEVGAKGVGKLNAGTPMSESAINDGLDGNYR
LRGGMATSNGQIVSYYQALSGDEVKLVITGEPKGRLQRVDVLDPQVATEWGNKLGTPFGDMYSKAFGS
CKPGSGEDAGKVECVAEQSKYVTYIFSGKWAGAQDIIPPDDTLKSWTVSKIIWHAKPQQ
>CORE_REP|Org46_Gene2343#
MKVAKDLVVSLAYQVRTEDGVLVDESPVSAPLDYLHGHGSLIAGLEKALEGHDVGDRFDVHVGANDAY
GNYDENLVQRVPKDVFMGVDELQVGMRFLADTDQGPVPVEITEVDGDHVVVDGNHMLAGQNLNFNVEV
VAIREATAEELAHGHVHGEHDHHHEHGDGCCGGHGHSHDHDHGHDHGKGGCGNGGCGCH
>CORE_REP|Org49_Gene2303#
MTTGIVITAAGRGERFIQAGGRGNKLNAGFADAAGERRSLFEHTLRQALASGLPVQVVTRPDNLPVLA
ACAANQVPVTLLASAGLGDSIAAGVAATPHWQGWLIHLADMPFVGADVFRQVADALRQHAIVRPCYAQ
QPGHPVGFSALLRKPLCQLRGDNGARELLQGAAVHLLPLEQPGVVQDIDLPSQLPASE
>CORE_REP|Org27_Gene798#
MPSTLLYLHGFNSSPQSAKAVAFKAWLAEHHPDIEMLVPQLPAFPAEAAEMLENMVLERAGQTLGLVG
SSLGGYYATWLSQCFSLPAVVVNPAVKPFELLVDYLGANENPYTGQQYVLESRHVYDLKVMQIDPLES
PDLIWLLQQTGDEILDYGQAVAYYTACRQTVESGGNHAFVGFEHYFPQIVDFLGLNAV
>CORE_REP|Org3_Gene4064#
MTSLYLASGSPRRRELLTLLGVSFDILLTHTEEQRREGEAAEAYVRRLAQDKAKAGVALAQEDRPVLG
ADTIVVLNGRVLEKPRDEAHAAEMLAALSGKQHQVMTAVAIADRDDVRCQLVVTDVTFRSLSQQDIRD
YIATGEPMDKAGAYGIQGKGGCFVRTITGSYHAVVGLPLVETHELLSNFVALRDVRTG
>CORE_REP|Org37_Gene784#
MFGYRSASPKVRLTTDRMVVRLVHERDAYRLADYYAENRTFLKPWEPVRDESHCYPSGWQARLGMITE
MQKQGSAYYFILLDPEEQEVRGVANFSNVLRGSFHACFLGYSLGEKWQGQGLMFEALQSAIRYMLRQQ
RMHRIMANYMPHNQRSGALLTRLGFEREGYAKDYLLIDGKWQDHVLTAYTNKEWLPPR
>CORE_REP|Org23_Gene685#
MAQLYFYYSAMNAGKSTALLQSSYNYQERGMRTLVFTAEIDHRFGVGKVSSRIGLSSQAQLYNNDSML
YAMIQQEHQQQPVHCVLLDESQFLTKAQVEQLCDVVDQLDIPVLCYGLRTDFLGELFTGSQYLLAWAD
KLVELKTICHCGRKANMVLRLDENGQAMHAGEQVVIGGNESYVSVCRKHYKEAIHSLE
>CORE_REP|Org25_Gene1525#
MADILLLDNVDSFTYNLVDQLRASGHQVVIYRNQIAAEVIIERLAQMEQPVLMLSPGPGAPSEAGCMP
ELLQRLRGRLPIIGICLGHQAIVEAYGGQVGQAGEILHGKASAIVHDGEGMFAGMANPLPVARYHSLV
GSNIPADLTVNARFGEMVMAVRDDRRRVCGFQFHPESILTTHGARLLEQTLAWALAKS
>CORE_REP|Org35_Gene2820#
MSNILIINAKKQFGHSNGELNQTLSDVAESFLRDLQHDVQVTVVDDGYDIEAEVKKYLWADAVIYQMP
GWWMGEPWILKKYIDEVFTAGHGSLYASDGRSRSDESKKYGSGGLIQGKKYLLSLTWNAPLEAFTDPD
QFFHGVGVDGVYLHFHKANQFLGMEALPTFICNDVIKQPDIEADIARYREHLAKIFA
>CORE_REP|Org1_Gene852#
MRLCDRDIEAWLDSEKLVISPRPPLERINGATVDVRLGNQFRVFSGHTAPFIDLSGPKDEVSAALDRV
MSDEIVLPEGEAFFLHPGELALAVTFESVTLPDDLVGWLDGRSSLARLGLMVHVTAHRIDPGWHGRIV
LEFYNSGKLPLALRPGMLIGALSFEPLSGPAARPYNSRQDAKYRDQQGAVASRIDKD
>CORE_REP|Org42_Gene115#
MYHDLIRSELNEAADTLAKFINDDANIDAIQRAAVLLADSFKAGGKVISCGNGGSHCDAMHFAEELTG
RYRENRPGYPAIAISDVSHLSCVSNDFGYEYVFSRYVEAVGREGDVLLGISTSGNSGNIIKAIDAARA
KGMKVITLTGKDGGKMAGSADVEIRVPHFGYADRIQEIHIKAIHILIQLIEKEMVKA
>CORE_REP|Org45_Gene4281#
MQRLLLASTSPYRKMLLGKLRLPFDCAAPEVDETPLPGESAEALVLRLAAAKAQALALAYPEHLIIGS

DQVCVIDGNITGKPHTEENARAQLRQASAQAVTFYTGLALYNGRSKQLQALCEPFHVHFRALSEAEIA
AYVRIEQPLNCAGSFKSEGLGIALFDRLEGRDPNALIGLPLIALLEMLRAEGINPLV
>CORE_REP|Org36_Gene3434#
MSLLTGFEQPIDQSQHAFRLILKALSEPGHRVTLPNGPAWAPLNAASTAALLTLADQETPLQLCAALN
SEQVLTNIRFHSAAPLATTAQKVCFALFDEQLQAADLQALPHGTEISPECGATVVVQLAELENGTVLR
LTGPGIESQRLVAPRLPPALLDYLVNRPQRFPLGLDILLTCGDRLLAIPRTTRVEVC
>CORE_REP|Org24_Gene4548#
MFDIGFSELLLVLVIGLIVLGPERLPVAVRTVSGWIRALRSLAASVQHELSQELKLQELQDSLKKAEQ
AGLQNLTPELKASMDELKDAAESLKRTYRGETEELANTIHNPQAIDPEALHDGVTPAEAATRASAPAA
VPKPAAEPEAVAAASPAASPAAPVAAQPAKAPVETAPAPVEPVADKIPASHQPSGDR
>CORE_REP|Org41_Gene1255#
MSIQNTFPSYQSLTVALNQQSVALTAAEMHGLISGLLCGGSRDAGWQALVHDLTNEGVAFPQALSQPL
QQLYEVTRETLEDDEFLFQLMLPEGEIVSVFDRADALAGWVNHFLLGLGMMQPKLAQVKDEVGEAIDD
LRNIAQLGYDEDEDQEELEQSLEEVAEYVRVAAIMCHGEFTRHKPTAPENIKPTLH
>CORE_REP|Org23_Gene256#
MSFELPALPYEKNALEPHISAETLEYHYGKHHNTYVVNLNNLVKGSEFEGKSLEEIIKTSNGGVFNNA
AQVWNHTFYWHCLSPQGGGEPQGELAAAIVKSFGSFAAFKEQFTDAAVKNFGAGWTWLVKKPDGALAI
VNTSNAATPLTGEDKPLLTVDVWEHAYYIDYRNARPKYLENFWALVNWTFAAENLA
>CORE_REP|Org2_Gene2819#
MNEFSIVCRVLGTLFYRQPQDPLLVPLFALIKEGKLQQHWPLEQDDLLKRLQQGCDVNQLATDFNAMF
VGSECSVSPFRSDYVEGASEADVRTFLQQRGMPLAEAPADHFGQLLLAASWLEDQSQEDETQAQIALF
DEFLLPWCGRFLGKVEAHATTGFYRTLALMTRDAIQAMRDELAEYEQDDEAGDEEA
>CORE_REP|Org43_Gene1549#
MRVISLAGSPRIPSRSAALLSLSQNWLRQQGVEVTAYTLHDFDAEDLLYANFNSPAIKAFADQLAQAD
GLLISTPVYKASFSGALKTLLDLLPERALDHKVVLPLATGGSVGHMLAVDYALKPVLAALKAQEVLHG
VFADDSQIQLTGESATLTDAVAARLEEALASFYLALGRRKPPALRVASPLTARQTA
>CORE_REP|Org34_Gene2630#
MLKLDAQTTALVLIDLQNGILPYAGGPHGAEQVVANAALLAARFRLLGAPVLLVRVGWSDSFAEALKQ
PVDKPAPAPAGGLPASWWEFPAPLAVCDSDILITKRQWGAFYGTDLDLQLRRRGIKSVVLGGISTNIG
VESTARAAWEHGYELVIAEDVCSAQNAEMHRFAFEHIFPRLARVRDTGEILAALDR
>CORE_REP|Org4_Gene2025#
MTADLNALPARQRILLTAHDLFYQEGIRATGIDRIIKESGVTKVTFYRHFPSKNDLITAFLAYRHQQW
LAWFSTALARHVAQTGGLLAALAPCLAEWFDDPRFRGCAFINTAVELADLLPESLHIASQHKRQMADE
LARHLPAGPQREQHSAMLAMLIDGAIVRVQIERLPQAALQVLNATLDMLAQGGFDQ
>CORE_REP|Org15_Gene750#
MLKKTVLGLTAGALLLSAGSALAADYKIDKQGQHAFIEFRIQHLGYSWLYGSFKDFDGGFTFDEKDPS
KDKVNVTINTASVDTNHAERDKHLRSAEFLNVEKNKQAKFESTEVKKSGDGYAVVGNLTLNGVTKPVT
LDAKLIGQGNDPWGGYRAGFEANGKIKLKDFGITTDLGPASQDVELIISVEGVREK
>CORE_REP|Org49_Gene2560#
MSKTKLWITILLTVIVLALIGWNMTDFSDDTAPGPVNDQDPTYQSQHTVTVVYNPAGKLNYKLVAEDA
KYYTAGELSWFTQPVMTLFDENAVATWSVRADRAKLTKDRMLYLYGHVEVNSLTTTSQLEKIKTDNAQ
VNLVTQDVTSDDEVTIYGTNFTSNGMKMRGNLRTKTAELIDKVKTNYEIQNQKPTP
>CORE_REP|Org36_Gene3245#
MLTLARGFTLPEVMLALVFGSVIALGAAKTYPLLRQQSVAVGQHFRLESTLRQLAFGIEKDLRRAGFC
AGQCAGRPLLIGQAAGEAAGSCVIVAYDIARSGQWLTSGEDAGYFGYRLRNGGLEGQRGVSQCDGGGW
ERLLDHDEVRIERFHVAIERGNHGATLARLTLAGRSASDARIGRSLSGSVEMAALP
>CORE_REP|Org26_Gene838#
MLKKLCLPLLAALLLAGCATSSNTLNVTPKIVLPQQDPTLQGITVSINGADQRTDQALAKVNRDGQLV
TLTPSRDLRFLLQEVLEKQMGARGYMIGADGPVALQIVVNNLYADVSEGNLRYNITTKADISIIAQAK
NGNKQVKNYRSTYNVQGAFTATNEKITDAVNSVLGDVIADMAQDTSVNDFIKQNAR
>CORE_REP|Org9_Gene2646#
MIPSNSPASLHAFISRFQLQLPQAAQISHNVRPAAVLIPIVCRAEPTLLLTRRADSLRKHAGQVAFPG
GKTDAEDGSAIVTALREAQEEVAIPPHAVTVLGQMAPLDSSTGFQVTPIVGLIPPDVQFRANEGEVAD
VFEMPLREALTLSRYYPLDIHRAGRTHRIYLSWYHSQFIWGLTAAIIRRLAQQVSI

>CORE_REP|Org10_Gene4090#
MSLYINNGALGAMAHTGAAASALALSVPAAAGALLLKTENLLGIGATLMLLTALALAFLGGAALARLR
PLRNRPALAILRYGFLLAAVGWLLTLLMLFGGHDWPALLLGIALGGLGQGVAYRTAVGEKAAFAVACR
LTTVVTIVAVVIAASLVQTYAAPGIKGACFALALLVDLALLGALMARVAERDSGDY
>CORE_REP|Org9_Gene1201#
MLLLIDNYDSFTYNLYQYFCELGAEVLVKRNDELQLADIERLAPQHLVISPGPCTPNEAGISLAAIRH
FAGKLPILGVCLGHQALGQAFGAEVVRARAVMHGKTSAIRHLGVGVFRGLSDPLTVTRYHSLVLKADT
LPDCFEVTAWSERDGVCDEIMGIRHRTLALEGVQFHPESVLSEQGHQLLDNFLQR
>CORE_REP|Org44_Gene966#
MIRITDAAQEHFAKLLANQEEGTQIRVFVINPGTPTAECGVSYCPPDAVEATDTELKFDKLSAYVDEL
SAPYLEDAEIDFVTDQLGSQLTLKAPNAKMRKVDDNAPLMERVEYVLQSQINPQLAGHGGRVTLMEIT
DDNMAILQFGGGCNGCSMVDVTLKEGIEKELLQKFPELKGVRDLTEHQRGEHSYY
>CORE_REP|Org47_Gene3946#
MNDKRPPHYDGGDLHRQGYRYRQDSERLTSRDKMQPVLLDMLTDDEPQKKQEAQVRNLVSHSELRRRV
LRDLQWLFNCVNSESNLDLGDFPQVRRSTLNYGIASLAGKRMSDIEWLDIQRALTESILHFEPRILPE
GLQVRCISDTGSLELHNVLSIEIKGRLWCVPYPLEFLFRTDVDLENGHFDLKDIG
>CORE_REP|Org45_Gene1407#
MQREHVLDTALGLLEQQGLATATLEMLAEKLDVQPGDLKRFWPDREALLYDCLRYHGQQIDTWRRQLL
LDENLSPQQKLLARYDVLAEYVQHDRYPGCLFIAACSFFPETDHPIHQLAEQQKLTSLELTRELLREM
DADDADMVAQQMELIQEGCLSKLLVKRQLQDVSVAKRLAEDILQVAQCRRNGALS
>CORE_REP|Org4_Gene3004#
MSNTAHNAKTDPPKVLQYLSSNLRGYRQQAGLSQMALAELSGVSRRMLAGIEAGDRNVSLAVLDKIAA
ALAISFTDLIQAPEARGSHMVGELAWQGAQPGSQALFAASVPARQRAELWEWTLMPGERYDSAPDAEG
WSEMIYVIAGTLTLELEQDTLTLTVGATQTFNSDQRYAYANHGELPLRFIRNVAF
>CORE_REP|Org11_Gene1672#
MVKSQPFLRYFLRVVPAIAAAVMLSACSSTHTSNLNNAQTEMRAVNDKDGLLLQASQDEFEAMVRNVD
VKSKIMDQYADWKGVRYRLGGDTKRGIDCSAFVQRTFREQFGMDLPRSTYEQEDLGKKIQRTKLRAGD
LVLFRAGSTGRHVGIYLGNDQFVHASTSSGVMISKLTDNYWNKRYREARRVLTNG
>CORE_REP|Org3_Gene1212#
MKLKATFAVLSSALLLQGCIAGVVVGSAAVATKTATDPRSVGTQVDDGTLEARVENALSKDQQLKKDA
RVVATAYQGKVLLTGQSPNADLTARAKQIAMGVEGTSEVYNEIRQGTPVSLSTASSDTWITTKVRSQL
LTSDTVKSSNVKVTTENGEVFLLGLVTQQEGQSAAQIASQVSGVKHVTTAFTYVK
>CORE_REP|Org24_Gene1065#
MSSKIENVKKELLSDNWYVLHKYTFDLKRKNGGSVQQMREVYDRGNGATILLYNRAKGTVVLTNQFRM
PTYVNGNESGMLLEACAGLLDADSPEQCARREAVEETGFQVGEVKKIFEAYMSPGGVTEIVHFFIAEY
HDDERRAAGGGIEDEDIEVIELPFSEAVAMIADGRIKDGKTIMLLQYLQIHRVMA
>CORE_REP|Org20_Gene68#
MSEQLTDQVLVERVQKGDQKSFNLLVVRYQHKVASLVSRYVPQGDVPDVVQESFIKAYRALESFRGDS
AFYTWLYRIAVNTAKNYLVAQGRRPPSSDVDANDAENYESAGALKEISNPENLMLSEELRQIVFRTIE
SLPEDLRMAITLRELDGLSYEEIAAIMDCPVGTVRSRIFRAREAIDNKVQPLIQR
>CORE_REP|Org32_Gene4120#
MTALWIAIAALSALGLLFGLVLGYAARRFEVEEDPVAEQVDAILPQSQCGQCGYPGCRPYAEAVANGE
MINKCAPGGEQVMLKLAELLNVEPQPLGSEAAAEPERKVAYIDEANCIGCTKCIQACPVDAIVGATRA
MHTVITDLCTGCDLCVAPCPTDCIEMRPVATITANWKWDMKTIPVQVIHVEQHA
>CORE_REP|Org7_Gene4105#
MRNNICVFCGASEGVNPAYAEQARQLGQLLAAQGRRLIYGGGKKGLMGIVADAVLAAGGEAVGIIPER
LVEAETAHRGLTELEVVPDMHTRKARMAALADCFIALPGGIGTLEELFEIWTWGQIGYHNKPVGLLNV
NGFYRPLSQFLEHVADQGFMRHDYLGTLHISESAQTLLQQFDDYQPKNYDRWAK
>CORE_REP|Org31_Gene327#
MARANEIKRGMAISYNGKLLLVKDIDVQSPSARGASTLYKMRFSDVRTGLKVEERFKGDDILDTISLS
RRKVNFSYIDGEEYVFMDDEDYTPYIFKKDQIEDELLFIPEGGLPGMQVLTLDGQVLALELPQTVDME
IVETAPGIKGASASARNKPATMATGLVIHVPEYLSAGDKIRIHIAERRYMSRAD
>CORE_REP|Org22_Gene3650#
MSAALIIIDLIEDLIGPKGRANHCREQVLATHLLANVNAAAAYARVRKIPVIWVRVGFADDYHDIPPH

SPLFNHLKQIGALRLNSPGCRWMPELHQEETDLLFEKTAVSAFSGNNLLAWLRQHRCHHLLLAGVSTP
LAIESTARQAHDAGFQVTVLHDLCAAPTQEIHQQSLDTLQNLAEITRSQAWMKG
>CORE_REP|Org41_Gene4339#
MNKEARVKTIQARRRPGGHGAIGLLLLTLGVNGAAYAVDNLRMHGALVAEPCVIQPGDEDIELDFGTL
VDKYLYLNQRTQGQPFELRLTECDLSLGKTVSITFKGTENAQLPGLLALAASSQARGIAIGMETPQGQ
PLPLNSAGGKYPLQNGSTVIGVQAYVRGEPEAIAKRAIGRGAFSAIATFSLDYQ
>CORE_REP|Org4_Gene2047#
MKRLIIGISGASGAIYGVRLLQVLRDVAEVETHLVMSNAARQTLALETPYSLREVQALADVVHDARDI
AAGISSGSFKTLGMAILPCSIKTLSGIVNSYSDGLLTRAADVVLKERRRLVLCVRETPLHLGHLRLMT
QAAEMGAVIMPPVPAFYHQPKSVEDIIDQTVNRVIDQFDIELPTDLFTRWQGVN
>CORE_REP|Org9_Gene3992#
MSTILTLDSARLRLRPWRDEDLPAFAALNADPQVMRYFPFPATMTAEESHAQAERIRAFMQQHGWGLW
AVEVKDGAPFIGFVGLARPGDDLPCSPCVEIGWRLAAAHWGNGYAAEAARAALACAFDTLHLPEVVSF
TAENNQPSRRVMARIGMQFDGETFLHPRLPAGHPLQKHVLYRLNRQTWRERRGD
>CORE_REP|Org11_Gene610#
MPQSDEHYLVITALGADRPGIVNTITRHVSSCGCNIEDSRLAMLGEEFTFIMLLSGSWNAITLIESTL
PQKGAELDLLIVMKRTNSHERPPMPATVWVQVEVKDSPHIIERFTDLFDSSQMNIAELVSRTQPADGD
LPPQLYIQITAHSSGDRDASNIEQAFHRLCTELNAQGSISVVNYPQHDEKDGE
>CORE_REP|Org41_Gene3146#
MDNGLETADLKLAQRLADLRQQQGWSLEALAQRTGLSRATLSRVERTETSPTASLLNRLCAAYGLTMS
RLLSEIEDEPPELLRPPQQPVWVDRASGFHRRSVSPPAALYKAEFIEARLDAGAQIDYDLPSIPALEH
HLWLLSGQLELTLEGRVFRLSPGDCLRYRLFGASRFHVPGDEPAHYTLVICRP
>CORE_REP|Org26_Gene2451#
MSKKKILMLVGDYAEDYETMVPFQALQMIGHWVDAVCPGKAVGDYIMTAIHDFDGAQTYSEKPGHRFT
LNADFATAKEEHYDALLIPGGRAPEYLRLNPDVIALVQAFDAAKKPIAAVCHGPQLLAAAGVLQGRTC
SAYPACAPEVRLGGGHYAEIGIDQAHVDGNLVTAPAWPAHPQWLAKFVEVLEQ
>CORE_REP|Org7_Gene2210#
MLNLDRNNLPLLSEELLKKQRTTLLIIAFLLLAGGILCLVNPFASGAALSIVVGILLLLSGAGLIIAM
IANRAQNTWPMIGGILLGVAYLIIGYVFITSPLAGILALAVYLAVLFALGGIARLAAGYMRRGLPGNW
LQFVIGVLDLIIAWMLIGSGPAASVTLVTAIVGIEMLVSSFALFQAANLFKRA
>CORE_REP|Org34_Gene1978#
MFKRTLVTFVALCSLTAMAPAALAAGETHVMLTTSAGNIELALDSQKAPVSTQNFVDYVNSGYYNNTI
FHRVIPGFMVQGGGFTADMQQKSTKAPIKNEADNGLRNLRGTISMARTADKDSATSQFFLNVADNAFL
DHGQRDFGYAVFGKVVKGMDVVDKIAQVPTGNVGPYQNVPTKPVVILSAKVLP
>CORE_REP|Org42_Gene3616#
MTYLRPALVMLILLTLITGIAYPLLTTGLAQLLFPAAANGSLLYQGDKAVGSALIGQNFTRADYFWGR
PSATGDSAYNPQASAGSNLAATNPAQDKAIAERAAQLRQANPAMSGPIPVDLLTASGSGLDPQISIAA
AQYQLARVAAARHLPPEQVAKLIEDNTDRATPNFMGESVVNVLKLNLALDALK
>CORE_REP|Org9_Gene3400#
MARLIYLMGPSGAGKDSLLAALRADADRAPLVAHRYITRPADAGCENHIALNEPEFLRRRAKGLFALD
WQAHQQRYAFGIEVDLWLLQGIDVAVNGSRAHLPQAQQRYGAQLLPVCLQVSTAILRRRLQDRGRENA
EQIEQRLARAAEYQQSLPAGCRVLHNDGPLDDTLAALLALLPTATRQAQDATP
>CORE_REP|Org29_Gene3042#
MNSELSPNLVSIIDALHQQQVIAYPTEAVFGLGCDPDSEQAVNALLALKQRPWEKGLILIAADYAQLK
PYIDDSALSEQQRATMFASWPGPVTWVLPARPETPRLLTGRFSSLAVRVSDHPLVQLLCRQYGKPLVS
TSANLSGLEPCRSADEVTRQFGTAFPVLAGEVGGRLNPSEIRDALTGEQIRQG
>CORE_REP|Org44_Gene2530#
MNIKLDNLNQLIGARIRLEREARHWSLSDLAEQAGVSRAMVHKIERGESSPTAMLLARLAGAFGLSMS
KLIARAETQEGRLLRREDQPVWVDPESGYVRRHVSPRTDLPLDLVRIELPAGATIPMPASVYAFKRQL
IWVLSGELVFVEGDARHDMAEGDCLELGPPADCRFENQSDQPCVYMVAVLSAA
>CORE_REP|Org36_Gene26#
MNLSATLILAFGMSMDAFAASIGKGASLHQPRFREAIRTGLIFGVVEAITPLIGWAIGLFASQYIMEW
DHWVAFSLLFILGMRMIVEGVRNRPDEEEKVKRHGFWILVATAIATSLDAMAIGVGLAFLQVNIVHTA
MAIGCATMIMATLGMMIGRFIGPLLGKRAEILGGVVLIGIGVNILLEHLGYLA

>CORE_REP|Org34_Gene3164#
MSDINTLKPHPAAAPGNAELFLGFLWLGLIGFGGVLPMARSMLVERRRWLSGEQFTELLGLCQFLPGG
NVINLSVAVGMEFRGLRGALCALLGLISAPTAIVVGLGVVYARFQNDPHVQHVFAGLAAAAAGLLLST
GIKMLLPLRGKWPALAIVALALIAIAWLRLPLLPTMLVLAPLSILLMWRWPS
>CORE_REP|Org48_Gene90#
MATYSSNDFRPGLKIMFEGEPYAVEASEFVKPGKGQAFARVKMRRLLTGTRVEKTFKSTDSCEGADVM
DTNMNYLYSDGEFYHFMHPESFEQHQVDGKTVGDAAKWLQDNAECIITLWDGRPIAVQPPNFIEAEIT
DTDPGLKGDTAGTGGKPATLSTGAVVKVPLFVQIGEVIKVDTRSGEYVSRVK
>CORE_REP|Org14_Gene2655#
MYDRYQGLIFDMDGTILDTEPTHRKAWREVLSRYGMTFDEAAMVALSGSPTWRIAQAIIASHQADLDP
HHLAAEKTRAVEAMLLDSVRPLPLIEVVKSYHGRRPMAVGTGSEHRMAEMLLRHLGLFNCFDAIVGAD
DVQRHKPEPDTFLRCAELIGVRPEKCVVFEDADFGIQAAKSAGMAVVDVRTL
>CORE_REP|Org42_Gene2058#
MTTPLQAIAELDDLKLDLPRFEQALHQFAAKLRLDLSAFTADHISLRCHQNATAERWRQGLMQCGTLL
SESMINGRPICLFDLSQPLAVGPWRIDCIELPYPGEKRYPHEGWEHVELVLSGDPQTLYARALSHLAD
EALLAPGIKLKQSSPKGEGERLPNPTLAITDGTVTIKFHPYSIRDIVASEQG
>CORE_REP|Org40_Gene3273#
MTLRRLLTLALLLSPLAASAHNFVHGRPVAPIAIADRGELLLRNGDFSYRPWNSAKLAGKVRVIQYIA
GRTSAKKKNSLLINAVKDANLPGDRFQPTTIVNTDDAIPGSGFFVRGKIEKNKRHYPWAQFIVDSDGL
GRMAWRLPEESSTIVVLDKAGRVQWAKDGALTPQEVDQVIALLRTLIAQETP
>CORE_REP|Org22_Gene1083#
MSDQALKIVTLLGSLRKGSYNAMVARALPGLAPQGVTIEALPSIRDIPLYDADMQQGEGFPAAVEAIA
EQIRQADGVIIVTPEYNYSVPGGLKNAIDWLSRLPNQPLAGKPVAIQTSSMGPIGGARCQYHLRQILV
FLDAMVMNKPEFMGGVIQSKVDEQAGELSDQGTLDFLTGQLSAFSDFIRRVE
>CORE_REP|Org20_Gene2193#
MTDLAHYITDYGYWALFIGCLAEGETITLLGGIAAHEGLLHWPWVIAVVALGGTLGDQLLYFAGRRFE
GRVISRLKGQEKRIARARKLIARHPMLFVIGVRFMYGFRIIGPVLIGASRLPPSRFVPLNILGAILWA
TIFVMLGYFGGQAIERFVTGFDKKLSSLLFVALAIAAILLVRFWWRKRHAE
>CORE_REP|Org43_Gene4615#
MKKILMGAAALLFAGVLAGCNQLTQYTLSEQEVNDYLQKHNDYQKQIGVPGLLDANIVLTQLQSQIGR
SEPGKVTLSGDAKVNITSILGPQTADLKLTLKAQPVYDRAQGAIFLKDMELTDYSVQQEKMQTVMKAL
TPYLNQSLKSYFDQKPAYVLNPDNSKTEALAKKLAKGLEVKPGELVIPFSD
>CORE_REP|Org9_Gene1292#
MRKVAKLMGISLLALGLAACDGDTKDTKAASDGAAASAPAGQQVSLLDGKLAFTLPVGMADQSGKLGN
QANNMHVYADSTGQRAVIVILGDKTADSLETLAKRLENTQRARDANLQVITNKALDVNGVPLRQLDSI
ITSGGEKAYSSVLIGSLNNNMLTIQVTLPADNQQQAQAEAEGIISTLKLKQ
>CORE_REP|Org35_Gene1582#
MQELAGHLAHTLRTLRAQRGWSLTQAAEYTGVSKAMLGQIERGESSPTVATLWKIATGFNVAFSAFLE
ASPAQQQATLHRYGELPVYDQDNADMRVVPLFPYDRQLGFDMFVIDLAPGALSESSPHEPGVIEHVIV
ISGRLELAIDGEWHSLAAGEAMRFQADRPHAYRNAGSHTVRIHDLIHYPQS
>CORE_REP|Org9_Gene1134#
MNLQHHFLIAMPTLQDPRFKRSVIYVCEHNEEGAMGLVINKPVEQFTVATVLSKLKIMPPARDPAISL
DKPVFAGGPLADDRGFILHTPRHGFGASIQISPNTMITTSKDVLETLGTPEQPDDVLVALGYAGWEKG
QLEQEVLENAWLTIEANTDILFRTPIASRWREAGNLLGIDIRSIANHAGHA
>CORE_REP|Org15_Gene3390#
MFKVNRHVTTLSAPVLSLLDATPNDMAAVLRIYTQHVLYGAASFEEQPPTLAEMQLRLSKVREAGLPW
LVAKSAGVIVGYCYATPYRPRPAYRFTVEDSVYIAEGQQGKGIGRALLSALIARCEQGPWRQMLAIVG
DSAANRGSLALHQSLGFTSAGTLKAVGFKLGEWRDTQIMQRALGAGDKRRP
>CORE_REP|Org6_Gene2783#
MHHLMLDIETLDIKPSAVILVVAAVFFDPRTGALGAEFETAVSSQKDQPGRTISLDTVAWWAKQSDEA
RKQAFGGTESLKRVLSSLSRFIHMNSTDTVKVWGNGKEFDCAILEHAFQQLEMPCPWKFWDTQDVRTV
ITLAELHGFNPKKARPFEGMPHRALDDARHQARYVADTVSALYYRQGAQR
>CORE_REP|Org22_Gene2009#
MTTCYPKIALLAAAVLLTGCAHNAAVPQLRHQVAALNQKVSVLTDQTTALERQNLLNQHSDNGVYLLP

AARSAARLQSSLGELSVSLSHIKSEANGTQAQLHVRILSQATLPPFNAVVEWGQLDEATGRPLTAEAL
SQPIASADSLLPKPGQDFELRFSGLMPEQLGYIRLHSLVSTAQPAVVQPH
>CORE_REP|Org27_Gene2058#
MRQRILTLLLGLAVLVTAGCGFHLRGTTQVPNEMKTLILDSADPYGPLTRSVREQLRLNDVTIVSDPK
RKDVPSLRIVGATESQDTASIFQDGKTAEYQLVLTVQAQVLIPGHDLYPLSVKVFRSFFDNPLTALAK
DSEQEIIRQEMREQAAQQLVRKLLAVHAAEEDNRQKAAAAGERAASQTAQ
>CORE_REP|Org2_Gene1537#
MTYQQAGRIAILKRILGWVVFIPALLSTLISVLGFVYQHSEKTKGINAVMLDFVHVMVDMVRFNTPFL
NLFWYNSPVPDVDKSLFSGANLMFIIIYILIFVGLALQASGARMSRQVKFIREGLEDQMILEQAKGSE
GHTRQQLEERITLPHHTIFLQYFPLYILPIVIAVIAWFVIRLLGQLAGAA
>CORE_REP|Org17_Gene1176#
MKLNKIMIAAVLAFGASSMMAQAANQGSGTVTFTGEIIDAPCSIAPGNIDQTVPLGQISNLSLKDGRE
SEVKEPFSIKLEDCTNATAQTVKTTFTGEAGGSAGTDKKMIALGSGSTAKGASIVITDTLNSNAVVEL
GTATTGQKIEIGDTGAELAFKAFLKGNGGTLDTIIPGAFTSVVNFALSYQ
>CORE_REP|Org3_Gene731#
MEIAFYLAGLIAVAATIRVITHTNPVHALLYLIVSLLAISAVFFSLGAYFAAALEIIVYAGAIMVLFV
FVVMMLNLGNVQQQERDWMKPTVWIGPGLLSLALLVVLIVAIRSVSDQGISGEMVDAKAVGISLFGPY
VLAVELASMLLLAGLVVAFHIGREHKPGEVLSNAPASGEMARRKSEEQA
>CORE_REP|Org14_Gene2099#
MSDTQTRHHYEQLIEIFNQCFSDDYNTRLVKGDDEPIYLPADDELPYHRIVFAHGFYASGLHEISHWC
IAGEARRQRVDFGYWYCPDGRDAQTQSEFEAVEIKPQALEWMFCVAAGFPFNVSCDNLNGDCEPDRIA
FQRKVRDRVLALLEQGIPTRPARFIQALQSFYNTPPLAAEHFPYPEDLN
>CORE_REP|Org21_Gene4528#
MKLNKIMLAAVMAFGVSSLAHAAVKDQGHGKVTFSGSIIDAPCSIAPESIDQTVELGAISNVALKDGG
KSMPRPFQIKLENCDLTVDANDPSKNNNKVSLTFTGSASEADSALLGITGTAKGAGIALTDGNGKNIT
LGTATDARLLQNGSNTLSFSAYLQGSGASGAQIVPGEFQSVADFTLAYQ
>CORE_REP|Org32_Gene2796#
MAISEEKRKMIAGELYDAGDDLLRSERRRARQLTHRYNHSSPEEGELRKQWLDELLGGYQGGTIEPTF
RCDYGYNIYLGKSFYANFDCVILDVCEVHIGDNCLLAPGVHIYTATHPLDAETRVGGAEFGKPVKIGD
NVWIGGRAVINPGVTIGDNAVVASGAVVTKDVPANCVVGGNPARVIKQL
>CORE_REP|Org5_Gene1632#
MSDVSLAPGKRLSQIRQQLGLSQRRVAELSGLTHSAISTIEQDKVSPAISTLQKLLTVYGLSLSAFFA
EPEKPAEPQIVIGSDDLIEIGSQGVSMKLIHNGDPNRTLAMMIETYEPGTTTGERIKHQGEEIGTLLE
GEVVLQVNGQSYHLLAGQSYAINTGIPHSFSNTSARICRIISAHTPTTF
>CORE_REP|Org3_Gene1862#
MLNATRLQLMNHFAYLQQFMASPRTVGTLAPSSPWLCQAMLNQIEWTRALSIAELGAADGVLTKRILG
RMRADAALEAFEIQPHFVHRLRGIDDRRLQVMAHSATRMATDYDVVFSCLPLLSIPVRISVRILQQAR
QRLLARNGTLVLFQYSHLSEKLLSRYFHWKRLRVVRNFPPALVYVCTPR
>CORE_REP|Org3_Gene1187#
MSIKTQPISLTINQKQYGPIEVPEGLMMIDFLHEYLDLTGSRLGCGQGICHACVAIVDHPSGTSEEVR
TCITGAHFFNGKKVRTVEGHARVDEQGEVVELSPIQQAFLEHYSFQCGYCTPGFVNAATIFVEKLKRE
PIAREQLESAIEQALDSHICRCTGYVRYYEAVRDVVLKTPGLLKETAQ
>CORE_REP|Org18_Gene1897#
MTMRASICGLLFCLPLSPAQSDTGVNDLTKSQPFTVNATVVKGCVLGSGVSDVTTFGTLNFGQLSSLS
NAVSVVSSSGAGSVLFRCNPGLSVTLALGVGNHVTGSIAGGRKLQNAATSETLLYQLYQDSNYATLWG
DGANGGAAQTVAATGSTQEIKVYARLFSTSTLPTSGVYSDTVLLTVTY
>CORE_REP|Org17_Gene1529#
MLWKNTADRFGHVSVLIHWLVALTVYGMFALGLWMVTLGYYDVWYHQAPEIHKSIGTLLFIVMVIRVV
WRFVSPPPKPLASYGRLTRVSAILAHLALYAVLFGILISGYLISTADGQPISVFGWFNVPATVTGMAE
QADTAGAIHLYLAWAVVVLSVLHGLAALKHHFIDRDVTLKRMLGSSAD
>CORE_REP|Org1_Gene4662#
MQNITPIGLLSAAIRRERERLNLSVTELAKRAGIAKSTLSQLEAGSGNPSLETLWALAMALDVPVSRL
ISQPSRPVQVIRAHEGTPALSEQGNYAATLLATCPPGAQRDIYRLRVQPGEAKLSRPHPPGTVEHVII
SSGRARLGPAEQPLELGAGDYISYSADREHVFEALEPETTAVMLIEQG

>CORE_REP|Org23_Gene715#
MDKHLQKPKILKVETVARSRLFNVESVDLEFSNGVRRVYERMRPSDREAVMIVPVIGDDLLLIREYAV
GTESYELGFPKGLIDPGEGVLEAANRELMEEVGYGAKRFDFLSKLTMAPSYFSSKMNIVLAHDLYPQS
LEGDEPEPLPQVRWPIANMMALLAEPDFREARNVSALFLSEAFLRASR
>CORE_REP|Org45_Gene985#
MKTLVVVTHPDMANSVVNKRWLEELRRYPERYTVHELHQAYPDWQIDVAQEQRLIEAHDNIVLQFPIF
WFSSPPLLKKWLDDVLTYGWAYGSRSGYKMQNKKLALAVTAGVRAEDYARDGRYRYSLEEIFRPFEVT
AGYVRADYASFFAFYGREAASDGTVEPLQSHELDRSAQGYQAFLAALN
>CORE_REP|Org18_Gene844#
MGMVETCYGPVEQEVMARAGNIRLLICDVDGVLSDGLIFMGNNGEELKAFNVRDGYGIRCLKTSDIEV
AIITGRSAKLLEDRAQTLGITHLYQGQSDKLLAFRELLDKLSLTADQVAYIGDDLIDWPVMAQVGLAV
AVADAHPLLTPRAHYVTRIAGGRGAVRELCDIILLAQNKLEDAKGLSI
>CORE_REP|Org12_Gene553#
MSSTASVRLRPLERDDLSFVHQMDNNASVMRYWFEEPYEAFVELSDLYDKHIHDQSERRFIIEHEGAK
VGLVELVEIDHIHRRAEFQIIIDPAHQGKGYASTAARLAMDYGFSVLNLYKLYLIVDKENPKAIHIYS
KLGFNVEGELIDEFFVNGEYRTVLRMCIFQPQYLAKFKTPNDKPLVK
>CORE_REP|Org8_Gene789#
MAKTVVVFHSGYGHTERLAKVVAEGAGAELIAIDQNGDISDEAWQTLDEADAIIFGSPTYMGGPSWQF
KKFADASSKAWFGRKWQDKVFGGFTNSASLNGDKQVTLIALQTLASQHGGLWVSLGLLPANTKSAQRT
DVNNLGGSVGLLVQTPADAGVDEMLSGDLATAKLYGQRVAGFAAKLA
>CORE_REP|Org44_Gene658#
MTATAQQLQFIKDSIKTIPDYPKPGILFRDVTSLLENPLAYAASIELLVERYREAGVTKVVGTEARGF
LFGAPVALALGVGFVPVRKPGKLPRATLSESYELEYGTDKLEIHTDAITAGDKVLVVDDLLATGGTIE
ATTKLIRRLGGEVNDAAFIINLPDLGGEARLNKLGIDCYCLVDFAGH
>CORE_REP|Org49_Gene2776#
MSKPIDNLPLQTIENQPTTLGAYRDKVLLVVNVASECGLTKQYEGLEALYEAYRDQGLEVLGFPSNEF
LGQEPGSNEEILAFCRGTFGVQFPMFAKIEVNGERRHPLYQALIAAQPEAVAPQGSEFLARMTSKGRA
PKHTGDILWNFEKFLIARDGTVIQRFSPDTTPEDPALVAAVKQALAG
>CORE_REP|Org42_Gene2020#
MKINVVGTSGSGKSTLARQLAERLDVPYIEMDRLYWRPEWQGTPDDAFLARLEQTLAEAGGGWVLDGN
YSRTQAIKWREVDYILWLDYGFCRTLWQAVRRACRRAASKRELWPGTGNRESFRRSFFSRESIVLWTI
RTYSKNRRKYLAEMARTDAGRRFKRLRSPRQAADFLRTLPQVHSSRR
>CORE_REP|Org6_Gene3535#
MKRKLLFIPLALFLLLVAALMVQLTRNAGGEDPTMLESALIGKPVPTFRLESLDQPGKTYDQAVLRDG
KPILLNVWATWCPTCRAEHQYLNTLAARGVRVVGLNYKDDRAKAVTWLNSLGNPYALSLYDGDGMLGL
DLGVYGAPETFLIDGQGIIRYRHAGDMNERVWQQEVLPLYKKYGGEA
>CORE_REP|Org20_Gene3413#
MKIDIFSECTYTIIGIRQLINQTWSEKNTPADFNRRRVCFIDVTLANFEARYRDEYANPKTYKIVIIT
DCPHEMVIVDNKTIILSNLISLSRFSHLIGDLYTRYSHYTEPPQLSRRESLFLSEWSTGKSLTDISNA
MNIRNKTANHYKSRIMKKLGASRIKPLLHITRVRCLTDRLNIKINKE
>CORE_REP|Org11_Gene1211#
MHWLNFKRYKSDVAKQAVPPHLNAAEFARHYADKPQTDTEEYLSLSGEMCWDAVVLCAHRSGALSKAK
YKQLWQTVFDKQYKHFVSPDDTEIRTMADMLRAPQGCFIGIFSLRDAAAPRLLHAMIGTGAGFAAGNK
NLCIGVGGAVGWENLNLARDLRWQPEGGFLRQGDNEVLRIFYRPFPA
>CORE_REP|Org2_Gene1919#
MTIHWRGALLIALSIGSASATAKTQGHGKVSLGGEIVETPCNIAGDSLDQTVDFGLVSMSDAGRDAQP
SLIGSRRHFAIRLVNCELASQIKPDFIYRAANLTFSGIADSQDPQWLAVHGEARGMAIELLTDAGTPI
PLGSTTADYLIVAGDNTLRFGAQLRIHPDRARAGGFSSLAKFTLSYL
>CORE_REP|Org36_Gene202#
MDALDLLLNRRSASRLAEPAPAGEVRQNIINAGLRAPDHGALQPWRFVMIENQGLERFSQLLQAAAKQ
DQLDEAAIEKATKAPFRAPLIITVIAHCTEETKVPRWEQVVSAGCAVQAMQMAALAQGFNGIWRTGAW
TEHALVREAFGCREQDEIVGFLYLGTPQLKAATKVTPPDSTPFVSYF
>CORE_REP|Org40_Gene3606#
MSQSALLIIDVQQSFQHRPFWQEDDLPAFQQALARLIAGCQRHGAALVDVLHVSPQGPFSLASGHVQR

LPFLTHQADITVHKHVHNALTESGLDAWLRERNINHLIVSGIRTEQCCETTTRVASDLGYRVTFVTEA
TLTFPMRHPDGEVFTPAQLKRHTETVLVDRFARIASVDEALAQLAQE
>CORE_REP|Org13_Gene4680#
MASRGVNKVILVGHLGQDPEVRYMPNGGAVANITLATSESWRDKATGEQKEKTEWHRVVLFGKLAEIA
GEYLRKGSQVYIEGSLQTRKWTDQAGVEKFTTEIVVNIGGTLQMLGGRSQPANAGQLGQGSWGKPQQP
QPSHSGTPAQQPQPAQSDGNAAPMDFDDDIPFLGFGYGIAKSAIYAL
>CORE_REP|Org9_Gene1900#
MPSFNPSAPYFSERLQMRPPVMADLERFYAIFGDPQTQLFNPAGPLTSEAQAASALQERLTGWRQHGY
GSWALALRERPDWVIGFGGLSWKPLGAQRTVNLGYRFDTRVWGMGLATEMARASLQYGFVGLGLGEIS
AIVRAENQASWRVLEKIGMQRVDTLDDVPGAAPSLVYTLKRGDYQG
>CORE_REP|Org7_Gene38#
MKIKPDDNWRWYFDAEHDRLMLDLANGMIFRSRFPAKMLTPDAFDECAFCVDDAALYFTYEEQCKQVK
LSHEQRAELVLNALVAYRFLKPLMPKSWHFSQQHYPLQPKNGELAAVKVMESGAEARLLVVEAGDNAS
LCLLAQNQLTVAGRTMVLGDAIKVMHDRLKPCAQDESAAPAYDRAV
>CORE_REP|Org44_Gene447#
MNKQPEDWLDDVPENENEDDDEIIWVSKSEIKRDAEALKDLGAEMVDLGKNALDRIPLDEDLRAAIEL
AQKIKKEGRRRQLQLIGKMLRARDIEPIQTALDKLKNRHNQQVSLFHKLEALRDRLVEEGDDVIPSIL
DLYPAADRQQLRSLVRNAQKEKAANKPPKAYRQIFQYLRELAEAAD
>CORE_REP|Org12_Gene182#
MTAEGHLIFSVACAIFAKKAEVTPELATGDWWHIIPAALLTSLLPDIDHPKSVLGQRLRWIALPIARA
FGHRGFTHSLLAIAGGMALFQLDVPRSWPIPADALHAMIIGYFSHLLADMLTPAGVPLLWPCRWRFRL
PLLNSQKGNQLERVLCLCLVAFAICWQGDFTLPVQSYVEQIRNIRL
>CORE_REP|Org36_Gene2424#
MQTKKSEIWVGAFMLIALCAIVFICLQVANLKSIGSEPTYRIYATFDNIGGLKPRSPVKIGGVVIGRV
ADIELDPKTYTPRVALDIQKKYDQIPDTSSLAIRTSGLLGEQYLALNVGFEDPDMGTTILKDGGTIQD
TKSAMVLEDLIGQFLYKSGGQDSAKSGDAAAEPAAGAAPQPAVPNH
>CORE_REP|Org14_Gene4048#
MDPQRQIIDWLQQDDARMAALRTVRRLGLNDWCLGAGFVRNLVWDRRHGYVEPTPLNDIDVIHFDAER
ADAERDRMLEARLQQWLPQPWSVKNQARMHLRGGRAPYRNSEEAISFWTEVETAIGARLNADDSLTLV
APFGLGALFGDTITFNVKNGDRAAYAQRVLDKGWLQRWPRLRQVKI
>CORE_REP|Org20_Gene715#
MSKQLKPVAPKQPIVLGKMGSAYGIRGWLRVFSSTENAESIFDYQPWFIQQAGQWQHIELEDWKRHSQ
DLIIKVKGIDDRDAANLLTNREIMVDSEQLPPLEGDDYYWKDLMGCQVVTTAGYELGKVIDMMETGSN
DVMVVKANLKDAFGMKERLIPFLHGQVIKKVDLTARVIEADWDPGF
>CORE_REP|Org19_Gene1538#
MDKIDHHRRKWLALGGAAMGIALLPGQAFASISTARPRILVLNNLNTGESIKAEFFDGKGYNKEELVR
LNHLFRDYRANKVKSIDPRLFDHLYRLQGLLGTSKPVQLISGYRSVDTNNELRAHSRGVAKHSYHTKG
QAMDFHIEGIQLSNIRKAALKMRAGGVGYYPRSNFVHIDTGPVRTW
>CORE_REP|Org39_Gene4340#
MAAVDPQLAAERAVSLPELLTSRECRQARQQAWLAQHECTLLVLTLVVPGPVKDSALTRGIFNLGWEA
LLRLCAEQGWPSPQAEALALATGCEGFVALRVDAQRVKDCAMQLEVSRPIGRLWDIDVLDTQGRILSR
RDIGLPERRCLLCGQPAKICARQRRHSSEQLLHEMERMFNDAISAD
>CORE_REP|Org40_Gene1811#
MDHDVSHALREFTQRYVELWQQERGHAPASEALYGVPSPCIVENREDDVLWLPQPFEPAATLEKVETA
LELRLQPDAHRFYTQQYAGDMSAQFGEHRLSLLQVWSEEDFIRLQENLIGHLVTQKRLKLSPTLFLAT
TESEMTMVSLCNVSGNVVLEQFGSDKRTLLAATLGNFLDALRPVLD
>CORE_REP|Org19_Gene515#
MIELITHGAEWFIGLFQKGGEVFVGMVTGILPLLISLLVIMNALIKFVGQERIERLAQRCAGNPVSRY
LLLPLIGTFVFCNPMTLSLGRFMPEKYKPSYYAAASYSCHSMNGLFPHINPGELFVYLGIASGLTTLG
LPLGPLAVSYFLVGLFTNFFRGWVTDLTTSLFERKMGIRLDRQVQL
>CORE_REP|Org19_Gene1561#
MQLNIPTWLTLFRVVLIPFFVLAFYLPFNWAPMVCAVIFVFAAVTDWFDGFLARRWKQTTRFGAFLDP
VADKVMVAVALVLVAEHYHSWWITLPAATMIAREIIISSLREWMAEIGKRSSVAVSWIGKVKTMAQMM
SLVGLLWRPDRSVEYVAIGLLYIAAVLTFWSMFQYLKAARNDLLEP

>CORE_REP|Org43_Gene2154#
MKIVPINAATLPIYRDELARLLTDAVTHGASVGYDTLIPHEDAESYFHSLRPALAKGELLLWIARDER
GVVGTVQLELCQKPNGRNRAEVVKLLVHSRARRNGVGLALMKTLEQAALQQQRGLLYLDTQAGSAAEA
LYRSLGYRCLGEMPDYAAGPDGYYHSTVIYYKRLFTVTPSSRAIAS
>CORE_REP|Org47_Gene4422#
MAHPQHVTLVITHRLAPGNEQAYEAWLNRIMPDAAGFTGHLGVNVIRPSGDEQAYTVLVRFDNLDNLY
HWIHSPLRKQYIEEVTPLLLENDHIEIRPGAEFWFTPPNPGVRQPPQWKQFIITLLVIFPSTNLVPWF
WGLVLPQLDGTLLGHFLNDATVVALVVYLWMPIVTRVFHQWLTRR
>CORE_REP|Org41_Gene830#
MTKNAIFAARQNEPCPECGAELVIRSGRHGPFLGCSQYPECQYIRPLKAQADGHIVKVLDGQQCPKCQ
ATLVLRQGRYGMFIGCSDYPQCDHTEVIDKPDETAITCPQCGQGKLLQRKSRYGKVFHSCDRYPECQF
ALNVKPVAGECAYCHYPLLMEKRTAKGPVLCCASKLCGKPVATTE
>CORE_REP|Org34_Gene1673#
MAEQGQAADTEWVDIVDEQNEVIAQSSRQQMRAERLRHRATYIVVHDGMGKILVQRRTEIKDFYPGWL
DATAGGVVQSGENVLDSARREAEEELGIAGVPFAEHGLFYFEEDQCRVWGALFSCVSHGPFALQEEEV
AEVCWLTPEEITARCDEFTPDSLKALSLWLTRNNEQDYGKPLDNH
>CORE_REP|Org35_Gene2899#
MNIRQARPTDYPAILQLQAENVPEALDERQKRQGFIVSRMNEAQLTAINRGIGIWVAEQEGQLAGFVC
LMPADAQPRPPVVDAMLATLATHSFAGRPLSEQRVFLYGPVCLGMAWRGKGVLKRLYAAVKAHTRHDY
DVGALFIDDDNPHSLAAHVQGLGMTALAPFHCGQKGYQLVVFATR
>CORE_REP|Org31_Gene102#
MSEAPKKRWYVVQAFSGFEGRVAQSLREHIKLHDMEELFGEVMVPTEEVVEIRGGQRRKSERKFFPGY
VLVQMVMNDASWHLVRSVPRVMGFIGGTSDRPAPISDKEVDAIMNRLQQVGDKPRPKTLFEPGELVRV
NDGPFADFNGVVEEVDYEKSRLKVSVSIFGRATPVELDFSQVEKG
>CORE_REP|Org41_Gene1082#
MTGNENNLIWIDLEMTGLDPERDRIIEIATLVTDANLNILAEGPVIAVHQSDEQLALMDEWNVRTHTG
SGLVERVKASRQDDRAAELETIAFLQQWVPAGKSPICGNSVGQDRRFLFRYMPELEAYFHYRYLDVST
LKELARRWKPEILAGFKKQGTHQALDDIRESVAELAYYREHFIQL
>CORE_REP|Org49_Gene3773#
MSGLTIFSDTAPQQPLWQSCDAQEIQRQLAQIGVRFERWQADRELGDNPQPEAVIAAYQHEIDRLVAE
KGYQSWDVISMRPDHEQRQALREKFLSEHTHGEDEVRFFVEGAGLFCLHLDGKIFQILCEKNDLISVP
ANTRHWFDMGSAPHFTAIRVFDNPEGWVAHFTGDKIADAYPRLD
>CORE_REP|Org24_Gene1468#
MKTIEVDEELYRYIASHTQHIGESASDILRRMLKFTAGQPVRALPAASAPQSVELEKAAPAQRPRDRV
RAMRELLLSDEYAEQNKAVNRFMLVLSTLYTLDAAGFAAATEALTGRTRTYFAGDQQTLLANGTHTKP
KHVPGTPYWVITNTNTGRKRSMIEHIMQAMQFPAELIEKVCGTV
>CORE_REP|Org43_Gene724#
MIIYLHGFDSTSPGNHEKVLQLQFIDPDVRFISYSTLHPRHDMQHLLKEVDKAVQQGGDAHPLICGVG
LGGFWAERIGFLCGIRQAMFNPNLYPEEHMHGKIDRPEEYRDIATKCVEDFREKNRDRCLVVLSRHDE
VLDNRRSAELLHHYYEIVWDEQQTHKFKNISPHLQRLKAFKALG
>CORE_REP|Org18_Gene1969#
MTPVIRIAAIEALPDDYLTRGDFGFTIRCYALPQFDTPVDSWPTRPVAPFRKQYPLAPFANEDSATFL
AYRQDQAVGHITLSKNWNGYTLIDEIAVSAHARRQGIAGALLDCAKQWARQQETSGMMLETQNNNLAA
CRCYQHYGFILGGIDRLLYRAEPEIADHEIALFWYLPFNSEIGY
>CORE_REP|Org36_Gene860#
MTLKELVVGFGTQVRSIWMIGMHAFAKRETQMYPEEPVYLPPRYRGRIVLTRDPDGEERCVACNLCAV
ACPVGCISLQKAEQKDGRWYPEFFRINFSRCIFCGLCEEACPTTAIQLTPDFELGEYKRQDLVYEKED
LLISGPGKYPEYNFYRMAGMAIDGKAKGEAENEAKPIDVKGLLP
>CORE_REP|Org11_Gene2500#
MKTKTIAAVLPLALLLSACTTVEPAYKDIGTRSGSCVEGGPDTVAQKFYDLHIQQGAGLPDSNRLAQL
QPYLSKVLYQDLVSAGQNPGKHRITGDLFSGNAQGPSSASVASASTIPNTDAKNIPLRVDLSYQKDAN
STVNWQDEVLMVREGTCWVVDDIRYLNVPAHATNGSVRQVLENQ
>CORE_REP|Org33_Gene4649#
MSNSRRLFFALSLPDALQQQVIRWRAEAFTPEAGRPVAAANLHLTLAFLGEVTAQKELALRKLAGRIV

QPGFSMRLDDLGHWPRPGVVWLGTRRAPRGLLQLAELLRSQAARNGCHQSALPFHPHITLLRAATQPV
AIPPATPGWAFSADAFSLYESVFENGRTRYQHLEQWPLAKQASA
>CORE_REP|Org6_Gene1674#
MNQPSKAPRGSAAKSKTKKKSRMELDQEARERKRLKKRRGHASGSRTQVESGSQKNKSAAEAKDPRIG
SKVPVALVVDETKVKAKPQPKPKAEAKPRLSPEEELAKLENDERLNALLDRIDDGETLNAQEQAYVDK
TLDRIDVLMDVLGIELGDEDDEEEEEKQEDILKLLKGGNPKDAF
>CORE_REP|Org24_Gene4104#
MSAPVLLQRQQLDRLWEIDRSEIIDTLYRLQDGKLQAYPDYYDVRGWDPHDRETYTPIHEACFDRGGA
FFARFEGEEIVAAAALDTEPRGPQRDLRQLLFFYVSAHKRGQGLGKQLFQLCLRQAAQEGAAGLYVSS
IPNKSTVDFYLAQGCRLIERPDAELFAREPEDIHLVCPLLAAGT
>CORE_REP|Org41_Gene92#
MAKLHDYYKDEVVKQLMSQFDYNSVMQVPRVEKITLNMGVGEAIADKKLLDNAAADLAAISGQKPLIT
KARKSVAGFKIRQGYPIGCKVTLRGERMWEFFERLISIAVPRIRDFRGLSAKSFDGRGNYSMGVREQI
IFPEIDYDKVDRVRGLDITITTTAKSDDEGRALLAAFNFPFRK
>CORE_REP|Org16_Gene1036#
MATVGIFFGSDTGNTENIAKMIQKILQKQFGDDVSEVHDIAKSSKEDLEGFDILLLGIPTWYYGEAQC
DWDDFFPTLEEVDFNGKLVALFGCGDQEDYAEYFCDAMGTIRDIIEPRGAAIVGHWPTKGYHFEASKG
LADDDHFIGLAIDEDRQPELTNERVDAWVKQIVEELSLADIVG
>CORE_REP|Org32_Gene2953#
MYQVNFLPWRQRRDRRRGCFWLGALLLQVTLLLLALFLVAGQLRHQQAQRQARLTMLGEELAALTLLA
HQQQQERAQRALHSARAERQARNGQHNRRYLQLLQQLSFSIPPPLWLTALDSDATNGLRLRGLSRSPA
AITQFERRLAGMPALPRLRLAEVTQRDDGLYSFHLAAQWGRDG
>CORE_REP|Org11_Gene372#
MTNNQNRIYVVMGVSGSGKSAVAAAAARQLSAGFLDGDFLHPRSNILKMAAGEALNDDDRAPWLAALN
DAAFAMQRTNNVSIIVCSALKKQYRDRLRAGNGNLSFIYLHGEFPVIESRLAARNGHFFKPQMLVTQF
AALEQPGADENDVMAIDINQPLDAVIADTVRHIQSFLPQDVCA
>CORE_REP|Org4_Gene1456#
MRLRALALLSCVPWWFTAMPATANTELIGAPVQFHGTVVSRPCNIEPQSADQLVEMGTVIVKTLYRYG
HTTPVPFSIKLTDCKTTVFKSVSVTFSGTEDGELPGKLAINNGANGAAIALFDHQGADIDLNKATSAV
ALQNGPNSLNFTAYVQGRPSAIQNQSITEGEFTSVANFVLAYQ
>CORE_REP|Org2_Gene3965#
MAYITTSVEYGIHCLLWLVGDNQRALSSRELAELQGISPSFLAKIFPKLEKAGIVAASEGVRGGYRLA
RPADEISFLQIIDAIEGHKPLFDCQEVRGRCAVFDDSPPDWAVSGKCAIHAVMLQAEKAMRDALAVQT
LGAVAARFGRKAPQGFFGEVNLWLDERMTERTARSGKTARAKT
>CORE_REP|Org29_Gene4208#
MKLVTERLSLQSITAEDWPLFLRLYQDPEVIRYISDPRSEAEIRTRFEERLSAWDKHGEQWLCLVMRE
KHSGEAVGITGFRPQWVPYRQAEVGYGSLPAGQGKGYGKESLRAVLDFAVNACGFHKLTATVTAGNLA
SRGLLESCGFQLEGTLRDNYRLAGQWCDDWLFGLLAAEFQGGK
>CORE_REP|Org21_Gene2104#
MKKTWVACAAGLLFVTGAANAISVSGEAGQHYTNLGVGMSTGSSGLGLTGNWARSDHDGNVGSVGLNF
GVPLGPLTATVGAKALYLSPKDGKSGGAVALGGGLEWEINRYFSLHGEGYFAPDSFTSGVKAYNEASG
GLRWKFRPLSVDVGYRYMQMEGKDGRRDNTLADGPYVGVGLSF
>CORE_REP|Org46_Gene448#
MKFRTKNQLRNLLLGSLVLAASAPALALKSDSSQPVSIDSLKQSLDMQSNVSTFTDNVVIKQGTIDIR
ADKVVVTRPGGDQNKTYIEAFGNPVTFYQMQDSGKPVKGHAQKVRYDVATQLVTLTGNAYLEQLDSNV
KGDRITYLVQQQQMQAFSDKGKRVTTVLVPSQLQDKNEQKKSN
>CORE_REP|Org34_Gene1847#
MTKLTLQEQMLKAGLVTSKKMAKVQRTAKKSRVQAREAREAVEENKKAQLERDKALNEQQKQAALSKE
YKAQVKQLIEMNRIVLAKGDIGFNFTDGNLIKKILVDKATQTQLINGRLAIARLVVENREECEYAIIP
ASVADKIAQRDAASIVLHSALSQEEQDEDDPYADFKVPDDLMW
>CORE_REP|Org39_Gene196#
MKQFLDFLPLIVFFAFYKLYDIYVASGALIVATALALVFTWVKYRKVEKMTLITFLMVLVFGTLTLVF
HNDLFIKWKVTVIYALFALALLISQWVLKKPLVQRMLGKELTLPDKVWSNLNLAWAVFFLACGLANIY
VAFWLPQSVWVNFKVFGLTVLTLVFTLLSGIYIYRHMPEEQKK

>CORE_REP|Org5_Gene3160#
MKHTVDVMISEQEVKTRIAELGRQITEHYRDSGSDMVLVGLLRGSFMFMADLCRAIDVPHEVDFMTAS
SYGSGMSTTRDVKILKDLDEDIRGKDVLIVEDIIDSGNTLNKVREILALRGPKSLAICTLLDKPERRE
VQVPVEYVGFSIPDEFVVGYGIDYAQRYRHLPYVGKVVLLDE
>CORE_REP|Org20_Gene1460#
MSTQRLLQVLAQQIAALAAEVTPRGDAPIPQARFDAALFANRGTRLRDYLAEVEKNFAQLQSAANDSR
TSQVAFLAEKLVAQIAALQRELATQALRRKNQPKEAPEADLYHKLAEHQDYERRLIAMIQDRESLLGR
QTTLAAQQKLQHELAALEGRLMRCRQALARIERNIERKENGF
>CORE_REP|Org5_Gene1823#
MHKQVIDFWFDEIEPIMWFKKDDDFDRLLHSRFGEIWRAAAAGELAHWRDTIEGRLAEVIVLDQFSRN
LFRGTPQSFACDGMALILAQEAIRSGECERLSREQRGFLYLPFMHSESPLIHQQALVLYTELNNGDQL
EFELRHKAIIDRFGRYPHRNAILGRTSTPEEEAFLQQPGSGF
>CORE_REP|Org6_Gene110#
MNKSMLAGVGIGIAAALGIAAVASLDVFSAGPQYAQVLAATPIKETIKTPRQECRNVTVTHRAPVQDE
NRIAGSVLGAVAGGVIGHQFGGGRGRDVATVVGALGGGYAGNQVQGAMQNNDVTTSVQQRCKTVYDKS
QKMLGYDVTYKIGNQQGKIRMDHDPGTQIPLDKNGQLVLNRA
>CORE_REP|Org44_Gene164#
MSRVAKAPVVIPAGVEVKLNGQVISIKGKNGELTRTIHDAVEVKQEANALTFAPREGFANAWAQAGTT
RALLNAMVVGVTEGFTKKLQLVGVGYRAAVKGNVVNLALGFSHPIDHQLPAGITAECPSQTEIVLKGA
DKQVIGQVAADLRAYRRPEPYKGKGVRYADEVVRTKEAKKK
>CORE_REP|Org9_Gene2392#
MSEFVTVARPYAKAAFDFAVEHQSVERWQEMLAFAAEVTRNEQISELLSGAVAPETLSKTFIAVCGDR
LDEHGQNFIRVMAENGRLLVLPAVLQQFIELRASLESTVEVEVLSASALSDEQQAKIAAAMEKRLSRK
VKLNCKIDKSVLAGIVVRAGDMVIDGSVRGRLERLTDVLQS
>CORE_REP|Org5_Gene2874#
MKALILYSSRDGQTRAIASYIASKLQDTLRCEVIDLLQAEQVDLNQYQLVMIGASIRYGHFNPALDKF
VKRHAEQLNRMPSAFFAVNLTARKPEKRSPQTNAYTRKFLLTSPWQPKQCAVFAGALRYPRYRWFDRI
MIQFIMRMTGGETDTSKEVEYTDWQQVDRFAQEFSHIPYEK
>CORE_REP|Org22_Gene2730#
MNDYGMIIEPGTLRIQRLLPGPIERVWAYLTESDKRATWLAAGAMTLENGAPLELEFRNSDLAGEHEP
PPAKYKQHGGCVSNRGHITCLFPPRLLSFTWAEQDQGRPSEVTFELTEQGSAVLLTVTHRRLANRDEM
LSVAGGWHTHLDILLDRLHDRAPQPFWSTHARLEEEYRARL
>CORE_REP|Org34_Gene271#
MSLNLVPAGKDLPEDIYVVIEIPANADPIKYEIDKETGALFVDRFMSTAMFYPCNYGYINHTLSLDGD
PVDVLVPTPYPLQPGSVIRCRPVGVLKMTDEAGEDAKLVAVPHSKLTKEYDHVKDVNDLPELLKAQIA
HFFEHYKDLEKGKWVKVEGWADAAAAKAEIIASFERAAKK
>CORE_REP|Org34_Gene1678#
MKNKPPLSKDEQQLFRESVAGAKKLRQDTIVHRPPKLKVKQVAPQRLLQEQVDASYYFSDEYQPQLEE
EGPTRYVRPGSSPYELKKLRRGDYSPELFLDLHGLTQLQAKQELGALIAACKREHVHCACVMHGHGKH
ILKQQTPLWLAQHPDVLAFHQAPKEWGGNAAVLLLVELAE
>CORE_REP|Org26_Gene897#
MLQFFNRCSQGRGAWLLMALTALVLELVALYFQHVMLLQPCVMCIYERCALFGILGASLVGAIAPKTP
LRYAAILLWIYSAWEGLQLAWKHTMIQLHPSPFNTCDFFVSFPSWLPLDKWLPAVFHASGDCSVRQWQ
FLTLEMPQWLVGIFGAYLLVALIVLIAQFVRPRRRDLFGR
>CORE_REP|Org49_Gene3134#
MIGILNRWRQFGRRYFWPHLLLGMVAATLGASSSLSGAPDQAALPNTSSSLNRQNAANGTFNSLALLQ
DAHRRPTFSVDYWQQHALRTVIRHLSFALAPQAVYARVQESETQAEPPLQVAQLALLSTLNALLTHEP
KPPTIIRHTHLEVLPTLARHQTGLWVAQVQGIRAGPAALV
>CORE_REP|Org10_Gene4032#
MMTTPIASRWCGMLFLLLGTLALGGCMSSAKSVPSRYSLVFDADRQVNAAAGAQPAPIKIRVLLLRSD
AEFMDADFFSLQNDAKSVLGNSLLDSDQFFLTPGQTGKTLGGQSALDARYIGVIAEYQNLDGKTWRIS
LPLPEPTETNFYKVWQFSPDELEAHIVAGVSGLRPVKKVD
>CORE_REP|Org16_Gene151#
MTTIVSVRRNGQVVIGGDGQATLGNTVMKGNVKKVRRLYNDKVIAGFAGGTADAFTLFELFERKLEMH

QGHLVKAAVELAKDWRTDRMLRKLEALLAVADETASLIITGNGDVVQPENDLIAIGSGGPYAQAAARA
MLENTELSARDIVEKSLNIAGDICIYTNHFHTIEELPSKA
>CORE_REP|Org33_Gene3101#
MTVINTAPTLITERLRLDAHTLDDFESLAALWADPQVVRYIGGTPRDREDSWGRLMRYVGHWALLGYG
YWAVRDKLSGEYLGSIGFSNFLRDITPALDAPEMGWTLVSSAHGKGYATEALRAALTWGKTHLPGEKT
VCIISPENQASLALAKKVGFCESHRSEYHQSPIVVMHCPL
>CORE_REP|Org41_Gene2055#
MQTFLIDRTATPVGELVLIADEQGRLRAIDWTDHEARLMKLLNTHYRADRFTLREQRDPSGLTDAMQR
YFAGELGIIDRLPVMTAGTEFQRTVWQQLRQIPCGEILTYGQLAQRIGRPTASRAVGMANGSNPISIV
VPCHRVIGSQGALTGYAGGVQRKQWLLQHEGYLPQDLLSR
>CORE_REP|Org6_Gene3725#
MTQMGDGKEFIVKKWLCAAGLGLAMAASAGVQAADKIAVVNVSSIFQQLPAREAVAKQLENEFKGRAS
ELQNMERSLQTKMQRLQRDGATMKASDRSKLEKDVMAQREQFSQKAQAFEQDNRRRQMEERNKILSRI
QDAVKSVASKGGYDVVIDANAVAYADSSKDITADVLKQVK
>CORE_REP|Org28_Gene1750#
MSATATASAPHTALSRLYATHHAWLQGWLRRRLGCAFDADDVAQDTFMRLLKSDAAATLREPKDFLVT
VAKRVMVDLFRRRTLERAYLEMLALIPDGYAPSPEQRQSLLESLQQIDAMLDGLGPKVKQAFLLSQLE
GLGYADIAVRLGVSISSVKKYMAKATEHCLLFSLENDVFS
>CORE_REP|Org6_Gene3004#
MNKRRTTLTVLALIASLGLSSAPALADKGGNGNGNGNGHGNSGNHGNNGNHGNNGNNGNHGNNGNNGN
HGNKGNKDKGGYRNDDNLVSVSLSRDRARSLAHNYGLTGYSSLPPGIAKNLARGKPLPPGIAKKVVPY
SMLRELPQYPGYEWRIAGDDLVLVALSTAIVASVINGVFD
>CORE_REP|Org43_Gene254#
MIDDDGYRPNVGIVICNRQGQVLWARRYGQHSWQFPQGGINPGETAEQAMYRELFEEVGLSKKDVRIL
ASTRNWLRYKLPKRLVRWDTKPVCIGQKQKWFLLQLLCNDADINMQRSSTPEFDGWRWVSFWYPVRQV
VSFKRDVYRRVMKEFAVTVMPMQEQAAPRQAPAYRRKRG
>CORE_REP|Org35_Gene799#
MPLNDLLTLPGVAAQPDAVTDGYVFNHTMIRVKDLTKALDFYTRVLGFTPVYLEEFKEAAFTICYLTR
SPREQIPQDDDERKRWALSQPGILELTHNHGTENQADFHYHNGNGEPRGFGHLCVTVPDVRAACERFE
RLGVTFQKRLHEGRMNYVAFIRDPDDYWIEILQPTPLQD
>CORE_REP|Org43_Gene3122#
MTQDTFIQTLPTDPLIAPVIEGLFGEYRQRYGDYFGDQEPEPLDLYAPPQGAFIVLLRAGTPIAMGAF
KRYDGQTAELKRIWTRGDLRRQGLAQRVLQQLETLALAQGYRRLYLTTGFRQPEAVGLYLSNGYQPQF
DPTVDSEVYSRPPYDGRLPFRKSLIAEDACCCASERKIA
>CORE_REP|Org7_Gene458#
MSQVENKAGVKPQDLSMENWVESRIARFEGRKYDWNALKFQADFDPKYRRAQMRYIGTGATGVASDAN
TIPAGNFTFSTMVLPSKCEGPLHLHDDVEEVFFMLKGSITLMIQDGEEYYETRLKERDLISVPAGVYR
GLFNHGEEEALMCVMLGTAKPEIPTYPADHPLSKVKRN
>CORE_REP|Org22_Gene1865#
MGANAASAATTGAKIAIVMGSKSDWATMQFAAEVLTQLEVPFHVEVVSAHRTPDKLFSFAEQATANGF
DVIIAGAGGAAHLPGMLAAKTLVPVLGVPVQSAALSGVDSLYSIVQMPRGIPVGTLAIGKAGAANAGL
LAMQILALHDAALAQRLADWRRAQTEDVLNHPDPREDA
>CORE_REP|Org31_Gene4559#
MPWQIRLHWLVAILLVITCVTIELRGFAEPGSAPWYVLVVTHFSCGVTVFALMIARLFLRWRHPSPAI
APKPPKWQTGLAHLTHTLIYLLLLTLPVLGVYSRYLGGKEWYLFGLPMPFADVADRPQARMIIGWHKT
LASFGYWLIGLHAAAALFHHYIVKDNALVRMLPLMKKR
>CORE_REP|Org21_Gene3117#
MTQTLFMIGARGAGKTTVGSALALALGYQFVDTDLFMQQAAQMSVAEMVEREGWLGFRRRETIALQTV
TRPSTIVATGGGAILAEENRQFMRQHGTVIYLRAPASVLAQRLAEYPEDAQRPTLTGRPIAEEMLEVL
AAREALYQDAAHYVIDGAADPQRVVEQILAVLPRETVK
>CORE_REP|Org1_Gene2987#
MSNRYARSQIVLHWLTLLMVILTYAAMLLKDSVPEAWAPLVKNLHFNFGVSVFALMLIRLAMRAFHAA
PPTTPPLEEWQEVGAKIFHWLLYVVFLMLPLLGMLTLAYGGKSWSLLGWLMPQWVTPDPVMRRLVKTV
HETLANIGYFIIGAHALAALYHHYLRKDDTLRRMMPGK

>CORE_REP|Org38_Gene2222#
MSGVLMALALIFTELSLLAFGGGMTILPEMQRQVVEVHQWMSAQEFSALFAMAQAAPGPNMMIVPLVG
WHVAGWAGLLVSSIAKFGPSSIVTLLVMGAWRRFKDRPWRRIVQAGLVPLTVGLVVASGLLIAEASAP
HWRLAAIVALATGLSMSKRLHPLWVLAGGALLGLLFA
>CORE_REP|Org2_Gene2712#
MKRYWYAALGLMACGAAQAATTDVEMHLVTGQGIGQDIGKVVISETPYGLLFTPSLKALPAGVHGFHV
HEKGSCEPGMKDGKAVAALAAGGHLDPQKTGKHLGPYADGHLGDLPAIYVAADGMANYPVLAPRLKKI
SDIEGKALMVHAGGDNHSDHPQPLGGGGERFACGVIK
>CORE_REP|Org20_Gene34#
MQKVKLPLTIDAVRTAQKRLDYAGVYAPEQVTRVADSVVSVDSDVEVSLSFNIDNQRLAVITGHADVT
VTLMCQRCGVPFEHQVHTTYCFSPVVNDEQAEALPEAYEPIEVDEFGEVDLLAMIEDEIILSLPVVPV
HESEHCEVSEADMVFGQLPPEAEKPNPFAVLASLKRK
>CORE_REP|Org16_Gene18#
MAEKRNIFLVGPMGAGKSTIGRQLAQQLNMEFFDSDQEIERRTGADVGWVFDVEGEEGFRDREEKVIN
ELTEKQGIVLATGGGSVKSRETRNRLSARGVVVYLETTIEKQLARTQRDKKRPLLQVDSPPREVLEAL
AKERNPLYEEIADVTIRTDDQSAKVVANQIINMLESN
>CORE_REP|Org24_Gene3493#
MLEQLRARLVRQGPSLLRIPLKFTPFALQRQLLQQVLSWQFRQALADGDLEFLESRWLKIEVRDLALQ
WFMTVENDKLVVSQHAEADVSFSGDANDLILIAARKQDPDTLFFQRRLQIEGDTELGLYVKNLMDAIE
LESMPAPLRMGLLQLADFVEAGLQEGTASASRVAVSC
>CORE_REP|Org26_Gene908#
MAIILGIDPGSRVTGYGLIRQQGRQLSYIASGCIRTVVDDMPTRLKLIYAGVSEIITQFQPDFFAVEQ
VFMAKNPDSALKLGQARGVAIVAAVNQNLEVFEYAARQVKQTVVGTGAAEKAQVQHMVRSLLKLSANP
QADAADALAIAITHCHLSQNVLRMSEGRLNLARGRLR
>CORE_REP|Org31_Gene2652#
MDYFTLFGLPVRYTVDGSLLASRFQDLQRQFHPDRFANQPERERLMALQQAATINEAYQSLKHPLKRA
EYMLSLHGFELGNEQHTMRDTAFLMEQLELREELDAIERKPEAESLLADFGARLAVSIKQRSALMLQQ
LDGELWADAADTVRKLRFLDKLQQQVEQLEEKLLGFE
>CORE_REP|Org6_Gene1083#
MIELQQWDAAAAQSAIADLAEMLHASVAHGASIGFVMPFTQEQAQAFWQGVLPALARGERAMLVALAN
GRPVGTVQLLLAMPDNGRHRAEVVKLMVHPQARRQGVARLLMQEVQALAARHRRSLLVLDTLSGSAAQ
GLYRQLGFEAAGDIPQYARASDGGALEATCYMYKLL
>CORE_REP|Org6_Gene540#
MYHVVAATTNPAKIKAIQLAFDDVFGAGQYRIESVDVASGVSLQPIGNHETRTGSRQRVMEARQVRPE
ADFWVGVEAGIEENMTFAWMTIENPHIRGESRSASLMLPEVILQGIRAGRELGSEMAAITGNAEVKRQ
GGAIGVFTDGRLSRTSVYHQALLLALVPFHNAIYQQ
>CORE_REP|Org6_Gene1075#
MKKIACLSAVAACVLAVSAGTAFAGQSTVSAGYAQGDLQGVANKANGFNLKYRYEFDNNPLGVIGSFT
HLEKNRSESGFYKKSQYDSITAGPAYRFNDWASIYGVIGVGYGKNIDNAQAGGNKGGNSDYGFTYGAG
LQFNPIENVALDVGYEQSRIRSVDVGSWNVGVGYRF
>CORE_REP|Org38_Gene38#
MVDKRDSYTKEDLEASGRGELFGAGGPPLPAGNMLMMDRVVKMTEDGGTHNKGYVEAELDINPDLWFF
GCHFIGDPVMPGCLGLDAMWQLVGFYLGWLGGEGKGRALGVGEVKFTGQVLPTAKKVTYRINFKRVIT
RKLIMGVADGEVLVDGEVIYTATDLKVGLFKDTTAF
>CORE_REP|Org37_Gene3888#
MIIRNATLNDSAAIAAIYNDAVLNSTAIWNEQTVDAANRAAWIGERQAAGYPVLVAVNGADEAIGYAS
FGDWRAWDGYRHTVEHSVYVHQAHRGEGIGKALLIALIARAQEIGKHVMVAGIESGNQASIKLHLALG
FREVGRMEQVGAKFGQWLDLTFLQLTLDERAAPPAR
>CORE_REP|Org6_Gene1446#
MRLNTWSKALLPLVVLACVSATQVRAAESDTGPIPKQLLGNWRVSKIVPTQTTGCWDQQQAQSLIGGK
ISYKADAFSWNGTALKSEGATVSTVEAQEFVEDNAGSSSYIDFPMLGISTPSVERVAIQHADTAIKGI
TDQGTDGVPGDNVLVKDANTLILSLCNVWFEAQREK
>CORE_REP|Org6_Gene1444#
MLTQEMTQKLNEQLNLEFYSANLYLQMSAWCSDKGFEGAAAFLKEHSQEEMQHMQRLFDYLSDTGSLP

LLGTIAAPPVAFESLADVFQQTYEHEQLITRQINELAHAAMTAHDYSTFNFLQWYVAEQHEEEKLFKS
VLDKLALVGTSGKGLFFIDKDLKKMGAMGQGGNDQA
>CORE_REP|Org6_Gene389#
MKIGLFYGSSTCYTEMAAEKIREILGEDLVDLHNLKDVSPKLMEDYSILILGIPTWDFGELQEDWEAI
WPQLAALDLKGKIVAMYGMGDQLGYGEWFLDALGMLHDHIAPLGVQFIGFWPTEGFEFTSPKPLSADG
KHFVGLALDEVNQYDLSEERLQQWCEQILLEMEPLL
>CORE_REP|Org29_Gene4501#
MSEIVIRHVETTDAQALHHLYSQTPVYRDTLHLPLPTVELWHKRLANPEPGTHNLAAFIDGQLAGQLA
VMLNQRVRRRHVATFGIGVDPRYHGKGVGSRLIQAMIDLCDNWAAIERIELTVFTDNPAAIALYRKFG
FEIEGTSRAYAMRDGVLVDAYHMARLRCGAVNAAQS
>CORE_REP|Org9_Gene4520#
MNVLFAIAVTTGILSGVWGWVAVSLGLIGWAGFLGCTAYFACPQGGLKGLLIGALTCCSGVFWAMAII
HGSELAPQWELLGYLLTGVVAFLMCIQAKQQWLGFVPGTFIGACATFAGGGDWPLVTLSLLVGLLFGY
AMKNSGLWWAARSEKAVPHSRSARMPAQTVPPSERR
>CORE_REP|Org2_Gene3876#
MPGLLMFYVGGTAPGANIELHDVQFAAADRPEEAYPLLREKWFGDKRKVHVDGYARIDWADGYDVSLE
PAPFAGEEKLFFVNVGGYRSDELAELHQFGLFVARSADEAKDKAKRMLLTDSAQQHKDDLAEVDDCLL
LQALQGYHVHLRANPHGKPARPLWQGYLPIGEPAL
>CORE_REP|Org35_Gene2546#
MSGIRDSILPPLEWLSEQDPPAPAAVSDWLMELGSMTRRFERHCAQVRVEPQRECFVTREALGDEANH
LPDSPRYWLREVVLLGDDQPWLLGRTVIPEHTLTGPDQALVDLGTLPLGRYLFSSGDLTRDYIHIGRQ
DALWARRSRLRLAGKPLLLTELFLPASPLYSAVPA
>CORE_REP|Org42_Gene716#
MPLLDSFTVDHTRMAAPAVRVAKTMKTPHGDTITVFDLRFCRPNLEVMPERGIHTLEHLFAGFMRDHL
NGQGVEIIDISPMGCRTGFYMSLIGVPEEQRVADAWKAAMADVLKVTDQRKIPELNEYQCGTYHMHSL
EEAQEIAKHILDNDVVVNHNDELALPKEKLQELHI
>CORE_REP|Org20_Gene1497#
MSHASSEFIAAHIAILTVSDSRGAAEDTSGHYLQEAAQEAGHQVVDRAIVKDDIYQIRARVSAWIADD
NVQAVLITGGTGFTARDNTPEALLPLFDREVEGFGELFRMVSYEEIGTATIQSRALAGLANRTVIFAM
PGSTRACRTAWERIIEEQLDARHRPCNFQPHLKKP
>CORE_REP|Org10_Gene639#
MKVWNKVLLASFIGLLVAGCDDSSKVDANLDKAKDSAEQMKDAAQKKADDLTDKAKATADEIKKEAAT
QADQLKDKASAIKEDAAKQADAITNDAKAKAAAIKDDAGKQSQQLVDQAKAIKNDAINGANDLTEQAK
AKTEAIKNSAENKAQELKQETDAAVNGNTQPATQQ
>CORE_REP|Org35_Gene759#
MSQENEQRLRFRDAMASLSAAVNIVTTDGPAGRCGITATAVCSVTDTPPTLLVCINRNSAMNPVFQEN
RRLCVNVLNHEQELMARHFAGMTGVSMEDRFRLEEWQLGALGQPVLRNTLASLEGEIEQIQSIGTHQM
YLVQIKQIALSEAGNGLIYFKRNFHPVIHQMAVPA
>CORE_REP|Org4_Gene1486#
MSAIVKRCSVAAVLAIAVLLPSFGELQTSEAGLRLIADLEGCRLSPYQCSAGVWTQGIGHTAGVIPGQ
AIDERQAAVDLVDDVRRTERGMAACLPQTLPQETYDAVIAFAFNVGISAACHSTLVTLLQQRQWQQAC
DQLPRWVYVNGKKNKGLEQRRATERALCLQGIASS
>CORE_REP|Org43_Gene304#
MRDSRPQLLDVLFDDASAGSKGPLHNVQQRAVALLKLNRAVKGLLPAPLHPWCRVANFRQGILVLETA
NASWMMRLRYEQPALLSALRAQILPSLSSIDIRINPALMAKGSNQVQNAEKAPEKPVPMRHLSLESAE
ELRGLASRSPEKLRKILERLAALAGEGTNTTSRDK
>CORE_REP|Org8_Gene757#
MQLNKVLKGLLLALPVLAVAACSSNKSANNDQSGMGAGAGTGMENGSSNLSSEEQARLQMQELQKNNI
VYFGLDKYDVSSEFAQMLDAHAAFLRNNPSYKVTVEGHADERGTPEYNIALGERRANAVKMYLQGKGV
SADQISIVSYGKEKPAVLGHDEAAYAKNRRAVLVY
>CORE_REP|Org38_Gene1270#
MNDLLSRAMRLLSQRDHSEAELRRKLAAQPFVAKARFGGKGPHAPTSSPPVAEEPVDPALIDQVIVYC
YQHNWLDDKRFAHSYIGSRSRKGYGAQRIRSELMQKGVDKALVQDALAECDIDWCEQAKQVAQRKFGD
ALPTDWKEKAKVQRYLLYRGFFQEEIQSIYRDFAQ

>CORE_REP|Org28_Gene811#
MNSTTQEKLLAQAERLCQQRNVRLTPQRLEVLRLMTQQPGAISAYDLLDLLRVAEPQAKPPTVYRALD
FLLEQGFIHRVESANSYVLCHHFEQPMHTSALFICDRCGQVTERTTEGVEETLQKLAQEAGFALRHSV
VEAHGLCAGCVEVEACDSKHDCAEHDHSIAIKKK
>CORE_REP|Org23_Gene4575#
MNKPRIPIALQQAVMRCLREKLQLARQHFAVEFPEPSIVYQQRGTSAGTAWLQSWEIRLNPVLLLENQ
QPFIDEVVPHELAHLLVFRQFGHVAPHGREWRWMMESVLLTPASRTHRFETASVQSKTFPYRCGCGQH
QLTIRRHNRVLRGESEYRCRRCGEKLKFLASENL
>CORE_REP|Org39_Gene3353#
MRSLEDQLAAYAAYHRDARNIATHFIGIPLIVISLLALLSRPAWGMGGLPFSPACAVVIVATLYYLRL
NLRLGAMMLALLLLCLGFGAWVASLSTAAWLSVGIGGFVIGWLFQFVGHFWEGRKPAFMDDVTGLIIG
PLFVLAEACFLAGGLRELQRNIEMRAGRVRNGRA
>CORE_REP|Org47_Gene1596#
MNKASVVFSGLLMAVSAGAMAATSGDDADISKQPLEKVAPYPKAEKGMNRQVIYLPKQEHEENYKVEL
LIGKTLEVDCNRHMIGGTLETKTLSGWGYDYLVLEKLSEPASTMMGCPDNTKTKQFIAANLGDAAMQR
YNSRLPIVVYAPKDAEVKYRIWKAEDTVSQAEQK
>CORE_REP|Org29_Gene3496#
MFTVRQALLEDLTQVRDIGIRTYRAHFGELWRYPHELEAFLAEDFSVSALERTLRDPDVCWLLAYEDD
TLVGYARVNFDSLLAATQRRGAELQKIYFLPDYAGRGFGRQFFEQVQRRAVGRRQPLLWLEVLKQNAD
AQRFYQRQGLAVCGEAQYTSEQGAIELWAMSKAL
>CORE_REP|Org25_Gene2720#
MHDIELAYLQGYPAHLQSQVQQLIHQNRLGDVLLQRYPQVHDCTTDKSLYQFTVDLKNQYLRNAQPLS
KVAYDSKIHVMKHALGLHTAISRVQGGKLKAKAEIRVATVFKVAPEPFLRMIVVHELAHLKEKDHNKA
FYSLCCHMEPDYHQLEFDTRLYLTHLSLFGELYA
>CORE_REP|Org2_Gene1392#
MGIKRLNHAVLYVSDVRQSADFYHQVLGFKLKPSDSPDRAVFTQAADSDNDHDLALFSKNLGQQRAGV
FRANNEPPAEHEPAAGLYHLAWEVDSLEELERIRHQLAERGILGLEEDHGVHKSIYGHDPDGLLFEVT
WFIPPALLTEEDKNQQGIRPLDFAAEKRRFAGR
>CORE_REP|Org1_Gene379#
MGLFDKLKSLVSDDKKDTGTIEIVAPLSGEIVNIEDVPDVVFAEKIVGDGIAIKPAGNKMVAPVDGTI
GKIFETNHAFSIESDSGIELFVHFGIDTVELKGEGFKRIAEEGQRVKKGDVVIEFNLPLLEEKAKSTL
TPVVISNMDEIKELIKLSGSVTVGETPIIRIKK
>CORE_REP|Org48_Gene1720#
MPGILLKKRPLSRYLKDYKHSQTHCSQCGKLLDRMALVFRGKIINKEAIARMDQPIDDAVWQNVQHEL
TALCRFCSEISCNSHPSYFDIMAFKQYLFEQTEMSHSTIREYVVRLRRLDEMLVARNYPADKFASSAS
HQRIIDDLPTAAHNNYRIALRKYDQYLAWQRSY
>CORE_REP|Org2_Gene584#
MSVLQVLHFPDERLRKVAAPVKEVNADIQRIVDDMFETMYAEEGIGLAATQVDIHQRIIVIDVSENRD
QRLVLINPELLEKSGETGIEEGCLSIPEQRALVPRAAQVKIRALDREGKPFELEADDLLAICIQHEMD
HLMGKLFVDYLSPLKRQRIRQKLEKMAKLQARA
>CORE_REP|Org4_Gene1656#
MSKSICSTGLRWLWVVVAVLALDFGSKQWILANFTLGQSQPLIPSFNLFYARNYGAAFSFLADHGGWQ
RWFFAGIAIAIVAVLLVMMYRSTAQQKLNNIAYAFIIGGALGNLFDRLWHGFVVDFIDFYVGDWHYPT
FNLADSFICVGAAMIVLEGFLSPANKDAKSKGE
>CORE_REP|Org8_Gene3768#
MDELTHIIAKYSPEPGMLLLLIFLFALGKSVIVVSSALPPASVTLLMGIVAGKHALPFGAVWAAIALG
AALGSILSFHCGARLHGHDLGCRLPARFHAPLRKAQRSLQKRGLPLLFASRFLAVMRYTVPLMAGMLQ
LPARRVYATAALSAAVWALLLMSAAHLFPRFLT
>CORE_REP|Org39_Gene1644#
MRTQSLYQPHFSHGSFTANSVAKNTNVGKENGLISELVYNERQPAVAQLLLPLLQQLGKQSRWLLWLT
PQQKLSKQWLQQSGLPVDKMVQLSQISPVNTVEAMEKALQTGNYSVVLGWLPELTEEDRLKLRRAAEL
GNAYGFIMRPQRDIDPTHGHCSTLKIHSSLYH
>CORE_REP|Org35_Gene1997#
MTEYNDEYWMRQALQLALRAQEEGEVPVGALLVLDNQVIGEGWNRPIGRHDPTAHAEIMALRQGGAVL

QNYRLLNATLYVTLEPCVMCAGAMVHSRIRRLVYGAADEKTGAAGSLVDILRHPGMNHQVEIVSGVLA
DECAATLSNFFRLRREQKKALKLAQRAADKPE
>CORE_REP|Org9_Gene4043#
MTTATPARLLVRSITAEDNTAIAHVIREVSAEHGLTADKGYTVSDPNLDALYQLYSLPRSAYWVIEVD
GQVAGGGGIAPLQGGADDICELQKMYFLPVLRGKGLAKRLALQALDFARQHGFRCCYLETTASLTQAV
ALYEHLGFEHIDHAMGATGHVDCEVTMLKTL
>CORE_REP|Org9_Gene4307#
MRHALCCGGLWACSLLALADSKTATLGVSATLLSACEAGSSSGGNVSFGTLNFGTLYFLSTATSVAGQ
QNAGAIRVKCTNGTSYSVLLGGGQSGNTAARYLQSAAGQRVNYNLYTNAAHSTIWDNLTGVSQTANGS
DNWLPVYGMIPAQSTPPTGSYTDTVQVTINW
>CORE_REP|Org9_Gene122#
MTQTVHFQGNPVSVAGKLPQQGEQAKAFSLVAKDLSDVALSSFAGKRKVLNIFPSIDTGVCATSVRKF
NQLASGLDNTVVLCISADLPFAQSRFCGAEGLSNVVTLSTLRGAEFKQAYGVEIAEGPLAGLTARAVV
VLDGQDNVLYSELVNEITTEPDYDAALAALK
>CORE_REP|Org14_Gene2222#
MLIRVEIPVDAAGIDALLRRAFGRDDEADLVQQLREDGLLTLGVVATDDEGGVVGYAAFSPVDVAGED
RQWVALAPLAVDESLRRQGLAEKLVYEGLDSLNEFSYAAVVVLGDPAYYGRFGFKPAAAYGLNCRWPD
TESAFQVYPLAEDALNGVSGEVAFSAPFNRF
>CORE_REP|Org15_Gene1377#
MDAIKSILVKEIEQINRREGRDGKPRFNSEFARTHRYLCLAMFAGYFAVIAVMYQVPYMGMYAFLGFT
AFVLFMFAMLLIEIRPVYRFEDIGVLDLRVCYNGEWYFTRALSPYAVQQLLDEPSIGAALKQKMRVII
GNKGEIDFYDVYDMAYGKKPQDEPPQLAAAN
>CORE_REP|Org30_Gene3579#
MTEKAALLTQRLIRNVERVDKRHDRRPPLYDSVGTRLGTGTAADKLGASFDCVAPGMRSCPYHFHYAQ
EEMFIILEGSGTLRVAGEMLPIVSGDVIFIPPGPEYPHQIINTSDAPLKYLSVSTREQPELVMYPDSG
KYQAMARGADGQQVRYLQRPSTSLDYWQDEP
>CORE_REP|Org43_Gene956#
MLNRLERLTQRVGGSNELVDQWLQARKQLLVAYCTLVGLKPNKEKHTPLNEKALENFCHNLVDYLSAG
HFHIYDRIIKQVEGAASPKMSLAVNIYPKLWANTEQIMAFHDRYTEVDIDQEVCLEFHQALSDIGETL
AARFALEDKLILLEAEAAQQPLPDQALDPAR
>CORE_REP|Org4_Gene589#
MSTAKLVKTKSSDLLYTRNDLDEKVKLAAIKALNHQVVQFIDLSLITKQAHWNMRGANFIAVHEMLDG
FRTAIIEHQDTFAERVVQLGGVALGTVQVVNDRTPLKSYPTNIHSVQEHLKALADRYGAVANDIRKAI
TEVEDEDTADMFTAASRDLDKFLWFIESNIE
>CORE_REP|Org11_Gene1590#
MKTRITLTLLTALTLAGCSTPPPPPPALNNDALISSEVNGVTLQHRAAVSAPKQFKPIGEEYRSLYAA
SIMSSPNYTGTAVGSLDNAAAFYALGEVENNWLAISAIRGGDLMGYIQANAGVPEARYKSTLRKDLPR
RARAAKQDCVKVGGDSKACKNAGSATWILQ
>CORE_REP|Org42_Gene3549#
MGAFIAFSTQDSLWGQILFTFFGILVGCVLLAAIGKTLLSASRAAALPARLLIFVALMLALLKYNQQV
MSAVNAPAELIGFLILPGFLMGYLGAAPTGRLALAIELPCFAALAALLILGTADVEFFAPYHLESAGQ
FIAKAIAAALSVAAVLLARGINGKDGALSS
>CORE_REP|Org6_Gene4164#
MIRVYIALGSNLAQPLQQVNAALEALEHLPRTRLVTCSSFYRTKPLGPQNQPDFLNAVVAMDTLLPPE
QLLDHTQAIERNQGRVRKDERWGPRTLDLDIMLYGDKVIHTDRLTVPHYGLKEREFMLYPLAEIAPDL
IFPDGEPLASCLKRVPENGMALWHSPKPQS
>CORE_REP|Org11_Gene1581#
MAGLAAVSKLAGETVGQEFLIFTLGNEEYGIDILKVQEIRGYDQVTRIANTPAFIKGVTNLRGVIVPI
IDLRVKFSQQSVSYDENTVVIVLNFGQRVVGIVVDGVSDVLSLTAEQIRPAPEFAVTLATEYLTGLGS
LGERMLILVDIEKLLSSEEMSLVDSVAKSV
>CORE_REP|Org17_Gene2138#
MDMSQMTPRRPYLLRAFYDWLLDNQLTPHLVVDVTRPDVQVPMEFARDGQIVLNIAPRAVGNLALGNE
DVQFNARFGGVPRQVSVPMAAVLAIYARENGAGTMFEPEAAYDESEGVFEGLDNETIPSETLMSVIDG
DRPDTAEDDGSDDEPPQPPRGGRPALRVVK

>CORE_REP|Org31_Gene86#
MAHIEKQAGELQEKLIAVNRVSKTVKGGRIFSFTALTVVGDGNGRVGFGYGKAREVPAAIQKAMEKAR
RNMMNVALNSGTLQHPVKGAHTGSRVFMQPAHEGTGIIAGGAMRAVLEVAGVHNVLAKAYGSTNPINV
VRATIDALANMKSPEMVAAKRGKSVADILG
>CORE_REP|Org33_Gene3271#
MEDSGLPLKRRKGATMSIIHRLAQPDDLNGLLALYRELRPQDAPLRTDDARRTLQRLLDDPAIRLVVA
ADEEQPIATCMLALIPGLAHQAQPFGVIEHVVTAEPYRGHGVALAMIEYALQLAWRKGCYKVMLLSGQ
QRTGAHQLYLKAGFDGDRERGFVIRRPEGR
>CORE_REP|Org11_Gene1816#
MTIEIYPAQKSDAKLILDMIIELAVYEKAREQVLASVEDIERSLFGPGACSEALICTVNGEPAGYAVF
FMSYSTWLGKNGIYLEDLYVAPKHRGAGAGKKLLRHIAQLACERQCGRLEWSVLDWNQPAIDFYLSIG
AQPQGEWVRYRMEGEALTHFAAHGAALPAQ
>CORE_REP|Org25_Gene3716#
MNGKITTFFEDKGFGFITDENGENRYFHVIKVQNPELIKKNAAVTFEPTNNTKGPSAYAVKVLAPSKY
IIIANERIKITSIKSFNTFTKEVPVKADVDKENTVLSVGLLMNRIRPQSEDQAQAMQTLRMLTITTFQ
NTTHTFSEHEIDMDETIKALKSLQASAYRK
>CORE_REP|Org9_Gene1621#
MTKEQFYAELKRDLSALLGGETNFIAALSNASALLNERLDDVNWVGFYLMDGGQLVLGPFQGKIACVR
IPVGKGVCGTAVAENRVQRVGDVHAFPGHIACDAASNAEIVLPLAVGGRAIGVLDIDSTVYQRFDEQD
EAGLKAVVAGLCEQLEQCDSAKYVTVAAS
>CORE_REP|Org17_Gene1231#
MIQQLFKGRFSLLTMAFVALLALAGCQSKPQGLTPEQVALLQSQGFKLTDNGWEFGLSDKVLFGNNIG
KLNPESTETVQKMGRALLSVGITKFRLDGHTDNYGEDSYNDQLSLRRADAVADLLASVGIPRANIETR
GMGKRDPVADNRTSSGRAENRRVAIVVTP
>CORE_REP|Org15_Gene106#
MALNLQDKQAIVAEVSEVAKGALSAVVADSRGVTVDKMTELRKAGREAGVYMRVVRNTLMRRVVEGTP
FECLKDTFVGPTLIAFSHEHPGAAARLFKEFAKANAKFEVKAAAFEGELIPAAQIDRLATLPTYEEAI
ARLMATMKEAAAGKLVRTLAAVRDQKEAA
>CORE_REP|Org23_Gene2350#
MLLMPSVFRDRKSILTTDTHTLDIAEILDLLPHRYPFLLVDRVLEFEEHKYLRAVKNVSVNEPFFQGH
FPGKPIFPGVLILEAMAQATGILAFKSVGKLEPGELYYFAGIDEARFKRPVVPGDQMVMEVTFEKTRR
GLTRFKGVATVDGKIVCEATMMCARSREA
>CORE_REP|Org44_Gene1826#
MKKPHAGFWLATLGLGVCWLLALLTRDRLNGILLAGAALALIFTPAARRKGAALAALGGIGMDGLWSY
SGVLHYSGQHGLPLWTIALWIGFGCWWYWLLDVVRAAPWPLAVLGAAAGPFAYAVSWKLDALLPGVPP
EFMLLLLAVGWSIYLPAVSWLQWKRRDS
>CORE_REP|Org30_Gene2433#
MTPDLLVRILLTGAGATLCMDLWALLLKRRFDIPSLDYALVGRWFLGMFDGRWFHATIVTAPPRQGER
EIGWILHYAIGIAFAFIPLGLAGSEWYAAPEPLIALLSGWLSLAAPFLVMQPALGFGVAAAKTAYPRK
ARLLSLLTHTVYGLGLLIAARLLATLCA
>CORE_REP|Org4_Gene500#
MNPRRKSRLYLAIVVLIGIALTATLMLYALRSNIDLFYTPGEILQGKGENHEKPEVGQRLRIGGMVMP
GSVKRDPNTLQVSFKIYDARGAIGVTYTGILPDLFREGQGVVAQGVLGEGNVVNAREVLAKHDEKYTP
PEVADAMKENHKGPAEAYNAPQAEGAKS
>CORE_REP|Org24_Gene49#
MADNKKRPGKDLDRIDRNILNELQKDGRISNVELSKRVGLSPTPCLERVRRLERQGFIHGYTALLNPH
YLDASLLVFVEITLNRGAPDVFEQFNSAVQKLEEIQECHLVSGDFDYLLKTRVPDMSAYRKLLGETLL
RLPGVNDTRTYVVMEEVKQSNRLVIKTR
>CORE_REP|Org13_Gene1024#
MLFKLCKTLAMACVALIIAQSSALSFTLEVAGKIDNVTDPVNKSYLFTDKQLLAMPVRSITTSTSWTP
QRKFEGISVADILERVGAKGETLTFYALNDYYIDVPLSDVKKYNMILAYKMDGEMLKLRNFGPLFLVY
PRDAAGPELNSPLYNSRFIWQVDRIVIK
>CORE_REP|Org6_Gene1254#
MRLTSKGRYAVTAMLDVALHSQEGPVPLADISERQGISLSYLEQLFSRLRKNGLVASVRGPGGGYLLG

KDAGEIAVGAVITAVDESVDATRCQGKEGCQGGDRCLTHALWRDLSERISGFLNNITLAELVNNQEVL
VVADRQNNDTRRTANGRPQETINVNLRA
>CORE_REP|Org13_Gene488#
MDEAKRRLRMSNPWHLLATGFGSGLSPVMPGTMGSLAAIPFWLLLIQLPWQLYSLAVMFSICIGVYIC
HRTAKDMKVHDHGSIVWDEFVGMWITLMALPVNDWRWVAAGFVIFRILDMWKPWPIRWFDRNVHGGMG
IMVDDIIAGVLSAGIIYLIGHHWPIGLF
>CORE_REP|Org13_Gene628#
MEMTNAQRLILSNQYKMMTLLDPDNGDRYRRLQTIVERGFGLQMRELDRDFGELSEEVCRTIINVMEM
HHALQVSWGNLKEKQDLDERRLAFLGFDAATEARYLSYVRFLVSTEGRYTHFDSGSHGFNAQTKMWEK
YQRMLAIWLACPRQYHLSAVEIAQIINA
>CORE_REP|Org9_Gene827#
MMRRILSVLLENESGALSRVVGLFSQRGYNIESLTVAPTDDPTLSRMTIQTVGDEKVLEQIEKQLHKL
VDVLRVSELVQGAHVEREIMLVKLQASGYGREEVKRCADIFRGQIVDVTATLYTVQLAGTSDKLDAFL
SAVREVAEIVEVARSGVVGVSRGDKIMR
>CORE_REP|Org2_Gene1807#
MKKALITCAIVGGLLASYPALSTDQAASDKSAGKGGYHHAQQKLKVVEDLYAGFFNRHDIGVAQKLIV
ENYKQHNPFVGDGIKPFLDFFSQTFKDNPQYSAKIYRSAVNGDLVYVHVKYQNNPQDRGTASVDIYRV
NDQGKITEHWDVNQDVPEKSANDNTMF
>CORE_REP|Org34_Gene204#
MATAKKAAKTHIGLDSKQSAKLAEALNALLANYQVLYMNVRGYHWNITGPQFFELHAKFEETYNDLLT
KVDELAERILTLGSQPRHAFSDYLKTADIKEHTNVTDDKGTLRGLLEGYSILLQQQRELLTVAADAGD
EGTASLMSDYIKEQEKQVWMLNAYLGK
>CORE_REP|Org31_Gene474#
MPSFDIVSEIDMQEVRNAVENATRDLGTRWDFRNVPASFELNEKNQSIKVATESDFQVQQLLDILREK
LSKRSIDGAALEIPEEFTHSGKTYSVEAKLKQGIETSVAKKIVKLIKDSKLKVQAQIQGEEVRVTGKS
RDDLQSVMALVRGGDLGQPFQFKNFRD
>CORE_REP|Org30_Gene3816#
MKLWQIVGGAAALTITLAGCAQKSADVPTHAAGNPAAAQTQIQGPAVTGSVNIRQRIALPPDAVLTVT
LSDASLADAPSKVIAQRAVRTEGQQAPFSFTLPYNPSDIQPNARIILSAAVTVNGRLMFITDTIQEVV
NRNGTRADLLLVPVQGVPIQAAPTAMQ
>CORE_REP|Org34_Gene2010#
MRKLTALFIASTLALGSASAAFAADTVAAPAADAAPMKMMHHKGEGKGGPFAGLNLTEQQRQQMRDIM
KESHQKRGPGMKEERQALHNLVASDSFDEAKAKAQIDAIGKAQSERMLERAKAENKMYNLLTPEQKKQ
YNENYQKREQKMMDHMNKMKEQMSSGQ
>CORE_REP|Org3_Gene410#
MNSYRSHGRWIIWLSFLVALVLQIMPWPEQIYMFRPSWLVLILIYWVMALPHRVNVGTGFVLGLIMDL
ILGSTLGVRALALGIIAYLVAFKFQLFRNMALWQQALIVVLLSLSMDVVVFWAEFLVINVSFRPEVFW
SSVVNGILWPWLFLLMRKIRRQFAVQ
>CORE_REP|Org22_Gene2630#
MELLAPGLRVVFCGINPGLSSAHQGYPFANGSNRFWKVIHQAGFTESQLAPEQWQQLKDNGCGITALV
ARPTVAASELSRDELRSGGEVLQEKILRYQPRALAILGKQAFATAFGVKNAPWGKQALTLGETEVWVL
PNPSGLNRATLEQLTASYRELFLALQ
>CORE_REP|Org47_Gene4392#
MPPDKWYYLYVLCLTGNDMPQISRSALVPFSAEQMYQLVNDVHSYPDFLPGCTGSRVLNATSNEMTAA
VDVAKAGISKTFTTRNTLLDNQSINMQLVDGPFRKLMGGWQFTPLSEEACKVELHLDFEFTNKLIELA
FGKVFKELAGSMVQAFTQRAKEVYSV
>CORE_REP|Org16_Gene2439#
MITKHRLLLALALSATLASGASQAAGLMDSLSSAAGELSKSGGDSSSGMSLSSLTGLLNGGDKALSSS
SMTNAAGILQYCVKNNVLSANGAEGVKDQLLSKLGITSTENAKSQDYQQGLGGLLQTGEGKSLDLNSL
GTSQITEKVKQKACDLVLKQGKSFIS
>CORE_REP|Org5_Gene4055#
MVTRAPDNADRHAQTDMPDEAEHALRFSRAREAQSNALIEDYVELIADLLQSTREARTTDIARRFGVS
HPTAIKNIARLKSAGLVESRPYRGVFLTEEGEQLAQKVRRRHRIVVDLLMCVGVPSETAELDSEGIEH
HISDETLAVFEHYLQKHAKPCPPERR

>CORE_REP|Org23_Gene1631#
MSQNTLPAAPKRSLLVILLVLISVVACGAAGYSWWLLQQHKNGAEPAAVKQQPPAAPVFMPLDTFTVN
LVTPDNNPDRVLYIGLTLRLPDESTRRQLNDFLPEVRSRLLMLLSRQEAGQLANEQGKQQLVAQIKDV
LSPPLVKGQPKQVVSDVLFTAFILR
>CORE_REP|Org40_Gene154#
MKYDTSELCDIYHEEVNVVEPLFSNFGGRTSFGGQITTVKCFEDNGLLFDLLEENGRGRVLLVDGGGS
VRRALINAELARLAAQNEWEGIVVYGAVRQVDDLEELDIGIQAMAAIPAGAVSEGVGESDIRVNFGGV
TFFSGDHLYADNTGIILSEDPLDIE
>CORE_REP|Org19_Gene153#
MTSKAIYPGTFDPMTNGHLDLVTRASLMFDHVILAIAASPSKKPLFSLDERVALATQVTSHLDNVEVL
GFSELMAHFAAHQNANILVRGLRAVSDFEYELQLANMNRHLMPTLESVFLMPSEEWSFISSSLVKEVA
RHGGDIAPFLPDVVTQALMAKLAAE
>CORE_REP|Org37_Gene1932#
MNIDDELQALSEQEATLAFTHFDAATAWELGAALKTAAERRGLSIAIEIQLAGQTLFYYAMPGATPDI
ADWVRRKRNLVNHFHKSSYAIGLRLQQRQTSLEERYGLSVRDYSVHGGAFPLTLAGLGCVGSISISGA
PQKEDHQLLISTLAHFLGLSLPALH
>CORE_REP|Org1_Gene2273#
MAEKLLHALPLFAAWETLIAEGNTAFNQRHDRQAMTSYQQALEGAQAIIAEKPWLNADYTARFGALNI
FESALAALIVTFNNITHLRLRQKPLRGVELHVHQARVAIEQLLADSSLCEQMQQIAERHLLRFRIETL
HLLREHRLPETSICAPATHHSATVH
>CORE_REP|Org47_Gene2398#
MIHSLLSIPCVTLQGEQKTLGDFPARAYLVVNTASKCGFTPQYRGLENLWQYYRERGLVVLGFPCNQF
GSQEPGSPLEIANFCSLNYGVSFPLFSKIDVNGPGAHPLFNELKRLAPGILGSRRIKWNFTKFLLTAD
GQRVTRFAPITKPERLFDRIETLLK
>CORE_REP|Org6_Gene3606#
MSDMLKSGQGMGSTSDAPVPVMVAGTAMVAIKCISVVLLLGELGVDGAQEFVNTSAQAWDSTFIFLAG
LMLLCLQISCGFAVMRGRNWGRWGYVACQCIVVLYLLLATIGSVFPEVFTVEGETSGQILHVLILQKI
PDVVILALLFVPAASRRFFTAQVRF
>CORE_REP|Org42_Gene1427#
MIISLIAALAADRVIGMENAMPWHLPADLAWFKRNTLNKPVIMGRKTFESIGRPLPGRHNIVLSSRPG
NAAGVTWVTSLDEALAAAGEVEEVMVIGGGRIYTQLLPRADRLYLTHIDAEVGGDTHFPDYEPDEWET
TFSEFHDADDLNSHSYCFEILQRR
>CORE_REP|Org1_Gene372#
MQVLIMRHGEAALEAASDAVRPLTLCGRDESRQMAAWLNTKSVDIERVLVSPYLRAEQTLATVREALT
LPEGEEVLPELTPGGNAEQVGSYLQALAMQGVSSVLIVSHLPLVGYLVAELCPGECPPMFATSAIANV
DLPADGSYGKFEWQVSPSQVMAKV
>CORE_REP|Org34_Gene1057#
MNKVAQYYRELVASLTERLKNGERDIDELVASAERRLNEAEDLTRDEVRQVTQAVRRDLEEFARSYQE
SRDEFTDSVFMRVIKESLWQELADITDKTQLEWREVFKDVSHHGVYHSGEVVGLGNLVCEQCHYHLAF
YTPEVLPLCPKCGHDQFQRRPFEP
>CORE_REP|Org21_Gene844#
MTKKKAHKPGSATIAQNKRARFEYFIEEEFEAGLSLQGWEVKSLRAGKANLSDSYVTFRDGEAYLFGA
TITPLNVASSHVVCDPTRTRKLLLNRRELDTLLGRVNRDGYTVVALSLYWKNAWSKIKIGVAKGKKEH
DKRDDIKDREWQTAKARIMKHANR
>CORE_REP|Org7_Gene356#
MAIDMFLKVEGASGESKDSNHKGWTDITSFSWGASQPGNMGVGGGGGAGKVCFNDLHVNALIDKSTPA
LLKHCSSGKHLTKIELSVCKAGGTQVEYAKITLEDVLVTAVQYTGAGGEDTVGVTYSFQAAKVKQQYW
EQSDKGGKGAESSAGWNIKENREA
>CORE_REP|Org6_Gene4107#
MWQGTSVEAILPRFSPDDYDQHGALRLPLGFWAVLILQARTWLLFVMAGVSREQGESLLALFYPDTQR
FWYGMLLGLPAALAFLLSGRRHQWPRLWRGWRWGLAVSLLASLGGLLFSLWRQDGDPPGLELALVLLD
ALALSYLLLNARLKACFAPAEQAD
>CORE_REP|Org41_Gene2219#
MAFRLYSNDLQDGGKLPQAQVFNGMGYHGDNLSPHLAWDGVPAGTKSFVIAVYDPDAPTGSGWWHWIV

ANIPADVRELPQGAGSGKAPLPAGALQTRTDFGSAGYGGAAPPEGESHRYQFTVHALDVERIEVDEGS
SGALVGFNVHFHSLGSATLTVTFN
>CORE_REP|Org28_Gene2966#
MLDFRFPTALQMVLSVAVAEKQGMRSTSATLAAALEANPSFIRKLMVPLTKDGIIVSTLGRNGSIHLG
RPAEEITLRDIYLAVIDDKRIWASRPEVPARCLVSANACWYFKSVVNEAEQASLAVLARHTVADSLAE
LERGDKRACAEYAAEQQEAEAAGK
>CORE_REP|Org32_Gene451#
MDSLIVPDLALLRRWLDQSGISFFECDSCQALHLPHMQNFDGVFDAKIDLVDNVILFSALAEVKPTAL
IPLVADLSQINASSLTIKAFVDIQDDNLPKLIVCQSLSVAVGITYEQFTHFMQQGEEQVSMVILEARA
NDLLFMGDEEEIPAGAVRQPMLH
>CORE_REP|Org22_Gene287#
MRKVTALVMASLLAIGSTAAFAADTIPDTAQPPGNDAMTRIPGQHHMFDGVSLSEQQRQQMRDLMRQA
RHDLPGVNVAEMEAMHKLVTAEKFDEAAVYAQAEKMAQQQVKRQVEMARVRNQMYNLLTPEQKSVLDQ
KHQQRMQQMEQQISGLQQASAQK
>CORE_REP|Org41_Gene1885#
MTQLTHINAAGEAHMVDVSAKAETVREARAEAFVEMLPATLAMIVDGSHHKGDVFATARIAGIQAAKR
TWELIPLCHPLMLSKVEVQLEAQTQHNRVRIETCCRLTGKTGVEMEALTAASVAALTIYDMCKAVQKD
MVIGPVRLLAKSGGKSGDFKVNV
>CORE_REP|Org21_Gene277#
MKGDKKIIAHLNKLLGNELVAINQYFLHARMFKNWGLMRLNDKEYHESIDEMKHADRYIERILFLEGI
PNLQDLGKLNIGEDIEEMLRSDLALELAGAKNLREGIAYADSIHDYVSRDLMIDILADEEEHIDWLET
ELDLIARLGIQNYAQAQILERKE
>CORE_REP|Org20_Gene1406#
MKKGWIFLFGLVCATVGSAQAEQVGSVDTVFKLLGPDHKIVVEAFDDPDVKNVTCYISRAKTGGIKGG
LGLAEDTADAAISCQQVGPIELSDKIKNGKSEGEVVFQKRTSLVFKKLQVVRFYDAKRNSLIYLSYSD
KVIDGSPKNALSAVPIMPWAETK
>CORE_REP|Org20_Gene3542#
MKCSISLLAGALLAVSFSASAMALDSQEVGYNIEARGARPVVAQLGKAGQFNAVENSIRLGDDNWIAL
APKLAGGGNAGFTAGIKSALSAALIYNPAAVLQALNKGNDLPLNDICTAPPEVQGDTAVTSFRQRATR
ALSTVRVGDLSAPRDACIAALKG
>CORE_REP|Org32_Gene789#
MIIIDTNVISETLRPSPYYNVINWLNEKDNDELYLSAIVLAELFSGVACMPDGKRQRDLKLKLADAIE
LKFEGQILPFDGLCAMQYAELTARNRLLGKAMSVPDTQIAATCLHYGAALATRNTKDFLHCGIELIDP
WQAPTGRRLHEDAAEYYVMSRKS
>CORE_REP|Org26_Gene256#
MNQIPMTLFGAEKLREELEYLKSVRRPKIIADIAEAREHGDLKENAEYHAAREQQGFCEGRIQEIEAK
LSNAQVIDITKMPNTGRVIFGATVSVMNLDSEEEVTYRIVGDDEADFKKNLISVNSPMARGLIGKEQD
DVVVIKTPGGDVEYEILKVEYL
>CORE_REP|Org25_Gene3141#
MMKEWATVVSWQQGVALLRCEPKAGCGSCTARSGCGARALNELVPETEHQLQVHIDQPLEPGQRVEVG
IAEGSLLRSAMLVYLTPLLGMMLGGALLQYCFGSDASAAVGAVLGGGAAFMLARRLARRLGEQADYQP
VVLQIGLPPTALRMQAENSPLI
>CORE_REP|Org42_Gene4407#
MHVLPKATVIRPCGPEDIDRLMALWLPSTIAAHPFVAEKYWRESATLVRENYLPRAQSWACWHDDEIV
GFISVLDEQFIGALFVERAFHGRGVAQALMTHVQQRYRRLSLEVYQQNLRACAFYHRHGFQVTQRLFN
DETQAYTLIMNWPAVENSTRYA
>CORE_REP|Org18_Gene2358#
MFDVLMYLFETYIHNEPEMRVDQDQLTDDLAQAGFHRDDIYNALNWLEKLADLQEGQNAPYFMDADPL
AMRIYTEEESARLDAGCRGFLLFLEQIQVLNLETREMVIDRVMALDNAEFDLEDLKWVVLMVLFNIPG
YESAYQQMEELLFEVNEGYLH
>CORE_REP|Org29_Gene2218#
MYNIDDFDMKILTLLQANGRLTNQELSELVGLSASQCSRRRISLEQAQLIRGYHARLAPEAVGLGLVG
LIEVSLITHAQEQIDSFHQMLEEVDAILDAYKTTGDADYLLKVAVADLPALSEFISQTLSPHKSVAHI
KTAVVLNRIKENGLLPVARES

>CORE_REP|Org4_Gene4805#
MKQITAHELTAMSEQAASVARLRAHRTLHEELSDPVQRLAIAMEPGTYIRPHRHPQTWELLTPLRGRF
VVLQFNDDGVVTQRTLLGEETKVLEMPAFTWHAVLSVDAGGVVFEVKQGPYQALTEEDCMQWAPQEGG
EGSEAVMRWYATAQVGDRYPH
>CORE_REP|Org38_Gene1104#
MRTQLITREGYDKLKQELDYLWREERPEVTKKVTWAASLGDRSENADYQYNKKRLREIDRRVRYLTKC
LEQLKIVDYSPQQEGKVFFGAWVEVENDDGETKRFRIVGYDEIFGRKDYISIDAPMARALLKKEVGDV
ATVNTPLGEAQWYVNEIDYPK
>CORE_REP|Org43_Gene2420#
MLNIVLFEPEIPPNTGNIIRLCANTGFNLHLIEPLGFPWDDKRLRRAGLDYHEFTRVHRHADYAAFLA
AEDPQRLFALTTKGTPAHSAVSYQAGDYLLFGPETRGLPADILDALPAQQKIRIPMQAQSRSMNLSNA
VAVVVYEAWRQLDYAGALIKA
>CORE_REP|Org27_Gene223#
MRIGHGFDVHKFGGEGPLVIGGVRIPYDKGLLAHSDGDVALHAATDALLGAAALGDIGKLFPDTDPAF
KGADSRELLREAWKRIRAKGYRLGNLDITIIAQAPKMAPHIPQMRVFLAEDLQCHMDDVNVKATTTEQ
LGFTGRGEGIACEAVALLIKE
>CORE_REP|Org3_Gene910#
MARITQHLWFEKDMEAALNCYVALIPGSSVNWFSDLPADTPSGPAGSVKLASFTLGDQRYAAIEAGPL
DPFNHSFSVMVECDDQAEVDRLWDALCVGGEAEQCGWLRDRWGLCWQIVPRRLTELMNDPDAARVRRV
TEAMLLMGKIDIAGLEAAARG
>CORE_REP|Org8_Gene1519#
MSQVILDLQIACESSDGLPDEATFQRWLEGVLPQFQEEAEVTVRLVDEAESHELNLTYRGKDKPTNVL
SFPFEAPPGIELPLLGDLIICRQVVEQEAIEQGKTLEAHWAHMVVHGSLHLLGYDHIEDDEAEEMESL
ETEIMHGLGYPDPYLAEKDPV
>CORE_REP|Org40_Gene250#
MVMNLLWALLQEMLLAAVPALGFAMVFNVPLRALRYCALLGAIGRGSRMLMMHAGMNIEWASLLAAIL
IGIIGIYWSRWLLAHPKVFTVAAVIPMFPGISAYTAMITVVEISHLGYSEALMETMITNFLKASFIVG
ALSIGLSLPGLWLYRKRPGV
>CORE_REP|Org27_Gene2572#
MKELVLPLPDEAATVALGTTLAQACDRASVIYLYGDLGAGKTTFSRGFLQALGHQGNVKSPTYTLVEP
YALQPLAVYHFDLYRLADPEELEFMGIRDYFAQDAICLVEWPQQGTGVLPEPDLALHLSYQGEGREAK
IEAISAYGSQLLDRIHGSQG
>CORE_REP|Org15_Gene2097#
MSTSTRFAVAIHILTNITLCRGQTVRSEDIARSVNTNPTVVRRILGALAEAGLTYSQMGQGGGALLAR
PAEAISLLDVYRAVEDQPYFTLHRTRPNEACYIGHAITPVLEQEFARVGHALEASLAQTSIAEMAGQV
ELRAGYPFVPCSPQYQADTQ
>CORE_REP|Org15_Gene160#
MSEQNSTEMAFQIQRIYTKDISFEAPNAPQVFQQEWQPEVKLDLDTASSQLADEVYEVVLRVTVTATL
GEETAFLCEVQQAGIFSVAGIEGTQLAHCLGAYCPNILFPYARECITSLVSRGTFPQLNLAPVNFDAL
FMNYLQQQAEGEGAAPHQDA
>CORE_REP|Org24_Gene1981#
MTHSLHNAVPRAALTDTIMAAKIRHNLTFDALAEGTGLSLAFVTAALLGQHALPEQAARTVAAKLGLD
EEAVQLLQTIPLRGSIPGGVPTDPTLYRFYEMLQVYGSTLKALVHEQFGDGIISAINFKLDIKKVADP
EGGERAVITLDGKYLPTKPF
>CORE_REP|Org15_Gene1948#
MSTTPLTLEAARRKIGEIFVYHMPFNRELGLELRRFDDDYVELSFTNQRKLVGNAAQEILHGGVIAAV
LDVAAGLACAGGVLTRLEPLVEEEIAAKLSRMGTIDLRVDYLRPGRGEHFVASASVLRSGNKVAVARV
ELHNHDGLHIATATATYLVG
>CORE_REP|Org9_Gene468#
MKLQLVAVGTKMPDWVQTGFMDYLHRFPKDMPFELTEIPAGKRGKNADIKRILDKEGEQMLAAVGKGN
RIVTLDIPGTPWETPQLAQQLERWKQDGRNVSLLIGGPEGLAPACKAAAEQSWSLSPLTLPHPLVRVL
VAESLYRAWSITTNHPYHRE
>CORE_REP|Org43_Gene866#
MRNPAKQEDLIKTFKALLKEEKFSSQGEIVLALQEEGFENINQSKVSRMLTKFGAVRTRNAKMEMVYC

LPAELGVPTTTSPLKNLVLDVDHNDAVVVIHTSPGAAQLIARLLDSLGKSQGILGTIAGDDTIFVTPS
SGFTAQKLHEAILGVFEQEL
>CORE_REP|Org43_Gene858#
MTAQVISDSAVLVHFTLKLEDGSTAESTRSSGKPALFRLGDGSLSAPLEEQLLGLRAGDKRAFTLQPE
AAFGAENPDLVQFFSRRDFAETGVPDVGTIMLFTAIDGSEMPGVVRAVAEDSITVDFNHPLAGHPVTF
DIEVLEIDPQQEETHANIAG
>CORE_REP|Org15_Gene299#
MNLNATILGQAIAFVLFVWFCMKYVWPPIMAAIEKRQKEIADGLASAERAKKDLDLAQANATDQLKTA
KAEAQVIIEQANKRKAQIMDEAKAEAEQERNKIVAQAQAEIEAERKRAREELRKQVAMLAIAGAEKII
ERSVDEAANSDIVDKLVAEL
>CORE_REP|Org27_Gene1502#
MMLATWLRRFQRSNGDVIFMRAALVVIFAAFGYAKWFDYEAQGLVPLIGNSPLLSWMHQAFGIRGASY
ALGVAEWSFGLLLLAGFWSRRLGLLGAIGSTVTYLTTLTLIFSTPGAWEPSAGGFPAMAGATSFLIKD
LVLLAGSLVLLKADLPAEQA
>CORE_REP|Org48_Gene579#
MPRRRVIGQRKILPDPKFGSELLAKFVNILMVDGKKSTAEAIVYTALETLAQRSGKDHLEAFEVALDN
VRPTVEVKSRRVGGSTYQVPVEVRPVRRNALAMRWIVDAARKRGDKSMALRLANELSDAAENKGSAVK
KREDVHRMAEANKAFAHYRW
>CORE_REP|Org9_Gene1820#
MIHNSLFNPRFGRGLAPALVSTLTELRRCDLPELALGHHPIDGDNIFMDVMTLTTVPAAEKRAEMHQE
YIALHLLISGEERIEYGLAGDWHREHPHAENSDLLLLDIKRHPQTLHMTHGMFAIFFQMEAHKTGCLW
QRPQHIKKAVVRIHHRLLL
>CORE_REP|Org38_Gene2067#
MSIKPPLPPFTRESAIEKVRLAEDAWNSRDPERVSQVYSLDTHWRNRAEFVDGRAAVVAFLQRKWAKE
LEYRLIKELWAFDGARIAVRFAYEWRDDSGNWYRSFGNENWEFNQEGLMINRHACINDMPIREQDRRF
HWPLGRRPDDVPALSDFGF
>CORE_REP|Org9_Gene1848#
MSSVITTRQAGIVDVDAGRNVTVVEPCNLYGCRLGDDVFVGPFVEIQRHVSIGARSKIQSHSFICEYV
TLGEACFVGHGVTFANDLFKDGAPNADPASWGRTRVGDRVSIGSGATILAVEICSDAVIGAGAVVTRN
ITRKGIYAGNPARLLRELP
>CORE_REP|Org5_Gene2494#
MSTSITMIVPIKNTVPGVSNVFSAPGHQGELLMAKPVNNQPEGGKAKKSEGANGNDEPANLNESRASA
RIKALNRQIQALQQKLVELKDSSADPKEIEKQKQLIEAQIKMLQAEIARIQKEEMEKQQQEQMEKAAA
QAGDGVNRPTPLNAVDVYI
>CORE_REP|Org11_Gene381#
MDIRKIKKLIELVEESGISELEISEGEESVRISRAAPAQAYPVMQQAYAMPAQQQPALATAVATAPAA
ETPAAPAAVSGHVVRSPMVGTFYRTPSPDAKAFVEVGQKVNAGDTLCIVEAMKMMNQIEADKSGVVKA
ILVENGQPVEYDEPLVVIE
>CORE_REP|Org38_Gene1454#
MENTRIRVGEAPFNVGDEYQWLAQCDADGAVVTFTGKVRNHNLGDDVSALTLEHYPGMTEKALAEIVA
EARSRWPLQRVTVIHRVGALYPGDEIVFVGVTGAHRGMAFAASEFIMDYLKTRAPFWKREATGQGDRW
VDARDSDRQAAQRWHDAAK
>CORE_REP|Org9_Gene1999#
MSVRIIDKAAEKVVGVRVVGPYPQTIPQGSQQLLAWRQRHGVPFGKWLVLYWDDPAEVAPEKLRADVV
MSVADDFALPAESEGVTVQTLPAGQYAVYHVRISDGDFERVWGEFYQKLLPASGYQPVEGVSYEHYLN
DCEADGYFDLDIYQTVKKW
>CORE_REP|Org4_Gene3894#
MNKRILVAIVIAVVIAVFSALRGNEHRAIGGNPAVVQAPAGQQQSIDRLTQQQTVVSYLQQHQRLPDY
YVTKKQAREQGWDPRSGNLCAVLPGKAIGGDRFSNREGQLPSAGGRVWREADINYQCGRRGADRLLYS
SDGLIFVTRDHYKNFIRVE
>CORE_REP|Org6_Gene1564#
MRLIVLLCAALLSWSAAAAIDTYRFNSVEQEQQYRELTEQLRCPKCQNNSIADSNAIIAADMRTKVYE
LMMQGQSKQQIIDYMVARYGNFVTYEPPVTPATLILWIGPLLFVLIGGAVVILRTRRKPDAAVDDAFS
ERERQRLAALLQETDRKKP

>CORE_REP|Org23_Gene4414#
MNVVTGVGVIIVNPQGEILLGKRCGSHAPFWSIPGGHLDAGETFEQCAQREIAEETGLIIDPPRFVGV
SNNLQTWHAEGKHTVSICLQVDHPGGEAELKEPEKCAEWRWCAPDSLPEPHFEASRTAIRLWLSGQAY
LPTADPSRARAAPPHRYIN
>CORE_REP|Org20_Gene1378#
MTHDNKLQVEAIKCGTVIDHIPAQIGFKLLTLFKLTATDQRITIGLNLPSNALGRKDLIKIENTFLTE
QQANQLAMYAPKATVNRIDNYEVVRKLTLSLPDHIDGVLTCPNSNCISRSEPVASSFSVKPRDGEVHL
KCRYCEKEFEHQVVLQAD
>CORE_REP|Org5_Gene4503#
MKSTDIEQPHAKNLLQLDQQLCFALYSANLALHKVYRKLLSQLELTYPQYLVMMVLWERDRVTVSDIG
ERLFLDSATLTPLLKRLETAGLLVRYRATADERQVIIALTEAGRALRERAQSVPEAVMCATDCSLDEI
VSLKQQLEKLRGSLIDQI
>CORE_REP|Org40_Gene873#
MIKRLLVVAVAAVTLAGCANESMSGDVYSSSQAKQVQNVTYGTLVSVRPVKIQGGEGSNALGMIGGAV
IGGLLGNTVGGGTGRTLATAAGAIAGGAAGNSIGEVAGRTSGYELEIKTDQKENIVVVQKAGDTKFSP
GQRVRMARMGDTITVSPL
>CORE_REP|Org19_Gene1349#
MNYHQYYPIDVVNGPGTRCTLFVAGCVHQCPGCYNKSTWRLNSGQPFTPALEDRIIADLNDTRVPRQG
LSLSGGDPLHPANVPSILTLVKRVRAECPGKDIWLWTGYRLAELDAQQMQVVDRINVLIDGKFVQDLK
DPALIWRGSSNQVVHRLR
>CORE_REP|Org3_Gene479#
MMNNDVLRSVRYMLSINNAKMVEIIKLDDFEVAVSAMDAYVIKEGEPGYEKCPDEVMAHFLNGLVFFK
RGKDDKFPAPAVELPITNNLVLKKLRVAFELKDTDMHQIFTAVDFRISKPELSALFRKEGTKNYRPCG
DQMLRYFLKGLAQRVRGE
>CORE_REP|Org2_Gene3447#
MKINGLMTLLLLSLFSAPLYAAVDYFYLKPLDGEKIKITRQMLEAMPSHTIKTSTNFTPEDTFTGVEF
AELVKAYHLKGQSVRAFAWDDYSYSMPIDEMTKYRVIVAYQRGGKPIDVGELGPFAIIYPRDSYAELN
NLDVNAKTVWQVKRLELK
>CORE_REP|Org28_Gene5100#
MFAILMLFIAPVVSRSLEHVRAGSAGTTVMADCGMEMPMHHGMHSPPPEPEKASQPLMQHGGGHHNMA
MMMDDSACGYCVLLLHAPLLDVSHAPLFWSRLLASRPPPIHYLVPLFAHVVHTELQPRAPPSLSSDLT
INPLPLSVLPLIASTRRN
>CORE_REP|Org28_Gene1706#
MSPLKAGDTAPKFSLPDQDGEEINLADFQGQRVLVYFYPKAMTPGCTVQACGLRDNMDELKKVGVEVL
GISTDKPEKLSRFAEKELLNFTLLSDEDHQVAQQFGVWGEKTFMGKTYDGIHRISFLIDGKGKIEKVF
DDFKTTNHHDIVLSYLQQ
>CORE_REP|Org20_Gene628#
MRMRVWFLLASLILAGCSSHAPPPSGRLADSIVVVAQLNEQLRQWYGTPYRYGGLDRGGVDCSGFVYR
TFRDRFDMQLPRSTEEQTSLGTKVSRDELMPGDLVFFKTGGGENGLHVGIYDTNDQFIHASTSRGVIR
SSLDNVYWKRVYWQARRI
>CORE_REP|Org42_Gene277#
MAEIYQIDNLDRGILNALMDNARTPYAELAKNFAVSPGTIHVRVEKMKQAGIITGARIDVSPKQLGYD
VCCFIGIILKSAKDYPSALKKLESLEEVVEAYYTTGHYSVFIKVMCRSIDALQQVLINKIQTIDEIQS
TETLISLQNPIMRTIVP
>CORE_REP|Org44_Gene555#
MLDKTDRKLLCMLQQDCTQSLQALADAVNLTSTPCWKRLKRLEDEGYIRGRVALLDNEKLGLGLTAFV
LIKTQQHSSEWYQAFVQLTSQMPEVLAFYRMAGEYDYLMQVEVADMKSYDGFYKRLVNGVPGLIDVTS
SFAMEKIKYTTALPVPE
>CORE_REP|Org42_Gene1090#
MDKIDRKILAELQADGRLSVTELAERIGLSVSPCHRRVRALEESGVIRGYRAQLDPGSLGYNFSALVF
VTMREGDRRAVETFENAMMDIPQVVQAQRLFGDPDYLLHVIARDLPAFQQLYDEKLSALPGVQRLSST
LVMKTVVPERSFLPLGK
>CORE_REP|Org42_Gene3323#
MSCIFCDIVAGKAPCHKIWEDDDHLAFLSIFPNTDGFSVVIPKAHHPSYAFDLPDEVLSALMLASKRV

AKQLDRAFDDVGRCGMVFEGYGVDHVHAKLIPLHGTASLEQWRPIESTSPKFFARYEGYISSHDAARA
DDEQLAALAARIRQSAV
>CORE_REP|Org41_Gene2306#
MDQRIIPLSECPQFADVCAAWAFGQWGSQRGGTLERTQLRFAQCSRPSDDHLTLMMIDGDRPVGMASL
WPSDDHQRQDLTPWLAGVFVHPDHRRKGIAHRLEMAIVEAARQRHHSILHLITDKSEALYAGWGWQPL
ERRRQYDEDVVVMVKTL
>CORE_REP|Org47_Gene4834#
MNNEYMQLSSVLNIECTKSSVHCTSKKRALEIISELAAKQLNLPPQVVFDAVLTRERMGSTGIGNGIA
IPHGKLEEDTLRAVGVFIRLDQPIAFDAIDNQPVDLLFALLVPADQCKTHLHTLSLVAKRLADKTVCR
RLRAAQSDEELYQIITE
>CORE_REP|Org43_Gene4857#
MDNQHLRFTKDKFTVTTDPAFFQLEAIHEYLSQSSWAPGIDAETVRISIQNSLCFALLDGTRQIGFAR
LVTDYATFGYLCDVYVLSDYQKSGLGRWLIECCHAHPLMSRLRRIMLVTDSAPWLYQKLGYNPLNRPD
FVWQINRPDIYRKNKSK
>CORE_REP|Org34_Gene4532#
MSLTDAVLMLFIALLLLYSLYDEFGMDLLKGKTRLKVPLKRRHRLDSLIFVGLIAILIYRNVTDNGAV
LTTYLLISLALLAIYISYIRAPKMVFKAHGFFFANVFVEYNRIKAMNLSEDGILAVDLEQRRLLVQVT
HLDDLEKIYHFLLIINK
>CORE_REP|Org8_Gene1413#
MELTTRTIAARKHIALVAHDHRKQALLEWVESHKTILAQHQLYATGTTGNLIQRASGIPVTSMLSGPM
GGDQQVGALIAEGKIDMLIFFWDPLNAVPHDPDVKALLRLATVWNIPVATNRSTADFLIDSPLFKNEV
EIAIPDYQRYLQDRLK
>CORE_REP|Org33_Gene2501#
MTGHQALRCALFGALLSFSAGSFAQQIVTTSDLIQQPGYQASWQNMVKGQARMPGWARKGVGTSTPAQ
NLSWKGKEYLVGNLCKPHDCGNNFLIVAFSADKSQAWGVRVAVEDRPEAVDHPKKYAKYQWLGKPNDD
MKALLKQQFENNPDWK
>CORE_REP|Org21_Gene2401#
MSEMMNENGAPVMEWSYGGRGDDISSSLSLDTVEEQPQGFLMTIDRPVQIFKDALSPMMAFVTLLRPL
YAKTGEGFQFVPVSNPWTDVQRLPPSAGYLGVQVNAGLFFHGAKPTHYKDRDGQMKPIDGGTVLQNIT
VLHQVAQPQGPGEGGI
>CORE_REP|Org20_Gene2998#
MIQLTVNEQPLTFEGDPHMPLLWFLRDEAGLTGTKFGCGIAMCGACTVHLDGVPVRSCMTPISAAVGK
KITTIEAVGATPEGKAVQEAWLDLDVVQCGYCQSGQIMSASALLAQSKNPSDADIDAAMGGNVCRCAT
YVRIRAAIHQAAKALG
>CORE_REP|Org26_Gene1411#
MSEKYVVTWDMLQMHARKLAHRLLPADKWTGIIAVSRGGLVPAALLARELGIRHVDTVCISSYDHDNQ
REMKVLKRAEGDGEGFIVVDDLVDTGGTAKAIRDMYPKAHFVTIFAKPAGRPLVDDYVVDIPQDTWIE
QPWDMGVSFVPPIGGR
>CORE_REP|Org48_Gene842#
MMKKIDVKILDPRIGQDFPLPTYATPGSAGLDLRACLDSAVELAPGETTLLPTGLAIHIADAGLAAVI
LPRSGLGHKHGVVLGNLVGLIDSDYQGQLMVSVWNRGQKHFTIEPGERIAQMVFVPVVQAEFNLVEEF
DSSERGEGGFGHSGRH
>CORE_REP|Org45_Gene116#
MKYQQLENLESGWKWKYLVKKHREGELITRYIENSAAQEAVNELLKLENEPVKVLAWIAAHMNPELDN
RMKQTIRARRKRHFNAEHQHTRKKSIDLEFLVWQRLAALARRRGVTLSETVVQLIEDAERKEKYANQM
SSLKEDLKAILGKDPK
>CORE_REP|Org44_Gene841#
MFRGATMVNLDSKGRLAVPTRYRELLNEESQGQMVCTIDLHQPCLLLYPLPEWEIIEQKLSRLSSMNP
AERRVQRLLLGHASECQMDSAGRLLLANTLRQHAGLTKEVMLVGQFNKFELWDEQTWYQQVKDDIDAE
QSTQEPLSERLQDLSL
>CORE_REP|Org23_Gene3463#
MGLSTLEQQLTEMLSAPVEALGFELVGIEFIRARQSTLRIYIDSDNGINVDDCADVSHQVSAVLDVED
PITVAYNLEVSSPGLDRPMFTAEHYTRYLGEEVSLVLRMAVQNRRKWQGIIKSVEGEMITVTVEGKDE
VFALSNIQKANLVPHF

>CORE_REP|Org2_Gene4349#
MQIWVDADACPNVIKEVLFRAADRTAITVTLVANQPLRTPPSKYIRSLQVAAGFDVADNGIVRRCEAG
DLVITADIPLAAEVIEKGAVALNPRGERYTPDTIRERLNMRDFMDTLRASGIQTGGPNALNQRDRQQF
ANELDKWLLQAKRAQ
>CORE_REP|Org34_Gene2901#
MSKESLVLAFPPAAPEESLAYLAAKLSRYADAWDVAEDLRNGVDGMVVIDTRAEALYAAGHIPGAFSL
PHRLMDEAGTAHLDRQKVYVTYCDGIGCNGSTKGAYKLATLGFRVKELIGGLDFWLRDGHPLAIGEQP
GSLRDRAAVADCGCA
>CORE_REP|Org33_Gene424#
MQEGQNRKTSSLSILAIAGVEPYQEKPGEEYMNDAQLSHFKRILEAWRNQLRDEVDRTVSHMQEEAAN
FPDPADRATQEEEFSLELRNRDRERKLIKKIEKTLKKVEDDDFGYCESCGVEIGIRRLEARPTADLCI
DCKTLAEIREKQMAG
>CORE_REP|Org12_Gene3842#
MYKTILVPVDITEPELTQQVIPHVNALARLEDSHVHFLAVIPSRATYAAFGFAATAAVETKDETIAVA
TEGLAKAAKQFSVPEDRVTTHVAVGDPKDQILELADALNAEIIVMGSNRPSAMTYLLGSNATAVVRHA
NCPVLVVRESANAAQ
>CORE_REP|Org48_Gene200#
MAQVGIFVGTVYGNSLLVAEEAQNILSEQGHEVKLFEEGTLEAWQFYRQHYALVITSTTGQGDLPDSI
APLFHAIRDQVGYQPELRYGLIALGDSSYDNFCGAGRAFDALLQEQGATRVGEVLEIDAMEQPEPEVA
ACPWVEQWGTLLQS
>CORE_REP|Org44_Gene345#
MQVILLDKVANLGSLGDQVNVKAGYARNFLVPQGKAVPATKKNVEFFEARRAELEAKLADVLAAAEAR
ATKINELGSVTIASKSGDEGKLFGSIGTRDIADAVTAAGVEVAKSEVRLPNGVLRTTGEHEVHFQVHS
DVFAQLNVVVVAEA
>CORE_REP|Org8_Gene50#
MRTVLNILNFVLGGFFTTLGWLIATVFSVLLVITLPLTRSCWEITKLSLVPFGNEAIHVDELYPEKSN
ALLSAGGSLLNIIWLVLFGWWLCLSHIAAGIVQCVSIIGIPVGIANFKIAAIALWPVGRRVVSVEMAQ
QARIENARRHYHQR
>CORE_REP|Org20_Gene1815#
MNLSNPEVTIRRINGDDKAQWLALWQGYLDFYRADVAPQVTDRTFERLGQDEQVYGLVAQDADGQLLG
LMNLVFHPSTWSAVGYCYIEDLYVSPQARGHKVSEKLFEQAYRLAETRGSDRVYWMTQEYNAPARSLY
DKIGRRSSFIVYSR
>CORE_REP|Org47_Gene4352#
MDMISVRQAPELAPRAIAYFQRHWATAETLMMYEDAINRSLGAANPLPQWYLLMENDQILGCAGLITN
DFISRGELYPWLCALYVEETQRGRGYGAKLIEHVAAESRRLGFPQLHLCTDLEGYYERSGFVYNGLGY
HPWGEASRVYSRVL
>CORE_REP|Org27_Gene2112#
MTPLSPLPYLQPGDRALLFDGECNLCHGLVRYLIRADRQRCILLATVQSVEGQAILQALGLPTDRFDS
VVYVEQGCYWLRSAALFQALRQLGWPYRVLALARYLPPRLADKVYDAVAGNRYRLFGRNDGTGLPGAD
QPGRYLPRRREPPA
>CORE_REP|Org12_Gene2908#
MHESLTIALLQARETAMGFFRPILKSHNLTEQQWRIIRVLANSRSIEFHELAAETCILRPSLTGILSR
MERDKLIFRLKPVNDQRKLYVSLTQQGQDLYEVARHQVEQGYAEIEAAFSRQKMDQLMTLLDELITLG
DSLPANVAAHPAKQ
>CORE_REP|Org43_Gene792#
MQLQNKSLIERIVHAVGFEAIAVMVCAPLGAWLLNRSMVQVGALAVMLSTVAMLWNMVYNTVFDRLWP
VSRVVRNLKVRALHAVGFEAGFILIGVPIAAWMLSISWAQAFMLEIGFFLFFLPYTMAYNWLYDTLRQ
RWFEARQPINSGAK
>CORE_REP|Org43_Gene476#
MADKFHILLLNGPNLNLLGTREPEKYGSTTLTEIVNGLENQASALDITLSHLQSNAEHQLIDRIHQAR
GNTDFILINPAAFTHTSVALRDALLAVQIPFIEIHLSNVHAREPFRHHSYLSDVAVGVICGLGADGYA
FALQAAVNRLSKTH
>CORE_REP|Org7_Gene4762#
MKTVKRTGIALAIALTFPLALPAATAAQTSLTNSKAATMTEKHGQFIAVGKVVQVTFGDFAFKLDFTD

DKTMTFTGIGEASQGITDTVQYTAVEIRPKVYMVYWHEPQSGDNVTHIEDFERGEVYTNIAAKDGSFT
HLKGQLKIVGHSGN
>CORE_REP|Org34_Gene2179#
MKQATFYLLDNAEPSGALSAHEAVACAVAASGFRSGKRVLIACESQEQAQRLDEALWQREPHEFVPHN
LAGEGPHYGAPVELCWPGKRGNAPRDLLIALLPQFADFATAFHEVVDFVPYEDTLKQLARDRYKAYRS
VGFHLTTATPPTH
>CORE_REP|Org44_Gene565#
MHCPFCAAVDTKVIDSRLVGDGSQVRRRRQCLVCNERFTTFEVAELVMPRVIKSDEVREPFNEDKLRR
GMLKALEKRPVSSDDVENALNHIKSQLRATGEREVPTKLVGNLVMDALKKLDKVAYIRFASVYRSFED
VREFGEEIARLQD
>CORE_REP|Org22_Gene1878#
MKKDHPEDVTLLRTQLMSLVRRLRRESRSDEKSWAQLMLLGAIDRHGGEATPSLLAESERMRSSNLAA
ALRELEADGLLVRTPDAEDKRRVRVRLTPAGLGLLQQSRSRREAWLLAAMESCLTEREQALLIEAGAL
MARLAAAPSTETE
>CORE_REP|Org1_Gene1861#
MFKPHVTVACVVHAAGKFLVVEETINGKALWNQPAGHLEADETLVQAAERELWEETGIRATPQAFLRL
HQWIAPDRTPFLRFCFVIELEQPLPTEPHDSDIDRCLWLSADEILQAPNLRSALVAESIRSYQQPERY
PLSLVGSFAWPF
>CORE_REP|Org32_Gene2470#
MKSQSPLITLRDLAQDAVEQAAQQLGQVRQAQQAAEQQLSMLLNYQDEYRQKLNHTLCDGMDSSSWQN
YQQFIGTLEQAIDQHRQQLLQWGQKVDHAVKQWQDKQQRLNAFETLHTRALNAEQQQENKRDQKLMDE
FAQRSAQRNINP
>CORE_REP|Org40_Gene225#
MSYKRPESILVVIYAKSSGRVLMLQRRDDTEFWQSVTGSLEQDESPPHAARREVMEEVGIDIEAEHLP
LFDCQRCVEFELFVHLRHRYAPGTTRNKEHWFCLALPEERDPVITEHHAYQWLEAAEAVKLTKSWSNQ
QAIEEFVINSVQ
>CORE_REP|Org19_Gene683#
MTDNNIALKKAGLKVTLPRLKILEVLQNPECHHVSAEDLYKKLIDMGEEIGLATVYRVLNQFDDAGIV
TRHNFEGGKSVFELTQQHHHDHLICLDCGKVIEFSDESIEVRQRDIAKQHGIKLTNHSLYLYGHCETG
DCREDETLHDKK
>CORE_REP|Org25_Gene3515#
MDSLITFEKLTAKHLPYLYEIRFSVEENLLHPHQIQYLQRKQALEDINQGGGWICKYGEDYAGVGFGL
FIPEPLIGGLFVKPEYQSMGMGSALLARVTAWMFEHGAEAIHLTTDPGSKAEGFYQHHGWDVIGRDEF
GQTELVKRKEGE
>CORE_REP|Org49_Gene714#
MSQLAFWQQKTLAEMSEQEWESLCDGCGQCCLNKLIDEDTDEIYFTNVACNQLNIKSCQCRNYERRFE
LEEDCIKLTRENLTTFDWLPPTCAYRLIGEGKPLFPWHPLLSGSKAAMHGERITVRHIAVRESEVVDW
QDHILNKPDWAR
>CORE_REP|Org14_Gene935#
MGLFNFVKEAGEKLWDTVTGNASAEDQGAKLKEHLDKSGLPGTDKVNVQVIDGKAVVTGDAVSQELKE
KILVAVGNVAGISGVEDKVAVTQPDAESRFYTVKKGDTLSAISKEMYGNANQYNKIFEANKPMLSSPD
KIYPGQVLRIPQ
>CORE_REP|Org21_Gene243#
MTWIILAVLIVVFIIGYRILTSDTRKAIDSLAHLLRVKPMLIESMIQEMGGRQSQTFIRMLNNGYTEE
MHQAAYLLFIYLTFIKQADDEQIALWRDVLLRAGLSPELHAEHTEAALFYFAELDIDAFELAQFRRAY
NERFNREALAHG
>CORE_REP|Org18_Gene1363#
MTVNAFIGSWALVSSAFENQDGELNYPLGEQVLGRIHYEANGTMAAQLYSAVRPRFAADDLAQGSERE
IRAAFINMICYFGRYQVEESEQRVVHQVEGCSFPNWVGSRQVRFYAFSGDRLTLRTVPLQLGNGVQVG
ELVWQRTGASL
>CORE_REP|Org10_Gene731#
MHALESRQRWMSVLAHSRPEQLRSHWQALNLSPGYRSIRAPEIGLAQLQGRMGATGRRFVLGDMTVTR
AVVQLDNGGYGYSYIAGRDKAHAELCALADALLQQPEHGERLQQQLIAPLAALQHEQRQLRARAIAAS
RVDFFTLVRGD

>CORE_REP|Org29_Gene3296#
MNTISTLTAADLATAFTIEQASHAFPWTETTFASNQGDRYLNLKLSADGEMAGFAITQIVLDEATLFN
IAIHPQHQRRGLGRLLLNAVIEQLESRGVVTLWLEVRASNQAAIALYEDLGFNEVTVRRNYYPSAQGR
EDAIVMALPLA
>CORE_REP|Org1_Gene737#
MITIRARAAEDNAQLADIWLRSVRATHHFLTEDNIAQLFPLVLNDYLPAVNVWVAEERPGHPCGFIGL
NGNKVEMLFIDADQRGKGVGKALLTHAETLHDELQLDVNEQNPQASGFYRHYGFVITGRSPLDGQGNP
FPLLHMKLEKR
>CORE_REP|Org49_Gene4025#
MKIGELAQRAGMAASAIRYYEQLGLLPKPVRGVNGYRVYGDSALERLHLIQIGQNLGFSLQAIQRVLA
LQGSAYQDGLIRGVDERLAEIELMMATLNEQRETLLTTRLTLLESGVAGLCQAKGEKQADASPAWPAK
LARMRNRIDTE
>CORE_REP|Org21_Gene2464#
MNMLLLLIAAGMGLVVQNLLMVRMTESVSTILITLVINSSVGLLLLVGLLLAKNGLGAVAEVTGAARW
WMLLPGLLGSLFVFAGILGYQKLGAAATISILVASQLCMGLLVDVYRAGPAALRENLPALFGALLLVA
GAYLVAKRSF
>CORE_REP|Org6_Gene515#
MLSPATHWRSAAADTFALVVYCFIAGMAIEVLISGMSFQQSLSSRLLSIPVNILIAWPYGRYRDLFIR
TARRCPRGQFLLRNLADLLAYVSFQSPVYAAILWSVGADGQQMLAAVTSNALVSMAMGVVYGYFLEYC
RRLFRVADYV
>CORE_REP|Org44_Gene2158#
MNTPEQRQQIADFIGKQHVLTLCAGDGLDMWCANCFYVFDAAAMALWLMTEPHTRHGGLMLNNGRVVG
TIAPKPKSIALIRGVQYRAEAVLLSGEEADAARARYCKRFPIARAMKASVWRLDLHEVKMTDNTLGFG
KKLHWARSIL
>CORE_REP|Org43_Gene4954#
MAHITLISGSTLGSAEYVAEHLAEKLEDAGFSTEMLHGPELDELPLNGRWLVVSSTHGAGELPDNLQP
LLEQIAEQQPDLSEVQFGAVGLGSSEYDTFCGAIKQIDDLLIARGAKRIGDRLEIDVTEHEIPEDPAE
EWVKNWINLL
>CORE_REP|Org39_Gene2286#
MIVNCDHDNLDAWLALRTALWPSSSPEDHRAEMREILASPHHTAFMARGLDGAFVGFAEVALRYDYVN
GCESSPVAFLEGIYTVERARRQGWAARLIAQVQEWAKQQGCSELASDTDIANLDSQRLHAALGFAETE
RVVFYRKTLG
>CORE_REP|Org24_Gene706#
MSQAEYSRIGGWLLAPMAYLIVTLLSASLMLLLYAMAIFMPESRDYLLTNAQAFTLQWYFSVLTTLLM
WCFTLWLLWLFCQRSQRFPKLFLLWLLITVLLAVKAFAFAPVPDEVAVRSLGWPLLMAALLVPYIKRS
QRVKGTFTER
>CORE_REP|Org15_Gene394#
MSTTTEVIAHHWALAVFLVVAIGLCGLMLLGAFFLGGRARARAKHTPFESGIDSVGTARMRLSAKFYL
VAMFFVIFDVEALYLYAWSVSIRESGWVGFIEAAIFILVLLAGLVYLVRIGALDWTPARSQRRSKPST
ITNTNSHPQ
>CORE_REP|Org1_Gene976#
METQRGFSLIELMVVIAIIAILSAIGIPAYQRYIQKAAMTDMLQTMAPYKLAVELCVLDEGAPAGCEA
GSKGIPTGGASRYVSGVKVVKGVITLTGAQTLQGLAVALTPKSNADGLTHWTRLCSSENATLVEVCRE
VFRFDDAAE
>CORE_REP|Org39_Gene1653#
MQGVKRKLVYVTAYELIGMTISALGLALLSGHAPSSTGPLAVVITTLAVSWNFIYNYLFEWWESRQAS
RTRTLKRRILHAVGFQLTLVVYLIPLIAWWMGITLWQALLLDMALIVIIPCYTFLFNWAFDKLFGLPA
SALPAGESA
>CORE_REP|Org45_Gene757#
MIALIQRVLNASVTVGGETVGKIGPGLLVLLGVEQGDNEQKAQRLCERVLGYRIFGDENDKMNLNVQQ
AGGSVLVVSQFTLAADTQKGMRPSFSRGAAPLVADRLYQYFVGQCRERGVETQTGEFAADMQVALVND
GPVTFWLQV
>CORE_REP|Org33_Gene4450#
MYSHYPAHYTFDSDSDAWQIGFHDFPEWQSACYKREDVELEAQESLLAAIAAAMDEGLPLPAPSLLQT

DDLRVHLPALVALKIELHNAMLRKNTGKAELARKLGFNSGQMERLLDLGYASKVEALEQALYLLGYEV
RVTISEVCQ
>CORE_REP|Org27_Gene1375#
MASKRDWLLQQLGITQWTLRRPGVLQGEVAVSLPPEARLLVVAQTLPAPDDPLFCDVLRSLGLTPAQT
YSLTPEQAAMLPEETACNSWRLGVAEPLAVAGAQLHSPALAELSQDAGAKRALWQQICHHEHDFYPDG
GRPGHGLHH
>CORE_REP|Org39_Gene132#
MAYKHILIAVDLSPESKILVEKAVSMARPYNAKVSLIHVDVNYSDLYTGLIDVNLGDMQKRISEETHQ
ALTELSQNAGYPITETLSGSGDLAQVLVDAIKKYDMDLVLCGHHQDFWSKLMSSARQLINTVHIDMLI
VPLRDEEDE
>CORE_REP|Org31_Gene3127#
MLAPHPFGREVTAEALIATFSTLKQWEDRYRQLIMLAKRLPPLPEALRSEEMALSGCENRVWLGHQLL
EDGTLHFYGDSEGRIVRGLLAVLLTEVEGKTPQQIAALDPLALFDRLALRAQLSATRAGGLAALAAAV
KAIAARYA
>CORE_REP|Org42_Gene1556#
MFDSILVVCVGNICRSPTGERLLKQHLPDKEIASAGIGALVGKTADKSAISIAEKHQLSLEGHEARQL
TKEMCREYSLILVMEKGHIDAVCRLAPEVRGKTMLFAHWLGQKEIPDPYRKSAEAFEFVYRLLDDAAQ
KWAQALNR
>CORE_REP|Org29_Gene1162#
MLQEIMPFVSRHPILSLAWIALLVAVIVMTFKSRFSKVKEIARGEAIRLINKEEAVVVDTRSRDDFRR
GHLANAINLTASEIKNGSLGELEKHKAQPIIVVCANGTASREPAENLSKAGFEKVTMLKDGISGWSGE
NLPLVRGK
>CORE_REP|Org13_Gene3376#
MDQQFAGLGVLFVAGFGPITRESDESKAFYVEALGLPLKPMPGNETYLLSEQGALDGVKHFALWPLAQ
AAQSCFGDDRWPADLAVPQAWIEFDVADMAAATQGLVDRGYRLLVANREEPWGQSVTRLLSPEGLLVG
VTYTPWLR
>CORE_REP|Org12_Gene92#
MRLNTLSPAEGAKHAPKRVGRGIGSGLGKTGGRGHKGQKSRSGGGVRRGFEGGQMPLYRRLPKFGFTS
RKAMITAEVRLSELALVEGDVIDLNALKAANVVGVQIEFAKVVLSGEVARPVTLRGLRVTKGARAAIE
AAGGKIEE
>CORE_REP|Org8_Gene1202#
MENLAQLLDKLLETLQALNGVLEEEHDLLCSGQLPGVALQRVTDAKSQLLATVAYLEQQRLGQEKTCG
QRAPYASQAPLADRWQRVQLLSQTLREKNQHNGLLLNQQIDHNAQALAILSKNNKSLYGPDGQSHAGS
LLGRKIGV
>CORE_REP|Org6_Gene1672#
MKNSPRLAAGAMMAALLLSGCVAPGHQAVSPTPACRTGDPLVQTTLYFGLNRPAGPAITAAEWQTFVD
SQVTPRFKDGLTVFDAKGQWLGHDGKLARENSKALLLIHAPGKESEANIEALRSGYKQQFAQDSVMRV
DAPVCVAF
>CORE_REP|Org31_Gene1963#
MDDIDRQILTLLAQDARASLKTLSAQVGLSSPSTSERLRRLEESGVIQGYTLNVNLQAVGYAFESLVR
IKPLPGMLKKVEQLIQAIPEVVECDKVTGEDCFIVRLVAHSMAQLDHTLDRLAEHAQSNTSIVKTTPV
KRRLPPLL
>CORE_REP|Org49_Gene2501#
MPGIVITGHGGFATGLLQAVEQVVGPQRHCAAVDFPEQMSTAQLNDALRSALAAVAQPDGVVFLTDML
GGSPFRSACELADAQGDCEVLTGVNMQLAAEMMLERDGLSLDEFREVALACGKRGLTSLWHERRRVKC
EDVPADGI
>CORE_REP|Org16_Gene3072#
MSALSRCAFSEGSVALPEGYADRTVNVLLAGDDVSPSVNISRDALQPEENLEGYVTRQLDALAQGLKG
WAFKSREPATLGDGLAAGEWVRASYLRDGKRIWQNQAVFALAEGRVLVFTLAMARKLTPQDDALLQQV
LSSYRAA
>CORE_REP|Org24_Gene160#
MESTLGSDLARLVRVWRALIDHRLKPLELTQTHWVTLHNINRLPPEQSQIQLAKAIGIEQPSLVRTLD
QLEEKGLITRHTCANDRRAKRIKLTEAADPIIREVDSVITSTRSEILSGITADEVHLLVGLIGKLEQN
ITELQNK

>CORE_REP|Org46_Gene2863#
MKHYAALLFASALLTGCATQAVPPQQAELPPPSRLMLYQNIPDTPYATVVVVRDSGMLAGSCRTGVYV
NGEFAASLEAKEKAEFRVPPGNVSVSIGQDMIENHLCIWRETGGSIPMTLRAGESRYLRIAGDRQRGF
VLQAGRP
>CORE_REP|Org44_Gene1295#
MFKIGQLAKLAEVTPDTVRYYEKQGMMDHNVRTEGGYRLYTEQDLQRLRFIRYAKQLGFTLETIAELL
SIRVDPEHHTCQESKSIVDARLSEVESKLAELTRMRESLKRLSDACCGTAHTSNYCSILEALEQGASD
EKGKKGC
>CORE_REP|Org25_Gene312#
MRNYDLSPLLRQWIGFDKLASSMGGQEPQGFPPYNIEKSDDNHYRISLALAGFRQSELNIEVEGPRLT
VSGKPTPPEKQVEYLHQGLVCKEFQLTFTLAEHLQVSEAKFENGLLHIDLVRQVPEALQPQRIAIGAT
PELEAK
>CORE_REP|Org15_Gene207#
MTQQRPLPSGELVLRTLAMPADTNANGDIFGGWLMSQMDIGGAIQAKEIAEGRVVTVRVDGMTFLKPV
AVGDVVCCYAHCIRTGRSSITINIEVWVKKVSSAPIGQRYRATEAVFTYVAVDAEGNSRALPEGKMNF
RVGFEE
>CORE_REP|Org41_Gene2048#
MRKTTVAALAALALAGCSMKPHTAVTPGDLLHHNFVLQSVDGETAKSPAGGGLLNLEFGESLHVSGTM
CNRFFGQGQLRDGVLTVKPLATTRRLCPDEQRNRWDRVIGTVLENGAEVTLNAQQLTLNGSGHTLIYT
LRDWVY
>CORE_REP|Org49_Gene1534#
MYKTILVPIDIEEDLLTEHALKHVEYLAKLSGAKVHFFHALPDASAFVTAYSFGLKEFENQAEVKAVD
KLKKIMSEIDLPLDRLNYTVSFGSARDQVLELAEEIDADLIIIGSRRPSVKTYLLGSNAAAIVRHANI
SVMVVR
>CORE_REP|Org28_Gene603#
MARVRGRSRRELKSEINIVPLLDVLLVLLLIFMATAPIITQSVEVDLPDATDSKTVSSDDNPPVILEV
SGVGQYTLVVDHNRMELLPPEQVAAEAKSRLAANPKTVFLIGGAKEVPYDEIIKALNILHQAGVASVG
LMTQPI
>CORE_REP|Org35_Gene426#
MKTFTAKPETVKRDWYVVDADGKTLGRLATELARRLRGKHKAEYTPHVDTGDYIIVLNADKVAVTGNK
RTDKVYYHHTGHIGGIKQATFEEMIARRPERVIEIAVKGMLPKGPLGRAMFRKLKVYAGTEHNHAAQQ
PQVLDI
>CORE_REP|Org43_Gene294#
MSSTLAVISLFGSSFLSATLLPGNSEIVLVALLTQSRVSPELLVLAATLGNTLGGLTNVIIGRLLPAL
KPQRGLATALGWLQRFGPAALLLSWVPVVGDLLCVLAGWLRMPWGSVALFLCIGKALRYIVLAMITVQ
GIAWWQ
>CORE_REP|Org8_Gene149#
MAKKVQAYVKLQVAAGMANPSPPVGPALGQQGVNIMEFCKAFNAKTDSIEKGLPIPVVITVYSDRSFT
FVTKTPPAAVLLKKAAGIKSGSGKPNKDKVGKVTRAQVREIAETKAADMTGSDVEAMTRSIEGTARSM
GLVVED
>CORE_REP|Org40_Gene4106#
MKKILLLVCIPLSLSLSLPLAAQADVSVDINVPGVSLHLGDQDRRGYYWDGYDWRPPQWWHAHQGRGL
GERNARGLYWDGGRWQPSPPRGYEHRQPARSGNPFHGDRDNRDHDRRHDDRGDHDRRDGGRGQPYPPG
NAGPHR
>CORE_REP|Org32_Gene2168#
MQLTSFTDYGLRALIYMASLPQDKMTNISEVTEVYGVSRNHMVKIINQLSRVGFVTAVRGKHGGIRLG
KPAESIRLGDVVRALEPLALVNCSSDFCHITPACRLKQVLQQGVQNFLEELDKHTLADMVEDNPPLYK
LLLVE
>CORE_REP|Org16_Gene2579#
MQIRPYQETDRPFLRTLYLASRKAAFGWRDTSNYQLEDFDGATLGEAIWVAEDGGTLLGFVSVYREDN
FIHNLYVDPHQPPRGVGSALLQAAQATFTATGSLKCLVKNENALAFYRKHGWRIISTGNDGEEDYYLM
HSPAR
>CORE_REP|Org47_Gene430#
MEIRVFRQDDFEEVITLWERCDLLRPWNDPEMDIERKLNHDPELFLVAEVGGEVVGSVMGGYDGHRGS

AYYLGVHPDYRGRGIANALINRLEKKLIARGCPKIQIMVREDNDTVVEMYEKLGYEIQGITSLGKRLI
EDQEY
>CORE_REP|Org14_Gene867#
MRQRIIVCPLIENDNAFLLCKMAAHKGVFPGQWALSGGGVEPGEQIEQALRREVREELGAALILDSIT
PWTFRDDVRIKTYADGSREQIYMIYLIFDCHAANRDVTINDEFDDYAWVPRERLADYDLNEATRFTLQ
QKGLL
>CORE_REP|Org32_Gene2192#
MANRELLEEQREETRLIIEELLEDGSDPDALYTIEHHLSAEKFEVLEQAAVEAFKLGYEVTDAEELEV
EDGSMVMCCDVISEVGLNAELIDAQVEQLVALAERCGVNYDGWGTYFEDPNGEDGEDDEDGDYIDEDD
DGKRH
>CORE_REP|Org32_Gene1390#
MTTQTTARPSAAAVAVYEWLNPIPYGFFTAALIFDIIYACTANIQWVNGASWLIAIGLIFAIIPRLIN
LVQVWFGSGRLLGSSVKLHFWLNLLAIVLAIINAFVHSRDAYAVVPQGLVLSAIVVALLSLANILLAL
GARAK
>CORE_REP|Org45_Gene2265#
MTVMTLPDAQQLLAMPDSDYMNSVQRAFFRQLLQDERQKLLLHIDELKKEIDGGEATGDEADKAAREE
DLRLLFRQLDRESRLLPKIDAALARLQNGEYGYCRETGEPIGLARLLLRPTAELSIEAKTAQEMREPH
MRKGG
>CORE_REP|Org24_Gene504#
MTVERTFSIVKPNAVANNDIGAIYARFERAGFKIIAAKMLRLTREQAEGFYAEHKGRPFFDGLVEFMT
SGPIMVQVLESENAVQRNRDIMGATNPDNALAGTLRADYADSFTANAVHGSDSVESAQREIAYFFNES
EICAR
>CORE_REP|Org1_Gene1397#
MTDETFGGAKIALLCDDRLLVYQRDDKPGIPWPGQWDLPGGGRENGETPLQCVQRETQEEFGVTIAEC
QVVWRRRYDGVLPGSPPTWFMAGEIAPAQIAAIRFGDEGQRWRMMPVAQFIEHPQGIEHLRRRVAAYW
RRRGG
>CORE_REP|Org41_Gene623#
MSNRTIIAFDFGTKSIGAAVGQELTGSARALPAFKAQDGSPDWLKIEKLLKEWQPDLVVVGLPLNMDG
TEQPVTAQARKFANRLHGRFGIQIDLHDERLSTVEARANLFDRGGFRALDKGSVDSASAVVILESWFE
RQLG
>CORE_REP|Org42_Gene1749#
MSLALRRVAPSLLTRLQVPIQVALYAALFLIADRLVQQFHLPLPANIVGMLMLLALILLRILPLNWVK
AGSRWLLAEMLLFFVPAVVAVVNYAQLLMVEGWKIFLVIAVSTMLTLGATGLVVDRVYRLEIWLQRRK
QRHE
>CORE_REP|Org46_Gene462#
MAMRLNEDLDDSGELHEINVTPFIDVMLVLLIIFMVAAPLATVDIRVDLPASSAKPQPRPEKPVFLSV
KADKQLYVGDQPVNADQLTSVLDQRTQANKETTIFFQADKSVDYETLMSVMDTLRKAGYLKVGLVGME
GAAK
>CORE_REP|Org5_Gene67#
MNTVCAACNATNRVPEERLADNAKCGRCGHELFDGEVINATAATLDQLLQDDLPVVVDFWAPWCGPCR
SFAPIFEDVAEERAGKVRFVKVNTEAEPELSARFRIRSIPTIMVFRQGKMVDMLNGAMPKAPFDNWLN
ELV
>CORE_REP|Org42_Gene3423#
MGYQNVLVTVAVAPDSHRLVEKAVSIVRPYGGSITLLSTLANPEMYNNFAGPMLGDLRSLMEEETRLF
MAELRQRAGYPIADALIVHGELGDSLEYASRRQPFDLLICGNHRDGMMNKVSCSAARFINISHIDVLI
VPL
>CORE_REP|Org46_Gene2079#
MKKGVLLNSDVSAVIARLGHTDQLTLCDAGLPIPAATQRIDLALTQGVPTFMQVFAAVTQEMQVESAI
LAEEIVKQNPSLHEALLAELTALGQRQGNTISVRYISHQAFKAQTEHSRAVIRSGECSPYANLILCAG
VTF
>CORE_REP|Org32_Gene296#
MAMTYHLDVVSAEKQMFSGLVQKIQVTGSEGELGIFPGHAPLLTAIKPGMVRIVKQHGEEEFIYLSGG
ILEVQPSVVTVLADTAIRGTDLDEARALEAKRKAEEHIRSSHGDVDYAQASAELAKAIAKLRVIELTR
KAM

>CORE_REP|Org48_Gene1272#
MDITVTDAIDEQTLDAIRQGLRAYNLPHIDASHRKPLSVYARDEAGTVIGGLTAETWGNWLSVEWLWV
ADTQRGSGLGGRLMRAAEREAQARGCRYARLDTFSFQARPFYEKLGYQLQMTLKDYPVEHECYFLTKT
LTD
>CORE_REP|Org16_Gene4356#
MAEQPQVRAQAQVRAQGPAKGVLDIGQRIITILVGMVETRVRLAVVELEEEKANLIQLLIMAGMTLLF
TAFGLMSLLILIFWAIDPVYRLVALGATTGMLLFLAVVGTIWTLVKARRTTLLGATRKQLELDRAELE
REP
>CORE_REP|Org38_Gene2629#
MTVRPFNLQQLDHVVLRVRDMQNSLRFYTQVIGCDIAKQRPDLGLVHLRAGASMIDLVDVNGVLGKKG
GEAPDLHRQNVDHVCLRIDPFNEDALLTYLRSQGIDADPAESRYGAEGDGPSIYFSDPDGNRVELKGP
ALD
>CORE_REP|Org26_Gene3611#
MELKIDKVIETVLYVSDIERADAFYRQVLKLPAMVANERFRAYNVGDRSVLLLFIEGDSLRGAQYLTG
FIPAHDGVGPAHIGLAVAKEQLPHWERHLVANGVEIEGRMRWEHGGESIYFRDPDAHLLELVTPGIWA
NY
>CORE_REP|Org14_Gene1668#
MANLPDKDKLVRNFSRCLNWEEKYLYVIELGAKLPPLDEAERQAGNLISGCQSQVWIVMRRDEQGQVE
FHGDSDAAIVKGLLAVVFILYRQLTPQQIVDLDVRPFFSELALSQHLTPSRSQGLEAMIRAIRSKAAQ
LA
>CORE_REP|Org39_Gene662#
MKLWKRETSLEQLNRAGDGCMVSHVGIEFTQLGEDFLEATMPVDGRTRQPFGLLHGGASVVLAESMGS
MAGYLCSEGEQKVVGLEINANHLRAVFDGQVRGVCRALHVGRRHQVWQIEIFDARDRLCCTSRLTTAV
ID
>CORE_REP|Org15_Gene1625#
MIKSAITHPPLLAALAQCGHKTQVLIADGNYACVTHAPKDATVVYLNLAPGTLAAPPILEKLLACINV
ESAALMACPPDFTNTIEAEYRQLLPEHCPIEHLPREAFYAAVKSDRTLLVIASGERRRFANLLLTVAP
VV
>CORE_REP|Org26_Gene3680#
MTLFILLRDTTVIGKIRSSCRLLSTVFVLFVGLSSQQALAHAHLKVETPKADASVSPAPKALTLSFSE
GIEPNFSGVKITGPDNAVVKTGKLQLDPNNNTQVNVPIEGELSAGKYNVSWHVVSVDGHKTKGQYSFT
VN
>CORE_REP|Org20_Gene493#
MKPAARRRARECAVQALYSWQLSKNDLADVEHQFLTEQDVKDVDVAYFRELLSGAAVNAGMLDELMAP
YLSRQLEELGQVERAVLRVALFELKMREDVPYKVAINEAIELAKTFGAEDSHKFVNGVLDKVAPTLRK
KK
>CORE_REP|Org46_Gene927#
MSMLKEFREFAMRGNVVDLAVGVIIGAAFGKIVSSFVADIIMPPLGLLIGGVDFKQFHLVLREAQGAV
PAVVMNYGSFIQTVFDFVIVAFAIFLAIKLMNKMRRKQEEAPAAPPAPTAEEKLLTEIRDLLSQQQQP
KL
>CORE_REP|Org43_Gene408#
MSDPKQPSGEGKESVDDLWADAFNEQQSSEKSGASTEGVFKSLEAQDALGSLQDIDLILDIPVKLTVE
LGRTKMTIKELLRLSQGSVVALDGLAGEPLDILINGYLIAQGEVVVVADKFGVRITDIITPSERMRRL
SR
>CORE_REP|Org5_Gene3268#
MYSDNEKKFYIILNRNHEPATLFNASCHLTAGITDLIEQRQFHHYPSSLDGVSANMSHYPIVILQAKN
SSQLSNLILKCKEEGVLSNFFTTTMLSHSAEQQIADTANTPYEQLDFVAVALYGDAEQLKPLTKKFSV
YR
>CORE_REP|Org47_Gene438#
MWHQQTLILSAKARGFHLVTDEIVNQLTHLHRLQTGLLHLLLQHTSASLTLNENCDPTVRADMEQHFL
RQVPENAPYQHDYEGPDDMPAHIKSSLLGASLTLPVRHGRLMLGTWQGIWLGEHRIHGGARRIVATLQ
GE
>CORE_REP|Org8_Gene1715#
MGDSSKDGVILLSRILLMVLFIIFGWMKLVNFGATVTAMEGYGTPMPYLAAIVAVVVEFIFGIALILG

LFTRPIAVIFALYVLGTAFIGHPFWKMTGMEMMGNEINFFKNISIIGGLLLLAVTGAGRYSLDYKIFN
K
>CORE_REP|Org40_Gene3060#
MQDQEIVELLQQVKTIALVGASDNPSRPSYGVMAYLLAQGYQVIPVSPKLAGQTLLGQPVYATLAAIP
QPVDMVDVFRNSEAAYGVAQEAIAIGAKALWLQIGVINDQAAELAQQAGLRVVMDRCPKIEIPRLGLE
R
>CORE_REP|Org19_Gene949#
MLDKLDAALRFGQEALNLRAQRQEILAANIANADTPGYQARDIDFASQLNKVLEQGRVNGNGMSLNLT
AARHIPAQTLQPPQLDLLYRVPDQPSMDGNTVDMDRERTNFADNSLKYQTDLTLLNGQIKGMMSVLQQ
G
>CORE_REP|Org5_Gene1447#
MQTFIKVRGYHLDVYQHVNNARYLEFLEEARWEWLENEAGFRWMTENNIAFIVVNININYRSPAVLGD
KLRIDSQMVQLNGKSGVLSQKVTQDPAGTPVADALLTFVCVDLKTQRALPLEGELRAHLEALVPQENS
R
>CORE_REP|Org38_Gene320#
MRNFDLSPLYRSAIGFDRLFNALEAGQSQGNGGYPPYNVELVDENHYRIAIAVAGFAEQELEITTQDN
LLIVRGAHNNEPAEKTYLYQGIAERNFERKFQLAEHIQIKGAKLENGLLYIDMQRIVPETLKPRRIEI
K
>CORE_REP|Org17_Gene3322#
MLELSRNVAIPDNELELTAIRAQGAGGQHVNKTSTAIHLRFDIRASSLPEYYKERLLALNHHLITADG
VVIIKAQEYRSQELNREAALARLVALIQQAMVVEKTRKATKPTKGAKLRRLEGKARKGATKALRGKVR
T
>CORE_REP|Org17_Gene1761#
MAKETTPAHPVTELNEIQRYVTQQRGTEAPFSGKLLHNKREGVYHCLCCNQPLFYSETKYDSGCGWPS
FYEPVSADAIRYLDDNSHNMHRVEIRCGHCDAHLGHVFPDGPQPTGDRYCVNSASLSFTDGENGDQTA
G
>CORE_REP|Org11_Gene3552#
MTSYRYWLGILSCFLLFSLVFLGQQTGLFGSTDHEHHGETGLLLFVVPGAIASYLSSRKRLLCPLLGA
LYALPLCLLIRHFWLTPSYSFWLTPSYSFWQELAYATSAVFWCVFGAMLMLFALGLLQTLQQLHRRQR
Q
>CORE_REP|Org6_Gene4225#
MWRSRNEHHPCLSHVSLGSNDFEAAAAFYDRALAALGCRRVLEHPGAIGYGRDYPEFWLQVPIDGRPA
SPGNGTHVGFFATSKQQVDEFHRQALLAGAVDEGAPGSRPHYGEAYYGCFVRDLDGHKIEASFWDESA
A
>CORE_REP|Org12_Gene102#
MLQPKRTKFRKVHKGRNRGLAQGTDVSFGTFGLKAVGRGRLTARQIEAARRAMTRAVKRQGKIWIRVF
PDKPITEKPLEVRMGKGKGNVEYWVALIQPGKVLYEMDGVPEEVAREAFKLAAAKLPIKTTFVTKTVM
>CORE_REP|Org31_Gene4251#
MFKPLLLAAILATTVLPAAQQAHAEGISIDLMPGVSLRIGDRDNRGRYWDGYDWRDRDWWQGHQGRYL
GDRSRRGYYWDGYRWRDRDYWRKHYYYHEGRYRKYDRHYDKHHWKKEKKHHHDHDRDRWRRHGDDDRD
>CORE_REP|Org45_Gene448#
MTKPIITINELDAERLDALLEQPAFANTDVAAALNDELDRAEILPPEKMPANVVTMNSRVRFRDLHTD
EEHVRTLVYPASLKDSHDQLSVMAPLGAALLGMHVGKQISWQLPNGEEARIEVLELLYQPEAAGEYHR
>CORE_REP|Org7_Gene1691#
MNSNHLASIARSEKICFDYMDFLSASCRKHWRFVDAIYGVMPIFGMVLKSRVTTSQTRKEQLKELALQ
VVSTQVSDETNIVRLIDLAQQQGLAVFDIQLPYALEAQQLAAIQKECAEGIAITLVGERMTVAIPPKS
>CORE_REP|Org8_Gene2170#
MNMQPQLAPVQPLLCWRKGVFHISTDRQLLDIEAIQRFLSLPDRAAAERLARHGLCFGLYRKRHQLGF
ARMVTDYATFASVSELFVSAEYRGVGLGSWLTRCCLAHPALRGLRVTLPALQAPWRADCAAHAPRALH
>CORE_REP|Org38_Gene351#
MYNRSGTQAYAQVSLESGAMSASPHQLIVMLFDGALSALLRARILMNQGDIAGKGMALSKAINIIDNG
LKSGLDPQQGGEIAENLAALYDYMKRRLMQANLHNDEAAIAEVVKLLENIADAWRQIGPNYQPSQDAV
>CORE_REP|Org43_Gene912#
MNINITDKPNPQDEEFVIDSLWAHNHKTQPVDIHPLFLTVTDDNQQIVGGLVARTWWGGLEVQYLWVG

DQCRKSGYGRQLMQLAEEEARKRGCHMAYVDTFDFQARGFYEKLGYRVYGELGDYAHRHTRHYLAKSL
>CORE_REP|Org28_Gene416#
MAKEFSRGQRVAQEMQKEIAIILQREVKDPRVGMATVSGVEVSRDLAYAKVYVTFLNVLTENHDPDLV
TNGIKALQDASGYIRTLLGKAMRLRVVPELTFAYDNSLVEGMRMSNLVTNVVKNDAERRSASGDDKED
>CORE_REP|Org31_Gene4274#
MSRQLQQRFTACIAIMAVLLLFVAPVVSKSLMHHQMMGGMAQTDAAMPHNMHAMTMPAAEHAGHHGMD
PAEMIFCGYCELLIHVPLLLWTFVPLLWLMARIARLPSAPRIVSTPLRRLVLRPSPRGPPGQPFFPIA
>CORE_REP|Org1_Gene4324#
MRNWNEPKKNKAHIELIPMIDVMMFLLVFFVLISLNVIPALGLKTQLPSAGSAQQLKPQKKAIITLGA
DEQLQLDGQPIALGALVTTLKQQQQANQTTTTIIVNSDKGVAVERLVAVMDNLRQGGFFSVSIATRKL
>CORE_REP|Org40_Gene3745#
MSGAAAGRRRFCQALDLIDSPALIAEYQQHHQRIWPGIAAHLRKHGILDMEIYRLGTRLFMIVEVSAD
FDAARFDAASLNNPEVQRWEALMWHYQAATPWTPQGEKWVEMARIFSLQQQERQHHHRQTIRGYHHAC
>CORE_REP|Org16_Gene2037#
MKEKATRDDAVRLDKWLWAARFYKTRALAREMIDGGKVHYNGQRGKPSKIVELNAELKLRQGNEERTV
IVLALTSQRRGANEAQQMYQETEASIANREKMALARKMNALTMPHPDRRPDKKERRDLMKFKFGEPE
>CORE_REP|Org17_Gene1455#
MANVLTLCWKYLRALLLIYLCLFVGNAVAALLPFAIPGSIIGMLLLFALLSTQILPAKWVKPGCHLLI
RYMVLLFVPIGVGVMKYYDQIVAHLGPLVISCLISTLMVLVVVGYTSHYFHRERRIAGKPDDTEGNS
>CORE_REP|Org18_Gene3421#
MLTGLNHLTLAVSDLDRSFDFYRHLLGFTPHARWQGGAYLSLGSLWLCLSLDERRTQQRERDYTHYAF
SIAPEHIEQASQRLRQAGVKEWKSNRSEGESLYFLDPDGHQLEIHAGDLASRLAACREKPYQGMVFY
>CORE_REP|Org34_Gene1938#
MATQIFVNLPVRDLPASMAFFTHLGFAFNPQFTDDTAACMVVSDTIYVMLLTHDKFRMFTPNPIGDAK
QATEVLVCLSQPSRAAVDELVRKAIAGGGNTYHQPRDYGMMYGHGFQDLDGHIWELMYMDPAAVQAQ
>CORE_REP|Org29_Gene594#
MAKTSRSIMIAKGLQRVLNVGLLLLAAILVVFLVKETIHLAKVLFINSEESSSYLLIEGIVIYFLYFE
FIALIVKYFESGYHFPLRYFIYIGITAIIRLIIVDHKNPIDTLIYAAAILVLVVTLYLANTDRLKRE
>CORE_REP|Org25_Gene3304#
MMPFHPEALWRRLQGSPFRARFRLNPKDQSYLDDKGLPLILSHARDFIDRRLAAAHPKNDGKQTPMRG
HPVFVAQHATATCCRSCLEKWHGIPQGIALDEQQKDYIVQAIALWLVRRGGAREESGANLFDPDRGL
>CORE_REP|Org34_Gene207#
MSEALKILNNIRTLRAQARECTLETLEEMLEKLEVVVNERREEDSQAQAEIEERTRKLQQYREMLIAD
GIDPNELLQTMAANKAAGKAKRAARPAKYQYKDENGELKTWTGQGRTPAVIKKAIEEQGKSLDDFLL
>CORE_REP|Org47_Gene190#
MQARVKWVEGLTFLGESASGHQVLMDGNAGDKAPSPMEMVLMSVGGCSAIDVVSILQKGRNDVRDCEV
KLTSERREEAPRLFTHINLHFIVTGQDLTDKIVERAVNLSAEKYCSVALMLNKAATVTHSFEIRQPA
>CORE_REP|Org38_Gene2894#
MRLLHTMIRVGDLQRSIDFYTKVLGMRLLRTSENPEYKYSLAFVGYTEESEGAVIELTYNWGTDSYDM
GTAFGHLALGVDDVAATCDNIRRAGGNVTREAGPVKGGTTVIAFVEDPDGYKIELIENKHAGQGLGH
>CORE_REP|Org19_Gene792#
MRVLPLCLLALALAGCSSQRIAPSSTNSTSKPTTTAPAKTTPAARPAPVKLYKSAEELVGKPFRDLGE
VSGESCQTTVQDSPPNLATARKRMQIRASYMKANAVLLHDCQIVSGVAGCYQQAVCQGSALNVSSK
>CORE_REP|Org42_Gene2097#
MKRCLFVLCLLAPLTAGAVSGSWVAEGAGVTLEQGGMRDESAGLRPPNVLPDANARITRVSWRYRLLG
PEPAGLQAQLCTVNRCIALGGGSGSSNGLQGEPANAELRFVYYVQSQGGLNPPLRVIGNQVIVNYQ
>CORE_REP|Org49_Gene2979#
MSQVSTFVMFQGEAQQAIDLYSQVFARFRLMQVQHYDPTPDGRRLIKHATIDFDRQNLVFIDSPISHD
FSFTPAVSLFINLPNEEALERAFHRLAEGGKVLMPLDDYGFSARFGWLNDRFGLSWQLNVPAGDLP
>CORE_REP|Org10_Gene66#
MNPLVYFSSSSENTHRFVEKLGLPAIRIPIAGARSKLLMEQPYILIVPSYGGGSAVGAVPIQVIRFLN
VPQNRSYLRGVIAAGNTNFGAAYGIAGDIIAKKCQVPFLYRFELLGTTQDVENVRQGVTAFWQRQN
>CORE_REP|Org39_Gene851#
MSNTLFRWPVRVYYEDTDASGVVYHARYVAFFERARTEMLRQHNFHQQQLLSEQVAFVVRRMTVDYLA

PARLDEQLEVQSEITCLRGASLTFAQRIVNSDGALLSQADVLIACIDPHQMKPRALPKSIVAEFKQ
>CORE_REP|Org22_Gene2003#
MSSTFIDKLALITLKDDKVALVRSHNKTLFYMPGGKREAGETDEQALCREIDEELTVALLPNSIAFYG
EFTGPADGKQDGTQVRIRCYQADFTGQLQPAAEIAELRWCDSRDLPHCSHVAALILHDLKARNLIK
>CORE_REP|Org11_Gene3698#
MKRLKSAALLLPLLALSACTHHLSSAEQHAKHYVYQTRDDFDPQFRTDVNGSIKNAVPMFEQFYQLGK
KDRAAGVARSEAQKKADYLASAEFQQNMEHKTIFINRAYSSADNPKRRQVLSQEAVGAYWDGYEGR
>CORE_REP|Org27_Gene316#
MSLLNIFDISGSALSAQSQRMNVSASNMANADSVTGPDGEPYRAKQVVFQVAAAPGQPTGGVRVAQVV
DDPAPERLVYQPGNPLADAKGYVRMPNVDVVGEMVNTISASRSYQANVEVLNTTKSMMMKTLTLGQ
>CORE_REP|Org39_Gene2481#
MTRTAHCLSAALLLTLALTGCQTAKRPVSTLSQPPSAEEIAEQDKRQREAERMQQCQRELDAMRGMDN
EKYQKFKREFDTLMGGAAQYAGVRQRVNTGTQETVDALYRYRTSRLCADISSAMMTGLAERGERAQ
>CORE_REP|Org42_Gene3444#
MKHLNIAVGIIRNAQREIFITRRAADAHMAGFWEFPGGKIEQGETPEQALSRELREETGIEAERAELL
EVVEHRFSDRIVTLNFYLVEAWAGEPFGREGQPMRWVKQAELREDEFPEANVGIIRLLVAQANAAQ
>CORE_REP|Org43_Gene4516#
MTSLKRINYPQLPTPGGPYVHAVRHGDRLYVSGLTAFATDAQGLSAPQQTQEILEQLATIAVSEGVNL
KALIKISVFLTDIADLASVRPVLFDYFDGALPACSLMAVSALFSPAVCVEIEAVMALQLGAARAQC
>CORE_REP|Org23_Gene1104#
MNISDVAKKTGLTSKTIRFYEEKALITAPIRSDNGYRHYSAKHVEELTLLRQARQVGFNLDECRELVA
LFNDPARHSADVKARTLQKVAEIEKHISELGNMRQRLLTLAEQCPGDEGAECPIINNLAGCCRSN
>CORE_REP|Org13_Gene373#
MTWEYALIGLVVGIVIGAVAMRFGNRKLRQQQVLQNELDKSKTELEEYRQELVGHFARSAELLDNMAR
DYRQLYQHMAKSSNNLLPDLPMQENPFRYRLTEAEADNDQAPVEMPRDYSEGASGLLRGQSARRD
>CORE_REP|Org8_Gene977#
MFSHITVGVSDLDAAAAFYDAILLPLGLQRREVTPDGGPAARCWVMPGQTLPRFYAYQPFDRQPASAG
NGSMLAFLAADELAVKRAYAAGLLAGGSSEGEPGERAHYGKGYFGAYLRDPDGNKVHVVYRGDLA
>CORE_REP|Org20_Gene640#
MISLKKRTPWFGWLRRWAKGIKRDIYALWLAARDKRTPWYAKLIALLVAGYAISPIDLIPDFIPVLGY
LDDVIIVPLGIMLAVRLIPKPLMAELREKAQKRVDNPTGRMAAVMIILLWLLCLGLLARYLDQHW
>CORE_REP|Org42_Gene757#
MLNDIEEIRFTARSEENLRGVHPDLVRVIRLALRYSLVPFSVSEGLRSMARQREMVRAGSSQTLRSRH
LTGHAVDVVAMPAGVVSWEWDYYAQIAVAVRRAARECGINVEWGGEWKTLKDGPHFQLAFRDYPA
>CORE_REP|Org7_Gene125#
MTLPSGHPKSRLMKRFASLGPYLREGQCENDRFFFDCLAVCVNVKPAPEKREFWGWWMELQAEEARFT
YSYQFGLFDKEGDWTAQNIKDDEVNAKLEDTLRDFHRRLGELLATMELGLEPADDFKEKLIKLSA
>CORE_REP|Org30_Gene2207#
MKIIDVVAAILEQNGRILLAQRGAGSDQAGLWEFPGGKVEAGESQPEALARELDEELGICARIGEHVG
SNQWQQGERLIRLHAWRVAAFSGELQLHCHSALVWVTPQQAQEYALAPADVPLLADYIAAQGDAR
>CORE_REP|Org27_Gene266#
MRHYEIVFMVHPDQSEQVPGMIERYSATITNAQGQIHRLEDWGRRQLAYPINKLHKAHYVLLNVEAPQ
EAIDELETNFRFNDAVIRSMVMRVKHAVTEASPMVKAKDERRGDRREDFANETADDADAGDSEE
>CORE_REP|Org1_Gene1737#
MSIAKAVLRIARPTDRLQEIATLYCRGLGFEKLGEFVDHQGFDGMMIGHPQHAYHLEFTQHRGVRVGQ
APTQDHLLVFYLPDENEWRAACERMQAAGFLSVAAYNPYWDRAGQTFEDPDGYRVVLQHQAWQA
>CORE_REP|Org11_Gene2594#
MKKLTLAALIALCSAPVLAQQGGFLDPAAPQAQTQTTAQGGFAGPSAALTTVDKVKSLSDDTWVMLQG
NIEQRIGDETYTFRDATGTLTVDIDRKRWNGQTVTPKDKVQLEGKVDKDWNSVEVDVKTVKKLP
>CORE_REP|Org37_Gene527#
MDILSALAAFASYFFSGFAMILVFLFVYTRITPHDEWALIKADNQSAAFAFIGACLGYVIPLASAAIN
SISLLDYLLWGVVALVVQLLLFAAVKIYMPRISDKIEANHLAAGIFLGGVSVSGGVLNAACMSY
>CORE_REP|Org43_Gene1619#
MTAYTALKHFHLLTVAISITLFVLRFYWQWRRSPIVGRRWVKIAPHLNDTLLFVSGIALVVTFGFYPL

LGMDSWLTEKLFGVIIYILLGYVALGKKTKSQRLRTVAFVLALGCLYLIIKLATTKIPFLMGYL
>CORE_REP|Org10_Gene3889#
MSDLVSASGKPVKIPGPDHPITLTRHPARVVVRAAGQTLADSRNVLILQEATYPPVFYFPREDVNRAL
LQKNEHVSYCPYKGDCSYFSLVLNGEQEANVAWSYEMPYNAVVAIKDHLAFYTDKVTKIRTEA
>CORE_REP|Org11_Gene220#
MVSALYVVLGALLLIKLSYDVVRLRMQYRVAYGDGGFYELQTAIRVHGNAVEYIPIAAVLLVIMEMNG
AEIWMIHLCGLMLMAGRLVHYYGLRNREVRWRRSGMAATYISLLLMVLANIFYLPWDLIFSLH
>CORE_REP|Org22_Gene516#
MKKLEDTGLLVARILMPILFIVAGYGKMGDAYAGTQQYMQSMGVPGFLLPLTILLEFGGGLAILFGFL
TRTVALFTAGFTILTALLFHTDFAEGVNQLMFMKNLTIAGGFIVLAVAGPGGFSIDRLLNKKW
>CORE_REP|Org28_Gene350#
MSSRSMLVFAAISGFVFVALGAFGAHVLSGTLGANEMAWIRTGLEYQGFHTLAILALAVAMQRRVSLW
FYWSGALLAFGTVLFSGSLYCLALSHLKVWVYITPVGGVCFLIGWILMLIGALRLRKKAERHE
>CORE_REP|Org21_Gene2012#
MRGKKTLRGLVALALALPAALLMGCSSPSGIAVNKRQTVVMDPSVLTAGILADTPSISNASGRVMATS
VLNNSQSTPVTVHYRFYWYDAQGLDIRPFEKPREIVVAPDSDAKIYSINGNLDAKSARLYLYL
>CORE_REP|Org44_Gene3325#
MTTQRKPYVRTMTPTWWQKLGFYRFYMLREGTSVLAVWFSIVLLYGVFALKGGAESWVGFVGFLQNPL
VLLINVVALLAAALHTKTWFDLAPKAANIVVNSEKMGPGPIVKGLWAVTIVVSVVILAVALI
>CORE_REP|Org43_Gene1700#
MAKELDIGAVARLSGVAPSALRHYEKKGLIASIGRHGLRRQYAAGVLDQLRLIALARLAGFTLDEMSA
LFDERGKIALDRVLLAARADELDRHIQRLIQVRDGLRHMVDCPEPEHLQCPQFRKILQQGEF
>CORE_REP|Org22_Gene452#
MFANSQMIGVDLAFPDVCLTPSPAPVPVPYPDIALAPTAIPNAFNILFVGTPAHNMATVTPLTNGDNP
GVATGVASGTVMGPSRHLTGAFTVLLKGTPATRLTSLSLQNSTNALGMRIVPSQLKVLLLAP
>CORE_REP|Org23_Gene2977#
MKGYAWGAASVLLVTLAQLLMKWGMVQIPLMSFADVSLALIGNYWLPLLAVGGGIFGYALSMLCWFFA
LRHLPLNRAYPLLSVSYALVYLAAVILPWFNESATLLKTLGTLFILFGVGLINSKSETKSPE
>CORE_REP|Org14_Gene1591#
MATAIPQKYEHLERWAFGDTEQQADEVVQLILNGTKTATCSNLDGEGIPQAGDRFLVVDGKGEPVCAV
ELTAVEMKTYDQVDEAHAFAEGEGDRSLAHWRKELQRFFEEYDLFSPDMTLVLMNFKVVDKF
>CORE_REP|Org22_Gene550#
MPTPLEIVRATYEGSSEENGRNLLAALAPDAEWTEAAGFPYAGTYIGPENIIKNVHQRLGSEWQGYRA
DVDHFYDAGDNVIAQGFYHGTYRATGKSFSASFAHIYTLRAGKIVKFVQIVDSAKVLEAMQP
>CORE_REP|Org26_Gene89#
MSMQDPIADMLTRIRNGQAANKVAVTMPSSKLKVAIANVLKEEGFIEDFKIEGDAKPVLELVLKYFQG
KAVVESIQRISRPGLRIYKKKDELPKVMAGLGIAVVSTSKGVMTDRAARQAGLGGEIICYVA
>CORE_REP|Org22_Gene509#
MAENQYYGTGRRKSSAARVFIKPGNGNIVINQRSLEQYFGRETARMVVRQPLELVDMVGKLDLYITVK
GGGISGQAGAIRHGITRALMEYDETLRSELRKAGFVTRDARQVERKKVGLRKARRRPQFSKR
>CORE_REP|Org12_Gene2180#
MKLTIERLTALSPQDLIDLGKIWPHQQPEQWQGWLSDDKALFAARFNERLLGAVKIALQDDRAELQDL
LVREVTRRRGVGLYLVQDAQRQLPQVAQWQLSTAGLAPRERGEVDNFMRACGFAPQGDLWQK
>CORE_REP|Org23_Gene3550#
MPFSRIALHQGKSADYLQTLSDSLHQALVEAFEVPPADKFQVFDQYRPGELIYDRDYLGGPRSADFVL
FYITAGRPRDSDTKRRFYRRLAALLMEKLRLNPEDVMVVIATTQLDEWSFGGGRASMIDPQA
>CORE_REP|Org12_Gene2700#
MVAIPSGYGRSGLLNPRASGAGYELLARRHLEHAGLTFCAANVAVRGGELDLIMRDGTTWVFVEVRYR
RNDAFGGAAASVTFRKQQRLLHAAAVWLAGRGASFDTSSCRFDVLAITGSQLEWIPNAFNAD
>CORE_REP|Org14_Gene90#
MAKAPVRTRKRVRKQVSDGVAHIHASFNNTIVTITDRQGNALGWATAGGSGFRGSRKSTPFAAQVAAE
RCADAVKEYGIKNLEVMVKGPGPGRESTIRALNAAGFRITNITDVTPIPHNGCRPPKKRRV
>CORE_REP|Org41_Gene2207#
MADKNLRFLVVDDFSTMRRIVRNLLKELGFNNVEEAEDGADALNKLRAGGFDFVVSDWNMPNMDGLEL

LQTIRADSVLAAMPVLMVTAEAKKENIIAAAQAGASGYVVKPFTAATLEEKLNKIFEKLGM
>CORE_REP|Org7_Gene202#
MYDNLKSLGINQPEDVDRYSLRQEANNDILKIYFRKDKGEFFAKSVKFKYPRQRKTVVADNAGQGYKE
IHEINPNLRYVIDELDQLCQRDQVEVDLKRKILDDLRHLEGVVSHKIAEIEADLEKLTRGK
>CORE_REP|Org24_Gene4160#
MNTGTLLRALANGVNPDTGELLRPASAACAPEAIRLLFALANEFDGEAELQKKARLTPEDKRQKNLAE
GRPANAYFPWPEEEKLRLRESHAAAATLEALSDEFERSTWSIAVQLQKMGLIDEEQAAAHR
>CORE_REP|Org12_Gene481#
MGKYVKKQRPVNLDLQTIRFPVTAIASILHRVSGVITFVAVGILLWLLGLSLSSQEGFLQAAAIMNSF
IVKFIFWGILTALAYHICGGIRHLLMDFGYIEESLAAGTRSAQVAIGLTVVLSVLAGVLVW
>CORE_REP|Org14_Gene88#
MRHRKSGRQLNRNSSHRQAMFRNMAGSLVRHEIIKTTLPKAKELRRVVEPLITLAKTDSVANRRLAFA
RTRDNEIVAKLFNELGPRFASRAGGYTRILKCGFRAGDNAPMAYIELVDRAESQAEVATAE
>CORE_REP|Org9_Gene1200#
MLDYCLLVTGPAYGTQQASSAYQFAQALLAKGHRLSSVFFYREGVLNANQLTAPASDEFDLVRGWTQL
AQQHGVALNVCVAAALRRGVTDEQEAAQQGLASANLQPGFTLSGLGSLAEASLSCDRLVQF
>CORE_REP|Org17_Gene4670#
MPHFYAECTDNIRRDADLPTLFAKVNEALAATGIFPLAGVRSRAIWLDTWQMADGKQDYAFVHMTLKI
GHGRSLESRQQVGEMLFALIKEHFAALMAQRYLALSFTMEELDPVLNYKQNNVHALFNKA
>CORE_REP|Org29_Gene3250#
MSRNISTELAPAAIGPYVQGVDLGSMIITSGQIPVDPKTGAVADDVATQARQSLENVKAIVEAAGLIV
ADIVKTTVFVKDLNDFATVNAAYEAFFTEHSAPFPARSCVEVARLPKDVKIEIEAIAVRR
>CORE_REP|Org45_Gene696#
MQNTLKALAALTLLAASSLAVAADLGDDMDTLAENYKTVLNTDSAATLKQSLQNMRAAAQDAKQGTPP
KLEDKAADSPEMKDFRSGLDTLIGQIDQALALANQGKVAEAKQLAQGFKQTRDANHKKFR
>CORE_REP|Org25_Gene3681#
MKRLLLDTHALLWWLIDDACLGVNAKRQIADPGNAVYVSAASIWEISIKQALGKLALPEDIFAIIEAE
DFLALPMDAFHCQQAGQLPPYHQDPFDRMLIAQAQAEGLTLISADTVFPQYGVRVADARR
>CORE_REP|Org29_Gene3059#
MWYYLPHSDSRRTTTMAYIPKNYAKLETGYREKALKLFPWVCGRCSREFVYSNLRELTVHHIDHDHSN
NPEDGSNWEMLCLYCHDHEHSKYTEADQYGSTVVAGEDAQKDVGVATHNPFANLKAMMKK
>CORE_REP|Org22_Gene183#
MIQEHHLSLVCALSKWVETHLGRVIHLEELAEYSGYSLWHMQKLFKEATGISLGKYIRERRLAGAVYQ
LRSSEASIFDIALDFGFGSQSHFTYMFRKRFNITPYDFRQDLSVDLHIDPPLHVIHQKSA
>CORE_REP|Org29_Gene2623#
MGLLDQWAERHILDAQDKGEFDNLPGQGQPLALEDDSAVPAELRAGYRLLKNAGYLPPELEDRKEALT
VARLLQEISSEHPDYVELSKRMALLEHRLRQAGMSTDFLHGEYQRALDDKFTREEKSCSR
>CORE_REP|Org29_Gene2864#
MSNVPTELKYASSHEWVRSEGNGVYTVGITEHAQELLGDMVFVDLPEVGRNVAAGEDCAVAESVKAAS
DIYAPISGEIVAVNGELESSPELVNSEPYGDGFLFQIKAADEGELANLLDAAAYQASIDE
>CORE_REP|Org35_Gene571#
MAYSEKVIDHYENPRNVGSFDNEDPTVGSGMVGAPACGDVMKLQIKVNDEGIIEDARFKTYGCGSAIA
SSSLVTEWMKGKSLDQAEAIKNTQIAEELELPPVKIHCSILAEDAIKAAIADYKSKHSAK
>CORE_REP|Org15_Gene4152#
MANKPQQTTLTMYGIKNCDTIKKARRWLEDQGVAYHFHDYRADGLDEQRLRGFVAQLGWEPLLNTRGT
TWRKLDEAQRNACDNADAAIALMLAQPAIIKRPLLDAGNGRALLGFNTDAYQQFIAEVAV
>CORE_REP|Org45_Gene4246#
MTSALAMLKNGGLLALLLSTLCLMGYSSLLSHRLESARQQAAELQKSLAQQAGLIATLQTQDAQNRAL
MAAQQQQEQQLRQQSDEYQRKYREAIKGNRCAAERMPDAVLELLRPAAGTGAAGGAAAAP
>CORE_REP|Org10_Gene2119#
MSVSLYSGKVARKLLFLQLMTFVLISVAFWLKSPEWSASALAGGLAAWLPSAMFMLFALRHQAQTPAP
GRVAWSFAIGEGLKVVITIVLLIVALGVFKAEFIPLGLTYLAVLVVQIVAPAVINSYRN
>CORE_REP|Org38_Gene72#
MITGIQITKANDQALVNSFWLLDDEKAEARCVCAKANYAEDQVVAVSDLGQIEYREVPLEMQPTVRVE

GGQHLNVNVLRRETLEDAVKHPEKYPQLTIRVSGYAVRFNSLTPEQQRDVIARTFTESL
>CORE_REP|Org29_Gene104#
MSANTEAQGSGRGLEAAKWLIVAVLLVVAIVGNYYYRDLSLPLRALAVVLIIAVAGAVALMTTKGKAT
VAFAREARTEVRKVIWPTRQETLHTTLIVAAVTAVMSLILWGLDGILVRLVSFITGLRF
>CORE_REP|Org28_Gene3347#
MKHTEKKSALSGYPHHIKAALTALLLCLAGPAAAVDKAEAPAPMLSAPASNGGQSVMAKTAEEAAVSI
NQATAEQLAAALSGVGLKKAEGIVRYREQNGPFTQVEQLQEVPGIGPALFEKNRTRLKM
>CORE_REP|Org33_Gene2485#
MLSSLLAVFIGGGVGSALRWAVSMKMNPLNAHIPLGTLMVNLIGGFIIGLAMAIFTRMTHLDPTWKLL
ITTGFCGGLTTFSTFSLEVVYLMQDGRFGWALANMLLNLAGSLAMTLLAFMLVMWVNGR
>CORE_REP|Org23_Gene2603#
MNPYFTEVIDAHIAIERWLGKGAGEEQALLARFTPEFSMIALNGAPLDFTALCAFFRAHRAAKPGLEI
EIEEMKLVAEWPTGAVVSYREKQSLPGQSATLRYSTVVFERLPDALGWRHLHETAAAQ
>CORE_REP|Org39_Gene422#
MIRTMLQGKLHRVKVTQADLHYEGSCAIDQDFLEAAGILEYEAIDIYNVDNGQRFSTYAIAAERGSRI
ISVNGAAARCACVGDKLIICSYVQMTDADARQHHPKVAYFEGDNNLQRKAKAVPVQVA
>CORE_REP|Org13_Gene1332#
MNDKTARPQDVVQQQLDAYNARDIDAFMACWADDAQYYEHPDTLLASGKAAIRERHLVRFQEPSLYGE
RIKRMAVGNMVVDQEVVTRNFPQGRGKMDVIAIYEVEQGQIAKAWFKIGPCVPDEGAL
>CORE_REP|Org33_Gene718#
MAVLGLGTDIVEMARIEAVVERSGDRLARRVLSDAEWALYQQHQQPIRFLAKRFAVKEAAAKAFGTGI
RNGLAFNQFEVFNDALGKPNIRLHGRAAELAGEMGVAAIHVSLADERRYACATVIVES
>CORE_REP|Org12_Gene4464#
MIKGISNTAETARGLVGTMKVRADLNSAAFSEMLGAADGKVRQNKADPLSRITAQTVQNLTALFAFAP
KAAATHTAAAAAGTLSFEQRVTLTQSAELLQTRTNYLRMALQLHNPGKAFSAPALLKL
>CORE_REP|Org6_Gene2105#
MSLDTADFPLVWMRRSGRDTQAELAEFSALLARAEPFVLMTDRSVGDEEQEHDRDARTQVALWKRDNR
EALKRWVKGMIMLEPDDAKRAAAEEFAEYGAKFWGYPVLVAADEAAGRVLAQTLLLK
>CORE_REP|Org48_Gene2900#
MSLYATLEEAIEAAREEFLDTAEGGSGDEPPVPQQFNLQKYVMQDGDTMWQAEFFEEEGEAVECLTLR
SGAAAQAIFDGDYDEVEITAEWIDENTLYEWEEGDFQLEPPLDTEEGQAAADEWDER
>CORE_REP|Org31_Gene631#
MIDSPRVCIQVQSIYVESQSIPEEERYVFAYTITIRNLGRTDVQLLGRYWLITNSNGRQTEVQGEGVI
GEQPVIPPGGEFQYTSGAILETPLGTMEGHYEMVDHQGQPFRTAIPVFRLAIPTLIH
>CORE_REP|Org7_Gene1020#
MKGTLTRAALAAGGMMVTSAVMAGSLALPTAQSLAGQWQVADSERQCQIEFLANEQSETNGYQLVDRQ
RCLQSVFAAEVVGWRPAPDGIALLQADGSTLAFFSRDGDLYRNQLGAGDALTLKALA
>CORE_REP|Org48_Gene3630#
MVTKIKSLCVASMIFLGCIQSAQAKETYTPEEYLKNYALSVCIAEGYSAKEVKNDAAAAARGYTEFGD
YSLEAHTAVRALAKEFLAKPYDSMSGEPMTMAKCIDLVHSQALQAIIKKYQGKDDN
>CORE_REP|Org21_Gene546#
MTAKRPRWQYVLMIALALLALATLLVPCMVRTESELRIRASQQGLSLPDGFYVYQRLDERGIRIKSIT
PEGDGLVIRLDSPEQQLLAREALQTILPPGYHIALSESPVPSHWVREFARSPLNLG
>CORE_REP|Org3_Gene2206#
MEKPGRENGTLLLALIAGLSINGSFAALFSSVVPFSIFPLIALVLAVYCLHQRYLNRAMPDGMPKLAA
ACFLLGLLMYSAIVRAEYPQIGSNFLPSVICVALVLWIGMKLKARKAPDAPTQES
>CORE_REP|Org10_Gene1961#
MKLRHSLLLLLPLCTLPALAAAAEGGCAAKAQNIQQQIDYATQHGNTHRVDGLKKALSEVQTHCTEAG
LQAERQKNIAEKQRKVDERRQELKETQQTGKLDKVAKKQQKLAEAQAELKRAQAE
>CORE_REP|Org12_Gene175#
MQTENVGTFSLDENVWQGISLSDSAVRQITKLMQQDPQVKGLQLGVKQSGCAGFAYVLDLTREPADDD
LLFERDGAKLYVPLKAMPFIDGTTVDYVREGLNQIFKFNNPKAQHACGCGESFGV
>CORE_REP|Org43_Gene8#
MIQEQTMLNVADNSGARRVMCIKVLGGSHRRYAGVGDIIKITIKEAIPRGKVKKGDVLKAVVVRTKKG

VRRPDGSVIRFDGNACVILNNNSEQPIGTRIFGPVTRELRNEKFMKIISLAPEVL
>CORE_REP|Org42_Gene1052#
MSFSQPDAVIRIKNLRLRTFIGIKEEEINNRQDILINVVIHYPADKARNSEDIADALNYRTITKAIIR
HVEDNRFALLEKLTQDVLDIVKEHAWVTYAEVEIDKLLALRYADSVSMTMSYRRD
>CORE_REP|Org25_Gene3802#
MIAVIFELQAADGQRDAYLGLAAELKPLLAQIDGFISIERFQSLADPARLLSLSFWRDEAAVQQWRNL
EQHRAAQARGRGEVLAQYRLRVAEVVRDYGLESREQAPQDSRCYHAQSKPASEAN
>CORE_REP|Org13_Gene200#
MIYWIFLGLAIMTEIIGTLSMKYASVNGGMIGHIVMYVMITASYVLLSVAVKRVALGVAYALWEGIGI
LFITLFSVLWFDEPISALKVLGLATLIAGIMLVKSGTRKERKQVSPRGDNHATV
>CORE_REP|Org45_Gene1714#
MTLPSMTLLYVDNPLNSAAFYQRLLGQAPVELSPGFALFVLNNGFKLGMWAKQGVKPAATLTGGGGEL
GFLCQDPQEVEARYNQWRELGLPIEQTPTEMAFGYTFVARDPDGHRLRVYALSE
>CORE_REP|Org20_Gene312#
MERQQQLLAAYQQIYSLSSQMIALAQTGRWEELVELEFAYVTAVEKTAAFTGQAGPSMALQEMLRNKL
QQILDNETELKRLLQQRMDELKMLIEQSTRQNVVNNTYGQFHDRALLLGEPQVR
>CORE_REP|Org41_Gene1338#
MKTFSTQRLLRGMLPVAMLAVMGAWQAPALAATCTQGSTCVTVDGKGSGAMSTEEARQSKEQWNDTKT
LRHKVNTRVEKEFDKADRAADDEDRCNDSNNVNAYWEPDTRKCLDRQTGRRIIP
>CORE_REP|Org34_Gene490#
MDEYSPKRHDIAQLKFLCENLYDEGIATLGDSHHGWVNDPTSSVNLQLNELIEHIASFVMSYKIKYMD
ESDLSELVEEYLDDTYTLFSSYGINDSDLRRWQKTKARLFRMFSGEDICTTMKT
>CORE_REP|Org39_Gene624#
MANQATGLTRIIKAAGYSYKGLSAAWQHEAAFRQELVVTLLAIILAVWLDVGAIARILLIGSVALVMI
VEILNSAIEAVVDRIGSEHHELSGRAKDMGSAAVSLAIVLALFVWGTVLWQHFG
>CORE_REP|Org4_Gene2623#
MSQANNAVVEALLADIGSTQGDLLAIVQQVRRIAAESGNGVTESVKYGGIMFSHSQFFCGVFAYRNHV
TVEFGQGHRLEDRYQQLEGSGQYRRHIKLHSPDEVKSKHLADYIQQAYTLTTG
>CORE_REP|Org23_Gene468#
MGQITVVIGDRLGKGQKVGQGVENAGGKAVVIPGVAADMKLGDVMKAENAQLGISFCGSGGAGAITAQ
TKYGYKAKYGMRSVEEGVTAINEGCNVLGFGFMDKEELGQKLVEAYVKKHGNP
>CORE_REP|Org2_Gene686#
MKPLLLKQPLPELLEIGSVSNLGATVIAGTPTVGVASIFGEPTDNLNCGVFSCTRGSFVMEYPFAEHA
TVWEGSATLTNERTGESVQYQAGDSWFVEKGTPVRWDITSDRFVKHYLAIVEG
>CORE_REP|Org16_Gene111#
MSITKDQILEAVAAMSVMDVVELVSAMEEKFGVSAAAAVAVAAGPAEAAEEKTEFDVILKAAGANKVA
VIKAVRGATGLGLKEAKDLVESAPAALKEGVSKDDAEALKKSLEEAGAEVEVK
>CORE_REP|Org37_Gene4353#
MSKRPGRGWWWLLWPWALCAEPRDPFAPPPLPACAQTAASPAGWRLKGVIGQPTLRYGWVVTPAGQWL
RLRPQQRVLAERWQVTQVQARSLTLTALAAEPDCPASQGDISLMMGDNKDGKP
>CORE_REP|Org28_Gene1716#
MHTIFHTTITQIGGCARDALLDNMLITFREGAPADIEEFCFIHRHGATAGELQVGGVMELAQHRYAIT
AVGSVATQNLRELGHITVRFDGEAEAEFPGSIHVSGPTPTDIPLGSTLSFIA
>CORE_REP|Org7_Gene861#
MKEKEKAEIKRLSDLLDALNHKDATVIQQGNPELIAQHTKEKEKLATEIERLKNVRGEKLSAEAQKLS
QLPFSREITKKEQADMGALKKSARGLIVVHPMTALGREMGLKAVTGYAKKAF
>CORE_REP|Org15_Gene455#
MDYEFLRDVTGQVIVRFSMGHEAIGHWINEEVKGDFNLLDRIEAGAAEVKGSERQWQLEGHEYTLLMD
GEEVMIRANQLEFEGDEMEEGMNYYDEESLSFCGVEDFLLVLKAYRSFMLKY
>CORE_REP|Org4_Gene3442#
MATTLFKDFQFEAAHRLPHVPEGHKCGRLHGHSFMVRLEVTGEVDPHTGWVMDFAELKAVFSPIWERL
DHHYLNDIPGLENPTSEVLAAWIWQQLKPQLPELTAVMVKETCTAGCVYKGD
>CORE_REP|Org37_Gene2403#
MKRVAFVFTHGPHGGAGGREGLDALLATSALSEDLGVFFVGDGVLQLLPGQQPEKILARNYIATFGVL

PLYDVERCYLCQASLQERGLSQVTDWVLNAEVLAPDELRRELAGYDAVMTF
>CORE_REP|Org17_Gene1787#
MKKTGLAIALLAMMGASTTVWAQDHEQRAAKVGQCAGLQPADIAAQVKRDFLQNRITRWESDKKLLGT
ATPIAWVSPDAISGKDQVWQVPLTVRGTKADKTYNVTLNCNTGEIAYSAPQ
>CORE_REP|Org27_Gene1698#
MSMTLGSKKLYRVPEEGMVKGVCAGLAHYFDVPVRLIRVMVVLSLFFGLFFFTLVAYIALVFVLDEAP
ASRFEGEHQKTPRQLLDQLEYELGSGEQQLRQVERYVTSDTFGVQSRFRKL
>CORE_REP|Org35_Gene3554#
MGNMGTSELLKHIYDINLSYLLLAQRLINDEKASAMFRLGIDETMADALAQLTLPQMVKLAETNQLVC
HFRFNDHQTIERLTKESRVDDLQQIHTGILLSSHLLQELSSKDASPTKKRA
>CORE_REP|Org36_Gene736#
MDIVFIEELTVITTIGVYEWEQGIRQKLVFDIEMGWDNRPAAASDDVTDCLSYADVSDAVIQHVESNR
FALVERVAEEISEILLQRFNSPWVRIKVSKPGAVAHASRVGVIIERGTRPA
>CORE_REP|Org9_Gene893#
MIKRVSFLAALLFSSMANAGFYSGSSLLAESGGYVKNKNGEATVAEALNGGMFMGYVAGVFDTYSMQG
NRNICPEAGMSIGTAADAVMRYLNEHPEQLHYSAPSVIMLALSAAYPCVRH
>CORE_REP|Org3_Gene327#
MVKLAFPRELRLLTPTHFTFVFQQPQRAGTPQITILGRLNQLGHPRIGLTVAKKHVKRAHERNRIKRL
TRESFRLRQHELPAMDFVVVAKKGIADLDNRALTEALDKLWRRHCRQAPAS
>CORE_REP|Org45_Gene4733#
MTAESHCSPTGISLEDTGFGYTLSVINGKYKMIILYWLALYKPVLRFNELQRCIGTISYKTLSSTLKE
LEKDRLVVRKEYPQIPPKVEYSLSERGRSLIPVIDMMCNWGEQHRPEQPVP
>CORE_REP|Org12_Gene933#
MSNIIKQLEQEQMKQDVPAFRPGDSVEVKVWVVEGSKKRLQAFEGVVIAIRNRGLHSAFTVRKISNGE
GVERVFQTHSPVIDSIAVKRRGAVRKAKLYYLRERTGKAARIKERLNRVG
>CORE_REP|Org10_Gene2347#
MNTTGFITDLKTWIDNNLEEKLDINTVADRAGYSKWHLQRMFKRQTGYALGEYIRMQKLKVSAERLAN
SGEPIVSVAISLGFDSQQSFNRSFKRQFGQTPGDWRRALAQPTAVRCTHH
>CORE_REP|Org39_Gene2295#
MTVNTRAGRISRNGFSLPEVLVAALLFSVSLLGLLQYHQVLLQSFQRQWHYRQAWGLAHQQLEAFAVT
GRLEAEPLPEGWRRETALDDAESDCRRLTVRIQTPQRQWATLSRWYCTRF
>CORE_REP|Org10_Gene2244#
MKNEWFAAKELTGIAGLPSSPQGINLMARREGWISRRRKGVQGKALEYHIDSLPSGVRNLLVLKEDGA
AYDVERQDPLAVWIEYYYHLTESEREKMVAFLMREGIGGLLARIDEESNT
>CORE_REP|Org11_Gene175#
MARVKRGVIARARHKKIMKQAKGYYGARSRVYRVAFQAVIKAGQYAYRDRRQRKRQFRQLWIARINAA
ARQNGLSYSKFINGLKKASIEIDRKILADIAVFDKVAFGALVEKAKAALA
>CORE_REP|Org33_Gene84#
MARIAGINIPDHKHTVIALTSIFGIGKTRSQAICASTGIAENVKISELSEEQIEKLRDAVAKYTVEGD
LRREITLSIKRLMDLGCYRGLRHRRGLPVRGQRTKTNARTRKGPRKPIKK
>CORE_REP|Org3_Gene4186#
MSKPPLFFVAVIALIAVLATQRYFKQRQREAENDRTPMRSLQVTVSDKRSFPVARTRAPQREPLVNEP
MYYEVVFSPNQGGEDITLRLKQWQYNPIEKGAQGTLNMQGTRFISFSVQP
>CORE_REP|Org6_Gene2454#
MKKVLALIVAATMGLSSVAFAADTAATAPATTTAPAATTTTTTAAPAAAAEKAPAKATHHKKAKHHKK
APAQKAQAAKKHHKKAPAQKAQAAKKHHKKAPVQKAQAAKKHHKKAGKKA
>CORE_REP|Org6_Gene3975#
MNSNHDRFLLVIVVSLLTCGGQLCQKQAAHCWERADEARLKQTLRWLALAVLLLGLGMAVWLNVLQRL
PLSLAYPMLSLNFVMVTLAARWLFHEPTTVRHWCGVASIMLGILLMSVHS
>CORE_REP|Org31_Gene87#
MDKKSARIRRATRARRKLQELGATRLVVHRTPRHIYAQVIAPNGSEVLVAASTLEKAIAEQLKYSGNK
DAAAAVGKALAERALEKGIAKVSFDRSGFQYHGRVQALADAAREAGLQF
>CORE_REP|Org48_Gene1103#
MLVTLACNLDVNALSVNKLKKVINDNIAELVPALTSGLSFYSESARYAEDSLEILDIVQQGGSDYSMS

YRYKWGIFNACLDINSEDTLSDSVRFRVTERGLIFDIIDNSRPSTADEL
>CORE_REP|Org9_Gene1956#
MTLFADILLLLIAALHLYILVLEMFLWRTPLGRRAFGTTAEFAAATRVLAANQGLYNGFLAVGLAWSY
WRGDMALQLFFLGCVLAAGVFGGLTASRKILWVQALPAAIAILAAWAAR
>CORE_REP|Org8_Gene1540#
MSSVVLIANGAAYGHESLFNALRLAIAMKEQQSDLDLRLFLMSDAVVAGLAGQQPHEGYHLQQMLEIL
TAQQVPVKLCKTCADARGVSRLPLADGVAIGTLVELAQWTLAAEKVLTF
>CORE_REP|Org26_Gene665#
MNNSALLEYCMAKPGAEQSDQNQWQASQIKVGDVMFAMVCEVEGRPALAVKSSPELAESLREHHPEIV
ACDGLNKAHWNTVFLDGNLPNSQFYTLIDSSYQLVVEGLPEHVRQELRA
>CORE_REP|Org19_Gene3310#
MSVLCSSALGSSLPLLEPAALKGDWQLAAIGDGKAVCEVQLSDEAVANTNAFRFSSSAQCLQPLALQT
LPVAWRPTPDGMTLTDAEGGMVAFLALTAPKRYELIDRNGPSRFMLIPR
>CORE_REP|Org12_Gene2235#
MAKIMLQRVYDFSAPAPEHCYLIDRLWPRGISKERLTGVQWLKQVAPDDELRKWFHQHTDQWTVFEER
YRQQLATNDAWQPLVALLRQGKTLTLLYGSKDTEHNQGVVLREFLLAQL
>CORE_REP|Org10_Gene1242#
MKIKALLFVALAALAGCSQEGTSMNQPANEKGGQTEVLLVNSALVDCVGVGPMKCMQVRRSAQQPWEL
FYTGIEGFTFEPGYQYRLKVLVTPVENVPADASSLRYTLIEQLEKNKA
>CORE_REP|Org30_Gene856#
MKYVDGFVVAVPAANKEAYHRLAAAAAPLFKEFGATRVVECWGDDVPDGKLTDFRGAVKAQEGEVVVF
SWIEYPSKAVRDAANEKMMNDPRMKALGEMPFDGKRMIFGGFAPILDT
>CORE_REP|Org7_Gene646#
MPVARIIASYSENENDTITLLCGVDAENQIRQGEWFGVVKNDDGRGDESNYPFTLHIDYQKDAFYLDY
GYDDADARQLQKTDISARPLAEKGFFTVFDEEEGEEFSYRINSIHLYD
>CORE_REP|Org37_Gene58#
MAEETIFSKIIRREIPADVVYQDELVTAFRDISPQAPTHVLIVPNVLIPTVNDVTVEHEAALGRMITA
AAKIAEQEGIAEDGYRLIVNCNRHAGQEVYHIHMHLVGGRSLGPLLSR
>CORE_REP|Org28_Gene332#
MTTTLEKIQHQIAENPILLYMKGSPKLPSCGFSAQAVQALSACGERFAYVDILQNPDIRAELPKYANW
PTFPQLWVDGELVGGCDIIIEMYQRGELQQLIKETAEKYKAQEDQQS
>CORE_REP|Org38_Gene1438#
MRLITALPLLAGLMLSTNLMAADATTATKTPSPAQAAQQKRMTDCNQQASTKSLKGADRSTFMSTCLK
SEGAAATGKTLTPQQQKMKSCNADAKTKDLKGDARKTFMSNCLKKSA
>CORE_REP|Org48_Gene437#
MKKTTLSMLLLAMLGFSNASLALNESEAEDLADLTAVFIYLKNDCGYNDLPNAQIKRAIVYFAQQNRW
DLSNYNSFNMKALGEDSYRDLSGIAIPTPKKCKSLARDSLSLLAYAN
>CORE_REP|Org2_Gene413#
MVELSSSQLDAIFHALADPTRRSMLRALAGGERSIGELAAPLQMSFAGASKHVKALEHAGLVQRTVQG
RNHICRLEPEPMAQAMHWLQTYEHFWTERLDALEQALLQPEQLPPKE
>CORE_REP|Org19_Gene4036#
MKLNVICYALLGLALVASLFSLHPVWMWLLLANLLTFLIYGGDKLAARKGWRRVPEATLLLFGALGGW
LGALAAQQLFRHKTQKQPFKTWFILSVALNLAAVLGLWYWVYGRWIF
>CORE_REP|Org27_Gene1275#
MTTMKAIATGCMLLATSGCSSVMSHTGPNQGYYPGTRASVDMLKDDNTSWAMMPLVALDLPFSAVMDT
VLLPYDYLRSGNDATADSPKARILRSEQQNLAATGNPGEGNDSAATH
>CORE_REP|Org28_Gene1343#
MRIIVYGSLRRKQGNSHWMTNAQWLGEHELEGYQIYNLGHYPAAIPGEGTIHCEVYRINSSILAELDE
LKSNTKDYKRELIQTPYGSAWIYLYKHSVDGYPRITSGDWLKRLDEQ
>CORE_REP|Org20_Gene451#
MVNNVSALGRNGVHDWLLLRASAIIITLYVLYILGFFVTAPELTYDIWRGFFATSITKVFTLLTLLSI
LVHAWIGLWQVLTDYVKPLAVRLVLQLAVVVALLVYLLYGTIVVWGA
>CORE_REP|Org37_Gene1555#
MNDGSASIKEIEQSDVSEAGAIAARVLADITAMLNAENIYTNAVQQQMLESHIRAMVLRSITGEPLPE

VDKSLFDEISAESMQMAERVVNQFGTLPIEEAYLLSVHFEVAKDNNA
>CORE_REP|Org35_Gene1029#
MSHSSHETSHGGASHGSVKSYLIGFILSIILTVIPFAMVMYGNDGSISHSTILAVVVGMAVIQVIVHL
VYFLHMNTSSEERWNLVALLFTAMIIGIVVVGSLWIMYNLNINMMVD
>CORE_REP|Org15_Gene221#
MNIERIDPDQRWSEAVVHNETVYYTSVPENLDDDATAQTANALAAIDVLLERVGSDKSRILDATIFLA
NTADFAAMNAAWDAWVVAGSAPVRCTVQAQLMNPKYKVEIKIIAAL
>CORE_REP|Org25_Gene1014#
MRIKALLGLSAAMLLAGCSTTHELTAAGQQIKFTDSKPAAECQLLGEVTGTQSNWLSGNGGEGSSMRG
AANDLRNKAAAMGGNVIYGANSPSQNLLSSFAPLDSKMVGQVYKCP
>CORE_REP|Org15_Gene382#
MESIQLSVVHRLPQSYRWLSGFTGVKVEPIPFNGIDEDNNLIGLKLLSHEGAEAWQVMQQLNLSLQEI
QVDCAIVEWEGEPCLFVQRSDESATMCRLKNVGAAIAEPLSAQYPF
>CORE_REP|Org7_Gene1208#
MSDETALPLQFTEAAANKVKYLIADEENPNLKLRVYITGGGCSGFQYGFTFDDKVNDGDMTIEKQGVA
LVVDPMSLQYLVGGSVDYTEGLEGSRFVVTNPNAKTTCGCGSSFSI
>CORE_REP|Org48_Gene863#
MKKSIIALSALLLASPVFAAETATDTAAHASNEVVAEAHKGADTAKEKLHQSQDKGEELKLKAKHAAE
GKSDSVGSKVSEGSQKAWHSTKQGTEKAWDKTKQGAESLKNKATE
>CORE_REP|Org24_Gene2713#
MKPLPTLNQDTVIELAREGGFAYIPKLAGQRRIALADITPEQRQRLNQLLNQTLPYAQEEGQPSSPGC
GDQRYYRVQINYTSPTLSTEIVLLIPESSAPQALVDLWKTGQVDE
>CORE_REP|Org31_Gene212#
MTLNITSKQMDITPAIRNHVEDRLTKLEKWQTQLINPHIVLSKEPQGFVADATITTPNGPLVASAKHD
DMYTAVNELIAKLERQLNKVQHKGEARRCNASVKDIVPEVTQEQE
>CORE_REP|Org19_Gene1559#
MAILVKALIGALVVVLIGLLAKTRNYYIAGLVPLFPTFALIAHYIVGNERSIAALKTTLIFGMWAVLP
YLVYLISLYFLINSLRLSLALGAAVLCWIAAAWLLITLWGWWQGR
>CORE_REP|Org24_Gene761#
MKSVLLGITLLATATGALAADKLVNITKLEYGKQWAFTKEEVTLQCRSGGALFVLNNSTLMQYPLNDA
AEQQVKKGHQRAQPLEVLLLDDPAEPGKKMSLAPFIERAEKLCAD
>CORE_REP|Org15_Gene3011#
MKKTLLLLAALLALPLASQADVSIGVNVPGLSLHIGDRDNRGHYWDGYRWRDPRWWADHRRYERPRGY
YYGPPPRVIYYGPPRRDWRDWRDRPHYRMPPPPPPGYYYRPGPRW
>CORE_REP|Org11_Gene1264#
MKSTPIALLLAALAAPFAAQAASFDCAKAANRDEQAICANRTLNDLDVQMATKYQFLRGLFAMGARGA
MQDSQQAWLAKRQRCGGDTACLLQSYRTRIAELDDIYRRIDKPL
>CORE_REP|Org1_Gene3210#
MYLRPDEVAKVLENTGFERDYVTDQAYGYRKGAHYVYVNREARMGRTALVIHPALKERSVHFATPTSP
VRFSEQYLEFPLDLSSDTPNQRYGIAHGFSSREALSRYLYSMFL
>CORE_REP|Org1_Gene23#
MKKIDAIIKPFKLDDVREALAEVGITGMTVTEVKGFGRQKGHTELYRGAEYMVDFLPKVKIEIVVADD
IVDTCVETIMQTAQTGKIGDGKIFVFDVARVVRIRTGEQDEEAI
>CORE_REP|Org28_Gene244#
MRCKTLTAAAVVLVMLTAGCSTFEKVVYRPDINQGNYLTSTDVAKIQKGMTQQQVAYTLGTPMLQDPF
GTQTWFYVFRQQPGHEDITQQTLTLTFDSAGVLTDIQNKPALTK
>CORE_REP|Org27_Gene1670#
MTEVSPEFESTIMELLVFSGSARSNALTALRQARAGDFAAAAKHMAESKEWVKKAHLIQTELIGLDEG
CGKLAINLITVHAQDHLMNAMVIQDLADDMIELYRRQSQTEGRP
>CORE_REP|Org27_Gene2609#
MAIGHYELKKAKNGQYHFNLKASNGESILASEMYASKASAENGIASVQTNSPHEAQYELKHSTSNQPY
FVLKAKNHQVIGVSEMYSSESAAKNGIQSVMKNGPTTDIRDLSA
>CORE_REP|Org19_Gene108#
MADSFTTTNRFFDNKHYPRGFSRHGDFTIKEAQLLERFGYAFNELDSGKRQPATEEEQLFVAVCRGER

EAATEQEKVWAKYLARTRRPKKFHTLSGGKPQADAVEDYSDSED
>CORE_REP|Org11_Gene546#
MPKIVFLPHQDLCPEGAVLEAEKGESILNVALRNGIEIEHACEKSCACTTCHCIVREGFDSLEESSEL
EDDMLDKAWGLEPESRLSCQALVADEDLVVEMPRYTVNHAREH
>CORE_REP|Org7_Gene1526#
MKRLVLVGVALCCACFAQARVDLEYHAAPPKDNKVPEEYQKKRSALRHKVWNVDSLTQANEQAIAREQ
QREAAEAAKRQQRHLEQQQKKQRCLRIHSKDPHREARCYMYNF
>CORE_REP|Org1_Gene282#
MYEALLVIFLLISIGLVALIMLQQGKGADMGASFGAGASGTLFGSSGSGNFMTRMTAVLATLFFVISL
ILGNLSSNQSKKGSEWENLGQPVKTEQTTAPAAPAKPSSDIPQ
>CORE_REP|Org48_Gene77#
MTMRELEIQFQNAMNELQSSFERQHREWQQSYQALQQLLEEAKQREAALRAQNEQLARKLSTASSVPE
QHALVKQIKMLGAHLDALAKDAATFNHHLRQQSAVGNFSGEQH
>CORE_REP|Org2_Gene457#
MQLPHCPKCNSEYTYQDNALFICPECAHEWSDSAPAEDQDALIVKDANGNLLADGDAVTVIKDLKVKG
SSSMLKIGTKVKNIRLVEGDHNIDCKIDGFGPMKLKSEFVKKN
>CORE_REP|Org7_Gene151#
MISTVALFWALCVVCVVNMVRYYSSLRALLVVLRGCDPLLYQYVDGGGFFTAHGQPSKQLRLVRYIFA
QRYVEHHDPEFIRRCERVRGQFMLTSALCGLVVICLIAMMIWY
>CORE_REP|Org2_Gene3003#
MKKLFAMMMCSALMLGVAGANAADGMSKDAMKKDGMSQMSHDGMAKDGMKKECMGKDCMKKDKMGKDA
MKKDGMAKDGMAKDAMKKDGMAKDGMAKDGMKKDEMKKDAMGH
>CORE_REP|Org22_Gene3973#
MLKVIAEDFIHPEHIETVMPWYRELVEKTRQEPLCISYELCIDQQDPGHFIFIESWPDRAALDVHCQT
EHFTRLVPQINQHQRKPCTFLFMQVFPGLNGDWMKKAPHGGRG
>CORE_REP|Org8_Gene298#
MQMSIHNQVRRSLQAIEQSMRDLALWQAAPPEHEAFSSTEPFCIDSMSAEAWLQWVFLPRMYALLDAE
APLPTRFAITPYFEEALKDREPSSLPLLVLLQQLDLMLNKEP
>CORE_REP|Org6_Gene1081#
MPSKSQAGQQAFGDIAPKLAQLSDSVLFDDVWQRPELSPRERSLITVAALVALNRVEQLPFHLQLAQR
NGVAHQQLAELITHLAFYAGWPAAASAVARLRELEPEAPHAV
>CORE_REP|Org24_Gene10#
METIAKHRHARSSAQKVRLVADLIRGKKVSQALETLTYTNKKAAGLVKKVLESAIANAEHNDGADIDD
LKVTKIFVDEGPSMKRIMPRAKGRADRILKRTSHITVVVSDR
>CORE_REP|Org33_Gene518#
MSFFISDAVASAGAPAQGSPYSLIIMLVVFGLIFYFMILRPQQKRAKDHKKLMDSIGKGDEVLTTGGL
IGRVTKVADTGIIAIALNDTTEVMIKRDFVAAVLPKGTMKAL
>CORE_REP|Org37_Gene613#
MQQFELYHIGFLGLAIVLEIIANIFLKMSDGFRKIWLGLLSLLSVLGAFSALAQAVKGIDLSIAYALW
GGFGIAATIAAGWIMFGQRLNAKGWIGLALLLTGMVILKLS
>CORE_REP|Org4_Gene216#
MLEFEGQVIDTDAQGYLKNSADWHEGLAPLLAAQEEIVLTEAHWEVVRFVRDFYQEFNTSPAIRMLVK
AMAQKYGEEKGNSRYLYRLFPKGPAKQATKIAGLPKPVKCI
>CORE_REP|Org3_Gene512#
MFGKGGLGNLMKQAQQMQEKMQQMQEEVAKLEVTGESGAGLVKVTINGAHNCRRVEIDPSLMEDDKEM
LEDLIAAAFNDAARRIEETQKEKMASVSSGMQLPPGFKMPF
>CORE_REP|Org46_Gene4740#
MKTTHILFVLLWLPGLAAAANGVTDLRSQQQLLNQNSQQAQQQRLLNNRQMDQRRLQQQRQFDNQRQQ
LLQTSPNGGQLLPNGNQPRDPFNRTAPAVPQAPMPATPARP
>CORE_REP|Org7_Gene770#
MTQLSLNDPALSEQGNEDWLRFVALLQNSFAQELHQPLLQLMLTPDERTALGTRVRIIQELMRGEMSQ
RELKNELGAGIATITRGSNSLKAASPALKQWLEQQLLHDAP
>CORE_REP|Org12_Gene3028#
MFSLGKLFGGRDSAKVCAIKRLPEVYAEMVGETGQCRLKRLRTDVGVFELHFVNADGEKYACQMTACV

TGIDLVFAVNNRSVLVSSPFTADKLRPVLDIAVADSPIPLI
>CORE_REP|Org11_Gene2400#
MKQAIKILALPCMGMALLFSASAMSAPEAPAAKALPPVAGEPMRMPPFPGGHPPKPGFCHGGELFCAT
SASDNPVETINKLTAVIPAGSAKHYEVRVAVVAVPDAPPAP
>CORE_REP|Org7_Gene380#
MTKKLVLAVGTAAVFSILAGCTAYDRATSYVNEPVVSDVKVGMTKQQVRAIAGPPSTSATLIHAQGTC
DTYAVAPRDGKVQTYFVSYNDTGHVMNKGYQTCSDYDSQPK
>CORE_REP|Org17_Gene468#
MAKNRSRRLRKKLHIEEFQELGFSVAWRFAEGTSVEDIDSTLDTFIDEVIEPNGLAFDGSGYLQWEGL
ICLQKIGHCTDEHRELVKNWLEARKLTDVKVSDLFDIWWD
>CORE_REP|Org30_Gene702#
MSQWITVCPVADILPGTGVCALIGDRQVAVFRPYADEQVFAISNIDPFAQASVLSRGLIAEHQGELWV
ASPLKKQHFRLYDGHCLEDDSRSVASFASRVVDGIVQVAA
>CORE_REP|Org17_Gene537#
MKKIMLMLAAAAALSACAQPAAPPEDAKLKQAYSACINTAEGSPERLQPCKAVLNVLKQEKQHQQFAA
QETVRVMDYQNCIMAVHSGNGQAYDAKCGKLWQEIRDNNN
>CORE_REP|Org26_Gene356#
MSSIEPDAVGLIVQRVCEYLQPVLNAILAGVMALLHGAYRNVGIRRRLLNAAMCALLAWTVRDALALM
GLELKWANLASVLIGFMGADYINALIKKFIGKKTGFKNVK
>CORE_REP|Org8_Gene475#
MSDKIIHLTDSSFEADVLKAEGPILVDFWAEWCGPCKMIAPILDEIAEEFEGKLTITKLNIDQNPATA
PKYGIRGIPTLLLFKNGEVAATKVGALSKGQLKDFLNANL
>CORE_REP|Org37_Gene123#
MEQFEAINVEQAYTRWKEGSAALVDIRDPQSFEAGHTPGAFHLTNATLSAFMQQNDFERPVMVMCYHG
NSSRSAAQYLLHQGFDAVYSIDGGFEAWARQYPQDVETSA
>CORE_REP|Org30_Gene2438#
MLKTTLLFFATALAEIIGCFLPYLWLKKQGSAWLLLPAAVSLMLFVWLLTLHPAASGRVYAAYGGVYV
ATALLWLRVVDGVKLSALDWVGAGVALAGMLIIVSGWRAA
>CORE_REP|Org12_Gene2315#
MQNVTPILIDSKQSKQEREEPSYSFLGGVSGFVFYWLAFALPFLVYGSNTLFFLLYTWPFFLALMPLS
VLIGITLSMLLRGRLILTLLLTGTIVLCLFWLVFSFLTGW
>CORE_REP|Org6_Gene3852#
MGKLTLLLLALLGWLQYSLWLGKNGIHDYVRVNEDVAVQQGNNAKLKARNDQLFAEIDDLNGGQEAIE
ERARNELGMIKPGETFYRLVPDQSRRNAASSSQNNAQNNA
>CORE_REP|Org2_Gene506#
MSITMSDSAAQRVQAFLTNRGKGLGLRLGVRTSGCSGMAYVLEFVDEANDDDIVFEDKGIKVIIDGKS
LVYLDGTELDFVKEGLNEGFKFNNPNVSSECGCGESFNV
>CORE_REP|Org25_Gene75#
MSDCEAVVILCTAPDEASAQELAARVLGDKLAACATLLPGATSLYYWEGKLEQEYEVQMLFKSDRRRQ
QALLDTLKQHHPYQTPELLVLPVMAGDKDYLLWINASLN
>CORE_REP|Org36_Gene2052#
MKKRYLWLAGAAWLFSGVVMALTLDEAKQQGRVGETLSGYIAPVKQDAETLALVKRINAGRAEKYQEV
ADGNHIAVDEVARMAGQKLVTRAQSGEYVRGINGQWLQK
>CORE_REP|Org40_Gene57#
MQLSTTPTLEGFTITEYCGVVTGEAILGANIFRDFFAGVRDIVGGRSGAYEKELRKARLIAFEELEDQ
AKELGANAVVGIDIDYETVGKDGSMLMVTVSGTAVKVSR
>CORE_REP|Org21_Gene366#
MDLNNRLTEDETLEQAYDIFLELAGDNLDPADILLFNLQFEERGGAELYDPSEDWSEHVDYDLNPDFF
AEVVIGLADSDGEPINDVFARVLICREKDHKLCHILWKE
>CORE_REP|Org43_Gene4616#
MAVEVKYVVVRNGEEKMTFASKKEADAYDKMLDLADNLGEWLQQAPLNLDDEQREGLSFFLAENKDVL
GLILRGASSAETLKKPAESKAKKAASTPKEGSASEKQAA
>CORE_REP|Org33_Gene50#
MGNNNDWLNFEHLAEEKQIDAVKPPSMYKVILNNDDYTPMEFVIDVLQKFFSYDIERATQLMLTVHYQ

GKAICGVFTAEVAETKVVHVNRYARENEHPLLCTLEKA
>CORE_REP|Org16_Gene915#
MIGNERHGLVGVIGGDLLRNAKIPMILLIASLVSAVFVVTTAHRTRLLTAEREQLVLERDALDIEWRN
LILEENALGDHSRVERIATEKLQMQHVDPSQENIIVKQ
>CORE_REP|Org45_Gene2098#
MNDSEFHQLADQLMLNIEETLDDFDGDADIDYETNGGVMTLSFENGTKIVINRQEPLHQVWLATKAGG
YHFNYRDGAWLCDRSGQPFYQLLSEAASAQAGEELRFS
>CORE_REP|Org14_Gene563#
MHKRTLASLHYENMPYDGVHFHILKRETPGHTALLKLDAGSRQPARFHPGWVKLMVLSGELKVDDQTL
QPHEMLIIPANTAYTVQAITEVICLAISELDGAELAR
>CORE_REP|Org42_Gene876#
MSWKNVCEVSQVKEDFPFSGNVEGKEIGVYLLDGNYYALEDVCPHAYALLSQGFVDDGKVECPLHEAL
FDVRTGQCLREPGGRDLQTYPTRVVDNQIQITFIAEE
>CORE_REP|Org33_Gene136#
MAEWNGEYVSPYAEHGKKSEQVKKITVSIPLKVLKILTDERTRRQVNNLRHATNSELLCEAFLHAFTG
QPLPNDEDLRKERSDEIPEAAKALMRELGVDPDTWEY
>CORE_REP|Org18_Gene749#
MQGKALQDFVIDKIDDLKGQDIIALDVQGKSSITDCMIICTGTSTRHVMSIASHVVQESRAAGMELYG
MKGQEASDWIVVDLGEVIVHVMQEESRRLYELEKLWS
>CORE_REP|Org17_Gene43#
MIASKFGIGQQVRHKLLGYLGVVIDIDPEYSLEQPKADEIAANDELRSAPWYHVVMEDEEGQPVHTYL
AEAQLDGEPQEAHPEQPSLDELAESIRHQLQAPRLRN
>CORE_REP|Org15_Gene2974#
MTANRLVLSGTVCKAPVRKVSPSGIPHCQFVLEHRSQQMEAGFSRQAWCRMPVVVSGQQSQALTQRLT
VGSQITVQGFVSCHQGRNGLSKLVLHAEQIELIDSGD
>CORE_REP|Org37_Gene1069#
MEKKKIYLFCSAGMSTSLLVSKMKAQAEKYEVPVIIAAYPEALAAEKGIEADLILLGPQIAYTLPEVQ
KQLPNKPVEVIDPLLYGKVDGLGVLKAAVAAIKKANQ
>CORE_REP|Org33_Gene76#
MAWIILLIAGLLEVVWAIGLKYTHGFTRLTPSIITIAAMVVSMLLLANAMKTLPAGTAYAVWTGIGAV
GAAIMGMVLLGESTNIARIISLCLIVVGILGLKFSSH
>CORE_REP|Org15_Gene2739#
MFIALYQFSVKTGHEHAFRQAWLELTQGIYQQRGSLGSRLHREVNGKYIGYAQWPSRSAWENAGDIQL
DERYRAARDRMRATLLADETLFEMDVTDDYLQIRPFD
>CORE_REP|Org35_Gene368#
MDNANKPSFQDVLEFVRMFRRKNKLQREIVDNEKKIRDNQKRVLLLDNLSEYIKPGMSIEDIQGIIAN
MRSDYEDRVDDYIIKNADLSKERRELSKKLKAMGEVK
>CORE_REP|Org32_Gene2245#
MDIDAMRKAAAQAGAMLRTLGNEDRLLLLCRLSQGEMAVSELAQALDIRQPTLSQQLGVLREEGLVST
RRAGKQIYYAVADAKALALLHTLYQLYCPQPENRDDH
>CORE_REP|Org21_Gene1094#
MKKPLIALTVTMMIAGCSTLKTDQAIPLLQAETAKMLGLGSSDEITVTNVNGAQPDALGGQKLSYRAT
TEKGRIFDCSSLMMPGILGSSPSLSAPTCTPVVTHK
>CORE_REP|Org17_Gene774#
MDLIIENVDSFKALAPRAPEPAPAPPPPVHHYDSFCENIALKVVLKVLLSSVDKIVKVQIANKVEQEI
AQIEQQVVRAGGGPVSPDDLAQMTHYVRRTVNDLLE
>CORE_REP|Org5_Gene485#
MIREQIEAKLRAAFEPAYLEVVDESYRHNVPAGSESHFKVVLVSDRFVGERFLTRHRSIYGVLSEELA
DGVHALALHTYTLKEWEGLQDTVPASPPCRGAGTLA
>CORE_REP|Org16_Gene1157#
MRSFGDLPRPVLVLEGLGIVMLVLAYLSIHGHLQLPGWLASQQAAVGMIFLGVALMVPAAAFLVWRVV
QGFGPLMRGGLPPENDRRKPRDADASKDNQDRDPRA
>CORE_REP|Org17_Gene753#
MKLFKTLSALCMAAVVAVAVSACAPTAKSEGTGGYIDDTVVTTKVKSALLADKNIKSREISVETFKGR

VQLSGFVTSSDDANRAVQVTRGVAGVKSVENVMQVK
>CORE_REP|Org36_Gene1640#
MNENDIFKALADPTRRTIFDKLAAGSMNASALREGLPISQSAMSQHLAVLRNAGLVREAKQGRCVNYQ
VDPDGLAQIAQWLAKYRAWWPARIDALQTLLKDMDQ
>CORE_REP|Org3_Gene4472#
MAIQGIEGVLQQMQTMAVQAGKMGQNTAPQGVSFASELTAALGKISETQQTARKQAQDFELGVPGISL
NDVMVDLQKSSVSLQMGVQVRNKLVAAYQDIMNMPV
>CORE_REP|Org8_Gene4527#
MTFVRTLIALTLAAQLSACGIMTTTPKPPPPPTAQAQAQEIVRAQTAKLVKIGTVTAVVRGSPMDVEA
EIQRKATAAGARYYVIIMNSETVVPGQWYSQALLYR
>CORE_REP|Org7_Gene1936#
MARHTDAEQTTLDDDLRMLTETLEEVLQYSGDRADQAYIDIKSHAEQALSEVKARWVNTGESYYARAK
EAVHRTDDYVRDKPWHSVGIGATVGLVLGLLLARK
>CORE_REP|Org27_Gene2252#
MSIDRTQRLQPVSTVQPRETPADNPLQPRKTAVAETAVSGTQVKLSEAQARLMQPGTQDIDMGRVEAI
KQAIRSGELKMDAGKIADALLQDAQSDIQWIAGRD
>CORE_REP|Org6_Gene593#
MYAVFQSGGKQHRVSEGQTVRLEKLDIATGEAVEFDQILMIANGEDIKIGVPFVDGGKIKAEVVAHGR
GEKIKIVKFRRRKHHRKQQGHRQWFTDVKITGISA
>CORE_REP|Org27_Gene6#
MQNQRIRIRLKAFDHRLIDQSTAEIVETAKRTGAQVRGPIPLPTRKERFTVLISPHVNKDARDQYEIR
THKRLVDIVEPTEKTVDALMRLDLAAGVDVQISLG
>CORE_REP|Org23_Gene4301#
MNHYLAVLSALVILPFALQAKPLSGSQVASGVVRFTGAITTPTCAITHQDEQFISNCFGKVTEYQNGY
ISSSLKEMPKELVSAVTSEMVNNNPHLKRVTISYK
>CORE_REP|Org49_Gene108#
MNDTIYQGIEENPRFKELVRKRGRFAWLLSLITLALYVGFILLIAFDPQWLGTPIAAGSTITRGIPVG
VGLIVISFVLTGIYVFRANGEFDRLTAEILREVKQ
>CORE_REP|Org10_Gene68#
MFKKAERTERDIDQDVTLLADTLDEVLRESGDKTKEELKELHSKAKGVLRDARARFNGSTSLTQHARD
AVDQADSYVRDKPWQGVGIGAAVGIVLGVLLARR
>CORE_REP|Org25_Gene22#
MSREITFFSRFEQDILAGRKTITIRDASESHFEPGEVLRVSRNEDGVFFCFIEVLSVTPVRLDALTER
HAQQENMSLGELKQVIKEIYPGLDALFVIEFVKR
>CORE_REP|Org14_Gene662#
MKIIREAMAGTLESSDVMVRIAPAEGPQHDLLIASSVEKQFGAAIRHTLLEVLQRYEVEPVQVIVDDK
GALDCVLRARLETALMRACEGGQLPWEAKDENAE
>CORE_REP|Org22_Gene1156#
MNTFRLFLIVGMLFSWTAVSHAGVAGGVIRFVGSIVESPCTVNIADSKANTQCYRNGQRYQAQQALSG
FDTTRKELPLNLGTTEMKWVDQQKKLAVMTVVYR
>CORE_REP|Org13_Gene1089#
MITVIAEIKVKPGHRATVLQAIEKLVPLVLAEEGCGEYTPMVDSHTQAPWQKLSPDSVFMLEKWQSQA
HLEKHLQIDHMLKHRETIKDYVLGTEIYVLENAL
>CORE_REP|Org6_Gene614#
MAKGQSLQDPFLNALRRERVPVSIYLVNGIKLQGQIESFDQFVILLKNTVSQMVYKHAISTVVPSRPV
SHHSNNPSGGSSNYHHGNNPSAQQQPQQESDDAE
>CORE_REP|Org31_Gene4662#
MTDTSPTLWHLYMLRLPSGMLYTGITTDVARRMAQHQAGKGAKALRGKGELTLAFHCQVGDRSTALKL
EYRVKQLSKIQKERLVNHPPLSLEYLLPVVVKSD
>CORE_REP|Org19_Gene94#
MAKQSMKAREVKRVKLADKFFAKRAELKAIISDVNASDEDRWNAVLKLQTLPRDSSPSRQRKRCRQTG
RPHGYVGKFGLSRIKLREAAMRGEVPGLKKASW
>CORE_REP|Org38_Gene144#
MMKKEITFTVVELCQRVEISEDELVEIVGLGVIVPLEPAQPRWEFDYPALSHLQRARRLRAELDLDWP

GIAMALTLLDRVDALQQENRQLRRQLARFLQTS
>CORE_REP|Org17_Gene456#
MAHDSNAENLRAELKSLADTLEEVLNSSTDKPKAELEKLRSKAEGALKETRARLSDAGDKLASQTKQI
AGQADDYVRDNPWTGIGIGAAVGVVLGVLLARR
>CORE_REP|Org48_Gene1469#
MTTFTAKTAFLPLDNGLARREGVMSDGSPAAEVRFEAGAFGQLQKPPHALQTRVISGEFEFTLGGDTR
TVRAGESLILPANVASGCFCLSAGVLLEIPQTR
>CORE_REP|Org42_Gene1814#
MAKEMAAIFLLLLLPLSQSAGAARYDRPCEGVSPVLFQKRLKALAQDLRQEMSEEATPTERDAEELAR
LAECGIEPGKAPVTGGQRAPHSEEKPPASPNGC
>CORE_REP|Org21_Gene331#
MKKVIFVAGALALSGCSYILPQSSQTMHYQCGTTPLTVALDGKASEVSLLMDGEQLHLKQVLALTGAK
YSDGKYTFWSKDRNAYLERNGKVVMSDCVLTE
>CORE_REP|Org3_Gene167#
MKYLLIFLLVLVIFVISVTLGAHNDQVVNFNYLVAQGDYRVSTLLATLFGAGFVLGWIICGLFYLRTR
IALGRAERKIKRLELQLEQPAEPAAQPVVSKE
>CORE_REP|Org30_Gene335#
MIPLQHGLILAAILFVLGLTGLLVRRNLLFMLISLEVMINAAALAFIVAGSYWGQADGQVMYILAISL
AAAEASIGLALLLQLYRRRHTLNIDTVSEMRG
>CORE_REP|Org16_Gene1850#
MAKPLSQLIDKLDPAVVAAARQKADKEIFELRLAMLREELAVSQVELAKRLGISQPSVANLEKRGSEI
KLSSLKRYIEAMGGTLSLDVQLPNGQHRRMTL
>CORE_REP|Org30_Gene27#
MIKWYEESDAEVNRSIALLTGENPDKWYPYGGVKGKDYCKNPSDAWPIICANKISLNPDNQSDSPQWQ
ARMSTQGGEWQADSASPLRAAMICFLMSRQAN
>CORE_REP|Org3_Gene565#
MIREERLLKVLRAPHVSEKASAAMEKSNTIVLKVAKDATKAEIKAAVQKLFEVEVEDVNTLVVKGKVK
RHGQRVGRRSDWKKAYVTLKEGQNLDFIGGAE
>CORE_REP|Org44_Gene3959#
MSKILAEIHQEVQGLHRAGFVDDVTMRTFDMLCLRPVKQFGPAEIRALRERENVSQPVFALYLNVSKK
AVQKWERGEAQPNSAAMKLLTLVERNGLAILA
>CORE_REP|Org26_Gene648#
MSAALSFESEQQTLILRGELDRETLQPLWRQRDALMADKTAIDVAQLQRVDSAGLALLLHLQEEQRER
GVALKIFGATERLKTLIALYNLQAIMPVDTAG
>CORE_REP|Org48_Gene1448#
MSQSATNCFVVTFRYQEAGLTDLAKLTGQLTREGFVTSVTDENGVHHELGSNSFAFITVLDQEDVLRL
AQGFGQLALGKQPEVTICACEDYLQRLHTDT
>CORE_REP|Org39_Gene1732#
MAKVEFDFEQIQDVPAFYRDFARKFALDEGFGANLDALWDVVTGDIGLPVEIEFTHLNARSKRRFGAI
ILLFEEAEEELEGSLRFNIRESSGEPAHHRG
>CORE_REP|Org37_Gene1344#
MPGHRFTITVEALSDRQGNPVEKAPLSFEVTNHDDILDIVERIRARDDLNFGPEQSAAFAVGLKLFSE
VMIENRKHPVFAPLRDAFKEFMVGLKKGPAA
>CORE_REP|Org18_Gene2118#
MKKTLILTAVLLAGAPLAALASCESVKADISQKIINNGVPESGFKLEIVPNDQADQAGGQVVGHCEND
TQKIVYTRLNNGDDRGDAAQTGSSQDTSNTQ
>CORE_REP|Org20_Gene634#
MSHSEQLQELLQRVAALEAREKALSAASNAYQAIITTMLGNMEKTERDRIIAMIDQAHEIAYARAIQR
SNEPQKQKIKQADDVAQRMFMFAQGKAAQPR
>CORE_REP|Org15_Gene1548#
MKDYKNTHGTRLLLIFTLAFYILLPPVLTACVFTRFNLNPFAIVKFLHFNPFFADRGIPGYQTFLYLL
MLWLGGNILLWLMVWGAGRLYRRWRSRRGE
>CORE_REP|Org13_Gene1412#
MSSSVIDKLYAVTDKGPIRALSLVLALILAGCVFWDPTRFAAKTSSLEIWEGLLLIWAVCAGVIHGFG

FRPQRSIWRAFFAPLPAIVILCAGLLYVSL
>CORE_REP|Org32_Gene3319#
MSDAINKCSAQETAACCCVDVGTVMDNTDCTASYSQVFSNQQDAEAMLAALSEKARAVESDPCDISSS
IKPVDGGVQLEADFTFACQAETLIFQLGLR
>CORE_REP|Org29_Gene1802#
MLYLIYAQDVPNSLENRLAVRPAHLARLQALRDAGRLVVAGPNPAIDSNDPGAAGFTGSTVIAEFESL
AEAQAWAQQDPYIAAGVYADVTVKPFKQVL
>CORE_REP|Org44_Gene239#
MALTKAEMSEHLFEKLGLSKRDAKDLVELFFEEVRRALENGEQVKLSGFGNFDLRDKNQRPGRNPKTG
EDIPITARRVVTFRPGQKLKSRVENASPKG
>CORE_REP|Org1_Gene402#
MKEQYTTSVKVEGKGDSKAKAFASALANVQGAVLKSTNNILLRIEPQDVSVVKAEEKITKEKFLFFFL
PRERKSYAVSLEITVNVTIINTEKVVFVTK
>CORE_REP|Org25_Gene2#
MFEQRVNSDVLTVSTVNSQDQVTQKPLRDSVKQALKNYFAQLNGQDVNDLYELVLAEVEQPLLDMVMQ
YTRGNQTRAALMMGINRGTLRKKLKKYGMN
>CORE_REP|Org10_Gene1483#
MKRGLNGVLAAAALAYCGLAAANRGDVDVVLPVSPEIWGTAKSGVNPPPAQPCNRCCVYQNQNYSEGA
VLKVEGEVLQCVREPNTIGTNPLIWVRLKK
>CORE_REP|Org40_Gene693#
MKFYGKTLLLTALLALSACSSSPEQGAGAQMADADDTCGASQYQNFVGKPMTSLEGVRIDAKVRAIPY
NSAVTMDFNLRRLNFLGDSDDKIIRVYCG
>CORE_REP|Org45_Gene516#
MHVTLVEINVKQDKIDEFIDVFRANHLGAIEEPGNLRFDVLQDEQIPTRFYIYEAYRDEQAVAAHKQT
PHYLQCVEKLEALMTGPRKKTTFIGLMPE
>CORE_REP|Org12_Gene55#
MKNAPAVTIHYCSQCNWLLRAGWMAQELLNTFGDDLASVTLMPGTGGVYQISVDGVMIWDRKIDGGFP
DAAQLKQRLRDHSFPDRSLGHSDGKKARH
>CORE_REP|Org44_Gene746#
MLQPQDKPQVILELAVRNHPGVMSHVCGLFARRAFNVEGILCLPLKDGQQSRIWLLVADDQRLEQMIS
QVEKLEDVLQVKRHSEDVRVFEQLEAFFQ
>CORE_REP|Org38_Gene287#
MNLNNKQKQHLKGLAHPLKPVVMLGNNGLTEGVLAEIEQALEHHELIKVKIAAEDRETKTLIADAIVR
ETGACNVQVIGSTLILYRPSKERKISLPR
>CORE_REP|Org12_Gene78#
MSIRPLHDRVIVKRKEVESKSAGGIVLTGSAAGKSTRGEVVAVGKGRVLENGNIQPLDVKVGDIVIFN
DGYGVKAEKIDNEEVLIMSESDILAIVEA
>CORE_REP|Org35_Gene1866#
MGDHFGKGLLAGLKADNLKPEAELSRFCSDYKRGFVLGYAHHLAQRCGDENRAAFEAGQLSRAYGLGS
EPMSEFFSGGDSRLAEKFFRAGYNRPAQG
>CORE_REP|Org6_Gene1756#
MSAVTPVLDGLAIRLYIQPKASRDQIIGLHGDELKVAITAPPVDGQANAHLIKFIAKQFKVAKSNVTI
EKGELGRHKQLRIVNPQQIPAVVAALYTK
>CORE_REP|Org25_Gene2858#
MRLPPTSVIPLLFTLLLAGCQYHKTVFPPPYIGTPQEIGTYEVKGLTFVTTVTFSGLFAEDLVREYAQ
KHHYRYYVVTMRSNGFKDRSERVSAMMYR
>CORE_REP|Org30_Gene4244#
MRNLLMLLVGLFSLPAMAAESHVCHSQAYDYAEVSKLRLSDDTLFICRDVGRLTIPELARKGWKVIHL
AQQTEYTDSVDSDGEVIKLYQEIVVYKE
>CORE_REP|Org6_Gene1204#
MLKVNEYFAGKVKSIGFDSSSIGLTSVGVMEEGEYTFSTAQPEEMTVITGALKVLLPGAPDWQVFTPG
EKFFVPGHSEFNLQVADATAYLCRYLSK
>CORE_REP|Org43_Gene1847#
MSRSTSRKSGAGLGRRIRSALLAQKQYWKMYFAMKLRRVRVPAPLVLLGGSLLGFFMLTLLMLSVVTI

DVISTLLLLCRKLLGRGDGEGRAFMPRS
>CORE_REP|Org46_Gene459#
MSRRRYLEWKKERLIRQIQQQRLDLAEHKSLWLEKTERIDRGWQTVFGLRRYLVLGSSVMALYGIRHP
SKMIRWSRRAFGAWSAIRLFKKTFSAK
>CORE_REP|Org42_Gene2469#
MAKVPKTFESRKSLMLDVLRGLCRDLVEGAGGLHPPPEQWPKTRELADKCGENIYTTRTLLLALEKEG
KVRCTHRSINNSLRWYSCDEQAAIKKE
>CORE_REP|Org41_Gene478#
MAKEQTDRTTLDLFADERRPGRPKTNPLSRDEQLRINKRNQLRRDKVRGLRRVELKINADAVDALNKL
AEQRNISRSELIEQMLLAQLAEEQPEH
>CORE_REP|Org47_Gene1299#
MLYTLSHSPNQCDLPALLRLTAEGDALLLLQDGVLAGLAGSAHLESLLAAPISLYALQDDLEARGLVG
HFSHKITVVGYNHFVELTEQHRSQMAW
>CORE_REP|Org28_Gene1924#
MQPYPYKEVEFDHELCLAIGKAVLDVVSQGDETSAPGVMNAIERSVEQGLNDDEIAAADDALDLMARL
IHGCRFVNLPGEEEASRALVEFQTLQE
>CORE_REP|Org7_Gene1170#
MLTAMIAACGLWGVSWCLGDRLASAWGVLLPCALMPLLALIDLDMVQLRTLIVIAMLATLVMLFNSRL
RHYLLLPSCMALAGGLAAVSLNFSFG
>CORE_REP|Org36_Gene3035#
MSDIQVEVVYALPERQYLRKVKLAEGSSVEQAIQTSGLLELRQDIDLKSNKIGIYSRPAKLGDTLNDG
DRVEIYRPLIADPKELRRQRAEKAKK
>CORE_REP|Org34_Gene145#
MKSLKMIIAAAVLGSVSFGSMAATLLTKEDLDKNPGKYEKIGTVTTTAETTSPMDAKEELSKLADEKG
GQYYVILAGREHGKFSAIAEVYKDKQ
>CORE_REP|Org30_Gene2127#
MKLIPSIAAVLIAAASFSTFAAPLSSVKQVNSEQAANLQSVGVVSVSGISGSPHDAITALKEKAAADG
ASHYRIIGLDTPGDSSNWRGNAEIYR
>CORE_REP|Org8_Gene545#
MFTINAEVRKDQGKGASRRLRAANKFPAIVYGGKEAAVSIELDHDSVKNMEAKPEFYSEAVTLVIDGK
ETKVKVQAVQRHPFKPKLAHIDFVRV
>CORE_REP|Org4_Gene456#
MAKTACALHILVDNEKLANELLAKLKRGVSFDTLARKYSSCPSKRNGGSLGEFNKGAMVPAFDKAVFS
IPLLKPYGPVKTQFGYHIIKVLYRN
>CORE_REP|Org12_Gene2300#
MSTIATGLVMMRWELLSAVMMFFASQLNVVCRKTSRNGMAFMFSSLGLFTACWFVMGLMGIHLTLEAF
TQFWSSAWDRYVDVVSTMPVDWPMP
>CORE_REP|Org17_Gene4301#
MIAEFEARILALIDDMVEHASDDELFAGGYLRGHLTLAVAEAEEQGEHTAEALKTRVEESLHKAIAAG
ELSPPDQILVRGMWENLYQAATPAA
>CORE_REP|Org25_Gene1380#
MAPDKVKSEDAMTFGELLALIADQKRRLNVLEVAFSYLAFSLDDKANQRLIHSLRLESQSQNRDTETQ
KHFAHLAEELEKCLHGSAPPTGGAE
>CORE_REP|Org24_Gene3422#
MTLNPEHTPVFRRGYRLQWEQVQNSHVILYPEGMAKLNDSAAAILQLVDGHTTLNGIIAQLNARFPGA
EGLAEDVLEFFQRAYEQKWVTFRD
>CORE_REP|Org24_Gene3613#
MKRRNADRMGNFFMGLGLVVMIGGVGYSIIAEVSQFNLPQFFAHGAIMSIFVGALLWLVGARIGGREQ
VADRYWWVKHFDKRCRNDQHRSSH
>CORE_REP|Org23_Gene1591#
MYDLGFGQNGLLSLALAVVALLAGLWVWFLVNRASVRANEQIRLLQEIAEQQRQQTALLKRLAHSAGA
DGAAAADDDDLSPALDFKGFIPER
>CORE_REP|Org7_Gene104#
MPRSLKKGPFIDLHLLKKVEKAVESGDKKPLRTWSRRSTIFPNMIGLTIAVHNGRQHVPVFVSDEMVG

HKLGEFAPTRTYRGHAADKKAKKR
>CORE_REP|Org2_Gene3975#
MGINPVFARRLYLCWLISHSERPNVPRLMALTGWPRRTLQDVLKALPGMGIELQFVQQGVRNNDGFYQ
LESWGPFNKSWVHQHHQALLSAIE
>CORE_REP|Org41_Gene2135#
MTQVCIAAYVYGVVQGVGFRYSTQRQAEALGVTGYARNLDDGSVQVVACGTQAQVDKLVAWLKQGGPR
SARVERVLVEPQGVVDYAGFGIRY
>CORE_REP|Org43_Gene2101#
MDHKILADEKRFYINDEHGKLIAEISFVPSGDKLTIIDHTWVDESLKGQGVGKKLVALVVEKMRAEQR
KIIPLCPFAKHEFDTTPAYQDIRA
>CORE_REP|Org30_Gene1689#
MKRSTALTSLLLSVGLLSTGAQSAELAPADRVTPLSEIAEITFNDLPGSPQEAEQAIARTAGQHGASY
YRILRMEEQAHPLGWRASAILYL
>CORE_REP|Org1_Gene3607#
MSLLKRLLGQGGINGGHLRASHGGGHHGNRYNQHGEPGPQCPECRAVNKSGARYCHRCGQPFAAASAT
CGQCAAPLPPGSRFCPQCGGGVP
>CORE_REP|Org6_Gene948#
MIDPKKIEQIARQVHESMPKGVREFGEDVEKKIRQVLQSQLTRLDLVNREEFDVQTQVLLRTREKLAL
LEQRMAELESKLSAAPAAKQEDE
>CORE_REP|Org26_Gene152#
MARVTVQDAVEKIGNRFDLVLVAARRARQIQTGGKDALVPEENDKYTVIALREIEEGLITSQILDVRE
RQEQQEQEAAEIQAVTAIAEGRR
>CORE_REP|Org43_Gene277#
MDVKDLIAAMTPEIYQRLVQAVELGKWPDGVALTPEQKENSLQAVMLWQSMNNVDPQHMSIGTDGQIV
MKSKQELKQQFVAEPLVKLKPQ
>CORE_REP|Org36_Gene415#
MSRTIFCTFLQRDAEGQDFQLYPGDVGKRIYNEISKEAWGEWMKKQTMLINEKKLNMMNVDDRKLLEE
EMIKFLFEGHDVHIEGYTPPSE
>CORE_REP|Org5_Gene505#
MRSDIQFIQQPEIDVHHYERGDTVRLTTANLRHEYQLDVYILRRDDKLIYGSVVAAAPKTDIPVASWE
VKNGEEVAFRQENIAKAVPAVN
>CORE_REP|Org46_Gene1029#
MLCVIYRSPKRDQTYLYVEKKDDFSRVPEDLMKSFGVPQFAMMLSLDERKKLASADIEKVKQALKDEG
YYLQFPPPVENLLNQHLAGDKA
>CORE_REP|Org40_Gene2607#
MTVKQTVEIKNKLGMHARPAMKLFELVQSFDAEVMLRNESGTEAEASSVIALLMLDSAQGRHIEVEAT
GPDEVNALTAVVELFNSGFDED
>CORE_REP|Org36_Gene505#
MNKSQLIDKIAAGADISKAAAGRALDAVIASVTDSLKAGDDVALVGFGSFTVRERSARTGRNPQTGKE
IKIAAAKVPAFRAGKALKDAVN
>CORE_REP|Org16_Gene309#
MLINLGRLLMLCVWGFLLSNLFHPFPKPLKYFIDVALFFMVVMHGLQLVLLKSTQPKDQPISYWQEAK
IFIFGVFELLAWQKKQPPIKKK
>CORE_REP|Org33_Gene441#
MQQKPVLDEARQLKRLNAATRRQSHMYKWIALIAALDALLVFWYDRDMRNVTFSGAVALVCGYLWIRD
RNKARGYRREFDRKFGKRSGD
>CORE_REP|Org18_Gene1923#
MTDYAFYNQILTRLAANHPGTLDEKTYELWKQDATSPHAFADPFAYLKTKGLIQAYVMSDIDENNYDI
DPHQTRITAAGLEFIRNGGFK
>CORE_REP|Org49_Gene211#
MALLDFFLSRKKQTANIAKERLQIIVAERRRGDSEPPYLPDLKRDILAVICKYIQIDPEMLHVQFEQK
GDDISVLELNVTLPESEEAAK
>CORE_REP|Org9_Gene258#
MSLSVEAKAQIVADFGRGTNDSGSTEVQVALLTAQINHLQGHFSEHKKDHHSRRGLLRMVSQRRKLLD

YLKRKDVARYTSLIERLGLRR
>CORE_REP|Org24_Gene2170#
MKKTLMLSLLAGMTMLQGCSVKSNDAPPPPPPQVKPIGMANPADVYCTQIGGKLNAKENAAGQYSTCT
LPSGQEIESWELFRRDHPVKK
>CORE_REP|Org36_Gene161#
MKTHRVNELIELLHPAWQEDPDLNLMQFLQKLAKEAGFQGELSELSDDILIYHLKMRGSAGTDQIPGL
KKDYEEDFKTALLRARGVIKD
>CORE_REP|Org41_Gene320#
MTPESVMALGTEAMKVALALAAPLLLAALISGLVVSLLQAATQINEMTLSFIPKILAVVATIIIAGPW
MLNLLLDYMRTLFSNLPTLIG
>CORE_REP|Org29_Gene3377#
MLKRLRHRLFTLMATLLFIGCLHSASLDESRMPRNLQPSLSALSSNMQDIREVLARCAGEEEEERLNG
GDMHIEARAAGEDEVEDKRLA
>CORE_REP|Org41_Gene4205#
MNKIDVANTAKTGGFKRGVAAMLKTLAKVIIIALLNYGPAGAAGWLLKTVGRKPVRFVLALVLEPLFR
KGLNKAFGRYVKESNETTAK
>CORE_REP|Org18_Gene251#
MDINNKARIHWACRRGMRELDISIMPFFEYEYDSLNDADKALFIRLLECDDPDLFNWLMNHGAPQDGE
LQRMVTLIQTRNKDRGPVAM
>CORE_REP|Org6_Gene608#
MKRIILATALAALFSANVMAATEITSHQADQRQSVGFVTLNQNVVSPDDASSQVSKIADQRGASSYRI
IALHEPGDNSTMHVSAELYR
>CORE_REP|Org21_Gene619#
MQKTKLNELLEFPCSFTYKVMGLAQPELVDQVVEVVQRHAPGDYNPQVKPSSKGNYHSVSITINATHI
EQVETLYEELGNIEIVRMVL
>CORE_REP|Org47_Gene518#
MSQHQSKDPLHGVTLEQLLNKLVDHYGWSELGARIRINCFRSDPSIKSSLKFLRRTPWARKEVEDLYI
DMVSHPAPASDNPWLRGRDG
>CORE_REP|Org32_Gene2267#
MNIESRNLRDEASQVYLEQVLVFDEMERLRLARQVLCGIFAVSVGVFAAHAYWEKNEALNQVFELVKI
GALPLVTLVVSFYFPNSASR
>CORE_REP|Org10_Gene465#
MSNNANAQAQLDNLRNVASQLKEMRHYAQANTETLSAHWLAFDQGECKNKAFAEAINDLLNKQGACLE
GLEKTIQDIEIELNRLDKAA
>CORE_REP|Org14_Gene1826#
MASGWAADGAVQDQIDSTVDDAVQRAREALGHGESERYCQECGEPIPEARRKALIGVRFCLACQTEQD
KKHAAASLYNRRGSKDSQLR
>CORE_REP|Org29_Gene4287#
MTEKKTYNLASLSREDMDKVNVDLAASGVAYKERMNMPIVPAQVEAEQPENLRDYFKERLQHYREQSA
RFPGPNDPRYQQMAEANGKK
>CORE_REP|Org15_Gene253#
MANIKSAKKRAVQSEKRRKHNASRRSMVRTFIKKVDAAIAAGDKEAAQNAFLVMQPLVDRQAAKGLIH
KNKAARHKSNLTARINAMQ
>CORE_REP|Org15_Gene205#
MLMNDEYQPINCDDYDNLELACQHKLILKLELRDGEVIEAKAIDLLQKKRVEYLTIEQNGQQRDLRLD
HISSFSHPEIGTVVVSLSD
>CORE_REP|Org15_Gene287#
MKIKATVATLSVLSMLSFGAFAAQSVDATQAAKLQPAGVITVSGVAAAPSDIRQALSDKADAKGATAY
RVIEARNDGNFHATAEIYK
>CORE_REP|Org2_Gene422#
MFAVIFGRPGCPYCVRAKELAEKLTEERDDFNFRYVDIHAEGITKADLEKTVGKPVETVPQIFLDEKH
IGGCTDFEAYAKEHLNLFQ
>CORE_REP|Org28_Gene1285#
MKKLTLKEMTESEQREVKTELDKARKSHGRPLTNAEQHKVKDEVVTRIMAARAKLAKAERAERKANRY

RPSGDTFSWSATIGSRPPR
>CORE_REP|Org49_Gene128#
MATITVRNLDDEIKELLRISAAKNGHSMEEEARMILKQALVKKPPRYGLGTWMHQHFAEFGGVELEIP
PRDAVPPRIVTFDDEDDNA
>CORE_REP|Org47_Gene1752#
MPKKPAQSASTEQTASFESALGELESIVTRLESGELPLEDALNEFERGVQLARQGQQKLQQAEQRVQI
LLNDSADDAALAPFTPDTE
>CORE_REP|Org21_Gene3421#
MRWQSKANIDIYTKATCPFCHRAKALLNSKGAAFNEIAIDGDNAKREVMIERSGRTTVPQIFIDGRHI
GGCDDLYELDARGGLDPLL
>CORE_REP|Org47_Gene4525#
MNNPAASQNMAPNGTIKSDWHRADIVAAVKKKGTTLAAISRAQGWHARTLNNALDRHWPKGENIIATV
IGVSPAEIWPTRYSDGVAQ
>CORE_REP|Org19_Gene2923#
MALLITRKCINCDMCEPECPNQAISMGDEIYQIDTDRCTECIGHYDTPTCQQVCPIDNTIITDPQHRE
TNEQLWDKFVVLHHADRI
>CORE_REP|Org49_Gene1170#
MKNVKIFATAALLATASFATFAADLPSSQPAADAQKIGVVSVSGASNLSALESELASKAAASGASSYR
IVAAGGQNKLYGTAEIFN
>CORE_REP|Org37_Gene1156#
MATPIVTLRAAGTQLSCPNSENCLLDVLELHDVQVEYQCRSGYCGACRLKLVKGEVAYRQQPLAFINA
GEILPCCCMPLTDIELEM
>CORE_REP|Org20_Gene1906#
MNERQNTIALSIDGEHRRVPVGLSVAAALSLCGDDRCRLSVSHQPRAPFCGMGVCQECRVNINGLRRL
ACQTLCQAGMRIERSDDE
>CORE_REP|Org20_Gene2102#
MKTIKTFAAAIAVATVSFTTFAAEHVSAQDAAQFEKAGVIVASGATDLSSLKSQLAAKADAAGAKAFT
ITSTSGNNLMHATAVIYK
>CORE_REP|Org42_Gene1074#
MFQQEVTITAPNGLHTRPAAQFVKEAKGFASDITVTSNGKSASAKSLFKLQTLGLTQGTVVTISAEGE
DEQKAVEHLVKLMAELE
>CORE_REP|Org5_Gene113#
MMQHQLSIQARFRPEMLERVLRVVRHRGFQVCAMNMVSPANADSINIELTVASPRPVALLSSQLSKLM
DVSCVEIQQPTSQQIRA
>CORE_REP|Org5_Gene270#
MAHKKAGGSTRNGRDSEAKRLGVKRFGGEAVLAGSIIVRQRGTKFHAGTNVGCGKDHTLFALKDGKVK
FEVKGPSNRKFISIEAE
>CORE_REP|Org48_Gene306#
MASPLSPGSRILIGLVRAYQLVISPLLGPRCRFQPTCSHYAIEALSRFGMIKGSWLALKRVLKCHPLN
PGGDDPVPPKTDDNREH
>CORE_REP|Org35_Gene4361#
MTTSKWLLASAVLLLAACSSNNNEEPVQQGNSARINTLQTSAGCAAVGGVTTIAHNLNGEALRMCQMP
NGKQCEERTLGLGSCAG
>CORE_REP|Org46_Gene990#
MDKHYVLDIQYAGKAFARLQMFTPWAADQLEQACALFPAAQGYQVQLSQVSERRIVYQSGAQGIAVLG
ESLALTPFTHAHEVKQ
>CORE_REP|Org46_Gene543#
MKPGIHPDYRTVVFHDVSANAYFKVGSTIKTDRTIELDGESWPYVTLDVSSASHPYYTGKQKDYSKEG
STARFQQRFGRFIGNK
>CORE_REP|Org17_Gene36#
MDRNDEVIQTHPLVGWDISTVDVYDAMMIRLHYLSSLDQTPEEAQVDRTLWLTTDVARQLINILEAGI
AKIEATDYQDLDRRKH
>CORE_REP|Org38_Gene102#
MTEVIRTLQGRVVSDKMEKSIVVAIERTVKHPIYGKFIKRTTKLHVHDENNECGTGDVVEIRECRPLS

KTKSWTLVRVVEKAIL
>CORE_REP|Org15_Gene1000#
METNEIKDVLMQALALDEAHVTGDGSHFQAIVVGELFAGMSRVKKQQTVYAPLMEYIADNRIHALSIK
AYTPEEWQRDRKLNGF
>CORE_REP|Org11_Gene793#
MDCRSDCGACCIAPSISSPIPGMPNGKPANTRCIHLDEQLRCGLFHSPLRPKVCGSLQPSRDMCHDHR
DQALIYLARLEADTAP
>CORE_REP|Org23_Gene1059#
MGISEEEHIRRLTQEKNDMGNTAKWVAIVSAVYFALMVFYGHPTGVLAMSGAVFIVSTTTWLKKRQKV
KSYRMALAKISDDQP
>CORE_REP|Org23_Gene2420#
MHKNTAPIGREALLAEANNIIRQHEDYLHGMIATDVEQKSGVLVFRGEYFLDADGLPTAKTTAVFNMF
KHLAHLLSEKYHLVD
>CORE_REP|Org3_Gene454#
MFGKAEDKANEAAGAVEEAFGKAVDSPEHQVRGAARKYASQASYAARDAADSVRTQVEANPLAGVAVA
AAVGIVFGFLLGRK
>CORE_REP|Org48_Gene137#
MLRDHLKINASDTLEKISNIHLQNQGQEEISEFVVKNAAGLQIGKVSVHDRFSTRRSYPTSYRITQTD
MSGRVVVDAMRDCL
>CORE_REP|Org22_Gene356#
MVTIRLARGGAKKRPFYQVVVTDSRNARDGRFIERVGFFNPIASGQAEALRLDLDRIEHWVGLGATVS
DRVHALIKDAKKAA
>CORE_REP|Org2_Gene902#
MSEEAKLPQLLEHMILNLRMIYARATLVEKALAHILASDAGLKNDIIKQLQVVTAANERDRIDLDEAR
IHLIDVLNSVPAKK
>CORE_REP|Org2_Gene945#
MGIISWIIFGLIAGILAKWIMPGKDGGGFIVTVILGVVGAVVGGYISTFFGMGRVDGFNLGSFVVAVI
GALVVLFVYRKIKG
>CORE_REP|Org25_Gene2792#
MFVELIFDQRNVKGLPDAAEIIKAELTRRVHRVFPDAEVKVKPMQTNGLISDANKSDREKLNRLLEDM
FEESEQWLMSDIYG
>CORE_REP|Org7_Gene1019#
MTDLFAQADQTLDALGLRCPEPVMMVRKTVRHMDNGETLLIIADDPATTRDIPGFCRFMEHTLVAQET
EQAPYRYLLRKGV
>CORE_REP|Org32_Gene1233#
MKLSSYLRLACVAAGVMVLTGCGSIISRTMPGAGHGHQYYPGVQWDLRDTPWRYVTVIDVPLSMVVDT
FMLPFDAQHGPYE
>CORE_REP|Org37_Gene2772#
MIDILFFAQVRELVGTGGLSMPADYPTVEALRQALCARGDRWALALESGKLLAAVNQSLVAADHPLRP
GDEVAFFPPVTGG
>CORE_REP|Org4_Gene2153#
MHAGMPLLSHAEQQEAADRIHQLMEQGMSSGEAIARVAQEIREKHQGAQVAVLFDDDDEDEQHDAAPG
ASRDDDAEEDEDY
>CORE_REP|Org29_Gene854#
MKIQLSLLFLSLLAGKAAAFQSKVPEPRPEQARAAEQNIENLFYGFHAMDAQPVNIGSCAAVPVAGCQ
CAFCTQLRQAGR
>CORE_REP|Org34_Gene2575#
MDKRFIQAHREARWALGLTLLYLLAWGLAAYLPDSAIGVTGLPHWFEMACLLVPLLFTGLCWLMVRTV
FRDISLEDRDAN
>CORE_REP|Org28_Gene5173#
MYSIEDNSKAEEYALLAFVCQWICANKRRLYRRHSLRCDGAGFYQLRSWEYSVSRENSPMFCRGGRKP
LAAVENRPGSAG
>CORE_REP|Org27_Gene1580#
MRVEVTIDKTRPLPSGAIEALTGELGKRVNRQFPDAVVHVRYAGANGLSVLGGAKTDRDLIEEILQET

```
WESADEWFSAE
>CORE_REP|Org33_Gene255#
MKETTIVPDYRLDMLGEPCPYPAVATLEAMPQLKPGEILEVISDCPQSINNIPLDARNHGYKVLDIQQ
DGPTIRYLIQR
>CORE_REP|Org14_Gene149#
MSFEVFEKLEAKVQQAIDTITLLQMEIEELKDKNNSLSQEVQAASGNHEALVRENQQLKEEQHVWQDR
LRALLGKMEEV
>CORE_REP|Org43_Gene192#
MAGLLQVRDALALRGSAQAQQLSQSLATPLPLVQAMLDRLTAMGKVERIEQDDGACLSGGCKSCPQGQ
GCSTVFYRLKA
>CORE_REP|Org1_Gene2462#
MSDMLSNEQELASDLVACQLVIKQILDVIDVIAPTEVRDKMAGQLKSIDFSTHPAGADPVTRRAIDKA
IALIEMKFTRS
>CORE_REP|Org31_Gene173#
MSKTVVKIGSFEVDDAHLHAGADRAGTLSIPCKSDPDLCMQLDGWDEHTSIPAILDGKQSLLYKQHYD
RQADAWVMRLA
>CORE_REP|Org46_Gene666#
MAQQLEFFDIPSPCRGICQADDRGFCRGCLRSREERFGWMNMSDAQKREVLRLCRQRFLRLQRANKAP
DEPLPEQPSLF
>CORE_REP|Org29_Gene17#
MENLSMDLLYMAAAVMMGLAAIGAAIGIGILGGKFLEGAARQPDLIPLLRTQFFIVMGLVDAIPMIAV
GLGLYVMFAVA
>CORE_REP|Org6_Gene20#
MSTIEERVKKIIVEQLGVKQEEVLNNASFVEDLGADSLDTVELVMALEEEFDTEIPDEEAEKITTVQA
AIDFINASQQ
>CORE_REP|Org30_Gene26#
MKRIIKGDKNLSHLVIAHAAIDRHAESFGQRRQGWPSTYLIKYQNDRVAVEVVTRSQSYVATLMIGAR
NLTKLCGMPG
>CORE_REP|Org14_Gene3793#
MNAAFDSWSAFFAMGGYAFYVWLAVAATLISLLGLVAHTVWQRRQLLAEIGRRQARERRIRHAQQSKQ
KSAALREKSL
>CORE_REP|Org28_Gene321#
MSRVCQVTGKRPVSGNNRSHAMNATKRRFLPNLHSHRFWVEAEKRFVTLRVSAKGMRVIDKKGIETVL
ADLRARGEKY
>CORE_REP|Org19_Gene169#
MNRTKLVLGAVILGSTLLAGCSSNAKIDQLSSDVQTLNAKVDQLSNDVNAMRSDVQAAKDDAARANQR
LDNQAHAYKK
>CORE_REP|Org43_Gene990#
MNTSWLHASSPLPDLVLGASLYFPPIFKAFLLGLVLWLLIHQLLRDWMYSGEIWHPMLMDLSIFVLTV
SGSLWILASW
>CORE_REP|Org39_Gene1440#
MSRLIKWVLVLAVIYGGFLISGYGVLIGSNKNVGGLGLQCKYLTARNVAIAQYVNGDNGFIGVADCPL
FKKIETVVD
>CORE_REP|Org9_Gene120#
MIIPWQELATDTLNSLIESFVLREGTDYGEHERSLEQKVEDVRRQLKNGEVVLVWSELHESVNIMPRG
QFRAGQEEI
>CORE_REP|Org37_Gene1678#
MAIQANMHEAKSNLSQLADRAAEGETVIIAKAGKPYVQLVAIQGEARRPGAAKGKFTVPADFNAGDAA
IAALFEGDE
>CORE_REP|Org38_Gene1865#
MIDYTLYGLNKNDVDEYHKQICCLLGKSVLLVLIANKPITKQNLLASLIQEVEQQHDEYFQKLHRAAI
EMIGVNGR
>CORE_REP|Org33_Gene61#
MSIIIYSKPDCVQCNATYRAFDKQGIDYQVIDLTQDQQALNHVKSLGYQQVPVIIAGDDHWSGFRPDK
```

IGALALTC
>CORE_REP|Org14_Gene1996#
MPYQDTNDLPDNVKHVLPAHAQAIYKEAFNSAWQQYRDPEDRRGDAGREETAHKVAWAAVKQSYRKGD
DERWHKK
>CORE_REP|Org10_Gene723#
MLTVFAATFVLFLLIVGGMSLGYVFKRKSLQGSCGGITALGMEKVCDCPEPCDARKKREAKAAQRREQ
LEKHRIL
>CORE_REP|Org31_Gene1417#
MMHCPLCGHVAHTRSSRYLSESTKERYHQCRNINCSCTFATHESVARVIVKPGDDIVPAQPHPPESQH
KQSAAAL
>CORE_REP|Org13_Gene318#
MPQSSRYSDEHVEQLLSELVNVLEKHHTPTDLSLMVLGNMVTNLINTSVAPAQRKTLARSFAEALQAS
VREDKAH
>CORE_REP|Org17_Gene2375#
MHNADIQSLLQRLEELESRQAFQELTIEELNQTVTQHQMEMLKMREHLRMLTDKLRSTQTSMIASQSE
ETPPPHY
>CORE_REP|Org35_Gene129#
MSALLLAIPLTIFVLFVAPIWLWLHYSNRQQTGIQLSQQEMQRLAQLAEDAKRMRERIQALEEILDAE
HPNWRQS
>CORE_REP|Org35_Gene1#
MARYFRRRKFCRFTAEGVQEIDYKDIATLKNYITESGKIVPSRITGTRAKYQRQLARAIKRARYLSLL
PYTDRHQ
>CORE_REP|Org15_Gene32#
MSNVDFTTSANPEILATEVACLKATLTLILKSIGQADAGKVIINMERFIAQIEDPTQAEIFKNSIQQI
KHAYRQ
>CORE_REP|Org47_Gene1633#
MTDKNTPAQEAIAENATLLKILGRLLSALDASISGKERSILVAELSNFNLDGISEAESKLASELIKRA
LESLDH
>CORE_REP|Org44_Gene2314#
MPASAHSNEQYETLLHDVSLALGDAVLQLIQNHKKVSGSNILSQLVNEIEREQDQQRFAALRSAIELV
GLAPKG
>CORE_REP|Org25_Gene2729#
MQLQPKHTYKIVGFSSEIAPAYRQKLLSLGMLPGSSFDVVRVAPLGDPIEIKTRRVSLVLRRKDLALL
QLDGQP
>CORE_REP|Org3_Gene838#
MKKTAAVLSALMLTFTLAACSSNYVMHTNDGRTIVADGKPKVDNDTGMISYKDANGVEQQINRADVKE
MVESNQ
>CORE_REP|Org10_Gene548#
MDAHCPHCRQTMNWVAGHYHCAACQRDYRQLASCPECGQPLQELKACGAVDYLCQNGHGLISKKRVNF
SYQPL
>CORE_REP|Org39_Gene357#
MKKNLSCISLNYRKVAGASAPVLVIDFCNENNERYTLRYNLPPDTPERTERRVSLLLYLLRKHRSAEI
DNLAD
>CORE_REP|Org39_Gene352#
MKKLIAVGLLSAVLAGCATDSPCVPVYDDQGRLVHTNTCMKGTTQDNWETAGAIAGGAAAVAGLTLGI
IALTK
>CORE_REP|Org39_Gene51#
METGTVKWFNNAKGFGFICPAGGGEDIFAHYSTIKMDGYRTLKAGQQVSFDVHQGPKGNHASLIVPVE
SEALS
>CORE_REP|Org18_Gene110#
MFKNTVLKMGRVKWFNQAEGYGFISPVDGSDEIYVNRNAIANTKNKSLNEGQNVEFSIYRSSHGLSAA
DVIAF
>CORE_REP|Org10_Gene1999#
MLEIFFLIGFVVMLMVTGISLLGIFAALLVAAAFMLLGGLFAVVIKLLPWLILAVVGVWIYRSMQKPQ

ARRY
>CORE_REP|Org19_Gene8#
MAKEDNIEMQGTVLDTLPNTMFRVELENGHVVTAHISGKMRKNYIRILTGDKVTVELTPYDLSKGRIV
FRSR
>CORE_REP|Org46_Gene1205#
MKKWVMAVSALVMVAGLAGCSSDYVMATKDGNMILTQGKPEIDEDTGLISYKDEKGNQRQINGDQVSQ
VIER
>CORE_REP|Org19_Gene523#
MMIINKRFATAALALTLAFSLSACSNMSKRDRNTAIGAGAGAVGGAVLTDGSALGTLGGAAVGGIIGH
QVGK
>CORE_REP|Org18_Gene2426#
MMIFNVFGRLLGVKRIGDEWRLFRVTLPERKYAPSCDIVLPADLREEEIAGYLGDIYHEAATPQRPEV
FRVE
>CORE_REP|Org48_Gene4259#
MTSGIMGLVKWFNEDKGFGFISPLDGSKDIFVHLSALNGDNFKTLFEGQKVEFAIHRGDKGPAAANVT
LCDK
>CORE_REP|Org37_Gene3125#
MEGISITKLLVIAVLVILLFGTSKLRTLGADLGAALKGFKKAVGDDSTTPPAAGNNTAESQSAQQSVE
KKDV
>CORE_REP|Org49_Gene138#
MKQGIHPKYEEVTANCSCGNVMKIRSTVGHDLNLDVCGACHPFYTGKQRDVATGGRVDRFNKRFSVPG
AKK
>CORE_REP|Org49_Gene6#
MPVIKVRENEPFDVALRRFKRSCEKAGVLAEVRRREFYEKPTTERKRAKASAVKRHAKKLARENARRT
RLY
>CORE_REP|Org49_Gene344#
MEKENLLEIANTVMPFGKYQGRVLIDLPEEYLLWFARKGEFPKGKLGMLMEMTLAIKIEGLDHLVKPL
KKS
>CORE_REP|Org48_Gene1738#
MTDTLEYLLTFRKCSSLDSLEKVYDKLNYSIENDTEMSNMYRAADHRRAELVAGKLFDLGKVPKTLWT
QVL
>CORE_REP|Org48_Gene1933#
MNSKPCEFDEAICLAIGRGVCEVVKIGDEISVFSLLEAIESFIERQALSESEIAAADDALDLITGLVR
RQC
>CORE_REP|Org49_Gene610#
MNWYQVALWTMIFLKSGAFLVFVWWWMFKRATKPYVRPQDVVVDDTPYDENLTMEEVMKNHGIVRTDM
SVK
>CORE_REP|Org6_Gene3501#
MTILILGLLYAILMISVGVNEIYFYSTGKSNFLTSLMLTFSGSMLLIAFVWQLSSKVKNKSPSECEQH
HAK
>CORE_REP|Org19_Gene1438#
MEIFNLDNHPHVELCDLLKFQGWCESGGAAKEVIAEGQVKVDGKVETRKRCKIVADQVVEFNGGKVMV
KP
>CORE_REP|Org19_Gene3798#
MANHNVKSWATVRETSVEIAEAIFELAGNDEVLAQKIWEEGSDEALEKAFAKTTSDQLYWGEETVERK
NV
>CORE_REP|Org4_Gene3645#
MQMKKQLRYALMAVLLTGLAGCGLKGPLYFPPADSAGQKPAKPPVQTGDQVQKNQQEQSGSQQKPSMV
DQ
>CORE_REP|Org37_Gene445#
MSTTYHAYSLHRILLRRSAVVIAGILALPVMLFRSDRGRFYSYLHRVWSKTSDKPVWLQQAELASCDF
Y
>CORE_REP|Org15_Gene1420#
MTFSEQEIKPVWDEVARLIGDSVMQLRHRGEALSVETLEKHLARQLAESNDIERRILLGAAINMLKGG

K
>CORE_REP|Org38_Gene422#
MNKDQADGNWKQLKGKVKEKWGKLTDDDLTVIEGKREQLVGKIQERYGYQKEAAEKEVKAWEDHTKHR
W
>CORE_REP|Org26_Gene738#
MLEHYYPANMGAKPVTDEQHKRLVAVQAALELIKATLSDTNDGNGVDFQLKAAEKHIGPMADAIQEAL
K
>CORE_REP|Org16_Gene2328#
MTKYRHTKGQIQDNAIEALLHDPLFRQRVEKNVKGKGSYRRKEKHNKGGNWEASGKLSSDNLPLAFWF
>CORE_REP|Org7_Gene288#
MRIFQRYNPLKVAKYVKTLFRGRLYIKDVGAFEFDEGKILPPKIRDKRHFSVMSEVNRQVLLLQTEMG
>CORE_REP|Org21_Gene4781#
MKTRQDWLFQLRKCQHRETLEKVIEKNERSLTAGELVTFYSAADHRLAEIIMNKLFDKIPASVWQHVR
>CORE_REP|Org25_Gene2005#
MLNLNDILQLILLCALVFIPLGYAFHRRFPHLRQYWQNLLLSPRYLKSAGLWVRTGSSSQIKRHKKQP
>CORE_REP|Org31_Gene580#
MSATRKRFIAGAVCPSCSAMDTLAVWREDQVEVVECVKCGHHQRQTEQQVEKHVRPQEQVIGIFQPK
>CORE_REP|Org33_Gene382#
MSLLPVMVIFGLSFPPVFFELLVSLALFFLLRRLLQPTGIYDFVWHPALFNTALYCCLFYLISCLFV
>CORE_REP|Org8_Gene891#
MFGNLGQAGKYLGQAARMLVGVPDYDTYVQHMKDNHPDKPVMTYKEFFRERQQARYGGDGKGGMRCC
>CORE_REP|Org18_Gene2526#
MAGDNQQSQHGVRSPCVSLCRIDDETQQCRGCRRTRAEIAAWPTAGDAEKRAIWRRLELRAAFKQNT
>CORE_REP|Org28_Gene686#
MGLKWTDSREIGEALYDQYPDTDPKTVRFTDMHQWICDLEEFDDDPQASNEKILEAILLVWLDEAE
>CORE_REP|Org27_Gene635#
MKIRLNDQPLELAQPLSVAALLTQLERHQPGTALAINQTIIPRADWADHQVQDGDDILLFQAIAGG
>CORE_REP|Org28_Gene359#
MQQYCELVRRTYAEIGSGDLGYVPDALACALKALDDVAANDALPSSVREQAAFAAANLLVSDYVDE
>CORE_REP|Org28_Gene3578#
MNSTIWLALGLVLVLEGLRPMLFPQAWRKMILAMSQLPDATLRRFGGGIVVAGCVIYYMLSGRTGL
>CORE_REP|Org46_Gene1907#
MDTDDQRREQIDREVGRAVRRLIEENKAIDRDHLVAMLQLIQRDEAGTPREAALAAAAQLVALGK
>CORE_REP|Org3_Gene3396#
MKIMSVSTLAALLCVSLLSGCVPRIEVATPKDPITINMNVKIEHEIHIKVDKDVENLLKTQSGLF
>CORE_REP|Org47_Gene700#
MYVCLCNAVTDKAIRNAVRQHNPHTMKQLRELVPIGTDCGKCIRQARQIMVEERGTIIPMHEVA
>CORE_REP|Org12_Gene56#
MEKKTARLTVLIDPDKKKALEELCLQQDVTPSQVVRQLIRDYLHKHQVDYPSQPTHANPRVESN
>CORE_REP|Org19_Gene97#
MKAQELREKSVEELNAELLNLLREQFNLRMQAASGQLQQTHLLKQVRRDVARVKTLLTQKAGV
>CORE_REP|Org28_Gene2142#
MYALVMFVCYLDGGCSEMVVDILRDEPQCLIAMKEQNLRHAGCYPMEEFIDGFWLPASEYSDF
>CORE_REP|Org9_Gene2509#
MNNAPTHFRAAAPGESQDDLQALSQAFSLPKLSYVDISRQERLTQMMTRWPLLAELAQTTGSH
>CORE_REP|Org9_Gene844#
MPIYEYACGACNHRLEKLQKFSDAPLADCPACGQPALTKLISSSGFQLKGTGWYATDFKPGNK
>CORE_REP|Org3_Gene2616#
MTTVVKCPTCGTGVVWGEISPFRPFCSKRCQLIDLGEWADEEKRIPSNSDLSESEDWSEMDDR
>CORE_REP|Org29_Gene2074#
MARKLDSLPQAQREKIETDLLAISVIYNERYGIASTQAETEQQIPDHLLPYFHQRLDYYRRA
>CORE_REP|Org34_Gene29#
MLILTRRVGETLMIGDEVTVTVLGVKGNQVRIGVNAPKEVSVHREEIYQRIQAEKSQQTTY
>CORE_REP|Org47_Gene595#

MQWLADYWWIILLVLVGMVVSGIKELRRVDVKSYLANKPEIPPHRDNNAQWDDEDDWPKKK
>CORE_REP|Org4_Gene1136#
MNSDIIVGRWKQLKGQVWQAWAEWSGSDCAWLAGSNDFLAGVLQEDYGRERDAVSSEKTSH
>CORE_REP|Org29_Gene842#
MEDGEMKKIILVFLMLMLSGCIGGGGGPSNAPSASPNEPYTCTKSSANSDCSQSVEPSMY
>CORE_REP|Org2_Gene47#
MDHRLLEIVACPVCNGKLYFNKENQELVCKADGLAYPLRDGIPVLLENEARALSLDEKHA
>CORE_REP|Org29_Gene867#
MAFNIGDFVQRTTGGPKMTVVAIDGETLVCSWNELGKEQRTEVQASDVALYHEDGDFGVC
>CORE_REP|Org11_Gene4037#
MRKKTTGQMTKIVLFISFIILVGRLLYAAVVAVPHHQEKKQAAAQNAPEVSAPQQDASSE
>CORE_REP|Org34_Gene2537#
MDTANTPTPAAAAPTEHSANAPPSYDSAQLLGTDGIAFITHQGQRYQLRQTKAGKLILTK
>CORE_REP|Org8_Gene2961#
MAKTIKVTQTRSSIGRLPKHKATLLGLGLRRIGHTVEREDTPAVRGMVNLVSYMVKVEE
>CORE_REP|Org15_Gene362#
MYNFSRYQAKELALAYMSGKKHDLSPQEFLQQLKSSERSFEHLLKHGSEMPRDVLVKSF
>CORE_REP|Org41_Gene871#
MQMTLMEYITRHFNGDLHRYAQSEGVSREQIISWINNECHVIKGRLFMPVKHLPVEQAQ
>CORE_REP|Org4_Gene850#
MSLENASPELQLAVDLIYLLECNEIDPATALAALDIVKRDYQEKLQRAGVTSPYLPAGQ
>CORE_REP|Org35_Gene1061#
MSSLWVATQYCYFIGLLVSMIFTYLVSRDTVKIRCISALTIGLTWPLSLPVVLLFSLF
>CORE_REP|Org18_Gene864#
MRRSRNEVGRWRMLRQSQRRRHRWLERQSCSNRHIIRVRRRLDDQHRRALLFAVSCEW
>CORE_REP|Org7_Gene3396#
MKAFLSKSFYQRYFSAVRRQHADWLSVVPEQARLAILAHLTQWDIKEMSDKQYREHL
>CORE_REP|Org46_Gene113#
MKRQKRDRLERAHSRGYQAGILGRSREHCPYQSSVDARSQWLGGWREAMEDRAVTA
>CORE_REP|Org30_Gene32#
MAVQQNKPTRSKRGMRRSHDALTTTTLSVDKVSGETHRRHHITADGFYRGRKVIG
>CORE_REP|Org42_Gene15#
MAKGVREKIKLVSSAGTGHFYTTTKNKRTKPEKLELKKFDPVVRQHVVYKEAKIK
>CORE_REP|Org15_Gene682#
MMPNKRSLLKLVVICATVISLAWITRSTLCELRIRSGTTEVAAILAYESER
>CORE_REP|Org29_Gene3070#
MQDKTATVAPAPWWKRIGWLVIIWSASVLGLFVVASLFRLLMTAAGMKSH
>CORE_REP|Org43_Gene2689#
MRLRHVFHSLTVVSITVLLFIWMIRDSLCELKIYQKNITILIRLACEVKR
>CORE_REP|Org16_Gene470#
MLELLKSLLFAVVMVPVVMALILGLIYGFGEVFNVFSKVGRSKENRTQH
>CORE_REP|Org1_Gene693#
MLRKKILVIMTAVAACLFFYLLALDSYCDDGGNFALGICSVTRFVPW
>CORE_REP|Org9_Gene4#
MKRTFQPSVLKRNRSHGFRARMATKNGRQVLARRRAKGRARLTVSK
>CORE_REP|Org1_Gene514#
MQVLSSLRSAKNRHPDCKVVRRRGRIYVICKSNPRFKAVQGRKKKR
>CORE_REP|Org34_Gene95#
MLKKSIIAIFSLMILSSLTACNTTRGVGEDIQAGGKAIQRSAE
>CORE_REP|Org33_Gene1911#
MFKKTLLAVLSLLFLFSLSGCNTFRGFGEDVQHLGGAISRTAG
>CORE_REP|Org41_Gene33#
MLNRILAAIVNNVREHLVLYLCLWLVLLALDVYFFFVM
>CORE_REP|Org6_Gene7#

MCGIFSKEVLSKDVSVEYRFSADPYLSASSSNDSSLSM
>CORE_REP|Org22_Gene107#
MKVRASVKKLCRNCKIVKRNGVVRVICSAEPKHKQRQG
>CORE_REP|Org36_Gene470#
MWYFAWILGTLLACAFGIITALALEQSEATKAQQDGK
>CORE_REP|Org14_Gene251#
MENNNRKMAHIRRTTHIMMMAHRSCFSFAFFNYR
>CORE_REP|Org1_Gene582#
MKAFAQVISLVVISVVVIIIPPCGAALGRRKA
>CORE_REP|Org2_Gene4689#
MIASSLTSTLLVTALPAAVVVVRVVVVVGNAP
>CORE_REP|Org31_Gene622#
MSFSVIGGALLVLLLLAYLVYALFNAEDF
>CORE_REP|Org47_Gene817#
MDTELKMSLFTTVCALAVIIAFSFVAALN
>CORE_REP|Org36_Gene3272#
MIRTHRLLGLLLNASCLRGMPVDEVRS
>CORE_REP|Org13_Gene4952#
MKMTENFLDELCRLAAIINEARVHDD
>CORE_REP|Org34_Gene4322#
MKYFLMGVSFMLVAWVGTFMLMVA
>CORE_REP|Org13_Gene2819#
MRNISLNTTIITTTDTTGNGAG
