# Supplementary material for: A Systematic Immuno-Informatic Approach to Design a Multiepitope-Based Vaccine Against Emerging Multiple Drug Resistant Serratia marcescens
Source: Front Immunol. 2022 Mar 14;13:768569. doi: 10.3389/fimmu.2022.768569 (PMC8967166; doi:10.3389/fimmu.2022.768569)
Supplement: Supplementary Data Sheet S4 — Virulence protein sequences. [file DataSheet_4.pdf]

>CORE_REP|Org44_Gene3968#
MGTTTMGVKLDEATRDRIKSAAQRIDRTPHWLIKQAIFNYLERLESGTDIPEIPALAAAGQPEADDIM
PQAQEESHQPFLDFAEQILPQSVTRAAITAAYRRPETEAVPMLLEQARLPADLAQATHKMAYGIAEKL
RNQKSANGRAGMVQGLLQEFSLSSQEGVALMCLAEALLRIPDKPTRDALIRDKISNGNWHSHLGRSPS
LFVNAATWGLLFTGKLVSTHNEANLSRSLNRIIGKSGEPLIRKGVDMAMRLMGEQFVTGETIAEALAN
ARKLEDKGFRYSYDMLGEAALTEADAQAYLVSYQQAIHAIGKASNGRGIYEGPGISIKLSALHPRYSR
AQYERVMEELYPRLLSLTLQARQYDIGINIDAEEADRLEISLDLLEKLCFEPQLAGWNGIGFVIQAYQ
KRCPFAIDAVIDMAQRSRRRLMIRLVKGAYWDSEIKRAQMDGLEGYPVYTRKVYTDVSYLACARKLLS
VPNLIYPQFATHNAHTLSAIYHLAGNNYYPGQYEFQCLHGMGEPLYEQVVGKVADGKLNRPCRIYAPV
GTHETLLAYLVRRLLENGANTSFVNRIADATLPLDELVADPVSAVEALAASEGQIGLPHPRIPLPREL
YGEKRTNSSGLDLSNEQRLASLSSALLTSASHPWRAEPIIDAELDQGVEQPVINPAEPGDVVGYVREA
TENEVSRALDAAAAAGPIWFATPPTERAAILERAAELMESQLQSLLGILVREAGKTFNNAIAEVREAV
DFLHYYAGQVRDDFANDSHRPLGPVVCISPWNFPLAIFTGQIAAALAAGNSVLAKPAEQTPLVAAQAV
RILLEAGIPQGVLQLLPGQGETVGSTLVNDARVRGVMFTGSTDVAGILQRSIAGRLDPQGRPTPLIAE
TGGLNAMIVDSSALTEQVVTDVVASAFDSAGQRCSALRILCIQEDVAEHTLQMLRGAMAECRMGNPER
LSTDVGPVIDADAKTGIERHIQAMRAKGRKVYQAAKGSAQDEKEWARGTFIKPTLIELDSFDELQKEI
FGPVLHVVRFQRNNLDALVDQINAAGYGLTLGIHTRIDETIARVTERAKVGNLYVNRNMVGAVVGVQP
FGGEGLSGTGPKAGGPLYLYRLLANRPDDALQRTLHRQDEERPMEATARPQLLGALQSLEKWAVTSQQ
GELAALAQRYAELGQGGTVRPLPGPTGERNTYALLPRERVLCLADNEADALIQLAAVLAVGSSALWPE
AELQRNLFRRLPNDVQARIAFSKDWQQDKVEFDAAIYHGDADQLRTLCEQIAQRGGAIVSVQGFAHGE
TNILLERLLIERSLSVNTAAAGGNASLMTIG
>CORE_REP|Org14_Gene4616#
MKYLASFRTTLKISRYLFRVLAILLWSLGALLTTFYILNILHQKESDIRQEYNLNFDQAQGYIRHSAD
IIRDIKYMAENRLNGSVSGLDMFSGVIPGKGSPPQFFPLYPESNCALSTTYRSSLDSLSGLIQYWKEN
FVAAYDLNRVFFIGGDSLCMAEFGGGNASANRENMLKLLHERILKYRNAKNLDKDNNLYWISPSAQRP
DVGYLYVLTPLYIGNKLEALLGIEQTVRLEDFVTAGNLPIGVTLLDENNEPVLRLADGERYAAALNSY
PEEHAYFGYVDNYRDLILKKALPPSSLSIVYALPVKSVVERFKMLILNALLLNLLSAIVLFTLAWLFE
RKMFLPAEDNAFRLEEHEQFNRKIVASAPVGICILRISDGTNILSNELAHNYINLLTHEDRDRITRII
CEQQANFVDVMTSNNNNLQISFVHSRYRNEEVAICVLVDVSARVKMEESLQEMAAAAEQASQSKSMFL
ATVSHELRTPLYGIIGNLDLLQTKALPQGVDRLVNAMNNSSGLLLKIISDILDFSKIESEQLKIEPRE
FSCLEVITHIAGNYLPLVVKKRLGLYCFIEQNVPERIFGDPVRLQQVLSNLVNNAIKFTDTGCIVLQV
CTRGSYLEFSVRDTGVGIPEKEISRLFDPFFQVGTGVQRHFQGTGLGLAICEKLVNLMDGDVSVESEP
GLGSLFSIRIPLFNAQFPIPQASDTWQGRRLWLDIRNQRLESYLMAILGGYGADIQRYDGQETAAGEV
LLSDHPLMLDAPLLAQIQFSTEHIGPSQETRPGYWMHSTSTPRETLTLLNRLFGVGVGSGAAEALVQL
PVPAKASAADNGDIHLLVVDDHPINRRLLSDQLGSLGYQVVTANDGVDAPGVLKQHRVDIVLTDVNMP
NMDGYRLTQALRQMQFSAPVIGVTANALAEEKQRCLEAGMDNCLSKPVTLETLEQTLAYYSQQVRYSR
SEA
>CORE_REP|Org2_Gene3277#
MTKYSLRARMMILILAPTLLIGLLLSTFFVVHRYNELQEQLVDAGASIIEPLAVASEYGMTFRSRESV
RQLVSLLHRRHSDIVRSITVFDAQNNLFVTSNYHHNFAQLQLPKGVPLPTELMLTRRGDSLILRTPIL
SESQYPDETADGGSHPDNNLGYVAIELDLQSVRLQQYKEVFVSTLLLLLCMCIAILFAYRLMRDVTGP
IRNMVNTVDRIRRGQLDSRVEGYMLGELHMLKNGINSMAMSLTAYHEEMQQNIDQATSDLRETLEQME
IQNVELDLAKKRAQEAARIKSEFLANMSHELRTPLNGVIGFTRQMLKTDLSATQTDYLQTIERSANNL
LTIINDVLDFSKLEAGKLVLEHIPFALRETLDEVVVLLAPSAHDKGLELTLDVHNDVPEQVIGDSLRL
QQIITNLLGNAIKFTETGNIDIRVELRKQLDRRVEVEVQIHDTGIGISERQQSQLFQAFRQADASISR
RHGGTGLGLVITQKLVKEMGGDICFHSQLNRGSTFWFHITLDLNEGMLSLAPSLPDLSGKTLAYIESN
PTAAQATLNMLSITQLVITHSPTLGQLPPGHYDFLLAGVPIPFRDNMAQHEDKLLASLKLADRVILAL
PCQAQIDAELLKQQGALGCLIKPITSTRLFPLLRMEAPARLTAQPERKRLPLTVMAVDDNPANLKLIG
TLLGEQVEKTLLCESGEEALALARDNVLDLILMDIQMPKMDGIHASELIRQLPHHNSTPIVAVTAHAA
SGEREHLLQAGMDDYLAKPIDEKMLTRVLSRYHSGDVENAIADDAPLSLDWPLALRQAANKPDLARDL
LQMLLDFLPQVRERVQALLDGQHDDEILDLVHKLHGSCSYSGVPRLKQLCFYLERQLRQGVTNDELEP
EWLELLDEIELVIHAARAHLTQPA
>CORE_REP|Org21_Gene3694#
MSHTTMLALQGLSCMNCAQRVKKALESRSDVEQADVNVHYAKVTGDAPDSALIDSVIAAGYQAEVAPH

ADTELQLSGLSCMHCVGTTRKALEAVPGVFATDVTIDGAKVYGDADPQTLIAAVEDAGYHAGVAGAVA
PKTEPLTDATPSLPDVQPAAQSPLPATDGADDSVQLLLSGMTCASCVNKVQLALQSVPGVEHARVNLA
ERSALVTGAADAQALVAAVEKAGYGAEMIQDETERRERQQQTARANMKRFSWQAALGLALGIPLMAWG
LFGGSMTLTPETQRPWLLVGVITLAVMVFAGGHFYRNAWRALMNGSATMDTLVALGTGAAWLYSIAVN
IWPDFFPMEARHLYYEASAMIIGLINLGHALEQRARQRSSQALERLLDLTPPTARLVTDDGERDIPLA
EVQLGMTLRLTTGDRVPVDGEIVQGEVWLDEAMLTGEAVPQQKGAGDTVHAGTVVDDGSVLFRAAAIG
SQTTLARIIKLVRQAQSSKPAIGQLADRVSAVFVPAVVGIALFSAAMWYFFGPQPQLVYTLVIATTVL
IIACPCALGLATPMSIISGVGRAAEFGVLVRDADALQQASQLDTLVFDKTGTLTEGKPQVVEILTFNQ
VSEQQAIGWAAALEQGSNHPLARAIMERAAGQTLPQVAQFRTLRGAGVSGEIDGVPVLLGNAALLEQH
QVATAELEAPMRALAERGVTPVLLAVNGKPAALFAIRDPLREDSVAALQRLHRQGYQLVMLTGDNPVT
ANAIAKEAGIDRVIAGVLPDGKAAAIKQLQAQGQRVAMVGDGINDAPALAQADVGIAMGGGSDIAIET
AAITLMRHSLHGVADAVALSKATLRNMKQNLFGAFIYNTLGIPIAAGVLYPLTGTLLSPVVAGAAMAL
SSITVVSNANRLLRFKPRK
>CORE_REP|Org38_Gene4244#
MKLKKLPRQLLGLFARGLPRRLVRRDSLLDSVGGAARDMPAGLAQQRLECAAAETMQLFERFHSHPEG
ITAHEAEQVRQRCGENVIDDQQKEAWWQHLWHCYRNPFNLLLTALGMISYATEDLTGALVIALMVLIS
TLLNFIQEARSNRAADALKAMVSNTATVIRSDALTGRSEHVELPIAQLVPGDIIKLAAGDMIPADLRV
LSAKDLFISQAALTGESLPVEKSAAPQALAADPLDCQNLCFMGTNVVSGTALAMVIGTGGGTYFGQLA
QRVTSQDEQPNAFQSGISKVSWLLIRFMLVMTPIVLLINGYTKGDWWEAALFALSVAVGLTPEMLPMI
VTSTLAKGAVKLSRQKVIVKRLDAIQNFGAMDILCTDKTGTLTQDKIVLERHTDVFGASSERVLRYAW
LNSFYQTGLKNLLDVAVLSCAEQNQQPQALQNYRKVDEIPFDFVRRRMSVVVAKDNEYHELVCKGALE
EMLAICSHVRHEDEVIPLSEALLVRIRRITDDLNQQGLRVVAVANKILPAQTHEYGVADESDLILEGY
VAFLDPPKESTAPALAALKQNGVTVKILTGDNELVAAKVCRDVGLEADHLLRGSEIEQMDDEQLAQAA
ARTTVFAKLTPLHKERIVKLLRRQGHVVGFMGDGINDAPALRAADIGISVDSAVDIAKEAADIILLEK
SLMVLEQGVIEGRRTFANMLKYIKMTASSNFGNVFSVLIASAFLPFLPMLPLHLLIQNLMYDISQIAI
PFDNVDDDQITQPQRWNSADLGRFMVFFGPISSIFDVLTFSLMWWVFKANTPEMQTLFQSGWFVEGLL
SQTLIVHMIRTRKIPFIQSRPSWPLCIMTLAVIATGIGLVFSPLAGFLQLQALPLGYFPWLVLILAGY
MVLTQCVKGWFVRRYGWQ
>CORE_REP|Org34_Gene4528#
MAVTNVAELNELVARVKKAQREYANFTQEQVDKIFRAAALAAADARIPLAKMAVEESGMGIVEDKVIK
NHFASEYIYNAYKDEKTCGILSEDDTFGTITIAEPIGLICGIVPTTNPTSTAIFKALISLKTRNGIIF
SPHPRAKNATNKAADIVLQAAIAAGAPKDIIGWIDQPTVELSNQLMHHPDINLILATGGPGMVKAAYS
SGKPAIGVGAGNTPVVVDETADIKRVVASILMSKTFDSGVICASEQSVIVVDAIYDAVRERFASHGGY
LLQGKELKAVQDIILKNGGLNAAIVGQSAPKIAEMAGIKVPANTKVLIGEVKLVDESEPFAHEKLSPT
LAMYRAKDFEDAVAKAEKLVAMGGIGHTSCLYTDQDNQTARIAYFGDKMKTARILINTPASQGGIGDL
YNFKLAPSLTLGCGSWGGNSISENVGPKHLINKKTVAKRAENMLWHKLPKSIYFRRGSLPIALEEVAT
DGAKRAFIVTDRFLFNNGYADQITKVLKSHGIETEVFFEVEADPTLSIVRKGAEQMNSFKPDVIIALG
GGSPMDAAKIMWVLYEHPETHFEDLALRFMDIRKRIYKFPKMGVKAKMIAITTTSGTGSEVTPFAVVT
DDTTGQKYPLADYALTPDMAIVDANLVMNMPKSLCAFGGLDAVTHALEAYVSVLANEYSDGQALQALK
LLKEYLPASYKEGAKNPVARERVHNAATIAGIAFANAFLGVCHSMAHKLGSEFHIPHGLANAMLISNV
IRYNANDNPTKQTAFSQYDRPQARRRYAEIADHLGLSAPGDRTAQKIEKLLAWLDELKTELGIPTSIR
EAGVQEADFLAKVDKLSEDAFDDQCTGANPRYPLIAELKQIMLDTFYGREFSEAVDEEAATPAAAKTA
VKKPRNNGSVNR
>CORE_REP|Org6_Gene4045#
MSEISRSVLFGKLDSLLFTSLESATAFCKLRGNPYVELAHWLHQLMQSPDGDLQQIVRHFALDEAQLA
RDIVEALDRLPRGASAISDLSEHIDSAVERAWVYGSLKFGAARIRGGHLQLGILKTYSLRHLLKAISP
QFERINADLLMEQFAAITAHSAENAEDAPAESRAENASGAARSGESVLAQYAQDLTARARDGEIDPVA
GRDEEIRQIIDILMRRRQNNPLLTGEAGVGKTAVVEGLALRIVAGDVPPQLRDVKLCLLDIGMLQAGA
GVKGEFEKRLQAVIDEVQSSPTPIILFIDEIHTLIGAGGAQGTGDAANLLKPALARGQLRTIGATTWS
EYKKYIEKDPALTRRFQVVQVHEPSEDKALLMLRSTVSPLEQHHRVLLLDEAVDAAVRLSHRYIPARQ
LPDKAVALLDTACARVAVSQHAEPAQVEDCRHRIDALQIELDIARREAKVGIGDPLRPQEIEAQLTAL
RLELEQLTERWQQELTLIQEIITLRAQLHRQEAEEPAGEETEAGPDADALRAQLGELQQQLSALQGEA
PLIFAAVDANIVAAVVADWTGIPLGRMVKNEIEAVLQLSDTLNQRVIGQRHALDLIARRVRTSRARLD
DPNKPVGVFLLAGPSGVGKTETALALAETLYGGEQNVITINMSEFQESHTVSTLKGAPPGYVGYGEGG

VLTEAVRRRPYSVVLLDEIEKAHPDVHEIFFQVFDKGWMEDGEGRHIDFRNTIIILTSNVGTDLIAGL
CSDPELLPEPEALSGALRQPLLSVFPAALLGRLLVVPYYPLTDATLGNIVRLQLGRIQRRLAENHDIV
CTFDDAVIEQIVSRCTEVESGGRMVDAILTNTLLPQISHTLLTGSANDQRYRQLHIALQNHEFICQFQ
A
>CORE_REP|Org37_Gene3116#
MRVSPAGKLMRLQTLGLCISLALGVPSMAVFAAGDIQFNTDVLDVHDRENIDLSQFSRGGYIMPGTYG
MVVHVNKNDLQEQQVPFYAPEDDPNGSRACVTQVLTGQLGLKEDALKGVTWWHQGECLDEASIPGMEV
RGDLATSALYLSIPQAFLEYTAENWDPPSRWDEGIPGLLFDYNVNARTQKQHQGNGSSYSLSGNGTTG
LNLGAWRLRADWQGNVDHTTGSGQSTAQKLDWSRYYAYRAIPALRSKLTVGENYLDSGIFDSFRFTGA
SLMSDDNMLPPNLRGYAPEVVGIAKTNAKVVISQQGRVLYETQVAAGPFRIQDINDAVSGEMNVRVEE
QDGSVQEFTMNTATIPYLTRPGSVRFKLASGKPSDFQHHSRGPMFGTGEFSWGVSNGWSLYGGALVGG
DYNALSLGLGRDLMALGALSFDATQSRARLPQADGTLSGGSYRLSYSKNFDEYDSQVTFAGYRFSQED
FMSMSEYLDARYYGTRTGNGKEMYTVTFNKHFRDWGLSTYLNYSHETFWDRPANDRYNLTLSRYLDIG
SFRNVSLSLSAYRNKYNGVNDDGGYLSLSLPWGNSGSVGYSATVNRSDVTHQANYYDRLDEHNNYSMS
AGSSRSGASLSGYYNHEGDMARMSANASYQEGRHSAMGLSLQGGATLTMEGGALHRAGIPGGTRMLID
TNGVADVPVRGYGRTSNTNAWGKVVIGDVNSYYRNKASIDLNKLGDNAEATTSVVQATLTEGAIGYRQ
FDVIAGEKAMAVIKLADGSQPPFGATVMNARKQETGIVNDGGSVYLSGINAGDTMTVHWAGNAQCEVR
MPTPLPAEMLMNSLLLPCHPLSAQAPTHDGQTTAEDAPGAVTSTVPGRTVQPPSLSDKNRELF
>CORE_REP|Org7_Gene1835#
MLDRIIAHTPLGQEQLLFRSLDGIEALSTPFDFSIELLSTDARLDRKALLGQPLTLEIPTQGFLSAPR
YLNGKITAIAVSSEEIGGTRYAVYNLHVQPDLWPMTKDRNFRIFQEQTVPQIVKTLLAEHNVQLEDQL
TGDYRLWGYCVQYNESSFNFISRLMEQEGIYYYFKHEMGKHTLVLGDAPHHHQPYPGYEMIPYHLTPS
GGSTSEEGISQWTLSDRVTPGIYSLDDYDFRKPNAWLFQARQNPVSPTPGQIDVYDWPGRYTEHQQGE
FYARVRQEAWQAEHQQIRGTATAMGIAPGSTFTLYNAPHADDNREYLTLQASYHLKENRYASGDDQSS
EHRIDFIVLPADVPWHPPQQATWPKTHGPQTARVVGPAGESIWTDKYGRIKVKFHWDRFGPKDDGSSC
WVRVSSAWAGQGYGGVQIPRVNDEVVVDFINGDPDRPIVTGRVYNEASMPPWALPAAATQMGFMSRTK
DGTADNANALRFEDKAGAEQVWIQAERNMDTQVKNDESHTIANDHTHLVGGNQIKRVVLNQATGVKGE
SSALTGKTRSDAVVNAFTLGSGESLRLECGESVIELLADGQINITGTSFNITVKEDGAINTGGQLDLN
QPGGAARTAAPGGGHQAAIQSAVDQLFPNEEASGTPGKPVNAAPRAAAAAPASITQNAQSTTKPGRID
NRVVESVMASEGSAGEQGGRRELYGFRKGNGNAYDKILAARNQYGQGSAEEFEEVSKAMSASAKSAGA
LNFSDPGKQGAITSLAHMRGSSGAQAILNSMESGRIVKADTLTSEAIAKIESMSAESFQDNLLKARVE
YDRAIYGDTITTQGGKQYNWWARYGNGLQKRYAREAEEFLKLSNE
>CORE_REP|Org17_Gene960#
MRLDRLTNKFQLALADAQSLALGHDNQFIEPLHLMSALLNQEGGTVRPLLTSAGIDAGRVRTEIEQAL
SRLPQVEGTGGDVQPSHELVRVLNLCDKLAQKRADKFISSELFVLAVLEDRGSLTDLLKAAGATADKI
SKAIEQMRGGDSVEDQGAEDQRQALKKYTIDLTERAEQGKLDPVIGRDEEIRRTIQVLQRRTKNNPVL
IGEPGVGKTAIVEGLAQRIINGEVPEGLKHKRVLSLDMGALIAGAKYRGEFEERLKGVLNDLAKQEGS
VILFIDELHTMVGAGKADGAMDAGNMLKPALARGELHCVGATTLDEYRQYIEKDAALERRFQKVYVAE
PSVEDTIAILRGLKERYELHHHVQITDPAIVAAATLSHRYIADRQLPDKAIDLIDEAASSIRMQMDSK
PESLDRLERRIIQLKLEQQALNKESDDASKKRLDMLSDELGQKEREYSELEEEWKAEKASLSGTQNIK
AELEQAKITLEQARRVGDLGRMSELQYGKIPELEKQLAAATQAEGKSMKLLRNRVTDAEIAEVLARAT
GIPVARMLEGERDKLLRLEQELHSRVIGQDEAVSAVSNAIRRSRAGLSDPNRPIGSFLFLGPTGVGKT
ELCKALASFLFDSDDAMVRIDMSEFMEKHSVSRLVGAPPGYVGYEEGGYLTEAVRRRPYSVILLDEVE
KAHPDVFNILLQVLDDGRLTDGQGRTVDFRNTVVIMTSNLGSDLIQEHFGQMNYAQMKESVMEMVSHH
FRPEFINRIDEVVVFHPLGEKHIAAIAKIQLSRLYKRLEERGYEVTMTEPALALLSKTGYDPVYGARP
LKRAIQQEIENPLAQQILSGKLIPGKLVTLDVENDHIVARQ
>CORE_REP|Org46_Gene4129#
MSDHSMTSLLTIKKTPIIVILSVLWAKPVFSATEFNTDVLDIGERSKVDLSRFSDADYVMPGTYLLDI
KINQKTLPQRSIQYFPSPDNKSGSQVCLPPDLVEKMALKEDAARKVTLWHDNQCADIRGIKGATVSDR
ISGGVLAITIPQAWMKYSDPDWTPPEQWDDGIPGVLLDYNLSGQIGKQHHDNGTAESLSSYGTLGANL
GAWRLRADYQTDFNQQYGRRDSNFDWNQIYAYRALPMQAARLTLGETYLNSPVFDAYRFTGLNLASDE
RMLPPNLQGYAPEVRGIAKSNARITVSQEGRTLYQTTVPAGPFAIQDLSSSVRGKLDVKVEEQDGSVS
TFQVDTASIPYLTRPGYVRYNMALGKPSAYDHRTQGPVFSAGDFSWGLSNAWSLYGGALLGGDYNAWA
LGLGRDLNLFGALSVDATQSIARLPDEPSAKGMSFKVNYAKRFDELNGQITFAGYRFSQRKFMTMSQY

```
LQARYGDIDDRYSGRQKELYTVTASKTFMAEDSAQAITAYLTYSHQTYWDAGAQNRYGMSTSKLFDFG
GISNITASLAAYRTHYRGRTDDSAMLNFTVPIGEHNRLGYALQVNNRDVSQTATYTDNSDINNTWQVG
SGVTQSGKPTASGYYTHNASFGTLNANASYQQGSYSSIGGTFRGGLTATRHGVAAHQNAGNGGSRMML
DTNGVAGVPINNGRAYSNRFGLAVISDITSYYNTDTRIDVNKLADDVEATRAVVQGTLTEGAIGYRHF
EVVKGSKLLATIKLADGSEPPFGATVLSATGREIAVVNDGGSVYLTGVQPEERLDVAWEGRRQCRIAI
PGAAKPLDRLLLPCAKP
>CORE_REP|Org37_Gene4361#
MKKLTIGLIGNPNSGKTTLFNQLTGARQRVGNWAGVTVERKEGHFTTPQSDVRLVDLPGTYSLTTISE
QTSLDEQIACHYILSGDADLLINVVDASNLERNLYLTLQLLELGIPCIVALNMLDIATSQHIDIDVAA
LSARLGCPVVPMVSTRADGIGVLKQMIDNHHINEQQALVNYPPLLLKAVATLSDAMPQTLPAVQRRWL
ALQMLEGDIYSHRLAGPAVALLPAAIQALQQQQQQEDPALVIADARYQSIAALCDAVSNSQQAMPNR
LTEMLDKVILNRWLGVPIFLLVMYLMFLLAINIGGALQPIFDIGSAAIFIQGIQWLGYTLHFPDWLTV
FLAQGIGGGINTVLPLVPQIGMMYLFLSFLEDSGYMARAAFVMDRLMQALGLPGKSFVPLIVGFGCNV
PSIMGARTLDAQRERLITIMMAPFMSCGARLAIFAVFAAAFFGQDGAGVVFSLYMLGIAVAILTGLVL
KYTIMRGEASPFVMELPVYHVPHLKSLLLQTWQRLKGFVLRAGKVIVVASMFIGGLNSFSFSGKTVDN
INDSALASVSKVLTPLLQPMGVHSDNWQATVGLVTGAMAKEVVVGTLNTLYTAEHINKEAFDAANFNL
LDELGGALNETWDGLKNTFSLSVLSNPIEASKGDGEMGVGSMGVMSSKFGSGISAYSYLIFVLLYVPC
VSVMGAIARESSRGWMTFSILWGLNVAYSLATLFYQVATFNQHPQYSLTAILVVLAVNLLVLFGLRRA
RSRVTVRLGNATPAACCQGAKGSCH
>CORE_REP|Org23_Gene1253#
MTELNEKLANAWEGFSKGDWQNEVNVRDFIQKNYTPYEGDESFLAGATQATTTLWDKVMEGIKLENRT
HAPVDFDTNVAATITSHDAGYIAKELETIVGLQTDAPLKRALIPFGGIKMVEGSCKVYGRELDPQLKK
VFTEYRKTHNQGVFDVYTKDILNCRKSGVLTGLPDAYGRGRIIGDYRRVALYGIDFLMADKLNQFKSL
QEKLENGEDLEMTIQLREEIAEQHRALAQIKEMAAKYGYDISGPATNAQQAVQWTYFGYLAAVKSQNG
AAMSFGRVSTFLDVFIERDIKAGKLTEEQAQELIDHLVMKLRMVRFLRTPEYDELFSGDPIWATESLA
GMGVDGRTLVTKNSFRFLNTLYTMGPSPEPNMTILWSEKLPLNFKKFAAKVSIDTSSVQYENDDLMRP
DFNNDDYAIACCVSPMIVGKQMQFFGARANLAKTMLYAINGGVDEKLKMQVGPKEAPMMDEVLDYDKV
MARMDHFMDWLAKQYVTALNIIHYMHDKYSYEAALMALHDRDVYRTMACGIAGLSVAADSLSAIKYAK
VTTIRDEDGLAIDFKVEGEYPQFGNNDARVDDIACDLVERFMKKIQKLRTYRNAVPTQSVLTITSNVV
YGKKTGNTPDGRRAGAPFGPGANPMHGRDQKGAVASLTSVAKLPFAYAKDGISYTFSIVPNALGKDDD
VRKANLAGLMDGYFHHEASIEGGQHLNVNVMNREMLLDAMENPEKYPQLTIRVSGYAVRFNSLTKEQQ
QDVITRTFTQTM
>CORE_REP|Org9_Gene770#
MLNQELELSLNMAFARAREHRHEFMTVEHLLLALLSNPAAREALEACTVDLAALRQELEAFIEQTTPT
LPAGEEERDTQPTLSFQRVLQRAVFHVQSSGRSEVSGANVLVAIFSEQESQAAYLLRKHDVSRLDVVN
FISHGTRKDEPGQAPNAENPVNEEQSGGEDRMENFTTNLNQLARVGGIDPLIGRDRELERAIQVLCRR
RKNNPLLVGESGVGKTAIAEGLAWRIVQGDVPEVMADCTLYSLDIGSLLAGTKYRGDFEKRFKALLKQ
LEQDQNSILFIDEIHTIIGAGAASGGQVDAANLIKPLLSSGKIRVIGSTTYQEFSNIFEKDRALARRF
QKIDITEPTAEETVQIINGLKTKYEAHHDVRYTAKAIRAAVELSVKYINDRHLPDKAIDVIDEAGARS
RLMPASKRKKTVNVADIESVVARIARIPEKTVSASDRDVLRNLGDRLKMLVFGQDQAIEALTEAIKMS
RAGLGHERKPVGSFLFAGPTGVGKTEVTVQLAKAMDIELLRFDMSEYMERHTVSRLIGAPPGYVGYDQ
GGLLTDAVIKHPHAVVLLDEIEKAHPDVFNLLLQVMDNGTLTDNNGRKADFRNVILVMTTNAGVRETE
RKSIGLVQQDNSTDAMEEIKKVFTPEFRNRLDNIIWFNHLSTEVIQQVVDKFIVELQAQLDAKGVSLE
VSDEARDWLSVKGYDRAMGARPMARVMQENLKKPLANELLFGSLVDGGSVKVELDKDKKQLTYHFLSA
AKRKADEGAVH
>CORE_REP|Org2_Gene3241#
MKRKHLWVLNPCLLAMLAPAAWAEDQKTGNEEQLVVSASRSHRSVAEMAQTTWVIESQEIEQQVQGGK
EIKDMLAQLIPGMDVSGQGRTNYGMNIRGRSMMVMIDGVRLNSSRSDSRQLDSIDPFNIDHIEVISGA
TSLYGGGSTGGLINIVTKKGQPEQQVELQIGGKTGFGGHNDHDENVAAAVSGGNDNASGRLSVSYQRY
GGWYDGKGNEVLIDNTQTSLQYSDRLDVMGTGTLNIDDHQQLQLTTQYYKSQSDGDHGLFLGENFAAV
TGNAKAYNSGSLDSDRIPGTERHLINLQYSNTDFLGQDLVAQVYYRDETLTFYPFPTLAGKAPNYYVS
SIGASQQKTDFYGGKLTLNSKPVDALTLTYGIDAEHESFNANQQFFNLAKAQQSGGMTLENAYSTGRY
PSYTTSNLASFLQASYDINPIFTLSGGVRYQYTENKIDDFVGYNQQQAIATGAAASADAIPGGKTDYN
NALFNAGLLAHLTERQQTWFNFSQGFEIPDPGKYYGNGTYALNGGHYQLLKSVNVGDSRLEGIKVNAY
```

ELGWRYTGDNLRTQIAAYYSLSDKSIAINKTDMTINVNADKRRIYGVEGAVDYFFEDSDWSAGTNFNV
IRSETKVNGEWKKLVVDTASPSKVTAYVGWAPGDWNLRLQSQQTFDVSDDGDYTKANSTQGRKIDGYN
TLDFLGSYALPVGKISFSVENLLDKEYTTVWGQRAPILYSPTYGSPELYSYKGRGRTFGLNYSVLF
>CORE_REP|Org16_Gene928#
MVAVRSAHLNTAGEFALDEWIAGLGLPNPQSCERLAATWRYCEQQTQNHPDASLLLWRGLEMVEILST
LSMDNDSMRAALLFPLVDAGIVQEETLTEAFGNGIVALVHGVRDMDAIRQLKATQNDSMASEQVDNVR
RMLLAMVEDFRCVVIKLAERIAHLREVKDAPEDERVLAAKECSNIYAPLANRLGIGQLKWELEDFCFR
YLHPEEYKRIAKLLHERRIDREQFIDDFVAGLRAEMAKEGIRVEIYGRPKHIYSIWRKMQKKHLAFDE
LFDVRAVRIVAERLQDCYAALGIVHTHFRHLPDEFDDYVANPKPNGYQSIHTVVLGPRGKTVEIQIRT
RQMHEDAELGVAAHWKYKEGAGVTVRSGYEERIAWLRKLIAWQEEMADSGEMLDEVRSQVFDDRVYVF
TPKGDVVDLPAGSTPLDFAYHIHSDVGHRCIGAKIGGRIVPFTYQLKMGDQIEIITQKQPNPSRDWLN
PNLGYVTTSRGRSKIHNWFRKQDRDKNILAGRQMLDNELEHLGISLKEAEKLLIPRYNMNSLDEVLAA
IGGGDIRLNQMVNYLQGKFNKPSAEEQDREALRQLVQQKAPPPTRNKDNGRVVVEGVGNLMHHIARCC
QPIPGDDIVGFITQGRGISIHRADCDQLVDLQSHAPERIVDAVWGESYSSGYSLVVRVMANDRSGLLR
DITTILANEKVNVLGVASRSDTKKQLATIDMDIEIYNQQVLSRVLAKLNQLPDVIDAKRLHGN
>CORE_REP|Org10_Gene3143#
MPTKRLSSSAAKQGRLPVSALAITVAAALGTLAMPAFSADAKPAAKEDTITVVGGSNSAQQESAWGPV
GTYVAKRSATGTKTDTPIEKNPQSVSVVTREEMDMRQPDTVKSALAYTPGVMIGNRGASTAYDAVNIR
GFSSVGTNMYLDGLKLQDDNYSIYQIDPYFLERAEVLRGPSSVLYGKSNPGGVVALVSKRPTTETLRE
VQFKMGTDNLFQTGFDFSDALDDAGVYSYRLTGVARDEDQQQVGEKSKRYAIAPSFSWRPDDRTSLTF
LSSFQDDPSVGFYGWLPKEGTVQNGVNGKLPTSFNDGEPGYNNISRKQQMVGYAFEHAFDDVWTVRQN
LRYSKMDVDYRSIYGLGIDPDNSAELKRGVMNSKEHMSSFAVDTQAQAKFATGQVDHIVLMGVDYMRM
RNDVVYQYGSASNLNVIAPQYGNRSYTITGGASQVNRQEQTGLYVQDQAEWNNWVLTMGGRYDWSDTN
STNRLNQNSVSKQQDKQFTGRAGLNYVFENGIAPYVSYSESFEPTSGTDFSGNTFAASKGKQYEAGVK
YAPKDRPITASLALYQLTKTNNKVADPNPEHAFASILGGEIRSRGVELEAKAALTANLNILGSYTYTN
TEYTKDTTLQGNTPAAIPKHMASLWADYTFHETAISGLTLGSGVRYVGSSYGDEANTFKVKDYTVFDA
AIKYDLARFNLPGSSIGINVNNLFDKEYVSSCFATYGCYWGAERQVVATATFRF
>CORE_REP|Org5_Gene3766#
MTTESKCPFSGGKQPAPQNGPTNQDWWPNQLSLKPLHQHSPLSDPMDKDFNYADAFNSLDLAAVKQDL
HALMTDSQEWWPADFGHYGGLFIRMAWHSAGTYRIGDGRGGAGEGQQRFAPLNSWPDNVSLDKARRLL
WPIKQKYGRNISWADLIILTGNVALESMGFKTFGYAGGRADTWEPDDVYWGSEKIWLELSGGPNSRYS
GDRDLENPLAAVQMGLIYVNPEGPDGNPDPVAAARDIRETFARMAMNDEETVALIAGGHTFGKTHGAG
PASNVGADPEAAGLESQGLGWHSTFGTGVGKDAITSGLEVTWTTTPTQWNHDFFRHLFEYEWELSQSP
AGAHQWVAKDIGETIPDAFDPNKKRRPTMLTTDLSLRFDPAYEKISRRFYEHPEELADAFARAWFKLT
HRDMGPRPRYLGPEVPQEELIWQDPIPAVDHPLIDEQDIAALKNAVLASGLPVSALVSTAWASASSFR
GSDKRGGANGARIRLAPQKDWAVNQPAQLAATLATLESIQRTFNDAQAGGKRVSLADLIVLAGAAGVE
QAAKNAGLALTVPFAPGRMDASQEQTDVDSFEAMEPLADGFRNFLKGKYRVPAETLLVDKAQLLTLTA
PEMTVLVGGLRVLGANVGGTPHGVFTQRPQALTNDFFVNLLDMGTTWHPVGEDGLFEGRDRRSGAVKW
TGTRVDLVFGSHAQLRALAEVYGSADAQEKFAHDFVAAWNKVMNLDRFDLA
>CORE_REP|Org40_Gene2964#
MKNNALSVMVAEQDEKLEWERLIGPLWDNRWRIAVVTGVAGMLGVAYALLATPVYQATAVVQVEKQLS
GDSLLRETLDSSMMGQNSATQDEVTLAKSRYVLGKTVDTLGLTVRVSPDYFPVFGKGFARLSGEKPPV
LSIATLTTPADMEGEALTLTVRDGQHYELSYDGSKLFSGVVGQPVAQGGWNMTVSALDASPGASFTVV
KVARQEAVDDLRKYLDVVPGGKDSGIMTFTLPSEDPQSAEAMLKNITDNYLQQNVDRKTEEAQRMLAF
LQEQLPQTQTSLNNAETQLNQFRQQNDSVDLSLEAKSVLDTQVQLEAQLNELTFKEAEISKLYTRAHP
AYRALLEKRATLEAEKARLGKQVQTLPKMQQEILRLTRDVQVDQQVYMQLMNKQQELSISKAGTVGNV
RIIDEAETALRPIKPQKMLIVLLALLLGAGGGAIVVLLRAAFHRGINDIDTLEKRGINVYATVPLSPW
QVKRNREQRQLLPRSGGRRLPILAVAEPTDLSVEAIRSLRTSLHFAMMEAKNNILMVSGASPESGKSF
TSTNLAVVVAQAGQRVLLIDADMRKGFLHRWLADDGHQGLSDMLVGNVMAEQAVRKTAIANLDFVPRG
QVPPNPSELLMHRRFADFLRWAGQNYDLVLIDTPPILAVTDAAIVGNHAGTSLLVVRFEVNTVKQIET
SMRRFEQNGVAIKGVILNGVVKKAATDMSYYNFAYPSHREDHPQAGE
>CORE_REP|Org6_Gene3182#
MKELKIATSASLVATIETLRQIVRVEQTDCTDVAAAVVSVADVNAGILARLQATGFDIPTFVAVEGDE
HLSPDYLPFVSGVFALLAGASKPFYMAQLEAAADAYEQALLPPFFKTLKTYVEMENSTFACPGHQGGE

FFRKHPAGRQFFDFYGETLFRSDMCNADVKLGDLLIHEGSAKDAQKHAARVFNADKTYFVLNGTSAAN
KVVTNALLTRGDLVLFDRNNHKSNHHGALIQAGATPIYLETARNPFGFIGGIDAHCFDERYLREQIRE
VAPEKAAAARPFRLAIIQLGTYDGTIYNARQVIDTIGHLCDYILFDSAWVGYEGFIPMLKECSPLLLE
LDEHDPGIFVTQSVHKQQAGFSQTSQIHKKDDHIKGQKRHCNHKHLNNAFMLHASTSPFYPLFAALDV
NAKMHAGPAGRRMWMDCVKLGIETRKQLLTRCSQLKPFIPQQVAGKDWQDYDTDLIANDARFFTFVPG
ETWHGFEGYAQDQYLVDPCKLLLTTPGIDAATGQYTEFGVPATILANFLRENGIVPEKCDLNSILFLL
TPAENPAKMEQLVEMLAQFERYVEEDAPLSVVLPTVYRKNEQRYRGYSIRRLCQEMHDLYVSFDVKQL
QKEMFRQDHFPPVVMNPQDANVEFIRDNVELVPIGQAEGRIAAEGALPYPPGVLCVVPGEVWGGAVQR
YFLALEEGINRLPGFSPELQGVYIEKKDHGWKRIFGYMIKQ
>CORE_REP|Org41_Gene4663#
MPDLSRRDILRAAAIGSAFSLLPASIRKALAIPANNRTGTLRDVEHVVILMQENRSFDHYFGTLPGVR
GFSDRFTIPLPGDRHVWQQQGAERLVLPYHLDSKRGNAQRVTGTPHSWVDEQAAWDHGRMSAWPTYKT
PASMGYYRQHELPFQFALANAFTLCDAYHCAIHAGTNTNRLFHWTGTNGPSAADVAVVVNEWDSPGPA
EIGYQWTTYPERLEASGVSWKVYQFLPDNFTDNPLAGFRQYRAASIQVGNPARPPKDFNAFVPYRDAL
NEAAPLYKGNGNTLPAADGNDLDAMLAGFRADIQQGKLPQVSWIIAPAAYSEHPDPSSPVQGGWFTQE
ILNALTDNPEVWSKTVLLVNYDENDGFFDHMPSPSAPSLREDGSFAGKSTVPFDTEIFQHVAPPGSQD
QPPPDGRIYGPGPRVPMLVLSPWSRGGWVNSQVFDHTSVLQFLEKRFQVHEPNISAWRRAVCGDLTSA
FNFVDPNSEALPSLPVTSRHAADGLRQRQEQLPQVPLPSPAHQRLPHQRRQARPSRALPYQLHVEATV
VAEQRRVTLNLFNTGEQGAVFHVYDRRDLAQIPRRFTVEAGKAVSDDWQTEDEYHLWLLGPNGFHREL
RGALNRPQPEVRLRPTGRSLQLQLNNPGTEAIAVTLERCPYTQQGPWHITLPAGGSHQQSFDAHASGG
WYDLTLQSPGGWLRRLAGRLEDGEHSVSDPLMGQE
>CORE_REP|Org38_Gene2989#
MNIIAIMGPTGVYYKDEPIRELHAALGAMGFQLVYPKNSGDLLKLIEANARICGVIFDWDDYSLELCS
EINELNEYLPLYAFINTHSTFDVSLHEMRMVLYFFEYGLNAADDIAQRIQQYTAEYIDTITPPLTKAL
FNYVREGKYTFCTPGHMAGTAFQKSPVGCLFYDFFGANTLKADISISVTELGSLLDHTGPHLEAEEYI
ARTFNAEQSYLVTNGTSTANKIVGMYSAPAGSTVLIDRNCHKSLCHLLMMSDIVPIYLRPLRNAYGIL
GGIPQREFTRASIAARVQETPNATWPVHAVITNSTYDGLLYNTDYIKQTLEVPSIHFDSAWVPYTNFH
PIYDGKSGMSGDRVPGKVFYETQSTHKLLAAFSQASMIHIKGDYDESTFNEAYMMHTTTSPHYGIVAS
METAAAMLRGNPGRRLINRSVERALHFRREVQRLREESDSWFFDIWQPEEIDEAQCWPLDPDDNWHGF
GQTDRDHMYLDPIKVTILTPGMNELGALEEEGIPAALVAKYLDERGIVVEKTGPYNLLFLFSIGIDKT
KAMSLLRGLTDFKRAYDLNLRVKNMLPDLYAEDPDFYRHMRIQDLAAGIHRLICQHDLPRLMQRAFDV
LPEMKLTPHQMFQEQVRGNVETCELDQLVGKVAANMILPYPPGVPLVMPGEMITEESRAVLDFLLMLC
SIGERYPGFETDIHGAKLTEDGRYLVKVLKAPQP
>CORE_REP|Org43_Gene425#
MKDNNLFERINEKLTFSLRKRVPSILQSESSECGLACLAMIASYYGFNVDMLSLRQRFGISTQGATLG
TISQIASQIQLKTRALSLDIDEINQLKTPCILHWNMNHFVVLVKVQRAGFVIHDPAFGRRVIGLQEMS
NHFTGIALELWPDRAFQKETLKTRLRLLDLMKNIEGLPGTLLKIFALSIVIESVNLLLPVGTQLVTDH
VIQAHDYSLLTVICLGLIFFTLFRAVVSIARAWISIVLGTLTDIQWKTTLFEHLMKLPLDFFEKRHLG
DIQSRFSSLDAIRTTFTNNIVSGIIDGIMTVGLFAMMMVYGGWLVWVVAGFTLIYILIRMMTYRTYRQ
FSEEQIVKAAKANSHFMETLYGISTVKALGIKETRSSYWLNLNVDAANTNIKITRFNMMFGGINTFIT
TLDQVAILWLGAMMVIDNSMTLGMFMAFNAYRGQFSQRASSLIDLAIGLRMLSLHNERISDIVFTDAE
TESAPRQVFPSGTGIAIEVKNLTYQYDALSRPIFKDLNMRIAAGESVAVVGASGAGKTTLLKVMCGLL
SPTSGQVLADAMDIHKVGVNNYRNAIACVLQDDRLFSGSIAENISGFEVNANKELIMACAIHSNIHDE
IMQMPMGYETLIGELGNGISGGQKQRLFIARALYRRPSVLFMDEATSHLDVENESAINRAISSLNITR
VIVAHRKSTIDSADRVVVLGAESGAPAGGGE
>CORE_REP|Org12_Gene3625#
MNNNKRGWWCALPLAACATLPTWAAEKVASKEESLTVIGRKDADGVQSYQPLTSVTGTRSETNLLNVP
QAIDVVPQQVITDQAVSSLDEALYNVSGITQANTLGGTQDAVMKRGFGDNRDGSILRDGVRSVQARNF
TPTTERVEVLKGPASMLYGMGEPGGMINMITKKPQLQQHTHVEGWGSSFNGGGGQLDVTGPLGTSGFA
YRMIVDHDETDYWRNFGRNRQTVIAPSLMWYGENTTVRLAYEHMEYLVPFDRGTIIDSRTGKPVNTPR
DRRFDEAYNATRGDQDSITLQIDQTLNERWKSSLTYAYSRNSYSDNQARATALNPVTGVLSRQADSTA
NAVSHANAVQLTLNGDVDWGSINHQMLFGFDFEDNRTYRGDMIRGKKNSDFNIYHPVYGLMPPSTAVS
AKDSDQRENLTSYGWFMQDSIQLTDKWLVMGGLRYDAFDVYAGKGRPFQTNTDSSDGKLVPRAGVVYK
LTPYVSLYSSYTESFKPNSSIATQIDSLPPEQGKSWEVGGKLALPNGVTGTLALFDITKRNVMVNELV

EGETVTRTAGRVRSQGVELDVAGNITDSLSLIGSYAYTDARVVDDPDNKGKEMTNVARHTASLFLTQN
LGSLGLYSGDEVRIGAGARYVGRRPGDAANSFYLDNYTVADAFAAYTMPINGYRVKWQLNVKNLFDKT
YYPSSGGNLRVAVGEPREVVLRGSIDF
>CORE_REP|Org36_Gene2938#
MRHSQIKTADDRVYSARFEGAGESQPPFCFSPISRRAGLRVRRSLTIKQMATVSGVALVTICIFIVIQ
LFHFVQQRRDDYAQQLENIAHSVRQPLAEAVLRMDVPEAKKVLNTLLPVGILSRADIVLPNEFQALHA
NFPPERPVPTLIARLFELPIQISVPLYSLERVPANQQPLAYLVLQADSFRMYQFILSILSTMLSTYLL
LALILSVAITWCMNRLMVHPLRAMAKELENISQDEAPYHQLMLPALHQDDELGLLVRNYNRNQQTLAK
AHADMSRLSTRHPVTELPNPALLNALLEQHIASSLRPERFNLLVIGIETLHEASGVMSPAMREALLLA
LAKKLRGCIDENGVLAQLSNTEFAILAKGTERPFHAMQLARRIMAEINAPLTLEGLALRPNASIGIAH
YLNQGESAEQLLRSATSAMMSAHREGKNQILFFEPSLTERTQKRLTQESEILHGIEQRHFTLFLQPQI
DMQSNEVIGAEALLRWQQYDGSYTLPADVIPLAEELGVIVPLGNWVLEESCRILADWQQRGIELPLAV
NVSGIQMQDEAFVPHLKNLLAQYRIDPRKLLLEITETVRIDDLDRALALLRELHDLGLSIALDDFGMG
YSSLEYLNRLKSLPIDLIKIDRSFIQGLPADDAMVRIVSSISEVLALPVMAEGVENAEQRDWLLKHGI
RSGQGFLFARPLPREAFEAEFCRAAP
>CORE_REP|Org16_Gene630#
MYLFESLNLLIQRYLPEEQIKRLKQAYLVARDAHEGQTRSSGEPYITHPVAVACILAEMRLDHETLMA
ALLHDVIEDTPATYQDMEQLFGKSVAELVEGVSKLDKLKFQDKKEAQAENFRKMIMAMVQDIRVVLIK
LADRTHNMRTLGSLRPDKRRRIARETLEIYSPLAHRLGIHHLKTELEELGFEALYPNRYRVIKEVVKA
ARGNRKEMIQKILSEIEGRLTEAGIACRVSGREKHLYSIYLKMHLKEQRFHSIMDIYAFRVIVKEVDT
CYRVLGQVHSLYKPRPGRVKDYIAIPKANGYQSLHTSLIGPHGVPVEVQIRTEDMDQMAEMGVAAHWA
YKEREQGETGTTAQIRAQRWMQSLLELQQSAGSSFEFIESVKSDLFPDEIYVFTPEGRIVELPAGATP
VDFAYAVHTDIGHACVGARVDRQPYPLSQSLTSGQTVEIITAPGARPNAAWLNFVVSSKARAKIRQML
KNLKRDDSVGLGRRLLNHALGGSRKLAEIPQENIQHELDRMKLATLDDLLAEIGLGNAMSVVVAKNLQ
GDQSSLGTTSGVRNLAIKGADGVLITFAKCCRPIPGDPIIAHVSPGKGLVIHHESCRNIRGYQKEPEK
FMAVEWDKETEQEFIAEIKVDMFNHQGALANLTAAINAAESNIQSLNTEEKDGRVYSAFIRLTTRDRI
HLANIMRKIRIMPDVIKVNRNRN
>CORE_REP|Org17_Gene4022#
MPLTFSTRLRFSALSLAIACALPTVALAQNTSTTPSSSPATPAKKAKAADETMTVVATGNQRSSFEAP
MMVTVIEGSSPESQTAGTAADMLRRVPGITVTGSGRSNGQDLMMRGYDRRGVLTLVDGIRQGTDTGHI
NGTFLDPALVKRIEIVRGPSALLYGSGALGGVVSYETVDAADLLLPGHDSGFRVYGTAGSGDHSLGMG
ASAYGKTDNLDGLLSFGTRDVGNLRQGNGFDAPNDETINNVLAKGTWKIDDNQSLGGNLRYYNNSAQE
PKNPQTPASSAGNLMTNRSTIQRDAALSYKLKPVGQDWLDAEAKVYYSDVKINAHASGSEDEARKQTT
KGAKLENRTRLFADTFASHLLTYGTEAYKQEQTPGGATESFPHAKINFASGWLQDEITLRDLPVTLLA
GTRYDNYKGSSDGYADVDANKWSSRGAVSITPTDWLMLFGSYSQAFRAPTMGEMYNDSKHFSIPMGPT
TITNYWVPNPNLKPETNETQEYGFGLRFDDLLLADDSLQFKASYFDTKAKDYITTDVTMELGRGPRGP
YCISCTTFSTNIDRAKIWGWDATLSYKTSWFGWDLAYNRTRGKNEATGDWLSSINPDTVTSSLDVPLG
ETGLSAGWVATFAERATRVQTGTPEQGGYGVNDFYLSYKGRDRLQGVTTTVVLGNAFDKEYYSPQGVP
QDGRNAKLLVSYQW
>CORE_REP|Org3_Gene1504#
MTRKQRALFEPALVRTALIDAVKKLDPRVQWRNPVMFVVYIGSILTTAIWLAILTGQTDGAAAFTGSV
ALWLWFTVLFANFAEALAEGRSKAQAESLKGTKKTSWAKKLAGPRRDGATEKVAAESLRKGDIVLVEA
GDTIPCDGEVLEGGASVDESAITGESAPVIRESGGDFSSVTGGTRVLSDWLVVQCSVNPGETFLDRMI
AMVEGAKRRKTPNEIALTILLVALTIVFVLATATLFPFSQYSVEAAGSGSVVTITVLVALLVCLIPTT
IGGLLSAIGVAGMSRMLGANVIATSGRAVEAAGDVDVLLLDKTGTITLGNRQASEFLPAPGVKEQELA
DAAQLASLADETPEGRSIVVLAKQRFNLRERDLQALNATFVPFSAQTRMSGVNVQERMIRKGAVDAIR
RHVETNQGHFPRAVDDLVESVARTGGTPLVVAEGARVLGVVALKDIVKGGIKERFNELRKMGIKTVMI
TGDNPLTAAAIAAEAGVDDFLSEATPEAKLALIRQYQGEGRLVAMTGDGTNDAPALAQADVAVAMNSG
TQAAKEAGNMVDLDSNPTKLIEVVHIGKQMLMTRGSLTTFSIANDVAKYFAIIPAAFAATYPQLNALN
VMHLHSPASAIMSAVIFNALVIVFLIPLALKGVSYKPMSAAALLRRNLWLYGVGGLLVPFVGIKLIDL
ILVALHVAG
>CORE_REP|Org25_Gene4140#
MSMDISDFYQTFFDEADELLADMEQHLLELDPLAPDIEPLNAIFRAAHSIKGGAATFGFSVLQETTHL
LENLLDGARRQEMSLSTEIINLFLETKDIMQEQLDAYKTSQQPDAESFEYICQALRQLALEAQQQQEA

PAAQPAVQQPVAQPSAAPAAIEGGMRISLNGLKASEIPLMLEELGNLGEVKDPHQTEHSLDVTLLTSA
SEDDISAVLCFVLEPEQISFSTPTQSAPAVAEANLPETVAAPEPEPEPAPAPIASAAAPAAKPAAAAA
EAPKARAKASESTSIRVAVEKVDQLINLVGELVITQSMLAQRSGTLDPVNHGDLLNSMSQLERNARDL
QESVMSIRMMPMEYVFSRYPRLVRDLAGKLNKQVELTLQGSSTELDKSLIERIIDPLTHLVRNSLDHG
IEEPATRIAAGKSAVGNLVLSAEHQGGNICIEVIDDGAGLNREKILAKAASQGLAVSDSMSDEEVGML
IFAPGFSTAEQVTDVSGRGVGMDVVKRNIQEMGGHVEIRSQAGKGTTIRILLPLTLAILDGMSVKVND
EVFILPLNAVMESLQPQAEDLHPLAGGERVLQVRGEYLPLVELYRVFEVEGAKTDATQGIVVILQSAG
RRYALLVDQLIGQHQVVVKNLESNYRKVPGISAATILGDGSVALIVDVSALQTLNREKRLTDAAA
>CORE_REP|Org13_Gene4551#
MPADQGPELLNAHFGTQSPHWRLAFDSNALELSAVKGKAHVAVAFSAMEAAKIRRLTGVTASLELTIT
LAGEPLHLHLVGRRVNNLEWAGTASAFSDTQSVARDLVHGLSFAEQVVSEANSVIVIVDQHGRIQRFN
RLSEEYTGLREHEVIGKNVFQLFMSPEEAAASRRNIAGFFRNGSSYEVERWVKTVKGERLFLFRNKFV
HSGSGKNEVYLICSGTDITEERRAQERLRVLANTDLITGLPNRNAIQDKINHAIATRGEESFGLVYLD
LDNFKKVNDAYGHMFGDRLLVEVALAILGCLSPDQVLARLGGDEFLVLAPQTDRERLQTLAPQTDRER
LQTLAQRIIDRLKTPFRIGLIEVYTGCSIGIALCPEHGNDLDSLIRSADTAMYVAKEHGKRTYTVFSP
EMNKRVAEYMWLDTNLRKGLEQNQLVLYYQPKIDARSGEVHSVEALVRWDSPERGLIPPLQFISYAEE
SGLIGPLGQWVLQTAAGQAAQWQEQGLNLRVAVNLSARQLADDSIVNDLLGVLRRHRMAPCLLDFELT
ESSLIEDENRARALITRLRELGAQVHLDDFGTGYSSLAQLARIPLDAIKLDKSFVRGVNFNPVSQSLV
RAIVAAAEALAFRVIAEGVETESENHFLDEVGVDEKQGFLFARPMLPEQLEHWLQSYRPHSPSA
>CORE_REP|Org13_Gene4198#
METPRYSKLAALVVASLSATAALAAPQNDTQDTMVVTASGFQQKIQDSAASISVIPRQQIEDKAYRDV
TDALKDVPGVVVTGGASSSDISIRGMSSKYTLILVDGKRVDTRSTRPNSDNAGIEQGWLPPLEAIERI
EVVRGPMSSLYGSDAMGGVINVITRKTSRTEWKGSLHGDATIQENRNSGDLFQTNAYASGPLVEGLLG
LRVNGLLSRRAEDKIVNGYNEQRMRSGTAVFTLTPDEKNEFDFEIGRSLQDRNSTPGKSVVAERCSKG
KCTPTEVSESLYTRTNYALTHNGYYDFGNSTSYVQREETGNPGRNMKAYNTIFNTQNQFELGSHMLNL
GGQYRYEKLGDGGNQLESAQGLSKLTRWSWALFAEDEWALTNDFSLTSGIRMDRDENFGSHWTPRMYG
VWHLTEQWTLKGGVSAGYKSPDLRQSSPNWGQVTGGGVRKGIIVGNPDLQPEKSLSEEIGLMWDSLKG
VNAGVTVFNTDFKDKITEVRRCEDTPDCKIGNDVYDFISDRVNVDKANMRGVEATFGWQINKDWKWNT
NYTYTSSEQKSGEFQGKALNQMPKHMLNTVLDWRATQDLSLWSRVNFRSKTSQYLSRTSMATSTPSYT
FVDAGLSYQAAKNLQLTGGVYNILDKTVDYDHFRTTLDGRRYTVGMTYNF
>CORE_REP|Org17_Gene1483#
MAALLQLNGIRRSYRSGEQTVEVLKGISLSIDAGEMVAIMGASGSGKSTLMNILGCLDKPSAGVYRVA
GQDVATLSDDALAQLRREHFGFIFQRYHLLPHLSAAHNVEVPAVYAGLGKAARRERAEALLRRLGLGE
RVNYRPSQLSGGQQQRVSIARALMNGGQVILADEPTGALDSHSGEEVMAILKQLCAQGHTVILVTHDP
AVARQAERIIEIRDGEIIADSRPAPSEDAQAKPLTLAAAAPSWRQMGGRFREALVMAWRAMAANKMRT
ALTMLGIIIGIASVVSILVIGDAAKQMVLADIKSIGTNTVDIYPGKDFGDDDPTYRQSLKYGDLDALR
EQPYISALSPSISSSMRLRLGNVDAAANVNGVSEQFFRVYGMSFTQGVGIDPMQVQSQAQTVVIDANT
QRRLFPHQKNVVGEVILVGNMPATVVGVAKEKQSMFGSSKTLNVWVPYSTMANRLMGNSYFDSITVRI
RDGYDSKEAEQQLSRLLTLRHGKKDFFTYNMDSLVQTAEKTTRTLQLFLTLVAVISLVVGGIGVMNIM
LVSVTERTREIGIRMAVGARSGDVLQQFLIEAVLVCLVGGALGITLSFAIGLAVQLVLPGWQISFPPA
ALLSAFLCSTGIGVVFGYLPARNAARLNPIDALARE
>CORE_REP|Org6_Gene4205#
MILRHPPIKFGCFSHDNYKNTLLAVVSSVTAFSGWAQDNTTATNGDNLVVTANRFPQPVSSVLAPTSV
VTRNDIDRWQAKSLTDVMRRLPGVDIAQNGGLGQQSSLFIRGTNSSHVLVLIDGIRLNQAGVSGSSDL
SQIPISLVQKVEYIRGPRSAVYGSDAIGGVVNIITTREKNGTTLAAGVGSNGYQSYDASTQQPLGDST
VATVAGNYTYAKGYNVIANLPDSFGNPAQPDRDGFMSKSLYGGIEHKFNEAFSGFVRGYGYDNRTAYD
GNYSYSDPAHLDALPDTRQLYSQSWDSGLRYQDGIYATQLIASYSHTKDYNYDPKYGPYSASATLDDS
TQYNVQWGNTFQVAQGHISTGVDWQNQKIEPGTAYITDSKSQRNTGLYLTAQQQVDAFTLEGAVRGDD
NSQFGWHGTWQTSVAWEFVEGYRAIASYGTAFKAPNLGQQYGSFGGNPDLKPEESKQWEGGFEGLTGP
VTWRVTGYRNDIDNLISYASSGSGSAYYNVNQARIKGVEATVSFDTGPLTHQIGYDYVDPRNAKTNEV
LLRRAKQQVKYELDWQLYDFDWAVTYQYLGERYDGDYSGYTTRTVKLGGVSLWDLAVSYPVTSHLTVR
GRIANLFDKDYETAYGYATPGREYYLTGSYTF
>CORE_REP|Org26_Gene2931#
MAALQALAGKFTHLTVGKKLGLGFALLLLLAVIIAGTGAQYLHIIESRADRIDFSNRLNEEINQAKYN

RAMYGQTYRPEYIQNNRANIENAVKLIDHGQALDWDAQSRKDLQRLVTLIGEYQQQQKEFEQAVTAKD
AVRQSWNMSEVQASLSQVERQLTNGDLQLAFIQLNQKLTQVRYGARGLLLSLNKETEAPLMAAIDDAR
DAASALSRRVSDAQRPQLQPLLAALDDYKNRIAAYLPAYEHEQQISRRLGERAQAIGMLVNAFMQDEL
AQTHNDINLAQLQMGITTLIAIIAGVLIAWRITLQITRPLHSTLAMAERIAGGDLRQAQTSTRRDELG
QLLNAVAAMSQNLRTMIEKIQMGVSQVSTASAEIAAGNTDLSSRTEQQAAAVEETAASMEQLTATVKQ
NADNAHHANQLATDASQTAQQGGKLVENVVSTMRDISSSSQRIAEITTLINGIAFQTNILALNAAVEA
ARAGEQGRGFSVVASEVRSLASRSAQAAKEIEGLIAESVSRVKTGTELVESTGNTMEQIVRSVTHVRD
IMAEIAAASDEQTRGIAQIGQAIVEMDHTTQQNAALVEESAAAADSLEEQADMLLQSVSVFRLAEQTE
PAAVKAAAPKAPAVKPAAANAAAEENWTTF
>CORE_REP|Org44_Gene1901#
MDSKLLDYYNRELAYLREMGAEFAEQYPKVAGRLGMRGIDVADPYIERLMEGFAFLTSRVQLKMDAEF
PRFSQRLLEIIYPNYLSPTPSMAIAELQPDSSKGDISNGFVVPRGTMMDSQTLKKSGITCSYATAHDV
TLQPVRIAAVELGGIPADIPLASLGLQHSGCVSALRIRLECYESVTLNNLQLDQLMFYLAGPDMQAQQ
LLELLMQHSVGLVCQTVEAQPQRRALALDGLRQEGFAAEQALLPNDLRNFEGYRLLQEYFAFPARFQF
FSVNGLRPLLQSVREGKKALRQFEIVVLLDRHDAALERVVDAAHLALHCTPVINLFPKVAERIAINEK
NHEYHLVVDNIRPLDYEVFSVQRLGGSASEKRYEQEFRPFYSTLSADDGNYGAYFSLRREQRTLSEHA
RRYGTRTGYAGSEVFVSLVDERQSPWHSDLKYLTADVLCTSRDLPLMLLQQDQGNFVMPDSIPIKQVS
LRKGPTPPRPALAEGMITWRLISQLQLNYLSMMDGDPEQGAASLRQLLGLYGNLSEPAIAKQIQGVRH
CNLRPVYRRVPEPGPIVFARGIAIDLTVDEQAFSGNSPYLLGSVLERLFSRLVAMNTFTEMTLSSQQR
GEIAHWQARMGKRTLI
>CORE_REP|Org20_Gene2054#
MTDVLLRPAASGGPSLPPQRVLTVRDLSISFPQPDGAVAAVRNLSFDLDRGETLAIVGESGSGKSVTS
LGLMRLVEQGGGRIVGGVMTLRRRDGALLDLAQASQSTLRTVRGADMAMIFQEPMTSLNPVFPVGEQI
AESLRLHQGMDRRSARQEALRMLDLVRIPEAKEVLGRYPHQLSGGMRQRVMIAMALSCKPALLIADEP
TTALDVTIQAQILQLIRVLQREMQMGVIFITHDMGVVAEIADRVLVMRRGEQVEQNRVRELFAAPQQA
YTRALLAAVPKLGAMADRPLPAKFPLPGGEGDTAPQDTVPPGAAPILQVEHLVTRFDLRGGLFNRVTR
RVHAVENVSFDLYPGETLGLVGESGCGKSTTGRSLLKLVDSQSGTITFAGRRIDQLKGPALQHLRRDI
QFIFQDPYASLDPRLTVGFSIMEPLLVHNVMRGREAEQRVAWLLERVGLLPEHARRYPHEFSGGQRQR
ICIARALALNPKVVIADEAVSALDVSIQAQIVNLLLDLQREFGVAFLFISHDMAVVERISHRVAVMYL
GQIVEIGPRQAVFDNPQHPYTRKLMAAVPVADPAHAHKRQPLPADEIPSPVRALGDEPVTAPLVQVGA
GHFVARHPIAGAF
>CORE_REP|Org23_Gene2753#
MEQNPQSQLKLLVTRGKEQGYLTYAEVNDHLPEDIVDSDQIEDIIQMINDMGIQVMEEAPDADDLLLA
ENSNSTDEDAEEAAAQVLSSVESEIGRTTDPVRMYMREMGTVELLTREGEIDIAKRIEDGINQVQCSV
AEYPEAITYLLEQYDRVEAGEARLSDLITGFVDPNAEEDIAPTATHIGSELSSEEQDDDEEDEDAEDD
DTEDDNSIDPELARQKFAELRDQYEATRLVIKKNGRSHASAADEILKLSEVFKQFRLVPKQFDFLVNS
MRTMMDRVRTQERIIMKLCVEQCKMPKKNFVTLFAGNETSDSWFEAAVAMAKPWSEKLKDVAEDVQRS
LQKLRQIEEETGLTIEQVKDINRRMSIGEAKARRAKKEMVEANLRLVISIAKKYTNRGLQFLDLIQEG
NIGLMKAVDKFEYRRGYKFSTYATWWIRQAITRSIADQARTIRIPVHMIETINKLNRISRQMLQEMGR
EPTPEELAERMLMPEDKIRKVLKIAKEPISMETPIGDDEDSHLGDFIEDTTLELPLDSATSESLRSAT
HDVLAGLTAREAKVLRMRFGIDMNTDHTLEEVGKQFDVTRERIRQIEAKALRKLRHPSRSEVLRSFLD
D
>CORE_REP|Org8_Gene3621#
MKNAVRRGEVMSVLAAYRRGFWGIALFTAVINLLMLAPALYMLQVYDRVLPSGNRMTLAMLTLMVVGL
YLFMGLLEWVRSQVVIRLGAQMDMRLNQRVYDAAFETNLKTGNPLAGQALNDLTNLRQFATGNALFAF
FDAPWFPVYLLVVFLLHPWLGALASAGVIVLVLLAWLNQRVSQAPLAEAGRVALSATQQANGNLRNAE
AIAAMGMLTDLRLRWLRQHQQFLLLQNRASEKIAAVTAWSKTVRLALQSLMLGCGALLAVSGDITPGM
MIAGSILIGRVLGPIDQLIGAWKQWSSARQSLQRLEVMLAANPPRIPSLPLPTPGGALTVSQLTASAP
GGTAPVLHGVSFRLEAGEVLGVIGASGSGKTLLMRQLVGALTPISGDVRLDGADIQQWDKQQLGPHIG
YLPQDIQLFAGTLTDNIARFGQVDAEKVVVAAALAGVHQLILHLPKGYETELGEGGSGLSGGQRQRVA
LARALYGSPALVVLDEPNANLDREGEEALQRAIEALKARGTTIVLVTHKPAILATTDKLLVLTAGQVQ
HFGPSDAILKKLPGFAPAAAAAPANTGRSNGGFNVNYANFAKTASGERKV
>CORE_REP|Org36_Gene3179#
MRFFQNLQNDGISPRAQIRLLDNTFMRLVFSFTAVPFVGIPFAIWIYLLGDELGPTITWIIVYLLCAV

AIRIWHRRYLHEAKENDEDAVLRRWLPRINKVAFIHGLGISSLYLITPQTHNFDFFLLLNISIAAIVA
ANATHLTPVISTFTRFFFASWGLLNLGIICRLEDVMFIVLMLNLLYGFAIYRHALTSHAFFIQQALLE
EQSSRLAEQFRQAKEEAEQALLDKNQFLTTASHDLRQPVHAMGFLIEAIIHKNRDDSLTPQLLDLQQS
VRSVHLMFNSLLDLSKIESGNVRTAATHVDIGALLDSVITLFREEANSRALALRTWRPKRRISVMGDP
LLVRQSLINLIQNALRYTQQGGVLIAIRPRGAECLVEVWDTGVGIADEEKSKIFSPYYRPELAWKIDS
AGHGLGLAVVARCAKLMKVKYGMHSVEGKGSRFWMRFTQYIGEDKAPETAAAYDNTATPIRYAPLRGA
CLVVDDDPLVTSAWESLMSTWGITVRCAASAEEAFAIVDDGFTPFAVLCDQRLRSGESGFDILKALFE
RLPDVSGAMVSGEFNSQILQEAEQEGYLVLRKPLEPARLHALLTQWGAAS
>CORE_REP|Org5_Gene3887#
MNKVQKLWPTLKRLLAYGSPYRKPLGLAVLMLWIAAAAEVAGPILVSYFIDNYVAKGQLPLTIVGGLA
AAYILLELLAAALHYFQALLFNQAAVGVVQRLRTDVMDAALRQPLSAFDTQPVGQLISRVTNDTEVIK
DLYVMVVSTVLKSAALIGAMLVAMFSLDWRMALVAVCIFPAVFVVMGIYQYYSTPIVRRVRSYLADIN
DGFNEVINGMGVIQQFRQQVRFGERMSAASQSHYLARMQTLRLDGFLLRPLLSLFSALVLCGLLMLFG
FSGEGVIGVGVLYAFINYLGRLNEPLIELTSQQSILQQAVVAGERIFELMDRSQQSYGADDRPLAGGR
IDITDLSFAYRADKKVLQHISLAVPSRGFVALVGHTGSGKSTLANLLMGYYPVSEGEVRLDGRPISSL
SHRTLRQGVAMVQQDPVVIADSVLANVTLGRNIEEDAVWRALETVQLASLVRGFPQGIHTRLGEQGNN
LSVGQKQLLAMARVLVQAPQILILDEATANIDSGTEQAIQRALRAIREHTTLVVIAHRLSTIVDADSI
LVLHRGQAVEQGNHQQLLAQQGRYYQMYQLQLAGEQLAEAVREESQPA
>CORE_REP|Org19_Gene2614#
MRLFAQIGWYFRREWRRYLGAVVLLIVIAILQLLPPKLVGIIVDGVTEKQMSTGVLMAWLGLMIGTAI
VVYLLRYVWRVLLFGASYQLAVELRENFYRQLSRQNPAFYLRHRTGDLMARATNDVDRVVFAAGEGVL
TLVDSLVMGLVVLVVMSTQISWQLTVLALIPMPLMAIAIKYYGDQLHQRFKSAQAAFSSLNDQAQESM
TSIRMIKAFGLEDHQSNRFADVAAQTGAKNMHVARVDARFDPTIYIAIGASNLLAIGGGSWMVVNGSL
TLGQLTSFVMYLGLMIWPMLALAWMFNIVERGSAAYSRIRSLLDEAPAVQDGPQALPAGRGVLDVDIR
AFHYPENPHPALHDVALTLKPGQMLGLCGPTGAGKSTLLSLIQRQFDVDQGQIRYHGLPLPQVKLDDW
RSRLSVVSQTPFLFSDTVANNIALGHPGATQAQIEQAARLASVHEDILRLPQGYDTEVGERGVMLSGG
QKQRISIARALLLDAEILILDDALSAVDGRTEHQILHNLRSWGQDRTVIISAHRLSALTEAGEILVMQ
HGGVAQRGDHAALAAQPGWYRDMYRYQQLEAALDEAPENGEEALADE
>CORE_REP|Org2_Gene2928#
MKKTRQQQLTRWLKTQSSLAQRWLRLSMLLGLFSGLLIVAQAWLLASLLHALIIEHTPREQLIPSFIW
LAAAFALRALLSWLRERVGFRCGQVIRQRMRQQVLDKLQQLGPAWIQGKPAGSWASIIVEQIEDMQDY
YSRYLPQMYLAVFIPLLILIAVFPINWAAGIILLATAPLIPLFMVLVGMGAADANRRNFVALARLSGN
FLDRLRGLDTLRLFDRAQAETAQIAKSSEDFRSRTMEVLRMAFLSSGVLEFFASISIAVVAVYFGFSY
LGELNFGSYGLGVTLFSGFLVLILAPEFFQPLRDLGTFYHAKAQAVGAAEALETFLSAEGEQMGNGTR
QLAADQPLTLQANALEILSPNGVLLAGPLSFTLQPQQRVALVGLSGAGKSSLLNLLLGFLPYRGSLTV
NGVELRDLSAENWRQQLSWVGQNPHLPAQTLRANILLGCPQADEAQLQQAVEHAYVSELLPYLPQGLD
TEVGDNAARLSVGQAQRVAVARALIGPRRLLLLDEPAASLDAHSEQRVMQALNAASHQQTTLLVTHQL
EDTEDYDQIWVMDNGRIVQQGDYATLSAQPGLFATLIAHRRGEL
>CORE_REP|Org38_Gene1111#
MFLQKWSKPLTMAALLVSGSLYAASNPAVEAKNGMVVTSQHLASQVGVDILKMGGNAIDAAVAVGYAQ
AVVNPCCGNIGGGGFMTVHLADGTDTFINFRETAPAAASANMYLDADGKVKKDASLYGYLAAGVPGTV
LGMETAREKYGKLSREQVLAPAIRLAREGFVLTRADTDILDTTVARFKQDPESAKIFLRPDGSPLQPG
DKLVQTDLANTLEAIAKGGTDAFYKGKIPQAVEAAAKQGGGILTAADFANYKVTETPPITCSYRGYKF
VSAPPPSSGGVTLCEILNVVEGYDLKSMGFNSAAAIHTMTEAMRHAYMDRNTYLGDPEFIKNPIDRLV
SKSYAEQIRKKIVADKATPSENVQPGMEPHEKPETTHYSIVDHDGNAVSTTYTVNGRFGAVVIAPGTG
FFLNDEMDDFTVKVGEKNLYGLVQGTANSIAPGKRPLSSMSPTLVTKDNKIFMVLGSPGGSRIITITL
QTALNVIDHGMAPQEAVDAPRIHHQWLPDEVYYEQRGVSADTLKLLSGMGYKMVEQTPWGAAELILVG
LPGAAGVSPANSGNDSAVSGKVREGYLYGANDVRRPAGSAVGY
>CORE_REP|Org13_Gene2952#
MMNDKDLSTWQTFRRLWPMITPFKTGLIVAAIALIMNAAGDTLMLSLLKPLLDDGFGKTDSSVLVWMP
LAVIALMLMRGVTSFVSSYCISWVSGMVVMQMRRRLFGHMMRMPVAFFDQQSTGTLLSRITYDSEQVA
SSSSSALVTVVREGASIIGLFIMMFYYSWQLSVILIVLAPIVSIAIRLVSKRFRNISKNMQNTMGQVT
TSAEQMLKGHKEVLIFGGQQVETERFNSVSNRMRQQGMKLVSASSISDPIIQLIASLALAFVLFAASF
PSVMSTLTAGTITVVFSSMIALMRPLKSLTNVNAQFQRGMAACQTLFSILDMEQEKDTGTREVMRAKG

DIEFRNVTFYYPSKETPALRDINLKIAEGKTVALVGRSGSGKSTIANLLTRFYDIQEGEILMDGHDLR
EYTLASLRNQVALVSQNVHLFNDTIANNIAYARESEYSREQIEKAAEMAYAMDFINKMENGLDTVIGE
NGVMLSGGQRQRIAIARALLRDCPILILDEATSALDTESERAIQAALDELQKDRTSLVIAHRLSTIEK
ADEILVVEDGRIVERGEHAELLERQGAYAQLHRMQFGQ
>CORE_REP|Org3_Gene3058#
MRVLLPFLALYRRHSLLISLGILLAIVTLLASIGLLALSGWFLAASSLAGLAGLLTFNYMLPAAGVRG
AAIFRTAGRYAERVVSHDATFRVLSHLRVFTFSKILPLTPGGIARFRQAELLNRLVADVDTLDHLYLR
VISPLISAAVVILVVTYGLSWLDPALALTLGGILLLLLLLVPPVFYCAGKPIGGQLTALRGQYRTDLT
AWLQGQAELVVFGAVNDFRQTLNATEQRWQRRQWQQASLSGMAQALMILASGLTVTLLLWLSAAGIGG
DTQPGALIALFVFAALASFEALMPVAGAFQHLGQVIASATRVKQIIDRQPEVTFPAAGPAAADRAQLS
LQQLSFTYPDQPQPVLRDVTLEVAAGEHIALLGRTGCGKSTLLQLLTRAWRTDGGKILLNGEPLEDYD
EATLRRMTTVVSQRVHIFSDTLRENLRLAAPDADDARLSEVLRQVGLDKLLDSDGGLNAWLGEGGRQL
SGGEQRRLGIARALLHPAPLLLLDEPTEGLDAETEQQILALLRRHCQGKTLILVTHRLYGLEHLDRIC
VMDDGRIVEQGDHATLMRRQGRYARFRNRISNLAP
>CORE_REP|Org26_Gene4103#
MSASITAGESRDNGLQAIWNRLRANPKIPLLVAASAAIAIVVALLLWVKSPDYRVLYSNLNDRDGGAI
VTQLTQMNIPYRFAENGAALLIPAEKVHETRLRLAQQGLPKGGAVGFELLDQEKFGISQFSEQINYQR
ALEGELSRTIESLGPVQNARVHLALPKPSLFVREQKSPSASVTLTLQPGRALDDGQINAIVYMVSSSV
AGLPPGNVTVVDQAGRLLTQSDGTGRDLNASQLKYANEVENGFQRRIEAILAPVVGSANVRAQVTAQI
DFATREQTDEQYQPNQQPDKAAIRSQQTSLSEQIGGPQVGGVPGALSNQPSAPATAPIETAKPATAAG
NNANANATATAQNAATTRSAAANGVPQNTRRDATTNYELDRTIRHTQQKAGTVQRLSVAVVVNYLGTD
KDGKPQPMSKEQLAQIEALVREAMGYSSSRGDTLNVVNTPFTDTQVTGGELPFWQSQSFIDRLIDAGR
YLLVLLVAWLLWRKLVRPQLQQRQAAQQAAVAAANAPAAKAVDSSKPSNEELAQRRKSQQRVSAEVQS
QRIRDLADKDPRVVALVIRQWMSNEI
>CORE_REP|Org6_Gene2773#
MDKVWLKRYPADVPAEIDADRYSSLIEMFEHAVQRYADQPAFINMGEVMTFRKLEERSRAFAAYLQNE
LGLKKGDRVALMMPNLLQYPIALFGILRAGMVVVNVNPLYTPRELEHQLNDSGASAIVIVSNFAHTLE
KVVFNTQVKHVILTRMGDQLSAAKGTLVNFVVKYVKRLVPKYNLPDAISFRSALQRGRRLQYVKPDII
NADLAFLQYTGGTTGVAKGAMLTHRNMQANLEQAKAAYSPLFREGQELVVTALPLYHIFALTVNCLLF
IDLGGRNLLITNPRDIPGLVKELGKYPFTAMSGVNTLFNALLNNEEFHKLDFSTLRFSVGGGMSVQKA
VAEKWEKTTGKHLLEGYGLTECAPLVAGNPYDLKHYSGSIGLPVPSTDIRLVDDNGQDVPPGEPGELW
VKGPQVMLGYWQRPGATDEVLKDGWLATGDVVTVDEQGFVRIVDRKKDMILVSGFNVYPNEIEDVVSQ
HPKVLECAAIGVPSEVSGETVKICVVKKDASLTKEELLTHCRRHLTGYKVPKIVEFRDELPKSNVGKI
LRRELRDELKTPKPADAA
>CORE_REP|Org8_Gene1820#
MIKKITALTLLVSTALSAETLPDSHMMQDMSMGESRRALQDSTREVNQLIEQRRYQQLKQQRLLAEPE
PAAPALPQSAQCLPIAGVYLQGVTLLSPADLSALSALPEQCISSNDINRLTRELTRLYVQKGYITARV
QIVRPNSQGELGLSVTEGFIEKIEGGDRWVNSRLLFPGLEGKPLKLTELDQGLDQANRLQSNTTKLDI
LPGRQVGGSVIRLRNQHAKPWLITAGTDNYGQKSTGRWLARATATLDSPFGLSDFVSLNANSTLENPA
HRYNRAYTLLYSLPYGAFTFSGFASFSSYENHQQLPHNVVKLHGQTQQYGLRSDYVFYRDHDQIDSLS
GQLTYKRIDNYFESVRLEVSSPTLTLAELSASHLQILPNGVFSANLSVEQGMPWLGAGRHPSSVHLDS
QFTKGKLFANLSQRLRLGDATYQLNNLFYGQYSRDPLPGVEWLSLTDRSAVRGFSRSTQSGDNGWYLQ
NTLSRSFNLGATTLTPRLGADVGRILPRQDNSGWRSSAGISTGATLRYQRALVDLEVSRGWILSNHAT
PEDPVQVLARFSYTF
>CORE_REP|Org12_Gene4030#
MLNRMKVVTSLLLVLVLFGALQLISGGLFFSSLKSDKENFTVLQTIRQQQLQLSESRVDLLQARNSLN
RAGIRYMMDTNKIGSGATIDELLAKAKEELGEAERHYAAYEKIPQDPRQDPQSAERVKQQYDILYGAL
SELIQLLGEGKINAFFDQPTQSYQDNFEQSYNVYLEQNGKLYQIAVDGSNSSYNSAIWTLIVILVVVL
AVIVLVWTGIHHILVRPLNRMIDHIKQIAAGDLTQQIVVNSRNEMGVLAASLKHMQGELIETVSGVRQ
GADAIYSGASEIAAGNNDLSSRTEQQAASLEETAASMEQLTATVKQNAENARQASQLALSASETAQKG
GKVVANVVQTMHDIAGSSQKIADITGVIDGIAFQTNILALNAAVEAARAGEQGRGFAVVAGEVRNLAQ
RSAQAAKEIKGLIEDSVSRVDMGSVLVESAGETMGDIVNAVTRVTDIMGEIASASDEQSRGIDQVGQA
VAEMDRVTQQNASLVEESASAAAALEEQASMLTQSVAVFRLRSEGQEEFKAPVTNKATVTPVINHKKM
NASDLQDNWETF

>CORE_REP|Org30_Gene3742#
MAAKDVKFGNDARVKMLRGVNVLADAVKVTLGPKGRNVVLDKSFGAPTITKDGVSVAREIELEDKFEN
MGAQMVKEVASKANDAAGDGTTTATVLAQSIITEGLKAVAAGMNPMDLKRGIDKAVVAAVEELKKLSV
PCSDSKAIAQVGTISANSDETVGKLIAEAMEKVGKEGVITVEEGTGLQDELDVVEGMQFDRGYLSPYF
INKPETGSVELESPFILLADKKISNIREMLPVLEAVAKAGKPLLIIAEDVEGEALATLVVNTMRGIVK
VAAVKAPGFGDRRKAMLQDIATLTAGTVISEEIGLELEKATLEDLGQAKRVVINKDTTIIIDGVGDEA
TIQGRVAQIRQQIEEATSDYDREKLQERVAKLAGGVAVIKVGAATEVEMKEKKARVEDALHATRAAVE
EGVVAGGGVALIRVAGKIAALKGDNEDQNVGIKVALRAMEAPLRQIVVNAGEEASVIANQVKAGEGSY
GYNAYSEEYGDMIAMGILDPTKVTRSALQYAASVAGLMITTECMVTDLPKADAPDMGAAGGMGGMGGM
GGMM
>CORE_REP|Org35_Gene3691#
MSIAFTPWPAEFASRYRERGYWIDKPLTEILDRQANNDAPAIIDAQGSLTYRELQQRSDRLAAALLRR
GVKSGDTALVQLGNVAEFYIVFFALLKIGVAPVNALFSHQRSELNAYAEQIKPALLIADRRHALFADD
QFLSAFRDAHPSLRAVALRSQPEGELALAAWLEEASDGFVAQPSAADRVAFFQLSGGSTGTPKLIPRT
HNDYYYSIRRSVEICRFDAQTRYLCALPVAHNYPMSSPGVLGVFYGAGLVVFAADPDAAQCFRLIEQH
QINVTALVPPAVTLWLQAIEEWGGNAQLASLKLLQVGGAKLGETLAARIQNEIGCQLQQVFGMAEGLV
NYTRLDDDERHILTTQGRPMSPDDEVWVADDDGNPLPAGEIGRLMTRGPYTFRGYYQSPAHNADAFDA
DGFYCSGDLISISEDGYITVQGRQKDQINRGGEKIAAEEIENLLLRHSDVINAALVSMPDALMGEKSC
AYIIANAPLKPVVLRRHLREQGVADFKLPDRFIQVDSLPLTPVGKVDKKRLREQLDAQLQTQAQGD
>CORE_REP|Org25_Gene4270#
MATPLLAIQDLSIAFRQGDAVTPVVNELSLQIAPAETLALVGESGSGKSVTALSILRLLPAPPVVYPG
GDILFNGNSLLHAPEAELRKVRGNQIAMIFQEPMVSLNPLHTIEKQLAEVLMLHRGLRRETARAEIVD
CLERVGIRQAKTRLQDYPHQLSGGERQRVMIAMAVLTRPKLLIADEPTTALDVTIQAQILTLLQELKQ
EMGMGLLFITHNLNIVRRLADNVAVMRQGRCVEQNGRAQLFSRPQHPYTRQLLAAEEVGEPLPLPAAA
SARPGDERPLLKVEDLQVRFPIRRGLLRRTVDYHYALKSLSFELRAGESVGLVGESGSGKSTTGLALL
RLLASQGAIWFDGEPLHPLTMKQMLPYRSRMQIVFQDPYSALNPRLNVQQIIAEGLEVHQRLNAEQRE
QRVIEVLQEVGLDPQLRHRYPTEFSGGQRQRIAIARALILQPQLLILDEPTSSLDKSVQAQILTLLKS
LQQRHRLAYLFISHDLQVVRSLCHQVIVLRQGEVVEQGDCRAIFAAPAADYTRQLLQLAD
>CORE_REP|Org25_Gene3782#
MSDHITPSRTPDKPVIWTVSITRLFDLFRDISLEFDHLATITPIRLGFEEAVQHIRARLATEPCDAII
AAGSNGAYLKSRLSVPVILVKPGGFDLLQALSQARRTADRIGVITYKTPLPALMEFQQTFDLPLEQRS
YVTEEDARGQIAELKAAGIQAVVGAGLISDLAEEAGLTAIFLYSAATLRAAFSDALDVTRLMLGGAKR
GGDYAARDTLQPRYGLSDLQGDSPQMEQTRRTIMLYARSPAAVLIEGETGTGKELAAQAIHREYFSRR
GAPSRGATPPFVAINCGAIAESLLEAELFGYEEGAFTGSRRGGRRGLLETANGGTLFLDEIGEMPLHL
QTRLLRALEEKTITRVGGQQPVKVDFRVISATHTRLEQAIQQGDFRADLFYRLSALRLQLPPLRARGD
DIAMLAEHFLKQSLAALDAPLTEPLRAALAGCYAALGHYAWPGNLRELRNMMERVALMLSTGVAPSGE
TLQWLLPELAVAPPPETPAAPVSAHEALARCGGDHAAAARLLGISRTTLWRRLKKPH
>CORE_REP|Org2_Gene2285#
MLMTHLAASRYRYYRWLLAGAVGAAILLVSLYTRYYQEVKSIELSQHTLATRTVGKLNQLLTPAQLQA
ERSMDMLNQSCENVSSTLRFRAAQNQALRAMLLVKNGIIYCSSLFGARHYQLAAVMPSFVNSDARLAL
RPSLAVSKGLPTLVLWTPSPRDKTSGVLHVFNIELLSNFLLEPQEPYVQRVVLNVADSSLEYGRREIL
SRDTLTNDLRYTAGSALYPFSISLFGPQIGMLALSALPRHIPLALLISLLAAYVVYLLTANRMSLSYH
IGHAITHREFRVYCQPIIHSDTGRCAGVEMLLRWKKKRQGWISPDVFIPLAEQHELIIPLTRYLMSTV
TENLQLFPPRPSFYISINVAAEHFKTLNIIDDIRQIWLPAHPMPSLMLELTERTALSAIQYDQIRTLK
DMGIMLAIDDFGTGHSSLSYLKNLSPDVLKIDRGFTAAIGTDAVNATVTDTIITLAQRLKLKLVAEGV
ETEEQADYLRSREVNALQGYYFAKPMPIHVFPLWLQQYESRVRKAEEDPPEA
>CORE_REP|Org8_Gene2192#
MRLEVFCEDRLGLTRELLDLLVSRSIDLRGIEIDPIGRIYLNFSQLDFDTFRALMAEIRRIAGVTDVR
TVSFMPSEREHRALRALLESMPEPVFSIDMKGKVELANPAAQALFSLSEDKIRNQTAGALIGGYNFSR
WLESEHTAPHAERVVIRSQDFLMDITPIYLEDQQQQPAAVGAVVMLKSTARMGRQLQNLSVNDDTEFD
HIVAVSAKMRHVLEQARKLAMLDAPLLIVGDTGTGKDILARACHLRSPRGKQPFLALNCAALPDDVVE
SELFGHAPGAYPNALEGKKGFFEQANGGSVLLDEIGEMSPRMQTKLLRFLNDGTFRRVGEEHEVHVDV
RVICATQKNLTELVQRGEFREDLYYRLNVLTITIPPLRERPQDIMPLTELFVARFADEQGVARPKLAS
DLGGFLSKYGWPGNVRQLKNAIYRALTQTEGYELRPQDIVLPEFEVEMSLGDEVLDGSLDDISKRFER

SVLTRLYRTYPSTRKLAKRLGVSHTAIANKLREYGLSSRKGGAEGEE
>CORE_REP|Org33_Gene1456#
MMNTKPQLTLLKAQASYRGDPTTIFHQLCGARPATLLLESAEINSKQNLQSLLVIDSALRITALGHTV
SVQALTANGAALLPLLDEALPPEVRNQARPNGRELTFPAIDAVQDEDARLRSLSVFDALRTLLTLVDS
PADEREAVMLGGLFAYDLVAGFEDLPALRQDQRCPDFCFYLAETLLVLDHQRGTARLQASVFSEQASE
AQRLQQRLEQLQAELQQTPQPIPHQTLENMQLSCNQSDEEYGAVVSGLQEAIRQGEIFQVVPSRRFSL
PCPAPLAAYQTLKDNNPSPYMFFMQDDDFTLFGASPESALKYDAGNRQIEIYPIAGTRPRGRRADGSL
DLDLDSRIELEMRTDHKELAEHLMLVDLARNDLARICQAGSRYVADLTKVDRYSFVMHLVSRVVGTLR
ADLDVLHAYQACMNMGTLSGAPKVRAMQLIAASEGTRRGSYGGAVGYFTATGDLDTCIVIRSAYVEDG
IATVQAGAGVVLDSIPQAEADETRNKARAVLRAIATAHHAKEVF
>CORE_REP|Org9_Gene1572#
MQSSVNKSESRTFFGHPYPLGSLFFTEMWERFSFYGIRPLLILFMAATVYDGGLGLARENASAIVGIF
AGSMYLAALPGGWLADNWLGQRKAVWYGSILIALGHLSIALSAVMGTNLFFIGLMFIVLGSGLFKTCI
SVMVGTLYKKGDARRDGGFSLFYMGINIGSFIAPLISGWLIKSHGWHWGFGIGGIGMLVALVIFRVFA
VPAMKRYDREVGLDSTWNSPVAKKKGVGAWLLALAVGLAAVIVLIAQGTIVINPVEVASVLVYVIAAS
VTLYFIYLFAFAGLSRKERARLLVCFILLISAAFFWSAFEQKPTSFNLFANDYTNRMVGGFEIPAVWF
QSINALFIILLAPVFSWAWPALARNNVRPSSITKFVIGILCAAGGFGLMMLAAQNVLSNGGAGVSPMW
LVGSILMLTLGELCLSPIGLATMTLLAPERMRGQMMGLWFCASALGNLAAGLIGGHVKADQLDMLPDL
FARCSVALLICAAVLIVLIVPIRRMLENTQTKSAQKPATSA
>CORE_REP|Org46_Gene1409#
MLKKITVKAGLIALLSLMTMLLIMVSVIGVNAINEGSRSIHTLNQILGEELGSLANSSNLTLRARTAA
SLAVRQREIGQTDVSDATVGRIYGYLEQSNKEMARFVGVGTVTERGRELSNRLQNSYRAYLDQGVKPM
AAAIKAGKIDEYYHIQETRISALSIAFEKDLSDFRSFAMKLGAQQVYDAESNASTKISLIVVAGLLSV
LLAVLAWFALRVIILRPLDESIAQLEHIAGGDLTHEIRGEGDTEMGRLVRAMQRMQQALASSVSKVRD
ASSQIDTGSRELAAGNLHLAQRTEESAASLEETAASMEQLTSTVKMNAENCEQANQLALSVSDIANQG
SEVVSQVMSKMQAITDSSRRIADIISVMDGIAFQTNILALNAAVEAARAGEQGRGFAVVAGEVRNLAQ
RSAQSAKEIKGLIEASQNRVQEGEQMVESAAQTMSGITGEVGRVTALMREISAATREQSSGIEQVNLA
VAQMDQVAQQNAALVEESAAATRSLEDQAQLLAQSMAAFKL
>CORE_REP|Org18_Gene4132#
MKIRSQVGMVLNLDKCIGCHTCSVTCKNVWTSREGMEYAWFNNVESKPGVGYPHAWEDQEKWKGGWIR
KINGKLEPRMGNRVGVLAKIFANPDVPALDDYYEPFDYDYQHLHTAKQGKHQPVARPRSLITGQRMNK
IESGPNWEEILGGEFEKRSQDKNFDNLQKAMYGQFENTFMMYLPRLCEHCLNPACVATCPSGAIYKRG
EDGIVLIDQDKCRGWRMCLTGCPYKKIYFNWKSGKSEKCIFCYPRIEAGQPTVCSETCVGRIRYLGVL
LYDADRIEQAAAVENDKDLYQSQLDIFLDPHDPKVIAQALADGVPQGVIEAAQQSPVYKMAMDWKLAL
PLHPEYRTLPMVWYVPPLSPIQSAADAGELAHSGVLPDVESLRIPVQYLANLLTAGDTEPVLLALKRM
LAMRHYKRAETVDGVVDTSALEQVGLSEAQAREMYRYLAIANYEDRFVVPSSHRELAREAFPESKGCG
FSFGDGCHGSDGKFNLFNSRRIDAIDVTAKTARPEDAS
>CORE_REP|Org43_Gene3942#
MQLNSTEISELIKQRIAQFNVVSEAHNEGTIVSVSDGIIRVHGLAEVMQGEMIALPGNRYAIALNLER
DSVGAVVMGPYADLAEGMKVKCTGRILEVPVGRGLLGRVVNTLGAPIDGKGPIENDGFSPVEAIAPGV
IERQSVDQPVQTGYKSVDAMIPIGRGQRELVIGDRQTGKTALAIDAIINQRDSGIKCVYVAIGQKAST
IANVVRKLEEHGALANTIVVVATASESAALQYLAPYAGCAMGEYFRDRGEDALIVYDDLSKQAVAYRQ
ISLLLRRPPGREAYPGDVFYLHSRLLERAARVNAEYVEAFTKGEVKGKTGSLTALPIIETQAGDVSAF
VPTNVISITDGQIFLESNLFNSGIRPAVNPGISVSRVGGAAQTKIMKKLSGGIRTALAQYRELAAFSQ
FASDLDDATRKQLSHGQKVTELLKQKQYAPMSVAQQSLVLFAAERGYLNDVEVAKVVSFEAALVAYAD
REHAELLNHINQTGNFNDEIEGKLKDILETFKKTQSW
>CORE_REP|Org28_Gene971#
MRARQRRYSAKQQATAVAEVETQETENELDGLLKRSFRPRTNEASEAVRRAIGTLSEYANQGKVKVSQ
DVVLTIESLIAQIDEQLSQQMNNILHHKEFQKLESAWQGLSYLVDNTNVSETLKIRVLNISQDELTRN
LRRYRGSAWDQSPVFKQIYEQEYGQFGGEPFGCIIGDFEFDHSPMSVTLLTELAKISAASHCPFISAA
SPSLLQMSKWNELGNPRDIGKIFTTPEYASWRRLRESNDSRYLVLTMPRFLSRLPYGAKTNPIEEFAF
EEAVRPDMDDDFSWANSAYAMGVNINRAFHEYGWCSKIRGIESGGSVEELPAYAFPSDEGGYELTCPT
EVAISDRREQELSDAGFLPLVYRKHSDFAAFIGSCTMHAPAKYEDPDATANAKLSSRLPYIFATCRFA
HYLKCIVRDKIGSFRSRDDMQLWLNDWLMNYVDGDPSVSTEATKARRPLAAAEVRVEDVEDDPGYYRA

HFYLRPHYQLEGMTVSLRLVSKLPSAKKDGSR
>CORE_REP|Org48_Gene195#
MSEQQVQGADQALDLNNELQSRREKLAGLRENGIAFPNDFRRDSTSDKLHAAYGDKDNEELEALGVEV
TVAGRMMTRRIMGKASFVTLQDVGGRIQLYVARDDLAEGVYNEQFKKWDLGDILGARGKLFKTKTGEL
SIHCTELRLLTKALRPLPDKFHGLADQETRYRQRYLDLIANEESRNTFKVRSQVMSAIRNFMVERGFM
EVETPMMQVIPGGASARPFITHHNALDIDMYLRIAPELYLKRLVVGGFERVFEINRNFRNEGVSPRHN
PEFTMMELYMAYADYKDLIELTETLFRTLTEKVLGTSQVQYGDEVFDFGKPFEKLTMTEAIKKYRPET
DLADLADMGKAVAIAESIGIKVEKSWGLGRVVTEIFEEVAESHLIQPTFITEYPAEVSPLARRNDVNP
EITDRFEFFIGGREIGNGFSELNDAEDQAQRFADQVNAKDAGDDEAMFYDEDYVTALEHGLPPTAGLG
IGIDRMVMLFTNSHTIRDVILFPAMRPQK
>CORE_REP|Org36_Gene2733#
MAISGNTATQGAPLIALQQLSMTFGGQRALNAISLALMPGEVHCLAGTNGCGKSTLIKAIAGVYQPDD
GSRITIDGQTFGRLSPDQARAFGIQVIYQDLSLFPNLTVAENIAFEHNLHGLLGWYRPARLRRTAERL
LQELSFHLDLDKRVAELPIAQRQQVAICRALVAEARLVIMDEPTASLTRTEVNQLLRTVDYLKAKGIC
VVFVSHRLDEVLEISDRVTVIRDGNKIGTWPAAEITGDRLTELMTGLKLDYRLKSPSMNKDRVMLEAD
RLSRTGQYQDVSFRLHQGEVLGLCGLLGSGRTELALSLFGMTRPDSGKLYLDSKPVRFRGHEDAIKAG
IGYVSEDRLTLGLVQQQSVADNAVLTILDKLRGRFRLIDDYRKNRIVAEWIAKLGVRVADPEQAVSTL
SGGNQQKIVLAKWVLTQPRILILDSPTVGVDVGAKASIYQLIHLLAQEGIAILLISDEVPEVYYNCDR
VLHFSGGSVIGEYLPGQVSQQQLAEAVNA
>CORE_REP|Org29_Gene3810#
MSSQSKTFAQWRQQAAGLQIEGRAYIAGRYQDSTDGATFVDASPIDGRPLANIADCDEATVNHAVAAA
EQAFKAGDWARRTPAQRKATLLRLADLVAQHQEELALLESLDTGKTIRESLDMDMQDVQTAIRYYAEA
IDKVSGEVAPTGDAFHGMITLVPLGVVAVMTPWNNPLMIACWKIAPALAIGNSVVFKPSEKAPLTGIR
LAELTRMAGIPDGVFNVVTGGAQVGKTLALHPRVRAMAFTGSTQVAKQLLIYAGQSNMKRTFLEGGGK
NAHIIFADTPDLARAARFAALGFCANQGAVCASGTRLLVQSSIKDRFLTLLLDELKKWQPGHPLDPAT
AMGPLIDAQHQANVLRYIESARAEGASIISGGQADNNLLPGGHVLPPTLIDNATPEMTASQNEIFGPV
ASLMTFEDEEQAIRLANDSEYGLTVGFWTPDVAKVHRVARQLEAGTVWVNHFLTRDILSPFGGFKQSG
IGRDLSLHALPQYGEMKATWIALQDVDAH
>CORE_REP|Org23_Gene1416#
MSTANNQHPESVSLNAFKQPKAFYLIFSIELWERFGYYGLQGIMAVYLVKMLGMSEADSITLFSSFSA
LVYGFVAIGGWLGDKVLGAKRVIVLGALVLAAGYAMVAYSGHDIFWVYMGMATIAVGSGLFKANPSSL
LSTCYEKDDPRLDGAFTMYYMSVNIGSFFSMLATPWLAAKYGWSVAFSLSVVGMLITLVNFMMCHKWV
KENGSKPDFKPLHLPKLLMVLVGIVALIAVSSWLLHNQVIARWALALVSAGIVLVFAKETFALHGAAR
RKMIVAFLLMLEAVVFFVLYSQMPTSLNFFAIHNVEHSIFGIAFEPEQYQALNPFWIMLASPILAALY
NKMGDRLPMPHKFAFGMILCSCAFLVLPWGASFANEQGIVSVNWLILSYALQSIGELMISGLGLAMVA
QLVPQRLMGFIMGSWFLTTAAAALIAGKVAGLTAVPSDINDAHASLAIYSHVFMQIGIVTAVIAILMM
LTAPKLYRMTLDTTEDANQKAQEATAAH
>CORE_REP|Org39_Gene3471#
MTITHWINGEPAAGGERSQPVYDPATGQSAQEVQLADRATVERAIAAAEQAYPAWRDTPPLKRARIMM
KLKDLLEQHADAICQLITAEHGKVLSDALGELQRGIENIEYAGYVPELLKGEHSKEAGPGIDSWSEFQ
PLGVVAGITPFNFPAMVPLWMWPMAVACGNTFVLKPSERVPSAALYIARLAAEAGLPPGVLNVVNGDR
EAVETLLHDGRVKAISFVGSTPVAEHIYHTGCGQNKRVQALGGAKNHAVVLPDADIPGAVGALMGAAF
GSCGQRCMAIPLLVAVGDGTAEALIAGLRQQMAAMRVGPGSDNRNDMGPLVTQQHYQKVKGYIDQGVA
EGAELLVDGRELSVIGADGRPSQGYFLGPTLFDRVTPGMRIYQEEIFGPVLGVVRAASLPEAMALIDA
HEYGNGTCLFTRDGEAARYFSSRIQVGMVGINVALPVPVAYHSFGGWKRSLFGDLHAYGPDAVRFYTK
RKTVTQRWPASGDARRASFSFPSGQG
>CORE_REP|Org8_Gene3095#
MKNLTLAQRLTLIFALLIVIGCAFSGWMQVRSSTQYSQAVIQRLSGNLAQHIADSNPLLGVNGPDPQA
VHTLFDQLMAVNPSVEVYLLDKQGAIIGNAAPAGHLKRQRVALAPLQALLDGAQMPVYGDDPRSADGR
KVFSVAPLKVDGRVEGYLYVVLLGEEYTALASNAQFNSAVRMALWTSGVMVLFSLLAGGFAFYWVTRP
IRRLTRQVNALDSGGIEAVQAYAALPAAPAGRDEVSQLQQAFHRMAQRLAEQWQTLAQQDRLRREFIA
NVSHDLRTPLTSLHGYLETLSVKAATLSDTERRRYLEIALAQSRKVGKLAQELFELARLEYGVVKPQK
EPFSLSELLQDVFQKFELAAEARNQRLHADIAPGIPPVFADLSMIERVLTNLLDNAIRHTPPGGDIGV
RLWRQEGRVMVQVSDSGPGIPQTLRADLFVRPSILSGARRPAGGLGLMIVRRILQLHDSDIQLIEQPQ

SGACFRFAIPPRESGTTVIARAGTAG
>CORE_REP|Org25_Gene2547#
MSDNTVAVLDSVSRFLDRQHGLYIDGQWRASAAEGRLAVYNPANGQQIATTADANEHDVAQAVESAHK
AFSEGVWAQRLPVERERILLRFADLVEQHAEELAQLETLEQGKSINIARAFEVGSTLNWMRYTAGLAT
KITGQTLDVSIPMPPGAKYQVYTRKEPIGVVAGIVPWNFPLMIGMWKVMPALAAGCSIVIKPSETTPL
TLLRIAELASEAGVPPGVFNVVTGRGTVCGKALTEHPLIAKVSFTGSTPVGKSIARAAADRLTRVTLE
LGGKNPAIVLQDADPQQVIEGLMLGSFLNQGQVCAASSRIYIEAPIYDRLVAGFEQAVKSLSVGPGMD
TGAQINPLVSLAHRNKVAAYLDDARAKNAELIGGAAGPDANGFYIPPTLVINPDDRLNLTREEVFGPV
VNLIRVASAEEALSKANDTDFGLTASLWTTSLQKAMAFTPRIQAGTVWVNTHTLIDPNMPFGGFKQSG
SGRDFGPDWLDAYTESKSVCIRY
>CORE_REP|Org42_Gene3971#
MSTSISATLKDPTLFREANYIDGQWLPAQAGRSIAIHNPANGELVGHVPAFGAEETARAIAAAKKALP
AWRALTAKERAGKLRRLFELMMENQDDLARIMTAEQGKPLAESRGEIAYAASFIEWFAEEGKRVYGDT
IPQPQAGRRIIVQKEPIGVFAAITPWNFPAAMITRKAGPGWAAGCTGVIRPASQTPFSALAIAVLAER
AGLPAGVCNVITGPSKGIGGELTANPDVRKLSFTGSTEVGAQLLAQCAPTIKKTSMELGGNAPFIVFD
DADLDAAVAGAVASKYRNAGQTCVCTNRFLVQDGVYDAFAAKLKAAVAKLKVGNGLDEGVTIGPLINP
DAVEKVREHIADAVEHGASVLLGGKPDALGGNFFTPTILTDVPRTAKIFREETFGPVAPLIRFNHEAD
AVELANDTPFGLAAYFYSRDIGRVMRVAEALEYGIVGINEGLISTEVAPFGGMKHSGLGREGSKYGIE
DYLEIKYLCLGDLGA
>CORE_REP|Org33_Gene2637#
MSRFGLQKLYIHGAYVDSTAGKTFNAINPANGEVLAEVQSAGAEDVDRAVASAASGQKVWAAMTAMAR
SRILRRAVDILRERNDELAALETLDTGKAMSETTAVDIVTGADVLEYYAGLIPAIEGEQIPLRETSFV
YTRREPLGVVAGIGAWNYPIQIALWKSAPALAAGNAMIFKPSEVTSLTALKLAEIYTEAGLPDGVFNV
VTGSGAEVGQYLTDHPGIAKVSFTGGVKTGKKVMANASGSTLKEVTMELGGKSPLIIFDDADLDRAAD
IAMMANFYSSGQVCTNGTRVFVPAALQAQFEAKILERVKRIRLGDPTDPQTNFGPLVSFAHMESVLRF
IESGKNSGARLLCGGERVTHGEFGKGAYVAPTVFSDCRDEMEIVREEIFGPVMSILSYQSEEEVVRRA
NDTTFGLAAGVVTNDLARAHRVIHQLEAGICWINTWGESAAEMPVGGYKQSGVGRENGLTTLEHYTQI
KSVQVELGEYASVF
>CORE_REP|Org15_Gene1885#
MERLNIFVAGRWREGRGEEMASVFPADGSVNARLRAANVEDVNEAVEAAEKAWRAPEWRGLVPHQRAS
ILYRVSNLILAQQEQLAELQTRDNGKPLAETRGLVASAAATARYFAAACEVLEGELPTPRSAEVMTLS
QYQPMGVIAAITPWNSPIASEMQKVAPALAAGNAVVLKPAEATPLMALKLAELFEQAGLPAGLLSVLP
GKGSVIGEALARHPLVKKIAFTGGTSTGRHLAHIAADKLIPTSLELGGKSPTIVLEDADLEQAARGIC
YGIFSSAGQACIAGSRLFVHRSLYQPLLARLTELTAGLRIGNPLVPGVHLGPLISAKHRQSVADYVAL
ARQEGGRVVIGGEAPADPQLASGSYYLPTIIEGLNNDARVCQEEIFGPVLVALPFDDERQLIEQANDS
VYGLAAGIWSRDFPRAMALAERLETGTVWVNTYKTFSISTPFGGFKESGLGREKGLNGIKAYMQQKSV
YLALSHQVNRWSD
>CORE_REP|Org39_Gene3379#
MQSTKKAIEITESNFAAAKTGYDAVADLLHYHERGNGIQINGKDSFSNEQAGLFITRENQTWNGYKVF
GQPVKLTFSFPDYKFSATNVAGDTGLSKFSAEQQQQAKLSLQSWADVANITFTEVAAGQKANITFGNY
SQDRPGHYDYGTQAYAFLPNTIWQGQDLGGQTWYNVNQSNVKHPATEDYGRQTFTHEIGHALGLSHPG
DYNAGEGNPTYRDASYAEDTRQFSLMSYWSETNTGGDNGGHYAAAPLLDDIAAIQHLYGANLSTRTGD
TVYGFNSNTGRDFLSTTSNSQKVIFAVWDAGGNDTFDFSGYTANQRINLNEKSFSDVGGLKGNVSIAA
GVTIENAIGGSGNDVIVGNAANNVLKGGAGNDVLFGGGGADELWGGSGQDTFVFSAASDSAPGASDWI
RDFQKGIDKIDLSFFNKEAQSSDFIHFVDHFSGAAGEALLSYNASNNVTDLSVNIGGHQAPDFLVKIV
GQVDVATDFIV
>CORE_REP|Org25_Gene4368#
MADVQVLSGLSCQQFINGQLIDGEGQQECIVNPANGETLIALTEASSAQVGSAVKAAQQAFSHWSRTT
PAQRATLLLRIADAIERQASQLAQLEALNCGKPLHQALNDDLPAAVDVFRFFAGAVRAQQGQLAGEYV
PGHTSMIRRDPIGVVASIAPWNYPLMMAAWKIAPALAAGNTVVFKPSEHTPLTILALVPALQEILPPG
VLNIVYGGGEGVGSQLVGHPQVRLVSVTGDIVTGQKILQAAAKSVKRTHLELGGKAPVIVCDDADLDE
VVNGIRTYGYYNAGQDCTAACRIYAQAGIYPKLVDALGEAVASLRFARKRDQDNEIGPLISSRQRDRV
ASFVERALSQPHIELITGAAAHSGPGFYYQPTLLAGCLQSDEIVQREVFGPVVSVTRFEHLAQAVEWA
NDSEYGLASSVWTQNIDRALHIAAHLQYGSTWINTHFTLASEMPHGGLKRSGYGKDLSSDSLQDYSVV

RHVMAKFKASF
>CORE_REP|Org47_Gene4880#
MQTNSAAVENFAQHHEERRSSAFQNEVAHYLERHPATQYVDILLTDLNGSFRGKRIPVSGLKKLEKGS
YFPASVFAMDILGNVVEETGLGQELGEPDRICLPVPGSLTPSAADPQHIGQVLLTMLDEDGTPFDVEP
RNVLNRVWQALRQRGLFPVAAVELEFYLIDRQRDAEGDLQPPCAPGTQERNTQSQVYSVDNLNHFAEV
LNDIDALAKLQGLPADGAVAEASPGQFEVNLRHTDDILLACDHALALKRLVRLVAENHDMHATFMAKP
YEDHAGSGMHVHVSMQDGAGNNLFADDEGEDSPLLKQALAGMITLMPASMALLAPNVNAYRRFQPGMY
VPIQAAWGHNNRTVALRIPCGEPENHRVEYRVAGADANPYLVMAAILAGMLYGLDNALPLPEPVTGNG
LEQEGLPLPIRQSDALYEFEHQHALTHYLGERFTQVYHACKTDELLQFERRVTETEIDWMCVSSHRYC
KYLRVNKGVT
>CORE_REP|Org42_Gene1651#
MFNKNKKPFSLRARFLMATAGVILALSLSYGLVAVVGYIVSFDKTAFRLLRGESNLFFSLAQWKDNKL
TIAIPPDIDLNFPTLVFIYDDKGNLLWSQRKVPELEKLINKEWLEESGFYEIDTDTRVSSEVLGDNPK
AQDQLKNYDDTDQNALTHSVAVNTYAATPRLPALTIVVVDSIPQELQRSDVVWEWFSYVLLANLLLVV
PLLWLAAYWSLRPIKALVNQVGELENGERDQLDENPPSELRGLVRNLNILVRNERQRYTKYRTTLSDL
THSLKTPLAVLQSTLRSLRSGKQTTIEEAEPIMLDQIGRISQQIGYYLHRASINSGQTVLTREIHSVP
ALLDSLVVALNKVYQRKGVVITLDISPEVTFMGEKNDFMEVMGNVLENACKYCLEFVEITSLHSEKNL
TIVIDDDGPGIPESKRQLIFQRGQRVDTLRPGQGLGLSVAAEIIEQYDGEIVISDSPLGGARMQVTFA
RQHDTHHNE
>CORE_REP|Org13_Gene1039#
MKTINHWINGKNVASKEYFTTTNPANGEVLAEVASGGQLEIDQAVAAAKEAFPKWANTPMKERARLMR
RLGELIDQNVPQIAELETADTGLPIHQTKNVLIPRASHNFEFFAEVCQQMNGKTYPVDDKMLNYTLVQ
PVGVCALVSPWNVPFMTATWKTAPCLALGNTAVLKMSELSPLTADRLGELALEAGIPAGVLNVVQGYG
ATAGDALVRHKDVRAVSFTGGTATGRRIIESAGLKKFSMELGGKSPVLIFEDADIERALDAALFTIFS
INGERCTAGSRIFIQESIYPEFVKRFAERANRLRVGDPQDPNTQVGALISPQHWEKVSGYIRLGVEEG
ATLLAGGPDKPAGLSHGNFLRPTVLADVDNRMRVAQEEIFGPVACLLPFKSEEDGLRMANDVEYGLAS
YIWTQDVSKVLRLARGIEAGMVFVNTQNVRDLRQPFGGVKASGTGREGGEYSFEVFAEMKNVCISMGD
HPIPKWGV
>CORE_REP|Org31_Gene1090#
MRSPFNWRFTPLFAVLLLAGCASTDNIAPQSTLMDPQSLQLAQPKVSSLAVSPQWWRALKDPQLDTLM
TQTLQSSPTLRQAAARVREAQSVVGEASAANGPNLDLNASTQRQRVPQNVNMGLGYPHKPIYSSSNSL
GLNLAYEFDWWGKYRNQVNAAKAQVNAARAEQEQAALTLTSSVASAYYQLQSNLALEKLLQQEVNNNE
RLTALRQQRYQAGLTGVDVPQQTQAQSDVAKQQILQLQSQIEQLRHQLAALAGQGPNAMQHLRQVPLP
ADNLMAPQGELTADLLGKRPDIAAQRQLVESYSQRVSAARKEFYPSLTISAFAGLMTTNTSGTSPNLF
EAASQAWNVMPAISLPIFHAGALRSKLGEESALYDEAVESYNQTILNAVQETADAITIQQSSAQQQLQ
AASAAQSMQQVYQVANARYQAGIIGRDDLLTSQTQLLQQQQAELNASSNLLQAKIGLIRALGGGYQAP
AAADSKA
>CORE_REP|Org16_Gene217#
MSRRLRRTKIVTTLGPATDRDNNLEKIIAAGANVVRLNFSHGSPEDHQARADKVREIAAKLGRHVAIL
GDLQGPKIRVSTFKEGKIFLNVGDKFLLDANLSKGEGDKEKVGIDYKGLPADVVPGDVLLLDDGRVQL
KVLEVQGMKVFTEVTVGGPLSNNKGINKLGGGLSAEALTEKDKADIVTAAKIGVDYLAVSFPRTGEDL
NYARRLARDAGCNAKIVSKVERAEAVCTDEAMDDIILASDVVMVARGDLGVEIGDPELVGIQKKLIRR
ARTLNRAVITATQMMESMITNPMPTRAEVMDVANAVLDGTDAVMLSAETAAGQYPAETVAAMARVCLG
AEKIPSINVSKHRLDVQFDNIEEAIAMSSMYAANHLKGVTALIAMTESGRTALMMSRISSGLPIFAMS
RHEHTLNLTALYRGVTPVYFDSHKDGVIAANEAVNRLRDKGFLVSGDLVIVTQGDVMETVGTTNTSRI
LRVE
>CORE_REP|Org4_Gene2540#
MYPDTQLYIDGQWRNALAGKTLPVTNPATDEIIGQVAHAATEDLDLALAATERGFTVWRDTAAHQRAN
LMRKAAALLRERANAIAAVMTQEQGKPVAQAKIEILNAADVIDWFAGEATRTYGQIIPSRARDVQQQT
LKLPVGPVAAFTPWNFPINQIVRKLSAALAAGCSIIVKGPEETPASPAELIKAFADAGIPAGVIALVY
GTPAEISEYLIPHPTIRKISFTGSTRVGKHLAALAGQHMKKATMELGGHAPVLIFDDADLDAAAKELA
QSKFRNAGQVCIAPTRFLIQQGVYEAFVEKFTAAVRELKLGNGLEDGVTMGPMVLGRSVDNIEALVQD
AVAHGAKACSGGKRVAGKGNFFEPTVLRDVPLSARAMSEEPFGPVALLRPFATYDEAIAEANRLPYGL
AAYAYSRNIATVNALGRDVESGMLSINHIGFGLPETPFGGVKDSGHGTEGGSEAIESYLETRFVTVAG

R
>CORE_REP|Org14_Gene1147#
MKVTLPDFRRAGVLVVGDVMLDRYWYGPTSRISPEAPVPVVKVDTIEERPGGAANVAMNIASLGANSR
LVGLTGIDDAARALSAKLNEVNVRCDFVSVPTHPTITKLRVLSRNQQLIRLDFEEGFSNVDPQPMLER
IQQALPQIGALVLSDYAKGALSQVQGMIQLARAAKVPVLIDPKGSDFERYRGATLLTPNLSEFEAVVG
HCKDEAELVARGMKLVADFELSALLVTRSEHGMTLLQPGVEPLHLPTQAQEVFDVTGAGDTVIGVLAA
SLAAGNSLEESCFLANAAAGVVVGKLGTSTVSPIELENAVRGRAETGFGVMTEAQLKTAVAQARQRGE
KVVMTNGIFDILHAGHVSYLANARKLGDRLIVAVNSDASTKRLKGEKRPVNALENRMIVLGALEAVDW
VVPFEEDTPQRLIADILPDLLVKGGDYKPEEIAGSAEVWANGGDVKVLNFEDGLSTTNIIKAIKDGRG
>CORE_REP|Org5_Gene4763#
MRVKMSNLYPVIMAGGTGSRLWPLSRELFPKQFLALCNEFSMLQTTVMRLKGLEIINPLVICNEEHRF
IVAEQLRQITRLSHNIILEPVGRNTAPAIALAALQAVSSGDDPLMLVLAADHVIQDEAIFRDAVNQAI
PYAEAGKLATFGIVPTGPETGYGYIQKGASVDGSSICGVSRFVEKPNLETAQQYLASGDYLWNSGMFL
FKASRYLEELGRFRPDILDACKQSLAHLTPDMDFIRVDRDAFIACPDESVDYAVMEQTADAVVVPLDA
GWNDVGSWSALWEISEKDTKGNSTFGDVLEHNCSNNYIRAEHKLVAAVGVTNLVVVETKDAVLIADKD
NVQDVKEIVNQLKRQKRSESKQHREVYRPWGKHDAIAQGDRFQVRRITVKPGEKLSLQMHHHRSEHWV
VVSGTAKVHTNGKMQLISENESVYIPLGVEHSLENPGKIPLDLIEIQSGAYLGEDDIVRIGDSAQHN
>CORE_REP|Org39_Gene2538#
MASISSLGIGSGLDLNGLLDKLTKAEQQRLTPYTTQQTSYNAQLTAYGTLKGALEKFDNLSKDLAKPE
FFNNTTATKHDQFTVTTTDKSVPGNYSIEVLKLAQPQTLTTQTPIADQQAKLGTPGSSDRSISITAGN
PPKETKIPLGDDQTSLVEMRDAINKSKSGVTASIMRVGDNDYQLALSSTTPGEKNTIAVQVNNDDKLG
AILNYDPKKPPKDGSTAMKQTVPGQDAEIIVNGTKIKRSTNSIADALQGVTLDLKTTTKSGEPQNLVI
GIDKSGSADKIKEWVDNYNSLLDTFNSLTKYTPVKSGEAQNAKNGALLGDNTLRGIQSSIKSALSSAQ
DNPELKGLGNLGITTNVKTGKLEIDSTKLNKAIDEKPEQVANFFAGNGKDTGMATQIHNDIQSYIKAG
GIIENSTKSINTNLDRLNIQITTVTASIQNTIDRYKQQFVQLDTMMSKLSSTGNYLQQQFSAK
>CORE_REP|Org24_Gene1650#
MKKTKIVCTIGPKTESEEMLTNLLNAGMNVMRLNFSHGDYEEHGNRIKNMRAVMAKTGINAGILLDTK
GPEIRTMKLEGGKDASLVAGQTFTFTTDQSVIGNSERVAVTYAGFAADLKIGNTVLVDDGLIGMEVTN
VTENEVVCKVLNNGDLGENKGVNLPGVSIQLPALAEKDKRDLIFGCEQGVDFVAASFIRKRSDVLEIR
EHLKAHGGEQIQIISKIENQEGLNNFDEILEASDGIMVARGDLGVEIPVEEVIFAQKMMIEKCNRARK
VVITATQMLDSMIKNPRPTRAEAGDVANAILDGTDAVMLSGESAKGKYPLEAVNIMATICERTDRVMP
SRIDALNDRRKLRITEAVCRGAVETAEKLDAPLIVVATSGGKSAKSVRKYFPNAVILALTTNETTAHQ
LVLSKGVIPQMVKEIASTDDFYRIGKEAALASGLAQKGDVVVMVSGALVPSGTTNTASVHVL
>CORE_REP|Org34_Gene2045#
MQRGIVWIVDDDSSIRWVLERALTGAGLSCATFEGGNDVLEALATQTPDVLLSDIRMPGIDGLALLKQ
IKQRHPMLPVIIMTAHSDLDAAVSAYQQGAFDYLPKPFDIDEAVALVERAISHYQEQQQPVRSQPASD
PAADIIGEAPAMQDVFRIIGRLSRSSISVLINGESGTGKELVAHALHRHSPRAKSPFIALNMAAIPKD
LIESELFGHEKGAFTGANQIRQGRFEQADGGTLFLDEIGDMPLDVQTRLLRVLADGQFYRVGGYAPVK
VDVRIIAATHQNLELRVQEGKFREDLFHRLNVIRVHLPPLRERREDIPRLARHFLQIAAKELGVEAKN
LHPETETALTRLPWPGNVRQLENTCRWLTVMAAGQEVLIQDLPSELFETAAPESPSHSLPDSWATLLA
QWADRALRSGHQNLLSEAQPEMERTLLTTALRHTQGHKQEAARLLGWGRNTLTRKLKELGME
>CORE_REP|Org15_Gene1015#
MSAEHVLTMLNEHEVKFVDLRFTDTKGKEQHVTIPAHQVNADFFEEGKMFDGSSIGGWKGINESDMVL
MPDASTAVLDPFFEEPTLIIRCDILEPGTMQGYDRDPRSISKRAEDFLRSSGIADTVLFGPEPEFFLF
DDIRFGSSIRGSHVAIDDIEGAWNSGTKYDGGNKGHRPAVKGGYFPVPPVDSSQDLRSTMCLTMEEMG
LVVEAHHHEVATAGQNEVATRFNTMTKKADEIQIYKYVVHNVAHAFGKTATFMPKPMFGDNGSGMHCH
MSLSKNGTNLFAGDKYGGLSETALFYIGGIIKHAKAINALANPTTNSYKRLVPGYEAPVMLAYSARNR
SASIRIPVVASPKARRIEARFPDPAANPYLCFAALLMAGLDGIINKIHPGDAMDKNLYDLPPEEEAEI
PKVAGSLDEAMAALNEDREFLTRGGVFTDDAIDAYIELRKEEMDRVRMTPHPVEFELYYSV
>CORE_REP|Org49_Gene4620#
MRHWKKKLGLTALTALVLSSMLGAGVFSLPQNMAQVASPAALLLGWGITGVGILFLAFAMLLLTRLRP
DLDGGIFTYAKEGFGELVGFCSAWGYWLCAVIANVSYLVIVFAALSIFTDRGGSVILGDGNTWQALIA
ESALLWIVHALVLRGVQTAASINLAATLAKLLPLGMFAVLAAIAFKMDVFTLDFKGIALGKPVWEQVK
DTMLITLWVFIGVEGAVVVSARARNKKDVGRATMLAVLSALAVYLMVTLLSLGVVPRSELAEMRNPSM

AVLMVELIGPWGDVLIAAGLIISVCGAYLSWTIMAAEVPLLAAQHGAFPRVFGKQNRHHAPSSSLWLT
NIAVQLALVLIWLTGSNYNSLLTIASEMILVPYFLVGAFLFKVAYRRRDKRLIFAATGACVYGLWLLY
ASGLMHLLMSVLLYAPGLLVFMYARRGHRDINLLNRLEKSSIFLLLAATLPAGWLMLH
>CORE_REP|Org12_Gene295#
MATGKIIQVIGAVVDVEFPQDAVPKVYDALEVENGTEKLVLEVQQQLGGGVVRCIAMGTSDGLRRGLK
VNNLDHPIEVPVGKATLGRIMNVLGQPIDMKGDIGEEERWAIHRAAPSYEELSSSQELLETGIKVMDL
ICPFAKGGKVGLFGGAGVGKTVNMMELIRNIAIEHSGYSVFAGVGERTREGNDFYHEMTDSNVLDKVS
LVYGQMNEPPGNRLRVALTGLTMAEKFRDEGRDVLLFVDNIYRYTLAGTEVSALLGRMPSAVGYQPTL
AEEMGVLQERITSTKTGSITSVQAVYVPADDLTDPSPATTFAHLDATVVLSRNIASLGIYPAVDPLDS
TSRQLDPLVVGQEHYDVARGVQSILQRYQELKDIIAILGMDELSEEDKLVVSRARKIQRFLSQPFFVA
EVFTGSPGKFVSLKDTIRGFKGIMDGDYDHLPEQAFYMVGTIEEAVEKAKKL
>CORE_REP|Org13_Gene3724#
MSEALTCFKAYDIRGKLGSELNEDIAYRIGRAYGEYLRPKTMVLGGDVRLTSESLKLALARGLQDSGT
DVIDIGLSGTEEIYFATSHLKVDGGIEVTASHNPMDYNGMKLVREESKPISGDTGLRDIQRLAENNSF
PAVNDAARGGYQQLSILDAYVQKLLSFVALDNFTRPLKLVINSGNGAAGHVIDAIEARFKNAGLPVEF
IKVHHAPDGNFPNGIPNPLLPECRQDTTDAVLKHGADMGIAFDGDFDRCFLFDERGNFIEGYYIVGLL
AEAFLEKSPGSRIIHDPRLSWNTIDIVEKAYGIPVMSKTGHAFIKERMRKEDAVYGGEMSAHHYFRDF
YYCDSGMIPWLLVAELLCIKGRSLGELVNDRVAAYPASGEINSSLNNPKEAIGRVLGKYEMEADAVDH
TDGISVEYDNWRFNLRSSNTEPVVRLNVESRANVELMQEKTEEILQLLRSE
>CORE_REP|Org1_Gene3914#
MSEQTIVWDLALIQKYNYSGPRYTSYPTALEFNQCYDEAAFQRAAARYPERPLSLYVHIPFCHKLCYF
CGCNKLVTRQTHKADEYLNVLAQEIASRAPLFAGRKVGQMHWGGGTPTYLDKAQISRLVALLREHFDF
LPDAEMSIEVDPREIELDVLDHLRAEGFNRLSMGVQDFNKQVQQLVNREQDEAFIFALIERAKALGFR
STNIDLIYGLPKQTPESFAFTLQRVAELNPDRLSVFNYAHMPNLFAAQRKIKDADLPGAQQKLDILQQ
SIAFLTDAGYQFIGMDHFAHPDDELAIAQREGKLHRNFQGYTTQGDSDLLGLGVSAISMLGDSYAQNQ
KELKRYYDSVQAQGNALWRGLALTDDDCLRRDLIKTLICNFRLAYQQLERQYGIDFTAYFAEDLQLLA
PFERDGLVERDEQGIRVTPRGRLLIRNICMCFDRYLRQQARSQQFSRVI
>CORE_REP|Org34_Gene2699#
MSTSLLLLIAVLGVVLLLLMVIKAKVQPFVALLVVSLLVALASGIPTGEVMKVMTAGMGGVLGSVTII
IGLGAMLGRMIEHSGGAESLAQRFSQGLGPKHTVAALTLAAFILGIPVFFDVGFIILAPIIYGFAKVA
KVSPLKFGLPMAGVMLTVHVALPPHPGPVAAAGLLNADIGWLTIIGLAICIPVGVIGYFAANYLNRKT
YPLSIEVLEQLQLAAPEPRPEGQAPLSDRINPPGAGLVAALIVIPIAIIMLGTVSATLLPAGSALRDA
LSLLGSPAVALMIALLLAFYFLALRRGWSLQHASDVMGAALPTAAVVILVTGAGGVFGKVLVESGVGK
ALAEVLTAIGLPLVPAAFIISLALRASQGSATVAILTTGGLLSEAVSGLNQLQLVLVTLATCFGGLGL
SHVNDSGFWIVTRYLGLSVADGLKTWTVLTTLLGLSGFLFTWLLWLAV
>CORE_REP|Org1_Gene1887#
MTIKAIDGGPAGKPQLRKSLKLWQVVMMGLAYLTPMTVFDTFGIVSGLTDGHVPTSYLLALAGVLFTA
ISYGKLVRQFPTAGSAYTYAQKAINPHVGFLVGWSSLLDYLFLPMINTLLAKIYLTALFPEVPPWVWV
VGFVILITAINLKSVNLVANFNTLFVLAQVAIILVFIYLVVRGLHNGEGMGTVWSLRPFLSENAHLLP
IITGATILCFSFLGFDAVTTLCEETPDAAKVIPRAIFLTALYGGVIFISVSFFIQLFFPSIQRFHQPD
AALPEIALYVGGKLFQSIFLCVTFINTLASGLASHASVSRLLYVMGRDNVFPEKFFGYIHPKWRTPAL
NVLMVGLVALSALSFDLVTATALINFGALVAFTFVNLSVISHFFIREGRNKSWKDRFNFLFLPLVGAL
TVGVLWLNLEKSSLTMGLIWATLGFGYLAWLTRRFRQPPPQLERQPQQ
>CORE_REP|Org18_Gene3234#
MKILSPLALSLAALLTAGCGNALKSDYRAPQVNYPTSWQHAADNAAPTPFDWRDFHDPELDRWLQQVM
DSNNDLAVAVLRVYRARLEAERVGISTAPDVNASLNSGINRPLSESSAWNKTSGATLSTSYEVDLWGK
LARQRDAAEWASQASEQDLQTARLTLLANAATNYWRIGFLNQQIGVSQASIAYAKQTLRLANARYRAG
SISALDVVNAEQNVLTQESRLLALQHDRQQALNEQAVLLGAPTGQATIAPARLPTTAMPQINTGIPAS
VLSRRPDLSAKELRLRAALANVDEKRLQYYPAFSLTGSLGASSSALLEFLRNPTGSLGASLTLPFLQW
RQMGVDIKIARNDYEQQVLEFRQALYKAMGDVNNALSLRAQLRAQETQLQASLALARKSERLNEVRYR
QGAVTITDWLNAQEQRRQAELAVDENRFAQYQNLAKIYLEFGGSSAP
>CORE_REP|Org14_Gene947#
MTARLGRWLSSLDTLEKRIARAPMVRRYGRLTRATGLVLEATGLQLPLGATCLIERHDAGEVQEVESE
VVGFNGQRLFLMPLEEVEGIVPGARVYARIAPEGQSAGKQLPLGPALLGRVLDGSAKPLDGLPSPETG

YRAPLITAPFNPLQRTPIEQVLDVGVRTINGLLTVGRGQRMGLFAGSGVGKSVLLGMMARYTQADVIV
VGLIGERGREVKDFIENILGAEGRARSVVIAAPADVSPLLRMQGAAYATRIAEDFRDRGQHVLLIMDS
LTRYAMAQREIALAIGEPPATKGYPPSVFAKLPALVERAGNGISGGGSITAFYTVLTEGDDQQDPIAD
SARAILDGHVVLSRRLAEAGHYPAIDIEASISRAMTSLIDEEHYRRVRTFKQMLASYQRNRDLISVGA
YAAGSDPLLDKAMTLYPQMEAYLQQGIFERSGYDEACQQLQQLIV
>CORE_REP|Org12_Gene4291#
MKRLSLRLRLILIFSLLALLTWCTASVVAWVMSRNTINEVFDTQQMLFAKRLATANLGDLLADESARS
LPKTKKLVHHGKRGEQDDDALAFAIFDRDGKMLLNDGENGADFLFDGEREGFTDGERKGDDDSWRLVW
LTSPDGRYRIVVGQEWDYRRDMALGMVTGQLVPWLATLPVLMLLIALMVGRELRPLRAVAAGLRRRAP
DDATPLDARQVPTEVRPLVDALNALFARINALLVRERRFTSDAAHELRSPLAALRVQTEVVQLAGDDA
PMREHALDNLTVGIDRATRLVDQLLTLSRLDSLLDLAELAPIDWNDLVTMTLAEQDRQAHAAGVTLRY
EHRGTPPPRQGETLLLSLLLRNLLDNAVRYTPQGGVVTVTLSERSLTVEDDGPGVTAEHLARLGERFY
RPPGQEQTGSGLGLSIVQRIAGLHGLQISFANRSAGGFVARLAL
>CORE_REP|Org16_Gene3986#
MKVTVFGIGYVGLVQAAVLAEVGHDVMCIDVDERKVKNLKKGNIPIFEPGLTPLVQQNYEAGRLHFTT
DAKAGVAHGNIQFIAVGTPPDEDGSADLKYVTAVARTIAEHMTDRKVVIDKSTVPVGTADKVRQVMAE
TLAKRGSNVAFDVVSNPEFLKEGAAVADCMRPERIVIGTDNKEVIEPIRELYEPFNRNHDRMIMMDIR
SAELTKYAANCMLATKISFMNEMSNLAEMLGADIEKVRQGIGSDSRIGYHFIYPGCGYGGSCFPKDVQ
ALIRTAEQIGYQPKLLQAVEQVNYQQKDKLNSFIKDYFGSDLKGKTFALWGLAFKPNTDDMREASSRV
LMEQLWAAGATVQAYDPEAMNEVQRIYGQRDDLKLMGTKEAALHGADALVICTEWQNFRAPDFDVIKS
ALKQPVIFDGRNLYDPERLENRGFTYYAIGRGASIKPVI
>CORE_REP|Org13_Gene871#
MDFFLQLAVILACLLYGARKGGIALGLLGGIGLMILVFGFHLQPGKPPVDVMLVIIAVVAASATLQAS
GGLDVMLQIAERMLRRNPRYVSIIAPFVTCILTILCGTGHVVYTILPIIYDVAIKNNIRPERPMAASS
IGAQMGIIASPVSVAVVSLVAMLSSYTFNGRHLEFLDLLSITIPSTLCGILAIGIFSWFRGKDLDKDP
EFQKFISVPENHRYVYGDAATLLDRVLPRSNWIAMWIFLATIALVAVLGAFSDLRPSFGGKPLSMVLV
IQMCMLMAGALIVIITRTNPASISKNEVFRSGMIAIVAVYGVAWMAETMFGAHLAQIEATLGVLVKEY
PWAYALILLLVSKFVNSQAAALAALVPVALAIGVNPAYIVASAPACYGYYILPTYPSDLAAIQFDRSG
TTRIGRFVINHSFILPGLIGVSVSCVFGWILAAAFGFL
>CORE_REP|Org1_Gene1537#
MSTHIGEPQDSYIEEIPQDERRFTRMGWLVVGIGLFGFLAWAAFAPLDKGVASPGSVTVSGNRKTVQA
PASGIIKNIAVKEGDKVKAGEVLVQLSQVQAQAQVDSLRDQYYTTLATEGRLLAERDGLSSVTFSPIF
TQIKDQPRVAEIIALQTQLFASRRQGLQSEIDGYKQSMDGIRFQLKGLQDSRVNKQIQLSSLREQMNS
MKQLAADGYLPRNRYLEVQRQFAEVNSSIDETVGRIGQLQKQLQESQQRIDQRFADYQREVRTQLAQT
QMDASEFRNKLQMADFDLGNTAITSPVDGTVVGLNIFTQGGVVGAGDHLMDVVPSQATLVVDSRLKVD
LIDKVYNGLPVDLMFTAFNQNKTPKIPGTVTLVSADRLVDKANGEPYYQMQVTVSPEGMKMLSGEDIK
PGMPVEVFVKTGSRSLLSYLFKPILDRAHTSLTEE
>CORE_REP|Org36_Gene3361#
MWSFLKSRPDAPQVTDQRQIDASYKYWRIQLMCTMYIGYAAFYFTRKSFNFIMPAMLSDLGLTMSDVG
ILGTLFYITYGCSKFISGMISDRSNPRYFMGLGLIMTGVLNIFFGLSSSLLMLGTLWILNAFFQGWGW
PPCSKILTSWYSRSERGSWWAIWNTSHNVGGALIPLLVGFISLHFSWRYGMIIPGIIGVVLGLLMCWR
LRDKPSTLGLPSVGKWRNDAMELVQESEGQGLSNREIIKRYVLTNKYIWLLAVSYVLVYIVRTAINDW
GNLYLTQEKGYSLMTANSAISLFEVGGFIGSLVAGWGSDKLFRGNRGPMNLIFAIGIFLSVAALWLMP
GVTYLLQACCFFAIGFFIFGPQMLIGMAAAECSHKDAAGAATGFVGLFAYLGAALSGYPIARVMEIWH
WNGFFVVISIAACLSALFLLPFLRAQTPALKTANA
>CORE_REP|Org1_Gene4394#
MIMSKSNNKMGVVQLTILTAVNMMGSGIIMLPTKLAEVGTISIVSWLVTAVGSMALAYAFAKCGMFSR
KSGGMGGYAEYAFGKSGNFMANYTYGVSLLIANIAIAISAVGYGTELFDTTLSPLGICIATIGVLWLA
TVANFGGARITGKISGITVWGVIIPVVGISVIGWYWFSGSAYVAAWNPHQVPTFEAIGASISMTLWAF
LGLESACANTDVVENPERNVPIAVLGGTLSAAVIYIISTNVIAGIVPNMDLANSTAPFGLAFSHMFNP
TVGKIIMALMVMSCVGSLLGWQFTIAQVFKSSADSGFFPKIFSKLSKADAPVKGMLTIVVIQSGLSLM
TISPSLNKQFNVLVNLAVVTNIIPYILSMAALVIIQKVAKVPDNKARIANIIAGIGALYSFYALYSSG
EEAMMWGAIATFLGWTLYGIVSPRFELAGKKG
>CORE_REP|Org31_Gene1229#

MPLVIVAGGVALLLLLMIRFKLNGFISLVLVALAVGIAQGMPVDKVIGSIKAGVGGTLGSLALIMGFG
AMLGKLLADCGGAQRIATTLIDKFGRKHIQWAVVLTGFTVGFALFYEVGFVLLLPLVFTIAASARIPL
LYVGVPMAAALSVTHGFLPPHPGPTAIATIFHADMGKTLLYGTLLAIPTVILAGPVYARFLKGIDKPV
PEGLYNPKTFTEAEMPSFGVSVATSLVPVILMALRAVAEMVLPKGHSLLRFAEFFGDPVMATLIAVLI
AIFTFGLNRGRTMDEVMGTITDSIKIIAMMLLIIGGGGAFKQVLVDSGVEQYIAGLMEGSNVSPILMA
WSIAAALRLALGSATVAAITAGGIVAPLIATTGVSPELMVIAVGSGSVIFSHVNDPGFWLFKEYFNLS
IMETIKSWSVLETIISVCGLVGCLLLATVV
>CORE_REP|Org11_Gene2002#
MSTSRSQQIQQLEQEWKSARWEGITRPYSAEDVINLRGSVNPECTLAQNGAAKLWALLNGKARKGYVN
CLGALTGGQALQQAKAGVEAIYLSGWQVAADANSAAAMYPDQSLYPVDSVPKVVERINNTFRRADQIQ
WANQIEPGSKGYTDYFLPIVADAEAGFGGVLNAFELMKAMITAGAAGVHFEDQLAAVKKCGHMGGKVL
VPTQEAIQKLVAARLAADVLGVPTLVIARTDADAADLLTSDCDPYDSAFVTGERTAEGFFRTHAGVEQ
AISRGLAYAPYADMVWCETSTPDLDAAQRFADAIHAKYPGKLLAYNCSPSFNWKKNLDDQTIARFQQA
LSDMGYKYQFITLAGIHSMWFNMFDLAHAYAQGEGMKHYVEKVQQAEFAAVDRGYTFASHQQEVGTGY
FDKVTTVIQGGASSVTALTGSTEEQQF
>CORE_REP|Org38_Gene3496#
MLLRFSQLTTHKGAELSAIEHAVPMIVFSPDGTVLRANDLFLSTLGFQRDDVIGRHHRIFCDPNYVAS
PLYREHWETLNKGQPITDTIKRIAKNGEAVWLQGTYAPVLNKQGKVVEIVKIASEVTERVTQAQEHRS
LLAALNRSMAMISFTPQGTIVSANDNMLALMGYRLEEACGQSHAVLCPPAFAASDDYRRHWQRLARGE
FITGRFERVNRRGERVWLEASYNPILDNDGQVVKVVKIAQDITRLMQQQQHEEEMVRNAHHLSLDTDR
QAAQGAVIVQQAVKGMQQVEAAARETSDVVTELGKCSQQIGTIVEAIRKIASQTNLLAINASIEAAHA
GEHGRGFAVVANEVRTLAEQSRKAATEIERMTKSIQQGVAAAIAGMATCVEQAGGGVALTHDAGEVIN
QVNIGMHDVVKLMQAFTSVKQGDALH
>CORE_REP|Org19_Gene2793#
MKAIITNALWLMLERVSLSLSGIFVSIYVARYLGPAQFGALNYLLATIAIVVPLVQLGADSIIFNRVA
RRQPSGIRLMLASIRLRRRLFLVVALPILIWSYFSQTPASQLMTLLLLVSAYFSIQDVYKIYYDARLQ
SKRNTLINNLALLLSIGLRLALVSAALPLVWFAVPYILSSAVPYLVRLWLFRGEAAASPRVTPRQARR
YGRYLLKVGLPLAISSLSIVIYTRIDQIMLGNLVGEQAVGWFSAATTLSQGWVFVPMALITSLMPGIA
SCRDPLEQEYRIRVLYLVVLGLSLPVLLGLWWFAHPAIALLYGAAFQPAASILAICTLTSLFSVMGTV
SYRSIVLFAGYRFIAIKMPLVAVANVVMNLLLIPRYGLIGAAVSTLLAEFISFFVLNSLFRGGKITRL
QLTCFYCLPRLVSKLRREHVKHPG
>CORE_REP|Org41_Gene2975#
MNQRLDIIGIGLGPSNLSLAALGSEIEGFTGQFLERKPHFSWHPGMILADCSMQTNFLKDLVSAVAPT
NRYSFLNYLVKNRKFYRFLTTEQRTASREEFADYLTWAAGGMDSLAFNQDVQQIEFDDRQRQFVVTTS
NKVFHAKHVSIGIGKKIKLPDCVTAQSDRCFHASEMMLRNPDLTGKRVAIVGGGQSGADLFLNIFKGE
WGQPDQLDWISRRNNYNALDEAAFANEYFTPDYVESFYSLDSAAKRHMLAEQKMTSDGITSESLLAIY
RAMYHRFDVLREKLWVRLLPSRSLTAVKHTLDNAYQLETRHHLDHGEEAFKADVVIFATGYQTATPEF
LEPLAHRLLTTADGEYRIAPDFTFEWEGPAENCLFAMNASMHNHGIADPQLSLMAWRSARILNRALDH
KPFDLGTTPTAIQWRSESVPHAF
>CORE_REP|Org18_Gene3825#
MLLRLYQVLLYLIQPLIWLRLLLRSRKAPAYRKRWAERYGFCAGKVVPGGIMLHSVSVGETLAAIPLV
RALRHRYPALPITVTTMTPTGSERVQSAFGKDVHHVYLPYDLPSSMNRFLDQVNPKLVIIMETELWPN
LINALHQRQIPLVIANARLSARSAAGYKKIGGFMRDMLRRITLIAAQNQEDGDRFIELGLKRSQLAVT
GSLKFDISVTPELAARAVTLRRQWAPRRPVWIATSTHDGEETILLEAHRKLLEKHPDLLLILVPRHPE
RFPTAKELVQKAGFSYTLRSSGEIPSGSTQVVIGDTMGELMLLYGIADLAFVGGSLVERGGHNPLEAA
AHAIPVLMGPHTFNFKDICAKLSQAEGLITVTDVDSLVKEVETLLTDEDYRRYYGRHAVEVLYQNQGA
LQRLLQLLEPHLPPRSH
>CORE_REP|Org13_Gene3315#
MAQVINTNSLSLMAQNNLNKSQSSLGTAIERLSSGLRINSAKDDAAGQAISNRFTANIKGLTQASRNA
NDGISLAQTTEGALNEVNDNLQNIRRLTVQSQNGSNSSSDLQSIQDEISQRLSEINRISEQTDFNGVK
VLSADQKLTIQVGANDGETIDIELKDINAKTLGLDKFSVADPIDTTKIGTTKLTAVDKMGAPTINADS
KAATPKNSDGKLYSTDDGAGNVAYFVKSTDGGIYDATVAADGKVSWDSSTATTKATAGMKETSQVKVG
TTNISGLTGSDELRTYTDPAGKSGYVVKGKDDNGNDAYFKATVDASGKVTKGAQQSTDPKTADPLATL
DKALSQVDSLRSSLGAVQNRFDSVINNLNSTVNNLSASQSRIQDADYATEVSNMSRANILQQAGTSVL

AQANQSTQNVLSLLR
>CORE_REP|Org20_Gene4243#
MSFDTISVIGLGYIGLPTAAAFASRKKKVVGVDVNQHAVDTINRGAIHIVEPDLDKVVKDAVDGGFLR
AVTKPLAADAFLIAVPTPFKGDHEPDLAYVEAAAKSLAPVLKKGDLVILESTSPVGATEQMADWLAQA
RSDLSFPQQAGEAADVNIAYCPERVLPGQVMVELIQNDRVIGGMTPKCSERASALYKIFLEGECVITN
SRTAEMCKLTENSFRDVNIAFANELSLICAEQGINVWELIRLANRHPRVNILQPGPGVGGHCIAVDPW
FIVAQNPQQARLIHTARLVNDGKPLWVVDRVKAAVADCLAATDKRASEVKIACFGLAFKPNIDDLRES
PAVEVVHLIAEWHVGETLAVEPNVEQLPKSLAGHVTLTPIAEALQQADVIVMLVDHQQFKAIRPEEIK
QSWVVDTKGVWR
>CORE_REP|Org31_Gene1596#
MSKRRVVVTGLGMLSPVGNTVESTWNALLAGQSGISLIDHFDTTAYATKFAGLVKNFNSEDFISRKDA
RKMDAFIQYGIAAGMQAMQDAGLDITEANASRIGAAIGSGIGGLGLIEENHSSLVNGGPRKISPFFVP
STIVNMIAGHLTIMYGMRGPSISIATACTSGVHNIGHAARIIAYNDADVMLAGGAEKASTPLGVGGFG
AARALSTRNDNPQAASRPWDKDRDGFVLGDGAGMMVLEEYEHAKKRGAKIYAEVVGFGMSSDAYHMTS
PPENGAGAALAMENALLDAGVTPSQIGYINAHGTSTPAGDQAEAQAVKSVFGADAERVLVSSTKSMTG
HLLGAAGAIESIFTVLALRDQAVPPTINLDNPDEGCDLDFVPHEARQVSDMEFSLCNSFGFGGTNGSL
IFRRV
>CORE_REP|Org18_Gene1127#
MAFSQAVSGLNAAATNLDVIGNNIANSATAGFKSGSVSFADMFAGSQVGLGVKVSGITQNFKGGTTTG
TSRALDIAINGNGFFRMQDKDGGIFYTRNGQFKLDENRNLTNMQGLQLTGYPAAGSPPTIQQGANPVP
LSIPEGMMNAKASTSGEMVTNLKSTHKVPENKTFDPTKQDSYNYVNTITAYDSLGNAHNINAYFVKTD
DNKWQVYTQDGSAAPVNAGTMEFSTSGNLVKTTSTNGAPGEFSMVIPMTAKDGAPAQNFTLSFAGSMQ
QNVGSDSVSKVAQDGYAAGEYTNFQINNDGTVVGIYSNQQTQVLGQIVMANFSNPEGLASQGDNVWQE
TGASGQPRVGLSGGGGFGKLTSGALESSNVDLSQELVNMIVAQRNYQSNAQTIKTQDSILQTLVSLR
>CORE_REP|Org41_Gene646#
MKRAVITGLGVVSSIGNNQQEVLASLQEGRSGITFSQELKDSGMRSHVWGQVKLDTTGLIDRKAVRFM
SDASIYAFLAMQEAIASSGLKEEEYQNNPRVGLIAGSGGGSPRFQVFGADAMRSPRGLKAVGPYVVTK
AMASGVSACLATPFKIHGVNYSISSACATSAHCIGNAVEQIQLGKQDIVFAGGGEELCWEMACEFDAM
GALSTKYNDTPEKASRTYDADRDGFVIAGGGGMVVVEELEHALARGAHIYAEIVGYGATSDGADMVAP
SGEGAVRCMKMAMQDLDAPIDYINVHGTSTPVGDVKELGAIREVFGDNTPAISSTKAMTGHSLGAAGV
QEAIYSLLMLEHGFIAPSINIETLDEQAAGMNIVTQPTQRELTTVMSNSFGFGGTNATLVMRKLAK
>CORE_REP|Org14_Gene4570#
MTQENSIAPAANPLVDAANPLLNAISQIRQSATHANPAQLRQQLIDEMRRFEIRGQRANLPYEVIIGA
RYCLCTALDEAAALTPWGSNSVWSGSGLLVTFHNETWGGEKFFQLLAKLSQSPREHINLLELINYCLL
LGFEGRYRVMENGRSQLETMKQRLLQLIRSVRGGYAPPLSPHALDLPVQQKLWRPLVPLWACVALTGF
LASLLFIALNWRLGDNTSPVLAAIYQTNLPQVAIGNPAPAAPPTLSLKSFLRKEIAEGLVVVRDEAQQ
SVVILKGDGLFDSAATTVRANYIPVIDRIAAAMNGVSGKILVTGYSDNVPIRSARFASNWELSLARAE
AVSARLQKHLANPQRVKAEGRGESNPVAPNDNKVNRALNRRVEITLLVAPENTQAEINGLPQGTGK
>CORE_REP|Org47_Gene2401#
MAIKLEVKNLYKIFGEHPERAFKLLDKGLTKDRLFEKTGLSLGVKDATLAIEEGEIFVIMGLSGSGKS
TLVRLLNRLIEPTRGQVLIDGEDIAKISDTALRTVRRNKISMVFQSFALMPHMNVLNNTAFGMELAGI
PLQERQEKALDALRQVGLENYALSYPDELSGGMRQRVGLARALANNPDILLMDEAFSALDPLIRTEMQ
DELVKLQAQHQRTIVFISHDLDEAMRIGDRIAIMQGGEVIQVGTPDEILNNPANDYVRTFFRGVDISH
VFSAKDIAQRRPVTLIRKTPGFGPRSALQLLRDEDRDYGYVVERGKKFIGVVSIESLKKALSANQTLD
DALLEAPAAVPADTPLSDLISLVAQAPCAVPVVCEEHNYLGIISKAMLLQALDKEGSANE
>CORE_REP|Org30_Gene2796#
MKARRLFLWQAFDAQGALRRGELMSDEKRQVSRLLIEQGMQPWRIGHGKRVTPGQWRGEPLIHFTRQL
ATLLQAGLPLVNTLQLLAAEHPSAAWRCLLRQLAEQVREGQPLSETLAAQPGVFPLIYRQLIAIGELT
GNLDRSCLQLAQQQEAQLLLRRKVTKALRYPLFICAVALLVSVLMLVMVLPEFAKVYQSFDAPLPWFT
QGLLGLSALLIAVGPYLALLLGALLFGYCRWLHPRPPWRRREQAALLRLPLIARLVSGGALSQTFRIL
TMTQRAGLTLVEGLNAAALAADHLLYRQALEQVQRQLAEGEAFHHALALQPLFPPLCRQLVRVGEESG
SLDVLLDKLAQWYERQTHELADTLAQTLEPLLMLVVGGIVGALVIAMYLPIFQLGSVLG
>CORE_REP|Org10_Gene158#
MSASFYQQLEQQLAATRSEGLFKEERIITSAQQADIAVADGSHVINFCANNYLGLANHPALIAAAKSG

MDSHGFGMASVRFICGTQDSHKQLEQKLAAFLGMEDAILYSSCFDANGGLFETLLGPEDAIISDALNH
ASIIDGVRLCKAKRYRYANNDMTELAAQLKQAKADGARHILIATDGVFSMDGVIANLKGVCDLADEYQ
ALVMVDDSHAVGFVGANGRGTHEYCEVMGRVDIITGTLGKALGGASGGYTAAKKEVVEWLRQRSRPYL
FSNSLAPAIVAASIKVLELLEEGDALRDRLWANARLFREKMTAAGFTLAGADHAIIPVMLGEAKLAQE
FANALLKEGIYVTGFFYPVVPKGQARIRTQMSADHTPEQIERAVAAFTRIGKDLGVIA
>CORE_REP|Org17_Gene4241#
MATLTTENQTFSGFEYAEQSTFLYRSEFRSLSAHGVFERIETPVFGGEQEGSALAQHIRQALARAKAA
GQAAPVVVGAIPFDTRRPSCLYIPEESRFVANDSFIRAARPMLQQPHRLVACTSIPDEPRFKHAVAEA
VSRFKQGKLDKAVLSRILDIELEQPVAGHRILNNLMVQNPTGYHFSLPLADGSVLIGASPELLIRKQG
GEIHTNPLAGSARRQDDPQQDRLGSERLMRSTKDKYEHKLVIDDIRRHLTPLCATLSVPSGPSLLSTG
TMWHLSTRIRGELLNPALNVMQLACLLHPTPALCGFPTESARQLIAALEPHDRGLFSGIVGWCDANGD
GEWAIVIRSGLLRGNRVRLFAGAGIVAASTPQSEWMETTAKLGTMLNAFGLNSGAL
>CORE_REP|Org2_Gene2691#
MFDFSTPIDRHGTWCTQWDYIADRFGSDDLLPFTISDMDFATAPCILDALQQRLQHGVLGYSRWQHED
FLGALRHWYQQRFNVGIDTATAVYGPSVIYMAAQLIRQWSVPGDYVVTHTPAYDAFYKVILANQRQLL
ACPLQKAGDDWRCDMAHLEALLARPQTKILLLCSPHNPTGKVWRRDELQQMAELCERHDVRVISDEIH
MDMVWGEHRHTPWSQVASGAWALLTSGSKSFNIPALTGAYGFISDAASREAYFQQLKARDGLSSPAVL
AVAAHVAAYRHGEPWLDALRDYLQDNLTYVAERLEQAFPALGWRPPQATYLAWIDLRPLAVDDRALQQ
VLIEREKVAIMPGFTYGEEGRGFLRLNVGCPRSKLEAGMDKLIAGLRLVLDEQ
>CORE_REP|Org12_Gene1014#
MDSLSVDELAQKKDRWYRIVEEMLAEAGVAINGPRAWDIRVHNPALFKRILQEGSLGFGESYMDGWWE
CERLDMLFTRILQAGVDERLPKSLSDIARIAYARLFNRQSRKRAWQVGKEHYDIGNDLFRAMLDPYMQ
YSCGYWKEAQTLEQAQQAKLRMICEKLQLKPGMTLLDIGCGWGGLAQFAAQNYGVSVHGVTISAEQQK
LAQARCAGLDVEILLQDYRDLDRQFDRIVSVGMFEHVGPKNYETYFSVAARNLKPDGLFLLHTIGSNQ
TDLNVDAWIDKYIFPNGCLPSVRHIAEASEGRFVMEDWHNFGADYDRTLMAWLENFKRAWPDLMGGYS
ERFERMFTYYLNACAGAFRSRNIQLWQVLFSPAGVEGGVRVYR
>CORE_REP|Org7_Gene2853#
MSWQQRIEQALAERRFNAAYRRRQTTEGGNGRQIRLGDRLYLNFSGNDYLGLSQDARVIAAWQQGAQR
YGVGSGGSGHVTGFSAAHQALEEQLAAWLGYPRALLFISGYAANQAVLAALMQKGDRILADRLSHASL
LEAAAQSPAELRRFQHNQPQALADLLAKPCDGQRLAVTEGVFSMDGDGAPLAELHRLTRAAGAWLMVD
DAHGIGVRGEQGRGSCWQQGVRPELLVATFGKAFGVSGAAVLCDEATAEYLLQFARHLIYSTAMPPAQ
ACALQAALVCIREGDDLRARLQDNIRRFRQGAAPLALTLTDSDTAIQPLLVGDNQRALDLATRLRECG
LWVSAIRPPTVPPGGARLRITLTAAHQPQDIDRLLEVLNDVSQ
>CORE_REP|Org29_Gene693#
MAEDSDLEKSEAPTPHRLEKAREDGQIPRSRELTSVLMLLSGLAIILMSGSNMAQQLAAMLTQGLNFD
HGMVSNDKQMLRQLGMLLRQAVLALLPIMAGLVLVALAAPMLLGGILFSGKSIKFDLKRLNPLSGLKR
IFSTQVLAELLKGILKATLVGWVTGLYLWHNWAAMLHLMTQQPLDALGNALQMILFCGFLVVLGLTPM
VAFDVFYQLWSHFKKLKMTKQDIRDEFKDQEGDPHVKGRIRQQQRAIARRRMMADVPKADVIVTNPTH
YAVALQYNDKKMSAPKVLAKGAGEIALRIRELGAEHRIPMLEAPPLARALYRHSEIGQHIPATLYAAV
AEVLAWVYQLRRWRREGGLIPKKPERLPVPEALDFARESDSDG
>CORE_REP|Org14_Gene3402#
MAFNFDQWVDRSHSDSVKWDKYRGSDIIPLWVADSDFTSPPAVIEALQRRVAHGVFGYTHPSPDLIEV
FTRRMVERYGWHIKPEWIIFLPGLVCGLNLCVRACTEEHQSTLAPSPIYPPFRKAAKFAGREHLAVPL
KATGQRWVLDFSSLHDRLSGNEKLLLLCNPQNPGGTVYRRDELLQHHQFAREHALIVCSDEIHCELLL
EPGVRHIPFATLNDDAAQRSVTLMSPSKTFNLAGLGASLAIVPNEALRQKLKRARSGIVPEVNLLALV
AAQAAYQYGQPWLDEQLIYLRANRDRLIKRINAMPGLTLLPVEATYLAWIDCSALPVDNPHQFFERAG
VGLSAGLDFGDRRFVRLNFGCRWALLDEALDRMARACAALPG
>CORE_REP|Org39_Gene1042#
MSNRPLTIGLVAGETSGDILGAGLIRALKAQIPDARFVGVAGPLMQAEGCEAWYEMEELAVMGVVEVL
ERLPRLLKIRKDLTRRFGELRPDVFVGIDAPDFNITLEGRLKQRGIRTIHYVSPSVWAWRQKRVFKIG
KATDLVLAFLPFEKAFYDRFNVPCRFIGHTMADAMPLQPDRLAARAQLGIDPQARCLALLPGSRGAEV
EMLSADFLKTAQLLRTRYPELEVVVPLVNAKRREQFERIKAEVAPDLTVHLLNGQGREAMIASDAALL
ASGTAALECMLAKCPMVVGYRMKPFTFWLAQKLVKTPYVSLPNLLAGREIVTELLQHDCVPDKLAAAV
MPLLEESPQTEALKQTFLTLHQSIRCGADEQAAQAVLELAKA

>CORE_REP|Org14_Gene1134#
MLKLPPLSLYIHIPWCVQKCPYCDFNSHALKGDVPHQEYVDHLLADLDADLPLAGGREISTIFIGGGT
PSLLSAEAMQALLDGVRARIRVADDAEITMEANPGTVEADRFSGYQRAGVNRISIGVQSFSAEKLTRL
GRIHGPEEAKRAATLATGLGLRSFNLDLMHGLPDQSLEEALDDLRQAIALNPPHLSWYQLTIEPNTLF
SSRPPVLPDDDALWDIFERGHQLLSAAGYQQYETSAYAKPGYQCQHNLNYWRFGDYLGIGCGAHGKVT
FSDGRILRTAKTKHPRGFMRGDYMDKQHEVAAADRPFEFFMNRFRLLEAAPRADFVNYTGLAESVIRP
QLDEALAKGYLEETAEHWQITEKGKLFLNSLLELFLADDE
>CORE_REP|Org5_Gene2948#
MIKLENLTKQFMQKNGTPFNAVDNINLDVPEGEICVLLGPSGCGKTTTLKMINRLIEPTGGTILVNGE
DTSALDTVSLRRKIGYVIQQIGLFPNMTIEENITVVPRMLGWDKKRCHDRAEELMSMVALDPKRFLHR
YPKEMSGGQQQRIGVIRALAADPPVLLMDEPFGAVDPINRETIQNEFLDMQRQLKKTVMLVSHDIDEA
LKLGDRIAVFRQGKIVQNASADELLARPANDFVASFVGQDRTLKRLLLVQAGDVADQQETVTVRRETP
LVEAFGLMDDIDARSVTVVDADGKPLGYVKRREARGAPGVCADSLHRFRVTARAEENLRVVLSKLYEH
NTSWMPIVDEDGRYSGEISQDYIADYLSSGRTRRVLTPQ
>CORE_REP|Org44_Gene4288#
MINPKVKALAIIVYGAMLTGCAIAPGQHLTTDSKNVVKQEDSDFQIDELVNIYPLTPSLIAKLRPVKV
VAQPNVLLEQATKNYEYRIGVGDVLNITVWDHPELTTPAGQYRSASDTGNWVQSDGTIFYPYIGKVKV
SGKTASQVRSEISSRLTQYIESPQVDVNIAAFRSQKAYITGEVEKSGQQPITNIPLTVLDAINAAGGL
SANADWRNVVLTHNGKEQILSLQKLMQNGDLTQNQLLYPGDIIYVPRNDDLKVFVMGEVKSPATLKMD
RSGMTLTEALGNSAGLDQNTADATGVFVIRPLRGTQGKKIADIYQLNMADATAMVMGTEFHLQPYDVV
YVTAAPVVRWNRVIVQLAPTISSFNNLTEASLRIRNWP
>CORE_REP|Org8_Gene2746#
MNDAIPRPQAKSQKVFTPLLEIRNLTKTFDGQNAVEDVSLTIYKGEIFALLGPSGCGKSTLLRMLAGF
EQPTEGQIVLDGQDMSHVPPYQRPINMMFQSYALFPHMTVEQNIAFGLKQDKMPRAEIAERVAEMLAL
VHMQEFAKRKPHQLSGGQRQRVALARSLAKRPKLLLLDEPMGALDKKLRDRMQLEVTDILERVGVTCV
MVTHDQEEAMTMAGRIAIMNRGKFVQIGEPEEIYEHPNSRFSAEFIGSVNVFDCVLQERHDDALILQS
PGLRHAIKVDPDASVVDGVPIQVALRPEKILLCEQVPEDGCNFAVGEVAHISYLGDLSIYHVKLHSGQ
IISAQLQNGHRFRKGMPTWGDEVRLCWETDSCVVLTV
>CORE_REP|Org49_Gene1267#
MIPFNAPPVVGTELEYMQAAMGSGKLCGDGGFTRRCQQWMEQRFGSAKVLLTPSCTASLEMAAILLDI
QPGDEVIMPSFTFVSTANAFVLRGAKVVFVDLRPDTMNIDETKIEAAITDKTRAIVPVHYAGVACEMD
TIMALAKKYNLFVVEDAAQGVMSTYKGKALGTIGHIGCFSFHETKNYTAGGEGGATLVNDPALIDRAE
VIREKGTNRSQFFRGQVDKYTWRDIGSSYLMSDLQAAYLWGQLEVAERINQRRLALWQKYYDSFLPLA
RSGRIELPVIPADCVHNAHMFYIKLRDIEERTAFIDYLKEAEIMAVFHYIPLHDCPAGERFGRFAGED
RYTTQESARLVRLPLFYNMSDVNQRTVINTILSFFA
>CORE_REP|Org16_Gene2773#
MKVLTVFGTRPEAIKMAPLVHALAQDEAFDARVCVTAQHREMLDQVLRLFEITPDYDLNIMKPGQGLT
EITCRILEGLKGVLEDFKPDVVLVHGDTTTTLATSLAAFYQRIPVGHVEAGLRTGNLYSPWPEEANRK
LTGHLAMYHFAPTENSRQNLLRELLPDNRIFVTGNTVIDALFWVRDRVMSDAALRAGLAQRYPFLDAD
KKLILVTGHRRESFGGGFERICSALAEIASNHPEVQVVYPVHLNPNVSEPVNRILKGIDNVMLIEPQD
YLPFVYLMTQAYMILTDSGGIQEEAPSLGKPVLVMRDTTERPEAVDAGTVRLVGTDVAKIVEAVTRLL
TDESEYHAMSRAHNPYGDGHACQRILEALKNHQVKL
>CORE_REP|Org3_Gene4268#
MKAFRLAIVRQKYRPDGGAERFVSRALEALEQQDLDLNVITREWQGDANPNWHIHLCNPLKLGRISRE
RGFAVAARALWQKEHFDLVQSHERIPGCDIYRAGDGVHRRWLLQRARLLPEWRRKWLFSNRYHRYVMC
AERAMYAAPELKAVICNAEMIKQEIIADFGVPAEKITVIYNAIDNQKFPPADEALRQRLREQYQIPQQ
AHCLIFVGSGFERKGLAAAIRAVAATDSHLLVVGKDKAEKRYRALAQSLGCGDRVHFMGVQKQTLPFY
QAADALLLPTLYDPFPNVILEAMSCGLPVITSTTCGGAEFITPGQNGFVTDALDVPAIAEAIRALPRQ
ALGAEMGAAARATILPHDAQRLSQQLISLYRKLLTP
>CORE_REP|Org36_Gene3978#
MTETSSLTPLVELHALSKAFDGKTIIADLELAINHGEFLTILGPSGCGKTTVLRLIAGLEDADRGRIV
LDGQDITAIPAEHRHVNTVFQSYALFPHMSVFDNVAFGLRMQKVPAAELTPRVEEALRMVQLDTFAKR
RPGQLSGGQQQRVAIARAVVNKPKVLLLDESLSALDYKLRKQMQNELKALQRKLGITFVFVTHDQEEA
LTMSDRIVVMREGRIEQDGTPREIYEEPKNLFVASFIGEINIFDAVVLQRLDAQRVRANVEGRECDIY

ADLPVEPGQKLKVLLRPEDLRVEEVNDSAQHDGLIGYVRERNYKGMTLESVVELESGKTVMVSEFFNE
DDPDVDHSLNQKMAVTWVESWEVVLADEEIA
>CORE_REP|Org35_Gene788#
MASVTLRSVYKAFGEAVISKDVNLTIEDGEFVVFVGPSGCGKSTLLRMIAGLEDITSGDLLIGEKRMN
EVPPSERGIGMVFQSYALYPHLSVADNMSFGLKLAGAKKAEINQRVNQVSEVLQLAHLLDRRPKALSG
GQRQRVAIGRTLVAEPDVFLLDEPLSNLDAALRVQMRIEISRLHKRLQRTMIYVTHDQVEAMTLADKI
VVLDAGRVAQVGKPLELYHYPANRFVAGFIGSPKMNFLPVKVTAAEPRQVQVELPNRQLVWLPVEGAG
VQPGANLSLGIRPEHLLPGEASEVRLTGDVQVVEQLGNETQIHIQIPAIRQNLVYRQNDVVLVEEGAT
FAIGLPPHRCHLFREDGTACKRLHQEPGV
>CORE_REP|Org46_Gene381#
MLKKWFIALCVGLVCLPAAAERIRDLVTVQGVRDNALIGYGLVVGLDGSGDQTMQTPFTTQSLSNMLS
QLGITVPPGTNMQLKNVAAVMVTAKLPPFSRAGQNIDVVVSSMGNAKSLRGGTLLMTPLKGVDNQVYA
LAQGNVLVGGAGAAAGGSSVQVNQLAGGRISNGATIERELPTTFGSGGVLNLQLNDEDFTLAQQISDA
INRQRGGGTATPLDARTIQVLVPQGNSSQVRFLAEIQNITVNVGAMDAKVIINSRTGSVVMNRDVILD
SCAVAQGNLSVVVDRQNTVSQPTTPFGGGQTVVTPNTQISVQQQGGSLQKVNASANLNNVIRALNALG
ATPIDLMSILQAMQSAGCLRAKLEII
>CORE_REP|Org29_Gene4570#
MKKTAIALAVALAGFATVAQAAPKDNTWYTGAKLGWSQYHDTGFYGNGYQNGIGNGPTHKDQLGAGAF
LGYQANQYLGFELGYDWLGRMPYKGSENNGAFKAQGVQLAAKLSYPITDDLDIYTRLGGMVWRADSKA
NYTTGVSAGQRLSAHDTGVSPLAAVGVEYALTKNWATRLDYQFVSNIGDAGTVGARPDNTMLSLGVSY
RFGQDDVVAPVAPAPAPAPVVETKRFTLKSDVLFNFNKATLKPQGQQALDQLYTQLSSMDPKDGSVVV
LGYTDAVGSAQYNQKLSEKRAQSVVDYLVSKGIPSDKISARGMGKADPVTGNTCGYKAGRATKAQIDC
LAPDRRVEIEVKGIKDVVTQPQG
>CORE_REP|Org32_Gene3251#
MSIEKLARANVRELTPYQSARRLGGKGDVWLNANEYPIAPEFQLTAQTFNRYPECQPAQVIERYAAYA
GVKKEQVLVSRGADEGIELLIRAFCEPGKDAILFCPPTYGMYAVSAETFGVERRTVAAKEDWQLDLPA
IADSLDNVKLIYVCSPNNPTGNLIDPDSLRSLLELAKGKAIVAVDEAYIEFCPQATVAGWLSDYPHLA
ILRTLSKAFALAGLRCGFTLANEDLIALLLKVIAPYPLSTPVADIAAQALSEEGIRTMRQRVTDIAAT
RSWLQQQLEKCACVEQVFASDSNYLLARFTAASNVFKSLWDQGIILRDQNKQPGLSGCLRITIGTRDE
CQRVVDALSALPGANPTRQEPM
>CORE_REP|Org45_Gene3841#
MSVSTDPMTDAKGQLNAGVMGRYQHILRHRLLMMGVLALAILGSLLLDFTMGPSGLSLSSLWQTLLDP
AAADAGTRVIVWDIRLPYALMAVVVGFALGLAGAEMQTILNNPLASPFTLGVSSAAAFGAALAIVLGI
GIPGIPDQWFISANAFIFALFAALMLDGITRWTRVATSGVVLFGIALVFTFNALVSMMQFIASEDTLQ
GLVFWTMGSLARASWDKLGILFGVFAVLLPLSMMSSWKLTALRLGEDRAVSFGIDVRRLRLTTLLRIS
ILSALAVAFVGPIGFIGLVAPHIARMIFGEDHRFYLPASALIGALVLSMASVASKNLVPGVIIPVGIV
TSLVGVPFFLSIILRHRGNV
>CORE_REP|Org46_Gene737#
MQKDALNNVHISAEQVLITPEELKNQFPLSADDENEIATARNTIANILQGRDHRLLVVCGPCSIHDPD
AALDYARRLKTLAADLSDQLYIVMRVYFEKPRTTVGWKGLINDPYMDGSFDVEAGLHIARRLLLDLVG
MGLPLATEALDPNSPQYLGDLFSWSAIGARTTESQTHREMASGLSMPVGFKNGTDGSLGTAINAMRAA
AMPHRFVGINQAGQVCLLQTQGNPDGHVILRGGKTPNYSAEHVAACEKQMLEAGLHPSLMIDCSHGNS
NKDYRRQPAVAESVVEQIKAGNRSITGIMLESHLHEGNQSSEQPRADMRYGVSVTDACINWESTETLL
RHMHQELGAALTARTGEK
>CORE_REP|Org45_Gene3361#
MAGLKLQAVTKSYDGKTPVIKQIDLDVADGEFIVMVGPSGCGKSTLLRMVAGLERTTSGDIYIDTRRV
TDLEPKDRGIAMVFQNYALYPHMSVYDNMAYGLKIRGFGKDHIRQRVEEAARILELEPLLKRKPRELS
GGQRQRVAMGRAIVREPAVFLFDEPLSNLDAKLRVQMRLELQQLHRRLKTTSLYVTHDQVEAMTLAQR
VIVMNKGVAEQIGTPSEVYQRPASLFVAGFIGSPAMNLLPGTLSADGGQLLLADGMALPLPAAKPQWA
GRPLTLGIRPEHIQLVAQGQGVPLQLQTLELLGADNLAHGQWGGHGVIARLSHETLPAAGSTLYLQLP
AQALHFFDTDSGLRMD
>CORE_REP|Org3_Gene3945#
MIELSVENLHLTYGDNPVLKGVSMDLKRGEVVSLLGPSGSGKTTLLRAVAGLEKPSQGRIVIGNNAVY
NGSARSEIPAEERNLGLVFQSYALWPHKTVFENVAYPLKLRKIASAEITLRVQAVLDQLGLGHLAKRH

PHQLSGGQQQRVAIGRALVYNPPVILLDEPLSNLDAKLREEARVFLRELIIKLGLSALMVTHDQNEAM
AISDRILLLNNGKIEQQGTPQEMYGSPTTLFTAEFMGSNNRLPGKIVALEGDRARIEGKDWALWGKAG
EGVQVGQEGSAVIRVERVRLGEDPQGNQLELPLLTSMYLGDRWEYLFRTVAEDFVVRAYGHEARDRAL
CRLSLPAEHLWIFPKA
>CORE_REP|Org20_Gene2219#
MLELDFSQQLGDLNLNVRADLPAQGITAIFGLSGAGKTSLINAIGGLTRLQQGRIALNGRTLVDTAAG
LCLPPEKRRIGYVFQDARLFPHYRVRGNLQYGMAAGMRAQFNTIVELLGIGPLLNRLPLTLSGGEKQR
VAIGRALLTAPELLLMDEPLASLDLPRKRELLPYLERLAQDVNIPILYVSHSLDEILRLAEQVMVLDR
GEVRAFGGLEAVWASSALRPWLQREDQSSVLRVSVIEHHQRYAMTALALGDQRLWVSGIDAELGTQLR
IRINAADVSLVLQPPVNSSIRNVLPAKVSESLDVDGQVEVKLAVGEHVLWARITPWARDELAIRPGQW
LYAQVKSVSISRESR
>CORE_REP|Org31_Gene3705#
MNALITKGKRAAFPLMLLSTLLFSNTLLAQTAPEVLRKPVGKGAYEMAYSPSENALYLATSQSRKLDK
GGIVYRLDPTTLDVTQIIHNDIKPFGAAVNAKTGTLFFGNTVNNSVTAIDAKTGDVKGRLVLDARKRS
ETVKPLAPRELVADADSDTLYITGLGESSVVWVVDGKDLTLRATVTDTGKYGTGLALDAAAKRLYVTN
ADGELVTIDTQSNKVLSRKKLDESKEHFFLNISLDTATHRAFITDSKQPQVLVVDTRNGNILSKIDVP
ESLAVLFNPARNEVYVTHRQAGEVSVIDAKSYKVLNTIKTPTHPNSLALSPDGQTLYVSIKQASSREK
EATAPDDVIRVALK
>CORE_REP|Org7_Gene1147#
MISMRRRLLLMLALILLVTQLISAFWLWHESQEQISFLVDETLSAKVRSERVDTEIAEAIASLLAPSL
IMMIVTLLASFWAISWITRPLNQLQQRLEKRSADNLTPLPITSDSQEMVAVTNALNQLFSRLDNTIQQ
ERLFTADAAHELRTPLAGIRLHLELMEKQGVKGSQALIARIDQLMHTVEQLLMLSRAGQDFASGHYQH
FDWVADVIQPLREELDEMTAQRGQTLAWQLPAAAAVNGDPVLLRLLLRNLVENAHRYGPEGGAIQVRL
TPQDRGYLLQVIDDGPGIKEEMVGELTQAFRRMDQRYGGSGLGLNIVIRIVQLHQGRLTLENRRDARG
LNAQCWLPEKALK
>CORE_REP|Org43_Gene3638#
MNVPTPTERTLSSAPHAAYASGFKRIAGFGIGLALLLLCIIASLMLGSKAIPFHTVWLSLQGAASGSD
STIILNARVPRTLAGILAGMALGAAGALIQALTRNPLADPGVLGINAGASFAVVIGIMFFGAATTESY
MAYAFVGAAVTTLLVYVIGTLAGGRINPVRLTLAGVAIGAVLLGITTGLSLIDPQTFDQLRFWQAGTL
DIRTLATLPVTAPAILLGCLLTLLIARPLNTIGMGEDLAIALGARVVLTQAIAVLAITLLCGAATATV
GPISFIGLMVPHIARWWVGPDQRWILPYSMLLAPILLLCADVVGRLLAAGELRVSIVAAFIGAPVLIW
LVRRKKTLGGL
>CORE_REP|Org38_Gene2549#
MKILVIGPSWVGDMMMSQSLYRTLKAEYPTAEIDVMAPAWCRPLLARMPEVNQALAMPLGHGALGLGE
RRRLGRALRANRYDRAYVLPNSFKSALVPFFADIPQRTGWRGEMRYGLLNDVRVLDKAAFPLMVQRYV
ALAYDKGRVQRADDLPQPLLWPQLRVSDEEIAETTSAFNLTDSRPIVGFCPGAEFGPAKRWPHYHYAA
LAQRLIESGYQIALFGSAKDHEAGEQIRAALQDDARDFCLNLAGKTQLEQAVILIAACRAVVSNDSGL
MHVAAALNKPLIALYGPSSPDFTPPLSDKARVIRLISGYHKVRKGDAEQGYHQSLIDIQPQQVLDALT
PLLVASEE
>CORE_REP|Org40_Gene4771#
MSQKNFVELRNVSKRFGSNTVIDNITLTIPRGQMVTLLGPSGCGKTTILRLVAGLEKPSDGQIFIDGE
DVTHRSIQQRDICMVFQSYALFPHMSLGENVGYGLKMLGIPRAEVKARVQEALAMVDLAGFDDRYVDQ
ISGGQQQRVALARALILKPKVLLFDEPLSNLDANLRRSMREKIRELQKQFDITSLYVTHDQSEAFAVS
DTVLVMNKGHIMQMGSPQDLYRQPASRFMASFMGDANLFPAGFSADHVDISGYRLPRPAHFAAEGAGT
VGVRPEAITLSEHGDESQRCVIQHVAYMGPQYEVTVAWHGQQILLQVNATRLQPNVGEQYYLEIHPYG
MFMLADAA
>CORE_REP|Org10_Gene4169#
MSLPHTLHIGRPGGVINWRMPLRLLLVNLSLLALCLAMAVAALCYGTLQLSLEQVFAALSGEAPKNLV
TVVTQWRLPRIAMALLLGGALGMSGAIFQSIIRNPLGSPDVIGFNMGAYTGALIAITLFNGGYYYIAG
GALAGGILAALAIYLLAWRQGIAGFRLIIVGIAISAVLVSTNTWLIITASLERAMDAAMWQAGSLNGM
TWQKAQPATAFIVLAAAAALLMGKRLQLLEMGDDTARALGVNAEGSRLWLMLFGVTLTAAVTATAGPI
SFIALAAPQIARRLAGQSSVTLTSSALMGAALLLSADVVSQHLFAPIQLPVGVVTVCIGGLYLIWLLI
REARR
>CORE_REP|Org48_Gene1493#

MSIKKITITDVAQQAGVSVTTVSLVLSGKGRISPTTVEKVNQAIEQLGYVRNRQAATLRGAESGVIGL
ILRDICEPFYAEMTAGLSEALEAHDKLLFLTQSGRDGQGLQRAFDALLAQGVDGIVLAGGIRAAAGLK
EKAAEQGVPLVCVARSSGLEGVDVVRPDNMQAAKLATEFLIKRGHSQIAYLGGQSDSLTRAERLGGFC
ATLVQYGLPFRSEWIVECDCRQREAAEAAEQLLRHYPNITAIVCHKASVALGAYFGLTRSGRSIGSDG
VDAYYGRQVALIGFGDVPEAELTEPPLTFVSSSAREVGRSAAARLLQRIGDADLPAQNVILPPTLIRR
GSA
>CORE_REP|Org33_Gene1231#
MTIAVQFIDVSRTFGDVRAVDRVSIDIQDGEFFSMLGPSGSGKTTCLRLIAGFEQLTSGSIRIHGQEA
ANLPPYQRDVNTVFQDYALFPHMSVLENVAYGLMVKGVAKRERLARAQEALESVALGFVAERKPAHLS
GGQRQRVALARALVNRPRVLLLDEPLGALDLKLREQMQGELKKLQRQLGITFIFVTHDQSEALSMSDR
VAVFNNGRIEQVDTPRELYMRPKTPFVAEFVGTSNVVRSELAQRLLGESRTFSIRPEHIRLLEHGGAA
QDEIQVQGTLQEIHYQGAATRYEIALNGGEKLLVSQANPQWIAEGQQRQIGQPIVACWPRAAMVPLLE
ER
>CORE_REP|Org28_Gene1284#
MATIKDVAKRAGVSTTTVSHVINKTRFVAEETKAAVWAAIKELHYSPSAVARSLKVNHTKSIGLLATS
SEAPYFAEVIEAVENSCYSKGYTLILCNSHNNLDKQRAYLAMLAQKRVDGLLVMCSEYPDQLLGMLED
YRNIPMVVMDWGAARGDFTDTIIDNAFEGGYLAGRYLIERGHRDIGAIPGQLSRNTGGGRHQGFMKAL
QEAHIDIREEWIVQGDFEPESGYKAMHQILSQKQRPTAVFCGGDIMAMGAICAADELGLRVPQDISVI
GYDNVRNARYFTPALTTIHQPKERLGEMAFTMLLDRIISKREESQVIEVHPKLIERRSVADGPFIDYR
R
>CORE_REP|Org39_Gene2443#
MPAQRMRSVIPPYMLRRIIEHGNAPQRDCALHTLNHVQSLLGNKPLRSPAEKNARAGEALRDIYDAQN
GTQLPGKQVRKEGQPSNHDVAVDEAYDYLGVTYDFFWQAYRRNSLDNQGLPLVGSVHYGKEYQNAFWN
GQQMVFGDGDGEIFNRFTIAIDVVGHELAHGVTESEAGLIYYQQSGALNESLSDVFGSLVKQFHLQQT
ADKADWLIGAGLLAKGIKGKGLRSMSAPGTAYDDPLLGKDPQPASMKDYIQTKEDNGGVHLNSGIPNR
AFYLAATALGGFAWEKAGYVWYDTVCDKALPQNADFATFARATVKHALARFDQSVADKVQQAWHQVGV
E
>CORE_REP|Org14_Gene1844#
MIVLSNVCKTFDSTQGRVVAVDNVSLAVEAGQIYGIIGYSGAGKSTLIRLLNGLETPTSGRIDVGGFD
IARAKGSHLRQARLKISMVFQHFNLLWSRTVSQNIAFSMQIAGVPKAQIAPRVAELIALVGLQGREDA
YPSQLSGGQKQRVGIARALANNPSVLLCDEATSALDPQTTDAILDLLLDINRQLKLTIVLITHEMHVV
RKICHRVAVMENGRIVEEGPVLDVFTRPQQPITRQFVKQVSQYADTEESFNPLLTAHLPGAIFKLTFV
GVQTHQAVISEVIRRYALTINILHGKISHTLNGSFGELYIHAEGNEQQVADMLSLLHERDIAVEVIQH
D
>CORE_REP|Org32_Gene2866#
MAVLVTGGAGYIGSHTVLALLEHGEDVVVLDNLSNSSDESLRRVEKLAGRSAQFYQGDILDAECLHRI
FEAHAISAVIHFAGLKAVGESTRKPLEYYQNNVTGTLVLLEEMRRAGVHKFIFSSSATVYGTPEQVPL
TETSRVGGTTNPYGTSKLMVEQILQDFAKAEPQFSITALRYFNPVGAHESGMIGEDPNGIPNNLMPYI
AQVAIGKLEKLSIFGDDYPTQDGTGVRDYIHVMDLAEGHLKAIEHIDEHQGFTVYNLGTGVGYSVLEM
LHAFEKASGRNVAYQIVPRREGDIAECWSAPELAFKELGWKATRDLDAMMRDAWNWQKNNPRGYRPG
>CORE_REP|Org45_Gene4005#
MNSLRLLISDSYDPWFNLAVEECIFREMTTQKILFLWRNAETVVIGQSQNPWKECNTRRMEQDGIRLA
RRSSGGGAVFHDLGNTCFTFMAGKPGYDKSVSTDIILQALRQLGVAAGASGRNDLVMETADGPRKISG
SAYRETQDRGFHHGTLLLNADLERLANYLNPDPKKLQAKGITSVRSRVANLAEFLPGISHEQVCDAIV
QAFFAHYGETAEPEIISPDVFPDLPDFAAQFAKQSSWEWNFGKAPAFSHLLNERFVWGGVDLFFDVEK
GAIVRAQVFTDSLNPAPLQRLADMLVGCPYRSEPVAACCDRLMADYPQQAAELAELRQWLSETIR
>CORE_REP|Org40_Gene2850#
MKFLVTGAAGFIGYHVAERLLTAGHQVVGIDNLNDYYDVGLKMARLDRLADKPGFRFIKLDLADREGM
AALFAEHQFQRVIHLGAQAGVRYSLVNPLAYADANLIGHLNVLEGCRHNKVEHLLYASSSSVYGLNRK
LPFATEDSVDHPVSLYAATKKANELMSHSYSHLYSLPTTGLRFFTVYGPWGRPDMALFKFTKAILAGE
SIDVYNHGEMHRDFTYIDDITEAIVRLQAVIPQADPSWSVEQGSPATSSAPYHVYNIGNNTPVKLMEY
ITALEQALGVTARKNMLPMQPGDVMDTSADTAELYRDIGFKPETSVEEGVKRFVDWYKAFYQVQ
>CORE_REP|Org2_Gene3995#
MTLSLRQLFIAFIATSSLLLSGCGPDDQSDGQKPSAPAADSSWPRTIDSAKGKFTLEKPPQRIVSTSV

TITGTLLAIDAPVVASAATSPNPLVADKQGFFTQWSEVAKQRHVERLYQVEPNAEAVAAAAPDLIVVA
ATGGDSALKLYDQLSAIAPTLVLDYGDKSWQQLASELGEITGHEAGAKQAEDRFEQRVEQVKQAIALP
PQPTTPLVYADNGREAMLWTPGSAQGKLLTQLGFQLATPPESAKGNTSMGKRHDIIQISGEKMAEGLN
GKTLILFATNERKVQEVLTNPFLKHLEPVEQRHVYAVGNDTFRLDYYSATNMLNQIERLFKKP
>CORE_REP|Org9_Gene2618#
MPTSRTFTLLLQHQRYRDKRQIGLLALCVAVALLFSLCAGDQWIWPSEWFSDRAQLFVWQLRLPRALA
VMLVGAALAVAGAVMQALFENPLAEPGLLGVANGAGVALVLTVLLGQGLLPVALMSAAAIAGALAMTF
LLLGFARRRRLTNARLLLVGVALGIVCSALMTWAVYFSTSLDLRQLMYWMMGGFGGVDWRQKWLVLAL
LPVLLWLCGQGKALNLMALGEVQARQLGLSLHLWRNLLVLAIGWLVGVSVALAGVIGFVGLVIPHILR
LIGLTDQRYLLPACALAGAGVLLVADVVARIALLAAELPIGVVTATLGAPLFIWLLTRAKGVR
>CORE_REP|Org48_Gene1728#
MGDSILSQAEIDALLNGDSAGDEPEAIVGKESEVKPYDPNTQRRVVRERLHALEIINERFARQFRMGL
FNLLRRSPDITVGPIKIQPYHEFARNLPVPTNLNLVHLNPLRGTALFVFAPSLVFIAVDNLFGGDGRF
PTKVEGREFTPTEQRVIKRMLRLALDAYGDAWSAIYKIDVEYVRAEMQVKFTNITTSPNDIVVTTPFQ
VEIGALTGEFNICIPFAMIEPLRELLTNPPLENSRQEDSHWRETLVKQVQHSELELIANFVDIPMRLS
KVLKLQPGDVLPIDKPERLIAHVDGVPVLTSQYGTLNGQYALRVEHLINPILNALSEEQPNE
>CORE_REP|Org49_Gene1607#
MRCRTSPQLAIIGLLVLLTLLALVAANLGALTLSFRTLWREPFSDAAWHIWLNIRLPRVLLAVVIGCA
LAVSGAVMQGLFRNPLADPSLLGISSGGALFVALFIVMPLALPVTIALYGHMLAAFLGSLLVSLLIYG
ISRSGHGNLSRLLLAGIAINALCMAAIGVLSYVSSDQQLRQFSLWMMGSLSQSQWPTLAVSASLILPA
ALLTLLQARRLNLLQLGDEEAHYLGVNVQRAKLQLLLLSALLIGAAVAMSGVIGFVGLVVPHLVRMRL
GGDHRWLLPCSALGGACLLLVSDTLARTLVAPAEMPVGLMTSLIGGPYFLWLVMRQRERAGG
>CORE_REP|Org14_Gene641#
MATMKDVARLAGVSTSTVSHVVNNNRFVSDSVRDKVMAAVEQLNYAPSALARSLKLNQTRTIGMLVTA
SNNPFYAEVVRGVERSCYERGYSLILCNTEEDAARMNRSMETLLQKRVDGLLLMCTENHRPSQDALSR
YPSLPIVMMDWAPFEGANDIIQDNSLLGGEMATDHLIACGYRKIACIAGPQDKTTARHRLEGYRNAMR
RAGLPVPPGYEVHCDFEFEGGVNAMRQLLALDEPPHAVFAGNDAVAVGVYQALYQAGLSVPQDMAVMG
YDDIELARYLAPPLSTIHQPKDSLGELAIDALINRLQNPERAPQVLVLTPELVERASVGRR
>CORE_REP|Org7_Gene812#
MTAVTDKKVLLEVADLKVHFDIHDDKQWFWQPPKTLKAVDGVTLRLFEGETLGVVGESGCGKSTFARA
IIGLVKATSGRVAWLGKDLLGMSDADWRKTRSDIQMIFQDPLASLNPRMTIGEIIAEPLRTYYPKMPR
QEVKDKVKAMMLKVGLLPNLINRYPHEFSGGQCQRIGIARALILEPKLVICDEPVSALDVSIQAQVVN
LLQQLQREMGLSLIFIAHDLAVVKHISDRVLVMYLGHAVELGTYDEVYHNPQHPYTKALMSAVPIPDP
DKEKEKQIQLLEGELPSPINPPSGCVFRTRCPIAGPECAKTRPLLEGSFRHAVSCLKVDPL
>CORE_REP|Org34_Gene1466#
MQPILEKLYRAESMSQQESQQLFSAIVRGELEPSQLAAALISMKVRGERPEEIAGAAKALLDDAQPFP
RPDYPFADIVGTGGDGTNSINISTASAFVAAACGAKIAKHGNRSVSSRSGSSDLLAAFGIRLDLPAEE
ARKALDDLGVCFLFAPQYHTGFRHAMPVRQQLKTRTLFNVLGPLINPARPPLALIGVYSPELVLPIAE
TLRVLGYQRAAVVHGGGMDEVAIHAPTHVAELNNGEISSYQLTPQSFGLETYPLEALLGGTPEENRDI
LARLLQGKGEPAHAAAVAANVALLLKLFGHEDLRQNARQALDMINSGQAYERVIALAARG
>CORE_REP|Org42_Gene2589#
MAYLNVTRLNKHYGQTQVFQDIDFTAEEGEFVTLLGPSGCGKSTLLRCLAGLTPVDSGQILLQGQDLV
PLAPQKRGIGMVFQSYALFPNMTVEGNVAFGLKMQKLAAGQIGQRVQEVLALVELSDLAKRYPHQLSG
GQCQRVALARSLVTRPRLLLLDEPLSALDARIRKHLREQIRRIQRELNLTAIFVTHDQEEALTLSDRI
VLMNKGQIVQSGDAETLYTQPADAFAAGFIGNYNLLTAEQAAQLTGRSYVGKVAIRPESIGLLPAGQG
IGGVILGHSLLGNVVRYRIQVRGVELLVDVLNRSVADLRPDGEQIGLHLEPVVLREVA
>CORE_REP|Org8_Gene900#
MSQNQPLLQAIDLKKHYPVKKGLFAPERLVKALDGVSFTLERGKTLAVVGESGCGKSTLGRLLTMIEV
PTGGELYYQGQDLLKPDVSAEKLRRQKIQIVFQNPYGSLNPRKKVGQILEEPLLINTSLSAAERREKA
LEMMAKVGLKTEHYDRYPHMFSGGQRQRIAIARGLMLNPDVVIADEPVSALDVSVRAQVLNLMMDLQQ
ELGLSYVFISHDLSVVEHIADEVMVMYLGRCVEKGSKEAIFNNPRHPYTQALLSATPRLNPDMRRERI
KLTGELPSPMNPPPGCAFNARCRRAFGTCVQLQPQLKQYGEQMVACFAVDQDEHPGA
>CORE_REP|Org2_Gene2735#
MPRVEPIKKVSVVIPVYNEQESLPALLERTTAACKQLSQPYEIILVDDGSSDNSADMLTAAAEKPDSH

VIAVLLNRNYGQHSAIMAGFNQVTGDLVITLDADLQNPPEEIPRLVSVAEEGYDVVGTVRANRQDSWF
RKSASRVINMMIQRATGKSMGDYGCMLRAYRRHIVEAMLHCHERSTFIPILANTFARRTTEIDVRHAE
REFGDSKYSLMKLINLINLMYDLITCLTTTPLRLLSVVGSIVALSGFVLALVLIALRLLLGPEWAAGG
VFTLFAVLFTFIGAQFVGMGLLGEYIGRIYTDVRARPRYFVQKVVGAQQGHNTQEEE
>CORE_REP|Org41_Gene1531#
MSNIELQPGFDFQQAGKEVLQIEREGLAQLDSYINADFTRACETIAACGGKVVVMGMGKSGHIGCKIA
ATFASTGTPSFFVHPAEASHGDLGMVTPQDIVLAISNSGESSEILALIPVLKRQQITLICMTNNPESS
MGKAADIHLCIKVPQEACPLGLAPTTSTTATLVMGDALAVALLKARGFTPEDFALSHPGGALGRKLLL
RVSDIMHSGDEMPHVSADASLRDALLEITRKNLGLTVICDDLMKIAGIFTDGDLRRVFDMGINLHEAK
IADVMTPGGVRVRPNILAVDALNLMQQRHITALLVADGDQLLGVVHMHDMLRAGVV
>CORE_REP|Org46_Gene3314#
MSETASWQPSAPIANLLKRAAILAEIRRFFADRGVLEVETPTMSQATVTDVHLFPFETRFVGPGAAEG
LTLYMMTSPEYHMKRLLAAGSGPIYQMGRSFRNEEAGRHHNPEFTMLEWYRPHYDMYRLMNEVDDLLQ
QVLDCENAETLSYQQAFLRHLDIDPLSAEKAQLREAAAKLDLSNIADTEEDRDTLLQLLFTVGVEPHI
GREKPAFVYHFPASQAALAEISTEDHRVAERFEVYFKGIELANGFRELTDGREQQQRFEQDNRKRAER
GLPQQPIDYNLLAALQHGMPECSGVALGVDRLVMLALGAESLSDVLAFPVGIA
>CORE_REP|Org1_Gene3115#
MSNANALLAFARETLEIELTEAQRLLARLDDNFVCACELLLNCRGKAVISGIGKSGHIGKKIAASLAS
TGTPSFFVHPAEALHGDLGMIGADDVVVFISYSGRAKELDLILPLLAENGIPVIAVTGGKESPLTQAA
ACVLDIGVEREACPMGLAPTSSAVNTLMMGDALAMALMRQRGFNAEDFARSHPGGSLGARLLNRVHHL
MRTGDRLPRVSESANVMEAMLELSRTGLGLVAVCDAQQRVVGVFTDGDLRRWLVKGNSLQDPLSPAIT
RPGYRLPEQWRAGEALEALHEQHISAAPVVDMDGVLVGALNLHDLHQAGIG
>CORE_REP|Org41_Gene1675#
MQVLIVKTSSMGDVLHTLPALTDALQAIPDIRFDWVVEEGFSQIPTWHPAVDRVIPVAIRRWRKNWFG
NDTRQQRCDFKRALQERRYDVVIDAQGLIKSAALITRIAKGNKHGPDCKSAREPFASWFYNVRHEIDK
QQHAVERTRELFAKSLGYDKPGSYGDYAIAARFLSRPPADAGQYLVFLHATTRDDKHWPEQNWRELIA
LTADSGLKIKLPWGAEHEHQRALRLAEGFSHVEVLPKLSLQQVAEVLAGAKGVVSVDTGLSHLTAALD
KPNITLFGPTDPGLIGGYGQNQHSLISPEKSMATIDADTAWQALQKVIA
>CORE_REP|Org18_Gene1250#
MKQIWFSVCLLTGSLLYSSIAPAQPTASGALLQQMSSASRSLNYELAYISISKQGIESLRYRHAVIGN
VPLGQLLHMDGPRREVLQRGGGISYFEPGLEPFTLTGDHIVDALPAIVYADFTRLAKYYDFISVGSTR
IADRPCEVLRVVARDGSRYSYIVWMDEDTKLPLRVDLLDRDGETLEQYRVISFAVGADVQGAMQGLLK
ANLPPLLSLPAVENVKLSWSTGWLPAGVDEVARNRRKLPNVAVPVESRLYSDGLFSFSVNVSPAGSGA
GQQYYRQGRRTIQTEVRAGNEITIVGELPPATAKRIADSISFKVSPQ
>CORE_REP|Org16_Gene3401#
MSNAITPEQRQALKMAAHWYALLCDEHVTERQRQQWQAWHQQHDDHRWAWQRVEALQSQLQGVPGKFS
YRALDRADRQSAIDRRTLLKSLLLLLGVGGGGFFYQSPLGRELRADYRTATGEIKPIVLSDGTQLVLN
TASAVDVHYDDRQRLIRLHAGEISLVTGRDPRPLWVQSPQGAMRALGTRFLVRESDGETRLAVLEHAV
EAQLAQDAQQKRRVNAGEQISFSTTAFSDKQPATTEDGWLRGVLSVSQWRLDRVIAELARYRRGHLSC
DPAVAGLRVSGSFPLNDTDRALALLSQTLPVRLQSFTRYWLQIVPA
>CORE_REP|Org34_Gene1075#
MSDKIPIGISACLLGDAVRFDGGHKRLAFAVEQLAPYVRFEPVCPEMAIGLPTPRPALRLVKQAQPWP
AMRYSNDAGVDLTEEMRSFSAQRVAALQHLCGYIVCAKSPSCGLERVRVYSENGKDSRKNGVGLFTAE
LLRQMPWLPVEEDGRLQDAALRENFIERVYALYELNMLWRQGLTRGGLIAFHSRYKLSLLAHSQPAYR
ELGRFVADIHRWDSLEAFAVEYRSRLMALLAHKATRRNHTNVLMHVQGYFRRQLSAAQRQELAHLIDR
YRQGMQPLLAPIALLKHYMAEYPDRYLAEQRYFEPYPEALRLRYGH
>CORE_REP|Org8_Gene3849#
MPLISCAFHRSRPARAAALLRPFTLACLLLGAALVSQNALAEKKLRVVTTFTIIQDIAQNVAGDAAVV
ESITKPGAEIHDYQPTPRDIVKAQHADLILWNGMNLERWFQRFFENIKQVPAAVVTEGITPLPIREGP
YNGNPNPHAWMSPSNALVYIENIRKALVEHDPAHAETYNRNAKAYAEKIGALDAPLRERLARIPAAQR
WLVTSEGAFSYLAQDYQLKEVYLWPINADEQGSPQQVRRVIDAVRAHHIPVVFSESTISDKPAKQVAK
ETGAKYGGVLYVDSLSTRDGPVPTYIDLLNTTVQTIAKGFDQ
>CORE_REP|Org30_Gene1499#
MIHFEQVSKIFQGKPAVDDLTLHIAEGEFTVLIGTSGSGKSTTLKMINRLIEHDRGKILFAGEEIQSF

KPQDLRRRMGYAIQSIGLFPHWTVEENIATVPQLLKWPRARIRDRVTELLELLHLEPDLFRRRYPHQL
SGGQQQRVGVARALAADPEVLLMDEPFGALDPVTRAALQAEIARIHQLSGRTIVLVTHDIDEALGLAD
RLVLLDQGRVVQQGTPLALLTAPANDFVRDFFGRSDRGIKLLSLGTVAERVRPGAAEGEPIAAAMSLR
EALSVFVARGSDCLPVVDERGEALGVLHFNDLIAGQALS
>CORE_REP|Org17_Gene4455#
MLLSHRHFICTLLALAIGFWLPTAVQARPDLERRIGVTVADSDSADYRFSDLRFTSADGQRRYRVRIA
QPRRAPAPDGYPTIYFLDGNAVLMELNASLLARLATAKRPPVLVMIGYDNDLRIDAAGRAYDYTLPLP
TGMTGMTGMKKSPQAGGGAEAFLQLIETRIKPAIAAKLAVDQQRQTLWGHSYGGLFVLHTLFAHPAAF
QHYIAVEPSLWWGNGMILQEAQQWAERHPTPAARLQLWVGLAERDRAAPPGVKSPALPANAAQMLAER
LAKLDGLTVGYREWPGLGHGAMLGAAIEPALNSVAYED
>CORE_REP|Org5_Gene2049#
MFPSKKHSQRATPLTSYQFSRLHTFECVARHLSFALAAQELSITPSAVSHRINLLEKELGFLLFQRFH
RRITLTPEGERMQWALDSSFNTLNQEILDIKNRELTGTLTLYSHPSLVQCLLLPRIGDFIAQHPTIHL
NILTGQEIINLANRGVDLAMYFGKLPSGRHLDEAFMQESMVPICTPQYAAAHSLYDAPENLAHCTLLH
DRYNSGEDEWQTWSQHFALGLDTDSKSMEFDRSDLAVLAATRHLGVAMGRLNLVQDWIKSGELIIPFT
DMTVPCEHCYFTSTISERQWPKILAFKQWIMKIAPLV
>CORE_REP|Org24_Gene644#
MIIVTGGAGMIGSNIIKALNDKGYRDILVVDNLKDGTKFVNLVDLDIADYIDKEDFIASIVAGDDLGD
IEAVFHEGACSATTEWDGKYMMDNNYQYSKDLLHYCLDREIPFLYASSAATYGGREEFIEEREYEAPL
NVYGYSKFLFDQYVREILPEADSQICGFRYFNVYGPREGHKGSMASVAFHLNTQINRGENPKLFAGSE
NFKRDFIYVGDVAAVNLWFWETGKSGIFNCGTGRAETFQAVADAVVDFHQKGAVEYIEFPEKLKGRYQ
AYTQADLTKLRAAGYDAPFKTVAEGVKEYMAWLNRTA
>CORE_REP|Org21_Gene3163#
METGKLVVLGSINADHILNIEQFPHPGETVIGKQYKVAFGGKGANQAVAAGRSGAEIAFIACVGADDI
GERVRRQLASDRIDTQPIEAIADSTTGVALIFVNAEGENVIGIDAGANAAVTPDYLARYQQKVIDADA
LLMQLESPLETVIAAARLAKQYHTQVILNPAPARELPDELLGMIDMITPNETEAQRLTGIAVDNDADA
ARAAQALHDKGIATVIITLGSRGVWLSENGNGKLVPGFKVQAVDTIAAGDTFNGALVTALLEGKIMAD
AVRFAHAAAAIAVTRPGAQPSVPWREEIDAFLQQQG
>CORE_REP|Org4_Gene4035#
MKGKVCVFGSFNLDIVAGMARFPQPGESLIARNSMMGAGGKGANQATAALRAGARVHYIGKVGRDDFG
TFARRHLATAGFDAVTLFSTGDCPTGNALIYVAGEEAENMIAVDPGANLTVSEDEVRQCRPAIAAADI
LLTQLENNLPAIEQVIAIAREAQTFIILNPAPFQPVPDSLLAQVDMLTPNATECTLLTGVPVRDVASA
RQAAQVLHAKGIRLLIVTLGTQGALFSDGENSELIPAFPAQPKDTTGAGDAFNGALAAQLANQVPLAD
AVRFAAAYAAVCVERAGAAGSMPSYEEALERQRAFA
>CORE_REP|Org40_Gene3137#
MPISLPSLDVLKTFVVVAQRLNFTHAARQLHLTQGAVSRQILGLEQRLGYPLFSRQARGLALTPQGAQ
LLAPVQQALGQLDEALTRAAAPPGALRIKCPTCAMRWVLPRIIRLQNERPDMHIELTASVSHGLDFST
EQFDAAVVFGRPPGKKLTAHLLFDEILTPVCTPTFLPPTPRLTDLTDKTLLHPTRDRRDWLRWLKAAG
ADALPSGKAQHFDTLDLAMSAALQGFGIAIGDLCLLEEDIQAQRIVTPFPLCVSSGAAYYLVYPERTV
APPTLTALVDFLAAEAADSRARLQNYLPMTCNAL
>CORE_REP|Org5_Gene1939#
MPAVNRKVRKAVIPVAGLGTRMLPATKAIPKEMLPLVDKPLIQYVVNECIAAGINEIVLVTHSSKNSI
ENHFDTSFELEAMLEKRVKRQLLDEVQSICPKGVTVMQVRQGNAKGLGHAIMCAYPMVGDEPVAVVLP
DVILDEYSADPKKDNLHEMLQRFETTGVSQIMVEPVPHKDVGNYGVADCKGVDLQPGESAPMVSVVEK
PSPDKAPSNLAIVGRYVLSADIWPLLAKTPPGAGDEIQLTDSIEMLMQQETVEAYHLKGVSHDCGNKL
GYMQAFVEYSMRHASLGKEFSQWLQQVVAADKK
>CORE_REP|Org20_Gene743#
MIKQRTLKRIVQATGVGLHTGKKVTLTMRPAPANTGVIYRRTDLNPPVDFPADAKSVRDTMLCTCLVN
EHDVRISTVEHLNAALAGLGIDNIVIEVDAAEIPIMDGSASPFVFLLLDAGIEELNSAKKFLRLKETV
RVEDGDKWAELSPHNGFRLDFTIDFNHPAIDASSQRYRLDFSAESFVRQISRARTFGFMRDIEYLQSR
GLALGGSFDCAIVVDDYRVLNEDGLRFEDEFVRHKMLDAIGDLFMCGHNIIGAFTAYKSGHALNNKLL
QAVLAKQEAWEYVTFQDEAEMPLAFKAPSTVLA
>CORE_REP|Org22_Gene2033#
MFFNLQRYSTHDGPGIRSVVFLKGCPLSCRWCQNPESRSRRADLLFDERLCLSGCTLCTERCPQGLRR

NEEALTLQRDVISADDYAALAAACPTGALSLCGSAVNPDDIMAEVMRDKPFYLRSGGGLTLSGGEPFM
QPEAAAELLRRGREAGIHTAVESCLHVPWRYIAPSLPWLDLLLADLKHTDEARFKAWTGGSARRVMNN
FRRLAAHGVPMTVRVPLIPDFNADRHSVRAIVDFAADEIGVSEIHFLPYHTLGINKYHLLGEPYRAAR
TPLDAPDLLAFAEAYAGAKGLTAILRG
>CORE_REP|Org2_Gene3088#
MHSPSRARLPKLSAILAFETAARTGSLARAADTLALTAAAVSQQIRQLEQHLGITLFIRAKSGVTLTE
QGADYLAYVQEAFETLRVAQQHVERQRGKQALTVFALPALASKWLNPALGDWLAQCPDGDLRLHATHA
AVDFAHSAADFALCFGDQDYPLLEKVRLFQDRVQPVCSPALRDRGDWTQLPLIHVDWGKESQFLPGWH
EWFTAADRMPPARRGLTYNLTSLAIDAAVQGRGVLLGQRRLIGRELAAGQLVTLAEPALPLSKPYYVV
YPPRTLEKPGAAAFLAWLQTLASTDQA
>CORE_REP|Org19_Gene3737#
MFATLPVNALRTFESAARLRSFKLAAAELAVTPTAISHQIKALEQQLGFALFERVPRGVRLTPKGETL
FAGVHGALLDVAATLEGLRPQPSTGSLCVSVTHSFAALWLVPRLGRFYQAYPHYLVRLEACAEVIDLQ
QDASVDVAVRYSRAQYPALHQTARLEESFGVYAAPGLAAAEPENPVLITVKWGDSALYDSGWRDWCRA
AGVDWWQRHAAMRSYHEEHYALQAAVAGQGIVLASSVMVSDMVDNGLLVAYRPEVRVPGAAYSVLCAP
GRERHPPVRAFLAWLQQELPQGNGTK
>CORE_REP|Org44_Gene1228#
MKRPDYRTLQALDAVIRERGFERAAQKLCITQSAVSQRIKQLENLFGQPLLVRTVPPRPTEQGQKLLA
LLHQVELLEEEWLGNDTGVDTPLLLSLAVNADSLATWLLPALKPVLADSPIRLNLQVEDETRTQERLR
RGEVVGAVSIQPQPLPSCLVDRLGALDYLFVASSAFAERYFPSGVTRSALLKAPAVAFDHLDDMHQAF
LQQNFDLSPGSVPCHIVNSSEAFVQLARQGTTCCMIPHLQIEKELASGELIDLTPGLYQRRMLYWHRF
APESRMMRKVTDALLEHGHQVLRQD
>CORE_REP|Org45_Gene2437#
MTNEDIFFIEELIEWVEIHLEKRPNLDEVARISGYSKWHLQRKFKRITGIQLATYIRSRILTRAAVAL
RITRRSIIDISDELGFDSQQTFTRMFKQRFGTTPNRYRSMTHWDVKNLMPRFNFDASYGAGYYPEVKR
LTLPEMQLVGFTRRLDFASEQELEYSSCMAMKDEIFNDFFKGLHVDCRRIYSIYSPHAGEGDELSSTL
VMAVDPEHKKDILSNYQIDTFHLPSREFISINHKGSAKECLQFFGYLMSHVMPGLKDEVRGSMEMEII
QTKEWNPESKLRQIDVDYTYLISID
>CORE_REP|Org45_Gene2599#
MLVILGYLVVLGAVFGGYLIVGGHLGALYQPAEFLIIGGAGVGAFIVGNNGKAIKATLRALPRLMRRS
KYNKDLYMDLMALLFRLLAKSRQQGMLSLEFDIDNPQESEIFSNYPRILADNTLVEFITDYLRLMVSG
NMNAFEIEALMDEEIETYEQESEVPAGSLAMVGDSLPAFGIVAAVMGVVHALASADRPAAELGALIAN
AMVGTFLGILLAYGFISPLATLLRQKSAENVKMMQCIKVTLLSSLNGYAPQIAVEFGRKTLYTTERPS
FVELEEHVRRVKAPAQQVTEEEEA
>CORE_REP|Org5_Gene3514#
MRKPRLPPLGALRAFHAVAGCRSFKLAAEALGVSATAVSHQIKLLESVLACRVCERSAQGVSLTETGE
ILYAGTQRAFAALEQSVAQITRAQQPPALTVTTTSNFLTHWLVPRLADFKAEFPAIDLRLHTSVERVD
LSQRTVDVAIRYRETPESDLHCTLLHEDRFIVVASPALALERSEDLQRVTLFHVEHRQVPADAPTWEN
WRRRYGPEGLNVEAGLTFSDETHALQAAVAGQGVVIASRLLARDLLQRGVLAAPFETALPGANYYLVA
TEETAQRPDIIALREWLLRQMAAG
>CORE_REP|Org35_Gene1585#
MRNRLPLNALRAFESSARHLNFTRAGLELRVTQAAVSQQVRMLEEQLGIQLFRRLPRGLDLTEEGQAL
LPVLSDAFDRIEAVLQQFEGGHFHEVLTVAVVGTFAVGWLMPRLAAFRAAHPFIDLRVLTHNNLVNLS
ADGMDFAIRFGEGLWPATRNIKLFDAPLTVLCSPAVAARLHTPKDLQHELLMRTYRQDEWERWFTAAQ
VTPWRINGPVFDSSRLMVEGALQCDGVALAPVSMFRRELAAGALQRPFAAEAALGAYWLTHLKSRDLT
PAMKAFIGWICREAEEEQRRD
>CORE_REP|Org41_Gene3095#
MKRLPISLAVAALLASPWAMAKTVDAVASFSILGDIVKQVGGDHVKVSTLVGPDGDPHSFEPSPQDGK
KLAQADVVFVSGLGLEGWIDRLVSASGYKGQVITASQGISTRQMEEDGKPITDPHAWNSMKNGVQYAT
NVMNALIAADPEDANYFRQRGADYIQQLQKLDLWAKTQFAAVPPQKRKVLTSHDAFGYFGQEYGVTFL
APVGFSTEAEASASDVAGLIKQIKQEKVNAYFIENQTDPRLVKQIAAATGAKAGGELYPEALSRAGGP
AATYEQAFKHNVDALLSSMK
>CORE_REP|Org16_Gene2285#
MDQAGIIRDLLSWLESHLDQPLSLDNVAAKAGYSKWHLQRMFKDITGNAIGAYIRARRLSKAAVALRL

TSRPILDIALQYRFDSQQTFTRAFKKQFAQTPALYRRAEDWNAFGICPPIRLGAFTLPQPEFVSLPDK
HLVGLTQSYSCTLEQITTVRTELRSQFWRQFLGDVETLPPVLYGLHHSRPSQEKDDEQEVLYTTALEP
DQVPDKVQEGQPLVLPGGEFAMFSYEGPTENLQDFILTVYGTCLPALQLTRRKGHDIERFYPKGERRP
HQAPIEIKCDYLIPIRR
>CORE_REP|Org36_Gene2971#
MGTQESHIKELLVWIEDNLTNPLSLDIVSAKSGYTKWYLQRMFKKQTGLSLASYIRARRLYLAAFALR
FTQKSILDISVEYQFDNQQTFSRCFKKHFAESPSVYRHARKQDFSNLVRSLAASQPGDIQVERVSIAR
GQYAFHGKQYAYHLDIEKLDKSHLPQRSALRGQFYTLLGERPTQTYSFTQLVPDGERVRVDYTLGVTT
EYPLREGVVLEPLPEIHGEFCRFRYSGKPVALNDHIIQIYTQVLPEMGLARGDGPDITVFSYSLSGKE
ELHLELQHLVPVVPLH
>CORE_REP|Org28_Gene4729#
MLKHWPPLSALRGFEAAARLSSFHQAAEELHLTQSAISQQIRSLEAFLEQPLFFRTGRSVTLTDAGHD
LFSTAQVMLQQLAVGIRRLDQYRKPNQLIVNTTPAFARHWLMPRLGDFNRQHPQADLWLFTSFEPPNM
ATDSIDLAIRDDLSAQADCTFNVLCSDRLYPACHPSLLALAAEQRMTLHGEREMDWSHWTVAGGAHVG
QRDSGLNFSDPGLLLDAACQGLGIALVSQLLAQQARDAGLLQPLTEQRVRGANWAWLLHRDSEHNPLT
RHFCQWLQSALPAGA
>CORE_REP|Org21_Gene2638#
MAIPKLNDYALPTADELPQNKVTWQVEPQRAALLIHDMQQYFLNFWGEDSALIKQVVENIANLRRYCK
QQDIPVFYTAQPNQQSDEDRALLNDMWGPGLNKHPEQQAVTAALAPDEDDTVLVKWRYSAFHRSPLQE
ILQESGRDQLIICGVYAHIGCLTTAIDAFMRNIQPFMVADGLADFSRDEHLMALRYTAGRCGRVVTTA
SLLPAAGIASIDALRQQILPLLDEDSEDMGNDENLIDYGLDSVRIMELATRWRKIRGDIDFIALARNP
TIDSWWALLSEEKA
>CORE_REP|Org13_Gene1299#
MTKEMQTLALVPQGSLEAYIRAANAYPMLTAEEERELAERLHYQGDLDAAKQLILSHLRFVAHIARNY
SGYGLPQADLIQEGNIGLMKAVRRFNPEVGVRLVSFAVHWIKAEIHEYVLRNWRIVKVATTKAQRKLF
FNLRKTKQRLGWFNQDEVELVARELGVTSKDVREMESRMAAQDMTFDPTPDDEARDGQAMAPVLYLQD
KSSDFAEGIEEDNWESNAADKLAYALEGLDERSQHIIRARWLDDDNKSTLQELADQYGVSAERVRQLE
KNAMKKLKMAIEA
>CORE_REP|Org19_Gene1426#
MMLWHGLIDPFLSFGFMRRALMACLALSLSAAPLGVFLLLRRMSLVGDALSHAVLPGAAIGYLISGMS
LVAMGVGGFIAGLAVAMLSGLVSRRTPLKEDASFAGFYLGSLALGVTLVSLRGSSVDLLHVLFGSILA
VDAQAMLMVGAIASVSLLALAALYRALVIESFDVTFLRVNAPRRLALIHGLFLALVVVNLVAGFQILG
TLMSVGLMMLPAASARFWARNLPQTLATAMGIGALSSLIGLVWSYYASLPAGPAIVLSASVIFFVSIL
FGTRGGIYAFARR
>CORE_REP|Org15_Gene3670#
MDTLYQLLSEPFAYPFMQRAIVAAIVTGVVCAVLSCYLVLKGWSLMGDAISHAVLPGIVVAFVIGIPL
AIGAFLSGIFCAVATGYLKENSRVKEDTVMGIVFSGMFAFGLVLFSRIDTDQHLSHILFGNMLGITDG
ELKQTLLIAGLTLAVVLLKRKDFMLYCFDPHHARVIGLPVKLLHYGLLCLLAMTIVASLQAVGVILVI
AMLIAPGIIAFMLCRRFDRMLMVATVVSVFSCVLGTLISFHIDGATGPCIVIVQAVLFVIALLYGKLR
PLQRNQTALSDS
>CORE_REP|Org12_Gene1894#
MIIIETLPMLRQQIRRWRQEGKRIALVPTMGNLHDGHMTLVDEARARADVVVVSIFVNPMQFDRPDDL
ARYPRTLQEDSEKLTRRGVDLVFAPAPAAVYPQGLEQQTYVDVPGISSILEGASRPGHFRGVSTIVSK
LFNLVQPDLACFGEKDYQQLALIRKMVADMGYDIDIVGVPTVRAKDGLALSSRNGYLTAEERKVAPQL
SKIMNALAQQLANGERQVEALLEQTAEQLRAAGFTPDELFIRDADSLQPLTVDSQRAVVLMAAWLGKA
RLIDNQQVDLTL
>CORE_REP|Org20_Gene2558#
MLNILVKNISKRLPDAWHHQVKYALYFKKLPHLSKPTGFSEKIMRRKIYPRSIYTTLSDKFKVREFIA
GLWGEEYLVELYAHGTELSYDMFRQLPNAFVLKANHGSGYNRLVFDKRQVSYAELYDLSNAWMRSNFY
EQSREKHYLDIEPCIMVERMLLDGEQVPNDIKFHCFNDNHEIRMFIQVDYQRFGTHRRDIFDVDWNRT
EIRISLPNADEPMPRPTRLDEMIRLARQTAQQFSYVRVDFYQVGEKVYFGELTFTPGAGLSKLMPKNI
EQEWGSYFTE
>CORE_REP|Org47_Gene769#
MKSLFKVTLLATTMAFALNATQVMAADAAKPAEAAKPADAAAAPSTGKFKNDDEQAAYALGASLGRYM

DNSLKEQEKLGIKLDKDQLIAGVQDAFANKSKLNDADIEKTLQGFEARVKASAQAKMEQDAKDNETKG
AKYRDSFAKEKGVKKTESGLLYQVEKPGAGEAPKDSDTVVVNYKGTLTDGTEFDNSYTRGEPLSFRLD
GVIPGWTEGLKHIKKGGKIKLVIPPALAYGKTGVPGIPANSTLVFDVELLDVKAAPKADAKAEKPADA
KADAKAK
>CORE_REP|Org44_Gene4808#
MQGVPQQFPFEKDCAQFRHLSHLPGVELYQAHIERYAFEPHTHDAFAIGTVDTGAERFRYRGAQHLAA
PGALVLMNPDELHTGEAETPGGWCYRMLYLAPEALEQLSGARSQWFTDAVRHDPRAAQRLSAILATLW
QTDDPLTLDGLLLEAVELLYPHIRTGQREKAEAAHRFEVVKSYLHDNFAEAVTLNQLAELVSLSPYHF
LRKFKAEYHVSPQQMLMAIRLSQAKRMLERGMPAAQVAAAAGLTDQAHLTRAFANRYGVTPVRFQKQV
KLG
>CORE_REP|Org48_Gene1124#
MSEAIDYYALPEQPVRNLTPVPARGLIEISNVSKFFGKHKALDDVSLTLQPGTVTVILGPSGSGKSTL
LRAINHLERVDEGFIRIDGDYVGYRRKGNRLYELKEKAILRQRINVGYVFQNFNLFPHLTVLENIIEA
PVVHKIHSRERAKAVAYELLDTVGLRHKADAYPRHLSGGQQQRIAIARALALNPKVILFDEPTSALDP
ELVGEVLDVIKGLADLGVTLVVVTHEIGFAREAADRVVFMVDGQIVEQGDARQVLSQPQHPRTVNFLN
KVL
>CORE_REP|Org23_Gene4353#
MKSLASTLQGQSIAAAITAVENDIKAKPADADLRAALVQLLCLSGNWTRANAQLKSWQALKPIAQPTT
LLLMQSVNAELQRQAVFAGAAAPALLRQDQPWLQLLVQALHQDAQGAAEQAQTLRDEALEAAPAGAGQ
LTLAEGNQERQLSFDWLTDGDGRLGPVCELALNGVYYWLPFADIAAIQFQAPQSAIDLVWSHALVRLT
DGREQVCQLPARYPLAEGSDDALLLGKRTEWQPLGDGTHYAGLGLKTWLSESDEFPLHSLRQLSFDAS
A
>CORE_REP|Org20_Gene834#
MLQKADNLVEVRDMSFSRGDRRIFEDINLTVPRGKVTAIMGPSGIGKTTLLRLIGGQLAPDSGEIWFD
GDNIPALSRRQLYDARKKMSMLFQSGALFTDLTVFENVAYPLREHSNLPEPLLRSTVLMKLEAVGLRG
AAQLMPNELSGGMARRAALARAIALDPEMIMFDEPFVGQDPITMGVLVKLIDELNHALGITCIVVSHD
VPEVLSIADYAYIVADHRVIAEGTTQQLQNNPDARVRQFLDGIADGPVPFRYPAGDYQTELLGLGSK
>CORE_REP|Org40_Gene798#
MGLMTPGSLPRLDVQHLDDEQTALAVNGLNLFYGDKQVLHDISLRIPKHRVTALIGPSGCGKSTLLRC
FNRMNDLVDNCRIEGDLQLNGAAISGAQIDVAALRRRVGMVFQRPNPFPKSIYENVVYGLRLQGVRDR
RLLDEAVERSLRAAALWHEVKDRLRENAFRLSSGQQQRLVIARAIAIEPEVLLLDEPTSALDPISTLT
IEELISALKQRYSVVLVTHNMQQAARVSDYTAFIHQGRLVEYNDTDAIFTSPRQRRTEDYITGRYG
>CORE_REP|Org6_Gene848#
MSHRLHASHLKLGYDNKIIADDLSVAIPDGAFTVIVGPNACGKSTLLRALCRLLKPSAGEVMLDGKNI
SSFATKALARELGLLPQTSIAPDSITVADLVSRGRYPHQSLLKQWTQADKQAVEAAMAATNVSQLADR
SVDELSGGQRQRVWVAMALAQQTPLLLLDEPTTYLDIAHQIELLDLFRQLNRERGQTLIAVLHDLNHA
CRYADHIIAMRDGKIVAEGKPAEIITAELVERVFGMPCMIIDDPLSHTPLVIPRGRYHCDAPQA
>CORE_REP|Org48_Gene2882#
MQDKLLNPGAAFALDNASFAVPGRVLLQPLSLSFPQGKVCGLIGHNGSGKSTLLKLLGRHQAPSGGQV
LLNRQPLAQWDSKSFARQVAYLPQQLPAAEGMTVRELVAVGRYPWHGALGRFGANDRQLVEEAISLVG
LKPFANRLVDSLSGGERQRAWLAMMVAQDSRCLLLDEPTSALDIAHQVEVLALIQRLSRERDLTVIAV
LHDINMAARYCDHLVALRGGEMIAQGGPLELMQGPVLEQIYGIPMGTLPHPSGGAPVSFVY
>CORE_REP|Org16_Gene4625#
MSATYAANAFAGQVVLVTGGAQGIGLAIVSAFARLGAEVTIADVQLPQAQAAAQTLRDEGLSVQALAC
DLAEPGQIAELVAAVGERHQRLDVVIHNAAYFPLTPFAAIDAALLQRTLSVNLMAPFFLAQAALPWMR
HRGGGCILVTSSVTGPRVAYPGLAHYAASKAGVNGFIRAAALELAAENIRVNGVEPGMIRTPAMANLG
DAQVNQAIAASVPLGRLGEPADIAAAMVFLASPAAAYITGQTLVVDGGALLPETNSLLT
>CORE_REP|Org42_Gene2137#
MSQGLRIEHFSAGYPKRQVIDDLSVPMLPRGQITVLLGPNGSGKSTLLRSLAGLNPAQGKLWLDDGDL
MQMPFARRAEKVVYLPQSLPAGVHLHVLESIIVAQRASGGRSNAGSEAEVMALLEQLGIAHLALSYLD
QLSGGQKQLVGLAQSLIRQPSLLLLDEPLSALDLNYQFHVMDLVRRETRKRNIVTVVVVHDINIALRH
GDHVLMLQDGDLIADGAPDQVITPQSLARVYGVRGRIERCSQGTPQVLIDGLVNQPTI
>CORE_REP|Org10_Gene1322#
MDNAACLTARELRYSLGTRRLINDVSLSLASGEMVAIIGPNGAGKSTLLRLLTGYLTPDCGECRLLDR

PLEHWAPQQLAKVRAVMRQYSDLAFPFSVEEVVSMGRSPHGKRDEHQAIQQVMEQTDCLALAQRDYRR
LSGGEQQRVQLARVLAQLWQPQPSPAWLFLDEPTSALDLYHQQHTLRLLRSLTRQQPLGVCCVLHDLN
LAALYADRILLLHQGRLVASGTPQEVLQTEILTRWYQADLGVVHHPEVSLPQVYLRQ
>CORE_REP|Org7_Gene348#
MIPSLWIAKTGLDAQQTNMDVIANNLANVSTNGFKRQRAVFEDLLYQTMRQPGAQSSEQTTLPSGLQI
GTGVRPVATERLHSQGNLSQTNNSKDVAIKGQGFFQVMLPDGTQAYTRDGSFQIDQNGQLVTSSGFQV
QPAITIPANALSITVGRDGIVSVTQQGQTAAQQVGQLTLTTFVNDSGLESVGENLYQETESSGAPNES
TPGLNGAGLLYQGYVETSNVNVAEELVNMIQTQRAYEINSKAVSTSDQMLQKLTQL
>CORE_REP|Org13_Gene968#
MLTFDSAQLTVWLSHYFWPLLRILALISTAPIFSEKQISKKVKIGLGGLIVILIAPTLPASNIPIFSA
AGLWLAIQQILIGVALGLTMQFAFAAVRLAGEVIGMQMGLSFATFFDPSGGPNMPVLARLLNLLAMLL
FLSFDGHLWLISLLADSFHTLPIQTQPLNGNGFLVLTQVGSLIFINGMMLALPLICLLLTLNMALGLL
NRMTPQLSVFVIGFPVTMTFGIMTLGMMMPMLAPFCEHLFGEIFDRLAAVIGGMTF
>CORE_REP|Org12_Gene3522#
MKKDKQIMTIGEGQAIVKEAQRLLSAPSRRRFLRNGLTLGGIAMLTGCDLSDNANVEQALSRMSRLND
RVQGWLFNGDRLAPVYPELMITRPFPFNAFYAEEDAPDINGDDYRLEVAGLVQDKRAWSLPQLHRMAQ
VSQVTRHICVEGWSAIGKWGGVPFATFLKAIGADLSARYVSFKCADDYYTSIDMATALHPQTIIALTY
DGQILPRKYGYPMKLRMPTKLGYKNPKHIQVIEVTNRFPGGYWEDQGYNWFGGS
>CORE_REP|Org27_Gene4360#
MSSRKSLSVVMIAKNEAGLLPDCLRSVEWADEIIVLDSGSEDDSVAIAESLGAKVFTHTDWQGFGKQR
QLAQSYASHDYVLMIDADERVTPELRQSIERVLNAPDDGAVYSCARRNLFLGRFMRHSGWYPDRVNRL
YANRRYRYNDDLVHESLNIGGAKVIPLNGDMLHLTCRDFFAFQRKQLRYAEEWATQRHRAGKRCGYLS
ILTHTLGAFVKTWLLRAGFLDGKQGLLLAVVNAQYTFNKYAALWALGRNYSEK
>CORE_REP|Org31_Gene1858#
MTLPARLAQGTPVTLESIGKGYGNRTVLDNIQLRISAGQFVAVVGRSGCGKSTLLRLLAGLEQPSSGA
LLSGNAPLAAAKEDTRLMFQDARLLPWKTVIDNVGLGLRGQWRDAALQALDAVGLADRARDWPAALSG
GQKQRVALARALIHRPRLLLLDEPLGALDALTRIEMQGLIETLWQQHGFTILLVTHDVSEAIALADRV
ILIEEGRIGLDLTLDLPRPRRKGSARLAELEAEVLERVLSPPATAASGRRAAN
>CORE_REP|Org24_Gene336#
MSENKLAVTELHKRYGDHEVLKGVSLAANAGDVISIIGSSGSGKSTFLRCINFLEKPSEGSISLNNED
IRMVRDKDGQLKVFDKKQLQLLRTRLTMVFQHFNLWSHMTVLENVMEAPVQVLGLSKADAHERAVRYL
DKVGIDERARGKYPVHLSGGQQQRVSIARALAMEPEVLLFDEPTSALDPELVGEVLRIMQKLAEEGKT
MVVVTHEMEFARHVSNHVIFLHKGLIEEQGPPAELFGNPKSPRLQQFLSGALK
>CORE_REP|Org47_Gene3539#
MLNVNHLSAEYQGRPALRDVSFQIAAGQLVVVLGPSGCGKTTLLNLIAGFIEPSAGSITLDGTPVHGP
SAERGVVFQHEGLLPWRNVVDNVEFGLQLAGVGKAQRRQVAEQMLQRVGLAGYEQHFIWQLSGGMRQR
VGIARALAADPRLLLLDEPFGALDAFTREQMQELLLTIWRDTGKQVLLITHDIEEAVFLASELLLLSP
GPGQVVERLSLNFGQRYADGEACRVIKSDPEFIAQREYVLGKVFQQREAML
>CORE_REP|Org13_Gene1701#
MMNLTMTRKTVFLVGPLAAALTIGAVSLPAHAAIALDRTRVIFDGDLKTVSLNISNQNKQLPYLAQGW
IEDDRGNKIQSPFTVLPPVQRVEPGKPSQVKIQSLPAARQLPQDRETLYYFNLREIPPRSNKPNTLQI
ALQTRIKMFYRPAALAPKKNAAPWQEQLTLTRQGDKYVVNNPTPYYVTIVEASAGKGGKGAAGFEPLM
VAPKASAPLNVSAASLGNGPSLAYINDYGGRPQLNFRCAGNACQVVPVAK
>CORE_REP|Org30_Gene769#
MTSTLHTLVRRPAVWLPAALLFISPAALAQLPGLISQPLANGGQSWSLPVQTLVLLTSLTFLPAMLLM
MTSFTRIIIVLGLLRNALGTPSAPPNQVMLGLALFLTFFIMSPVFDKVYQDAYLPFSQDKIGLEVALD
KGAQPLREFMLRQTRETDLALYARLANQPPLAGPEAVPMRILLPAYVTSELKTAFQIGFTVFIPFLII
DLVVASVLMALGMMMVPPATISLPFKLMLFVLVDGWQLLLGSLAQSFYS
>CORE_REP|Org22_Gene4202#
MSIQMDFTGKRVWVTGAARGIGEQIARHFLTQGAEVVGFDREFANPDQPYPCVMLDISRPEQVEAVCR
QQLAENPRLDVLVNAAGILRMGNTEDLSVDDWHQCINVNASGAFYLFRAVLPHFKAQRSGAIVSIGSN
AAHVPRAQMAAYCASKAALTSLNHCVGLEMAPFGVRCNLVSPGSTDTPMQRGMWQTDDAQQRTIAGFP
EMFKLGIPLGKIARPDEIANAVLFLASDLASHITMQDIVIDGGATLAA
>CORE_REP|Org48_Gene3986#

MLQLVEVGVAGRLAPFTAQIDGGLQVHLIGPNGAGKSTLLARAAGMLPGQGEVCLDGRALSCYSGDEL
AHRRGYLSQQQPPVSLMPVFQYLALHRPAGAVQTEVEQAILYLCQRLKLVDKLSRMLTQLSGGEWQRV
RLAAVLLQVWPSVNPHSRLLLLDEPTNSLDVAQKVALDRLLREFCQSGRSALVCAHDLNHTLQQADRV
WLLHAGQLVAQGITREVMAPGLLSQIYEVDFHLQWVGDQRWIMTRTA
>CORE_REP|Org12_Gene1280#
MRFDNKVVVITGAGNGMGEAAARRFSAEGAIVVLADWAKEAVDKVAASLPKGRAMAVHIDVSDHVAVE
KMMNEVAEKLGRIDVLLNNAGVHVAGSVLETSVDDWRRIAGVDIDGVVFCSKFALPHLLKTKGCIVNT
ASVSGLGGDWGAAYYCAAKGAVVNLTRAMALDHGGDGVRINSVCPSLVKTNMTNGWPQEIRDKFNERI
ALGRAAEPEEVAAVMAFLASDDASFINGANIPVDGGATASDGQPKIV
>CORE_REP|Org1_Gene2546#
MDHAIYTAMGAARQTLEQQSITANNLANASTPGFRAQLAALRAVPVDGPSLATRTLVTASTPGADMSQ
GALNYTARPLDVALQQDGFLAVSLPGGGEAYTRNGNIQISSTGQLTVQGLPVMGDGGPIEVPPSAEIT
IAADGTISALNAGDPPNTIAQIGRLKLVKADAREVMRGDDGLFRLTPETQQQRGNQLQNDPQVRVMPG
VLEGSNVKPMETMVDMIANARRFEMQMKVIHSVDENEQRANSLLSMS
>CORE_REP|Org6_Gene1423#
MSIQFWRMSIQLNGINCYYGAHQALFDITLECPAGETLVLLGPSGAGKSSLLRVLNLLEMPRSGQLQI
AGNQFDFRQAPGEKAIRELRQNVGMVFQQYNLWPHLTVVQNLIEAPCRVLGLTKAQAMERADKLLKRL
RLTDFADRFPLHLSGGQQQRVAIARALMMEPQVLLFDEPTAALDPEITAQIVSIIRELAGTGITQVIV
THEVEVARKTASRVVYMENGHVVEQGDSSHFTQPRTTEFANYLSH
>CORE_REP|Org7_Gene2423#
MNKHPITLLTAAGLALSAVLPTADAAISLDRTRAVYVSDAKSISLNIVNENKELPFLAQSWLENEQHQ
KITSPLVVLPPLQRVEPSERSVVRITKTPEADRLPQDRESVFYFNLREIPPKSTKTNVMQLALQTQIK
LFYRPKAIVAPKGQVWQEKLVFRKSGGAITVDNPTPFYITLTGMTRQTQKQGGGAIGGFQPLMLKPKS
SESLKLQETGMNSFVITYINDYGGHPELRFVCNGGVCTAVPEKK
>CORE_REP|Org32_Gene1615#
MTSPLLPPGIQVRDLSLRFGQQIVFDRLSFDIAGGSFVALLGASGAGKTSLLKIIAGLAQASSGTVTG
SDGLPIAGRIAYMGQKDLLYPWLTVEENVALGSRLRGEVADRAWVAHLLERVGLAAHGRSLPAALSGG
MRQRAAIARTLYERQPIVLMDEPFSALDAITRAEIQSLAAELLAQNTVLLITHDPMEACRLSHRLLVL
SPWPLGLDDTHRISGQPPRAPDDADLLKSQAELLQQLVRAAQ
>CORE_REP|Org34_Gene2364#
MDLTGKRVLITAAGQGIGFTTARLFAAAGAEVIASDINLERLQGSAGIRALTLNVTDPAAIAAAAEAI
GPIDVLFNCAGVVHSGSILDCSEDQWAFALDLNVTAMFRMIRAFLPGMLARGKGSIINMSSVASSVKG
VPNRFAYSASKAAVIGLTRSVAADYVTQGIRCNAICPGTVESPSLRQRIAEQAREQGRSEQEVYQAFV
ARQPIGRIGTTEEIAQLALYLASDASSYTTGTVQIIDGGWSN
>CORE_REP|Org40_Gene4448#
MWKWLHQLARPERLYHVCGRFIPWLGLAAAACLLLGWAWGFGFAPKDYQQGDSFRIIYIHVPAAMWSM
GIYASMAVAAFIGLVWQMKMSDTVVAAMAPIGAVFTFIALVTGSAWGKPMWGSWWVWDARLTSELVLL
FLYMGVIALYNAFEDRRLAGRAAGILVLVGVVNIPIIHFSVEWWNTLHQGSTNMQQSIAPSMRTPLRW
AILGYLLLFVTLTLMRLRNLILFQERQRPWVAGLVNKERQS
>CORE_REP|Org48_Gene782#
MSFEGKIVLVTGASRGIGRAIAETFVARGAKVIGTATSESGAEAISSYLGANGKGFMLNVVDAQSIDS
VLASIRAEFGEIDILVNNAGITRDNLLMRMKDDEWEDILDTNLTSVFRLSKAVMRAMMKKRFGRIITI
GSVVGTMGNAGQANYAAAKAGLIGFSKSLAREVASRGITVNVVAPGFIETDMTRALTDDQRAGILSSV
PANRLGDAKEIASAVAFLASDEAGYITGETLHVNGGMYMI
>CORE_REP|Org21_Gene531#
MATLIAENLAKAYKGRKVVEDVSLKVKSGEIVGLLGPNGAGKTTTFYMVVGIVPRDAGRIVIDEEDIS
LLPLHARARRGIGYLPQEASIFRRLSVYDNLMAVLEIRPDLTSEQREDRAKELMEEFHISHLRDSLGQ
ALSGGERRRVEIARALAANPKFILLDEPFAGVDPISVIDIKKIIEHLRDSGLGVLITDHNVRETLDVC
ERAYIVSQGKLIAHGTPDAILADEQVKRVYLGEEFRL
>CORE_REP|Org27_Gene864#
MHKIVFVEDDPEVGKLIAAYLGKHDIEVLIEPRGDSAQARIAHEQPDLVLLDIMLPGKDGMTLCRDLR
PTFPGPIVLLTSLDSDMNHILSLEMGANDYILKTTPPAVLLARLRLHLRQHGQQPKEESVQPLTQHNA
LHFGLLCIDPVNRQVTLGEETVTLSTSDFDLLWELATHAGQIMDREALLQNLRGVSYDGMDRSIDVAI
SRLRRKLYDNALEPFRIKTVRNKGYLFAPNAWASVQQ

>CORE_REP|Org36_Gene774#
MISLKNVSKWYGHFQVLTDCTTEVKKGEVVVVCGPSGSGKSTLIKTVNGLEPIQQGDILVNGTPVNDK
KTNLAQLRAKVGMVFQHFELFPHLSIIDNLTLAQVKVLKRDKTASREKGLKLLERVGLSAHANKFPGQ
LSGGQQQRVAIARALCMDPIAMLFDEPTSALDPEMINEVLDVMVELANEGMTMMVVTHEMGFARKVAN
RVIFMDEGKIVEDRNKDDFFNNPESERAKDFLAKILH
>CORE_REP|Org6_Gene19#
MQENHKILVVDDDMRLRALLERYLTEQGFQVRSVANAEQMDRLLTRESFHLMVLDLMLPGEDGLSICR
RLRSQSNPMPIIMVTAKGEEVDRIVGLEIGADDYIPKPFNPRELLARIRAVLRRQANELPGAPSQEEA
VIAFGKFKLNLGTREMFREDEPMPLTSGEFAVLKALVSHPREPLSRDKLMNLARGREYSAMERSIDVQ
ISRLRRMVEEDPAHPRYIQTVWGLGYVFVPDGSKA
>CORE_REP|Org21_Gene2589#
MEDEYNLSNIIGKRLEAALGELGDLLWAYVVLSKKDIACIFGVTNYPSEWVKKYQEQGLQYIDPVVLT
ARNRLTPFAWDEQIMADAGLHFPELFEQARGFGVTHGYTFVLHDYNDNLVTLSFAFNVEQRAEAIQAL
TERKGDISVLLSSLHESYLALSPLSAKNAAALERNVRFTDRENEILYWASVGKTYQETAMILGIKTGT
IKFHMSNIVKKLGVTNARHAVRLGMELRLIKPVEY
>CORE_REP|Org35_Gene254#
MQTPHILIVEDELVTRNTLKSIFEAEGYIVHEANDGAEMHNILSENDINLVIMDINLPGKNGLLLARE
LREQASVALMFLTGRDNEVDKILGLEIGADDYITKPFNPRELTIRARNLLSRTMNLGSLGEERRLVES
YKFNGWELDINSRSLISPAGEQYKLPRSEFRAMLHFCENPGKIQSRGELLKKMTGRELKPHDRTVDVT
IRRIRKHFESTPDTPEIIATIHGEGYRFCGDLEE
>CORE_REP|Org7_Gene2013#
MDIQNQPVQIMIVEDEPKLGQLLVDYLQAAGYATRWLTNGNEVVPTVHQHPPALILLDLMLPGADGLT
VCRELRRFSDVPIVMVTAKIEEIDRLLGLEIGADDYICKPYSPREVVARVKTILRRSYRPQENAREDD
LLHIDEPRFQASYQGQLLDLTPAEFRLLKTLASQPGNVFSREQLLNNLYDDYRVVTDRTIDSHIKNLR
RKLELIDGQKSFIRSVYGVGYRWEAEPCRLVNGV
>CORE_REP|Org46_Gene1803#
MDKPKRILIVEDDGDIAELLQLHLRDEGYAISHAADGNQGMAMLEQGGWDALILDLMLPGVDGLEICR
RARTMTRYTPIIIISARSSEVHRVLGLELGADDYLAKPFSMLELVARVKALFRRQEAMSRNLRMDAGV
LSFNDLTIDPIAREVHLHQQPVELTPREFDLLYFFARHPGQVFSRLSLLNQVWGYQHEGYEHTVNTHI
NRLRIKIERNPAEPERILTVWGMGYKFAAAPQE
>CORE_REP|Org38_Gene3476#
MISLRQLAIGYGATPLFPPLSGQFSAGSLTAVVGVNGAGKSTLLKTLAGLLPPVAGRLDFSGEKPPRK
AYLPQQAELDRQFPIAVSDLVAMGCWPQSGMFGGMNQRAASQVNEALASVGMSALAHSPVGELSGGQL
QRVLFARLLVQQAPLILLDEPFTGIDSATTQILLQVIAQLHQQGRTVIAVLHDMSMVAEHFPQVLLLT
PQACHWGAAERVLEQVPRYLAAERQPGLRVVAP
>CORE_REP|Org37_Gene3786#
MTTETAATILLIDDHPMLRNGVKQLIGMDARLQVIAEASNGEQGVTLAEQHDPDLILLDLNMPGINGL
ETLDRLRQTDLSGRVVVFSVSNHEDDVVSALKRGADGYLLKDMEPEDLLKALHQAAAGQMVLSETLTP
ILAASLRENRPSADRDIQQLTPRERDILKLIAQGLPNKLIARRLTITESTVKVHVKHLLKKMKLKSRV
EAAVWVLQGKTVNRRRTAARFAPEWAAASGF
>CORE_REP|Org17_Gene3023#
MLTLEKLTYLYEHLPMRFDLRIQPGERVAVLGPSGAGKSTLLSLIAGFLPAASGRLLLNGEDHTATPP
AKRPVSMLFQENNLFAHLTVAQNIGLGLDPGLRLTAQQRQQREHIARQVGLEEHLDRLPAQLSGGQRQ
RAALARCLIRRRPILLLDEPFSALDPALRNEMLQLLQTVCEQRDLTLLMVSHNLDDAARIAPRTLLVV
DGRIYYDGPTQALLDGSAPEARVLGISGKA
>CORE_REP|Org8_Gene519#
MAILPRQGQRWLAGMVMLTLSGCAYIPHKPLVDGATTAQPAPASAPMPNGSIFQTVQPMNYGYQPLFE
DRRPRNVGDTLTIVLQENVSASKSSSANASRNGASKFGVATSPRYLDGLLGNARADMDISGDSTFGGK
GGANANNTFNGTITVTVNQVLANGNLHVVGEKQIAINQGTEFIRFSGVVNPRTISGNNSVTSTQVADA
RIEYVGNGYINEAQTMGWLQRFFLNVSPF
>CORE_REP|Org7_Gene165#
MNKILLVDDDRELTSLLKELLEMEGFNIVVAHDGEQALSLLDSSVDLLLLDIMMPKKNGIDTLKELRQ
HHQTPVIMLTARGSELDRVLGLELGADDYLPKPFNDRELVARIRAILRRSNWSEQQQQVDSGAPTLDV
DGLQLNPGRQEASFDGQVLDLTGTEFTLLYLLAQHLGQVVSRELLSQEVLGKRLTPFDRAIDMHISNL

RRKLPDRKDGHPWFKTLRGRGYLMVSAT
>CORE_REP|Org22_Gene620#
MARRILVVEDEAPIREMVCFVLEQNGYQPLEAEDYDSAVTRLSEPFPDLVLLDWMLPGGSGIQFIKHM
KREALTRDIPVMMLTARGEEEDRVRGLEVGADDYITKPFSPKELVARIKAVMRRISPMAVEEVIEMQG
LSLDPSSHRVMANDQALDMGPTEFKLLHFFMTHPERVYSREQLLNHVWGTNVYVEDRTVDVHIRRLRK
ALETSGHDKMVQTVRGTGYRFSTRY
>CORE_REP|Org49_Gene1435#
MSITPTNILIVEDEKEIRRFVRTALESEGLRVFESETLQRGLIEAGTRKPDLIILDLGLPDGDGLSYI
RDLRQWSAIPVIVLSARNAEEDKIAALDAGADDYLSKPFGIGELLARVRVALRRHSASQQESPLVSFS
AITVDLVNRRVLRNDEDLHLTPIEFRLLAELLANAGKVITQRQLLSHVWGPNYVEHSHYLRIYMGHLR
QKLEADPARPKHLLTETGVGYRFMP
>CORE_REP|Org5_Gene817#
MSNPIVACWPGALAPREKLLMQGAAALSDAELLAIFLRTGLPGVHVMQLAEQLLRRFGSLYHLMSADH
QAFCSQKGLGDASYTQLQAIAELALRFFSSHLSQENAMLNPRVTQHYLQSLLAHREREVFLVLFLDNQ
HRVIRHQEMFAGTISSVVVYPREIVREALKANAAALILAHNHPSGKAEPSHADRLITEQVVKACQLLE
IRVLDHLVIGRGECVSFAERGWL
>CORE_REP|Org42_Gene4092#
MHNIDLEDRLAALSAAIADRTRARMLCLLMDGRAYTATELSAAVEVAPSTASAHLAKLLEQRLIACVK
QGRYRYFRLAGQPVAEALEGLMALAGVPRPSVKSSTPTTLQYARTCYDHMAGEVAVKLHDRLHALNWL
NGEEDYRLSDAGQAALARLGVDCSPAPTRRRFACGCLDWSERRSHLGGALGAALLAAFIHRGWIVRRL
DSRELQLTPAGKKALAAHFDLTV
>CORE_REP|Org9_Gene1038#
MKILLVDDDLELGTMLSEYLTGEGFDATLVLTGKAGVEGALSGDYTAMILDIMLPDMSGIDVLRDVRK
KSRLPIIMLTAKGDNIDRVIGLEMGADDYMPKPCYPRELVARLRAVLRRFEERPQEADDEAAISFGEL
TLNPSTRSSEWRGKAFDLTASEFNLLELLLRAPDRVVSKDELSEKGLGRPREAYDRSVDVHISNIRQK
LSALAGSKLIIETVRSIGYRIR
>CORE_REP|Org4_Gene59#
MQRILIVEDEQKTGRYLQQGLVEEGYQADLFNNGRDGLGAASKGQYDLIILDVMLPFLDGWQIISALR
ESGHEEPVLFLTAKDNVRDKVKGLELGADDYLIKPFDFTELVARVRTLLRRARSQAATVCTIADMTVD
MVRRTVIRSGKKIHLTGKEYVLLELLLQRTGEVLPRSLISSLVWNMNFDSDTNVIDVAVRRLRSKIDD
DFEPKLIHTVRGAGYVLEIREE
>CORE_REP|Org47_Gene894#
MQFLNQFFFDIYPYLAGAVFLIGSWLRYDYGQYSWRAGSSQMLDKKGMRLASNLFHIGIIGIFAGHFL
GMLTPHWMYEAFLPIDVKQKLAMIAGGACGLMTLIGGALLLKRRLTNPRVRATSSFADIMILTLLVVQ
VCLGLLTIPFSAQHMDGSEMMKLVAWAQAVVTFHAGASAHLEGVAIIFKLHMVLGMTLFVLFPFCRLV
HIWSAPVEYLTRRYQLVRNRR
>CORE_REP|Org11_Gene655#
MAIAATTNESLDNTVIGNNSKNTNSQDLHNSFLTLLVAQLKNQDPTNPMQNNELTSQLAQINTVQGIE
KLNTTLGSISGQINSNQSLQATALIGHGVMVPGNNILVGSKDGKVSTTPFGVELERAADQVTATITNA
SGQVVRTIEIGGLTAGVHAFTWDGSLDDGSTAPDGAYKVAINAKGNGEQLVARSLHFGLVNGVIRDGN
GAKLDLGLAGNATLEDVRQIL
>CORE_REP|Org1_Gene324#
MIRFEQVSKAYLGGRQALQGVDFHLRPAEMAFLTGHSGAGKSTLLKLICGIERPSAGHIWFGGHDISR
LKNREVPFLRRQIGMIFQDHHLLLDRTVYDNVAMPLIIAGASTEDIRRRVSAALDKVGLLDKAKNFPI
QLSGGEQQRVGIARAVVNKPAVLLADEPTGNLDDALSEGILRLFEEFNRVGVTVLMATHDTGLIARRN
YRILTLSQGRMQGGAHHGQ
>CORE_REP|Org17_Gene594#
MRVLVVEDNGLLRHHLSVQMREMGHQVDAAEDAKEADYFLQEHAPDIAIVDLGLPGEDGLSLIRRWRA
HQTKLPILVLTARESWQDKVAVLEAGADDYVTKPFHLEEVIARMQALMRRNSGLASQVIVLPPFQIDL
SRRELSVNDQQIKLTAFEYTIIETLIRNAGKVVSKDSLMLQLYPDAELRESHTIDVLMGRLRKKVQAE
YPHEVITTVRGQGYRFDAK
>CORE_REP|Org22_Gene2188#
MSVIALENLSVSHRQGYELRTVVHEVNLRIEPGECFGLVGPSGCGKSSLLWVLAGLNGSWQGGFELLG
RRLQPGQAFTGELRREVQMVFQDPYASLHPKHRLLRTLSEPLKLLKESDIERKVSAGFRQVGLDPRLL

DRYPHQLSGGQRQRVAIVRALLLRPKLLLLDEPTSALDMSVQAEILNLLNELKQAGDLTMVLVSHDAD
VIDHMCDRSVAMAHGRIIV
>CORE_REP|Org15_Gene2222#
MRILLIEDDKLIGDGIKAGLTKLGFNLDWFTDGAVGKNALGSAPYDAVILDLSLPGLDGLDLLRQWRQ
AGQDVPVLILTARDALEQRVSGLQSGADDYLCKPFALAEVAARLQALIRRRHGQLMPQLTHGNVVFDS
ATRSVSCNGEPVTLTPRELAVLELFLHNKGRVLARPLIQEKLYNWDDEVSSNAVEVHIHHLRRKLGNG
FIRTIHGVGYTLGDAP
>CORE_REP|Org37_Gene701#
MKLLVVEDDELLQQGLALALTGEGYVCDCAATAAEANSLLITSQYSMVILDLGLPDMDGAALLRQWRR
QQIDLPVLILTARDALEDRVDGLDAGADDYLVKPFALVELQARVRALLRRYQGHSDNLMQVDDLQLNL
SSQQVYLQQQPVEVTPKEFAILARLIMRAGQTVNRELLQQDLYTWQDDLGSNTLEVHIHNLRRKLGKD
RIRTVRGIGYRLEPSS
>CORE_REP|Org46_Gene107#
MISVLLVDDHELVRAGIRRILEDIKGIKVVGEAQCGEDAVKWCRGNAVDIVLMDMNMPGIGGLEATRK
IVRYAPDVKVIMLTIHTENPLPAKVMQAGAAGYLSKGAAPQEVINALRSVHAGQRYIASDIAQQMALS
QLEPQAETPFSCLSERELQIMLMITKGKKVNEISEQLSLSPKTVNSYRYRMFSKLNISGDVELTHLAI
RHGLFNAETLLSSE
>CORE_REP|Org22_Gene3251#
MKGKMLLLIGLLCSLNARADDLATQIDSFIKGKFTGEPVQVKVRVRTPPAQWPACELPQLSLPPNARI
GGNVSISARCGQERRFIQTQVQVFGRYLVSARGISAGSRLTAADLTLKEGRLDTLPPRALTEASKALD
AVSLRNISPGQPLTLAMLRRAWIIKAGQPVQVTAQGEGFNISGAGKAMNNAAAEDSVRVRMASGQIVS
GVVGDDGAIRITL
>CORE_REP|Org45_Gene513#
MSKFQLLDKDNSALIFIDHQPQMAFGVANIDRQQLKNNVVGLAKAGKIFNVPTLFTSVETESFSGYIW
PELLAVHPEITPIERTSMNSWEDAAFVKAVEATGRKKLVISALWTEVCLTFPALMALEAGYEVYVVTD
TSGGTSVDAHERSIDRMVQAGAVPVTWQQVLLEYQRDWARKDTYDAVMALVREHSGAYGMGVDYAYTM
VHHAPARTVK
>CORE_REP|Org8_Gene887#
MVMKQYRVMIVDDHPLMRRGIKQLLGLDARFGVVAEAGNGSEAVALALQHAPDVILLDLNMKGMSGLD
TLRALRDEGVDARIIVLTVSDARSDLYALIDAGADGYLLKDSEPEQLLEHISAAAEGQNVISDAMADY
LLARSEQRDPFTALTERELDVLQEVARGLSNKQVAAQLHISEETVKVHIRNILRKLDVRSRVAATVMY
LEYKSH
>CORE_REP|Org4_Gene1541#
MNEKIDYHIEKYHFAPLDEAPRLAHQWSEVLNECRETQAGAEERLRIALLNVDYVTSFELPFRLLLVR
APQLIAGIREELPLSQKNVVFNGKRFGCVYSLKSDLSGVPEAFQYSLSTRIHRRAASGVDALPYREIA
KALKAPRERLRLALEQGLPVTALDGLFWFGIQRIAAEVRRLRKTGMAIVTAETEIFDTLTGTTRKVPV
YRLAES
>CORE_REP|Org28_Gene2514#
MIRVILVDDHVVVRSGFAQLLNLEDDLDVVGQYSSAAAAWPALLRGDVNVAVMDIAMPDENGLSLLKR
LRAQKPQFRAIILSIYDSPTFVQSALDAGASGYLTKRCGPEELVQAVRSVDMGGHYLCADALRALRGG
ERPATALEVLTPREREIFDLLVKGDSVKEIAFKLDLSHKTVHVHRANVLGKLQCNSTIELVHFALDHQ
LLAGH
>CORE_REP|Org34_Gene4021#
MTFKYSRLDKAQAAVLLVDHQTGLLSLVRDQDPDKFKNNVLALADLAKYFNLPTILTTSFENGPNGPL
VPELKQTFPDAPYIARPGNINAWDNEDFVKAVKATGKKQLIIAGVVTEVCVAFPALSALEEGYEVFVI
TDASGTFNAITRDAAWDRMSQAGAQLMSWFGAACELHRDWRNDIEGLGNLFSQHIPDYRNLMTSFSLL
TSGKQ
>CORE_REP|Org5_Gene483#
MSYSGERDQFAPNMALVPMVVEQTSRGERSYDIYSRLLKERIIFLTGQVEDHMANLIVAQMLFLEAES
PEKDIYLYINSPGGVITAGMSIYDTMKFIKPDVSTICMGQACSMGSFLLTAGAKGKRFCLPNSRVMIH
QPLGGYQGQATDIEIHAREILKVKARMNELMAEHTGQPLEQIERDTERDRFMTAEEAVEYGLVDGILT
HRS
>CORE_REP|Org24_Gene262#
MTTPSFDSVEAQASYGIGLQVGQQLQESGLEGLQPEALLAGLRDALEGNAPAVPVDVVHRALREIHER

ADAVRRERQQAMAVEGQKFLDDNAKRDDVTLTESGLQFSVLEQGNGPIPSRQDRVRVHYTGRLINGDV
FDSSVERGQPAEFPVSGVIPGWIEALTLMPVGSKWQLYIPHNLAYGERGAGASIPPFSALVFDVELLE
IL
>CORE_REP|Org35_Gene2480#
MSLTLTPEQRQTVHPLWLRLTHWLNALAMLIMVTSGWRIYNASPLFNFSFFNELTLGGWLGGALQWHF
AGMWLFGVNGLCYLLLNLASGRFKRKYWPLSPKQFLADVGAALRGRLQHADLRHYNMVQRVAYLSVML
LGVLSALSGLVLWKSVQFPLLRTLLGGYEAARYIHFFAMSALVAFVAIHLVMVALVPRTLLAMLRGR
>CORE_REP|Org42_Gene764#
MAKFTQHTGLVVPLDAANVDTDAIIPKQFLQKVTRTGFGQHLFNDWRFLDDAGQQPNPEFVLNKPRYK
GASILLARENFGCGSSREHAPWALTDYGFKVVIAPSFADIFYGNSFNNQLLPVTLSEQQVDELFKLVD
ANEGTEFVVDLENQTVNAGGKSYPFEIDSFRRHCMINGLDSIGLTLQHEADISRYEAQQPAFLN
>CORE_REP|Org40_Gene522#
MVLVTRQAPDFTAAAVLGSGEIVENFNLKKHLNGKPAVLFFWPMDFTFVCPSELIAFDHRYEEFQKRG
VEVVGVSFDSEFVHNAWRKTPVDKGGIGEVKYAMVADVKREIQKAYGIEHPEAGVALRGSFLIDKDGI
VRAQVVNDLPIGRNIDEMIRTVDALQFHEEHGEVCPAQWEKGKAGMGASPDGVAKYLSENAAKL
>CORE_REP|Org26_Gene381#
MLDRIKVCFTESIQTQIAAAEALPDAISRAAMTLVQSLLNGNKILCCGNGTSAANAQHFAASMINRFE
TERPSLPAIALNADNVVLTAISNDRLHDEVYAKQVRALGHTGDVLLAISTRGNSRDIVKAVEAAVTRD
MTIVALTGYDGGELAGLLGQQDVEIRIPSHRSSRIQEMHMLTVNCLCDLIDNTLFPHQDD
>CORE_REP|Org4_Gene1977#
MTLRVAFIDDHDIVRSGFVQLLSLEADIQVVGEFSSAAQARAGLPGLEAEICICDISMPDGSGLDLLA
DIPSGIRVVMLSMHDNPALVEMALDRGASGFLSKRCKPEDLITAVRTVAGGGVYLMPEIAQQLARVRV
DPLTRREREIALLLAQGQEVREIAAALGLSPKTVHVHRANLFAKLGINNNVELARRMLNL
>CORE_REP|Org42_Gene115#
MYHDLIRSELNEAADTLAKFINDDANIDAIQRAAVLLADSFKAGGKVISCGNGGSHCDAMHFAEELTG
RYRENRPGYPAIAISDVSHLSCVSNDFGYEYVFSRYVEAVGREGDVLLGISTSGNSGNIIKAIDAARA
KGMKVITLTGKDGGKMAGSADVEIRVPHFGYADRIQEIHIKAIHILIQLIEKEMVKA
>CORE_REP|Org23_Gene256#
MSFELPALPYEKNALEPHISAETLEYHYGKHHNTYVVNLNNLVKGSEFEGKSLEEIIKTSNGGVFNNA
AQVWNHTFYWHCLSPQGGGEPQGELAAAIVKSFGSFAAFKEQFTDAAVKNFGAGWTWLVKKPDGALAI
VNTSNAATPLTGEDKPLLTVDVWEHAYYIDYRNARPKYLENFWALVNWTFAAENLA
>CORE_REP|Org47_Gene3946#
MNDKRPPHYDGGDLHRQGYRYRQDSERLTSRDKMQPVLLDMLTDDEPQKKQEAQVRNLVSHSELRRRV
LRDLQWLFNCVNSESNLDLGDFPQVRRSTLNYGIASLAGKRMSDIEWLDIQRALTESILHFEPRILPE
GLQVRCISDTGSLELHNVLSIEIKGRLWCVPYPLEFLFRTDVDLENGHFDLKDIG
>CORE_REP|Org20_Gene68#
MSEQLTDQVLVERVQKGDQKSFNLLVVRYQHKVASLVSRYVPQGDVPDVVQESFIKAYRALESFRGDS
AFYTWLYRIAVNTAKNYLVAQGRRPPSSDVDANDAENYESAGALKEISNPENLMLSEELRQIVFRTIE
SLPEDLRMAITLRELDGLSYEEIAAIMDCPVGTVRSRIFRAREAIDNKVQPLIQR
>CORE_REP|Org21_Gene4528#
MKLNKIMLAAVMAFGVSSLAHAAVKDQGHGKVTFSGSIIDAPCSIAPESIDQTVELGAISNVALKDGG
KSMPRPFQIKLENCDLTVDANDPSKNNNKVSLTFTGSASEADSALLGITGTAKGAGIALTDGNGKNIT
LGTATDARLLQNGSNTLSFSAYLQGSGASGAQIVPGEFQSVADFTLAYQ
>CORE_REP|Org13_Gene4680#
MASRGVNKVILVGHLGQDPEVRYMPNGGAVANITLATSESWRDKATGEQKEKTEWHRVVLFGKLAEIA
GEYLRKGSQVYIEGSLQTRKWTDQAGVEKFTTEIVVNIGGTLQMLGGRSQPANAGQLGQGSWGKPQQP
QPSHSGTPAQQPQPAQSDGNAAPMDFDDDIPFLGFGYGIAKSAIYAL
>CORE_REP|Org28_Gene1750#
MSATATASAPHTALSRLYATHHAWLQGWLRRRLGCAFDADDVAQDTFMRLLKSDAAATLREPKDFLVT
VAKRVMVDLFRRRTLERAYLEMLALIPDGYAPSPEQRQSLLESLQQIDAMLDGLGPKVKQAFLLSQLE
GLGYADIAVRLGVSISSVKKYMAKATEHCLLFSLENDVFS
>CORE_REP|Org6_Gene3004#
MNKRRTTLTVLALIASLGLSSAPALADKGGNGNGNGNGHGNSGNHGNNGNHGNNGNNGNHGNNGNNGN
HGNKGNKDKGGYRNDDNLVSVSLSRDRARSLAHNYGLTGYSSLPPGIAKNLARGKPLPPGIAKKVVPY

SMLRELPQYPGYEWRIAGDDLVLVALSTAIVASVINGVFD
>CORE_REP|Org2_Gene2712#
MKRYWYAALGLMACGAAQAATTDVEMHLVTGQGIGQDIGKVVISETPYGLLFTPSLKALPAGVHGFHV
HEKGSCEPGMKDGKAVAALAAGGHLDPQKTGKHLGPYADGHLGDLPAIYVAADGMANYPVLAPRLKKI
SDIEGKALMVHAGGDNHSDHPQPLGGGGERFACGVIK
>CORE_REP|Org6_Gene1075#
MKKIACLSAVAACVLAVSAGTAFAGQSTVSAGYAQGDLQGVANKANGFNLKYRYEFDNNPLGVIGSFT
HLEKNRSESGFYKKSQYDSITAGPAYRFNDWASIYGVIGVGYGKNIDNAQAGGNKGGNSDYGFTYGAG
LQFNPIENVALDVGYEQSRIRSVDVGSWNVGVGYRF
>CORE_REP|Org48_Gene1720#
MPGILLKKRPLSRYLKDYKHSQTHCSQCGKLLDRMALVFRGKIINKEAIARMDQPIDDAVWQNVQHEL
TALCRFCSEISCNSHPSYFDIMAFKQYLFEQTEMSHSTIREYVVRLRRLDEMLVARNYPADKFASSAS
HQRIIDDLPTAAHNNYRIALRKYDQYLAWQRSY
>CORE_REP|Org9_Gene122#
MTQTVHFQGNPVSVAGKLPQQGEQAKAFSLVAKDLSDVALSSFAGKRKVLNIFPSIDTGVCATSVRKF
NQLASGLDNTVVLCISADLPFAQSRFCGAEGLSNVVTLSTLRGAEFKQAYGVEIAEGPLAGLTARAVV
VLDGQDNVLYSELVNEITTEPDYDAALAALK
>CORE_REP|Org23_Gene1631#
MSQNTLPAAPKRSLLVILLVLISVVACGAAGYSWWLLQQHKNGAEPAAVKQQPPAAPVFMPLDTFTVN
LVTPDNNPDRVLYIGLTLRLPDESTRRQLNDFLPEVRSRLLMLLSRQEAGQLANEQGKQQLVAQIKDV
LSPPLVKGQPKQVVSDVLFTAFILR
>CORE_REP|Org19_Gene153#
MTSKAIYPGTFDPMTNGHLDLVTRASLMFDHVILAIAASPSKKPLFSLDERVALATQVTSHLDNVEVL
GFSELMAHFAAHQNANILVRGLRAVSDFEYELQLANMNRHLMPTLESVFLMPSEEWSFISSSLVKEVA
RHGGDIAPFLPDVVTQALMAKLAAE
>CORE_REP|Org7_Gene356#
MAIDMFLKVEGASGESKDSNHKGWTDITSFSWGASQPGNMGVGGGGGAGKVCFNDLHVNALIDKSTPA
LLKHCSSGKHLTKIELSVCKAGGTQVEYAKITLEDVLVTAVQYTGAGGEDTVGVTYSFQAAKVKQQYW
EQSDKGGKGAESSAGWNIKENREA
>CORE_REP|Org32_Gene2470#
MKSQSPLITLRDLAQDAVEQAAQQLGQVRQAQQAAEQQLSMLLNYQDEYRQKLNHTLCDGMDSSSWQN
YQQFIGTLEQAIDQHRQQLLQWGQKVDHAVKQWQDKQQRLNAFETLHTRALNAEQQQENKRDQKLMDE
FAQRSAQRNINP
>CORE_REP|Org19_Gene683#
MTDNNIALKKAGLKVTLPRLKILEVLQNPECHHVSAEDLYKKLIDMGEEIGLATVYRVLNQFDDAGIV
TRHNFEGGKSVFELTQQHHHDHLICLDCGKVIEFSDESIEVRQRDIAKQHGIKLTNHSLYLYGHCETG
DCREDETLHDKK
>CORE_REP|Org8_Gene1202#
MENLAQLLDKLLETLQALNGVLEEEHDLLCSGQLPGVALQRVTDAKSQLLATVAYLEQQRLGQEKTCG
QRAPYASQAPLADRWQRVQLLSQTLREKNQHNGLLLNQQIDHNAQALAILSKNNKSLYGPDGQSHAGS
LLGRKIGV
>CORE_REP|Org43_Gene408#
MSDPKQPSGEGKESVDDLWADAFNEQQSSEKSGASTEGVFKSLEAQDALGSLQDIDLILDIPVKLTVE
LGRTKMTIKELLRLSQGSVVALDGLAGEPLDILINGYLIAQGEVVVVADKFGVRITDIITPSERMRRL
SR
>CORE_REP|Org19_Gene949#
MLDKLDAALRFGQEALNLRAQRQEILAANIANADTPGYQARDIDFASQLNKVLEQGRVNGNGMSLNLT
AARHIPAQTLQPPQLDLLYRVPDQPSMDGNTVDMDRERTNFADNSLKYQTDLTLLNGQIKGMMSVLQQ
G
>CORE_REP|Org38_Gene351#
MYNRSGTQAYAQVSLESGAMSASPHQLIVMLFDGALSALLRARILMNQGDIAGKGMALSKAINIIDNG
LKSGLDPQQGGEIAENLAALYDYMKRRLMQANLHNDEAAIAEVVKLLENIADAWRQIGPNYQPSQDAV
>CORE_REP|Org42_Gene2097#
MKRCLFVLCLLAPLTAGAVSGSWVAEGAGVTLEQGGMRDESAGLRPPNVLPDANARITRVSWRYRLLG

PEPAGLQAQLCTVNRCIALGGGSGSSNGLQGEPANAELRFVYYVQSQGGLNPPLRVIGNQVIVNYQ
>CORE_REP|Org27_Gene316#
MSLLNIFDISGSALSAQSQRMNVSASNMANADSVTGPDGEPYRAKQVVFQVAAAPGQPTGGVRVAQVV
DDPAPERLVYQPGNPLADAKGYVRMPNVDVVGEMVNTISASRSYQANVEVLNTTKSMMMKTLTLGQ
>CORE_REP|Org41_Gene2207#
MADKNLRFLVVDDFSTMRRIVRNLLKELGFNNVEEAEDGADALNKLRAGGFDFVVSDWNMPNMDGLEL
LQTIRADSVLAAMPVLMVTAEAKKENIIAAAQAGASGYVVKPFTAATLEEKLNKIFEKLGM
>CORE_REP|Org22_Gene183#
MIQEHHLSLVCALSKWVETHLGRVIHLEELAEYSGYSLWHMQKLFKEATGISLGKYIRERRLAGAVYQ
LRSSEASIFDIALDFGFGSQSHFTYMFRKRFNITPYDFRQDLSVDLHIDPPLHVIHQKSA
>CORE_REP|Org39_Gene422#
MIRTMLQGKLHRVKVTQADLHYEGSCAIDQDFLEAAGILEYEAIDIYNVDNGQRFSTYAIAAERGSRI
ISVNGAAARCACVGDKLIICSYVQMTDADARQHHPKVAYFEGDNNLQRKAKAVPVQVA
>CORE_REP|Org20_Gene312#
MERQQQLLAAYQQIYSLSSQMIALAQTGRWEELVELEFAYVTAVEKTAAFTGQAGPSMALQEMLRNKL
QQILDNETELKRLLQQRMDELKMLIEQSTRQNVVNNTYGQFHDRALLLGEPQVR
>CORE_REP|Org35_Gene3554#
MGNMGTSELLKHIYDINLSYLLLAQRLINDEKASAMFRLGIDETMADALAQLTLPQMVKLAETNQLVC
HFRFNDHQTIERLTKESRVDDLQQIHTGILLSSHLLQELSSKDASPTKKRA
>CORE_REP|Org10_Gene2347#
MNTTGFITDLKTWIDNNLEEKLDINTVADRAGYSKWHLQRMFKRQTGYALGEYIRMQKLKVSAERLAN
SGEPIVSVAISLGFDSQQSFNRSFKRQFGQTPGDWRRALAQPTAVRCTHH
>CORE_REP|Org3_Gene4472#
MAIQGIEGVLQQMQTMAVQAGKMGQNTAPQGVSFASELTAALGKISETQQTARKQAQDFELGVPGISL
NDVMVDLQKSSVSLQMGVQVRNKLVAAYQDIMNMPV
>CORE_REP|Org41_Gene320#
MTPESVMALGTEAMKVALALAAPLLLAALISGLVVSLLQAATQINEMTLSFIPKILAVVATIIIAGPW
MLNLLLDYMRTLFSNLPTLIG
>CORE_REP|Org43_Gene4415#
MIERGKFRSLTLVNWNGFFARTFDLDELVTTLSGGNGAGKSTTMAAFVTALIPDLTLLHFRNTTEAGA
TSGSRDKGLHGKLRAGVCYSTLDVVNSRHQRVVVGVRLQQVAGRDRKVDIKPFTIQGLPTAVQPTELL
TQTVGERQARVLSLQELKERVEEMEGVQFKQFNSITDYHSLMFDLGVIPKRLRSSADRSKFYRLIEAS
LYGGISSAITRSLRDYLLPENSGVRKAFQDMEAALRENRMTLEAIRVTQSDRDLFKHLISEATSYVAA
DYMRHANERRIHLDGALALRSDLLGSRKQLAAEQYRHVEMARELSEQSGAESDLETDYQAASDHLNLV
QTAMRQQEKIERYEADLEELTYRLEEQNEVVAEASEQQAENEARAEAAELEVDELKSQLADYQQALDV
QQTRAIQYQQALQALERARALCQLPDLTADNAEQWLDTFQAREQEATEALLMLEQKLSVADAAHGQFE
QAYQLVGKIAGQVSRSEAWQCARELLRDWPSQQHLAECVQPLRLRLSELEQRLRSQQDAERLLQEFCK
RHGQEYQPDDLDMLQQELEERLEALSQNVSEAGERRMEMRQELEQIQQRIRELTARAPVWLAAQDALS
QLSDQSGEPLENSQQVTEYMQQLLERERETTVERDEVAARKREVEAQIERLSQPGGAEDQRLVTLAER
FGGVLLSEIYDDVTIDDAPYFSALYGPSRHAIVVPDLSLVREMLEGLEDCPEDLYLIEGDPQSFDDSV
FAVEEQDKAVVVKIADRQWRYSRYPEVPLFGRAARENRLEVLHAERETLAERYATLSFDVQKTQRSHQ
AFSRFIGTHLAVAFDADPEAEIRNLNARRGEIERALNNHEAQNQQQRQQYDQAKEGISALNRLMPLVS
VLNDETLQDRVDEIREELEEAQDAARHIQQHGVSLTKLEPLLSVLQSDPQQHEQLQQDYAQAQSVQRQ
AKQQAFALTEVVQRRAHFSYTDSAGMQNANNDLNDKLRQRLEQAEAERARAREQLRQYQTQFTQYSQV
LASLKSSYDAKRDMLKELSQELVDIGVQADANAEARARQRRDELHAALSNNRARRNQLEKQLTFCEAE
MDGLQKKLRKLERDYHQLREQVVTAKAGWCAVMRLVKDNGVERRLHRRELAYMDGDELRSMSDKALGA
LRLAVADNEHLRDVLRLSEDPKRPERKIQFYIAVYQHLRERIRQDIIRTDDPVEAIEQMEIELGRLTE
ELTAREQKLAISSKSVANIIRKTIQREQNRIRMLNQGLQAVAFGQVKSVRLNVNVREAHATLLDVLSE
QQEQHQDLFNSNRLTFSEALAKLYQRLNPQIDMGQRTPQTIGEELLDYRNYLEMEVEVYRGSDGWLRA
ESGALSTGEAIGTGMSILVMVVQSWEEESRRLRGKDISPCRLLFLDEAARLDAKSIATLFELCDRLEM
QLIIAAPENISPEKGTTYKLVRKVFQNHEHVHVVGLRGFASEPPALGTAPVETP
>CORE_REP|Org11_Gene2189#
MKDLLKFLKAQTKTEEFDAIKIALASPDMIRSWSFGEVKKPETINYRTFKPERDGLFCARIFGPVKDY
ECLCGKYKRLKHRGVICEKCGVEVTQTKVRRERMGHIELASPTAHIWFLKSLPSRIGLLLDMPLRDIE

RVLYFESYVVVEGGMTNLERRQILTEEQYLDALEEFGDEFDAKMGAEAIQALLKNMDLEAECEQLREE
LNETNSETKRKKLTKRIKLLEAFVQSGNKPEWMILTVLPVLPPDLRPLVPLDGGRFATSDLNDLYRRV
INRNNRLKRLLDLAAPDIIVRNEKRMLQEAVDALLDNGRRGRAITGSNKRPLKSLADMIKGKQGRFRQ
NLLGKRVDYSGRSVITVGPYLRLHQCGLPKKMALELFKPFIYGKLELRGLATTIKAAKKMVEREEAVV
WDILDEVIREHPVLLNRAPTLHRLGIQAFEPVLIEGKAIQLHPLVCAAYNADFDGDQMAVHVPLTLEA
QLEARALMMSTNNILSPANGEPIIVPSQDVVLGLYYMTRDCVNAKGEGMVLNGSKEAERVYRAGLASL
HARVKVRITEDVKNAEGEWTSQTSIIDTTIGRAILWMIVPKGLPYSIVNQPLGKKAISKMLNTCYRIL
GLKPTVIFADQIMYTGFAYAARSGASVGIDDMVIPAKKAEIIEEAETEVAEIQEQFQSGLVTAGERYN
KVIDIWAAANERVAKAMMENLSVEDVVNRDGEVEQQVSFNSIFMMADSGARGSAAQIRQLAGMRGLMA
KPDGSIIETPITANFREGLNVLQYFISTHGARKGLADTALKTANSGYLTRRLVDVAQDLVVTEDDCGT
HDGILMTPVIEGGDVKEPLRERVLGRVTAEDVLKPGTADILVPRNTLLNEKACDLLEENSVDSVKVRS
VVSCETDFGVCANCYGRDLARGHIINKGEAIGVIAAQSIGEPGTQLTMRTFHIGGAASRAAAESSIQV
KNKGSLKLSNVKFVMNAAGKLVITSRNTELKLIDEFGRTKESYKVPYGAVMGKGDGEEVNGGETVANW
DPHTMPVISEVSGFIRFADMVDGQTITRQTDELTGLSSLVVLDSAERTGSGKDLRPALKIVDAQGEDV
LIPGTDMPAQYFLPGKAIVQLEDGIQIGAGDTLARIPQESGGTKDITGGLPRVADLFEARRPKEPAIL
AEISGIISFGKETKGKRRLVISPLDGSDAYEEMIPKWRQLNVFEGEVVERGDVVSDGPESPHDILRLR
GVHAVTRYITNEVQEVYRLQGVKINDKHIEVIVRQMLRKGTIVSAGGSEFLEGEQAEVSRVKIANRQL
EAEGKIAATFSRDLLGITKASLATESFISAASFQETTRVLTEAAVAGKRDELRGLKENVIVGRLIPAG
TGYAYHQDRMRRRAQGEAPVVPQVSAEEATANLAELLNAGLGGSDDE
>CORE_REP|Org37_Gene101#
MVYSYTEKKRIRKDFGKRPQVLDIPYLLSIQLDSFQKFIEQDPEGQYGLEAAFRSVFPIQSYSGNSEL
QYVSYRLGEPVFDVKECQIRGVTFSAPLRVKLRLVIYEREAPEGTVKDIKEQEVYMGEIPLMTENGTF
VINGTERVIVSQLHRSPGVFFDSDKGKTHSSGKVLYNARIIPYRGSWLDFEFDPKDNLFVRIDRRRKL
PATIILRALNYTTEQILDLFFDKIVFEIRDNKLQMELVPERLRGETASFDIEANGKIYVEKGRRITAR
HIRQLEKDDIQSIEVPVEYIAGKVVAKDYIDTNTGELICAANMELSLDLLAKLSQSGHKRIETLFTND
LDHGAYISETLRVDPTNDRLSSLVEIYRMMRPGEPPTREAAESLFENLFFSEDRYDLSAVGRMKFNRS
LLRDEIEGSGILSKDDIIEVMKKLIDIRNGKGEVDDIDHLGNRRIRSVGEMAENQFRVGLVRVERAVK
ERLSLGDLDTLMPQDMINAKPISAAVKEFFGSSQLSQFMDQNNPLSEITHKRRISALGPGGLTRERAG
FEVRDVHPTHYGRVCPIETPEGPNIGLINSLSVYAQTNEYGFLETPYRRVRDGVVTDEINYLSAIEEG
NFVIAQANSNLDEEGRFVEDLVTCRSKGESSLFSRDQVDYMDVSTQQVVSVGASLIPFLEHDDANRAL
MGANMQRQAVPTLRADKPLVGTGMERAVAVDSGVTAVAKRGGVIQYVDASRIVIKVNEDEMYPGEAGI
DIYNLTKYTRSNQNTCINQMPCVNLGEPIERGDVLADGPSTDLGELALGQNMRVAFMPWNGYNFEDSI
LVSERVVQEDRFTTIHIQELACVSRDTKLGPEEITADIPNVGEAALSKLDESGIVYIGAEVTGGDILV
GKVTPKGETQLTPEEKLLRAIFGEKASDVKDSSLRVPNGVSGTVIDVQVFTRDGVEKDKRALEIEEMQ
LKQAKKDLTEELQILEAGLFARIHAVLVAGGIEADKLSKLPRDRWLELGLTDEEKQNQLEQLAEQYDE
LKSDFEKKLEAKRRKITQGDDLAPGVLKIVKVYLAVKRQIQPGDKMAGRHGNKGVISKINPIEDMPYD
ENGTPVDIVLNPLGVPSRMNIGQILETHLGMAAKGIGEKINQMLKQQQEVAKLREFIQKAYDLGDDVC
QKVDLNTFSDDEVLRLAENLKKGMPIATPVFDGAKETEIKKLLEMGGIPTSGQITLFDGRTGEQFERQ
VTVGYMYMLKLNHLVDDKMHARSTGSYSLVTQQPLGGKAQFGGQRFGEMEVWALEAYGAAYTLQEMLT
VKSDDVNGRTKMYKNIVDGDHRMEPGMPESFNVLLKEIRSLGINIELEDE
>CORE_REP|Org31_Gene4064#
MITTDGNNAVASVAYRTNEVIAIYPITPSSTMAEQADAWSGDGRQNIWGDIPRVVEMQSEGGAIATVH
GALQTGALSTSFTSSQGLLLMIPTLYKLAGELTPFVLHVAARTVATHALSIFGDHSDVMAVRQTGCAM
LCAGSVQEAQDFALISQTATLNARVPFIHFFDGFRTSHEINKIVPLSDDTLRQMLPQAAIDAHRSRAL
SPDHPVVRGTSANPDTYFQSREATNPWYDATGQHVIDAMAAFAALTGRHYRPFDYYGHPQAERVVVVM
GSAAGTCEEVIDTLLTRGEKVGVLKVRLFRPFSAQHMLGALPDSVRSVAVLDRTKEPGALAEPLYLDV
MTALAEAYSRGERATLPRVIGGRYGLSSKEFGPDCALAVFRELAQPQPRPRFTVGIFDDVTGLSLPLS
DEILPQRASLEALFYGLGSDGSVSATKNNIKIIGNATPLYAQGYFVYDSKKAGGLTVSHLRVSEQPIN
SAYLVSHADFIGCHQLQFIDKYQMVERLKPGGTFLLNTPYGADEVWSRLPQEVQALLHQRQARFYIIN
AAKLARECRLGARINTVMQMAFFHLTQILPSDVALQQLQDAIARSYSSKGQEIVERNWQALGATRAAL
TEIPLQPVDVSSPMRPPVVSDAAPDFVKTVTAAMLAGLGDALPVSAFPPDGTWPVGTTQWEKRNIAEA
IPIWQPDLCTQCNHCVAACPHSAIRAKVVQPAEMEHAPASLQSLDVKARDMRGQKYVLQVAPEDCTGC
NLCVEVCPAKDRQNPEIKAINMASRLDNLTAEKDNYDFFLQLPEIDPTQLERIDIRTSQLITPLFEYS
GACSGCGETPYIKLLTQLYGDRLLIANATGCSSIYGGNLPTTPYTTNAEGRGPAWANSLFEDNAEFGL

GFRLTVDQHRARVLRLLNALAPQLPERLVNALQMGDVAPEPRRKQIAELRTLLANLEGEDARQLAAGA
DYLVDKSIWLIGGDGWAYDIGFGGLDHVLSLTENVNVLVLDTQCYSNTGGQQSKATPLGAVTKFGEHG
KRKARKDLGVSMMMYGHVYVAQISLGAQLNQTVKAIQEAEAYPGPSLIIAYSPCEEHGYDLALSHDQM
KQLTATGFWPLYRFDPRRSAEGKAALALDSRPPNSSLSETLLKEQRFRRLNAQQPEVASALYQAAEKE
LQEKYDFLSLLAGKAEKSAAE
>CORE_REP|Org9_Gene3622#
MPKRTDIKSILILGAGPIVIGQACEFDYSGAQACKALREEGYRVILVNSNPATIMTDPEMADATYIEP
IHWEVVRKIIEKERPDAVLPTMGGQTALNCALELERQGVLAEFGVTMIGATADAIDKAEDRRRFDVAM
KKIGLDTARSGIAHTMEEALAVAADVGFPCIIRPSFTMGGTGGGIAYNREEFEEICERGLDLSPTNEL
LIDESLIGWKEYEMEVVRDKNDNCIIVCSIENFDAMGIHTGDSITVAPAQTLTDKEYQIMRNASMAVL
REIGVETGGSNVQFSVNPKTGRLIVIEMNPRVSRSSALASKATGFPIAKIAAKLAVGYTLDELMNDIT
GGRTPASFEPSIDYVVTKIPRFNFEKFAGANDRLTTQMKSVGEVMAIGRTQQESLQKALRGLEVGATG
FDPKVSLDDPEALTKIRRELKDAGSDRIWYIADAFRAGLSVDGVFNLTNIDRWFLVQIEELVRLEEQV
ADAGINGLNKAFLRTLKRKGFADARLAKLAGVAESEIRKLRHSHGLHPVYKRVDTCAAEFATDTAYMY
STYEEECESNPTNDRPKVMVLGGGPNRIGQGIEFDYCCVHASLALREDGYETIMVNCNPETVSTDYDT
SDRLYFEPVTLEDVLEIVRIEKPKGVIVQYGGQTPLKLARELEAAGVPIIGTSPDAIDRAEDRERFQQ
AVNRLGLKQPANATVTAIEQAVEKAAGIGYPLVVRPSYVLGGRAMEIVYDETDLRRYFQTAVSVSNDA
PVLLDRFLDDAVEVDVDAICDGERVLIGGIMEHIEQAGVHSGDSACSLPAYTLSQEIQDVMRRQVEKL
AFELQVRGLMNVQFAVKDNEVYLIEVNPRAARTVPFVSKATGVPLAKVAARVMAGKTLVEQGVTEEII
PPYYSVKEVVLPFNKFPGVDPILGPEMRSTGEVMGVGRTFAEAFSKAMLGSNSGMKKQGRALLSVREG
DKARVVDLAASLLKQGFELDATHGTAVVLGEAGINPRLVNKVHEGRPHIQDRIKNGEYTYIVNTTAGR
QAIEDSKLIRRSALQYKVHYDTTLNGGFATAMALKADPTEQVTSVQEMHARIGK
>CORE_REP|Org10_Gene3917#
MTFPRFFIKRPIFAIVLSILTLIAGIVALFQLPLSEYPAVTPPTVQVTASYPGANPNVIAETVAAPLE
QAITGVEGMLYMSSQAATDGRMTLTVTFAQGTNADMAQIQVQNRVARALPRLPAEVQHQGVVTQKTSP
DILMVVHLLSPDQRYDPLYISNYAYLQVRDELSRIPGVSDVQVWGAGEYSMRLWLDPDLIAARGLTAG
DVIAAVREQNVQVAAGSVGQAPDTNAAFQVTVNTLGRLADEKQFGDIIIRTGSDGQVTRLRDVARIDM
GADAYALRSLLDGEPAVALQIIQSPGANALDVAQAVRATVKRLEGDFPAGLSSRIAYDPTVFVRASLE
SVVTTLLEAILLVVIVVVVFLRSWRASLIPLLAVPVSLVGTFAIMHLMGFSLNTLSLFGLVLSIGIVV
DDAIVVVENVERHIENGKTPQQAARLAMDEVTGPIVAITSVLAAVFIPTAFLSGLQGEFYRQFALTIA
ISTLLSALNSLTLSPALAGLLLRPHPAEHRAPGRIQRILQAAVRPFQRAPDAYANAVRKTVRVSGVAL
AIYGGLLVLTFFGFQAVPPGFVPMQDKYYLVGIAQLPNSASLDRTDAVVKQMSKIALAEPGVESVVAF
PGLSINGFVNVPNAAVMFVMLDPFKERATPDLAASAIAGRLQAKFADIPDGFLGVFPPPPVPGLGATG
GFKMQVEDRGGVGLESLVEHTRLLMVKATESGQVAGLMTSLDINAPQLDVVIDRTQAKSQGVSLADVF
ESLQIYLGSLYINDFNRFGRTYKVTAQADADHRMQAEAIGRLQVRNAAGDMLPLSSFVTVTPGSGPDR
IIRYNGYPSADISGGAAIGVSSGQAVALMEQLAKETLPEGMTVEWTDLTYQQKLAGNAALFIFPLCVL
LAYLILATQYNSWLLPLAVLLIVPMCLLSAMIGVWLLGGDNNVFVQIGLIVLVGLAAKNAILIVEFAR
GLEDEGANTLEAVIKACRLRLRPIVMTSIAFIAGVIPLIFASGAGAEMRHAMGVAVFAGMLGVTLFGL
FLTPVFYVVIRGLTARFEQYRTKGKANVRSESDEKSS
>CORE_REP|Org40_Gene1002#
MAKFFIDRPIFAWVIAIIVMLAGVLAIMKLPIAQYPTIAPPAVSISANYPGADAKTVQDTVTQIIEQN
MNGIDNLMYMSSTSDSSGSVTITLTFDSGTDPDIAQVQVQNKLSLATPLLPQEVQQQGLKVEKSSSSF
LMVAGFVSDDPNMTQDDIADYVASNIKDPISRSSGVGEVQLFGAQYAMRIWLDPNKLNNFQLTTTDVT
SAITEQNNQIAAGQLGGLPPVPGQQLNASIIAQTRLTSPEEFGKILLKVNTDGSQVRLRDVAHIERGA
ESYAVTARYNGKPAAGLGIKLATGANALNTAKGVKDELAKMAPFFPQGMKVVYPYDTTPFVKISINEV
VKTLIEAIILVFLVMYLFLQNFRATLIPTIAVPVVLLGTFAILAAFGFSINTLTMFGMVLAIGLLVDD
AIVVVENVERVMSEEGLPPKEATRKSMGQIQGALVGIAMVLSAVFVPMAFFGGSTGAIYRQFSITIVS
AMALSVLVALILTPALCATLLKPIPKGDHGVKTGFFGWFNRMFEKSTHHYTDSVGNILRSTGRYLIIY
LLIVVGMGLLFLRLPSSFLPDEDQGILLTMVQLPAGATESRTNKVLEEVSDYFLNKEKDNVVSVFTVA
GFGFNGNGQNNGLAFVSLKDWGERPGAGNKVEAIAGRAMGAFSQIKEGLVFPFNLPAIIELGTATGFD
FELIDQGGLGHEKLTEARNQLLGMVAQHPDVLVGVRPNGLEDTPQFKLIVDQEKAKALGVSITTINST
LSTALGGSYVNDFIDRGRVKKVYVQADAPFRMLPEDINKWYVRGTSGQMVPFSAFSSAKWEYGSPRLE
RYNGLPSMEILGQAAPGKSTGEAMNLMEQLASKLPSGIGYDWTGMSYQERLSGNQAPALYAISILVVF
LCLAALYESWSVPFSVMLVLPLGVIGALLAATMRGMNNDVYFQVGLLTTIGLSAKNAILIVEFAKDLM

EKEGKGLIEATLEAVRMRLRPILMTSLAFILGVLPLVISSGAGSGAQNAVGTGVMGGMITATVLAIFF
VPVFFVVVRRRFSKKNEDLEHSHPVEHH
>CORE_REP|Org28_Gene4189#
MQVMPPNAGGGPSRLFILRPVATTLLMVAILLAGIIGYRALPVSALPEVDYPTIQVVTLYPGASPDVV
TSAITAPLERQFGQMSGLKQMASQSSGGASVVTLQFQLALPLDVAEQEVQAAINAATNLLPSDLPYPP
IYSKVNPADPPILTLAVTSTAMPMTQVEDMVETRVAQKISQVTGVGLVTLAGGQRPAVRVKLNAAAVA
AYGLNSETIRAAISNANVNSAKGSLDGPTRSVTLSANDQMKSADDYRQLIVAYQNGAAIRLQDIATIE
QGAENTRLAAWANKQQAIVLNIQRQPGVNVITTADSIREMLPTLIKSLPKSVDVKVLTDRTTTIRASV
SDVQFELLLAIALVVMVIYVFLRNVPATIIPSVAVPLSLVGTFAAMYFLGFSINNLTLMALTIATGFV
VDDAIVVIENISRYIEKGEKPLDAALKGAGEIGFTIISLTFSLVAVLIPLLFMGDIVGRLFREFAVTL
AVAILISAVVSLTLTPMMCARMLSHESLRKQNRFSAASERFFDRVIAQYGQWLKTVLNHPWLTLGVAV
GTLALTVLLYLLIPKGFFPVQDNGIIQGTLEAPQSVSFSNMAERQQQVAAQILKDPAVESLTSFVGVD
GSNATLNSGRLQINLKPLSERSDRIPAIISRLQQQTAQFPGVKLYLQPVQDLTIDTQVSRTQYQFTLQ
AMSLDDLSLWVPQLMNELKQTPQLADVTSDWQDQGLVAYVNVDRDSASRLGVTMSDVDNALYNAFGQR
LISTIYTQANQYRVVLEHDVSATPGLAALNEIRLSGNDGAVVPLSAIAKIEERFGPLSVNHLDQFPSA
TVSFNVADGYSLGEAVDAVTQAEKNLNMPRDITTQFQGATLAFQAALGSTLWLILAAVVAMYIVLGVL
YESFIHPVTILSTLPTAGVGALLALMLAGSELDVIAIIGIILLIGIVKKNAIMMIDFALAAEREQGLS
ARDAIYQACLLRFRPILMTTLAALLGALPLMLSTGVGAELRHPLGVCMVGGLIMSQILTLFTTPVIYL
LFDKLARNTHRQPDTQELP
>CORE_REP|Org7_Gene4479#
MDNIEVRGARTHNLKNINLIIPRDKLIVVTGLSGSGKSSLAFDTLYAEGQRRYVESLSAYARQFLSLM
EKPDVDHIEGLSPAISIEQKSTSHNPRSTVGTITEIHDYLRLLFARVGEPRCPDHHVPLAAQTVSQMV
DNVLSQPEGKRLMLLAPVVKDRKGEHTKTLENLSAQGYIRARIDGEVCDLSDPPKLELQKKHTIEVVV
DRFKVRDDMAQRLAESFETALELSGGTAVVADMDDEKADELLFSANFACPICGYSMRELEPRLFSFNN
PAGACPTCDGLGVQQFFDPDRVVQNPELSLAGGAIRGWDRRNFYYFQMLRSLAEHYEFDVEAPFNTLS
ANVQKAVLSGSGKESIEFKYINDRGDTTVRRHPFEGVLHNMERRYKETESSAVREELAKFISNRPCAS
CHGTRLREEARNVFVEDTTLPEISDLSIGHAMTFFQNMKLSGQRAKIAEKVLKEIGDRLKFLVNVGLN
YLSLSRSAETLSGGEAQRIRLASQIGAGLVGVMYVLDEPSIGLHQRDNERLLETLIHLRNLGNTVIVV
EHDEDAIRAADHVIDIGPGAGVHGGQVVAEGTVDDIMAQPESLTGQFLSGKREIAIPAQRVQADPTKV
LKLSGARGNNLKDVTLTLPVGLFTCITGVSGSGKSTLINDTLFPIAQRQLNGATIAEPAPFREVTGLE
HFDKVIDIDQSPIGRTPRSNPATYTGIFTPVRELFAGVPESRSRGYTPGRFSFNVKGGRCEACQGDGV
IKVEMHFLPDIYVPCDQCKGKRYNRETLEVKYKGKSIHEVLEMTIEEARDFFDAVPALARKLQTLMDV
GLSYIRLGQSATTLSGGEAQRVKLARELSKRGTGQTLYILDEPTTGLHFADIQQLLAVLHQLRDQGNT
IVVIEHNLDVIKTADWIVDLGPEGGSGGGEILVAGTPETVAECEKSHTARFLKPLLEK
>CORE_REP|Org46_Gene1471#
MLVKLLTKVFGSRNDRTLRRMRKVVEQINRMEPDMEKLSDDELKAKTNEFRARLEKGESLESLIPEAF
AVVREASKRVFGMRHFDVQLLGGMVLNDRCIAEMRTGEGKTLTATLPAYLNALSGRGVHVVTVNDYLA
QRDAENNRPLFEFLGLSIGINLPGMPAPAKREAYAADITYGTNNEYGFDYLRDNMAFSPEERVQRKLH
YALVDEVDSILIDEARTPLIISGPAEDSSEMYIKVNKLIPKLIRQEKEDSDTFKGEGHFSVDEKARQV
HLTERGLILIEEMLVEAGIMDEGESLYSPTNIMLMHHVTAALRAHVLFTRDVDYIVKDGEVIIVDEHT
GRTMQGRRWSDGLHQAVEAKEGVEIQNENQTLASITFQNYFRLYEKLAGMTGTADTEAFEFSSIYKLD
TIVVPTNRPMIRKDMPDLVYMTEKEKIGAIIEDIRERTAKGQPVLVGTISIEKSEVVSRELTKAGIDH
KVLNAKFHAMEADIVAQAGQSGAVTIATNMAGRGTDIVLGGSWQAEVALLEAPTEEQIEAIKAAWKER
HDAVLAAGGLHIIGTERHESRRIDNQLRGRSGRQGDAGSSRFYLSMEDALMRIFASDRVSGMMRKLGM
KEGEAIEHPWVTKAIANAQRKVESRNFDIRKQLLEYDDVANDQRRAIYSQRNELLDVSDVSETIASIR
EDVFKSTIDNYITPQSLEEEWDIQGLEERLKNDFDLEMPIAQWLDKEPELHEETLRERILENAKEQYQ
RKEDVVGSEMMRNFEKGVMLQTLDSLWKEHLAAMDYLRQGIHLRGYAQKDPKQEYKRESFNMFATMLE
SLKYEVISVLSKVQVRMPEEVEALEQQRREEAERLAQHQQLSHHDENALVTEDPNAPATAERKVGRND
PCPCGSGKKYKQCHGRLQ
>CORE_REP|Org26_Gene4376#
MQQETPTTPTEARVKNKRRISPFWLLPFIALLIAGWLVYNNVQERGTTVTIDFQSAAGIVAGRTPVRY
QGVEVGTVQKISLSKDLRSIVVEASIKSDLEDSLREGTQFWLVTPKASLAGVSGLDALVGGNYIGMMP
GSGKEQTHFTALDTQPKYRLNTGELMIHLHADDLGSLNSGSLVYYRKIPVGKVYDYTISEGNKGVTID
VLIDRRFANLVKSNSRFWNVSGFKGDFSLSGATVQMESLAALVNGAIAFDSPADGQQAKGDQSYTLYP

DLAHSQRGVNILLDLPNGNSLSENRTPLMYQGLQVGTLTKLTLQQDSKVVGELTIDPSVVDLMRSGTR
IVMRSPRISLNDAKLSQLLTGTTLELVPGEGEPQQRFNVLDSSETLLQQPGVLTVTLNAPQSYGIDVG
QPLVVHGVKVGQILSRTLTAGGVVFTAAIDAQYRGLLHKDSKFVVNSRLDVKLGIDGMEVLGASAQEW
VDGGVRIIPGSKGEPGGQYPLYANSEKAEEGIVGNAPSTTLTLSATSLPDVQAGSVVLYRKFQVGEIV
NVRPKANEFEVDVYISPEYRKLLTRESIFWAEGGAKVQLNGSGLTVQASPLNRALKGAISFDNLQGVT
LNKGANRVLYASETAARAVGSQIMLRTYDASKLSAGMPLRYLGIDVGQVESLQLAPERNEVLAKAVLY
PEYVHTFARLGSRFSIVSPEISAAGVSNLDTLLQPYINVEPGRGRELRTFELQQASITDSRYLDGLSV
VLDAAETGSLQIGTPVLFRGVEVGTITGFYLGAMSDRVHVALRISKKYQHLVRNNSVFWLASGYNLQF
GLTGGVIKSGTFQQFIRGGIAFATPPTIPLAPKATPNKHFLLNPEEPKDWKTWGTAIPRD
>CORE_REP|Org38_Gene3259#
MTQQPQAKYRHDYRAPDYTTTDIDLDFSLDAETTRVTAVSKIKRQGAAGAPLVLDGEDLTLVSIQVDG
QPWSAYRQQDNQLIVEALPAQFTLTIVNDIHPAKNTALEGLYLSGDALCTQCEAEGFHHITYYQDRPD
VLARFTTRIVADKARYPFLLSNGNRIGQGELADGRHWVQWQDPFPKPCYLFALVAGDFDVLRDSFTTR
SGRKVALELFVDRGNLDRADWAMTSLKNSMKWDETRFGLEYDLDIYMIVAVDFFNMGAMENKGLNIFN
SKYVLAKAETATDKDYLNIEAVIGHEYFHNWTGNRVTCRDWFQLSLKEGLTVFRDQEFSSDLGSRSVN
RIDNVRVMRGAQFAEDASPMAHAIRPDKVIEMNNFYTLTVYEKGSEVIRMMHTLLGEENFQKGMQLYF
ERHDGSAATCDDFVQAMEDASNVDLSRFRRWYSQSGTPLLTVRDEYDAETQQYRLHVSQKTPPTADQP
EKLPLHIPLDIELYDSEGNVIALQKGGLPVNNVLNVTEAEQTFVFDGVAHKPVPSLLREFSAPVKLDY
PYSDQQLTFLMQHARNEFARWDAAQSLLATYIKLNVARHQQKQPLSLPLHVADAFRAVLLNETLDPAL
AAQILTLPSENEIAELFATIDPEAIAAVHEAIVRCLARELADEWLAVYHANKTDGYRVEHADIAKRAL
RNVCLGYLAFGEDAALADQLVSEQYRQADNMTDSLAALSAAVAAQLPCRDALLAAFDERWHQDGLVMD
KWFVLQGSSPSADVLSKVRALLQHRSFSLSNPNRTRSLIGGFASGNPAAFHAADGSGYQFLVEILSDL
NQRNPQIAARLIEPLIRLKRYDAGRQALMRKALEQLKGLENLSGDLYEKISKALDA
>CORE_REP|Org11_Gene135#
MGKALVIVESPAKAKTINKYLGSDYVVKSSVGHIRDLPTSGSASKKSADSTEDKAKKKVKKDEKAALV
NRMGVDPYHGWKAHYEILPGKEKVVAELKSLAENADHIYLATDLDREGEAIAWHLREVIGGDDKRFSR
VVFNEITKNAIQQAFKQPGELNIDRVNAQQARRFMDRVVGYMVSPLLWKKIARGLSAGRVQSVAVRLV
VERERDIKAFVPEEYWELHADLLAKGETALQMEVTHAHDKPFKPVNREQTHAAVKLLEKARYTVLDRE
DKPTSSKPGAPFITSTLQQAASTRLSFGVKKTMMMAQRLYEAGHITYMRTDSTNLSQDALNMVRGYIG
DNFGDKYLPKAPNQYSSKENSQEAHEAIRPSDVNVLAEQLKDMEADAQKLYQLIWRQFVACQMTPAQY
DSTTLTVKAGDYQLRAKGRTLRFDGWTKVMPALRKGDEDRTLPYVEIGSELDLQKLIPSQHFTKPPAR
YSEASLVKELEKRGIGRPSTYASIISTIQDRGYVRVESRRFYAEKMGEIVTDRLEENFRELMNYDFTA
RMEDGLDEVANNQAEWKAVLDEFFVDFSEQLETAEKDPEEGGMRPNQMVMTSIDCPTCGRKMGIRTAS
TGVFLGCSGYALPPKERCKTTINLVPEAEVLNILEGDDAETNALRARRRCQKCGTAMDSYLIDNQRKL
HVCGNNPACDGYEIEEGEFRLKGYDGPVVECDKCGSEMHLKMGRFGKYMGCTNENCKNTRKILRNGDV
APPKEDPVPLPELPCEKSDAYFVLRDGAAGVFLAANTFPKSRETRAPLVEELARFKDRLPEKLRYLAD
APVADAEGNKTLVRFSRKTKQQYVSSEKDGKATGWSAFYVDGKWVEGKK
>CORE_REP|Org44_Gene2350#
MKFVKYFLILAVCCIVLGAASIFGLYKYVEPQLPDVATLKDVRLQIPMQVYSADGELIAQYGEKRRIP
LKLDQIPPVMVHAFIATEDSRFYDHHGVDPVGIFRAASIALVSGHASQGASTITQQLARNFFLSPERT
LMRKIKEAFLAVRIEQMLTKDEILELYLNKIYLGYRAYGVGAAAQVYFGKDVSQLTLSEMATIAGLPK
APSTFNPLYSHDRAVARRNVVLSRMLDEHYITQAQYDQARSEDLVANYHAPEISFSAPYLSEMVRQEM
IKRYGENAYTDGYKVYTTVTKRLQLAAQESVRNNVLAYDMRHGYRGPSNVLWKVGEAAWDRKQIVDSL
KNLPNYGPLAPAVITAANPQEATAMLADGSSIALPMATMRWARPYRSDTQQGPTPKRVTDVVQAGQQV
WVRKVNDAWWLSQVPDVNSALVSINPNDGAVKALVGGFDFNQSKFNRVTQALRQVGSNIKPFLYTAAM
DKGLTLATILNDLPITRWDAGAGTDWRPKNSPPTYDGPIRLRQGLGQSKNVVMVRAMRAMGVDYAAEY
LQRFGFPAQNIVHTESLALGSASFTPMQLVRGYAVLANGGYLVDPYFITKIEDDNGNTVFEAKPKVVC
SSCNLPVIYGDTHRSAVLSDDNIENVATSQEGNNSTVPMPQLEQVTPAQVQQDGDQQYAPHVISTQLA
FLIHDALNSNIFGEPGWMGTAWRAGRDLKRHDIGGKTGTTNSSKDAWFSGYGPDTVTSVWIGFDDHRR
DLGRSTVSGAIPDQISGGEGGAKSAQPAWDDFMKTALEGIPEQKVTPPPGIISVTIDKSSGKLSGGGG
GSRSEYFIEGTQPTDYPSRDTGTTLTDPGGESHELF
>CORE_REP|Org29_Gene3641#
MNKPTQPAQDYLAALPLTAERSEALNPQTADDAQALEALHRQMGAADANVNSLSADDVALASVKPRIE
SAWPDAVSDDDFDTDAEGRAILKATPPIKRTTMFPEAWRTNPVARFWDSLLGRSPHNRHATKEEAEAE

NRWRVVGSMRRYVLLVLMLVQTGIATWYMKTILPYQGWALIDPIAMLDQDLMQSVLQLLPYVLQTGIL
ILFAVLFCWVSAGFWTALMGFLQLLIGKDKYSISSTIKGDEPINPAHRTALIMPICNEDVERVFAGLR
ATYESVAATGQLEHFDIYVLSDSYDPDICVAEQKAWMELCRDVDGHGRIFYRRRRRRVKRKSGNIDDF
CRRWGGEYSYMVILDADSVMSGECLTGLVRLMEANPNAGIIQSAPKASGMDTLYARVQQFATRVYGPL
FTAGLHFWQLGESHYWGHNAIIRVKPFIEHCALAPLPGEGSFAGSILSHDFVEAALMRRAGWGVWIAY
DLPGSYEELPPNLLDELKRDRRWCHGNLMNFRLFLVKGMHPVHRAVFLTGVMSYLSAPLWFMFLALST
ALQVVHTLMEPQYFLQPRQLFPVWPQWRPELAIALFSTTLVLLFLPKLLSIVLIWAKGAKEYGGAFRL
FISMLMEMLFSVLLAPVRMLFHTVFVVSAFLGWEVVWNSPQRDDDDTPWGEAFRRHGSQMLLGLVWAG
GMAWLDLRFLWWLSPIVFSLILSPFVSVLSSRATLGMKSKRAKLFLIPEEYNPPRELLATEEYLHLNR
NRALTNGFMHAVVNPSFNALATALATARHHLRATLDRNREERVNEALQLGPEKLVKGKRLELLSDPVT
LARLHQRVWLLPEGAAWREHYQQLPHNPLAHPTGRR
>CORE_REP|Org37_Gene3353#
MKISLDLAIFAVICGILPLLILPRLPEPWLQWPMLFVACLLLRTRWPICRYLACLGLGFIWAVFNAGS
LLGQMERLSCMPDVTAVAQVSSVALEPAASKQTLMRIERVDGHWLVPALAFTTTWAPERQRLCAGQRW
QLKLRLRPVHGKLNEGGFDSQRWAIAQRQPLTAQVRQARLLDGDCGLRQRIISHAETNIGELRYKAVL
LALAFGERTALEQALRTLMLKTGIAHLMAISGLHVAMVAILFWAVLRALQFFLPAHLIGYRFPLVAGW
VATLIYVWLVGAQPPAVRTVLAMTLWMLLRLRGVHCSSWQVWLWCVGLILLCDPLAVLSDSFWLSVLA
VGCLIFWFEWAPLGERFRSAWYWAPVRWLHIQLGMTLLLVPMQVALFLGLTLTSLPANLWAVPIVSLV
TVPLILLAVIGGVFPSLSYGLWWLADFTLSGVFVPLHYLQRGWVDLGAASLLASIAGWLIVICWRFHW
WWRYAPGLATIAICCVLWRGKEPGYRWRVDMLDVGHGLAMVIEQNGKGILYDTGDRWPAGSAAERHIL
PMLNWRGIELEQIIISHAHLDHIGGLSTVQSAFPQATVRSPIRGEGHLPCVAGERWRWQSLQFEVLWP
PKTLKRPVNDDSCVIRIDDGKYSLLLTGDAEKKAEAQLIRLRRDRLAATVLQVGHHGSRTSSTPPFLR
AVNPEVALASASRYNKWRLPARKVVARYRANGITWRDTTRSGQLSVLFFDNDWQIKGFREQLMPRWYH
QRFGVEGDNE
>CORE_REP|Org48_Gene2830#
MLTRLREIVEKVAAAASLTDALDLLVNETCLAMDTEVCSIYLADNDRRCYYLMATRGLKKPRGRTIAL
AFDEGVVGLVGRRAEPINLADAQSHPSFKYVPQVKEDRFRSFLGVPIIHRRQLLGVLVVQQRELRQFD
ESEESFMVTLATQMAGILSQSQLNAIFGQYRQTRVRALAASPGVAVAEGWQDSSQPSLDQVYRASTLD
TASERERLTLALEEAGAEFRRFSKRFAASSQKESAAIFDLYSHLLNDARLKRELFAEIDNGSVAEWAV
KQVIEAFAEQFAKLQDTYMRERGSDLRALGQRLLFHLDDTTQGATQWPARFVLVADELTATLLAEVPQ
DRLVGVVVRDGAANSHAAILVRAMGVPTVMGADIQPSLLSQRLLIVDGYRGELLVDPEPVLVQEYQRL
ISEEQELSKLAEDDVEQPAQLKSGERVQVMLNAGLSPEHEQLLGGRVDGVGLYRTEIPFMLQSGFPSE
EEQVAQYQGMLQLYPNKPVTLRTLDIGADKQLPYMPISEENPCLGWRGIRITLDQPEIFLIQVRAMLR
ANAGTGNLGILLPMVTSLEEVDEAKRLIDRAGREVEEVLGYAIPKPKIGVMLEVPSMIFLIPHLAGRV
DFISVGTNDLTQYLLAVDRNNTRVASLYDSLHPAMLQVLKLIAEQGKAAGLQLSLCGELAGDPMGALL
LVGMGYRNLSMNGRSVARIKYLLRHIDLADAEVLALRVLNTQMTTEVRHLVAAFMERRGMGGLIRGGR
>CORE_REP|Org17_Gene2248#
MLYQGETLQLHWLDNGIAELVFNAPGSVNKLDTRTVASLGEALTVLENQPELKGLLLRSTKAAFIVGA
DITEFLSLFAAPAEKLQEWLVFANNVFNRLEDLPVPTISAINGYALGGGCECILATDFRVASPDARIG
LPETKLGIMPGFGGSVRLPRLLGNDSALEIIAAGKDVSAKDALKVGLVDAVVAPEKLAEAALNMLQQA
IDGKLNWRAARQPKLEPLKLSPIEAAMSFTTAKGMVLQTAGKHYPAPMTAVKTIEAAAKLGRDEALKL
ETASFVPLARSNEARALVGIFLNDQFVKGQAKKLAKNVDAPKQAAVLGAGIMGGGIAYQSALKGVPVI
MKDISDKSLTLGMNEAAKLLNKQLERGKLDGLKMAQVLSTIQPTLDYAGIERAQVIVEAVVENPKVKA
AVLSEVENLIGEDTVLASNTSTIPINHLAKSLKRPQNFCGMHFFNPVHRMPLVEIIRGEQTSDETIAK
VVAYASRMGKTPIVVNDCPGFFVNRVLFPYFAGFSLLLRDGADFRQIDKVMEKQFGWPMGPAYLLDVV
GIDTAHHAQAVMAAGFPERMSKDYRDAIDVMFDNQRFGQKNQLGFYRYSQDNKGKPRKDNDEQTDALL
AEVSQPRQTISDEEIVARMMIPMINEVVRCLEEKIVASPAEADMALVYGIGFPPFHGGAFRYLDTLGT
ANYVELAQRYAHLGALYQVPAGLRAKAERNESYYPVATPLSDVATRQPA
>CORE_REP|Org28_Gene2061#
MSFENALHEQRAKPSAFQLTIRPDNIGVITIDVPGDKVNTLKAEFVEQVNDVLIRAQQHTALEGLVIV
SGKPDSFIAGADITMIAACTSAKEAETLAKKGQSTLAQIAAFPVPVVAAIHGACLGGGLELALACHGR
VCSLDDKTALGLPEVQLGLLPGSGGTQRLPRLVGAAKALDMILTGKHIRARQALRMGLVDDAVPQSIL
LQTAIERVKQGWKHQRELPWQDRLLNGPLGKNLLFSIVRKKTLAKTHGNYPAAERIIQVVRSGLDHGS
ASGYEAEARAFGELAMTPQSAALRSLFFASTALKKERGGNAQPRALHRVGILGGGLMGGGIACVTATR

GGLPVRIKDINETGINHALKYSWDVLGKRVRSKRMRPAERQKQMMLISGSTDYTGFEQVDIVVEAVFE
DLALKQQMVAEVEANCATHTVFASNTSSLPIGRIAEKAQRPQQVIGLHYFSPVDKMPLVEVIPHAGTS
EETIATTVALAHKQGKTAIVVGDSAGFYVNRILAPYINEAARCLLEGEPIESLDKALVDFGFPVGPIT
LLDEVGIDVGTKIIPVLVEALGPRFAAPAAFDAVLKDGRKGRKNGRGFYLYPSEGQQRQRRKRADTSL
YTLLGVTPKAHMLPATVAQRCVMMMLNEAARCLDEGVIRSARDGDVGAVFGIGFPPFLGGPFRYMDEL
GAEKVVKTLDYLRQQHGEHFAPCERLQRMAQQGERFYPLGS
>CORE_REP|Org29_Gene4668#
MRNNFLFGDKHDMNSLFASTARGLEELLKSELEALGAHDCKVVQGGVHFQGDDRLLYQSLLWSRLASR
ILLPLNEFRVHSDLDLYLGVQAIDWPSIFGVDKTFAVHFSGVNEEIRNSQYGALKVKDAIVDSFTRKL
DQRPTVAKQQPDIRVNVFLQRDMASVALDLSGEGLHQRGYRDLTGQAPLKENLAAAIVLRSGWQPGTP
MLDPMCGSGTLLIEAAMIAADRAPGLHRQHWGFTAWNGHNAELWREVTTEAQVRARRGLQETASRFFG
SDIDRRVIEMARGNARRAGVAELITFNVGDVARLTNPLPEGPHGTVISNPPYGERLESEPALIALHNM
LGRVMKSAFGGWQLSLFSASPELLSCLQLRAERQFKAKNGPLECVQKNYQLAANPAGGTTGGVQVAED
FANRLRKNLKKLDKWAKQQGIECYRLYDADLPEYNVAVDRYGSKVVVQEYAPPKTVDAQKARQRLFDV
INATLAVLELPSNQLILKTRERQKGKNQYEKLAQKGEFLLVEEYNAKLWVNLTDYLDTGLFLDHRIAR
RMLGEMSNGKDFLNLFAYTGTASVHAGLGGARSTTTVDMSRTYLEWAEKNLRANGLTGRQHRLIQADC
LSWLSNANEQFDVIFIDPPTFSNSKRMENTFDVQRDHLALMKDLKRLLRRNGTIMFSNNKRGFQMDMA
GLSALGLEAKEITAKTLSQDFARNRQIHNCWLVTHAGEGK
>CORE_REP|Org24_Gene1133#
MIFSLLRALFRLLFRVRVEGDVQHFERQKLLITPNHVSFLDGVLLALFLPIKPVFAVYASIGESWFMR
WLRPYIDFVSLDPTKPMAIKQLVRMVEQGRPIVVFPEGRITVTGALMKIYDGAAFIAAKSGATVVPVR
IDGPEFSPFGRMAGVFKIRWFPQISIRILPPTTLPMPEAPRARERRALAGDRLMQIMMRARMDTREPQ
TLFHALLAAQHRYGRRKPCIEDIAFKEDSYQTLIKKSLGVSRILQRFTAEGEHVGLLLPNATITAAAI
FGASLRNRIPAMLNYTAGANGLNSAMLAAGIKTIVTSRQFLEKGKLTHLPEQVTQANWVYLEDLKDTV
TLADKLWILRHLLQPQRAALPQRPEDAALILFTSGSEGHPKGVVHSHASLLANVEQIRTIADFTPRDR
FMSSLPLFHSFGLTVGLLTPLITGSRIFLYPSPLHYRVVPELVYDRNCTVLFGTATFLNNYARFAHPY
DFARLRYVVAGAEKLADSTKQIYQDKYGIRILEGYGVTECAPVVSINVPLATKVGTVGRIMPQMEARL
IAVPGIDNGGRLQLKGPNIMKGYLRVERPGELEPPAAEDANGVLQPGWYDTGDIVSLDEQGYCTIRGR
VKRFAKLAGEMVSLESVELLAQRLSPEKMHAATAKGDSSKGEALVLFTTDPAITREALLRVARELGSP
ELAVPRDIRLLKTLPVLGSGKPDFVTLRHMAEQPESAR
>CORE_REP|Org8_Gene4201#
MNPLLFTSRRRIAAALWLSLGVAGAALAQPQPQPPLAERAPKALTAHGETRTDDYYWLRDDSRKEQKV
LNYLKAENRYTEQMMAPYQNLRATLYQEMLGRMSPDDRSVPYQLNGYRYQESYAAGKEFARYQRQALT
ADAPWQTLLDANQRAAGHAYYRLGAMDISRDNRRLAVAEDLQGRRQYRISLRELGSERWSPETLENTS
GNMVWANDNQTLFYVRNHPQTLLPYQVYRHQYGTPPAEDKLVYQENDPAFYLSLSRSSSRDYLILTIS
GNTTSEVRLIDASQPQREPQLFAARQNGREYYLDHYRGEFYLRSNHQDPNFGLYRTAAAGKPWQTLIA
PQAQHEVESFSLFRDWLVVQERANGLVQLRQISWDGKTERAIPFDDASYMAWLGYNPEPDSDRLRYGY
SAMTTPTRTYEWDLNKGERTLLKQQEVKGVDPSLYHSERIWIAARDGVKVPVSLVYRTSLFKNGHNPL
LVYGYGAYGMSMDPAFSANRISLLDRGFVYALIHVRGGGELGQRWYKQGKLTHKPNSFNDFIDATQAL
INDGYGQPGRIYAMGGSAGGLLMGAVINQAPQLYNAVVAQVPFVDVVTTMLDDSIPLTTGEYEEWGNP
HQPAAYALMKSYSPYDNVRRQHYPNLLVTSGLYDSQVQYWEPAKWVAKLRRFKQGDSLLLLSTDMTAG
HGGKSGRLARLENGALEYAFILAADRQAQK
>CORE_REP|Org7_Gene773#
MLTPIIRKFQYGQHTVTIETGMMARQATAAVMVSMDDTAVFVTVVGQKKAKPGQSFFPLTVNYQERTY
AAGRIPGSFFRREGRPSEGETLTSRLIDRPIRPLFPDSFLNEVQVIATVVSVNPQVNPDIVAMIGASA
ALSLSGIPFNGPIGAARVGYINDQYVLNPTTDELKESRLDLVVAGTAGAVLMVESEADVLSEDQMLGA
VVFGHDQQQVVIENINALVAEAGKPKWDWQAPAVNEALHARVAELAEARLGDAYHITEKQERYAQVDA
IKDSVVETLLAQDETLDAGEIQDILGTVEKNVVRSRVLRGEPRIDGREKDMIRGLDVRTGVLPRTHGS
ALFTRGETQALVTATLGTARDAQNLDELMGEKTDSFLFHYNFPPYSVGETGMVGSPKRREIGHGRLAK
RGVLAMMPKPEDFPYTVRVVSEITESNGSSSMASVCGASLALMDAGVPIKAAVAGIAMGLVKEADNYV
VLSDILGDEDHLGDMDFKVAGSRDGITALQMDIKIEGITREIMQVALNQAKGARLHILGVMEQAISTP
RGDISEFAPRIHTIRINPDKIKDVIGKGGSVIRALTEETGTTIEIEDDGTVKIAATDGEKAKFAIRRI
EEITAEIEVGRIYQGKVTRIVDFGAFVAIGGGKEGLVHISQIADKRVEKVTDYLQMGQEVPVKVLEVD
RQGRVRLSIKEAMAPEAGSPAPEAE

>CORE_REP|Org36_Gene4690#
MARTTPIARYRNIGISAHIDAGKTTTTERILFYTGVNHKIGEVHDGAATMDWMEQEQERGITITSAAT
TAFWSGMAKQFEPHRVNIIDTPGHVDFTIEVERSMRVLDGAVMVYCAVGGVQPQSETVWRQANKYKVP
RIAFVNKMDRMGANFLKVVGQIKSRLGANPVPLQLAIGAEDKFTGVIDLVKMKAINWNEEDAGVTFEY
EDVPADMMDLAEEWRQNLIESAAEASEELMEKYLGGEELTEAEIKSALRQRVLNNEIILVTCGSAFKN
KGVQAMLDAVIEYLPAPTDVPAINGILDDGKDTPAERHASDDEPFSALAFKIATDPFVGNLTFFRVYS
GVVNSGDTVLNSVKSARERFGRIVQMHANKREEIKEVRAGDIAAAIGLKDVTTGDTLCDPDSPIILER
MEFPEPVISIAVEPKTKADQEKMGLALGRLAKEDPSFRVWTDEESNQTIIAGMGELHLDIIVDRMKRE
FNVEANVGKPQVAYREAIRAKITDVEGKHAKQSGGRGQYGHVVIDMYPLEPGSNPKGYEFINDIKGGV
IPGEYIPAVDKGIQEQLKSGPLAGYPVVDMGIRLHFGSYHDVDSSELAFKLAASIAFKEGFKKAKPVL
LEPIMKVEVETPEENTGDVIGDLSRRRGMLRGQESEVTGVKIHAEVPLSEMFGYATQLRSLTKGRASY
TMEFLKYDDAPNNVAQAVIEARGK
>CORE_REP|Org2_Gene3654#
MWRRLIYHPEINYALRQTLVLCLPVLFGLLIGQLQLGLMFSLVPACCNIAGLDTPHKRFFKRLVVGGS
LFAFSSVLLQQALLWHVPLPALMLGLALLLGVTGEISPLHARLLPAALVAAIFALSTAGTVPIWQAPL
LYAIGTVWYGLFTWFWFKLWKEQPMRETLSQLYLELADYFEAKYSLLTQHTDPQTALPPLLVRQQKVM
DLISLLYQQLNFLPHANNLEQKRLQRAFQVAMDLQEHITVSLHLPEEVQKLVEQSQAEAIIRRNAQVI
AGRLRVVAHDILYHQHSKRFSMAHELAALEKMAAQHPDNPVGQFCYYHFSRIARLLRTQHPLYRRDLM
PGQHRLPFWPALASYLSFKSTALRNAARLGVTLAVGSSLGAVFNLPKPYWILLTIMLVSQNGYNATRV
RIQHRALGTIAGLLLAAGLLQLQLPEGETLSIMLVITLLAYLVSRKNYGLSVIGFTVTAVYTLQLLAL
NGSHFLVPRLIDTLIGCVLAFGGTIWLWPQWQSGLLRKNAHQALEHDQTALRLMLEQPEPDATALAYT
RMQVNQAHNALFTSLNQAMQEPGFASNYLADMRLWVTHSQFIVEHLNAMTILAREHYMLTPKLAEAYL
QTCEIALQSCQQRLEYDGPSSGNSGIMQPPDLHPEMPVTEMERHLRRILSHLSVMHTISSLAWRQRPH
HGIWLKRKLRDQ
>CORE_REP|Org45_Gene1678#
MNKFVRLTAIAGLLWAGVSYGAETANIRIGQLPQLQQEPQHATVSERVTSRFTRSHYRQFALDAEFSG
KIFDRYLNMLDYSHNVLLASDVAQFAGKRNQVGEELKTGKLDTFYALFNLAQKRRFERYTYALSLLDK
PMNFTGNGTIDLDRSKAPWPKDKAELDSLWDAKVTYDELNLKLTGKTDKEIRDTLTKRYQFAIKRLTQ
SNSEDVFQLAMNAFAHEIDPHTNYLSPRNTEQFNTEMSLSLEGIGAVLQMDDDYTLINSMVPGGPAAK
SKAITVGDRIVGVGQAGKPMVDVIGWRLDDVVSLIKGPKGSKVRLEILPAGKGTKTRVVTLTRERIRL
EDRAVKMTIKTVGKEKVAVFDIPGFYVGLTDDVKVQLQKMAKQNVKSVIIDLRTNGGGALTEAVSLSG
LFIPSGPVVQVRDNNGKVREDADTDGVTYYKGPLVVLVDRFSASASEIFAAAMQDYGRALIVGEPTFG
KGTVQQYRSLNRIYDQMLRPEWPALGSVQYTIQKFYRVNGGSTQRKGVTPDILMPTGVDPAETGEAFE
DNAMPWDSINAATYSKTGDMAPFEPELLKDHQQRIAQNPEFQYIAQDIAHYKALKDKRNIVSLNLAVR
EKENHDDDATRLKRINERLERAGKKPLKSLDDLPKDYQEPDPYLDETVHIALELAHLEKDRPAQQPTP
AK
>CORE_REP|Org22_Gene2298#
MNILSYLQKVGRALMVPVATLPAAAILMGVGYWIDPVGWGGDNALAALFIKSGSAIIDHMAVLFAIGV
AYGMSKDKDGSAALTGFVGFLVLTTLCSPAAVSMIQKIPLDQVPAAFGKIENQFVGILVGIISAEVYN
RFSGVELPKALSFFSGRRLVPILISFLMILVAYILMFVWPVVFGALVSFGEHIQKLGSVGAGIYAFFN
RLLIPVGLHHALNSVFWFDVAGINDIPNFLGGQQSIEAGKAVVGITGRYQAGFFPIMMFGLPGAALAI
YHCARPENKAKVLGIMMAGAFAAFFTGITEPLEFSFMFVAPVLYVLHAILTGISVFIAASMHWIAGFG
FSAGLVDMVLSSRNPLATHWYMLIPQGLVFFVIYYVVFRFTINKFNLMTPGRELAVAGDETDGYDVNV
NSNAGKDENETTTLARRYVGAIGGSDNLTGIDACITRLRLNVKDSALVNDALAKRLGASGVIRLNKQS
VQVIVGTRAELIASAMRNVIAAGPVAAAAAPAAAPAAEAKSQAVPNAPKTALETLVAPVTGEVVALDQ
VPDEAFASKAVGDGLAIRPTDNIVVAPADGTVVKIFNTNHAFCLETDKGAEIVVHMGIDTVALEGQGF
KRLVEEGAEVKAGQPILELDLDYLNANARSMISPVVVSNSDDYAGLAALASGSVVAGQTKLYEIQK
>CORE_REP|Org22_Gene3407#
MRLNPSQQQAVEFVTGPCLVLAGAGSGKTRVITNKIAHLIHHCGYQARHIAAVTFTNKAAREMKERVS
QTLGRKEARGLMISTFHTLGLEIIKREYVALGMKSNFSLFDDQDQLALLKELTEKWLESDKTLVAQLI
STISNWKNDLIDPQRAAELARSERDKLFAHCYGLYHAHMRACNVLDFDDLILLPTLLLQRNEEVRERW
QQRIRYLLVDEYQDTNTSQYELVKLLVGNRARFTVVGDDDQSIYSWRGARPQNLVLLKEDFPALQVIK
LEQNYRSSERILKAANILIANNPHVFEKRLFSELGYGEELKVVTANNEDHEAERVVGELIAHHFVKKT
NYGDYAILYRGNHQSRVFEKMLMQNRIPYKISGGTSFFSRPEIKDLLAYLRVLTNPDDDSAFLRIVNT

PKREIGPATLQKLGEWANQRNKSLFHASFDLGLSQHLTGRGLESLQRFTHWLGGIAQQAEREPVAAVR
DLIRGVDYESWLFETSTSPKAAEMRMKNVNTLFGWMTEMLEGNDLDEPMTLTQVVTRFTLRDMMERGE
SEEELDQVQLMTLHASKGLEFPYVFLVGMEEGLLPHQSSIDEDNVDEERRLAYVGITRAQKELIFTLC
RERRQYGELVRPEPSRFLLELPQDDLAWETERKVVSPQERMQKGQSHLANIRAQLAKAKGGN
>CORE_REP|Org37_Gene4277#
MSNYPHLLAPLDLGFTTLKNRVLMGSMHTGLEELPDGPQRLAAFYAERAAAGVALIVTGGIAPNDKGV
VYRGGSTLNSEAQLPHHRPVTEAVHRAGGKIALQILHAGRYSYQPHPVGPSALQAPINPFAPSALSEA
EIEQTIADFARCAALAQQAGYDGVEVMGSEGYLINQFLAARTNQRDDRWGGSFTNRMRFAVEIVRAVR
QAVGAKFILIYRLSMLDLVEDGSSWQEIEQLALAVEQAGATIINTGIGWHEARIPTIATMVPRAGFSW
VTRKLMGKVGIPLITTNRINDPAVAEQVLADGCADMVSMARPFLADAAFVQKAAEGRADEINTCIGCN
QACLDQIFEGKLTSCLVNPRACRETEMPLTMAEKPKTLAVIGAGPAGLAFATTAASRGHQVTLFDAAD
QIGGQFNIAKQIPGKEEFHETLRYFRRQLALREVKVRLGVKVEAADLSEFDEVILACGIMPRTPDIPG
IGHAKVLSYLDVLRDKKPVGQRVAIVGAGGIGFDTAEYLSQHGVSSSQDQAEFNREWGIDGRLEQRGG
LAAQGPQAPRAARQIYLLQRKTSKVGEGLGKTTGWIHRASLAMRGVKMLNSVSYRLIDDEGLHITRAE
QDSCLPVDTVVICAGQEPRRELQQPLQAMGKTVHLIGGADVAAELDARRAIDQGTRLAMAL
>CORE_REP|Org16_Gene4255#
MSSRKELANAIRALSMDAVQKANSGHPGAPMGMADIAEVLWRDYLNHNPTNPHWADRDRFVLSNGHGS
MLIYSLLHLTGYDLPMRELENFRQLHSKTPGHPEYGYTPGVETTTGPLGQGIANAVGFAIAERTLAAQ
FNRPGHDIVDHHTYAFMGDGCMMEGISHEVCSLAGTLKLGKLTAFYDDNGISIDGHVDGWFTDDTALR
FEAYGWHVVRNVDGHNPDAIKAAIEAARKVTDKPSLLMCKTVIGFGSPNKAGTHDVHGAALGAAEVAA
TREALGWKYAAFEIPQDIYAQWDAKEAGQAKEAAWNDKFAAYAKAFPELAAEFKRRMNGELPADWKAD
AKAFVEKLQANPANIASRKASQNALEAFGKVLPEFLGGSADLAPSNLTMWSGSKALNVDPAGNYIHYG
VREFGMTAITNGIALHGGFLPYSATFLMFVEYARNAVRMAALMKLRNVFVYTHDSIGLGEDGPTHQPV
EQLASLRVTPNMSTWRPCDQVESAVAWQYGIERNDGPTTLVFSRQNLTQQPRSAEQLANVYRGGYVLK
DCAGTPDVILIATGSEVGITVEAADKLTAAGRKVRVVSMPSTDAFDKQDAAYRESVLPAAVTARVAVE
AGIADYWYKYVGLNGAIVGMTTFGESAPAEQLFAEFGFTVDNVVAKAQALLK
>CORE_REP|Org1_Gene4227#
MTTEFETSFADLGLSAPIISALNDLGYEKPSPIQAECIPHLLNGRDVLGMAQTGSGKTAAFSLPLLHN
LQADLKAPQILVLAPTRELAVQVAEAMTDFSKHMNGVNVVALYGGQRYDVQLRALRQGPQIVVGTPGR
LLDHLKRGTLNLSNLSGLVLDEADEMLRMGFIEDVETIMAEIPAEHQTALFSATMPEAIRRITRRFMK
EPQEVRIQSSVTTRPDISQSYWSVYGMRKNEALVRFLEAEDFDAAIIFVRTKNATLEVAEALERSGYS
SAALNGDMNQALREQTLERLKDGRLDILIATDVAARGLDVERISLVVNYDIPMDSESYVHRIGRTGRA
GRAGRALLFVENRERRLLRNIERTMKLTIPEVELPNAELLGERRLAKFAAKVQQQLESSDLDLYRALL
TKLQPEEELDMETLAAALLKMAQGERPLILPPDPVFKPRQRREFNDRDDRRGDRGDRRDSRDSRDGDR
PRRERRDVGEMQLYRIEVGRDDGVEVRHIVGAIANEGDISSRYIGNIKLFASHSTIELPKGMPGEILN
HFTRTRILNKPMNMQLLGDAQPFERRERRDGGNGGERRGNGNGRPFNGERREGGPRRSFGERREGGNG
GERRGGNYNRDGKPAPRRDDSAPAAPRRRFGDA
>CORE_REP|Org31_Gene3983#
MALLKETIRDHSAEERLFIRRAGVALALVVVCFGALIVNLYRLQIRQHGFYQTRSNQNDIKMLPIAPS
RGLIFDRNGTPLVRNVTLYRIEITPSKISDMAALLQALTPIVDLTPEDISAFRDDMHHNSRYKPVTLK
AGLSDTEVARFAVNQYRFDGVTIDTYQQREYPYGAQLAHVLGYVSKINDSDLKRLDKAGLSENYAADR
NIGKQGIEAYYEAELHGTTGYQEVEVDNHGRVIRLLKEQPPKAGKNIYLTLDLPLQQYIESVLKGQRA
AVVVEDPRDGGILAMVSSPSYDPNPFVKGIGYQAYKALLTNPDLPLINRVTQGLYPPASTVKPYMAVS
ALFAGVITPTTTFFGAPTWTLPGTERRYRDWLKTGHGMLNVTKAIEESADTFFYQVAYEMGIDRIHHW
LSQFGYGQSTGIDLNEEYRGVLPSRDWKLKVHKKGWYQGDTVSVGIGQGYWVATPIQMVKALTTLINN
GQVKTPHLLYSLQQGNRVTRYPPPAKTAQIGDPNSPYWGIVRNGMYGMANLPNGTGYKLFHTAPYQIA
AKSGTSQVFSLKQNQTYNAKMIPVRLRDHIFYTLFAPYKNPRVAMALILENGGGNGVVAGPTARAILD
HIFDPANAPQPGDAGQSKPQLNDSADVQR
>CORE_REP|Org5_Gene3577#
MIVFSSLQIRRGIRVLLDNATATVNPGQKVGLVGKNGCGKSTLLSLLKGEIAADGGSFTFPGNWALAW
VNQETPALDVPAIEYVIDGDREFRQLEAELQAANDRNDGHAIATLHGKLDAIDAWTIRSRAASLLHGL
GFSNEQLQSPVRDFSGGWRMRLNLAQALVCRSDLLLLDEPTNHLDLDAVIWLERWLKSYPGTLVLISH
DRDFLDPIVDKILHIEQQTINEYTGNYSSFERQRATKLAQQQSLYQHQQEKVAHLQSYIDRFRAQATK
AKQAQSRIKMLERMELIAPAHVDNPFTFSFRPPESLPNPLLRMDKVSAGYGDKVILKSIKLNLVPGSR

IGLLGRNGAGKSTLIKLLAGTLEPLSGEIGLAKGIKLGYFAQHQLEFLRADESPLQHLSRIAPRVLEQ
QLRDYLGGFGFQGDKVSEVTERFSGGEKARLVLALIVWQRPNLLLLDEPTNHLDLDMRQALTEALIDF
EGALVVVSHDRHLLRSTTDDLYLVHDGQVEPFEGDLDDYQQWLVDLQRQESQQDAPEKESGGNSAQAR
KEQKRREAEFRTQTQPLRKQIAKLEQQMEKLGAELAAVEEQLADPALYDISRKAELTDCLQKQSQAKS
ALEETEMTWLDAQEQLEQLTQAFEA
>CORE_REP|Org40_Gene254#
MGKIIGIDLGTTNSCVAIMDGAKARVLENAEGDRTTPSIIAYTQDGETLVGQPAKRQAVTNPENTLFA
IKRLIGRRFQDEEAQRDKAIMPYKIVEADNGDAWLEVKGQKMAPPQISAEVLKKMKKTAEDYLGEPVT
EAVITVPAYFNDAQRQATKDAGRIAGLEVKRIINEPTAAALAYGLDKEVGNRTIAVYDLGGGTFDISI
IEIDDVDGEKTFEVLATNGDTHLGGEDFDSRLINYLVEEFKKDQGIDLRNDPLAMQRLKEAAEKAKIE
LSSAQQTDVNLPYITADATGPKHMNIKVTRAKLESLVEDLVARSIEPLKVALKDAGLSVSDIQDVILV
GGQTRMPMVQKKVADFFGKEPRKDVNPDEAVAVGAAVQGGVLAGDVKDVLLLDVTPLSLGIETMGSVM
TPLITKNTTIPTKHSQVFSTAEDNQSAVTIHVLQGERKRASDNKSLGQFNLDGIQAAPRGMAQIEVTF
DIDADGILHVSAKDKNTGREQKITIKASSGLNEDEIQKMVRDAELNAEADRKFEELVQTRNQADHLIH
GTRKQLEEAGDKLPAEDKTAIEAALKDLEAAVKGEDKAEIEAKTQALVQVSGKLLEMAQAQQAQQGAD
AGADNAAQKDDDVVDAEFEEVKDKK
>CORE_REP|Org2_Gene2907#
MSLISMSGAWLSFSDAPLLDNTEIHIEDNERVCLVGRNGAGKSTLLKILGKEIPLDDGRVIYEQDLIV
ARLQQDPPRNIGGSVFDFVAEGVAEQAEHLKAYHAISHLVESDPSEKNLARMAQIMEILDHQGLWQLD
SRISEVLLQLGLNGDAELSSLSGGWLRKAALGRALVSSPRVLLLDEPTNHLDIETIDWLEGFLKEFDG
SIVFISHDRSFIRNMATRIVDLDRGKLVSWPGNYDLYLQSKEEALRVEELQNAEFDRKLAQEEVWIRQ
GIKARRTRNEGRVRALKALRVERSERREVMGTAKMQVEEATRSGKIVFELEDVNYQVGEKVLVRGFSA
QVQRGDKIALVGPNGCGKTTLLKLMLGQLKADSGRVHCGTKLEVAYFDQHRADLDPERTVMDNLAEGK
QEVVVNGRPRHVLGYLQDFLFHPKRAMTPVKALSGGERNRLLLAKLFLKPSNLLILDEPTNDLDVETL
ELLEELIDGYQGTVLLVSHDRQFVDNSVTECWIFEGNGVINAFVGGYYDAHHQRATAKPIRQAAPSAS
KPAAEKKAEQPKKAAAKLSYNLLRELEQLPQRLEQLEAEIEALQAQMSDADFFTRPHSETQQVLTALA
NAEQALEQAFARWEELEAMKNG
>CORE_REP|Org26_Gene4567#
MSMKGQETRGFQSEVKQLLHLMIHSLYSNKEIFLRELISNASDAADKLRFRALSAPELYAGDGELRVR
LSFDKEQRTLTIADNGIGMRREEVIENLGTIAKSGTKAFLESIGSDQAKDSQLIGQFGVGFYSAFIVA
DKVTVRTRAAGAADDEGVFWESAGEGDYTIADITKETRGTEITLHLREGEDEYLDAWRLRSVIGKYSD
HIALPVEIESKNEEDDTVTWEKINKAQALWTRSKADVTDEEYKEFYKHIAHDFTDPLSWSHNRVEGKQ
EYTSLLYIPAQAPWDMWNRDHKHGLKLYVQRVFIMDDAEQFMPNYLRFVRGLIDSNDLPLNVSREILQ
DSRVTQNLRGALTKRVLQMLDKLAKDDAEGYQKFWQQFGLVLKEGPAEDHGNQEAIAKLLRFASTHGD
SSAQTVSLEEYVGRMAEGQEKIYYITADSYAAAKSSPHLELFRKKGIEVLLLSDRIDEWMMSYLTEFD
GKPFQSVSKADETLDKLADETEEQKAAEKQLEPFIERVKTLLGERVKDVRLTHRLTDTPAIVVTDADE
MSTQMAKLFAAAGQQAPEVKYIFELNPEHALVKRASDVGDNEHFAEWIDLLLDQALLAERGTLEDPNL
FIRRMNKLLSA
>CORE_REP|Org15_Gene3079#
MKYDTLASEILAGVGGRDNVKSLVHCATRLRFKLRDDRRANAAALKKNPGVIMVVESGGQFQVVVGNH
VAEVFDAVNRVGGLAEGASSGSDADGKKDNLLSRFIDVVSGIFTPLLGVMAASGVLKGLLALSLACGW
LLESSGAFKMLFAASDALFYFFPIMLGYTAGKKFGGNPFVTMAIGGALTHPLMMAAFEAAQQPGAVRE
YFFGIPLTFINYSSSVIPIIFAAWVSCRLEPLFNRVIHSALRNFITPLLCLAITVPLTFLLIGPAATW
LSHLLANGYQAIYAFNPIIAGAFMGAMWQVCVIFGLHWGLVPLMINNLSVLGRDTMVPLLLPAVMGQV
GATLGVMLRTRDAKLRALSGSAIGAGIFGITEPAVYGVTLPNKRPFIFGCIGGALGGAVIGYFHTSVY
SFGLVNVFTFAQIIPNGGIDATVWGAIGGTLLSFVFAALASYLFGVAPAEETAQPEAAAPLNRKQAIL
SPIAGDIVPLEQVNDATFASGLLGKGVAIAPLQGRVVAPVSGSVASLFKTKHAIGIESDDGAEILIHV
GIDTVKLDGAHFTAHVREGERIAPGDLLIEFDQAAIHAAGYDTTTPIIISNSDDYVDVLTSGLSPVQE
QAPLLTLLR
>CORE_REP|Org48_Gene4550#
MDNHHMMIEGLIYLGSAALFVPIAVRLGLGSVLGYLIAGCIIGPWGLKLVSDAESILTFAEIGVVLML
FIIGLELDPKRLWTLRASVFGGGSIQMVGCGLALSAFCYFLGLNWKVALLIGLTLALSSTAIAMQAMS
ERNLTPSPIGRSAFAVLLFQDIAAIPLVAMIPLLASSGATTTLGAFVLSAAKVVGALTMVVLLGRYVT
RPLLHFVARSGMREVFSAVALFLVFGFGILLEMAGLSMAMGAFLAGVLLASSEYRHALESDIQPFKGL

LLGLFFIGVGMSIDFGTLFHHPLLIASLLLGFMLIKAALLWLIGPLLGVPKRQRGLFAILLGQGSEFA
FVIFSAAQLAGVLPVEWAKSLTLAVALSMAATPLLLVIAAQLEKNAPKEERPADVIDDENASVIIAGF
GRFGQIAGRLLLANGVHTVVLDHDPDHIETLRKFDTKVFYGDATRADLLEAAGAAHAKVLINAIDDVE
DSLALTELARQHFPHLKVVARARDVDHWYQLRQLGVEKPERETFESSLRIGRETLELLGLDAYEAREK
ADMFRRYNLKMLEDTLENYQDTEFRIASLQRAKEMLSAAIEQDQNRLSRVQQTGWRGSIDGKAPEDEV
VEAKG
>CORE_REP|Org49_Gene2135#
MALLQISEPGLSAAPHQRRLAAGIDLGTTNSLVATVRSGQAETLADEQGRHLLPSVVHYQADAQRVGW
EARQQAAQDPANTVSSIKRMMGRSLADVQQRYPNLPYQFQASDNGLPLIVTAGGPVNPVGVSADILRA
LSARAQAALEGELDGVVITVPAYFDDAQRQGTKDAARLAGLHVLRLLNEPTAAAIAYGLDSGQEGVIA
VYDLGGGTFDISILRLSRGVFEVLATGGDSALGGDDFDHLLADWLREQAGVADRSDHGVQRQLLDAAI
AAKIALSDADSVRVEVAGWQGEVTRAQFEALIAPLVKRTLMACRRALKDAGVAADEVLEAVMVGGSTR
VPLVREQVGAFFGRTPLTSIDPDKVVAIGAAIQADILVGNKPDSDMLLLDVIPLSLGLETMGGLVEKV
IPRNTTIPVARAQEFTTFKDGQSAMMIHVLQGERELVQDCRSLARFTLRGLPPLPAGGAHIRVTFQVD
ADGLLSVTAMEKSTGVEASIQVKPSYGLSDSEIAGMIKDSMANAQSDVGARKLAEQRVEAARVLESLQ
GALASDAALLSEAESQAIAAATQALQQAVQGEDPAAIEDAIKTLDAQTQDFAARRMDASIRRALAGHS
VDEV
>CORE_REP|Org25_Gene3079#
MLNRYPLWKYLMLIVVILVGLLYALPNIYGEDPAVQITGARGVAASETTLDQVRTVLEKDNIASKSIA
LENGAILARFKDPDVQLRAREALVTELGDKFVVALNLAPATPTWLAMLGAEPMKLGLDLRGGVHFLME
VDMDTALSKLQEQTMDTLRSELREKGIPYASIRKLDNNGVEVRFRDDAARDQAISYIGPRQRDLVLSA
NGANTMKASLTDARLSEAREYAVQQNITILRNRVNQLGVAEPLVQRQGSDRIVVELPGIQDTARAKEI
LGATATLEFRLVNTNADATAAANGRVPGDSEVKYTRDGQPIVLYKRVILTGDHITDSTSSTDEYNQPQ
VNISLDSAGGTSMSNFTKDNIGKPMATLFVEYKDSGKKDANGRAVLVKQEEVINVANIQSRLGNSFRI
TGIGNPNEARQLSLLLRAGALIAPIQIVEERTIGPTLGQQNITQGLEACLWGLVASIVFMVVWYRKFG
VIATTALVANLVLIVGVMSLLPGATLTMPGIAGIVLTLAVAVDANVLINERIKEELKNGRSVQQAIHE
GYKGAFSSIVDANITTLITAVILYAVGTGSIKGFAITTAIGVATSMFTAIVGTRAIVNLLYGGKRINK
LSI
>CORE_REP|Org30_Gene154#
MIENLRNIAIIAHVDHGKTTLVDKLLQQSGTFGERAEATERVMDSNDLEKERGITILAKNTAINWNGY
RINIVDTPGHADFGGEVERVMSMVDSVLLVVDAMDGPMPQTRFVTKKAFANGLKPIVVINKVDRPGAR
PDWVVDQVFDLFVNLDATDEQLDFPIIYASALNGIAGVDHTDMAEDMTPLYQAIVDHVSAPQVELEAP
FQMQISQLDYNNYLGVIGIGRIKRGKVKPNQQVTIIDSEGKTRNGKVGKVLGHLGLERIDSTLAEAGD
IIAITGLGELNISDTICDTNAVEALPALSVDEPTVTMFFNVNTSPFCGKEGKYVTSRQILDRLNKELV
HNVALRVEETDDADAFRVSGRGELHLSVLIENMRREGFELAVSRPKVIFREIDGRKQEPFENVTLDIE
EQHQGSVMQAMGERKADLKNMDPDGKGRVRLDYVIPSRGLIGFRNEFMTMTSGTGLLYSTFSHYDDVR
PGEVGQRQNGVLISNGQGKAVAFALFGLQDRGKLFLGHGAEVYEGQIIGIHSRSNDLTVNCLTGKKLT
NMRASGTDEATTLVPAIKMTLEQALEFIDDDELVEVTPTSIRIRKRHLTENDRKRASRGPKDA
>CORE_REP|Org47_Gene4848#
MINPTLSRVTQRIIHRSQASRAAYLARIEAARSQTVHRAQLACGNLAHGFAACQPNDKTALKNMVRSD
IAIITAYNDMLSAHQPYEHYPQRLKQALKAVGAVGQVAGGVPAMCDGVTQGQDGMELSLMSRDVIAMS
AAVGLSHNMFDGALFLGICDKIVPGLVMAALSFGHLPALFVPAGPMSSGLPNKEKVRVRQLYAEGKAD
RLALLEAEAASYHGIGTCTFYGTANTNQMVMEVMGLHLPGASFVHPDTPLRDALNDAAARQVTRLTDT
AGNYLPIGRLVDEKVVVNGIVSLLATGGSTNLTMHLVAMARAAGIIITWDDFSELSEAVPLLCRIYPN
GPADINQFQAAGGVPLVVRELLQHGLLHEDVHTVAGFGLHRYTQEPWLDNGQLVWREGVAGSLDASVI
ASVAQPFEHHGGTKVMAGNLGRAVMKTSAVPADNQIIEAPAVVFDSQHDIVPAFEAGKLDRDCVVVVR
FQGPQANGMPELHKLMPPLGVLMDRGFKVALVTDGRLSGASGKVPSAIHVTPEAYTGGLLAKVRDGDP
IRVNGRSGELQVLVDADELAQRTPCQPDLSAEHIGCGRELFGALRSQLSGAEQGACCITF
>CORE_REP|Org47_Gene4278#
MLLKKGNSLRRLALSGAIACSLVSSFSASATVPALPVASAGMSVAQSRSELLAALPRGMDLHYLSTLA
PLYAANHMQPMWQDREAVQQFQQQLAELAMSGVQPQFTQWVKMLTDPALSEAGRDAVLSDAMLGYLQF
VSAIGANGNNWLYSNIPYKLGLPPTAVINQWQLAVRQARTLSYVNSLAPQHPQYAKMHQALRDMLADN
RPWPQVGSGPSLRPGQMSNDIPALREILTRTGMLAASAPEADPEPAVVSAKINEPDDGGLTVDEEKSR
VTVSPSAAPVTELTAEQTPPQIGSVVSDNLYTDELVEGVKRFQKWQGLTADGVIGVRTREWLNVSPKT

RAALLALNIQRLRILPGHVGTGIMVNIPNYSLTYYQNGNEVLSSRVIVGRPSRKTPLMSSALNNVVVN
PPWNVPTTLVREDIVPKAMRDGNYFQKHGYTVLSGWSNDAEVINPAMIDWSMISARNFPYRVRQAPGA
TNSLGRFKFNMPSSDAIYLHDTPNHSLFQKDIRALSSGCVRVNKASDLANMLLQDAGWNNSRVSSTLK
EGNTTYVNIRQRIPVKLYYLTAWVSDDGQPQFRTDIYNYDNTVRSGAQILAQAKKLMQ
>CORE_REP|Org28_Gene1320#
MEGSTLLTAILLFLFAAVVTVPIARRLGIGAVLGYLIAGIAIGPWGLGFIRDVDEILHFSELGVVFLM
FIIGLELNPSKLWELRRSIFGAGAGQVLITAAVLGALLYLTHFAWQAAVIGGVGLAMSSTAMALQLMR
EKGMNRNEGGQLGFSVLLFQDMAVIPALALIPILAGAGGTSDDWAKIALKVAAFGGMLIGGRFLLRPL
FRYIAASGVREIFTAAALLLVLGSALFMEALGLSMALGTFIAGVLLAESEYRHELEISIEPFKGLLLG
LFFISVGMVLNIGVLYTHLAEVLIGVLVLVTVKSGVLYGVSRLFGLRSSVRLQFAGVLSQGGEFAFVL
FSAAGAQKVLQPDQLSLLLVVVTLSMMTTPLLMQAIDRILARRYNAKDEDEETPYVEDDDPQVIIVGF
GRFGQVIGRLLMANKMRITVLERDVSAVGVLRRYGYKVYYGDATELELLRAAGAEKAKSIVITCNEPE
DTMEIVRLCQQHFPNLSILARARGRVEAHELLQAGVKQFSRETFSSALELGRKALMELGMHPHQAFRA
QQHFRRLDMRMLRELMPPHQGDVAQISRVKEARRELEELFHREMQKESRQFDGWDEYE
>CORE_REP|Org9_Gene2216#
MKHIRNFSIIAHIDHGKSTLSDRIIQICGGLSDREMAAQVLDSMDLERERGITIKAQSVTLDYKALDG
QTYQLNFIDTPGHVDFSYEVSRSLAACEGALLVVDAGQGVEAQTLANCYTAIEMDLEVVPVLNKIDLP
AADPDRAAQEIEDIVGIDATDAVRCSAKTGVGVPDVLERLVRDIPPPQGDPDAPLQALIIDSWFDNYL
GVVSLVRVKNGTLRKGDKIKVMSTGQVYNADRLGIFTPKQVDRDVLNCGEVGWLVCAIKDILGAPVGD
TLTQARQPADKALPGFKKVKPQVYAGLFPISSDDYESFRDALGKLSLNDASLFYEPESSTALGFGFRC
GFLGLLHMEIIQERLEREYDLDLITTAPTVVYEVETTGKEVIYVDSPSKLPPLNNIQELREPIAECHM
LMPQEYLGNVITLCVEKRGVQTNMVYHGNQVALTYEIPMAEVVLDFFDRLKSTSRGYASLDYNFKRFQ
ASDMVRVDVLINNERVDALALITHRDNSQYRGRELVEKMKDLIPRQQFDIAIQAAIGTHIIARSTVKQ
LRKNVLAKCYGGDVSRKKKLLQKQKDGKKRMKQVGNVELPQEAFLAILHVGKDGK
>CORE_REP|Org20_Gene235#
MKAARPGKLRRQEDQASFVSWRFALLCGCILLAMVGLMLRVAYLQVINPDRLVKEGDMRSLRVQEVPT
ARGMISDRAGRPLAVSVPVNAVWADPKELNERGGITLDSRWKALSDALNIPLDQLSNRINANPKGRFV
YLARQVNPAIGDYIHKLKLPGIYLRQESRRYYPAGQVTSHIIGVTNIDGQGIEGVEKSFDRWLTGQPG
ERTVRKDRFGRVIEDISSVDSQAAHNLVLSVDERLQALVYRELNNAVAFNKAESGTAVLIDVNTGEVL
AMANSPSYNPNNMAGTPKETMRNRAITDIFEPGSTVKPMVVMTALQNGVVRENSVLNTIPYRIQGHEI
KDVARYSELSLTGILQKSSNVGVSKLALAMPSSALVDTYSRFGLGKATNLGLVGESSGIYPKKQRWSD
IERATFSFGYGLMVTPLQLARVYATIGSLGVYRPLSITKVDPPVAGERVFPEPLVRTVVHMMESVALP
GGGGVKAAIKGYRIAIKTGTAKKVGPDGKYVNRYIAYTAGVAPASNPRFALVVVINDPQGGKYYGGAI
SAPVFGAIMGGVLRTMNVEPDALPTGDKSELVINKKEGSGGRS
>CORE_REP|Org28_Gene3029#
MTQTFIPGKDAALEDSIARFQQKLSDLGFNIEEASWLNPVPHVWSVHIRDRDCPLCFTNGKGASKKAA
LASALGEYFERLSTNYFFADFYLGRQIAEGDFVHYPNEKWFPIPEDDALPAGILDERLHAFYDPQQEL
SASDLVDLQSGNADRGVCALPFTRQSDQQTVYIPMNIIGNLYVSNGMSAGNTANEARVQGLSEVFERY
VKNRIIAESISLPAIPDEVLNRYPGVVEAIAKLEEEGFPILSYDASLGGNYPVICVVLFNPTNGTCFA
SFGAHPDFGVALERTVTELLQGRSLKDLDVFTAPTFDDEEVAEHTNLETHFIDSSGLISWDLFKQDAD
YPFVDWNFSGSTQEEFATLMSIFDKEDAEVYIADYEHLGVYACRIIVPGMSDIYPAEDLLLANNSMGA
HLRDTLLALPGSEWKPEEYLALIEQLDDEGLDDFTRVRELLGIASGKDNAWHTLRVGELKSMLALAGG
DLDQALIWTEWTQDFNASVLSPARSNYYRCLQTLLLLAQEPEREAAQYYTAFVKMYGQEAVDAASAAI
SGEERFNGLFAVDADLKALPAHQALLAAYEKLQAAKRRHWAKA
>CORE_REP|Org35_Gene3748#
MASNTLTNNRLEWQSLLPDVTPYQAIFDTAAQLAPVPFSAIQPRLENALTLFCHPQSPPRFMLLKAQE
TREYLELIANAVKPLLPQNTACRGSHYVIQDGKVSVEPASHGDEPFAAGGACVFQEWIEPEQLFGCVR
IHNGDITLQPGLVHQANGGILILSARALLAQPLLWLRLKQMIGQRQFHWVSPDETRPLPVAIPPMPLD
LRLIVVGDRHGLADFHDIEPELSEQAVYGEYEDDLQLTEVDDMAQWCGYVNGVIAERQLPMLAADAWL
PLIVQAVRYSGDQGILPLSPVWLGQQLSEAALYAEEDRITAKAFEAALNAREWRESYLAERMQDEIEL
GQILIETEGEVVGQINGLSVLDYPGHPRSFGEPSRISCVVHLGDGEFTDVERKAELGGNLHAKGMMIM
QAFVIAELDLDQQLPFSASIVFEQSYGEVDGDSASLAELCALISALSQQPITQQIAVTGSVDQFGNVQ
PIGGVNEKVEGFFEVCLRRGLTGKQGVILPVTNVRHLCLRQDVVDAVREGQFHLWAVESAAEALPLLT
GCLYSDEQQPNLLAAIQERIAQVSLQERRRPWPLRWLNWFNHG

>CORE_REP|Org1_Gene2813#
MAFTLRPYQLEAVEATINHFRRHPEPALIVLPTGAGKSLVIAELAKRARGRVLVLAHVKELVAQNHAK
YCAYGLEADIFAAGLQQKESAGKVVFGSVQSVARNLPLFDGAFSLLIVDECHRISDDDDSQYQQIIQH
LQKTNPQLRLLGLTATPYRLGKGWIYQYHYHGFTRGDSASLFRDCIYELPLRYMIKNGFLVPPERLDM
PIVQYDFSRLEARSNGLFSEAELNRELKRQNRVTPHIISQIVEYAEDRKGVMIFAATVEHAREIHGLL
PNGEAALVSAETPPAERDALIDAFKQQRLRYLVNVAVLTTGFDAPHVDLIAILRPTESVSLYQQIVGR
GLRLAPNKKDCLILDYAGNPHDLFTPEVGVSKPHGDSQPVQVFCPACGFANLFWGKCTENGDIIEHYG
RRCQGWLEDDDGHREQCDYRFRFKSCPHCGAENDIAARRCHQCQEVLVDPDDMLKAALKLKDALVLRC
GGMELQSGRDDKGEWLKATYYDEDGTSTSERFRLQTPAQRKAFEMLFLRPHQRAPGVPFAWHTAADVL
AQQQALRHPDFVVARKRGQFWQVREKVFDYQGRFRRANQLA
>CORE_REP|Org37_Gene3654#
MSTLLSAQSVGYDNAFGVLLSEISFSLKKGDRIGLIGDNGCGKSTLLQLLSGALPIHSGTVTLSHQCL
MARIEQHLPPELHASTLLDAVLAQLPAGQHLSERWRCEALLAELGFEPTSWTLTAGTLSGGQHTRLLL
ARALIRQPDLLLLDEPSNHLDLPTLLWLEQFLRSWSGSFVLVSHDRYLLDQVTNCTWILRDKTLQFFR
LPCSAARAALAEQDAADEHRRQAEQKEIDRVEKSAKRLATWGKVYDNEDLARKAKQMEKRVDRLKEEQ
TTLTAGSPWRLRLQGEALDADRLLALPQWAVRPAPDAPVLFSLEHLRVKSGDRIAIVGRNGCGKSSLL
RLLWQAYQHPAERPAIFHPRVRIGYYDQSLQQLRDEDTLSEALAQFAPLTEEQRKMALIGAGFPYLRH
HQQIRSLSGGERSRLLFVGLTLANHSLLLLDEPTNHLDMAGKEELAETLRQFAGAVILVTHDRMLIEQ
SCNRFWLIDQQKLDEWHDLAPVYQRLAGEAPALPTADKANAGGPTPDERLEGEEALLTTLFALESKLE
DDLARKPKHQKPALQARWRREIADITARLNLG
>CORE_REP|Org1_Gene3313#
MNATPLQKHAVWQLIKPFWVSEERWRAWMMLIAIVILSLGLVYISVLINQWNQVFYDALQNKNYPVFK
AQLWRFTYLALIFIVLAVYKIYLTQGLQMRWRRWMTEKFMGKWLAHQAYYHTEQQQIVDNPDQRIAED
LNVLTQYTLSLSLGLLSSLVTLFSFIDILWHVSGPMTFALGQHAITLSGYMVWFALLYAVLGSLLIWW
VGKPLVMLGFNQERYEANFRFGLIRIRENNDAIALYHGEPREAQQLGDRFDTIRSNWWAIMRITRRLN
IATNFYSQFAIVFPLLVAAPRYFSGAIQMGGLMQIASAFGQVQGALSWFIDAFNDLATWKACVNRLAG
FNAAVDQVHHQPRGIQLREEAAHPLTLDNLSLNLPDGQPLLAGAKMTLQRGDRLLIVGPSGCGKSTLL
RAIAGIWPYGAGAIGLPANANTLFLPQRSYIPIGTLREALSYPSQATQYSDEQLMRVLENCRLKHLQR
WLDTAANWSHRLSPGEQQRLAFARALLIRPSILFLDEATSALDDETEQLMYCLLVDELPDVTLISVAH
RNSVAKYHQTCWRFSRSEDQPARLALSPLPV
>CORE_REP|Org45_Gene427#
MISGILVSPGIAFGKALLLKEDDIVINRKKISADQVEQEVSRFLAGRAKASEQLEAIKTKAGETFGEE
KEAIFEGHIMLLEDEELEQEIIALIKDDLASADAAAYTVIEGQAKALEELDDEYLKERAADVRDIGKR
LLQNILGMPIVDLGSIQDEVILVATDLTPSETAQLNLDKVLGFITDLGGRTSHTSIMARSLELPAIVG
TSDVTKQVKNDDYLILDAVNNQIYVNPTADVIDQLKAAQNQYITEKNDLAKLKDLPAITLDGHQVEVC
ANIGTVRDVAGAERNGAEGVGLYRTEFLFMDRDSLPTEDEQFQAYKAVAEAMGSQAVIVRTMDIGGDK
DLPYMNLPKEENPFLGWRAIRIAMDRREILHAQLRAILRASAFGKLRIMFPMIISVEEVRDLKGEIET
LKAQLREEGKAFDESIEVGVMVETPAAAVIAHHLAKEVDFFSIGTNDLTQYTLAVDRGNELISHLYNP
MSPSVLGLIKQVIDASHAEGKWTGMCGELAGDERATLLLLGMGLDEFSMSAISIPRIKKIIRNTNFED
VKALAAQALAQPTAQDLMNCVNKFIEEKTLC
>CORE_REP|Org23_Gene2786#
MAPSTKKSGKTYSTVRFGWICAGMLVCFFLLAFRVGYLQLLEHQQLADQADQRSIRTQVVPTNRAMIT
DRNDEALAVSVSSKDIVLDPKHILDTQTDTGNERWQSMANVLKIPLADIQHLIQSNAHKRFVYLARKV
EDDNAAYISKLHLTGVSAEQDFSRFYPMGQDAAGLIGIVGQDNQGLEGIELGFNPLLQGKNGLRVYQK
DGSGAVIGVLKSVDPVPPPNVTLSIDKFIQYVLYAQIRDGVVANQADSGCAVLVKIDTGEILGMASYP
SFNPNNYGSTPAKDIRNVCSSDSFEPGSTVKPVVVMVGLEHKLIRPDTVLDTTPYRVNGHLIKDVGHW
SKLTITGVLQKSSDIAVSHIALALPATVLPAVYRSFGLGRPTELGIGNESSGYLPQHRERWADIERAT
FSFGYGLRVTPLQMAREYAAIGSFGIYRPLSITKVTPPVMGQRILPADTVRSVVHMMESDALPGGSGV
SAAVPGYRLAIKTGTAEKMGPSGKYDGGYINYTAGVAPASDPQVALVVMVNNPKAGKHFGGSVAGPVF
GKIMAQVLEHMNILPDAQPLNVVSSVKS
>CORE_REP|Org42_Gene1552#
MELEYESKRPLYIPYAGPILLEFPLLNKGSAFTEEERSHFNLHGLLPEAVETIEEQVERAYRQYQDFK
NDNDKHIYLRNIQDTNETLFYRLLDSHLSEMMPIIYTPTVGEACEHFSDIYRRARGLFISYPNRDRID
DMLQNATKQNVKIVVTDGERILGLGDQGIGGMGIPIGKLSLYTACGGISPAYTLPVVLDVGTNNPQR

LNDPLYMGWRHPRISGEEYHAFVEEFIQAVKRRWPNVLLQFEDFAQNNATPLLNRYRDEICCFNDDIQ
GTAAVTLGSLIAASRAAGSQLRDQTVTFLGAGSAGCGIAEQIIAQMKSEGLSEDEARARVFMVDRFGL
LTDKLPNLLDFQSKLVQKSDNLAGWETASDAISLLDVVRNAKPTILIGVSGQPGLFTEELIREMHKHC
ERPIVMPLSNPTSRVEARPEDIINWTDGAALVATGSPFAPVSYKDQLYPIAQCNNSYIFPGIGLGVLA
SGATRVTDAMLMAASRALADCSPLATDGHGALLPNIDDIQGVSKCIAMEVGKAAQLQGVAIVTSEDAL
SKAIEHNFWRPQYRSYKRTSF
>CORE_REP|Org5_Gene2562#
MNKVKSLSQQNLSLLLAIYIGIFLNLSVFYRRFDSLAHGIQGIKVISAVTEVIAIVLFTFFIMRLVSL
GGRLFYRIVASLLVLISVAASYYMTFFNVVIGYGIVVSVMTTDIDLSKEVVGLHFVLWMVALSALPLL
LIWKNSLRYTLIEQLKTPGHRIKPLLVLLAVVALVWLPLRMLDDEQSVQEKLSNVDLPSYGGVVAHSY
LPSNWLSALGLFAYTRYDESQDQSTMFDPGKHFTYVPPADIDDTYVVFIIGETTRWDHMGMLGYERDT
TPRLSKEKNLVAFRGESCDTSTKLSLRCMFVREGGTEDNPQRTLKEQNVFAVLKDLGFSSELFAMQSE
VWFYNNTEVNNYSFREMIASEKRNDGKAVDDMLLVDEMKESLARYPKGKHLVILHTKGSHYLYSQRYP
RSYARYQPECMGVDDSCTKAQLINAFDNTVLYTDSFIANVIDQVRDKKAIVFYAADHGESIGENTHLH
GTPREMAPPEQFRVPMIVWASDKFLENPQHLSAFEQLQAQQRIGKTHRHVELFDTILGCLGYTSPDGG
IVDKNNWCHLPQDKTAPASL
>CORE_REP|Org14_Gene52#
MTESFAQLFEESLKEIETRPGSIVRGVVVAIDKDIVLVDAGLKSESAIPAEQFKNAQGELEIQVGDEV
DVALDAVEDGFGETLLSREKAKRHEAWITLEKAYEEAETVTGVINGKVKGGFTVELNGIRAFLPGSLV
DVRPVRDTLHLEGKELEFKVIKLDQKRNNVVVSRRAVIESENSAERDQLLENLQEGMEVKGIVKNLTD
YGAFVDLGGVDGLLHITDMAWKRVKHPSEIVNVGDEITVKVLKFDRERTRVSLGLKQLGEDPWVAIAK
RYPEGTKLTGRVTNLTDYGCFVEIEEGVEGLVHVSEMDWTNKNIHPSKVVNVGDVVEVMVLDIDEERR
RISLGLKQCKSNPWQQFAETHNKGDRVEGKIKSITDFGIFIGLDGGIDGLVHLSDISWNVAGEEAVRE
YKKGDEIAAVVLQVDAERERISLGVKQLAEDPFNNYLSMNKKGAIVTGKVTAVDAKGATVELAGGVEG
YLRASEASRDRIEDATLVLNVGDEVEAKFTGVDRKNRVVSLSVRAKDEADEKDAIATVNNKQEEGNFS
NAMAEAFKAAKGE
>CORE_REP|Org49_Gene2198#
MAQYVYTMHRVGKVVPPKRHILKNISLSFFPGAKIGVLGLNGAGKSTLLRIMAGIDTDIEGEARPQPG
IKIGYLPQEPQLNLEHTVRESVEEALAEVVGALKRLDEVYALYAEEGADFDKLAAEQGRLEEIIQAHD
GHNLNAQLERAADALRLPDWDAKIAHLSGGERRRVALCRLLLEKPDMLLLDEPTNHLDAESVAWLERF
LHDFEGTVVAITHDRYFLDNVAGWILELDRGEGIPWEGNYSSWLEQKDARLAQEASAEAARRKSIEKE
LEWVRQGTKGRQSKGKARLARFEELNNTEYQKRNETNELFIPPGARLGDKVVEVSNLRKSYGDRLLID
DLSFSVPKGAIVGIIGPNGAGKSTLFRMMSGQEQPDSGSIVLGDTVKLASVDQFRDSMDGSKTVWEEV
SGGQDIMRIGNTEMPSRAYVGRFNFKGVDQGKRVGELSGGERGRLHLAKLLQVGGNVLLLDEPTNDLD
IETLRALENALLEFPGCAMVISHDRWFLDRIATHILDYQDEGKVEFFEGNFTEYEEYKKRTLGADALE
PHRIKYKKIAK
>CORE_REP|Org13_Gene4747#
MSNPTPWWQNGVIYQIYPKSFQDSTGNGYGDLAGVTRRLDYLQELGVDAIWLTPVYVSPQVDNGYDVA
DYCAIDPAYGTMADFEQLVAAAHRRGIRIVMDMVFNHTSTEHPWFKAAQDRHSPYRQFYVWRDGEGDT
PPNNWRSKFGGNAWQWHADSGQYYLHLFAVEQADLNWEHPPVREELKKVCQFWADKGVDGLRLDVINL
VSKQQDFPSDSQGDGRRFYTDGPRIHEFLQEMSRDVFQPRGLMTVGEMSSTTLEYCQQYAAQSGEELS
MTFNFHHLKVDYANGEKWTRAAPDYVELKQIFRHWQQGMHNRAWNALFWCNHDQPRIVSRFGDEGALR
VPAAKMLAMVLHGMQGTPYIYQGEEIGMTNPGFRAIEQYRDVESLNMYAELSAQGRSDAELLAILADK
SRDNGRTPMQWSAAPHAGFTTGTPWIGCAENYPQINADAALADLDSVFYAYRQLIILRKQYPLLTHGD
YQDLAPDHPALWCYQRSWNGQRLLVVANLSREPLAWAAEGVEASAQWRPLMSNYSDSADQPQALTLRP
FEAVWWLLED
>CORE_REP|Org22_Gene1819#
MTENNHSVADVEKIKRWSPVWIIPIVTALIGAWILFYHFSHQGPVVTLVTTTAEGLEAGKTKIKSRSV
DVGVVETVTLSDDLSKVMVQARLNAGMEKLLRQDSAFWVVKPQIGREGVSGLGTLLSGAYIELQPGSK
GKDGKDNYQLLDAPPLASPDAKGLRIVLDSEKSGQLNAGDPVLFRGYRVGSVETSYFDPKERAMRYQL
FITAPYDQLVTTNVRFWKDSGVAFDMSAQGMRVEMGSLTTLFSGGVSFDVPDGWDRGEPAKEKAEYQL
FDNQRSTQDSLYTVHKDYLLFFSDSVRGLQPGAPVEFRGIRLGTVAQVPFYKDGMAQRLDNDYRIPVL
IRIEPDRLHKQLGDNVDIEAHLKDAESRGMRASMKSANLLTGSLYIDLDFYPQEKPWKGPRELFGYPL
MPTTSGGLAQIQQKLMQTLDKINAMPINPMLNEATKTLAESQKTMKSTQQTMKSLNDIIASKEMQALP

QDMQKTLLELNRSMKGFQPGSPAYNKMVGDMQRLDQVLRELQPVLRTLNEKSNALVFEAAGSTDPQPK
KATK
>CORE_REP|Org33_Gene227#
MTTNYIFVTGGVVSSLGKGIAAASLAAILEARGLNVTIMKLDPYINVDPGTMSPIQHGEVFVTEDGAE
TDLDLGHYERFIRTKMSRRNNFTTGRIYSDVLRKERRGDYLGATVQVIPHITNAIKERIIEGGEGHDV
VLVEIGGTVGDIESLPFLEAIRQMAVEVGREHTLYMHLTLVPYMAAAGEVKTKPTQHSVKELLSIGIQ
PDVLICRSDRAVPANERAKIALFCNVPEKAVISLKDVDSIYKIPGLLKSQGLDDYICKRFSLNAPEAN
LAEWEQVIYEEANPGGEVTIGMVGKYVELPDAYKSVIEALKHGGLKNRLTVNIKLIDSQDVETRGVEV
LKGLDAILIPGGFGYRGVEGKVMTARYARENNIPYLGICLGMQVALMEFARNVAGMENANSTEFMPDC
KYPVVALITEWRDEDGNVEVRSEESDLGGTMRVGGQQCNLSDNSLVRQLYGEPTIVERHRHRYEVNNM
LLKQIEAAGLRVAGRSADNKLVEIIELPNHPWFVACQFHPEFTSTPRDGHPLFAGFVKAAGEHQKRQV
K
>CORE_REP|Org39_Gene4197#
MQQTLKRTSLTLLISGALGAGAVNSSLAAEVPAGVQLAQQQNIVINNGSEVASLDPHKVEGVPESNII
LNLLEGLVSTDANGHVVPAAATSWENQNYQQWTFHLRPGAVWSDGSPVIAQDFVYSWQRLADPKIASP
YASYLQYTKVENIDDILTGKKSPQTLGVKALDDQTLQVTLSEPVPYFISMLSHTSLKPVKQAVVEKFG
DKWTLPANYVGNGAYRLKEWVVNERIVLERSPSYWNNKQTVINQATFLPITSEVSDVNRFRSGEIDIT
NSAIPPYLYVKMKREMPEQLHVNPYLCTFYYELNNKRAPFTDPRVRTAVKMTLDRDIIANKIMGQGQI
PAYSFTPTFTEGASFTQPAWAGWSQEQRNAEARKLLAEAGYSDAKPLKFSLLYNTSDQNKQQAIAAAS
MWKKNLGAEVTLRNQEWKTSLESRHQGQYDVARATWCGDYNEPSAFLNLVLSNSSINTVFYKSPAFDA
IMAATLKAPDEAARTALYQQAEAQLDKDSALVPVYYRVSARLIKPTVGGFTGKDPLDYTDVKNLYIIK
Q
>CORE_REP|Org29_Gene2085#
MDSQRNLLLIALLFVSFMIWQAWQTDNAPQPAAQTTQQTSNAVAGDAASQAVPASGQGKLITVNTDVL
SLTINTRGGDIEQAKLLAYPDTLGSSTPFQLLETTPSFVYQAQSGLTGKNGPDNPANGERPLYQAAQD
SYTLPEGQDELRIPLTYTDKDGAVYTKTFVLKRDHYAVGVDYSIDNKGATPLELTLFGQLKQTTELPK
HRDTGSSNFALHTFRGAAYSSSDDKYQKYAFDKDENLSVTTKDGWVAMLQQYFATAWVPATKGDNTFY
TAKPGDNLSTIGFKSTPVVVQPGAQQQLNATLWVGPELQDQMAQLAPHLDLTVDYGWLWFISQPLFKL
LKFIHGFIGNWGFSIIIITFIVRGIMYPLTKAQYTSMAKMRMLQPKLQAMRERIGDDKQRMSQEMMAL
YKAEKVNPLGGCLPLIIQMPIFLALYYMLMGSVELRHAPFALWIHDLSAQDPYYILPILMGVTMFFIQ
KMSPTTVTDPMQQKIMTFMPVIFTVFFLWFPSGLVLYYIVSNLVTILQQQLIYRGLEKRGLHSRDKKK
S
>CORE_REP|Org2_Gene2715#
MWLRQCLRCNSLGFTLATALFFTLFQNALFLHRAWSYITFDSVHSVIFAASMPVVIFCALNIIFSVLT
VPYLRKPLIIFFLLGSAAANYFMFSYGVVIDGNMMQNAFETNPQEATALLTPRMGLWLALLGILPAVA
VCFTQIRQTRPWWYMVGLRAANVMLSLAVILIVAALFYKDYASLIRNNKSVVKMLTPSNFVAGTIKFT
EQRYFTRNLPLVKIGEDARKGPLIAGQAKKTLVILVVGETARAENFSLGGYQRETNPRLKQDNVVYFK
NASSCGTETAISVPCMFSNMPRKEYDATQATHQEGMLDVLAHAGVSVLWRDNDGGCKGACDRVPHIDM
TKLKLPQDCDGEVCMDNALLYKLNDYINGLKDDGVIVLHQMGSHGPAYYRRSTPEFQTFSPTCNSNQI
QDCSHEQLVNTYDNSILYTDAMLDATIKLLRQYDDQFNTALVYLSDHGESLGENGMYLHGTPYVFAPS
QQTHVPFLMWMSADYQRNFGVDRQCLNALAEKDDVSQDNLFHTLLGMLNVQTREYQSRLDILQRCRNA
A
>CORE_REP|Org24_Gene4315#
MTGRKIQRGFRLALCAAAIGACMSSAMAAQVPPGTALAAKQEIVRHIKDEPASLDPIKAVGLPEAQLA
RDLFEGLVNQDANGKVIPGVATRWQTSDNQTYIFHLRKDARWSNGDPVTAKDFVYSWQRLVDPKNLSP
FAWFAQLAGIQNAEQIISGKLPADRLGVSAPDDYTLKVQLDKPVPYFVSLTANFSLFPVNKAVVEKYG
NDWTKVGNLVGNGAFKLQERVVNEKLVLTPNDHYWDHARTVLTKVTFVPINQESNATKRYLAGDIDIT
ESFPKNMYQKLLKDIPGQVYTPDQLGTYYYAFNTQRAPTNDVRVRQALSYAIDRKIIAEKVLGTGEKP
AYHFTPDVTAGFKPEVSLLQQQSQAELDAQAKALLQAAGYGPNNPLKLTLLYNTSESHQKIAIAVASM
WKKKLGIDVKLQNQEWKTYIDSRNTGNFDVIRASWVGDYNEASTFLSLLTSTHSGNIAKFKNADYDKL
LAQAGRETNPAAVTADYNKMEQIIADQAPIAPIYQYTNGRLIKPWVKGYPITNPEDVAYSQTMYIIKH
>CORE_REP|Org31_Gene2710#
MAHFAQSPYFVLHQLTCQFADGETLFGPLDLAFDRQRCGLVGRNGVGKTQLLRLIAGRDRPGNGHVES
HAALAYVAQQPEIAADTTLAQLLGYGEVFAALARIEQGRPLADDIDRLEGRWDLNDRLQSAFAAAGLP

AFDPLRSACDLSGGERMRAALCGAFLGEADYLLLDEPTNHLDSAGRAWLYQQLERWQGGLLIASHDRQ
LLGRMERIVELTPGALRSYGGNYDDYRRQRDTEQQAARADLEHAREERRRTRARQQKEHDMSQRRSAQ
TLRVVDTLNIASFERVAYKSAAKESLGTLRKQHQDQRDSLDAAVREAYQRVEEEQPVLLALPGSEVSA
NKQVLVLEQLQLPFVSAPPLDLRIDGPMRVALTGPNGCGKSTLLKTVLGQLAPLAGHCHCPLSTAYLD
QTLSQLDPSLSVMEHLGLQDSPLVEGALRTRLAQLQLGADRIALPLGSLSGGERLKAALACALWRRQP
AQLLLLDEPTNHLDLASSLAIETALADFPGAMLVVSHDEDFLQALRPTHRLHRQADGWRLQAW
>CORE_REP|Org3_Gene4776#
MDLNDLSTRIGGDVLVNILSGQPRAASVRWLGATVLFTLFSSPAWAFSIDDVAKQAQDLAAKGFEAPK
SNLPSQFREMKFADYQQIQFNHDKAYWSKLKTPFKLEFYHQGMYFDTPVKINEVTSTSVKQIKYSPDY
FNFGSVKHDPESVKNLGFAGFKVLYPVNSADKNDEIMSLLGASYFRVVGKGQVYGLSARGLAIDTALP
SGEEFPRFREFWVERPKQGDKHLVIYALLDSPRATGAYRFTVIPGRDTTVDVESKVFLRDKVGKLGLA
PLTSMFLFGPNQPSPTLNYRPALHDSNGLSIHAGNGEWIWRPLNNPKHLSVSTYTVENPKGFGLLQRG
RNFKEYEDLDDRYDLRPSAWIEPKGDWGKGKVELVEIPTADETNDNIVAFWTPDTLPEAKKPLTLSYR
LNFTRDEDKLHSQDIAYVARTMRSTGDVKQSNLIREPDGSVAFLVDFVGPVLKGLDANTPVASQISIG
DNGEMVENNVRYNPVTKGWRLTVRLKVKDDKKPVEMRAALVNGDKTLSETWSYQLPANE
>CORE_REP|Org37_Gene1031#
MLSTNNITMQFGSKPLFENISVKFGGGNRYGLIGANGCGKSTFMKILGGDLAPTGGNVFLDPNERLGK
LRQDQFAFEQYSVLDTVIMGHTELWAVKEERDRIYAMAEMSEEDGYKVADLEVAYGEMDGYTAEARAG
ELLLGVGIPVEQHYGPMSEIAPGWKLRVLLAQALFSDPEILLLDEPTNNLDIDTIRWLEQVLNERNST
MIIISHDRHFLNMVCTHMADLDYGELRVYPGNYDEYMTAATQARERLLADNAKKKAQINELQSFVSRF
SANASKSKQATSRARQIDKIQLEEVKASSRQNPFIRFDQDKKLFRNALEVEALTKGFDNGPLFSKLNL
MVEVGEKVAVLGANGIGKTTLLKTLVGDAQPDSGTVKWSENARIGYYAQDHEYEFDDTLTVFDWMSQW
KQEKDDEQAVRSVLGRLLFSQDDIKKKVKVLSGGEKGRMLFGKLMMQRPNILVMDEPTNHLDMESIES
LNMALEMYEGTLIFVSHDREFVSSLATRILEITPNKVIDFTGNYEDYLRSQGIV
>CORE_REP|Org17_Gene4674#
MSPSEFAREVSKRRTFAIISHPDAGKTTITEKVLLFGQAIQTAGTVKGRGSSQHAKSDWMEMERQRGI
SITTSVMQFPYRDSLVNLLDTPGHEDFSEDTYRTLTAVDCCLMVIDAAKGVEDRTRKLMEVTRLRDTP
ILTFMNKLDRDIRDPMEVMDEVERELKIACSPITWPIGCGKLFKGVYHLYKDETYLYQTGKGHTIQEV
RIVKGLNNPELDVAVGEDLAAQLRDELELVQGASHEFDQAAFLSGELTPVFFGTALGNFGVDHMLDGL
VAWAPAPMPRKTDTREVTAAEEKFTGFVFKIQANMDPKHRDRVAFMRVVSGRYEKGMKLRQVRTGKDV
VISDALTFMAGDRSHVEEAYPGDIIGLHNHGTIQIGDTFTQGEDMKFTGIPNFAPELFRRIRLRDPLK
QKQLLKGLVQLSEEGAVQVFRPIANNDLIVGAVGVLQFDVVVARLKSEYNVEALYESVNVSTARWVEC
DDVKKFEEFKRKNEINLALDGGDNLSYIAPTMVNLNLTQERYPDVTFRKTREH
>CORE_REP|Org37_Gene721#
MFDIVELSRLQFALTAMYHFLFVPLTLGMAFLLAIMETVYVLSGKQIYKDMTKFWGKLFAINFALGVA
TGLTMEFQFGTNWSYFSHYVGDIFGAPLAIEGLMAFFLESTLVGLFFFGWDRLSKVQHMAVTWFVALG
SNLSALWILVANGWMQNPIASDFNFETMRMEMVSFSELVLNPVAQVKFVHTVASGYTCGAMFVLGISS
YYLLKGRDIAFAKRSFAIAASFGMAAILSVIVLGDESGYEMGDVQKTKLAAIEAEWDTQPAPASFTLF
GIPDQDKMENSFSIQIPYALGLIATRSTDTQVTGLKDLMAQHEVRIRNGMKAYQLLEELRGGNTDPAV
RAEFNKTKQDLGYGMLLKRYTPNVTDATEAQIQLATKDSIPRVAPLYFAFRIMVACGVIMLLIIGLSF
WNVIRGRIGQKKWLHRAALYGLPLPWIAIESGWFVAEYGRQPWAIGEVLPTAVANSSLTAGDILFSMG
LICGLYTLFLVAELYLMFKFARLGPSSLKTGRYHFEQPTAAVQEAR
>CORE_REP|Org3_Gene1399#
MKPEDFRADSKRPFTGAEYLKSLQDSREIYIYGERVKDVTTHPAFRNAAASVGQLYDALHDPASQDRL
CWNTDTGNGGYTHKFFRYARSPEEMRQQRDAIADWSRQSYGWMGRTPDYKAAFGCALGAYPEFYGQFA
DNARHWYKRIQETGLYFNHAIVNPPIDRHKPVNEVKDVYIQVEKETDAGIVVSGAKVVATNSALTHYN
FIGFGSAQVMGDNPDFALMFVAPMDAEGVKLISRASYELVAGATGSPFDYPLSSRFDENDAILIMDHV
LIPWENVLIYRDFDRCRRWSTQGGFARLFPLQACVRLAVKMDFITALLQKSLSCTGVLEFRGVQADLG
EVVAWRNLFWSLSDAMCAEATKWENGAYLPDSAALQTYRVMAPMAYTKVKHIIEKNVTSGLIYLPSSV
RDMNNPEIDKYLARYVRGSDGMDHVERIKILKLMWDAIGSEFGGRHELYEINYAGSQDEIRLQCLRHA
QGSGNMDRMMQMVDKCLADYDQHGWKVPHLRNNDDINQLDNLLK
>CORE_REP|Org47_Gene3450#
MSSNKKPMVLVILDGYGHREERQDNAILNAGTPVMDRLWREQPHTLIAASGLDVGLPDGQMGNSEVGH
VNLGAGRIVYQDLTRLDKAIADGDFFANPVLTAAVDKAVAAGKAVHIMGLLSPGGVHSHDEHILAMIK

LAAQRGAKAVYLHAFLDGRDTPPRSAEAPLQRCRDAFAALGVGRIASLIGRYYAMDRDNRWDRVQLAY
DLLTAAKGDAVAEDAIAGLQAAYQRGENDEFVRPTVIRAAGEADAAMQDGDALIFMNFRADRARQITR
AFVNADFDGFPRAKQVQFGDFVMLTEYAADIATACAYPPASLANTFGEWLMKHDKTQLRISETEKYAH
VTFFYNGGVEAPFKGEDRVLVNSPKVATYDLQPEMSAAELTDKLLSAIRSGKYDAIICNYPNGDMVGH
TGVYEAAVKAVETLDACIAQVVDAVRDVDGQLLITADHGNAEQMRDPATGQAHTAHTSLPVPLIYVGK
PARAVEGGKLSDIAPTLLTLMGMEIPQEMTGKPLFIVE
>CORE_REP|Org27_Gene3371#
MIKIVVYLILAVTIAIIAARVLFRLPDISQRLPQAALPADPAAQLPARAAELMAAHPGLSGVVPLASG
HDAFASRLALARMAERSIDAQYYIWHNDTSGQILLKTLYDAAQRGVRVRLLLDDNGVAMDETLAALNA
QENVEIRLFNPSTVRTPKLAGYAFDFMRMNRRMHNKSYIVDGAVAIIGGRNIGDEYFQVGDENYFLDL
DVLSVGSVVAETAEVFDRYWNSASVFGVEQIIRGKGNLSAFLTQATATESSERARKLAVQLETSAVRF
RDGAVQPEFTQVELVADDPAKGLGKASRDRLMVTQLGKIIGGVGRQLDLVSAYFVPGREGASFFESLA
KQGKSIRVLTNAMNTTDVLVVHAGYAKYRRELLQAGVELFELKLRAGQPTGRKELKPLGLSGAALHAK
TFAIDDKRVFIGSFNFDPRSAHLNCEMGFLIDSPTLAADTRQLFDGPLEYAAYRPVLTPEGKMVWKEA
FEDGHTEVHQQEPGAGWVKRIILTVAGWLPIEWML
>CORE_REP|Org22_Gene3723#
MKHTFKRNALLAAVLLAAGTGPVWAAKDAVIAVASNFTTLDPYDANDTLSQAVAKSFYQGLFGFDKDM
KLVNVLADSYEVSKDGLTYTVKLRQGIKFHDGTAFNAEAVKINLDRASNPDSHLKRYNLFKMIDKTEA
VDADTVKIVLKAPFSAFVNNLAHPAAAIISPAALKQYGKEIGFHPVGTGPYQFVTWNQTDFVKVKKFD
GYWQPGLPKLDSITWRPVVDNNTRAAMLQTGEATFAFPIPYEQAKVLEGNAKLDVVAAPSILQRYISM
NVTQKPFDNPKIRQALNYAINKDALIKVAFAGYAVPAEGPVPPAIDFAARYKPWPYDPAKARELLKEA
GYPNGFTTTLWSSHNHSTAQKVLQFTQQQLAQVGVKVTVTAMDAGQRAAQVESVGVQDTGVRLFYTGW
SASTGEADWALSPLFSTQAAPPKQFNTAFYSNPQVDKDLTDALATTDRAEKQKLYQDAQDRIWADAPW
IFLATERLLSANSKQLSGFYVMPDTSFNFDNADLK
>CORE_REP|Org28_Gene2160#
MNLLKSLAAVSSMTMFSRVLGFARDAIVARVFGAGMATDAFFVAFKLPNLLRRIFAEGAFSQAFVPIL
AEYKSQQGEEATRTFIAYVSGLLTLVLAVVTVLGMLAAPWVIYITAPGFTDTPDKFALTSALLRITFP
YILLISLASLVGAILNTWNRFSIPAFAPTLLNVSMIGFALFAAPYFNPPVLALAWAVVVGGVLQLGYQ
LPHLRKIGMLVLPRLKLGDAGVWRVMRQMGPAILGVSVSQISLIINTIFASFLVSGSVSWMYYADRLM
EFPSGVLGVALGTILLPSLAKSFSSGNHDEYSRLMDWGLRLCFLLALPSAIALGILAKPLTVSLFQYG
KFSAFDAAMTQRALVAYSVGLMGLIVVKVLAPGFYSRQDIKTPVKIAIITLIMTQVMNLAFIGPLKHA
GLALSIGLAACLNASLLYWQLRKQKIFQPQPGWALFLTKLVIAVLVMSAVLIGVMWLMPAWDQGNMLE
RLLRLAAVVVAGVVAYFGVLAGLGFRPRDFARRVA
>CORE_REP|Org25_Gene2809#
MKKLLPLLIGLSLGGFSAMSQAENLLQVYKQARESNPDLRKSAADRDAAFEKINEARSPLLPQLGLTA
GYDYTNGYRDSNGVNSNVTSGSLALTQTLFDMSKWRQLTLQEKSAGISDVTFQTAEQSLILNTATAYF
NVLKAIDTLSYTQAQKDAVYRTLDQTTQRFNVGLVAITDVQNARSNYDTVLAAEVSARNDLDNALETL
RQVTGAFYPELASLNTDRFSTQRPEAVNNLLKEAEARNLSLLSARLSQDLAREQIRAAQTGYMPTIDV
SASTGISNTKYNGSNTGGANAARYSDSDAGQNKVGISFNLPLYSGGATNSQVKQAQYGFVGASEQLES
AHRSVVQTVRSSFNNVNASISSINAYKQAVISAQSSLDAMEAGYQVGTRTIVDVLDATTTLYNAKRQL
SDARYTYLINQLNIKSALGTLNQNDLLLLNGALGKPVSTAPDAVAPQNRAQDAYADGYQDNAPMQQTA
APAPAATRASAPAVTTSQPARHSGNPFRN
>CORE_REP|Org43_Gene333#
MEFSVKSGSPEKQRSACIVVGVFEPRRLSPIAEQLDKISDGYISALLRRGELEGKVGQTLLLHHVPNI
LSERILLIGCGKERELDERQYKQVIQKTINTLNDTGSMEAVCFLTELHVKGRNTYWKVRQAVETAKET
LYTFDQLKSNKVEPRRPLRKMVFNVPTRRELTSGERAIQHGLAVASGIKAAKDLGNMPPNICNAGYLA
SQARQLADAFSTNITTRVIGEQQMKELGMNAYLAVGAGSQNESLMSVMEYKGNPNPDAKPIVLVGKGL
TFDSGGISIKPADGMDEMKYDMCGAATVYGVMRVVAELNLPLNVIGVLAGCENMPGGRAYRPGDVLTT
MSGQTVEVLNTDAEGRLVLCDTLTYVERFEPELVIDIATLTGACVIALGHHITGLMSNHNPLAHELLG
ASEQAGDRAWRLPMADEYYEQLDSNFADMANIGGRPGGAITAACFLSRFTRKYTWAHLDIAGTAWRSG
KAKGATGRPVALLSQFLLNRAGLNGDD
>CORE_REP|Org37_Gene3954#
MSTATPSRLEMRNISIAFAGFNALQDVDFTLQGGSIHALVGANGAGKSTLMAILSGAHDHYRGEILID
GQAVAIHSPLQARRHGIHVVQQEVDVALIPTLSVAENIMLDWLNEPGHWLNWAELHRRAAQLLQQWAL

PLNPRRRLADCTLAEKQQVLLARALSHRCRFLVLDEPTAPLDRAESERLFNVVRRLQSEGIGIVFISH
RIHELSDICDRLTVLRDGRRVSEDPMRGLSGEQIVEKMLGHRLDDIFPPPRPPHAKRTLLQVQGLRDR
HKLRDVSLRLHEGEILGIAGLAGAGKTELCKALFGASAVQLERGELRGQPWAPRAPHLSVEQGLALVP
EERRKEGIFIDEAIPMNLSVSADDSFSRWSLFSRRQELRWAREIMQRLNIRASGPQQRLARLSGGNQQ
KVAIGKWLRGDAEVLIFDEPTKGVDIKAKQELFGLIDGLARAGKGVIYASGEFAELVGLCDRICVLWD
GRIVAELNAADIDEETLLLYSTGGTPA
>CORE_REP|Org31_Gene1519#
MADSSREFLLEMTDICKSFPGVKALDNVNLRVRPHSIHALMGENGAGKSTLLKCLFGIYKKDSGSIVF
QGREIDFKSSKEALEHGVSMVHQELNLVLQRTVMDNMWLGRYPTKGLFVDQEKMLKDTQAIFDELDID
INPREKVGNLSVSQMQMIEIAKAFSYDAKIVIMDEPTSSLTEKEVNHLFTIIRKLKERGCGIVYISHK
MEEIFQLCDEITVLRDGQWIATQPLEGLDMDKIIAMMVGRSLSQRFPDRQNTPGEVILEVKNLTSLRQ
PSIRDVSFDLHQGEILGIAGLVGAKRTDIVETLFGIREKVAGTIKLHGKAIDNHSANEAINHGFALVT
EERRSTGIYAYLDVGFNSLISNIRNYKNKLGLLDNARMKSDTQWVIDAMRVKTPGHHTHIGSLSGGNQ
QKVIIGRWLLTQPEILMLDEPTRGIDVGAKFEIYQLMTELAKKGKGIIIVSSEMPELLGITDRILVMS
NGQVAGIVNTKQTSQNEILRLASLHL
>CORE_REP|Org37_Gene1051#
MTRSNVEMPNEVQAWVSEGRYKEGFFTQLATDELAKGINEEVVRAISAKRNEPEWMLEFRLEAYRAWL
QMEEPHWLKANYDRLNYQDYSYYSAPSCGSCDDACGSQPGAEQQPGAATEKDYLTSEVELAFNQLGVP
VREGSEVAVDAIFDSVSVATTYREKLAESGVIFCSFGEAIQEYPDLVRQYLGRVVPSNDNFFAALNAA
VASDGTFVYVPKGVRCPMELSTYFRINAAKTGQFERTILIADEGSYVSYIEGCSAPVRDSYQLHAAVV
EVILHKDAEVKYSTVQNWFSGGESKGGILNFVTKRALCEGAGSKMSWTQSETGSAITWKYPSVILQGD
NSIGEFFSVALTSGHQQADTGTKMIHIGKNTKSTIIAKGISAGHSENTYRGLVKILPGAENARNFTQC
DSMLIGPDSGAHTFPYVEARNNSAQLEHEATTSKIGDDQLFYCLQRGISEDDAISMIVNGFCKDVFSE
LPLEFAVEAQKLLAISLEHSVG
>CORE_REP|Org7_Gene3966#
MTNNASPAPIAHRPLILIACMLAMFMSAIEATIVATAMPTIIGDLGGFSLLGWVFAVYLLSQAITIPI
YGRLADLYGRKRVFFFGATLFLLGSVLCGFAPDMYWLIGFRLLQGLGAGAIMPIASTIIGDIYSATER
PKVMGYLSSVWGVSAIIGPLLGAFIVQHLPWALVFWVNLPIGLLAMFFLWRYLPAHQPLRQHALDLAG
TAWLTLFVSALLLALLQMESLGWWVVPLFALAAAALALLVRQERRAVEPLFPLALWQSRVIVAGNIGG
LVIGAAMMGISAFLPTFIQGVMGGSPLEAGTTLALMSIGWPLASTLSGRLMLMTSYRATALLGALLLV
AGGLILLLLQPEGGLLWGRVAAFMVGAGMGLCNTTFLVSVQNAAHYSIRGIATACTVFTRMVGSAIGT
AILGATLNLNLQWRLPEIDDPVQRLMEPAVRQSMGSEALAQLTQQVAASLHWVFLVSALVSLLALAAA
MLIPARCRPQGEEEEAEQA
>CORE_REP|Org17_Gene1818#
MTMSTPMLVTFLVYIFGMVLIGLLAYRATNNFDDYILGGRSLGSVVTALSAGASDMSGWLLMGLPGAI
FLSGISESWIAIGLTIGAYLNWKLVAGRLRVHTEANNNALTLPDYFTSRFEDNSKLLRVISAIVILVF
FTIYCASGIVAGARLFESTFGMSYETALWAGAAATILYTFIGGFLAVSWTDTVQASLMIFALILTPVI
VIFAVGGIDTSMLVIQAQNPANLDMLKGLNFVAILSLLGWGLGYFGQPHILARFMAADSHRTIRSARR
ISMTWMILCLAGTIAVGFFGIAYFANNPDQAGNVSQNGERVFIELAMLLFNPWVAGVLLSAILAAVMS
TLSCQLLVCSSAITEDLYKAFLRKGASQRELVWVGRVMVLVVALVAIALAANPENRVLGLVSYAWAGF
GAAFGPVVLISVMWSRMTRNGALAGMLVGAVTVIVWKQYEWLGLYEIIPGFILGCLAIVVVSLMGRQP
SSTMTERFDQAEAEYKTV
>CORE_REP|Org31_Gene3285#
MSKHYLNIFTQRNSAILLLLGFASGLPLALTSGTLQAWMTVENIDLKTIGIFSLVGQAYVFKFLWSPF
MDRYTPPFLGRRRGWLLVSQLLLVAAIVAMGFMQPAQHLWWLAALAVLVAFCSASQDIVFDAYKTDLL
KAEERGAGAAISVLGYRLAMLVSGGLALWLADRYFGWQATYWLMAGLMLIGVAATLLAPEPDESIPAP
RTMEQAVVAPLRDFFGRNNAWLILLLIVMYKMGDAFAGSLSTTFLIRGVGFDAGEVGLVNKTLGLFAT
IVGALFGGVLMQRLSLFRALMLFGVLQAVSNLGYWILAVTDKSLLTMGSAIFLENLCGGMGTAAFVAL
LMTLCNRSFSATQFALLSALSAVGRVYVGPIAGWFVEAHGWPLFYLFSIAAALPGLLLLGICRQTLEH
TQQHGDFMPRTEFSESYRWALRLLTLGCSLLGLWLLLLIANALDWTQAPLLADRLLQVGAALSLLGVA
MGSTLDYLALRRTRLA
>CORE_REP|Org5_Gene1012#
MAQQDIKTSGQQAPGLRRELKARHLTMIAIGGSIGTGLFVASGATVSQAGPGGALLSYALIGLMVYFL
MTSLGELAAFMPVSGSFSTYGAKYVEEGFGFALGWNYWYNWAVTIAVDLVASQLVMSYWFPDTPGWIW

SALFLGLMFLLNYISVKGFGEAEYWFALIKVSTVIIFIAVGVLMIVGILKGGEHAGWQNWTIGDAPFA
GGFSAMIGVAMIVGFSFQGTELIGIAAGESENPGKNIPRAVRQVFWRILLFYIFAILIISLIIPYTDP
SLLRNDVKDISVSPFTLVFQHAGLLSAAAVMNAVILTAVLSAGNSGMYASTRMLYTLASEGKAPRIFA
KLSKGGVPRNALYATCVVAGLCFLTSMFGNQSVYLWLLNTSGMTGFIAWLGIAISHYRFRRGYMLQGR
DLNDLPYRSGFFPLGPIFAFVLCLIITLGQNYQAFLQDKIDWYGVTATYIGIPLFLLIWFGYKLSRGT
RVVKYSEMEFPKMDVK
>CORE_REP|Org21_Gene3032#
MTLLDETPDTRYLQLADTLAEAIRRGTLQPGDRLPSVRRCAQTHRVSINTVVSAYRTLEDRGLIEARP
QSGFYVRSTLPALKMASAPSSRIEPPADDVLALIDTVFAAQQNPAFTNIALACPQTSDFYPGGKLGRM
LSSQLRRQPGLIGQYPLPPGSLRLRQQIARRSMTLGMLLEPGDVVLTHGCMEALQLALRVTTKPGDCV
GLESPTYFYLLPLLASLGLKALEIPTDPQLGLSLDALELLLNEKRLNAVIAMPTVQNPLGCTMPLAAK
KRLARLMNDHQVPLIEDGLYAEIQFGGALSPAVKAFDRDGWVLFCSSFTKTLAPDFRVGWICGGRFHE
ALRKLKAVSSMSESQLLSETLATFLESGGYDHHLRNLRKRYAAQVDEARALIARHFPRGTLATQPAGG
FVFWVEFPPGVDSVALFHQLLEEQICLTPGTLYSPSGRYRNALRLSCCYPFNARYTQALARLGARACE
MSGLPPGIAQDG
>CORE_REP|Org20_Gene1502#
MHSQKKSADDKHAARRRWLDSHESGYHKSMGNRQIQMIAIGGSIGTGLFLGTGGRLELAGPALALVYL
VCGIFSFFILRALGELVLHRPSSGSFVSYAREFLGEKASYVAGWMYFLNWAMTGIVDITAVALYMHYW
GTFADVPQWLFALGALGIVATMNMIGVKWFAEMEFWFALIKVVAIALFLIVGVVFLGTGTPVAGHTTG
MHLITENGGMFPHGLLPALVLVQGVIFAFAGIELIGTAAGECKDPAKMMPKAINSVIWRIGLFYVGSV
VLLVLLLPWNAYQAGQSPFVTFFSKLGVPYIGTIMNIVVLTAALSSLNSGLYSTGRILRSLAMGGSAP
KLMAKMSSQQVPYAGILVTCGIYVIGVVLNYLVPSQVFEIVLNIASLGIIASWAFIIVCQMRLRKAVR
EGRAQPVSFKMPGAPFTSWLTLAFLLIVVVMMAFDYPNGTWTIATIPVLAVLLTLGWFGLRKRAQEVK
REQQAHEEQNPH
>CORE_REP|Org17_Gene3814#
MITHDDSRWSDLFSGKNAASAIALSLGVALHAINILVATTILPSVVQDIGGLDLYAWNTTLFVVASIL
GSALSARLLSGYGARNAYLVASLFFIAGAGLCALAPSMPVMLVGRTVQGFGGGLIFALSYAMINLVFE
QRLWPRAMALISAMWGIATLVGPAVGGIFAELHAWRWAFGILLPIMALYAAFTFLILPKGQAQQAAAP
LPTAQLLLLTVAVLVVSAGSLAHSVWINLAGIALSLALMAWLMKREARSRTRLLPHGALRRGSSLAAL
YITVSLLVIGMTSEIFVPYFLQLLHGQSPLISGYIAATMAAGWTLSEILSSGWRGAGIRRAIVSGPLF
VLVGLLALAILMPTPSGGHWQALTPIVIALSLVGFGIGFGWPHLLTRILQVAPEADKDIAGASITTVQ
LFATAFGAALAGMIANLAGLNDPGGAAGAAGAASAARWLFLAFALAPLLAVFSAWRCAAIAPPAAETG
NFVPNPSSREC
>CORE_REP|Org12_Gene1018#
MGKAMNARINARHGLPLTVQLINLFMLAFLLSLVGILSRPIGSLSLFWPVNAILLGLLLRKPIYGTPL
GWLTTYLGMVAADLSTGEGWSLALWLNACNMSLIAVGYGIMLMLPQSQRRMGKPQAILYMFSASLAGA
AVASTLSVLRNDSLYNNTVVIAWLAWFSEQFSTTLLLLPVLMAAPRLKQLLRMQVRWRLKGCLPLLAL
LLSLAFSVYIGGPGAIAFPIPALLWCAVRYPLFPVTLLTLLTGMTEISSISANLVLYETPNNHNAFLD
TLMSARLGIAMLVMGPLILASSIAANRKLMRRLEHSANHDFLTGVLARSAMTRKAGELLEHKHRSKEA
VSLLLIDIDHFKQINDTHGHSAGDQVLASFAHIVRRELRHDQLFGRLGGEEFAIMLPRALAAQGVALG
EHLRRLVEQTELQAEGKQTLKITISVGVASLAMNEVKSLEQLMNMADIALYRAKSQGRNRVESFNVIN
GNSVEHILFR
>CORE_REP|Org29_Gene3840#
MTKNHCDRLNPGSAEGPNCAQTLQRGLSARHIQLISIGGAIGTGLFMGSGKTIALSGTSIVLTYAIVG
FFMFMVMRAMGELLLTRLDYRSFADFVSEYLGPRASFFLGWSYWLSWVVTCIADVVVCGGYVQYWLPN
VSPWLPALLTLGFLCLFNMLSVKMFGEAEFWFAMIKVVAIVALIATGAWMVFSGWTSPDGVTASLHNV
TDPAIFMPHGIFGFFAGFQIAIFSCTGIELLGTMSAETKNPEKVLPKAINVIPARIIVFYVCSMLTII
AVTSWSHISPDSSPFVMLFDRAGLPAAAAVINFVVLTSAMSSANSGVYSSTRMLYSLSMEKHAHGQFR
ILSRTTAIPIRSLLFSCFCMVAGTLLLVLVPNVMTLFTIVSTVAAILVVYSWGMILVAYLVYRQKRPD
LHADSNFKMPGGIAMAWLTLAFFAFTLVLMVFDRDTLIALCSMPLWFTTLGLIWRYRVRDSVTRESYV
FYQRGAEAE
>CORE_REP|Org38_Gene1257#
MKKTALVLSALAFSIGMAMGPVTASAAETASSSTQQLPSLAPMLEKVMPSVVSINVEGSTTVNTPRMP
QQFQQFFGEDSPFCQDGSPFQGSPMCQGAEQPGPDGQQPQGTQQKFQALGAGVVIDAAKGYVVTNNHV

VDNANKIQVQLSDGRRFDAKVIGKDPRSDIALIQLKDFKNLTAIKMADSDQLRVGDYTVAIGNPYGLG
ETATSGIVSALGRSGLNIENYENFIQTDAAINRGNSGGALVNLNGELIGINTAILAPDGGNIGIGFAI
PSNMVKNLTAQMVEYGQVKRGELGIMGTELNSELAKAMKVDAQRGAFVSQVMPKSSAAKAGIKAGDVI
VTMNGKAISSFASFRAEIGTLPVGSKMSLGIIRDGKPITIDVTLEQSAQTQVASGNIYTGIEGAELSN
TQVGNVKGVKVDSVKAGSAAARIGLKKGDVILGVNQQPIQNLGELRKILDSKPSVLALNIQRGDSQLY
LLAQ
>CORE_REP|Org14_Gene1836#
MTRYATLAAILSQRIQQGLYPAGHRLPSVRALSQEHGVSISTVQQAYRLLEEQRLVEARPKSGYFVHT
RRAQAELPAMTAPVQRPVDISQWEQVLELVRSRPREGLIQLGRGMPDIAEPTMKPLIVALRNAARHGD
LRSLYYDSIQGVAALREQVARLLLDSGCQIGPDQLLITTGCQEAISAGLRAVCQPGDIVAVDSPCFHG
TMQTLKGLGIKALEIPTDPLTGVSLAALEMALEQWPIKAILLTPNCNNPLGYIMPDAHKQRLLTLAQR
HDAAIIEDDVYGDIAYHYPRPRTIKSFDEDGRVLLCSSFSKTLAPGLRVGWIAPGRYLERVLHMKFIG
SGATATQPQLAIAEFIRGGHYLQHLRRMRARYQQNRDRMTDLILKHFPAGTRVSRPRGGFMLWIELDE
AFDTLRLNRHLEQQGVQIAVGSIFSAAGKYRNCLRINYAPKLTAEIEQAVQRVGATIQALMPSGVLQP
QAD
>CORE_REP|Org43_Gene2089#
MISLKKWRLFPRSLRQLVLLAFLLVLLPLLVLAYQAYQSLDHLSAQAADINRTTLVDARRSEAMTSVA
LEMERSYRQYCVLVEPTLQKLYQNQRKQYSQMLDAHAPILPDERYYQTLRQLLTQLAAIKCHNSGPDQ
EASALLESFSRSNAEMVQATRAVVFSRGQQLQQAIAERGQFFGWQALLLFLVSVLLVVLFTRMIIGPV
KAVERMINRLGEGRALGSTASFKGPRELRSLAQRIIWLSERLAWLESQRHEFLRHISHELKTPLASMR
EGTELLADEVAGPLTSDQKEVVTILDNSSRHLQQLIEQLLDYNRKLADGPAEHENVELREMVDLVVAA
HSLPARAKMISTEIALEAEICWAEPTLLMRVLDNLYSNAVHYGKESGNIWIRSRQVGQRVQIDVANTG
TPIPEAERAMIFEPFFQGSHQRKGAVKGSGLGLSIAQDCIRRMRGELQLATVAGADVCFRIELPLTAE
NE
>CORE_REP|Org15_Gene2303#
MSKKGLTTAAGAPVVDNNNVITAGKRGPMLLQDVWFLEKLAHFDREVIPERRMHAKGSGAYGTFTVTH
DITRYTRAKIFSEIGKQTDMFIRFSTVAGERGAADAERDIRGFAMKFYTEEGNWDLVGNDTPVFYLRD
PLKFPDLNHVVKRDPHTNLRNPVYKWDFFSHLPESLHQLTIDFSDRGIPKSYRHMHGFGSHTFSFINA
ANERFWVKFHFRCEQGIENLMDEEAEAIIAKDRESSQRDLFDAIKRGDFPRWKLQIQIMPEHEASQTP
YNPFDLTKVWPHGDYPLIDVGFFELNRNPDNYFSEVEQVAMNPANVVPGISFSPDKMLQGRLFSYGDA
HRYRLGVNHHQIPVNGAKCPFHNYHRDGAMRVDGNSGNGATYEPNSFGLFQEQPDFSEPPLSIEGAAD
HWNHREDDDYYSQPRALFNLLSAEEHQRMFTRIAGELSQVPEHIQRRQVELFTKVHPDYGAGVAKALG
LK
>CORE_REP|Org41_Gene2421#
MKQGLQLRLSQQLAMTPQLQQAIRLLQLSTLELQQEIQLALESNPLLEQTDLHDEIDAKEIQETEGLD
TREALEQKDMPEELPLDATWDEIYTAGTPSGTGTDYSDDELPVYQGETTQTLQDYLMWQVDLTPFSDT
DAAIATSIVDAVDDTGYLTVPLEDILESLGDENVTLEEVEAVLKRVQRFDPIGVAARDLRDCLLVQLS
QYAKDTPYLAEARLIISDHLDLLANHDFRSLMRSTRLKEDTLKEAMLLIQSLDPRPGQSINTGESEYV
IPDVLVRKTQNTWTVELNGDSIPRLKINQQYAALGNSARSEADGQFIRSNLQEAKWLIKSLESRNETL
LKVTRCIVSQQQAFFEQGEEFMKPMVLADIAQAVEMHESTISRVTTQKFLHSPRGIFELKYFFSSHVN
TDSGGEASSTAIRALVKKLIAAENPAKPLSDSKLATLLSDQGIIVARRTVAKYRESLSIPPSNQRKQL
V
>CORE_REP|Org45_Gene4692#
MTTHLVWLRNDLRITDNKALHAACSDPEARVLAVFIATPQQWRQHEMAPRQAALIHASLQAVQQALAH
KGIALHCHSCADFAASIDWLADYCEREQVDALFYNRQYELNERRRDARLEQRLSGRVRCHGFDDSLLL
PPGSVLTGGGEMYKVYTPFRNAFLQRLTESDVSCLPAPKIRAGGALPAPEAPAAFDYPTAETGDGYPA
GEEAALQRLRAFCREQVQDYLRQRDLPALAGTSSLSPYLAIGTLSPRQCFNRLRAECPQLLEDRESGA
FAWLNELIWREFYRHLLMAYPDLCRHRPFIAWTDKVRWCDDAAKLHAWQRGETGYPIVDAAMRQLNAT
GWMHNRLRMISASFLVKDLLIDWRAGERYFMSQLLDGDLAANNGGWQWAASTGTDAAPYFRIFNPTTQ
GERFDPQGTFIRKWLPELADVPDNDIHHPHRWAEKQRCTLNYPLPIVDHKQARLETLAAFEAAKRGES
>CORE_REP|Org16_Gene2983#
MINIVVVSHSALLARGVEQLARQMMRGDGCKLALAAGVDDEQHPIGTDAVKVMEAIEAVADGDGVLVL
MDLGSALLSAETALDLLDPDLAAKVRLCAAPLVEGTLAAVVAANSGASLEQVVAEAQGALQAKQAQLG
EASPTAKSVALPLAQGKSVTWTVQNPHGLHARPAARLVETLAPFKAELVLEKQGQCVDPRSLNQLALL

QVRHGDTVRLIADGAQADEALAAFKALAEQHFGETVSERQQPSLHGIPVAESVTSGPVFQAHSFWPPT
VDRRIGADEVLGEQQRLREALQHTLSDLNRLAERTGTLIGKPQAAIFGAHSMLLDDPDLQQAAYTRIA
QQLCCAEQAWRQVLGAIAEEYRELDDDYMRARELDVRDMLRRTLCHLQGLPLPAMALAEPSILVMDEL
MPSEVVMLDRRLVLGICLSGGNALSHSAILAKAMGIPMVVGMQDCLSKTRSGQKAMLDAARGVLQLSH
>CORE_REP|Org49_Gene720#
MAGKKPTNKTNADETRERSRDRQMEGLKMPPHSLEAEQSVLGGLMLDNERWDNVAERVVANDFFSRPH
RLIFTEMQRLLEMSKPIDLITLSESLEQKGELDSVGGFAYLAELSKNTPSAANIGAYADIVRERAVVR
EMISVANEIADAGYDPQGRSSEDLLDLAESRVFQIAENRASKDEGPKGIERILEDTVSRIEQLYQQPH
DGVTGVDTGYQDLNKKTAGMQKSDLIIVAARPSMGKTTFAMNLCENAAMTQEKPVLIFSLEMPGEQIM
MRMLASLSRVDQTRIRTGQLDDEDWARISSTMGILLEKRNMYIDDSSGLTPTEVRSRARRIFREHDGL
SLIMIDYLQLMRVPALSDNRTLEIAEISRSLKALAKELQVPVVALSQLNRSLEQRADKRPVNSDLRES
GSIEQDADLIMFIYRDEVYHENSDLKGIAEIIIGKQRNGPIGTVRLTFNGQWSRFDNYAGPQYDDE
>CORE_REP|Org22_Gene4669#
MFPVPPTTKWVGVVNTELQSESSRLLASSNAGSPGWWLTVARRGTPWVEPAGNGRWRTTFFWRDPQGC
ELTSAYRRVWININCLTDHHQPNPPQSLQRLAGTDVWYWQTELSGAWRGSYCFIPCFDERPPAFSGDD
AHANMHNLRHWWHQVFASATPDLLNPYRSWQSASGHSVSGLHMPDAPPQPVWRSFDEYEIASGRCTPP
LPARLQRHTWQSERLGNSRDVWIYTTGDSKPAERPLAILLDGQFWAKQMPVWEPLMQLTREGALPEAV
YVLIDIIDLPHRSRELTCKDDFWLAVQEELMPQLADWAPHSGKPADTVVAGQSFGGLASLYAGLRWPQ
RFGAVIAQSGSYWWPRRDMLQLPSIPDDACWLMQQVERHGLGNHGALKVFMEAGSQEKLVHRVSGEMA
ARLSDAGHRVHYRVVEGGHDALCWRSGLTDGLQAVWASAFATAYPASATATATARGTHDGKPESVR
>CORE_REP|Org46_Gene2007#
MSQNVYQFIDLQRVDPPKKPLKIRKIEFVEIYEPFSETQAKAQADRCLSCGNPYCEWKCPVHNYIPNW
LKLANEGRIMEAADLAHQTNSLPEVCGRVCPQDRLCEGSCTLNDEFGAVTIGNIERYISDKAIEMGWK
PDMSHVQPTGKRVAIVGAGPAGLACADVLTRNGVKAVVYDRHPEIGGLLTFGIPAFKLEKEVMVKRRG
IFSEMGIEFQLNTEVGKDVSMETLLSEYDAVFLGVGTYQSMRGGLENEEAQGVYDALPFLIANTKQLM
GYEADQHEPYVSMEGKRVVVLGGGDTAMDCVRTSVRQGATQVICAYRRDEANMPGSKREVKNAREEGV
DFQFNLQPLSIELNSAGRVAGVKMVRTQLGAPDANGRQAAEQVPGSEHVIDADAVVMAFGFRPHRMDW
LAAHDVQLDKQGRILAPEGSDNAFQTSNPKIFAGGDAVRGSDLVVTAIAEGRKAADGIMNYLEV
>CORE_REP|Org11_Gene3175#
MTQSARSMAGLPWIAAMAFFMQALDATILNTALPAIAQSLGRSPLAMQSAVISYTLTVAMLIPVSGWL
ADRFGTRRVFIFAVTLFTLGSLLCALSPTLSALVASRVLQGIGGAMMMPVARLALLRAYPRSELLPVL
NFVTMPGLVGPILGPLLGGWLVTYATWHWIFLINIPIGLLGIFYARKYMPDFTTPKRRFDFLGFMLFG
LSLVLISTGLELFGERVLASYVSLGILLSGFVMLFGYITHARRHPQPLIGLDLFKTRTFSVGIAGNVA
SRLGTGCVPFLMPLMLQVGFGYTAIVAGCMMAPTAIGSLMAKSTVTQVLRWFGYRKTLVGITVIIGVL
IAQFALQSPGMPLWLMILPLFVLGMAMSTQFTAMNTISLADLNDANASAGNSVLAVTQQLSISFGVAI
SAAVLRFYESLSLGTMIDHFHYTFITMGIVTVASALVFMLLRRKDGRNLISGQESKKEAKAAS
>CORE_REP|Org21_Gene1305#
MKIKTRFAPSPTGYLHVGGARTALYSWLFSRHAGGEFVLRIEDTDLERSTQDAIDAIMDGMNWLNLDW
DEGPYFQTKRFDRYNAVIDEMLEQGTAYKCYCSKERLEALREKQMENGEKPRYDGHCRDSQCSHTDDE
PHVVRFRNPQEGSVIFDDKIRGPIEFSNQELDDLIIRRTDGSPTYNFCVVVDDWDMEITHVIRGEDHI
NNTPRQINILKALGAPVPEYAHVSMILGDDGKKLSKRHGAVGVMQYRDDGYLPQALLNYLVRLGWSHG
DQEIFSIDEMKEFFTLEAINKSASAFNTEKLQWLNHHYINHMPAEEVAVHLAWHVEQLGIETRNGPEL
KDIVKLLGERCKTLKEMAESCRYFYEDFSEFDADAAKKHLRPVARQPLEAVRAKLAAITVWTPENVHD
AIQGTADELGVGMGKVGMPLRVAVTGAGQSPGMDVTVHAIGQKRSLQRIDMALAYIAEREAQA
>CORE_REP|Org45_Gene4578#
MSKVKQQDIDRLIVLVGGRENIATVSHCITRLRFVLNDPSKASPKEIEELPMVKGCFTNAGQFQVVIG
TDVGDYYQALIASTGVNEADKEQAKVAARQNMTWTERTISHFAEIFFPLLPALISGGLILGFRNVIGD
IPMSGGQTLAQMHPAWKTIYDFLWLLGEAIFMFLPVAICWSTVKKMGGTPVLGIVLGVTLVSPQLMNS
YLLGQQTPEVWNFGWFVIQKVGYQAQVIPSILAGMALGWIETRLKKIVPDYLYLVVVPVVSLLLAVFL
AHALIGPFGRMIGDGVAWAVKAVMTGSFAPVGAALFGFLYAPLVITGVHQTTLAIDMQMIQSMGGTPV
WPLIALSNIAQASAVLGIIIISRKANEREISVPAAISAYLGVTEPAMYGINLKYRFPMLCAMIGSAIA
GLFCGLDGVMANGIGVGGLPGILSIKPQFWLIYSLAILVAIVIPLVLTIMVYKRKAARGELPV
>CORE_REP|Org27_Gene3126#
MQPSAPAAGQFKRSMKARHLVMLSLGGVIGTGLFFNTGYIISTTGALGTLLAYLIGALVVYLVMLCLG

ELSVAMPETGAFHVYASRYLGPATGYTVAWLYWLTWTVALGSSLTAAGFCMQYWFPQSPVWLWCLIFC
VAIFLLNVVTTRFFAESEFWFSLIKVVTILAFIILGGAAMFGLLPMKDGTPAPFLHNLTASGWLPHGT
LPILMTMVAVNFAFSGTELIGIAAGETENPEKVVPLAIRTTVIRLMLFFIGTVFVLAALIPMDQAGIV
KSPFVLVFERIGVPYAADIFNFVILTAILSAANSGLYASGRMLWSLAHQRTLPAYFARVNARGIPINA
LTFSMLGGVLALLTSVIAPDTVFVALSAISGFAVVAVWLSICAAHFAFRRAYLRSGQPISGLKYRAPG
YPLTPILGFALCLLACIGLAFDPEQRIALYCGLPFVALCYLTYFLTRRAGQKTALGEQHVG
>CORE_REP|Org47_Gene3139#
MQQQSPTTAPDNKLKRGLSTRHIRFMALGSAIGTGLFYGSADAIKMAGPSVLLAYLIGGIVAFIIMRA
LGEMSVNNPQASSFSRYAQDYLGPMAGYITGWTYCFEILIVAIADVTAFGIYMGVWFPEVPHWIWVLS
VVLIIGAINLISVKVFGELEFWFSFFKVATIIIMIAAGIGIIIWGIGNGGQPTGIHNLWSNGGFFSNG
FIGMILSLQLVMFAYGGIEIIGITAGEAKDPKKSIPKAINSVPWRILVFYVGTLFVIMSIYPWNQVGT
NGSPFVLTFQHMGITVAAGILNFVVITASLSAINSDVFGVGRMLHGMAEQGHAPKMFSKVSKRGIPWV
TVVVMMLALLLAVYLNYIMPESVFLVIASLATFATVWVWIMILFSQIAFRRSLSKEQVKQLAFPLRGG
VFTSVVAIVFLVFIIGLIGYFPTTRVSLYAGLVWVVLLLAGYWFKVNHQKKRAPLATQQD
>CORE_REP|Org41_Gene316#
MLGLDALELARIQFAFTVSFHIIFPAITIGLASYLAVLEGLWLKTHNEAYRELYHFWSKIFAVNFGMG
VVSGLVMAYQFGTNWSFFSEFAGSITGPLLTYEVLTAFFLEAGFLGVMLFGWNRVGPGLHFFATCMVA
LGTLISTFWILASNSWMQTPQGHEIINGQVVPVDWLKVIFNPSFPYRLLHMSTAAFLSSAFFVGASAA
WHLLRGRDTPAMRKMLSMAMWMALIVAPVQALIGDAHGLNTLKHQPAKIAAIEGHWENPPGEATPLIL
VGWPDMQREETRFKLEVPYLGSLILTHSLTEQVPALKSFPPEDRPNSTVVFWSFRIMVGLGMLMILAG
VWSLWLRWRGGLYQSRPFLYFILWMGPSGLLALLAGWFTTEIGRQPWVVYGLLRTKDAVSAHGDLHMS
ISLLAFIIVYCSVFGVGYSYMMRLIRKGPQPHEHQEDNTEGRPARPLSAVNDTLDDRS
>CORE_REP|Org5_Gene1561#
MSVVPVVDVLQGRAAVDSEVTVRGWVRTRRDSKAGISFLAVYDGSCFDPLQAVVNNSLPNYQDEVLHL
TTGCSVEVTGKVVASPGEGQSFELQATAINVVGWVDDPDTYPMAAKRHSIEYLREVAHLRPRTNLIGA
VARVRHTLAQAIHRFFHENGYFWVSTPLITASDTEGAGEMFRVSTLDLENLPRTDKGAVDFSQDFFGK
EAFLTVSGQLNGETYACALSKVYTFGPTFRAENSNTSRHLAEFWMIEPEVAFATLDDVAGLAESMLKY
VFQAVLDERADDLKFFAERVDKDAISRLERFVSSDFAQVDYTDAIEILLASGQTFENPVSWGIDLSSE
HERYLAEKHFQAPVVVKNYPKDIKAFYMRMNEDGKTVAAMDVLAPGIGEIIGGSQREERLDMLDQRLE
EMGLNKEDYWWYRDLRRYGTVPHSGFGLGFERLIAYVTGVQNVRDVIPFPRTPRNASF
>CORE_REP|Org17_Gene161#
MQQHYQFDAIVIGSGPGGEGAAMGLVKQGARVAVIERYNNVGGGCTHWGTIPSKALRHAVSRIIEFNQ
NPLYNNSRTLSATFPDILRHADNVISQQTRMRQGFYERNQCKLFAGDARFIDANTVSVSYMDGTQDTI
RADHIVIACGSRPYHPASVDFNHPRIYDSDSILELSHEPRHVIIYGAGVIGCEYASIFRGLNVKVDLI
NTRDRLLAFLDQEMSDSLSYHFWNNGVVIRHNEEFEKIEGTEDGVIVHLKSGKKVKADCLLYANGRTG
NTDSLGLENVGLESDSRGLLKVNSMYQTALSHIYAVGDVIGYPSLASAAYDQGRIAAQAIASGEASGH
LIEDIPTGIYTIPEISSVGKTEQELTAMKVPYEVGRAQFKHLARAQIAGMNVGSLKILFHRDTLQILG
IHCFGERAAEIIHIGQAIMEQKGEGNTIEYFVNTTFNYPTMAEAYRVAALNGLNRLF
>CORE_REP|Org8_Gene458#
MSLSLWQQCLARLQDELPATEFSMWIRPLQAELSDNTLALYAPNRFVLDWVRDKYLNNINGLLNDFCG
TDAPLLRFEVGSKPITQVISQTVTASVSSAPAAPAARTAAPSRPSWDNAAAQPELSYRSNVNPKHTFD
NFVEGKSNQLARAAARQVADNPGGAYNPLFLYGGTGLGKTHLLHAVGNGIMARKANAKVVYMHSERFV
QDMVKALQNNAIEEFKRYYRSVDALLIDDIQFFANKERSQEEFFHTFNALLEGNQQIILTSDRYPKEI
NGVEDRLKSRFGWGLTVAIEPPELETRVAILMKKADENDIRLPGEVAFFIAKRLRSNVRELEGALNRV
IANANFTGRAITIDFVREALRDLLALQEKLVTIDNIQKTVAEYYKIKVADLLSKRRSRSVARPRQMAM
ALAKELTNHSLPEIGDAFGGRDHTTVLHACRKIEQLREESHDIKEDFSNLIRTLSS
>CORE_REP|Org25_Gene687#
MINSLTARIFAIFWFTLALVLMLVLMVPKLDSRQMTSLLDSEQRQGLMLEQHVEAELQNDPANDLMWW
RRLFRAIDKWAPPGQRLLLVTSEGRVIGAQRNEMQIVRNFIGQSDNSDHPKKKKYGRVELVGPFAVRD
GEDNYQLYLIRPANSPQSDFINLMFDRPLLLLIVTMLISAPLLLWLAWSLAKPARKLKNAADDVARGN
LKQHPELEAGPQEFLATGASFNQMVSALERMMNAQQRLISDISHELRTPLTRLQLATALMRRRHGEGH
ELARIETEAQRLDSMINDLLALSRGQQKGELAREQLKANELWADVLDNARFEAEQMGKQLEIAAPPGP
WTLFGNASALDSALENIVRNALRYSHTRIAVAFSADNQGVTIQVDDDGPGVSAEDREQIFRPFYRTDE
ARDRESGGTGLGLAIVEAAVNQHRGWVKAEDSPLGGLRLVLWLPLHHQRLSSKTEQ

>CORE_REP|Org37_Gene1287#
MLKIFNTLSRQKEEFKPIHAGKVGMYVCGVTIYDLCHIGHGRTFVAFDVVARYLRYLGYSLNYVRNVT
DVDDKIIRRAAENHETCDQLTERMLAEMHADFDALLIDRPDQEPRATQHIAEIIEITQRLIDRDHAYV
ASNGDVMFSIDSDPQYGLLSRQDLDQLQAGARVEIDDVKRNPMDFVLWKMSKPGEPSWQSPWGPGRPG
WHIECSAMNCKQLGTHFDIHGGGSDLMFPHHENEIAQSSCAHDGPYVNYWMHSGMVMIDKEKMSKSLD
NFFTIRDVLGHYDAETVRYFLMSGHYRSQLNYSEENLKQARTALERLYTALRGTDADAAPAGGEAFEA
RFREAMDDDFNTPEAYSALFDLAREVNRLKGEDMAAANGMAAELRKLANVLGLLQQEPEQFLQGGAQV
DDGEVAEIEALIKQRNEARAAKDWALADAARDRLNEMNIVLEDGPQGTTWRRK
>CORE_REP|Org14_Gene3002#
MSYRSKVAIVYLLGFFVDLINMFIANVAYPAIGQAMRASVSQLAWVSNGYILGLTLVIPLSAWLAQRI
GGRRVFLLSLALFMLATFGAGNADSIGALIGWRTLQGMGGGLLIPIGQTLTYQLYRSHERAGLSAAIM
LVGLLAPALSPALGGWLVDRLDWRWVFFANLPLAALALALAALWLRAETSATAVRKPLDGKGLLSACA
ALTLLLLGLTRLSEAGHQASGAALLAAGLLVLAYYLRHSLRTPQPLLNLRLVGDPLLRNAMGVYLCIP
GLFIGVSLVAMLYLQNQLGMPAAQVGGLMLPWALASFLAITLTGKTFNRLGPRPLLIAGCLLQGAGML
TLAQIDQAGQHAWQIAAFALMGFGGSLCSSTAQSSAFLQIPDAQLADASALWNINRQLSFCLGVALLS
LLLNLLLTGLPPAAAYRTCFILAGASVFIPLLLCLRLANRAIVRQLNAQQDAL
>CORE_REP|Org46_Gene2224#
MSFETLGLSAEIVRAVEEQGYREPTPIQRQAIPVVLEGRDLMASAQTGTGKTAGFTLPLLQLLSKHDH
PVKGRRPVRALILTPTRELAAQIGENVDAYSKHLRLRSLVVFGGVSINPQMMKLRGGVDILVATPGRL
LDLEHQNAVDLSKIEILVLDEADRMLDMGFIHDIRRVLAKLPAKRQNLLFSATFSDDIKALANKLLHN
PASVEVARRNTASEQIEQSVHFVDKKRKRELLSQMIGEGDWKQVLVFTRTKHGANHLAEQLNKDGITA
AAIHGNKSQGARTRALADFKDGRIRVLVATDIAARGLDIDQLPHVVNYELPNVPEDYVHRIGRTGRAE
RTGEAISLVCVDEHKLLRDIERLLKREIPRIALPGYEPDPTIKAEPIINGRQGGGRGAPRGNGGGQRS
GNGGGQRSGNANGGQRENRGNGNGNGNARPQGDGQRRSGAPSRRPRNRKPAE
>CORE_REP|Org12_Gene2801#
MSTVSFSSLPLPAEQLANLNELGYAEMTPVQAAALPAILQGRDVRAKAKTGSGKTAAFGIGLLNSIVV
GQVATQALVLCPTRELADQVSKELRRLARFTQNIKILTLCGGQPMGPQLDSLVHAPHIVVGTPGRIQE
HLRKKTLQLDELKVLVLDEADRMLDMGFADDIDDVISYTPPQRQTLLFSATYPAGIERISARVQRQPL
SVEVDDGEAQASIEQRFYETTRDQRPALLVSAIRYHQPASCVVFCNTKRDCQTVLEALEARGISALAL
HGDLEQRDRDQVLVRFANRSCRVLVATDVAARGLDIKELELVVNYELAFDPEVHVHRIGRTGRAGMSG
LAISLCTPQEMARAHAIEDYLQMSVDWSPVSELSGAANGSLEAEMVTLCIDGGRKAKIRPGDILGALT
GDAGLTAAEVGKIDMFPVHAYVAIRKASARKALQQLQQGKIKGKSCKVRLLK
>CORE_REP|Org33_Gene3049#
MKKTLGVFLPLYTTTLLLLLGSGLLTTYVSLRLASIHVSSALIGAIIAANYIGLVIGGKVGHFLIARV
GHIRAYVACAGIITAAVLGHGLTEFIPAWVALRLVIGLCMMCQYMVLESWLNDQAESNQRGMVFGFYM
AATYLGMSLGQIVLMLQSNLGITTLLVIALCFALCLVPIALTTRTNARHMSPAPMELRYFVGAIPKVL
ATTLVIGMVVGSFYGLAPVYASLQSLTTQQTGLFMALAIFAGLVAQFPLSWLSDRYSRPLLMRLNAIL
LIVAALPLALLPHIDFPLLLAVGFVVSMLQFTLYPLVVALANDLIEPERRVSLAACLLMAFGVGASIG
PLAVGALIEPLGGNILYAFFALCGVLLAALSRTAKAEEPQLAQDAPVPHIPLPDSLASSPLSPALNPT
FDEQIIHDTMPPPEAAPDVDEPQPEEQEAEPLPQGADPQEDTGLKKAHAML
>CORE_REP|Org40_Gene1872#
MDGQQQGDQLKRGLKNRHIQLIALGGAIGTGLFLGIAQTIKMAGPSVLLGYAIGGFIAFLIMRQLGEM
VVEEPVAGSFSHFAYKYWGNFAGFASGWNYWVLYVLVAMAELTAVGIYVQYWWPEIPTWVSAAVFFLA
INAINLANVKVYGEMEFWFAIIKVVAIIGMIVFGAYLLFSGMGGPEATVTNLWAQGGFFPNGVMGLVM
AMAVIMFSFGGLELVGITAAEADNPQKSIPKATNQVIYRILIFYIGSLAILLSLYPWGKVVEGGSPFV
LIFHALNSNLVATVLNVVVLTAALSVYNSCVYCNSRMLYGLAQQGNGPKSLLKVDGRGVPVVAIGISA
LATALCVLINYLIPGRAFELLMALVVSALVINWAMISLAHLKFRAAKNREGVVPKFKAFWYPFSNYLC
LLFMAGILVIMYLTPGIQISVLLIPVWVAILAVGYAIKQRSQRVDGVTSR
>CORE_REP|Org5_Gene2995#
MALWGGRFTQAADQRFKQLNDSLRFDYRLAEQDIVGSVAWSKALVTVNVLTATEQQQLEQALNALLTE
VQADPLAIVQSDAEDIHSWVEQKLIEKVGDLGKKLHTGRSRNDQVATDLKLWCKQQIGDLHQAIVQLQ
QALVETAEANQDAVMPGYTHLQRAQPVTFAHWCLAYVEMLARDESRLQDTLKRLDVSPLGSGALAGTA
YPIDREQLAGWLGFASATRNSLDSVSDRDHVLELLSNAAISMVHLSRFAEDLIFFNSGEAAFVELSDR
VTSGSSLMPQKKNPDALELIRGKCGRVQGALTGMMMTLKGLPLAYNKDMQEDKEGLFDALDTWMDCLQ

MAALVLDGIQVKRPRCQEAAEQGYANATELADYLVAKGVPFREAHHIVGEAVVEAIRQGKPLEALPLA
DLQQFSATIGDDVYPILALQSCLDKRAAKGGVAPQQVAAAIAAAKQRLA
>CORE_REP|Org7_Gene2401#
MRRLRFSPRSSFARTLLLIVTLLFVSLVTTYLVVLNFAILPSLQQFNKVLAYEVRMLMTDRLQLEDGT
LLEVPPAFRREIYRELGISLYTNSAAEESGLRWAQHYQFLSQQMAQQLGGPTDVRVEVNKNSPVVWLK
TWLQPDIWVRVPLTEIHQGDFSPLFRYTLAIMLLAIGGAWLFIRIQNRPLVELEHAALQVGKGIIPPP
LREYGASEVRSVTRAFNQMASGVKQLADDRTLLMAGVSHDLRTPLTRIRLATEMMSAEDGYLAESINK
DIEECNAIIEQFIDYLRTGQEMPTESSDLNAILGEVVAAESGYERVIETALSPGELMMNVHPLSIKRA
AVNMVVNAARYGNGWIKVSSGRELQRGWFQVEDDGPGIKPDELKHLLQPFVRGDSARSTSGTGLGLAI
VQRIIDAHDGELDIGTSERGGLLIRAYIPLPMEKKESTNGHQTARETA
>CORE_REP|Org5_Gene4424#
MDITATANALLPLLGGKENIASAAHCATRLRLVLVDDSKVDKEAIGKLDGVKGCFSNAGQIQVIFGTG
LVNKVHAEFIKAAGVSESSKAEAADIAAKKLNPLQRIARLLSNIFVPIIPAIVASGLLMGLLGMVKTY
GWADANSALFIMLDMFSSAAFIILPILIGFTAAREFGGNPYLGATLGGILTHPALTNAWGVAEGFHTM
NFFGLEIAMIGYQGTVFPVLLTVWFMSLLEKRLRKVIPNALDLILTPFLTVVISGFVALLFIGPAGRA
LGDGISFVLSTLIAHAGWFAGLLFGGLYSAIVITGIHHSFHAVEAGLLGNPNIGVNFLLPIWSMANIA
QGGACLAVYFKTRDAKIKAIAVPSAFSAMLGITEAALFGINLRFVKPFLAALAGGALGGAWVVANHVG
MNAVGLTAIPGMAIVQASSLVSYIIGLAIAFGSAFALSLLLKYKTDAQ
>CORE_REP|Org41_Gene3508#
MIPVSLQTLAEVLSAELIGADCQIVEVTTDTRKVTAGCLFVALKGERFDAHDFAADAVAAGAGALLVS
KRLLVDAPQLVVQDTRLALGQLGAWVRQQVPARVVALTGSSGKTSVKEMTAAILRECGEVLYTAGNFN
NDIGVPLTLLRLEPQHDFAVIELGANHIGEIAYTTALTRPQTALVNNLAAAHLEGFGSLAGVAQAKGE
IFTGLPADGVAIINADNNDWPHWQSMLGGKTVWRFSPQAAAGVDFFADNVRVNGAGTQFTLHSPFGTA
EIALPLPGRHNVANALAATALATSVGATLEAVRQGLKQLQAVPGRLFPVALAEGKLLLDDSYNANVGS
MTAAAQVLAEMPGYRVMVVGDMAELGAEAEECHRQVGEAARLAGVDKVISVGGLSRVLSEASGNGEHY
QDKTAVIARVAELLSEHAVITVLIKGSRSAAMEQVVRALQEKAPC
>CORE_REP|Org9_Gene4194#
MTKHYDYLAIGGGSGGIASINRAAMYGQKCALIEAKELGGTCVNVGCVPKKVMWHAAQIAEAIHQYGP
DYGFDTIVNAFDWKKLVANRTAYIDRIHNSYDNVLGKNKVDVIKGFARFVDAHTVEVNGELITADHIL
IATGGRPSHPAIPGAEYGIDSDGFFELDAMPKRVAVVGAGYIAVEIAGVLNALGAETHLFVRKHAPLR
SFDPMIVETLVEVMNTEGPSLHTESVPKAIVKNADGSLTLQLENGKAFTVDCLIWAIGREPATDNLNL
GVTGVKTNEQGYIDVDKFQNTNVKGIYAVGDNTGAVELTPVAVAAGRRLSERLFNNKPDEHLDYSNIA
TVVFSHPPIGTVGLTEPEAIEKFGADNVKVYKSSFTAMYSAVTQHRQPCRMKLVCAGKEEKIVGLHGI
GFGMDEILQGFAVAVKMGATKKDFDNTVAIHPTAAEEFVTMR
>CORE_REP|Org38_Gene883#
MEYAVSVESFLSSLQRHNPHQPEYLQAVREVFTSLWPFIERNPRYREQALLERLVEPERIIQFRVSWV
DDRGQVQVNRAFRVQFNSAIGPYKGGMRFHPSVNLSILKFLGFEQTFKNALTTLPMGGGKGGSDFDPK
GKSQGEIMRFCQALMTELYRHLGPDTDVPAGDIGVGGREVGFMAGMMKKLSNNTACVFTGKGLSFGGS
LIRPEATGYGLVYFTDAMLQRHGLGFEGMRVAVSGSGNVAQYTIEKALELDARVITVSDSGGTLVDED
GFTTEKLAHLAEIKNQRYGRVADYARERGLTYLAGQQPWNVPVDIALPCATQNELDLEAAQTLIRNGV
KAVAEGANMPTTIQATDAFLDAGVLFAPGKAANAGGVATSGLEMAQNAARIGWRAEKVDVRLQHIMAD
IHHACVEYGGEGKQTHYVHGANIAGFVKVAEAMLAQGVL
>CORE_REP|Org22_Gene3871#
MKAFVPALVLSAVSFSVWAQDATVSSELIKRGEYLARAGDCVACHTDGKSGKTFAGGLAMETPIGTIY
STNITPDKKTGIGDYSFEDFDNAVRKGVAKNGSTLYPAMPYPSFALVKEEDMRAMYAYFMHGVQPVEQ
ANKDSDIPWPLSMRWPLSIWRGMFAPSPADFVADAKADPVIERGRYLVEGLGHCGACHTPRSITMQEK
ALSNGESDDYLSGSNAPIDGWVASSLRSDRKDGLGSWSEAELTEFLKTGRNDKAIVFGGMSDVVEHSL
QYLSDDDLTAIARYLKSLPPKDGKQQAAPVEDSVAKDLWRGDDSKPGAALYVDNCAACHRTDGVGYKR
AFPSLKGNPVVQTEDATSLIHIVLTGNTTPAVQGAVSNITMPPFGWRLNDQQVADVVNFIRTSWGNSA
KPVSASDVADVRKDRSMIRDEKAMGSAEVPDHPDAKK
>CORE_REP|Org12_Gene282#
MLNSILLILFLIAVSAFFSLSEISLAASRKIKLKLMADEGNVNAARVLKLQETPGIFFTVVQIGLNAV
AILGGIVGDAAFSPTFKVLFDRFLSPELAEQVSFICSFVLVTSLFILFADLTPKRIGMIAPETVAVRI
INPMRFSIMIFRPLVWFFNGMANLIFRMFKLPMVRKDDITSDDIYAVVEAGALAGVLRKQEHELIENV

FELESRTVPSSMTSRESVVYFDLRESEESIKEKVSTHPHSKFLVCDGHIDQVVGYVDSKDLLNRVLGN
QSLVLSSGVQIRSALIVPDTLTLSEALESFKTAGEDFAVILNEYALVVGIITLNDVMTTLMGDLVGQG
QEEQIVARDESSWLIEGGTPIDDVMRVLDIDEFPQAGNYETIGGFMMYMLRKIPKRTDFVKYAGYKFE
VVDIDSYKIDQLLVTRLSDKPAAVLPKAPDDTPAA
>CORE_REP|Org12_Gene91#
MAKQPGLDFQSAKGGLGELKRRLLFVIGALIVFRIGSFIPIPGIDATVLAKLLEQQRGTIIEMFNMFS
GGALSRASIFALGIMPYISASIIIQLLTVVHPALAEIKKEGEAGRRKISQYTRYGTLVLAIFQSIGIA
TGLPNMPGMQGLVLNPGFAFYFTAVVSLVTGTMFLMWLGEQITERGIGNGISIIIFAGIVAGLPPAVA
HTIEQARQGDLHFLLLLLVAVLVFAVTFFVVFIERGQRRIVVNYAKRQQGRRVYAAQSTHLPLKVNMA
GVIPAIFASSIILFPATIASWFGGGTGWNWLTTISLYLQPGQPLYVLLYASAIIFFCFFYTALVFNPR
ETADNLKKSGAFVPGIRPGEQTAKYIDKVMTRLTLVGAMYITFICLIPEFMRDAMKVPFYFGGTSLLI
VVVVIMDFMAQVQTLMMSSQYESALKKANLKGYNR
>CORE_REP|Org11_Gene3941#
MRGRLFWKILLGFWLTFLIMTQALWVAFSLYGDRYVPPENAMARRVIGLQLTSAATQLRSGGMPALEA
LMRDWPEDDRRLLSVTPMTQPPPPAPEEPVFEGRRMPKAISAWVQTGEGQGYWLSYDVRGLREEYRPE
RRSHFFNIPAPMLWVGGLGGLLFSAVLAWNLTRPMRQLRGGLDRVAQGDLSVRLFPNMRRRHDELSDV
ARDFDTMAERLELLVSAREQLLHDVSHELRSPLARLQLAIGLARQNAGNVEASLKRIEHESGRLDKMI
GELLALSRTEHSSLPDEEYFDLYGLVDAVVSDARYEAQVPGVDIVLQAESDVEYTVKGNAELMRRAVD
NIVRNALRFSSHGQRVTVTVALSRVDNQFQIAVSDQGPGVEEAKLSSIFDPFVRVKSAQSGKGYGLGL
AITRKVVLAHGGQVEARNGDREGLVITLRIPRWSS
>CORE_REP|Org27_Gene3797#
MSDNATNQRQLRLGAILHGASGNMSAWRHPDATADASINLEFNIATAKKAEQGKFDFVFVADGLYINE
KSIPHFLNRFEPLTLLAALSAATDKIGLVGTLSTSYSDPFTVARQFASLDHLSNGRAGWNVVTSPLEG
SAKNFSRKEHPEHSLRYRIAGEFLDVAKGLWDSWEDDAFVRNKASGEFFRAGKLHTLNHQGEFFSVQG
PLNIGRTPQGRPILFQAGASEDGKRLAAQHADAIFTHHDTLEQAQDFYQDVKRQLVEQGREPDDLRIF
QGVSVIVGDDDADVERQYQETARLVSIENALNYLGRYFEHYDFARHPLDAPFPDIGDLGQNSFRSTTD
AIKRSARERHLTLRQVALEAASPRPVFSGTPEAVADGLQRWFDGEAADGFIISGGTPNAFGHFVDRVV
PVLQQRGLFRQAYHGDTLREHLGLKRPLNRFTQ
>CORE_REP|Org25_Gene39#
MSEFSQTVPELVAWARKNDFSISLPTERLAFLLAIATLNGERLDGEMSEGELVDAFRHVSKGFEQTHE
TVAMRANNAINDMVRQRLLNRFTSELADGNAIYRLTPLGIGITDYYIRQREFSTLRLSMQLSIVAQEL
KRAADAADEGGDDFHWHRNVFAPLKYSVAEIFDSIDMTQRVMDEQQQSVKNDIAALLSKDWRAAISSC
EMLLSETSGTLRELQDTLDAAGDKLQANLLRIQDATLGNVELGFVDKLVFDLQSKLDRIISWGQQAID
LWIGYDRHVHKFIRTAIDMDKNRVFAQRLRQSVQTYFDHPWALTHANADRLLDMRDEELALRSEEVTG
ELPPDLEFEEFSEIREQLAAMIEEALKVYQEQQMPLNLAAVMRDYLAQYPRARHFDVARLVVDQAVRL
GVAEADFSGLPAEWQAINDYGAKVQAHVIDKY
>CORE_REP|Org14_Gene489#
MLERLSWKRLALELALFCLPALLLGLIFGYLPWFLLASALAALVWNFYNQLKLSHWLWIDRSMTPPPG
RWSWEPLFYGLYQMQQRNRRRRRELALLIKRFRSGAESLPDAVVMTTVEGNIFWCNGLAQHLLGFRWP
EDNGQHILNLLRYPEFSHYLQQQEFSRPLTLQLNNEHYVEFRVMPYSEGQLLMVARDVTQMRQLEGAR
RNFFANVSHELRTPLTVLQGYLEMMGDEEQDGSLRSKALSTMQEQTRRMDGLVKQLLTLSRIEAAPNV
DMNERVDIPLMLRVLQREAQSLSGGNHEITFRVNEQLNVFGNEDQLRSAVSNLVYNAVNHTPKGTHIE
VSWQQTAHGAQFQVSDNGPGIAAEHLPRLTERFYRVDKARSRQTGGSGLGLAIVKHALSHHDARLEIL
SEPGIGTRFIFTLPNRLIVPAALSENAVKN
>CORE_REP|Org8_Gene817#
MQVSVETTQGLGRRLSITVPADTIKQAVKKELINAAKSVRIDGFRKGKVPMNIVEQRYGASVRQDVLG
EAMQRSFVDAIIKEKINPAGAPNYVPGEYKEGEDFTFAVEFEVYPEVELKGLENIEVEKPVVEVNDED
VDAMLDTLRKQQATWKETDRAAEAEDRVTVDFTGSIDGEEFEGGKASDFVLAMGQGRMIPGFEEGLVG
HKAGEEFSIDVNFPEDYHAENLKGKAAKFAIVLKKVEERELPELTEEFIKRFGVADGSVAGLRTEVRK
NMERELKGAVRNRIKSQAIDGLVSANEIDVPAALIDGEIDVLRRQAAQRFGGNEKQALELPRELFEEQ
AKRRVVVGLLLGEVISTNDLKADEDRVKTLIEEMASAYEDPSEVIEFYSKNKELMNNMRNVALEEQAV
EALLAKAKVTEKATTFSELMNQTQQA
>CORE_REP|Org26_Gene1535#
MLDPNLLRNELDAVAVKLARRGFKLDLDLLRSQEERRKVLQVETETLQAERNSRSKSIGAAKARGEDI

EPLRREVNELGDKLDAAKAALDALQSEIRDYALTLPNLPDDAVPDGKDDSENLEVARWGEPRQYDFAV
RDHVDLGEMAGGLDFAAAVKLTGSRFVVMKGQIARMHRALSQFMLDLHTEQHGYLEAYVPYLVNHATL
YGTGQLPKFGEDLFHTRPLEEEADSSNYALIPTAEVPLTNLVRDEIVEEETLPLKMTAHTPCFRAEAG
SYGRDTRGLIRMHQFDKVEMVQIVRPEDSMDALEELTGHAEKVLQLLNLPYRKVLLCTGDMGFGACKT
YDLEVWLPAQDTYREISSCSNMWDFQARRMQARCRSKADKKPRLVHTLNGSGLAVGRTLVAVLENYQQ
ADGRIQVPEVLRPYMGGLEYIG
>CORE_REP|Org25_Gene1949#
MSKTHLTEQKFSDFALHPLVLEALEKKGFHNCTPIQALALPLTLSGRDVAGQAQTGTGKTLAFLASTF
HYLLTHPAKQDRQTNQPRALIMAPTRELAVQIHSDAEALSQSTGLKLGLAYGGDGYDKQLKVLESGVD
ILIGTTGRLIDYTKQNYVDLGAMQVVVLDEADRMYDLGFIKDIRWLFRRMPAADQRLNMLFSATLSYR
VRELAFEQMNNAEYVEVEPEQKTGHRIKEELFYPSNEEKMRLLQTLIEEEWPDRAIIFANTKHRCEDI
WGHLAADGHRVGLLTGDVAQKKRLRILDDFTKGNLDILVATDVAARGLHIPAVTHVFNYDLPDDCEDY
VHRIGRTGRAGASGHSISLACEEYALNLPAIETYIDHSIPVSKYNSDALLTDLPAPKRLSRPRGGNGP
RRNSAPRRGGAPRSNRKRSS
>CORE_REP|Org37_Gene3352#
MVDSLTLHPVALVNGTVNLPGSKSVSNRALLLAALAKGTTRLTNLLDSDDVRHMLNALQTLGVNYQLS
ADRTVCEVTGVAGPLVAGQPLELFLGNAGTAMRPLAAALCLGEGDVVLTGEPRMKERPIGHLVDALRQ
GGAQIDYLEQTDYPPIRLRGGFQGGDVTVDGSVSSQFLTALLMTAPLAPQDTQIHIKGELVSKPYIDI
TLHLMRTFGVSVSHDNYRVFHIQGRQTYLAPGDYLVEGDASSASYFLAAAAIKGGTVRVTGIGRKSVQ
GDTKFADVLEKMGARITWGDDFIECSRGELRGIDMDMNHIPDAAMTIATAALFAEGPTTIRNIYNWRV
KETDRLAAMATELRKVGAEVDEGEDYIHVVPPAKLQFAEIGTYNDHRMAMCFSLVALSDTPVTILDPK
CTAKTFPDYFEQLARISQPA
>CORE_REP|Org26_Gene3371#
MARPSFFLDFSLLRSNAHFRAIFCARMLSVFSLGMLAVGVPIQIQAMTGSTLQVGVAVALDGVGMFIG
LMLGGVLADRYDRRKLILFARGTCGLGFVALSLNAFAPAPSLLALYLLAAWDGFFGALGMTALMAVIP
LLVGRENLAAAGALSMVTVRIGAILAPALGGIIIVFGGVGLAFAVAAAGTLGTLVPLVRLPTLLPQQQ
EPEHPLRALASGFQFVWRNKVVGSVVLLGMLMSIVGAVRVLFPALAQDAYHVGASSIGLMYSAVPLGA
MLGALTSGWVGRFSRPGVLILVAAIVAFTAIASLGLFSHLAPALLALVCYGYANAIASLLQFMLIQSN
TPDHLLGRVNSLGTAQDVTGDSIGALGLGVLGRVFTPLMSVLSFGAFAAVLGVLVAFSVRTLRQCRPA
DALVEHDEPAPATSAAADN
>CORE_REP|Org2_Gene1799#
MASSNLIKQLQERGLVAQVTDEEALAERLAQGPIALYCGFDPTADSLHLGHLVPLLCLKRFQLAGHKP
VALVGGATGLIGDPSFKAAERKLNTTDTVNEWVEKIRKQVSPFLDFDCGSNSAIAANNYDWFGGMNVL
TFLRDIGKHFSVNQMINKEAVKQRLNRDDSGISFTEFSYNLLQGYDFSELYNRHQVELQIGGSDQWGN
ITSGIDLTRRQHQKQVFGLTVPLITKADGTKFGKTEGGAVWLAPEKTSPYKFYQFWINTADADVYRFL
KFFTFMSLEDINALEEEDKNSGKAPRAQYVLAEEVTGMVHGAEGLAAAKRITQSLFSGALHDMTEADF
AQLAQDGMPTIKLERDADLQQALVNAELVPSRGQARTMIGSNAVTINGEKQSDAEYRFSDADRLFGRY
TLLRRGKKHYCLVDWQ
>CORE_REP|Org5_Gene2120#
MEKLSYASDSSTTAWATYLQQIDRVAPYLGELSRWVDTLRHPKRALIVDIPLQMDDGTIRHFEGFRVQ
HNLSRGPGKGGIRFHPDVDLNEVMALSAWMTIKCAAVNLPYGGAKGGIRVDPFKLSEGELERLTRRYT
SEIGFIIGPQKDIPAPDVGTNAKVMAWMMDTYSMNHGTTITGVVTGKPIHLGGSLGREKATGRGVFVT
GSEVAKRLGVQIEGAKVAVQGFGNVGSEAARLFVGVGARVVTIQDHSATLFNADGIDLAALTEYQTKH
KQIAGFPGASEIESEAFWSVDMDILIPAALEGQITRERAEILSAKLVLEGANGPTFPEADDILRSRNI
TVVPDVICNAGGVTVSYFEWVQDMASYFWSESEINERMDKIMTDAMVHVWNKAAEKECSLRTAAYIVA
CERILTARKERGIYPG
>CORE_REP|Org19_Gene479#
MTDKRKDGSGKLLYCSFCGKSQHEVRKLIAGPSVYICDECVDLCNDIIREEIKEVAPHRERSALPTPH
EIRHHLDDYVIGQEQAKKVLAVAVYNHYKRLRNGDTSNGIELGKSNILLIGPTGSGKTLLAETLARFL
DVPFTMADATTLTEAGYVGEDVENIIQKLLQKCDYDVQKAQRGIVYIDEIDKISRKSDNPSITRDVSG
EGVQQALLKLIEGTIAAVPPQGGRKHPQQEFLQVDTSKILFICGGAFAGLDKVIGQRVNTGSGIGFGA
TVKGESEKATEGELLLQAEPEDLIKFGLIPEFIGRLPVVATLSELSEDALIQILKEPKNALTKQYQAL
FNLEGVELEFRDEALNAIAKKAMARKTGARGLRSIVEGALLDTMYDLPSMDSVDKVVIDESVIAGQSK
PLLIYGKPEAQASGE

>CORE_REP|Org33_Gene2400#
MNKATVAAKRWWYIMPIVFITYSLAYLDRANFSFASAAGINEDLGITKGMASLLGALFFLGYFFFQIP
GAIYAERRSVKKLIFWCLILWGGCASLTGVVSNIPMLAAIRFILGVVEAAVMPAMLIYISNWFTKSER
SRANTFLILGNPVTVLWMSVVSGYLIHAFGWREMFIIEGIPAVIWAFCWWVLAKDKPAQAGWLSAEEK
LALQQQLDEEQKGIKAVRNYGEAFRSRNVILLCVQYFAWSIGVYGFVLWLPSILRSGMQMGMVEAGWL
SAVPYLAATIAMIVVSWASDKMQNRKLFVWPLLLIGALAFFGSYAVGTNHFWISYGLLVVAGAAMYAP
YGPFFAIIPEMLPKNVAGGAMALINSMGALGSFFGSWFVGYLNGATGSPAASYMFMAIALVVAVVLTL
IVKPARNEIQPQLA
>CORE_REP|Org25_Gene896#
MSMLEQMGKAAKQASWQLAVLSTAKKNQVLSVMADRLEANSEAILLANEQDMAQARATGMSEALLDRL
LLTPARLAAIANDVRQVCRLNDPVGHVLDGNLLDSGLKLERRRVPLGVIGVIYEARPNVTIDVASLCL
KTGNAVILRGGKETHNTNQATVKVIQQALEQCGLPAAAVQAIDSPDRALVNELLRLDRYVDMLIPRGG
AGLHKLCREQSTIPVITGGIGVCHTYVDADVDFDKALTVIENAKIQRPSACNSLETLLVNRSIAAEFL
PALSAKMAAVGVTLHAAENALPLLQGGPATVVPVNAEDYDDEWLSLDLNVLLVDDIDQAIDHIRTHGT
NHSDAILTRSLSSAEHFVRAVDSSAVYVNASTRFTDGGQFGLGAEVAVSTQKLHARGPMGLDALTTYK
WIGYGDDLVRS
>CORE_REP|Org45_Gene2458#
MPNGHPVRRPLGRLYTALWGGCLLMLSQSAAARFAIPGYELVYTAPVETALQADDLRNTAEVWREMFD
AAKTRIDLGQFYVANQDGSLLDGVLQHLKAAGERGVKIRFLLEEKGLRMSTAETLEQLKAIPNLELRI
IPYQKLSGGILHAKYLLVDGEQAFVGSQNFDWRALEHIHETGLRISDAKVVGQIQAIFEQDWQAQALL
AQDKPVPALPDSPPTAQPQGNYLAASPRAYNPAGVIDSQAELPRLLAGAKRRVRVQVMDYAPLSFGPE
RSRPFYAVIDNALRSAAARGVQIELMVANWNTKKPDIAWLKSLALVPNVQIKVVTIPPASSGFIPFAR
VIHSKLMTIDDEIAWVGTSNWTGGYLDNSRNLELVMHSAAMSGRLDKLYQQLWNSVYAEPLRLDYDYP
PPKPGGES
>CORE_REP|Org8_Gene2144#
MKIHAITAPLSKARHQRCCECDLLFMLPPLSGNQAAYCPRCNAKVVHGRDWSMTRLTAMAITMLLLMP
FAFTEPLISIRLLGTRIDASLLEGIWQMSRQGDPLTASMVAFCTLGAPLTLALSLLYLRFGHALGMNL
RPVLLMLERLKEWVMLDIYLIGMAVAAIKVQDYADIQAGSALIAYLSLTLLSILTLIHANLEQLWERY
YPQEQPEGPPAALHICLSCHYTGYPDARGRCPRCHVPMCHRQPYSLQKTWAALIAAMILLIPANLLPI
SIIYANGVRLEDTIFSGVVSLATSGNVPIAAIVFIASVLVPFTKVIVLITLLLSIHFKTSHSLKTRIR
LLRLVTWIGRWSMLDLFVIALMMSLVNRDQLLSFTMGPAAFYFGSAVILTILAVEWLDSRLIWDAHAT
GNADYTD
>CORE_REP|Org40_Gene2806#
MKTTLPPAARLGRQALLFPLCLVLFEFATYIGNDMIQPGMLAVVADFNAGEEWVPTSMTAYLAGGIFL
QWLLGPLSDRRGRRPVMLAGVAFFIVSCLAILLVTTIEQFIAMRFLQGIGLCFIGAVGYATIQESFEE
SVCIKITALMANVALIAPLLGPLAGAALIHVAPWQSMFVLFAALAAIAFYGLWKAMPETATLQGEAFS
AANLWRDYRQVLGNRRFLCGALAIGFASLPLLAWIAQSPVILISGESLSTLDYGLLQIPVFGALILGN
LTLARLTGKNSVERLIKLGAGPMLLGLLIAALATQFSSHAYLWMTAGLSLYAFGIGLANAGLYRLTLF
SSNVSKGTVSATMGMLSMMVFTVGIELAKVAYVWGGSGLFNLFNLISGLCWLTLVALFLGKRRNGDPT
PQPTGAV
>CORE_REP|Org11_Gene2984#
MSVITEKKNHATPGKAMLASVTGYAMDGFDLLILGFMLPAISIELGLTSSAAGSLVTWTLIGAVLGGV
IFGHLSDRFGRIRVLTITILMFSLFTGLCAVAQGYWDLLAYRTLAGIGLGGEFGIGMALIAEAWPAEK
RNRASAYVGMGWQLGVLAAAFLTPLLLEHIGWRGMFLVGLLPALASFLIRRTLGEPEAFVRQKDAGQP
LSFLQRLRLLFKDRATSKASIGIFILCSVQNFGYYGLMIWMPTYLAKNFGFSLTKSGLWTAVTVVGMT
FGIWLFGMLADRFARWKIFVLYQVGAVVMVIGYAQLSDPMLMLFAGAVMGMFVNGMIGGYGALISDTY
PVQARATAQNILFNLGRGVGGLGPLVIGALVTQVSFTAAISLLAAIYLLDIYATLFLLPKKQGAGDTL
GAIG
>CORE_REP|Org30_Gene792#
MAKVSLEKDRIKFLLVEGVHQSTVDNLRAAGYTNIEYHKGALDTESLKASIRDAHFVGIRSRTHLTEE
VFAAAEKLVAVGCFCIGTNQVDLKAATKRGIPVFNAPFSNTRSVAEMVLGELLLMLRGIPAANAKAHR
GVWHKLAVGSYEARGKKLGIIGYGHIGTQLGILAEGLGMKVFFYDIENKLPLGNAQQVRHLSDLLNMS
DVVTLHVPETLATKNMMGAEELALMKPGAILINASRGTVVDIPALCDALASNHLAGAAIDVFPEEPAT
NSDPFNSPLCEFDNVLLTPHIGGSTQEAQENIGDEVAGKLAKYSDNGSTLSAVNFPEVSLPAHGPNAS

RLLHIHENRPGVLTQINQIFAEEGVNIAAQYLQTGPEIGYVVIDIEAETARADAALQRMKAIDGTIRA
RLLF
>CORE_REP|Org21_Gene3877#
MASVAEPVNWKRNFFVAWVGCFLTGAAFSLVMPFLPLYVETLGVTGHQALNMWSGLLFSITFLFSAIA
APFWGALADRRGRKLMLLRSALGMAIVMVLMGMAQTVWQFLALRAVLGLLGGFIPNANALIATQVPRN
RSGWALGTLSTGGVGGALIGPLIGGLLADLYGLRPVFYITAAVLFVCFVLTLLYVKEQFTPVQKRDML
HARQVFASLKNPKLVLSLFVTTMIIQIATGSIAPILTLYVRDLAGATHNLAFISGLIASVPGVAALMS
APRLGKLGDRIGPERILICMLIVSVLLLIPMAFVQTPWQLGVLRFLLGAADGALLPAVQTLLIYNCTN
QVAGRIFSYNQSFRDVGNVSGPLLGAAVSAGYGFRAVFGVTALVVLFNAGYSWWCLRRRPGYMREDTL
QEEQ
>CORE_REP|Org2_Gene3767#
MKKRLAVLILLVAIVVIALLWWRENRRYDGPVQQVTAGAEQIARGRYLAQAADCAACHTASGGAPLAG
GYPLETPFGTIYGSNLTPSADHGIGRWTRDDFFLALTQGVAPGGRHLYPAMPYTSYKGMSRQDADDIY
AYLMTRPAVDVAIPANEMPFPFNQRMALIGWNLLFRSQDPLPASSQGSSPQWQRGRYLVDVLGHCGEC
HTPRGALGQMDLAKPMQGGDLGRFMAPDITPHGLAQRGWTPQDVSRFLSTGLAPQGSAFSEMHMVVDL
STRHLTPEDHQALALYLMGEQPPAAVPVKMGQGSDAGRMAYLDQCAGCHAREGEGKPHVAPAMRDNAT
LRQVDGKNLIVSVLDGLPAQQFPNGESMQSMPGFGERLSDADVAELVNYLRVTWGGLPADITAEQVKA
LRK
>CORE_REP|Org42_Gene3550#
MFGWTPLQRNAAIASFSSWTLDAFDFFVLVFLLSDIAQSFHVGLEQVTLAILLTLAVRPIGALIFGRA
AEKYGRKPILMLNIVFFSVFELLSAAAPSLTVFLLLRVLYGVAMGGIWGVASSLAMETIPDRSRGLMS
GIFQAGYPFGYLLAAVVYGLLFETVGWRGMFVIGAAPILLLPFIYYCVQESPVWLAARERKESSALLP
VLKSHWKLCCYLVLLMAAFNFFSHGTQDLYPVFLKVQHGFDPKTVSIIAISYNIASIIGGVFFGSLSE
KIGRKKAIIIAALLALPVIPLWAFSSGSLMLGIGAFLMQFMVQGAWGVVPTYLTELVPANTRAVLPGF
VYQLGNLIASVNATLQATIAEHHGHNYGLAMAIVAGTVAVAIALLVFFGKDTRGKAITDAVKNPGVRA
NV
>CORE_REP|Org43_Gene3254#
MRKKTLLLCLPLLFTGNALADAGGYQLEQVLVMSRHNLRAPLANNGSVLAQSTPKAWPAWETPGGQLT
TKGGVLEVYMGHYFNAWLKQTGLLPQEGCPTAGSVYVYANSLQRTVATAQFFSNGAFPGCDVSVHHQD
KMGEMDPTFNPIITDTGEAFNQQALAAMNAALGSLKLDASYQQLAKIIDYKDSAACKTDKHCDLTKEA
SVMSAVPGKEPGVSGPLRVGNSLVDAFMLQYYEGFPMKEVAWGKIATPHQWQQLAQLKDGYQDSLFTS
PVVAQNVAKPLLTYINNALLGERKPDAPKLTVLVGHDSNIASLLSAMQFQPYQLPQQYEKTPIGGKLV
FQRWRDAQNDRELLKIEYVYQSTEQLRKATPLTLQTPPQRVTLALKGCPIDKDGFCAWSDFEKTMKGI
L
>CORE_REP|Org49_Gene3307#
MSDKLLDPPCAALGRLPAPLVLLLAAASAFSVANVYYAQPLLDAIAHDFSISLAAVGMVITVTQLGCA
LALLLVVPLGDRLNRHWLLAGQQLGLIGALLLVGWAHSAPWLLAGMLLVGLLGTAMTQGLIAFAAALA
APQERGRVVGAAQGGVVLGLLLARTLSGALADVGGWRTVYFFSAGVTLVLLPILSRLLPAPRTAPSTL
SYPALLRSMLTLLLHDRTLQIRGMLALLMFGAFSLFWSSLVLPLSQAPFNFTHAAVGAFGLVGAVGAL
AAVRAGHLADRGLGQAASGVCLLLLTLAWLPLGLLGSGLVWLVAGIVLLDLAGQAIHVLNQSMIFSAH
PQSHSRLVGCYMLFYAVGSGLGAFAGTHMYAWAGWSGVCWLGAGVSLSALLFWRLTLRGMPPSAAAVE
Q
>CORE_REP|Org37_Gene3355#
MTTPIISLTAARALHLAAQGLLSPLKRQAKPDDVVSAIQRMGLLQIDTISVVARSPYLVLFSRLGAYQ
SEWLEQALAGRKLFEYWAHEACFLPIEDFGLLRHRMLAPHDMGWKYSADWVQQHQAAMDSLLRHIEQQ
GPVRSADFSAEKKGNSGWWDWKPEKRHLEILFTAGKLMVAERRNFHRVYDLTERLLPAWDDARHTLPA
ERARRQMLRRTCRYLGIFRAEWLADYYRLKRVAPKALLAELQEQGEITPVQVEGLEGPFYLHESLAEL
LPLAEQSKLKSTVTSLLSPFDPVVWDRRRALELFNFDYRLECYTPKEKRRYGYFTLPVLHRGELVGRI
DAKAHRRQGVFEIISFHAEPQVRFGKQRAQDIRQAIARTAKWHGAQRVALGDIPAALAAEWGAGWEVG
>CORE_REP|Org46_Gene4169#
MTDSSQSAMPPAKGSAVKGTAFSILGAISVSHLLNDMIQSLILAIYPILQADFHLSFVQIGMITLTYQ
LTASLLQPLIGYYTDKHPQPYSLPIGMGFTLSGLLLLSVASTFPLVLLAAALVGTGSSVFHPESSRVA
RMASGGRHGLAQSLFQVGGNFGSSLGPLLAALIIAPYGKGNVAWFTLAALLAIVVLLQVSKWYQHQHR
ATKGQPKSPSTLKALPKRTVVYSLGILLVLIFSKYFYLASISSYYTFYLIHKFGVSVQNAQIHLFAFL

FAVAAGTIIGGPLGDKIGRKYVIWGSILGAAPFTLVLPYASLYWTGILTVIIGVILASAFSAILVYAQ
ELIPGKVGMVSGLFFGFAFGMGGLGAAVLGYVADLTSIELVYQICAFLPLIGIITALLPNMEHKPQ
>CORE_REP|Org36_Gene712#
MQNQGIKKIVLAYSGGLDTSAIIPWLKENYGGCEVVAFVADIGQERSDLEGVEQKALQSGASECHVVD
LREEFIRDYVYPVLQTGALYEGSYLLGTSMARPIIAKAQVELALKVGADALCHGATGKGNDQVRFETT
YTALAPQLKVVAPWREWNLRSREALLDYLKERNIPTTASLEKIYSRDENAWHISTEGGVLESPWNAPN
KDCWVWTVDPQEAPDQPEQVTVTVEKGRVVAVNGKALSPYQCLETLNALGAKHGVGRIDIVENRLVGI
KSRGCYETPGGTIMVAALRAVEQLVLDRDSFKWREQLGLEMSYVVYDGRWFAPLRRSLQASAEALAEE
VNGEVVLQLYKGQVTATQKKSANSLYSEEFATFGEDEVYDHSHAGGFIRLFSLSSRIRALNEKKNK
>CORE_REP|Org42_Gene666#
MKLPIYLDYSATTPVDPRVAEKMMQFLTLDGTFGNPASRSHRFGWQAEEAVDIARNQIAELVGADPRE
IVFTSGATESDNLAIKGAANFYQKKGKHIITSKTEHKAVLDTCRQLEREGFEVTYLAPQSNGIISLQD
LEAALRDDTILVSIMHVNNEIGVVQDIEAIGEMCRARGIIYHVDATQSVGKLPIDLSKLKVDLMSFSG
HKIYGPKGIGALYVRRKPRIRIEAQVHGGGHERGMRSGTLPVHQIVGMGEAYRIAKEEMTEEMARLRT
LRDRLWNGVKDMEEVYLNGDLEHGAPNILNVSFNYVEGESLIMALKDLAVSSGSACTSASLEPSYVLR
ALGMSDELAHSSIRFSLGRFTTEEEIDYTIQLVRKSIGRLRDLSPLWEMFKQGVDINSIEWAHH
>CORE_REP|Org4_Gene5062#
MENLGMPSLKLTPRRLTLIAVIVFIAVAIAIALTLYWQRPPQQDYVTAPARLGDIENAVLATGRLDAV
ERVNVGARVSGEVKSLKVKLGDRVTKGQPIADIDDLQQRNDLRNAEAALNVIKAELQAKQAQLKQAES
RFKRQRRMLNDEASSREDFETAEATLATTRAELLSLNARLVQAQIEVDKKKIDLGYTRVVAPMDGIVI
AVVTQQGQTVNSTQSAPTIVKLARLDMMTIKAQISEADITRISPGQKAYFTIFSDPDKRYDATLRTIE
LAPESVMKDDSLAGTSSASGSGTSNASVYYNALLDVPNPENRLRIAMTAQVSLLLGEAKNALLVPIQA
VHKTEGKVQQVQVLTQDQRLETREVTTGITNNVDIQILSGLKAGETVVLSQPAAKSAEDGIFL
>CORE_REP|Org7_Gene3115#
MTQYASPILTSLLDTDAYKLHMQQAVFHRYPAISVAAEFRCRGDELLGEYADEIRAQVALMSQLTLTD
AEFAYLSSLPFFRQDYLSWLRTFRYDPQQVTIDNRDGKLQIRIAGPWREVIMWEVPLLAVISEVVHRR
RSPLATPEQAVAHLQTKLAQFKTLAGDLDLSRFKLMDFGTRRRFSQGVQQAIVSTLQAEFPYLSGTSN
YDLAHQLGLAPVGTQAHEWFQAHQQISPVLANSQRAALQAWLDEYPDQLGIALTDCITMDAFLRDFGP
QFAERYQGLRHDSGDPVEWGEKAIAHYQKLGIDPMSKTLVFSDNLDLEKALALYRHFYQRINLSFGIG
TRLTCDIPGVKPLNIVIKLVECKGKPVAKLSDSPGKTICQDQAFVRALRKAFDLPLVKKAS
>CORE_REP|Org15_Gene4578#
MSALHADGGAKAWLATFAVGLSTFTVVTAEMLPVGLLTPIVSTLNASIGRAGLLISLPALFAALFAPL
VVLGARRTDRRNLLAGFLLLLIAANLLAAAATSLALLFAARILLGFCIGGIWAIAGGLAERLVPPASV
GLALSIIFGGVAAASVFGVPLGVFLGEALGWRMAFLAVAVLAALTLLLLVCVLPPLPVTQAIGWRSFT
ALRANRRLLTGLLLTFLLVAGHFMAYTFVRPLLQTVAGIESRWVGPLLFAYGVAGIFGNFIAGQAAAK
RLRRTLALIALGLALAVLLLPLLGHAPLSGGAFLLLWGIAYGGVSVALMAWMLKAAPDAVEVASSLYI
ALFNLAISCGSLAGGLVVDAGGLTINGALSGLVLLLALAILMGTRRQRPKTAAKADSPPG
>CORE_REP|Org40_Gene547#
MLEPITSEHTVSENNSLTTPSVNVEQPAAAKINLLDLNRQQMREFFAEMGEKPFRADQVMKWIYHYCC
DDFEQMTDINKVLRGKLQRVAEIRAPEVAEEQRSADGTIKWAIKVGDQQVETVYIPEADRATLCVSSQ
VGCALECKFCSTAQQGFNRNLRVSEIIGQVWRAAKIIGALKVTGQRPITNVVMMGMGEPLLNLNNVVP
AMEIMLDDFGFGLSKRRVTLSTSGVVPALDKLGDMIDVALAISLHAPNDTIRDEIVPINRKYNIETFL
SAVRRYLEKSNANQGRVTVEYVMLDHINDSTDDAHQLAEVLKDTPCKINLIPWNPFPGAPYGRSSNSR
VDRFSKVLMEYGFTTIVRKTRGDDIDAACGQLAGEVIDRTKRTLKKKMAGEPINVRAV
>CORE_REP|Org5_Gene782#
MQQNRTSHLGLIFILGLLSMLMPLAIDMYLPSMPVIAAQFGVESGSVQMTLSAYMLGFAFGQLFYGPM
SDSIGRKPVILWGTLIFAIAGCACAMAQSIDQLIGLRFLHGLAAAAASVVINALMRDMFTKDEFSRMM
SFVILVMTIAPLLAPMIGGALLLWFSWHAIFWTMGAAALIGSLLVALFIKETLPKERRQRFHLRTTLG
NFGSLFRHKRVLSYMLASAFSFAGMFSFLSAGPFVYIELNHVSPQHFGYYFALNIVFLFLTTLINSRN
VRRFGAVKMFKLGLLVQLAMGLWLLAVSAVGLGFWALVIGVAVYLGCIAMISSNAMAVILDDFPHMAG
TASSLAGTLRFSIGALVGAVLSMAPGKSAWPMVTSMALCSIVAVLFYVYASRPRDRAA
>CORE_REP|Org22_Gene1065#
MTRSITLARTLALSALATLVLSSSAFAKIEEGKLVIWINGDKGYNGLAEVGKKFEKDTGIKVTVEHPD
KLEEKYPQVAATGDGPDIIFWAHDRFGGYAQSGLLAEIHPSKAFQDKLFPFTWDAVRYDGKLIGYPIA

VEALSLIYNKDLVKQPPKTWEEIPALDKQLRANGKSAIMWNLQEPYFTWPIIAADGGYAFKYENGKYN
IKDVGVANAGSQAGLQFIVDLVKNKHINADTDYSIAEAAFNKGQTAMTINGPWAWNNIEQSKINYGVT
LLPTFKGKPSKPFVGVLTAGINAASPNKELATEFLENYLLTNEGLADVNKDKPLGAVALKSYQEALAK
DPKIAATMQNSQNGEIMPNIPQMSAFWYAERSAVINAVSGRQTVKAALDDVQTRITK
>CORE_REP|Org16_Gene1641#
MEHAPVSRSTAWLRVVILAVSAFIFNTTEFIPVGLLSDIAASFSMQTEQVGLIITIYAWIVAAASLAC
MLLTSKIERRKLLIGVFMLFIASHVLTAVAWDFTTLVISRAGVALAHSVFWSITASLAIRVAPPGKKA
QALSLLAGGTALAMVLGLPLGRVVGQLLGWRMTFIGIAVCATLALVLLWRLLPVLKSEHSGSLASVPL
LFKRPALVALYMLTIIVVTAHFTAYSYIEPFIQTVAGLSENFTTLMLLLFGAAGIVGSLLFSRYSERF
PSGFFIGAIVLLALSLLLLLPAAGSESHLTVLCIFWGMAIMAIGLSMQAKVLSLAPDATDVAMAIFSG
LYNFGIGSGALLGNQVSLHLGMGNIGFVAAPLALIALGWCLLSVYRSERLQQHHSR
>CORE_REP|Org7_Gene3423#
MTVRLFLAKGREKSLLRRHPWVFSGAVQRVEGKALSGETIDILDSQGKWLARGAYSPESQIRARVWTF
QQDEEINIDFFIRRLQQAQSWRDWVAQRDGLDGYRLIAGESDGLPGITIDRFQNFLVLQLLSAGAEYQ
RPALLSALQHCYPECSIYDRSDVAVRKKEGLPLAQGPVLGDLPPELLPITEHGMKLLVDIQQGHKTGF
YLDQRDSRLAARNYSAGRRVLNCFSYTGAFAVSALMGGCAQVISVDTSQAALDIARQNVELNKLELNK
AEFVRDDVFQLLRNYRAQGEKFDLIIMDPPKFVENKNQLASACRGYKDINMLALQLLNPGGILLSFSC
SGLMPTDLFQKILADAAVDAGRDVQFIEQFRQAADHPVIATYPEGLYLKGFACRVM
>CORE_REP|Org34_Gene3799#
MLDSQTIATVKSTIPLLAATGPKLTAHFYDRMFAHNPELKDIFNMSNQRNGDQRQALFDAICAYAANI
ENLAALLPAVERIAQKHTSFNIQPEQYQIVGGHLLATLDEMFSPGQEVLDAWGKAYGVLANVFIQREE
QIYQQSETDNGGWRDLRAFRILKKQPQSDVICSFVLAPVDGGRVADFKPGQYLAVYIKHDSLEHQEIR
QYSLTTSPNGEFYRIAVKREDQGKVSNYLHQQAQEGDVIDIAPPHGDFFLDVATTTPVALISAGVGQT
PMLGMLNTLHDSQHQAQVHWLHAAENGSVHAFADEVADIAGRMPNLSRHVWYREPGADDVEGRDYHSR
GLMDLSALQGSLADPQMHYYFCGPVAFMQFVGKQLLEMGVEAERIHYECFGPHKVL
>CORE_REP|Org34_Gene3464#
MTKHLARQRLVYAVVLGLLAALGPLCTDLYLPALPEMAGELNTSTAAAQLSLTTGLLGLGVGQLIFGP
YSDKLGRMRPLLLSLILLLGASLWCALAPTIDQLLIARLLQGIAGAGGAVISRAIARDLYAGHELTRF
FALLMLVNGLAPIVAPVLGGVMLQVMNWRGIFGVLAAIAVLLFSLSALKLRESLPVERRSQGGILAML
MSLGGLLTQRYFMGLCLTQGFVMAGMFAYIGASPFVLQQIYGLSPQMFSLCFAINGVGLIIAAQLASR
LSARWGERRVLRGGLTLAAVASLLLLLAAALHAPLVVLLVPLFFSVAVIGIVGPTASSLAMQSQGDKA
GSASALIGVCMFALGACAVPLTGLGGTSGLSMALTIVGCYAIAILLFGLLARRNDA
>CORE_REP|Org38_Gene4435#
MPNQPNSSFNAGGRTRAFALGQRLSGVALLAALLAGCDNSVAHNAPPPPPVVSAASVVVKPISQWDAF
NGRVEAVQSVQLRPRVSGYIERVNYTEGDEVKKGQVLFIIDDRTYRAAREQAQAELVRARNQAALARS
ESSRTEKLIGTQAISQEVWEQRRSSAAQAQSNVLAAQAQLDMAQLNLDFTRVTAPIDGRASRAMITAG
NLVTAGDSASVLTTLVSLDKVYVYFDVDEATFLRYQQQGRHDVRLPVKVGLVGEDGTPHQGLVDFTDN
QLNAGTGTIRMRALLDNRDRRFTPGLFARVQMPGSAEFNAMLIDDKAVMTDQNRKFVYIVDKDGKAQR
RDIDVGRMAEGLRIVQKGLVNGDRVIVDGMQKVFMPGMPVDAKNVAMTTTASALN
>CORE_REP|Org35_Gene1276#
MNKNRGLTPLAAVLMLSGSLVLTGCNDKETQQQGAQQQAPEVGVVTLKAEPLNITTDLPGRTAAYRIA
EVRPQVSGIILKRNFVEGSDIKAGTSLYQIDPATYQASYDSAKGDLAKAQASASIARVTVNRYKPLLG
TSYISKQDYDNAVSTLQQADAAVVAAKAAVETARINLAYTKVTSPISGRIGKSAVTEGALVSNGQATA
LSTVQQLDPMYVDVTQSSTDFLRLKQELASGALKQENGKAKVKLMLENGTEYAQEGTLEFSDVTVDET
TGSITIRALFPNPNDTLLPGMFVRARLDEGVRSDALLVPQQGVTRNPRGDATALVVGADNKVELRTLK
ADQAIGDKWLVTDGLKAGDRVIVTGLMKVHPGAQVKVQEVDTQAQKQPQSEAQKS
>CORE_REP|Org39_Gene141#
MRKLENFHLLVMLILLVAVGQMAQTIYVPVIADIAHDLSVRTGAVQRVMAAYLLTYGFSQLIYGPISD
RIGRRPVILTGMMIFLVGALGALLSTNLTMLVAASAIQGMGTGVAGVMARTMPRDLYAGTALRYANSL
LNMGILVSPLLAPVIGGALAMVFGWRACYAFLLALCACVAFAMFRWLPETRPVQTEKRRMLASFRQLL
GDSTFSCYLVMLIGALAGIAVFEASCGVLMGGVLGLSGLTVSILFILPIPAAFFGAWYAGRDGKTFHT
LMWHSVISCLLAGAMMWIPGWFGVMNIWTLIVPAALFFFGAGMLFPLATTGAMEPFPYLAGAAGALVG
GMQNMGSGLATWLSAMLPQTGQFSLGLLMFAMALLILLCWWPLSNRMQHQGHTA
>CORE_REP|Org29_Gene2914#

MSLRSLYLLSLLAAGGSAQAMSAGEYVAKAGDCTACHTAPGGAELAGGMKFPTPLGAIYATNITPDKL
HGIGAYSFEEFDRAMRQGVAKDGHRLYPAMPYTSYAKMSAEDMRALYDYLMNEVPAQNVANRDSDISW
PLSMRWPLAVWNQLFHDDQPYQADPQQSAEWNRGAYLVQGAGHCGSCHTPRGWAMQEKGLDGKEPVFL
SGAELDGWYASNLRGLPPEEVTALLKTGRSRHAAVAGPMSEVVTHSTQYLSDGDLNAIAVYLRSLAPE
TAAKAAAPAVQANNPGGQATYAMYCSTCHGNKGEGTDFAIPALAGNATVTADNPLTALRVVLEGAHTP
ATQHAMAFDMPAYGWALNDRQAADLMSYLRGSWGNQAAPVTVQQVQDARQLQAK
>CORE_REP|Org28_Gene2530#
MLNNKDKPASSPWPAIFSLTVACFVMVTTEFLPIGLLTNIAPSLGVSTGTAGLMVTMPGIVAAVAAPA
LSLISGRLDRRLLMLGLSLLLIVSNLVAALAVNFPMMLLGRVLLGICVGGFWSFAANYGRHLVPEANQ
GRATALILSGISVGAVCGVPAGALIGDLFGWRAAFFGGAALAVGVLLAQLRLLTSVPPSRPVTPRDLV
LPLRLPMARIGLIAIVLLFIGHFAAYTYLRPLLQQVFVLSPSAISLQLLAYGAIGLLGTFLGERLGEY
SLRATFILIAAMLAAILIVSPLLSGLGGATLMVMVWGLAFGAVPVCATNWMFAAVPQAPEAGQALLVC
VVQIALASGALLGGEVVDWQGVSSAMLFGGALILSAALVFGLSLRSGAIGAKQC
>CORE_REP|Org47_Gene2881#
MTLADYNGHLVTLCLMATGTFAIGTDAFIVAGVLSDISDTFAVSPAQAGQLISVFALAYMLFAPLTAW
LLGNVNRKHILQLALVLFIAGNLACAWATSYLQISLGRVLAALGAACYTPQAAAAAVGLVAEKRRGLA
ISIVYGGMTLAIALGIPFGTFLAKLIGWREIFLFIALLGAIALLGLSLALRAIAPPGKHSLKERLAPL
RQKAVLTTLLITFFAVCSEHIVYSYVSVLLKNTQFGPQAILPLALLVFGIGAVIGNFASGALTDALGS
KFVLLFSVAIQTLSLFLLAFYVTSPWWVLAIFLVWGITGWMYLVPIQHHLLSLSKRFGALTVSLNSSV
LYAGIAAGGMLGGLTLYALPAHYLPLFSLPLGAIALLLTLLFFRGETGNE
>CORE_REP|Org25_Gene4390#
MSASAETQNPQQPSGKKKQRKFWLLLLTVIFIVIGVAYLVYWFLVLRHHQETDDAYVSGNQVQIMAQV
SGSVNSVNFDNTDYVKQGDVLLTLDPTDAEQAFERAKTGLANSVRQTHQLIINSKQYQANIALRKTDL
SKAENDLKRRVVLGSVDAIGREELQHARDAVDSAKAALEVAVQQYNANQAMVLNTPLEQQPAIQQAAA
QMRDAWLALQRTKVISPITGYVSRRSVQVGAQIAAGSPLMAVVPADHIWVDANFKETQIANMRIGQPA
KVVSDVYGDDVVYQGKVVGIDMGTGSAFSLLPAQNATGNWIKVVQRLPVRIELDAKQVADHPLRIGLS
TLVTVDTANLDGRVLSDVVRDKPLYQSDALALNLAPVNQLIADVIHANAG
>CORE_REP|Org47_Gene663#
MNLHEYQAKQLFARYGMPAPTGYACTTPREAEEAASKIGSGPWVVKCQVHAGGRGKAGGVKVVNSKED
IRAFAEAWLGKRLVTYQTDALGQPVNQILVEAATDIDKELYLGAVVDRATRRIVFMASTEGGVEIEKV
AEETPELIHKMTIDPLAGPQPYQGRELAFKLGLTGKQVSQFAKIFMGLATLFLERDLAMVEINPLVIT
KQGDLVCLDGKLGADGNALFRQPELREMRDPSQEDERESRAAQWELNYVALDGNIGCMVNGAGLAMGT
MDIVKLHGGEPANFLDVGGGATKERVTEAFKIILSDDKVKAVLVNIFGGIVRCDLIADGIIGAVAEVG
VNVPVVVRLEGNNAELGAKKLADSGLNIIAATSLTDAAQQVVAAVEGK
>CORE_REP|Org27_Gene3222#
MKVNYPLLALAVGAFGIGTTEFSPMGLLPTIAKGVDVSIPMAGMLISAYAVGVMVGAPLMTLLLSHRA
RRSALIFLMAIFTLGNVLSAIAPDYTTLMLSRIITSLNHGAFFGLGSVVAASVVPKEKQASAVATMFM
GLTIANIGGVPAATWLGETIGWRMSFLATAGLGVIAMLGLWFSLPKGSAGARPDVKRELSVLVRPQVL
TALLTTVLGAGAMFTLYTYISPVLQHITEATPLFVTTMLVLIGVGFSIGNYLGGKFADRSESATLKGF
LLLLVAIMLLIPLLARSDIGAAVSMMIWGAATFAVVPPLQMRVMRVASEAPGLSSSVNIGAFNLGNAL
GAAAGGAVVSAGLGYSFVPVMGAIIAGLALLLVLFTSRTAAKVVYANG
>CORE_REP|Org11_Gene3606#
MTIAVKHRVLLTLFMLLLLAAFGLPFLSYAPNRLLSGKSLSLFSLLHGPALWLLLPLAALAILSLLPP
TRGRALLAALAACGVLTLAFWISGQAAGHLAQEGSRLARTSWGSGCWLTMALSLLIAADAMARLTASH
LWRMLGNALVLVPPALLLFGHQLDQLSLLKEYHNRQEVFDAALLQHLTILLATMAPALAIGVPLGVLC
FRSERWQRPIFSALNIIQTVPSIALFGLLIAPLAGLATAVPWLAEHGVSGIGMAPAIVALVLYALLPL
VRSVVAGLQSVPAGVIESATGMGLTRGQIFLRVQLPLALPLFLTGVRILAVQTVGMAVVAALIGAGGF
GAIVFQGLLSSALDLVLLGVIPVVMMAVIVDSLFKFWVSILDVSRR
>CORE_REP|Org40_Gene2960#
MQSACSSRSKLPDVGTTIFTVIGQLSAEHQALNLSQGAPNFAGDPQLIEATAQAMRAGHNQYAPMSGV
AALRAALAEKAERLYGARYDADEEITVIASASEGLYSAISALVHPGDEVIYFEPAFDSYAPIVRLQGA
TPVAIKLSLQDFRVDWDEVAAAINGKTRMIIVNTPHNPTGAVFDAQDIDRLTALTRDTDIVILSDEVY
EHVVFDGDIHHSMARYPQLAERSVIVSSFGKTYHVTGWRVGYCLAPAALMDEIRKVHQFMVFSADTPM
QYAFAAALANPQSYLGLAAFYQQKRDLLASALQDSRFELLPSRGSFFMLARFSGFSHESDNDFAVRLI

REAKVATIPLSAFYSDGTDTGLIRLSFSKDNETLLEGARRLSQV
>CORE_REP|Org7_Gene1444#
MSAYSRPVLLLLCGLLLLTVSIAVLNTLVPLWLTHAQLSTWQVGMVSSSYFSGNLLGTLVAGKLIQRV
GFTRSYHLSCLLFAAATAGMVLSIDFWSWLGWRFFAGVGCAWIWVIVESALLRSGNLSNRGQLLAAYM
IVYYLGTVTGQLLLSMTSTELLHVVPWVTAIVISAMLPMLFARVNRHEDEPQQAAVWTMLRRRSARLG
INGCIISGIVLGSLYGLMPLYLSHQGMSDANVGYWMALLVSSGIVGQWPVGRLADRYGRLLVLRIQVF
VVILASVAMLGNYAMAPSLFILGCAGFTLYPVAMSWACEKAMPHELVAMNQALLMSYTIGSLLGPSMT
ALLMQNYSDRVLFVMIAAVALVYLLMLLKKQKPDRHHTPFAAA
>CORE_REP|Org9_Gene4300#
MIKSALLVLEDGTQFHGRAIGAEGTAVGEVVFNTSMTGYQEILTDPSYSRQIVTLTYPHIGNVGTNAS
DEESSAVHAQGLVIRDLPLIASNYRNEESLSDYLKRHNIVAIADIDTRKLTRLLREKGAQNGCIIAAD
SPDAALALAKAQGFPGLKGMDLAKEVTTQEAYSWQQGSWTLEGDLPEAKTAAELPFHVVAYDYGAKRN
ILRMLVDRGCRLTVVPAQTPADDVLKMNPDGIFLSNGPGDPEPCDYAIAAIKQFLETDIPVFGICLGH
QLLALASGAKTMKMKLGHHGGNHPVKDLDNNTVMITAQNHGFAVDENNLPANLRVTHKSLFDHTVQGI
HRTDKAAFSFQGHPEASPGPHDAAPLFDHFIELIETYRSNAK
>CORE_REP|Org9_Gene697#
MFDYEVLRFIWWVLVGVLLIGFAVTDGFDMGVGILVRIIGKTDTERRVMINSIAPHWDGNQVWLITAG
GALFAAWPMVYAAAFSGFYVAMILVLAALFFRPVGFDYRSKLESSRWRNMWDWGIFIGSFVPAVVFGV
AFGNLLQGVPFHMDEYMRLFYTGNFFQLLNPFGLLAGVVSLTMLVTQGATYLQMRTTGEIHLRSRAAA
QIATLIMAVCFLLAGVWLVKGIDGFVVTSALDTLAESNPMRKEVAHQAGAWLINFNKYPLLWALPALG
VVLPLFTILFSRLEKGALAFVTSSLTIACVILTAGVTMFPFVMPSSTVPNVSLTMWDATSSLLTLKVM
TVVAAIFVPIVLAYTSWSYYKMFGRLDKNYIENNKHSLY
>CORE_REP|Org29_Gene2782#
MKRNILAVVIPALLAAGAANAAEIYNKDGNKLDLYGKVDGLHYFSKDKGNDGDQTYVRFGFKGETQIT
DQLTGYGQWEYNVQSNHSESQGTEGTKTRLGFAGLKFADYGSFDYGRNYGVLYDVEGWTDMLPEFGGD
TYTNSDNFMTGRTNGVATYRNNNFFGLVDGLNFALQYQGKNQNDGRDIKKQNGDGWGISSTYDIGEGV
SFGAAYASSNRTDAQKNKSNERGDKADAWTVGAKYDANNVYLAAMYAETRNMTPYGGNNSLKDGTTSC
ADTQNNSCGGFASKTQNFEVTAQYQFDFGLRPEVSYLQSKGKNMNVPGAGSDQDLVKYVSVGTTYYFN
KNMSTYVDYKINLLDDNAFTKAAGIATDDIVAVGLVYQF
>CORE_REP|Org22_Gene1273#
MKYELQTTDGRARRGRLIFDRGVVETPAFMPVGTYGTVKGMTPEEVKETGAQILLGNTFHLWLRPGQE
IMKLHGDLHDFMQWHGPILTDSGGFQVFSLGAMRKIKEEGVYFRNPINGDKVFLSPEKSMEIQYDLGS
DIVMIFDECTPYPADWDYAKRSMEMSLRWAERSRKRFDELENKNALFGIIQGGVYEDLRDVSVKGLVD
IGFDGYAVGGLAVGEPKEDMHRILEHVCPQIPEDKPRYLMGVGKPEDLVEGVRRGIDMFDCVMPTRNA
RNGHLFVTDGVVKIRNAKHKDDTSPLDKDCDCYTCRHYSRAYLHHLDRCNEILGARLNTIHNLRHYQR
LMAGLRQAIEEGKLEQFVADFYGRIGKPIPPLNA
>CORE_REP|Org3_Gene1416#
MAKKDYYEILGVSKTADEREIKKAYKRLAMKYHPDRNQEQDAEIKFKEVKEAYEVLTDDQKRAAYDQY
GHAAFEQGGMGGGGFGGGADFSDIFGDVFGDIFGGGRRQRASRGSDLRYNMELTLEEAVRGVTKEIRI
PTLEECDVCHGSGAKPGSSPVTCPTCHGQGQVQMRQGFFTVQQACPHCHGRGQIIKDPCNKCHGHGRV
EKSKTLSVKIPAGVDTGDRIRLAGEGEAGEHGAPAGDLYVQVQVKAHPIFEREGNNLYCEVPINFAMA
ALGGEIEVPTLDGRVKLKVPSETQTGKLFRMRGKGVKSVRGGSQGDLLCRVVVETPVNLNDKQKQLLR
ELEESLGGPSGDKNSPRSKSFFDGVKKFFDDLTR
>CORE_REP|Org48_Gene4084#
MNYQLITTDAGLQQVCEQARKHAQIALDTEFVRTRTYYPQLGLIQLYDGEQLSLIDPLPIKQWQPFID
LLADTAVVKFLHAGSEDLEVFLNAFKTLPTPMIDTQILAAFTGRPLSCGFATLVAEYMKVELDKSESR
TDWLARPLTERQCVYAAADVFYLLPMAKQLVQETEEAGWTAAAHNECLLLCQRRSETLAPEVAYREIS
NAWQLRPRQLGCLQKLAEWRLRQARERDLAVNFVVREENLWQVARYMPSSLGELDSLGLSGPEIRYHG
KTLLALVAEAEALEESELPAPLANLIDQPGYKKVFKDIKAAIATVSEQSGLSSELLASRRQINQLLNW
HWKLKDGESRPELISGWRGDLLMAPLQDILKDY
>CORE_REP|Org19_Gene4738#
MRVITLSRLYVHPVKSLRGLQLSYAQVGSSGLAFDRNFMITEPDGTFITARQYPQMVLFTPALLPDGL
FLSAPDGESAAIRFSDFTAAPQPTEVWGNHFTALIAPDEINRWLSGYFQRDVQLRWLGPELTRRVKKH
PEIPLTFADGYPYLLINQASFNDLQRRCPGSIKLEQFRPNLVVSGAAAWAEDGWQVIRVGDVMFDLVK

PCSRCVLTTVSTERGRKHPSGEPLSTLQKFRSADNGDIDFGQNMIARSSGIIRVGDTVEVLSTKPPRP
YGAGKVVESVQAPQDSEHSVTIEYEGKVFTGNNQQILLEQLEQQGIRVPYSCRAGICGSCRITLLSGE
VAPLKKSALGDNGTILCCSCIPKSDLTLA
>CORE_REP|Org12_Gene3248#
MSDTTQTQDPWATAPADAGAAPAHDAAANAGDAWSSAPPPAAHDAAGQGADWLSSAPAQPEHFSLLDP
FHKAWVPFDSWVTQGIDWLVLHFRPLFQGIRVPVDMILSGFQQLLLGMPAPIAILVFSLLAWQVSGLG
MGAATLLSLVAIGAIGAWSQAMVTLALVLTALFFCILIGLPLGIWLARSKHAAKVIRPLLDAMQTTPA
FVYLVPIVMLFGIGNVPGVVVTIIFALPPIVRLTILGIKQVPEDLIEAAESFGASPRQLLFKVQLPLA
MPTIMAGVNQTLMLALSMVVIASMIAVGGLGQMVLRGIGRLDMGLAAVGGVGIVILAIILDRLTQSLG
RDRRSKGIGRWYRRGPIGLLTRPFIKQA
>CORE_REP|Org22_Gene3622#
MIRCHDTYQGDAGCILPPPIFDARKSGLSFAKRTYLPRVAGLGLGFICVCAALYPLAPPTAVWLLLAF
HGFLWPHLAYRLACRAKDPFKAEIRNLLIDSAFGGFWAAMMAFNALPAIVILSMMSMNNIASAGKALF
VKGLAIQLAAAALTGALLGFPFHPHSTPLQIYLCLPMIYLYPTLLGLVTYRTAKRLAEKKQELQRIST
RDGLTGLYNRRHWEHLLHRQFDSCRRYQDNATLILMDIDRFKTINDTFGHALGDEALAALAEELLIGL
RNVDIVGRYGGDEFGAVLPNTSAEQAETVLRRIQQRLDVVIFKEAPQLRLQISAGIANYHPALGGYLD
WLKAADGALYRAKQNGRNRLETAAPTGD
>CORE_REP|Org1_Gene1987#
MKTEKLLSPLKVGAVTLPNRVFMAPLTRLRSIEPGDIPTPLMAEYYAQRASAGLIVTEATQISFQAKG
YAGAPGLHTPEQIAAWKHITQAVHDKNGHIAVQLWHVGRISHASLQPGGQAPVAPSAINADTRTTVRD
ETGAWVRVPTSTPRALETSEIPGIVNDFRQATANARDAGFDFIELHAAHGYLLHQFMSPASNQRTDQY
GGSIENRTRLTLEVVDATIAEWGSEHIGIRISPLGPFNGLDNGEDQEEAALYLVEELNKRNIAYLHIS
EPDWAGGKPYSDAFRDSVRAHFKGVIVGAGAYTAEKAEALIEKGFIDAVAFGRSYIANPDLVERFRQH
APLNEPKPETFYGGGAEGYTDYPFLAK
>CORE_REP|Org39_Gene576#
MNGSQTLVVKLGTSVLTGGSLRLNRAHIVELVRQCAQQHAAGHRIVIVTSGAIAAGREHLGYPELPAT
IASKQLLAAVGQSRLIQLWEQLFSIYGIHVGQMLLTRADLEDRERFLNARDTMTALLDNRIVPVINEN
DAVATAEIKVGDNDNLSALAAILAGADKLLLLTDQQGLYTADPRNNPQAELIREVHGIDDALRAIAGD
SVSGLGTGGMGTKLQAADVACRAGIDVVIAAGSKPGVVADVIEGKPVGTRFHALETPLENRKRWIFGA
PPAGEITVDDGAVEAMMARGSSLLPKGIREVKGDFSRGEVIRIRNLAGRDLAHGVSRYNSDAMRMIAG
HHSQEISEILGYEYGPVAVHRDDMIVS
>CORE_REP|Org45_Gene2965#
MPRLTLLAGLLLCSGLLHAAPTVSQLQDGLEHPWSLAFLPAEQGLLITERPGRLRLWQQGKGLSPPIA
GVPQVYAEGQGGLLEVLPAPDFAASRRVYLSFAEPGDGGKAGTAVGYGRLSDDGARLENFKVIFRQLP
KLSVGNHFGGKLAFDRQGYLFIALGENNQRPTAQETDKLQGKLVRLTAEGAVPPDNPWVGQAGKRPEV
WSYGHRNPQGLALNPWSGAIWEHEHGPRGGDELNLPLPGKNYGWPLATYGINYSGQPIPEAKGERVPG
TEQPLHYWRVSPGLSGMAFYDGQRFPAWRHSLFIGALAQKELIRLTLEGDKVVAEERLLGDRGERIRE
VRSGPDGYLYLLTDERNGKLLKVGAS
>CORE_REP|Org27_Gene1708#
MPLPQSRYDLPRIIFGVLFIAIMIVACFWVIQPFILGFAWAGMVVIATWPLLLKLQKLLWGRRSLAVL
VMTLLLILLFILPISLLISSVVDNSAPLIAWASSPGKLHIPDLAWLQSVPMIGDRLYTSYHTLVNAGG
AALLAKVQPYFGQTATWFVAQAAHIGRLLLHCALMLLFSALLYARGEQVALGIRHFAVRLGSARGDAA
VLLGGQAIRAVALGVVVTALVQSVLGGIGLAVSGIPAATLLTMLIFICCVAQLGPLLVLVPAIIWLYW
HGDTTWGTVLLVWSCVVATLDNVLRPVLIRMGADLPLLLILSGVIGGLLAFGMIGLFIGPVVLAVSYR
LLTAWMDEAPEPTTAPEQVIEDLEKR
>CORE_REP|Org33_Gene2599#
MSDNSQKKVIVGMSGGVDSSVTAYLLQQQGYQVAGLFMKNWEEDDDEEYCSAATDLADAQAVCDKLGI
ELHTVNFAAEYWDNVFELFLEEYKAGRTPNPDILCNKEIKFKAFLEFAAEDLGADFIATGHYVRRQDV
DGKSRLLRGVDGNKDQSYFLYTLSHEQVAQSLFPVGELEKPEVRRIAEQLELVTAKKKDSTGICFIGE
RKFRDFLGRYLPAQPGPIVSVDGQTVGEHQGLMYHTLGQRKGLGIGGMKDSSEDPWYVVDKDVANNVL
VVAQGHDHPRLMSVGLIAQQLHWVDRLPLSGPFRCTVKTRYRQQDIPCTVTPLDDERIEVRFDEPVSA
VTPGQSAVFYQGEICLGGGIIEQRLA
>CORE_REP|Org15_Gene4579#
MNARSYQELLNSKQRLALFLFLIMNAASSVFTLLFPFRDTPAFTLPLLCIPLFCLVAALFSLQTPRKY

LCKLNLFAGVLGLLWAAHIYVKSQYCLPNNQDFLLISLFSIFFISAISLTDNFTAFCLHAVPSAMVIL
ALDGMHNTLRILFTTLLPIIAFSIHHLMLKRSEIFTHALVANLYNERDKFNNLSMIDPLTGLYNRRGL
ENKITMLLEPQTGHHYVLLLDIDHFKVYNDSYGHAMGDRALVQVAVAIRDAVRSRDIVVRYGGEEFLV
LLTNVHEGYAAQLAERVRQRVAELNIPHGASPQHSGTLTLSAGISALEKLDIESAIGAADAALYLAKH
SGRNNIQLAQNVEPALLQPQELTR
>CORE_REP|Org45_Gene2934#
MTQVYNFSSGPAMLPVEVLRRAEQELCNWHGLGTSVMEISHRSKEFIAVAEQAEQDLRDLLKVPSNYK
VLFCHGGARAQFAALPLNLLGDKATADYIDGGYWAHSAIKEAEKYCAPNVIDVKTRIDGLSGIKPMKE
WQLSDDAAYVHYCPNETIDGVAIDETPDFGDKVVIGDYSSTILSRPLDVSRFGVIYAGAQKNIGPAGL
TLVIVRDDLLGKARKEVPSILDYTVLAENDSMFNTPPTFAWYLSGLVFKWLKEQGGLVEMQKRNQAKA
ELLYATIDKSDFYRSQVAIANRSWMNVPFQLVDAALDKVFLSEAEAIGLQALKGHRVVGGMRASIYNA
MPLAGVQALTDFMADFERRHG
>CORE_REP|Org36_Gene2461#
MKAATAVIDRRALRHNLQQVRRQAPQSRLIAVVKANAYGHGLLETAHTLQDADCYGVARIGEALMLRS
GGIVKPILLLEGFFSAEDLPVLVANNIETAVHSIEQLEALEQAELARPVPVWMKLDTGMHRLGVRPEH
AEAFYQRLCACRNVAQPVNIMSHFSRADEPESDTTLKQIACFEQFARGKPGQRSVAASGGTLLWPDAH
NEWVRPGIILYGVSPLDNGSGAEHGLQPAMTLKSSLIAVREHKAGEAVGYGGTWVSPRDTRLGVVAMG
YGDGYPRSAPTGTPILINGREVPIVGRVSMDMISVDLGPAAADKVGDEAVLWGPALPVERIAVCTGIS
AYELITKLTQRVAMEYIGD
>CORE_REP|Org5_Gene1945#
MPRPITATMHLGAIENNLQVVRRFAPGAKVWAVVKANAYGHGIKHVWRSMAQTDGFAMLDLAEAVLLR
ESGWQGPILLLEGFFQPQDLALLDRYRLTTAVHSDWQLAAIADATLSAPLNVYLKVNSGMNRLGFAPE
RLHEVWRRAQAIANIGELTLMSHFATADGPEGVTQQMATIEAAAADIPLPRCLANSAATLWHSSTHGS
WVRPGIILYGASPSGCWNDVAATGLQPAMTLSSEIIGIQQLKSGDRVGYGGRYSAAGAQRIGVVACGY
ADGYPRHAPTGTPVWVDGVLTRTLGTVSMDMLAVDLTPCPQVELGAEVELWGKRLPVDEVATAAGTLG
YELLSALAARVPVAIEA
>CORE_REP|Org39_Gene3369#
MKRILVTGGAGFIGSAVVRHIIEATDDSVVVVDKLTYAGNLESLAVVAESERYAFEQVDICDRAELDR
VFAQYQPDVVMHLAAESHVDRSIDGPAAFIETNVVGTYTLLEAARHYWQPLAAEKKQAFRFHHISTDE
VYGDLHGTDDLFTETTPYAPSSPYSASKASSDHLVRAWLRTYGLPTLVTNCSNNYGPYHFPEKLIPLV
ILNAVAGKPLPVYGNGAQVRDWLYVEDHARALYQVVTEGVVGETYNIGGHNERKNIDVVQTICELLEE
LAPNKPQGVANYRDLITYVKDRPGHDMRYAIDAGKIDRELDWRPQETFESGLRKTVVWYLNNETWWRR
VQDGSYAGERLGLSE
>CORE_REP|Org24_Gene1188#
MFAKLLRSVVIGLIVAGLLLAALPVLRSSNGLFAEKTENTSDETPVSYNKAVRRAAPAVVNIYNRNLN
GAANVLSLGSGVIMNERGYIITNRHVIKDAQQITVVLQDGRRYEALLVGSDGLTDLAVLKIDPGNLPV
IPTNKNRVAHVGDVVLAIGNPYNLGQTVTQGILSATGRISMSTTGRQTFLQTDASINRGNSGGALVNS
LGELIGINTLTYDKITDGETPEGLGFAIPIELATKIMDKLIRDGRVIRGYFGIQGKEIIPLRSSNSGI
DRLQGIIVTEITPNGPASSAGFQINDIIINVDNKPAVSVLETMDQVAEIRPGTEIPVIVLRDGKRITL
KMTVGEFPEDNN
>CORE_REP|Org6_Gene1135#
MNVATQEILLEPADNQRLLSLCGPFDDNIKQLERRLGIEINRRDNRFKLVGKNLCVVAAADILRHLYV
DTAPIRGVIPDIDPEQIHLAIKESRVLEQVADSVPDYGKAVTIKTKRGMVKPRTPNQAQYIANILDHD
ITFGIGPAGTGKTYLAVAAAVDALERQEIRRILLTRPAVEAGEKLGFLPGDLSQKVDPYLRPLYDALF
EMLGFERVEKLIERNVIEVAPLAYMRGRTLNDAFIILDESQNTTIEQMKMFLTRIGFNSKAVITGDVT
QIDLPRNQKSGLRHAVEVLSDVEELSFNFFHSEDVVRHPVVARVVIAYEAWEAAEQKRKDAIAEQRKR
EALAASEQETP
>CORE_REP|Org47_Gene4611#
MAAELSASAGAARRYWRWGGRLLGGALSLALTLLGLLLFTFMLSHLAPIDPALQVAGDHASEATYAQV
RHELGLDQPLPVQFWRYLVHLAHGDLGISRITAQPVLSDLLRTFPATVELATCAIILGALCGITLAFL
AVLKPGSWLDNAARLLSLIGYSVPIFWLSLLGLLLFYATLHWSAGPGRLDDIYLYSMEPRSGFVLIDS
WLSGDRDMFYNAIGHLWLPVVALALLSMAGITRLLRAAMLEECNKEYVTLARSKGAGRLRILLRHVFP
NVLGTLITVLSLSYASLLEGAVLTETVFAWPGVGRYLTSALFAADTPAILGATLLIGTCFVLLNALAD
ALTYLVDPRTR

>CORE_REP|Org20_Gene4467#
MSKELALRPALPWRQQLFDFLYKWGMLLTVAALIALFGLASDNFLDANNIINILRSIAIVTVIAIGVS
ISLSVGGFDLSVGSTASLANALVISLFVWHGFGTTGAIVVTLLLCTLVGLFNALLIVVFRIPDMLATL
ASLFVIQGVAMTYSYGGSITQNMVLPNGDMAEGLIPEVFSALGQVPVIVLIMLAVTVAVQLFLSLTKH
GRRMYAIGGNPEAARLSGIRTVRYRVAAYVISSWLAALGGILLASRIGSSQVNAGGGYLMDAVAAAYI
GFSLAGAGKPNALGTLIGAVILGVLQNGLVMLSVPYYAMDIIKGLVLALALAITYIQKRCSSPAHARC
AAFPFNSSG
>CORE_REP|Org29_Gene14#
MFKKFRGMFSNDLSIDLGTANTLIYVKGQGIVLNEPSVVAIRQDRAGSPKSVAAVGHDAKQMLGRTPG
NIAAIRPMKDGVIADFFVTEKMLQHFIKQVHSNSFMRPSPRVLVCVPVGATQVERRAIRESAQGAGAR
EVFLIEEPMAAAIGAGLPVSEATGSMVVDIGGGTTEVAVISLNGVVYSSSVRIGGDRFDEAIINYVRR
NYGSLIGEATAERIKHEIGSAYPGDEVREIEVRGRNLAEGVPRGFTLNSNEILEALQEPLTGIVSAVM
VALEQCPPELASDISERGMVLTGGGALLRNLDRLLMEETGIPVVVAEDPLTCVARGGGKALEMIDMHG
GDLFSEE
>CORE_REP|Org39_Gene2294#
MQYHRIPHSSLEVSVLGLGTMTFGEQNTEADAHAQLDYALAAGVNLIDTAELYPVPPRPETQGLTESY
IGSWIKARGNREKIVLASKVSGPVRGTDSSIRPQQALDRKNIRAALDASLKRLNTDYLDLYQLHWPQR
ATNCFGKLNYQYTDDKATVTLLETLEALTEQVRAGKIRYIGVSNETPWGVMRYLQLAEKHELPRIVSI
QNPYSLLNRSFEIGLAEISQHEGVELLAYSSLAFGTLSGKYLNGAKPAGARNTLFTRFNRYSGQQTQL
AIAEYVALAKKHGLDPSQMALAFVRQQPFVASTLLGATTVEQLKINIDSLDVVLDEDVLQALEEIHTR
FTIPAP
>CORE_REP|Org40_Gene2778#
MSNVKIEKPLSADSTGKGGLFSGLSGKMPKDTGIFIVMIGIALIFEILGWYMRDQSFLLNPNRLLLIV
LQVAIIGIIAVGVTQVIITTGIDLSSGSLIALTAVVAASLAQTSDSISPMYPGLLDLPAAIPIGAGIG
VGIVCGFINGFLITRTGIPPFIATLGMMVSARGLAQYYTKGNPVSFLSDGFTSIGQGAMPVIIFLVIA
VIFHIALKHTRYGKYIYAIGGNMTSARVSGINVNKYLVTVYTIAGGLAGLAGVVLAARVSSGQSSMGM
SYELDAIAAAVIGGSSLMGGVGRITGTLIGAVILGLIKSGFTFIGVDSYIQDIIKGVIIVAAVSIDMR
RNRKKH
>CORE_REP|Org18_Gene4103#
MANIRDVARLAGVSISSVSNLLNNRSHQMSAQTRERIEQAMATLGYRPARTAALPAPQAKIIGLLLPS
IVNPSFSALAHAVDGAARAHRYRVLLGNAYRQEQEEAAFIDDMFLHGVRGIIVAASDIRQTHFVRAAE
RGMKIVSYDSPFAEPMATDTRLFDSVSMDNIAAGRLAAQHLLERGCRHIVFATEATLTVGRSHKIDGF
LSALGHSLSERQRVIEGKANSAYGDTEMFELGLTLAPRVLALTPRPDGIVAINDALGIGLMVGLRAAG
VQVPADISVIGIDNIALADLAEPGLTSVRPPLAEMAQLMVERLIGRINDDAQPPGEFLFPPTVISRRS
VKAAG
>CORE_REP|Org33_Gene3792#
MPPVHPITIRDVAKRAGVSVATVSRVLNHSALTSKETREQVLQAVAELGYRPNANAQALATQSSDTLG
VVVMDVSDPFFGALVKAVDTVAQKHHKYLLIGNSYHQAGKERHAIEVLIRQRCNALIVHAKALSDAEL
IGFLEQVPGMVLINRIIPGYEPRCVGLDNVCGAEMAMRLLLSQGHRRIGYLGSNHPIEDGPLRQQGYA
QAMAAAGLATPDNWRAYGSPDLQGGEAAMVELLGRNLQLSAVFAYNDAMAAGAMAVLKENGITVPQHF
SLIGFDDIPIARYTSPKLTTVRYPIVSMATLATELALQGAAGLAEPQAAHLFMPTLVRRHSVAPWQSE
ATVTL
>CORE_REP|Org26_Gene2881#
MGNYLKFRPDGTAVGLLALLVAVVLAFSLLMPGRFFSGATFTSVAFQLPELGLLTLAMFIPILSGGLN
LCIIASANLTSLLMAWLFISYLPPDAGLGLQALWLVLALAAAMLLAVTIGAATGALVAYVGAHPILVT
LATMTTVNGIGIYLTRGAALSGMPEIVRFIGAERVLGVPVPLLIFLAVAVLLALFLQKTRLGKCIYMS
GSNINATHFSGVNTHRVLIAIYTLSSLLCVIAGLVMMARFNSARMGYGDSYLLLTVLAIILGGTDPFG
GFGRVSGVVLALIVLQVIATGLNLMNVSPHFSLAMWGAVLIAVLALKFFRHRYRQRRAMRRSAAQARA
AAGH
>CORE_REP|Org22_Gene2198#
MMIIRPIERRDLADLLTLAGKSGIGLTSLPQNEDTLSARIERALKTWQGELPQSDQCYLFVLEDSERR
QAVGVCAIEVAVGLAEPWYSFRVGTQVHASKQLNVYKSVPTLFLSNDHTGHSELCTLFLDPDYRHGEN
GKLLSKVRFLFIAAFRERFSRRLIAEMRGFSDENGRSPFWESVGHHFFSIEFAKADYLSGTGQKAFIA
ELMPKHPLYVDFLAEDAQKVIGEVHPQTLPARRLLEAEGLSYQGYVDIFDGGPTLEAEIDHIRAVKQS

RLVKVVLDDTPMRADAPVHLVANDNYQNYRALLVNADLYDDRLHINAATAAALGVEQGSPVRVLPLIA
QEKA
>CORE_REP|Org13_Gene3324#
MKRINRYYDAAKAHHTPEGFRNPEPSQRQEGDLQRWQDERKRQGLPRPPQQGYAQFTERWWQPADLSG
SDDSIWWLGHASMLLRLGGRYILIDPVLSERASPLSFYGPKRRTPAPLTVEQLPAVDAVLISHNHYDH
LDRRTVRQLARRFPQAEFIVPLGLKRWFRRYRLKVHELDWWQSLSLGELTVYATPARHWSMRTLWDRN
RSLWCGWVIHHPALRFYFSGDSGYSARLAEIGQRLGPFDVAALPIGAYAPRWFMQEQHMDPQQSVALY
RELNQPRAIPIHWGVFELADESLDEPPQQLNLALSEAGLEQHQFHPLKIGERIALQDSQQALSIRPVV
ERKE
>CORE_REP|Org24_Gene2291#
MSPSSQQNRRFLLASRPHGEPTAANFRLDTVPAPQPGAGQLVLRTVYLSLDPYMRGRMSDAPSYAPPV
EIGQVMVGGTVSRVAASQHPDFNVGDWVLGYDGWQDYALSDGSGLRNLGPHLPQPSRLLGVLGMPGFT
AYMGLLDIGQPQAGETLVVAAASGAVGSVVGQIGKLKGCRVVGVAGGAEKCRYVVEELGFDACIDHRA
PDFAEQLAAACPKGIDIYYENVGGAVFDAVLPLLNTKARIPVCGIIAHYNATGLPAGPDRLPLLEGLI
LRKRIRMQGFIIFDDYGSRFDEFLQQMSSWVEEGKIKFREDIVDGLEQAPQAFIGLLQGKNFGKLVIR
VADE
>CORE_REP|Org12_Gene1068#
MKKTWVTTLIASGIALATLSGAAHAKGRLVVYCSATNEMCEAETKAFGEKYDVKTAFIRNGSGSTLAK
VDAEKKNPQADVWYGGTLDPQSQAGEMGLLQPYKSPNLEQVMTQFRDPAKLKGNYSSAVYVGILGFGV
NTQRLKEKNLPVPKCWKDLTKPEYKGEIQIADPQSSGTAYTALATFAQLWGDDQAFAYLKQLNANVSQ
YTKSGIAPARNAARGETAIGIGFLHDYSLEKEQGAPLELISPCEGTGYEIGGVSILKGARNLDNAKLF
VDWVLSKEAQELAWKKGKSYQILTNTTADTSPNSLKLDDLKLINYDMDKYGSTEVRKALINKWVSEVK
MGK
>CORE_REP|Org42_Gene845#
MKALSKLKAEEGIWMTDVPQPELGHNDIMIKIRKTAICGTDVHIYNWDEWSQKTIPVPMVVGHEYVGE
VVAIGQEVKGFSIGDRVSGEGHITCGHCRNCRGGRTHLCRNTVGVGVNRPGSFAEYLVIPAFNAFKIP
DNISDELASIFDPFGNAVHTALSFDLVGEDVLVSGAGPIGIMAAAVCKHVGARHVVITDVNEYRLELA
RKMGVTRAVNVSKENLNDVMAELGMTEGFDVGLEMSGAPPAFRTLLNAMNHGGRIAMLGIPPSDMSID
WNQVIFKGLFIKGIYGREMFETWYKMAALIQSGLDLTPIITHRFSIDEFQQGFDAMRSGKSGKVVLSW
D
>CORE_REP|Org33_Gene2736#
MLSIRLADLAQQLDAQLHGDGDLVITGIASMHSAQPGQITFLSNSRYQEQLSSCQASAVVLTEADLPH
CRTAALVVKNPYLTYARMAQLMDTTPAPAQDIAPSAVISPEAQLGHNVAIGANAVIESGAVLGDNVVI
GPGCFIGKHARIGAGTRLWANVTIYHAVEIGQRCLIQSGTVIGADGFGYANERGEWIKIPQLGTVIIG
DRVEIGACTTIDRGALDNTQIGNGVIIDNQCQIAHNVVIGDNTAVAGGVIMAGSLKIGRYCQIGGASV
INGHMEIADKVVVTGMGMVMRPITEPGVYSSGIPLQPNKVWRKTAALVMNIDEISKRLKAVERKVGKD
>CORE_REP|Org31_Gene3765#
MSTGNAFYQRHFLRLMDFTPAELQALLRLSADLKQAKKQGQEQRRLQGKNIALIFEKDSTRTRCSFEV
AAFDQGAQVTYLGPSGSQIGHKESMKDTARVLGRLYDGIQYRGYGQALVETLAEYAGVPVWNGLTDEF
HPTQLLADLLTVQEHLPGKALSEVKLAYIGDARNNMGNTLLEAAALAGMDLRLVAPKACWPQPELVAE
CQALAQQTGAKLTLTEDIAEGVQDADFLYTDVWVSMGEPKETWQERIALLRPYQVNMAMLKLTGNPNV
KFLHCLPAFHDDQTTLGKQMAQQYDLHGGMEVTDEVFESAHSVVFDQAENRLHTIKAVLVATLSETL
>CORE_REP|Org10_Gene2404#
MLQFILRRLGLVIPTFIGITLLTFAFVHMIPGDPVTIMAGERGISAERHAQLMAEMGLDKPLYQQYFS
YVSNVLHGDLGTSLKSRISVWSEFVPRFQATLELGFCAMLFAVLVGIPVGVLAAVKRGSVFDHTAVGI
SLTGYSMPIFWWGMMLIMLVSVQLNLTPVSGRISDTVFLDDSQPLTGFMLIDTLIWGEPGDFIDAVMH
MILPAIVLGTIPLAVIVRMTRSSMLEVLGEDYIRTARAKGVSRMRVIVVHALRNALLPVVTVIGLQVG
TMLAGAILTETIFSWPGLGRWLIDALQRRDYPVVQGGVLLVACMIILVNLLVDVLYGVVNPRIRHKK
>CORE_REP|Org37_Gene944#
MSTIEHPQLQTGAGVKTSPLLDVKDLRVTFSTPDGDVTAVNDLNFDLRAGETLGIVGESGSGKSQTAF
ALMGLLASNGRIGGSAKFNGREILNLPENQLNKLRAEEISMIFQDPMTSLNPYMRVGEQLMEVLMLHK
KMSKSEAFEESVRMLDAVKMPEARKRMRMYPHEFSGGMRQRVMIAMALLCRPKLLIADEPTTALDVTV
QAQIMTLLNELKREFNTAIIMITHDLGVVAGICNKVLVMYAGRTMEYGSAREVFYQPSHPYSIGLLNA
VPRLDAEGEALLTIPGNPPNLLRLPKGCPFQPRCPYAMEQCASAPPLEQFGEGRLRACFKPVEALV

>CORE_REP|Org11_Gene2791#
MQKKSIYVAYTGGTIGMQRSDHGYIPVSGHLQRQLALMPEFHRPEMPDFTIHEYAPLIDSSDMTPEDW
QHIADDIKQNYDRYDGFVILHGTDTMAFTASALSFMLENLAKPVIVTGSQIPLAELRSDGQTNLLNAL
YLAANHPVNEVSLFFNNKLFRGNRTTKAHADGFDAFASPNLPPLLEAGIHIRRQHGIDSPACNGALRV
HDITPQPIGVVTIYPGISGAVVRNFLLQPVKALILRSYGVGNAPQKAELIDELRAASERGIVVVNLTQ
CISGRVNMEGYATGNALAHAGVISGFDMTVEAALTKLHYLLSQPLTPEQIRALMQQDLRGELSING
>CORE_REP|Org45_Gene984#
MKAAVVTKNHTVDIQDKVLRPLKHGEAALKMECCGVCHTDLHVKNGDFGEVPGITLGHEGIGVVSAVG
EGVTSLKVGDRASVAWFYQGCGHCEYCVSGNETLCRSVKNAGYSVDGGMAEECIVVADYAVKVPDGLD
SFAASSITCAGVTTYKAVKISDIKPGQWIAIYGLGGLGNLALQYAKNVFNAKVIAIDVNDGQLEFAKQ
IGADLAINSKTQNAEEIIQQQTGGAHAAVVTAVAKAAFNSAVNAVRAGGKVVAVGLPPESMDLSIPRL
VLDGIQVVGSLVGTREDLKEAFQFAAEGKVTPKVTKRPLGDINAIFDEMKAGTIRGRMVIDLGMAK
>CORE_REP|Org5_Gene1792#
MYYPLIRKALFQLDPERAHEVTFRQLSRITGTPLAFLVRQSVPTKPVSCMGLSFKNPLGLAAGLDKNG
ECIDAFGAMGFGHVEVGTVTPRPQPGNDKPRLFRVIEAEGLINRMGFNNHGVDNLVENVKKSHFGGIL
GINIGKNKDTPVEQGKDDYLICMDKVYPYAGYIAINISSPNTPGLRSLQYGEALDDLLAAIKNKQQEL
HARHHKYVPVAVKIAPDLSEDELIQIADSLVRHNIDGVIATNTTLDRKLIQGLNYCEQAGGLSGRPLQ
SRSTEVIRRLSTELQGRLPIIGVGGIDSLTAAREKMEAGASLLQIYSGFIYHGPRLIKDIVTHI
>CORE_REP|Org36_Gene1608#
MSKANPNATIVDIARRARVTNITVSRAFNKPELVKPETRERIHAIAKELNYVPNAFAQGLKSSSSQII
GIVTSSMYNPFYSGLIKTVSRIARQQGYQIMLFDTDGSEEAEMRAIQALFGYKARGILLSAVRDDKRY
RPAYLELAEVYGVPLILIDRDLYDQQLSGVFLDNREIGVLAGRYLAEQPEQKLLIIGGPADSEITLTR
TAGIVAALQGSGREIHIINGDYDFTSQESEVRAYLAQPENRPDYIIGLNGIITLGAIAICHEMGLYEQ
VKFFSIDEPPRAGAYGLHIPGVYHDTQKLGEIAAELLFSAINSPRGELPVRREFFTGSLLNR
>CORE_REP|Org45_Gene2110#
MDNHSARRVTRADVARVAGTSVAVVSYVINNGPRPVAEATRLRVLAAIEQTGYRPNDIARALASGSTQ
TYGLVVPDISNPFFATLARALQQEAFSRGRVLLLGDAGDDRQREYELINNLLRRQVDGLLYTSVDRHP
WFDLIRASGTPCVMIDTIDSQAGVCAIRVDERDAACQATRHLLQHGYRDIGIFIGPLTMLNAQDRLNG
WRDALLEAGIAPRDAWIFEAPYTRQGGYQATQRLVQGPRPRAVFTSNEQQALGCLSALAEHGLRAPDD
LALICFNGTQQSEFSVPPLSAVEQPIDAMAKRAIAMLAAGAAPAELHEFAFQLRIRRSCGC
>CORE_REP|Org17_Gene4548#
MKKKRPVLQDVADKVGVTKMTVSRYLRNPDQVSAALQQKIAVALDELGYIPNRAPDILSNATSRAIGV
LLPSLTNQVFAEVLRGIESVTDAHNYQTMLAHYGYLPEREEERLTSLLSYNIDGLILSERHHTPRTLK
MIEVAGIPVVELMDCVSPCIDLAVGFNNFEAARQMTQQIIAHGHRHVVYFGARQDERTLIKQQGYEQA
MRESGLEPHSIMTARSSSYSAGGELLRVAQRDYPQIDSIFCTNDDLAIGAAFECQRQGLSIPQDMAIA
GFHGHDIGQVMVPKLASVLTPRERMGQIGAERLLARLRGETVCPRMVDVGFTVIPGGSI
>CORE_REP|Org2_Gene127#
MPLLDIRNLTIEFMTAEGPVKAVDRVSMTLTEGEVRGLVGESGSGKSLIAKAICGVTKDNWRVTADRF
RFDDIDLLQLSPRERRRLVGHNVSMIFQEPQSCLDPSESIGRQLAQAIPGWTYKGHWWQRFNWRKRRA
IELLHRVGIKDHDDIMGSFPYELTEGECQKVMIAIALANQPRLLIADEPTNAMEPTTQAQIFRLLARL
NQNNNTTILLISHDLQMMSKWADRVNVLYCGQTVESAQCEELLAAPHHPYTQALIRAMPDFGRSLPHK
SRLNTLPGAIPSLEHLPIGCRLGPRCPYAQKKCIETPRLRPVKNHFFACHFPLNMEEQ
>CORE_REP|Org30_Gene2750#
MQQRKLGSHGPLVSALGLGCMGMSDFYSTGADRQEAIATLHRALELGVTLLDTADMYGPHTNEELVGE
AIKGKRQQVFLATKFGILRDPADPSARGVSSRPEYIRRSVEGSLRRLGVEEIDLYYQHRVDPQVPIED
VVGTMADLIREGKIRHIGLSEASVATLERAHKVHPITALQTEYSLWTRDAEQGVLAACERLGIGFVPY
SPLGRGFLTGAIRRPEDLAEDDFRRGNPRFQGENFARNLALVEKVGELAAQKGVKPSQLALAWVLAQG
EHIVPIPGTKRRRYLEENVAAAEITLSAAELAAIDAVFPLSAAAGDRYGAESMTYING
>CORE_REP|Org31_Gene600#
MKLAIYSTKQYDRKYLELVNQQFGYELEFFDFLLSKKTAKTAAGCKAVCIFVNDDGSREVLEELAALG
VEILALRCAGFNNVDLDAAKELGIKVVRVPAYSPEAVAEHAVGMMMCLNRRIHRAYQRTRDANFSLEG
LIGFNMHNRTAGVIGTGKIGVATMRILKGFGMKLLAYDPFPSEQALELGAEYVDLKTLYAQSDVITLH
CPLTPENHHLLNADAFAMMKNGVMVINTSRGALIDSTAAIDALKQQKIGALGMDVYENERDLFFEDKS
NDVIQDDVFRRLSACHNVLFTGHQAFLTEEALTSISQTTLQNISQLDRGEACPNQLNA

>CORE_REP|Org20_Gene2905#
MSYPASPSRYQDMEYRRCGRSGLKLPAVSLGLWHNFGDATLYDNARGLIRCAFDRGITHFDLANNYGP
PPGAAEENFGRILNADLRAWRDELIVSSKAGYTMWPGPYGDWGSKKYLVASLDQSLRRMGLEYVDIFY
HHRPDPQTPLEETMAALDLLVRQGKALYVGLSNYPAERARQAFDILQRLGTPCVIHQPKYSMLERGPE
TALLDTLAEHGVGSIAFSPLAGGLLTDRYLHGVPQDSRAASGSRFLQPEQLTAERLDKVRRLDALARQ
RGQKLSQMALAWVLRGDRVTSVLIGASKNAQIEDAVGMLANRHFSEEELAQIEKILL
>CORE_REP|Org21_Gene2443#
MIGIEQPAVARRAAGPSLGKRWEKLLHHPAVLPFIGFAVLFVLMSLLNDSFLSVNNLTNVARQVSINA
IIAVGMTCAILTGGIDLSVGPVMALAGSVAAGLMLAAVPIPLAMVAALAVGALFGLANGACIAYLRMP
PIIVTLASMGIARGLALLYTGGYPISGLPDVFSFFGRGTVLGIQVPILIMLGVYVLAWLMLNQLPFGR
YVYAMGGNEEAARLSGIRVPRYKMLVYVISGLTAALAGLVLTSRLMSGQPNAGEGFELDAIAAVVLGG
AAISGGRGAIVGTLVGAMMLGVLNNGLNLMNVSPYIQNVVKGGIILAAIYLSSVRRK
>CORE_REP|Org26_Gene583#
MDKSLLARLAGRHEFYLGLLVLLLAIGLSAGTDEFLTLGNLTDVATSYAILGILACGLFVVLIAGGID
ISFPAVTAIAQYVMASWVIQHGGSFPLAFALAIGVGLLLGLVNGFLVYWLKVPAIIITIATLNLFYGL
LVYATNGTWLYGFPDWFMNGINWFSFQGADGYDYGLTLPLLCLLATIVVTGVLMNRTRLGRQIYAMGG
NRDAASRLGLNLLRLHFCVYGYMGILAGVAAVVQAQITQSVAPNSLLGFELTVLAAVVLGGTSMSGGR
GSLTGTLLGVVLLAFLQNGLTLLSVSAYWHQVFSGAIILISISTTAWNEKRKLLKEI
>CORE_REP|Org20_Gene1136#
MRRFSLKPRGNEGYLAWVLLLTVIVFSLLSEQFLTVQNLLDLCESYAVSGIFALGLFVVLVTGGIDIS
FAAVASVVQYLIATLATHYGLASPAGSILLALAIGAALGMVNALLIYCLRIVSIIVTISMQALLFGML
MWLTNGRSLYALPDWWTLPRSVLPFQLGEQSYQLGLPTLVMLAVALLTWLLLNKTHLGRQLFAVGGDA
ESARRIGIRVGLLHLFAYGYLGVMAAIGGLVQVYRMGEVVPNALVGGELDVLAAAVLGGASLNGGKGS
VIGTLMGVFLIGVLKNGLNLIGVSSYFMNVVIGLVIVAAITVTHYKKRKETDVGFA
>CORE_REP|Org11_Gene3964#
MAKRIQFSATGGPEVLQYVDFTPLDPAAGEVQIENKAIGINYIDTYVRSGLYAPASLPSGLGTEAAGV
VTKVGAGVSAIKPGDRVVYAQSALGAYSEIHNVSAERVALLPGNLSFEQGAASFLKGLTVYYLLRQTY
DVQPGEVFLFHAASGGVGLIACQWAKALGARLIGSVGSDEKAALAKQAGAWATINYHKEDIAQRVAEL
TQGEKVGVVYDSVGKSTWLASLDSLKRRGLMVSFGNASGPVTGVDLALLNQKGSLYVTRPSLNGYITN
RAELQYASNELFSLIGSGAIRVEVKDEQKFALADAQRAHQVLESRSTSGSSLLIP
>CORE_REP|Org24_Gene288#
MALLNVDKLSVHFGDEGTPFRAVDRISYSVEQGQVVGIVGESGSGKSVSSLAIMGLIDFPGKVMADKL
EFNGQDLRKISEKERRQLVGSEVAMIFQDPMTSLNPCYTVGYQIMEALKVHQGGNRRTRRQRAIDLLT
QVGIPDPASRLDVYPHQLSGGMSQRVMIAMAIACRPKLLIADEPTTALDVTIQAQIIELLLDLQQREN
MALLLITHDLALVAEAAHHIIVMYAGQVVESGKAAEIFRAPRHPYTQALLRALPEFAADKARLASLPG
VVPGKYDRPTGCLLNPRCPYANERCRNEEPELRSIPGRQVKCHTPLDDAGRPTV
>CORE_REP|Org22_Gene2986#
MIERIWSGGSLLYLALLPFSWLYGLLSWLIRLSYRCGLRKSWRAPVPVVVVGNLTAGGNGKTPMVIWL
VEHLQQRGYRVGVVSRGYGGKSAVYPLVLNQNTSTREAGDEPVLIYQRTGAPVAIAPKRAEAVQALLQ
QQPLDAIITDDGLQHYALQRDFELVVIDGVRRFGNGWWLPAGPMRERAARLGSVDACVANGGVAQAGE
IAMRLQARDAVNLLSGERRPAAELPRVVAMAGIGHPPRFFATLEKLNVEVVQEVAFADHQEYQQPQLT
GLVTAEQTLLMTEKDAVKCRAFAQPNWWYLPVDAVLPSAQAEQLLQDIESLLTK
>CORE_REP|Org43_Gene3526#
MKTAGKNLNQGSFGQGRAQWGKAFGRSLMASMVLVVGLAGSAQAAPASNPAVAESVAPTTAPAPAAAA
APESITPVNPAPTIQPPETRGMDLSVWGMYQHADAVVKAVMIGLVLASIVTWTILFSKGSELLRAKRR
LRREQLALAEARSLDEASELAQNFAPESVSAVLLNDAQNELELSAESNDNNGIKERTGFRLERRVAAY
SRNMGRGNGFLATIGAISPFVGLFGTVWGIMNSFIGIAHSQTTNLAVVAPGIAEALLATALGLVAAIP
AVVIYNIFARVISGHRAQVGDVAAQVMLLQGRDLDLAATAEAKRSQHAHQLRAG
>CORE_REP|Org22_Gene2067#
MFNVVTQLENDAVEMRKVYAGAVRRQIEAGAPIIALEADLMSSMAMDGVHKDHPQHVINCGIMEANVI
GVAAGLSLTGRVPFVHTFTAFASRRCFDQLFMSLDYQRNNVKVIASDAGVSACHNGGTHMSFEDMGIV
RGLAHSVVLEVTDATMFADILRQLMDLRGFYWVRTIRKQATRIYQEGSRFTIGKGNLLRDGDDITLIA
NGIMVAEALKAAQMLAQQGVSAAVIDMFTLKPIDRELIKTYAAKTGRIVTCENHSIHNGLGSAVAEVL
AEECPAPMRRVGVKERYGQVGTQAFLQQEYGLTAEHILEAAGQLLQKQFSSQ

>CORE_REP|Org3_Gene1465#
MKYLKLGNTDLNVSRICLGCMTYGEPNRGNHAWTLPEESSRPLLKQALEAGINFFDTANSYSDGSSEE
ILGRALRDYARREDVVVATKVYFPLSNLERGLSRANIMQSIDDSLRRLGTDYVDLLQIHRWDYETPLE
ETLEALHDVVKAGKARYIGASSMYAWQFAKALYTADLHGWTRFVSMQDQYNLIQREEEREMHPLCTAE
GIAVLPWSPLARGRLTRPWGETTARLVSDQFGKSLYEETEGIDAIIAERVASLADERGVSRAQIALAW
LLNKPAVSAPIVGASRSEQLDDAIAAVDLSLSPQEVAELETAYVPHRVTGFE
>CORE_REP|Org27_Gene2677#
MMNSVGTPWLWGSFAAVIVVMLAIDLLLQGRKGAHTMSMKQAASWSLVWVSLSLLFNFGFWYYLNETA
GRAVADTQALAFLTGYLIEKALAVDNVFVWLMLFSYFAVPANLQRRVLIYGVLGAIVLRTIMIFAGSW
LVSQFQWLLYLFGAFLLFTGIKMALAKEDDSAIGDKPLVKWLRSHLRMTDNLEGERFFVRRNGILFAT
PLVLVLILVELSDVIFAVDSIPAIFAVTTDPFIVLTSNLFAIMGLRAMYFLLANVAERFSMLKYGLSV
ILVFIGIKMLIIDFFHIPIGVSLGVVAGILTLTLLINAWVNRRNDRLANKQP
>CORE_REP|Org1_Gene4215#
MNLEQITELTAQDMAAVNATILEQLNSDVTLINQLGYYIISGGGKRIRPMIAVLAARALGYEGNKHVT
VAALIEFIHTATLLHDDVVDESDMRRGKATANAAFGNAASVLVGDFIYTRAFQMMTSLESLRVLALMS
EAVNVIAEGEVLQLMNVHDPDISEESYMRVIYSKTARLFEAAAQSSAILSGASAEQEKALQDYGRYLG
TAFQLIDDLLDYSADGSTLGKNTGDDLNEGKPTLPLLHAMHNGDDAQRDMIRGAIEQGNGRHLLEPVL
QAMQQCGSLEYTRQRAEEEADKAIAALQVLPASEHRTALEGLAHLAVQRDF
>CORE_REP|Org47_Gene1607#
MGTAKHSKLLILGSGPAGYTAAVYAARANLSPVLITGMEQGGQLTTTTEVENWPGDAEGLTGPALMER
MREHAEKFQTEIVFDHINSVDLQQRPFRLFGDSGEYSCDALIIATGASARYLGLPSEEAFKGKGVSAC
ATCDGFFYRNQKVAVVGGGNTAVEEALYLSNIAAEVHLIHRRDSFRSEKILIDRLMEKVKSGNIVLHT
DHTLDEVLGDEMGVTGVRIRSTKAENETRELELAGVFIAIGHSPNTGIFGGQLELENGYIKVQSGIHG
NATQTTIPGVFAAGDVMDHIYRQAITSAGTGCMAALDAERYLDGIAGAEVC
>CORE_REP|Org12_Gene517#
MAQDYTVEQLNYGRKVYDFMRWDYLAFGISLLLLVASIVTMSVRGFNWGLDFTGGTVIEINLEKPANL
DLMRDTLEKAGFQDPIIQNFGSSRDVMVRMPPATGTAGQELGNKVIGVINDSVDKNATVKRIEFVGPS
VGSELAQTGGMALLVALICILIYVGFRFEWRLALGAVIALAHDVIITLGVLSLFHIEIDLTIVASLMS
VIGYSLNDSIVVSDRIRENFRKIRRGTPYEIMNVSLTQTLSRTLMTSGTTLMVVLMLYIFGGAMLQGF
SLAMLIGVSIGTVSSIYVASALALKLGMKREHMLQQKVEKEGADQPSILP
>CORE_REP|Org7_Gene2835#
MQNEKKSNVEFIPQFQKAFLYPRYWGVWLGTGLMAGVSLVPARLRDPVLGAIGTLAGKLAKGARRRAR
INLLYCLPELPESEREHIIDQMFACAPQSMVLMAELACTKPEKVLKRVRWHGEEVLDKIRAEGRNVIF
LVPHGWAVDVPAMLMAARGQPMAAMFHNQRNQLIDYLWNAVRRKFGGRMHARNDGIKPFISSVRQGYW
GYYLPDQDHGAEHSEFVDFFATYKATLPAVGRLMKVCRAAIVPLFPVYDGKTSMLDIYIREPMDDLAE
ADDPRIARRMNEEVENLVGPNPEQYTWILKLLKTRKEGEIEPYSRDDLYR
>CORE_REP|Org28_Gene3714#
MSSQTIAAKRWFSKEWLLEQKSLIALLVLIAVVSSMSPNFFTLNNLFNILQQTSVNAIMAVGMTLVIL
TSGIDLSVGSLLALTGAVAASIVGFEVNALVAVAAALALGAAVGACTGMIVAKGKVQAFIATLVMMLL
LRGVTMVYTNGSPVNTGFTDVADTFGWFGIGRPLGVPTPIWIMAIVFIAAWYMLHHTRLGRYIYALGG
NEAATRLSGISVDKVKIIVYSLCGLLAALAGVIEVARLSSAQPTAGTGYELDAIAAVVLGGTSLAGGK
GRIVGTLIGALILGFLNNGLNLLGVSSYYQMIVKAVVILLAVLVDNKSNK
>CORE_REP|Org1_Gene136#
MIIFTLRRILLLLITLFFLTLVSFSLSYFTPRAPLNGAALLDAYQFYFVSLLHWDFGVSSINGQAISE
QLREVFPATMELCLLAFALALFIGIPLGIIAGVLRGKWQDTAISTFALLGFSMPVFWLALLLMLFFSL
HLGWLPVSGRFDLLYQVKPITGFALIDAWLSDSPYRAEMIGSALRHMILPIAALAVAPTTEVVRLMRI
STDDVLSQNYIKAAATRGLSRFTIIRRHVLHNALPPIVPKLGLQFSTMLTLAMITEVVFSWPGLGRWL
INAIRQQDYAAISAGVMVVGTLVITINVLADILGAATNPLKHKEWYALR
>CORE_REP|Org29_Gene475#
MKQEKHGVLLVNLGTPDAPTSSAVKRYLKEFLSDDRVVDTAPLIWWPILNGAILPIRSPRVAKLYQSV
WMEEGSPLLVYSRRQQRALAARMPNTPVELGMSYGSPSLAEAIDKLLAQGVTNLVVLPLYPQYSCSTS
AAVWDGVARVLKGYRRLPSVAFIRDYAEHPAYIAALQQSVERSFAEHGQPDRLVLSFHGIPKRYARLG
DDYPQRCEDTLRALSATLPLAPERVMMTYQSRFGREPWLTPYTDETLKGLPAQGVKHIQLICPGFSAD
CLETLEEIKEQNREIFLKAGGEKFEYISALNDEPAHIDMMQQLVAQRL

>CORE_REP|Org46_Gene1306#
MEFKDYYATMGVEPNADLKTIKTAYRRLARKYHPDVSTEEDAESKFKELAEAYEVLKDEERRAEYDQI
RLHRNDPNFGRQARGDRGGYQQSASWHGGGADAQDFSDFFESMFGGRAAGGHRSASHSHGGHGFRGQD
LEMEVPLFLEETLHGQSREISYKLPVYDELGRQVSEASKTLNVKIPAGVGDGERIRLKGQGVAGVGGG
QNGDLYLVIRLAPHPLFEIDGHNLSIVAPLAPWEAALGASIEVPTLTGKIALTVPAGSQSGKRLRVKG
KGLAGKKEPGDLYVILKVVMPPKPNEKASALWRELAEQAAFNPRAEWE
>CORE_REP|Org17_Gene317#
MAEYKPTIKAPGKNGDIIFSALVRLAALITLLLLGGIIVSLIFASWPSMQKFGFAFLWTKEWDAPAEQ
FGALVPIYGTVVTSLIALIIAVPVSFGIALFLTELAPNWLKRPLGIAIELLAAIPSIVYGMWGLFVFA
PLFAEYFQTPVGEVLSGIPIVGELFSGPAFGIGILAAGVILAIMIIPYIAAVMRDVFEQTPVMMKESA
YGIGCTTWEVIWRIVLPFTKNGVIGGVMLGLGRALGETMAVTFIIGNTYQLDSASLYMPGNSITSALA
NEFAEAESGVHTAALMELGLILFVITFIVLALSKLMIMRLAKNEGR
>CORE_REP|Org14_Gene3071#
MRLRRSPLMIALLAAMALAGCHSKTATPVAATPATVNVQHLNGSTEVKKHPQRIVVLDYASLETLQLL
GVEPLALPGNRKNLPDSLKRYQDDKYLNAGTLFEPDMAVLRAAKPDLILIAGRASKAYDELNALAPTL
NMSVDPQDQLGSLKQRTLQLGELFDKQQQAQAAVDKLDAQIAAVKPQAAQAGRGLVVLFSGGKISAYA
PKSRFSFVYDALGFSSALQSDEKDVRGNKLTPEQVAKLNPDWLFVIDRDAATGRPNAVAPQKILTGTA
LKKTTAVKKGQVVYLPAAEVYLSGGIVTAQHVVERVSEALNHAAR
>CORE_REP|Org3_Gene542#
MPDMKLFAGNATPELAQRIANRLYTSLGDAAVGRFSDGEVSVQINENVRGGDIFIIQSTCAPTNDNLM
ELVVMVDALRRASAGRITAVIPYFGYARQDRRVRSARVPITAKVVADFLSSVGVDRVLTVDLHAEQIQ
GFFDVPVDNVFGSPILLEDMLQQNLENPIVVSPDIGGVVRARAIAKLLNDTDMAIIDKRRPRANVSQV
MHIIGDVAGRDCVLVDDMIDTGGTLCKAAEALKERGAKRVFAYATHPIFSGNAVDNIKNSVIDEVIVC
DTIPLSPEIKALKNVRTLTLSGMLAEAIRRISNEESISAMFEH
>CORE_REP|Org2_Gene2309#
MAKKNKWLQRTLLASALLMAGPLSSASAAVVTSIRPLGFIASAIADGVTPTEVLLPDGASPHDFALRP
SDIQRLRSADLVLWVGPDMEAFLNKALVPISATRKLAISELPAVKPLLMKGEEDDDHDHAGEAHNHAD
DDHGHHHGEYNMHVWLSPEIAKVTAIAIHDRLLELMPQNKDKLDANLRQFENLLTQTDKNVGNMLTPV
QGKGYFVFHDAYGYFEKHYGLSPLGHFTVNPEIQPGAQRLHQIRTQLVEQKAVCVFAEPQFRPAVINA
VAKGTKVRSGTLDPLGIGIALGKDSYGKFLTQLSNQYVSCLK
>CORE_REP|Org33_Gene2258#
MDQVQAMRIFTRIVELGSFSRAAERLQLPRATVSNALKRLEQRLGVRLLIRTTRQVQVTSEGSLYYQR
CVQLLGALEEADTLFSHHKLQPSGKVRIDMPHSLARQIVIPALGDFYRRYPDITLALGANDTHVDLLR
EGVDCVLRAWETEDDSLVARRIAQLPQITCASPAYLQASGTPLDIDSLAPHRAVGYFSLASNRDYPLE
FCRGGKVELRELPARLSVSGADAYIAGARAGMGLIQAARYSLAPWLERGELVEVLADTPPPPMPIYIM
YPPGRFLAPRVRVLIDWLIWLFDQQKSGDMAVFPANARKAGK
>CORE_REP|Org35_Gene2368#
MSDSLRIIFAGTPDFAARHLDALLSSEHQIVGVFTQPDRPAGRGNKLTPSPVKMLAEQHQLPVFQPKS
LRPEENQRLVADLNADVMVVVAYGLILPQAVLDMPRLGCINVHGSLLPRWRGAAPIQRSLWAGDSETG
VTIMQMDVGLDTGDMMHKIACPIESSDTSASLYDKLAQLGPQGMLTTLRQMADGSATREVQDESQVTY
AEKLSKEEARLDWTLSAAQLERCIRAFNPWPISYFTIDEQPVKVWQASVMAESANAEPGTVVHADKHG
IQVATADGILNLIQLQPAGKKPMSAQDLLNSRREWFTPGNRL
>CORE_REP|Org31_Gene2245#
MSQSRPTLSSPTRECPVVDGVRQIQRIAVRNAEVGGIPINRALPTRERRTVGAWCFLDHAGPTVFNGT
SPGMDVGPHPHIGLQTFTWMIEGEVLHRDSLGSEQVIRPGQVNLMTAGRGIAHTEQSVGEQRRLHAAQ
LWIALPAEHADMAPRFDHYPDLPQWQNNGVNHRLLVGEFGAYRSPVFTLSPLIAIDLEWQEAARIELP
LRDDDEIGFLPLIGAFELNGETFSPDEFAYLGMKNNSIGLNAQKGSRGLLIGGAPLNEEILIWWNFVG
HSKAEITRAQHDWEQGAPRFPAVSGYSGERMTAPRLPWSDV
>CORE_REP|Org40_Gene4471#
MSVNNPVARLLAEHPTLLLDGALATELEARGCDLTDPLWSAKVLIENPELIYQIHLDYFNAGAQCAIT
ASYQATPQGFLRRGLDQDQSLALIAKSVQLAQRARHDYLAAHPQAAPLLIAGSVGPYGAYLADGSEYR
GDYRLAQDDMIAFHRPRLAALAAAGVDLLACETLPSFAELQALLTLLQEFPTLGAWFAFTLRDSQHLS
DGTPLTEVLSALRGNPQVLAIGINCIALDKVAPALRQLGALADKPLLVYPNSGEHYDAVSKTWHACGG
EHGSLADQATEWSTLGAQLIGGCCRTTPQDIRAIAARCKK

>CORE_REP|Org26_Gene3903#
MFRGLKAFLLLTLSLLFCQRAFADCATTNGTVTLPGSSSFVVYNGQINAQGTAGLNCTGLGLSLLSQN
TVTVKVASTTNGMAVANTDGSGDKIAYLIYPDANYQYPYSIGQTIDYSSLNLLSLILISSNVNFPLYI
KTTAGANVRSGTYTDTINLIWNYHICGLGVLGLCIWWDGVNKVSTVSVSVAITKDCLIGTAPNVNFGS
MALVGQFNPVNQSITLTCTKTEGYNTYFTNGNNPVSGWRRMKSGTSNFMQYQIYLPNTTTVWDSTNKQ
SGAGTGLAQSIPYKAAVNAAQTEVAVGSYQDNLSFVVEY
>CORE_REP|Org28_Gene4384#
MSTNLSYALLPEMAVFVQVVESGSFSAAARKLGTSPSAVSRSVAKLEQALALQLLHRTTRKLRLSESG
EEAFAHCRTLLAAADAVMAIGGRGAVEPEGLVSVSVPKAVGRFVLHPHMPEFLRRYPKVDVRLRLEDR
YMDLIDDRVDLALRITDRPSPGLIGRQLMRIDHLLCATPHYLAQHGTPQHPHALAAHSCIYLGETPSD
AQWKFRRSGKTVTVNVRGRYAANHTGVRLDAVKQHIGIGSLPYFTARQALDDGEIVQVLPEWDFLSSY
HGGLWLLYAPNQYLPPKLRVFIDYLVACLAQEPQLKRLA
>CORE_REP|Org26_Gene1351#
MANPLYHKHIISINDLSREDLELVLRTAAGLKANPQPELLKHKVIASCFFEASTRTRLSFETSMHRLG
ASVVGFADGSNTSLGKKGETLADTISVISTYVDAIVMRHPQEGAARMAAEFSGNVPVLNAGDGANQHP
TQTLLDLFTIQETQGRLSNLSIAMVGDLKYGRTVHSLTQALAKFEGNRFYFIAPDALAMPAYILKMLE
EKGIEYSLHSSIEEVVPELDILYMTRVQKERLDPSEYANVKAQFVLRAADLAGARANLKVLHPLPRID
EIATDVDKTPHAYYFQQAGNGIFARQALLALVLNADLAL
>CORE_REP|Org15_Gene1826#
MKNFSIKITRIAITLILVLLGIAAVFKAWVFYTESPWTRDAKFTADVVAIAPDVSGLLTDVPVVDNQL
VKKGQVLFVVDRPRYEQALAEAGADVAYYQTLAAEKRREAGRRVKLGVQAMSQEEIDQSNNSLQTVQH
QLAKAIAARELAQLDLERTTVRAPADGWITNLNVHAGEYITRGSVAVALVKKDSFYILAYLEETKLNG
LNKGDRAEITPLGSNRIMHGTVDSVAAAVNNSSSTVNNKGLASIDSNLEWVRLAQRVPVKILLDAKDQ
QHPYPAGTTATVVIVGKNDRNADSGSPFVRLMHRLREFG
>CORE_REP|Org15_Gene3972#
MKSDLSALPAFVAVAEGGSFAAAAEKLHLTRSAVSKIVSRLEARLGVMLFMRTTRSLSLTDEGALYYE
HCRQALANVQAAENQLDSGKMQVSGRLRVSVPVLFGHLCIAPLLTALANEHPLLTLEISFSDRRIDLV
DEGFDLAVRIGELADSGSLVARRLGEHGMLLCASPDYVRRCGEPSTVEALSRHQAVGYLHAGAVLPWQ
LRGENGELQSFSPPAKMMMDDMQGIVDAISAGAGAGIAWLPEWLVRERLMAGTLVEIMRGESNLSFPV
NVVWPYMPYQPLKVRLAVDKLVAELPAKLALVPPPLSQR
>CORE_REP|Org5_Gene1608#
MKLKKLIAASVLMCMLPASVLAKDIKIGVSMAYFDDNFLTILRQSMQNKMKADGNVSGQFEDAKGDIA
QQIQQIENFVSQGVDAIILNPVDTQGVKPMIKLAEKAKIPLVFVNRKPEVALPAGMAYVGSDSKLAGK
LQMEELAKLMNGKGNVMILMGELSSEATRDRTRGVEEVAANYPGIKIIDKQTAKFFRKEAVDVTTDWI
LSGQQIDAIASNNDEMAIGAILALKQAKKSGVLVAGVDGTPDALEFIKKGDLALSVFQDAKGQGEGAV
QTAVQLVKGEKVESNVLIPYQLITQANYQQFADKNKK
>CORE_REP|Org13_Gene567#
MTQFAFVFPGQGSQTVGMLAELAAQFPIVEETFGEASSALGYDLWQLVQQGPAEELNKTWQTQPALLA
ASVAIFRVWQQQGGKAPALMAGHSLGEYSALVCAGVLDFKAAIRLVELRGKLMQEAVPEGTGAMYAII
GLDNDAIAKACEESAQGQVVSPVNFNSPGQVVIAGNKEAVERAGAACKAAGAKRALPLPVSVPSHCAL
MKPAADKLAVALQDITFNAPQVPVVNNVDVRTENDPEAIRSALVRQLYSPVRWTESVEFIAAQGVTSL
LEVGPGKVLTGLTKRIVDTLTAAAVNDTASLSAALEQ
>CORE_REP|Org18_Gene2648#
MMTLRQIRHFIAVAETGSISAGAQAVFVSQSSLTLAIQQLETEIGVRLFDRHAKGMTLTHQGHQFLRQ
SYLILATVDNAKRSLQIGTESLTGKLTVGVTSLVAGYFLVELLTRFKSAYPNVTVQVVEDERPYIEHL
LVSGEIDIGVLILSNIEDRDALQTEVLMHSPYRLWLPPLHPLLEHESISLADVAKQPLIQLNADEMDV
HARRIWSRAGLKPEIAMKTASTEAVRSLVAAGMGVSIQPDMAYRAWSLEGNMIEARKLDDLLEPLDIG
LAWRRGSARPELVTPFLTIARENGSKHAAGLKHSI
>CORE_REP|Org26_Gene1133#
MNYALELAQLTKTYAGGVKALRGIDLSVEAGDFYALLGPNGAGKSTTIGIISSLVNKTAGSVRVFGYD
IDKDIVNAKRQLGLVPQEFNFNPFETVLQIVVNQAGYYGVTRREAMARAEKYLNQLDLWGKRNERARM
LSGGMKRRLMIARALMHQPKLLILDEPTAGVDIELRRSMWGFLKELNAQGTTIILTTHYLEEAEMLCR
NIGIIQNGELVENTSMKGLLAKLKSETFILDLAAKSPLPKLDGYHSRLTDTSTLEVEVMREQGLNGLF
TQLSAQGVQVLSMRNKANRLEELFVTLVNGNGEKA

>CORE_REP|Org33_Gene2755#
MLATHEYANDLILFALIVDCGSFSKAAESAGITSSVVSKRIGRLEKSLGARLLYRTTRSLTLTESGQA
LYQQAKEIGAKVQEALYAVSEKSEELTGTIRMSVPTISGELLLSESVAEFCALHPSLKVEMRLENRFV
DLVEEGIDLAIRTGTMPDSSLIARPIFDSRWVIVCSPGYLESHPEPRSAEDLLGHNCLTYTYQESGTA
NWLMKRPGRNEIYELQVNGNLSANNARAIRKAVIGGHGIAMVPRCMVYEDLQDGKLTEILAGHCGKVL
GIYAVYPYTRNLPLKTRLLIEHIIGSYQNISHYF
>CORE_REP|Org40_Gene785#
MKSAKAFQLALLHPRYWLTWFGLALLFLLVQLPYPLLNRLGVWMGRTSMRFLKRRVTITRRNLELCFP
EMDEAQRERKVVGNFESLGMGLLETGMAWFWSDKRVKRWFNVSGINHLKMAQRDDRGVLVIGVHFMSL
ELGGRAMGLCQPMMAMYRPHNNKAMEWAQTKGRMRSNKAMIDRKDLRGMVHALKRGEAVWFAPDQDYG
PRGSVFAPLFAVDQAATTSGTFMLARMANPALVPVVLIRREGGRGYDLLIQPALEDYPLSDEQAAAAY
MNKVIEKEIMRAPEQYMWLHRRFKTRPAGAPSLY
>CORE_REP|Org32_Gene2582#
MNYLKGLWLAVALCASTSAWAQTIGVSMAYFDQNFLTIIRQAIDKEAKARGITVQFEDARGDVGRQTD
QVQSFISAGVDAIIVDPVNSASTPVMTKMVQAAGVPLVYVNRTPGDAKLPQGVVFVGSDERESGTLQM
EELARLANYQGNVAVMIGNLTDAGALQRTKDVEQVVAKYPKMKVVQKQSANYSRSEGMDLMMNWLTNG
EAIDIVAANNDEMAIGAIMALQQAGKADKKVLIGGIDATPDGLKALASGKMQVTVFQDAVGQGKASVD
VAQRMINGEKLEPYYWIPFELVTPANQGKYAARP
>CORE_REP|Org47_Gene1024#
MLKFILRRLLEAIPTLFILITISFFMMRLAPGSPFTGERALPPEVMANIEAKYHLNDPIWKQYGHYLA
QLAQGDFGPSFKYKDYSVNDLVAGSFPVSAKLGLAAFLLAVVLGVSAGVVAALNQNTKWDYTVMGFAM
TGVVIPSFVVAPLLVLIFAITLKWLPGGGWNGGAPKFIILPMVALSLAYIASIARITRGSMIEVLHSN
FIRTARAKGLPMRRIIFRHALKPALLPVLSYMGPAFVGIITGSMVIETIYGLPGIGQLFVNGALNRDY
SLVLSLTILVGALTILFNAIVDVLYAVIDPKIRY
>CORE_REP|Org7_Gene3795#
MSHKTLSWSGVFPAVTTQFRNDFSLDLDATHTVIKNLVRDGVSGLVVCGTVGENTSMTVQEKLAVIEV
ARDAADGQVPVIAGIAEFTTAFAQNMAREAQKAGVDGIMVMPALVYSAKPHETAAHFRSVAGATDLPI
MVYNNPPIYKNDVTPDILTSLVDCENIVCFKDSSGDTRRFIDLRNEVGDRFVLFAGLDDVVLESIAVG
AQGWISGMSNAFPREGETLFRLAKEKRYEEALALYSWFMPLLHLDARPDLVQCIKLCEQRVGRGSAVT
RPPRLALQGETLSEINAVIDKALATRPALPDVGL
>CORE_REP|Org11_Gene2832#
MDHHLLAIRVFNRVVETGGFTRAAESLGMPKATVTKLIQNLEDHLQTKLFQRTTRSVSVTREGECYYQ
NTVKWLADLEQMEGCLTESQSSPQGVLRIDTGGGTARRLLLPALPDFLARYPQIQIDLSVGDRVIDLI
SDSTDCVIRSGPLADSSLIARRLFDLDWVSCATPAYLALHGTPRHPCDLEQGFPMVHYRHPLNDRIHP
QRYAEHGKEIAIQQRSPVSINEGNALLAASLAGLGIIQIYRFMAQPHLDSGELVSLLHDWQPPPEQMY
VVYPSNRHLSGKLRAFIDWAVETFDSGKMSRTL
>CORE_REP|Org13_Gene586#
MFSLFKKTLPFIVAGGMLAASHGALAKQITIGMSFQEMNNDYFVTMKQALDQAAADIGAKVYVADARH
DVAKQIGDVEDMLQKKVDILLINPTDSVGVQSAVISAHKAGAVVVAIDAQAEGPLDSFVGSENYDAGF
QAGEYLAKALGGKGKVAILDGIPVVPILERVRGFEAAMKKYPDIKIVTKQNGKQERDTALTVTENMLQ
SAPDLAGIFSVNDVGALGALAAIESNGAKVKLVSVDGQPEAIKEILKPNSPFIATSAQFPRDQLRIAL
GIALARYWGATVPKTVPVKVKLIDRSNAAGFSW
>CORE_REP|Org8_Gene1071#
MNIRDLEYLVALAEHRHFRRAADSCHVSQPTLSGQIRKLEDELGVMLLERTSRKVLFTQAGLLLVEQA
RTVLREVKVLKEMASQQGEAMSGPLHIGLIPTVGPYLLPQIIPTLHKTFPKLEMYLHEAQTHQLLAQL
DSGKLDCAILALVKETEAFIEVPLFDEPMKLAVYSDHPWAQRERVAMPDLAGEKLLMLEDGHCLRDQA
MGFCFQAGADEDTHFRATSLETLRNMVAAGSGITLLPSLAVPPQRERDGVCYLDCYKPEPKRTIALVY
RPGSPLRSRYEQLAEAIREHMQGYIDSALKQAV
>CORE_REP|Org23_Gene3194#
MSLPFDVHRLLPAFLAAAQAQNFSAAARQLGVTPAAVSKNIRALEEKLALRLFQRNTHNVLLTDEGKA
LLAQVAPLWQALAATLESAGGERQAPAGVVRVTMIPGFGRQMLMPLIPQFLARYPQIDLDLSLDARVV
NLVGEGFDVGIGSRVDPDSRLVARPLYPMHMVLAASPDYLARRGEPQTPHDLLRHDCLLHRNPANGRH
VKWQLRHQGETLALDLNGRLVVSRPEMLLDAALAGLGIVNLAHWYVEKHFVQGTLRPVLAECWPRPVQ
LWLYYASADLPPRVRVWVDFLLEHFRDRPTGD

>CORE_REP|Org39_Gene3100#
MKITLEELLAFTAVVDSGSVTAAADRLGQTTSGVSRALSRLETKLDATLLRRTTRRLSLTEEGLSFLA
PAREILRSVDQAEELMALRRRLPAGRLRVNAAAPFMAHVLVPMVAEFRRRYPQIELELDTDDRNIDLL
EKRADIAIRIGALRDSTLHARLLGNSRLRILASPDYLQRHGEPRGVEDLHRHCLLGFTYPESLNQWPL
RHRQARHFAIEPTISASSGETLRELALRGAGIVQLADFMTRRDREAGRLVPLLVRETLDVRQPIHAVY
YHDAQLAARLTCFLDYVSARLEGEPPEAAEGL
>CORE_REP|Org19_Gene4376#
MQESHYVGRFAPSPSGDLHFGSLIAALGSYLQARAQRGQWLVRIEDIDPPREVPGAAARILSALEHYG
LHWDGQVIYQSQRHDAYRAALDLLQRQGLSYYCTCTRSRIQQIGGLYDGHCRDLQLGPQGAAIRLRQT
APVYGFHDRLQGELHADPALAGEDFIIRRRDGLFAYNLAVVIDDHFHGVTEIVRGADLIEPTVRQIAL
YRQLQAPVPAYVHLPLALGANGIKLSKQNHAPALPAGDPRPVLIAVLKFLRQPLPESWQDLDLPLLLS
WAVAHWRLENVPRQEAIPLDENTPAFSKEPW
>CORE_REP|Org6_Gene1333#
MEFKQLQDMALFALVAECGSFTAAAQRVGLPKSSVSQRISQLEQTLGLRLLNRTTRQLNLTFAGERYL
EHCQVMMSAAERADLALQRLRDNPSGRLRISTPAGLGATLVARLAADFQRQYPDVSLEVSVSDAMVDL
VQEGFDAALRTGKPQDSSLIGRRLGYAPRYLLAAPSYLAQHPPIEHPQQLQQHRCIAHRAWTAWNLRC
GDDYYRWQLPLAHTTDNLLYARECAIAGAGITLLPAFLSREVVAQKLLVEVLPAWRAEGNELYLVYPS
RKLNSAALACFIDVVLQHPAFDDYARELARE
>CORE_REP|Org6_Gene1631#
MERLKRMSVFAKVVEFGSFTAAARQLDMSVSSISQTVSKLENELQVKLLNRSTRSIGLTEAGKIYYQG
CRRMLQEVSEVHEQLYAFNNTPAGTLRIGSSSTMAQNVLANMTAEMMKEYPGLTVNLVTGIPAPDLIT
DGLDLVIRTGALQDSSLFSRRLGQMPMVVCAAKSYLIQHGTPQKPSDMVNFSWLEYSVRPDSEFELMS
PEGITTRISPQGRFVTNDSSTMIRWLKNGAGIAYAPLMWVIEEIKRGEIEILFKSYHSDPRPIYALYT
EKDKLPLKVQVCINYLTDYFERVAAVYQGYR
>CORE_REP|Org4_Gene3643#
MTPIAPSLQQPVDAFLRYLKVERRLSPLTQLSYSRQLAALMRLAQEIGVTDWTALDAARVRMLAARSK
RAGLQSASLALRLSSLRSFLDWLVSQGVLHANPAKGIRTPRSGRHLPKNIDVDEMNQLLEIDLNDPLA
VRDRAMLEVMYGAGLRLSELVGLDCRHVDMAAGEVWVMGKGSKERKLPIGRTAVTWLEHWLAMRDLFG
PEDDAMFLSNQGRRISTRNVQKRFAEWGVKQGVNSHIHPHKLRHSFATHMLESSGDLRAVQELLGHAN
LTTTQIYTHLDFQHLANVYDAAHPRAKRGKS
>CORE_REP|Org46_Gene1493#
MLTDLNDLFFFASVVDHQGFAPAGRALGIPKSKLSRRVALLEERLGVRLIQRSTRRFSVTEVGQNYYA
HCKAMLVEAEAAQQAIEQTRAEPCGTVRMSCPVAILHTRVGSMVAAFMADYPKVTVHLEATNRRVDVV
GEGLDLAIRVRPPPLEDSDLVLKILAQRTWCVAASPALVRTLGPAHAPEDLRKYPTLDLGPARAQHQW
RLTGPQGERVEWEHTPRLVTDDMLMLRTAAIAGAGIVQLPAMMMRDDMLRGELVQLLPGWQPQGGVVH
AVYPSRRGLLPAVRLLLDYLGEQFTSIEEE
>CORE_REP|Org10_Gene4419#
MDRFNQYRVFVQVAEMGSFIRAAHALEVPRASVSAAVQQLETQLGVRLLHRTTRQVRLTADGEQLLER
LRPLLAEVEDIDQSFQASQRQASGRLSVDVPSRIARRLIAPALPSLLRRHPHLQLVLGSADRAIDLVQ
EGVDCAVRVGDLHDSSLVMRPLGHIALINCASPAYLSEFGHPRQPADLAEGHWSIGYASPKTGRESPW
EYLTDDGHTQRLELPSRVVVNNAESYIACCSAGLGLMQIPRYDVQHLLDAGELVEVLPGYRAASMPIA
LIYPHRRQRSRRLAVFHEWFESLLQPHLER
>CORE_REP|Org41_Gene4645#
MSSAFTSSHDPLRRRLLMALALSPLLGSLPGRAADAPPDITRVAALEWLPIELLLALGVTPLAVADVH
NYNLWVAEPKLPATVVDVGQRTEPNLELLQQLQPSLVLLSQGYGPTPRKIQPIAPTMSFGFNDGSGKP
LTVARQSLLALGQRLGIESRAVNHLAQFDRFMQDARQRLQSYTRQPLLLFSLIDTRHALIIGQKSLFQ
EAMDQLGIRNAWQEQTDFWGTAVVGIERLATVRNARVIYLDHGNQAMMDKVSATPLWQSLPFVRQNQL
RQVPAVWFYGATLSTMRFCRLLAQAQERAA
>CORE_REP|Org18_Gene686#
MDRITAAEVFVTIVDRGSMIAAAETLEMSRAMVTRYLAQMEQWAGARLLHRTTRKLSLTDAGERTLER
CRQMLALAGEIDLVEEGQSDELRGLLRITCSQSLGQTALVGAVAQYLKRHPQVAVDLQMNNRAVNLVE
ERIDLALRITNELDPNLIARPLSTCASVVCAAPAYLAAHGTPRQPQDLALHNCLTYSYFGKSLWHFDA
QGVKSAVAVSGNLSANESVVLMAGTVQGAGISMQPYYSAAPLLASGELVELLPDYRPQSMGIYGIYTS
RRQMPATLRTMLDFLVEWFATDPQWQATLR

>CORE_REP|Org31_Gene884#
MMLTKKNSEALEHFSEKLEVEGRSLWQDARRRFMHNRAAVSSLFILVLITLFVVLAPMLSQFAYDDTD
WAMMSAAPSVESGHYFGTDSSGRDLLVRVAIGGRISLMVGVAAALVAVIVGTLYGAMSGYLGGKVDSV
MMRLLEILNSFPFMFFVILLVTFFGQNILLIFVAIGMVSWLDMARIVRGQTLGLKRKEFIEAALVCGV
STRNIVLRHIVPNVLGVVVVYASLLVPSMILFESFLSFLGLGTQEPLSSWGALLSDGANSMEVSPWLL
LFPAGFLVVTLFCFNFIGDGLRDALDPKDR
>CORE_REP|Org2_Gene2686#
MSSLLQLLPYFEAVARLGNFTRAASQLGVTPPAVSQNIQALENQLGVRLFHRTSRSVRLSDEGRLFYQ
KVSPAMSQIDVAADDVRALGAQPAGLLRITLPQLAASLLVMPHLAEFQRRYPDVQLELFTEDRFSDLV
LGSFDAGIRMHAMLQKDMIAVPIDNGQRRVLVASPDYLARCGVPATPDDLPVHHCLRYRFPGSGKLEP
WYFSLGDDERALDVSGSLIFNEDRLIKDAALAGLGIAQRFQGTVLQELAQGQLVEVLPDYASEASGFF
IYFPAGRHLPLKLRAFIDFMREQRERQHRW
>CORE_REP|Org41_Gene2769#
MKHWRRNAALKAMPLIDPNAVRTPWGEFWRRFRRQRAALVAGLFVLLLIAAALLAPYLAPFDAENYFD
YDRLNEGPSLMHWLGVDSLGRDIFSRILMGTRISLAAGVFSVLAGGAIGTLLGLLAGYYEGWWDRLTM
RVCDVLFAFPGILLAIGVVAIMGSGMANVIVAVAIFSIPAFARLVRGNTLVLKHLTYIESARSIGASD
WTIILRHILPGTLSSIVVYFTLRIGTSIITAASLSFLGLGAQPPTPEWGAMLNEARADMVIAPHVAIF
PSLAIFITVLAFNLLGDGLRDALDPKLKG
>CORE_REP|Org30_Gene1294#
MSQITESAVKGAPKPMTPFQEFWHYFKRNKGAVVGLVYIVLMLVIALGAGVLAPHAPADQFRDALLKP
PVWQEGGSWQYILGTDDVGRDVLSRLMYGARLSLLVGCLVVVLSLIMGVIFGLLAGYFGGVVDAIIMR
VVDIMLALPSLLLALVLVAVFGPSIVNASLALTFVALPHYVRLTRAAVLVEVNRDYVTASRVAGAGAL
RQMFVNILPNCLAPLIVQASLGFSNAILDMAALGFLGMGAQPPTPEWGTMLSDVLQFAQSAWWVVTFP
GLAILLTVLAFNLMGDGLRDALDPKLKQ
>CORE_REP|Org6_Gene3537#
MNDARYVEHLPIFLDVARLGSFSAAARRLGMVPSSLVRHIDALESALGATLFVRSTRGLLLTDAGELL
LTRAAALMTDITGIHAELSALNETPQGTLRISCLPTFGKTYVLPLLPTLAERYPQLSIDLDLTERQTD
PTQERLDAALRIGEQKDSALYASRIATQRWVMCASPAYVARYGLPSDLEALPQHRLIARYHKQQPACW
AQILDAALMSRCTMALRCDDFTAQRQAALLGLGIAFLPNWVVGPDVQNGQLVQMLEDPRHEQQGIYLL
RPMAKVSARLAAFTALLQQTLGQPPSWG
>CORE_REP|Org40_Gene887#
MQQQDNALIEQFLDALWLERNLAENTLASYRLDLQALGAWLGQQNTTLLQAQALDLQAFLAERVDGGY
KATSSARLLSAMRRLFQYLYREKLRADDPTAQLASPKLPQRLPKDLSEAQVDALLQAPCVDQPLELRD
KAMLEVLYATGLRVSELVGLSISDVSLRQGVVRVIGKGNKERLVPLGEEAVYWIENYLEHGRPWLVNG
QTLDVLFPSTRCQQMTRQTFWHRIKHYAILAGIDSERLSPHVLRHAFATHLLNHGADLRVVQMLLGHS
DLSTTQIYTHVATERLKQLHQQHHPRA
>CORE_REP|Org23_Gene336#
MRKSTGFIANIDICKEYDARYAADEVHYETFAGLAAFFGRDMQVHWHDCFFQVHFLETGKIELQLDDQ
HYSVQAPLFILTPPSVPHAFFTEPDSDGHVLTVRQELIWPLLERLYPGSNLALDMPGICLSLADAPQE
LTALSHYWALIRREFAQNLAGREQTLALLAQAVFTLLLRNTALEDSANSGVRGELQLFQRFNKMVDER
FREHLPVPEYAQALGVTESRLNDLCRRFANRPPKRLIFDRLLREAKRMLLFSACTVHETAYSLGFKDP
AYFARFFNRLEGCSPSTYRAAQHALS
>CORE_REP|Org9_Gene2634#
MEQLRAELSIVLGESISRLERVSEQPYAHMYSLYDRQGNAIPLMAKSFICQGIAQQEAYKLSMLARDG
DIRLPTVYGVVCTHQAPYKEILLIERLRGVSAEAPTRSPDRWNMLMEQIVDGILAWHRIDSHGSVGSV
DSTQENDWFCWYQQRVEVLWATVVNLTTPQLTMADRRLLYRTREALTHFFVGFDDPCVLVHGNLSLRS
MLKDPKSDQLLAMLNPGVVLWAPREYDLFRLCEAGMPSQLLFSYLRRAPVADAFLARRWLYVVWEAVG
RLIHTGKLERRPFDYASQQLLPWLAG
>CORE_REP|Org38_Gene139#
MPFDNVYREKKMPSPLRYTWRIFYGDALAMIGFYGVIALLLLSLFGSLLAPYALDQQFLGYQLLPPSW
SRYGNVSFFLGTDDLGRDILSRLLTGTAATFGSALAVTLAAAFCGVILGVFAGVTHGLRSAVLNHILD
TLLSIPSLLLAIVVVAFIGPKLEHAMLAVWLALLPRMVRTIYSAVHDELEKEYVVAARLDGASTLQIL
WYAVMPNIAAVLVTEFTRALSMAILDIAALGFLDLGAQLPSPEWGAMLGDSLELVYVAPWTVMLPGAA
ILVSVLLVNLLGDGMRRAINAGVE

>CORE_REP|Org17_Gene124#
MKANSDELITFVTVVESGSFSRAAERLEQANSVVSRTVKKLESKLGVTLLNRTTRQISLTQEGENYFR
QVQKVLNDMAAAENALMESRQRPQGLLRVDAATPVVLHMLTPLVAEFRERYPEMSLSLVSSENFINLI
ERKVDIAIRVGELTDSTLKARKLMTSYRHVLASPAYLAQHGTPLTVEDLAHHCCIGFNDLPSLNRWPL
ACSDGSQLEITPGLTTNSGETQRHLCLHGNGIACLSDFMSDEDIKRGDLVPILVEATLPVAMPINAVY
YSDSAVSNRLRSFIDFVSEYLKR
>CORE_REP|Org14_Gene1282#
MDAKQTRQGIFFALAAYFMWGIAPAYFKLIQQVSADEILTHRIIWSFFFMLALITLGRNWPKVRAACQ
NRKRLLLLAVTALLIGGNWLLFIWAVNNHHMLEASLGYFINPLVNVLLGMLFLGERFRRMQWVAVALA
FTGVLVQLWQFGSLPIIGLGLAFSFAFYGLLRKKIAIDAQTGMLIETLWLLPVAAAYLFLFADSPTSH
LSANPWSLNLLLVAAGIVTTVPLLCFTAAATRLRLSTLGFFQYLGPTLMFLLAITFYGETVGQDKLVT
FGFIWAALILFTLDALYTQRKLR
>CORE_REP|Org1_Gene3926#
MKMKKLATLASAIALSATLSANAMAKDTIALVVSTLNNPFFVSMKDGAQQEANKLGYNLVVLDSQNNP
AKELANVQDLMVRAPKLLLINPTDSDAVGNAIKMANQAKIPVITLDRVASKGDVVSHIASDNRVGGKM
AGDFIAKKAGADAKVIQLEGIAGTSAARERGEGFKQSLDQNKFKLLASQPADFDRTKGLNVMQNLLTA
HPDVQAVFAQNDEMALGALRALQTAGKTDVIVVGFDGTADGVKAVEGGKLAATVAQRPDQIGVIGVET
ADKVLKGEKVPATIPVDLKLVTQ
>CORE_REP|Org48_Gene3990#
MASFSGIWVAMVTPFNQDAVDLPAVKRLARHLLDAGIDGLVVCGSTGEAAALSKEEQLAVLDAVLEVA
PAHQVVMGLSGNNMAATLQMQQAIQLRDIAGVLIPAPYYIRPSQCGLIDYFTQLADASTVPVILYNIP
QRTGIAMELATLRRLARHPRITAIKDCGGNPDATMALIADGEIDVMTGEDNLILTTLCLGGTGAISAA
AHVHPERFVQLVQQVATGDLAAARSNFYELLPMIHQMFSFPNPAPVKTVLAQQGLIANELRSPMQVAP
QALQQQIAATQAQLQTAEALIG
>CORE_REP|Org49_Gene2811#
MFKKSLLTLAFTGVATLSTYATAADTLTMEVYNPGEKSVFPVSSEIISGKHEVALIDAQFQRNDAEEL
VKKIKATGKKLTTVYISHSDPDFYFGLDVIKAAFPEAKIIASPGTIKDINATKDGKVAYWGPILKDNA
PKTVIVPQPLQGDSFTIDGQKVEVKGLNGPTPDRTFVWIPALKAVVGGVAVAGDNIHPWIADNQSVES
RQHWQQTLKNIEALKPQVVVPGHFLPGAAQTLASVHFTQKYLTTLEAELPKAKDSAALIEAMKKHYPT
LKDESSLELSAKVLKGEMKWPQ
>CORE_REP|Org24_Gene1069#
MFTGSIVALVTPMDDKGAVDRASLKKLIDYHVASGTAAIVSVGTTGESATLAHDEHVDVVLQTLELAD
GRIPVIAGTGANATAEAIALTTRFANTGVVGCLTVTPYYNKPTQEGLYQHFKAIAESTELPQILYNVP
SRTGCDMLPPTIARLAKIKNIVAVKEATGNLSRVSQIQVLVDDEDFILLSGDDASGLDFMQLGGKGVI
SVTANVAAREMAELCALAAQGKFAQARRLNQRLMPLHQDLFVEANPIPVKWACKALGLMATDTLRLPM
TPLSEAARPVVERALKSVGLL
>CORE_REP|Org48_Gene4426#
MKGIILAGGSGTRLHPITRGVSKQLLPIYDKPMIYYPMSVLMLAGIRDILIISTPEDLPSFERLLGNG
EQFGVNLSYAAQPKPEGLAQAFLIGEEFINGDSCCLVLGDNIFFGQSFSPKLKTVAARTEGATVFGYQ
VMDPERFGVVEFDGNFRALSIEEKPKKPKSDWAVTGLYFYDSQVVEFAKQVKPSERGELEITSINQMY
LERRELTVELLGRGFAWLDTGTHDSLLEASSFVQTVEKRQGFKIACLEEIAWRNGWLDDEGVKRAAQT
LAKTGYGKYLLDLLHARPRQY
>CORE_REP|Org19_Gene503#
MKKIALAAGVLLAASYSASSMADSKDSQYVSDWWHQSVNVVGSYHTRFGPQLNNDVYLEYEAFAKKDW
FDFYGYVDVPKFFGVGNTPDRGIWDKGSPLFMEIEPRFSIDKLTGTDLSFGPFKEWYFANNYIYDLGH
NADGRQNTWYMGLGTDIDTGLPMSLSMNIYAKYQWENYQAANENSWDGYRFKVKYFVPLTQVWGGNLS
YIGFTNFDFGSDLGKDSHWVDGTGKQVRTSNSIASSHILALNYDHWHYSFVARYFHNGGQWQDGADIG
TPQGPIKSTGWGYYLVVGYNF
>CORE_REP|Org38_Gene534#
MSDDHSQSNDSPSPKKGFFTLILNQLFHGEPKNRGDLVELIRDSEQNDLIDPDTRDMLEGVMDIAEQR
VRDIMIPRSQMVTLKRNQTLEECLDVIIDSAHSRFPVISEDKDHIEGILMAKDLLPFMRADSEPFSID
KVLRTAVVVPESKRVDRMLKEFRSQRYHMAIVIDEFGGVSGLVTIEDILELIVGEIEDEYDDEDDLDI
RQLSRHMYTVRALAPIEDFNEAFGTHFSDDEVDTIGGLVMQAFGHLPARGETIEIEGYLFKVAMADSR
RIIQVHVKIPDDSPPPKLED

>CORE_REP|Org18_Gene1164#
MSDAILRVEHLMMRFGGIKALNDVNLEVERGSITALIGPNGAGKTTVFNCLTGFYRASGGAILLNTHK
RPTDVIQVLGQKFRAGDWIRPKRLGSRLYYKMFGGTHLVNRAGLARTFQNIRLFREMSVVENLLVAQH
MQSNRNLIAGVLNTPGYRRAESAALDHAFYWLEVVDLVDCANRLAGEMSYGQQRRLEIARAMCTAPEM
ICLDEPAAGLNPVETATLSRIIRFLRQHHGITVLLIEHDMGMVMEISDRVIVLDHGDVIARGTPQEIQ
HNEAVIAAYLGADEEELAG
>CORE_REP|Org32_Gene4334#
MMNERIPLHVLPTFAIAARLENLRAAAQQVHLTHGAVSQQIQLLEQAVGYPLFERRGRGVRLNAAGRE
LLAAVEPALQALLQGVARARRAATSQTLRISVLPSFAHYWLLPRLPAFHEACADIALDIDASLALQDL
SQRGFDAAIRIGSGQWTGLQAQRIATGDVLPVASPDMAREWRAAFESGGDIPLLEHDVSPWRDWFNAQ
GRPLCGRQQALFNDAGLLIRAAEQGFGIALAKKLLVQDALDAGRLVALAAPRRLSDDDVYLVWPQTAG
LTPAVTRLLQWLQRQLAAI
>CORE_REP|Org30_Gene906#
MNTLDKIQSHLELLSKSERKVAEVILASPQTAIHSSIATLARMADVSEPTVNRFCRRLDTKGFPDFKL
HLAQSLANGTPYVNRNVEEDDSVDAYTSKIFESVMASLDTVKANLDIAAINRAVDLLTQAKKISFFGL
GASAAVAHDAMNKFFRFNIPVVYFDDIVMQRMSCMNSGEGDVVVLISHTGRTKNLVEMAHLARENDAT
VLAITSRDTPLAQAATLALLLDVPEDTDVYMPMVSRIAQLTLIDVLATGFTLRRGAKFRDNLKRVKEA
LKESRFDKGVVIPNSFDS
>CORE_REP|Org17_Gene530#
MSILIDKNTKVICQGFTGSQGTFHSEQAIAYGTKMVGGVTPGKGGTQHLGLPVFNTVREAVEATGATA
SVIYVPAPFCKDSILEAIDAGIKLIITITEGIPTLDMLTVKVKLDEAGVRMIGPNCPGVITPGECKIG
IMPGHIHLPGKVGIVSRSGTLTYEAVKQTTDAGLGQSTCVGIGGDPIPGSNFIDILKMFQQDPQTEAI
VMIGEIGGSAEEEAAAYIKEHVTKPVVGYIAGVTAPKGKRMGHAGAIIAGGKGTADEKFAALEAAGVK
TVRSLADIGDAVKAVLKR
>CORE_REP|Org23_Gene2399#
MWQAVSRLLSEHLGSAEIRERIELPGGDIHPAWRVSYGDNEVFVKCDAREQLPIFTAEADQLALLARS
KSVRVPEVYGVGSDRDYSFLLLEYQQLKPLDAHGAYCLGQQLAHLHQWSEQPQFGLDFDSDLTTTPQP
NAWQRRWSEFFAEQRIGWQLQLAAEKGMTFGDIDDIVDRVYLRLQHHQPQPSLLHGNLWPGNCAMTAN
GPILFDPASYWGDRECDLAMLPLYPELPPQIYDGYQSVWPLGAGFIERQPLYQLYYLLNRSNLFGGQH
LVAAQRAVEALLQPEAS
>CORE_REP|Org23_Gene3070#
MIRQWPSPAKLNLFLYITGRREDGYHLLQTLFQFLDYGDTLTIDPRQDDRIHLLTPVDGVPDEQNLIV
RAARLLQRYCDERGLQTAPRGADISIDKRLPMGGGLGGGSSNAATVLVALNELWRCGLGDDQLAALGL
SLGADVPVFVRGHAAFAEGIGERLQPAEPQEKWYLVAHPGVGIPTPVIFGDPELKRNTPVRSLSELLQ
APYANDCEPIARKRFREVEQLLSWLLEYAPSRLTGTGACVFAEFDTEIAARQVLNQAPEWLCGFVARG
VNVSPLHRIRSGRFES
>CORE_REP|Org44_Gene232#
MQHKTLKYFSTVIVCAIAVCAGWWLWNYYMQSPWTRDGKVRAELVNITPEVSGRLEKISANDNQFVPA
GSLIFTLDPVPYQIALDNAEAAVAKAQSDLAKADHEAARRRGLPRNVISAEDLDESNLAAQAMKAAYK
AALANLEQAKWNLSKTKIYAPTDGYITNLQARVGNYANAGTPLVALVDVHSFYVLGYFEETKLKHIKE
GNKADIVLYNGNTPLQGEVESIGRAIYDQSVDSSNDLLMDVKPNVPWVRLAQRVPVRIKLLNVPADLT
LVAGTTCTISIHQRN
>CORE_REP|Org21_Gene1636#
MEQQLLCYKTLPEWNSDTLPEAFRQRHNTQSGTWAKLTVLSGSLTFAMMTEDGATTETWQFSPESQPP
FIAPQQWHRIVSFSDDMICRLAFYCTPEDYYHKKYELTRTHSEVIEAAARIAPGKALDLGCGGGRNSL
YLNLKGFDVTAWDKHAPSIDRLNQIIDAEQLTRLSARVQDLNTHRFSGEYDFILSTVVMMFLERQQIP
PIVQNMQDSTVRGGHNLIVAAMDTEDYPCPLPFPFTFSPGELKHYYRDWGILKYNEDVGQLHKTDAAG
NRISLRFATLLARKL
>CORE_REP|Org45_Gene805#
MKTDSPFDLILPAATAKIAEDAGVYKATKHPLKTFYLAITAGVFISIAFVFYITATTGTAGVPFGLAK
LVGGICFSLGLMLVVVSGADLFTSTVLIVIAKASGRISWGQLGANWLNVYLGNLVGALFFVALIWFSG
EYMVANGQWGLNVLQTADHKLHHTFIEAVCLGILANLMVCLAVWMSYSGRTLTDKMLAMVLPVGMFVA
SGFEHSIANMFMIPMGIVVKHFATPEFWQAVGAVPEQFAHLTVSNFIIDNLIPVTIGNIIGGGLLVGL
TYWVIYLRGGREQH

>CORE_REP|Org3_Gene3521#
MERTINLCPGIGASAHIIQHTELLFPSVYFEQPHLYLIQQGHKRVRWQQREVVAHAGELLIIDGGQTV
DIINGPSEEGVFSCQLLTCDPLLLTVQPPAEDSPAPMPFDAVLALRSLPCALKHSFETTSLALALRQR
FPTIIVRHKMLEILLWLAQFGIRFIHNEAKDLTQRVRRCLATDPHSIWTAAKVAESLSMSEVMLRRKL
SMENTALRNLMIDVRMSSALALLQSTDWPISAIAQHVGYESASRFAERFRKRFGFAPTAIRGHQRIME
PTSQGVETMATGET
>CORE_REP|Org34_Gene1366#
MNPLFTPYLQRWQLEQDGKAFETHSSLLMPVRYRGEAAMLKIAREQEERFGGQLMCWWRGEGAAQVLA
WHEDGILLERAQGESSLAQLVRDGDDEQATAILCRAIAALHAPRAAPLPELIPLQEWFSSLWPAAQAH
GGMLRLSATTAAELLSSPRDESVLHGDIHHDNVLDFGERGWLAIDPKRLYGERGFDYANIFCNPNYGI
ATDPAIFQRRVEQVCRLAGLERRRLLQWILAWAGLSAAWFMEDGQAADIDFRVAELAARALDLPLPAG
DSGFILPVIERG
>CORE_REP|Org34_Gene1435#
MYLIANREMLLKAQRQGYAVPAFNVHNLETVQVVAETAAELRSPVIMAGTPGTFSYAGTDYLIGICQS
AAHRYDLPLALHLDHHEELDDIEHKVKSGIRSVMIDGSHLPFEQNIAKVAAAVALCHRYGASVEAELG
RLGGQEDDLIVDTADSFYTDPMAAREFVAATGIDSLAVAIGSAHGLYHGEPKLDFERLALIREQVDVP
LVLHGASGIPEAMVKRAISLGVCKVNVATELKIAFADAVKSYFSQHPDANDPRKYIVPGKLAMKEVVA
EKIRICGSSGML
>CORE_REP|Org40_Gene2379#
MSTYLIGDVHGCFDELKSLLAQAAFDPERDQLWLTGDLVARGPASLDVLRYVRSLGPAVRMVLGNHDL
HLLAVYAGISRNKPKDRITPLLEAPDADELINWLRRQPVLQVDDEQKLVMAHAGITPQWDIDTAKMCA
REVEAVLSSDSYPLFLDAMYGDMPNNWAPELSGLARLRFSTNALTRMRYCFPNGQLDMICKDAPGSAP
APLKPWFELPRLVDPEYTIIFGHWASLEGKGTPEGVIGLDTGCCWGGDLTMLRWEDRRYFTQPANRGE
APDHAGRLAAS
>CORE_REP|Org47_Gene2007#
MSTLLRIRQMYPTLAQNDRKLADFLLNNAEQARHLSSQKLAELAGISQSSVVKFAQKLGYKGFPALKL
ALSETLAQPQAEPVVTVHNHILSSDTLKIVGEKLLAEKQAALRATLDINSEERLHQALDMLRQARRVM
LIGIGASGLVAKDFSFKLLKIGVMAVAEPDMHVQLAAVQALDKRDLLLAISFSGERREINLAAEEARQ
AGARVLALTSFSPNGLQQRADHCLYTIAEEPHTRSAAISSSTAQYALTDLLFMALIQHDLDHARDRIK
HSEQLMKKLV
>CORE_REP|Org40_Gene2389#
MKLLRYGEPGQERPGMLDEQGRLRDLSQHIADVGGAALSPASLAKLRTLDSAALPLVEGQPRLGACVG
GIGKFICIGLNYADHAAETGAAIPEEPVVFNKWTSAVVGPYDRVEIPRGSQKTDWEVELGVVIGLGGR
YISEADAMRHVAGYCVINDVSEREYQIERGGTWDKGKGCDTFGPIGPWLVTADEIADPHSLNLWLEVD
GKRYQDGNTSTMIFRIPQIVSYLSRFMSLQPGDVISTGTPPGVGMGQKPQPIYLRAGQTMRLGIEGLG
EQRQQTVQA
>CORE_REP|Org34_Gene1189#
MNNLPVVRSPWRIAILTVGFTFLYAPMLMLVIYSFNSSKLVTVWAGWSTRWYTELFHDSAMISAVGLS
LTIAAASATAAVVLGAIAAVVMVRFGRFRGSTGFAFMLTAPLVMPDVITGLSLLLLFVAMGHAFGWPS
ERGMFTIWLAHVTFCTAYVAVVISSRLREVDRSIEEAAMDLGAPPLKVFFVITLPMIAPALISGWMLA
FTLSLDDLVIASFVSGPGATTLPMLVFSSVRMGVNPEINALASLILLVVGILGLIAWWFMARSEKQRS
RELQRAARS
>CORE_REP|Org21_Gene3288#
MGTDNTLLTVERLAIGVPEPQPVALVKNISFSMGRERLALVGESGSGKSLTARALMGLLPPPLQLQAH
RLTLGDEDLTRLSERQWSRLRGDRVAMVMQDPKHALNPNQPIGRQVEEPLVLHTKLSRAERREKVLEM
LAAVGLPDPAALCRRYPHQLSGGMGQRVMLAIALINDPQLLIADEPTSALDHQMRDQVLQLIDNLVAQ
RNMGLILISHDLQQVAHHCERVLVMYKGELLDQLPAAELAQATHPYTRTLWACRPSRETRGKPLPVLD
RALLETLK
>CORE_REP|Org24_Gene4551#
MQLTVRDMTLNLSHPQVMGILNVTPDSFSDGGRHNTLNQALLHAHALILAGATMIDIGGESTRPGAAE
VSEEEELERVVPVVEALAQRFEVFISVDTSKAGVIRESAHAGAHLINDIRSLQEPGALAAAAESGLPV
CLMHMQGQPRTMQQAPHYDDLIADVQAFFEHHIRRCNEAGITNQKLLLDPGFGFGKNLAHNYQLLARL
SEFHRFGLPLLVGMSRKSMIGQLLNVPPDQRVIGSVACAVIAAMQGAQIVRVHDVKETVEAMRVVEAT
LSAKGQ

>CORE_REP|Org11_Gene813#
MAQALLKLAQHDFPGQHAAASRKVLSVKGLGKAYKAQQRVLDDINFDLHAGEFVAVIGRSGAGKSTLL
HTLNGTIPSSCGEMLHFEDDGVAQDIAQLAGRQMRQWRARCGMIFQDFCLVPRLDVMTNVLLGRLSHT
STLKSFFKLFDDADRARAIELLQWLNMLPHALQRAEHLSGGQMQRVAICRALMQNPQILLADEPVASL
DPKNTRRIMDALQKISEDGIAVMVNLHSVELVKEYCSRVIGIAHGKIVFDGHPSQLNERILHQLYGEE
ANQIH
>CORE_REP|Org31_Gene2716#
MTDMHSLFIAFVLGVVEGLTEFLPVSSTGHMIIVGEWLGFTGDKAKTFEVIIQLGSILAVVVMFWRRL
FGLIGIHFGGKPVEHEGKTHGRLKLGHILLGMIPAVVLGLIFHDVIKSLFAPKNVMYALVVGGLLLLT
AEWLKPKKPRAEGLDDITYRQAFLIGCFQCLALWPGFSRSGATIAGGMLVGVNRYAASEFSFILAVPM
MIGASGLDLYKSLHFLTWGDLPMFAVGFVTAFVVALIAIKTFLSLIKRISFVPFAIYRFIVAAVVYMV
FL
>CORE_REP|Org34_Gene2028#
MSSEELEQVWNSIKSEARALADCEPMLASFFHATLLKHENLGSALSYMLANKLATPIMPAIAVREVVE
EAYKSDNQMIVSAARDILAVRLRDPAVDKYSTPLLYLKGFHALQAYRIGHWLWQQGRQALAIYLQNQI
SVAFGVDIHPAATIGCGIMLDHATGIVIGETAVVENNVSILQSVTLGGTGKTSGDRHPKIREGVMIGA
GAKILGNIEVGKGAKIGAGSVVLQAVPPHTTAAGVPARIVGRPESDTPSMDMDQYFNGTNHGFEYGDG
I
>CORE_REP|Org22_Gene3649#
MWGVLAASLFFLPFNRLIAWVILAASAGMGLYHGVLTPLSLSYLLAIVALAGLRHHFREQRNLAIAFE
GLVVAGCIALFLHLVPGIHNQLMIDGDKAGPLSAPFTMYYNFDKAMVPFLLFACLPTLFRTDKAEKSV
ASGSWIALIISVPALLLLAVALGGLKIELHAPAWILPFVMANLFFVCMAEEALFRGYLQQRLSQWLGA
WPALIVAALVFGAAHLAGGMLMVIFATLAGVIYGLAWMWSGRLWVPILFHFGLNLIHLLFFTYPLYQH
P
>CORE_REP|Org9_Gene1383#
MNNRVHQGHFARKRFGQNFLTDQFVIDSIVSAIHPQPGEAVVEIGPGLGALTEPVGARMDRMTVIELD
RDLATRLENHPRLKDKLTIHQQDAMTVNFAELAEEAGQPLRVFGNLPYNISTPLMFHLFSYTQAIRDM
HFMLQKEVVNRLVAGPNSKAYGRLTVMAQYYCNVIPVLEVPPTAFAPPPKVDSAVVRLVPHSVLPNPV
GDVRMLSRITTQAFNQRRKTIRNSLGDLFTPEQLTELGVDPSLRAENISVAQYCKLANWLSANPAPQQ
>CORE_REP|Org48_Gene2755#
MKKHLLMLAFASVATLASYGAAAATKLVVGASNVPHAEILEQAKPILAKEGIDLQIKRFQDYILPNTA
LASHDIDANYFQHVPYLNSVLKDHADDKSYDFVSAGAIHIEPIGIYSKKYKSLKDLPENGKIIMRDAV
AEEGRILSIFEQQGVIKLKPGVSKVDARITDVVENPKHLKFQANVEGALLPQMYNNNEGDAVVINANY
AIDAGLNPTKDPIAVESGENNPYANIITVHKADVNKPEIVALVKVLHSKPIQDFIREKYQGAVIPVNQ
>CORE_REP|Org10_Gene1819#
MSLTFKSIATIGALIGTLALAGCGQDEKNPNHIKVGVIVGAEQQVAEVAQKVAKEKYGLDVELVTFND
YVLPNEALSKGDIDLNAFQHKPYLDQQIKDRGYKLVPVGSTFVYPIAGYSKKIKSLDELKEGSQIALP
NDPTNLGRSLLLLQKVGLIKLKDGVGLLPTVLDVTENPKNLKLVELEAPQLPRSLDDQQIALAVINTT
YASQIGLTPAKDGLFVEDKDSPYVNLLVAREDNKDAENVKKFVQAYQSDEVDAAANKIFNGGAVKGW
>CORE_REP|Org34_Gene2792#
MGVGAPPFQPTKKEHPLNFRWEIIQEYAPLFMEGAWMTIKCTIICVLLGTTWGLILGLGRLAQAPHGI
WKPILHYGVQWPVRIYISAFRGTPLFVQIMVVHFALVPLFINPRDGLLVTSGLMSVDFARALRADYGA
FLSCVVAITLNAGAYVSEIFRAGIQSIDRGQMEASRSLGMSYGKTMRQVILPQAFRRMLPPLGNNAIA
IVKDSSLASAIGLADLAYAARTVSGAYATYWEPYLTISLVYWVITFLLSLLVQHMEKRFGKSDSRT
>CORE_REP|Org9_Gene117#
MKKNRAFLKWAGGKYPLVDEIRRHLPAGDCLIEPFVGAGSVFLNTDYDAYILADINSDLINLYNIVKL
RTDDFVRDARTLFADEFNNSDQFYLLREEFNTSTEPYRRALLFLYLNRHCYNGLCRYNLRGEFNVPFG
RYKKPYFPEEELYWFAEKSRNATFVCEHYRDTMAKAVAGAVVYCDPPYAPLSATANFTAYHTNSFSIA
DQQSLAHLAHQLSVESQVPVLISNHDTELTRDWYQHAALYVVKARRTISRNILGRSKVNELLALYR
>CORE_REP|Org27_Gene597#
MARIIVVTSGKGGVGKTTSSAAIATGLAQKGKKTVVIDFDIGLRNLDLIMGCERRVVYDFVNVIQGDA
TLNQALIKDKRTENLYILPASQTRDKDALTREGVEKILNDLGEMDFDFVVCDSPAGIETGALMALYFA
DEAIITTNPEVSSVRDSDRILGILSSKSRRAEKGESPIKEHLLLTRYNPGRVSRGDMLSMEDVLEILR
IPLVGVIPEDQSVLRASNQGEPVILDAESDAGKAYDDTVCRLLGEERPFRFIEEEKKGFLKRLFGG

>CORE_REP|Org23_Gene1532#
MERYQQLFTRLESSKEGAFVPFVTLGDPNPTLSLQIIDTLIEAGADALELGIPFSDPLADGPTIQSAT
LRAFAAGVTPTQCFEMLAAIRQKHPTIPIGLLMYANLVFHKGIDAFYQRCAEVGVDSVLVADVPFEES
APFRAAAIRHGIAPIFICPPNADDDLLREIASHGRGYTYLLSRAGVTGTESRAQLPLHHLVNKLREYH
AAPPLQGFGISEPEQVKAALQAGAAGAISGSAIVKIIEQHHANPAEMLTKLAAFVSNMKRATRA
>CORE_REP|Org37_Gene3756#
MIELLLPGWLAGVLLAGAAGPLGSFVVWRRMSYFGDTLAHASLLGVAFGLLLDINPFYAVIAITLLLA
LALVWLERRPQLSVDTLLGILAHSALSLGLVVVALMSNVRVDLMAYLFGDLLSVTLSDILMIAGGVAV
VLLVLWWQWRDLLSMTISPELAHVDGVNLVRARTVLMLVTALTIGLAMKFVGALIITSLLIIPAATAR
RFARTPEQMAGVAVLLGMVAVTGGLTFSAFYDTPAGPSVVLCAAVLFTLSLFKKTTGLIKAGI
>CORE_REP|Org22_Gene4310#
MKRINALTIAGTDPSGGAGIQADLKAFSALGAYGTSVITALVAQNTRGVQSVYYIDPAFVAAQLDSVF
SDVRIDSVKIGMLANADIVQAVAERLRHYRPEFVVLDTVMLAKSGDPLLAPEAVASIRRELLPLVSII
TPNLPEAAALLACAPAEDEAQMREQGRALLAMGCRAVLMKGGHLSESESPDWLFSAEGEQRFTAPRVA
TRHTHGTGCTLSAALAALRPRHADWAATVAAAKDYLQQALQQAGTLEVGHGIGPVHHFHAWW
>CORE_REP|Org2_Gene4668#
MMKPTTVTHLRQCKQEQRKFATLTAYDASFAKLFEEQGIKVLLVGDSLGMTLQGHDSTLPVTVADVAY
HTRAVRRGAPACLLLADLPFMSYATPEQTFANAAELMRAGANMVKLEGGSWLCDTVKMLAERAVPVCG
HLGLTPQSVNVFGGYKVQGRDELAAKQLLQDAQNLELAGIQLLVLECVPTELARQITEALSIPVIGIG
AGNGTDGQILVMHDAFGITGGHTPKFAKNFLAQSGDIRTAVQHYIQEVEQGLYPAAEHSFN
>CORE_REP|Org19_Gene410#
MADEQACKYLIPGLDRGLQLLLAFGEQHKEMTFAELHRLVDMPKATAYRVVQTLEHLGFLERNPRTNT
FALGIKVLRLGFEYIASLDVAQAGQPVIEQLRDRSQCSSHLAIRDGRDVIYIARVSAAGSQINQVSVG
TRLPVHQTSLGRMLLTSATRSEFEQLYPDAQLPGNAPGTPADRETLWQMVQQDKARGYVIGESFFRHG
ISSIVYPIFNREQRVEAVVSIMVPSDEIPKADRERLRMEVRDAAEKISGFLGAPPQANVG
>CORE_REP|Org23_Gene4113#
MQDRNFDDIAEKFARNIYGTTKGKIRQAVVWQDLTGLLAQLPQLPQRPLRILDAGGGEGHMACQLAEL
GHQVLLCDLSGEMIQRAAQLAEQKGVSQNMQFVQSSAQDIAQHLEQPVDLILFHAVLEWIAEPEAALQ
ALCDCLTPGGALSLMFFNANGLLMRNVVLGNFQLVDPEVRRRRKRSLSPQYPHDPLLVYGWLEQLGMR
ISGKTGVRVFHDYLQSRQLQTQKFEELLALEQHYCRQEPYVSLGRYIHVMAHKPNLKDEL
>CORE_REP|Org35_Gene1535#
MANADSDKQPDAVSSVMKVFGILQALGDEREIGITELSQRVMMSKSTVYRFLQTMKALGYVSQEGETE
KYALTLKLFELGAKSLQNVDLIRSADVQMRELSNHTRETIHLGALDEDGIVYIHKIDAMYNLRMYSRI
GRRNPLHSTAIGKVLLAWRERDEVAQILSQIEFTRSTEHTLTSAAELLPVLDRVRAQGYGEDAEEQEA
GIRCIAVPVFDRFGVAIAGLSISFPTLRFSEAAREEYVALLHVAARRISEQQGYHDYPF
>CORE_REP|Org36_Gene1514#
MTSTPLSTTPLLSVNRLTHLYAPGKGFSDVSFDIYPGEVLGIVGESGSGKTTLLKSISARLAPQRGQI
LYRPQAGQEQDLYAMAESDRRRLLRTDWGVVHQHPLDGLRPQVSAGGNIGERLMAIGQRHYGDIRRQA
GQWLEDVEIPLSRLDDLPTTFSGGMQQRLQIARNLVTHPKLVFMDEPTGGLDVSVQARLLDLLRNLVV
EMQLAAVIVTHDLGVARLLAHRLLVMKQGEVVESGLTDRVLDDPHHPYTQLLVSSVLS
>CORE_REP|Org37_Gene4403#
MRHPLVMGNWKLNGSTHMVNELIAGLRNELSSVDGCGVAIAPPVMYLDQAKHALAGSRIALGAQNVDV
NLSGAFTGEVSANMLKDVGAQYIIIGHSERRTYHKESDEVIAEKFAVLKEAGLIPVLCIGETEAENAA
GKTEEVCARQIDAVLKTLGAPAMKGTVIAYEPVWAIGTGKSATPAQAQAVHKFIRDHIAKHDAAVAAE
IIIQYGGSVNDKNAAELFSQPDIDGALVGGASLKADAFAVIVKAAAAAKSLIALGLP
>CORE_REP|Org29_Gene3298#
MSYAFTDTHCHFDFPPFTGHEAESLARAASAGVQRIIVPTVTADRFARVLRLAQEHAPLFAALGLHPL
YIAQHHEPQLDQLATLLAERPRKLVAVGEIGLDLYMENPQFERQQSVLLAQLKLAKQHDLPVILHSRR
THDQLAAALRRMQLPRCGVVHGFAGSLSQAQAFIRLGYYIGVGGTITYERAQKTRGVMAQLPLEALLL
ETDAPDMPLAGYQGQPNRPERAAEVFQTLCELRPEPADEIAAHLQRNTQALFAMPDL
>CORE_REP|Org43_Gene1624#
MIGRLLRGGFMTLVYAYLYIPIVILIVNSFNASRFGINWQGFTTKWYSTLLNNDSLLQAAGHSLTMAV
LSATFATLIGSLTAVALYRYRFRGKPFVGGMLFVVMMSPDIVMAISLLVLFMLLGISLGFWSLLFSHI
TFCLPFVVVTVYARLKGFDVKMLEAARDLGASEFTILRKIILPLAMPAVAAGWLLSFTLSMDDVVVSS

FVTGPSYEILPLKIYSMVKVGVSPEVNALATILLLLSLTLVIASQWVMRDRSPKAE
>CORE_REP|Org43_Gene3845#
MFDIGVNLTSSQFAKDRETVVERARAAGVTGMLITGTDLPESREAAKLAQQHAGYCWSTAGVHPHNAG
SWDERSTEQIYALAAGPEVVAIGECGLDFNRNFSTPEQQEAAFSAQLALAAELALPVFLHCRDAHARF
AELLTPWLDKLPAAVVHCFTGTAEELASCLSLGLSIGITGWVCDERRGLELRALLPQIPAERLLLETD
APYLLPRDLQPKPASRRNEPCFLPHLVHQVAVWRQEEPQWLGQKTDENARRLFRLV
>CORE_REP|Org37_Gene4465#
MIFDAVQPEKFFGAMLMPLPLLMLLMGLALLLLWFTRWQKAVRRFYAELAVLLLFSLQPVADRLLRPI
EAQYQTYRGNDPVSYIVVLGGGYTYNPDWAPSSNLLGNSLPRVTEGVRLYLAHPGARMVFTGASAGSM
QSNAATAALVAESLGVPRSDMVILREPRDTEEEAAQVAKLVGEQPFILVTSANHLPRAMRFFEAKGLH
PIPAPANQLAIDSPLNIWDRATPSSMFLGHTERAWYETLGSLWQWLKGADRAGAE
>CORE_REP|Org32_Gene2590#
MLLVDSHCHLDSLDYQTLHQNVDDALAKAKARDVGYVLAVATTLPGYRSMTELIGERNDVAFSCGVHP
LNLEEGYDYAELRRLAAAEQVVALGETGLDYFYQKDNLELQQDSFREHIRIGRDLNKPVIVHTREARA
DTLAILREENAQDCGGVLHCFTEDLTTAEALLDLGFYISFSGIVTFRNAEQLREVARYVPLDRILVET
DSPYLAPVPHRGKENQPAYVRDVAEYMAVLKGVSLEQLAEATTANFSRLFHLDL
>CORE_REP|Org8_Gene1470#
MSGRIGRLLRDPLPWTLALLLALVFGMDHLRGLFAAWFPDLERPIYQQDSFIALVGAHLSLVAISSLI
AVAIGVAAGVAVTRRSGREFRSLVETVVAVGQTFPPVAVLAVAVPVMGFSEQPAIIALVLYGLLPILQ
GTLAGIESVPPATREIARGVGMSAWQILWRVELPLAAPVIVAGIRTSVIINIGTAAIASTVGTKTLGS
PIIIGLSGFNTAYVIQGAAVVALLAIITDMLFERWVRYLTAWRQQTLAATSAG
>CORE_REP|Org43_Gene2476#
MNQHNITNESLGLSMVLVVVAILISHREKLALEKDIIWSICRAVVQLIIVGYVLKYIFDLDNAVLTVL
MVLFICFNAAYNAKKRSKYVEHAFVTSFIAITTGAVLTLAVLVLTGSIEFTPMQVIPISGMIAGNAMV
AVGLCYTNLGQRFKSEQQKIQEMLSLGATPKFASAALIRDSIRASLIPTVDSAKTVGLVSLPGMMSGL
IFAGIDPVKAIKYQIMVTFMLLSTASLSTIIACYLAYRKFYNERHQLVVGNLK
>CORE_REP|Org31_Gene2550#
MSVQGKTALVTGSTSGIGLGIASVLAAAGARVILNGFGDVAQAQAQVAQLGAAPGYHGADLGDAAQIA
DMMQYAESEFGGVDILVNNAGIQHVAPLDQFPVEKWNAILAINLSAVFHTCRLALPGMRERNWGRIIN
VASVHGLVASKDKSAYVAAKHGVVGLTKTLALETARTPVTCNAICPGWVLTPLVQQQIDKRIAAGTDP
QRARDELLAEKQPSQEFVTPEQLGELALFLCSDAAAQVRGAAWNMDGGWLAQ
>CORE_REP|Org39_Gene1904#
MRSTLLRLPLLAGLLLAGQPQAFAAASILIWPIDPAIEDNQQATALWLENRDSKPVYMQIRVLGWQQT
GGKDDYRNQSEVVASPPVATILPGKRQLIRLIKQTPVAAGQERAYRILVDEVPIKDKDGAAPDKGAQM
GLKFQMRYSVPLFVSGKGVWTKQDFEHPRDYATANQPKLSYRLLQQNSQRWLDVRNDGIVHARLSQVS
IQGKPLNNGLLGYVLPGSQMRFALPPSGSFAAGKLQAMVNDNKQPVTIPSY
>CORE_REP|Org43_Gene1989#
MNNLFSLDNKTVLITGASRGIGFLLARGLAQQGAHILVNATTEEHARHAADRLREEGLRADAAAFDVT
DSQAVHAAIGRIETDIGAIDVLINNAGIQRRHPFTEFPEQDWDDIIAVNQKAVFIVSQTVARYMVPRR
RGKIVNIGSMQSELGRDTITPYAASKGAVKMLTRGMCVELARYNIQVNGIAPGYFKTEMTQALADDPA
FTAWLTQRTPAARWGDPQELIGAAVFLAADASSFVNGQLLFVDGGMSAAV
>CORE_REP|Org39_Gene1977#
MKHARIRHHGQVFNVQVDEQLRVTLPNGEVLQEREVEWLPPAQGTVFALGLNYADHASELEFKAPEEP
LVFLKAPNTLTGHRQVSVRPAGVEYMHYEAELVAVIGKTARNVSREHAMEYVAGYTLCNDYAIRDYLE
NYYRPNLRVKSRDTLTPIGPYIVDRDDVADPHRLALSTYVNGELRQRGSTADMIFDIPFLVAYLSEFM
TLQPGDMIATGTPKGLADVQPGDEVVVEIEGIGRLVNHIISEKDYEESLR
>CORE_REP|Org37_Gene3755#
MSTLITLKNISVAFGNRKVLSNISLSLQPGRILTLLGPNGAGKSTLVRVVLGLVKPTAGTLEREPDLR
IGYVPQKLHLDATLPLTVSRFMRLKPGVKKADILPALKRVHAAHLLDQPMQKLSGGENQRVLLARALL
NKPQLLVLDEPTQGVDVNGQLALYDLIDQLRKELGCAVLMVSHDLHLVMAKTDEVLCLNQHICCSGAP
EVVSMHPEFIAMFGNRGAEQLAVYRHHHNHRHDLQGRIVLKKTGSREA
>CORE_REP|Org34_Gene2871#
MSSANQQEANPSSQGPRHVAIIMDGNGRWAKRQGKLRVFGHKAGVKSVRRAVSFAASHHLDALTLYAF
SSENWNRPVQEVSALMELFVRALDSEVKSLHKHNVRLRVIGDISRFSARLQERIRRSEALTENNDGLT

LNIAANYGGRWDIIQGVRELAEQVRVGELHPDQISEELLNERVCMSDLAPVDLVIRTGGEHRISNFLL
WQIAYAELYFTDVLWPDFDELVFEGALNAFAQRERRFGGTTPNGADAS
>CORE_REP|Org2_Gene1095#
MKIRTSLIALGIATLATGCQNLNTETLMQSGAQAFQAATLSNDDVKALSDKSCAEMDSKAQIAPADST
YAKRLNKIAAALGDNINGTPANYKVYVTKDVNAWAMANGCIRVYSGLMDMMNDNEVEGVLGHEMGHVA
LGHTRKAMQVAYGTVALRTAASSAGGVIGSLSQSQLADIGEKLVSAQFSQKQESEADDYSFDLLKKRG
IDPNGLATSFEKLAQMEAGRQSSMFDDHPSSQARAQHIRDRIAAEK
>CORE_REP|Org1_Gene4545#
MRTALVTGASAGFGKAICHRLIAEGYRVIGAARRMEKLLLLRQELGEQFIPLPLDVTDPLSLDKAFEQ
VSEAPFSIDILVNNAGLALGIERAQQSNVQNWERMIATNITGLALVTHRVLPGMVEANSGLIINIGSI
AGTYPYPGGNVYGASKAFVKQFSLNLRADLAGTHVRVTNIEPGLCSGTEFSVVRLNGNMEAVEALYKD
VKAIAPEDIANTVFWIIQQPEHLNINTIEIMPVAQSSAALNVVRNL
>CORE_REP|Org26_Gene3049#
MTTAQPLHGKVAFVQGGSRGIGAAIVKRLASEGAAVAFTYAASADRAEAVASAVTAAGGKALAIKADS
ADAAALQQAVRQAVSQFGNLDILVNNAGVFTLGSTEELALDDLDRMLAVNVRSVFVASQEAARHMNDG
GRIIHIGSTNAERVPFGGAAVYAMSKSALVGLTKGMARDLGPRGITVNNVQPGPVDTEMNPDAGEFAE
QLKQLMAIGRYGKDEEIAGFVAYLAGPQAGYITGASLSIDGGFSA
>CORE_REP|Org15_Gene1348#
MSFIAIIPARYASTRLPGKPLADIHGKPMVVHVMERARESGASRVIVATDHPEVAKAVEAAGGEVCMT
SPDHHSGTERLAEVIAHYGFADDQIIVNVQGDEPLIPPVIVRQVAENLAGSQAGMATLAVPIDSAEEA
FNPNAVKVVMDAQGYALYFSRATIPWDRERFAASKESIGDSLLRHIGIYAYRAGFVRRYVSWAPSQLE
QIELLEQLRVLWYGEKIHVAVAKAVPSVGVDTPEDLQRVRDSIQP
>CORE_REP|Org43_Gene4812#
MMKVALVTGASRGVGRATALLLARQGYAVGVNYLRDESAARQVVAEIEAQGGKALALQADVADEAQVM
AMFSALDAGLGTLSALVNNAGILFRQANIEQLTAERINKVLGTNVTGYFLCCREAVKRMARRHGGQGG
AIVNVSSAASRLGAAGEYVDYAASKGAVDTLTIGLSREVAAQGIRVNGVRPGFIYTEMHASGGEPGRV
DRVKSSLPMQRGGQPQEVAEAIAWLLSDAASYVTGTFIEAAGGR
>CORE_REP|Org45_Gene722#
MLSIKNLKVSVEGNEILKGLDLEIKPGEVHAIMGPNGSGKSTLSATLAGREEYEVTEGEVTFKGKDLL
ELDPEDRAGEGVFLAFQYPVEIPGVSNHFFLQTSVNAVRKYREQEPLDRFDFADFIEEKIALLDMPAD
LLTRSVNVGFSGGEKKRNDILQMAALEPDLCILDETDSGLDIDALKIVANGVNSLRDGKRAFIIVTHY
QRILDYIQPDYVHVLSQGRIVKSGDFSLVKQLEEQGYGWLTDQQ
>CORE_REP|Org37_Gene4141#
MQHATQRVAIVTGASRGIGAAIAERLAADGFTVIINYSGNPAPADELVRKIEQAGGRALGAKADVSDA
AAVSRLFASAEQAFGGVDVLVNNAGVMALAPVADMRDEDADRLIDINLKGSFNTMREAAKRLRDNGRI
INFSSSVVGLLQPGYGMYAASKAAIEALTSVLAKELRGRNITVNAVAPGPTATGLFLDGKTPELIERL
AKMAPLERLGTPEDIAAAVAFLAGADGGWINGQTLRANGGII
>CORE_REP|Org41_Gene591#
MSVTGRIHSFESCGTVDGPGIRFIVFFQGCLMRCLYCHNRDTWDTHGGKEVTVEELMKDAVAYRHFMN
ASGGGVTASGGEAILQAEFVRDWFRACHAEGINTCLDTNGFVRRYDPVIDELLDTTDLVMLDLKQMND
EIHQNLVGVSNHRTLEFARYLAKRNQRTWIRYVVVPGWSDDDKSAHLLGEFTKDMTNIEKIELLPYHE
LGKHKWVAMGEEYKLDGVHPPKAETMDRVKGILESYGHKVIY
>CORE_REP|Org29_Gene4084#
MSRIFITGSVDGLGRAAAQTLLDEGHQVILHARAPGRLDAVRDLLERGAQAVIGDLSDVQQIRQLAEQ
VNRLGRPDAVIHNAGMFTGPQVMPVNVIAPYLLTALIERPKRIVYLSSSMHFDGVPELNGVDWLGGAA
GSYSDSKLFVTALAAAVARLWPDVISSAVDPGWVPTKMGGADAPDDLALGHVTQAWLVTSDEPQALAS
GGYWHHQQRFEPHAAVHDEAFQTALLAQLARAGGVSLPQA
>CORE_REP|Org11_Gene1725#
MKAIIVEDEFLAQEELSYLIKKHSNIDIVATFEDGLDVLKYLQTHQVDAIFLDINIPSLDGVLLAQNI
SKFAHRPSIVFITAYKEHAVEAFEIEAFDYILKPYHEARIVTMLQKLEALHHRPAGATEPASAPSRGS
HSINLIKDERIIVTDINDIYYAAADEKVTRVYTRREEFVMPMNITEFYGRLPEEHFFRCHRSYCVNLA
KIREIVPWFNNTYILRLSDLEFEVPVSRSKVKEFRKLMRL
>CORE_REP|Org31_Gene266#
MSEIIYGIHAVKALLERDPQRFLEVFILKGREDRRLQPLIAELEATGIVIQVANRQWLDDKVEGAVHQ

GIIARVREGRQYQENDLPGLLESVETPFLLVLDGVTDPHNLGACLRSADAAGVHAVIVPRDRSAQLNA
TAKKVACGAAENVPLIRVTNLARTLRLLQEMNVWVVGTAGEADHTLYQSKMTGPMALVMGAEGEGMRR
LTREHCDELISIPMAGTVSSLNVSVATGICLFEAVRQRG
>CORE_REP|Org36_Gene1111#
MSSTNIEQVMPVKLAKALSNSLFPALDSQLRAGRHIGIDELDNHAFLMDFQDELEEFYTRYSVELIRA
PEGFFYLRPRSTTLIPRSVLSELDMMVGKILCYLYLSPERLAHEGIFSHQELYDELLSLADENKLLKF
VNQRSTGSDLDRQKLHEKVRTSLNRLRRLGMVYFMGNDSSKFRITEAVFRFGADVRSGDDPREAQLRM
IRDGEAMPVETSLSLNDENEAEDQQVDNAPDGAEDEQE
>CORE_REP|Org37_Gene1550#
MLNALIVDDEPSARDNLRHLLEAEAEIAIIGECANAIEAISQIHRLQPDVVFLDIQMPRISGLEMVGM
LDPNRMPHIVFLTAYDEYAVQAFEEHAFDYLLKPAEPKRLSKTLQRLRQRSAPQDVAALEESAGYLKY
IPCTGHSRIYLLRFDEVLAIRSKLSGVFVVRSDGMECFTELTLRTLESRTPLVRCHRQYLVNLEQVRE
IRFEEGGAAEMIMSAGDPVPVSRRYLKALKEQLGLRG
>CORE_REP|Org15_Gene1664#
MSETMLEFREVDVFYGPIQALRQVSLQVNAGETVALIGANGAGKSTLLMSIFGQPRIAGGQILFRGED
ISRRSTHFVASSGIAQAPEGRRIFPDMSVEENLLMGTITVGNRYLEEDLPRMFELFPRLKERRNQRAM
TMSGGEQQMLAIARALMSRPKLLLLDEPSLGLAPIVVRQIFGVLRELTRSGMTLFLVEQNANHALKLS
DRGYVMVNGQIRLTGSGEELLNDPQVRKAYLGGG
>CORE_REP|Org16_Gene1693#
MIEILQQYWQSLLWSDGYRFTGVAVTLWLLIASVVMGGLLAIPMAVARVSSLRWVRFPVWLYTYVFRG
TPLYVQLLVFYSGMYSLEIVRGTEFLNAFFRSGLNCTILALTLNTCAYTTEIFAGAIRAVPHGEIEAA
NAYGFSRFKMYRCIILPSALRTALPAYSNEVILMLHSTALAFTATVPDLLKIARDINAATYQPFYAFG
IAAVLYLIISYVLISLFRKAEKRWMAHVSH
>CORE_REP|Org1_Gene4257#
MTMQIRVEHISKTFVLHQQYGTRLPVLHDANLTVHGGECVVLHGHSGSGKSTLLRSLYANYLPDSGHI
WINHQGDWLDMVSADARQILAVRRHTLGWVSQFLRVIPRISALEVVMQPLLEQGVERAECRDRAEALL
AALNVPQRLWPLAPSTFSGGEQQRVNIARGFIVDYPILLLDEPTASLDSRNSAAVVQLIERAKARGAA
IVGIFHDEGVRQQVADRLYDMQAPQALEAL
>CORE_REP|Org14_Gene3426#
MKAQVRIALVGDYNPQAVAHQAIPVALQLTAAHLDIDVQPQWLPTETLTTPDILQNFDAIWVVPGSPY
RYDDGAFMAIRHARENDVPFLGSCGGFQYAIVEYARNVMGWHDAGHAETDSGGRLVIAPLSCSLVEKT
GDIVFQPDTRVAQAYGSLNTHEGYHCNFGVNPEFVADLERFPLIISGHDTEGDVRAIELPGRRFYVAT
LFQSERAALRGELSPLVVELVKTAANG
>CORE_REP|Org22_Gene200#
MTATAPVITVDGPSGAGKGTLCKALAESLGWRLLDSGAIYRVLALAALHHQVDITSEEALVPLAAHLD
VRFVAQDGKLQVILEGEDVSNEIRTETVGNTASQAAAFPRVREALLRRQRAFREAPGLIADGRDMGTV
VFPDAPVKIFLDASSEERAHRRMLQLQEKGFNVNFERLLAEIKERDDRDRNRPIAPLVPASDALVLDS
TSMSIEEVIRQALTYAQKVLALPQQ
>CORE_REP|Org2_Gene1619#
MTGILQLFQAIGLGLVLLLPLANPLTTVALLLGLSGNMTREERNQQSLMASVYYVFCIMTVAFYAGQVV
MNTFGISIPGLRIAGGLIVAFIGFRMLFPQQSADEAPEVESKSHELRHKTSANIAFVPLAMPSTAGPG
TIAMIISSASSVKDNTLGFEPWVLTVAPVAIFLTVAVILWGCLLSSGAIMRLVGKSGIEAISRLMGFL
LVCMGVQFIINGVLEIISTYTPAAA
>CORE_REP|Org21_Gene1456#
MKEKKDILRLDDIHYQIDNQVILDSVSFTLGEGEFKLITGPSGCGKSTLLKIISSLMDPTRGSLYFDG
QAIAEMSPEAYRKQVSYCFQTPALFGNTVYDNLALPYQIRQQSPDERKMKADLTRFGLPEAMLTKSIN
ELSGGEKQRVSLIRNLQFMPRVLLLDEITSALDEENKRNVNEIVHQLVAEHRLAVLWVTHDTEEIAHA
DEVITLRAHGAEQQEQQEQQHESA
>CORE_REP|Org25_Gene499#
MTDMNILDLFLKASLLVKLIMLILICFSVASWAIIIQRTRILNAATRDAEAFEDKFWSGIELSRLYQE
SQARRDSLTGSEQIFHSGFKEFARLHRANNHAPESVIEGASRAMRISMNRELETLETHIPFLGTVGSI
SPYIGLFGTVWGIMHAFIALGAVKQATLQMVAPGIAEALIATAIGLFAAIPAVMAYNRLNQRVNKLEQ
NYDNFMEEFTAILHRQAFSSDSK
>CORE_REP|Org13_Gene804#

```
MKKFLIAPSILSADFARLGEDTANVLAAGGDVVHFDVMDNHYVPNLTIGPMVCEALRNYGITAPIDVH
LMVKPVDRIVPDFAKAGASYISFHPEASEHVDRTIQLIKEHGCKAGLVFNPATPLSYLDYVMDKIDVI
LLMSVNPGFGGQSFIHGTLDKLRQVRKLIDDSGRDIRLEVDGGVKVDNIAEIAAAGADMFVAGSAIFG
QPDYRKVIDEMRSELAKVSHG
>CORE_REP|Org4_Gene1430#
MGKTDVVKAGNNKIVAAAYPWAEEIANSISHGIGLVFGIVGLVLLLVQAVNTGADATAITSYSLYGGS
MILLFLASTLYHAIPHQKAKHWLKKFDHCAIYLLIAGTYTPFLLVGLDSPLAKGLMAVIWGLALLGVL
FKLAFAHRFEALSLVTYLTMGWLSLIVIYQLVTRLEAGGVTLLAIGGVVYTLGVIFYASKRIRFGHAI
WHAFVLGGSACHFMAIYLYV
>CORE_REP|Org13_Gene460#
MYEFDWASIVPSFPYLLQGMVITLKITVTAIVVGILWGTVLAVMRLSPFKPISWFATLYVNLFRSVPL
VMVLLWFYLVVPSLLQQVLGLSPKTDIRLISAMVAFSLFEAAYYSEIIRAGIISISRGQSSAALALGM
THWQSMRLVILPQAFRAMVPLLLTQGIVLFQDTSLVYVLSLADFFRTASTIGERDGTQVEMILFAGFV
YFVISLAASALVSYLKKRTV
>CORE_REP|Org23_Gene2642#
MIIVHHLNHSRSQRILWFLEELGVPYQVQRYERDPQTLLAPAALKKIHPLGKSPVIVDGDLTLAESGA
IIEYLQEAYDAQGMFMPTDFHARQQYRYWLHYAEGSLMPLLVMKLVFSRLGQPPIPWLLRPVAGAIGK
GVQREYLDKQIAPHCEYLEQHLNKGSWFVGNDFSAADIQMSFPLEAMAARGALDNCPKLRGFLQRIHA
RPAYQRALEQGGPYNLLS
>CORE_REP|Org3_Gene1527#
MIESLFPHLRLDQLWDATWETLYMTGIAGLATLVLGIVLGVLLFLTSKGQLWQNRAVYSLISVLVNVF
RSIPFIILIVLLIPFTKSLIGTILGADAALPALIVGAAPFYARLVEIALREVDKGVIEAARSMGAKNR
TLIFRVLLPESSPALVSGITVTLIALVSYTAMAGVIGAGGLGNLAYLEGFQRNHSDVTLVATLTILLI
VFVIQFIGDTLTRTLDKR
>CORE_REP|Org42_Gene3883#
MKFGLVVAALLSISMPAAATTLKLSPDIDLLVVDGKKMTGSLLKGADSLELDGGQHQLLFKVTKTVRS
GQHTQAYASLPLVATFNTQKISQVAIELPRIENDRDAQRFDRTLNYLVVDKDGNALPFRHDVLHPDSV
TFNTDLEKVMTDYNRQNRPASVPSFVQANAGNASALTLAGAPINAPTVTLKGENVSEQMLQYWFQQAD
KETQKRFLRWANKQPIR
>CORE_REP|Org37_Gene713#
MDIIKFIIDFILHIDVHLAELVAQYGMWVYAILFLILFCETGLVVTPFLPGDSLLFVAGALAALPTND
LNVHTMVALMVVAAILGDAVNYTIGRLFGEKLFSNPNSKIFRRSYLDKTHQFYEKHGGKTIILARFVP
IVRTFAPFVAGMGHMSYRHFAAYNVIGALVWVLLFTYAGYLFGDLPVVQENLKLLIVGIIIVSILPGV
IEIWRHKRAAARQQKQ
>CORE_REP|Org19_Gene942#
MHESIQLALDSAPFLLKGAILTLQLSLGGMAFGLLLGFLLALMRLSPLWPLAWLSRIYVSLFRGTPLI
AQLFMIYYGLPQFGIEFDPFPAALIGLSLNTAAYTSETLRAAISSIDKGQWEAAASIGMTRWQTLRRV
ILPQAARTALPPLGNSFIGLVKDTSLAATIQVPELFRQAQLITSRTLEVFTMYLAASLIYWVMATLLS
ALQNRLEAHVNRQDQE
>CORE_REP|Org1_Gene782#
MKKRIAIIAGAVSVALTLSACTTNPYTGESEVGKSGIGAGLGAALGAGVGVLSSSKKDRGKGALIGAA
AGAALGGGAGYYMDVQEAKLRDKMKGTGVSVTRQGDNIVLNMPNNVTFDSSSATLKPAGANTLTGVAM
VLKEYPKTAVNVVGYTDSTGSRSLNMNLSQQRADGVASALITQGVAANRIRTTGAGPDNPIASNSTAE
GKAQNRRVEITLSPLQ
>CORE_REP|Org30_Gene425#
MQFDWSAIWPAIPILLEGAKMTLWISVLGLIGGLIIGLVAGFARTYGGWIANHIALVFIEVIRGTPIV
VQVMFIYFALPMAFTDLRIDPFTAAVVTIMINSGAYIAEITRGAVLSIHNGFREAGLALGLSRRETIR
YVIMPLALRRMLPPLGNQWIISIKDTSLFIVIGVAELTRQGQEIIAGNFRALEIWSAVAVIYLIITLV
LSFVLRRLERRMKIL
>CORE_REP|Org43_Gene4420#
MKRMSTRRIAQAKNCFAALGAITTRSQFGGYGLLAEGVMFAVIAEGELYLRATASMEPAFRARGMVNM
VYSKRGVPITLRYYWVDESLWRERNELVGLAWQAVREARREQRLKAGDHGRLKALPNIDVNMERLLWR
AGIRNAYDLRLHGAKRSYLRLLKQQTNLGLRVLLSLGGAIAGYHQAALPAELRSELVRWFDHTMAMRR
HGHEPVIQGPSSGPE
```

>CORE_REP|Org45_Gene114#
MDRIVVSSSSRDSLLSTHKVLRNTYFLLSLTLAFSALTATASTMLGLPAPGLLLMLVGFYGLMFLTHK
LANSPAGILAAFALTGFMGYALGPILSSFLNAGAGDLIMLALGGTAAVFFCCSAYVLTTRKDMSFLSG
MMMAGFVVLLVAVIANLFLQIPALHLAISALFILFSAGAILWETSNIIHGGETNYIRATVSLYVSLYN
MFISLLSILGFARSN
>CORE_REP|Org36_Gene207#
MYQHRDWQGSLLDFPVNKVVCVGSNYADHIKEMGSAVSVEPVVFIKPETALCDIRQPVAIPKEFGAVH
HEVELAVLIGTPLKQANEDRVARAIAGYGVALDLTLRDLQAGFKKAGQPWEKAKAFDGSCPMSGFIPV
AEFGDPQNAELSLTVNDQLRQQGNTRDMITPILPLISYMSRFFTLRAGDIILTGTPQGVGPMASGDML
KISLNGKTLSTRVI
>CORE_REP|Org18_Gene267#
MSEAMMWLMARGVWETVMMTFVSGFFGFVLGLPVGVLLYVTRPGQIIANNSLYKILSGLVNIFRSIPF
IILLVWMIPFTRMIVGTSIGLQAAIVPLTVGAAPFIARMVENALLEIPSGLVEAARAMGATPMQIIKK
VLLPEALPGLVNAATITLITLVGYSAMGGAVGAGGLGQIGYQYGYIGYNATVMNTVLVLLVVLVYLIQ
FCGDRIVKAVTHK
>CORE_REP|Org6_Gene1037#
MNTLLYAWQNWAYIAGLTLEHLLLVGIAVGLAILIGVPLGVLIVRHKWLATPVLSLATLVLTVPSIAL
FGLMIPLFSLIGHGIGYVPAITAVFLYSLLPIVRNTHTALDNLPGGLREAGRGIGMTFWQRLRWVEIP
VALPVIFGGIRTAVVMNIGVMAIAAVIGAGGLGLLLLNGISSSDIRQLITGAVMISLLAIVLDWLLHR
LQIALTPKGIRS
>CORE_REP|Org38_Gene1576#
MKYHLIPVTAFSQNCSLIWCENTQQAALVDPGGEAEKIKAEVAKQGVTITQILLTHGHLDHVGAAAEL
AEHYQVPIYGPDKEDAFWLDGLPAQSRMFGLEECAPLTPTRWLSEGDEMQVGEMKLKVLHCPGHTPGH
IVFINEQARLALVGDVLFNGGVGRSDFPRGDHQALIASIRTKLLPLGDDMRFIPGHGPMSTFGHERQT
NPFLREEPAVW
>CORE_REP|Org31_Gene553#
MGQSLLDLSGFIKFFVGLFALVNPVGILPVFISMTSYQAEAGRNKTNLTANLSVAIILWTSLFLGEGI
LRMFGISIDSFRIAGGILVVTIAMSMISGKLGEDKQNKQEKSESAIRESIGVVPLALPLMAGPGAISS
TIVWSSRYHNWQSLLGFTVAIALFAFCCWLLFRAAPLLVRLLGQTGINVITRIMGLLLMALGIEFIVT
GIKAIFPGLL
>CORE_REP|Org4_Gene2280#
MSLMLKGEKIDRNRFTGEKIENGSFMLCDFSGADLTGTEFIGCQFYDRESRQGGNFSRAILKDASFRS
CDLSMADFRHVDALGVEIRECRAQGADFRGASFMNMITSRTWFCSAYITKSNLSYANFAKVVLEKCEL
WENRWHGAQVLGASFSGSDLSGGEFSGFDWRAADVTQCDLSNAELGELDLRTTDLQGVKMDSHQAAQL
LERLGIAIG
>CORE_REP|Org30_Gene1505#
MTTTIEQQAREEMVRLGASFFQRGYATGSAGNLSLLLPDGALLATPTGSCLGELQADRLSKVSLNGDW
LSGDKPSKEISFHRALYLNNPECKAVVHLHCTYLTALSCLQGLDVDNAIKPFTPYVVMRVGKVPVVPY
YRPGDERLAQDLARLAPTHRAFLLANHGPVVTGKDLRAAADNTEEMEDAARLIFTLGDRPIRYLTDDE
IAELRS
>CORE_REP|Org24_Gene15#
MVLGKPQTDPTLEWFLSHCHIHKYPSKSTLIHQGEKAETLYYIVKGSVAVLIKDEEGKEMILSYLNQG
DFIGELGLFEEGQERSAWVRAKTACEVAEISYKKFRQLIQVNPDILMRLSAQMASRLQVTSEKVGNLA
FLDVTGRIAQTLLNLAKQPDAMTHPDGMQIKITRQEIGQIVGCSRETVGRILKMLEDQNLISAHGKTI
VVYGTR
>CORE_REP|Org48_Gene4541#
MKGTVFSVALNHRSQLDAWDQAFHQPPYQTPPKTPVWFIKPRNTHLANGGAIPFPAGETVQSGGTLAV
IIGDTVRKVPAAQVSRYLAGYALANDVSLPESSFYRPAIKAKCRDGFCPLGEIGLLENADRLEIVTEI
NGVEQDRWSTADLVRSVPELIAAISDFITLQPGDAVLIGTPHQRVDIKPGDEVTVRAAGLPTLTNRVT
QAGAAS
>CORE_REP|Org11_Gene3912#
MSKKHWSNTELLHQTVTNPNIIVKGTHSYYSDCWDNGFERSVVRYLHGDAVSRQWQPLGDIDRLLIGD
YVCIAAEAVILMGGNHTHRIDWLSLYPFMETIKRAYRPKGDTRLGDGCWIGMRAMLMPGVSIGEGAIV
AAGSVVVGDVEPYAIVGGNPARFIRWRFAPEVIARLLALRLYDLSEADFAVVQPLLVDNDIAAVERAI

```
CNIKRY
>CORE_REP|Org31_Gene2423#
MLGTMMTKSTHCNVDTREHLLATGETLSLRLGFTGMGLSELLATAGVPKGSFYHYFRSKEAFGEAMLQ
RYFAHYDAQMQALFADRRGDARHQLLGYYAQAISYHCRSECHNACLAVKLSAEVSDLSEPMRHALETG
TARVIGHLQEAIERGIAEGSLSVAMSPAATAETLYSLWLGASLRAKIRHSLAPLTSALESIELLLRPP
QA
>CORE_REP|Org20_Gene1585#
MTKVLVLYYSMYGHIESLAQAVAEGANRVNGVDVTIKRVPETMTPEAFAKAGGKQHQQAPVATPQELA
DYDGIIFGTPTRFGNMAGQMRTFLDQTGGLWASGALYGKVGSVFSSTGTGGGQEHTISSTWTTLAHHG
FIIVPIGYATPELFDVSQVRGGTPYGATTIAGADGSRQPSNEELTIARYQGEHVAKITAKLKS
>CORE_REP|Org19_Gene4712#
MMKWIPVALALLLSACSSSPQKTYYQLPALGAPAAVSSSSGASTRQLWLEHVSVADYLAQSGVVYQTN
DVQYVIAQNNLWASPLDQQLQQTLVTNLSSALPGWVVSSQPMSSDQDVLNVTISGFHGRFDGKAIVRG
EWVLNRQGRLIKRPFSLELKQGEDGYDALVRTLAEGWQKEAKSIALQLKKICFIFGRDIKAL
>CORE_REP|Org24_Gene4548#
MFDIGFSELLLVLVIGLIVLGPERLPVAVRTVSGWIRALRSLAASVQHELSQELKLQELQDSLKKAEQ
AGLQNLTPELKASMDELKDAAESLKRTYRGETEELANTIHNPQAIDPEALHDGVTPAEAATRASAPAA
VPKPAAEPEAVAAASPAASPAAPVAAQPAKAPVETAPAPVEPVADKIPASHQPSGDR
>CORE_REP|Org7_Gene4105#
MRNNICVFCGASEGVNPAYAEQARQLGQLLAAQGRRLIYGGGKKGLMGIVADAVLAAGGEAVGIIPER
LVEAETAHRGLTELEVVPDMHTRKARMAALADCFIALPGGIGTLEELFEIWTWGQIGYHNKPVGLLNV
NGFYRPLSQFLEHVADQGFMRHDYLGTLHISESAQTLLQQFDDYQPKNYDRWAK
>CORE_REP|Org6_Gene2783#
MHHLMLDIETLDIKPSAVILVVAAVFFDPRTGALGAEFETAVSSQKDQPGRTISLDTVAWWAKQSDEA
RKQAFGGTESLKRVLSSLSRFIHMNSTDTVKVWGNGKEFDCAILEHAFQQLEMPCPWKFWDTQDVRTV
ITLAELHGFNPKKARPFEGMPHRALDDARHQARYVADTVSALYYRQGAQR
>CORE_REP|Org2_Gene1537#
MTYQQAGRIAILKRILGWVVFIPALLSTLISVLGFVYQHSEKTKGINAVMLDFVHVMVDMVRFNTPFL
NLFWYNSPVPDVDKSLFSGANLMFIIIYILIFVGLALQASGARMSRQVKFIREGLEDQMILEQAKGSE
GHTRQQLEERITLPHHTIFLQYFPLYILPIVIAVIAWFVIRLLGQLAGAA
>CORE_REP|Org8_Gene789#
MAKTVVVFHSGYGHTERLAKVVAEGAGAELIAIDQNGDISDEAWQTLDEADAIIFGSPTYMGGPSWQF
KKFADASSKAWFGRKWQDKVFGGFTNSASLNGDKQVTLIALQTLASQHGGLWVSLGLLPANTKSAQRT
DVNNLGGSVGLLVQTPADAGVDEMLSGDLATAKLYGQRVAGFAAKLA
>CORE_REP|Org7_Gene38#
MKIKPDDNWRWYFDAEHDRLMLDLANGMIFRSRFPAKMLTPDAFDECAFCVDDAALYFTYEEQCKQVK
LSHEQRAELVLNALVAYRFLKPLMPKSWHFSQQHYPLQPKNGELAAVKVMESGAEARLLVVEAGDNAS
LCLLAQNQLTVAGRTMVLGDAIKVMHDRLKPCAQDESAAPAYDRAV
>CORE_REP|Org19_Gene1538#
MDKIDHHRRKWLALGGAAMGIALLPGQAFASISTARPRILVLNNLNTGESIKAEFFDGKGYNKEELVR
LNHLFRDYRANKVKSIDPRLFDHLYRLQGLLGTSKPVQLISGYRSVDTNNELRAHSRGVAKHSYHTKG
QAMDFHIEGIQLSNIRKAALKMRAGGVGYYPRSNFVHIDTGPVRTW
>CORE_REP|Org18_Gene1969#
MTPVIRIAAIEALPDDYLTRGDFGFTIRCYALPQFDTPVDSWPTRPVAPFRKQYPLAPFANEDSATFL
AYRQDQAVGHITLSKNWNGYTLIDEIAVSAHARRQGIAGALLDCAKQWARQQETSGMMLETQNNNLAA
CRCYQHYGFILGGIDRLLYRAEPEIADHEIALFWYLPFNSEIGY
>CORE_REP|Org44_Gene164#
MSRVAKAPVVIPAGVEVKLNGQVISIKGKNGELTRTIHDAVEVKQEANALTFAPREGFANAWAQAGTT
RALLNAMVVGVTEGFTKKLQLVGVGYRAAVKGNVVNLALGFSHPIDHQLPAGITAECPSQTEIVLKGA
DKQVIGQVAADLRAYRRPEPYKGKGVRYADEVVRTKEAKKK
>CORE_REP|Org43_Gene254#
MIDDDGYRPNVGIVICNRQGQVLWARRYGQHSWQFPQGGINPGETAEQAMYRELFEEVGLSKKDVRIL
ASTRNWLRYKLPKRLVRWDTKPVCIGQKQKWFLLQLLCNDADINMQRSSTPEFDGWRWVSFWYPVRQV
VSFKRDVYRRVMKEFAVTVMPMQEQAAPRQAPAYRRKRG
```

>CORE_REP|Org38_Gene38#
MVDKRDSYTKEDLEASGRGELFGAGGPPLPAGNMLMMDRVVKMTEDGGTHNKGYVEAELDINPDLWFF
GCHFIGDPVMPGCLGLDAMWQLVGFYLGWLGGEGKGRALGVGEVKFTGQVLPTAKKVTYRINFKRVIT
RKLIMGVADGEVLVDGEVIYTATDLKVGLFKDTTAF
>CORE_REP|Org6_Gene1444#
MLTQEMTQKLNEQLNLEFYSANLYLQMSAWCSDKGFEGAAAFLKEHSQEEMQHMQRLFDYLSDTGSLP
LLGTIAAPPVAFESLADVFQQTYEHEQLITRQINELAHAAMTAHDYSTFNFLQWYVAEQHEEEKLFKS
VLDKLALVGTSGKGLFFIDKDLKKMGAMGQGGNDQA
>CORE_REP|Org42_Gene716#
MPLLDSFTVDHTRMAAPAVRVAKTMKTPHGDTITVFDLRFCRPNLEVMPERGIHTLEHLFAGFMRDHL
NGQGVEIIDISPMGCRTGFYMSLIGVPEEQRVADAWKAAMADVLKVTDQRKIPELNEYQCGTYHMHSL
EEAQEIAKHILDNDVVVNHNDELALPKEKLQELHI
>CORE_REP|Org35_Gene759#
MSQENEQRLRFRDAMASLSAAVNIVTTDGPAGRCGITATAVCSVTDTPPTLLVCINRNSAMNPVFQEN
RRLCVNVLNHEQELMARHFAGMTGVSMEDRFRLEEWQLGALGQPVLRNTLASLEGEIEQIQSIGTHQM
YLVQIKQIALSEAGNGLIYFKRNFHPVIHQMAVPA
>CORE_REP|Org4_Gene1486#
MSAIVKRCSVAAVLAIAVLLPSFGELQTSEAGLRLIADLEGCRLSPYQCSAGVWTQGIGHTAGVIPGQ
AIDERQAAVDLVDDVRRTERGMAACLPQTLPQETYDAVIAFAFNVGISAACHSTLVTLLQQRQWQQAC
DQLPRWVYVNGKKNKGLEQRRATERALCLQGIASS
>CORE_REP|Org8_Gene757#
MQLNKVLKGLLLALPVLAVAACSSNKSANNDQSGMGAGAGTGMENGSSNLSSEEQARLQMQELQKNNI
VYFGLDKYDVSSEFAQMLDAHAAFLRNNPSYKVTVEGHADERGTPEYNIALGERRANAVKMYLQGKGV
SADQISIVSYGKEKPAVLGHDEAAYAKNRRAVLVY
>CORE_REP|Org47_Gene1596#
MNKASVVFSGLLMAVSAGAMAATSGDDADISKQPLEKVAPYPKAEKGMNRQVIYLPKQEHEENYKVEL
LIGKTLEVDCNRHMIGGTLETKTLSGWGYDYLVLEKLSEPASTMMGCPDNTKTKQFIAANLGDAAMQR
YNSRLPIVVYAPKDAEVKYRIWKAEDTVSQAEQK
>CORE_REP|Org1_Gene379#
MGLFDKLKSLVSDDKKDTGTIEIVAPLSGEIVNIEDVPDVVFAEKIVGDGIAIKPAGNKMVAPVDGTI
GKIFETNHAFSIESDSGIELFVHFGIDTVELKGEGFKRIAEEGQRVKKGDVVIEFNLPLLEEKAKSTL
TPVVISNMDEIKELIKLSGSVTVGETPIIRIKK
>CORE_REP|Org39_Gene1644#
MRTQSLYQPHFSHGSFTANSVAKNTNVGKENGLISELVYNERQPAVAQLLLPLLQQLGKQSRWLLWLT
PQQKLSKQWLQQSGLPVDKMVQLSQISPVNTVEAMEKALQTGNYSVVLGWLPELTEEDRLKLRRAAEL
GNAYGFIMRPQRDIDPTHGHCSTLKIHSSLYH
>CORE_REP|Org4_Gene589#
MSTAKLVKTKSSDLLYTRNDLDEKVKLAAIKALNHQVVQFIDLSLITKQAHWNMRGANFIAVHEMLDG
FRTAIIEHQDTFAERVVQLGGVALGTVQVVNDRTPLKSYPTNIHSVQEHLKALADRYGAVANDIRKAI
TEVEDEDTADMFTAASRDLDKFLWFIESNIE
>CORE_REP|Org9_Gene1621#
MTKEQFYAELKRDLSALLGGETNFIAALSNASALLNERLDDVNWVGFYLMDGGQLVLGPFQGKIACVR
IPVGKGVCGTAVAENRVQRVGDVHAFPGHIACDAASNAEIVLPLAVGGRAIGVLDIDSTVYQRFDEQD
EAGLKAVVAGLCEQLEQCDSAKYVTVAAS
>CORE_REP|Org17_Gene1231#
MIQQLFKGRFSLLTMAFVALLALAGCQSKPQGLTPEQVALLQSQGFKLTDNGWEFGLSDKVLFGNNIG
KLNPESTETVQKMGRALLSVGITKFRLDGHTDNYGEDSYNDQLSLRRADAVADLLASVGIPRANIETR
GMGKRDPVADNRTSSGRAENRRVAIVVTP
>CORE_REP|Org24_Gene49#
MADNKKRPGKDLDRIDRNILNELQKDGRISNVELSKRVGLSPTPCLERVRRLERQGFIHGYTALLNPH
YLDASLLVFVEITLNRGAPDVFEQFNSAVQKLEEIQECHLVSGDFDYLLKTRVPDMSAYRKLLGETLL
RLPGVNDTRTYVVMEEVKQSNRLVIKTR
>CORE_REP|Org34_Gene204#
MATAKKAAKTHIGLDSKQSAKLAEALNALLANYQVLYMNVRGYHWNITGPQFFELHAKFEETYNDLLT

KVDELAERILTLGSQPRHAFSDYLKTADIKEHTNVTDDKGTLRGLLEGYSILLQQQRELLTVAADAGD
EGTASLMSDYIKEQEKQVWMLNAYLGK
>CORE_REP|Org42_Gene1427#
MIISLIAALAADRVIGMENAMPWHLPADLAWFKRNTLNKPVIMGRKTFESIGRPLPGRHNIVLSSRPG
NAAGVTWVTSLDEALAAAGEVEEVMVIGGGRIYTQLLPRADRLYLTHIDAEVGGDTHFPDYEPDEWET
TFSEFHDADDLNSHSYCFEILQRR
>CORE_REP|Org41_Gene2219#
MAFRLYSNDLQDGGKLPQAQVFNGMGYHGDNLSPHLAWDGVPAGTKSFVIAVYDPDAPTGSGWWHWIV
ANIPADVRELPQGAGSGKAPLPAGALQTRTDFGSAGYGGAAPPEGESHRYQFTVHALDVERIEVDEGS
SGALVGFNVHFHSLGSATLTVTFN
>CORE_REP|Org21_Gene277#
MKGDKKIIAHLNKLLGNELVAINQYFLHARMFKNWGLMRLNDKEYHESIDEMKHADRYIERILFLEGI
PNLQDLGKLNIGEDIEEMLRSDLALELAGAKNLREGIAYADSIHDYVSRDLMIDILADEEEHIDWLET
ELDLIARLGIQNYAQAQILERKE
>CORE_REP|Org15_Gene160#
MSEQNSTEMAFQIQRIYTKDISFEAPNAPQVFQQEWQPEVKLDLDTASSQLADEVYEVVLRVTVTATL
GEETAFLCEVQQAGIFSVAGIEGTQLAHCLGAYCPNILFPYARECITSLVSRGTFPQLNLAPVNFDAL
FMNYLQQQAEGEGAAPHQDA
>CORE_REP|Org43_Gene866#
MRNPAKQEDLIKTFKALLKEEKFSSQGEIVLALQEEGFENINQSKVSRMLTKFGAVRTRNAKMEMVYC
LPAELGVPTTTSPLKNLVLDVDHNDAVVVIHTSPGAAQLIARLLDSLGKSQGILGTIAGDDTIFVTPS
SGFTAQKLHEAILGVFEQEL
>CORE_REP|Org28_Gene1706#
MSPLKAGDTAPKFSLPDQDGEEINLADFQGQRVLVYFYPKAMTPGCTVQACGLRDNMDELKKVGVEVL
GISTDKPEKLSRFAEKELLNFTLLSDEDHQVAQQFGVWGEKTFMGKTYDGIHRISFLIDGKGKIEKVF
DDFKTTNHHDIVLSYLQQ
>CORE_REP|Org42_Gene1090#
MDKIDRKILAELQADGRLSVTELAERIGLSVSPCHRRVRALEESGVIRGYRAQLDPGSLGYNFSALVF
VTMREGDRRAVETFENAMMDIPQVVQAQRLFGDPDYLLHVIARDLPAFQQLYDEKLSALPGVQRLSST
LVMKTVVPERSFLPLGK
>CORE_REP|Org8_Gene1413#
MELTTRTIAARKHIALVAHDHRKQALLEWVESHKTILAQHQLYATGTTGNLIQRASGIPVTSMLSGPM
GGDQQVGALIAEGKIDMLIFFWDPLNAVPHDPDVKALLRLATVWNIPVATNRSTADFLIDSPLFKNEV
EIAIPDYQRYLQDRLK
>CORE_REP|Org20_Gene2998#
MIQLTVNEQPLTFEGDPHMPLLWFLRDEAGLTGTKFGCGIAMCGACTVHLDGVPVRSCMTPISAAVGK
KITTIEAVGATPEGKAVQEAWLDLDVVQCGYCQSGQIMSASALLAQSKNPSDADIDAAMGGNVCRCAT
YVRIRAAIHQAAKALG
>CORE_REP|Org26_Gene1411#
MSEKYVVTWDMLQMHARKLAHRLLPADKWTGIIAVSRGGLVPAALLARELGIRHVDTVCISSYDHDNQ
REMKVLKRAEGDGEGFIVVDDLVDTGGTAKAIRDMYPKAHFVTIFAKPAGRPLVDDYVVDIPQDTWIE
QPWDMGVSFVPPIGGR
>CORE_REP|Org45_Gene116#
MKYQQLENLESGWKWKYLVKKHREGELITRYIENSAAQEAVNELLKLENEPVKVLAWIAAHMNPELDN
RMKQTIRARRKRHFNAEHQHTRKKSIDLEFLVWQRLAALARRRGVTLSETVVQLIEDAERKEKYANQM
SSLKEDLKAILGKDPK
>CORE_REP|Org8_Gene50#
MRTVLNILNFVLGGFFTTLGWLIATVFSVLLVITLPLTRSCWEITKLSLVPFGNEAIHVDELYPEKSN
ALLSAGGSLLNIIWLVLFGWWLCLSHIAAGIVQCVSIIGIPVGIANFKIAAIALWPVGRRVVSVEMAQ
QARIENARRHYHQR
>CORE_REP|Org12_Gene2908#
MHESLTIALLQARETAMGFFRPILKSHNLTEQQWRIIRVLANSRSIEFHELAAETCILRPSLTGILSR
MERDKLIFRLKPVNDQRKLYVSLTQQGQDLYEVARHQVEQGYAEIEAAFSRQKMDQLMTLLDELITLG
DSLPANVAAHPAKQ

>CORE_REP|Org7_Gene4762#
MKTVKRTGIALAIALTFPLALPAATAAQTSLTNSKAATMTEKHGQFIAVGKVVQVTFGDFAFKLDFTD
DKTMTFTGIGEASQGITDTVQYTAVEIRPKVYMVYWHEPQSGDNVTHIEDFERGEVYTNIAAKDGSFT
HLKGQLKIVGHSGN
>CORE_REP|Org44_Gene2158#
MNTPEQRQQIADFIGKQHVLTLCAGDGLDMWCANCFYVFDAAAMALWLMTEPHTRHGGLMLNNGRVVG
TIAPKPKSIALIRGVQYRAEAVLLSGEEADAARARYCKRFPIARAMKASVWRLDLHEVKMTDNTLGFG
KKLHWARSIL
>CORE_REP|Org39_Gene2286#
MIVNCDHDNLDAWLALRTALWPSSSPEDHRAEMREILASPHHTAFMARGLDGAFVGFAEVALRYDYVN
GCESSPVAFLEGIYTVERARRQGWAARLIAQVQEWAKQQGCSELASDTDIANLDSQRLHAALGFAETE
RVVFYRKTLG
>CORE_REP|Org35_Gene426#
MKTFTAKPETVKRDWYVVDADGKTLGRLATELARRLRGKHKAEYTPHVDTGDYIIVLNADKVAVTGNK
RTDKVYYHHTGHIGGIKQATFEEMIARRPERVIEIAVKGMLPKGPLGRAMFRKLKVYAGTEHNHAAQQ
PQVLDI
>CORE_REP|Org46_Gene462#
MAMRLNEDLDDSGELHEINVTPFIDVMLVLLIIFMVAAPLATVDIRVDLPASSAKPQPRPEKPVFLSV
KADKQLYVGDQPVNADQLTSVLDQRTQANKETTIFFQADKSVDYETLMSVMDTLRKAGYLKVGLVGME
GAAK
>CORE_REP|Org46_Gene2079#
MKKGVLLNSDVSAVIARLGHTDQLTLCDAGLPIPAATQRIDLALTQGVPTFMQVFAAVTQEMQVESAI
LAEEIVKQNPSLHEALLAELTALGQRQGNTISVRYISHQAFKAQTEHSRAVIRSGECSPYANLILCAG
VTF
>CORE_REP|Org46_Gene927#
MSMLKEFREFAMRGNVVDLAVGVIIGAAFGKIVSSFVADIIMPPLGLLIGGVDFKQFHLVLREAQGAV
PAVVMNYGSFIQTVFDFVIVAFAIFLAIKLMNKMRRKQEEAPAAPPAPTAEEKLLTEIRDLLSQQQQP
KL
>CORE_REP|Org40_Gene3060#
MQDQEIVELLQQVKTIALVGASDNPSRPSYGVMAYLLAQGYQVIPVSPKLAGQTLLGQPVYATLAAIP
QPVDMVDVFRNSEAAYGVAQEAIAIGAKALWLQIGVINDQAAELAQQAGLRVVMDRCPKIEIPRLGLE
R
>CORE_REP|Org18_Gene3421#
MLTGLNHLTLAVSDLDRSFDFYRHLLGFTPHARWQGGAYLSLGSLWLCLSLDERRTQQRERDYTHYAF
SIAPEHIEQASQRLRQAGVKEWKSNRSEGESLYFLDPDGHQLEIHAGDLASRLAACREKPYQGMVFY
>CORE_REP|Org29_Gene594#
MAKTSRSIMIAKGLQRVLNVGLLLLAAILVVFLVKETIHLAKVLFINSEESSSYLLIEGIVIYFLYFE
FIALIVKYFESGYHFPLRYFIYIGITAIIRLIIVDHKNPIDTLIYAAAILVLVVTLYLANTDRLKRE
>CORE_REP|Org34_Gene207#
MSEALKILNNIRTLRAQARECTLETLEEMLEKLEVVVNERREEDSQAQAEIEERTRKLQQYREMLIAD
GIDPNELLQTMAANKAAGKAKRAARPAKYQYKDENGELKTWTGQGRTPAVIKKAIEEQGKSLDDFLL
>CORE_REP|Org42_Gene757#
MLNDIEEIRFTARSEENLRGVHPDLVRVIRLALRYSLVPFSVSEGLRSMARQREMVRAGSSQTLRSRH
LTGHAVDVVAMPAGVVSWEWDYYAQIAVAVRRAARECGINVEWGGEWKTLKDGPHFQLAFRDYPA
>CORE_REP|Org22_Gene509#
MAENQYYGTGRRKSSAARVFIKPGNGNIVINQRSLEQYFGRETARMVVRQPLELVDMVGKLDLYITVK
GGGISGQAGAIRHGITRALMEYDETLRSELRKAGFVTRDARQVERKKVGLRKARRRPQFSKR
>CORE_REP|Org17_Gene4670#
MPHFYAECTDNIRRDADLPTLFAKVNEALAATGIFPLAGVRSRAIWLDTWQMADGKQDYAFVHMTLKI
GHGRSLESRQQVGEMLFALIKEHFAALMAQRYLALSFTMEELDPVLNYKQNNVHALFNKA
>CORE_REP|Org25_Gene3681#
MKRLLLDTHALLWWLIDDACLGVNAKRQIADPGNAVYVSAASIWEISIKQALGKLALPEDIFAIIEAE
DFLALPMDAFHCQQAGQLPPYHQDPFDRMLIAQAQAEGLTLISADTVFPQYGVRVADARR
>CORE_REP|Org38_Gene72#

MITGIQITKANDQALVNSFWLLDDEKAEARCVCAKANYAEDQVVAVSDLGQIEYREVPLEMQPTVRVE
GGQHLNVNVLRRETLEDAVKHPEKYPQLTIRVSGYAVRFNSLTPEQQRDVIARTFTESL
>CORE_REP|Org29_Gene104#
MSANTEAQGSGRGLEAAKWLIVAVLLVVAIVGNYYYRDLSLPLRALAVVLIIAVAGAVALMTTKGKAT
VAFAREARTEVRKVIWPTRQETLHTTLIVAAVTAVMSLILWGLDGILVRLVSFITGLRF
>CORE_REP|Org7_Gene1020#
MKGTLTRAALAAGGMMVTSAVMAGSLALPTAQSLAGQWQVADSERQCQIEFLANEQSETNGYQLVDRQ
RCLQSVFAAEVVGWRPAPDGIALLQADGSTLAFFSRDGDLYRNQLGAGDALTLKALA
>CORE_REP|Org39_Gene624#
MANQATGLTRIIKAAGYSYKGLSAAWQHEAAFRQELVVTLLAIILAVWLDVGAIARILLIGSVALVMI
VEILNSAIEAVVDRIGSEHHELSGRAKDMGSAAVSLAIVLALFVWGTVLWQHFG
>CORE_REP|Org27_Gene2609#
MAIGHYELKKAKNGQYHFNLKASNGESILASEMYASKASAENGIASVQTNSPHEAQYELKHSTSNQPY
FVLKAKNHQVIGVSEMYSSESAAKNGIQSVMKNGPTTDIRDLSA
>CORE_REP|Org1_Gene282#
MYEALLVIFLLISIGLVALIMLQQGKGADMGASFGAGASGTLFGSSGSGNFMTRMTAVLATLFFVISL
ILGNLSSNQSKKGSEWENLGQPVKTEQTTAPAAPAKPSSDIPQ
>CORE_REP|Org4_Gene216#
MLEFEGQVIDTDAQGYLKNSADWHEGLAPLLAAQEEIVLTEAHWEVVRFVRDFYQEFNTSPAIRMLVK
AMAQKYGEEKGNSRYLYRLFPKGPAKQATKIAGLPKPVKCI
>CORE_REP|Org8_Gene475#
MSDKIIHLTDSSFEADVLKAEGPILVDFWAEWCGPCKMIAPILDEIAEEFEGKLTITKLNIDQNPATA
PKYGIRGIPTLLLFKNGEVAATKVGALSKGQLKDFLNANL
>CORE_REP|Org17_Gene43#
MIASKFGIGQQVRHKLLGYLGVVIDIDPEYSLEQPKADEIAANDELRSAPWYHVVMEDEEGQPVHTYL
AEAQLDGEPQEAHPEQPSLDELAESIRHQLQAPRLRN
>CORE_REP|Org6_Gene614#
MAKGQSLQDPFLNALRRERVPVSIYLVNGIKLQGQIESFDQFVILLKNTVSQMVYKHAISTVVPSRPV
SHHSNNPSGGSSNYHHGNNPSAQQQPQQESDDAE
>CORE_REP|Org38_Gene144#
MMKKEITFTVVELCQRVEISEDELVEIVGLGVIVPLEPAQPRWEFDYPALSHLQRARRLRAELDLDWP
GIAMALTLLDRVDALQQENRQLRRQLARFLQTS
>CORE_REP|Org44_Gene239#
MALTKAEMSEHLFEKLGLSKRDAKDLVELFFEEVRRALENGEQVKLSGFGNFDLRDKNQRPGRNPKTG
EDIPITARRVVTFRPGQKLKSRVENASPKG
>CORE_REP|Org25_Gene2#
MFEQRVNSDVLTVSTVNSQDQVTQKPLRDSVKQALKNYFAQLNGQDVNDLYELVLAEVEQPLLDMVMQ
YTRGNQTRAALMMGINRGTLRKKLKKYGMN
>CORE_REP|Org4_Gene456#
MAKTACALHILVDNEKLANELLAKLKRGVSFDTLARKYSSCPSKRNGGSLGEFNKGAMVPAFDKAVFS
IPLLKPYGPVKTQFGYHIIKVLYRN
>CORE_REP|Org41_Gene2135#
MTQVCIAAYVYGVVQGVGFRYSTQRQAEALGVTGYARNLDDGSVQVVACGTQAQVDKLVAWLKQGGPR
SARVERVLVEPQGVVDYAGFGIRY
>CORE_REP|Org36_Gene505#
MNKSQLIDKIAAGADISKAAAGRALDAVIASVTDSLKAGDDVALVGFGSFTVRERSARTGRNPQTGKE
IKIAAAKVPAFRAGKALKDAVN
>CORE_REP|Org9_Gene258#
MSLSVEAKAQIVADFGRGTNDSGSTEVQVALLTAQINHLQGHFSEHKKDHHSRRGLLRMVSQRRKLLD
YLKRKDVARYTSLIERLGLRR
>CORE_REP|Org46_Gene543#
MKPGIHPDYRTVVFHDVSANAYFKVGSTIKTDRTIELDGESWPYVTLDVSSASHPYYTGKQKDYSKEG
STARFQQRFGRFIGNK
>CORE_REP|Org25_Gene2792#

MFVELIFDQRNVKGLPDAAEIIKAELTRRVHRVFPDAEVKVKPMQTNGLISDANKSDREKLNRLLEDM
FEESEQWLMSDIYG
>CORE_REP|Org19_Gene169#
MNRTKLVLGAVILGSTLLAGCSSNAKIDQLSSDVQTLNAKVDQLSNDVNAMRSDVQAAKDDAARANQR
LDNQAHAYKK
>CORE_REP|Org25_Gene2729#
MQLQPKHTYKIVGFSSEIAPAYRQKLLSLGMLPGSSFDVVRVAPLGDPIEIKTRRVSLVLRRKDLALL
QLDGQP
>CORE_REP|Org19_Gene8#
MAKEDNIEMQGTVLDTLPNTMFRVELENGHVVTAHISGKMRKNYIRILTGDKVTVELTPYDLSKGRIV
FRSR
>CORE_REP|Org48_Gene4259#
MTSGIMGLVKWFNEDKGFGFISPLDGSKDIFVHLSALNGDNFKTLFEGQKVEFAIHRGDKGPAAANVT
LCDK
>CORE_REP|Org2_Gene47#
MDHRLLEIVACPVCNGKLYFNKENQELVCKADGLAYPLRDGIPVLLENEARALSLDEKHA
>CORE_REP|Org41_Gene3684#
MKKIIFASVLGLSGNAMAAETVNITLLGTSDLHGTFVPWDYASDTENLAGSLSQIATQVKKVRAEQPN
LILVDAGDTIQGNFVETFKHEAVSPMMLGLNALNYDVWVMGNHEFDFGLPVLATPLKQFKGAALAGNI
VWDNGKPYLPAYTIVERQGVKIGIIGMDTPMTAEFAKGTDRIKGLNFTDPVQAVKQVIRQIDGQVDAI
VLVAHMGIDNENQRPGTGVADIANANPELAAIVAGHMHVKIDKAVVNGVIITEPDKYGRALSRIDLQF
ERRDGKFTLIDKNSYTYSIKGMTPDSAMQALYQPYHDILRANANRVVAKLSGSDLVPTDEFRGIPQVH
VQDTGISALFQQAARHYAPLAQVIALQIDNDRAKLDVGDIKAKDIAFNYQYAGGEITVYQLNGKALKR
YMEWSAGYFNQLQPGDVTYSFNPARRSSKYSTNDFFDGVTYTIDLRQPAGSRIVDLRLADGTPVTDDM
PIRLGMNSYRMGHLTQKGGALEGQSFPVLFDSKAQYGEEEGTIRHLTLRYLTEVKHGHYQGVPPQRWK
LIGMEGYEPQRAIVKQLLNEGVIQVPTTDDGRYTNVASINVKDALFSNADDYRATLTSLEQQRQAATD
PVQQRRLQDRIALIKALNDF
>CORE_REP|Org30_Gene2089#
MRFSLKTTACALAVSLTLLSGAASAWEKDKTYAITILHTNDHHGHFWQNDHGEYGLGAQKTLVDGIRQ
EVAAQGGSLLLLSGGDINTGVPESDLQDAEPDFRGMNLVGYDAMAIGNHEFDNPLSVLRQQEKWATFP
LLSANIYQKSTGQRLFKPYALFDKQGIKIAVIGLTTDDTAKIGNPEYFTDMEFRVPAQEAKQVVEQLR
KDEKPDVIIAATHMGHYDNGEHGSNAPGDVEMARSLPAGYLDMIVGGHSQDPVCMAGDNRKQADYVPG
TPCSPDRQNGTWIVQAHEWGKYVGRADFEFRNGELKLVHYQLIPVNLKKKVEKADGTSERVYYTQQIA
EDPTMMKLLTPFQEKGKAQLGVKIGSVNGKLEGDRSKVRFVQTNLARVMLAAQRERADADFAVMSGGG
VRDSIESGDITYKNVLKVQPFGNTLVHVDMKGSEVEQYLAVVANMKPDSGAYAQFANVSLVADGKGVS
EVKINGQPLQADKTYRMATLNFNALGGDGYPKLDGLPSYVNTGFIDAEVLKQYIEKHSPLDAAAYEPK
GEIVYR
>CORE_REP|Org29_Gene2094#
MKQNHPVVLVRKRKSHHAAHHGGSWKIAYADFMTAMMAFFLVMWLLAIASPQELTQIAEYFRTPLKVA
LTSGDKSSSESSPIPGGGDDPTQQHGLVRKQVDSPDKRAEELRLNKLREKLDELIESDPRLKALRPHL
LINMMDEGLRIQIIDSQNRPMFKTGSAQVESYMRDILRAIAPILNDLPNKISLSGHTDDIPYATGERG
YSNWELSADRANASRRELIAGGLAEGKVLRVVGMAATMSLKQHGADDAINRRITVLVLNKQTQEGIEH
ENAESNAMDIAQPDSLKQLAPSATAPASQTPESQAVTPTDQATLPPATDPVAQSQASPAIAPAGQAPE
QPVAAPAPTNRDSQPEVTP
>CORE_REP|Org30_Gene945#
MSKIRVLCVDDSALMRQLMTEIVNGHADMEMVATAPDPLVARDLIKKFNPQVLTLDVEMPRMDGLDFL
EKLMRLRPMPVVMVSSLTGKGSEITLRALELGAVDFVTKPQLGIREGMLAYSELIAEKIRTAARARLP
QRSNSPAPAILSHAPLLSSEKLIAIGASTGGTEAIRQVLQPLPATSPALLITQHMPPGFTRSFAERLN
KLCQITVKEAEDGERVLPGHAYIAPGDRHLELARSGANYQVKLHDGPAVNRHRPSVDVLFRSVAQYAG
RNAVGVILTGMGNDGAAGMLEMHRAGAYTLAQNEASCVVFGMPREAIAGGGVSEVVELDRMSQRMLAQ
IAGGQALRI
>CORE_REP|Org10_Gene328#
MRDIPMPASDAATAGEIISRIGQLTRMLRDSMRELGLDQAIAQAAEAIPDARDRLDYVVTMTAQAAER
ALNCVEAAQPRQAELESGANALKGRWDEWFANPIELDDARSLVNDTRQYLDQVPGHTAFTNAQLLEIM

MAQDFQDLTGQVIKRMMDVVQEIEKQLLMVLMENMPEQPVKEKRPNDSLLNGPQLDQNGVGVIANQAQ
VDDLLDSLGF
>CORE_REP|Org11_Gene1581#
MAGLAAVSKLAGETVGQEFLIFTLGNEEYGIDILKVQEIRGYDQVTRIANTPAFIKGVTNLRGVIVPI
IDLRVKFSQQSVSYDENTVVIVLNFGQRVVGIVVDGVSDVLSLTAEQIRPAPEFAVTLATEYLTGLGS
LGERMLILVDIEKLLSSEEMSLVDSVAKSV
