# Supplementary material for: A Systematic Immuno-Informatic Approach to Design a Multiepitope-Based Vaccine Against Emerging Multiple Drug Resistant Serratia marcescens
Source: Front Immunol. 2022 Mar 14;13:768569. doi: 10.3389/fimmu.2022.768569 (PMC8967166; doi:10.3389/fimmu.2022.768569)
Supplement: Supplementary Data Sheet S5 — Resistance protein sequences. [file DataSheet_5.pdf]

>CORE_REP|Org10_Gene3917#
MTFPRFFIKRPIFAIVLSILTLIAGIVALFQLPLSEYPAVTPPTVQVTASYPGANPNVIAETVAAPLE
QAITGVEGMLYMSSQAATDGRMTLTVTFAQGTNADMAQIQVQNRVARALPRLPAEVQHQGVVTQKTSP
DILMVVHLLSPDQRYDPLYISNYAYLQVRDELSRIPGVSDVQVWGAGEYSMRLWLDPDLIAARGLTAG
DVIAAVREQNVQVAAGSVGQAPDTNAAFQVTVNTLGRLADEKQFGDIIIRTGSDGQVTRLRDVARIDM
GADAYALRSLLDGEPAVALQIIQSPGANALDVAQAVRATVKRLEGDFPAGLSSRIAYDPTVFVRASLE
SVVTTLLEAILLVVIVVVVFLRSWRASLIPLLAVPVSLVGTFAIMHLMGFSLNTLSLFGLVLSIGIVV
DDAIVVVENVERHIENGKTPQQAARLAMDEVTGPIVAITSVLAAVFIPTAFLSGLQGEFYRQFALTIA
ISTLLSALNSLTLSPALAGLLLRPHPAEHRAPGRIQRILQAAVRPFQRAPDAYANAVRKTVRVSGVAL
AIYGGLLVLTFFGFQAVPPGFVPMQDKYYLVGIAQLPNSASLDRTDAVVKQMSKIALAEPGVESVVAF
PGLSINGFVNVPNAAVMFVMLDPFKERATPDLAASAIAGRLQAKFADIPDGFLGVFPPPPVPGLGATG
GFKMQVEDRGGVGLESLVEHTRLLMVKATESGQVAGLMTSLDINAPQLDVVIDRTQAKSQGVSLADVF
ESLQIYLGSLYINDFNRFGRTYKVTAQADADHRMQAEAIGRLQVRNAAGDMLPLSSFVTVTPGSGPDR
IIRYNGYPSADISGGAAIGVSSGQAVALMEQLAKETLPEGMTVEWTDLTYQQKLAGNAALFIFPLCVL
LAYLILATQYNSWLLPLAVLLIVPMCLLSAMIGVWLLGGDNNVFVQIGLIVLVGLAAKNAILIVEFAR
GLEDEGANTLEAVIKACRLRLRPIVMTSIAFIAGVIPLIFASGAGAEMRHAMGVAVFAGMLGVTLFGL
FLTPVFYVVIRGLTARFEQYRTKGKANVRSESDEKSS
>CORE_REP|Org40_Gene1002#
MAKFFIDRPIFAWVIAIIVMLAGVLAIMKLPIAQYPTIAPPAVSISANYPGADAKTVQDTVTQIIEQN
MNGIDNLMYMSSTSDSSGSVTITLTFDSGTDPDIAQVQVQNKLSLATPLLPQEVQQQGLKVEKSSSSF
LMVAGFVSDDPNMTQDDIADYVASNIKDPISRSSGVGEVQLFGAQYAMRIWLDPNKLNNFQLTTTDVT
SAITEQNNQIAAGQLGGLPPVPGQQLNASIIAQTRLTSPEEFGKILLKVNTDGSQVRLRDVAHIERGA
ESYAVTARYNGKPAAGLGIKLATGANALNTAKGVKDELAKMAPFFPQGMKVVYPYDTTPFVKISINEV
VKTLIEAIILVFLVMYLFLQNFRATLIPTIAVPVVLLGTFAILAAFGFSINTLTMFGMVLAIGLLVDD
AIVVVENVERVMSEEGLPPKEATRKSMGQIQGALVGIAMVLSAVFVPMAFFGGSTGAIYRQFSITIVS
AMALSVLVALILTPALCATLLKPIPKGDHGVKTGFFGWFNRMFEKSTHHYTDSVGNILRSTGRYLIIY
LLIVVGMGLLFLRLPSSFLPDEDQGILLTMVQLPAGATESRTNKVLEEVSDYFLNKEKDNVVSVFTVA
GFGFNGNGQNNGLAFVSLKDWGERPGAGNKVEAIAGRAMGAFSQIKEGLVFPFNLPAIIELGTATGFD
FELIDQGGLGHEKLTEARNQLLGMVAQHPDVLVGVRPNGLEDTPQFKLIVDQEKAKALGVSITTINST
LSTALGGSYVNDFIDRGRVKKVYVQADAPFRMLPEDINKWYVRGTSGQMVPFSAFSSAKWEYGSPRLE
RYNGLPSMEILGQAAPGKSTGEAMNLMEQLASKLPSGIGYDWTGMSYQERLSGNQAPALYAISILVVF
LCLAALYESWSVPFSVMLVLPLGVIGALLAATMRGMNNDVYFQVGLLTTIGLSAKNAILIVEFAKDLM
EKEGKGLIEATLEAVRMRLRPILMTSLAFILGVLPLVISSGAGSGAQNAVGTGVMGGMITATVLAIFF
VPVFFVVVRRRFSKKNEDLEHSHPVEHH
>CORE_REP|Org28_Gene4189#
MQVMPPNAGGGPSRLFILRPVATTLLMVAILLAGIIGYRALPVSALPEVDYPTIQVVTLYPGASPDVV
TSAITAPLERQFGQMSGLKQMASQSSGGASVVTLQFQLALPLDVAEQEVQAAINAATNLLPSDLPYPP
IYSKVNPADPPILTLAVTSTAMPMTQVEDMVETRVAQKISQVTGVGLVTLAGGQRPAVRVKLNAAAVA
AYGLNSETIRAAISNANVNSAKGSLDGPTRSVTLSANDQMKSADDYRQLIVAYQNGAAIRLQDIATIE
QGAENTRLAAWANKQQAIVLNIQRQPGVNVITTADSIREMLPTLIKSLPKSVDVKVLTDRTTTIRASV
SDVQFELLLAIALVVMVIYVFLRNVPATIIPSVAVPLSLVGTFAAMYFLGFSINNLTLMALTIATGFV
VDDAIVVIENISRYIEKGEKPLDAALKGAGEIGFTIISLTFSLVAVLIPLLFMGDIVGRLFREFAVTL
AVAILISAVVSLTLTPMMCARMLSHESLRKQNRFSAASERFFDRVIAQYGQWLKTVLNHPWLTLGVAV
GTLALTVLLYLLIPKGFFPVQDNGIIQGTLEAPQSVSFSNMAERQQQVAAQILKDPAVESLTSFVGVD
GSNATLNSGRLQINLKPLSERSDRIPAIISRLQQQTAQFPGVKLYLQPVQDLTIDTQVSRTQYQFTLQ
AMSLDDLSLWVPQLMNELKQTPQLADVTSDWQDQGLVAYVNVDRDSASRLGVTMSDVDNALYNAFGQR
LISTIYTQANQYRVVLEHDVSATPGLAALNEIRLSGNDGAVVPLSAIAKIEERFGPLSVNHLDQFPSA
TVSFNVADGYSLGEAVDAVTQAEKNLNMPRDITTQFQGATLAFQAALGSTLWLILAAVVAMYIVLGVL
YESFIHPVTILSTLPTAGVGALLALMLAGSELDVIAIIGIILLIGIVKKNAIMMIDFALAAEREQGLS
ARDAIYQACLLRFRPILMTTLAALLGALPLMLSTGVGAELRHPLGVCMVGGLIMSQILTLFTTPVIYL
LFDKLARNTHRQPDTQELP
>CORE_REP|Org10_Gene3237#
MEKTNSQLDTAYDPKQIEQKLYDHWENQGYFKPNGDTSQESFCIMIPPPNVTGSLHMGHAFQQTIMDT
MIRYQRMQGKNTLWQAGTDHAGIATQMVVERKIAAEEGKTRHDYGRDAFIDKIWQWKAESGGTITRQM

```
RRLGNSVDWERERFTMDEGLSNAVREVFVRLHKEDLIYRGKRLVNWDPKLRTAISDLEVENRESKGSM
WHLRYPLADGAKTAEGKDYLVVATTRPETVLGDTGVAVNPEDPRYKDLIGKEIILPLVGRRIRIVGDE
HADMEKGTGCVKITPAHDFNDYEVGKRHGLPMINILTFDGDIRQEAEVFNTLGEVCTDYCNEIPAEFR
GLERFAARKAVVAAFDQLGLLDEVKPHDLTVPYGDRGGVVIEPMLTDQWYVRTAPLAKVAVEAVEQGD
IQFVPKQYENMYFSWMRDIQDWCISRQLWWGHRIPAWYDVNGKVYVGRSEEEVRSENNLGADVVLTQD
EDVLDTWFSSGLWTFSTLGWPEQTEALKTFHPTSVMVSGFDIIFFWIARMIMLTMHFIKDENGKPQVP
FKTVYMTGLIRDDEGQKMSKSKGNVIDPLDMVDGISLEDLLEKRTGNMMQPQLAEKIRKRTEKQFPNG
IEPHGTDALRFTLAALASTGRDINWDMKRLEGYRNFCNKLWNASRFVLMNTEAHDCGFNGGEKVLSLA
DRWILAEFNRTVKAYREALDTYRFDLAANILYEFTWNQFCDWYLELTKPVVSNGSEAEQRGTRHTLIT
VLEALLRLAHPIIPFITETIWQRVKPLTGTTADTIMLQPFPAYDAALEDEQALNDLEWIKQTIIAVRN
IRAEMNIAPSKALDVLLRNCSADAQRRVQENQSFIARLARLESIALLPAGEKGPVSVTKLVDGAELLI
PMAGFIDKDAEIARLAKEMGKLDAEIASIEGKLANEGFVARAPEAVVAKERDRLAACKEGKVKLQEQQ
ATIAAL
>CORE_REP|Org14_Gene4616#
MKYLASFRTTLKISRYLFRVLAILLWSLGALLTTFYILNILHQKESDIRQEYNLNFDQAQGYIRHSAD
IIRDIKYMAENRLNGSVSGLDMFSGVIPGKGSPPQFFPLYPESNCALSTTYRSSLDSLSGLIQYWKEN
FVAAYDLNRVFFIGGDSLCMAEFGGGNASANRENMLKLLHERILKYRNAKNLDKDNNLYWISPSAQRP
DVGYLYVLTPLYIGNKLEALLGIEQTVRLEDFVTAGNLPIGVTLLDENNEPVLRLADGERYAAALNSY
PEEHAYFGYVDNYRDLILKKALPPSSLSIVYALPVKSVVERFKMLILNALLLNLLSAIVLFTLAWLFE
RKMFLPAEDNAFRLEEHEQFNRKIVASAPVGICILRISDGTNILSNELAHNYINLLTHEDRDRITRII
CEQQANFVDVMTSNNNNLQISFVHSRYRNEEVAICVLVDVSARVKMEESLQEMAAAAEQASQSKSMFL
ATVSHELRTPLYGIIGNLDLLQTKALPQGVDRLVNAMNNSSGLLLKIISDILDFSKIESEQLKIEPRE
FSCLEVITHIAGNYLPLVVKKRLGLYCFIEQNVPERIFGDPVRLQQVLSNLVNNAIKFTDTGCIVLQV
CTRGSYLEFSVRDTGVGIPEKEISRLFDPFFQVGTGVQRHFQGTGLGLAICEKLVNLMDGDVSVESEP
GLGSLFSIRIPLFNAQFPIPQASDTWQGRRLWLDIRNQRLESYLMAILGGYGADIQRYDGQETAAGEV
LLSDHPLMLDAPLLAQIQFSTEHIGPSQETRPGYWMHSTSTPRETLTLLNRLFGVGVGSGAAEALVQL
PVPAKASAADNGDIHLLVVDDHPINRRLLSDQLGSLGYQVVTANDGVDAPGVLKQHRVDIVLTDVNMP
NMDGYRLTQALRQMQFSAPVIGVTANALAEEKQRCLEAGMDNCLSKPVTLETLEQTLAYYSQQVRYSR
SEA
>CORE_REP|Org7_Gene4479#
MDNIEVRGARTHNLKNINLIIPRDKLIVVTGLSGSGKSSLAFDTLYAEGQRRYVESLSAYARQFLSLM
EKPDVDHIEGLSPAISIEQKSTSHNPRSTVGTITEIHDYLRLLFARVGEPRCPDHHVPLAAQTVSQMV
DNVLSQPEGKRLMLLAPVVKDRKGEHTKTLENLSAQGYIRARIDGEVCDLSDPPKLELQKKHTIEVVV
DRFKVRDDMAQRLAESFETALELSGGTAVVADMDDEKADELLFSANFACPICGYSMRELEPRLFSFNN
PAGACPTCDGLGVQQFFDPDRVVQNPELSLAGGAIRGWDRRNFYYFQMLRSLAEHYEFDVEAPFNTLS
ANVQKAVLSGSGKESIEFKYINDRGDTTVRRHPFEGVLHNMERRYKETESSAVREELAKFISNRPCAS
CHGTRLREEARNVFVEDTTLPEISDLSIGHAMTFFQNMKLSGQRAKIAEKVLKEIGDRLKFLVNVGLN
YLSLSRSAETLSGGEAQRIRLASQIGAGLVGVMYVLDEPSIGLHQRDNERLLETLIHLRNLGNTVIVV
EHDEDAIRAADHVIDIGPGAGVHGGQVVAEGTVDDIMAQPESLTGQFLSGKREIAIPAQRVQADPTKV
LKLSGARGNNLKDVTLTLPVGLFTCITGVSGSGKSTLINDTLFPIAQRQLNGATIAEPAPFREVTGLE
HFDKVIDIDQSPIGRTPRSNPATYTGIFTPVRELFAGVPESRSRGYTPGRFSFNVKGGRCEACQGDGV
IKVEMHFLPDIYVPCDQCKGKRYNRETLEVKYKGKSIHEVLEMTIEEARDFFDAVPALARKLQTLMDV
GLSYIRLGQSATTLSGGEAQRVKLARELSKRGTGQTLYILDEPTTGLHFADIQQLLAVLHQLRDQGNT
IVVIEHNLDVIKTADWIVDLGPEGGSGGGEILVAGTPETVAECEKSHTARFLKPLLEK
>CORE_REP|Org44_Gene2039#
MSDYKNTLNLPETGFPMRGDLAKREPGMLQRWYEQDLYGIIRTAKKGKKTFILHDGPPYANGSIHIGH
SVNKILKDIIIKSKGMAGFDSPYVPGWDCHGLPIELKVEQLYGKPGEKLTAAEFRQKCREYAAEQVEG
QKKDFIRLGVLGDWDRPYLTMDFKTEANIIRALGKIISNGHLLKGAKPVHWCTDCGSSLAEAEVEYYD
KTSPSIDVTFHAADAAAVAAKFGVSNFSGAISLVIWTTTPWTLPANRAISLHPDFTYQLVQVDGQCLI
LAAELVESVMKRAGITEWTVLGSCKGADLELLRFKHPFMGFDVPAIMGEHVTLDAGTGAVHTAGGHGP
DDFVISQKYGLEIANPVGPNGCYLTGTHPLLDGKFVFKANDLIVDLLREKGALLHVEKFLHSYPCCWR
HKTPIIFRATPQWFISMDQKGLRQQSLEEIKGVQWIPDWGQARIEMMVANRPDWCISRQRTWGVPMSL
FVHKETEQLHPRSVELMEEVAKRVEQDGIQAWWDLDAADILGADAADYVKVPDTLDVWFDSGSTHASV
VDVRPEFHGHSADMYLEGSDQHRGWFMSSLMISTAMKGKAPYKEVLTHGFTVDGQGRKMSKSIGNTVS
```

PQDVMNKLGGDILRLWVASTDYTGEIAVSDEILKRSADSYRRIRNTARFLLANLNGFEPSTDCVAPED
MVVLDRWAVGRALAAQQDIEQAYANYDFHEVVQRLMQFCSVEMGSFYLDIIKDRQYTAKSDSVARRSC
QTALYHIVEALVRWMAPIMSFTADEIWGFMPGKRAQYVFTEEWYDGLFGLAEGEPMNDAFWAELLKVR
GEVNKVLEQARADKRLGGSLEAAVTLYADSELAARLNSLQDELRFVLLTSAASVAPLAEAPADAQASE
LLKGLKIAFSTAPGEKCPRCWHYTTDIGLVAEHADICGRCVSNVAGDGEKRNFA
>CORE_REP|Org2_Gene3277#
MTKYSLRARMMILILAPTLLIGLLLSTFFVVHRYNELQEQLVDAGASIIEPLAVASEYGMTFRSRESV
RQLVSLLHRRHSDIVRSITVFDAQNNLFVTSNYHHNFAQLQLPKGVPLPTELMLTRRGDSLILRTPIL
SESQYPDETADGGSHPDNNLGYVAIELDLQSVRLQQYKEVFVSTLLLLLCMCIAILFAYRLMRDVTGP
IRNMVNTVDRIRRGQLDSRVEGYMLGELHMLKNGINSMAMSLTAYHEEMQQNIDQATSDLRETLEQME
IQNVELDLAKKRAQEAARIKSEFLANMSHELRTPLNGVIGFTRQMLKTDLSATQTDYLQTIERSANNL
LTIINDVLDFSKLEAGKLVLEHIPFALRETLDEVVVLLAPSAHDKGLELTLDVHNDVPEQVIGDSLRL
QQIITNLLGNAIKFTETGNIDIRVELRKQLDRRVEVEVQIHDTGIGISERQQSQLFQAFRQADASISR
RHGGTGLGLVITQKLVKEMGGDICFHSQLNRGSTFWFHITLDLNEGMLSLAPSLPDLSGKTLAYIESN
PTAAQATLNMLSITQLVITHSPTLGQLPPGHYDFLLAGVPIPFRDNMAQHEDKLLASLKLADRVILAL
PCQAQIDAELLKQQGALGCLIKPITSTRLFPLLRMEAPARLTAQPERKRLPLTVMAVDDNPANLKLIG
TLLGEQVEKTLLCESGEEALALARDNVLDLILMDIQMPKMDGIHASELIRQLPHHNSTPIVAVTAHAA
SGEREHLLQAGMDDYLAKPIDEKMLTRVLSRYHSGDVENAIADDAPLSLDWPLALRQAANKPDLARDL
LQMLLDFLPQVRERVQALLDGQHDDEILDLVHKLHGSCSYSGVPRLKQLCFYLERQLRQGVTNDELEP
EWLELLDEIELVIHAARAHLTQPA
>CORE_REP|Org3_Gene2841#
MQEQYRPEDIESNVQLHWQEKQTFKVTEDDSKEKYYCLSMLPYPSGRLHMGHVRNYTIGDVISRYQRM
LGKNVLQPIGWDAFGLPAEGAAVKNNTAPAPWTYDNIEYMKNQLKLLGFGYDWDREIATCQPEYYRWE
QWFFTKLYEKGLVYKKTSAVNWCPHDLTVLANEQVIDGCCWRCDTKVERKEIPQWFIKITAYADQLLN
DLDTLESWPEQVKTMQRNWIGRSEGVEITFDVADSEEKLTVYTTRPDTFMGATYVAVAAGHPLAQQGA
RNNPALTDFIDECRNTKVAEAEMATMEKKGMPTGLFVVHPLSGEKLPVWVANFVLMEYGTGAVMAVPA
HDQRDWEFATKYDLPIKPVILNLDGSQPDVSAEAMTDKGALFKSGEFDGLDNEAGFNAIADKLVAKGV
GQRKVNYRLRDWGVSRQRYWGAPIPMVTLEDGTVMPTPEDQLPVILPEDVVMDGITSPIKADPEWAKT
TVDGQPALRETDTFDTFMESSWYYARYTCPQYDQGMLDPAAANYWLPVDQYIGGIEHAIMHLMYFRFF
HKLMRDAGLVDSDEPAKRLLCQGMVLADAFYYTGNSGERVWVSPVDATVERDDKGRIIKATDPQGREL
VYAGMSKMSKSKNNGIDPQEMVEKYGADTVRLFMMFASPAEMTLEWQESGVEGANRFLKRVWKLAYDH
VEKGAVQPLDVAALNEDQKALRRDLHKTIAKVTDDIGRRQTFNTAIAAVMELMNKLARAPQESEQDRA
LLQEALLAVVRMLYPFTPHVCFTLWQALGGEGDVDTAPWPVADEQAMVEDSKLVVVQVNGKVRAKITV
SADATEEQVRARAAEEHLVAKYLDGVTIRKVIYVPGKLLNLVVG
>CORE_REP|Org44_Gene2350#
MKFVKYFLILAVCCIVLGAASIFGLYKYVEPQLPDVATLKDVRLQIPMQVYSADGELIAQYGEKRRIP
LKLDQIPPVMVHAFIATEDSRFYDHHGVDPVGIFRAASIALVSGHASQGASTITQQLARNFFLSPERT
LMRKIKEAFLAVRIEQMLTKDEILELYLNKIYLGYRAYGVGAAAQVYFGKDVSQLTLSEMATIAGLPK
APSTFNPLYSHDRAVARRNVVLSRMLDEHYITQAQYDQARSEDLVANYHAPEISFSAPYLSEMVRQEM
IKRYGENAYTDGYKVYTTVTKRLQLAAQESVRNNVLAYDMRHGYRGPSNVLWKVGEAAWDRKQIVDSL
KNLPNYGPLAPAVITAANPQEATAMLADGSSIALPMATMRWARPYRSDTQQGPTPKRVTDVVQAGQQV
WVRKVNDAWWLSQVPDVNSALVSINPNDGAVKALVGGFDFNQSKFNRVTQALRQVGSNIKPFLYTAAM
DKGLTLATILNDLPITRWDAGAGTDWRPKNSPPTYDGPIRLRQGLGQSKNVVMVRAMRAMGVDYAAEY
LQRFGFPAQNIVHTESLALGSASFTPMQLVRGYAVLANGGYLVDPYFITKIEDDNGNTVFEAKPKVVC
SSCNLPVIYGDTHRSAVLSDDNIENVATSQEGNNSTVPMPQLEQVTPAQVQQDGDQQYAPHVISTQLA
FLIHDALNSNIFGEPGWMGTAWRAGRDLKRHDIGGKTGTTNSSKDAWFSGYGPDTVTSVWIGFDDHRR
DLGRSTVSGAIPDQISGGEGGAKSAQPAWDDFMKTALEGIPEQKVTPPPGIISVTIDKSSGKLSGGGG
GSRSEYFIEGTQPTDYPSRDTGTTLTDPGGESHELF
>CORE_REP|Org43_Gene425#
MKDNNLFERINEKLTFSLRKRVPSILQSESSECGLACLAMIASYYGFNVDMLSLRQRFGISTQGATLG
TISQIASQIQLKTRALSLDIDEINQLKTPCILHWNMNHFVVLVKVQRAGFVIHDPAFGRRVIGLQEMS
NHFTGIALELWPDRAFQKETLKTRLRLLDLMKNIEGLPGTLLKIFALSIVIESVNLLLPVGTQLVTDH
VIQAHDYSLLTVICLGLIFFTLFRAVVSIARAWISIVLGTLTDIQWKTTLFEHLMKLPLDFFEKRHLG
DIQSRFSSLDAIRTTFTNNIVSGIIDGIMTVGLFAMMMVYGGWLVWVVAGFTLIYILIRMMTYRTYRQ

FSEEQIVKAAKANSHFMETLYGISTVKALGIKETRSSYWLNLNVDAANTNIKITRFNMMFGGINTFIT
TLDQVAILWLGAMMVIDNSMTLGMFMAFNAYRGQFSQRASSLIDLAIGLRMLSLHNERISDIVFTDAE
TESAPRQVFPSGTGIAIEVKNLTYQYDALSRPIFKDLNMRIAAGESVAVVGASGAGKTTLLKVMCGLL
SPTSGQVLADAMDIHKVGVNNYRNAIACVLQDDRLFSGSIAENISGFEVNANKELIMACAIHSNIHDE
IMQMPMGYETLIGELGNGISGGQKQRLFIARALYRRPSVLFMDEATSHLDVENESAINRAISSLNITR
VIVAHRKSTIDSADRVVVLGAESGAPAGGGE
>CORE_REP|Org36_Gene4690#
MARTTPIARYRNIGISAHIDAGKTTTTERILFYTGVNHKIGEVHDGAATMDWMEQEQERGITITSAAT
TAFWSGMAKQFEPHRVNIIDTPGHVDFTIEVERSMRVLDGAVMVYCAVGGVQPQSETVWRQANKYKVP
RIAFVNKMDRMGANFLKVVGQIKSRLGANPVPLQLAIGAEDKFTGVIDLVKMKAINWNEEDAGVTFEY
EDVPADMMDLAEEWRQNLIESAAEASEELMEKYLGGEELTEAEIKSALRQRVLNNEIILVTCGSAFKN
KGVQAMLDAVIEYLPAPTDVPAINGILDDGKDTPAERHASDDEPFSALAFKIATDPFVGNLTFFRVYS
GVVNSGDTVLNSVKSARERFGRIVQMHANKREEIKEVRAGDIAAAIGLKDVTTGDTLCDPDSPIILER
MEFPEPVISIAVEPKTKADQEKMGLALGRLAKEDPSFRVWTDEESNQTIIAGMGELHLDIIVDRMKRE
FNVEANVGKPQVAYREAIRAKITDVEGKHAKQSGGRGQYGHVVIDMYPLEPGSNPKGYEFINDIKGGV
IPGEYIPAVDKGIQEQLKSGPLAGYPVVDMGIRLHFGSYHDVDSSELAFKLAASIAFKEGFKKAKPVL
LEPIMKVEVETPEENTGDVIGDLSRRRGMLRGQESEVTGVKIHAEVPLSEMFGYATQLRSLTKGRASY
TMEFLKYDDAPNNVAQAVIEARGK
>CORE_REP|Org34_Gene2953#
MAQVAKKLLVTCALPYANGSIHLGHMLEHIQADIWVRYQRMRGHEVHFICADDAHGTPIMLKAQQLGV
KPEEMIAEMSQEHQQDFAGFGISYDNYHSTHSDENRELSTLIYSRLKENGFIKNRTISQLYDPEKGMF
LPDRFVKGTCPKCKSPDQYGDNCEVCGATYSPTELIDPKSVVSGATPVMRDSEHFFFDLPAFSEMLQA
WTRSGALQEQVANKMQEWFESGLQQWDISRDAPYFGFEIPDAPGKYFYVWLDAPIGYMGSFKNLCDKR
GDLDFDEFWRKDATTELYHFIGKDIVYFHSLFWPAMLEGSNFRKPTNLFVHGYVTVNGAKMSKSRGTF
IKAGTYLQHLDADCLRYYYAAKLSSRIDDIDLNLEDFVQRVNADIVNKVVNLASRNAGFINKRFGGKL
ADSLADPALYQTFVDAAQSIAEAYASREFSRAIREIMALADLANRYVDEQAPWVVAKEEGRDADLQAI
CSMGINLFRVLMTYLKPVLPSLTERAEAFLNAELSWDAIPQPLLGHQVNAFKALFNRIDLDKVSEMVN
ASKEDMAAAKPVTGPLADDPIQETITFDDFAKVDMRIALIKSADFVEGSDKLLKLQLDLGGELRQIFS
GIRSAYPDPKALEGRLTIMVANLAPRKMRFGVSEGMVMAAGPGGKEIFLLSPDSGAQPGMQVK
>CORE_REP|Org17_Gene1483#
MAALLQLNGIRRSYRSGEQTVEVLKGISLSIDAGEMVAIMGASGSGKSTLMNILGCLDKPSAGVYRVA
GQDVATLSDDALAQLRREHFGFIFQRYHLLPHLSAAHNVEVPAVYAGLGKAARRERAEALLRRLGLGE
RVNYRPSQLSGGQQQRVSIARALMNGGQVILADEPTGALDSHSGEEVMAILKQLCAQGHTVILVTHDP
AVARQAERIIEIRDGEIIADSRPAPSEDAQAKPLTLAAAAPSWRQMGGRFREALVMAWRAMAANKMRT
ALTMLGIIIGIASVVSILVIGDAAKQMVLADIKSIGTNTVDIYPGKDFGDDDPTYRQSLKYGDLDALR
EQPYISALSPSISSSMRLRLGNVDAAANVNGVSEQFFRVYGMSFTQGVGIDPMQVQSQAQTVVIDANT
QRRLFPHQKNVVGEVILVGNMPATVVGVAKEKQSMFGSSKTLNVWVPYSTMANRLMGNSYFDSITVRI
RDGYDSKEAEQQLSRLLTLRHGKKDFFTYNMDSLVQTAEKTTRTLQLFLTLVAVISLVVGGIGVMNIM
LVSVTERTREIGIRMAVGARSGDVLQQFLIEAVLVCLVGGALGITLSFAIGLAVQLVLPGWQISFPPA
ALLSAFLCSTGIGVVFGYLPARNAARLNPIDALARE
>CORE_REP|Org31_Gene3983#
MALLKETIRDHSAEERLFIRRAGVALALVVVCFGALIVNLYRLQIRQHGFYQTRSNQNDIKMLPIAPS
RGLIFDRNGTPLVRNVTLYRIEITPSKISDMAALLQALTPIVDLTPEDISAFRDDMHHNSRYKPVTLK
AGLSDTEVARFAVNQYRFDGVTIDTYQQREYPYGAQLAHVLGYVSKINDSDLKRLDKAGLSENYAADR
NIGKQGIEAYYEAELHGTTGYQEVEVDNHGRVIRLLKEQPPKAGKNIYLTLDLPLQQYIESVLKGQRA
AVVVEDPRDGGILAMVSSPSYDPNPFVKGIGYQAYKALLTNPDLPLINRVTQGLYPPASTVKPYMAVS
ALFAGVITPTTTFFGAPTWTLPGTERRYRDWLKTGHGMLNVTKAIEESADTFFYQVAYEMGIDRIHHW
LSQFGYGQSTGIDLNEEYRGVLPSRDWKLKVHKKGWYQGDTVSVGIGQGYWVATPIQMVKALTTLINN
GQVKTPHLLYSLQQGNRVTRYPPPAKTAQIGDPNSPYWGIVRNGMYGMANLPNGTGYKLFHTAPYQIA
AKSGTSQVFSLKQNQTYNAKMIPVRLRDHIFYTLFAPYKNPRVAMALILENGGGNGVVAGPTARAILD
HIFDPANAPQPGDAGQSKPQLNDSADVQR
>CORE_REP|Org5_Gene3577#
MIVFSSLQIRRGIRVLLDNATATVNPGQKVGLVGKNGCGKSTLLSLLKGEIAADGGSFTFPGNWALAW
VNQETPALDVPAIEYVIDGDREFRQLEAELQAANDRNDGHAIATLHGKLDAIDAWTIRSRAASLLHGL

GFSNEQLQSPVRDFSGGWRMRLNLAQALVCRSDLLLLDEPTNHLDLDAVIWLERWLKSYPGTLVLISH
DRDFLDPIVDKILHIEQQTINEYTGNYSSFERQRATKLAQQQSLYQHQQEKVAHLQSYIDRFRAQATK
AKQAQSRIKMLERMELIAPAHVDNPFTFSFRPPESLPNPLLRMDKVSAGYGDKVILKSIKLNLVPGSR
IGLLGRNGAGKSTLIKLLAGTLEPLSGEIGLAKGIKLGYFAQHQLEFLRADESPLQHLSRIAPRVLEQ
QLRDYLGGFGFQGDKVSEVTERFSGGEKARLVLALIVWQRPNLLLLDEPTNHLDLDMRQALTEALIDF
EGALVVVSHDRHLLRSTTDDLYLVHDGQVEPFEGDLDDYQQWLVDLQRQESQQDAPEKESGGNSAQAR
KEQKRREAEFRTQTQPLRKQIAKLEQQMEKLGAELAAVEEQLADPALYDISRKAELTDCLQKQSQAKS
ALEETEMTWLDAQEQLEQLTQAFEA
>CORE_REP|Org2_Gene2907#
MSLISMSGAWLSFSDAPLLDNTEIHIEDNERVCLVGRNGAGKSTLLKILGKEIPLDDGRVIYEQDLIV
ARLQQDPPRNIGGSVFDFVAEGVAEQAEHLKAYHAISHLVESDPSEKNLARMAQIMEILDHQGLWQLD
SRISEVLLQLGLNGDAELSSLSGGWLRKAALGRALVSSPRVLLLDEPTNHLDIETIDWLEGFLKEFDG
SIVFISHDRSFIRNMATRIVDLDRGKLVSWPGNYDLYLQSKEEALRVEELQNAEFDRKLAQEEVWIRQ
GIKARRTRNEGRVRALKALRVERSERREVMGTAKMQVEEATRSGKIVFELEDVNYQVGEKVLVRGFSA
QVQRGDKIALVGPNGCGKTTLLKLMLGQLKADSGRVHCGTKLEVAYFDQHRADLDPERTVMDNLAEGK
QEVVVNGRPRHVLGYLQDFLFHPKRAMTPVKALSGGERNRLLLAKLFLKPSNLLILDEPTNDLDVETL
ELLEELIDGYQGTVLLVSHDRQFVDNSVTECWIFEGNGVINAFVGGYYDAHHQRATAKPIRQAAPSAS
KPAAEKKAEQPKKAAAKLSYNLLRELEQLPQRLEQLEAEIEALQAQMSDADFFTRPHSETQQVLTALA
NAEQALEQAFARWEELEAMKNG
>CORE_REP|Org20_Gene2054#
MTDVLLRPAASGGPSLPPQRVLTVRDLSISFPQPDGAVAAVRNLSFDLDRGETLAIVGESGSGKSVTS
LGLMRLVEQGGGRIVGGVMTLRRRDGALLDLAQASQSTLRTVRGADMAMIFQEPMTSLNPVFPVGEQI
AESLRLHQGMDRRSARQEALRMLDLVRIPEAKEVLGRYPHQLSGGMRQRVMIAMALSCKPALLIADEP
TTALDVTIQAQILQLIRVLQREMQMGVIFITHDMGVVAEIADRVLVMRRGEQVEQNRVRELFAAPQQA
YTRALLAAVPKLGAMADRPLPAKFPLPGGEGDTAPQDTVPPGAAPILQVEHLVTRFDLRGGLFNRVTR
RVHAVENVSFDLYPGETLGLVGESGCGKSTTGRSLLKLVDSQSGTITFAGRRIDQLKGPALQHLRRDI
QFIFQDPYASLDPRLTVGFSIMEPLLVHNVMRGREAEQRVAWLLERVGLLPEHARRYPHEFSGGQRQR
ICIARALALNPKVVIADEAVSALDVSIQAQIVNLLLDLQREFGVAFLFISHDMAVVERISHRVAVMYL
GQIVEIGPRQAVFDNPQHPYTRKLMAAVPVADPAHAHKRQPLPADEIPSPVRALGDEPVTAPLVQVGA
GHFVARHPIAGAF
>CORE_REP|Org30_Gene154#
MIENLRNIAIIAHVDHGKTTLVDKLLQQSGTFGERAEATERVMDSNDLEKERGITILAKNTAINWNGY
RINIVDTPGHADFGGEVERVMSMVDSVLLVVDAMDGPMPQTRFVTKKAFANGLKPIVVINKVDRPGAR
PDWVVDQVFDLFVNLDATDEQLDFPIIYASALNGIAGVDHTDMAEDMTPLYQAIVDHVSAPQVELEAP
FQMQISQLDYNNYLGVIGIGRIKRGKVKPNQQVTIIDSEGKTRNGKVGKVLGHLGLERIDSTLAEAGD
IIAITGLGELNISDTICDTNAVEALPALSVDEPTVTMFFNVNTSPFCGKEGKYVTSRQILDRLNKELV
HNVALRVEETDDADAFRVSGRGELHLSVLIENMRREGFELAVSRPKVIFREIDGRKQEPFENVTLDIE
EQHQGSVMQAMGERKADLKNMDPDGKGRVRLDYVIPSRGLIGFRNEFMTMTSGTGLLYSTFSHYDDVR
PGEVGQRQNGVLISNGQGKAVAFALFGLQDRGKLFLGHGAEVYEGQIIGIHSRSNDLTVNCLTGKKLT
NMRASGTDEATTLVPAIKMTLEQALEFIDDDELVEVTPTSIRIRKRHLTENDRKRASRGPKDA
>CORE_REP|Org9_Gene2216#
MKHIRNFSIIAHIDHGKSTLSDRIIQICGGLSDREMAAQVLDSMDLERERGITIKAQSVTLDYKALDG
QTYQLNFIDTPGHVDFSYEVSRSLAACEGALLVVDAGQGVEAQTLANCYTAIEMDLEVVPVLNKIDLP
AADPDRAAQEIEDIVGIDATDAVRCSAKTGVGVPDVLERLVRDIPPPQGDPDAPLQALIIDSWFDNYL
GVVSLVRVKNGTLRKGDKIKVMSTGQVYNADRLGIFTPKQVDRDVLNCGEVGWLVCAIKDILGAPVGD
TLTQARQPADKALPGFKKVKPQVYAGLFPISSDDYESFRDALGKLSLNDASLFYEPESSTALGFGFRC
GFLGLLHMEIIQERLEREYDLDLITTAPTVVYEVETTGKEVIYVDSPSKLPPLNNIQELREPIAECHM
LMPQEYLGNVITLCVEKRGVQTNMVYHGNQVALTYEIPMAEVVLDFFDRLKSTSRGYASLDYNFKRFQ
ASDMVRVDVLINNERVDALALITHRDNSQYRGRELVEKMKDLIPRQQFDIAIQAAIGTHIIARSTVKQ
LRKNVLAKCYGGDVSRKKKLLQKQKDGKKRMKQVGNVELPQEAFLAILHVGKDGK
>CORE_REP|Org8_Gene3621#
MKNAVRRGEVMSVLAAYRRGFWGIALFTAVINLLMLAPALYMLQVYDRVLPSGNRMTLAMLTLMVVGL
YLFMGLLEWVRSQVVIRLGAQMDMRLNQRVYDAAFETNLKTGNPLAGQALNDLTNLRQFATGNALFAF
FDAPWFPVYLLVVFLLHPWLGALASAGVIVLVLLAWLNQRVSQAPLAEAGRVALSATQQANGNLRNAE

AIAAMGMLTDLRLRWLRQHQQFLLLQNRASEKIAAVTAWSKTVRLALQSLMLGCGALLAVSGDITPGM
MIAGSILIGRVLGPIDQLIGAWKQWSSARQSLQRLEVMLAANPPRIPSLPLPTPGGALTVSQLTASAP
GGTAPVLHGVSFRLEAGEVLGVIGASGSGKTLLMRQLVGALTPISGDVRLDGADIQQWDKQQLGPHIG
YLPQDIQLFAGTLTDNIARFGQVDAEKVVVAAALAGVHQLILHLPKGYETELGEGGSGLSGGQRQRVA
LARALYGSPALVVLDEPNANLDREGEEALQRAIEALKARGTTIVLVTHKPAILATTDKLLVLTAGQVQ
HFGPSDAILKKLPGFAPAAAAAPANTGRSNGGFNVNYANFAKTASGERKV
>CORE_REP|Org36_Gene3179#
MRFFQNLQNDGISPRAQIRLLDNTFMRLVFSFTAVPFVGIPFAIWIYLLGDELGPTITWIIVYLLCAV
AIRIWHRRYLHEAKENDEDAVLRRWLPRINKVAFIHGLGISSLYLITPQTHNFDFFLLLNISIAAIVA
ANATHLTPVISTFTRFFFASWGLLNLGIICRLEDVMFIVLMLNLLYGFAIYRHALTSHAFFIQQALLE
EQSSRLAEQFRQAKEEAEQALLDKNQFLTTASHDLRQPVHAMGFLIEAIIHKNRDDSLTPQLLDLQQS
VRSVHLMFNSLLDLSKIESGNVRTAATHVDIGALLDSVITLFREEANSRALALRTWRPKRRISVMGDP
LLVRQSLINLIQNALRYTQQGGVLIAIRPRGAECLVEVWDTGVGIADEEKSKIFSPYYRPELAWKIDS
AGHGLGLAVVARCAKLMKVKYGMHSVEGKGSRFWMRFTQYIGEDKAPETAAAYDNTATPIRYAPLRGA
CLVVDDDPLVTSAWESLMSTWGITVRCAASAEEAFAIVDDGFTPFAVLCDQRLRSGESGFDILKALFE
RLPDVSGAMVSGEFNSQILQEAEQEGYLVLRKPLEPARLHALLTQWGAAS
>CORE_REP|Org5_Gene3887#
MNKVQKLWPTLKRLLAYGSPYRKPLGLAVLMLWIAAAAEVAGPILVSYFIDNYVAKGQLPLTIVGGLA
AAYILLELLAAALHYFQALLFNQAAVGVVQRLRTDVMDAALRQPLSAFDTQPVGQLISRVTNDTEVIK
DLYVMVVSTVLKSAALIGAMLVAMFSLDWRMALVAVCIFPAVFVVMGIYQYYSTPIVRRVRSYLADIN
DGFNEVINGMGVIQQFRQQVRFGERMSAASQSHYLARMQTLRLDGFLLRPLLSLFSALVLCGLLMLFG
FSGEGVIGVGVLYAFINYLGRLNEPLIELTSQQSILQQAVVAGERIFELMDRSQQSYGADDRPLAGGR
IDITDLSFAYRADKKVLQHISLAVPSRGFVALVGHTGSGKSTLANLLMGYYPVSEGEVRLDGRPISSL
SHRTLRQGVAMVQQDPVVIADSVLANVTLGRNIEEDAVWRALETVQLASLVRGFPQGIHTRLGEQGNN
LSVGQKQLLAMARVLVQAPQILILDEATANIDSGTEQAIQRALRAIREHTTLVVIAHRLSTIVDADSI
LVLHRGQAVEQGNHQQLLAQQGRYYQMYQLQLAGEQLAEAVREESQPA
>CORE_REP|Org19_Gene2614#
MRLFAQIGWYFRREWRRYLGAVVLLIVIAILQLLPPKLVGIIVDGVTEKQMSTGVLMAWLGLMIGTAI
VVYLLRYVWRVLLFGASYQLAVELRENFYRQLSRQNPAFYLRHRTGDLMARATNDVDRVVFAAGEGVL
TLVDSLVMGLVVLVVMSTQISWQLTVLALIPMPLMAIAIKYYGDQLHQRFKSAQAAFSSLNDQAQESM
TSIRMIKAFGLEDHQSNRFADVAAQTGAKNMHVARVDARFDPTIYIAIGASNLLAIGGGSWMVVNGSL
TLGQLTSFVMYLGLMIWPMLALAWMFNIVERGSAAYSRIRSLLDEAPAVQDGPQALPAGRGVLDVDIR
AFHYPENPHPALHDVALTLKPGQMLGLCGPTGAGKSTLLSLIQRQFDVDQGQIRYHGLPLPQVKLDDW
RSRLSVVSQTPFLFSDTVANNIALGHPGATQAQIEQAARLASVHEDILRLPQGYDTEVGERGVMLSGG
QKQRISIARALLLDAEILILDDALSAVDGRTEHQILHNLRSWGQDRTVIISAHRLSALTEAGEILVMQ
HGGVAQRGDHAALAAQPGWYRDMYRYQQLEAALDEAPENGEEALADE
>CORE_REP|Org2_Gene2928#
MKKTRQQQLTRWLKTQSSLAQRWLRLSMLLGLFSGLLIVAQAWLLASLLHALIIEHTPREQLIPSFIW
LAAAFALRALLSWLRERVGFRCGQVIRQRMRQQVLDKLQQLGPAWIQGKPAGSWASIIVEQIEDMQDY
YSRYLPQMYLAVFIPLLILIAVFPINWAAGIILLATAPLIPLFMVLVGMGAADANRRNFVALARLSGN
FLDRLRGLDTLRLFDRAQAETAQIAKSSEDFRSRTMEVLRMAFLSSGVLEFFASISIAVVAVYFGFSY
LGELNFGSYGLGVTLFSGFLVLILAPEFFQPLRDLGTFYHAKAQAVGAAEALETFLSAEGEQMGNGTR
QLAADQPLTLQANALEILSPNGVLLAGPLSFTLQPQQRVALVGLSGAGKSSLLNLLLGFLPYRGSLTV
NGVELRDLSAENWRQQLSWVGQNPHLPAQTLRANILLGCPQADEAQLQQAVEHAYVSELLPYLPQGLD
TEVGDNAARLSVGQAQRVAVARALIGPRRLLLLDEPAASLDAHSEQRVMQALNAASHQQTTLLVTHQL
EDTEDYDQIWVMDNGRIVQQGDYATLSAQPGLFATLIAHRRGEL
>CORE_REP|Org20_Gene235#
MKAARPGKLRRQEDQASFVSWRFALLCGCILLAMVGLMLRVAYLQVINPDRLVKEGDMRSLRVQEVPT
ARGMISDRAGRPLAVSVPVNAVWADPKELNERGGITLDSRWKALSDALNIPLDQLSNRINANPKGRFV
YLARQVNPAIGDYIHKLKLPGIYLRQESRRYYPAGQVTSHIIGVTNIDGQGIEGVEKSFDRWLTGQPG
ERTVRKDRFGRVIEDISSVDSQAAHNLVLSVDERLQALVYRELNNAVAFNKAESGTAVLIDVNTGEVL
AMANSPSYNPNNMAGTPKETMRNRAITDIFEPGSTVKPMVVMTALQNGVVRENSVLNTIPYRIQGHEI
KDVARYSELSLTGILQKSSNVGVSKLALAMPSSALVDTYSRFGLGKATNLGLVGESSGIYPKKQRWSD
IERATFSFGYGLMVTPLQLARVYATIGSLGVYRPLSITKVDPPVAGERVFPEPLVRTVVHMMESVALP

GGGGVKAAIKGYRIAIKTGTAKKVGPDGKYVNRYIAYTAGVAPASNPRFALVVVINDPQGGKYYGGAI
SAPVFGAIMGGVLRTMNVEPDALPTGDKSELVINKKEGSGGRS
>CORE_REP|Org13_Gene2952#
MMNDKDLSTWQTFRRLWPMITPFKTGLIVAAIALIMNAAGDTLMLSLLKPLLDDGFGKTDSSVLVWMP
LAVIALMLMRGVTSFVSSYCISWVSGMVVMQMRRRLFGHMMRMPVAFFDQQSTGTLLSRITYDSEQVA
SSSSSALVTVVREGASIIGLFIMMFYYSWQLSVILIVLAPIVSIAIRLVSKRFRNISKNMQNTMGQVT
TSAEQMLKGHKEVLIFGGQQVETERFNSVSNRMRQQGMKLVSASSISDPIIQLIASLALAFVLFAASF
PSVMSTLTAGTITVVFSSMIALMRPLKSLTNVNAQFQRGMAACQTLFSILDMEQEKDTGTREVMRAKG
DIEFRNVTFYYPSKETPALRDINLKIAEGKTVALVGRSGSGKSTIANLLTRFYDIQEGEILMDGHDLR
EYTLASLRNQVALVSQNVHLFNDTIANNIAYARESEYSREQIEKAAEMAYAMDFINKMENGLDTVIGE
NGVMLSGGQRQRIAIARALLRDCPILILDEATSALDTESERAIQAALDELQKDRTSLVIAHRLSTIEK
ADEILVVEDGRIVERGEHAELLERQGAYAQLHRMQFGQ
>CORE_REP|Org3_Gene3058#
MRVLLPFLALYRRHSLLISLGILLAIVTLLASIGLLALSGWFLAASSLAGLAGLLTFNYMLPAAGVRG
AAIFRTAGRYAERVVSHDATFRVLSHLRVFTFSKILPLTPGGIARFRQAELLNRLVADVDTLDHLYLR
VISPLISAAVVILVVTYGLSWLDPALALTLGGILLLLLLLVPPVFYCAGKPIGGQLTALRGQYRTDLT
AWLQGQAELVVFGAVNDFRQTLNATEQRWQRRQWQQASLSGMAQALMILASGLTVTLLLWLSAAGIGG
DTQPGALIALFVFAALASFEALMPVAGAFQHLGQVIASATRVKQIIDRQPEVTFPAAGPAAADRAQLS
LQQLSFTYPDQPQPVLRDVTLEVAAGEHIALLGRTGCGKSTLLQLLTRAWRTDGGKILLNGEPLEDYD
EATLRRMTTVVSQRVHIFSDTLRENLRLAAPDADDARLSEVLRQVGLDKLLDSDGGLNAWLGEGGRQL
SGGEQRRLGIARALLHPAPLLLLDEPTEGLDAETEQQILALLRRHCQGKTLILVTHRLYGLEHLDRIC
VMDDGRIVEQGDHATLMRRQGRYARFRNRISNLAP
>CORE_REP|Org37_Gene3654#
MSTLLSAQSVGYDNAFGVLLSEISFSLKKGDRIGLIGDNGCGKSTLLQLLSGALPIHSGTVTLSHQCL
MARIEQHLPPELHASTLLDAVLAQLPAGQHLSERWRCEALLAELGFEPTSWTLTAGTLSGGQHTRLLL
ARALIRQPDLLLLDEPSNHLDLPTLLWLEQFLRSWSGSFVLVSHDRYLLDQVTNCTWILRDKTLQFFR
LPCSAARAALAEQDAADEHRRQAEQKEIDRVEKSAKRLATWGKVYDNEDLARKAKQMEKRVDRLKEEQ
TTLTAGSPWRLRLQGEALDADRLLALPQWAVRPAPDAPVLFSLEHLRVKSGDRIAIVGRNGCGKSSLL
RLLWQAYQHPAERPAIFHPRVRIGYYDQSLQQLRDEDTLSEALAQFAPLTEEQRKMALIGAGFPYLRH
HQQIRSLSGGERSRLLFVGLTLANHSLLLLDEPTNHLDMAGKEELAETLRQFAGAVILVTHDRMLIEQ
SCNRFWLIDQQKLDEWHDLAPVYQRLAGEAPALPTADKANAGGPTPDERLEGEEALLTTLFALESKLE
DDLARKPKHQKPALQARWRREIADITARLNLG
>CORE_REP|Org1_Gene3313#
MNATPLQKHAVWQLIKPFWVSEERWRAWMMLIAIVILSLGLVYISVLINQWNQVFYDALQNKNYPVFK
AQLWRFTYLALIFIVLAVYKIYLTQGLQMRWRRWMTEKFMGKWLAHQAYYHTEQQQIVDNPDQRIAED
LNVLTQYTLSLSLGLLSSLVTLFSFIDILWHVSGPMTFALGQHAITLSGYMVWFALLYAVLGSLLIWW
VGKPLVMLGFNQERYEANFRFGLIRIRENNDAIALYHGEPREAQQLGDRFDTIRSNWWAIMRITRRLN
IATNFYSQFAIVFPLLVAAPRYFSGAIQMGGLMQIASAFGQVQGALSWFIDAFNDLATWKACVNRLAG
FNAAVDQVHHQPRGIQLREEAAHPLTLDNLSLNLPDGQPLLAGAKMTLQRGDRLLIVGPSGCGKSTLL
RAIAGIWPYGAGAIGLPANANTLFLPQRSYIPIGTLREALSYPSQATQYSDEQLMRVLENCRLKHLQR
WLDTAANWSHRLSPGEQQRLAFARALLIRPSILFLDEATSALDDETEQLMYCLLVDELPDVTLISVAH
RNSVAKYHQTCWRFSRSEDQPARLALSPLPV
>CORE_REP|Org23_Gene2786#
MAPSTKKSGKTYSTVRFGWICAGMLVCFFLLAFRVGYLQLLEHQQLADQADQRSIRTQVVPTNRAMIT
DRNDEALAVSVSSKDIVLDPKHILDTQTDTGNERWQSMANVLKIPLADIQHLIQSNAHKRFVYLARKV
EDDNAAYISKLHLTGVSAEQDFSRFYPMGQDAAGLIGIVGQDNQGLEGIELGFNPLLQGKNGLRVYQK
DGSGAVIGVLKSVDPVPPPNVTLSIDKFIQYVLYAQIRDGVVANQADSGCAVLVKIDTGEILGMASYP
SFNPNNYGSTPAKDIRNVCSSDSFEPGSTVKPVVVMVGLEHKLIRPDTVLDTTPYRVNGHLIKDVGHW
SKLTITGVLQKSSDIAVSHIALALPATVLPAVYRSFGLGRPTELGIGNESSGYLPQHRERWADIERAT
FSFGYGLRVTPLQMAREYAAIGSFGIYRPLSITKVTPPVMGQRILPADTVRSVVHMMESDALPGGSGV
SAAVPGYRLAIKTGTAEKMGPSGKYDGGYINYTAGVAPASDPQVALVVMVNNPKAGKHFGGSVAGPVF
GKIMAQVLEHMNILPDAQPLNVVSSVKS
>CORE_REP|Org5_Gene2562#
MNKVKSLSQQNLSLLLAIYIGIFLNLSVFYRRFDSLAHGIQGIKVISAVTEVIAIVLFTFFIMRLVSL

GGRLFYRIVASLLVLISVAASYYMTFFNVVIGYGIVVSVMTTDIDLSKEVVGLHFVLWMVALSALPLL
LIWKNSLRYTLIEQLKTPGHRIKPLLVLLAVVALVWLPLRMLDDEQSVQEKLSNVDLPSYGGVVAHSY
LPSNWLSALGLFAYTRYDESQDQSTMFDPGKHFTYVPPADIDDTYVVFIIGETTRWDHMGMLGYERDT
TPRLSKEKNLVAFRGESCDTSTKLSLRCMFVREGGTEDNPQRTLKEQNVFAVLKDLGFSSELFAMQSE
VWFYNNTEVNNYSFREMIASEKRNDGKAVDDMLLVDEMKESLARYPKGKHLVILHTKGSHYLYSQRYP
RSYARYQPECMGVDDSCTKAQLINAFDNTVLYTDSFIANVIDQVRDKKAIVFYAADHGESIGENTHLH
GTPREMAPPEQFRVPMIVWASDKFLENPQHLSAFEQLQAQQRIGKTHRHVELFDTILGCLGYTSPDGG
IVDKNNWCHLPQDKTAPASL
>CORE_REP|Org49_Gene2198#
MAQYVYTMHRVGKVVPPKRHILKNISLSFFPGAKIGVLGLNGAGKSTLLRIMAGIDTDIEGEARPQPG
IKIGYLPQEPQLNLEHTVRESVEEALAEVVGALKRLDEVYALYAEEGADFDKLAAEQGRLEEIIQAHD
GHNLNAQLERAADALRLPDWDAKIAHLSGGERRRVALCRLLLEKPDMLLLDEPTNHLDAESVAWLERF
LHDFEGTVVAITHDRYFLDNVAGWILELDRGEGIPWEGNYSSWLEQKDARLAQEASAEAARRKSIEKE
LEWVRQGTKGRQSKGKARLARFEELNNTEYQKRNETNELFIPPGARLGDKVVEVSNLRKSYGDRLLID
DLSFSVPKGAIVGIIGPNGAGKSTLFRMMSGQEQPDSGSIVLGDTVKLASVDQFRDSMDGSKTVWEEV
SGGQDIMRIGNTEMPSRAYVGRFNFKGVDQGKRVGELSGGERGRLHLAKLLQVGGNVLLLDEPTNDLD
IETLRALENALLEFPGCAMVISHDRWFLDRIATHILDYQDEGKVEFFEGNFTEYEEYKKRTLGADALE
PHRIKYKKIAK
>CORE_REP|Org2_Gene2715#
MWLRQCLRCNSLGFTLATALFFTLFQNALFLHRAWSYITFDSVHSVIFAASMPVVIFCALNIIFSVLT
VPYLRKPLIIFFLLGSAAANYFMFSYGVVIDGNMMQNAFETNPQEATALLTPRMGLWLALLGILPAVA
VCFTQIRQTRPWWYMVGLRAANVMLSLAVILIVAALFYKDYASLIRNNKSVVKMLTPSNFVAGTIKFT
EQRYFTRNLPLVKIGEDARKGPLIAGQAKKTLVILVVGETARAENFSLGGYQRETNPRLKQDNVVYFK
NASSCGTETAISVPCMFSNMPRKEYDATQATHQEGMLDVLAHAGVSVLWRDNDGGCKGACDRVPHIDM
TKLKLPQDCDGEVCMDNALLYKLNDYINGLKDDGVIVLHQMGSHGPAYYRRSTPEFQTFSPTCNSNQI
QDCSHEQLVNTYDNSILYTDAMLDATIKLLRQYDDQFNTALVYLSDHGESLGENGMYLHGTPYVFAPS
QQTHVPFLMWMSADYQRNFGVDRQCLNALAEKDDVSQDNLFHTLLGMLNVQTREYQSRLDILQRCRNA
A
>CORE_REP|Org31_Gene2710#
MAHFAQSPYFVLHQLTCQFADGETLFGPLDLAFDRQRCGLVGRNGVGKTQLLRLIAGRDRPGNGHVES
HAALAYVAQQPEIAADTTLAQLLGYGEVFAALARIEQGRPLADDIDRLEGRWDLNDRLQSAFAAAGLP
AFDPLRSACDLSGGERMRAALCGAFLGEADYLLLDEPTNHLDSAGRAWLYQQLERWQGGLLIASHDRQ
LLGRMERIVELTPGALRSYGGNYDDYRRQRDTEQQAARADLEHAREERRRTRARQQKEHDMSQRRSAQ
TLRVVDTLNIASFERVAYKSAAKESLGTLRKQHQDQRDSLDAAVREAYQRVEEEQPVLLALPGSEVSA
NKQVLVLEQLQLPFVSAPPLDLRIDGPMRVALTGPNGCGKSTLLKTVLGQLAPLAGHCHCPLSTAYLD
QTLSQLDPSLSVMEHLGLQDSPLVEGALRTRLAQLQLGADRIALPLGSLSGGERLKAALACALWRRQP
AQLLLLDEPTNHLDLASSLAIETALADFPGAMLVVSHDEDFLQALRPTHRLHRQADGWRLQAW
>CORE_REP|Org25_Gene4270#
MATPLLAIQDLSIAFRQGDAVTPVVNELSLQIAPAETLALVGESGSGKSVTALSILRLLPAPPVVYPG
GDILFNGNSLLHAPEAELRKVRGNQIAMIFQEPMVSLNPLHTIEKQLAEVLMLHRGLRRETARAEIVD
CLERVGIRQAKTRLQDYPHQLSGGERQRVMIAMAVLTRPKLLIADEPTTALDVTIQAQILTLLQELKQ
EMGMGLLFITHNLNIVRRLADNVAVMRQGRCVEQNGRAQLFSRPQHPYTRQLLAAEEVGEPLPLPAAA
SARPGDERPLLKVEDLQVRFPIRRGLLRRTVDYHYALKSLSFELRAGESVGLVGESGSGKSTTGLALL
RLLASQGAIWFDGEPLHPLTMKQMLPYRSRMQIVFQDPYSALNPRLNVQQIIAEGLEVHQRLNAEQRE
QRVIEVLQEVGLDPQLRHRYPTEFSGGQRQRIAIARALILQPQLLILDEPTSSLDKSVQAQILTLLKS
LQQRHRLAYLFISHDLQVVRSLCHQVIVLRQGEVVEQGDCRAIFAAPAADYTRQLLQLAD
>CORE_REP|Org37_Gene1031#
MLSTNNITMQFGSKPLFENISVKFGGGNRYGLIGANGCGKSTFMKILGGDLAPTGGNVFLDPNERLGK
LRQDQFAFEQYSVLDTVIMGHTELWAVKEERDRIYAMAEMSEEDGYKVADLEVAYGEMDGYTAEARAG
ELLLGVGIPVEQHYGPMSEIAPGWKLRVLLAQALFSDPEILLLDEPTNNLDIDTIRWLEQVLNERNST
MIIISHDRHFLNMVCTHMADLDYGELRVYPGNYDEYMTAATQARERLLADNAKKKAQINELQSFVSRF
SANASKSKQATSRARQIDKIQLEEVKASSRQNPFIRFDQDKKLFRNALEVEALTKGFDNGPLFSKLNL
MVEVGEKVAVLGANGIGKTTLLKTLVGDAQPDSGTVKWSENARIGYYAQDHEYEFDDTLTVFDWMSQW
KQEKDDEQAVRSVLGRLLFSQDDIKKKVKVLSGGEKGRMLFGKLMMQRPNILVMDEPTNHLDMESIES

LNMALEMYEGTLIFVSHDREFVSSLATRILEITPNKVIDFTGNYEDYLRSQGIV
>CORE_REP|Org17_Gene4674#
MSPSEFAREVSKRRTFAIISHPDAGKTTITEKVLLFGQAIQTAGTVKGRGSSQHAKSDWMEMERQRGI
SITTSVMQFPYRDSLVNLLDTPGHEDFSEDTYRTLTAVDCCLMVIDAAKGVEDRTRKLMEVTRLRDTP
ILTFMNKLDRDIRDPMEVMDEVERELKIACSPITWPIGCGKLFKGVYHLYKDETYLYQTGKGHTIQEV
RIVKGLNNPELDVAVGEDLAAQLRDELELVQGASHEFDQAAFLSGELTPVFFGTALGNFGVDHMLDGL
VAWAPAPMPRKTDTREVTAAEEKFTGFVFKIQANMDPKHRDRVAFMRVVSGRYEKGMKLRQVRTGKDV
VISDALTFMAGDRSHVEEAYPGDIIGLHNHGTIQIGDTFTQGEDMKFTGIPNFAPELFRRIRLRDPLK
QKQLLKGLVQLSEEGAVQVFRPIANNDLIVGAVGVLQFDVVVARLKSEYNVEALYESVNVSTARWVEC
DDVKKFEEFKRKNEINLALDGGDNLSYIAPTMVNLNLTQERYPDVTFRKTREH
>CORE_REP|Org36_Gene2733#
MAISGNTATQGAPLIALQQLSMTFGGQRALNAISLALMPGEVHCLAGTNGCGKSTLIKAIAGVYQPDD
GSRITIDGQTFGRLSPDQARAFGIQVIYQDLSLFPNLTVAENIAFEHNLHGLLGWYRPARLRRTAERL
LQELSFHLDLDKRVAELPIAQRQQVAICRALVAEARLVIMDEPTASLTRTEVNQLLRTVDYLKAKGIC
VVFVSHRLDEVLEISDRVTVIRDGNKIGTWPAAEITGDRLTELMTGLKLDYRLKSPSMNKDRVMLEAD
RLSRTGQYQDVSFRLHQGEVLGLCGLLGSGRTELALSLFGMTRPDSGKLYLDSKPVRFRGHEDAIKAG
IGYVSEDRLTLGLVQQQSVADNAVLTILDKLRGRFRLIDDYRKNRIVAEWIAKLGVRVADPEQAVSTL
SGGNQQKIVLAKWVLTQPRILILDSPTVGVDVGAKASIYQLIHLLAQEGIAILLISDEVPEVYYNCDR
VLHFSGGSVIGEYLPGQVSQQQLAEAVNA
>CORE_REP|Org37_Gene3954#
MSTATPSRLEMRNISIAFAGFNALQDVDFTLQGGSIHALVGANGAGKSTLMAILSGAHDHYRGEILID
GQAVAIHSPLQARRHGIHVVQQEVDVALIPTLSVAENIMLDWLNEPGHWLNWAELHRRAAQLLQQWAL
PLNPRRRLADCTLAEKQQVLLARALSHRCRFLVLDEPTAPLDRAESERLFNVVRRLQSEGIGIVFISH
RIHELSDICDRLTVLRDGRRVSEDPMRGLSGEQIVEKMLGHRLDDIFPPPRPPHAKRTLLQVQGLRDR
HKLRDVSLRLHEGEILGIAGLAGAGKTELCKALFGASAVQLERGELRGQPWAPRAPHLSVEQGLALVP
EERRKEGIFIDEAIPMNLSVSADDSFSRWSLFSRRQELRWAREIMQRLNIRASGPQQRLARLSGGNQQ
KVAIGKWLRGDAEVLIFDEPTKGVDIKAKQELFGLIDGLARAGKGVIYASGEFAELVGLCDRICVLWD
GRIVAELNAADIDEETLLLYSTGGTPA
>CORE_REP|Org31_Gene1519#
MADSSREFLLEMTDICKSFPGVKALDNVNLRVRPHSIHALMGENGAGKSTLLKCLFGIYKKDSGSIVF
QGREIDFKSSKEALEHGVSMVHQELNLVLQRTVMDNMWLGRYPTKGLFVDQEKMLKDTQAIFDELDID
INPREKVGNLSVSQMQMIEIAKAFSYDAKIVIMDEPTSSLTEKEVNHLFTIIRKLKERGCGIVYISHK
MEEIFQLCDEITVLRDGQWIATQPLEGLDMDKIIAMMVGRSLSQRFPDRQNTPGEVILEVKNLTSLRQ
PSIRDVSFDLHQGEILGIAGLVGAKRTDIVETLFGIREKVAGTIKLHGKAIDNHSANEAINHGFALVT
EERRSTGIYAYLDVGFNSLISNIRNYKNKLGLLDNARMKSDTQWVIDAMRVKTPGHHTHIGSLSGGNQ
QKVIIGRWLLTQPEILMLDEPTRGIDVGAKFEIYQLMTELAKKGKGIIIVSSEMPELLGITDRILVMS
NGQVAGIVNTKQTSQNEILRLASLHL
>CORE_REP|Org8_Gene3095#
MKNLTLAQRLTLIFALLIVIGCAFSGWMQVRSSTQYSQAVIQRLSGNLAQHIADSNPLLGVNGPDPQA
VHTLFDQLMAVNPSVEVYLLDKQGAIIGNAAPAGHLKRQRVALAPLQALLDGAQMPVYGDDPRSADGR
KVFSVAPLKVDGRVEGYLYVVLLGEEYTALASNAQFNSAVRMALWTSGVMVLFSLLAGGFAFYWVTRP
IRRLTRQVNALDSGGIEAVQAYAALPAAPAGRDEVSQLQQAFHRMAQRLAEQWQTLAQQDRLRREFIA
NVSHDLRTPLTSLHGYLETLSVKAATLSDTERRRYLEIALAQSRKVGKLAQELFELARLEYGVVKPQK
EPFSLSELLQDVFQKFELAAEARNQRLHADIAPGIPPVFADLSMIERVLTNLLDNAIRHTPPGGDIGV
RLWRQEGRVMVQVSDSGPGIPQTLRADLFVRPSILSGARRPAGGLGLMIVRRILQLHDSDIQLIEQPQ
SGACFRFAIPPRESGTTVIARAGTAG
>CORE_REP|Org45_Gene2871#
MKLRKKRHKPMHINDITIIDDSKLKKAITAAALGNAMEWFDFGVYGFVAYALGQVFFPGASPGVQMIA
ALATFSVPFLVRPLGGLFFGAMGDKFGRQKVLSITIIIMAVSTFCIGLIPSYASIGIWAPILLLLAKL
AQGFSVGGEYSGAAIFVAEYSPDRKRGFMGSWLDFGSIAGFVMGAGVVVLISSIVGEANFLDWGWRIP
FFIAAPLGLIGLYLRHALEETPAFQQHVDKMEKEDRNAIENPPKTSFKEIAAKHWKSLLVCVGIVIST
NVTYYMLLTYMPSYLSHNLHYSEDHGVLIIIAIMIGMLFVQPVIGLTSDRIGRKPFIIGGSIGLLALA
IPCFILINSNVIGLIFVGLLVLAVLLNSFTGVMASILPAMFPTHIRYSALAISFNISVLIAGATPTAA
AWLVEATGNLYMPAYYLMVVAVIGLITGLYMKETANKPLRGATPAASDRSEAKELLQETYDNIEQKVE

DINAQIAELEKKKQILIDQHPKLD
>CORE_REP|Org15_Gene4594#
MSSLHISQGSFRLSDTRTLTLEALAIQAGESWAFVGANGSGKSALARALADELVLLRGERRSDFQHAV
RISFEQLQKMVSDEWQRNNTDLLSADEDDTGRTAAEIIQEEVKDAARCERLAAQFGITALLTRRFKYL
STGETRKTLLCRALMPQPDLLILDEPFDGLDVHSRAQLAALLSELSAQGQTVVLVLNRFDEIPDFVRQ
VGVLADCTLTNRGPRSQVMADALVAQLAHSENLSGLALPETEDPAHKVALPADRPLIVLRDGVVSYND
RPILNHLDWQVNPGEHWQIVGPNGAGKSTLLSLITGDHPQGYSNDLTLFGRRRGSGETIWEIKRHIGY
VSSSLHLDYRVNTSVRNVVLSGFFDSIGIYQAVSDRQRQLTEQWLALLGLDGARGDAPFHSLSWGQQR
LALIARALVKHPALLILDEPLQGLDPLNRQLVRRFIDVLIGQGATQLLFVSHHAEDAPQCITHRLSFV
PDGEGGYGYQHQRLEAASV
>CORE_REP|Org7_Gene3966#
MTNNASPAPIAHRPLILIACMLAMFMSAIEATIVATAMPTIIGDLGGFSLLGWVFAVYLLSQAITIPI
YGRLADLYGRKRVFFFGATLFLLGSVLCGFAPDMYWLIGFRLLQGLGAGAIMPIASTIIGDIYSATER
PKVMGYLSSVWGVSAIIGPLLGAFIVQHLPWALVFWVNLPIGLLAMFFLWRYLPAHQPLRQHALDLAG
TAWLTLFVSALLLALLQMESLGWWVVPLFALAAAALALLVRQERRAVEPLFPLALWQSRVIVAGNIGG
LVIGAAMMGISAFLPTFIQGVMGGSPLEAGTTLALMSIGWPLASTLSGRLMLMTSYRATALLGALLLV
AGGLILLLLQPEGGLLWGRVAAFMVGAGMGLCNTTFLVSVQNAAHYSIRGIATACTVFTRMVGSAIGT
AILGATLNLNLQWRLPEIDDPVQRLMEPAVRQSMGSEALAQLTQQVAASLHWVFLVSALVSLLALAAA
MLIPARCRPQGEEEEAEQA
>CORE_REP|Org17_Gene3814#
MITHDDSRWSDLFSGKNAASAIALSLGVALHAINILVATTILPSVVQDIGGLDLYAWNTTLFVVASIL
GSALSARLLSGYGARNAYLVASLFFIAGAGLCALAPSMPVMLVGRTVQGFGGGLIFALSYAMINLVFE
QRLWPRAMALISAMWGIATLVGPAVGGIFAELHAWRWAFGILLPIMALYAAFTFLILPKGQAQQAAAP
LPTAQLLLLTVAVLVVSAGSLAHSVWINLAGIALSLALMAWLMKREARSRTRLLPHGALRRGSSLAAL
YITVSLLVIGMTSEIFVPYFLQLLHGQSPLISGYIAATMAAGWTLSEILSSGWRGAGIRRAIVSGPLF
VLVGLLALAILMPTPSGGHWQALTPIVIALSLVGFGIGFGWPHLLTRILQVAPEADKDIAGASITTVQ
LFATAFGAALAGMIANLAGLNDPGGAAGAAGAASAARWLFLAFALAPLLAVFSAWRCAAIAPPAAETG
NFVPNPSSREC
>CORE_REP|Org42_Gene1651#
MFNKNKKPFSLRARFLMATAGVILALSLSYGLVAVVGYIVSFDKTAFRLLRGESNLFFSLAQWKDNKL
TIAIPPDIDLNFPTLVFIYDDKGNLLWSQRKVPELEKLINKEWLEESGFYEIDTDTRVSSEVLGDNPK
AQDQLKNYDDTDQNALTHSVAVNTYAATPRLPALTIVVVDSIPQELQRSDVVWEWFSYVLLANLLLVV
PLLWLAAYWSLRPIKALVNQVGELENGERDQLDENPPSELRGLVRNLNILVRNERQRYTKYRTTLSDL
THSLKTPLAVLQSTLRSLRSGKQTTIEEAEPIMLDQIGRISQQIGYYLHRASINSGQTVLTREIHSVP
ALLDSLVVALNKVYQRKGVVITLDISPEVTFMGEKNDFMEVMGNVLENACKYCLEFVEITSLHSEKNL
TIVIDDDGPGIPESKRQLIFQRGQRVDTLRPGQGLGLSVAAEIIEQYDGEIVISDSPLGGARMQVTFA
RQHDTHHNE
>CORE_REP|Org19_Gene4298#
MSYQRKHNTGYILRICGIAALGGILFGYDTAVISGAIEALKTYFNLSPAETGWAVSNVVIGCVVGAFA
AGPLAARWGRKKALMLAALLFTVSAVGAALAPTFTWFVIYRIIGGLAVGIAATVSPMYMSEVSPKDMR
GRALSMQQFAIVFGQIVIFYVNFKIASLASEAWLVEMGWRWMFASGVIPCILFCILVFVIPESPRWNV
MMGRDDQALAMLTKVSNAAHAQNLLKEIKDSLQQDQQQRHRKLNYGDVRVRFILFVGCMIAMLQQVTG
VNVMMYYAPVVLKTVTENAQEALFQTIWIGVLQLVGSVIGAMLMDRMGRIPLMRYGTLGAIAGLLLTS
YALYTQATGYFALFGMLFFMVFYALSWGVGAWVLVSEIFPNRMRAQGMSIAVGCMWVANFAVSQSFPM
INDHPYLFSHFHGAFPMWIFAACCLFSYWFIGRYIPETKGVSLEKMEQVVLAKRHRHRHPLPDGKPLP
LENGKS
>CORE_REP|Org43_Gene2089#
MISLKKWRLFPRSLRQLVLLAFLLVLLPLLVLAYQAYQSLDHLSAQAADINRTTLVDARRSEAMTSVA
LEMERSYRQYCVLVEPTLQKLYQNQRKQYSQMLDAHAPILPDERYYQTLRQLLTQLAAIKCHNSGPDQ
EASALLESFSRSNAEMVQATRAVVFSRGQQLQQAIAERGQFFGWQALLLFLVSVLLVVLFTRMIIGPV
KAVERMINRLGEGRALGSTASFKGPRELRSLAQRIIWLSERLAWLESQRHEFLRHISHELKTPLASMR
EGTELLADEVAGPLTSDQKEVVTILDNSSRHLQQLIEQLLDYNRKLADGPAEHENVELREMVDLVVAA
HSLPARAKMISTEIALEAEICWAEPTLLMRVLDNLYSNAVHYGKESGNIWIRSRQVGQRVQIDVANTG
TPIPEAERAMIFEPFFQGSHQRKGAVKGSGLGLSIAQDCIRRMRGELQLATVAGADVCFRIELPLTAE

NE
>CORE_REP|Org34_Gene4434#
MNNAIAQQIADQGGVESYLHAQQHKSLLRFLTCGSVDDGKSTLIGRLLHDTRQIYEDQLSTLHSDSKR
IGTQGEKKLDLALLVDGLQAEREQGITIDVAYRYFSTEKRKFIIADTPGHEQYTRNMATGASTCDLAI
LLIDARKGVLDQTRRHSFIATLLGIRHLVVAVNKMDLVDYQEAVFEQFKQDYLTFAQQLPGDLDIKFV
PLSALDGDNVASESAHMPWYSGPTLLEVLESVDVISERENQPLRFPVQYVNRPNLDFRGYAGTLSAGV
VRVGQRVKVLPSGVESSVARIVTFDGDLQEAVPGEAITLVLKDEVDISRGDLLVDAGESLQAAQSALV
DVVWMAEQPLVPGQSYDIKIAGKKTRARVESIRHQVEINTLTQHPADTLPLNGIGLVELTFDEPLVLD
SYQNNHDTGGLIFIDRMSNVTVGAGLVRETLQAASAARGEFSAFELELNALVRKHFPHWGARDLLGGR
>CORE_REP|Org11_Gene3175#
MTQSARSMAGLPWIAAMAFFMQALDATILNTALPAIAQSLGRSPLAMQSAVISYTLTVAMLIPVSGWL
ADRFGTRRVFIFAVTLFTLGSLLCALSPTLSALVASRVLQGIGGAMMMPVARLALLRAYPRSELLPVL
NFVTMPGLVGPILGPLLGGWLVTYATWHWIFLINIPIGLLGIFYARKYMPDFTTPKRRFDFLGFMLFG
LSLVLISTGLELFGERVLASYVSLGILLSGFVMLFGYITHARRHPQPLIGLDLFKTRTFSVGIAGNVA
SRLGTGCVPFLMPLMLQVGFGYTAIVAGCMMAPTAIGSLMAKSTVTQVLRWFGYRKTLVGITVIIGVL
IAQFALQSPGMPLWLMILPLFVLGMAMSTQFTAMNTISLADLNDANASAGNSVLAVTQQLSISFGVAI
SAAVLRFYESLSLGTMIDHFHYTFITMGIVTVASALVFMLLRRKDGRNLISGQESKKEAKAAS
>CORE_REP|Org34_Gene2045#
MQRGIVWIVDDDSSIRWVLERALTGAGLSCATFEGGNDVLEALATQTPDVLLSDIRMPGIDGLALLKQ
IKQRHPMLPVIIMTAHSDLDAAVSAYQQGAFDYLPKPFDIDEAVALVERAISHYQEQQQPVRSQPASD
PAADIIGEAPAMQDVFRIIGRLSRSSISVLINGESGTGKELVAHALHRHSPRAKSPFIALNMAAIPKD
LIESELFGHEKGAFTGANQIRQGRFEQADGGTLFLDEIGDMPLDVQTRLLRVLADGQFYRVGGYAPVK
VDVRIIAATHQNLELRVQEGKFREDLFHRLNVIRVHLPPLRERREDIPRLARHFLQIAAKELGVEAKN
LHPETETALTRLPWPGNVRQLENTCRWLTVMAAGQEVLIQDLPSELFETAAPESPSHSLPDSWATLLA
QWADRALRSGHQNLLSEAQPEMERTLLTTALRHTQGHKQEAARLLGWGRNTLTRKLKELGME
>CORE_REP|Org25_Gene687#
MINSLTARIFAIFWFTLALVLMLVLMVPKLDSRQMTSLLDSEQRQGLMLEQHVEAELQNDPANDLMWW
RRLFRAIDKWAPPGQRLLLVTSEGRVIGAQRNEMQIVRNFIGQSDNSDHPKKKKYGRVELVGPFAVRD
GEDNYQLYLIRPANSPQSDFINLMFDRPLLLLIVTMLISAPLLLWLAWSLAKPARKLKNAADDVARGN
LKQHPELEAGPQEFLATGASFNQMVSALERMMNAQQRLISDISHELRTPLTRLQLATALMRRRHGEGH
ELARIETEAQRLDSMINDLLALSRGQQKGELAREQLKANELWADVLDNARFEAEQMGKQLEIAAPPGP
WTLFGNASALDSALENIVRNALRYSHTRIAVAFSADNQGVTIQVDDDGPGVSAEDREQIFRPFYRTDE
ARDRESGGTGLGLAIVEAAVNQHRGWVKAEDSPLGGLRLVLWLPLHHQRLSSKTEQ
>CORE_REP|Org18_Gene4752#
MAWFLPRFDVNDNSMTPQERRATWGLGTVFSLRMLGMFMVLPVLTTYGMALNGASEALIGIAIGIYGL
AQAVFQIPFGLVSDRIGRKPLIVGGLLIFALGSVIAAATDSIWGVILGRALQGSGAIAAAVMALLSDL
TREQNRTKAMAFIGVSFGITFAIAMVLGPIITHALGLHALFWMIAVLALAGIVITLAVVPSADTHLLN
RESSIVRGSFRKVLSNSRLLKLNFGIMCLHILLMSSFVALPLAMEKAGLAASEHWIVYLVTMLVSFAA
VVPFIIYAEKYRRMKQVFMGCVAVLFCAEVLLWLSGARLWGIIAGVQLFFIAFNVMEAILPSLISKES
PAGYKGTAMGVYSTSQFIGVAIGGSLGGWLYGLQGAGLVFIAGAVLAAVWFLVSSTMKEPPYVSSLRI
TLSELAVKDSALESRLKAQPGVAEAIVVPEERSAYVKVDTKQTNRGQLEALVNSL
>CORE_REP|Org14_Gene3002#
MSYRSKVAIVYLLGFFVDLINMFIANVAYPAIGQAMRASVSQLAWVSNGYILGLTLVIPLSAWLAQRI
GGRRVFLLSLALFMLATFGAGNADSIGALIGWRTLQGMGGGLLIPIGQTLTYQLYRSHERAGLSAAIM
LVGLLAPALSPALGGWLVDRLDWRWVFFANLPLAALALALAALWLRAETSATAVRKPLDGKGLLSACA
ALTLLLLGLTRLSEAGHQASGAALLAAGLLVLAYYLRHSLRTPQPLLNLRLVGDPLLRNAMGVYLCIP
GLFIGVSLVAMLYLQNQLGMPAAQVGGLMLPWALASFLAITLTGKTFNRLGPRPLLIAGCLLQGAGML
TLAQIDQAGQHAWQIAAFALMGFGGSLCSSTAQSSAFLQIPDAQLADASALWNINRQLSFCLGVALLS
LLLNLLLTGLPPAAAYRTCFILAGASVFIPLLLCLRLANRAIVRQLNAQQDAL
>CORE_REP|Org7_Gene2401#
MRRLRFSPRSSFARTLLLIVTLLFVSLVTTYLVVLNFAILPSLQQFNKVLAYEVRMLMTDRLQLEDGT
LLEVPPAFRREIYRELGISLYTNSAAEESGLRWAQHYQFLSQQMAQQLGGPTDVRVEVNKNSPVVWLK
TWLQPDIWVRVPLTEIHQGDFSPLFRYTLAIMLLAIGGAWLFIRIQNRPLVELEHAALQVGKGIIPPP
LREYGASEVRSVTRAFNQMASGVKQLADDRTLLMAGVSHDLRTPLTRIRLATEMMSAEDGYLAESINK

DIEECNAIIEQFIDYLRTGQEMPTESSDLNAILGEVVAAESGYERVIETALSPGELMMNVHPLSIKRA
AVNMVVNAARYGNGWIKVSSGRELQRGWFQVEDDGPGIKPDELKHLLQPFVRGDSARSTSGTGLGLAI
VQRIIDAHDGELDIGTSERGGLLIRAYIPLPMEKKESTNGHQTARETA
>CORE_REP|Org12_Gene4291#
MKRLSLRLRLILIFSLLALLTWCTASVVAWVMSRNTINEVFDTQQMLFAKRLATANLGDLLADESARS
LPKTKKLVHHGKRGEQDDDALAFAIFDRDGKMLLNDGENGADFLFDGEREGFTDGERKGDDDSWRLVW
LTSPDGRYRIVVGQEWDYRRDMALGMVTGQLVPWLATLPVLMLLIALMVGRELRPLRAVAAGLRRRAP
DDATPLDARQVPTEVRPLVDALNALFARINALLVRERRFTSDAAHELRSPLAALRVQTEVVQLAGDDA
PMREHALDNLTVGIDRATRLVDQLLTLSRLDSLLDLAELAPIDWNDLVTMTLAEQDRQAHAAGVTLRY
EHRGTPPPRQGETLLLSLLLRNLLDNAVRYTPQGGVVTVTLSERSLTVEDDGPGVTAEHLARLGERFY
RPPGQEQTGSGLGLSIVQRIAGLHGLQISFANRSAGGFVARLAL
>CORE_REP|Org49_Gene2784#
MPHDNNVEIRGFIDDQPFSRYQWLILVLCFLAVALDGFDTAIIGFIATSLVQEWGIEKTSLGPVMSAA
LVGLAVGALAAGPLADRIGRKKVLVISLLLFGGFSLLTAFAGSLSTLTLLRFLTGLGLGAAMPNAATL
MSEYAPQRCRALMVNLMFCGFPLGSSLGGFSSAWLIPHFGWQSVMVLGGVMPLLLALVLIAALPESAR
FMVARGYAAERIAKVLKRIAPLPKTPHFMLQEEGQIKAASPLGMIFSRRYLLGTLMLCLTYFMGLMIF
YLLTSWLPLLIRETGASVTQASLITALFPLGGGLGVLIIGWLMDRMNPHKVVAVGYLLTGLFVGIIGF
VYSYPPLMAITVFIAGTCMNGAQSSMPALAAGFYPTQSRATGVAWMLGLGRFGGILGAMSGGALMQMQ
LSFSTIFTLLAIPALIAALALIAKHLSGYPALPAPLNKNAVRE
>CORE_REP|Org19_Gene367#
MLDKIVIANRGEIALRILRACKELGIKTVAVHSAADRDLKHVLLADETVCIGPAPSVKSYLNIPAIIS
AAEITGAVAIHPGYGFLSENADFAEQVERSGFIFIGPKAETIRLMGDKVSAINAMKKAGVPCVPGSDG
PLTDDMDKNRAFAKRIGYPVIIKASGGGGGRGMRVVRSDKDLEQSINMTKAEAKAAFNNDMVYMEKYL
ENPRHIEIQVLADGQGNAIYLAERDCSMQRRHQKVVEEAPAPGITSEMRRYIGERCSKACVEIGYRGA
GTFEFLYENGEFYFIEMNTRIQVEHPVTEMITGVDLIKEQLRIAAGQPLSIKQEEVKIHGHAVECRIN
AEDPNTFLPSPGKITRFHAPGGFGVRWESHIYAGYTVPPYYDSMIGKLITFGENRDVAIARMKNALAE
LIIDGIKTNVELQQKIMNDENFQHGGTNIHYLEKKLGLQET
>CORE_REP|Org36_Gene3361#
MWSFLKSRPDAPQVTDQRQIDASYKYWRIQLMCTMYIGYAAFYFTRKSFNFIMPAMLSDLGLTMSDVG
ILGTLFYITYGCSKFISGMISDRSNPRYFMGLGLIMTGVLNIFFGLSSSLLMLGTLWILNAFFQGWGW
PPCSKILTSWYSRSERGSWWAIWNTSHNVGGALIPLLVGFISLHFSWRYGMIIPGIIGVVLGLLMCWR
LRDKPSTLGLPSVGKWRNDAMELVQESEGQGLSNREIIKRYVLTNKYIWLLAVSYVLVYIVRTAINDW
GNLYLTQEKGYSLMTANSAISLFEVGGFIGSLVAGWGSDKLFRGNRGPMNLIFAIGIFLSVAALWLMP
GVTYLLQACCFFAIGFFIFGPQMLIGMAAAECSHKDAAGAATGFVGLFAYLGAALSGYPIARVMEIWH
WNGFFVVISIAACLSALFLLPFLRAQTPALKTANA
>CORE_REP|Org11_Gene3941#
MRGRLFWKILLGFWLTFLIMTQALWVAFSLYGDRYVPPENAMARRVIGLQLTSAATQLRSGGMPALEA
LMRDWPEDDRRLLSVTPMTQPPPPAPEEPVFEGRRMPKAISAWVQTGEGQGYWLSYDVRGLREEYRPE
RRSHFFNIPAPMLWVGGLGGLLFSAVLAWNLTRPMRQLRGGLDRVAQGDLSVRLFPNMRRRHDELSDV
ARDFDTMAERLELLVSAREQLLHDVSHELRSPLARLQLAIGLARQNAGNVEASLKRIEHESGRLDKMI
GELLALSRTEHSSLPDEEYFDLYGLVDAVVSDARYEAQVPGVDIVLQAESDVEYTVKGNAELMRRAVD
NIVRNALRFSSHGQRVTVTVALSRVDNQFQIAVSDQGPGVEEAKLSSIFDPFVRVKSAQSGKGYGLGL
AITRKVVLAHGGQVEARNGDREGLVITLRIPRWSS
>CORE_REP|Org14_Gene489#
MLERLSWKRLALELALFCLPALLLGLIFGYLPWFLLASALAALVWNFYNQLKLSHWLWIDRSMTPPPG
RWSWEPLFYGLYQMQQRNRRRRRELALLIKRFRSGAESLPDAVVMTTVEGNIFWCNGLAQHLLGFRWP
EDNGQHILNLLRYPEFSHYLQQQEFSRPLTLQLNNEHYVEFRVMPYSEGQLLMVARDVTQMRQLEGAR
RNFFANVSHELRTPLTVLQGYLEMMGDEEQDGSLRSKALSTMQEQTRRMDGLVKQLLTLSRIEAAPNV
DMNERVDIPLMLRVLQREAQSLSGGNHEITFRVNEQLNVFGNEDQLRSAVSNLVYNAVNHTPKGTHIE
VSWQQTAHGAQFQVSDNGPGIAAEHLPRLTERFYRVDKARSRQTGGSGLGLAIVKHALSHHDARLEIL
SEPGIGTRFIFTLPNRLIVPAALSENAVKN
>CORE_REP|Org10_Gene3824#
MNQQTLDARRSRQALLAGSVGNFIEWYEFGVYGFLATVIAANFFTLQGENEVTSLILTYAAFALAFFC
RPIGAVIFGRIGDRIGRRPTLIAVLLLMTLATALIGVMPTYASIGVAAPLLLTLLRMFQGLFAGGEFG

GAVSLMTEFAPKGKRGLFGAWQSLTVALGLLAGAGLVALLAALLSVQQLHDWGWRIPFLLALPMGAVA
LWLRLKLEETPTFTQAQQAAEHSAAPQEASLGGVAKTILIGIGRMMGWSAAGYTFLVVMPSYLQTSLH
ATFQQALVATVLANVGFALTILPAGIISDKLGRKTVMLTAVAAVILFTFPLLHLLQDAQSSLWAKGLV
VMIAGAVVGLLAGPGPAMLAEMFPTRVRYTGLGLAYSLSNAVFSGSAGLIITGLIKQTGNIDIPAYYV
VATSVVSLFALMTLRRDDHLRSLNER
>CORE_REP|Org30_Gene1669#
MTTPVRSAAAMPSTLAANDDAAAAPKVKRSTLNNKLPYIERGTPQFMRVTLALFSAGLATFALLYCVQ
PILPVLSQDFGVSPAESSLSLSVSTGLLAIGLMFTGPLSDAIGRKSVMVVALLLAAVCTLICAFMTSW
HGILLMRALIGLSLSGVAAVGMTYLSEEIHPSFVAFSMGLYISGNSIGGMSGRLVTGVLTDFFSWRVS
LGVIGLFALAAACMFWRILPASRHFRASSLRPRTLLINFKLHWHDKGLPLLFAEGFLLMGSFVTLFNY
IGYRLLADPYHLSQAIVGLLSVVYLTGSYSSPKAGALTSRFGRGPVLLASIVIMLIGILITALPQVPA
IFIGMMLFTAGFFAAHSVASSWIGRRARRAKGQASSLYLFCYYVGSSVAGTLGGVFWHSFGWNGVAAF
ISLMLLLALLVVHYLKRLPEAARL
>CORE_REP|Org27_Gene4319#
MRKIKGLRWYMIGLVTIGTVLGYLTRNAIAVAAPTLEDTLHITTQQYSYIVAAYSACYTVMQPVAGYV
LDVLGTKVGYAMFAILWALFCMGTALANSWGGLALARGAVGMAEAAMIPAGLKASSEWFPAKERSIAV
GYFNVGSSIGGMIAPPLVVWAIVAHSWEMAFVITGVLSLIWAICWLLFYKHPKDQKKLSQEERRYILE
GQEAQHQTSNAKKMSAWQIVRNRQFWGIALPRFLAEPAWGTFNAWIPLFMFKAYGFNLKEIAMFAWMP
MLFADLGCIVGGYLPPLFQKYFKVNLIVSRKLVVTMGAVLMIGPGMIGLFTSPYAAIALLCVGGFAHQ
ALSGALITLSSDVFGRNEVATANGLTGMAAWTASTLFALVVGALADTLGFSPLFAALSVFDILGAIVI
WTVLQNRPAAEPQPATLQPSPARS
>CORE_REP|Org26_Gene3371#
MARPSFFLDFSLLRSNAHFRAIFCARMLSVFSLGMLAVGVPIQIQAMTGSTLQVGVAVALDGVGMFIG
LMLGGVLADRYDRRKLILFARGTCGLGFVALSLNAFAPAPSLLALYLLAAWDGFFGALGMTALMAVIP
LLVGRENLAAAGALSMVTVRIGAILAPALGGIIIVFGGVGLAFAVAAAGTLGTLVPLVRLPTLLPQQQ
EPEHPLRALASGFQFVWRNKVVGSVVLLGMLMSIVGAVRVLFPALAQDAYHVGASSIGLMYSAVPLGA
MLGALTSGWVGRFSRPGVLILVAAIVAFTAIASLGLFSHLAPALLALVCYGYANAIASLLQFMLIQSN
TPDHLLGRVNSLGTAQDVTGDSIGALGLGVLGRVFTPLMSVLSFGAFAAVLGVLVAFSVRTLRQCRPA
DALVEHDEPAPATSAAADN
>CORE_REP|Org34_Gene4576#
MSENTASQIAAPQNAQASGRTILLFLALALMSALLNSSAPTPLYPLYQQQLTLSSVSLTVIYGAYAAG
VLISLFGVGNLAGKVKDLRSMIVPALLVVLSGALLFAQADTFAMMLMARLLAGVGTGALTGAANIALV
RFGPRDGGKNAALIATLSFTTGLALGPIFSGIALQTGFHPTTLPFVFIMVMAAVAALGVMFSWPRGVV
TAPSHVTSAETEKSSLLDGLRATGGKFFVCAGALFICWALAASILAIGPGVAETLLGLHARGVFGYAI
AVYLLIAGISQILSRRVNARHSLLFGCLAQVLAAVVFTMAIQWHSLGLAAVGLVVAGYAYGAIFVGSA
TLVNLISPPTSHARLLSLFYVIAYIANWVPILLGVVVDRVDLNQATHLLFLGSTVVCLLLAWKTSRVG
FLANYFVMNIYFYN
>CORE_REP|Org29_Gene2731#
MNNSPGQRSRLSEENRLLIILFFVFGCVFVDRLTISFLFPMIAADLKLSNVHLGTLSAVLALTWALSG
AGLGAIADRFNIRKPMLILSILVFSLFSALSGLVSGFAMLLIFRALMGIAEGPVLPIAQSLMVEKSQP
QRRGFNMGLIQGAAPGLLGGIIAPPLIIYLAQKWGWSMAFHLTAVPGIILAWLIYRYVNGKKDPAFSA
APAAGKAANKAAYGELFKIKNVALCILISCVFVTWFMIIITFTPNFLVTDRGFSEGTMGGIMSAIGAA
WVFWGVAVPAISDRLGRKPTLIFFSLLAVCCPLFLSYVDNPWLLGVLVFLSYTGLGCFTLFMATIPSE
TVSPARIATALGLVMGIGEVIGGCLAPFIAGLIADRYGLVSVMWLAAAGAVCAGVLSCFLDETAPAVV
SRRAVKPASVTDV
>CORE_REP|Org2_Gene3295#
MAAVATPGHVYPMLAIARHLIAQGHQVRVMTGALFRERAEVAGASFVPFDAQVDFDYRHLEEHFPERA
ALPPGNAQMALALKDFFAAPIPLLDRQLRATIAAEKTDLLMVENCFYGVLPLLQSAARPPVFGIGVTP
LSYSSRDAIFYGPRIPPALLPQALTREQLVDEETRVLIDDVQQSFDAALLQAGGRALDRPFTDALIGG
CERFLQLATTALEYERDDLPSGVRFVGPLRSGGQPAAEETLWEADDRRPLVIVTQGTLANVDLHQLIV
PTLQALAHLPVRVLATTGGRATEGLMDALPGNARVREFISFERWLPETALLITNGGYGSISYALDSGV
PLIVAGTGEDKLEAAARVVAAGCGISLHTSTPSAEQILAAATRILQQPIYRQRAALVREDYARHDALT
AIANEVAAITA
>CORE_REP|Org7_Gene4391#

MTTQDIDARAGRAGETVAENPQQRVRWSVPIALFACVLLAFFDKISIAALFSDSEFQQALGIGFDPAR
LGLLMSAFLFSYGISSMLLSGIGDRLNPVKVLIGMMVVWGVLMVLMGLVRSYHAMMTLRILLGIAEGP
LLPMAYAIIRQAFPPQLQARATMLWLLGTPLGAALGFPVTLYILNTFDWQATFFFMAFLTLPVMLLVL
FGMRHLNVSRPAAAAKPAVSERQQHRRELLRSPHFWMICLFNIAFLTYLWGMNGWLPSYLIKGKGIHL
EHAGYLSSLPFIAMLLGEVLGAWLSDKLDRRALACFLSLCGAGLGLAVVLHLQGTYSVIAAMAFSTFM
WGAGAPNIFALLAKATSSKVSATAGGIFNGLGNFAGALAPVLMGALIAATGNMDNGLLFLVVMAFVGC
LILLPLLRKY
>CORE_REP|Org40_Gene2806#
MKTTLPPAARLGRQALLFPLCLVLFEFATYIGNDMIQPGMLAVVADFNAGEEWVPTSMTAYLAGGIFL
QWLLGPLSDRRGRRPVMLAGVAFFIVSCLAILLVTTIEQFIAMRFLQGIGLCFIGAVGYATIQESFEE
SVCIKITALMANVALIAPLLGPLAGAALIHVAPWQSMFVLFAALAAIAFYGLWKAMPETATLQGEAFS
AANLWRDYRQVLGNRRFLCGALAIGFASLPLLAWIAQSPVILISGESLSTLDYGLLQIPVFGALILGN
LTLARLTGKNSVERLIKLGAGPMLLGLLIAALATQFSSHAYLWMTAGLSLYAFGIGLANAGLYRLTLF
SSNVSKGTVSATMGMLSMMVFTVGIELAKVAYVWGGSGLFNLFNLISGLCWLTLVALFLGKRRNGDPT
PQPTGAV
>CORE_REP|Org11_Gene2984#
MSVITEKKNHATPGKAMLASVTGYAMDGFDLLILGFMLPAISIELGLTSSAAGSLVTWTLIGAVLGGV
IFGHLSDRFGRIRVLTITILMFSLFTGLCAVAQGYWDLLAYRTLAGIGLGGEFGIGMALIAEAWPAEK
RNRASAYVGMGWQLGVLAAAFLTPLLLEHIGWRGMFLVGLLPALASFLIRRTLGEPEAFVRQKDAGQP
LSFLQRLRLLFKDRATSKASIGIFILCSVQNFGYYGLMIWMPTYLAKNFGFSLTKSGLWTAVTVVGMT
FGIWLFGMLADRFARWKIFVLYQVGAVVMVIGYAQLSDPMLMLFAGAVMGMFVNGMIGGYGALISDTY
PVQARATAQNILFNLGRGVGGLGPLVIGALVTQVSFTAAISLLAAIYLLDIYATLFLLPKKQGAGDTL
GAIG
>CORE_REP|Org30_Gene792#
MAKVSLEKDRIKFLLVEGVHQSTVDNLRAAGYTNIEYHKGALDTESLKASIRDAHFVGIRSRTHLTEE
VFAAAEKLVAVGCFCIGTNQVDLKAATKRGIPVFNAPFSNTRSVAEMVLGELLLMLRGIPAANAKAHR
GVWHKLAVGSYEARGKKLGIIGYGHIGTQLGILAEGLGMKVFFYDIENKLPLGNAQQVRHLSDLLNMS
DVVTLHVPETLATKNMMGAEELALMKPGAILINASRGTVVDIPALCDALASNHLAGAAIDVFPEEPAT
NSDPFNSPLCEFDNVLLTPHIGGSTQEAQENIGDEVAGKLAKYSDNGSTLSAVNFPEVSLPAHGPNAS
RLLHIHENRPGVLTQINQIFAEEGVNIAAQYLQTGPEIGYVVIDIEAETARADAALQRMKAIDGTIRA
RLLF
>CORE_REP|Org21_Gene3877#
MASVAEPVNWKRNFFVAWVGCFLTGAAFSLVMPFLPLYVETLGVTGHQALNMWSGLLFSITFLFSAIA
APFWGALADRRGRKLMLLRSALGMAIVMVLMGMAQTVWQFLALRAVLGLLGGFIPNANALIATQVPRN
RSGWALGTLSTGGVGGALIGPLIGGLLADLYGLRPVFYITAAVLFVCFVLTLLYVKEQFTPVQKRDML
HARQVFASLKNPKLVLSLFVTTMIIQIATGSIAPILTLYVRDLAGATHNLAFISGLIASVPGVAALMS
APRLGKLGDRIGPERILICMLIVSVLLLIPMAFVQTPWQLGVLRFLLGAADGALLPAVQTLLIYNCTN
QVAGRIFSYNQSFRDVGNVSGPLLGAAVSAGYGFRAVFGVTALVVLFNAGYSWWCLRRRPGYMREDTL
QEEQ
>CORE_REP|Org42_Gene3550#
MFGWTPLQRNAAIASFSSWTLDAFDFFVLVFLLSDIAQSFHVGLEQVTLAILLTLAVRPIGALIFGRA
AEKYGRKPILMLNIVFFSVFELLSAAAPSLTVFLLLRVLYGVAMGGIWGVASSLAMETIPDRSRGLMS
GIFQAGYPFGYLLAAVVYGLLFETVGWRGMFVIGAAPILLLPFIYYCVQESPVWLAARERKESSALLP
VLKSHWKLCCYLVLLMAAFNFFSHGTQDLYPVFLKVQHGFDPKTVSIIAISYNIASIIGGVFFGSLSE
KIGRKKAIIIAALLALPVIPLWAFSSGGSLMLGIGAFLMQFMVQGAWGVVPTYLTELVPANTRAVLPGF
VYQLGNLIASVNATLQATIAEHHGHNYGLAMAIVAGTVAVAIALLVFFGKDTRGKAITDAVKNPGVRA
NV
>CORE_REP|Org37_Gene4035#
MNTKPANRSLIVLGTIICQMGLGTIYTWSLFNQPLVDKFHWGLADVATTFSITSFFLAFATLFAGKLQ
ERFGIRNLTLCSGILVGLGLIASAHVSSLDMIYLLAGVVVGFAVGIAYISTLSNLIKWFPANKGLISG
ISVGAFGSGSLLFKYVNAALIADVGVSGAFFYWGAIVMGLIVVGSLLLKEPVLATNAAQQGANGLGND
YSVRQMLATKEAYLLFTIFFAACMSGLYLIGIVKDMGVQLAGMDLATAANTVSAVAIFNTAGRIILGT
LSDKVGRMRVISFTMLVTVLAIVALSFMTLNHTLFFICVGAVAFCFGGNITVFPAIVGDFFGLKNHSK
NYGIIYQGFGLGALAGSFVAKYFGGFHATFMVIGVLSAASLLITLFIKAPKAVEAETAETTQTAELAK

A
>CORE_REP|Org49_Gene3307#
MSDKLLDPPCAALGRLPAPLVLLLAAASAFSVANVYYAQPLLDAIAHDFSISLAAVGMVITVTQLGCA
LALLLVVPLGDRLNRHWLLAGQQLGLIGALLLVGWAHSAPWLLAGMLLVGLLGTAMTQGLIAFAAALA
APQERGRVVGAAQGGVVLGLLLARTLSGALADVGGWRTVYFFSAGVTLVLLPILSRLLPAPRTAPSTL
SYPALLRSMLTLLLHDRTLQIRGMLALLMFGAFSLFWSSLVLPLSQAPFNFTHAAVGAFGLVGAVGAL
AAVRAGHLADRGLGQAASGVCLLLLTLAWLPLGLLGSGLVWLVAGIVLLDLAGQAIHVLNQSMIFSAH
PQSHSRLVGCYMLFYAVGSGLGAFAGTHMYAWAGWSGVCWLGAGVSLSALLFWRLTLRGMPPSAAAVE
Q
>CORE_REP|Org43_Gene3862#
MKGFPSLINVLLASSLVLTIGRGVTLPFITIYLTEHFQLLPKSVGVILGVSFTLGIIASLYGGYLVDK
FSKNRLILLSIVLFALSFFAIPWIPRPGGVIVVLAILHTCYSVLSITIKACFADGLPVEQRIKAFSIN
YTLVNVGWAIGSALGVLVAGLSPLLPFYLSGGLALATVAALSLRLRGGEQRPAPSAAAPAALANFRQT
LAILRCDRRLIYFTLGSTLGAVVFGQFTGYLSQYLITVSSAEFAYKIIGLVMIVNAGIVIALQYLLSR
GMRQENMLRWLALGTLFFIVGLLGFMAAGQAVWLWLAAMAVFTLGEIIVIPVEYMFIDFIAPPHLKGS
YYGVQNLSALGGAINPVLCGVLLSYAAPPLMFVMLIAAALLSLLFFFLGHRLAEHAAAAAEDVR
>CORE_REP|Org4_Gene5062#
MENLGMPSLKLTPRRLTLIAVIVFIAVAIAIALTLYWQRPPQQDYVTAPARLGDIENAVLATGRLDAV
ERVNVGARVSGEVKSLKVKLGDRVTKGQPIADIDDLQQRNDLRNAEAALNVIKAELQAKQAQLKQAES
RFKRQRRMLNDEASSREDFETAEATLATTRAELLSLNARLVQAQIEVDKKKIDLGYTRVVAPMDGIVI
AVVTQQGQTVNSTQSAPTIVKLARLDMMTIKAQISEADITRISPGQKAYFTIFSDPDKRYDATLRTIE
LAPESVMKDDSLAGTSSASGSGTSNASVYYNALLDVPNPENRLRIAMTAQVSLLLGEAKNALLVPIQA
VHKTEGKVQQVQVLTQDQRLETREVTTGITNNVDIQILSGLKAGETVVLSQPAAKSAEDGIFL
>CORE_REP|Org15_Gene4578#
MSALHADGGAKAWLATFAVGLSTFTVVTAEMLPVGLLTPIVSTLNASIGRAGLLISLPALFAALFAPL
VVLGARRTDRRNLLAGFLLLLIAANLLAAAATSLALLFAARILLGFCIGGIWAIAGGLAERLVPPASV
GLALSIIFGGVAAASVFGVPLGVFLGEALGWRMAFLAVAVLAALTLLLLVCVLPPLPVTQAIGWRSFT
ALRANRRLLTGLLLTFLLVAGHFMAYTFVRPLLQTVAGIESRWVGPLLFAYGVAGIFGNFIAGQAAAK
RLRRTLALIALGLALAVLLLPLLGHAPLSGGAFLLLWGIAYGGVSVALMAWMLKAAPDAVEVASSLYI
ALFNLAISCGSLAGGLVVDAGGLTINGALSGLVLLLALAILMGTRRQRPKTAAKADSPPG
>CORE_REP|Org47_Gene2401#
MAIKLEVKNLYKIFGEHPERAFKLLDKGLTKDRLFEKTGLSLGVKDATLAIEEGEIFVIMGLSGSGKS
TLVRLLNRLIEPTRGQVLIDGEDIAKISDTALRTVRRNKISMVFQSFALMPHMNVLNNTAFGMELAGI
PLQERQEKALDALRQVGLENYALSYPDELSGGMRQRVGLARALANNPDILLMDEAFSALDPLIRTEMQ
DELVKLQAQHQRTIVFISHDLDEAMRIGDRIAIMQGGEVIQVGTPDEILNNPANDYVRTFFRGVDISH
VFSAKDIAQRRPVTLIRKTPGFGPRSALQLLRDEDRDYGYVVERGKKFIGVVSIESLKKALSANQTLD
DALLEAPAAVPADTPLSDLISLVAQAPCAVPVVCEEHNYLGIISKAMLLQALDKEGSANE
>CORE_REP|Org32_Gene1843#
MQSFDVIIAGGGMVGLALACGLQGSGLRVAVLEQRQPEMAPPSEQPALRVSAINAASERLLQHIGVWD
DILQLRASAYNAMEVWDRDSFGKIAFRGDECGFSHLGHIIENSVIQQALWKRAESLSDITLITPAALK
QVAWGENDAFVTLEDGRMLTARLVIGADGAQSWLRQHADIPLTFWDYRHHALVATVRTEEPHQATARQ
IFHGDGILAFLPFSDPHLSSIVWSVTPEEAERLKRLEPEQFNRELAMTFDMRLGACSLESERLAFPLT
GRYARSFAAHRLALVGDAAHTVHPLAGQGVNLGFMDAAELISELRRLQRQGKDIGQHLYLRRYERRRK
HGAAVMLASMQGFRELFDGNHPAKKLLRDVGLRLADSLPGVKPKLVRQAMGLNDLPEWLA
>CORE_REP|Org40_Gene547#
MLEPITSEHTVSENNSLTTPSVNVEQPAAAKINLLDLNRQQMREFFAEMGEKPFRADQVMKWIYHYCC
DDFEQMTDINKVLRGKLQRVAEIRAPEVAEEQRSADGTIKWAIKVGDQQVETVYIPEADRATLCVSSQ
VGCALECKFCSTAQQGFNRNLRVSEIIGQVWRAAKIIGALKVTGQRPITNVVMMGMGEPLLNLNNVVP
AMEIMLDDFGFGLSKRRVTLSTSGVVPALDKLGDMIDVALAISLHAPNDTIRDEIVPINRKYNIETFL
SAVRRYLEKSNANQGRVTVEYVMLDHINDSTDDAHQLAEVLKDTPCKINLIPWNPFPGAPYGRSSNSR
VDRFSKVLMEYGFTTIVRKTRGDDIDAACGQLAGEVIDRTKRTLKKKMAGEPINVRAV
>CORE_REP|Org5_Gene782#
MQQNRTSHLGLIFILGLLSMLMPLAIDMYLPSMPVIAAQFGVESGSVQMTLSAYMLGFAFGQLFYGPM
SDSIGRKPVILWGTLIFAIAGCACAMAQSIDQLIGLRFLHGLAAAAASVVINALMRDMFTKDEFSRMM

SFVILVMTIAPLLAPMIGGALLLWFSWHAIFWTMGAAALIGSLLVALFIKETLPKERRQRFHLRTTLG
NFGSLFRHKRVLSYMLASAFSFAGMFSFLSAGPFVYIELNHVSPQHFGYYFALNIVFLFLTTLINSRN
VRRFGAVKMFKLGLLVQLAMGLWLLAVSAVGLGFWALVIGVAVYLGCIAMISSNAMAVILDDFPHMAG
TASSLAGTLRFSIGALVGAVLSMAPGKSAWPMVTSMALCSIVAVLFYVYASRPRDRAA
>CORE_REP|Org16_Gene1641#
MEHAPVSRSTAWLRVVILAVSAFIFNTTEFIPVGLLSDIAASFSMQTEQVGLIITIYAWIVAAASLAC
MLLTSKIERRKLLIGVFMLFIASHVLTAVAWDFTTLVISRAGVALAHSVFWSITASLAIRVAPPGKKA
QALSLLAGGTALAMVLGLPLGRVVGQLLGWRMTFIGIAVCATLALVLLWRLLPVLKSEHSGSLASVPL
LFKRPALVALYMLTIIVVTAHFTAYSYIEPFIQTVAGLSENFTTLMLLLFGAAGIVGSLLFSRYSERF
PSGFFIGAIVLLALSLLLLLPAAGSESHLTVLCIFWGMAIMAIGLSMQAKVLSLAPDATDVAMAIFSG
LYNFGIGSGALLGNQVSLHLGMGNIGFVAAPLALIALGWCLLSVYRSERLQQHHSR
>CORE_REP|Org34_Gene3464#
MTKHLARQRLVYAVVLGLLAALGPLCTDLYLPALPEMAGELNTSTAAAQLSLTTGLLGLGVGQLIFGP
YSDKLGRMRPLLLSLILLLGASLWCALAPTIDQLLIARLLQGIAGAGGAVISRAIARDLYAGHELTRF
FALLMLVNGLAPIVAPVLGGVMLQVMNWRGIFGVLAAIAVLLFSLSALKLRESLPVERRSQGGILAML
MSLGGLLTQRYFMGLCLTQGFVMAGMFAYIGASPFVLQQIYGLSPQMFSLCFAINGVGLIIAAQLASR
LSARWGERRVLRGGLTLAAVASLLLLLAAALHAPLVVLLVPLFFSVAVIGIVGPTASSLAMQSQGDKA
GSASALIGVCMFALGACAVPLTGLGGTSGLSMALTIVGCYAIAILLFGLLARRNDA
>CORE_REP|Org38_Gene4435#
MPNQPNSSFNAGGRTRAFALGQRLSGVALLAALLAGCDNSVAHNAPPPPPVVSAASVVVKPISQWDAF
NGRVEAVQSVQLRPRVSGYIERVNYTEGDEVKKGQVLFIIDDRTYRAAREQAQAELVRARNQAALARS
ESSRTEKLIGTQAISQEVWEQRRSSAAQAQSNVLAAQAQLDMAQLNLDFTRVTAPIDGRASRAMITAG
NLVTAGDSASVLTTLVSLDKVYVYFDVDEATFLRYQQQGRHDVRLPVKVGLVGEDGTPHQGLVDFTDN
QLNAGTGTIRMRALLDNRDRRFTPGLFARVQMPGSAEFNAMLIDDKAVMTDQNRKFVYIVDKDGKAQR
RDIDVGRMAEGLRIVQKGLVNGDRVIVDGMQKVFMPGMPVDAKNVAMTTTASALN
>CORE_REP|Org35_Gene1276#
MNKNRGLTPLAAVLMLSGSLVLTGCNDKETQQQGAQQQAPEVGVVTLKAEPLNITTDLPGRTAAYRIA
EVRPQVSGIILKRNFVEGSDIKAGTSLYQIDPATYQASYDSAKGDLAKAQASASIARVTVNRYKPLLG
TSYISKQDYDNAVSTLQQADAAVVAAKAAVETARINLAYTKVTSPISGRIGKSAVTEGALVSNGQATA
LSTVQQLDPMYVDVTQSSTDFLRLKQELASGALKQENGKAKVKLMLENGTEYAQEGTLEFSDVTVDET
TGSITIRALFPNPNDTLLPGMFVRARLDEGVRSDALLVPQQGVTRNPRGDATALVVGADNKVELRTLK
ADQAIGDKWLVTDGLKAGDRVIVTGLMKVHPGAQVKVQEVDTQAQKQPQSEAQKS
>CORE_REP|Org39_Gene141#
MRKLENFHLLVMLILLVAVGQMAQTIYVPVIADIAHDLSVRTGAVQRVMAAYLLTYGFSQLIYGPISD
RIGRRPVILTGMMIFLVGALGALLSTNLTMLVAASAIQGMGTGVAGVMARTMPRDLYAGTALRYANSL
LNMGILVSPLLAPVIGGALAMVFGWRACYAFLLALCACVAFAMFRWLPETRPVQTEKRRMLASFRQLL
GDSTFSCYLVMLIGALAGIAVFEASCGVLMGGVLGLSGLTVSILFILPIPAAFFGAWYAGRDGKTFHT
LMWHSVISCLLAGAMMWIPGWFGVMNIWTLIVPAALFFFGAGMLFPLATTGAMEPFPYLAGAAGALVG
GMQNMGSGLATWLSAMLPQTGQFSLGLLMFAMALLILLCWWPLSNRMQHQGHTA
>CORE_REP|Org28_Gene2530#
MLNNKDKPASSPWPAIFSLTVACFVMVTTEFLPIGLLTNIAPSLGVSTGTAGLMVTMPGIVAAVAAPA
LSLISGRLDRRLLMLGLSLLLIVSNLVAALAVNFPMMLLGRVLLGICVGGFWSFAANYGRHLVPEANQ
GRATALILSGISVGAVCGVPAGALIGDLFGWRAAFFGGAALAVGVLLAQLRLLTSVPPSRPVTPRDLV
LPLRLPMARIGLIAIVLLFIGHFAAYTYLRPLLQQVFVLSPSAISLQLLAYGAIGLLGTFLGERLGEY
SLRATFILIAAMLAAILIVSPLLSGLGGATLMVMVWGLAFGAVPVCATNWMFAAVPQAPEAGQALLVC
VVQIALASGALLGGEVVDWQGVSSAMLFGGALILSAALVFGLSLRSGAIGAKQC
>CORE_REP|Org39_Gene558#
MSKEKFERTKPHVNVGTIGHVDHGKTTLTAAITTVLAKTYGGSARAFDQIDNAPEEKARGITINTSHV
EYDTPTRHYAHVDCPGHADYVKNMITGAAQMDGAILVVAATDGPMPQTREHILLGRQVGVPFIIVFMN
KCDMVDDEELLELVEMEVRELLSAYDFPGDDLPVIRGSALKALEGEAEWEAKIIELAEALDSYIPEPE
RAIDKPFLLPIEDVFSISGRGTVVTGRVERGIIKVGEEVEIVGIKDTVKSTCTGVEMFRKLLDEGRAG
ENVGVLLRGIKREEIERGQVLAKPGSIKPHTQFESEVYILSKDEGGRHTPFFKGYRPQFYFRTTDVTG
TIELPEGVEMVMPGDNVNMVVTLIHPIAMDDGLRFAIREGGRTVGAGVVAKVIA
>CORE_REP|Org39_Gene3651#

MKTSQNRYDAVVVGGGMVGAAAALGLAQAGWSVALLEYQAPQAFEAQSLPDLRISAIGCTSVGLLKQL
GAWQAVTAMRTAPYRRLETWEWASSRVAFDAVSLGLPELGFMVENRILQLALWQQFAQCANLTLLCPA
RLQSLQRADNAWQLTLDGGEALQARLVVGADGANSQVRKLAAIGTNGWQYRQACMLITVDTGAPQQDV
TWQRFFPSGPRAFLPLYDSWASLVWYDSPQRIRQLQAMPPAQLEREIAAAFPARLGPVKVHAAGSFPL
TRRHAQRYVLPGLALLGDAAHTINPLAGQGVNLGYRDVDALLNVLSDAREQGEDWSSEAVLLRYQRRR
RTDNLLMQSGMDLFYTAFSNNLAPLNVARNLALMAAQRAGKLKEHALKYALGL
>CORE_REP|Org46_Gene1185#
MTAENNLQLNRRILSVVMFTFVCYLTIGLPLAVLPGFVHDHLGYNSVLAGLIISAQYFATLFSRPHAG
RYADQLGPKKVVLFGLACCGASGLFYALAFGVDGYPWLSLLLLCVGRVFLGVGESFASTGSTLWGIGR
VGAMHTARVISWNGVATYGAMAAGAPLGVYLNQQWGLAGVAALIVLAVAVALLLASGKPDVSIAAGQR
IAFRAVFGRIWAYGLGLAMGTVGFGVIATFITLYYADKGWSGAAFSLTLFSCAFVGIRLIFSNVINRH
GGLKVTLASFLVEIVGLLLIWQAGEPWMVQTGALLAGAGFSLVFPALGVEAVKQVPPQNQGTALGTYS
AFLDLALGITGPLAGLLIGQAGVPSIYLAAALLVALGVLLTLRLLQRSRAIQE
>CORE_REP|Org12_Gene3114#
MLTIGTALRPSATRVMLLGSGELGKEVAIECQRLGLEVIAVDRYPDAPAMHVAHRSHVINMLDGDALK
AVIEQERPDYIVPEIEAIATAMLVELERQGHRVVPCAEATRLTMNREGIRRLAAEELALPTSSYRFAD
SEIAFRQAVEHIGYPCIVKPVMSSSGKGQSLIRTPEQLQSAWDYAQQGGRAGGGRVIVEGLVRFDFEI
TLLTISAVDGVHFCEPIGHRQEDGDYRESWQPQRMSATALSRAQAIAEKVVKALGGFGLFGVELFVCG
DDVIFSEVSPRPHDTGMVTLISQDLSEFALHVRAFLGLPIGAIRQFGPSASAVILPQLTSTDVRFSGL
ENALRGHNQLRLFGKPEIAGQRRLGVALATADTTEQAVEQAKQAATAVVVNG
>CORE_REP|Org47_Gene2881#
MTLADYNGHLVTLCLMATGTFAIGTDAFIVAGVLSDISDTFAVSPAQAGQLISVFALAYMLFAPLTAW
LLGNVNRKHILQLALVLFIAGNLACAWATSYLQISLGRVLAALGAACYTPQAAAAAVGLVAEKRRGLA
ISIVYGGMTLAIALGIPFGTFLAKLIGWREIFLFIALLGAIALLGLSLALRAIAPPGKHSLKERLAPL
RQKAVLTTLLITFFAVCSEHIVYSYVSVLLKNTQFGPQAILPLALLVFGIGAVIGNFASGALTDALGS
KFVLLFSVAIQTLSLFLLAFYVTSPWWVLAIFLVWGITGWMYLVPIQHHLLSLSKRFGALTVSLNSSV
LYAGIAAGGMLGGLTLYALPAHYLPLFSLPLGAIALLLTLLFFRGETGNE
>CORE_REP|Org27_Gene3222#
MKVNYPLLALAVGAFGIGTTEFSPMGLLPTIAKGVDVSIPMAGMLISAYAVGVMVGAPLMTLLLSHRA
RRSALIFLMAIFTLGNVLSAIAPDYTTLMLSRIITSLNHGAFFGLGSVVAASVVPKEKQASAVATMFM
GLTIANIGGVPAATWLGETIGWRMSFLATAGLGVIAMLGLWFSLPKGSAGARPDVKRELSVLVRPQVL
TALLTTVLGAGAMFTLYTYISPVLQHITEATPLFVTTMLVLIGVGFSIGNYLGGKFADRSESATLKGF
LLLLVAIMLLIPLLARSDIGAAVSMMIWGAATFAVVPPLQMRVMRVASEAPGLSSSVNIGAFNLGNAL
GAAAGGAVVSAGLGYSFVPVMGAIIAGLALLLVLFTSRTAAKVVYANG
>CORE_REP|Org14_Gene4877#
MTTVEELCLRFIAGAGPVRRAAGRAGHPPQLASQIVDQYAEHIFYNSGATGMALVVIDGNQVVNRSFG
DTKPGNNLRPRPDSLIRIASITKLMTSEVMVKMAAAGQVKLTDPLRKYAPKGAYVPAYNAGQPITLLN
LATHTSSLPREQPGKKPPKTPVFTWPTKAQRWQWLAHANVTVPPGVRAAYSNLAYDLLADALSRAAGK
PYNALLKEKITAPLGMVDTTLTPSPEQCSRLMVAAAGPSACRDTTAAAGSGGVYSTPRDMQRWMQQFL
SSSASGPRKATAASEQTMYFQRHDLVSLKGMDVPGQADALGLGWVYMAPKDGLPGIIQKTGGGGGFIT
YMAMIPAKNVGVFVVVTRSELSKFTNMSDPVNRLVSDLAANKS
>CORE_REP|Org5_Gene2948#
MIKLENLTKQFMQKNGTPFNAVDNINLDVPEGEICVLLGPSGCGKTTTLKMINRLIEPTGGTILVNGE
DTSALDTVSLRRKIGYVIQQIGLFPNMTIEENITVVPRMLGWDKKRCHDRAEELMSMVALDPKRFLHR
YPKEMSGGQQQRIGVIRALAADPPVLLMDEPFGAVDPINRETIQNEFLDMQRQLKKTVMLVSHDIDEA
LKLGDRIAVFRQGKIVQNASADELLARPANDFVASFVGQDRTLKRLLLVQAGDVADQQETVTVRRETP
LVEAFGLMDDIDARSVTVVDADGKPLGYVKRREARGAPGVCADSLHRFRVTARAEENLRVVLSKLYEH
NTSWMPIVDEDGRYSGEISQDYIADYLSSGRTRRVLTPQ
>CORE_REP|Org8_Gene2746#
MNDAIPRPQAKSQKVFTPLLEIRNLTKTFDGQNAVEDVSLTIYKGEIFALLGPSGCGKSTLLRMLAGF
EQPTEGQIVLDGQDMSHVPPYQRPINMMFQSYALFPHMTVEQNIAFGLKQDKMPRAEIAERVAEMLAL
VHMQEFAKRKPHQLSGGQRQRVALARSLAKRPKLLLLDEPMGALDKKLRDRMQLEVTDILERVGVTCV
MVTHDQEEAMTMAGRIAIMNRGKFVQIGEPEEIYEHPNSRFSAEFIGSVNVFDCVLQERHDDALILQS
PGLRHAIKVDPDASVVDGVPIQVALRPEKILLCEQVPEDGCNFAVGEVAHISYLGDLSIYHVKLHSGQ

IISAQLQNGHRFRKGMPTWGDEVRLCWETDSCVVLTV
>CORE_REP|Org36_Gene3978#
MTETSSLTPLVELHALSKAFDGKTIIADLELAINHGEFLTILGPSGCGKTTVLRLIAGLEDADRGRIV
LDGQDITAIPAEHRHVNTVFQSYALFPHMSVFDNVAFGLRMQKVPAAELTPRVEEALRMVQLDTFAKR
RPGQLSGGQQQRVAIARAVVNKPKVLLLDESLSALDYKLRKQMQNELKALQRKLGITFVFVTHDQEEA
LTMSDRIVVMREGRIEQDGTPREIYEEPKNLFVASFIGEINIFDAVVLQRLDAQRVRANVEGRECDIY
ADLPVEPGQKLKVLLRPEDLRVEEVNDSAQHDGLIGYVRERNYKGMTLESVVELESGKTVMVSEFFNE
DDPDVDHSLNQKMAVTWVESWEVVLADEEIA
>CORE_REP|Org35_Gene788#
MASVTLRSVYKAFGEAVISKDVNLTIEDGEFVVFVGPSGCGKSTLLRMIAGLEDITSGDLLIGEKRMN
EVPPSERGIGMVFQSYALYPHLSVADNMSFGLKLAGAKKAEINQRVNQVSEVLQLAHLLDRRPKALSG
GQRQRVAIGRTLVAEPDVFLLDEPLSNLDAALRVQMRIEISRLHKRLQRTMIYVTHDQVEAMTLADKI
VVLDAGRVAQVGKPLELYHYPANRFVAGFIGSPKMNFLPVKVTAAEPRQVQVELPNRQLVWLPVEGAG
VQPGANLSLGIRPEHLLPGEASEVRLTGDVQVVEQLGNETQIHIQIPAIRQNLVYRQNDVVLVEEGAT
FAIGLPPHRCHLFREDGTACKRLHQEPGV
>CORE_REP|Org6_Gene4336#
MLLRHIRYFLAVAEQGNFTRAAEALHVSQPTLSQQIKQLEDALGAPLFDRSGRRVQLTDAGEAWMRYA
RLALQDLDAGARAIHDVATLARGHLRLAMTPTFTAYLVGPAIDAFYRRYPGITLSIEEMAQERIEVLL
AQDRLDLGIAFEMAQSAEVEATPLFSETLELMVGADHPLAARRRPLTLAEWRHLPLALLSGDFATRQF
IDRYCTQLGFRPLVAVEANALGAIVEIVRRGQLATLLPAAIARENRQLKKVALVNAMPARQAVLLQRQ
GAYRSAAAQAFIAVLQQQGVTPTPPALHHPQQMHQSETEANQRADDDEALAAAGIAQGERAGDAAQQV
DKGDDKQRGPQKRQRRDQARAG
>CORE_REP|Org36_Gene2461#
MKAATAVIDRRALRHNLQQVRRQAPQSRLIAVVKANAYGHGLLETAHTLQDADCYGVARIGEALMLRS
GGIVKPILLLEGFFSAEDLPVLVANNIETAVHSIEQLEALEQAELARPVPVWMKLDTGMHRLGVRPEH
AEAFYQRLCACRNVAQPVNIMSHFSRADEPESDTTLKQIACFEQFARGKPGQRSVAASGGTLLWPDAH
NEWVRPGIILYGVSPLDNGSGAEHGLQPAMTLKSSLIAVREHKAGEAVGYGGTWVSPRDTRLGVVAMG
YGDGYPRSAPTGTPILINGREVPIVGRVSMDMISVDLGPAAADKVGDEAVLWGPALPVERIAVCTGIS
AYELITKLTQRVAMEYIGD
>CORE_REP|Org5_Gene1945#
MPRPITATMHLGAIENNLQVVRRFAPGAKVWAVVKANAYGHGIKHVWRSMAQTDGFAMLDLAEAVLLR
ESGWQGPILLLEGFFQPQDLALLDRYRLTTAVHSDWQLAAIADATLSAPLNVYLKVNSGMNRLGFAPE
RLHEVWRRAQAIANIGELTLMSHFATADGPEGVTQQMATIEAAAADIPLPRCLANSAATLWHSSTHGS
WVRPGIILYGASPSGCWNDVAATGLQPAMTLSSEIIGIQQLKSGDRVGYGGRYSAAGAQRIGVVACGY
ADGYPRHAPTGTPVWVDGVLTRTLGTVSMDMLAVDLTPCPQVELGAEVELWGKRLPVDEVATAAGTLG
YELLSALAARVPVAIEA
>CORE_REP|Org45_Gene3361#
MAGLKLQAVTKSYDGKTPVIKQIDLDVADGEFIVMVGPSGCGKSTLLRMVAGLERTTSGDIYIDTRRV
TDLEPKDRGIAMVFQNYALYPHMSVYDNMAYGLKIRGFGKDHIRQRVEEAARILELEPLLKRKPRELS
GGQRQRVAMGRAIVREPAVFLFDEPLSNLDAKLRVQMRLELQQLHRRLKTTSLYVTHDQVEAMTLAQR
VIVMNKGVAEQIGTPSEVYQRPASLFVAGFIGSPAMNLLPGTLSADGGQLLLADGMALPLPAAKPQWA
GRPLTLGIRPEHIQLVAQGQGVPLQLQTLELLGADNLAHGQWGGHGVIARLSHETLPAAGSTLYLQLP
AQALHFFDTDSGLRMD
>CORE_REP|Org3_Gene3945#
MIELSVENLHLTYGDNPVLKGVSMDLKRGEVVSLLGPSGSGKTTLLRAVAGLEKPSQGRIVIGNNAVY
NGSARSEIPAEERNLGLVFQSYALWPHKTVFENVAYPLKLRKIASAEITLRVQAVLDQLGLGHLAKRH
PHQLSGGQQQRVAIGRALVYNPPVILLDEPLSNLDAKLREEARVFLRELIIKLGLSALMVTHDQNEAM
AISDRILLLNNGKIEQQGTPQEMYGSPTTLFTAEFMGSNNRLPGKIVALEGDRARIEGKDWALWGKAG
EGVQVGQEGSAVIRVERVRLGEDPQGNQLELPLLTSMYLGDRWEYLFRTVAEDFVVRAYGHEARDRAL
CRLSLPAEHLWIFPKA
>CORE_REP|Org13_Gene3304#
MTSKPEQIRQRVKRGELIAGEDLHGLSFAGMDLAGGMFNELNLSGVNFSDCDLRDSVFSDCRLEHAQF
ARANLKQTAFNQCAMPGGRFCESHIELTMFNDCRLEQSDFSRLPLNQSHWMSCQLAGANFSATQHDRT
TFYESPLDGAALNHARLSLVTFFRLNLCKTEFEGVDFDRVTFFECDHRGKSYAGQRLIACQFTDNQLD

DVDFSQATLRQSNFKGASLRRANLTGVQAQQSLWLEANLTQAQCRSGQFDQAIFSEATLDAANFSQAR
LYQCVFQRSRAARCDFSDSDLTYADFCYADIGAADFRRARFMRTRMHRAHQQQTRWGDRSGILERDEE
LYAAETWSAQRQSRI
>CORE_REP|Org20_Gene2219#
MLELDFSQQLGDLNLNVRADLPAQGITAIFGLSGAGKTSLINAIGGLTRLQQGRIALNGRTLVDTAAG
LCLPPEKRRIGYVFQDARLFPHYRVRGNLQYGMAAGMRAQFNTIVELLGIGPLLNRLPLTLSGGEKQR
VAIGRALLTAPELLLMDEPLASLDLPRKRELLPYLERLAQDVNIPILYVSHSLDEILRLAEQVMVLDR
GEVRAFGGLEAVWASSALRPWLQREDQSSVLRVSVIEHHQRYAMTALALGDQRLWVSGIDAELGTQLR
IRINAADVSLVLQPPVNSSIRNVLPAKVSESLDVDGQVEVKLAVGEHVLWARITPWARDELAIRPGQW
LYAQVKSVSISRESR
>CORE_REP|Org7_Gene1147#
MISMRRRLLLMLALILLVTQLISAFWLWHESQEQISFLVDETLSAKVRSERVDTEIAEAIASLLAPSL
IMMIVTLLASFWAISWITRPLNQLQQRLEKRSADNLTPLPITSDSQEMVAVTNALNQLFSRLDNTIQQ
ERLFTADAAHELRTPLAGIRLHLELMEKQGVKGSQALIARIDQLMHTVEQLLMLSRAGQDFASGHYQH
FDWVADVIQPLREELDEMTAQRGQTLAWQLPAAAAVNGDPVLLRLLLRNLVENAHRYGPEGGAIQVRL
TPQDRGYLLQVIDDGPGIKEEMVGELTQAFRRMDQRYGGSGLGLNIVIRIVQLHQGRLTLENRRDARG
LNAQCWLPEKALK
>CORE_REP|Org30_Gene945#
MSKIRVLCVDDSALMRQLMTEIVNGHADMEMVATAPDPLVARDLIKKFNPQVLTLDVEMPRMDGLDFL
EKLMRLRPMPVVMVSSLTGKGSEITLRALELGAVDFVTKPQLGIREGMLAYSELIAEKIRTAARARLP
QRSNSPAPAILSHAPLLSSEKLIAIGASTGGTEAIRQVLQPLPATSPALLITQHMPPGFTRSFAERLN
KLCQITVKEAEDGERVLPGHAYIAPGDRHLELARSGANYQVKLHDGPAVNRHRPSVDVLFRSVAQYAG
RNAVGVILTGMGNDGAAGMLEMHRAGAYTLAQNEASCVVFGMPREAIAGGGVSEVVELDRMSQRMLAQ
IAGGQALRI
>CORE_REP|Org40_Gene4771#
MSQKNFVELRNVSKRFGSNTVIDNITLTIPRGQMVTLLGPSGCGKTTILRLVAGLEKPSDGQIFIDGE
DVTHRSIQQRDICMVFQSYALFPHMSLGENVGYGLKMLGIPRAEVKARVQEALAMVDLAGFDDRYVDQ
ISGGQQQRVALARALILKPKVLLFDEPLSNLDANLRRSMREKIRELQKQFDITSLYVTHDQSEAFAVS
DTVLVMNKGHIMQMGSPQDLYRQPASRFMASFMGDANLFPAGFSADHVDISGYRLPRPAHFAAEGAGT
VGVRPEAITLSEHGDESQRCVIQHVAYMGPQYEVTVAWHGQQILLQVNATRLQPNVGEQYYLEIHPYG
MFMLADAA
>CORE_REP|Org33_Gene1231#
MTIAVQFIDVSRTFGDVRAVDRVSIDIQDGEFFSMLGPSGSGKTTCLRLIAGFEQLTSGSIRIHGQEA
ANLPPYQRDVNTVFQDYALFPHMSVLENVAYGLMVKGVAKRERLARAQEALESVALGFVAERKPAHLS
GGQRQRVALARALVNRPRVLLLDEPLGALDLKLREQMQGELKKLQRQLGITFIFVTHDQSEALSMSDR
VAVFNNGRIEQVDTPRELYMRPKTPFVAEFVGTSNVVRSELAQRLLGESRTFSIRPEHIRLLEHGGAA
QDEIQVQGTLQEIHYQGAATRYEIALNGGEKLLVSQANPQWIAEGQQRQIGQPIVACWPRAAMVPLLE
ER
>CORE_REP|Org14_Gene1844#
MIVLSNVCKTFDSTQGRVVAVDNVSLAVEAGQIYGIIGYSGAGKSTLIRLLNGLETPTSGRIDVGGFD
IARAKGSHLRQARLKISMVFQHFNLLWSRTVSQNIAFSMQIAGVPKAQIAPRVAELIALVGLQGREDA
YPSQLSGGQKQRVGIARALANNPSVLLCDEATSALDPQTTDAILDLLLDINRQLKLTIVLITHEMHVV
RKICHRVAVMENGRIVEEGPVLDVFTRPQQPITRQFVKQVSQYADTEESFNPLLTAHLPGAIFKLTFV
GVQTHQAVISEVIRRYALTINILHGKISHTLNGSFGELYIHAEGNEQQVADMLSLLHERDIAVEVIQH
D
>CORE_REP|Org37_Gene944#
MSTIEHPQLQTGAGVKTSPLLDVKDLRVTFSTPDGDVTAVNDLNFDLRAGETLGIVGESGSGKSQTAF
ALMGLLASNGRIGGSAKFNGREILNLPENQLNKLRAEEISMIFQDPMTSLNPYMRVGEQLMEVLMLHK
KMSKSEAFEESVRMLDAVKMPEARKRMRMYPHEFSGGMRQRVMIAMALLCRPKLLIADEPTTALDVTV
QAQIMTLLNELKREFNTAIIMITHDLGVVAGICNKVLVMYAGRTMEYGSAREVFYQPSHPYSIGLLNA
VPRLDAEGEALLTIPGNPPNLLRLPKGCPFQPRCPYAMEQCASAPPLEQFGEGRLRACFKPVEALV
>CORE_REP|Org7_Gene812#
MTAVTDKKVLLEVADLKVHFDIHDDKQWFWQPPKTLKAVDGVTLRLFEGETLGVVGESGCGKSTFARA
IIGLVKATSGRVAWLGKDLLGMSDADWRKTRSDIQMIFQDPLASLNPRMTIGEIIAEPLRTYYPKMPR

QEVKDKVKAMMLKVGLLPNLINRYPHEFSGGQCQRIGIARALILEPKLVICDEPVSALDVSIQAQVVN
LLQQLQREMGLSLIFIAHDLAVVKHISDRVLVMYLGHAVELGTYDEVYHNPQHPYTKALMSAVPIPDP
DKEKEKQIQLLEGELPSPINPPSGCVFRTRCPIAGPECAKTRPLLEGSFRHAVSCLKVDPL
>CORE_REP|Org2_Gene127#
MPLLDIRNLTIEFMTAEGPVKAVDRVSMTLTEGEVRGLVGESGSGKSLIAKAICGVTKDNWRVTADRF
RFDDIDLLQLSPRERRRLVGHNVSMIFQEPQSCLDPSESIGRQLAQAIPGWTYKGHWWQRFNWRKRRA
IELLHRVGIKDHDDIMGSFPYELTEGECQKVMIAIALANQPRLLIADEPTNAMEPTTQAQIFRLLARL
NQNNNTTILLISHDLQMMSKWADRVNVLYCGQTVESAQCEELLAAPHHPYTQALIRAMPDFGRSLPHK
SRLNTLPGAIPSLEHLPIGCRLGPRCPYAQKKCIETPRLRPVKNHFFACHFPLNMEEQ
>CORE_REP|Org31_Gene600#
MKLAIYSTKQYDRKYLELVNQQFGYELEFFDFLLSKKTAKTAAGCKAVCIFVNDDGSREVLEELAALG
VEILALRCAGFNNVDLDAAKELGIKVVRVPAYSPEAVAEHAVGMMMCLNRRIHRAYQRTRDANFSLEG
LIGFNMHNRTAGVIGTGKIGVATMRILKGFGMKLLAYDPFPSEQALELGAEYVDLKTLYAQSDVITLH
CPLTPENHHLLNADAFAMMKNGVMVINTSRGALIDSTAAIDALKQQKIGALGMDVYENERDLFFEDKS
NDVIQDDVFRRLSACHNVLFTGHQAFLTEEALTSISQTTLQNISQLDRGEACPNQLNA
>CORE_REP|Org42_Gene2589#
MAYLNVTRLNKHYGQTQVFQDIDFTAEEGEFVTLLGPSGCGKSTLLRCLAGLTPVDSGQILLQGQDLV
PLAPQKRGIGMVFQSYALFPNMTVEGNVAFGLKMQKLAAGQIGQRVQEVLALVELSDLAKRYPHQLSG
GQCQRVALARSLVTRPRLLLLDEPLSALDARIRKHLREQIRRIQRELNLTAIFVTHDQEEALTLSDRI
VLMNKGQIVQSGDAETLYTQPADAFAAGFIGNYNLLTAEQAAQLTGRSYVGKVAIRPESIGLLPAGQG
IGGVILGHSLLGNVVRYRIQVRGVELLVDVLNRSVADLRPDGEQIGLHLEPVVLREVA
>CORE_REP|Org8_Gene900#
MSQNQPLLQAIDLKKHYPVKKGLFAPERLVKALDGVSFTLERGKTLAVVGESGCGKSTLGRLLTMIEV
PTGGELYYQGQDLLKPDVSAEKLRRQKIQIVFQNPYGSLNPRKKVGQILEEPLLINTSLSAAERREKA
LEMMAKVGLKTEHYDRYPHMFSGGQRQRIAIARGLMLNPDVVIADEPVSALDVSVRAQVLNLMMDLQQ
ELGLSYVFISHDLSVVEHIADEVMVMYLGRCVEKGSKEAIFNNPRHPYTQALLSATPRLNPDMRRERI
KLTGELPSPMNPPPGCAFNARCRRAFGTCVQLQPQLKQYGEQMVACFAVDQDEHPGA
>CORE_REP|Org24_Gene288#
MALLNVDKLSVHFGDEGTPFRAVDRISYSVEQGQVVGIVGESGSGKSVSSLAIMGLIDFPGKVMADKL
EFNGQDLRKISEKERRQLVGSEVAMIFQDPMTSLNPCYTVGYQIMEALKVHQGGNRRTRRQRAIDLLT
QVGIPDPASRLDVYPHQLSGGMSQRVMIAMAIACRPKLLIADEPTTALDVTIQAQIIELLLDLQQREN
MALLLITHDLALVAEAAHHIIVMYAGQVVESGKAAEIFRAPRHPYTQALLRALPEFAADKARLASLPG
VVPGKYDRPTGCLLNPRCPYANERCRNEEPELRSIPGRQVKCHTPLDDAGRPTV
>CORE_REP|Org41_Gene1020#
MNFQQLKIIRESARCNYNLTEVANTLFTSQSGVSRHIRELEEELGIEIFIRRGKRLLGMTEPGKELLV
VAERILNDANNIRRLADVFSSNDSGQLHIATTHTQARYSLPGVIKEFRALYPRVRVVLNQGSPEEIVS
MLAAGEADIGIASERLMSDESLAAFPYYRWHHTILVPEGHELTRQPQVTLEMLSTLPLITYRQGITGR
AKLDAAFKAAGLTPDIALSAQDSDVIKTYVELGLGVGVLADMSYEKERDRGLVSLNAEHLFEPNTVWL
GLKRSQLQRNYAWRFIQLCNPTLSLTEIKDKVFSSQLDAVIDYQI
>CORE_REP|Org43_Gene4535#
MAMIELRRLRAFVTVVEEGNITRAAERLFIQQPPLTRLLQGLEDELGVKLLQRLPRGVRVTEAGDVLF
EEARALLARAERLREAVQRAARGEQGHIAIGFTSSAALHPFVPNLLRRYRDILPGITTQLEEAGSGEL
MEALLEQRLDAAFVRSPANGIPGLSVEPVLSEPMIVALPLGHRLAQETQQPLPLAELAHEAFILYRRP
AGQGLYDAILAACHRAGFSPRIVQEAPRLPATLSLVGAGLGVSIVPGSMRRLGGDGIVYRTLAAEAQL
SAPLYLALRRSPASPIVERFRQLVLETVGAPDADVTTTARTANKK
>CORE_REP|Org6_Gene518#
MAEYDSEIAMVKEPADIHLRSVDLNLLTVFDAVMQMQNITRAANSLGMSQPAVSNAVARLKVMFNDEL
FVRCGRGIQPTMRARQLFGPVRQALQLVQNELPGSEFEPLTSTRAFSLSLCSPLDLRLGAGIINHVKQ
IAPQLNLQIKSYINNNIERQLRYQDVEFVIGYSRFESAEFRSLAMFDDELVLAVAQAHPRIGEEVTPE
HMLAEQHAAVSLESFGSFSKPFYLDEPMLRAVTQQCTDLYSVLNMVSQTEMVAIAPAWLVRQQTEALK
IKAVPLCGNDNKATCYLSWHESSERDKGHQWMKSVLIEAGNPK
>CORE_REP|Org33_Gene2258#
MDQVQAMRIFTRIVELGSFSRAAERLQLPRATVSNALKRLEQRLGVRLLIRTTRQVQVTSEGSLYYQR
CVQLLGALEEADTLFSHHKLQPSGKVRIDMPHSLARQIVIPALGDFYRRYPDITLALGANDTHVDLLR

EGVDCVLRAWETEDDSLVARRIAQLPQITCASPAYLQASGTPLDIDSLAPHRAVGYFSLASNRDYPLE
FCRGGKVELRELPARLSVSGADAYIAGARAGMGLIQAARYSLAPWLERGELVEVLADTPPPPMPIYIM
YPPGRFLAPRVRVLIDWLIWLFDQQKSGDMAVFPANARKAGK
>CORE_REP|Org41_Gene3376#
MIELKHLRTLQALRNSGSLAGAAAQLHQTQSALSHQFSDLEQRLGFKLFVRKSQPLRFTAQGEILLQL
AEQVLPQIQQALQACHEPHQTTLRIAIECHSCIQWLTPALDNFRRRFPQVVMDFTSGVTFDPQPALQQ
GELDLVMTSDILPRSGLHYSPMFDFEVRLVLAPDHPLAGRPHIEPEDLSDETLLIYPVQRQRLDIWRH
FLQPAGVSPALKNVDNTLLLIQMVSARMGIAALPHWVVESFEQQGLVVTKTLGDGLWSRLYAAVRDGE
QRQAVTEAFIRSARQHACDHLPFVRDAARPGATCAKALAAGV
>CORE_REP|Org5_Gene3088#
MFWKRCLLGAALAVMSLQAGAAAPQAKTPTPGVYRIMLGSFEVTALSDGIIRLPADKLLLNTTPQQIA
AGLAERHQSLPVVTSVNAYLINTGDKLVMIDSGAGQLLGDGLGKLVDNLRAAGYQPEQVDEIYLTHMH
PDHLGGLTHDGKAVFPNAVVRAASQDADFWLSAERLKQAKAQNKGNFEKAMAAIKPYQAAGHFKPFNG
DGELSPGIAAFAAHGHTPGHSVYQVTSQGKKLLLLGDLIHVAAVQMPHPKVAISFDSDAKAAVAQRLR
VFSDSARQSELVGGAHLSFPGLGYLNRQGEGYSWVPLNYGAL
>CORE_REP|Org31_Gene1780#
MNIIYYHPLFNAQEWLAGIKQRLPQAEIREWQRGDERPADYALVWRPPHEMLANRRDLKAVFALGAGV
DAILDQERKHPGTLPAGVPLLRLEDTGMAQQMQEYALSYVLRYFRRFDEYQALQQRQEWQPLDPHSLD
DFTIGILGAGVLGQSVARKLTEFGFSVRCWSRSAKQIDGVQSFAGEAQRAAFLDGVKLLINLLPNTPE
TVGILNRELFAQLSSGAYLINIARGAHLVEADLLAALEQGQLAAATLDVFAREPLPQDHPFWRHPRVT
ITPHIAAITLPQQAMDQIAANIRALEAGHAPAGVVDRQRGY
>CORE_REP|Org30_Gene1499#
MIHFEQVSKIFQGKPAVDDLTLHIAEGEFTVLIGTSGSGKSTTLKMINRLIEHDRGKILFAGEEIQSF
KPQDLRRRMGYAIQSIGLFPHWTVEENIATVPQLLKWPRARIRDRVTELLELLHLEPDLFRRRYPHQL
SGGQQQRVGVARALAADPEVLLMDEPFGALDPVTRAALQAEIARIHQLSGRTIVLVTHDIDEALGLAD
RLVLLDQGRVVQQGTPLALLTAPANDFVRDFFGRSDRGIKLLSLGTVAERVRPGAAEGEPIAAAMSLR
EALSVFVARGSDCLPVVDERGEALGVLHFNDLIAGQALS
>CORE_REP|Org28_Gene4384#
MSTNLSYALLPEMAVFVQVVESGSFSAAARKLGTSPSAVSRSVAKLEQALALQLLHRTTRKLRLSESG
EEAFAHCRTLLAAADAVMAIGGRGAVEPEGLVSVSVPKAVGRFVLHPHMPEFLRRYPKVDVRLRLEDR
YMDLIDDRVDLALRITDRPSPGLIGRQLMRIDHLLCATPHYLAQHGTPQHPHALAAHSCIYLGETPSD
AQWKFRRSGKTVTVNVRGRYAANHTGVRLDAVKQHIGIGSLPYFTARQALDDGEIVQVLPEWDFLSSY
HGGLWLLYAPNQYLPPKLRVFIDYLVACLAQEPQLKRLA
>CORE_REP|Org15_Gene1826#
MKNFSIKITRIAITLILVLLGIAAVFKAWVFYTESPWTRDAKFTADVVAIAPDVSGLLTDVPVVDNQL
VKKGQVLFVVDRPRYEQALAEAGADVAYYQTLAAEKRREAGRRVKLGVQAMSQEEIDQSNNSLQTVQH
QLAKAIAARELAQLDLERTTVRAPADGWITNLNVHAGEYITRGSVAVALVKKDSFYILAYLEETKLNG
LNKGDRAEITPLGSNRIMHGTVDSVAAAVNNSSSTVNNKGLASIDSNLEWVRLAQRVPVKILLDAKDQ
QHPYPAGTTATVVIVGKNDRNADSGSPFVRLMHRLREFG
>CORE_REP|Org15_Gene3972#
MKSDLSALPAFVAVAEGGSFAAAAEKLHLTRSAVSKIVSRLEARLGVMLFMRTTRSLSLTDEGALYYE
HCRQALANVQAAENQLDSGKMQVSGRLRVSVPVLFGHLCIAPLLTALANEHPLLTLEISFSDRRIDLV
DEGFDLAVRIGELADSGSLVARRLGEHGMLLCASPDYVRRCGEPSTVEALSRHQAVGYLHAGAVLPWQ
LRGENGELQSFSPPAKMMMDDMQGIVDAISAGAGAGIAWLPEWLVRERLMAGTLVEIMRGESNLSFPV
NVVWPYMPYQPLKVRLAVDKLVAELPAKLALVPPPLSQR
>CORE_REP|Org5_Gene2049#
MFPSKKHSQRATPLTSYQFSRLHTFECVARHLSFALAAQELSITPSAVSHRINLLEKELGFLLFQRFH
RRITLTPEGERMQWALDSSFNTLNQEILDIKNRELTGTLTLYSHPSLVQCLLLPRIGDFIAQHPTIHL
NILTGQEIINLANRGVDLAMYFGKLPSGRHLDEAFMQESMVPICTPQYAAAHSLYDAPENLAHCTLLH
DRYNSGEDEWQTWSQHFALGLDTDSKSMEFDRSDLAVLAATRHLGVAMGRLNLVQDWIKSGELIIPFT
DMTVPCEHCYFTSTISERQWPKILAFKQWIMKIAPLV
>CORE_REP|Org48_Gene2513#
MQVKKRALLGQLSDMDLRLLRVFKAVVDCGGMSAAELELNISLSTISKHIKDLEQRLGLTLCQRGREG
FAVTDEGLLIYQETVNLLAATEAFRRGVDEVHQRMGGQLHVAIFDHTVSNPQAQIGRAIALFSERAPE

VSLQMYVEPINTIERGVIDGQFQVGVIPMHRSAESLSYHSLFSERMFLYCGAQHELFSGPHETLNWDL
LHNYAFAGLGYHSPNMELSLQQHLHRKATGFAQESIATLILSGKYVGFLPDHYAAFFVAQNMMRAIKP
ALFRYHCEYSSVLRRSPVPQRVVKLFHECLLAAHGTA
>CORE_REP|Org17_Gene4464#
MNPPHALPDPGRINFRLLHYFRVVAEEMNFTQAARRLNMSQPPLSKHIKELESQLGVVLFKRTTRSMT
LTPAGRTLLRNVERLLDQADSALHQVQQMGRGEGGHMVVGMVGTSAWGGLIAALRRFSEQSVGATWSL
NELTPSQQITALQKRHIDIGVWREAQQQTLPGLTCQRLARESIAVVLPLDHPLAQQENIPLAALQNDS
FIVLPPHEASLGLYLHNLCLQQGFLPDVAYQVNEPQTLLALVAEGCGITLLPDSYGRIPWPGVRFCSL
QQAPPADLYAVYRTDSVTPVVQAFLATLTPSSSAAR
>CORE_REP|Org47_Gene2528#
MNFRRLKYFVKIVDIGSLTQAAEVLHIAQPALSQQLATLEGELKQQLLIRTKRGVQPTEAGNILYAHA
QTILRQCEQAQSAVNSAGQAMSGQVSLGLASGSTAAQLALPLLQSLRDQQPGILLSLHENGGAALAGQ
VANQTLDMAMVYGAKMPAGLHAIALMREDLYLVATRAVPHPGNSVELLDVARLNLFLPREGDAVRNQL
EEAMALRKLAVNVVGEIESSGALSAAIASGLGATVLPESVARAMIGPAKAWMARINAPTMSVPLSLCM
SGQQALSAPALLVKDLLLSIAGGRSQEKRALALVR
>CORE_REP|Org26_Gene1133#
MNYALELAQLTKTYAGGVKALRGIDLSVEAGDFYALLGPNGAGKSTTIGIISSLVNKTAGSVRVFGYD
IDKDIVNAKRQLGLVPQEFNFNPFETVLQIVVNQAGYYGVTRREAMARAEKYLNQLDLWGKRNERARM
LSGGMKRRLMIARALMHQPKLLILDEPTAGVDIELRRSMWGFLKELNAQGTTIILTTHYLEEAEMLCR
NIGIIQNGELVENTSMKGLLAKLKSETFILDLAAKSPLPKLDGYHSRLTDTSTLEVEVMREQGLNGLF
TQLSAQGVQVLSMRNKANRLEELFVTLVNGNGEKA
>CORE_REP|Org40_Gene3137#
MPISLPSLDVLKTFVVVAQRLNFTHAARQLHLTQGAVSRQILGLEQRLGYPLFSRQARGLALTPQGAQ
LLAPVQQALGQLDEALTRAAAPPGALRIKCPTCAMRWVLPRIIRLQNERPDMHIELTASVSHGLDFST
EQFDAAVVFGRPPGKKLTAHLLFDEILTPVCTPTFLPPTPRLTDLTDKTLLHPTRDRRDWLRWLKAAG
ADALPSGKAQHFDTLDLAMSAALQGFGIAIGDLCLLEEDIQAQRIVTPFPLCVSSGAAYYLVYPERTV
APPTLTALVDFLAAEAADSRARLQNYLPMTCNAL
>CORE_REP|Org33_Gene2755#
MLATHEYANDLILFALIVDCGSFSKAAESAGITSSVVSKRIGRLEKSLGARLLYRTTRSLTLTESGQA
LYQQAKEIGAKVQEALYAVSEKSEELTGTIRMSVPTISGELLLSESVAEFCALHPSLKVEMRLENRFV
DLVEEGIDLAIRTGTMPDSSLIARPIFDSRWVIVCSPGYLESHPEPRSAEDLLGHNCLTYTYQESGTA
NWLMKRPGRNEIYELQVNGNLSANNARAIRKAVIGGHGIAMVPRCMVYEDLQDGKLTEILAGHCGKVL
GIYAVYPYTRNLPLKTRLLIEHIIGSYQNISHYF
>CORE_REP|Org11_Gene2832#
MDHHLLAIRVFNRVVETGGFTRAAESLGMPKATVTKLIQNLEDHLQTKLFQRTTRSVSVTREGECYYQ
NTVKWLADLEQMEGCLTESQSSPQGVLRIDTGGGTARRLLLPALPDFLARYPQIQIDLSVGDRVIDLI
SDSTDCVIRSGPLADSSLIARRLFDLDWVSCATPAYLALHGTPRHPCDLEQGFPMVHYRHPLNDRIHP
QRYAEHGKEIAIQQRSPVSINEGNALLAASLAGLGIIQIYRFMAQPHLDSGELVSLLHDWQPPPEQMY
VVYPSNRHLSGKLRAFIDWAVETFDSGKMSRTL
>CORE_REP|Org8_Gene1071#
MNIRDLEYLVALAEHRHFRRAADSCHVSQPTLSGQIRKLEDELGVMLLERTSRKVLFTQAGLLLVEQA
RTVLREVKVLKEMASQQGEAMSGPLHIGLIPTVGPYLLPQIIPTLHKTFPKLEMYLHEAQTHQLLAQL
DSGKLDCAILALVKETEAFIEVPLFDEPMKLAVYSDHPWAQRERVAMPDLAGEKLLMLEDGHCLRDQA
MGFCFQAGADEDTHFRATSLETLRNMVAAGSGITLLPSLAVPPQRERDGVCYLDCYKPEPKRTIALVY
RPGSPLRSRYEQLAEAIREHMQGYIDSALKQAV
>CORE_REP|Org3_Gene2035#
MKMSIKQLRAFLAVAHTLNFAQASERLNISQPALSLAIRGLEDALGGPLLLRTTRRVTLTPEGETFFP
MARQLLADWDNAEEAMRQRFTLQMGKVAIAAMPSFAGNPLPPILKAFRDRYAGINVAVHDVINEQVFE
MIREGRVEMGIAFEPEPSDTLHFTPLCRDRFLAVVPKDSALARKAQVSWKELLTLDFITLQRPSAVRL
LLEQELARSGRTLEVAFESHQLVTVGRMVANGLGASAVPALCEQQMDELGAVCVPLIGPIIERRVGLI
RLAQHQLSSAAQALATVIEREMAGSGAQPALRP
>CORE_REP|Org23_Gene3194#
MSLPFDVHRLLPAFLAAAQAQNFSAAARQLGVTPAAVSKNIRALEEKLALRLFQRNTHNVLLTDEGKA
LLAQVAPLWQALAATLESAGGERQAPAGVVRVTMIPGFGRQMLMPLIPQFLARYPQIDLDLSLDARVV

NLVGEGFDVGIGSRVDPDSRLVARPLYPMHMVLAASPDYLARRGEPQTPHDLLRHDCLLHRNPANGRH
VKWQLRHQGETLALDLNGRLVVSRPEMLLDAALAGLGIVNLAHWYVEKHFVQGTLRPVLAECWPRPVQ
LWLYYASADLPPRVRVWVDFLLEHFRDRPTGD
>CORE_REP|Org39_Gene3100#
MKITLEELLAFTAVVDSGSVTAAADRLGQTTSGVSRALSRLETKLDATLLRRTTRRLSLTEEGLSFLA
PAREILRSVDQAEELMALRRRLPAGRLRVNAAAPFMAHVLVPMVAEFRRRYPQIELELDTDDRNIDLL
EKRADIAIRIGALRDSTLHARLLGNSRLRILASPDYLQRHGEPRGVEDLHRHCLLGFTYPESLNQWPL
RHRQARHFAIEPTISASSGETLRELALRGAGIVQLADFMTRRDREAGRLVPLLVRETLDVRQPIHAVY
YHDAQLAARLTCFLDYVSARLEGEPPEAAEGL
>CORE_REP|Org6_Gene1333#
MEFKQLQDMALFALVAECGSFTAAAQRVGLPKSSVSQRISQLEQTLGLRLLNRTTRQLNLTFAGERYL
EHCQVMMSAAERADLALQRLRDNPSGRLRISTPAGLGATLVARLAADFQRQYPDVSLEVSVSDAMVDL
VQEGFDAALRTGKPQDSSLIGRRLGYAPRYLLAAPSYLAQHPPIEHPQQLQQHRCIAHRAWTAWNLRC
GDDYYRWQLPLAHTTDNLLYARECAIAGAGITLLPAFLSREVVAQKLLVEVLPAWRAEGNELYLVYPS
RKLNSAALACFIDVVLQHPAFDDYARELARE
>CORE_REP|Org6_Gene1631#
MERLKRMSVFAKVVEFGSFTAAARQLDMSVSSISQTVSKLENELQVKLLNRSTRSIGLTEAGKIYYQG
CRRMLQEVSEVHEQLYAFNNTPAGTLRIGSSSTMAQNVLANMTAEMMKEYPGLTVNLVTGIPAPDLIT
DGLDLVIRTGALQDSSLFSRRLGQMPMVVCAAKSYLIQHGTPQKPSDMVNFSWLEYSVRPDSEFELMS
PEGITTRISPQGRFVTNDSSTMIRWLKNGAGIAYAPLMWVIEEIKRGEIEILFKSYHSDPRPIYALYT
EKDKLPLKVQVCINYLTDYFERVAAVYQGYR
>CORE_REP|Org46_Gene1493#
MLTDLNDLFFFASVVDHQGFAPAGRALGIPKSKLSRRVALLEERLGVRLIQRSTRRFSVTEVGQNYYA
HCKAMLVEAEAAQQAIEQTRAEPCGTVRMSCPVAILHTRVGSMVAAFMADYPKVTVHLEATNRRVDVV
GEGLDLAIRVRPPPLEDSDLVLKILAQRTWCVAASPALVRTLGPAHAPEDLRKYPTLDLGPARAQHQW
RLTGPQGERVEWEHTPRLVTDDMLMLRTAAIAGAGIVQLPAMMMRDDMLRGELVQLLPGWQPQGGVVH
AVYPSRRGLLPAVRLLLDYLGEQFTSIEEE
>CORE_REP|Org10_Gene4419#
MDRFNQYRVFVQVAEMGSFIRAAHALEVPRASVSAAVQQLETQLGVRLLHRTTRQVRLTADGEQLLER
LRPLLAEVEDIDQSFQASQRQASGRLSVDVPSRIARRLIAPALPSLLRRHPHLQLVLGSADRAIDLVQ
EGVDCAVRVGDLHDSSLVMRPLGHIALINCASPAYLSEFGHPRQPADLAEGHWSIGYASPKTGRESPW
EYLTDDGHTQRLELPSRVVVNNAESYIACCSAGLGLMQIPRYDVQHLLDAGELVEVLPGYRAASMPIA
LIYPHRRQRSRRLAVFHEWFESLLQPHLER
>CORE_REP|Org18_Gene686#
MDRITAAEVFVTIVDRGSMIAAAETLEMSRAMVTRYLAQMEQWAGARLLHRTTRKLSLTDAGERTLER
CRQMLALAGEIDLVEEGQSDELRGLLRITCSQSLGQTALVGAVAQYLKRHPQVAVDLQMNNRAVNLVE
ERIDLALRITNELDPNLIARPLSTCASVVCAAPAYLAAHGTPRQPQDLALHNCLTYSYFGKSLWHFDA
QGVKSAVAVSGNLSANESVVLMAGTVQGAGISMQPYYSAAPLLASGELVELLPDYRPQSMGIYGIYTS
RRQMPATLRTMLDFLVEWFATDPQWQATLR
>CORE_REP|Org2_Gene2686#
MSSLLQLLPYFEAVARLGNFTRAASQLGVTPPAVSQNIQALENQLGVRLFHRTSRSVRLSDEGRLFYQ
KVSPAMSQIDVAADDVRALGAQPAGLLRITLPQLAASLLVMPHLAEFQRRYPDVQLELFTEDRFSDLV
LGSFDAGIRMHAMLQKDMIAVPIDNGQRRVLVASPDYLARCGVPATPDDLPVHHCLRYRFPGSGKLEP
WYFSLGDDERALDVSGSLIFNEDRLIKDAALAGLGIAQRFQGTVLQELAQGQLVEVLPDYASEASGFF
IYFPAGRHLPLKLRAFIDFMREQRERQHRW
>CORE_REP|Org6_Gene3537#
MNDARYVEHLPIFLDVARLGSFSAAARRLGMVPSSLVRHIDALESALGATLFVRSTRGLLLTDAGELL
LTRAAALMTDITGIHAELSALNETPQGTLRISCLPTFGKTYVLPLLPTLAERYPQLSIDLDLTERQTD
PTQERLDAALRIGEQKDSALYASRIATQRWVMCASPAYVARYGLPSDLEALPQHRLIARYHKQQPACW
AQILDAALMSRCTMALRCDDFTAQRQAALLGLGIAFLPNWVVGPDVQNGQLVQMLEDPRHEQQGIYLL
RPMAKVSARLAAFTALLQQTLGQPPSWG
>CORE_REP|Org2_Gene3088#
MHSPSRARLPKLSAILAFETAARTGSLARAADTLALTAAAVSQQIRQLEQHLGITLFIRAKSGVTLTE
QGADYLAYVQEAFETLRVAQQHVERQRGKQALTVFALPALASKWLNPALGDWLAQCPDGDLRLHATHA

AVDFAHSAADFALCFGDQDYPLLEKVRLFQDRVQPVCSPALRDRGDWTQLPLIHVDWGKESQFLPGWH
EWFTAADRMPPARRGLTYNLTSLAIDAAVQGRGVLLGQRRLIGRELAAGQLVTLAEPALPLSKPYYVV
YPPRTLEKPGAAAFLAWLQTLASTDQA
>CORE_REP|Org19_Gene3737#
MFATLPVNALRTFESAARLRSFKLAAAELAVTPTAISHQIKALEQQLGFALFERVPRGVRLTPKGETL
FAGVHGALLDVAATLEGLRPQPSTGSLCVSVTHSFAALWLVPRLGRFYQAYPHYLVRLEACAEVIDLQ
QDASVDVAVRYSRAQYPALHQTARLEESFGVYAAPGLAAAEPENPVLITVKWGDSALYDSGWRDWCRA
AGVDWWQRHAAMRSYHEEHYALQAAVAGQGIVLASSVMVSDMVDNGLLVAYRPEVRVPGAAYSVLCAP
GRERHPPVRAFLAWLQQELPQGNGTK
>CORE_REP|Org44_Gene1228#
MKRPDYRTLQALDAVIRERGFERAAQKLCITQSAVSQRIKQLENLFGQPLLVRTVPPRPTEQGQKLLA
LLHQVELLEEEWLGNDTGVDTPLLLSLAVNADSLATWLLPALKPVLADSPIRLNLQVEDETRTQERLR
RGEVVGAVSIQPQPLPSCLVDRLGALDYLFVASSAFAERYFPSGVTRSALLKAPAVAFDHLDDMHQAF
LQQNFDLSPGSVPCHIVNSSEAFVQLARQGTTCCMIPHLQIEKELASGELIDLTPGLYQRRMLYWHRF
APESRMMRKVTDALLEHGHQVLRQD
>CORE_REP|Org38_Gene118#
MDLRDLKLFLHLAESHHFGRTAKAMHVSPSTLSRQIQRLEEILGQPLFLRDNRTVQLTDAGEQLKEFA
QQTLLQYQQLKHSLGQHGPSLSGELRLFCSVTAAYSHLPPILDRFRAQHPLVEIKLTTGDAADAVDKV
QSNEADLGIAGRPETLPASVAFTKIGEIPLVLIAPALPCAVRSQAFADKPDWAEIPFILPEHGPSRKR
IELWFRRHRISNPLIYATVGGHEAIVSMVALGCGIALIPSVVVDNSPEPVRNRISQLDNISMVEPFEL
GVCVQKKRLSDPLIDAFWRLLHPR
>CORE_REP|Org23_Gene3099#
MNIELRHLRYFIAVAEELHFGRAAERLRISQPPLSQQIQALEEMVGARLLARNNRNVSLTQAGEMFLK
EAYQVLDQVGRAAEKAARLDRGELGEMTIGFTSSAPFIGVVARSLRTFRQQSPQVHIKMREINTKQQI
EPLLNGELDLGVMRNTRLPEALHYQLLLREPLVAVVPEGHPLAETPGGGLRFQHLAQEPFVFFSREVG
TALYDEILLLLSKAGITPYITQEVGEAMTIIGLVSAGLGVSILPASFARVRVDGVRYLPLAEPDATTE
VWLVHHRRRPLTAAAQALMALMLK
>CORE_REP|Org5_Gene3514#
MRKPRLPPLGALRAFHAVAGCRSFKLAAEALGVSATAVSHQIKLLESVLACRVCERSAQGVSLTETGE
ILYAGTQRAFAALEQSVAQITRAQQPPALTVTTTSNFLTHWLVPRLADFKAEFPAIDLRLHTSVERVD
LSQRTVDVAIRYRETPESDLHCTLLHEDRFIVVASPALALERSEDLQRVTLFHVEHRQVPADAPTWEN
WRRRYGPEGLNVEAGLTFSDETHALQAAVAGQGVVIASRLLARDLLQRGVLAAPFETALPGANYYLVA
TEETAQRPDIIALREWLLRQMAAG
>CORE_REP|Org17_Gene124#
MKANSDELITFVTVVESGSFSRAAERLEQANSVVSRTVKKLESKLGVTLLNRTTRQISLTQEGENYFR
QVQKVLNDMAAAENALMESRQRPQGLLRVDAATPVVLHMLTPLVAEFRERYPEMSLSLVSSENFINLI
ERKVDIAIRVGELTDSTLKARKLMTSYRHVLASPAYLAQHGTPLTVEDLAHHCCIGFNDLPSLNRWPL
ACSDGSQLEITPGLTTNSGETQRHLCLHGNGIACLSDFMSDEDIKRGDLVPILVEATLPVAMPINAVY
YSDSAVSNRLRSFIDFVSEYLKR
>CORE_REP|Org28_Gene1805#
MDVRTLRYFVEVVRQQSFTRAAEKLFVTQPTISKMLRHLEEELECTLLIREGRKLRLTDSGQALYQRG
LTILDEFRQLEAELEDISSLKKGVLRLGIPPMVGRQIADLIRRFRQTYPGIELKISELGGLSVEQAVM
SGELDLAMTVLPFDSEQPLTFLPLLGHPMCVVAPRTPQWLNRTSINIAELADSPILIYNEDFALYKML
MKAFRQAGFEPQIAVRSGQWDFLASMVQAGVGIAMLPEPVCRWLDKENLVWLPLEPRMEWKIGLIWRQ
GSYLSHGAQAWIACCRDYWPPLK
>CORE_REP|Org49_Gene2811#
MFKKSLLTLAFTGVATLSTYATAADTLTMEVYNPGEKSVFPVSSEIISGKHEVALIDAQFQRNDAEEL
VKKIKATGKKLTTVYISHSDPDFYFGLDVIKAAFPEAKIIASPGTIKDINATKDGKVAYWGPILKDNA
PKTVIVPQPLQGDSFTIDGQKVEVKGLNGPTPDRTFVWIPALKAVVGGVAVAGDNIHPWIADNQSVES
RQHWQQTLKNIEALKPQVVVPGHFLPGAAQTLASVHFTQKYLTTLEAELPKAKDSAALIEAMKKHYPT
LKDESSLELSAKVLKGEMKWPQ
>CORE_REP|Org35_Gene1585#
MRNRLPLNALRAFESSARHLNFTRAGLELRVTQAAVSQQVRMLEEQLGIQLFRRLPRGLDLTEEGQAL
LPVLSDAFDRIEAVLQQFEGGHFHEVLTVAVVGTFAVGWLMPRLAAFRAAHPFIDLRVLTHNNLVNLS

ADGMDFAIRFGEGLWPATRNIKLFDAPLTVLCSPAVAARLHTPKDLQHELLMRTYRQDEWERWFTAAQ
VTPWRINGPVFDSSRLMVEGALQCDGVALAPVSMFRRELAAGALQRPFAAEAALGAYWLTHLKSRDLT
PAMKAFIGWICREAEEEQRRD
>CORE_REP|Org18_Gene1164#
MSDAILRVEHLMMRFGGIKALNDVNLEVERGSITALIGPNGAGKTTVFNCLTGFYRASGGAILLNTHK
RPTDVIQVLGQKFRAGDWIRPKRLGSRLYYKMFGGTHLVNRAGLARTFQNIRLFREMSVVENLLVAQH
MQSNRNLIAGVLNTPGYRRAESAALDHAFYWLEVVDLVDCANRLAGEMSYGQQRRLEIARAMCTAPEM
ICLDEPAAGLNPVETATLSRIIRFLRQHHGITVLLIEHDMGMVMEISDRVIVLDHGDVIARGTPQEIQ
HNEAVIAAYLGADEEELAG
>CORE_REP|Org32_Gene4334#
MMNERIPLHVLPTFAIAARLENLRAAAQQVHLTHGAVSQQIQLLEQAVGYPLFERRGRGVRLNAAGRE
LLAAVEPALQALLQGVARARRAATSQTLRISVLPSFAHYWLLPRLPAFHEACADIALDIDASLALQDL
SQRGFDAAIRIGSGQWTGLQAQRIATGDVLPVASPDMAREWRAAFESGGDIPLLEHDVSPWRDWFNAQ
GRPLCGRQQALFNDAGLLIRAAEQGFGIALAKKLLVQDALDAGRLVALAAPRRLSDDDVYLVWPQTAG
LTPAVTRLLQWLQRQLAAI
>CORE_REP|Org28_Gene2548#
MGRITFDLEDLRSFVTGVELGSFAKAAERLGRSTSAVSAHLKKLEQQVGAPILRKAGRGMVMTEAGET
LLGYARRLLELNDEAAAAVRGLDLQGTVRLGLQEDFGETFLPQVLGSFARANPKVRIEARIARNAELI
DWVLKGQLDLSLAWDGGLSTPFHQALGQRQLHWIASPGFALAPWREGDEPLSLVMFDAPCLMRSAATQ
ALDRAGIPWRIAFTSRSLNGVWAAVNAGLGVTVRTAAGLPPGLAPLAPELLPALGQLGVVLHRAEDQP
SAAVQRLAQIVVERIGTNL
>CORE_REP|Org44_Gene232#
MQHKTLKYFSTVIVCAIAVCAGWWLWNYYMQSPWTRDGKVRAELVNITPEVSGRLEKISANDNQFVPA
GSLIFTLDPVPYQIALDNAEAAVAKAQSDLAKADHEAARRRGLPRNVISAEDLDESNLAAQAMKAAYK
AALANLEQAKWNLSKTKIYAPTDGYITNLQARVGNYANAGTPLVALVDVHSFYVLGYFEETKLKHIKE
GNKADIVLYNGNTPLQGEVESIGRAIYDQSVDSSNDLLMDVKPNVPWVRLAQRVPVRIKLLNVPADLT
LVAGTTCTISIHQRN
>CORE_REP|Org28_Gene4729#
MLKHWPPLSALRGFEAAARLSSFHQAAEELHLTQSAISQQIRSLEAFLEQPLFFRTGRSVTLTDAGHD
LFSTAQVMLQQLAVGIRRLDQYRKPNQLIVNTTPAFARHWLMPRLGDFNRQHPQADLWLFTSFEPPNM
ATDSIDLAIRDDLSAQADCTFNVLCSDRLYPACHPSLLALAAEQRMTLHGEREMDWSHWTVAGGAHVG
QRDSGLNFSDPGLLLDAACQGLGIALVSQLLAQQARDAGLLQPLTEQRVRGANWAWLLHRDSEHNPLT
RHFCQWLQSALPAGA
>CORE_REP|Org9_Gene2151#
MKLSQLKFFCTVVEHKTIAAAARELHCVPSNVTLRLRELEESLGGELFFRDKNRLYVNPKGRLFYQQA
RDIVAQAERSKQLFAGEHQHGLLNLGALDFSLVSHLPARIARLRRLQPHLHINVLSRDSLVLERMLID
SDLDLAITDGPIEHPLLASQKAFDERLVLLMPADAGEPDAATLAPLEFYTFSRECSFRLKVDHWLASR
GLKPRMTLEMESYAAMAACVQAGCGVACVPGSLLPLILPAPGLKVVEMGEEGVSDLYFVWRRHQLSDE
LQTILAILARPG
>CORE_REP|Org34_Gene1366#
MNPLFTPYLQRWQLEQDGKAFETHSSLLMPVRYRGEAAMLKIAREQEERFGGQLMCWWRGEGAAQVLA
WHEDGILLERAQGESSLAQLVRDGDDEQATAILCRAIAALHAPRAAPLPELIPLQEWFSSLWPAAQAH
GGMLRLSATTAAELLSSPRDESVLHGDIHHDNVLDFGERGWLAIDPKRLYGERGFDYANIFCNPNYGI
ATDPAIFQRRVEQVCRLAGLERRRLLQWILAWAGLSAAWFMEDGQAADIDFRVAELAARALDLPLPAG
DSGFILPVIERG
>CORE_REP|Org6_Gene1996#
MNRYPMFNPQLLLSFVAVCDSNSFTRAAERVFLSQSTVSQQVRRLEEMLGKPLFERSSHQVLLTEEGV
KLLSYARRIIALNEEAHDALTGIWRDGVLRIGMPEDFAVPTTELLAEFSREHPHLRLDVASGLSADLH
SAYAREELDLILVKQRRQQPPRAARPEPLLWLDSLAFPAIEQSPVPLAVFPLSGLYRDELCQALDNLG
KRWRIGYSSASLAALTAASAAGLGVTLLPAGCRLPTHRVLGAAEGLPPIDSFELALYYRDGAPAATLA
LAQRLTVFCGLI
>CORE_REP|Org22_Gene2790#
MHNDGRFDYHNALRYSSDELAKILSHCFENYIVRFVLDGPTFAARFGAEDISLNDSLIVTHRHEPVAV
ALIARRGQHSRVAAFSVRPEMRGQGLGKALMQRLVADARQRGDRRLSLEVIEGNEAALALYHRAGLRI

VRTLTGHQAPAEAPPGTTAGLQAVDPLTVSHRLTAEGATDLPWLIAPESLFKLPGKPQAYTLNRQAYA
VVMPGAEHCWLRLIYVPPQHRGQGHARALLAALQTRFAPLPLTANVFVPEVAAPFFTHLGWRQDPLRQ
FEMDMLLDSPQE
>CORE_REP|Org21_Gene3288#
MGTDNTLLTVERLAIGVPEPQPVALVKNISFSMGRERLALVGESGSGKSLTARALMGLLPPPLQLQAH
RLTLGDEDLTRLSERQWSRLRGDRVAMVMQDPKHALNPNQPIGRQVEEPLVLHTKLSRAERREKVLEM
LAAVGLPDPAALCRRYPHQLSGGMGQRVMLAIALINDPQLLIADEPTSALDHQMRDQVLQLIDNLVAQ
RNMGLILISHDLQQVAHHCERVLVMYKGELLDQLPAAELAQATHPYTRTLWACRPSRETRGKPLPVLD
RALLETLK
>CORE_REP|Org24_Gene4551#
MQLTVRDMTLNLSHPQVMGILNVTPDSFSDGGRHNTLNQALLHAHALILAGATMIDIGGESTRPGAAE
VSEEEELERVVPVVEALAQRFEVFISVDTSKAGVIRESAHAGAHLINDIRSLQEPGALAAAAESGLPV
CLMHMQGQPRTMQQAPHYDDLIADVQAFFEHHIRRCNEAGITNQKLLLDPGFGFGKNLAHNYQLLARL
SEFHRFGLPLLVGMSRKSMIGQLLNVPPDQRVIGSVACAVIAAMQGAQIVRVHDVKETVEAMRVVEAT
LSAKGQ
>CORE_REP|Org11_Gene813#
MAQALLKLAQHDFPGQHAAASRKVLSVKGLGKAYKAQQRVLDDINFDLHAGEFVAVIGRSGAGKSTLL
HTLNGTIPSSCGEMLHFEDDGVAQDIAQLAGRQMRQWRARCGMIFQDFCLVPRLDVMTNVLLGRLSHT
STLKSFFKLFDDADRARAIELLQWLNMLPHALQRAEHLSGGQMQRVAICRALMQNPQILLADEPVASL
DPKNTRRIMDALQKISEDGIAVMVNLHSVELVKEYCSRVIGIAHGKIVFDGHPSQLNERILHQLYGEE
ANQIH
>CORE_REP|Org23_Gene2132#
MDTELLKTFLEVSRTRHFGRAAESLYLTQSAVSFRIRQLENQLGANLFTRHRNNIRLTPAGERLLPYA
ESLMNTWQLAKKEVVRSLQHTELSIGATASLWEAYLTPWLQALYQQREALQLEARVALRHSLVKQLHE
RQLDLLITTEPPKMDELASQQLSNFSLRLFSSAYRDKQAPMPYIKLEWGADFHQQESRMLEGDNVPVL
TTTSAHLTRQLLETTGGCAFLPSQWEKEYPQLVATTEIPPIIRPLYAVWLQNSDQQPLIRQLLKIPLN
TAA
>CORE_REP|Org48_Gene1124#
MSEAIDYYALPEQPVRNLTPVPARGLIEISNVSKFFGKHKALDDVSLTLQPGTVTVILGPSGSGKSTL
LRAINHLERVDEGFIRIDGDYVGYRRKGNRLYELKEKAILRQRINVGYVFQNFNLFPHLTVLENIIEA
PVVHKIHSRERAKAVAYELLDTVGLRHKADAYPRHLSGGQQQRIAIARALALNPKVILFDEPTSALDP
ELVGEVLDVIKGLADLGVTLVVVTHEIGFAREAADRVVFMVDGQIVEQGDARQVLSQPQHPRTVNFLN
KVL
>CORE_REP|Org34_Gene2028#
MSSEELEQVWNSIKSEARALADCEPMLASFFHATLLKHENLGSALSYMLANKLATPIMPAIAVREVVE
EAYKSDNQMIVSAARDILAVRLRDPAVDKYSTPLLYLKGFHALQAYRIGHWLWQQGRQALAIYLQNQI
SVAFGVDIHPAATIGCGIMLDHATGIVIGETAVVENNVSILQSVTLGGTGKTSGDRHPKIREGVMIGA
GAKILGNIEVGKGAKIGAGSVVLQAVPPHTTAAGVPARIVGRPESDTPSMDMDQYFNGTNHGFEYGDG
I
>CORE_REP|Org26_Gene2222#
MSYQCPLCHQPLHFSSQRWRCDGNHQFDQAKEGYVNLLPVQHKRSKQPGDSAEMMQARRAFLDGGFYQ
PLQQQVAEWLDLALAADAGALLDIGCGEGYYTAAVAARLAQARNMAVYGLDVAKVAIRYAAKRYPAVS
FCVASSHRLPFADAALDAVLRIYAPCKAAELARVVKPGGVVVTVSPGPRHLYQLKEQVYQQAQLHAEQ
DEQFDGFECERKEALAYTMALPGAQAANLLQMTPFAWRATPEVQLRLAHGGEFECETDFVLALYRRRA
>CORE_REP|Org9_Gene1383#
MNNRVHQGHFARKRFGQNFLTDQFVIDSIVSAIHPQPGEAVVEIGPGLGALTEPVGARMDRMTVIELD
RDLATRLENHPRLKDKLTIHQQDAMTVNFAELAEEAGQPLRVFGNLPYNISTPLMFHLFSYTQAIRDM
HFMLQKEVVNRLVAGPNSKAYGRLTVMAQYYCNVIPVLEVPPTAFAPPPKVDSAVVRLVPHSVLPNPV
GDVRMLSRITTQAFNQRRKTIRNSLGDLFTPEQLTELGVDPSLRAENISVAQYCKLANWLSANPAPQQ
>CORE_REP|Org20_Gene834#
MLQKADNLVEVRDMSFSRGDRRIFEDINLTVPRGKVTAIMGPSGIGKTTLLRLIGGQLAPDSGEIWFD
GDNIPALSRRQLYDARKKMSMLFQSGALFTDLTVFENVAYPLREHSNLPEPLLRSTVLMKLEAVGLRG
AAQLMPNELSGGMARRAALARAIALDPEMIMFDEPFVGQDPITMGVLVKLIDELNHALGITCIVVSHD
VPEVLSIADYAYIVADHRVIAEGTTQQLQNNPDARVRQFLDGIADGPVPFRYPAGDYQTELLGLGSK

>CORE_REP|Org40_Gene798#
MGLMTPGSLPRLDVQHLDDEQTALAVNGLNLFYGDKQVLHDISLRIPKHRVTALIGPSGCGKSTLLRC
FNRMNDLVDNCRIEGDLQLNGAAISGAQIDVAALRRRVGMVFQRPNPFPKSIYENVVYGLRLQGVRDR
RLLDEAVERSLRAAALWHEVKDRLRENAFRLSSGQQQRLVIARAIAIEPEVLLLDEPTSALDPISTLT
IEELISALKQRYSVVLVTHNMQQAARVSDYTAFIHQGRLVEYNDTDAIFTSPRQRRTEDYITGRYG
>CORE_REP|Org45_Gene4078#
MKAMQVTDELFTHPLPADKYRQQTDPVLQLDSVNVSFDGFRALTDLSLRIGVGELRCVIGPNGAGKTT
LMDVITGKTRPDSGRVFYDQTVDLTRLAPMQIAHAGIGRKFQKPTVFEALTVFENLEIAQKTRKSVWA
CLRARLSSEQRDRIDEMLKTLRLGHERHRPAGLLSHGQKQFLEIGMLLVQEPHLLLLDEPAAGMTDAE
TDYTAELFRELAGKHSLMVVEHDMGFVETIADRVTVLHQGQVLAEGSLAQVQADERVIEVYLGR
>CORE_REP|Org6_Gene848#
MSHRLHASHLKLGYDNKIIADDLSVAIPDGAFTVIVGPNACGKSTLLRALCRLLKPSAGEVMLDGKNI
SSFATKALARELGLLPQTSIAPDSITVADLVSRGRYPHQSLLKQWTQADKQAVEAAMAATNVSQLADR
SVDELSGGQRQRVWVAMALAQQTPLLLLDEPTTYLDIAHQIELLDLFRQLNRERGQTLIAVLHDLNHA
CRYADHIIAMRDGKIVAEGKPAEIITAELVERVFGMPCMIIDDPLSHTPLVIPRGRYHCDAPQA
>CORE_REP|Org48_Gene2882#
MQDKLLNPGAAFALDNASFAVPGRVLLQPLSLSFPQGKVCGLIGHNGSGKSTLLKLLGRHQAPSGGQV
LLNRQPLAQWDSKSFARQVAYLPQQLPAAEGMTVRELVAVGRYPWHGALGRFGANDRQLVEEAISLVG
LKPFANRLVDSLSGGERQRAWLAMMVAQDSRCLLLDEPTSALDIAHQVEVLALIQRLSRERDLTVIAV
LHDINMAARYCDHLVALRGGEMIAQGGPLELMQGPVLEQIYGIPMGTLPHPSGGAPVSFVY
>CORE_REP|Org42_Gene2137#
MSQGLRIEHFSAGYPKRQVIDDLSVPMLPRGQITVLLGPNGSGKSTLLRSLAGLNPAQGKLWLDDGDL
MQMPFARRAEKVVYLPQSLPAGVHLHVLESIIVAQRASGGRSNAGSEAEVMALLEQLGIAHLALSYLD
QLSGGQKQLVGLAQSLIRQPSLLLLDEPLSALDLNYQFHVMDLVRRETRKRNIVTVVVVHDINIALRH
GDHVLMLQDGDLIADGAPDQVITPQSLARVYGVRGRIERCSQGTPQVLIDGLVNQPTI
>CORE_REP|Org36_Gene1514#
MTSTPLSTTPLLSVNRLTHLYAPGKGFSDVSFDIYPGEVLGIVGESGSGKTTLLKSISARLAPQRGQI
LYRPQAGQEQDLYAMAESDRRRLLRTDWGVVHQHPLDGLRPQVSAGGNIGERLMAIGQRHYGDIRRQA
GQWLEDVEIPLSRLDDLPTTFSGGMQQRLQIARNLVTHPKLVFMDEPTGGLDVSVQARLLDLLRNLVV
EMQLAAVIVTHDLGVARLLAHRLLVMKQGEVVESGLTDRVLDDPHHPYTQLLVSSVLS
>CORE_REP|Org10_Gene1322#
MDNAACLTARELRYSLGTRRLINDVSLSLASGEMVAIIGPNGAGKSTLLRLLTGYLTPDCGECRLLDR
PLEHWAPQQLAKVRAVMRQYSDLAFPFSVEEVVSMGRSPHGKRDEHQAIQQVMEQTDCLALAQRDYRR
LSGGEQQRVQLARVLAQLWQPQPSPAWLFLDEPTSALDLYHQQHTLRLLRSLTRQQPLGVCCVLHDLN
LAALYADRILLLHQGRLVASGTPQEVLQTEILTRWYQADLGVVHHPEVSLPQVYLRQ
>CORE_REP|Org7_Gene4574#
MTTPTHSIHHAAAEGYQANADRYVKGRPDYPPEIAAWLRDVIGLHAGMTVIDLGAGTGKFTPRLLETG
AQVIAVEPVPQMLEKLSAALPQVKTLAGTADAIPLPDESVDAVVCAQSFHWFATPQALAEIQRILKPG
GKLGLVWNMRDARVGWVRKLNQIVDSHEGDAPRFYTGEWRKFFPFKGFEPLQEQVFMLGHRGAVEDVI
YNRVRSTSFIAALPQPQQEQVIDRLRQLVAEEEELRGKDTVTVPYQTKAYFTTKV
>CORE_REP|Org31_Gene1858#
MTLPARLAQGTPVTLESIGKGYGNRTVLDNIQLRISAGQFVAVVGRSGCGKSTLLRLLAGLEQPSSGA
LLSGNAPLAAAKEDTRLMFQDARLLPWKTVIDNVGLGLRGQWRDAALQALDAVGLADRARDWPAALSG
GQKQRVALARALIHRPRLLLLDEPLGALDALTRIEMQGLIETLWQQHGFTILLVTHDVSEAIALADRV
ILIEEGRIGLDLTLDLPRPRRKGSARLAELEAEVLERVLSPPATAASGRRAAN
>CORE_REP|Org24_Gene336#
MSENKLAVTELHKRYGDHEVLKGVSLAANAGDVISIIGSSGSGKSTFLRCINFLEKPSEGSISLNNED
IRMVRDKDGQLKVFDKKQLQLLRTRLTMVFQHFNLWSHMTVLENVMEAPVQVLGLSKADAHERAVRYL
DKVGIDERARGKYPVHLSGGQQQRVSIARALAMEPEVLLFDEPTSALDPELVGEVLRIMQKLAEEGKT
MVVVTHEMEFARHVSNHVIFLHKGLIEEQGPPAELFGNPKSPRLQQFLSGALK
>CORE_REP|Org47_Gene3539#
MLNVNHLSAEYQGRPALRDVSFQIAAGQLVVVLGPSGCGKTTLLNLIAGFIEPSAGSITLDGTPVHGP
SAERGVVFQHEGLLPWRNVVDNVEFGLQLAGVGKAQRRQVAEQMLQRVGLAGYEQHFIWQLSGGMRQR
VGIARALAADPRLLLLDEPFGALDAFTREQMQELLLTIWRDTGKQVLLITHDIEEAVFLASELLLLSP

GPGQVVERLSLNFGQRYADGEACRVIKSDPEFIAQREYVLGKVFQQREAML
>CORE_REP|Org37_Gene3755#
MSTLITLKNISVAFGNRKVLSNISLSLQPGRILTLLGPNGAGKSTLVRVVLGLVKPTAGTLEREPDLR
IGYVPQKLHLDATLPLTVSRFMRLKPGVKKADILPALKRVHAAHLLDQPMQKLSGGENQRVLLARALL
NKPQLLVLDEPTQGVDVNGQLALYDLIDQLRKELGCAVLMVSHDLHLVMAKTDEVLCLNQHICCSGAP
EVVSMHPEFIAMFGNRGAEQLAVYRHHHNHRHDLQGRIVLKKTGSREA
>CORE_REP|Org48_Gene3986#
MLQLVEVGVAGRLAPFTAQIDGGLQVHLIGPNGAGKSTLLARAAGMLPGQGEVCLDGRALSCYSGDEL
AHRRGYLSQQQPPVSLMPVFQYLALHRPAGAVQTEVEQAILYLCQRLKLVDKLSRMLTQLSGGEWQRV
RLAAVLLQVWPSVNPHSRLLLLDEPTNSLDVAQKVALDRLLREFCQSGRSALVCAHDLNHTLQQADRV
WLLHAGQLVAQGITREVMAPGLLSQIYEVDFHLQWVGDQRWIMTRTA
>CORE_REP|Org6_Gene1423#
MSIQFWRMSIQLNGINCYYGAHQALFDITLECPAGETLVLLGPSGAGKSSLLRVLNLLEMPRSGQLQI
AGNQFDFRQAPGEKAIRELRQNVGMVFQQYNLWPHLTVVQNLIEAPCRVLGLTKAQAMERADKLLKRL
RLTDFADRFPLHLSGGQQQRVAIARALMMEPQVLLFDEPTAALDPEITAQIVSIIRELAGTGITQVIV
THEVEVARKTASRVVYMENGHVVEQGDSSHFTQPRTTEFANYLSH
>CORE_REP|Org45_Gene722#
MLSIKNLKVSVEGNEILKGLDLEIKPGEVHAIMGPNGSGKSTLSATLAGREEYEVTEGEVTFKGKDLL
ELDPEDRAGEGVFLAFQYPVEIPGVSNHFFLQTSVNAVRKYREQEPLDRFDFADFIEEKIALLDMPAD
LLTRSVNVGFSGGEKKRNDILQMAALEPDLCILDETDSGLDIDALKIVANGVNSLRDGKRAFIIVTHY
QRILDYIQPDYVHVLSQGRIVKSGDFSLVKQLEEQGYGWLTDQQ
>CORE_REP|Org32_Gene1615#
MTSPLLPPGIQVRDLSLRFGQQIVFDRLSFDIAGGSFVALLGASGAGKTSLLKIIAGLAQASSGTVTG
SDGLPIAGRIAYMGQKDLLYPWLTVEENVALGSRLRGEVADRAWVAHLLERVGLAAHGRSLPAALSGG
MRQRAAIARTLYERQPIVLMDEPFSALDAITRAEIQSLAAELLAQNTVLLITHDPMEACRLSHRLLVL
SPWPLGLDDTHRISGQPPRAPDDADLLKSQAELLQQLVRAAQ
>CORE_REP|Org11_Gene1725#
MKAIIVEDEFLAQEELSYLIKKHSNIDIVATFEDGLDVLKYLQTHQVDAIFLDINIPSLDGVLLAQNI
SKFAHRPSIVFITAYKEHAVEAFEIEAFDYILKPYHEARIVTMLQKLEALHHRPAGATEPASAPSRGS
HSINLIKDERIIVTDINDIYYAAADEKVTRVYTRREEFVMPMNITEFYGRLPEEHFFRCHRSYCVNLA
KIREIVPWFNNTYILRLSDLEFEVPVSRSKVKEFRKLMRL
>CORE_REP|Org19_Gene2545#
MRVHASIEPLVWESDFFQLESAKLHFDSSAAPVAEADLDAYALVQAKIPAYRLGWADALSTLGFRLVE
GEVDLVVNVAPESAMADAASAVAVRQAVPEDIPSLRAAAGEVFAASRFRAPWYDRADSGRFYAAWIEK
AVQGTFDHQCLLVLDSQGQPEGFVSLRDIGGQEMRIGLLAAFPGASGRGVGARLMTAAIAECRQQGMQ
RLRVATQVGNIAALRLYQRQGAVIESTAYWLYRGRHDSI
>CORE_REP|Org4_Gene3913#
MRKSLGGWRRLRPGYWLKRGVIAILGLWVLGIAAFAFLPVPFSAVMVERQVSAWLSGDFGYVAHSDWV
SMDDISPQMALAVMAAEDQKFPDHWGFDVAAIEKALSHNEKRPTRIRGASTLSQQTAKNLFLWDGRSW
LRKGLEAGLTSGIELVWTKRRILTVYLNIVEFGDGVFGVEEASQRFFHKPAKRLTAAEAALLAAVLPN
PHRFRADAPSGYVIQRQQWIMRQMRQLGGEAFLSENKLD
>CORE_REP|Org21_Gene531#
MATLIAENLAKAYKGRKVVEDVSLKVKSGEIVGLLGPNGAGKTTTFYMVVGIVPRDAGRIVIDEEDIS
LLPLHARARRGIGYLPQEASIFRRLSVYDNLMAVLEIRPDLTSEQREDRAKELMEEFHISHLRDSLGQ
ALSGGERRRVEIARALAANPKFILLDEPFAGVDPISVIDIKKIIEHLRDSGLGVLITDHNVRETLDVC
ERAYIVSQGKLIAHGTPDAILADEQVKRVYLGEEFRL
>CORE_REP|Org37_Gene1550#
MLNALIVDDEPSARDNLRHLLEAEAEIAIIGECANAIEAISQIHRLQPDVVFLDIQMPRISGLEMVGM
LDPNRMPHIVFLTAYDEYAVQAFEEHAFDYLLKPAEPKRLSKTLQRLRQRSAPQDVAALEESAGYLKY
IPCTGHSRIYLLRFDEVLAIRSKLSGVFVVRSDGMECFTELTLRTLESRTPLVRCHRQYLVNLEQVRE
IRFEEGGAAEMIMSAGDPVPVSRRYLKALKEQLGLRG
>CORE_REP|Org27_Gene864#
MHKIVFVEDDPEVGKLIAAYLGKHDIEVLIEPRGDSAQARIAHEQPDLVLLDIMLPGKDGMTLCRDLR
PTFPGPIVLLTSLDSDMNHILSLEMGANDYILKTTPPAVLLARLRLHLRQHGQQPKEESVQPLTQHNA

LHFGLLCIDPVNRQVTLGEETVTLSTSDFDLLWELATHAGQIMDREALLQNLRGVSYDGMDRSIDVAI
SRLRRKLYDNALEPFRIKTVRNKGYLFAPNAWASVQQ
>CORE_REP|Org36_Gene774#
MISLKNVSKWYGHFQVLTDCTTEVKKGEVVVVCGPSGSGKSTLIKTVNGLEPIQQGDILVNGTPVNDK
KTNLAQLRAKVGMVFQHFELFPHLSIIDNLTLAQVKVLKRDKTASREKGLKLLERVGLSAHANKFPGQ
LSGGQQQRVAIARALCMDPIAMLFDEPTSALDPEMINEVLDVMVELANEGMTMMVVTHEMGFARKVAN
RVIFMDEGKIVEDRNKDDFFNNPESERAKDFLAKILH
>CORE_REP|Org26_Gene2019#
MLSLRSVNQFYGQNHTLWDINLELPRGQCTVLLGRNGVGKTTLVNCIMGHVPVVSGSMTWQPADQPPQ
NLLLQPMERRAALGISHVPQGRQLFSQLSVEENLQVAQMAGRGAPRRIPPLIYSLFPHLRQMRARRAG
DLCVGAQRQLAIGRALAQEPALLILDEPTAGVPPSIAADIGNVIRRLNRELGMTILLVEHQLPFVRRV
ADRFCLLDSGRTVAHGALAQLDEALIGAGLAGQEEG
>CORE_REP|Org6_Gene19#
MQENHKILVVDDDMRLRALLERYLTEQGFQVRSVANAEQMDRLLTRESFHLMVLDLMLPGEDGLSICR
RLRSQSNPMPIIMVTAKGEEVDRIVGLEIGADDYIPKPFNPRELLARIRAVLRRQANELPGAPSQEEA
VIAFGKFKLNLGTREMFREDEPMPLTSGEFAVLKALVSHPREPLSRDKLMNLARGREYSAMERSIDVQ
ISRLRRMVEEDPAHPRYIQTVWGLGYVFVPDGSKA
>CORE_REP|Org18_Gene1779#
MINVLIVDDDPMVAELNKYYLSQVGGFHCQATVATLSQARALLADAGVSIDLVLLDIYMQQENGLDLL
PGLRELGEKTDVIIISSASDVNTVQKALHYGVVDYLIKPFQFSRFKEALSHYRQQSQLLAQREFSQAD
VDSLLRRQPGGQESKKLPKGLTSITLSTVCEWIEQQHDNEFSTDNLANAIGISRVSCRKYLIYLAESG
ILGTRILYGATGRPVYLYQLKPDAIAMLKEHCRPA
>CORE_REP|Org35_Gene254#
MQTPHILIVEDELVTRNTLKSIFEAEGYIVHEANDGAEMHNILSENDINLVIMDINLPGKNGLLLARE
LREQASVALMFLTGRDNEVDKILGLEIGADDYITKPFNPRELTIRARNLLSRTMNLGSLGEERRLVES
YKFNGWELDINSRSLISPAGEQYKLPRSEFRAMLHFCENPGKIQSRGELLKKMTGRELKPHDRTVDVT
IRRIRKHFESTPDTPEIIATIHGEGYRFCGDLEE
>CORE_REP|Org15_Gene1664#
MSETMLEFREVDVFYGPIQALRQVSLQVNAGETVALIGANGAGKSTLLMSIFGQPRIAGGQILFRGED
ISRRSTHFVASSGIAQAPEGRRIFPDMSVEENLLMGTITVGNRYLEEDLPRMFELFPRLKERRNQRAM
TMSGGEQQMLAIARALMSRPKLLLLDEPSLGLAPIVVRQIFGVLRELTRSGMTLFLVEQNANHALKLS
DRGYVMVNGQIRLTGSGEELLNDPQVRKAYLGGG
>CORE_REP|Org7_Gene2013#
MDIQNQPVQIMIVEDEPKLGQLLVDYLQAAGYATRWLTNGNEVVPTVHQHPPALILLDLMLPGADGLT
VCRELRRFSDVPIVMVTAKIEEIDRLLGLEIGADDYICKPYSPREVVARVKTILRRSYRPQENAREDD
LLHIDEPRFQASYQGQLLDLTPAEFRLLKTLASQPGNVFSREQLLNNLYDDYRVVTDRTIDSHIKNLR
RKLELIDGQKSFIRSVYGVGYRWEAEPCRLVNGV
>CORE_REP|Org46_Gene1803#
MDKPKRILIVEDDGDIAELLQLHLRDEGYAISHAADGNQGMAMLEQGGWDALILDLMLPGVDGLEICR
RARTMTRYTPIIIISARSSEVHRVLGLELGADDYLAKPFSMLELVARVKALFRRQEAMSRNLRMDAGV
LSFNDLTIDPIAREVHLHQQPVELTPREFDLLYFFARHPGQVFSRLSLLNQVWGYQHEGYEHTVNTHI
NRLRIKIERNPAEPERILTVWGMGYKFAAAPQE
>CORE_REP|Org38_Gene3476#
MISLRQLAIGYGATPLFPPLSGQFSAGSLTAVVGVNGAGKSTLLKTLAGLLPPVAGRLDFSGEKPPRK
AYLPQQAELDRQFPIAVSDLVAMGCWPQSGMFGGMNQRAASQVNEALASVGMSALAHSPVGELSGGQL
QRVLFARLLVQQAPLILLDEPFTGIDSATTQILLQVIAQLHQQGRTVIAVLHDMSMVAEHFPQVLLLT
PQACHWGAAERVLEQVPRYLAAERQPGLRVVAP
>CORE_REP|Org37_Gene3786#
MTTETAATILLIDDHPMLRNGVKQLIGMDARLQVIAEASNGEQGVTLAEQHDPDLILLDLNMPGINGL
ETLDRLRQTDLSGRVVVFSVSNHEDDVVSALKRGADGYLLKDMEPEDLLKALHQAAAGQMVLSETLTP
ILAASLRENRPSADRDIQQLTPRERDILKLIAQGLPNKLIARRLTITESTVKVHVKHLLKKMKLKSRV
EAAVWVLQGKTVNRRRTAARFAPEWAAASGF
>CORE_REP|Org17_Gene3023#
MLTLEKLTYLYEHLPMRFDLRIQPGERVAVLGPSGAGKSTLLSLIAGFLPAASGRLLLNGEDHTATPP

AKRPVSMLFQENNLFAHLTVAQNIGLGLDPGLRLTAQQRQQREHIARQVGLEEHLDRLPAQLSGGQRQ
RAALARCLIRRRPILLLDEPFSALDPALRNEMLQLLQTVCEQRDLTLLMVSHNLDDAARIAPRTLLVV
DGRIYYDGPTQALLDGSAPEARVLGISGKA
>CORE_REP|Org1_Gene4257#
MTMQIRVEHISKTFVLHQQYGTRLPVLHDANLTVHGGECVVLHGHSGSGKSTLLRSLYANYLPDSGHI
WINHQGDWLDMVSADARQILAVRRHTLGWVSQFLRVIPRISALEVVMQPLLEQGVERAECRDRAEALL
AALNVPQRLWPLAPSTFSGGEQQRVNIARGFIVDYPILLLDEPTASLDSRNSAAVVQLIERAKARGAA
IVGIFHDEGVRQQVADRLYDMQAPQALEAL
>CORE_REP|Org7_Gene165#
MNKILLVDDDRELTSLLKELLEMEGFNIVVAHDGEQALSLLDSSVDLLLLDIMMPKKNGIDTLKELRQ
HHQTPVIMLTARGSELDRVLGLELGADDYLPKPFNDRELVARIRAILRRSNWSEQQQQVDSGAPTLDV
DGLQLNPGRQEASFDGQVLDLTGTEFTLLYLLAQHLGQVVSRELLSQEVLGKRLTPFDRAIDMHISNL
RRKLPDRKDGHPWFKTLRGRGYLMVSAT
>CORE_REP|Org22_Gene620#
MARRILVVEDEAPIREMVCFVLEQNGYQPLEAEDYDSAVTRLSEPFPDLVLLDWMLPGGSGIQFIKHM
KREALTRDIPVMMLTARGEEEDRVRGLEVGADDYITKPFSPKELVARIKAVMRRISPMAVEEVIEMQG
LSLDPSSHRVMANDQALDMGPTEFKLLHFFMTHPERVYSREQLLNHVWGTNVYVEDRTVDVHIRRLRK
ALETSGHDKMVQTVRGTGYRFSTRY
>CORE_REP|Org49_Gene1435#
MSITPTNILIVEDEKEIRRFVRTALESEGLRVFESETLQRGLIEAGTRKPDLIILDLGLPDGDGLSYI
RDLRQWSAIPVIVLSARNAEEDKIAALDAGADDYLSKPFGIGELLARVRVALRRHSASQQESPLVSFS
AITVDLVNRRVLRNDEDLHLTPIEFRLLAELLANAGKVITQRQLLSHVWGPNYVEHSHYLRIYMGHLR
QKLEADPARPKHLLTETGVGYRFMP
>CORE_REP|Org38_Gene2185#
MSHQTLDVLIVEDEPQLATLHAEFIEKNFNLRVVAYAATLAEARAKANEHQPRLILLDNFLPDGQGIE
LMEEPAVKNPACSVIFITAASDMHTCSQAIRNGAFDYIIKPVSYKRLRNSLERFMQFVQTQRTFKIID
QDNVDALYNLQSKQFSSEPSAKGIETNTLELVQALFIAQPAVAHAVEDVVEQVGISKTTARRYLEYCV
ATQFVRVEMLYGNIGHPRRLYRKA
>CORE_REP|Org21_Gene1456#
MKEKKDILRLDDIHYQIDNQVILDSVSFTLGEGEFKLITGPSGCGKSTLLKIISSLMDPTRGSLYFDG
QAIAEMSPEAYRKQVSYCFQTPALFGNTVYDNLALPYQIRQQSPDERKMKADLTRFGLPEAMLTKSIN
ELSGGEKQRVSLIRNLQFMPRVLLLDEITSALDEENKRNVNEIVHQLVAEHRLAVLWVTHDTEEIAHA
DEVITLRAHGAEQQEQQEQQHESA
>CORE_REP|Org9_Gene1038#
MKILLVDDDLELGTMLSEYLTGEGFDATLVLTGKAGVEGALSGDYTAMILDIMLPDMSGIDVLRDVRK
KSRLPIIMLTAKGDNIDRVIGLEMGADDYMPKPCYPRELVARLRAVLRRFEERPQEADDEAAISFGEL
TLNPSTRSSEWRGKAFDLTASEFNLLELLLRAPDRVVSKDELSEKGLGRPREAYDRSVDVHISNIRQK
LSALAGSKLIIETVRSIGYRIR
>CORE_REP|Org4_Gene59#
MQRILIVEDEQKTGRYLQQGLVEEGYQADLFNNGRDGLGAASKGQYDLIILDVMLPFLDGWQIISALR
ESGHEEPVLFLTAKDNVRDKVKGLELGADDYLIKPFDFTELVARVRTLLRRARSQAATVCTIADMTVD
MVRRTVIRSGKKIHLTGKEYVLLELLLQRTGEVLPRSLISSLVWNMNFDSDTNVIDVAVRRLRSKIDD
DFEPKLIHTVRGAGYVLEIREE
>CORE_REP|Org17_Gene1995#
MINAEMLTGRSTEHLAPLSGNHRLQPEAVNAFLAMQQAARAAGFDLQPASTFRDFDRQLAIWNGKFCG
QRPVLDKDSQPIDVAPLSAAERCEAILRWSALPGASRHHWGSDLDVYDPSLLPEGQKLQLEPWEYEEG
GYFAPLNQWLTAHMAEFGFYRPFTEDCGGVAVEPWHLSYRPLAQEAEHLLTPALLLAAWQDKEVAGAE
WLERHLPSIFSRFIRSKGKE
>CORE_REP|Org1_Gene324#
MIRFEQVSKAYLGGRQALQGVDFHLRPAEMAFLTGHSGAGKSTLLKLICGIERPSAGHIWFGGHDISR
LKNREVPFLRRQIGMIFQDHHLLLDRTVYDNVAMPLIIAGASTEDIRRRVSAALDKVGLLDKAKNFPI
QLSGGEQQRVGIARAVVNKPAVLLADEPTGNLDDALSEGILRLFEEFNRVGVTVLMATHDTGLIARRN
YRILTLSQGRMQGGAHHGQ
>CORE_REP|Org17_Gene594#

MRVLVVEDNGLLRHHLSVQMREMGHQVDAAEDAKEADYFLQEHAPDIAIVDLGLPGEDGLSLIRRWRA
HQTKLPILVLTARESWQDKVAVLEAGADDYVTKPFHLEEVIARMQALMRRNSGLASQVIVLPPFQIDL
SRRELSVNDQQIKLTAFEYTIIETLIRNAGKVVSKDSLMLQLYPDAELRESHTIDVLMGRLRKKVQAE
YPHEVITTVRGQGYRFDAK
>CORE_REP|Org22_Gene2188#
MSVIALENLSVSHRQGYELRTVVHEVNLRIEPGECFGLVGPSGCGKSSLLWVLAGLNGSWQGGFELLG
RRLQPGQAFTGELRREVQMVFQDPYASLHPKHRLLRTLSEPLKLLKESDIERKVSAGFRQVGLDPRLL
DRYPHQLSGGQRQRVAIVRALLLRPKLLLLDEPTSALDMSVQAEILNLLNELKQAGDLTMVLVSHDAD
VIDHMCDRSVAMAHGRIIV
>CORE_REP|Org15_Gene2222#
MRILLIEDDKLIGDGIKAGLTKLGFNLDWFTDGAVGKNALGSAPYDAVILDLSLPGLDGLDLLRQWRQ
AGQDVPVLILTARDALEQRVSGLQSGADDYLCKPFALAEVAARLQALIRRRHGQLMPQLTHGNVVFDS
ATRSVSCNGEPVTLTPRELAVLELFLHNKGRVLARPLIQEKLYNWDDEVSSNAVEVHIHHLRRKLGNG
FIRTIHGVGYTLGDAP
>CORE_REP|Org37_Gene701#
MKLLVVEDDELLQQGLALALTGEGYVCDCAATAAEANSLLITSQYSMVILDLGLPDMDGAALLRQWRR
QQIDLPVLILTARDALEDRVDGLDAGADDYLVKPFALVELQARVRALLRRYQGHSDNLMQVDDLQLNL
SSQQVYLQQQPVEVTPKEFAILARLIMRAGQTVNRELLQQDLYTWQDDLGSNTLEVHIHNLRRKLGKD
RIRTVRGIGYRLEPSS
>CORE_REP|Org46_Gene107#
MISVLLVDDHELVRAGIRRILEDIKGIKVVGEAQCGEDAVKWCRGNAVDIVLMDMNMPGIGGLEATRK
IVRYAPDVKVIMLTIHTENPLPAKVMQAGAAGYLSKGAAPQEVINALRSVHAGQRYIASDIAQQMALS
QLEPQAETPFSCLSERELQIMLMITKGKKVNEISEQLSLSPKTVNSYRYRMFSKLNISGDVELTHLAI
RHGLFNAETLLSSE
>CORE_REP|Org38_Gene1576#
MKYHLIPVTAFSQNCSLIWCENTQQAALVDPGGEAEKIKAEVAKQGVTITQILLTHGHLDHVGAAAEL
AEHYQVPIYGPDKEDAFWLDGLPAQSRMFGLEECAPLTPTRWLSEGDEMQVGEMKLKVLHCPGHTPGH
IVFINEQARLALVGDVLFNGGVGRSDFPRGDHQALIASIRTKLLPLGDDMRFIPGHGPMSTFGHERQT
NPFLREEPAVW
>CORE_REP|Org4_Gene2280#
MSLMLKGEKIDRNRFTGEKIENGSFMLCDFSGADLTGTEFIGCQFYDRESRQGGNFSRAILKDASFRS
CDLSMADFRHVDALGVEIRECRAQGADFRGASFMNMITSRTWFCSAYITKSNLSYANFAKVVLEKCEL
WENRWHGAQVLGASFSGSDLSGGEFSGFDWRAADVTQCDLSNAELGELDLRTTDLQGVKMDSHQAAQL
LERLGIAIIG
>CORE_REP|Org8_Gene887#
MVMKQYRVMIVDDHPLMRRGIKQLLGLDARFGVVAEAGNGSEAVALALQHAPDVILLDLNMKGMSGLD
TLRALRDEGVDARIIVLTVSDARSDLYALIDAGADGYLLKDSEPEQLLEHISAAAEGQNVISDAMADY
LLARSEQRDPFTALTERELDVLQEVARGLSNKQVAAQLHISEETVKVHIRNILRKLDVRSRVAATVMY
LEYKSH
>CORE_REP|Org41_Gene2158#
MLEAKSLSCVRDERILFSELSFSVQPGDIIQVEGPNGAGKTSLLRILAGLARPDGGEVCWRGRSTLRD
RAGYQQDLLFIGHQPGIKAVLTPFENLQFYQAVRGTTDHPAIWRALEQVGLVGYEDLPVAQLSAGQQR
RVALARLWLSAAPLWILDEPLTAIDKQGVAELISLFEQHAQRGGMVLLTTHQDLAGVSQTVGKVRLAE
HDAGSL
>CORE_REP|Org11_Gene3912#
MSKKHWSNTELLHQTVTNPNIIVKGTHSYYSDCWDNGFERSVVRYLHGDAVSRQWQPLGDIDRLLIGD
YVCIAAEAVILMGGNHTHRIDWLSLYPFMETIKRAYRPKGDTRLGDGCWIGMRAMLMPGVSIGEGAIV
AAGSVVVGDVEPYAIVGGNPARFIRWRFAPEVIARLLALRLYDLSEADFAVVQPLLVDNDIAAVERAI
CNIKRY
>CORE_REP|Org28_Gene2514#
MIRVILVDDHVVVRSGFAQLLNLEDDLDVVGQYSSAAAAWPALLRGDVNVAVMDIAMPDENGLSLLKR
LRAQKPQFRAIILSIYDSPTFVQSALDAGASGYLTKRCGPEELVQAVRSVDMGGHYLCADALRALRGG
ERPATALEVLTPREREIFDLLVKGDSVKEIAFKLDLSHKTVHVHRANVLGKLQCNSTIELVHFALDHQ
LLAGH

>CORE_REP|Org49_Gene3493#
MNDFAQPKIGDNVTLNRTRLGQYVHLADDAILEEVEMGDYSYTAGHNQIFYATIGKFVSIASYARINP
GNHPTYQRIAQHHFTYRASEYGLGEDDAAFFDWRREHHVAVGHDVWIGHNAILMPGVSVGNGAVIGSA
AVVTKDVEPYSIVAGVAAKKIGMRFDDALIERIERSQWWHWDHATLQARLADFRDINRFAQKYL
>CORE_REP|Org4_Gene1977#
MTLRVAFIDDHDIVRSGFVQLLSLEADIQVVGEFSSAAQARAGLPGLEAEICICDISMPDGSGLDLLA
DIPSGIRVVMLSMHDNPALVEMALDRGASGFLSKRCKPEDLITAVRTVAGGGVYLMPEIAQQLARVRV
DPLTRREREIALLLAQGQEVREIAAALGLSPKTVHVHRANLFAKLGINNNVELARRMLNL
>CORE_REP|Org37_Gene784#
MFGYRSASPKVRLTTDRMVVRLVHERDAYRLADYYAENRTFLKPWEPVRDESHCYPSGWQARLGMITE
MQKQGSAYYFILLDPEEQEVRGVANFSNVLRGSFHACFLGYSLGEKWQGQGLMFEALQSAIRYMLRQQ
RMHRIMANYMPHNQRSGALLTRLGFEREGYAKDYLLIDGKWQDHVLTAYTNKEWLPPR
>CORE_REP|Org32_Gene2796#
MAISEEKRKMIAGELYDAGDDLLRSERRRARQLTHRYNHSSPEEGELRKQWLDELLGGYQGGTIEPTF
RCDYGYNIYLGKSFYANFDCVILDVCEVHIGDNCLLAPGVHIYTATHPLDAETRVGGAEFGKPVKIGD
NVWIGGRAVINPGVTIGDNAVVASGAVVTKDVPANCVVGGNPARVIKQL
>CORE_REP|Org12_Gene553#
MSSTASVRLRPLERDDLSFVHQMDNNASVMRYWFEEPYEAFVELSDLYDKHIHDQSERRFIIEHEGAK
VGLVELVEIDHIHRRAEFQIIIDPAHQGKGYASTAARLAMDYGFSVLNLYKLYLIVDKENPKAIHIYS
KLGFNVEGELIDEFFVNGEYRTVLRMCIFQPQYLAKFKTPNDKPLVK
>CORE_REP|Org18_Gene1969#
MTPVIRIAAIEALPDDYLTRGDFGFTIRCYALPQFDTPVDSWPTRPVAPFRKQYPLAPFANEDSATFL
AYRQDQAVGHITLSKNWNGYTLIDEIAVSAHARRQGIAGALLDCAKQWARQQETSGMMLETQNNNLAA
CRCYQHYGFILGGIDRLLYRAEPEIADHEIALFWYLPFNSEIGY
>CORE_REP|Org29_Gene4208#
MKLVTERLSLQSITAEDWPLFLRLYQDPEVIRYISDPRSEAEIRTRFEERLSAWDKHGEQWLCLVMRE
KHSGEAVGITGFRPQWVPYRQAEVGYGSLPAGQGKGYGKESLRAVLDFAVNACGFHKLTATVTAGNLA
SRGLLESCGFQLEGTLRDNYRLAGQWCDDWLFGLLAAEFQGGK
>CORE_REP|Org5_Gene3160#
MKHTVDVMISEQEVKTRIAELGRQITEHYRDSGSDMVLVGLLRGSFMFMADLCRAIDVPHEVDFMTAS
SYGSGMSTTRDVKILKDLDEDIRGKDVLIVEDIIDSGNTLNKVREILALRGPKSLAICTLLDKPERRE
VQVPVEYVGFSIPDEFVVGYGIDYAQRYRHLPYVGKVVLLDE
>CORE_REP|Org37_Gene3888#
MIIRNATLNDSAAIAAIYNDAVLNSTAIWNEQTVDAANRAAWIGERQAAGYPVLVAVNGADEAIGYAS
FGDWRAWDGYRHTVEHSVYVHQAHRGEGIGKALLIALIARAQEIGKHVMVAGIESGNQASIKLHLALG
FREVGRMEQVGAKFGQWLDLTFLQLTLDERAAPPAR
>CORE_REP|Org29_Gene4501#
MSEIVIRHVETTDAQALHHLYSQTPVYRDTLHLPLPTVELWHKRLANPEPGTHNLAAFIDGQLAGQLA
VMLNQRVRRRHVATFGIGVDPRYHGKGVGSRLIQAMIDLCDNWAAIERIELTVFTDNPAAIALYRKFG
FEIEGTSRAYAMRDGVLVDAYHMARLRCGAVNAAQS
>CORE_REP|Org33_Gene3271#
MEDSGLPLKRRKGATMSIIHRLAQPDDLNGLLALYRELRPQDAPLRTDDARRTLQRLLDDPAIRLVVA
ADEEQPIATCMLALIPGLAHQAQPFGVIEHVVTAEPYRGHGVALAMIEYALQLAWRKGCYKVMLLSGQ
QRTGAHQLYLKAGFDGDRERGFVIRRPEGR
>CORE_REP|Org42_Gene1427#
MIISLIAALAADRVIGMENAMPWHLPADLAWFKRNTLNKPVIMGRKTFESIGRPLPGRHNIVLSSRPG
NAAGVTWVTSLDEALAAAGEVEEVMVIGGGRIYTQLLPRADRLYLTHIDAEVGGDTHFPDYEPDEWET
TFSEFHDADDLNSHSYCFEILQRR
>CORE_REP|Org42_Gene4407#
MHVLPKATVIRPCGPEDIDRLMALWLPSTIAAHPFVAEKYWRESATLVRENYLPRAQSWACWHDDEIV
GFISVLDEQFIGALFVERAFHGRGVAQALMTHVQQRYRRLSLEVYQQNLRACAFYHRHGFQVTQRLFN
DETQAYTLIMNWPAVENSTRYA
>CORE_REP|Org9_Gene1848#
MSSVITTRQAGIVDVDAGRNVTVVEPCNLYGCRLGDDVFVGPFVEIQRHVSIGARSKIQSHSFICEYV

TLGEACFVGHGVTFANDLFKDGAPNADPASWGRTRVGDRVSIGSGATILAVEICSDAVIGAGAVVTRN
ITRKGIYAGNPARLLRELP
>CORE_REP|Org26_Gene1411#
MSEKYVVTWDMLQMHARKLAHRLLPADKWTGIIAVSRGGLVPAALLARELGIRHVDTVCISSYDHDNQ
REMKVLKRAEGDGEGFIVVDDLVDTGGTAKAIRDMYPKAHFVTIFAKPAGRPLVDDYVVDIPQDTWIE
QPWDMGVSFVPPIGGR
>CORE_REP|Org20_Gene1815#
MNLSNPEVTIRRINGDDKAQWLALWQGYLDFYRADVAPQVTDRTFERLGQDEQVYGLVAQDADGQLLG
LMNLVFHPSTWSAVGYCYIEDLYVSPQARGHKVSEKLFEQAYRLAETRGSDRVYWMTQEYNAPARSLY
DKIGRRSSFIVYSR
>CORE_REP|Org29_Gene3296#
MNTISTLTAADLATAFTIEQASHAFPWTETTFASNQGDRYLNLKLSADGEMAGFAITQIVLDEATLFN
IAIHPQHQRRGLGRLLLNAVIEQLESRGVVTLWLEVRASNQAAIALYEDLGFNEVTVRRNYYPSAQGR
EDAIVMALPLA
>CORE_REP|Org39_Gene2286#
MIVNCDHDNLDAWLALRTALWPSSSPEDHRAEMREILASPHHTAFMARGLDGAFVGFAEVALRYDYVN
GCESSPVAFLEGIYTVERARRQGWAARLIAQVQEWAKQQGCSELASDTDIANLDSQRLHAALGFAETE
RVVFYRKTLG
>CORE_REP|Org47_Gene430#
MEIRVFRQDDFEEVITLWERCDLLRPWNDPEMDIERKLNHDPELFLVAEVGGEVVGSVMGGYDGHRGS
AYYLGVHPDYRGRGIANALINRLEKKLIARGCPKIQIMVREDNDTVVEMYEKLGYEIQGITSLGKRLI
EDQEY
>CORE_REP|Org38_Gene2629#
MTVRPFNLQQLDHVVLRVRDMQNSLRFYTQVIGCDIAKQRPDLGLVHLRAGASMIDLVDVNGVLGKKG
GEAPDLHRQNVDHVCLRIDPFNEDALLTYLRSQGIDADPAESRYGAEGDGPSIYFSDPDGNRVELKGP
ALD
>CORE_REP|Org26_Gene3611#
MELKIDKVIETVLYVSDIERADAFYRQVLKLPAMVANERFRAYNVGDRSVLLLFIEGDSLRGAQYLTG
FIPAHDGVGPAHIGLAVAKEQLPHWERHLVANGVEIEGRMRWEHGGESIYFRDPDAHLLELVTPGIWA
NY
>CORE_REP|Org18_Gene3421#
MLTGLNHLTLAVSDLDRSFDFYRHLLGFTPHARWQGGAYLSLGSLWLCLSLDERRTQQRERDYTHYAF
SIAPEHIEQASQRLRQAGVKEWKSNRSEGESLYFLDPDGHQLEIHAGDLASRLAACREKPYQGMVFY
>CORE_REP|Org38_Gene2894#
MRLLHTMIRVGDLQRSIDFYTKVLGMRLLRTSENPEYKYSLAFVGYTEESEGAVIELTYNWGTDSYDM
GTAFGHLALGVDDVAATCDNIRRAGGNVTREAGPVKGGTTVIAFVEDPDGYKIELIENKHAGQGLGH
>CORE_REP|Org41_Gene2207#
MADKNLRFLVVDDFSTMRRIVRNLLKELGFNNVEEAEDGADALNKLRAGGFDFVVSDWNMPNMDGLEL
LQTIRADSVLAAMPVLMVTAEAKKENIIAAAQAGASGYVVKPFTAATLEEKLNKIFEKLGM
>CORE_REP|Org11_Gene2189#
MKDLLKFLKAQTKTEEFDAIKIALASPDMIRSWSFGEVKKPETINYRTFKPERDGLFCARIFGPVKDY
ECLCGKYKRLKHRGVICEKCGVEVTQTKVRRERMGHIELASPTAHIWFLKSLPSRIGLLLDMPLRDIE
RVLYFESYVVVEGGMTNLERRQILTEEQYLDALEEFGDEFDAKMGAEAIQALLKNMDLEAECEQLREE
LNETNSETKRKKLTKRIKLLEAFVQSGNKPEWMILTVLPVLPPDLRPLVPLDGGRFATSDLNDLYRRV
INRNNRLKRLLDLAAPDIIVRNEKRMLQEAVDALLDNGRRGRAITGSNKRPLKSLADMIKGKQGRFRQ
NLLGKRVDYSGRSVITVGPYLRLHQCGLPKKMALELFKPFIYGKLELRGLATTIKAAKKMVEREEAVV
WDILDEVIREHPVLLNRAPTLHRLGIQAFEPVLIEGKAIQLHPLVCAAYNADFDGDQMAVHVPLTLEA
QLEARALMMSTNNILSPANGEPIIVPSQDVVLGLYYMTRDCVNAKGEGMVLNGSKEAERVYRAGLASL
HARVKVRITEDVKNAEGEWTSQTSIIDTTIGRAILWMIVPKGLPYSIVNQPLGKKAISKMLNTCYRIL
GLKPTVIFADQIMYTGFAYAARSGASVGIDDMVIPAKKAEIIEEAETEVAEIQEQFQSGLVTAGERYN
KVIDIWAAANERVAKAMMENLSVEDVVNRDGEVEQQVSFNSIFMMADSGARGSAAQIRQLAGMRGLMA
KPDGSIIETPITANFREGLNVLQYFISTHGARKGLADTALKTANSGYLTRRLVDVAQDLVVTEDDCGT
HDGILMTPVIEGGDVKEPLRERVLGRVTAEDVLKPGTADILVPRNTLLNEKACDLLEENSVDSVKVRS
VVSCETDFGVCANCYGRDLARGHIINKGEAIGVIAAQSIGEPGTQLTMRTFHIGGAASRAAAESSIQV

KNKGSLKLSNVKFVMNAAGKLVITSRNTELKLIDEFGRTKESYKVPYGAVMGKGDGEEVNGGETVANW
DPHTMPVISEVSGFIRFADMVDGQTITRQTDELTGLSSLVVLDSAERTGSGKDLRPALKIVDAQGEDV
LIPGTDMPAQYFLPGKAIVQLEDGIQIGAGDTLARIPQESGGTKDITGGLPRVADLFEARRPKEPAIL
AEISGIISFGKETKGKRRLVISPLDGSDAYEEMIPKWRQLNVFEGEVVERGDVVSDGPESPHDILRLR
GVHAVTRYITNEVQEVYRLQGVKINDKHIEVIVRQMLRKGTIVSAGGSEFLEGEQAEVSRVKIANRQL
EAEGKIAATFSRDLLGITKASLATESFISAASFQETTRVLTEAAVAGKRDELRGLKENVIVGRLIPAG
TGYAYHQDRMRRRAQGEAPVVPQVSAEEATANLAELLNAGLGGSDDE
>CORE_REP|Org37_Gene101#
MVYSYTEKKRIRKDFGKRPQVLDIPYLLSIQLDSFQKFIEQDPEGQYGLEAAFRSVFPIQSYSGNSEL
QYVSYRLGEPVFDVKECQIRGVTFSAPLRVKLRLVIYEREAPEGTVKDIKEQEVYMGEIPLMTENGTF
VINGTERVIVSQLHRSPGVFFDSDKGKTHSSGKVLYNARIIPYRGSWLDFEFDPKDNLFVRIDRRRKL
PATIILRALNYTTEQILDLFFDKIVFEIRDNKLQMELVPERLRGETASFDIEANGKIYVEKGRRITAR
HIRQLEKDDIQSIEVPVEYIAGKVVAKDYIDTNTGELICAANMELSLDLLAKLSQSGHKRIETLFTND
LDHGAYISETLRVDPTNDRLSSLVEIYRMMRPGEPPTREAAESLFENLFFSEDRYDLSAVGRMKFNRS
LLRDEIEGSGILSKDDIIEVMKKLIDIRNGKGEVDDIDHLGNRRIRSVGEMAENQFRVGLVRVERAVK
ERLSLGDLDTLMPQDMINAKPISAAVKEFFGSSQLSQFMDQNNPLSEITHKRRISALGPGGLTRERAG
FEVRDVHPTHYGRVCPIETPEGPNIGLINSLSVYAQTNEYGFLETPYRRVRDGVVTDEINYLSAIEEG
NFVIAQANSNLDEEGRFVEDLVTCRSKGESSLFSRDQVDYMDVSTQQVVSVGASLIPFLEHDDANRAL
MGANMQRQAVPTLRADKPLVGTGMERAVAVDSGVTAVAKRGGVIQYVDASRIVIKVNEDEMYPGEAGI
DIYNLTKYTRSNQNTCINQMPCVNLGEPIERGDVLADGPSTDLGELALGQNMRVAFMPWNGYNFEDSI
LVSERVVQEDRFTTIHIQELACVSRDTKLGPEEITADIPNVGEAALSKLDESGIVYIGAEVTGGDILV
GKVTPKGETQLTPEEKLLRAIFGEKASDVKDSSLRVPNGVSGTVIDVQVFTRDGVEKDKRALEIEEMQ
LKQAKKDLTEELQILEAGLFARIHAVLVAGGIEADKLSKLPRDRWLELGLTDEEKQNQLEQLAEQYDE
LKSDFEKKLEAKRRKITQGDDLAPGVLKIVKVYLAVKRQIQPGDKMAGRHGNKGVISKINPIEDMPYD
ENGTPVDIVLNPLGVPSRMNIGQILETHLGMAAKGIGEKINQMLKQQQEVAKLREFIQKAYDLGDDVC
QKVDLNTFSDDEVLRLAENLKKGMPIATPVFDGAKETEIKKLLEMGGIPTSGQITLFDGRTGEQFERQ
VTVGYMYMLKLNHLVDDKMHARSTGSYSLVTQQPLGGKAQFGGQRFGEMEVWALEAYGAAYTLQEMLT
VKSDDVNGRTKMYKNIVDGDHRMEPGMPESFNVLLKEIRSLGINIELEDE
>CORE_REP|Org29_Gene3008#
MSTSDSRNRSSSPRYSLPDRAGDLRQLGQLTGAACAVECAEIVERHPGPVMLIAPDMQNALRLRDEIQ
QFTDQMVTTLSDWETLPYDSFSPHQEIISDRLSSLYHLPTMARGVIILPVNTLMQRVCPHEFLHGHAL
VMKKGQRLSRDKLRAQLEQAGYRSVDQVMEHGEFATRGALLDLYPMGSDEPYRIDFFDDEIDSLRIFD
VDSQRTLSEVEAINLLPAHEFPTDKNAIELFRSQWREQFEVRRDAEHIYQQVSKSAWPAGIEYWQPLF
FSQPLPSLFSYLPANTLIVNTGDLESAAERFWQDVNQRYESRRVDPMRPLLAPDTLWLRVDALFGELK
AWPRIALKTDELPAKAGNTNLDYHALPDLAVQAQHKSPLDNLRRFIEGFDGSVIFSVESEGRRETLQD
LLGRIKLAPALIQRLDQAETASRYMMVGAAEHGFLDGLRQRALICESDLLGERVSRRRQDNRRTINTD
TLIRNLAELHPGQPVVHLEHGVGRYVGLTTLEAGGIKAEYLILSYAGEDKLYVPVSSLHLISRYAGGA
DENAPLHKLGGDAWTRARQKAAERVRDVAAELLDIYAQRAAKAGFAFKHNREQYQLFCQSFPFETTPD
QEQAINAVLSDMCQPLAMDRLVCGDVGFGKTEVAMRAAFLAVENGKQVAVLVPTTLLAQQHFDNFRDR
FATWPIRIEMMSRFRSAKEQQQVLDDAAEGKVDIIIGTHKLLQSDLRWKDLGLLIVDEEHRFGVRHKE
RIKAMRADVDILTLTATPIPRTLNMAMSGMRDLSIIATPPARRLAVKTFVREYDNLVVREAILREVLR
GGQVYYLYNDVENIEKAAQRLAELVPEARIAIGHGQMRERDLERVMNDFHHQRFNVLVCTTIIETGID
IPSANTIIIERADRFGLAQLHQLRGRVGRSHHQAYAYLLTPNPKAMGTDAHKRLEAIASLEDLGAGFA
LATHDLEIRGAGELLGEDQSGQMTTVGFSLYMELLESAVDALKNGREPSLEDLTSSQTEVELRMPALL
PEDFIPDVNTRLSLYKRIASAKNDGELDELKVELIDRFGQLPDAARNLLQCAALRLHAQKLGIKRIES
NERGGFIEFGDNNRVDPGYLIGLLQGNPQVYRLDGPSKLKFTLDLADRQKRLTFTEDLLDAFREHTLA
A
>CORE_REP|Org38_Gene4841#
MLIPSKLSRPVRLQNTVIRDRLLAKLASAGNYRLTLVNCPAGYGKTTLIAQWAAGKADLGWYSLDESD
NQPERFASYLIAALQQASGGRCVKSEALSQKHQYASLSALFAQLFIELADWHQPLYLVIDDYHLITND
AIHEAMRFFLRHQPENLTLILLSRTLPPLGIANLRVRDQLLEMGTQQLAFTHQEAKQFFDCRLAAPME
QQDSSRLCDEVEGWATALQLIALSARQSASSAQLSAKRLAGLNASHLSDYLVDEVLDHVDADARAFLL
RCSVLRSMNDALIVRLTGEDNGQQRLEELERQGLFIHRMDDTGEWFNFHPLFASFLRQRCQWELALEL
PGLHRAAAEGWLALGYPAEAIHHALAASDVSMLRDILLQHAWSLFHHSELALLEECLNALPYERLIQN

PKLALLQAWLAQSQHRYSEVNTLLERAERTMREQKIEIDQTLHAEFDALRAQVAINAGKPEEAERLAT
EALKFLPLSSYYSRIVATSVTGEVHHCKGELARALPMMQQTEQMARRHQANHYALWALLQQSEILIAQ
GFLQAAYETQDKAFELIREQHLEQLPMHEFLLRIRAQILWSWSRLDEAEDAARTGLKILANYQPQQQL
QCIAMLAKCSLARGDLDNANTHLQRCETLLHGAHYHRDWLTNTDKSRVIHWQMTGDTTAAAQWLRHTE
KPGMADNHFTQGQWRNIARVQILLGQYDEAGVVLDELNENARRLRLVSDLNRNLLLSNQLYWLQERKG
EAQQALIEALSLANRTGFISHFVIEGEAMAQQLRQLIQLNTLPELEQHRAQRILRDINQHHRHKFAHF
DENFVDKLLTHPQVPELIRTSPLTQREWQVLGLIYSGYSNDQIAGELDVAATTIKTHIRNLYQKLGVA
HRQEAVQQAQQLLKMMGYGA
>CORE_REP|Org3_Gene3709#
MQNKQLTISSSNITRCFLLFIVLLTIGIGLYGYNYTNAWLAEKKYALNSIAGSLQKRIDTYRYMTYQV
YDKFGNAPAQNVDPGLQETRLRPDVYYIEKPHKKTDAVIFGSHDESTLAMIANISDYLDTRWGAKTEN
YAMYYLNGQDNSLSLITTQPLKELASRFRESYLTTSADERRAEMLQQANMLDERESFSDLRKQRFQNA
YSFSIRTTFNQPGHLATVIAFDLPINDIIPANLARANFLLQPDDVDLDDSTIPAETVLGTHATMSGGW
VEFSAALPNAPLKVVYRVSAINLAIDLLRNNIWLIAVNLLLLALSMLSIYFIRRQYIRPSENMAVELE
AERALNQEIVSSLPSGLLVYSFANNAVIASNKIAEHLLPHLSLQKIAHMAEQHHGVIQATVNNEVYEI
RIFRSQLSPDTYLFLMHDQDKEVMVNKRLQQARREYDKNVQARKLMLHNLGIELNQPVRQMHDLVDRL
HGRPDEEQQQALLGQLTAASASVLELIDNITLLTRLETQDWQPSREPFSPTAMIDELLLEALPALNQK
GLALFKHFQLDVEQNYIGDANALRKVISLLVHYAIITTACGKISLVVDHEPEHPDRLIFQINDTGSGI
SNEEISNLNYPFLSQTLVDRFNHGSGLTFFLCNQLCKKLNGQLDIRSKVDIGTRYTIRVAMEMEKKEP
QEQEKLFDGVTALLDVTSDEVRGIVTRLLQAYGADCLVADDRAVNRDYDVLLTDNPQRADDYTLLLAT
DEPGWQALDKRYIRVNYNLNGALIDAVLILIEQQMAALEQEESPLSLSSEDIQLYEKQLKSSDYYGLF
VDTVPDDVKKLYTEAGSSDFNALSQTAHRLKGVFAMLNLLPGKQLCESLEQRIAEGDAPEIENNISQI
DFFVSRLLKQGSQQHE
>CORE_REP|Org12_Gene4140#
MSDLAREITPVNIEDELKNSYLDYAMSVIVGRALPDVRDGLKPVHRRVLYAMSVLGNDWNKPYKKSAR
VVGDVIGKYHPHGDSAVYDTIVRMAQPFSLRYMLVDGQGNFGSVDGDSAAAMRYTEVRMSKIAHELLA
DLEKETVDFVPNYDGTEQIPAVMPTKIPNLLVNGSSGIAVGMATNIPPHNLAEVVNGCLAYIDDENIS
IEGLMEHIPGPDFPTAAIINGRRGIEEAYRTGRGKIYLRARAEVEADAKTGRETIIVHEIPYQVNKAR
LIEKIAELVKEKRVEGISALRDESDKDGMRIVIEVKRDAVGEVVLNNLYALTQLQVTFGINMVALHQG
QPKLLNLKDILEAFVRHRREVVTRRTIFELRKARDRAHILEALAIALANIDPIIELIRRAPTPAEAKV
ALVAQPWDLGNVSAMLERAGDDAARPEWLEPEFGIRDGKYYLTEQQAQAILDLRLQKLTGLEHEKLLE
EYKELLNFIAELIFILESPERLMEVIREELVAVKELYNDGRRTEITANTSDINIEDLINQEDVVVTLS
HQGYVKYQPLSDYEAQRRGGKGKSAARIKEEDFIDRLLVANTHDTILCFSSRGRLYWMKVYQLPEASR
GARGRPIVNLLPLEADERITAILPVREYEEGRHVFMATASGTVKKTALTEFSRPRSAGIIAVNLNEGD
ELIGVDLTDGSNEVMLFSANGKVVRFPEAQVRSMGRTATGVRGINLGEGDSVISLIVPRGEGDILTVT
QNGFGKRTAVTEYPTKSRATQGVISIKVSERNGQVVGAVQVETSDQIMMITDAGTLVRTRVSEVSVVG
RNTQGVTLIRTAEDENVVGLQRVAEPVEDEELDSLEPGAEAVEEDTTPLDDGDDAAEPMDDENV
>CORE_REP|Org44_Gene3424#
MSNSYDSSSIKVLKGLDAVRKRPGMYIGDTDDGTGLHHMVFEVVDNAIDEALAGHCSDIQVTIHADNS
VSVQDDGRGIPTGIHPEEGVSAAEVIMTVLHAGGKFDDNSYKVSGGLHGVGVSVVNALSEKLELVIRR
EGKVHEQTYSHGEPQAPLTVVGETEQTGTMVRFWPSHQTFTNVTDFEYDILAKRLRELSFLNSGVSIR
LKDKRTDREDHFHYEGGIKAFVEYLNKNKTPIHPNVFYFSTVKDDIGVEVALQWNDGFQENIYCFTNN
IPQRDGGTHLVGFRTAMTRTLNSYMEKEGYSKKAKVSATGDDAREGLIAVVSVKVPDPKFSSQTKDKL
VSSEVKTAVETLMNEKLVDYLMENPGDAKIVVGKIIDAARAREAARKAREMTRRKGALDLAGLPGKLA
DCQERDPALSELYLVEGDSAGGSAKQGRNRKNQAILPLKGKILNVEKARFDKMLSSQEVATLITALGC
GIGRDEYSPDKLRYHSIIIMTDADVDGSHIRTLLLTFFYRQMPEIIERGHVFIAQPPLYKVKKGKQEQ
YIKDDEAMDQYQIAIAMDGATLHTNASAPALGGEQLEKLVAEHYAVQKLIGRMERRYPRALLNNLIYQ
PTLNEGDLSDEAKVKTWIASLVQALNDKEQHGSSYDFVIFENRERQMFEPALRIRTHGVDTDYKLDFD
FIHGGEYRKICQLGEKLRGLIEDGAFIERGERRQPVDSFEQALEWLVKESRRGLSVQRYKGLGEMNPE
QLWETTMDPESRRMLRVTVKDAIAADQLFTTLMGDAVEPRRAFIEENALKAANIDI
>CORE_REP|Org13_Gene2736#
MKSFNRSGIYLAVMSAMLPGAALAADATDVGTISVKGQSLGGGMMVQDDSAKARSTVTKEAMDKMPSA
ANAIDKLKYTPGLNVNSNDASGLSGVDYTMRGMNSDQIGLSMDGIPINDSGNYAVYPNLLGDAENLEE
VFVTQGSSEADGPHIGSSGGNIGLVTRRPAKDFGGFVKQTLGSNSLSKTFARLDTGEYNGFSNWLSYS

HTEAKKWRGEGRLYSDKFEMNSLYEDGNGNSSNLVMKYNRQNNTNYNTLSKAQFQNDGRDTDYVTTPE
YNNKGQLNKYYKIERNPFENFTLSFTQKLQLRDNLSLTLQPYYYWGNGGSFNGQTASVLSNTSSKAGQ
YDLSNLKSNTYYRPSWTQTWRPGITTKLKWDINEQHSLDVGYWYERARQLQTQPFISIKGDGNPSQIW
GQPGGSDQVKDANGNTVQGRNQYTITPAQKVWLQDTWFATPDWTFVGGLAYQYVERKGDNRGSLYNVP
EKRKATYHEFLPNFSASYKVNQENQVFYNLTRNMRTPPNYVLYNVGDSLSTKPELSWNHELGWRFQQE
DMLLSATLFYMRYSDRQISTTNSAGDYEMMNIGNVENKGLELEWSGQLPHNFNYYTSYTYTESKQKSD
IVSNGGLPLPTSGKEVPNVPKNLLNMTLGYDDGLYYGSVSGKYVSSFYGDLTNDEKIGGRTVFDLAAG
VHLPVDKKIVKSAALRFGISNLFDKEYLTSVRTTTFNAAPYGGVKASTPYYNVGEERTFSVSLEATF
>CORE_REP|Org2_Gene3241#
MKRKHLWVLNPCLLAMLAPAAWAEDQKTGNEEQLVVSASRSHRSVAEMAQTTWVIESQEIEQQVQGGK
EIKDMLAQLIPGMDVSGQGRTNYGMNIRGRSMMVMIDGVRLNSSRSDSRQLDSIDPFNIDHIEVISGA
TSLYGGGSTGGLINIVTKKGQPEQQVELQIGGKTGFGGHNDHDENVAAAVSGGNDNASGRLSVSYQRY
GGWYDGKGNEVLIDNTQTSLQYSDRLDVMGTGTLNIDDHQQLQLTTQYYKSQSDGDHGLFLGENFAAV
TGNAKAYNSGSLDSDRIPGTERHLINLQYSNTDFLGQDLVAQVYYRDETLTFYPFPTLAGKAPNYYVS
SIGASQQKTDFYGGKLTLNSKPVDALTLTYGIDAEHESFNANQQFFNLAKAQQSGGMTLENAYSTGRY
PSYTTSNLASFLQASYDINPIFTLSGGVRYQYTENKIDDFVGYNQQQAIATGAAASADAIPGGKTDYN
NALFNAGLLAHLTERQQTWFNFSQGFEIPDPGKYYGNGTYALNGGHYQLLKSVNVGDSRLEGIKVNAY
ELGWRYTGDNLRTQIAAYYSLSDKSIAINKTDMTINVNADKRRIYGVEGAVDYFFEDSDWSAGTNFNV
IRSETKVNGEWKKLVVDTASPSKVTAYVGWAPGDWNLRLQSQQTFDVSDDGDYTKANSTQGRKIDGYN
TLDFLGSYALPVGKISFSVENLLDKEYTTVWGQRAPILYSPTYGSPELYSYKGRGRTFGLNYSVLF
>CORE_REP|Org10_Gene3143#
MPTKRLSSSAAKQGRLPVSALAITVAAALGTLAMPAFSADAKPAAKEDTITVVGGSNSAQQESAWGPV
GTYVAKRSATGTKTDTPIEKNPQSVSVVTREEMDMRQPDTVKSALAYTPGVMIGNRGASTAYDAVNIR
GFSSVGTNMYLDGLKLQDDNYSIYQIDPYFLERAEVLRGPSSVLYGKSNPGGVVALVSKRPTTETLRE
VQFKMGTDNLFQTGFDFSDALDDAGVYSYRLTGVARDEDQQQVGEKSKRYAIAPSFSWRPDDRTSLTF
LSSFQDDPSVGFYGWLPKEGTVQNGVNGKLPTSFNDGEPGYNNISRKQQMVGYAFEHAFDDVWTVRQN
LRYSKMDVDYRSIYGLGIDPDNSAELKRGVMNSKEHMSSFAVDTQAQAKFATGQVDHIVLMGVDYMRM
RNDVVYQYGSASNLNVIAPQYGNRSYTITGGASQVNRQEQTGLYVQDQAEWNNWVLTMGGRYDWSDTN
STNRLNQNSVSKQQDKQFTGRAGLNYVFENGIAPYVSYSESFEPTSGTDFSGNTFAASKGKQYEAGVK
YAPKDRPITASLALYQLTKTNNKVADPNPEHAFASILGGEIRSRGVELEAKAALTANLNILGSYTYTN
TEYTKDTTLQGNTPAAIPKHMASLWADYTFHETAISGLTLGSGVRYVGSSYGDEANTFKVKDYTVFDA
AIKYDLARFNLPGSSIGINVNNLFDKEYVSSCFATYGCYWGAERQVVATATFRF
>CORE_REP|Org5_Gene3766#
MTTESKCPFSGGKQPAPQNGPTNQDWWPNQLSLKPLHQHSPLSDPMDKDFNYADAFNSLDLAAVKQDL
HALMTDSQEWWPADFGHYGGLFIRMAWHSAGTYRIGDGRGGAGEGQQRFAPLNSWPDNVSLDKARRLL
WPIKQKYGRNISWADLIILTGNVALESMGFKTFGYAGGRADTWEPDDVYWGSEKIWLELSGGPNSRYS
GDRDLENPLAAVQMGLIYVNPEGPDGNPDPVAAARDIRETFARMAMNDEETVALIAGGHTFGKTHGAG
PASNVGADPEAAGLESQGLGWHSTFGTGVGKDAITSGLEVTWTTTPTQWNHDFFRHLFEYEWELSQSP
AGAHQWVAKDIGETIPDAFDPNKKRRPTMLTTDLSLRFDPAYEKISRRFYEHPEELADAFARAWFKLT
HRDMGPRPRYLGPEVPQEELIWQDPIPAVDHPLIDEQDIAALKNAVLASGLPVSALVSTAWASASSFR
GSDKRGGANGARIRLAPQKDWAVNQPAQLAATLATLESIQRTFNDAQAGGKRVSLADLIVLAGAAGVE
QAAKNAGLALTVPFAPGRMDASQEQTDVDSFEAMEPLADGFRNFLKGKYRVPAETLLVDKAQLLTLTA
PEMTVLVGGLRVLGANVGGTPHGVFTQRPQALTNDFFVNLLDMGTTWHPVGEDGLFEGRDRRSGAVKW
TGTRVDLVFGSHAQLRALAEVYGSADAQEKFAHDFVAAWNKVMNLDRFDLA
>CORE_REP|Org2_Gene2457#
MPVVHVALPVPLARTFDYLLPPGMQPVAGARVGVPWGRQHAIGIVTGCSDTSELPLDKLKPIDSVIDA
ESLFSPSLWRILRWASDYYHYPIGEVLFHALPILLRQGKPAEAAPLWQWFATEEGRATPPESLKRAPK
QQQALAALLQRPVYRHQVSQLELTESALQALRAKGLIDLRAQVADTHDWRPNFAVLGERLRLNTEQAT
AVGAIRSEDEQFAAWLLAGVTGSGKTEVYLSVLENVLAKGRQALVLVPEIGLTPQTIARFRERFNAPV
DVLHSGLNDSERLAVWLRARSGEAAIVIGTRSALFTPFRQLGVIIIDEEHDSSYKQQEGWRYHARDLA
VFRAREEDIPMVMGSATPALETLHNVQLGKYRQLKLTQRAGNAKPATQHLIDLKGLPLKVGLSQPLLK
SMQHHLKAGNQVMLFLNRRGYAPALLCHECGWIAECQRCDHYYTFHQHQRQLRCHHCDSQRPVPHQCP
QCGSTHLVSVGVGTEQLEQELAPLFPDTPITRIDRDTTSRKGALEQHLADIHRGEARILIGTQMLAKG
HHFPDVTLVALLDVDGALFSADFRSAERFAQLYTQVSGRAGRAGKQGEVLLQTHHPEHPLLQVLLQQG

YDAFAKQTLAERNSVFLPPYTSHIIVRAEDHDNQQAPLFLQQLRNLLEASPLKDDSLWVMGPVPALQS
KRGGRFRWQLLLQHPTRRVLQQLMKSSLPLIGTLPQTRKVKWTLDVDPIDS
>CORE_REP|Org12_Gene3625#
MNNNKRGWWCALPLAACATLPTWAAEKVASKEESLTVIGRKDADGVQSYQPLTSVTGTRSETNLLNVP
QAIDVVPQQVITDQAVSSLDEALYNVSGITQANTLGGTQDAVMKRGFGDNRDGSILRDGVRSVQARNF
TPTTERVEVLKGPASMLYGMGEPGGMINMITKKPQLQQHTHVEGWGSSFNGGGGQLDVTGPLGTSGFA
YRMIVDHDETDYWRNFGRNRQTVIAPSLMWYGENTTVRLAYEHMEYLVPFDRGTIIDSRTGKPVNTPR
DRRFDEAYNATRGDQDSITLQIDQTLNERWKSSLTYAYSRNSYSDNQARATALNPVTGVLSRQADSTA
NAVSHANAVQLTLNGDVDWGSINHQMLFGFDFEDNRTYRGDMIRGKKNSDFNIYHPVYGLMPPSTAVS
AKDSDQRENLTSYGWFMQDSIQLTDKWLVMGGLRYDAFDVYAGKGRPFQTNTDSSDGKLVPRAGVVYK
LTPYVSLYSSYTESFKPNSSIATQIDSLPPEQGKSWEVGGKLALPNGVTGTLALFDITKRNVMVNELV
EGETVTRTAGRVRSQGVELDVAGNITDSLSLIGSYAYTDARVVDDPDNKGKEMTNVARHTASLFLTQN
LGSLGLYSGDEVRIGAGARYVGRRPGDAANSFYLDNYTVADAFAAYTMPINGYRVKWQLNVKNLFDKT
YYPSSGGNLRVAVGEPREVVLRGSIDF
>CORE_REP|Org36_Gene2938#
MRHSQIKTADDRVYSARFEGAGESQPPFCFSPISRRAGLRVRRSLTIKQMATVSGVALVTICIFIVIQ
LFHFVQQRRDDYAQQLENIAHSVRQPLAEAVLRMDVPEAKKVLNTLLPVGILSRADIVLPNEFQALHA
NFPPERPVPTLIARLFELPIQISVPLYSLERVPANQQPLAYLVLQADSFRMYQFILSILSTMLSTYLL
LALILSVAITWCMNRLMVHPLRAMAKELENISQDEAPYHQLMLPALHQDDELGLLVRNYNRNQQTLAK
AHADMSRLSTRHPVTELPNPALLNALLEQHIASSLRPERFNLLVIGIETLHEASGVMSPAMREALLLA
LAKKLRGCIDENGVLAQLSNTEFAILAKGTERPFHAMQLARRIMAEINAPLTLEGLALRPNASIGIAH
YLNQGESAEQLLRSATSAMMSAHREGKNQILFFEPSLTERTQKRLTQESEILHGIEQRHFTLFLQPQI
DMQSNEVIGAEALLRWQQYDGSYTLPADVIPLAEELGVIVPLGNWVLEESCRILADWQQRGIELPLAV
NVSGIQMQDEAFVPHLKNLLAQYRIDPRKLLLEITETVRIDDLDRALALLRELHDLGLSIALDDFGMG
YSSLEYLNRLKSLPIDLIKIDRSFIQGLPADDAMVRIVSSISEVLALPVMAEGVENAEQRDWLLKHGI
RSGQGFLFARPLPREAFEAEFCRAAP
>CORE_REP|Org9_Gene1317#
MGKHFAAQRHESVGNGEKAGMKAIGAFSSVFLGVCSLAIGNVNAAETKSNETYQDAETLLVTGEKVKR
SIFDTSSSVQVFDSNRIASMPDAVQIPDLLRMTPNVVDLGIGNELPTVRGIDGSGPNVGANAFLSGTR
PRLNLSLDGRSLTYNEQAFGPQSLWDLDRVEVFLGPQSYIQGRNAIAGAIVMASKDPTFEWESAFKGG
AGNQHSSQLAAMASGPLVEDQLAFRVSVDRQRRRSEADLPAYAPVGDPREVEATTARAKLLFNPAGLR
DLTTKLTFNHFGSTAPQNESLNPQPHPTNPRHDPRRAVFKSNMNSTIWDLAWEASDALTLENRVIYTD
FNINRPTAYNIQYAEIDGQEVHVEPVVRFGGADSRLHGLAGLRYFHGTQDEFVNIFGGSTFKDKTDTH
SAFAELTYALTPQVDVTAASRLEREHRRRDGGSQAVRIDFDETYTVFLPKLDVAWKPTDTQTYGAKIA
RGYNAGGGGITIGTPVVSYTYGSEYVWNYELYTRHHLKDANVVLTGNIFYNDYKDMQLPYSLGENSSV
IRNADKVETYGAEIGATWQPRWDFELFGNLGLLKTDIKKFSGSGVEGHELARAPAYTANMGAKYQFLK
GWELSSNVAFSDSYYSAYDNDSRGRIGSYWTANAQLAYTFDYGRATLYAKNLFDSDRREMVRSNDIYT
ATLQRGRLVGAAVELNF
>CORE_REP|Org17_Gene4022#
MPLTFSTRLRFSALSLAIACALPTVALAQNTSTTPSSSPATPAKKAKAADETMTVVATGNQRSSFEAP
MMVTVIEGSSPESQTAGTAADMLRRVPGITVTGSGRSNGQDLMMRGYDRRGVLTLVDGIRQGTDTGHI
NGTFLDPALVKRIEIVRGPSALLYGSGALGGVVSYETVDAADLLLPGHDSGFRVYGTAGSGDHSLGMG
ASAYGKTDNLDGLLSFGTRDVGNLRQGNGFDAPNDETINNVLAKGTWKIDDNQSLGGNLRYYNNSAQE
PKNPQTPASSAGNLMTNRSTIQRDAALSYKLKPVGQDWLDAEAKVYYSDVKINAHASGSEDEARKQTT
KGAKLENRTRLFADTFASHLLTYGTEAYKQEQTPGGATESFPHAKINFASGWLQDEITLRDLPVTLLA
GTRYDNYKGSSDGYADVDANKWSSRGAVSITPTDWLMLFGSYSQAFRAPTMGEMYNDSKHFSIPMGPT
TITNYWVPNPNLKPETNETQEYGFGLRFDDLLLADDSLQFKASYFDTKAKDYITTDVTMELGRGPRGP
YCISCTTFSTNIDRAKIWGWDATLSYKTSWFGWDLAYNRTRGKNEATGDWLSSINPDTVTSSLDVPLG
ETGLSAGWVATFAERATRVQTGTPEQGGYGVNDFYLSYKGRDRLQGVTTTVVLGNAFDKEYYSPQGVP
QDGRNAKLLVSYQW
>CORE_REP|Org13_Gene4551#
MPADQGPELLNAHFGTQSPHWRLAFDSNALELSAVKGKAHVAVAFSAMEAAKIRRLTGVTASLELTIT
LAGEPLHLHLVGRRVNNLEWAGTASAFSDTQSVARDLVHGLSFAEQVVSEANSVIVIVDQHGRIQRFN
RLSEEYTGLREHEVIGKNVFQLFMSPEEAAASRRNIAGFFRNGSSYEVERWVKTVKGERLFLFRNKFV

HSGSGKNEVYLICSGTDITEERRAQERLRVLANTDLITGLPNRNAIQDKINHAIATRGEESFGLVYLD
LDNFKKVNDAYGHMFGDRLLVEVALAILGCLSPDQVLARLGGDEFLVLAPQTDRERLQTLAPQTDRER
LQTLAQRIIDRLKTPFRIGLIEVYTGCSIGIALCPEHGNDLDSLIRSADTAMYVAKEHGKRTYTVFSP
EMNKRVAEYMWLDTNLRKGLEQNQLVLYYQPKIDARSGEVHSVEALVRWDSPERGLIPPLQFISYAEE
SGLIGPLGQWVLQTAAGQAAQWQEQGLNLRVAVNLSARQLADDSIVNDLLGVLRRHRMAPCLLDFELT
ESSLIEDENRARALITRLRELGAQVHLDDFGTGYSSLAQLARIPLDAIKLDKSFVRGVNFNPVSQSLV
RAIVAAAEALAFRVIAEGVETESENHFLDEVGVDEKQGFLFARPMLPEQLEHWLQSYRPHSPSA
>CORE_REP|Org42_Gene1946#
MSKLFKLHSEFKPAGDQPEAIRKLEEGLEDGLAHQTLLGVTGSGKTFTIANVIADLNRPTMVLAPNKT
LAAQLYGEMKEFFPENAVEYFVSYYDYYQPEAYVPSSDTFIEKDASVNEHIEQMRLSATKALLERRDV
VVVASVSAIYGLGDPDLYLKMMLHLTQGMIIDQRSILRRLAELQYSRNDQAFQRATFRVRGEVIDIYP
AESDELALRVELFDEEVERLSLFDPLTGQIEQVVPRFTIYPKSHYVTPRERIMQAMEEIKVDLADRRK
VLLANNKLLEEQRLTQRTQFDLEMMNELGYCSGIENYSRYLSGRAEGEPPPTLFDYLPADGLLVVDES
HVTIPQIGAMFKGDRARKETLVEYGFRLPSALDNRPLRFEEFEALAPQTIYVSATPGKYELEKSGGDL
IDQVVRPTGLLDPIVEVRPVATQVDDLLSEIRKRAAINERVLVTTLTKRMAEDLTEYLEEHGERVRYL
HSDIDTVERVEIIRDLRLGEFDVLVGINLLREGLDMPEVSLVAILDADKEGFLRSERSLIQTIGRAAR
NLNGKAILYGDRITDSMAKAIGETERRRAKQQAYNEANGIVPQGLNKKIGDILQIGQPVNRAKSKGKG
KAADGGASLQNLTPKALDQKIRDLEAQMYTHAQNLEFEQAAALRDQIHQLREQFIAIS
>CORE_REP|Org13_Gene4198#
METPRYSKLAALVVASLSATAALAAPQNDTQDTMVVTASGFQQKIQDSAASISVIPRQQIEDKAYRDV
TDALKDVPGVVVTGGASSSDISIRGMSSKYTLILVDGKRVDTRSTRPNSDNAGIEQGWLPPLEAIERI
EVVRGPMSSLYGSDAMGGVINVITRKTSRTEWKGSLHGDATIQENRNSGDLFQTNAYASGPLVEGLLG
LRVNGLLSRRAEDKIVNGYNEQRMRSGTAVFTLTPDEKNEFDFEIGRSLQDRNSTPGKSVVAERCSKG
KCTPTEVSESLYTRTNYALTHNGYYDFGNSTSYVQREETGNPGRNMKAYNTIFNTQNQFELGSHMLNL
GGQYRYEKLGDGGNQLESAQGLSKLTRWSWALFAEDEWALTNDFSLTSGIRMDRDENFGSHWTPRMYG
VWHLTEQWTLKGGVSAGYKSPDLRQSSPNWGQVTGGGVRKGIIVGNPDLQPEKSLSEEIGLMWDSLKG
VNAGVTVFNTDFKDKITEVRRCEDTPDCKIGNDVYDFISDRVNVDKANMRGVEATFGWQINKDWKWNT
NYTYTSSEQKSGEFQGKALNQMPKHMLNTVLDWRATQDLSLWSRVNFRSKTSQYLSRTSMATSTPSYT
FVDAGLSYQAAKNLQLTGGVYNILDKTVDYDHFRTTLDGRRYTVGMTYNF
>CORE_REP|Org6_Gene4205#
MILRHPPIKFGCFSHDNYKNTLLAVVSSVTAFSGWAQDNTTATNGDNLVVTANRFPQPVSSVLAPTSV
VTRNDIDRWQAKSLTDVMRRLPGVDIAQNGGLGQQSSLFIRGTNSSHVLVLIDGIRLNQAGVSGSSDL
SQIPISLVQKVEYIRGPRSAVYGSDAIGGVVNIITTREKNGTTLAAGVGSNGYQSYDASTQQPLGDST
VATVAGNYTYAKGYNVIANLPDSFGNPAQPDRDGFMSKSLYGGIEHKFNEAFSGFVRGYGYDNRTAYD
GNYSYSDPAHLDALPDTRQLYSQSWDSGLRYQDGIYATQLIASYSHTKDYNYDPKYGPYSASATLDDS
TQYNVQWGNTFQVAQGHISTGVDWQNQKIEPGTAYITDSKSQRNTGLYLTAQQQVDAFTLEGAVRGDD
NSQFGWHGTWQTSVAWEFVEGYRAIASYGTAFKAPNLGQQYGSFGGNPDLKPEESKQWEGGFEGLTGP
VTWRVTGYRNDIDNLISYASSGSGSAYYNVNQARIKGVEATVSFDTGPLTHQIGYDYVDPRNAKTNEV
LLRRAKQQVKYELDWQLYDFDWAVTYQYLGERYDGDYSGYTTRTVKLGGVSLWDLAVSYPVTSHLTVR
GRIANLFDKDYETAYGYATPGREYYLTGSYTF
>CORE_REP|Org11_Gene1936#
MTQSSYNADAIEVLSGLEPVRRRPGMYTDTTRPNHLGQEVIDNSVDEALAGHAKRIDVILHADQSLEV
IDDGRGMPVDIHPEEGVPAVELILCRLHAGGKFSNKNYQFSGGLHGVGISVVNALSKRVEVNVRRDGN
VYGIAFENGDKVQDLTVTGTCGKRNTGTSVHFWPDEQFFDSPRFSVSRLTHLLKAKAVLCPGVEIYFI
DKVNNTEQRWCYQDGLTDYLMEAVNGLITLPEAPFVGNFAGDTEAVDWALLWLPEGGELLTESYVNLI
PTMQGGTHVNGLRQGLLDAMREFCEFRNILPRGVKLSAEDIWDRCAYVLSVKMQDPQFAGQTKERLSS
RQCAAFVSGVVKDAFSLWLNQNVQAAEQLAELAISSAQRRLRAAKKVVRKKLTSGPALPGKLADCTSQ
DLAMTELFLVEGDSAGGSAKQARDREYQAIMPLKGKILNTWEVSSDEVLASQEVHDISVAIGIDPDSE
DLSQLRYGKICILADADSDGLHIATLLCALFVRHFRSLVKGGHVYVAMPPLYRIDLGKEVFYALDEEE
KAGVLEQLKRKKGKPNVQRFKGLGEMNPLQLRETTLDPNTRRLVQLTVAEDDVDQTLAVMDMLLAKKR
SEDRRNWLQDKGDMAELAV
>CORE_REP|Org48_Gene4550#
MDNHHMMIEGLIYLGSAALFVPIAVRLGLGSVLGYLIAGCIIGPWGLKLVSDAESILTFAEIGVVLML
FIIGLELDPKRLWTLRASVFGGGSIQMVGCGLALSAFCYFLGLNWKVALLIGLTLALSSTAIAMQAMS

ERNLTPSPIGRSAFAVLLFQDIAAIPLVAMIPLLASSGATTTLGAFVLSAAKVVGALTMVVLLGRYVT
RPLLHFVARSGMREVFSAVALFLVFGFGILLEMAGLSMAMGAFLAGVLLASSEYRHALESDIQPFKGL
LLGLFFIGVGMSIDFGTLFHHPLLIASLLLGFMLIKAALLWLIGPLLGVPKRQRGLFAILLGQGSEFA
FVIFSAAQLAGVLPVEWAKSLTLAVALSMAATPLLLVIAAQLEKNAPKEERPADVIDDENASVIIAGF
GRFGQIAGRLLLANGVHTVVLDHDPDHIETLRKFDTKVFYGDATRADLLEAAGAAHAKVLINAIDDVE
DSLALTELARQHFPHLKVVARARDVDHWYQLRQLGVEKPERETFESSLRIGRETLELLGLDAYEAREK
ADMFRRYNLKMLEDTLENYQDTEFRIASLQRAKEMLSAAIEQDQNRLSRVQQTGWRGSIDGKAPEDEV
VEAKG
>CORE_REP|Org28_Gene1320#
MEGSTLLTAILLFLFAAVVTVPIARRLGIGAVLGYLIAGIAIGPWGLGFIRDVDEILHFSELGVVFLM
FIIGLELNPSKLWELRRSIFGAGAGQVLITAAVLGALLYLTHFAWQAAVIGGVGLAMSSTAMALQLMR
EKGMNRNEGGQLGFSVLLFQDMAVIPALALIPILAGAGGTSDDWAKIALKVAAFGGMLIGGRFLLRPL
FRYIAASGVREIFTAAALLLVLGSALFMEALGLSMALGTFIAGVLLAESEYRHELEISIEPFKGLLLG
LFFISVGMVLNIGVLYTHLAEVLIGVLVLVTVKSGVLYGVSRLFGLRSSVRLQFAGVLSQGGEFAFVL
FSAAGAQKVLQPDQLSLLLVVVTLSMMTTPLLMQAIDRILARRYNAKDEDEETPYVEDDDPQVIIVGF
GRFGQVIGRLLMANKMRITVLERDVSAVGVLRRYGYKVYYGDATELELLRAAGAEKAKSIVITCNEPE
DTMEIVRLCQQHFPNLSILARARGRVEAHELLQAGVKQFSRETFSSALELGRKALMELGMHPHQAFRA
QQHFRRLDMRMLRELMPPHQGDVAQISRVKEARRELEELFHREMQKESRQFDGWDEYE
>CORE_REP|Org41_Gene4075#
MDIKQKVKNMTLEEKIGQKIMLDFRYWDRNGSSNQDMTVPDEAIGKLIADNHVGGVILFANNLKDKQQ
INTLTAWYAAMKTHAGIRLFIGTDNEGGNVFRLPRGDYASFPGNMALAAAIEGGADEQLAVEQGKLMA
QDMRALHINTNFAPVVDVNTNPFNPVINVRAFSDDKNTVSRLAEKMVAGMKHQGLITAYKHFPGHGST
STDSHTGLPRVDRTREEAFAIDIAPYKQAIDRCAAPDMVMTAHIQYPALDNRQIDTRSGEKITVPATM
SHEIQTQILRNELGYAGVTISDALDMGAIAEHFSQQAAAENVFAAGVDIALMPVSIASPAQASLLPAL
IRYLADRVKTGHLSEADIDASVERILRLKLRHSLMDHSDRPCSNDVASSAHKLEKCIADRSITVVINR
HSLLPLKDKALRYFILTPWGEQASGIARVMAQEGYQNVVAAKETELSDAQVRKHIAGCDVFLLGTLST
RFTPAEQDGVVTSATGASNDSSPYPGWLKYAAEQGKKRVHLSLRAPYDIVNYAAEVEAAVVTYSYYGY
DSGVWRGPSMVSLAQVLTGKIKPQGKLPVNTWHDYDVETNTGKVAFPRGTGLSW
>CORE_REP|Org40_Gene4041#
MPKQRVPLKLSTSITLMVSAIIASVLLVVFALFFVQMSREGQDQLQQKAIAVANTLALSNTVIDGLQR
RDQSGAIQRFAEQVRHQNELLFVVVVDMQGIRYSHPKPWLIGKHFIGDDLAPALQGNVNSAINRGTLA
PALRVFVPVYDDQKQQIGVVALGIALDTVQRVVAESRWIIYWTIAFAALVGSLGTFFLVSALKRIMLG
FEPYEISNLFEQRNAMLQSIKEGVIAVDNESRITIVNDEAKRLLRQSGPVENLLLEAASKHWPAQLHL
AEVLASGEPLRDRQISFNGSELLTNTVPVIVNGQVTGAIATFRDKTEVSRLLQRLSGMAHYADALRVQ
SHEFMNKLHVILGMLHMKAYQQLENYIINTASNYQEEIGALLRKIHSPEVAGFFIGKISRAHEAGVEL
TIEENSLLPETDDAETTHVLISVLGNLIENAIDAIDGVEGHEIGLSFHHHDDQLHCIVSDDGPGIDPA
IAARIFEHGFSTKGTGRGIGLALIRSHLEKLGGSIDFESEPGELTQFFVHLPYQAKSRAHD
>CORE_REP|Org29_Gene4329#
MRLKLSFQIKLFLCLVAFSCLLLTCIGAYTYYQLDAQLHRDLGARAQVQAREIALIPSLVDAVENNDA
ARIAALMKKIRASSDASYIVIGDNHARHLYHSEYEGRLGTPMIGGDNKEVLEGKSIISIRKGGIGVSL
RSKAPIVDENNRVIGIVSVGYLKSHIDNLNARTLTQIIGSIILLLIALFVFSWLLSKNLKRQMFWLEP
KEIALLVRQQKALLEAIYEGVIAIDPQLRIITINHAARELLDLHQPAAGLLGRPIGDVIQAQPNFFAA
AQLGQDTHDEVCRFNHVRVIASRVRIMQEQELQGWVISFRDKNDINTLSSQLSQVKRYADNLRIMRHE
QLNWTATLAGLLHMQRYDEAIRYVEAQSEGAQEILDFISQRFSSAALCGLLLGKYSSAREKGIELRFD
PACQLRQIPAALNETELMSIVGNLLDNAVEATLHYPAPHEAIELYISDGSDELVIEVADHGTGIAEEI
RDTLFEQGVTTKADKSDHGIGLHLVASHVAQAHGSIEVSDNEPHGAIFSIFIPK
>CORE_REP|Org2_Gene2285#
MLMTHLAASRYRYYRWLLAGAVGAAILLVSLYTRYYQEVKSIELSQHTLATRTVGKLNQLLTPAQLQA
ERSMDMLNQSCENVSSTLRFRAAQNQALRAMLLVKNGIIYCSSLFGARHYQLAAVMPSFVNSDARLAL
RPSLAVSKGLPTLVLWTPSPRDKTSGVLHVFNIELLSNFLLEPQEPYVQRVVLNVADSSLEYGRREIL
SRDTLTNDLRYTAGSALYPFSISLFGPQIGMLALSALPRHIPLALLISLLAAYVVYLLTANRMSLSYH
IGHAITHREFRVYCQPIIHSDTGRCAGVEMLLRWKKKRQGWISPDVFIPLAEQHELIIPLTRYLMSTV
TENLQLFPPRPSFYISINVAAEHFKTLNIIDDIRQIWLPAHPMPSLMLELTERTALSAIQYDQIRTLK
DMGIMLAIDDFGTGHSSLSYLKNLSPDVLKIDRGFTAAIGTDAVNATVTDTIITLAQRLKLKLVAEGV

ETEEQADYLRSREVNALQGYYFAKPMPIHVFPLWLQQYESRVRKAEEDPPEA
>CORE_REP|Org27_Gene3371#
MIKIVVYLILAVTIAIIAARVLFRLPDISQRLPQAALPADPAAQLPARAAELMAAHPGLSGVVPLASG
HDAFASRLALARMAERSIDAQYYIWHNDTSGQILLKTLYDAAQRGVRVRLLLDDNGVAMDETLAALNA
QENVEIRLFNPSTVRTPKLAGYAFDFMRMNRRMHNKSYIVDGAVAIIGGRNIGDEYFQVGDENYFLDL
DVLSVGSVVAETAEVFDRYWNSASVFGVEQIIRGKGNLSAFLTQATATESSERARKLAVQLETSAVRF
RDGAVQPEFTQVELVADDPAKGLGKASRDRLMVTQLGKIIGGVGRQLDLVSAYFVPGREGASFFESLA
KQGKSIRVLTNAMNTTDVLVVHAGYAKYRRELLQAGVELFELKLRAGQPTGRKELKPLGLSGAALHAK
TFAIDDKRVFIGSFNFDPRSAHLNCEMGFLIDSPTLAADTRQLFDGPLEYAAYRPVLTPEGKMVWKEA
FEDGHTEVHQQEPGAGWVKRIILTVAGWLPIEWML
>CORE_REP|Org25_Gene2809#
MKKLLPLLIGLSLGGFSAMSQAENLLQVYKQARESNPDLRKSAADRDAAFEKINEARSPLLPQLGLTA
GYDYTNGYRDSNGVNSNVTSGSLALTQTLFDMSKWRQLTLQEKSAGISDVTFQTAEQSLILNTATAYF
NVLKAIDTLSYTQAQKDAVYRTLDQTTQRFNVGLVAITDVQNARSNYDTVLAAEVSARNDLDNALETL
RQVTGAFYPELASLNTDRFSTQRPEAVNNLLKEAEARNLSLLSARLSQDLAREQIRAAQTGYMPTIDV
SASTGISNTKYNGSNTGGANAARYSDSDAGQNKVGISFNLPLYSGGATNSQVKQAQYGFVGASEQLES
AHRSVVQTVRSSFNNVNASISSINAYKQAVISAQSSLDAMEAGYQVGTRTIVDVLDATTTLYNAKRQL
SDARYTYLINQLNIKSALGTLNQNDLLLLNGALGKPVSTAPDAVAPQNRAQDAYADGYQDNAPMQQTA
APAPAATRASAPAVTTSQPARHSGNPFRN
>CORE_REP|Org46_Gene4641#
MSQSVLASETHRGHLQQIISGLSDGVILTDTDRTLLWANEAALAMHGVSHQKALGANAGEYAARFALR
YRNNHPLALEQYPLNRVADGETFTDVVVEVRQADDPDSFWVHRLRSLIITDAQGQPELLALILSDATE
WASAEQRFEKTFNANPAPAVICRLSDLRYVKVNQGFLDMTGYQREQVMGRSVYELDVLEQAEHKDLAI
QRLGEGATIPQMEAELKLPGGGSKLVVVAGQPLDINEDDCMLFTFTDLEPRRQAESALRESEERFAKA
FRLSPVPTLLCTAHERRVLDVNEAFTRTTEYDAEALIGKTVDEIQFIDDPEASRRLFAALEKSGNVEG
LDIRVRKKGSESIDCVASADAVSIHNAPCYLLVLMDITERKRSELELVSAIEEVMQDASWFSQTLIEK
LANVKSINRPDQAGLTASDLTPRERDVLELICEGLPDKKIAARLNLALNTIRNHVATVYSKLGVHSRS
EAIVWARERGLFTGGLAARNGK
>CORE_REP|Org4_Gene2195#
MTTFYTVISWLMVFGYWLLIAGVTMRILMKRRAVPSAMAWLLVIYILPLFGIVAYLSFGELHLGKRRA
ERAKAMWPSTARWLKELKESRRIFATEYSEVAEPLFQLCNRRQGIDGVKGNQLQLLTTTDDTLKALIR
DIELARHNIEMVFYIWQPGGLVDQVAESLMAAARRGVHCRLMLDSAGSLQFFRSPYPAMMRNAGIEVV
EALKVNLLRVFLRRMDLRQHRKVVLIDNYIAYTGSMNMVDPRYFKQDAGVGQWIDLMARMEGPVATTM
GIVYACDWEIETGKRILPPPPDVNIMPFEQESGHTIQVIASGPGFPEEMIHQALLTAVYSAREQLIMT
TPYFVPSDDLLHAICTAALRGVEVSIIVPRDNDSMMVRWASRAFFSELLEAGVKIYQFEGGLLHTKSV
LVDGQLSLVGTVNLDMRSLWLNFEITLVIDDDGFGSDLACVQEDYIARSQLLNAKEWLKRPFWHRLVE
RLFYFFSPLL
>CORE_REP|Org31_Gene1090#
MRSPFNWRFTPLFAVLLLAGCASTDNIAPQSTLMDPQSLQLAQPKVSSLAVSPQWWRALKDPQLDTLM
TQTLQSSPTLRQAAARVREAQSVVGEASAANGPNLDLNASTQRQRVPQNVNMGLGYPHKPIYSSSNSL
GLNLAYEFDWWGKYRNQVNAAKAQVNAARAEQEQAALTLTSSVASAYYQLQSNLALEKLLQQEVNNNE
RLTALRQQRYQAGLTGVDVPQQTQAQSDVAKQQILQLQSQIEQLRHQLAALAGQGPNAMQHLRQVPLP
ADNLMAPQGELTADLLGKRPDIAAQRQLVESYSQRVSAARKEFYPSLTISAFAGLMTTNTSGTSPNLF
EAASQAWNVMPAISLPIFHAGALRSKLGEESALYDEAVESYNQTILNAVQETADAITIQQSSAQQQLQ
AASAAQSMQQVYQVANARYQAGIIGRDDLLTSQTQLLQQQQAELNASSNLLQAKIGLIRALGGGYQAP
AAADSKA
>CORE_REP|Org14_Gene1836#
MTRYATLAAILSQRIQQGLYPAGHRLPSVRALSQEHGVSISTVQQAYRLLEEQRLVEARPKSGYFVHT
RRAQAELPAMTAPVQRPVDISQWEQVLELVRSRPREGLIQLGRGMPDIAEPTMKPLIVALRNAARHGD
LRSLYYDSIQGVAALREQVARLLLDSGCQIGPDQLLITTGCQEAISAGLRAVCQPGDIVAVDSPCFHG
TMQTLKGLGIKALEIPTDPLTGVSLAALEMALEQWPIKAILLTPNCNNPLGYIMPDAHKQRLLTLAQR
HDAAIIEDDVYGDIAYHYPRPRTIKSFDEDGRVLLCSSFSKTLAPGLRVGWIAPGRYLERVLHMKFIG
SGATATQPQLAIAEFIRGGHYLQHLRRMRARYQQNRDRMTDLILKHFPAGTRVSRPRGGFMLWIELDE
AFDTLRLNRHLEQQGVQIAVGSIFSAAGKYRNCLRINYAPKLTAEIEQAVQRVGATIQALMPSGVLQP

QAD
>CORE_REP|Org31_Gene850#
MMTDKVRIDTLVANSLNGNNETYLARQAEFESNVRSYPRKLPLAIAKAQGVWITDVENNQYLDCLAGA
GTLALGHNHPDVLQSIQNVITSGLPLHTLDLTTPLKDRFSDYLLSLLPGEGKEYCLQFCGPSGADAVE
AALKLAKKHTGRSGVISFSGGYHGMTHGALSVTGNLSPKAAINGMMPEVQFMPYPHEYRCPLGIGGEA
GVKALTYYFDNLINDVESGVRKPAAVILEAVQGEGGVNPAPAEWLQRIRKVTQEHGILLIIDEVQAGF
ARTGKLFAFEHAGIEPDIIVMSKAVGGGLPLAVLGIKKEFDAWEPGHHTGTFRGNQLAMATGLTTLQY
LKEHQVADKVAAQGEWLKGKLAELQKRYPVIGHVRGLGLMIGIEIVKPNEAQDHMGCYPADGELSALL
QKKCFESGLILERGGRNGCVLRLLPSLLITNDELGIFLDKFEQALLAAGVKPV
>CORE_REP|Org18_Gene3234#
MKILSPLALSLAALLTAGCGNALKSDYRAPQVNYPTSWQHAADNAAPTPFDWRDFHDPELDRWLQQVM
DSNNDLAVAVLRVYRARLEAERVGISTAPDVNASLNSGINRPLSESSAWNKTSGATLSTSYEVDLWGK
LARQRDAAEWASQASEQDLQTARLTLLANAATNYWRIGFLNQQIGVSQASIAYAKQTLRLANARYRAG
SISALDVVNAEQNVLTQESRLLALQHDRQQALNEQAVLLGAPTGQATIAPARLPTTAMPQINTGIPAS
VLSRRPDLSAKELRLRAALANVDEKRLQYYPAFSLTGSLGASSSALLEFLRNPTGSLGASLTLPFLQW
RQMGVDIKIARNDYEQQVLEFRQALYKAMGDVNNALSLRAQLRAQETQLQASLALARKSERLNEVRYR
QGAVTITDWLNAQEQRRQAELAVDENRFAQYQNLAKIYLEFGGSSAP
>CORE_REP|Org16_Gene3986#
MKVTVFGIGYVGLVQAAVLAEVGHDVMCIDVDERKVKNLKKGNIPIFEPGLTPLVQQNYEAGRLHFTT
DAKAGVAHGNIQFIAVGTPPDEDGSADLKYVTAVARTIAEHMTDRKVVIDKSTVPVGTADKVRQVMAE
TLAKRGSNVAFDVVSNPEFLKEGAAVADCMRPERIVIGTDNKEVIEPIRELYEPFNRNHDRMIMMDIR
SAELTKYAANCMLATKISFMNEMSNLAEMLGADIEKVRQGIGSDSRIGYHFIYPGCGYGGSCFPKDVQ
ALIRTAEQIGYQPKLLQAVEQVNYQQKDKLNSFIKDYFGSDLKGKTFALWGLAFKPNTDDMREASSRV
LMEQLWAAGATVQAYDPEAMNEVQRIYGQRDDLKLMGTKEAALHGADALVICTEWQNFRAPDFDVIKS
ALKQPVIFDGRNLYDPERLENRGFTYYAIGRGASIKPVI
>CORE_REP|Org30_Gene3692#
MAAENHPLNSLDAEGLEAHWMPFTGNRNFKAQPRIITQAAGAYYTSHDGRKIFDGLSGLWCCGLGHGR
QEITDAAQRQLATLDYSPAFQFGHPLSFELANKIKALTPAGLDYVFFTGSGSEAADTSLKMARAYWRA
KGQAGKTCFIGREKGYHGVNFGGISVGGIAGNRKTFGAGAEADHLPHTLLAGNAFSRGMPQQGAELAE
ELNRIIALRDASTIAAVIVEPFSGSAGVIVPPVGYLQRLREICTQHDILLIFDEVITAFGRCGAMTGA
EAFGVTPDIMNIAKQVTNGAQPMGAVVVRPEIYQTFMNGGEPDYQLEFPHGYTYSAHPVSCAVALATL
DILQREQMVERVQALAPYFERAVHSLQGAKHVADIRNIGLAAGITLAARPGEPARRPFEAAMRCWESG
FYVRYGGDTLQLAPPFISSEAQVDALINAVGDALNATE
>CORE_REP|Org19_Gene1398#
MSLHSKSESLYAQAQQLIPGGVNSPVRAFTGVGGVPLFIERADGAYLFDADGKAYIDYVGSWGPMVLG
HNHPAIRDAVIEAAQRGLSFGAPTEMEVKMAQLVTELVPTMDMVRMVNSGTEATMSAIRLARGYTNRD
KIIKFEGCYHGHADCLLVKAGSGALTLGQPNSPGVPADFAKHTLTCTYNDLDSVRAAFEQYPSEIACI
IVEPVAGNMNCVPPLPEFLPGLRALCDKYGALLIIDEVMTGFRVALAGAQSYYGVEPDLTCLGKIIGG
GMPVGAFGGRRDVMDALAPTGPVYQAGTLSGNPIAMAAGYACLTEVSQVGVHQTLTELTEMLAAGLLH
AAQEENIPLVVNNVGGMFGLFFTDAPAVTCYQDVMQCDVERFKRFFHLMLEEGVYLAPSAFEAGFMSV
AHSKEDIQRTIDAARRCFAKL
>CORE_REP|Org37_Gene3352#
MVDSLTLHPVALVNGTVNLPGSKSVSNRALLLAALAKGTTRLTNLLDSDDVRHMLNALQTLGVNYQLS
ADRTVCEVTGVAGPLVAGQPLELFLGNAGTAMRPLAAALCLGEGDVVLTGEPRMKERPIGHLVDALRQ
GGAQIDYLEQTDYPPIRLRGGFQGGDVTVDGSVSSQFLTALLMTAPLAPQDTQIHIKGELVSKPYIDI
TLHLMRTFGVSVSHDNYRVFHIQGRQTYLAPGDYLVEGDASSASYFLAAAAIKGGTVRVTGIGRKSVQ
GDTKFADVLEKMGARITWGDDFIECSRGELRGIDMDMNHIPDAAMTIATAALFAEGPTTIRNIYNWRV
KETDRLAAMATELRKVGAEVDEGEDYIHVVPPAKLQFAEIGTYNDHRMAMCFSLVALSDTPVTILDPK
CTAKTFPDYFEQLARISQPA
>CORE_REP|Org39_Gene3672#
MSVTASDLAFDQRHIWHPYTSMSRPLPCYPIESASGVELQLADGRRLVDGMSSWWAAIHGYNHPHLNQ
AASRQLEKMSHVMFGGITHPAAISLCRRLVAMTPEALQCVFLADSGSVAVEVSLKMALQYWQARGERR
QRILTLRHGYHGDTFGAMSVCDPDNSMHSLYQGYLAPHLFATAPQCRFDEEWREEDIAPFAALLEQHA
GEVAAVILEPVVQGAGGMRIYHPTYLKRVRELCDRHQVLLIADEIATGFGRTGKLFACEHAQVVPDIL

CLGKALTGGYMTLSATLTTRHVAETISNGAAGCFMHGPTFMGNPLACAVADASLALLAENRWQAQVSA
IEAQLKQELLPLAALPKVADVRVLGAIGVVEMREPVDVAGLQRGFVERGVWIRPFGKLIYLMPPYIIE
AEQLSRLTAAVAAAAR
>CORE_REP|Org33_Gene2400#
MNKATVAAKRWWYIMPIVFITYSLAYLDRANFSFASAAGINEDLGITKGMASLLGALFFLGYFFFQIP
GAIYAERRSVKKLIFWCLILWGGCASLTGVVSNIPMLAAIRFILGVVEAAVMPAMLIYISNWFTKSER
SRANTFLILGNPVTVLWMSVVSGYLIHAFGWREMFIIEGIPAVIWAFCWWVLAKDKPAQAGWLSAEEK
LALQQQLDEEQKGIKAVRNYGEAFRSRNVILLCVQYFAWSIGVYGFVLWLPSILRSGMQMGMVEAGWL
SAVPYLAATIAMIVVSWASDKMQNRKLFVWPLLLIGALAFFGSYAVGTNHFWISYGLLVVAGAAMYAP
YGPFFAIIPEMLPKNVAGGAMALINSMGALGSFFGSWFVGYLNGATGSPAASYMFMAIALVVAVVLTL
IVKPARNEIQPQLA
>CORE_REP|Org36_Gene4636#
MKNAELNQRRQDATPRGVGVMCGFYAERAENATLWDVEGKEVIDFASGIAVLNTGHRHPKVIAAIEKQ
LQAFTHTAYQIVPYESYVSLAERINQRAPIAGPCKTAFFTTGAEAVENAVKIARAYTGRPGLITFGGG
FHGRTYMTMALTGKVAPYKLGFGPFPGSVFHGQYPNALYGVTTEDAMNSLDRLFKADIDPKQVAAIVL
EPVQGEGGFNVAPAEFMQALRALCDQHGILLIADEVQTGFARTGKLFAMEHYSVKPDLITMAKSLAGG
MPLSAVAGRAEVMDAPAPGGLGGTYAGNPLAVAAAHAVLDVIEEEQLCQRAQRLGQHLVEVLQQARKT
SPAIADVRAQGSMVAVEFNDPATGKPSADITRQVQQKAMEEGLLLLSCGVNGNVIRFLYPLTIPDDQF
TKAMGILSRALAH
>CORE_REP|Org20_Gene4243#
MSFDTISVIGLGYIGLPTAAAFASRKKKVVGVDVNQHAVDTINRGAIHIVEPDLDKVVKDAVDGGFLR
AVTKPLAADAFLIAVPTPFKGDHEPDLAYVEAAAKSLAPVLKKGDLVILESTSPVGATEQMADWLAQA
RSDLSFPQQAGEAADVNIAYCPERVLPGQVMVELIQNDRVIGGMTPKCSERASALYKIFLEGECVITN
SRTAEMCKLTENSFRDVNIAFANELSLICAEQGINVWELIRLANRHPRVNILQPGPGVGGHCIAVDPW
FIVAQNPQQARLIHTARLVNDGKPLWVVDRVKAAVADCLAATDKRASEVKIACFGLAFKPNIDDLRES
PAVEVVHLIAEWHVGETLAVEPNVEQLPKSLAGHVTLTPIAEALQQADVIVMLVDHQQFKAIRPEEIK
QSWVVDTKGVWR
>CORE_REP|Org24_Gene371#
MDKFRVQGRTRLSGEVSISGAKNAALPILFAALLAEEPVELQNVPKLKDIDTTIKLLNQLGTKIERNG
SVFVDASGVNEFCAPYDLVKTMRASIWALGPLVARFGRGQVSLPGGCAIGARPVDLHITGLEQLGAEI
KLEEGYVKASVEGRLKGAHIVMDKVSVGATVTIMSAATLATGTTVIENAAREPEIVDTANFLNTLGAK
ISGAGSDKITIEGVERLGGGVYRVLPDRIETGTFLIAAAVSGGKVMCRNTRPDTLDAVLAKLREAGAD
IEVGEDWISLDMHGKRPKAVTVRTAPHPGFPTDMQAQFSLLNLVAEGTGVITETIFENRFMHVPELIR
MGAHAEIESNTVICHGVEQLSGAQVMATDLRASASLVIAGCIADGVTVVDRIYHIDRGYERIEDKLRA
LGANIERVKGE
>CORE_REP|Org45_Gene2458#
MPNGHPVRRPLGRLYTALWGGCLLMLSQSAAARFAIPGYELVYTAPVETALQADDLRNTAEVWREMFD
AAKTRIDLGQFYVANQDGSLLDGVLQHLKAAGERGVKIRFLLEEKGLRMSTAETLEQLKAIPNLELRI
IPYQKLSGGILHAKYLLVDGEQAFVGSQNFDWRALEHIHETGLRISDAKVVGQIQAIFEQDWQAQALL
AQDKPVPALPDSPPTAQPQGNYLAASPRAYNPAGVIDSQAELPRLLAGAKRRVRVQVMDYAPLSFGPE
RSRPFYAVIDNALRSAAARGVQIELMVANWNTKKPDIAWLKSLALVPNVQIKVVTIPPASSGFIPFAR
VIHSKLMTIDDEIAWVGTSNWTGGYLDNSRNLELVMHSAAMSGRLDKLYQQLWNSVYAEPLRLDYDYP
PPKPGGES
>CORE_REP|Org31_Gene1596#
MSKRRVVVTGLGMLSPVGNTVESTWNALLAGQSGISLIDHFDTTAYATKFAGLVKNFNSEDFISRKDA
RKMDAFIQYGIAAGMQAMQDAGLDITEANASRIGAAIGSGIGGLGLIEENHSSLVNGGPRKISPFFVP
STIVNMIAGHLTIMYGMRGPSISIATACTSGVHNIGHAARIIAYNDADVMLAGGAEKASTPLGVGGFG
AARALSTRNDNPQAASRPWDKDRDGFVLGDGAGMMVLEEYEHAKKRGAKIYAEVVGFGMSSDAYHMTS
PPENGAGAALAMENALLDAGVTPSQIGYINAHGTSTPAGDQAEAQAVKSVFGADAERVLVSSTKSMTG
HLLGAAGAIESIFTVLALRDQAVPPTINLDNPDEGCDLDFVPHEARQVSDMEFSLCNSFGFGGTNGSL
IFRRV
>CORE_REP|Org41_Gene646#
MKRAVITGLGVVSSIGNNQQEVLASLQEGRSGITFSQELKDSGMRSHVWGQVKLDTTGLIDRKAVRFM
SDASIYAFLAMQEAIASSGLKEEEYQNNPRVGLIAGSGGGSPRFQVFGADAMRSPRGLKAVGPYVVTK

AMASGVSACLATPFKIHGVNYSISSACATSAHCIGNAVEQIQLGKQDIVFAGGGEELCWEMACEFDAM
GALSTKYNDTPEKASRTYDADRDGFVIAGGGGMVVVEELEHALARGAHIYAEIVGYGATSDGADMVAP
SGEGAVRCMKMAMQDLDAPIDYINVHGTSTPVGDVKELGAIREVFGDNTPAISSTKAMTGHSLGAAGV
QEAIYSLLMLEHGFIAPSINIETLDEQAAGMNIVTQPTQRELTTVMSNSFGFGGTNATLVMRKLAK
>CORE_REP|Org46_Gene4169#
MTDSSQSAMPPAKGSAVKGTAFSILGAISVSHLLNDMIQSLILAIYPILQADFHLSFVQIGMITLTYQ
LTASLLQPLIGYYTDKHPQPYSLPIGMGFTLSGLLLLSVASTFPLVLLAAALVGTGSSVFHPESSRVA
RMASGGRHGLAQSLFQVGGNFGSSLGPLLAALIIAPYGKGNVAWFTLAALLAIVVLLQVSKWYQHQHR
ATKGQPKSPSTLKALPKRTVVYSLGILLVLIFSKYFYLASISSYYTFYLIHKFGVSVQNAQIHLFAFL
FAVAAGTIIGGPLGDKIGRKYVIWGSILGAAPFTLVLPYASLYWTGILTVIIGVILASAFSAILVYAQ
ELIPGKVGMVSGLFFGFAFGMGGLGAAVLGYVADLTSIELVYQICAFLPLIGIITALLPNMEHKPQ
>CORE_REP|Org26_Gene2805#
MTEKSAVSRSTFDQVILPVYAPAQFVPVRGQGSRVWDQQGKEYIDFSGGIAVTALGHCHPALVEALKR
QGETLWHTSNVFTNEPALRLASKLIDATFADRVFFANSGAEANEAAFKLARHYAITRHSPYKTKIIAF
YNAFHGRTLFTVSVGGQAKYSDGFGPKPADIVHVPFNDLAAVKAVMDDHTCAVVMEPIQGEGGITPVD
AGFLKGVRELCDQHQALLVFDEVQSGMGRSGKLFAYMHYGVTPDILTTAKALGGGFPVSAMLTTEDIA
SVMQVGTHGTTYGGNPLACAVAEAALDVINTPEVLSGIEQRHALYVQALQHIGDKYGIFTAIRGMGLL
IGAELTPHYHGRARDFLTAAAARGLMILNAGPNVIRFAPSLVVELQDIEAGMALFELAVQDVINA
>CORE_REP|Org2_Gene2691#
MFDFSTPIDRHGTWCTQWDYIADRFGSDDLLPFTISDMDFATAPCILDALQQRLQHGVLGYSRWQHED
FLGALRHWYQQRFNVGIDTATAVYGPSVIYMAAQLIRQWSVPGDYVVTHTPAYDAFYKVILANQRQLL
ACPLQKAGDDWRCDMAHLEALLARPQTKILLLCSPHNPTGKVWRRDELQQMAELCERHDVRVISDEIH
MDMVWGEHRHTPWSQVASGAWALLTSGSKSFNIPALTGAYGFISDAASREAYFQQLKARDGLSSPAVL
AVAAHVAAYRHGEPWLDALRDYLQDNLTYVAERLEQAFPALGWRPPQATYLAWIDLRPLAVDDRALQQ
VLIEREKVAIMPGFTYGEEGRGFLRLNVGCPRSKLEAGMDKLIAGLRLVLDEQ
>CORE_REP|Org25_Gene4390#
MSASAETQNPQQPSGKKKQRKFWLLLLTVIFIVIGVAYLVYWFLVLRHHQETDDAYVSGNQVQIMAQV
SGSVNSVNFDNTDYVKQGDVLLTLDPTDAEQAFERAKTGLANSVRQTHQLIINSKQYQANIALRKTDL
SKAENDLKRRVVLGSVDAIGREELQHARDAVDSAKAALEVAVQQYNANQAMVLNTPLEQQPAIQQAAA
QMRDAWLALQRTKVISPITGYVSRRSVQVGAQIAAGSPLMAVVPADHIWVDANFKETQIANMRIGQPA
KVVSDVYGDDVVYQGKVVGIDMGTGSAFSLLPAQNATGNWIKVVQRLPVRIELDAKQVADHPLRIGLS
TLVTVDTANLDGRVLSDVVRDKPLYQSDALALNLAPVNQLIADVIHANAG
>CORE_REP|Org14_Gene3265#
MSTSALIPESKLPSLGTTIFTQMSALAQQHQAINLSQGFPDFDGPDYLKERLAWHVAQGANQYAPMTG
VAPLREAIADKTAELYGWQPDAGSEVTVTAGATEALFAAISALVRPCDEVVCFDPSYDSYAPAVTLAG
GILKRIALQPPAFAVDWPAFAAALSPRTRLVIVNTPHNPSATAWQAEDMQQLWHAIAEREIYVLSDEV
YEHICFAKGGHASVLAHPQLRQRAIAVSSFGKTFHMTGWKVGYCVAPAALSAEVRKVHQYLTFSVNTP
AQLALADSLRAEPEHWRQLPAFYRAKRDRFVQALASSRLEILPCAGTYFLLADYGAISDLDDVAFCHW
LTEHVGVAAIPLSVFCADPFPHKLIRLCFAKQDATLDAAAERLCRL
>CORE_REP|Org40_Gene2960#
MQSACSSRSKLPDVGTTIFTVIGQLSAEHQALNLSQGAPNFAGDPQLIEATAQAMRAGHNQYAPMSGV
AALRAALAEKAERLYGARYDADEEITVIASASEGLYSAISALVHPGDEVIYFEPAFDSYAPIVRLQGA
TPVAIKLSLQDFRVDWDEVAAAINGKTRMIIVNTPHNPTGAVFDAQDIDRLTALTRDTDIVILSDEVY
EHVVFDGDIHHSMARYPQLAERSVIVSSFGKTYHVTGWRVGYCLAPAALMDEIRKVHQFMVFSADTPM
QYAFAAALANPQSYLGLAAFYQQKRDLLASALQDSRFELLPSRGSFFMLARFSGFSHESDNDFAVRLI
REAKVATIPLSAFYSDGTDTGLIRLSFSKDNETLLEGARRLSQV
>CORE_REP|Org14_Gene3402#
MAFNFDQWVDRSHSDSVKWDKYRGSDIIPLWVADSDFTSPPAVIEALQRRVAHGVFGYTHPSPDLIEV
FTRRMVERYGWHIKPEWIIFLPGLVCGLNLCVRACTEEHQSTLAPSPIYPPFRKAAKFAGREHLAVPL
KATGQRWVLDFSSLHDRLSGNEKLLLLCNPQNPGGTVYRRDELLQHHQFAREHALIVCSDEIHCELLL
EPGVRHIPFATLNDDAAQRSVTLMSPSKTFNLAGLGASLAIVPNEALRQKLKRARSGIVPEVNLLALV
AAQAAYQYGQPWLDEQLIYLRANRDRLIKRINAMPGLTLLPVEATYLAWIDCSALPVDNPHQFFERAG
VGLSAGLDFGDRRFVRLNFGCRWALLDEALDRMARACAALPG
>CORE_REP|Org29_Gene2782#

```
MKRNILAVVIPALLAAGAANAAEIYNKDGNKLDLYGKVDGLHYFSKDKGNDGDQTYVRFGFKGETQIT
DQLTGYGQWEYNVQSNHSESQGTEGTKTRLGFAGLKFADYGSFDYGRNYGVLYDVEGWTDMLPEFGGD
TYTNSDNFMTGRTNGVATYRNNNFFGLVDGLNFALQYQGKNQNDGRDIKKQNGDGWGISSTYDIGEGV
SFGAAYASSNRTDAQKNKSNERGDKADAWTVGAKYDANNVYLAAMYAETRNMTPYGGNNSLKDGTTSC
ADTQNNSCGGFASKTQNFEVTAQYQFDFGLRPEVSYLQSKGKNMNVPGAGSDQDLVKYVSVGTTYYFN
KNMSTYVDYKINLLDDNAFTKAAGIATDDIVAVGLVYQF
>CORE_REP|Org5_Gene1821#
MEMIKTRAAVAWGPNQPLKIEEVELMPPQKGEVLVRIVATGVCHTDAYTLSGKDPEGVFPAILGHEGG
GVVEAVGEGVTSVAVGDHVIPLYTPECGECKFCKSGKTNLCQAIRATQGKGLMPDGTTRFFKDGKPIF
HYMGTSTFSEYTVVPEISLAKINKEAPLEEVCLLGCGVTTGMGAVMNTAKVQPGDTVAIFGLGGIGLS
AIIGAQMAGAGRIIGIDINTSKFELARKLGATDLINPKDYDKPIQEVIVELTDGGVDFSFECIGNVNV
MRSALECCHKGWGESVIIGVAGAGEEISTRPFQLVTGRVWRGSAFGGVKGRSQLPGIVERYLDGEFAL
NDFITHTMGLEQINEAFDLMHEGKSIRSVIHFDQ
>CORE_REP|Org32_Gene1732#
MCQTCMTEAKMAAGIPEIMKAVVAYAPKDYRLEQVPVPKIGPKEILVKIEACGICAGDVKAFEGAPSF
WGDEKQPAYIKAPMIPGHEFIGHVVGYGEGVEGFNLGDRVISEQIVPCWQCRFCNRGQYWMCEKHDLY
GFQKNVNGGMAEYMKFTKEAINYHVPADLPIEKAILIEPYACSFHAVQRANIKLGDVVVLAGAGTLGL
GMIGAIKKSGPSKLVVLDLSDERLALAKRFGADVTLNPTRDDVPAAVKAMTDGYGCDIYIEATGAQKS
VEQGLTLIRKLGTFVEFSVFKDPVTVDWSIISDRKELDVLGSHLGPYCYPLVIEGIANGDLPTEGVVT
HTLPLEQFAEGFELMKRGIGSIKVVLNPNL
>CORE_REP|Org39_Gene3369#
MKRILVTGGAGFIGSAVVRHIIEATDDSVVVVDKLTYAGNLESLAVVAESERYAFEQVDICDRAELDR
VFAQYQPDVVMHLAAESHVDRSIDGPAAFIETNVVGTYTLLEAARHYWQPLAAEKKQAFRFHHISTDE
VYGDLHGTDDLFTETTPYAPSSPYSASKASSDHLVRAWLRTYGLPTLVTNCSNNYGPYHFPEKLIPLV
ILNAVAGKPLPVYGNGAQVRDWLYVEDHARALYQVVTEGVVGETYNIGGHNERKNIDVVQTICELLEE
LAPNKPQGVANYRDLITYVKDRPGHDMRYAIDAGKIDRELDWRPQETFESGLRKTVVWYLNNETWWRR
VQDGSYAGERLGLSE
>CORE_REP|Org24_Gene2291#
MSPSSQQNRRFLLASRPHGEPTAANFRLDTVPAPQPGAGQLVLRTVYLSLDPYMRGRMSDAPSYAPPV
EIGQVMVGGTVSRVAASQHPDFNVGDWVLGYDGWQDYALSDGSGLRNLGPHLPQPSRLLGVLGMPGFT
AYMGLLDIGQPQAGETLVVAAASGAVGSVVGQIGKLKGCRVVGVAGGAEKCRYVVEELGFDACIDHRA
PDFAEQLAAACPKGIDIYYENVGGAVFDAVLPLLNTKARIPVCGIIAHYNATGLPAGPDRLPLLEGLI
LRKRIRMQGFIIFDDYGSRFDEFLQQMSSWVEEGKIKFREDIVDGLEQAPQAFIGLLQGKNFGKLVIR
VADE
>CORE_REP|Org33_Gene2736#
MLSIRLADLAQQLDAQLHGDGDLVITGIASMHSAQPGQITFLSNSRYQEQLSSCQASAVVLTEADLPH
CRTAALVVKNPYLTYARMAQLMDTTPAPAQDIAPSAVISPEAQLGHNVAIGANAVIESGAVLGDNVVI
GPGCFIGKHARIGAGTRLWANVTIYHAVEIGQRCLIQSGTVIGADGFGYANERGEWIKIPQLGTVIIG
DRVEIGACTTIDRGALDNTQIGNGVIIDNQCQIAHNVVIGDNTAVAGGVIMAGSLKIGRYCQIGGASV
INGHMEIADKVVVTGMGMVMRPITEPGVYSSGIPLQPNKVWRKTAALVMNIDEISKRLKAVERKVGKD
>CORE_REP|Org18_Gene4598#
MLDVAGYELDAEEREILKHPLVGGLILFTRNFHDAEQLRELVRQIRAASHDRLVVAVDQEGGRVQRFR
EGFTRLPAAQSFAALHDAQEGGRLAQEAGWLMAAEMIAQDIDISFAPVLDIGHGSAAIGERSFHSDPQ
QALAMAERFILGMHSAGMKTTGKHFPGHGAVSADSHKETPRDPRPLAQIREHDMAIFRELINRQLLDA
VMPAHVIYTEADPRPASGSPYWLQQILRRELGFDGVIFSDDLSMEGAAIMGSYAERGQAALDAGCDMI
LVCNHREGAVSVLDNLSPVKAEKVKRLYHRGQFTRQELRDSERWQQAHKALSALSERWEEHKQRSQG
>CORE_REP|Org25_Gene3878#
MPPLFPRLSFNRFGDPAQVLELQQTSRPLLRPGQRLLQMRYAPINPSDLIPIHGQYAHRIALPQVPGY
EGVGVVVNPQNGHSTGRRALAVAGNGSWQTFVTLPEDRVVWVPDDIDDACAAQIYINPLTCWVLLTQW
LPLSAGDVLLLNGGGSAVSQLLAQLTALRGIRLAVVVRNAAHRQALLAAGAWRVIEAPQLAEMTNFGA
RAAIDCIGGEDGLQLARAVRTGGDFVALGLLSGRQVDWRRVVDELKLRASLFHLRKWNAQAAPAQWQT
AFYQLFQLLRRGQLALRPPAAIYPLRQYAAALHHAAQPGVNGKIFLTPTTSETIAVDELLFNGVPNK
>CORE_REP|Org32_Gene2866#
MAVLVTGGAGYIGSHTVLALLEHGEDVVVLDNLSNSSDESLRRVEKLAGRSAQFYQGDILDAECLHRI
```

FEAHAISAVIHFAGLKAVGESTRKPLEYYQNNVTGTLVLLEEMRRAGVHKFIFSSSATVYGTPEQVPL
TETSRVGGTTNPYGTSKLMVEQILQDFAKAEPQFSITALRYFNPVGAHESGMIGEDPNGIPNNLMPYI
AQVAIGKLEKLSIFGDDYPTQDGTGVRDYIHVMDLAEGHLKAIEHIDEHQGFTVYNLGTGVGYSVLEM
LHAFEKASGRNVAYQIVPRREGDIAECWSAPELAFKELGWKATRDLDAMMRDAWNWQKNNPRGYRPG
>CORE_REP|Org40_Gene2850#
MKFLVTGAAGFIGYHVAERLLTAGHQVVGIDNLNDYYDVGLKMARLDRLADKPGFRFIKLDLADREGM
AALFAEHQFQRVIHLGAQAGVRYSLVNPLAYADANLIGHLNVLEGCRHNKVEHLLYASSSSVYGLNRK
LPFATEDSVDHPVSLYAATKKANELMSHSYSHLYSLPTTGLRFFTVYGPWGRPDMALFKFTKAILAGE
SIDVYNHGEMHRDFTYIDDITEAIVRLQAVIPQADPSWSVEQGSPATSSAPYHVYNIGNNTPVKLMEY
ITALEQALGVTARKNMLPMQPGDVMDTSADTAELYRDIGFKPETSVEEGVKRFVDWYKAFYQVQ
>CORE_REP|Org41_Gene3489#
MSIKAIAVDPQNPAGFIEISPGMPVPGQYDLLVEVKAVSVNPVDTKVHAGLQKSGLQQPRILGWDASG
IVVGVGSGVSGFKPGDEVWYAGDITRPGSNSSHQLIDSRIAAHKPRSLNWAESAALPLTALTAWEALF
EHLNIQDAPEHKTLLIIGGAGGVGSLAIPLAALRSKVKVIATASRPESAAWCRERGADLVVDYRDLKG
NLAQHGIEQVDYILCLNDTDGHWPAMAELVAPLGHICTIVENAQPLDQNALKLKSAALHWEFMFTRSM
FTTPDIAQQGKILQQMAQLLDEGKLSTTLSETLHGLSVDTLTAAHRQLLGGHMQGKLVIAY
>CORE_REP|Org2_Gene2735#
MPRVEPIKKVSVVIPVYNEQESLPALLERTTAACKQLSQPYEIILVDDGSSDNSADMLTAAAEKPDSH
VIAVLLNRNYGQHSAIMAGFNQVTGDLVITLDADLQNPPEEIPRLVSVAEEGYDVVGTVRANRQDSWF
RKSASRVINMMIQRATGKSMGDYGCMLRAYRRHIVEAMLHCHERSTFIPILANTFARRTTEIDVRHAE
REFGDSKYSLMKLINLINLMYDLITCLTTTPLRLLSVVGSIVALSGFVLALVLIALRLLLGPEWAAGG
VFTLFAVLFTFIGAQFVGMGLLGEYIGRIYTDVRARPRYFVQKVVGAQQGHNTQEEE
>CORE_REP|Org11_Gene3964#
MAKRIQFSATGGPEVLQYVDFTPLDPAAGEVQIENKAIGINYIDTYVRSGLYAPASLPSGLGTEAAGV
VTKVGAGVSAIKPGDRVVYAQSALGAYSEIHNVSAERVALLPGNLSFEQGAASFLKGLTVYYLLRQTY
DVQPGEVFLFHAASGGVGLIACQWAKALGARLIGSVGSDEKAALAKQAGAWATINYHKEDIAQRVAEL
TQGEKVGVVYDSVGKSTWLASLDSLKRRGLMVSFGNASGPVTGVDLALLNQKGSLYVTRPSLNGYITN
RAELQYASNELFSLIGSGAIRVEVKDEQKFALADAQRAHQVLESRSTSGSSLLIP
>CORE_REP|Org19_Gene877#
MKLQQLRYIVEVVNHNLNVSSTAEGLYTSQPGISKQVRMLEDELGIQIFARSGKHLTQVTPAGQEIIR
IAREVLSKVDAIKAVAGEHTYPDKGSLYVATTHTQARYALPNVIKGFIERYPRVSLHMHQGSPTQIAE
AVSKGTADFAIATEALHLYDDLIMLPCYHWNRAVVVKPDHPLAGKSSISIEELAAYPIVTYTFGFTGR
SELDTAFNRAGLTPRIVFTATDADVIKTYVRLGLGVGVIASMAVDPVQDPDLVTVDASDIFTYSTTKI
GFRRSTFLRSYMYDFIQRFAPHLTRDVVDSAVALRSNEEIEAMFKDIKLPIK
>CORE_REP|Org21_Gene1286#
MKKKTLFTLLLMLAAIALAILFRAHNQDLLLQGEVDAPEVIVASKAKGRVVERLIERGDDVKSGQLII
QLDSPELMAQLRSAQATRDEAKAQLELSLHGTREESIRNLRANLAQAEAQYRNAQNDYNRNLSVAGKG
YISKSELDASRRSRDTAFQQVQAAKANLDEGINGDRVEQRQQYAAALRAAEENLLQIQAQSDDLQVKA
PVDGEVGPIPAEVGELLNAGSPLVTLIRVPDAYFVFNLREDILAHVRKGDKVKLRVPALKDKMIDTEV
RYIAPLGDYATKRATRATGDFDLKTFEVRLYPSQPVDGLRPGMSTLWQWKE
>CORE_REP|Org35_Gene2368#
MSDSLRIIFAGTPDFAARHLDALLSSEHQIVGVFTQPDRPAGRGNKLTPSPVKMLAEQHQLPVFQPKS
LRPEENQRLVADLNADVMVVVAYGLILPQAVLDMPRLGCINVHGSLLPRWRGAAPIQRSLWAGDSETG
VTIMQMDVGLDTGDMMHKIACPIESSDTSASLYDKLAQLGPQGMLTTLRQMADGSATREVQDESQVTY
AEKLSKEEARLDWTLSAAQLERCIRAFNPWPISYFTIDEQPVKVWQASVMAESANAEPGTVVHADKHG
IQVATADGILNLIQLQPAGKKPMSAQDLLNSRREWFTPGNRL
>CORE_REP|Org44_Gene3052#
MKTIGFVVFPGFNLLDFAGPLAAFDNVSQFTDPPAYRCVAISPQGGMVASSAGVEIATQPCGDERFDT
LVVAGGSGNVMAAQSPALVAFLTTHSRRARRIASVCTGAFILAACGLLDGKRATTHWYHAARLQQSYP
RIRVDSNRIFIRDGDIWTSAGISAGIDLALALIEDDLGATLAAVVARQLVVYHRRPGGQSQYSLLLAL
NPSSDRMRAALSFAREHLHLPLSVADLADAACLSERQFGRLFRAETGQTPAKVIEQLRVEAARVRIEE
SAEPLEAIARSVGFSDPERMRRAFIRVFGLSPQAIRRLGRAG
>CORE_REP|Org13_Gene1498#
MTRLSLDAIKIISTIKSTGSFSMAAEALHKTPSAISYRVSNIESKLCVKLFHRNGPMITLTDEGEFLL

QEGSWILNAVQDLESRVRNIPKLDNNIRLAVDTFFPLETLTQDIRDYIQHCPNANISVQREALNGTWD
ALKNNRADLIIAIGQIPDSVQAKTLMLGKLNFVLCVSPSHPFAAQRKPVCKKQRLNDIVVVIADSSHE
LPKRNHGTLPLQRQLVVCDVESNLALLKRGIGHAFLPPALIEKELASGELVTVPVEMQKGDEMIWLAW
HPASKGAGFSWWHERLTRKSDVYSLMGREVVRDGGYPWCHN
>CORE_REP|Org8_Gene3935#
MTDPDFNLLIALDALLTAGSVAGAARRLGLSPSAMSRTLSRLRAATGDPLLVRAGRHMVLTPYAETLR
ERARHAAFEARAVLRPAQGALDPAALDRTFTLRANDGFVEAFGPALIAAAAEQAPRVRLRFAPKPEKS
DRPLREGLVDLEVGVLGDMGPEIRLQALFRDRFVGVMRTAHPLAQQPEIDVADYAACGHVVASRSGRI
LGPVDAALAELGLARHIAAVVPSFPAALAVAQASNLLALLPASFLQAQPADGPLRVFELPVKTPPITV
SQMWHPRLDAEADHRWLRQLVLSVCRRQAQPPASDNVT
>CORE_REP|Org18_Gene2648#
MMTLRQIRHFIAVAETGSISAGAQAVFVSQSSLTLAIQQLETEIGVRLFDRHAKGMTLTHQGHQFLRQ
SYLILATVDNAKRSLQIGTESLTGKLTVGVTSLVAGYFLVELLTRFKSAYPNVTVQVVEDERPYIEHL
LVSGEIDIGVLILSNIEDRDALQTEVLMHSPYRLWLPPLHPLLEHESISLADVAKQPLIQLNADEMDV
HARRIWSRAGLKPEIAMKTASTEAVRSLVAAGMGVSIQPDMAYRAWSLEGNMIEARKLDDLLEPLDIG
LAWRRGSARPELVTPFLTIARENGSKHAAGLKHSI
>CORE_REP|Org13_Gene2245#
MHSISLRQIEIFRAVMTTGNLTEAAALLQTSQPTVSRELARFEKLIRLQLFDRVRGRLSPTVQGLRLF
EEVQRSYYGLDRIVNAAAGIRQFQQAQLSIVCLPVFSQSLLPAVCRPFIERYPEVSFSVIPQESPLLE
EWLSAQRHDLGLTETTLTPAGTERVTLMTLNEVCVLPTGHPLLVKDRLTPQDFAGQNFISLSSTDSYR
HLLDALFGEQGVERRMVMETHSAASVCAMVRAGVGVSIVNPLTALDYAGNGVHVRPFSIDVPFTVSLI
RPLHRPSSALVTAFIDHLHQQAAAFAARLAAAVRR
>CORE_REP|Org14_Gene622#
MDLTQLRMFCCVAETGSVARAAEQLHRVPSNLTTRLRQLEQELGADLFIREKQRLRLSPMGHNFLCYA
NRILALSDEAMSITHAGEPAGNFALGSMESTAATRLPSLLAAYHQRFSQVSLSLTTGTSGEIADRVRA
GTLAAALVDGPVPYDELNGCIAYPEHMVVISCLDHAPIHSAKDANGETLFAFRASCSYRLRLEAWFKR
EGARPGQIMEIQSYHAMLACVASGAGLAMIPHSVLSLLPGHERVRVHTLPPDVADTATWLLWRRDAFG
PNVRALKELIIEQTETAAVDESTPNDLSDVVDIA
>CORE_REP|Org3_Gene2084#
MIEIDAQRSRMARQAMAIATGNGYTPSPVPRVKILYVDRHCPRQPVMYEPGIVIIFQGHKVGYCGSKV
FQYDPRNYLLMTVPLPFECETFASPELPLVGLAVNIDTQMLQDLLIDIGDDDYLMQPRAESNGVNLAE
LTEALLCATERLLDVMAKPLDARVLGPQIVREILYYVLRGTCGASLQELVNRHTHFSQIAKALRRIEH
QYADNLNVEQLAGEVNMSVSAFHHNFKAVTNTSPLQYVKSYRLHKARLLMVHDGLKASTAAIRVGYES
ASQFSREFKRLFGMTPSDEVARLREANPLLLEG
>CORE_REP|Org20_Gene743#
MIKQRTLKRIVQATGVGLHTGKKVTLTMRPAPANTGVIYRRTDLNPPVDFPADAKSVRDTMLCTCLVN
EHDVRISTVEHLNAALAGLGIDNIVIEVDAAEIPIMDGSASPFVFLLLDAGIEELNSAKKFLRLKETV
RVEDGDKWAELSPHNGFRLDFTIDFNHPAIDASSQRYRLDFSAESFVRQISRARTFGFMRDIEYLQSR
GLALGGSFDCAIVVDDYRVLNEDGLRFEDEFVRHKMLDAIGDLFMCGHNIIGAFTAYKSGHALNNKLL
QAVLAKQEAWEYVTFQDEAEMPLAFKAPSTVLA
>CORE_REP|Org41_Gene1437#
MDFSIVIPIYNSARYLPKTLKRVFNACQGFDYQVILVDDCSDEDDIKYIRQIAATNSNVMLHEKKKKS
NAAVSRNLGIKLAQSEIVFFLDSDDYFTTNYIIRRMNKHEDRHIDIIFGNYAEVSGDSVRDFNFHYKS
GSSGEDFLFLEMGDIRSSTISIRKKSDRRFLFPEFLNKHQDWGFLVNATNLGAHVAHDAGGGVFLDVG
RYGRMTAKLNLEASDRFINTFLSPSDRHISGFACKHLLASLYSENAQAFNYYSSRIVRRSLSGKFKMI
KWYGDCLARLGLFSVGGRLLRNVRQGFRR
>CORE_REP|Org44_Gene4817#
MTALATLRDVGFEEWLGKINTACGRFCAKTLGPGFSGAMQEFRAHALRLSVVDVSQARLYRTPREIAR
SDGAHFFTVFQLRGSALMEQGESQTVLSPGDITLIDASRPSSFTFQRDSRQISLLLPRGCLPVPPPCA
QRLGAELSAVRLSRRLVLSSMQDPQLAAAESEAVLNALAALLRPALALEQARPEGQQPVFDKALALID
RHIQSAQLRPEWVAAELGVSLRSLYRVFARQGLVVAQYIRNRRLDLCAQALRSAAGQEKLAGVGLDWG
FADHSHFSTAFKQRFGMSPSEYRRQYQ
>CORE_REP|Org23_Gene336#
MRKSTGFIANIDICKEYDARYAADEVHYETFAGLAAFFGRDMQVHWHDCFFQVHFLETGKIELQLDDQ

HYSVQAPLFILTPPSVPHAFFTEPDSDGHVLTVRQELIWPLLERLYPGSNLALDMPGICLSLADAPQE
LTALSHYWALIRREFAQNLAGREQTLALLAQAVFTLLLRNTALEDSANSGVRGELQLFQRFNKMVDER
FREHLPVPEYAQALGVTESRLNDLCRRFANRPPKRLIFDRLLREAKRMLLFSACTVHETAYSLGFKDP
AYFARFFNRLEGCSPSTYRAAQHALS
>CORE_REP|Org45_Gene2437#
MTNEDIFFIEELIEWVEIHLEKRPNLDEVARISGYSKWHLQRKFKRITGIQLATYIRSRILTRAAVAL
RITRRSIIDISDELGFDSQQTFTRMFKQRFGTTPNRYRSMTHWDVKNLMPRFNFDASYGAGYYPEVKR
LTLPEMQLVGFTRRLDFASEQELEYSSCMAMKDEIFNDFFKGLHVDCRRIYSIYSPHAGEGDELSSTL
VMAVDPEHKKDILSNYQIDTFHLPSREFISINHKGSAKECLQFFGYLMSHVMPGLKDEVRGSMEMEII
QTKEWNPESKLRQIDVDYTYLISID
>CORE_REP|Org32_Gene2606#
MKNVALFVGNDIFSWLVCQDLIAALRDECTFTVYFPLAKSAGRTQEPAVRRLGLYEREVLNDFVFPFV
GRNAAACEGAYQPPALFLAAAGVKAHRVLDINDAAFISSLGHMDGVISLRCYQKFSADYVRAFSRRGK
LLWNLHPGDLPRYRGVMTLFRAMMNGDRDCAVTLHEMDEHWDAGPVIARLPAELRHDLSFLENMMLLG
VQSGTFLARQLMRAEHSEAITAEKQGDSRYWGFPDAQTLAQAEQAGIELVDHDAVREQYLGLFVGDRF
HPLAGQFCAGFDDFVRAHGHD
>CORE_REP|Org21_Gene2881#
MDIKQLRALVALAEQGNYRQAASLLCISQPALSKQIQALETQLGVRLFERGRQGAVLTAGGQRLYPEA
QALVEQYQQFQRRARRVALGEAGRLALGFGLSSFHLAPQLVAAFRRRFPEVAIGLEDMPSERQYQLLL
QGELQVGFVRLPVTTPLCGAALLSDRLVLAAPGALALRADDLMARFNQLPLLQLTPKRGRGLSDQSLR
FIAAHRLTPNVVQQAGDIQTLLALVAAGVGVALLPHSITHIAPAGIDILPLSGEETEWQVGIAWDPQR
ADALRDNFIQTALAVQRA
>CORE_REP|Org16_Gene2285#
MDQAGIIRDLLSWLESHLDQPLSLDNVAAKAGYSKWHLQRMFKDITGNAIGAYIRARRLSKAAVALRL
TSRPILDIALQYRFDSQQTFTRAFKKQFAQTPALYRRAEDWNAFGICPPIRLGAFTLPQPEFVSLPDK
HLVGLTQSYSCTLEQITTVRTELRSQFWRQFLGDVETLPPVLYGLHHSRPSQEKDDEQEVLYTTALEP
DQVPDKVQEGQPLVLPGGEFAMFSYEGPTENLQDFILTVYGTCLPALQLTRRKGHDIERFYPKGERRP
HQAPIEIKCDYLIPIRR
>CORE_REP|Org36_Gene2971#
MGTQESHIKELLVWIEDNLTNPLSLDIVSAKSGYTKWYLQRMFKKQTGLSLASYIRARRLYLAAFALR
FTQKSILDISVEYQFDNQQTFSRCFKKHFAESPSVYRHARKQDFSNLVRSLAASQPGDIQVERVSIAR
GQYAFHGKQYAYHLDIEKLDKSHLPQRSALRGQFYTLLGERPTQTYSFTQLVPDGERVRVDYTLGVTT
EYPLREGVVLEPLPEIHGEFCRFRYSGKPVALNDHIIQIYTQVLPEMGLARGDGPDITVFSYSLSGKE
ELHLELQHLVPVVPLH
>CORE_REP|Org3_Gene3521#
MERTINLCPGIGASAHIIQHTELLFPSVYFEQPHLYLIQQGHKRVRWQQREVVAHAGELLIIDGGQTV
DIINGPSEEGVFSCQLLTCDPLLLTVQPPAEDSPAPMPFDAVLALRSLPCALKHSFETTSLALALRQR
FPTIIVRHKMLEILLWLAQFGIRFIHNEAKDLTQRVRRCLATDPHSIWTAAKVAESLSMSEVMLRRKL
SMENTALRNLMIDVRMSSALALLQSTDWPISAIAQHVGYESASRFAERFRKRFGFAPTAIRGHQRIME
PTSQGVETMATGET
>CORE_REP|Org25_Gene2382#
MTQTYAKRFAQVFDYIDRHLDEALTVDKLSEVAHFSRFHFQRQFSAYCGISVWRYIQWMRLKRASYRL
AYNPLEPVIDIALDAGFQNPESFSRAFKQAFSQTPSQFRKQPAWIDWQQRFPEPKHRRKHPMKVDIVD
CPATPVAMLEHRGPSALVNETAARFIEWRKTSGLSPVRSSRTYGIAPHDPATTEAQDFRFYLCGEVTA
PIPEDNAFGVVNSMLPAGRCAVLRHLGSLDGLSESARYLYSEWLPASGEELRDFPLYFHYHNFVHEVA
EYELVTDLYLPLK
>CORE_REP|Org20_Gene1909#
MRSTSDYQPPVSLSPPSLVGLPEQEDVFAVEHLSRLCDGLAQQRPNNLRDLLNTLALIAPLLNAIPNV
VFFIKDAQARYLLANLTLARRCGFKTVTPLLGKTSADVFPAQLGSDYTEQDLRVLRHGVLIQDQLEMH
LYNGRETGWCLTQKLALYDAQGKIIGMAGISHDLQEARANHPAYQRLAAIDVHIRRHYARPIALEELT
ALTGLSVAQIERYCKRIFHLTPRQMIHKVRLEKATELLAGDLPITDIALQCGYTDHSAFSRQFKAMTG
STPRDFRLTLSA
>CORE_REP|Org28_Gene3430#
MSDNRISLSANDVKLIREQDFFNCKDFHLFIYNKVESATGLHQHDYYEFTIVLSGKCYQEINGKRVLL

ERGDFVFIPIGSHHQSFYEFGATKIFNVAVSKAFFEEHYLHQLPRCFVASQAYSLRSEFLAYIESVVS
SPQFREDDFAEFLETLTFYVISRIRHYKEENDGGDDIPQWLKNTLAGMHDKAMFGERALANMVALSGK
TQEYLTRAMRRYYHKTPMQVINEIRINFAKTQLEVTNSSVSDIAFDSGYGDVSLFIKNFKRLTDVTPG
NYRKKCYGPL
>CORE_REP|Org1_Gene218#
MPHQNVQRKVLRTICPDAKGLIAKITNICYKHELNIVQNNEFVDHRTGRFFMRTELEGIFNDNTLLAD
LDSALPEGSLRELHSTGRRRIVVLVTKEAHCLGDLLMKSAYGGLDVEIAAVIGNHDTLQTLVERFDIP
FHLVSHEGLTREQHDREMTAKIDQYQPDYVVLAKYMRVLTPAFVQHYPNQVINIHHSFLPAFIGARPY
HQAYERGVKIIGATAHYVNDNLDEGPIIMQDVIHVDHTYSAEDMMRAGRDVEKNVLSRALYQVLAQRV
FVYGNRTVIL
>CORE_REP|Org44_Gene4808#
MQGVPQQFPFEKDCAQFRHLSHLPGVELYQAHIERYAFEPHTHDAFAIGTVDTGAERFRYRGAQHLAA
PGALVLMNPDELHTGEAETPGGWCYRMLYLAPEALEQLSGARSQWFTDAVRHDPRAAQRLSAILATLW
QTDDPLTLDGLLLEAVELLYPHIRTGQREKAEAAHRFEVVKSYLHDNFAEAVTLNQLAELVSLSPYHF
LRKFKAEYHVSPQQMLMAIRLSQAKRMLERGMPAAQVAAAAGLTDQAHLTRAFANRYGVTPVRFQKQV
KLG
>CORE_REP|Org23_Gene4045#
MSEKTPQFWRDPQLPFVEARAIADGRQACYSLHSHEFFSIGAITGGVSTYVNGERRMQVSAGDLVVIN
PQQAHACNPIADRRWSYIMFYLDLAWIGALQQELLGGEGERFVPFSRPLSRDPALFHGLNRLYALLTD
PLCSALEKQIAMVEYFSALQLGLGSGRQPETPPHARLEAAAAFIDAHCTRPLTLDDICRAAALSPSYL
IRAFRQRYGMTPHAYLVNRRVQHGHRLLKSGLPIAAAASESGFADQAHFQRTFKQLLAATPGQYQKPS
ANR
>CORE_REP|Org31_Gene2716#
MTDMHSLFIAFVLGVVEGLTEFLPVSSTGHMIIVGEWLGFTGDKAKTFEVIIQLGSILAVVVMFWRRL
FGLIGIHFGGKPVEHEGKTHGRLKLGHILLGMIPAVVLGLIFHDVIKSLFAPKNVMYALVVGGLLLLT
AEWLKPKKPRAEGLDDITYRQAFLIGCFQCLALWPGFSRSGATIAGGMLVGVNRYAASEFSFILAVPM
MIGASGLDLYKSLHFLTWGDLPMFAVGFVTAFVVALIAIKTFLSLIKRISFVPFAIYRFIVAAVVYMV
FL
>CORE_REP|Org12_Gene2905#
MKVQIPRRNLDIDQVPRPLFAVQSSTIEQDWEVEPHRHQKAQLIYTVRGMIRCEVENGLWLVPPQCAL
WMPGNVLHNAQGAGSTEAYCLFVDQHVVAGLPQSCCTLSVSPLLRELLLQATTFEPLYDEQGAEGRLT
AVLLDQLVAAPIENLHLPVSDDARIRQLTEGMLSCPADKSTLGQWAQRIGMSERSLSRTLQQQMGMSF
GHWRRQLHVMLALQRLTQGESVQTVALDLGYESASGFVTMFRKAVGKPPARYLAERNASGQPLGGAIS
M
>CORE_REP|Org34_Gene1812#
MKQYLDLMNKVLAEGTPKADRTGTGTLSIFGHQMRFNLQDGFPLVTTKKCHLRSIIHELLWFLNGDTN
TAYLRDNKVTIWDEWADENGDLGPVYGKQWRAWGAADGRQIDQLSNVLQQLKQDPDSRRIIVSAWNVG
ELDQMALAPCHAFFQFYVADGKLSCQLYQRSCDVFLGLPFNIASYALLVHMMAQQCDLEVGDFVWTGG
DTHLYSNHMEQTRLQLTREPRPLPKLVIKRKPASLFDYRFEDFEIEGYDPHPAIKAPVAI
>CORE_REP|Org36_Gene2477#
MITNLISGLLAPCFALYACAKSRRYWRQCRRLYTFQPIYRTSGALLAVELLTAVYHPNEPDKRQSPEQ
YFASLGVAQRLRVIQEQLALLQRWQALFIRHAVMVSVNIDGIALQALQRHSELQRQIAEMPYLRFELV
EHAETASNHPLQQIVGGERLWLDDFGSGLANFSAVGAWRYQYIKVARELFTLLKQSEEGVQLLGTLIT
MMNQHSDGVIVEGVETEQEWRLVQRSGALAAQGYYLSRPACFETLHSVPTLFAAPGAPA
>CORE_REP|Org18_Gene263#
MIDQTAFIHPSAIVEEGAVIGANVHIGPFCYVGSQVEIGAGTVLKSHVVVNGITKIGCDNQIYQFASI
GEVNQDLKYAGEPTRVEVGDRNRIRESVTIHRGTAQGTGLTKVGNDNLLMVNVHVAHDCVVGNACVLA
NNATLAGHVEIDDHAIIGGMTAIHQFCIIGAHVMVGGCSGVAQDVPPFVIAQGNHATPFGVNAVGLKR
RGFDKDEMQAIRNAYKILYRSEKTLDEAKAEIEALAKEQPVVQQYLDFFTRSTRGIIR
>CORE_REP|Org19_Gene3014#
MRNVRIDDIDHVTRAVIAIGTDYPPGHLLPMHSHRRAQLLYGATGVMHVFTQQGNWVVPPQHAVWLPP
QMPHAVRMVGVTTRSLYLEPGALPAERPQVCQVVSVTPLMRQLLMAAVDMPLEYAQEGRDGALATLLL
HELARLQPLPLHIPLPADPRLGELCRAFLQHPDAHDSAQRWAPRLYMSIRTFSRFFRAQTGLPFSQWR
QRACVVLALALLAEGRSVTQVAMEMGYDSSAAFSTMFRRVLGQAPSSYLTEDGRDG

>CORE_REP|Org25_Gene3739#
MTSANDTVNKQAVASAFSRAAGSYDAAAELQRDVGERLLGMGSSHPGEQLLDAGCGTGYFSRMWRERG
KRVTALDLAPGMLEVARQRQAAHHYLLGDIEQVPLPDAAMDICFSSLVVQWCSDLPAALAELYRVTRP
GGVILFSTLAAGSLQELGDAWQQVDGERHVNAFLPLTQIRTACAAYRHELVTELRTLNYPDVMTLMRS
LKGIGATHLHQGREGGLMSRGRFAALQAAYPCRQGQFPLSYHLAYGVIYRE
>CORE_REP|Org20_Gene874#
MIPEKRVIRRIQSGGCAIHCQDCSISQLCIPFTLNAHELDQLDNIIERKKPIQKGQTLFKAGDELKSL
YAIRSGTIKSYTITEQGDEQITGFHLAGDLVGFDAIGGLKHPSFAQALETSMVCEIPFETLDDLSGKM
PNLRQQIMRLMSGEIKGDQDMILLLSKKNAEERLAAFVYNLSRRFAERGFSPREFRLTMTRGDIGNYL
GLTVETISRLLGRFQKSEILSVKGKYITIENADALSVLAGTPRINVSVNA
>CORE_REP|Org22_Gene4202#
MSIQMDFTGKRVWVTGAARGIGEQIARHFLTQGAEVVGFDREFANPDQPYPCVMLDISRPEQVEAVCR
QQLAENPRLDVLVNAAGILRMGNTEDLSVDDWHQCINVNASGAFYLFRAVLPHFKAQRSGAIVSIGSN
AAHVPRAQMAAYCASKAALTSLNHCVGLEMAPFGVRCNLVSPGSTDTPMQRGMWQTDDAQQRTIAGFP
EMFKLGIPLGKIARPDEIANAVLFLASDLASHITMQDIVIDGGATLAA
>CORE_REP|Org44_Gene3272#
MDIQVERLSAVIDAVASPRFYPSLLNWLEGFFAFDNAIVYAFERGRPPRCLIKTERDNSDAVNQIYQQ
GAYLQDPFYRALNDGGAGEVLTLRQLAPCGFYHSDYYRNFYRKTGWHDEAGVLLQLTPERGLGVFFGS
ARRTVAVRYPQRADLRSALTLVKSVARLHGEVVAAPAEADTGNDDGAQARYLLTPREREIVDLILAGC
GSQQIADRLFISLGTVKNHRKNIYGKLNIGSQAELFSLLLTAPQRRSA
>CORE_REP|Org30_Gene1131#
MNGLLNGKRIVVTGAARGLGYSFAAAIAAAGAQVVMCDILADELAASGAALREQGAQVETQTIDLASP
DSIRSAFEKIAAGGGIDGLVNNAALATGVGGKTMMEYDIDLWDRVMQVNVRGTWLVSQAAVPLLARSP
HAKIVNVASDTALWGAPRLMAYVASKGALIAMTRSMARELGPQGICVNAIAPGLTRVEATEYVPAERH
QLYEQGRALAGAQHPDDVNGTVLYLLSPLADFVTGQLLPVNGGFVFN
>CORE_REP|Org14_Gene4232#
MMQIRSQRLQHEKKHLTPWHRHEGGQIYLLTRGMLAMELPGRQWAITGGTLGWLPPGCAHQALACGDV
AGWSLYLPVESVPEMPPLPQLPQLFTASALLQALVERIAQFPAGPLSAPQRRLLQVLLDEMHAASSAP
LQLPLPQDARLLNIARALLNDPASPRSQCDWAIWAGLSPRTLSRRFLQETGISFALWRQQARVLRSLE
GLSRGKAVGEVADACGYDNVSAYIAAFRHRFGVTPGAYFAPARQTE
>CORE_REP|Org31_Gene266#
MSEIIYGIHAVKALLERDPQRFLEVFILKGREDRRLQPLIAELEATGIVIQVANRQWLDDKVEGAVHQ
GIIARVREGRQYQENDLPGLLESVETPFLLVLDGVTDPHNLGACLRSADAAGVHAVIVPRDRSAQLNA
TAKKVACGAAENVPLIRVTNLARTLRLLQEMNVWVVGTAGEADHTLYQSKMTGPMALVMGAEGEGMRR
LTREHCDELISIPMAGTVSSLNVSVATGICLFEAVRQRG
>CORE_REP|Org21_Gene2589#
MEDEYNLSNIIGKRLEAALGELGDLLWAYVVLSKKDIACIFGVTNYPSEWVKKYQEQGLQYIDPVVLT
ARNRLTPFAWDEQIMADAGLHFPELFEQARGFGVTHGYTFVLHDYNDNLVTLSFAFNVEQRAEAIQAL
TERKGDISVLLSSLHESYLALSPLSAKNAAALERNVRFTDRENEILYWASVGKTYQETAMILGIKTGT
IKFHMSNIVKKLGVTNARHAVRLGMELRLIKPVEY
>CORE_REP|Org11_Gene4378#
MSPERYARICEMLATRQPDLTVCLEQVHKPHNVSAIIRTADAVGVHQVHAVWPTTRMRTLVSSAAGSN
SWVSVKTHPTIGDAVGHLKAQGMQILATNLSARAVDFREVDYTRPTCVLLGQEKTGITEEALALADQD
IVIPMIGMVQSLNVSVASALILYEAQRQRQNAGLYRRDNSMLDEEEQQRLLFEGGYPVLANVAKRKGL
PRPKIDEQGQVVASAEWWAAMQATVRK
>CORE_REP|Org38_Gene3396#
MHSTASDAFINSCLATITHLIPVSAGVFYLVDRDLRPDHYILHGMPDKTHQQYLNHFQQIDPLQPANF
HRQDITMVGMSPAAIADNRRYYHDFMLPNDMRDMTEIFIRQRKRIVAGVSLIRDTPFTDVERGRLRAV
LPLIELATRDLLPDSEAQLLTAKEQEIVNLVREGASNKRIALKLGISLSTVKTHMRNIFAKTDVVNRT
ELVAGGFLAHG
>CORE_REP|Org5_Gene2965#
MKKIVVLVSGQGSNLQALIDACQQDRIAAEIVAVFSNKAQAYGLQRAEAADIATQALDAKAYADRTAF
DAALADAIDQYQPDLVVLAGYMRILSPQFVQRYAGRMLNIHPSLLPKYPGLHTHRQAIDNGDSEHGTS
VHFVTEQLDGGPVILQAKVPIFADDEEDDVVERVQTQEHTIYPLVVSWFVDGRLAMRDGAAWLDGERL

PEQGHAAD
>CORE_REP|Org24_Gene15#
MVLGKPQTDPTLEWFLSHCHIHKYPSKSTLIHQGEKAETLYYIVKGSVAVLIKDEEGKEMILSYLNQG
DFIGELGLFEEGQERSAWVRAKTACEVAEISYKKFRQLIQVNPDILMRLSAQMASRLQVTSEKVGNLA
FLDVTGRIAQTLLNLAKQPDAMTHPDGMQIKITRQEIGQIVGCSRETVGRILKMLEDQNLISAHGKTI
VVYGTR
>CORE_REP|Org23_Gene2880#
MKTALLLIDLQNDFCPGGALAVTAGDAVIPVANQAIAACLARGEPVVASQDWHPANHRSFAVNSDAQV
GTLGELEGLPQVWWPVHCVQGSHGADFHPQLQRQHINAVFRKGQDTNIDSYSAFFDNGHRAQTELHGW
LQSQGVRRLAVMGLATDYCVKFSVLDALAAGYPTQVIVDGCRGVNLQPDDSERALQDMARAGAQLVTL
SQFLAN
>CORE_REP|Org12_Gene2927#
MGQDYALVVDDHPLVASGIANFLSTHCRFKQAHVVTNEENCYRHIRENGPPRLLVIDFWLSSGTALKL
LKEVKQRYPQVRILVVSGDENNDIWQKVHNAGGHGFVLKNEPPELFARAVFALNNNQEWFPEGNEAAI
KNNHDHLNKFNLTPRQLDVLTMMLRGLPNKRIATQLSISEPTVKEHISNILKKIGVNSRVEAITLLHG
KRDPSS
>CORE_REP|Org33_Gene329#
MPTIIMDSCSYTRLGLTDYLTSHGVKKRHINAIEDIDSLHEKCSKLNPSLVFINEDCFIHEANATERI
KRVISLHPDTLFFIFMAITNVHFDDYLYVRKNVIISSKSIKPETMNQLLSHYLERTSLRTEKSSLDQT
PVTLSQTESNMLRMWMSGQGTIQISDQMQIKAKTVSSHKGNIKRKIKTHNKQIIYHVVRLTDTLTSGI
FVNSR
>CORE_REP|Org26_Gene1779#
MKLLVVDECCFTRVGIASYFADSGITTIKCCHSIEYATPLLASFQPSHILVNLSNQCRYNEADAQLLA
FMEASQSALLFIYLDTPYPYSETPMRIADNAFLFNKSILPLTLRTLRENPLALADDGEERSLFSPQEL
TVMKYWMAEMPNYRIAKKLQISSHTVYVHKRHITEKINARNRLEFYSLYNVLRYFYPPNTPTNSTPLA
LLAV
>CORE_REP|Org48_Gene2344#
MQKKLDSLLAAAGIELPDQQKQQLLGYVGMLDKWNKAYNLTSVRDPQQMLVRHILDSIVVNPHLQGSR
FIDVGTGPGLPGIPLAIVRPDAHFTLLDSLGKRVRFLRQVQHELGLNNIEPVQSRVEAFPAEPPFDGV
ISRAFASLQDMLSWCHHLPAKGQGRFYALKGVRPDEELAHLPEGVSLESIVRLQVPELEGERHLVVLK
AN
>CORE_REP|Org31_Gene2423#
MLGTMMTKSTHCNVDTREHLLATGETLSLRLGFTGMGLSELLATAGVPKGSFYHYFRSKEAFGEAMLQ
RYFAHYDAQMQALFADRRGDARHQLLGYYAQAISYHCRSECHNACLAVKLSAEVSDLSEPMRHALETG
TARVIGHLQEAIERGIAEGSLSVAMSPAATAETLYSLWLGASLRAKIRHSLAPLTSALESIELLLRPP
QA
>CORE_REP|Org21_Gene2928#
MEQLNHFLFAWINATPASPEWMIDFATFLARDLIIIVPLLIVGLWLWGPQSQLASQRQVVAKTTIALL
FAMLAAATIGALLPHERPFVAGVGYTFLAHAPDSSFPSDHGTAIFTFALAFLFWHRVWSGVLLMIVAV
GIAWSRVYLGVHWPLDMVGGFLLGLVGCLFAQLVWNLFGDAIADKLSRLYRFLFAFAIRRGWVKE
>CORE_REP|Org44_Gene507#
MPKVGMQPIRRQQLIDATLAAVNEVGMHDATIAQIARRAGVSNGIISHYFKDKNGLLEATMRYLISHL
GEAVKLRLQALTDNSPAARLQAIVAGNFDDSQINSAAMKTWLAFWASSLHQPQLNRLQQVNGRRLYSN
LCAEFRRVMPQPQARLAAKGLAALIDGLWLRSALRGSAFNQAQALALTTEYITFQLRGQTPPGG
>CORE_REP|Org12_Gene2101#
MAEKENTKRNRREEILQALAQMLESSDGSQRITTAKLAANVGVSEAALYRHFPSKTRMFDSLIEFIED
SLITRINLILQDEKETFNRLRLILLLILGFAERNPGLTRIMTGHALMFEQDRLQGRINQLFERIEAQL
RQVLKERKLREGKGFIVDETLLASQLLAFCEGMLSRYVRSEFRYRPTQEFDARWPLLAAQLQ
>CORE_REP|Org11_Gene1861#
MYRRDIPEQRKEQLINAAFETISVVGLAGVTLSQVAKEAGLSTGIVSHYFGDKEGLLSATMRKILRDL
RDAVAECRAQAASDSQSQLCAIIQGNFHPSQTNAISMRAWLDFWAASMHQPVLRRLQRANDRRLYSNI
CSQFRRELPLQQARDAARGLAAMIDGLWLRGSLAGDDTDLQQDYRIACDYVIQRLRAAPSAE
>CORE_REP|Org14_Gene2680#
MLSPNDSKQAKVQARRDQIVEAAKTSFRRHGFHAASMAEIAQGSQLSVGQIYRYFANKDAIIEEIVNR

IIASKMQRLENLGDHINLIAGTLAARTLFQQPGESETDHMLMLEVTAEATRNPVVAKMLSDAEARLFR
HVCHNLQRLYPDFSAEEIAARVEFIAVMSEGTGYRILTTQKADASLLRDLYQQAISHLFRKR
>CORE_REP|Org4_Gene2025#
MTADLNALPARQRILLTAHDLFYQEGIRATGIDRIIKESGVTKVTFYRHFPSKNDLITAFLAYRHQQW
LAWFSTALARHVAQTGGLLAALAPCLAEWFDDPRFRGCAFINTAVELADLLPESLHIASQHKRQMADE
LARHLPAGPQREQHSAMLAMLIDGAIVRVQIERLPQAALQVLNATLDMLAQGGFDQ
>CORE_REP|Org22_Gene3650#
MSAALIIIDLIEDLIGPKGRANHCREQVLATHLLANVNAAAAYARVRKIPVIWVRVGFADDYHDIPPH
SPLFNHLKQIGALRLNSPGCRWMPELHQEETDLLFEKTAVSAFSGNNLLAWLRQHRCHHLLLAGVSTP
LAIESTARQAHDAGFQVTVLHDLCAAPTQEIHQQSLDTLQNLAEITRSQAWMKG
>CORE_REP|Org19_Gene1561#
MQLNIPTWLTLFRVVLIPFFVLAFYLPFNWAPMVCAVIFVFAAVTDWFDGFLARRWKQTTRFGAFLDP
VADKVMVAVALVLVAEHYHSWWITLPAATMIAREIIISSLREWMAEIGKRSSVAVSWIGKVKTMAQMM
SLVGLLWRPDRSVEYVAIGLLYIAAVLTFWSMFQYLKAARNDLLEP
>CORE_REP|Org43_Gene2420#
MLNIVLFEPEIPPNTGNIIRLCANTGFNLHLIEPLGFPWDDKRLRRAGLDYHEFTRVHRHADYAAFLA
AEDPQRLFALTTKGTPAHSAVSYQAGDYLLFGPETRGLPADILDALPAQQKIRIPMQAQSRSMNLSNA
VAVVVYEAWRQLDYAGALIKA
>CORE_REP|Org5_Gene4503#
MKSTDIEQPHAKNLLQLDQQLCFALYSANLALHKVYRKLLSQLELTYPQYLVMMVLWERDRVTVSDIG
ERLFLDSATLTPLLKRLETAGLLVRYRATADERQVIIALTEAGRALRERAQSVPEAVMCATDCSLDEI
VSLKQQLEKLRGSLIDQI
>CORE_REP|Org12_Gene2908#
MHESLTIALLQARETAMGFFRPILKSHNLTEQQWRIIRVLANSRSIEFHELAAETCILRPSLTGILSR
MERDKLIFRLKPVNDQRKLYVSLTQQGQDLYEVARHQVEQGYAEIEAAFSRQKMDQLMTLLDELITLG
DSLPANVAAHPAKQ
>CORE_REP|Org49_Gene4025#
MKIGELAQRAGMAASAIRYYEQLGLLPKPVRGVNGYRVYGDSALERLHLIQIGQNLGFSLQAIQRVLA
LQGSAYQDGLIRGVDERLAEIELMMATLNEQRETLLTTRLTLLESGVAGLCQAKGEKQADASPAWPAK
LARMRNRIDTE
>CORE_REP|Org24_Gene160#
MESTLGSDLARLVRVWRALIDHRLKPLELTQTHWVTLHNINRLPPEQSQIQLAKAIGIEQPSLVRTLD
QLEEKGLITRHTCANDRRAKRIKLTEAADPIIREVDSVITSTRSEILSGITADEVHLLVGLIGKLEQN
ITELQNK
>CORE_REP|Org44_Gene1295#
MFKIGQLAKLAEVTPDTVRYYEKQGMMDHNVRTEGGYRLYTEQDLQRLRFIRYAKQLGFTLETIAELL
SIRVDPEHHTCQESKSIVDARLSEVESKLAELTRMRESLKRLSDACCGTAHTSNYCSILEALEQGASD
EKGKKGC
>CORE_REP|Org8_Gene1715#
MGDSSKDGVILLSRILLMVLFIIFGWMKLVNFGATVTAMEGYGTPMPYLAAIVAVVVEFIFGIALILG
LFTRPIAVIFALYVLGTAFIGHPFWKMTGMEMMGNEINFFKNISIIGGLLLLAVTGAGRYSLDYKIFN
K
>CORE_REP|Org34_Gene207#
MSEALKILNNIRTLRAQARECTLETLEEMLEKLEVVVNERREEDSQAQAEIEERTRKLQQYREMLIAD
GIDPNELLQTMAANKAAGKAKRAARPAKYQYKDENGELKTWTGQGRTPAVIKKAIEEQGKSLDDFLL
>CORE_REP|Org23_Gene1104#
MNISDVAKKTGLTSKTIRFYEEKALITAPIRSDNGYRHYSAKHVEELTLLRQARQVGFNLDECRELVA
LFNDPARHSADVKARTLQKVAEIEKHISELGNMRQRLLTLAEQCPGDEGAECPIINNLAGCCRSN
>CORE_REP|Org43_Gene1700#
MAKELDIGAVARLSGVAPSALRHYEKKGLIASIGRHGLRRQYAAGVLDQLRLIALARLAGFTLDEMSA
LFDERGKIALDRVLLAARADELDRHIQRLIQVRDGLRHMVDCPEPEHLQCPQFRKILQQGEF
>CORE_REP|Org22_Gene183#
MIQEHHLSLVCALSKWVETHLGRVIHLEELAEYSGYSLWHMQKLFKEATGISLGKYIRERRLAGAVYQ
LRSSEASIFDIALDFGFGSQSHFTYMFRKRFNITPYDFRQDLSVDLHIDPPLHVIHQKSA

```
>CORE_REP|Org13_Gene200#
MIYWIFLGLAIMTEIIGTLSMKYASVNGGMIGHIVMYVMITASYVLLSVAVKRVALGVAYALWEGIGI
LFITLFSVLWFDEPISALKVLGLATLIAGIMLVKSGTRKERKQVSPRGDNHATV
>CORE_REP|Org10_Gene2347#
MNTTGFITDLKTWIDNNLEEKLDINTVADRAGYSKWHLQRMFKRQTGYALGEYIRMQKLKVSAERLAN
SGEPIVSVAISLGFDSQQSFNRSFKRQFGQTPGDWRRALAQPTAVRCTHH
>CORE_REP|Org37_Gene613#
MQQFELYHIGFLGLAIVLEIIANIFLKMSDGFRKIWLGLLSLLSVLGAFSALAQAVKGIDLSIAYALW
GGFGIAATIAAGWIMFGQRLNAKGWIGLALLLTGMVILKLS
>CORE_REP|Org33_Gene76#
MAWIILLIAGLLEVVWAIGLKYTHGFTRLTPSIITIAAMVVSMLLLANAMKTLPAGTAYAVWTGIGAV
GAAIMGMVLLGESTNIARIISLCLIVVGILGLKFSSH
>CORE_REP|Org27_Gene6#
MQNQRIRIRLKAFDHRLIDQSTAEIVETAKRTGAQVRGPIPLPTRKERFTVLISPHVNKDARDQYEIR
THKRLVDIVEPTEKTVDALMRLDLAAGVDVQISLG
>CORE_REP|Org34_Gene4528#
MAVTNVAELNELVARVKKAQREYANFTQEQVDKIFRAAALAAADARIPLAKMAVEESGMGIVEDKVIK
NHFASEYIYNAYKDEKTCGILSEDDTFGTITIAEPIGLICGIVPTTNPTSTAIFKALISLKTRNGIIF
SPHPRAKNATNKAADIVLQAAIAAGAPKDIIGWIDQPTVELSNQLMHHPDINLILATGGPGMVKAAYS
SGKPAIGVGAGNTPVVVDETADIKRVVASILMSKTFDSGVICASEQSVIVVDAIYDAVRERFASHGGY
LLQGKELKAVQDIILKNGGLNAAIVGQSAPKIAEMAGIKVPANTKVLIGEVKLVDESEPFAHEKLSPT
LAMYRAKDFEDAVAKAEKLVAMGGIGHTSCLYTDQDNQTARIAYFGDKMKTARILINTPASQGGIGDL
YNFKLAPSLTLGCGSWGGNSISENVGPKHLINKKTVAKRAENMLWHKLPKSIYFRRGSLPIALEEVAT
DGAKRAFIVTDRFLFNNGYADQITKVLKSHGIETEVFFEVEADPTLSIVRKGAEQMNSFKPDVIIALG
GGSPMDAAKIMWVLYEHPETHFEDLALRFMDIRKRIYKFPKMGVKAKMIAITTTSGTGSEVTPFAVVT
DDTTGQKYPLADYALTPDMAIVDANLVMNMPKSLCAFGGLDAVTHALEAYVSVLANEYSDGQALQALK
LLKEYLPASYKEGAKNPVARERVHNAATIAGIAFANAFLGVCHSMAHKLGSEFHIPHGLANAMLISNV
IRYNANDNPTKQTAFSQYDRPQARRRYAEIADHLGLSAPGDRTAQKIEKLLAWLDELKTELGIPTSIR
EAGVQEADFLAKVDKLSEDAFDDQCTGANPRYPLIAELKQIMLDTFYGREFSEAVDEEAATPAAAKTA
VKKPRNNGSVNR
>CORE_REP|Org10_Gene2637#
MSETTLAPSQTADAALAADERLATKEGRSQFWRATFSCWLGTAMEYVDFALYGLAAGMVFGDVFFPEA
TPLVALLASFATYSVGFVARPIGALVFGWIGDRKGRRVVLITTVALMGLSTTLIGLIPSYAQIGVWAP
ACLVILRFAQGFGAGAELSGGAVMLAEYAPAKRRGLVASIIAIGSNSGTLLASLVWLLVLQLDKEDLM
SWGWRIPFLASILIAGAALYLRRHVRETPVFERELQQNHQRMLDAAQAAPDTRSYLQRTKAFWVMLGL
RIGENGPSYLCQGFIVGYVAKVLMVDKSVPALAVLIASLCGFLVIPLAGWLSDRFGRRITYRWFCLLL
VLYAFPAFWLLDSREPAIVISVIVVGMCIASLGIFGVQAAYGVELFGVKNRYSKMAFAKELGSILSGG
TAPLIATALLSGFGHWWPVACYFVVMAAIGLITTFFAPETRGRDLNLPQDAA
>CORE_REP|Org1_Gene4681#
MQSISRIILAFYSACGPEEHSVKRSVSVVIGGILLPLAAQAAETTPHFPTMTPPAIDAASYVLMDYTT
GQVLAAGNADERRNPASLTKLMTGLVIDHALDQHKIGLDDVVTVGKDAWAQGNPVFKGSSLMFLKPGD
RVTVRDLSRGIIIDSGNDACVAMADYVAGSQANFVKLMNEKSAQLGLQNTHFETVHGLDAPGQFTTAG
DLVVIARAIIMSEPAEYHMYSEKSLTWNGITQQNRNGLLWDKTLHVDGLKTGHTASAGFNIIASATEG
DRRLIAVVMGGKSSKGREEQARKLLSWGLRDFTTVHLFSAGQSLGEEPVWYGENHRLPVGSVQEQSLS
LPKNEADKLKAQYVINTARLEAPIGKGQTVGEIRISDNGQVVKTLPLVALQAVPQGGVFSRLVDYVKL
RL
>CORE_REP|Org24_Gene4604#
MNGHSGDVMMKKNLSTSLKKLTFSVGILLLAAPAVHAAEPPAPPQVDAKAYILMDYNSGKVLTEGNAD
TRLDPASLTKIMSSYVIGQAIKAGKIKPEDLVTVGKDAWAPGNPALRGSSLMFIKPGDQVPVLELNKG
IVIQSGNDASIALADYVAGSQDSFVGLMNNYAKSLGLQNTHFLTVHGLDAEGQYSTARDMALLSQALI
RDVPDEYALHKEKEFTFNKIRQINRNRLLWSSNLNVDGIKTGYTSGAGHNLVASATDGPMRLISVVLG
APSDRVRFSESEKLLTWGFRFYETATPIKADKPFVTQKVWFGDVSEVPLGVAKDASVTIPKGQMKNLK
ASYKLTQPTLEAPLAKNQVVGTIDFQLDGKTIEQHPLVVMQEVKEGNFFSRIWDMVMMKLSQWFGGIF
G
```

>CORE_REP|Org12_Gene4273#
MSVIIVGGGMAGATLALAISSLTQGRMAVDLVEATRPDDRSHPGFDARAIALAQGTCQQLARIGVWPA
LRDCATPITQVHVSDRGHAGFVNLQAQDYQVDALGQVIELHDAGQRLFALLAKAPGVTLHCPARVVDV
IRTAERAEVLLDNGQRLRGQLLVAADGSRSALAQACNMQWRQEDYPQFATIANVTTAEDPQGRAFERF
TRYGPLALLPMSQGRSSLVWCHAREDRAQVDAWDDERFIAELQQAFGWRLGRILKAGKRHSYPLGLLT
ADRHVSHRLALVGNAAQTLHPIAGQGFNLGLRDVMSLAETLAEVADSREDAGGYALLSRYQQRRQNDQ
RATIGVTDGLIHLFANRYGPLVIGRNLGLMAMARLPAIRDAFAKRTLGWVER
>CORE_REP|Org30_Gene3769#
MSVSHIIANRQTWFGHGSIRQLPPLLLADPQPTLLFSCRSFLNGPVYAGLRESLAPLFIGTEIVSHEA
SPQEIDAWVARWRGQARRVVAIGGGSVLDAAKAFSALVEHPLPTLRYMEKVGDSKISGATLPLIAIPT
TAGTGSEVTQNAVITDTQVSKVKASLRHNNFVPHTAILDPQLLAGAPDKVLAYCAIDAFTHLFEAYLS
KTAGAMTRDMSLSGIRHFLAAWPALNRSDAAREAIMQASYLGGLTLSATGLGVIHGIAGEIGALRDYH
HGQVCGRLLLPFLALLENSEQPQQRALMAELAARLYPHWQGSPESYLTDFITRHAIAPFWQDDLPISG
QELAVALDKSNSKNSWIDYAPAQRQRMIEEAFRVE
>CORE_REP|Org13_Gene3324#
MKRINRYYDAAKAHHTPEGFRNPEPSQRQEGDLQRWQDERKRQGLPRPPQQGYAQFTERWWQPADLSG
SDDSIWWLGHASMLLRLGGRYILIDPVLSERASPLSFYGPKRRTPAPLTVEQLPAVDAVLISHNHYDH
LDRRTVRQLARRFPQAEFIVPLGLKRWFRRYRLKVHELDWWQSLSLGELTVYATPARHWSMRTLWDRN
RSLWCGWVIHHPALRFYFSGDSGYSARLAEIGQRLGPFDVAALPIGAYAPRWFMQEQHMDPQQSVALY
RELNQPRAIPIHWGVFELADESLDEPPQQLNLALSEAGLEQHQFHPLKIGERIALQDSQQALSIRPVV
ERKE
>CORE_REP|Org6_Gene1086#
MRLNLEALLILDALDRHGSFAAAAAALFKTPSALSYMVQKLENDLDITLLDRSGHRAKFTDTGKLMLE
KGRVLLRAAQDLEQQARYVENGWESEITLGIDASFPFARLLPLIDEFYRQHHHTRLRFSHEVLAGSWE
SLVYGCADIIIGAICEPPSRVGYAFSRLGQLDYVFAVAPQHPLAALPEPLPKDEIRQHRAVVVRDTSR
VNAPQSLNLLEEQDTLTVFGFDAKLQAQLAGLGCGYLPRSLAEPYLNSGELVAKRVESERCSDIAYFG
WRESASGLAAKWWRERLQRYADDGEAYPAAQ
>CORE_REP|Org24_Gene3483#
MAFNDSDDLLAQKAEQRMAQALATSQVEQDDYLDNQPAEALTRGDINRMAWRSLLLQASFNYERMQAG
GWLYQLIPGLRKIHRNPQDLANSMKMHMEFINVHPFDVTFLSGLVLAMEQNKEKISTIRAVKVALMGP
LGGIGDALFWLTLLPICAGIGASLALEGSLFGPIVFLLLFNLFHFGLRFGLAHYGYQAGTSALALLKT
HTRRISHAASIVGMTVIGALVASYVHLSTPLVMHAGKARVALQTDVLDKLMPNLLPLCFTLLIFFLMK
RGFSPVKLIGVTVAIGVAGKFIGIL
>CORE_REP|Org46_Gene1042#
MVDTTAQKKLTPADIRGVFLRSNLFQGSWNFERMQALGFCFSMVPVIRRLYPENNDDRKQAIKRHLEF
FNTHPYVAAPVLGVTMAMEEQRANGAPIDDGAINGIKVGLMGPLAGVGDPIFWGTVRPVFAALGAGIA
MSGSLLGPILFFVLFNLVRLLTRYYGVAYGYRKGVDIVNDMGGGFLQKLTEGASILGLFVMGALVNKW
THVNIPLVVSKITDQTGHTNVTTVQTILDQLMPGLVPLLLTFACMWLLRKKVNALWIIIGFFVIGIFG
YWIGLLGL
>CORE_REP|Org1_Gene4517#
MNLISIPAFQDNYIWLLDDRQGRCIIVDPGEAQPVLEALQRLQLAPAAILLTHHHHDHVGGVAQIVAK
YPGLAVYGPQETADKGANHIVRDGDTFDIDGRQYRTIAVPGHTLGHVAFYSAPYLFCGDTIFSAGCGR
LFEGTAKQMYDSFQQLAQLPDNTLICCAHEYTLSNLKFARAILPEDREIETYQQHVEALRAKGQASVP
TTLQLERKINLFLRCHDADLQKKLGFNSPPESLHSVFSELRLRKDNF
>CORE_REP|Org29_Gene3496#
MFTVRQALLEDLTQVRDIGIRTYRAHFGELWRYPHELEAFLAEDFSVSALERTLRDPDVCWLLAYEDD
TLVGYARVNFDSLLAATQRRGAELQKIYFLPDYAGRGFGRQFFEQVQRRAVGRRQPLLWLEVLKQNAD
AQRFYQRQGLAVCGEAQYTSEQGAIELWAMSKAL
>CORE_REP|Org9_Gene4043#
MTTATPARLLVRSITAEDNTAIAHVIREVSAEHGLTADKGYTVSDPNLDALYQLYSLPRSAYWVIEVD
GQVAGGGGIAPLQGGADDICELQKMYFLPVLRGKGLAKRLALQALDFARQHGFRCCYLETTASLTQAV
ALYEHLGFEHIDHAMGATGHVDCEVTMLKTL
>CORE_REP|Org11_Gene1816#
MTIEIYPAQKSDAKLILDMIIELAVYEKAREQVLASVEDIERSLFGPGACSEALICTVNGEPAGYAVF

FMSYSTWLGKNGIYLEDLYVAPKHRGAGAGKKLLRHIAQLACERQCGRLEWSVLDWNQPAIDFYLSIG
AQPQGEWVRYRMEGEALTHFAAHGAALPAQ
