# Supplementary material for: A Systematic Immuno-Informatic Approach to Design a Multiepitope-Based Vaccine Against Emerging Multiple Drug Resistant Serratia marcescens
Source: Front Immunol. 2022 Mar 14;13:768569. doi: 10.3389/fimmu.2022.768569 (PMC8967166; doi:10.3389/fimmu.2022.768569)
Supplement: Supplementary Data Sheet S6 — Human non-homologue protein sequences. [file DataSheet_6.pdf]

```
>CORE_REP|Org10_Gene3917#
MTFPRFFIKRPIFAIVLSILTLIAGIVALFQLPLSEYPAVTPPTVQVTASYPGANPNVIAETVAAPLE
QAITGVEGMLYMSSQAATDGRMTLTVTFAQGTNADMAQIQVQNRVARALPRLPAEVQHQGVVTQKTSP
DILMVVHLLSPDQRYDPLYISNYAYLQVRDELSRIPGVSDVQVWGAGEYSMRLWLDPDLIAARGLTAG
DVIAAVREQNVQVAAGSVGQAPDTNAAFQVTVNTLGRLADEKQFGDIIIRTGSDGQVTRLRDVARIDM
GADAYALRSLLDGEPAVALQIIQSPGANALDVAQAVRATVKRLEGDFPAGLSSRIAYDPTVFVRASLE
SVVTTLLEAILLVVIVVVVFLRSWRASLIPLLAVPVSLVGTFAIMHLMGFSLNTLSLFGLVLSIGIVV
DDAIVVVENVERHIENGKTPQQAARLAMDEVTGPIVAITSVLAAVFIPTAFLSGLQGEFYRQFALTIA
ISTLLSALNSLTLSPALAGLLLRPHPAEHRAPGRIQRILQAAVRPFQRAPDAYANAVRKTVRVSGVAL
AIYGGLLVLTFFGFQAVPPGFVPMQDKYYLVGIAQLPNSASLDRTDAVVKQMSKIALAEPGVESVVAF
PGLSINGFVNVPNAAVMFVMLDPFKERATPDLAASAIAGRLQAKFADIPDGFLGVFPPPPVPGLGATG
GFKMQVEDRGGVGLESLVEHTRLLMVKATESGQVAGLMTSLDINAPQLDVVIDRTQAKSQGVSLADVF
ESLQIYLGSLYINDFNRFGRTYKVTAQADADHRMQAEAIGRLQVRNAAGDMLPLSSFVTVTPGSGPDR
IIRYNGYPSADISGGAAIGVSSGQAVALMEQLAKETLPEGMTVEWTDLTYQQKLAGNAALFIFPLCVL
LAYLILATQYNSWLLPLAVLLIVPMCLLSAMIGVWLLGGDNNVFVQIGLIVLVGLAAKNAILIVEFAR
GLEDEGANTLEAVIKACRLRLRPIVMTSIAFIAGVIPLIFASGAGAEMRHAMGVAVFAGMLGVTLFGL
FLTPVFYVVIRGLTARFEQYRTKGKANVRSESDEKSS
>CORE_REP|Org40_Gene1002#
MAKFFIDRPIFAWVIAIIVMLAGVLAIMKLPIAQYPTIAPPAVSISANYPGADAKTVQDTVTQIIEQN
MNGIDNLMYMSSTSDSSGSVTITLTFDSGTDPDIAQVQVQNKLSLATPLLPQEVQQQGLKVEKSSSSF
LMVAGFVSDDPNMTQDDIADYVASNIKDPISRSSGVGEVQLFGAQYAMRIWLDPNKLNNFQLTTTDVT
SAITEQNNQIAAGQLGGLPPVPGQQLNASIIAQTRLTSPEEFGKILLKVNTDGSQVRLRDVAHIERGA
ESYAVTARYNGKPAAGLGIKLATGANALNTAKGVKDELAKMAPFFPQGMKVVYPYDTTPFVKISINEV
VKTLIEAIILVFLVMYLFLQNFRATLIPTIAVPVVLLGTFAILAAFGFSINTLTMFGMVLAIGLLVDD
AIVVVENVERVMSEEGLPPKEATRKSMGQIQGALVGIAMVLSAVFVPMAFFGGSTGAIYRQFSITIVS
AMALSVLVALILTPALCATLLKPIPKGDHGVKTGFFGWFNRMFEKSTHHYTDSVGNILRSTGRYLIIY
LLIVVGMGLLFLRLPSSFLPDEDQGILLTMVQLPAGATESRTNKVLEEVSDYFLNKEKDNVVSVFTVA
GFGFNGNGQNNGLAFVSLKDWGERPGAGNKVEAIAGRAMGAFSQIKEGLVFPFNLPAIIELGTATGFD
FELIDQGGLGHEKLTEARNQLLGMVAQHPDVLVGVRPNGLEDTPQFKLIVDQEKAKALGVSITTINST
LSTALGGSYVNDFIDRGRVKKVYVQADAPFRMLPEDINKWYVRGTSGQMVPFSAFSSAKWEYGSPRLE
RYNGLPSMEILGQAAPGKSTGEAMNLMEQLASKLPSGIGYDWTGMSYQERLSGNQAPALYAISILVVF
LCLAALYESWSVPFSVMLVLPLGVIGALLAATMRGMNNDVYFQVGLLTTIGLSAKNAILIVEFAKDLM
EKEGKGLIEATLEAVRMRLRPILMTSLAFILGVLPLVISSGAGSGAQNAVGTGVMGGMITATVLAIFF
VPVFFVVVRRRFSKKNEDLEHSHPVEHH
>CORE_REP|Org28_Gene4189#
MQVMPPNAGGGPSRLFILRPVATTLLMVAILLAGIIGYRALPVSALPEVDYPTIQVVTLYPGASPDVV
TSAITAPLERQFGQMSGLKQMASQSSGGASVVTLQFQLALPLDVAEQEVQAAINAATNLLPSDLPYPP
IYSKVNPADPPILTLAVTSTAMPMTQVEDMVETRVAQKISQVTGVGLVTLAGGQRPAVRVKLNAAAVA
AYGLNSETIRAAISNANVNSAKGSLDGPTRSVTLSANDQMKSADDYRQLIVAYQNGAAIRLQDIATIE
QGAENTRLAAWANKQQAIVLNIQRQPGVNVITTADSIREMLPTLIKSLPKSVDVKVLTDRTTTIRASV
SDVQFELLLAIALVVMVIYVFLRNVPATIIPSVAVPLSLVGTFAAMYFLGFSINNLTLMALTIATGFV
VDDAIVVIENISRYIEKGEKPLDAALKGAGEIGFTIISLTFSLVAVLIPLLFMGDIVGRLFREFAVTL
AVAILISAVVSLTLTPMMCARMLSHESLRKQNRFSAASERFFDRVIAQYGQWLKTVLNHPWLTLGVAV
GTLALTVLLYLLIPKGFFPVQDNGIIQGTLEAPQSVSFSNMAERQQQVAAQILKDPAVESLTSFVGVD
GSNATLNSGRLQINLKPLSERSDRIPAIISRLQQQTAQFPGVKLYLQPVQDLTIDTQVSRTQYQFTLQ
AMSLDDLSLWVPQLMNELKQTPQLADVTSDWQDQGLVAYVNVDRDSASRLGVTMSDVDNALYNAFGQR
LISTIYTQANQYRVVLEHDVSATPGLAALNEIRLSGNDGAVVPLSAIAKIEERFGPLSVNHLDQFPSA
TVSFNVADGYSLGEAVDAVTQAEKNLNMPRDITTQFQGATLAFQAALGSTLWLILAAVVAMYIVLGVL
YESFIHPVTILSTLPTAGVGALLALMLAGSELDVIAIIGIILLIGIVKKNAIMMIDFALAAEREQGLS
ARDAIYQACLLRFRPILMTTLAALLGALPLMLSTGVGAELRHPLGVCMVGGLIMSQILTLFTTPVIYL
LFDKLARNTHRQPDTQELP
>CORE_REP|Org2_Gene3277#
MTKYSLRARMMILILAPTLLIGLLLSTFFVVHRYNELQEQLVDAGASIIEPLAVASEYGMTFRSRESV
RQLVSLLHRRHSDIVRSITVFDAQNNLFVTSNYHHNFAQLQLPKGVPLPTELMLTRRGDSLILRTPIL
```

SESQYPDETADGGSHPDNNLGYVAIELDLQSVRLQQYKEVFVSTLLLLLCMCIAILFAYRLMRDVTGP
IRNMVNTVDRIRRGQLDSRVEGYMLGELHMLKNGINSMAMSLTAYHEEMQQNIDQATSDLRETLEQME
IQNVELDLAKKRAQEAARIKSEFLANMSHELRTPLNGVIGFTRQMLKTDLSATQTDYLQTIERSANNL
LTIINDVLDFSKLEAGKLVLEHIPFALRETLDEVVVLLAPSAHDKGLELTLDVHNDVPEQVIGDSLRL
QQIITNLLGNAIKFTETGNIDIRVELRKQLDRRVEVEVQIHDTGIGISERQQSQLFQAFRQADASISR
RHGGTGLGLVITQKLVKEMGGDICFHSQLNRGSTFWFHITLDLNEGMLSLAPSLPDLSGKTLAYIESN
PTAAQATLNMLSITQLVITHSPTLGQLPPGHYDFLLAGVPIPFRDNMAQHEDKLLASLKLADRVILAL
PCQAQIDAELLKQQGALGCLIKPITSTRLFPLLRMEAPARLTAQPERKRLPLTVMAVDDNPANLKLIG
TLLGEQVEKTLLCESGEEALALARDNVLDLILMDIQMPKMDGIHASELIRQLPHHNSTPIVAVTAHAA
SGEREHLLQAGMDDYLAKPIDEKMLTRVLSRYHSGDVENAIADDAPLSLDWPLALRQAANKPDLARDL
LQMLLDFLPQVRERVQALLDGQHDDEILDLVHKLHGSCSYSGVPRLKQLCFYLERQLRQGVTNDELEP
EWLELLDEIELVIHAARAHLTQPA
>CORE_REP|Org44_Gene2350#
MKFVKYFLILAVCCIVLGAASIFGLYKYVEPQLPDVATLKDVRLQIPMQVYSADGELIAQYGEKRRIP
LKLDQIPPVMVHAFIATEDSRFYDHHGVDPVGIFRAASIALVSGHASQGASTITQQLARNFFLSPERT
LMRKIKEAFLAVRIEQMLTKDEILELYLNKIYLGYRAYGVGAAAQVYFGKDVSQLTLSEMATIAGLPK
APSTFNPLYSHDRAVARRNVVLSRMLDEHYITQAQYDQARSEDLVANYHAPEISFSAPYLSEMVRQEM
IKRYGENAYTDGYKVYTTVTKRLQLAAQESVRNNVLAYDMRHGYRGPSNVLWKVGEAAWDRKQIVDSL
KNLPNYGPLAPAVITAANPQEATAMLADGSSIALPMATMRWARPYRSDTQQGPTPKRVTDVVQAGQQV
WVRKVNDAWWLSQVPDVNSALVSINPNDGAVKALVGGFDFNQSKFNRVTQALRQVGSNIKPFLYTAAM
DKGLTLATILNDLPITRWDAGAGTDWRPKNSPPTYDGPIRLRQGLGQSKNVVMVRAMRAMGVDYAAEY
LQRFGFPAQNIVHTESLALGSASFTPMQLVRGYAVLANGGYLVDPYFITKIEDDNGNTVFEAKPKVVC
SSCNLPVIYGDTHRSAVLSDDNIENVATSQEGNNSTVPMPQLEQVTPAQVQQDGDQQYAPHVISTQLA
FLIHDALNSNIFGEPGWMGTAWRAGRDLKRHDIGGKTGTTNSSKDAWFSGYGPDTVTSVWIGFDDHRR
DLGRSTVSGAIPDQISGGEGGAKSAQPAWDDFMKTALEGIPEQKVTPPPGIISVTIDKSSGKLSGGGG
GSRSEYFIEGTQPTDYPSRDTGTTLTDPGGESHELF
>CORE_REP|Org31_Gene3983#
MALLKETIRDHSAEERLFIRRAGVALALVVVCFGALIVNLYRLQIRQHGFYQTRSNQNDIKMLPIAPS
RGLIFDRNGTPLVRNVTLYRIEITPSKISDMAALLQALTPIVDLTPEDISAFRDDMHHNSRYKPVTLK
AGLSDTEVARFAVNQYRFDGVTIDTYQQREYPYGAQLAHVLGYVSKINDSDLKRLDKAGLSENYAADR
NIGKQGIEAYYEAELHGTTGYQEVEVDNHGRVIRLLKEQPPKAGKNIYLTLDLPLQQYIESVLKGQRA
AVVVEDPRDGGILAMVSSPSYDPNPFVKGIGYQAYKALLTNPDLPLINRVTQGLYPPASTVKPYMAVS
ALFAGVITPTTTFFGAPTWTLPGTERRYRDWLKTGHGMLNVTKAIEESADTFFYQVAYEMGIDRIHHW
LSQFGYGQSTGIDLNEEYRGVLPSRDWKLKVHKKGWYQGDTVSVGIGQGYWVATPIQMVKALTTLINN
GQVKTPHLLYSLQQGNRVTRYPPPAKTAQIGDPNSPYWGIVRNGMYGMANLPNGTGYKLFHTAPYQIA
AKSGTSQVFSLKQNQTYNAKMIPVRLRDHIFYTLFAPYKNPRVAMALILENGGGNGVVAGPTARAILD
HIFDPANAPQPGDAGQSKPQLNDSADVQR
>CORE_REP|Org36_Gene3179#
MRFFQNLQNDGISPRAQIRLLDNTFMRLVFSFTAVPFVGIPFAIWIYLLGDELGPTITWIIVYLLCAV
AIRIWHRRYLHEAKENDEDAVLRRWLPRINKVAFIHGLGISSLYLITPQTHNFDFFLLLNISIAAIVA
ANATHLTPVISTFTRFFFASWGLLNLGIICRLEDVMFIVLMLNLLYGFAIYRHALTSHAFFIQQALLE
EQSSRLAEQFRQAKEEAEQALLDKNQFLTTASHDLRQPVHAMGFLIEAIIHKNRDDSLTPQLLDLQQS
VRSVHLMFNSLLDLSKIESGNVRTAATHVDIGALLDSVITLFREEANSRALALRTWRPKRRISVMGDP
LLVRQSLINLIQNALRYTQQGGVLIAIRPRGAECLVEVWDTGVGIADEEKSKIFSPYYRPELAWKIDS
AGHGLGLAVVARCAKLMKVKYGMHSVEGKGSRFWMRFTQYIGEDKAPETAAAYDNTATPIRYAPLRGA
CLVVDDDPLVTSAWESLMSTWGITVRCAASAEEAFAIVDDGFTPFAVLCDQRLRSGESGFDILKALFE
RLPDVSGAMVSGEFNSQILQEAEQEGYLVLRKPLEPARLHALLTQWGAAS
>CORE_REP|Org20_Gene235#
MKAARPGKLRRQEDQASFVSWRFALLCGCILLAMVGLMLRVAYLQVINPDRLVKEGDMRSLRVQEVPT
ARGMISDRAGRPLAVSVPVNAVWADPKELNERGGITLDSRWKALSDALNIPLDQLSNRINANPKGRFV
YLARQVNPAIGDYIHKLKLPGIYLRQESRRYYPAGQVTSHIIGVTNIDGQGIEGVEKSFDRWLTGQPG
ERTVRKDRFGRVIEDISSVDSQAAHNLVLSVDERLQALVYRELNNAVAFNKAESGTAVLIDVNTGEVL
AMANSPSYNPNNMAGTPKETMRNRAITDIFEPGSTVKPMVVMTALQNGVVRENSVLNTIPYRIQGHEI
KDVARYSELSLTGILQKSSNVGVSKLALAMPSSALVDTYSRFGLGKATNLGLVGESSGIYPKKQRWSD

IERATFSFGYGLMVTPLQLARVYATIGSLGVYRPLSITKVDPPVAGERVFPEPLVRTVVHMMESVALP
GGGGVKAAIKGYRIAIKTGTAKKVGPDGKYVNRYIAYTAGVAPASNPRFALVVVINDPQGGKYYGGAI
SAPVFGAIMGGVLRTMNVEPDALPTGDKSELVINKKEGSGGRS
>CORE_REP|Org23_Gene2786#
MAPSTKKSGKTYSTVRFGWICAGMLVCFFLLAFRVGYLQLLEHQQLADQADQRSIRTQVVPTNRAMIT
DRNDEALAVSVSSKDIVLDPKHILDTQTDTGNERWQSMANVLKIPLADIQHLIQSNAHKRFVYLARKV
EDDNAAYISKLHLTGVSAEQDFSRFYPMGQDAAGLIGIVGQDNQGLEGIELGFNPLLQGKNGLRVYQK
DGSGAVIGVLKSVDPVPPPNVTLSIDKFIQYVLYAQIRDGVVANQADSGCAVLVKIDTGEILGMASYP
SFNPNNYGSTPAKDIRNVCSSDSFEPGSTVKPVVVMVGLEHKLIRPDTVLDTTPYRVNGHLIKDVGHW
SKLTITGVLQKSSDIAVSHIALALPATVLPAVYRSFGLGRPTELGIGNESSGYLPQHRERWADIERAT
FSFGYGLRVTPLQMAREYAAIGSFGIYRPLSITKVTPPVMGQRILPADTVRSVVHMMESDALPGGSGV
SAAVPGYRLAIKTGTAEKMGPSGKYDGGYINYTAGVAPASDPQVALVVMVNNPKAGKHFGGSVAGPVF
GKIMAQVLEHMNILPDAQPLNVVSSVKS
>CORE_REP|Org5_Gene2562#
MNKVKSLSQQNLSLLLAIYIGIFLNLSVFYRRFDSLAHGIQGIKVISAVTEVIAIVLFTFFIMRLVSL
GGRLFYRIVASLLVLISVAASYYMTFFNVVIGYGIVVSVMTTDIDLSKEVVGLHFVLWMVALSALPLL
LIWKNSLRYTLIEQLKTPGHRIKPLLVLLAVVALVWLPLRMLDDEQSVQEKLSNVDLPSYGGVVAHSY
LPSNWLSALGLFAYTRYDESQDQSTMFDPGKHFTYVPPADIDDTYVVFIIGETTRWDHMGMLGYERDT
TPRLSKEKNLVAFRGESCDTSTKLSLRCMFVREGGTEDNPQRTLKEQNVFAVLKDLGFSSELFAMQSE
VWFYNNTEVNNYSFREMIASEKRNDGKAVDDMLLVDEMKESLARYPKGKHLVILHTKGSHYLYSQRYP
RSYARYQPECMGVDDSCTKAQLINAFDNTVLYTDSFIANVIDQVRDKKAIVFYAADHGESIGENTHLH
GTPREMAPPEQFRVPMIVWASDKFLENPQHLSAFEQLQAQQRIGKTHRHVELFDTILGCLGYTSPDGG
IVDKNNWCHLPQDKTAPASL
>CORE_REP|Org2_Gene2715#
MWLRQCLRCNSLGFTLATALFFTLFQNALFLHRAWSYITFDSVHSVIFAASMPVVIFCALNIIFSVLT
VPYLRKPLIIFFLLGSAAANYFMFSYGVVIDGNMMQNAFETNPQEATALLTPRMGLWLALLGILPAVA
VCFTQIRQTRPWWYMVGLRAANVMLSLAVILIVAALFYKDYASLIRNNKSVVKMLTPSNFVAGTIKFT
EQRYFTRNLPLVKIGEDARKGPLIAGQAKKTLVILVVGETARAENFSLGGYQRETNPRLKQDNVVYFK
NASSCGTETAISVPCMFSNMPRKEYDATQATHQEGMLDVLAHAGVSVLWRDNDGGCKGACDRVPHIDM
TKLKLPQDCDGEVCMDNALLYKLNDYINGLKDDGVIVLHQMGSHGPAYYRRSTPEFQTFSPTCNSNQI
QDCSHEQLVNTYDNSILYTDAMLDATIKLLRQYDDQFNTALVYLSDHGESLGENGMYLHGTPYVFAPS
QQTHVPFLMWMSADYQRNFGVDRQCLNALAEKDDVSQDNLFHTLLGMLNVQTREYQSRLDILQRCRNA
A
>CORE_REP|Org45_Gene2871#
MKLRKKRHKPMHINDITIIDDSKLKKAITAAALGNAMEWFDFGVYGFVAYALGQVFFPGASPGVQMIA
ALATFSVPFLVRPLGGLFFGAMGDKFGRQKVLSITIIIMAVSTFCIGLIPSYASIGIWAPILLLLAKL
AQGFSVGGEYSGAAIFVAEYSPDRKRGFMGSWLDFGSIAGFVMGAGVVVLISSIVGEANFLDWGWRIP
FFIAAPLGLIGLYLRHALEETPAFQQHVDKMEKEDRNAIENPPKTSFKEIAAKHWKSLLVCVGIVIST
NVTYYMLLTYMPSYLSHNLHYSEDHGVLIIIAIMIGMLFVQPVIGLTSDRIGRKPFIIGGSIGLLALA
IPCFILINSNVIGLIFVGLLVLAVLLNSFTGVMASILPAMFPTHIRYSALAISFNISVLIAGATPTAA
AWLVEATGNLYMPAYYLMVVAVIGLITGLYMKETANKPLRGATPAASDRSEAKELLQETYDNIEQKVE
DINAQIAELEKKKQILIDQHPKLD
>CORE_REP|Org7_Gene3966#
MTNNASPAPIAHRPLILIACMLAMFMSAIEATIVATAMPTIIGDLGGFSLLGWVFAVYLLSQAITIPI
YGRLADLYGRKRVFFFGATLFLLGSVLCGFAPDMYWLIGFRLLQGLGAGAIMPIASTIIGDIYSATER
PKVMGYLSSVWGVSAIIGPLLGAFIVQHLPWALVFWVNLPIGLLAMFFLWRYLPAHQPLRQHALDLAG
TAWLTLFVSALLLALLQMESLGWWVVPLFALAAAALALLVRQERRAVEPLFPLALWQSRVIVAGNIGG
LVIGAAMMGISAFLPTFIQGVMGGSPLEAGTTLALMSIGWPLASTLSGRLMLMTSYRATALLGALLLV
AGGLILLLLQPEGGLLWGRVAAFMVGAGMGLCNTTFLVSVQNAAHYSIRGIATACTVFTRMVGSAIGT
AILGATLNLNLQWRLPEIDDPVQRLMEPAVRQSMGSEALAQLTQQVAASLHWVFLVSALVSLLALAAA
MLIPARCRPQGEEEEAEQA
>CORE_REP|Org17_Gene3814#
MITHDDSRWSDLFSGKNAASAIALSLGVALHAINILVATTILPSVVQDIGGLDLYAWNTTLFVVASIL
GSALSARLLSGYGARNAYLVASLFFIAGAGLCALAPSMPVMLVGRTVQGFGGGLIFALSYAMINLVFE

QRLWPRAMALISAMWGIATLVGPAVGGIFAELHAWRWAFGILLPIMALYAAFTFLILPKGQAQQAAAP
LPTAQLLLLTVAVLVVSAGSLAHSVWINLAGIALSLALMAWLMKREARSRTRLLPHGALRRGSSLAAL
YITVSLLVIGMTSEIFVPYFLQLLHGQSPLISGYIAATMAAGWTLSEILSSGWRGAGIRRAIVSGPLF
VLVGLLALAILMPTPSGGHWQALTPIVIALSLVGFGIGFGWPHLLTRILQVAPEADKDIAGASITTVQ
LFATAFGAALAGMIANLAGLNDPGGAAGAAGAASAARWLFLAFALAPLLAVFSAWRCAAIAPPAAETG
NFVPNPSSREC
>CORE_REP|Org42_Gene1651#
MFNKNKKPFSLRARFLMATAGVILALSLSYGLVAVVGYIVSFDKTAFRLLRGESNLFFSLAQWKDNKL
TIAIPPDIDLNFPTLVFIYDDKGNLLWSQRKVPELEKLINKEWLEESGFYEIDTDTRVSSEVLGDNPK
AQDQLKNYDDTDQNALTHSVAVNTYAATPRLPALTIVVVDSIPQELQRSDVVWEWFSYVLLANLLLVV
PLLWLAAYWSLRPIKALVNQVGELENGERDQLDENPPSELRGLVRNLNILVRNERQRYTKYRTTLSDL
THSLKTPLAVLQSTLRSLRSGKQTTIEEAEPIMLDQIGRISQQIGYYLHRASINSGQTVLTREIHSVP
ALLDSLVVALNKVYQRKGVVITLDISPEVTFMGEKNDFMEVMGNVLENACKYCLEFVEITSLHSEKNL
TIVIDDDGPGIPESKRQLIFQRGQRVDTLRPGQGLGLSVAAEIIEQYDGEIVISDSPLGGARMQVTFA
RQHDTHHNE
>CORE_REP|Org43_Gene2089#
MISLKKWRLFPRSLRQLVLLAFLLVLLPLLVLAYQAYQSLDHLSAQAADINRTTLVDARRSEAMTSVA
LEMERSYRQYCVLVEPTLQKLYQNQRKQYSQMLDAHAPILPDERYYQTLRQLLTQLAAIKCHNSGPDQ
EASALLESFSRSNAEMVQATRAVVFSRGQQLQQAIAERGQFFGWQALLLFLVSVLLVVLFTRMIIGPV
KAVERMINRLGEGRALGSTASFKGPRELRSLAQRIIWLSERLAWLESQRHEFLRHISHELKTPLASMR
EGTELLADEVAGPLTSDQKEVVTILDNSSRHLQQLIEQLLDYNRKLADGPAEHENVELREMVDLVVAA
HSLPARAKMISTEIALEAEICWAEPTLLMRVLDNLYSNAVHYGKESGNIWIRSRQVGQRVQIDVANTG
TPIPEAERAMIFEPFFQGSHQRKGAVKGSGLGLSIAQDCIRRMRGELQLATVAGADVCFRIELPLTAE
NE
>CORE_REP|Org11_Gene3175#
MTQSARSMAGLPWIAAMAFFMQALDATILNTALPAIAQSLGRSPLAMQSAVISYTLTVAMLIPVSGWL
ADRFGTRRVFIFAVTLFTLGSLLCALSPTLSALVASRVLQGIGGAMMMPVARLALLRAYPRSELLPVL
NFVTMPGLVGPILGPLLGGWLVTYATWHWIFLINIPIGLLGIFYARKYMPDFTTPKRRFDFLGFMLFG
LSLVLISTGLELFGERVLASYVSLGILLSGFVMLFGYITHARRHPQPLIGLDLFKTRTFSVGIAGNVA
SRLGTGCVPFLMPLMLQVGFGYTAIVAGCMMAPTAIGSLMAKSTVTQVLRWFGYRKTLVGITVIIGVL
IAQFALQSPGMPLWLMILPLFVLGMAMSTQFTAMNTISLADLNDANASAGNSVLAVTQQLSISFGVAI
SAAVLRFYESLSLGTMIDHFHYTFITMGIVTVASALVFMLLRRKDGRNLISGQESKKEAKAAS
>CORE_REP|Org25_Gene687#
MINSLTARIFAIFWFTLALVLMLVLMVPKLDSRQMTSLLDSEQRQGLMLEQHVEAELQNDPANDLMWW
RRLFRAIDKWAPPGQRLLLVTSEGRVIGAQRNEMQIVRNFIGQSDNSDHPKKKKYGRVELVGPFAVRD
GEDNYQLYLIRPANSPQSDFINLMFDRPLLLLIVTMLISAPLLLWLAWSLAKPARKLKNAADDVARGN
LKQHPELEAGPQEFLATGASFNQMVSALERMMNAQQRLISDISHELRTPLTRLQLATALMRRRHGEGH
ELARIETEAQRLDSMINDLLALSRGQQKGELAREQLKANELWADVLDNARFEAEQMGKQLEIAAPPGP
WTLFGNASALDSALENIVRNALRYSHTRIAVAFSADNQGVTIQVDDDGPGVSAEDREQIFRPFYRTDE
ARDRESGGTGLGLAIVEAAVNQHRGWVKAEDSPLGGLRLVLWLPLHHQRLSSKTEQ
>CORE_REP|Org14_Gene3002#
MSYRSKVAIVYLLGFFVDLINMFIANVAYPAIGQAMRASVSQLAWVSNGYILGLTLVIPLSAWLAQRI
GGRRVFLLSLALFMLATFGAGNADSIGALIGWRTLQGMGGGLLIPIGQTLTYQLYRSHERAGLSAAIM
LVGLLAPALSPALGGWLVDRLDWRWVFFANLPLAALALALAALWLRAETSATAVRKPLDGKGLLSACA
ALTLLLLGLTRLSEAGHQASGAALLAAGLLVLAYYLRHSLRTPQPLLNLRLVGDPLLRNAMGVYLCIP
GLFIGVSLVAMLYLQNQLGMPAAQVGGLMLPWALASFLAITLTGKTFNRLGPRPLLIAGCLLQGAGML
TLAQIDQAGQHAWQIAAFALMGFGGSLCSSTAQSSAFLQIPDAQLADASALWNINRQLSFCLGVALLS
LLLNLLLTGLPPAAAYRTCFILAGASVFIPLLLCLRLANRAIVRQLNAQQDAL
>CORE_REP|Org10_Gene2637#
MSETTLAPSQTADAALAADERLATKEGRSQFWRATFSCWLGTAMEYVDFALYGLAAGMVFGDVFFPEA
TPLVALLASFATYSVGFVARPIGALVFGWIGDRKGRRVVLITTVALMGLSTTLIGLIPSYAQIGVWAP
ACLVILRFAQGFGAGAELSGGAVMLAEYAPAKRRGLVASIIAIGSNSGTLLASLVWLLVLQLDKEDLM
SWGWRIPFLASILIAGAALYLRRHVRETPVFERELQQNHQRMLDAAQAAPDTRSYLQRTKAFWVMLGL
RIGENGPSYLCQGFIVGYVAKVLMVDKSVPALAVLIASLCGFLVIPLAGWLSDRFGRRITYRWFCLLL

VLYAFPAFWLLDSREPAIVISVIVVGMCIASLGIFGVQAAYGVELFGVKNRYSKMAFAKELGSILSGG
TAPLIATALLSGFGHWWPVACYFVVMAAIGLITTFFAPETRGRDLNLPQDAA
>CORE_REP|Org7_Gene2401#
MRRLRFSPRSSFARTLLLIVTLLFVSLVTTYLVVLNFAILPSLQQFNKVLAYEVRMLMTDRLQLEDGT
LLEVPPAFRREIYRELGISLYTNSAAEESGLRWAQHYQFLSQQMAQQLGGPTDVRVEVNKNSPVVWLK
TWLQPDIWVRVPLTEIHQGDFSPLFRYTLAIMLLAIGGAWLFIRIQNRPLVELEHAALQVGKGIIPPP
LREYGASEVRSVTRAFNQMASGVKQLADDRTLLMAGVSHDLRTPLTRIRLATEMMSAEDGYLAESINK
DIEECNAIIEQFIDYLRTGQEMPTESSDLNAILGEVVAAESGYERVIETALSPGELMMNVHPLSIKRA
AVNMVVNAARYGNGWIKVSSGRELQRGWFQVEDDGPGIKPDELKHLLQPFVRGDSARSTSGTGLGLAI
VQRIIDAHDGELDIGTSERGGLLIRAYIPLPMEKKESTNGHQTARETA
>CORE_REP|Org12_Gene4291#
MKRLSLRLRLILIFSLLALLTWCTASVVAWVMSRNTINEVFDTQQMLFAKRLATANLGDLLADESARS
LPKTKKLVHHGKRGEQDDDALAFAIFDRDGKMLLNDGENGADFLFDGEREGFTDGERKGDDDSWRLVW
LTSPDGRYRIVVGQEWDYRRDMALGMVTGQLVPWLATLPVLMLLIALMVGRELRPLRAVAAGLRRRAP
DDATPLDARQVPTEVRPLVDALNALFARINALLVRERRFTSDAAHELRSPLAALRVQTEVVQLAGDDA
PMREHALDNLTVGIDRATRLVDQLLTLSRLDSLLDLAELAPIDWNDLVTMTLAEQDRQAHAAGVTLRY
EHRGTPPPRQGETLLLSLLLRNLLDNAVRYTPQGGVVTVTLSERSLTVEDDGPGVTAEHLARLGERFY
RPPGQEQTGSGLGLSIVQRIAGLHGLQISFANRSAGGFVARLAL
>CORE_REP|Org11_Gene3941#
MRGRLFWKILLGFWLTFLIMTQALWVAFSLYGDRYVPPENAMARRVIGLQLTSAATQLRSGGMPALEA
LMRDWPEDDRRLLSVTPMTQPPPPAPEEPVFEGRRMPKAISAWVQTGEGQGYWLSYDVRGLREEYRPE
RRSHFFNIPAPMLWVGGLGGLLFSAVLAWNLTRPMRQLRGGLDRVAQGDLSVRLFPNMRRRHDELSDV
ARDFDTMAERLELLVSAREQLLHDVSHELRSPLARLQLAIGLARQNAGNVEASLKRIEHESGRLDKMI
GELLALSRTEHSSLPDEEYFDLYGLVDAVVSDARYEAQVPGVDIVLQAESDVEYTVKGNAELMRRAVD
NIVRNALRFSSHGQRVTVTVALSRVDNQFQIAVSDQGPGVEEAKLSSIFDPFVRVKSAQSGKGYGLGL
AITRKVVLAHGGQVEARNGDREGLVITLRIPRWSS
>CORE_REP|Org10_Gene3824#
MNQQTLDARRSRQALLAGSVGNFIEWYEFGVYGFLATVIAANFFTLQGENEVTSLILTYAAFALAFFC
RPIGAVIFGRIGDRIGRRPTLIAVLLLMTLATALIGVMPTYASIGVAAPLLLTLLRMFQGLFAGGEFG
GAVSLMTEFAPKGKRGLFGAWQSLTVALGLLAGAGLVALLAALLSVQQLHDWGWRIPFLLALPMGAVA
LWLRLKLEETPTFTQAQQAAEHSAAPQEASLGGVAKTILIGIGRMMGWSAAGYTFLVVMPSYLQTSLH
ATFQQALVATVLANVGFALTILPAGIISDKLGRKTVMLTAVAAVILFTFPLLHLLQDAQSSLWAKGLV
VMIAGAVVGLLAGPGPAMLAEMFPTRVRYTGLGLAYSLSNAVFSGSAGLIITGLIKQTGNIDIPAYYV
VATSVVSLFALMTLRRDDHLRSLNER
>CORE_REP|Org26_Gene3371#
MARPSFFLDFSLLRSNAHFRAIFCARMLSVFSLGMLAVGVPIQIQAMTGSTLQVGVAVALDGVGMFIG
LMLGGVLADRYDRRKLILFARGTCGLGFVALSLNAFAPAPSLLALYLLAAWDGFFGALGMTALMAVIP
LLVGRENLAAAGALSMVTVRIGAILAPALGGIIIVFGGVGLAFAVAAAGTLGTLVPLVRLPTLLPQQQ
EPEHPLRALASGFQFVWRNKVVGSVVLLGMLMSIVGAVRVLFPALAQDAYHVGASSIGLMYSAVPLGA
MLGALTSGWVGRFSRPGVLILVAAIVAFTAIASLGLFSHLAPALLALVCYGYANAIASLLQFMLIQSN
TPDHLLGRVNSLGTAQDVTGDSIGALGLGVLGRVFTPLMSVLSFGAFAAVLGVLVAFSVRTLRQCRPA
DALVEHDEPAPATSAAADN
>CORE_REP|Org34_Gene4576#
MSENTASQIAAPQNAQASGRTILLFLALALMSALLNSSAPTPLYPLYQQQLTLSSVSLTVIYGAYAAG
VLISLFGVGNLAGKVKDLRSMIVPALLVVLSGALLFAQADTFAMMLMARLLAGVGTGALTGAANIALV
RFGPRDGGKNAALIATLSFTTGLALGPIFSGIALQTGFHPTTLPFVFIMVMAAVAALGVMFSWPRGVV
TAPSHVTSAETEKSSLLDGLRATGGKFFVCAGALFICWALAASILAIGPGVAETLLGLHARGVFGYAI
AVYLLIAGISQILSRRVNARHSLLFGCLAQVLAAVVFTMAIQWHSLGLAAVGLVVAGYAYGAIFVGSA
TLVNLISPPTSHARLLSLFYVIAYIANWVPILLGVVVDRVDLNQATHLLFLGSTVVCLLLAWKTSRVG
FLANYFVMNIYFYN
>CORE_REP|Org40_Gene2806#
MKTTLPPAARLGRQALLFPLCLVLFEFATYIGNDMIQPGMLAVVADFNAGEEWVPTSMTAYLAGGIFL
QWLLGPLSDRRGRRPVMLAGVAFFIVSCLAILLVTTIEQFIAMRFLQGIGLCFIGAVGYATIQESFEE
SVCIKITALMANVALIAPLLGPLAGAALIHVAPWQSMFVLFAALAAIAFYGLWKAMPETATLQGEAFS

AANLWRDYRQVLGNRRFLCGALAIGFASLPLLAWIAQSPVILISGESLSTLDYGLLQIPVFGALILGN
LTLARLTGKNSVERLIKLGAGPMLLGLLIAALATQFSSHAYLWMTAGLSLYAFGIGLANAGLYRLTLF
SSNVSKGTVSATMGMLSMMVFTVGIELAKVAYVWGGSGLFNLFNLISGLCWLTLVALFLGKRRNGDPT
PQPTGAV
>CORE_REP|Org1_Gene4681#
MQSISRIILAFYSACGPEEHSVKRSVSVVIGGILLPLAAQAAETTPHFPTMTPPAIDAASYVLMDYTT
GQVLAAGNADERRNPASLTKLMTGLVIDHALDQHKIGLDDVVTVGKDAWAQGNPVFKGSSLMFLKPGD
RVTVRDLSRGIIIDSGNDACVAMADYVAGSQANFVKLMNEKSAQLGLQNTHFETVHGLDAPGQFTTAG
DLVVIARAIIMSEPAEYHMYSEKSLTWNGITQQNRNGLLWDKTLHVDGLKTGHTASAGFNIIASATEG
DRRLIAVVMGGKSSKGREEQARKLLSWGLRDFTTVHLFSAGQSLGEEPVWYGENHRLPVGSVQEQSLS
LPKNEADKLKAQYVINTARLEAPIGKGQTVGEIRISDNGQVVKTLPLVALQAVPQGGVFSRLVDYVKL
RL
>CORE_REP|Org24_Gene4604#
MNGHSGDVMMKKNLSTSLKKLTFSVGILLLAAPAVHAAEPPAPPQVDAKAYILMDYNSGKVLTEGNAD
TRLDPASLTKIMSSYVIGQAIKAGKIKPEDLVTVGKDAWAPGNPALRGSSLMFIKPGDQVPVLELNKG
IVIQSGNDASIALADYVAGSQDSFVGLMNNYAKSLGLQNTHFLTVHGLDAEGQYSTARDMALLSQALI
RDVPDEYALHKEKEFTFNKIRQINRNRLLWSSNLNVDGIKTGYTSGAGHNLVASATDGPMRLISVVLG
APSDRVRFSESEKLLTWGFRFYETATPIKADKPFVTQKVWFGDVSEVPLGVAKDASVTIPKGQMKNLK
ASYKLTQPTLEAPLAKNQVVGTIDFQLDGKTIEQHPLVVMQEVKEGNFFSRIWDMVMMKLSQWFGGIF
G
>CORE_REP|Org49_Gene3307#
MSDKLLDPPCAALGRLPAPLVLLLAAASAFSVANVYYAQPLLDAIAHDFSISLAAVGMVITVTQLGCA
LALLLVVPLGDRLNRHWLLAGQQLGLIGALLLVGWAHSAPWLLAGMLLVGLLGTAMTQGLIAFAAALA
APQERGRVVGAAQGGVVLGLLLARTLSGALADVGGWRTVYFFSAGVTLVLLPILSRLLPAPRTAPSTL
SYPALLRSMLTLLLHDRTLQIRGMLALLMFGAFSLFWSSLVLPLSQAPFNFTHAAVGAFGLVGAVGAL
AAVRAGHLADRGLGQAASGVCLLLLTLAWLPLGLLGSGLVWLVAGIVLLDLAGQAIHVLNQSMIFSAH
PQSHSRLVGCYMLFYAVGSGLGAFAGTHMYAWAGWSGVCWLGAGVSLSALLFWRLTLRGMPPSAAAVE
Q
>CORE_REP|Org43_Gene3862#
MKGFPSLINVLLASSLVLTIGRGVTLPFITIYLTEHFQLLPKSVGVILGVSFTLGIIASLYGGYLVDK
FSKNRLILLSIVLFALSFFAIPWIPRPGGVIVVLAILHTCYSVLSITIKACFADGLPVEQRIKAFSIN
YTLVNVGWAIGSALGVLVAGLSPLLPFYLSGGLALATVAALSLRLRGGEQRPAPSAAAPAALANFRQT
LAILRCDRRLIYFTLGSTLGAVVFGQFTGYLSQYLITVSSAEFAYKIIGLVMIVNAGIVIALQYLLSR
GMRQENMLRWLALGTLFFIVGLLGFMAAGQAVWLWLAAMAVFTLGEIIVIPVEYMFIDFIAPPHLKGS
YYGVQNLSALGGAINPVLCGVLLSYAAPPLMFVMLIAAALLSLLFFFLGHRLAEHAAAAAEDVR
>CORE_REP|Org4_Gene5062#
MENLGMPSLKLTPRRLTLIAVIVFIAVAIAIALTLYWQRPPQQDYVTAPARLGDIENAVLATGRLDAV
ERVNVGARVSGEVKSLKVKLGDRVTKGQPIADIDDLQQRNDLRNAEAALNVIKAELQAKQAQLKQAES
RFKRQRRMLNDEASSREDFETAEATLATTRAELLSLNARLVQAQIEVDKKKIDLGYTRVVAPMDGIVI
AVVTQQGQTVNSTQSAPTIVKLARLDMMTIKAQISEADITRISPGQKAYFTIFSDPDKRYDATLRTIE
LAPESVMKDDSLAGTSSASGSGTSNASVYYNALLDVPNPENRLRIAMTAQVSLLLGEAKNALLVPIQA
VHKTEGKVQQVQVLTQDQRLETREVTTGITNNVDIQILSGLKAGETVVLSQPAAKSAEDGIFL
>CORE_REP|Org15_Gene4578#
MSALHADGGAKAWLATFAVGLSTFTVVTAEMLPVGLLTPIVSTLNASIGRAGLLISLPALFAALFAPL
VVLGARRTDRRNLLAGFLLLLIAANLLAAAATSLALLFAARILLGFCIGGIWAIAGGLAERLVPPASV
GLALSIIFGGVAAASVFGVPLGVFLGEALGWRMAFLAVAVLAALTLLLLVCVLPPLPVTQAIGWRSFT
ALRANRRLLTGLLLTFLLVAGHFMAYTFVRPLLQTVAGIESRWVGPLLFAYGVAGIFGNFIAGQAAAK
RLRRTLALIALGLALAVLLLPLLGHAPLSGGAFLLLWGIAYGGVSVALMAWMLKAAPDAVEVASSLYI
ALFNLAISCGSLAGGLVVDAGGLTINGALSGLVLLLALAILMGTRRQRPKTAAKADSPPG
>CORE_REP|Org40_Gene547#
MLEPITSEHTVSENNSLTTPSVNVEQPAAAKINLLDLNRQQMREFFAEMGEKPFRADQVMKWIYHYCC
DDFEQMTDINKVLRGKLQRVAEIRAPEVAEEQRSADGTIKWAIKVGDQQVETVYIPEADRATLCVSSQ
VGCALECKFCSTAQQGFNRNLRVSEIIGQVWRAAKIIGALKVTGQRPITNVVMMGMGEPLLNLNNVVP
AMEIMLDDFGFGLSKRRVTLSTSGVVPALDKLGDMIDVALAISLHAPNDTIRDEIVPINRKYNIETFL

SAVRRYLEKSNANQGRVTVEYVMLDHINDSTDDAHQLAEVLKDTPCKINLIPWNPFPGAPYGRSSNSR
VDRFSKVLMEYGFTTIVRKTRGDDIDAACGQLAGEVIDRTKRTLKKKMAGEPINVRAV
>CORE_REP|Org5_Gene782#
MQQNRTSHLGLIFILGLLSMLMPLAIDMYLPSMPVIAAQFGVESGSVQMTLSAYMLGFAFGQLFYGPM
SDSIGRKPVILWGTLIFAIAGCACAMAQSIDQLIGLRFLHGLAAAAASVVINALMRDMFTKDEFSRMM
SFVILVMTIAPLLAPMIGGALLLWFSWHAIFWTMGAAALIGSLLVALFIKETLPKERRQRFHLRTTLG
NFGSLFRHKRVLSYMLASAFSFAGMFSFLSAGPFVYIELNHVSPQHFGYYFALNIVFLFLTTLINSRN
VRRFGAVKMFKLGLLVQLAMGLWLLAVSAVGLGFWALVIGVAVYLGCIAMISSNAMAVILDDFPHMAG
TASSLAGTLRFSIGALVGAVLSMAPGKSAWPMVTSMALCSIVAVLFYVYASRPRDRAA
>CORE_REP|Org16_Gene1641#
MEHAPVSRSTAWLRVVILAVSAFIFNTTEFIPVGLLSDIAASFSMQTEQVGLIITIYAWIVAAASLAC
MLLTSKIERRKLLIGVFMLFIASHVLTAVAWDFTTLVISRAGVALAHSVFWSITASLAIRVAPPGKKA
QALSLLAGGTALAMVLGLPLGRVVGQLLGWRMTFIGIAVCATLALVLLWRLLPVLKSEHSGSLASVPL
LFKRPALVALYMLTIIVVTAHFTAYSYIEPFIQTVAGLSENFTTLMLLLFGAAGIVGSLLFSRYSERF
PSGFFIGAIVLLALSLLLLLPAAGSESHLTVLCIFWGMAIMAIGLSMQAKVLSLAPDATDVAMAIFSG
LYNFGIGSGALLGNQVSLHLGMGNIGFVAAPLALIALGWCLLSVYRSERLQQHHSR
>CORE_REP|Org34_Gene3464#
MTKHLARQRLVYAVVLGLLAALGPLCTDLYLPALPEMAGELNTSTAAAQLSLTTGLLGLGVGQLIFGP
YSDKLGRMRPLLLSLILLLGASLWCALAPTIDQLLIARLLQGIAGAGGAVISRAIARDLYAGHELTRF
FALLMLVNGLAPIVAPVLGGVMLQVMNWRGIFGVLAAIAVLLFSLSALKLRESLPVERRSQGGILAML
MSLGGLLTQRYFMGLCLTQGFVMAGMFAYIGASPFVLQQIYGLSPQMFSLCFAINGVGLIIAAQLASR
LSARWGERRVLRGGLTLAAVASLLLLLAAALHAPLVVLLVPLFFSVAVIGIVGPTASSLAMQSQGDKA
GSASALIGVCMFALGACAVPLTGLGGTSGLSMALTIVGCYAIAILLFGLLARRNDA
>CORE_REP|Org38_Gene4435#
MPNQPNSSFNAGGRTRAFALGQRLSGVALLAALLAGCDNSVAHNAPPPPPVVSAASVVVKPISQWDAF
NGRVEAVQSVQLRPRVSGYIERVNYTEGDEVKKGQVLFIIDDRTYRAAREQAQAELVRARNQAALARS
ESSRTEKLIGTQAISQEVWEQRRSSAAQAQSNVLAAQAQLDMAQLNLDFTRVTAPIDGRASRAMITAG
NLVTAGDSASVLTTLVSLDKVYVYFDVDEATFLRYQQQGRHDVRLPVKVGLVGEDGTPHQGLVDFTDN
QLNAGTGTIRMRALLDNRDRRFTPGLFARVQMPGSAEFNAMLIDDKAVMTDQNRKFVYIVDKDGKAQR
RDIDVGRMAEGLRIVQKGLVNGDRVIVDGMQKVFMPGMPVDAKNVAMTTTASALN
>CORE_REP|Org35_Gene1276#
MNKNRGLTPLAAVLMLSGSLVLTGCNDKETQQQGAQQQAPEVGVVTLKAEPLNITTDLPGRTAAYRIA
EVRPQVSGIILKRNFVEGSDIKAGTSLYQIDPATYQASYDSAKGDLAKAQASASIARVTVNRYKPLLG
TSYISKQDYDNAVSTLQQADAAVVAAKAAVETARINLAYTKVTSPISGRIGKSAVTEGALVSNGQATA
LSTVQQLDPMYVDVTQSSTDFLRLKQELASGALKQENGKAKVKLMLENGTEYAQEGTLEFSDVTVDET
TGSITIRALFPNPNDTLLPGMFVRARLDEGVRSDALLVPQQGVTRNPRGDATALVVGADNKVELRTLK
ADQAIGDKWLVTDGLKAGDRVIVTGLMKVHPGAQVKVQEVDTQAQKQPQSEAQKS
>CORE_REP|Org39_Gene141#
MRKLENFHLLVMLILLVAVGQMAQTIYVPVIADIAHDLSVRTGAVQRVMAAYLLTYGFSQLIYGPISD
RIGRRPVILTGMMIFLVGALGALLSTNLTMLVAASAIQGMGTGVAGVMARTMPRDLYAGTALRYANSL
LNMGILVSPLLAPVIGGALAMVFGWRACYAFLLALCACVAFAMFRWLPETRPVQTEKRRMLASFRQLL
GDSTFSCYLVMLIGALAGIAVFEASCGVLMGGVLGLSGLTVSILFILPIPAAFFGAWYAGRDGKTFHT
LMWHSVISCLLAGAMMWIPGWFGVMNIWTLIVPAALFFFGAGMLFPLATTGAMEPFPYLAGAAGALVG
GMQNMGSGLATWLSAMLPQTGQFSLGLLMFAMALLILLCWWPLSNRMQHQGHTA
>CORE_REP|Org28_Gene2530#
MLNNKDKPASSPWPAIFSLTVACFVMVTTEFLPIGLLTNIAPSLGVSTGTAGLMVTMPGIVAAVAAPA
LSLISGRLDRRLLMLGLSLLLIVSNLVAALAVNFPMMLLGRVLLGICVGGFWSFAANYGRHLVPEANQ
GRATALILSGISVGAVCGVPAGALIGDLFGWRAAFFGGAALAVGVLLAQLRLLTSVPPSRPVTPRDLV
LPLRLPMARIGLIAIVLLFIGHFAAYTYLRPLLQQVFVLSPSAISLQLLAYGAIGLLGTFLGERLGEY
SLRATFILIAAMLAAILIVSPLLSGLGGATLMVMVWGLAFGAVPVCATNWMFAAVPQAPEAGQALLVC
VVQIALASGALLGGEVVDWQGVSSAMLFGGALILSAALVFGLSLRSGAIGAKQC
>CORE_REP|Org46_Gene1185#
MTAENNLQLNRRILSVVMFTFVCYLTIGLPLAVLPGFVHDHLGYNSVLAGLIISAQYFATLFSRPHAG
RYADQLGPKKVVLFGLACCGASGLFYALAFGVDGYPWLSLLLLCVGRVFLGVGESFASTGSTLWGIGR

VGAMHTARVISWNGVATYGAMAAGAPLGVYLNQQWGLAGVAALIVLAVAVALLLASGKPDVSIAAGQR
IAFRAVFGRIWAYGLGLAMGTVGFGVIATFITLYYADKGWSGAAFSLTLFSCAFVGIRLIFSNVINRH
GGLKVTLASFLVEIVGLLLIWQAGEPWMVQTGALLAGAGFSLVFPALGVEAVKQVPPQNQGTALGTYS
AFLDLALGITGPLAGLLIGQAGVPSIYLAAALLVALGVLLTLRLLQRSRAIQE
>CORE_REP|Org27_Gene3222#
MKVNYPLLALAVGAFGIGTTEFSPMGLLPTIAKGVDVSIPMAGMLISAYAVGVMVGAPLMTLLLSHRA
RRSALIFLMAIFTLGNVLSAIAPDYTTLMLSRIITSLNHGAFFGLGSVVAASVVPKEKQASAVATMFM
GLTIANIGGVPAATWLGETIGWRMSFLATAGLGVIAMLGLWFSLPKGSAGARPDVKRELSVLVRPQVL
TALLTTVLGAGAMFTLYTYISPVLQHITEATPLFVTTMLVLIGVGFSIGNYLGGKFADRSESATLKGF
LLLLVAIMLLIPLLARSDIGAAVSMMIWGAATFAVVPPLQMRVMRVASEAPGLSSSVNIGAFNLGNAL
GAAAGGAVVSAGLGYSFVPVMGAIIAGLALLLVLFTSRTAAKVVYANG
>CORE_REP|Org30_Gene3769#
MSVSHIIANRQTWFGHGSIRQLPPLLLADPQPTLLFSCRSFLNGPVYAGLRESLAPLFIGTEIVSHEA
SPQEIDAWVARWRGQARRVVAIGGGSVLDAAKAFSALVEHPLPTLRYMEKVGDSKISGATLPLIAIPT
TAGTGSEVTQNAVITDTQVSKVKASLRHNNFVPHTAILDPQLLAGAPDKVLAYCAIDAFTHLFEAYLS
KTAGAMTRDMSLSGIRHFLAAWPALNRSDAAREAIMQASYLGGLTLSATGLGVIHGIAGEIGALRDYH
HGQVCGRLLLPFLALLENSEQPQQRALMAELAARLYPHWQGSPESYLTDFITRHAIAPFWQDDLPISG
QELAVALDKSNSKNSWIDYAPAQRQRMIEEAFRVE
>CORE_REP|Org6_Gene4336#
MLLRHIRYFLAVAEQGNFTRAAEALHVSQPTLSQQIKQLEDALGAPLFDRSGRRVQLTDAGEAWMRYA
RLALQDLDAGARAIHDVATLARGHLRLAMTPTFTAYLVGPAIDAFYRRYPGITLSIEEMAQERIEVLL
AQDRLDLGIAFEMAQSAEVEATPLFSETLELMVGADHPLAARRRPLTLAEWRHLPLALLSGDFATRQF
IDRYCTQLGFRPLVAVEANALGAIVEIVRRGQLATLLPAAIARENRQLKKVALVNAMPARQAVLLQRQ
GAYRSAAAQAFIAVLQQQGVTPTPPALHHPQQMHQSETEANQRADDDEALAAAGIAQGERAGDAAQQV
DKGDDKQRGPQKRQRRDQARAG
>CORE_REP|Org36_Gene2461#
MKAATAVIDRRALRHNLQQVRRQAPQSRLIAVVKANAYGHGLLETAHTLQDADCYGVARIGEALMLRS
GGIVKPILLLEGFFSAEDLPVLVANNIETAVHSIEQLEALEQAELARPVPVWMKLDTGMHRLGVRPEH
AEAFYQRLCACRNVAQPVNIMSHFSRADEPESDTTLKQIACFEQFARGKPGQRSVAASGGTLLWPDAH
NEWVRPGIILYGVSPLDNGSGAEHGLQPAMTLKSSLIAVREHKAGEAVGYGGTWVSPRDTRLGVVAMG
YGDGYPRSAPTGTPILINGREVPIVGRVSMDMISVDLGPAAADKVGDEAVLWGPALPVERIAVCTGIS
AYELITKLTQRVAMEYIGD
>CORE_REP|Org5_Gene1945#
MPRPITATMHLGAIENNLQVVRRFAPGAKVWAVVKANAYGHGIKHVWRSMAQTDGFAMLDLAEAVLLR
ESGWQGPILLLEGFFQPQDLALLDRYRLTTAVHSDWQLAAIADATLSAPLNVYLKVNSGMNRLGFAPE
RLHEVWRRAQAIANIGELTLMSHFATADGPEGVTQQMATIEAAAADIPLPRCLANSAATLWHSSTHGS
WVRPGIILYGASPSGCWNDVAATGLQPAMTLSSEIIGIQQLKSGDRVGYGGRYSAAGAQRIGVVACGY
ADGYPRHAPTGTPVWVDGVLTRTLGTVSMDMLAVDLTPCPQVELGAEVELWGKRLPVDEVATAAGTLG
YELLSALAARVPVAIEA
>CORE_REP|Org13_Gene3304#
MTSKPEQIRQRVKRGELIAGEDLHGLSFAGMDLAGGMFNELNLSGVNFSDCDLRDSVFSDCRLEHAQF
ARANLKQTAFNQCAMPGGRFCESHIELTMFNDCRLEQSDFSRLPLNQSHWMSCQLAGANFSATQHDRT
TFYESPLDGAALNHARLSLVTFFRLNLCKTEFEGVDFDRVTFFECDHRGKSYAGQRLIACQFTDNQLD
DVDFSQATLRQSNFKGASLRRANLTGVQAQQSLWLEANLTQAQCRSGQFDQAIFSEATLDAANFSQAR
LYQCVFQRSRAARCDFSDSDLTYADFCYADIGAADFRRARFMRTRMHRAHQQQTRWGDRSGILERDEE
LYAAETWSAQRQSRI
>CORE_REP|Org7_Gene1147#
MISMRRRLLLMLALILLVTQLISAFWLWHESQEQISFLVDETLSAKVRSERVDTEIAEAIASLLAPSL
IMMIVTLLASFWAISWITRPLNQLQQRLEKRSADNLTPLPITSDSQEMVAVTNALNQLFSRLDNTIQQ
ERLFTADAAHELRTPLAGIRLHLELMEKQGVKGSQALIARIDQLMHTVEQLLMLSRAGQDFASGHYQH
FDWVADVIQPLREELDEMTAQRGQTLAWQLPAAAAVNGDPVLLRLLLRNLVENAHRYGPEGGAIQVRL
TPQDRGYLLQVIDDGPGIKEEMVGELTQAFRRMDQRYGGSGLGLNIVIRIVQLHQGRLTLENRRDARG
LNAQCWLPEKALK
>CORE_REP|Org30_Gene945#

MSKIRVLCVDDSALMRQLMTEIVNGHADMEMVATAPDPLVARDLIKKFNPQVLTLDVEMPRMDGLDFL
EKLMRLRPMPVVMVSSLTGKGSEITLRALELGAVDFVTKPQLGIREGMLAYSELIAEKIRTAARARLP
QRSNSPAPAILSHAPLLSSEKLIAIGASTGGTEAIRQVLQPLPATSPALLITQHMPPGFTRSFAERLN
KLCQITVKEAEDGERVLPGHAYIAPGDRHLELARSGANYQVKLHDGPAVNRHRPSVDVLFRSVAQYAG
RNAVGVILTGMGNDGAAGMLEMHRAGAYTLAQNEASCVVFGMPREAIAGGGVSEVVELDRMSQRMLAQ
IAGGQALRI
>CORE_REP|Org41_Gene1020#
MNFQQLKIIRESARCNYNLTEVANTLFTSQSGVSRHIRELEEELGIEIFIRRGKRLLGMTEPGKELLV
VAERILNDANNIRRLADVFSSNDSGQLHIATTHTQARYSLPGVIKEFRALYPRVRVVLNQGSPEEIVS
MLAAGEADIGIASERLMSDESLAAFPYYRWHHTILVPEGHELTRQPQVTLEMLSTLPLITYRQGITGR
AKLDAAFKAAGLTPDIALSAQDSDVIKTYVELGLGVGVLADMSYEKERDRGLVSLNAEHLFEPNTVWL
GLKRSQLQRNYAWRFIQLCNPTLSLTEIKDKVFSSQLDAVIDYQI
>CORE_REP|Org43_Gene4535#
MAMIELRRLRAFVTVVEEGNITRAAERLFIQQPPLTRLLQGLEDELGVKLLQRLPRGVRVTEAGDVLF
EEARALLARAERLREAVQRAARGEQGHIAIGFTSSAALHPFVPNLLRRYRDILPGITTQLEEAGSGEL
MEALLEQRLDAAFVRSPANGIPGLSVEPVLSEPMIVALPLGHRLAQETQQPLPLAELAHEAFILYRRP
AGQGLYDAILAACHRAGFSPRIVQEAPRLPATLSLVGAGLGVSIVPGSMRRLGGDGIVYRTLAAEAQL
SAPLYLALRRSPASPIVERFRQLVLETVGAPDADVTTTARTANKK
>CORE_REP|Org6_Gene518#
MAEYDSEIAMVKEPADIHLRSVDLNLLTVFDAVMQMQNITRAANSLGMSQPAVSNAVARLKVMFNDEL
FVRCGRGIQPTMRARQLFGPVRQALQLVQNELPGSEFEPLTSTRAFSLSLCSPLDLRLGAGIINHVKQ
IAPQLNLQIKSYINNNIERQLRYQDVEFVIGYSRFESAEFRSLAMFDDELVLAVAQAHPRIGEEVTPE
HMLAEQHAAVSLESFGSFSKPFYLDEPMLRAVTQQCTDLYSVLNMVSQTEMVAIAPAWLVRQQTEALK
IKAVPLCGNDNKATCYLSWHESSERDKGHQWMKSVLIEAGNPK
>CORE_REP|Org33_Gene2258#
MDQVQAMRIFTRIVELGSFSRAAERLQLPRATVSNALKRLEQRLGVRLLIRTTRQVQVTSEGSLYYQR
CVQLLGALEEADTLFSHHKLQPSGKVRIDMPHSLARQIVIPALGDFYRRYPDITLALGANDTHVDLLR
EGVDCVLRAWETEDDSLVARRIAQLPQITCASPAYLQASGTPLDIDSLAPHRAVGYFSLASNRDYPLE
FCRGGKVELRELPARLSVSGADAYIAGARAGMGLIQAARYSLAPWLERGELVEVLADTPPPPMPIYIM
YPPGRFLAPRVRVLIDWLIWLFDQQKSGDMAVFPANARKAGK
>CORE_REP|Org41_Gene3376#
MIELKHLRTLQALRNSGSLAGAAAQLHQTQSALSHQFSDLEQRLGFKLFVRKSQPLRFTAQGEILLQL
AEQVLPQIQQALQACHEPHQTTLRIAIECHSCIQWLTPALDNFRRRFPQVVMDFTSGVTFDPQPALQQ
GELDLVMTSDILPRSGLHYSPMFDFEVRLVLAPDHPLAGRPHIEPEDLSDETLLIYPVQRQRLDIWRH
FLQPAGVSPALKNVDNTLLLIQMVSARMGIAALPHWVVESFEQQGLVVTKTLGDGLWSRLYAAVRDGE
QRQAVTEAFIRSARQHACDHLPFVRDAARPGATCAKALAAGV
>CORE_REP|Org5_Gene3088#
MFWKRCLLGAALAVMSLQAGAAAPQAKTPTPGVYRIMLGSFEVTALSDGIIRLPADKLLLNTTPQQIA
AGLAERHQSLPVVTSVNAYLINTGDKLVMIDSGAGQLLGDGLGKLVDNLRAAGYQPEQVDEIYLTHMH
PDHLGGLTHDGKAVFPNAVVRAASQDADFWLSAERLKQAKAQNKGNFEKAMAAIKPYQAAGHFKPFNG
DGELSPGIAAFAAHGHTPGHSVYQVTSQGKKLLLLGDLIHVAAVQMPHPKVAISFDSDAKAAVAQRLR
VFSDSARQSELVGGAHLSFPGLGYLNRQGEGYSWVPLNYGAL
>CORE_REP|Org28_Gene4384#
MSTNLSYALLPEMAVFVQVVESGSFSAAARKLGTSPSAVSRSVAKLEQALALQLLHRTTRKLRLSESG
EEAFAHCRTLLAAADAVMAIGGRGAVEPEGLVSVSVPKAVGRFVLHPHMPEFLRRYPKVDVRLRLEDR
YMDLIDDRVDLALRITDRPSPGLIGRQLMRIDHLLCATPHYLAQHGTPQHPHALAAHSCIYLGETPSD
AQWKFRRSGKTVTVNVRGRYAANHTGVRLDAVKQHIGIGSLPYFTARQALDDGEIVQVLPEWDFLSSY
HGGLWLLYAPNQYLPPKLRVFIDYLVACLAQEPQLKRLA
>CORE_REP|Org15_Gene1826#
MKNFSIKITRIAITLILVLLGIAAVFKAWVFYTESPWTRDAKFTADVVAIAPDVSGLLTDVPVVDNQL
VKKGQVLFVVDRPRYEQALAEAGADVAYYQTLAAEKRREAGRRVKLGVQAMSQEEIDQSNNSLQTVQH
QLAKAIAARELAQLDLERTTVRAPADGWITNLNVHAGEYITRGSVAVALVKKDSFYILAYLEETKLNG
LNKGDRAEITPLGSNRIMHGTVDSVAAAVNNSSSTVNNKGLASIDSNLEWVRLAQRVPVKILLDAKDQ
QHPYPAGTTATVVIVGKNDRNADSGSPFVRLMHRLREFG

>CORE_REP|Org15_Gene3972#
MKSDLSALPAFVAVAEGGSFAAAAEKLHLTRSAVSKIVSRLEARLGVMLFMRTTRSLSLTDEGALYYE
HCRQALANVQAAENQLDSGKMQVSGRLRVSVPVLFGHLCIAPLLTALANEHPLLTLEISFSDRRIDLV
DEGFDLAVRIGELADSGSLVARRLGEHGMLLCASPDYVRRCGEPSTVEALSRHQAVGYLHAGAVLPWQ
LRGENGELQSFSPPAKMMMDDMQGIVDAISAGAGAGIAWLPEWLVRERLMAGTLVEIMRGESNLSFPV
NVVWPYMPYQPLKVRLAVDKLVAELPAKLALVPPPLSQR
>CORE_REP|Org5_Gene2049#
MFPSKKHSQRATPLTSYQFSRLHTFECVARHLSFALAAQELSITPSAVSHRINLLEKELGFLLFQRFH
RRITLTPEGERMQWALDSSFNTLNQEILDIKNRELTGTLTLYSHPSLVQCLLLPRIGDFIAQHPTIHL
NILTGQEIINLANRGVDLAMYFGKLPSGRHLDEAFMQESMVPICTPQYAAAHSLYDAPENLAHCTLLH
DRYNSGEDEWQTWSQHFALGLDTDSKSMEFDRSDLAVLAATRHLGVAMGRLNLVQDWIKSGELIIPFT
DMTVPCEHCYFTSTISERQWPKILAFKQWIMKIAPLV
>CORE_REP|Org48_Gene2513#
MQVKKRALLGQLSDMDLRLLRVFKAVVDCGGMSAAELELNISLSTISKHIKDLEQRLGLTLCQRGREG
FAVTDEGLLIYQETVNLLAATEAFRRGVDEVHQRMGGQLHVAIFDHTVSNPQAQIGRAIALFSERAPE
VSLQMYVEPINTIERGVIDGQFQVGVIPMHRSAESLSYHSLFSERMFLYCGAQHELFSGPHETLNWDL
LHNYAFAGLGYHSPNMELSLQQHLHRKATGFAQESIATLILSGKYVGFLPDHYAAFFVAQNMMRAIKP
ALFRYHCEYSSVLRRSPVPQRVVKLFHECLLAAHGTA
>CORE_REP|Org17_Gene4464#
MNPPHALPDPGRINFRLLHYFRVVAEEMNFTQAARRLNMSQPPLSKHIKELESQLGVVLFKRTTRSMT
LTPAGRTLLRNVERLLDQADSALHQVQQMGRGEGGHMVVGMVGTSAWGGLIAALRRFSEQSVGATWSL
NELTPSQQITALQKRHIDIGVWREAQQQTLPGLTCQRLARESIAVVLPLDHPLAQQENIPLAALQNDS
FIVLPPHEASLGLYLHNLCLQQGFLPDVAYQVNEPQTLLALVAEGCGITLLPDSYGRIPWPGVRFCSL
QQAPPADLYAVYRTDSVTPVVQAFLATLTPSSSAAR
>CORE_REP|Org47_Gene2528#
MNFRRLKYFVKIVDIGSLTQAAEVLHIAQPALSQQLATLEGELKQQLLIRTKRGVQPTEAGNILYAHA
QTILRQCEQAQSAVNSAGQAMSGQVSLGLASGSTAAQLALPLLQSLRDQQPGILLSLHENGGAALAGQ
VANQTLDMAMVYGAKMPAGLHAIALMREDLYLVATRAVPHPGNSVELLDVARLNLFLPREGDAVRNQL
EEAMALRKLAVNVVGEIESSGALSAAIASGLGATVLPESVARAMIGPAKAWMARINAPTMSVPLSLCM
SGQQALSAPALLVKDLLLSIAGGRSQEKRALALVR
>CORE_REP|Org40_Gene3137#
MPISLPSLDVLKTFVVVAQRLNFTHAARQLHLTQGAVSRQILGLEQRLGYPLFSRQARGLALTPQGAQ
LLAPVQQALGQLDEALTRAAAPPGALRIKCPTCAMRWVLPRIIRLQNERPDMHIELTASVSHGLDFST
EQFDAAVVFGRPPGKKLTAHLLFDEILTPVCTPTFLPPTPRLTDLTDKTLLHPTRDRRDWLRWLKAAG
ADALPSGKAQHFDTLDLAMSAALQGFGIAIGDLCLLEEDIQAQRIVTPFPLCVSSGAAYYLVYPERTV
APPTLTALVDFLAAEAADSRARLQNYLPMTCNAL
>CORE_REP|Org33_Gene2755#
MLATHEYANDLILFALIVDCGSFSKAAESAGITSSVVSKRIGRLEKSLGARLLYRTTRSLTLTESGQA
LYQQAKEIGAKVQEALYAVSEKSEELTGTIRMSVPTISGELLLSESVAEFCALHPSLKVEMRLENRFV
DLVEEGIDLAIRTGTMPDSSLIARPIFDSRWVIVCSPGYLESHPEPRSAEDLLGHNCLTYTYQESGTA
NWLMKRPGRNEIYELQVNGNLSANNARAIRKAVIGGHGIAMVPRCMVYEDLQDGKLTEILAGHCGKVL
GIYAVYPYTRNLPLKTRLLIEHIIGSYQNISHYF
>CORE_REP|Org11_Gene2832#
MDHHLLAIRVFNRVVETGGFTRAAESLGMPKATVTKLIQNLEDHLQTKLFQRTTRSVSVTREGECYYQ
NTVKWLADLEQMEGCLTESQSSPQGVLRIDTGGGTARRLLLPALPDFLARYPQIQIDLSVGDRVIDLI
SDSTDCVIRSGPLADSSLIARRLFDLDWVSCATPAYLALHGTPRHPCDLEQGFPMVHYRHPLNDRIHP
QRYAEHGKEIAIQQRSPVSINEGNALLAASLAGLGIIQIYRFMAQPHLDSGELVSLLHDWQPPPEQMY
VVYPSNRHLSGKLRAFIDWAVETFDSGKMSRTL
>CORE_REP|Org8_Gene1071#
MNIRDLEYLVALAEHRHFRRAADSCHVSQPTLSGQIRKLEDELGVMLLERTSRKVLFTQAGLLLVEQA
RTVLREVKVLKEMASQQGEAMSGPLHIGLIPTVGPYLLPQIIPTLHKTFPKLEMYLHEAQTHQLLAQL
DSGKLDCAILALVKETEAFIEVPLFDEPMKLAVYSDHPWAQRERVAMPDLAGEKLLMLEDGHCLRDQA
MGFCFQAGADEDTHFRATSLETLRNMVAAGSGITLLPSLAVPPQRERDGVCYLDCYKPEPKRTIALVY
RPGSPLRSRYEQLAEAIREHMQGYIDSALKQAV

>CORE_REP|Org3_Gene2035#
MKMSIKQLRAFLAVAHTLNFAQASERLNISQPALSLAIRGLEDALGGPLLLRTTRRVTLTPEGETFFP
MARQLLADWDNAEEAMRQRFTLQMGKVAIAAMPSFAGNPLPPILKAFRDRYAGINVAVHDVINEQVFE
MIREGRVEMGIAFEPEPSDTLHFTPLCRDRFLAVVPKDSALARKAQVSWKELLTLDFITLQRPSAVRL
LLEQELARSGRTLEVAFESHQLVTVGRMVANGLGASAVPALCEQQMDELGAVCVPLIGPIIERRVGLI
RLAQHQLSSAAQALATVIEREMAGSGAQPALRP
>CORE_REP|Org23_Gene3194#
MSLPFDVHRLLPAFLAAAQAQNFSAAARQLGVTPAAVSKNIRALEEKLALRLFQRNTHNVLLTDEGKA
LLAQVAPLWQALAATLESAGGERQAPAGVVRVTMIPGFGRQMLMPLIPQFLARYPQIDLDLSLDARVV
NLVGEGFDVGIGSRVDPDSRLVARPLYPMHMVLAASPDYLARRGEPQTPHDLLRHDCLLHRNPANGRH
VKWQLRHQGETLALDLNGRLVVSRPEMLLDAALAGLGIVNLAHWYVEKHFVQGTLRPVLAECWPRPVQ
LWLYYASADLPPRVRVWVDFLLEHFRDRPTGD
>CORE_REP|Org39_Gene3100#
MKITLEELLAFTAVVDSGSVTAAADRLGQTTSGVSRALSRLETKLDATLLRRTTRRLSLTEEGLSFLA
PAREILRSVDQAEELMALRRRLPAGRLRVNAAAPFMAHVLVPMVAEFRRRYPQIELELDTDDRNIDLL
EKRADIAIRIGALRDSTLHARLLGNSRLRILASPDYLQRHGEPRGVEDLHRHCLLGFTYPESLNQWPL
RHRQARHFAIEPTISASSGETLRELALRGAGIVQLADFMTRRDREAGRLVPLLVRETLDVRQPIHAVY
YHDAQLAARLTCFLDYVSARLEGEPPEAAEGL
>CORE_REP|Org6_Gene1333#
MEFKQLQDMALFALVAECGSFTAAAQRVGLPKSSVSQRISQLEQTLGLRLLNRTTRQLNLTFAGERYL
EHCQVMMSAAERADLALQRLRDNPSGRLRISTPAGLGATLVARLAADFQRQYPDVSLEVSVSDAMVDL
VQEGFDAALRTGKPQDSSLIGRRLGYAPRYLLAAPSYLAQHPPIEHPQQLQQHRCIAHRAWTAWNLRC
GDDYYRWQLPLAHTTDNLLYARECAIAGAGITLLPAFLSREVVAQKLLVEVLPAWRAEGNELYLVYPS
RKLNSAALACFIDVVLQHPAFDDYARELARE
>CORE_REP|Org6_Gene1631#
MERLKRMSVFAKVVEFGSFTAAARQLDMSVSSISQTVSKLENELQVKLLNRSTRSIGLTEAGKIYYQG
CRRMLQEVSEVHEQLYAFNNTPAGTLRIGSSSTMAQNVLANMTAEMMKEYPGLTVNLVTGIPAPDLIT
DGLDLVIRTGALQDSSLFSRRLGQMPMVVCAAKSYLIQHGTPQKPSDMVNFSWLEYSVRPDSEFELMS
PEGITTRISPQGRFVTNDSSTMIRWLKNGAGIAYAPLMWVIEEIKRGEIEILFKSYHSDPRPIYALYT
EKDKLPLKVQVCINYLTDYFERVAAVYQGYR
>CORE_REP|Org6_Gene1086#
MRLNLEALLILDALDRHGSFAAAAAALFKTPSALSYMVQKLENDLDITLLDRSGHRAKFTDTGKLMLE
KGRVLLRAAQDLEQQARYVENGWESEITLGIDASFPFARLLPLIDEFYRQHHHTRLRFSHEVLAGSWE
SLVYGCADIIIGAICEPPSRVGYAFSRLGQLDYVFAVAPQHPLAALPEPLPKDEIRQHRAVVVRDTSR
VNAPQSLNLLEEQDTLTVFGFDAKLQAQLAGLGCGYLPRSLAEPYLNSGELVAKRVESERCSDIAYFG
WRESASGLAAKWWRERLQRYADDGEAYPAAQ
>CORE_REP|Org46_Gene1493#
MLTDLNDLFFFASVVDHQGFAPAGRALGIPKSKLSRRVALLEERLGVRLIQRSTRRFSVTEVGQNYYA
HCKAMLVEAEAAQQAIEQTRAEPCGTVRMSCPVAILHTRVGSMVAAFMADYPKVTVHLEATNRRVDVV
GEGLDLAIRVRPPPLEDSDLVLKILAQRTWCVAASPALVRTLGPAHAPEDLRKYPTLDLGPARAQHQW
RLTGPQGERVEWEHTPRLVTDDMLMLRTAAIAGAGIVQLPAMMMRDDMLRGELVQLLPGWQPQGGVVH
AVYPSRRGLLPAVRLLLDYLGEQFTSIEEE
>CORE_REP|Org10_Gene4419#
MDRFNQYRVFVQVAEMGSFIRAAHALEVPRASVSAAVQQLETQLGVRLLHRTTRQVRLTADGEQLLER
LRPLLAEVEDIDQSFQASQRQASGRLSVDVPSRIARRLIAPALPSLLRRHPHLQLVLGSADRAIDLVQ
EGVDCAVRVGDLHDSSLVMRPLGHIALINCASPAYLSEFGHPRQPADLAEGHWSIGYASPKTGRESPW
EYLTDDGHTQRLELPSRVVVNNAESYIACCSAGLGLMQIPRYDVQHLLDAGELVEVLPGYRAASMPIA
LIYPHRRQRSRRLAVFHEWFESLLQPHLER
>CORE_REP|Org18_Gene686#
MDRITAAEVFVTIVDRGSMIAAAETLEMSRAMVTRYLAQMEQWAGARLLHRTTRKLSLTDAGERTLER
CRQMLALAGEIDLVEEGQSDELRGLLRITCSQSLGQTALVGAVAQYLKRHPQVAVDLQMNNRAVNLVE
ERIDLALRITNELDPNLIARPLSTCASVVCAAPAYLAAHGTPRQPQDLALHNCLTYSYFGKSLWHFDA
QGVKSAVAVSGNLSANESVVLMAGTVQGAGISMQPYYSAAPLLASGELVELLPDYRPQSMGIYGIYTS
RRQMPATLRTMLDFLVEWFATDPQWQATLR

```
>CORE_REP|Org2_Gene2686#
MSSLLQLLPYFEAVARLGNFTRAASQLGVTPPAVSQNIQALENQLGVRLFHRTSRSVRLSDEGRLFYQ
KVSPAMSQIDVAADDVRALGAQPAGLLRITLPQLAASLLVMPHLAEFQRRYPDVQLELFTEDRFSDLV
LGSFDAGIRMHAMLQKDMIAVPIDNGQRRVLVASPDYLARCGVPATPDDLPVHHCLRYRFPGSGKLEP
WYFSLGDDERALDVSGSLIFNEDRLIKDAALAGLGIAQRFQGTVLQELAQGQLVEVLPDYASEASGFF
IYFPAGRHLPLKLRAFIDFMREQRERQHRW
>CORE_REP|Org6_Gene3537#
MNDARYVEHLPIFLDVARLGSFSAAARRLGMVPSSLVRHIDALESALGATLFVRSTRGLLLTDAGELL
LTRAAALMTDITGIHAELSALNETPQGTLRISCLPTFGKTYVLPLLPTLAERYPQLSIDLDLTERQTD
PTQERLDAALRIGEQKDSALYASRIATQRWVMCASPAYVARYGLPSDLEALPQHRLIARYHKQQPACW
AQILDAALMSRCTMALRCDDFTAQRQAALLGLGIAFLPNWVVGPDVQNGQLVQMLEDPRHEQQGIYLL
RPMAKVSARLAAFTALLQQTLGQPPSWG
>CORE_REP|Org2_Gene3088#
MHSPSRARLPKLSAILAFETAARTGSLARAADTLALTAAAVSQQIRQLEQHLGITLFIRAKSGVTLTE
QGADYLAYVQEAFETLRVAQQHVERQRGKQALTVFALPALASKWLNPALGDWLAQCPDGDLRLHATHA
AVDFAHSAADFALCFGDQDYPLLEKVRLFQDRVQPVCSPALRDRGDWTQLPLIHVDWGKESQFLPGWH
EWFTAADRMPPARRGLTYNLTSLAIDAAVQGRGVLLGQRRLIGRELAAGQLVTLAEPALPLSKPYYVV
YPPRTLEKPGAAAFLAWLQTLASTDQA
>CORE_REP|Org19_Gene3737#
MFATLPVNALRTFESAARLRSFKLAAAELAVTPTAISHQIKALEQQLGFALFERVPRGVRLTPKGETL
FAGVHGALLDVAATLEGLRPQPSTGSLCVSVTHSFAALWLVPRLGRFYQAYPHYLVRLEACAEVIDLQ
QDASVDVAVRYSRAQYPALHQTARLEESFGVYAAPGLAAAEPENPVLITVKWGDSALYDSGWRDWCRA
AGVDWWQRHAAMRSYHEEHYALQAAVAGQGIVLASSVMVSDMVDNGLLVAYRPEVRVPGAAYSVLCAP
GRERHPPVRAFLAWLQQELPQGNGTK
>CORE_REP|Org44_Gene1228#
MKRPDYRTLQALDAVIRERGFERAAQKLCITQSAVSQRIKQLENLFGQPLLVRTVPPRPTEQGQKLLA
LLHQVELLEEEWLGNDTGVDTPLLLSLAVNADSLATWLLPALKPVLADSPIRLNLQVEDETRTQERLR
RGEVVGAVSIQPQPLPSCLVDRLGALDYLFVASSAFAERYFPSGVTRSALLKAPAVAFDHLDDMHQAF
LQQNFDLSPGSVPCHIVNSSEAFVQLARQGTTCCMIPHLQIEKELASGELIDLTPGLYQRRMLYWHRF
APESRMMRKVTDALLEHGHQVLRQD
>CORE_REP|Org24_Gene3483#
MAFNDSDDLLAQKAEQRMAQALATSQVEQDDYLDNQPAEALTRGDINRMAWRSLLLQASFNYERMQAG
GWLYQLIPGLRKIHRNPQDLANSMKMHMEFINVHPFDVTFLSGLVLAMEQNKEKISTIRAVKVALMGP
LGGIGDALFWLTLLPICAGIGASLALEGSLFGPIVFLLLFNLFHFGLRFGLAHYGYQAGTSALALLKT
HTRRISHAASIVGMTVIGALVASYVHLSTPLVMHAGKARVALQTDVLDKLMPNLLPLCFTLLIFFLMK
RGFSPVKLIGVTVAIGVAGKFIGIL
>CORE_REP|Org38_Gene118#
MDLRDLKLFLHLAESHHFGRTAKAMHVSPSTLSRQIQRLEEILGQPLFLRDNRTVQLTDAGEQLKEFA
QQTLLQYQQLKHSLGQHGPSLSGELRLFCSVTAAYSHLPPILDRFRAQHPLVEIKLTTGDAADAVDKV
QSNEADLGIAGRPETLPASVAFTKIGEIPLVLIAPALPCAVRSQAFADKPDWAEIPFILPEHGPSRKR
IELWFRRHRISNPLIYATVGGHEAIVSMVALGCGIALIPSVVVDNSPEPVRNRISQLDNISMVEPFEL
GVCVQKKRLSDPLIDAFWRLLHPR
>CORE_REP|Org23_Gene3099#
MNIELRHLRYFIAVAEELHFGRAAERLRISQPPLSQQIQALEEMVGARLLARNNRNVSLTQAGEMFLK
EAYQVLDQVGRAAEKAARLDRGELGEMTIGFTSSAPFIGVVARSLRTFRQQSPQVHIKMREINTKQQI
EPLLNGELDLGVMRNTRLPEALHYQLLLREPLVAVVPEGHPLAETPGGGLRFQHLAQEPFVFFSREVG
TALYDEILLLLSKAGITPYITQEVGEAMTIIGLVSAGLGVSILPASFARVRVDGVRYLPLAEPDATTE
VWLVHHRRRPLTAAAQALMALMLK
>CORE_REP|Org5_Gene3514#
MRKPRLPPLGALRAFHAVAGCRSFKLAAEALGVSATAVSHQIKLLESVLACRVCERSAQGVSLTETGE
ILYAGTQRAFAALEQSVAQITRAQQPPALTVTTTSNFLTHWLVPRLADFKAEFPAIDLRLHTSVERVD
LSQRTVDVAIRYRETPESDLHCTLLHEDRFIVVASPALALERSEDLQRVTLFHVEHRQVPADAPTWEN
WRRRYGPEGLNVEAGLTFSDETHALQAAVAGQGVVIASRLLARDLLQRGVLAAPFETALPGANYYLVA
TEETAQRPDIIALREWLLRQMAAG
```

>CORE_REP|Org17_Gene124#
MKANSDELITFVTVVESGSFSRAAERLEQANSVVSRTVKKLESKLGVTLLNRTTRQISLTQEGENYFR
QVQKVLNDMAAAENALMESRQRPQGLLRVDAATPVVLHMLTPLVAEFRERYPEMSLSLVSSENFINLI
ERKVDIAIRVGELTDSTLKARKLMTSYRHVLASPAYLAQHGTPLTVEDLAHHCCIGFNDLPSLNRWPL
ACSDGSQLEITPGLTTNSGETQRHLCLHGNGIACLSDFMSDEDIKRGDLVPILVEATLPVAMPINAVY
YSDSAVSNRLRSFIDFVSEYLKR
>CORE_REP|Org28_Gene1805#
MDVRTLRYFVEVVRQQSFTRAAEKLFVTQPTISKMLRHLEEELECTLLIREGRKLRLTDSGQALYQRG
LTILDEFRQLEAELEDISSLKKGVLRLGIPPMVGRQIADLIRRFRQTYPGIELKISELGGLSVEQAVM
SGELDLAMTVLPFDSEQPLTFLPLLGHPMCVVAPRTPQWLNRTSINIAELADSPILIYNEDFALYKML
MKAFRQAGFEPQIAVRSGQWDFLASMVQAGVGIAMLPEPVCRWLDKENLVWLPLEPRMEWKIGLIWRQ
GSYLSHGAQAWIACCRDYWPPLK
>CORE_REP|Org49_Gene2811#
MFKKSLLTLAFTGVATLSTYATAADTLTMEVYNPGEKSVFPVSSEIISGKHEVALIDAQFQRNDAEEL
VKKIKATGKKLTTVYISHSDPDFYFGLDVIKAAFPEAKIIASPGTIKDINATKDGKVAYWGPILKDNA
PKTVIVPQPLQGDSFTIDGQKVEVKGLNGPTPDRTFVWIPALKAVVGGVAVAGDNIHPWIADNQSVES
RQHWQQTLKNIEALKPQVVVPGHFLPGAAQTLASVHFTQKYLTTLEAELPKAKDSAALIEAMKKHYPT
LKDESSLELSAKVLKGEMKWPQ
>CORE_REP|Org35_Gene1585#
MRNRLPLNALRAFESSARHLNFTRAGLELRVTQAAVSQQVRMLEEQLGIQLFRRLPRGLDLTEEGQAL
LPVLSDAFDRIEAVLQQFEGGHFHEVLTVAVVGTFAVGWLMPRLAAFRAAHPFIDLRVLTHNNLVNLS
ADGMDFAIRFGEGLWPATRNIKLFDAPLTVLCSPAVAARLHTPKDLQHELLMRTYRQDEWERWFTAAQ
VTPWRINGPVFDSSRLMVEGALQCDGVALAPVSMFRRELAAGALQRPFAAEAALGAYWLTHLKSRDLT
PAMKAFIGWICREAEEEQRRD
>CORE_REP|Org32_Gene4334#
MMNERIPLHVLPTFAIAARLENLRAAAQQVHLTHGAVSQQIQLLEQAVGYPLFERRGRGVRLNAAGRE
LLAAVEPALQALLQGVARARRAATSQTLRISVLPSFAHYWLLPRLPAFHEACADIALDIDASLALQDL
SQRGFDAAIRIGSGQWTGLQAQRIATGDVLPVASPDMAREWRAAFESGGDIPLLEHDVSPWRDWFNAQ
GRPLCGRQQALFNDAGLLIRAAEQGFGIALAKKLLVQDALDAGRLVALAAPRRLSDDDVYLVWPQTAG
LTPAVTRLLQWLQRQLAAI
>CORE_REP|Org28_Gene2548#
MGRITFDLEDLRSFVTGVELGSFAKAAERLGRSTSAVSAHLKKLEQQVGAPILRKAGRGMVMTEAGET
LLGYARRLLELNDEAAAAVRGLDLQGTVRLGLQEDFGETFLPQVLGSFARANPKVRIEARIARNAELI
DWVLKGQLDLSLAWDGGLSTPFHQALGQRQLHWIASPGFALAPWREGDEPLSLVMFDAPCLMRSAATQ
ALDRAGIPWRIAFTSRSLNGVWAAVNAGLGVTVRTAAGLPPGLAPLAPELLPALGQLGVVLHRAEDQP
SAAVQRLAQIVVERIGTNL
>CORE_REP|Org44_Gene232#
MQHKTLKYFSTVIVCAIAVCAGWWLWNYYMQSPWTRDGKVRAELVNITPEVSGRLEKISANDNQFVPA
GSLIFTLDPVPYQIALDNAEAAVAKAQSDLAKADHEAARRRGLPRNVISAEDLDESNLAAQAMKAAYK
AALANLEQAKWNLSKTKIYAPTDGYITNLQARVGNYANAGTPLVALVDVHSFYVLGYFEETKLKHIKE
GNKADIVLYNGNTPLQGEVESIGRAIYDQSVDSSNDLLMDVKPNVPWVRLAQRVPVRIKLLNVPADLT
LVAGTTCTISIHQRN
>CORE_REP|Org28_Gene4729#
MLKHWPPLSALRGFEAAARLSSFHQAAEELHLTQSAISQQIRSLEAFLEQPLFFRTGRSVTLTDAGHD
LFSTAQVMLQQLAVGIRRLDQYRKPNQLIVNTTPAFARHWLMPRLGDFNRQHPQADLWLFTSFEPPNM
ATDSIDLAIRDDLSAQADCTFNVLCSDRLYPACHPSLLALAAEQRMTLHGEREMDWSHWTVAGGAHVG
QRDSGLNFSDPGLLLDAACQGLGIALVSQLLAQQARDAGLLQPLTEQRVRGANWAWLLHRDSEHNPLT
RHFCQWLQSALPAGA
>CORE_REP|Org9_Gene2151#
MKLSQLKFFCTVVEHKTIAAAARELHCVPSNVTLRLRELEESLGGELFFRDKNRLYVNPKGRLFYQQA
RDIVAQAERSKQLFAGEHQHGLLNLGALDFSLVSHLPARIARLRRLQPHLHINVLSRDSLVLERMLID
SDLDLAITDGPIEHPLLASQKAFDERLVLLMPADAGEPDAATLAPLEFYTFSRECSFRLKVDHWLASR
GLKPRMTLEMESYAAMAACVQAGCGVACVPGSLLPLILPAPGLKVVEMGEEGVSDLYFVWRRHQLSDE
LQTILAILARPG

>CORE_REP|Org34_Gene1366#
MNPLFTPYLQRWQLEQDGKAFETHSSLLMPVRYRGEAAMLKIAREQEERFGGQLMCWWRGEGAAQVLA
WHEDGILLERAQGESSLAQLVRDGDDEQATAILCRAIAALHAPRAAPLPELIPLQEWFSSLWPAAQAH
GGMLRLSATTAAELLSSPRDESVLHGDIHHDNVLDFGERGWLAIDPKRLYGERGFDYANIFCNPNYGI
ATDPAIFQRRVEQVCRLAGLERRRLLQWILAWAGLSAAWFMEDGQAADIDFRVAELAARALDLPLPAG
DSGFILPVIERG
>CORE_REP|Org6_Gene1996#
MNRYPMFNPQLLLSFVAVCDSNSFTRAAERVFLSQSTVSQQVRRLEEMLGKPLFERSSHQVLLTEEGV
KLLSYARRIIALNEEAHDALTGIWRDGVLRIGMPEDFAVPTTELLAEFSREHPHLRLDVASGLSADLH
SAYAREELDLILVKQRRQQPPRAARPEPLLWLDSLAFPAIEQSPVPLAVFPLSGLYRDELCQALDNLG
KRWRIGYSSASLAALTAASAAGLGVTLLPAGCRLPTHRVLGAAEGLPPIDSFELALYYRDGAPAATLA
LAQRLTVFCGLI
>CORE_REP|Org46_Gene1042#
MVDTTAQKKLTPADIRGVFLRSNLFQGSWNFERMQALGFCFSMVPVIRRLYPENNDDRKQAIKRHLEF
FNTHPYVAAPVLGVTMAMEEQRANGAPIDDGAINGIKVGLMGPLAGVGDPIFWGTVRPVFAALGAGIA
MSGSLLGPILFFVLFNLVRLLTRYYGVAYGYRKGVDIVNDMGGGFLQKLTEGASILGLFVMGALVNKW
THVNIPLVVSKITDQTGHTNVTTVQTILDQLMPGLVPLLLTFACMWLLRKKVNALWIIIGFFVIGIFG
YWIGLLGL
>CORE_REP|Org24_Gene4551#
MQLTVRDMTLNLSHPQVMGILNVTPDSFSDGGRHNTLNQALLHAHALILAGATMIDIGGESTRPGAAE
VSEEEELERVVPVVEALAQRFEVFISVDTSKAGVIRESAHAGAHLINDIRSLQEPGALAAAAESGLPV
CLMHMQGQPRTMQQAPHYDDLIADVQAFFEHHIRRCNEAGITNQKLLLDPGFGFGKNLAHNYQLLARL
SEFHRFGLPLLVGMSRKSMIGQLLNVPPDQRVIGSVACAVIAAMQGAQIVRVHDVKETVEAMRVVEAT
LSAKGQ
>CORE_REP|Org23_Gene2132#
MDTELLKTFLEVSRTRHFGRAAESLYLTQSAVSFRIRQLENQLGANLFTRHRNNIRLTPAGERLLPYA
ESLMNTWQLAKKEVVRSLQHTELSIGATASLWEAYLTPWLQALYQQREALQLEARVALRHSLVKQLHE
RQLDLLITTEPPKMDELASQQLSNFSLRLFSSAYRDKQAPMPYIKLEWGADFHQQESRMLEGDNVPVL
TTTSAHLTRQLLETTGGCAFLPSQWEKEYPQLVATTEIPPIIRPLYAVWLQNSDQQPLIRQLLKIPLN
TAA
>CORE_REP|Org34_Gene2028#
MSSEELEQVWNSIKSEARALADCEPMLASFFHATLLKHENLGSALSYMLANKLATPIMPAIAVREVVE
EAYKSDNQMIVSAARDILAVRLRDPAVDKYSTPLLYLKGFHALQAYRIGHWLWQQGRQALAIYLQNQI
SVAFGVDIHPAATIGCGIMLDHATGIVIGETAVVENNVSILQSVTLGGTGKTSGDRHPKIREGVMIGA
GAKILGNIEVGKGAKIGAGSVVLQAVPPHTTAAGVPARIVGRPESDTPSMDMDQYFNGTNHGFEYGDG
I
>CORE_REP|Org26_Gene2222#
MSYQCPLCHQPLHFSSQRWRCDGNHQFDQAKEGYVNLLPVQHKRSKQPGDSAEMMQARRAFLDGGFYQ
PLQQQVAEWLDLALAADAGALLDIGCGEGYYTAAVAARLAQARNMAVYGLDVAKVAIRYAAKRYPAVS
FCVASSHRLPFADAALDAVLRIYAPCKAAELARVVKPGGVVVTVSPGPRHLYQLKEQVYQQAQLHAEQ
DEQFDGFECERKEALAYTMALPGAQAANLLQMTPFAWRATPEVQLRLAHGGEFECETDFVLALYRRRA
>CORE_REP|Org7_Gene4574#
MTTPTHSIHHAAAEGYQANADRYVKGRPDYPPEIAAWLRDVIGLHAGMTVIDLGAGTGKFTPRLLETG
AQVIAVEPVPQMLEKLSAALPQVKTLAGTADAIPLPDESVDAVVCAQSFHWFATPQALAEIQRILKPG
GKLGLVWNMRDARVGWVRKLNQIVDSHEGDAPRFYTGEWRKFFPFKGFEPLQEQVFMLGHRGAVEDVI
YNRVRSTSFIAALPQPQQEQVIDRLRQLVAEEEELRGKDTVTVPYQTKAYFTTKV
>CORE_REP|Org11_Gene1725#
MKAIIVEDEFLAQEELSYLIKKHSNIDIVATFEDGLDVLKYLQTHQVDAIFLDINIPSLDGVLLAQNI
SKFAHRPSIVFITAYKEHAVEAFEIEAFDYILKPYHEARIVTMLQKLEALHHRPAGATEPASAPSRGS
HSINLIKDERIIVTDINDIYYAAADEKVTRVYTRREEFVMPMNITEFYGRLPEEHFFRCHRSYCVNLA
KIREIVPWFNNTYILRLSDLEFEVPVSRSKVKEFRKLMRL
>CORE_REP|Org19_Gene2545#
MRVHASIEPLVWESDFFQLESAKLHFDSSAAPVAEADLDAYALVQAKIPAYRLGWADALSTLGFRLVE
GEVDLVVNVAPESAMADAASAVAVRQAVPEDIPSLRAAAGEVFAASRFRAPWYDRADSGRFYAAWIEK

AVQGTFDHQCLLVLDSQGQPEGFVSLRDIGGQEMRIGLLAAFPGASGRGVGARLMTAAIAECRQQGMQ
RLRVATQVGNIAALRLYQRQGAVIESTAYWLYRGRHDSI
>CORE_REP|Org4_Gene3913#
MRKSLGGWRRLRPGYWLKRGVIAILGLWVLGIAAFAFLPVPFSAVMVERQVSAWLSGDFGYVAHSDWV
SMDDISPQMALAVMAAEDQKFPDHWGFDVAAIEKALSHNEKRPTRIRGASTLSQQTAKNLFLWDGRSW
LRKGLEAGLTSGIELVWTKRRILTVYLNIVEFGDGVFGVEEASQRFFHKPAKRLTAAEAALLAAVLPN
PHRFRADAPSGYVIQRQQWIMRQMRQLGGEAFLSENKLD
>CORE_REP|Org37_Gene1550#
MLNALIVDDEPSARDNLRHLLEAEAEIAIIGECANAIEAISQIHRLQPDVVFLDIQMPRISGLEMVGM
LDPNRMPHIVFLTAYDEYAVQAFEEHAFDYLLKPAEPKRLSKTLQRLRQRSAPQDVAALEESAGYLKY
IPCTGHSRIYLLRFDEVLAIRSKLSGVFVVRSDGMECFTELTLRTLESRTPLVRCHRQYLVNLEQVRE
IRFEEGGAAEMIMSAGDPVPVSRRYLKALKEQLGLRG
>CORE_REP|Org27_Gene864#
MHKIVFVEDDPEVGKLIAAYLGKHDIEVLIEPRGDSAQARIAHEQPDLVLLDIMLPGKDGMTLCRDLR
PTFPGPIVLLTSLDSDMNHILSLEMGANDYILKTTPPAVLLARLRLHLRQHGQQPKEESVQPLTQHNA
LHFGLLCIDPVNRQVTLGEETVTLSTSDFDLLWELATHAGQIMDREALLQNLRGVSYDGMDRSIDVAI
SRLRRKLYDNALEPFRIKTVRNKGYLFAPNAWASVQQ
>CORE_REP|Org18_Gene1779#
MINVLIVDDDPMVAELNKYYLSQVGGFHCQATVATLSQARALLADAGVSIDLVLLDIYMQQENGLDLL
PGLRELGEKTDVIIISSASDVNTVQKALHYGVVDYLIKPFQFSRFKEALSHYRQQSQLLAQREFSQAD
VDSLLRRQPGGQESKKLPKGLTSITLSTVCEWIEQQHDNEFSTDNLANAIGISRVSCRKYLIYLAESG
ILGTRILYGATGRPVYLYQLKPDAIAMLKEHCRPA
>CORE_REP|Org7_Gene2013#
MDIQNQPVQIMIVEDEPKLGQLLVDYLQAAGYATRWLTNGNEVVPTVHQHPPALILLDLMLPGADGLT
VCRELRRFSDVPIVMVTAKIEEIDRLLGLEIGADDYICKPYSPREVVARVKTILRRSYRPQENAREDD
LLHIDEPRFQASYQGQLLDLTPAEFRLLKTLASQPGNVFSREQLLNNLYDDYRVVTDRTIDSHIKNLR
RKLELIDGQKSFIRSVYGVGYRWEAEPCRLVNGV
>CORE_REP|Org37_Gene3786#
MTTETAATILLIDDHPMLRNGVKQLIGMDARLQVIAEASNGEQGVTLAEQHDPDLILLDLNMPGINGL
ETLDRLRQTDLSGRVVVFSVSNHEDDVVSALKRGADGYLLKDMEPEDLLKALHQAAAGQMVLSETLTP
ILAASLRENRPSADRDIQQLTPRERDILKLIAQGLPNKLIARRLTITESTVKVHVKHLLKKMKLKSRV
EAAVWVLQGKTVNRRRTAARFAPEWAAASGF
>CORE_REP|Org22_Gene620#
MARRILVVEDEAPIREMVCFVLEQNGYQPLEAEDYDSAVTRLSEPFPDLVLLDWMLPGGSGIQFIKHM
KREALTRDIPVMMLTARGEEEDRVRGLEVGADDYITKPFSPKELVARIKAVMRRISPMAVEEVIEMQG
LSLDPSSHRVMANDQALDMGPTEFKLLHFFMTHPERVYSREQLLNHVWGTNVYVEDRTVDVHIRRLRK
ALETSGHDKMVQTVRGTGYRFSTRY
>CORE_REP|Org49_Gene1435#
MSITPTNILIVEDEKEIRRFVRTALESEGLRVFESETLQRGLIEAGTRKPDLIILDLGLPDGDGLSYI
RDLRQWSAIPVIVLSARNAEEDKIAALDAGADDYLSKPFGIGELLARVRVALRRHSASQQESPLVSFS
AITVDLVNRRVLRNDEDLHLTPIEFRLLAELLANAGKVITQRQLLSHVWGPNYVEHSHYLRIYMGHLR
QKLEADPARPKHLLTETGVGYRFMP
>CORE_REP|Org38_Gene2185#
MSHQTLDVLIVEDEPQLATLHAEFIEKNFNLRVVAYAATLAEARAKANEHQPRLILLDNFLPDGQGIE
LMEEPAVKNPACSVIFITAASDMHTCSQAIRNGAFDYIIKPVSYKRLRNSLERFMQFVQTQRTFKIID
QDNVDALYNLQSKQFSSEPSAKGIETNTLELVQALFIAQPAVAHAVEDVVEQVGISKTTARRYLEYCV
ATQFVRVEMLYGNIGHPRRLYRKA
>CORE_REP|Org9_Gene1038#
MKILLVDDDLELGTMLSEYLTGEGFDATLVLTGKAGVEGALSGDYTAMILDIMLPDMSGIDVLRDVRK
KSRLPIIMLTAKGDNIDRVIGLEMGADDYMPKPCYPRELVARLRAVLRRFEERPQEADDEAAISFGEL
TLNPSTRSSEWRGKAFDLTASEFNLLELLLRAPDRVVSKDELSEKGLGRPREAYDRSVDVHISNIRQK
LSALAGSKLIIETVRSIGYRIR
>CORE_REP|Org4_Gene59#
MQRILIVEDEQKTGRYLQQGLVEEGYQADLFNNGRDGLGAASKGQYDLIILDVMLPFLDGWQIISALR

ESGHEEPVLFLTAKDNVRDKVKGLELGADDYLIKPFDFTELVARVRTLLRRARSQAATVCTIADMTVD
MVRRTVIRSGKKIHLTGKEYVLLELLLQRTGEVLPRSLISSLVWNMNFDSDTNVIDVAVRRLRSKIDD
DFEPKLIHTVRGAGYVLEIREE
>CORE_REP|Org17_Gene1995#
MINAEMLTGRSTEHLAPLSGNHRLQPEAVNAFLAMQQAARAAGFDLQPASTFRDFDRQLAIWNGKFCG
QRPVLDKDSQPIDVAPLSAAERCEAILRWSALPGASRHHWGSDLDVYDPSLLPEGQKLQLEPWEYEEG
GYFAPLNQWLTAHMAEFGFYRPFTEDCGGVAVEPWHLSYRPLAQEAEHLLTPALLLAAWQDKEVAGAE
WLERHLPSIFSRFIRSKGKE
>CORE_REP|Org15_Gene2222#
MRILLIEDDKLIGDGIKAGLTKLGFNLDWFTDGAVGKNALGSAPYDAVILDLSLPGLDGLDLLRQWRQ
AGQDVPVLILTARDALEQRVSGLQSGADDYLCKPFALAEVAARLQALIRRRHGQLMPQLTHGNVVFDS
ATRSVSCNGEPVTLTPRELAVLELFLHNKGRVLARPLIQEKLYNWDDEVSSNAVEVHIHHLRRKLGNG
FIRTIHGVGYTLGDAP
>CORE_REP|Org46_Gene107#
MISVLLVDDHELVRAGIRRILEDIKGIKVVGEAQCGEDAVKWCRGNAVDIVLMDMNMPGIGGLEATRK
IVRYAPDVKVIMLTIHTENPLPAKVMQAGAAGYLSKGAAPQEVINALRSVHAGQRYIASDIAQQMALS
QLEPQAETPFSCLSERELQIMLMITKGKKVNEISEQLSLSPKTVNSYRYRMFSKLNISGDVELTHLAI
RHGLFNAETLLSSE
>CORE_REP|Org4_Gene2280#
MSLMLKGEKIDRNRFTGEKIENGSFMLCDFSGADLTGTEFIGCQFYDRESRQGGNFSRAILKDASFRS
CDLSMADFRHVDALGVEIRECRAQGADFRGASFMNMITSRTWFCSAYITKSNLSYANFAKVVLEKCEL
WENRWHGAQVLGASFSGSDLSGGEFSGFDWRAADVTQCDLSNAELGELDLRTTDLQGVKMDSHQAAQL
LERLGIAIIG
>CORE_REP|Org8_Gene887#
MVMKQYRVMIVDDHPLMRRGIKQLLGLDARFGVVAEAGNGSEAVALALQHAPDVILLDLNMKGMSGLD
TLRALRDEGVDARIIVLTVSDARSDLYALIDAGADGYLLKDSEPEQLLEHISAAAEGQNVISDAMADY
LLARSEQRDPFTALTERELDVLQEVARGLSNKQVAAQLHISEETVKVHIRNILRKLDVRSRVAATVMY
LEYKSH
>CORE_REP|Org11_Gene3912#
MSKKHWSNTELLHQTVTNPNIIVKGTHSYYSDCWDNGFERSVVRYLHGDAVSRQWQPLGDIDRLLIGD
YVCIAAEAVILMGGNHTHRIDWLSLYPFMETIKRAYRPKGDTRLGDGCWIGMRAMLMPGVSIGEGAIV
AAGSVVVGDVEPYAIVGGNPARFIRWRFAPEVIARLLALRLYDLSEADFAVVQPLLVDNDIAAVERAI
CNIKRY
>CORE_REP|Org28_Gene2514#
MIRVILVDDHVVVRSGFAQLLNLEDDLDVVGQYSSAAAAWPALLRGDVNVAVMDIAMPDENGLSLLKR
LRAQKPQFRAIILSIYDSPTFVQSALDAGASGYLTKRCGPEELVQAVRSVDMGGHYLCADALRALRGG
ERPATALEVLTPREREIFDLLVKGDSVKEIAFKLDLSHKTVHVHRANVLGKLQCNSTIELVHFALDHQ
LLAGH
>CORE_REP|Org49_Gene3493#
MNDFAQPKIGDNVTLNRTRLGQYVHLADDAILEEVEMGDYSYTAGHNQIFYATIGKFVSIASYARINP
GNHPTYQRIAQHHFTYRASEYGLGEDDAAFFDWRREHHVAVGHDVWIGHNAILMPGVSVGNGAVIGSA
AVVTKDVEPYSIVAGVAAKKIGMRFDDALIERIERSQWWHWDHATLQARLADFRDINRFAQKYL
>CORE_REP|Org4_Gene1977#
MTLRVAFIDDHDIVRSGFVQLLSLEADIQVVGEFSSAAQARAGLPGLEAEICICDISMPDGSGLDLLA
DIPSGIRVVMLSMHDNPALVEMALDRGASGFLSKRCKPEDLITAVRTVAGGGVYLMPEIAQQLARVRV
DPLTRREREIALLLAQGQEVREIAAALGLSPKTVHVHRANLFAKLGINNNVELARRMLNL
>CORE_REP|Org37_Gene784#
MFGYRSASPKVRLTTDRMVVRLVHERDAYRLADYYAENRTFLKPWEPVRDESHCYPSGWQARLGMITE
MQKQGSAYYFILLDPEEQEVRGVANFSNVLRGSFHACFLGYSLGEKWQGQGLMFEALQSAIRYMLRQQ
RMHRIMANYMPHNQRSGALLTRLGFEREGYAKDYLLIDGKWQDHVLTAYTNKEWLPPR
>CORE_REP|Org32_Gene2796#
MAISEEKRKMIAGELYDAGDDLLRSERRRARQLTHRYNHSSPEEGELRKQWLDELLGGYQGGTIEPTF
RCDYGYNIYLGKSFYANFDCVILDVCEVHIGDNCLLAPGVHIYTATHPLDAETRVGGAEFGKPVKIGD
NVWIGGRAVINPGVTIGDNAVVASGAVVTKDVPANCVVGGNPARVIKQL

>CORE_REP|Org12_Gene553#
MSSTASVRLRPLERDDLSFVHQMDNNASVMRYWFEEPYEAFVELSDLYDKHIHDQSERRFIIEHEGAK
VGLVELVEIDHIHRRAEFQIIIDPAHQGKGYASTAARLAMDYGFSVLNLYKLYLIVDKENPKAIHIYS
KLGFNVEGELIDEFFVNGEYRTVLRMCIFQPQYLAKFKTPNDKPLVK
>CORE_REP|Org18_Gene1969#
MTPVIRIAAIEALPDDYLTRGDFGFTIRCYALPQFDTPVDSWPTRPVAPFRKQYPLAPFANEDSATFL
AYRQDQAVGHITLSKNWNGYTLIDEIAVSAHARRQGIAGALLDCAKQWARQQETSGMMLETQNNNLAA
CRCYQHYGFILGGIDRLLYRAEPEIADHEIALFWYLPFNSEIGY
>CORE_REP|Org29_Gene4208#
MKLVTERLSLQSITAEDWPLFLRLYQDPEVIRYISDPRSEAEIRTRFEERLSAWDKHGEQWLCLVMRE
KHSGEAVGITGFRPQWVPYRQAEVGYGSLPAGQGKGYGKESLRAVLDFAVNACGFHKLTATVTAGNLA
SRGLLESCGFQLEGTLRDNYRLAGQWCDDWLFGLLAAEFQGGK
>CORE_REP|Org37_Gene3888#
MIIRNATLNDSAAIAAIYNDAVLNSTAIWNEQTVDAANRAAWIGERQAAGYPVLVAVNGADEAIGYAS
FGDWRAWDGYRHTVEHSVYVHQAHRGEGIGKALLIALIARAQEIGKHVMVAGIESGNQASIKLHLALG
FREVGRMEQVGAKFGQWLDLTFLQLTLDERAAPPAR
>CORE_REP|Org29_Gene3496#
MFTVRQALLEDLTQVRDIGIRTYRAHFGELWRYPHELEAFLAEDFSVSALERTLRDPDVCWLLAYEDD
TLVGYARVNFDSLLAATQRRGAELQKIYFLPDYAGRGFGRQFFEQVQRRAVGRRQPLLWLEVLKQNAD
AQRFYQRQGLAVCGEAQYTSEQGAIELWAMSKAL
>CORE_REP|Org33_Gene3271#
MEDSGLPLKRRKGATMSIIHRLAQPDDLNGLLALYRELRPQDAPLRTDDARRTLQRLLDDPAIRLVVA
ADEEQPIATCMLALIPGLAHQAQPFGVIEHVVTAEPYRGHGVALAMIEYALQLAWRKGCYKVMLLSGQ
QRTGAHQLYLKAGFDGDRERGFVIRRPEGR
>CORE_REP|Org42_Gene4407#
MHVLPKATVIRPCGPEDIDRLMALWLPSTIAAHPFVAEKYWRESATLVRENYLPRAQSWACWHDDEIV
GFISVLDEQFIGALFVERAFHGRGVAQALMTHVQQRYRRLSLEVYQQNLRACAFYHRHGFQVTQRLFN
DETQAYTLIMNWPAVENSTRYA
>CORE_REP|Org9_Gene1848#
MSSVITTRQAGIVDVDAGRNVTVVEPCNLYGCRLGDDVFVGPFVEIQRHVSIGARSKIQSHSFICEYV
TLGEACFVGHGVTFANDLFKDGAPNADPASWGRTRVGDRVSIGSGATILAVEICSDAVIGAGAVVTRN
ITRKGIYAGNPARLLRELP
>CORE_REP|Org26_Gene1411#
MSEKYVVTWDMLQMHARKLAHRLLPADKWTGIIAVSRGGLVPAALLARELGIRHVDTVCISSYDHDNQ
REMKVLKRAEGDGEGFIVVDDLVDTGGTAKAIRDMYPKAHFVTIFAKPAGRPLVDDYVVDIPQDTWIE
QPWDMGVSFVPPIGGR
>CORE_REP|Org20_Gene1815#
MNLSNPEVTIRRINGDDKAQWLALWQGYLDFYRADVAPQVTDRTFERLGQDEQVYGLVAQDADGQLLG
LMNLVFHPSTWSAVGYCYIEDLYVSPQARGHKVSEKLFEQAYRLAETRGSDRVYWMTQEYNAPARSLY
DKIGRRSSFIVYSR
>CORE_REP|Org39_Gene2286#
MIVNCDHDNLDAWLALRTALWPSSSPEDHRAEMREILASPHHTAFMARGLDGAFVGFAEVALRYDYVN
GCESSPVAFLEGIYTVERARRQGWAARLIAQVQEWAKQQGCSELASDTDIANLDSQRLHAALGFAETE
RVVFYRKTLG
>CORE_REP|Org26_Gene3611#
MELKIDKVIETVLYVSDIERADAFYRQVLKLPAMVANERFRAYNVGDRSVLLLFIEGDSLRGAQYLTG
FIPAHDGVGPAHIGLAVAKEQLPHWERHLVANGVEIEGRMRWEHGGESIYFRDPDAHLLELVTPGIWA
NY
>CORE_REP|Org18_Gene3421#
MLTGLNHLTLAVSDLDRSFDFYRHLLGFTPHARWQGGAYLSLGSLWLCLSLDERRTQQRERDYTHYAF
SIAPEHIEQASQRLRQAGVKEWKSNRSEGESLYFLDPDGHQLEIHAGDLASRLAACREKPYQGMVFY
>CORE_REP|Org38_Gene4841#
MLIPSKLSRPVRLQNTVIRDRLLAKLASAGNYRLTLVNCPAGYGKTTLIAQWAAGKADLGWYSLDESD
NQPERFASYLIAALQQASGGRCVKSEALSQKHQYASLSALFAQLFIELADWHQPLYLVIDDYHLITND

AIHEAMRFFLRHQPENLTLILLSRTLPPLGIANLRVRDQLLEMGTQQLAFTHQEAKQFFDCRLAAPME
QQDSSRLCDEVEGWATALQLIALSARQSASSAQLSAKRLAGLNASHLSDYLVDEVLDHVDADARAFLL
RCSVLRSMNDALIVRLTGEDNGQQRLEELERQGLFIHRMDDTGEWFNFHPLFASFLRQRCQWELALEL
PGLHRAAAEGWLALGYPAEAIHHALAASDVSMLRDILLQHAWSLFHHSELALLEECLNALPYERLIQN
PKLALLQAWLAQSQHRYSEVNTLLERAERTMREQKIEIDQTLHAEFDALRAQVAINAGKPEEAERLAT
EALKFLPLSSYYSRIVATSVTGEVHHCKGELARALPMMQQTEQMARRHQANHYALWALLQQSEILIAQ
GFLQAAYETQDKAFELIREQHLEQLPMHEFLLRIRAQILWSWSRLDEAEDAARTGLKILANYQPQQQL
QCIAMLAKCSLARGDLDNANTHLQRCETLLHGAHYHRDWLTNTDKSRVIHWQMTGDTTAAAQWLRHTE
KPGMADNHFTQGQWRNIARVQILLGQYDEAGVVLDELNENARRLRLVSDLNRNLLLSNQLYWLQERKG
EAQQALIEALSLANRTGFISHFVIEGEAMAQQLRQLIQLNTLPELEQHRAQRILRDINQHHRHKFAHF
DENFVDKLLTHPQVPELIRTSPLTQREWQVLGLIYSGYSNDQIAGELDVAATTIKTHIRNLYQKLGVA
HRQEAVQQAQQLLKMMGYGA
>CORE_REP|Org3_Gene3709#
MQNKQLTISSSNITRCFLLFIVLLTIGIGLYGYNYTNAWLAEKKYALNSIAGSLQKRIDTYRYMTYQV
YDKFGNAPAQNVDPGLQETRLRPDVYYIEKPHKKTDAVIFGSHDESTLAMIANISDYLDTRWGAKTEN
YAMYYLNGQDNSLSLITTQPLKELASRFRESYLTTSADERRAEMLQQANMLDERESFSDLRKQRFQNA
YSFSIRTTFNQPGHLATVIAFDLPINDIIPANLARANFLLQPDDVDLDDSTIPAETVLGTHATMSGGW
VEFSAALPNAPLKVVYRVSAINLAIDLLRNNIWLIAVNLLLLALSMLSIYFIRRQYIRPSENMAVELE
AERALNQEIVSSLPSGLLVYSFANNAVIASNKIAEHLLPHLSLQKIAHMAEQHHGVIQATVNNEVYEI
RIFRSQLSPDTYLFLMHDQDKEVMVNKRLQQARREYDKNVQARKLMLHNLGIELNQPVRQMHDLVDRL
HGRPDEEQQQALLGQLTAASASVLELIDNITLLTRLETQDWQPSREPFSPTAMIDELLLEALPALNQK
GLALFKHFQLDVEQNYIGDANALRKVISLLVHYAIITTACGKISLVVDHEPEHPDRLIFQINDTGSGI
SNEEISNLNYPFLSQTLVDRFNHGSGLTFFLCNQLCKKLNGQLDIRSKVDIGTRYTIRVAMEMEKKEP
QEQEKLFDGVTALLDVTSDEVRGIVTRLLQAYGADCLVADDRAVNRDYDVLLTDNPQRADDYTLLLAT
DEPGWQALDKRYIRVNYNLNGALIDAVLILIEQQMAALEQEESPLSLSSEDIQLYEKQLKSSDYYGLF
VDTVPDDVKKLYTEAGSSDFNALSQTAHRLKGVFAMLNLLPGKQLCESLEQRIAEGDAPEIENNISQI
DFFVSRLLKQGSQQHE
>CORE_REP|Org13_Gene2736#
MKSFNRSGIYLAVMSAMLPGAALAADATDVGTISVKGQSLGGGMMVQDDSAKARSTVTKEAMDKMPSA
ANAIDKLKYTPGLNVNSNDASGLSGVDYTMRGMNSDQIGLSMDGIPINDSGNYAVYPNLLGDAENLEE
VFVTQGSSEADGPHIGSSGGNIGLVTRRPAKDFGGFVKQTLGSNSLSKTFARLDTGEYNGFSNWLSYS
HTEAKKWRGEGRLYSDKFEMNSLYEDGNGNSSNLVMKYNRQNNTNYNTLSKAQFQNDGRDTDYVTTPE
YNNKGQLNKYYKIERNPFENFTLSFTQKLQLRDNLSLTLQPYYYWGNGGSFNGQTASVLSNTSSKAGQ
YDLSNLKSNTYYRPSWTQTWRPGITTKLKWDINEQHSLDVGYWYERARQLQTQPFISIKGDGNPSQIW
GQPGGSDQVKDANGNTVQGRNQYTITPAQKVWLQDTWFATPDWTFVGGLAYQYVERKGDNRGSLYNVP
EKRKATYHEFLPNFSASYKVNQENQVFYNLTRNMRTPPNYVLYNVGDSLSTKPELSWNHELGWRFQQE
DMLLSATLFYMRYSDRQISTTNSAGDYEMMNIGNVENKGLELEWSGQLPHNFNYYTSYTYTESKQKSD
IVSNGGLPLPTSGKEVPNVPKNLLNMTLGYDDGLYYGSVSGKYVSSFYGDLTNDEKIGGRTVFDLAAG
VHLPVDKKIVKSAALRFGISNLFDKEYLTSVRTTTFNAAPYGGVKASTPYYNVGEERTFSVSLEATF
>CORE_REP|Org2_Gene3241#
MKRKHLWVLNPCLLAMLAPAAWAEDQKTGNEEQLVVSASRSHRSVAEMAQTTWVIESQEIEQQVQGGK
EIKDMLAQLIPGMDVSGQGRTNYGMNIRGRSMMVMIDGVRLNSSRSDSRQLDSIDPFNIDHIEVISGA
TSLYGGGSTGGLINIVTKKGQPEQQVELQIGGKTGFGGHNDHDENVAAAVSGGNDNASGRLSVSYQRY
GGWYDGKGNEVLIDNTQTSLQYSDRLDVMGTGTLNIDDHQQLQLTTQYYKSQSDGDHGLFLGENFAAV
TGNAKAYNSGSLDSDRIPGTERHLINLQYSNTDFLGQDLVAQVYYRDETLTFYPFPTLAGKAPNYYVS
SIGASQQKTDFYGGKLTLNSKPVDALTLTYGIDAEHESFNANQQFFNLAKAQQSGGMTLENAYSTGRY
PSYTTSNLASFLQASYDINPIFTLSGGVRYQYTENKIDDFVGYNQQQAIATGAAASADAIPGGKTDYN
NALFNAGLLAHLTERQQTWFNFSQGFEIPDPGKYYGNGTYALNGGHYQLLKSVNVGDSRLEGIKVNAY
ELGWRYTGDNLRTQIAAYYSLSDKSIAINKTDMTINVNADKRRIYGVEGAVDYFFEDSDWSAGTNFNV
IRSETKVNGEWKKLVVDTASPSKVTAYVGWAPGDWNLRLQSQQTFDVSDDGDYTKANSTQGRKIDGYN
TLDFLGSYALPVGKISFSVENLLDKEYTTVWGQRAPILYSPTYGSPELYSYKGRGRTFGLNYSVLF
>CORE_REP|Org10_Gene3143#
MPTKRLSSSAAKQGRLPVSALAITVAAALGTLAMPAFSADAKPAAKEDTITVVGGSNSAQQESAWGPV
GTYVAKRSATGTKTDTPIEKNPQSVSVVTREEMDMRQPDTVKSALAYTPGVMIGNRGASTAYDAVNIR

GFSSVGTNMYLDGLKLQDDNYSIYQIDPYFLERAEVLRGPSSVLYGKSNPGGVVALVSKRPTTETLRE
VQFKMGTDNLFQTGFDFSDALDDAGVYSYRLTGVARDEDQQQVGEKSKRYAIAPSFSWRPDDRTSLTF
LSSFQDDPSVGFYGWLPKEGTVQNGVNGKLPTSFNDGEPGYNNISRKQQMVGYAFEHAFDDVWTVRQN
LRYSKMDVDYRSIYGLGIDPDNSAELKRGVMNSKEHMSSFAVDTQAQAKFATGQVDHIVLMGVDYMRM
RNDVVYQYGSASNLNVIAPQYGNRSYTITGGASQVNRQEQTGLYVQDQAEWNNWVLTMGGRYDWSDTN
STNRLNQNSVSKQQDKQFTGRAGLNYVFENGIAPYVSYSESFEPTSGTDFSGNTFAASKGKQYEAGVK
YAPKDRPITASLALYQLTKTNNKVADPNPEHAFASILGGEIRSRGVELEAKAALTANLNILGSYTYTN
TEYTKDTTLQGNTPAAIPKHMASLWADYTFHETAISGLTLGSGVRYVGSSYGDEANTFKVKDYTVFDA
AIKYDLARFNLPGSSIGINVNNLFDKEYVSSCFATYGCYWGAERQVVATATFRF
>CORE_REP|Org5_Gene3766#
MTTESKCPFSGGKQPAPQNGPTNQDWWPNQLSLKPLHQHSPLSDPMDKDFNYADAFNSLDLAAVKQDL
HALMTDSQEWWPADFGHYGGLFIRMAWHSAGTYRIGDGRGGAGEGQQRFAPLNSWPDNVSLDKARRLL
WPIKQKYGRNISWADLIILTGNVALESMGFKTFGYAGGRADTWEPDDVYWGSEKIWLELSGGPNSRYS
GDRDLENPLAAVQMGLIYVNPEGPDGNPDPVAAARDIRETFARMAMNDEETVALIAGGHTFGKTHGAG
PASNVGADPEAAGLESQGLGWHSTFGTGVGKDAITSGLEVTWTTTPTQWNHDFFRHLFEYEWELSQSP
AGAHQWVAKDIGETIPDAFDPNKKRRPTMLTTDLSLRFDPAYEKISRRFYEHPEELADAFARAWFKLT
HRDMGPRPRYLGPEVPQEELIWQDPIPAVDHPLIDEQDIAALKNAVLASGLPVSALVSTAWASASSFR
GSDKRGGANGARIRLAPQKDWAVNQPAQLAATLATLESIQRTFNDAQAGGKRVSLADLIVLAGAAGVE
QAAKNAGLALTVPFAPGRMDASQEQTDVDSFEAMEPLADGFRNFLKGKYRVPAETLLVDKAQLLTLTA
PEMTVLVGGLRVLGANVGGTPHGVFTQRPQALTNDFFVNLLDMGTTWHPVGEDGLFEGRDRRSGAVKW
TGTRVDLVFGSHAQLRALAEVYGSADAQEKFAHDFVAAWNKVMNLDRFDLA
>CORE_REP|Org2_Gene2457#
MPVVHVALPVPLARTFDYLLPPGMQPVAGARVGVPWGRQHAIGIVTGCSDTSELPLDKLKPIDSVIDA
ESLFSPSLWRILRWASDYYHYPIGEVLFHALPILLRQGKPAEAAPLWQWFATEEGRATPPESLKRAPK
QQQALAALLQRPVYRHQVSQLELTESALQALRAKGLIDLRAQVADTHDWRPNFAVLGERLRLNTEQAT
AVGAIRSEDEQFAAWLLAGVTGSGKTEVYLSVLENVLAKGRQALVLVPEIGLTPQTIARFRERFNAPV
DVLHSGLNDSERLAVWLRARSGEAAIVIGTRSALFTPFRQLGVIIIDEEHDSSYKQQEGWRYHARDLA
VFRAREEDIPMVMGSATPALETLHNVQLGKYRQLKLTQRAGNAKPATQHLIDLKGLPLKVGLSQPLLK
SMQHHLKAGNQVMLFLNRRGYAPALLCHECGWIAECQRCDHYYTFHQHQRQLRCHHCDSQRPVPHQCP
QCGSTHLVSVGVGTEQLEQELAPLFPDTPITRIDRDTTSRKGALEQHLADIHRGEARILIGTQMLAKG
HHFPDVTLVALLDVDGALFSADFRSAERFAQLYTQVSGRAGRAGKQGEVLLQTHHPEHPLLQVLLQQG
YDAFAKQTLAERNSVFLPPYTSHIIVRAEDHDNQQAPLFLQQLRNLLEASPLKDDSLWVMGPVPALQS
KRGGRFRWQLLLQHPTRRVLQQLMKSSLPLIGTLPQTRKVKWTLDVDPIDS
>CORE_REP|Org12_Gene3625#
MNNNKRGWWCALPLAACATLPTWAAEKVASKEESLTVIGRKDADGVQSYQPLTSVTGTRSETNLLNVP
QAIDVVPQQVITDQAVSSLDEALYNVSGITQANTLGGTQDAVMKRGFGDNRDGSILRDGVRSVQARNF
TPTTERVEVLKGPASMLYGMGEPGGMINMITKKPQLQQHTHVEGWGSSFNGGGGQLDVTGPLGTSGFA
YRMIVDHDETDYWRNFGRNRQTVIAPSLMWYGENTTVRLAYEHMEYLVPFDRGTIIDSRTGKPVNTPR
DRRFDEAYNATRGDQDSITLQIDQTLNERWKSSLTYAYSRNSYSDNQARATALNPVTGVLSRQADSTA
NAVSHANAVQLTLNGDVDWGSINHQMLFGFDFEDNRTYRGDMIRGKKNSDFNIYHPVYGLMPPSTAVS
AKDSDQRENLTSYGWFMQDSIQLTDKWLVMGGLRYDAFDVYAGKGRPFQTNTDSSDGKLVPRAGVVYK
LTPYVSLYSSYTESFKPNSSIATQIDSLPPEQGKSWEVGGKLALPNGVTGTLALFDITKRNVMVNELV
EGETVTRTAGRVRSQGVELDVAGNITDSLSLIGSYAYTDARVVDDPDNKGKEMTNVARHTASLFLTQN
LGSLGLYSGDEVRIGAGARYVGRRPGDAANSFYLDNYTVADAFAAYTMPINGYRVKWQLNVKNLFDKT
YYPSSGGNLRVAVGEPREVVLRGSIDF
>CORE_REP|Org36_Gene2938#
MRHSQIKTADDRVYSARFEGAGESQPPFCFSPISRRAGLRVRRSLTIKQMATVSGVALVTICIFIVIQ
LFHFVQQRRDDYAQQLENIAHSVRQPLAEAVLRMDVPEAKKVLNTLLPVGILSRADIVLPNEFQALHA
NFPPERPVPTLIARLFELPIQISVPLYSLERVPANQQPLAYLVLQADSFRMYQFILSILSTMLSTYLL
LALILSVAITWCMNRLMVHPLRAMAKELENISQDEAPYHQLMLPALHQDDELGLLVRNYNRNQQTLAK
AHADMSRLSTRHPVTELPNPALLNALLEQHIASSLRPERFNLLVIGIETLHEASGVMSPAMREALLLA
LAKKLRGCIDENGVLAQLSNTEFAILAKGTERPFHAMQLARRIMAEINAPLTLEGLALRPNASIGIAH
YLNQGESAEQLLRSATSAMMSAHREGKNQILFFEPSLTERTQKRLTQESEILHGIEQRHFTLFLQPQI
DMQSNEVIGAEALLRWQQYDGSYTLPADVIPLAEELGVIVPLGNWVLEESCRILADWQQRGIELPLAV

NVSGIQMQDEAFVPHLKNLLAQYRIDPRKLLLEITETVRIDDLDRALALLRELHDLGLSIALDDFGMG
YSSLEYLNRLKSLPIDLIKIDRSFIQGLPADDAMVRIVSSISEVLALPVMAEGVENAEQRDWLLKHGI
RSGQGFLFARPLPREAFEAEFCRAAP
>CORE_REP|Org9_Gene1317#
MGKHFAAQRHESVGNGEKAGMKAIGAFSSVFLGVCSLAIGNVNAAETKSNETYQDAETLLVTGEKVKR
SIFDTSSSVQVFDSNRIASMPDAVQIPDLLRMTPNVVDLGIGNELPTVRGIDGSGPNVGANAFLSGTR
PRLNLSLDGRSLTYNEQAFGPQSLWDLDRVEVFLGPQSYIQGRNAIAGAIVMASKDPTFEWESAFKGG
AGNQHSSQLAAMASGPLVEDQLAFRVSVDRQRRRSEADLPAYAPVGDPREVEATTARAKLLFNPAGLR
DLTTKLTFNHFGSTAPQNESLNPQPHPTNPRHDPRRAVFKSNMNSTIWDLAWEASDALTLENRVIYTD
FNINRPTAYNIQYAEIDGQEVHVEPVVRFGGADSRLHGLAGLRYFHGTQDEFVNIFGGSTFKDKTDTH
SAFAELTYALTPQVDVTAASRLEREHRRRDGGSQAVRIDFDETYTVFLPKLDVAWKPTDTQTYGAKIA
RGYNAGGGGITIGTPVVSYTYGSEYVWNYELYTRHHLKDANVVLTGNIFYNDYKDMQLPYSLGENSSV
IRNADKVETYGAEIGATWQPRWDFELFGNLGLLKTDIKKFSGSGVEGHELARAPAYTANMGAKYQFLK
GWELSSNVAFSDSYYSAYDNDSRGRIGSYWTANAQLAYTFDYGRATLYAKNLFDSDRREMVRSNDIYT
ATLQRGRLVGAAVELNF
>CORE_REP|Org17_Gene4022#
MPLTFSTRLRFSALSLAIACALPTVALAQNTSTTPSSSPATPAKKAKAADETMTVVATGNQRSSFEAP
MMVTVIEGSSPESQTAGTAADMLRRVPGITVTGSGRSNGQDLMMRGYDRRGVLTLVDGIRQGTDTGHI
NGTFLDPALVKRIEIVRGPSALLYGSGALGGVVSYETVDAADLLLPGHDSGFRVYGTAGSGDHSLGMG
ASAYGKTDNLDGLLSFGTRDVGNLRQGNGFDAPNDETINNVLAKGTWKIDDNQSLGGNLRYYNNSAQE
PKNPQTPASSAGNLMTNRSTIQRDAALSYKLKPVGQDWLDAEAKVYYSDVKINAHASGSEDEARKQTT
KGAKLENRTRLFADTFASHLLTYGTEAYKQEQTPGGATESFPHAKINFASGWLQDEITLRDLPVTLLA
GTRYDNYKGSSDGYADVDANKWSSRGAVSITPTDWLMLFGSYSQAFRAPTMGEMYNDSKHFSIPMGPT
TITNYWVPNPNLKPETNETQEYGFGLRFDDLLLADDSLQFKASYFDTKAKDYITTDVTMELGRGPRGP
YCISCTTFSTNIDRAKIWGWDATLSYKTSWFGWDLAYNRTRGKNEATGDWLSSINPDTVTSSLDVPLG
ETGLSAGWVATFAERATRVQTGTPEQGGYGVNDFYLSYKGRDRLQGVTTTVVLGNAFDKEYYSPQGVP
QDGRNAKLLVSYQW
>CORE_REP|Org13_Gene4551#
MPADQGPELLNAHFGTQSPHWRLAFDSNALELSAVKGKAHVAVAFSAMEAAKIRRLTGVTASLELTIT
LAGEPLHLHLVGRRVNNLEWAGTASAFSDTQSVARDLVHGLSFAEQVVSEANSVIVIVDQHGRIQRFN
RLSEEYTGLREHEVIGKNVFQLFMSPEEAAASRRNIAGFFRNGSSYEVERWVKTVKGERLFLFRNKFV
HSGSGKNEVYLICSGTDITEERRAQERLRVLANTDLITGLPNRNAIQDKINHAIATRGEESFGLVYLD
LDNFKKVNDAYGHMFGDRLLVEVALAILGCLSPDQVLARLGGDEFLVLAPQTDRERLQTLAPQTDRER
LQTLAQRIIDRLKTPFRIGLIEVYTGCSIGIALCPEHGNDLDSLIRSADTAMYVAKEHGKRTYTVFSP
EMNKRVAEYMWLDTNLRKGLEQNQLVLYYQPKIDARSGEVHSVEALVRWDSPERGLIPPLQFISYAEE
SGLIGPLGQWVLQTAAGQAAQWQEQGLNLRVAVNLSARQLADDSIVNDLLGVLRRHRMAPCLLDFELT
ESSLIEDENRARALITRLRELGAQVHLDDFGTGYSSLAQLARIPLDAIKLDKSFVRGVNFNPVSQSLV
RAIVAAAEALAFRVIAEGVETESENHFLDEVGVDEKQGFLFARPMLPEQLEHWLQSYRPHSPSA
>CORE_REP|Org13_Gene4198#
METPRYSKLAALVVASLSATAALAAPQNDTQDTMVVTASGFQQKIQDSAASISVIPRQQIEDKAYRDV
TDALKDVPGVVVTGGASSSDISIRGMSSKYTLILVDGKRVDTRSTRPNSDNAGIEQGWLPPLEAIERI
EVVRGPMSSLYGSDAMGGVINVITRKTSRTEWKGSLHGDATIQENRNSGDLFQTNAYASGPLVEGLLG
LRVNGLLSRRAEDKIVNGYNEQRMRSGTAVFTLTPDEKNEFDFEIGRSLQDRNSTPGKSVVAERCSKG
KCTPTEVSESLYTRTNYALTHNGYYDFGNSTSYVQREETGNPGRNMKAYNTIFNTQNQFELGSHMLNL
GGQYRYEKLGDGGNQLESAQGLSKLTRWSWALFAEDEWALTNDFSLTSGIRMDRDENFGSHWTPRMYG
VWHLTEQWTLKGGVSAGYKSPDLRQSSPNWGQVTGGGVRKGIIVGNPDLQPEKSLSEEIGLMWDSLKG
VNAGVTVFNTDFKDKITEVRRCEDTPDCKIGNDVYDFISDRVNVDKANMRGVEATFGWQINKDWKWNT
NYTYTSSEQKSGEFQGKALNQMPKHMLNTVLDWRATQDLSLWSRVNFRSKTSQYLSRTSMATSTPSYT
FVDAGLSYQAAKNLQLTGGVYNILDKTVDYDHFRTTLDGRRYTVGMTYNF
>CORE_REP|Org6_Gene4205#
MILRHPPIKFGCFSHDNYKNTLLAVVSSVTAFSGWAQDNTTATNGDNLVVTANRFPQPVSSVLAPTSV
VTRNDIDRWQAKSLTDVMRRLPGVDIAQNGGLGQQSSLFIRGTNSSHVLVLIDGIRLNQAGVSGSSDL
SQIPISLVQKVEYIRGPRSAVYGSDAIGGVVNIITTREKNGTTLAAGVGSNGYQSYDASTQQPLGDST
VATVAGNYTYAKGYNVIANLPDSFGNPAQPDRDGFMSKSLYGGIEHKFNEAFSGFVRGYGYDNRTAYD

GNYSYSDPAHLDALPDTRQLYSQSWDSGLRYQDGIYATQLIASYSHTKDYNYDPKYGPYSASATLDDS
TQYNVQWGNTFQVAQGHISTGVDWQNQKIEPGTAYITDSKSQRNTGLYLTAQQQVDAFTLEGAVRGDD
NSQFGWHGTWQTSVAWEFVEGYRAIASYGTAFKAPNLGQQYGSFGGNPDLKPEESKQWEGGFEGLTGP
VTWRVTGYRNDIDNLISYASSGSGSAYYNVNQARIKGVEATVSFDTGPLTHQIGYDYVDPRNAKTNEV
LLRRAKQQVKYELDWQLYDFDWAVTYQYLGERYDGDYSGYTTRTVKLGGVSLWDLAVSYPVTSHLTVR
GRIANLFDKDYETAYGYATPGREYYLTGSYTF
>CORE_REP|Org41_Gene4075#
MDIKQKVKNMTLEEKIGQKIMLDFRYWDRNGSSNQDMTVPDEAIGKLIADNHVGGVILFANNLKDKQQ
INTLTAWYAAMKTHAGIRLFIGTDNEGGNVFRLPRGDYASFPGNMALAAAIEGGADEQLAVEQGKLMA
QDMRALHINTNFAPVVDVNTNPFNPVINVRAFSDDKNTVSRLAEKMVAGMKHQGLITAYKHFPGHGST
STDSHTGLPRVDRTREEAFAIDIAPYKQAIDRCAAPDMVMTAHIQYPALDNRQIDTRSGEKITVPATM
SHEIQTQILRNELGYAGVTISDALDMGAIAEHFSQQAAAENVFAAGVDIALMPVSIASPAQASLLPAL
IRYLADRVKTGHLSEADIDASVERILRLKLRHSLMDHSDRPCSNDVASSAHKLEKCIADRSITVVINR
HSLLPLKDKALRYFILTPWGEQASGIARVMAQEGYQNVVAAKETELSDAQVRKHIAGCDVFLLGTLST
RFTPAEQDGVVTSATGASNDSSPYPGWLKYAAEQGKKRVHLSLRAPYDIVNYAAEVEAAVVTYSYYGY
DSGVWRGPSMVSLAQVLTGKIKPQGKLPVNTWHDYDVETNTGKVAFPRGTGLSW
>CORE_REP|Org40_Gene4041#
MPKQRVPLKLSTSITLMVSAIIASVLLVVFALFFVQMSREGQDQLQQKAIAVANTLALSNTVIDGLQR
RDQSGAIQRFAEQVRHQNELLFVVVVDMQGIRYSHPKPWLIGKHFIGDDLAPALQGNVNSAINRGTLA
PALRVFVPVYDDQKQQIGVVALGIALDTVQRVVAESRWIIYWTIAFAALVGSLGTFFLVSALKRIMLG
FEPYEISNLFEQRNAMLQSIKEGVIAVDNESRITIVNDEAKRLLRQSGPVENLLLEAASKHWPAQLHL
AEVLASGEPLRDRQISFNGSELLTNTVPVIVNGQVTGAIATFRDKTEVSRLLQRLSGMAHYADALRVQ
SHEFMNKLHVILGMLHMKAYQQLENYIINTASNYQEEIGALLRKIHSPEVAGFFIGKISRAHEAGVEL
TIEENSLLPETDDAETTHVLISVLGNLIENAIDAIDGVEGHEIGLSFHHHDDQLHCIVSDDGPGIDPA
IAARIFEHGFSTKGTGRGIGLALIRSHLEKLGGSIDFESEPGELTQFFVHLPYQAKSRAHD
>CORE_REP|Org29_Gene4329#
MRLKLSFQIKLFLCLVAFSCLLLTCIGAYTYYQLDAQLHRDLGARAQVQAREIALIPSLVDAVENNDA
ARIAALMKKIRASSDASYIVIGDNHARHLYHSEYEGRLGTPMIGGDNKEVLEGKSIISIRKGGIGVSL
RSKAPIVDENNRVIGIVSVGYLKSHIDNLNARTLTQIIGSIILLLIALFVFSWLLSKNLKRQMFWLEP
KEIALLVRQQKALLEAIYEGVIAIDPQLRIITINHAARELLDLHQPAAGLLGRPIGDVIQAQPNFFAA
AQLGQDTHDEVCRFNHVRVIASRVRIMQEQELQGWVISFRDKNDINTLSSQLSQVKRYADNLRIMRHE
QLNWTATLAGLLHMQRYDEAIRYVEAQSEGAQEILDFISQRFSSAALCGLLLGKYSSAREKGIELRFD
PACQLRQIPAALNETELMSIVGNLLDNAVEATLHYPAPHEAIELYISDGSDELVIEVADHGTGIAEEI
RDTLFEQGVTTKADKSDHGIGLHLVASHVAQAHGSIEVSDNEPHGAIFSIFIPK
>CORE_REP|Org2_Gene2285#
MLMTHLAASRYRYYRWLLAGAVGAAILLVSLYTRYYQEVKSIELSQHTLATRTVGKLNQLLTPAQLQA
ERSMDMLNQSCENVSSTLRFRAAQNQALRAMLLVKNGIIYCSSLFGARHYQLAAVMPSFVNSDARLAL
RPSLAVSKGLPTLVLWTPSPRDKTSGVLHVFNIELLSNFLLEPQEPYVQRVVLNVADSSLEYGRREIL
SRDTLTNDLRYTAGSALYPFSISLFGPQIGMLALSALPRHIPLALLISLLAAYVVYLLTANRMSLSYH
IGHAITHREFRVYCQPIIHSDTGRCAGVEMLLRWKKKRQGWISPDVFIPLAEQHELIIPLTRYLMSTV
TENLQLFPPRPSFYISINVAAEHFKTLNIIDDIRQIWLPAHPMPSLMLELTERTALSAIQYDQIRTLK
DMGIMLAIDDFGTGHSSLSYLKNLSPDVLKIDRGFTAAIGTDAVNATVTDTIITLAQRLKLKLVAEGV
ETEEQADYLRSREVNALQGYYFAKPMPIHVFPLWLQQYESRVRKAEEDPPEA
>CORE_REP|Org27_Gene3371#
MIKIVVYLILAVTIAIIAARVLFRLPDISQRLPQAALPADPAAQLPARAAELMAAHPGLSGVVPLASG
HDAFASRLALARMAERSIDAQYYIWHNDTSGQILLKTLYDAAQRGVRVRLLLDDNGVAMDETLAALNA
QENVEIRLFNPSTVRTPKLAGYAFDFMRMNRRMHNKSYIVDGAVAIIGGRNIGDEYFQVGDENYFLDL
DVLSVGSVVAETAEVFDRYWNSASVFGVEQIIRGKGNLSAFLTQATATESSERARKLAVQLETSAVRF
RDGAVQPEFTQVELVADDPAKGLGKASRDRLMVTQLGKIIGGVGRQLDLVSAYFVPGREGASFFESLA
KQGKSIRVLTNAMNTTDVLVVHAGYAKYRRELLQAGVELFELKLRAGQPTGRKELKPLGLSGAALHAK
TFAIDDKRVFIGSFNFDPRSAHLNCEMGFLIDSPTLAADTRQLFDGPLEYAAYRPVLTPEGKMVWKEA
FEDGHTEVHQQEPGAGWVKRIILTVAGWLPIEWML
>CORE_REP|Org25_Gene2809#
MKKLLPLLIGLSLGGFSAMSQAENLLQVYKQARESNPDLRKSAADRDAAFEKINEARSPLLPQLGLTA

GYDYTNGYRDSNGVNSNVTSGSLALTQTLFDMSKWRQLTLQEKSAGISDVTFQTAEQSLILNTATAYF
NVLKAIDTLSYTQAQKDAVYRTLDQTTQRFNVGLVAITDVQNARSNYDTVLAAEVSARNDLDNALETL
RQVTGAFYPELASLNTDRFSTQRPEAVNNLLKEAEARNLSLLSARLSQDLAREQIRAAQTGYMPTIDV
SASTGISNTKYNGSNTGGANAARYSDSDAGQNKVGISFNLPLYSGGATNSQVKQAQYGFVGASEQLES
AHRSVVQTVRSSFNNVNASISSINAYKQAVISAQSSLDAMEAGYQVGTRTIVDVLDATTTLYNAKRQL
SDARYTYLINQLNIKSALGTLNQNDLLLLNGALGKPVSTAPDAVAPQNRAQDAYADGYQDNAPMQQTA
APAPAATRASAPAVTTSQPARHSGNPFRN
>CORE_REP|Org46_Gene4641#
MSQSVLASETHRGHLQQIISGLSDGVILTDTDRTLLWANEAALAMHGVSHQKALGANAGEYAARFALR
YRNNHPLALEQYPLNRVADGETFTDVVVEVRQADDPDSFWVHRLRSLIITDAQGQPELLALILSDATE
WASAEQRFEKTFNANPAPAVICRLSDLRYVKVNQGFLDMTGYQREQVMGRSVYELDVLEQAEHKDLAI
QRLGEGATIPQMEAELKLPGGGSKLVVVAGQPLDINEDDCMLFTFTDLEPRRQAESALRESEERFAKA
FRLSPVPTLLCTAHERRVLDVNEAFTRTTEYDAEALIGKTVDEIQFIDDPEASRRLFAALEKSGNVEG
LDIRVRKKGSESIDCVASADAVSIHNAPCYLLVLMDITERKRSELELVSAIEEVMQDASWFSQTLIEK
LANVKSINRPDQAGLTASDLTPRERDVLELICEGLPDKKIAARLNLALNTIRNHVATVYSKLGVHSRS
EAIVWARERGLFTGGLAARNGK
>CORE_REP|Org4_Gene2195#
MTTFYTVISWLMVFGYWLLIAGVTMRILMKRRAVPSAMAWLLVIYILPLFGIVAYLSFGELHLGKRRA
ERAKAMWPSTARWLKELKESRRIFATEYSEVAEPLFQLCNRRQGIDGVKGNQLQLLTTTDDTLKALIR
DIELARHNIEMVFYIWQPGGLVDQVAESLMAAARRGVHCRLMLDSAGSLQFFRSPYPAMMRNAGIEVV
EALKVNLLRVFLRRMDLRQHRKVVLIDNYIAYTGSMNMVDPRYFKQDAGVGQWIDLMARMEGPVATTM
GIVYACDWEIETGKRILPPPPDVNIMPFEQESGHTIQVIASGPGFPEEMIHQALLTAVYSAREQLIMT
TPYFVPSDDLLHAICTAALRGVEVSIIVPRDNDSMMVRWASRAFFSELLEAGVKIYQFEGGLLHTKSV
LVDGQLSLVGTVNLDMRSLWLNFEITLVIDDDGFGSDLACVQEDYIARSQLLNAKEWLKRPFWHRLVE
RLFYFFSPLL
>CORE_REP|Org31_Gene1090#
MRSPFNWRFTPLFAVLLLAGCASTDNIAPQSTLMDPQSLQLAQPKVSSLAVSPQWWRALKDPQLDTLM
TQTLQSSPTLRQAAARVREAQSVVGEASAANGPNLDLNASTQRQRVPQNVNMGLGYPHKPIYSSSNSL
GLNLAYEFDWWGKYRNQVNAAKAQVNAARAEQEQAALTLTSSVASAYYQLQSNLALEKLLQQEVNNNE
RLTALRQQRYQAGLTGVDVPQQTQAQSDVAKQQILQLQSQIEQLRHQLAALAGQGPNAMQHLRQVPLP
ADNLMAPQGELTADLLGKRPDIAAQRQLVESYSQRVSAARKEFYPSLTISAFAGLMTTNTSGTSPNLF
EAASQAWNVMPAISLPIFHAGALRSKLGEESALYDEAVESYNQTILNAVQETADAITIQQSSAQQQLQ
AASAAQSMQQVYQVANARYQAGIIGRDDLLTSQTQLLQQQQAELNASSNLLQAKIGLIRALGGGYQAP
AAADSKA
>CORE_REP|Org18_Gene3234#
MKILSPLALSLAALLTAGCGNALKSDYRAPQVNYPTSWQHAADNAAPTPFDWRDFHDPELDRWLQQVM
DSNNDLAVAVLRVYRARLEAERVGISTAPDVNASLNSGINRPLSESSAWNKTSGATLSTSYEVDLWGK
LARQRDAAEWASQASEQDLQTARLTLLANAATNYWRIGFLNQQIGVSQASIAYAKQTLRLANARYRAG
SISALDVVNAEQNVLTQESRLLALQHDRQQALNEQAVLLGAPTGQATIAPARLPTTAMPQINTGIPAS
VLSRRPDLSAKELRLRAALANVDEKRLQYYPAFSLTGSLGASSSALLEFLRNPTGSLGASLTLPFLQW
RQMGVDIKIARNDYEQQVLEFRQALYKAMGDVNNALSLRAQLRAQETQLQASLALARKSERLNEVRYR
QGAVTITDWLNAQEQRRQAELAVDENRFAQYQNLAKIYLEFGGSSAP
>CORE_REP|Org37_Gene3352#
MVDSLTLHPVALVNGTVNLPGSKSVSNRALLLAALAKGTTRLTNLLDSDDVRHMLNALQTLGVNYQLS
ADRTVCEVTGVAGPLVAGQPLELFLGNAGTAMRPLAAALCLGEGDVVLTGEPRMKERPIGHLVDALRQ
GGAQIDYLEQTDYPPIRLRGGFQGGDVTVDGSVSSQFLTALLMTAPLAPQDTQIHIKGELVSKPYIDI
TLHLMRTFGVSVSHDNYRVFHIQGRQTYLAPGDYLVEGDASSASYFLAAAAIKGGTVRVTGIGRKSVQ
GDTKFADVLEKMGARITWGDDFIECSRGELRGIDMDMNHIPDAAMTIATAALFAEGPTTIRNIYNWRV
KETDRLAAMATELRKVGAEVDEGEDYIHVVPPAKLQFAEIGTYNDHRMAMCFSLVALSDTPVTILDPK
CTAKTFPDYFEQLARISQPA
>CORE_REP|Org24_Gene371#
MDKFRVQGRTRLSGEVSISGAKNAALPILFAALLAEEPVELQNVPKLKDIDTTIKLLNQLGTKIERNG
SVFVDASGVNEFCAPYDLVKTMRASIWALGPLVARFGRGQVSLPGGCAIGARPVDLHITGLEQLGAEI
KLEEGYVKASVEGRLKGAHIVMDKVSVGATVTIMSAATLATGTTVIENAAREPEIVDTANFLNTLGAK

ISGAGSDKITIEGVERLGGGVYRVLPDRIETGTFLIAAAVSGGKVMCRNTRPDTLDAVLAKLREAGAD
IEVGEDWISLDMHGKRPKAVTVRTAPHPGFPTDMQAQFSLLNLVAEGTGVITETIFENRFMHVPELIR
MGAHAEIESNTVICHGVEQLSGAQVMATDLRASASLVIAGCIADGVTVVDRIYHIDRGYERIEDKLRA
LGANIERVKGE
>CORE_REP|Org46_Gene4169#
MTDSSQSAMPPAKGSAVKGTAFSILGAISVSHLLNDMIQSLILAIYPILQADFHLSFVQIGMITLTYQ
LTASLLQPLIGYYTDKHPQPYSLPIGMGFTLSGLLLLSVASTFPLVLLAAALVGTGSSVFHPESSRVA
RMASGGRHGLAQSLFQVGGNFGSSLGPLLAALIIAPYGKGNVAWFTLAALLAIVVLLQVSKWYQHQHR
ATKGQPKSPSTLKALPKRTVVYSLGILLVLIFSKYFYLASISSYYTFYLIHKFGVSVQNAQIHLFAFL
FAVAAGTIIGGPLGDKIGRKYVIWGSILGAAPFTLVLPYASLYWTGILTVIIGVILASAFSAILVYAQ
ELIPGKVGMVSGLFFGFAFGMGGLGAAVLGYVADLTSIELVYQICAFLPLIGIITALLPNMEHKPQ
>CORE_REP|Org25_Gene4390#
MSASAETQNPQQPSGKKKQRKFWLLLLTVIFIVIGVAYLVYWFLVLRHHQETDDAYVSGNQVQIMAQV
SGSVNSVNFDNTDYVKQGDVLLTLDPTDAEQAFERAKTGLANSVRQTHQLIINSKQYQANIALRKTDL
SKAENDLKRRVVLGSVDAIGREELQHARDAVDSAKAALEVAVQQYNANQAMVLNTPLEQQPAIQQAAA
QMRDAWLALQRTKVISPITGYVSRRSVQVGAQIAAGSPLMAVVPADHIWVDANFKETQIANMRIGQPA
KVVSDVYGDDVVYQGKVVGIDMGTGSAFSLLPAQNATGNWIKVVQRLPVRIELDAKQVADHPLRIGLS
TLVTVDTANLDGRVLSDVVRDKPLYQSDALALNLAPVNQLIADVIHANAG
>CORE_REP|Org29_Gene2782#
MKRNILAVVIPALLAAGAANAAEIYNKDGNKLDLYGKVDGLHYFSKDKGNDGDQTYVRFGFKGETQIT
DQLTGYGQWEYNVQSNHSESQGTEGTKTRLGFAGLKFADYGSFDYGRNYGVLYDVEGWTDMLPEFGGD
TYTNSDNFMTGRTNGVATYRNNNFFGLVDGLNFALQYQGKNQNDGRDIKKQNGDGWGISSTYDIGEGV
SFGAAYASSNRTDAQKNKSNERGDKADAWTVGAKYDANNVYLAAMYAETRNMTPYGGNNSLKDGTTSC
ADTQNNSCGGFASKTQNFEVTAQYQFDFGLRPEVSYLQSKGKNMNVPGAGSDQDLVKYVSVGTTYYFN
KNMSTYVDYKINLLDDNAFTKAAGIATDDIVAVGLVYQF
>CORE_REP|Org33_Gene2736#
MLSIRLADLAQQLDAQLHGDGDLVITGIASMHSAQPGQITFLSNSRYQEQLSSCQASAVVLTEADLPH
CRTAALVVKNPYLTYARMAQLMDTTPAPAQDIAPSAVISPEAQLGHNVAIGANAVIESGAVLGDNVVI
GPGCFIGKHARIGAGTRLWANVTIYHAVEIGQRCLIQSGTVIGADGFGYANERGEWIKIPQLGTVIIG
DRVEIGACTTIDRGALDNTQIGNGVIIDNQCQIAHNVVIGDNTAVAGGVIMAGSLKIGRYCQIGGASV
INGHMEIADKVVVTGMGMVMRPITEPGVYSSGIPLQPNKVWRKTAALVMNIDEISKRLKAVERKVGKD
>CORE_REP|Org18_Gene4598#
MLDVAGYELDAEEREILKHPLVGGLILFTRNFHDAEQLRELVRQIRAASHDRLVVAVDQEGGRVQRFR
EGFTRLPAAQSFAALHDAQEGGRLAQEAGWLMAAEMIAQDIDISFAPVLDIGHGSAAIGERSFHSDPQ
QALAMAERFILGMHSAGMKTTGKHFPGHGAVSADSHKETPRDPRPLAQIREHDMAIFRELINRQLLDA
VMPAHVIYTEADPRPASGSPYWLQQILRRELGFDGVIFSDDLSMEGAAIMGSYAERGQAALDAGCDMI
LVCNHREGAVSVLDNLSPVKAEKVKRLYHRGQFTRQELRDSERWQQAHKALSALSERWEEHKQRSQG
>CORE_REP|Org19_Gene877#
MKLQQLRYIVEVVNHNLNVSSTAEGLYTSQPGISKQVRMLEDELGIQIFARSGKHLTQVTPAGQEIIR
IAREVLSKVDAIKAVAGEHTYPDKGSLYVATTHTQARYALPNVIKGFIERYPRVSLHMHQGSPTQIAE
AVSKGTADFAIATEALHLYDDLIMLPCYHWNRAVVVKPDHPLAGKSSISIEELAAYPIVTYTFGFTGR
SELDTAFNRAGLTPRIVFTATDADVIKTYVRLGLGVGVIASMAVDPVQDPDLVTVDASDIFTYSTTKI
GFRRSTFLRSYMYDFIQRFAPHLTRDVVDSAVALRSNEEIEAMFKDIKLPIK
>CORE_REP|Org21_Gene1286#
MKKKTLFTLLLMLAAIALAILFRAHNQDLLLQGEVDAPEVIVASKAKGRVVERLIERGDDVKSGQLII
QLDSPELMAQLRSAQATRDEAKAQLELSLHGTREESIRNLRANLAQAEAQYRNAQNDYNRNLSVAGKG
YISKSELDASRRSRDTAFQQVQAAKANLDEGINGDRVEQRQQYAAALRAAEENLLQIQAQSDDLQVKA
PVDGEVGPIPAEVGELLNAGSPLVTLIRVPDAYFVFNLREDILAHVRKGDKVKLRVPALKDKMIDTEV
RYIAPLGDYATKRATRATGDFDLKTFEVRLYPSQPVDGLRPGMSTLWQWKE
>CORE_REP|Org13_Gene1498#
MTRLSLDAIKIISTIKSTGSFSMAAEALHKTPSAISYRVSNIESKLCVKLFHRNGPMITLTDEGEFLL
QEGSWILNAVQDLESRVRNIPKLDNNIRLAVDTFFPLETLTQDIRDYIQHCPNANISVQREALNGTWD
ALKNNRADLIIAIGQIPDSVQAKTLMLGKLNFVLCVSPSHPFAAQRKPVCKKQRLNDIVVVIADSSHE
LPKRNHGTLPLQRQLVVCDVESNLALLKRGIGHAFLPPALIEKELASGELVTVPVEMQKGDEMIWLAW

HPASKGAGFSWWHERLTRKSDVYSLMGREVVRDGGYPWCHN
>CORE_REP|Org8_Gene3935#
MTDPDFNLLIALDALLTAGSVAGAARRLGLSPSAMSRTLSRLRAATGDPLLVRAGRHMVLTPYAETLR
ERARHAAFEARAVLRPAQGALDPAALDRTFTLRANDGFVEAFGPALIAAAAEQAPRVRLRFAPKPEKS
DRPLREGLVDLEVGVLGDMGPEIRLQALFRDRFVGVMRTAHPLAQQPEIDVADYAACGHVVASRSGRI
LGPVDAALAELGLARHIAAVVPSFPAALAVAQASNLLALLPASFLQAQPADGPLRVFELPVKTPPITV
SQMWHPRLDAEADHRWLRQLVLSVCRRQAQPPASDNVT
>CORE_REP|Org18_Gene2648#
MMTLRQIRHFIAVAETGSISAGAQAVFVSQSSLTLAIQQLETEIGVRLFDRHAKGMTLTHQGHQFLRQ
SYLILATVDNAKRSLQIGTESLTGKLTVGVTSLVAGYFLVELLTRFKSAYPNVTVQVVEDERPYIEHL
LVSGEIDIGVLILSNIEDRDALQTEVLMHSPYRLWLPPLHPLLEHESISLADVAKQPLIQLNADEMDV
HARRIWSRAGLKPEIAMKTASTEAVRSLVAAGMGVSIQPDMAYRAWSLEGNMIEARKLDDLLEPLDIG
LAWRRGSARPELVTPFLTIARENGSKHAAGLKHSI
>CORE_REP|Org13_Gene2245#
MHSISLRQIEIFRAVMTTGNLTEAAALLQTSQPTVSRELARFEKLIRLQLFDRVRGRLSPTVQGLRLF
EEVQRSYYGLDRIVNAAAGIRQFQQAQLSIVCLPVFSQSLLPAVCRPFIERYPEVSFSVIPQESPLLE
EWLSAQRHDLGLTETTLTPAGTERVTLMTLNEVCVLPTGHPLLVKDRLTPQDFAGQNFISLSSTDSYR
HLLDALFGEQGVERRMVMETHSAASVCAMVRAGVGVSIVNPLTALDYAGNGVHVRPFSIDVPFTVSLI
RPLHRPSSALVTAFIDHLHQQAAAFAARLAAAVRR
>CORE_REP|Org14_Gene622#
MDLTQLRMFCCVAETGSVARAAEQLHRVPSNLTTRLRQLEQELGADLFIREKQRLRLSPMGHNFLCYA
NRILALSDEAMSITHAGEPAGNFALGSMESTAATRLPSLLAAYHQRFSQVSLSLTTGTSGEIADRVRA
GTLAAALVDGPVPYDELNGCIAYPEHMVVISCLDHAPIHSAKDANGETLFAFRASCSYRLRLEAWFKR
EGARPGQIMEIQSYHAMLACVASGAGLAMIPHSVLSLLPGHERVRVHTLPPDVADTATWLLWRRDAFG
PNVRALKELIIEQTETAAVDESTPNDLSDVVDIA
>CORE_REP|Org3_Gene2084#
MIEIDAQRSRMARQAMAIATGNGYTPSPVPRVKILYVDRHCPRQPVMYEPGIVIIFQGHKVGYCGSKV
FQYDPRNYLLMTVPLPFECETFASPELPLVGLAVNIDTQMLQDLLIDIGDDDYLMQPRAESNGVNLAE
LTEALLCATERLLDVMAKPLDARVLGPQIVREILYYVLRGTCGASLQELVNRHTHFSQIAKALRRIEH
QYADNLNVEQLAGEVNMSVSAFHHNFKAVTNTSPLQYVKSYRLHKARLLMVHDGLKASTAAIRVGYES
ASQFSREFKRLFGMTPSDEVARLREANPLLLEG
>CORE_REP|Org20_Gene743#
MIKQRTLKRIVQATGVGLHTGKKVTLTMRPAPANTGVIYRRTDLNPPVDFPADAKSVRDTMLCTCLVN
EHDVRISTVEHLNAALAGLGIDNIVIEVDAAEIPIMDGSASPFVFLLLDAGIEELNSAKKFLRLKETV
RVEDGDKWAELSPHNGFRLDFTIDFNHPAIDASSQRYRLDFSAESFVRQISRARTFGFMRDIEYLQSR
GLALGGSFDCAIVVDDYRVLNEDGLRFEDEFVRHKMLDAIGDLFMCGHNIIGAFTAYKSGHALNNKLL
QAVLAKQEAWEYVTFQDEAEMPLAFKAPSTVLA
>CORE_REP|Org44_Gene4817#
MTALATLRDVGFEEWLGKINTACGRFCAKTLGPGFSGAMQEFRAHALRLSVVDVSQARLYRTPREIAR
SDGAHFFTVFQLRGSALMEQGESQTVLSPGDITLIDASRPSSFTFQRDSRQISLLLPRGCLPVPPPCA
QRLGAELSAVRLSRRLVLSSMQDPQLAAAESEAVLNALAALLRPALALEQARPEGQQPVFDKALALID
RHIQSAQLRPEWVAAELGVSLRSLYRVFARQGLVVAQYIRNRRLDLCAQALRSAAGQEKLAGVGLDWG
FADHSHFSTAFKQRFGMSPSEYRRQYQ
>CORE_REP|Org23_Gene336#
MRKSTGFIANIDICKEYDARYAADEVHYETFAGLAAFFGRDMQVHWHDCFFQVHFLETGKIELQLDDQ
HYSVQAPLFILTPPSVPHAFFTEPDSDGHVLTVRQELIWPLLERLYPGSNLALDMPGICLSLADAPQE
LTALSHYWALIRREFAQNLAGREQTLALLAQAVFTLLLRNTALEDSANSGVRGELQLFQRFNKMVDER
FREHLPVPEYAQALGVTESRLNDLCRRFANRPPKRLIFDRLLREAKRMLLFSACTVHETAYSLGFKDP
AYFARFFNRLEGCSPSTYRAAQHALS
>CORE_REP|Org45_Gene2437#
MTNEDIFFIEELIEWVEIHLEKRPNLDEVARISGYSKWHLQRKFKRITGIQLATYIRSRILTRAAVAL
RITRRSIIDISDELGFDSQQTFTRMFKQRFGTTPNRYRSMTHWDVKNLMPRFNFDASYGAGYYPEVKR
LTLPEMQLVGFTRRLDFASEQELEYSSCMAMKDEIFNDFFKGLHVDCRRIYSIYSPHAGEGDELSSTL
VMAVDPEHKKDILSNYQIDTFHLPSREFISINHKGSAKECLQFFGYLMSHVMPGLKDEVRGSMEMEII

QTKEWNPESKLRQIDVDYTYLISID
>CORE_REP|Org32_Gene2606#
MKNVALFVGNDIFSWLVCQDLIAALRDECTFTVYFPLAKSAGRTQEPAVRRLGLYEREVLNDFVFPFV
GRNAAACEGAYQPPALFLAAAGVKAHRVLDINDAAFISSLGHMDGVISLRCYQKFSADYVRAFSRRGK
LLWNLHPGDLPRYRGVMTLFRAMMNGDRDCAVTLHEMDEHWDAGPVIARLPAELRHDLSFLENMMLLG
VQSGTFLARQLMRAEHSEAITAEKQGDSRYWGFPDAQTLAQAEQAGIELVDHDAVREQYLGLFVGDRF
HPLAGQFCAGFDDFVRAHGHD
>CORE_REP|Org21_Gene2881#
MDIKQLRALVALAEQGNYRQAASLLCISQPALSKQIQALETQLGVRLFERGRQGAVLTAGGQRLYPEA
QALVEQYQQFQRRARRVALGEAGRLALGFGLSSFHLAPQLVAAFRRRFPEVAIGLEDMPSERQYQLLL
QGELQVGFVRLPVTTPLCGAALLSDRLVLAAPGALALRADDLMARFNQLPLLQLTPKRGRGLSDQSLR
FIAAHRLTPNVVQQAGDIQTLLALVAAGVGVALLPHSITHIAPAGIDILPLSGEETEWQVGIAWDPQR
ADALRDNFIQTALAVQRA
>CORE_REP|Org16_Gene2285#
MDQAGIIRDLLSWLESHLDQPLSLDNVAAKAGYSKWHLQRMFKDITGNAIGAYIRARRLSKAAVALRL
TSRPILDIALQYRFDSQQTFTRAFKKQFAQTPALYRRAEDWNAFGICPPIRLGAFTLPQPEFVSLPDK
HLVGLTQSYSCTLEQITTVRTELRSQFWRQFLGDVETLPPVLYGLHHSRPSQEKDDEQEVLYTTALEP
DQVPDKVQEGQPLVLPGGEFAMFSYEGPTENLQDFILTVYGTCLPALQLTRRKGHDIERFYPKGERRP
HQAPIEIKCDYLIPIRR
>CORE_REP|Org36_Gene2971#
MGTQESHIKELLVWIEDNLTNPLSLDIVSAKSGYTKWYLQRMFKKQTGLSLASYIRARRLYLAAFALR
FTQKSILDISVEYQFDNQQTFSRCFKKHFAESPSVYRHARKQDFSNLVRSLAASQPGDIQVERVSIAR
GQYAFHGKQYAYHLDIEKLDKSHLPQRSALRGQFYTLLGERPTQTYSFTQLVPDGERVRVDYTLGVTT
EYPLREGVVLEPLPEIHGEFCRFRYSGKPVALNDHIIQIYTQVLPEMGLARGDGPDITVFSYSLSGKE
ELHLELQHLVPVVPLH
>CORE_REP|Org3_Gene3521#
MERTINLCPGIGASAHIIQHTELLFPSVYFEQPHLYLIQQGHKRVRWQQREVVAHAGELLIIDGGQTV
DIINGPSEEGVFSCQLLTCDPLLLTVQPPAEDSPAPMPFDAVLALRSLPCALKHSFETTSLALALRQR
FPTIIVRHKMLEILLWLAQFGIRFIHNEAKDLTQRVRRCLATDPHSIWTAAKVAESLSMSEVMLRRKL
SMENTALRNLMIDVRMSSALALLQSTDWPISAIAQHVGYESASRFAERFRKRFGFAPTAIRGHQRIME
PTSQGVETMATGET
>CORE_REP|Org25_Gene2382#
MTQTYAKRFAQVFDYIDRHLDEALTVDKLSEVAHFSRFHFQRQFSAYCGISVWRYIQWMRLKRASYRL
AYNPLEPVIDIALDAGFQNPESFSRAFKQAFSQTPSQFRKQPAWIDWQQRFPEPKHRRKHPMKVDIVD
CPATPVAMLEHRGPSALVNETAARFIEWRKTSGLSPVRSSRTYGIAPHDPATTEAQDFRFYLCGEVTA
PIPEDNAFGVVNSMLPAGRCAVLRHLGSLDGLSESARYLYSEWLPASGEELRDFPLYFHYHNFVHEVA
EYELVTDLYLPLK
>CORE_REP|Org20_Gene1909#
MRSTSDYQPPVSLSPPSLVGLPEQEDVFAVEHLSRLCDGLAQQRPNNLRDLLNTLALIAPLLNAIPNV
VFFIKDAQARYLLANLTLARRCGFKTVTPLLGKTSADVFPAQLGSDYTEQDLRVLRHGVLIQDQLEMH
LYNGRETGWCLTQKLALYDAQGKIIGMAGISHDLQEARANHPAYQRLAAIDVHIRRHYARPIALEELT
ALTGLSVAQIERYCKRIFHLTPRQMIHKVRLEKATELLAGDLPITDIALQCGYTDHSAFSRQFKAMTG
STPRDFRLTLSA
>CORE_REP|Org28_Gene3430#
MSDNRISLSANDVKLIREQDFFNCKDFHLFIYNKVESATGLHQHDYYEFTIVLSGKCYQEINGKRVLL
ERGDFVFIPIGSHHQSFYEFGATKIFNVAVSKAFFEEHYLHQLPRCFVASQAYSLRSEFLAYIESVVS
SPQFREDDFAEFLETLTFYVISRIRHYKEENDGGDDIPQWLKNTLAGMHDKAMFGERALANMVALSGK
TQEYLTRAMRRYYHKTPMQVINEIRINFAKTQLEVTNSSVSDIAFDSGYGDVSLFIKNFKRLTDVTPG
NYRKKCYGPL
>CORE_REP|Org44_Gene4808#
MQGVPQQFPFEKDCAQFRHLSHLPGVELYQAHIERYAFEPHTHDAFAIGTVDTGAERFRYRGAQHLAA
PGALVLMNPDELHTGEAETPGGWCYRMLYLAPEALEQLSGARSQWFTDAVRHDPRAAQRLSAILATLW
QTDDPLTLDGLLLEAVELLYPHIRTGQREKAEAAHRFEVVKSYLHDNFAEAVTLNQLAELVSLSPYHF
LRKFKAEYHVSPQQMLMAIRLSQAKRMLERGMPAAQVAAAAGLTDQAHLTRAFANRYGVTPVRFQKQV

KLG
>CORE_REP|Org23_Gene4045#
MSEKTPQFWRDPQLPFVEARAIADGRQACYSLHSHEFFSIGAITGGVSTYVNGERRMQVSAGDLVVIN
PQQAHACNPIADRRWSYIMFYLDLAWIGALQQELLGGEGERFVPFSRPLSRDPALFHGLNRLYALLTD
PLCSALEKQIAMVEYFSALQLGLGSGRQPETPPHARLEAAAAFIDAHCTRPLTLDDICRAAALSPSYL
IRAFRQRYGMTPHAYLVNRRVQHGHRLLKSGLPIAAAASESGFADQAHFQRTFKQLLAATPGQYQKPS
ANR
>CORE_REP|Org31_Gene2716#
MTDMHSLFIAFVLGVVEGLTEFLPVSSTGHMIIVGEWLGFTGDKAKTFEVIIQLGSILAVVVMFWRRL
FGLIGIHFGGKPVEHEGKTHGRLKLGHILLGMIPAVVLGLIFHDVIKSLFAPKNVMYALVVGGLLLLT
AEWLKPKKPRAEGLDDITYRQAFLIGCFQCLALWPGFSRSGATIAGGMLVGVNRYAASEFSFILAVPM
MIGASGLDLYKSLHFLTWGDLPMFAVGFVTAFVVALIAIKTFLSLIKRISFVPFAIYRFIVAAVVYMV
FL
>CORE_REP|Org12_Gene2905#
MKVQIPRRNLDIDQVPRPLFAVQSSTIEQDWEVEPHRHQKAQLIYTVRGMIRCEVENGLWLVPPQCAL
WMPGNVLHNAQGAGSTEAYCLFVDQHVVAGLPQSCCTLSVSPLLRELLLQATTFEPLYDEQGAEGRLT
AVLLDQLVAAPIENLHLPVSDDARIRQLTEGMLSCPADKSTLGQWAQRIGMSERSLSRTLQQQMGMSF
GHWRRQLHVMLALQRLTQGESVQTVALDLGYESASGFVTMFRKAVGKPPARYLAERNASGQPLGGAIS
M
>CORE_REP|Org36_Gene2477#
MITNLISGLLAPCFALYACAKSRRYWRQCRRLYTFQPIYRTSGALLAVELLTAVYHPNEPDKRQSPEQ
YFASLGVAQRLRVIQEQLALLQRWQALFIRHAVMVSVNIDGIALQALQRHSELQRQIAEMPYLRFELV
EHAETASNHPLQQIVGGERLWLDDFGSGLANFSAVGAWRYQYIKVARELFTLLKQSEEGVQLLGTLIT
MMNQHSDGVIVEGVETEQEWRLVQRSGALAAQGYYLSRPACFETLHSVPTLFAAPGAPA
>CORE_REP|Org18_Gene263#
MIDQTAFIHPSAIVEEGAVIGANVHIGPFCYVGSQVEIGAGTVLKSHVVVNGITKIGCDNQIYQFASI
GEVNQDLKYAGEPTRVEVGDRNRIRESVTIHRGTAQGTGLTKVGNDNLLMVNVHVAHDCVVGNACVLA
NNATLAGHVEIDDHAIIGGMTAIHQFCIIGAHVMVGGCSGVAQDVPPFVIAQGNHATPFGVNAVGLKR
RGFDKDEMQAIRNAYKILYRSEKTLDEAKAEIEALAKEQPVVQQYLDFFTRSTRGIIR
>CORE_REP|Org19_Gene3014#
MRNVRIDDIDHVTRAVIAIGTDYPPGHLLPMHSHRRAQLLYGATGVMHVFTQQGNWVVPPQHAVWLPP
QMPHAVRMVGVTTRSLYLEPGALPAERPQVCQVVSVTPLMRQLLMAAVDMPLEYAQEGRDGALATLLL
HELARLQPLPLHIPLPADPRLGELCRAFLQHPDAHDSAQRWAPRLYMSIRTFSRFFRAQTGLPFSQWR
QRACVVLALALLAEGRSVTQVAMEMGYDSSAAFSTMFRRVLGQAPSSYLTEDGRDG
>CORE_REP|Org20_Gene874#
MIPEKRVIRRIQSGGCAIHCQDCSISQLCIPFTLNAHELDQLDNIIERKKPIQKGQTLFKAGDELKSL
YAIRSGTIKSYTITEQGDEQITGFHLAGDLVGFDAIGGLKHPSFAQALETSMVCEIPFETLDDLSGKM
PNLRQQIMRLMSGEIKGDQDMILLLSKKNAEERLAAFVYNLSRRFAERGFSPREFRLTMTRGDIGNYL
GLTVETISRLLGRFQKSEILSVKGKYITIENADALSVLAGTPRINVSVNA
>CORE_REP|Org44_Gene3272#
MDIQVERLSAVIDAVASPRFYPSLLNWLEGFFAFDNAIVYAFERGRPPRCLIKTERDNSDAVNQIYQQ
GAYLQDPFYRALNDGGAGEVLTLRQLAPCGFYHSDYYRNFYRKTGWHDEAGVLLQLTPERGLGVFFGS
ARRTVAVRYPQRADLRSALTLVKSVARLHGEVVAAPAEADTGNDDGAQARYLLTPREREIVDLILAGC
GSQQIADRLFISLGTVKNHRKNIYGKLNIGSQAELFSLLLTAPQRRSA
>CORE_REP|Org14_Gene4232#
MMQIRSQRLQHEKKHLTPWHRHEGGQIYLLTRGMLAMELPGRQWAITGGTLGWLPPGCAHQALACGDV
AGWSLYLPVESVPEMPPLPQLPQLFTASALLQALVERIAQFPAGPLSAPQRRLLQVLLDEMHAASSAP
LQLPLPQDARLLNIARALLNDPASPRSQCDWAIWAGLSPRTLSRRFLQETGISFALWRQQARVLRSLE
GLSRGKAVGEVADACGYDNVSAYIAAFRHRFGVTPGAYFAPARQTE
>CORE_REP|Org21_Gene2589#
MEDEYNLSNIIGKRLEAALGELGDLLWAYVVLSKKDIACIFGVTNYPSEWVKKYQEQGLQYIDPVVLT
ARNRLTPFAWDEQIMADAGLHFPELFEQARGFGVTHGYTFVLHDYNDNLVTLSFAFNVEQRAEAIQAL
TERKGDISVLLSSLHESYLALSPLSAKNAAALERNVRFTDRENEILYWASVGKTYQETAMILGIKTGT
IKFHMSNIVKKLGVTNARHAVRLGMELRLIKPVEY

>CORE_REP|Org38_Gene3396#
MHSTASDAFINSCLATITHLIPVSAGVFYLVDRDLRPDHYILHGMPDKTHQQYLNHFQQIDPLQPANF
HRQDITMVGMSPAAIADNRRYYHDFMLPNDMRDMTEIFIRQRKRIVAGVSLIRDTPFTDVERGRLRAV
LPLIELATRDLLPDSEAQLLTAKEQEIVNLVREGASNKRIALKLGISLSTVKTHMRNIFAKTDVVNRT
ELVAGGFLAHG
>CORE_REP|Org23_Gene2880#
MKTALLLIDLQNDFCPGGALAVTAGDAVIPVANQAIAACLARGEPVVASQDWHPANHRSFAVNSDAQV
GTLGELEGLPQVWWPVHCVQGSHGADFHPQLQRQHINAVFRKGQDTNIDSYSAFFDNGHRAQTELHGW
LQSQGVRRLAVMGLATDYCVKFSVLDALAAGYPTQVIVDGCRGVNLQPDDSERALQDMARAGAQLVTL
SQFLAN
>CORE_REP|Org12_Gene2927#
MGQDYALVVDDHPLVASGIANFLSTHCRFKQAHVVTNEENCYRHIRENGPPRLLVIDFWLSSGTALKL
LKEVKQRYPQVRILVVSGDENNDIWQKVHNAGGHGFVLKNEPPELFARAVFALNNNQEWFPEGNEAAI
KNNHDHLNKFNLTPRQLDVLTMMLRGLPNKRIATQLSISEPTVKEHISNILKKIGVNSRVEAITLLHG
KRDPSS
>CORE_REP|Org33_Gene329#
MPTIIMDSCSYTRLGLTDYLTSHGVKKRHINAIEDIDSLHEKCSKLNPSLVFINEDCFIHEANATERI
KRVISLHPDTLFFIFMAITNVHFDDYLYVRKNVIISSKSIKPETMNQLLSHYLERTSLRTEKSSLDQT
PVTLSQTESNMLRMWMSGQGTIQISDQMQIKAKTVSSHKGNIKRKIKTHNKQIIYHVVRLTDTLTSGI
FVNSR
>CORE_REP|Org26_Gene1779#
MKLLVVDECCFTRVGIASYFADSGITTIKCCHSIEYATPLLASFQPSHILVNLSNQCRYNEADAQLLA
FMEASQSALLFIYLDTPYPYSETPMRIADNAFLFNKSILPLTLRTLRENPLALADDGEERSLFSPQEL
TVMKYWMAEMPNYRIAKKLQISSHTVYVHKRHITEKINARNRLEFYSLYNVLRYFYPPNTPTNSTPLA
LLAV
>CORE_REP|Org48_Gene2344#
MQKKLDSLLAAAGIELPDQQKQQLLGYVGMLDKWNKAYNLTSVRDPQQMLVRHILDSIVVNPHLQGSR
FIDVGTGPGLPGIPLAIVRPDAHFTLLDSLGKRVRFLRQVQHELGLNNIEPVQSRVEAFPAEPPFDGV
ISRAFASLQDMLSWCHHLPAKGQGRFYALKGVRPDEELAHLPEGVSLESIVRLQVPELEGERHLVVLK
AN
>CORE_REP|Org31_Gene2423#
MLGTMMTKSTHCNVDTREHLLATGETLSLRLGFTGMGLSELLATAGVPKGSFYHYFRSKEAFGEAMLQ
RYFAHYDAQMQALFADRRGDARHQLLGYYAQAISYHCRSECHNACLAVKLSAEVSDLSEPMRHALETG
TARVIGHLQEAIERGIAEGSLSVAMSPAATAETLYSLWLGASLRAKIRHSLAPLTSALESIELLLRPP
QA
>CORE_REP|Org21_Gene2928#
MEQLNHFLFAWINATPASPEWMIDFATFLARDLIIIVPLLIVGLWLWGPQSQLASQRQVVAKTTIALL
FAMLAAATIGALLPHERPFVAGVGYTFLAHAPDSSFPSDHGTAIFTFALAFLFWHRVWSGVLLMIVAV
GIAWSRVYLGVHWPLDMVGGFLLGLVGCLFAQLVWNLFGDAIADKLSRLYRFLFAFAIRRGWVKE
>CORE_REP|Org44_Gene507#
MPKVGMQPIRRQQLIDATLAAVNEVGMHDATIAQIARRAGVSNGIISHYFKDKNGLLEATMRYLISHL
GEAVKLRLQALTDNSPAARLQAIVAGNFDDSQINSAAMKTWLAFWASSLHQPQLNRLQQVNGRRLYSN
LCAEFRRVMPQPQARLAAKGLAALIDGLWLRSALRGSAFNQAQALALTTEYITFQLRGQTPPGG
>CORE_REP|Org12_Gene2101#
MAEKENTKRNRREEILQALAQMLESSDGSQRITTAKLAANVGVSEAALYRHFPSKTRMFDSLIEFIED
SLITRINLILQDEKETFNRLRLILLLILGFAERNPGLTRIMTGHALMFEQDRLQGRINQLFERIEAQL
RQVLKERKLREGKGFIVDETLLASQLLAFCEGMLSRYVRSEFRYRPTQEFDARWPLLAAQLQ
>CORE_REP|Org11_Gene1861#
MYRRDIPEQRKEQLINAAFETISVVGLAGVTLSQVAKEAGLSTGIVSHYFGDKEGLLSATMRKILRDL
RDAVAECRAQAASDSQSQLCAIIQGNFHPSQTNAISMRAWLDFWAASMHQPVLRRLQRANDRRLYSNI
CSQFRRELPLQQARDAARGLAAMIDGLWLRGSLAGDDTDLQQDYRIACDYVIQRLRAAPSAE
>CORE_REP|Org14_Gene2680#
MLSPNDSKQAKVQARRDQIVEAAKTSFRRHGFHAASMAEIAQGSQLSVGQIYRYFANKDAIIEEIVNR
IIASKMQRLENLGDHINLIAGTLAARTLFQQPGESETDHMLMLEVTAEATRNPVVAKMLSDAEARLFR

HVCHNLQRLYPDFSAEEIAARVEFIAVMSEGTGYRILTTQKADASLLRDLYQQAISHLFRKR
>CORE_REP|Org4_Gene2025#
MTADLNALPARQRILLTAHDLFYQEGIRATGIDRIIKESGVTKVTFYRHFPSKNDLITAFLAYRHQQW
LAWFSTALARHVAQTGGLLAALAPCLAEWFDDPRFRGCAFINTAVELADLLPESLHIASQHKRQMADE
LARHLPAGPQREQHSAMLAMLIDGAIVRVQIERLPQAALQVLNATLDMLAQGGFDQ
>CORE_REP|Org22_Gene3650#
MSAALIIIDLIEDLIGPKGRANHCREQVLATHLLANVNAAAAYARVRKIPVIWVRVGFADDYHDIPPH
SPLFNHLKQIGALRLNSPGCRWMPELHQEETDLLFEKTAVSAFSGNNLLAWLRQHRCHHLLLAGVSTP
LAIESTARQAHDAGFQVTVLHDLCAAPTQEIHQQSLDTLQNLAEITRSQAWMKG
>CORE_REP|Org43_Gene2420#
MLNIVLFEPEIPPNTGNIIRLCANTGFNLHLIEPLGFPWDDKRLRRAGLDYHEFTRVHRHADYAAFLA
AEDPQRLFALTTKGTPAHSAVSYQAGDYLLFGPETRGLPADILDALPAQQKIRIPMQAQSRSMNLSNA
VAVVVYEAWRQLDYAGALIKA
>CORE_REP|Org5_Gene4503#
MKSTDIEQPHAKNLLQLDQQLCFALYSANLALHKVYRKLLSQLELTYPQYLVMMVLWERDRVTVSDIG
ERLFLDSATLTPLLKRLETAGLLVRYRATADERQVIIALTEAGRALRERAQSVPEAVMCATDCSLDEI
VSLKQQLEKLRGSLIDQI
>CORE_REP|Org12_Gene2908#
MHESLTIALLQARETAMGFFRPILKSHNLTEQQWRIIRVLANSRSIEFHELAAETCILRPSLTGILSR
MERDKLIFRLKPVNDQRKLYVSLTQQGQDLYEVARHQVEQGYAEIEAAFSRQKMDQLMTLLDELITLG
DSLPANVAAHPAKQ
>CORE_REP|Org49_Gene4025#
MKIGELAQRAGMAASAIRYYEQLGLLPKPVRGVNGYRVYGDSALERLHLIQIGQNLGFSLQAIQRVLA
LQGSAYQDGLIRGVDERLAEIELMMATLNEQRETLLTTRLTLLESGVAGLCQAKGEKQADASPAWPAK
LARMRNRIDTE
>CORE_REP|Org24_Gene160#
MESTLGSDLARLVRVWRALIDHRLKPLELTQTHWVTLHNINRLPPEQSQIQLAKAIGIEQPSLVRTLD
QLEEKGLITRHTCANDRRAKRIKLTEAADPIIREVDSVITSTRSEILSGITADEVHLLVGLIGKLEQN
ITELQNK
>CORE_REP|Org44_Gene1295#
MFKIGQLAKLAEVTPDTVRYYEKQGMMDHNVRTEGGYRLYTEQDLQRLRFIRYAKQLGFTLETIAELL
SIRVDPEHHTCQESKSIVDARLSEVESKLAELTRMRESLKRLSDACCGTAHTSNYCSILEALEQGASD
EKGKKGC
>CORE_REP|Org8_Gene1715#
MGDSSKDGVILLSRILLMVLFIIFGWMKLVNFGATVTAMEGYGTPMPYLAAIVAVVVEFIFGIALILG
LFTRPIAVIFALYVLGTAFIGHPFWKMTGMEMMGNEINFFKNISIIGGLLLLAVTGAGRYSLDYKIFN
K
>CORE_REP|Org34_Gene207#
MSEALKILNNIRTLRAQARECTLETLEEMLEKLEVVVNERREEDSQAQAEIEERTRKLQQYREMLIAD
GIDPNELLQTMAANKAAGKAKRAARPAKYQYKDENGELKTWTGQGRTPAVIKKAIEEQGKSLDDFLL
>CORE_REP|Org23_Gene1104#
MNISDVAKKTGLTSKTIRFYEEKALITAPIRSDNGYRHYSAKHVEELTLLRQARQVGFNLDECRELVA
LFNDPARHSADVKARTLQKVAEIEKHISELGNMRQRLLTLAEQCPGDEGAECPIINNLAGCCRSN
>CORE_REP|Org43_Gene1700#
MAKELDIGAVARLSGVAPSALRHYEKKGLIASIGRHGLRRQYAAGVLDQLRLIALARLAGFTLDEMSA
LFDERGKIALDRVLLAARADELDRHIQRLIQVRDGLRHMVDCPEPEHLQCPQFRKILQQGEF
>CORE_REP|Org22_Gene183#
MIQEHHLSLVCALSKWVETHLGRVIHLEELAEYSGYSLWHMQKLFKEATGISLGKYIRERRLAGAVYQ
LRSSEASIFDIALDFGFGSQSHFTYMFRKRFNITPYDFRQDLSVDLHIDPPLHVIHQKSA
>CORE_REP|Org13_Gene200#
MIYWIFLGLAIMTEIIGTLSMKYASVNGGMIGHIVMYVMITASYVLLSVAVKRVALGVAYALWEGIGI
LFITLFSVLWFDEPISALKVLGLATLIAGIMLVKSGTRKERKQVSPRGDNHATV
>CORE_REP|Org10_Gene2347#
MNTTGFITDLKTWIDNNLEEKLDINTVADRAGYSKWHLQRMFKRQTGYALGEYIRMQKLKVSAERLAN

SGEPIVSVAISLGFDSQQSFNRSFKRQFGQTPGDWRRALAQPTAVRCTHH
>CORE_REP|Org37_Gene613#
MQQFELYHIGFLGLAIVLEIIANIFLKMSDGFRKIWLGLLSLLSVLGAFSALAQAVKGIDLSIAYALW
GGFGIAATIAAGWIMFGQRLNAKGWIGLALLLTGMVILKLS
>CORE_REP|Org33_Gene76#
MAWIILLIAGLLEVVWAIGLKYTHGFTRLTPSIITIAAMVVSMLLLANAMKTLPAGTAYAVWTGIGAV
GAAIMGMVLLGESTNIARIISLCLIVVGILGLKFSSH
>CORE_REP|Org37_Gene3116#
MRVSPAGKLMRLQTLGLCISLALGVPSMAVFAAGDIQFNTDVLDVHDRENIDLSQFSRGGYIMPGTYG
MVVHVNKNDLQEQQVPFYAPEDDPNGSRACVTQVLTGQLGLKEDALKGVTWWHQGECLDEASIPGMEV
RGDLATSALYLSIPQAFLEYTAENWDPPSRWDEGIPGLLFDYNVNARTQKQHQGNGSSYSLSGNGTTG
LNLGAWRLRADWQGNVDHTTGSGQSTAQKLDWSRYYAYRAIPALRSKLTVGENYLDSGIFDSFRFTGA
SLMSDDNMLPPNLRGYAPEVVGIAKTNAKVVISQQGRVLYETQVAAGPFRIQDINDAVSGEMNVRVEE
QDGSVQEFTMNTATIPYLTRPGSVRFKLASGKPSDFQHHSRGPMFGTGEFSWGVSNGWSLYGGALVGG
DYNALSLGLGRDLMALGALSFDATQSRARLPQADGTLSGGSYRLSYSKNFDEYDSQVTFAGYRFSQED
FMSMSEYLDARYYGTRTGNGKEMYTVTFNKHFRDWGLSTYLNYSHETFWDRPANDRYNLTLSRYLDIG
SFRNVSLSLSAYRNKYNGVNDDGGYLSLSLPWGNSGSVGYSATVNRSDVTHQANYYDRLDEHNNYSMS
AGSSRSGASLSGYYNHEGDMARMSANASYQEGRHSAMGLSLQGGATLTMEGGALHRAGIPGGTRMLID
TNGVADVPVRGYGRTSNTNAWGKVVIGDVNSYYRNKASIDLNKLGDNAEATTSVVQATLTEGAIGYRQ
FDVIAGEKAMAVIKLADGSQPPFGATVMNARKQETGIVNDGGSVYLSGINAGDTMTVHWAGNAQCEVR
MPTPLPAEMLMNSLLLPCHPLSAQAPTHDGQTTAEDAPGAVTSTVPGRTVQPPSLSDKNRELF
>CORE_REP|Org7_Gene1835#
MLDRIIAHTPLGQEQLLFRSLDGIEALSTPFDFSIELLSTDARLDRKALLGQPLTLEIPTQGFLSAPR
YLNGKITAIAVSSEEIGGTRYAVYNLHVQPDLWPMTKDRNFRIFQEQTVPQIVKTLLAEHNVQLEDQL
TGDYRLWGYCVQYNESSFNFISRLMEQEGIYYYFKHEMGKHTLVLGDAPHHHQPYPGYEMIPYHLTPS
GGSTSEEGISQWTLSDRVTPGIYSLDDYDFRKPNAWLFQARQNPVSPTPGQIDVYDWPGRYTEHQQGE
FYARVRQEAWQAEHQQIRGTATAMGIAPGSTFTLYNAPHADDNREYLTLQASYHLKENRYASGDDQSS
EHRIDFIVLPADVPWHPPQQATWPKTHGPQTARVVGPAGESIWTDKYGRIKVKFHWDRFGPKDDGSSC
WVRVSSAWAGQGYGGVQIPRVNDEVVVDFINGDPDRPIVTGRVYNEASMPPWALPAAATQMGFMSRTK
DGTADNANALRFEDKAGAEQVWIQAERNMDTQVKNDESHTIANDHTHLVGGNQIKRVVLNQATGVKGE
SSALTGKTRSDAVVNAFTLGSGESLRLECGESVIELLADGQINITGTSFNITVKEDGAINTGGQLDLN
QPGGAARTAAPGGGHQAAIQSAVDQLFPNEEASGTPGKPVNAAPRAAAAAPASITQNAQSTTKPGRID
NRVVESVMASEGSAGEQGGRRELYGFRKGNGNAYDKILAARNQYGQGSAEEFEEVSKAMSASAKSAGA
LNFSDPGKQGAITSLAHMRGSSGAQAILNSMESGRIVKADTLTSEAIAKIESMSAESFQDNLLKARVE
YDRAIYGDTITTQGGKQYNWWARYGNGLQKRYAREAEEFLKLSNE
>CORE_REP|Org46_Gene4129#
MSDHSMTSLLTIKKTPIIVILSVLWAKPVFSATEFNTDVLDIGERSKVDLSRFSDADYVMPGTYLLDI
KINQKTLPQRSIQYFPSPDNKSGSQVCLPPDLVEKMALKEDAARKVTLWHDNQCADIRGIKGATVSDR
ISGGVLAITIPQAWMKYSDPDWTPPEQWDDGIPGVLLDYNLSGQIGKQHHDNGTAESLSSYGTLGANL
GAWRLRADYQTDFNQQYGRRDSNFDWNQIYAYRALPMQAARLTLGETYLNSPVFDAYRFTGLNLASDE
RMLPPNLQGYAPEVRGIAKSNARITVSQEGRTLYQTTVPAGPFAIQDLSSSVRGKLDVKVEEQDGSVS
TFQVDTASIPYLTRPGYVRYNMALGKPSAYDHRTQGPVFSAGDFSWGLSNAWSLYGGALLGGDYNAWA
LGLGRDLNLFGALSVDATQSIARLPDEPSAKGMSFKVNYAKRFDELNGQITFAGYRFSQRKFMTMSQY
LQARYGDIDDRYSGRQKELYTVTASKTFMAEDSAQAITAYLTYSHQTYWDAGAQNRYGMSTSKLFDFG
GISNITASLAAYRTHYRGRTDDSAMLNFTVPIGEHNRLGYALQVNNRDVSQTATYTDNSDINNTWQVG
SGVTQSGKPTASGYYTHNASFGTLNANASYQQGSYSSIGGTFRGGLTATRHGVAAHQNAGNGGSRMML
DTNGVAGVPINNGRAYSNRFGLAVISDITSYYNTDTRIDVNKLADDVEATRAVVQGTLTEGAIGYRHF
EVVKGSKLLATIKLADGSEPPFGATVLSATGREIAVVNDGGSVYLTGVQPEERLDVAWEGRRQCRIAI
PGAAKPLDRLLLPCAKP
>CORE_REP|Org37_Gene4361#
MKKLTIGLIGNPNSGKTTLFNQLTGARQRVGNWAGVTVERKEGHFTTPQSDVRLVDLPGTYSLTTISE
QTSLDEQIACHYILSGDADLLINVVDASNLERNLYLTLQLLELGIPCIVALNMLDIATSQHIDIDVAA
LSARLGCPVVPMVSTRADGIGVLKQMIDNHHINEQQALVNYPPLLLKAVATLSDAMPQTLPAVQRRWL
ALQMLEGDIYSHRLAGPAVALLPAAIQALQQQQQQEDPALVIADARYQSIAALCDAVSNSQQAMPNR

LTEMLDKVILNRWLGVPIFLLVMYLMFLLAINIGGALQPIFDIGSAAIFIQGIQWLGYTLHFPDWLTV
FLAQGIGGGINTVLPLVPQIGMMYLFLSFLEDSGYMARAAFVMDRLMQALGLPGKSFVPLIVGFGCNV
PSIMGARTLDAQRERLITIMMAPFMSCGARLAIFAVFAAAFFGQDGAGVVFSLYMLGIAVAILTGLVL
KYTIMRGEASPFVMELPVYHVPHLKSLLLQTWQRLKGFVLRAGKVIVVASMFIGGLNSFSFSGKTVDN
INDSALASVSKVLTPLLQPMGVHSDNWQATVGLVTGAMAKEVVVGTLNTLYTAEHINKEAFDAANFNL
LDELGGALNETWDGLKNTFSLSVLSNPIEASKGDGEMGVGSMGVMSSKFGSGISAYSYLIFVLLYVPC
VSVMGAIARESSRGWMTFSILWGLNVAYSLATLFYQVATFNQHPQYSLTAILVVLAVNLLVLFGLRRA
RSRVTVRLGNATPAACCQGAKGSCH
>CORE_REP|Org23_Gene1253#
MTELNEKLANAWEGFSKGDWQNEVNVRDFIQKNYTPYEGDESFLAGATQATTTLWDKVMEGIKLENRT
HAPVDFDTNVAATITSHDAGYIAKELETIVGLQTDAPLKRALIPFGGIKMVEGSCKVYGRELDPQLKK
VFTEYRKTHNQGVFDVYTKDILNCRKSGVLTGLPDAYGRGRIIGDYRRVALYGIDFLMADKLNQFKSL
QEKLENGEDLEMTIQLREEIAEQHRALAQIKEMAAKYGYDISGPATNAQQAVQWTYFGYLAAVKSQNG
AAMSFGRVSTFLDVFIERDIKAGKLTEEQAQELIDHLVMKLRMVRFLRTPEYDELFSGDPIWATESLA
GMGVDGRTLVTKNSFRFLNTLYTMGPSPEPNMTILWSEKLPLNFKKFAAKVSIDTSSVQYENDDLMRP
DFNNDDYAIACCVSPMIVGKQMQFFGARANLAKTMLYAINGGVDEKLKMQVGPKEAPMMDEVLDYDKV
MARMDHFMDWLAKQYVTALNIIHYMHDKYSYEAALMALHDRDVYRTMACGIAGLSVAADSLSAIKYAK
VTTIRDEDGLAIDFKVEGEYPQFGNNDARVDDIACDLVERFMKKIQKLRTYRNAVPTQSVLTITSNVV
YGKKTGNTPDGRRAGAPFGPGANPMHGRDQKGAVASLTSVAKLPFAYAKDGISYTFSIVPNALGKDDD
VRKANLAGLMDGYFHHEASIEGGQHLNVNVMNREMLLDAMENPEKYPQLTIRVSGYAVRFNSLTKEQQ
QDVITRTFTQTM
>CORE_REP|Org16_Gene928#
MVAVRSAHLNTAGEFALDEWIAGLGLPNPQSCERLAATWRYCEQQTQNHPDASLLLWRGLEMVEILST
LSMDNDSMRAALLFPLVDAGIVQEETLTEAFGNGIVALVHGVRDMDAIRQLKATQNDSMASEQVDNVR
RMLLAMVEDFRCVVIKLAERIAHLREVKDAPEDERVLAAKECSNIYAPLANRLGIGQLKWELEDFCFR
YLHPEEYKRIAKLLHERRIDREQFIDDFVAGLRAEMAKEGIRVEIYGRPKHIYSIWRKMQKKHLAFDE
LFDVRAVRIVAERLQDCYAALGIVHTHFRHLPDEFDDYVANPKPNGYQSIHTVVLGPRGKTVEIQIRT
RQMHEDAELGVAAHWKYKEGAGVTVRSGYEERIAWLRKLIAWQEEMADSGEMLDEVRSQVFDDRVYVF
TPKGDVVDLPAGSTPLDFAYHIHSDVGHRCIGAKIGGRIVPFTYQLKMGDQIEIITQKQPNPSRDWLN
PNLGYVTTSRGRSKIHNWFRKQDRDKNILAGRQMLDNELEHLGISLKEAEKLLIPRYNMNSLDEVLAA
IGGGDIRLNQMVNYLQGKFNKPSAEEQDREALRQLVQQKAPPPTRNKDNGRVVVEGVGNLMHHIARCC
QPIPGDDIVGFITQGRGISIHRADCDQLVDLQSHAPERIVDAVWGESYSSGYSLVVRVMANDRSGLLR
DITTILANEKVNVLGVASRSDTKKQLATIDMDIEIYNQQVLSRVLAKLNQLPDVIDAKRLHGN
>CORE_REP|Org40_Gene2964#
MKNNALSVMVAEQDEKLEWERLIGPLWDNRWRIAVVTGVAGMLGVAYALLATPVYQATAVVQVEKQLS
GDSLLRETLDSSMMGQNSATQDEVTLAKSRYVLGKTVDTLGLTVRVSPDYFPVFGKGFARLSGEKPPV
LSIATLTTPADMEGEALTLTVRDGQHYELSYDGSKLFSGVVGQPVAQGGWNMTVSALDASPGASFTVV
KVARQEAVDDLRKYLDVVPGGKDSGIMTFTLPSEDPQSAEAMLKNITDNYLQQNVDRKTEEAQRMLAF
LQEQLPQTQTSLNNAETQLNQFRQQNDSVDLSLEAKSVLDTQVQLEAQLNELTFKEAEISKLYTRAHP
AYRALLEKRATLEAEKARLGKQVQTLPKMQQEILRLTRDVQVDQQVYMQLMNKQQELSISKAGTVGNV
RIIDEAETALRPIKPQKMLIVLLALLLGAGGGAIVVLLRAAFHRGINDIDTLEKRGINVYATVPLSPW
QVKRNREQRQLLPRSGGRRLPILAVAEPTDLSVEAIRSLRTSLHFAMMEAKNNILMVSGASPESGKSF
TSTNLAVVVAQAGQRVLLIDADMRKGFLHRWLADDGHQGLSDMLVGNVMAEQAVRKTAIANLDFVPRG
QVPPNPSELLMHRRFADFLRWAGQNYDLVLIDTPPILAVTDAAIVGNHAGTSLLVVRFEVNTVKQIET
SMRRFEQNGVAIKGVILNGVVKKAATDMSYYNFAYPSHREDHPQAGE
>CORE_REP|Org6_Gene3182#
MKELKIATSASLVATIETLRQIVRVEQTDCTDVAAAVVSVADVNAGILARLQATGFDIPTFVAVEGDE
HLSPDYLPFVSGVFALLAGASKPFYMAQLEAAADAYEQALLPPFFKTLKTYVEMENSTFACPGHQGGE
FFRKHPAGRQFFDFYGETLFRSDMCNADVKLGDLLIHEGSAKDAQKHAARVFNADKTYFVLNGTSAAN
KVVTNALLTRGDLVLFDRNNHKSNHHGALIQAGATPIYLETARNPFGFIGGIDAHCFDERYLREQIRE
VAPEKAAAARPFRLAIIQLGTYDGTIYNARQVIDTIGHLCDYILFDSAWVGYEGFIPMLKECSPLLLE
LDEHDPGIFVTQSVHKQQAGFSQTSQIHKKDDHIKGQKRHCNHKHLNNAFMLHASTSPFYPLFAALDV
NAKMHAGPAGRRMWMDCVKLGIETRKQLLTRCSQLKPFIPQQVAGKDWQDYDTDLIANDARFFTFVPG
ETWHGFEGYAQDQYLVDPCKLLLTTPGIDAATGQYTEFGVPATILANFLRENGIVPEKCDLNSILFLL

TPAENPAKMEQLVEMLAQFERYVEEDAPLSVVLPTVYRKNEQRYRGYSIRRLCQEMHDLYVSFDVKQL
QKEMFRQDHFPPVVMNPQDANVEFIRDNVELVPIGQAEGRIAAEGALPYPPGVLCVVPGEVWGGAVQR
YFLALEEGINRLPGFSPELQGVYIEKKDHGWKRIFGYMIKQ
>CORE_REP|Org41_Gene4663#
MPDLSRRDILRAAAIGSAFSLLPASIRKALAIPANNRTGTLRDVEHVVILMQENRSFDHYFGTLPGVR
GFSDRFTIPLPGDRHVWQQQGAERLVLPYHLDSKRGNAQRVTGTPHSWVDEQAAWDHGRMSAWPTYKT
PASMGYYRQHELPFQFALANAFTLCDAYHCAIHAGTNTNRLFHWTGTNGPSAADVAVVVNEWDSPGPA
EIGYQWTTYPERLEASGVSWKVYQFLPDNFTDNPLAGFRQYRAASIQVGNPARPPKDFNAFVPYRDAL
NEAAPLYKGNGNTLPAADGNDLDAMLAGFRADIQQGKLPQVSWIIAPAAYSEHPDPSSPVQGGWFTQE
ILNALTDNPEVWSKTVLLVNYDENDGFFDHMPSPSAPSLREDGSFAGKSTVPFDTEIFQHVAPPGSQD
QPPPDGRIYGPGPRVPMLVLSPWSRGGWVNSQVFDHTSVLQFLEKRFQVHEPNISAWRRAVCGDLTSA
FNFVDPNSEALPSLPVTSRHAADGLRQRQEQLPQVPLPSPAHQRLPHQRRQARPSRALPYQLHVEATV
VAEQRRVTLNLFNTGEQGAVFHVYDRRDLAQIPRRFTVEAGKAVSDDWQTEDEYHLWLLGPNGFHREL
RGALNRPQPEVRLRPTGRSLQLQLNNPGTEAIAVTLERCPYTQQGPWHITLPAGGSHQQSFDAHASGG
WYDLTLQSPGGWLRRLAGRLEDGEHSVSDPLMGQE
>CORE_REP|Org38_Gene2989#
MNIIAIMGPTGVYYKDEPIRELHAALGAMGFQLVYPKNSGDLLKLIEANARICGVIFDWDDYSLELCS
EINELNEYLPLYAFINTHSTFDVSLHEMRMVLYFFEYGLNAADDIAQRIQQYTAEYIDTITPPLTKAL
FNYVREGKYTFCTPGHMAGTAFQKSPVGCLFYDFFGANTLKADISISVTELGSLLDHTGPHLEAEEYI
ARTFNAEQSYLVTNGTSTANKIVGMYSAPAGSTVLIDRNCHKSLCHLLMMSDIVPIYLRPLRNAYGIL
GGIPQREFTRASIAARVQETPNATWPVHAVITNSTYDGLLYNTDYIKQTLEVPSIHFDSAWVPYTNFH
PIYDGKSGMSGDRVPGKVFYETQSTHKLLAAFSQASMIHIKGDYDESTFNEAYMMHTTTSPHYGIVAS
METAAAMLRGNPGRRLINRSVERALHFRREVQRLREESDSWFFDIWQPEEIDEAQCWPLDPDDNWHGF
GQTDRDHMYLDPIKVTILTPGMNELGALEEEGIPAALVAKYLDERGIVVEKTGPYNLLFLFSIGIDKT
KAMSLLRGLTDFKRAYDLNLRVKNMLPDLYAEDPDFYRHMRIQDLAAGIHRLICQHDLPRLMQRAFDV
LPEMKLTPHQMFQEQVRGNVETCELDQLVGKVAANMILPYPPGVPLVMPGEMITEESRAVLDFLLMLC
SIGERYPGFETDIHGAKLTEDGRYLVKVLKAPQP
>CORE_REP|Org26_Gene2931#
MAALQALAGKFTHLTVGKKLGLGFALLLLLAVIIAGTGAQYLHIIESRADRIDFSNRLNEEINQAKYN
RAMYGQTYRPEYIQNNRANIENAVKLIDHGQALDWDAQSRKDLQRLVTLIGEYQQQQKEFEQAVTAKD
AVRQSWNMSEVQASLSQVERQLTNGDLQLAFIQLNQKLTQVRYGARGLLLSLNKETEAPLMAAIDDAR
DAASALSRRVSDAQRPQLQPLLAALDDYKNRIAAYLPAYEHEQQISRRLGERAQAIGMLVNAFMQDEL
AQTHNDINLAQLQMGITTLIAIIAGVLIAWRITLQITRPLHSTLAMAERIAGGDLRQAQTSTRRDELG
QLLNAVAAMSQNLRTMIEKIQMGVSQVSTASAEIAAGNTDLSSRTEQQAAAVEETAASMEQLTATVKQ
NADNAHHANQLATDASQTAQQGGKLVENVVSTMRDISSSSQRIAEITTLINGIAFQTNILALNAAVEA
ARAGEQGRGFSVVASEVRSLASRSAQAAKEIEGLIAESVSRVKTGTELVESTGNTMEQIVRSVTHVRD
IMAEIAAASDEQTRGIAQIGQAIVEMDHTTQQNAALVEESAAAADSLEEQADMLLQSVSVFRLAEQTE
PAAVKAAAPKAPAVKPAAANAAAEENWTTF
>CORE_REP|Org44_Gene1901#
MDSKLLDYYNRELAYLREMGAEFAEQYPKVAGRLGMRGIDVADPYIERLMEGFAFLTSRVQLKMDAEF
PRFSQRLLEIIYPNYLSPTPSMAIAELQPDSSKGDISNGFVVPRGTMMDSQTLKKSGITCSYATAHDV
TLQPVRIAAVELGGIPADIPLASLGLQHSGCVSALRIRLECYESVTLNNLQLDQLMFYLAGPDMQAQQ
LLELLMQHSVGLVCQTVEAQPQRRALALDGLRQEGFAAEQALLPNDLRNFEGYRLLQEYFAFPARFQF
FSVNGLRPLLQSVREGKKALRQFEIVVLLDRHDAALERVVDAAHLALHCTPVINLFPKVAERIAINEK
NHEYHLVVDNIRPLDYEVFSVQRLGGSASEKRYEQEFRPFYSTLSADDGNYGAYFSLRREQRTLSEHA
RRYGTRTGYAGSEVFVSLVDERQSPWHSDLKYLTADVLCTSRDLPLMLLQQDQGNFVMPDSIPIKQVS
LRKGPTPPRPALAEGMITWRLISQLQLNYLSMMDGDPEQGAASLRQLLGLYGNLSEPAIAKQIQGVRH
CNLRPVYRRVPEPGPIVFARGIAIDLTVDEQAFSGNSPYLLGSVLERLFSRLVAMNTFTEMTLSSQQR
GEIAHWQARMGKRTLI
>CORE_REP|Org23_Gene2753#
MEQNPQSQLKLLVTRGKEQGYLTYAEVNDHLPEDIVDSDQIEDIIQMINDMGIQVMEEAPDADDLLLA
ENSNSTDEDAEEAAAQVLSSVESEIGRTTDPVRMYMREMGTVELLTREGEIDIAKRIEDGINQVQCSV
AEYPEAITYLLEQYDRVEAGEARLSDLITGFVDPNAEEDIAPTATHIGSELSSEEQDDDEEDEDAEDD
DTEDDNSIDPELARQKFAELRDQYEATRLVIKKNGRSHASAADEILKLSEVFKQFRLVPKQFDFLVNS

MRTMMDRVRTQERIIMKLCVEQCKMPKKNFVTLFAGNETSDSWFEAAVAMAKPWSEKLKDVAEDVQRS
LQKLRQIEEETGLTIEQVKDINRRMSIGEAKARRAKKEMVEANLRLVISIAKKYTNRGLQFLDLIQEG
NIGLMKAVDKFEYRRGYKFSTYATWWIRQAITRSIADQARTIRIPVHMIETINKLNRISRQMLQEMGR
EPTPEELAERMLMPEDKIRKVLKIAKEPISMETPIGDDEDSHLGDFIEDTTLELPLDSATSESLRSAT
HDVLAGLTAREAKVLRMRFGIDMNTDHTLEEVGKQFDVTRERIRQIEAKALRKLRHPSRSEVLRSFLD
D
>CORE_REP|Org26_Gene4103#
MSASITAGESRDNGLQAIWNRLRANPKIPLLVAASAAIAIVVALLLWVKSPDYRVLYSNLNDRDGGAI
VTQLTQMNIPYRFAENGAALLIPAEKVHETRLRLAQQGLPKGGAVGFELLDQEKFGISQFSEQINYQR
ALEGELSRTIESLGPVQNARVHLALPKPSLFVREQKSPSASVTLTLQPGRALDDGQINAIVYMVSSSV
AGLPPGNVTVVDQAGRLLTQSDGTGRDLNASQLKYANEVENGFQRRIEAILAPVVGSANVRAQVTAQI
DFATREQTDEQYQPNQQPDKAAIRSQQTSLSEQIGGPQVGGVPGALSNQPSAPATAPIETAKPATAAG
NNANANATATAQNAATTRSAAANGVPQNTRRDATTNYELDRTIRHTQQKAGTVQRLSVAVVVNYLGTD
KDGKPQPMSKEQLAQIEALVREAMGYSSSRGDTLNVVNTPFTDTQVTGGELPFWQSQSFIDRLIDAGR
YLLVLLVAWLLWRKLVRPQLQQRQAAQQAAVAAANAPAAKAVDSSKPSNEELAQRRKSQQRVSAEVQS
QRIRDLADKDPRVVALVIRQWMSNEI
>CORE_REP|Org8_Gene1820#
MIKKITALTLLVSTALSAETLPDSHMMQDMSMGESRRALQDSTREVNQLIEQRRYQQLKQQRLLAEPE
PAAPALPQSAQCLPIAGVYLQGVTLLSPADLSALSALPEQCISSNDINRLTRELTRLYVQKGYITARV
QIVRPNSQGELGLSVTEGFIEKIEGGDRWVNSRLLFPGLEGKPLKLTELDQGLDQANRLQSNTTKLDI
LPGRQVGGSVIRLRNQHAKPWLITAGTDNYGQKSTGRWLARATATLDSPFGLSDFVSLNANSTLENPA
HRYNRAYTLLYSLPYGAFTFSGFASFSSYENHQQLPHNVVKLHGQTQQYGLRSDYVFYRDHDQIDSLS
GQLTYKRIDNYFESVRLEVSSPTLTLAELSASHLQILPNGVFSANLSVEQGMPWLGAGRHPSSVHLDS
QFTKGKLFANLSQRLRLGDATYQLNNLFYGQYSRDPLPGVEWLSLTDRSAVRGFSRSTQSGDNGWYLQ
NTLSRSFNLGATTLTPRLGADVGRILPRQDNSGWRSSAGISTGATLRYQRALVDLEVSRGWILSNHAT
PEDPVQVLARFSYTF
>CORE_REP|Org12_Gene4030#
MLNRMKVVTSLLLVLVLFGALQLISGGLFFSSLKSDKENFTVLQTIRQQQLQLSESRVDLLQARNSLN
RAGIRYMMDTNKIGSGATIDELLAKAKEELGEAERHYAAYEKIPQDPRQDPQSAERVKQQYDILYGAL
SELIQLLGEGKINAFFDQPTQSYQDNFEQSYNVYLEQNGKLYQIAVDGSNSSYNSAIWTLIVILVVVL
AVIVLVWTGIHHILVRPLNRMIDHIKQIAAGDLTQQIVVNSRNEMGVLAASLKHMQGELIETVSGVRQ
GADAIYSGASEIAAGNNDLSSRTEQQAASLEETAASMEQLTATVKQNAENARQASQLALSASETAQKG
GKVVANVVQTMHDIAGSSQKIADITGVIDGIAFQTNILALNAAVEAARAGEQGRGFAVVAGEVRNLAQ
RSAQAAKEIKGLIEDSVSRVDMGSVLVESAGETMGDIVNAVTRVTDIMGEIASASDEQSRGIDQVGQA
VAEMDRVTQQNASLVEESASAAAALEEQASMLTQSVAVFRLRSEGQEEFKAPVTNKATVTPVINHKKM
NASDLQDNWETF
>CORE_REP|Org25_Gene3782#
MSDHITPSRTPDKPVIWTVSITRLFDLFRDISLEFDHLATITPIRLGFEEAVQHIRARLATEPCDAII
AAGSNGAYLKSRLSVPVILVKPGGFDLLQALSQARRTADRIGVITYKTPLPALMEFQQTFDLPLEQRS
YVTEEDARGQIAELKAAGIQAVVGAGLISDLAEEAGLTAIFLYSAATLRAAFSDALDVTRLMLGGAKR
GGDYAARDTLQPRYGLSDLQGDSPQMEQTRRTIMLYARSPAAVLIEGETGTGKELAAQAIHREYFSRR
GAPSRGATPPFVAINCGAIAESLLEAELFGYEEGAFTGSRRGGRRGLLETANGGTLFLDEIGEMPLHL
QTRLLRALEEKTITRVGGQQPVKVDFRVISATHTRLEQAIQQGDFRADLFYRLSALRLQLPPLRARGD
DIAMLAEHFLKQSLAALDAPLTEPLRAALAGCYAALGHYAWPGNLRELRNMMERVALMLSTGVAPSGE
TLQWLLPELAVAPPPETPAAPVSAHEALARCGGDHAAAARLLGISRTTLWRRLKKPH
>CORE_REP|Org8_Gene2192#
MRLEVFCEDRLGLTRELLDLLVSRSIDLRGIEIDPIGRIYLNFSQLDFDTFRALMAEIRRIAGVTDVR
TVSFMPSEREHRALRALLESMPEPVFSIDMKGKVELANPAAQALFSLSEDKIRNQTAGALIGGYNFSR
WLESEHTAPHAERVVIRSQDFLMDITPIYLEDQQQQPAAVGAVVMLKSTARMGRQLQNLSVNDDTEFD
HIVAVSAKMRHVLEQARKLAMLDAPLLIVGDTGTGKDILARACHLRSPRGKQPFLALNCAALPDDVVE
SELFGHAPGAYPNALEGKKGFFEQANGGSVLLDEIGEMSPRMQTKLLRFLNDGTFRRVGEEHEVHVDV
RVICATQKNLTELVQRGEFREDLYYRLNVLTITIPPLRERPQDIMPLTELFVARFADEQGVARPKLAS
DLGGFLSKYGWPGNVRQLKNAIYRALTQTEGYELRPQDIVLPEFEVEMSLGDEVLDGSLDDISKRFER
SVLTRLYRTYPSTRKLAKRLGVSHTAIANKLREYGLSSRKGGAEGEE

>CORE_REP|Org33_Gene1456#
MMNTKPQLTLLKAQASYRGDPTTIFHQLCGARPATLLLESAEINSKQNLQSLLVIDSALRITALGHTV
SVQALTANGAALLPLLDEALPPEVRNQARPNGRELTFPAIDAVQDEDARLRSLSVFDALRTLLTLVDS
PADEREAVMLGGLFAYDLVAGFEDLPALRQDQRCPDFCFYLAETLLVLDHQRGTARLQASVFSEQASE
AQRLQQRLEQLQAELQQTPQPIPHQTLENMQLSCNQSDEEYGAVVSGLQEAIRQGEIFQVVPSRRFSL
PCPAPLAAYQTLKDNNPSPYMFFMQDDDFTLFGASPESALKYDAGNRQIEIYPIAGTRPRGRRADGSL
DLDLDSRIELEMRTDHKELAEHLMLVDLARNDLARICQAGSRYVADLTKVDRYSFVMHLVSRVVGTLR
ADLDVLHAYQACMNMGTLSGAPKVRAMQLIAASEGTRRGSYGGAVGYFTATGDLDTCIVIRSAYVEDG
IATVQAGAGVVLDSIPQAEADETRNKARAVLRAIATAHHAKEVF
>CORE_REP|Org46_Gene1409#
MLKKITVKAGLIALLSLMTMLLIMVSVIGVNAINEGSRSIHTLNQILGEELGSLANSSNLTLRARTAA
SLAVRQREIGQTDVSDATVGRIYGYLEQSNKEMARFVGVGTVTERGRELSNRLQNSYRAYLDQGVKPM
AAAIKAGKIDEYYHIQETRISALSIAFEKDLSDFRSFAMKLGAQQVYDAESNASTKISLIVVAGLLSV
LLAVLAWFALRVIILRPLDESIAQLEHIAGGDLTHEIRGEGDTEMGRLVRAMQRMQQALASSVSKVRD
ASSQIDTGSRELAAGNLHLAQRTEESAASLEETAASMEQLTSTVKMNAENCEQANQLALSVSDIANQG
SEVVSQVMSKMQAITDSSRRIADIISVMDGIAFQTNILALNAAVEAARAGEQGRGFAVVAGEVRNLAQ
RSAQSAKEIKGLIEASQNRVQEGEQMVESAAQTMSGITGEVGRVTALMREISAATREQSSGIEQVNLA
VAQMDQVAQQNAALVEESAAATRSLEDQAQLLAQSMAAFKL
>CORE_REP|Org18_Gene4132#
MKIRSQVGMVLNLDKCIGCHTCSVTCKNVWTSREGMEYAWFNNVESKPGVGYPHAWEDQEKWKGGWIR
KINGKLEPRMGNRVGVLAKIFANPDVPALDDYYEPFDYDYQHLHTAKQGKHQPVARPRSLITGQRMNK
IESGPNWEEILGGEFEKRSQDKNFDNLQKAMYGQFENTFMMYLPRLCEHCLNPACVATCPSGAIYKRG
EDGIVLIDQDKCRGWRMCLTGCPYKKIYFNWKSGKSEKCIFCYPRIEAGQPTVCSETCVGRIRYLGVL
LYDADRIEQAAAVENDKDLYQSQLDIFLDPHDPKVIAQALADGVPQGVIEAAQQSPVYKMAMDWKLAL
PLHPEYRTLPMVWYVPPLSPIQSAADAGELAHSGVLPDVESLRIPVQYLANLLTAGDTEPVLLALKRM
LAMRHYKRAETVDGVVDTSALEQVGLSEAQAREMYRYLAIANYEDRFVVPSSHRELAREAFPESKGCG
FSFGDGCHGSDGKFNLFNSRRIDAIDVTAKTARPEDAS
>CORE_REP|Org28_Gene971#
MRARQRRYSAKQQATAVAEVETQETENELDGLLKRSFRPRTNEASEAVRRAIGTLSEYANQGKVKVSQ
DVVLTIESLIAQIDEQLSQQMNNILHHKEFQKLESAWQGLSYLVDNTNVSETLKIRVLNISQDELTRN
LRRYRGSAWDQSPVFKQIYEQEYGQFGGEPFGCIIGDFEFDHSPMSVTLLTELAKISAASHCPFISAA
SPSLLQMSKWNELGNPRDIGKIFTTPEYASWRRLRESNDSRYLVLTMPRFLSRLPYGAKTNPIEEFAF
EEAVRPDMDDDFSWANSAYAMGVNINRAFHEYGWCSKIRGIESGGSVEELPAYAFPSDEGGYELTCPT
EVAISDRREQELSDAGFLPLVYRKHSDFAAFIGSCTMHAPAKYEDPDATANAKLSSRLPYIFATCRFA
HYLKCIVRDKIGSFRSRDDMQLWLNDWLMNYVDGDPSVSTEATKARRPLAAAEVRVEDVEDDPGYYRA
HFYLRPHYQLEGMTVSLRLVSKLPSAKKDGSR
>CORE_REP|Org39_Gene3379#
MQSTKKAIEITESNFAAAKTGYDAVADLLHYHERGNGIQINGKDSFSNEQAGLFITRENQTWNGYKVF
GQPVKLTFSFPDYKFSATNVAGDTGLSKFSAEQQQQAKLSLQSWADVANITFTEVAAGQKANITFGNY
SQDRPGHYDYGTQAYAFLPNTIWQGQDLGGQTWYNVNQSNVKHPATEDYGRQTFTHEIGHALGLSHPG
DYNAGEGNPTYRDASYAEDTRQFSLMSYWSETNTGGDNGGHYAAAPLLDDIAAIQHLYGANLSTRTGD
TVYGFNSNTGRDFLSTTSNSQKVIFAVWDAGGNDTFDFSGYTANQRINLNEKSFSDVGGLKGNVSIAA
GVTIENAIGGSGNDVIVGNAANNVLKGGAGNDVLFGGGGADELWGGSGQDTFVFSAASDSAPGASDWI
RDFQKGIDKIDLSFFNKEAQSSDFIHFVDHFSGAAGEALLSYNASNNVTDLSVNIGGHQAPDFLVKIV
GQVDVATDFIV
>CORE_REP|Org5_Gene4763#
MRVKMSNLYPVIMAGGTGSRLWPLSRELFPKQFLALCNEFSMLQTTVMRLKGLEIINPLVICNEEHRF
IVAEQLRQITRLSHNIILEPVGRNTAPAIALAALQAVSSGDDPLMLVLAADHVIQDEAIFRDAVNQAI
PYAEAGKLATFGIVPTGPETGYGYIQKGASVDGSSICGVSRFVEKPNLETAQQYLASGDYLWNSGMFL
FKASRYLEELGRFRPDILDACKQSLAHLTPDMDFIRVDRDAFIACPDESVDYAVMEQTADAVVVPLDA
GWNDVGSWSALWEISEKDTKGNSTFGDVLEHNCSNNYIRAEHKLVAAVGVTNLVVVETKDAVLIADKD
NVQDVKEIVNQLKRQKRSESKQHREVYRPWGKHDAIAQGDRFQVRRITVKPGEKLSLQMHHHRSEHWV
VVSGTAKVHTNGKMQLISENESVYIPLGVEHSLENPGKIPLDLIEIQSGAYLGEDDIVRIGDSAQHN
>CORE_REP|Org39_Gene2538#

MASISSLGIGSGLDLNGLLDKLTKAEQQRLTPYTTQQTSYNAQLTAYGTLKGALEKFDNLSKDLAKPE
FFNNTTATKHDQFTVTTTDKSVPGNYSIEVLKLAQPQTLTTQTPIADQQAKLGTPGSSDRSISITAGN
PPKETKIPLGDDQTSLVEMRDAINKSKSGVTASIMRVGDNDYQLALSSTTPGEKNTIAVQVNNDDKLG
AILNYDPKKPPKDGSTAMKQTVPGQDAEIIVNGTKIKRSTNSIADALQGVTLDLKTTTKSGEPQNLVI
GIDKSGSADKIKEWVDNYNSLLDTFNSLTKYTPVKSGEAQNAKNGALLGDNTLRGIQSSIKSALSSAQ
DNPELKGLGNLGITTNVKTGKLEIDSTKLNKAIDEKPEQVANFFAGNGKDTGMATQIHNDIQSYIKAG
GIIENSTKSINTNLDRLNIQITTVTASIQNTIDRYKQQFVQLDTMMSKLSSTGNYLQQQFSAK
>CORE_REP|Org49_Gene4620#
MRHWKKKLGLTALTALVLSSMLGAGVFSLPQNMAQVASPAALLLGWGITGVGILFLAFAMLLLTRLRP
DLDGGIFTYAKEGFGELVGFCSAWGYWLCAVIANVSYLVIVFAALSIFTDRGGSVILGDGNTWQALIA
ESALLWIVHALVLRGVQTAASINLAATLAKLLPLGMFAVLAAIAFKMDVFTLDFKGIALGKPVWEQVK
DTMLITLWVFIGVEGAVVVSARARNKKDVGRATMLAVLSALAVYLMVTLLSLGVVPRSELAEMRNPSM
AVLMVELIGPWGDVLIAAGLIISVCGAYLSWTIMAAEVPLLAAQHGAFPRVFGKQNRHHAPSSSLWLT
NIAVQLALVLIWLTGSNYNSLLTIASEMILVPYFLVGAFLFKVAYRRRDKRLIFAATGACVYGLWLLY
ASGLMHLLMSVLLYAPGLLVFMYARRGHRDINLLNRLEKSSIFLLLAATLPAGWLMLH
>CORE_REP|Org13_Gene3724#
MSEALTCFKAYDIRGKLGSELNEDIAYRIGRAYGEYLRPKTMVLGGDVRLTSESLKLALARGLQDSGT
DVIDIGLSGTEEIYFATSHLKVDGGIEVTASHNPMDYNGMKLVREESKPISGDTGLRDIQRLAENNSF
PAVNDAARGGYQQLSILDAYVQKLLSFVALDNFTRPLKLVINSGNGAAGHVIDAIEARFKNAGLPVEF
IKVHHAPDGNFPNGIPNPLLPECRQDTTDAVLKHGADMGIAFDGDFDRCFLFDERGNFIEGYYIVGLL
AEAFLEKSPGSRIIHDPRLSWNTIDIVEKAYGIPVMSKTGHAFIKERMRKEDAVYGGEMSAHHYFRDF
YYCDSGMIPWLLVAELLCIKGRSLGELVNDRVAAYPASGEINSSLNNPKEAIGRVLGKYEMEADAVDH
TDGISVEYDNWRFNLRSSNTEPVVRLNVESRANVELMQEKTEEILQLLRSE
>CORE_REP|Org34_Gene2699#
MSTSLLLLIAVLGVVLLLLMVIKAKVQPFVALLVVSLLVALASGIPTGEVMKVMTAGMGGVLGSVTII
IGLGAMLGRMIEHSGGAESLAQRFSQGLGPKHTVAALTLAAFILGIPVFFDVGFIILAPIIYGFAKVA
KVSPLKFGLPMAGVMLTVHVALPPHPGPVAAAGLLNADIGWLTIIGLAICIPVGVIGYFAANYLNRKT
YPLSIEVLEQLQLAAPEPRPEGQAPLSDRINPPGAGLVAALIVIPIAIIMLGTVSATLLPAGSALRDA
LSLLGSPAVALMIALLLAFYFLALRRGWSLQHASDVMGAALPTAAVVILVTGAGGVFGKVLVESGVGK
ALAEVLTAIGLPLVPAAFIISLALRASQGSATVAILTTGGLLSEAVSGLNQLQLVLVTLATCFGGLGL
SHVNDSGFWIVTRYLGLSVADGLKTWTVLTTLLGLSGFLFTWLLWLAV
>CORE_REP|Org13_Gene871#
MDFFLQLAVILACLLYGARKGGIALGLLGGIGLMILVFGFHLQPGKPPVDVMLVIIAVVAASATLQAS
GGLDVMLQIAERMLRRNPRYVSIIAPFVTCILTILCGTGHVVYTILPIIYDVAIKNNIRPERPMAASS
IGAQMGIIASPVSVAVVSLVAMLSSYTFNGRHLEFLDLLSITIPSTLCGILAIGIFSWFRGKDLDKDP
EFQKFISVPENHRYVYGDAATLLDRVLPRSNWIAMWIFLATIALVAVLGAFSDLRPSFGGKPLSMVLV
IQMCMLMAGALIVIITRTNPASISKNEVFRSGMIAIVAVYGVAWMAETMFGAHLAQIEATLGVLVKEY
PWAYALILLLVSKFVNSQAAALAALVPVALAIGVNPAYIVASAPACYGYYILPTYPSDLAAIQFDRSG
TTRIGRFVINHSFILPGLIGVSVSCVFGWILAAAFGFL
>CORE_REP|Org1_Gene1537#
MSTHIGEPQDSYIEEIPQDERRFTRMGWLVVGIGLFGFLAWAAFAPLDKGVASPGSVTVSGNRKTVQA
PASGIIKNIAVKEGDKVKAGEVLVQLSQVQAQAQVDSLRDQYYTTLATEGRLLAERDGLSSVTFSPIF
TQIKDQPRVAEIIALQTQLFASRRQGLQSEIDGYKQSMDGIRFQLKGLQDSRVNKQIQLSSLREQMNS
MKQLAADGYLPRNRYLEVQRQFAEVNSSIDETVGRIGQLQKQLQESQQRIDQRFADYQREVRTQLAQT
QMDASEFRNKLQMADFDLGNTAITSPVDGTVVGLNIFTQGGVVGAGDHLMDVVPSQATLVVDSRLKVD
LIDKVYNGLPVDLMFTAFNQNKTPKIPGTVTLVSADRLVDKANGEPYYQMQVTVSPEGMKMLSGEDIK
PGMPVEVFVKTGSRSLLSYLFKPILDRAHTSLTEE
>CORE_REP|Org31_Gene1229#
MPLVIVAGGVALLLLLMIRFKLNGFISLVLVALAVGIAQGMPVDKVIGSIKAGVGGTLGSLALIMGFG
AMLGKLLADCGGAQRIATTLIDKFGRKHIQWAVVLTGFTVGFALFYEVGFVLLLPLVFTIAASARIPL
LYVGVPMAAALSVTHGFLPPHPGPTAIATIFHADMGKTLLYGTLLAIPTVILAGPVYARFLKGIDKPV
PEGLYNPKTFTEAEMPSFGVSVATSLVPVILMALRAVAEMVLPKGHSLLRFAEFFGDPVMATLIAVLI
AIFTFGLNRGRTMDEVMGTITDSIKIIAMMLLIIGGGGAFKQVLVDSGVEQYIAGLMEGSNVSPILMA
WSIAAALRLALGSATVAAITAGGIVAPLIATTGVSPELMVIAVGSGSVIFSHVNDPGFWLFKEYFNLS

IMETIKSWSVLETIISVCGLVGCLLLATVV
>CORE_REP|Org11_Gene2002#
MSTSRSQQIQQLEQEWKSARWEGITRPYSAEDVINLRGSVNPECTLAQNGAAKLWALLNGKARKGYVN
CLGALTGGQALQQAKAGVEAIYLSGWQVAADANSAAAMYPDQSLYPVDSVPKVVERINNTFRRADQIQ
WANQIEPGSKGYTDYFLPIVADAEAGFGGVLNAFELMKAMITAGAAGVHFEDQLAAVKKCGHMGGKVL
VPTQEAIQKLVAARLAADVLGVPTLVIARTDADAADLLTSDCDPYDSAFVTGERTAEGFFRTHAGVEQ
AISRGLAYAPYADMVWCETSTPDLDAAQRFADAIHAKYPGKLLAYNCSPSFNWKKNLDDQTIARFQQA
LSDMGYKYQFITLAGIHSMWFNMFDLAHAYAQGEGMKHYVEKVQQAEFAAVDRGYTFASHQQEVGTGY
FDKVTTVIQGGASSVTALTGSTEEQQF
>CORE_REP|Org38_Gene3496#
MLLRFSQLTTHKGAELSAIEHAVPMIVFSPDGTVLRANDLFLSTLGFQRDDVIGRHHRIFCDPNYVAS
PLYREHWETLNKGQPITDTIKRIAKNGEAVWLQGTYAPVLNKQGKVVEIVKIASEVTERVTQAQEHRS
LLAALNRSMAMISFTPQGTIVSANDNMLALMGYRLEEACGQSHAVLCPPAFAASDDYRRHWQRLARGE
FITGRFERVNRRGERVWLEASYNPILDNDGQVVKVVKIAQDITRLMQQQQHEEEMVRNAHHLSLDTDR
QAAQGAVIVQQAVKGMQQVEAAARETSDVVTELGKCSQQIGTIVEAIRKIASQTNLLAINASIEAAHA
GEHGRGFAVVANEVRTLAEQSRKAATEIERMTKSIQQGVAAAIAGMATCVEQAGGGVALTHDAGEVIN
QVNIGMHDVVKLMQAFTSVKQGDALH
>CORE_REP|Org19_Gene2793#
MKAIITNALWLMLERVSLSLSGIFVSIYVARYLGPAQFGALNYLLATIAIVVPLVQLGADSIIFNRVA
RRQPSGIRLMLASIRLRRRLFLVVALPILIWSYFSQTPASQLMTLLLLVSAYFSIQDVYKIYYDARLQ
SKRNTLINNLALLLSIGLRLALVSAALPLVWFAVPYILSSAVPYLVRLWLFRGEAAASPRVTPRQARR
YGRYLLKVGLPLAISSLSIVIYTRIDQIMLGNLVGEQAVGWFSAATTLSQGWVFVPMALITSLMPGIA
SCRDPLEQEYRIRVLYLVVLGLSLPVLLGLWWFAHPAIALLYGAAFQPAASILAICTLTSLFSVMGTV
SYRSIVLFAGYRFIAIKMPLVAVANVVMNLLLIPRYGLIGAAVSTLLAEFISFFVLNSLFRGGKITRL
QLTCFYCLPRLVSKLRREHVKHPG
>CORE_REP|Org41_Gene2975#
MNQRLDIIGIGLGPSNLSLAALGSEIEGFTGQFLERKPHFSWHPGMILADCSMQTNFLKDLVSAVAPT
NRYSFLNYLVKNRKFYRFLTTEQRTASREEFADYLTWAAGGMDSLAFNQDVQQIEFDDRQRQFVVTTS
NKVFHAKHVSIGIGKKIKLPDCVTAQSDRCFHASEMMLRNPDLTGKRVAIVGGGQSGADLFLNIFKGE
WGQPDQLDWISRRNNYNALDEAAFANEYFTPDYVESFYSLDSAAKRHMLAEQKMTSDGITSESLLAIY
RAMYHRFDVLREKLWVRLLPSRSLTAVKHTLDNAYQLETRHHLDHGEEAFKADVVIFATGYQTATPEF
LEPLAHRLLTTADGEYRIAPDFTFEWEGPAENCLFAMNASMHNHGIADPQLSLMAWRSARILNRALDH
KPFDLGTTPTAIQWRSESVPHAF
>CORE_REP|Org18_Gene3825#
MLLRLYQVLLYLIQPLIWLRLLLRSRKAPAYRKRWAERYGFCAGKVVPGGIMLHSVSVGETLAAIPLV
RALRHRYPALPITVTTMTPTGSERVQSAFGKDVHHVYLPYDLPSSMNRFLDQVNPKLVIIMETELWPN
LINALHQRQIPLVIANARLSARSAAGYKKIGGFMRDMLRRITLIAAQNQEDGDRFIELGLKRSQLAVT
GSLKFDISVTPELAARAVTLRRQWAPRRPVWIATSTHDGEETILLEAHRKLLEKHPDLLLILVPRHPE
RFPTAKELVQKAGFSYTLRSSGEIPSGSTQVVIGDTMGELMLLYGIADLAFVGGSLVERGGHNPLEAA
AHAIPVLMGPHTFNFKDICAKLSQAEGLITVTDVDSLVKEVETLLTDEDYRRYYGRHAVEVLYQNQGA
LQRLLQLLEPHLPPRSH
>CORE_REP|Org13_Gene3315#
MAQVINTNSLSLMAQNNLNKSQSSLGTAIERLSSGLRINSAKDDAAGQAISNRFTANIKGLTQASRNA
NDGISLAQTTEGALNEVNDNLQNIRRLTVQSQNGSNSSSDLQSIQDEISQRLSEINRISEQTDFNGVK
VLSADQKLTIQVGANDGETIDIELKDINAKTLGLDKFSVADPIDTTKIGTTKLTAVDKMGAPTINADS
KAATPKNSDGKLYSTDDGAGNVAYFVKSTDGGIYDATVAADGKVSWDSSTATTKATAGMKETSQVKVG
TTNISGLTGSDELRTYTDPAGKSGYVVKGKDDNGNDAYFKATVDASGKVTKGAQQSTDPKTADPLATL
DKALSQVDSLRSSLGAVQNRFDSVINNLNSTVNNLSASQSRIQDADYATEVSNMSRANILQQAGTSVL
AQANQSTQNVLSLLR
>CORE_REP|Org18_Gene1127#
MAFSQAVSGLNAAATNLDVIGNNIANSATAGFKSGSVSFADMFAGSQVGLGVKVSGITQNFKGGTTTG
TSRALDIAINGNGFFRMQDKDGGIFYTRNGQFKLDENRNLTNMQGLQLTGYPAAGSPPTIQQGANPVP
LSIPEGMMNAKASTSGEMVTNLKSTHKVPENKTFDPTKQDSYNYVNTITAYDSLGNAHNINAYFVKTD
DNKWQVYTQDGSAAPVNAGTMEFSTSGNLVKTTSTNGAPGEFSMVIPMTAKDGAPAQNFTLSFAGSMQ

QNVGSDSVSKVAQDGYAAGEYTNFQINNDGTVVGIYSNQQTQVLGQIVMANFSNPEGLASQGDNVWQE
TGASGQPRVGLSGGGGFGKLTSGALESSNVDLSQELVNMIVAQRNYQSNAQTIKTQDSILQTLVSLR
>CORE_REP|Org14_Gene4570#
MTQENSIAPAANPLVDAANPLLNAISQIRQSATHANPAQLRQQLIDEMRRFEIRGQRANLPYEVIIGA
RYCLCTALDEAAALTPWGSNSVWSGSGLLVTFHNETWGGEKFFQLLAKLSQSPREHINLLELINYCLL
LGFEGRYRVMENGRSQLETMKQRLLQLIRSVRGGYAPPLSPHALDLPVQQKLWRPLVPLWACVALTGF
LASLLFIALNWRLGDNTSPVLAAIYQTNLPQVAIGNPAPAAPPTLSLKSFLRKEIAEGLVVVRDEAQQ
SVVILKGDGLFDSAATTVRANYIPVIDRIAAAMNGVSGKILVTGYSDNVPIRSARFASNWELSLARAE
AVSARLQKHLANPQRVKAEGRGESNPVAPNDNKVNRALNRRVEITLLVAPENTQAEINGLPQGTGK
>CORE_REP|Org30_Gene2796#
MKARRLFLWQAFDAQGALRRGELMSDEKRQVSRLLIEQGMQPWRIGHGKRVTPGQWRGEPLIHFTRQL
ATLLQAGLPLVNTLQLLAAEHPSAAWRCLLRQLAEQVREGQPLSETLAAQPGVFPLIYRQLIAIGELT
GNLDRSCLQLAQQQEAQLLLRRKVTKALRYPLFICAVALLVSVLMLVMVLPEFAKVYQSFDAPLPWFT
QGLLGLSALLIAVGPYLALLLGALLFGYCRWLHPRPPWRRREQAALLRLPLIARLVSGGALSQTFRIL
TMTQRAGLTLVEGLNAAALAADHLLYRQALEQVQRQLAEGEAFHHALALQPLFPPLCRQLVRVGEESG
SLDVLLDKLAQWYERQTHELADTLAQTLEPLLMLVVGGIVGALVIAMYLPIFQLGSVLG
>CORE_REP|Org17_Gene4241#
MATLTTENQTFSGFEYAEQSTFLYRSEFRSLSAHGVFERIETPVFGGEQEGSALAQHIRQALARAKAA
GQAAPVVVGAIPFDTRRPSCLYIPEESRFVANDSFIRAARPMLQQPHRLVACTSIPDEPRFKHAVAEA
VSRFKQGKLDKAVLSRILDIELEQPVAGHRILNNLMVQNPTGYHFSLPLADGSVLIGASPELLIRKQG
GEIHTNPLAGSARRQDDPQQDRLGSERLMRSTKDKYEHKLVIDDIRRHLTPLCATLSVPSGPSLLSTG
TMWHLSTRIRGELLNPALNVMQLACLLHPTPALCGFPTESARQLIAALEPHDRGLFSGIVGWCDANGD
GEWAIVIRSGLLRGNRVRLFAGAGIVAASTPQSEWMETTAKLGTMLNAFGLNSGAL
>CORE_REP|Org12_Gene1014#
MDSLSVDELAQKKDRWYRIVEEMLAEAGVAINGPRAWDIRVHNPALFKRILQEGSLGFGESYMDGWWE
CERLDMLFTRILQAGVDERLPKSLSDIARIAYARLFNRQSRKRAWQVGKEHYDIGNDLFRAMLDPYMQ
YSCGYWKEAQTLEQAQQAKLRMICEKLQLKPGMTLLDIGCGWGGLAQFAAQNYGVSVHGVTISAEQQK
LAQARCAGLDVEILLQDYRDLDRQFDRIVSVGMFEHVGPKNYETYFSVAARNLKPDGLFLLHTIGSNQ
TDLNVDAWIDKYIFPNGCLPSVRHIAEASEGRFVMEDWHNFGADYDRTLMAWLENFKRAWPDLMGGYS
ERFERMFTYYLNACAGAFRSRNIQLWQVLFSPAGVEGGVRVYR
>CORE_REP|Org29_Gene693#
MAEDSDLEKSEAPTPHRLEKAREDGQIPRSRELTSVLMLLSGLAIILMSGSNMAQQLAAMLTQGLNFD
HGMVSNDKQMLRQLGMLLRQAVLALLPIMAGLVLVALAAPMLLGGILFSGKSIKFDLKRLNPLSGLKR
IFSTQVLAELLKGILKATLVGWVTGLYLWHNWAAMLHLMTQQPLDALGNALQMILFCGFLVVLGLTPM
VAFDVFYQLWSHFKKLKMTKQDIRDEFKDQEGDPHVKGRIRQQQRAIARRRMMADVPKADVIVTNPTH
YAVALQYNDKKMSAPKVLAKGAGEIALRIRELGAEHRIPMLEAPPLARALYRHSEIGQHIPATLYAAV
AEVLAWVYQLRRWRREGGLIPKKPERLPVPEALDFARESDSDG
>CORE_REP|Org39_Gene1042#
MSNRPLTIGLVAGETSGDILGAGLIRALKAQIPDARFVGVAGPLMQAEGCEAWYEMEELAVMGVVEVL
ERLPRLLKIRKDLTRRFGELRPDVFVGIDAPDFNITLEGRLKQRGIRTIHYVSPSVWAWRQKRVFKIG
KATDLVLAFLPFEKAFYDRFNVPCRFIGHTMADAMPLQPDRLAARAQLGIDPQARCLALLPGSRGAEV
EMLSADFLKTAQLLRTRYPELEVVVPLVNAKRREQFERIKAEVAPDLTVHLLNGQGREAMIASDAALL
ASGTAALECMLAKCPMVVGYRMKPFTFWLAQKLVKTPYVSLPNLLAGREIVTELLQHDCVPDKLAAAV
MPLLEESPQTEALKQTFLTLHQSIRCGADEQAAQAVLELAKA
>CORE_REP|Org44_Gene4288#
MINPKVKALAIIVYGAMLTGCAIAPGQHLTTDSKNVVKQEDSDFQIDELVNIYPLTPSLIAKLRPVKV
VAQPNVLLEQATKNYEYRIGVGDVLNITVWDHPELTTPAGQYRSASDTGNWVQSDGTIFYPYIGKVKV
SGKTASQVRSEISSRLTQYIESPQVDVNIAAFRSQKAYITGEVEKSGQQPITNIPLTVLDAINAAGGL
SANADWRNVVLTHNGKEQILSLQKLMQNGDLTQNQLLYPGDIIYVPRNDDLKVFVMGEVKSPATLKMD
RSGMTLTEALGNSAGLDQNTADATGVFVIRPLRGTQGKKIADIYQLNMADATAMVMGTEFHLQPYDVV
YVTAAPVVRWNRVIVQLAPTISSFNNLTEASLRIRNWP
>CORE_REP|Org49_Gene1267#
MIPFNAPPVVGTELEYMQAAMGSGKLCGDGGFTRRCQQWMEQRFGSAKVLLTPSCTASLEMAAILLDI
QPGDEVIMPSFTFVSTANAFVLRGAKVVFVDLRPDTMNIDETKIEAAITDKTRAIVPVHYAGVACEMD

TIMALAKKYNLFVVEDAAQGVMSTYKGKALGTIGHIGCFSFHETKNYTAGGEGGATLVNDPALIDRAE
VIREKGTNRSQFFRGQVDKYTWRDIGSSYLMSDLQAAYLWGQLEVAERINQRRLALWQKYYDSFLPLA
RSGRIELPVIPADCVHNAHMFYIKLRDIEERTAFIDYLKEAEIMAVFHYIPLHDCPAGERFGRFAGED
RYTTQESARLVRLPLFYNMSDVNQRTVINTILSFFA
>CORE_REP|Org3_Gene4268#
MKAFRLAIVRQKYRPDGGAERFVSRALEALEQQDLDLNVITREWQGDANPNWHIHLCNPLKLGRISRE
RGFAVAARALWQKEHFDLVQSHERIPGCDIYRAGDGVHRRWLLQRARLLPEWRRKWLFSNRYHRYVMC
AERAMYAAPELKAVICNAEMIKQEIIADFGVPAEKITVIYNAIDNQKFPPADEALRQRLREQYQIPQQ
AHCLIFVGSGFERKGLAAAIRAVAATDSHLLVVGKDKAEKRYRALAQSLGCGDRVHFMGVQKQTLPFY
QAADALLLPTLYDPFPNVILEAMSCGLPVITSTTCGGAEFITPGQNGFVTDALDVPAIAEAIRALPRQ
ALGAEMGAAARATILPHDAQRLSQQLISLYRKLLTP
>CORE_REP|Org46_Gene381#
MLKKWFIALCVGLVCLPAAAERIRDLVTVQGVRDNALIGYGLVVGLDGSGDQTMQTPFTTQSLSNMLS
QLGITVPPGTNMQLKNVAAVMVTAKLPPFSRAGQNIDVVVSSMGNAKSLRGGTLLMTPLKGVDNQVYA
LAQGNVLVGGAGAAAGGSSVQVNQLAGGRISNGATIERELPTTFGSGGVLNLQLNDEDFTLAQQISDA
INRQRGGGTATPLDARTIQVLVPQGNSSQVRFLAEIQNITVNVGAMDAKVIINSRTGSVVMNRDVILD
SCAVAQGNLSVVVDRQNTVSQPTTPFGGGQTVVTPNTQISVQQQGGSLQKVNASANLNNVIRALNALG
ATPIDLMSILQAMQSAGCLRAKLEII
>CORE_REP|Org29_Gene4570#
MKKTAIALAVALAGFATVAQAAPKDNTWYTGAKLGWSQYHDTGFYGNGYQNGIGNGPTHKDQLGAGAF
LGYQANQYLGFELGYDWLGRMPYKGSENNGAFKAQGVQLAAKLSYPITDDLDIYTRLGGMVWRADSKA
NYTTGVSAGQRLSAHDTGVSPLAAVGVEYALTKNWATRLDYQFVSNIGDAGTVGARPDNTMLSLGVSY
RFGQDDVVAPVAPAPAPAPVVETKRFTLKSDVLFNFNKATLKPQGQQALDQLYTQLSSMDPKDGSVVV
LGYTDAVGSAQYNQKLSEKRAQSVVDYLVSKGIPSDKISARGMGKADPVTGNTCGYKAGRATKAQIDC
LAPDRRVEIEVKGIKDVVTQPQG
>CORE_REP|Org45_Gene3841#
MSVSTDPMTDAKGQLNAGVMGRYQHILRHRLLMMGVLALAILGSLLLDFTMGPSGLSLSSLWQTLLDP
AAADAGTRVIVWDIRLPYALMAVVVGFALGLAGAEMQTILNNPLASPFTLGVSSAAAFGAALAIVLGI
GIPGIPDQWFISANAFIFALFAALMLDGITRWTRVATSGVVLFGIALVFTFNALVSMMQFIASEDTLQ
GLVFWTMGSLARASWDKLGILFGVFAVLLPLSMMSSWKLTALRLGEDRAVSFGIDVRRLRLTTLLRIS
ILSALAVAFVGPIGFIGLVAPHIARMIFGEDHRFYLPASALIGALVLSMASVASKNLVPGVIIPVGIV
TSLVGVPFFLSIILRHRGNV
>CORE_REP|Org29_Gene2094#
MKQNHPVVLVRKRKSHHAAHHGGSWKIAYADFMTAMMAFFLVMWLLAIASPQELTQIAEYFRTPLKVA
LTSGDKSSSESSPIPGGGDDPTQQHGLVRKQVDSPDKRAEELRLNKLREKLDELIESDPRLKALRPHL
LINMMDEGLRIQIIDSQNRPMFKTGSAQVESYMRDILRAIAPILNDLPNKISLSGHTDDIPYATGERG
YSNWELSADRANASRRELIAGGLAEGKVLRVVGMAATMSLKQHGADDAINRRITVLVLNKQTQEGIEH
ENAESNAMDIAQPDSLKQLAPSATAPASQTPESQAVTPTDQATLPPATDPVAQSQASPAIAPAGQAPE
QPVAAPAPTNRDSQPEVTP
>CORE_REP|Org46_Gene737#
MQKDALNNVHISAEQVLITPEELKNQFPLSADDENEIATARNTIANILQGRDHRLLVVCGPCSIHDPD
AALDYARRLKTLAADLSDQLYIVMRVYFEKPRTTVGWKGLINDPYMDGSFDVEAGLHIARRLLLDLVG
MGLPLATEALDPNSPQYLGDLFSWSAIGARTTESQTHREMASGLSMPVGFKNGTDGSLGTAINAMRAA
AMPHRFVGINQAGQVCLLQTQGNPDGHVILRGGKTPNYSAEHVAACEKQMLEAGLHPSLMIDCSHGNS
NKDYRRQPAVAESVVEQIKAGNRSITGIMLESHLHEGNQSSEQPRADMRYGVSVTDACINWESTETLL
RHMHQELGAALTARTGEK
>CORE_REP|Org31_Gene3705#
MNALITKGKRAAFPLMLLSTLLFSNTLLAQTAPEVLRKPVGKGAYEMAYSPSENALYLATSQSRKLDK
GGIVYRLDPTTLDVTQIIHNDIKPFGAAVNAKTGTLFFGNTVNNSVTAIDAKTGDVKGRLVLDARKRS
ETVKPLAPRELVADADSDTLYITGLGESSVVWVVDGKDLTLRATVTDTGKYGTGLALDAAAKRLYVTN
ADGELVTIDTQSNKVLSRKKLDESKEHFFLNISLDTATHRAFITDSKQPQVLVVDTRNGNILSKIDVP
ESLAVLFNPARNEVYVTHRQAGEVSVIDAKSYKVLNTIKTPTHPNSLALSPDGQTLYVSIKQASSREK
EATAPDDVIRVALK
>CORE_REP|Org43_Gene3638#

MNVPTPTERTLSSAPHAAYASGFKRIAGFGIGLALLLLCIIASLMLGSKAIPFHTVWLSLQGAASGSD
STIILNARVPRTLAGILAGMALGAAGALIQALTRNPLADPGVLGINAGASFAVVIGIMFFGAATTESY
MAYAFVGAAVTTLLVYVIGTLAGGRINPVRLTLAGVAIGAVLLGITTGLSLIDPQTFDQLRFWQAGTL
DIRTLATLPVTAPAILLGCLLTLLIARPLNTIGMGEDLAIALGARVVLTQAIAVLAITLLCGAATATV
GPISFIGLMVPHIARWWVGPDQRWILPYSMLLAPILLLCADVVGRLLAAGELRVSIVAAFIGAPVLIW
LVRRKKTLGGL
>CORE_REP|Org38_Gene2549#
MKILVIGPSWVGDMMMSQSLYRTLKAEYPTAEIDVMAPAWCRPLLARMPEVNQALAMPLGHGALGLGE
RRRLGRALRANRYDRAYVLPNSFKSALVPFFADIPQRTGWRGEMRYGLLNDVRVLDKAAFPLMVQRYV
ALAYDKGRVQRADDLPQPLLWPQLRVSDEEIAETTSAFNLTDSRPIVGFCPGAEFGPAKRWPHYHYAA
LAQRLIESGYQIALFGSAKDHEAGEQIRAALQDDARDFCLNLAGKTQLEQAVILIAACRAVVSNDSGL
MHVAAALNKPLIALYGPSSPDFTPPLSDKARVIRLISGYHKVRKGDAEQGYHQSLIDIQPQQVLDALT
PLLVASEE
>CORE_REP|Org10_Gene4169#
MSLPHTLHIGRPGGVINWRMPLRLLLVNLSLLALCLAMAVAALCYGTLQLSLEQVFAALSGEAPKNLV
TVVTQWRLPRIAMALLLGGALGMSGAIFQSIIRNPLGSPDVIGFNMGAYTGALIAITLFNGGYYYIAG
GALAGGILAALAIYLLAWRQGIAGFRLIIVGIAISAVLVSTNTWLIITASLERAMDAAMWQAGSLNGM
TWQKAQPATAFIVLAAAAALLMGKRLQLLEMGDDTARALGVNAEGSRLWLMLFGVTLTAAVTATAGPI
SFIALAAPQIARRLAGQSSVTLTSSALMGAALLLSADVVSQHLFAPIQLPVGVVTVCIGGLYLIWLLI
REARR
>CORE_REP|Org48_Gene1493#
MSIKKITITDVAQQAGVSVTTVSLVLSGKGRISPTTVEKVNQAIEQLGYVRNRQAATLRGAESGVIGL
ILRDICEPFYAEMTAGLSEALEAHDKLLFLTQSGRDGQGLQRAFDALLAQGVDGIVLAGGIRAAAGLK
EKAAEQGVPLVCVARSSGLEGVDVVRPDNMQAAKLATEFLIKRGHSQIAYLGGQSDSLTRAERLGGFC
ATLVQYGLPFRSEWIVECDCRQREAAEAAEQLLRHYPNITAIVCHKASVALGAYFGLTRSGRSIGSDG
VDAYYGRQVALIGFGDVPEAELTEPPLTFVSSSAREVGRSAAARLLQRIGDADLPAQNVILPPTLIRR
GSA
>CORE_REP|Org28_Gene1284#
MATIKDVAKRAGVSTTTVSHVINKTRFVAEETKAAVWAAIKELHYSPSAVARSLKVNHTKSIGLLATS
SEAPYFAEVIEAVENSCYSKGYTLILCNSHNNLDKQRAYLAMLAQKRVDGLLVMCSEYPDQLLGMLED
YRNIPMVVMDWGAARGDFTDTIIDNAFEGGYLAGRYLIERGHRDIGAIPGQLSRNTGGGRHQGFMKAL
QEAHIDIREEWIVQGDFEPESGYKAMHQILSQKQRPTAVFCGGDIMAMGAICAADELGLRVPQDISVI
GYDNVRNARYFTPALTTIHQPKERLGEMAFTMLLDRIISKREESQVIEVHPKLIERRSVADGPFIDYR
R
>CORE_REP|Org39_Gene2443#
MPAQRMRSVIPPYMLRRIIEHGNAPQRDCALHTLNHVQSLLGNKPLRSPAEKNARAGEALRDIYDAQN
GTQLPGKQVRKEGQPSNHDVAVDEAYDYLGVTYDFFWQAYRRNSLDNQGLPLVGSVHYGKEYQNAFWN
GQQMVFGDGDGEIFNRFTIAIDVVGHELAHGVTESEAGLIYYQQSGALNESLSDVFGSLVKQFHLQQT
ADKADWLIGAGLLAKGIKGKGLRSMSAPGTAYDDPLLGKDPQPASMKDYIQTKEDNGGVHLNSGIPNR
AFYLAATALGGFAWEKAGYVWYDTVCDKALPQNADFATFARATVKHALARFDQSVADKVQQAWHQVGV
E
>CORE_REP|Org2_Gene3995#
MTLSLRQLFIAFIATSSLLLSGCGPDDQSDGQKPSAPAADSSWPRTIDSAKGKFTLEKPPQRIVSTSV
TITGTLLAIDAPVVASAATSPNPLVADKQGFFTQWSEVAKQRHVERLYQVEPNAEAVAAAAPDLIVVA
ATGGDSALKLYDQLSAIAPTLVLDYGDKSWQQLASELGEITGHEAGAKQAEDRFEQRVEQVKQAIALP
PQPTTPLVYADNGREAMLWTPGSAQGKLLTQLGFQLATPPESAKGNTSMGKRHDIIQISGEKMAEGLN
GKTLILFATNERKVQEVLTNPFLKHLEPVEQRHVYAVGNDTFRLDYYSATNMLNQIERLFKKP
>CORE_REP|Org9_Gene2618#
MPTSRTFTLLLQHQRYRDKRQIGLLALCVAVALLFSLCAGDQWIWPSEWFSDRAQLFVWQLRLPRALA
VMLVGAALAVAGAVMQALFENPLAEPGLLGVANGAGVALVLTVLLGQGLLPVALMSAAAIAGALAMTF
LLLGFARRRRLTNARLLLVGVALGIVCSALMTWAVYFSTSLDLRQLMYWMMGGFGGVDWRQKWLVLAL
LPVLLWLCGQGKALNLMALGEVQARQLGLSLHLWRNLLVLAIGWLVGVSVALAGVIGFVGLVIPHILR
LIGLTDQRYLLPACALAGAGVLLVADVVARIALLAAELPIGVVTATLGAPLFIWLLTRAKGVR
>CORE_REP|Org48_Gene1728#

```
MGDSILSQAEIDALLNGDSAGDEPEAIVGKESEVKPYDPNTQRRVVRERLHALEIINERFARQFRMGL
FNLLRRSPDITVGPIKIQPYHEFARNLPVPTNLNLVHLNPLRGTALFVFAPSLVFIAVDNLFGGDGRF
PTKVEGREFTPTEQRVIKRMLRLALDAYGDAWSAIYKIDVEYVRAEMQVKFTNITTSPNDIVVTTPFQ
VEIGALTGEFNICIPFAMIEPLRELLTNPPLENSRQEDSHWRETLVKQVQHSELELIANFVDIPMRLS
KVLKLQPGDVLPIDKPERLIAHVDGVPVLTSQYGTLNGQYALRVEHLINPILNALSEEQPNE
>CORE_REP|Org49_Gene1607#
MRCRTSPQLAIIGLLVLLTLLALVAANLGALTLSFRTLWREPFSDAAWHIWLNIRLPRVLLAVVIGCA
LAVSGAVMQGLFRNPLADPSLLGISSGGALFVALFIVMPLALPVTIALYGHMLAAFLGSLLVSLLIYG
ISRSGHGNLSRLLLAGIAINALCMAAIGVLSYVSSDQQLRQFSLWMMGSLSQSQWPTLAVSASLILPA
ALLTLLQARRLNLLQLGDEEAHYLGVNVQRAKLQLLLLSALLIGAAVAMSGVIGFVGLVVPHLVRMRL
GGDHRWLLPCSALGGACLLLVSDTLARTLVAPAEMPVGLMTSLIGGPYFLWLVMRQRERAGG
>CORE_REP|Org14_Gene641#
MATMKDVARLAGVSTSTVSHVVNNNRFVSDSVRDKVMAAVEQLNYAPSALARSLKLNQTRTIGMLVTA
SNNPFYAEVVRGVERSCYERGYSLILCNTEEDAARMNRSMETLLQKRVDGLLLMCTENHRPSQDALSR
YPSLPIVMMDWAPFEGANDIIQDNSLLGGEMATDHLIACGYRKIACIAGPQDKTTARHRLEGYRNAMR
RAGLPVPPGYEVHCDFEFEGGVNAMRQLLALDEPPHAVFAGNDAVAVGVYQALYQAGLSVPQDMAVMG
YDDIELARYLAPPLSTIHQPKDSLGELAIDALINRLQNPERAPQVLVLTPELVERASVGRR
>CORE_REP|Org34_Gene1466#
MQPILEKLYRAESMSQQESQQLFSAIVRGELEPSQLAAALISMKVRGERPEEIAGAAKALLDDAQPFP
RPDYPFADIVGTGGDGTNSINISTASAFVAAACGAKIAKHGNRSVSSRSGSSDLLAAFGIRLDLPAEE
ARKALDDLGVCFLFAPQYHTGFRHAMPVRQQLKTRTLFNVLGPLINPARPPLALIGVYSPELVLPIAE
TLRVLGYQRAAVVHGGGMDEVAIHAPTHVAELNNGEISSYQLTPQSFGLETYPLEALLGGTPEENRDI
LARLLQGKGEPAHAAAVAANVALLLKLFGHEDLRQNARQALDMINSGQAYERVIALAARG
>CORE_REP|Org41_Gene1531#
MSNIELQPGFDFQQAGKEVLQIEREGLAQLDSYINADFTRACETIAACGGKVVVMGMGKSGHIGCKIA
ATFASTGTPSFFVHPAEASHGDLGMVTPQDIVLAISNSGESSEILALIPVLKRQQITLICMTNNPESS
MGKAADIHLCIKVPQEACPLGLAPTTSTTATLVMGDALAVALLKARGFTPEDFALSHPGGALGRKLLL
RVSDIMHSGDEMPHVSADASLRDALLEITRKNLGLTVICDDLMKIAGIFTDGDLRRVFDMGINLHEAK
IADVMTPGGVRVRPNILAVDALNLMQQRHITALLVADGDQLLGVVHMHDMLRAGVV
>CORE_REP|Org1_Gene3115#
MSNANALLAFARETLEIELTEAQRLLARLDDNFVCACELLLNCRGKAVISGIGKSGHIGKKIAASLAS
TGTPSFFVHPAEALHGDLGMIGADDVVVFISYSGRAKELDLILPLLAENGIPVIAVTGGKESPLTQAA
ACVLDIGVEREACPMGLAPTSSAVNTLMMGDALAMALMRQRGFNAEDFARSHPGGSLGARLLNRVHHL
MRTGDRLPRVSESANVMEAMLELSRTGLGLVAVCDAQQRVVGVFTDGDLRRWLVKGNSLQDPLSPAIT
RPGYRLPEQWRAGEALEALHEQHISAAPVVDMDGVLVGALNLHDLHQAGIG
>CORE_REP|Org41_Gene1675#
MQVLIVKTSSMGDVLHTLPALTDALQAIPDIRFDWVVEEGFSQIPTWHPAVDRVIPVAIRRWRKNWFG
NDTRQQRCDFKRALQERRYDVVIDAQGLIKSAALITRIAKGNKHGPDCKSAREPFASWFYNVRHEIDK
QQHAVERTRELFAKSLGYDKPGSYGDYAIAARFLSRPPADAGQYLVFLHATTRDDKHWPEQNWRELIA
LTADSGLKIKLPWGAEHEHQRALRLAEGFSHVEVLPKLSLQQVAEVLAGAKGVVSVDTGLSHLTAALD
KPNITLFGPTDPGLIGGYGQNQHSLISPEKSMATIDADTAWQALQKVIA
>CORE_REP|Org18_Gene1250#
MKQIWFSVCLLTGSLLYSSIAPAQPTASGALLQQMSSASRSLNYELAYISISKQGIESLRYRHAVIGN
VPLGQLLHMDGPRREVLQRGGGISYFEPGLEPFTLTGDHIVDALPAIVYADFTRLAKYYDFISVGSTR
IADRPCEVLRVVARDGSRYSYIVWMDEDTKLPLRVDLLDRDGETLEQYRVISFAVGADVQGAMQGLLK
ANLPPLLSLPAVENVKLSWSTGWLPAGVDEVARNRRKLPNVAVPVESRLYSDGLFSFSVNVSPAGSGA
GQQYYRQGRRTIQTEVRAGNEITIVGELPPATAKRIADSISFKVSPQ
>CORE_REP|Org16_Gene3401#
MSNAITPEQRQALKMAAHWYALLCDEHVTERQRQQWQAWHQQHDDHRWAWQRVEALQSQLQGVPGKFS
YRALDRADRQSAIDRRTLLKSLLLLLGVGGGGFFYQSPLGRELRADYRTATGEIKPIVLSDGTQLVLN
TASAVDVHYDDRQRLIRLHAGEISLVTGRDPRPLWVQSPQGAMRALGTRFLVRESDGETRLAVLEHAV
EAQLAQDAQQKRRVNAGEQISFSTTAFSDKQPATTEDGWLRGVLSVSQWRLDRVIAELARYRRGHLSC
DPAVAGLRVSGSFPLNDTDRALALLSQTLPVRLQSFTRYWLQIVPA
>CORE_REP|Org34_Gene1075#
```

MSDKIPIGISACLLGDAVRFDGGHKRLAFAVEQLAPYVRFEPVCPEMAIGLPTPRPALRLVKQAQPWP
AMRYSNDAGVDLTEEMRSFSAQRVAALQHLCGYIVCAKSPSCGLERVRVYSENGKDSRKNGVGLFTAE
LLRQMPWLPVEEDGRLQDAALRENFIERVYALYELNMLWRQGLTRGGLIAFHSRYKLSLLAHSQPAYR
ELGRFVADIHRWDSLEAFAVEYRSRLMALLAHKATRRNHTNVLMHVQGYFRRQLSAAQRQELAHLIDR
YRQGMQPLLAPIALLKHYMAEYPDRYLAEQRYFEPYPEALRLRYGH
>CORE_REP|Org8_Gene3849#
MPLISCAFHRSRPARAAALLRPFTLACLLLGAALVSQNALAEKKLRVVTTFTIIQDIAQNVAGDAAVV
ESITKPGAEIHDYQPTPRDIVKAQHADLILWNGMNLERWFQRFFENIKQVPAAVVTEGITPLPIREGP
YNGNPNPHAWMSPSNALVYIENIRKALVEHDPAHAETYNRNAKAYAEKIGALDAPLRERLARIPAAQR
WLVTSEGAFSYLAQDYQLKEVYLWPINADEQGSPQQVRRVIDAVRAHHIPVVFSESTISDKPAKQVAK
ETGAKYGGVLYVDSLSTRDGPVPTYIDLLNTTVQTIAKGFDQ
>CORE_REP|Org17_Gene4455#
MLLSHRHFICTLLALAIGFWLPTAVQARPDLERRIGVTVADSDSADYRFSDLRFTSADGQRRYRVRIA
QPRRAPAPDGYPTIYFLDGNAVLMELNASLLARLATAKRPPVLVMIGYDNDLRIDAAGRAYDYTLPLP
TGMTGMTGMKKSPQAGGGAEAFLQLIETRIKPAIAAKLAVDQQRQTLWGHSYGGLFVLHTLFAHPAAF
QHYIAVEPSLWWGNGMILQEAQQWAERHPTPAARLQLWVGLAERDRAAPPGVKSPALPANAAQMLAER
LAKLDGLTVGYREWPGLGHGAMLGAAIEPALNSVAYED
>CORE_REP|Org5_Gene1939#
MPAVNRKVRKAVIPVAGLGTRMLPATKAIPKEMLPLVDKPLIQYVVNECIAAGINEIVLVTHSSKNSI
ENHFDTSFELEAMLEKRVKRQLLDEVQSICPKGVTVMQVRQGNAKGLGHAIMCAYPMVGDEPVAVVLP
DVILDEYSADPKKDNLHEMLQRFETTGVSQIMVEPVPHKDVGNYGVADCKGVDLQPGESAPMVSVVEK
PSPDKAPSNLAIVGRYVLSADIWPLLAKTPPGAGDEIQLTDSIEMLMQQETVEAYHLKGVSHDCGNKL
GYMQAFVEYSMRHASLGKEFSQWLQQVVAADKK
>CORE_REP|Org22_Gene2033#
MFFNLQRYSTHDGPGIRSVVFLKGCPLSCRWCQNPESRSRRADLLFDERLCLSGCTLCTERCPQGLRR
NEEALTLQRDVISADDYAALAAACPTGALSLCGSAVNPDDIMAEVMRDKPFYLRSGGGLTLSGGEPFM
QPEAAAELLRRGREAGIHTAVESCLHVPWRYIAPSLPWLDLLLADLKHTDEARFKAWTGGSARRVMNN
FRRLAAHGVPMTVRVPLIPDFNADRHSVRAIVDFAADEIGVSEIHFLPYHTLGINKYHLLGEPYRAAR
TPLDAPDLLAFAEAYAGAKGLTAILRG
>CORE_REP|Org45_Gene2599#
MLVILGYLVVLGAVFGGYLIVGGHLGALYQPAEFLIIGGAGVGAFIVGNNGKAIKATLRALPRLMRRS
KYNKDLYMDLMALLFRLLAKSRQQGMLSLEFDIDNPQESEIFSNYPRILADNTLVEFITDYLRLMVSG
NMNAFEIEALMDEEIETYEQESEVPAGSLAMVGDSLPAFGIVAAVMGVVHALASADRPAAELGALIAN
AMVGTFLGILLAYGFISPLATLLRQKSAENVKMMQCIKVTLLSSLNGYAPQIAVEFGRKTLYTTERPS
FVELEEHVRRVKAPAQQVTEEEEA
>CORE_REP|Org41_Gene3095#
MKRLPISLAVAALLASPWAMAKTVDAVASFSILGDIVKQVGGDHVKVSTLVGPDGDPHSFEPSPQDGK
KLAQADVVFVSGLGLEGWIDRLVSASGYKGQVITASQGISTRQMEEDGKPITDPHAWNSMKNGVQYAT
NVMNALIAADPEDANYFRQRGADYIQQLQKLDLWAKTQFAAVPPQKRKVLTSHDAFGYFGQEYGVTFL
APVGFSTEAEASASDVAGLIKQIKQEKVNAYFIENQTDPRLVKQIAAATGAKAGGELYPEALSRAGGP
AATYEQAFKHNVDALLSSMK
>CORE_REP|Org21_Gene2638#
MAIPKLNDYALPTADELPQNKVTWQVEPQRAALLIHDMQQYFLNFWGEDSALIKQVVENIANLRRYCK
QQDIPVFYTAQPNQQSDEDRALLNDMWGPGLNKHPEQQAVTAALAPDEDDTVLVKWRYSAFHRSPLQE
ILQESGRDQLIICGVYAHIGCLTTAIDAFMRNIQPFMVADGLADFSRDEHLMALRYTAGRCGRVVTTA
SLLPAAGIASIDALRQQILPLLDEDSEDMGNDENLIDYGLDSVRIMELATRWRKIRGDIDFIALARNP
TIDSWWALLSEEKA
>CORE_REP|Org13_Gene1299#
MTKEMQTLALVPQGSLEAYIRAANAYPMLTAEEERELAERLHYQGDLDAAKQLILSHLRFVAHIARNY
SGYGLPQADLIQEGNIGLMKAVRRFNPEVGVRLVSFAVHWIKAEIHEYVLRNWRIVKVATTKAQRKLF
FNLRKTKQRLGWFNQDEVELVARELGVTSKDVREMESRMAAQDMTFDPTPDDEARDGQAMAPVLYLQD
KSSDFAEGIEEDNWESNAADKLAYALEGLDERSQHIIRARWLDDDNKSTLQELADQYGVSAERVRQLE
KNAMKKLKMAIEA
>CORE_REP|Org19_Gene1426#

MMLWHGLIDPFLSFGFMRRALMACLALSLSAAPLGVFLLLRRMSLVGDALSHAVLPGAAIGYLISGMS
LVAMGVGGFIAGLAVAMLSGLVSRRTPLKEDASFAGFYLGSLALGVTLVSLRGSSVDLLHVLFGSILA
VDAQAMLMVGAIASVSLLALAALYRALVIESFDVTFLRVNAPRRLALIHGLFLALVVVNLVAGFQILG
TLMSVGLMMLPAASARFWARNLPQTLATAMGIGALSSLIGLVWSYYASLPAGPAIVLSASVIFFVSIL
FGTRGGIYAFARR
>CORE_REP|Org15_Gene3670#
MDTLYQLLSEPFAYPFMQRAIVAAIVTGVVCAVLSCYLVLKGWSLMGDAISHAVLPGIVVAFVIGIPL
AIGAFLSGIFCAVATGYLKENSRVKEDTVMGIVFSGMFAFGLVLFSRIDTDQHLSHILFGNMLGITDG
ELKQTLLIAGLTLAVVLLKRKDFMLYCFDPHHARVIGLPVKLLHYGLLCLLAMTIVASLQAVGVILVI
AMLIAPGIIAFMLCRRFDRMLMVATVVSVFSCVLGTLISFHIDGATGPCIVIVQAVLFVIALLYGKLR
PLQRNQTALSDS
>CORE_REP|Org12_Gene1894#
MIIIETLPMLRQQIRRWRQEGKRIALVPTMGNLHDGHMTLVDEARARADVVVVSIFVNPMQFDRPDDL
ARYPRTLQEDSEKLTRRGVDLVFAPAPAAVYPQGLEQQTYVDVPGISSILEGASRPGHFRGVSTIVSK
LFNLVQPDLACFGEKDYQQLALIRKMVADMGYDIDIVGVPTVRAKDGLALSSRNGYLTAEERKVAPQL
SKIMNALAQQLANGERQVEALLEQTAEQLRAAGFTPDELFIRDADSLQPLTVDSQRAVVLMAAWLGKA
RLIDNQQVDLTL
>CORE_REP|Org20_Gene2558#
MLNILVKNISKRLPDAWHHQVKYALYFKKLPHLSKPTGFSEKIMRRKIYPRSIYTTLSDKFKVREFIA
GLWGEEYLVELYAHGTELSYDMFRQLPNAFVLKANHGSGYNRLVFDKRQVSYAELYDLSNAWMRSNFY
EQSREKHYLDIEPCIMVERMLLDGEQVPNDIKFHCFNDNHEIRMFIQVDYQRFGTHRRDIFDVDWNRT
EIRISLPNADEPMPRPTRLDEMIRLARQTAQQFSYVRVDFYQVGEKVYFGELTFTPGAGLSKLMPKNI
EQEWGSYFTE
>CORE_REP|Org23_Gene4353#
MKSLASTLQGQSIAAAITAVENDIKAKPADADLRAALVQLLCLSGNWTRANAQLKSWQALKPIAQPTT
LLLMQSVNAELQRQAVFAGAAAPALLRQDQPWLQLLVQALHQDAQGAAEQAQTLRDEALEAAPAGAGQ
LTLAEGNQERQLSFDWLTDGDGRLGPVCELALNGVYYWLPFADIAAIQFQAPQSAIDLVWSHALVRLT
DGREQVCQLPARYPLAEGSDDALLLGKRTEWQPLGDGTHYAGLGLKTWLSESDEFPLHSLRQLSFDAS
A
>CORE_REP|Org7_Gene348#
MIPSLWIAKTGLDAQQTNMDVIANNLANVSTNGFKRQRAVFEDLLYQTMRQPGAQSSEQTTLPSGLQI
GTGVRPVATERLHSQGNLSQTNNSKDVAIKGQGFFQVMLPDGTQAYTRDGSFQIDQNGQLVTSSGFQV
QPAITIPANALSITVGRDGIVSVTQQGQTAAQQVGQLTLTTFVNDSGLESVGENLYQETESSGAPNES
TPGLNGAGLLYQGYVETSNVNVAEELVNMIQTQRAYEINSKAVSTSDQMLQKLTQL
>CORE_REP|Org13_Gene968#
MLTFDSAQLTVWLSHYFWPLLRILALISTAPIFSEKQISKKVKIGLGGLIVILIAPTLPASNIPIFSA
AGLWLAIQQILIGVALGLTMQFAFAAVRLAGEVIGMQMGLSFATFFDPSGGPNMPVLARLLNLLAMLL
FLSFDGHLWLISLLADSFHTLPIQTQPLNGNGFLVLTQVGSLIFINGMMLALPLICLLLTLNMALGLL
NRMTPQLSVFVIGFPVTMTFGIMTLGMMMPMLAPFCEHLFGEIFDRLAAVIGGMTF
>CORE_REP|Org12_Gene3522#
MKKDKQIMTIGEGQAIVKEAQRLLSAPSRRRFLRNGLTLGGIAMLTGCDLSDNANVEQALSRMSRLND
RVQGWLFNGDRLAPVYPELMITRPFPFNAFYAEEDAPDINGDDYRLEVAGLVQDKRAWSLPQLHRMAQ
VSQVTRHICVEGWSAIGKWGGVPFATFLKAIGADLSARYVSFKCADDYYTSIDMATALHPQTIIALTY
DGQILPRKYGYPMKLRMPTKLGYKNPKHIQVIEVTNRFPGGYWEDQGYNWFGGS
>CORE_REP|Org27_Gene4360#
MSSRKSLSVVMIAKNEAGLLPDCLRSVEWADEIIVLDSGSEDDSVAIAESLGAKVFTHTDWQGFGKQR
QLAQSYASHDYVLMIDADERVTPELRQSIERVLNAPDDGAVYSCARRNLFLGRFMRHSGWYPDRVNRL
YANRRYRYNDDLVHESLNIGGAKVIPLNGDMLHLTCRDFFAFQRKQLRYAEEWATQRHRAGKRCGYLS
ILTHTLGAFVKTWLLRAGFLDGKQGLLLAVVNAQYTFNKYAALWALGRNYSEK
>CORE_REP|Org13_Gene1701#
MMNLTMTRKTVFLVGPLAAALTIGAVSLPAHAAIALDRTRVIFDGDLKTVSLNISNQNKQLPYLAQGW
IEDDRGNKIQSPFTVLPPVQRVEPGKPSQVKIQSLPAARQLPQDRETLYYFNLREIPPRSNKPNTLQI
ALQTRIKMFYRPAALAPKKNAAPWQEQLTLTRQGDKYVVNNPTPYYVTIVEASAGKGGKGAAGFEPLM
VAPKASAPLNVSAASLGNGPSLAYINDYGGRPQLNFRCAGNACQVVPVAK

>CORE_REP|Org30_Gene769#
MTSTLHTLVRRPAVWLPAALLFISPAALAQLPGLISQPLANGGQSWSLPVQTLVLLTSLTFLPAMLLM
MTSFTRIIIVLGLLRNALGTPSAPPNQVMLGLALFLTFFIMSPVFDKVYQDAYLPFSQDKIGLEVALD
KGAQPLREFMLRQTRETDLALYARLANQPPLAGPEAVPMRILLPAYVTSELKTAFQIGFTVFIPFLII
DLVVASVLMALGMMMVPPATISLPFKLMLFVLVDGWQLLLGSLAQSFYS
>CORE_REP|Org1_Gene2546#
MDHAIYTAMGAARQTLEQQSITANNLANASTPGFRAQLAALRAVPVDGPSLATRTLVTASTPGADMSQ
GALNYTARPLDVALQQDGFLAVSLPGGGEAYTRNGNIQISSTGQLTVQGLPVMGDGGPIEVPPSAEIT
IAADGTISALNAGDPPNTIAQIGRLKLVKADAREVMRGDDGLFRLTPETQQQRGNQLQNDPQVRVMPG
VLEGSNVKPMETMVDMIANARRFEMQMKVIHSVDENEQRANSLLSMS
>CORE_REP|Org7_Gene2423#
MNKHPITLLTAAGLALSAVLPTADAAISLDRTRAVYVSDAKSISLNIVNENKELPFLAQSWLENEQHQ
KITSPLVVLPPLQRVEPSERSVVRITKTPEADRLPQDRESVFYFNLREIPPKSTKTNVMQLALQTQIK
LFYRPKAIVAPKGQVWQEKLVFRKSGGAITVDNPTPFYITLTGMTRQTQKQGGGAIGGFQPLMLKPKS
SESLKLQETGMNSFVITYINDYGGHPELRFVCNGGVCTAVPEKK
>CORE_REP|Org40_Gene4448#
MWKWLHQLARPERLYHVCGRFIPWLGLAAAACLLLGWAWGFGFAPKDYQQGDSFRIIYIHVPAAMWSM
GIYASMAVAAFIGLVWQMKMSDTVVAAMAPIGAVFTFIALVTGSAWGKPMWGSWWVWDARLTSELVLL
FLYMGVIALYNAFEDRRLAGRAAGILVLVGVVNIPIIHFSVEWWNTLHQGSTNMQQSIAPSMRTPLRW
AILGYLLLFVTLTLMRLRNLILFQERQRPWVAGLVNKERQS
>CORE_REP|Org8_Gene519#
MAILPRQGQRWLAGMVMLTLSGCAYIPHKPLVDGATTAQPAPASAPMPNGSIFQTVQPMNYGYQPLFE
DRRPRNVGDTLTIVLQENVSASKSSSANASRNGASKFGVATSPRYLDGLLGNARADMDISGDSTFGGK
GGANANNTFNGTITVTVNQVLANGNLHVVGEKQIAINQGTEFIRFSGVVNPRTISGNNSVTSTQVADA
RIEYVGNGYINEAQTMGWLQRFFLNVSPF
>CORE_REP|Org5_Gene817#
MSNPIVACWPGALAPREKLLMQGAAALSDAELLAIFLRTGLPGVHVMQLAEQLLRRFGSLYHLMSADH
QAFCSQKGLGDASYTQLQAIAELALRFFSSHLSQENAMLNPRVTQHYLQSLLAHREREVFLVLFLDNQ
HRVIRHQEMFAGTISSVVVYPREIVREALKANAAALILAHNHPSGKAEPSHADRLITEQVVKACQLLE
IRVLDHLVIGRGECVSFAERGWL
>CORE_REP|Org42_Gene4092#
MHNIDLEDRLAALSAAIADRTRARMLCLLMDGRAYTATELSAAVEVAPSTASAHLAKLLEQRLIACVK
QGRYRYFRLAGQPVAEALEGLMALAGVPRPSVKSSTPTTLQYARTCYDHMAGEVAVKLHDRLHALNWL
NGEEDYRLSDAGQAALARLGVDCSPAPTRRRFACGCLDWSERRSHLGGALGAALLAAFIHRGWIVRRL
DSRELQLTPAGKKALAAHFDLTV
>CORE_REP|Org47_Gene894#
MQFLNQFFFDIYPYLAGAVFLIGSWLRYDYGQYSWRAGSSQMLDKKGMRLASNLFHIGIIGIFAGHFL
GMLTPHWMYEAFLPIDVKQKLAMIAGGACGLMTLIGGALLLKRRLTNPRVRATSSFADIMILTLLVVQ
VCLGLLTIPFSAQHMDGSEMMKLVAWAQAVVTFHAGASAHLEGVAIIFKLHMVLGMTLFVLFPFCRLV
HIWSAPVEYLTRRYQLVRNRR
>CORE_REP|Org11_Gene655#
MAIAATTNESLDNTVIGNNSKNTNSQDLHNSFLTLLVAQLKNQDPTNPMQNNELTSQLAQINTVQGIE
KLNTTLGSISGQINSNQSLQATALIGHGVMVPGNNILVGSKDGKVSTTPFGVELERAADQVTATITNA
SGQVVRTIEIGGLTAGVHAFTWDGSLDDGSTAPDGAYKVAINAKGNGEQLVARSLHFGLVNGVIRDGN
GAKLDLGLAGNATLEDVRQIL
>CORE_REP|Org22_Gene3251#
MKGKMLLLIGLLCSLNARADDLATQIDSFIKGKFTGEPVQVKVRVRTPPAQWPACELPQLSLPPNARI
GGNVSISARCGQERRFIQTQVQVFGRYLVSARGISAGSRLTAADLTLKEGRLDTLPPRALTEASKALD
AVSLRNISPGQPLTLAMLRRAWIIKAGQPVQVTAQGEGFNISGAGKAMNNAAAEDSVRVRMASGQIVS
GVVGDDGAIRITL
>CORE_REP|Org10_Gene328#
MRDIPMPASDAATAGEIISRIGQLTRMLRDSMRELGLDQAIAQAAEAIPDARDRLDYVVTMTAQAAER
ALNCVEAAQPRQAELESGANALKGRWDEWFANPIELDDARSLVNDTRQYLDQVPGHTAFTNAQLLEIM
MAQDFQDLTGQVIKRMMDVVQEIEKQLLMVLMENMPEQPVKEKRPNDSLLNGPQLDQNGVGVIANQAQ

VDDLLDSLGF
>CORE_REP|Org45_Gene513#
MSKFQLLDKDNSALIFIDHQPQMAFGVANIDRQQLKNNVVGLAKAGKIFNVPTLFTSVETESFSGYIW
PELLAVHPEITPIERTSMNSWEDAAFVKAVEATGRKKLVISALWTEVCLTFPALMALEAGYEVYVVTD
TSGGTSVDAHERSIDRMVQAGAVPVTWQQVLLEYQRDWARKDTYDAVMALVREHSGAYGMGVDYAYTM
VHHAPARTVK
>CORE_REP|Org4_Gene1541#
MNEKIDYHIEKYHFAPLDEAPRLAHQWSEVLNECRETQAGAEERLRIALLNVDYVTSFELPFRLLLVR
APQLIAGIREELPLSQKNVVFNGKRFGCVYSLKSDLSGVPEAFQYSLSTRIHRRAASGVDALPYREIA
KALKAPRERLRLALEQGLPVTALDGLFWFGIQRIAAEVRRLRKTGMAIVTAETEIFDTLTGTTRKVPV
YRLAES
>CORE_REP|Org34_Gene4021#
MTFKYSRLDKAQAAVLLVDHQTGLLSLVRDQDPDKFKNNVLALADLAKYFNLPTILTTSFENGPNGPL
VPELKQTFPDAPYIARPGNINAWDNEDFVKAVKATGKKQLIIAGVVTEVCVAFPALSALEEGYEVFVI
TDASGTFNAITRDAAWDRMSQAGAQLMSWFGAACELHRDWRNDIEGLGNLFSQHIPDYRNLMTSFSLL
TSGKQ
>CORE_REP|Org35_Gene2480#
MSLTLTPEQRQTVHPLWLRLTHWLNALAMLIMVTSGWRIYNASPLFNFSFFNELTLGGWLGGALQWHF
AGMWLFGVNGLCYLLLNLASGRFKRKYWPLSPKQFLADVGAALRGRLQHADLRHYNMVQRVAYLSVML
LGVLSALSGLVLWKSVQFPLLRTLLGGYEAARYIHFFAMSALVAFVAIHLVMVALVPRTLLAMLRGR
>CORE_REP|Org42_Gene764#
MAKFTQHTGLVVPLDAANVDTDAIIPKQFLQKVTRTGFGQHLFNDWRFLDDAGQQPNPEFVLNKPRYK
GASILLARENFGCGSSREHAPWALTDYGFKVVIAPSFADIFYGNSFNNQLLPVTLSEQQVDELFKLVD
ANEGTEFVVDLENQTVNAGGKSYPFEIDSFRRHCMINGLDSIGLTLQHEADISRYEAQQPAFLN
>CORE_REP|Org26_Gene381#
MLDRIKVCFTESIQTQIAAAEALPDAISRAAMTLVQSLLNGNKILCCGNGTSAANAQHFAASMINRFE
TERPSLPAIALNADNVVLTAISNDRLHDEVYAKQVRALGHTGDVLLAISTRGNSRDIVKAVEAAVTRD
MTIVALTGYDGGELAGLLGQQDVEIRIPSHRSSRIQEMHMLTVNCLCDLIDNTLFPHQDD
>CORE_REP|Org42_Gene115#
MYHDLIRSELNEAADTLAKFINDDANIDAIQRAAVLLADSFKAGGKVISCGNGGSHCDAMHFAEELTG
RYRENRPGYPAIAISDVSHLSCVSNDFGYEYVFSRYVEAVGREGDVLLGISTSGNSGNIIKAIDAARA
KGMKVITLTGKDGGKMAGSADVEIRVPHFGYADRIQEIHIKAIHILIQLIEKEMVKA
>CORE_REP|Org47_Gene3946#
MNDKRPPHYDGGDLHRQGYRYRQDSERLTSRDKMQPVLLDMLTDDEPQKKQEAQVRNLVSHSELRRRV
LRDLQWLFNCVNSESNLDLGDFPQVRRSTLNYGIASLAGKRMSDIEWLDIQRALTESILHFEPRILPE
GLQVRCISDTGSLELHNVLSIEIKGRLWCVPYPLEFLFRTDVDLENGHFDLKDIG
>CORE_REP|Org20_Gene68#
MSEQLTDQVLVERVQKGDQKSFNLLVVRYQHKVASLVSRYVPQGDVPDVVQESFIKAYRALESFRGDS
AFYTWLYRIAVNTAKNYLVAQGRRPPSSDVDANDAENYESAGALKEISNPENLMLSEELRQIVFRTIE
SLPEDLRMAITLRELDGLSYEEIAAIMDCPVGTVRSRIFRAREAIDNKVQPLIQR
>CORE_REP|Org21_Gene4528#
MKLNKIMLAAVMAFGVSSLAHAAVKDQGHGKVTFSGSIIDAPCSIAPESIDQTVELGAISNVALKDGG
KSMPRPFQIKLENCDLTVDANDPSKNNNKVSLTFTGSASEADSALLGITGTAKGAGIALTDGNGKNIT
LGTATDARLLQNGSNTLSFSAYLQGSGASGAQIVPGEFQSVADFTLAYQ
>CORE_REP|Org28_Gene1750#
MSATATASAPHTALSRLYATHHAWLQGWLRRRLGCAFDADDVAQDTFMRLLKSDAAATLREPKDFLVT
VAKRVMVDLFRRRTLERAYLEMLALIPDGYAPSPEQRQSLLESLQQIDAMLDGLGPKVKQAFLLSQLE
GLGYADIAVRLGVSISSVKKYMAKATEHCLLFSLENDVFS
>CORE_REP|Org6_Gene3004#
MNKRRTTLTVLALIASLGLSSAPALADKGGNGNGNGNGHGNSGNHGNNGNHGNNGNNGNHGNNGNNGN
HGNKGNKDKGGYRNDDNLVSVSLSRDRARSLAHNYGLTGYSSLPPGIAKNLARGKPLPPGIAKKVVPY
SMLRELPQYPGYEWRIAGDDLVLVALSTAIVASVINGVFD
>CORE_REP|Org6_Gene1075#
MKKIACLSAVAACVLAVSAGTAFAGQSTVSAGYAQGDLQGVANKANGFNLKYRYEFDNNPLGVIGSFT

HLEKNRSESGFYKKSQYDSITAGPAYRFNDWASIYGVIGVGYGKNIDNAQAGGNKGGNSDYGFTYGAG
LQFNPIENVALDVGYEQSRIRSVDVGSWNVGVGYRF
>CORE_REP|Org48_Gene1720#
MPGILLKKRPLSRYLKDYKHSQTHCSQCGKLLDRMALVFRGKIINKEAIARMDQPIDDAVWQNVQHEL
TALCRFCSEISCNSHPSYFDIMAFKQYLFEQTEMSHSTIREYVVRLRRLDEMLVARNYPADKFASSAS
HQRIIDDLPTAAHNNYRIALRKYDQYLAWQRSY
>CORE_REP|Org9_Gene122#
MTQTVHFQGNPVSVAGKLPQQGEQAKAFSLVAKDLSDVALSSFAGKRKVLNIFPSIDTGVCATSVRKF
NQLASGLDNTVVLCISADLPFAQSRFCGAEGLSNVVTLSTLRGAEFKQAYGVEIAEGPLAGLTARAVV
VLDGQDNVLYSELVNEITTEPDYDAALAALK
>CORE_REP|Org11_Gene1581#
MAGLAAVSKLAGETVGQEFLIFTLGNEEYGIDILKVQEIRGYDQVTRIANTPAFIKGVTNLRGVIVPI
IDLRVKFSQQSVSYDENTVVIVLNFGQRVVGIVVDGVSDVLSLTAEQIRPAPEFAVTLATEYLTGLGS
LGERMLILVDIEKLLSSEEMSLVDSVAKSV
>CORE_REP|Org23_Gene1631#
MSQNTLPAAPKRSLLVILLVLISVVACGAAGYSWWLLQQHKNGAEPAAVKQQPPAAPVFMPLDTFTVN
LVTPDNNPDRVLYIGLTLRLPDESTRRQLNDFLPEVRSRLLMLLSRQEAGQLANEQGKQQLVAQIKDV
LSPPLVKGQPKQVVSDVLFTAFILR
>CORE_REP|Org19_Gene153#
MTSKAIYPGTFDPMTNGHLDLVTRASLMFDHVILAIAASPSKKPLFSLDERVALATQVTSHLDNVEVL
GFSELMAHFAAHQNANILVRGLRAVSDFEYELQLANMNRHLMPTLESVFLMPSEEWSFISSSLVKEVA
RHGGDIAPFLPDVVTQALMAKLAAE
>CORE_REP|Org7_Gene356#
MAIDMFLKVEGASGESKDSNHKGWTDITSFSWGASQPGNMGVGGGGGAGKVCFNDLHVNALIDKSTPA
LLKHCSSGKHLTKIELSVCKAGGTQVEYAKITLEDVLVTAVQYTGAGGEDTVGVTYSFQAAKVKQQYW
EQSDKGGKGAESSAGWNIKENREA
>CORE_REP|Org32_Gene2470#
MKSQSPLITLRDLAQDAVEQAAQQLGQVRQAQQAAEQQLSMLLNYQDEYRQKLNHTLCDGMDSSSWQN
YQQFIGTLEQAIDQHRQQLLQWGQKVDHAVKQWQDKQQRLNAFETLHTRALNAEQQQENKRDQKLMDE
FAQRSAQRNINP
>CORE_REP|Org19_Gene683#
MTDNNIALKKAGLKVTLPRLKILEVLQNPECHHVSAEDLYKKLIDMGEEIGLATVYRVLNQFDDAGIV
TRHNFEGGKSVFELTQQHHHDHLICLDCGKVIEFSDESIEVRQRDIAKQHGIKLTNHSLYLYGHCETG
DCREDETLHDKK
>CORE_REP|Org8_Gene1202#
MENLAQLLDKLLETLQALNGVLEEEHDLLCSGQLPGVALQRVTDAKSQLLATVAYLEQQRLGQEKTCG
QRAPYASQAPLADRWQRVQLLSQTLREKNQHNGLLLNQQIDHNAQALAILSKNNKSLYGPDGQSHAGS
LLGRKIGV
>CORE_REP|Org43_Gene408#
MSDPKQPSGEGKESVDDLWADAFNEQQSSEKSGASTEGVFKSLEAQDALGSLQDIDLILDIPVKLTVE
LGRTKMTIKELLRLSQGSVVALDGLAGEPLDILINGYLIAQGEVVVVADKFGVRITDIITPSERMRRL
SR
>CORE_REP|Org19_Gene949#
MLDKLDAALRFGQEALNLRAQRQEILAANIANADTPGYQARDIDFASQLNKVLEQGRVNGNGMSLNLT
AARHIPAQTLQPPQLDLLYRVPDQPSMDGNTVDMDRERTNFADNSLKYQTDLTLLNGQIKGMMSVLQQ
G
>CORE_REP|Org38_Gene351#
MYNRSGTQAYAQVSLESGAMSASPHQLIVMLFDGALSALLRARILMNQGDIAGKGMALSKAINIIDNG
LKSGLDPQQGGEIAENLAALYDYMKRRLMQANLHNDEAAIAEVVKLLENIADAWRQIGPNYQPSQDAV
>CORE_REP|Org42_Gene2097#
MKRCLFVLCLLAPLTAGAVSGSWVAEGAGVTLEQGGMRDESAGLRPPNVLPDANARITRVSWRYRLLG
PEPAGLQAQLCTVNRCIALGGGSGSSNGLQGEPANAELRFVYYVQSQGGLNPPLRVIGNQVIVNYQ
>CORE_REP|Org27_Gene316#
MSLLNIFDISGSALSAQSQRMNVSASNMANADSVTGPDGEPYRAKQVVFQVAAAPGQPTGGVRVAQVV

DDPAPERLVYQPGNPLADAKGYVRMPNVDVVGEMVNTISASRSYQANVEVLNTTKSMMMKTLTLGQ
>CORE_REP|Org39_Gene422#
MIRTMLQGKLHRVKVTQADLHYEGSCAIDQDFLEAAGILEYEAIDIYNVDNGQRFSTYAIAAERGSRI
ISVNGAAARCACVGDKLIICSYVQMTDADARQHHPKVAYFEGDNNLQRKAKAVPVQVA
>CORE_REP|Org20_Gene312#
MERQQQLLAAYQQIYSLSSQMIALAQTGRWEELVELEFAYVTAVEKTAAFTGQAGPSMALQEMLRNKL
QQILDNETELKRLLQQRMDELKMLIEQSTRQNVVNNTYGQFHDRALLLGEPQVR
>CORE_REP|Org35_Gene3554#
MGNMGTSELLKHIYDINLSYLLLAQRLINDEKASAMFRLGIDETMADALAQLTLPQMVKLAETNQLVC
HFRFNDHQTIERLTKESRVDDLQQIHTGILLSSHLLQELSSKDASPTKKRA
>CORE_REP|Org3_Gene4472#
MAIQGIEGVLQQMQTMAVQAGKMGQNTAPQGVSFASELTAALGKISETQQTARKQAQDFELGVPGISL
NDVMVDLQKSSVSLQMGVQVRNKLVAAYQDIMNMPV
>CORE_REP|Org41_Gene320#
MTPESVMALGTEAMKVALALAAPLLLAALISGLVVSLLQAATQINEMTLSFIPKILAVVATIIIAGPW
MLNLLLDYMRTLFSNLPTLIG
>CORE_REP|Org43_Gene4415#
MIERGKFRSLTLVNWNGFFARTFDLDELVTTLSGGNGAGKSTTMAAFVTALIPDLTLLHFRNTTEAGA
TSGSRDKGLHGKLRAGVCYSTLDVVNSRHQRVVVGVRLQQVAGRDRKVDIKPFTIQGLPTAVQPTELL
TQTVGERQARVLSLQELKERVEEMEGVQFKQFNSITDYHSLMFDLGVIPKRLRSSADRSKFYRLIEAS
LYGGISSAITRSLRDYLLPENSGVRKAFQDMEAALRENRMTLEAIRVTQSDRDLFKHLISEATSYVAA
DYMRHANERRIHLDGALALRSDLLGSRKQLAAEQYRHVEMARELSEQSGAESDLETDYQAASDHLNLV
QTAMRQQEKIERYEADLEELTYRLEEQNEVVAEASEQQAENEARAEAAELEVDELKSQLADYQQALDV
QQTRAIQYQQALQALERARALCQLPDLTADNAEQWLDTFQAREQEATEALLMLEQKLSVADAAHGQFE
QAYQLVGKIAGQVSRSEAWQCARELLRDWPSQQHLAECVQPLRLRLSELEQRLRSQQDAERLLQEFCK
RHGQEYQPDDLDMLQQELEERLEALSQNVSEAGERRMEMRQELEQIQQRIRELTARAPVWLAAQDALS
QLSDQSGEPLENSQQVTEYMQQLLERERETTVERDEVAARKREVEAQIERLSQPGGAEDQRLVTLAER
FGGVLLSEIYDDVTIDDAPYFSALYGPSRHAIVVPDLSLVREMLEGLEDCPEDLYLIEGDPQSFDDSV
FAVEEQDKAVVVKIADRQWRYSRYPEVPLFGRAARENRLEVLHAERETLAERYATLSFDVQKTQRSHQ
AFSRFIGTHLAVAFDADPEAEIRNLNARRGEIERALNNHEAQNQQQRQQYDQAKEGISALNRLMPLVS
VLNDETLQDRVDEIREELEEAQDAARHIQQHGVSLTKLEPLLSVLQSDPQQHEQLQQDYAQAQSVQRQ
AKQQAFALTEVVQRRAHFSYTDSAGMQNANNDLNDKLRQRLEQAEAERARAREQLRQYQTQFTQYSQV
LASLKSSYDAKRDMLKELSQELVDIGVQADANAEARARQRRDELHAALSNNRARRNQLEKQLTFCEAE
MDGLQKKLRKLERDYHQLREQVVTAKAGWCAVMRLVKDNGVERRLHRRELAYMDGDELRSMSDKALGA
LRLAVADNEHLRDVLRLSEDPKRPERKIQFYIAVYQHLRERIRQDIIRTDDPVEAIEQMEIELGRLTE
ELTAREQKLAISSKSVANIIRKTIQREQNRIRMLNQGLQAVAFGQVKSVRLNVNVREAHATLLDVLSE
QQEQHQDLFNSNRLTFSEALAKLYQRLNPQIDMGQRTPQTIGEELLDYRNYLEMEVEVYRGSDGWLRA
ESGALSTGEAIGTGMSILVMVVQSWEEESRRLRGKDISPCRLLFLDEAARLDAKSIATLFELCDRLEM
QLIIAAPENISPEKGTTYKLVRKVFQNHEHVHVVGLRGFASEPPALGTAPVETP
>CORE_REP|Org31_Gene4064#
MITTDGNNAVASVAYRTNEVIAIYPITPSSTMAEQADAWSGDGRQNIWGDIPRVVEMQSEGGAIATVH
GALQTGALSTSFTSSQGLLLMIPTLYKLAGELTPFVLHVAARTVATHALSIFGDHSDVMAVRQTGCAM
LCAGSVQEAQDFALISQTATLNARVPFIHFFDGFRTSHEINKIVPLSDDTLRQMLPQAAIDAHRSRAL
SPDHPVVRGTSANPDTYFQSREATNPWYDATGQHVIDAMAAFAALTGRHYRPFDYYGHPQAERVVVVM
GSAAGTCEEVIDTLLTRGEKVGVLKVRLFRPFSAQHMLGALPDSVRSVAVLDRTKEPGALAEPLYLDV
MTALAEAYSRGERATLPRVIGGRYGLSSKEFGPDCALAVFRELAQPQPRPRFTVGIFDDVTGLSLPLS
DEILPQRASLEALFYGLGSDGSVSATKNNIKIIGNATPLYAQGYFVYDSKKAGGLTVSHLRVSEQPIN
SAYLVSHADFIGCHQLQFIDKYQMVERLKPGGTFLLNTPYGADEVWSRLPQEVQALLHQRQARFYIIN
AAKLARECRLGARINTVMQMAFFHLTQILPSDVALQQLQDAIARSYSSKGQEIVERNWQALGATRAAL
TEIPLQPVDVSSPMRPPVVSDAAPDFVKTVTAAMLAGLGDALPVSAFPPDGTWPVGTTQWEKRNIAEA
IPIWQPDLCTQCNHCVAACPHSAIRAKVVQPAEMEHAPASLQSLDVKARDMRGQKYVLQVAPEDCTGC
NLCVEVCPAKDRQNPEIKAINMASRLDNLTAEKDNYDFFLQLPEIDPTQLERIDIRTSQLITPLFEYS
GACSGCGETPYIKLLTQLYGDRLLIANATGCSSIYGGNLPTTPYTTNAEGRGPAWANSLFEDNAEFGL
GFRLTVDQHRARVLRLLNALAPQLPERLVNALQMGDVAPEPRRKQIAELRTLLANLEGEDARQLAAGA

DYLVDKSIWLIGGDGWAYDIGFGGLDHVLSLTENVNVLVLDTQCYSNTGGQQSKATPLGAVTKFGEHG
KRKARKDLGVSMMMYGHVYVAQISLGAQLNQTVKAIQEAEAYPGPSLIIAYSPCEEHGYDLALSHDQM
KQLTATGFWPLYRFDPRRSAEGKAALALDSRPPNSSLSETLLKEQRFRRLNAQQPEVASALYQAAEKE
LQEKYDFLSLLAGKAEKSAAE
>CORE_REP|Org46_Gene1471#
MLVKLLTKVFGSRNDRTLRRMRKVVEQINRMEPDMEKLSDDELKAKTNEFRARLEKGESLESLIPEAF
AVVREASKRVFGMRHFDVQLLGGMVLNDRCIAEMRTGEGKTLTATLPAYLNALSGRGVHVVTVNDYLA
QRDAENNRPLFEFLGLSIGINLPGMPAPAKREAYAADITYGTNNEYGFDYLRDNMAFSPEERVQRKLH
YALVDEVDSILIDEARTPLIISGPAEDSSEMYIKVNKLIPKLIRQEKEDSDTFKGEGHFSVDEKARQV
HLTERGLILIEEMLVEAGIMDEGESLYSPTNIMLMHHVTAALRAHVLFTRDVDYIVKDGEVIIVDEHT
GRTMQGRRWSDGLHQAVEAKEGVEIQNENQTLASITFQNYFRLYEKLAGMTGTADTEAFEFSSIYKLD
TIVVPTNRPMIRKDMPDLVYMTEKEKIGAIIEDIRERTAKGQPVLVGTISIEKSEVVSRELTKAGIDH
KVLNAKFHAMEADIVAQAGQSGAVTIATNMAGRGTDIVLGGSWQAEVALLEAPTEEQIEAIKAAWKER
HDAVLAAGGLHIIGTERHESRRIDNQLRGRSGRQGDAGSSRFYLSMEDALMRIFASDRVSGMMRKLGM
KEGEAIEHPWVTKAIANAQRKVESRNFDIRKQLLEYDDVANDQRRAIYSQRNELLDVSDVSETIASIR
EDVFKSTIDNYITPQSLEEEWDIQGLEERLKNDFDLEMPIAQWLDKEPELHEETLRERILENAKEQYQ
RKEDVVGSEMMRNFEKGVMLQTLDSLWKEHLAAMDYLRQGIHLRGYAQKDPKQEYKRESFNMFATMLE
SLKYEVISVLSKVQVRMPEEVEALEQQRREEAERLAQHQQLSHHDENALVTEDPNAPATAERKVGRND
PCPCGSGKKYKQCHGRLQ
>CORE_REP|Org26_Gene4376#
MQQETPTTPTEARVKNKRRISPFWLLPFIALLIAGWLVYNNVQERGTTVTIDFQSAAGIVAGRTPVRY
QGVEVGTVQKISLSKDLRSIVVEASIKSDLEDSLREGTQFWLVTPKASLAGVSGLDALVGGNYIGMMP
GSGKEQTHFTALDTQPKYRLNTGELMIHLHADDLGSLNSGSLVYYRKIPVGKVYDYTISEGNKGVTID
VLIDRRFANLVKSNSRFWNVSGFKGDFSLSGATVQMESLAALVNGAIAFDSPADGQQAKGDQSYTLYP
DLAHSQRGVNILLDLPNGNSLSENRTPLMYQGLQVGTLTKLTLQQDSKVVGELTIDPSVVDLMRSGTR
IVMRSPRISLNDAKLSQLLTGTTLELVPGEGEPQQRFNVLDSSETLLQQPGVLTVTLNAPQSYGIDVG
QPLVVHGVKVGQILSRTLTAGGVVFTAAIDAQYRGLLHKDSKFVVNSRLDVKLGIDGMEVLGASAQEW
VDGGVRIIPGSKGEPGGQYPLYANSEKAEEGIVGNAPSTTLTLSATSLPDVQAGSVVLYRKFQVGEIV
NVRPKANEFEVDVYISPEYRKLLTRESIFWAEGGAKVQLNGSGLTVQASPLNRALKGAISFDNLQGVT
LNKGANRVLYASETAARAVGSQIMLRTYDASKLSAGMPLRYLGIDVGQVESLQLAPERNEVLAKAVLY
PEYVHTFARLGSRFSIVSPEISAAGVSNLDTLLQPYINVEPGRGRELRTFELQQASITDSRYLDGLSV
VLDAAETGSLQIGTPVLFRGVEVGTITGFYLGAMSDRVHVALRISKKYQHLVRNNSVFWLASGYNLQF
GLTGGVIKSGTFQQFIRGGIAFATPPTIPLAPKATPNKHFLLNPEEPKDWKTWGTAIPRD
>CORE_REP|Org29_Gene3641#
MNKPTQPAQDYLAALPLTAERSEALNPQTADDAQALEALHRQMGAADANVNSLSADDVALASVKPRIE
SAWPDAVSDDDFDTDAEGRAILKATPPIKRTTMFPEAWRTNPVARFWDSLLGRSPHNRHATKEEAEAE
NRWRVVGSMRRYVLLVLMLVQTGIATWYMKTILPYQGWALIDPIAMLDQDLMQSVLQLLPYVLQTGIL
ILFAVLFCWVSAGFWTALMGFLQLLIGKDKYSISSTIKGDEPINPAHRTALIMPICNEDVERVFAGLR
ATYESVAATGQLEHFDIYVLSDSYDPDICVAEQKAWMELCRDVDGHGRIFYRRRRRRVKRKSGNIDDF
CRRWGGEYSYMVILDADSVMSGECLTGLVRLMEANPNAGIIQSAPKASGMDTLYARVQQFATRVYGPL
FTAGLHFWQLGESHYWGHNAIIRVKPFIEHCALAPLPGEGSFAGSILSHDFVEAALMRRAGWGVWIAY
DLPGSYEELPPNLLDELKRDRRWCHGNLMNFRLFLVKGMHPVHRAVFLTGVMSYLSAPLWFMFLALST
ALQVVHTLMEPQYFLQPRQLFPVWPQWRPELAIALFSTTLVLLFLPKLLSIVLIWAKGAKEYGGAFRL
FISMLMEMLFSVLLAPVRMLFHTVFVVSAFLGWEVVWNSPQRDDDDTPWGEAFRRHGSQMLLGLVWAG
GMAWLDLRFLWWLSPIVFSLILSPFVSVLSSRATLGMKSKRAKLFLIPEEYNPPRELLATEEYLHLNR
NRALTNGFMHAVVNPSFNALATALATARHHLRATLDRNREERVNEALQLGPEKLVKGKRLELLSDPVT
LARLHQRVWLLPEGAAWREHYQQLPHNPLAHPTGRR
>CORE_REP|Org37_Gene3353#
MKISLDLAIFAVICGILPLLILPRLPEPWLQWPMLFVACLLLRTRWPICRYLACLGLGFIWAVFNAGS
LLGQMERLSCMPDVTAVAQVSSVALEPAASKQTLMRIERVDGHWLVPALAFTTTWAPERQRLCAGQRW
QLKLRLRPVHGKLNEGGFDSQRWAIAQRQPLTAQVRQARLLDGDCGLRQRIISHAETNIGELRYKAVL
LALAFGERTALEQALRTLMLKTGIAHLMAISGLHVAMVAILFWAVLRALQFFLPAHLIGYRFPLVAGW
VATLIYVWLVGAQPPAVRTVLAMTLWMLLRLRGVHCSSWQVWLWCVGLILLCDPLAVLSDSFWLSVLA
VGCLIFWFEWAPLGERFRSAWYWAPVRWLHIQLGMTLLLVPMQVALFLGLTLTSLPANLWAVPIVSLV

TVPLILLAVIGGVFPSLSYGLWWLADFTLSGVFVPLHYLQRGWVDLGAASLLASIAGWLIVICWRFHW
WWRYAPGLATIAICCVLWRGKEPGYRWRVDMLDVGHGLAMVIEQNGKGILYDTGDRWPAGSAAERHIL
PMLNWRGIELEQIIISHAHLDHIGGLSTVQSAFPQATVRSPIRGEGHLPCVAGERWRWQSLQFEVLWP
PKTLKRPVNDDSCVIRIDDGKYSLLLTGDAEKKAEAQLIRLRRDRLAATVLQVGHHGSRTSSTPPFLR
AVNPEVALASASRYNKWRLPARKVVARYRANGITWRDTTRSGQLSVLFFDNDWQIKGFREQLMPRWYH
QRFGVEGDNE
>CORE_REP|Org48_Gene2830#
MLTRLREIVEKVAAAASLTDALDLLVNETCLAMDTEVCSIYLADNDRRCYYLMATRGLKKPRGRTIAL
AFDEGVVGLVGRRAEPINLADAQSHPSFKYVPQVKEDRFRSFLGVPIIHRRQLLGVLVVQQRELRQFD
ESEESFMVTLATQMAGILSQSQLNAIFGQYRQTRVRALAASPGVAVAEGWQDSSQPSLDQVYRASTLD
TASERERLTLALEEAGAEFRRFSKRFAASSQKESAAIFDLYSHLLNDARLKRELFAEIDNGSVAEWAV
KQVIEAFAEQFAKLQDTYMRERGSDLRALGQRLLFHLDDTTQGATQWPARFVLVADELTATLLAEVPQ
DRLVGVVVRDGAANSHAAILVRAMGVPTVMGADIQPSLLSQRLLIVDGYRGELLVDPEPVLVQEYQRL
ISEEQELSKLAEDDVEQPAQLKSGERVQVMLNAGLSPEHEQLLGGRVDGVGLYRTEIPFMLQSGFPSE
EEQVAQYQGMLQLYPNKPVTLRTLDIGADKQLPYMPISEENPCLGWRGIRITLDQPEIFLIQVRAMLR
ANAGTGNLGILLPMVTSLEEVDEAKRLIDRAGREVEEVLGYAIPKPKIGVMLEVPSMIFLIPHLAGRV
DFISVGTNDLTQYLLAVDRNNTRVASLYDSLHPAMLQVLKLIAEQGKAAGLQLSLCGELAGDPMGALL
LVGMGYRNLSMNGRSVARIKYLLRHIDLADAEVLALRVLNTQMTTEVRHLVAAFMERRGMGGLIRGGR
>CORE_REP|Org29_Gene4668#
MRNNFLFGDKHDMNSLFASTARGLEELLKSELEALGAHDCKVVQGGVHFQGDDRLLYQSLLWSRLASR
ILLPLNEFRVHSDLDLYLGVQAIDWPSIFGVDKTFAVHFSGVNEEIRNSQYGALKVKDAIVDSFTRKL
DQRPTVAKQQPDIRVNVFLQRDMASVALDLSGEGLHQRGYRDLTGQAPLKENLAAAIVLRSGWQPGTP
MLDPMCGSGTLLIEAAMIAADRAPGLHRQHWGFTAWNGHNAELWREVTTEAQVRARRGLQETASRFFG
SDIDRRVIEMARGNARRAGVAELITFNVGDVARLTNPLPEGPHGTVISNPPYGERLESEPALIALHNM
LGRVMKSAFGGWQLSLFSASPELLSCLQLRAERQFKAKNGPLECVQKNYQLAANPAGGTTGGVQVAED
FANRLRKNLKKLDKWAKQQGIECYRLYDADLPEYNVAVDRYGSKVVVQEYAPPKTVDAQKARQRLFDV
INATLAVLELPSNQLILKTRERQKGKNQYEKLAQKGEFLLVEEYNAKLWVNLTDYLDTGLFLDHRIAR
RMLGEMSNGKDFLNLFAYTGTASVHAGLGGARSTTTVDMSRTYLEWAEKNLRANGLTGRQHRLIQADC
LSWLSNANEQFDVIFIDPPTFSNSKRMENTFDVQRDHLALMKDLKRLLRRNGTIMFSNNKRGFQMDMA
GLSALGLEAKEITAKTLSQDFARNRQIHNCWLVTHAGEGK
>CORE_REP|Org2_Gene3654#
MWRRLIYHPEINYALRQTLVLCLPVLFGLLIGQLQLGLMFSLVPACCNIAGLDTPHKRFFKRLVVGGS
LFAFSSVLLQQALLWHVPLPALMLGLALLLGVTGEISPLHARLLPAALVAAIFALSTAGTVPIWQAPL
LYAIGTVWYGLFTWFWFKLWKEQPMRETLSQLYLELADYFEAKYSLLTQHTDPQTALPPLLVRQQKVM
DLISLLYQQLNFLPHANNLEQKRLQRAFQVAMDLQEHITVSLHLPEEVQKLVEQSQAEAIIRRNAQVI
AGRLRVVAHDILYHQHSKRFSMAHELAALEKMAAQHPDNPVGQFCYYHFSRIARLLRTQHPLYRRDLM
PGQHRLPFWPALASYLSFKSTALRNAARLGVTLAVGSSLGAVFNLPKPYWILLTIMLVSQNGYNATRV
RIQHRALGTIAGLLLAAGLLQLQLPEGETLSIMLVITLLAYLVSRKNYGLSVIGFTVTAVYTLQLLAL
NGSHFLVPRLIDTLIGCVLAFGGTIWLWPQWQSGLLRKNAHQALEHDQTALRLMLEQPEPDATALAYT
RMQVNQAHNALFTSLNQAMQEPGFASNYLADMRLWVTHSQFIVEHLNAMTILAREHYMLTPKLAEAYL
QTCEIALQSCQQRLEYDGPSSGNSGIMQPPDLHPEMPVTEMERHLRRILSHLSVMHTISSLAWRQRPH
HGIWLKRKLRDQ
>CORE_REP|Org45_Gene1678#
MNKFVRLTAIAGLLWAGVSYGAETANIRIGQLPQLQQEPQHATVSERVTSRFTRSHYRQFALDAEFSG
KIFDRYLNMLDYSHNVLLASDVAQFAGKRNQVGEELKTGKLDTFYALFNLAQKRRFERYTYALSLLDK
PMNFTGNGTIDLDRSKAPWPKDKAELDSLWDAKVTYDELNLKLTGKTDKEIRDTLTKRYQFAIKRLTQ
SNSEDVFQLAMNAFAHEIDPHTNYLSPRNTEQFNTEMSLSLEGIGAVLQMDDDYTLINSMVPGGPAAK
SKAITVGDRIVGVGQAGKPMVDVIGWRLDDVVSLIKGPKGSKVRLEILPAGKGTKTRVVTLTRERIRL
EDRAVKMTIKTVGKEKVAVFDIPGFYVGLTDDVKVQLQKMAKQNVKSVIIDLRTNGGGALTEAVSLSG
LFIPSGPVVQVRDNNGKVREDADTDGVTYYKGPLVVLVDRFSASASEIFAAAMQDYGRALIVGEPTFG
KGTVQQYRSLNRIYDQMLRPEWPALGSVQYTIQKFYRVNGGSTQRKGVTPDILMPTGVDPAETGEAFE
DNAMPWDSINAATYSKTGDMAPFEPELLKDHQQRIAQNPEFQYIAQDIAHYKALKDKRNIVSLNLAVR
EKENHDDDATRLKRINERLERAGKKPLKSLDDLPKDYQEPDPYLDETVHIALELAHLEKDRPAQQPTP
AK

>CORE_REP|Org22_Gene2298#
MNILSYLQKVGRALMVPVATLPAAAILMGVGYWIDPVGWGGDNALAALFIKSGSAIIDHMAVLFAIGV
AYGMSKDKDGSAALTGFVGFLVLTTLCSPAAVSMIQKIPLDQVPAAFGKIENQFVGILVGIISAEVYN
RFSGVELPKALSFFSGRRLVPILISFLMILVAYILMFVWPVVFGALVSFGEHIQKLGSVGAGIYAFFN
RLLIPVGLHHALNSVFWFDVAGINDIPNFLGGQQSIEAGKAVVGITGRYQAGFFPIMMFGLPGAALAI
YHCARPENKAKVLGIMMAGAFAAFFTGITEPLEFSFMFVAPVLYVLHAILTGISVFIAASMHWIAGFG
FSAGLVDMVLSSRNPLATHWYMLIPQGLVFFVIYYVVFRFTINKFNLMTPGRELAVAGDETDGYDVNV
NSNAGKDENETTTLARRYVGAIGGSDNLTGIDACITRLRLNVKDSALVNDALAKRLGASGVIRLNKQS
VQVIVGTRAELIASAMRNVIAAGPVAAAAAPAAAPAAEAKSQAVPNAPKTALETLVAPVTGEVVALDQ
VPDEAFASKAVGDGLAIRPTDNIVVAPADGTVVKIFNTNHAFCLETDKGAEIVVHMGIDTVALEGQGF
KRLVEEGAEVKAGQPILELDLDYLNANARSMISPVVVSNSDDYAGLAALASGSVVAGQTKLYEIQK
>CORE_REP|Org37_Gene4277#
MSNYPHLLAPLDLGFTTLKNRVLMGSMHTGLEELPDGPQRLAAFYAERAAAGVALIVTGGIAPNDKGV
VYRGGSTLNSEAQLPHHRPVTEAVHRAGGKIALQILHAGRYSYQPHPVGPSALQAPINPFAPSALSEA
EIEQTIADFARCAALAQQAGYDGVEVMGSEGYLINQFLAARTNQRDDRWGGSFTNRMRFAVEIVRAVR
QAVGAKFILIYRLSMLDLVEDGSSWQEIEQLALAVEQAGATIINTGIGWHEARIPTIATMVPRAGFSW
VTRKLMGKVGIPLITTNRINDPAVAEQVLADGCADMVSMARPFLADAAFVQKAAEGRADEINTCIGCN
QACLDQIFEGKLTSCLVNPRACRETEMPLTMAEKPKTLAVIGAGPAGLAFATTAASRGHQVTLFDAAD
QIGGQFNIAKQIPGKEEFHETLRYFRRQLALREVKVRLGVKVEAADLSEFDEVILACGIMPRTPDIPG
IGHAKVLSYLDVLRDKKPVGQRVAIVGAGGIGFDTAEYLSQHGVSSSQDQAEFNREWGIDGRLEQRGG
LAAQGPQAPRAARQIYLLQRKTSKVGEGLGKTTGWIHRASLAMRGVKMLNSVSYRLIDDEGLHITRAE
QDSCLPVDTVVICAGQEPRRELQQPLQAMGKTVHLIGGADVAAELDARRAIDQGTRLAMAL
>CORE_REP|Org15_Gene3079#
MKYDTLASEILAGVGGRDNVKSLVHCATRLRFKLRDDRRANAAALKKNPGVIMVVESGGQFQVVVGNH
VAEVFDAVNRVGGLAEGASSGSDADGKKDNLLSRFIDVVSGIFTPLLGVMAASGVLKGLLALSLACGW
LLESSGAFKMLFAASDALFYFFPIMLGYTAGKKFGGNPFVTMAIGGALTHPLMMAAFEAAQQPGAVRE
YFFGIPLTFINYSSSVIPIIFAAWVSCRLEPLFNRVIHSALRNFITPLLCLAITVPLTFLLIGPAATW
LSHLLANGYQAIYAFNPIIAGAFMGAMWQVCVIFGLHWGLVPLMINNLSVLGRDTMVPLLLPAVMGQV
GATLGVMLRTRDAKLRALSGSAIGAGIFGITEPAVYGVTLPNKRPFIFGCIGGALGGAVIGYFHTSVY
SFGLVNVFTFAQIIPNGGIDATVWGAIGGTLLSFVFAALASYLFGVAPAEETAQPEAAAPLNRKQAIL
SPIAGDIVPLEQVNDATFASGLLGKGVAIAPLQGRVVAPVSGSVASLFKTKHAIGIESDDGAEILIHV
GIDTVKLDGAHFTAHVREGERIAPGDLLIEFDQAAIHAAGYDTTTPIIISNSDDYVDVLTSGLSPVQE
QAPLLTLLR
>CORE_REP|Org25_Gene3079#
MLNRYPLWKYLMLIVVILVGLLYALPNIYGEDPAVQITGARGVAASETTLDQVRTVLEKDNIASKSIA
LENGAILARFKDPDVQLRAREALVTELGDKFVVALNLAPATPTWLAMLGAEPMKLGLDLRGGVHFLME
VDMDTALSKLQEQTMDTLRSELREKGIPYASIRKLDNNGVEVRFRDDAARDQAISYIGPRQRDLVLSA
NGANTMKASLTDARLSEAREYAVQQNITILRNRVNQLGVAEPLVQRQGSDRIVVELPGIQDTARAKEI
LGATATLEFRLVNTNADATAAANGRVPGDSEVKYTRDGQPIVLYKRVILTGDHITDSTSSTDEYNQPQ
VNISLDSAGGTSMSNFTKDNIGKPMATLFVEYKDSGKKDANGRAVLVKQEEVINVANIQSRLGNSFRI
TGIGNPNEARQLSLLLRAGALIAPIQIVEERTIGPTLGQQNITQGLEACLWGLVASIVFMVVWYRKFG
VIATTALVANLVLIVGVMSLLPGATLTMPGIAGIVLTLAVAVDANVLINERIKEELKNGRSVQQAIHE
GYKGAFSSIVDANITTLITAVILYAVGTGSIKGFAITTAIGVATSMFTAIVGTRAIVNLLYGGKRINK
LSI
>CORE_REP|Org47_Gene4848#
MINPTLSRVTQRIIHRSQASRAAYLARIEAARSQTVHRAQLACGNLAHGFAACQPNDKTALKNMVRSD
IAIITAYNDMLSAHQPYEHYPQRLKQALKAVGAVGQVAGGVPAMCDGVTQGQDGMELSLMSRDVIAMS
AAVGLSHNMFDGALFLGICDKIVPGLVMAALSFGHLPALFVPAGPMSSGLPNKEKVRVRQLYAEGKAD
RLALLEAEAASYHGIGTCTFYGTANTNQMVMEVMGLHLPGASFVHPDTPLRDALNDAAARQVTRLTDT
AGNYLPIGRLVDEKVVVNGIVSLLATGGSTNLTMHLVAMARAAGIIITWDDFSELSEAVPLLCRIYPN
GPADINQFQAAGGVPLVVRELLQHGLLHEDVHTVAGFGLHRYTQEPWLDNGQLVWREGVAGSLDASVI
ASVAQPFEHHGGTKVMAGNLGRAVMKTSAVPADNQIIEAPAVVFDSQHDIVPAFEAGKLDRDCVVVVR
FQGPQANGMPELHKLMPPLGVLMDRGFKVALVTDGRLSGASGKVPSAIHVTPEAYTGGLLAKVRDGDP
IRVNGRSGELQVLVDADELAQRTPCQPDLSAEHIGCGRELFGALRSQLSGAEQGACCITF

>CORE_REP|Org47_Gene4278#
MLLKKGNSLRRLALSGAIACSLVSSFSASATVPALPVASAGMSVAQSRSELLAALPRGMDLHYLSTLA
PLYAANHMQPMWQDREAVQQFQQQLAELAMSGVQPQFTQWVKMLTDPALSEAGRDAVLSDAMLGYLQF
VSAIGANGNNWLYSNIPYKLGLPPTAVINQWQLAVRQARTLSYVNSLAPQHPQYAKMHQALRDMLADN
RPWPQVGSGPSLRPGQMSNDIPALREILTRTGMLAASAPEADPEPAVVSAKINEPDDGGLTVDEEKSR
VTVSPSAAPVTELTAEQTPPQIGSVVSDNLYTDELVEGVKRFQKWQGLTADGVIGVRTREWLNVSPKT
RAALLALNIQRLRILPGHVGTGIMVNIPNYSLTYYQNGNEVLSSRVIVGRPSRKTPLMSSALNNVVVN
PPWNVPTTLVREDIVPKAMRDGNYFQKHGYTVLSGWSNDAEVINPAMIDWSMISARNFPYRVRQAPGA
TNSLGRFKFNMPSSDAIYLHDTPNHSLFQKDIRALSSGCVRVNKASDLANMLLQDAGWNNSRVSSTLK
EGNTTYVNIRQRIPVKLYYLTAWVSDDGQPQFRTDIYNYDNTVRSGAQILAQAKKLMQ
>CORE_REP|Org28_Gene3029#
MTQTFIPGKDAALEDSIARFQQKLSDLGFNIEEASWLNPVPHVWSVHIRDRDCPLCFTNGKGASKKAA
LASALGEYFERLSTNYFFADFYLGRQIAEGDFVHYPNEKWFPIPEDDALPAGILDERLHAFYDPQQEL
SASDLVDLQSGNADRGVCALPFTRQSDQQTVYIPMNIIGNLYVSNGMSAGNTANEARVQGLSEVFERY
VKNRIIAESISLPAIPDEVLNRYPGVVEAIAKLEEEGFPILSYDASLGGNYPVICVVLFNPTNGTCFA
SFGAHPDFGVALERTVTELLQGRSLKDLDVFTAPTFDDEEVAEHTNLETHFIDSSGLISWDLFKQDAD
YPFVDWNFSGSTQEEFATLMSIFDKEDAEVYIADYEHLGVYACRIIVPGMSDIYPAEDLLLANNSMGA
HLRDTLLALPGSEWKPEEYLALIEQLDDEGLDDFTRVRELLGIASGKDNAWHTLRVGELKSMLALAGG
DLDQALIWTEWTQDFNASVLSPARSNYYRCLQTLLLLAQEPEREAAQYYTAFVKMYGQEAVDAASAAI
SGEERFNGLFAVDADLKALPAHQALLAAYEKLQAAKRRHWAKA
>CORE_REP|Org35_Gene3748#
MASNTLTNNRLEWQSLLPDVTPYQAIFDTAAQLAPVPFSAIQPRLENALTLFCHPQSPPRFMLLKAQE
TREYLELIANAVKPLLPQNTACRGSHYVIQDGKVSVEPASHGDEPFAAGGACVFQEWIEPEQLFGCVR
IHNGDITLQPGLVHQANGGILILSARALLAQPLLWLRLKQMIGQRQFHWVSPDETRPLPVAIPPMPLD
LRLIVVGDRHGLADFHDIEPELSEQAVYGEYEDDLQLTEVDDMAQWCGYVNGVIAERQLPMLAADAWL
PLIVQAVRYSGDQGILPLSPVWLGQQLSEAALYAEEDRITAKAFEAALNAREWRESYLAERMQDEIEL
GQILIETEGEVVGQINGLSVLDYPGHPRSFGEPSRISCVVHLGDGEFTDVERKAELGGNLHAKGMMIM
QAFVIAELDLDQQLPFSASIVFEQSYGEVDGDSASLAELCALISALSQQPITQQIAVTGSVDQFGNVQ
PIGGVNEKVEGFFEVCLRRGLTGKQGVILPVTNVRHLCLRQDVVDAVREGQFHLWAVESAAEALPLLT
GCLYSDEQQPNLLAAIQERIAQVSLQERRRPWPLRWLNWFNHG
>CORE_REP|Org45_Gene427#
MISGILVSPGIAFGKALLLKEDDIVINRKKISADQVEQEVSRFLAGRAKASEQLEAIKTKAGETFGEE
KEAIFEGHIMLLEDEELEQEIIALIKDDLASADAAAYTVIEGQAKALEELDDEYLKERAADVRDIGKR
LLQNILGMPIVDLGSIQDEVILVATDLTPSETAQLNLDKVLGFITDLGGRTSHTSIMARSLELPAIVG
TSDVTKQVKNDDYLILDAVNNQIYVNPTADVIDQLKAAQNQYITEKNDLAKLKDLPAITLDGHQVEVC
ANIGTVRDVAGAERNGAEGVGLYRTEFLFMDRDSLPTEDEQFQAYKAVAEAMGSQAVIVRTMDIGGDK
DLPYMNLPKEENPFLGWRAIRIAMDRREILHAQLRAILRASAFGKLRIMFPMIISVEEVRDLKGEIET
LKAQLREEGKAFDESIEVGVMVETPAAAVIAHHLAKEVDFFSIGTNDLTQYTLAVDRGNELISHLYNP
MSPSVLGLIKQVIDASHAEGKWTGMCGELAGDERATLLLLGMGLDEFSMSAISIPRIKKIIRNTNFED
VKALAAQALAQPTAQDLMNCVNKFIEEKTLC
>CORE_REP|Org22_Gene1819#
MTENNHSVADVEKIKRWSPVWIIPIVTALIGAWILFYHFSHQGPVVTLVTTTAEGLEAGKTKIKSRSV
DVGVVETVTLSDDLSKVMVQARLNAGMEKLLRQDSAFWVVKPQIGREGVSGLGTLLSGAYIELQPGSK
GKDGKDNYQLLDAPPLASPDAKGLRIVLDSEKSGQLNAGDPVLFRGYRVGSVETSYFDPKERAMRYQL
FITAPYDQLVTTNVRFWKDSGVAFDMSAQGMRVEMGSLTTLFSGGVSFDVPDGWDRGEPAKEKAEYQL
FDNQRSTQDSLYTVHKDYLLFFSDSVRGLQPGAPVEFRGIRLGTVAQVPFYKDGMAQRLDNDYRIPVL
IRIEPDRLHKQLGDNVDIEAHLKDAESRGMRASMKSANLLTGSLYIDLDFYPQEKPWKGPRELFGYPL
MPTTSGGLAQIQQKLMQTLDKINAMPINPMLNEATKTLAESQKTMKSTQQTMKSLNDIIASKEMQALP
QDMQKTLLELNRSMKGFQPGSPAYNKMVGDMQRLDQVLRELQPVLRTLNEKSNALVFEAAGSTDPQPK
KATK
>CORE_REP|Org39_Gene4197#
MQQTLKRTSLTLLISGALGAGAVNSSLAAEVPAGVQLAQQQNIVINNGSEVASLDPHKVEGVPESNII
LNLLEGLVSTDANGHVVPAAATSWENQNYQQWTFHLRPGAVWSDGSPVIAQDFVYSWQRLADPKIASP
YASYLQYTKVENIDDILTGKKSPQTLGVKALDDQTLQVTLSEPVPYFISMLSHTSLKPVKQAVVEKFG

DKWTLPANYVGNGAYRLKEWVVNERIVLERSPSYWNNKQTVINQATFLPITSEVSDVNRFRSGEIDIT
NSAIPPYLYVKMKREMPEQLHVNPYLCTFYYELNNKRAPFTDPRVRTAVKMTLDRDIIANKIMGQGQI
PAYSFTPTFTEGASFTQPAWAGWSQEQRNAEARKLLAEAGYSDAKPLKFSLLYNTSDQNKQQAIAAAS
MWKKNLGAEVTLRNQEWKTSLESRHQGQYDVARATWCGDYNEPSAFLNLVLSNSSINTVFYKSPAFDA
IMAATLKAPDEAARTALYQQAEAQLDKDSALVPVYYRVSARLIKPTVGGFTGKDPLDYTDVKNLYIIK
Q
>CORE_REP|Org24_Gene4315#
MTGRKIQRGFRLALCAAAIGACMSSAMAAQVPPGTALAAKQEIVRHIKDEPASLDPIKAVGLPEAQLA
RDLFEGLVNQDANGKVIPGVATRWQTSDNQTYIFHLRKDARWSNGDPVTAKDFVYSWQRLVDPKNLSP
FAWFAQLAGIQNAEQIISGKLPADRLGVSAPDDYTLKVQLDKPVPYFVSLTANFSLFPVNKAVVEKYG
NDWTKVGNLVGNGAFKLQERVVNEKLVLTPNDHYWDHARTVLTKVTFVPINQESNATKRYLAGDIDIT
ESFPKNMYQKLLKDIPGQVYTPDQLGTYYYAFNTQRAPTNDVRVRQALSYAIDRKIIAEKVLGTGEKP
AYHFTPDVTAGFKPEVSLLQQQSQAELDAQAKALLQAAGYGPNNPLKLTLLYNTSESHQKIAIAVASM
WKKKLGIDVKLQNQEWKTYIDSRNTGNFDVIRASWVGDYNEASTFLSLLTSTHSGNIAKFKNADYDKL
LAQAGRETNPAAVTADYNKMEQIIADQAPIAPIYQYTNGRLIKPWVKGYPITNPEDVAYSQTMYIIKH
>CORE_REP|Org3_Gene4776#
MDLNDLSTRIGGDVLVNILSGQPRAASVRWLGATVLFTLFSSPAWAFSIDDVAKQAQDLAAKGFEAPK
SNLPSQFREMKFADYQQIQFNHDKAYWSKLKTPFKLEFYHQGMYFDTPVKINEVTSTSVKQIKYSPDY
FNFGSVKHDPESVKNLGFAGFKVLYPVNSADKNDEIMSLLGASYFRVVGKGQVYGLSARGLAIDTALP
SGEEFPRFREFWVERPKQGDKHLVIYALLDSPRATGAYRFTVIPGRDTTVDVESKVFLRDKVGKLGLA
PLTSMFLFGPNQPSPTLNYRPALHDSNGLSIHAGNGEWIWRPLNNPKHLSVSTYTVENPKGFGLLQRG
RNFKEYEDLDDRYDLRPSAWIEPKGDWGKGKVELVEIPTADETNDNIVAFWTPDTLPEAKKPLTLSYR
LNFTRDEDKLHSQDIAYVARTMRSTGDVKQSNLIREPDGSVAFLVDFVGPVLKGLDANTPVASQISIG
DNGEMVENNVRYNPVTKGWRLTVRLKVKDDKKPVEMRAALVNGDKTLSETWSYQLPANE
>CORE_REP|Org37_Gene721#
MFDIVELSRLQFALTAMYHFLFVPLTLGMAFLLAIMETVYVLSGKQIYKDMTKFWGKLFAINFALGVA
TGLTMEFQFGTNWSYFSHYVGDIFGAPLAIEGLMAFFLESTLVGLFFFGWDRLSKVQHMAVTWFVALG
SNLSALWILVANGWMQNPIASDFNFETMRMEMVSFSELVLNPVAQVKFVHTVASGYTCGAMFVLGISS
YYLLKGRDIAFAKRSFAIAASFGMAAILSVIVLGDESGYEMGDVQKTKLAAIEAEWDTQPAPASFTLF
GIPDQDKMENSFSIQIPYALGLIATRSTDTQVTGLKDLMAQHEVRIRNGMKAYQLLEELRGGNTDPAV
RAEFNKTKQDLGYGMLLKRYTPNVTDATEAQIQLATKDSIPRVAPLYFAFRIMVACGVIMLLIIGLSF
WNVIRGRIGQKKWLHRAALYGLPLPWIAIESGWFVAEYGRQPWAIGEVLPTAVANSSLTAGDILFSMG
LICGLYTLFLVAELYLMFKFARLGPSSLKTGRYHFEQPTAAVQEAR
>CORE_REP|Org3_Gene1399#
MKPEDFRADSKRPFTGAEYLKSLQDSREIYIYGERVKDVTTHPAFRNAAASVGQLYDALHDPASQDRL
CWNTDTGNGGYTHKFFRYARSPEEMRQQRDAIADWSRQSYGWMGRTPDYKAAFGCALGAYPEFYGQFA
DNARHWYKRIQETGLYFNHAIVNPPIDRHKPVNEVKDVYIQVEKETDAGIVVSGAKVVATNSALTHYN
FIGFGSAQVMGDNPDFALMFVAPMDAEGVKLISRASYELVAGATGSPFDYPLSSRFDENDAILIMDHV
LIPWENVLIYRDFDRCRRWSTQGGFARLFPLQACVRLAVKMDFITALLQKSLSCTGVLEFRGVQADLG
EVVAWRNLFWSLSDAMCAEATKWENGAYLPDSAALQTYRVMAPMAYTKVKHIIEKNVTSGLIYLPSSV
RDMNNPEIDKYLARYVRGSDGMDHVERIKILKLMWDAIGSEFGGRHELYEINYAGSQDEIRLQCLRHA
QGSGNMDRMMQMVDKCLADYDQHGWKVPHLRNNDDINQLDNLLK
>CORE_REP|Org47_Gene3450#
MSSNKKPMVLVILDGYGHREERQDNAILNAGTPVMDRLWREQPHTLIAASGLDVGLPDGQMGNSEVGH
VNLGAGRIVYQDLTRLDKAIADGDFFANPVLTAAVDKAVAAGKAVHIMGLLSPGGVHSHDEHILAMIK
LAAQRGAKAVYLHAFLDGRDTPPRSAEAPLQRCRDAFAALGVGRIASLIGRYYAMDRDNRWDRVQLAY
DLLTAAKGDAVAEDAIAGLQAAYQRGENDEFVRPTVIRAAGEADAAMQDGDALIFMNFRADRARQITR
AFVNADFDGFPRAKQVQFGDFVMLTEYAADIATACAYPPASLANTFGEWLMKHDKTQLRISETEKYAH
VTFFYNGGVEAPFKGEDRVLVNSPKVATYDLQPEMSAAELTDKLLSAIRSGKYDAIICNYPNGDMVGH
TGVYEAAVKAVETLDACIAQVVDAVRDVDGQLLITADHGNAEQMRDPATGQAHTAHTSLPVPLIYVGK
PARAVEGGKLSDIAPTLLTLMGMEIPQEMTGKPLFIVE
>CORE_REP|Org22_Gene3723#
MKHTFKRNALLAAVLLAAGTGPVWAAKDAVIAVASNFTTLDPYDANDTLSQAVAKSFYQGLFGFDKDM
KLVNVLADSYEVSKDGLTYTVKLRQGIKFHDGTAFNAEAVKINLDRASNPDSHLKRYNLFKMIDKTEA

VDADTVKIVLKAPFSAFVNNLAHPAAAIISPAALKQYGKEIGFHPVGTGPYQFVTWNQTDFVKVKKFD
GYWQPGLPKLDSITWRPVVDNNTRAAMLQTGEATFAFPIPYEQAKVLEGNAKLDVVAAPSILQRYISM
NVTQKPFDNPKIRQALNYAINKDALIKVAFAGYAVPAEGPVPPAIDFAARYKPWPYDPAKARELLKEA
GYPNGFTTTLWSSHNHSTAQKVLQFTQQQLAQVGVKVTVTAMDAGQRAAQVESVGVQDTGVRLFYTGW
SASTGEADWALSPLFSTQAAPPKQFNTAFYSNPQVDKDLTDALATTDRAEKQKLYQDAQDRIWADAPW
IFLATERLLSANSKQLSGFYVMPDTSFNFDNADLK
>CORE_REP|Org28_Gene2160#
MNLLKSLAAVSSMTMFSRVLGFARDAIVARVFGAGMATDAFFVAFKLPNLLRRIFAEGAFSQAFVPIL
AEYKSQQGEEATRTFIAYVSGLLTLVLAVVTVLGMLAAPWVIYITAPGFTDTPDKFALTSALLRITFP
YILLISLASLVGAILNTWNRFSIPAFAPTLLNVSMIGFALFAAPYFNPPVLALAWAVVVGGVLQLGYQ
LPHLRKIGMLVLPRLKLGDAGVWRVMRQMGPAILGVSVSQISLIINTIFASFLVSGSVSWMYYADRLM
EFPSGVLGVALGTILLPSLAKSFSSGNHDEYSRLMDWGLRLCFLLALPSAIALGILAKPLTVSLFQYG
KFSAFDAAMTQRALVAYSVGLMGLIVVKVLAPGFYSRQDIKTPVKIAIITLIMTQVMNLAFIGPLKHA
GLALSIGLAACLNASLLYWQLRKQKIFQPQPGWALFLTKLVIAVLVMSAVLIGVMWLMPAWDQGNMLE
RLLRLAAVVVAGVVAYFGVLAGLGFRPRDFARRVA
>CORE_REP|Org37_Gene1051#
MTRSNVEMPNEVQAWVSEGRYKEGFFTQLATDELAKGINEEVVRAISAKRNEPEWMLEFRLEAYRAWL
QMEEPHWLKANYDRLNYQDYSYYSAPSCGSCDDACGSQPGAEQQPGAATEKDYLTSEVELAFNQLGVP
VREGSEVAVDAIFDSVSVATTYREKLAESGVIFCSFGEAIQEYPDLVRQYLGRVVPSNDNFFAALNAA
VASDGTFVYVPKGVRCPMELSTYFRINAAKTGQFERTILIADEGSYVSYIEGCSAPVRDSYQLHAAVV
EVILHKDAEVKYSTVQNWFSGGESKGGILNFVTKRALCEGAGSKMSWTQSETGSAITWKYPSVILQGD
NSIGEFFSVALTSGHQQADTGTKMIHIGKNTKSTIIAKGISAGHSENTYRGLVKILPGAENARNFTQC
DSMLIGPDSGAHTFPYVEARNNSAQLEHEATTSKIGDDQLFYCLQRGISEDDAISMIVNGFCKDVFSE
LPLEFAVEAQKLLAISLEHSVG
>CORE_REP|Org12_Gene1018#
MGKAMNARINARHGLPLTVQLINLFMLAFLLSLVGILSRPIGSLSLFWPVNAILLGLLLRKPIYGTPL
GWLTTYLGMVAADLSTGEGWSLALWLNACNMSLIAVGYGIMLMLPQSQRRMGKPQAILYMFSASLAGA
AVASTLSVLRNDSLYNNTVVIAWLAWFSEQFSTTLLLLPVLMAAPRLKQLLRMQVRWRLKGCLPLLAL
LLSLAFSVYIGGPGAIAFPIPALLWCAVRYPLFPVTLLTLLTGMTEISSISANLVLYETPNNHNAFLD
TLMSARLGIAMLVMGPLILASSIAANRKLMRRLEHSANHDFLTGVLARSAMTRKAGELLEHKHRSKEA
VSLLLIDIDHFKQINDTHGHSAGDQVLASFAHIVRRELRHDQLFGRLGGEEFAIMLPRALAAQGVALG
EHLRRLVEQTELQAEGKQTLKITISVGVASLAMNEVKSLEQLMNMADIALYRAKSQGRNRVESFNVIN
GNSVEHILFR
>CORE_REP|Org41_Gene2421#
MKQGLQLRLSQQLAMTPQLQQAIRLLQLSTLELQQEIQLALESNPLLEQTDLHDEIDAKEIQETEGLD
TREALEQKDMPEELPLDATWDEIYTAGTPSGTGTDYSDDELPVYQGETTQTLQDYLMWQVDLTPFSDT
DAAIATSIVDAVDDTGYLTVPLEDILESLGDENVTLEEVEAVLKRVQRFDPIGVAARDLRDCLLVQLS
QYAKDTPYLAEARLIISDHLDLLANHDFRSLMRSTRLKEDTLKEAMLLIQSLDPRPGQSINTGESEYV
IPDVLVRKTQNTWTVELNGDSIPRLKINQQYAALGNSARSEADGQFIRSNLQEAKWLIKSLESRNETL
LKVTRCIVSQQQAFFEQGEEFMKPMVLADIAQAVEMHESTISRVTTQKFLHSPRGIFELKYFFSSHVN
TDSGGEASSTAIRALVKKLIAAENPAKPLSDSKLATLLSDQGIIVARRTVAKYRESLSIPPSNQRKQL
V
>CORE_REP|Org16_Gene2983#
MINIVVVSHSALLARGVEQLARQMMRGDGCKLALAAGVDDEQHPIGTDAVKVMEAIEAVADGDGVLVL
MDLGSALLSAETALDLLDPDLAAKVRLCAAPLVEGTLAAVVAANSGASLEQVVAEAQGALQAKQAQLG
EASPTAKSVALPLAQGKSVTWTVQNPHGLHARPAARLVETLAPFKAELVLEKQGQCVDPRSLNQLALL
QVRHGDTVRLIADGAQADEALAAFKALAEQHFGETVSERQQPSLHGIPVAESVTSGPVFQAHSFWPPT
VDRRIGADEVLGEQQRLREALQHTLSDLNRLAERTGTLIGKPQAAIFGAHSMLLDDPDLQQAAYTRIA
QQLCCAEQAWRQVLGAIAEEYRELDDDYMRARELDVRDMLRRTLCHLQGLPLPAMALAEPSILVMDEL
MPSEVVMLDRRLVLGICLSGGNALSHSAILAKAMGIPMVVGMQDCLSKTRSGQKAMLDAARGVLQLSH
>CORE_REP|Org49_Gene720#
MAGKKPTNKTNADETRERSRDRQMEGLKMPPHSLEAEQSVLGGLMLDNERWDNVAERVVANDFFSRPH
RLIFTEMQRLLEMSKPIDLITLSESLEQKGELDSVGGFAYLAELSKNTPSAANIGAYADIVRERAVVR
EMISVANEIADAGYDPQGRSSEDLLDLAESRVFQIAENRASKDEGPKGIERILEDTVSRIEQLYQQPH

DGVTGVDTGYQDLNKKTAGMQKSDLIIVAARPSMGKTTFAMNLCENAAMTQEKPVLIFSLEMPGEQIM
MRMLASLSRVDQTRIRTGQLDDEDWARISSTMGILLEKRNMYIDDSSGLTPTEVRSRARRIFREHDGL
SLIMIDYLQLMRVPALSDNRTLEIAEISRSLKALAKELQVPVVALSQLNRSLEQRADKRPVNSDLRES
GSIEQDADLIMFIYRDEVYHENSDLKGIAEIIIGKQRNGPIGTVRLTFNGQWSRFDNYAGPQYDDE
>CORE_REP|Org22_Gene4669#
MFPVPPTTKWVGVVNTELQSESSRLLASSNAGSPGWWLTVARRGTPWVEPAGNGRWRTTFFWRDPQGC
ELTSAYRRVWININCLTDHHQPNPPQSLQRLAGTDVWYWQTELSGAWRGSYCFIPCFDERPPAFSGDD
AHANMHNLRHWWHQVFASATPDLLNPYRSWQSASGHSVSGLHMPDAPPQPVWRSFDEYEIASGRCTPP
LPARLQRHTWQSERLGNSRDVWIYTTGDSKPAERPLAILLDGQFWAKQMPVWEPLMQLTREGALPEAV
YVLIDIIDLPHRSRELTCKDDFWLAVQEELMPQLADWAPHSGKPADTVVAGQSFGGLASLYAGLRWPQ
RFGAVIAQSGSYWWPRRDMLQLPSIPDDACWLMQQVERHGLGNHGALKVFMEAGSQEKLVHRVSGEMA
ARLSDAGHRVHYRVVEGGHDALCWRSGLTDGLQAVWASAFATAYPASATATATARGTHDGKPESVR
>CORE_REP|Org45_Gene4578#
MSKVKQQDIDRLIVLVGGRENIATVSHCITRLRFVLNDPSKASPKEIEELPMVKGCFTNAGQFQVVIG
TDVGDYYQALIASTGVNEADKEQAKVAARQNMTWTERTISHFAEIFFPLLPALISGGLILGFRNVIGD
IPMSGGQTLAQMHPAWKTIYDFLWLLGEAIFMFLPVAICWSTVKKMGGTPVLGIVLGVTLVSPQLMNS
YLLGQQTPEVWNFGWFVIQKVGYQAQVIPSILAGMALGWIETRLKKIVPDYLYLVVVPVVSLLLAVFL
AHALIGPFGRMIGDGVAWAVKAVMTGSFAPVGAALFGFLYAPLVITGVHQTTLAIDMQMIQSMGGTPV
WPLIALSNIAQASAVLGIIIISRKANEREISVPAAISAYLGVTEPAMYGINLKYRFPMLCAMIGSAIA
GLFCGLDGVMANGIGVGGLPGILSIKPQFWLIYSLAILVAIVIPLVLTIMVYKRKAARGELPV
>CORE_REP|Org41_Gene316#
MLGLDALELARIQFAFTVSFHIIFPAITIGLASYLAVLEGLWLKTHNEAYRELYHFWSKIFAVNFGMG
VVSGLVMAYQFGTNWSFFSEFAGSITGPLLTYEVLTAFFLEAGFLGVMLFGWNRVGPGLHFFATCMVA
LGTLISTFWILASNSWMQTPQGHEIINGQVVPVDWLKVIFNPSFPYRLLHMSTAAFLSSAFFVGASAA
WHLLRGRDTPAMRKMLSMAMWMALIVAPVQALIGDAHGLNTLKHQPAKIAAIEGHWENPPGEATPLIL
VGWPDMQREETRFKLEVPYLGSLILTHSLTEQVPALKSFPPEDRPNSTVVFWSFRIMVGLGMLMILAG
VWSLWLRWRGGLYQSRPFLYFILWMGPSGLLALLAGWFTTEIGRQPWVVYGLLRTKDAVSAHGDLHMS
ISLLAFIIVYCSVFGVGYSYMMRLIRKGPQPHEHQEDNTEGRPARPLSAVNDTLDDRS
>CORE_REP|Org8_Gene458#
MSLSLWQQCLARLQDELPATEFSMWIRPLQAELSDNTLALYAPNRFVLDWVRDKYLNNINGLLNDFCG
TDAPLLRFEVGSKPITQVISQTVTASVSSAPAAPAARTAAPSRPSWDNAAAQPELSYRSNVNPKHTFD
NFVEGKSNQLARAAARQVADNPGGAYNPLFLYGGTGLGKTHLLHAVGNGIMARKANAKVVYMHSERFV
QDMVKALQNNAIEEFKRYYRSVDALLIDDIQFFANKERSQEEFFHTFNALLEGNQQIILTSDRYPKEI
NGVEDRLKSRFGWGLTVAIEPPELETRVAILMKKADENDIRLPGEVAFFIAKRLRSNVRELEGALNRV
IANANFTGRAITIDFVREALRDLLALQEKLVTIDNIQKTVAEYYKIKVADLLSKRRSRSVARPRQMAM
ALAKELTNHSLPEIGDAFGGRDHTTVLHACRKIEQLREESHDIKEDFSNLIRTLSS
>CORE_REP|Org33_Gene3049#
MKKTLGVFLPLYTTTLLLLLGSGLLTTYVSLRLASIHVSSALIGAIIAANYIGLVIGGKVGHFLIARV
GHIRAYVACAGIITAAVLGHGLTEFIPAWVALRLVIGLCMMCQYMVLESWLNDQAESNQRGMVFGFYM
AATYLGMSLGQIVLMLQSNLGITTLLVIALCFALCLVPIALTTRTNARHMSPAPMELRYFVGAIPKVL
ATTLVIGMVVGSFYGLAPVYASLQSLTTQQTGLFMALAIFAGLVAQFPLSWLSDRYSRPLLMRLNAIL
LIVAALPLALLPHIDFPLLLAVGFVVSMLQFTLYPLVVALANDLIEPERRVSLAACLLMAFGVGASIG
PLAVGALIEPLGGNILYAFFALCGVLLAALSRTAKAEEPQLAQDAPVPHIPLPDSLASSPLSPALNPT
FDEQIIHDTMPPPEAAPDVDEPQPEEQEAEPLPQGADPQEDTGLKKAHAML
>CORE_REP|Org5_Gene4424#
MDITATANALLPLLGGKENIASAAHCATRLRLVLVDDSKVDKEAIGKLDGVKGCFSNAGQIQVIFGTG
LVNKVHAEFIKAAGVSESSKAEAADIAAKKLNPLQRIARLLSNIFVPIIPAIVASGLLMGLLGMVKTY
GWADANSALFIMLDMFSSAAFIILPILIGFTAAREFGGNPYLGATLGGILTHPALTNAWGVAEGFHTM
NFFGLEIAMIGYQGTVFPVLLTVWFMSLLEKRLRKVIPNALDLILTPFLTVVISGFVALLFIGPAGRA
LGDGISFVLSTLIAHAGWFAGLLFGGLYSAIVITGIHHSFHAVEAGLLGNPNIGVNFLLPIWSMANIA
QGGACLAVYFKTRDAKIKAIAVPSAFSAMLGITEAALFGINLRFVKPFLAALAGGALGGAWVVANHVG
MNAVGLTAIPGMAIVQASSLVSYIIGLAIAFGSAFALSLLLKYKTDAQ
>CORE_REP|Org41_Gene3508#
MIPVSLQTLAEVLSAELIGADCQIVEVTTDTRKVTAGCLFVALKGERFDAHDFAADAVAAGAGALLVS

KRLLVDAPQLVVQDTRLALGQLGAWVRQQVPARVVALTGSSGKTSVKEMTAAILRECGEVLYTAGNFN
NDIGVPLTLLRLEPQHDFAVIELGANHIGEIAYTTALTRPQTALVNNLAAAHLEGFGSLAGVAQAKGE
IFTGLPADGVAIINADNNDWPHWQSMLGGKTVWRFSPQAAAGVDFFADNVRVNGAGTQFTLHSPFGTA
EIALPLPGRHNVANALAATALATSVGATLEAVRQGLKQLQAVPGRLFPVALAEGKLLLDDSYNANVGS
MTAAAQVLAEMPGYRVMVVGDMAELGAEAEECHRQVGEAARLAGVDKVISVGGLSRVLSEASGNGEHY
QDKTAVIARVAELLSEHAVITVLIKGSRSAAMEQVVRALQEKAPC
>CORE_REP|Org22_Gene3871#
MKAFVPALVLSAVSFSVWAQDATVSSELIKRGEYLARAGDCVACHTDGKSGKTFAGGLAMETPIGTIY
STNITPDKKTGIGDYSFEDFDNAVRKGVAKNGSTLYPAMPYPSFALVKEEDMRAMYAYFMHGVQPVEQ
ANKDSDIPWPLSMRWPLSIWRGMFAPSPADFVADAKADPVIERGRYLVEGLGHCGACHTPRSITMQEK
ALSNGESDDYLSGSNAPIDGWVASSLRSDRKDGLGSWSEAELTEFLKTGRNDKAIVFGGMSDVVEHSL
QYLSDDDLTAIARYLKSLPPKDGKQQAAPVEDSVAKDLWRGDDSKPGAALYVDNCAACHRTDGVGYKR
AFPSLKGNPVVQTEDATSLIHIVLTGNTTPAVQGAVSNITMPPFGWRLNDQQVADVVNFIRTSWGNSA
KPVSASDVADVRKDRSMIRDEKAMGSAEVPDHPDAKK
>CORE_REP|Org12_Gene91#
MAKQPGLDFQSAKGGLGELKRRLLFVIGALIVFRIGSFIPIPGIDATVLAKLLEQQRGTIIEMFNMFS
GGALSRASIFALGIMPYISASIIIQLLTVVHPALAEIKKEGEAGRRKISQYTRYGTLVLAIFQSIGIA
TGLPNMPGMQGLVLNPGFAFYFTAVVSLVTGTMFLMWLGEQITERGIGNGISIIIFAGIVAGLPPAVA
HTIEQARQGDLHFLLLLLVAVLVFAVTFFVVFIERGQRRIVVNYAKRQQGRRVYAAQSTHLPLKVNMA
GVIPAIFASSIILFPATIASWFGGGTGWNWLTTISLYLQPGQPLYVLLYASAIIFFCFFYTALVFNPR
ETADNLKKSGAFVPGIRPGEQTAKYIDKVMTRLTLVGAMYITFICLIPEFMRDAMKVPFYFGGTSLLI
VVVVIMDFMAQVQTLMMSSQYESALKKANLKGYNR
>CORE_REP|Org27_Gene3797#
MSDNATNQRQLRLGAILHGASGNMSAWRHPDATADASINLEFNIATAKKAEQGKFDFVFVADGLYINE
KSIPHFLNRFEPLTLLAALSAATDKIGLVGTLSTSYSDPFTVARQFASLDHLSNGRAGWNVVTSPLEG
SAKNFSRKEHPEHSLRYRIAGEFLDVAKGLWDSWEDDAFVRNKASGEFFRAGKLHTLNHQGEFFSVQG
PLNIGRTPQGRPILFQAGASEDGKRLAAQHADAIFTHHDTLEQAQDFYQDVKRQLVEQGREPDDLRIF
QGVSVIVGDDDADVERQYQETARLVSIENALNYLGRYFEHYDFARHPLDAPFPDIGDLGQNSFRSTTD
AIKRSARERHLTLRQVALEAASPRPVFSGTPEAVADGLQRWFDGEAADGFIISGGTPNAFGHFVDRVV
PVLQQRGLFRQAYHGDTLREHLGLKRPLNRFTQ
>CORE_REP|Org25_Gene39#
MSEFSQTVPELVAWARKNDFSISLPTERLAFLLAIATLNGERLDGEMSEGELVDAFRHVSKGFEQTHE
TVAMRANNAINDMVRQRLLNRFTSELADGNAIYRLTPLGIGITDYYIRQREFSTLRLSMQLSIVAQEL
KRAADAADEGGDDFHWHRNVFAPLKYSVAEIFDSIDMTQRVMDEQQQSVKNDIAALLSKDWRAAISSC
EMLLSETSGTLRELQDTLDAAGDKLQANLLRIQDATLGNVELGFVDKLVFDLQSKLDRIISWGQQAID
LWIGYDRHVHKFIRTAIDMDKNRVFAQRLRQSVQTYFDHPWALTHANADRLLDMRDEELALRSEEVTG
ELPPDLEFEEFSEIREQLAAMIEEALKVYQEQQMPLNLAAVMRDYLAQYPRARHFDVARLVVDQAVRL
GVAEADFSGLPAEWQAINDYGAKVQAHVIDKY
>CORE_REP|Org8_Gene817#
MQVSVETTQGLGRRLSITVPADTIKQAVKKELINAAKSVRIDGFRKGKVPMNIVEQRYGASVRQDVLG
EAMQRSFVDAIIKEKINPAGAPNYVPGEYKEGEDFTFAVEFEVYPEVELKGLENIEVEKPVVEVNDED
VDAMLDTLRKQQATWKETDRAAEAEDRVTVDFTGSIDGEEFEGGKASDFVLAMGQGRMIPGFEEGLVG
HKAGEEFSIDVNFPEDYHAENLKGKAAKFAIVLKKVEERELPELTEEFIKRFGVADGSVAGLRTEVRK
NMERELKGAVRNRIKSQAIDGLVSANEIDVPAALIDGEIDVLRRQAAQRFGGNEKQALELPRELFEEQ
AKRRVVVGLLLGEVISTNDLKADEDRVKTLIEEMASAYEDPSEVIEFYSKNKELMNNMRNVALEEQAV
EALLAKAKVTEKATTFSELMNQTQQA
>CORE_REP|Org8_Gene2144#
MKIHAITAPLSKARHQRCCECDLLFMLPPLSGNQAAYCPRCNAKVVHGRDWSMTRLTAMAITMLLLMP
FAFTEPLISIRLLGTRIDASLLEGIWQMSRQGDPLTASMVAFCTLGAPLTLALSLLYLRFGHALGMNL
RPVLLMLERLKEWVMLDIYLIGMAVAAIKVQDYADIQAGSALIAYLSLTLLSILTLIHANLEQLWERY
YPQEQPEGPPAALHICLSCHYTGYPDARGRCPRCHVPMCHRQPYSLQKTWAALIAAMILLIPANLLPI
SIIYANGVRLEDTIFSGVVSLATSGNVPIAAIVFIASVLVPFTKVIVLITLLLSIHFKTSHSLKTRIR
LLRLVTWIGRWSMLDLFVIALMMSLVNRDQLLSFTMGPAAFYFGSAVILTILAVEWLDSRLIWDAHAT
GNADYTD

>CORE_REP|Org2_Gene3767#
MKKRLAVLILLVAIVVIALLWWRENRRYDGPVQQVTAGAEQIARGRYLAQAADCAACHTASGGAPLAG
GYPLETPFGTIYGSNLTPSADHGIGRWTRDDFFLALTQGVAPGGRHLYPAMPYTSYKGMSRQDADDIY
AYLMTRPAVDVAIPANEMPFPFNQRMALIGWNLLFRSQDPLPASSQGSSPQWQRGRYLVDVLGHCGEC
HTPRGALGQMDLAKPMQGGDLGRFMAPDITPHGLAQRGWTPQDVSRFLSTGLAPQGSAFSEMHMVVDL
STRHLTPEDHQALALYLMGEQPPAAVPVKMGQGSDAGRMAYLDQCAGCHAREGEGKPHVAPAMRDNAT
LRQVDGKNLIVSVLDGLPAQQFPNGESMQSMPGFGERLSDADVAELVNYLRVTWGGLPADITAEQVKA
LRK
>CORE_REP|Org43_Gene3254#
MRKKTLLLCLPLLFTGNALADAGGYQLEQVLVMSRHNLRAPLANNGSVLAQSTPKAWPAWETPGGQLT
TKGGVLEVYMGHYFNAWLKQTGLLPQEGCPTAGSVYVYANSLQRTVATAQFFSNGAFPGCDVSVHHQD
KMGEMDPTFNPIITDTGEAFNQQALAAMNAALGSLKLDASYQQLAKIIDYKDSAACKTDKHCDLTKEA
SVMSAVPGKEPGVSGPLRVGNSLVDAFMLQYYEGFPMKEVAWGKIATPHQWQQLAQLKDGYQDSLFTS
PVVAQNVAKPLLTYINNALLGERKPDAPKLTVLVGHDSNIASLLSAMQFQPYQLPQQYEKTPIGGKLV
FQRWRDAQNDRELLKIEYVYQSTEQLRKATPLTLQTPPQRVTLALKGCPIDKDGFCAWSDFEKTMKGI
L
>CORE_REP|Org37_Gene3355#
MTTPIISLTAARALHLAAQGLLSPLKRQAKPDDVVSAIQRMGLLQIDTISVVARSPYLVLFSRLGAYQ
SEWLEQALAGRKLFEYWAHEACFLPIEDFGLLRHRMLAPHDMGWKYSADWVQQHQAAMDSLLRHIEQQ
GPVRSADFSAEKKGNSGWWDWKPEKRHLEILFTAGKLMVAERRNFHRVYDLTERLLPAWDDARHTLPA
ERARRQMLRRTCRYLGIFRAEWLADYYRLKRVAPKALLAELQEQGEITPVQVEGLEGPFYLHESLAEL
LPLAEQSKLKSTVTSLLSPFDPVVWDRRRALELFNFDYRLECYTPKEKRRYGYFTLPVLHRGELVGRI
DAKAHRRQGVFEIISFHAEPQVRFGKQRAQDIRQAIARTAKWHGAQRVALGDIPAALAAEWGAGWEVG
>CORE_REP|Org7_Gene3115#
MTQYASPILTSLLDTDAYKLHMQQAVFHRYPAISVAAEFRCRGDELLGEYADEIRAQVALMSQLTLTD
AEFAYLSSLPFFRQDYLSWLRTFRYDPQQVTIDNRDGKLQIRIAGPWREVIMWEVPLLAVISEVVHRR
RSPLATPEQAVAHLQTKLAQFKTLAGDLDLSRFKLMDFGTRRRFSQGVQQAIVSTLQAEFPYLSGTSN
YDLAHQLGLAPVGTQAHEWFQAHQQISPVLANSQRAALQAWLDEYPDQLGIALTDCITMDAFLRDFGP
QFAERYQGLRHDSGDPVEWGEKAIAHYQKLGIDPMSKTLVFSDNLDLEKALALYRHFYQRINLSFGIG
TRLTCDIPGVKPLNIVIKLVECKGKPVAKLSDSPGKTICQDQAFVRALRKAFDLPLVKKAS
>CORE_REP|Org22_Gene1065#
MTRSITLARTLALSALATLVLSSSAFAKIEEGKLVIWINGDKGYNGLAEVGKKFEKDTGIKVTVEHPD
KLEEKYPQVAATGDGPDIIFWAHDRFGGYAQSGLLAEIHPSKAFQDKLFPFTWDAVRYDGKLIGYPIA
VEALSLIYNKDLVKQPPKTWEEIPALDKQLRANGKSAIMWNLQEPYFTWPIIAADGGYAFKYENGKYN
IKDVGVANAGSQAGLQFIVDLVKNKHINADTDYSIAEAAFNKGQTAMTINGPWAWNNIEQSKINYGVT
LLPTFKGKPSKPFVGVLTAGINAASPNKELATEFLENYLLTNEGLADVNKDKPLGAVALKSYQEALAK
DPKIAATMQNSQNGEIMPNIPQMSAFWYAERSAVINAVSGRQTVKAALDDVQTRITK
>CORE_REP|Org7_Gene3423#
MTVRLFLAKGREKSLLRRHPWVFSGAVQRVEGKALSGETIDILDSQGKWLARGAYSPESQIRARVWTF
QQDEEINIDFFIRRLQQAQSWRDWVAQRDGLDGYRLIAGESDGLPGITIDRFQNFLVLQLLSAGAEYQ
RPALLSALQHCYPECSIYDRSDVAVRKKEGLPLAQGPVLGDLPPELLPITEHGMKLLVDIQQGHKTGF
YLDQRDSRLAARNYSAGRRVLNCFSYTGAFAVSALMGGCAQVISVDTSQAALDIARQNVELNKLELNK
AEFVRDDVFQLLRNYRAQGEKFDLIIMDPPKFVENKNQLASACRGYKDINMLALQLLNPGGILLSFSC
SGLMPTDLFQKILADAAVDAGRDVQFIEQFRQAADHPVIATYPEGLYLKGFACRVM
>CORE_REP|Org34_Gene3799#
MLDSQTIATVKSTIPLLAATGPKLTAHFYDRMFAHNPELKDIFNMSNQRNGDQRQALFDAICAYAANI
ENLAALLPAVERIAQKHTSFNIQPEQYQIVGGHLLATLDEMFSPGQEVLDAWGKAYGVLANVFIQREE
QIYQQSETDNGGWRDLRAFRILKKQPQSDVICSFVLAPVDGGRVADFKPGQYLAVYIKHDSLEHQEIR
QYSLTTSPNGEFYRIAVKREDQGKVSNYLHQQAQEGDVIDIAPPHGDFFLDVATTTPVALISAGVGQT
PMLGMLNTLHDSQHQAQVHWLHAAENGSVHAFADEVADIAGRMPNLSRHVWYREPGADDVEGRDYHSR
GLMDLSALQGSLADPQMHYYFCGPVAFMQFVGKQLLEMGVEAERIHYECFGPHKVL
>CORE_REP|Org29_Gene2914#
MSLRSLYLLSLLAAGGSAQAMSAGEYVAKAGDCTACHTAPGGAELAGGMKFPTPLGAIYATNITPDKL
HGIGAYSFEEFDRAMRQGVAKDGHRLYPAMPYTSYAKMSAEDMRALYDYLMNEVPAQNVANRDSDISW

PLSMRWPLAVWNQLFHDDQPYQADPQQSAEWNRGAYLVQGAGHCGSCHTPRGWAMQEKGLDGKEPVFL
SGAELDGWYASNLRGLPPEEVTALLKTGRSRHAAVAGPMSEVVTHSTQYLSDGDLNAIAVYLRSLAPE
TAAKAAAPAVQANNPGGQATYAMYCSTCHGNKGEGTDFAIPALAGNATVTADNPLTALRVVLEGAHTP
ATQHAMAFDMPAYGWALNDRQAADLMSYLRGSWGNQAAPVTVQQVQDARQLQAK
>CORE_REP|Org11_Gene3606#
MTIAVKHRVLLTLFMLLLLAAFGLPFLSYAPNRLLSGKSLSLFSLLHGPALWLLLPLAALAILSLLPP
TRGRALLAALAACGVLTLAFWISGQAAGHLAQEGSRLARTSWGSGCWLTMALSLLIAADAMARLTASH
LWRMLGNALVLVPPALLLFGHQLDQLSLLKEYHNRQEVFDAALLQHLTILLATMAPALAIGVPLGVLC
FRSERWQRPIFSALNIIQTVPSIALFGLLIAPLAGLATAVPWLAEHGVSGIGMAPAIVALVLYALLPL
VRSVVAGLQSVPAGVIESATGMGLTRGQIFLRVQLPLALPLFLTGVRILAVQTVGMAVVAALIGAGGF
GAIVFQGLLSSALDLVLLGVIPVVMMAVIVDSLFKFWVSILDVSRR
>CORE_REP|Org7_Gene1444#
MSAYSRPVLLLLCGLLLLTVSIAVLNTLVPLWLTHAQLSTWQVGMVSSSYFSGNLLGTLVAGKLIQRV
GFTRSYHLSCLLFAAATAGMVLSIDFWSWLGWRFFAGVGCAWIWVIVESALLRSGNLSNRGQLLAAYM
IVYYLGTVTGQLLLSMTSTELLHVVPWVTAIVISAMLPMLFARVNRHEDEPQQAAVWTMLRRRSARLG
INGCIISGIVLGSLYGLMPLYLSHQGMSDANVGYWMALLVSSGIVGQWPVGRLADRYGRLLVLRIQVF
VVILASVAMLGNYAMAPSLFILGCAGFTLYPVAMSWACEKAMPHELVAMNQALLMSYTIGSLLGPSMT
ALLMQNYSDRVLFVMIAAVALVYLLMLLKKQKPDRHHTPFAAA
>CORE_REP|Org9_Gene697#
MFDYEVLRFIWWVLVGVLLIGFAVTDGFDMGVGILVRIIGKTDTERRVMINSIAPHWDGNQVWLITAG
GALFAAWPMVYAAAFSGFYVAMILVLAALFFRPVGFDYRSKLESSRWRNMWDWGIFIGSFVPAVVFGV
AFGNLLQGVPFHMDEYMRLFYTGNFFQLLNPFGLLAGVVSLTMLVTQGATYLQMRTTGEIHLRSRAAA
QIATLIMAVCFLLAGVWLVKGIDGFVVTSALDTLAESNPMRKEVAHQAGAWLINFNKYPLLWALPALG
VVLPLFTILFSRLEKGALAFVTSSLTIACVILTAGVTMFPFVMPSSTVPNVSLTMWDATSSLLTLKVM
TVVAAIFVPIVLAYTSWSYYKMFGRLDKNYIENNKHSLY
>CORE_REP|Org12_Gene3248#
MSDTTQTQDPWATAPADAGAAPAHDAAANAGDAWSSAPPPAAHDAAGQGADWLSSAPAQPEHFSLLDP
FHKAWVPFDSWVTQGIDWLVLHFRPLFQGIRVPVDMILSGFQQLLLGMPAPIAILVFSLLAWQVSGLG
MGAATLLSLVAIGAIGAWSQAMVTLALVLTALFFCILIGLPLGIWLARSKHAAKVIRPLLDAMQTTPA
FVYLVPIVMLFGIGNVPGVVVTIIFALPPIVRLTILGIKQVPEDLIEAAESFGASPRQLLFKVQLPLA
MPTIMAGVNQTLMLALSMVVIASMIAVGGLGQMVLRGIGRLDMGLAAVGGVGIVILAIILDRLTQSLG
RDRRSKGIGRWYRRGPIGLLTRPFIKQA
>CORE_REP|Org22_Gene3622#
MIRCHDTYQGDAGCILPPPIFDARKSGLSFAKRTYLPRVAGLGLGFICVCAALYPLAPPTAVWLLLAF
HGFLWPHLAYRLACRAKDPFKAEIRNLLIDSAFGGFWAAMMAFNALPAIVILSMMSMNNIASAGKALF
VKGLAIQLAAAALTGALLGFPFHPHSTPLQIYLCLPMIYLYPTLLGLVTYRTAKRLAEKKQELQRIST
RDGLTGLYNRRHWEHLLHRQFDSCRRYQDNATLILMDIDRFKTINDTFGHALGDEALAALAEELLIGL
RNVDIVGRYGGDEFGAVLPNTSAEQAETVLRRIQQRLDVVIFKEAPQLRLQISAGIANYHPALGGYLD
WLKAADGALYRAKQNGRNRLETAAPTGD
>CORE_REP|Org1_Gene1987#
MKTEKLLSPLKVGAVTLPNRVFMAPLTRLRSIEPGDIPTPLMAEYYAQRASAGLIVTEATQISFQAKG
YAGAPGLHTPEQIAAWKHITQAVHDKNGHIAVQLWHVGRISHASLQPGGQAPVAPSAINADTRTTVRD
ETGAWVRVPTSTPRALETSEIPGIVNDFRQATANARDAGFDFIELHAAHGYLLHQFMSPASNQRTDQY
GGSIENRTRLTLEVVDATIAEWGSEHIGIRISPLGPFNGLDNGEDQEEAALYLVEELNKRNIAYLHIS
EPDWAGGKPYSDAFRDSVRAHFKGVIVGAGAYTAEKAEALIEKGFIDAVAFGRSYIANPDLVERFRQH
APLNEPKPETFYGGGAEGYTDYPFLAK
>CORE_REP|Org27_Gene1708#
MPLPQSRYDLPRIIFGVLFIAIMIVACFWVIQPFILGFAWAGMVVIATWPLLLKLQKLLWGRRSLAVL
VMTLLLILLFILPISLLISSVVDNSAPLIAWASSPGKLHIPDLAWLQSVPMIGDRLYTSYHTLVNAGG
AALLAKVQPYFGQTATWFVAQAAHIGRLLLHCALMLLFSALLYARGEQVALGIRHFAVRLGSARGDAA
VLLGGQAIRAVALGVVVTALVQSVLGGIGLAVSGIPAATLLTMLIFICCVAQLGPLLVLVPAIIWLYW
HGDTTWGTVLLVWSCVVATLDNVLRPVLIRMGADLPLLLILSGVIGGLLAFGMIGLFIGPVVLAVSYR
LLTAWMDEAPEPTTAPEQVIEDLEKR
>CORE_REP|Org15_Gene4579#

MNARSYQELLNSKQRLALFLFLIMNAASSVFTLLFPFRDTPAFTLPLLCIPLFCLVAALFSLQTPRKY
LCKLNLFAGVLGLLWAAHIYVKSQYCLPNNQDFLLISLFSIFFISAISLTDNFTAFCLHAVPSAMVIL
ALDGMHNTLRILFTTLLPIIAFSIHHLMLKRSEIFTHALVANLYNERDKFNNLSMIDPLTGLYNRRGL
ENKITMLLEPQTGHHYVLLLDIDHFKVYNDSYGHAMGDRALVQVAVAIRDAVRSRDIVVRYGGEEFLV
LLTNVHEGYAAQLAERVRQRVAELNIPHGASPQHSGTLTLSAGISALEKLDIESAIGAADAALYLAKH
SGRNNIQLAQNVEPALLQPQELTR
>CORE_REP|Org6_Gene1135#
MNVATQEILLEPADNQRLLSLCGPFDDNIKQLERRLGIEINRRDNRFKLVGKNLCVVAAADILRHLYV
DTAPIRGVIPDIDPEQIHLAIKESRVLEQVADSVPDYGKAVTIKTKRGMVKPRTPNQAQYIANILDHD
ITFGIGPAGTGKTYLAVAAAVDALERQEIRRILLTRPAVEAGEKLGFLPGDLSQKVDPYLRPLYDALF
EMLGFERVEKLIERNVIEVAPLAYMRGRTLNDAFIILDESQNTTIEQMKMFLTRIGFNSKAVITGDVT
QIDLPRNQKSGLRHAVEVLSDVEELSFNFFHSEDVVRHPVVARVVIAYEAWEAAEQKRKDAIAEQRKR
EALAASEQETP
>CORE_REP|Org47_Gene4611#
MAAELSASAGAARRYWRWGGRLLGGALSLALTLLGLLLFTFMLSHLAPIDPALQVAGDHASEATYAQV
RHELGLDQPLPVQFWRYLVHLAHGDLGISRITAQPVLSDLLRTFPATVELATCAIILGALCGITLAFL
AVLKPGSWLDNAARLLSLIGYSVPIFWLSLLGLLLFYATLHWSAGPGRLDDIYLYSMEPRSGFVLIDS
WLSGDRDMFYNAIGHLWLPVVALALLSMAGITRLLRAAMLEECNKEYVTLARSKGAGRLRILLRHVFP
NVLGTLITVLSLSYASLLEGAVLTETVFAWPGVGRYLTSALFAADTPAILGATLLIGTCFVLLNALAD
ALTYLVDPRTR
>CORE_REP|Org20_Gene4467#
MSKELALRPALPWRQQLFDFLYKWGMLLTVAALIALFGLASDNFLDANNIINILRSIAIVTVIAIGVS
ISLSVGGFDLSVGSTASLANALVISLFVWHGFGTTGAIVVTLLLCTLVGLFNALLIVVFRIPDMLATL
ASLFVIQGVAMTYSYGGSITQNMVLPNGDMAEGLIPEVFSALGQVPVIVLIMLAVTVAVQLFLSLTKH
GRRMYAIGGNPEAARLSGIRTVRYRVAAYVISSWLAALGGILLASRIGSSQVNAGGGYLMDAVAAAYI
GFSLAGAGKPNALGTLIGAVILGVLQNGLVMLSVPYYAMDIIKGLVLALALAITYIQKRCSSPAHARC
AAFPFNSSG
>CORE_REP|Org40_Gene2778#
MSNVKIEKPLSADSTGKGGLFSGLSGKMPKDTGIFIVMIGIALIFEILGWYMRDQSFLLNPNRLLLIV
LQVAIIGIIAVGVTQVIITTGIDLSSGSLIALTAVVAASLAQTSDSISPMYPGLLDLPAAIPIGAGIG
VGIVCGFINGFLITRTGIPPFIATLGMMVSARGLAQYYTKGNPVSFLSDGFTSIGQGAMPVIIFLVIA
VIFHIALKHTRYGKYIYAIGGNMTSARVSGINVNKYLVTVYTIAGGLAGLAGVVLAARVSSGQSSMGM
SYELDAIAAAVIGGSSLMGGVGRITGTLIGAVILGLIKSGFTFIGVDSYIQDIIKGVIIVAAVSIDMR
RNRKKH
>CORE_REP|Org18_Gene4103#
MANIRDVARLAGVSISSVSNLLNNRSHQMSAQTRERIEQAMATLGYRPARTAALPAPQAKIIGLLLPS
IVNPSFSALAHAVDGAARAHRYRVLLGNAYRQEQEEAAFIDDMFLHGVRGIIVAASDIRQTHFVRAAE
RGMKIVSYDSPFAEPMATDTRLFDSVSMDNIAAGRLAAQHLLERGCRHIVFATEATLTVGRSHKIDGF
LSALGHSLSERQRVIEGKANSAYGDTEMFELGLTLAPRVLALTPRPDGIVAINDALGIGLMVGLRAAG
VQVPADISVIGIDNIALADLAEPGLTSVRPPLAEMAQLMVERLIGRINDDAQPPGEFLFPPTVISRRS
VKAAG
>CORE_REP|Org33_Gene3792#
MPPVHPITIRDVAKRAGVSVATVSRVLNHSALTSKETREQVLQAVAELGYRPNANAQALATQSSDTLG
VVVMDVSDPFFGALVKAVDTVAQKHHKYLLIGNSYHQAGKERHAIEVLIRQRCNALIVHAKALSDAEL
IGFLEQVPGMVLINRIIPGYEPRCVGLDNVCGAEMAMRLLLSQGHRRIGYLGSNHPIEDGPLRQQGYA
QAMAAAGLATPDNWRAYGSPDLQGGEAAMVELLGRNLQLSAVFAYNDAMAAGAMAVLKENGITVPQHF
SLIGFDDIPIARYTSPKLTTVRYPIVSMATLATELALQGAAGLAEPQAAHLFMPTLVRRHSVAPWQSE
ATVTL
>CORE_REP|Org26_Gene2881#
MGNYLKFRPDGTAVGLLALLVAVVLAFSLLMPGRFFSGATFTSVAFQLPELGLLTLAMFIPILSGGLN
LCIIASANLTSLLMAWLFISYLPPDAGLGLQALWLVLALAAAMLLAVTIGAATGALVAYVGAHPILVT
LATMTTVNGIGIYLTRGAALSGMPEIVRFIGAERVLGVPVPLLIFLAVAVLLALFLQKTRLGKCIYMS
GSNINATHFSGVNTHRVLIAIYTLSSLLCVIAGLVMMARFNSARMGYGDSYLLLTVLAIILGGTDPFG
GFGRVSGVVLALIVLQVIATGLNLMNVSPHFSLAMWGAVLIAVLALKFFRHRYRQRRAMRRSAAQARA

AAGH
>CORE_REP|Org22_Gene2198#
MMIIRPIERRDLADLLTLAGKSGIGLTSLPQNEDTLSARIERALKTWQGELPQSDQCYLFVLEDSERR
QAVGVCAIEVAVGLAEPWYSFRVGTQVHASKQLNVYKSVPTLFLSNDHTGHSELCTLFLDPDYRHGEN
GKLLSKVRFLFIAAFRERFSRRLIAEMRGFSDENGRSPFWESVGHHFFSIEFAKADYLSGTGQKAFIA
ELMPKHPLYVDFLAEDAQKVIGEVHPQTLPARRLLEAEGLSYQGYVDIFDGGPTLEAEIDHIRAVKQS
RLVKVVLDDTPMRADAPVHLVANDNYQNYRALLVNADLYDDRLHINAATAAALGVEQGSPVRVLPLIA
QEKA
>CORE_REP|Org12_Gene1068#
MKKTWVTTLIASGIALATLSGAAHAKGRLVVYCSATNEMCEAETKAFGEKYDVKTAFIRNGSGSTLAK
VDAEKKNPQADVWYGGTLDPQSQAGEMGLLQPYKSPNLEQVMTQFRDPAKLKGNYSSAVYVGILGFGV
NTQRLKEKNLPVPKCWKDLTKPEYKGEIQIADPQSSGTAYTALATFAQLWGDDQAFAYLKQLNANVSQ
YTKSGIAPARNAARGETAIGIGFLHDYSLEKEQGAPLELISPCEGTGYEIGGVSILKGARNLDNAKLF
VDWVLSKEAQELAWKKGKSYQILTNTTADTSPNSLKLDDLKLINYDMDKYGSTEVRKALINKWVSEVK
MGK
>CORE_REP|Org10_Gene2404#
MLQFILRRLGLVIPTFIGITLLTFAFVHMIPGDPVTIMAGERGISAERHAQLMAEMGLDKPLYQQYFS
YVSNVLHGDLGTSLKSRISVWSEFVPRFQATLELGFCAMLFAVLVGIPVGVLAAVKRGSVFDHTAVGI
SLTGYSMPIFWWGMMLIMLVSVQLNLTPVSGRISDTVFLDDSQPLTGFMLIDTLIWGEPGDFIDAVMH
MILPAIVLGTIPLAVIVRMTRSSMLEVLGEDYIRTARAKGVSRMRVIVVHALRNALLPVVTVIGLQVG
TMLAGAILTETIFSWPGLGRWLIDALQRRDYPVVQGGVLLVACMIILVNLLVDVLYGVVNPRIRHKK
>CORE_REP|Org36_Gene1608#
MSKANPNATIVDIARRARVTNITVSRAFNKPELVKPETRERIHAIAKELNYVPNAFAQGLKSSSSQII
GIVTSSMYNPFYSGLIKTVSRIARQQGYQIMLFDTDGSEEAEMRAIQALFGYKARGILLSAVRDDKRY
RPAYLELAEVYGVPLILIDRDLYDQQLSGVFLDNREIGVLAGRYLAEQPEQKLLIIGGPADSEITLTR
TAGIVAALQGSGREIHIINGDYDFTSQESEVRAYLAQPENRPDYIIGLNGIITLGAIAICHEMGLYEQ
VKFFSIDEPPRAGAYGLHIPGVYHDTQKLGEIAAELLFSAINSPRGELPVRREFFTGSLLNR
>CORE_REP|Org45_Gene2110#
MDNHSARRVTRADVARVAGTSVAVVSYVINNGPRPVAEATRLRVLAAIEQTGYRPNDIARALASGSTQ
TYGLVVPDISNPFFATLARALQQEAFSRGRVLLLGDAGDDRQREYELINNLLRRQVDGLLYTSVDRHP
WFDLIRASGTPCVMIDTIDSQAGVCAIRVDERDAACQATRHLLQHGYRDIGIFIGPLTMLNAQDRLNG
WRDALLEAGIAPRDAWIFEAPYTRQGGYQATQRLVQGPRPRAVFTSNEQQALGCLSALAEHGLRAPDD
LALICFNGTQQSEFSVPPLSAVEQPIDAMAKRAIAMLAAGAAPAELHEFAFQLRIRRSCGC
>CORE_REP|Org17_Gene4548#
MKKKRPVLQDVADKVGVTKMTVSRYLRNPDQVSAALQQKIAVALDELGYIPNRAPDILSNATSRAIGV
LLPSLTNQVFAEVLRGIESVTDAHNYQTMLAHYGYLPEREEERLTSLLSYNIDGLILSERHHTPRTLK
MIEVAGIPVVELMDCVSPCIDLAVGFNNFEAARQMTQQIIAHGHRHVVYFGARQDERTLIKQQGYEQA
MRESGLEPHSIMTARSSSYSAGGELLRVAQRDYPQIDSIFCTNDDLAIGAAFECQRQGLSIPQDMAIA
GFHGHDIGQVMVPKLASVLTPRERMGQIGAERLLARLRGETVCPRMVDVGFTVIPGGSI
>CORE_REP|Org21_Gene2443#
MIGIEQPAVARRAAGPSLGKRWEKLLHHPAVLPFIGFAVLFVLMSLLNDSFLSVNNLTNVARQVSINA
IIAVGMTCAILTGGIDLSVGPVMALAGSVAAGLMLAAVPIPLAMVAALAVGALFGLANGACIAYLRMP
PIIVTLASMGIARGLALLYTGGYPISGLPDVFSFFGRGTVLGIQVPILIMLGVYVLAWLMLNQLPFGR
YVYAMGGNEEAARLSGIRVPRYKMLVYVISGLTAALAGLVLTSRLMSGQPNAGEGFELDAIAAVVLGG
AAISGGRGAIVGTLVGAMMLGVLNNGLNLMNVSPYIQNVVKGGIILAAIYLSSVRRK
>CORE_REP|Org26_Gene583#
MDKSLLARLAGRHEFYLGLLVLLLAIGLSAGTDEFLTLGNLTDVATSYAILGILACGLFVVLIAGGID
ISFPAVTAIAQYVMASWVIQHGGSFPLAFALAIGVGLLLGLVNGFLVYWLKVPAIIITIATLNLFYGL
LVYATNGTWLYGFPDWFMNGINWFSFQGADGYDYGLTLPLLCLLATIVVTGVLMNRTRLGRQIYAMGG
NRDAASRLGLNLLRLHFCVYGYMGILAGVAAVVQAQITQSVAPNSLLGFELTVLAAVVLGGTSMSGGR
GSLTGTLLGVVLLAFLQNGLTLLSVSAYWHQVFSGAIILISISTTAWNEKRKLLKEI
>CORE_REP|Org20_Gene1136#
MRRFSLKPRGNEGYLAWVLLLTVIVFSLLSEQFLTVQNLLDLCESYAVSGIFALGLFVVLVTGGIDIS
FAAVASVVQYLIATLATHYGLASPAGSILLALAIGAALGMVNALLIYCLRIVSIIVTISMQALLFGML

MWLTNGRSLYALPDWWTLPRSVLPFQLGEQSYQLGLPTLVMLAVALLTWLLLNKTHLGRQLFAVGGDA
ESARRIGIRVGLLHLFAYGYLGVMAAIGGLVQVYRMGEVVPNALVGGELDVLAAAVLGGASLNGGKGS
VIGTLMGVFLIGVLKNGLNLIGVSSYFMNVVIGLVIVAAITVTHYKKRKETDVGFA
>CORE_REP|Org22_Gene2986#
MIERIWSGGSLLYLALLPFSWLYGLLSWLIRLSYRCGLRKSWRAPVPVVVVGNLTAGGNGKTPMVIWL
VEHLQQRGYRVGVVSRGYGGKSAVYPLVLNQNTSTREAGDEPVLIYQRTGAPVAIAPKRAEAVQALLQ
QQPLDAIITDDGLQHYALQRDFELVVIDGVRRFGNGWWLPAGPMRERAARLGSVDACVANGGVAQAGE
IAMRLQARDAVNLLSGERRPAAELPRVVAMAGIGHPPRFFATLEKLNVEVVQEVAFADHQEYQQPQLT
GLVTAEQTLLMTEKDAVKCRAFAQPNWWYLPVDAVLPSAQAEQLLQDIESLLTK
>CORE_REP|Org43_Gene3526#
MKTAGKNLNQGSFGQGRAQWGKAFGRSLMASMVLVVGLAGSAQAAPASNPAVAESVAPTTAPAPAAAA
APESITPVNPAPTIQPPETRGMDLSVWGMYQHADAVVKAVMIGLVLASIVTWTILFSKGSELLRAKRR
LRREQLALAEARSLDEASELAQNFAPESVSAVLLNDAQNELELSAESNDNNGIKERTGFRLERRVAAY
SRNMGRGNGFLATIGAISPFVGLFGTVWGIMNSFIGIAHSQTTNLAVVAPGIAEALLATALGLVAAIP
AVVIYNIFARVISGHRAQVGDVAAQVMLLQGRDLDLAATAEAKRSQHAHQLRAG
>CORE_REP|Org27_Gene2677#
MMNSVGTPWLWGSFAAVIVVMLAIDLLLQGRKGAHTMSMKQAASWSLVWVSLSLLFNFGFWYYLNETA
GRAVADTQALAFLTGYLIEKALAVDNVFVWLMLFSYFAVPANLQRRVLIYGVLGAIVLRTIMIFAGSW
LVSQFQWLLYLFGAFLLFTGIKMALAKEDDSAIGDKPLVKWLRSHLRMTDNLEGERFFVRRNGILFAT
PLVLVLILVELSDVIFAVDSIPAIFAVTTDPFIVLTSNLFAIMGLRAMYFLLANVAERFSMLKYGLSV
ILVFIGIKMLIIDFFHIPIGVSLGVVAGILTLTLLINAWVNRRNDRLANKQP
>CORE_REP|Org12_Gene517#
MAQDYTVEQLNYGRKVYDFMRWDYLAFGISLLLLVASIVTMSVRGFNWGLDFTGGTVIEINLEKPANL
DLMRDTLEKAGFQDPIIQNFGSSRDVMVRMPPATGTAGQELGNKVIGVINDSVDKNATVKRIEFVGPS
VGSELAQTGGMALLVALICILIYVGFRFEWRLALGAVIALAHDVIITLGVLSLFHIEIDLTIVASLMS
VIGYSLNDSIVVSDRIRENFRKIRRGTPYEIMNVSLTQTLSRTLMTSGTTLMVVLMLYIFGGAMLQGF
SLAMLIGVSIGTVSSIYVASALALKLGMKREHMLQQKVEKEGADQPSILP
>CORE_REP|Org7_Gene2835#
MQNEKKSNVEFIPQFQKAFLYPRYWGVWLGTGLMAGVSLVPARLRDPVLGAIGTLAGKLAKGARRRAR
INLLYCLPELPESEREHIIDQMFACAPQSMVLMAELACTKPEKVLKRVRWHGEEVLDKIRAEGRNVIF
LVPHGWAVDVPAMLMAARGQPMAAMFHNQRNQLIDYLWNAVRRKFGGRMHARNDGIKPFISSVRQGYW
GYYLPDQDHGAEHSEFVDFFATYKATLPAVGRLMKVCRAAIVPLFPVYDGKTSMLDIYIREPMDDLAE
ADDPRIARRMNEEVENLVGPNPEQYTWILKLLKTRKEGEIEPYSRDDLYR
>CORE_REP|Org28_Gene3714#
MSSQTIAAKRWFSKEWLLEQKSLIALLVLIAVVSSMSPNFFTLNNLFNILQQTSVNAIMAVGMTLVIL
TSGIDLSVGSLLALTGAVAASIVGFEVNALVAVAAALALGAAVGACTGMIVAKGKVQAFIATLVMMLL
LRGVTMVYTNGSPVNTGFTDVADTFGWFGIGRPLGVPTPIWIMAIVFIAAWYMLHHTRLGRYIYALGG
NEAATRLSGISVDKVKIIVYSLCGLLAALAGVIEVARLSSAQPTAGTGYELDAIAAVVLGGTSLAGGK
GRIVGTLIGALILGFLNNGLNLLGVSSYYQMIVKAVVILLAVLVDNKSNK
>CORE_REP|Org1_Gene136#
MIIFTLRRILLLLITLFFLTLVSFSLSYFTPRAPLNGAALLDAYQFYFVSLLHWDFGVSSINGQAISE
QLREVFPATMELCLLAFALALFIGIPLGIIAGVLRGKWQDTAISTFALLGFSMPVFWLALLLMLFFSL
HLGWLPVSGRFDLLYQVKPITGFALIDAWLSDSPYRAEMIGSALRHMILPIAALAVAPTTEVVRLMRI
STDDVLSQNYIKAAATRGLSRFTIIRRHVLHNALPPIVPKLGLQFSTMLTLAMITEVVFSWPGLGRWL
INAIRQQDYAAISAGVMVVGTLVITINVLADILGAATNPLKHKEWYALR
>CORE_REP|Org17_Gene317#
MAEYKPTIKAPGKNGDIIFSALVRLAALITLLLLGGIIVSLIFASWPSMQKFGFAFLWTKEWDAPAEQ
FGALVPIYGTVVTSLIALIIAVPVSFGIALFLTELAPNWLKRPLGIAIELLAAIPSIVYGMWGLFVFA
PLFAEYFQTPVGEVLSGIPIVGELFSGPAFGIGILAAGVILAIMIIPYIAAVMRDVFEQTPVMMKESA
YGIGCTTWEVIWRIVLPFTKNGVIGGVMLGLGRALGETMAVTFIIGNTYQLDSASLYMPGNSITSALA
NEFAEAESGVHTAALMELGLILFVITFIVLALSKLMIMRLAKNEGR
>CORE_REP|Org14_Gene3071#
MRLRRSPLMIALLAAMALAGCHSKTATPVAATPATVNVQHLNGSTEVKKHPQRIVVLDYASLETLQLL
GVEPLALPGNRKNLPDSLKRYQDDKYLNAGTLFEPDMAVLRAAKPDLILIAGRASKAYDELNALAPTL

NMSVDPQDQLGSLKQRTLQLGELFDKQQQAQAAVDKLDAQIAAVKPQAAQAGRGLVVLFSGGKISAYA
PKSRFSFVYDALGFSSALQSDEKDVRGNKLTPEQVAKLNPDWLFVIDRDAATGRPNAVAPQKILTGTA
LKKTTAVKKGQVVYLPAAEVYLSGGIVTAQHVVERVSEALNHAAR
>CORE_REP|Org2_Gene2309#
MAKKNKWLQRTLLASALLMAGPLSSASAAVVTSIRPLGFIASAIADGVTPTEVLLPDGASPHDFALRP
SDIQRLRSADLVLWVGPDMEAFLNKALVPISATRKLAISELPAVKPLLMKGEEDDDHDHAGEAHNHAD
DDHGHHHGEYNMHVWLSPEIAKVTAIAIHDRLLELMPQNKDKLDANLRQFENLLTQTDKNVGNMLTPV
QGKGYFVFHDAYGYFEKHYGLSPLGHFTVNPEIQPGAQRLHQIRTQLVEQKAVCVFAEPQFRPAVINA
VAKGTKVRSGTLDPLGIGIALGKDSYGKFLTQLSNQYVSCLK
>CORE_REP|Org26_Gene3903#
MFRGLKAFLLLTLSLLFCQRAFADCATTNGTVTLPGSSSFVVYNGQINAQGTAGLNCTGLGLSLLSQN
TVTVKVASTTNGMAVANTDGSGDKIAYLIYPDANYQYPYSIGQTIDYSSLNLLSLILISSNVNFPLYI
KTTAGANVRSGTYTDTINLIWNYHICGLGVLGLCIWWDGVNKVSTVSVSVAITKDCLIGTAPNVNFGS
MALVGQFNPVNQSITLTCTKTEGYNTYFTNGNNPVSGWRRMKSGTSNFMQYQIYLPNTTTVWDSTNKQ
SGAGTGLAQSIPYKAAVNAAQTEVAVGSYQDNLSFVVEY
>CORE_REP|Org5_Gene1608#
MKLKKLIAASVLMCMLPASVLAKDIKIGVSMAYFDDNFLTILRQSMQNKMKADGNVSGQFEDAKGDIA
QQIQQIENFVSQGVDAIILNPVDTQGVKPMIKLAEKAKIPLVFVNRKPEVALPAGMAYVGSDSKLAGK
LQMEELAKLMNGKGNVMILMGELSSEATRDRTRGVEEVAANYPGIKIIDKQTAKFFRKEAVDVTTDWI
LSGQQIDAIASNNDEMAIGAILALKQAKKSGVLVAGVDGTPDALEFIKKGDLALSVFQDAKGQGEGAV
QTAVQLVKGEKVESNVLIPYQLITQANYQQFADKNKK
>CORE_REP|Org40_Gene785#
MKSAKAFQLALLHPRYWLTWFGLALLFLLVQLPYPLLNRLGVWMGRTSMRFLKRRVTITRRNLELCFP
EMDEAQRERKVVGNFESLGMGLLETGMAWFWSDKRVKRWFNVSGINHLKMAQRDDRGVLVIGVHFMSL
ELGGRAMGLCQPMMAMYRPHNNKAMEWAQTKGRMRSNKAMIDRKDLRGMVHALKRGEAVWFAPDQDYG
PRGSVFAPLFAVDQAATTSGTFMLARMANPALVPVVLIRREGGRGYDLLIQPALEDYPLSDEQAAAAY
MNKVIEKEIMRAPEQYMWLHRRFKTRPAGAPSLY
>CORE_REP|Org32_Gene2582#
MNYLKGLWLAVALCASTSAWAQTIGVSMAYFDQNFLTIIRQAIDKEAKARGITVQFEDARGDVGRQTD
QVQSFISAGVDAIIVDPVNSASTPVMTKMVQAAGVPLVYVNRTPGDAKLPQGVVFVGSDERESGTLQM
EELARLANYQGNVAVMIGNLTDAGALQRTKDVEQVVAKYPKMKVVQKQSANYSRSEGMDLMMNWLTNG
EAIDIVAANNDEMAIGAIMALQQAGKADKKVLIGGIDATPDGLKALASGKMQVTVFQDAVGQGKASVD
VAQRMINGEKLEPYYWIPFELVTPANQGKYAARP
>CORE_REP|Org47_Gene1024#
MLKFILRRLLEAIPTLFILITISFFMMRLAPGSPFTGERALPPEVMANIEAKYHLNDPIWKQYGHYLA
QLAQGDFGPSFKYKDYSVNDLVAGSFPVSAKLGLAAFLLAVVLGVSAGVVAALNQNTKWDYTVMGFAM
TGVVIPSFVVAPLLVLIFAITLKWLPGGGWNGGAPKFIILPMVALSLAYIASIARITRGSMIEVLHSN
FIRTARAKGLPMRRIIFRHALKPALLPVLSYMGPAFVGIITGSMVIETIYGLPGIGQLFVNGALNRDY
SLVLSLTILVGALTILFNAIVDVLYAVIDPKIRY
>CORE_REP|Org13_Gene586#
MFSLFKKTLPFIVAGGMLAASHGALAKQITIGMSFQEMNNDYFVTMKQALDQAAADIGAKVYVADARH
DVAKQIGDVEDMLQKKVDILLINPTDSVGVQSAVISAHKAGAVVVAIDAQAEGPLDSFVGSENYDAGF
QAGEYLAKALGGKGKVAILDGIPVVPILERVRGFEAAMKKYPDIKIVTKQNGKQERDTALTVTENMLQ
SAPDLAGIFSVNDVGALGALAAIESNGAKVKLVSVDGQPEAIKEILKPNSPFIATSAQFPRDQLRIAL
GIALARYWGATVPKTVPVKVKLIDRSNAAGFSW
>CORE_REP|Org4_Gene3643#
MTPIAPSLQQPVDAFLRYLKVERRLSPLTQLSYSRQLAALMRLAQEIGVTDWTALDAARVRMLAARSK
RAGLQSASLALRLSSLRSFLDWLVSQGVLHANPAKGIRTPRSGRHLPKNIDVDEMNQLLEIDLNDPLA
VRDRAMLEVMYGAGLRLSELVGLDCRHVDMAAGEVWVMGKGSKERKLPIGRTAVTWLEHWLAMRDLFG
PEDDAMFLSNQGRRISTRNVQKRFAEWGVKQGVNSHIHPHKLRHSFATHMLESSGDLRAVQELLGHAN
LTTTQIYTHLDFQHLANVYDAAHPRAKRGKS
>CORE_REP|Org41_Gene4645#
MSSAFTSSHDPLRRRLLMALALSPLLGSLPGRAADAPPDITRVAALEWLPIELLLALGVTPLAVADVH
NYNLWVAEPKLPATVVDVGQRTEPNLELLQQLQPSLVLLSQGYGPTPRKIQPIAPTMSFGFNDGSGKP

LTVARQSLLALGQRLGIESRAVNHLAQFDRFMQDARQRLQSYTRQPLLLFSLIDTRHALIIGQKSLFQ
EAMDQLGIRNAWQEQTDFWGTAVVGIERLATVRNARVIYLDHGNQAMMDKVSATPLWQSLPFVRQNQL
RQVPAVWFYGATLSTMRFCRLLAQAQERAA
>CORE_REP|Org31_Gene884#
MMLTKKNSEALEHFSEKLEVEGRSLWQDARRRFMHNRAAVSSLFILVLITLFVVLAPMLSQFAYDDTD
WAMMSAAPSVESGHYFGTDSSGRDLLVRVAIGGRISLMVGVAAALVAVIVGTLYGAMSGYLGGKVDSV
MMRLLEILNSFPFMFFVILLVTFFGQNILLIFVAIGMVSWLDMARIVRGQTLGLKRKEFIEAALVCGV
STRNIVLRHIVPNVLGVVVVYASLLVPSMILFESFLSFLGLGTQEPLSSWGALLSDGANSMEVSPWLL
LFPAGFLVVTLFCFNFIGDGLRDALDPKDR
>CORE_REP|Org41_Gene2769#
MKHWRRNAALKAMPLIDPNAVRTPWGEFWRRFRRQRAALVAGLFVLLLIAAALLAPYLAPFDAENYFD
YDRLNEGPSLMHWLGVDSLGRDIFSRILMGTRISLAAGVFSVLAGGAIGTLLGLLAGYYEGWWDRLTM
RVCDVLFAFPGILLAIGVVAIMGSGMANVIVAVAIFSIPAFARLVRGNTLVLKHLTYIESARSIGASD
WTIILRHILPGTLSSIVVYFTLRIGTSIITAASLSFLGLGAQPPTPEWGAMLNEARADMVIAPHVAIF
PSLAIFITVLAFNLLGDGLRDALDPKLKG
>CORE_REP|Org30_Gene1294#
MSQITESAVKGAPKPMTPFQEFWHYFKRNKGAVVGLVYIVLMLVIALGAGVLAPHAPADQFRDALLKP
PVWQEGGSWQYILGTDDVGRDVLSRLMYGARLSLLVGCLVVVLSLIMGVIFGLLAGYFGGVVDAIIMR
VVDIMLALPSLLLALVLVAVFGPSIVNASLALTFVALPHYVRLTRAAVLVEVNRDYVTASRVAGAGAL
RQMFVNILPNCLAPLIVQASLGFSNAILDMAALGFLGMGAQPPTPEWGTMLSDVLQFAQSAWWVVTFP
GLAILLTVLAFNLMGDGLRDALDPKLKQ
>CORE_REP|Org40_Gene887#
MQQQDNALIEQFLDALWLERNLAENTLASYRLDLQALGAWLGQQNTTLLQAQALDLQAFLAERVDGGY
KATSSARLLSAMRRLFQYLYREKLRADDPTAQLASPKLPQRLPKDLSEAQVDALLQAPCVDQPLELRD
KAMLEVLYATGLRVSELVGLSISDVSLRQGVVRVIGKGNKERLVPLGEEAVYWIENYLEHGRPWLVNG
QTLDVLFPSTRCQQMTRQTFWHRIKHYAILAGIDSERLSPHVLRHAFATHLLNHGADLRVVQMLLGHS
DLSTTQIYTHVATERLKQLHQQHHPRA
>CORE_REP|Org9_Gene2634#
MEQLRAELSIVLGESISRLERVSEQPYAHMYSLYDRQGNAIPLMAKSFICQGIAQQEAYKLSMLARDG
DIRLPTVYGVVCTHQAPYKEILLIERLRGVSAEAPTRSPDRWNMLMEQIVDGILAWHRIDSHGSVGSV
DSTQENDWFCWYQQRVEVLWATVVNLTTPQLTMADRRLLYRTREALTHFFVGFDDPCVLVHGNLSLRS
MLKDPKSDQLLAMLNPGVVLWAPREYDLFRLCEAGMPSQLLFSYLRRAPVADAFLARRWLYVVWEAVG
RLIHTGKLERRPFDYASQQLLPWLAG
>CORE_REP|Org38_Gene139#
MPFDNVYREKKMPSPLRYTWRIFYGDALAMIGFYGVIALLLLSLFGSLLAPYALDQQFLGYQLLPPSW
SRYGNVSFFLGTDDLGRDILSRLLTGTAATFGSALAVTLAAAFCGVILGVFAGVTHGLRSAVLNHILD
TLLSIPSLLLAIVVVAFIGPKLEHAMLAVWLALLPRMVRTIYSAVHDELEKEYVVAARLDGASTLQIL
WYAVMPNIAAVLVTEFTRALSMAILDIAALGFLDLGAQLPSPEWGAMLGDSLELVYYVAPWTVMLPGAA
ILVSVLLVNLLGDGMRRAINAGVE
>CORE_REP|Org14_Gene1282#
MDAKQTRQGIFFALAAYFMWGIAPAYFKLIQQVSADEILTHRIIWSFFFMLALITLGRNWPKVRAACQ
NRKRLLLLAVTALLIGGNWLLFIWAVNNHHMLEASLGYFINPLVNVLLGMLFLGERFRRMQWVAVALA
FTGVLVQLWQFGSLPIIGLGLAFSFAFYGLLRKKIAIDAQTGMLIETLWLLPVAAAYLFLFADSPTSH
LSANPWSLNLLLVAAGIVTTVPLLCFTAAATRLRLSTLGFFQYLGPTLMFLLAITFYGETVGQDKLVT
FGFIWAALILFTLDALYTQRKLR
>CORE_REP|Org1_Gene3926#
MKMKKLATLASAIALSATLSANAMAKDTIALVVSTLNNPFFVSMKDGAQQEANKLGYNLVVLDSQNNP
AKELANVQDLMVRAPKLLLINPTDSDAVGNAIKMANQAKIPVITLDRVASKGDVVSHIASDNRVGGKM
AGDFIAKKAGADAKVIQLEGIAGTSAARERGEGFKQSLDQNKFKLLASQPADFDRTKGLNVMQNLLTA
HPDVQAVFAQNDEMALGALRALQTAGKTDVIVVGFDGTADGVKAVEGGKLAATVAQRPDQIGVIGVET
ADKVLKGEKVPATIPVDLKLVTQ
>CORE_REP|Org19_Gene503#
MKKIALAAGVLLAASYSASSMADSKDSQYVSDWWHQSVNVVGSYHTRFGPQLNNDVYLEYEAFAKKDW
FDFYGYVDVPKFFGVGNTPDRGIWDKGSPLFMEIEPRFSIDKLTGTDLSFGPFKEWYFANNYIYDLGH

NADGRQNTWYMGLGTDIDTGLPMSLSMNIYAKYQWENYQAANENSWDGYRFKVKYFVPLTQVWGGNLS
YIGFTNFDFGSDLGKDSHWVDGTGKQVRTSNSIASSHILALNYDHWHYSFVARYFHNGGQWQDGADIG
TPQGPIKSTGWGYYLVVGYNF
>CORE_REP|Org30_Gene906#
MNTLDKIQSHLELLSKSERKVAEVILASPQTAIHSSIATLARMADVSEPTVNRFCRRLDTKGFPDFKL
HLAQSLANGTPYVNRNVEEDDSVDAYTSKIFESVMASLDTVKANLDIAAINRAVDLLTQAKKISFFGL
GASAAVAHDAMNKFFRFNIPVVYFDDIVMQRMSCMNSGEGDVVVLISHTGRTKNLVEMAHLARENDAT
VLAITSRDTPLAQAATLALLLDVPEDTDVYMPMVSRIAQLTLIDVLATGFTLRRGAKFRDNLKRVKEA
LKESRFDKGVVIPNSFDS
>CORE_REP|Org23_Gene3070#
MIRQWPSPAKLNLFLYITGRREDGYHLLQTLFQFLDYGDTLTIDPRQDDRIHLLTPVDGVPDEQNLIV
RAARLLQRYCDERGLQTAPRGADISIDKRLPMGGGLGGGSSNAATVLVALNELWRCGLGDDQLAALGL
SLGADVPVFVRGHAAFAEGIGERLQPAEPQEKWYLVAHPGVGIPTPVIFGDPELKRNTPVRSLSELLQ
APYANDCEPIARKRFREVEQLLSWLLEYAPSRLTGTGACVFAEFDTEIAARQVLNQAPEWLCGFVARG
VNVSPLHRIRSGRFES
>CORE_REP|Org21_Gene1636#
MEQQLLCYKTLPEWNSDTLPEAFRQRHNTQSGTWAKLTVLSGSLTFAMMTEDGATTETWQFSPESQPP
FIAPQQWHRIVSFSDDMICRLAFYCTPEDYYHKKYELTRTHSEVIEAAARIAPGKALDLGCGGGRNSL
YLNLKGFDVTAWDKHAPSIDRLNQIIDAEQLTRLSARVQDLNTHRFSGEYDFILSTVVMMFLERQQIP
PIVQNMQDSTVRGGHNLIVAAMDTEDYPCPLPFPFTFSPGELKHYYRDWGILKYNEDVGQLHKTDAAG
NRISLRFATLLARKL
>CORE_REP|Org45_Gene805#
MKTDSPFDLILPAATAKIAEDAGVYKATKHPLKTFYLAITAGVFISIAFVFYITATTGTAGVPFGLAK
LVGGICFSLGLMLVVVSGADLFTSTVLIVIAKASGRISWGQLGANWLNVYLGNLVGALFFVALIWFSG
EYMVANGQWGLNVLQTADHKLHHTFIEAVCLGILANLMVCLAVWMSYSGRTLTDKMLAMVLPVGMFVA
SGFEHSIANMFMIPMGIVVKHFATPEFWQAVGAVPEQFAHLTVSNFIIDNLIPVTIGNIIGGGLLVGL
TYWVIYLRGGREQH
>CORE_REP|Org34_Gene1435#
MYLIANREMLLKAQRQGYAVPAFNVHNLETVQVVAETAAELRSPVIMAGTPGTFSYAGTDYLIGICQS
AAHRYDLPLALHLDHHEELDDIEHKVKSGIRSVMIDGSHLPFEQNIAKVAAAVALCHRYGASVEAELG
RLGGQEDDLIVDTADSFYTDPMAAREFVAATGIDSLAVAIGSAHGLYHGEPKLDFERLALIREQVDVP
LVLHGASGIPEAMVKRAISLGVCKVNVATELKIAFADAVKSYFSQHPDANDPRKYIVPGKLAMKEVVA
EKIRICGSSGML
>CORE_REP|Org40_Gene2379#
MSTYLIGDVHGCFDELKSLLAQAAFDPERDQLWLTGDLVARGPASLDVLRYVRSLGPAVRMVLGNHDL
HLLAVYAGISRNKPKDRITPLLEAPDADELINWLRRQPVLQVDDEQKLVMAHAGITPQWDIDTAKMCA
REVEAVLSSDSYPLFLDAMYGDMPNNWAPELSGLARLRFSTNALTRMRYCFPNGQLDMICKDAPGSAP
APLKPWFELPRLVDPEYTIIFGHWASLEGKGTPEGVIGLDTGCCWGGDLTMLRWEDRRYFTQPANRGE
APDHAGRLAAS
>CORE_REP|Org47_Gene2007#
MSTLLRIRQMYPTLAQNDRKLADFLLNNAEQARHLSSQKLAELAGISQSSVVKFAQKLGYKGFPALKL
ALSETLAQPQAEPVVTVHNHILSSDTLKIVGEKLLAEKQAALRATLDINSEERLHQALDMLRQARRVM
LIGIGASGLVAKDFSFKLLKIGVMAVAEPDMHVQLAAVQALDKRDLLLAISFSGERREINLAAEEARQ
AGARVLALTSFSPNGLQQRADHCLYTIAEEPHTRSAAISSSTAQYALTDLLFMALIQHDLDHARDRIK
HSEQLMKKLV
>CORE_REP|Org34_Gene1189#
MNNLPVVRSPWRIAILTVGFTFLYAPMLMLVIYSFNSSKLVTVWAGWSTRWYTELFHDSAMISAVGLS
LTIAAASATAAVVLGAIAAVVMVRFGRFRGSTGFAFMLTAPLVMPDVITGLSLLLLFVAMGHAFGWPS
ERGMFTIWLAHVTFCTAYVAVVISSRLREVDRSIEEAAMDLGAPPLKVFFVITLPMIAPALISGWMLA
FTLSLDDLVIASFVSGPGATTLPMLVFSSVRMGVNPEINALASLILLVVGILGLIAWWFMARSEKQRS
RELQRAARS
>CORE_REP|Org22_Gene3649#
MWGVLAASLFFLPFNRLIAWVILAASAGMGLYHGVLTPLSLSYLLAIVALAGLRHHFREQRNLAIAFE
GLVVAGCIALFLHLVPGIHNQLMIDGDKAGPLSAPFTMYYNFDKAMVPFLLFACLPTLFRTDKAEKSV

ASGSWIALIISVPALLLLAVALGGLKIELHAPAWILPFVMANLFFVCMAEEALFRGYLQQRLSQWLGA
WPALIVAALVFGAAHLAGGMLMVIFATLAGVIYGLAWMWSGRLWVPILFHFGLNLIHLLFFTYPLYQH
P
>CORE_REP|Org48_Gene2755#
MKKHLLMLAFASVATLASYGAAAATKLVVGASNVPHAEILEQAKPILAKEGIDLQIKRFQDYILPNTA
LASHDIDANYFQHVPYLNSVLKDHADDKSYDFVSAGAIHIEPIGIYSKKYKSLKDLPENGKIIMRDAV
AEEGRILSIFEQQGVIKLKPGVSKVDARITDVVENPKHLKFQANVEGALLPQMYNNNEGDAVVINANY
AIDAGLNPTKDPIAVESGENNPYANIITVHKADVNKPEIVALVKVLHSKPIQDFIREKYQGAVIPVNQ
>CORE_REP|Org10_Gene1819#
MSLTFKSIATIGALIGTLALAGCGQDEKNPNHIKVGVIVGAEQQVAEVAQKVAKEKYGLDVELVTFND
YVLPNEALSKGDIDLNAFQHKPYLDQQIKDRGYKLVPVGSTFVYPIAGYSKKIKSLDELKEGSQIALP
NDPTNLGRSLLLLQKVGLIKLKDGVGLLPTVLDVTENPKNLKLVELEAPQLPRSLDDQQIALAVINTT
YASQIGLTPAKDGLFVEDKDSPYVNLLVAREDNKDAENVKKFVQAYQSDEVDAAANKIFNGGAVKGW
>CORE_REP|Org34_Gene2792#
MGVGAPPFQPTKKEHPLNFRWEIIQEYAPLFMEGAWMTIKCTIICVLLGTTWGLILGLGRLAQAPHGI
WKPILHYGVQWPVRIYISAFRGTPLFVQIMVVHFALVPLFINPRDGLLVTSGLMSVDFARALRADYGA
FLSCVVAITLNAGAYVSEIFRAGIQSIDRGQMEASRSLGMSYGKTMRQVILPQAFRRMLPPLGNNAIA
IVKDSSLASAIGLADLAYAARTVSGAYATYWEPYLTISLVYWVITFLLSLLVQHMEKRFGKSDSRT
>CORE_REP|Org9_Gene117#
MKKNRAFLKWAGGKYPLVDEIRRHLPAGDCLIEPFVGAGSVFLNTDYDAYILADINSDLINLYNIVKL
RTDDFVRDARTLFADEFNNSDQFYLLREEFNTSTEPYRRALLFLYLNRHCYNGLCRYNLRGEFNVPFG
RYKKPYFPEEELYWFAEKSRNATFVCEHYRDTMAKAVAGAVVYCDPPYAPLSATANFTAYHTNSFSIA
DQQSLAHLAHQLSVESQVPVLISNHDTELTRDWYQHAALYVVKARRTISRNILGRSKVNELLALYR
>CORE_REP|Org23_Gene1532#
MERYQQLFTRLESSKEGAFVPFVTLGDPNPTLSLQIIDTLIEAGADALELGIPFSDPLADGPTIQSAT
LRAFAAGVTPTQCFEMLAAIRQKHPTIPIGLLMYANLVFHKGIDAFYQRCAEVGVDSVLVADVPFEES
APFRAAAIRHGIAPIFICPPNADDDLLREIASHGRGYTYLLSRAGVTGTESRAQLPLHHLVNKLREYH
AAPPLQGFGISEPEQVKAALQAGAAGAISGSAIVKIIEQHHANPAEMLTKLAAFVSNMKRATRA
>CORE_REP|Org37_Gene3756#
MIELLLPGWLAGVLLAGAAGPLGSFVVWRRMSYFGDTLAHASLLGVAFGLLLDINPFYAVIAITLLLA
LALVWLERRPQLSVDTLLGILAHSALSLGLVVVALMSNVRVDLMAYLFGDLLSVTLSDILMIAGGVAV
VLLVLWWQWRDLLSMTISPELAHVDGVNLVRARTVLMLVTALTIGLAMKFVGALIITSLLIIPAATAR
RFARTPEQMAGVAVLLGMVAVTGGLTFSAFYDTPAGPSVVLCAAVLFTLSLFKKTTGLIKAGI
>CORE_REP|Org22_Gene4310#
MKRINALTIAGTDPSGGAGIQADLKAFSALGAYGTSVITALVAQNTRGVQSVYYIDPAFVAAQLDSVF
SDVRIDSVKIGMLANADIVQAVAERLRHYRPEFVVLDTVMLAKSGDPLLAPEAVASIRRELLPLVSII
TPNLPEAAALLACAPAEDEAQMREQGRALLAMGCRAVLMKGGHLSESESPDWLFSAEGEQRFTAPRVA
TRHTHGTGCTLSAALAALRPRHADWAATVAAAKDYLQQALQQAGTLEVGHGIGPVHHFHAWW
>CORE_REP|Org2_Gene4668#
MMKPTTVTHLRQCKQEQRKFATLTAYDASFAKLFEEQGIKVLLVGDSLGMTLQGHDSTLPVTVADVAY
HTRAVRRGAPACLLLADLPFMSYATPEQTFANAAELMRAGANMVKLEGGSWLCDTVKMLAERAVPVCG
HLGLTPQSVNVFGGYKVQGRDELAAKQLLQDAQNLELAGIQLLVLECVPTELARQITEALSIPVIGIG
AGNGTDGQILVMHDAFGITGGHTPKFAKNFLAQSGDIRTAVQHYIQEVEQGLYPAAEHSFN
>CORE_REP|Org19_Gene410#
MADEQACKYLIPGLDRGLQLLLAFGEQHKEMTFAELHRLVDMPKATAYRVVQTLEHLGFLERNPRTNT
FALGIKVLRLGFEYIASLDVAQAGQPVIEQLRDRSQCSSHLAIRDGRDVIYIARVSAAGSQINQVSVG
TRLPVHQTSLGRMLLTSATRSEFEQLYPDAQLPGNAPGTPADRETLWQMVQQDKARGYVIGESFFRHG
ISSIVYPIFNREQRVEAVVSIMVPSDEIPKADRERLRMEVRDAAEKISGFLGAPPQANVG
>CORE_REP|Org35_Gene1535#
MANADSDKQPDAVSSVMKVFGILQALGDEREIGITELSQRVMMSKSTVYRFLQTMKALGYVSQEGETE
KYALTLKLFELGAKSLQNVDLIRSADVQMRELSNHTRETIHLGALDEDGIVYIHKIDAMYNLRMYSRI
GRRNPLHSTAIGKVLLAWRERDEVAQILSQIEFTRSTEHTLTSAAELLPVLDRVRAQGYGEDAEEQEA
GIRCIAVPVFDRFGVAIAGLSISFPTLRFSEAAREEYVALLHVAARRISEQQGYHDYPF
>CORE_REP|Org43_Gene1624#

MIGRLLRGGFMTLVYAYLYIPIVILIVNSFNASRFGINWQGFTTKWYSTLLNNDSLLQAAGHSLTMAV
LSATFATLIGSLTAVALYRYRFRGKPFVGGMLFVVMMSPDIVMAISLLVLFMLLGISLGFWSLLFSHI
TFCLPFVVVTVYARLKGFDVKMLEAARDLGASEFTILRKIILPLAMPAVAAGWLLSFTLSMDDVVVSS
FVTGPSYEILPLKIYSMVKVGVSPEVNALATILLLLSLTLVIASQWVMRDRSPKAE
>CORE_REP|Org37_Gene4465#
MIFDAVQPEKFFGAMLMPLPLLMLLMGLALLLLWFTRWQKAVRRFYAELAVLLLFSLQPVADRLLRPI
EAQYQTYRGNDPVSYIVVLGGGYTYNPDWAPSSNLLGNSLPRVTEGVRLYLAHPGARMVFTGASAGSM
QSNAATAALVAESLGVPRSDMVILREPRDTEEEAAQVAKLVGEQPFILVTSANHLPRAMRFFEAKGLH
PIPAPANQLAIDSPLNIWDRATPSSMFLGHTERAWYETLGSLWQWLKGADRAGAE
>CORE_REP|Org8_Gene1470#
MSGRIGRLLRDPLPWTLALLLALVFGMDHLRGLFAAWFPDLERPIYQQDSFIALVGAHLSLVAISSLI
AVAIGVAAGVAVTRRSGREFRSLVETVVAVGQTFPPVAVLAVAVPVMGFSEQPAIIALVLYGLLPILQ
GTLAGIESVPPATREIARGVGMSAWQILWRVELPLAAPVIVAGIRTSVIINIGTAAIASTVGTKTLGS
PIIIGLSGFNTAYVIQGAAVVALLAIITDMLFERWVRYLTAWRQQTLAATSAG
>CORE_REP|Org43_Gene2476#
MNQHNITNESLGLSMVLVVVAILISHREKLALEKDIIWSICRAVVQLIIVGYVLKYIFDLDNAVLTVL
MVLFICFNAAYNAKKRSKYVEHAFVTSFIAITTGAVLTLAVLVLTGSIEFTPMQVIPISGMIAGNAMV
AVGLCYTNLGQRFKSEQQKIQEMLSLGATPKFASAALIRDSIRASLIPTVDSAKTVGLVSLPGMMSGL
IFAGIDPVKAIKYQIMVTFMLLSTASLSTIIACYLAYRKFYNERHQLVVGNLK
>CORE_REP|Org39_Gene1904#
MRSTLLRLPLLAGLLLAGQPQAFAAASILIWPIDPAIEDNQQATALWLENRDSKPVYMQIRVLGWQQT
GGKDDYRNQSEVVASPPVATILPGKRQLIRLIKQTPVAAGQERAYRILVDEVPIKDKDGAAPDKGAQM
GLKFQMRYSVPLFVSGKGVWTKQDFEHPRDYATANQPKLSYRLLQQNSQRWLDVRNDGIVHARLSQVS
IQGKPLNNGLLGYVLPGSQMRFALPPSGSFAAGKLQAMVNDNKQPVTIPSY
>CORE_REP|Org15_Gene1348#
MSFIAIIPARYASTRLPGKPLADIHGKPMVVHVMERARESGASRVIVATDHPEVAKAVEAAGGEVCMT
SPDHHSGTERLAEVIAHYGFADDQIIVNVQGDEPLIPPVIVRQVAENLAGSQAGMATLAVPIDSAEEA
FNPNAVKVVMDAQGYALYFSRATIPWDRERFAASKESIGDSLLRHIGIYAYRAGFVRRYVSWAPSQLE
QIELLEQLRVLWYGEKIHVAVAKAVPSVGVDTPEDLQRVRDSIQP
>CORE_REP|Org41_Gene591#
MSVTGRIHSFESCGTVDGPGIRFIVFFQGCLMRCLYCHNRDTWDTHGGKEVTVEELMKDAVAYRHFMN
ASGGGVTASGGEAILQAEFVRDWFRACHAEGINTCLDTNGFVRRYDPVIDELLDTTDLVMLDLKQMND
EIHQNLVGVSNHRTLEFARYLAKRNQRTWIRYVVVPGWSDDDKSAHLLGEFTKDMTNIEKIELLPYHE
LGKHKWVAMGEEYKLDGVHPPKAETMDRVKGILESYGHKVIY
>CORE_REP|Org36_Gene1111#
MSSTNIEQVMPVKLAKALSNSLFPALDSQLRAGRHIGIDELDNHAFLMDFQDELEEFYTRYSVELIRA
PEGFFYLRPRSTTLIPRSVLSELDMMVGKILCYLYLSPERLAHEGIFSHQELYDELLSLADENKLLKF
VNQRSTGSDLDRQKLHEKVRTSLNRLRRLGMVYFMGNDSSKFRITEAVFRFGADVRSGDDPREAQLRM
IRDGEAMPVETSLSLNDENEAEDQQVDNAPDGAEDEQE
>CORE_REP|Org16_Gene1693#
MIEILQQYWQSLLWSDGYRFTGVAVTLWLLIASVVMGGLLAIPMAVARVSSLRWVRFPVWLYTYVFRG
TPLYVQLLVFYSGMYSLEIVRGTEFLNAFFRSGLNCTILALTLNTCAYTTEIFAGAIRAVPHGEIEAA
NAYGFSRFKMYRCIILPSALRTALPAYSNEVILMLHSTALAFTATVPDLLKIARDINAATYQPFYAFG
IAAVLYLIISYVLISLFRKAEKRWMAHVSH
>CORE_REP|Org22_Gene200#
MTATAPVITVDGPSGAGKGTLCKALAESLGWRLLDSGAIYRVLALAALHHQVDITSEEALVPLAAHLD
VRFVAQDGKLQVILEGEDVSNEIRTETVGNTASQAAAFPRVREALLRRQRAFREAPGLIADGRDMGTV
VFPDAPVKIFLDASSEERAHRRMLQLQEKGFNVNFERLLAEIKERDDRDRNRPIAPLVPASDALVLDS
TSMSIEEVIRQALTYAQKVLALPQQ
>CORE_REP|Org2_Gene1619#
MTGILQLFQAIGLGLVLLLPLANPLTTVALLLGLSGNMTREERNQQSLMASVYVFCIMTVAFYAGQVV
MNTFGISIPGLRIAGGLIVAFIGFRMLFPQQSADEAPEVESKSHELRHKTSANIAFVPLAMPSTAGPG
TIAMIISSASSVKDNTLGFEPWVLTVAPVAIFLTVAVILWGCLLSSGAIMRLVGKSGIEAISRLMGFL
LVCMGVQFIINGVLEIISTYTPAAA

>CORE_REP|Org25_Gene499#
MTDMNILDLFLKASLLVKLIMLILICFSVASWAIIIQRTRILNAATRDAEAFEDKFWSGIELSRLYQE
SQARRDSLTGSEQIFHSGFKEFARLHRANNHAPESVIEGASRAMRISMNRELETLETHIPFLGTVGSI
SPYIGLFGTVWGIMHAFIALGAVKQATLQMVAPGIAEALIATAIGLFAAIPAVMAYNRLNQRVNKLEQ
NYDNFMEEFTAILHRQAFSSDSK
>CORE_REP|Org13_Gene460#
MYEFDWASIVPSFPYLLQGMVITLKITVTAIVVGILWGTVLAVMRLSPFKPISWFATLYVNLFRSVPL
VMVLLWFYLVVPSLLQQVLGLSPKTDIRLISAMVAFSLFEAAYYSEIIRAGIISISRGQSSAALALGM
THWQSMRLVILPQAFRAMVPLLLTQGIVLFQDTSLVYVLSLADFFRTASTIGERDGTQVEMILFAGFV
YFVISLAASALVSYLKKRTV
>CORE_REP|Org3_Gene1527#
MIESLFPHLRLDQLWDATWETLYMTGIAGLATLVLGIVLGVLLFLTSKGQLWQNRAVYSLISVLVNVF
RSIPFIILIVLLIPFTKSLIGTILGADAALPALIVGAAPFYARLVEIALREVDKGVIEAARSMGAKNR
TLIFRVLLPESSPALVSGITVTLIALVSYTAMAGVIGAGGLGNLAYLEGFQRNHSDVTLVATLTILLI
VFVIQFIGDTLTRTLDKR
>CORE_REP|Org42_Gene3883#
MKFGLVVAALLSISMPAAATTLKLSPDIDLLVVDGKKMTGSLLKGADSLELDGGQHQLLFKVTKTVRS
GQHTQAYASLPLVATFNTQKISQVAIELPRIENDRDAQRFDRTLNYLVVDKDGNALPFRHDVLHPDSV
TFNTDLEKVMTDYNRQNRPASVPSFVQANAGNASALTLAGAPINAPTVTLKGENVSEQMLQYWFQQAD
KETQKRFLRWANKQPIR
>CORE_REP|Org37_Gene713#
MDIIKFIIDFILHIDVHLAELVAQYGMWVYAILFLILFCETGLVVTPFLPGDSLLFVAGALAALPTND
LNVHTMVALMVVAAILGDAVNYTIGRLFGEKLFSNPNSKIFRRSYLDKTHQFYEKHGGKTIILARFVP
IVRTFAPFVAGMGHMSYRHFAAYNVIGALVWVLLFTYAGYLFGDLPVVQENLKLLIVGIIIVSILPGV
IEIWRHKRAAARQQKQ
>CORE_REP|Org19_Gene942#
MHESIQLALDSAPFLLKGAILTLQLSLGGMAFGLLLGFLLALMRLSPLWPLAWLSRIYVSLFRGTPLI
AQLFMIYYGLPQFGIEFDPFPAALIGLSLNTAAYTSETLRAAISSIDKGQWEAAASIGMTRWQTLRRV
ILPQAARTALPPLGNSFIGLVKDTSLAATIQVPELFRQAQLITSRTLEVFTMYLAASLIYWVMATLLS
ALQNRLEAHVNRQDQE
>CORE_REP|Org1_Gene782#
MKKRIAIIAGAVSVALTLSACTTNPYTGESEVGKSGIGAGLGAALGAGVGVLSSSKKDRGKGALIGAA
AGAALGGGAGYYMDVQEAKLRDKMKGTGVSVTRQGDNIVLNMPNNVTFDSSSATLKPAGANTLTGVAM
VLKEYPKTAVNVVGYTDSTGSRSLNMNLSQQRADGVASALITQGVAANRIRTTGAGPDNPIASNSTAE
GKAQNRRVEITLSPLQ
>CORE_REP|Org30_Gene425#
MQFDWSAIWPAIPILLEGAKMTLWISVLGLIGGLIIGLVAGFARTYGGWIANHIALVFIEVIRGTPIV
VQVMFIYFALPMAFTDLRIDPFTAAVVTIMINSGAYIAEITRGAVLSIHNGFREAGLALGLSRRETIR
YVIMPLALRRMLPPLGNQWIISIKDTSLFIVIGVAELTRQGQEIIAGNFRALEIWSAVAVIYLIITLV
LSFVLRRLERRMKIL
>CORE_REP|Org43_Gene4420#
MKRMSTRRIAQAKNCFAALGAITTRSQFGGYGLLAEGVMFAVIAEGELYLRATASMEPAFRARGMVNM
VYSKRGVPITLRYYWVDESLWRERNELVGLAWQAVREARREQRLKAGDHGRLKALPNIDVNMERLLWR
AGIRNAYDLRLHGAKRSYLRLLKQQTNLGLRVLLSLGGAIAGYHQAALPAELRSELVRWFDHTMAMRR
HGHEPVIQGPSSGPE
>CORE_REP|Org18_Gene267#
MSEAMMWLMARGVWETVMMTFVSGFFGFVLGLPVGVLLYVTRPGQIIANNSLYKILSGLVNIFRSIPF
IILLVWMIPFTRMIVGTSIGLQAAIVPLTVGAAPFIARMVENALLEIPSGLVEAARAMGATPMQIIKK
VLLPEALPGLVNAATITLITLVGYSAMGGAVGAGGLGQIGYQYGYIGYNATVMNTVLVLLVVLVYLIQ
FCGDRIVKAVTHK
>CORE_REP|Org6_Gene1037#
MNTLLYAWQNWAYIAGLTLEHLLLVGIAVGLAILIGVPLGVLIVRHKWLATPVLSLATLVLTVPSIAL
FGLMIPLFSLIGHGIGYVPAITAVFLYSLLPIVRNTHTALDNLPGGLREAGRGIGMTFWQRLRWVEIP
VALPVIFGGIRTAVVMNIGVMAIAAVIGAGGLGLLLLNGISSSDIRQLITGAVMISLLAIVLDWLLHR

LQIALTPKGIRS
>CORE_REP|Org31_Gene553#
MGQSLLDLSGFIKFFVGLFALVNPVGILPVFISMTSYQAEAGRNKTNLTANLSVAIILWTSLFLGEGI
LRMFGISIDSFRIAGGILVVTIAMSMISGKLGEDKQNKQEKSESAIRESIGVVPLALPLMAGPGAISS
TIVWSSRYHNWQSLLGFTVAIALFAFCCWLLFRAAPLLVRLLGQTGINVITRIMGLLLMALGIEFIVT
GIKAIFPGLL
>CORE_REP|Org20_Gene1585#
MTKVLVLYYSMYGHIESLAQAVAEGANRVNGVDVTIKRVPETMTPEAFAKAGGKQHQQAPVATPQELA
DYDGIIFGTPTRFGNMAGQMRTFLDQTGGLWASGALYGKVGSVFSSTGTGGGQEHTISSTWTTLAHHG
FIIVPIGYATPELFDVSQVRGGTPYGATTIAGADGSRQPSNEELTIARYQGEHVAKITAKLKS
>CORE_REP|Org19_Gene4712#
MMKWIPVALALLLSACSSSPQKTYYQLPALGAPAAVSSSSGASTRQLWLEHVSVADYLAQSGVVYQTN
DVQYVIAQNNLWASPLDQQLQQTLVTNLSSALPGWVVSSQPMSSDQDVLNVTISGFHGRFDGKAIVRG
EWVLNRQGRLIKRPFSLELKQGEDGYDALVRTLAEGWQKEAKSIALQLKKICFIFGRDIKAL
>CORE_REP|Org24_Gene4548#
MFDIGFSELLLVLVIGLIVLGPERLPVAVRTVSGWIRALRSLAASVQHELSQELKLQELQDSLKKAEQ
AGLQNLTPELKASMDELKDAAESLKRTYRGETEELANTIHNPQAIDPEALHDGVTPAEAATRASAPAA
VPKPAAEPEAVAAASPAASPAAPVAAQPAKAPVETAPAPVEPVADKIPASHQPSGDR
>CORE_REP|Org7_Gene4105#
MRNNICVFCGASEGVNPAYAEQARQLGQLLAAQGRRLIYGGGKKGLMGIVADAVLAAGGEAVGIIPER
LVEAETAHRGLTELEVVPDMHTRKARMAALADCFIALPGGIGTLEELFEIWTWGQIGYHNKPVGLLNV
NGFYRPLSQFLEHVADQGFMRHDYLGTLHISESAQTLLQQFDDYQPKNYDRWAK
>CORE_REP|Org6_Gene2783#
MHHLMLDIETLDIKPSAVILVVAAVFFDPRTGALGAEFETAVSSQKDQPGRTISLDTVAWWAKQSDEA
RKQAFGGTESLKRVLSSLSRFIHMNSTDTVKVWGNGKEFDCAILEHAFQQLEMPCPWKFWDTQDVRTV
ITLAELHGFNPKKARPFEGMPHRALDDARHQARYVADTVSALYYRQGAQR
>CORE_REP|Org2_Gene1537#
MTYQQAGRIAILKRILGWVVFIPALLSTLISVLGFVYQHSEKTKGINAVMLDFVHVMVDMVRFNTPFL
NLFWYNSPVPDVDKSLFSGANLMFIIIYILIFVGLALQASGARMSRQVKFIREGLEDQMILEQAKGSE
GHTRQQLEERITLPHHTIFLQYFPLYILPIVIAVIAWFVIRLLGQLAGAA
>CORE_REP|Org8_Gene789#
MAKTVVVFHSGYGHTERLAKVVAEGAGAELIAIDQNGDISDEAWQTLDEADAIIFGSPTYMGGPSWQF
KKFADASSKAWFGRKWQDKVFGGFTNSASLNGDKQVTLIALQTLASQHGGLWVSLGLLPANTKSAQRT
DVNNLGGSVGLLVQTPADAGVDEMLSGDLATAKLYGQRVAGFAAKLA
>CORE_REP|Org7_Gene38#
MKIKPDDNWRWYFDAEHDRLMLDLANGMIFRSRFPAKMLTPDAFDECAFCVDDAALYFTYEEQCKQVK
LSHEQRAELVLNALVAYRFLKPLMPKSWHFSQQHYPLQPKNGELAAVKVMESGAEARLLVVEAGDNAS
LCLLAQNQLTVAGRTMVLGDAIKVMHDRLKPCAQDESAAPAYDRAV
>CORE_REP|Org19_Gene1538#
MDKIDHHRRKWLALGGAAMGIALLPGQAFASISTARPRILVLNNLNTGESIKAEFFDGKGYNKEELVR
LNHLFRDYRANKVKSIDPRLFDHLYRLQGLLGTSKPVQLISGYRSVDTNNELRAHSRGVAKHSYHTKG
QAMDFHIEGIQLSNIRKAALKMRAGGVGYYPRSNFVHIDTGPVRTW
>CORE_REP|Org44_Gene164#
MSRVAKAPVVIPAGVEVKLNGQVISIKGKNGELTRTIHDAVEVKQEANALTFAPREGFANAWAQAGTT
RALLNAMVVGVTEGFTKKLQLVGVGYRAAVKGNVVNLALGFSHPIDHQLPAGITAECPSQTEIVLKGA
DKQVIGQVAADLRAYRRPEPYKGKGVRYADEVVRTKEAKKK
>CORE_REP|Org43_Gene254#
MIDDDGYRPNVGIVICNRQGQVLWARRYGQHSWQFPQGGINPGETAEQAMYRELFEEVGLSKKDVRIL
ASTRNWLRYKLPKRLVRWDTKPVCIGQKQKWFLLQLLCNDADINMQRSSTPEFDGWRWVSFWYPVRQV
VSFKRDVYRRVMKEFAVTVMPMQEQAAPRQAPAYRRKRG
>CORE_REP|Org38_Gene38#
MVDKRDSYTKEDLEASGRGELFGAGGPPLPAGNMLMMDRVVKMTEDGGTHNKGYVEAELDINPDLWFF
GCHFIGDPVMPGCLGLDAMWQLVGFYLGWLGGEGKGRALGVGEVKFTGQVLPTAKKVTYRINFKRVIT
RKLIMGVADGEVLVDGEVIYTATDLKVGLFKDTTAF

>CORE_REP|Org6_Gene1444#
MLTQEMTQKLNEQLNLEFYSANLYLQMSAWCSDKGFEGAAAFLKEHSQEEMQHMQRLFDYLSDTGSLP
LLGTIAAPPVAFESLADVFQQTYEHEQLITRQINELAHAAMTAHDYSTFNFLQWYVAEQHEEEKLFKS
VLDKLALVGTSGKGLFFIDKDLKKMGAMGQGGNDQA
>CORE_REP|Org42_Gene716#
MPLLDSFTVDHTRMAAPAVRVAKTMKTPHGDTITVFDLRFCRPNLEVMPERGIHTLEHLFAGFMRDHL
NGQGVEIIDISPMGCRTGFYMSLIGVPEEQRVADAWKAAMADVLKVTDQRKIPELNEYQCGTYHMHSL
EEAQEIAKHILDNDVVVNHNDELALPKEKLQELHI
>CORE_REP|Org35_Gene759#
MSQENEQRLRFRDAMASLSAAVNIVTTDGPAGRCGITATAVCSVTDTPPTLLVCINRNSAMNPVFQEN
RRLCVNVLNHEQELMARHFAGMTGVSMEDRFRLEEWQLGALGQPVLRNTLASLEGEIEQIQSIGTHQM
YLVQIKQIALSEAGNGLIYFKRNFHPVIHQMAVPA
>CORE_REP|Org4_Gene1486#
MSAIVKRCSVAAVLAIAVLLPSFGELQTSEAGLRLIADLEGCRLSPYQCSAGVWTQGIGHTAGVIPGQ
AIDERQAAVDLVDDVRRTERGMAACLPQTLPQETYDAVIAFAFNVGISAACHSTLVTLLQQRQWQQAC
DQLPRWVYVNGKKNKGLEQRRATERALCLQGIASS
>CORE_REP|Org8_Gene757#
MQLNKVLKGLLLALPVLAVAACSSNKSANNDQSGMGAGAGTGMENGSSNLSSEEQARLQMQELQKNNI
VYFGLDKYDVSSEFAQMLDAHAAFLRNNPSYKVTVEGHADERGTPEYNIALGERRANAVKMYLQGKGV
SADQISIVSYGKEKPAVLGHDEAAYAKNRRAVLVY
>CORE_REP|Org47_Gene1596#
MNKASVVFSGLLMAVSAGAMAATSGDDADISKQPLEKVAPYPKAEKGMNRQVIYLPKQEHEENYKVEL
LIGKTLEVDCNRHMIGGTLETKTLSGWGYDYLVLEKLSEPASTMMGCPDNTKTKQFIAANLGDAAMQR
YNSRLPIVVYAPKDAEVKYRIWKAEDTVSQAEQK
>CORE_REP|Org1_Gene379#
MGLFDKLKSLVSDDKKDTGTIEIVAPLSGEIVNIEDVPDVVFAEKIVGDGIAIKPAGNKMVAPVDGTI
GKIFETNHAFSIESDSGIELFVHFGIDTVELKGEGFKRIAEEGQRVKKGDVVIEFNLPLLEEKAKSTL
TPVVISNMDEIKELIKLSGSVTVGETPIIRIKK
>CORE_REP|Org39_Gene1644#
MRTQSLYQPHFSHGSFTANSVAKNTNVGKENGLISELVYNERQPAVAQLLLPLLQQLGKQSRWLLWLT
PQQKLSKQWLQQSGLPVDKMVQLSQISPVNTVEAMEKALQTGNYSVVLGWLPELTEEDRLKLRRAAEL
GNAYGFIMRPQRDIDPTHGHCSTLKIHSSLYH
>CORE_REP|Org4_Gene589#
MSTAKLVKTKSSDLLYTRNDLDEKVKLAAIKALNHQVVQFIDLSLITKQAHWNMRGANFIAVHEMLDG
FRTAIIEHQDTFAERVVQLGGVALGTVQVVNDRTPLKSYPTNIHSVQEHLKALADRYGAVANDIRKAI
TEVEDEDTADMFTAASRDLDKFLWFIESNIE
>CORE_REP|Org9_Gene1621#
MTKEQFYAELKRDLSALLGGETNFIAALSNASALLNERLDDVNWVGFYLMDGGQLVLGPFQGKIACVR
IPVGKGVCGTAVAENRVQRVGDVHAFPGHIACDAASNAEIVLPLAVGGRAIGVLDIDSTVYQRFDEQD
EAGLKAVVAGLCEQLEQCDSAKYVTVAAS
>CORE_REP|Org17_Gene1231#
MIQQLFKGRFSLLTMAFVALLALAGCQSKPQGLTPEQVALLQSQGFKLTDNGWEFGLSDKVLFGNNIG
KLNPESTETVQKMGRALLSVGITKFRLDGHTDNYGEDSYNDQLSLRRADAVADLLASVGIPRANIETR
GMGKRDPVADNRTSSGRAENRRVAIVVTP
>CORE_REP|Org24_Gene49#
MADNKKRPGKDLDRIDRNILNELQKDGRISNVELSKRVGLSPTPCLERVRRLERQGFIHGYTALLNPH
YLDASLLVFVEITLNRGAPDVFEQFNSAVQKLEEIQECHLVSGDFDYLLKTRVPDMSAYRKLLGETLL
RLPGVNDTRTYVVMEEVKQSNRLVIKTR
>CORE_REP|Org34_Gene204#
MATAKKAAKTHIGLDSKQSAKLAEALNALLANYQVLYMNVRGYHWNITGPQFFELHAKFEETYNDLLT
KVDELAERILTLGSQPRHAFSDYLKTADIKEHTNVTDDKGTLRGLLEGYSILLQQQRELLTVAADAGD
EGTASLMSDYIKEQEKQVWMLNAYLGK
>CORE_REP|Org41_Gene2219#
MAFRLYSNDLQDGGKLPQAQVFNGMGYHGDNLSPHLAWDGVPAGTKSFVIAVYDPDAPTGSGWWHWIV

ANIPADVRELPQGAGSGKAPLPAGALQTRTDFGSAGYGGAAPPEGESHRYQFTVHALDVERIEVDEGS
SGALVGFNVHFHSLGSATLTVTFN
>CORE_REP|Org21_Gene277#
MKGDKKIIAHLNKLLGNELVAINQYFLHARMFKNWGLMRLNDKEYHESIDEMKHADRYIERILFLEGI
PNLQDLGKLNIGEDIEEMLRSDLALELAGAKNLREGIAYADSIHDYVSRDLMIDILADEEEHIDWLET
ELDLIARLGIQNYAQAQILERKE
>CORE_REP|Org15_Gene160#
MSEQNSTEMAFQIQRIYTKDISFEAPNAPQVFQQEWQPEVKLDLDTASSQLADEVYEVVLRVTVTATL
GEETAFLCEVQQAGIFSVAGIEGTQLAHCLGAYCPNILFPYARECITSLVSRGTFPQLNLAPVNFDAL
FMNYLQQQAEGEGAAPHQDA
>CORE_REP|Org43_Gene866#
MRNPAKQEDLIKTFKALLKEEKFSSQGEIVLALQEEGFENINQSKVSRMLTKFGAVRTRNAKMEMVYC
LPAELGVPTTTSPLKNLVLDVDHNDAVVVIHTSPGAAQLIARLLDSLGKSQGILGTIAGDDTIFVTPS
SGFTAQKLHEAILGVFEQEL
>CORE_REP|Org42_Gene1090#
MDKIDRKILAELQADGRLSVTELAERIGLSVSPCHRRVRALEESGVIRGYRAQLDPGSLGYNFSALVF
VTMREGDRRAVETFENAMMDIPQVVQAQRLFGDPDYLLHVIARDLPAFQQLYDEKLSALPGVQRLSST
LVMKTVVPERSFLPLGK
>CORE_REP|Org8_Gene1413#
MELTTRTIAARKHIALVAHDHRKQALLEWVESHKTILAQHQLYATGTTGNLIQRASGIPVTSMLSGPM
GGDQQVGALIAEGKIDMLIFFWDPLNAVPHDPDVKALLRLATVWNIPVATNRSTADFLIDSPLFKNEV
EIAIPDYQRYLQDRLK
>CORE_REP|Org45_Gene116#
MKYQQLENLESGWKWKYLVKKHREGELITRYIENSAAQEAVNELLKLENEPVKVLAWIAAHMNPELDN
RMKQTIRARRKRHFNAEHQHTRKKSIDLEFLVWQRLAALARRRGVTLSETVVQLIEDAERKEKYANQM
SSLKEDLKAILGKDPK
>CORE_REP|Org8_Gene50#
MRTVLNILNFVLGGFFTTLGWLIATVFSVLLVITLPLTRSCWEITKLSLVPFGNEAIHVDELYPEKSN
ALLSAGGSLLNIIWLVLFGWWLCLSHIAAGIVQCVSIIGIPVGIANFKIAAIALWPVGRRVVSVEMAQ
QARIENARRHYHQR
>CORE_REP|Org7_Gene4762#
MKTVKRTGIALAIALTFPLALPAATAAQTSLTNSKAATMTEKHGQFIAVGKVVQVTFGDFAFKLDFTD
DKTMTFTGIGEASQGITDTVQYTAVEIRPKVYMVYWHEPQSGDNVTHIEDFERGEVYTNIAAKDGSFT
HLKGQLKIVGHSGN
>CORE_REP|Org44_Gene2158#
MNTPEQRQQIADFIGKQHVLTLCAGDGLDMWCANCFYVFDAAAMALWLMTEPHTRHGGLMLNNGRVVG
TIAPKPKSIALIRGVQYRAEAVLLSGEEADAARARYCKRFPIARAMKASVWRLDLHEVKMTDNTLGFG
KKLHWARSIL
>CORE_REP|Org46_Gene462#
MAMRLNEDLDDSGELHEINVTPFIDVMLVLLIIFMVAAPLATVDIRVDLPASSAKPQPRPEKPVFLSV
KADKQLYVGDQPVNADQLTSVLDQRTQANKETTIFFQADKSVDYETLMSVMDTLRKAGYLKVGLVGME
GAAK
>CORE_REP|Org46_Gene927#
MSMLKEFREFAMRGNVVDLAVGVIIGAAFGKIVSSFVADIIMPPLGLLIGGVDFKQFHLVLREAQGAV
PAVVMNYGSFIQTVFDFVIVAFAIFLAIKLMNKMRRKQEEAPAAPPAPTAEEKLLTEIRDLLSQQQQP
KL
>CORE_REP|Org40_Gene3060#
MQDQEIVELLQQVKTIALVGASDNPSRPSYGVMAYLLAQGYQVIPVSPKLAGQTLLGQPVYATLAAIP
QPVDMVDVFRNSEAAYGVAQEAIAIGAKALWLQIGVINDQAAELAQQAGLRVVMDRCPKIEIPRLGLE
R
>CORE_REP|Org29_Gene594#
MAKTSRSIMIAKGLQRVLNVGLLLLAAILVVFLVKETIHLAKVLFINSEESSSYLLIEGIVIYFLYFE
FIALIVKYFESGYHFPLRYFIYIGITAIIRLIIVDHKNPIDTLIYAAAILVLVVTLYLANTDRLKRE
>CORE_REP|Org42_Gene757#

MLNDIEEIRFTARSEENLRGVHPDLVRVIRLALRYSLVPFSVSEGLRSMARQREMVRAGSSQTLRSRH
LTGHAVDVVAMPAGVVSWEWDYYAQIAVAVRRAARECGINVEWGGEWKTLKDGPHFQLAFRDYPA
>CORE_REP|Org17_Gene4670#
MPHFYAECTDNIRRDADLPTLFAKVNEALAATGIFPLAGVRSRAIWLDTWQMADGKQDYAFVHMTLKI
GHGRSLESRQQVGEMLFALIKEHFAALMAQRYLALSFTMEELDPVLNYKQNNVHALFNKA
>CORE_REP|Org25_Gene3681#
MKRLLLDTHALLWWLIDDACLGVNAKRQIADPGNAVYVSAASIWEISIKQALGKLALPEDIFAIIEAE
DFLALPMDAFHCQQAGQLPPYHQDPFDRMLIAQAQAEGLTLISADTVFPQYGVRVADARR
>CORE_REP|Org38_Gene72#
MITGIQITKANDQALVNSFWLLDDEKAEARCVCAKANYAEDQVVAVSDLGQIEYREVPLEMQPTVRVE
GGQHLNVNVLRRETLEDAVKHPEKYPQLTIRVSGYAVRFNSLTPEQQRDVIARTFTESL
>CORE_REP|Org29_Gene104#
MSANTEAQGSGRGLEAAKWLIVAVLLVVAIVGNYYYRDLSLPLRALAVVLIIAVAGAVALMTTKGKAT
VAFAREARTEVRKVIWPTRQETLHTTLIVAAVTAVMSLILWGLDGILVRLVSFITGLRF
>CORE_REP|Org7_Gene1020#
MKGTLTRAALAAGGMMVTSAVMAGSLALPTAQSLAGQWQVADSERQCQIEFLANEQSETNGYQLVDRQ
RCLQSVFAAEVVGWRPAPDGIALLQADGSTLAFFSRDGDLYRNQLGAGDALTLKALA
>CORE_REP|Org39_Gene624#
MANQATGLTRIIKAAGYSYKGLSAAWQHEAAFRQELVVTLLAIILAVWLDVGAIARILLIGSVALVMI
VEILNSAIEAVVDRIGSEHHELSGRAKDMGSAAVSLAIVLALFVWGTVLWQHFG
>CORE_REP|Org27_Gene2609#
MAIGHYELKKAKNGQYHFNLKASNGESILASEMYASKASAENGIASVQTNSPHEAQYELKHSTSNQPY
FVLKAKNHQVIGVSEMYSSESAAKNGIQSVMKNGPTTDIRDLSA
>CORE_REP|Org1_Gene282#
MYEALLVIFLLISIGLVALIMLQQGKGADMGASFGAGASGTLFGSSGSGNFMTRMTAVLATLFFVISL
ILGNLSSNQSKKGSEWENLGQPVKTEQTTAPAAPAKPSSDIPQ
>CORE_REP|Org4_Gene216#
MLEFEGQVIDTDAQGYLKNSADWHEGLAPLLAAQEEIVLTEAHWEVVRFVRDFYQEFNTSPAIRMLVK
AMAQKYGEEKGNSRYLYRLFPKGPAKQATKIAGLPKPVKCI
>CORE_REP|Org17_Gene43#
MIASKFGIGQQVRHKLLGYLGVVIDIDPEYSLEQPKADEIAANDELRSAPWYHVVMEDEEGQPVHTYL
AEAQLDGEPQEAHPEQPSLDELAESIRHQLQAPRLRN
>CORE_REP|Org38_Gene144#
MMKKEITFTVVELCQRVEISEDELVEIVGLGVIVPLEPAQPRWEFDYPALSHLQRARRLRAELDLDWP
GIAMALTLLDRVDALQQENRQLRRQLARFLQTS
>CORE_REP|Org44_Gene239#
MALTKAEMSEHLFEKLGLSKRDAKDLVELFFEEVRRALENGEQVKLSGFGNFDLRDKNQRPGRNPKTG
EDIPITARRVVTFRPGQKLKSRVENASPKG
>CORE_REP|Org25_Gene2#
MFEQRVNSDVLTVSTVNSQDQVTQKPLRDSVKQALKNYFAQLNGQDVNDLYELVLAEVEQPLLDMVMQ
YTRGNQTRAALMMGINRGTLRKKLKKYGMN
>CORE_REP|Org36_Gene505#
MNKSQLIDKIAAGADISKAAAGRALDAVIASVTDSLKAGDDVALVGFGSFTVRERSARTGRNPQTGKE
IKIAAAKVPAFRAGKALKDAVN
>CORE_REP|Org9_Gene258#
MSLSVEAKAQIVADFGRGTNDSGSTEVQVALLTAQINHLQGHFSEHKKDHHSRRGLLRMVSQRRKLLD
YLKRKDVARYTSLIERLGLRR
>CORE_REP|Org46_Gene543#
MKPGIHPDYRTVVFHDVSANAYFKVGSTIKTDRTIELDGESWPYVTLDVSSASHPYYTGKQKDYSKEG
STARFQQRFGRFIGNK
>CORE_REP|Org25_Gene2792#
MFVELIFDQRNVKGLPDAAEIIKAELTRRVHRVFPDAEVKVKPMQTNGLISDANKSDREKLNRLLEDM
FEESEQWLMSDIYG
>CORE_REP|Org19_Gene169#

MNRTKLVLGAVILGSTLLAGCSSNAKIDQLSSDVQTLNAKVDQLSNDVNAMRSDVQAAKDDAARANQR
LDNQAHAYKK
>CORE_REP|Org25_Gene2729#
MQLQPKHTYKIVGFSSEIAPAYRQKLLSLGMLPGSSFDVVRVAPLGDPIEIKTRRVSLVLRRKDLALL
QLDGQP
>CORE_REP|Org19_Gene8#
MAKEDNIEMQGTVLDTLPNTMFRVELENGHVVTAHISGKMRKNYIRILTGDKVTVELTPYDLSKGRIV
FRSR
>CORE_REP|Org9_Gene4123#
MLYDKSQERDNCGFGLIAHIEGEPSHKVVRTAIHALARMQHRGAILADGKTGDGCGLLLQKPDRFFRM
VAEERGWRLAKNYAVGMMFLSQNEEEARLSRRIVEEELQNETLSIVGWREVPTNPDVLGEIALSSLPR
IEQIFVNAPAGWRPRDMERRLFVARRRIEKRVQDNSFYVCSFSNLVTIYKGLCMPADLPRFYLDLADL
RLESAICLFHQRFSTNTVPRWPLAQPFRYLAHNGEINTITGNRQWARARTYKFQTPLIPDLQAAAPFV
NETGSDSSSLDNMLELLLAGGMDLIRAMRLLVPPAWQNNPDMDGDLRAFFDFNSMHMEPWDGPAGIVM
SDGRYAACNLDRNGLRPARYVITKDKLITCASEVGIWDYQPDEVVEKGRVGPGELMVIDTRSGRILHS
AETDNDLKSRHPYKEWMEKNVKRLVPFEDLPDDQVGSRELDDAQLETYQKQFGYSSEELDQVIRVLGE
IGQEATGSMGDDTPFAVLSSRPRIVYDYFRQQFAQVTNPPIDPLREAHVMSLATSIGREMNVFCEAEG
QAHRLSFKSPILLYSDFKQLTTLEGEYYRAETLDLTFDPQQQDLEQTIRALCDEAERKVREGAVLLVL
SDRAIAPGRLPVPAPMAVGAVQTRLVEKSLRCDANLIVETASARDPHHFAVLLGFGATAIYPYLAYET
LAKLVDSQAIDKKYRDVMLNYRNGINKGLYKIMSKMGISTIASYRCSKLFEAVGLHRDLADLCFQGVV
SRIGGASFSDFQQDLQNLSKRAWLKRKPLEQGGLLKFVHGGEYHAYNPDVVNSLQKAVHSGEYSDYQA
YAKLVNERPVAMLRDLLAITPKGEPIPVDQVEPAESLFKRFDTAAMSIGALSPEAHESLAIAMNGLGG
FSNSGEGGEDPARYRTNKVSRIKQVASGRFGVTPAYLVNADVIQIKVAQGAKPGEGGQLPGDKVTPYI
ARLRYSVPGVTLISPPPHHDIYSIEDLAQLIFDLKQVNPKAVISVKLVSEPGVGTIATGVAKAYADLI
TIAGYDGGTGASPLSSVKYAGCPWELGLVETQQALVANGLRHKIRLQVDGGLKTGVDIVKAAILGAES
FGFGTGPMVALGCKYLRICHLNNCATGVATQDDKLRRDHYHGLPERVTNYFQFIARETREIMAQLGVS
QLVDLIGRTEFLTSLPGISAKQNKLDLSPLLKTATPHPGKAVYCTESSNPAFDKGLLNKELLAQAQPH
IEAKQGKTFYFDIRNTDRSVGAMLSGAIADVHGDQGMAADPIKAHFSGTAGQSFGVWNAGGVELTLTG
DANDYVGKGMAGGSIAVRPPIGSAFRSHEASIVGNTCLYGATGGKLFAAGRAGERFAVRNSGAITVVE
GIGDNGCEYMTGGIVCVLGKTGINFGAGMTGGFAYVLDEDGEFRKRVNPELVEVLDVDQLAIHEEHLR
GLITEHVQATGSSRAEEILANWPEWAPKFALVKPKSSDVKALLGHRSRSAAELRVQAQ
>CORE_REP|Org6_Gene4143#
MTKIAPQRLEPLSLPLFGERLIEASAGTGKTFTIGALYLRLLLGLGGDAAFPRPLTVEEILVVTFTEA
ATEELRGRIRDNIHGLRIACVRGKDAECKNPLFIALMEEIDDLSDAASQLLAAERQMDEAAIYTIHGF
CQRMLTHNAFESGMLFEQTLVQDELPLRRQACADFWRRHCYPLPLGVARAVSQEWSGPEALLADLSGY
LHGEAPALRRPPKDEETVLMRHEQIVARIDAIKAQWRAEAGDLEALIAQSGVDKRSYSSKHLPNWLNK
VGEWSGQETQDYQLPKELDKFRQSVLLEKTKKGEPPRHALFTAIDQLFDEPLTLRDLIMARALSEIRT
SIQQEKRQRAELGFDDLLSRLDGALQSGGGEQLALAIRQRYPVAMIDEFQDTDPQQYRIFQKLYVGRP
DCGLLLIGDPKQAIYAFRGADIFTYMRARSEVSAHYTLETNWRSSPSMVASVNHLFSQVEKPFLFGQI
PFIEVNAAEKNQGLAFELHGKPQPAMQFWLQQGEGAGVSDYQQLMARLCATQIRDWLSAGQQGLARLQ
NGKESRPVQASDITVLVRSRNEAALVRDALSALSIPSVYLSNRDSVFDTPEAKDLLWLLQAVLAPEQE
RTLRSAMATGLMGLDAPTLDGLSRDERAWDALVNEFDNYRTLWLRRGVLPMLSELMKARQLAENLLAS
AGGERRLTDVLHLGELLQEAAAQLDSEHALVRWLAQQIAQPNRQSDNQQLRLESDRHLVQVITIHKSK
GLEFDLVWLPFVGNFRQQQQALYHDRHSFQALLDLDANEESQAWAEEERLAEDLRLLYVALTRSVYHC
SIGIAPLFQGTRKKQGDTDLHRSALGYLVQGGQAGDAVYLQERLQQLAGGGIALSLVEPPDEEPWHPQ
AALAEALAAKSFTRRIQDFWRVTSYTGLQQHGASLMQDLLPRLDVDAAGERSEESEPALTPHTFPRGA
TPGTFLHSLFETLDFTQPLDEQWLLAQLQQQGFAEHWQPILLAWMQVLLNTPLTDSGVTLAALTPQHK
QAELQFYLPINRLLQAKELDALVKRYDPLSARCPALDFHQVQGMLKGFIDLVFCWQGKYYLLDYKSNW
LGEDSSAYTRPAMEQAMAEHRYDLQYQLYTLALHRYLRHRLPDYDYRRHFGGVIYLFLRGVDTAHPGN
GIFTCLPEFELVAGMDRLFSGEAAATEDGS
>CORE_REP|Org49_Gene3454#
MFTVYHSNQLDLLKTLTSALIARDPLADPFQPEVVLVQSPGMAQWLQMQLAEQFGIAANIAFPLPATF
IWDMFTRVLPDIPKESAFSKDAMTWKLMWLLPEMLTQPAFAPLQHYLTDDGDKRKIHQLAGRVADLFD
QYLVYRPQWLESWQRGERIDGLAEAQQWQAPLWARLVEYTRELGQPEWHRANLYSRFIHALEQAKTCP

PGLPPRVFICGISALPPVYLEALQALGRHIDIHLMFTNPCRYYWGDIQDYAFLARLQSRKRRHYHQAR
EHGLFRQPDDAARLFDAEGQQQLSNPLLASWGKLGRDHLYLLSQMEGAQEVDAFVDIPADTMLHAVQR
DMLELEDHAVIGITAETLESSFSKRPLDENDRSLSLHACHSPQREVEVLHDQLLTMLAQDPSLTPRDI
IVMVADIDSYTPYIQAVFGNAPAERYLPFAISDRKARQAHPALQAFISLLDLPQSRFTSEQVLALLEV
PALAARFAIGEEGLRLLRHWVGESGVRWGLDDDNVRELDLPATGQHTWRFGITRMLLGYAMDSNAGDW
QGILPYDESSGLVAELAGQLADLLAQLSHWRQILSEARPLEAWLPLCRQLLDAFFAADSDTEVVLALI
EQQWQQAINFGLAARYPDEVPLTILRDDLAARLDQERISQRFLAGQINFCTLMPMRSIPFKVVCLLGM
NDGVYPRTLPPLGFDLMAQQVKRGDRSRRDDDRYLFLEAILSAQQRLYISFIGRSIQDNSPRYPSVLV
TELLEYLEQSYCLPGDEELSADDSARRVGEHLLKWHARMPFAAENFLPGSEEQSYAAEWLPAADGRGA
AHPAFNQPLPAEALQQISLEELLRFYRHPIRAFFQLRLGVSFILEETELPDEEPFTLDNLSRYQFNSQ
LLNTLIDGDDPERLFQRVRAAGGLPYGAFGEIYWQKQQEEMSELAEQVRAERAESHSLELDIDIAGVR
LSGWLHQVQDDGLLRWRPATLSAVDGILLWLEHLVYCCAGGTGESRMYGRKNSAWRFAALAPEEAQAQ
LAELLTGYQRGLSQPLLLLNKSGWAWLSQCYLPETQQIDWEEEAQIKARAKLLQAWQGDQRIPGEGED
PYVQRVFRQLDNEYLAQILAETERYLLPVARHNLG
>CORE_REP|Org2_Gene3732#
MSPLSAALQAQAQQVVQRFQEVHGADSAFSEQEQWVLASSDFVSDALLAQPAWLATLREQPPAPGEWQ
HYAAWLQDELEEVRDEAQLMRTLRLFRRETLVRIAWAQAQGLCSTEETLLQLSGLAETLIVSARDWLY
QTCCREWGTPCNAAGEPQPLLILGMGKLGGGELNFSSDIDLIFAYPENGQTQGGRRELDNAQFFTRLG
QRLIKALDQQTIDGFVYRVDMRLRPFGDSGPLVMSFAALEDYYQEQGRDWERYAMVKARLMGGAEDAY
SQELRKTLRPFVFRRYIDFSVIQSLRNMKGMIAREVRRRGLKDNIKLGAGGIREIEFITQVFQLIRGG
REPALQGRSLLPTLQAVGELGLLEAEQVRALSAAYLFLRRLENLLQAIGDQQTQTLPQDALDQARLAY
GMGLADWPALMATLEVHMQAVRAVFDDLIGDDSPDVGEDPDYQHYHSLWQDALEENELAPLTPHLDEE
GRRQMLRTIADFRHDVDKRTIGPRGRDVLDQLMPRLLAEVCPRQDAPTALVRLAQLLLSIVTRTTYLE
LLVEYHAALSHLIRLCAASPMVANQLSRYPLLLDELLDPATLYQPVALDAYRSELRQYLLRVPEDDEE
QKLEALRQFKQAQQLRIAAADIAGALPVMKVSDHLTYLAEAIIDAVVQQAWSDMVARYGQPTHLQERE
GRGFAVIGYGKLGGWELGYSSDLDLVFLLDCPPEVMTDGDRCIDGRQFYLRLAQRVMHLFSTRTSSGI
LYEVDARLRPSGAAGMLVSTVEAFADYQQNEAWTWEHQALVRARIVHGDPALHQQFDAIRREILCKTR
DAETLKREVREMREKMRNHLGNKQRDLFDIKTDEGGITDIEFIAQYLVLRYAPGEPRLTRWSDNVRIF
ELMANYVIMPEEEARALTQAYVTMRDEIHHLALQEHSGKVGSELFTAEREQVRASWAKWLD
>CORE_REP|Org45_Gene2134#
MSDNLLQQAAPAVPMPPASPSTYGDEELTCPMLKQRLEQFQLWLAAAFDAGSSAESLVAARSDFIDRL
LRRLWTFYGFEDIPETALVAVGGYGRGELHPLSDIDVLVLSQRRLTEQQSQRVGEFITLLWDLKLEVG
HSVRTLEECLLEGLADLTVATNLIESRMICGDVALFLQMQKHIFSDGFWPSPQFFHAKINEQQERHQR
YHGTSYNLEPDIKSSPGGLRDIHTLLWVARRHFGATSLDEMVGFGFLTQAERNELNECQSFLWRIRFA
LHLVLPRYDNRLLFDRQLSVAQLLRYEGEGNEPVERMMKDFYRMTRRVSELNHMLLQLFDEAILALDA
TEKPRPLNDDFQLRGDLIDLRDETLFIREPQAIMRMFYLMVRNREIKGIYSTTVRQLRHARRHLKQPL
CTIPEARDLFMAILRHPGAVSRALVPMHRHSVLWAYMPQWGKIVGQMQFDLFHAYTVDEHTIRVLQKL
ESFADDQTRPRHPLCVELYPRLPHPELLLLAALFHDIAKGRGGDHSILGAEDVVEFAELHGLNSRETQ
LVAWLVRCHLLMSVTAQRRDIQDPTVIQQFSSEVQSETRLRYLVCLTVADICATNETLWNSWKQSLLR
ELYFATEKQLRRGMQNSPDLRERVRHHRLQALALLRMDNIDEEALHRIWSRCRADYFLRHSPNQLAWH
ARHLLAHDSTQPLVLVSRQATRGGTEIFIWSPDRPYLFAAVAGEMDRRNLSVHDAQIFTNRDGMAMDT
FIVLEPDGSPLAQDRHAAIRQALLQAITQREYQPPRVRRPSSKLRHFSVPTEVTFLPTHTDRRSYLEL
TALDQPGLLARVGEVFADLGLSLHGARISTIGERVEDLFILADGERRALDQETRRKLEQRLTEALTPN
DKM
>CORE_REP|Org20_Gene4791#
MNEQYSAMRSNVSMLGKLLGDTIKEALGEHILDRVETIRKLSKSSRAGNEAHRQELLSTLQNLSNDEL
LPVARAFSQFLNLTNVAEQYHSISPNGEAASNPEALAQLFSRLKDKKLSDKELQHAVSQLSIELVLTA
HPTEITRRTLIHKLVEVNTCLSQLDHNDLADYERNKIMRRLRQLVAQSWHTDEIRKHRPSPIDEAKWG
FAVVENSLWEGVPAFLREFNEQLENSIDYSLPAEAVPVRFTSWMGGDRDGNPNVTAEITRHVLLLSRW
KACDLFTRDIQVLVSELSMTECTPELRARAGGDEVQEPYREIMKQLRSQLMSSQAYLEGRLKGERVLK
PHDLLVNNEQLWEPLYACYQSLQACGMGIIANGQLLDTLRRVRCFGVPLVRIDVRQESTRHTEAIAEL
TRYLGLGDYESWSEADKQAFLIRELNSKRPLVPLKWQPSADTQEVLETCRVIAEAPQGSIAAYVISMA
RTPSDVLAVHLLLKEAGCPFALPVAPLFETLDDLNNADDVMTQLLNIDWYRGFIQGKQMVMIGYSDSA
KDAGVMAASWAQYRAQDALIKTCEKAGVALTLFHGRGGSIGRGGAPAHAALLSQPPGSLKGGLRVTEQ

GEMIRFKFGLPEATISSLALYAGAILEANLLPPPEPKKEWRALMDDLSDTSCRMYRGYVRENPDFVPY
FRAATPELELGKLPLGSRPAKRKPNGGVESLRAIPWIFAWTQNRLMLPAWLGAGAGLQEAVKAGKQAE
LEAMCRDWPFFSTRIAMLEMVFAKADLWLAEYYDQRLVDKSLWPLGQQLRDQLESDIKVVLTIANDAH
LMEDLPWIAESIALRNVYTDPLNVLQAELLHRSRQQEQPDARVEQALMVTIAGVAAGMRNTG
>CORE_REP|Org15_Gene823#
MYLYIETLKQRLDAINQLRVDRALAAMKPAFQRVYSLLPTLLHHHHPLMPGYLNGNVPHGICLYTPDE
TQQDYLNDLEDKWGSPFDKPASGELPITGVYSMGSTSSIGQSCSSDLDIWVCHQSWLDNEERTRLQQK
CSLLEKWAASMGVEVSFFLIDENRFRHNESGSLGGEDCGSTQHILLLDEFYRTAVRLAGKRILWNMVP
GEEEAHYDEYVLSLYAQGALTPNEWLDLGGLSSLSAEEYFGASLWQLYKSIDSPYKAVLKTLLLEAYS
WEYPNTQLLATDIKHRLHQGEIVSFGLDAYCMMLERVTRYLTDINDTTRLDLARRCFYLKVCEKLSLA
KACVGWRREILSQLVSEWGWSEERLAMLDNRANWKIERVREAHNELLDAMMQSYRNLIRFARRNNLSV
SASPQDIGVLTRKLYAAFEALPGKVTLVNPQISPDLSENDLTFIHVPVGRANRTGWYLYNQAPAMDSI
VSHQPLEYNRYLNKLVAWAYFNGLLTPQTRLHIKSGNLCDTAKLQELVADVSHHFPLRLPAPTPKALY
SPCEIRHLAIIVNLENDPTAAFRNQVVHFDFRKLDVFSFGQQQQCLVGSIDLLYRNSWNEVRTLHFSG
EQSVLEALKTILGKMHQDAAPPESVEVFCYSQHLRGLIRTRIQQLVSECIELRLSSTRLEPGRFKAVR
VAGQTWGLFFERLSVSVQKLENAVEFYGAISNNKLHGLSIKVETDQVHLPPVVDGFASEGIIQFFFED
TSDDKGFNIYILDESNRVEVYHHCEGSKEELVRDVSRFYSSSHDRFTYGSSFINFNLPQFYQIVQLDG
RTQVIPFRSNVLSSLCVTVADGAAQPLKQQFQLH
>CORE_REP|Org18_Gene3790#
MRVLKFGGTSVANAERFLRVADIMESNACQGQVATVLSAPAKITNHLVAMIDKTVAGQDILPNISDAE
RIFADLLSGLAQALPGFEYDRLKGVVDQEFAQLKQVLHGVSLLGQCPDSVNAAIICRGEKLSIAIMEG
VFRAKGYPVTVINPVEKLLAQGHYLESTVDIAESTLRIAAAAIPADHIVLMAGFTAGNDKGELVVLGR
NGSDYSAAVLAACLRADCCEIWTDVDGVYTCDPRTVPDARLLKSMSYQEAMELSYFGAKVLHPRTITP
IAQFQIPCLIKNTSNPQAPGTLIGKDSTDDAMPVKGITNLNNMAMINVSGPGMKGMVGMAARVFAVMS
RAGISVVLITQSSSEYSISFCVPQGELLRARRALEEEFYLELKDGVLDPLDVMERLAIISVVGDGMRT
LRGISARFFSALARANINIVAIAQGSSERSISVVVSNESATTGVRVSHQMLFNTDQVIEVFVIGVGGV
GGALIEQIYRQQPWLKQKHIDLRVCGIANSRVMLTNVHGIALDSWRDELAGAQEPFNLGRLIRLVKEY
HLLNPVIVDCTSSQAVADQYVDFLADGFHVVTPNKKANTSSMNYYQQLRAAAAGSHRKFLYDTNVGAG
LPVIENLQNLLNAGDELVRFSGILSGSLSFIFGKLDEGLSLSAATLQARANGYTEPDPRDDLSGMDVA
RKLLILAREAGYKLELSDIEVESVLPPSFDASGDVDQFLARLPELDKEFARNVANAAEQGKVLRYVGL
IDEGRCKVRIEAVDGNDPLYKVKNGENALAFYSRYYQPLPLVLRGYGAGNDVTAAGVFADLLRTLSWK
LGV
>CORE_REP|Org26_Gene3156#
MNAIAVAGPVSGRQLHKFGGSSLADVKCYLRVAGIMAEYSQPGDMMVVSAAGSTTNQLINWLKLSQSD
RLSAHQVQQTLRRYHSDLISGLLPPESAEPLIAEFIQDLERLAVLLDGKVDEVCYAEVVGHGEIWSAR
LMAAVLNHLDMQAAWLDARDFLRAERAAQPQVDEGRSYPLLQQLLAQHPGKRLVVTGFISRNDAGETV
LLGRNGSDYSATQVGALAGAARVTIWSDVAGVYSADPRKVKDACLLPLLRLDEASELARLAAPVLHTR
TLQPVSGSDIDLQLRCSYQPEQGSTRIERVLASGTGAKIVTSHDDVCLIELHVAAQHDFKLAQKELDL
VLKRAQIKPLAVGIHPDRNRVQLCYTSEVVNSALAILQASALPGELHLREGLALVAMVGAGVCKNPLH
SHRFYQQLKDQPVEFIWQAEDGISLVAVLRQGPTALLIQGLHQSLFRAEKRIGLVLFGKGNIGSRWLE
LFAREQTNISARSGFEFILAGVVDSRRSLLNYEGLDASRALAFFEDEAQALDEESLFLWMRAHPFDDL
VVLDVTASEELAGQYLDFASYGFHVISANKLAGASCSDTYRQIRDAFAKTGRHWLYNATVGAGLPVNH
TVRDLRDSGDSILAISGIFSGTLSWLFLQYDGTVPFTELVDQAWQQGLTEPDPRVDLSGQDVMRKLVI
LAREAGYDIEPNQVRVESLVPAGCEQGSVDQFFENGEALNQQMQQRFEAASEMGLVLRHVARFDANGK
ARVGVEAVRPEHPLASLLPCDNVFAIESRWYRDNPLVIRGPGAGRDVTAGAIQSDLNRLAQLL
>CORE_REP|Org21_Gene1674#
MKKSFPTLLATMIWTALYSQHALADLAEQCMLGVPVYDKPLVSGDPNSQPVTINADDSRADYPKSALF
SGNVHIEQGNSTLTAKEVELNQTQKPGQTEPVRTVTATGDVHYSDNQIKLKGPKAWSNLNTKDTDVYE
GDYQMVGRQGRGDADKMKMRGANRYTILENGTFTSCLPGDDSWSVVGSEVIHDREEQVAEVWNARFRI
GGVPVFYSPYLQLPVGDKRRSGFLIPNAKYGSNNGFEFMLPYYWNIAPNYDATITPHYMSKRGLQWQT
EFRYLVQPGLGLMEFDWLPDDKEYGKDNDDSKRWLFYWNHNGVMDQVWRFNVDYTKVSDYKYFTDLDS
KYGSTTDGYATQKFSLGYANENWNATLSSKQFQIFDTTDRTRSDTYKVQPQLDLNYYKNDLGPFDFHI
YGQAAKFTSVNPYSPDATRLHMEPTLNLPLTNGWASLNTEAKLMATHYQQDIPDGFAANYESRKSTQN
NPVTAPNLDNSVNRVLPQFKVDGKLVFERPMIWAEGATQTLEPRVQYLYVPYRDQSNIYTYDTTLLQT

DYSGLFRDRTYSGLDRIASQNRVSTGLTTRIYDDALVERFNASVGQIYYFSRSRTGDQVTGYDNNDDT
GSVAWAGDTYWKIDDRWGLRGGLQYDTRLNSVSLGNGVVEYRQDAERVVQLNYRYATPEYIQTALNTK
TVPAFQDGISQVGITGSWPIADRWAVVGAYYYDTRAKQSADQLVGLKYNTCCWAVTLGYERKITDWNN
SNNTSVYDNRVSFNVELRGLSSDHSLGSAEMLRSGILPYQRAF
>CORE_REP|Org12_Gene2380#
MKREGILKHIPWMLLGILGAACLGVVALRRGEHISALWIVVASVAVYLVAYRYYSLYIATKVMKLDAG
RATPAVVNNDGLNYVPTNKNVLFGHHFAAIAGAGPLVGPVLAAQVGYLPGTLWLLGGVVLAGAVQDFM
VLFISSRRNGASLGEIIKKEMGPIPGTIALFGCFLIMIIILAVLALIVVKALAESPWGVFTVCSTVPI
ALFMGIYMRYLRPGRVGEVSIIGIVLLVAAIWFGGVVAHDPYWGPALTFKDTTITFTLIGYAFVSALL
PVWLILAPRDYLATFLKIGVIVGLAIGIVILNPELKMPAVTQFVDGTGPVWKGTLFPFLFITIACGAV
SGFHALIASGTTPKLLANETDARFIGYGAMLMESFVAIMALVAASIIEPGLYFAMNTPPAALGITMPD
LHRLGTEDAPMIMASLKDVTVHAAAAVSSWGFVISPEQILQTATDIGEPSVLNRAGGAPTLAVGIAHV
FHQIIPGANMGFWYHFGILFEALFILTALDAGTRSGRFMLQDLLGNFVPFLKKTDSLVAGIVGTAGCV
GLWGYLLYQGVVDPLGGVKSLWPLFGISNQMLAAVALVLGTVVLIKMKRTQYIWVTVLPAVWLLICTT
YALGLKLFSDNPQLEGFFFLAGEYKRKIAEGGAELSAQQIANMNHIVVNNYTNAGLSILFLLVVYSII
FYGVKTAMAAHKNPKRTDQETPYVPVPQAAPTDGVTEGEVKVSTQH
>CORE_REP|Org34_Gene2798#
MTQTTANQHPRDRMRGRIDRGVQAAVTASGLMVLMTLMLIFVYLLFAVLPLFKPASLGQAQPLPIAVS
APALALGMDVQQRVGYRIDAQGAGQFYRLTPAPSGQAQTPLVQQTLLAKPALLAQAAGERDLFALAQA
NGRLVVARADFATAENGRPQWQFPLGQQPLALDPQNKPLKLLSLADAHRGQYLLAGVTDDRRLVFGRF
SPDRPPQFSERPLEHDGEQLVLTPDGRQLYLLTGNRLARYQIDGAQLQLRETRTLGEHAPYQMTALPG
GSALLIKGADGNLREWFEVEKDRRWRLTPVQHFDHGADGQELTVAEPYRRVFATLRPDGGFSLFSAIQ
PQPLLNTRLGAEVRQMAFAPRGDGLLLESAQGWQRYALDNPYPDVTWRSLWGKVWYENYPQPAYVWQS
TSGEDSYQPKFSLMPVIFGTFKAAAYAMLFAIPLALAGAIYTAYFMTAGLRRVIKPAIEVMGALPTVV
IGLVAGIWLAPIIEQYLLAVLALPLLLAAAVLLCGALTHRFMPRCRPGVDLLLLLPLLALTVWLAFSL
GPWLEVALFGEPLHFWLGDNYDQRNALVVGVAMGFALVPIIFSLAEDALFSVPATLSQGSLALGATQW
QTVIKVVLPSASAGIFSALMIGFGRAVGETMIVLMATGNTPIIDGSLFQGLRALAANIAIEMPEAVSG
SSHYRVLFLTALVLFVFTFVFNTLAEAVRLRLRKRYTPNQEAP
>CORE_REP|Org40_Gene2515#
MSRTIMLIPTGTSVGLTSVSLGVIRSMEQKGVRLSVFKPIAQPRTGDNALDQTTTIIRSSNSTITAAE
PLRMDYVEGLLSSNQQDVLMEEIVARYHENTKDAEVVLIEGLVPTRKHQFANALNYEIAKTLNAEIVF
VLALGNDSPAQLKERIELARTSFGGSKNKNITGVIINKLNAPVDDQGRTRPDLSEIFDDSTKASIAHV
DPAQLFANSPLPVLGCVPWSFDLIATRAIDMARHLKARVVNEGDIMTRRVKSVTFCARSIPHMLEHFR
PGSLLVTSADRPDVLVSACLAAMNGVEIGAILLTGGYAIDEPIKKLCERAFQTGLPVFMVDTNTWQTS
LSLQSFNLEVPADDHQRIEKVQNYVASHINTEWIDSLTATSERSRRLSPPAFRYELTELARKAGKRIV
LPEGDEPRTVKAAAICAERGIAECVLLGNPDEIQRVAAAQGVELGKGIEIVDPVAVRENYVPRLVELR
KSKGMTEVVAREQLEDNVVLGTLMLEQGEVDGLVSGAVHTTANTIRPPLQLIKTAPGSSLVSSVFFML
LPDQVLVYGDCAINPDPTAEQLSEIAIQSADSAAAFGIEPRVAMISYSTGNSGAGSDVEKVREATRLA
QEKRPDLIIDGPLQYDAAIMADVAKSKAPNSPVAGQATVFIFPDLNTGNTTYKAVQRSADLVSIGPML
QGMRKPVNDLSRGALVDDIVYTVALTAIQSSQADAAAAKA
>CORE_REP|Org44_Gene2080#
MTQQTFLVEIGTEELPPKALRSLAESFAANFTAELDNAGLEHGDVSWFAAPRRLALKVANLSAAQADR
EIEKRGPAIAQAFDAEGKPSKAAEGWARGCGITVDQAERLVTDKGEWLMYRAHVKGQSAQALLAGMVS
TALAKLPIPKLMRWGDSDVQFVRPVHTVTMLLGADLIPGTVLGIDSARTVRGHRFMGEAEFTLDNADQ
YPQILLERGKVVADYEARKALIKRDAELAAQKIGGKADLSDSLLEEVASLVEWPVVLTAKFEEKFLAV
PAEALVYTMKGDQKYFPVYDAAGKLLPNFIFVANIESKDPQQIISGNEKVVRPRLADAEFFFNTDRKK
RLEDNLPRLETVLFQQQLGTLRDKTDRIQALAGWVAGQIGADVNHATRAGLLSKCDLMTNMVFEFTDT
QGVMGMHYARHDGEAEDVAVALNEQYQPRYAGDALPQSLVACSLAIADKMDTLAGIFGIGQHPKGDKD
PFALRRAALGVLRIIVEKNLPLDLQTLTEEAVRLYGSKLTNAKVVDEVVEFMLGRFRAWYQEEGHAVD
TIQAVLARRPTKPADFDARVKAVSHFRTLEAAAALAAANKRVSNILAKSTETLNDSVRASVLKDAAEI
QLATHLVVLRDKLQPYFAAGNYQEALVELAALREPVDAFFDNVMVMADDAEVRVNRLTLLSKLRELFL
QVADISVLQ
>CORE_REP|Org34_Gene3734#
MLSSTFVRTKAGRSKPVRLTAVIAAALFLAGCPSRAPQTPPANIQDEASASSDYYLQQLQQSSDDNKA

DWQLLAIRALLREGKLPQAGDQLNQLPKNLSGAQQTERQLLTAELQIANKSYVSARSSLGHLDSGALS
PNQKVRYYQAQIAANQGKASLPLIRAYIAQEPLLTGKPHQDNLDQTWQALLQLTPQEMNSLVINADEN
VLQGWLDLLRVYQDNKQDPDLLKAGIKDWQNRYPKNPAAKTLPARLNQVLNFTQASTSKIALLLPLNG
QAKVFADAIQQGFEAAKNGGSMPAPQPQAAQPASAPAQTAPADQAAAGDINANGAVSPSAQESQPAVT
AAQPAAPSSAPITPLQAANAQVKVYDTTSQPLAALLSQAQQDGATLVVGPLLKENVDQLSASTTTLNV
LALNQPETPKDNLNICYFALSPEDEARDAARHIWEQQKRQPLLLVPRGAFGDRVAKAFNQEWQKQGGQ
TVLRQDIGSAGELRQMVNSGGIRMTGTPMSSAPAPQSVTIAGLTIPAPPSDVPAATGGSVDAVYIVAT
QSQLTLIKPMIDMATSSRGKPAMYASSRSYQAGAGPDFRLEMEGLQFSDIPLLAGSNPQLLQQASARF
RNDYSLVRLYAMGMDAWTLANHFAEMRQLPGFQVSGTTGTLSASPNCVINRKLPWLQYRQGTVVPVS
>CORE_REP|Org15_Gene2698#
MTTRETSSKPQQDRLNPVVFFTSAGLILAFSLMTIFFTDFSGQWITRTLNWVSTTFGWYYLLAATLYI
VFVVFIAASRFGAIKLGPEQSKPEFSLMSWAAMLFAAGIGIDLMFFSVAEPVTQYMMPPEGDAQTLEA
ARQAMVWTLFHYGLTGWSMYALMGIALGYFSYRYNLPLTIRSALYPIFGKRINGPIGHSVDIAAVLGT
IFGIATTLGIGVVQLNYGLKVLFEIPENLTVQGSLILLSVIMATISVTSGVNKGIRILSELNVLLALG
LILFVLFFGDTEFLLNALVLNVGDYVNRFMGMTLNSFAFDRPVEWMNNWTLFFWAWWVAWSPFVGLFL
ARISRGRTIRQFVVGTLIIPFVFTLLWLSIFGNSALYQIIHGNAEFAQEVMQFPERGFYSLLAQYPGF
TFSASVATITGLLFYVTSADSGSLVLGNFTSRLADINNDAPNWLRIFWSVAIGLLTIGMLMTDGVPAL
QKTTVIMGLPFSFVIFFVMAGLYKSLRVEDYRKASALSTLAPVPVSSHDVLNWKQRLSRVMNYPGTQY
TQKMLDKVCRPAMQDVARELELRGAKVEFSEVPPTEDERLNHLELLVHLGEEQNFIYQIWPMRYSVPG
FTYRARSGKSHYYRLETFLMEGTQGNDLMDYSKEQVIGDILDQYEKHLNFLHIHREAPGGTLTFPDM
>CORE_REP|Org16_Gene430#
MSDDLLIHRPPVAGESLSLRSMQEVAMNDRNASKMLRTYNVAYWGNNYYDVNELGHISVCPDPDVPQA
RVDLAELVKTRQQDGQRLPALFCFPQILQHRLRSINAAFKRARESFGYQGGYFLVYPIKVNQHRRVIE
SLVNSGEPLGLEAGSKAELMAVLAHAGMTRSVIVCNGYKDREYIRLALIGEKLGHKVYLVIEKMSEIN
LVLEEAERLNVIPRLGVRARLASQGSGKWQSSGGEKSKFGLAAVQVLKLVETLREAGRLDSLQLLHFH
LGSQLANIRDIATGVRESARFYVELHKLGVNIQCFDVGGGLGVDYEGTRSQSDCSVNYGLNEYANNVI
WGIGDACNEHGLPHPTVITESGRAVTAHHTVLVSNVIGVERNEFSEPQPPEADAPRALESMWETWLEM
NEPENRRSLREWLHDSQMDLHDVHTQYAHGMLDLTKRAWAEQLYLNICNKIQQQLDPSNRAHRPIIDE
LQERMADKFYVNFSLFQSMPDAWGIDQLFPVLPLEGLDKPPEGRAVLLDITCDSDGTIDHYVDGDGVA
TTMPMPPYDPENPPALGFFMVGAYQEILGNMHNLFGDTASVDVYVFPDGSVETELSDEGDTVADMLEY
VQLDPSALLSKFRDQVKETDLDSELQAQFLEEFEAGLYGYTYLEDE
>CORE_REP|Org2_Gene1789#
MMPELGSFLLCLALAIALLLSIYPQWGAARQDSRMMAVARPLTYGMFAAIALAFLCLVHAFVVNDFTV
AYVATNSNTQLPVYYRIAATWGAHEGSLLLWVLLLSCWSLAVALCSRAMPQDAVARVLSVMGMITAGF
LLFIIMTSNPFTRTLPNFPIDGSDLNPLLQDIGLIFHPPLLYMGYVGFSVAFAFAIASLMAGRLDTAW
ARWSRPWTTAAWVFLTLGIVLGSAWAYYELGWGGWWFWDPVENASFMPWLAGTALMHSLAVTEKRGTF
KAWTVLLAITAFSLCLLGTFLVRSGVLVSVHSFASDPARGMFILAYLVIVIGGSLLLYAVKGGQVRSR
VQHETFSRETFLLGNNVLLIAAMLVVLLGTLLPLVHKQLGLGSISIGEPFFNTMFTWLMAPLALLLGI
GPLVRWRRDEPSKLWRRLGVALLATLALSILLPWLLQDSIAGMTVVGLIMALWVIILTLMELHERATH
RHGFWRGLRQLSRSHWGMVLGHLGVAVTVIGIAFSQNYSVERDVRMKAGDSVDIHNYHFVFRDVHDIR
GPNYSGGVGIIDVTRNGKPEATLRAEKRYYSVARSMMTEAAIDGGFSRDLYAALGEELEDGSWAVRLY
YKPFVRWIWFGGVFMAIGGLLCILDPRYRMSKKLKREGKLETQP
>CORE_REP|Org36_Gene3393#
MSNVNPPRARKAQREAAQQFIDTLQGMAFPNSRRIYLQGSHSDIQVPMREIQLSPTLVGGSKDNPQYE
QNEAIPVYDTAGPYGDPQAELDVHAGLAKLRAGWIDARGDTATLSGASSGFTQQRLADEGLDHLRFEH
LPLPRKALPGKCVTQLHYARAGIVTPEMEFIAIRENMGRERIRGEVLRHQHPGQSWGANLPDNITPEF
VRREVAAGRAIIPANINHPEAEPMIIGRNFLVKVNANIGNSAVTSSIEEEVEKLVWSTRWGADTVMDL
STGRYIHETREWILRNSPVPIGTVPIYQALEKVNGVAENLTWAMFRDTLLEQAEQGVDYFTIHAGVLL
RYVPMTAKRLTGIVSRGGSIMAKWCLSHHQENFLYQHFREICEICAAYDVSLSLGDGLRPGSIQDAND
EAQFAELHTLGELTKIAWEYDVQVMIEGPGHVPMQMIRRNMTEELEHCHEAPFYTLGPLTTDIAPGYD
HFTSGIGAAMIGWFGCAMLCYVTPKEHLGLPNKEDVKQGLITYKIAAHAADLAKGHPGAQIRDNAMSK
ARFEFRWEDQFNLALDPATARAYHDETLPQESGKVAHFCSMCGPKFCSMKISQEVRDYAAAQEAAKPI
EVQLTGMEKMSAEFRARGSELYHSAGASTLQEETSND
>CORE_REP|Org5_Gene1386#

```
MVKVDKRRLLAMGLCLTAFSGAALADSLDAQRQRYQQIKQAWDGNQMGVVAQLMPTLRDYPLYPYLEY
RELTQDLSQAGFSEVNDFIKRNPTLPPAKSLAPRFVNELARREDWRTLLAFSPQPPKPVAARCNYYYA
KWATGDQQAAWSGADELWLNGKTLPGACDRLFSVWRGAGKQTPLDILARMKLALKEGNSSLVSNLYSQ
LPADYQTMGNALVRLQNDPTTVEAFARSVGPTDFTRAATGIAFERLARQDVENARAMIPTLARLQKMS
DDERLGLEEAVAWRLMGSDATYEQAQWRDRVILRSRSPSLLERRVRMALGNGDRQGVATWLARLPEAS
RNKDEWRYWRASQLMDEGKRAEGEEILRNLMTERGFYPMAAAQKLNATYPVMVAVAAKPRASLVDGPE
VARVRELMYWNMDNLARSEWGSYVASRSRPEQEALARYAFEQKWADLSVQATIVGKLWDHLEERFPVA
WPQEFRRATDDKGITTSYAMAIARQESAWNPKAQSPVGASGLMQVMPRTAQHTVQMFNIPGYVGPSQL
FDPQTNITIGTSYLESVYQQFGRNRILSSAAYNAGPSRVNTWLGNSAGRIDPVAFIESIPFSETRGYV
KNVLAYDAFYRYLTHRPAKVLTDAEWQRRY
>CORE_REP|Org19_Gene3023#
MFSPDIKVKVQNFGRFLSNMVMPNIGAFIAWGIITALFIPTGWLPNETLAKLVGPMITYLLPLLIGYT
GGRLVGGERGGVVGAITTMGVIVGADMPMFLGSMIAGPLGGWAIKHFDRWVDGKIKSGFEMLVNNFSA
GIIGMLLAILAFLGIGPLVEVLSKLLAAGVHVMVKNNLLPLASIFVEPAKILFLNNAINHGIFSPLGI
QQATEVGKSVFFLIEANPGPGMGVLMAYMFFGRGSAKQSAGGAAIIHFLGGIHEIYFPYVLMNPRLLL
AVILGGMTGVFTLTMLNGGLVSPASPGSILAVLAMTPKGAYFANIAAVCAAFAVSFVVSAFLLKTSKV
KEDDDLEAATRRMQEMKSQSKGGAATPASVDGDLSTVRKIIVACDAGMGSSAMGAGVLRKKVADAGLK
NISVTNSAINSLPDDVDLVITHRDLTERAMRHAPQAQHISLTNFLDSKLYSDLVDRLLAANKTSDNQQ
KVLGALDDSFEAGESNLFKLSESNVFLNLQASDKEQAIRFAGEQLVKGGYVEAEYVPAMLEREKLTST
YLGESIAVPHGTIEAKDRVLRTGVVFCQYPQGVRFGEEEDEVARLVIGIAARNNEHIQVITSLTNALD
DESVIERLANTTSVQEVLDLLGGKKAG
>CORE_REP|Org35_Gene218#
MTDDFATDGALAQAIKGFKPREPQRQMAQAVTEAINFKQELVVEAGTGTGKTFAYLAPALRADRKVII
STGSKALQDQLYARDLPTVAKALKYKGKLALLKGRSNYLCLERLEQQSMAGGELAGQTLIDLVQLRKW
SSQTKEGDISTCSEVAEDSFVWPLVTSTNDNCLGSDCPLYQDCFVVKARRRAMDADVVVVNHHLFLAD
MVVKEGGFAELIPEAEVMIFDEAHQIPDIASQYFGQQLTSRQLLDLAKDITIAYRTEVRDAAQLQKSA
DRLSLSTQDFRLNLGEPGFRGNLRDVLGEPNVQRALLLLDDALELCYDVMKLSLGRSALLDAAFERAT
LYRARLKRLKAVTEPGYSYWYECNSRHFVLALTPLTVADRFREMLDEKPGSWIFTSATLSVNDQLGHF
TERLGLTKAKTLLLPSPFDYAKQALLCVPRFLPSPNQPGGARQLARMLRPLIEANNGRCFFLCTSHQM
MRELAEEFRATMTLPVLLQGETSKGQLLAQFVAAGNALLVATSSFWEGVDVRGDALSCVIIDKLPFTS
PDDPLLKARIEDCRLRGGDPFNDVQLPDAVITLKQGVGRLIRDTDDRGVLVICDNRLVMRPYGEVFLN
SLPPTPRTRDLKQAIAFLQAADTSAT
>CORE_REP|Org42_Gene1473#
MMDNLRAAANHVVLKIILALIILSFVLTGVGNYLIGGSGDYAAKVNGQTIERAQLEQAFQSERSRMQQ
QLGDQFSALAGNEGYMQQMRRQVLSQLIDNMLLDQYAKKLGLAVSDDQIKDAIRKAPYFQTNGQFDNA
KYLDLIGRMGYTADNFAQSMRQQLVNQQVIQAFGDSGFVLPSESQAMAALVLQERDVRLATIDLKALQ
AKQSAGDDELKAYYDQNKNSFIAPEQVKVSYIPLDAASMQDKVKVSEEDISAYYDQHKSSYGQPERKN
YSVIQLKTEAEANAALDELKKGADFATLAKEKSTDIISRRTGGELGWLEPETTADELKQANLTEKGQL
SGVVKSSVGYLIVRLNDIEPEKVKPLSEVHDAIAKQVQQEKAVDAYYALQQKVSEAATSDNESLASAE
EAAGVKAAQSDWFTRDNIPAALNFKPVVQAIFDGSLIGENGAPGSNSDVITVDGDRAFVVRVSGHKPE
GIEPFDQVKDRVAELVKRNKALQAAKLQGEKLLVELKQGKGDEAMKAAGLSFGAVQKMARAPEDSQLV
ESVFALPHPQDGKPVYGMSQDRQDNVVLIALDAVKPGTLPEDEMKTFVGKMEEGATGVSFDSLLASLR
KEAEIKMGAAEQQPQ
>CORE_REP|Org14_Gene3724#
MNHHASLIITNGKFHTVDRQNPTAQAVAIRDGKFLAVGSESEVMQHAGPETQVIDLHGHTAIPGLNDS
HLHLIRGGLNYNLELRWEGVPSLADALRMLKEQALRTPSPQWVRVVGGWTEFQFAERRMPTLDEINQA
APDTPVFILHLYDRALLNRAALKVVGYTKDTPNPPGGEIQRDANGNPTGMLIAKPNAMILYATLAKGP
KLPLEQQVNSTRQFMRELNRLGLTSAIDAGGGFQNYPEDYQVIAELHEKKQLTIRIAYNLFTQRPKQE
LEDFELWTDMLKPGQGTDFYRHNGAGEMLVFSAADFEDFLQPRPDLPPGTEDELERVVRHLVEHRWPF
RLHATYDESISRMLDVFEKVNRDIPFNGLHWLFDHAETITERNIERVKALGGGIAVQHRMAFQGEYFA
DRYGIEATKHTPPVAKMLAAELPVGLGTDATRVASYNPWTALYWLVSGRTVGGMAMYDDSARLDRETA
LMLWTQGSAWFSTEQGKKGQIKVGQLADVAVLSQDYFSVPEEQIKGIESVMTVVDGKVVYAAGSFSPL
APPPLPVLPEWSPVTQVPGHYRSAPPSAAAKVGVLSQAHQCCGPCGVHAHQHDIARRSSIPVSDENAF
WGAFGCSCFSF
```

>CORE_REP|Org1_Gene3287#
MIALLEQAVALGALRPLDVQFARVVANEDEPDILLAAACLSAEAGAGHVCLMLEQLQADTLFEGRQPA
LALAVWEAVGRPDSARWQQRLAASAAVGDGSGATPLVLRGPRLYLQRMWQNEGEVAAFIGGEGESLAV
PEEALRAILDRLFGTASDEPDWQKIAAAVAATRRIAVISGGPGTGKTTTVAKLLAALVQLDESARLRI
QLAAPTGKAAARLTESLGSASRQLALTPAQQALFPTEAATLHRLLGAQPNSQRMRYHRGNRLHLDVLV
VDEASMVDLPMMARLIAALPDRARVIFLGDRDQLASVEAGAVLGDICRFAEQGYSDARAAELSRLTGC
SIEGRQADAEAVVRDSLCLLRKSYRFDARSGIGQLALAVNAGAGDRALAALNGSFGDVAGYALATSEE
YQALLDACVAGYRDYLRLTAEGADAATVLAAFGRFQVLCALREGPFGVAGLNERIELGLQRAGLIDRK
PGVLGRWYRGRPVMIGRNDSALGLFNGDIGITLPDEHGDLRVHFQLPDGSIKSVQPSRLPAHETAYAM
TVHKSQGSEFDHTVLVLPNHFLPVLTRELVYTAITRARKQLSLYATETVLLRAIRTPTQRRSGLAERL
QATE
>CORE_REP|Org46_Gene2862#
MNDRFDAKAFLSTVTSQPGVYRMYDATGTVIYVGKAKDLKKRLASYFRQQVSSRKTETLVKNIAQIDV
TVTHTETEALLLEHNYIKLYQPRYNVLLRDDKSYPLIFLSADTHPRLAVHRGAKHAKGEYFGPFPNSY
AVRETLALLQKLFPIRQCENSVYRNRSRPCLQYQIGRCLGPCVAGLVSEEEYRQQVDYVRLFLSGKDQ
QVLHQLIARMEEASKLLNFEEAARIRDQIQAVRRVTERQFVSGDSDDLDVIGVAFDAGMACLHVLFIR
QGKVLGSRSYFPKVPGGTDMGEVVQTFVGQFYLQGSQARTLPGEILLDFSLPEKDLLAESLSELAGRK
IQIQSKPRGDRARYLKLARTNAATALTTKLSQQSTIHQRLAELAKVLNLTEINRMECFDISHTMGEQT
VASCVVFDGNGPVRAEYRRYNISGITPGDDYAAMTQVLKRRYGKALEEKKIPDVIFIDGGKGQLGMAI
EVFKSLNVTWDKNKPLLIGIAKGADRKAGLETLFFVPEGEGISLPPDSPALHVIQHIRDDSHNHAITG
HRQRRAKVRNTSALELIEGVGPKRRQVLLKYMGGLQPLLNASVEEIAKVPGISQALAEKIYNALKH
>CORE_REP|Org43_Gene1979#
MVTNRQRYREKVSQMISWGHWFALFNILLALGLGSRYLFVTDWPASLLGRVYALVSLLGHFSFIVFAG
YLLVIFPLTFVVMSQRLLRFISAALATAGLTLLLVDSEVFSHFHLHLNPVVWDLVVNPDQSELSRDWQ
LMFICVPVIFLVEMLFGTWSWQKLRSLNRRRFGKPLAALFISAFFASHLIYIWADANFYRPITMQRAN
LPLSYPMTARKFLEKHGLLDQQEYERRLVQQGNPEAVAVEYPLSDLSYGDKGSGYNLLMIVVDGIRAK
DVAQDMPTLTRFAQENVRFSDHYSSGNHADTGLFGLFYGISPTYLDSVLAGRKPSALINALGEQGYQL
GLFSSDGFNASLYRQALLTDFSLPTPAPQSDAQTTQQWQRWLTDQGDKEPWFSYINFSGAEPAEGAKT
PAPADFIQRYRTGAQDVDSQIAQVLDTLKQRGLLDKTVVVITAEHGVEFNDSGKGQWGAGTAFNQAQL
QVPLVIHWPGTPAQTINKLTGHNDVMRTLMQRLLHVKTAPKDYSQGEDLFTAQRRNNWIATGDGNQLV
ITTPTQTLMLDNSGNYRVYDQNGDEIKDEKPQLALLLQVLTDVKRFIAN
>CORE_REP|Org37_Gene887#
MAGRIPRVFINDLLARTDIVDLIDARVKLKKQGKNYHACCPFHHEKTPSFTVNGEKQFYHCFGCGAHG
NAVDFLMNYDRLEFVETIEELATMHGLEVPYEAGTGPTQIERHQRQSLYQLMEQLSAFYQQSLQQSSG
APARSYLQQRGLSDDVIRHFAIGFAPAGWDNALKRFGRDADSRRALNDAGMLVTNDQGRSYDRFRERV
MFPIRDKRGRVIAFGGRVLGDGMPKYLNSPETEVFHKGRQLYGLYEAQQNHPTLQRLLVVEGYMDVVA
LAQFGIDYAVASLGTSTTAEHIQLLFRATDNVVCCYDGDRAGREAAWRALETALPYLNDGRQLRFMFL
PDGEDPDTLVRKEGKEAFEQRMEQAQPLSTFLFESLLPQVDLSSPDGRAKLSTLALPLITQVPGETLR
LYLRQELGNKLGLLDDSQLDKLMPKQAENANPYQAPQLKRTTMRILIGLLVQNPQLATLIPSLEGLEQ
TKQAGLPLFVELVQTCLAQPGLTTGQLLELYRDNKFSQQLETLATWNHMIVEDMVEQTFLDTLASLYD
SVLEQRLETLIAQARTRGLSPEEREEVRSLNQVLAKKN
>CORE_REP|Org13_Gene3486#
MSDKHPGPLVVEGKLADAERMKKESHFLRGTIAEDLNDGLTGGFNGDNFLLIRFHGMYQQDDRDIRAE
RAEQKLEPRHAMMLRCRLPGGIISPQQWLGIDKFAQESTLYGSIRITNRQTFQFHGILKGNVKPVHQL
LNRLGLDALATANDVNRNVLCTSNPVESELHQEAYEWAKKISEHLLPRTRAYAEVWLDQEKVATTDEE
PILGPTYLPRKFKTTVVIPPQNDVDLHANDMNFVAIAENGKLVGFNLLVGGGLSIEHGNKKTYARQAS
EFGYIPLEHTLPVAEAVVTTQRDWGNRTDRKNAKTKYTLERVGVDVFRAEVEKRAGITFAPVRPYEFT
GRGDRIGWVKGIDDQWHLTLFIENGRLLDYPGRPLKTGMAEIAKIHKGDFRLTANQNVIIAGVPESEK
AKIEALARDHGLIDDEISEQRKNSMACVSFPTCPLAMAEAERFLPQFVTKVEGIMHRHGVGDEHIVLR
ITGCPNGCGRALLAELGLVGKAVGRYNLHLGGNREGTRIPRMYRENINEDEILSEIDLLVGRWAKERN
AGEGFGDFTVRAGIVKPVLDPARDFWE
>CORE_REP|Org13_Gene4429#
MLRRDFIKLTAALGAASALPLWSRAAWAADRPALPVPPLLTPDAQGKIALALQAGETRWLPGAATKTW
GFNGALLGPAVKLQRGQPVTVDIKNSLAEASTVHWHGLEIPGDVDGGPQALIHPGATRTVNFTVDQPA

ATCWFHPHTHGKTGSQVIMGLAGLVLLEDEESAKLPLPKTWGQDDIPVILQDKRLGKDAQIEYRLDVM
SAAVGWFGDRMFTNGAQYPQHLAPRGWLRLRFLNGCNARSLNLAASDNRPLYVIASDGGFLAEPVKLT
ELPMLMGERFEVLVDASDGKAFDIVTLPVKQMGMTLAPFDQALPVLRIQPSLAQGIKTMPDSLVKLPT
LPATTGIQERWLQLMMDPQLDMLGMQALMDRYGHQAMAGMSMNHGATGGADMKGMEKGGMQSMDHGNM
KGMEKGGMQGMDHGNMGNMGNMKGMDHGNMAGMDHGGAQGKAKSFDFSHGNMINGKAFDMTKPMFAAK
RGQYEKWTISGEGDMMLHPFHIHGTQFRILSENGKPPAAHRSGWKDTVRVEGWRSEVLVRFDHPASSE
HAYMAHCHLLEHEDTGMMMGFTVAD
>CORE_REP|Org20_Gene1894#
MLIDPSSKYRPFPPVALPDRQWPARTLRQAPRWCSSDLRDGNQALAEPMDNARKREFYQLLLQCGFKE
IEVAFPSASQTDFDFVRTLIDEQLIPDDVTIQVLTQSRDDLIDRTFEALQGAPRAIVHLYNATAPMFR
DIVFRQDKAATVALAVNGARRIRRQCEAQPDTAWCFEYSPETFCFTELEFALEICEAVAAVWQPGPQR
PMIINLPATVEVSTPNVYADQIEWFCRHFSRRADVTISVHPHNDRGTGVACAELALLAGADRVEGCLF
GNGERTGNVDLVTLALNLYTQGVAPGLDFSRLKQVVEVVEVVELCNQLPVHPRHPYAGELVFTAFSGS
HQDAIKKGFAAQRQRQDGWWQVPYLPLDPADVGCSYEAVIRVNSQSGKSGAAWLLEQNHGLALPRGLQ
IDFSQVVQRATDGSGKEMSGAQLWRLFRDTYGLVEQPRLQLLSYQTESHGVEAYSFNARVACEGEPLR
LQGAGNGLLSSAVDALRQRFGLPLAIEDYHEHTLGHQSDSRAVAYIRCSLPQGEATYGVGIDVDSASA
SLQALLNVAGRYLASTSARPG
>CORE_REP|Org20_Gene424#
MNINVASLLNGNYILLLFVVLALGLCLGKLRLGSVQLGNSIGVLVVSLLLGQQHFAINTEALNLGFML
FIFCVGVEAGPNFFSIFFRDGKNYLMLALVMVGSAMVIAIGLGKLFHWDIGLTAGMLAGSMTSTPVLV
GAGDTLRNTIVNGPALLAAQDHLSLGYALTYLIGLVSLIFGARYLPKLQHQDLSTSAQQIARERGLDT
DSQRKVYLPVIRAYRVGPELVAWADGKNLRELGIYRQTGCYIERIRRNGILANPDGDAVLQVGDEISL
VGYPDAHARLDPSFRNGKEVFDRDLLDMRIVTEEIVVKNSNAVNKRLSQLKLTDHGCFLNRVIRSQIE
MPIDDSIVLNKGDVLQVSGDARRVKSVAEKIGFISIHSQVTDLLAFCAFFIIGLLIGQITIQFSNFSF
GIGNAAGLLMSGIMLGFLRANHPTFGYIPQGALNMVKEFGLMVFMAGVGLSAGAGIGHSLGAVGGQML
IAGLIVSLVPVIICFLFGAYVLRMNRALLFGAIMGARTCAPAMEIISDTARSNIPALGYAGTYAIANV
LLTLAGSLIVVLWPGILG
>CORE_REP|Org44_Gene4221#
MKTLLMVDSSLGQARGHLAKRMLEAAAAKTGLTLVESLQDAELVAVAGQSAPADAGLNGKLVYVGNVE
QAVREPDAFLARAKAEAETYQAPQAAVPVKAGGQKRIVAITACPTGVAHTFMAAEAIESEAKKRGWWV
KVETRGSVGAGNAITPEEVAAADLVIVAADIEVDLDKFAGKPMYRTSTGLALKKTAQELDKALAEAEV
FQPQQRGSAAPAGKKKEGNGPYRHLLTGVSYMLPMVVAGGLCIALSFVFGIKAFEVKGTLAAALMQIG
GGSAFALMVPVLAGFIAFSIADRPGLTPGLIGGMLAVSTGAGFLGGIIAGFLAGYVAKAISSKLRLPQ
SMEALKPILIIPLVASLITGLIMIYVVGTPVAKIMEGLTHWLQSLGTANAVLLGAILGGMMCTDMGGP
VNKAAYAFGVALLSSSVYAPMAAIMAAGMVPPLAMGLATLLARRKFPKSEQEGGKAALVLGLCFITEG
AIPFAARDPMRVLPCCIAGGALTGALSMAFGAKLMAPHGGLFVLLIPGAISPVLLYLVAIAAGTLLAG
VAYALLKRAEVPAASVA
>CORE_REP|Org19_Gene3632#
MSTSVFNRRWAAVLLEALARHGVRHVCIAPGSRSTPLTLAAAANRSFICHTHFDERGLGHLALGLAKA
AREPVAVIVTSGTAAANLYPALIEAGLTGERLVFLTADRPPELIDCGANQAIRQNGLYSSHPTLAIDL
PRPTPDIPAAWLASSVDSAMARLQHGALHINCPFAEPLYGGDERHYADWSAALGDWWQSDRPWLQESE
THAAPLPQPDWFFWRQKRGVVLAGRMSAQEGAQVAEWAATLGWPLIGDVLSQTGQPLPCADLWLAHPQ
AQRVLQDAQLVVQFGGSLTGKRLLQWQAQCRPEEYWIIDELPGRLDPAQHRGRRLRAGVAQWLAQHPA
QPRQPWAAVLAVLADKALTAASTHLHDSFGEAQLAHRLPELLPENGQLFLGNSLVVRLIDALTPLPVA
YPVFSNRGASGIDGLISTAAGVQRATARPTLAVVGDLSALYDLNALALLRQSSAPTVLIVVNNNGGQV
FSLLPTPEEDRQRFYCMPQNVEFSHAAAMFQLGYARPENWNQLQQAVEQGWRRGGATLIELQVPPSAG
AESLQYLVQQMAVQ
>CORE_REP|Org5_Gene3280#
MSTSRLQQQFIRLWQRCHGETTDTTLQDLAEVLSCSRRHVRSLLSAMQREGWLTWQAESGRGKRSRLT
FHYTGLALQQQRAEELLEQDRIDQLVQLVGDKNVVRQMLLSQLGRSFRQGKHILRVLYYRQLYNLLPG
SALRRSETHLARQIFNGLTRINEENGELESDLSHHWQALTPLHWRFYLRPAIHFHHGRELEMADVITS
LSRLTSQPLFSHIESVTSPTPFVIDVQLRSPDHWLPWLLGSVQAMILPREWRELPDFARHPVGTGPYR
MVRNHPSQMKIHAFDDYFGYRALIDEVNIWVLPEFSEELVHSGVQLQGDETGNSELESRLEEGCYFLL
FDQRSPLATDPAIRSWLCELINPISLLSHAGPLYQRYWSPAYGLLPRWHHNRTLAQQPKPAGLTELTM

THFNEHSEFHAIRQAIEPLLAQHGIRLIVQSVDYATWHQGDARSDLWLGSANFYLPLEFSLFATLYEL
PLVQHCMNEDLAQDAALWRANRLPLAEFCQRLVSNHQLHPLFHHWLQLHGQRSMRGVRMNTLGWFDFK
SAWFAPPEA
>CORE_REP|Org8_Gene2744#
MFLERIEIVGFRGINRLSLMLDDNTLLLGENAWGKSSLLDALTLLLAPEQKLYRFEAHDFHFPPGEES
AKERHLQVVFTFCEKDIGHAHLPRYRHLTPLWVKGEDGLSRIHYRCEGELADDGTVCTWRGFLDADGN
AFQLHHIEQLAHAIIRIHPVLRLRDARFIRRLRPSSLGDERKPDTQALAQQLDQLTRELVRNPQKLTN
GELRQGLAAMQQLLEHYFAEQSSQVTRPRRRGGEPEQDAWRALDGINRMVAEPNSRSMRLILLGMFST
LLQAKGDVKLDPHARPLLLVEDPETRLHPIMLSVAWGLLNQLPLQRITTTNSSELVSLPVEHVCRLV
RESGRVATYRLGPRGLSAEDGRRIAFHIRFNRPSSLFARCWLLVEGETEVWLLNELARQCGYHFEAEG
VRVIEFAQCGLKPLLRFARRMGIEWHALVDGDEAGKKYANTVRSLLDNHEDNERDRLTALPAPDMEHF
MFREGFAPVYHRMASVPINAQMPVRKVILKAVHHSSKPDLAIEVAMQAGEWGTDSVPPLLKKMFSRVI
WLARGRAD
>CORE_REP|Org30_Gene2974#
MSDIALTVSMLALAAVIGLWMGNWKLYGVGLGIGGVLFGGILVGHFAQSGQINLNGDMLHFIQEFGLI
LFVYTIGIQVGPGFFSSLRVSGLRLNAFAVLLVLTGGVVAAAVHKLFDVPLPIILGVFSGAVTNTPAL
GAGQQILTDLGSDPALVDGMGMGYAMAYPFGICGILLVMWLIRLFFRINIEREAQAFESSLGNQRELL
HAINVAVRNPNLQGMAIKQVPLLNGEAIVCSRLKRGELLMVPAPHERLELGDYLHLVGKREDLENARL
VIGEEVDASLSTRGTALQVVRAVVTNEQVLGKKIRDLNLKQKYDVVISRLNRAGVELVAGSNVTLQFG
DILNLVGRPEAIDAVTAIVGNAQQKLQQVQMLPVFIGIGLGVLLGSIPLFVPGFPAALRLGLAGGPLV
AALILGRIGSIGKLYWFMPPSANLALRELGIVLFLAVVGLKSGGNFIDTLLHGEGLTWVGYGALITAI
PLLSVGILARTVGKMNYLTLSGMLAGSMTDPPALAFANGLHPTSGAAALSYATVYPLAMFLRIMSPQL
LAVLFWTL
>CORE_REP|Org18_Gene1231#
MKNWVKSGSPWIWLTAGSVAVSLLALIGILLLLAGQGMRYFWPSPVYQFELNQNGAGPVTVIGELYQQ
QSISRRQLTEAGVTPPGEAQSVERYLIKVGNREREGQDFRTLLASDIRSQSTPRSLLVLERDSHGTAY
GYLAGLLEDGQPLTGRNLGQALQQRLPQIAALSRQAHDIQFRDMARINQQFDALRLREKRLQRDDKLD
ARAQDAIKAERLELQRQYQLLSERLAGLNRDRQRDALLLRDMHGQTLTIPLSQVRDAWYPNAMNTSEK
LAHWGEQVKKFLTDSPREANTEGGVFPAIFGTVLMVILMSIVVMPFGVIAAVYLHEYAGNNLLTRVIR
IAVVNLAGVPSIVYGVFGLGFFVYMIGGTLDQLFYPESLPNPTFGTPGVLWAALTLALLTLPVVIVAT
EEGLSRIPTSLRQGSMALGASRAETLWRIVLPMAAPAMMTGLILAVARAAGETAPLMLVGVVKSVPVL
PVDEIFPYLHLERKFMHLSFQIYDMAFQSPSVEAARPLVFATAFLLVTIVVSLNLAAMGIRHSLRERY
RAWSQ
>CORE_REP|Org27_Gene2983#
MSNKPFHYQDPFPLKKDDTEYYLLSRDHVSVSEFEGQEILKVAPEALTLLAQHAFHDASFMLRPAHQQ
QVADILKDPDASENDKYVALQFLRNSEIAAKGILPTCQDTGTAIIVGKKGQRVWTGGGDEAALSRGVY
NTYIQENLRYSQNAALDMYKEVNTGSNLPAQIDLYSVDGEEYKFLCIAKGGGSANKTYLYQETKALLS
PGKLKNYLVDKMRTLGTAACPPYHVAFVIGGTSAEATLKTVKLASTKYYDGLPTEGNEHGQAFRDLEL
EAELLQEAQNLGLGAQFGGKYFAHDIRVVRLPRHGASCPVGMGVSCSADRNIKGKINRDGIWLEKLEH
NPGKFIPQELRQAGEGEAIKVDLNRPMAEILKQLSQYPVSTRLSLSGTIIVGRDIAHAKLKERLDRGE
GLPQYVKDHPIYYAGPAKTPEGYASGSLGPTTAGRMDSYVDLLQSHGGSMIMLAKGNRSQQVTDACHK
HGGFYLGSIGGPAAVLAQQSIKSLECVEYPELGMEAIWKIEVEDFPAFILVDDKGNDFFQKIQAGQCS
SCLK
>CORE_REP|Org25_Gene478#
MKSSLFSRYTCFVLSILLTLVFLIMMHNHPWFWLPALACGCLMVLGIYDLTQQRHAICRNYPIIGRLR
FFFEFIRPELRQYFLEQDNEEIPFSRTQRTLVYRRAKNEMGDKPFGTLLDVYQTGYECIGHSMRPVEA
ADPTSFRITIGGADCRQPYSASIFNISAMSFGALSANAIRALNLGAAKGNFYHDTGEGSISRYHRENN
GDLVWELGSGYFGCRTADGHFDPRRFAEQAQSPQVKMIEIKLSQGAKPGHGGILPAKKVDAEIAATRG
VPEGVDCISPASHSAFTTPLEMMQFIQQLRELSGGKPVGFKLCIGHPWEFVAIVKAMLHTRILPDFIV
VDGKEGGTGAAPLELSNYMGMPLREGLLFVHNTLVGCGLRDQIKIGASGKIISAFDIASVLVLGADWV
NSARGFMFAVGCIQSQSCHTNHCPTGVATQDPLRQKALVVPNKAERVYHFHQNTVKALADMLAAAGVS
RPEQLTSHHMLRRITPTEIKVYADIYYYLEPGALLQPEIKSEFYARMWRMATPNSFDQAISLPAA
>CORE_REP|Org29_Gene4497#
MRVKGITPQDLAAYGIHDVSEIVHNPSYELLFKEETDPSLEGFERGVVTKLGAVSVDTGIFTGRSPKD

KYIVRDDITRDTVWWADQGKGKNDNKPLSQEVWADLKHLVTEQLSGKRLFVVDTFCGANADSRLKVRF
ITEVAWQAHFVKNMFIRPSDEELADFEPDFVVMNGAKCTNPNWQQQGLNSENFVAFNLTERMQLIGGT
WYGGEMKKGMFSMMNYLLPLKGIASMHCSANVGEKGDVAVFFGLSGTGKTTLSTDPKRQLIGDDEHGW
DDDGVFNFEGGCYAKTIKLSEEAEPDIYHAIKRDALLENVTVLADGSIDFNDGSKTENTRVSYPIYHI
QNIVKPVSKAGHATKVIFLTADAFGVLPPVSRLTANQTQYHFLSGFTAKLAGTERGVTEPTPTFSACF
GAAFLSLHPTQYAEVLVKRMQAAGAQAYLVNTGWNGTGKRISIKDTRGIIDAILSGEIDKAETVTLPI
FDLAMPTALPGVNPEILDPRNTYASLEQWQEKAQDLAERFITNFDKYTDTPAGAALVSAGPKL
>CORE_REP|Org8_Gene3880#
MSAANKPKITLWEFFQSLGKTFMLPVALLSFCGIMLGIGSSLSSRDVITLMPFIGHPIFQLIFTWMSK
VGSFAFSFLPVMFAIAIPLGMARENKGVAGFSGFVGFAVLNLGTNFYLTAAGVLPTSDPLVLKANNIQ
NILGIQSIDTGILGAVIVGIIVYRLHERFHTIRLPDALAFFGGTRFVPIVTTVVLGLCGLVIPLIWPW
FAAGITGLGWVINSAGAFGPMLFGTGERLLLPFGLQHILVALIRFTEAGGTMEVCGHSVSGALTIFQA
QLSCPTTTGFAESATRFLSQGKMPAFLGGLPGAALAMYHCAKPENRHKIKGLLISGVVACVVGGTTEP
IEFLFLFVAPFLYLIHALLTGLGFTVMALLGVTIGNTDGNIIDFVVFGILHGTATKWYLVPVVAAVWF
VGYYAIFRFAIQRFNIKTPGRESDSAVSQSAPTGAVGKSGYNVPAILAALGGPDNIITLDNCITRLRL
SVNDMSRVDDAVLKANRAIGVVHLNDHNLQVVIGPQVQSVKDELDSLIATAQPAAALQGATHV
>CORE_REP|Org17_Gene3634#
MANRRQPLIPRWLWPGLLAAGMILLVAALAMGSLWRHSPDSGWRGLWQDSYLWHVVRFTFLQALLSAL
ISVLPAILLARALYRRRFPGRQLLLRLCAMTLVLPVLVAVFGLLSVYGRQGWLATLCGWLGVEYDFSP
YGLQGILLAHLFFNLPLATRLLLQALENIPVEQRQLAAQLGMNGWQQFRFVEWPALRRQILPSGALIF
MLCFASFATVLSLGGGPQATTIELAIYQALSYDYDLGRAALLALIQLGCCLGLVLISQRLSQALPVGH
THAQRWRNPEDSLWRRISDFLLIAAALLLLLPPLLAVIADGANQAIISVLRQPVLWQALFTSLRIALG
AGALCVALTMMLLWSSRELKLRQQLRGGQALELSGMVILAMPGIVLATGFFLLLNDTIGLPQSPYALV
ILTNALMAVPYALKVLENPMRDLAERYNPLCLSLDIRGWRRLRLIELRALRRPLAQALAFACVLSIGD
FGVVALFGNEHFRTLPFYLYQQIGAYRSQDGAVTALLLLLLCFLLFTLIERLPGRHADA
>CORE_REP|Org41_Gene1672#
MTRSLGKSGVLKFGIGLIALTVAASVQAKTLVYCSEGSPEGFNPQLFTSGTTYDASSVPIYNRLVEFK
IGTTELQPGLAEKWDVSEDGKTYTFHLRKGVKWQSSKDFKPTRDFNADDVVFSFERQLDANNAYHKVS
GGSYEYFEGMDMPKLIAKIEKVDDNTVRFVLNRPEAPFLADLGMDFASILSAEYADVMMKAGTPEKVD
LNPIGTGPFQLLQYQKDSKILYKAFDGFWGTKPKIDRLVFSITPDASVRYAKLQKNECQVMPYPNPAD
IARMKQDKSINLMEQPGLNVGYLSFNVEKKPLDNLKVRQALTMAVNKQAIIDAVYQGAGQAAKNLIPP
TMWGYNDAVQDYAYDPAKAKALLKEAGMADGFSIDLWAMPVQRPYNPNARRMAEMIQADWAKIGVKAK
IVTYEWGEYLKRAKAGEHQTVMMGWTGDNGDPDNFFATLFSCAAAKDGSNYSRWCYKPFEDLIQPARA
ESNHDKRIELYKQAQVVMHDQAPALIVAHSTVYEPVRKEVKGYVVDPLGKHHFENVSVD
>CORE_REP|Org12_Gene2579#
MPLLFSIAPIVLLIWMMTKRNGVPSYLALPLTAAAVYAVQLLWFDASLRLLHANIITALVSTLTPITI
IAGAILLNKLMQVSGAENVVRRWLETISPNPVAQLMIIGWAFAFMIEGASGFGTPAAIAAPILVGLGF
NPLRVALLTLVMNSVPVSFGAVGTPTWFGFANLGLSDASLLEIGRQTALIHFIAGFVIPLLALRFIVS
WQDIRRNLPFILLSVLSCTLPYLLLAQVNYEFPALVGGAIGLALSVLLARGGIGLARSDKLQNAGQAV
PFLQVVKAMTPTLLLIAILIVTRVHQLGLKALLNNTTLLWQENLGWLGELRISQALIVELQQVLGTSA
AAGYKTLYVPALIPFLLVVVLCIPLFRLNGGQVRQMFSETGGRIARPFIALFGALVMVNLMMQGGDNA
PVILIGKALAALTGESWLLFSSFLGALGSFFSGSNTVSNLTFGGIQQSIAQSSGLDVNLTLALQSVGG
AMGNMVCLNNIIAVCSILGIGNAEGKIIRKTVLPMLAYGGIAAGMAAILTL
>CORE_REP|Org35_Gene3082#
MIPDVSQALSWLEAHPQALKGIRRGIERETLRVTPNGTLATTGHPEKLGAALTHHWITTDFAEALLEF
ITPVDDNIDHLLTFLRDIHRYVARNIGDERMWPLSMPCFIEAEQDIELAQFGSSNIGRMKTLYREGLK
NRYGALMQTISGVHYNFSLPLEFWQAWAGVQDAESGKEQISAGYFRLIRNYYRFGWVIPYLFGASPAI
CSSFLKGRETNLPFERTEQGMCYLPYATSLRLSDLGYTNKSQSNLGITFNDLHTYVAGLKRAIATPSE
EFAKLGVKEGDRYLQLNSNVLQIENELYAPIRPKRVTQSGETPSDALLRGGIEYIEVRSLDINPFSPI
GVDAVQARFLDLFLVWCALADAPEMSSDELLCTRKNWNRVILEGRKPGQTIGIGCGASHEPLEKVGKA
LFADLRRVAEVLDGEAGDRQYQQVCDELVAAFDDPELTFSARILKAMKAEGTGRVGLQLAEQYRQTLL
EEPLEILNEAELDKEREASWQRQRDIEANDTLSFEAFLKQNGGS
>CORE_REP|Org43_Gene4940#
MSLAVIYSRAIIGVQAPSVTVEVHISNGLPGLTLVGLPETTVKEARDRVRSALINNGFTFPARRITVN

LAPADLPKEGGRYDLPIALAILAASEQLPLAPLARYEFLGELALSGALRAVRGAIPAALAAADAGRQL
VLSTDNAAEVGLIAQSQSHTAQHLLEVCAFLLGQGELPVAITPPAADNPHENADLRDIIGQEQAKRAL
EIAAAGGHNLLLIGPPGTGKTMLASRLTGLLPPLTEPEALESLAVASLQHPVLTALPWRQRPFRAPHH
SASMAALVGGGSLPRPGEISMAHNGVLFLDELPEFERKVLDALPEFERKVLDALREPLESGEIVISRA
NAKVCFPAKVQLIAAMNPSPTGHYQGMHNRASPQQVLRYLARLSGPFLDRFDLSIEVPLLPPGTLSKR
QTQGESSEQVRERVRQARTRQLERAGKINALLNNREVERDCVLQAADAEFLEVTLNALGLSVRAWQRI
LKVARTLADLAGDAEIGRRHLSEALSYRSMDRLLLQLHRSLE
>CORE_REP|Org24_Gene3686#
MPLKNTATNKPQEIAAIDLGSNSFHMVIARVVNGALQVLGRLKQRVHLADGLDSNNVLSEEAIERGLA
CLALFAERLQGFPADNVTIVGTHTLRQAVNAEVFLKRAAKVIPYPIEIIAGQEEARLIFMGVEHTQPE
KGRKLVIDIGGGSTELVIGEDFEPLLAESRRMGCVSFAQLFFPGGEISKNNFRRARLAAAQKLETLAW
QYRIQGWQYALGASGTIKAAHEVLVAMGEKDGLITLDRLEMLAEQVLQFKSFSSLSLPGLSEDRQSVF
VPGLAILCGVFDALAIRDLRLSDGALREGVLYEMEGRFRHQDIRSRTAKSLADHYNIDREQAKRVLET
TELLYSQWMAQNTKLAHPQLEALLKWAAMLHEVGLSINHSGMHRHSAYILQNSNLPGFNQEQQLLLSA
LVRFHRKAIKLEELPRLNLFKKKHYLPLIQLLRLSTLLNNQRQSTTTPETLRLTTDDNHWTLRFPAGY
LAQNNLVQLDFEREQAYWNDVVGWKLLIEEEGSQNEQRSA
>CORE_REP|Org19_Gene2732#
MDTSLIYGIGGVAIGMLLGWLIASLRVQQANAQHETELRLLEQALQQAQQETAARQEALQRHEQQLRQ
SELELRNLHSQLAAGHEKLQQLNHWRNECELLNQELRAQREVNSAQEAELREVTIRLEETRMAAEEKQ
RLLINSEQRLTTQFENLANRIFEHSGRKVDEQNKQSLDRLLLPLREQLDGFRRQVQDSFGQEARERHT
LTHEIRNLQQLNAQMAREAINLTKALKGDNKTQGNWGEVVLSRVLEASGLREGHEYETQVNVRVDHQS
RMQPDVIVRLPQGKDVVIDAKMSLIAYERYFNGEDEVEREAALSEHIASLRGHIRMLGRKDYQQLPGL
RSLDYVLMFIPVEPAFLLAIDREPELISEALKHNIMLVSPTTLLVALRTITNLWRYEHQSQNAQRIAD
RAAKLYDKMRLFVDDMSALGQSLDKAQGSYRQAMNKLSEGRGNLIGQIEGFRALGVEVKRPINPLLAQ
QAGAQHDEAEEANDDDVAALPQTKDDDDTAGEPGFVSHG
>CORE_REP|Org12_Gene3056#
MTMKTPPSLDEEPVRLNVPVFFGSAAVILLLGSLVVLFPAASKQWLNVAQSWVADVFGWYYMLLMVAC
MAFVFWLALSRFGHIRLSQDDEPPQFSYPSWVAMLFSSGIGIALVYYGAYEPLDHFLSPPEGSGGSVP
AARQAMALTFLHWGLHGWALYALIATALAYFAYCRGLPLALRSALYPIFGERIHGGVGHLVDSFGILV
TVISMVTNLGIGALLVNSGLFYLFDIPQSTGVLLALIVVMMVVATLAAVTGVEKGIAMLSNINVGFLC
LLLLFVFLAGPTLNLLNGMLQNLGDYLTSLVSRSFDMYLYGKARQWQGAWTLFYWAWWVAWAPFVGLF
IARISKGRTIRELIFGVLLIPLGFTLAWLSIFGNTAISLVLEGGQAILGQVAQSDPPMAVFKLFEYLP
YTQLTAGFVVVISFVLFLTPVDSGTLMIANLSCQGGSAHDDAPAWLRVFWAAVTTLVCAGLLYAGSFS
AMQTAVVLCGLPFSAVIVLYMVSLRKDLRGYAMTPAIP
>CORE_REP|Org22_Gene1710#
MKKSLVAVSVIVILGAAWTGASWYTGKQIEQHMGEVVDNANGQLKAYLPKAGVKLSYENYQRGLFSSK
VRYVLRSDGTDTSENAALKTGEEVAFLETIDHGPFPFAQLKKFNLLPSMASVHTELENTPAVKGLFDT
TKGKSLFTADSRISYSGDTSSAIDIIPLDYQKDKSSLKFSGATINADVSSDLKKFVLDANSDNIVFAS
PNEFGQNEQITFQGFNLKGNSNESKFGVKLGDQTMTLKQFKLTIDGKDTVALDGFNLVSKFGEQGSSN
IGGQIDYTMDALKVQGNDFGAGKLALKIDNVDGKALKDFADSYNRQTMALLQQGENLDPAVYEQQNAE
LLQKNLPLLLKGNPSLSIAPLSWKNSKGESTFTLDLALTDPSKAASPAQSPDQLIAQAVKKLDINLTI
PEAMATEVTAKTALLQGYNEEDAQKLAQQQVQGLAAMGQMFKLTTQKDGVIASTFHYADNQVDLNGNK
MSLQEFIGQFGLLGAPAEDGEPAQDAQPAPAPAQ
>CORE_REP|Org38_Gene3633#
MAKASLLERQWVRALLALLSGAGGTLAFSPYDFWPAAIVSLFGLLAVTLNRTTKQSALLGFVWGFGLF
GSGINWVYVSIADFGGMPFAVNVFLVALLAAYLSLYTGLFAGLLTRLWPATRWWRLAIAAPALWQVTE
FLRGWVLTGFPWLQFGYSQINGPLKGIAPLLGVDAITFVLMAIAGLLVYAVNQRRLSAAVIAAALLLL
PWPLRQLQWFTPQTDKAVNVAMVQGNIAQSMKWDPKALVSTLQTYLDETRPYMGKAPIVIWPESAIPD
YEANQNGFLTMMDDLMRAKNSSLITGIVDVRATPQGQQIYNSAIVLGEPTPYRYPANDRYNKHHLVPF
GEFVPLETLLRPLAPFFDLPMSSFSRGDYVQPQLSVRGYNLTAAICYEIVLGQQVRDNFRPDTNFLLT
ISNDAWFGHSIGPWQHFQMARMRALELGRPLLRSTNNGVTAAVDANGDVIAEIPQFTRQVLEVKVTPT
TGVTPYARFGAAPLWVITLLLGGWALMLGLRRK
>CORE_REP|Org46_Gene2457#
MSEPRIIAKAMKDGKPEQDLVILPALANRHGLITGATGTGKTVTLQKMAEQFSRIGVPVFLADVKGDL

SGIGAEGVPSEKLQARLAAIGVSDWQPQACTIIPWDIYGEKGHPIRATISDLGPLLLGRLLDLNEVQS
GVLQLVFKIADDNALLLLDMKDLRTMLQFVGDNAKQFQTQYGNISAASIGAIQRGLLTLEEQGADQFF
GEPMLDINDLMKTDANGHGVINLLAADKLINQPKLYSVFLLWLLAELFEHLPEVGDPEQPKLVFFFDE
AHLLFNDAPAALLTKIEQVVRLIRSKGVGIYFVTQNPLDIPDSVLGQLGNRVQHALRAFTPRDQKAVK
TAAQTLRANPAFDAETAITELGVGEALVSFLDEKGRPNVVERAMVIAPESKMGMLGAEGLNSAINKSP
LYGRYEDMIDRESAYEKLSSGGFATVGTPQQGQQPQAQQQQQQAGGGLMDGLNELLFGSTGPRGGKRD
GIVQTAAKSMARDLGRQILRGVLGSITGGRKK
>CORE_REP|Org15_Gene4380#
MPSPLAAIMISMKYRDLRDFLSLLEKRGELKRISQPIDPYLEMTEIADRTLRAGGPALLFENPKGYDM
PVLCNLFGTANRVAMGMGQEDISALREVGKLLAFLKEPEPPKGFRDLFDKIPKFKQVLNMPTKVLGSA
PCQEQVWQGDDVDLGRIPVMHCWPEDAAPLITWGLTVTRGPHKERQNLGIYRQQVLGKNKVIMRWLSH
RGGALDYQEWCQAHPGERFPVAVALGADPATILGAVTPVPDTLSEYAFAGLLRGNKTEVVKCLSNDLE
VPASAEIVLEGYIEPGEMAPEGPYGDHTGYYNEIDQFPVFTVTHITQRRNAIYHSTYTGRPPDEPAIL
GVALNEVFVPILQKQFPEIVDFYLPPEGCSYRLAVVTMKKQYAGHAKRVMMGVWSFLRQFMYTKFVIV
CDHDVNARDWNDVIWAITTRMDPARDTVLVENTPIDYLDFASPVSGLGSKMGLDATNKWPGETDREWG
RPIQMDEKVRARVDEIWDELAIFSDREPTL
>CORE_REP|Org26_Gene3602#
MNRQQRLMTFANPADLPGYQDVSKANLQARKPRDLKLCDSLQEAVRRSGLQDGMTISFHHAFRGGDLT
LNQVMETLAAMGFRNLTLASSSLTDCHAPLVEHIRHGVVSRIYTSGLRGPLADAVSRGLLAEPVQIHS
HGGRVNLIESGELRIDVAFLGVPACDEFGNANGYSGEACCGSLGYARVDAEAAGTVVLLTEQLVPYPH
HPASLAQDRVDLIVQLERVGDADKIGADATRMTSNPRELLIARRAAEVIAGSGYFTEGFSLQTGTGGA
SLAVTRFLEDKMRARGIRAAFALGGITSTMVDLHEKGLIGKLLDVQSFDRAAATSLARNPRHIEISAN
QYANFSSKGASVDRLDVVVLSALEIDTGFNVNVLTGSDGVLRGASGGHCDTAAAARLAIIVAPLVRGR
IPTLVEQVTTCVTPGSSIDILVTDHGIAVNPARPELAQRLREAGLEVVSIDWLRARALQLTGEPQPIA
FTDKVVAVVRYRDGSVIDVVHQVAE
>CORE_REP|Org33_Gene2051#
MTSAHSNTSAYDHLRALFSRLSRFGHLSAIAGWDMQTMMPPGGSKARSEALAELSVLQHQILTAQSTG
ELLDRAQQETLDELDRANLREMRRQYEDAVLLPASLVEAKSLAGARCEHAWRAQRPANDWDGFVENLR
EVVKLSREEAQIRAQAAGTSRYDALLNLYEPGMRSSDLDRIFGDLKTWLPDLLQRVVAKQANEPCQTP
QGPFNVDTQRQLSLSVMKLLGFNFDNGRVDVSAHPFCGGVPEDVRITTRYNEKEFLTALLGIVHETGH
ARYEQNLPRDLLGQPVALARSTAIHESQSLLFEMQLARGNEFLKILRPLVTAQFGEQPALEEANFIRL
NQRVKPGLIRVDADEVSYPAHVILRYEIEKALIEGDIEVEDIPALWNEKMHAYLGLDTIGNYRNGCMQ
DIHWTDGAFGYFPTYTLGAMYAAQLFHSVRQALPSLGEDIAAGNLQPLFHWLQHNIWRHGSRFPTETL
IANATGEALNPRYFRQHLENRYL
>CORE_REP|Org46_Gene1367#
MLSSSTLYAAIDLGSNSFHMLVVREVAGSIQTLARIKRKVRLAAGLDQNNHLSHEAMQRGWQCLRLFS
ERLQDIPREQIRVVATATLRLASNADEFLQAAEQILGCPVQVISGEEEARLIYHGVAHTTGGPDRRLV
VDIGGGSTELVTGTGAQASQLYSLSMGCVTWLERYFSDRNLGQENFERAEQAAREMVRPIAPQLRQQG
WQICVGASGTVQALQEIMVAQGMDERITLPKLRQLKQRAIQCGKLEELEIEGLTLERALVFPSGLSIL
LAIFQELGIESMMLAGGALREGLVYGMLHLPVEQDIRIRTIRNLQRRYLIDTEQAERVSQLAANFSQQ
VANEWQLDARCRELLHSASLIHELGLSVDFKQAPQHAAYLIRHLDLPGFTPAQKKLLATLLQNQSNPL
DLSLLNQQNALPPRTAQRLCRILRLAIIFASRRRDDTLPAVRLRANQDDELTVILPPGWLEQHPLRAE
ALEQESHWQSYVHWTLRLEEQR
>CORE_REP|Org8_Gene3394#
MADRNLRDLLAPWVPTAPGRALREMTLDSRVAAAGDLFVAVVGHQTDGRRYIPQAIAQGVAAVIAEAD
GQAEDGAIVEMHGVPVIYLSQLNQRLSALAGRFYHQPGERLRLVGVTGTNGKTTTTQLLAQWSQLLGE
TSAVMGTVGNGLLGQVCPTENTTGSAVDVQHVLNELAEQGATFAAMEVSSHGLVQHRVAALPFAAAVF
TNLSRDHLDYHGDMANYEAAKWSLFAAHNVGQAIINADDEVGQRWLSKLPDAVAVTMQDNLQPGCHGR
WLKTTAVDYHDNGATVRFSSSWGDGEIESRLMGAFNVSNLLLALATLLSLGYPLEALVETGSRLQPVC
GRMEVFNAPGKPTVVVDYAHTPDALEKALEAARLHCQGQLWCVFGCGGDRDKGKRPLMGGIAEQFADR
VVITDDNPRTEEPRAIINDILTGLLDAGQALVIHGRAEAVTSAIMQAQEQDVVLVAGKGHEDYQLVGN
RRLDYSDRTTVARLLGVLA
>CORE_REP|Org1_Gene3838#
MANYFNTLNLRQQLAQLGKCRFMARDEFADEAGYLKGKKVVIVGCGAQGLNQGLNMRDSGLDVAYALR

KEAIDEKRASWRKATENGFKVGTYEDLIPQADLVVNLTPDKQHSSVVRAVQPLMKDGAALGYSHGFNI
VEVGEQVRKDITVVMVAPKCPGTEVREEYKRGFGVPTLIAVHPENDPKGEGMAIAKAWAAATGGHRAG
VLESSFVAEVKSDLMGEQTILCGMLQAGSLLCFDKLVAEGTDPAYAEKLIQFGWETITEALKQGGITL
MMDRLSNPAKLRAYALSEQLKTIMAPLFQKHMDDIISGAFSSGMMADWAEDDVKLLTWREETGKTAFE
NAPQFEGKIGEQEYFDHGVLMVAMVKAGVELAFETMVDAGIIEESAYYESLHELPLIANTIARKRLYE
MNVVISDTAEYGNYLFANAAVPLLKDFMTTLQAGDLGKAVAGTAVDNAQLRDVNEAVRSHPIETVGRK
LRGYMTDMKRIAVAG
>CORE_REP|Org9_Gene4419#
MNTQQLAKLRTIVPEMRRVRHIHFVGIGGAGMGGIAEVLANEGYQISGSDLAPNPVTQQLSALGATIY
FNHRPENVLDASVVVVSTAISADNPEIVAAREARIPVIRRAEMLAELMRFRHGIAIAGTHGKTTTTAM
VSNIYAEARLDPTFVNGGLVKAAGTHARLGSSRYLIAEADESDASFLHLQPMVAVVTNIEADHMDTYQ
GDFENLKQTFINFLHNLPFYGRAVMCIDDPVVRELLPRVGRHITTYGFSEDADVRIEDYRQIGPQGHF
TLSRQDKPLLTVTLNAPGRHNALNAAAAVAVATEEGIDDEDILRALAGFQGTGRRFDFLGEFPLEPVN
GKAGSAMLVDDYGHHPTEVDATLKAARAGWPDKRLVMIFQPHRYTRTRDLYDDFANVLSQVDVLLMLD
VYAAGEAPIPGADSRSLCRTIRSRGKLDPILVSDADTVPETLAQLLQDEDLVLVQGAGNVGKIARKLA
ELKLQPPKKEEEHHG
>CORE_REP|Org6_Gene902#
MTAELLVNITPSETRVAYIDGGILQEIHIERESKRGIVGNIYKGRVSRVLPGMQAAFVDIGLDKAAFL
HASDIMPHTECVAGDERKNFHVRDIAELVRQGQDLMVQVVKDPLGTKGARLTTDITLPSRYLVFMPGA
AHVGVSQRIDSEAERERLKAIVAPHCDELGGFIIRTAAEGIGEEELAQDAAFLKRLWTKVMERKKRNQ
TKYKLYGELALAQRILRDFAGAALDRIRVDSRLTHDLLVEFTGEYIPDITNKLELYTGSQPIFDLYDV
ENEIQRALERKVELKSGGYLIIDQTEAMTTVDINTGAFVGHRNLDETIFNTNIEATQAIARQLRLRNL
GGIIIIDFIDMNNEEHRRRVLHSLEQALSKDRVKTTINGFSQLGLVEMTRKRTRESIEHVLCHDCPTC
NGRGTVKTVETVCYEILREIVRVHHAYDSDRFLVYASAAVGEALKSEESHALAEVEIFVGKQVKVQIE
PLYSQEQFDVVMM
>CORE_REP|Org3_Gene2772#
MLKTAGWLLPAILALAGCSSSGQRHEQTLSQALTALGQDKALIGASNGVMVRDAESGTVLYQAHAQQR
LAPASNMKMFTSLAAFGVLGADYRFETRLLTTGEQRGDTLRGDLYLQGSGDPTLHPDDLDTFAATLAQ
RGIRHIHGRLILDASAFDQTPFGAGWSWDDEPFAFAAPISALNYAFTPGGDINVVRVDVQPGARAGAP
GRVSFYPANDAVTLVNRTTTTGGDTALTFDRQPGSNRIVVSGTVAAQAEASSRLITVDQPARVVGALL
QNALRAHGITLRGNAEEGVTPAGARLLAEKTSPPLSRLAVTFLKVSNNGYGEVLTKAMGRKTQGKGDW
AAGLQAIGRFVQSQGIEAGAYRQVDGSGLSRMNQITPQQLTTLLLAARKQPWFADWYNALPIAGQPGL
LVGGTLRSRMVKSAAAGRAHAKSGSMTGVSSLSGYVDSATGRPLAFAIISNNYLVPGAEVKALEDRLV
ETLAACDATVVCR
>CORE_REP|Org18_Gene2077#
MSYAPPRSGLSADQALDQLEALYDAAVDALRQAVSDFISHGTLPDAQARAAGLFVYPELRVSWDGQQS
GPNKTRAFGRFTHPGSYSTTVTRPQLFRHYLAEQLAMLEHDYAAHIEVAPSQQEIPFPYVIDGSSLAL
DRSMSAGIAQHFPTTELAQIGDETADGLYHATDSHFPLSHFDALRADFSLARLRHYTGTPVEHFQPFV
LFTNYTRYVDEFVRWACAQIADPASPYIALSSAGGTYITPETRAPEQAVSDLAWKNHQMPAYHLISRT
GQGITLINIGVGPSNAKTICDHLAVLRPSAWLMIGHCGGLRESQKIGDYVLAHAYLRDDHVLDAVLPP
DIPIPSIAEVQRALYDATKMVSGMPGEEVKQRLRTGTVVTTDDRNWELRYSASARRFNLSRAVAVDME
SATIAAQGYRFRVPYGTLLCVSDKPLHGEIKLPGQANRFYEGAISEHLQIGICAIDLLRAEGDRLHSR
KLRTFNEPPFR
>CORE_REP|Org18_Gene1162#
MSQTQEKIWKAIAPLAVLAILLLIPVPDGMPPQAWHYFAIFVAMIVGMILEPIPATAISFIAVTVSVL
SANWVLFGAQELAEPGFKAGKEALKWGLAGFSSTTVWLVFGAFIFALGYEATGLGRRIALFLVKFMGK
RTLTLGYAVVIIDILLAPFTPSNTARTGGTVFPVVKNLPPLFDSFPNDPSSRRIGGYLMWMMVVGTSI
SSSMFVTGAAPNVLGIEFVGKIAGVHISWMQWFLAFLPVGLLLLIVAPLISYYLYKPGVTHSSEVAAW
ADTALGEMGKLTRKEYTLIGLVLLSLCLWVFGGKVLDATAVCLLAVSLMLALHVVSWKEITKYSSAWN
TLVNLATLVVMANGLTRSGFIDWFAQTMSTHLDGFSPNMTVVALVLVFYFAHYLFASLSAHTATMLPV
ILAVGKGLPGVPMEQLSMLLVLSIGIMGVLTPYATGPGVIIYGCGYVKSKDYWRLGGILGVVYIAALL
LIGWPIMSLWY
>CORE_REP|Org38_Gene3792#
MSELSQLSPQPLWDIFAKICSIPHPSYHEEALAQHILTWAKEKNLHAERDQVGNILLRKPATKGMENR

KPVALQAHLDMVPQKNNDTVHDFAKDPIQPYIAGEWVKARGTTLGADNGIGMASALAVLADDSVEHGP
LEVLLTMTEEAGMDGAFGLQPNWLQADILINTDSEEEGEIYMGCAGGIDFITTLPLQREAVPAGYQTL
KLTLKGLKGGHSGAEIHVGLGNANKLLARFLFAHAEALNLRVLDLNGGTLRNAIPREASAVVAVPAEK
ADALKALSQEFLAVLQNELSAKEKNITVLLEPTTSASQALSADSQQRFLALLNGTPNGVIRMSDAVKG
VVETSLNVGVVTTSENEAEIICLIRSLIDSGKDYVVEMLAALGQLAGANVAPKGGYPGWQPDADSPVM
HLVRELYQELFNKTPNIMVIHAGLECGLFKKPYPNMDMVSIGPTITGPHSPDEQVHIESVGLYWKLLT
SLLKAIPERA
>CORE_REP|Org12_Gene2435#
MKFIIKLFPEITIKSQSVRLRFIKILSTNIRNVLKQYDETLAVVRHWDHIEVRAKDENQRPIIADALT
RIPGIHHILEVEDRAYTDIHHIFEQTLEAYRAQLEGKTFCVRVKRRGKQAFNSQDVERYVGGGLNQHI
ESARVNLSRPQVTVNLEIEDDKLMLVKRRLEGIGGYPVGTQEDVLSLISGGFDSGVSSYMLMRRGCRV
HFCFFNLGGAAHEIGVKQVAHYLWNRFASSHKVRFVAIDFEPVVGEILEKVDDGQMGVVLKRMMVRAA
SQVAERYGVQALVTGEALGQVSSQTLTNLRLIDNASDTLILRPLISHDKEHIIKLAREIGTEDFAKTM
PEYCGVISKSPTVKAVKAKIEEEESHFDFSILDRVVSEAKNVDIRSIAEQTQEQVTEVETVAAFGADE
VILDIRSNDEQEEKPLQLEQVEVKALPFYKLSTQFGNLDQSKTYLLYCERGVMSRLQALYLLEQGFNN
VKVYRP
>CORE_REP|Org9_Gene2225#
MSLTFVSEQLLATNKLSHQDLYQVLGQLAERRIDYADLYFQSSYHEAWVIEDGIIKDGSYNIDQGVGV
RAVSGEKTGFAYADQITLNALQQSAQAARSIVREQGDGRAHTLGEIGYRALYPLLDPLQSLPREEKIA
LLHRVDKVARAADARVQEVNASITGVYEQVLVAATDGTLAADVRPLVRLSVSVLVEQDGKRERGSSGG
GGRFGYDYFLESVDGDVRADAYAKEAVRMALVNLGAVAAPAGNMPVVLGAGWPGVLLHEAVGHGLEGD
FNRRGTSVFSGHMGELVASELCTVVDDGTLQGRRGSLAIDDEGVPGQYNVLIENGVLKGYMQDKLNAR
LMGVAPTGNGRRESYAHLPMPRMTNTYMLAGQSTPEEIIASVEYGLYAPNFGGGQVDITSGKFVFSTT
EAYLIEKGRITKPVKGATLIGSGIEAMQQISMVGNDLALDKGVGVCGKEGQSVPVGVGQPTLKLDTLT
VGGTA
>CORE_REP|Org38_Gene899#
MRFSRIVSALACAFVLNANAAPVEEYTQYLPDGANLALVVQKIGAGSPTIDYHSQQMALPASTQKVLT
ALAALLQLGPDYRFTTTLESQGDIRDGVLRGNLIARFSGDPTFKRQSLRNMVAVLKKQGVRQISGDVL
VDTSVFASHDKAPGWPWNDLTQCFSAPPAAAIVDRNCFSVSLYSAPNPGDMAFIRVASYYPVNMFSQV
RTLARGSADAQYCELDVVPGELNRFTLTGCLTQRSEPLPLAFAIQDGASYAGAILKDELTQAGIQIDG
HLKRQTRPGITGTVIAQTQSAPLHDLLKIMLKKSDNMIADTVFRTIGHERFGVPGTWRAGSDAVRQVL
RQKAGIDLGNTIIADGSGLSRHNLLAPATMMQALQYIAQHDNELNFISMLPLSGYDGTLRYRGGLHEA
GVDGKVSAKTGALQGVYNLAGFITTASGQRMAFVQYLSGYAVPPEDQKQRRAPLVRFESRLYRDIYQN
N
>CORE_REP|Org18_Gene2559#
MSAATHNVKKVAEYRQRILTLLLNNKELVDGILGRPGDEHALSQSELLNQTAEITGLLDDMHAADLAD
LLEALPQDERMALWRLVGNSKRGQTLVEVAEPVWDSLIEEMSDKDLLKAIKTLDVDEQAYLAQYLPRN
LMGRLLTSLEPEQRAQVREMSQYAKDSVGWMMDFELVTVRPDVTLGAVHRFLRMRKTIPDATDKLFVT
DRKNTLLGELPLTAVLLNDPEIPVREVMDSDPATFQPEDKADEAAGAFERYDLISAPVVDAKGKLMGR
LTIEEIVDAVNEESDTNLRRMGGLSPEEDVFAPVSKAVKTRWAWLAINLCTAFIASRVIGLFEHTISQ
LVALAALMPIVAGIGGNTGNQTITMIVRALALHQIEVGNISRLMLRELGVAIINGVVWGGIMGVITWL
LYGDAAMGGVMTLAMLLNLLLAALMGVVIPMTMLKVGRDPAVGSSVLITALTDTGGFFIFLGLATLFL
L
>CORE_REP|Org21_Gene1518#
MDYLPIFADLKQRPVLVVGGGDVAARKVDLLQRAGAEIRIVAQSLSPELELQRQRGQLLWLGKTFDPP
QLDDVFLAIAATDDNALNAAVFAEADKRRVLANVVDDQPRCSFIFPSIIDRSPLVVAVSSSGQAPVLA
RLLREKLEALLPASLGQMAQVAGRWRGQVKQRLASIGERRRFWEKTFGGRFATLVANGQTAQAERQLE
QDLQSFAAGDEGAQGEIALVGAGPGDVGLLTLRGLQVMQQADVVLYDHLVSGEILDLVRRDAERICVG
KRAGAHSVIQEETNRLLVELAQQGKRVVRLKGGDPFIFGRGGEELQVAAAAGIPFQVVPGVTAAAGAT
AYAGIPLTHRDHAQSVTFITGHCRPDGDGLDWADLARARQTLAIYMGTMKAADISQRLIAHGRAADTP
VAVISRGTRADQQVQIGTLDQLEHLAQRAPLPALLVIGEVVELHHQIAWFGHQSQTEGAARPAVVNLA
>CORE_REP|Org25_Gene4261#
MKDNDRQNTFYIHDYETFGKSPSLDRPAQFAGVRTDMDFNIIEEPLVIYCAPADDYLPEPEAVMITGI
TPQVARAKGVNEAEFTRQIHQAFSVAGTCILGYNNIRFDDEVSRNIFYRNFYDPYAYSWQNGNSRWDL

LDVMRACYALRPDGIVWPENEDGFPSFRLEHLTRANGVEHTQAHDAMSDVYATIAMAKLVKQAQPRLF
DFLLQHRNKHKLNALIDVADMTPLVHVSGMFGAARGNTSWVSPLAWHPDNKNAVIMCDLAGDMTPLLT
LSAEQLRERLYTRRDDLAPDQAPVPIKLVHINKCPVLAPAKTLLTENAERLGIDRQACLQNLQLLKQH
PEVREKVVALFAEAEPFKGSEDVDARLYDGFFSDADKAAMRIIQQTKPQNLPALDLAFSDGRMKELLF
RFRARNYPNTLDDAEQRRWLQHRQEALSAERVQSYLLQLESLYNLHEGDKEKTALLKALFDYGKELVG
>CORE_REP|Org8_Gene2092#
MIARLLSNYIDTHMKAKAIFLASVLLVGCQSSRQDAPAPEQHAQSLSSAGQDGEAGEYTANGRASSAR
WLDNNSPAAQQDLWNFISDELKMEVPENSRIRDQKRKYLKSKSYLHDVTLRAEPYMYWIVGQIKKRNM
PMELVLLPIVESAFDPHATSSANAAGLWQIVPQTGRNYGLKNNQWYDGRRDVVASTTAALNMMQRLNR
MFNGDWLLTVAAYNSGEGRVMQAVKANKRQGKPTNFWALSLPRETSIYVPKMLALSDIIKNSKKYGVK
LPKTDETRALARIDVGQQIQLTQAAEMAGLSVTKMKAYNPGYKKGVTAPNGPHYIMVPKGHAEQLKDS
LADGQIAVTQPTTQLAKNSGLTGGSSYKVRSGDTLSGIAKRLNVKTSDLQSWNNLRAKSAIKVGQTLQ
VASNTGSNSSITYQVRKGDSLASIARRHGVDINDVMRWNSTLAKGNLQPGLKLTLFVGNKLTPDT
>CORE_REP|Org34_Gene4429#
MRIHILGICGTFMGGLAMLARSLGHDVTGSDANVYPPMSTLLENQGIDLIQGYDPAQLDPAPDLVIIG
NAMTRGNPCVEAVLERGIPYVSGPQWLHDAVLRDRWVLAVAGTHGKTTTAGMATWILEACGYQPGFVI
GGVPGNFDVSARLGGSPFFVIEADEYDCAFFDKRSKFVHYSPRTLIMNNSPRTLIMNNLEFDHADIFD
DLKAIQKQFHHLVRLVPGKGKIILPDNDSHLKQVMAMGCWSEQELVGEEGTWRAQKLTPDASHYAVFL
DGEQVGEVNWALVGEHNMHNGLMAIAATRHVGVQPADACRALGDFINARRRLELRGEANGVTVYDDFA
HHPTAILATLAALRGKVGGTARILAVLEPRSNTMKMGISKNDLAPSLGRADEVFLFQPHHIPWQVAEV
ADACVQPAHWSADLDTLVDMVVKTAQPGDHILVMSNGGFGGIHDRLLDALAKKAGTEGDLLIVQ
>CORE_REP|Org40_Gene1896#
MSLSRRQFLQASGLALCAGAVPLRAEASGTQTPLPIPPLLESRRGQPLFLTLQRAHWAFMDNRKAAVW
GINGMYLGPTVRVYSGDDVKLIYSNRLQEPVAMTISGLQVPGTLMGGAPRMMSPNVDWSPVLPIRQAA
ATCWYHANTPNRMAPHVYNGLAGLWLVEDAVSKALPLPNHYGVDDFPLIIQDKRFDNFGTPQYDAPSQ
GGFVGDTLLVNGVQNPYVDVSRGWVRLRLLNASNARRYTLQLSDGRPFNVIASDQGFLPAPVAVQQLS
LAPGERREVLIDMSKGEEVTITAGEAAGIMDRLRGLFEPSSILVSTQVLTLRPTGLLPLVTDNLPMRL
LADQLLDGSASRTRDFRLGDGGAGINGAIWDMNRIDVQAQQGTWERWNIHADTPQSFHIQGVQFLIKR
VNGAQPMAEDRGWKDTVWVDGDVELLVYFNQPTSEHFPFLYYSQTLEMADRGTAGQLMVQPTM
>CORE_REP|Org18_Gene4521#
MEELSLIKQALRAVMNGQPRALRDPHRPAWHLAPSVGLLNDPNGFIQHNGVYHLFYQWNPLGCDHRNK
CWGHWQSTDLLRWEHQPIALAPGACYDSHGCYSGSAVVAEDKITLIYTGNVKFPDGSRTAYQCLAQES
DRSEYRKLGPVLPLPEGYSGHVRDPKVWRHQDAWYMVLGARDLQDRGKVLLLRSCDLRDWQALGEIAG
SGLNDLGEFGYMWECPDLFSLDGDDVLICCPQGLAPQPERYLNRYQAGYLLGKLDYRQAAFSHGEFRE
LDAGFEFYAPQTTQAEDGRRLLFGWMGVPEQDEEAHPTRRYGWIHTMTCPRELSLRHGRLYQRPAREL
QQLRGERAGWQGHADDAPAYALGAAELQLTPQGAFSAAFGDAMTLSWDGERLQLTRASLADGRPEHRY
WHGPVTHLQLLFDRSSVEIFINHGEAVMSARYFPAAEPQLRLNGSAPLALEYWPLTPCMLE
>CORE_REP|Org48_Gene3914#
MENPQQPGRRAFLSQTGKLTTACAVIGLTGGMAQAASPGGEPCAPTPMTLTDRHYCLSEVRLEDGFEY
DGETVIGTRTALYTLEIKDGKIAAIHAANAALPAGVPRYKAQGRLLLPAFRDMHIHLDKTFYSGPWQA
PRPRQGKTIMDMIALEQTLIPTLLPTSQQRAENLIALLQSKGSTVARSHCNIDPVSGLKSLEHLQRAL
ENHRADFSCEIVAFPQHGLLHSKVDALMREAMQMGVQYVGGLDPTNVDGAMEKSLDAMFQIALDTGKG
VDIHLHETSPAGVAAINYMIATVEKNPALRGKVTISHAFALTTLTPGELAETATRLAAQQITIASTVP
IGGLMMPLPQLSEKGVFVMTGTDSVIDHWSPFGTGDILEKANLYAQLYRGSDEYHLSRAMAISTGGVL
PLDDKGQRAWPKAGDAAEFVLVNASCSAEAVARLPARSATFHQGRLVAGQVSKA
>CORE_REP|Org32_Gene89#
MKIIILGAGQVGGTLAENLVGENNDITVVDTDSGRLRQLQDKFDLRVVQGHGSHPRVLREAGAEDADM
LVAVTNSDETNMIACQIAYSLFNTPNRIARIRAPEYIRESEKLFLPEAVPIDHLISPEQLVIDYIYKL
IEYPGALQVVNFAEGKVSIAAVKAYYGGPLVGNALSSMREHMPHIDTRVAAIFRQDRPIRPQGSTIIE
AGDEVFFVAASQHIRAVMSELQRLEKPYKRIMIVGGGNVGAGLAAKLEKDYNVKLIERNQQRAAELAE
QLHDTIVFYGDASDQELLAEEHVEQVDVFIAITNDDEANIMSAMLAKRMGAKKVMVLIQRRAYVDLVQ
GSVIDIAISPQQATISALLGHVRKADIVSVSSLRRGVAEAIEAIAHGDESTSKVVGRIVEDIKLPPGT
TIGAIVRGDDVIIANGNSKIEQGDHVIMFITDKKFVPDVERLFQPSPFFL
>CORE_REP|Org22_Gene2005#

MSNSAMSVVILAAGKGTRMYSDLPKVLHPLAGKPMVQHVIDAAMKLGAQHVHLVYGHGGDLLKSTLTD
GALNWVLQAEQLGTGHAMQQAAPHFADDEDVLMLYGDVPLISVDTLQRLLAAKPQGGIGLLTVKLDDP
SGYGRIVREQGNVVGIVEHKDASEAQRQINEINTGILVANGRDLKRWLGMLNNDNAQGEFYITDIIAL
AHADGKKIETVHPSRLSEVEGVNNRLQLATLERVFQSEQSEKLLLAGVMLLDPARFDLRGELVHGRDI
TIDANVIIEGHVKLGDRVKIGAGCVLKNCVIGDDCEISPYSVLEDSVLEADCTVGPFARLRPGAELAA
GAHVGNFVEMKKARLGKGSKAGHLSYLGDAEIGDDVNIGAGTITCNYDGANKHKTVIGDGVFVGSDTQ
LVAPVSVGKGSTIAAGTTVTRDIAENELVLSRVKQVHIQGWQRPVKKKS
>CORE_REP|Org6_Gene3716#
MVSVFDIFKIGIGPSSSHTVGPMKAGKQFVDDLIAHQQLQDTTRVVVDVYGSLSLTGKGHHTDIAIIM
GLAGNLPHDVDIDSIPGFIRDVEQRGRLPLANGHHEVDFPLHGGMNFHSDNLPLHENGMRIRAFADER
LLHSKTYYSIGGGFIVDEEHFGQSAEGATPVPYPFKSAHDLQQHCKETGLSLSGLVMQNELALRSKAD
IDAHFADVWQVMSAGIERGINTEGLLPGPMKVPRRAAALRRILVTGDKNNIDPMNVVDWINMFALAVN
EENAAGGRVVTAPTNGACGIIPAVLAYYDKFIRPVNANSYTRYFLASGVIGALYKMNASISGAEVGCQ
GEVGVACSMAAAGLTELLGGSPAQVCIAAEIAMEHHLGLTCDPLAGQVQVPCIERNAISAVKAVNAAR
MAMRRTSEPRVCLDKVIETMYETGKDMNAKYRETSQGGLAIKVVACN
>CORE_REP|Org48_Gene2921#
MTLAQFGGLFVVYLVSLTFILTLTYQEFRRVRFNFNVFFSLLYLLTFYFGFPLTCLLVFQFDVEVVPV
EFLLYALLSATAFYAIYYVTYKTRLRKRRSQPRAALFTMNRVETHMTWVLLALVAIGTVGIFFMQNGF
LLFKLNSYSQIFSSDVSGVALKRFFYFFIPAMLVVYFLRQDLRAWFLFLVATVAFGILTYVIVGGTRA
NIIIAFALFLFIGIVRGWITLWMLAAAGIFGVVGMFWLALKRYSLDVSGAEAFYTFLYLTRDTFSPWE
NLALLLQNYDKIDFQGLAPILRDFYVFIPTWLWPGRPDVVLNSANYFTWEVLNNHSGLAISPTLIGSL
VVMGGALFIPLGAILVGMIIKWFDWLYELGKTEPNRYKAAILQGFCFGAVFNIIVLAREGVDSFVSRV
VFFCIIFGACLVLAKLLYWLFDTAGLIKARVTRARALASPPRANGLL
>CORE_REP|Org32_Gene2304#
MMQETVLHKIVRDKAQWIAARQQQQPLAGFQNDIVPSERSFYHALQGTRTAFILECKKASPSKGLIRE
SFDPVEIATVYKDFASAISVLTDEKYFQGSFDFLPLVSKTVSQPVLCKDFIIDPYQIYLARYYQADAI
LLMLSVLTDEQYRQLAAVAHSLNMGVLTEVISEEELQRAIALEARVVGINNRDLRDLSIDLDRTRQLA
PRVPHGVTVISESGINNYGQIRELSHYANGFLIGSALMSEPDLRAAVRRVILGDNKVCGLTRPQDAAA
AYQAGAIYGGLIFVGRSPRYVDITRAREVISGAPLKYVGVFCDAQVETVALTVERLGLHAVQLHGAED
QAYISALRARLPADCRIWKALSVKDRVPARDLQYVDRYLLDNGAGGTGQRFDWSVLQDEDLTNVMLAG
GLRADNCVEAAKLGCAGLDFNSGVESEPGIKDPARLASVFQTLRAY
>CORE_REP|Org3_Gene230#
MVSVLWNLVAFLIALGILITVHEFGHFWVARRCGVRVERFSIGFGRALWRRTDRQGTEYVIALIPLGG
YVKMLDERVESVAPEMRHQAFNNKTVWQRAAIISAGPIANFLFAILAYWLVFIIGVPSFRPVIGEIAP
QSIAAKAEISSGMELKSVDGIETPDWESVRLALVAKIGDAQTEVGVAPFGSSQVVTKTLDLRQWNFEP
DKQDPVVALGIMPRGPQIESVLAEVQPNSAAQKAGLQAGDRIVKVDGQPLGRWQTLVKRIHDGPGQPL
ALEIERNGAPLSLTLIPDTKPVGKDKSVGFAGIIPKVLPLPDEYKTIRQYGPFPALYQAGDKTWQLMR
LTVNMLGKLITGDVKLNNLSGPISIAQGAGASAGVGFVYYLMFLALISVNLGIINLFPLPVLDGGHLL
FLAIEKLKGGPVSERVQDYSYRIGSIVLVLLMGLALFNDFSRL
>CORE_REP|Org43_Gene4143#
MHNTKTRLALLVGCMALSANLWADAQPVQATLAGHALLPVKSAVSTPKDAPSDLQQSGKYTSGKRVTE
LGSVAGKSADRLTGFGLPIDGQPLQGHSGIKHMPDGTYWVLTDNGFGSKANSPDAMLYLNHYKIDFKD
GTVAPLKTVFLHDPDKKVPFHIINESTEKRYLTGSDFDPESFQFADDALWIGEEFGPYLIKADLNGKV
LAVFDTQVDGKVVKSPDNPTLTLPGAPDGKQNFQVARSKGFEGMAASPDGSKLYPLLEGALWDGEQFE
NVGGKRYLRVLEFDVKQQAWTGRSWQYVLEDNQNAIGDFNMIDATHGLVIERDNGEGTPDKACAAGAP
TDNCFSQVAKFKRVYKIAFSDANVGKPVEKLGYIDLMNIQDPNKLARKPLNDGVLTFPFFTIENVDVV
DANHIIVGNDNNFPFSSSRQPNMADDNEFILLDVKDFLK
>CORE_REP|Org39_Gene3088#
MSQKNLLTKSALAAAVAIISSNVSAAGFQLNEFSAAGLGRSYSGEGAMADTAASASRNPALLMMYTRP
ELSIGAVFIDPDVDITGKSPSGASLDAKNIAPTAWVPNLHYVHPINDQFAVGGSVTSNYGLATEFNDG
YTAGAYGGKTDLETLNLNLSGAYRLDQHFSFGLGFDAVYARAKLERYAGELPKLLAGQGLQTGQLTPQ
QAGMIAQIPGDTQISHLKGDKWGFGWNAGLLYEVDENNRYGFTYRSEVKIKFDGDYKSSLPAQYNPIT
QALGVGLPWGTSGSTIPGSLDLNLPEMWELSGYNKVAPQWAIHYSLAYTSWSQFQELRATGSNGQTLF
QKHEGFRDAYRIALGTTYFYDDNWTFRGGVAFDDSPVPAQNRSISIPDQDRFWVSAGTSYAFNKDASV

DVGVSYMHGQKVTINEGPYTFNSVGKAWLYGANFNYRF
>CORE_REP|Org47_Gene2010#
MKVVTQVAEQRKALEQAVSQALELARAGSDAAEVAVTKSTGISVSTRFGEVENVEFNSDGALGITVYH
RQRKGSASSTDLSPDAIARTVQAALDIARYTSEDPCAGPAEKDLLAFEAPDLDLFHPIELDAERGIEL
AARAEQASLAADKRITNTEGGSFNSHYGIKVFGNSHGMLQSYCSSRHSLSSCVIAEQDGDMERDYAYT
IGRAMGDLQSPEWVGQECARRTLARLAPRKLSTMKAPVLFASEVATGLFGHLVGAISGSSVYRKSTFL
LDSLGKQILPAWLTVEEHPHLLKGLASTPFDSEGVRTQRRDIVKDGVLQTWLMTSYSARKLGLHSTGH
AGGIHNWRIAGQGADFAGMLKQLGTGLVVTELMGQGVSGVTGDYSRGAAGFWVENGEIQYPVSEITIA
GNLKDMLRNIVSVGSDIETRSNIQCGSVLLPEMKIAGQ
>CORE_REP|Org31_Gene3088#
MREKAFFALSRVTRSANAGSRPLPERTAPRTFTAMCASVFCAGVLLLPLAGQAAEDNKSQLKDIQQSI
AEKEKAVKQQQQQRSSLQDQLRQQEKTIAQASRQLRDTQSTLTQLGKDIAGLNASIAKLQKQQSTQQN
LLAKQLDAAFRQGQHSAVQLILSGEESQRSERILAYFGYLNEARQKTIEELKQTRAELAKQKTTLVAK
QGQQKSLLGEQQTQQQKLEQARGARKKTLTALEASLEKDQQRLVELRQNEARMRDKIARAEREARARA
EREAREAAKVREQVRIKEQQAKKTGTTYKPSEADRSLMARTGGLGRPAGQLMWPVRGRTLHGFGEQQQ
GELRWKGMVIEAREGSEVKAVADGRVLLADWLQGYGLMVVVEHGKGDMSVYGYNQSALVNVGAQVRAG
QPIALVGTSGGQGTPSLYFEIRRQGQAVNPLPWLGR
>CORE_REP|Org29_Gene813#
MKERSTELVQGFRHSVPYINAHRGKTFVIMLGGEAIEHENFSNIVNDIGLLHSLGIRLVVVYGARPQI
DANLAQHNYEPIYHKHTRVTDAHTLELVKQAAGLLQLDITARLSMSLNNTPLQGAHINVVSGNFIIAQ
PLGVDDGIDYCHSGRIRRIDEDAIHRQLDSNAIVLIGPVAVSVTGESFNLTSEEVATQLAIKLKAEKM
IGFCSSQGVTDAEGNILSELFPNDAQKRLEELEEGGDYHSGTVRFLRGAVKGCRSGVRRSHLISYQED
GALVQELFSRDGIGTQIVMESAEQVRRATINDIGGILELIRPLEQQGILVRRSREQLEMEIDKFTIIE
RDNLTIACAALYPFLEEKIGEMACVAVHPDYRSSSRGEMLLQRVENQARQMGLKKLFVLTTRSIHWFQ
ERGFTPAEVDVLPMQKQALYNYQRRSKILLADL
>CORE_REP|Org17_Gene3316#
MTKSLSPKDIIALGFMTFALFVGAGNIIFPPLVGLQSGEHLWPAALGFMVTAVALPVIAVIALARVGG
SISLLTGPIGRTAGLLLATVCYLALGPLFATPRTANVSFALGIAPFTGDGALPQFIYSLLFFTLAMVV
SLYPGRLLDNVGHILAPLKILALAALGIAALVWPAGAPVSAVGSYQAAAFSTGFVQGYQTMDTLSALM
FGSIIVTAARSRGVSDSGLLLRYTLWASLIAGVGLTLVYICMFKLGAGSGSLVGSGAQDGAAILHAYV
QHTFGDLGSVFMAVLMFIACLVTAVGMTCACADFFSRYLPLSYRALVVILALFAMLVSNMGLANLIRV
SLPVLTAIYPPCIALVLLSFSQNRWRSARRVFAPVIATSLVFGLADGLKASSFSGLLPAWFDKLPLAE
QGLVWLQPTLLVLLLAAAYDRLRSAESVKAAS
>CORE_REP|Org34_Gene3838#
MADYQGRKVVIIGLGLTGLSCVDFFMARGVTPRVMDTRIAPPGLDKLPESVERHLGDLNQDWLLAADL
IVASPGVALATPALSAAADAGVEIVGDVELFCREAQAPIVAITGSNGKSTVTTLVGEMAKAAGWAVGV
GGNIGLPALSLLRQECQLYVLELSSFQLETTYSLRAAAATILNVTEDHMDRYPFGLQQYRGAKLRVYE
NAAVCVVNADDALTMPVRGADERCVSFGADVGDYHLNRQQGETWLRVRGEKVLNTREMKLTGRHNYTN
ALAALALADAVNIPRASSLKALTTFTGLAHRFQLAWEHNGVRWINDSKATNVGSTEAALNGLQVDGTL
HLLLGGDGKSADFSPLARYLQGDNVRLYCFGHDGAQLAQLRPEVATLTETMEQAMRTIAGRVQPGDMV
LLSPACASLDQFRNFEVRGDEFARLAQELGG
>CORE_REP|Org35_Gene4197#
MSTFNTPIDWATCSAERQAELLMRPAIAASDSITRTVNEILDNVKANGDRALRDYSAHFDKAEVSALR
VSAEQIAAAAARLGDDIKQAMAVAVANVETFHNAQRLPPVDVETQPGVRCQQVTRPIDSVGLYIPGGS
APLFSTVLMLATPARIAGCRRVVLCSPPPIADEILYAAQLCGVQEVFQVGGAQAIAALAFGTESVPRV
AKIFGPGNAFVTEAKRQVSQRLDGAAIDMPAGPSEVLVIADAGATPAFVASDLLSQAEHGPDSQVILL
TPDAAMAQAVADAVESQLAELPRADTARQALASSRLIVARDLPECVAISNRYGPEHLIIQTRNARDLV
ESITSAGSVFLGDWSPESAGDYASGTNHVLPTYGYTATCSSLGLADFQKRMTVQELTPQGFSNLAATI
ETLAAAEQLIAHKNAVTLRVAALKEQA
>CORE_REP|Org46_Gene2700#
MKLSSIAIALSAVLALAGCDNSAVISPEQQMGPDPTLPAAQDFLMPPMQVPKGVGWQQNQMPKVAEGL
KIDKVADGLLHPRQLLTLPNGDVLVVEANGPGTEAVSTPKQLIAGLVKGQSGKGGKGGNRITLLRPTA
DGSWEKHVFLEGLDSPFGVQLIGNTLYVANTGNIMQYAYQPGETRISDAGKELADLPDTINHHWTKAL
LASPDGKKLYVGVGSNSNITENGLAVEYRRAAVLEVDTASGASRIFASGLRNPTGLQWEPHSGKLWAI

VNERDEIGADLVPDYLTSVQDGGFYGWPYSYFGQHVDRRVQPARPDLVAKAIKPDYALSSHVAPLGLL
FYTANALPAEYRGGAFVSEHGSWDRSPLNGYRVSYVAFEQGKPVGKLKAVVTGFVSDDEKELYGAPVG
LAIDKTGALLIADDVGNTVWRVKSK
>CORE_REP|Org47_Gene2030#
MIKIEDYPLTRVPQDKRVSFLSVAIVHMGMLTALDQFMLGAVLGNSMTLIDAFTAIFVGSLIFGVVTY
GLGLAGMREGISGSLLARWCGFGRLGSVLIGVVVAVSLLGWFGIQNAIFAKSLDFALGNKLGFGLAAG
LSGTLLTILVAFGFKALRIAARIAVPMFIMLVAYISVTALSGHNLQEIIQLAPPGEPLTISAGITIVV
GGAIVASLMTPDLTRYSKNGKHVLGVTLFTIIAGEFVVNGLAILIAKTLGTADVVTIMSQAAGGAGLL
VVVFSTLRVNDLNLYSSSLGIVNAVEGITGKKLKYTYTTLVIGILGTTLSVLGILDRFVDFLTVLGVV
FPPIIGIMLVDYYLLRSHRKILDESRRTGQLPSETPTIGWAAIVASIVGGAVGLATEWGVPTINSLVA
ASLLYWVLKLAFSRAQKPLASQKSL
>CORE_REP|Org41_Gene448#
MDTTQTGTIASAASGSSSTWRKTDTMWMLGLYGTAIGAGVLFLPINAGIGGLIPLIIMAIIAFPMTFF
AHRGLCRFVLSGKNPGEDITAVVEEHFGITAGKLITLLYFFAIYPILLVYSVAITNTVDSFITHQLGM
TSPPRAILSLILIVGLMTIVRFGEQAIVKTMSILVFPFVAVLMLLAVYLIPNWTGAIFENVSLSGSGT
GMGHGLIMTLWLAIPVMVFSFNHSPIISAFAVAKREEYGADAEKKCSRILAYAHIMMVLTVMFFVFSC
VLSLTPENLAEAKAQNISILSYLANHFNNPMIEYIAPVIAFVAITKSFLGHYLGAREGFNGLVAKSMK
SRGKTVSTAKLNRMTAIFMLVTTWIVATLNPSILGMIETLGGPIIAMLLFLMPMYAIRKVPAMRKYSG
HISNVFVVVMGLIAISAIVFSLLG
>CORE_REP|Org19_Gene444#
MKQAFRVALGFLILWASVLHAEVRIEITQGVDSARPIGVVPFKWAGPGTPPEDIGKIVGADLRNSGKF
NPIDVARMPQQPTSASEVTPAAWTALGIDAVVVGQVQPGADGSYLISYQLVDTSGSPGTVLAQNQYKV
TKQWLRYSAHTASDEVFEKLTGIKGAFRTRIAYVVQTNGGKFPYELRVADYDGYNQFTVHRSPEPLMS
PAWSPDGSKLAYVTFESGRSALVVQTLANGAIRQIASFPRHNGAPAFSPDGSKLAFALSKSGSLNLYV
MNLGSGQITQLTDGRNNNTEPTWFPDGQSLAFTSDQGGRPQIYKISASGGAAQRLTWEGSQNQDSEVS
SDGKFLVMVSTNSGAQHIAKQDLGSGAVQVLTGTFLDETPSIAPNGTMVIYSSTQGMGSVLQLVSTDG
RFKARLPATDGQVKFPAWSPYL
>CORE_REP|Org13_Gene3894#
MAGLPTNSNSNALQQLYRLFEGRGGERSPHALAHWQQALRLGWPTRKHENWKYTPLESLLEQQFLDPQ
PAPVSAEQFEALALGIDACRLVFIDGRYSAALSDGDLGDYQFELTAYGTPQALPEPIQPEIFLHLTES
LAQETSLIRLPAGKAPARPLYLLHISSGRGATGEVNTVHHRHHLEIGRGAEAEVIEHYVSLGEAAHFT
GARLTANVADNAGLLHCKLAFESQPSYHFAHNDLVIGRDARVKSDSFLLGAGLTRHNTSAQLNGEGAN
LVINSLVLPVGKEICDTRTYLEHNKGYCESRQLHKTVVSDRGKAVFNGMIKVAKHAIKTDGQMTNHNL
LLGKVAEVDTKPQLEIYADDVKCSHGATVGRIDEEQLFYLQSRGIDKHAAQQMIIFAFAAELTEGIAN
DTIRERVLARIAQRLPGEAA
>CORE_REP|Org43_Gene4524#
MPTGATKLGGWLLMAAMLGVSGCAKPPEPEKPPQPAPPVTPMRGIWLATVMGLDWPPAASLKAETAPE
RIRLQQQALTDALDDMVKTGINTVYFQVKPDGTALWRSDILPWSEVLTGTVGQDPGYDPLAFMLKEAH
RRGIKVHAWLNPYRVSMNTRQQTIDALNQTLQSPPASVYVLHPDWIRTANDRFALDPGLPDVRNWITG
VVAEVVKNYDVDGIQFDDYFYYETPQSPLDDEKTYREYGKGFADKASWRRDNTLQLIKQVSATVRALK
PAVAFGVSPAGVWRNKADDPAGSATQAGAPSYDAAYADTRQWVKLGLLDYIAPQLYWPFDREIVRYDV
LANWWAEVVKDTPVRLYAGVALYKVGTPSASEPAWTVDGGVPELKRQLDLNESLPGMGGTILFRQRYL
TEPQTDKAVEYLRTRWKTGQ
>CORE_REP|Org24_Gene1642#
MSTISPDSGTLTAAQPAKWNKTDTVWMFGLYATAVGAGTLFLPINAGLNGPLVLLLMALFAFPLTYLP
HRALSRFVLSGSSRDGNIHDVVVEHFGVLAGKIIMMLYLMAFFPIVLVYSISITNALDSFLIHQFHVA
PLPRIWLSLAVVVVLNLVLLRGKDSIVAAMGMLVFPLLVFLMGISLYLMPSWNTANFVHGLAATRFDT
PGLWHSLWLAVPVMVFSFSHAPIISSFASTQKSLYGDKAERRCARIMRYSYVLICVTVLFFVFSCVLS
LSHEDMQQAKDQNITVLTTLANKFSNPLIAYLGPVMAMLAMAKSYLGTSLGVTEGATSLIDGVTRAVG
KPLSSRMTHRISAVSLFLLTWAATVWNPSALHIIETISGPLIAAILFILPMYAVRAVPAMRKYRAASN
VFVLVMGLIALSALIYGLV
>CORE_REP|Org36_Gene128#
MVRFENKDPLMLARQLPIKSVALILAGGRGSRLKDLTSTRAKPAVHFGGKFRIIDFALSNCLNSGIRR
IGVITQYQSHTLVQHIQRGWSFLNEEMNEFVDLLPAQQRLSTEHWYKGTADAVYQNLDIIRRYEAEYV

VILAGDHIYKMDYSRMLIDHVEKGAQCTVACLPVPRSEAGEFGVMKVDESDRIIEFLEKPADPPAMPG
NPDMSLASMGIYIFNAAYLFQLLEEDMSTPGSSHDFGKDLIPKITAQQAAWAHPFTLSCVTSNPDLPP
YWRDVGTLDAYWRANLDLASVTPELDMYDRAWPIRTHMEPLPPAKFVQDRSGSHGMTMNSLVSGGCIV
SGSVVVHSVLFPRVRVNSFCTIDSTVLLPDVNVGRSCRLRRCIIDRACHIPEGMVIGENADEDSKRFY
RSEGGIVLVTREMLSKL
>CORE_REP|Org7_Gene1179#
MPQFDYLKTSIKQKGCTLQQVADASGMTKGYLSQLLNDKIKSPSAQKLEALHRFLGLEFPRKEVKVGV
VFGKFYPLHTGHIYLIQRACSQVDELHVILCHDEPRDRELFENSSMSQQPTVSDRLRWLLQTFKYQKN
IHIHSFDEQGIEPYPHGWNVWSDGMKAFMEQKGIVPSFIYSSEAQDAPRYREHLGIETILVDPERSFM
NISGNQIRQDPFRYWDYIPTEVKPFFVRTVAILGGESSGKSTLVNKLANIFNTTSAWEYGRDYVFSHL
GGDEMALQYSDYDKIALGQAQYVDFAVKYANKVAFIDTDFVTTQAFCKKYEGREHPFVQALIDEYRFD
LVILLENNTPWVADGLRSLGSTADRLAFQRLLEEMLRANNIEYVHVESSDYEERFLRCVELVQQLLAA
DAGRLANAPAQRHAAG
>CORE_REP|Org21_Gene3509#
MRFDVVVIGGGLAGLSCAIAVAEQGKRCAVVSSGQSALYFSSGSLDLLARLPDGTPVEMPLAALPQLA
QQAPQHPYALIGPGRVAALSAAAQRLLARCGLQLQGEGANNHLRITPLGTRRATWLSPQAIPTLPLTG
QLPWRRIAVIGIEGFLDFQPQMAADSLSRELGVETEVAYLHMPALDRLRNNPSEFRAVNIARVLDLME
SLPPMAEELRRLAGEADALFLPACLGLEDDASLAVLQDAVGKPIRLLPTLPPSVPGMRLHQALRRRFQ
QLGGVFIPGDSVLRAECEAGRVTGLYTRNHGDIPLRAQQVVLASGSFFSNGLVADFDGVREPIFGLDV
HSRADRADWSRRELFAPQPYLQFGVRTDGRLRAMKQGMPFDNLYAIGAVAGGYDPLQQGCGAGVSLIG
ALHVAQQIAAEENA
>CORE_REP|Org38_Gene942#
MQRRRFIKAFALSAAAVGLGLAWSAQAADTIKVGILSSLSGTMAISETPLKDVALMTIDEINAKGGVL
GKKLEPVVVDPASNWPLFAEKARQLLSQDKVAAVFGCWTSVSRKSVLPVFEELNGLLFYPVQYEGEEM
SPNVFYTGAAPNQQAIPAVEYLLSEDGGGAKRFFLLGTDYVYPRTTNKILRAFLHSKGVQDKDIEEVY
TPFGYSDYQTIVANIKKFAAGGNTAVISTINGDSNVPFYKELANQGVKATDVPVIAFSVGEEELRGID
TKPLVGNLAAWNYFESLDNPTNKQFVSQWKAYAKAHNLPNYATAVTNDPMEATYVGLHMWAQAVEKAG
TTDVDKVRAAMAGQTFAAPSGFTLTMDKTNHHLHKPVMIGEIEGNGQFNVVWQTEAPVRAQPWSPYIA
GNDKKPDYPVKGGK
>CORE_REP|Org36_Gene1379#
MTTVSTLGALVALAVAIVLILRKVPPAYGMIAGALAGGLCGGADLVQTVTLMIGGAQGITNAVMRILA
AGVLAGVLIESGAAHTIAETIVRKVGETRALLALAVATLILTAVGVFIDVAVITVAPIALSIAQKAGI
SRAAILLAMIGGGKAGNVMSPNPNTIAAADNFHVPLTSVMMAGIVPGLCGLVVAYLLARRLSDKGSKV
MAEELTQHAEGARPGFAAAISAPLVAILLLSLRPIAGIAVDPLIALPAGGLAGALLMGRIRQCNHFMV
SGLSRMAPVAIMLLGTGTLAGIIANSALKDVLINGLTHTGLPAWLLAPLSGALMSMATASTTAGTAVA
SGVFSSTLLELGVSGLAGAAMIHAGATVLDHLPHGSFFHATGGSVNMAVHERLKLLPYETLVGFTIAA
ISALMFGVFNLAG
>CORE_REP|Org44_Gene49#
MQLGVIADDFTGATDIASFLVRNGMPTVQLNGVPTRDLPLTSEAVVISLKTRSCPAEMAVSQSLAALR
WLQAQGCQQFYFKYCSTFDSTAQGNIGPVLDALLAELGETRTVISPALPVNGRTVYQGYLFVGEQLLN
ESGMRHHPVTPMEDAHLGRLIERQGRGKAALIAWPIVARGPEAVAAALAAVNDPAVRYVVLDALSEQD
LLTQGVALREMKLVSGGSGLAIGLARDLAQRHGARGESAQAGMPLVGPAVVLSGSCSVMTNSQVAAYR
QQAPARAVDLSACFTDLESYVRTLTDWVDAQRDAPLAPMIYATTEPQTLQRIQAQYGDKASSERVEQL
FAALAAALKAKGFTRFIVAGGETSSIVAQTLGVEAFHIGPTISPGVPWVRDTRQPLSLALKSGNFGDI
QFFARAQQEFRHD
>CORE_REP|Org22_Gene1226#
MTLLALGINHKTAPVSLRERVTFSPESIDEALTSLLQQPLVQGGVVLSTCNRTELYLSVEQQEHMHEQ
LVAWLCAYHNLRPEEVKKSLYWHQGNDAVSHLMRVASGLDSLVLGEPQILGQVKKAFAESQRGQSLSG
ELERLFQKSFSVAKRVRTETDIGASAVSVAFAACTLARQIFESLADLNVLLVGAGETIELVARHLREH
KVRHMIIANRTRERAQLLADEVGAEVITLPEIDERLADADIIISSTASPLPIIGKGMVERALKARRNQ
PMLLVDIAVPRDIEPEVGKLANAYLYSVDDLHAIIQSNLAQRKAAAVQAESIVQQESTNFMAWLRSQG
AVETIRDYRSQADQIRAEMEAKALAAIAQGANVEQVIHELAHKLTNRLIHAPTKSLQQAAGDGDVERL
QLLRDSLGLDQH
>CORE_REP|Org21_Gene461#

MNLTELKNTPVSELITLGENMGLENLARMRKQDIIFSILKQHAKSGEDIFGDGVLEILQDGFGFLRSG
DSSYLAGPDDIYVSPSQIRRFNLRTGDTISGKIRPPKEGERYFALLKVNEVNYDKPENARSKILFENL
TPLHANSRLRMERGNGSTEDLTARVLDLAAPIGRGQRGLIVAPPKAGKTMLLQNIAQSIAYNHPDCVL
MVLLIDERPEEVTEMQRLVKGEVIASTFDEPASRHVQVAEMVIEKAKRLVEHKKDVIILLDSITRLAR
AYNTVVPASGKVLTGGVDANALHRPKRFFGAARNVEEGGSLTIIATALVDTGSKMDEVIYEEFKGTGN
MELHLARKIAEKRVFPAIDYNRSGTRKEELLTTSEELQKMWILRKIIHPMGEIDAMEFLINKLAMTKT
NDEFFDMMKRS
>CORE_REP|Org8_Gene364#
MIKSTDRKLVVGLEIGTAKVSALVGEVLPDGMVNIIGVGSCPSRGMDKGGVNDLESVVKCVQRAIDQA
ELMADCQISSVYLALSGKHISCQNEIGMVPISEEEVTQDDVENVVHTAKSVRVRDEHRILHVIPQEYA
IDYQEGIKNPVGLSGVRMQAKVHLITCHNDMAKNIVKAVERCGLKVDQLIFAGLAASYAVLTEDEREL
GVCVVDIGGGTMDMAVYTGGALRHTKVIPYAGNVVTSDIAYAFGTPPTDAEAIKVRHGCALGSIVSKD
ENVEVPSVGGRPPRSLQRQTLAEVIEPRYTELLNLVNDEILQLQEQLRQQGVKHHLAAGIVLTGGAAQ
IDGLAACAQRVFHTQVRIGQPLNITGLTDYAQEPYYSTAVGLLHYGKESHLSGETEVEKRASVGNWFK
RINSWLRKEF
>CORE_REP|Org17_Gene1564#
MAGASLSFLTALRFSRGRKRGGMVSLISVISTIGIALGVAVLIVGLSAMNGFERELKNRILAVVPHGE
LEPVNQPFSGWPSILQRVEKVPGIVAAAPYINFTGLMENGAQLRAVEVKGVDPQQESRLSALPQYVQG
DAWANFKPGEQQVILGKGVADALGVKQGAYVTVMIPNSDPEMKLLQPKRIRLHVTGILQLSGQLDHSL
ALVPLADAQQYLDMGDSVTGIALKMNDVFAANKLVRDAGEVTNAYIYIKSWIGKYGYMYRDIQMIRAI
MYLAMVLVIGVACFNIVSTLVMAVKDKSADIAVLRTLGAKDGFIRAIFIWYGLLAGLLGSLSGVVIGV
IASLQLTNIIRGIEKLVGHSFLSGDIYFIDFLPSELHWLDVLIVLATAIVLSLLASWYPARRASRIDP
ARVLSGQ
>CORE_REP|Org37_Gene2232#
MSTDTIQKLARPSVLGGAMIIAGTAVGAGMFSIPIVTSGVWFSGSVALLVYTWACMLLSGLMILEATL
HYPSGASFNTMVKDLLGKGWNAVNGLSVAFVLYILTYAYISAGGSIIAHTLEGIVGVGQTTAGLVFAL
VVAFIVWLSTRAVDRLSTILIGGMVITFVMSVGDMFTHVQPAVLFNTGDDQASYLPYALAALPYLLTS
FGYHGNIPGLVKYYHKDSGSVVRSLVYGTLLALAIYILWQYVIQGNIARDAFKQVIAEGGNIGSLLKQ
MGNVSSSQTVSQLLNAFSYMALASSFLGVSLGLFDYLADFCKFKDDAVGRSKTALVTFVPPTLAALLF
PNGFLYAIGFAGLAATIWAVIVPALMARASRRRYPQAGYRAPGGNGVILFVILFGLINAAAHILSLFG
LLPVFH
>CORE_REP|Org49_Gene2373#
MGLKNYFEKIEHHFTPGGKLEKWYPLYEATTTVFYTPGTVTRGASHVRDAIDLKRMMILVWLAVFPAM
FWGMYNVGQQAIPALHHLYSGDELQQVLAGDWHYRLAQWLGASLAADAGWVSKMVLGACYFLPIYAVV
FVVGGFWEVLFAIIRKHEVNEGFFVTSILFALIVPPTLPLWQAALGITFGVVVAKEIFGGTGRNFLNP
ALAGRAFLFFAYPAQISGDLVWTSADGFSGATPLAQWSAGGAHSLSNVATGQSISWMDAFLGNIPGSI
GEVSTLMILIGGAIILFGRVASWRIVAGVMLGMVASALLFNAIGSDTNPMFAMPWYWHLVLGGFAFGM
IFMATDPVSASFTNKGKWWYGILIGVMCVLIRVVNPAYPEGMMLAILFANLFAPLFDYLVVQANIKRR
KARGE
>CORE_REP|Org5_Gene3733#
MKRTFIMVLDSFGIGASEDAERFGDQGSDTLGHIAEVCARGEANVGRQGPLTLPNLSRLGLGKAAEES
TGNFPQGLDRNADIIGAYAYASELSSGKDTPSGHWEIAGVPVLFDWGYFKDEHNSFPQALLDKLVERA
KLPGYLGNCHSSGTVILDQLGEEHMKTGKPIFYTSADSVFQIACHEETFGLDRLYELCEIAREELTEG
GYNIGRVIARPFVGDKPGNFQRTGNRHDLAVEPPAPTVLKKLVDEKSGEVVSIGKIADIYANVGITKK
VKATGIDALFDATLIEMEKAGDNTIVFTNFVDFDSSYGHRRDVAGYAAALELFDRRLPELLKLVKDED
IIIFTADHGCDPTWPGTDHTREHIPVLVYGPKVKPGSLGHRETFADIGQTVANYFGLSPMDYGKSMF
>CORE_REP|Org42_Gene2299#
MSWQQFKSQYLVRFWAPLPAVIAAGILSTYYFGMTGTFWAVTGEFTRWGGHVLQWFGLHPEQWGYFKV
IGLQGTPLERIDGRMIIGMFAGCIAAALWANNIKLRQPQHRIRIVQALLGGIIAGFGARLAMGCNLAA
FFTGIPQFSLHAWFFALATAAGSYFGAKFTLLPMFRIPVKLQKVKAAAPLTQKPEQARRRFRLGMAVF
GLAVAWSLWTLFDAPKLGIAMLFGIGFGLLIERAQICFTSAFRDLWITGRTHMAKAIIIGMAVSAIGI
FSYVQLGVAPKIMWAGPNAVLGGLLFGFGIVLAGGCETGWMYRAVEGQVHYWWVGLGNIIGATLLAYY
WDDLAPALATDYDKINLLDTFGPIGGLLVTYLLLALAFAAMLWWEKRFFRARPEAQVVNLRSLP
>CORE_REP|Org36_Gene339#

MKNRTLGSVFIVAGTTIGAGMLAMPLAAAGVGFGVTLALLVGLWLLMCYTALLLVEVYQHEQADTGLG
TLAKRYLGGGGQWLTSFSMMFLMYALTAAYISGAGELLATSISQWTSQDFPVSLGVLLFTLVAGGVVC
IGTHSVDLFNRILFSAKVVFLVVMLGLMLPNIHQTNLMTLPLEQGLALSAIPVIFTSFGFHGSVPSIV
NYMGGNIRKLRWVFIIGSAIPLIAYIFWQLATLGSISSDTFVGILAQQAGLNGLLQAVRDAVASPHVE
LAVHLFADLALATSFLGVALGLFDFLADLFKRQDNVRGRLQTGAITFLPPLAFALFYPRGFVLALGFA
AIALSVLALLLPSLLVWKTRQKHQAQYRVWGGTPALALVFVCGVTVIAIQLGIASGMLPAVG
>CORE_REP|Org5_Gene2945#
MYQPVALFIGLRYMRGRASDRFGRFVSWLSTIGITLGVMALVTVLSVMNGFEKDLENNILGLMPQALI
TSPQGSVNPQQLPASEVQKLQGVTRVAPLTTGDVVLQSARSVAVGVMLGVNPDEADPLTPYLVNVKQQ
QLQPGQYNIIIGEQLAGQLGVKRGDSLRVMVPSASQFTPMGRIPSQRLFTLIGTFHANSEVDGYQLLV
NQQDASRLMRYPAGNITGWRLFLQQPLTVDTLSQQPLPAGTVWKDWRDRKGELFQAVRMEKNMMGLLL
SLIVAVAAFNIITSLGLLVMEKQGEVAILQTQGLTRRQIMSVFMVQGASAGIIGSLLGTLLGVLLATN
LNNLMPILGALIDGASLPVAVDPLQVTIIAVVAMAVSLLSTLYPSWRAAAVQPAEALRYE
>CORE_REP|Org33_Gene2721#
MSLHQDHSFESCIKCTVCTTYCPVAKVNPLYPGPKQAGPDGERLRLKDPALYDEALKYCTNCKRCEVA
CPSDVKIGDIIQRARADFAQSKPTLRDAILSHTDIMGSLSTPFAPIVNAATGLKPVRKLLDKALKIDH
RRELPKYSFGTFRRWYRQQAQAQQRYAEQVAFFHGCFVNYNHPQLGKDLIRVFNALDIGVQLLKREKC
CGVPLIANGFIEQAKKQARVNAESLHETVLERGIPVVATSSSCTFTLRDEYPHLLDVDTTPVRDRVEL
ATRYLYRLINQGRSLPLKHTPLRVAYHTPCHMEKMGWTAYTLELLRQIPGLELVVLDSQCCGIAGTYG
FKSENYATSQGIGAPLFRQIEESGVDLVVTDCETCKWQIEMSTSKRCEHPITLLAQALA
>CORE_REP|Org14_Gene889#
MTLLNPYFGEFGGQYVPQILMPALKQLEEAFVSAQRDPAFQAEFIDLLKNYAGRPTALTLCKNLTAGS
NTKLYLKREDLLHGGAHKTNQVLGQALLAKRMGKTEIIAETGAGQHGVASALACALLGLKCRIYMGAK
DVERQSPNVFRMRLMGAEVIPVHSGSATLKDACNEALRDWSGSYETAHYMLGTAAGPHPYPTIVREFQ
RMIGEETKAQVLEREGRLPDAVIACVGGGSNAIGMFADFIDDADVGLIGVEPAGLGIETGQHGAPLKH
GHVGIYFGMKAPMMQTAEGQIEESYSISAGLDFPSVGPQHAYLNSTGRAEYVSITDDEALEAFKALSR
HEGIIPALESSHALAHALKMIRETPQKEQILVVNLSGRGDKDIFTVHDILKARGEI
>CORE_REP|Org6_Gene1492#
MLELLFLLLPVAAAYGWYMGRRSAQQDKQQEANRLSREYVAGVNFLLSNQQDKAVDLFLDMLKEDSNT
VEAHLTLGNLFRSRGEVDRAIRIHQALMESASLTFEQRLLAVQQLGRDYMAAGLYDRAEDMFSQLTDE
ADFRVSALQQLLVIHQATSDWQKAIDVAEKLVKLGKEKQRVEIAHFYCELALQAMGSDDLDRAMSLLK
RADAADKQCARVSIMFGRIYMAQNDYAKAAESLQRVLSQDKELVSETLPMLQECYTHLPEQQHNWADF
LKRCVEENTGATADLMLAEIIEQHEGRDVVQVYINRQLQRHPTMRVFYRLMDYHLADAEDGRAKESLL
LLRDMVGEQIRTKPRYRCHKCGFTAHSLYWHCPSCRAWSSVKPIRGLDGQ
>CORE_REP|Org37_Gene3603#
MILHWLTGEATENHRRQIATRTIFFLAGLGMAAWAPLIPFVKARLGIDDGTLGLLLFCLAAGSMAIMP
FAGYLIAKLGCRTVLLGAGALLCIDLPLLALLDAPLLMGAALGVFGAVNGIMDVAMNSQAIVVERESG
QAKMSGFHGFYSLGGIAGAGGVSLLLLLGIVPALAIGLIALLIAILLPIVASDLLAQGGIGERCRGGV
RLALAHGKILFIALLCFFVFLTEGAMLDWSALFLHAERGVAKSQAGMGFTLYAVAVACGRLYGDRLIG
IIGRYRTLLFGSLCAATGLLLTVTVPLAWAAFGGLMMIGLGIANIVPILFNAVGNQKQVPPGQAFPAV
TLVGYIGLLTGPALIGFIAKYTTLTLAFGCTLLCLVLVSISARAVTRSSH
>CORE_REP|Org27_Gene2744#
MTNIIRQFLRQEAAGGIILIAAAIVALIMANTPAQGIYQAFLNLPVMVKIASLEIAKPLLLWINDGLM
AIFFLVVGLEVKRELMQGSLSGRDKAVFPAIAALGGMLAPALIYLLFNGADEVTRQGWAIPAATDIAF
ALGVMALLGNRVPTSLKVFLLALAIIDDLGVIIIIALFYTHEVSMVALGVAAAATVLLAVMNWRGVGK
TSLYMMVGLVLWVAILKSGVHATLAGVIVGFMIPLNVKKGPSPSETLEHELHPWVAFMILPLFAFANA
GVSLQGVSLEGLTSLLPVGIAAGLFIGKPLGIFLFSLLAVKMGIARLPEGIGFKQVFAVSVLCGIGFT
MSIFIASLAFGDADAALSTYSRLGILLGSTAAAVVGYGLLRLALPRVR
>CORE_REP|Org41_Gene2469#
MTDNPLLVLRERISALDLKLLALLAERRELAIEVGKTKLHSHRPIRDKERERDLLDALIAAAKPYDLD
GFYVTRLFQLIIEDSVLTQQALLQHQLNPVSQHSARIAFLGPKGSYSHLAARQYAARHFDQLIECGCQ
KFQDIFTQVETGQADYAILPIENTSSGSINEVYDLLQHTSLSIVGELTNPINHCVLIAGDSDLSQIET
VYSHPQPFQQCSQFLNRFPHWKIEYTESTAAAMEKVAKLNSPKVAALGSEAGGALYGLQVLEHNLANQ
QQNITRFIVLARKAIDVSEQVPAKTTLIMATGQQSGALVEALLVLRDNGIIMTKLESRPINGNPWEEM

FYIDVQANLRADAMQKALRDLAPITRSLKVLGCYPSDTVVPVNPS
>CORE_REP|Org21_Gene393#
MFEPMELTNDAVIKVIGVGGGGGNAVEHMVRERIEGVEFFAVNTDAQALRKTAVGQTIQIGSGITKGL
GAGANPEVGRNSAEEDREALRAALDGADMVFIAAGMGGGTGTGAAPVVAEVAKDLGILTVAVVTKPFN
FEGKKRMAFAEQGIAELSKHVDSLITIPNDKLLKVLGRGISLLDAFGAANDVLKGAVQGIAELITRPG
LMNVDFADVRTVMSEMGYAMMGSGVACGEDRAEEAAEMAISSPLLEDIDLSGARGVLVNITAGFDLRL
DEFETVGNTIRAFASDNATVVIGTSLDPEMNDELRVTVVATGIGMDKRPEITLVTNKQASQPVMDHRY
QQHGMSPLPQEVKPAAKVVNDQAAQPNKEPDYLDIPAFLRKQAD
>CORE_REP|Org6_Gene3980#
MKSGRYIGVMSGTSLDGIDVVLAAIDDRMVAQQASYSHPMPMALKKEILGMCQGQQTTLAAVGRLDAQ
LGTLFGEAVLGLLKQTGIDAEQVTAIGCHGQTVWHEPEGDARFSMQLGDNNRIAALTNITTVGDFRRR
DMAYGGQGAPLVPAFHQALLGHPVERRMVLNVGGIANLSLLLPGTAVRGFDTGPGNMLMDAWVWRHRA
QPYDQDGGWAMQGRVCLPLLQQMLADPYFAQPAPKSTGREYFNIAWLERQLAGLPAMAPVDVQATLTE
LTAVSISEQVQLAGGCERLLVCGGGARNTLLMARLSALLPGTEVGLTDDFGVSGDDMEALAFAWLAFR
TLSGQAGNLPSVTGASRETVLGGIYPVLPLGAVSSVRIAGEGFF
>CORE_REP|Org37_Gene2802#
MKALHFGAGNIGRGFIGKLLADAGVELTFADVNQTVLDLLNSRKSYAVHVVGEQERVENVNNVSAVNS
GSEAAVALIAEADLVTTAVGPQILGKIAGTIARGLVLRHQQGNVQPLNIIACENMVRGTSQLKQHVFA
ALPQDEQAWVEQHVGFVDSAVDRIVPPADSSDPLEVTVETFSEWIVDQTQFKGQPPAIAGMELTDNLM
AFVERKLFTLNTGHAITAYLGQQAGLQTIRDAILDPAIRRVVKGAMEESGAVLIKRYGFDADKHAAYI
NKILGRFENPYLHDDVERVGRQPLRKLSAGDRLIKPLLGTLEYGLPHANLIQGIAAAMSYRSEQDPQA
LELAELLNTLGPKAALAQISGLPAESEVVEEAVAVYNAMHK
>CORE_REP|Org1_Gene2196#
MSTRRTFIKQLSAVAGVSLTASLGIPLRGHAKAALNPAWRMPDEGEPQQRAFLAFGAQRAIWGGFTAD
VQAAQGRIARAIADFQPLTVFCRGNERQLAEATCGSHNVSYVVTELDDIWMRDIGANFVVNDAGELGA
VDFNFNGWGDKQQHARDARLAGFVARRYGVAQPRRSALVGEGGGIEVDGHGTGIMTESSWVNANRNPG
WSRDRVEQELKAMLGLRKIIWLPGIKGRDITDAHVDFYARFVRPGVVVANLDTDPASYDHAVTQAHLA
ILRAATDADGRTLQVHTLSPPRAPRESRFSRRNPDFAAGYINYFVINGAVIAPEFGDLQADKAAFELL
SALYPQRKVVQLEIDAIAAGGGGGGIHCVTSQLPVHGKPDQ
>CORE_REP|Org10_Gene2654#
MKGRWGKYLLGGLLVAVLAGCSSKPTDRGQQYKDGRLDQSLELVNQPNAKGSPVNAKDYSDQLMEIKY
ASPSLFNRNNSTYQAVQSWMASGADTRMLSQYGLSAYQMEGVDNYGNVQFTGYYTPVVQARYTQQGEF
RYPLYRMPPKGRGRLPDRAGIYSGALDDRYIIAYTNSLMDNFMMEVQGSGYVDYGNGQPLVFFGYGGK
NGHAYRSIGKVLIDRGEVAKADMSMQAIRQWADTHSAAEVRELLEQNPSFVFFRPEAFAPVKGASAVP
LIAKASVASDRSLIPAGTTLLAEVPLLDNKGKFTGKYEMRLMVALDVGGAIKGQHFDMYQGIGPDAGH
SAGYYNHYGRVWVLKNNGGGQLFSANQSNGSGSLLATR
>CORE_REP|Org21_Gene1915#
MLRQTNLLAEATARQIVQRAMGIISHSVNVMDSNGVIIASGNPQRLFQRHEGAVLALAENRVVEIDRA
TAEHLKGVRPGINLPFSFRNQRVGVIGISGEPAEVRAYAELVKMAAEMMVEQAALLDQHQWEKRYREE
LANQLLQPQPNNASLEAMAAYLGLDLRQARIVWIVELQEAQPHLLRELLAELEATQRDALIAITGFNE
MTLLRPACMAQGEWSLKLERQQAQRLQNQLKHRFRVRLIVGGFYDDPQSAYRSSLTARATQAMAQRLK
LRHATLFYHDYPLPSLLCDLGEDWRAQELGRPWRTLGEQDEKGVLRGTLRHYFSQNCDQTQTAAQLHI
HVNTLRYRLQRIEAITGMKINQLTDALRLYIGMLMHD
>CORE_REP|Org35_Gene2070#
MTDDFSSRWQQLDWDDITLRINGKTARDVERALNADKLTRDDFMALISPAAAPYLEPLAQRAQLLTRQ
RFGNVVSFYVPLYLSNLCANDCTYCGFSMSNRIKRKTLDAAEIARECEAIKALGFEHLLLVTGEHQTK
VGMDYFRQHIPAIRRHFSSLMMEVQPLAQEEYAELKTLGLDGVLVYQETYHPATYLQHHLRGQKQDFH
WRLATPDRLGRAGIDKIGLGALIGLSHSWRTDCYLLAEHLFYLQQTYWQSRYSISFPRLRPCAGGIEP
ASIMSEPQLVQLICAFRLFAPDVELSLSTRESPYFRDHMIPVAINSVSAGSKTQPGGYADDVPPELEQ
FEPHDGRTPQQVAEAISNAGLQPVWKDWDDYLGRSAQ
>CORE_REP|Org48_Gene2807#
MRPEEIALRMRGVTGLQTVVASRIVQQLSQMGCEPRRVLHELGLNERQQTQFNQLDPGYLSASLRWLE
LPAHRMLNYGAAGYPERLAQIDDAPLFLLIEGDPQALLHPQLAMVGSRQFSHYGERWANHFAEELARC
GFTITSGLAIGIDGICHRAALAAGGCTIAVLGSGLGNIYPRRHRRLAEQIVEQGGAVISDHLVTDLPL

ADHFPRRNRIISGLSLGVLVIEASLRSGTLVTARYALEQGREVFALPGPLGNPMSEGTHWLIQQGAHL
VTGPKDIAELLGSGLQWLSLNENTTICASQAEVELPFADVLANVGDEVTPVDVVAERAGQPVPVVVIK
LLELELAGWIAAVPGGYVRIRRASHVRRTHVLV
>CORE_REP|Org1_Gene791#
MHNQAPINRRKSTRIYVGKVPIGDGAPIAVQSMTNTRTTDVEATVNQIKALERVGVDIVRVSVPTMDA
AEAFKLIKQQVNVPLVADIHFDYRIALQVAEYGVDCLRINPGNIGNESRIRSVVDCARDKNIPIRIGV
NGGSLEKDLQEKYGEPTPEALLESAMRHVDILDRLNFDQFKVSVKASDVFLAVQSYRLLASRIDQPLH
LGITEAGGARSGSVKSAIGLGMLLSEGIGDTLRISLAADPVEEVKVGFDILKSLRIRARGINFIACPT
CSRQEFDVIGTVNALEQRLEDIITPMDVSIIGCVVNGPGEALVSTMGVTGGHKKSGFYEDGVRQKERF
DNEQMIDQLEAKIRAKAAMMDESNRITVNLLEK
>CORE_REP|Org25_Gene326#
MKLTTGKALLAGCIAMAMSQAAMAKDIKVAIVGAMSGPVAQYGDMEFTGARQAIADINAKGGIKGDKL
VGVEYDDACDPKQAVAVANKVINDGIRYVIGHLCSSSTQPASDIYEDEGVIMITPAATNADLTTRGYK
MILRTTGLDSDQGPTAAKYILSDIKPKRIAVVHDKQQYGEGLARSVRDSLKKQGTEVAMFEGITAGDK
DFSTLVARLKKENIDFVYFGGYYPEMGQILRQAKQAGLTTRFMGPEGVGNSSLSNIAGAASEGMLVTL
PKRYDQVPANQPIVDALKAKKLDPTGPFVWTTYAALQSLTTGMERSGSQEPADIVKDLKTGKPVDTVM
GPLTWDDKGDLKGFEFGVFEWHANGTSTPIK
>CORE_REP|Org35_Gene519#
MTESQQKGSIWTKIHIDPTFLLLILALLVYSAFVMWSASGQDIGMMERKIGQIVMGLIVMAVMAQIPP
RVYESWAPYLYIFCVILLILVDAFGQISKGAQRWLDLGVVRFQPSEIAKIAVPLMVARFMNRDVCPPS
LKNTAIALVLIFLPTLLVAAQPDLGTSILIAASGLFVLFLSGMSWKLIAVAAVALAAFIPVLWFFLMH
GYQRDRVMMLLDPESDPLGAGYHIIQSKIAIGSGGLSGKGWLHGTQSQLEFLPERHTDFIFAVLAEEL
GLIGVLVLLALYLLVIIRGLMIAAKAQTTFGRVMVGGLMLILFVYVFVNIGMVSGILPVVGVPLPLVS
YGGSALIVLMAGFGIIMSIHTHRKMLSKSL
>CORE_REP|Org21_Gene2062#
MYHDEFYMARAFELARLGRFTTAPNPNVGCVIVRDGEIVGEGYHLRAGEPHAEVHALRMAGDKARGAT
AYVTLEPCSHHGRTPPCADALVAAGVTRVVAAMQDPNPQVAGRGLYKLQQAGVEVRHGLMLAEAEAVN
LGFLKRMRTGFPYVQLKLGASLDGRTAMASGESQWITSPEARQDVQRLRAQSAAILSTSATVLADDPS
LTVRWDELDAETQRLYPRDNLRQPLRILLDSQNRITPQHRVVQQPGATWLARLQADAQTWPQDVEQFI
CPAHGGGVDLVVMMMLLAKRQVNSIWVEAGASLAGALLQAGLVDELILYIAPKLLGDNGRGLCHLPGL
ERLADAPEFVFSDVRQVGPDLRLRLRAKH
>CORE_REP|Org4_Gene2616#
MVTQRKKWLSGVVAGLLMAASVTASAEEKTLHVYNWSDYIAPDTLAKFQKETGIKVVYDVFDSNEVLE
GKLMAGSTGYDLVVPSSNFLERQSQAGIFEPLDKSKIPNYKNLDPEMLKLVAHNDKDNKYGIPYMMVT
TGIGYNVDKVKAVLGKDAPVNSWDLIFKPENLEKLKSCGVSFLDAPSEVYATVLHYLGKDPNSTNAAD
YTGAANDLLLKLRPNIRYFHSSQYINDLANGDICVAIGWSGDVMQAANRAKEAKNGVNVAYAIPKEGA
LTYFDMFAMPADAKNKDVAYQFLNFLLKPDVMADISNHVYYANAVKDSTPLVNAEVRDNPNVYPPADV
RAKLFTLNVQSPKLDRVITRAWTKVKSGK
>CORE_REP|Org29_Gene2996#
MERITVTLGERSYPITIAAGLFNDPASFMPLKAGEQAMLVTNQTLAPLYLERVRQVLEQGGVVVDQVI
LPDGEQYKSLAVLEQVFSALLEKPHGRDTTLIALGGGVVGDLTGFAAACYQRGVRFIQVPTTLLSQVD
SSVGGKTAVNHPLGKNMIGAFYQPASVVVDLDCLKTLPTRELSSGLAEVIKYGIILDRAFFVWLENNI
DALMALDMQALAYCIRRCCELKAEVVAADERESGLRALLNLGHTYGHAIEAEMGYGVWLHGEAVAAGM
VMAAETAHRLGQFSVEDIERIKTLLLRAGLPVCGPQEMTPESYLPHMLRDKKVLAGELRLVLPTAIGA
AEVRGGVGHELVLASIAACLPEQQRN
>CORE_REP|Org2_Gene4375#
MNLLTMSTEILFVFLFSLAFLFVARKAAKRIGLVDKPNYRKRHQGLIPLVGGISVYAGLCFAFWISEQ
PIAHAKLYLTCAGILVFVGALDDRFDISVKIRALVQALVGIAMMVFAGLYLRSFGHVLGDWEMLLGPF
GYLVTLFAVWAAINAFNMVDGIDGLLGGLSCVSFGALGLLLYLSGHHEMAFWCFAMIATIVPYILLNL
GILGRRYKVFMGDAGSTLIGFTAIWLLLQSSQGKAHSINPVTALWIIAIPLMDMIAIMYRRLRKGMSP
FSPDRQHIHHLIMRAGFTPRQAFVLITLAAALLAAVGVIGERLTFIPEWVMLALFLLAFFLYGYCIKR
AWRVARYIKRIKRRLRRSSDNKQVS
>CORE_REP|Org23_Gene2628#
MIIIRYLVRETLKSQIAILFILLLIFFCQNLVRVLGDAVDGNIPTNLVLSLLALGVPKMAQLILPLSL

FLGLLMTLGRLYTESEITVMHACGLGKRTLIIAAMILALLTSAIAAVNVFWAGPWASRYQDVVVNEAK
ANPSIAGLAEGQFKPSQDGNAVLFIGNVKGSTFNDVFLAQLRPNGNQRPSVVVAEHGNIVQQKDGSQV
VTLDKGTRFEGTALLRDFRITDFTDYKAVIGHRTVAADNTESEQMSMQTLWESDDPDARAELHWRLTL
VVSVALMALLVVPLSVVNPRQGRVLSMLPAILLYLIFFLLQTSLRSNAGKGKLDPMLWLWLVNGVYFA
IALALNLWDTVPMRKLRARLRGAA
>CORE_REP|Org25_Gene1948#
MTTLIHVLGSDIPHHNQTVLRFFNDVLAPRLPAEQTRHFMVAAKDVAALGDFPALNIEPYADKKSLAA
AVIARAQADRDARFFLHGQFNPGLWLALLSGKIKAHQVSWHIWGADLYEDATSWKFRLFYLLRRIAQG
RVGNVFATRGDVIHYQQRHARVPASLLYFPTRMDPALTDVHVEKNLAGPMTILVGNSGDRSNRHIEAL
QAIHQQFGADVRVILPMGYPANNDAYIEQVRAAGLPLFGEKNLQLLTQQVAFEDYLNILRACDLGYFI
FNRQQGIGTLCLLIQFGVPFVLSRQNPFWQDLAEQHLPVLFYGDSLDEAVVREAQRQLAAVDKQTIAF
FNPNYVDGWQQALALAAGEHS
>CORE_REP|Org21_Gene1210#
MAGNSIGQIFRVTTFGESHGVALGCIVDGVPPGIPLTEADLQHDLDRRRPGTSRYTTQRREPDQVRIL
SGVFEGVTTGTSIGLIIENTDQRSQDYSAIKDVFRPGHADYTYEQKYGLRDYRGGGRSSARETAMRVA
AGAIAKKYLQQKFGVQVRGYLAQIGDVTCELKDWDQVEQNPFFCPDPDKLEALDELMRALKKEGDSIG
AKVSVIAENVPVGLGEPVFDRLDADLAHALMSINAVKGVEIGDGFAVVTKRGSENRDEITPEGFQSNH
AGGILGGISSGQPVVAHLALKPTSSIMVPGRTINRQGEAVEMVTRGRHDPCVGIRAVPIAEAMMAIVL
MDHLLRQRAQNGDVVSDVPRW
>CORE_REP|Org40_Gene4117#
MKFRPVSPTAAKGLHIASSPFTHNQQSTSRIMLWVMLACIPGIAAQIWFFGYGVLIQVALAAIVALAA
EGAILKLRKLPVRSRLADNSALLTALLLGISLPPLAPWWMIVIGTFFAIVIAKQLYGGLGQNPFNPAM
VGYVVLLISFPVQMTSWLPPDELRATALPFHDTLLAIFSGHTSQGATLHALQMGVDGISQATPLDGFK
TGLRSGHSVEQVLQQPLFGGALAGIGWQWVNLGFLAGGLFMLARRLIHWQIPFSMLAAIAFCSGLAWW
LDPAHQASPLIHLFSGASMLGAFFIATDPVSASTTPKGRLIYGALIGVLVWLIRVYGGYPDGVAFAVL
LANITVPLIDHYTQPRVYGHR
>CORE_REP|Org23_Gene2182#
MALTRLLIKDFRNIEAADLALAPGFNFLVGANGSGKTSVLEAVYTLGHGRAFRSLQAGRVIRHDQPEF
VLHGRIEGAERELSVGLSKSRQGDSKVRIDGSDGHKVAELAQLLPMQLITPEGFTLLNGGPKFRRAFL
DWGCFHNEPGFFTAWSNLKRLLKQRNAALRQVSRYAQIRAWDQELIPLAERISEWRAEYSDAIAADIT
ATCAQFLPEFGLSFSFQRGWDKESDYGELLERQFERDRALTYTAVGPHKADFRIRADGTPVEDLLSRG
QLKLLMCALRLAQGEFLTRQSGRRCLYLIDDFASELDTGRRRLLADRLKATQAQVFVSAVSAEQVTDM
AGEKGKMFRVEQGKIEVQPQD
>CORE_REP|Org2_Gene234#
MLVWLAEHLVKYYSGFNVFSYLTFRAIVSLLTALFLSLWMGPRVIKRLQEMSFGQVVRNDGPESHFSK
RGTPTMGGIMILTSITISVLMWAYPSNPYVWCVLFVLVGYGIVGFVDDYRKVVRKDTKGLIARWKYFW
QSVIALIVAFAMYAVGKDTPATELVVPFFKDVMPQLGLLYILLAYFVIVGTSNAVNLTDGLDGLAIMP
TVFVAAGFALVAWATGNMNFANYLHIPYLRHAGELVIVCTAIVGAGLGFLWFNTYPAQVFMGDVGSLA
LGGALGTIAVLLRQEFLLVIMGGVFVVETLSVILQVGSFKLRGQRIFRMAPIHHHYELKGWPEPRVIV
RFWIISLMLVLIGLATLKVR
>CORE_REP|Org19_Gene798#
MSKIFDFVKPGVITGDDVQKVFAVAKENNFALPAVNCVGTDSINAVLEAAAKVRAPVIVQFSNGGAAF
IAGKGVKTDVPQGAAILGAISGAHHVHQMAEHYGVPVILHTDHCAKKLLPWLDGLLDAGEKHFAATGK
PLFSSHMIDLSEESLEENIEICSAYLKRMAKIGMTLEIELGCTGGEEDGVDNSHMDASALYTQPEDVA
YAYEKLNAISPRFTIAASFGNVHGVYKPGNVKLTPTILRDSQDYVSKKFNLPHNSLNFVFHGGSGSTD
AEIKESVGYGVIKMNIDTDTQWATWDGILQYYKANEAYLQGQLGNPKGADQPNKKYYDPRVWLRAAQT
SMVTRLEQAFKDLNAVDVL
>CORE_REP|Org8_Gene3145#
MSQPLTVTGVQKAPRLALSIGALALLALLVMPFMTLLPADHPLAVSTYTLTLAGKILCYAVVAVALDL
VWGYAGLLSLGHGLFFALGGYAMGMYLMRQAAGDGLPAFMAFLSWNELPWFWSGTQHFAWALCLIVLV
PGLLAFLFGYFAFRSKIKGVYFSIMTQALTYAGMLLFFRNETGFGGNNGFTGFTTLLGFPITAAGTRV
ALFLATVLLLAASLAIGFALARSKFGRVLTAVRDAENRLTFCGYDPKGFKLFVWTLSAVLCGLAGALY
VPQVGIINPGEMSPTNSIEAAIWVALGGRGTLVGPLLGAGIVNGAKSWFTMAIPEYWQFFLGLMFIVV
TLFLPKGVIGLLRRRKSS

>CORE_REP|Org47_Gene4637#
MTAPSAFARELGLRYPIVQGPMNGASPPALAVAVSNAGALGSCAAALFSPAVILERVQQIRAQTAAPF
NINLFLLDEQHPDLAELKRAQHLLRPFREALGLSEPPIPTQFAENNRDQIAALLEAAPPVASFTFGVL
PRATVTQFKKAGSRVIGTATTVAEARAWEAAGADFVCVSGAEAGGHRPTFLGDIEQSCVGLMALLPQV
AAAVKIPVIAAGGIMNGRGIAAARLLGAQAARLLGAQAAQLGTAFLCSPESGIAEAWRAALSNAGDDS
TRLTRAFSGRPARGIVNDFMRQMRAEEAQILPYPVQNALTGDIRQAAAKAGRGDFMSLWAGQGVGLAR
PMPAAELVATLAAELEAV
>CORE_REP|Org14_Gene2709#
MATDTTHTYPERRFPLFGLPRLVPGLALTGALTALAVWAGDIPWVAELGLGALTLAILFGILVGNTLY
PRWQTVCHGGVQLAKQRLLRLGIILYGFRLTFQQIADVGASGIIIDALTLTTTFLLACWLGKKVFGID
SQTAMLIGAGSSICGAAAVMATEPVLKADSSKVAVAVSTVVVFGTLAIFAYPWLYQLNEHFQWLPFSQ
ETFGIYAGSTIHEVAQVVAAGHAIGPDAENAAVIAKMIRVMMLAPFLLLLSGYISRGGAGKAEKSAIT
IPWFAVLFIAVAGLNSFNLLPATLVQHLITADTWMLAMAMAALGLTTHISAVRQAGMKPILLATLLFV
WLLVGGGAINQLVQHWL
>CORE_REP|Org29_Gene701#
MFGVLDRYIGKTIFNTIIMTLFMLVSLSGIIKFVDQLRKVGQGEYTALSAGMYTLLSVPKDIEIFFPM
AALLGALLGLGQLATRSELVVMQASGFTRLQIAGSVMKTAIPLVLLTMAIGEWVAPQGEQMARNYRAQ
QMYGGSLLSTKSGLWAKDGNDFIYIERVSGDKELSGVNIYHFNDQRRLETVRYAATASFENGLWQLSQ
VDTSDLTNPKQVTGTQTLTGEWKTNLTPDKLGVVALDPDSLSISGLHNYVKYLKQSGQESNRYQLNMW
SKIFSPLSVAVMMLMALSFIFGPLRSVPMGIRVVTGISFGFLFYVLDQIFGPLSMVYSMPPVLGALLP
SMLFLLISVYMLLKRK
>CORE_REP|Org14_Gene463#
MKPVCVLGNGQLGRMLRQAGEPLGIAVYPVGIDAEPEAVPYQNSVITAEIERWPETALTRELATHSAF
VNRDIFPRLADRLTQKQLLDQLGLATAPWQLLASAAEWPQVFAALGELAIVKRRVGGYDGRGQWRLRP
GQEAELPADAYGECIVEQGINFSGEVSLVGARGHDGRSVFYPLTHNLHEDGILRTSVALPQPNPALQQ
QAEQMLAAILNELNYVGVMAMECFIVGDRLLINELAPRVHNSGHWTQNGASISQFELHLRAILGLPLP
QPVVSTPSVMVNLIGTAVNEQWLSLPLVHLHWYEKEVRPGRKVGHLNLNDPSAADLRQALQALAPLLP
GEYQSGLAWAQQKLA
>CORE_REP|Org39_Gene1106#
MSQKILFIDRDGTLIAEPPEDFQVDRLDKLALEPDVIPSLLALQQAGYQLVMITNQDGLGTASFPQET
FDPPHNLMMQILSSQGIQFADVLICPHLPADNCDCRKPKTALVKGYLEPGVLNAAHSYVIGDRPTDVQ
LAENMGIQGLRYQRGVLGWKEIVRQLTLRDRHARVNRVTKETQIDVNVWLDREGGSKIKTGVGFFDHM
LDQIATHGGFRMEIDVKGDLYIDDHHTVEDTGLALGEALNKALGDKRGIARFGFVLPMDECLARCALD
ISGRPHLEYKAEFNYQRVGDLSTEMVEHFFRSLSYTMGCTLHLKTKGKNDHHRVESLFKVFGRTLRQA
IRVEGNTLPSSKGVL
>CORE_REP|Org34_Gene239#
MSGKPKRLMVMAGGTGGHVFPGLAVAHHLMAQGWQVRWLGTADRMEADLVPKHGIEIDFIRISGLRGK
GLKAQLTAPLRIWQAVRQAKAIMRSYQPDVVLGMGGYVSGPGGLAAWQCGIPVVLHEQNGIAGLTNRW
LARIAAKVMQAFPGAFPNAEVVGNPVRTDVLALPLPAERLQGREGPIRVLVIGGSQGARVLNQTVPEV
AARLGDRITLWHQVGKGALETVLRDYERVGQTQHKVTEFIDDMAAAYAWADVVVCRSGALTVSEIAAA
GLPAIFVPFMHKDRQQYWNARPLEEAGAAKIIEQPQFNADVVAELLASWDRKTLLAMAEKARAVAIPD
ATERVAAELVRLAK
>CORE_REP|Org25_Gene1048#
MKRLCASVLTSAIVLSSQMSWAADTDLAALEQAAKKEGEVNSVGMPDSWANWKGTWQDLLSKYGLKHV
DTDMSSAQEIAKFDAEKNNATADIGDVGAAFGPVAVQKGVTQPYKPSTWDQVPEWAKDKDGHWALAYT
GTIAFIINKQQVKDIPHSWADLLKGSYQVTIGDVGTASQAASGVLAATYAMGGNEKNLKPGLEFFGKL
AKAGRLSLSNPVIASLEKGEVQVGVVWDFNGLNYRDQIDKTRFEVLIPSDGSITSGYTTIINKYAKHP
NAAKLAREYIFSDAGQINLARGYARPIRAEHLTLPDDVKAKLLPAEQYKNAHPIADPAAWEQSAKALP
RLWQENVIMFMQQ
>CORE_REP|Org5_Gene1767#
MGRTRVTDSSLRLAVLLAMLVIILAGVKAAADIVVPFLLAVFLAMVLNPLVTMLERRRVPRILGVTLL
VTAVIVVVMLFIGMLGASLNEFARSLPQYRGMMIEKLRELQHYADRFNISLSSEAMLQYVDPSAAMNL
VTRMLGHLSGAMTNVFLLLMTVVFMLFEVQLLPYKLQQALDKPNEGLAAMRRALDGVTRYLVIKTIIS
LATGVIVWIFLAAVGVRFAFIWGLLAFLLNYIPNIGSVLAAIPPLIQALLFNGLGDALVVAGGFIAVN

MVIGNILEPRVMGRGLGLSTLVVFLSLIFWGWLLGPVGMLLSVPLTIVARIALETTEGGYRLAVILGD
GRPPRQPPAAPE
>CORE_REP|Org15_Gene2366#
MNPETTSDKHTPAVDNELDIRGLCCTLWRGKPWIIGIAVLFAAVALIVSYLVKQEWSATAITDRPTVN
ALGGYYSQQQFLRNLDVRTLPAAAAGDQPSIADEAYNEFIMQLAAYDTRRDFWLQSDYYKQRQEGDAR
ADAALLDELINNILFTPRDDKKVPNDGVKLTAETAADANRLLRQYVAFASHRAALHLNEEIQGAWAAR
TTSMKAQVKRQEAVAESVYKRELNTTQQALKIAESQGISRTQTDTPAEQLPDSDLFLLGRPMLQARLE
GLQASGPTYDLDYDQNRAMLATLNVGPTLDEKFQTYRYLRTPEEPVKRDSPRRVFWLILWGAMGALVG
AGVALARRPRS
>CORE_REP|Org26_Gene3305#
MSKNKLSKGQQRRVQANHQRRLKRADNKPEPDDSQLGEPQEGVVISRFGMHADVEAPDGTQHRCNIRR
TLRSLVTGDRVVWRPGLGAHEGVKGIVEAVHERTSVLTRPDFYDGVKPIAANINQIVIVSAILPELSL
NIIDRYLVACETLEVEPLIVLNKIDLLDAEARKLVDGMMDIYRKIGYRVLEVSSQTREGMPEFEQALA
GRISIFAGQSGVGKSSLLNALLPPSEEQILVNQVSDVSGLGQHTTTAARLYHFQHGGDVIDSPGVREF
GLWHLEPEQITRGFVEFRDYLGGCKFRDCRHDTDPGCAIRAAMEKGDIAEERFDNYHRILESMAQVKV
RKNFTDAAD
>CORE_REP|Org33_Gene2369#
MLYQLSVIFVLAVAWVLIARRAALVVGLVDKPNARKQHLGHIPLVGGIAVYLTLTLMTLWQPAWLPDS
AVYLLCVTALVVLGVLDDRFDLPVAPRVMVQGGIALAMMLAAGMQLSSLGYVWGHQEVMLGYGALLLT
PLAVWGAINAYNMVDGIDGQLGALSCVTFVALAILFGLGGREDLALWCLGLIVALAAYLLFNLSLFGA
RNKIFMGDAGSMVIGFSVLWLVLLATQGPQSVMRPVTALWLIAIPLMDMVTVMVRRLLRRQSPFKAGR
DHLHHILMRRGLNARQALAMSTMLAVTLACVGICSEVLKLQESLMLVAFLLCFCGYFAMLREPRQATV
LRDNPSVGR
>CORE_REP|Org12_Gene3606#
MTLVAKLRALAMKNWLTGVALSATLLLTGCGPEQVDLMGKTMGTSYSIRYVTGEDTPSAREMQAEIDK
RLEQVNDQMSTYRPDSELSRFNASRDIDRPFPVSPATAEVVREALRINRVTDGALDVTVGPLVNLWGF
GPEGRPDKVPSEAELAHRRAWTGADKLSVQGNALVKNIPELYVDLSSIAKGYGVDVVAEYLQSQHVQN
YMVDIGGEVRTRGRNGEQKPWRIAIERPTAGTQQQAQLVIQPGEMSIATSGDYRNYFEQDGVRYSHTI
DPITGRPINHRLVSITVLSPTCMTADGLSTGLNVMGPERGLALANLLGIPVFMIVKTADGFEERYSDA
FKPYLKKRS
>CORE_REP|Org46_Gene2850#
MTAQPQVLKIRRPDDWHIHLRDDEMLKTVVPYTSQVFGRAIVMPNLVPPVTTVAAARAYRDRILAAVP
QGHNFTPLMTCYLTNSLAASELVNGFEQGVFTAAKLYPANATTNSSHGVSDVTGIYPLFEQMQKIGMP
LLIHGEVTDPAVDIFDREARFIEQVMEPIRQQFPELKIVFEHITTKEAAQYVQAGNRFLGATITPQHL
MFNRNHMLVGGIRPHLFCLPILKRNVHQEALRQAVASGSDRFFLGTDSAPHLKHRKESSCGCAGCFNA
PNAIPAYAAVFEQLGALAHFEAFCSLNGPRFYGLPLNEDFIELQRVPTTQPEEIALGNESVIPFLAGE
TLNWSLKD
>CORE_REP|Org5_Gene898#
MEFISVYGLFLAKVATVVLAIAALAILAVSLGQRKSRQKGELQLTDLGEQYREMQRDMRLARMDAAEQ
KVWLKQFKKQTKADDKLKKQRAKSGAVEVAKPCLYVLDFKGSMDAHEVTSLREEISAVLAVASAQDEV
LLRLESPGGVVHGYGLAASQLERLRKGGIRLTVAVDKVAASGGYMMACVADRIVAAPFAIIGSIGVVA
QIPNFHRLLKKNDIDVELHTAGQFKRTLTLFGENTEQGREKFREDLNETHELFKRFVHEQRPSLDIDS
VATGEHWFGSQAKEKGLIDAIGTSDDLLIAELDNHEVIAVRYSRRKRLMDRFTGSAAESVDRLLLRWW
QRGEKPLL
>CORE_REP|Org17_Gene2268#
MTSFTLSSDDWLTRETQFAAFATGPLLDFWRQREEGEFSGVDGVPIRFVRFRSARHQRVVVVSPGRIE
SYVKYPEVAYDLFHCGYDVVIVDHRGQGRSGRLLADTHRGHVVNFADYVDDFEQLWLREVESRGYRQR
FALAHSMGGAILAQFLQRRPQAFDAAAFCAPMFGIQLPMPGWLADRILDWVETRPAIRDYYAVGTGQW
RPLPYVVNVLTHSRERYRRSLRYYADYPELQVGGPTYHWVRESIRAGRQIIAQAGKITTPLLLLQAGE
ERVVDNRSHQAFCQALSDAGRPCEGGLPWVINGARHEILFERDAMRAEALNAILRFFAQHLGGALPPT
TPSEVRT
>CORE_REP|Org15_Gene3628#
MKKWSHLLAAGMMALSFCSANASDGKTLYFYNWTEYVPPGLLEQFTKETGIKVIYSTYESNESMYAKL
KTYKDGAYDLVVPSTYFIAKMSKEGMLQKIDKSKLSHFKDLDPTLLNKPFDPNNDYSIPYIWGATAIG

VNSDAQDPSTVTSWADLWQPQYKGRLLLTDDAREVFQMALLKLGYSGNTTDPKEIEAAYNELQKLMPN
VLAFNSDNPGNPFMEGEVNVGMVWNGSAFVARQAGTPLEIVWPKEGGIFWMDSLAIPANARNVEGALK
LIDFLLRPEIAVQVAETIGYPTPNLAAKKLLSPEIANDPSLYPDKTVIEHGEWQNDVGDASTLYESYF
QKLKAGR
>CORE_REP|Org8_Gene1779#
MASVKKNKRITISDIATLAGVSKSTASLVLNGRSKEYRVSDDTRDRVLALAHEHHYQPSIHARSLRSN
RSHTLGLVVPEMTNYGFAVISRELETLCREAGLQLLIACTDENPAQEMMAVNSLVQRQVDGLIVASSQ
LNDAEYQKINAGLPVVQMDRLIAGSELPLVITDSVNSTADLVEKVARQHPDEIYFLGGQPRISPTRDR
LAGFQLGLERAGITCKPEWIINGNYHPSSGYEMFAQLCAQLGRPPKALFTAACGLLEGVLRYLTQHQL
MESDIHLCSFDDHYLFDCMTLKIDTVAQDCLALAQHSFDQVTALIDERPLEQSALYLPGRIHWRHAGS
RALLAGE
>CORE_REP|Org47_Gene3432#
MKLMRTTVASIVAATFSLTTVSAFAAASLTGAGATFPAPVYAKWADSYQKETGNKVNYQGIGSSGGVK
QIVANTVDFGASDAPLSDDKLAADGLFQFPTVIGGVVLAVNIPGIKSGELTLDGKTLGDIYLGNVKKW
NDPAITKLNPGVKLPDQNIAVVRRADGSGTSFVFTSYLSKANAQWKEKIGAGSTVNWPTGLGGKGNDG
IAAFVQRLPGSIGYVEYAYAKQNNLAYTKLVSADGKPVSPTEESFSNAAKGVDWSKTFAQDLTDQKGD
NVWPITSTTFILVHKEQKNPAQGAEVLKFFDWAYETGAKQANELDYATLPAEVVEQVRAAWKTNVKDS
SGKALY
>CORE_REP|Org1_Gene4118#
MSTESASLKNHNTFALPVNAAHLIMADRIELMLKVWQQTRKRQEPLLILGEGSNVLFLEDFSGTVMVN
QLKGIDVREDNDAWYLHVSSGENWHDLVQYTLQAGICGLENLALIPGLAGSAPIQNIGAYGVELKDVC
EYVDLLDFSTGAIDRIPAAECGFGYRESIFKHRFQTGHVIVGLGLRLNKQWQPKLSYGDLAKLEPTTV
TPLQVFESVCAMRRSKLPDPRETGNAGSFFKNPLVNAEKAAELVAKYPGMPHYPQQDGQVKLAAGWLI
DQCELKGYRIGGAAVHRQQALVLVNIDNAHSQDVVALARHVRKTVADKFGVWLEPEVRFIGATGELNA
VAVLS
>CORE_REP|Org22_Gene1341#
MADRIHWTVGQAQALFDKPLLELLFEAQTVHRQHFDPRQVQVSTLLSIKTGACPEDCKYCPQSSRYKT
GLESERLMQVEQVLESARKAKANGSTRFCMGAAWKNPHERDMPYLQQMVQGVKAMGMETCMTLGTLDG
TQAERLAEAGLDYYNHNLDTSPEFYGSIITTRSYQERLDTLDKVRDAGIKVCSGGIVGLGETVRDRAG
LLVQLANLPKPPESVPINMLVKVKGTPLADNDDVDPFDFIRTIAVARIMMPSSYVRLSAGREQMNEQT
QAMCFMAGANSIFYGCKLLTTPNPEEDKDLQLFRKLGLNPQQTATEHGDNQQQQALAKQLLNADTAEF
YNAAP
>CORE_REP|Org43_Gene3170#
MIRIYPEQLAAQLREGLRACYLLSGNEPLLLQESQDLLRQAAQQQQFSEHYSISLDAHTDWDAIFGIC
QAMSLFASRQTLLLIFPENGPTAPIGEQLTKLATLLHEDILLILRGPRLTKAQENSAWFKALSPHGAL
VSCQTPEQAQLPRWVATRAKAMKLELDDAANQLLCYCYEGNLLALSQALERLSLLHPDGKLTLPRVEQ
AVNDAAHFTPFHWLDALLAGKSKRAWHILQQLQQEDVEPVILLRTLQRELLLLLTLQRRMASAPLRTL
FDQHKVWQNRRPLVTQALQRLSSAQLQQAVQLLTQIELTLKQDYGQSVWPELETLSMLLCGKPLATSF
TDAH
>CORE_REP|Org23_Gene1311#
MTRLTLALDAMGGDFGPCVTVPASLQALASNLQLHLLLVGNPDVISPLLAHADPVLLERLQVVPAESV
IAGDAKPSQAIRASRGTSMRIALEQLSSGNAQGCVSAGNTGALMGLAKLLVKPLDGIERPALMTAIPN
QQRSKTVVLDLGANVECDSTMLVQFAVMGAVMAEEVIGIAQPRVALLNIGEEETKGLDNIREAAAVLK
NTPAINYIGYLEGNELLTGKTDVLVCDGFVGNVTLKTMEGVVRVFLSLLKSSGDGNKQAWWLKLLGRW
LQKRVVKRFGHLNPDQYNGACLLGLRSTVVKSHGAANPHAFAVAIEQAVQAVQRQVPERIAARLEAVL
PKSD
>CORE_REP|Org47_Gene1406#
MAHIITQSTAHREDWLHQLADVITDPDELLQLLSLNTHPELPQGRDARRLFALRVPRAFAARMRPGDA
NDPLLRQVLTAREEFINAPGFTTDPLDEQRSVVPGLLHKYRNRALLLVKGGCAVNCRYCFRRHFPYQD
NQGNKNNWRQALDYIRQHPELDEIIFSGGDPLMAKDSELEWLVGELEAIPHLKRLRIHTRLPVVIPAR
ITPALCRLLSASRLQVLMVTHINHANEIDRDLQSAMAQLRLAGVTLLNQSVLLRDVNDDADTLAALSN
ALFDAGILPYYIHVLDKVQGAAHFMVSDDEARAIMQALLSKVSGYLVPRLTREVGGEPSKTPIDLRLM
QE
>CORE_REP|Org44_Gene3565#

MKPIFSRGPSLQLRLFLAVIAAIGLIVADSRLGTFVKIRNYMDTAVSPFYFLANGPRKVLDSVSETLA
TRQQLELENRALRQELLLKNSDILLLGQFKQENARLRELLGSPLRQDEHKMVTQVISTGSDPYSDQVV
IDKGSDNGVYEGQPVISDKGVVGQVVAVAKVTSRVLLICDASHALPIQVLRNDIRVIAAGSGCADDLQ
LEHLPNNTDIRVGDVLVTSGLGGRFPEGYPVAVVSSVKVDNQRAYTVIQARPTAGLQRLRYLLLLWGA
DRNGDMPLPPDEVHRVANERLMQMMPQVLPPAGSVGPQLPAPATGVAPQTTAPASVQPQAQPAAAGVV
P
>CORE_REP|Org3_Gene3147#
MKKRKLKVLSIIVVLVLALLFWGYQKIERFADTPLAIQQETIFKLPAGTGRVALEGLLVRDKLVRNGR
WFQWLLKLEPELAEFKAGTYRFTPGMTVRQMLKLLASGKEAQFTARFIEGSRLRDWQQVLQQSKYLKH
TLAGKSEAEIAAALGIPAGETPEGHLYPDTYQYTAGMSDIALLKRAHVRMNKALQAAWAGRDTSLPYK
TPEELLTMASIVEKETAVPEERSKVASVFVNRLRIGMRLQTDPTVIYGMGESYNGNITRKDLETPTPY
NTYVIAGLPPTPIAMPGEASLQAAANPAKTPYLYFVADGKGGHTFTTNLASHNQAVRMYRQALKEKNE
K
>CORE_REP|Org49_Gene4201#
MKQNRVKNLVLKGWLAAALLASGAASAAELLNSSYDVSRELFVALNPGFEQQWNQQHPNDKLTIKQSH
AGSSKQALAILQGLRADVVTYNQVTDVQILHDRGQLIPADWQARLPNNSSPFYSTMAFLVRKDNPKGI
HTWNDLVRDDVKLVFPNPKTSGNGRYTYLAAWGAASQADGNDAAKTRAFMTRFLKNVLVFDTGGRGAT
TTFVERGLGDVLISFESEVNNIRKQYGEDKYEVIVPPVDILAEFPVAWIDKNVERNGTEQAAKAYLNY
LYSPAAQQVITSFYYRVYDQKAMVAAKGQFPDTQLFRVEDQFGGWPQVMKTHFATGGELDQLLAAGRK
>CORE_REP|Org4_Gene4172#
MSLNFSPPTTSRRHWVFGLWPLLPFTLALAAAALLAWHYWPQLLMQSVVWQKALHQQMAGLLQQVKAA
PQQAGLALMLFSLGYGILHALGPGHGKVVIATYLATHPARLKSSLKLTFAASLVQGGVAIALVTLMLV
VLQLSSRQLHQSSFWLEKGSFILVMLLGVLLSWRALKRLFAAIKAMRPAPALRINSLTPLAADHVHSA
HCGCGHRHLPSDSELQAGSDWRTQAAIVLAMGMRPCSGAILVLLFSKVIGVFGWGVISALAMAFGTSL
TISMLALLVHYSRRLAVRLSRSRARAAWSAVAWGALALAGGLILLAAGLLLYVSAQPEFGGGIRPFSR
>CORE_REP|Org49_Gene3579#
MSTGSPMITLRRVAVCTMVSLWLAGCTNNASTSAPISSVGGGGAVPSGNNNGGAQQASPEGRIVYNRS
YNAIPKGSYSGGDTYTVKRGDTLFYIAWITGNDFRDLAQRNNIPEPYSLNVGQTIQLGNGSANGGGGM
LATTDATQGGVPKPPSTSQIQTATVDSQSTNAYSENSGKQNVGKMLPAAGAAAVGTAAAPVTAPEAAP
PVSSTVSNSAPVSTWRWPTDGKVIDNFSSSEGGNKGVDIAGSRGQPIFATADGRVVYAGNALRGYGNL
IIIKHNDDYLSAYAHNDTMLVREQQEVKAGQKIATMGSTGTSSVRLHFEIRYKGKSVNPLRYLPQR
>CORE_REP|Org47_Gene3808#
MSDGWNIALLGATGAVGEALLELLQERQFPVGELYPLASERSAGANVRFNGKSLLVQNAEEFDWSQAQ
LAFFVAGSEASARYAEEAGNMGCLVIDTSGLFAMEPDVPLVVPGVNPQVLADYRNRNIVAVADSMVSQ
LLTAIKPLTEQAGLSRLHVTTLMSVSSRGKAAVDDLAGQSARLLNGIPAEEGVFGKQLAFNLLPLIAD
EQGSVREERLIVDQVRKVLQDEGLPISVSCVQSPVFYGHAQVVHLEALRPLSAEEARSELEEVEDIQL
SEEDDYPTQVTDASGSDALSIGCLRNDYGIPELLQFWTVADNVRFGGALMAVETAERLVQEQMY
>CORE_REP|Org24_Gene3190#
MKRELAIEFSRVTEAAALAGYKWLGRGDKNAADGAAVHAMRIMLNNVDIDGRIVIGEGEIDEAPMLYI
GEQVGTGQGDAVDIAVDPIEGTRMTAMGQSNALAVLAVGDRGAFLHAPDMYMEKLVVGPAARGAIDLN
LPLAENLQNVAARLGKPLSQLTVIVLAKPRHDGVIAQMQQLGVRVFAIPDGDVAASILTCMPESEVDV
MYGIGGAPEGVISAAVIRALDGDMQARLLPRHEVKGDSAENRRIGEQELARCREMGIEAGQALRLDQM
ARNDNVIFSATGITKGDLLEGISRQGNMATTETLLIRGKSRTIRRIRSTHYLDRKDPALHEFLL
>CORE_REP|Org10_Gene1229#
MNWYPWLNGPYRQLIGQYADGRGHHALLLHAAAGNGDDALAYGLSRWLICQQRNGEKSCGECHSCRLM
LAGNHPDYHVLAPEKGKSNLGIEPIRQVIETLYAHAQQGGAKVIWLPQAEQLTEAAANALLKTLEEPP
EKTYFLLGCREPSRLMATLRSRCLYWHLASPDEQLSLQWLGRQAAGSQTDRLTALRLHDGAPLAAEQL
LQPQQWQQRSALCTALSAALPQRDMLSLLPVLNHEDVAERLHWLCALLVDAMKWQQGAHHYVLNQDQQ
PLVHQLASVLSSASLQQIVQQWLTCRHQLLSVVGVNRELLLTEQLLRWEQMLGAAGYSHPHSL
>CORE_REP|Org47_Gene3979#
MTTQCRSPELLPLKIIATGAALPPNRVASSTLDARLGKPAGYVEKRSGIVYRYHADDDASQAELAAAA
LQDALARSTIPAASIDLLISASAIAVQALPCSAAHILKIAGLAPGTPGFDINSSCVSFISALQVAAGL
LNAGTYRRIAIVSADLASRGIDWQHEESSLIFGDGAACAIVERGDGTGGILASLVETYPAGSELCEIR
AGGTRRNPRAGMCEQDFLFHMQGKPLFRQASALIEDYLDRLLSASGLTLGQIATVVPHQASHLSLEHM

RKRLHVSSEALVDIYRHHGNQVAASIPTALHAAVTTGRFNPGQPVMLIGTAAGLALAGMVLLP
>CORE_REP|Org23_Gene2135#
MLNTLIVGASGYAGAELTAYLNRHPHMNITALAVSAQSADAGKLLSDLHPQLKGIVDLPLQPLTDVAK
AAQGIDVVFLATAHEVSHDIAPAFLAAGCVVFDLSGAFRVQDAGFYSQYYGFEHQHGALLEQAVYGLA
EWQSDKIKQAQLIAVPGCYPTAAQLALKPLIEKQLLNLDQWPVINATSGVSGAGRKASMTTSFCEVSL
QPYGIFTHRHQPEIAAHLGVPVIFTPHLGNFPRGILETITCRLKAGVTAQDVAAAYHAAYDDKPLVRL
YDQGVPALKAVVGLPFCDIGFAVQGEHLIAVAVEDNLLKGAAAQAVQCLNIRFGFPETQSLL
>CORE_REP|Org43_Gene2703#
MASLKDVAKLAGVSLMTVSRAINDPGKLRPETYRRVKQAIDRLDYVPDLSARRIRGDGNRVQTLGVLA
LDTATTPFSVEMILSIEKTARERGWNSFVVNLFADDNAEQTVDLLLAHRPDGVIFTTMGLREVTLPAK
LLDKKLVLANCVSPAHSIASYIPDDEQGQYDATRTLIAKGYRAPLCIHLPADTLAAGLRRRGLERAWR
EAGRDVEQLRQYHLDLSAGDQSYRDCVALLERHFSAGRRDCDVVVCGNDRIAFLAYQVLLAQGWRIPQ
QVAVLGYDNMVGTGELFLPALTTVQLPHYELGRLAALHVIERREQRDTVKVPCPLLERGSL
>CORE_REP|Org21_Gene3842#
MTQPTPLCPNRMQVHSIRRETADVWTLNLICDVFYPYQAGQFALVSIRNSEETLRAYTLSSSPGQSRF
LSISVRCLPDGVGSRWLTQEVKPGNTLWLSDAQGEFSCERHPADRYLMLAAGCGVTPIVSMCRWLTAN
RPACDIAAIVNVRTPADTIFADQWRALCAAHPQLRLTLMAERDLQPGYLSGRIDEQTLRQAAPDIAER
TVMTCGPAPYMEQVEQLCRQLGVPAERFHKEQFHTPTTQADATEGLTLRAARPLREFRVPVGSTLLAA
MEANALPVNAACRAGVCGSCKTRILEGDYTTTSTMTLSAEEVAQGYVLACSCRLQGDVTLA
>CORE_REP|Org46_Gene2042#
MHSNKRVVITPGEPAGVGPDLVAALAQQDWPVELVVCADPALLLERAKRLGLPLTLRDYQPQQPAEAQ
RAGTLTVLPVPLAHPVTAGELNVGNSAYVVETLARACDGCLNGEFAALITGPVNKGVINDAGVPFIGH
TEFFADRSRCDRVVMMLATEELRVALATTHLPLLAVPGAITQQSLFEVIRILDHDLKTKFGIARPHIY
VCGLNPHAGEGGHMGHEEIDTIIPALDALRAEGIHLVGPLPADTLFQPKYLQDADAVLAMYHDQGLPV
LKYQGFGRAVNITLGLPFIRTSVDHGTALELAGTGTADVGSFQTALNLAIKMIINCNE
>CORE_REP|Org47_Gene1511#
MACGEFDLIARYFDRFKRVRRDVQLGIGDDCALLAVPEKQLVAVSTDTLVAGVHFLPDIDPADLGYKA
LAVNLSDLAAMGADPAWLSLALTLPEVNESWLKAFSDSLFDQLNYYGMQLIGGDTTRGPLSMTLTIQG
LIPAGRALTRSGARIGDWIYVTGTLGDSAAGLAILQDRLAVTETTARDYLVARHLRPQPRVLQGQALR
DLASSAIDISDGLISDLKHILKASDCGARIVLDELPMSQALSSHADAEQALRWALAGGEDYELCFTVP
EINRGALEVALSHLGADYTCIGQIGPLSEGIRYYRDDEAVELDWAGFDHFNAEPGTHG
>CORE_REP|Org5_Gene1524#
MNKKVFTLAAMAAAMMFGAAAHADTRIGVTIYKYDDNFMSVVRKAIEKDAKASPDVTLLMNDSQNDQS
KQNDQIDVLLAKGVKALAINLVDPAAAPVVIDKARANDIPVVFYNKEPSRKALDSYEKAYYVGTDSKE
SGVIQGELIAKHWQANPAWDLNKDGQIQFVLLKGEPGHPDAEARTTYVVKTLNEKGIKTQQLQMDTAM
WDTAQAKDKMDAWLSGPNANKIEVVIANNDAMAMGAVEALKAHNKTTIPVFGVDALPEALALVKSGAM
AGTVLNDADNQAKATFELAKNLAAGKPATEGTQYKIENKVVRIPYVGVDKDNLSQFVK
>CORE_REP|Org22_Gene2932#
MKKQFIQKQQQISQVKSFFSRQLEQQLGLIEVQAPILSRLGDGTQDNLSGSEKAVQVKVKTLPDATFE
VVHSLAKWKRKTLGSYDFGAGEGLYTHMKALRPDEDRLTPIHSVYVDQWDWERVMGDGERSLDYLKST
VRSIYAAIKATEAEVSREHGLTPFLPEQIHFVHSETLLQRYPDLDAKGRERAIAKELGAVFLIGIGGK
LSHGKSHDVRAPDYDDWTTPAADGLAGLNGDIVVWNPVLQDAFELSSMGIRVDAAALKHQLALTGDEE
RMKLEWHQSLLRGDMPQTIGGGIGQSRLVMLLLQLSHIGQVQCGVWSPEVRGAVEGLL
>CORE_REP|Org47_Gene1344#
MRKWGVGLTLVLLASGAMAKDIQLLNVSYDPTREFYQAYNTAFSKHYQAETGDKVTVRQSHGGSGKQA
TSVINGIEADVVTLALAYDVDAIAERGRIEKDWIKRLPDNSAPYTSTIVFLVRKGNPKQIHDWPDLIK
PGVSVITPNPKTSGGARWNYLAAWGYALHHNNNDKAKAQDFVKNLYKNVEVLDSGARGATNTFVERGI
GDVLIAWENEALLAEKELGKDKFEIITPSESILAEPTVSVVDKVVDKRGTRDVATAYLKYLYSPEGQT
IAAQHYYRPRDAAVAAKFAGQFPQLKLFTVDDTFGGWTEAQKVHFATGGVFDEISKR
>CORE_REP|Org44_Gene57#
MQGSVTEFLKPRLVDIEQVSSTHAKVTLEPLERGFGHTLGNALRRILLSSMPGCAVTEVEIDGVLHEY
STKEGVQEDILEILLNLKGLAVRVQGKDEVILTLNKSGIGPVTAADITHDGDVEIVKPQHVICHLTDE
NAAISMRIKVQRGRGYVPASARIHSEEDERPIGRLLVDACYSPVERIAYNVEAARVEQRTDLDKLVIE
METNGTIDPEEAIRRAATILAEQLEAFVDLRDVRQPEVKEEKPEFDPILLRPVDDLELTVRSANCLKA

EAIHYIGDLVQRTEVELLKTPNLGKKSLTEIKDVLASRGLSLGMRLENWPPASIADE
>CORE_REP|Org25_Gene4545#
MVTLEDVAALAGVSRATVSRVVNGDSNVKAPTREKVERAVAQLGYTPNPAARALASSHSNTLGLVTTS
YRGGFFGALMDFVQTEAESHGKQLLVTQGRNSAENEWQAIQRLFSLRCDGVILHVRFLSDDRLRQLAA
EQRDFVLLDRLVPGLEARCVTFDHPLASRMATQQLLDAGHRRIACISGPRERPSSRLRLQGFEEAMQA
ANIEPVACLEGVYDLESGYRCADRLLRQAAPPSAIYCCNEEMAIGALLAINEHRLRVPQDISLICYDS
GERAPFVRPALSSVHFPISEMAQYAARRLIDPATPTHRFEPTIINRDSIVTVRK
>CORE_REP|Org23_Gene3300#
MNRDFTFTIKSSSFNEDYNPSESTRITTNFANLARGENRRENLRNTLVMINNRFNTLAHWDNPKADRY
SVELEIISVEMRVEDQGASFPVIEILKTNIIDKKTQKRIEGIVGNNFSSYVRDYDFSVLLPAHNKNTA
EFTIPDDFGDLHGNIFKHFVNSNEYHENFSKPPVICLSVSSKDTYHRTGNQHPVLGDEYRQDGASLTD
RYFKKMGLQVRYFMPKNSVAPLAFYFPGDLLSDYTDLELIGTISTMETFQKIYRPEIYNANSAAGQCY
QPSLNNQDHSLTKIVYDREERSQLAIEQGKFTEEQFIKPYKPLLEQWSAHYAL
>CORE_REP|Org13_Gene3194#
MNYQFENLVFEGGGVKGIAYGGALELLEAKGIMPQIKQTSGASAGAIAALLVGLGCSSADVTKILSAM
DFKKFLDYNGGFFGTLQDAYRLFNQYGIAPGDYFYQWSRDIIKQYTGKPDITFEQFEAMKAAKGFKSI
YFIGANLNSGQREVYSHRTTPRMKVADGLRISMSFPFAFVAKNNTLGDLCIDGGMIDNYPVRLFDYDF
SATPPYIDSSSQRINTRTLGLRLDSAGEIAQAAGQAGPRTPVNNLFDFTLAVANVMLDIQTKVHLDSD
DWKRTVYVDTLDVGTLEFGISEEKKRALIESGRRGVERYFAWYDTAMKQAA
>CORE_REP|Org32_Gene1497#
MIEFGDFYQHIAKSPLSHWLDTLPAQLSAWQRESLHGKFKQWFNSVEHLPTLTPTRLDLLHGVRAEME
PGLSPGQLEGIEKMLRTLMPWRKGPFSLYGIDIDTEWHSDWKWDRVLPHISPLAGRTILDVGCGSGYH
LWRMIGAGAHFAVGIDPMQLFLCQFEAVRKLLGGDQRAHLLPLGIEQLPDLAAFDTVFSMGVLYHRRS
PLDHLYQLKNQLVAEGELVLETLVVEGDRHQVLVPGDRYAQMRNVYFIPSAEALKCWLEKCGFVDVKI
ADMCVTSLEEQRRTDWMTSESLAEFLDPNDRSKTVEGYPAPLRAVLTAKKP
>CORE_REP|Org11_Gene964#
MTRITRGLLLCLALLAGRGALAQPDGMLAGNLSSVGSDTLANLMALWAQDFSQHYPNVNLQIQAAGSS
TAPTALAAGAAQLGPMSRPMKAAEVSAFEHRYGYAPLAVPVAVDALVVLVHQDNPLRGLNLQQLDRIF
SATRRCGESKPLTRWGELGLSGDWATRSLQRFGRNSASGTYGYFKLRALCGGDFMPRVNELPGSASVV
QAVAGSLNGIGYASIGFRASGVRLLPLAESGEDYVAPTAANVRNDRYPLSRYLYIYINKAPNQPLEPL
TAAFLDRVLSTAGQSLVNHDGYLPLPPGALQKTRQALGLPPLAPAMMK
>CORE_REP|Org42_Gene1867#
MTLLSERTPEPPAKTPGTLKALFHRLLMAHGRKLVIALPYLWLTLLFMLPFLIVFKISLAELALAVPP
YTELMSWVDGKLNIALNFANYLQLTDDPLYIDAYLQSLRVAAVSTLCCLIIGYPLAWAVAHSKASTRN
ILLLLVILPSWTSFLIRVYAWMGILKNNGILNNFLMWLGVIDQPLVILHTNLAVYIGIVYSYLPFMVL
PIYTALIRLDYSLVEASLDLGARPLKTFFSVIVPLTRGGIIAGSMLVFIPAVGEFVIPELLGGPDSIM
IGRVLWQEFFNNRDWPVASAVATIMLLLLIVPILWFHKHQNKEMGGQA
>CORE_REP|Org18_Gene1916#
MEQLRGLYPPLAAYDSGWLDTGDGHRIYWELSGNPNGKPAVFIHGGPGGGISPHHRQLFDPERYKVLL
FDQRGCGRSRPHASLDNNTTWHLVADIERLREMAGVDQWLVFGGSWGSTLALAYAQTHPERVSEMVLR
GIFTLRKQELHWYYQDGASRFFPDKWERVLSILSDEERKDVIAAYRQRLTSADPQVQLEAAKLWSVWE
GETVTLLPSRESASFGEDDFALAFARIENHYFTHLGFLESDDQLLRNVPLIRHIPAVIVHGRYDMACQ
VQNAWDLAKAWPEAELHIVEGAGHSYDEPGILHQLMIATDRFAGK
>CORE_REP|Org28_Gene1656#
MQILLANPRGFCAGVDRAISIVERALELYGAPIYVRHEVVHNRYVVDSLRERGAVFIEEIAEVPDGSI
LIFSAHGVSQAVRAEAKARDLTMLFDATCPLVTKVHMEVARASRRGTEAILIGHAGHPEVEGTMGQYS
NPQGGMYLVESPEDVWKLQVKDESNLCFMTQTTLSVDDTSDVIDALRQRFPSIIGPRKDDICYATTNR
QEAVRNLAGDADVVLVVGSKNSSNSNRLAELAQRVGKPAYLIDSAADIQESWLSGARHIGVTAGASAP
DVLVQEVISRLKALGGMDVHEISGREENIVFEVPKELRVDVRQID
>CORE_REP|Org38_Gene1107#
MANNAKTAKSKALRGGWREQWRYAWMNAIKDMLRQPLATLLTVMVIAISLTLPSVCYIVWKNVSTAAS
QWYPTPQLTVYLDKSLDDDAALKVLDAIKAEAGVEKVNYLSREEARGEFRNWSGFGGALDMLEENPLP
AVAIVTPKMSFQSSDTLNTLRDRVAAVQGVEEVRMDDSWFARLAALTGLAGQIAAIIGVLMIVAVFLV
IGNSVRLSIFSRRDTINVMKLIGATDGFILRPFLNGGAMLGFAGALLSLVLSGALVWQLESVVAGVAK

VFGTTFTLHGLGWDEALLLLIISAMIGWIAAWLATVQHLRRFTPQ
>CORE_REP|Org33_Gene3861#
MSQLSYPLASARPLNGWQLMTALINGEKAPSNAWKKTSFRLKFLGRSLLNWRTTSGLLSTLASNPLLE
EILSAQPNLPCKLHRPYLAANMSKIECLFALRDHYDLIAQRMPLKMRLGHLGPQPFVLASAMGKNEAP
IALELAAIDKLNKEGEATLLLRNANGVMLAEITFALMHYQQQPTLFIGGLQGANHEVPHAEIQHTTKE
CHGLFPKRLVLEGICTLARHLGIRQIVAVGNATHIYQNWRYQSKKKDKLHADYDQFWLSMGAKPLDSG
YFLLPERIARKPIEEIASKKRAEYRRRYQLLDELEQGLAAHFCAR
>CORE_REP|Org40_Gene2591#
MYTKILGTGSYLPVQVRTNADLEKMVDTSDEWIVTRTGIRERRIAAADETVATMSFQAAEKALEMAGV
AKEDIGLIVVATTTTTHAFPSAACLVQQMLGIKDCAAFDLAAACAGFTYALSVADQYVKNGAVKHALV
IGADVLSRTLDPEDRGTIILFGDGAGAVVLGASEAPGILSTHLHADGSYGSLLTLPYKDRQNQDKPAY
VTMAGNEVFKVAVTELARIVDETLQANNMDRSELDWLVPHQANLRIISATAKKLGMGMDKVVVTLDRH
GNTSAASVPSALDEAVRDGRIQRGQLVLLEAFGGGFTWGSALVRF
>CORE_REP|Org28_Gene3967#
MIKLGIVMDPIDSINIKKDTSFAMLLEAQRRGYELHYMEMNDLYLHAGDGRARTRLLSVKEDKENWFS
FGSEQDLALHDLDVILMRKDPPFDTEYIYATYILERAEVKGTLVVNKPQSLRDCNEKLFTAWFPELTP
DTLVSRSAAHIRKFHQQHGDVILKPLDGMGGASIFRVKQDDPNLSVIIETLTEHGSRFCMAQNFLPAI
KDGDKRILVVDGEPVPYCLARIPAQGETRGNLAAGGRGEARPLSESDWKIARAVAPTLKEKGLIFVGL
DVIGDRLTEINVTSPTCAREIEAAFPISITGMLMDAIEKRLAAK
>CORE_REP|Org39_Gene1077#
MIKRDQSLATPYLQFDRTQWAALRDSVPLTLSEEEIVKLKGINEDLSLEEVAQIYLPLSRLLNFYISS
NLRRQAVLEQFLGTDGQKIPYVIGIAGSVAVGKSTTARLLQALLSRWPEHRSVELITTDGFLHPNKVL
NERGLMKKKGFPQSYDMHSLVKFVSEVKSGAKRVTAPVYSHLIYDVVPEGNKVIEQPDILILEGLNVL
QSGMDYPHDPHRVFVSDFVDFSIYVDAPETLLQSWYINRFLKFRQGAFSNPDSYFHHYSKLPEPEAIN
IATQLWNEINGLNLQQNILPTRERASLIMTKSANHAVESVRLRK
>CORE_REP|Org21_Gene463#
MLSAFKLDNRRLSRLELDDSDDLTSSLWVDLVEPEEGERERVQNELGQSLATRPELDDIEASARFFED
EDGLHIHSFFYFEDAEDHAGNSTVAFTIRDGRLYTLRERELPAFRLYRMRARNQTMLEGNAYELLLDL
FETKIEQLADEIENIYSDLEQLSRVIMEGHQGDEYDAALSTLAELEDIGWKVRLCLMDTQRALNFLVR
KARLPTGQLEQAREVLRDIESLLPHNESLFQKVNFLMQAAMGFINIEQNRIIKIFSVVSVVFLPPTLV
ASSYGMNFEFMPELKWSFGYPGAITLMILAGLAPYLYFKRKNWL
>CORE_REP|Org15_Gene2458#
MQNRLTIKDIARLSGVGKSTVSRVLNNEGSVSPQTRERVEAVIRQQGFTPSKSARAMRGQSDKVVGII
VSRLDSPSENQAVRTMLPLLYQQGFDPIVMESQFETRLVQEHLHVLHQRNVDGVILFGFTGLTAAMLK
PWQEKMVVMVREYDGFSSVCYDDAGAVNLLMDRLYQQGHRHIGYLGVQLSDATTGQRRYQAYLDACER
LTLTPRATLGELSYQSGFQHAAEVIDAHTSALICASDSIALGAIKYLQQQPTRAIQVCAIGNTPLLSF
LFPDTLSVEFGYGSAGLLAAQQLLAQLSGEQGIRRLVVPSKLS
>CORE_REP|Org40_Gene167#
MYHLRVPVTEQELKEYYQFRWEMLRKPLHQPVGSEKDAYDAMAHHQMVVDEAGKIVAIGRLYINADNE
AAIRFLAVDPTLQDKGLGTLVAMTLESVARQEGVKRVVCSAREDAVDFFAKLGFVNQGEITAPQTTPI
RHFLMIKPVATLDDILHRPDWCGQLQQAWYEHIPLSEKMGVRISQYTGQRFVTTMPEIGNQNPHHTLF
AGSLFSLATLTAWGLIWLLLRERHLGGTIILADAHIRYSKPITGRPRAVADLGSLSGDLARLARGRRA
RVHAEVHLFGDEDKGAVFEGTYMVLPAEPDVPLDQGGSEALEE
>CORE_REP|Org9_Gene4148#
MMKKIILDCDPGHDDAIALLLAWGNPQIDLLAVTTVVGNQTLDKVTRNALAVARIANITGVPFAAGCP
RPLVRNIEVAPDIHGDSGLDGPVLPEPHLQLDSRHAVDLIIDTVMAHPPGSVTLVPTGGLTNIAMAVR
KEPRIAERVKEVVLMGGGYHVGNWSAVAEFNIKIDPEAAHIVFNEKWPLTMVGLDLTHQALATPAVCA
RIAALGTRPAAFVGELLAFFGRMYQQAQGFSAPPVHDPCAVAYVIDPSVMTVRKAPVDIELTGTLTLG
MTVADFRAPPPPDCHTQVAVKLDQDKFWDLVVDALERISEVE
>CORE_REP|Org30_Gene3715#
MAQKDYVSRGRAAGAKRKTPSRKKRSSPKVSKTVLALAAALLVVFVGGLYFITHNKPEDAPLLPAHTT
RPGNGLPPKPEERWRYIKELENRQIGVQTPTEPTAGGELNSKTQLTAEQRQLLEQMQADMQQRPTQLN
EVPYNDPGQANARSTRQQQQMQQQQMQQQPVQQQQQVSQPPRNPFNNGATTAPVQQHPQPKPTTQQPV
QVKQPEPKPQPKPEPKPEVKQETAKQETKPESKQKWMVQCGSFRATDQAESVRARLAFEGIESRITAG

GGWNRVVLGPYSSRAAADKTLSRLKGVGMSSCIPLSVGG
>CORE_REP|Org28_Gene1290#
MSIRIVPKEQLGAQREKSTTAENIPPLLFANLKSLYSRRADRLRQLAVDNPLGDYLNFAAELAQAQQH
ALHDNPLQLDLSEALAQGAASGKPPLDLSVFPRSEHWRKLLTSLIAELRPQAPEHILAVLDNLEKASA
HELELMADALLNREFGKVGSEKAPFLWAALSLYWAQMASLIPGKARAEYGEQRQFCPVCGSIPVSSMV
HIGTVNGLRYLHCNLCESEWHVVRVKCSNCEQTRDLNYWSLDSEQAAVKAESCGDCGTYLKILYQEKD
PQVEAVADDLASLVLDARMEEEGFGRSSINPFLFPAE
>CORE_REP|Org1_Gene911#
MVKVYAPASIGNVSVGFDVLGAAVSPIDGTLLGDCVSVEAAETFTLQNAGRFVSKLPAEPKENIVYQC
WERFCQEIGREVPVAMRLEKNMPIGSGLGSSACSVVAGLMAMNEFCDRPLDKMTLLGLMGELEGRISG
SVHYDNVAPCYLGGLQLMLEEEGIISQEVPCFDDWLWVMAYPGIKVSTAEARAILPAQYRRQDCISHG
RYLAGFIHACHTRQPQLAAKLMQDVIAEPYRTRLLPGFAEARKAAQDIGALACGISGSGPTLFAVCND
GATAQRMAAWLQQHYLQNDEGFVHICRLDTAGARLLG
>CORE_REP|Org36_Gene4684#
MPIRVPDELPAVNFLRGENVFVMTSSRAKTQEIRPLKVLILNLMPKKIETENQFLRLLSNSPLQIDIQ
LLRIDSRESKNTPAEHLNNFYCDFEDIQNENFDGLIVTGAPLGLVDFCDVAYWPQIERVIDWAKHHVT
STLFVCWAVQAALNILYGIPKMTREVKLSGVYPHQTLQQHALLTRGFDESFLAPHSRYADFPTEVIRQ
YTDLDILAESEQAGAYLFASRDKRLAFVTGHPEYDTLTLAGEYCRDNEAGLDPAVPLNYFPDDNPALT
PKATWRSHGHLLFSNWLNYYVYQITPYDLRHMNPTLD
>CORE_REP|Org17_Gene4172#
MSKLRVGVVGLGSIAQKAYLPILSQAADWTLVGCFSPNQQKAQPLCDSYRMACFPRLDSLAEQCDAVF
VHSSTASHFSVIGELLNRGVHVYVDKPLAETLEQGEQLLELAERRGKTLMVGFNRRFAPLYRQLKQQM
NQPASIRMDKHRADSVGPHDLRFTLLDDYLHVVDTTLWLAGGGEQLLSGSVRANAAGEMLYAEHHFAC
GDTLVTTSMHRRGGSQRESVQAVTDGARYQLTDMRHWLREDAQGELEQPVPGWQSTLVQRGFDGAVRH
FLCAVANQSAPETGGEQALVAQRVVERLLRDNSM
>CORE_REP|Org48_Gene2865#
MQKFDTKTFQGLILTLQDYWARQGCTIVQPLDMEVGAGTSHPMTCLRALGPEPMATAYVQPSRRPTDG
RYGENPNRLQHYYQFQVVIKPSPDNIQELYLGSLKELGLDPTIHDIRFVEDNWENPTLGAWGLGWEVW
LNGMEVTQFTYFQQVGGLECKPVTGEITYGLERLAMYIQGVDSVYDLVWSNGPLGVTTYGDVFHQNEV
EQSTYNFEYADVDFLFSCFEQYEKEAQSLLALEKPLPLPAYERILKAGHTFNLLDARKAISVTERQRY
ILRIRTLTKAVAEAYYASREALGFPMCNKKNEN
>CORE_REP|Org44_Gene1547#
MRNRTLADLDRVVALGGGHGLGRVMSALSSLGSRLTGIVTTTDNGGSTGRIRRSEGGIAWGDTRNCLN
QLITEPSVASAMFEYRFSGNGELAGHNLGNLMLKALDHLSVRPLEAINLVRSLLKVDAALIPMSEQPV
DLMAHDHEGNHVYGEVNVDQLAHMPQELMLSPPVSATREALDAIAQADVILIGPGSFLTSLMPLLLLD
DLTQALRRSSASMIYIGNLGRELSVAAAALSLQDKLTLMEEKIGRRMIDALIVGPAVDASEVQDRVVI
QQPLEASDIPYRHDRQLLRQALDRALVALAARR
>CORE_REP|Org43_Gene617#
MLWFKNLMVYRLSREVALNADEMEKQLSAFAFTPCGSQDMAKTGWVSPMGSHSDALTHAVNGQIVICA
RKEEKILPSPVIKQELQAKIERLEAEQHRKLKKTEKDALKDEVLHSLLPRAFSRFNQTFMWIDTVNDL
IMVDAASAKRAEDTLALLRKSLGSLPVVPLTMESPIELTLTEWVRSGEMPAGFAIQDEAELKAILEEG
GVIRCKKQNLISDEIAVHIEAGKLVTKLAVDWQERIQLMLSDDGSLKRLKFADTLREQNDDIDRDDFA
QRFDADFILMTSELAALIKNTIEALGGEAQR
>CORE_REP|Org26_Gene2883#
MMRHFLGVDVGGTNTRLLLMDDDGEFSGYRKIATADWARQADPLAALGRLIAGHCQDRQVAQVMLGLP
GILSRDRSRVLSLPFIPALDAQPVAALLADLLALPVRMDKDVNHLLWWDLQQLPALPQVAVGLYLGTG
MGNSLWLNGNFYHGAHGAAGELGHIPWPGHQGECPCGKRGCVESLTSGHWLTGWARANAAQTPFERLF
ERHGEHPDLRRFVERLAQTIAIEMNVLDPERLILGGGVIAMSGFPLAQLEQEIRRHLREPQPAQGLAI
SISRLSDETGSKGACLAARRHFQLSREYPQ
>CORE_REP|Org38_Gene1834#
MSEKLQKVLARAGHGSRREIETMIEAGRVSVDGKVAKLGDRVEVTPAMKIRLDGHVLSIKESEEVVCR
VLAYYKPEGELCTRSDPEGRPTVFDRLPKLRGSRWVAVGRLDVNTSGLLLFTTDGELANRLMHPSREV
EREYAVRVFGQVDDAKVKQLSKGVQLEDGPAAFRTISFQGGEGLNQWYNVTLTEGRNREVRRLWEAVG
VQVSRLIRVRYGDIDLPKGLPRGGWAELDLPAINYLRELVELKPETVSKMPVERERRRVKANQIRRAV

KRHSQVAGSGRRSAPGSKPSKSGKPSKRS
>CORE_REP|Org13_Gene2381#
MPQHSHAPALLILNGKGAGNEELRQAVKRLRAERITLHVRVTWEHGDAARYVAEAAQLGVGTVVAGGG
DGTINEVAAALVQLPAHNRPVLGILPLGTANDFAMACNIPPSPEQALQLAIKGRSVPIDLAKVNGERY
FINMATGGFGTRITTETPEKLKAALGGVSYFVHGLLRMDTLQADRCEIRGPDFRWAGEALVIGIGNGK
QAGGGQELCPSALINDGLLQLRLLIADELLPALVAALFNDEESNSILSAALPWLEIDAPHEMTFNLDG
EPLKGRHFRIEVLPQAIECRLPPNCALLG
>CORE_REP|Org34_Gene2652#
MKKVGLRVDVDTFSGTREGVPQLLDLFDKYDIQASFFFSVGPDNMGRHLWRLLRPKFLWKMLRSNAAS
LYGLDILLAGTAWPGRNISRALGPLMKRTAEAGHEVGLHAWDHQGWQAKVGKWSEAQLTEQVQRGVDA
LSASTGQPVKCSAVAGWRADTRVLEVKQRFGFHYNSDCRGTHPFRPVLSDGRHGTVQIPVTLPTFDEV
IGSEVSMADFNDYILRAIENDRGVPVYTIHTEVEGMSQAAMFEQLLQRARQQGIEFCPLSALLPQDLA
SLPLGRIKRAPFPGREGWLGCQTDVKDVT
>CORE_REP|Org25_Gene625#
MLDKTRLRIAMQKSGRLSDESQELLARCGIKINLQQQRLIAFAENMPIDILRVRDDDIPGLVMDGVVD
LGIIGENVLEEELLSRRAQGEDPRYFTLRRLDFGGCRLSLATPLDAEYAGPQSLQDARIATSYPHLLK
QYLDKQGVRFKSCLLNGSVEVAPRAGLADAICDLVSTGATLEANGLREVEVIYRSKACLIQRDGEMPE
AKQQLIDRLMTRIQGVIQARESKYIMLHAPSEKLDEIVALLPGAERPTILPLAGAQNRVAMHMVSSET
LFWETMEKLKALGASSILVLPIEKMME
>CORE_REP|Org18_Gene1667#
MIAGTPASAETLRQLKQQHPAIFAQQGRYLRTVGLIALAIVLYYVFFFLVFGITWPQFINGCQQLGRY
FLRMFVWHDFVNWPFMYYFQQIGITIAIVFAGTITASLIALPLSFFAARNVMSTPLLRPISVLVRRLL
DVLRGIDMAIWGLIFVRAVGMGPLAGVLAIVMQDVGLLGKLYAEGHEAVDKSPSRGLTAVGANGLQKH
RYGIFTQSFPTFLALSLYQIESNTRSAAVLGFVGAGGIGLVYAENMRLWNWDVVMFITLILVVVVMIM
DKVSSMLRNKYIIGEDIPLYQQKSQID
>CORE_REP|Org46_Gene1345#
MSFFRRKKLPSAIKPGVTFGRLLYQRIDHDGLTMLAGHLAYVSLLSLVPLVTVVFALFAAFPMFSDIS
EQLKSFIFSNFVPAAGNVIQNYLEQFVANSNKMTAVGTCGLIVTALLLISSVDSVLNTIWRSKNKRPI
VFSFAVYWMVLTLGPLLVGASMAISSYLLSLNWLAQTGVNGLVDQVLRIFPLILSCASFWLLYCIVPT
VRVPPKDALIGALVAGLLFELGKKGFALYVTMFPSYQLIYGVLAVIPILFLWVYWSWCIVLLGAEITV
TIGEYRDYRRQKAEQQEQKPEGQQE
>CORE_REP|Org37_Gene4414#
MPELPEVETSRRGIEPYLVGHSIQYAVVRNARLRWPVSEQILTLSDRPVLSVQRRAKYLLIELENGWI
IVHLGMSGSLRMLREENEDEAGKHDHVDLVISNGMILRYTDPRRFGAWLWCEDLATSSVLAHLGPEPL
SEAFNGDYLYEKSRNKRTLIKPWLMDNKLVVGVGNIYASESLFSAGILPDRPAGSLSKAEAELLVKTI
KAVLQRSIEQGGTTLRDFLQSDGKPGYFAQELQVYGRAGEPCRACGTPIESAKHGQRSTFFAAAASAE
HHSAASFAISAWVTKSGRKGAISPP
>CORE_REP|Org4_Gene959#
MATMDMQNAVVLAESRRKMQAWRRQKNRLALFLSMATMAFGLFWLIWILIATVTKGFDGMSLALFTEM
TPPPNTAGGGLANAIAGSGLLILWATIFGTPLGIMAGIYLAEYGRKSWLAEVIRFINDILLSAPSIVV
GLFVYTIVVAKMEHFSGWAGIVALALLQVPIVIRTTENMLKLVPDTLREAAYALGTPKWRMISAITLK
ASVSGIITGVLLAIARIAGETAPLLFTSLSNQFWSTDLMQPIANLPVTIFKFAMSPFAEWQQLAWAGV
LLITLCVLLLNILARVIFAKKKHS
>CORE_REP|Org29_Gene3419#
MPKQDVLKVVPARYPLRLIGALFSLFILAAIVQSVAGNARWEWGVFAEWFFAPAVLAGLGQTLLLTLL
GTLFSILFGTLLALARLSRSYLLASLAWGYIWLFRSLPLILVLIILYNFSYLYDAISLGIPFTSVVFA
SYPTIDILGQFAVAVLGLTLVQSAYTAEIIRGGILGVDYGQHEAAAALGLPGYRRTFRIILPQALRSI
IPTGFNEIISLAKGTSIVYVLALPELFYTIQVIYNRTQQVIPLLMVATVWYLFITTALSVIQYYIERY
FARGAVRELPPTPWQKLAGWLKR
>CORE_REP|Org24_Gene4547#
MSSSRPGFGCSWLPYALVLPQLLITAVFFLWPAGEALWYSVQSLDPFGLSSQFVGLDNFKQLFQDPYY
LDSFYTTLIFSFLVAGIGLAVSLFFAALVDYVLRGSRLYRTLMILPYAVATAVAAVLWIFLFNPGLGL
ITHFLNGLGYNWNHAQNSGQAMFLVVLASVWQQISYNFLFFLAALQSIPRSLVEAAAIDGAGPVRRFF
HLVLPLIAPVSFFLLVVNLVYAFFDTFPVIDAATGGGPVQSTTTLIYKIYREGFAGLDLSSSAAQSVI

LMLLVIGLTVIQFRFVERKVRYQ
>CORE_REP|Org46_Gene2357#
MKKQGKPFLNFKPLAVSPTRRQLLLSGLAVALLGVKSQRARAEGLLRNQHSKPAAKPAGAKKLVMIDP
GHGGIDSGAVGHEGSQEKHIVLEIANHVRRFLHERDHVEARLTREEDEFIPLFQRVEIAHQHQADLFI
SIHADGFTSPSASGASVFALSNRGASSAMARYLSNRENAADDVAGGKYKDQDNYLQQVLFDLVQTDTI
NNSLTLGRHVLGQIRPVHHLHSDSTEQAAFAVLKSPSIPSVLVETSFITNPNEERLLGTTAFREKIAR
AIADGIVNFFDYFDAHQRKPR
>CORE_REP|Org46_Gene2619#
MTDSNPPSTAFTLYDLHSHTTASDGYLTPTQLVQRAVEMRVGVLAITDHDTTAGLAEAAAAIAEQALP
LRLVNGVEISTLWENHEIHIVGLGMDVAHPALVALLAEQTERRNRRAQEIGVRLGKARIPDAYAGAQR
LAGAGAVTRGHFARYLVEIGVAGNMAQVFKKFLAKGKTGYVPPQWCTIEQAIDAIHQSGGQAVMAHPG
RYDLTAKWLKRLLAHFAEHGGDAMEVAQCQQAPHERSQLAKYAQDYRLLASQGSDFHQPCSWIELGRK
LWLPGGVEPVWRDWPQPGEAV
>CORE_REP|Org20_Gene2724#
MIKGITLSVVASILFGAMYYFTSTLTPLNGEQVYGWRTLLTLPFLTLFMALSGDWRKVGDTLGWIGQR
PQRLLGLLLTSALLGVQLWLFLWAPLHGKALDVSLGYFLLPLTMVLAGRLIYRDRLSLLQKLAVACAM
VGVGNELYQAGGVSWPTLVVALGYPLYFILRRRFGTDNLGGLWCELALMLPAAAWFAFGDGGAAALPT
NAELYWRIPLLGVISAVALVCYILASRLLPFSLFGLLSYVEPVLLVIVALLLGESIGQSEWPTYLAIW
LAVLLLAAEGAQHLLRRQRV
>CORE_REP|Org35_Gene1137#
MKYALGPVLYYWPKNDIATFYQQAADSSADIIYLGESVCAKRREMKVGDWLALAREIARSGKQVVIST
LALLQAPSELNELKRYVENGEFLFEANDLGAVNMAAERGLPFVAGHALNCYNAYTLRLLRRQGMIRWC
MPVELSRDWLANLLTQCDELGFRHDFEVEVLSYGHLPLAYSARCFTARSENRGKDECETCCIKYPQGR
MMRSQEQQQVFVLNGIQTMSGYCYNLGNELPNMQGLVDIVRLSPQGAETLAQIDAFRANERGEQPLAL
TDHADCNGYWRRVAGLALVG
>CORE_REP|Org33_Gene634#
MSNHDQLHRYLFENYAVRGELVTVSETYQQILNNHDYPAPVQKLLGELLVATSLLTATLKFDGDITVQ
LQGDGPLKLAVINGNNRQEMRGVARTQAPIADDSTLHQMIGNGVMVITISPTEGERYQGVVGLEGETL
AECLEAYFRQSEQLPTRLFIRTGEAEGNAAAAGMLLQVLPAQDGNLDDFDHLVQLTNTVKSEELFGLP
ANEVLYRLYHQEEVTLYEPQDVQFRCTCSRQRCADALLTLPTDEVADMLEQDGNIDMHCDYCGSHYVF
DPVDVAALYAGNTGESDQLH
>CORE_REP|Org22_Gene2240#
MNEKIFTLPVSEQISPYISQRQLDELGVVVVSHPKVRAAVALQGAHLLAWQPSGEQPVIWLSNNTPFA
KGKAIRGGVPICWPWFGPVAQPSHGFARNQPWSLTAHDEDDNGVILTFTLKDNEQTRKLWPHAFTLIA
RFKLGEECEIELESHGDYQATAALHSYFQVGDIDRVSVAGLGEPYIDKVAGGAEARQTGEVTFVGQTD
RVYTRPEAFSLIRDPAFTRTIEVHHHHMSDVIAWNPGVELSCSMGDMPNDGYKTMVCVETGRVSKPLV
AAGEQPARLGVTFRSRKQA
>CORE_REP|Org23_Gene1786#
MADVSAFNGAERPRVNWGKWTLIAIGTLFSVLLLVVPMMSIFAEAFSKGFGAMWSNLLDPDMLHAIWL
TVLIALITVPFNLVFGTLLAWLVTRFTFPGRQLLLTLIDIPFAVSPVVAGLIYLLFYGSNGLLGGWLD
AHNIQIMFSWPGMVLVTIFVTCPFVVRELVPMMLSQGSQEDEAAILLGASGWQMFRRVTLPNIRWALL
YGVVLTNARAIGEFGAVSVVSGSIRGETYSLPLQVELLQQDYNTVGSFTAAALLTLMAIVTLFLKSAL
QWRLERQNARLEREENHEH
>CORE_REP|Org4_Gene806#
MSNSYLAFPKFDPVIFSIGPVSLHWYGLMYLVGFVFAMWLAVRRANKPGSGWTKDEVENLLYAGFLGV
FVGGRVGYVLFYNLPLFLDNPLYLFKVWDGGMSFHGGLMGVILVMFWFARRTKRTFFQVSDFIAPLIP
FGLGAGRLGNFINGELWGRVTTDTPWAMLFPSSRSEDVALAAADPSLLPLLNQYGVLPRHPSQLYELL
LEGVVLFIILNLFIRKPRPMGAVSGLFLIGYGAFRIIVEAFRQPDAQLGLFDGVISMGQILSIPMVVA
GVIMMIWAYRRRPQQQLS
>CORE_REP|Org6_Gene3586#
MLTNSSIRLNKYISESGICSRRDADRYIEQGNVFINGKRATVGAQVFAGDVVKVNGQLIEPRNEEDLV
LIALNKPVGIVTTTEDGERDNIADFVNHSKRIFPIGRLDKDSQGLIFLTNHGDLVNKILRAGNNHEKE
YLVTVNKPVTDEFIRGMGAGVPMLGTVTKKCKVKKEAPFVFRITLVQGLNRQIRRMCEHFGYEVTKLE
RTRIMNVSLKGLPLGEWRDLTDDELIELFKLIEGSSSEAKPAKKAPAKSAAARKPSAGGPKSADKAAA

PAGRKRFTQPGRKKKGR
>CORE_REP|Org29_Gene904#
MATLLQEENTTSLEAIPSNSTATPRPTVLVFDSGVGGLSVYQEIRQLLPDLHYIYAFDNVAFPYGEKS
EEFIVERVLEIVGAVQQRHPLAIVVIACNTASTVSLPALRERFSFPVVGVVPAIKPAARLTVNGIVGL
LATRGTVQRSYTHELISRFATDCKIELLGSSELVELAEAKLHGEAVPLPVLKKILHPWLSMREPPDTV
VLGCTHFPLLAEELMQVLPEGTRLVDSGAAIARRTAWLISTQENLVSSQEENLAYCMALNEDTDALLP
VLQGYGFKSLKKLPL
>CORE_REP|Org10_Gene845#
MNTDFAHYYQRIRSKQKREALLWSLGLVVLYLGAGNLAEFNLHTVWVSIPHFFDYLAETVPTLHWHLL
FADGRTEGSLAYWGYRLNIQLPLIWETLQLALAATIFSVLVATVLAFLAAGNTYTPASVRLAIRTLVA
FLRTMPELAWAVMFVMAFGIGAIPGFLALALHTIGSLTKLFYESIETASNKPVRGLAACGATPLQRMR
FGLWPQVKPVFLSYSFMRLEINFRQSTILGLVGAGGIGQELMTNIKLDRYDQVSMTLLLIIVVVSVLD
YVSGELRKRVVEGAK
>CORE_REP|Org12_Gene3831#
MKKSRKVFQNVVIVGVVAWLLLFVFMPNLMIIGTSFLTRDDANLVQMVFTLDNYRRLFDPLYAQVLLH
SLNMALIATLCCLVIGYPFAFILARLPQKVRPLLLFLLIVPFWTNSLIRIYGLKLFLSTRGYLNDALL
WIGVIDKPLRIMYTSEAVILGLVYILLPFMVMPLYSSIEKLDKSCLEASRDLGASKLQTFIRIIVPLT
MPGIIAGCLLVVLPAMGLFFVADLMGGAKNLLIGNVIKSQFLNIRDWPFGAATSICLTLVMGLLLLVY
YRAARLLNKKEDLA
>CORE_REP|Org48_Gene3876#
MTAGTPFSGKEPRVERSVFYISDGTAITAEVLGHAVLSQFPVTATTFTLPFVETEARARAVRQQIDDI
YNQTGVRPLVFYSIISPEVRDVIVQSQGFCQDIVQALVGPLQGELEVEPTPVPNRTHGLTASNLGKYD
ARIAAIDYTLAHDDGISLRNLDQAQVILLGVSRCGKTPTSLYLAMQFGIRAANYPFIADDMDNLHLPA
SLKPFQHKLFGLTIDPERLAAIREERRENSRYASLRQCRMEIAEVEALFRKNQIRYLNTTNYSVEEIS
TKILDILGMSRRMF
>CORE_REP|Org45_Gene695#
MVLMIVSGRSGSGKSVALRALEDMGFYCVDNLPVVLLPQLANTLAERNISAAVSIDVRNMPESPEVFE
YAMTQLPDSFSPQLLFLDADRNTLIRRYSDTRRLHPLSSKNLSLESAIDEESDLLEPLRSRADLIIDT
SEMSVHELAEMLRTRLLGKRERELTMVFESFGFKHGIPIDADYVFDVRFLPNPHWDPKLRPMTGLDKP
VASFLDRHTEVHNFIYQTRSYLEQWLPMLETNNRSYLTVAIGCTGGKHRSVYVAEQLADYFRSRGKNV
QSRHRTLEKRKQ
>CORE_REP|Org8_Gene365#
MSQAALNARDREVDNGPRRSNGTQLAGMIFLLMVLGTVVWSGWAVLGWMKDASRLPLSRLVVTGERHY
TTNDDIRQAILALGAPGTFMTQDVDVIQQQIERLPWIKQASVRKQWPDELKIHLVEYVPVARWNDLHM
VDAEGKAFSVPAERAGKQKLPLLYGPEGSEQDVLEGYRAMSATLAASKYTLKMAAMTARHSWQLALDN
DVRLELGRDDRNGRLQRFIELYPVLQQQGQAESKRVSYVDLRYESGASVGWAPVLVDPQALGGQQNSN
QQQNQAQAKQQ
>CORE_REP|Org19_Gene2883#
MMPNRLEPELDIYQYPEHLRACLAPLPKGPGVYLFHGESESLPLYIGKSVNVRSRVMAHFRAQDEAKM
LRQTRRISFIETAGELGALLLEAQLIKQQQPLFNKRLRRSKQLCALRLQADTVTIAHAKEIDFAVTPH
LYGLFANRHAALEKLRAIADEHRLCYGKLGIDKLPAGRACFRYSLRKCAGACCGVESAQEHAQRLGAA
LEQLRIACWPFAGRVALEEQGETLRQYHVIHNWFYLGSVSSLAQAQRLQSAASHFDSDGYKILCKPLM
AGDYRIIELP
>CORE_REP|Org17_Gene2376#
MNERLNITPLGPYIGALVENVELARPLGDGQFEQLYHALLKHQVLFFRNQPITPLQQRDLAGRFGDLH
IHPVYPHATDVEEIIVLDTHDDNPPDNDNWHTDVTFIENPPLGAILAAKTLPATGGDTLWASGIAAYE
ALSAPFRTLLAGLRAEHDFTKSFPEHKHRGSEEEHQRWQLAVQKNPPLLHPVVRTHPVSGRQALFVNE
GFTTRIVDLAPKESDALLNFLFAHITKPEFQVRWRWQENDVAIWDNRVTQHYANADYLPQRRIMHRAT
ILGDKPFYKA
>CORE_REP|Org30_Gene693#
MIENRRGLDIFSHVMLIIGVLVVLFPLYVAFVAATLDDKQVFQVPMTLVPGGHLWENIRNIWQGGVGN
LKVPFSLLLLNSVIMALAITFGKIAVSVLSAYAIVYFRFPLRSLFFWLIFLTLMLPVEVRIFPTVEVI
SNLNLLDSYTGLTLPLMASATATFLLRQFFMTLPDELLEAARIDGAGPMRFFWDIVLPLSKTNLAALF
VITFIYGWNQYLWPILITSDASMGTAVAGIKSMISTSGAPTQWNQVMAAMILTLLPPLAVVLLMQRWF

VRGLVDSEK
>CORE_REP|Org24_Gene3142#
MSSYQDHQALSGLTLGKPTAYRDRYDASLLQAVPRSMNREPLGLYPDNLPFHGADIWTLYELSWLNAN
GLPQVAVGEISLNADSLNLIESKSFKLYLNSFNQTPFADWETVRSTLQRDLSACAQGEVSVTLFSVEQ
LEGTPIARLAGDCIDQQDIRIDNYEFNADYLLNAAGEEVVEEQLVSHLLKSNCLITHQPDWGSVQISY
RGGKIDREALLRYLVSFRHHNEFHEQCVERIFNDLMRYCRPQSLTVYARYTRRGGLDINPWRSNVEFA
PERGRLARQ
>CORE_REP|Org44_Gene792#
MLALSSSKRVLPGFGLSLGSSLFYTCLILLLPLTALVMQLAQMSLAQYWEVISNPQVVAAYKVTLLAA
GVASLFNAVFGMLMAWILTRYRFPGRSLLDGLIDLPFALPTAVAGLTLAGLFSTTGWYGQWLAHFDIK
VTFTWLGIAVAMAFTSLPFVVRTVQPVLEELGPEYEEAAETLGATRWQSFRRVVLPEVAPALLAGTAI
SFTRSLGEFGAVIFIAGNIAWKTEVTSLMIFVRLQEFDYPAASAIASVILAASLLLLFSINVLQSRFG
RRLGGGH
>CORE_REP|Org41_Gene649#
MKFVGAHVSASGGVDQAVIRAHELEATAFALFTKNQRQWKAAPLAADVIDKFKSACAQYGFGPGQILP
HDSYLINLGHPVAEALEKSREAFIDELQRCEQLGLTLLNFHPGSHLLQIDEDKCLARIAESINIALDK
TAGVTAVIENTAGQGSNLGFKFEHLAAIIDGVEDKSRVGVCIDTCHAFAAGYDLRTEEECERTFKQLG
DIVGFNYLRGMHLNDAKSEFNSRVDRHHSLGEGNIGKTVFSYIMRDPRFDNIPLILETVNPDIWAEEI
AWLKAQQ
>CORE_REP|Org28_Gene2350#
MVRVIAISNPRLALAFVDYMATQGIRLELRNSGEAAEIWLADDGHLEQVQHELQQFLVDPLNRRYQAA
SWQTGHTDAGLHYQSESYLHTLRSKAGPLTLGVMVLCIAVYILMQALGDDTVMYWLSWPQDSSQYTQL
WRWVSHAFLHFSLLHILFNLMWWWYLGGQMEKRLGAGKLFVLAVVSAFFSGWAQSLFSGALFGGLSGV
VYALMGYVWLTGERAPERGLMLPRGLMVFSVLWLVAGYFDILGMSIANAAHVAGLVLGLLMAFWDTRH
RAHNEQ
>CORE_REP|Org41_Gene130#
MNNPTQLSLLQDEIRHRYETLSKRLKQVARYILDNSNSIAFDTVASIAAQASVPPSTLIRFANAFGFS
GFNEMKQVFRQHLMEETVNYTERARLFRQTSTDDNVAPEKPAEILNVFTMVNAQALQQLAMQIAPEQL
DRAVELLNNAENIYVIGLRRSFSVASYLTYALRHLERRAFLIDGLGGMFTEQLSMVKPKDVVIAISYS
PYAREAVELVELGAKRGAQQIAITDSQVSPLAAFSDVCFVVREAQVDGFRSQVASMCLAQTLAVSLAL
NNARDE
>CORE_REP|Org13_Gene3749#
MKIWPGIIAAALLAGCQSPQQDTLVDRGAYQLETLHQAQGADQRIRFLVMHYTAEDFHSSLKTLTDEH
VSAHYLLPAHPQREHGKPTVYRLVPEAMRAWHAGTSAWRGRSNLNDTSIGIEIVNKGFTRSMLFTHWQ
PYTAEQIAVLIPLSRDIIQRYGIQPQDVVGHSDIAPQRKQDPGPLFPWRQLAQAGIGAWPDERDVQRL
LAGRDRHAPVPMAPLLEKLARYGYAIDPSWDARQQRNVVAAFQMHFRPDDVRGEPDAESEAIVDALLV
KYGAAR
>CORE_REP|Org6_Gene4274#
MFRKKCRRAEERPDGKERATHPVSAGPWALPLALVIIWQIAVETGWLSNRILPAPSAVLAAFWRLSQS
GELWQHLSISSQRALIGFGIGGSIGLILGFITGLSRWGERLLDSSVQMIRNVPHLALIPLVILWFGID
ESAKIFLVALGTLFPIYLNTYHGIKNIDRGLLEMARSYGLSGFRLFTQVVLPGALPSIMVGVRFALGF
MWLTLIVAETISANSGIGYLAMNAREFLQTDVVVVAIVLYALLGKLADVGAQLLERVWLRWHPAYQLK
QGEAL
>CORE_REP|Org36_Gene4626#
MESLFKLPVASGAAVRILQITDTHLFAGEHETLLGINTYRSYHAVLDAIQAQRRDVDLIVATGDLAQD
HSQEAYRHFAAGIAQLPAPCVWLPGNHDFQPAMVDALAAAGIAPSKQVLLGDRWQVLMLDSQVFGVPH
GELSEYQLEWMERCLQAHPERYTLLLLHHHPLPSGCTWLDQHSLRNPHMLGAILLRYPKVNTVVCGHI
HQDLDLEWQGRRLLATPSTCVQFKPHCTNFTIDDVSPGWRYLDLLPDGRVETQVFRLENDDFRPDMDS
DGY
>CORE_REP|Org14_Gene303#
MQFSKMHGLGNDFMVVDAVTQNVYFSPELIRRLADRHLGVGFDQMLVVEPPYDPELDFHYRIFNADGS
EVAQCGNGARCFARFVRLKGLTNKRDIRVSTQTGRMVLSVTDDDLVCVNMGEPNFDPQAVPFRAAKAE
KTYIMRAAEHTVLCGVVSMGNPHCVLQVDDVKTAKVELLGPVLEGHERFPERANIGFMQVVSRDHIKL
RVYERGAGETQACGSGACAAVAVGIQQELLSEEVHVELPGGSLHIRWKGPGNPLFMTGPATHVYDGFI

HL
>CORE_REP|Org38_Gene206#
MQQLQNVIESAFERRADITPANVDTVTREAVNQVIGLLDSGALRVAEKIDGQWVTHQWLKKAVLLSFR
INDNKVMDGAETRYYDKVPMKFADYDEARFQKEGFRVVPPATVRQGAFIARNTVLMPSYVNIGAYVDE
GTMVDTWATVGSCAQIGKNVHLSGGVGIGGVLEPLQANPTIIEDNCFIGARSEVVEGVIVEEGSVISM
GVYLGQSTRIYDRETGEIHYGRVPAGSVVVSGNLPSKDGSYSLYCAVIVKKVDAKTRGKVGINELLRT
ID
>CORE_REP|Org34_Gene3451#
MSYRVIALDLDGTLLDNQKRILPQSLEALALAQARAAGVQVVVVTGRHHVAIHPFYQALQIDTPAICC
NGTYLYDFQQKKVLAADPLAKDQAKQVLQMLKQTDIHGLMYVDDAMLYQEPSGHVTRSLAWAETLPAA
QRPTLLQVGSLAQAADDAQAIWKFATSHADIPALREFADTVEKELGLACEWSWHDQVDIAKGGNSKGK
RLRQWVESQGLNMDQVVAFGDNYNDISMLEAVGLGVAMGNADDAIKERADLVIADNLQPGIAEVIRTR
VL
>CORE_REP|Org22_Gene1610#
MSDSQIRIAIAGAGGRMGRQLIQAVQQAEGVVLGAALSRPGSSLVGVDAGELAGIGALGVKVSDSLEK
VANEFDILIDFTRPESTRGYLDFCVAHHKAMVIGTTGFDDAGKQAIRDAAQHIGIVFAANFSVGVNLV
LKLLEKAAQVMGNYTDIEIVEAHHRHKVDAPSGTALAMGEAIAGALGRDLKSCAVYAREGHTGERDPK
SIGFATIRAGDIVGEHTAMFADIGERVEITHKASSRMTFASGAVRAAAWLHNRDKGLFDMRDVLNLDQ
L
>CORE_REP|Org8_Gene4403#
MEKFAVFGNPIGHSKSPRIHALFAAQTDIEHPYGTVLAPLDGFEISLQEFIRAGGQGANVTVPFKERA
YSAASELSERAAMAGAVNTLKVLPNGGLLGDNTDGIGLLTDLQRQQLIRPQDRILLVGAGGAARGVIL
PLLSFGCRLTITNRTFSRAQELADAFRHLGEISAVPMDQLGQQAFDLVINATASGISGEIPALPTGVV
NAQTRCYDMFYQQGVTPFLAWAQQQGVTEYADGLGMLVGQAAHAFLLWHGVMPEIEPVLRQLRCELAA
>CORE_REP|Org27_Gene1013#
MDWFVIGPEMLGVLFAVALLAGFIDSIAGGGGLLTVPALLAVGVPPAQALATNKLQSVGGSFSASLYF
IRRRAVNLNDQKLTIFLTLIGSIAGAILVQHMRADLLRQMLPLLVIGIGLYFLLMPRLGEEDRQRRLG
ALPFGLVAGGCVGFYDGFFGPGAGSFYALAYVTLCGFNLAKSTAHAKVLNFTSNVGGLALFIIGGKVV
WSIGLVMLVGQVLGARLGAHMVLTRGQKLIRPMIVIVSLVMSLKLLYDNHGAEIQQWLSALVHG
>CORE_REP|Org25_Gene785#
MHSERAPLGLKLAAWGGLVFLHFPLAIIAIYAFNTEDAAFSFPPKGFTLHWFNVAAGRQDIIDAVLLS
AQIACLATAIALVLGTLAAAALYRRDFFGKDSISLLLLLPIALPGIVTGLALLAAFKALNIEPGILTI
VVGHATFCVVIVFNNVIARFRRTSYSLIEASMDLGADGWQTFRYVILPNLGSALLAGGMLAFALSFDE
IIVTTFTAGHERTLPLWLLNQLGRPRDVPITNVVALSVMLLTMLPILGAYYLTKGGESVAGSGK
>CORE_REP|Org21_Gene1917#
MGQLLRRLAAFLGLISPKRYAYPALDITLPGDRRLHLVGSIHMGTVDMSPLPSRLAARLQQADALIVE
ADITDSASPFDHAELQPALEQRLSAEEYQRLLALCHELGADPEAFVTLPGWQVALMMQARQAQRLGLR
AEYGVDYQLLQAARAQDKPVIELEGAQQQLAMLEQLPEGGIALLRDTLEHWHTNARLLQTMVSWWLDA
KPRGTLDTLPATFSAGLYDVLMHQRNRDWRRQLEALPAGDYVVAVGALHLYGEDNLPAMLQPQG
>CORE_REP|Org48_Gene4298#
MLHCAADAYQNRKDDWGQDLSTGSKLARWRRIAVCLTLGLLLAGCSGKNTYNRDYDKLPKGSYTGKSY
TVKRGDTLYYIAWITDSEVSDLARINKIRPPYSLEVGQKLRLSGSAPTKTAATRRKTSSSAIAKQTPP
PGAARCWRWPTSGRIVQAYSNADGGNKGIDIGGKRGQPIYASAKGKVVYVGNQLRGYGNLIMIKHGED
FITAYAHNDTTLVRNGQDVKAGQKIGTMGSTGTDSVFLHFQIRYRATALDPQRYLPPQGSSPSC
>CORE_REP|Org8_Gene1598#
MQQTTTTATDRPEPHRQQREITRLCIQCALLLLQHGAESTVVEQLSTRLGLALGMDSVESSISANAVV
LTTLSHGACLTTTRKNVDRGINMQVVTEVQHIVILAEHRLADAHDVARRFERIRPLRYPRWLVVLMVG
LSCGCFSMLNGGGGDAFLVTFIASGAAMLVRQILTARQMNPLINFCLTAFVATSISGLLLRLPAFKDT
SSVAMAASVLLLVPGFPLINAVADMFKGHVNTGLARWAMASLLTLATCIGVVMAMSLWDLRGWS
>CORE_REP|Org25_Gene835#
MCELLGMSANVPTDICFSFTGLVQRGGRTGPHKDGWGITFYEGNGCRTFKDPQPSFNSPIARLVQDYP
IKSCAVVSHIRQANRGEVALENTHPFTRELWGRNWTYAHNGQLKGYRQLDTGTFRPVGQTDSEYAFCW
LLHQLALKYPRTPSQWPAVFRYIGLLASQLRKKGVFNMLLSDGRFVMAYCSTNLYWITRRAPFGKATL
LDQDVEIDFQQQTTPNDVVTVIATQPLTANETWHKIEPGEFALFHFGERLVLSEGIGVGRRAG

>CORE_REP|Org48_Gene1788#
MIKWPWKATQPSQPQADTQAQWQDALAIPLLSPLNEQEQQRLVAVAGQILQQKRIVPLQGLQLTSQMQ
ARIALLFALPVLELGAECLDGFNEILLYPTPFVVEDEWQDEIGLVHSGPVVQSGQSWEQGPIVLNWQD
VQDSFDLSGFNLVIHEAVHKLDMRNGGVATGVPPIPLREVAAWEHDLHAAMESLQDEIDMVGEEAASM
DAYAATDAAECFAVLSEYFFSAPELLAERFPALYQHFCRFYRQDPLARLLRGQVENDAQWAD
>CORE_REP|Org36_Gene2125#
MYWINGQRHDALAPSDRGLQFGDGCFTTARVIDGNIELLPWHLERLQQAAQRLMLPATDWLAFEREMA
LAAESIPLGVVKAILTRGSGGRGYSPTGCENPTRIVARSSYPAHYLQWREQGITLALSPVALARNPLL
AGLKHLNRLEQVLIRAHLDQTAADEALVLDTAGMLVECCAANLFWRKGKAVFTPDLSQAGVAGLMRRR
VIALLAGSEYRLQCVSEPLETLADADEVLVSNALMPLLPVNAAQSWRYASRQLYDFLRPHC
>CORE_REP|Org13_Gene4492#
MSRAERLYHRTVTGLLLLILLILLLPLAATLIYALATQWGATILPDGFTLKWLTALWSDPRFLQALWH
SLLICFGTLLLSVVVILPAMFVIAYYFPKLDAVMNVLILLPFAVPPVVSAVGLMQLFAADPLPLLGTP
WILVGCYFTIALPFIYRAISNNMQAINLRDLMDAAHLLGASTWQAALLVVLPNLRKGGTIAVLLSFSF
LIGEFVFANLLVGSQYETLQVYLFNMRNGSGHFTSALVISYFAVVLLVTWLANLLNKNKG
>CORE_REP|Org6_Gene3678#
MSKPVHVNSWDSLRAFTDARIALGRTGASLPTDELLRFGLAHAQARDAVHQPFDSERLAADLHQAGWP
SLAVHSQAADRAAYLRRPDLGRRLASDSRSLLLGSPSRRVDLLLAVADGLSSKAVHRQALPLLQALRP
YLDTLGLSVSPVVLAHQARVALGDEIGECLQARAVAVLIGERPGLSSPDSLGIYLTWEPNARRTDAER
NCISNVRPEGLDYPQAAFRLAWLLEQAFQRRLSGIELKDESDNPALHNRVTPLYPQLGG
>CORE_REP|Org15_Gene3441#
MIPVERHQQILALVSERGVVSIAELTERLGVSHMTIRRDVQKLEEQGAVQSVSGGVQAPERVASEPSH
QTKEGMFGRQKIAIGRLAARQIPANSCIYLDAGTTTLALAKQIGERDDLTVVTNDFVIAGFLIEHSQC
RIIHTGGTVCRENRSCVGEAAAQALRGLFIDLAFISASSWSMRGLSTPNEDKVMVKKAIVEASRRRIL
LSDTSKYGKVATYLALPIAAFDAIITDEGLPAAAREAIEQAGIALLTAEKEEEEEEE
>CORE_REP|Org37_Gene363#
MLLRALASLGRSGINTSASFGRAGLMLFNALIGRPEPGKQWPLLLKQLYSVGVQSLLIIMVSGLFIGM
VLGLQGYIVLTTYSAEASLGMMVALSLLRELGPVVTALLFAGRAGSALTAEIGLMKATEQISSLEMMA
VDPLRRIVAPRFWAGLISMPLLTIIFVAIGIWGGSVVGVDWKGIDSGFFWSAMQGAVEWKKDLLNCLI
KSVVFAITVTWIAIFNGYDAVPTSEGISRATTRTVVHSSLAVLGLDFVLTALMFGN
>CORE_REP|Org36_Gene4482#
MLRQFYSQVVSVSKRSKADFTPRRGGFTFKQFFVAHDRCAMKVGTDGVLLGAWAPLGQARRVLDIGSG
SGLIALMLAQRSGDEVTIDAVELDEAAAGQARENAAESPWPQRIRVHAQDIHHYAQQHAAEYDLIVSN
PPYFEPAVACRDQARHNARYTETLTHDALLACAAQLLVEQGTFCVVLPHDIGAEFERLAQQNGWQTAA
KVNVSDRADTPLHRVLLALTRRETPLREQALAIKQADGCYTDDFLRLIADFYLFY
>CORE_REP|Org32_Gene2148#
MLKIADTTFTSRLFTGTGKFATPALMLEALAASGSQLVTMAMKRVDLRGGNDAILAPLQQLGVRLLPN
TSGAKTAAEAVFAARLAREALGTHWVKLEIHPDVKYLLPDPIETLKAAETLVKDGFVVLPYCGADPVL
CKRLEEVGCAAVMPLGAPIGSNRGLRTRDFLEIIIEQAKVPVVVDAGIGAPSHALAAMELGADAVLVN
TAIAVARDPVQMARAFRLALEAGELARSAGLGSSQRGAVASSPLTAFLSQPEEAQ
>CORE_REP|Org15_Gene1076#
MKALKTLFAAGCLLAAGSSLAAENSLRFGLEALYPPFESKSASGKLEGFDIELGDAVCAAAQLQCSWV
ETSFDSLIPALQARKFDAINSAMNVTEQRRQAIAFTDAIYQVPNRLIAKADSGLLPDAKALAGKHVGV
LQGSIQEIYAKTHWAPAGVDVVSYQDQNQVYLDLAAGRLDATLVMAPSGQSGFLSQPDGKGFAFVGEA
VRDDKILGEGIAFGLRKGDEALKKKLDAAIAKVKQQGTVTALSKKYFGDIDVTVK
>CORE_REP|Org14_Gene1496#
MLAKRIIPCLDVKDGQVVKGVQFRNHEIIGDIVPLAQRYAQEGADELVFYDITASSDGRVVDKSWVSR
VAEVIDIPFCVAGGIKSAEDASQILSFGADKISINSPALADPELISRLAERFGVQCIVVGIDTWFDSE
TGKYHVNQYTGDESRTRITQWETLDWVQEVQRRGAGEIVLNMMNQDGVRNGYDLQQLRRVREACKVPL
IASGGAGTMEHFLEAFRDADVDGALAASVFHKQIINIGELKRFLVEQGVEIRVC
>CORE_REP|Org13_Gene2734#
MLVIISPAKTLDYDSPLATERFTQPELLDKSQRLIKICRELTPAQIASLMSISDKLAGLNAARFSEWQ
PKFTPDNARQALLAFKGDVYTGLQAQDFNEADFDFAQQHLRMLSGLYGVLRPLDLMMPYRLEMGIKLE
NPKGKDLYSFWGDQITQKLNEALEQQGDDVVVNLASDEYFKAVKPAKLHGALIKPVFLDEKNGKFKVI

SFYAKKARGLMSRFIIKNRLTRSEQLVDFNLEGYAFDEAASQGNELVFKRPEQA
>CORE_REP|Org48_Gene1952#
MKPKQRQAAILEYLQRHGKTAVDALAEHFSTTGTTIRKDLTLLEDEGEVIRTYGGVVLSRDDGDQPID
RKTHINTEKKRHIASAAVALIADGDSLIFDAGSTVLQMVPHLAQFNNITVMTNSLTIVNALVELDNDQ
TILMPGGTYRKKSASFHGSLAESAFQQFSFDKLFIGADGVDLNAGVTTFNEVHNVSKAMCEAAGRIIL
LVDSSKFGRKSPNVVCELSAVDTLITDRDINPDYLAALQAKGINILLVGDPDE
>CORE_REP|Org46_Gene1332#
MNPLIIKLGGVLLDSEEALERLFTALDSYRQQHQRPLVIVHGGGCVVDELMKQLSLPVVKKNGLRVTP
ADQIDIITGALAGTANKTLLAWAIKHQINAVGLSLADGGSAVVTPLDPALGHVGNAQPGSPALLNTLL
SAGYLPVVSSIGITADGQLMNVNADQAATALAATLGADLILLSDVSGILDGKGQRIAEMTAQKAEQLI
AQGIITDGMVVKVNAALDAARTLGRPVDIASWRHADQLPALFNGVSIGTRILA
>CORE_REP|Org16_Gene2512#
MMLATRILQQGEAGRPWLVWLHGLLGNNNEWRVIAARCPEWPSLAIDLPGHGDSVAVGCRGFDDISAQ
IAATLQLRNIERYWLVGYSLGGRIAMYHACHGRHDGLQGVIVEGGNPGLEDEQQRRDRCEQDARWAAR
FRSELIAEVLADWYQQPVFKELSHVHRQALIAARSVNSGPAIADMLEATSLGRQPYLAPQLRQLTVPL
RVLCGENDPKFQRLARDAGLPLRIVPQAGHNAHLANPQDFVAELQTFLVNPG
>CORE_REP|Org22_Gene536#
MTRLYWVALQSIWAKEVNRFARIWIQTLVPPVITMTLYFIIFGNLIGSRIGDMHGFSYMQFIVPGLIM
MAVITNSYANVASSFFSAKFQRNIEELLVAPVPTHVVIAGYVGGGVARGICVGVLVTIISLFFVPLQV
HAWWVIALTLLLTAILFSLAGLINAVFATTFDDISLIPTFVLTPLTYLGGVFYSLSLLPPFWQAVSKL
NPIVYMISGFRYGFLGINDVPLAFTMAVLVAFIAVFYLLSWYLIERGRGLRS
>CORE_REP|Org20_Gene2782#
MYKIDYNSYRSVASFGHRVRFLVLHYTAQNFADSVKSLTGKSVSAHYLVPDPTEATYQAAGFSGVRIF
NLVDENERAWHAGASQWGTRSNINDTSIGIEIVNLASGDGGNITFPPFNPQQIEAVTQLAQNILQRYP
DISPVNVVAHSDIAPGRKSDPGPQFPWQQLYQAGVGAWYDEATKQQRQQEYCRQGLPAQAELLKLFAQ
YGYDTSAANTAEGYRQLVRAFQLHFRQQKYDGVMDVETAAALRALVDKYAA
>CORE_REP|Org27_Gene2316#
MNIAKERGLTRLRRGLTVFAGLLLLWWLAAQSGIPAFLLPTPSAVAQALWDGRGYLAWHTLITASEIV
SGLALGVLLGAALALCMIFSPRLQRWLMPLVLTSQAIPVFALAPLLVLWFGFGMSAKVAMAVLVIFFP
VVSAFFDGLRRVNNDYLDLARTMRASRWAQLRHVRLMAALPAFGSGLRMAAAVAPIGAIIGEWVGSAE
GLGYVMLNANARMQTDVCFAALFILVLMTVLLWVAVDALLRRLIAWAPEND
>CORE_REP|Org33_Gene417#
MAYSKIRQPKLSDVIEQQLEYLILEGTLRPGEKLPPERELAKQFDVSRPSLREAIQRLEAKGLLLRRQ
GGGTFVQTNLWQSFSDPLAELLADHPESQFDLLETRHALEGIAAYYAALRGTDEDLARIRDCHIVIQQ
AQDSGDLDAEADAVMQYQIAVTEAAHNVVLLHLLRCMGPMLEQNVRQNFELLYSRREMLAKVSSHRAG
IFEAIVAREPEKAREASHRHLAFIEEILLDLSREHTRRERSLRRLQQRKD
>CORE_REP|Org21_Gene4158#
MYEIAKRTAGGALLLLLMPLTVWVSGWHWQPGGNEPLLKALYWVTETVTSPWGILTSAILCGWFLWCL
RFRLKAAIGLALLLFAALVIGQGVKSLIKDRVQEPRPFVVWLEQTHGVDGKYFYSLHRKERSALVREQ
LQDQTLVPNWLRKHWQFETGFAFPSGHTMFAATWALLGVGLLWPRRHYKTVALLMVWATGVMGSRLLL
GMHWPRDLAMATLISWLLVVVTCWLAQRWFGPLTPPPQEQQEIAARKPEI
>CORE_REP|Org39_Gene565#
MTYQSLLAPILSFLHCETPDAWIDAARRPENLQLLLTDHLVCELKAAQTGMWLIRRYVADKESGDALL
ALLRPYEAFLHEAQGAPDTLFRQGQFTRKILPKNGSAYGQDLADRMVLLIKEELHHFSQVLEIMQARG
IPYRKITASRYAKGMIREIRTHDPATLIDKLICGAYIEARSCERFAKLAPHLDDELNRFYVSLLRSEA
RHYQDYLTLAEQIAGGDISERVAHFGRLEAELILSPDSELRFHSGVPAAA
>CORE_REP|Org33_Gene3522#
MMRILLSNDDGVSAPGIQVLAAALREFAEVQVVAPDRNRSGSSNALTLESPLRTQTLANGDIAVLQGT
PTDCVYLGVNALMHPAPDIVVSGINAGPNLGDDVIYSGTVAAAMEGRHLGLPALAVSLNGHQHYATAA
AITCRILRALQREPLRTGKILNINVPDLPLAEIRGLRVTRCGSRHPADKVFCQQDPRGQNLYWIGPPG
DKFDVGPDTDFAAVEQGYVAITPLQVDLTAYAAQEVVKTWLTKAEVSGEW
>CORE_REP|Org37_Gene1653#
MSYTQPPARSTSGIHYFAEGWRLISRPGIKRYVVLPLLVNVLLMGSAFWWLFSRLGDWIPAMMSHVPD
WLQWLSYLLWPLAVISVLLVFSYLFSTITNLIAAPFCGLLAEQLEGSLTGKPLPDTGLLGIAKDLPRI

MAREWRKLMYYLPRALLLLALYFVPGIGQTVAPVLWFLFSAWMLAIQYCDYPFDNHKVSFADMRRALR
QHKTDNLQFGALVSLFTMIPILNLVILPVAVCGATAMWVDRYRSQFVR
>CORE_REP|Org30_Gene1016#
MNFRLTGVAFATVLLVGCASAPDNDPQGRSDPLEGFNRTMFDFNYNVLDPYILRPVAVAWRDYVPMPA
RNGISNFTSNLEEPASMVNAFLKGDPYRGMIHFNRFFLNTLLGMGGLIDVAGMANPKLAREEPNRFGS
TLGHYDVGYGPYVMLPGYGSFTLREDGGDFADTLYPMLSYLTFWMSAGKWVVEGIETRAQLLDSDGLL
RNSSDPYIMVREAYFQRHDFIANGGSLKPEENPNAKAIQGELDEIDSQ
>CORE_REP|Org48_Gene151#
MKQTQRHDAIIELVRLQGYVSTEELVEHFDVSPQTIRRDLNDLADQNKIQRHHGGAALPSSSVNAAYN
DRKVMWSEEKARIAQRVASQIPDGATLFIDIGTTPEAVAHALMNHKNLRVVTNNLNVATLLTAKEDFR
LILAGGEVRTRDGGIMGEATLDFISQFRLDYGILGISGIDMDGSLLEFDYHEVRTKRAIIENSRCVML
VTDHSKFGRNAMVNLGNMNLIDYLFTDQLPPPSVMKIIEQYDVQLELC
>CORE_REP|Org33_Gene2987#
MSILEKVEEMKSENNTNNNDLKSSPIVVALDYADKNAALAFADRIDPQDCRLKVGKEMFTLFGPQLVR
DLHGRGFDVFLDLKFHDIPNTTAHAVAAAAELGVWMVNVHASGGARMMTAAKEALASFGVDAPLLIAV
TVLTSMEAEDLRGIGIEASPAEHAERLARLTRDCGLDGVVCSAHEAQRLKAACGQAFQLVTPGIRPEG
SAAGDQRRIMTPVQAQAAGVDYMVIGRPITQSADPAATLRAIRASLA
>CORE_REP|Org26_Gene3950#
MHKAARQRHLLDLLSERGQAAVAELAGAIGVSVDTVRRDLADLERQGLAQKHHGGAIALEPSDMPRQA
RAALLPQVKQRLGRAVAAQIPPGSTLMLDAGSTLLAVAQALRGPATVITASLDIAQCLSDRPEINLIL
LGGQWDARQRLFAGGATLALLARYRADIALLGACAVHAQLGLSAGEEADAEVKRAMLANSGERWLVAD
HMKLDRCEPHHVADLAQIQRLFTDRPWDNLDEQSLIELCVVADDR
>CORE_REP|Org12_Gene812#
METKEIRRNNLRELMARYARQGVNQNEFATLVESSAPTLSQIIGEKSSRNLGDNLARRIEARLNLPKG
WFDVFHEKQLVRPFDNVAAESDFQPARLKPVVWEDTEQDKEEFVEIPLLDIDFSAGDGCYEIVDREEF
SLIFRRYYLHKMGVAVNAARIIRISGSSMEPRLQDGDVVGINTDDTRIREGKTYAIRHGNLLRVKVLI
EQPDGGVIIRSLNREEYQDEHLSYQQRKEQLVVLGRVFWSSSSW
>CORE_REP|Org14_Gene1934#
MNLRQQTILQLVNDRRRISVNELARASGVSEVTIRQDLNLLEKRSYLKRVHGSAVALESDDVDARMMS
NFTLKQRLAQYAAAQVNDGETIFIESGSANALLARYIAERKRITLITVSHYIANLLKETDCDVIVLGG
MYQKKSETVVGPLTRLCIQQVHFNKAFIGIDGFQAETGFTGRDMMRADVVSAVLAKGVENIVLTDSSK
FGQIQPNPLAQTGQISRVITDSRLALEYQHQLKRQGVQVELVNE
>CORE_REP|Org2_Gene2300#
MWKRLIISLFIIIAVLMGSAIALDRWISWKTAPYVYDELQALPHRQVGVVLGTAKYYRTGVINQYYRY
RIQGAINAYNSGKVKYLLLSGDNAQQSYNEPMTMRRDLIAAGVAPSDIVLDYAGFRTLDSIVRTRKVF
DTNDFIIITQRFHCERALFIALHMGIQAQCYAVPSPKDMMTVRAREIFARLGALTDLYILKREPRFLG
PLIPISAMHTVPEDAQGYPAVSPEQLVELEHKLKEEKQKAKQP
>CORE_REP|Org28_Gene1440#
MISTTTRQIVLDTETTGMNKLGVHYEGHRIIEIGAVEVINRRLTGRNFHVYIKPDRLVDPEAYGVHGI
SDDFLADKPTFDQVADEFLDFIRGGELVIHNAAFDIGFMDHEFRMLQQGIPKTETFCTITDSLLMARR
LFPGKRNNLDALCSRYEIDNSKRTLHGALLDAEILAEVYLAMTGGQTSIAFQMEGDTQQNDAAQEIQR
IVRPATAMKVVYASDEEVKAHEARLDLVAKKGGSCLWRGAPAE
>CORE_REP|Org31_Gene1303#
MTILVTRPSPSGEQLVSRLRALGRVAYHAPLIDFAPGGDLPQLPQALQQLNAGDLVFVLSQHSVNYAD
SVIGRAGLSWPAHLTYYAIGRTTGLALHRISSLPVEYPREREISETLLLLPALQKLAGKRALILRGNG
GRELLGTTLSERGADVSYYECYQRSPVHYDGSEQSAHWQRAGVDTLVVTSGEMLQQLYTLVPDYYRSS
WLLRCRLVVVSERLATLARDLGWRTIRVADNADNDALIRALQ
>CORE_REP|Org37_Gene2847#
MEAKISVPQYELRGFSLWGFRDMAHCMDFLFDGGRVKQGTLVAMNAEKILKAEEDPALHALLDEAEYK
YADGISMVRSIRRKYPAADVSRVAGADLWEALMQRAGREGTPVFLVGGKPEVLAETEQKLRSQWNVNL
VGSQDGYFKPDQREALFERIRASGAVIVTVAMGSPKQEILMRDCRKVHPQALYMGVGGTFDVFTGHVK
RAPKVWQNLGLEWLYRLLSQPSRIGRQLKLLKFVGYYYSGKM
>CORE_REP|Org34_Gene1265#
MYPVDLHMHTVASTHAYSTLHDYIAEAQQKGIKLFAITDHGPDMADAPHYWHFMNMHVWPRRVNGVGI

LRGIEANIKNLQGDIDCTGPMLTATDVIIAGFHEPVFAPQDKASNTEAMIAAMAQGDVHIISHPGNPR
YPIDIPAVAAAAAKYEVALELNNSSFTHSRKGSEANCRAIAAAVRDAGGWLALGSDSHVAFSLGNFEH
CERIIDEVGFPQERILNVSPRRLLDFLERRGKPAIAELADL
>CORE_REP|Org15_Gene2513#
MIIPALDLIDGNVVRLHQGDYGQQRDYGNDPLLRLQDYQQQGAQVLHLVDLTGAKDPAARQIPLLRKL
LAGVNVPVQVGGGIRNEQDVSALLEAGATRVVIGSTAVKQPQLVQSWFERYGADALVLALDVRIDAQG
VKRVAISGWQEDSDATLEQVVEQFLPYGLKHVLCTDISRDGTLAGSNVALYQAISRRYPQVAFQASGG
IGNLDDIAQLRGSGVAGVIVGRALLEGKFSVEEAIACWQNG
>CORE_REP|Org29_Gene862#
MLHNIRIVLVETSHTGNMGSTARAMKTMGLTNLYLVNPLIKPDSQAIALAAGASDVIGNATIVDTLDD
AIAGCSLVVGTSARSRTLPWPMLEPRECGVRAVHEGEHAPVALVFGRERVGLTNDELQKCHYHVAIPA
NPDYSSLNLAMAVQILAYEVRVAYLDRQQAGAPQLEETPYPLVDDLERFYQHLEQTLQRTGFIRPSHP
GQVMSRLRRLFTRARPEGQELNILRGMLTSIEKQDKHQGN
>CORE_REP|Org23_Gene3523#
MVSSMSNSLLSSEASELDLLNERPFTQTDHEILKSYEAVVDGLAMLIGGHCEIVLHALEDLNSSAVRI
ANGEHTGRKIGSPITDLALRMLHDMAGDDSSVSKAYFTRAKSGVLMKSVTIAIRNREQRVIGLLCINM
NLDVPFSQIMQTFMPPATQDVPSSVNFASSVDDLVAQTLEFTIEEVNADRSVSNNAKNRQVVLNLYEK
GIFDIKDAINQVADRLNISKHTVYLYIRQFKSGDLLGSDR
>CORE_REP|Org33_Gene684#
MAEFDLAALNALPKSGQALALAVVNGQLETLSAEQRVAWALEHLPGEFVLSSSFGIQAAVCLHLVTRI
RPDIPVILTDTGYLFPETYRFIDQLTDQLKLNLQVFRAEQSPAWQEARYGKLWEQGVEGIEKYNQINK
VEPMNRALETLGAQSWFAGLRREQSGSRANLPVLAVQRGVFKILPIIDWDNRKIYQYLTEHGLSYHPL
WEQGYLSVGDTHTTQKWEPGMSEEETRFFGLKRECGLHEG
>CORE_REP|Org23_Gene669#
MKKLLLAATMLAGMTFNATAAETIRFAASATYPPFESLDANNQIVGFDIDLANALCKQMQAQCTFTNQ
AFDSLIAALKFKKYDAVISGMDITPERSKQVAFTQPYYANSAIVIAQKGKFSSLADLKGKKLGMENGT
THQKYMQDKHPEINTVSYDSYQNAILELKNGRIDGVFGDTAVVNEWLKTNPQLAPVGEHITDAQYFGT
GLGIAVRPDNQALLAKLNAALDAIKADGTYKAINDKWFPQ
>CORE_REP|Org23_Gene2366#
MDNLNLNKHISGQFNAELEHIRTQVLTMGGLVEQQLTDAITAMHNQDGELAKRVIEGDAKVNMMEVAI
DEACVRIIAKRQPTASDLRLVMAIIKTISELERIGDVADKICRTALEKFSHQHQPLLVSLESLGRHTV
QMLHDVLDAFARMDLDEAIRIYREDKKVDQEYEGIVRQLMTYMMEDSRTIPSVLTALFCARSIERIGD
RCQNICEFIFYFVKGQDFRHLGGDALEKLLSPGGKDDKAD
>CORE_REP|Org23_Gene3458#
MALPDTTSSGMCVLSIDVASKTTIIKRNRVMSATALGMIIFAYLCGSISSAILVCRIARLPDPREHGS
GNPGATNVLRIGGRLAAAAVLVFDILKGMLPVWLAYKLDVPPLYLGLTAIAACLGHIYPVFFHFRGGK
GVATAFGAIAPIGWDLTGLMTGTWLLTVLLSGYSSLGAIVSALIAPFYVWWFKPQFTFPVAMLSCLIL
MRHHDNIQRLWRGQEGKIWGKFRKKKNQADEDNGDQPKDE
>CORE_REP|Org42_Gene3472#
MADLLLGVNIDHIATLRNARGTQYPDPVQAAFIAEQAGADGITVHLREDRRHITDRDVRLLRQTIQTR
MNLEMAVTDEMLDIAIELKPHFCCLVPEKREEVTTEGGLDVAGQQDKMSVAVERLAQAGILVSLFIDP
DHRQIDAAVAVGAPYIEIHTGAYAEAQGELAVQAELRRIAVAAAYAAEKGLKVNAGHGLTYHNVQPIA
ALPEMHELNIGHAIIGQAVMGGLPAAVADMKVLMREARR
>CORE_REP|Org45_Gene2094#
MRIPRIYHPQPLTDRAEIALSEDAANHVGRVLRMSAGQALQLFDGSNQVFDAEIVRVDKKSVLVRLSD
GRVDDIESPLNLHLGQVISRGEKMEFTIQKSIELGVNVITPLFSERCGVKLDGERLAKKIQQWQKIAI
AACEQCGRNRIPEIREAMSLEAWCAEQDGSLKLNLHPRASHSINTLPQPVDRVRLLIGPEGGLSADEI
AMTTGHGFTDILLGPRVLRTETTALTAITALQVRFGDLG
>CORE_REP|Org19_Gene248#
MATPHINAEMGDFADVVLMPGDPLRAKYIAETFLEGAVEVNNVRGMLGFTGTYKGRRISVMGHGMGIP
SCSIYARELIAEFGVKKIIRVGSCGAVRDDIKLRDVVIGMGACTDSKVNRLRFKDNDYAAIADFDMVR
NAVDAAAAQGIPARVGNIFSADLFYTPDPDMFQVMKKYGILGVEMEAAGIYGVAAELYEEFGCKALTI
CTVSDHILRHEATTAAERQTTFNEMIVIALESVLLGDKA
>CORE_REP|Org19_Gene218#

MTRMKYLVAAATLSLALAGCSTSKDAVPDNPPSEIYATAQQKLQDGNFKGAITQLEALDNRYPFGPYS
QQVQLDLIYAYYKAADLPMAQASIDRFMRLNPTHPNIDYVMYMRGLTDMALDDSALQGFFGVDRSDRD
PQHARAAFRDFSQLIQQYPNSQYATDANKRLVYLKDRLAKYELSVAEYYTKRGAYVAVVNRAEQMLRE
FPDTKATHDVLPLMENAYKQLQLNGQADKVAKVIAANPQ
>CORE_REP|Org29_Gene191#
MATNAKPVYQRILLKLSGEALQGAEGFGIDASVLDRMAQEVKELVELGIQVGVVIGGGNLFRGAGLAQ
AGMNRVVGDHMGMLATVMNGLAMRDALHRAYVNARLMSAIPLNGVCDNYSWAEAISLLRNNRVVIFSA
GTGNPFFTTDSAACLRGIEIEADVVLKATKVDGVYSADPVKNPDATLYEQLTYQDVLERELKVMDLAA
FTLARDHGLPIRVFNMNKPGALRRVVMGENEGTLISK
>CORE_REP|Org24_Gene2256#
MISLKVIARLLDYPEQVLFDHQQALIEALEPASELDLHHSAQLILFIRRLCARPLLDAQADYCELFDR
GRATSLLLFEHVHGESRDRGQAMVDLMAQYRAAGLEIDSRELPDFLPLYLEYLASRSAAQAREGLQDI
APILALLGARLQQRESPYAVLFDLLLLLSGSEVQAQTLETQVAQEARDDTPQALDAVWEEEQVKFLGE
QGCASAQQTAHQRRFAGAVAPQYLDLTDALTGTKGR
>CORE_REP|Org49_Gene391#
MINDVISPEFDENGRAMRRIRSFVRRQGRLTKGQQHALENYWPVMGVEYQADAVDLAALFGREAPTVL
EIGFGMGASLVTMAGNNPQQNFLGIEVHSPGVGACLADAHEAKLSNLRVMCHDAVEVLENMIPDGSLD
MVQLFFPDPWHKARHNKRRIVQTPFVELVLRKLKTGGVFHMATDWQPYAEHMLEVMNGVAGYRNLSSD
NDYVPRPDSRPLTKFELRGQRLGHGVWDLMFERKE
>CORE_REP|Org45_Gene2275#
MKEHQGEMPHYLQIKDQLQARITRGALQAGDKLPSERELCAIFSTTRVTIRESLAQLEATGAIYRADR
RGWFVTPERLWLDPTQNTNFHRLCQEQGRAPRTALLSGEKTTVPLDVMQPLALEPFDQVYLLRRVRYA
DGRAICYCENHCLPQRVPELLSHDLNGSLTEVYQQHYALIYSNMHLSFYPTALPYRAANALGAMVGLP
ALLLRRLNYDQHGRILDFDIEYWRHDSLRIEVDTL
>CORE_REP|Org25_Gene885#
MNELQPLASAAGMTVGLAVCALILGLILAMLFAVWESSRWKAVSWLGTAWVTVLRGLPEILVVLFIYF
GSSQLLLMLSDGFTLNLGLFQLPIQLAIDNFEVSPFLCGVIALALLYSAYASQTLRGALKAVPQGQWE
SGQALGLGKAAIFFRLIMPQMWRHALPGLGNQWLVLLKDTALVSLISVNDLMLQTKSIATRTQEPFTW
YVIAAAIYLLVTLFSQYVIKRIELRATRFERGPV
>CORE_REP|Org21_Gene1433#
MKKGLMLLSLLVASVTGAAHADDAAIKKALASLGIQQADVQPSPVNGLKTVLTDSGVLYASEDGKHIL
QGPLFDVSGKEPVNVTNQLLSSKMDALKDQMIVYKAPKEKHVITVFTDITCGYCHKLHQQMKEYNDLG
ITVRYLAFPRQGLASQAEKDMKSIWCTADKAKAFDAAMKGDAISPATCKTDISKHYELGVQFGIQGTP
AIILENGMMIPGYQGPKEMAAMLDAHQAATKAGG
>CORE_REP|Org7_Gene2713#
MRLDKFLSQQLGISRALVARELRAKRVTVDGEVVKSGAVKLTPEQEVAFDGNPLQQQNGPRYFMLNKP
QGYVCSTDDPDHPTVLYFLDEPVAYKLHAAGRLDIDTTGLVLMTDDGQWSHRVTSPRHHCEKTYLVTL
EHPLAEDTAQRFAAGVQLHNEKDLTRPATLEQVDEHVVRLTISEGRYHQVKRMFAAVGNRVIELHRER
IGAIVLDDDLAPGEYRPLTEEEIASVGAPHLQD
>CORE_REP|Org43_Gene2400#
MNLDWLLAPQYLSWLWHGFLLTLWLSACAGLAATLLGFVLAAMRDSSLRPLRWLAMGYSSLFRNTPLL
VQLFFWYFAAGQILPSAAMQWLNSAHQVGPLEWPSFEFLAGFFGLTLYSTAFIAEEIRSGIRGVAGGQ
KYAAQALGLTGWQAMRYVVLPQALKIALPPLLGQYMNVIKNSSLTMAIGVAELSYASRQVETETLRTF
QAFGVATVLYIAIIALLEGWGMWRQQRKPLGGH
>CORE_REP|Org49_Gene4380#
MSEAPTTVATICQTLNARIAAGEFAVGGKLPSERALSEQFATTRITLQEALGQLEAQGVIYRQVRRGW
FISPPRLIYNPLQRSHFHAMAQQQGRAAHTEAIDSSVVTLDAPLAGRLALPPGAEAYRIRRLRYIDGR
AVLYCEHYLNPAYFPGILDEDLTQSLTALYAARYGIHYGRVRFDMLPTLLPQQAAAMLKVTYGSPALF
ITRVNRDQHDRVIDCDLEYWRYDALHIDVEAQ
>CORE_REP|Org25_Gene3724#
MSRVLVLKSSILGEYSQSGKLVDFFVEQWREAHPEDTFTVRDLANPTLPELDGEVMAGFTAGDKPLTP
HQQSTLALSDELIAELKSHDTLIISAPMYNFNIPTQLKIYFDLIARAGQTFRYTSAGAEGLVTGKKAI
VISSRGGIHADTPTDLITPYVKLFLGFIGITDVEFVLAEGFAYGPEAAEKAAQDSRIAVAQKIPAGVA
VPASAPAPAPANVAQEAVSGGFLSNLLKKLFR

>CORE_REP|Org37_Gene3701#
MTEHRPRSERGQLAVAGENYGSSLLGAPLLYFPAAVSGPETGLIIAGTHGDESAAIVTLSCALRSIAP
ERLRHHVVLAVNPDGCQLGLRANANGVDLNRNFPAANWRSGDTVYRWNSAAPVRDVKLSTGGRPGSEP
ETQALCHLIHRLKPHWVVSFHEPLACIEDPASSRLGVWLAHKFELPLVTSVGYETPGSFGSWCADLSL
PCITAEFPPISADDASERYIDAMTELLTYPN
>CORE_REP|Org47_Gene838#
MNTPAQLSLPLYLPDDETFASFYPGENPSLLAAIQSAVRQEHGSYIYFWSREGGGRSHLLHAACAELS
QKGEAVGYVPLDKRAYFVPEVLDGMEQLALVCIDNIECIAGDEEWEMAIFNLYNRILETGRTRLFITG
DRPPRQLNLRLPDLASRLDWGQIYKLQPLSDEEKLLALQLRGKLRGFELPEDVGRFLLKRLDREMRTL
FMTLDQLDRASITAQRKLTIPFVKEILGL
>CORE_REP|Org8_Gene359#
MKVGIIGAMEQEVTLLRDQIENRQTIQRAGCEIYTGQIGGVDVALLKSGIGKVSAAMGTTLLLEHCSP
DVVINTGSAGGLASTLRVGDIVVSEEVRYHDADVTAFGYEPGQMAGCPAAFVADDALIALAESCIKQL
DLHAVRGLICSGDAFINGAEPLARIRATFPRVAAVEMEAAAIAHVCHLFGTPFVVVRAISDVADSESH
MSFDEFLVVAAKQSTLMVNAMLQTLAKRG
>CORE_REP|Org29_Gene2561#
MSQSPIELKGSSFTLSVVHLHNSQPEVIRQALQEKVEQAPAFLKNAPVVINVATLDGDANWKELQQAV
AAAGLRVVGISGCRDERQKRAIARAGLPLLSEGKGQKMAAPEPAPAPVPAVADNAPAKTRIISTPVRS
GQQIYARNCDLIVTNSVSAGAELIADGNIHVYGMMRGRALAGASGDTQCQIFCTHLAAELVSIAGQYW
LSDQIPSDYVGQAVRLSLLDNALTIQPLN
>CORE_REP|Org44_Gene1704#
MSEAKELIVQGLWKNNSALVQLLGMCPLLAVTSTVTNALGLGLATTLVLVLTNASISAVRRWVPSEVR
IPIYVMIIAAVVSIVQMLINAYAFGLYQSLGIFIPLIVTNCIVVGRAEAVAARKPVGLSALDGLAIGL
GATGVMVTLGSMRELLGNGTLFDGADQLLGGWAKSLRIEVVHFDSPFLLAMLPPGAFIGLGLLLAVKY
LIDEKMKARKARAVAVEPLLEQGRAEKA
>CORE_REP|Org41_Gene786#
MIHEIWWSLPLTLAVYFAARWLARKLNMPLLNPLLVSMAVIIPLLLLTGIPYERYFQGSKILNDLLQP
AVVALAFPLYEQLHQIRARWKSIIAVCFIGSLTAMISGGAIALWLGATPEIAASILPKSVTTPIAMAV
ADSLGGIPAISAVCVIFVGILGAVFGHTLFNLLKITTHSARGLAMGTASHALGTARCAEMDYQEGAFG
SLALVICGIITSLLAPFLFPVLLHLFG
>CORE_REP|Org21_Gene749#
MILSEYEWQAVELSLKVSGLAVVCSLPFGILMAWVLVRCRFPGKALLDGVIHLPLVLPPVVVGYLLLI
AMGRRGVIGEWLYDWFGFSFSFSWRGAALASAVVAFPLMVRAIRLALEAVDTRLEQAARTLGANPWRV
FFTITLPLSLPGVIVGVVLAFARSLGEFGATITFVSNIPGETRTIPLAMYTLIETPGAEAAAARLCVI
AIVLSLASLMVSEWLARWGRKRMGV
>CORE_REP|Org21_Gene518#
MNDFILSLACFLATLALYFANKKLYRRRRTLLLMPLVLTPMILVLLLVVTHISYQDYIGETHWLLWLL
GPATIAFAVPVYENLHIIRRHWLSLTAGVTTAVLVAVYSSVWLARLLTLPEEVQRSLAVRSITTPFAL
EAAKQMGGQPDLVALFVVITGVFGMAVGDILFLRLAVRSRLAKGAGFGASSHGAGTARAYELGPQEGV
VSSLVMMLAGIITVVAAPLIGRLMW
>CORE_REP|Org11_Gene1703#
MLQGYSQLIFEGALVTLELALSSVLLAVVIGLIGAGGKLSHNPFISGLFGAYTTLIRGVPDLVLMLLI
FYGLQIALNNITTLLGFSQIDIDPLGAGIITLGFIYGAYFTETFRGAYLAVPKGQIEAATAYGFSGAQ
IFRRILFPAMMRFALPGIGNNWQVILKATALVSILGLNDVVKATQLAGKGTYQPFFFAIVAGVVYLIF
TTVSNGVLLWLERRYSLGVKRAEL
>CORE_REP|Org46_Gene714#
MRAAFDEDKKLMAEKSDLNALSGRFRGFYPVVIDVETAGFNANTDALLEIAAVTLKMDEDGWLQRDET
LHFHVEPFEGANLQPEALAFNGIDPHNPLRGAVSEYDALHAIFKAVRKGLKDRGCNRAIIVAHNANFD
HSFLMAAAERAGLKRNPFHPFATFDTAALSGLVLGQTVLAKACIAAGMPFDSSQAHSALYDTEQTALL
FCELVNRWKRLGGWPLPAGDLAE
>CORE_REP|Org9_Gene1153#
MDIIKELIHALWQQDFETLANPSLVWTLYILLFMILFLENGLLPAAFLPGDSLLILVGVLIAKGTMGF
PLTIVILTVAASLGCWVSYIQGRWLGNTRTVQGWLSHLPAHYHQRAHNLFHRHGLSALLVGRFLAFVR
TLLPTIAGLSGLSNARFQFFNWMSGLLWVLILTTLGFALGKTPVFRKYEDQLMFCLMMLPLVLLVVGL

IGSLIVLWRKKRASGQNPGKGA
>CORE_REP|Org45_Gene1603#
MIEYLPEILKGLHTSLTLTVAALIVALVLSLLLTVILTLKTPILTPLVKIYVTLFTGTPLLVQIFLIY
YGPGQFPAIRDYPWLWNLLSQPWLCAMIALALNSAAYTTQLFYGAVRAIPAGQWQSCEALGMSRRQTL
RILLPFAFKRALSSYSNEVVLVFKSTSLAYTITLMEVMGYSQLMYGRTYDVMVFGAAGLVYLCVNGLL
TLLMRLVERRALAFERRN
>CORE_REP|Org14_Gene1933#
MELYLDTADVTAVKRLARILPLHGVTTNPSIVAKEGKPIWEVLPALRDALGGTGKLFAQVMAADAERM
VAEAALLSQRVPGLVVKIPATAEGLAAIKKLKTMSIPTLGTAVYGAGQGLLAALAGAEYVAPYVNRVD
AQGGDGIEMVHELQQLLSLHAPSAQVLAASFKTPRQALECLLAGCQAITLPVDVAEQFLSAPAVQAAV
EKFEQDWQGAFGSNLLG
>CORE_REP|Org25_Gene616#
MPTNPEHPTVLHALFGGTFDPIHYGHLRPVEALAAEVGLNRVTLLPNHVPPHRPQPEANAQQRLKMVE
LAIAGNPLFAVDDRELHRTTPSYTIETLETIRKERGAAQPLAFIIGQDSLLTLHKWHRWQALLDVCHL
LVLARPGYNDRMDTPELQQWLERHRTADPALLSRRPHGHIYLADTPELEISATEIRQRRHQGLNCDDL
LPRPVQRYIELQGLYR
>CORE_REP|Org20_Gene4050#
MYSFTAQQRFTALVWLSLFHIAIITSSNYLVQLPITVFGFHTTWGAFTFPFIFLATDLTVRIFGAPLA
RRIILAVMVPALFISYVISTVTYQGEWQGFAALGSFNLFVARIAVASFMAYVLGQILDVHVFNRLRQR
SAWWVAPAAAMFFGNISDTLAFFFIAFYKSSDPFMANNWVEIALVDYSFKVMICLLFFLPMYGVLLNM
LLKRIAARSGNLQPG
>CORE_REP|Org36_Gene2160#
MFLTLVRRELKIACRKGSEIVNPLWFFLIVITLFPLGVGPEPQLLARIAPGIVWVAALLASLLSLERL
FRDDFLDGSLEQLLLLPTPLPMTVLGKVCAHWVVTGLPLLILSPLVALLLSLDMQTWLAVAGTLLLGT
PTLSLIGAIGVGLTVGLRKGGVLLSLLVLPLYIPVLIFATGAIDAASMGMPIDGYLAILGAMLAGSVT
LAPFATAAALRVSVH
>CORE_REP|Org32_Gene1151#
MDLALFDLDETLIDDDSASLWIRWLVGQGFAPAELELQEQQLMQLYYQGKLSMEDYMQATLAPLTGLS
VQTVAGWVQRYIRRDILPRVYPAARERLQWHRERGDCILVISATGEHLVAPIAEQLGADDALAIGVEI
SDGRFTGHTYGTMTYQQGKVIRLQHWLAQHPHLKFEHSHGYSDSLNDKAMLQFVDSATVINPDSELSA
LAAEHGWEVCRWER
>CORE_REP|Org24_Gene444#
MQQARYYLLGERAVVLELSPPVTLPSQQRIWALAEKLNHHPDVREVVPGMNNLTLLLHTPQADAEAML
ALLQQGWESKESLTPESRQVDIPVVYGGEQGPDLDEVARHTGMTPRQVVECHAAAAYVVYFLGFQPGF
SYLGGMPEQLATPRRAEPRLAVAAGSVGIGGGQTGIYPLVTPGGWQLIGRTPLALFNPHEMPPTLLRP
GDNVRFVPQKEGVC
>CORE_REP|Org23_Gene4202#
MFTGIVQGTAPLVAIDEKPNFRTHVIEMPTELLPGLELGASVAHNGCCLTVTAVEGNRVSFDLIKETL
RLTNLGDLALGDIVNIERAAKFNDEIGGHLMSGHIICTAEVAKIYTSENNRQVWLRMPDAELMKYVLH
KGFIGIDGISLTIGEVVNNRFCVHLIPETLDRTTLGKKRLGDKVNIEIDPQTQAVVDTVERVLANREA
TLAAAAAVAPAHKD
>CORE_REP|Org37_Gene1721#
MQKEKLSALMDGESFDSELLSSLSQDRTLQQSWQSYHLIRDTLRGDVGQVMHLDIADRVAAALEKEPA
RLVPSAVQESQPQPHTWQKMPFWDKVRPWASQITQIGMAACVSLAVIVGVQHYNQPAASSNASESPAF
TTLPIMGQASPVSLGVPADSFSTGSGQQQQVQEQRKRINAMLQDYELQRRLHSDQLQLEQSNPQQAAI
QVPGTQSLGMQQQ
>CORE_REP|Org10_Gene2915#
MKEVIEGFLKFQREAFVERTALFQQLATRQNPRTLFISCSDSRLVPELVTQREPGDLFVIRNAGNIVP
SFGPEPGGVTASVEYAVAALGVEDIVICGHSDCGAMTAIATCQCLDHLPTVAGWLRYADSAKAVNQAY
PHASDAARVASMVRENVIAQLNNIKTHPSVALALDQGRLALHGWVYDIASGAIEALDGETRRFVPLAT
HPEVTATPAIARF
>CORE_REP|Org22_Gene1173#
MNQTLLSDFGTPTERVERAIDALRNGRGVMVLDDENRENEGDMIFAAETMTVEQMALTIRHGSGIVCL
CITEERRQQLELPMMVTNNSSQFQTAFTVTIEAAQGVTTGVSASDRLTTIRAAVADSAKPSDLNRPGH

VFPLRAQPGGVLSRRGHTEATIDLVSMAGFKPAGVLCELTNDDGSMAHAPEVIAFAKQHDMVVLTIED
LVAYRQAHEKKAS
>CORE_REP|Org7_Gene3327#
MKSNWMQQIQTLIGQKAGAMGGAEGIGKLLAPTALGGLVGVLLANKSSRKLVGKFGKNALIIGGSAAV
GAVLWNKYKQRVKETHQDEPQFGLQTTPVDLRAKRLVQALVFAAKSDGHIDADEQRAIEHSLAQLQVG
EEAQGWVQEALDQPLNPALIARSVQNEDEALEVYYLSCLVIDVDHFMERGYLDALAQALKIPADVKQG
IESDVNEKKRELA
>CORE_REP|Org15_Gene3097#
MTDITTPFPATPHKLGLYPVVDSVAWIARLLEAGVTTIQLRIKDLPDEQVEEDIAAAITLGRRYQARL
FINDYWRLAIRHGAYGVHLGQEDLDTTDLAAIHRAGLRLGVSTHDDAELARALAVKPSYIALGHIFPT
QTKDMPSAPQGLAELKRHIAGLADYPTVAIGGISIDRVPAVLACGVGSVAVVSAITQAPDWRAATAEL
LRLIEGKEPNDA
>CORE_REP|Org10_Gene1505#
MKNWKTSAEQILTAGPVVPVIVINKLEQAVPLAKALVAGGVRVLEVTLRTACGLEAIRAIAKEVPEAI
IGAGTVINPQQLREVTEAGAQFAISPGLTDALLQAATAGSIPLIPGISTVSELMLGMDYGLREFKFFP
AEANGGVKALQAIGGPFPQVRFCPTGGITPNNYRDYLALKSVLCIGGSWLVPADALESGDYARITELA
RSAVSGAAL
>CORE_REP|Org46_Gene2477#
MLANVKRSFAAAVVLMLALPAVQAADYRAGEQYTRLDKPVAAAPAVVEFFSFYCGPCYQFAETYRVGS
TVAQALPAGEKVTKYHVSLMGKLGNELTEAWAVATVLGVEDKIEGAMFDAVQKQRAVNSAEDIQRVFT
AAGIDAATYENARHSLLVKGLIAKQNEAVKAFEVRGTPSFYVAGKYKIDNAGMASTSVEGYAKEYAAV
VRHLLDTQP
>CORE_REP|Org6_Gene3942#
MKSKFVVIEGLEGAGKTTARDTVVNVLREHGVSDIVFTREPGGTPLAEKLRDLFKRGIDGELPTIKAE
VLMLYAARVQLVETVIKPALARGAWVVGDRHDLSSQAYQGGGRGVDPQLMASLRDTVLGDFRPDLTVY
LDLPPLVGLQRAQARGQLDRIEQEALPFFERTRARYLELAAQDETIVTVNAAQPLEQVTAAIRDCVGH
WLRQQEGAL
>CORE_REP|Org40_Gene2220#
MMKLRRLLAAFGAVFSAGAIAHPHSFIDMNTTFVAKDQRLVGLKMVWVMDEITSADLLYDAKNAKSDS
EVWKKLAAEVMANVLGQHYFTDLYRDGKPVKYLNLPSEYHLSRQGNQAVLEFVLPLAEPQPLAGKPFE
LSTYDPTYFVDMTYKDQNALHLPPEMAQQCSYKLMTPQPNASLQAYALSLDKNDSPGEDLALGQQFAQ
RVTLQCR
>CORE_REP|Org10_Gene148#
MGVRAQQKERTRRSLIEAAFSQLSAERSFASLSLREVSREAGIAPTSFYRHFRDVDELGLTMVDESGL
MLRQLMRQARQRIAKGGSVIRTSVSTFMEFIGNNPNAFRLLLRERSGTSAAFRAAVAREIQHFIAELA
DYLELENHMPRSFTEAQAEAMVTIVFSAGAEALDIDVEQRQQLEERLVLQLRMISKGAYYWYRREQEK
ASVSHV
>CORE_REP|Org29_Gene2289#
MLNSMRKHGTTLAVFAAVTTGLTAVVYTLTKSTIAHQAALQQKALLDQVVPPENYDNVMQNECFLVSD
PALGNGAPHRLYLARKNGQPTAAALETTAPDGYSGAIKLLVGADFNGTVLGTRVIEHHETPGLGDKIE
LRISDWISFFSGKKIEGPDDKRWAVKKDGGMFDQFTGATITPRAVVNAVRRTALYMETLPPKLESLPA
CGASE
>CORE_REP|Org26_Gene111#
MADSKEIKRVLLGPLFDNNPIALQVLGVCSALAVTTKLETAVVMTIAVTLVTAFSSFFISLIRHHIPN
SVRIIVQMAIIASLVIVVDQLLRAYAFEISKQLSVFVGLIITNCIVMGRAEAYAMKSPPIESFMDGIG
NGLGYGVILVLVGFLRELIGSGKLFGVPVLETVQNGGWYQPNGLFLLAPSAFFIIGLLIWVLRTLKPA
QIEKE
>CORE_REP|Org33_Gene3157#
MAKNYYDITLAMAGISQAARLVQQLAHEGQCNREAFQTSLKSLLQMDPPSTLAVFGGEERNLLVGLET
LMGVLNANNKGPGAELTRYTISLMVLERKLNANKPAMNTLGERLGQLERQLAHFDLESDTIISALAGI
YVDVVSPLGPRIQVTGSPAILQNPQVQAKVRATLLAGIRAAVLWQQVGGSRLQLMFSRNRLFKQAQNI
VAHC
>CORE_REP|Org38_Gene4476#
MPIRKVSLLRLIPLASLVLAACTTTKPTGPATSPTSPQWRAHEQAVQQLSTYQTRGSFAYLSDQKKVY

ARFFWQQFSPERYRLLLTNPLGSTEMDLNVQKNVVQLTDNQGKRYVSDNPEEMIRKLTGMAIPLNNLR
QWMLGLPGEASDFALDDQYRLSKLTYQQGGQTWKVDYQGYSNDVQPTLPNRLELQQGDQRIKLKMDNW
TFK
>CORE_REP|Org32_Gene2065#
MKKIWLALVGMVMAFSASAAQFSDGTQYVTLDKPVTGEPQVLEFFSFYCPHCYQFEQVYHVSENVKKA
LPAGTKMTKYHVEFLGPLGKQLTQAWAVAMALGVEDKVSPLMFEAVQKTQTVQTPDDIRNVFVKAGVT
AADYDAAWNSFVVKSLVVQQEKAAEDLQLRGVPAVFVNGKYMVKNDGLDTSSMDAYVKQFADVVKFLS
QQK
>CORE_REP|Org5_Gene3276#
MLTEQQNNQLDWKKTDGLMPAIVQHAVSGEVLMLGYMTPEALAATEQSGNVTFFSRTKQRLWTKGESS
GHFLKVVSITPDCDNDTLLVLANPIGPTCHLGNSSCFHPAASDWTFLYQLEQLLAERKHASPDSSYTA
SLYASGTKRIAQKVGEEGVETALAATVNDREELTNEASDLIYHLLVLLQDQDLNLSAVIGRLRERHQK
KA
>CORE_REP|Org22_Gene1084#
MTLDWWLTYLLTTLILSLSPGSGAINTMSTGISHGYRGAAASIAGLQVGLSAHIVLVGIGLGALISQS
LLAFELLKWLGAAYLVWLGIQQWRAAGALDLHALAGSMPRRRLFRRAVLVNLTNPKSIVFLAALFPQF
ILPNQPQAEQYLVLGVTTVVVDILVMIGYATLATRIAGWLRTPRQMQLLNRVFGSLFILVAGLLATAR
KA
>CORE_REP|Org35_Gene1069#
MEVYTTENEQVDALRRFFAENGKALAVGVVLGIGALVGWRYWQSHENSNMMAASQSYQEASDRLAAGK
PDDVAAAEKFVQANGNSYGVLAALQLAKHFVEQNDFAKAEQQLALAQGQTKDDNLLAMIDLRLARVQL
QEKKLDEALKTLDGVKGEGWAAMMQDVRGDVLLAKGDAKGAREAYSKGIESNASQALQVLLRMKLNNL
SS
>CORE_REP|Org14_Gene638#
MLMLFLTVALVHLIALMSPGPDFFFVSQTAASRSRREAMMGVVGISLGIVVWAGVALMGLHLILQKMA
WLHQIIMVGGGIYLCWMGWQLLRSARAQQAQPAAEAQVALPKAGRSFIRGFLTNLSNPKAVIYFGSVF
SLFVGDSVGAGARWGLFLLIVAETFVWFSLVAVVFALPAMRRGYQRLAKWIDGVAGVLFTGFGLHLIF
TR
>CORE_REP|Org43_Gene141#
MARYLGPKLKLSRREGTDLFLKSGVRAIDSKCKIEQAPGQHGARKPRLSDYGVQLREKQKVRRMYGIL
ERQFRNYYKEATRLKGNTGENLLQLLEGRLDNVVYRMGFGATRAESRQLVSHKAVMVNGRVVNIASYQ
VSPNDVVSIREKAKKQSRVKASLELAEQREKPTWLEVDAAKMEGVFKRMPERTDLSADINEHLIVELY
SK
>CORE_REP|Org33_Gene738#
MQKLRLICLAALSFSITWAAHAEDKRYISDELSTYVHSGPGNQYRIVGTLNAGEEVTLLSVNDSTNYG
QIRDPKGRTTWIPLDQLSQTPSLRTRVPELEQQVKTLTDKLANIDNTWNQRTSEMQEKVAGSDSTISS
LQKENQDLKNQLVVAQKKVNAVNLQLDDKQRTIILQWFMYGGGVAGVGLLLGLLLPHLIPRRKNNNRW
MN
>CORE_REP|Org41_Gene1077#
MTRLSPRAAAKKPPQAAAGQIRIIGGQWRGRKLPVPNSPGLRPTTDRVRETLFNWLAPVIQGARCLDC
FAGSGALGLEALSRYAGSATLLEFERPVAQQLEKNLALLQGKGVVINTNALSWLAGEGQPFDVVFLDP
PFRKGLLAETALLLEQRGWLADEAWIYVEAEAESAAADVPASWQLHREKVAGQVAYRLYIRSQEKTDH
AD
>CORE_REP|Org25_Gene188#
MLAVFLQGFALSAAMILPLGPQNVFVMNQGIRRQYHLMIASLCALSDIVLICAGIFGGSALLTRSPLL
LALVTWGGVAFLLWYGWGAFRSAFSPQPAQAAAQELAQSRWRIVVTMLAVTWLNPHVYLDTFVVLGSL
GGQLTADVRSWFALGAVSASAVWFFGLALLASWLAPWLNTQRAQRIINALVGLVMWGIALQLAWQGAN
L
>CORE_REP|Org10_Gene1508#
MIGRLRGNILEKQPPLVLLEANGVGYEVHMPMTCFYELPELGQEAIVFTHFVVREDAQLLYGFNDKQE
RALFRELIKVNGVGPKLALAILSGMSAQQFVSAVEREEITALVKLPGVGKKTAERLVVEMKDRFKGLN
GDLFNSSSEISLPSAADNAPEADAEAEAVSALVALGYKPQEASRMVSKIAKPGADCETLIRDALRAAL
>CORE_REP|Org33_Gene3379#
MLFTPLLTGALETSLNNLLFRDRSMKAARQRLAGKVLRIELEELASPLVLVFSELRVDVLGQSEDSAD

CTVRSRIPDLLKLRDRQQLPVLMRSGELTVEGDIQVVQQLVGLLDLAEWDPAEWLAPYIGDIAAQGIT
QALGKGASLLKTGFMRRQQDMAEALTEEWRLAPRPLEVVWFNEEVDALARSAEALSARMDKLEGKR
>CORE_REP|Org37_Gene798#
MKALTTRQQEVYDLIRDHISQTGMPPTRAEIAMRLGFRSPNAAEEHLKALARKGVIEIVSGASRGIRL
LMEEEEGLPLIGRVAAGEPLLAQQHIEGHYQVDPSLFKPSADFLLRVNGMSMRDIGILDGDLLAVHKT
QDVRNGQVVVARIEDEVTVKRLKKHGNVVELLPENNEFQPIVVDLRQQNFTIEGLAVGVIRNGDWI
>CORE_REP|Org28_Gene634#
MQTSPLLESLMEALRCLPGVGPKSAQRMAFQLLQRDRSGGMRLAQALTRAMSEIGHCADCRTFTEQDV
CTICANPRRQQNGQICVVESPADIHAIEQTGQFAGRYFVLMGHLSPLDGIGPGDIGLDRLEQRLEKES
ITEVILATNPTVEGEATANYIAEMCGQYGVLASRIAHGVPVGGELEMVDGTTLSHSLAGRHAIKF
>CORE_REP|Org37_Gene2799#
MADERCGWVTADPLYLEYHDKEWGAPTTDARELFEMLCLEGQQAGLSWITVLKKRENYRRAFHGFDPQ
RVAAMTTEDVENLLQDSGIIRHRGKIEAIITNAKAYLAMEAAGEDFVAFIWDFVGGRPQLNRWQALNQ
VPAKTEQSDAMSKALKKRGFKFIGSTICYAFMQASGLVNDHLTGCICYPKPRCSAPVARRISNG
>CORE_REP|Org23_Gene1627#
MKKILVLKSSIMGNDSQTNNLIDHYLAARRAKGYEDQIVEHDLTALDLPVLDGELFDALRGAENISPR
AKATVALSDRLIAELKGSDLLLIGAPMYNLNVPTQLKNWFDLVARARVTFNYTATYPVGLVEGVNALV
FSSRGGIHAGQPTDAVTPYLRSVMGLMGIGDVQFIYAEGLDMKPHGLAQGLANARERIAELAG
>CORE_REP|Org49_Gene112#
MEHYISLFVRAVFVENMALAFFLGMCTFLAVSKKVSTAFGLGIAVTIVLGISVPVNNLVYNLILRDGA
LVEGVDLSFLNFITFIGVIAALVQILEMILDRFFPSLYNALGIFLPLITVNCAIFGGVSFMVQRDYNF
AESVVYGFGSGTGWMLAIVAMAGIREKLKYANVPAGLRGLGITFITTGLMALGFMSFSGVQL
>CORE_REP|Org14_Gene667#
MQLKRVAEAKLPTPWGDFLMVGFEELATGHDHLALVFGDISGEAPVLARVHSECLTGDALFSLRCDCG
FQLEAALEQIAEEGRGILLYHRQEGRNIGLLNKIRAYALQDKGADTVEANHQLGFAADERDFTLCADM
FKLLGVDAVRLLTNNPKKVEILTEAGINISERVPLIVGRNPKNEHYLATKAAKMGHLLDQK
>CORE_REP|Org24_Gene1121#
MIEPFIYPAATLIAGVDEVGRGPLVGAVVTAAVILDPAQPIVGLADSKKLSEKRRLALYDEIVAKALS
WSLGRAEPAEIDQLNILHATMLAMQRAVAGLHIAPDMVLIDGNRCPNLPMRSQAVVKGDSRVAEISAA
SILAKVTRDREMAALDSEFPDYGFAQHKGYPTAFHLERLAALGATEHHRRSFAPVKRALAL
>CORE_REP|Org14_Gene1789#
MNVVILDTGCANLASVTYAVRRLGYQPEVSRDPEIVLRADKLFLPGVGTAQAAMDQLRERDLVELIKA
CTQPVLGICLGMQLLAASSEENGGVTTLGLIDTPVKQMTDFGLPLPHMGWNQVSAQAGHHLFRGIDDG
AYFYFVHSYAMPICPSTIAQANYGEPFTAAVQKDNFFGVQFHPERSGAAGAQLLKNFLEM
>CORE_REP|Org4_Gene2206#
MAEDRHQQRQQRLKEQVDARIAAAQDTRGLLLVFTGNGKGKTTAAFGTVTRAVGHGMRAGVIQFIKGE
WPNGEKNLLQQHGVEFQVMATGFTWETQNKAGDTAACQAVWQHGKRMLADSSLDLVLLDEVTYMLTYD
YLELEELKAALLGRPAHQTVILTGRGCHRDLLELADTVTEMRPVKHAFDAGVKAQQGIDW
>CORE_REP|Org46_Gene2343#
MKVAKDLVVSLAYQVRTEDGVLVDESPVSAPLDYLHGHGSLIAGLEKALEGHDVGDRFDVHVGANDAY
GNYDENLVQRVPKDVFMGVDELQVGMRFLADTDQGPVPVEITEVDGDHVVVDGNHMLAGQNLNFNVEV
VAIREATAEELAHGHVHGEHDHHHEHGDGCCGGHGHSHDHDHGHDHGKGGCGNGGCGCH
>CORE_REP|Org23_Gene685#
MAQLYFYYSAMNAGKSTALLQSSYNYQERGMRTLVFTAEIDHRFGVGKVSSRIGLSSQAQLYNNDSML
YAMIQQEHQQQPVHCVLLDESQFLTKAQVEQLCDVVDQLDIPVLCYGLRTDFLGELFTGSQYLLAWAD
KLVELKTICHCGRKANMVLRLDENGQAMHAGEQVVIGGNESYVSVCRKHYKEAIHSLE
>CORE_REP|Org41_Gene1255#
MSIQNTFPSYQSLTVALNQQSVALTAAEMHGLISGLLCGGSRDAGWQALVHDLTNEGVAFPQALSQPL
QQLYEVTRETLEDDEFLFQLMLPEGEIVSVFDRADALAGWVNHFLLGLGMMQPKLAQVKDEVGEAIDD
LRNIAQLGYDEDEDQEELEQSLEEVAEYVRVAAIMCHGEFTRHKPTAPENIKPTLH
>CORE_REP|Org34_Gene2630#
MLKLDAQTTALVLIDLQNGILPYAGGPHGAEQVVANAALLAARFRLLGAPVLLVRVGWSDSFAEALKQ
PVDKPAPAPAGGLPASWWEFPAPLAVCDSDILITKRQWGAFYGTDLDLQLRRRGIKSVVLGGISTNIG
VESTARAAWEHGYELVIAEDVCSAQNAEMHRFAFEHIFPRLARVRDTGEILAALDR

>CORE_REP|Org15_Gene750#
MLKKTVLGLTAGALLLSAGSALAADYKIDKQGQHAFIEFRIQHLGYSWLYGSFKDFDGGFTFDEKDPS
KDKVNVTINTASVDTNHAERDKHLRSAEFLNVEKNKQAKFESTEVKKSGDGYAVVGNLTLNGVTKPVT
LDAKLIGQGNDPWGGYRAGFEANGKIKLKDFGITTDLGPASQDVELIISVEGVREK
>CORE_REP|Org49_Gene2560#
MSKTKLWITILLTVIVLALIGWNMTDFSDDTAPGPVNDQDPTYQSQHTVTVVYNPAGKLNYKLVAEDA
KYYTAGELSWFTQPVMTLFDENAVATWSVRADRAKLTKDRMLYLYGHVEVNSLTTTSQLEKIKTDNAQ
VNLVTQDVTSDDEVTIYGTNFTSNGMKMRGNLRTKTAELIDKVKTNYEIQNQKPTP
>CORE_REP|Org44_Gene966#
MIRITDAAQEHFAKLLANQEEGTQIRVFVINPGTPTAECGVSYCPPDAVEATDTELKFDKLSAYVDEL
SAPYLEDAEIDFVTDQLGSQLTLKAPNAKMRKVDDNAPLMERVEYVLQSQINPQLAGHGGRVTLMEIT
DDNMAILQFGGGCNGCSMVDVTLKEGIEKELLQKFPELKGVRDLTEHQRGEHSYY
>CORE_REP|Org11_Gene1672#
MVKSQPFLRYFLRVVPAIAAAVMLSACSSTHTSNLNNAQTEMRAVNDKDGLLLQASQDEFEAMVRNVD
VKSKIMDQYADWKGVRYRLGGDTKRGIDCSAFVQRTFREQFGMDLPRSTYEQEDLGKKIQRTKLRAGD
LVLFRAGSTGRHVGIYLGNDQFVHASTSSGVMISKLTDNYWNKRYREARRVLTNG
>CORE_REP|Org3_Gene1212#
MKLKATFAVLSSALLLQGCIAGVVVGSAAVATKTATDPRSVGTQVDDGTLEARVENALSKDQQLKKDA
RVVATAYQGKVLLTGQSPNADLTARAKQIAMGVEGTSEVYNEIRQGTPVSLSTASSDTWITTKVRSQL
LTSDTVKSSNVKVTTENGEVFLLGLVTQQEGQSAAQIASQVSGVKHVTTAFTYVK
>CORE_REP|Org31_Gene327#
MARANEIKRGMAISYNGKLLLVKDIDVQSPSARGASTLYKMRFSDVRTGLKVEERFKGDDILDTISLS
RRKVNFSYIDGEEYVFMDDEDYTPYIFKKDQIEDELLFIPEGGLPGMQVLTLDGQVLALELPQTVDME
IVETAPGIKGASASARNKPATMATGLVIHVPEYLSAGDKIRIHIAERRYMSRAD
>CORE_REP|Org4_Gene2047#
MKRLIIGISGASGAIYGVRLLQVLRDVAEVETHLVMSNAARQTLALETPYSLREVQALADVVHDARDI
AAGISSGSFKTLGMAILPCSIKTLSGIVNSYSDGLLTRAADVVLKERRRLVLCVRETPLHLGHLRLMT
QAAEMGAVIMPPVPAFYHQPKSVEDIIDQTVNRVIDQFDIELPTDLFTRWQGVN
>CORE_REP|Org11_Gene610#
MPQSDEHYLVITALGADRPGIVNTITRHVSSCGCNIEDSRLAMLGEEFTFIMLLSGSWNAITLIESTL
PQKGAELDLLIVMKRTNSHERPPMPATVWVQVEVKDSPHIIERFTDLFDSSQMNIAELVSRTQPADGD
LPPQLYIQITAHSSGDRDASNIEQAFHRLCTELNAQGSISVVNYPQHDEKDGE
>CORE_REP|Org41_Gene3146#
MDNGLETADLKLAQRLADLRQQQGWSLEALAQRTGLSRATLSRVERTETSPTASLLNRLCAAYGLTMS
RLLSEIEDEPPELLRPPQQPVWVDRASGFHRRSVSPPAALYKAEFIEARLDAGAQIDYDLPSIPALEH
HLWLLSGQLELTLEGRVFRLSPGDCLRYRLFGASRFHVPGDEPAHYTLVICRP
>CORE_REP|Org29_Gene3042#
MNSELSPNLVSIIDALHQQQVIAYPTEAVFGLGCDPDSEQAVNALLALKQRPWEKGLILIAADYAQLK
PYIDDSALSEQQRATMFASWPGPVTWVLPARPETPRLLTGRFSSLAVRVSDHPLVQLLCRQYGKPLVS
TSANLSGLEPCRSADEVTRQFGTAFPVLAGEVGGRLNPSEIRDALTGEQIRQG
>CORE_REP|Org44_Gene2530#
MNIKLDNLNQLIGARIRLEREARHWSLSDLAEQAGVSRAMVHKIERGESSPTAMLLARLAGAFGLSMS
KLIARAETQEGRLLRREDQPVWVDPESGYVRRHVSPRTDLPLDLVRIELPAGATIPMPASVYAFKRQL
IWVLSGELVFVEGDARHDMAEGDCLELGPPADCRFENQSDQPCVYMVAVLSAA
>CORE_REP|Org48_Gene90#
MATYSSNDFRPGLKIMFEGEPYAVEASEFVKPGKGQAFARVKMRRLLTGTRVEKTFKSTDSCEGADVM
DTNMNYLYSDGEFYHFMHPESFEQHQVDGKTVGDAAKWLQDNAECIITLWDGRPIAVQPPNFIEAEIT
DTDPGLKGDTAGTGGKPATLSTGAVVKVPLFVQIGEVIKVDTRSGEYVSRVK
>CORE_REP|Org40_Gene3273#
MTLRRLLTLALLLSPLAASAHNFVHGRPVAPIAIADRGELLLRNGDFSYRPWNSAKLAGKVRVIQYIA
GRTSAKKKNSLLINAVKDANLPGDRFQPTTIVNTDDAIPGSGFFVRGKIEKNKRHYPWAQFIVDSDGL
GRMAWRLPEESSTIVVLDKAGRVQWAKDGALTPQEVDQVIALLRTLIAQETP
>CORE_REP|Org35_Gene1582#
MQELAGHLAHTLRTLRAQRGWSLTQAAEYTGVSKAMLGQIERGESSPTVATLWKIATGFNVAFSAFLE

ASPAQQQATLHRYGELPVYDQDNADMRVVPLFPYDRQLGFDMFVIDLAPGALSESSPHEPGVIEHVIV
ISGRLELAIDGEWHSLAAGEAMRFQADRPHAYRNAGSHTVRIHDLIHYPQS
>CORE_REP|Org9_Gene1134#
MNLQHHFLIAMPTLQDPRFKRSVIYVCEHNEEGAMGLVINKPVEQFTVATVLSKLKIMPPARDPAISL
DKPVFAGGPLADDRGFILHTPRHGFGASIQISPNTMITTSKDVLETLGTPEQPDDVLVALGYAGWEKG
QLEQEVLENAWLTIEANTDILFRTPIASRWREAGNLLGIDIRSIANHAGHA
>CORE_REP|Org15_Gene3390#
MFKVNRHVTTLSAPVLSLLDATPNDMAAVLRIYTQHVLYGAASFEEQPPTLAEMQLRLSKVREAGLPW
LVAKSAGVIVGYCYATPYRPRPAYRFTVEDSVYIAEGQQGKGIGRALLSALIARCEQGPWRQMLAIVG
DSAANRGSLALHQSLGFTSAGTLKAVGFKLGEWRDTQIMQRALGAGDKRRP
>CORE_REP|Org27_Gene2058#
MRQRILTLLLGLAVLVTAGCGFHLRGTTQVPNEMKTLILDSADPYGPLTRSVREQLRLNDVTIVSDPK
RKDVPSLRIVGATESQDTASIFQDGKTAEYQLVLTVQAQVLIPGHDLYPLSVKVFRSFFDNPLTALAK
DSEQEIIRQEMREQAAQQLVRKLLAVHAAEEDNRQKAAAAGERAASQTAQ
>CORE_REP|Org36_Gene202#
MDALDLLLNRRSASRLAEPAPAGEVRQNIINAGLRAPDHGALQPWRFVMIENQGLERFSQLLQAAAKQ
DQLDEAAIEKATKAPFRAPLIITVIAHCTEETKVPRWEQVVSAGCAVQAMQMAALAQGFNGIWRTGAW
TEHALVREAFGCREQDEIVGFLYLGTPQLKAATKVTPPDSTPFVSYF
>CORE_REP|Org44_Gene447#
MNKQPEDWLDDVPENENEDDDEIIWVSKSEIKRDAEALKDLGAEMVDLGKNALDRIPLDEDLRAAIEL
AQKIKKEGRRRQLQLIGKMLRARDIEPIQTALDKLKNRHNQQVSLFHKLEALRDRLVEEGDDVIPSIL
DLYPAADRQQLRSLVRNAQKEKAANKPPKAYRQIFQYLRELAEAAD
>CORE_REP|Org20_Gene715#
MSKQLKPVAPKQPIVLGKMGSAYGIRGWLRVFSSTENAESIFDYQPWFIQQAGQWQHIELEDWKRHSQ
DLIIKVKGIDDRDAANLLTNREIMVDSEQLPPLEGDDYYWKDLMGCQVVTTAGYELGKVIDMMETGSN
DVMVVKANLKDAFGMKERLIPFLHGQVIKKVDLTARVIEADWDPGF
>CORE_REP|Org41_Gene830#
MTKNAIFAARQNEPCPECGAELVIRSGRHGPFLGCSQYPECQYIRPLKAQADGHIVKVLDGQQCPKCQ
ATLVLRQGRYGMFIGCSDYPQCDHTEVIDKPDETAITCPQCGQGKLLQRKSRYGKVFHSCDRYPECQF
ALNVKPVAGECAYCHYPLLMEKRTAKGPVLCCASKLCGKPVATTE
>CORE_REP|Org31_Gene102#
MSEAPKKRWYVVQAFSGFEGRVAQSLREHIKLHDMEELFGEVMVPTEEVVEIRGGQRRKSERKFFPGY
VLVQMVMNDASWHLVRSVPRVMGFIGGTSDRPAPISDKEVDAIMNRLQQVGDKPRPKTLFEPGELVRV
NDGPFADFNGVVEEVDYEKSRLKVSVSIFGRATPVELDFSQVEKG
>CORE_REP|Org24_Gene1468#
MKTIEVDEELYRYIASHTQHIGESASDILRRMLKFTAGQPVRALPAASAPQSVELEKAAPAQRPRDRV
RAMRELLLSDEYAEQNKAVNRFMLVLSTLYTLDAAGFAAATEALTGRTRTYFAGDQQTLLANGTHTKP
KHVPGTPYWVITNTNTGRKRSMIEHIMQAMQFPAELIEKVCGTV
>CORE_REP|Org43_Gene724#
MIIYLHGFDSTSPGNHEKVLQLQFIDPDVRFISYSTLHPRHDMQHLLKEVDKAVQQGGDAHPLICGVG
LGGFWAERIGFLCGIRQAMFNPNLYPEEHMHGKIDRPEEYRDIATKCVEDFREKNRDRCLVVLSRHDE
VLDNRRSAELLHHYYEIVWDEQQTHKFKNISPHLQRLKAFKALG
>CORE_REP|Org11_Gene2500#
MKTKTIAAVLPLALLLSACTTVEPAYKDIGTRSGSCVEGGPDTVAQKFYDLHIQQGAGLPDSNRLAQL
QPYLSKVLYQDLVSAGQNPGKHRITGDLFSGNAQGPSSASVASASTIPNTDAKNIPLRVDLSYQKDAN
STVNWQDEVLMVREGTCWVVDDIRYLNVPAHATNGSVRQVLENQ
>CORE_REP|Org16_Gene1036#
MATVGIFFGSDTGNTENIAKMIQKILQKQFGDDVSEVHDIAKSSKEDLEGFDILLLGIPTWYYGEAQC
DWDDFFPTLEEVDFNGKLVALFGCGDQEDYAEYFCDAMGTIRDIIEPRGAAIVGHWPTKGYHFEASKG
LADDDHFIGLAIDEDRQPELTNERVDAWVKQIVEELSLADIVG
>CORE_REP|Org46_Gene448#
MKFRTKNQLRNLLLGSLVLAASAPALALKSDSSQPVSIDSLKQSLDMQSNVSTFTDNVVIKQGTIDIR
ADKVVVTRPGGDQNKTYIEAFGNPVTFYQMQDSGKPVKGHAQKVRYDVATQLVTLTGNAYLEQLDSNV
KGDRITYLVQQQQMQAFSDKGKRVTTVLVPSQLQDKNEQKKSN

>CORE_REP|Org39_Gene196#
MKQFLDFLPLIVFFAFYKLYDIYVASGALIVATALALVFTWVKYRKVEKMTLITFLMVLVFGTLTLVF
HNDLFIKWKVTVIYALFALALLISQWVLKKPLVQRMLGKELTLPDKVWSNLNLAWAVFFLACGLANIY
VAFWLPQSVWVNFKVFGLTVLTLVFTLLSGIYIYRHMPEEQKK
>CORE_REP|Org5_Gene2874#
MKALILYSSRDGQTRAIASYIASKLQDTLRCEVIDLLQAEQVDLNQYQLVMIGASIRYGHFNPALDKF
VKRHAEQLNRMPSAFFAVNLTARKPEKRSPQTNAYTRKFLLTSPWQPKQCAVFAGALRYPRYRWFDRI
MIQFIMRMTGGETDTSKEVEYTDWQQVDRFAQEFSHIPYEK
>CORE_REP|Org34_Gene271#
MSLNLVPAGKDLPEDIYVVIEIPANADPIKYEIDKETGALFVDRFMSTAMFYPCNYGYINHTLSLDGD
PVDVLVPTPYPLQPGSVIRCRPVGVLKMTDEAGEDAKLVAVPHSKLTKEYDHVKDVNDLPELLKAQIA
HFFEHYKDLEKGKWVKVEGWADAAAAKAEIIASFERAAKK
>CORE_REP|Org34_Gene1678#
MKNKPPLSKDEQQLFRESVAGAKKLRQDTIVHRPPKLKVKQVAPQRLLQEQVDASYYFSDEYQPQLEE
EGPTRYVRPGSSPYELKKLRRGDYSPELFLDLHGLTQLQAKQELGALIAACKREHVHCACVMHGHGKH
ILKQQTPLWLAQHPDVLAFHQAPKEWGGNAAVLLLVELAE
>CORE_REP|Org49_Gene3134#
MIGILNRWRQFGRRYFWPHLLLGMVAATLGASSSLSGAPDQAALPNTSSSLNRQNAANGTFNSLALLQ
DAHRRPTFSVDYWQQHALRTVIRHLSFALAPQAVYARVQESETQAEPPLQVAQLALLSTLNALLTHEP
KPPTIIRHTHLEVLPTLARHQTGLWVAQVQGIRAGPAALV
>CORE_REP|Org16_Gene151#
MTTIVSVRRNGQVVIGGDGQATLGNTVMKGNVKKVRRLYNDKVIAGFAGGTADAFTLFELFERKLEMH
QGHLVKAAVELAKDWRTDRMLRKLEALLAVADETASLIITGNGDVVQPENDLIAIGSGGPYAQAAARA
MLENTELSARDIVEKSLNIAGDICIYTNHFHTIEELPSKA
>CORE_REP|Org6_Gene3725#
MTQMGDGKEFIVKKWLCAAGLGLAMAASAGVQAADKIAVVNVSSIFQQLPAREAVAKQLENEFKGRAS
ELQNMERSLQTKMQRLQRDGATMKASDRSKLEKDVMAQREQFSQKAQAFEQDNRRRQMEERNKILSRI
QDAVKSVASKGGYDVVIDANAVAYADSSKDITADVLKQVK
>CORE_REP|Org31_Gene4559#
MPWQIRLHWLVAILLVITCVTIELRGFAEPGSAPWYVLVVTHFSCGVTVFALMIARLFLRWRHPSPAI
APKPPKWQTGLAHLTHTLIYLLLLTLPVLGVYSRYLGGKEWYLFGLPMPFADVADRPQARMIIGWHKT
LASFGYWLIGLHAAAALFHHYIVKDNALVRMLPLMKKR
>CORE_REP|Org1_Gene2987#
MSNRYARSQIVLHWLTLLMVILTYAAMLLKDSVPEAWAPLVKNLHFNFGVSVFALMLIRLAMRAFHAA
PPTTPPLEEWQEVGAKIFHWLLYVVFLMLPLLGMLTLAYGGKSWSLLGWLMPQWVTPDPVMRRLVKTV
HETLANIGYFIIGAHALAALYHHYLRKDDTLRRMMPGK
>CORE_REP|Org20_Gene34#
MQKVKLPLTIDAVRTAQKRLDYAGVYAPEQVTRVADSVVSVDSDVEVSLSFNIDNQRLAVITGHADVT
VTLMCQRCGVPFEHQVHTTYCFSPVVNDEQAEALPEAYEPIEVDEFGEVDLLAMIEDEIILSLPVVPV
HESEHCEVSEADMVFGQLPPEAEKPNPFAVLASLKRK
>CORE_REP|Org26_Gene908#
MAIILGIDPGSRVTGYGLIRQQGRQLSYIASGCIRTVVDDMPTRLKLIYAGVSEIITQFQPDFFAVEQ
VFMAKNPDSALKLGQARGVAIVAAVNQNLEVFEYAARQVKQTVVGTGAAEKAQVQHMVRSLLKLSANP
QADAADALAIAITHCHLSQNVLRMSEGRLNLARGRLR
>CORE_REP|Org6_Gene389#
MKIGLFYGSSTCYTEMAAEKIREILGEDLVDLHNLKDVSPKLMEDYSILILGIPTWDFGELQEDWEAI
WPQLAALDLKGKIVAMYGMGDQLGYGEWFLDALGMLHDHIAPLGVQFIGFWPTEGFEFTSPKPLSADG
KHFVGLALDEVNQYDLSEERLQQWCEQILLEMEPLL
>CORE_REP|Org9_Gene4520#
MNVLFAIAVTTGILSGVWGWVAVSLGLIGWAGFLGCTAYFACPQGGLKGLLIGALTCCSGVFWAMAII
HGSELAPQWELLGYLLTGVVAFLMCIQAKQQWLGFVPGTFIGACATFAGGGDWPLVTLSLLVGLLFGY
AMKNSGLWWAARSEKAVPHSRSARMPAQTVPPSERR
>CORE_REP|Org23_Gene4575#
MNKPRIPIALQQAVMRCLREKLQLARQHFAVEFPEPSIVYQQRGTSAGTAWLQSWEIRLNPVLLLENQ

QPFIDEVVPHELAHLLVFRQFGHVAPHGREWRWMMESVLLTPASRTHRFETASVQSKTFPYRCGCGQH
QLTIRRHNRVLRGESEYRCRRCGEKLKFLASENL
>CORE_REP|Org4_Gene1656#
MSKSICSTGLRWLWVVVAVLALDFGSKQWILANFTLGQSQPLIPSFNLFYARNYGAAFSFLADHGGWQ
RWFFAGIAIAIVAVLLVMMYRSTAQQKLNNIAYAFIIGGALGNLFDRLWHGFVVDFIDFYVGDWHYPT
FNLADSFICVGAAMIVLEGFLSPANKDAKSKGE
>CORE_REP|Org14_Gene2222#
MLIRVEIPVDAAGIDALLRRAFGRDDEADLVQQLREDGLLTLGVVATDDEGGVVGYAAFSPVDVAGED
RQWVALAPLAVDESLRRQGLAEKLVYEGLDSLNEFSYAAVVVLGDPAYYGRFGFKPAAAYGLNCRWPD
TESAFQVYPLAEDALNGVSGEVAFSAPFNRF
>CORE_REP|Org43_Gene956#
MLNRLERLTQRVGGSNELVDQWLQARKQLLVAYCTLVGLKPNKEKHTPLNEKALENFCHNLVDYLSAG
HFHIYDRIIKQVEGAASPKMSLAVNIYPKLWANTEQIMAFHDRYTEVDIDQEVCLEFHQALSDIGETL
AARFALEDKLILLEAEAAQQPLPDQALDPAR
>CORE_REP|Org6_Gene4164#
MIRVYIALGSNLAQPLQQVNAALEALEHLPRTRLVTCSSFYRTKPLGPQNQPDFLNAVVAMDTLLPPE
QLLDHTQAIERNQGRVRKDERWGPRTLDLDIMLYGDKVIHTDRLTVPHYGLKEREFMLYPLAEIAPDL
IFPDGEPLASCLKRVPENGMALWHSPKPQS
>CORE_REP|Org17_Gene2138#
MDMSQMTPRRPYLLRAFYDWLLDNQLTPHLVVDVTRPDVQVPMEFARDGQIVLNIAPRAVGNLALGNE
DVQFNARFGGVPRQVSVPMAAVLAIYARENGAGTMFEPEAAYDESEGVFEGLDNETIPSETLMSVIDG
DRPDTAEDDGSDDEPPQPPRGGRPALRVVK
>CORE_REP|Org15_Gene106#
MALNLQDKQAIVAEVSEVAKGALSAVVADSRGVTVDKMTELRKAGREAGVYMRVVRNTLMRRVVEGTP
FECLKDTFVGPTLIAFSHEHPGAAARLFKEFAKANAKFEVKAAAFEGELIPAAQIDRLATLPTYEEAI
ARLMATMKEAAAGKLVRTLAAVRDQKEAA
>CORE_REP|Org23_Gene2350#
MLLMPSVFRDRKSILTTDTHTLDIAEILDLLPHRYPFLLVDRVLEFEEHKYLRAVKNVSVNEPFFQGH
FPGKPIFPGVLILEAMAQATGILAFKSVGKLEPGELYYFAGIDEARFKRPVVPGDQMVMEVTFEKTRR
GLTRFKGVATVDGKIVCEATMMCARSREA
>CORE_REP|Org4_Gene500#
MNPRRKSRLYLAIVVLIGIALTATLMLYALRSNIDLFYTPGEILQGKGENHEKPEVGQRLRIGGMVMP
GSVKRDPNTLQVSFKIYDARGAIGVTYTGILPDLFREGQGVVAQGVLGEGNVVNAREVLAKHDEKYTP
PEVADAMKENHKGPAEAYNAPQAEGAKS
>CORE_REP|Org6_Gene1254#
MRLTSKGRYAVTAMLDVALHSQEGPVPLADISERQGISLSYLEQLFSRLRKNGLVASVRGPGGGYLLG
KDAGEIAVGAVITAVDESVDATRCQGKEGCQGGDRCLTHALWRDLSERISGFLNNITLAELVNNQEVL
VVADRQNNDTRRTANGRPQETINVNLRA
>CORE_REP|Org13_Gene488#
MDEAKRRLRMSNPWHLLATGFGSGLSPVMPGTMGSLAAIPFWLLLIQLPWQLYSLAVMFSICIGVYIC
HRTAKDMKVHDHGSIVWDEFVGMWITLMALPVNDWRWVAAGFVIFRILDMWKPWPIRWFDRNVHGGMG
IMVDDIIAGVLSAGIIYLIGHHWPIGLF
>CORE_REP|Org9_Gene827#
MMRRILSVLLENESGALSRVVGLFSQRGYNIESLTVAPTDDPTLSRMTIQTVGDEKVLEQIEKQLHKL
VDVLRVSELVQGAHVEREIMLVKLQASGYGREEVKRCADIFRGQIVDVTATLYTVQLAGTSDKLDAFL
SAVREVAEIVEVARSGVVGVSRGDKIMR
>CORE_REP|Org31_Gene474#
MPSFDIVSEIDMQEVRNAVENATRDLGTRWDFRNVPASFELNEKNQSIKVATESDFQVQQLLDILREK
LSKRSIDGAALEIPEEFTHSGKTYSVEAKLKQGIETSVAKKIVKLIKDSKLKVQAQIQGEEVRVTGKS
RDDLQSVMALVRGGDLGQPFQFKNFRD
>CORE_REP|Org3_Gene410#
MNSYRSHGRWIIWLSFLVALVLQIMPWPEQIYMFRPSWLVLILIYWVMALPHRVNVGTGFVLGLIMDL
ILGSTLGVRALALGIIAYLVAFKFQLFRNMALWQQALIVVLLSLSMDVVVFWAEFLVINVSFRPEVFW
SSVVNGILWPWLFLLMRKIRRQFAVQ

>CORE_REP|Org5_Gene4055#
MVTRAPDNADRHAQTDMPDEAEHALRFSRAREAQSNALIEDYVELIADLLQSTREARTTDIARRFGVS
HPTAIKNIARLKSAGLVESRPYRGVFLTEEGEQLAQKVRRRHRIVVDLLMCVGVPSETAELDSEGIEH
HISDETLAVFEHYLQKHAKPCPPERR
>CORE_REP|Org6_Gene3606#
MSDMLKSGQGMGSTSDAPVPVMVAGTAMVAIKCISVVLLLGELGVDGAQEFVNTSAQAWDSTFIFLAG
LMLLCLQISCGFAVMRGRNWGRWGYVACQCIVVLYLLLATIGSVFPEVFTVEGETSGQILHVLILQKI
PDVVILALLFVPAASRRFFTAQVRF
>CORE_REP|Org1_Gene372#
MQVLIMRHGEAALEAASDAVRPLTLCGRDESRQMAAWLNTKSVDIERVLVSPYLRAEQTLATVREALT
LPEGEEVLPELTPGGNAEQVGSYLQALAMQGVSSVLIVSHLPLVGYLVAELCPGECPPMFATSAIANV
DLPADGSYGKFEWQVSPSQVMAKV
>CORE_REP|Org21_Gene844#
MTKKKAHKPGSATIAQNKRARFEYFIEEEFEAGLSLQGWEVKSLRAGKANLSDSYVTFRDGEAYLFGA
TITPLNVASSHVVCDPTRTRKLLLNRRELDTLLGRVNRDGYTVVALSLYWKNAWSKIKIGVAKGKKEH
DKRDDIKDREWQTAKARIMKHANR
>CORE_REP|Org26_Gene256#
MNQIPMTLFGAEKLREELEYLKSVRRPKIIADIAEAREHGDLKENAEYHAAREQQGFCEGRIQEIEAK
LSNAQVIDITKMPNTGRVIFGATVSVMNLDSEEEVTYRIVGDDEADFKKNLISVNSPMARGLIGKEQD
DVVVIKTPGGDVEYEILKVEYL
>CORE_REP|Org38_Gene1104#
MRTQLITREGYDKLKQELDYLWREERPEVTKKVTWAASLGDRSENADYQYNKKRLREIDRRVRYLTKC
LEQLKIVDYSPQQEGKVFFGAWVEVENDDGETKRFRIVGYDEIFGRKDYISIDAPMARALLKKEVGDV
ATVNTPLGEAQWYVNEIDYPK
>CORE_REP|Org27_Gene223#
MRIGHGFDVHKFGGEGPLVIGGVRIPYDKGLLAHSDGDVALHAATDALLGAAALGDIGKLFPDTDPAF
KGADSRELLREAWKRIRAKGYRLGNLDITIIAQAPKMAPHIPQMRVFLAEDLQCHMDDVNVKATTTEQ
LGFTGRGEGIACEAVALLIKE
>CORE_REP|Org40_Gene250#
MVMNLLWALLQEMLLAAVPALGFAMVFNVPLRALRYCALLGAIGRGSRMLMMHAGMNIEWASLLAAIL
IGIIGIYWSRWLLAHPKVFTVAAVIPMFPGISAYTAMITVVEISHLGYSEALMETMITNFLKASFIVG
ALSIGLSLPGLWLYRKRPGV
>CORE_REP|Org27_Gene2572#
MKELVLPLPDEAATVALGTTLAQACDRASVIYLYGDLGAGKTTFSRGFLQALGHQGNVKSPTYTLVEP
YALQPLAVYHFDLYRLADPEELEFMGIRDYFAQDAICLVEWPQQGTGVLPEPDLALHLSYQGEGREAK
IEAISAYGSQLLDRIHGSQG
>CORE_REP|Org24_Gene1981#
MTHSLHNAVPRAALTDTIMAAKIRHNLTFDALAEGTGLSLAFVTAALLGQHALPEQAARTVAAKLGLD
EEAVQLLQTIPLRGSIPGGVPTDPTLYRFYEMLQVYGSTLKALVHEQFGDGIISAINFKLDIKKVADP
EGGERAVITLDGKYLPTKPF
>CORE_REP|Org9_Gene468#
MKLQLVAVGTKMPDWVQTGFMDYLHRFPKDMPFELTEIPAGKRGKNADIKRILDKEGEQMLAAVGKGN
RIVTLDIPGTPWETPQLAQQLERWKQDGRNVSLLIGGPEGLAPACKAAAEQSWSLSPLTLPHPLVRVL
VAESLYRAWSITTNHPYHRE
>CORE_REP|Org15_Gene299#
MNLNATILGQAIAFVLFVWFCMKYVWPPIMAAIEKRQKEIADGLASAERAKKDLDLAQANATDQLKTA
KAEAQVIIEQANKRKAQIMDEAKAEAEQERNKIVAQAQAEIEAERKRAREELRKQVAMLAIAGAEKII
ERSVDEAANSDIVDKLVAEL
>CORE_REP|Org9_Gene1999#
MSVRIIDKAAEKVVGVRVVGPYPQTIPQGSQQLLAWRQRHGVPFGKWLVLYWDDPAEVAPEKLRADVV
MSVADDFALPAESEGVTVQTLPAGQYAVYHVRISDGDFERVWGEFYQKLLPASGYQPVEGVSYEHYLN
DCEADGYFDLDIYQTVKKW
>CORE_REP|Org6_Gene1564#
MRLIVLLCAALLSWSAAAAIDTYRFNSVEQEQQYRELTEQLRCPKCQNNSIADSNAIIAADMRTKVYE

LMMQGQSKQQIIDYMVARYGNFVTYEPPVTPATLILWIGPLLFVLIGGAVVILRTRRKPDAAVDDAFS
ERERQRLAALLQETDRKKP
>CORE_REP|Org20_Gene1378#
MTHDNKLQVEAIKCGTVIDHIPAQIGFKLLTLFKLTATDQRITIGLNLPSNALGRKDLIKIENTFLTE
QQANQLAMYAPKATVNRIDNYEVVRKLTLSLPDHIDGVLTCPNSNCISRSEPVASSFSVKPRDGEVHL
KCRYCEKEFEHQVVLQAD
>CORE_REP|Org20_Gene628#
MRMRVWFLLASLILAGCSSHAPPPSGRLADSIVVVAQLNEQLRQWYGTPYRYGGLDRGGVDCSGFVYR
TFRDRFDMQLPRSTEEQTSLGTKVSRDELMPGDLVFFKTGGGENGLHVGIYDTNDQFIHASTSRGVIR
SSLDNVYWKRVYWQARRI
>CORE_REP|Org47_Gene4834#
MNNEYMQLSSVLNIECTKSSVHCTSKKRALEIISELAAKQLNLPPQVVFDAVLTRERMGSTGIGNGIA
IPHGKLEEDTLRAVGVFIRLDQPIAFDAIDNQPVDLLFALLVPADQCKTHLHTLSLVAKRLADKTVCR
RLRAAQSDEELYQIITE
>CORE_REP|Org44_Gene841#
MFRGATMVNLDSKGRLAVPTRYRELLNEESQGQMVCTIDLHQPCLLLYPLPEWEIIEQKLSRLSSMNP
AERRVQRLLLGHASECQMDSAGRLLLANTLRQHAGLTKEVMLVGQFNKFELWDEQTWYQQVKDDIDAE
QSTQEPLSERLQDLSL
>CORE_REP|Org23_Gene3463#
MGLSTLEQQLTEMLSAPVEALGFELVGIEFIRARQSTLRIYIDSDNGINVDDCADVSHQVSAVLDVED
PITVAYNLEVSSPGLDRPMFTAEHYTRYLGEEVSLVLRMAVQNRRKWQGIIKSVEGEMITVTVEGKDE
VFALSNIQKANLVPHF
>CORE_REP|Org33_Gene424#
MQEGQNRKTSSLSILAIAGVEPYQEKPGEEYMNDAQLSHFKRILEAWRNQLRDEVDRTVSHMQEEAAN
FPDPADRATQEEEFSLELRNRDRERKLIKKIEKTLKKVEDDDFGYCESCGVEIGIRRLEARPTADLCI
DCKTLAEIREKQMAG
>CORE_REP|Org43_Gene476#
MADKFHILLLNGPNLNLLGTREPEKYGSTTLTEIVNGLENQASALDITLSHLQSNAEHQLIDRIHQAR
GNTDFILINPAAFTHTSVALRDALLAVQIPFIEIHLSNVHAREPFRHHSYLSDVAVGVICGLGADGYA
FALQAAVNRLSKTH
>CORE_REP|Org34_Gene2179#
MKQATFYLLDNAEPSGALSAHEAVACAVAASGFRSGKRVLIACESQEQAQRLDEALWQREPHEFVPHN
LAGEGPHYGAPVELCWPGKRGNAPRDLLIALLPQFADFATAFHEVVDFVPYEDTLKQLARDRYKAYRS
VGFHLTTATPPTH
>CORE_REP|Org44_Gene565#
MHCPFCAAVDTKVIDSRLVGDGSQVRRRRQCLVCNERFTTFEVAELVMPRVIKSDEVREPFNEDKLRR
GMLKALEKRPVSSDDVENALNHIKSQLRATGEREVPTKLVGNLVMDALKKLDKVAYIRFASVYRSFED
VREFGEEIARLQD
>CORE_REP|Org40_Gene225#
MSYKRPESILVVIYAKSSGRVLMLQRRDDTEFWQSVTGSLEQDESPPHAARREVMEEVGIDIEAEHLP
LFDCQRCVEFELFVHLRHRYAPGTTRNKEHWFCLALPEERDPVITEHHAYQWLEAAEAVKLTKSWSNQ
QAIEEFVINSVQ
>CORE_REP|Org49_Gene714#
MSQLAFWQQKTLAEMSEQEWESLCDGCGQCCLNKLIDEDTDEIYFTNVACNQLNIKSCQCRNYERRFE
LEEDCIKLTRENLTTFDWLPPTCAYRLIGEGKPLFPWHPLLSGSKAAMHGERITVRHIAVRESEVVDW
QDHILNKPDWAR
>CORE_REP|Org14_Gene935#
MGLFNFVKEAGEKLWDTVTGNASAEDQGAKLKEHLDKSGLPGTDKVNVQVIDGKAVVTGDAVSQELKE
KILVAVGNVAGISGVEDKVAVTQPDAESRFYTVKKGDTLSAISKEMYGNANQYNKIFEANKPMLSSPD
KIYPGQVLRIPQ
>CORE_REP|Org21_Gene2464#
MNMLLLLIAAGMGLVVQNLLMVRMTESVSTILITLVINSSVGLLLLVGLLLAKNGLGAVAEVTGAARW
WMLLPGLLGSLFVFAGILGYQKLGAAATISILVASQLCMGLLVDVYRAGPAALRENLPALFGALLLVA
GAYLVAKRSF

>CORE_REP|Org27_Gene1375#
MASKRDWLLQQLGITQWTLRRPGVLQGEVAVSLPPEARLLVVAQTLPAPDDPLFCDVLRSLGLTPAQT
YSLTPEQAAMLPEETACNSWRLGVAEPLAVAGAQLHSPALAELSQDAGAKRALWQQICHHEHDFYPDG
GRPGHGLHH
>CORE_REP|Org39_Gene132#
MAYKHILIAVDLSPESKILVEKAVSMARPYNAKVSLIHVDVNYSDLYTGLIDVNLGDMQKRISEETHQ
ALTELSQNAGYPITETLSGSGDLAQVLVDAIKKYDMDLVLCGHHQDFWSKLMSSARQLINTVHIDMLI
VPLRDEEDE
>CORE_REP|Org31_Gene3127#
MLAPHPFGREVTAEALIATFSTLKQWEDRYRQLIMLAKRLPPLPEALRSEEMALSGCENRVWLGHQLL
EDGTLHFYGDSEGRIVRGLLAVLLTEVEGKTPQQIAALDPLALFDRLALRAQLSATRAGGLAALAAAV
KAIAARYA
>CORE_REP|Org29_Gene1162#
MLQEIMPFVSRHPILSLAWIALLVAVIVMTFKSRFSKVKEIARGEAIRLINKEEAVVVDTRSRDDFRR
GHLANAINLTASEIKNGSLGELEKHKAQPIIVVCANGTASREPAENLSKAGFEKVTMLKDGISGWSGE
NLPLVRGK
>CORE_REP|Org12_Gene92#
MRLNTLSPAEGAKHAPKRVGRGIGSGLGKTGGRGHKGQKSRSGGGVRRGFEGGQMPLYRRLPKFGFTS
RKAMITAEVRLSELALVEGDVIDLNALKAANVVGVQIEFAKVVLSGEVARPVTLRGLRVTKGARAAIE
AAGGKIEE
>CORE_REP|Org31_Gene1963#
MDDIDRQILTLLAQDARASLKTLSAQVGLSSPSTSERLRRLEESGVIQGYTLNVNLQAVGYAFESLVR
IKPLPGMLKKVEQLIQAIPEVVECDKVTGEDCFIVRLVAHSMAQLDHTLDRLAEHAQSNTSIVKTTPV
KRRLPPLL
>CORE_REP|Org41_Gene2048#
MRKTTVAALAALALAGCSMKPHTAVTPGDLLHHNFVLQSVDGETAKSPAGGGLLNLEFGESLHVSGTM
CNRFFGQGQLRDGVLTVKPLATTRRLCPDEQRNRWDRVIGTVLENGAEVTLNAQQLTLNGSGHTLIYT
LRDWVY
>CORE_REP|Org16_Gene2579#
MQIRPYQETDRPFLRTLYLASRKAAFGWRDTSNYQLEDFDGATLGEAIWVAEDGGTLLGFVSVYREDN
FIHNLYVDPHQPPRGVGSALLQAAQATFTATGSLKCLVKNENALAFYRKHGWRIISTGNDGEEDYYLM
HSPAR
>CORE_REP|Org41_Gene623#
MSNRTIIAFDFGTKSIGAAVGQELTGSARALPAFKAQDGSPDWLKIEKLLKEWQPDLVVVGLPLNMDG
TEQPVTAQARKFANRLHGRFGIQIDLHDERLSTVEARANLFDRGGFRALDKGSVDSASAVVILESWFE
RQLG
>CORE_REP|Org42_Gene3423#
MGYQNVLVTVAVAPDSHRLVEKAVSIVRPYGGSITLLSTLANPEMYNNFAGPMLGDLRSLMEEETRLF
MAELRQRAGYPIADALIVHGELGDSLEYASRRQPFDLLICGNHRDGMMNKVSCSAARFINISHIDVLI
VPL
>CORE_REP|Org32_Gene296#
MAMTYHLDVVSAEKQMFSGLVQKIQVTGSEGELGIFPGHAPLLTAIKPGMVRIVKQHGEEEFIYLSGG
ILEVQPSVVTVLADTAIRGTDLDEARALEAKRKAEEHIRSSHGDVDYAQASAELAKAIAKLRVIELTR
KAM
>CORE_REP|Org14_Gene1668#
MANLPDKDKLVRNFSRCLNWEEKYLYVIELGAKLPPLDEAERQAGNLISGCQSQVWIVMRRDEQGQVE
FHGDSDAAIVKGLLAVVFILYRQLTPQQIVDLDVRPFFSELALSQHLTPSRSQGLEAMIRAIRSKAAQ
LA
>CORE_REP|Org39_Gene662#
MKLWKRETSLEQLNRAGDGCMVSHVGIEFTQLGEDFLEATMPVDGRTRQPFGLLHGGASVVLAESMGS
MAGYLCSEGEQKVVGLEINANHLRAVFDGQVRGVCRALHVGRRHQVWQIEIFDARDRLCCTSRLTTAV
ID
>CORE_REP|Org20_Gene493#
MKPAARRRARECAVQALYSWQLSKNDLADVEHQFLTEQDVKDVDVAYFRELLSGAAVNAGMLDELMAP

YLSRQLEELGQVERAVLRVALFELKMREDVPYKVAINEAIELAKTFGAEDSHKFVNGVLDKVAPTLRK
KK
>CORE_REP|Org45_Gene448#
MTKPIITINELDAERLDALLEQPAFANTDVAAALNDELDRAEILPPEKMPANVVTMNSRVRFRDLHTD
EEHVRTLVYPASLKDSHDQLSVMAPLGAALLGMHVGKQISWQLPNGEEARIEVLELLYQPEAAGEYHR
>CORE_REP|Org28_Gene416#
MAKEFSRGQRVAQEMQKEIAIILQREVKDPRVGMATVSGVEVSRDLAYAKVYVTFLNVLTENHDPDLV
TNGIKALQDASGYIRTLLGKAMRLRVVPELTFAYDNSLVEGMRMSNLVTNVVKNDAERRSASGDDKED
>CORE_REP|Org25_Gene3304#
MMPFHPEALWRRLQGSPFRARFRLNPKDQSYLDDKGLPLILSHARDFIDRRLAAAHPKNDGKQTPMRG
HPVFVAQHATATCCRSCLEKWHGIPQGIALDEQQKDYIVQAIALWLVRRGGAREESGANLFDPDRGL
>CORE_REP|Org47_Gene190#
MQARVKWVEGLTFLGESASGHQVLMDGNAGDKAPSPMEMVLMSVGGCSAIDVVSILQKGRNDVRDCEV
KLTSERREEAPRLFTHINLHFIVTGQDLTDKIVERAVNLSAEKYCSVALMLNKAATVTHSFEIRQPA
>CORE_REP|Org10_Gene66#
MNPLVYFSSSSENTHRFVEKLGLPAIRIPIAGARSKLLMEQPYILIVPSYGGGSAVGAVPIQVIRFLN
VPQNRSYLRGVIAAGNTNFGAAYGIAGDIIAKKCQVPFLYRFELLGTTQDVENVRQGVTAFWQRQN
>CORE_REP|Org39_Gene851#
MSNTLFRWPVRVYYEDTDASGVVYHARYVAFFERARTEMLRQHNFHQQQLLSEQVAFVVRRMTVDYLA
PARLDEQLEVQSEITCLRGASLTFAQRIVNSDGALLSQADVLIACIDPHQMKPRALPKSIVAEFKQ
>CORE_REP|Org13_Gene373#
MTWEYALIGLVVGIVIGAVAMRFGNRKLRQQQVLQNELDKSKTELEEYRQELVGHFARSAELLDNMAR
DYRQLYQHMAKSSNNLLPDLPMQENPFRYRLTEAEADNDQAPVEMPRDYSEGASGLLRGQSARRD
>CORE_REP|Org27_Gene266#
MRHYEIVFMVHPDQSEQVPGMIERYSATITNAQGQIHRLEDWGRRQLAYPINKLHKAHYVLLNVEAPQ
EAIDELETNFRFNDAVIRSMVMRVKHAVTEASPMVKAKDERRGDRREDFANETADDADAGDSEE
>CORE_REP|Org43_Gene1619#
MTAYTALKHFHLLTVAISITLFVLRFYWQWRRSPIVGRRWVKIAPHLNDTLLFVSGIALVVTFGFYPL
LGMDSWLTEKLFGVIIYILLGYVALGKKTKSQRLRTVAFVLALGCLYLIIKLATTKIPFLMGYL
>CORE_REP|Org26_Gene89#
MSMQDPIADMLTRIRNGQAANKVAVTMPSSKLKVAIANVLKEEGFIEDFKIEGDAKPVLELVLKYFQG
KAVVESIQRISRPGLRIYKKKDELPKVMAGLGIAVVSTSKGVMTDRAARQAGLGGEIICYVA
>CORE_REP|Org12_Gene481#
MGKYVKKQRPVNLDLQTIRFPVTAIASILHRVSGVITFVAVGILLWLLGLSLSSQEGFLQAAAIMNSF
IVKFIFWGILTALAYHICGGIRHLLMDFGYIEESLAAGTRSAQVAIGLTVVLSVLAGVLVW
>CORE_REP|Org9_Gene1200#
MLDYCLLVTGPAYGTQQASSAYQFAQALLAKGHRLSSVFFYREGVLNANQLTAPASDEFDLVRGWTQL
AQQHGVALNVCVAAALRRGVTDEQEAAQQGLASANLQPGFTLSGLGSLAEASLSCDRLVQF
>CORE_REP|Org15_Gene4152#
MANKPQQTTLTMYGIKNCDTIKKARRWLEDQGVAYHFHDYRADGLDEQRLRGFVAQLGWEPLLNTRGT
TWRKLDEAQRNACDNADAAIALMLAQPAIIKRPLLDAGNGRALLGFNTDAYQQFIAEVAV
>CORE_REP|Org33_Gene2485#
MLSSLLAVFIGGGVGSALRWAVSMKMNPLNAHIPLGTLMVNLIGGFIIGLAMAIFTRMTHLDPTWKLL
ITTGFCGGLTTFSTFSLEVVYLMQDGRFGWALANMLLNLAGSLAMTLLAFMLVMWVNGR
>CORE_REP|Org23_Gene2603#
MNPYFTEVIDAHIAIERWLGKGAGEEQALLARFTPEFSMIALNGAPLDFTALCAFFRAHRAAKPGLEI
EIEEMKLVAEWPTGAVVSYREKQSLPGQSATLRYSTVVFERLPDALGWRHLHETAAAQ
>CORE_REP|Org33_Gene718#
MAVLGLGTDIVEMARIEAVVERSGDRLARRVLSDAEWALYQQHQQPIRFLAKRFAVKEAAAKAFGTGI
RNGLAFNQFEVFNDALGKPNIRLHGRAAELAGEMGVAAIHVSLADERRYACATVIVES
>CORE_REP|Org48_Gene2900#
MSLYATLEEAIEAAREEFLDTAEGGSGDEPPVPQQFNLQKYVMQDGDTMWQAEFFEEEGEAVECLTLR
SGAAAQAIFDGDYDEVEITAEWIDENTLYEWEEGDFQLEPPLDTEEGQAAADEWDER
>CORE_REP|Org37_Gene2403#

MKRVAFVFTHGPHGGAGGREGLDALLATSALSEDLGVFFVGDGVLQLLPGQQPEKILARNYIATFGVL
PLYDVERCYLCQASLQERGLSQVTDWVLNAEVLAPDELRRELAGYDAVMTF
>CORE_REP|Org27_Gene1698#
MSMTLGSKKLYRVPEEGMVKGVCAGLAHYFDVPVRLIRVMVVLSLFFGLFFFTLVAYIALVFVLDEAP
ASRFEGEHQKTPRQLLDQLEYELGSGEQQLRQVERYVTSDTFGVQSRFRKL
>CORE_REP|Org36_Gene736#
MDIVFIEELTVITTIGVYEWEQGIRQKLVFDIEMGWDNRPAAASDDVTDCLSYADVSDAVIQHVESNR
FALVERVAEEISEILLQRFNSPWVRIKVSKPGAVAHASRVGVIIERGTRPA
>CORE_REP|Org3_Gene327#
MVKLAFPRELRLLTPTHFTFVFQQPQRAGTPQITILGRLNQLGHPRIGLTVAKKHVKRAHERNRIKRL
TRESFRLRQHELPAMDFVVVAKKGIADLDNRALTEALDKLWRRHCRQAPAS
>CORE_REP|Org12_Gene933#
MSNIIKQLEQEQMKQDVPAFRPGDSVEVKVWVVEGSKKRLQAFEGVVIAIRNRGLHSAFTVRKISNGE
GVERVFQTHSPVIDSIAVKRRGAVRKAKLYYLRERTGKAARIKERLNRVG
>CORE_REP|Org30_Gene856#
MKYVDGFVVAVPAANKEAYHRLAAAAAPLFKEFGATRVVECWGDDVPDGKLTDFRGAVKAQEGEVVVF
SWIEYPSKAVRDAANEKMMNDPRMKALGEMPFDGKRMIFGGFAPILDT
>CORE_REP|Org48_Gene437#
MKKTTLSMLLLAMLGFSNASLALNESEAEDLADLTAVFIYLKNDCGYNDLPNAQIKRAIVYFAQQNRW
DLSNYNSFNMKALGEDSYRDLSGIAIPTPKKCKSLARDSLSLLAYAN
>CORE_REP|Org28_Gene1343#
MRIIVYGSLRRKQGNSHWMTNAQWLGEHELEGYQIYNLGHYPAAIPGEGTIHCEVYRINSSILAELDE
LKSNTKDYKRELIQTPYGSAWIYLYKHSVDGYPRITSGDWLKRLDEQ
>CORE_REP|Org35_Gene1029#
MSHSSHETSHGGASHGSVKSYLIGFILSIILTVIPFAMVMYGNDGSISHSTILAVVVGMAVIQVIVHL
VYFLHMNTSSEERWNLVALLFTAMIIGIVVVGSLWIMYNLNINMMVD
>CORE_REP|Org15_Gene221#
MNIERIDPDQRWSEAVVHNETVYYTSVPENLDDDATAQTANALAAIDVLLERVGSDKSRILDATIFLA
NTADFAAMNAAWDAWVVAGSAPVRCTVQAQLMNPKYKVEIKIIAAL
>CORE_REP|Org24_Gene761#
MKSVLLGITLLATATGALAADKLVNITKLEYGKQWAFTKEEVTLQCRSGGALFVLNNSTLMQYPLNDA
AEQQVKKGHQRAQPLEVLLLDDPAEPGKKMSLAPFIERAEKLCAD
>CORE_REP|Org1_Gene23#
MKKIDAIIKPFKLDDVREALAEVGITGMTVTEVKGFGRQKGHTELYRGAEYMVDFLPKVKIEIVVADD
IVDTCVETIMQTAQTGKIGDGKIFVFDVARVVRIRTGEQDEEAI
>CORE_REP|Org2_Gene457#
MQLPHCPKCNSEYTYQDNALFICPECAHEWSDSAPAEDQDALIVKDANGNLLADGDAVTVIKDLKVKG
SSSMLKIGTKVKNIRLVEGDHNIDCKIDGFGPMKLKSEFVKKN
>CORE_REP|Org8_Gene298#
MQMSIHNQVRRSLQAIEQSMRDLALWQAAPPEHEAFSSTEPFCIDSMSAEAWLQWVFLPRMYALLDAE
APLPTRFAITPYFEEALKDREPSSLPLLVLLQQLDLMLNKEP
>CORE_REP|Org33_Gene518#
MSFFISDAVASAGAPAQGSPYSLIIMLVVFGLIFYFMILRPQQKRAKDHKKLMDSIGKGDEVLTTGGL
IGRVTKVADTGIIAIALNDTTEVMIKRDFVAAVLPKGTMKAL
>CORE_REP|Org3_Gene512#
MFGKGGLGNLMKQAQQMQEKMQQMQEEVAKLEVTGESGAGLVKVTINGAHNCRRVEIDPSLMEDDKEM
LEDLIAAAFNDAARRIEETQKEKMASVSSGMQLPPGFKMPF
>CORE_REP|Org17_Gene468#
MAKNRSRRLRKKLHIEEFQELGFSVAWRFAEGTSVEDIDSTLDTFIDEVIEPNGLAFDGSGYLQWEGL
ICLQKIGHCTDEHRELVKNWLEARKLTDVKVSDLFDIWWD
>CORE_REP|Org17_Gene537#
MKKIMLMLAAAAALSACAQPAAPPEDAKLKQAYSACINTAEGSPERLQPCKAVLNVLKQEKQHQQFAA
QETVRVMDYQNCIMAVHSGNGQAYDAKCGKLWQEIRDNNN
>CORE_REP|Org30_Gene2438#

MLKTTLLFFATALAEIIGCFLPYLWLKKQGSAWLLLPAAVSLMLFVWLLTLHPAASGRVYAAYGGVYV
ATALLWLRVVDGVKLSALDWVGAGVALAGMLIIVSGWRAA
>CORE_REP|Org6_Gene3852#
MGKLTLLLLALLGWLQYSLWLGKNGIHDYVRVNEDVAVQQGNNAKLKARNDQLFAEIDDLNGGQEAIE
ERARNELGMIKPGETFYRLVPDQSRRNAASSSQNNAQNNA
>CORE_REP|Org40_Gene57#
MQLSTTPTLEGFTITEYCGVVTGEAILGANIFRDFFAGVRDIVGGRSGAYEKELRKARLIAFEELEDQ
AKELGANAVVGIDIDYETVGKDGSMLMVTVSGTAVKVSR
>CORE_REP|Org21_Gene366#
MDLNNRLTEDETLEQAYDIFLELAGDNLDPADILLFNLQFEERGGAELYDPSEDWSEHVDYDLNPDFF
AEVVIGLADSDGEPINDVFARVLICREKDHKLCHILWKE
>CORE_REP|Org43_Gene4616#
MAVEVKYVVVRNGEEKMTFASKKEADAYDKMLDLADNLGEWLQQAPLNLDDEQREGLSFFLAENKDVL
GLILRGASSAETLKKPAESKAKKAASTPKEGSASEKQAA
>CORE_REP|Org33_Gene50#
MGNNNDWLNFEHLAEEKQIDAVKPPSMYKVILNNDDYTPMEFVIDVLQKFFSYDIERATQLMLTVHYQ
GKAICGVFTAEVAETKVVHVNRYARENEHPLLCTLEKA
>CORE_REP|Org16_Gene915#
MIGNERHGLVGVIGGDLLRNAKIPMILLIASLVSAVFVVTTAHRTRLLTAEREQLVLERDALDIEWRN
LILEENALGDHSRVERIATEKLQMQHVDPSQENIIVKQ
>CORE_REP|Org15_Gene2974#
MTANRLVLSGTVCKAPVRKVSPSGIPHCQFVLEHRSQQMEAGFSRQAWCRMPVVVSGQQSQALTQRLT
VGSQITVQGFVSCHQGRNGLSKLVLHAEQIELIDSGD
>CORE_REP|Org37_Gene1069#
MEKKKIYLFCSAGMSTSLLVSKMKAQAEKYEVPVIIAAYPEALAAEKGIEADLILLGPQIAYTLPEVQ
KQLPNKPVEVIDPLLYGKVDGLGVLKAAVAAIKKANQ
>CORE_REP|Org6_Gene593#
MYAVFQSGGKQHRVSEGQTVRLEKLDIATGEAVEFDQILMIANGEDIKIGVPFVDGGKIKAEVVAHGR
GEKIKIVKFRRRKHHRKQQGHRQWFTDVKITGISA
>CORE_REP|Org25_Gene22#
MSREITFFSRFEQDILAGRKTITIRDASESHFEPGEVLRVSRNEDGVFFCFIEVLSVTPVRLDALTER
HAQQENMSLGELKQVIKEIYPGLDALFVIEFVKR
>CORE_REP|Org31_Gene4662#
MTDTSPTLWHLYMLRLPSGMLYTGITTDVARRMAQHQAGKGAKALRGKGELTLAFHCQVGDRSTALKL
EYRVKQLSKIQKERLVNHPPLSLEYLLPVVVKSD
>CORE_REP|Org3_Gene167#
MKYLLIFLLVLVIFVISVTLGAHNDQVVNFNYLVAQGDYRVSTLLATLFGAGFVLGWIICGLFYLRTR
IALGRAERKIKRLELQLEQPAEPAAQPVVSKE
>CORE_REP|Org30_Gene335#
MIPLQHGLILAAILFVLGLTGLLVRRNLLFMLISLEVMINAAALAFIVAGSYWGQADGQVMYILAISL
AAAEASIGLALLLQLYRRRHTLNIDTVSEMRG
>CORE_REP|Org32_Gene3319#
MSDAINKCSAQETAACCCVDVGTVMDNTDCTASYSQVFSNQQDAEAMLAALSEKARAVESDPCDISSS
IKPVDGGVQLEADFTFACQAETLIFQLGLR
>CORE_REP|Org6_Gene1204#
MLKVNEYFAGKVKSIGFDSSSIGLTSVGVMEEGEYTFSTAQPEEMTVITGALKVLLPGAPDWQVFTPG
EKFFVPGHSEFNLQVADATAYLCRYLSK
>CORE_REP|Org47_Gene1299#
MLYTLSHSPNQCDLPALLRLTAEGDALLLLQDGVLAGLAGSAHLESLLAAPISLYALQDDLEARGLVG
HFSHKITVVGYNHFVELTEQHRSQMAW
>CORE_REP|Org36_Gene3035#
MSDIQVEVVYALPERQYLRKVKLAEGSSVEQAIQTSGLLELRQDIDLKSNKIGIYSRPAKLGDTLNDG
DRVEIYRPLIADPKELRRQRAEKAKK
>CORE_REP|Org8_Gene545#

MFTINAEVRKDQGKGASRRLRAANKFPAIVYGGKEAAVSIELDHDSVKNMEAKPEFYSEAVTLVIDGK
ETKVKVQAVQRHPFKPKLAHIDFVRV
>CORE_REP|Org24_Gene3613#
MKRRNADRMGNFFMGLGLVVMIGGVGYSIIAEVSQFNLPQFFAHGAIMSIFVGALLWLVGARIGGREQ
VADRYWWVKHFDKRCRNDQHRSSH
>CORE_REP|Org2_Gene3975#
MGINPVFARRLYLCWLISHSERPNVPRLMALTGWPRRTLQDVLKALPGMGIELQFVQQGVRNNDGFYQ
LESWGPFNKSWVHQHHQALLSAIE
>CORE_REP|Org26_Gene152#
MARVTVQDAVEKIGNRFDLVLVAARRARQIQTGGKDALVPEENDKYTVIALREIEEGLITSQILDVRE
RQEQQEQEAAEIQAVTAIAEGRR
>CORE_REP|Org36_Gene415#
MSRTIFCTFLQRDAEGQDFQLYPGDVGKRIYNEISKEAWGEWMKKQTMLINEKKLNMMNVDDRKLLEE
EMIKFLFEGHDVHIEGYTPPSE
>CORE_REP|Org16_Gene309#
MLINLGRLLMLCVWGFLLSNLFHPFPKPLKYFIDVALFFMVVMHGLQLVLLKSTQPKDQPISYWQEAK
IFIFGVFELLAWQKKQPPIKKK
>CORE_REP|Org49_Gene211#
MALLDFFLSRKKQTANIAKERLQIIVAERRRGDSEPPYLPDLKRDILAVICKYIQIDPEMLHVQFEQK
GDDISVLELNVTLPESEEAAK
>CORE_REP|Org18_Gene251#
MDINNKARIHWACRRGMRELDISIMPFFEYEYDSLNDADKALFIRLLECDDPDLFNWLMNHGAPQDGE
LQRMVTLIQTRNKDRGPVAM
>CORE_REP|Org21_Gene619#
MQKTKLNELLEFPCSFTYKVMGLAQPELVDQVVEVVQRHAPGDYNPQVKPSSKGNYHSVSITINATHI
EQVETLYEELGNIEIVRMVL
>CORE_REP|Org15_Gene253#
MANIKSAKKRAVQSEKRRKHNASRRSMVRTFIKKVDAAIAAGDKEAAQNAFLVMQPLVDRQAAKGLIH
KNKAARHKSNLTARINAMQ
>CORE_REP|Org42_Gene1074#
MFQQEVTITAPNGLHTRPAAQFVKEAKGFASDITVTSNGKSASAKSLFKLQTLGLTQGTVVTISAEGE
DEQKAVEHLVKLMAELE
>CORE_REP|Org7_Gene1019#
MTDLFAQADQTLDALGLRCPEPVMMVRKTVRHMDNGETLLIIADDPATTRDIPGFCRFMEHTLVAQET
EQAPYRYLLRKGV
>CORE_REP|Org33_Gene255#
MKETTIVPDYRLDMLGEPCPYPAVATLEAMPQLKPGEILEVISDCPQSINNIPLDARNHGYKVLDIQQ
DGPTIRYLIQR
>CORE_REP|Org14_Gene149#
MSFEVFEKLEAKVQQAIDTITLLQMEIEELKDKNNSLSQEVQAASGNHEALVRENQQLKEEQHVWQDR
LRALLGKMEEV
>CORE_REP|Org46_Gene666#
MAQQLEFFDIPSPCRGICQADDRGFCRGCLRSREERFGWMNMSDAQKREVLRLCRQRFLRLQRANKAP
DEPLPEQPSLF
>CORE_REP|Org28_Gene321#
MSRVCQVTGKRPVSGNNRSHAMNATKRRFLPNLHSHRFWVEAEKRFVTLRVSAKGMRVIDKKGIETVL
ADLRARGEKY
>CORE_REP|Org33_Gene61#
MSIIIYSKPDCVQCNATYRAFDKQGIDYQVIDLTQDQQALNHVKSLGYQQVPVIIAGDDHWSGFRPDK
IGALALTC
>CORE_REP|Org49_Gene138#
MKQGIHPKYEEVTANCSCGNVMKIRSTVGHDLNLDVCGACHPFYTGKQRDVATGGRVDRFNKRFSVPG
AKK
>CORE_REP|Org49_Gene6#

MPVIKVRENEPFDVALRRFKRSCEKAGVLAEVRRREFYEKPTTERKRAKASAVKRHAKKLARENARRT
RLY
>CORE_REP|Org19_Gene97#
MKAQELREKSVEELNAELLNLLREQFNLRMQAASGQLQQTHLLKQVRRDVARVKTLLTQKAGV
>CORE_REP|Org34_Gene29#
MLILTRRVGETLMIGDEVTVTVLGVKGNQVRIGVNAPKEVSVHREEIYQRIQAEKSQQTTY
>CORE_REP|Org8_Gene2961#
MAKTIKVTQTRSSIGRLPKHKATLLGLGLRRIGHTVEREDTPAVRGMVNLVSYMVKVEE
