# Supplementary material for: A Systematic Immuno-Informatic Approach to Design a Multiepitope-Based Vaccine Against Emerging Multiple Drug Resistant Serratia marcescens
Source: Front Immunol. 2022 Mar 14;13:768569. doi: 10.3389/fimmu.2022.768569 (PMC8967166; doi:10.3389/fimmu.2022.768569)
Supplement: Supplementary Data Sheet S7 — Gut flora non-homologue protein sequences. [file DataSheet_7.pdf]

>CORE_REP|Org34_Gene4576#
MSENTASQIAAPQNAQASGRTILLFLALALMSALLNSSAPTPLYPLYQQQLTLSSVSLTVIYGAYAAG
VLISLFGVGNLAGKVKDLRSMIVPALLVVLSGALLFAQADTFAMMLMARLLAGVGTGALTGAANIALV
RFGPRDGGKNAALIATLSFTTGLALGPIFSGIALQTGFHPTTLPFVFIMVMAAVAALGVMFSWPRGVV
TAPSHVTSAETEKSSLLDGLRATGGKFFVCAGALFICWALAASILAIGPGVAETLLGLHARGVFGYAI
AVYLLIAGISQILSRRVNARHSLLFGCLAQVLAAVVFTMAIQWHSLGLAAVGLVVAGYAYGAIFVGSA
TLVNLISPPTSHARLLSLFYVIAYIANWVPILLGVVVDRVDLNQATHLLFLGSTVVCLLLAWKTSRVG
FLANYFVMNIYFYN
>CORE_REP|Org34_Gene1366#
MNPLFTPYLQRWQLEQDGKAFETHSSLLMPVRYRGEAAMLKIAREQEERFGGQLMCWWRGEGAAQVLA
WHEDGILLERAQGESSLAQLVRDGDDEQATAILCRAIAALHAPRAAPLPELIPLQEWFSSLWPAAQAH
GGMLRLSATTAAELLSSPRDESVLHGDIHHDNVLDFGERGWLAIDPKRLYGERGFDYANIFCNPNYGI
ATDPAIFQRRVEQVCRLAGLERRRLLQWILAWAGLSAAWFMEDGQAADIDFRVAELAARALDLPLPAG
DSGFILPVIERG
>CORE_REP|Org9_Gene1317#
MGKHFAAQRHESVGNGEKAGMKAIGAFSSVFLGVCSLAIGNVNAAETKSNETYQDAETLLVTGEKVKR
SIFDTSSSVQVFDSNRIASMPDAVQIPDLLRMTPNVVDLGIGNELPTVRGIDGSGPNVGANAFLSGTR
PRLNLSLDGRSLTYNEQAFGPQSLWDLDRVEVFLGPQSYIQGRNAIAGAIVMASKDPTFEWESAFKGG
AGNQHSSQLAAMASGPLVEDQLAFRVSVDRQRRRSEADLPAYAPVGDPREVEATTARAKLLFNPAGLR
DLTTKLTFNHFGSTAPQNESLNPQPHPTNPRHDPRRAVFKSNMNSTIWDLAWEASDALTLENRVIYTD
FNINRPTAYNIQYAEIDGQEVHVEPVVRFGGADSRLHGLAGLRYFHGTQDEFVNIFGGSTFKDKTDTH
SAFAELTYALTPQVDVTAASRLEREHRRRDGGSQAVRIDFDETYTVFLPKLDVAWKPTDTQTYGAKIA
RGYNAGGGGITIGTPVVSYTYGSEYVWNYELYTRHHLKDANVVLTGNIFYNDYKDMQLPYSLGENSSV
IRNADKVETYGAEIGATWQPRWDFELFGNLGLLKTDIKKFSGSGVEGHELARAPAYTANMGAKYQFLK
GWELSSNVAFSDSYYSAYDNDSRGRIGSYWTANAQLAYTFDYGRATLYAKNLFDSDRREMVRSNDIYT
ATLQRGRLVGAAVELNF
>CORE_REP|Org41_Gene4663#
MPDLSRRDILRAAAIGSAFSLLPASIRKALAIPANNRTGTLRDVEHVVILMQENRSFDHYFGTLPGVR
GFSDRFTIPLPGDRHVWQQQGAERLVLPYHLDSKRGNAQRVTGTPHSWVDEQAAWDHGRMSAWPTYKT
PASMGYYRQHELPFQFALANAFTLCDAYHCAIHAGTNTNRLFHWTGTNGPSAADVAVVVNEWDSPGPA
EIGYQWTTYPERLEASGVSWKVYQFLPDNFTDNPLAGFRQYRAASIQVGNPARPPKDFNAFVPYRDAL
NEAAPLYKGNGNTLPAADGNDLDAMLAGFRADIQQGKLPQVSWIIAPAAYSEHPDPSSPVQGGWFTQE
ILNALTDNPEVWSKTVLLVNYDENDGFFDHMPSPSAPSLREDGSFAGKSTVPFDTEIFQHVAPPGSQD
QPPPDGRIYGPGPRVPMLVLSPWSRGGWVNSQVFDHTSVLQFLEKRFQVHEPNISAWRRAVCGDLTSA
FNFVDPNSEALPSLPVTSRHAADGLRQRQEQLPQVPLPSPAHQRLPHQRRQARPSRALPYQLHVEATV
VAEQRRVTLNLFNTGEQGAVFHVYDRRDLAQIPRRFTVEAGKAVSDDWQTEDEYHLWLLGPNGFHREL
RGALNRPQPEVRLRPTGRSLQLQLNNPGTEAIAVTLERCPYTQQGPWHITLPAGGSHQQSFDAHASGG
WYDLTLQSPGGWLRRLAGRLEDGEHSVSDPLMGQE
>CORE_REP|Org13_Gene3724#
MSEALTCFKAYDIRGKLGSELNEDIAYRIGRAYGEYLRPKTMVLGGDVRLTSESLKLALARGLQDSGT
DVIDIGLSGTEEIYFATSHLKVDGGIEVTASHNPMDYNGMKLVREESKPISGDTGLRDIQRLAENNSF
PAVNDAARGGYQQLSILDAYVQKLLSFVALDNFTRPLKLVINSGNGAAGHVIDAIEARFKNAGLPVEF
IKVHHAPDGNFPNGIPNPLLPECRQDTTDAVLKHGADMGIAFDGDFDRCFLFDERGNFIEGYYIVGLL
AEAFLEKSPGSRIIHDPRLSWNTIDIVEKAYGIPVMSKTGHAFIKERMRKEDAVYGGEMSAHHYFRDF
YYCDSGMIPWLLVAELLCIKGRSLGELVNDRVAAYPASGEINSSLNNPKEAIGRVLGKYEMEADAVDH
TDGISVEYDNWRFNLRSSNTEPVVRLNVESRANVELMQEKTEEILQLLRSE
>CORE_REP|Org41_Gene2975#
MNQRLDIIGIGLGPSNLSLAALGSEIEGFTGQFLERKPHFSWHPGMILADCSMQTNFLKDLVSAVAPT
NRYSFLNYLVKNRKFYRFLTTEQRTASREEFADYLTWAAGGMDSLAFNQDVQQIEFDDRQRQFVVTTS
NKVFHAKHVSIGIGKKIKLPDCVTAQSDRCFHASEMMLRNPDLTGKRVAIVGGGQSGADLFLNIFKGE
WGQPDQLDWISRRNNYNALDEAAFANEYFTPDYVESFYSLDSAAKRHMLAEQKMTSDGITSESLLAIY
RAMYHRFDVLREKLWVRLLPSRSLTAVKHTLDNAYQLETRHHLDHGEEAFKADVVIFATGYQTATPEF
LEPLAHRLLTTADGEYRIAPDFTFEWEGPAENCLFAMNASMHNHGIADPQLSLMAWRSARILNRALDH
KPFDLGTTPTAIQWRSESVPHAF

>CORE_REP|Org23_Gene4353#
MKSLASTLQGQSIAAAITAVENDIKAKPADADLRAALVQLLCLSGNWTRANAQLKSWQALKPIAQPTT
LLLMQSVNAELQRQAVFAGAAAPALLRQDQPWLQLLVQALHQDAQGAAEQAQTLRDEALEAAPAGAGQ
LTLAEGNQERQLSFDWLTDGDGRLGPVCELALNGVYYWLPFADIAAIQFQAPQSAIDLVWSHALVRLT
DGREQVCQLPARYPLAEGSDDALLLGKRTEWQPLGDGTHYAGLGLKTWLSESDEFPLHSLRQLSFDAS
A
>CORE_REP|Org42_Gene4092#
MHNIDLEDRLAALSAAIADRTRARMLCLLMDGRAYTATELSAAVEVAPSTASAHLAKLLEQRLIACVK
QGRYRYFRLAGQPVAEALEGLMALAGVPRPSVKSSTPTTLQYARTCYDHMAGEVAVKLHDRLHALNWL
NGEEDYRLSDAGQAALARLGVDCSPAPTRRRFACGCLDWSERRSHLGGALGAALLAAFIHRGWIVRRL
DSRELQLTPAGKKALAAHFDLTV
>CORE_REP|Org4_Gene1541#
MNEKIDYHIEKYHFAPLDEAPRLAHQWSEVLNECRETQAGAEERLRIALLNVDYVTSFELPFRLLLVR
APQLIAGIREELPLSQKNVVFNGKRFGCVYSLKSDLSGVPEAFQYSLSTRIHRRAASGVDALPYREIA
KALKAPRERLRLALEQGLPVTALDGLFWFGIQRIAAEVRRLRKTGMAIVTAETEIFDTLTGTTRKVPV
YRLAES
>CORE_REP|Org26_Gene3903#
MFRGLKAFLLLTLSLLFCQRAFADCATTNGTVTLPGSSSFVVYNGQINAQGTAGLNCTGLGLSLLSQN
TVTVKVASTTNGMAVANTDGSGDKIAYLIYPDANYQYPYSIGQTIDYSSLNLLSLILISSNVNFPLYI
KTTAGANVRSGTYTDTINLIWNYHICGLGVLGLCIWWDGVNKVSTVSVSVAITKDCLIGTAPNVNFGS
MALVGQFNPVNQSITLTCTKTEGYNTYFTNGNNPVSGWRRMKSGTSNFMQYQIYLPNTTTVWDSTNKQ
SGAGTGLAQSIPYKAAVNAAQTEVAVGSYQDNLSFVVEY
>CORE_REP|Org6_Gene2783#
MHHLMLDIETLDIKPSAVILVVAAVFFDPRTGALGAEFETAVSSQKDQPGRTISLDTVAWWAKQSDEA
RKQAFGGTESLKRVLSSLSRFIHMNSTDTVKVWGNGKEFDCAILEHAFQQLEMPCPWKFWDTQDVRTV
ITLAELHGFNPKKARPFEGMPHRALDDARHQARYVADTVSALYYRQGAQR
>CORE_REP|Org8_Gene789#
MAKTVVVFHSGYGHTERLAKVVAEGAGAELIAIDQNGDISDEAWQTLDEADAIIFGSPTYMGGPSWQF
KKFADASSKAWFGRKWQDKVFGGFTNSASLNGDKQVTLIALQTLASQHGGLWVSLGLLPANTKSAQRT
DVNNLGGSVGLLVQTPADAGVDEMLSGDLATAKLYGQRVAGFAAKLA
>CORE_REP|Org7_Gene4762#
MKTVKRTGIALAIALTFPLALPAATAAQTSLTNSKAATMTEKHGQFIAVGKVVQVTFGDFAFKLDFTD
DKTMTFTGIGEASQGITDTVQYTAVEIRPKVYMVYWHEPQSGDNVTHIEDFERGEVYTNIAAKDGSFT
HLKGQLKIVGHSGN
>CORE_REP|Org42_Gene757#
MLNDIEEIRFTARSEENLRGVHPDLVRVIRLALRYSLVPFSVSEGLRSMARQREMVRAGSSQTLRSRH
LTGHAVDVVAMPAGVVSWEWDYYAQIAVAVRRAARECGINVEWGGEWKTLKDGPHFQLAFRDYPA
>CORE_REP|Org25_Gene3681#
MKRLLLDTHALLWWLIDDACLGVNAKRQIADPGNAVYVSAASIWEISIKQALGKLALPEDIFAIIEAE
DFLALPMDAFHCQQAGQLPPYHQDPFDRMLIAQAQAEGLTLISADTVFPQYGVRVADARR
>CORE_REP|Org3_Gene2772#
MLKTAGWLLPAILALAGCSSSGQRHEQTLSQALTALGQDKALIGASNGVMVRDAESGTVLYQAHAQQR
LAPASNMKMFTSLAAFGVLGADYRFETRLLTTGEQRGDTLRGDLYLQGSGDPTLHPDDLDTFAATLAQ
RGIRHIHGRLILDASAFDQTPFGAGWSWDDEPFAFAAPISALNYAFTPGGDINVVRVDVQPGARAGAP
GRVSFYPANDAVTLVNRTTTTGGDTALTFDRQPGSNRIVVSGTVAAQAEASSRLITVDQPARVVGALL
QNALRAHGITLRGNAEEGVTPAGARLLAEKTSPPLSRLAVTFLKVSNNGYGEVLTKAMGRKTQGKGDW
AAGLQAIGRFVQSQGIEAGAYRQVDGSGLSRMNQITPQQLTTLLLAARKQPWFADWYNALPIAGQPGL
LVGGTLRSRMVKSAAAGRAHAKSGSMTGVSSLSGYVDSATGRPLAFAIISNNYLVPGAEVKALEDRLV
ETLAACDATVVCR
>CORE_REP|Org1_Gene2196#
MSTRRTFIKQLSAVAGVSLTASLGIPLRGHAKAALNPAWRMPDEGEPQQRAFLAFGAQRAIWGGFTAD
VQAAQGRIARAIADFQPLTVFCRGNERQLAEATCGSHNVSYVVTELDDIWMRDIGANFVVNDAGELGA
VDFNFNGWGDKQQHARDARLAGFVARRYGVAQPRRSALVGEGGGIEVDGHGTGIMTESSWVNANRNPG
WSRDRVEQELKAMLGLRKIIWLPGIKGRDITDAHVDFYARFVRPGVVVANLDTDPASYDHAVTQAHLA

ILRAATDADGRTLQVHTLSPPRAPRESRFSRRNPDFAAGYINYFVINGAVIAPEFGDLQADKAAFELL
SALYPQRKVVQLEIDAIAAGGGGGGIHCVTSQLPVHGKPDQ
>CORE_REP|Org13_Gene3194#
MNYQFENLVFEGGGVKGIAYGGALELLEAKGIMPQIKQTSGASAGAIAALLVGLGCSSADVTKILSAM
DFKKFLDYNGGFFGTLQDAYRLFNQYGIAPGDYFYQWSRDIIKQYTGKPDITFEQFEAMKAAKGFKSI
YFIGANLNSGQREVYSHRTTPRMKVADGLRISMSFPFAFVAKNNTLGDLCIDGGMIDNYPVRLFDYDF
SATPPYIDSSSQRINTRTLGLRLDSAGEIAQAAGQAGPRTPVNNLFDFTLAVANVMLDIQTKVHLDSD
DWKRTVYVDTLDVGTLEFGISEEKKRALIESGRRGVERYFAWYDTAMKQAA
>CORE_REP|Org17_Gene537#
MKKIMLMLAAAAALSACAQPAAPPEDAKLKQAYSACINTAEGSPERLQPCKAVLNVLKQEKQHQQFAA
QETVRVMDYQNCIMAVHSGNGQAYDAKCGKLWQEIRDNNN
>CORE_REP|Org2_Gene3975#
MGINPVFARRLYLCWLISHSERPNVPRLMALTGWPRRTLQDVLKALPGMGIELQFVQQGVRNNDGFYQ
LESWGPFNKSWVHQHHQALLSAIE
