# Supplementary material for: A Systematic Immuno-Informatic Approach to Design a Multiepitope-Based Vaccine Against Emerging Multiple Drug Resistant Serratia marcescens
Source: Front Immunol. 2022 Mar 14;13:768569. doi: 10.3389/fimmu.2022.768569 (PMC8967166; doi:10.3389/fimmu.2022.768569)
Supplement: Supplementary Data Sheet S8 — Cleavage sites predicted by NetChop 3.1. [file DataSheet_8.pdf]

MHC-I Ligands input sequence:
EAAAKAKFVAAWTLKAAAGGGSTPFGAGWSWGGGSLEDRLVETLGGGSSSNVNFPLYGGGSSEYVWNY
ELGGGSYQFLKGWELGPGPG

NetChop 3.0 predictions using version C-term. Threshold 0.900000

pos	AA	C	score	Ident
1	E	.	0.026290	Sequence
2	A	.	0.308160	Sequence
3	A	.	0.072641	Sequence
4	A	.	0.055348	Sequence
5	K	.	0.111006	Sequence
6	A	.	0.316249	Sequence
7	K	.	0.061281	Sequence
8	F	.	0.215252	Sequence
9	V	.	0.086420	Sequence
10	A	.	0.160237	Sequence
11	A	.	0.113622	Sequence
12	W	.	0.783911	Sequence
13	T	.	0.038419	Sequence
14	L	S	0.974618	Sequence
15	K	S	0.926387	Sequence
16	A	S	0.945674	Sequence
17	A	.	0.152781	Sequence
18	A	.	0.442851	Sequence
19	G	.	0.066943	Sequence
20	G	.	0.047954	Sequence
21	G	.	0.030822	Sequence
22	S	.	0.022822	Sequence
23	T	.	0.025300	Sequence
24	P	.	0.152624	Sequence
25	F	S	0.948856	Sequence
26	G	.	0.049691	Sequence
27	A	.	0.158143	Sequence
28	G	.	0.066917	Sequence
29	W	S	0.966769	Sequence
30	S	.	0.028708	Sequence
31	W	S	0.978238	Sequence
32	G	.	0.029521	Sequence
33	G	.	0.045344	Sequence
34	G	.	0.028986	Sequence
35	S	.	0.049729	Sequence
36	L	S	0.954228	Sequence
37	E	.	0.042397	Sequence
38	D	.	0.183098	Sequence
39	R	.	0.040214	Sequence
40	L	.	0.437333	Sequence
41	V	.	0.412908	Sequence
42	E	.	0.046222	Sequence
43	T	.	0.558735	Sequence
44	L	S	0.962129	Sequence

```
  45   G  .   0.049580   Sequence
  46   G  .   0.061192   Sequence
  47   G  .   0.134164   Sequence
  48   S  .   0.024867   Sequence
  49   S  .   0.033410   Sequence
  50   S  .   0.047141   Sequence
  51   N  .   0.102129   Sequence
  52   V  .   0.800465   Sequence
  53   N  .   0.031831   Sequence
  54   F  .   0.564182   Sequence
  55   P  .   0.030785   Sequence
  56   L  S   0.974094   Sequence
  57   Y  S   0.963198   Sequence
  58   G  .   0.058038   Sequence
  59   G  .   0.040819   Sequence
  60   G  .   0.034061   Sequence
  61   S  .   0.023547   Sequence
  62   S  .   0.058961   Sequence
  63   E  .   0.072302   Sequence
  64   Y  S   0.972669   Sequence
  65   V  .   0.343498   Sequence
  66   W  .   0.865409   Sequence
  67   N  .   0.025009   Sequence
  68   Y  S   0.978128   Sequence
  69   E  .   0.024615   Sequence
  70   L  S   0.967892   Sequence
  71   G  .   0.040622   Sequence
  72   G  .   0.149110   Sequence
  73   G  .   0.022318   Sequence
  74   S  .   0.023939   Sequence
  75   Y  S   0.967480   Sequence
  76   Q  .   0.148050   Sequence
  77   F  S   0.975848   Sequence
  78   L  .   0.788368   Sequence
  79   K  .   0.868170   Sequence
  80   G  .   0.105512   Sequence
  81   W  S   0.975757   Sequence
  82   E  .   0.024729   Sequence
  83   L  S   0.944942   Sequence
  84   G  .   0.073319   Sequence
  85   P  .   0.110796   Sequence
  86   G  .   0.064196   Sequence
  87   P  .   0.200242   Sequence
  88   G  .   0.112335   Sequence
______________________________________

Number of cleavage sites 17. Number of amino acids 88. Protein name
Sequence

______________________________________
```
